# Adaptive Evolution of Human-Isolated H5Nx Avian Influenza A Viruses

**DOI:** 10.3389/fmicb.2019.01328

**Published:** 2019-06-12

**Authors:** Fucheng Guo, Yiliang Li, Shu Yu, Lu Liu, Tingting Luo, Zhiqing Pu, Dan Xiang, Xuejuan Shen, David M. Irwin, Ming Liao, Yongyi Shen

**Affiliations:** ^1^College of Veterinary Medicine, South China Agricultural University, Guangzhou, China; ^2^Joint Influenza Research Centre (SUMC/HKU), Shantou University Medical College, Shantou, China; ^3^Department of Laboratory Medicine and Pathobiology, University of Toronto, Toronto, ON, Canada; ^4^Banting and Best Diabetes Centre, University of Toronto, Toronto, ON, Canada; ^5^Key Laboratory of Zoonosis Prevention and Control of Guangdong Province, Guangzhou, China

**Keywords:** H5Nx, Avian influenza A virus, convergent evolution, human infection, host shift

## Abstract

Avian influenza A viruses (AIVs) H5N1, first identified in 1996, are highly pathogenic in domestic poultry and continue to occasionally infect humans. In this study, we sought to identify genetic changes that occurred during their multiple invasions to humans. We evaluated all available H5Nx AIV genomes. Significant signals of positive selection were detected in 29 host-shift branches. 126 parallel evolution sites were detected on these branches, including 17 well-known sites (such as T271A, A274T, T339M, Q591K, E627K, and D701N in PB2; A134V, D154N, S223N, and R497K in HA) that play roles in allowing AIVs to cross species barriers. Our study suggests that during human infections, H5Nx viruses have experienced adaptive evolution (positive selection and convergent evolution) that allowed them to adapt to their new host environments. Analyses of adaptive evolution should be useful in identifying candidate sites that play roles in human infections, which can be tested by functional experiments.

## Introduction

Avian influenza viruses (AIVs) pose a continuous threat to public health due to their pandemic potential (Widdowson et al., 2017). The H5N1 subtype of highly pathogenic avian virus (HPAIV) is highly pathogenic in domestic poultry, since its initial detection in China in 1996 (Peiris et al., 2007). Since its first discovery in humans during 1997 in Hong Kong (Yuen et al., 1998), H5N1 HPAIVs continue to result in occasional human infections with an high fatality rate of more than 60% (Yu et al., 2008; Cowling et al., 2013). H5 viruses evolved into different clades, and have reassorted with different NA subtypes, including N1, N2, N3, N5, N6, and N8, resulting in outbreaks in poultry and lethal human infections (Smith and Donis, 2015). H5 is the predominate AIV subtype that infect human populations, and thus, pose great threat to public health (Peiris et al., 2007).

Human infections of H5Nx are mainly through direct avian-to-human transmission. The human-isolated H5Nx viruses are distributed into different phylogenetic clades, that is, these host-shift events occurred independently multiple times. During the adaptation of an organism to a new environment, adaptive evolution occurs. When similar morphological or physiological changes are observed on multiple evolutionary lineages, convergent or parallel amino acid changes in key genes occur (Zhang, 2006; Shen et al., 2012). Viruses face great challenges when they emerge in new hosts. Our previous study showed that during the multiple invasions of humans by H7N9 AIVs, convergent evolution occurred to allow these human-isolated viruses to adapt their new hosts (Xiang et al., 2018).

Although H5Nx viruses do not have the ability to be transmitted efficiently in a sustained manner from person-to-person, these HPAIVs, which are panzootic in poultry, continue to spread, and their interspecific transmission poses a major challenge to human health. In this study, we conducted a comprehensive evolutionary analysis of H5Nx viruses by collecting all available sequence data to examine molecular mechanisms used by H5Nx viruses to frequently infect humans.

## Materials and Methods

### Data Source and Preliminary Treatment

All available sequences of H5Nx viruses were downloaded from three databases: the Influenza Virus Resource at the National Center for Biotechnology Information (NCBI)^1^, the Global Initiative on Sharing Avian Influenza Data ^2^, and the Influenza Research Database (IRD) ^3^. Redundant sequences, laboratory strains and short (<80% of the corresponding gene) sequences were removed. Sequences from egg isolations from human hosts were excluded, as these sequences might carry additional *in vitro* adaptive mutations (Bush et al., 2000). Our final dataset contains 9945, 6719, 6845, 7966, 6454, 6401, 6466, and 6423 HA, NS, M, NA, NP, PA, PB1, and PB2 sequences, respectively (Supplementary Table 1). The sequences in each dataset were aligned by MAFFT v7.221, separately (Katoh and Toh, 2010). Initial phylogenetic trees for the eight genes were constructed separately, using the maximum likelihood method RAxML v.8.0.14 (Stamatakis, 2006). Best-fit evolutionary models for the sequences in each datasets were identified using ModelTest (Posada and Crandall, 1998).

### Selection Analyses

The CODEML program in the PAML package (Yang, 2007) was used to identify signals of potential positive selection. The branch-site model, which was used to determine whether a gene had undergone positive selection on a foreground branch, was used to assess selective pressure. Bayes Empirical Bayes (BEB) analysis was used to calculate the Bayesian posterior probability of any positively selected site or branch. Finally, LRT statistics were calculated between the branch-site model and the branch-site model with fixed ω_0_ = 1. The significance of the difference between the models was determined using twice the difference in the log-likelihood values of LRTs (2ΔlnL) between the two models, which follows a chi-squared (χ2) distribution with degrees of freedom equaling the difference in the number of parameter estimated (Zhang et al., 2005).

### Convergent Evolution Analyses

Ancestral amino acid sequences for target nodes of each dataset were inferred using PAML4.0 (Yang, 2007). The statistical significance of the number of convergent/parallel evolutionary substitutions between pairs of branches was tested using the method of Zhang and Kumar (1997). Candidate substitutions were defined if (i) the topology of each lineage consisting of human isolate and its genetically related isolates had high bootstrap support values (≥90), and (ii) the posterior probabilities of the character states at each ancestral node was ≥0.90. The corresponding sites in HA protein were mapped onto a published three-dimensional (3-D) structure of A/duck/Egypt/10185SS/2010 (H5N1) virus (Protein Data Bank code: 5E2Y) using PyMOL (Molecular Graphics System, version 2.0.7.0 Schrödinger, LLC, accessed on 19-Jan-2018)^4^ (Delano, 2002).

## Results

### Phylogenetic Analyses

**FIGURE 1:**
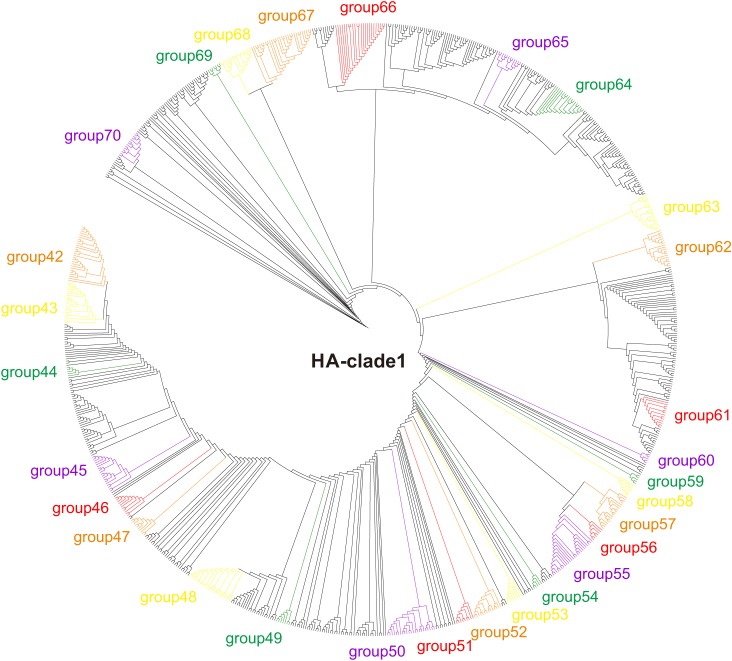
Phylogenetic tree of H5 clade 1 of H5Nx viruses. Maximum likelihood tree of H5Nx viral sequences generated using RAxML v.8.0.14 with the best fitting sequence evolutionary model identified by ModelTest.

The HA phylogeny reconstructed using RAxML v.8.0.14 (Stamatakis, 2006) revealed that the H5 sequences are grouped into 10 clades (clades 0–9), and that the human-isolated sequences distribute to clades 0 (16 human-isolated sequences), 1 (101 human-isolated sequences), 2 (360 human-isolated sequences), 3 (one human-isolated sequences), and 7 (two human-isolated sequences) (Figure 1, Supplementary Figures 1, 2, and Supplementary Table 2). Similarly, phylogenetic trees were reconstructed, separately, for other genes.

In order to simplify the calculations that focused on the human-isolated viruses, we divided the HA, NS, M, NA, NP, PA, PB1, and PB2 sequences into 132, 101, 98, 114, 87, 80, 90, and 92 datasets, respectively, based on the initial phylogenetic trees. Each dataset contains the human isolates and their closely related avian isolates (Supplementary Figures 3–10). These HA, NS, M, NA, NP, PA, PB1, and PB2 gene datasets contained 266, 147, 206, 260, 186, 155, 164, and 171 host-shift branches, respectively.

### Positive Selection on Host-Shift Branches

We used the CODEML program from the PAML package (Yang, 2007) to identify signals of positive selection on host-shift (avian-to-human) branches. In total, 29 branches with 38 sites (H5 numbering) were identified as having experienced significant positive selection, including branches HA-107b, HA-18c (473R), HA-6a, HA-64b (11N, 15Q, 20M, 314K, 315T, 522T, 529L, 546L, 547Q, and 548C), HA-68a (212R and 500R), HA-72b, HA-74a, HA-75a, HA-76a, HA-77a, HA-83a, and HA-107b in HA; PB2-14d and PB2-74b in PB2; MP-46e, MP-50a (5T, 6E, 7V, 8E, 257T, 258E, 259V, and 260E), and MP-85a (277P, 279V, 282A, 283N, 284I, 285I, 287I, 292L, 328Y, 330Q, 336V, 339D, 340D, and 344V) in MP; NA1-15b (188N) in NA1, NA6-2b in NA6; NP-32a, NP-65a (486S and 487Y) and NP-66c in NP; PA-11a, PA-25d, and PA-72b in PA; NS-31a, NS-72c, and NS-89a in NS, and PB1-31b in PB1 (Supplementary Table 3). Other host-shift branches did not show significant signals of positive selection (Supplementary Table 4).

### Convergent/Parallel Evolution of Human-Isolated H5Nx Viruses

To determine whether convergent/parallel evolution occurred during the multiple avian-to-human transmissions of H5Nx HPAIVs, ancestral amino acid sequences for the target nodes that lead to host-shift branches were reconstructed for convergent evolutionary analyses. In total, we identified 126 parallel evolution substitutions (34 in HA, 20 sites in PB2, seven in PB1, 13 in PA, eight in NP, 20 in NA, six in MP, and 18 in NS) that occurred on the host-shift branches (Table 1).

**Table 1:** Convergent/parallel evolution sites for eight genes in H5 human isolates.

Gene	Convergent/Parallel sites	*P* values	Phenotype
PB2	I64M	*P* < 0.05*	Human host marker
	V108A	*P* < 0.05*	
	I147M	*P* < 0.05*	
	E192K	*P* < 0.05*	
	T271A	*P* < 0.05*	Human host marker (host-specific polymerase activity)
	A274T	*P* < 0.05*	Increased polymerase activity, increased virulence in mammals and birds
	T339M	*P* < 0.05*	Enhanced polymerase activity, increased virulence in mice
	R369K	*P* < 0.05*	
	V444L	*P* > 0.05	
	V451I	*P* < 0.05*	
	N456D	*P* < 0.05*	
	I461V	*P* < 0.05*	
	S489P	*P* > 0.05	
	M570V	*P* < 0.05*	
	Q591K	*P* < 0.05*	Increased virulence in mammals
	I615V	*P* < 0.05*	
	E627K	*P* < 0.05*	Human host marker, enhanced polymerase activity, increased virulence in mammals
	V649I	*P* < 0.05*	
	D701N	*P* < 0.05*	Increased polymerase activity, increased virulence in mammals, mammalian host marker
	G727R	*P* < 0.05*	
	D740N	*P* < 0.05*	
	S741F	*P* < 0.05*	
HA	Q30P	*P* > 0.05	
	D31N	*P* < 0.05*	
	K35R	*P* < 0.05*	
	D45N	*P* < 0.05*	
	A86V	*P* > 0.05	
	D88G	*P* < 0.05*	
	A127T	*P* < 0.05*	
	A/T127S	*P* < 0.05*	
	A134V	*P* < 0.05*	Increased virus binding to 2,6
	R140K	*P* < 0.05*	
	M140T	*P* < 0.05*	
	S141P	*P* < 0.05*	
	D154N	*P* < 0.05*	Airborne transmissibility in mammals
	R162I	*P* < 0.05*	
	V174I	*P* < 0.05*	
	N182S	*P* < 0.05*	Increased virus binding to 2,6
	A184E	*P* > 0.05	
	E184G	*P* < 0.05*	Increased virulence in mammals
	T195A	*P* < 0.05*	
	T/N195I	*P* < 0.05*	
	K189R	*P* < 0.05*	
	V210A	*P* > 0.05	
	V219I	*P* < 0.05*	
	S223N	*P* < 0.05*	Increased virus binding to 2,6
	R310K	*P* < 0.05*	
	R323K	*P* < 0.05*	
	R326K	*P* < 0.05*	
	I375M	*P* < 0.05*	
	D376N	*P* < 0.05*	Increased virulence in mammals
	D387N	*P* < 0.05*	
	E433G	*P* < 0.05*	
	N476D	*P* < 0.05*	
	E477K	*P* < 0.05*	
	M479I	*P* < 0.05*	
	R497K	*P* < 0.05*	Increased virus binding to 2,6
	E502G	*P* < 0.05*	
	M532I	*P* < 0.05*	
	V533I	*P* < 0.05*	
PB1	I57T	*P* < 0.05*	
	E172D	*P* < 0.05*	
	M179I	*P* < 0.05*	
	S361G	*P* < 0.05*	
	N375S	*P* < 0.05*	Increased polymerase activity, increased virulence in mammals, human host marker
	K387R	*P* < 0.05*	
	L598P	*P* < 0.05*	Increased polymerase activity and replication efficiency
PA	F4C	*P* < 0.05*	
	M12I	*P* < 0.05*	
	M86V	*P* < 0.05*	
	T97I	*P* < 0.05*	Enhanced polymerase activity, increased virulence in mice
	F105L	*P* < 0.05*	
	K142E	*P* > 0.05	
	L226F	*P* < 0.05*	
	E237K	*P* < 0.05*	
	C241Y	*P* > 0.05	Enhance the replicative ability of an H5N1 virus in A549 cells and enhance its pathogenicity in mice
	P275L	*P* < 0.05*	
	N321K	*P* < 0.05*	Increased polymerase activity
	T369A	*P* < 0.05*	
	V387I	*P* < 0.05*	
	K615R	*P* < 0.05*	Increased polymerase activity, increased virulence in mammals, mammalian host marker
NP	R100I	*P* < 0.05*	
	A284T	*P* < 0.05*	Increased virulence in mice
	V343I	*P* < 0.05*	Highly BNP-sensitive to moderately BNP-resistant
	R384K	*P* < 0.05*	
	S413L	*P* < 0.05*	
	P419S	*P* < 0.05*	
	R452K	*P* < 0.05*	
	P453S	*P* < 0.05*	
NA1	I8T	*P* < 0.05*	
	I/V16A	*P* < 0.05*	
	V16I	*P* < 0.05*	
	V33I	*P* < 0.05*	
	N39S	*P* < 0.05*	
	P45T	*P* < 0.05*	
	K55R	*P* < 0.05*	
	A58T	*P* < 0.05*	
	K241R	*P* < 0.05*	
	I243V	*P* < 0.05*	
	N305T	*P* < 0.05*	
	G318S	*P* < 0.05*	
	P323S	*P* < 0.05*	
	S364N	*P* < 0.05*	
	G365E	*P* < 0.05*	
	I380V	*P* < 0.05*	
	V404I	*P* < 0.05*	
	N430S	*P* < 0.05*	
	N430D	*P* < 0.05*	
	G435S	*P* < 0.05*	
	T441D	*P* < 0.05*	
NA2, NA3, NA4, NA5, NA6, NA7, NA8, NA9	NA	
MP	T137A	*P* < 0.05*	Human host marker
	A239T	*P* < 0.05*	
	C269Y	*P* < 0.05*	
	V280I	*P* < 0.05*	
	S283N	*P* < 0.05*	
	D340N	*P* < 0.05*	
NS1	G47S	*P* < 0.05*	
	N48S	*P* < 0.05*	
	R59H	*P* < 0.05*	
	R67Q	*P* < 0.05*	
	E70K	*P* < 0.05*	
	T81I	*P* < 0.05*	
	R88C	*P* < 0.05*	
	V136A	*P* < 0.05*	
	I137V	*P* < 0.05*	
	D139N	*P* < 0.05*	
	L185F	*P* < 0.05*	
	S205N	*P* < 0.05*	Decreased IFN antagonism, conferred enhanced in-contact transmissibility in guinea pigs
	D209N	*P* < 0.05*	
	V209I	*P* < 0.05*	
	L212F	*P* < 0.05*	
NEP/NS2	M/A14V	*P* < 0.05*	
	A48T	*P* < 0.05*	
	T/V115A	*P* < 0.05*	


**FIGURE 2:**
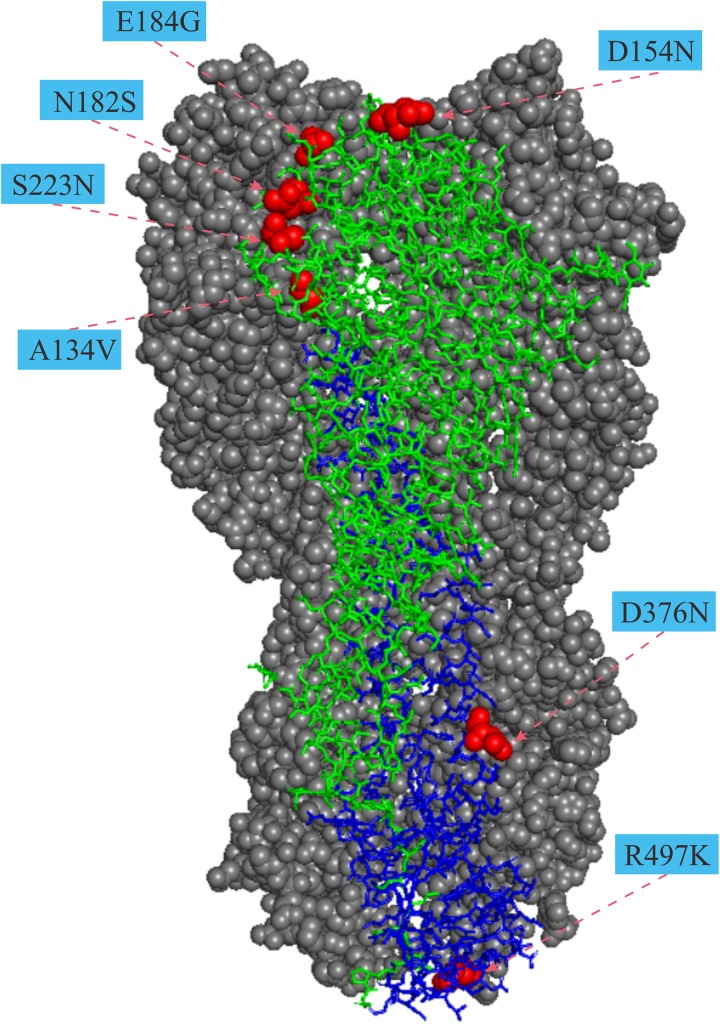
Mapping of parallel evolution sites on the structure of HA protein. Parallel evolution sites were mapped to know HA structure for the A/duck/Egypt/10185SS/2010 (H5N1) virus (Protein Data Bank code: 5e2y). The monomer shows the HA1 (A134V, D154N, N182S, and E184G) and the HA2 (D376N and R497K) subunits. Numbers in colored rectangle represents codon alignment number (H5 numbering) and their locations in the three-dimensional structure are shown with red spheres.

For the HA gene, the parallel amino acid substitutions S223N (H5 numbering) occurred on seven host-shift branches, A134V on four branches, and six mutations (D31N, D154N, T/N195I, V219I, I375M, and E502G, H5 numbering) were each discovered on three branches. The remaining 26 parallel mutations occurred each on pairs of branches. Of these convergent/parallel amino acid mutations, four substitutions (A134V, D154N, S223N, and R497K) had been identified in earlier studies as having functional roles and were mapped to the three-dimensional (3-D) structure of the HA protein (Figure 2).

For the PB2 gene, the well-known mutations E627K and D701N (Hatta et al., 2001; Li et al., 2005; Song et al., 2014; Zhu et al., 2015) were observed on 30 and 10 host-shift branches, respectively. N456D and G727R were detected on five and four branches, respectively. An additional four substitutions (T339M, I451V, S471F, and R369K) occur in parallel on three sets of branches and the remaining 12 parallel substitutions were shared by pairs of host-shift branches.

For the PB1 gene, there were seven parallel-evolved variations (I57T, E172D, M179I, S361G, N375S, K387R, and L598P) that occur on pairs of host-shift branches.

For the PA gene, three mutations K142E, N321K, and K615R were each detected on three host-shift branches and ten mutations (F4C, M12I, M86V, T97I, F105L, L226F, E237K, P275L, T369A, and V387I) parallelly occurred on pairs of host-shift branches.

For the NA gene, 20 parallel-evolved variations in the NA1 subtype, including three mutations I/V16A, V33I, and I243V occurred on each of three host-shift branches, and 17 mutations (V16I, N39S, P45T, K55R, A58T, K241R, N305T, G318S, P323S, S364N, G365E, I380V, V404I, N430S, N430D, G435S, and T441D) were shared by pairs of branches. No parallel substitutions were detected in the other NA subtypes.

For the NP gene, two mutations A284T and V343I were shared by four and three human-isolated branches, respectively, and five mutations (R100I, R384K, S413L, P419S, R452K, and P453S) were shared by pairs of human-isolated branches.

For the MP gene, six mutations were detected as parallel-evolved variations, including T137A, A239T, C269Y, V280I, S283N, and D340N. Each of these was separately shared by two human-isolated branches.

For the NS gene, 18 parallel-evolved variations were detected, 15 of which were in NS1 and three in NS2. Of the 15 NS1 mutations, four (R67Q, T81I, L212F, and L185F) were each shared by three branches, and 11 (G47S, N48S, R59H, E70K, R88C, V136A, I137V, D139N, S205N, D209N, and V209I) were identified in pairs of human-isolated branches. For the NEP/NS2 genes, three mutations M/A14V, A48T, and T/V115A were shared by pairs of branches.

## Discussion

Since their first detection in 1996, H5 AIVs have had a substantial impact on veterinary and human health (Harfoot and Webby, 2017). Although the direct transmission of H5Nx viruses from avian species to humans remains a relatively rare event, they still pose a serious pandemic threat due to their high virulence and mortality, and their increasingly expanding host range, as well as the significant ongoing evolution toward efficient transmission in mammals (Guan and Smith, 2013). Sporadic human infections continue to occur in countries where H5Nx have become endemic in birds, providing a persistent threat to global health due to the possibility of virus adaptation to humans. Thus, the study of their genetic mechanisms of human adaptation remains essential.

Adaptive evolution of functional important genes is essential for the invasion of new niches (Zhang and Kumar, 1997; Zhang, 2006; Shen et al., 2012). AIVs should face great challenges when they emerge in humans from their avian sources. During the host-shift process of the multiple human H7N9 AIV invasions, convergent evolution occurred for the human-isolated viruses to adapt to their new hosts (Xiang et al., 2018). Similar to H7N9, human-isolated H5 sequences are distributed in multiple phylogenetic positions (Figure 1 and Supplementary Figures 1, 2), suggesting that there were multiple independent invasions of H5Nx AIVs into humans. Here, we found that adaptive evolution (positive selection, and convergent/parallel evolution) occurred on the independent host-shift branches in H5Nx AIVs, and that some of the adaptive sites have functional importance (Table 1).

Avian influenza viruses preferentially bind α2-3 sialic acid receptors. In contrast, human-adapted influenza viruses preferentially bind α2-6 sialic acid receptors. The switch of preference, from avian to human type sialic acid receptors, is considered to be a key element necessary for AIVs to cause human pandemics (Matrosovich et al., 2000; Parrish and Kawaoka, 2005). The receptor-binding domain (RBD) of HA is formed by four loops and one helix in the RBS, which contact the base and potentially the extension region of the human receptor. Among the 34 parallel amino acid substitutions detected in the HA gene (Table 1), two of them A134V and S223N, have previously been shown to associate with the switch of preference from avian to human type sialic acid receptors (Yamada et al., 2006; Auewarakul et al., 2007; Imai et al., 2010; Chen et al., 2012). In addition, three others, S141P (130-loop), R162I (150-loop), and T/N195A/I(190-loop), are in or around the RBD and may dramatically alter the receptor binding preference of the H5Nx influenza viruses. Furthermore, S223N has previously been detected by *in silico* prediction and experimentally confirmed to enhance human receptor specificity of H5N1 influenza A viruses (Schmier et al., 2015), indicating that mutations detected by several methods might have greater potential functional effects and that the combination of multiple methods is recommended for selecting potential functional mutations for experimental studies. S223N is located close to the sialic-acid-binding site (Figure 2), and this mutation in H5N1 AIVs has weakened affinity toward α-2, 3 and an increased affinity toward α-2, 6 sialic acid receptors (Yamada et al., 2006; Chen et al., 2012). Alanine at position 134 (A134) is in the 130-loop of the receptor binding domain (Figure 2). This site is highly conserved in avian H5N1 viruses. The Ala to Val substitution at position 134 could change the receptor-binding preference of H5-HA from α-2,3 to both α-2,3 and α-2,6-sialic acid binding (Auewarakul et al., 2007;Imai et al., 2010). To initiate influenza virus infection, hemagglutinin (HA), which is the major surface glycoprotein of influenza viruses, binds to the host cell surface complex glycans via a terminal sialic acid. The preference of HA for particular sialic acid moieties on host cells is a key determinant of host range and tissue tropism (Matrosovich et al., 2000). Parallel evolution of amino acid sites that play important roles in the change of receptor-binding preference in host-shift branches suggests that during human invasion by AIVs that they have adapted to the common challenge of a difference in the surface glycoprotein between birds and humans.

After binding and entering human cells, efficient replication (efficient production of new viruses) is a critical factor that influences viral infection. A series of parallel evolution sites with roles in increasing polymerase activity and replication efficiency in mammals were identified, such as T271A (Finkelstein et al., 2007), A274T (Leung et al., 2010), Q591K (Yamada et al., 2010), E627K (Hatta et al., 2001; Song et al., 2014), and D701N (Li et al., 2005; Zhu et al., 2015), in PB2 (Table 1). The E627K and D701N mutations might be especially important as 17.5% (30 of 171) and 5.85% (10 of 171) of the host-shift lineages in the PB2 gene share these two mutations. In addition, some parallel sites in other genes are also suggested to be associated with enhanced polymerase activity and/or increased virulence in mammals (Table 1), such as L598P in PB1 (Xu et al., 2012), T97I and N321K in PA (Cheng et al., 2014; Yu et al., 2014; Wu et al., 2016; Zhao et al., 2016; Nam et al., 2017), and A284T in NP (Zhao et al., 2016). Parallel evolution at these sites suggests that during the host-shift of H5Nx AIVs, these changes have allowed more efficient replication in human cells. Compared with the adaptive evolution of human-isolated H7N9 AIVs (Xiang et al., 2018), human-isolated H5Nx also have the E627K and D701N mutations in PB2 genes in the host-shift branches. This suggests that these two mutations play important roles in human adaptation of AIVs in both subtypes. Other adaptive sites are not shared by these two viruses, suggesting that there are some differences in the adaptation of H7N9 and H5Nx to humans.

Positive selection is the force that drives an increase in the prevalence of advantageous new mutations. In this study, 29 host-shift branches had significant signals of positive selection, with 12 in HA, two in PB2, three in MP, one in NA1, one in NA6, three in NP, three in PA, three in NS, and one in PB1. The frequency of positive selection in host-shift branches is quite low, indeed, only a few adaptive events were detected by the PAML package. This is an unavoidable limitation of the current approaches for positive selection analyses. A total of 38 positively selected amino acid sites were identified. The 340D mutation in the MP gene had significant signals in both the positive selection and the convergent evolution analyses, implying that this site might be important for adaptation to new host environments. More attention on this positive selection site, and its functional roles, is needed.

To determine whether the human-adaptive sites were actually part of human adaptation following zoonosis, we calculated the prevalence of the adaptive mutations (positive selection and convergent/parallel evolution sites) in the human- and avian-isolated strains (Supplementary Table 5). It is expected that the adaptive-evolution substitutions should be more prevalent in the human viruses than in the avian viruses (Arai et al., 2016). For most of the adaptive mutations, their proportions in the human isolates are higher, with many tending to be fixed, than in the avian isolates, suggesting that these mutations have higher fitness in humans (Supplementary Table 5).

Host barriers restrict interspecies transmission of AIVs. Factors that contribute to AIVs infection and transmission in humans are complex. Although some amino acid changes associated with receptor affinity, temperature tolerance, viral replication, and mammalian adaptation have been found to play a role (Widdowson et al., 2017), the genetic basis for host shifts is not fully understood. In this study, we identified a series of adaptive changes at sites during the multiple human invasions by H5Nx viruses. Some of the adaptive mutations are known to have functional importance in cross-species transmission from avian to humans, while others are useful candidates for further experimental studies, especially those located in critical domains. Analyses of adaptive evolution should identify useful additional candidate sites that might play roles in human infections for functional studies.

## Author Contributions

YS conceived, designed, and supervised the study. FG, YL, SY, LL, TL, ZP, DX, and XS collected and analyzed the data. YS and DI wrote the drafts of the manuscript. ML commented on and revised the drafts of the manuscript. All authors read and approved the final draft of the manuscript.

## Conflict of Interest Statement

The authors declare that the research was conducted in the absence of any commercial or financial relationships that could be construed as a potential conflict of interest.

## Footnotes

1 www.ncbi.nlm.nih.gov/genomes/FLU

2 www.gisaid.org

3 www.fludb.org/brc/home.spg?decorator=influenza

4 http://www.pymol.org/
