# Supplementary material for: Adaptive Evolution of Human-Isolated H5Nx Avian Influenza A Viruses
Source: Front Microbiol. 2019 Jun 12;10:1328. doi: 10.3389/fmicb.2019.01328 (PMC6582624; doi:10.3389/fmicb.2019.01328)

Supplementary Figure 1. Phylogenetic tree of H5 clade 2 of H5Nx viruses. The human isolated sequences of clade 2 were divided into 94 phylogeny groups. Abbreviation, HA, hemagglutinin.

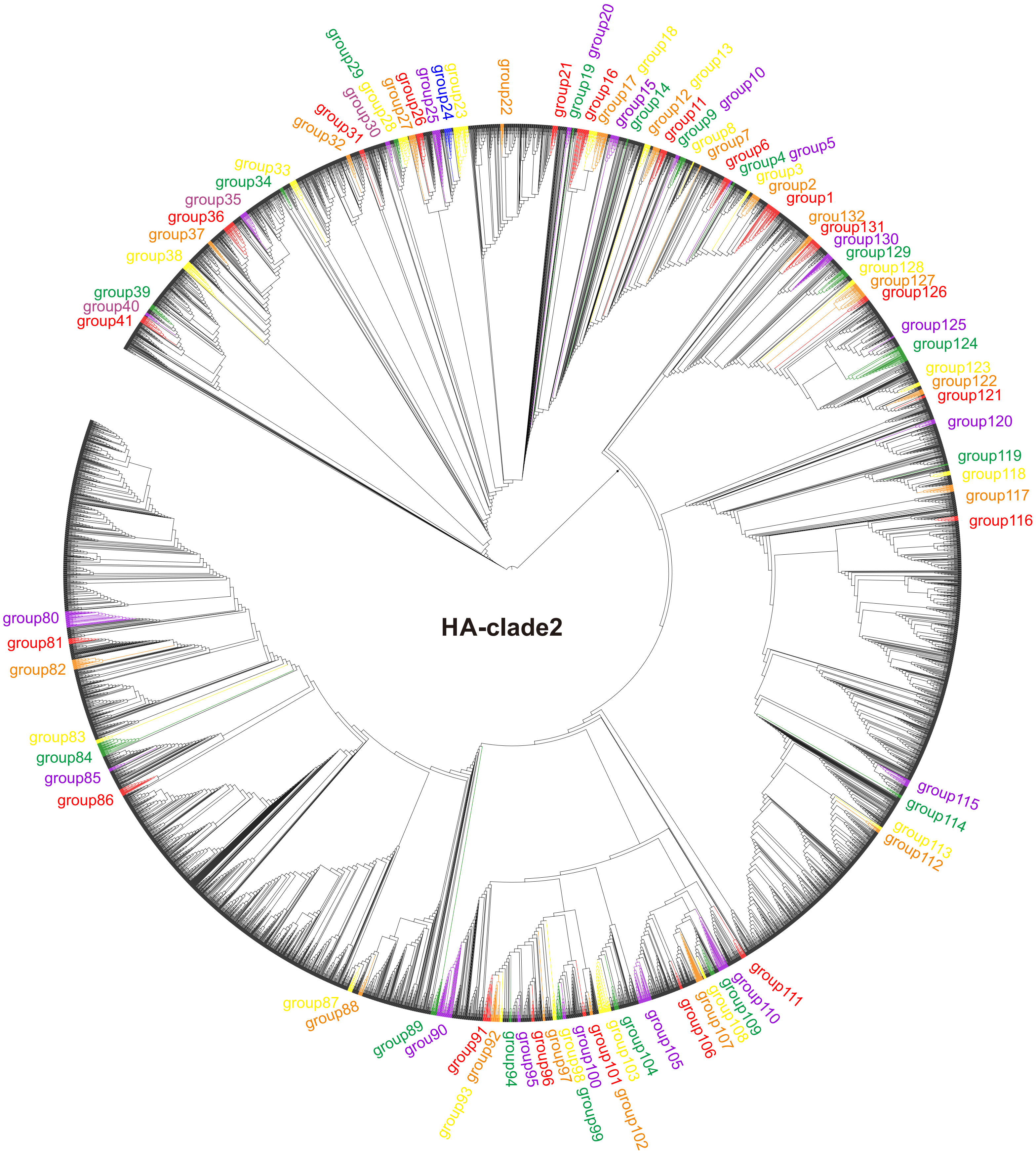

Supplement: Supplementary file 1 [file Data_Sheet_1.PDF]
