# Supplementary material for: Adaptive Evolution of Human-Isolated H5Nx Avian Influenza A Viruses
Source: Front Microbiol. 2019 Jun 12;10:1328. doi: 10.3389/fmicb.2019.01328 (PMC6582624; doi:10.3389/fmicb.2019.01328)

Supplementary Figure 2. Phylogenetic tree of all HA sequences. The human isolated sequences belong to non-clade2 and non-clade 1 were divided into 9 phylogeny groups.Abbreviation, HA, hemagglutinin.

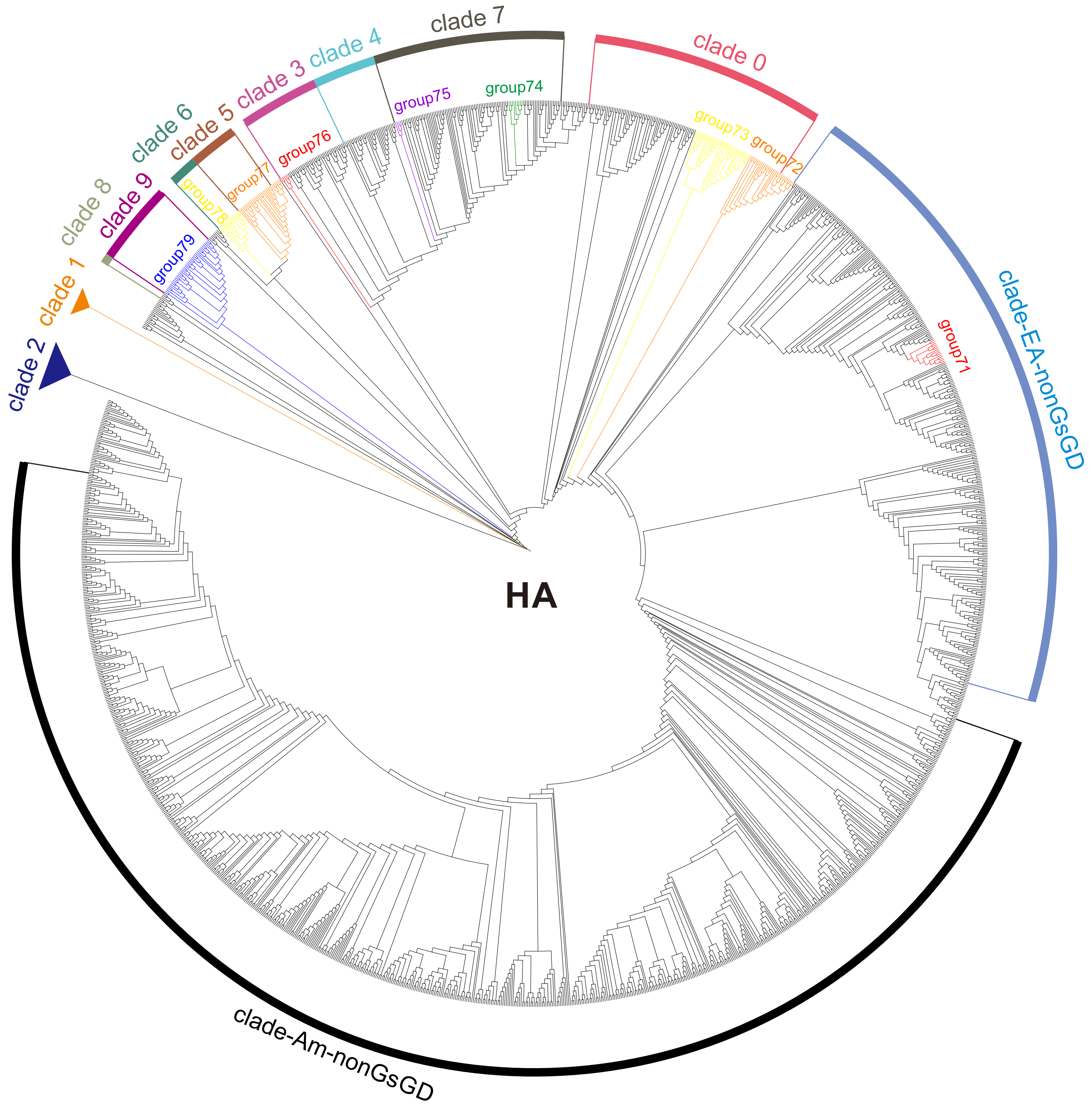

Supplement: Supplementary file 2 [file Data_Sheet_2.PDF]
