# Supplementary material for: Adaptive Evolution of Human-Isolated H5Nx Avian Influenza A Viruses
Source: Front Microbiol. 2019 Jun 12;10:1328. doi: 10.3389/fmicb.2019.01328 (PMC6582624; doi:10.3389/fmicb.2019.01328)
Supplement: Supplementary file 3 [file Data_Sheet_3.PDF]

HA-Group1

Supplementary Figure 3. 132 phylogenetic trees of HA used for the adaptive evolution analyses. Human strains are marked in red. Branches which have significant signals of positive selection are marked with *.

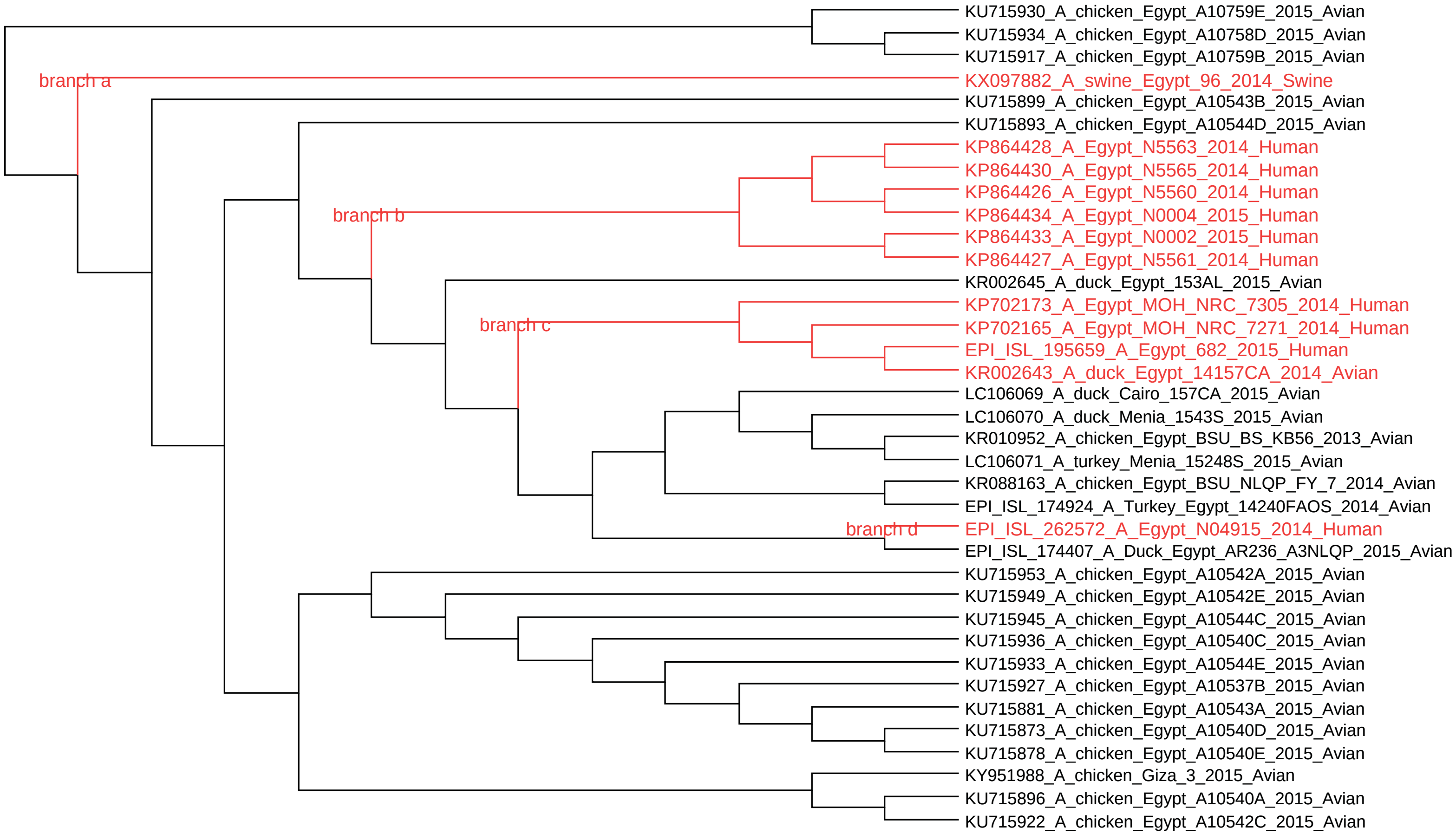

HA-Group2

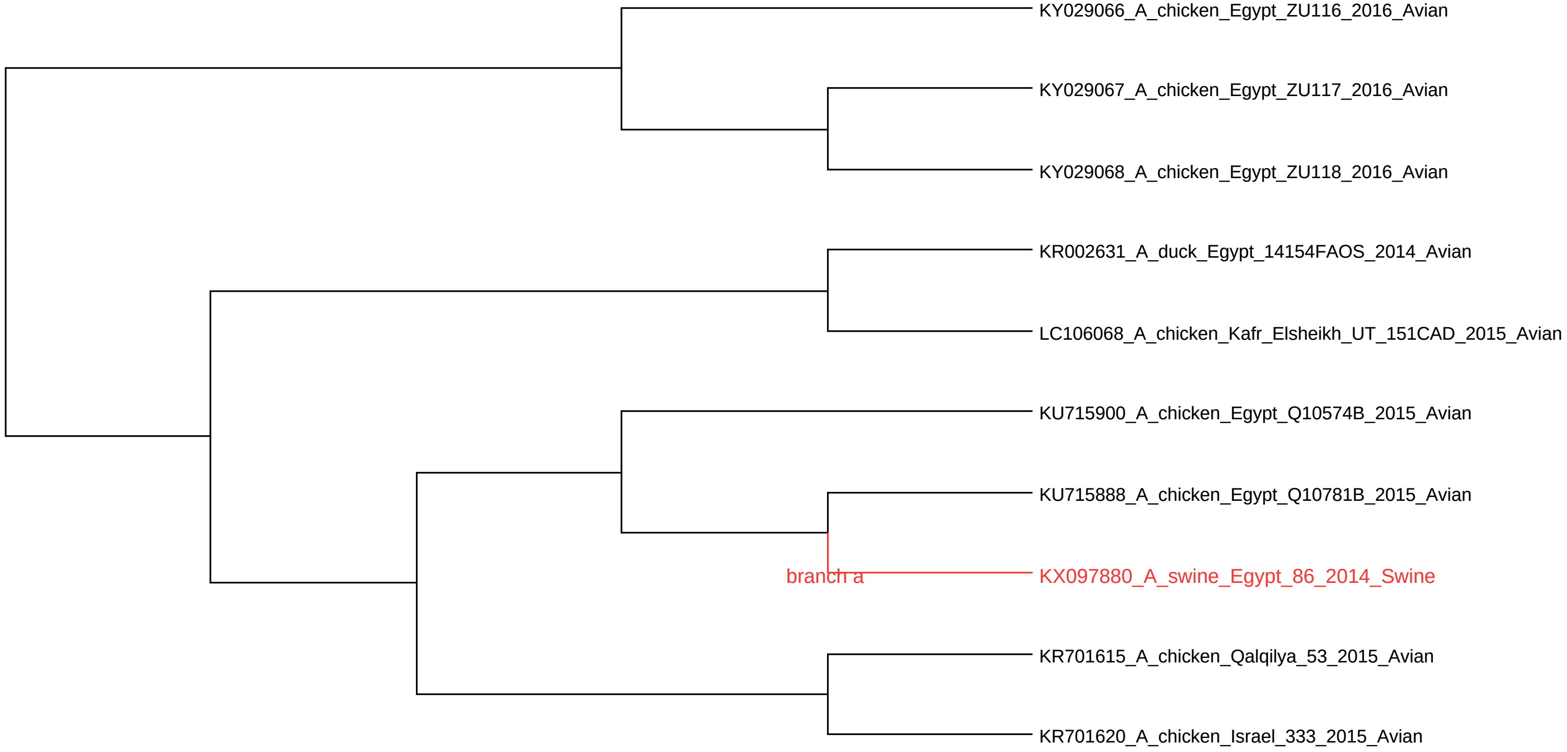
KY029066_A_chicken_Egypt_ZU116_2016_Avian
KY029067_A_chicken_Egypt_ZU117_2016_Avian
KY029068_A_chicken_Egypt_ZU118_2016_Avian
KR002631_A_duck_Egypt_14154FAOS_2014_Avian
LC106068_A_chicken_Kafr_Elsheikh_UT_151CAD_2015_Avian
KU715900_A_chicken_Egypt_Q10574B_2015_Avian
KU715888_A_chicken_Egypt_Q10781B_2015_Avian
branch a
KX097880_A_swine_Egypt_86_2014_Swine
KR701615_A_chicken_Qalqilya_53_2015_Avian
KR701620_A_chicken_Israel_333_2015_Avian

HA-Group3

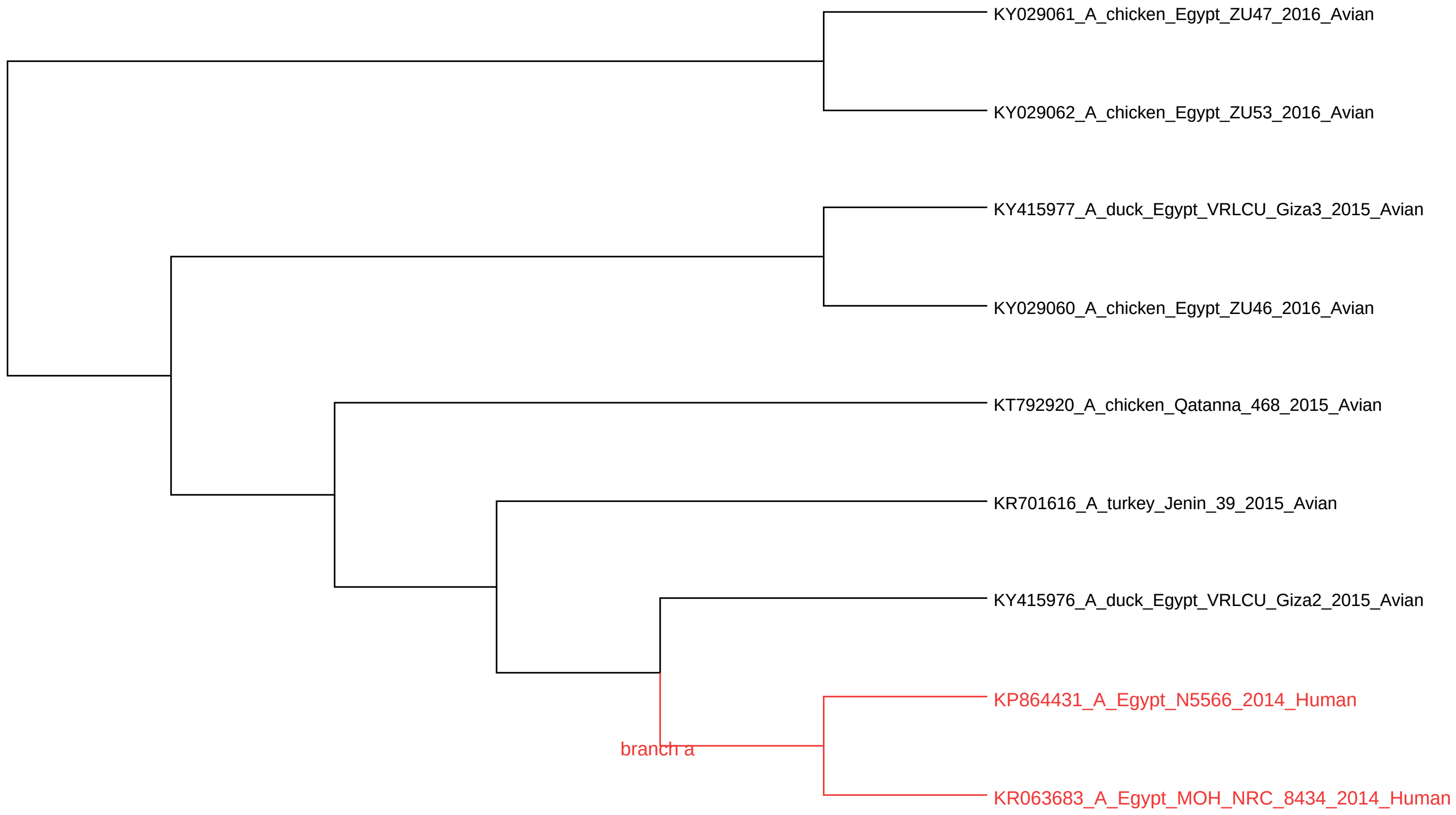

KY029061_A_chicken_Egypt_ZU47_2016_Avian
KY029062_A_chicken_Egypt_ZU53_2016_Avian
KY415977_A_duck_Egypt_VRLCU_Giza3_2015_Avian
KY029060_A_chicken_Egypt_ZU46_2016_Avian
KT792920_A_chicken_Qatanna_468_2015_Avian
KR701616_A_turkey_Jenin_39_2015_Avian
KY415976_A_duck_Egypt_VRLCU_Giza2_2015_Avian
KP864431_A_Egypt_N5566_2014_Human
branch a
KR063683_A_Egypt_MOH_NRC_8434_2014_Human

HA-Group4

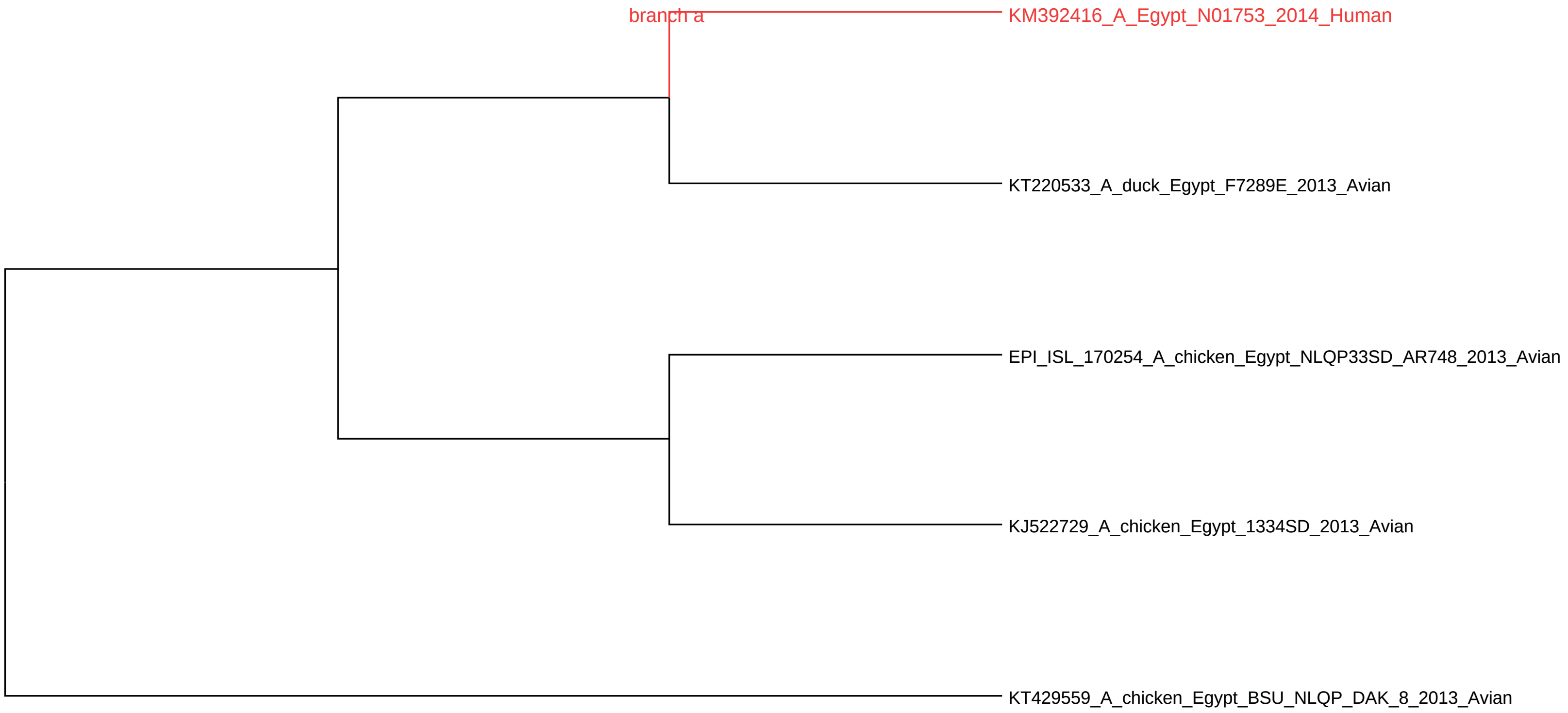

branch a
KM392416_A_Egypt_N01753_2014_Human
KT220533_A_duck_Egypt_F7289E_2013_Avian
EPI_ISL_170254_A_chicken_Egypt_NLQP33SD_AR748_2013_Avian
KJ522729_A_chicken_Egypt_1334SD_2013_Avian
KT429559_A_chicken_Egypt_BSU_NLQP_DAK_8_2013_Avian

HA-Group5

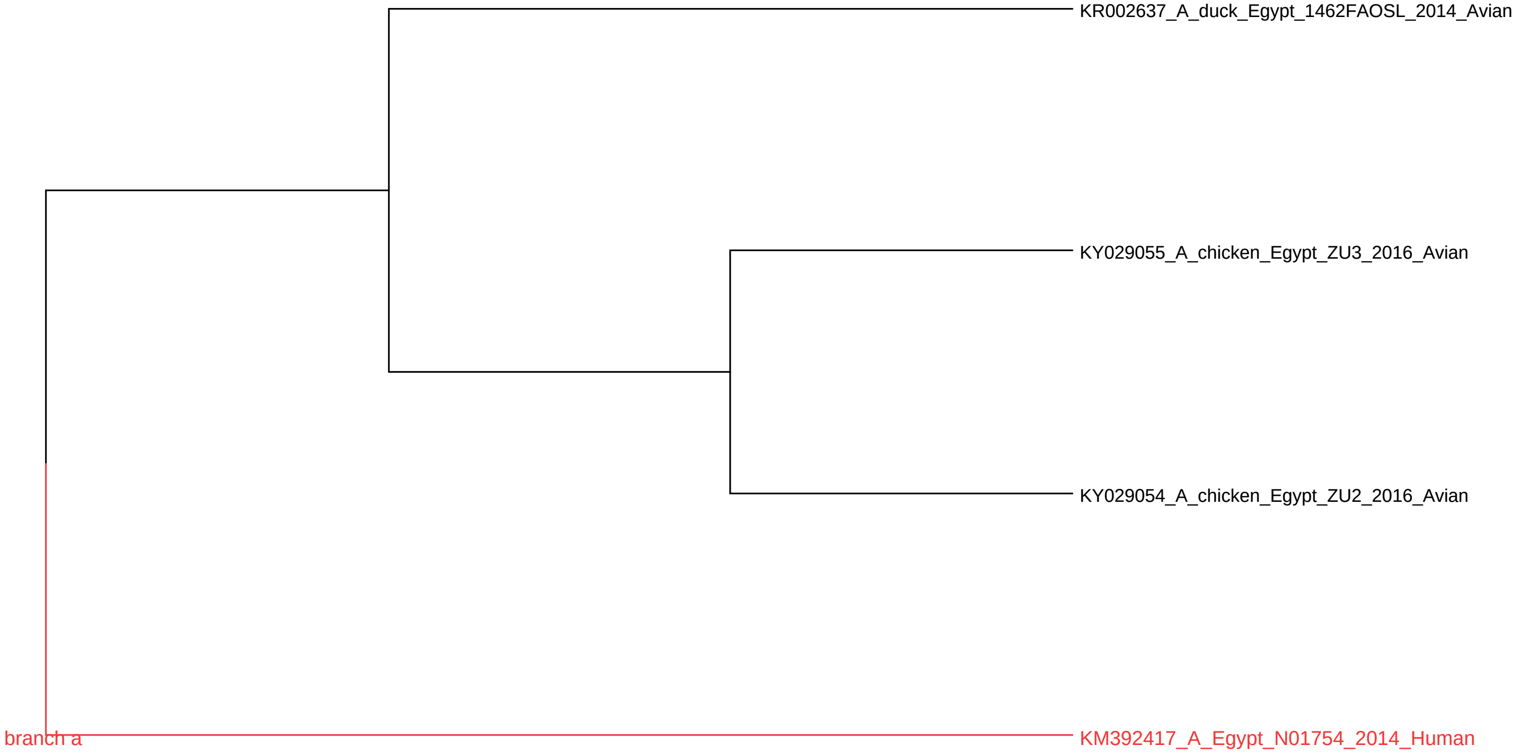
KR002637_A_duck_Egypt_1462FAOSL_2014_Avian
KY029055_A_chicken_Egypt_ZU3_2016_Avian
KY029054_A_chicken_Egypt_ZU2_2016_Avian
branch a
KM392417_A_Egypt_N01754_2014_Human

HA-Group6

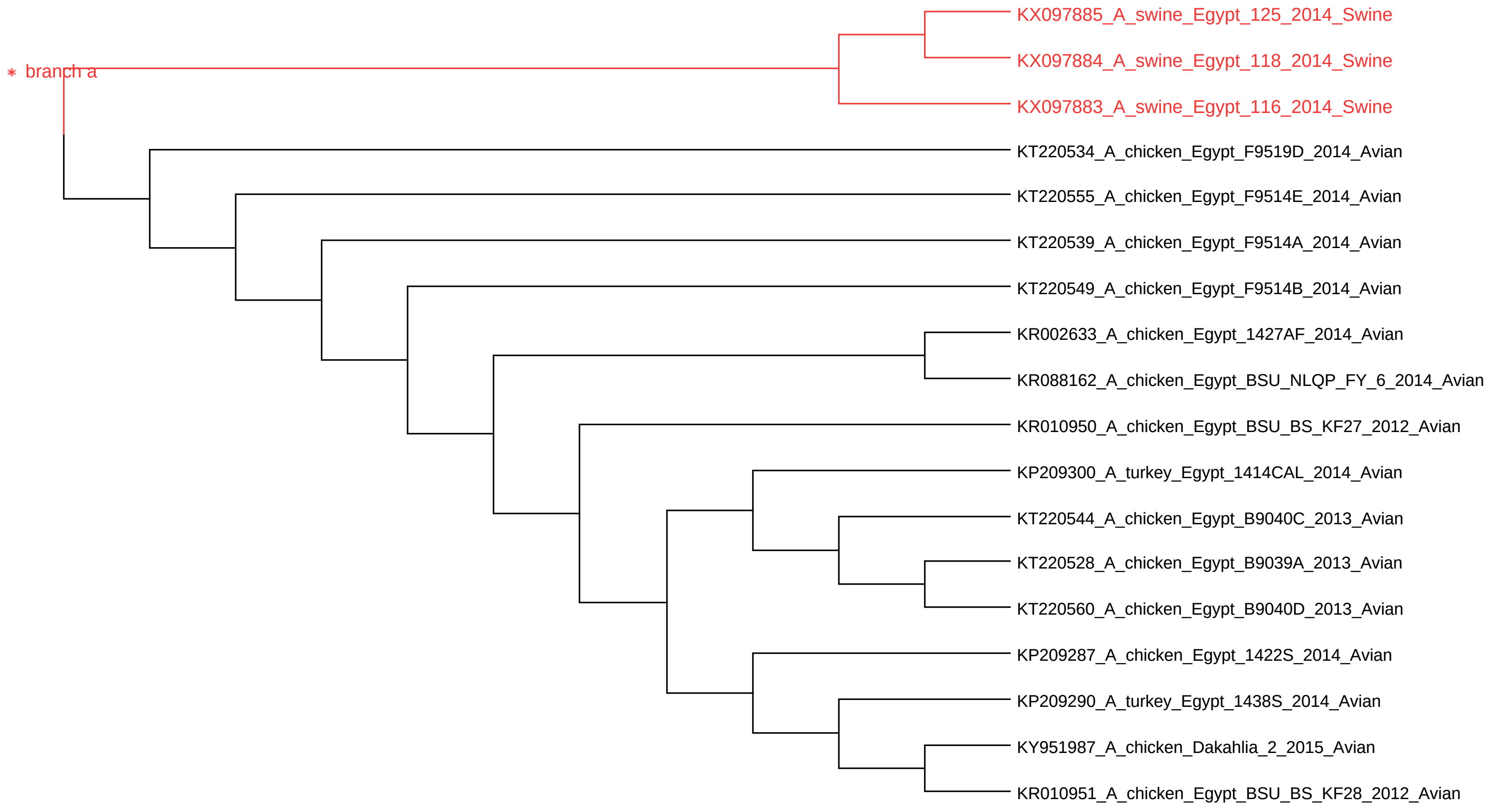

* branch a
KX097885_A_swine_Egypt_125_2014_Swine
KX097884_A_swine_Egypt_118_2014_Swine
KX097883_A_swine_Egypt_116_2014_Swine
KT220534_A_chicken_Egypt_F9519D_2014_Avian
KT220555_A_chicken_Egypt_F9514E_2014_Avian
KT220539_A_chicken_Egypt_F9514A_2014_Avian
KT220549_A_chicken_Egypt_F9514B_2014_Avian
KR002633_A_chicken_Egypt_1427AF_2014_Avian
KR088162_A_chicken_Egypt_BSU_NLQP_FY_6_2014_Avian
KR010950_A_chicken_Egypt_BSU_BS_KF27_2012_Avian
KP209300_A_turkey_Egypt_1414CAL_2014_Avian
KT220544_A_chicken_Egypt_B9040C_2013_Avian
KT220528_A_chicken_Egypt_B9039A_2013_Avian
KT220560_A_chicken_Egypt_B9040D_2013_Avian
KP209287_A_chicken_Egypt_1422S_2014_Avian
KP209290_A_turkey_Egypt_1438S_2014_Avian
KY951987_A_chicken_Dakahlia_2_2015_Avian
KR010951_A_chicken_Egypt_BSU_BS_KF28_2012_Avian

HA-Group7

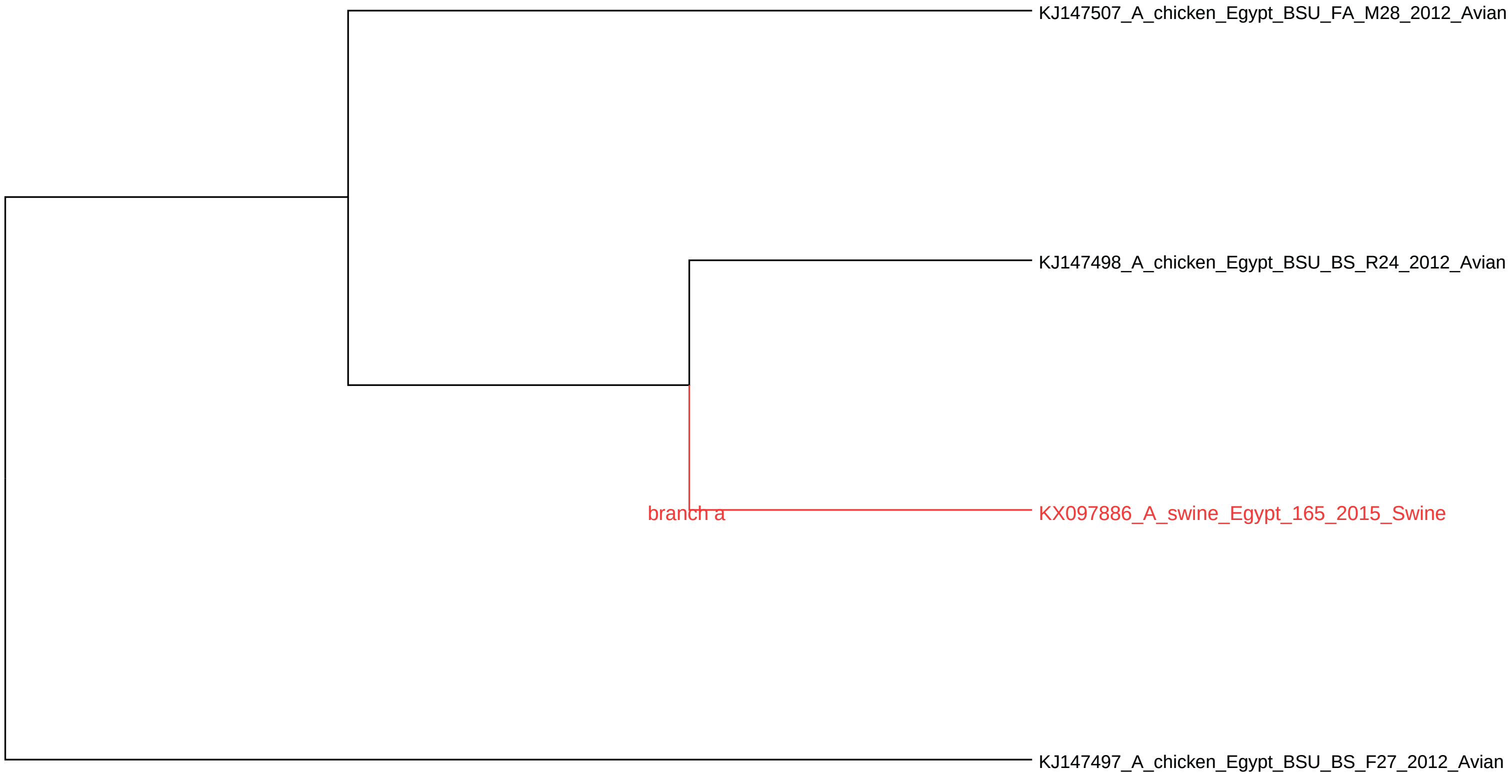

KJ147507_A_chicken_Egypt_BSU_FA_M28_2012_Avian
KJ147498_A_chicken_Egypt_BSU_BS_R24_2012_Avian
branch a
KX097886_A_swine_Egypt_165_2015_Swine
KJ147497_A_chicken_Egypt_BSU_BS_F27_2012_Avian

HA-Group8

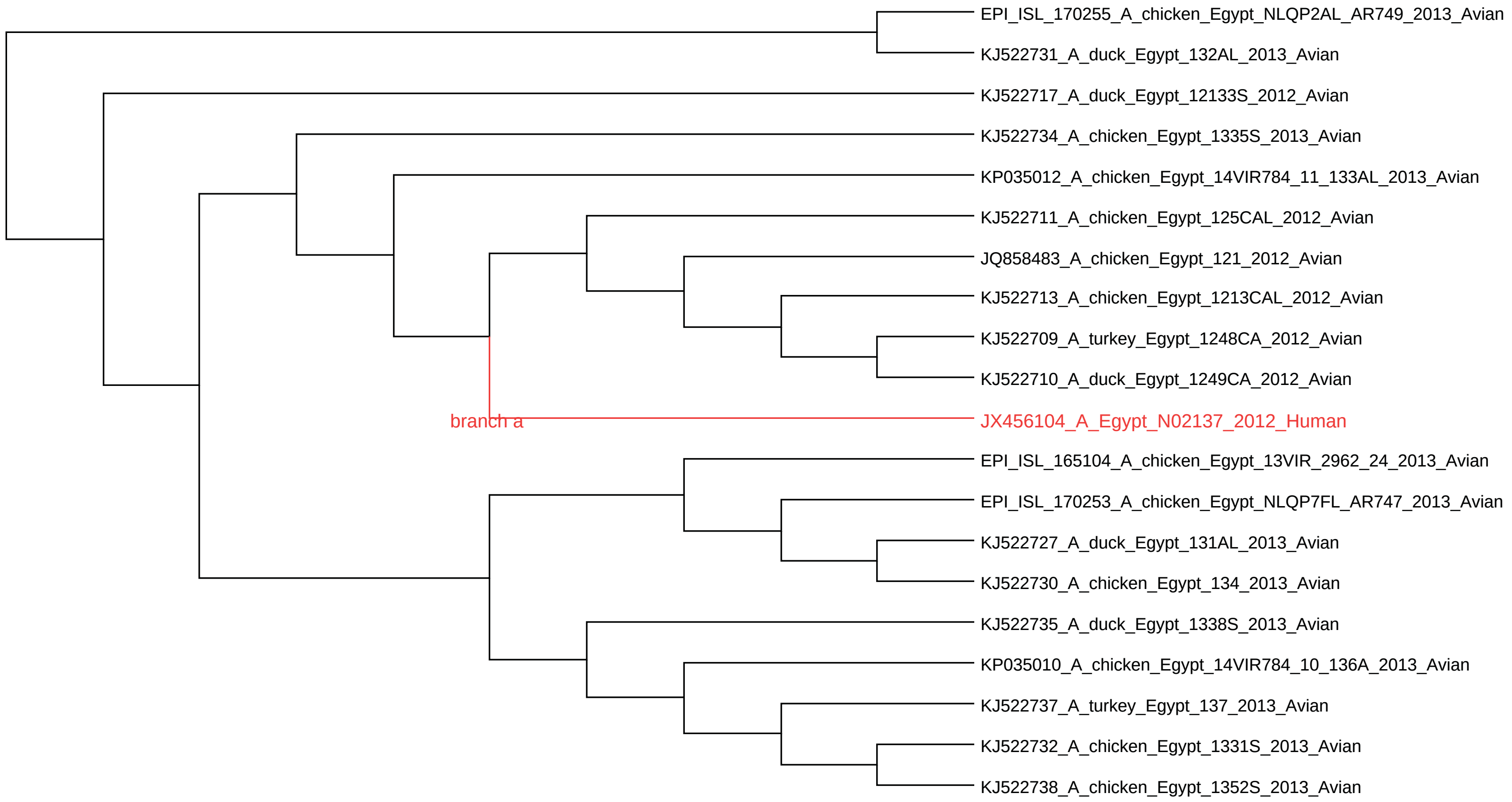

EPI_ISL_170255_A_chicken_Egypt_NLQP2AL_AR749_2013_Avian
KJ522731_A_duck_Egypt_132AL_2013_Avian
KJ522717_A_duck_Egypt_12133S_2012_Avian
KJ522734_A_chicken_Egypt_1335S_2013_Avian
KP035012_A_chicken_Egypt_14VIR784_11_133AL_2013_Avian
KJ522711_A_chicken_Egypt_125CAL_2012_Avian
JQ858483_A_chicken_Egypt_121_2012_Avian
KJ522713_A_chicken_Egypt_1213CAL_2012_Avian
KJ522709_A_turkey_Egypt_1248CA_2012_Avian
KJ522710_A_duck_Egypt_1249CA_2012_Avian
branch a
JX456104_A_Egypt_N02137_2012_Human
EPI_ISL_165104_A_chicken_Egypt_13VIR_2962_24_2013_Avian
EPI_ISL_170253_A_chicken_Egypt_NLQP7FL_AR747_2013_Avian
KJ522727_A_duck_Egypt_131AL_2013_Avian
KJ522730_A_chicken_Egypt_134_2013_Avian
KJ522735_A_duck_Egypt_1338S_2013_Avian
KP035010_A_chicken_Egypt_14VIR784_10_136A_2013_Avian
KJ522737_A_turkey_Egypt_137_2013_Avian
KJ522732_A_chicken_Egypt_1331S_2013_Avian
KJ522738_A_chicken_Egypt_1352S_2013_Avian

HA-Group9

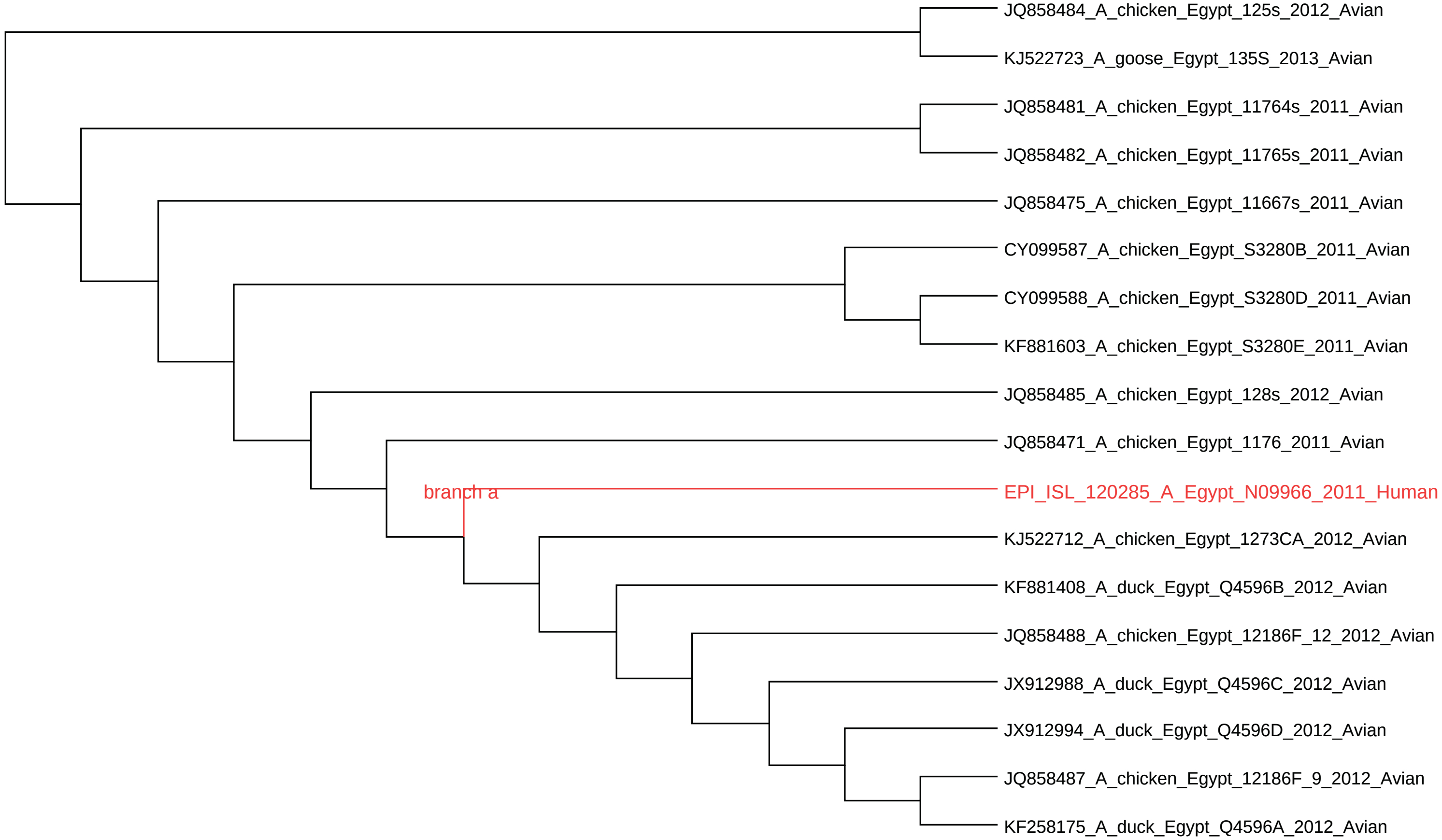

JQ858484_A_chicken_Egypt_125s_2012_Avian
KJ522723_A_goose_Egypt_135S_2013_Avian
JQ858481_A_chicken_Egypt_11764s_2011_Avian
JQ858482_A_chicken_Egypt_11765s_2011_Avian
JQ858475_A_chicken_Egypt_11667s_2011_Avian
CY099587_A_chicken_Egypt_S3280B_2011_Avian
CY099588_A_chicken_Egypt_S3280D_2011_Avian
KF881603_A_chicken_Egypt_S3280E_2011_Avian
JQ858485_A_chicken_Egypt_128s_2012_Avian
JQ858471_A_chicken_Egypt_1176_2011_Avian
branch a
EPI_ISL_120285_A_Egypt_N09966_2011_Human
KJ522712_A_chicken_Egypt_1273CA_2012_Avian
KF881408_A_duck_Egypt_Q4596B_2012_Avian
JQ858488_A_chicken_Egypt_12186F_12_2012_Avian
JX912988_A_duck_Egypt_Q4596C_2012_Avian
JX912994_A_duck_Egypt_Q4596D_2012_Avian
JQ858487_A_chicken_Egypt_12186F_9_2012_Avian
KF258175_A_duck_Egypt_Q4596A_2012_Avian

HA-Group10

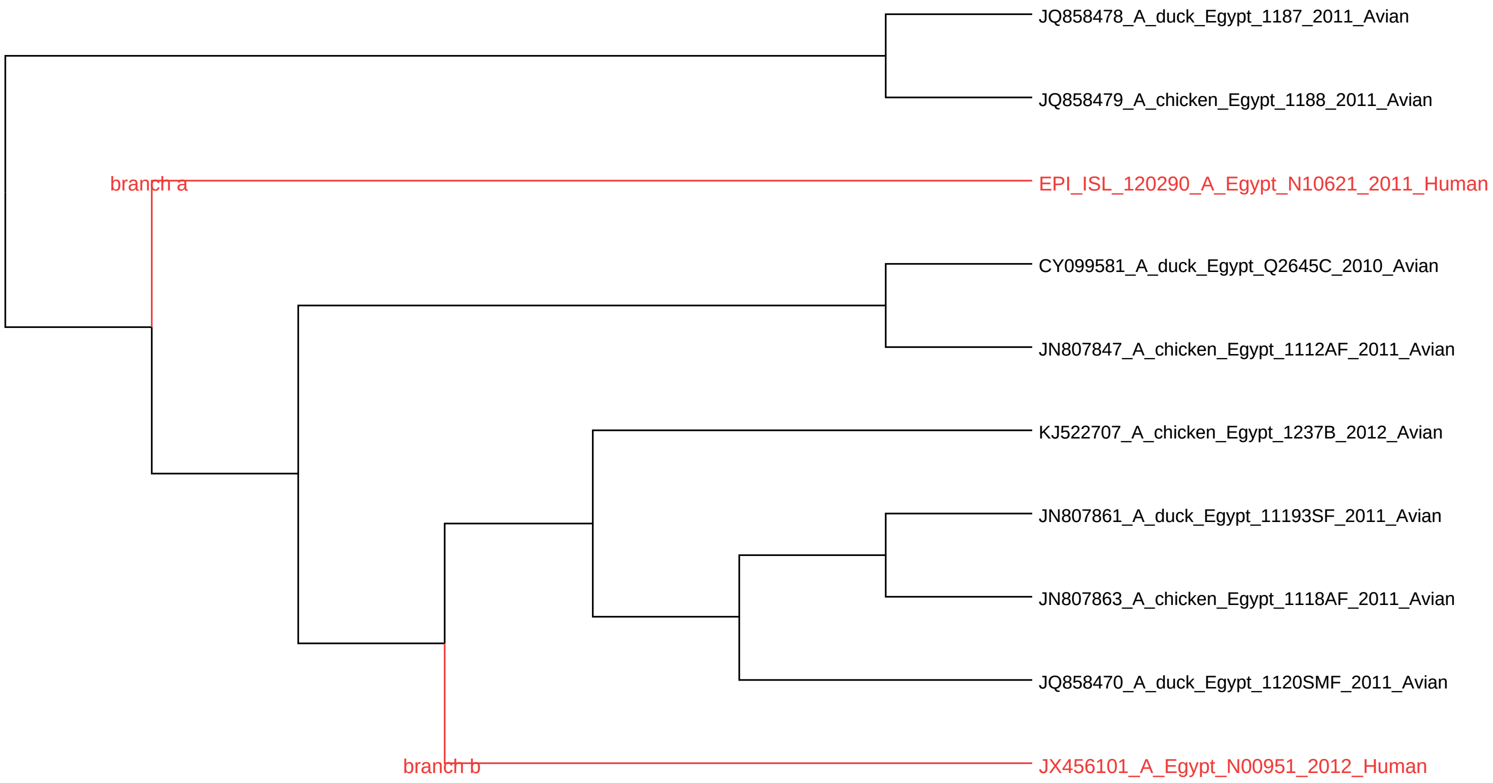

JQ858478_A_duck_Egypt_1187_2011_Avian
JQ858479_A_chicken_Egypt_1188_2011_Avian
branch a
EPI_ISL_120290_A_Egypt_N10621_2011_Human
CY099581_A_duck_Egypt_Q2645C_2010_Avian
JN807847_A_chicken_Egypt_1112AF_2011_Avian
KJ522707_A_chicken_Egypt_1237B_2012_Avian
JN807861_A_duck_Egypt_11193SF_2011_Avian
JN807863_A_chicken_Egypt_1118AF_2011_Avian
JQ858470_A_duck_Egypt_1120SMF_2011_Avian
branch b
JX456101_A_Egypt_N00951_2012_Human

HA-Group11

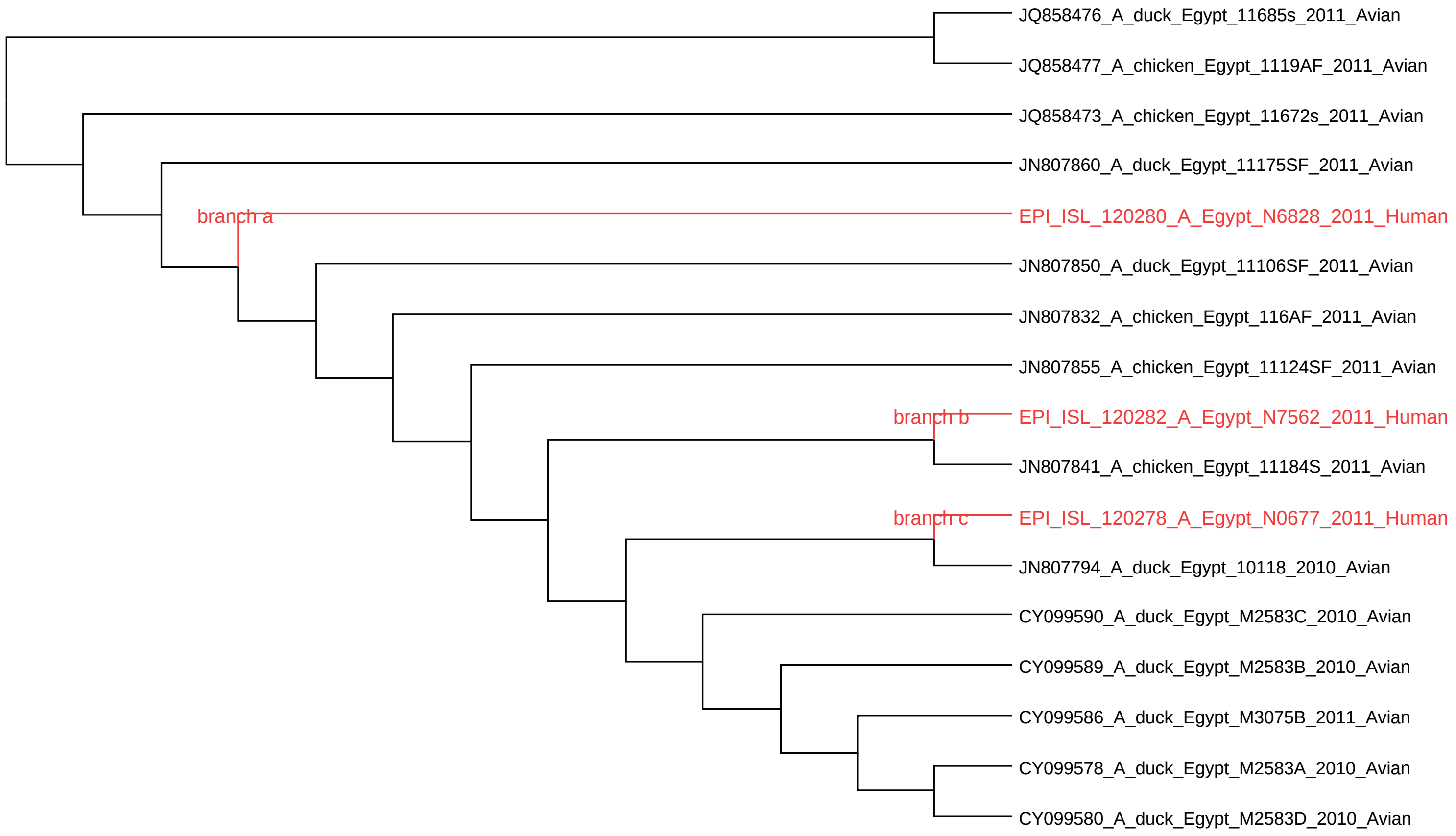

JQ858476_A_duck_Egypt_11685s_2011_Avian
JQ858477_A_chicken_Egypt_1119AF_2011_Avian
JQ858473_A_chicken_Egypt_11672s_2011_Avian
JN807860_A_duck_Egypt_11175SF_2011_Avian
branch a
EPI_ISL_120280_A_Egypt_N6828_2011_Human
JN807850_A_duck_Egypt_11106SF_2011_Avian
JN807832_A_chicken_Egypt_116AF_2011_Avian
JN807855_A_chicken_Egypt_11124SF_2011_Avian
branch b
EPI_ISL_120282_A_Egypt_N7562_2011_Human
JN807841_A_chicken_Egypt_11184S_2011_Avian
branch c
EPI_ISL_120278_A_Egypt_N0677_2011_Human
JN807794_A_duck_Egypt_10118_2010_Avian
CY099590_A_duck_Egypt_M2583C_2010_Avian
CY099589_A_duck_Egypt_M2583B_2010_Avian
CY099586_A_duck_Egypt_M3075B_2011_Avian
CY099578_A_duck_Egypt_M2583A_2010_Avian
CY099580_A_duck_Egypt_M2583D_2010_Avian

HA-Group12

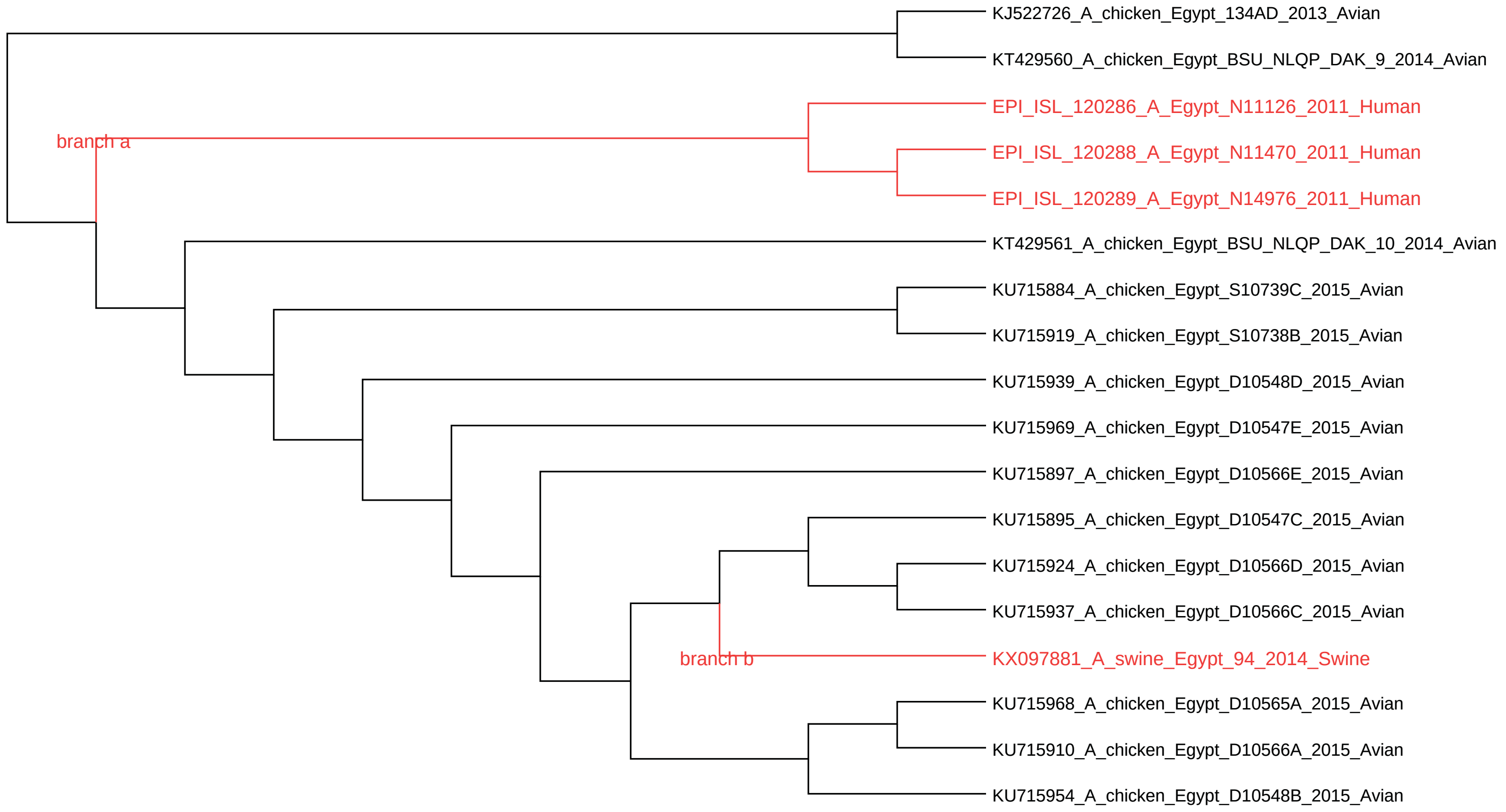
KJ522726_A_chicken_Egypt_134AD_2013_Avian
KT429560_A_chicken_Egypt_BSU_NLQP_DAK_9_2014_Avian
branch a
EPI_ISL_120286_A_Egypt_N11126_2011_Human
EPI_ISL_120288_A_Egypt_N11470_2011_Human
EPI_ISL_120289_A_Egypt_N14976_2011_Human
KT429561_A_chicken_Egypt_BSU_NLQP_DAK_10_2014_Avian
KU715884_A_chicken_Egypt_S10739C_2015_Avian
KU715919_A_chicken_Egypt_S10738B_2015_Avian
KU715939_A_chicken_Egypt_D10548D_2015_Avian
KU715969_A_chicken_Egypt_D10547E_2015_Avian
KU715897_A_chicken_Egypt_D10566E_2015_Avian
KU715895_A_chicken_Egypt_D10547C_2015_Avian
KU715924_A_chicken_Egypt_D10566D_2015_Avian
KU715937_A_chicken_Egypt_D10566C_2015_Avian
branch b
KX097881_A_swine_Egypt_94_2014_Swine
KU715968_A_chicken_Egypt_D10565A_2015_Avian
KU715910_A_chicken_Egypt_D10566A_2015_Avian
KU715954_A_chicken_Egypt_D10548B_2015_Avian

HA-Group13

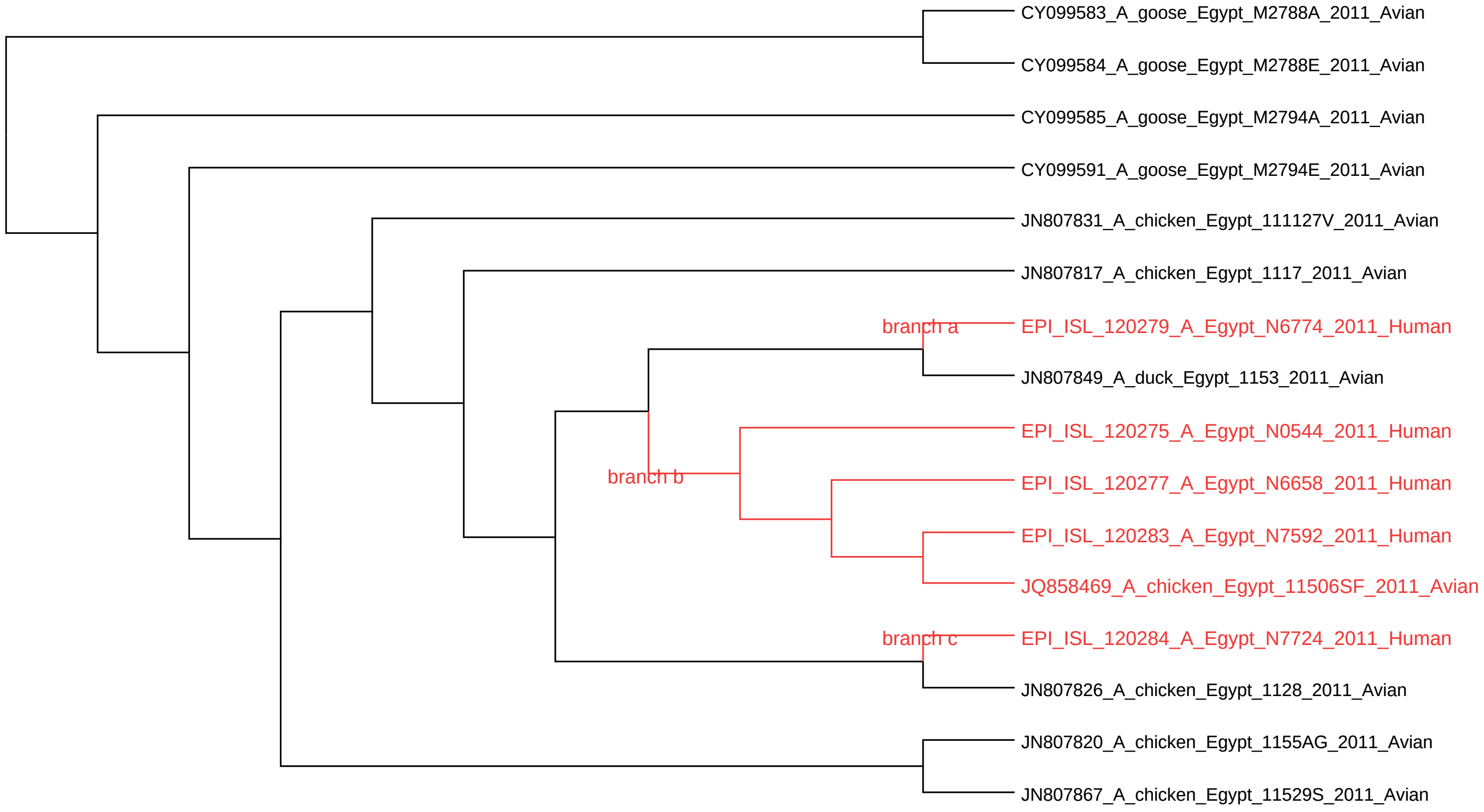
CY099583_A_goose_Egypt_M2788A_2011_Avian
CY099584_A_goose_Egypt_M2788E_2011_Avian
CY099585_A_goose_Egypt_M2794A_2011_Avian
CY099591_A_goose_Egypt_M2794E_2011_Avian
JN807831_A_chicken_Egypt_111127V_2011_Avian
JN807817_A_chicken_Egypt_1117_2011_Avian
branch a
EPI_ISL_120279_A_Egypt_N6774_2011_Human
JN807849_A_duck_Egypt_1153_2011_Avian
EPI_ISL_120275_A_Egypt_N0544_2011_Human
branch b
EPI_ISL_120277_A_Egypt_N6658_2011_Human
EPI_ISL_120283_A_Egypt_N7592_2011_Human
JQ858469_A_chicken_Egypt_11506SF_2011_Avian
branch c
EPI_ISL_120284_A_Egypt_N7724_2011_Human
JN807826_A_chicken_Egypt_1128_2011_Avian
JN807820_A_chicken_Egypt_1155AG_2011_Avian
JN807867_A_chicken_Egypt_11529S_2011_Avian

HA-Group14

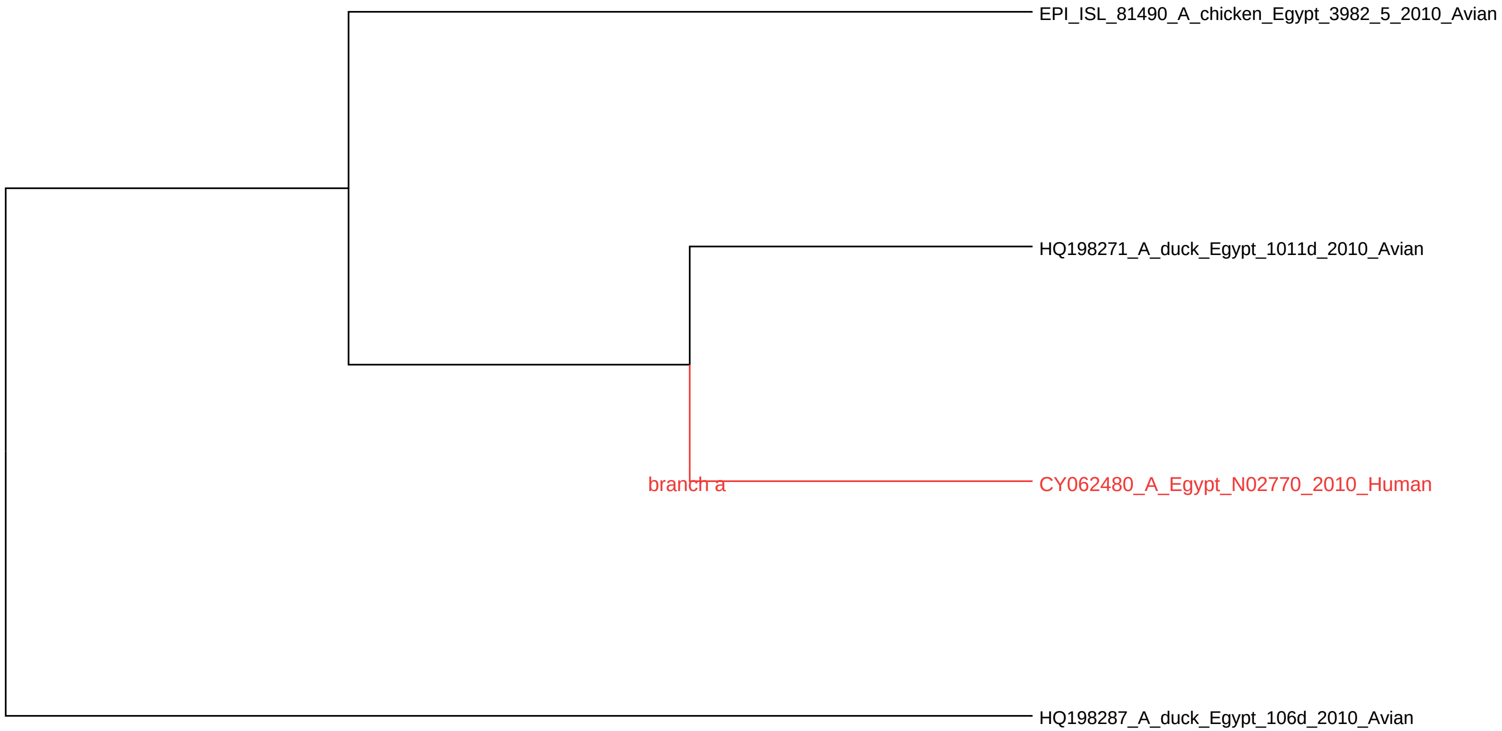

EPI_ISL_81490_A_chicken_Egypt_3982_5_2010_Avian
HQ198271_A_duck_Egypt_1011d_2010_Avian
branch a
CY062480_A_Egypt_N02770_2010_Human
HQ198287_A_duck_Egypt_106d_2010_Avian

HA-Group15

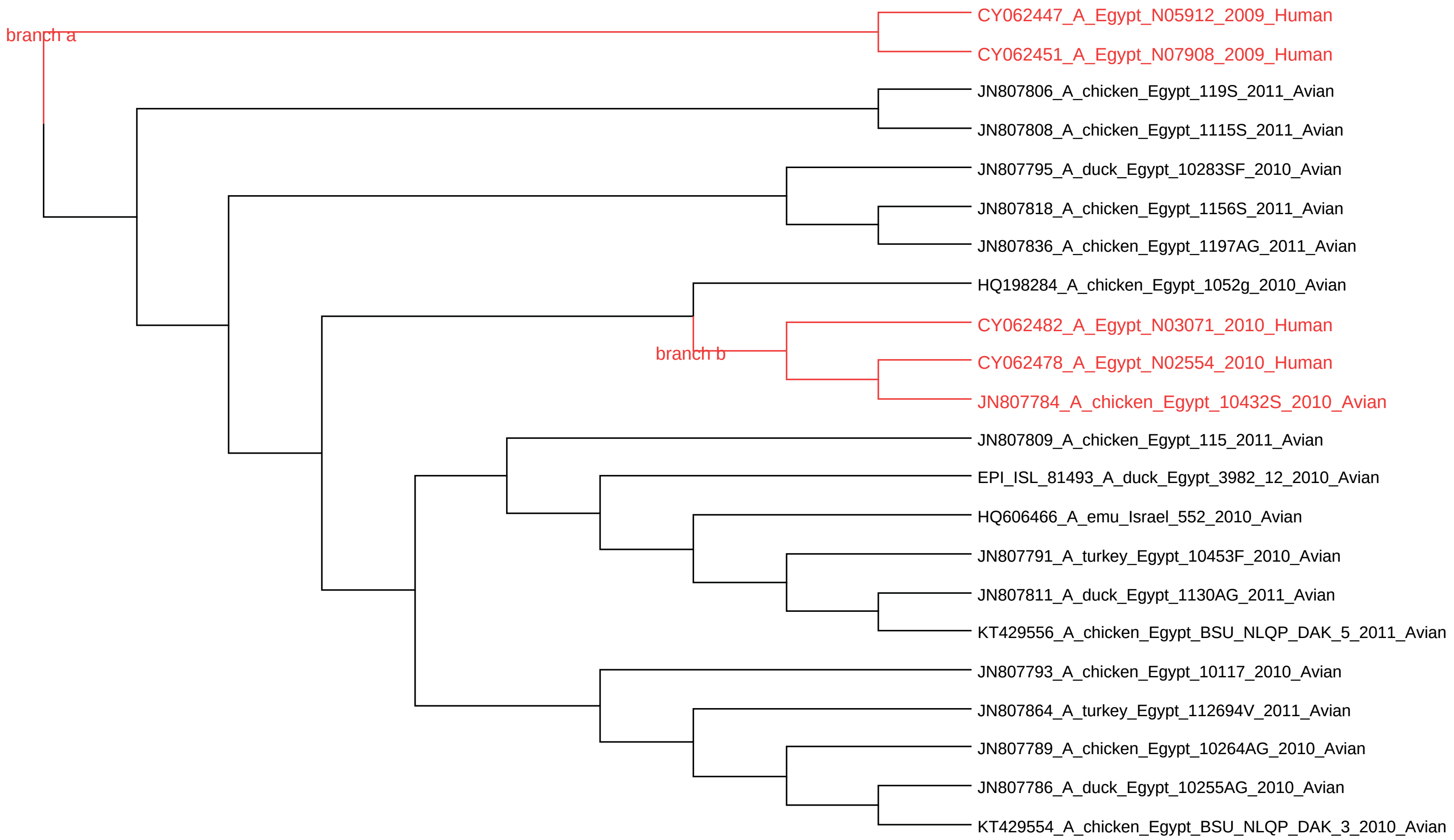
branch a
CY062447_A_Egypt_N05912_2009_Human
CY062451_A_Egypt_N07908_2009_Human
JN807806_A_chicken_Egypt_119S_2011_Avian
JN807808_A_chicken_Egypt_1115S_2011_Avian
JN807795_A_duck_Egypt_10283SF_2010_Avian
JN807818_A_chicken_Egypt_1156S_2011_Avian
JN807836_A_chicken_Egypt_1197AG_2011_Avian
HQ198284_A_chicken_Egypt_1052g_2010_Avian
CY062482_A_Egypt_N03071_2010_Human
branch b
CY062478_A_Egypt_N02554_2010_Human
JN807784_A_chicken_Egypt_10432S_2010_Avian
JN807809_A_chicken_Egypt_115_2011_Avian
EPI_ISL_81493_A_duck_Egypt_3982_12_2010_Avian
HQ606466_A_emu_Israel_552_2010_Avian
JN807791_A_turkey_Egypt_10453F_2010_Avian
JN807811_A_duck_Egypt_1130AG_2011_Avian
KT429556_A_chicken_Egypt_BSU_NLQP_DAK_5_2011_Avian
JN807793_A_chicken_Egypt_10117_2010_Avian
JN807864_A_turkey_Egypt_112694V_2011_Avian
JN807789_A_chicken_Egypt_10264AG_2010_Avian
JN807786_A_duck_Egypt_10255AG_2010_Avian
KT429554_A_chicken_Egypt_BSU_NLQP_DAK_3_2010_Avian

HA-Group16

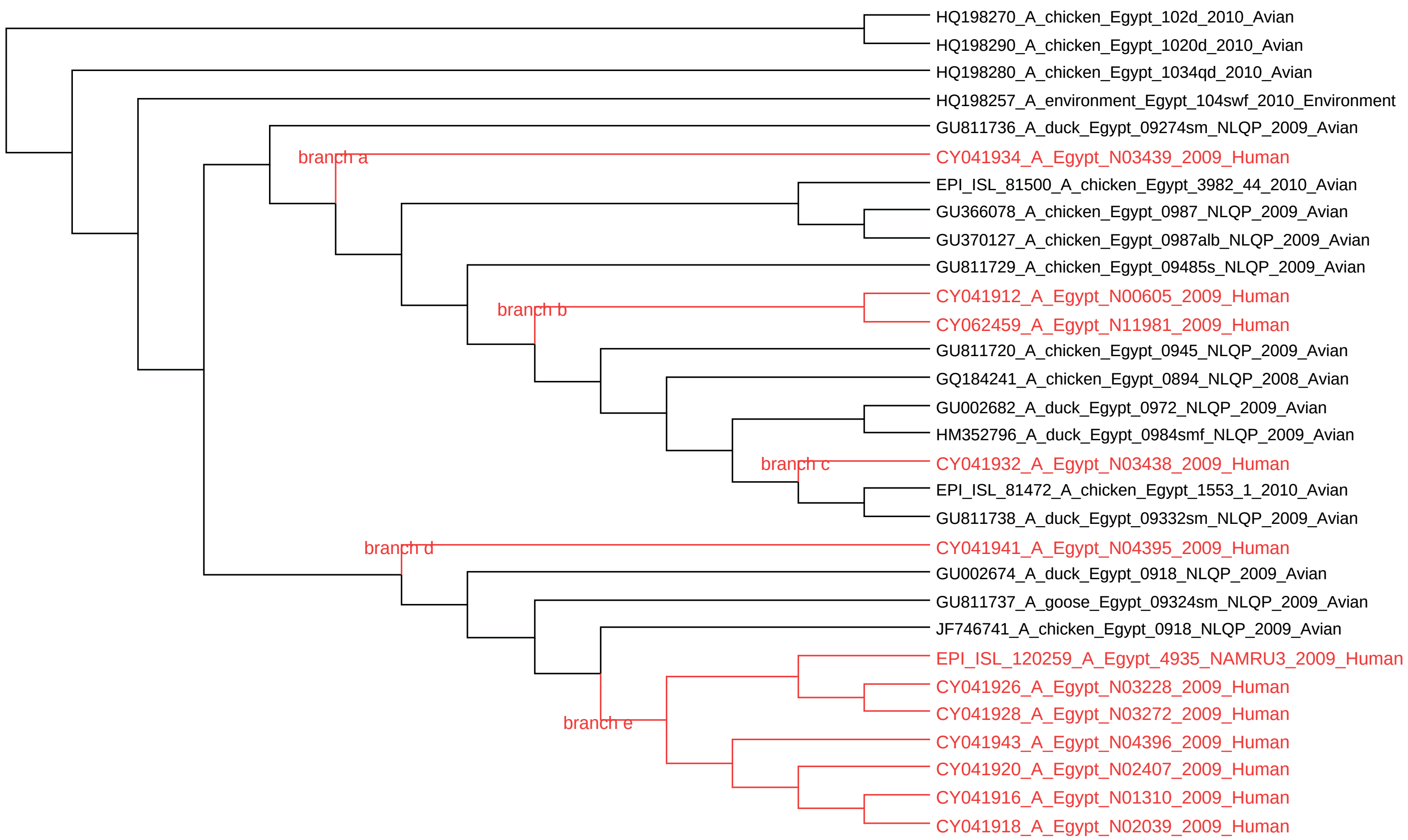

HQ198270_A_chicken_Egypt_102d_2010_Avian
HQ198290_A_chicken_Egypt_1020d_2010_Avian
HQ198280_A_chicken_Egypt_1034qd_2010_Avian
HQ198257_A_environment_Egypt_104swf_2010_Environment
GU811736_A_duck_Egypt_09274sm_NLQP_2009_Avian
branch a
CY041934_A_Egypt_N03439_2009_Human
EPI_ISL_81500_A_chicken_Egypt_3982_44_2010_Avian
GU366078_A_chicken_Egypt_0987_NLQP_2009_Avian
GU370127_A_chicken_Egypt_0987alb_NLQP_2009_Avian
GU811729_A_chicken_Egypt_09485s_NLQP_2009_Avian
branch b
CY041912_A_Egypt_N00605_2009_Human
CY062459_A_Egypt_N11981_2009_Human
GU811720_A_chicken_Egypt_0945_NLQP_2009_Avian
GQ184241_A_chicken_Egypt_0894_NLQP_2008_Avian
GU002682_A_duck_Egypt_0972_NLQP_2009_Avian
HM352796_A_duck_Egypt_0984smf_NLQP_2009_Avian
branch c
CY041932_A_Egypt_N03438_2009_Human
EPI_ISL_81472_A_chicken_Egypt_1553_1_2010_Avian
GU811738_A_duck_Egypt_09332sm_NLQP_2009_Avian
branch d
CY041941_A_Egypt_N04395_2009_Human
GU002674_A_duck_Egypt_0918_NLQP_2009_Avian
GU811737_A_goose_Egypt_09324sm_NLQP_2009_Avian
JF746741_A_chicken_Egypt_0918_NLQP_2009_Avian
EPI_ISL_120259_A_Egypt_4935_NAMRU3_2009_Human
CY041926_A_Egypt_N03228_2009_Human
CY041928_A_Egypt_N03272_2009_Human
branch e
CY041943_A_Egypt_N04396_2009_Human
CY041920_A_Egypt_N02407_2009_Human
CY041916_A_Egypt_N01310_2009_Human
CY041918_A_Egypt_N02039_2009_Human

HA-Group17

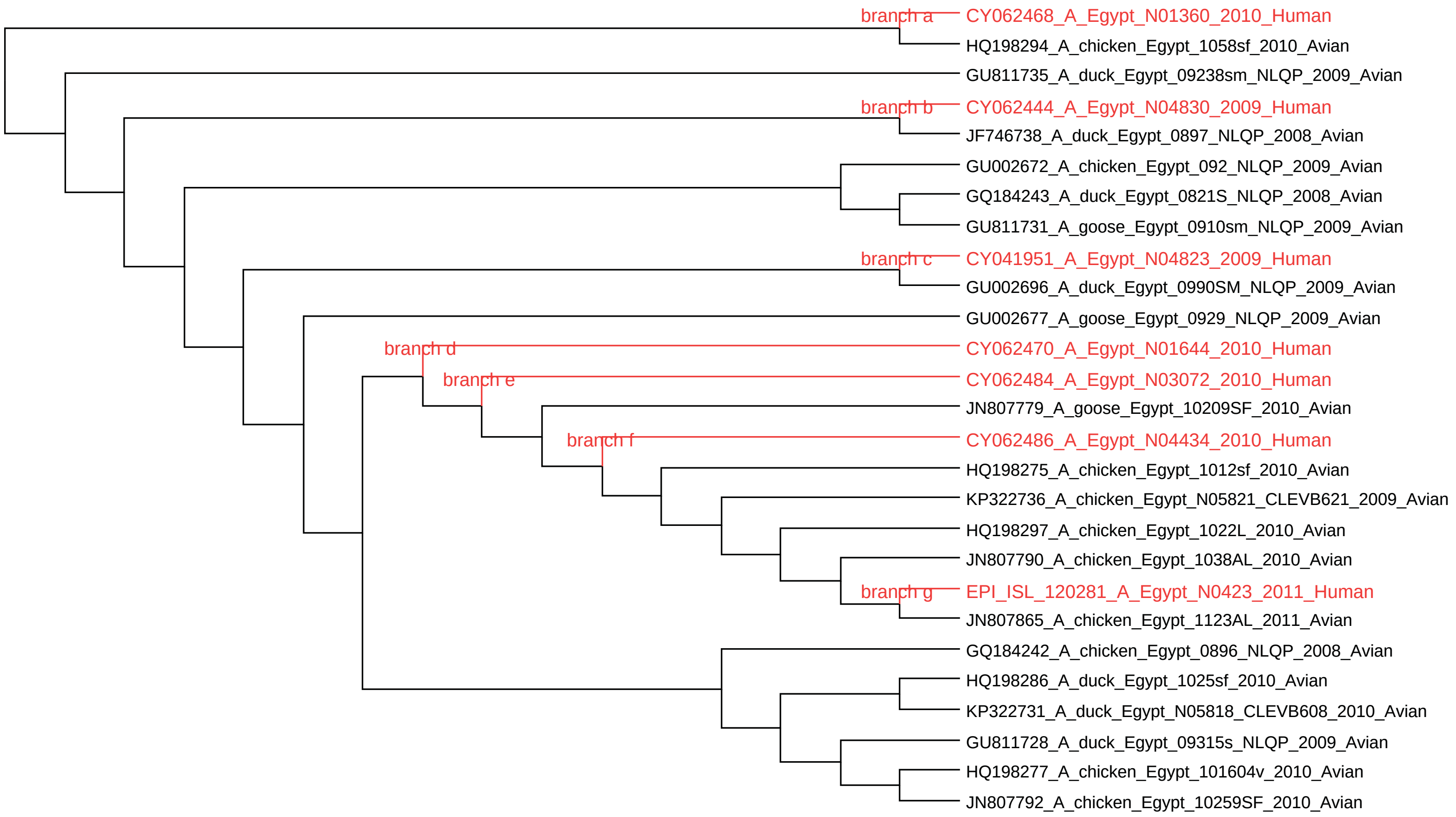

branch a
CY062468_A_Egypt_N01360_2010_Human
HQ198294_A_chicken_Egypt_1058sf_2010_Avian
GU811735_A_duck_Egypt_09238sm_NLQP_2009_Avian
branch b
CY062444_A_Egypt_N04830_2009_Human
JF746738_A_duck_Egypt_0897_NLQP_2008_Avian
GU002672_A_chicken_Egypt_092_NLQP_2009_Avian
GQ184243_A_duck_Egypt_0821S_NLQP_2008_Avian
GU811731_A_goose_Egypt_0910sm_NLQP_2009_Avian
branch c
CY041951_A_Egypt_N04823_2009_Human
GU002696_A_duck_Egypt_0990SM_NLQP_2009_Avian
GU002677_A_goose_Egypt_0929_NLQP_2009_Avian
branch d
CY062470_A_Egypt_N01644_2010_Human
branch e
CY062484_A_Egypt_N03072_2010_Human
JN807779_A_goose_Egypt_10209SF_2010_Avian
branch f
CY062486_A_Egypt_N04434_2010_Human
HQ198275_A_chicken_Egypt_1012sf_2010_Avian
KP322736_A_chicken_Egypt_N05821_CLEVB621_2009_Avian
HQ198297_A_chicken_Egypt_1022L_2010_Avian
JN807790_A_chicken_Egypt_1038AL_2010_Avian
branch g
EPI_ISL_120281_A_Egypt_N0423_2011_Human
JN807865_A_chicken_Egypt_1123AL_2011_Avian
GQ184242_A_chicken_Egypt_0896_NLQP_2008_Avian
HQ198286_A_duck_Egypt_1025sf_2010_Avian
KP322731_A_duck_Egypt_N05818_CLEVB608_2010_Avian
GU811728_A_duck_Egypt_09315s_NLQP_2009_Avian
HQ198277_A_chicken_Egypt_101604v_2010_Avian
JN807792_A_chicken_Egypt_10259SF_2010_Avian

HA-Group18

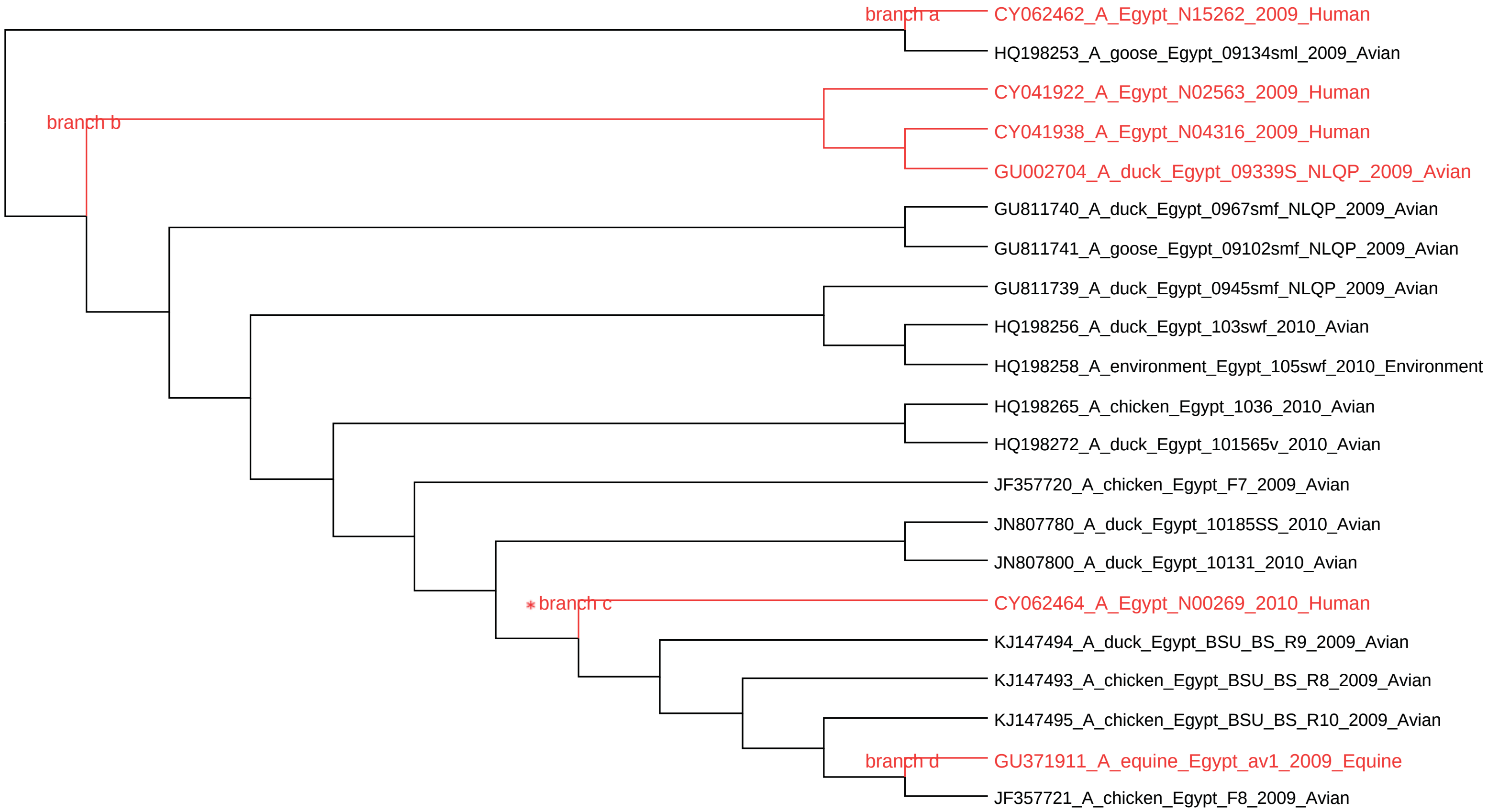

branch a
CY062462_A_Egypt_N15262_2009_Human
HQ198253_A_goose_Egypt_09134sml_2009_Avian
branch b
CY041922_A_Egypt_N02563_2009_Human
CY041938_A_Egypt_N04316_2009_Human
GU002704_A_duck_Egypt_09339S_NLQP_2009_Avian
GU811740_A_duck_Egypt_0967smf_NLQP_2009_Avian
GU811741_A_goose_Egypt_09102smf_NLQP_2009_Avian
GU811739_A_duck_Egypt_0945smf_NLQP_2009_Avian
HQ198256_A_duck_Egypt_103swf_2010_Avian
HQ198258_A_environment_Egypt_105swf_2010_Environment
HQ198265_A_chicken_Egypt_1036_2010_Avian
HQ198272_A_duck_Egypt_101565v_2010_Avian
JF357720_A_chicken_Egypt_F7_2009_Avian
JN807780_A_duck_Egypt_10185SS_2010_Avian
JN807800_A_duck_Egypt_10131_2010_Avian
* branch c
CY062464_A_Egypt_N00269_2010_Human
KJ147494_A_duck_Egypt_BSU_BS_R9_2009_Avian
KJ147493_A_chicken_Egypt_BSU_BS_R8_2009_Avian
KJ147495_A_chicken_Egypt_BSU_BS_R10_2009_Avian
branch d
GU371911_A_equine_Egypt_av1_2009_Equine
JF357721_A_chicken_Egypt_F8_2009_Avian

HA-Group19

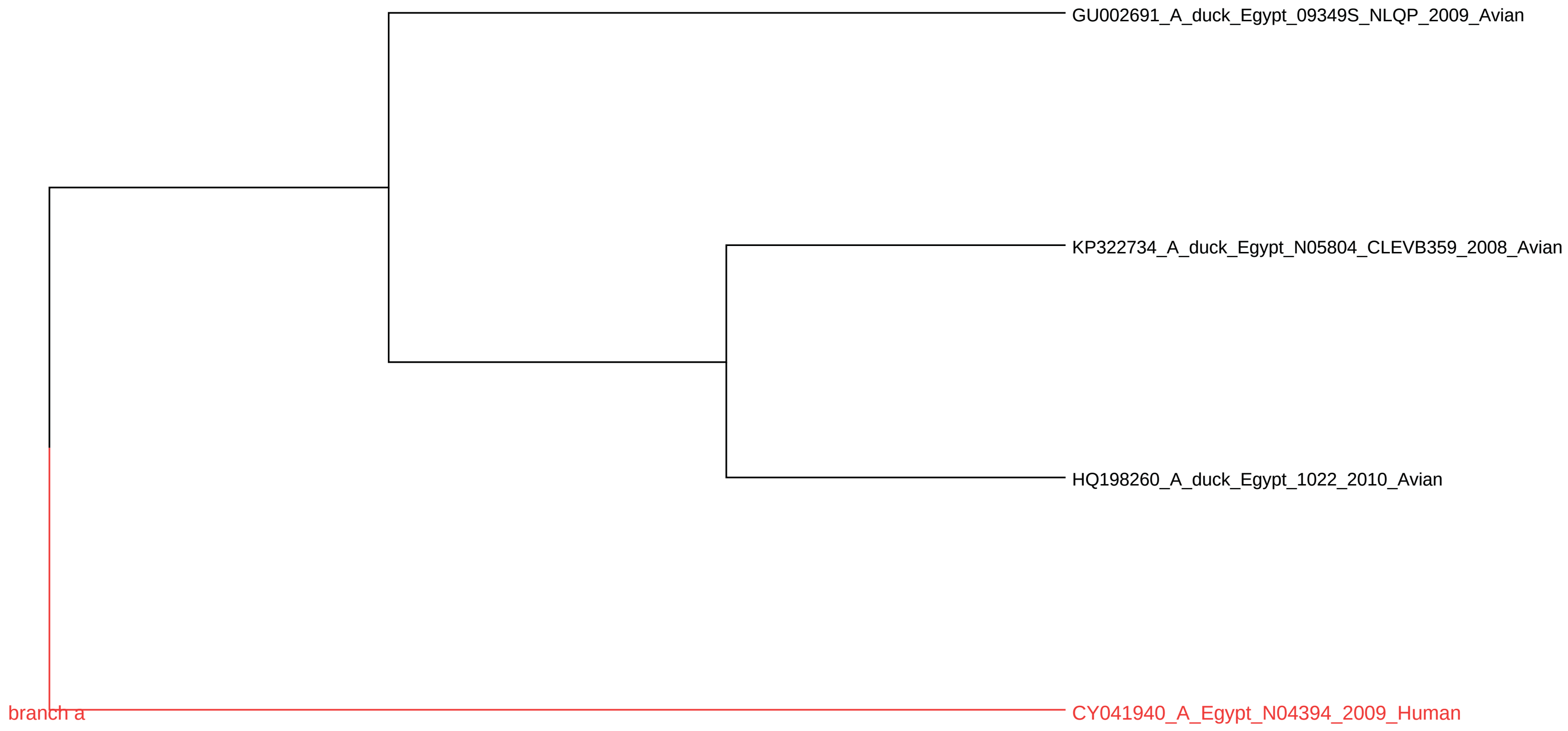
GU002691_A_duck_Egypt_09349S_NLQP_2009_Avian
KP322734_A_duck_Egypt_N05804_CLEVB359_2008_Avian
HQ198260_A_duck_Egypt_1022_2010_Avian
branch a
CY041940_A_Egypt_N04394_2009_Human

HA-Group20

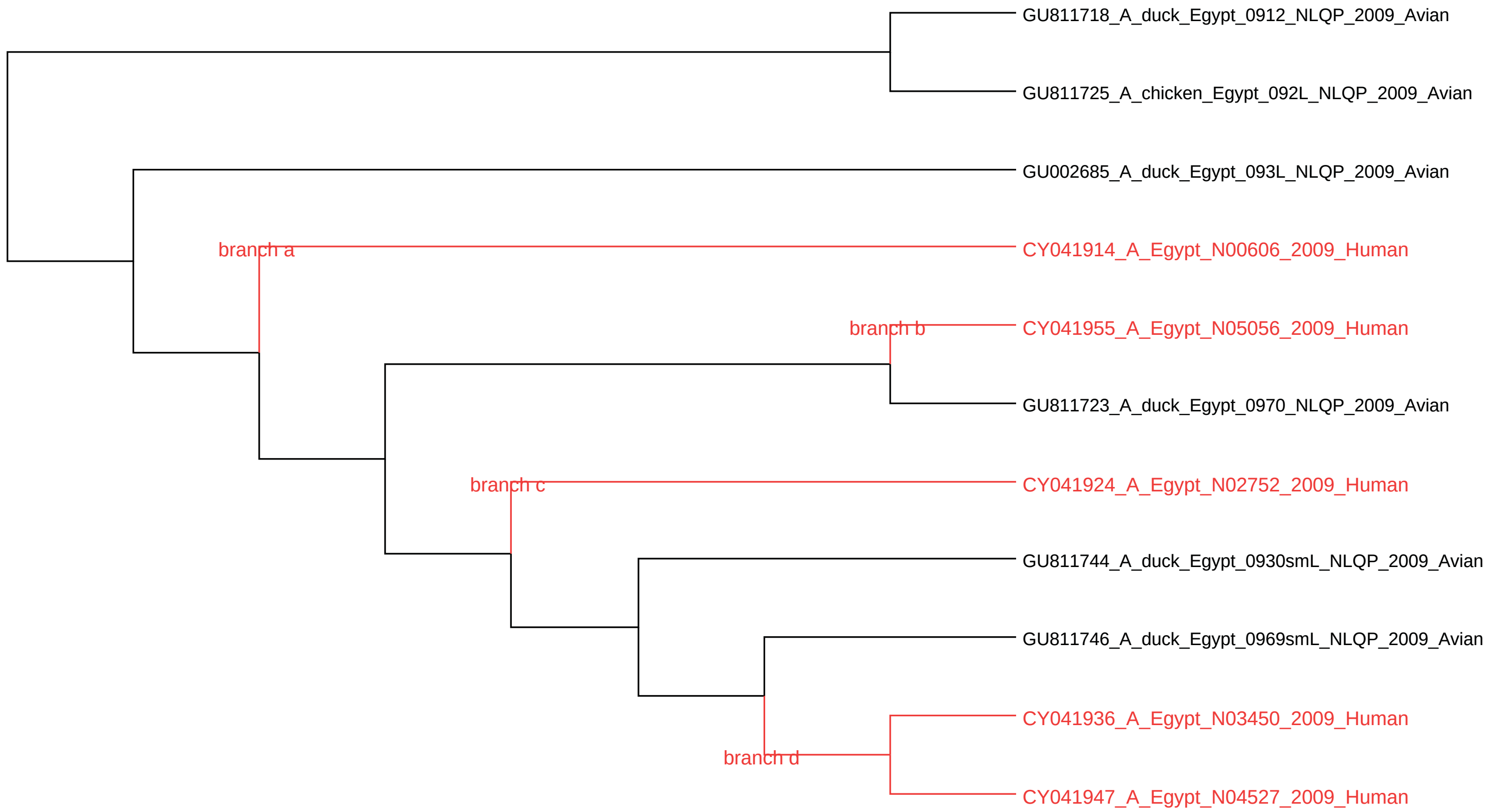
GU811718_A_duck_Egypt_0912_NLQP_2009_Avian
GU811725_A_chicken_Egypt_092L_NLQP_2009_Avian
GU002685_A_duck_Egypt_093L_NLQP_2009_Avian
branch a
CY041914_A_Egypt_N00606_2009_Human
branch b
CY041955_A_Egypt_N05056_2009_Human
GU811723_A_duck_Egypt_0970_NLQP_2009_Avian
branch c
CY041924_A_Egypt_N02752_2009_Human
GU811744_A_duck_Egypt_0930smL_NLQP_2009_Avian
GU811746_A_duck_Egypt_0969smL_NLQP_2009_Avian
CY041936_A_Egypt_N03450_2009_Human
branch d
CY041947_A_Egypt_N04527_2009_Human

HA-Group21

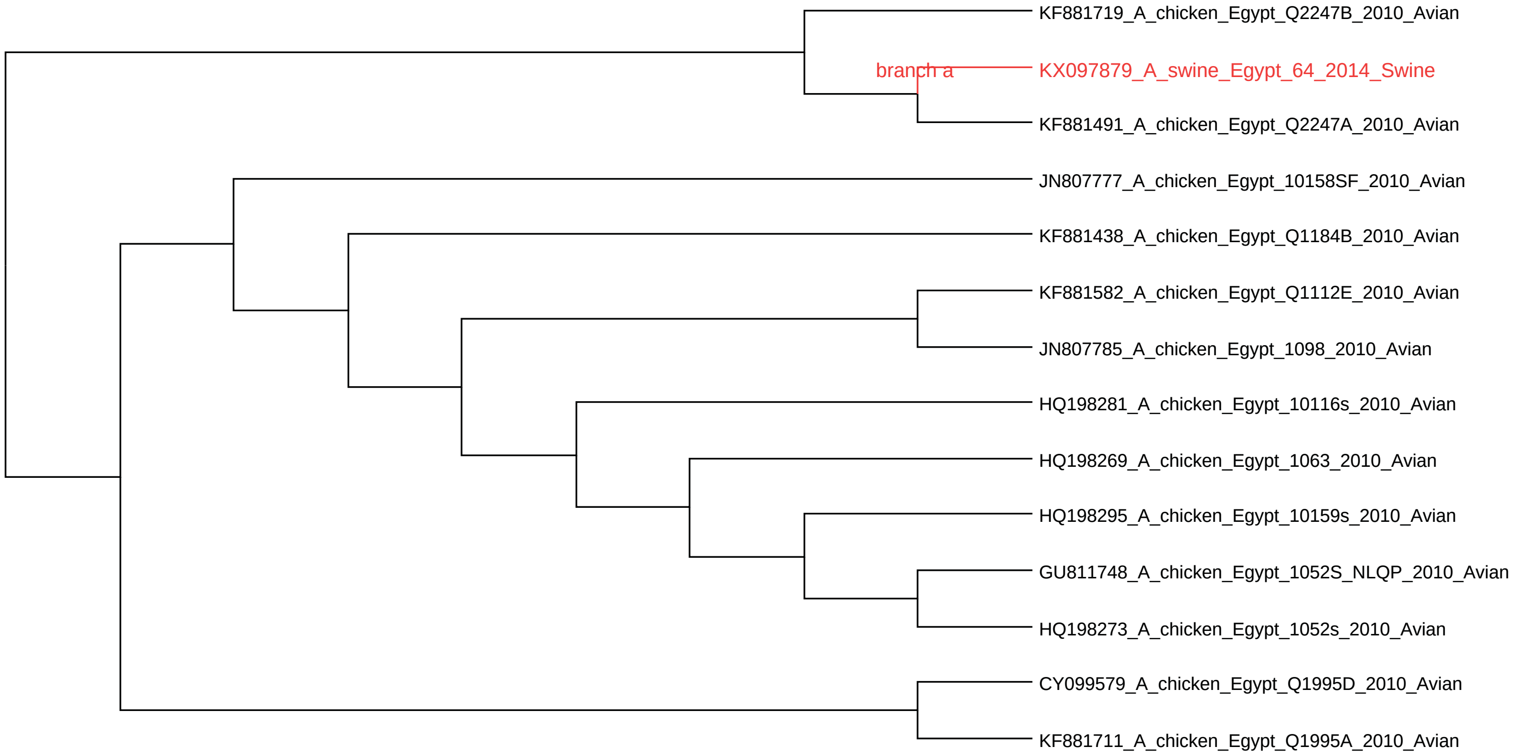

KF881719_A_chicken_Egypt_Q2247B_2010_Avian
branch a
KX097879_A_swine_Egypt_64_2014_Swine
KF881491_A_chicken_Egypt_Q2247A_2010_Avian
JN807777_A_chicken_Egypt_10158SF_2010_Avian
KF881438_A_chicken_Egypt_Q1184B_2010_Avian
KF881582_A_chicken_Egypt_Q1112E_2010_Avian
JN807785_A_chicken_Egypt_1098_2010_Avian
HQ198281_A_chicken_Egypt_10116s_2010_Avian
HQ198269_A_chicken_Egypt_1063_2010_Avian
HQ198295_A_chicken_Egypt_10159s_2010_Avian
GU811748_A_chicken_Egypt_1052S_NLQP_2010_Avian
HQ198273_A_chicken_Egypt_1052s_2010_Avian
CY099579_A_chicken_Egypt_Q1995D_2010_Avian
KF881711_A_chicken_Egypt_Q1995A_2010_Avian

HA-Group22

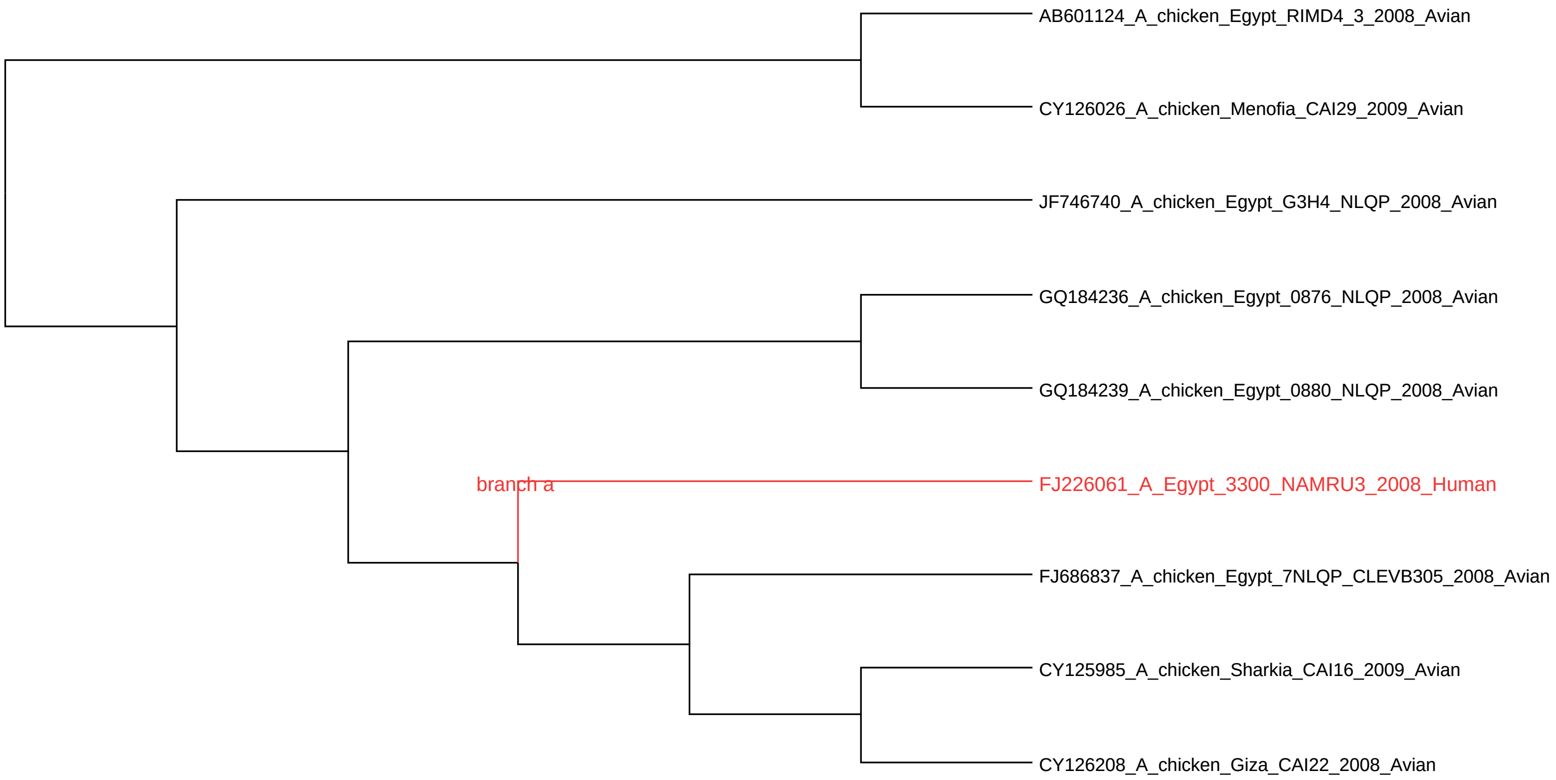

AB601124_A_chicken_Egypt_RIMD4_3_2008_Avian
CY126026_A_chicken_Menofia_CAI29_2009_Avian
JF746740_A_chicken_Egypt_G3H4_NLQP_2008_Avian
GQ184236_A_chicken_Egypt_0876_NLQP_2008_Avian
GQ184239_A_chicken_Egypt_0880_NLQP_2008_Avian
branch a
FJ226061_A_Egypt_3300_NAMRU3_2008_Human
FJ686837_A_chicken_Egypt_7NLQP_CLEVB305_2008_Avian
CY125985_A_chicken_Sharkia_CAI16_2009_Avian
CY126208_A_chicken_Giza_CAI22_2008_Avian

HA-Group23

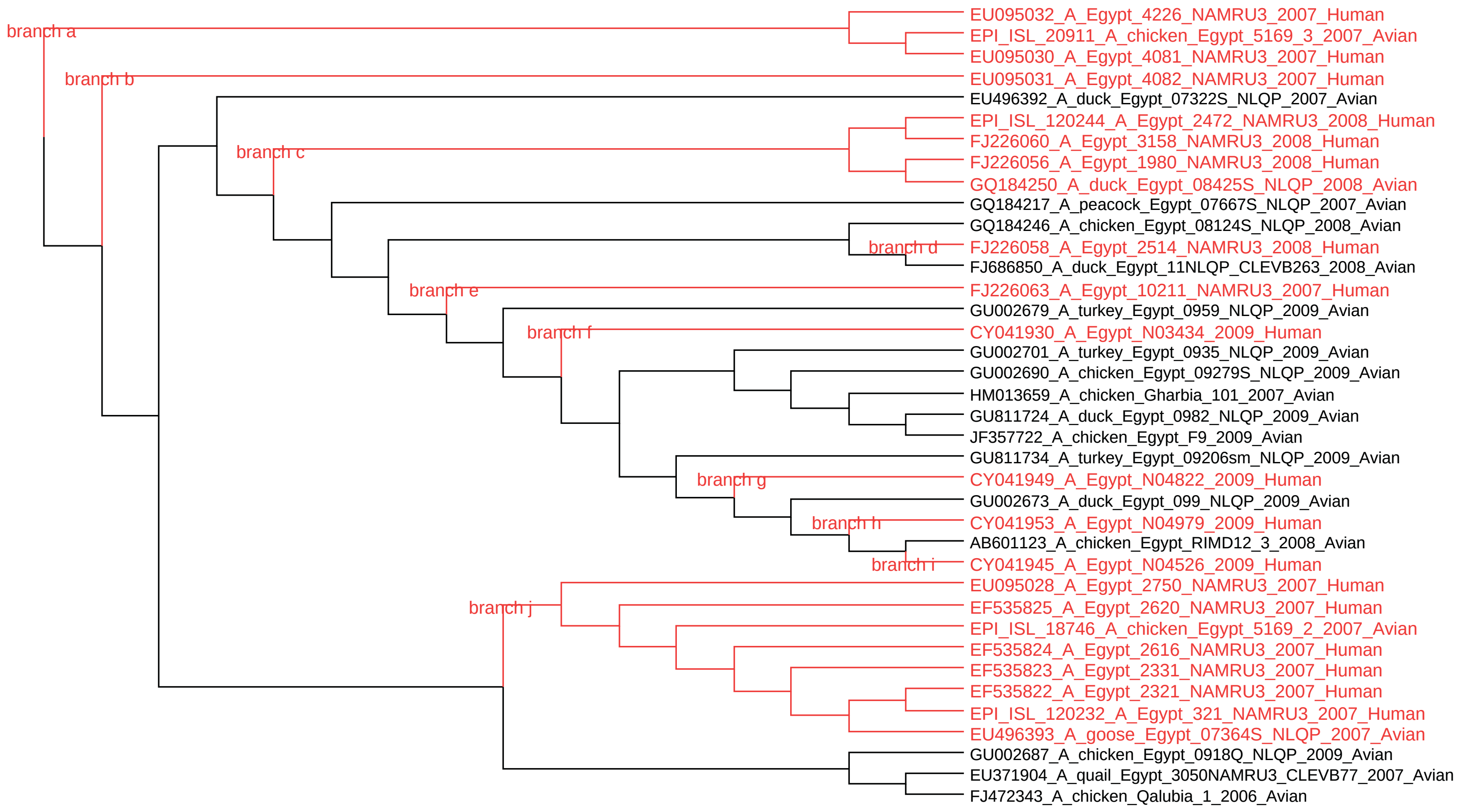

branch a
branch b
branch c
branch d
branch e
branch f
branch g
branch h
branch i
branch j
EU095032_A_Egypt_4226_NAMRU3_2007_Human
EPI_ISL_20911_A_chicken_Egypt_5169_3_2007_Avian
EU095030_A_Egypt_4081_NAMRU3_2007_Human
EU095031_A_Egypt_4082_NAMRU3_2007_Human
EU496392_A_duck_Egypt_07322S_NLQP_2007_Avian
EPI_ISL_120244_A_Egypt_2472_NAMRU3_2008_Human
FJ226060_A_Egypt_3158_NAMRU3_2008_Human
FJ226056_A_Egypt_1980_NAMRU3_2008_Human
GQ184250_A_duck_Egypt_08425S_NLQP_2008_Avian
GQ184217_A_peacock_Egypt_07667S_NLQP_2007_Avian
GQ184246_A_chicken_Egypt_08124S_NLQP_2008_Avian
FJ226058_A_Egypt_2514_NAMRU3_2008_Human
FJ686850_A_duck_Egypt_11NLQP_CLEVB263_2008_Avian
FJ226063_A_Egypt_10211_NAMRU3_2007_Human
GU002679_A_turkey_Egypt_0959_NLQP_2009_Avian
CY041930_A_Egypt_N03434_2009_Human
GU002701_A_turkey_Egypt_0935_NLQP_2009_Avian
GU002690_A_chicken_Egypt_09279S_NLQP_2009_Avian
HM013659_A_chicken_Gharbia_101_2007_Avian
GU811724_A_duck_Egypt_0982_NLQP_2009_Avian
JF357722_A_chicken_Egypt_F9_2009_Avian
GU811734_A_turkey_Egypt_09206sm_NLQP_2009_Avian
CY041949_A_Egypt_N04822_2009_Human
GU002673_A_duck_Egypt_099_NLQP_2009_Avian
CY041953_A_Egypt_N04979_2009_Human
AB601123_A_chicken_Egypt_RIMD12_3_2008_Avian
CY041945_A_Egypt_N04526_2009_Human
EU095028_A_Egypt_2750_NAMRU3_2007_Human
EF535825_A_Egypt_2620_NAMRU3_2007_Human
EPI_ISL_18746_A_chicken_Egypt_5169_2_2007_Avian
EF535824_A_Egypt_2616_NAMRU3_2007_Human
EF535823_A_Egypt_2331_NAMRU3_2007_Human
EF535822_A_Egypt_2321_NAMRU3_2007_Human
EPI_ISL_120232_A_Egypt_321_NAMRU3_2007_Human
EU496393_A_goose_Egypt_07364S_NLQP_2007_Avian
GU002687_A_chicken_Egypt_0918Q_NLQP_2009_Avian
EU371904_A_quail_Egypt_3050NAMRU3_CLEVB77_2007_Avian
FJ472343_A_chicken_Qalubia_1_2006_Avian

HA-Group24

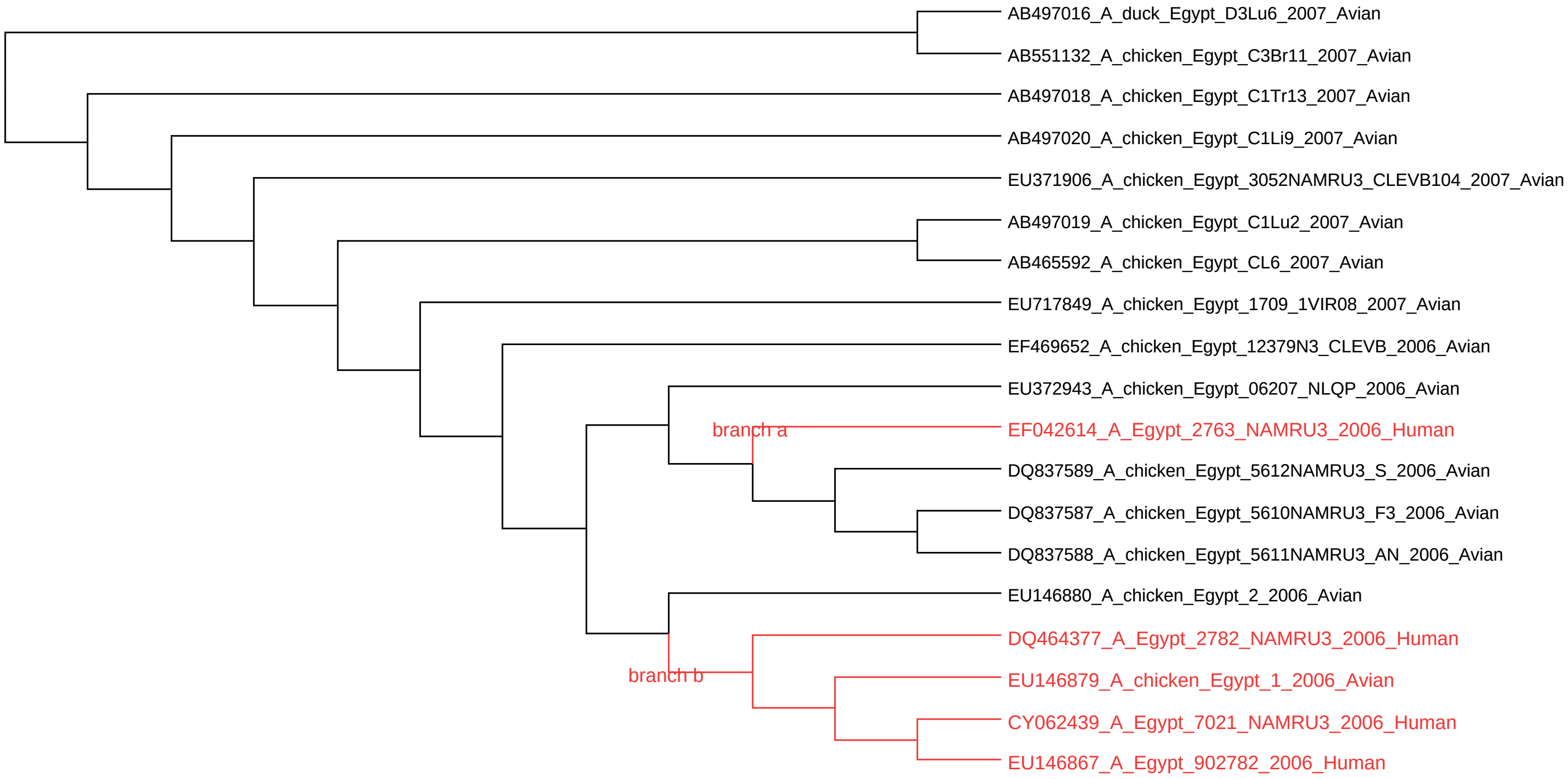

AB497016_A_duck_Egypt_D3Lu6_2007_Avian
AB551132_A_chicken_Egypt_C3Br11_2007_Avian
AB497018_A_chicken_Egypt_C1Tr13_2007_Avian
AB497020_A_chicken_Egypt_C1Li9_2007_Avian
EU371906_A_chicken_Egypt_3052NAMRU3_CLEVB104_2007_Avian
AB497019_A_chicken_Egypt_C1Lu2_2007_Avian
AB465592_A_chicken_Egypt_CL6_2007_Avian
EU717849_A_chicken_Egypt_1709_1VIR08_2007_Avian
EF469652_A_chicken_Egypt_12379N3_CLEVB_2006_Avian
EU372943_A_chicken_Egypt_06207_NLQP_2006_Avian
branch a
EF042614_A_Egypt_2763_NAMRU3_2006_Human
DQ837589_A_chicken_Egypt_5612NAMRU3_S_2006_Avian
DQ837587_A_chicken_Egypt_5610NAMRU3_F3_2006_Avian
DQ837588_A_chicken_Egypt_5611NAMRU3_AN_2006_Avian
EU146880_A_chicken_Egypt_2_2006_Avian
DQ464377_A_Egypt_2782_NAMRU3_2006_Human
branch b
EU146879_A_chicken_Egypt_1_2006_Avian
CY062439_A_Egypt_7021_NAMRU3_2006_Human
EU146867_A_Egypt_902782_2006_Human

HA-Group25

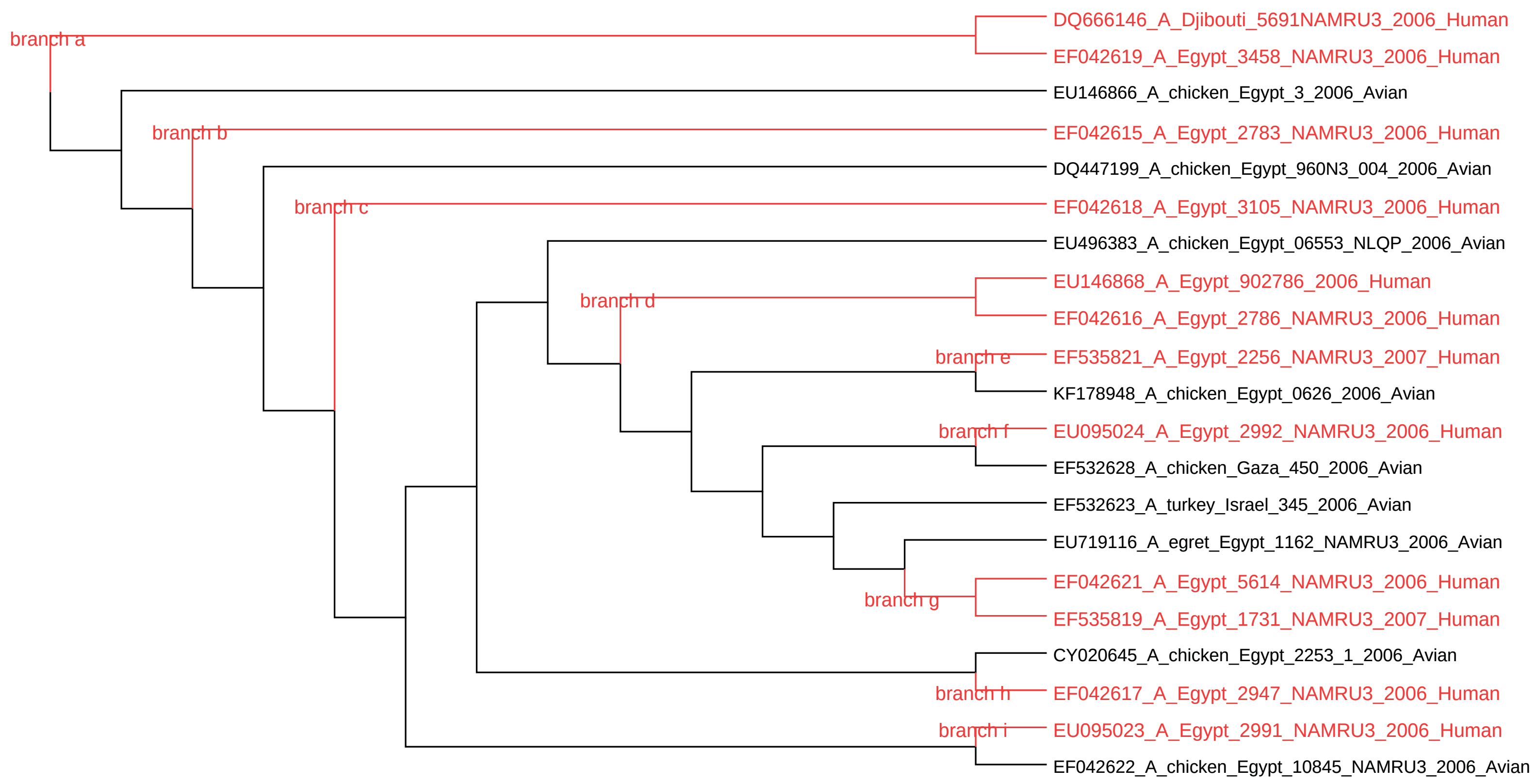

branch a
branch b
branch c
branch d
branch e
branch f
branch g
branch h
branch i
DQ666146_A_Djibouti_5691NAMRU3_2006_Human
EF042619_A_Egypt_3458_NAMRU3_2006_Human
EU146866_A_chicken_Egypt_3_2006_Avian
EF042615_A_Egypt_2783_NAMRU3_2006_Human
DQ447199_A_chicken_Egypt_960N3_004_2006_Avian
EF042618_A_Egypt_3105_NAMRU3_2006_Human
EU496383_A_chicken_Egypt_06553_NLQP_2006_Avian
EU146868_A_Egypt_902786_2006_Human
EF042616_A_Egypt_2786_NAMRU3_2006_Human
EF535821_A_Egypt_2256_NAMRU3_2007_Human
KF178948_A_chicken_Egypt_0626_2006_Avian
EU095024_A_Egypt_2992_NAMRU3_2006_Human
EF532628_A_chicken_Gaza_450_2006_Avian
EF532623_A_turkey_Israel_345_2006_Avian
EU719116_A_egret_Egypt_1162_NAMRU3_2006_Avian
EF042621_A_Egypt_5614_NAMRU3_2006_Human
EF535819_A_Egypt_1731_NAMRU3_2007_Human
CY020645_A_chicken_Egypt_2253_1_2006_Avian
EF042617_A_Egypt_2947_NAMRU3_2006_Human
EU095023_A_Egypt_2991_NAMRU3_2006_Human
EF042622_A_chicken_Egypt_10845_NAMRU3_2006_Avian

HA-Group26

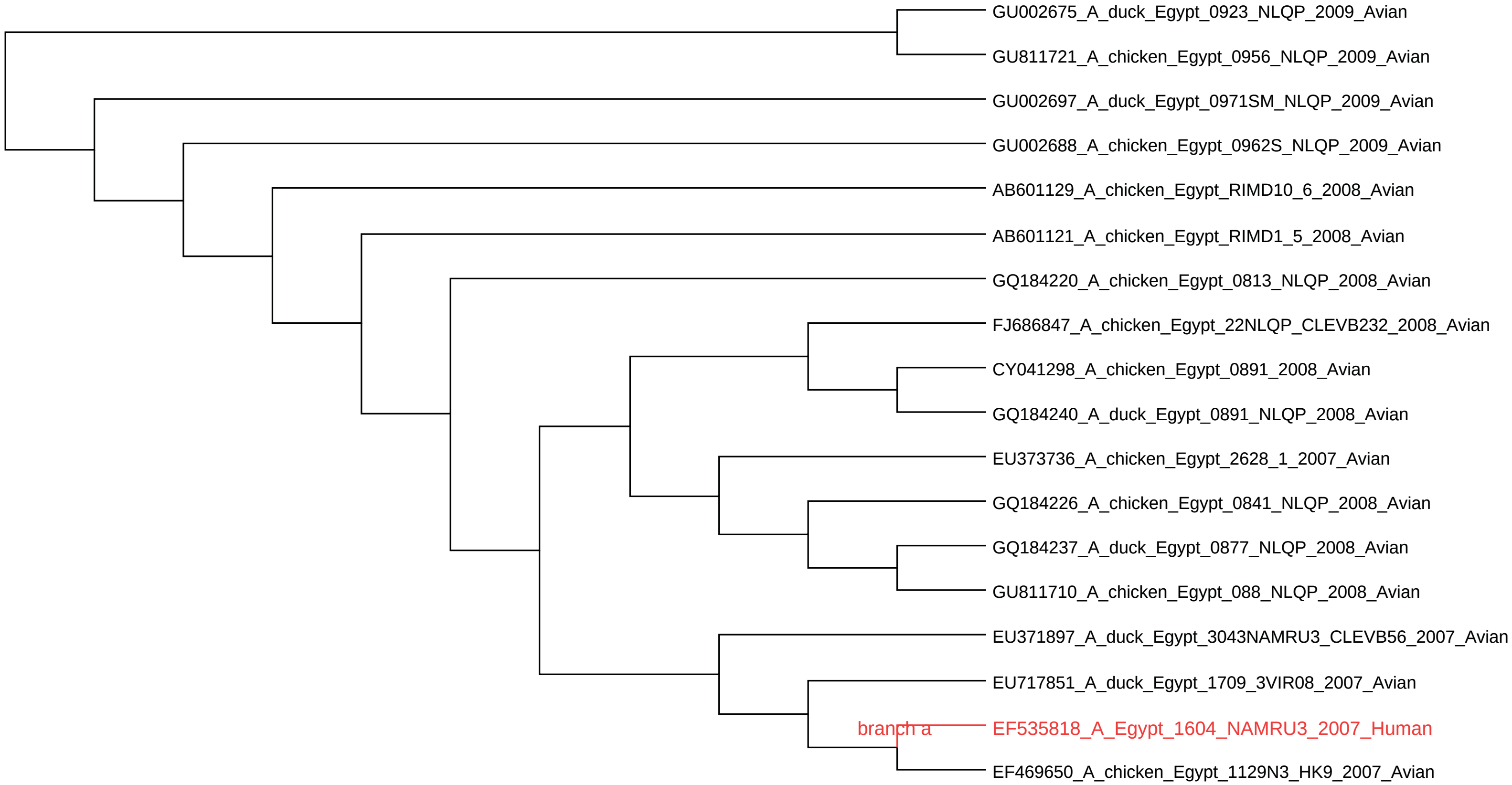
GU002675_A_duck_Egypt_0923_NLQP_2009_Avian
GU811721_A_chicken_Egypt_0956_NLQP_2009_Avian
GU002697_A_duck_Egypt_0971SM_NLQP_2009_Avian
GU002688_A_chicken_Egypt_0962S_NLQP_2009_Avian
AB601129_A_chicken_Egypt_RIMD10_6_2008_Avian
AB601121_A_chicken_Egypt_RIMD1_5_2008_Avian
GQ184220_A_chicken_Egypt_0813_NLQP_2008_Avian
FJ686847_A_chicken_Egypt_22NLQP_CLEVB232_2008_Avian
CY041298_A_chicken_Egypt_0891_2008_Avian
GQ184240_A_duck_Egypt_0891_NLQP_2008_Avian
EU373736_A_chicken_Egypt_2628_1_2007_Avian
GQ184226_A_chicken_Egypt_0841_NLQP_2008_Avian
GQ184237_A_duck_Egypt_0877_NLQP_2008_Avian
GU811710_A_chicken_Egypt_088_NLQP_2008_Avian
EU371897_A_duck_Egypt_3043NAMRU3_CLEVB56_2007_Avian
EU717851_A_duck_Egypt_1709_3VIR08_2007_Avian
branch a
EF535818_A_Egypt_1604_NAMRU3_2007_Human
EF469650_A_chicken_Egypt_1129N3_HK9_2007_Avian

HA-Group27

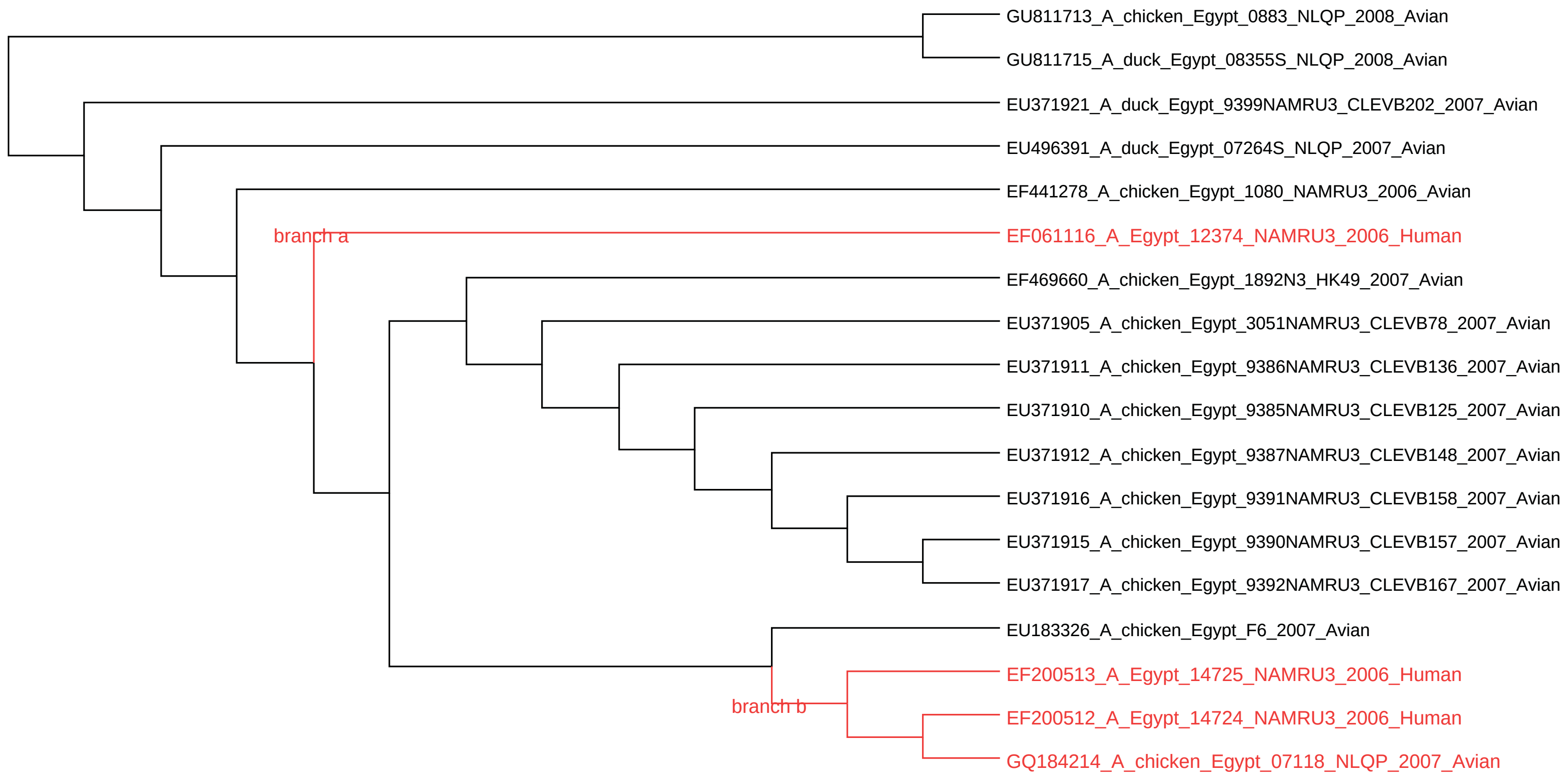
GU811713_A_chicken_Egypt_0883_NLQP_2008_Avian
GU811715_A_duck_Egypt_08355S_NLQP_2008_Avian
EU371921_A_duck_Egypt_9399NAMRU3_CLEVB202_2007_Avian
EU496391_A_duck_Egypt_07264S_NLQP_2007_Avian
EF441278_A_chicken_Egypt_1080_NAMRU3_2006_Avian
branch a
EF061116_A_Egypt_12374_NAMRU3_2006_Human
EF469660_A_chicken_Egypt_1892N3_HK49_2007_Avian
EU371905_A_chicken_Egypt_3051NAMRU3_CLEVB78_2007_Avian
EU371911_A_chicken_Egypt_9386NAMRU3_CLEVB136_2007_Avian
EU371910_A_chicken_Egypt_9385NAMRU3_CLEVB125_2007_Avian
EU371912_A_chicken_Egypt_9387NAMRU3_CLEVB148_2007_Avian
EU371916_A_chicken_Egypt_9391NAMRU3_CLEVB158_2007_Avian
EU371915_A_chicken_Egypt_9390NAMRU3_CLEVB157_2007_Avian
EU371917_A_chicken_Egypt_9392NAMRU3_CLEVB167_2007_Avian
EU183326_A_chicken_Egypt_F6_2007_Avian
EF200513_A_Egypt_14725_NAMRU3_2006_Human
branch b
EF200512_A_Egypt_14724_NAMRU3_2006_Human
GQ184214_A_chicken_Egypt_07118_NLQP_2007_Avian

HA-Group28

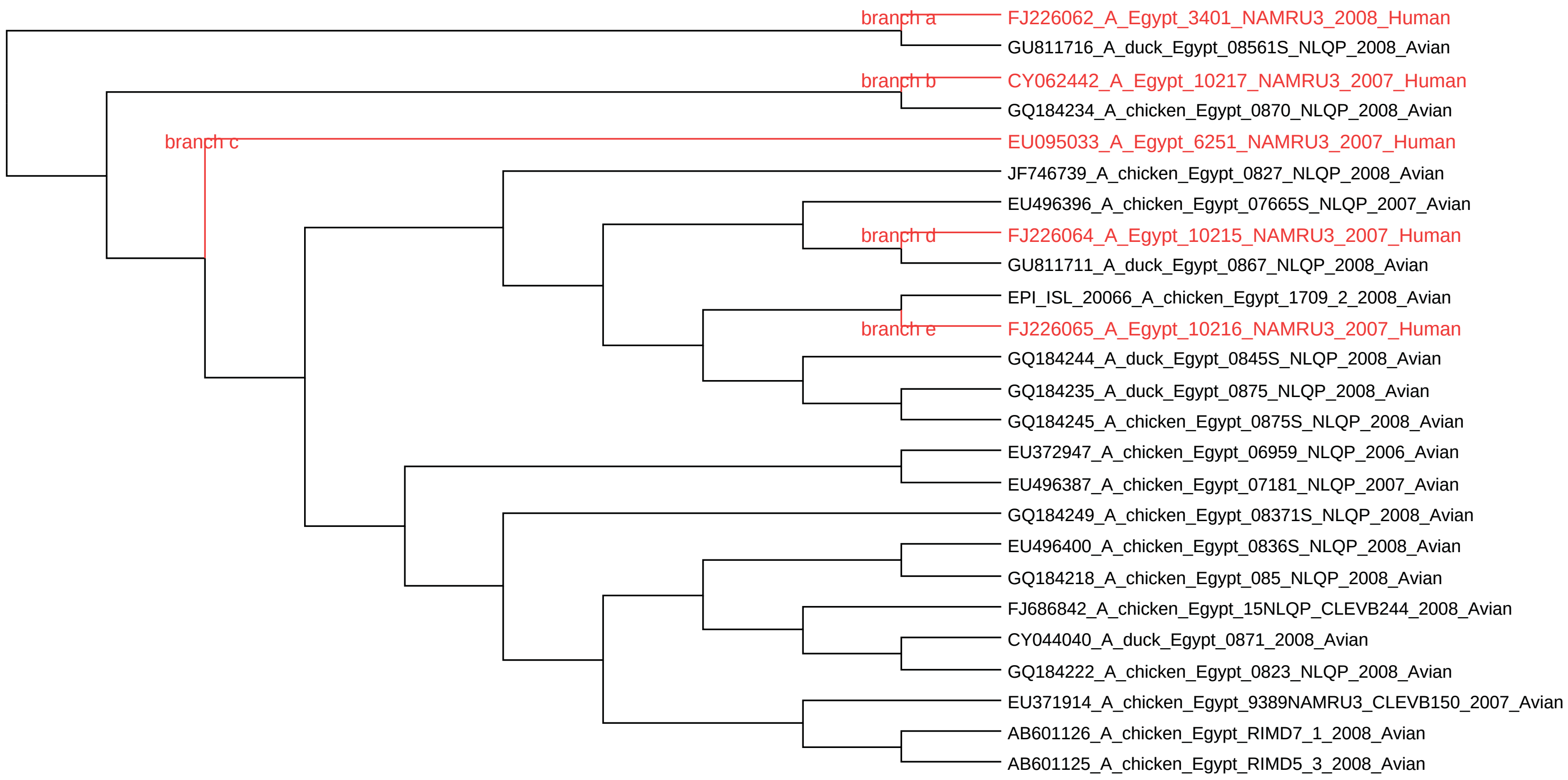
branch a
FJ226062_A_Egypt_3401_NAMRU3_2008_Human
GU811716_A_duck_Egypt_08561S_NLQP_2008_Avian
branch b
CY062442_A_Egypt_10217_NAMRU3_2007_Human
GQ184234_A_chicken_Egypt_0870_NLQP_2008_Avian
branch c
EU095033_A_Egypt_6251_NAMRU3_2007_Human
JF746739_A_chicken_Egypt_0827_NLQP_2008_Avian
EU496396_A_chicken_Egypt_07665S_NLQP_2007_Avian
branch d
FJ226064_A_Egypt_10215_NAMRU3_2007_Human
GU811711_A_duck_Egypt_0867_NLQP_2008_Avian
EPI_ISL_20066_A_chicken_Egypt_1709_2_2008_Avian
branch e
FJ226065_A_Egypt_10216_NAMRU3_2007_Human
GQ184244_A_duck_Egypt_0845S_NLQP_2008_Avian
GQ184235_A_duck_Egypt_0875_NLQP_2008_Avian
GQ184245_A_chicken_Egypt_0875S_NLQP_2008_Avian
EU372947_A_chicken_Egypt_06959_NLQP_2006_Avian
EU496387_A_chicken_Egypt_07181_NLQP_2007_Avian
GQ184249_A_chicken_Egypt_08371S_NLQP_2008_Avian
EU496400_A_chicken_Egypt_0836S_NLQP_2008_Avian
GQ184218_A_chicken_Egypt_085_NLQP_2008_Avian
FJ686842_A_chicken_Egypt_15NLQP_CLEVB244_2008_Avian
CY044040_A_duck_Egypt_0871_2008_Avian
GQ184222_A_chicken_Egypt_0823_NLQP_2008_Avian
EU371914_A_chicken_Egypt_9389NAMRU3_CLEVB150_2007_Avian
AB601126_A_chicken_Egypt_RIMD7_1_2008_Avian
AB601125_A_chicken_Egypt_RIMD5_3_2008_Avian

HA-Group29

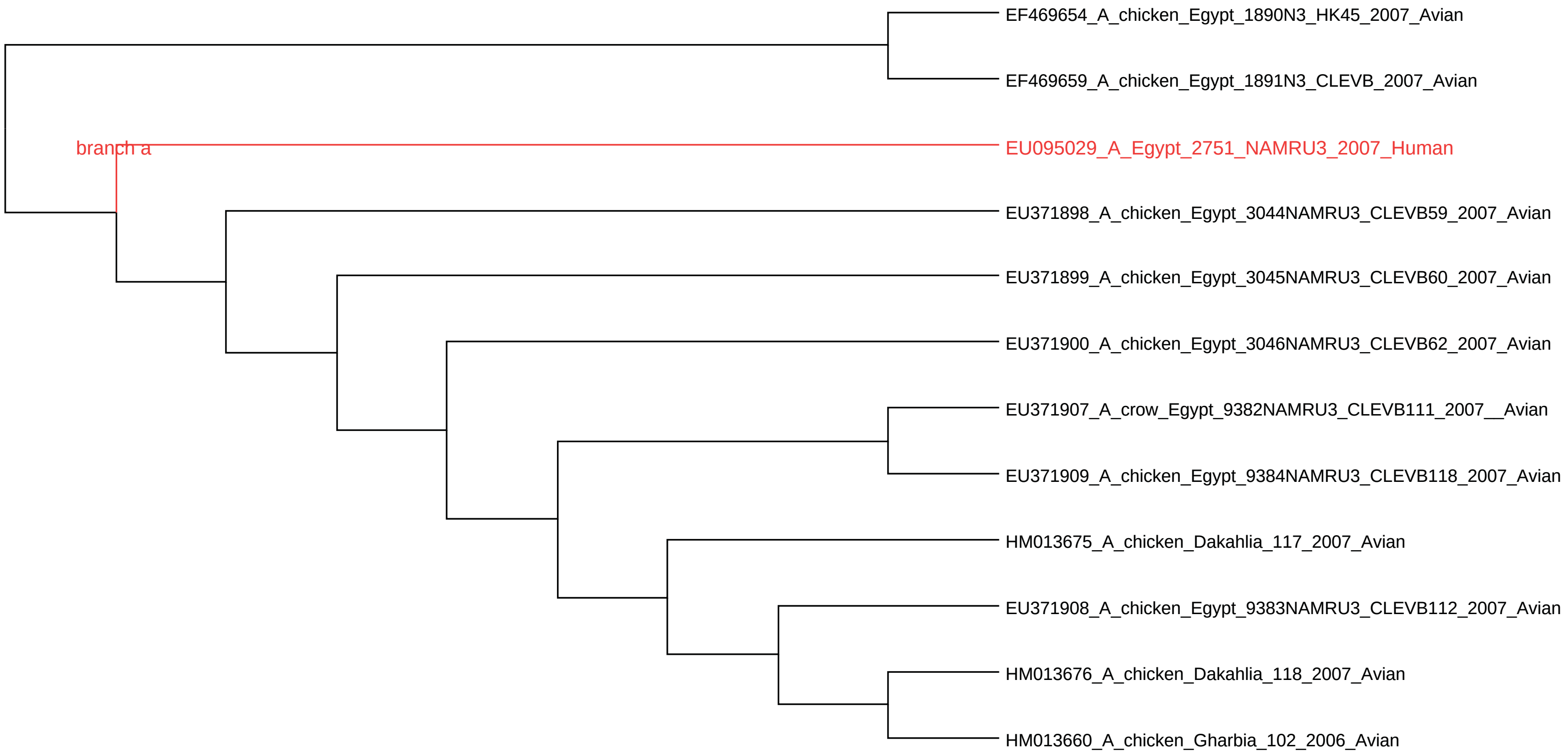
EF469654_A_chicken_Egypt_1890N3_HK45_2007_Avian
EF469659_A_chicken_Egypt_1891N3_CLEVB_2007_Avian
branch a
EU095029_A_Egypt_2751_NAMRU3_2007_Human
EU371898_A_chicken_Egypt_3044NAMRU3_CLEVB59_2007_Avian
EU371899_A_chicken_Egypt_3045NAMRU3_CLEVB60_2007_Avian
EU371900_A_chicken_Egypt_3046NAMRU3_CLEVB62_2007_Avian
EU371907_A_crow_Egypt_9382NAMRU3_CLEVB111_2007__Avian
EU371909_A_chicken_Egypt_9384NAMRU3_CLEVB118_2007_Avian
HM013675_A_chicken_Dakahlia_117_2007_Avian
EU371908_A_chicken_Egypt_9383NAMRU3_CLEVB112_2007_Avian
HM013676_A_chicken_Dakahlia_118_2007_Avian
HM013660_A_chicken_Gharbia_102_2006_Avian

HA-Group30

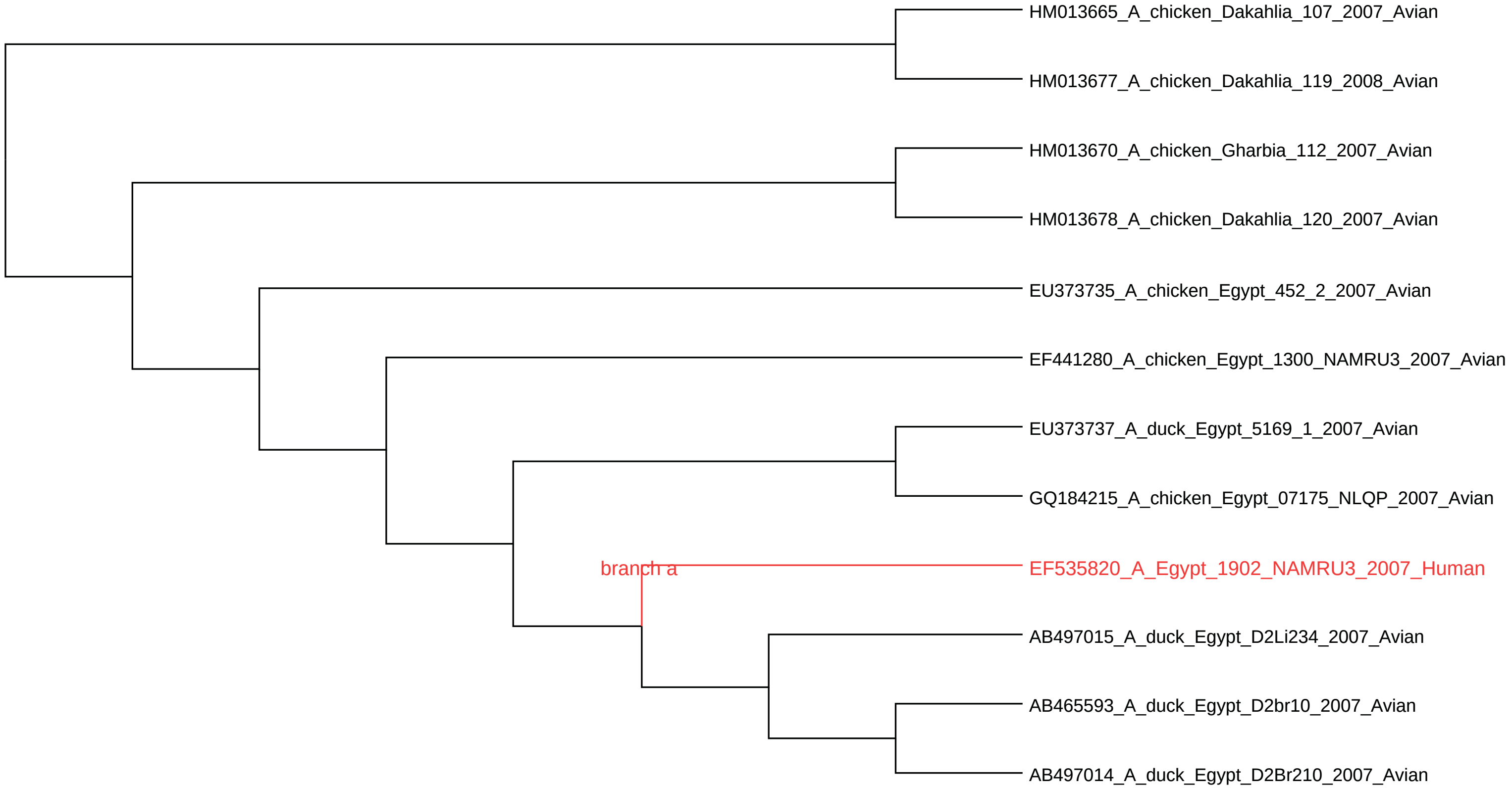

HM013665_A_chicken_Dakahlia_107_2007_Avian
HM013677_A_chicken_Dakahlia_119_2008_Avian
HM013670_A_chicken_Gharbia_112_2007_Avian
HM013678_A_chicken_Dakahlia_120_2007_Avian
EU373735_A_chicken_Egypt_452_2_2007_Avian
EF441280_A_chicken_Egypt_1300_NAMRU3_2007_Avian
EU373737_A_duck_Egypt_5169_1_2007_Avian
GQ184215_A_chicken_Egypt_07175_NLQP_2007_Avian
branch a
EF535820_A_Egypt_1902_NAMRU3_2007_Human
AB497015_A_duck_Egypt_D2Li234_2007_Avian
AB465593_A_duck_Egypt_D2br10_2007_Avian
AB497014_A_duck_Egypt_D2Br210_2007_Avian

HA-Group31

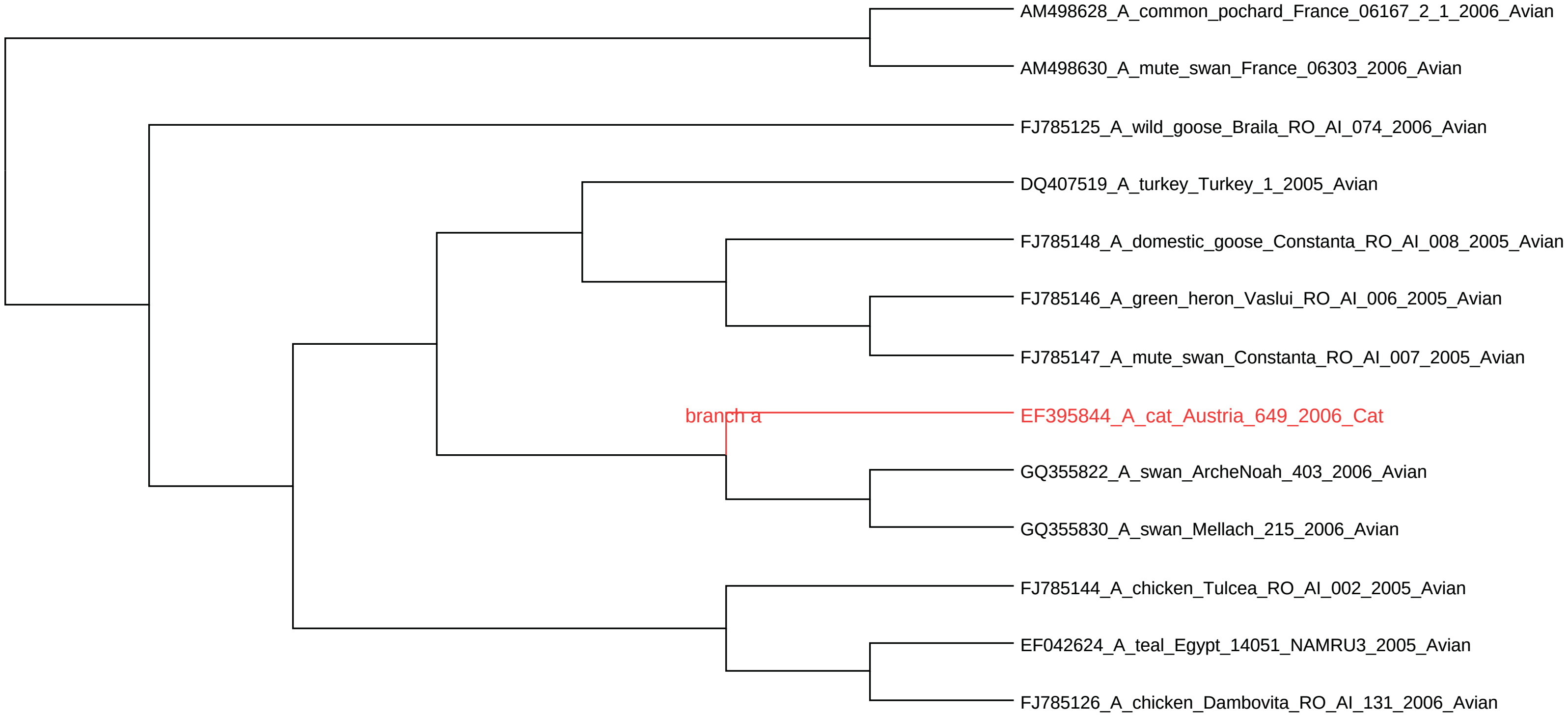
AM498628_A_common_pochard_France_06167_2_1_2006_Avian
AM498630_A_mute_swan_France_06303_2006_Avian
FJ785125_A_wild_goose_Braila_RO_AI_074_2006_Avian
DQ407519_A_turkey_Turkey_1_2005_Avian
FJ785148_A_domestic_goose_Constanta_RO_AI_008_2005_Avian
FJ785146_A_green_heron_Vaslui_RO_AI_006_2005_Avian
FJ785147_A_mute_swan_Constanta_RO_AI_007_2005_Avian
branch a
EF395844_A_cat_Austria_649_2006_Cat
GQ355822_A_swan_ArcheNoah_403_2006_Avian
GQ355830_A_swan_Mellach_215_2006_Avian
FJ785144_A_chicken_Tulcea_RO_AI_002_2005_Avian
EF042624_A_teal_Egypt_14051_NAMRU3_2005_Avian
FJ785126_A_chicken_Dambovita_RO_AI_131_2006_Avian

HA-Group32

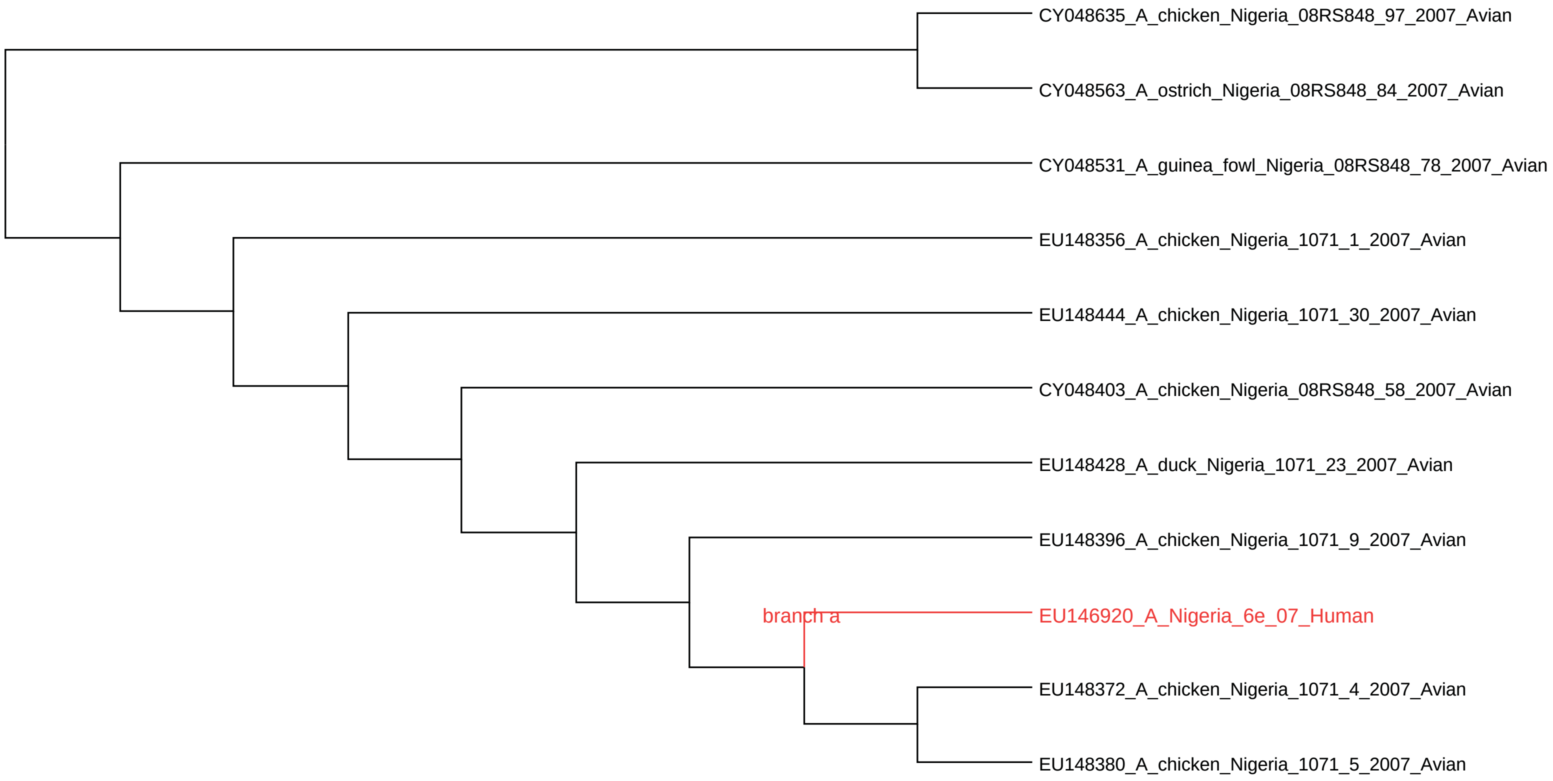

CY048635_A_chicken_Nigeria_08RS848_97_2007_Avian
CY048563_A_ostrich_Nigeria_08RS848_84_2007_Avian
CY048531_A_guinea_fowl_Nigeria_08RS848_78_2007_Avian
EU148356_A_chicken_Nigeria_1071_1_2007_Avian
EU148444_A_chicken_Nigeria_1071_30_2007_Avian
CY048403_A_chicken_Nigeria_08RS848_58_2007_Avian
EU148428_A_duck_Nigeria_1071_23_2007_Avian
EU148396_A_chicken_Nigeria_1071_9_2007_Avian
branch a
EU146920_A_Nigeria_6e_07_Human
EU148372_A_chicken_Nigeria_1071_4_2007_Avian
EU148380_A_chicken_Nigeria_1071_5_2007_Avian

HA-Group33

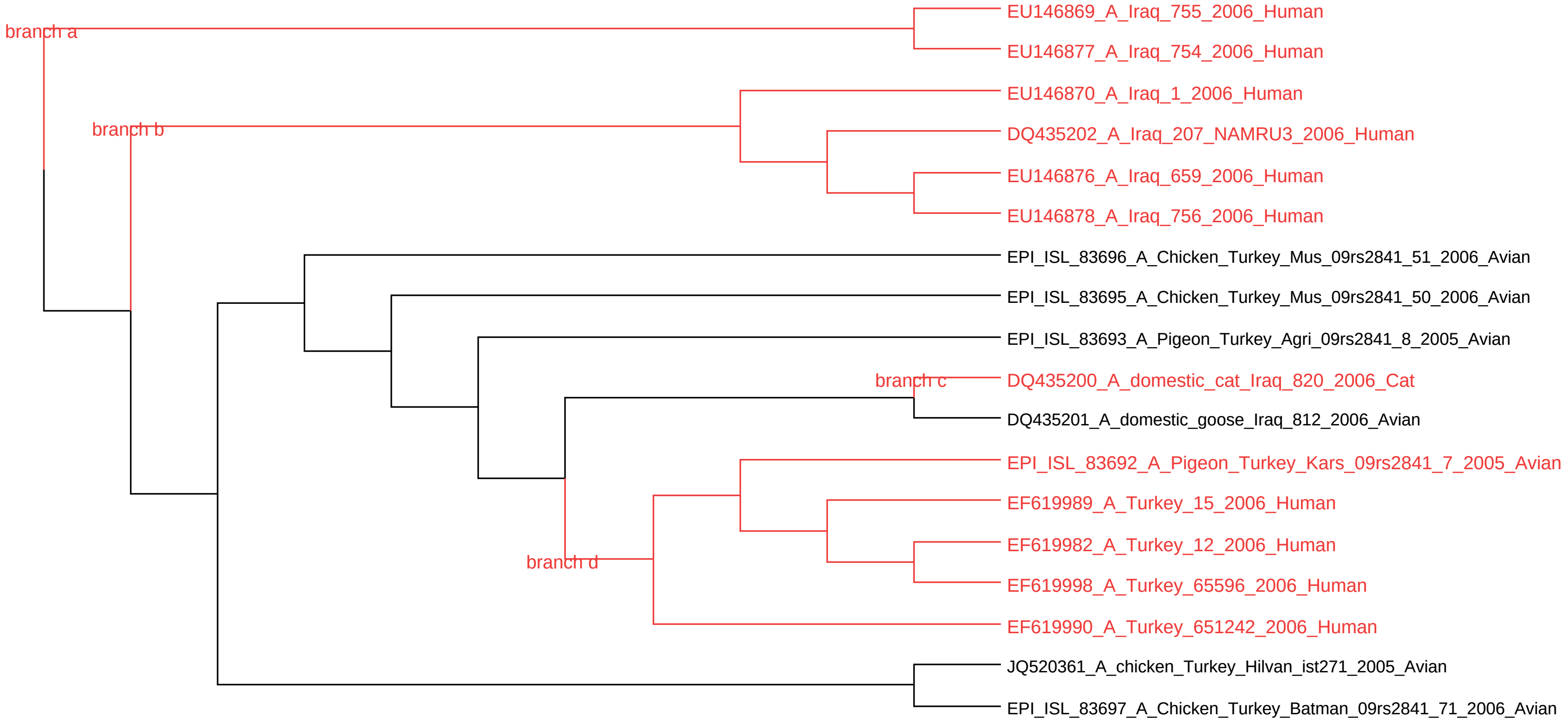

branch a
branch b
branch c
branch d
EU146869_A_Iraq_755_2006_Human
EU146877_A_Iraq_754_2006_Human
EU146870_A_Iraq_1_2006_Human
DQ435202_A_Iraq_207_NAMRU3_2006_Human
EU146876_A_Iraq_659_2006_Human
EU146878_A_Iraq_756_2006_Human
EPI_ISL_83696_A_Chicken_Turkey_Mus_09rs2841_51_2006_Avian
EPI_ISL_83695_A_Chicken_Turkey_Mus_09rs2841_50_2006_Avian
EPI_ISL_83693_A_Pigeon_Turkey_Agri_09rs2841_8_2005_Avian
DQ435200_A_domestic_cat_Iraq_820_2006_Cat
DQ435201_A_domestic_goose_Iraq_812_2006_Avian
EPI_ISL_83692_A_Pigeon_Turkey_Kars_09rs2841_7_2005_Avian
EF619989_A_Turkey_15_2006_Human
EF619982_A_Turkey_12_2006_Human
EF619998_A_Turkey_65596_2006_Human
EF619990_A_Turkey_651242_2006_Human
JQ520361_A_chicken_Turkey_Hilvan_ist271_2005_Avian
EPI_ISL_83697_A_Chicken_Turkey_Batman_09rs2841_71_2006_Avian

HA-Group34

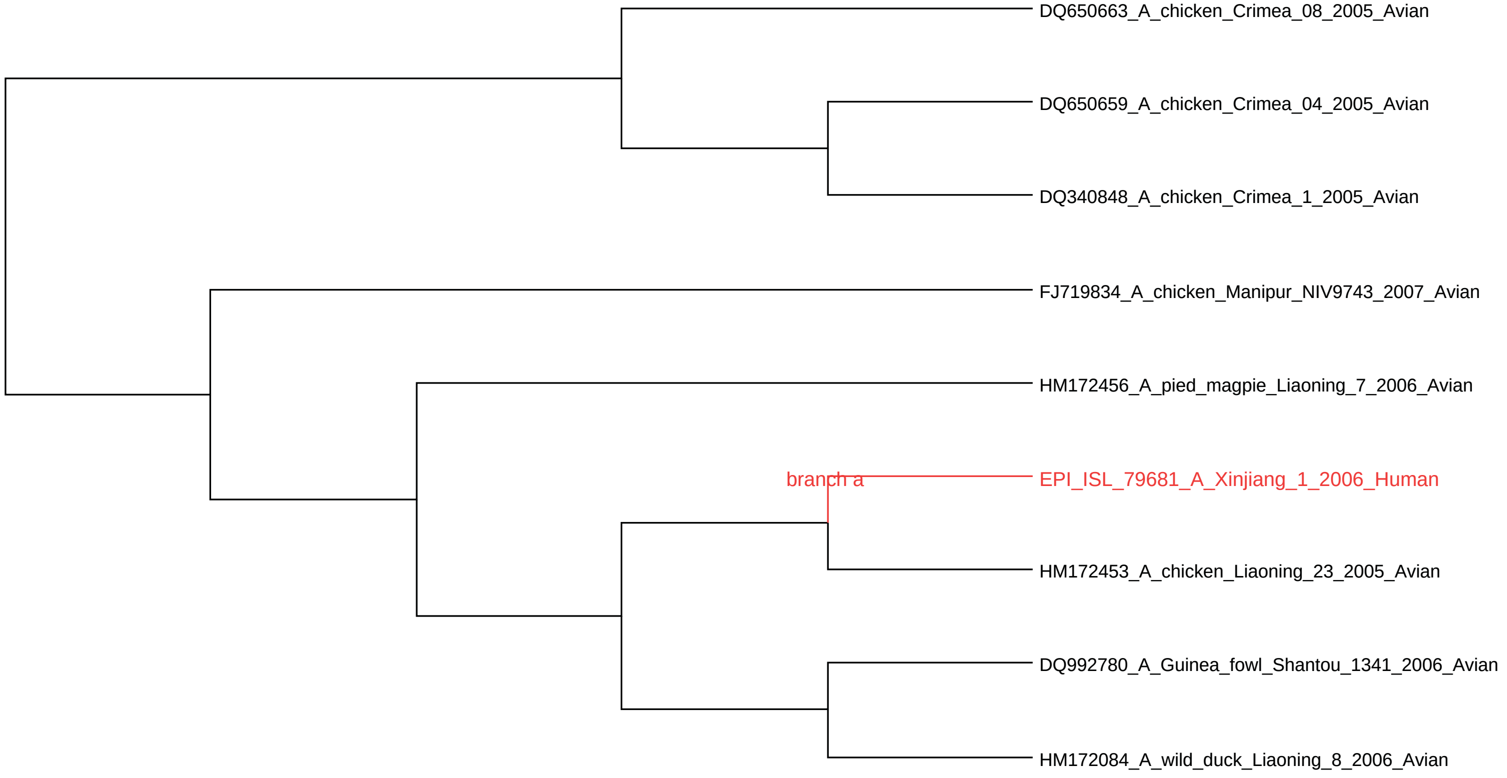
DQ650663_A_chicken_Crimea_08_2005_Avian
DQ650659_A_chicken_Crimea_04_2005_Avian
DQ340848_A_chicken_Crimea_1_2005_Avian
FJ719834_A_chicken_Manipur_NIV9743_2007_Avian
HM172456_A_pied_magpie_Liaoning_7_2006_Avian
branch a
EPI_ISL_79681_A_Xinjiang_1_2006_Human
HM172453_A_chicken_Liaoning_23_2005_Avian
DQ992780_A_Guinea_fowl_Shantou_1341_2006_Avian
HM172084_A_wild_duck_Liaoning_8_2006_Avian

HA-Group35

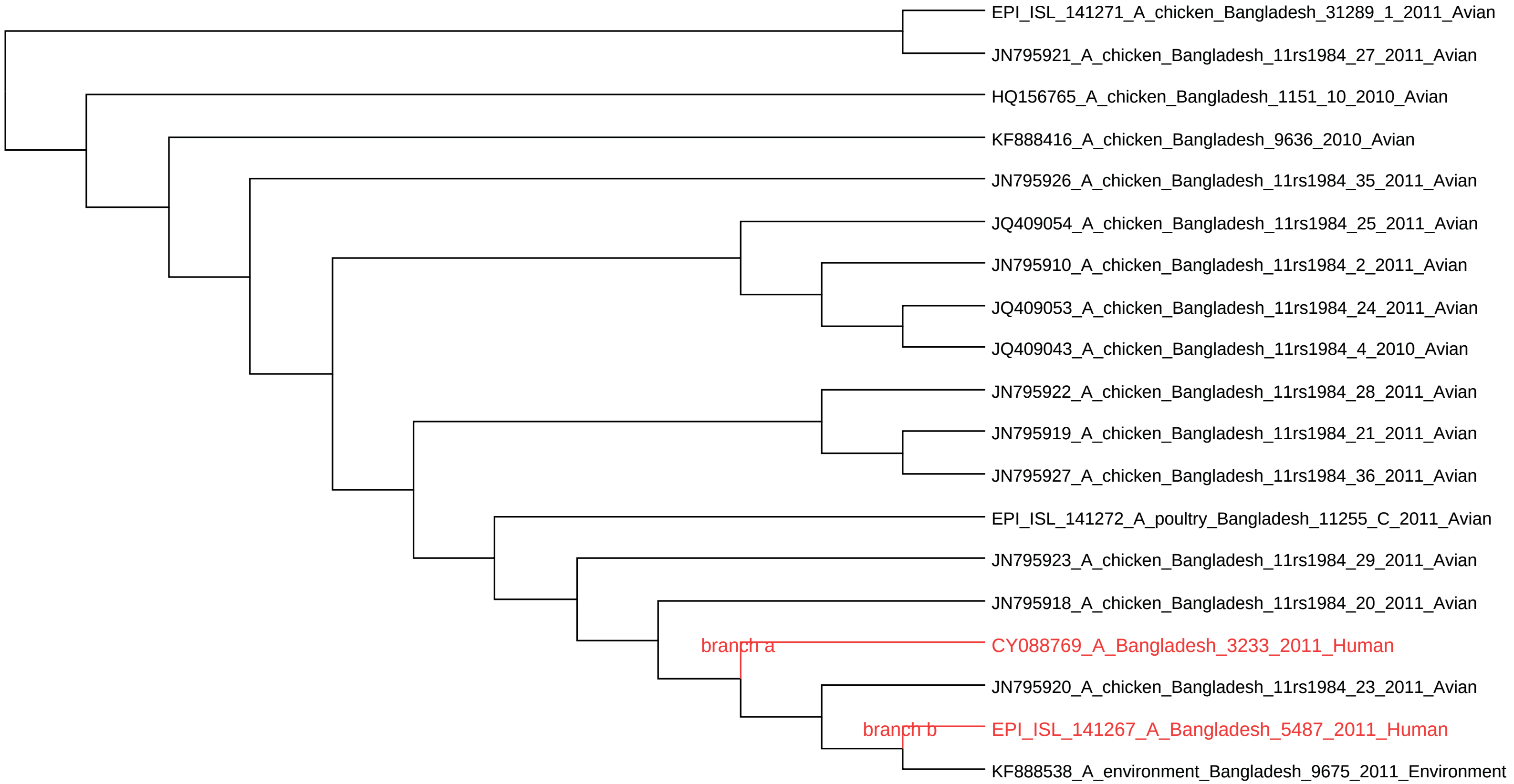

EPI_ISL_141271_A_chicken_Bangladesh_31289_1_2011_Avian
JN795921_A_chicken_Bangladesh_11rs1984_27_2011_Avian
HQ156765_A_chicken_Bangladesh_1151_10_2010_Avian
KF888416_A_chicken_Bangladesh_9636_2010_Avian
JN795926_A_chicken_Bangladesh_11rs1984_35_2011_Avian
JQ409054_A_chicken_Bangladesh_11rs1984_25_2011_Avian
JN795910_A_chicken_Bangladesh_11rs1984_2_2011_Avian
JQ409053_A_chicken_Bangladesh_11rs1984_24_2011_Avian
JQ409043_A_chicken_Bangladesh_11rs1984_4_2010_Avian
JN795922_A_chicken_Bangladesh_11rs1984_28_2011_Avian
JN795919_A_chicken_Bangladesh_11rs1984_21_2011_Avian
JN795927_A_chicken_Bangladesh_11rs1984_36_2011_Avian
EPI_ISL_141272_A_poultry_Bangladesh_11255_C_2011_Avian
JN795923_A_chicken_Bangladesh_11rs1984_29_2011_Avian
JN795918_A_chicken_Bangladesh_11rs1984_20_2011_Avian
branch a
CY088769_A_Bangladesh_3233_2011_Human
JN795920_A_chicken_Bangladesh_11rs1984_23_2011_Avian
branch b
EPI_ISL_141267_A_Bangladesh_5487_2011_Human
KF888538_A_environment_Bangladesh_9675_2011_Environment

HA-Group36

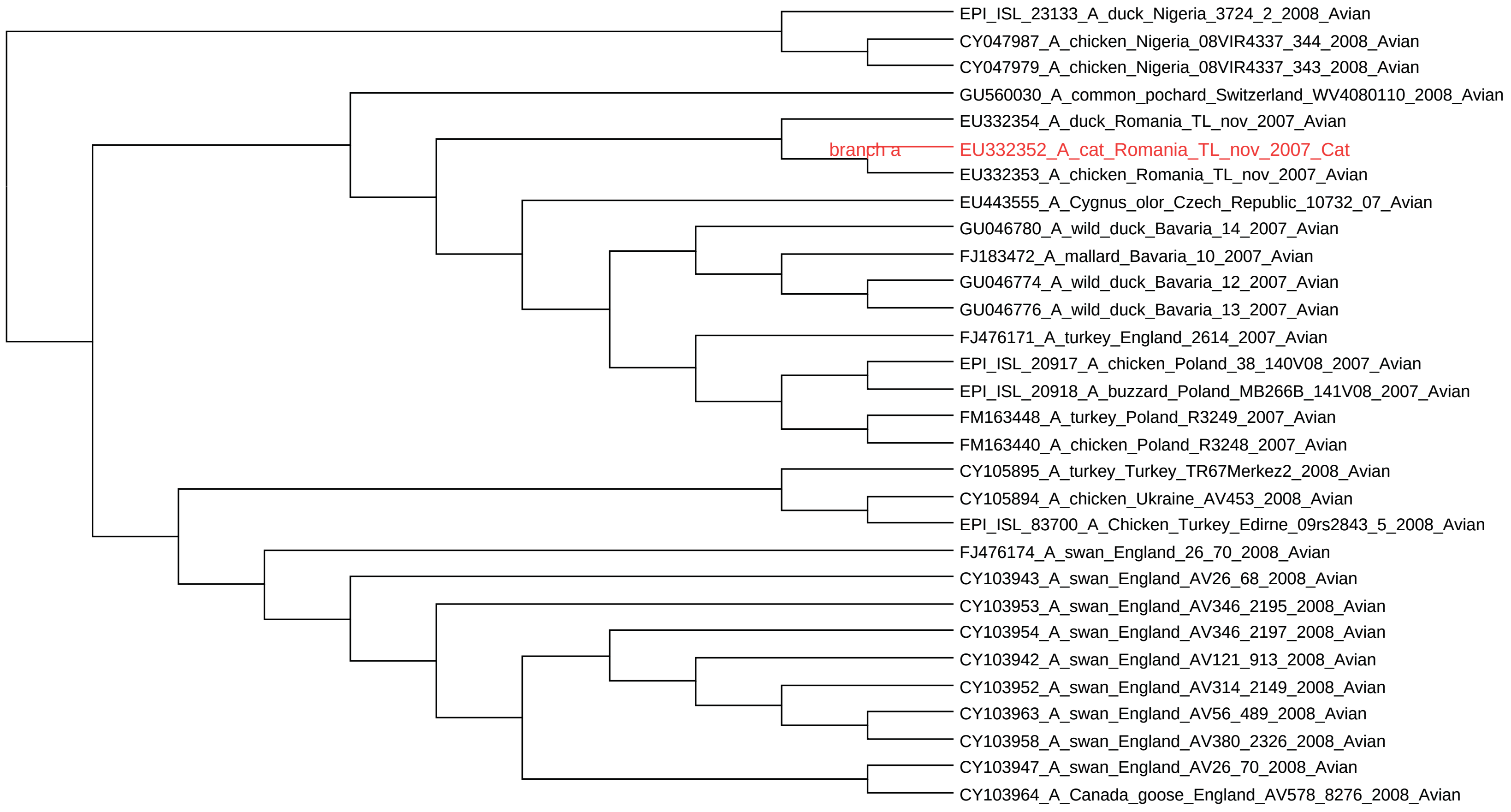
EPI_ISL_23133_A_duck_Nigeria_3724_2_2008_Avian
CY047987_A_chicken_Nigeria_08VIR4337_344_2008_Avian
CY047979_A_chicken_Nigeria_08VIR4337_343_2008_Avian
GU560030_A_common_pochard_Switzerland_WV4080110_2008_Avian
EU332354_A_duck_Romania_TL_nov_2007_Avian
branch a
EU332352_A_cat_Romania_TL_nov_2007_Cat
EU332353_A_chicken_Romania_TL_nov_2007_Avian
EU443555_A_Cygnus_olor_Czech_Republic_10732_07_Avian
GU046780_A_wild_duck_Bavaria_14_2007_Avian
FJ183472_A_mallard_Bavaria_10_2007_Avian
GU046774_A_wild_duck_Bavaria_12_2007_Avian
GU046776_A_wild_duck_Bavaria_13_2007_Avian
FJ476171_A_turkey_England_2614_2007_Avian
EPI_ISL_20917_A_chicken_Poland_38_140V08_2007_Avian
EPI_ISL_20918_A_buzzard_Poland_MB266B_141V08_2007_Avian
FM163448_A_turkey_Poland_R3249_2007_Avian
FM163440_A_chicken_Poland_R3248_2007_Avian
CY105895_A_turkey_Turkey_TR67Merkez2_2008_Avian
CY105894_A_chicken_Ukraine_AV453_2008_Avian
EPI_ISL_83700_A_Chicken_Turkey_Edirne_09rs2843_5_2008_Avian
FJ476174_A_swan_England_26_70_2008_Avian
CY103943_A_swan_England_AV26_68_2008_Avian
CY103953_A_swan_England_AV346_2195_2008_Avian
CY103954_A_swan_England_AV346_2197_2008_Avian
CY103942_A_swan_England_AV121_913_2008_Avian
CY103952_A_swan_England_AV314_2149_2008_Avian
CY103963_A_swan_England_AV56_489_2008_Avian
CY103958_A_swan_England_AV380_2326_2008_Avian
CY103947_A_swan_England_AV26_70_2008_Avian
CY103964_A_Canada_goose_England_AV578_8276_2008_Avian

HA-Group37

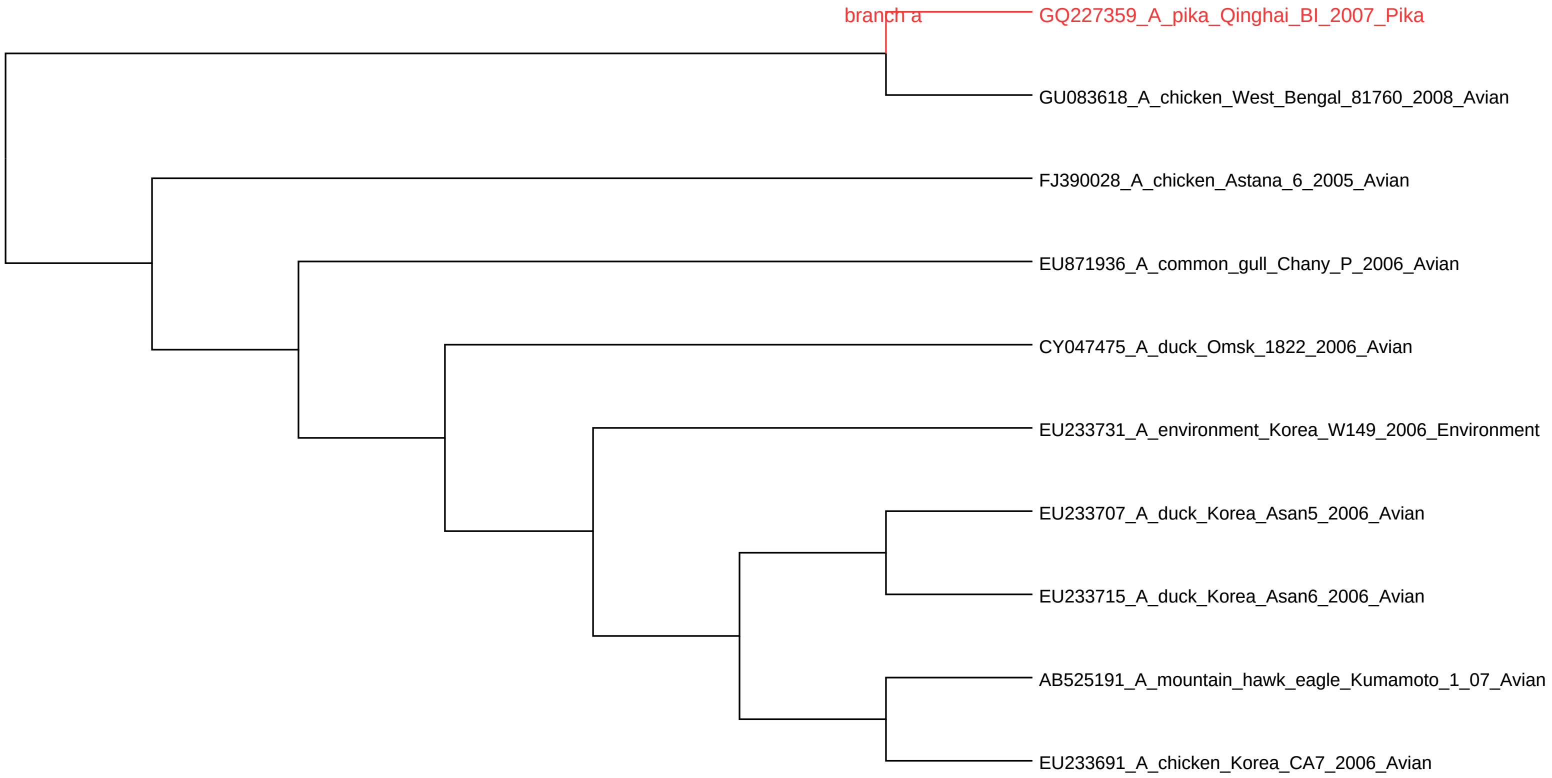
branch a
GQ227359_A_pika_Qinghai_BI_2007_Pika
GU083618_A_chicken_West_Bengal_81760_2008_Avian
FJ390028_A_chicken_Astana_6_2005_Avian
EU871936_A_common_gull_Chany_P_2006_Avian
CY047475_A_duck_Omsk_1822_2006_Avian
EU233731_A_environment_Korea_W149_2006_Environment
EU233707_A_duck_Korea_Asan5_2006_Avian
EU233715_A_duck_Korea_Asan6_2006_Avian
AB525191_A_mountain_hawk_eagle_Kumamoto_1_07_Avian
EU233691_A_chicken_Korea_CA7_2006_Avian

HA-Group38

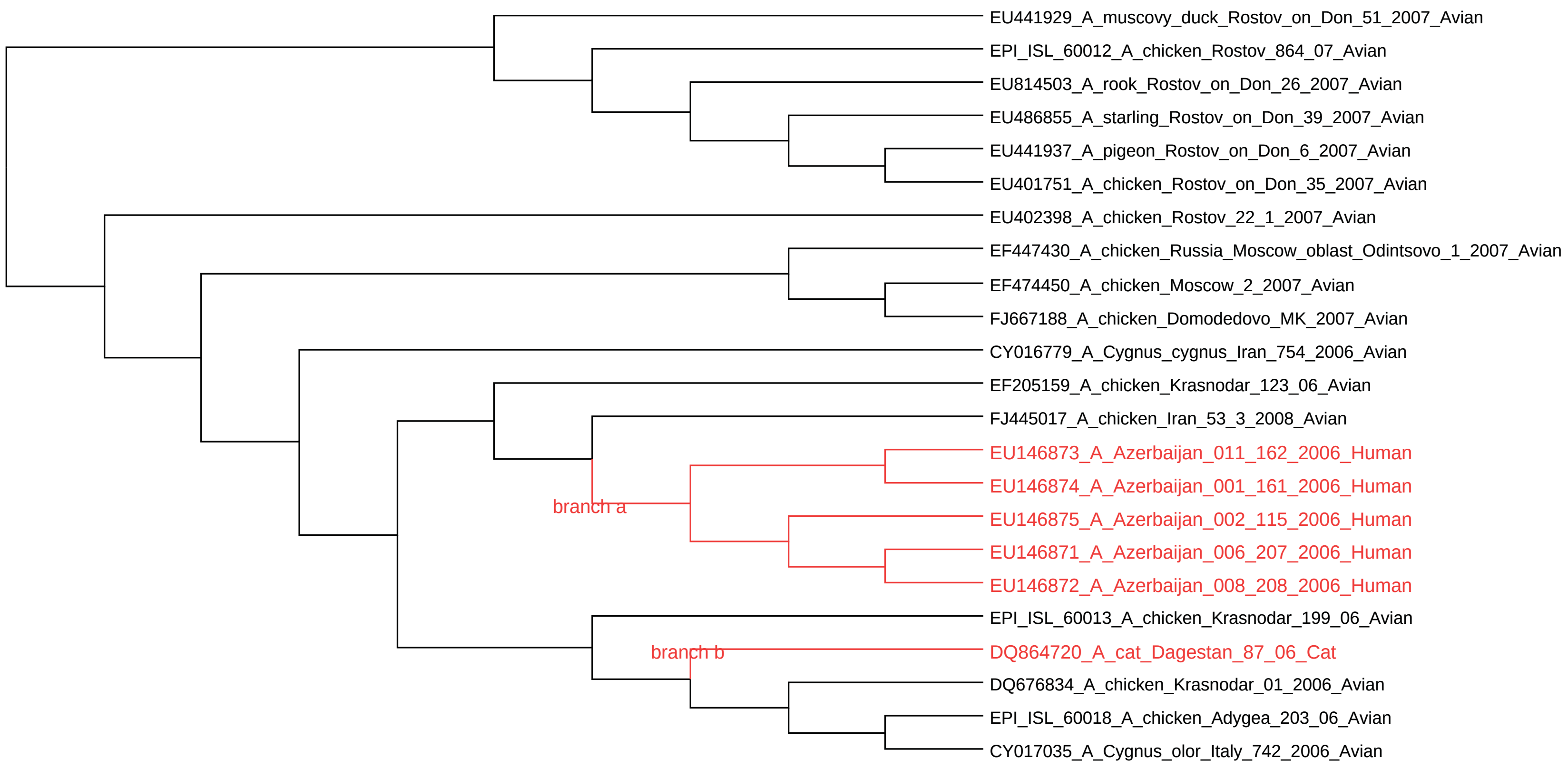

EU441929_A_muscovy_duck_Rostov_on_Don_51_2007_Avian
EPI_ISL_60012_A_chicken_Rostov_864_07_Avian
EU814503_A_rook_Rostov_on_Don_26_2007_Avian
EU486855_A_starling_Rostov_on_Don_39_2007_Avian
EU441937_A_pigeon_Rostov_on_Don_6_2007_Avian
EU401751_A_chicken_Rostov_on_Don_35_2007_Avian
EU402398_A_chicken_Rostov_22_1_2007_Avian
EF447430_A_chicken_Russia_Moscow_oblast_Odintsovo_1_2007_Avian
EF474450_A_chicken_Moscow_2_2007_Avian
FJ667188_A_chicken_Domodedovo_MK_2007_Avian
CY016779_A_Cygnus_cygnus_Iran_754_2006_Avian
EF205159_A_chicken_Krasnodar_123_06_Avian
FJ445017_A_chicken_Iran_53_3_2008_Avian
EU146873_A_Azerbaijan_011_162_2006_Human
EU146874_A_Azerbaijan_001_161_2006_Human
branch a
EU146875_A_Azerbaijan_002_115_2006_Human
EU146871_A_Azerbaijan_006_207_2006_Human
EU146872_A_Azerbaijan_008_208_2006_Human
EPI_ISL_60013_A_chicken_Krasnodar_199_06_Avian
branch b
DQ864720_A_cat_Dagestan_87_06_Cat
DQ676834_A_chicken_Krasnodar_01_2006_Avian
EPI_ISL_60018_A_chicken_Adygea_203_06_Avian
CY017035_A_Cygnus_olor_Italy_742_2006_Avian

HA-Group39

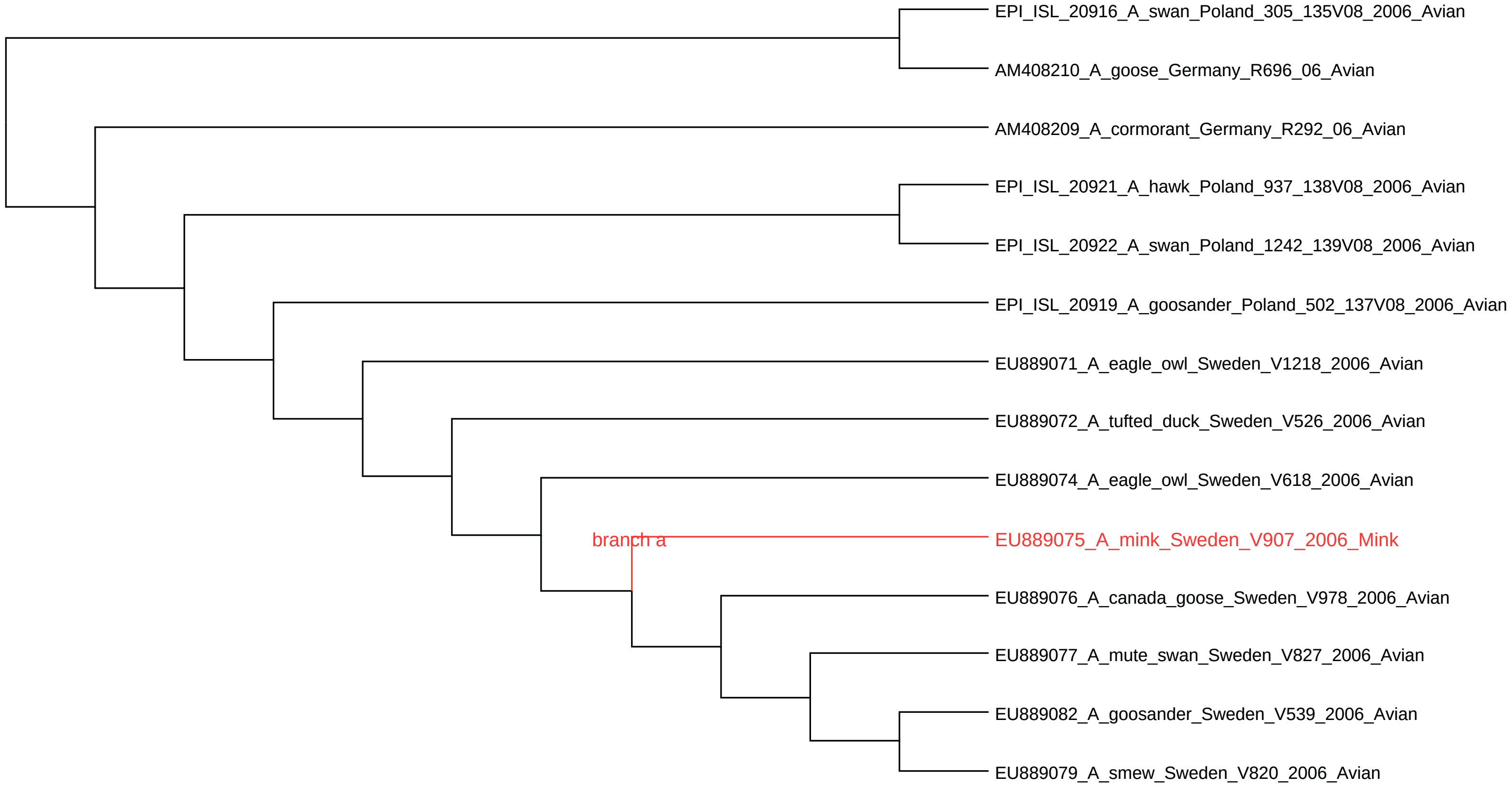

EPI_ISL_20916_A_swan_Poland_305_135V08_2006_Avian
AM408210_A_goose_Germany_R696_06_Avian
AM408209_A_cormorant_Germany_R292_06_Avian
EPI_ISL_20921_A_hawk_Poland_937_138V08_2006_Avian
EPI_ISL_20922_A_swan_Poland_1242_139V08_2006_Avian
EPI_ISL_20919_A_goosander_Poland_502_137V08_2006_Avian
EU889071_A_eagle_owl_Sweden_V1218_2006_Avian
EU889072_A_tufted_duck_Sweden_V526_2006_Avian
EU889074_A_eagle_owl_Sweden_V618_2006_Avian
branch a
EU889075_A_mink_Sweden_V907_2006_Mink
EU889076_A_canada_goose_Sweden_V978_2006_Avian
EU889077_A_mute_swan_Sweden_V827_2006_Avian
EU889082_A_goosander_Sweden_V539_2006_Avian
EU889079_A_smew_Sweden_V820_2006_Avian

HA-Group40

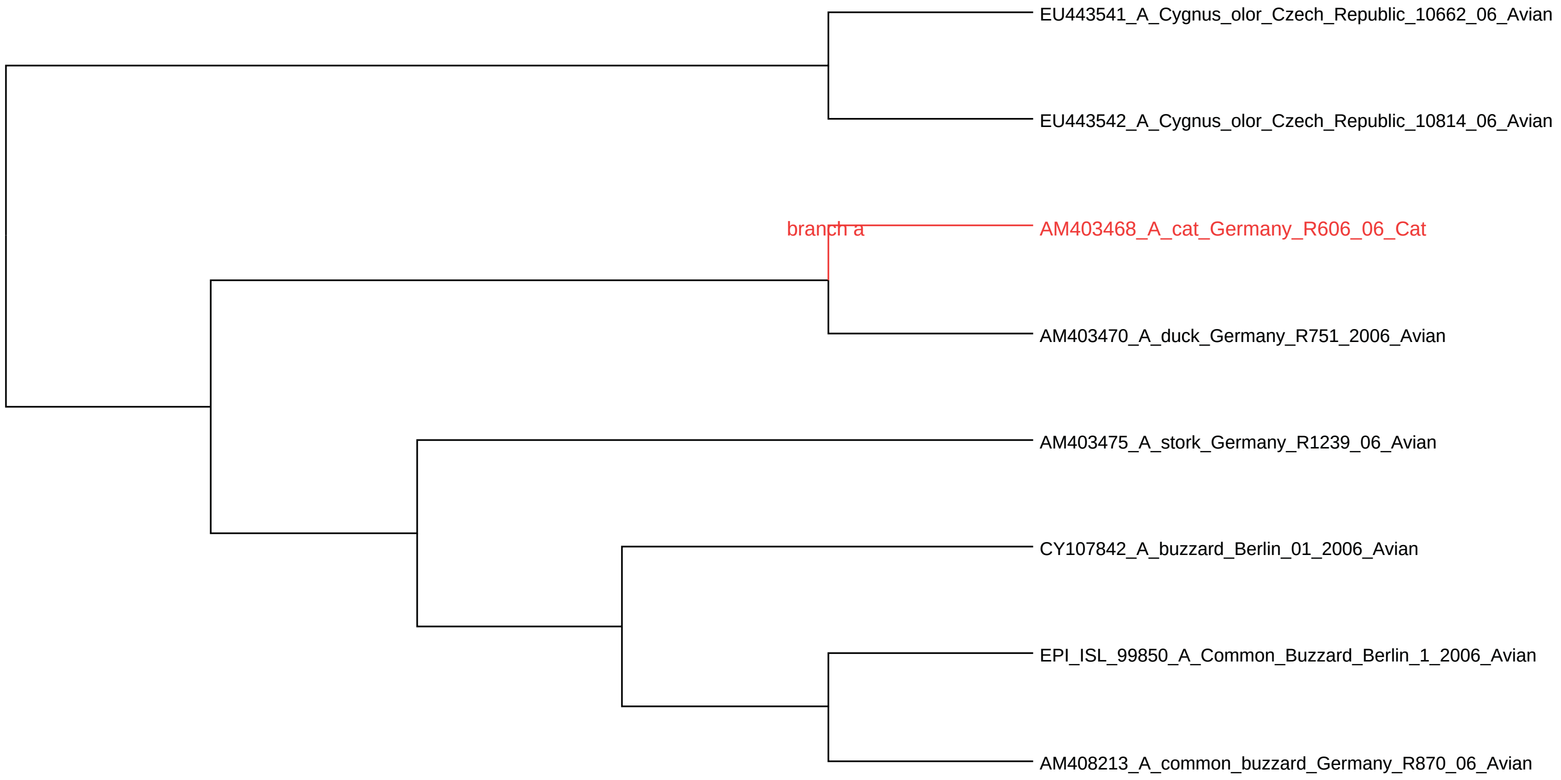
EU443541_A_Cygnus_olor_Czech_Republic_10662_06_Avian
EU443542_A_Cygnus_olor_Czech_Republic_10814_06_Avian
branch a
AM403468_A_cat_Germany_R606_06_Cat
AM403470_A_duck_Germany_R751_2006_Avian
AM403475_A_stork_Germany_R1239_06_Avian
CY107842_A_buzzard_Berlin_01_2006_Avian
EPI_ISL_99850_A_Common_Buzzard_Berlin_1_2006_Avian
AM408213_A_common_buzzard_Germany_R870_06_Avian

HA-Group41

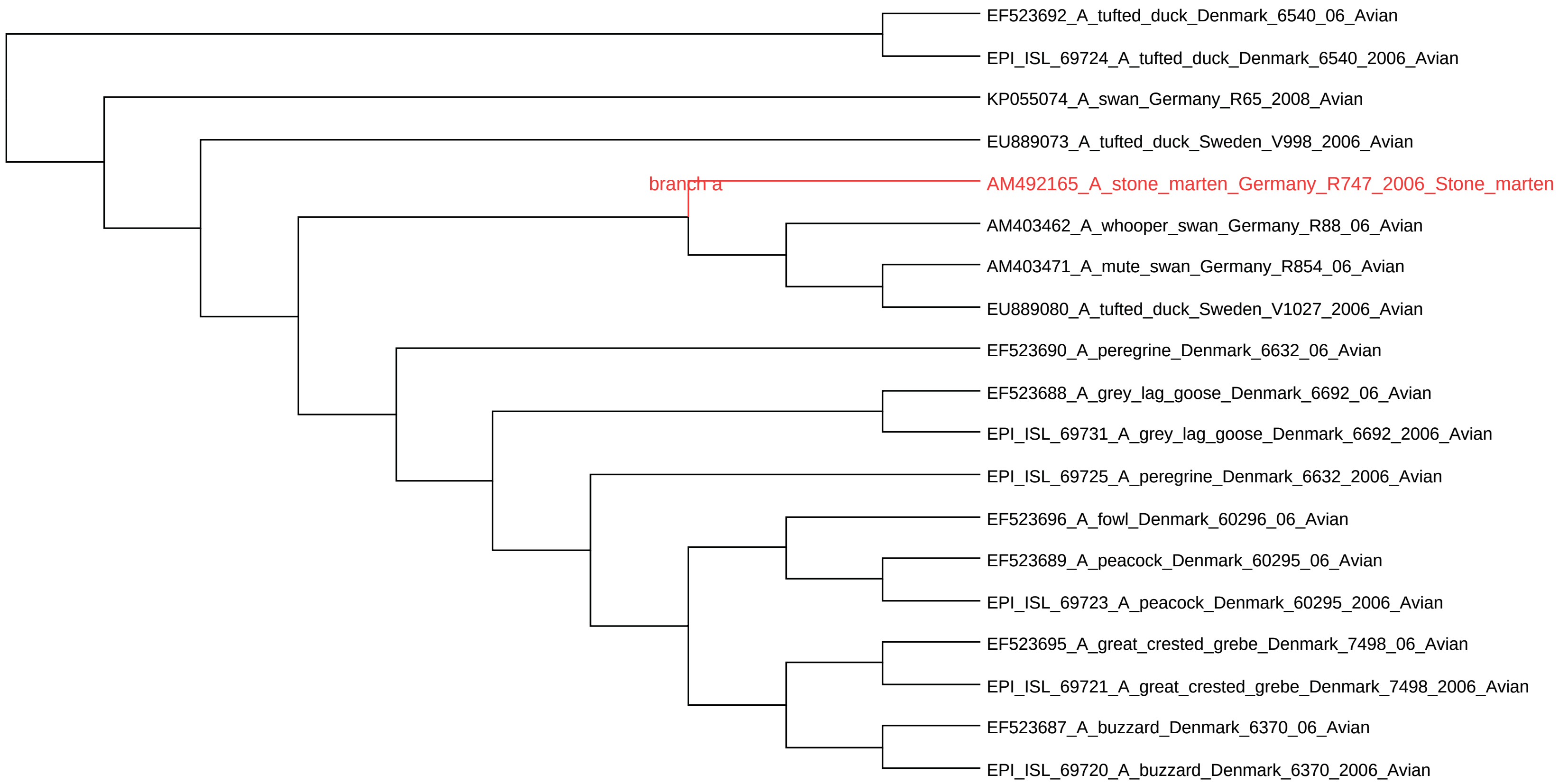

EF523692_A_tufted_duck_Denmark_6540_06_Avian
EPI_ISL_69724_A_tufted_duck_Denmark_6540_2006_Avian
KP055074_A_swan_Germany_R65_2008_Avian
EU889073_A_tufted_duck_Sweden_V998_2006_Avian
branch a
AM492165_A_stone_marten_Germany_R747_2006_Stone_marten
AM403462_A_whooper_swan_Germany_R88_06_Avian
AM403471_A_mute_swan_Germany_R854_06_Avian
EU889080_A_tufted_duck_Sweden_V1027_2006_Avian
EF523690_A_peregrine_Denmark_6632_06_Avian
EF523688_A_grey_lag_goose_Denmark_6692_06_Avian
EPI_ISL_69731_A_grey_lag_goose_Denmark_6692_2006_Avian
EPI_ISL_69725_A_peregrine_Denmark_6632_2006_Avian
EF523696_A_fowl_Denmark_60296_06_Avian
EF523689_A_peacock_Denmark_60295_06_Avian
EPI_ISL_69723_A_peacock_Denmark_60295_2006_Avian
EF523695_A_great_crested_grebe_Denmark_7498_06_Avian
EPI_ISL_69721_A_great_crested_grebe_Denmark_7498_2006_Avian
EF523687_A_buzzard_Denmark_6370_06_Avian
EPI_ISL_69720_A_buzzard_Denmark_6370_2006_Avian

HA-Group42

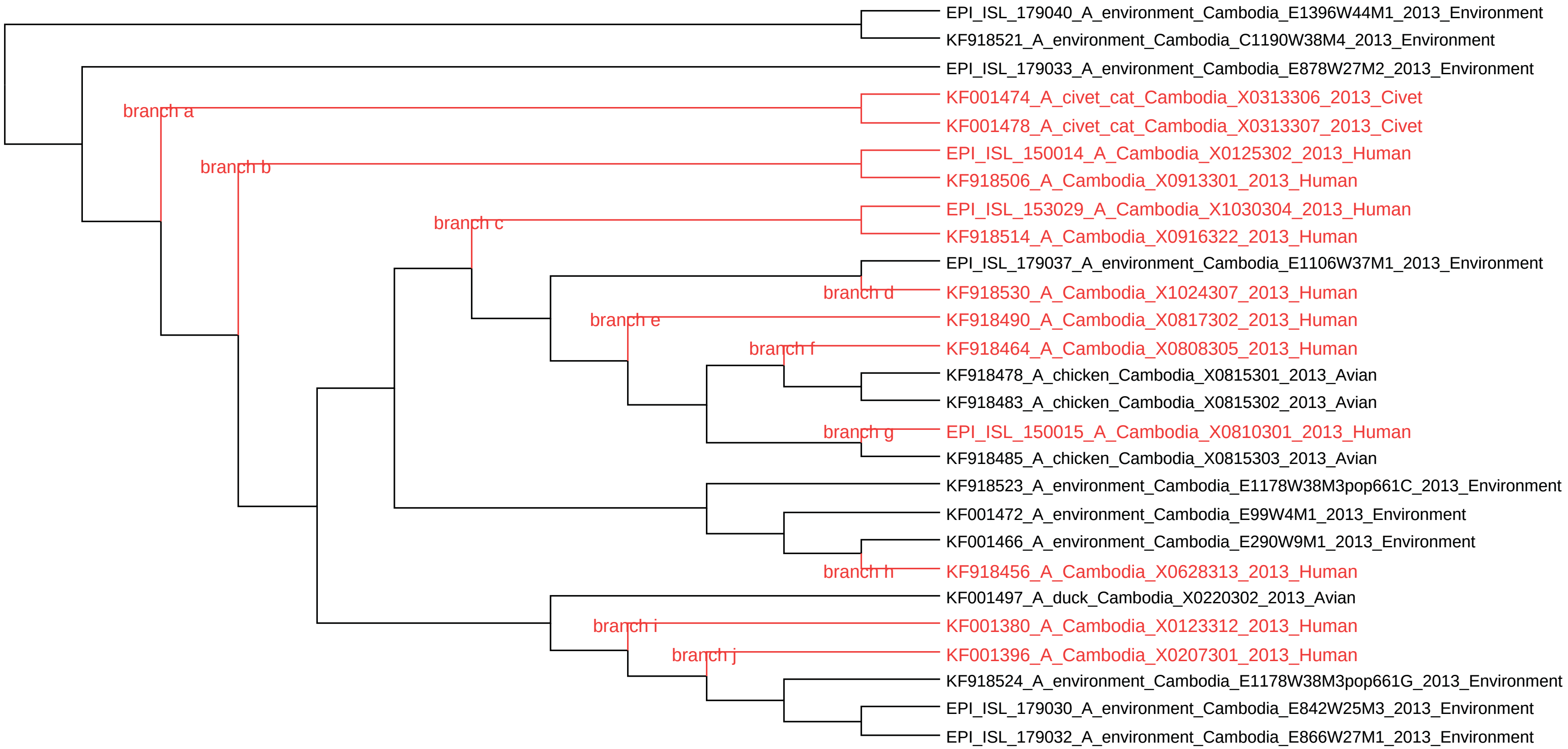

EPI_ISL_179040_A_environment_Cambodia_E1396W44M1_2013_Environment
KF918521_A_environment_Cambodia_C1190W38M4_2013_Environment
EPI_ISL_179033_A_environment_Cambodia_E878W27M2_2013_Environment
branch a
KF001474_A_civet_cat_Cambodia_X0313306_2013_Civet
KF001478_A_civet_cat_Cambodia_X0313307_2013_Civet
branch b
EPI_ISL_150014_A_Cambodia_X0125302_2013_Human
KF918506_A_Cambodia_X0913301_2013_Human
branch c
EPI_ISL_153029_A_Cambodia_X1030304_2013_Human
KF918514_A_Cambodia_X0916322_2013_Human
EPI_ISL_179037_A_environment_Cambodia_E1106W37M1_2013_Environment
branch d
KF918530_A_Cambodia_X1024307_2013_Human
branch e
KF918490_A_Cambodia_X0817302_2013_Human
branch f
KF918464_A_Cambodia_X0808305_2013_Human
KF918478_A_chicken_Cambodia_X0815301_2013_Avian
KF918483_A_chicken_Cambodia_X0815302_2013_Avian
branch g
EPI_ISL_150015_A_Cambodia_X0810301_2013_Human
KF918485_A_chicken_Cambodia_X0815303_2013_Avian
KF918523_A_environment_Cambodia_E1178W38M3pop661C_2013_Environment
KF001472_A_environment_Cambodia_E99W4M1_2013_Environment
KF001466_A_environment_Cambodia_E290W9M1_2013_Environment
branch h
KF918456_A_Cambodia_X0628313_2013_Human
KF001497_A_duck_Cambodia_X0220302_2013_Avian
branch i
KF001380_A_Cambodia_X0123312_2013_Human
branch j
KF001396_A_Cambodia_X0207301_2013_Human
KF918524_A_environment_Cambodia_E1178W38M3pop661G_2013_Environment
EPI_ISL_179030_A_environment_Cambodia_E842W25M3_2013_Environment
EPI_ISL_179032_A_environment_Cambodia_E866W27M1_2013_Environment

HA-Group43

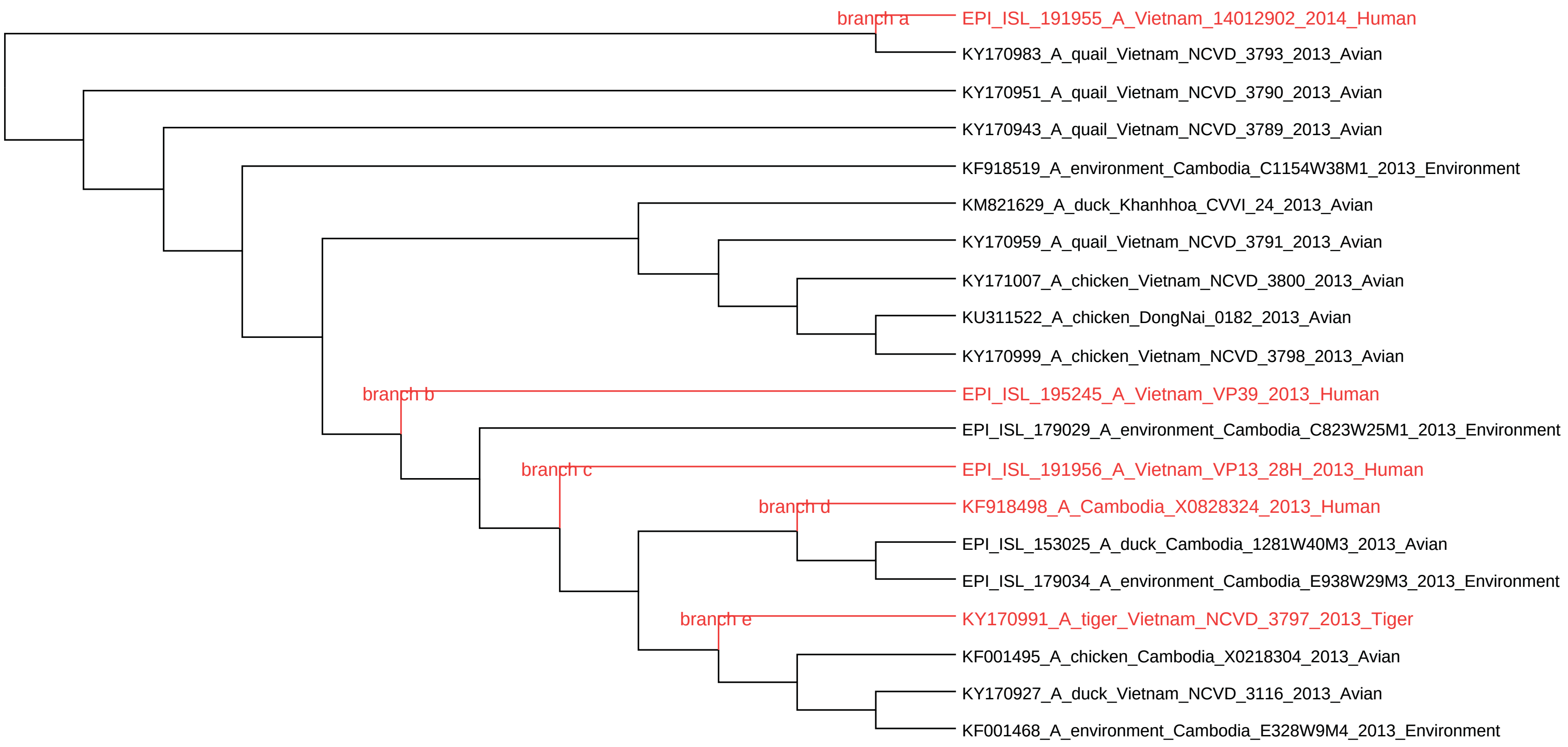

branch a
EPI_ISL_191955_A_Vietnam_14012902_2014_Human
KY170983_A_quail_Vietnam_NCVD_3793_2013_Avian
KY170951_A_quail_Vietnam_NCVD_3790_2013_Avian
KY170943_A_quail_Vietnam_NCVD_3789_2013_Avian
KF918519_A_environment_Cambodia_C1154W38M1_2013_Environment
KM821629_A_duck_Khanhhoa_CVVI_24_2013_Avian
KY170959_A_quail_Vietnam_NCVD_3791_2013_Avian
KY171007_A_chicken_Vietnam_NCVD_3800_2013_Avian
KU311522_A_chicken_DongNai_0182_2013_Avian
KY170999_A_chicken_Vietnam_NCVD_3798_2013_Avian
branch b
EPI_ISL_195245_A_Vietnam_VP39_2013_Human
EPI_ISL_179029_A_environment_Cambodia_C823W25M1_2013_Environment
branch c
EPI_ISL_191956_A_Vietnam_VP13_28H_2013_Human
branch d
KF918498_A_Cambodia_X0828324_2013_Human
EPI_ISL_153025_A_duck_Cambodia_1281W40M3_2013_Avian
EPI_ISL_179034_A_environment_Cambodia_E938W29M3_2013_Environment
branch e
KY170991_A_tiger_Vietnam_NCVD_3797_2013_Tiger
KF001495_A_chicken_Cambodia_X0218304_2013_Avian
KY170927_A_duck_Vietnam_NCVD_3116_2013_Avian
KF001468_A_environment_Cambodia_E328W9M4_2013_Environment

HA-Group44

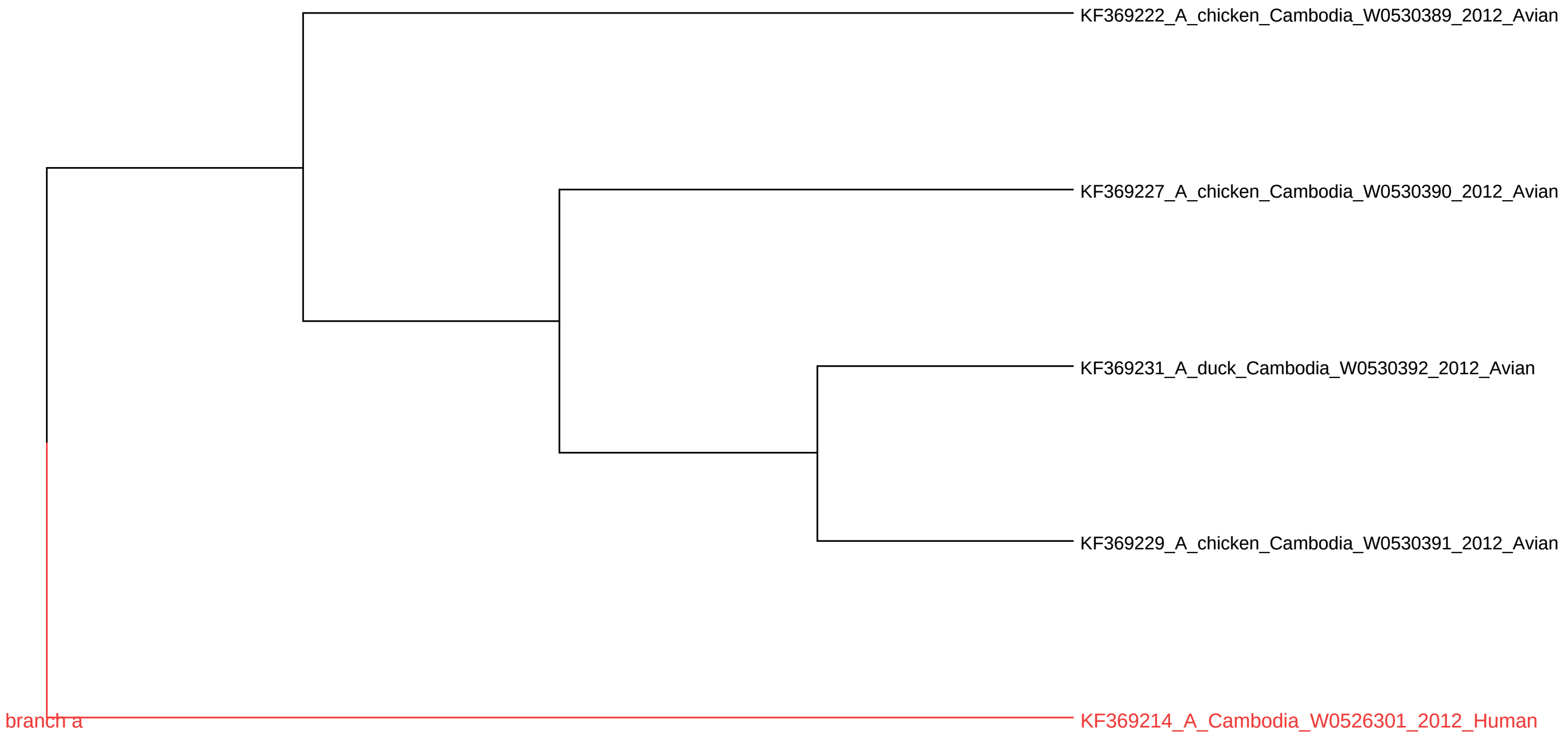

KF369222_A_chicken_Cambodia_W0530389_2012_Avian
KF369227_A_chicken_Cambodia_W0530390_2012_Avian
KF369231_A_duck_Cambodia_W0530392_2012_Avian
KF369229_A_chicken_Cambodia_W0530391_2012_Avian
branch a
KF369214_A_Cambodia_W0526301_2012_Human

HA-Group45

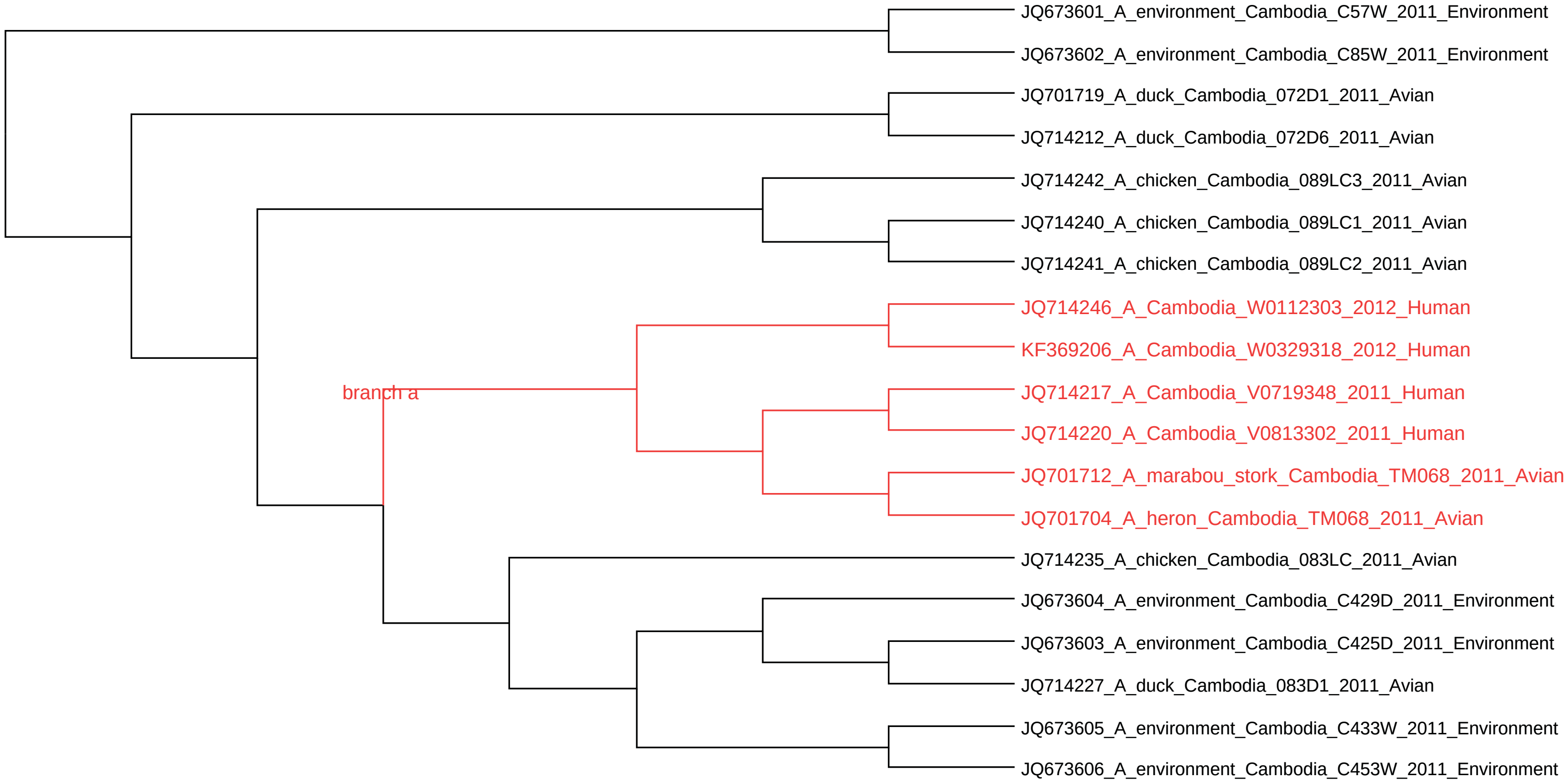

JQ673601_A_environment_Cambodia_C57W_2011_Environment
JQ673602_A_environment_Cambodia_C85W_2011_Environment
JQ701719_A_duck_Cambodia_072D1_2011_Avian
JQ714212_A_duck_Cambodia_072D6_2011_Avian
JQ714242_A_chicken_Cambodia_089LC3_2011_Avian
JQ714240_A_chicken_Cambodia_089LC1_2011_Avian
JQ714241_A_chicken_Cambodia_089LC2_2011_Avian
JQ714246_A_Cambodia_W0112303_2012_Human
KF369206_A_Cambodia_W0329318_2012_Human
branch a
JQ714217_A_Cambodia_V0719348_2011_Human
JQ714220_A_Cambodia_V0813302_2011_Human
JQ701712_A_marabou_stork_Cambodia_TM068_2011_Avian
JQ701704_A_heron_Cambodia_TM068_2011_Avian
JQ714235_A_chicken_Cambodia_083LC_2011_Avian
JQ673604_A_environment_Cambodia_C429D_2011_Environment
JQ673603_A_environment_Cambodia_C425D_2011_Environment
JQ714227_A_duck_Cambodia_083D1_2011_Avian
JQ673605_A_environment_Cambodia_C433W_2011_Environment
JQ673606_A_environment_Cambodia_C453W_2011_Environment

HA-Group46

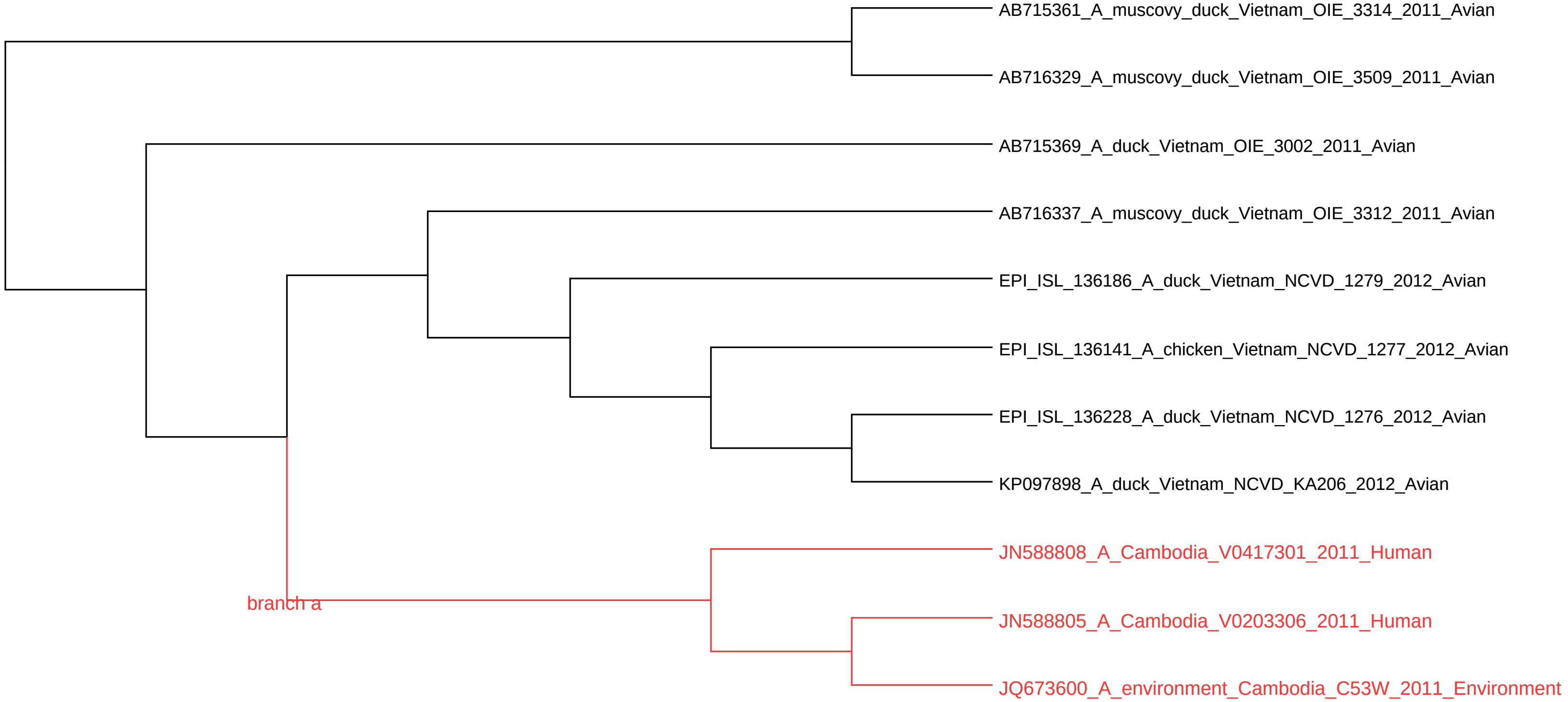
AB715361_A_muscovy_duck_Vietnam_OIE_3314_2011_Avian
AB716329_A_muscovy_duck_Vietnam_OIE_3509_2011_Avian
AB715369_A_duck_Vietnam_OIE_3002_2011_Avian
AB716337_A_muscovy_duck_Vietnam_OIE_3312_2011_Avian
EPI_ISL_136186_A_duck_Vietnam_NCVD_1279_2012_Avian
EPI_ISL_136141_A_chicken_Vietnam_NCVD_1277_2012_Avian
EPI_ISL_136228_A_duck_Vietnam_NCVD_1276_2012_Avian
KP097898_A_duck_Vietnam_NCVD_KA206_2012_Avian
JN588808_A_Cambodia_V0417301_2011_Human
branch a
JN588805_A_Cambodia_V0203306_2011_Human
JQ673600_A_environment_Cambodia_C53W_2011_Environment

HA-Group47

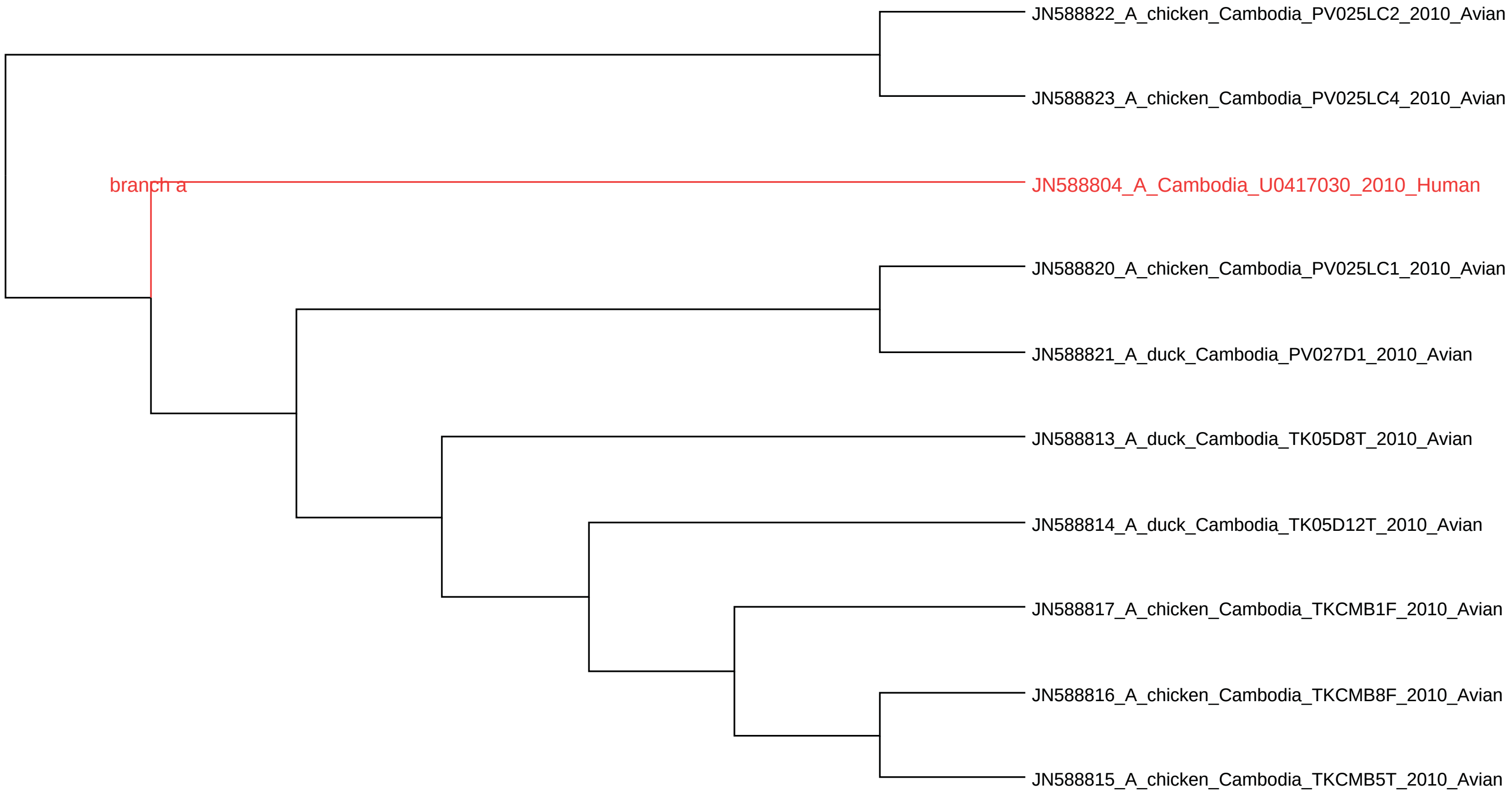

branch a
JN588822_A_chicken_Cambodia_PV025LC2_2010_Avian
JN588823_A_chicken_Cambodia_PV025LC4_2010_Avian
JN588804_A_Cambodia_U0417030_2010_Human
JN588820_A_chicken_Cambodia_PV025LC1_2010_Avian
JN588821_A_duck_Cambodia_PV027D1_2010_Avian
JN588813_A_duck_Cambodia_TK05D8T_2010_Avian
JN588814_A_duck_Cambodia_TK05D12T_2010_Avian
JN588817_A_chicken_Cambodia_TKCMB1F_2010_Avian
JN588816_A_chicken_Cambodia_TKCMB8F_2010_Avian
JN588815_A_chicken_Cambodia_TKCMB5T_2010_Avian

HA-Group48

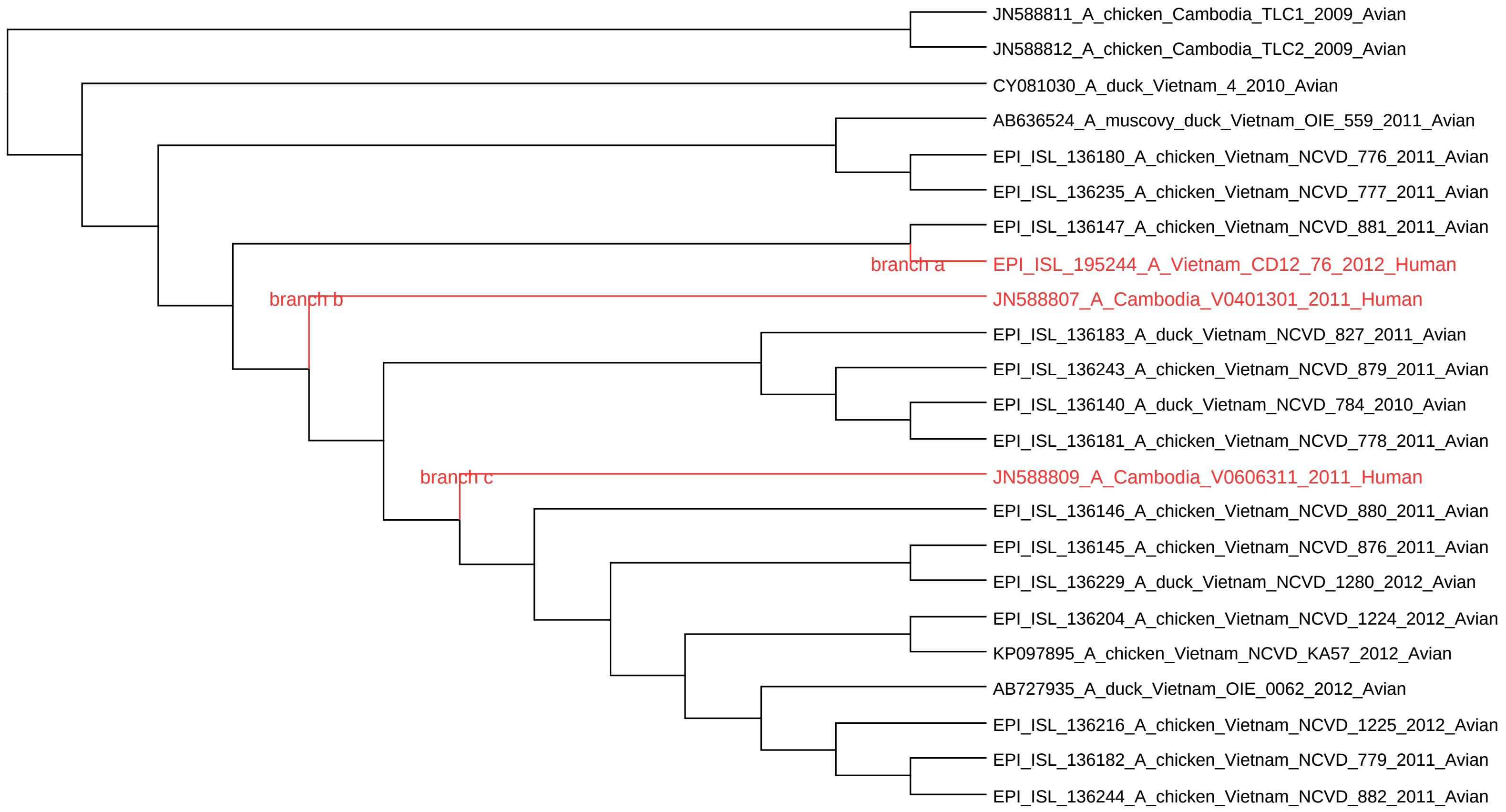

JN588811_A_chicken_Cambodia_TLC1_2009_Avian
JN588812_A_chicken_Cambodia_TLC2_2009_Avian
CY081030_A_duck_Vietnam_4_2010_Avian
AB636524_A_muscovy_duck_Vietnam_OIE_559_2011_Avian
EPI_ISL_136180_A_chicken_Vietnam_NCVD_776_2011_Avian
EPI_ISL_136235_A_chicken_Vietnam_NCVD_777_2011_Avian
EPI_ISL_136147_A_chicken_Vietnam_NCVD_881_2011_Avian
branch a
EPI_ISL_195244_A_Vietnam_CD12_76_2012_Human
branch b
JN588807_A_Cambodia_V0401301_2011_Human
EPI_ISL_136183_A_duck_Vietnam_NCVD_827_2011_Avian
EPI_ISL_136243_A_chicken_Vietnam_NCVD_879_2011_Avian
EPI_ISL_136140_A_duck_Vietnam_NCVD_784_2010_Avian
EPI_ISL_136181_A_chicken_Vietnam_NCVD_778_2011_Avian
branch c
JN588809_A_Cambodia_V0606311_2011_Human
EPI_ISL_136146_A_chicken_Vietnam_NCVD_880_2011_Avian
EPI_ISL_136145_A_chicken_Vietnam_NCVD_876_2011_Avian
EPI_ISL_136229_A_duck_Vietnam_NCVD_1280_2012_Avian
EPI_ISL_136204_A_chicken_Vietnam_NCVD_1224_2012_Avian
KP097895_A_chicken_Vietnam_NCVD_KA57_2012_Avian
AB727935_A_duck_Vietnam_OIE_0062_2012_Avian
EPI_ISL_136216_A_chicken_Vietnam_NCVD_1225_2012_Avian
EPI_ISL_136182_A_chicken_Vietnam_NCVD_779_2011_Avian
EPI_ISL_136244_A_chicken_Vietnam_NCVD_882_2011_Avian

HA-Group49

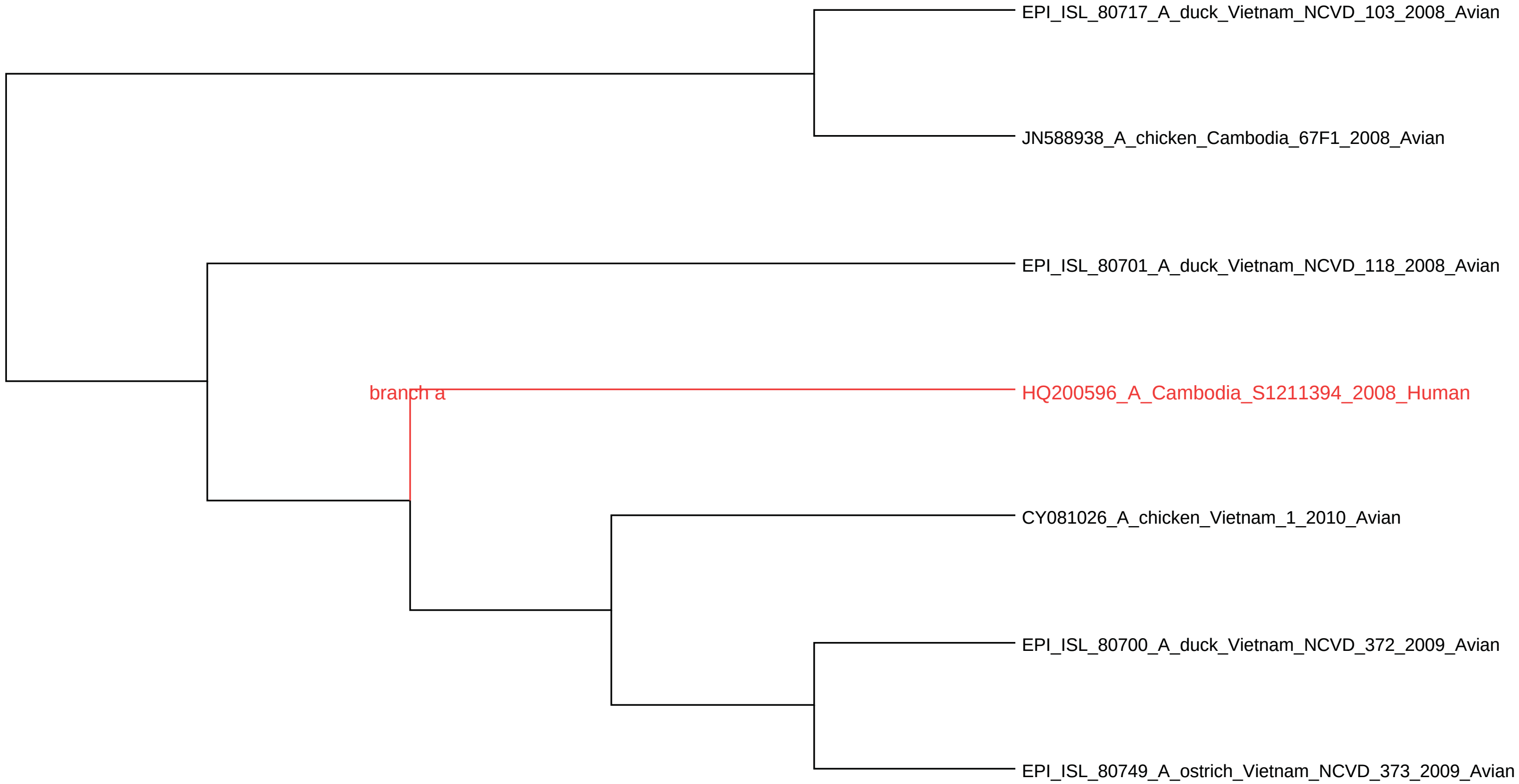
EPI_ISL_80717_A_duck_Vietnam_NCVD_103_2008_Avian
JN588938_A_chicken_Cambodia_67F1_2008_Avian
EPI_ISL_80701_A_duck_Vietnam_NCVD_118_2008_Avian
branch a
HQ200596_A_Cambodia_S1211394_2008_Human
CY081026_A_chicken_Vietnam_1_2010_Avian
EPI_ISL_80700_A_duck_Vietnam_NCVD_372_2009_Avian
EPI_ISL_80749_A_ostrich_Vietnam_NCVD_373_2009_Avian

HA-Group50

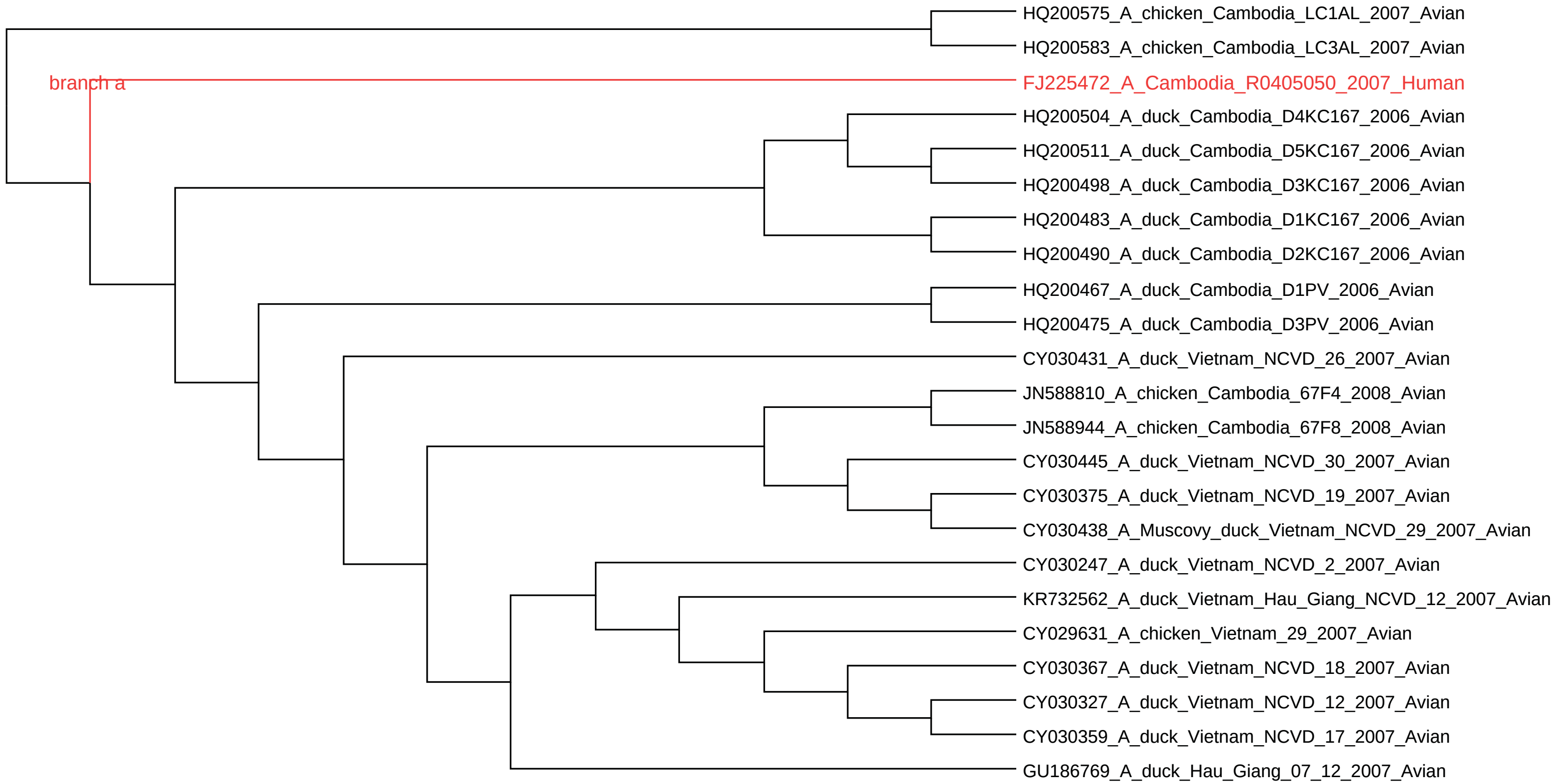

branch a
HQ200575_A_chicken_Cambodia_LC1AL_2007_Avian
HQ200583_A_chicken_Cambodia_LC3AL_2007_Avian
FJ225472_A_Cambodia_R0405050_2007_Human
HQ200504_A_duck_Cambodia_D4KC167_2006_Avian
HQ200511_A_duck_Cambodia_D5KC167_2006_Avian
HQ200498_A_duck_Cambodia_D3KC167_2006_Avian
HQ200483_A_duck_Cambodia_D1KC167_2006_Avian
HQ200490_A_duck_Cambodia_D2KC167_2006_Avian
HQ200467_A_duck_Cambodia_D1PV_2006_Avian
HQ200475_A_duck_Cambodia_D3PV_2006_Avian
CY030431_A_duck_Vietnam_NCVD_26_2007_Avian
JN588810_A_chicken_Cambodia_67F4_2008_Avian
JN588944_A_chicken_Cambodia_67F8_2008_Avian
CY030445_A_duck_Vietnam_NCVD_30_2007_Avian
CY030375_A_duck_Vietnam_NCVD_19_2007_Avian
CY030438_A_Muscovy_duck_Vietnam_NCVD_29_2007_Avian
CY030247_A_duck_Vietnam_NCVD_2_2007_Avian
KR732562_A_duck_Vietnam_Hau_Giang_NCVD_12_2007_Avian
CY029631_A_chicken_Vietnam_29_2007_Avian
CY030367_A_duck_Vietnam_NCVD_18_2007_Avian
CY030327_A_duck_Vietnam_NCVD_12_2007_Avian
CY030359_A_duck_Vietnam_NCVD_17_2007_Avian
GU186769_A_duck_Hau_Giang_07_12_2007_Avian

HA-Group51

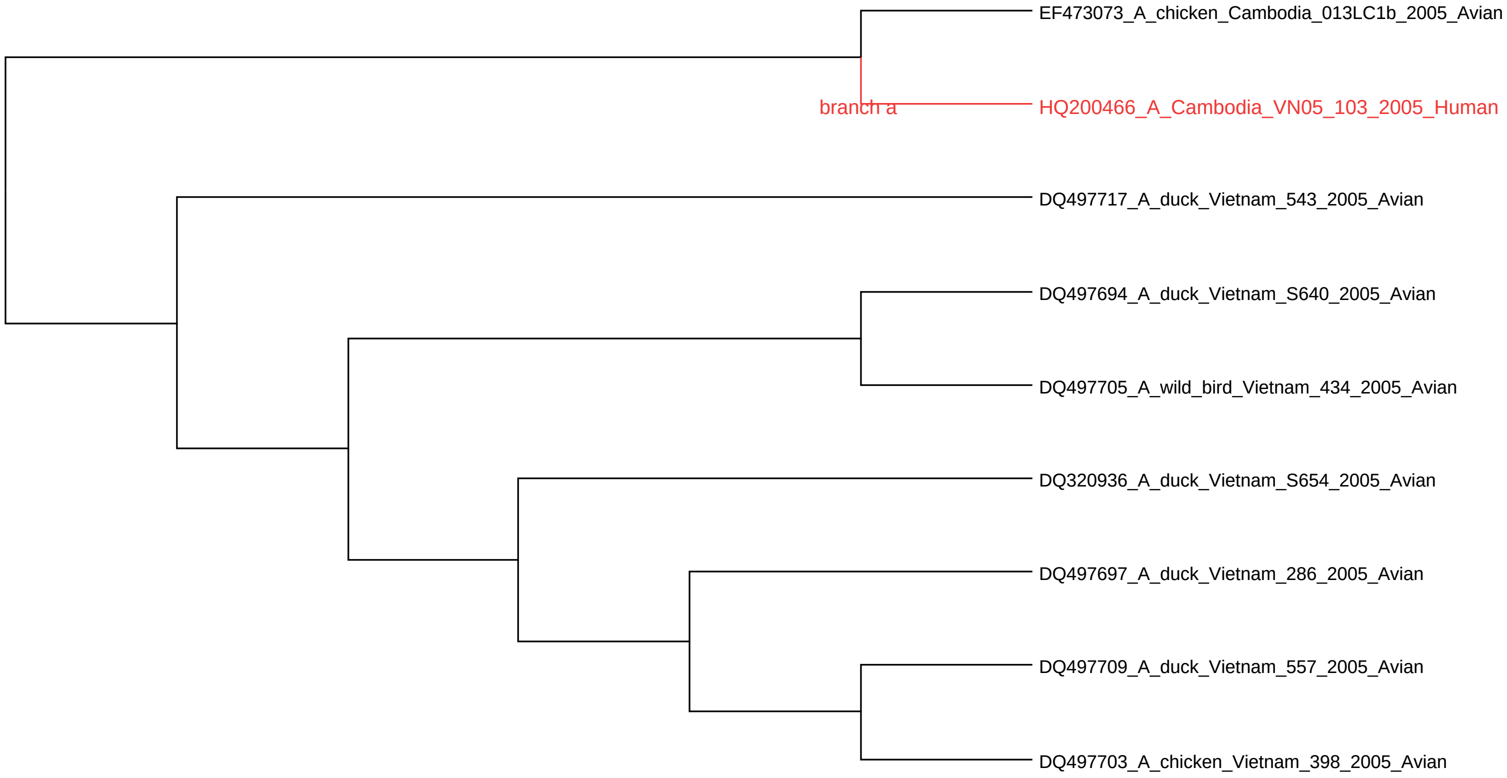

EF473073_A_chicken_Cambodia_013LC1b_2005_Avian
branch a
HQ200466_A_Cambodia_VN05_103_2005_Human
DQ497717_A_duck_Vietnam_543_2005_Avian
DQ497694_A_duck_Vietnam_S640_2005_Avian
DQ497705_A_wild_bird_Vietnam_434_2005_Avian
DQ320936_A_duck_Vietnam_S654_2005_Avian
DQ497697_A_duck_Vietnam_286_2005_Avian
DQ497709_A_duck_Vietnam_557_2005_Avian
DQ497703_A_chicken_Vietnam_398_2005_Avian

HA-Group52

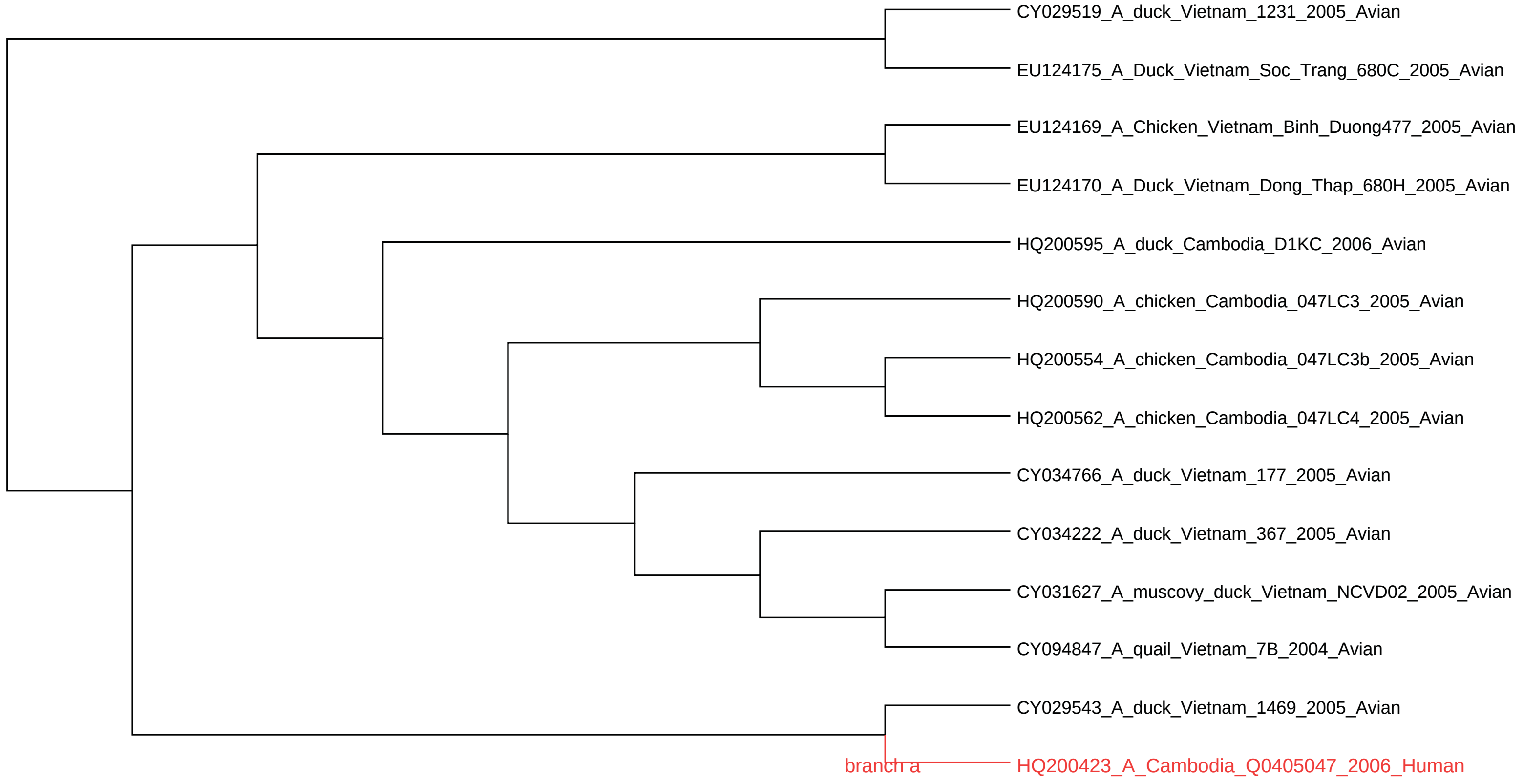
CY029519_A_duck_Vietnam_1231_2005_Avian
EU124175_A_Duck_Vietnam_Soc_Trang_680C_2005_Avian
EU124169_A_Chicken_Vietnam_Binh_Duong477_2005_Avian
EU124170_A_Duck_Vietnam_Dong_Thap_680H_2005_Avian
HQ200595_A_duck_Cambodia_D1KC_2006_Avian
HQ200590_A_chicken_Cambodia_047LC3_2005_Avian
HQ200554_A_chicken_Cambodia_047LC3b_2005_Avian
HQ200562_A_chicken_Cambodia_047LC4_2005_Avian
CY034766_A_duck_Vietnam_177_2005_Avian
CY034222_A_duck_Vietnam_367_2005_Avian
CY031627_A_muscovy_duck_Vietnam_NCVD02_2005_Avian
CY094847_A_quail_Vietnam_7B_2004_Avian
CY029543_A_duck_Vietnam_1469_2005_Avian
branch a
HQ200423_A_Cambodia_Q0405047_2006_Human

HA-Group53

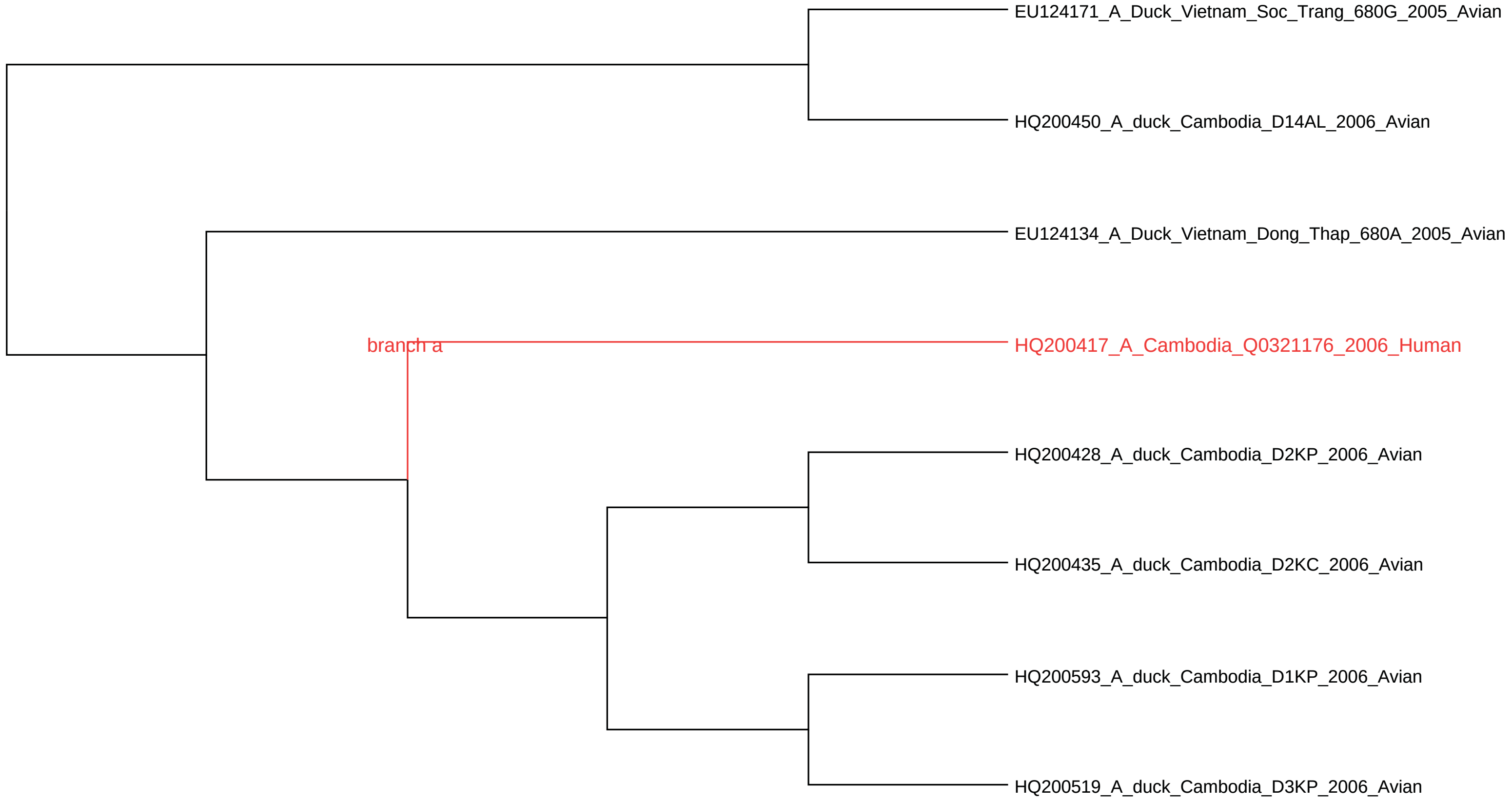

EU124171_A_Duck_Vietnam_Soc_Trang_680G_2005_Avian
HQ200450_A_duck_Cambodia_D14AL_2006_Avian
EU124134_A_Duck_Vietnam_Dong_Thap_680A_2005_Avian
branch a
HQ200417_A_Cambodia_Q0321176_2006_Human
HQ200428_A_duck_Cambodia_D2KP_2006_Avian
HQ200435_A_duck_Cambodia_D2KC_2006_Avian
HQ200593_A_duck_Cambodia_D1KP_2006_Avian
HQ200519_A_duck_Cambodia_D3KP_2006_Avian

HA-Group54

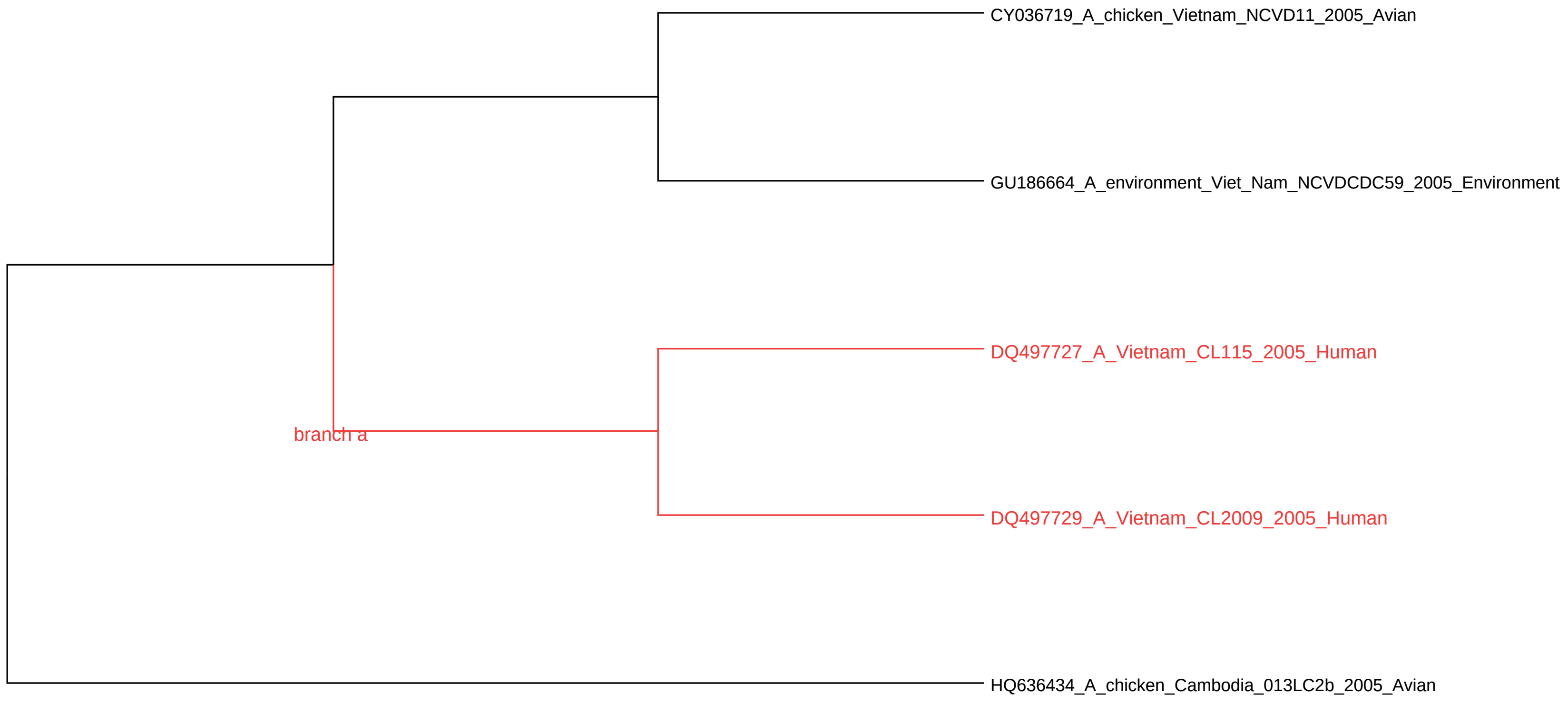

CY036719_A_chicken_Vietnam_NCVD11_2005_Avian
GU186664_A_environment_Viet_Nam_NCVDCDC59_2005_Environment
DQ497727_A_Vietnam_CL115_2005_Human
branch a
DQ497729_A_Vietnam_CL2009_2005_Human
HQ636434_A_chicken_Cambodia_013LC2b_2005_Avian

HA-Group55

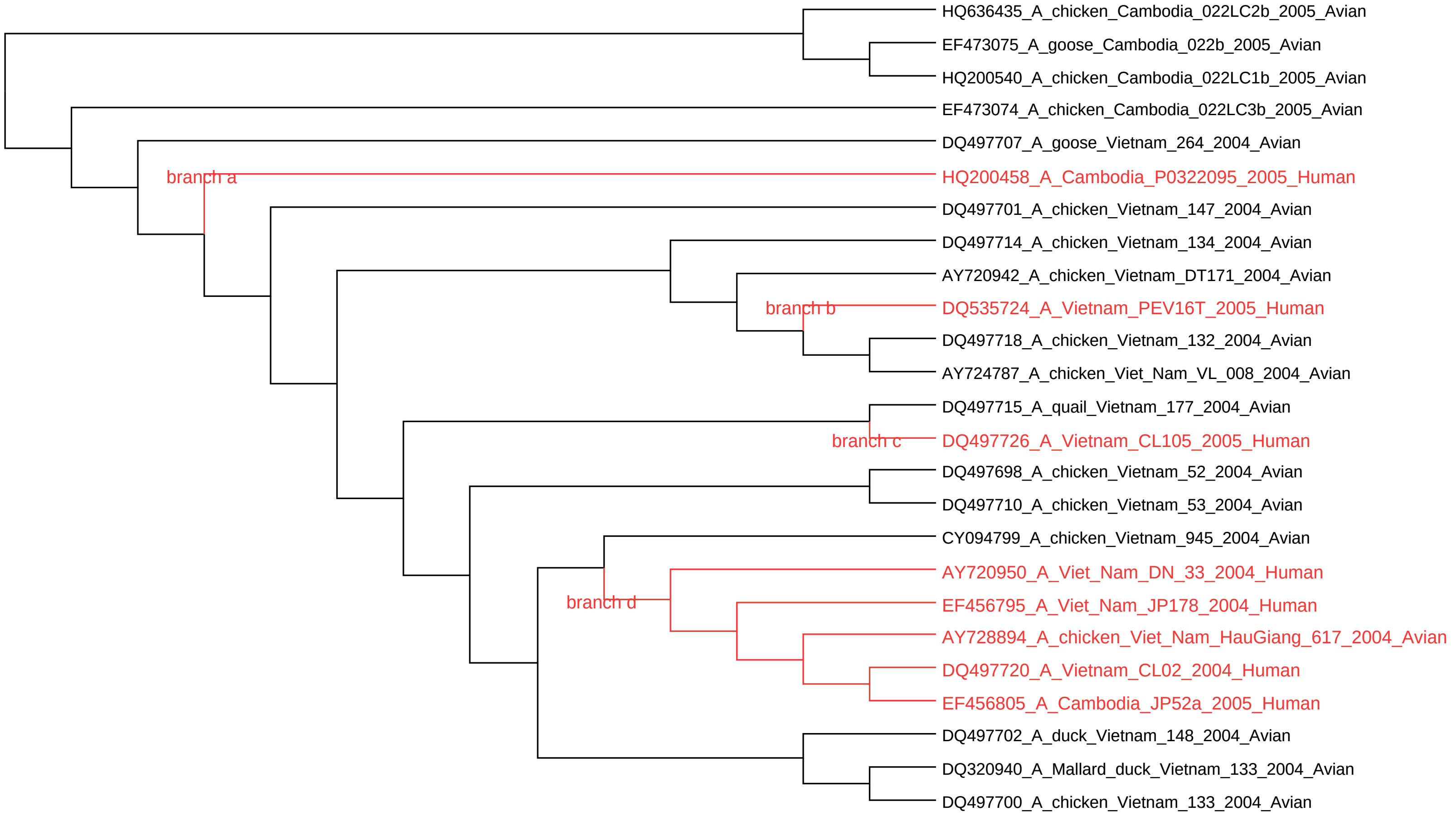

HQ636435_A_chicken_Cambodia_022LC2b_2005_Avian
EF473075_A_goose_Cambodia_022b_2005_Avian
HQ200540_A_chicken_Cambodia_022LC1b_2005_Avian
EF473074_A_chicken_Cambodia_022LC3b_2005_Avian
DQ497707_A_goose_Vietnam_264_2004_Avian
branch a
HQ200458_A_Cambodia_P0322095_2005_Human
DQ497701_A_chicken_Vietnam_147_2004_Avian
DQ497714_A_chicken_Vietnam_134_2004_Avian
AY720942_A_chicken_Vietnam_DT171_2004_Avian
branch b
DQ535724_A_Vietnam_PEV16T_2005_Human
DQ497718_A_chicken_Vietnam_132_2004_Avian
AY724787_A_chicken_Viet_Nam_VL_008_2004_Avian
DQ497715_A_quail_Vietnam_177_2004_Avian
branch c
DQ497726_A_Vietnam_CL105_2005_Human
DQ497698_A_chicken_Vietnam_52_2004_Avian
DQ497710_A_chicken_Vietnam_53_2004_Avian
CY094799_A_chicken_Vietnam_945_2004_Avian
AY720950_A_Viet_Nam_DN_33_2004_Human
branch d
EF456795_A_Viet_Nam_JP178_2004_Human
AY728894_A_chicken_Viet_Nam_HauGiang_617_2004_Avian
DQ497720_A_Vietnam_CL02_2004_Human
EF456805_A_Cambodia_JP52a_2005_Human
DQ497702_A_duck_Vietnam_148_2004_Avian
DQ320940_A_Mallard_duck_Vietnam_133_2004_Avian
DQ497700_A_chicken_Vietnam_133_2004_Avian

HA-Group56

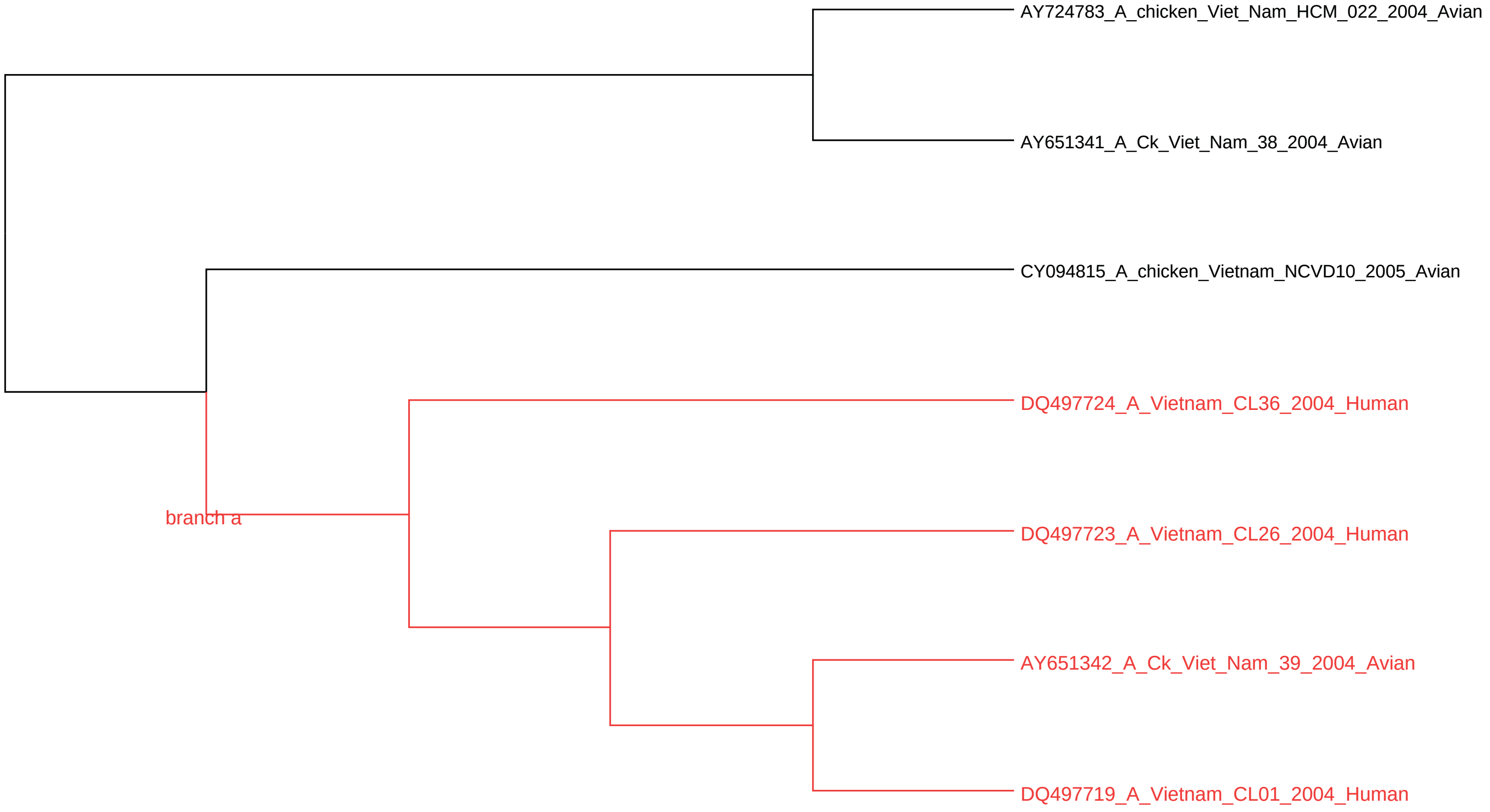
AY724783_A_chicken_Viet_Nam_HCM_022_2004_Avian
AY651341_A_Ck_Viet_Nam_38_2004_Avian
CY094815_A_chicken_Vietnam_NCVD10_2005_Avian
DQ497724_A_Vietnam_CL36_2004_Human
branch a
DQ497723_A_Vietnam_CL26_2004_Human
AY651342_A_Ck_Viet_Nam_39_2004_Avian
DQ497719_A_Vietnam_CL01_2004_Human

HA-Group57

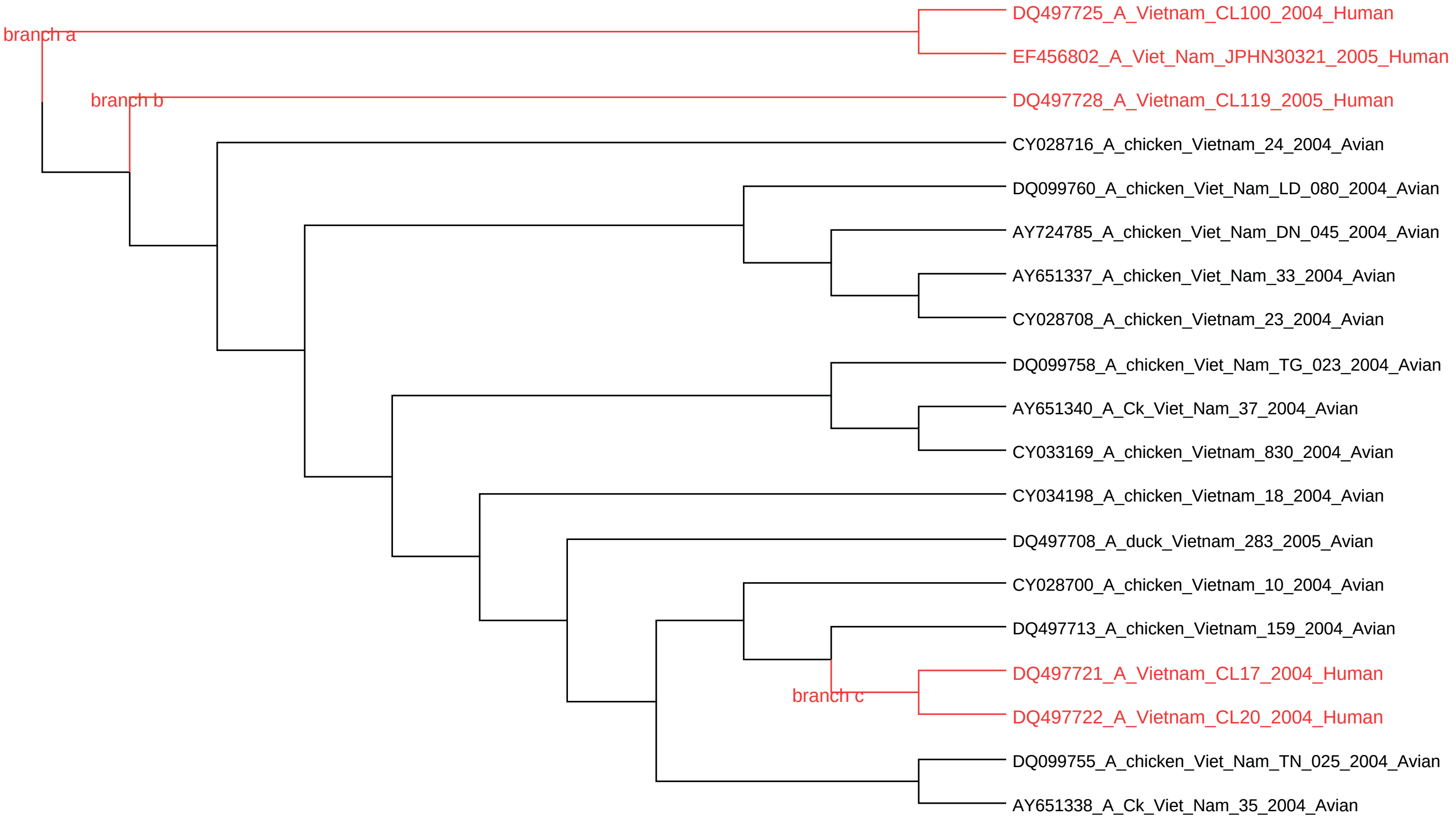

branch a
branch b
branch c
DQ497725_A_Vietnam_CL100_2004_Human
EF456802_A_Viet_Nam_JPHN30321_2005_Human
DQ497728_A_Vietnam_CL119_2005_Human
CY028716_A_chicken_Vietnam_24_2004_Avian
DQ099760_A_chicken_Viet_Nam_LD_080_2004_Avian
AY724785_A_chicken_Viet_Nam_DN_045_2004_Avian
AY651337_A_chicken_Viet_Nam_33_2004_Avian
CY028708_A_chicken_Vietnam_23_2004_Avian
DQ099758_A_chicken_Viet_Nam_TG_023_2004_Avian
AY651340_A_Ck_Viet_Nam_37_2004_Avian
CY033169_A_chicken_Vietnam_830_2004_Avian
CY034198_A_chicken_Vietnam_18_2004_Avian
DQ497708_A_duck_Vietnam_283_2005_Avian
CY028700_A_chicken_Vietnam_10_2004_Avian
DQ497713_A_chicken_Vietnam_159_2004_Avian
DQ497721_A_Vietnam_CL17_2004_Human
DQ497722_A_Vietnam_CL20_2004_Human
DQ099755_A_chicken_Viet_Nam_TN_025_2004_Avian
AY651338_A_Ck_Viet_Nam_35_2004_Avian

HA-Group58

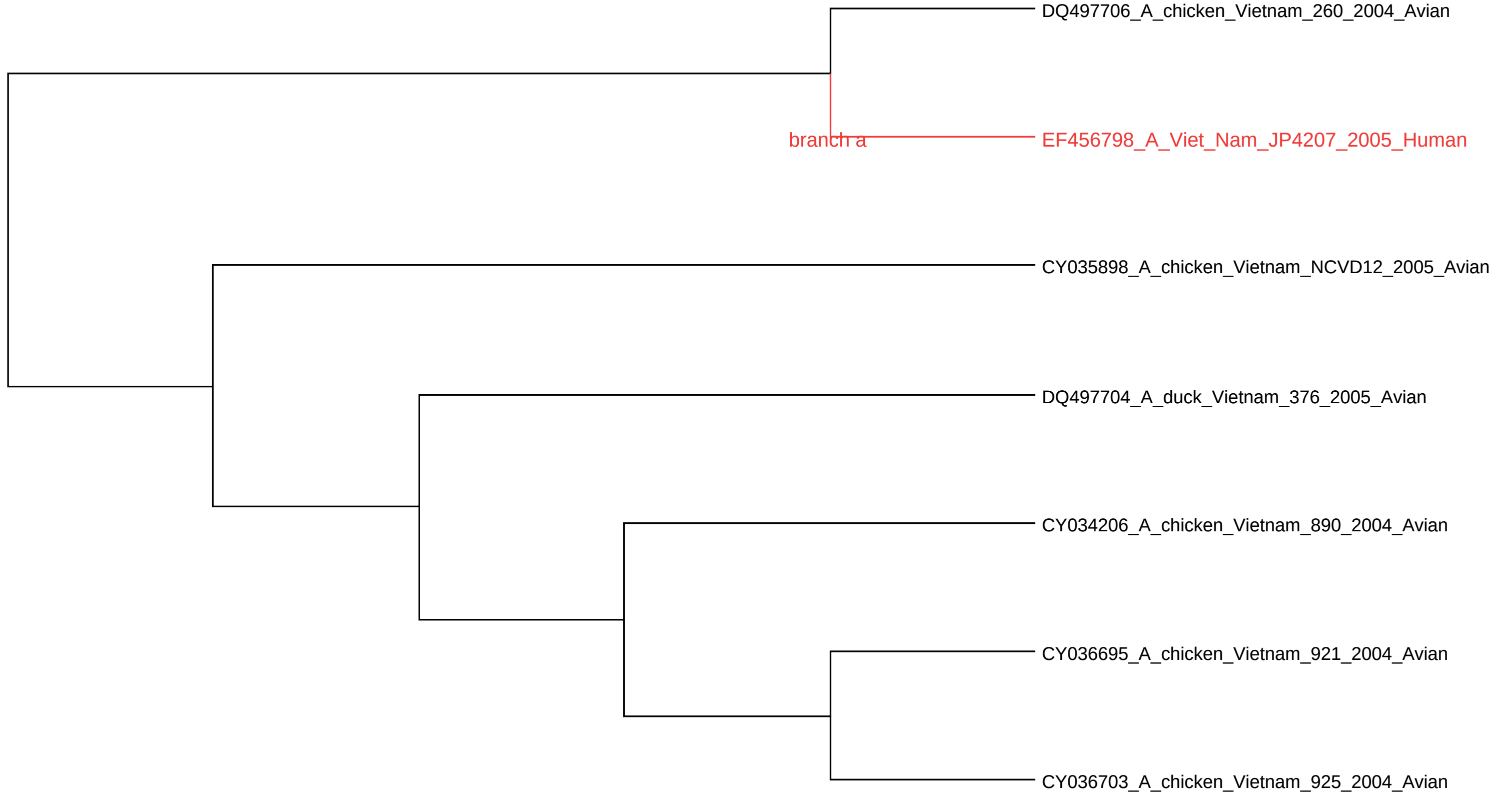

DQ497706_A_chicken_Vietnam_260_2004_Avian
branch a
EF456798_A_Viet_Nam_JP4207_2005_Human
CY035898_A_chicken_Vietnam_NCVD12_2005_Avian
DQ497704_A_duck_Vietnam_376_2005_Avian
CY034206_A_chicken_Vietnam_890_2004_Avian
CY036695_A_chicken_Vietnam_921_2004_Avian
CY036703_A_chicken_Vietnam_925_2004_Avian

HA-Group59

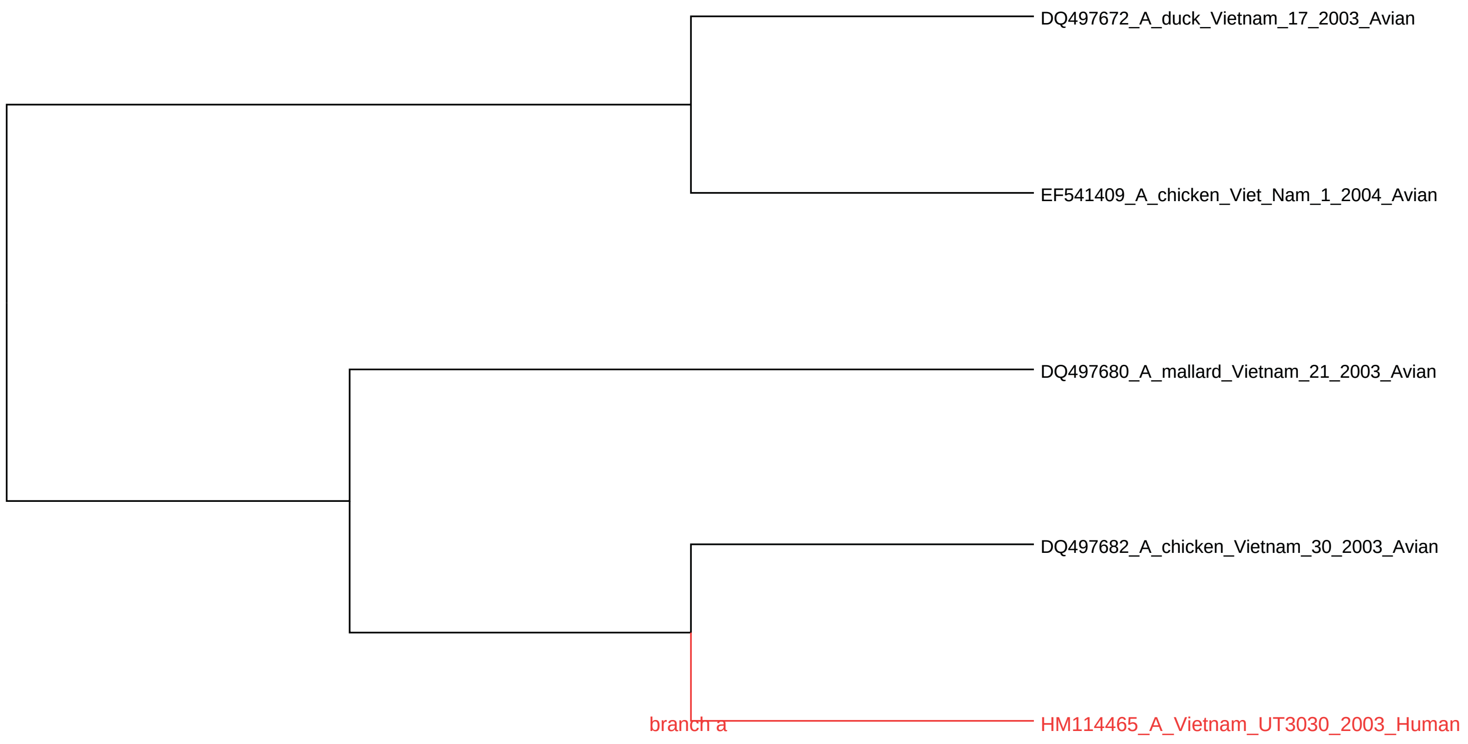

DQ497672_A_duck_Vietnam_17_2003_Avian
EF541409_A_chicken_Viet_Nam_1_2004_Avian
DQ497680_A_mallard_Vietnam_21_2003_Avian
DQ497682_A_chicken_Vietnam_30_2003_Avian
branch a
HM114465_A_Vietnam_UT3030_2003_Human

HA-Group60

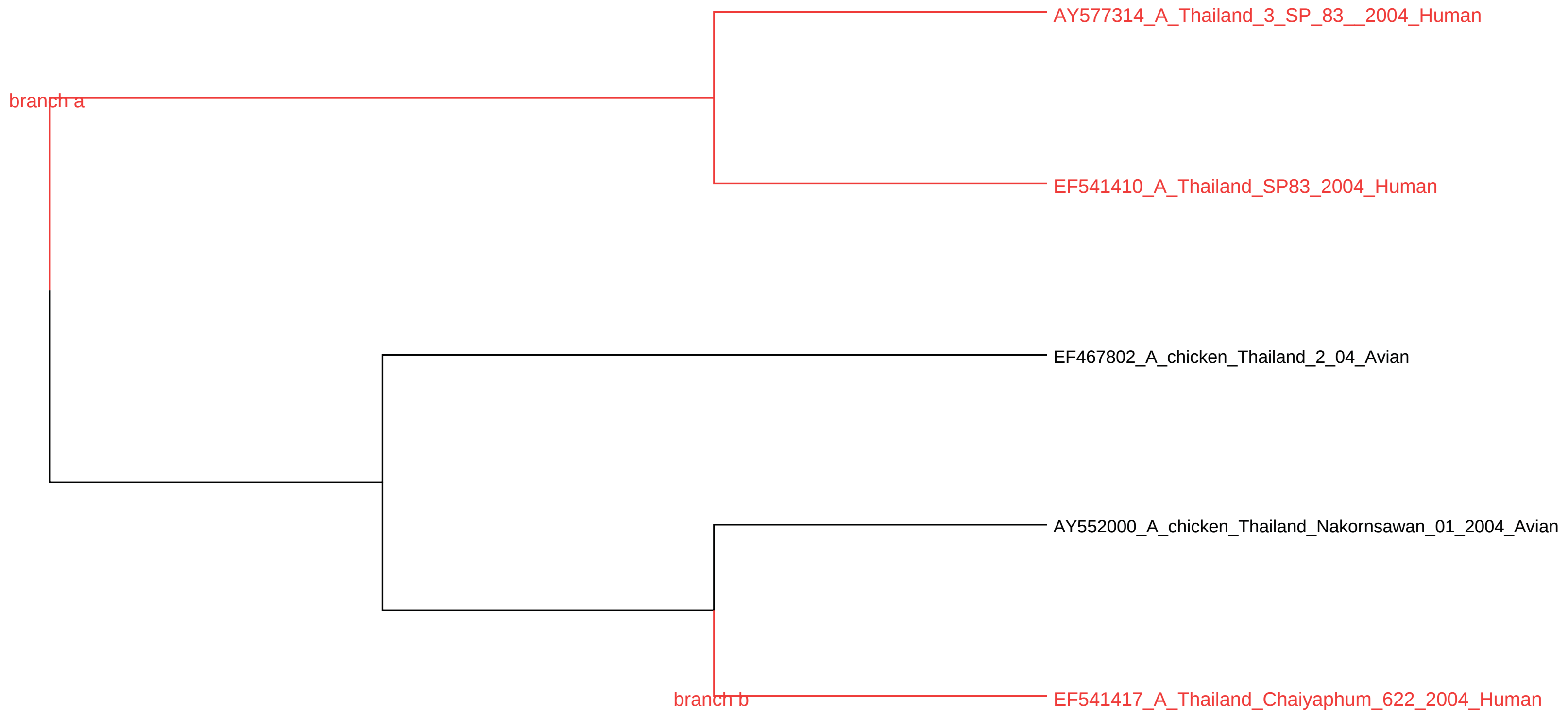

AY577314_A_Thailand_3_SP_83__2004_Human
branch a
EF541410_A_Thailand_SP83_2004_Human
EF467802_A_chicken_Thailand_2_04_Avian
AY552000_A_chicken_Thailand_Nakornsawan_01_2004_Avian
branch b
EF541417_A_Thailand_Chaiyaphum_622_2004_Human

HA-Group61

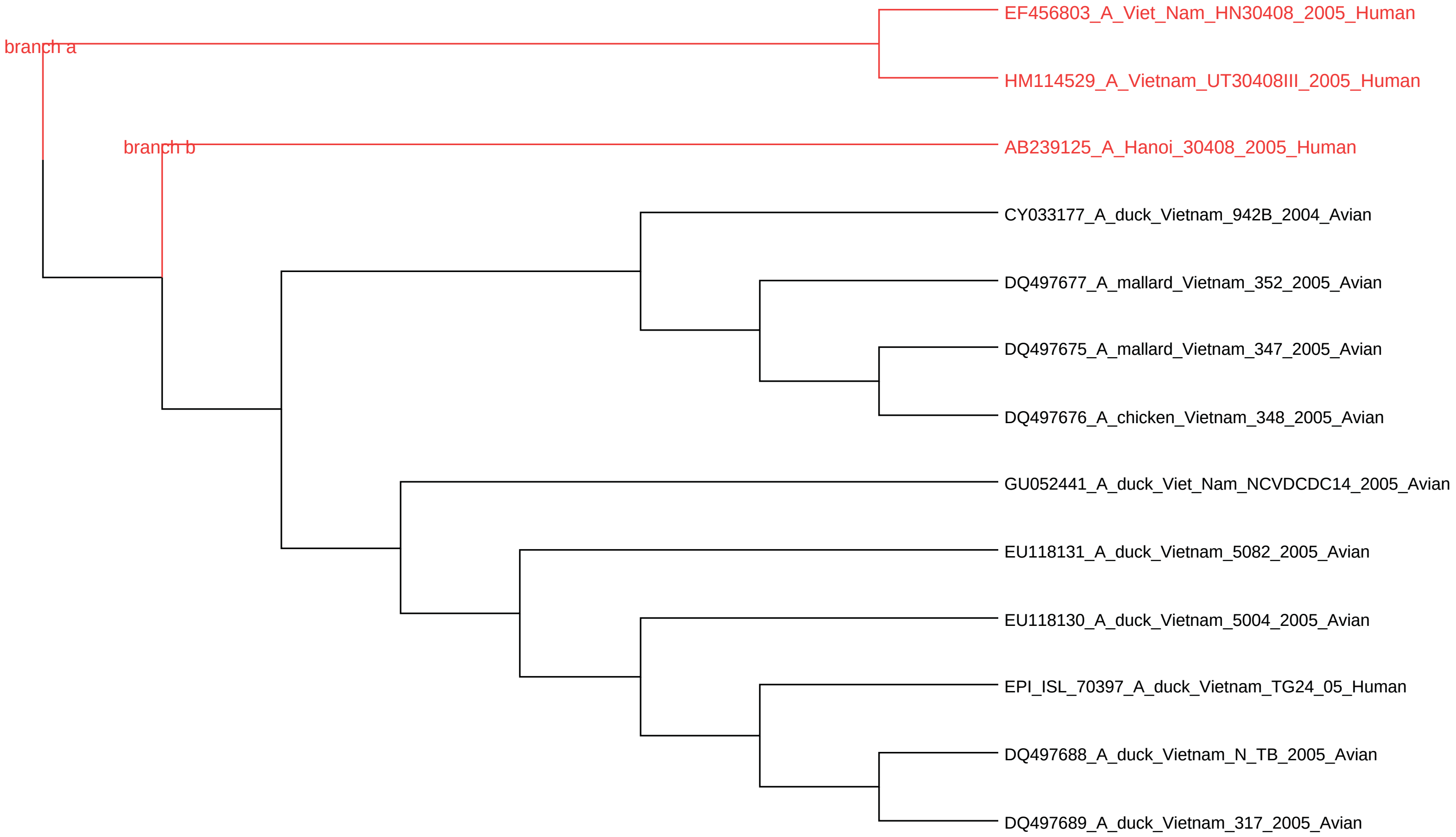

branch a
branch b
EF456803_A_Viet_Nam_HN30408_2005_Human
HM114529_A_Vietnam_UT30408III_2005_Human
AB239125_A_Hanoi_30408_2005_Human
CY033177_A_duck_Vietnam_942B_2004_Avian
DQ497677_A_mallard_Vietnam_352_2005_Avian
DQ497675_A_mallard_Vietnam_347_2005_Avian
DQ497676_A_chicken_Vietnam_348_2005_Avian
GU052441_A_duck_Viet_Nam_NCVDCDC14_2005_Avian
EU118131_A_duck_Vietnam_5082_2005_Avian
EU118130_A_duck_Vietnam_5004_2005_Avian
EPI_ISL_70397_A_duck_Vietnam_TG24_05_Human
DQ497688_A_duck_Vietnam_N_TB_2005_Avian
DQ497689_A_duck_Vietnam_317_2005_Avian

HA-Group62

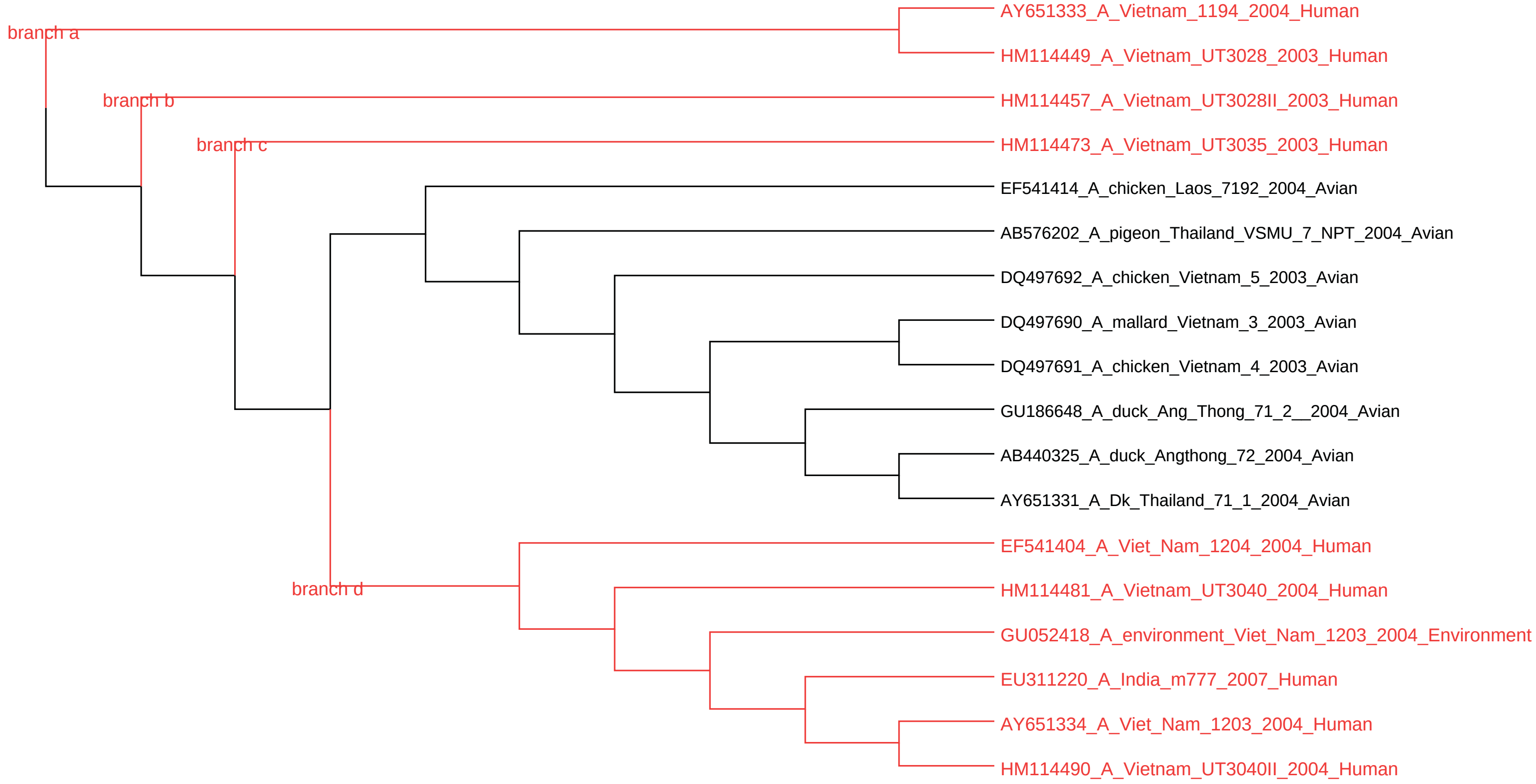

branch a
branch b
branch c
branch d
AY651333_A_Vietnam_1194_2004_Human
HM114449_A_Vietnam_UT3028_2003_Human
HM114457_A_Vietnam_UT3028II_2003_Human
HM114473_A_Vietnam_UT3035_2003_Human
EF541414_A_chicken_Laos_7192_2004_Avian
AB576202_A_pigeon_Thailand_VSMU_7_NPT_2004_Avian
DQ497692_A_chicken_Vietnam_5_2003_Avian
DQ497690_A_mallard_Vietnam_3_2003_Avian
DQ497691_A_chicken_Vietnam_4_2003_Avian
GU186648_A_duck_Ang_Thong_71_2__2004_Avian
AB440325_A_duck_Angthong_72_2004_Avian
AY651331_A_Dk_Thailand_71_1_2004_Avian
EF541404_A_Viet_Nam_1204_2004_Human
HM114481_A_Vietnam_UT3040_2004_Human
GU052418_A_environment_Viet_Nam_1203_2004_Environment
EU311220_A_India_m777_2007_Human
AY651334_A_Viet_Nam_1203_2004_Human
HM114490_A_Vietnam_UT3040II_2004_Human

HA-Group63

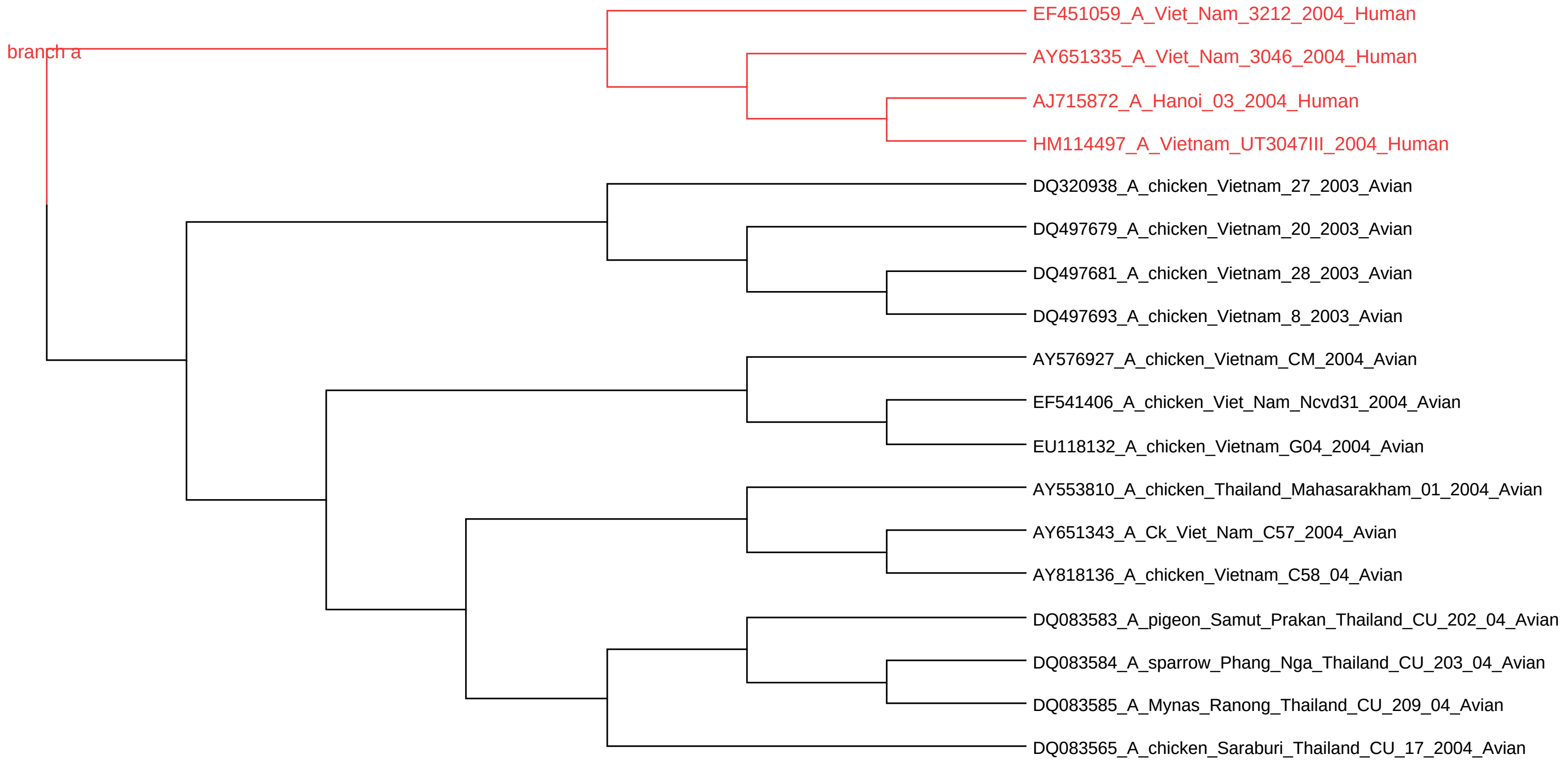
branch a
EF451059_A_Viet_Nam_3212_2004_Human
AY651335_A_Viet_Nam_3046_2004_Human
AJ715872_A_Hanoi_03_2004_Human
HM114497_A_Vietnam_UT3047III_2004_Human
DQ320938_A_chicken_Vietnam_27_2003_Avian
DQ497679_A_chicken_Vietnam_20_2003_Avian
DQ497681_A_chicken_Vietnam_28_2003_Avian
DQ497693_A_chicken_Vietnam_8_2003_Avian
AY576927_A_chicken_Vietnam_CM_2004_Avian
EF541406_A_chicken_Viet_Nam_Ncvd31_2004_Avian
EU118132_A_chicken_Vietnam_G04_2004_Avian
AY553810_A_chicken_Thailand_Mahasarakham_01_2004_Avian
AY651343_A_Ck_Viet_Nam_C57_2004_Avian
AY818136_A_chicken_Vietnam_C58_04_Avian
DQ083583_A_pigeon_Samut_Prakan_Thailand_CU_202_04_Avian
DQ083584_A_sparrow_Phang_Nga_Thailand_CU_203_04_Avian
DQ083585_A_Mynas_Ranong_Thailand_CU_209_04_Avian
DQ083565_A_chicken_Saraburi_Thailand_CU_17_2004_Avian

HA-Group64

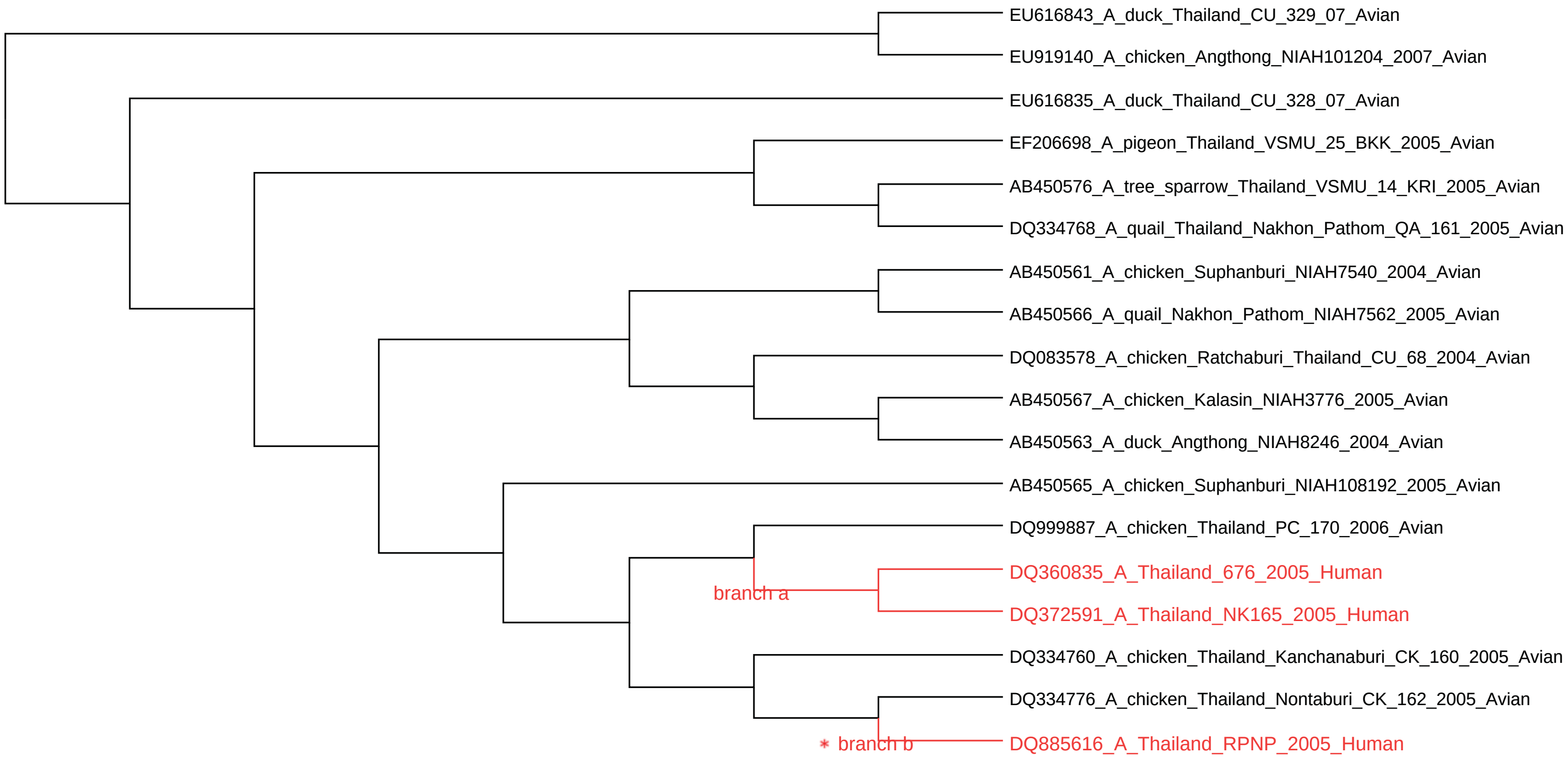
EU616843_A_duck_Thailand_CU_329_07_Avian
EU919140_A_chicken_Angthong_NIAH101204_2007_Avian
EU616835_A_duck_Thailand_CU_328_07_Avian
EF206698_A_pigeon_Thailand_VSMU_25_BKK_2005_Avian
AB450576_A_tree_sparrow_Thailand_VSMU_14_KRI_2005_Avian
DQ334768_A_quail_Thailand_Nakhon_Pathom_QA_161_2005_Avian
AB450561_A_chicken_Suphanburi_NIAH7540_2004_Avian
AB450566_A_quail_Nakhon_Pathom_NIAH7562_2005_Avian
DQ083578_A_chicken_Ratchaburi_Thailand_CU_68_2004_Avian
AB450567_A_chicken_Kalasin_NIAH3776_2005_Avian
AB450563_A_duck_Angthong_NIAH8246_2004_Avian
AB450565_A_chicken_Suphanburi_NIAH108192_2005_Avian
DQ999887_A_chicken_Thailand_PC_170_2006_Avian
DQ360835_A_Thailand_676_2005_Human
branch a
DQ372591_A_Thailand_NK165_2005_Human
DQ334760_A_chicken_Thailand_Kanchanaburi_CK_160_2005_Avian
DQ334776_A_chicken_Thailand_Nontaburi_CK_162_2005_Avian
* branch b
DQ885616_A_Thailand_RPNP_2005_Human

HA-Group65

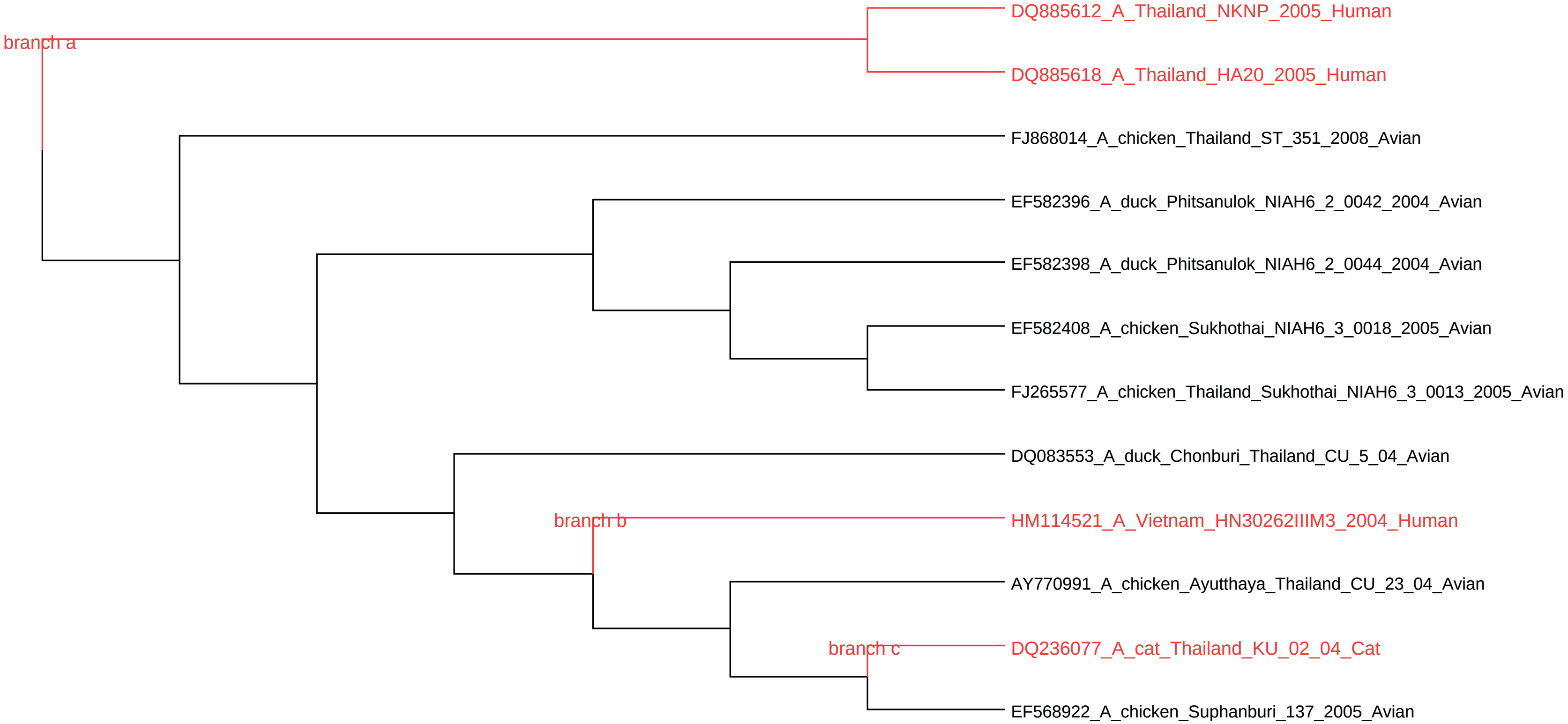
branch a
DQ885612_A_Thailand_NKNP_2005_Human
DQ885618_A_Thailand_HA20_2005_Human
FJ868014_A_chicken_Thailand_ST_351_2008_Avian
EF582396_A_duck_Phitsanulok_NIAH6_2_0042_2004_Avian
EF582398_A_duck_Phitsanulok_NIAH6_2_0044_2004_Avian
EF582408_A_chicken_Sukhothai_NIAH6_3_0018_2005_Avian
FJ265577_A_chicken_Thailand_Sukhothai_NIAH6_3_0013_2005_Avian
DQ083553_A_duck_Chonburi_Thailand_CU_5_04_Avian
branch b
HM114521_A_Vietnam_HN30262IIIM3_2004_Human
AY770991_A_chicken_Ayutthaya_Thailand_CU_23_04_Avian
branch c
DQ236077_A_cat_Thailand_KU_02_04_Cat
EF568922_A_chicken_Suphanburi_137_2005_Avian

HA-Group66

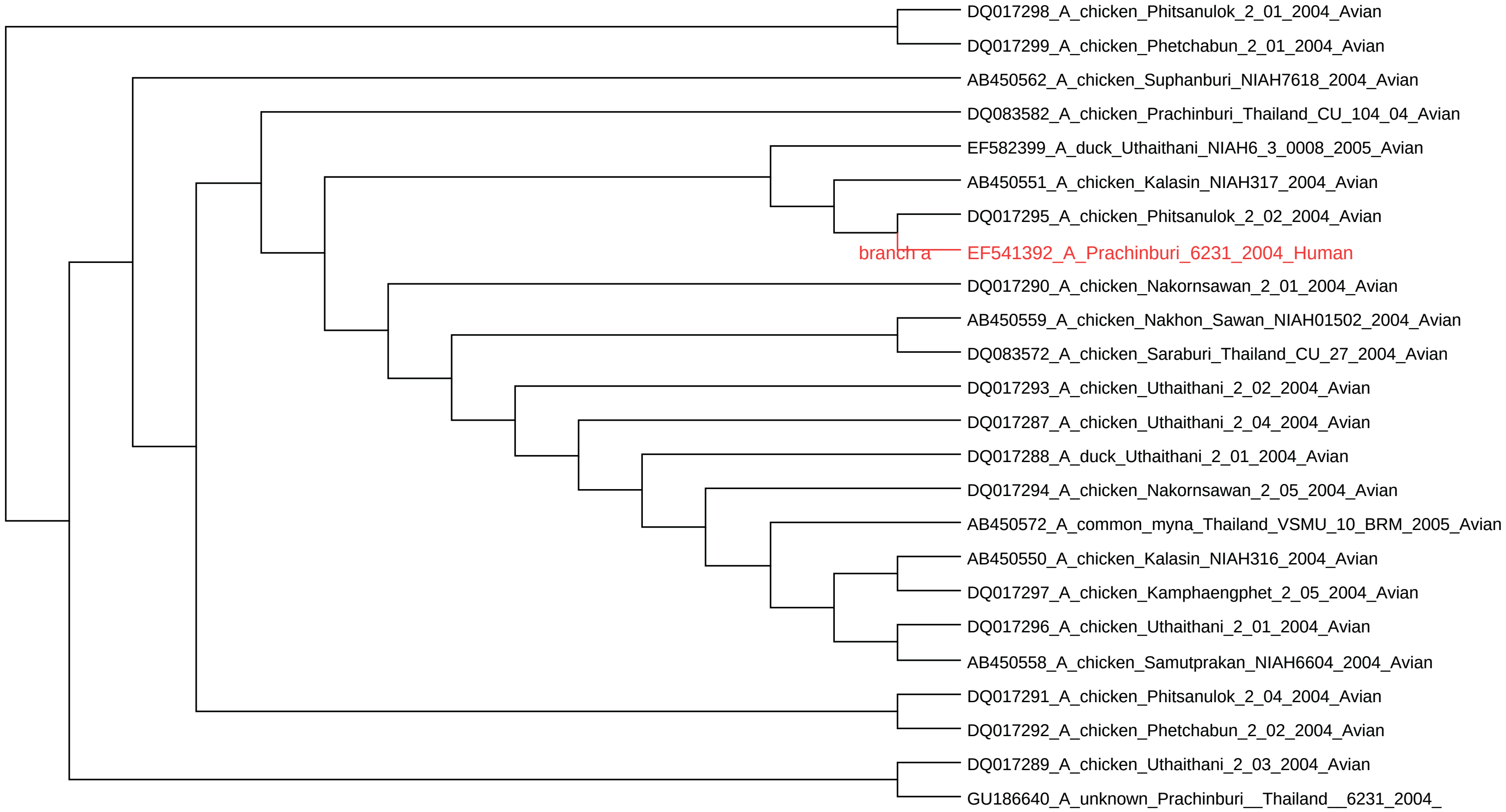

DQ017298_A_chicken_Phitsanulok_2_01_2004_Avian
DQ017299_A_chicken_Phetchabun_2_01_2004_Avian
AB450562_A_chicken_Suphanburi_NIAH7618_2004_Avian
DQ083582_A_chicken_Prachinburi_Thailand_CU_104_04_Avian
EF582399_A_duck_Uthaithani_NIAH6_3_0008_2005_Avian
AB450551_A_chicken_Kalasin_NIAH317_2004_Avian
DQ017295_A_chicken_Phitsanulok_2_02_2004_Avian
branch a
EF541392_A_Prachinburi_6231_2004_Human
DQ017290_A_chicken_Nakornsawan_2_01_2004_Avian
AB450559_A_chicken_Nakhon_Sawan_NIAH01502_2004_Avian
DQ083572_A_chicken_Saraburi_Thailand_CU_27_2004_Avian
DQ017293_A_chicken_Uthaithani_2_02_2004_Avian
DQ017287_A_chicken_Uthaithani_2_04_2004_Avian
DQ017288_A_duck_Uthaithani_2_01_2004_Avian
DQ017294_A_chicken_Nakornsawan_2_05_2004_Avian
AB450572_A_common_myna_Thailand_VSMU_10_BRM_2005_Avian
AB450550_A_chicken_Kalasin_NIAH316_2004_Avian
DQ017297_A_chicken_Kamphaengphet_2_05_2004_Avian
DQ017296_A_chicken_Uthaithani_2_01_2004_Avian
AB450558_A_chicken_Samutprakan_NIAH6604_2004_Avian
DQ017291_A_chicken_Phitsanulok_2_04_2004_Avian
DQ017292_A_chicken_Phetchabun_2_02_2004_Avian
DQ017289_A_chicken_Uthaithani_2_03_2004_Avian
GU186640_A_unknown_Prachinburi__Thailand__6231_2004_

HA-Group67

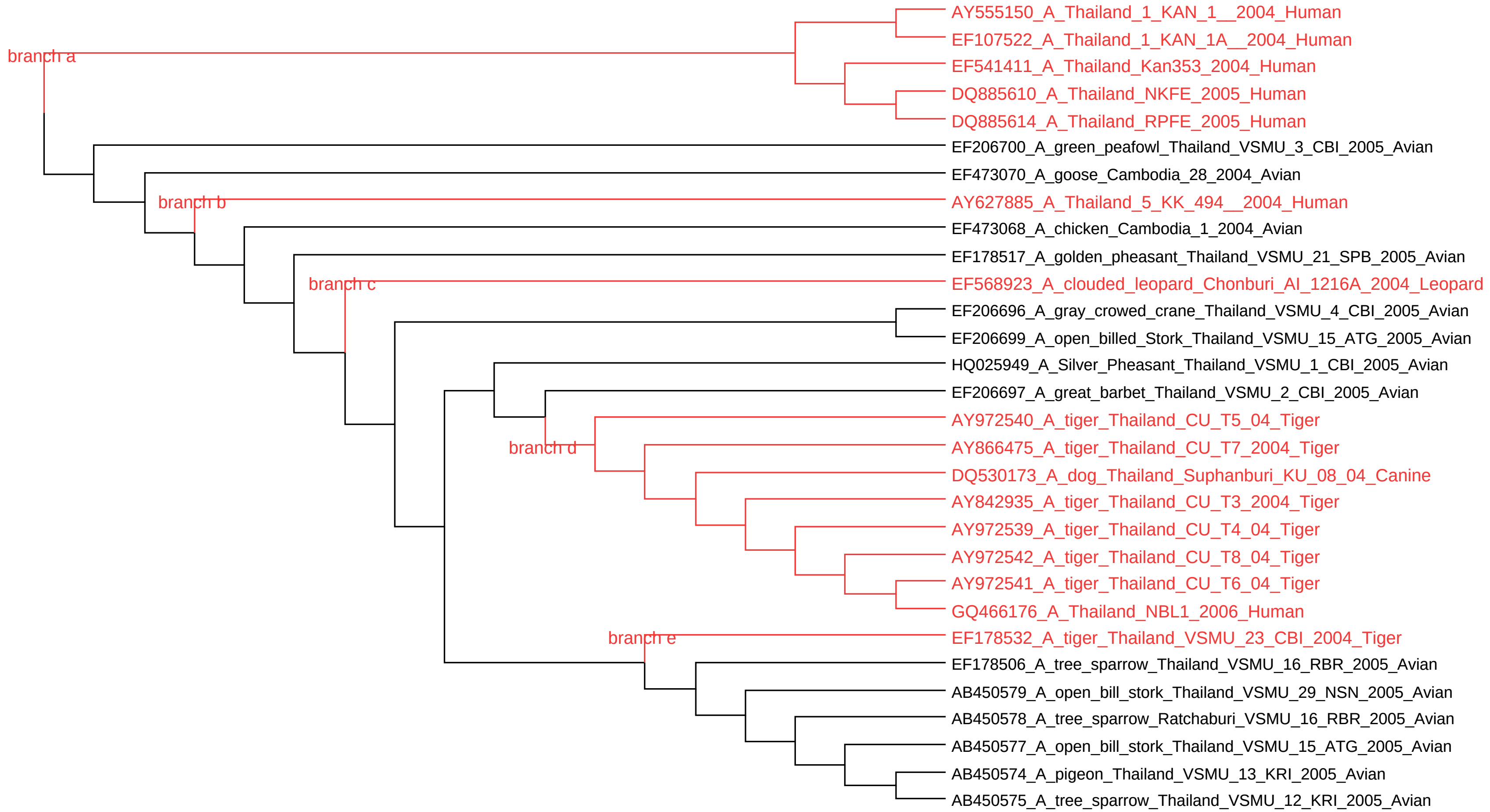

branch a
branch b
branch c
branch d
branch e
AY555150_A_Thailand_1_KAN_1__2004_Human
EF107522_A_Thailand_1_KAN_1A__2004_Human
EF541411_A_Thailand_Kan353_2004_Human
DQ885610_A_Thailand_NKFE_2005_Human
DQ885614_A_Thailand_RPFE_2005_Human
EF206700_A_green_peafowl_Thailand_VSMU_3_CBI_2005_Avian
EF473070_A_goose_Cambodia_28_2004_Avian
AY627885_A_Thailand_5_KK_494__2004_Human
EF473068_A_chicken_Cambodia_1_2004_Avian
EF178517_A_golden_pheasant_Thailand_VSMU_21_SPB_2005_Avian
EF568923_A_clouded_leopard_Chonburi_AI_1216A_2004_Leopard
EF206696_A_gray_crowed_crane_Thailand_VSMU_4_CBI_2005_Avian
EF206699_A_open_billed_Stork_Thailand_VSMU_15_ATG_2005_Avian
HQ025949_A_Silver_Pheasant_Thailand_VSMU_1_CBI_2005_Avian
EF206697_A_great_barbet_Thailand_VSMU_2_CBI_2005_Avian
AY972540_A_tiger_Thailand_CU_T5_04_Tiger
AY866475_A_tiger_Thailand_CU_T7_2004_Tiger
DQ530173_A_dog_Thailand_Suphanburi_KU_08_04_Canine
AY842935_A_tiger_Thailand_CU_T3_2004_Tiger
AY972539_A_tiger_Thailand_CU_T4_04_Tiger
AY972542_A_tiger_Thailand_CU_T8_04_Tiger
AY972541_A_tiger_Thailand_CU_T6_04_Tiger
GQ466176_A_Thailand_NBL1_2006_Human
EF178532_A_tiger_Thailand_VSMU_23_CBI_2004_Tiger
EF178506_A_tree_sparrow_Thailand_VSMU_16_RBR_2005_Avian
AB450579_A_open_bill_stork_Thailand_VSMU_29_NSN_2005_Avian
AB450578_A_tree_sparrow_Ratchaburi_VSMU_16_RBR_2005_Avian
AB450577_A_open_bill_stork_Thailand_VSMU_15_ATG_2005_Avian
AB450574_A_pigeon_Thailand_VSMU_13_KRI_2005_Avian
AB450575_A_tree_sparrow_Thailand_VSMU_12_KRI_2005_Avian

HA-Group68

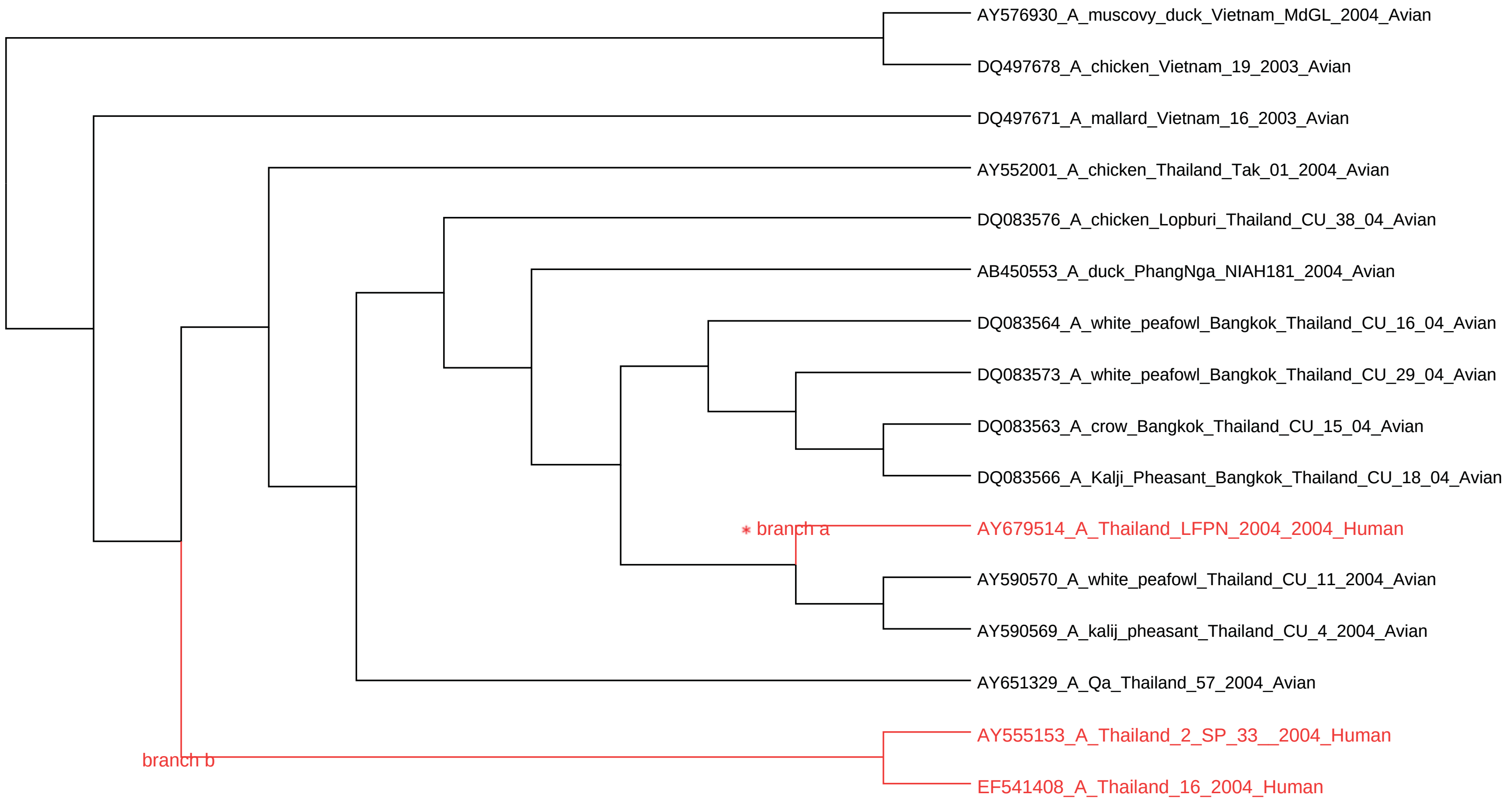

AY576930_A_muscovy_duck_Vietnam_MdGL_2004_Avian
DQ497678_A_chicken_Vietnam_19_2003_Avian
DQ497671_A_mallard_Vietnam_16_2003_Avian
AY552001_A_chicken_Thailand_Tak_01_2004_Avian
DQ083576_A_chicken_Lopburi_Thailand_CU_38_04_Avian
AB450553_A_duck_PhangNga_NIAH181_2004_Avian
DQ083564_A_white_peafowl_Bangkok_Thailand_CU_16_04_Avian
DQ083573_A_white_peafowl_Bangkok_Thailand_CU_29_04_Avian
DQ083563_A_crow_Bangkok_Thailand_CU_15_04_Avian
DQ083566_A_Kalji_Pheasant_Bangkok_Thailand_CU_18_04_Avian
* branch a
AY679514_A_Thailand_LFPN_2004_2004_Human
AY590570_A_white_peafowl_Thailand_CU_11_2004_Avian
AY590569_A_kalij_pheasant_Thailand_CU_4_2004_Avian
AY651329_A_Qa_Thailand_57_2004_Avian
AY555153_A_Thailand_2_SP_33__2004_Human
branch b
EF541408_A_Thailand_16_2004_Human

HA-Group69

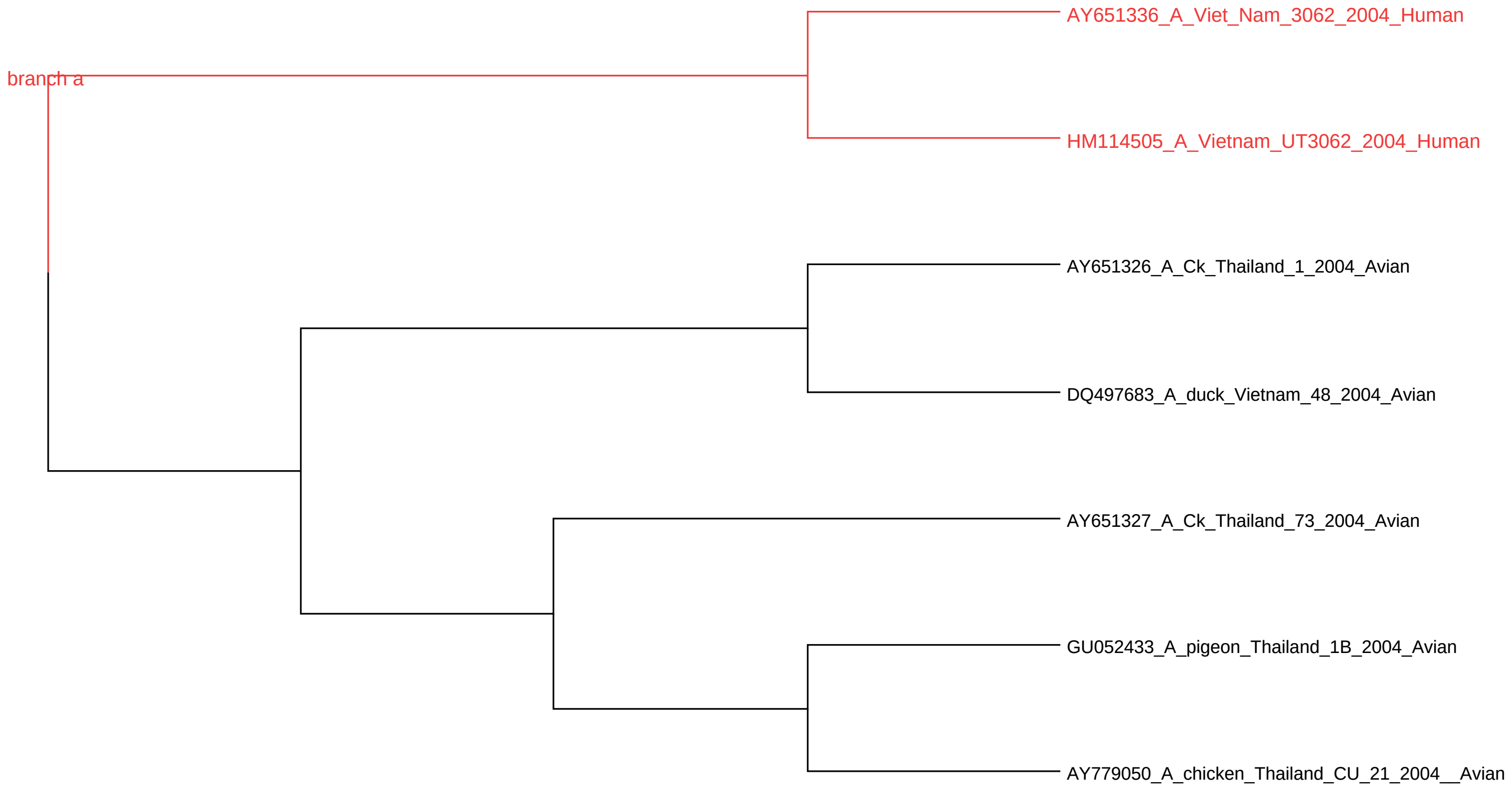

branch a
AY651336_A_Viet_Nam_3062_2004_Human
HM114505_A_Vietnam_UT3062_2004_Human
AY651326_A_Ck_Thailand_1_2004_Avian
DQ497683_A_duck_Vietnam_48_2004_Avian
AY651327_A_Ck_Thailand_73_2004_Avian
GU052433_A_pigeon_Thailand_1B_2004_Avian
AY779050_A_chicken_Thailand_CU_21_2004__Avian

HA-Group70

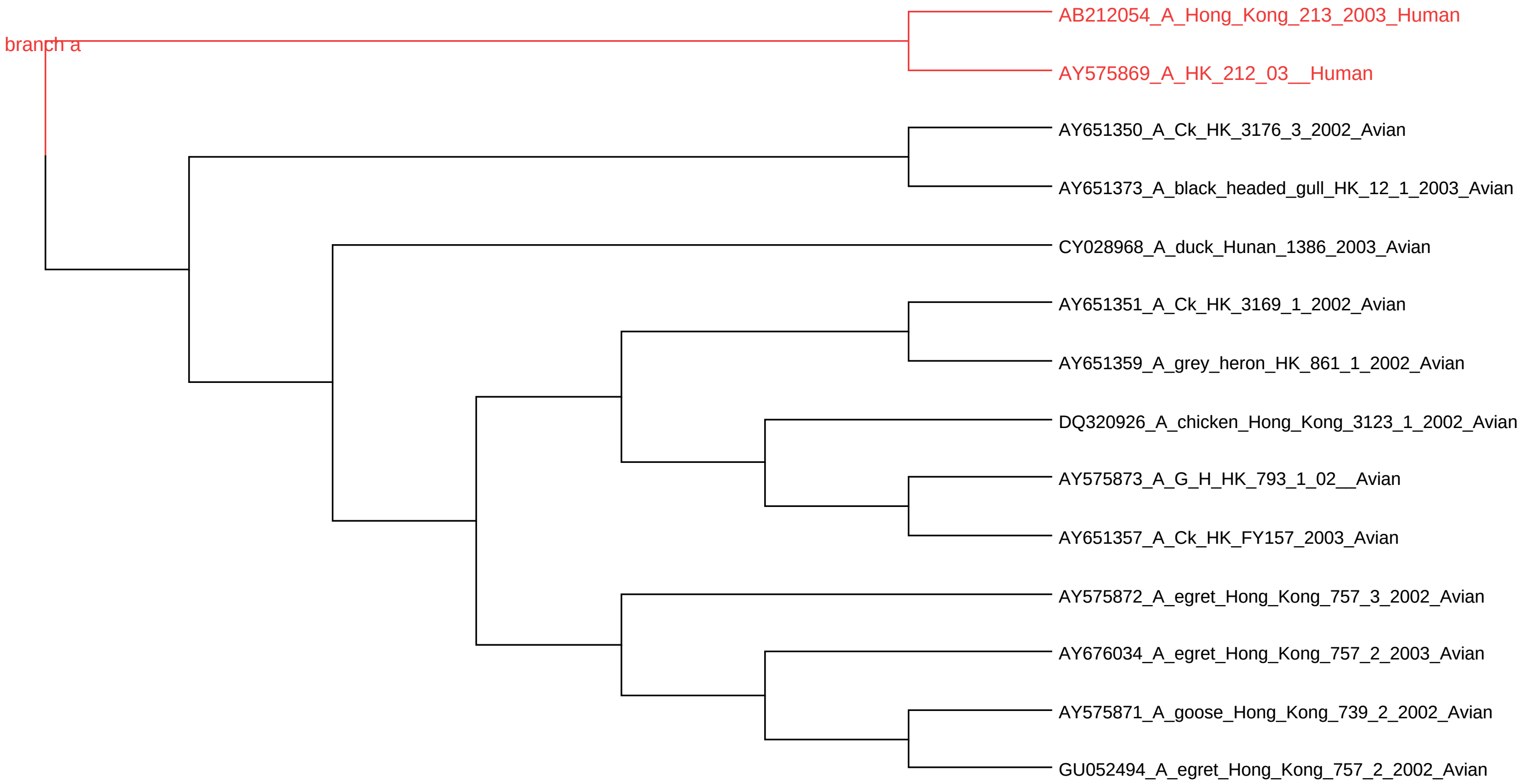

branch a
AB212054_A_Hong_Kong_213_2003_Human
AY575869_A_HK_212_03__Human
AY651350_A_Ck_HK_3176_3_2002_Avian
AY651373_A_black_headed_gull_HK_12_1_2003_Avian
CY028968_A_duck_Hunan_1386_2003_Avian
AY651351_A_Ck_HK_3169_1_2002_Avian
AY651359_A_grey_heron_HK_861_1_2002_Avian
DQ320926_A_chicken_Hong_Kong_3123_1_2002_Avian
AY575873_A_G_H_HK_793_1_02__Avian
AY651357_A_Ck_HK_FY157_2003_Avian
AY575872_A_egret_Hong_Kong_757_3_2002_Avian
AY676034_A_egret_Hong_Kong_757_2_2003_Avian
AY575871_A_goose_Hong_Kong_739_2_2002_Avian
GU052494_A_egret_Hong_Kong_757_2_2002_Avian

HA-Group71

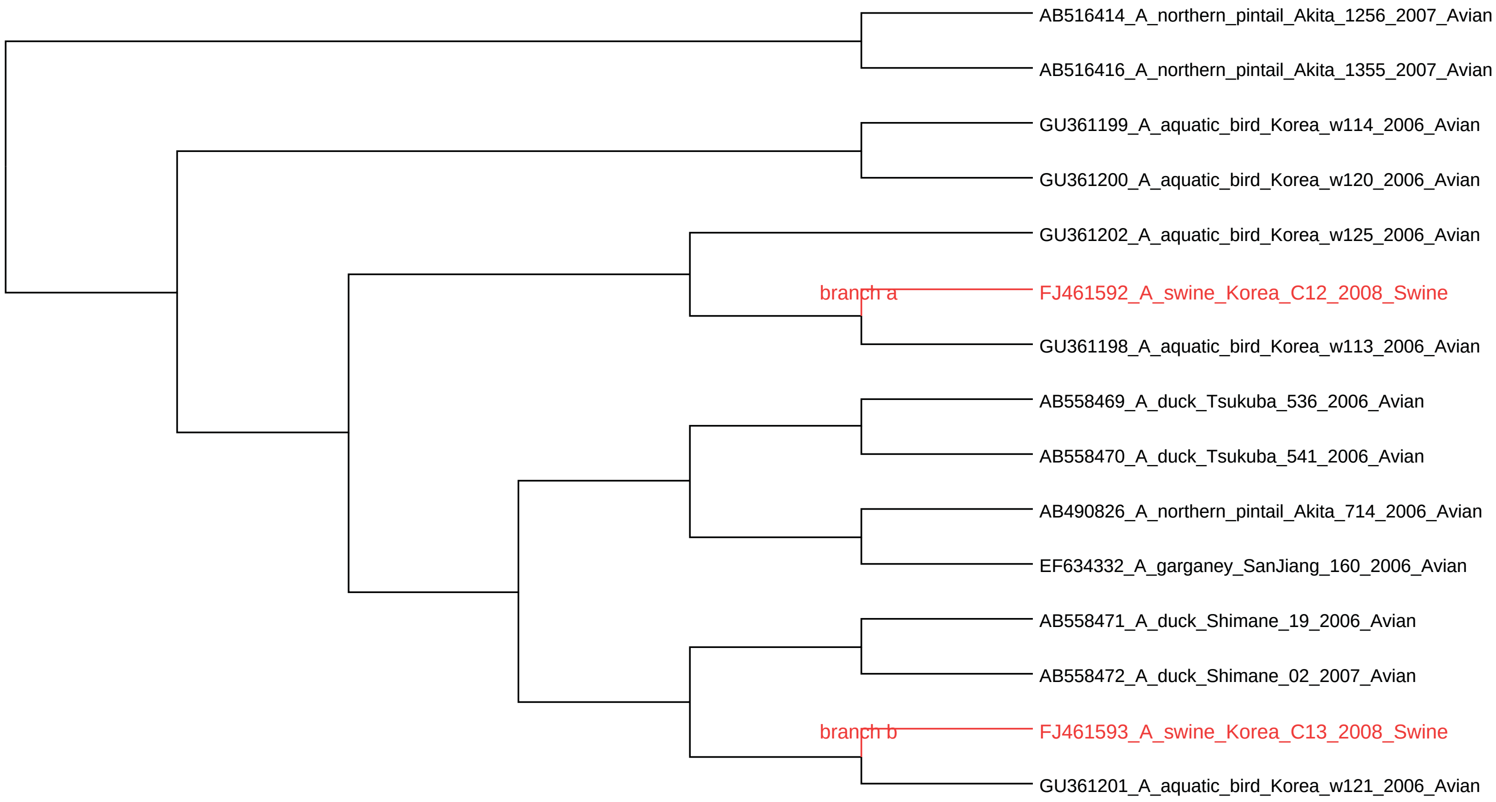
AB516414_A_northern_pintail_Akita_1256_2007_Avian
AB516416_A_northern_pintail_Akita_1355_2007_Avian
GU361199_A_aquatic_bird_Korea_w114_2006_Avian
GU361200_A_aquatic_bird_Korea_w120_2006_Avian
GU361202_A_aquatic_bird_Korea_w125_2006_Avian
branch a
FJ461592_A_swine_Korea_C12_2008_Swine
GU361198_A_aquatic_bird_Korea_w113_2006_Avian
AB558469_A_duck_Tsukuba_536_2006_Avian
AB558470_A_duck_Tsukuba_541_2006_Avian
AB490826_A_northern_pintail_Akita_714_2006_Avian
EF634332_A_garganey_SanJiang_160_2006_Avian
AB558471_A_duck_Shimane_19_2006_Avian
AB558472_A_duck_Shimane_02_2007_Avian
branch b
FJ461593_A_swine_Korea_C13_2008_Swine
GU361201_A_aquatic_bird_Korea_w121_2006_Avian

HA-Group72

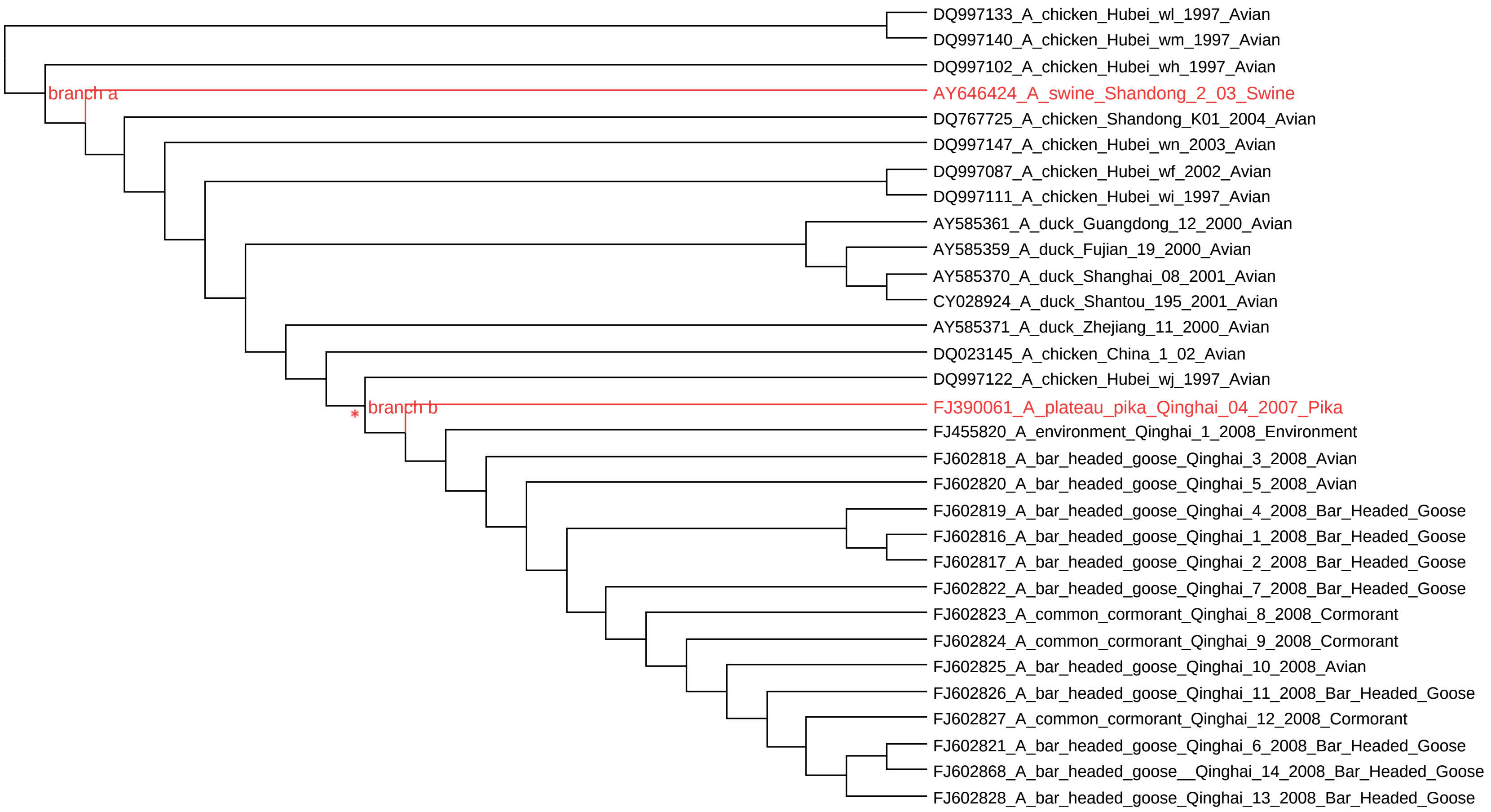
DQ997133_A_chicken_Hubei_wl_1997_Avian
DQ997140_A_chicken_Hubei_wm_1997_Avian
DQ997102_A_chicken_Hubei_wh_1997_Avian
branch a
AY646424_A_swine_Shandong_2_03_Swine
DQ767725_A_chicken_Shandong_K01_2004_Avian
DQ997147_A_chicken_Hubei_wn_2003_Avian
DQ997087_A_chicken_Hubei_wf_2002_Avian
DQ997111_A_chicken_Hubei_wi_1997_Avian
AY585361_A_duck_Guangdong_12_2000_Avian
AY585359_A_duck_Fujian_19_2000_Avian
AY585370_A_duck_Shanghai_08_2001_Avian
CY028924_A_duck_Shantou_195_2001_Avian
AY585371_A_duck_Zhejiang_11_2000_Avian
DQ023145_A_chicken_China_1_02_Avian
DQ997122_A_chicken_Hubei_wj_1997_Avian
*
branch b
FJ390061_A_plateau_pika_Qinghai_04_2007_Pika
FJ455820_A_environment_Qinghai_1_2008_Environment
FJ602818_A_bar_headed_goose_Qinghai_3_2008_Avian
FJ602820_A_bar_headed_goose_Qinghai_5_2008_Avian
FJ602819_A_bar_headed_goose_Qinghai_4_2008_Bar_Headed_Goose
FJ602816_A_bar_headed_goose_Qinghai_1_2008_Bar_Headed_Goose
FJ602817_A_bar_headed_goose_Qinghai_2_2008_Bar_Headed_Goose
FJ602822_A_bar_headed_goose_Qinghai_7_2008_Bar_Headed_Goose
FJ602823_A_common_cormorant_Qinghai_8_2008_Cormorant
FJ602824_A_common_cormorant_Qinghai_9_2008_Cormorant
FJ602825_A_bar_headed_goose_Qinghai_10_2008_Avian
FJ602826_A_bar_headed_goose_Qinghai_11_2008_Bar_Headed_Goose
FJ602827_A_common_cormorant_Qinghai_12_2008_Cormorant
FJ602821_A_bar_headed_goose_Qinghai_6_2008_Bar_Headed_Goose
FJ602868_A_bar_headed_goose__Qinghai_14_2008_Bar_Headed_Goose
FJ602828_A_bar_headed_goose_Qinghai_13_2008_Bar_Headed_Goose

HA-Group73

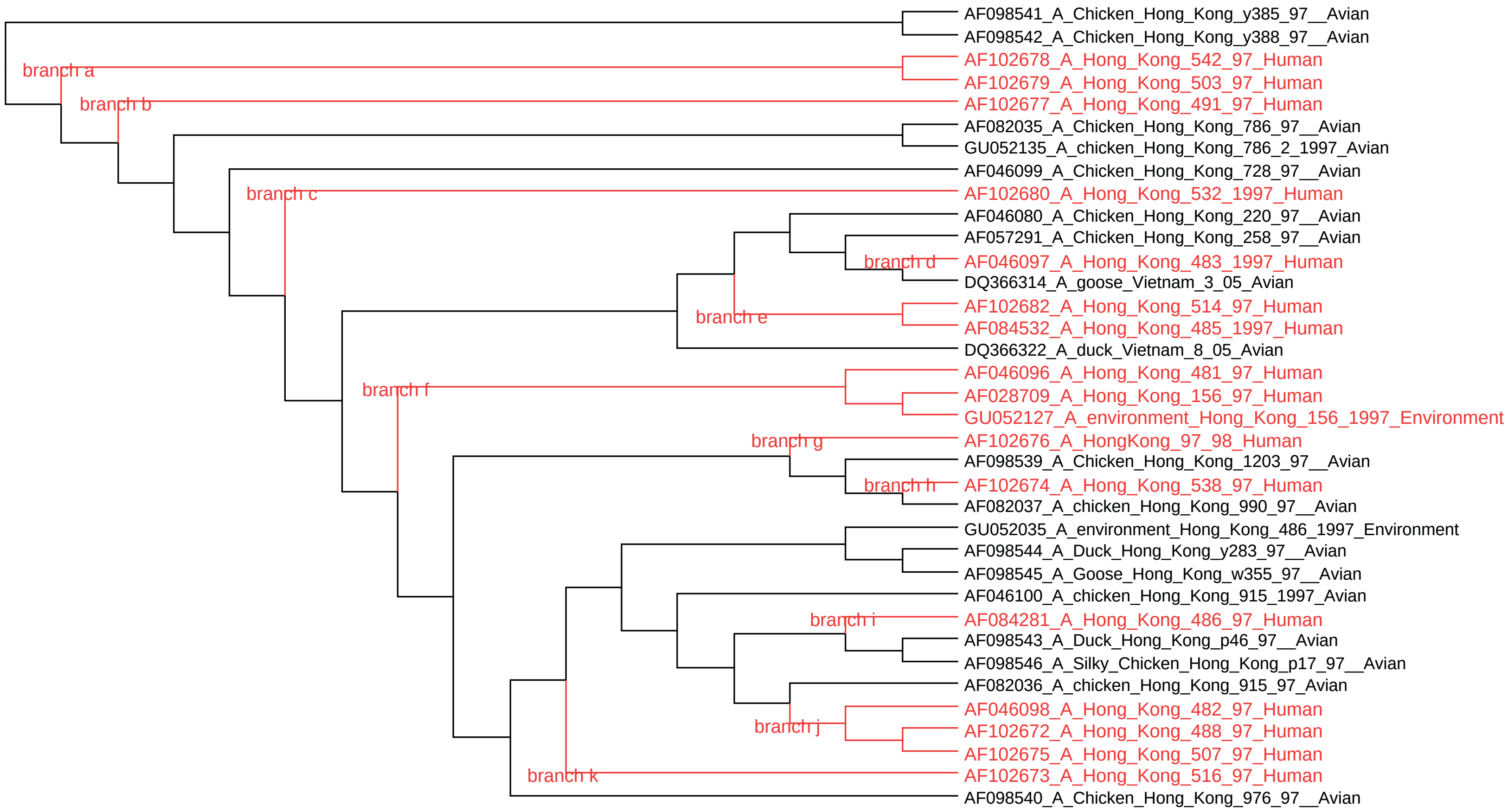

AF098541_A_Chicken_Hong_Kong_y385_97__Avian
AF098542_A_Chicken_Hong_Kong_y388_97__Avian
branch a
AF102678_A_Hong_Kong_542_97_Human
AF102679_A_Hong_Kong_503_97_Human
branch b
AF102677_A_Hong_Kong_491_97_Human
AF082035_A_Chicken_Hong_Kong_786_97__Avian
GU052135_A_chicken_Hong_Kong_786_2_1997_Avian
AF046099_A_Chicken_Hong_Kong_728_97__Avian
branch c
AF102680_A_Hong_Kong_532_1997_Human
AF046080_A_Chicken_Hong_Kong_220_97__Avian
AF057291_A_Chicken_Hong_Kong_258_97__Avian
branch d
AF046097_A_Hong_Kong_483_1997_Human
DQ366314_A_goose_Vietnam_3_05_Avian
branch e
AF102682_A_Hong_Kong_514_97_Human
AF084532_A_Hong_Kong_485_1997_Human
DQ366322_A_duck_Vietnam_8_05_Avian
branch f
AF046096_A_Hong_Kong_481_97_Human
AF028709_A_Hong_Kong_156_97_Human
GU052127_A_environment_Hong_Kong_156_1997_Environment
branch g
AF102676_A_HongKong_97_98_Human
AF098539_A_Chicken_Hong_Kong_1203_97__Avian
branch h
AF102674_A_Hong_Kong_538_97_Human
AF082037_A_chicken_Hong_Kong_990_97__Avian
GU052035_A_environment_Hong_Kong_486_1997_Environment
AF098544_A_Duck_Hong_Kong_y283_97__Avian
AF098545_A_Goose_Hong_Kong_w355_97__Avian
AF046100_A_chicken_Hong_Kong_915_1997_Avian
branch i
AF084281_A_Hong_Kong_486_97_Human
AF098543_A_Duck_Hong_Kong_p46_97__Avian
AF098546_A_Silky_Chicken_Hong_Kong_p17_97__Avian
AF082036_A_chicken_Hong_Kong_915_97_Avian
branch j
AF046098_A_Hong_Kong_482_97_Human
AF102672_A_Hong_Kong_488_97_Human
AF102675_A_Hong_Kong_507_97_Human
branch k
AF102673_A_Hong_Kong_516_97_Human
AF098540_A_Chicken_Hong_Kong_976_97__Avian

HA-Group74

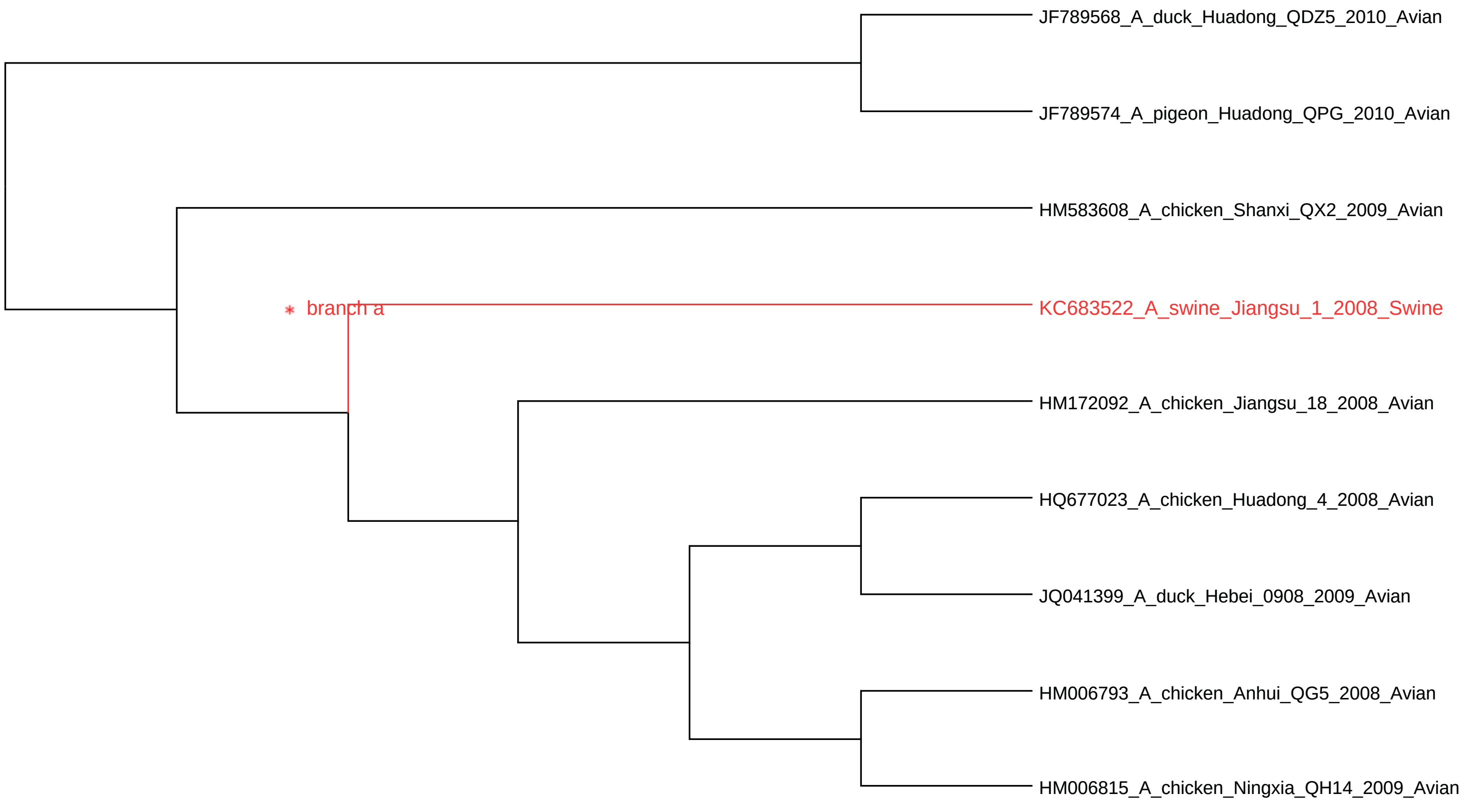
JF789568_A_duck_Huadong_QDZ5_2010_Avian
JF789574_A_pigeon_Huadong_QPG_2010_Avian
HM583608_A_chicken_Shanxi_QX2_2009_Avian
* branch a
KC683522_A_swine_Jiangsu_1_2008_Swine
HM172092_A_chicken_Jiangsu_18_2008_Avian
HQ677023_A_chicken_Huadong_4_2008_Avian
JQ041399_A_duck_Hebei_0908_2009_Avian
HM006793_A_chicken_Anhui_QG5_2008_Avian
HM006815_A_chicken_Ningxia_QH14_2009_Avian

HA-Group75

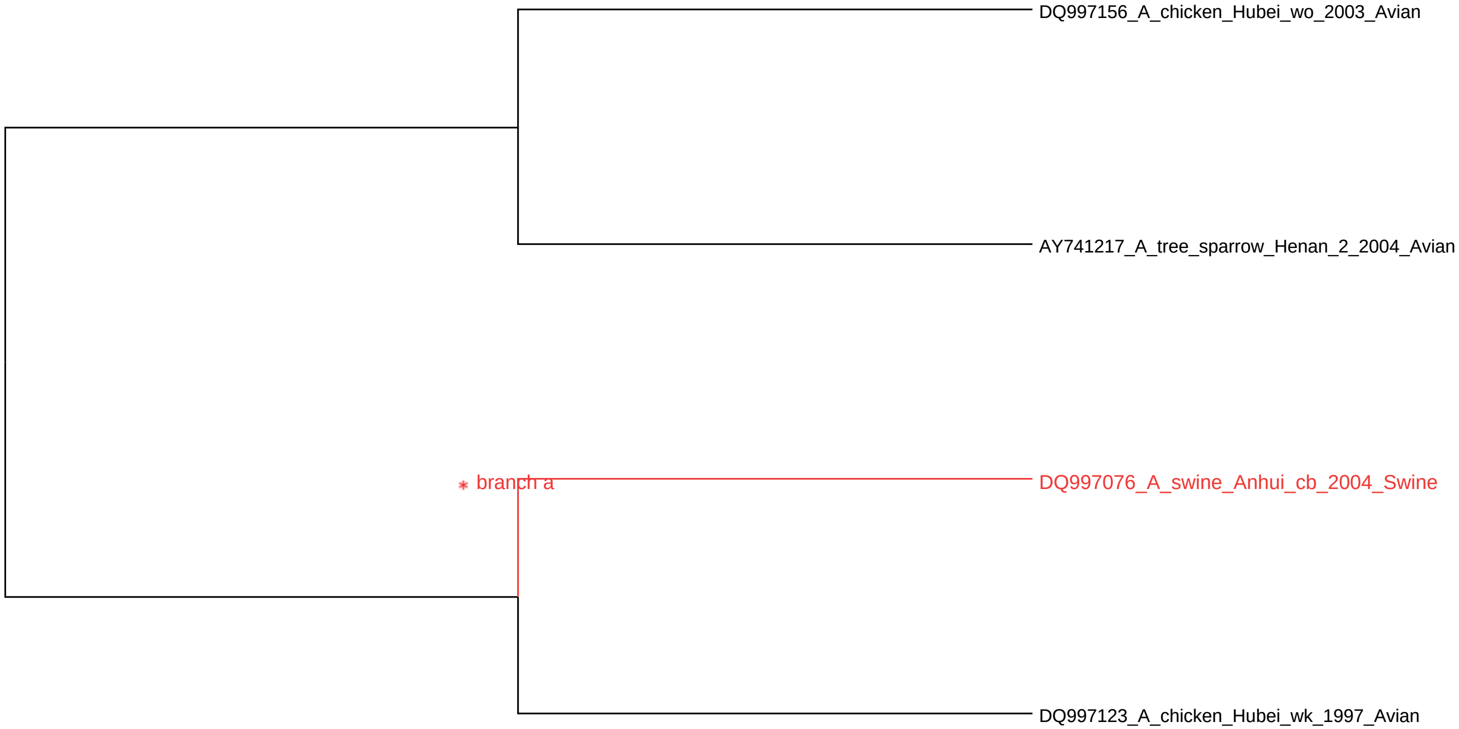
DQ997156_A_chicken_Hubei_wo_2003_Avian
AY741217_A_tree_sparrow_Henan_2_2004_Avian
* branch a
DQ997076_A_swine_Anhui_cb_2004_Swine
DQ997123_A_chicken_Hubei_wk_1997_Avian

HA-Group76

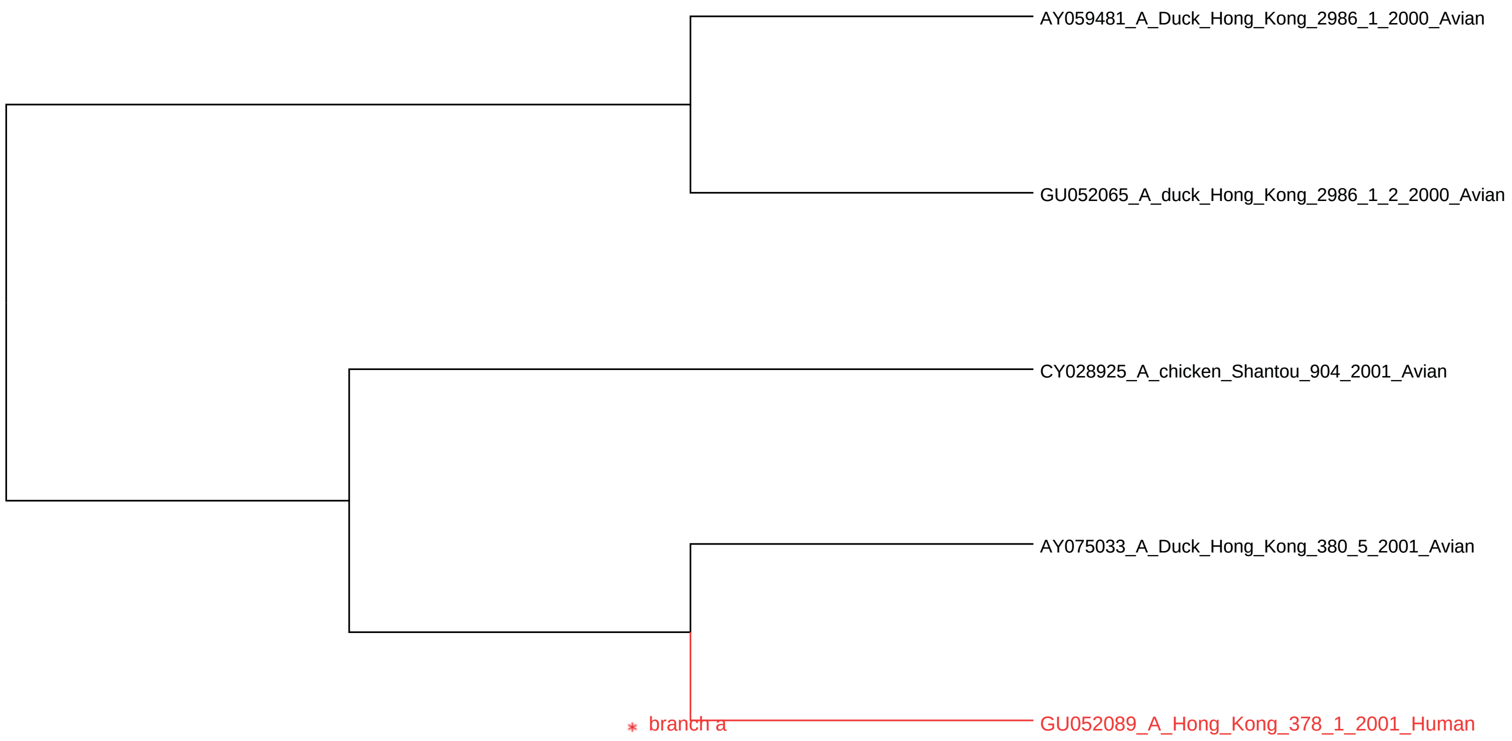
AY059481_A_Duck_Hong_Kong_2986_1_2000_Avian
GU052065_A_duck_Hong_Kong_2986_1_2_2000_Avian
CY028925_A_chicken_Shantou_904_2001_Avian
AY075033_A_Duck_Hong_Kong_380_5_2001_Avian
* branch a
GU052089_A_Hong_Kong_378_1_2001_Human

HA-Group77

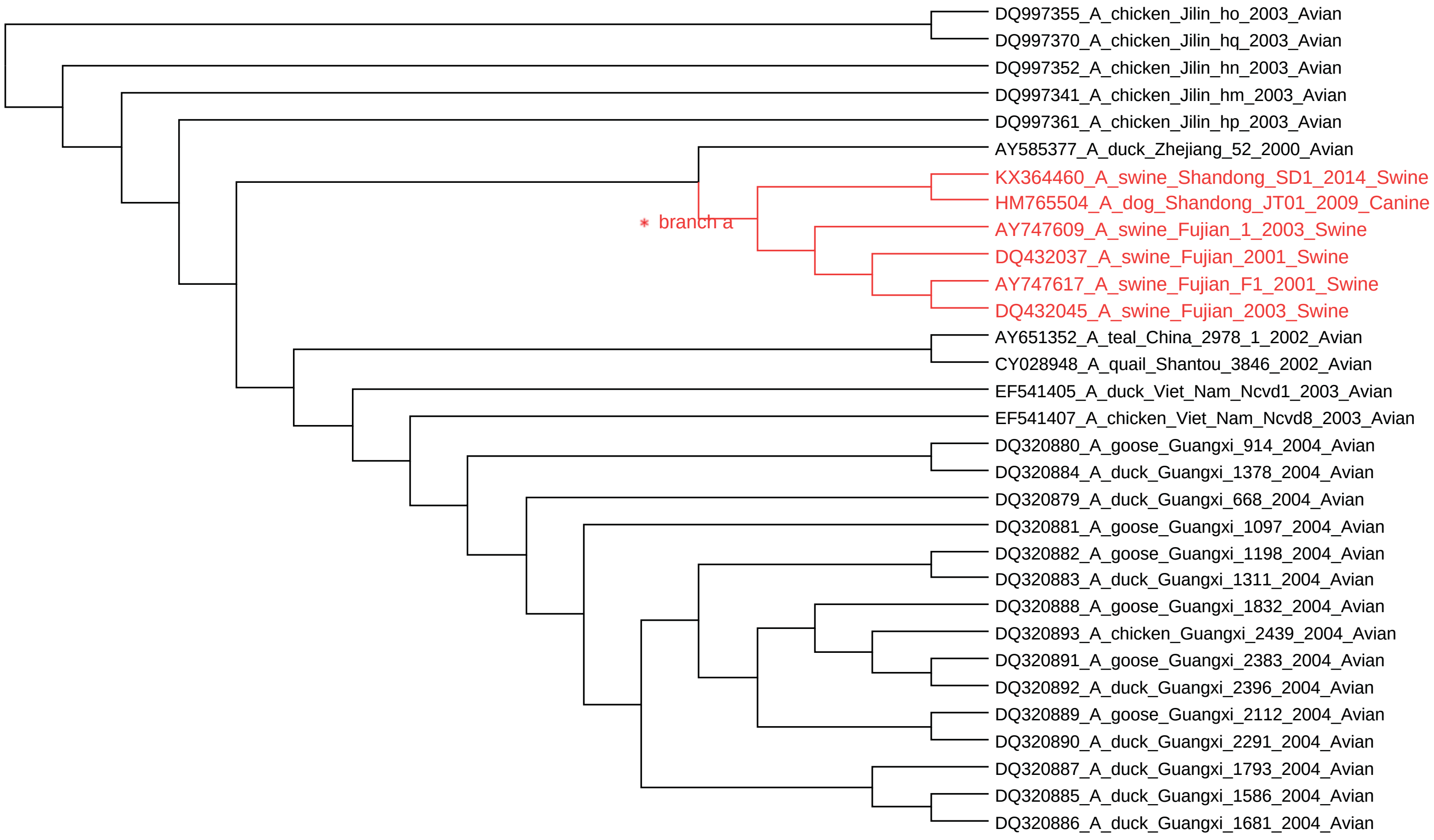

DQ997355_A_chicken_Jilin_ho_2003_Avian
DQ997370_A_chicken_Jilin_hq_2003_Avian
DQ997352_A_chicken_Jilin_hn_2003_Avian
DQ997341_A_chicken_Jilin_hm_2003_Avian
DQ997361_A_chicken_Jilin_hp_2003_Avian
AY585377_A_duck_Zhejiang_52_2000_Avian
KX364460_A_swine_Shandong_SD1_2014_Swine
HM765504_A_dog_Shandong_JT01_2009_Canine
* branch a
AY747609_A_swine_Fujian_1_2003_Swine
DQ432037_A_swine_Fujian_2001_Swine
AY747617_A_swine_Fujian_F1_2001_Swine
DQ432045_A_swine_Fujian_2003_Swine
AY651352_A_teal_China_2978_1_2002_Avian
CY028948_A_quail_Shantou_3846_2002_Avian
EF541405_A_duck_Viet_Nam_Ncvd1_2003_Avian
EF541407_A_chicken_Viet_Nam_Ncvd8_2003_Avian
DQ320880_A_goose_Guangxi_914_2004_Avian
DQ320884_A_duck_Guangxi_1378_2004_Avian
DQ320879_A_duck_Guangxi_668_2004_Avian
DQ320881_A_goose_Guangxi_1097_2004_Avian
DQ320882_A_goose_Guangxi_1198_2004_Avian
DQ320883_A_duck_Guangxi_1311_2004_Avian
DQ320888_A_goose_Guangxi_1832_2004_Avian
DQ320893_A_chicken_Guangxi_2439_2004_Avian
DQ320891_A_goose_Guangxi_2383_2004_Avian
DQ320892_A_duck_Guangxi_2396_2004_Avian
DQ320889_A_goose_Guangxi_2112_2004_Avian
DQ320890_A_duck_Guangxi_2291_2004_Avian
DQ320887_A_duck_Guangxi_1793_2004_Avian
DQ320885_A_duck_Guangxi_1586_2004_Avian
DQ320886_A_duck_Guangxi_1681_2004_Avian

HA-Group78

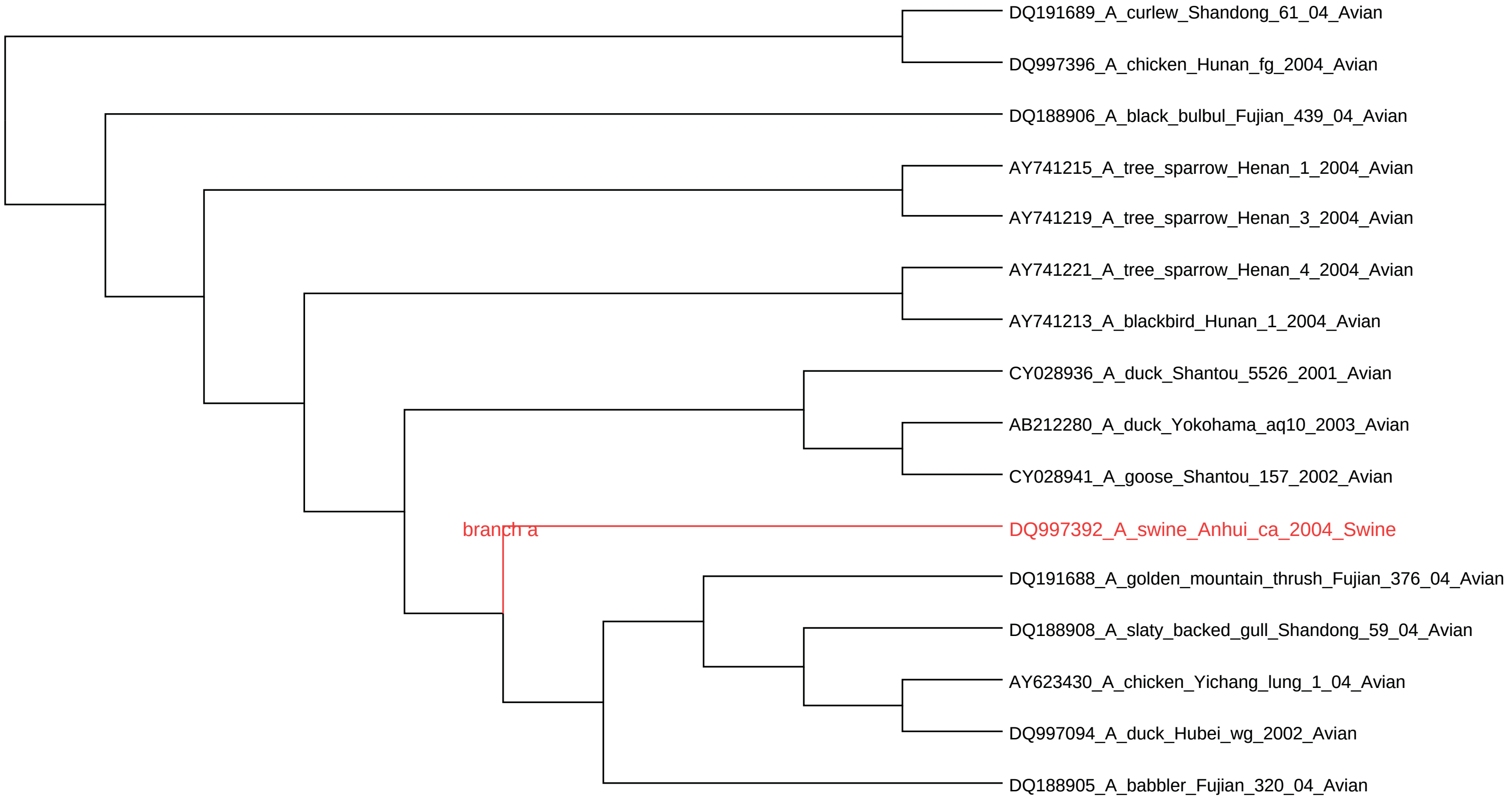

DQ191689_A_curlew_Shandong_61_04_Avian
DQ997396_A_chicken_Hunan_fg_2004_Avian
DQ188906_A_black_bulbul_Fujian_439_04_Avian
AY741215_A_tree_sparrow_Henan_1_2004_Avian
AY741219_A_tree_sparrow_Henan_3_2004_Avian
AY741221_A_tree_sparrow_Henan_4_2004_Avian
AY741213_A_blackbird_Hunan_1_2004_Avian
CY028936_A_duck_Shantou_5526_2001_Avian
AB212280_A_duck_Yokohama_aq10_2003_Avian
CY028941_A_goose_Shantou_157_2002_Avian
branch a
DQ997392_A_swine_Anhui_ca_2004_Swine
DQ191688_A_golden_mountain_thrush_Fujian_376_04_Avian
DQ188908_A_slaty_backed_gull_Shandong_59_04_Avian
AY623430_A_chicken_Yichang_lung_1_04_Avian
DQ997094_A_duck_Hubei_wg_2002_Avian
DQ188905_A_babbler_Fujian_320_04_Avian

HA-Group79

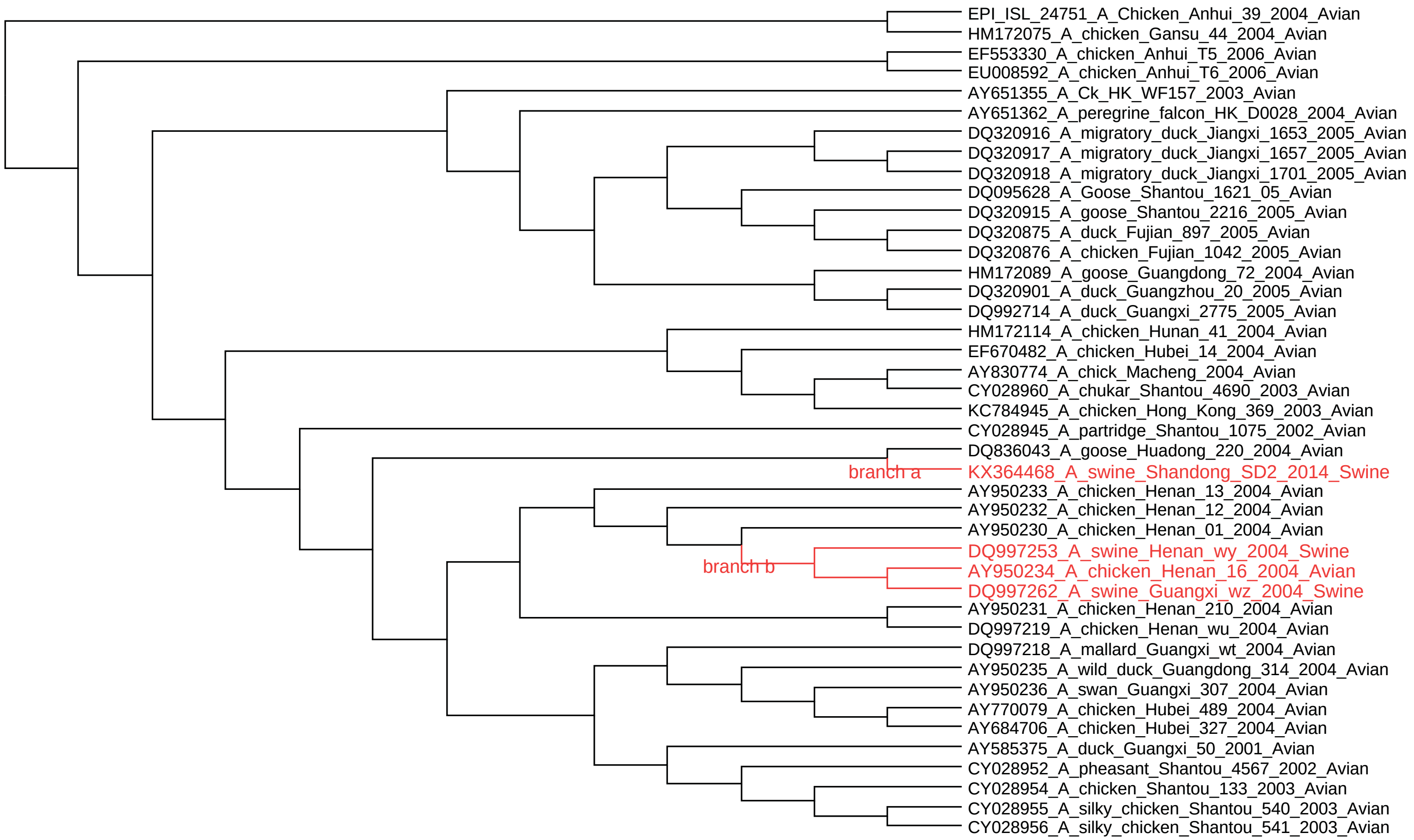

EPI_ISL_24751_A_Chicken_Anhui_39_2004_Avian
HM172075_A_chicken_Gansu_44_2004_Avian
EF553330_A_chicken_Anhui_T5_2006_Avian
EU008592_A_chicken_Anhui_T6_2006_Avian
AY651355_A_Ck_HK_WF157_2003_Avian
AY651362_A_peregrine_falcon_HK_D0028_2004_Avian
DQ320916_A_migratory_duck_Jiangxi_1653_2005_Avian
DQ320917_A_migratory_duck_Jiangxi_1657_2005_Avian
DQ320918_A_migratory_duck_Jiangxi_1701_2005_Avian
DQ095628_A_Goose_Shantou_1621_05_Avian
DQ320915_A_goose_Shantou_2216_2005_Avian
DQ320875_A_duck_Fujian_897_2005_Avian
DQ320876_A_chicken_Fujian_1042_2005_Avian
HM172089_A_goose_Guangdong_72_2004_Avian
DQ320901_A_duck_Guangzhou_20_2005_Avian
DQ992714_A_duck_Guangxi_2775_2005_Avian
HM172114_A_chicken_Hunan_41_2004_Avian
EF670482_A_chicken_Hubei_14_2004_Avian
AY830774_A_chick_Macheng_2004_Avian
CY028960_A_chukar_Shantou_4690_2003_Avian
KC784945_A_chicken_Hong_Kong_369_2003_Avian
CY028945_A_partridge_Shantou_1075_2002_Avian
DQ836043_A_goose_Huadong_220_2004_Avian
branch a
KX364468_A_swine_Shandong_SD2_2014_Swine
AY950233_A_chicken_Henan_13_2004_Avian
AY950232_A_chicken_Henan_12_2004_Avian
AY950230_A_chicken_Henan_01_2004_Avian
DQ997253_A_swine_Henan_wy_2004_Swine
branch b
AY950234_A_chicken_Henan_16_2004_Avian
DQ997262_A_swine_Guangxi_wz_2004_Swine
AY950231_A_chicken_Henan_210_2004_Avian
DQ997219_A_chicken_Henan_wu_2004_Avian
DQ997218_A_mallard_Guangxi_wt_2004_Avian
AY950235_A_wild_duck_Guangdong_314_2004_Avian
AY950236_A_swan_Guangxi_307_2004_Avian
AY770079_A_chicken_Hubei_489_2004_Avian
AY684706_A_chicken_Hubei_327_2004_Avian
AY585375_A_duck_Guangxi_50_2001_Avian
CY028952_A_pheasant_Shantou_4567_2002_Avian
CY028954_A_chicken_Shantou_133_2003_Avian
CY028955_A_silky_chicken_Shantou_540_2003_Avian
CY028956_A_silky_chicken_Shantou_541_2003_Avian

HA-Group80

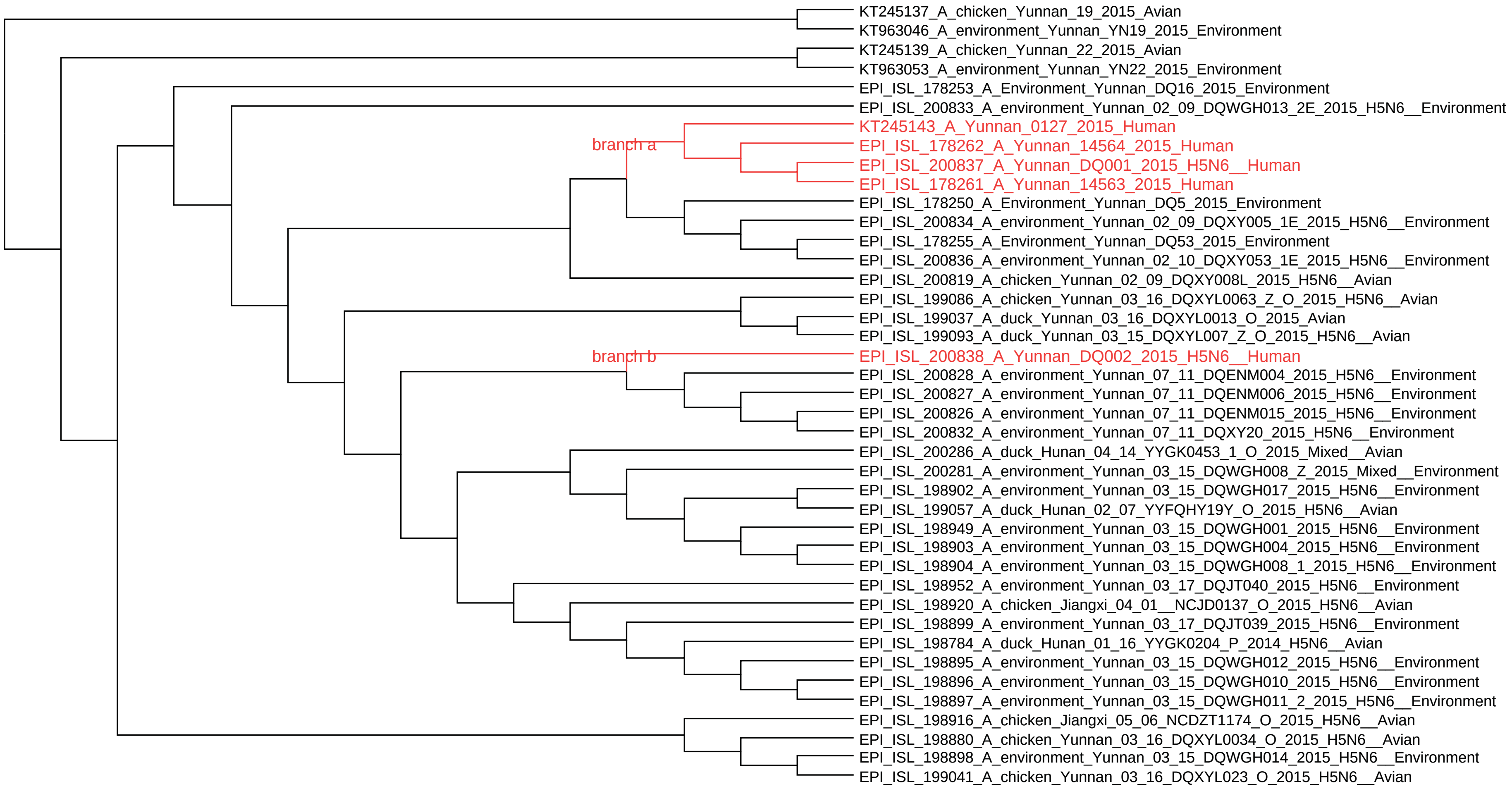

KT245137_A_chicken_Yunnan_19_2015_Avian
KT963046_A_environment_Yunnan_YN19_2015_Environment
KT245139_A_chicken_Yunnan_22_2015_Avian
KT963053_A_environment_Yunnan_YN22_2015_Environment
EPI_ISL_178253_A_Environment_Yunnan_DQ16_2015_Environment
EPI_ISL_200833_A_environment_Yunnan_02_09_DQWGH013_2E_2015_H5N6__Environment
branch a
KT245143_A_Yunnan_0127_2015_Human
EPI_ISL_178262_A_Yunnan_14564_2015_Human
EPI_ISL_200837_A_Yunnan_DQ001_2015_H5N6__Human
EPI_ISL_178261_A_Yunnan_14563_2015_Human
EPI_ISL_178250_A_Environment_Yunnan_DQ5_2015_Environment
EPI_ISL_200834_A_environment_Yunnan_02_09_DQXY005_1E_2015_H5N6__Environment
EPI_ISL_178255_A_Environment_Yunnan_DQ53_2015_Environment
EPI_ISL_200836_A_environment_Yunnan_02_10_DQXY053_1E_2015_H5N6__Environment
EPI_ISL_200819_A_chicken_Yunnan_02_09_DQXY008L_2015_H5N6__Avian
EPI_ISL_199086_A_chicken_Yunnan_03_16_DQXYL0063_Z_O_2015_H5N6__Avian
EPI_ISL_199037_A_duck_Yunnan_03_16_DQXYL0013_O_2015_Avian
EPI_ISL_199093_A_duck_Yunnan_03_15_DQXYL007_Z_O_2015_H5N6__Avian
branch b
EPI_ISL_200838_A_Yunnan_DQ002_2015_H5N6__Human
EPI_ISL_200828_A_environment_Yunnan_07_11_DQENM004_2015_H5N6__Environment
EPI_ISL_200827_A_environment_Yunnan_07_11_DQENM006_2015_H5N6__Environment
EPI_ISL_200826_A_environment_Yunnan_07_11_DQENM015_2015_H5N6__Environment
EPI_ISL_200832_A_environment_Yunnan_07_11_DQXY20_2015_H5N6__Environment
EPI_ISL_200286_A_duck_Hunan_04_14_YYGK0453_1_O_2015_Mixed__Avian
EPI_ISL_200281_A_environment_Yunnan_03_15_DQWGH008_Z_2015_Mixed__Environment
EPI_ISL_198902_A_environment_Yunnan_03_15_DQWGH017_2015_H5N6__Environment
EPI_ISL_199057_A_duck_Hunan_02_07_YYFQHY19Y_O_2015_H5N6__Avian
EPI_ISL_198949_A_environment_Yunnan_03_15_DQWGH001_2015_H5N6__Environment
EPI_ISL_198903_A_environment_Yunnan_03_15_DQWGH004_2015_H5N6__Environment
EPI_ISL_198904_A_environment_Yunnan_03_15_DQWGH008_1_2015_H5N6__Environment
EPI_ISL_198952_A_environment_Yunnan_03_17_DQJT040_2015_H5N6__Environment
EPI_ISL_198920_A_chicken_Jiangxi_04_01__NCJD0137_O_2015_H5N6__Avian
EPI_ISL_198899_A_environment_Yunnan_03_17_DQJT039_2015_H5N6__Environment
EPI_ISL_198784_A_duck_Hunan_01_16_YYGK0204_P_2014_H5N6__Avian
EPI_ISL_198895_A_environment_Yunnan_03_15_DQWGH012_2015_H5N6__Environment
EPI_ISL_198896_A_environment_Yunnan_03_15_DQWGH010_2015_H5N6__Environment
EPI_ISL_198897_A_environment_Yunnan_03_15_DQWGH011_2_2015_H5N6__Environment
EPI_ISL_198916_A_chicken_Jiangxi_05_06_NCDZT1174_O_2015_H5N6__Avian
EPI_ISL_198880_A_chicken_Yunnan_03_16_DQXYL0034_O_2015_H5N6__Avian
EPI_ISL_198898_A_environment_Yunnan_03_15_DQWGH014_2015_H5N6__Environment
EPI_ISL_199041_A_chicken_Yunnan_03_16_DQXYL023_O_2015_H5N6__Avian

HA-Group81

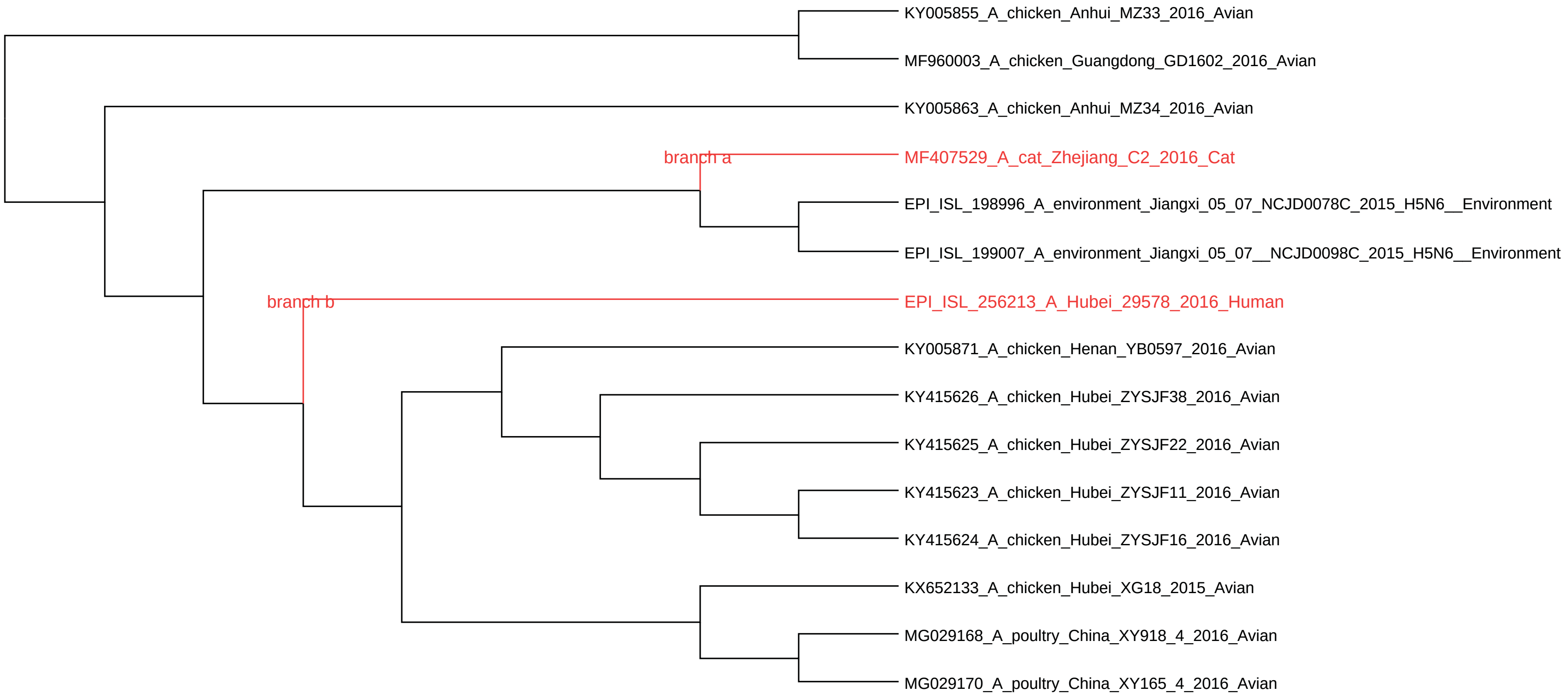

KY005855_A_chicken_Anhui_MZ33_2016_Avian
MF960003_A_chicken_Guangdong_GD1602_2016_Avian
KY005863_A_chicken_Anhui_MZ34_2016_Avian
branch a
MF407529_A_cat_Zhejiang_C2_2016_Cat
EPI_ISL_198996_A_environment_Jiangxi_05_07_NCJD0078C_2015_H5N6__Environment
EPI_ISL_199007_A_environment_Jiangxi_05_07__NCJD0098C_2015_H5N6__Environment
branch b
EPI_ISL_256213_A_Hubei_29578_2016_Human
KY005871_A_chicken_Henan_YB0597_2016_Avian
KY415626_A_chicken_Hubei_ZYSJF38_2016_Avian
KY415625_A_chicken_Hubei_ZYSJF22_2016_Avian
KY415623_A_chicken_Hubei_ZYSJF11_2016_Avian
KY415624_A_chicken_Hubei_ZYSJF16_2016_Avian
KX652133_A_chicken_Hubei_XG18_2015_Avian
MG029168_A_poultry_China_XY918_4_2016_Avian
MG029170_A_poultry_China_XY165_4_2016_Avian

HA-Group82

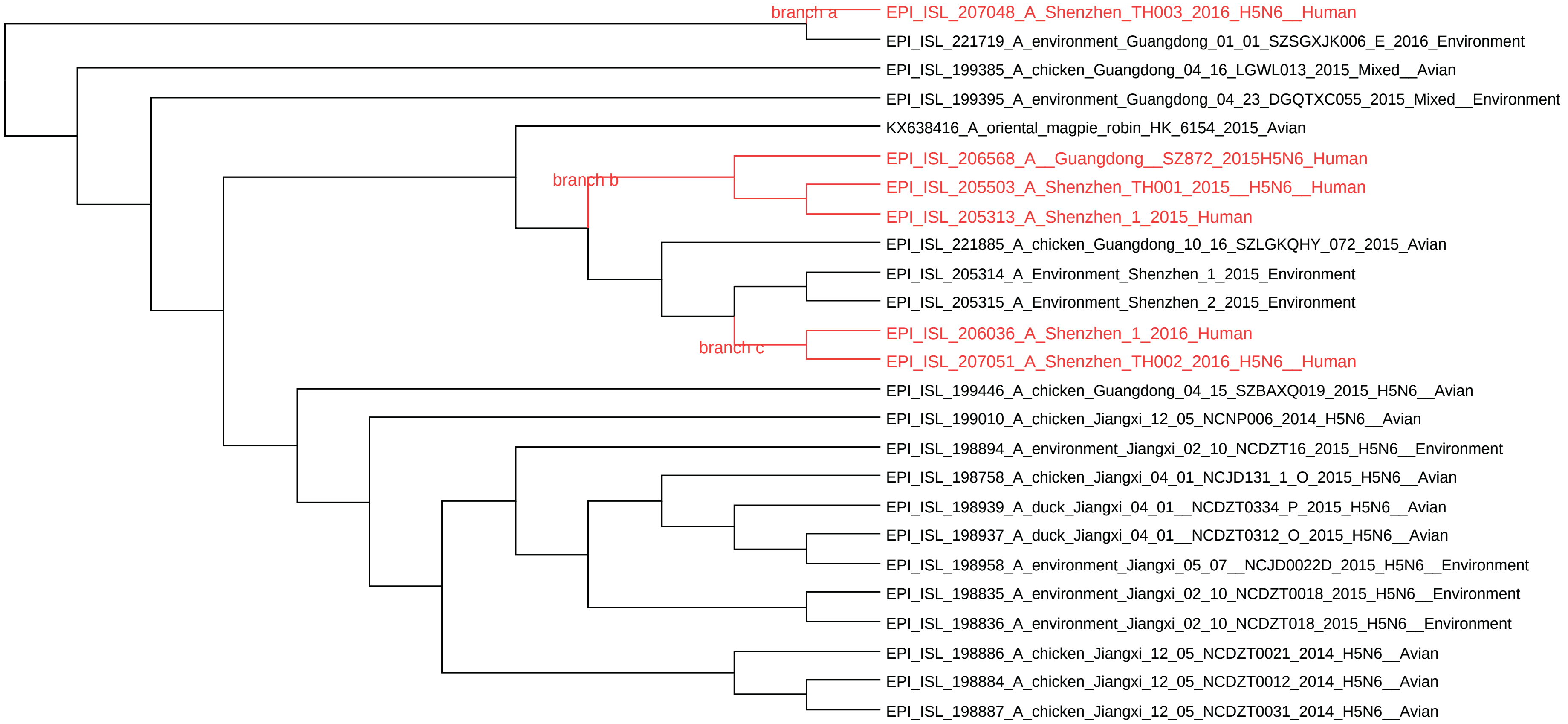
branch a
EPI_ISL_207048_A_Shenzhen_TH003_2016_H5N6__Human
EPI_ISL_221719_A_environment_Guangdong_01_01_SZSGXJK006_E_2016_Environment
EPI_ISL_199385_A_chicken_Guangdong_04_16_LGWL013_2015_Mixed__Avian
EPI_ISL_199395_A_environment_Guangdong_04_23_DGQTXC055_2015_Mixed__Environment
KX638416_A_oriental_magpie_robin_HK_6154_2015_Avian
EPI_ISL_206568_A__Guangdong__SZ872_2015H5N6_Human
branch b
EPI_ISL_205503_A_Shenzhen_TH001_2015__H5N6__Human
EPI_ISL_205313_A_Shenzhen_1_2015_Human
EPI_ISL_221885_A_chicken_Guangdong_10_16_SZLGKQHY_072_2015_Avian
EPI_ISL_205314_A_Environment_Shenzhen_1_2015_Environment
EPI_ISL_205315_A_Environment_Shenzhen_2_2015_Environment
EPI_ISL_206036_A_Shenzhen_1_2016_Human
branch c
EPI_ISL_207051_A_Shenzhen_TH002_2016_H5N6__Human
EPI_ISL_199446_A_chicken_Guangdong_04_15_SZBAXQ019_2015_H5N6__Avian
EPI_ISL_199010_A_chicken_Jiangxi_12_05_NCNP006_2014_H5N6__Avian
EPI_ISL_198894_A_environment_Jiangxi_02_10_NCDZT16_2015_H5N6__Environment
EPI_ISL_198758_A_chicken_Jiangxi_04_01_NCJD131_1_O_2015_H5N6__Avian
EPI_ISL_198939_A_duck_Jiangxi_04_01__NCDZT0334_P_2015_H5N6__Avian
EPI_ISL_198937_A_duck_Jiangxi_04_01__NCDZT0312_O_2015_H5N6__Avian
EPI_ISL_198958_A_environment_Jiangxi_05_07__NCJD0022D_2015_H5N6__Environment
EPI_ISL_198835_A_environment_Jiangxi_02_10_NCDZT0018_2015_H5N6__Environment
EPI_ISL_198836_A_environment_Jiangxi_02_10_NCDZT018_2015_H5N6__Environment
EPI_ISL_198886_A_chicken_Jiangxi_12_05_NCDZT0021_2014_H5N6__Avian
EPI_ISL_198884_A_chicken_Jiangxi_12_05_NCDZT0012_2014_H5N6__Avian
EPI_ISL_198887_A_chicken_Jiangxi_12_05_NCDZT0031_2014_H5N6__Avian

HA-Group83

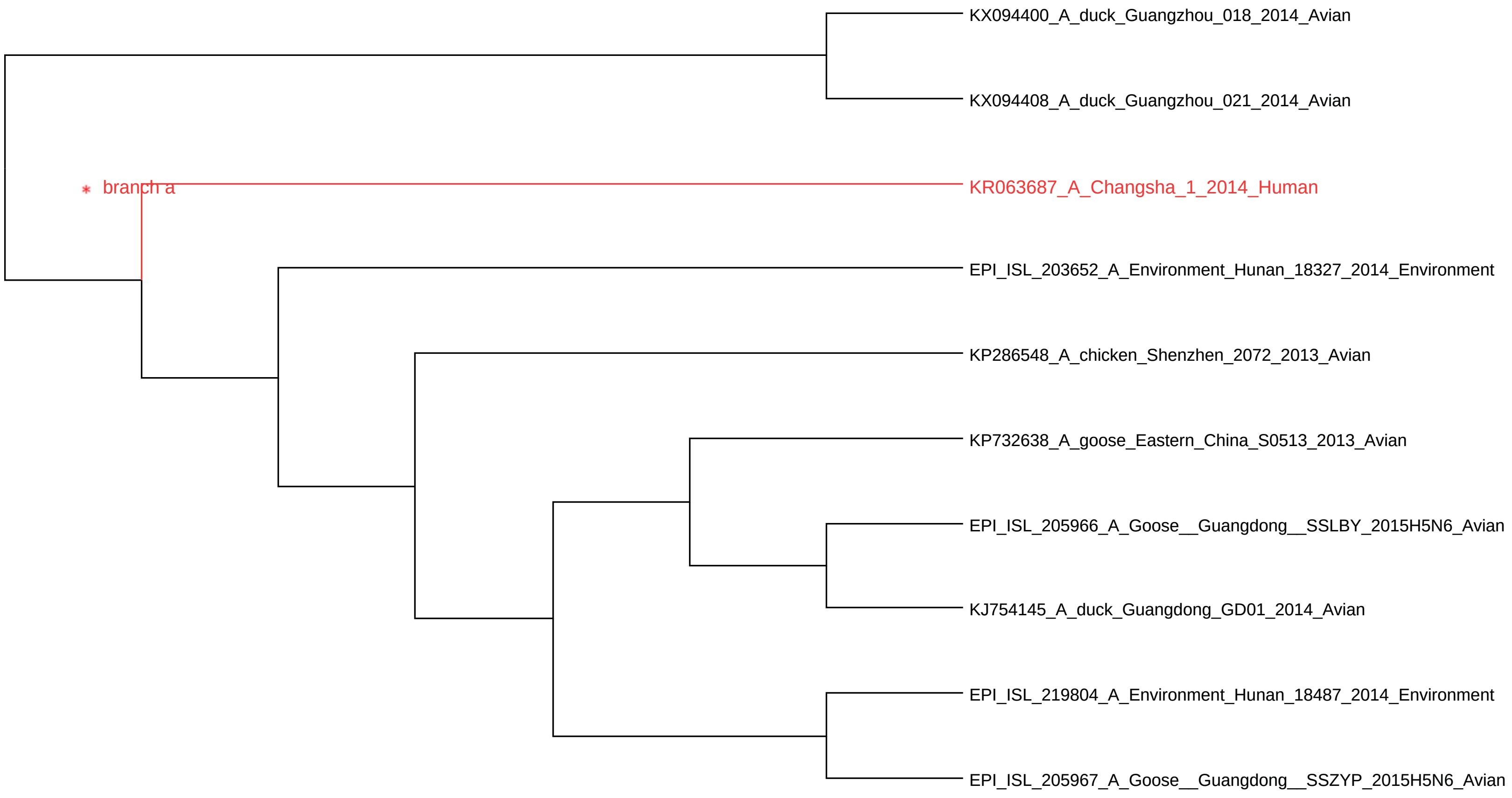

KX094400_A_duck_Guangzhou_018_2014_Avian
KX094408_A_duck_Guangzhou_021_2014_Avian
* branch a
KR063687_A_Changsha_1_2014_Human
EPI_ISL_203652_A_Environment_Hunan_18327_2014_Environment
KP286548_A_chicken_Shenzhen_2072_2013_Avian
KP732638_A_goose_Eastern_China_S0513_2013_Avian
EPI_ISL_205966_A_Goose__Guangdong__SSLBY_2015H5N6_Avian
KJ754145_A_duck_Guangdong_GD01_2014_Avian
EPI_ISL_219804_A_Environment_Hunan_18487_2014_Environment
EPI_ISL_205967_A_Goose__Guangdong__SSZYP_2015H5N6_Avian

HA-Group84

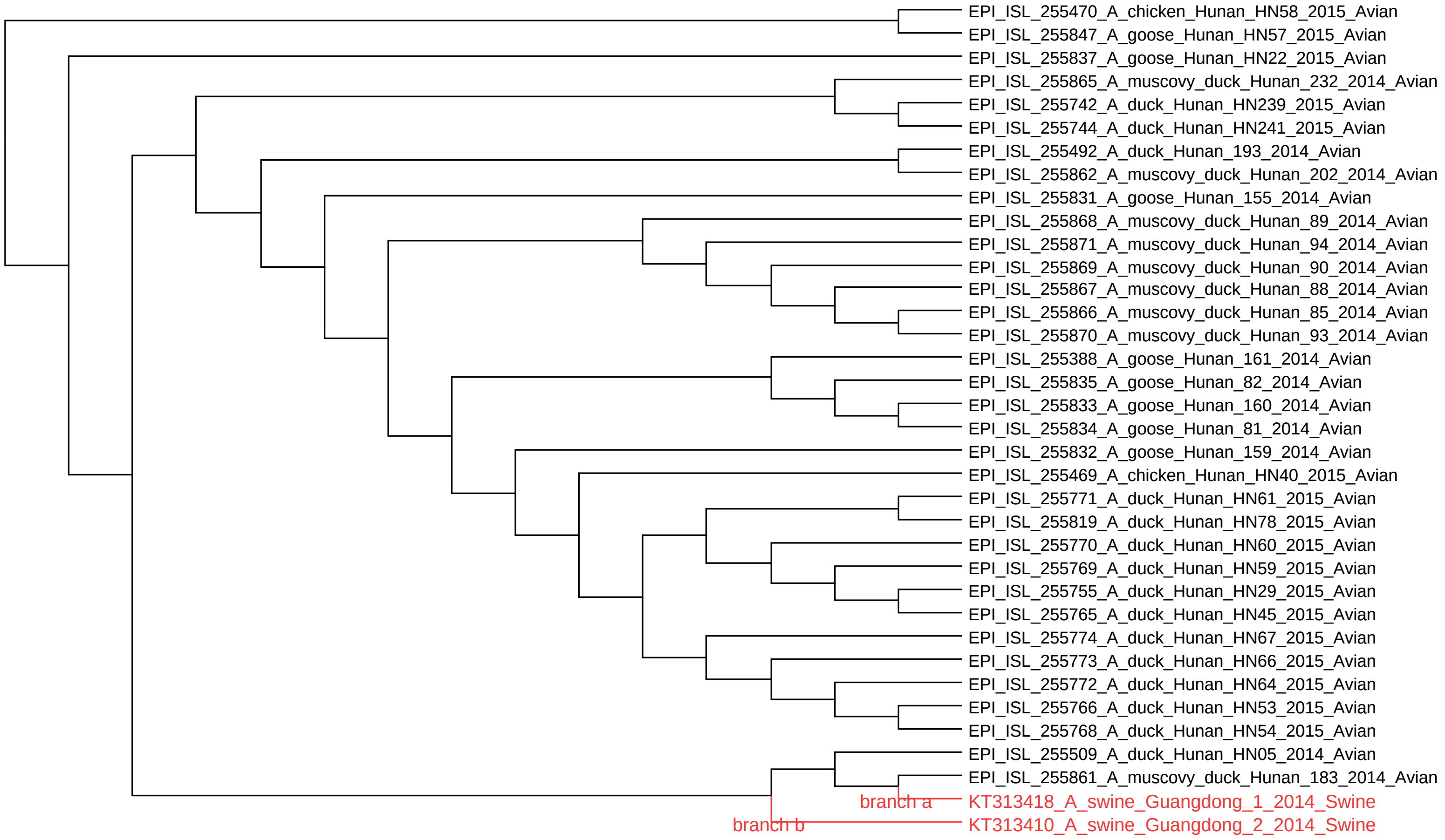
EPI_ISL_255470_A_chicken_Hunan_HN58_2015_Avian
EPI_ISL_255847_A_goose_Hunan_HN57_2015_Avian
EPI_ISL_255837_A_goose_Hunan_HN22_2015_Avian
EPI_ISL_255865_A_muscovy_duck_Hunan_232_2014_Avian
EPI_ISL_255742_A_duck_Hunan_HN239_2015_Avian
EPI_ISL_255744_A_duck_Hunan_HN241_2015_Avian
EPI_ISL_255492_A_duck_Hunan_193_2014_Avian
EPI_ISL_255862_A_muscovy_duck_Hunan_202_2014_Avian
EPI_ISL_255831_A_goose_Hunan_155_2014_Avian
EPI_ISL_255868_A_muscovy_duck_Hunan_89_2014_Avian
EPI_ISL_255871_A_muscovy_duck_Hunan_94_2014_Avian
EPI_ISL_255869_A_muscovy_duck_Hunan_90_2014_Avian
EPI_ISL_255867_A_muscovy_duck_Hunan_88_2014_Avian
EPI_ISL_255866_A_muscovy_duck_Hunan_85_2014_Avian
EPI_ISL_255870_A_muscovy_duck_Hunan_93_2014_Avian
EPI_ISL_255388_A_goose_Hunan_161_2014_Avian
EPI_ISL_255835_A_goose_Hunan_82_2014_Avian
EPI_ISL_255833_A_goose_Hunan_160_2014_Avian
EPI_ISL_255834_A_goose_Hunan_81_2014_Avian
EPI_ISL_255832_A_goose_Hunan_159_2014_Avian
EPI_ISL_255469_A_chicken_Hunan_HN40_2015_Avian
EPI_ISL_255771_A_duck_Hunan_HN61_2015_Avian
EPI_ISL_255819_A_duck_Hunan_HN78_2015_Avian
EPI_ISL_255770_A_duck_Hunan_HN60_2015_Avian
EPI_ISL_255769_A_duck_Hunan_HN59_2015_Avian
EPI_ISL_255755_A_duck_Hunan_HN29_2015_Avian
EPI_ISL_255765_A_duck_Hunan_HN45_2015_Avian
EPI_ISL_255774_A_duck_Hunan_HN67_2015_Avian
EPI_ISL_255773_A_duck_Hunan_HN66_2015_Avian
EPI_ISL_255772_A_duck_Hunan_HN64_2015_Avian
EPI_ISL_255766_A_duck_Hunan_HN53_2015_Avian
EPI_ISL_255768_A_duck_Hunan_HN54_2015_Avian
EPI_ISL_255509_A_duck_Hunan_HN05_2014_Avian
EPI_ISL_255861_A_muscovy_duck_Hunan_183_2014_Avian
branch a
KT313418_A_swine_Guangdong_1_2014_Swine
branch b
KT313410_A_swine_Guangdong_2_2014_Swine

HA-Group85

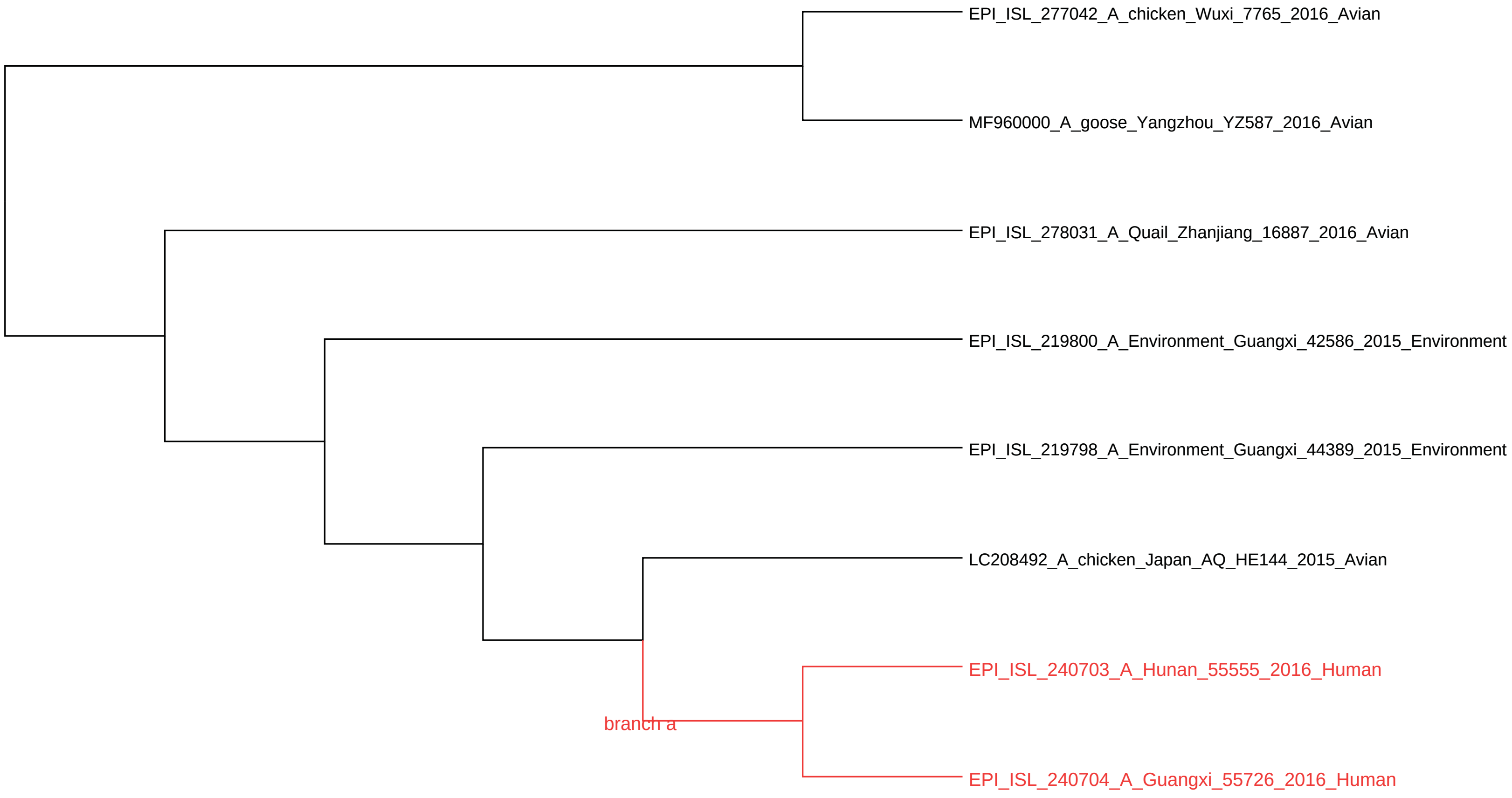
EPI_ISL_277042_A_chicken_Wuxi_7765_2016_Avian
MF960000_A_goose_Yangzhou_YZ587_2016_Avian
EPI_ISL_278031_A_Quail_Zhanjiang_16887_2016_Avian
EPI_ISL_219800_A_Environment_Guangxi_42586_2015_Environment
EPI_ISL_219798_A_Environment_Guangxi_44389_2015_Environment
LC208492_A_chicken_Japan_AQ_HE144_2015_Avian
EPI_ISL_240703_A_Hunan_55555_2016_Human
branch a
EPI_ISL_240704_A_Guangxi_55726_2016_Human

HA-Group86

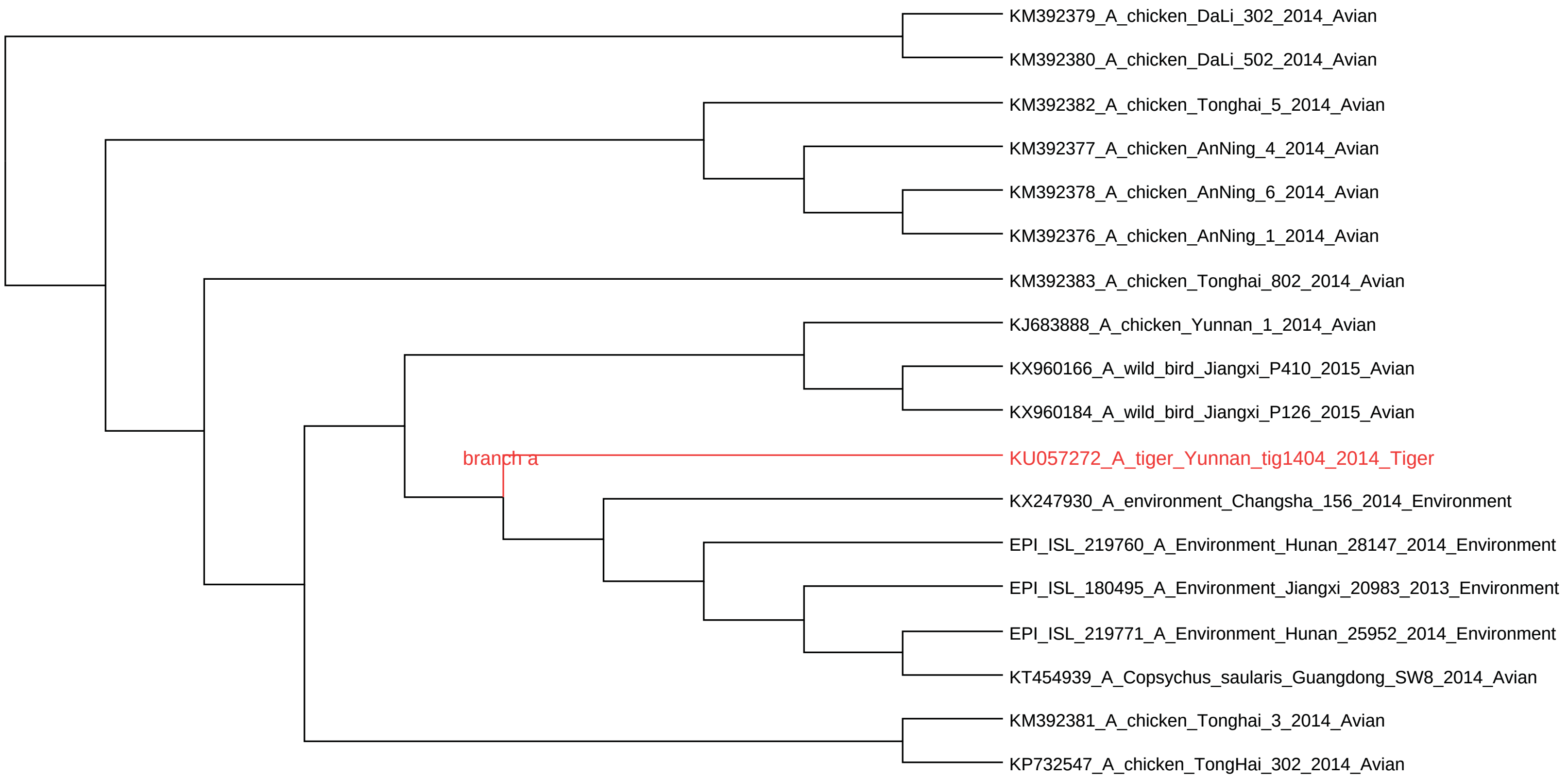
KM392379_A_chicken_DaLi_302_2014_Avian
KM392380_A_chicken_DaLi_502_2014_Avian
KM392382_A_chicken_Tonghai_5_2014_Avian
KM392377_A_chicken_AnNing_4_2014_Avian
KM392378_A_chicken_AnNing_6_2014_Avian
KM392376_A_chicken_AnNing_1_2014_Avian
KM392383_A_chicken_Tonghai_802_2014_Avian
KJ683888_A_chicken_Yunnan_1_2014_Avian
KX960166_A_wild_bird_Jiangxi_P410_2015_Avian
KX960184_A_wild_bird_Jiangxi_P126_2015_Avian
branch a
KU057272_A_tiger_Yunnan_tig1404_2014_Tiger
KX247930_A_environment_Changsha_156_2014_Environment
EPI_ISL_219760_A_Environment_Hunan_28147_2014_Environment
EPI_ISL_180495_A_Environment_Jiangxi_20983_2013_Environment
EPI_ISL_219771_A_Environment_Hunan_25952_2014_Environment
KT454939_A_Copsychus_saularis_Guangdong_SW8_2014_Avian
KM392381_A_chicken_Tonghai_3_2014_Avian
KP732547_A_chicken_TongHai_302_2014_Avian

HA-Group87

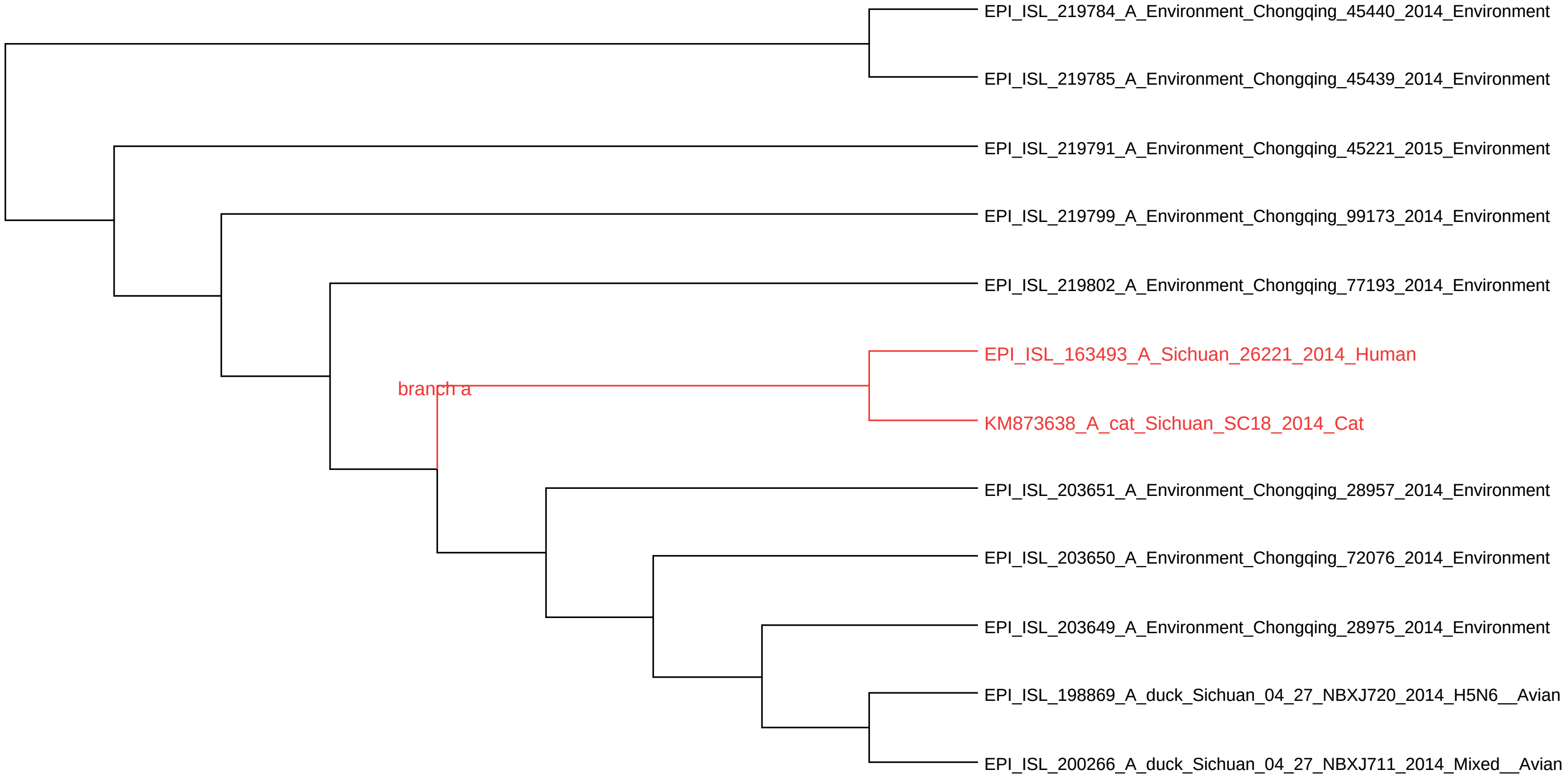

EPI_ISL_219784_A_Environment_Chongqing_45440_2014_Environment
EPI_ISL_219785_A_Environment_Chongqing_45439_2014_Environment
EPI_ISL_219791_A_Environment_Chongqing_45221_2015_Environment
EPI_ISL_219799_A_Environment_Chongqing_99173_2014_Environment
EPI_ISL_219802_A_Environment_Chongqing_77193_2014_Environment
branch a
EPI_ISL_163493_A_Sichuan_26221_2014_Human
KM873638_A_cat_Sichuan_SC18_2014_Cat
EPI_ISL_203651_A_Environment_Chongqing_28957_2014_Environment
EPI_ISL_203650_A_Environment_Chongqing_72076_2014_Environment
EPI_ISL_203649_A_Environment_Chongqing_28975_2014_Environment
EPI_ISL_198869_A_duck_Sichuan_04_27_NBXJ720_2014_H5N6__Avian
EPI_ISL_200266_A_duck_Sichuan_04_27_NBXJ711_2014_Mixed__Avian

HA-Group88

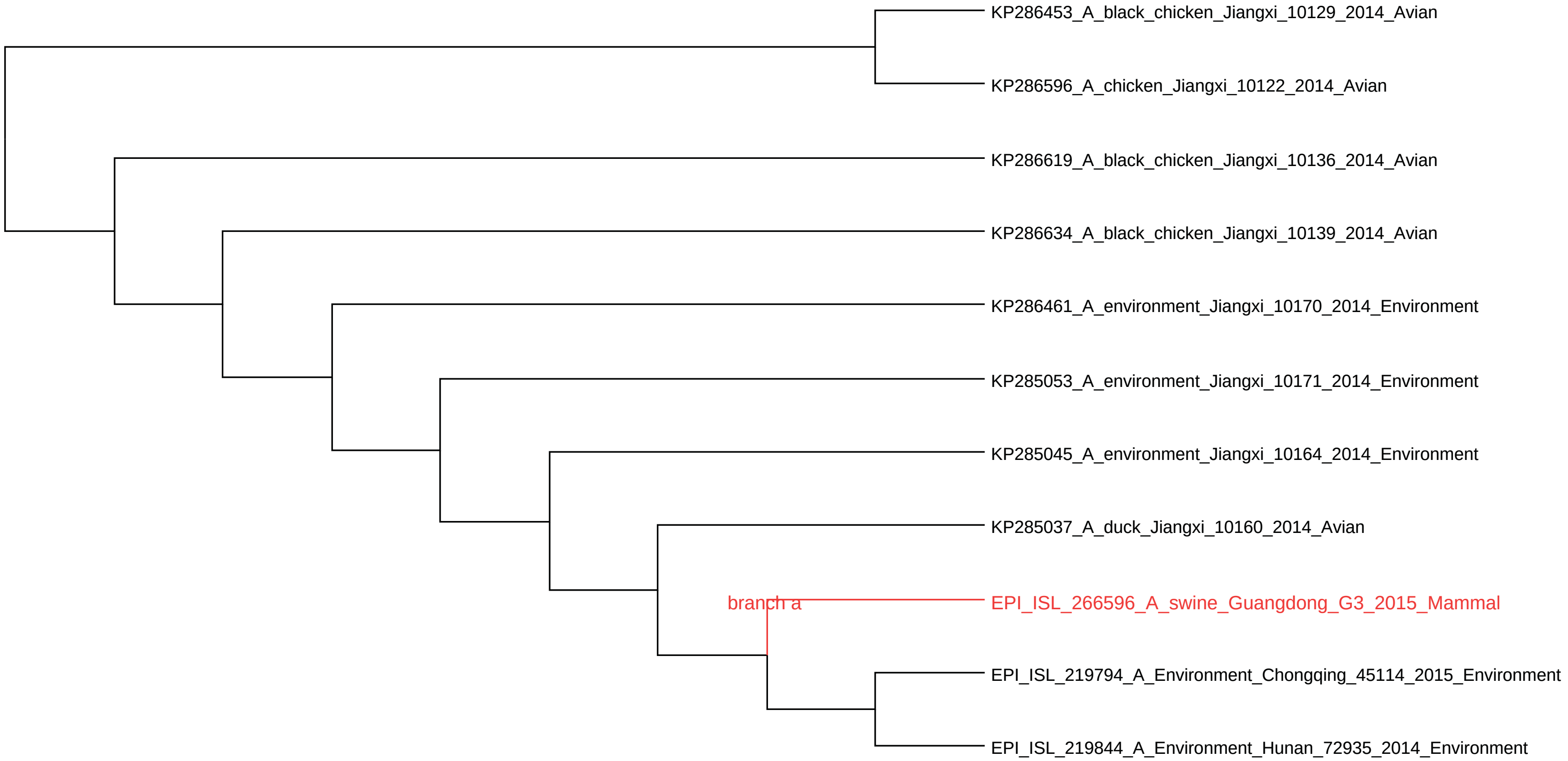

KP286453_A_black_chicken_Jiangxi_10129_2014_Avian
KP286596_A_chicken_Jiangxi_10122_2014_Avian
KP286619_A_black_chicken_Jiangxi_10136_2014_Avian
KP286634_A_black_chicken_Jiangxi_10139_2014_Avian
KP286461_A_environment_Jiangxi_10170_2014_Environment
KP285053_A_environment_Jiangxi_10171_2014_Environment
KP285045_A_environment_Jiangxi_10164_2014_Environment
KP285037_A_duck_Jiangxi_10160_2014_Avian
branch a
EPI_ISL_266596_A_swine_Guangdong_G3_2015_Mammal
EPI_ISL_219794_A_Environment_Chongqing_45114_2015_Environment
EPI_ISL_219844_A_Environment_Hunan_72935_2014_Environment

HA-Group89

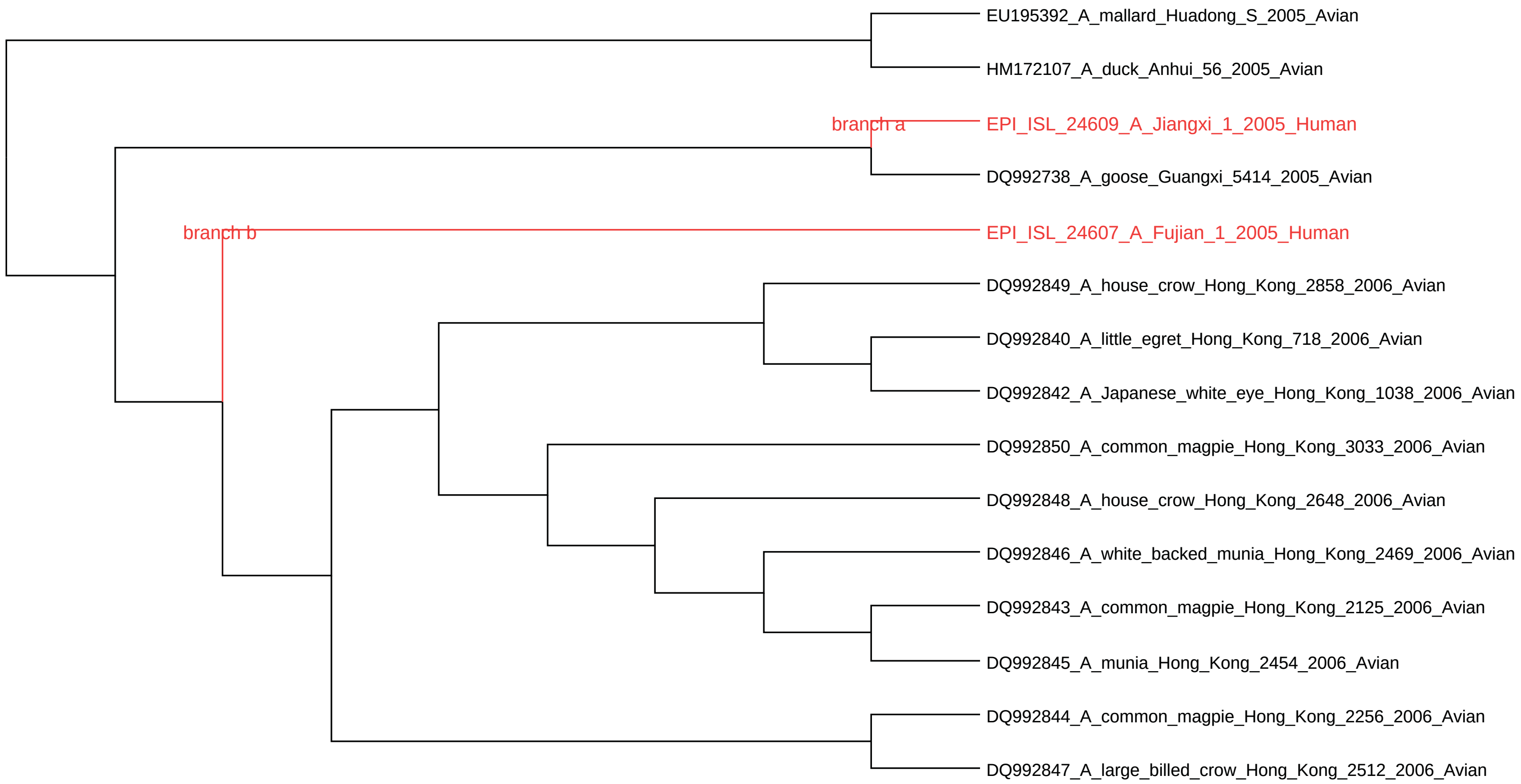

EU195392_A_mallard_Huadong_S_2005_Avian
HM172107_A_duck_Anhui_56_2005_Avian
branch a
EPI_ISL_24609_A_Jiangxi_1_2005_Human
DQ992738_A_goose_Guangxi_5414_2005_Avian
branch b
EPI_ISL_24607_A_Fujian_1_2005_Human
DQ992849_A_house_crow_Hong_Kong_2858_2006_Avian
DQ992840_A_little_egret_Hong_Kong_718_2006_Avian
DQ992842_A_Japanese_white_eye_Hong_Kong_1038_2006_Avian
DQ992850_A_common_magpie_Hong_Kong_3033_2006_Avian
DQ992848_A_house_crow_Hong_Kong_2648_2006_Avian
DQ992846_A_white_backed_munia_Hong_Kong_2469_2006_Avian
DQ992843_A_common_magpie_Hong_Kong_2125_2006_Avian
DQ992845_A_munia_Hong_Kong_2454_2006_Avian
DQ992844_A_common_magpie_Hong_Kong_2256_2006_Avian
DQ992847_A_large_billed_crow_Hong_Kong_2512_2006_Avian

HA-Group90

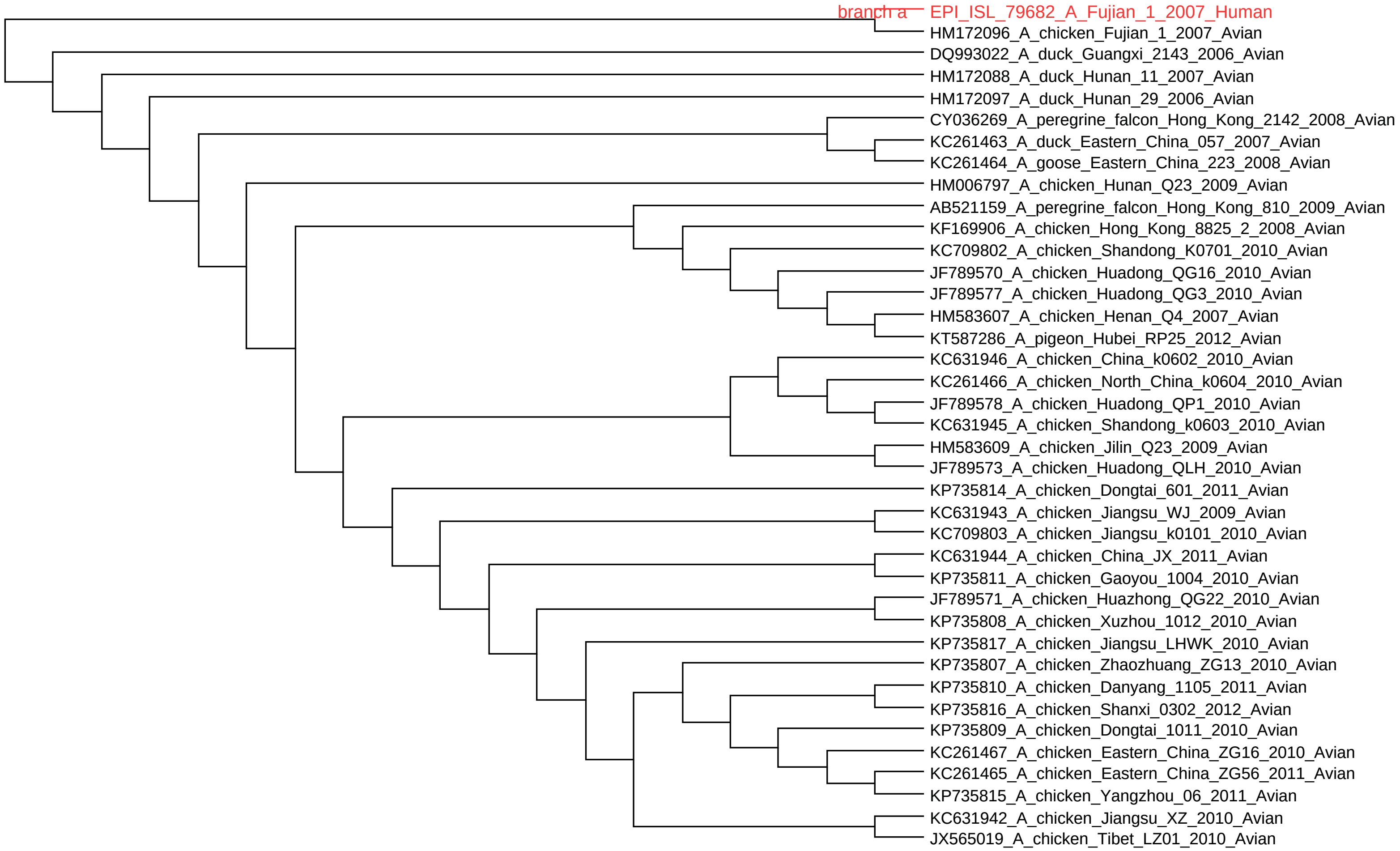
branch a
EPI_ISL_79682_A_Fujian_1_2007_Human
HM172096_A_chicken_Fujian_1_2007_Avian
DQ993022_A_duck_Guangxi_2143_2006_Avian
HM172088_A_duck_Hunan_11_2007_Avian
HM172097_A_duck_Hunan_29_2006_Avian
CY036269_A_peregrine_falcon_Hong_Kong_2142_2008_Avian
KC261463_A_duck_Eastern_China_057_2007_Avian
KC261464_A_goose_Eastern_China_223_2008_Avian
HM006797_A_chicken_Hunan_Q23_2009_Avian
AB521159_A_peregrine_falcon_Hong_Kong_810_2009_Avian
KF169906_A_chicken_Hong_Kong_8825_2_2008_Avian
KC709802_A_chicken_Shandong_K0701_2010_Avian
JF789570_A_chicken_Huadong_QG16_2010_Avian
JF789577_A_chicken_Huadong_QG3_2010_Avian
HM583607_A_chicken_Henan_Q4_2007_Avian
KT587286_A_pigeon_Hubei_RP25_2012_Avian
KC631946_A_chicken_China_k0602_2010_Avian
KC261466_A_chicken_North_China_k0604_2010_Avian
JF789578_A_chicken_Huadong_QP1_2010_Avian
KC631945_A_chicken_Shandong_k0603_2010_Avian
HM583609_A_chicken_Jilin_Q23_2009_Avian
JF789573_A_chicken_Huadong_QLH_2010_Avian
KP735814_A_chicken_Dongtai_601_2011_Avian
KC631943_A_chicken_Jiangsu_WJ_2009_Avian
KC709803_A_chicken_Jiangsu_k0101_2010_Avian
KC631944_A_chicken_China_JX_2011_Avian
KP735811_A_chicken_Gaoyou_1004_2010_Avian
JF789571_A_chicken_Huazhong_QG22_2010_Avian
KP735808_A_chicken_Xuzhou_1012_2010_Avian
KP735817_A_chicken_Jiangsu_LHWK_2010_Avian
KP735807_A_chicken_Zhaozhuang_ZG13_2010_Avian
KP735810_A_chicken_Danyang_1105_2011_Avian
KP735816_A_chicken_Shanxi_0302_2012_Avian
KP735809_A_chicken_Dongtai_1011_2010_Avian
KC261467_A_chicken_Eastern_China_ZG16_2010_Avian
KC261465_A_chicken_Eastern_China_ZG56_2011_Avian
KP735815_A_chicken_Yangzhou_06_2011_Avian
KC631942_A_chicken_Jiangsu_XZ_2010_Avian
JX565019_A_chicken_Tibet_LZ01_2010_Avian

HA-Group91

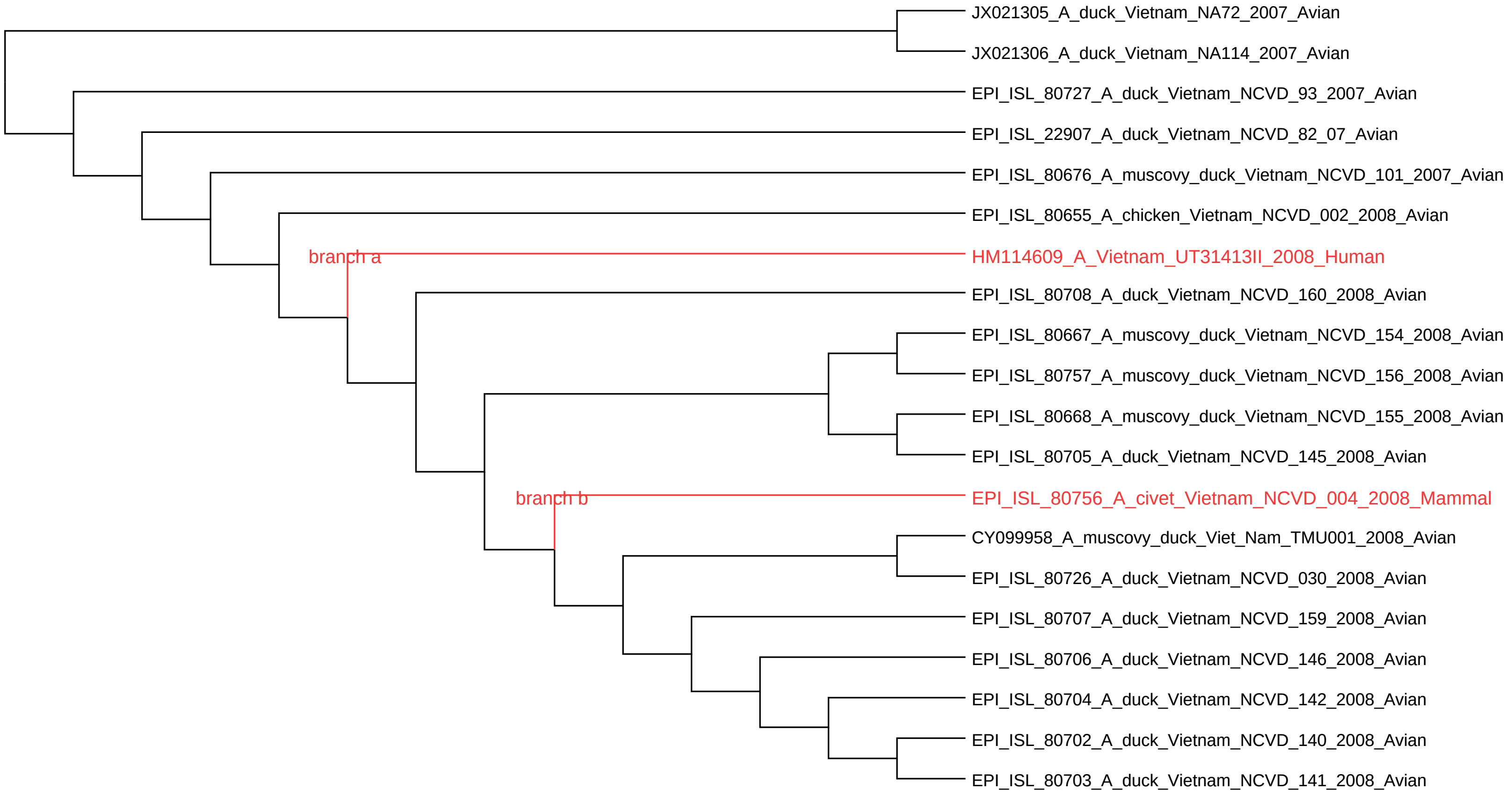
JX021305_A_duck_Vietnam_NA72_2007_Avian
JX021306_A_duck_Vietnam_NA114_2007_Avian
EPI_ISL_80727_A_duck_Vietnam_NCVD_93_2007_Avian
EPI_ISL_22907_A_duck_Vietnam_NCVD_82_07_Avian
EPI_ISL_80676_A_muscovy_duck_Vietnam_NCVD_101_2007_Avian
EPI_ISL_80655_A_chicken_Vietnam_NCVD_002_2008_Avian
branch a
HM114609_A_Vietnam_UT31413II_2008_Human
EPI_ISL_80708_A_duck_Vietnam_NCVD_160_2008_Avian
EPI_ISL_80667_A_muscovy_duck_Vietnam_NCVD_154_2008_Avian
EPI_ISL_80757_A_muscovy_duck_Vietnam_NCVD_156_2008_Avian
EPI_ISL_80668_A_muscovy_duck_Vietnam_NCVD_155_2008_Avian
EPI_ISL_80705_A_duck_Vietnam_NCVD_145_2008_Avian
branch b
EPI_ISL_80756_A_civet_Vietnam_NCVD_004_2008_Mammal
CY099958_A_muscovy_duck_Viet_Nam_TMU001_2008_Avian
EPI_ISL_80726_A_duck_Vietnam_NCVD_030_2008_Avian
EPI_ISL_80707_A_duck_Vietnam_NCVD_159_2008_Avian
EPI_ISL_80706_A_duck_Vietnam_NCVD_146_2008_Avian
EPI_ISL_80704_A_duck_Vietnam_NCVD_142_2008_Avian
EPI_ISL_80702_A_duck_Vietnam_NCVD_140_2008_Avian
EPI_ISL_80703_A_duck_Vietnam_NCVD_141_2008_Avian

HA-Group92

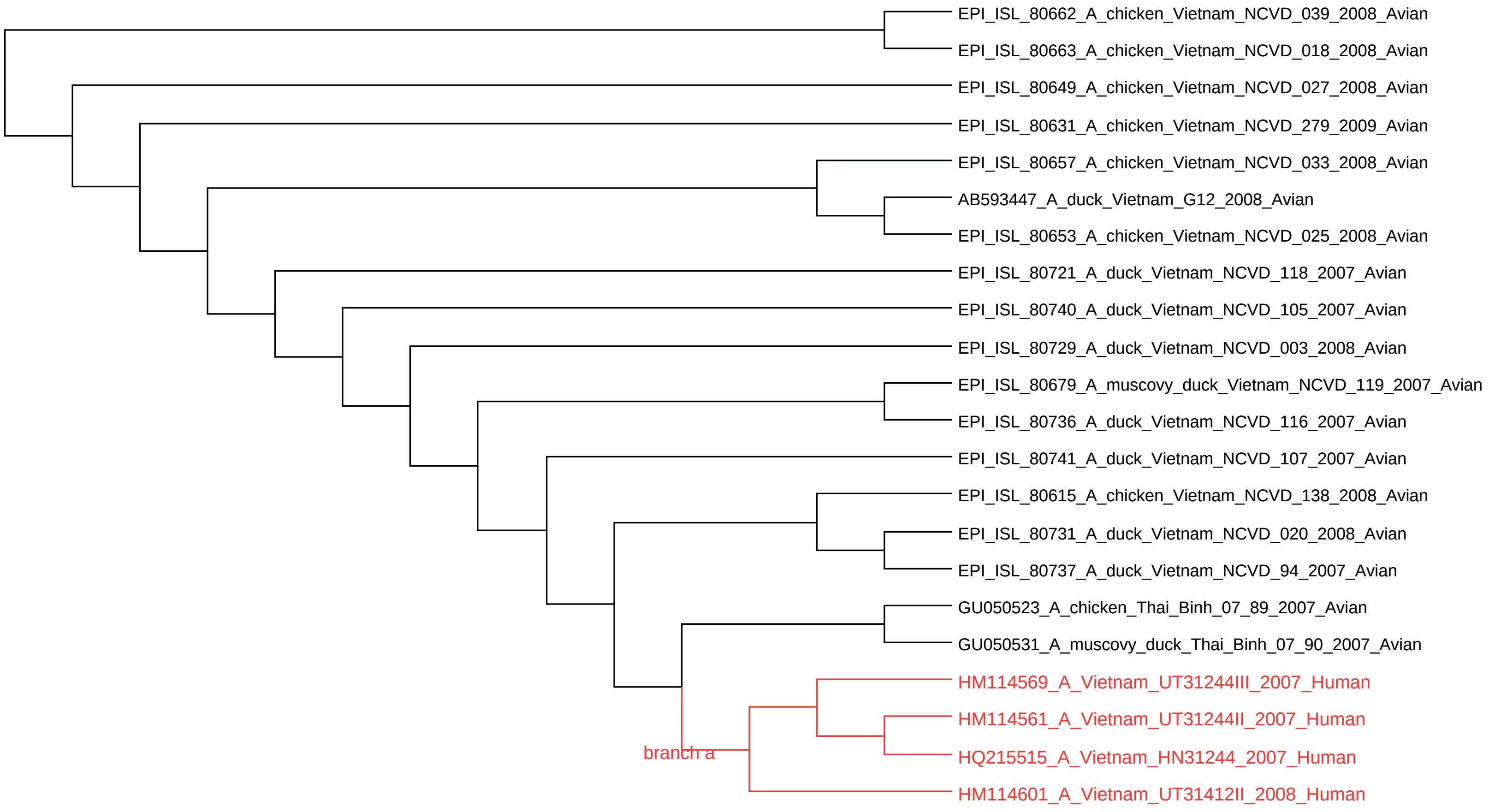
EPI_ISL_80662_A_chicken_Vietnam_NCVD_039_2008_Avian
EPI_ISL_80663_A_chicken_Vietnam_NCVD_018_2008_Avian
EPI_ISL_80649_A_chicken_Vietnam_NCVD_027_2008_Avian
EPI_ISL_80631_A_chicken_Vietnam_NCVD_279_2009_Avian
EPI_ISL_80657_A_chicken_Vietnam_NCVD_033_2008_Avian
AB593447_A_duck_Vietnam_G12_2008_Avian
EPI_ISL_80653_A_chicken_Vietnam_NCVD_025_2008_Avian
EPI_ISL_80721_A_duck_Vietnam_NCVD_118_2007_Avian
EPI_ISL_80740_A_duck_Vietnam_NCVD_105_2007_Avian
EPI_ISL_80729_A_duck_Vietnam_NCVD_003_2008_Avian
EPI_ISL_80679_A_muscovy_duck_Vietnam_NCVD_119_2007_Avian
EPI_ISL_80736_A_duck_Vietnam_NCVD_116_2007_Avian
EPI_ISL_80741_A_duck_Vietnam_NCVD_107_2007_Avian
EPI_ISL_80615_A_chicken_Vietnam_NCVD_138_2008_Avian
EPI_ISL_80731_A_duck_Vietnam_NCVD_020_2008_Avian
EPI_ISL_80737_A_duck_Vietnam_NCVD_94_2007_Avian
GU050523_A_chicken_Thai_Binh_07_89_2007_Avian
GU050531_A_muscovy_duck_Thai_Binh_07_90_2007_Avian
HM114569_A_Vietnam_UT31244III_2007_Human
HM114561_A_Vietnam_UT31244II_2007_Human
HQ215515_A_Vietnam_HN31244_2007_Human
HM114601_A_Vietnam_UT31412II_2008_Human
branch a

HA-Group93

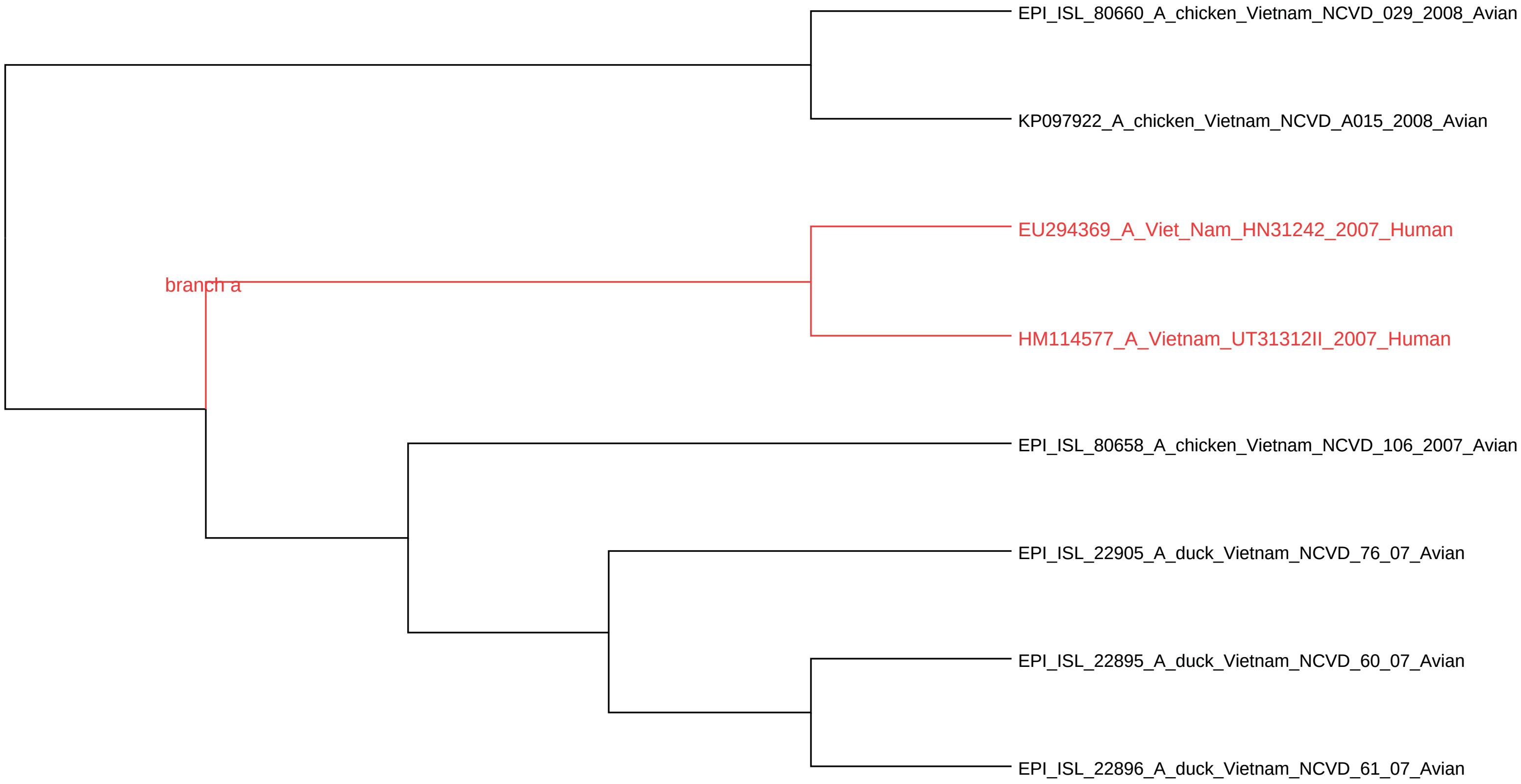

EPI_ISL_80660_A_chicken_Vietnam_NCVD_029_2008_Avian
KP097922_A_chicken_Vietnam_NCVD_A015_2008_Avian
EU294369_A_Viet_Nam_HN31242_2007_Human
branch a
HM114577_A_Vietnam_UT31312II_2007_Human
EPI_ISL_80658_A_chicken_Vietnam_NCVD_106_2007_Avian
EPI_ISL_22905_A_duck_Vietnam_NCVD_76_07_Avian
EPI_ISL_22895_A_duck_Vietnam_NCVD_60_07_Avian
EPI_ISL_22896_A_duck_Vietnam_NCVD_61_07_Avian

HA-Group94

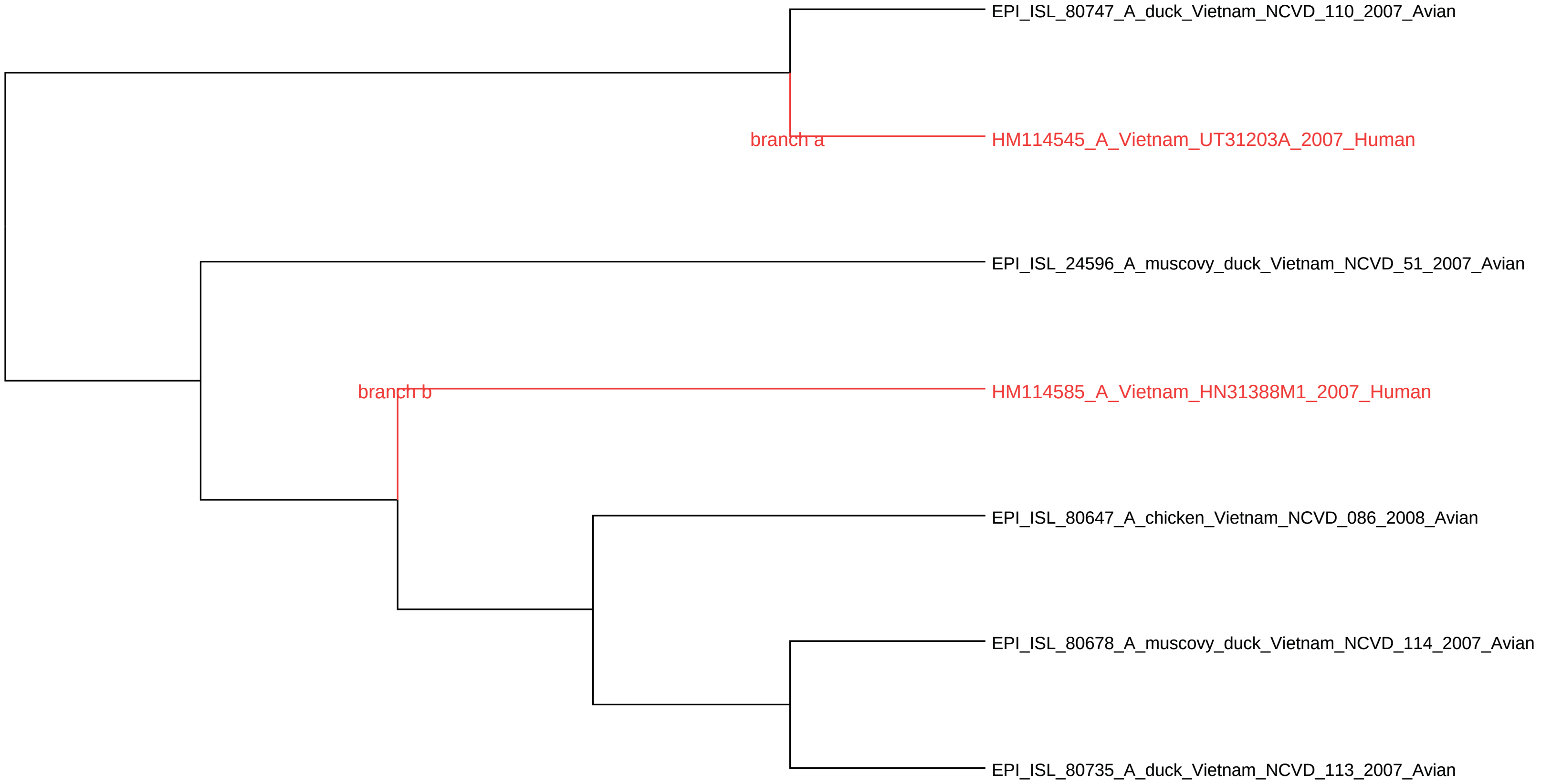

EPI_ISL_80747_A_duck_Vietnam_NCVD_110_2007_Avian
branch a
HM114545_A_Vietnam_UT31203A_2007_Human
EPI_ISL_24596_A_muscovy_duck_Vietnam_NCVD_51_2007_Avian
branch b
HM114585_A_Vietnam_HN31388M1_2007_Human
EPI_ISL_80647_A_chicken_Vietnam_NCVD_086_2008_Avian
EPI_ISL_80678_A_muscovy_duck_Vietnam_NCVD_114_2007_Avian
EPI_ISL_80735_A_duck_Vietnam_NCVD_113_2007_Avian

HA-Group95

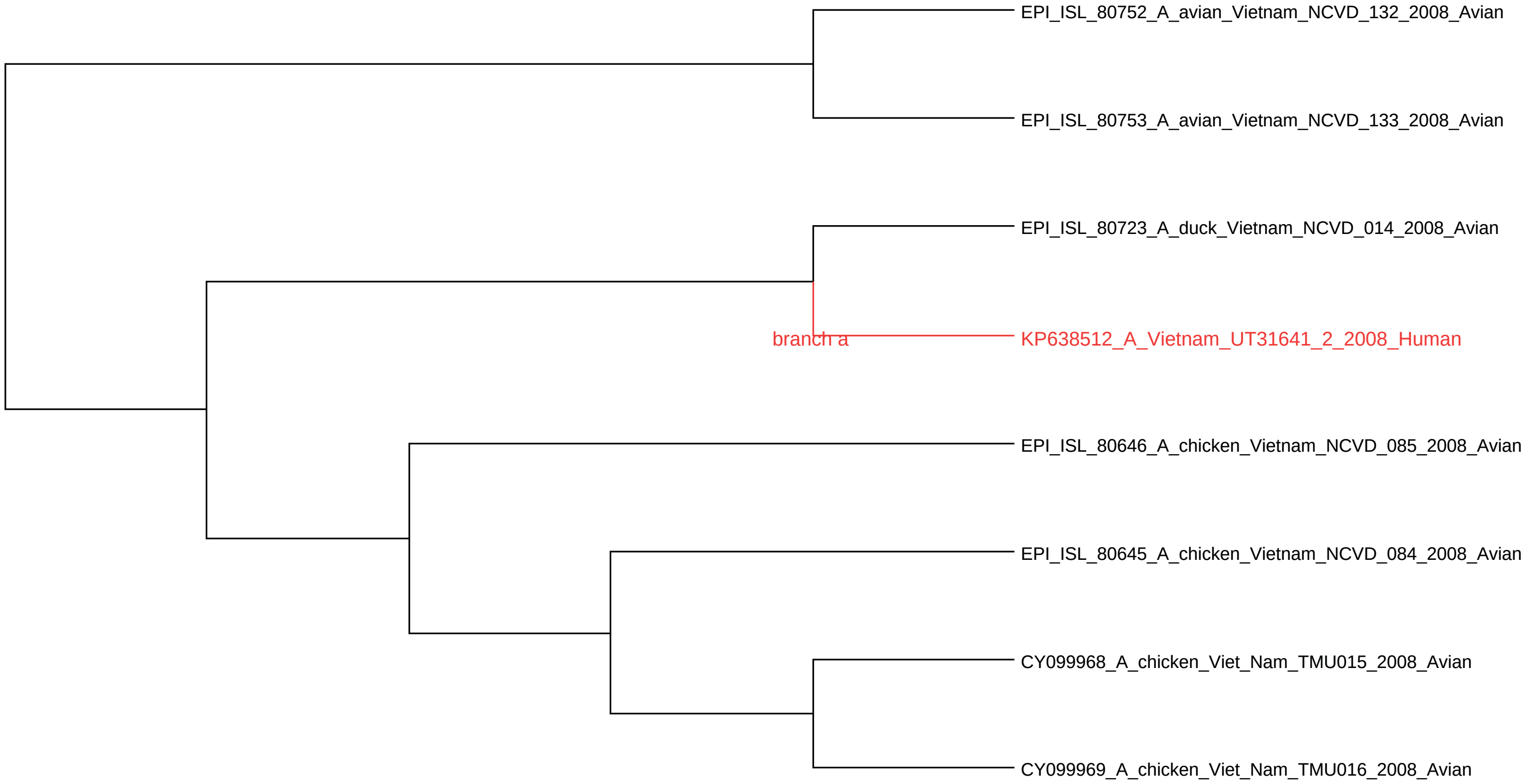
EPI_ISL_80752_A_avian_Vietnam_NCVD_132_2008_Avian
EPI_ISL_80753_A_avian_Vietnam_NCVD_133_2008_Avian
EPI_ISL_80723_A_duck_Vietnam_NCVD_014_2008_Avian
branch a
KP638512_A_Vietnam_UT31641_2_2008_Human
EPI_ISL_80646_A_chicken_Vietnam_NCVD_085_2008_Avian
EPI_ISL_80645_A_chicken_Vietnam_NCVD_084_2008_Avian
CY099968_A_chicken_Viet_Nam_TMU015_2008_Avian
CY099969_A_chicken_Viet_Nam_TMU016_2008_Avian

HA-Group96

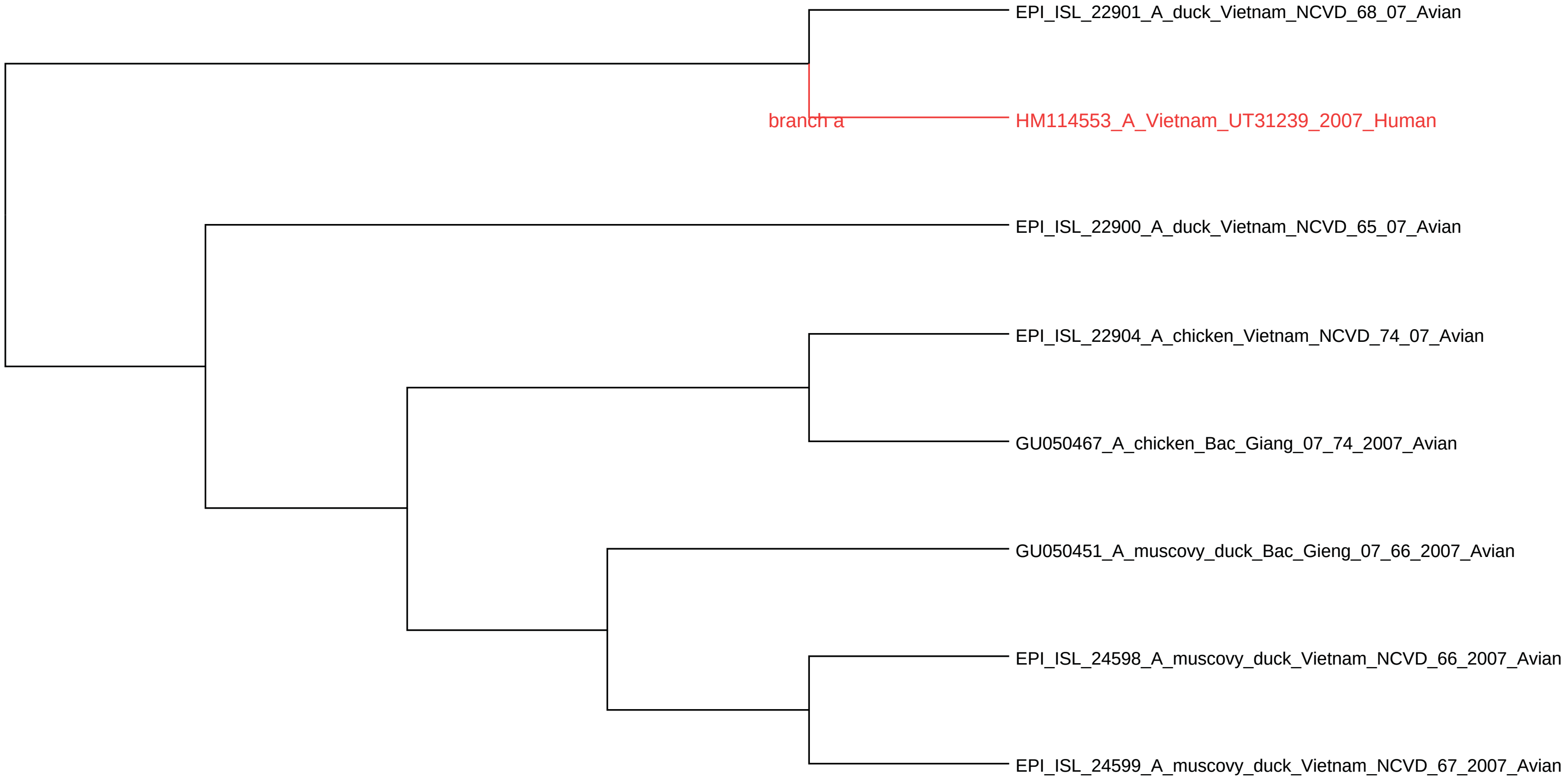
EPI_ISL_22901_A_duck_Vietnam_NCVD_68_07_Avian
branch a
HM114553_A_Vietnam_UT31239_2007_Human
EPI_ISL_22900_A_duck_Vietnam_NCVD_65_07_Avian
EPI_ISL_22904_A_chicken_Vietnam_NCVD_74_07_Avian
GU050467_A_chicken_Bac_Giang_07_74_2007_Avian
GU050451_A_muscovy_duck_Bac_Gieng_07_66_2007_Avian
EPI_ISL_24598_A_muscovy_duck_Vietnam_NCVD_66_2007_Avian
EPI_ISL_24599_A_muscovy_duck_Vietnam_NCVD_67_2007_Avian

HA-Group97

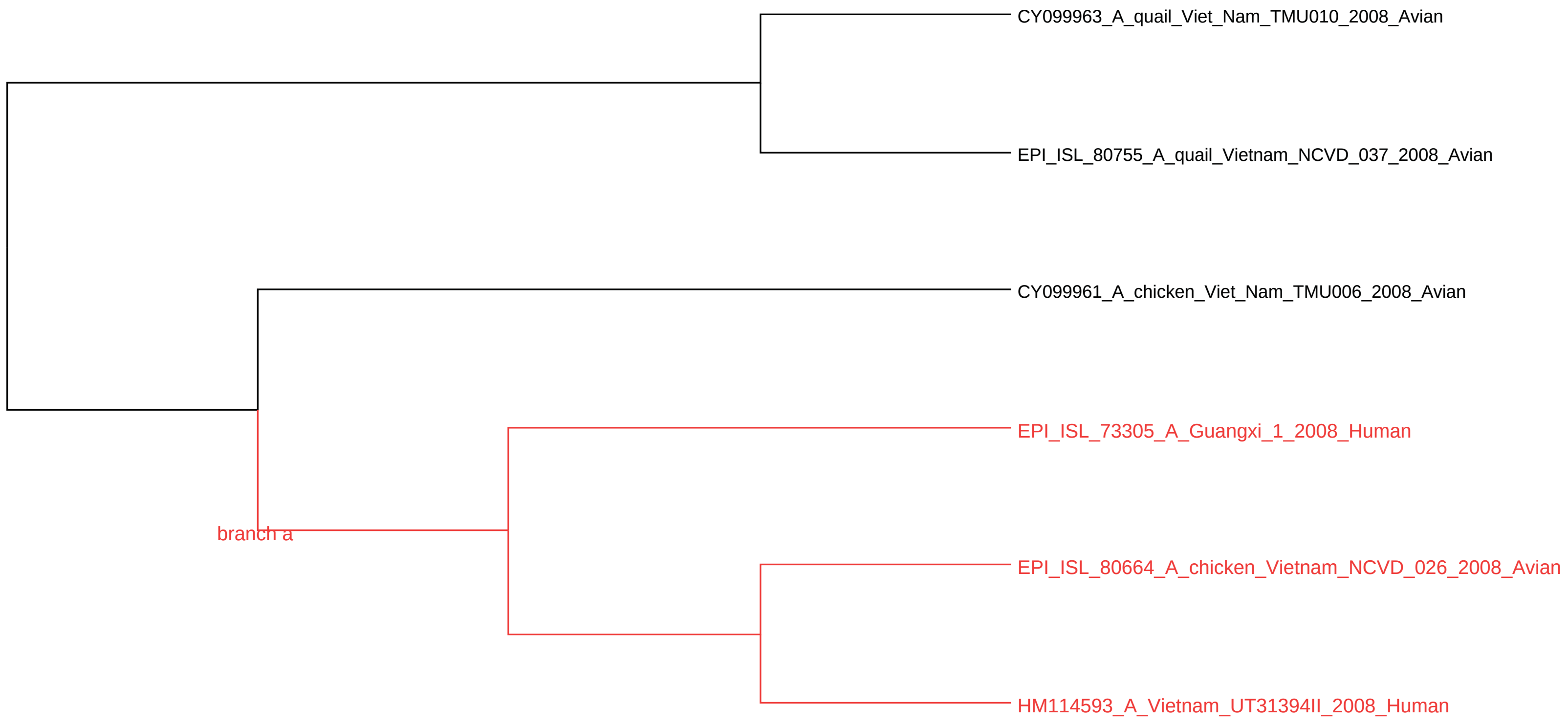
CY099963_A_quail_Viet_Nam_TMU010_2008_Avian
EPI_ISL_80755_A_quail_Vietnam_NCVD_037_2008_Avian
CY099961_A_chicken_Viet_Nam_TMU006_2008_Avian
EPI_ISL_73305_A_Guangxi_1_2008_Human
branch a
EPI_ISL_80664_A_chicken_Vietnam_NCVD_026_2008_Avian
HM114593_A_Vietnam_UT31394II_2008_Human

HA-Group98

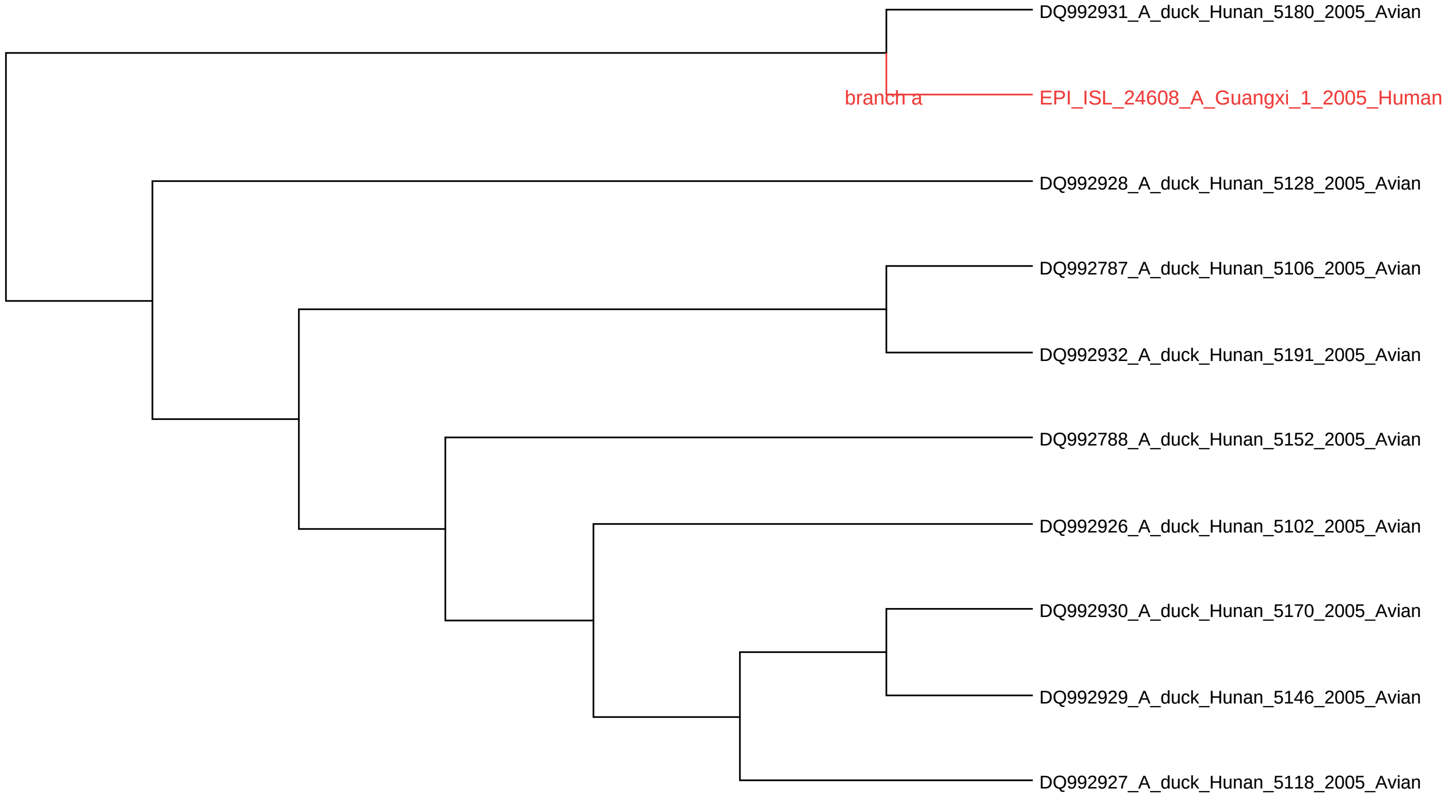

DQ992931_A_duck_Hunan_5180_2005_Avian
branch a
EPI_ISL_24608_A_Guangxi_1_2005_Human
DQ992928_A_duck_Hunan_5128_2005_Avian
DQ992787_A_duck_Hunan_5106_2005_Avian
DQ992932_A_duck_Hunan_5191_2005_Avian
DQ992788_A_duck_Hunan_5152_2005_Avian
DQ992926_A_duck_Hunan_5102_2005_Avian
DQ992930_A_duck_Hunan_5170_2005_Avian
DQ992929_A_duck_Hunan_5146_2005_Avian
DQ992927_A_duck_Hunan_5118_2005_Avian

HA-Group99

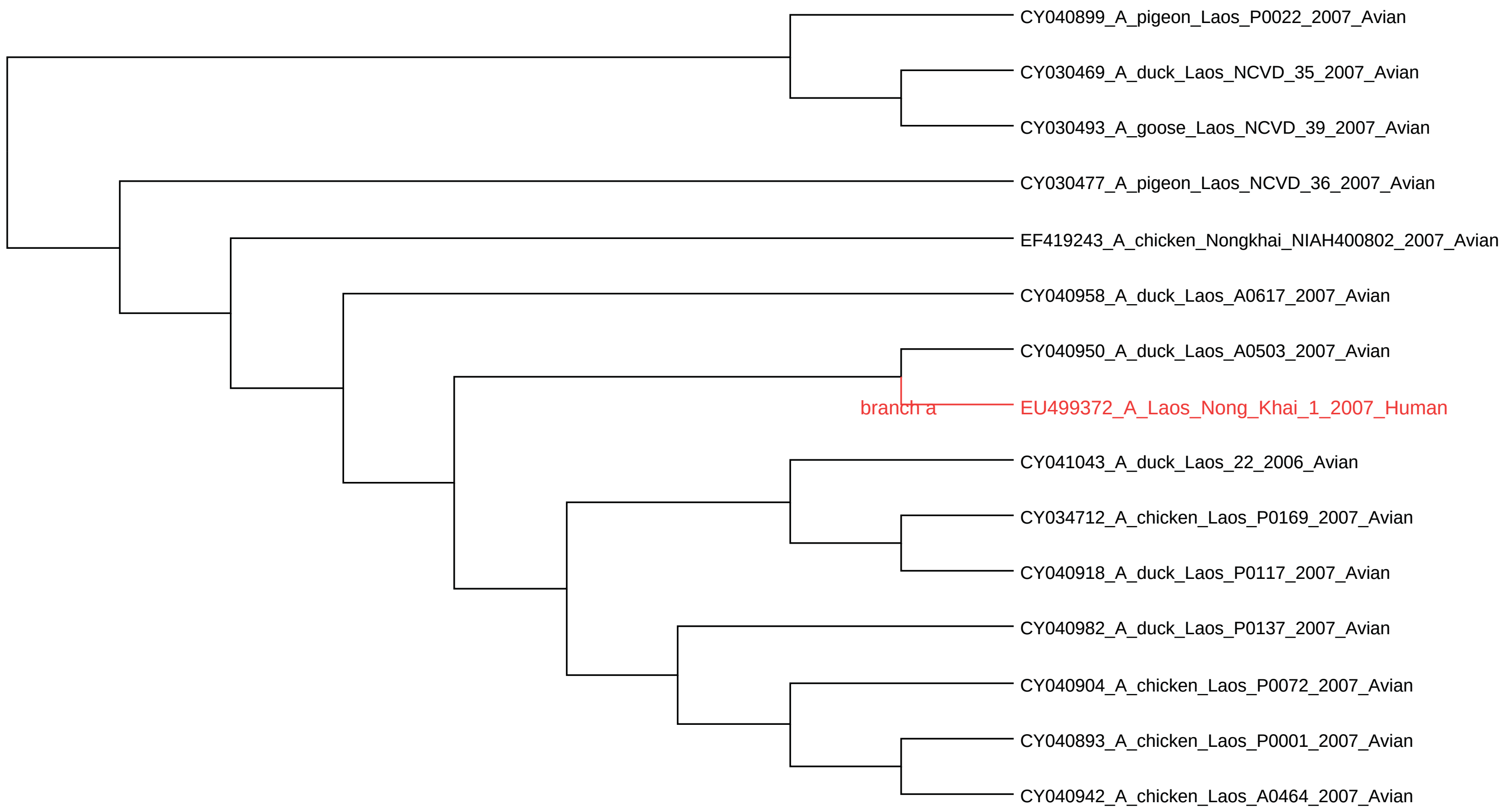

CY040899_A_pigeon_Laos_P0022_2007_Avian
CY030469_A_duck_Laos_NCVD_35_2007_Avian
CY030493_A_goose_Laos_NCVD_39_2007_Avian
CY030477_A_pigeon_Laos_NCVD_36_2007_Avian
EF419243_A_chicken_Nongkhai_NIAH400802_2007_Avian
CY040958_A_duck_Laos_A0617_2007_Avian
CY040950_A_duck_Laos_A0503_2007_Avian
branch a
EU499372_A_Laos_Nong_Khai_1_2007_Human
CY041043_A_duck_Laos_22_2006_Avian
CY034712_A_chicken_Laos_P0169_2007_Avian
CY040918_A_duck_Laos_P0117_2007_Avian
CY040982_A_duck_Laos_P0137_2007_Avian
CY040904_A_chicken_Laos_P0072_2007_Avian
CY040893_A_chicken_Laos_P0001_2007_Avian
CY040942_A_chicken_Laos_A0464_2007_Avian

HA-Group100

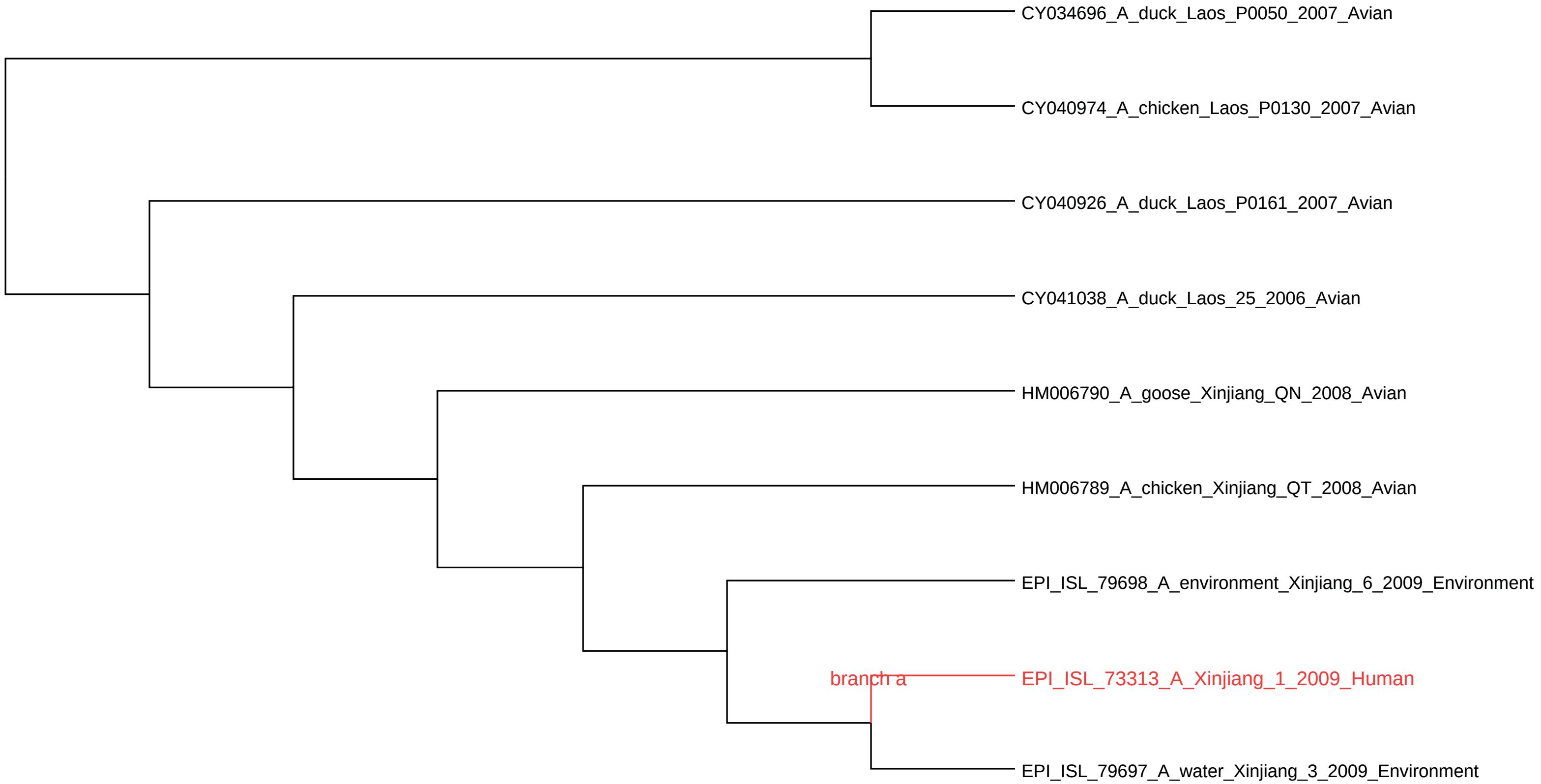
CY034696_A_duck_Laos_P0050_2007_Avian
CY040974_A_chicken_Laos_P0130_2007_Avian
CY040926_A_duck_Laos_P0161_2007_Avian
CY041038_A_duck_Laos_25_2006_Avian
HM006790_A_goose_Xinjiang_QN_2008_Avian
HM006789_A_chicken_Xinjiang_QT_2008_Avian
EPI_ISL_79698_A_environment_Xinjiang_6_2009_Environment
branch a
EPI_ISL_73313_A_Xinjiang_1_2009_Human
EPI_ISL_79697_A_water_Xinjiang_3_2009_Environment

HA-Group101

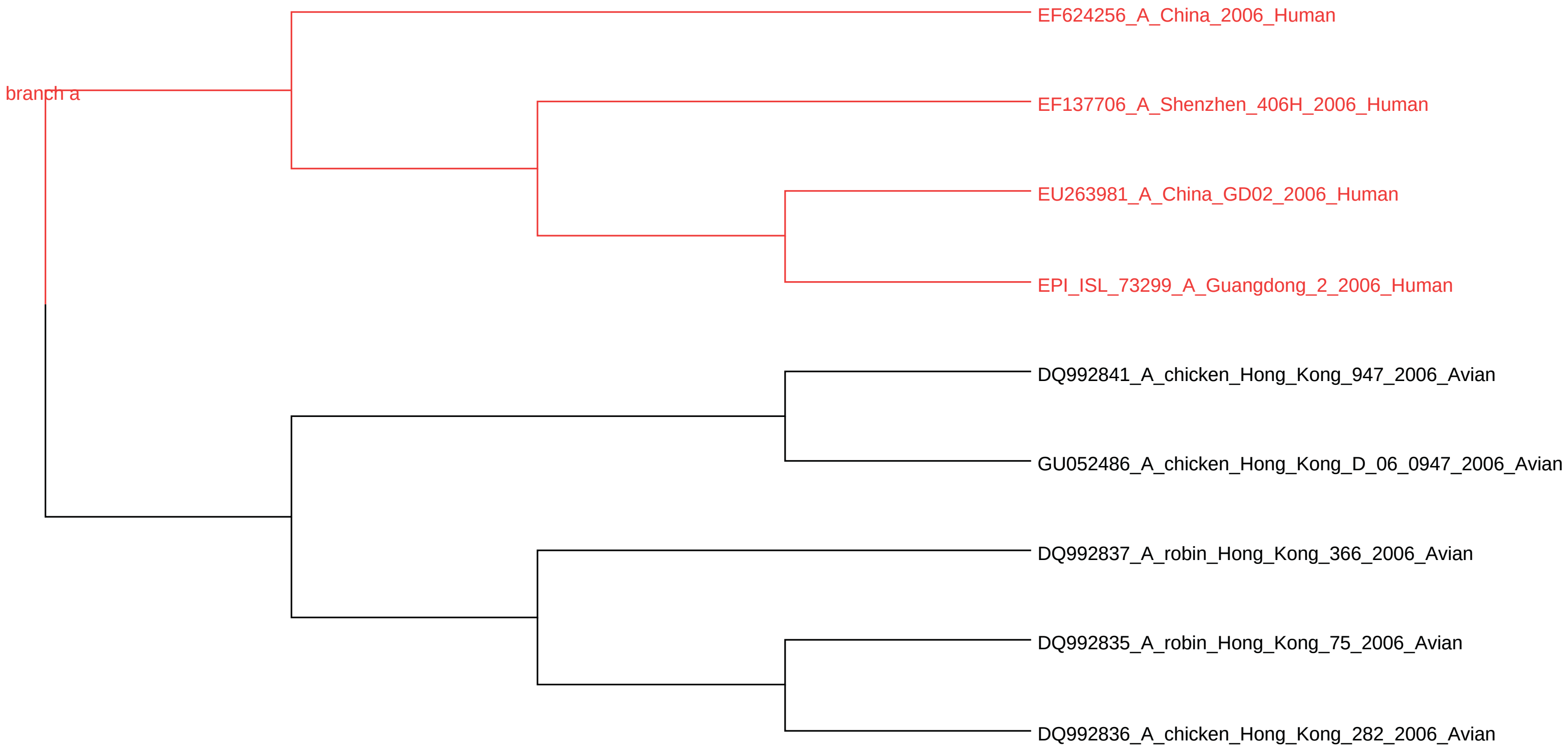

branch a
EF624256_A_China_2006_Human
EF137706_A_Shenzhen_406H_2006_Human
EU263981_A_China_GD02_2006_Human
EPI_ISL_73299_A_Guangdong_2_2006_Human
DQ992841_A_chicken_Hong_Kong_947_2006_Avian
GU052486_A_chicken_Hong_Kong_D_06_0947_2006_Avian
DQ992837_A_robin_Hong_Kong_366_2006_Avian
DQ992835_A_robin_Hong_Kong_75_2006_Avian
DQ992836_A_chicken_Hong_Kong_282_2006_Avian

HA-Group102

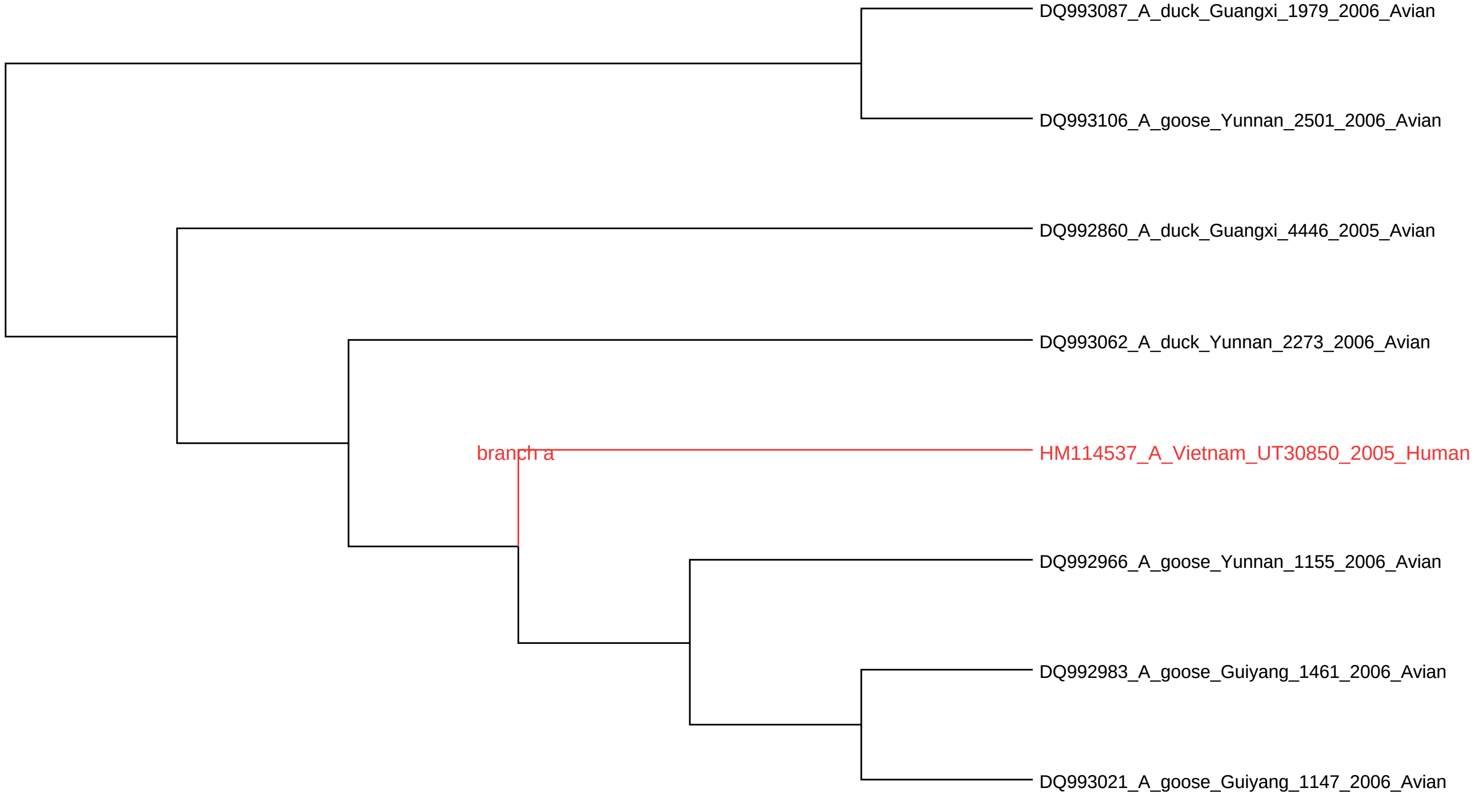

DQ993087_A_duck_Guangxi_1979_2006_Avian
DQ993106_A_goose_Yunnan_2501_2006_Avian
DQ992860_A_duck_Guangxi_4446_2005_Avian
DQ993062_A_duck_Yunnan_2273_2006_Avian
branch a
HM114537_A_Vietnam_UT30850_2005_Human
DQ992966_A_goose_Yunnan_1155_2006_Avian
DQ992983_A_goose_Guiyang_1461_2006_Avian
DQ993021_A_goose_Guiyang_1147_2006_Avian

HA-Group103

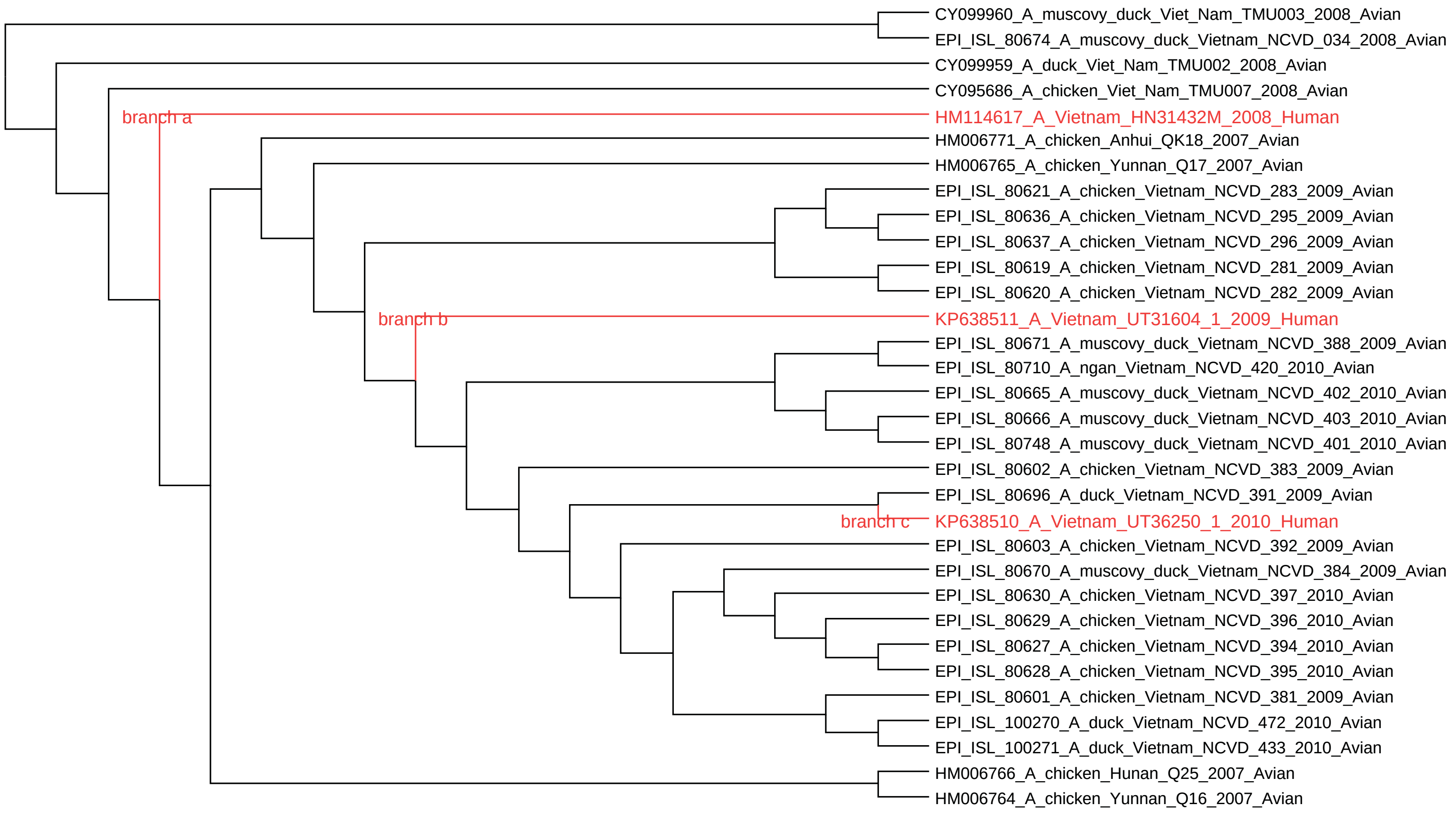

CY099960_A_muscovy_duck_Viet_Nam_TMU003_2008_Avian
EPI_ISL_80674_A_muscovy_duck_Vietnam_NCVD_034_2008_Avian
CY099959_A_duck_Viet_Nam_TMU002_2008_Avian
CY095686_A_chicken_Viet_Nam_TMU007_2008_Avian
branch a
HM114617_A_Vietnam_HN31432M_2008_Human
HM006771_A_chicken_Anhui_QK18_2007_Avian
HM006765_A_chicken_Yunnan_Q17_2007_Avian
EPI_ISL_80621_A_chicken_Vietnam_NCVD_283_2009_Avian
EPI_ISL_80636_A_chicken_Vietnam_NCVD_295_2009_Avian
EPI_ISL_80637_A_chicken_Vietnam_NCVD_296_2009_Avian
EPI_ISL_80619_A_chicken_Vietnam_NCVD_281_2009_Avian
EPI_ISL_80620_A_chicken_Vietnam_NCVD_282_2009_Avian
branch b
KP638511_A_Vietnam_UT31604_1_2009_Human
EPI_ISL_80671_A_muscovy_duck_Vietnam_NCVD_388_2009_Avian
EPI_ISL_80710_A_ngan_Vietnam_NCVD_420_2010_Avian
EPI_ISL_80665_A_muscovy_duck_Vietnam_NCVD_402_2010_Avian
EPI_ISL_80666_A_muscovy_duck_Vietnam_NCVD_403_2010_Avian
EPI_ISL_80748_A_muscovy_duck_Vietnam_NCVD_401_2010_Avian
EPI_ISL_80602_A_chicken_Vietnam_NCVD_383_2009_Avian
EPI_ISL_80696_A_duck_Vietnam_NCVD_391_2009_Avian
branch c
KP638510_A_Vietnam_UT36250_1_2010_Human
EPI_ISL_80603_A_chicken_Vietnam_NCVD_392_2009_Avian
EPI_ISL_80670_A_muscovy_duck_Vietnam_NCVD_384_2009_Avian
EPI_ISL_80630_A_chicken_Vietnam_NCVD_397_2010_Avian
EPI_ISL_80629_A_chicken_Vietnam_NCVD_396_2010_Avian
EPI_ISL_80627_A_chicken_Vietnam_NCVD_394_2010_Avian
EPI_ISL_80628_A_chicken_Vietnam_NCVD_395_2010_Avian
EPI_ISL_80601_A_chicken_Vietnam_NCVD_381_2009_Avian
EPI_ISL_100270_A_duck_Vietnam_NCVD_472_2010_Avian
EPI_ISL_100271_A_duck_Vietnam_NCVD_433_2010_Avian
HM006766_A_chicken_Hunan_Q25_2007_Avian
HM006764_A_chicken_Yunnan_Q16_2007_Avian

HA-Group104

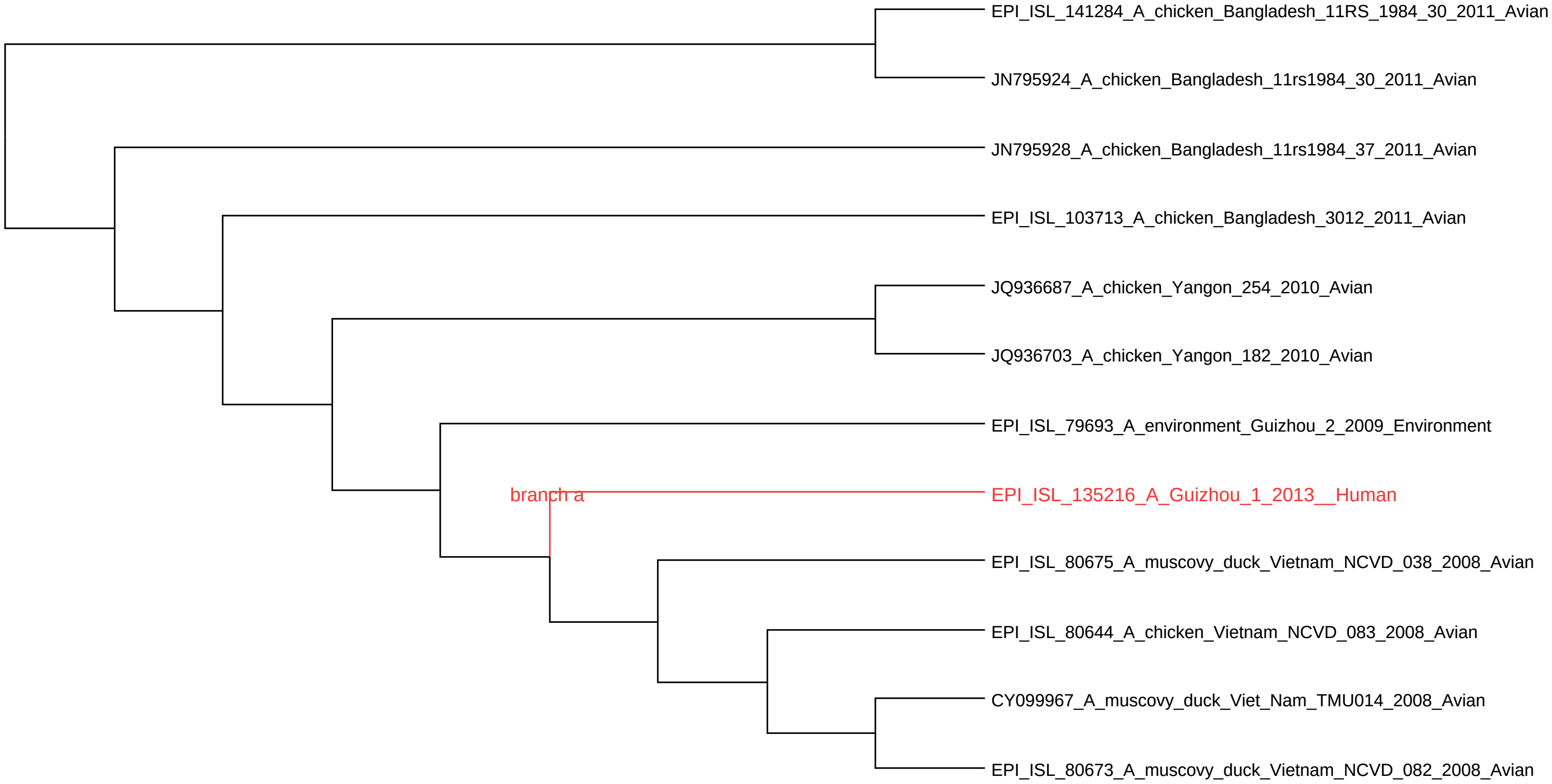

EPI_ISL_141284_A_chicken_Bangladesh_11RS_1984_30_2011_Avian
JN795924_A_chicken_Bangladesh_11rs1984_30_2011_Avian
JN795928_A_chicken_Bangladesh_11rs1984_37_2011_Avian
EPI_ISL_103713_A_chicken_Bangladesh_3012_2011_Avian
JQ936687_A_chicken_Yangon_254_2010_Avian
JQ936703_A_chicken_Yangon_182_2010_Avian
EPI_ISL_79693_A_environment_Guizhou_2_2009_Environment
branch a
EPI_ISL_135216_A_Guizhou_1_2013__Human
EPI_ISL_80675_A_muscovy_duck_Vietnam_NCVD_038_2008_Avian
EPI_ISL_80644_A_chicken_Vietnam_NCVD_083_2008_Avian
CY099967_A_muscovy_duck_Viet_Nam_TMU014_2008_Avian
EPI_ISL_80673_A_muscovy_duck_Vietnam_NCVD_082_2008_Avian

HA-Group105

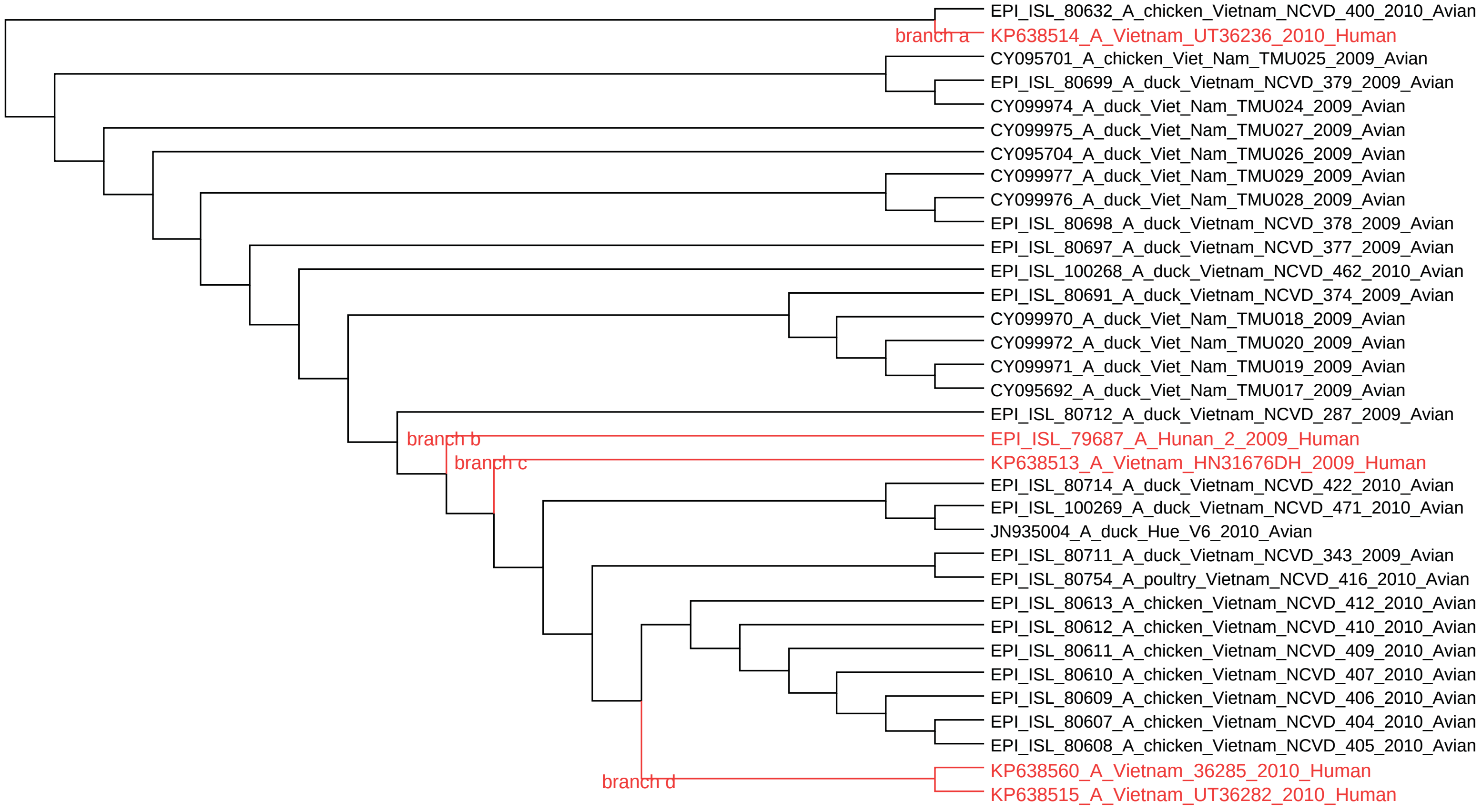

EPI_ISL_80632_A_chicken_Vietnam_NCVD_400_2010_Avian
branch a
KP638514_A_Vietnam_UT36236_2010_Human
CY095701_A_chicken_Viet_Nam_TMU025_2009_Avian
EPI_ISL_80699_A_duck_Vietnam_NCVD_379_2009_Avian
CY099974_A_duck_Viet_Nam_TMU024_2009_Avian
CY099975_A_duck_Viet_Nam_TMU027_2009_Avian
CY095704_A_duck_Viet_Nam_TMU026_2009_Avian
CY099977_A_duck_Viet_Nam_TMU029_2009_Avian
CY099976_A_duck_Viet_Nam_TMU028_2009_Avian
EPI_ISL_80698_A_duck_Vietnam_NCVD_378_2009_Avian
EPI_ISL_80697_A_duck_Vietnam_NCVD_377_2009_Avian
EPI_ISL_100268_A_duck_Vietnam_NCVD_462_2010_Avian
EPI_ISL_80691_A_duck_Vietnam_NCVD_374_2009_Avian
CY099970_A_duck_Viet_Nam_TMU018_2009_Avian
CY099972_A_duck_Viet_Nam_TMU020_2009_Avian
CY099971_A_duck_Viet_Nam_TMU019_2009_Avian
CY095692_A_duck_Viet_Nam_TMU017_2009_Avian
EPI_ISL_80712_A_duck_Vietnam_NCVD_287_2009_Avian
branch b
EPI_ISL_79687_A_Hunan_2_2009_Human
branch c
KP638513_A_Vietnam_HN31676DH_2009_Human
EPI_ISL_80714_A_duck_Vietnam_NCVD_422_2010_Avian
EPI_ISL_100269_A_duck_Vietnam_NCVD_471_2010_Avian
JN935004_A_duck_Hue_V6_2010_Avian
EPI_ISL_80711_A_duck_Vietnam_NCVD_343_2009_Avian
EPI_ISL_80754_A_poultry_Vietnam_NCVD_416_2010_Avian
EPI_ISL_80613_A_chicken_Vietnam_NCVD_412_2010_Avian
EPI_ISL_80612_A_chicken_Vietnam_NCVD_410_2010_Avian
EPI_ISL_80611_A_chicken_Vietnam_NCVD_409_2010_Avian
EPI_ISL_80610_A_chicken_Vietnam_NCVD_407_2010_Avian
EPI_ISL_80609_A_chicken_Vietnam_NCVD_406_2010_Avian
EPI_ISL_80607_A_chicken_Vietnam_NCVD_404_2010_Avian
EPI_ISL_80608_A_chicken_Vietnam_NCVD_405_2010_Avian
branch d
KP638560_A_Vietnam_36285_2010_Human
KP638515_A_Vietnam_UT36282_2010_Human

HA-Group106

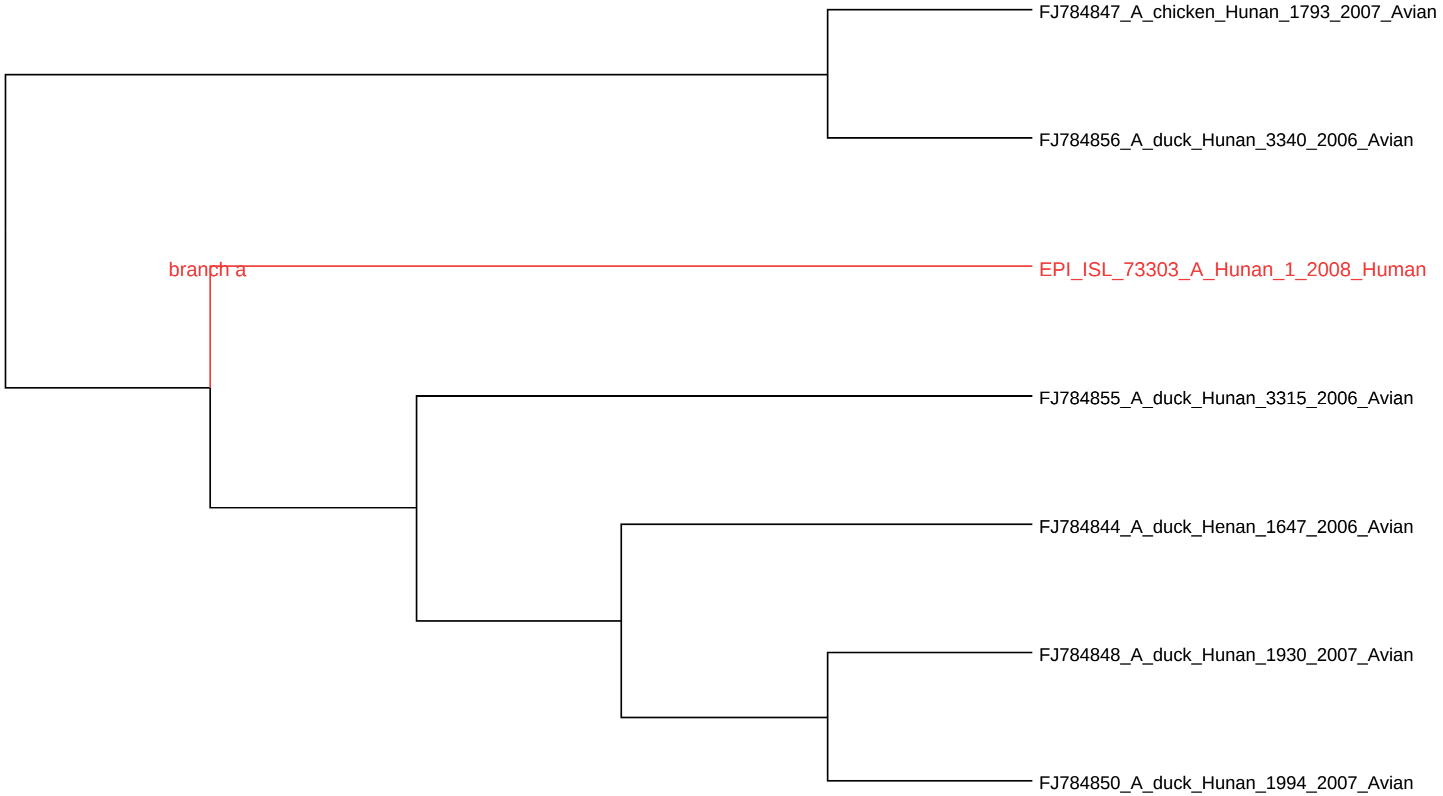

FJ784847_A_chicken_Hunan_1793_2007_Avian
FJ784856_A_duck_Hunan_3340_2006_Avian
branch a
EPI_ISL_73303_A_Hunan_1_2008_Human
FJ784855_A_duck_Hunan_3315_2006_Avian
FJ784844_A_duck_Henan_1647_2006_Avian
FJ784848_A_duck_Hunan_1930_2007_Avian
FJ784850_A_duck_Hunan_1994_2007_Avian

HA-Group107

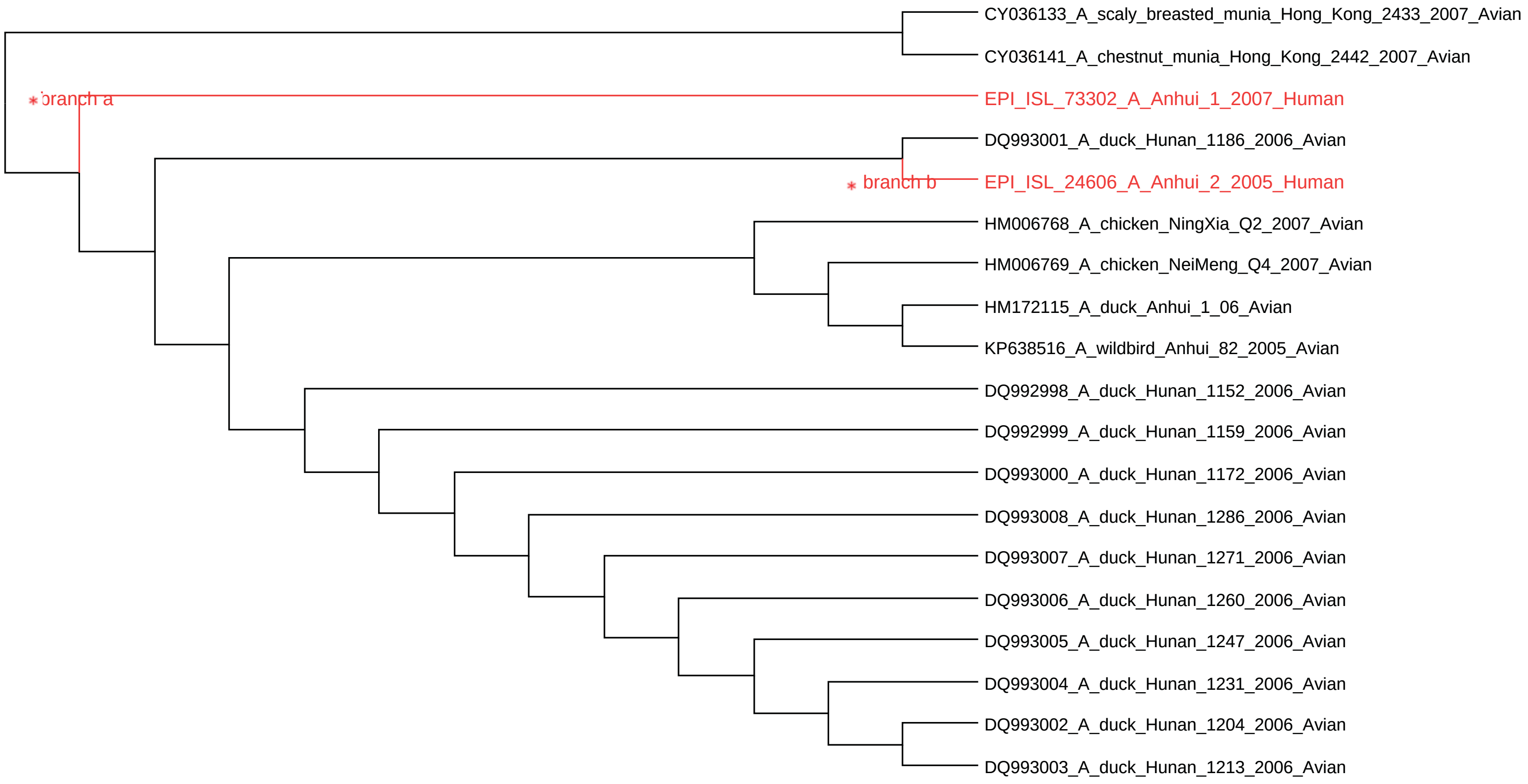
CY036133_A_scaly_breasted_munia_Hong_Kong_2433_2007_Avian
CY036141_A_chestnut_munia_Hong_Kong_2442_2007_Avian
branch a
EPI_ISL_73302_A_Anhui_1_2007_Human
DQ993001_A_duck_Hunan_1186_2006_Avian
branch b
EPI_ISL_24606_A_Anhui_2_2005_Human
HM006768_A_chicken_NingXia_Q2_2007_Avian
HM006769_A_chicken_NeiMeng_Q4_2007_Avian
HM172115_A_duck_Anhui_1_06_Avian
KP638516_A_wildbird_Anhui_82_2005_Avian
DQ992998_A_duck_Hunan_1152_2006_Avian
DQ992999_A_duck_Hunan_1159_2006_Avian
DQ993000_A_duck_Hunan_1172_2006_Avian
DQ993008_A_duck_Hunan_1286_2006_Avian
DQ993007_A_duck_Hunan_1271_2006_Avian
DQ993006_A_duck_Hunan_1260_2006_Avian
DQ993005_A_duck_Hunan_1247_2006_Avian
DQ993004_A_duck_Hunan_1231_2006_Avian
DQ993002_A_duck_Hunan_1204_2006_Avian
DQ993003_A_duck_Hunan_1213_2006_Avian

HA-Group108

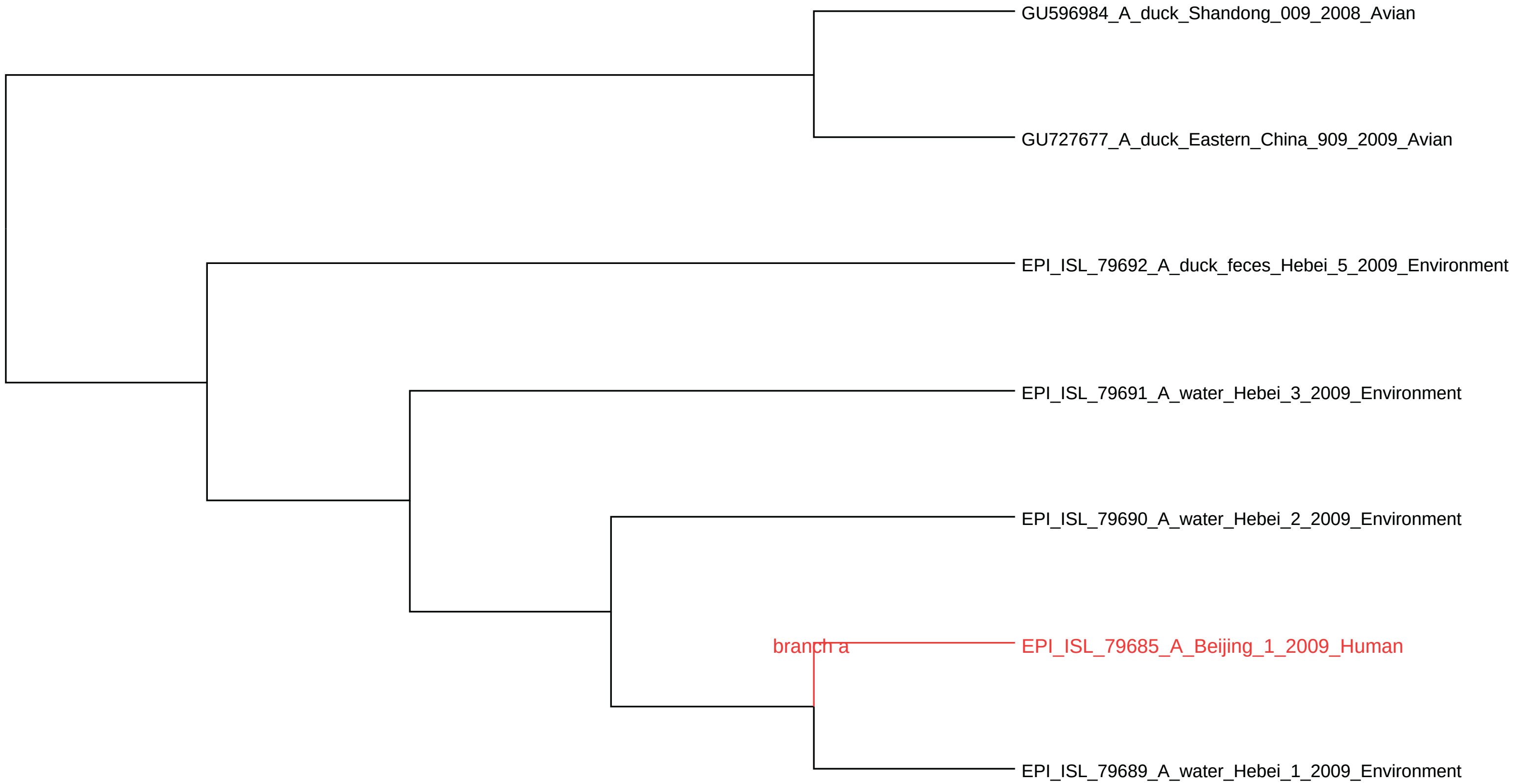

GU596984_A_duck_Shandong_009_2008_Avian
GU727677_A_duck_Eastern_China_909_2009_Avian
EPI_ISL_79692_A_duck_feces_Hebei_5_2009_Environment
EPI_ISL_79691_A_water_Hebei_3_2009_Environment
EPI_ISL_79690_A_water_Hebei_2_2009_Environment
branch a
EPI_ISL_79685_A_Beijing_1_2009_Human
EPI_ISL_79689_A_water_Hebei_1_2009_Environment

HA-Group109

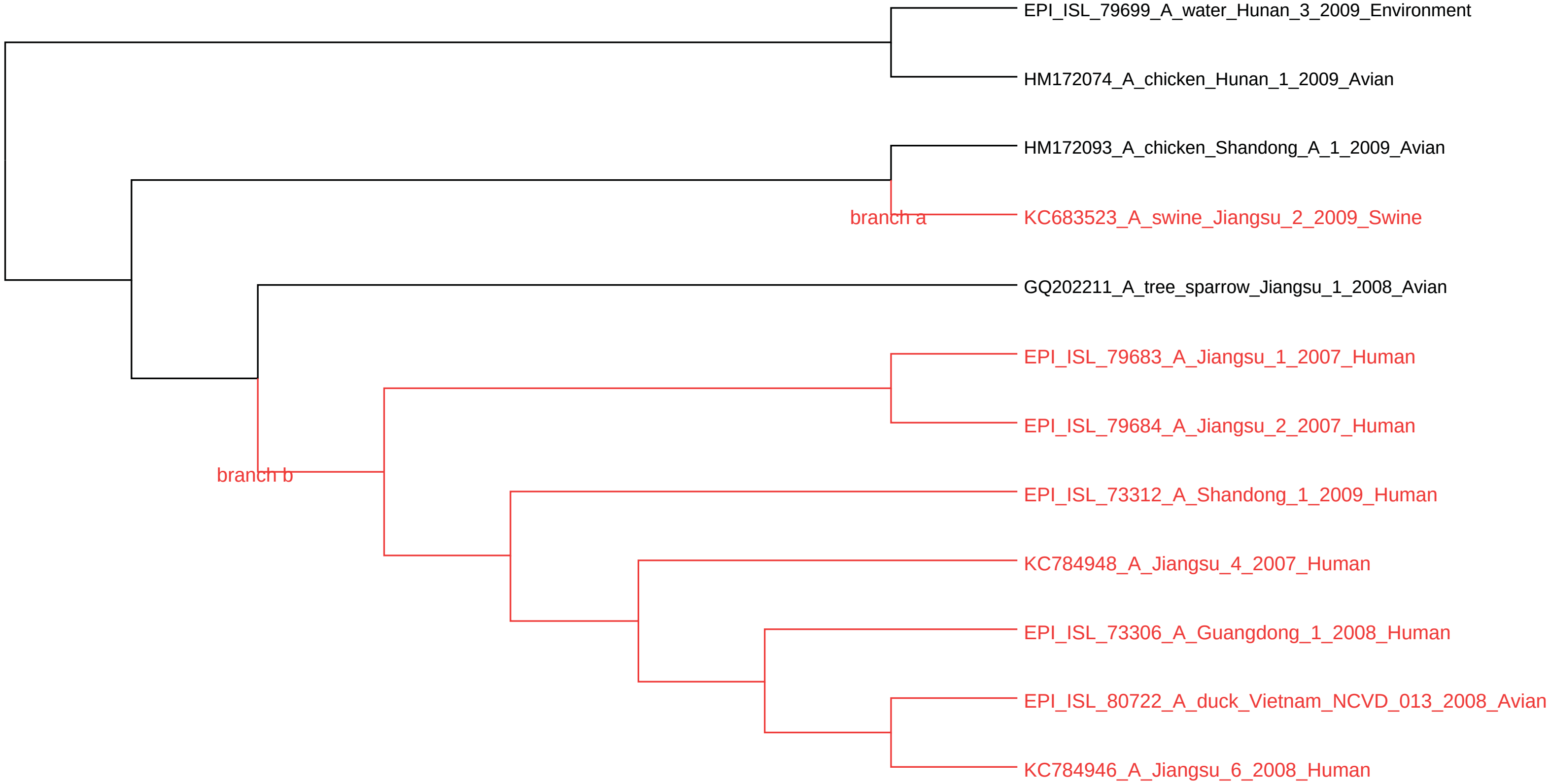

EPI_ISL_79699_A_water_Hunan_3_2009_Environment
HM172074_A_chicken_Hunan_1_2009_Avian
HM172093_A_chicken_Shandong_A_1_2009_Avian
branch a
KC683523_A_swine_Jiangsu_2_2009_Swine
GQ202211_A_tree_sparrow_Jiangsu_1_2008_Avian
EPI_ISL_79683_A_Jiangsu_1_2007_Human
EPI_ISL_79684_A_Jiangsu_2_2007_Human
branch b
EPI_ISL_73312_A_Shandong_1_2009_Human
KC784948_A_Jiangsu_4_2007_Human
EPI_ISL_73306_A_Guangdong_1_2008_Human
EPI_ISL_80722_A_duck_Vietnam_NCVD_013_2008_Avian
KC784946_A_Jiangsu_6_2008_Human

HA-Group110

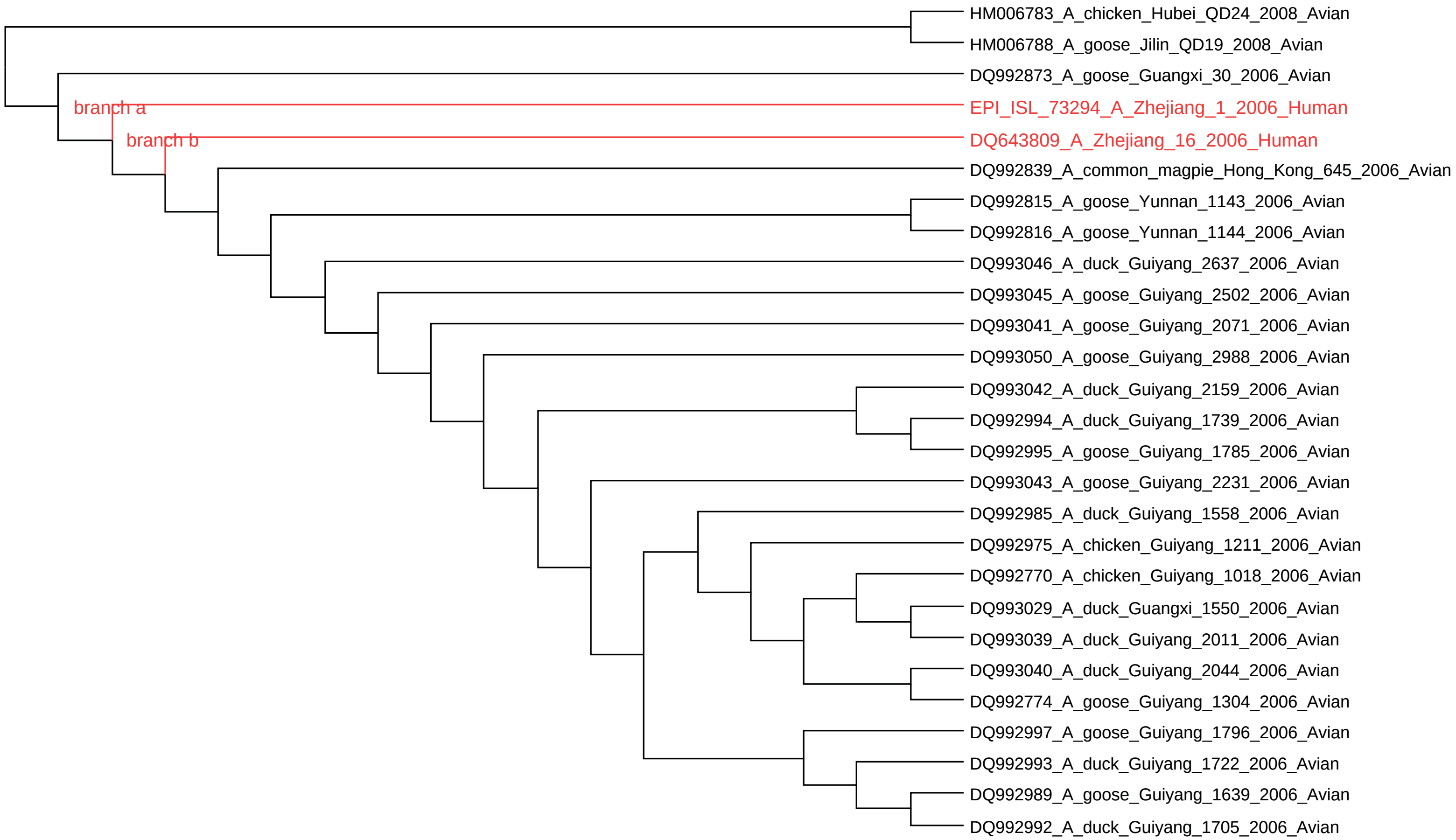
HM006783_A_chicken_Hubei_QD24_2008_Avian
HM006788_A_goose_Jilin_QD19_2008_Avian
DQ992873_A_goose_Guangxi_30_2006_Avian
branch a
EPI_ISL_73294_A_Zhejiang_1_2006_Human
branch b
DQ643809_A_Zhejiang_16_2006_Human
DQ992839_A_common_magpie_Hong_Kong_645_2006_Avian
DQ992815_A_goose_Yunnan_1143_2006_Avian
DQ992816_A_goose_Yunnan_1144_2006_Avian
DQ993046_A_duck_Guiyang_2637_2006_Avian
DQ993045_A_goose_Guiyang_2502_2006_Avian
DQ993041_A_goose_Guiyang_2071_2006_Avian
DQ993050_A_goose_Guiyang_2988_2006_Avian
DQ993042_A_duck_Guiyang_2159_2006_Avian
DQ992994_A_duck_Guiyang_1739_2006_Avian
DQ992995_A_goose_Guiyang_1785_2006_Avian
DQ993043_A_goose_Guiyang_2231_2006_Avian
DQ992985_A_duck_Guiyang_1558_2006_Avian
DQ992975_A_chicken_Guiyang_1211_2006_Avian
DQ992770_A_chicken_Guiyang_1018_2006_Avian
DQ993029_A_duck_Guangxi_1550_2006_Avian
DQ993039_A_duck_Guiyang_2011_2006_Avian
DQ993040_A_duck_Guiyang_2044_2006_Avian
DQ992774_A_goose_Guiyang_1304_2006_Avian
DQ992997_A_goose_Guiyang_1796_2006_Avian
DQ992993_A_duck_Guiyang_1722_2006_Avian
DQ992989_A_goose_Guiyang_1639_2006_Avian
DQ992992_A_duck_Guiyang_1705_2006_Avian

HA-Group111

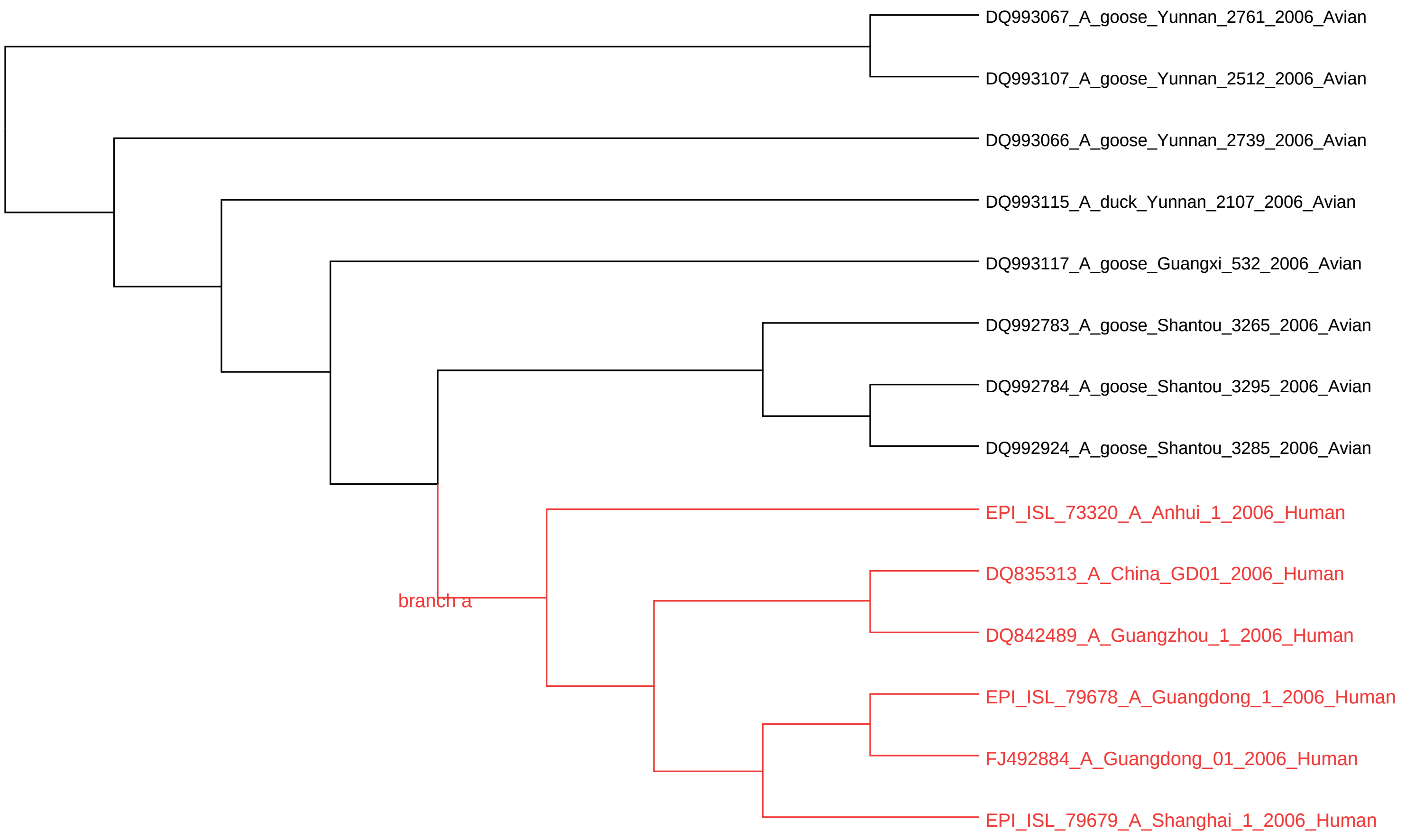
DQ993067_A_goose_Yunnan_2761_2006_Avian
DQ993107_A_goose_Yunnan_2512_2006_Avian
DQ993066_A_goose_Yunnan_2739_2006_Avian
DQ993115_A_duck_Yunnan_2107_2006_Avian
DQ993117_A_goose_Guangxi_532_2006_Avian
DQ992783_A_goose_Shantou_3265_2006_Avian
DQ992784_A_goose_Shantou_3295_2006_Avian
DQ992924_A_goose_Shantou_3285_2006_Avian
EPI_ISL_73320_A_Anhui_1_2006_Human
branch a
DQ835313_A_China_GD01_2006_Human
DQ842489_A_Guangzhou_1_2006_Human
EPI_ISL_79678_A_Guangdong_1_2006_Human
FJ492884_A_Guangdong_01_2006_Human
EPI_ISL_79679_A_Shanghai_1_2006_Human

HA-Group112

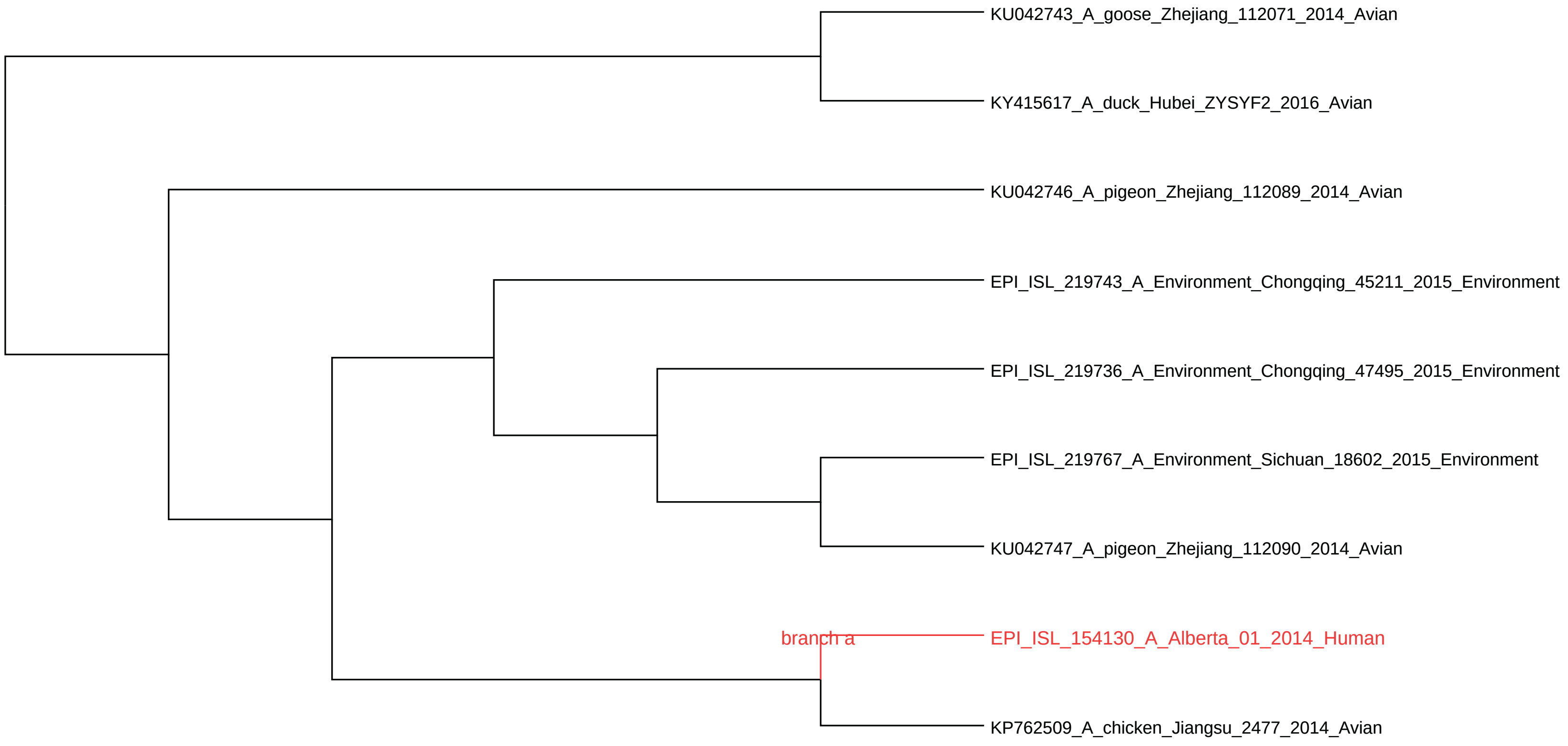

KU042743_A_goose_Zhejiang_112071_2014_Avian
KY415617_A_duck_Hubei_ZYSYF2_2016_Avian
KU042746_A_pigeon_Zhejiang_112089_2014_Avian
EPI_ISL_219743_A_Environment_Chongqing_45211_2015_Environment
EPI_ISL_219736_A_Environment_Chongqing_47495_2015_Environment
EPI_ISL_219767_A_Environment_Sichuan_18602_2015_Environment
KU042747_A_pigeon_Zhejiang_112090_2014_Avian
branch a
EPI_ISL_154130_A_Alberta_01_2014_Human
KP762509_A_chicken_Jiangsu_2477_2014_Avian

HA-Group113

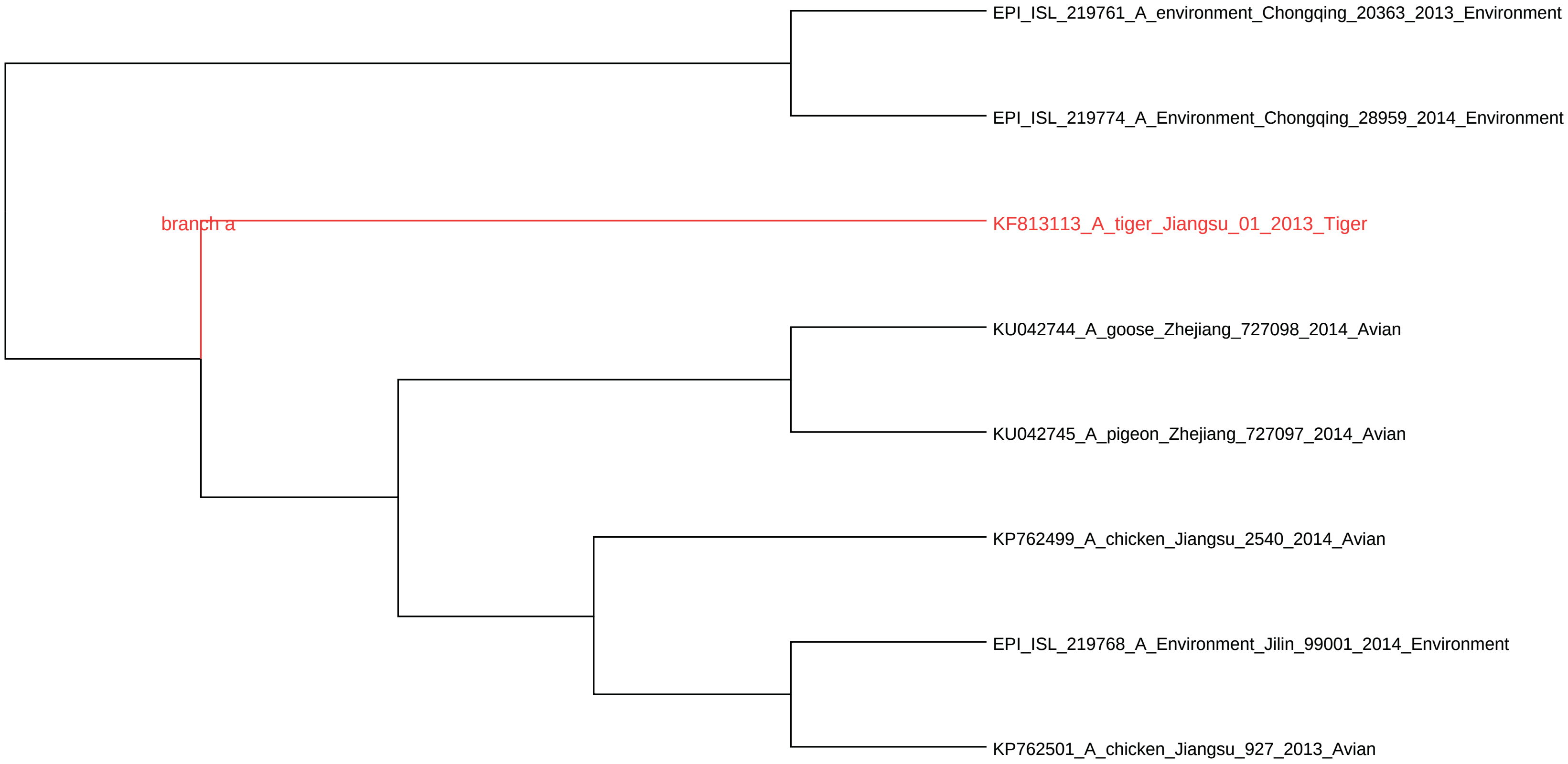
EPI_ISL_219761_A_environment_Chongqing_20363_2013_Environment
EPI_ISL_219774_A_Environment_Chongqing_28959_2014_Environment
branch a
KF813113_A_tiger_Jiangsu_01_2013_Tiger
KU042744_A_goose_Zhejiang_727098_2014_Avian
KU042745_A_pigeon_Zhejiang_727097_2014_Avian
KP762499_A_chicken_Jiangsu_2540_2014_Avian
EPI_ISL_219768_A_Environment_Jilin_99001_2014_Environment
KP762501_A_chicken_Jiangsu_927_2013_Avian

HA-Group114

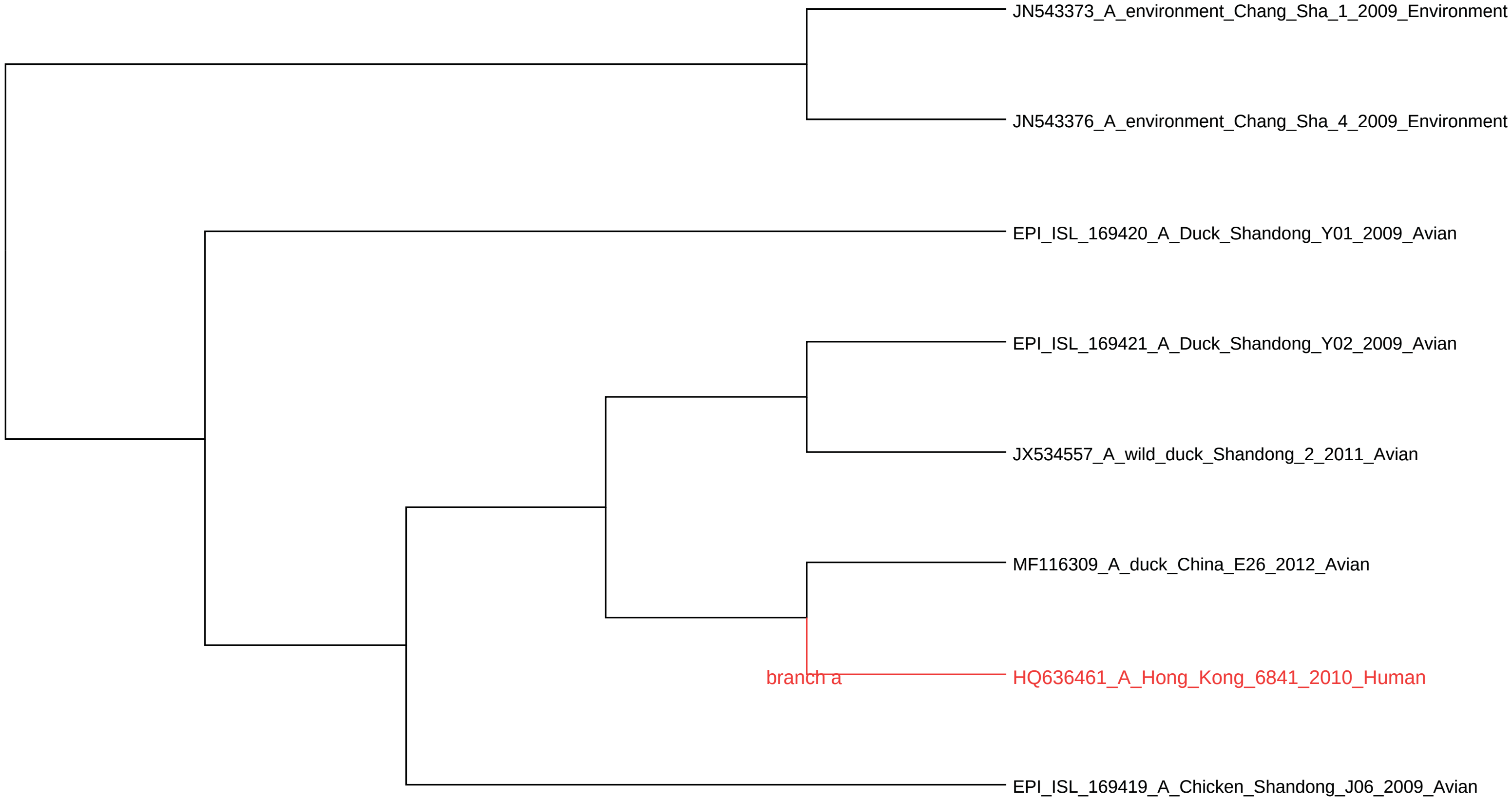
JN543373_A_environment_Chang_Sha_1_2009_Environment
JN543376_A_environment_Chang_Sha_4_2009_Environment
EPI_ISL_169420_A_Duck_Shandong_Y01_2009_Avian
EPI_ISL_169421_A_Duck_Shandong_Y02_2009_Avian
JX534557_A_wild_duck_Shandong_2_2011_Avian
MF116309_A_duck_China_E26_2012_Avian
branch a
HQ636461_A_Hong_Kong_6841_2010_Human
EPI_ISL_169419_A_Chicken_Shandong_J06_2009_Avian

HA-Group115

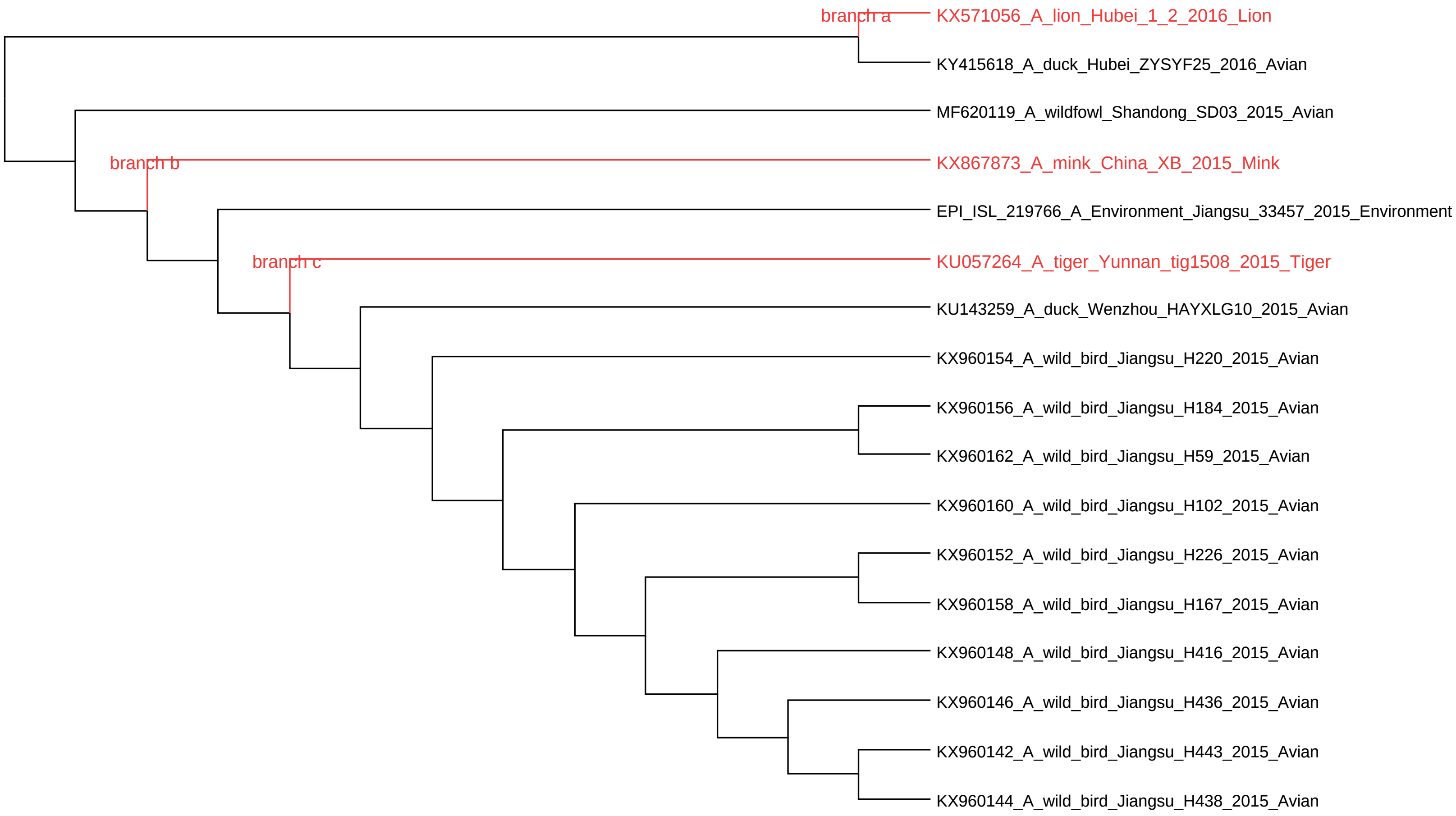

branch a
KX571056_A_lion_Hubei_1_2_2016_Lion
KY415618_A_duck_Hubei_ZYSYF25_2016_Avian
MF620119_A_wildfowl_Shandong_SD03_2015_Avian
branch b
KX867873_A_mink_China_XB_2015_Mink
EPI_ISL_219766_A_Environment_Jiangsu_33457_2015_Environment
branch c
KU057264_A_tiger_Yunnan_tig1508_2015_Tiger
KU143259_A_duck_Wenzhou_HAYXLG10_2015_Avian
KX960154_A_wild_bird_Jiangsu_H220_2015_Avian
KX960156_A_wild_bird_Jiangsu_H184_2015_Avian
KX960162_A_wild_bird_Jiangsu_H59_2015_Avian
KX960160_A_wild_bird_Jiangsu_H102_2015_Avian
KX960152_A_wild_bird_Jiangsu_H226_2015_Avian
KX960158_A_wild_bird_Jiangsu_H167_2015_Avian
KX960148_A_wild_bird_Jiangsu_H416_2015_Avian
KX960146_A_wild_bird_Jiangsu_H436_2015_Avian
KX960142_A_wild_bird_Jiangsu_H443_2015_Avian
KX960144_A_wild_bird_Jiangsu_H438_2015_Avian

HA-Group116

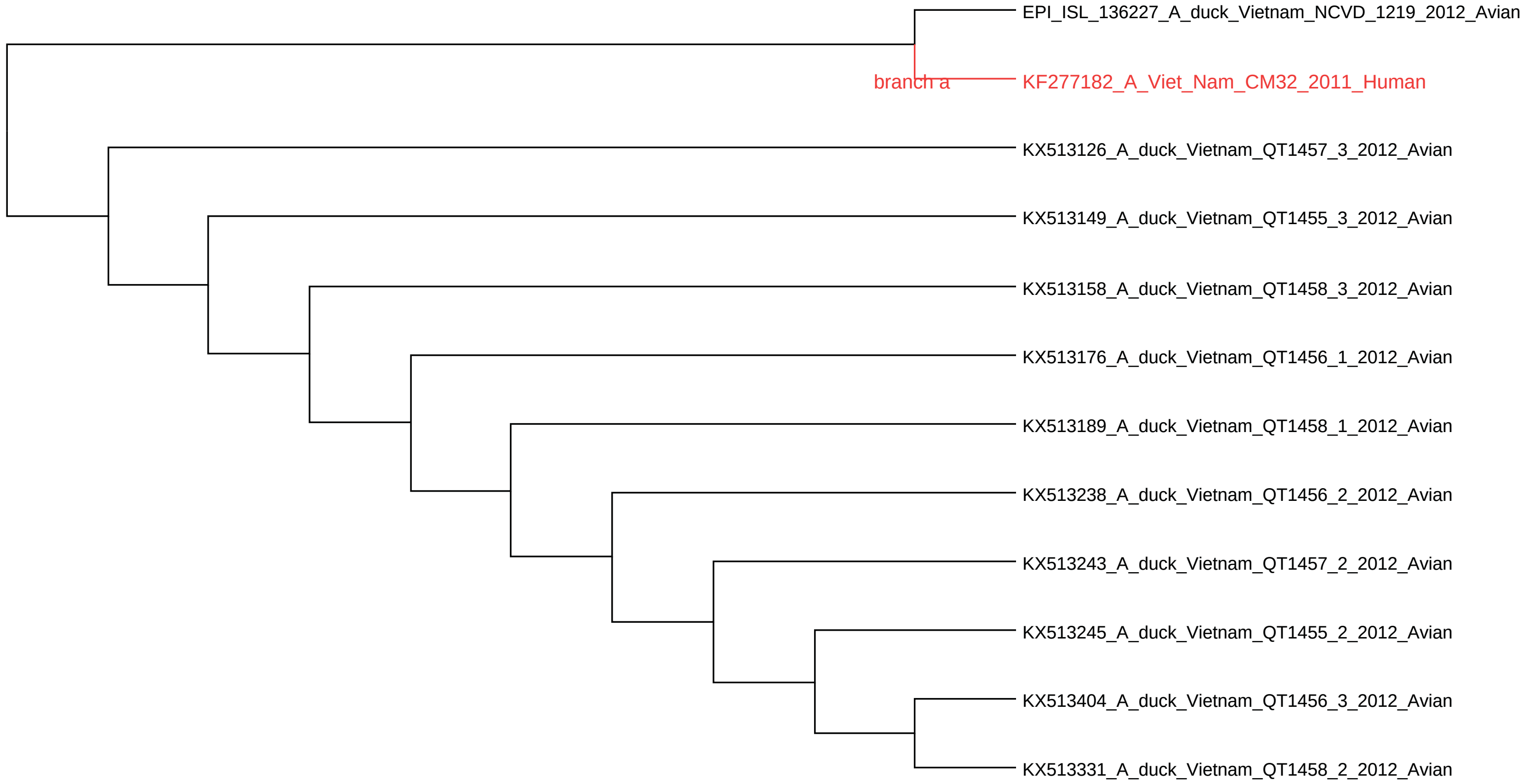

EPI_ISL_136227_A_duck_Vietnam_NCVD_1219_2012_Avian
branch a
KF277182_A_Viet_Nam_CM32_2011_Human
KX513126_A_duck_Vietnam_QT1457_3_2012_Avian
KX513149_A_duck_Vietnam_QT1455_3_2012_Avian
KX513158_A_duck_Vietnam_QT1458_3_2012_Avian
KX513176_A_duck_Vietnam_QT1456_1_2012_Avian
KX513189_A_duck_Vietnam_QT1458_1_2012_Avian
KX513238_A_duck_Vietnam_QT1456_2_2012_Avian
KX513243_A_duck_Vietnam_QT1457_2_2012_Avian
KX513245_A_duck_Vietnam_QT1455_2_2012_Avian
KX513404_A_duck_Vietnam_QT1456_3_2012_Avian
KX513331_A_duck_Vietnam_QT1458_2_2012_Avian

HA-Group117

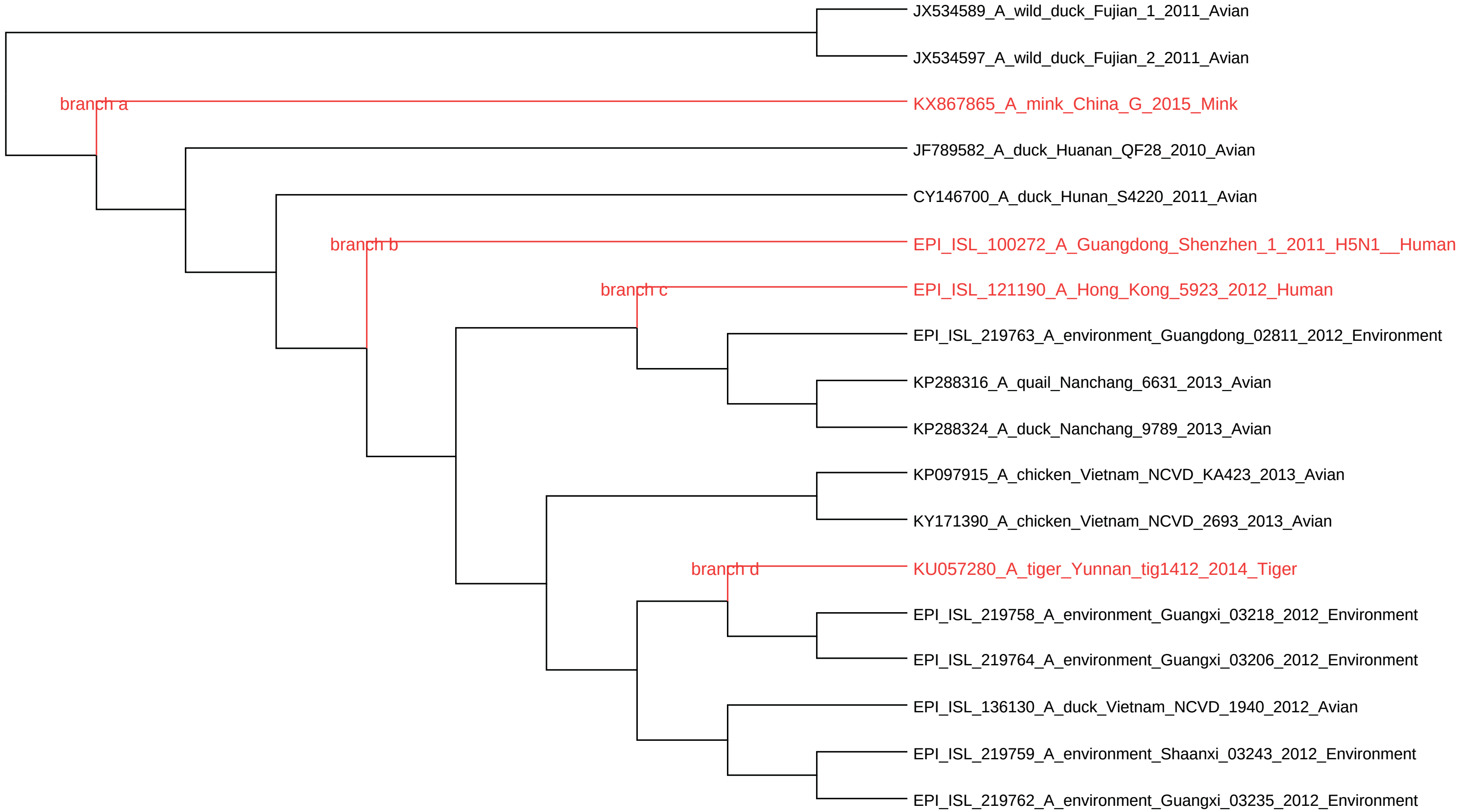

JX534589_A_wild_duck_Fujian_1_2011_Avian
JX534597_A_wild_duck_Fujian_2_2011_Avian
branch a
KX867865_A_mink_China_G_2015_Mink
JF789582_A_duck_Huanan_QF28_2010_Avian
CY146700_A_duck_Hunan_S4220_2011_Avian
branch b
EPI_ISL_100272_A_Guangdong_Shenzhen_1_2011_H5N1__Human
branch c
EPI_ISL_121190_A_Hong_Kong_5923_2012_Human
EPI_ISL_219763_A_environment_Guangdong_02811_2012_Environment
KP288316_A_quail_Nanchang_6631_2013_Avian
KP288324_A_duck_Nanchang_9789_2013_Avian
KP097915_A_chicken_Vietnam_NCVD_KA423_2013_Avian
KY171390_A_chicken_Vietnam_NCVD_2693_2013_Avian
branch d
KU057280_A_tiger_Yunnan_tig1412_2014_Tiger
EPI_ISL_219758_A_environment_Guangxi_03218_2012_Environment
EPI_ISL_219764_A_environment_Guangxi_03206_2012_Environment
EPI_ISL_136130_A_duck_Vietnam_NCVD_1940_2012_Avian
EPI_ISL_219759_A_environment_Shaanxi_03243_2012_Environment
EPI_ISL_219762_A_environment_Guangxi_03235_2012_Environment

HA-Group118

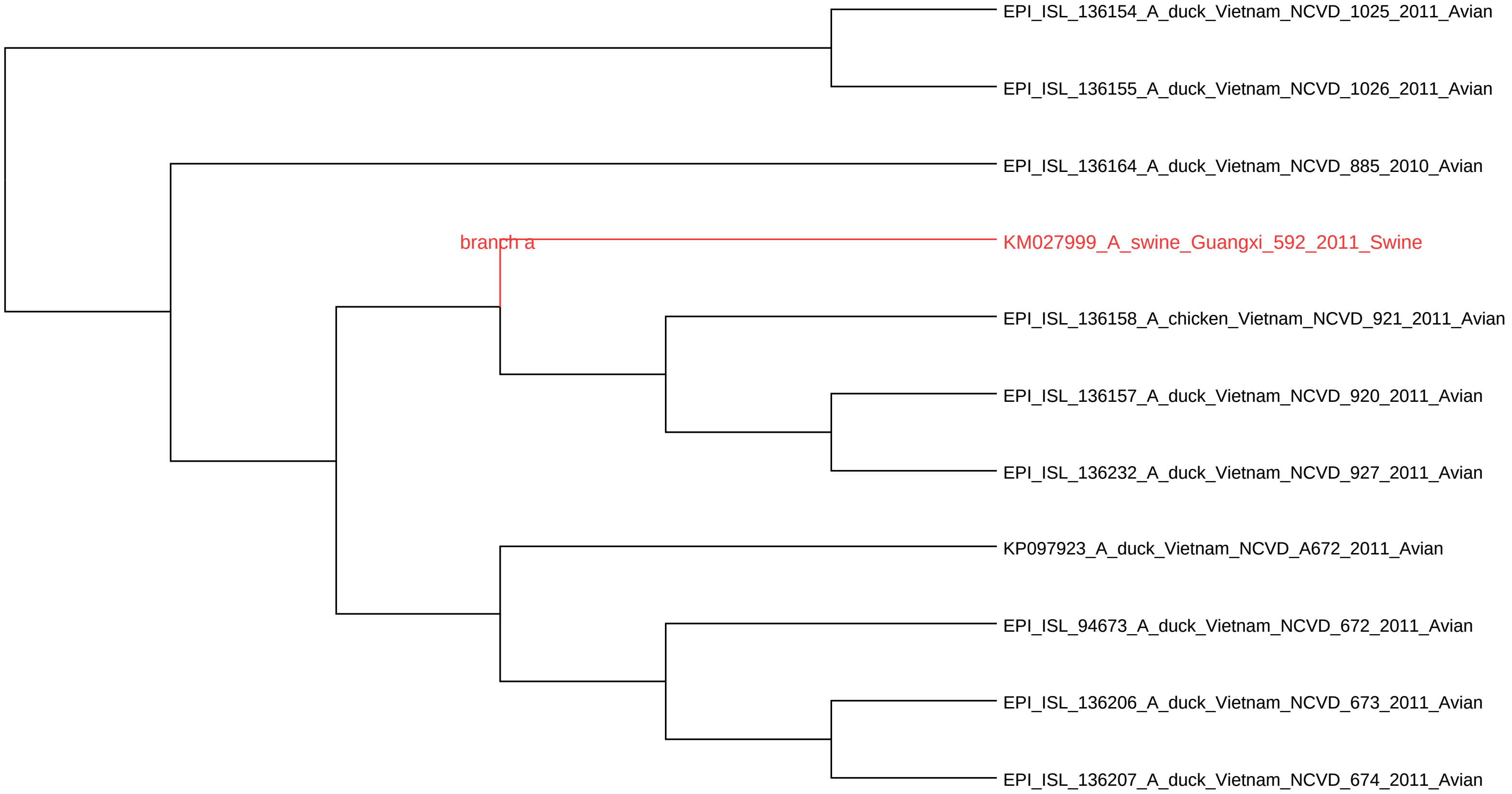

EPI_ISL_136154_A_duck_Vietnam_NCVD_1025_2011_Avian
EPI_ISL_136155_A_duck_Vietnam_NCVD_1026_2011_Avian
EPI_ISL_136164_A_duck_Vietnam_NCVD_885_2010_Avian
branch a
KM027999_A_swine_Guangxi_592_2011_Swine
EPI_ISL_136158_A_chicken_Vietnam_NCVD_921_2011_Avian
EPI_ISL_136157_A_duck_Vietnam_NCVD_920_2011_Avian
EPI_ISL_136232_A_duck_Vietnam_NCVD_927_2011_Avian
KP097923_A_duck_Vietnam_NCVD_A672_2011_Avian
EPI_ISL_94673_A_duck_Vietnam_NCVD_672_2011_Avian
EPI_ISL_136206_A_duck_Vietnam_NCVD_673_2011_Avian
EPI_ISL_136207_A_duck_Vietnam_NCVD_674_2011_Avian

HA-Group119

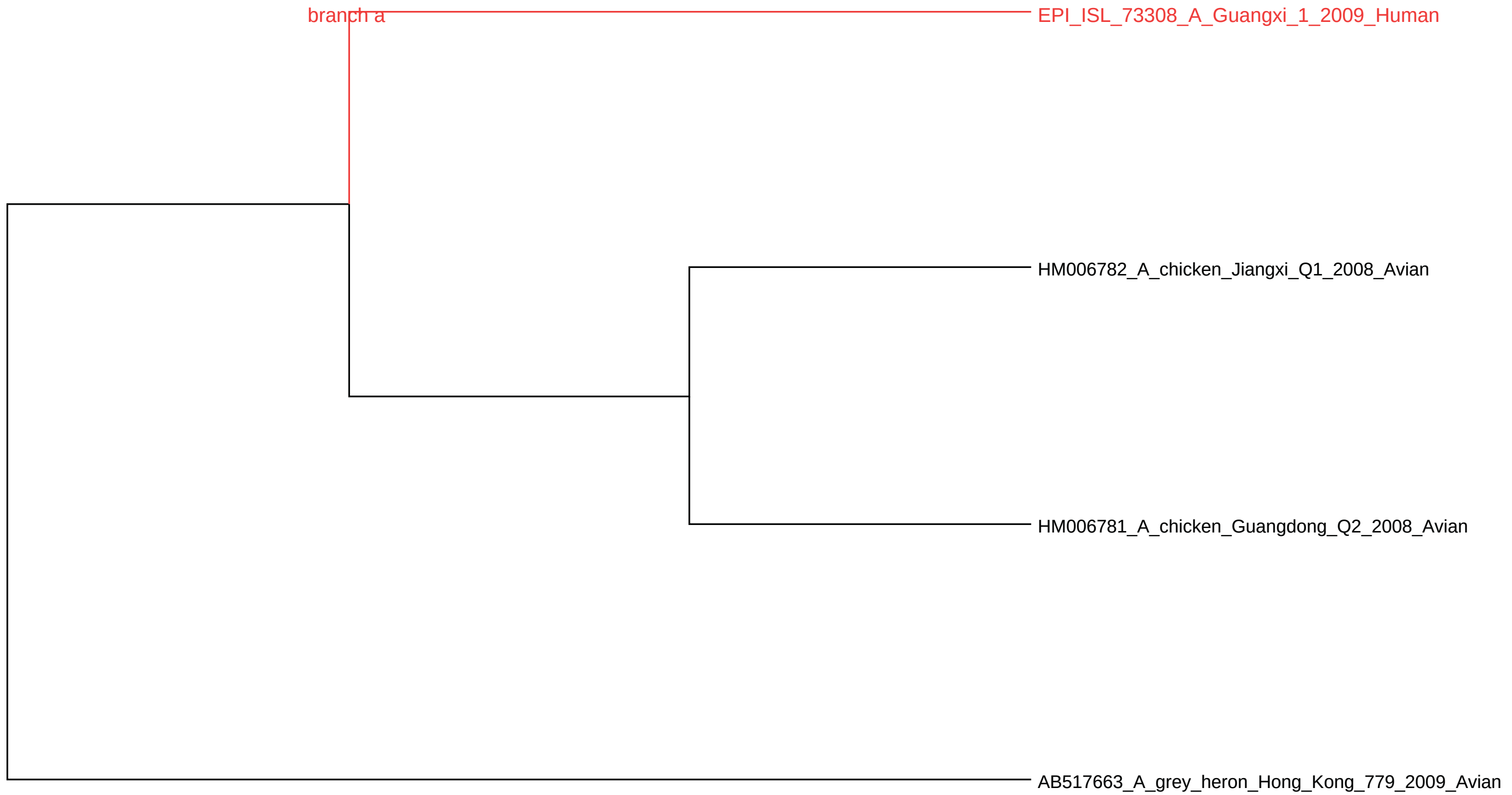

branch a
EPI_ISL_73308_A_Guangxi_1_2009_Human
HM006782_A_chicken_Jiangxi_Q1_2008_Avian
HM006781_A_chicken_Guangdong_Q2_2008_Avian
AB517663_A_grey_heron_Hong_Kong_779_2009_Avian

HA-Group120

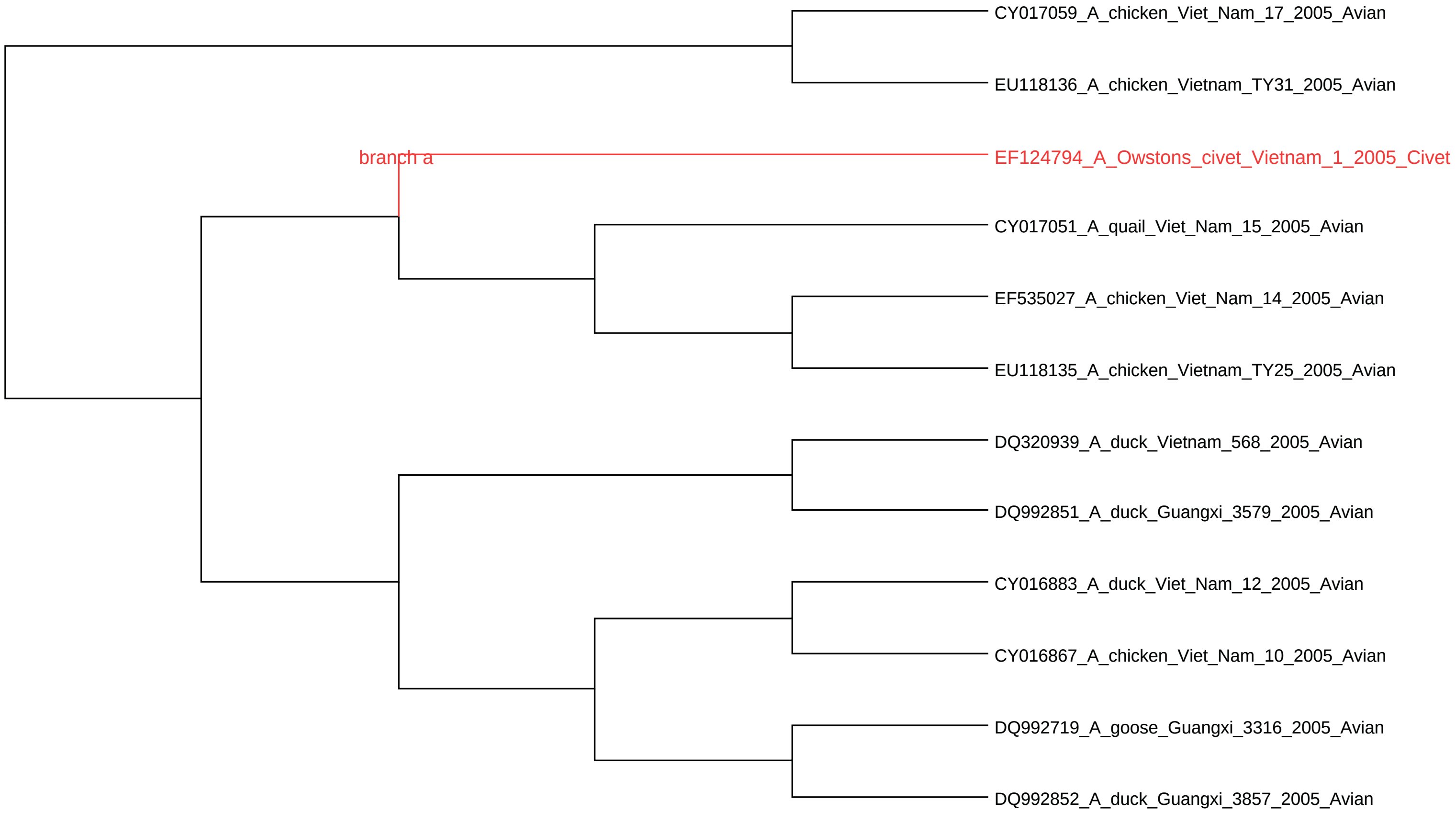
CY017059_A_chicken_Viet_Nam_17_2005_Avian
EU118136_A_chicken_Vietnam_TY31_2005_Avian
branch a
EF124794_A_Owstons_civet_Vietnam_1_2005_Civet
CY017051_A_quail_Viet_Nam_15_2005_Avian
EF535027_A_chicken_Viet_Nam_14_2005_Avian
EU118135_A_chicken_Vietnam_TY25_2005_Avian
DQ320939_A_duck_Vietnam_568_2005_Avian
DQ992851_A_duck_Guangxi_3579_2005_Avian
CY016883_A_duck_Viet_Nam_12_2005_Avian
CY016867_A_chicken_Viet_Nam_10_2005_Avian
DQ992719_A_goose_Guangxi_3316_2005_Avian
DQ992852_A_duck_Guangxi_3857_2005_Avian

HA-Group121

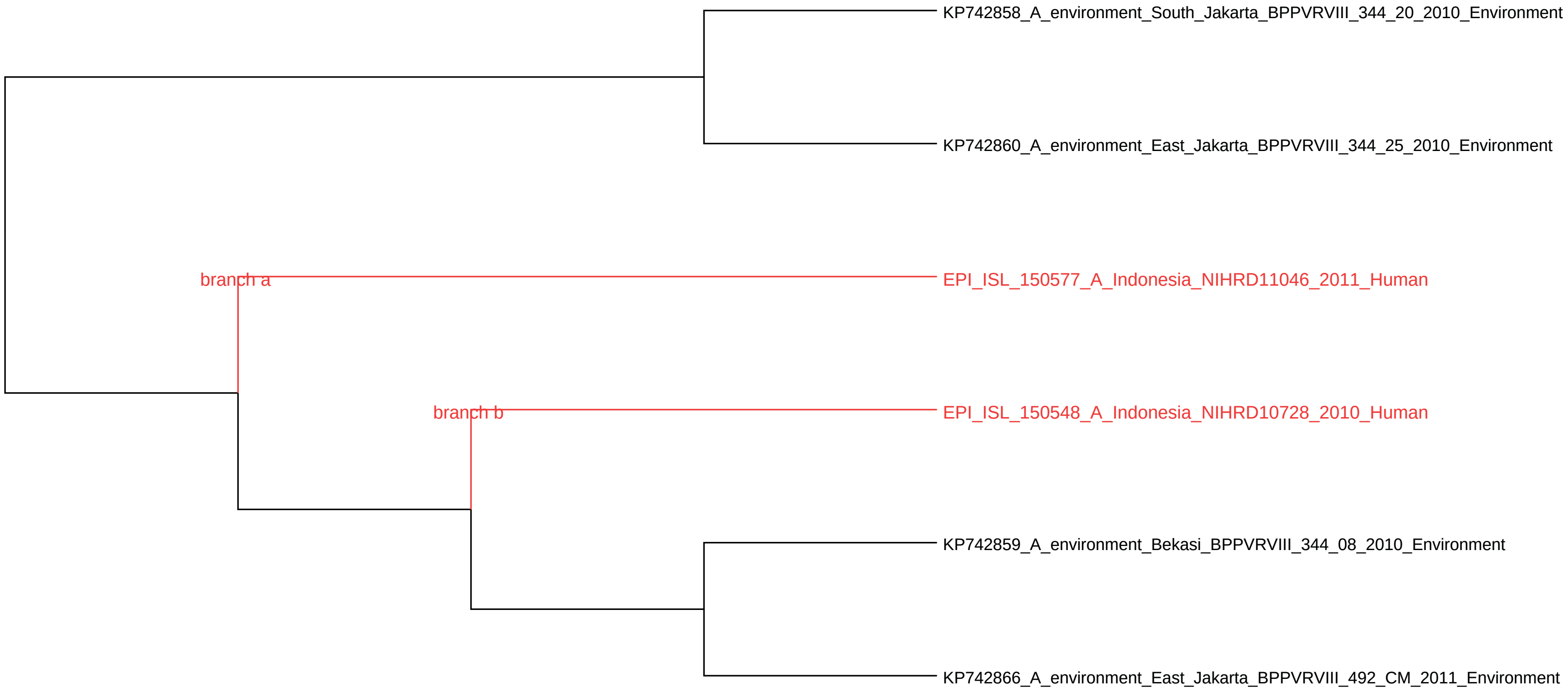

KP742858_A_environment_South_Jakarta_BPPVRVIII_344_20_2010_Environment
KP742860_A_environment_East_Jakarta_BPPVRVIII_344_25_2010_Environment
branch a
EPI_ISL_150577_A_Indonesia_NIHRD11046_2011_Human
branch b
EPI_ISL_150548_A_Indonesia_NIHRD10728_2010_Human
KP742859_A_environment_Bekasi_BPPVRVIII_344_08_2010_Environment
KP742866_A_environment_East_Jakarta_BPPVRVIII_492_CM_2011_Environment

HA-Group122

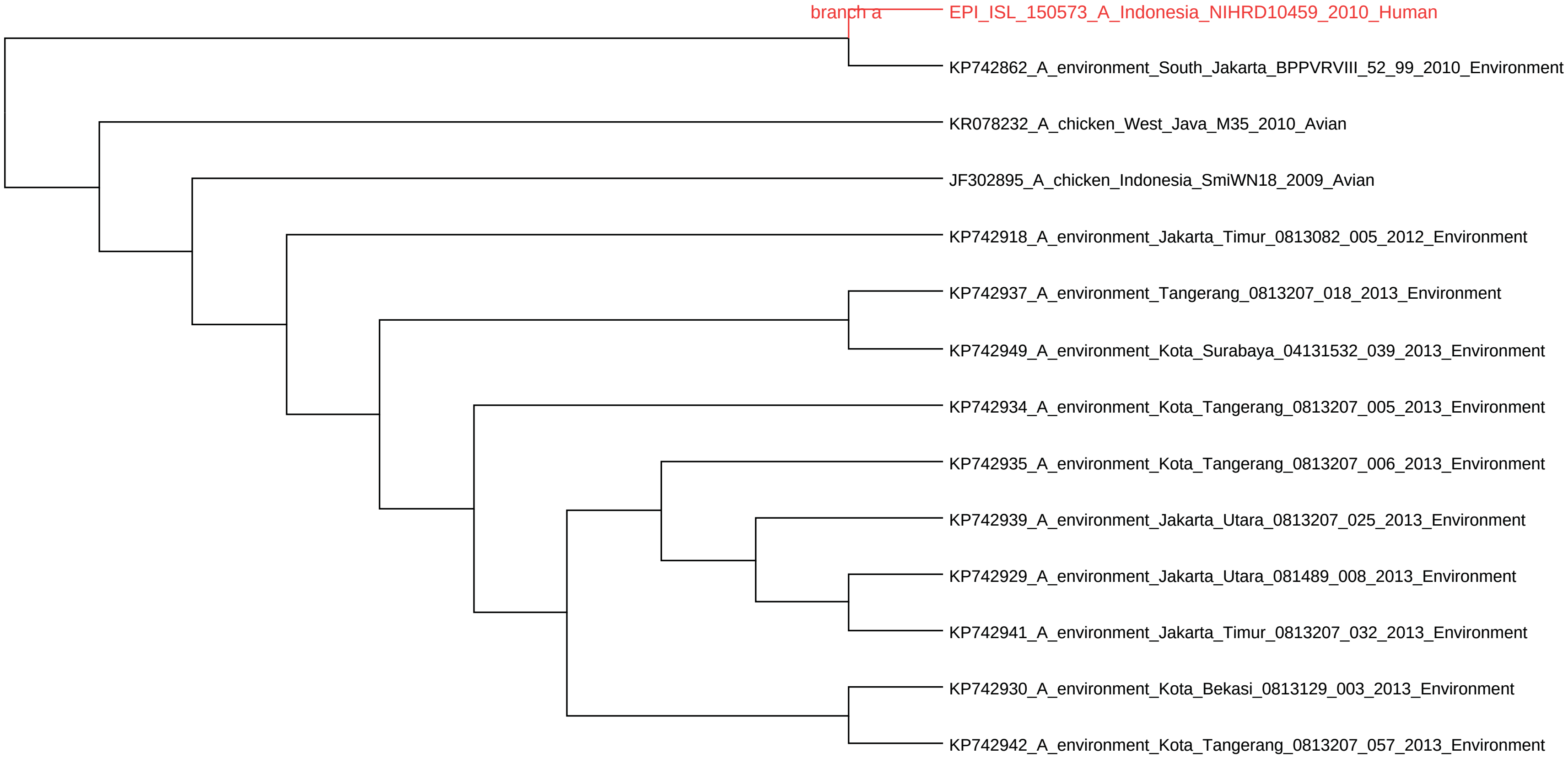
branch a
EPI_ISL_150573_A_Indonesia_NIHRD10459_2010_Human
KP742862_A_environment_South_Jakarta_BPPVRVIII_52_99_2010_Environment
KR078232_A_chicken_West_Java_M35_2010_Avian
JF302895_A_chicken_Indonesia_SmiWN18_2009_Avian
KP742918_A_environment_Jakarta_Timur_0813082_005_2012_Environment
KP742937_A_environment_Tangerang_0813207_018_2013_Environment
KP742949_A_environment_Kota_Surabaya_04131532_039_2013_Environment
KP742934_A_environment_Kota_Tangerang_0813207_005_2013_Environment
KP742935_A_environment_Kota_Tangerang_0813207_006_2013_Environment
KP742939_A_environment_Jakarta_Utara_0813207_025_2013_Environment
KP742929_A_environment_Jakarta_Utara_081489_008_2013_Environment
KP742941_A_environment_Jakarta_Timur_0813207_032_2013_Environment
KP742930_A_environment_Kota_Bekasi_0813129_003_2013_Environment
KP742942_A_environment_Kota_Tangerang_0813207_057_2013_Environment

HA-Group123

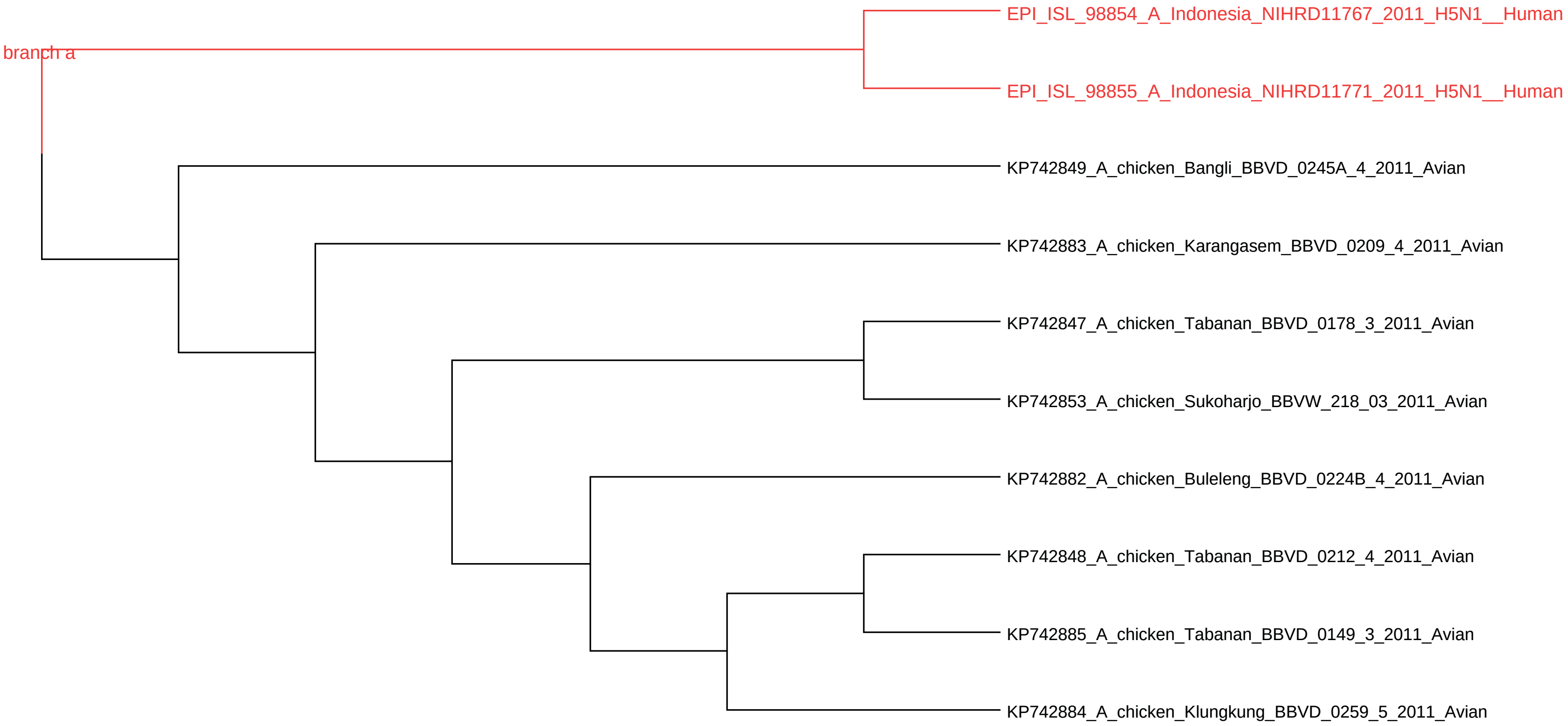

branch a
EPI_ISL_98854_A_Indonesia_NIHRD11767_2011_H5N1__Human
EPI_ISL_98855_A_Indonesia_NIHRD11771_2011_H5N1__Human
KP742849_A_chicken_Bangli_BBVD_0245A_4_2011_Avian
KP742883_A_chicken_Karangasem_BBVD_0209_4_2011_Avian
KP742847_A_chicken_Tabanan_BBVD_0178_3_2011_Avian
KP742853_A_chicken_Sukoharjo_BBVW_218_03_2011_Avian
KP742882_A_chicken_Buleleng_BBVD_0224B_4_2011_Avian
KP742848_A_chicken_Tabanan_BBVD_0212_4_2011_Avian
KP742885_A_chicken_Tabanan_BBVD_0149_3_2011_Avian
KP742884_A_chicken_Klungkung_BBVD_0259_5_2011_Avian

HA-Group124

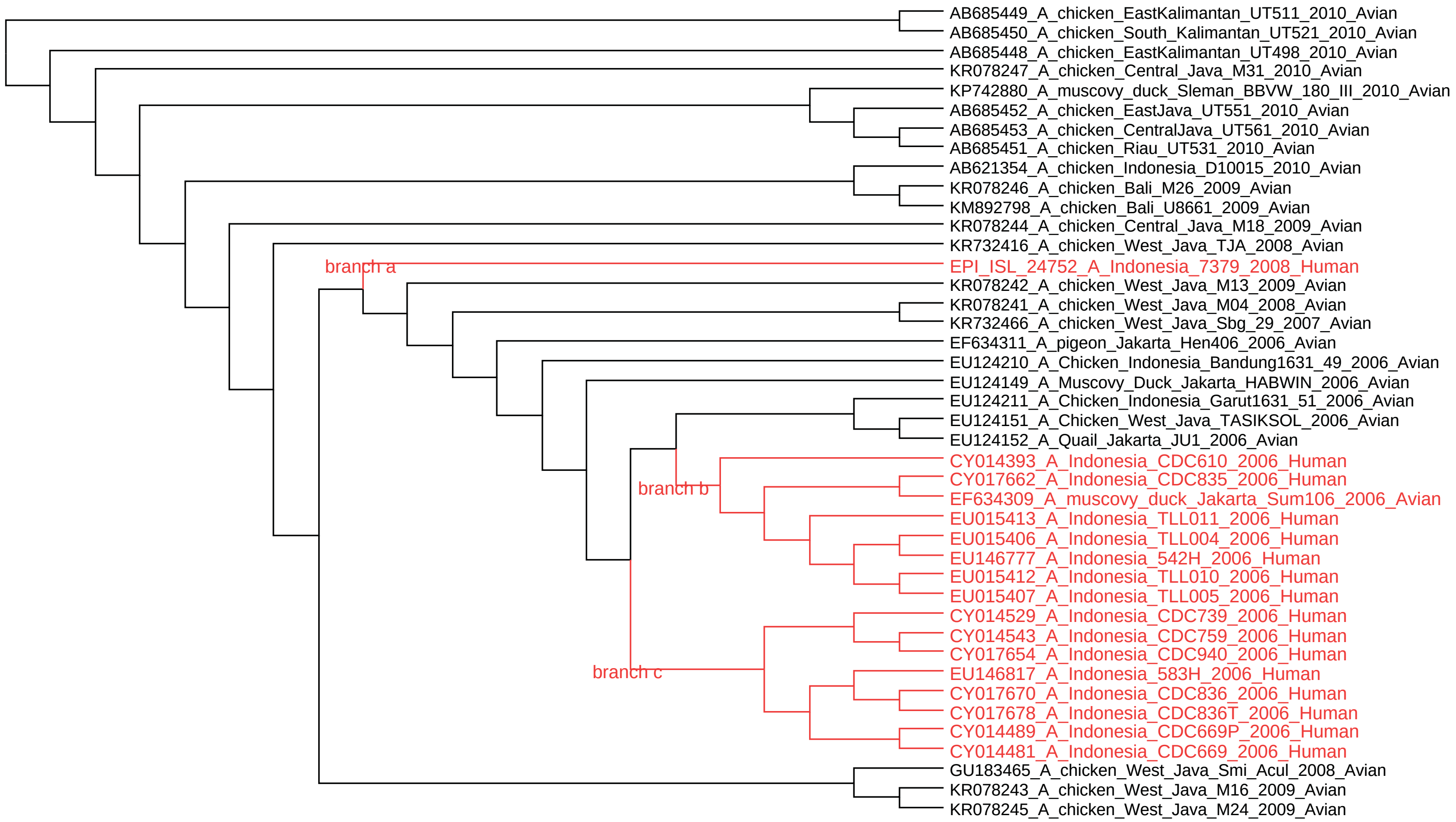

AB685449_A_chicken_EastKalimantan_UT511_2010_Avian
AB685450_A_chicken_South_Kalimantan_UT521_2010_Avian
AB685448_A_chicken_EastKalimantan_UT498_2010_Avian
KR078247_A_chicken_Central_Java_M31_2010_Avian
KP742880_A_muscovy_duck_Sleman_BBVW_180_III_2010_Avian
AB685452_A_chicken_EastJava_UT551_2010_Avian
AB685453_A_chicken_CentralJava_UT561_2010_Avian
AB685451_A_chicken_Riau_UT531_2010_Avian
AB621354_A_chicken_Indonesia_D10015_2010_Avian
KR078246_A_chicken_Bali_M26_2009_Avian
KM892798_A_chicken_Bali_U8661_2009_Avian
KR078244_A_chicken_Central_Java_M18_2009_Avian
KR732416_A_chicken_West_Java_TJA_2008_Avian
branch a
EPI_ISL_24752_A_Indonesia_7379_2008_Human
KR078242_A_chicken_West_Java_M13_2009_Avian
KR078241_A_chicken_West_Java_M04_2008_Avian
KR732466_A_chicken_West_Java_Sbg_29_2007_Avian
EF634311_A_pigeon_Jakarta_Hen406_2006_Avian
EU124210_A_Chicken_Indonesia_Bandung1631_49_2006_Avian
EU124149_A_Muscovy_Duck_Jakarta_HABWIN_2006_Avian
EU124211_A_Chicken_Indonesia_Garut1631_51_2006_Avian
EU124151_A_Chicken_West_Java_TASIKSOL_2006_Avian
EU124152_A_Quail_Jakarta_JU1_2006_Avian
CY014393_A_Indonesia_CDC610_2006_Human
branch b
CY017662_A_Indonesia_CDC835_2006_Human
EF634309_A_muscovy_duck_Jakarta_Sum106_2006_Avian
EU015413_A_Indonesia_TLL011_2006_Human
EU015406_A_Indonesia_TLL004_2006_Human
EU146777_A_Indonesia_542H_2006_Human
EU015412_A_Indonesia_TLL010_2006_Human
EU015407_A_Indonesia_TLL005_2006_Human
CY014529_A_Indonesia_CDC739_2006_Human
CY014543_A_Indonesia_CDC759_2006_Human
CY017654_A_Indonesia_CDC940_2006_Human
branch c
EU146817_A_Indonesia_583H_2006_Human
CY017670_A_Indonesia_CDC836_2006_Human
CY017678_A_Indonesia_CDC836T_2006_Human
CY014489_A_Indonesia_CDC669P_2006_Human
CY014481_A_Indonesia_CDC669_2006_Human
GU183465_A_chicken_West_Java_Smi_Acul_2008_Avian
KR078243_A_chicken_West_Java_M16_2009_Avian
KR078245_A_chicken_West_Java_M24_2009_Avian

HA-Group125

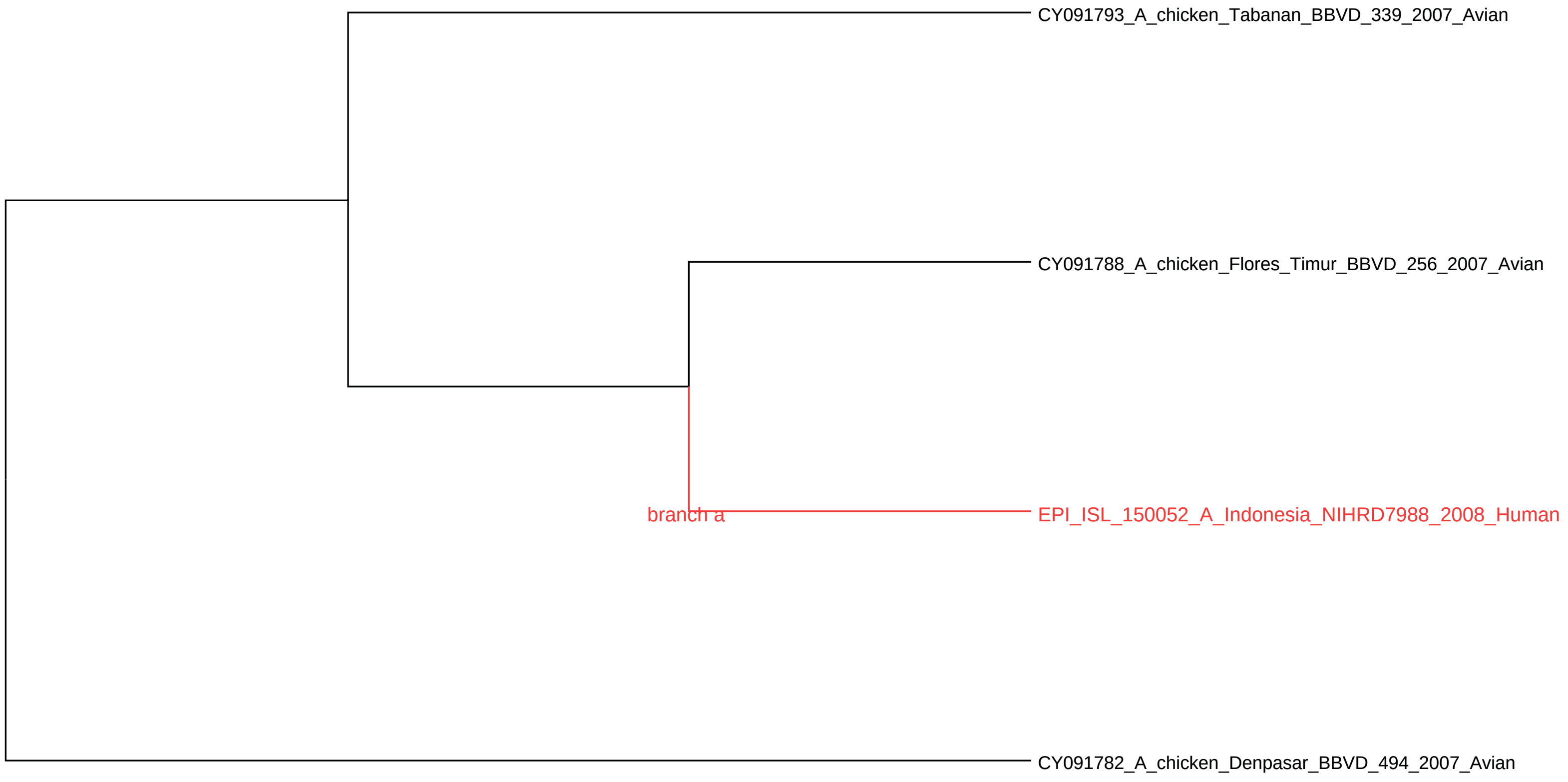

CY091793_A_chicken_Tabanan_BBVD_339_2007_Avian
CY091788_A_chicken_Flores_Timur_BBVD_256_2007_Avian
branch a
EPI_ISL_150052_A_Indonesia_NIHRD7988_2008_Human
CY091782_A_chicken_Denpasar_BBVD_494_2007_Avian

HA-Group126

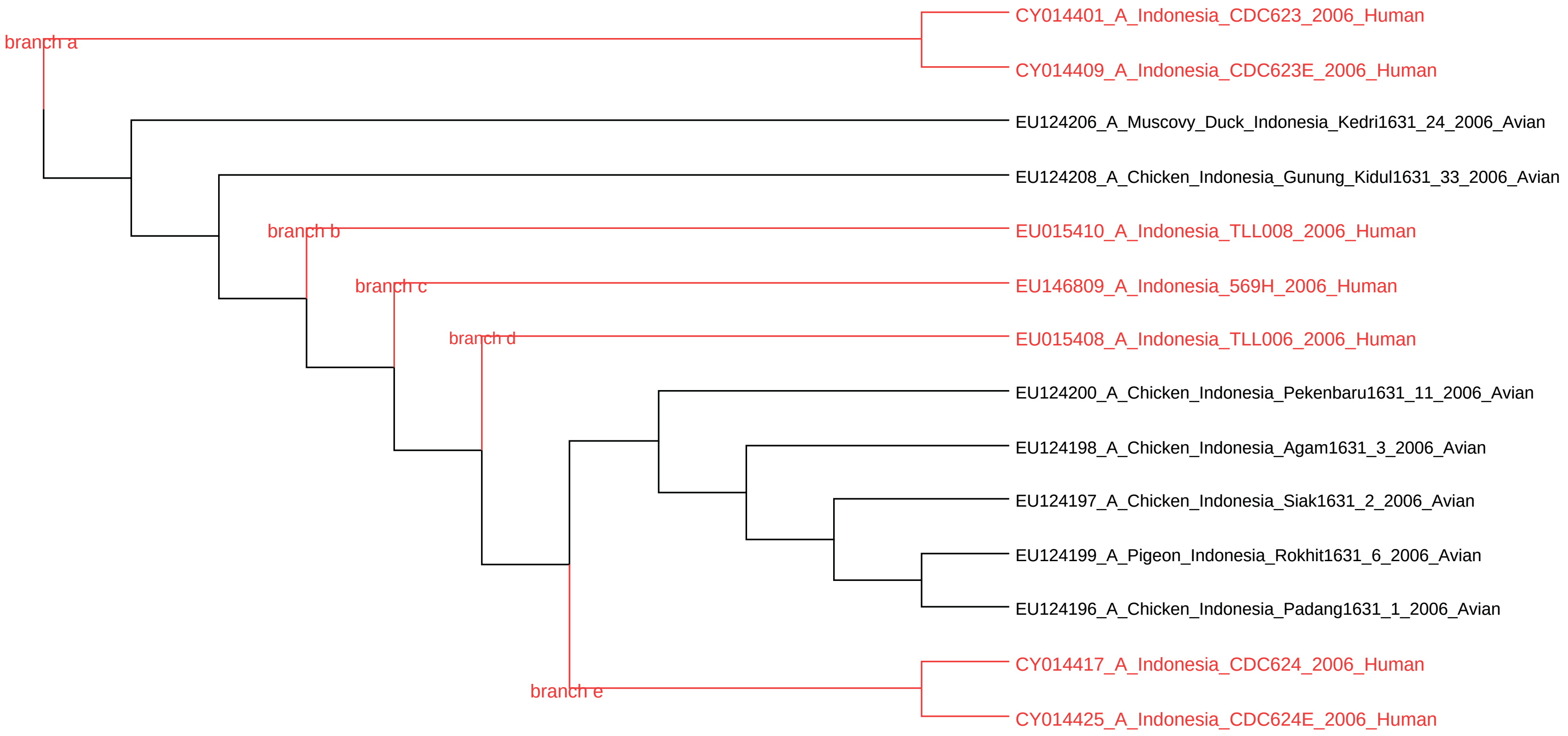
branch a
branch b
branch c
branch d
branch e
CY014401_A_Indonesia_CDC623_2006_Human
CY014409_A_Indonesia_CDC623E_2006_Human
EU124206_A_Muscovy_Duck_Indonesia_Kedri1631_24_2006_Avian
EU124208_A_Chicken_Indonesia_Gunung_Kidul1631_33_2006_Avian
EU015410_A_Indonesia_TLL008_2006_Human
EU146809_A_Indonesia_569H_2006_Human
EU015408_A_Indonesia_TLL006_2006_Human
EU124200_A_Chicken_Indonesia_Pekenbaru1631_11_2006_Avian
EU124198_A_Chicken_Indonesia_Agam1631_3_2006_Avian
EU124197_A_Chicken_Indonesia_Siak1631_2_2006_Avian
EU124199_A_Pigeon_Indonesia_Rokhit1631_6_2006_Avian
EU124196_A_Chicken_Indonesia_Padang1631_1_2006_Avian
CY014417_A_Indonesia_CDC624_2006_Human
CY014425_A_Indonesia_CDC624E_2006_Human

HA-Group127

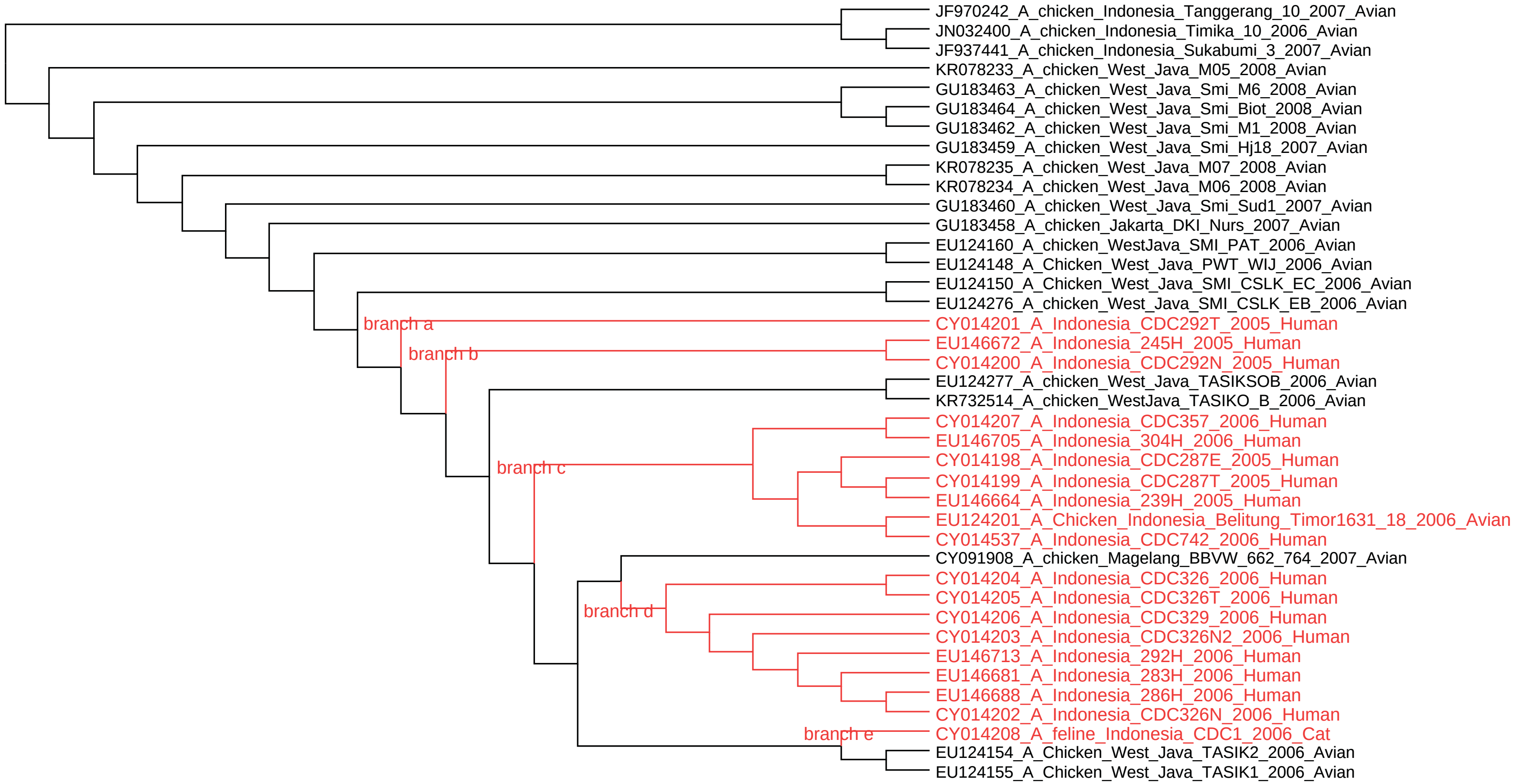
JF970242_A_chicken_Indonesia_Tanggerang_10_2007_Avian
JN032400_A_chicken_Indonesia_Timika_10_2006_Avian
JF937441_A_chicken_Indonesia_Sukabumi_3_2007_Avian
KR078233_A_chicken_West_Java_M05_2008_Avian
GU183463_A_chicken_West_Java_Smi_M6_2008_Avian
GU183464_A_chicken_West_Java_Smi_Biot_2008_Avian
GU183462_A_chicken_West_Java_Smi_M1_2008_Avian
GU183459_A_chicken_West_Java_Smi_Hj18_2007_Avian
KR078235_A_chicken_West_Java_M07_2008_Avian
KR078234_A_chicken_West_Java_M06_2008_Avian
GU183460_A_chicken_West_Java_Smi_Sud1_2007_Avian
GU183458_A_chicken_Jakarta_DKI_Nurs_2007_Avian
EU124160_A_chicken_WestJava_SMI_PAT_2006_Avian
EU124148_A_Chicken_West_Java_PWT_WIJ_2006_Avian
EU124150_A_Chicken_West_Java_SMI_CSLK_EC_2006_Avian
EU124276_A_chicken_West_Java_SMI_CSLK_EB_2006_Avian
branch a
CY014201_A_Indonesia_CDC292T_2005_Human
branch b
EU146672_A_Indonesia_245H_2005_Human
CY014200_A_Indonesia_CDC292N_2005_Human
EU124277_A_chicken_West_Java_TASIKSOB_2006_Avian
KR732514_A_chicken_WestJava_TASIKO_B_2006_Avian
CY014207_A_Indonesia_CDC357_2006_Human
EU146705_A_Indonesia_304H_2006_Human
branch c
CY014198_A_Indonesia_CDC287E_2005_Human
CY014199_A_Indonesia_CDC287T_2005_Human
EU146664_A_Indonesia_239H_2005_Human
EU124201_A_Chicken_Indonesia_Belitung_Timor1631_18_2006_Avian
CY014537_A_Indonesia_CDC742_2006_Human
CY091908_A_chicken_Magelang_BBVW_662_764_2007_Avian
CY014204_A_Indonesia_CDC326_2006_Human
CY014205_A_Indonesia_CDC326T_2006_Human
branch d
CY014206_A_Indonesia_CDC329_2006_Human
CY014203_A_Indonesia_CDC326N2_2006_Human
EU146713_A_Indonesia_292H_2006_Human
EU146681_A_Indonesia_283H_2006_Human
EU146688_A_Indonesia_286H_2006_Human
CY014202_A_Indonesia_CDC326N_2006_Human
branch e
CY014208_A_feline_Indonesia_CDC1_2006_Cat
EU124154_A_Chicken_West_Java_TASIK2_2006_Avian
EU124155_A_Chicken_West_Java_TASIK1_2006_Avian

HA-Group128

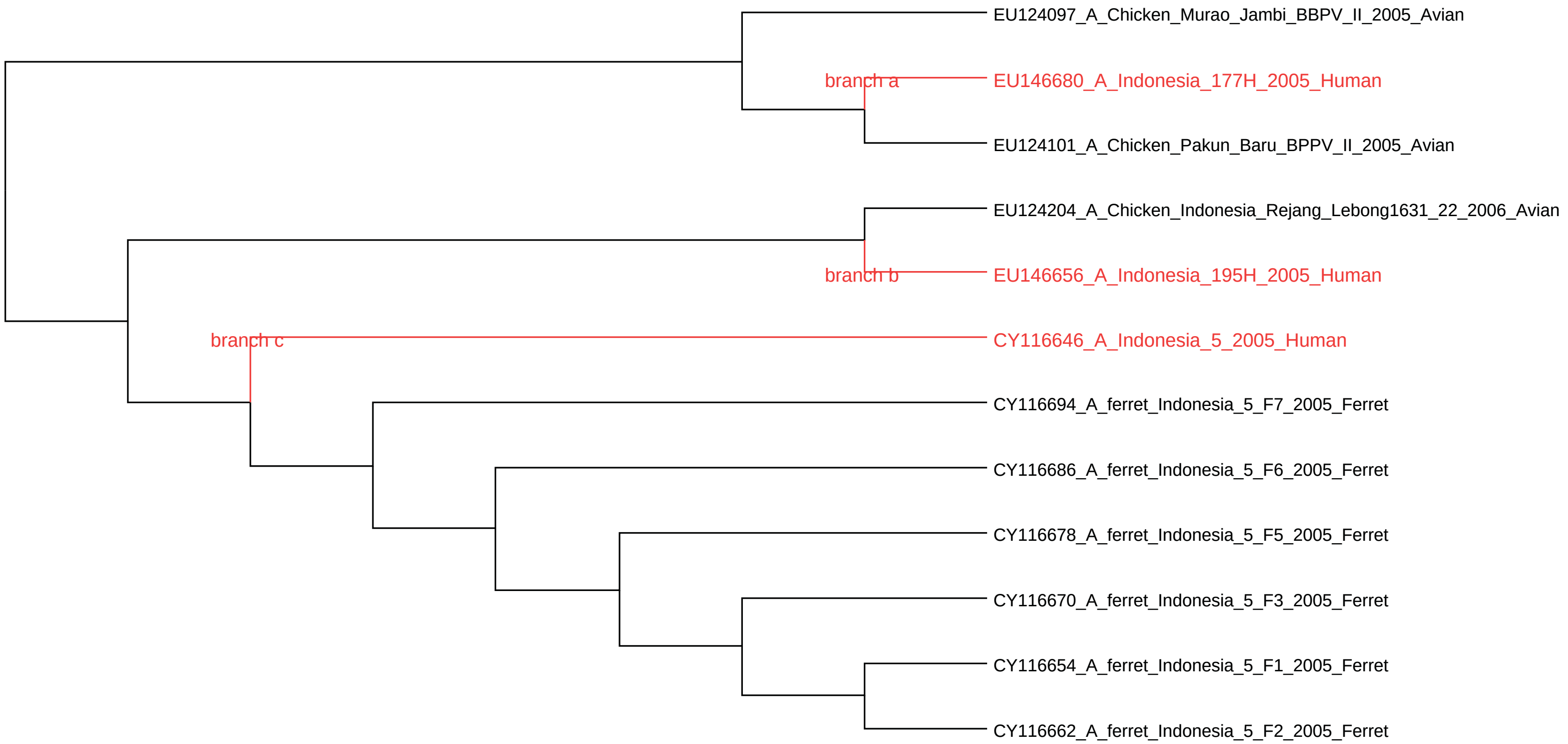

EU124097_A_Chicken_Murao_Jambi_BBPV_II_2005_Avian
branch a
EU146680_A_Indonesia_177H_2005_Human
EU124101_A_Chicken_Pakun_Baru_BPPV_II_2005_Avian
EU124204_A_Chicken_Indonesia_Rejang_Lebong1631_22_2006_Avian
branch b
EU146656_A_Indonesia_195H_2005_Human
branch c
CY116646_A_Indonesia_5_2005_Human
CY116694_A_ferret_Indonesia_5_F7_2005_Ferret
CY116686_A_ferret_Indonesia_5_F6_2005_Ferret
CY116678_A_ferret_Indonesia_5_F5_2005_Ferret
CY116670_A_ferret_Indonesia_5_F3_2005_Ferret
CY116654_A_ferret_Indonesia_5_F1_2005_Ferret
CY116662_A_ferret_Indonesia_5_F2_2005_Ferret

HA-Group129

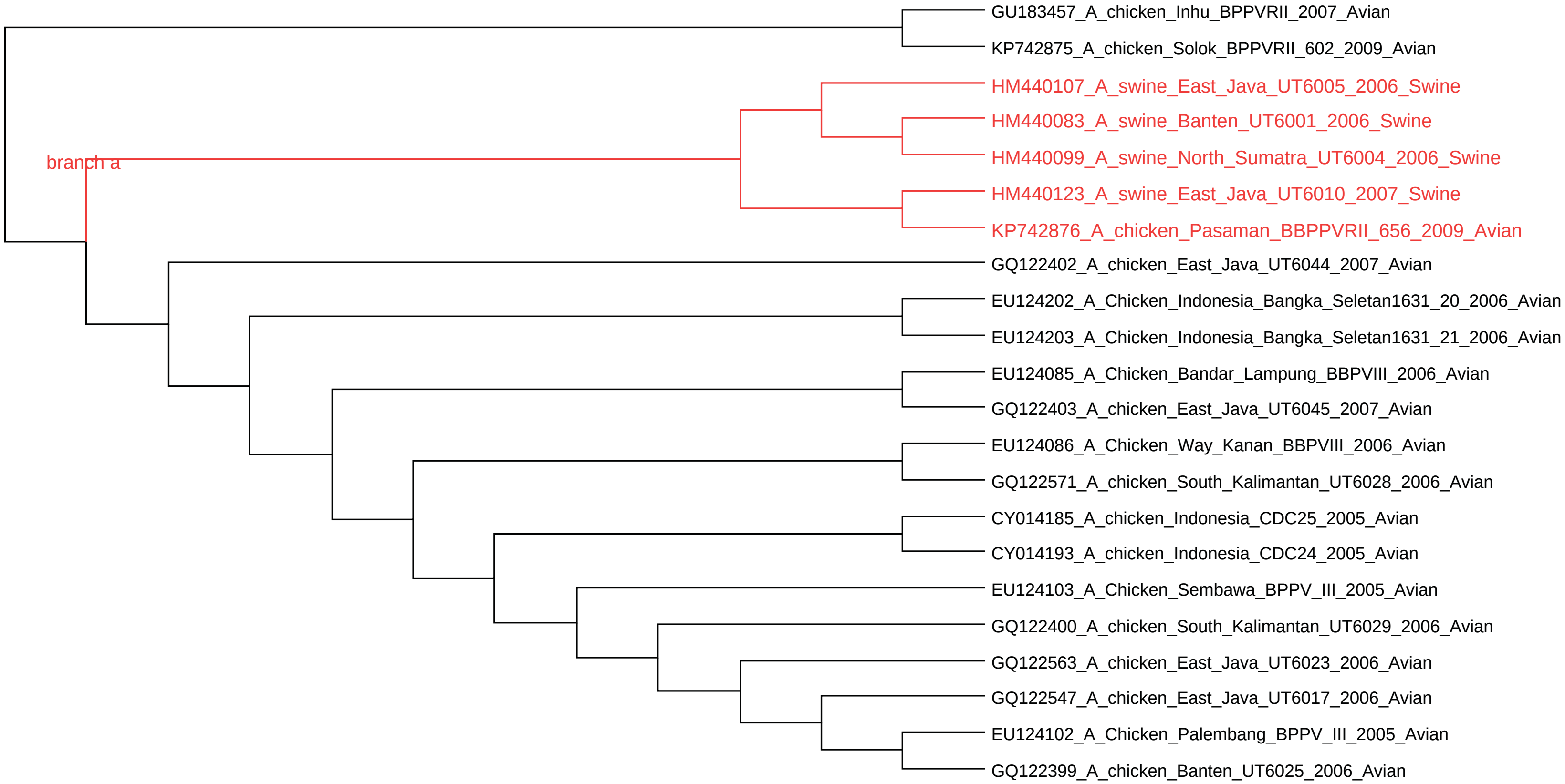

GU183457_A_chicken_Inhu_BPPVRII_2007_Avian
KP742875_A_chicken_Solok_BPPVRII_602_2009_Avian
HM440107_A_swine_East_Java_UT6005_2006_Swine
HM440083_A_swine_Banten_UT6001_2006_Swine
HM440099_A_swine_North_Sumatra_UT6004_2006_Swine
HM440123_A_swine_East_Java_UT6010_2007_Swine
KP742876_A_chicken_Pasaman_BBPPVRII_656_2009_Avian
branch a
GQ122402_A_chicken_East_Java_UT6044_2007_Avian
EU124202_A_Chicken_Indonesia_Bangka_Seletan1631_20_2006_Avian
EU124203_A_Chicken_Indonesia_Bangka_Seletan1631_21_2006_Avian
EU124085_A_Chicken_Bandar_Lampung_BBPVIII_2006_Avian
GQ122403_A_chicken_East_Java_UT6045_2007_Avian
EU124086_A_Chicken_Way_Kanan_BBPVIII_2006_Avian
GQ122571_A_chicken_South_Kalimantan_UT6028_2006_Avian
CY014185_A_chicken_Indonesia_CDC25_2005_Avian
CY014193_A_chicken_Indonesia_CDC24_2005_Avian
EU124103_A_Chicken_Sembawa_BPPV_III_2005_Avian
GQ122400_A_chicken_South_Kalimantan_UT6029_2006_Avian
GQ122563_A_chicken_East_Java_UT6023_2006_Avian
GQ122547_A_chicken_East_Java_UT6017_2006_Avian
EU124102_A_Chicken_Palembang_BPPV_III_2005_Avian
GQ122399_A_chicken_Banten_UT6025_2006_Avian

HA-Group130

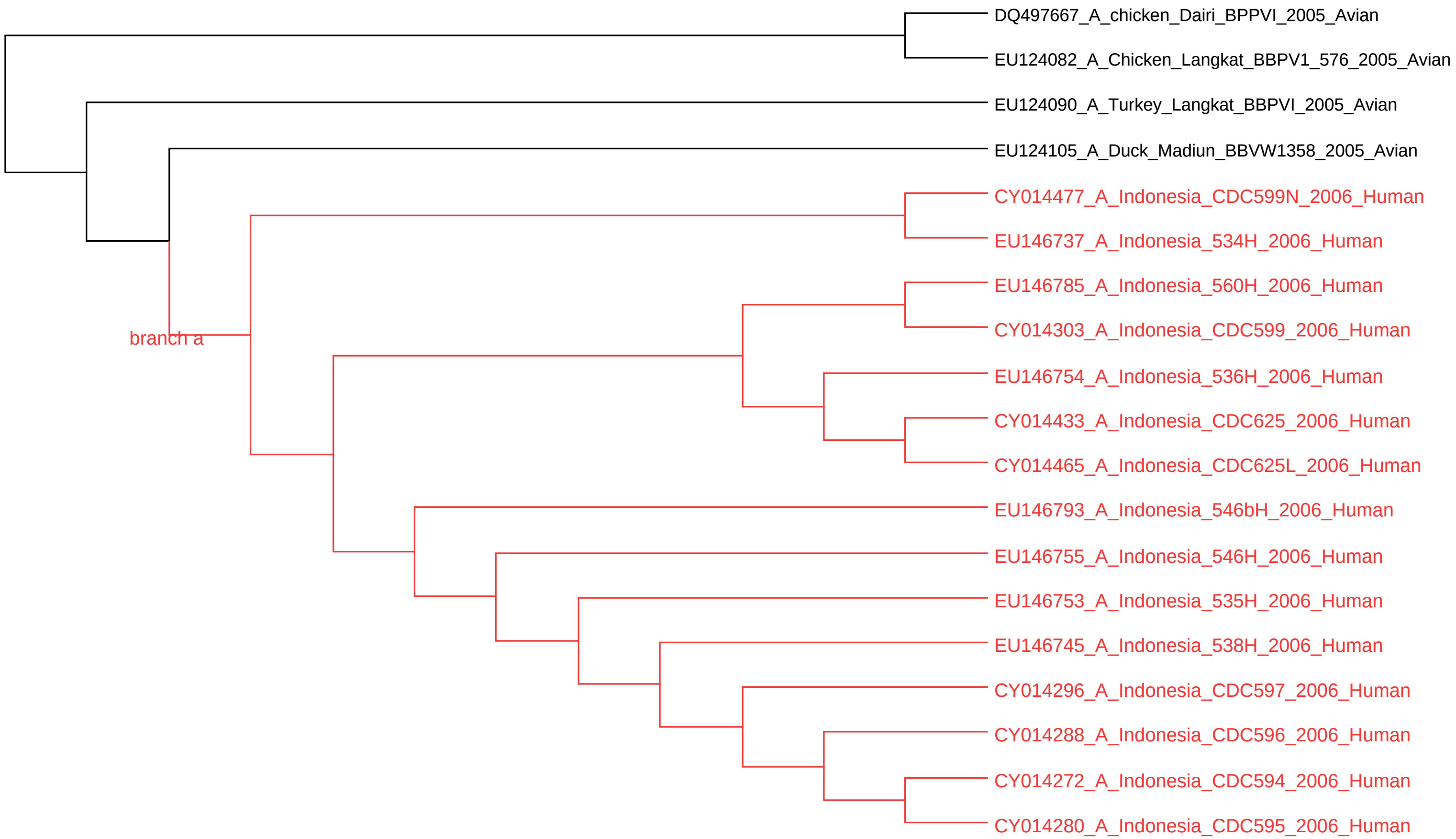

DQ497667_A_chicken_Dairi_BPPVI_2005_Avian
EU124082_A_Chicken_Langkat_BBPV1_576_2005_Avian
EU124090_A_Turkey_Langkat_BBPVI_2005_Avian
EU124105_A_Duck_Madiun_BBVW1358_2005_Avian
CY014477_A_Indonesia_CDC599N_2006_Human
EU146737_A_Indonesia_534H_2006_Human
EU146785_A_Indonesia_560H_2006_Human
CY014303_A_Indonesia_CDC599_2006_Human
EU146754_A_Indonesia_536H_2006_Human
CY014433_A_Indonesia_CDC625_2006_Human
CY014465_A_Indonesia_CDC625L_2006_Human
EU146793_A_Indonesia_546bH_2006_Human
EU146755_A_Indonesia_546H_2006_Human
EU146753_A_Indonesia_535H_2006_Human
EU146745_A_Indonesia_538H_2006_Human
CY014296_A_Indonesia_CDC597_2006_Human
CY014288_A_Indonesia_CDC596_2006_Human
CY014272_A_Indonesia_CDC594_2006_Human
CY014280_A_Indonesia_CDC595_2006_Human
branch a

HA-Group131

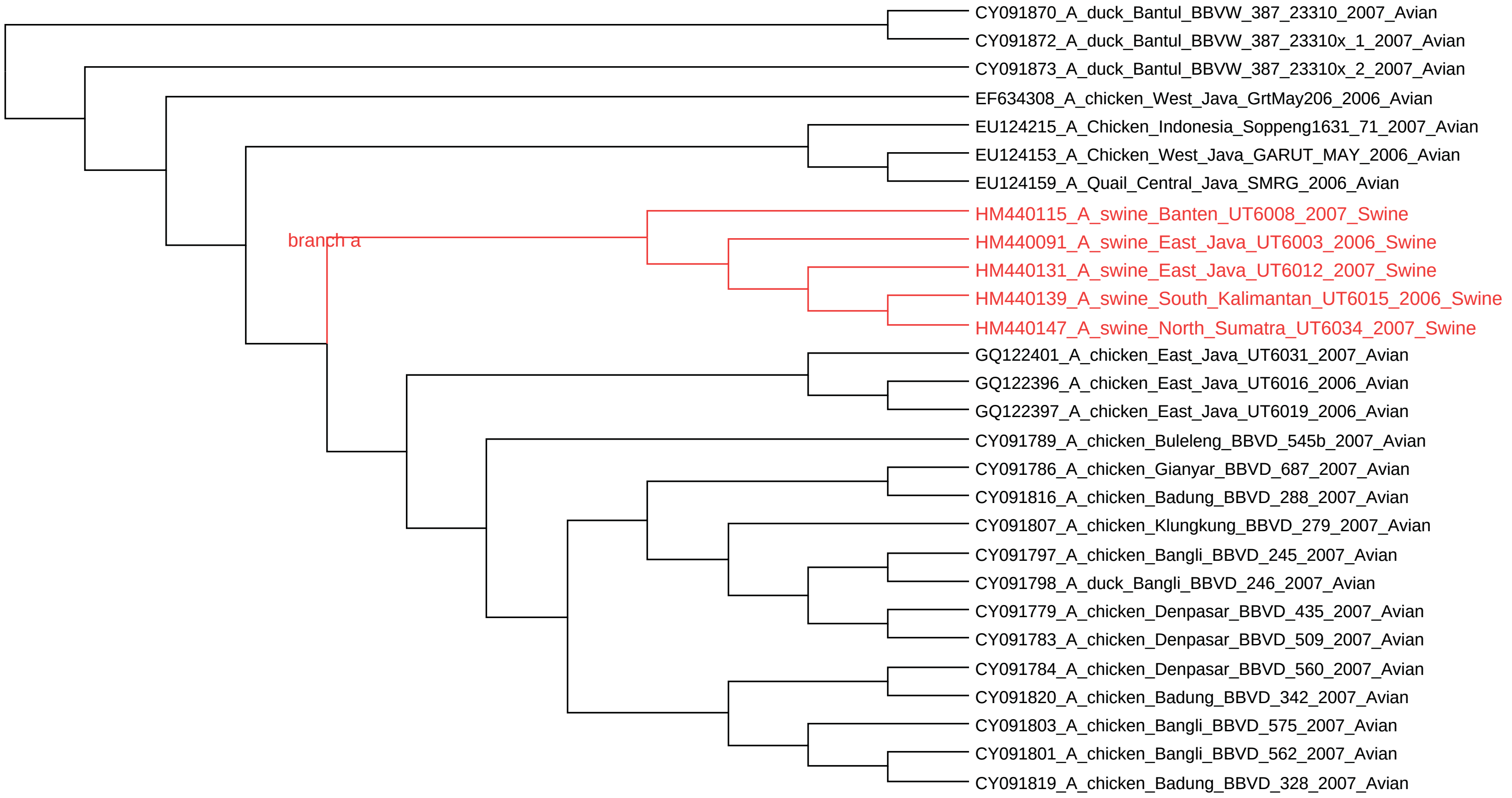

CY091870_A_duck_Bantul_BBVW_387_23310_2007_Avian
CY091872_A_duck_Bantul_BBVW_387_23310x_1_2007_Avian
CY091873_A_duck_Bantul_BBVW_387_23310x_2_2007_Avian
EF634308_A_chicken_West_Java_GrtMay206_2006_Avian
EU124215_A_Chicken_Indonesia_Soppeng1631_71_2007_Avian
EU124153_A_Chicken_West_Java_GARUT_MAY_2006_Avian
EU124159_A_Quail_Central_Java_SMRG_2006_Avian
branch a
HM440115_A_swine_Banten_UT6008_2007_Swine
HM440091_A_swine_East_Java_UT6003_2006_Swine
HM440131_A_swine_East_Java_UT6012_2007_Swine
HM440139_A_swine_South_Kalimantan_UT6015_2006_Swine
HM440147_A_swine_North_Sumatra_UT6034_2007_Swine
GQ122401_A_chicken_East_Java_UT6031_2007_Avian
GQ122396_A_chicken_East_Java_UT6016_2006_Avian
GQ122397_A_chicken_East_Java_UT6019_2006_Avian
CY091789_A_chicken_Buleleng_BBVD_545b_2007_Avian
CY091786_A_chicken_Gianyar_BBVD_687_2007_Avian
CY091816_A_chicken_Badung_BBVD_288_2007_Avian
CY091807_A_chicken_Klungkung_BBVD_279_2007_Avian
CY091797_A_chicken_Bangli_BBVD_245_2007_Avian
CY091798_A_duck_Bangli_BBVD_246_2007_Avian
CY091779_A_chicken_Denpasar_BBVD_435_2007_Avian
CY091783_A_chicken_Denpasar_BBVD_509_2007_Avian
CY091784_A_chicken_Denpasar_BBVD_560_2007_Avian
CY091820_A_chicken_Badung_BBVD_342_2007_Avian
CY091803_A_chicken_Bangli_BBVD_575_2007_Avian
CY091801_A_chicken_Bangli_BBVD_562_2007_Avian
CY091819_A_chicken_Badung_BBVD_328_2007_Avian

HA-Group132

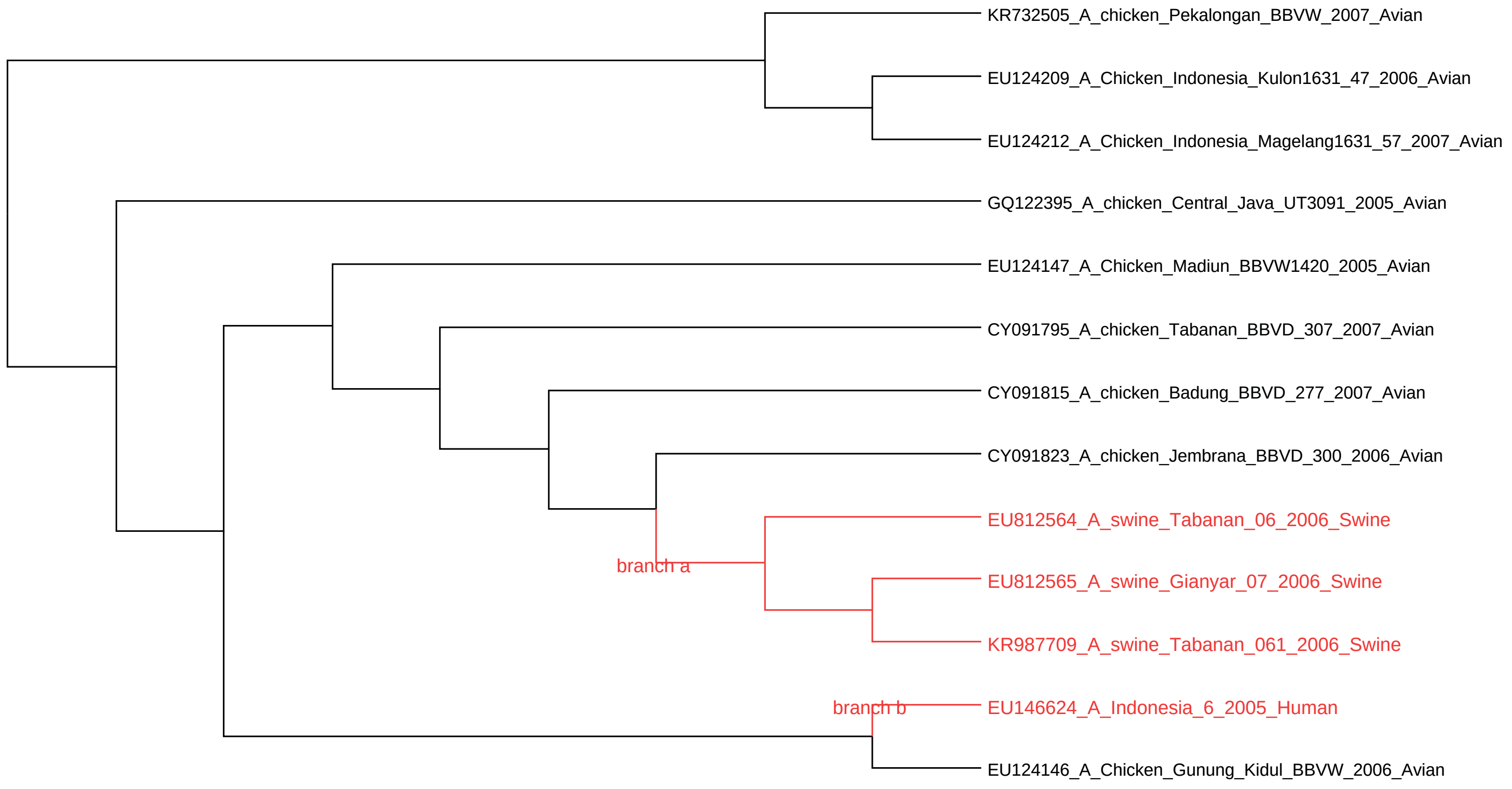
KR732505_A_chicken_Pekalongan_BBVW_2007_Avian
EU124209_A_Chicken_Indonesia_Kulon1631_47_2006_Avian
EU124212_A_Chicken_Indonesia_Magelang1631_57_2007_Avian
GQ122395_A_chicken_Central_Java_UT3091_2005_Avian
EU124147_A_Chicken_Madiun_BBVW1420_2005_Avian
CY091795_A_chicken_Tabanan_BBVD_307_2007_Avian
CY091815_A_chicken_Badung_BBVD_277_2007_Avian
CY091823_A_chicken_Jembrana_BBVD_300_2006_Avian
EU812564_A_swine_Tabanan_06_2006_Swine
branch a
EU812565_A_swine_Gianyar_07_2006_Swine
KR987709_A_swine_Tabanan_061_2006_Swine
branch b
EU146624_A_Indonesia_6_2005_Human
EU124146_A_Chicken_Gunung_Kidul_BBVW_2006_Avian
