# Supplementary material for: Adaptive Evolution of Human-Isolated H5Nx Avian Influenza A Viruses
Source: Front Microbiol. 2019 Jun 12;10:1328. doi: 10.3389/fmicb.2019.01328 (PMC6582624; doi:10.3389/fmicb.2019.01328)
Supplement: Supplementary file 4 [file Data_Sheet_4.PDF]

Supplementary Figure 4. 114 phylogenetic trees of NA used for the adaptive evolution analyses. Human strains are marked in red. Branches which have significant signals of positive selection are marked with *.

NA1-Group1

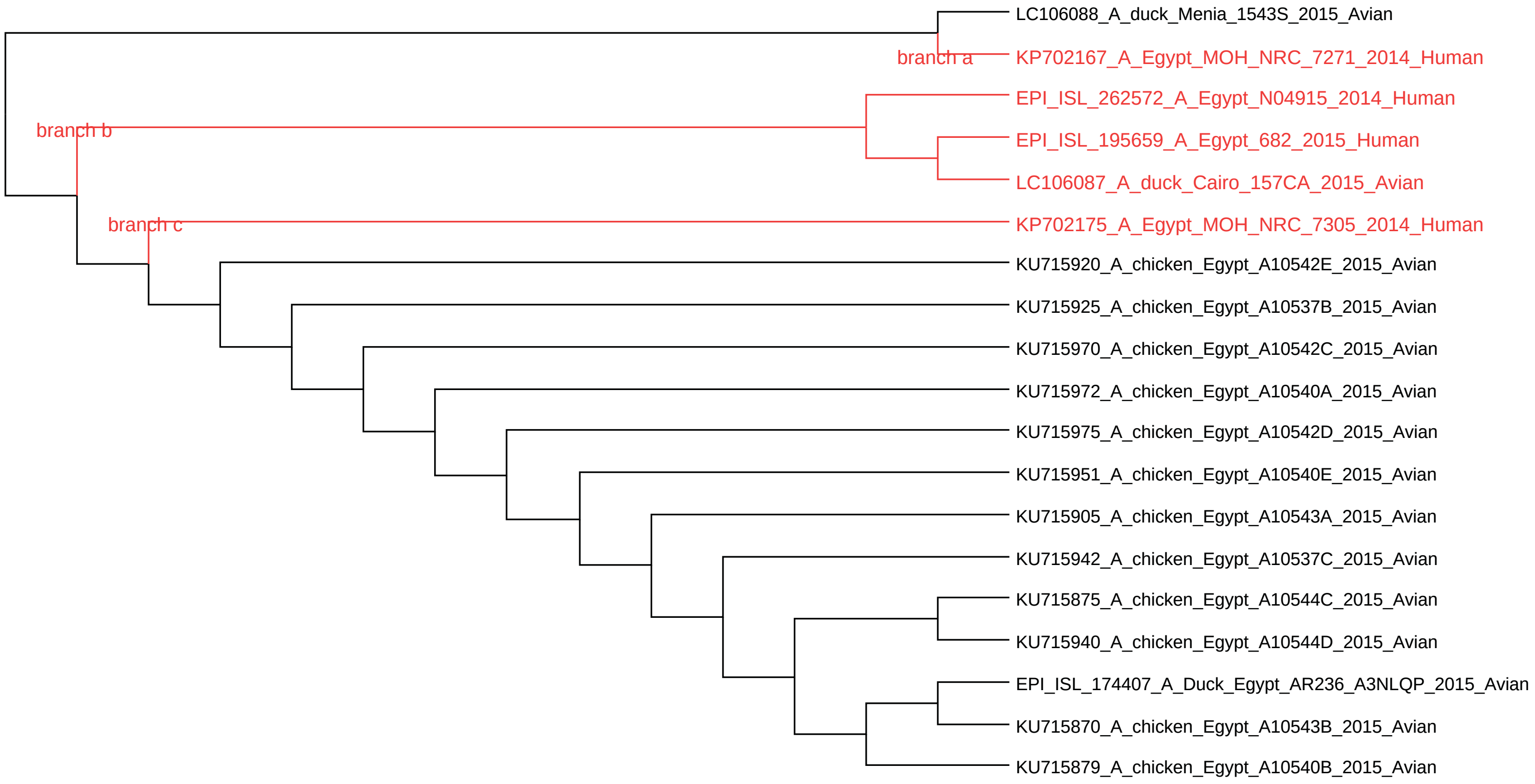

NA1-Group2

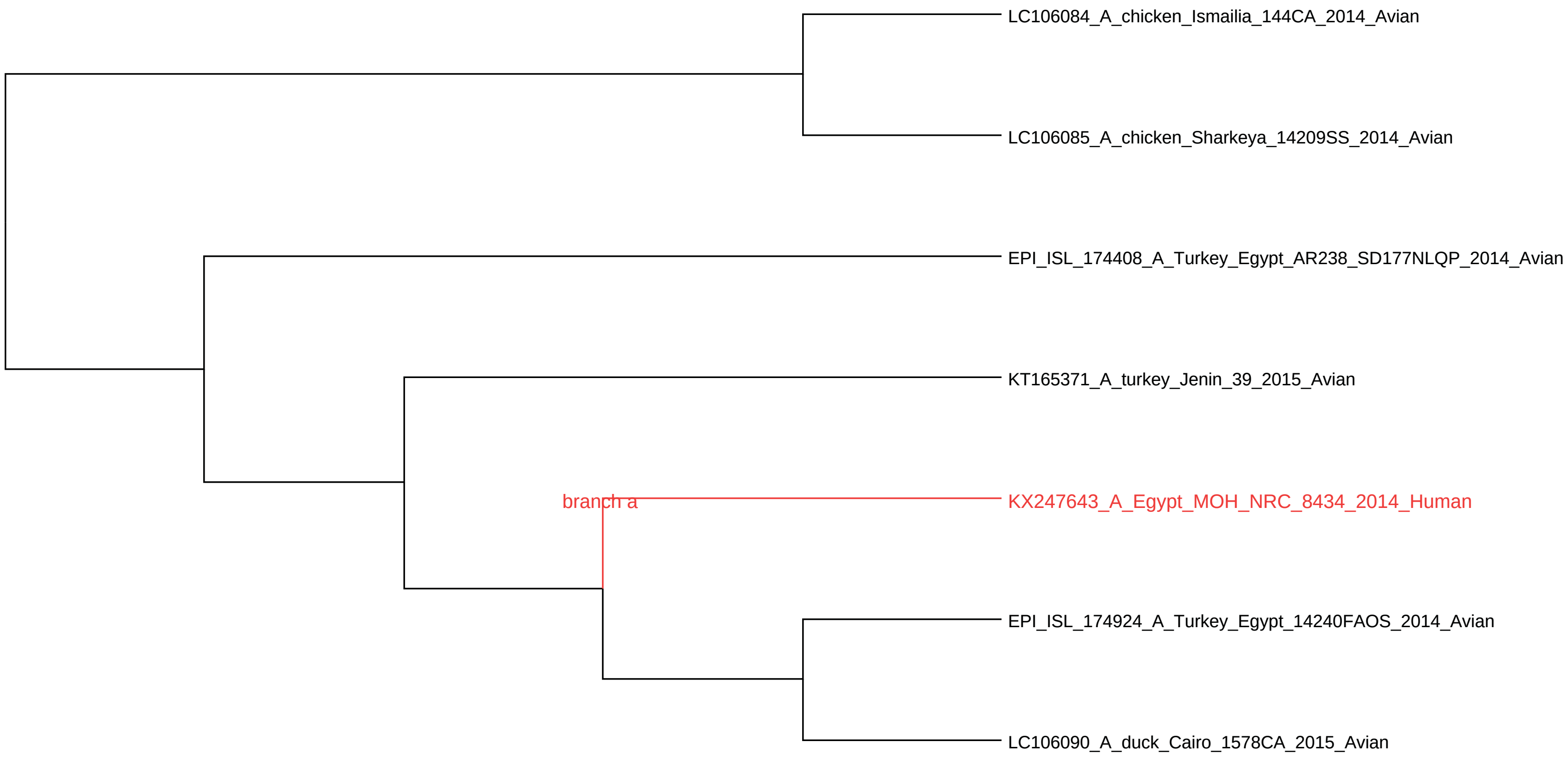
LC106084_A_chicken_Ismailia_144CA_2014_Avian
LC106085_A_chicken_Sharkeya_14209SS_2014_Avian
EPI_ISL_174408_A_Turkey_Egypt_AR238_SD177NLQP_2014_Avian
KT165371_A_turkey_Jenin_39_2015_Avian
branch a
KX247643_A_Egypt_MOH_NRC_8434_2014_Human
EPI_ISL_174924_A_Turkey_Egypt_14240FAOS_2014_Avian
LC106090_A_duck_Cairo_1578CA_2015_Avian

NA1-Group3

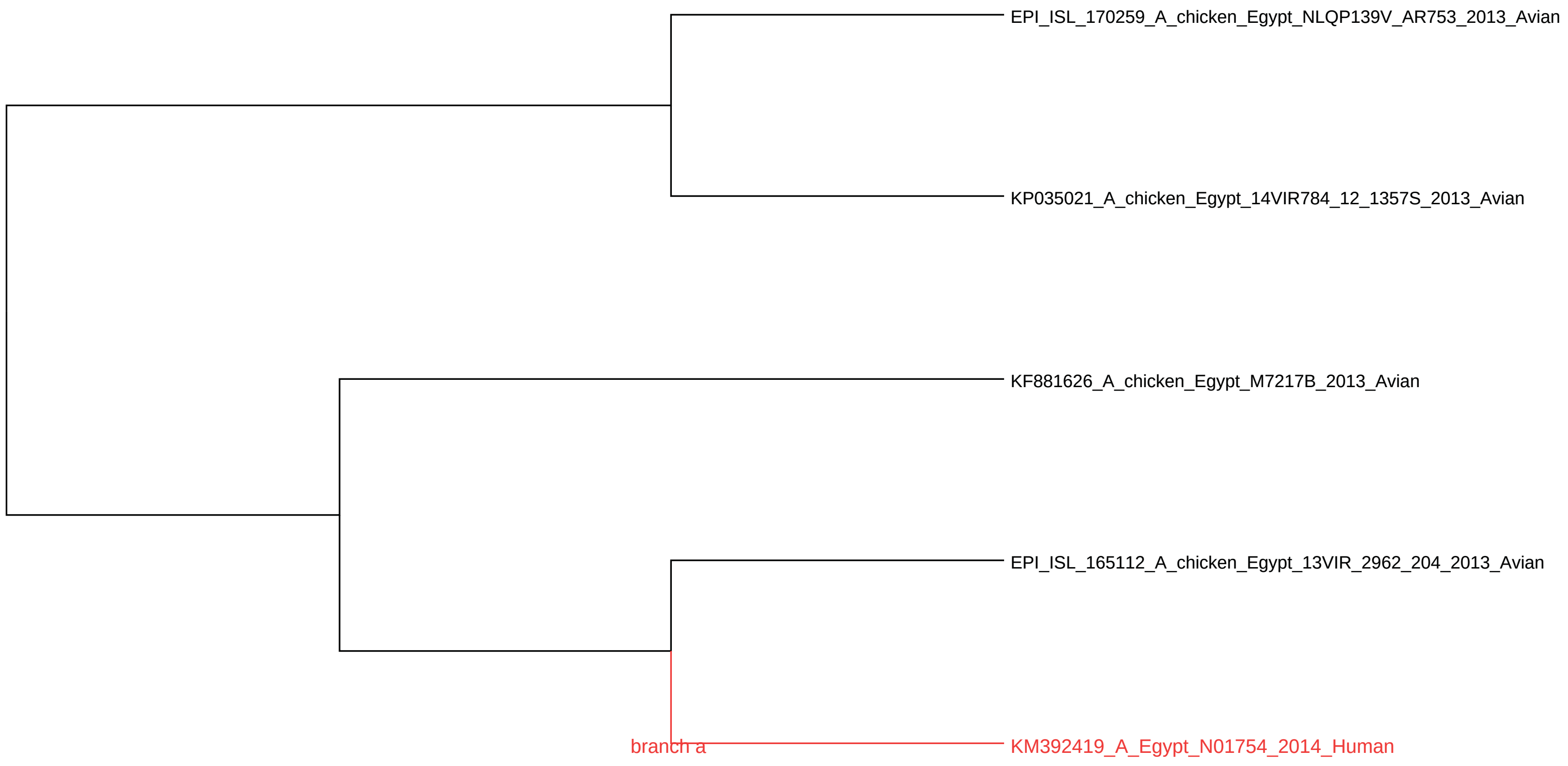

EPI_ISL_170259_A_chicken_Egypt_NLQP139V_AR753_2013_Avian
KP035021_A_chicken_Egypt_14VIR784_12_1357S_2013_Avian
KF881626_A_chicken_Egypt_M7217B_2013_Avian
EPI_ISL_165112_A_chicken_Egypt_13VIR_2962_204_2013_Avian
branch a
KM392419_A_Egypt_N01754_2014_Human

NA1-Group4

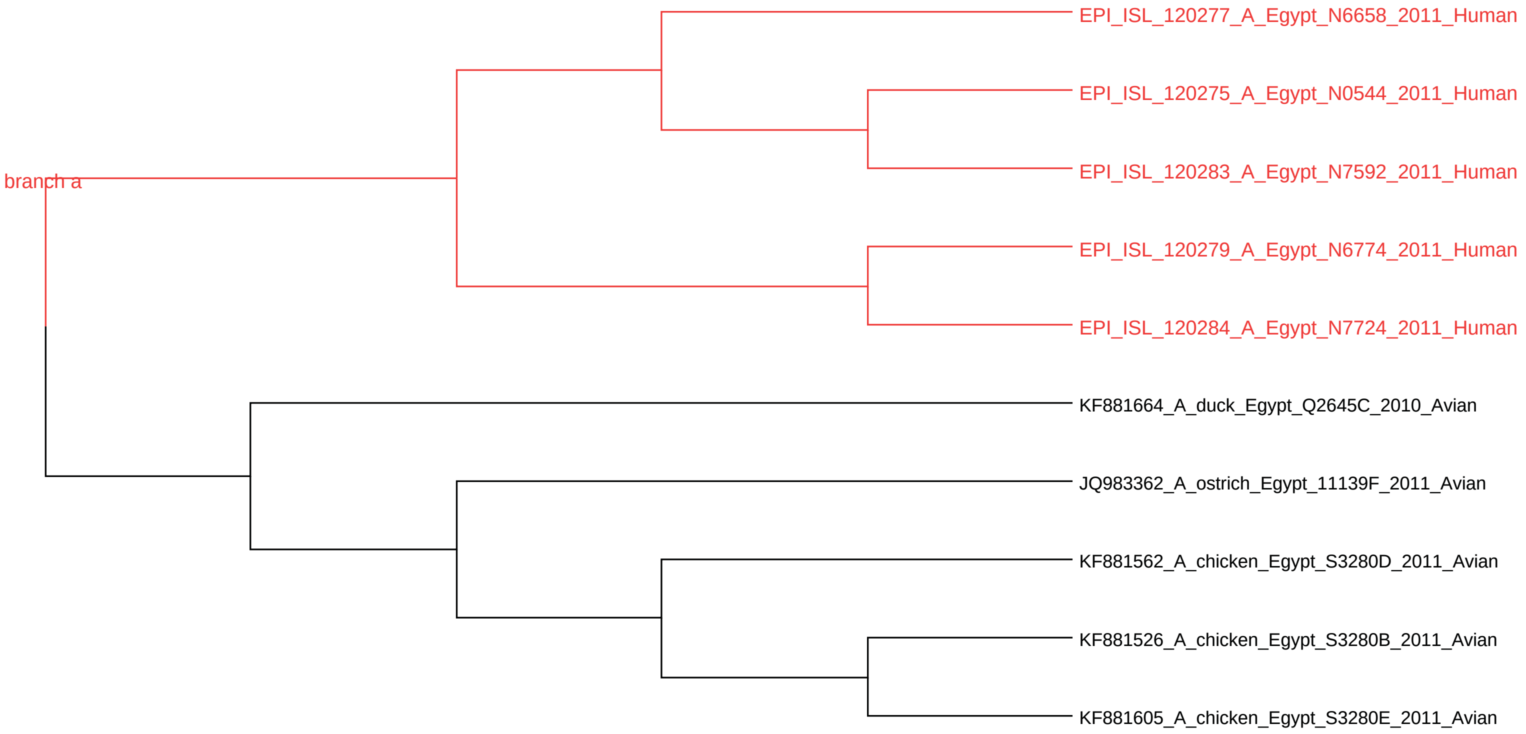

branch a
EPI_ISL_120277_A_Egypt_N6658_2011_Human
EPI_ISL_120275_A_Egypt_N0544_2011_Human
EPI_ISL_120283_A_Egypt_N7592_2011_Human
EPI_ISL_120279_A_Egypt_N6774_2011_Human
EPI_ISL_120284_A_Egypt_N7724_2011_Human
KF881664_A_duck_Egypt_Q2645C_2010_Avian
JQ983362_A_ostrich_Egypt_11139F_2011_Avian
KF881562_A_chicken_Egypt_S3280D_2011_Avian
KF881526_A_chicken_Egypt_S3280B_2011_Avian
KF881605_A_chicken_Egypt_S3280E_2011_Avian

NA1-Group5

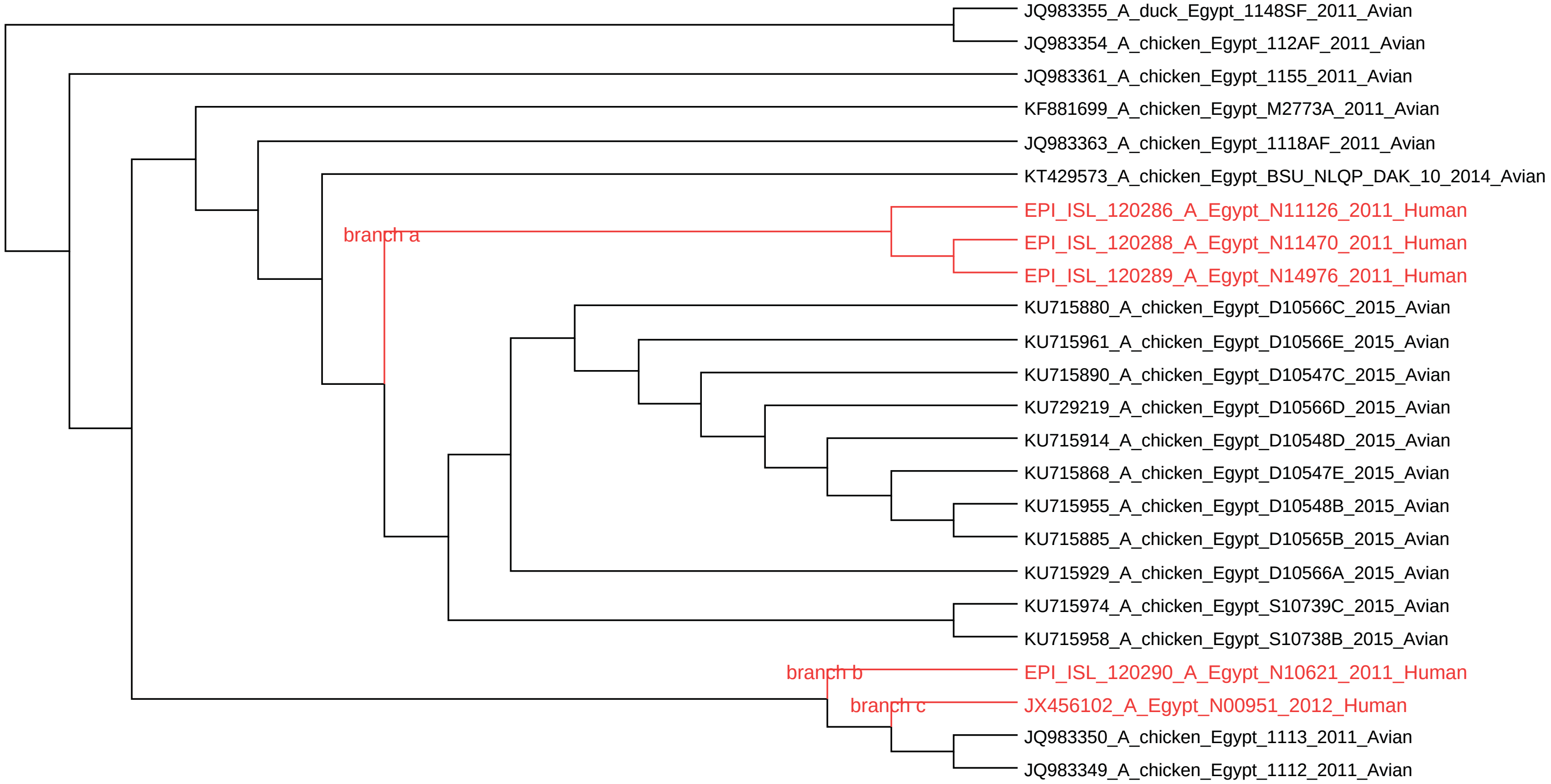
JQ983355_A_duck_Egypt_1148SF_2011_Avian
JQ983354_A_chicken_Egypt_112AF_2011_Avian
JQ983361_A_chicken_Egypt_1155_2011_Avian
KF881699_A_chicken_Egypt_M2773A_2011_Avian
JQ983363_A_chicken_Egypt_1118AF_2011_Avian
KT429573_A_chicken_Egypt_BSU_NLQP_DAK_10_2014_Avian
branch a
EPI_ISL_120286_A_Egypt_N11126_2011_Human
EPI_ISL_120288_A_Egypt_N11470_2011_Human
EPI_ISL_120289_A_Egypt_N14976_2011_Human
KU715880_A_chicken_Egypt_D10566C_2015_Avian
KU715961_A_chicken_Egypt_D10566E_2015_Avian
KU715890_A_chicken_Egypt_D10547C_2015_Avian
KU729219_A_chicken_Egypt_D10566D_2015_Avian
KU715914_A_chicken_Egypt_D10548D_2015_Avian
KU715868_A_chicken_Egypt_D10547E_2015_Avian
KU715955_A_chicken_Egypt_D10548B_2015_Avian
KU715885_A_chicken_Egypt_D10565B_2015_Avian
KU715929_A_chicken_Egypt_D10566A_2015_Avian
KU715974_A_chicken_Egypt_S10739C_2015_Avian
KU715958_A_chicken_Egypt_S10738B_2015_Avian
branch b
EPI_ISL_120290_A_Egypt_N10621_2011_Human
branch c
JX456102_A_Egypt_N00951_2012_Human
JQ983350_A_chicken_Egypt_1113_2011_Avian
JQ983349_A_chicken_Egypt_1112_2011_Avian

NA1-Group6

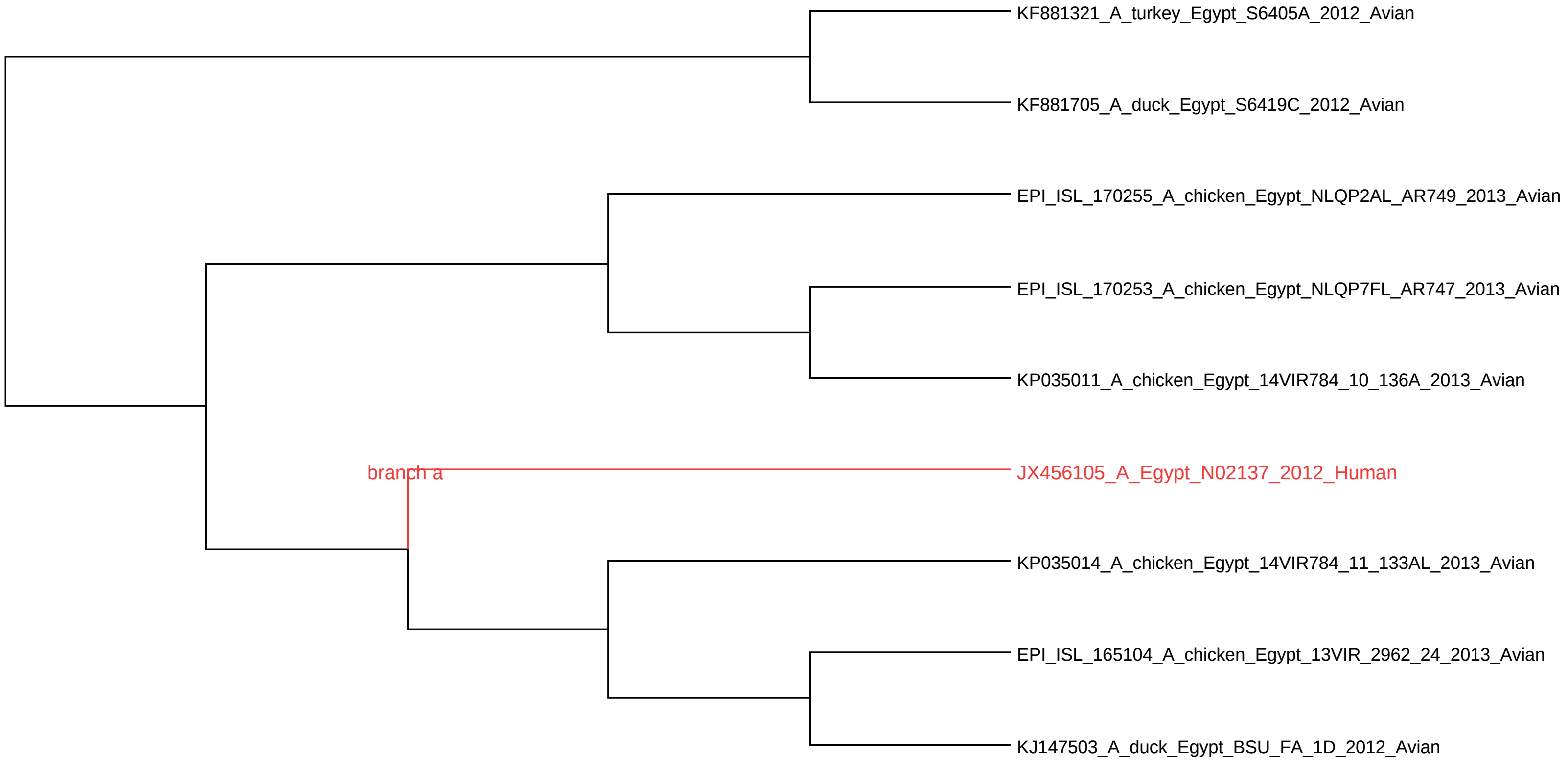

KF881321_A_turkey_Egypt_S6405A_2012_Avian
KF881705_A_duck_Egypt_S6419C_2012_Avian
EPI_ISL_170255_A_chicken_Egypt_NLQP2AL_AR749_2013_Avian
EPI_ISL_170253_A_chicken_Egypt_NLQP7FL_AR747_2013_Avian
KP035011_A_chicken_Egypt_14VIR784_10_136A_2013_Avian
branch a
JX456105_A_Egypt_N02137_2012_Human
KP035014_A_chicken_Egypt_14VIR784_11_133AL_2013_Avian
EPI_ISL_165104_A_chicken_Egypt_13VIR_2962_24_2013_Avian
KJ147503_A_duck_Egypt_BSU_FA_1D_2012_Avian

NA1-Group7

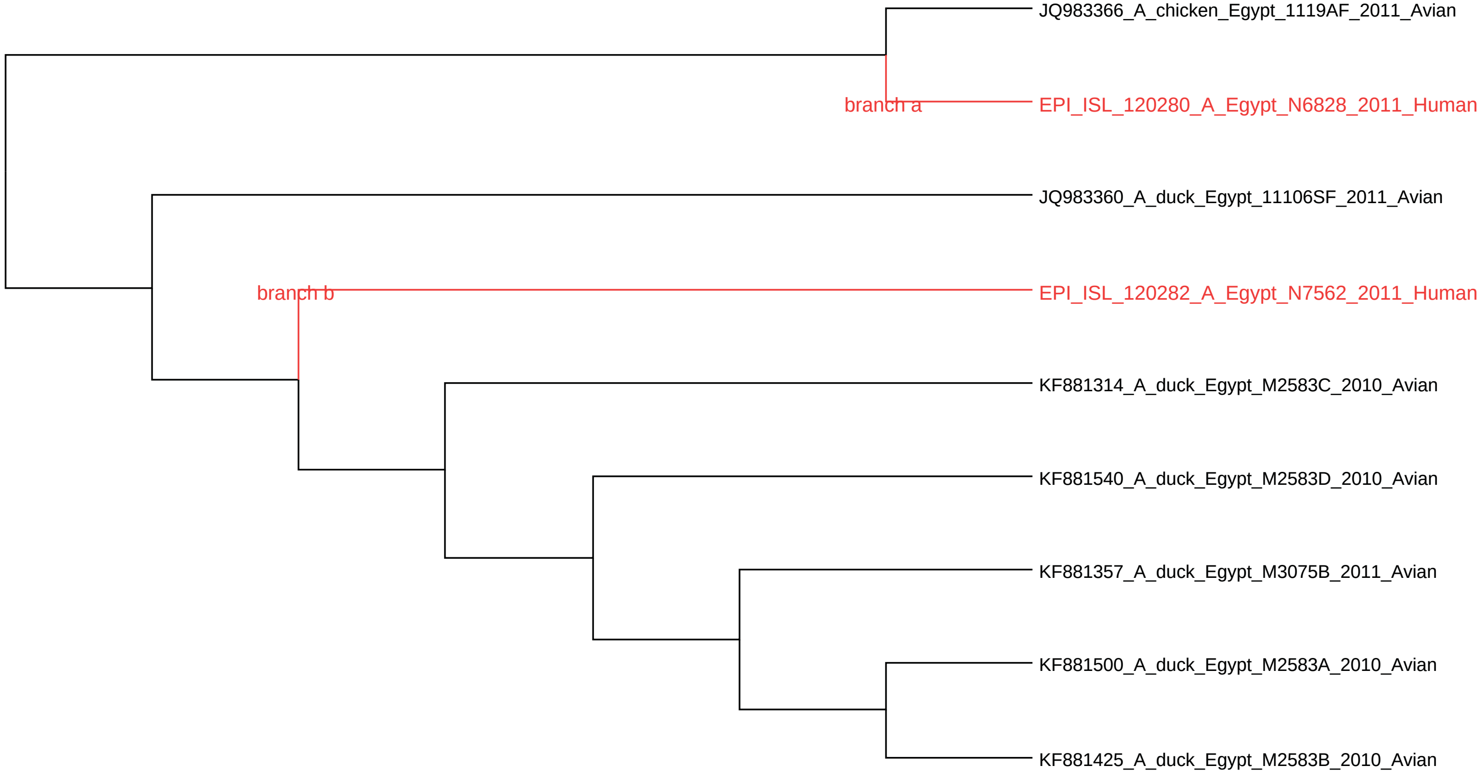

JQ983366_A_chicken_Egypt_1119AF_2011_Avian
branch a
EPI_ISL_120280_A_Egypt_N6828_2011_Human
JQ983360_A_duck_Egypt_11106SF_2011_Avian
branch b
EPI_ISL_120282_A_Egypt_N7562_2011_Human
KF881314_A_duck_Egypt_M2583C_2010_Avian
KF881540_A_duck_Egypt_M2583D_2010_Avian
KF881357_A_duck_Egypt_M3075B_2011_Avian
KF881500_A_duck_Egypt_M2583A_2010_Avian
KF881425_A_duck_Egypt_M2583B_2010_Avian

NA1-Group8

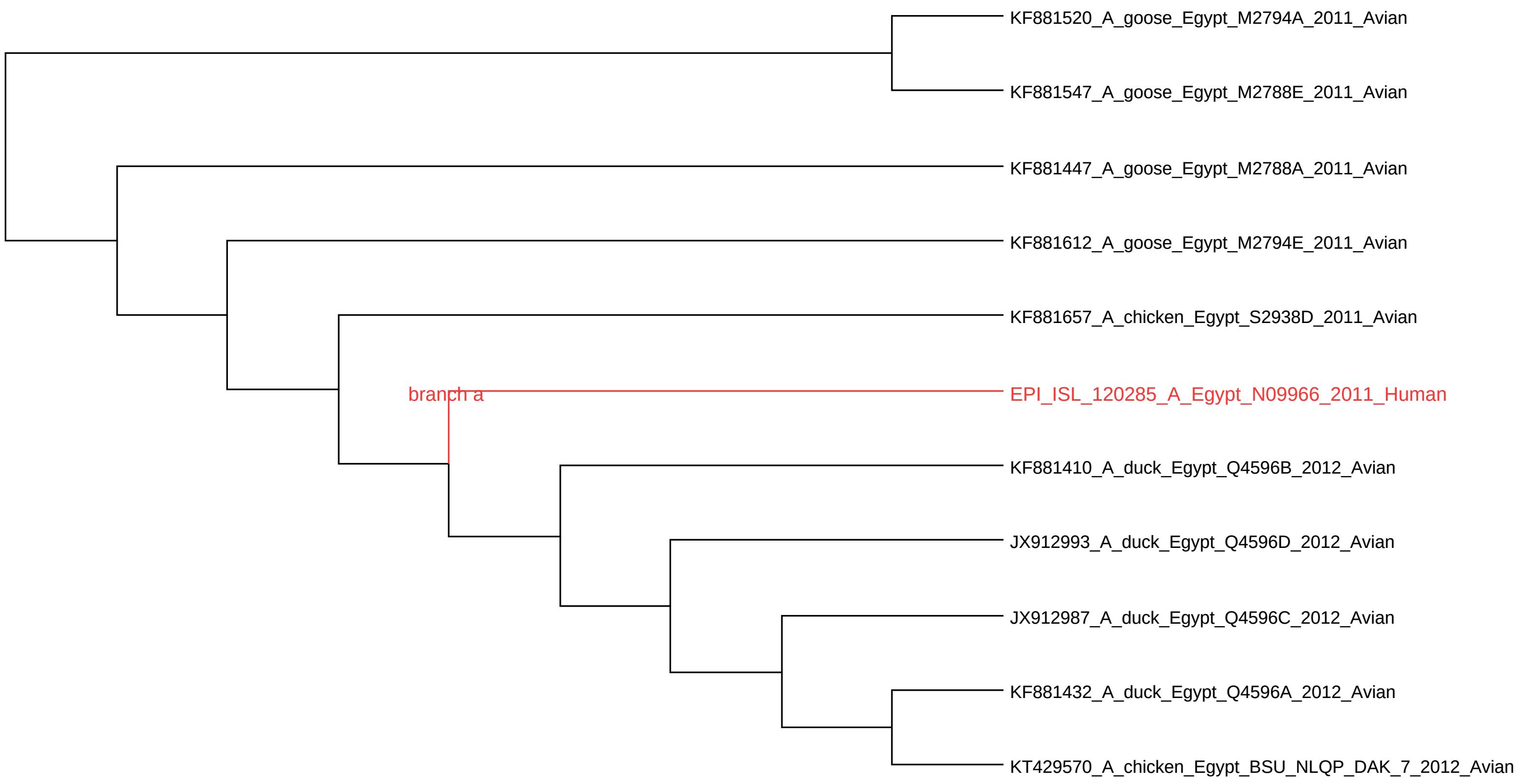

KF881520_A_goose_Egypt_M2794A_2011_Avian
KF881547_A_goose_Egypt_M2788E_2011_Avian
KF881447_A_goose_Egypt_M2788A_2011_Avian
KF881612_A_goose_Egypt_M2794E_2011_Avian
KF881657_A_chicken_Egypt_S2938D_2011_Avian
branch a
EPI_ISL_120285_A_Egypt_N09966_2011_Human
KF881410_A_duck_Egypt_Q4596B_2012_Avian
JX912993_A_duck_Egypt_Q4596D_2012_Avian
JX912987_A_duck_Egypt_Q4596C_2012_Avian
KF881432_A_duck_Egypt_Q4596A_2012_Avian
KT429570_A_chicken_Egypt_BSU_NLQP_DAK_7_2012_Avian

NA1-Group9

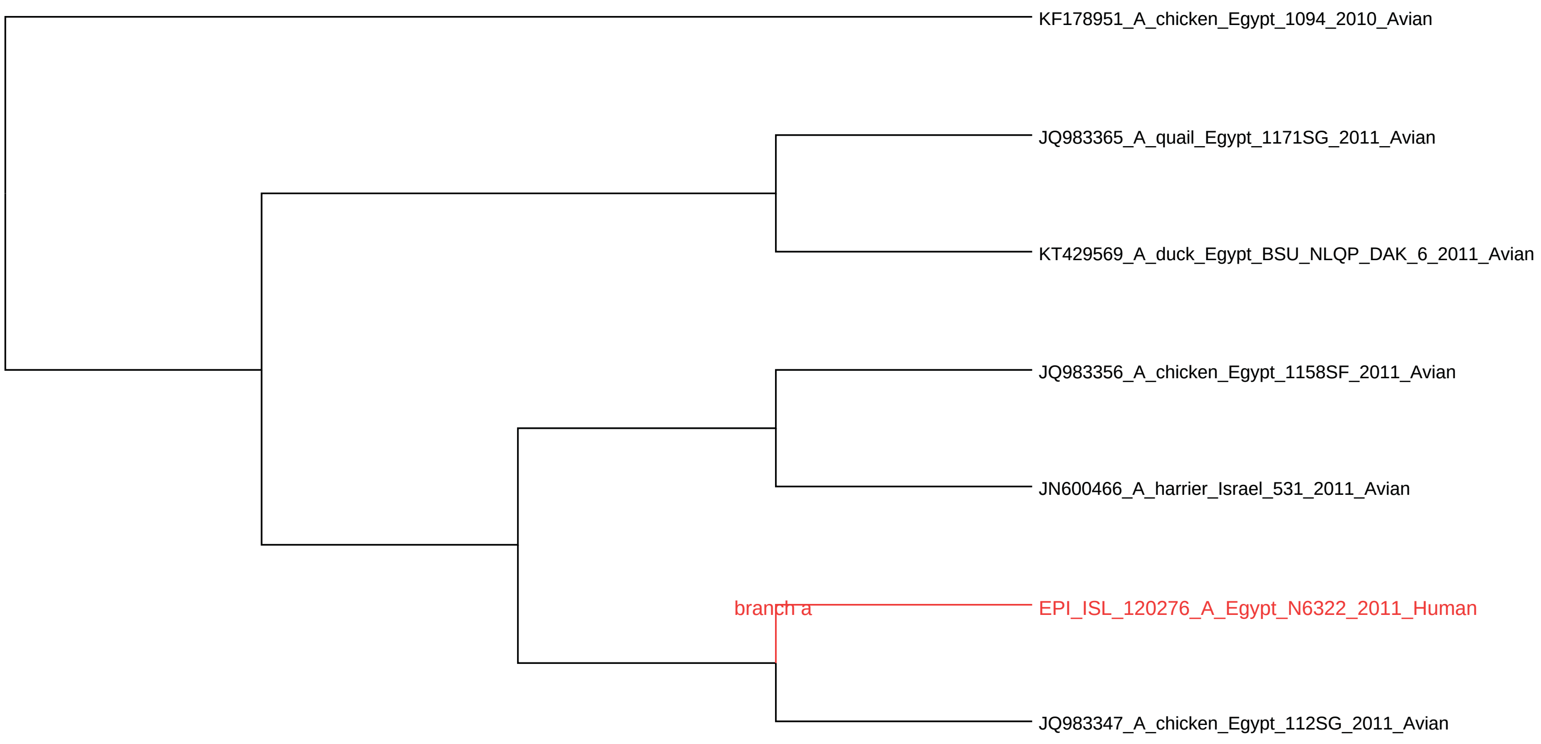

KF178951_A_chicken_Egypt_1094_2010_Avian
JQ983365_A_quail_Egypt_1171SG_2011_Avian
KT429569_A_duck_Egypt_BSU_NLQP_DAK_6_2011_Avian
JQ983356_A_chicken_Egypt_1158SF_2011_Avian
JN600466_A_harrier_Israel_531_2011_Avian
branch a
EPI_ISL_120276_A_Egypt_N6322_2011_Human
JQ983347_A_chicken_Egypt_112SG_2011_Avian

NA1-Group10

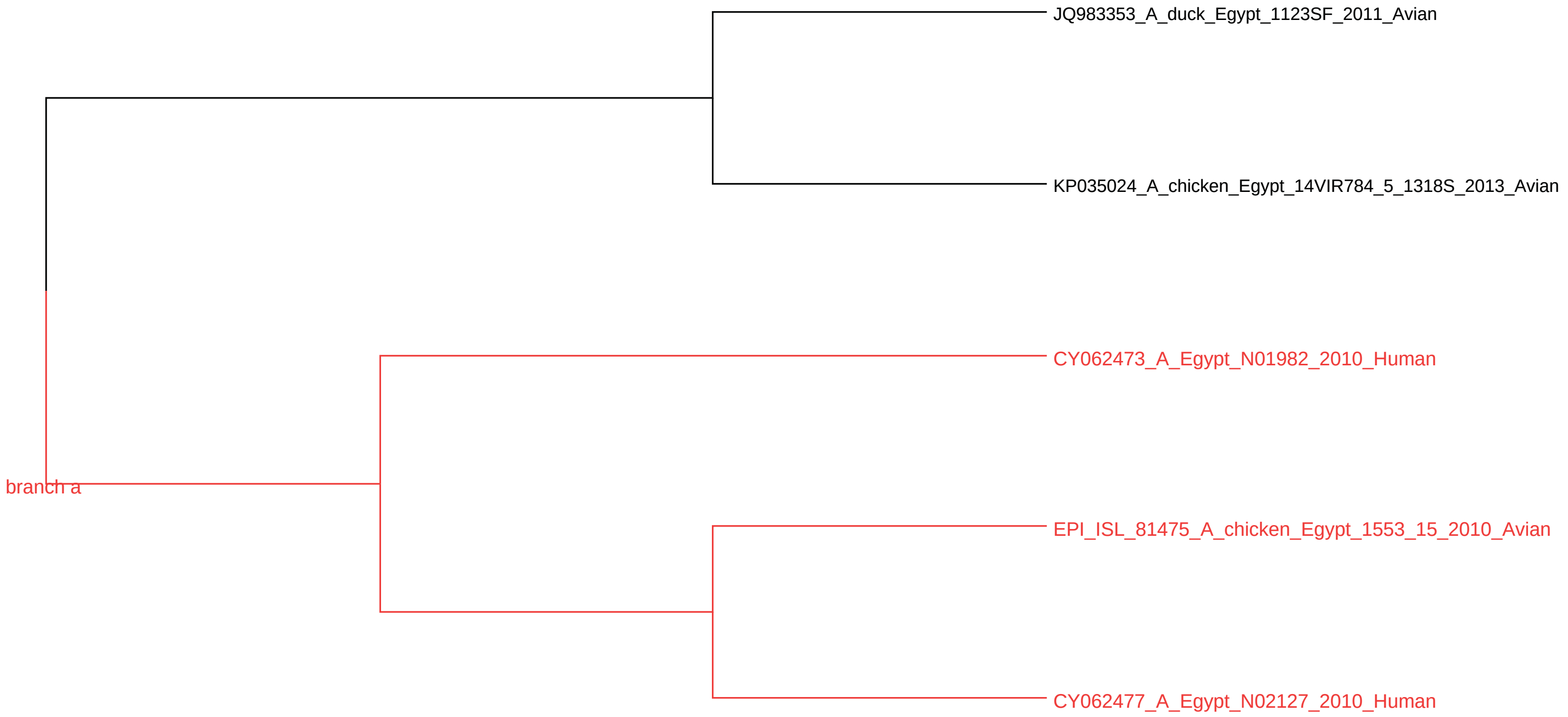
JQ983353_A_duck_Egypt_1123SF_2011_Avian
KP035024_A_chicken_Egypt_14VIR784_5_1318S_2013_Avian
CY062473_A_Egypt_N01982_2010_Human
branch a
EPI_ISL_81475_A_chicken_Egypt_1553_15_2010_Avian
CY062477_A_Egypt_N02127_2010_Human

NA1-Group11

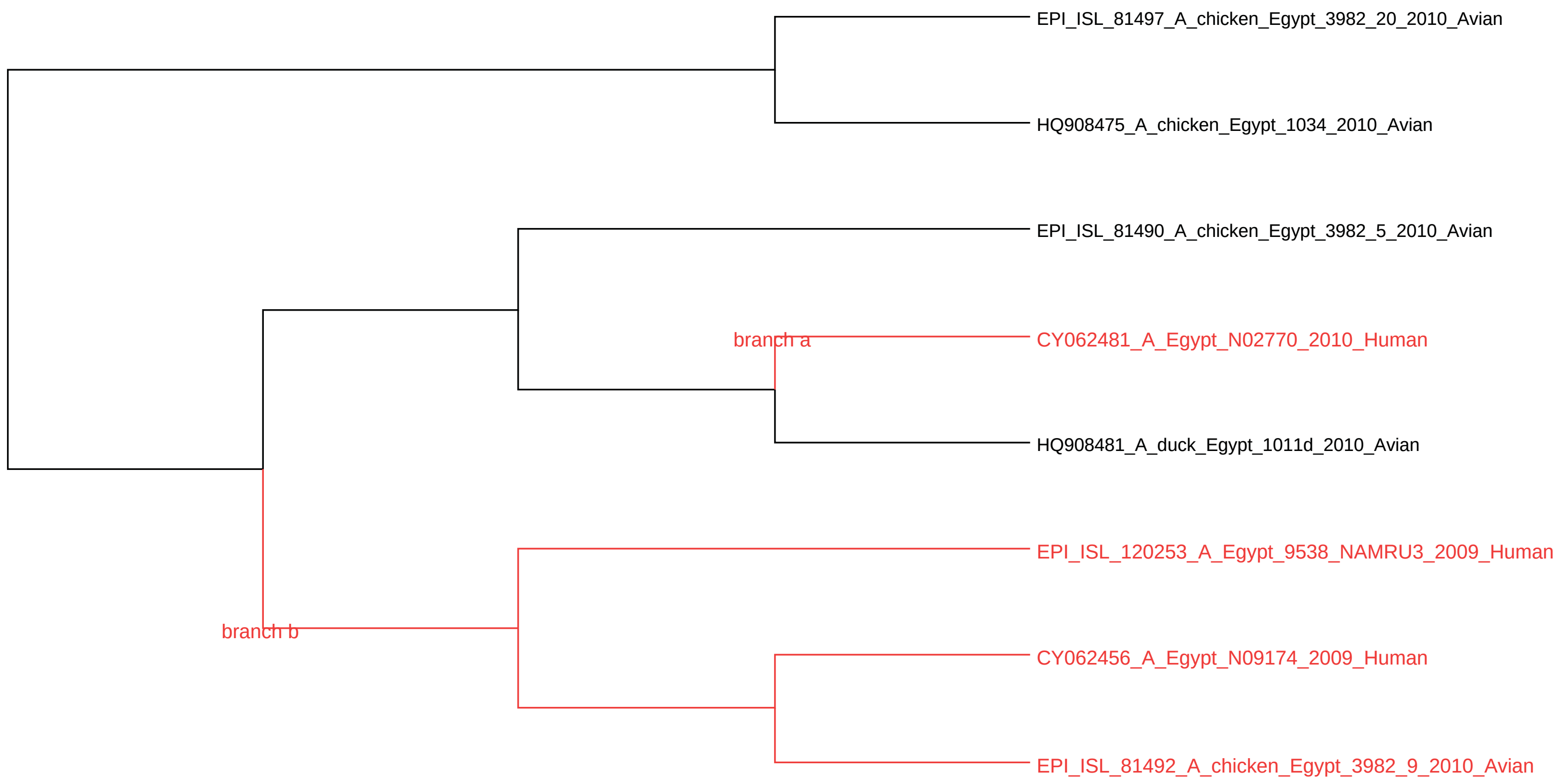

EPI_ISL_81497_A_chicken_Egypt_3982_20_2010_Avian
HQ908475_A_chicken_Egypt_1034_2010_Avian
EPI_ISL_81490_A_chicken_Egypt_3982_5_2010_Avian
branch a
CY062481_A_Egypt_N02770_2010_Human
HQ908481_A_duck_Egypt_1011d_2010_Avian
EPI_ISL_120253_A_Egypt_9538_NAMRU3_2009_Human
branch b
CY062456_A_Egypt_N09174_2009_Human
EPI_ISL_81492_A_chicken_Egypt_3982_9_2010_Avian

NA1-Group12

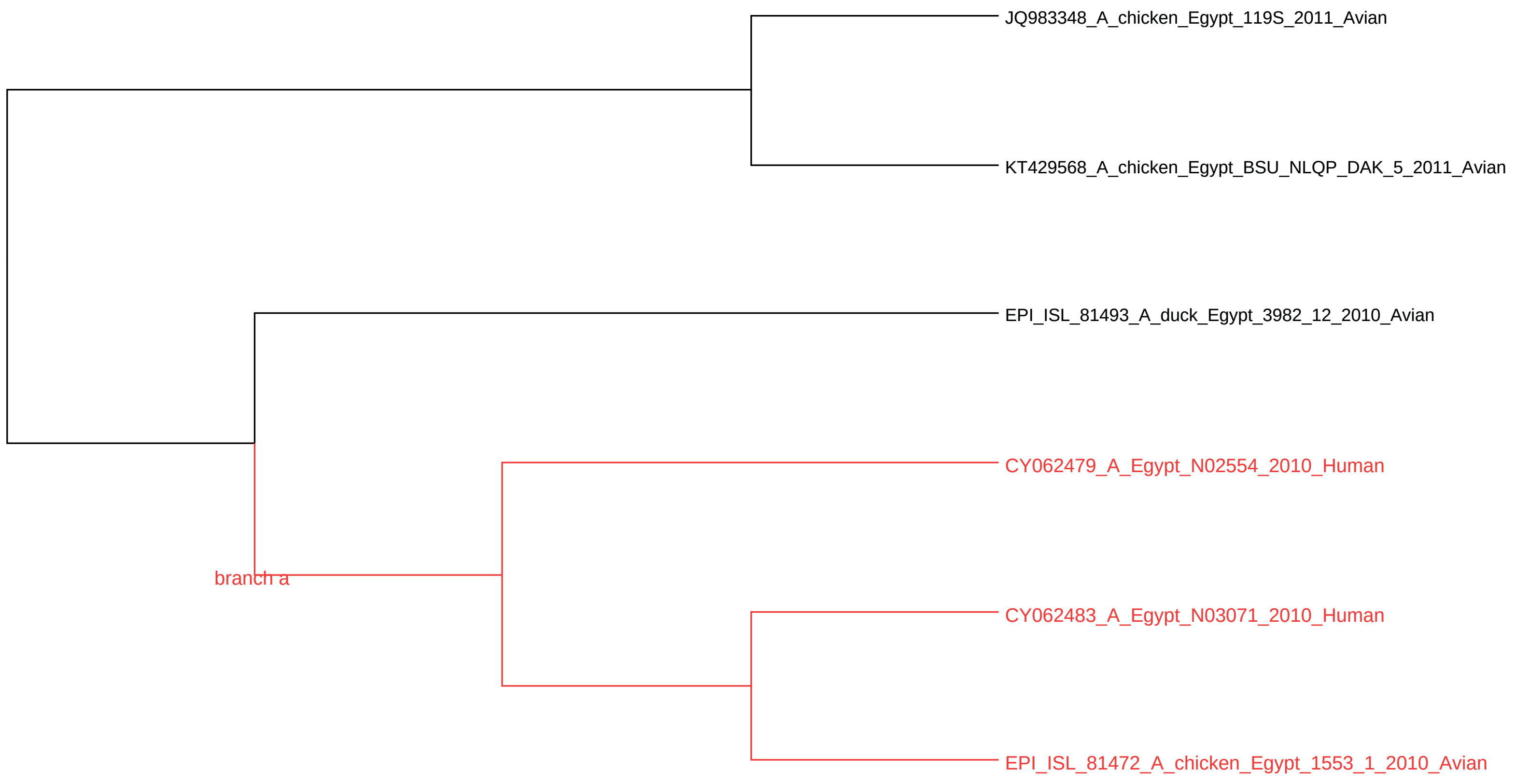

JQ983348_A_chicken_Egypt_119S_2011_Avian
KT429568_A_chicken_Egypt_BSU_NLQP_DAK_5_2011_Avian
EPI_ISL_81493_A_duck_Egypt_3982_12_2010_Avian
CY062479_A_Egypt_N02554_2010_Human
branch a
CY062483_A_Egypt_N03071_2010_Human
EPI_ISL_81472_A_chicken_Egypt_1553_1_2010_Avian

NA1-Group13

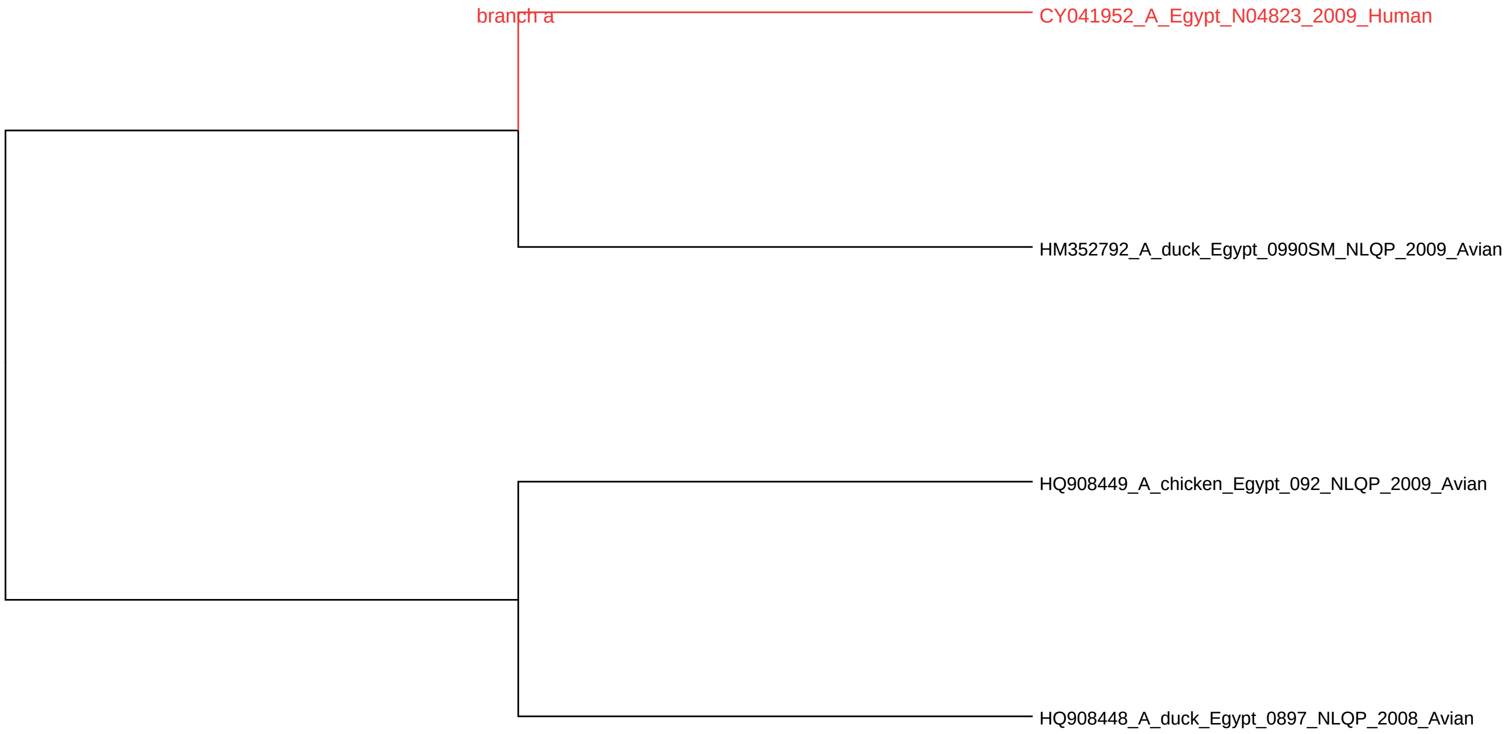
branch a
CY041952_A_Egypt_N04823_2009_Human
HM352792_A_duck_Egypt_0990SM_NLQP_2009_Avian
HQ908449_A_chicken_Egypt_092_NLQP_2009_Avian
HQ908448_A_duck_Egypt_0897_NLQP_2008_Avian

NA1-Group14

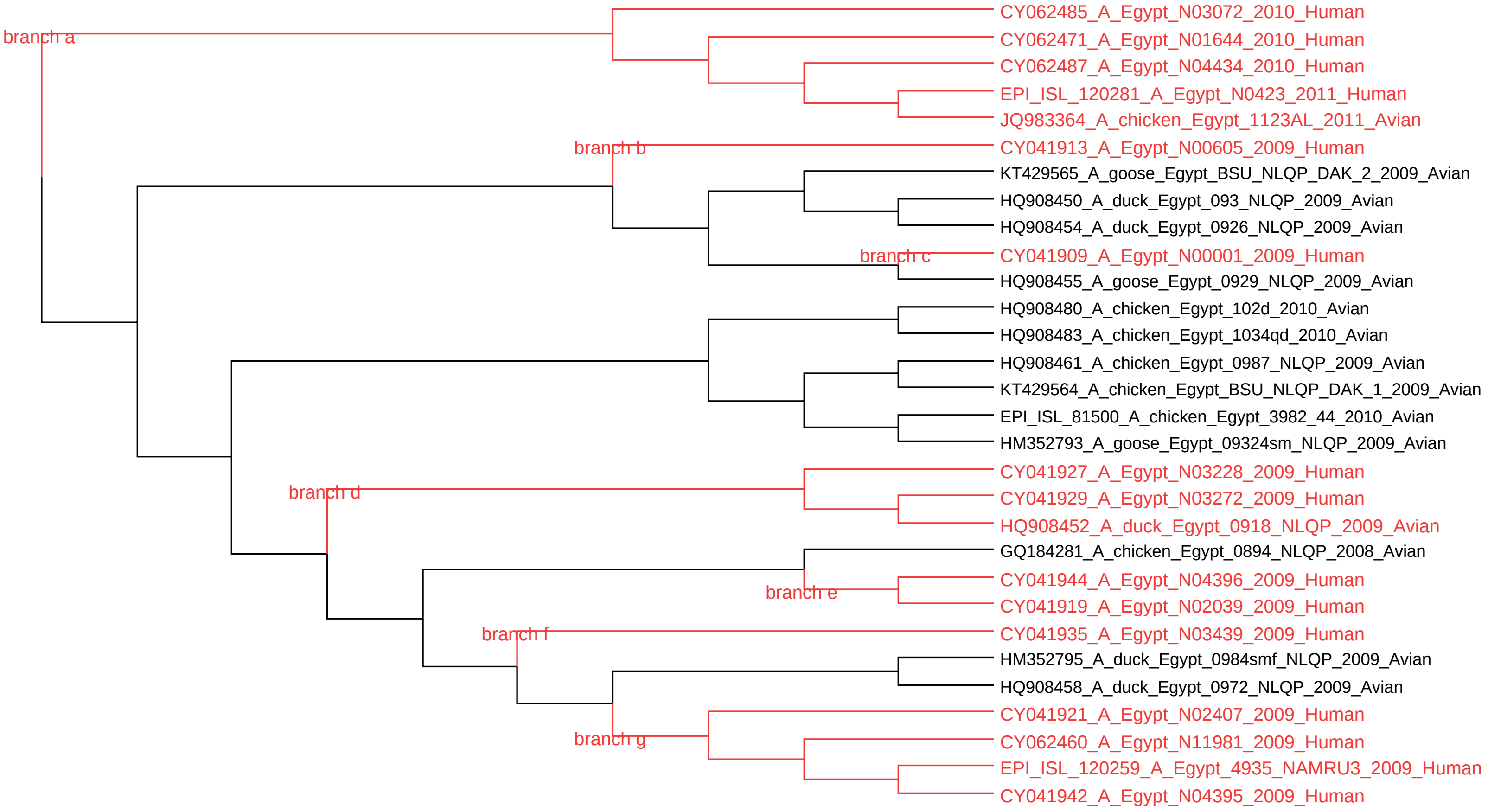

branch a
branch b
branch c
branch d
branch e
branch f
branch g
CY062485_A_Egypt_N03072_2010_Human
CY062471_A_Egypt_N01644_2010_Human
CY062487_A_Egypt_N04434_2010_Human
EPI_ISL_120281_A_Egypt_N0423_2011_Human
JQ983364_A_chicken_Egypt_1123AL_2011_Avian
CY041913_A_Egypt_N00605_2009_Human
KT429565_A_goose_Egypt_BSU_NLQP_DAK_2_2009_Avian
HQ908450_A_duck_Egypt_093_NLQP_2009_Avian
HQ908454_A_duck_Egypt_0926_NLQP_2009_Avian
CY041909_A_Egypt_N00001_2009_Human
HQ908455_A_goose_Egypt_0929_NLQP_2009_Avian
HQ908480_A_chicken_Egypt_102d_2010_Avian
HQ908483_A_chicken_Egypt_1034qd_2010_Avian
HQ908461_A_chicken_Egypt_0987_NLQP_2009_Avian
KT429564_A_chicken_Egypt_BSU_NLQP_DAK_1_2009_Avian
EPI_ISL_81500_A_chicken_Egypt_3982_44_2010_Avian
HM352793_A_goose_Egypt_09324sm_NLQP_2009_Avian
CY041927_A_Egypt_N03228_2009_Human
CY041929_A_Egypt_N03272_2009_Human
HQ908452_A_duck_Egypt_0918_NLQP_2009_Avian
GQ184281_A_chicken_Egypt_0894_NLQP_2008_Avian
CY041944_A_Egypt_N04396_2009_Human
CY041919_A_Egypt_N02039_2009_Human
CY041935_A_Egypt_N03439_2009_Human
HM352795_A_duck_Egypt_0984smf_NLQP_2009_Avian
HQ908458_A_duck_Egypt_0972_NLQP_2009_Avian
CY041921_A_Egypt_N02407_2009_Human
CY062460_A_Egypt_N11981_2009_Human
EPI_ISL_120259_A_Egypt_4935_NAMRU3_2009_Human
CY041942_A_Egypt_N04395_2009_Human

NA1-Group15

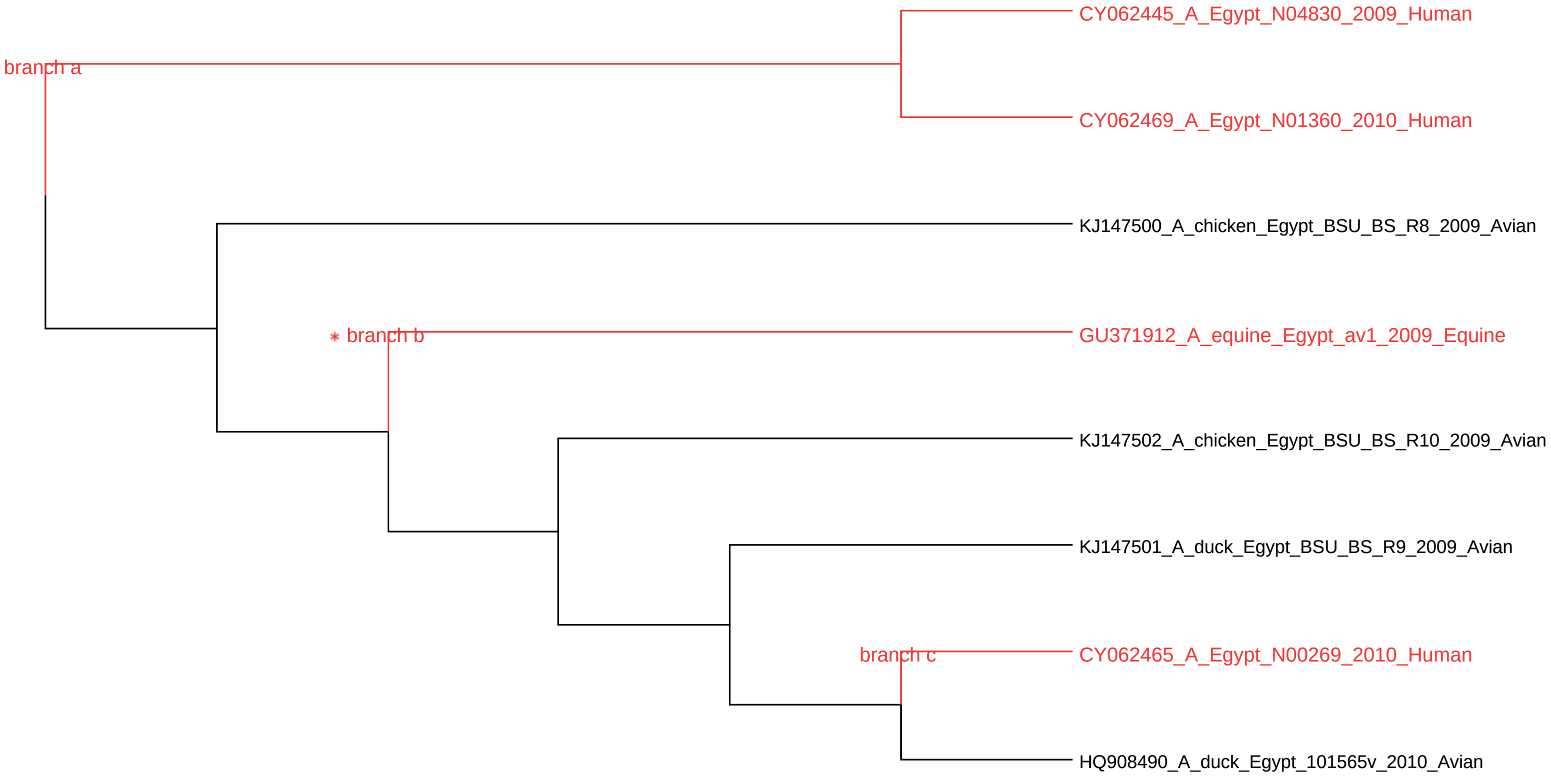

branch a
CY062445_A_Egypt_N04830_2009_Human
CY062469_A_Egypt_N01360_2010_Human
KJ147500_A_chicken_Egypt_BSU_BS_R8_2009_Avian
* branch b
GU371912_A_equine_Egypt_av1_2009_Equine
KJ147502_A_chicken_Egypt_BSU_BS_R10_2009_Avian
KJ147501_A_duck_Egypt_BSU_BS_R9_2009_Avian
branch c
CY062465_A_Egypt_N00269_2010_Human
HQ908490_A_duck_Egypt_101565v_2010_Avian

NA1-Group16

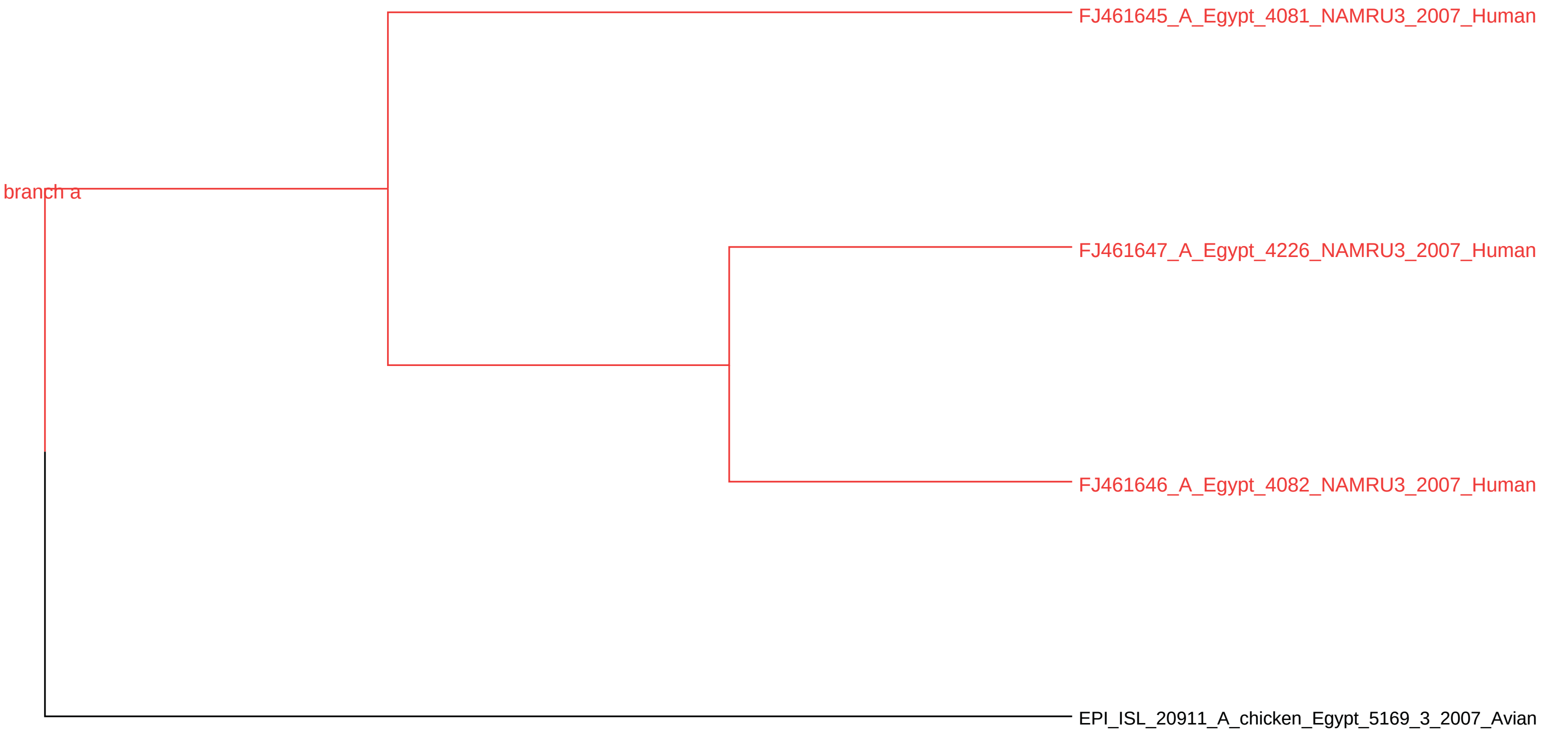

FJ461645_A_Egypt_4081_NAMRU3_2007_Human
branch a
FJ461647_A_Egypt_4226_NAMRU3_2007_Human
FJ461646_A_Egypt_4082_NAMRU3_2007_Human
EPI_ISL_20911_A_chicken_Egypt_5169_3_2007_Avian

NA1-Group17

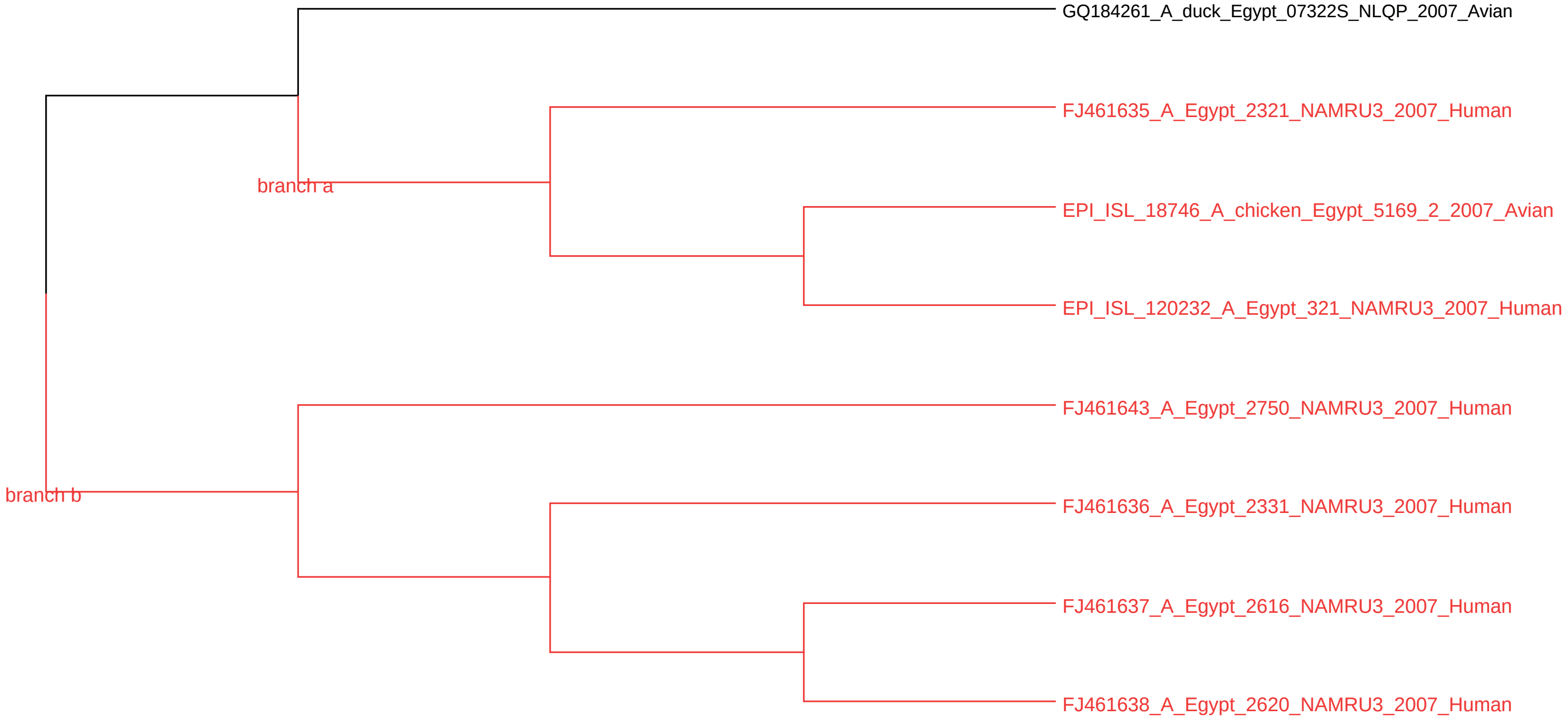

GQ184261_A_duck_Egypt_07322S_NLQP_2007_Avian
FJ461635_A_Egypt_2321_NAMRU3_2007_Human
branch a
EPI_ISL_18746_A_chicken_Egypt_5169_2_2007_Avian
EPI_ISL_120232_A_Egypt_321_NAMRU3_2007_Human
FJ461643_A_Egypt_2750_NAMRU3_2007_Human
branch b
FJ461636_A_Egypt_2331_NAMRU3_2007_Human
FJ461637_A_Egypt_2616_NAMRU3_2007_Human
FJ461638_A_Egypt_2620_NAMRU3_2007_Human

NA1-Group18

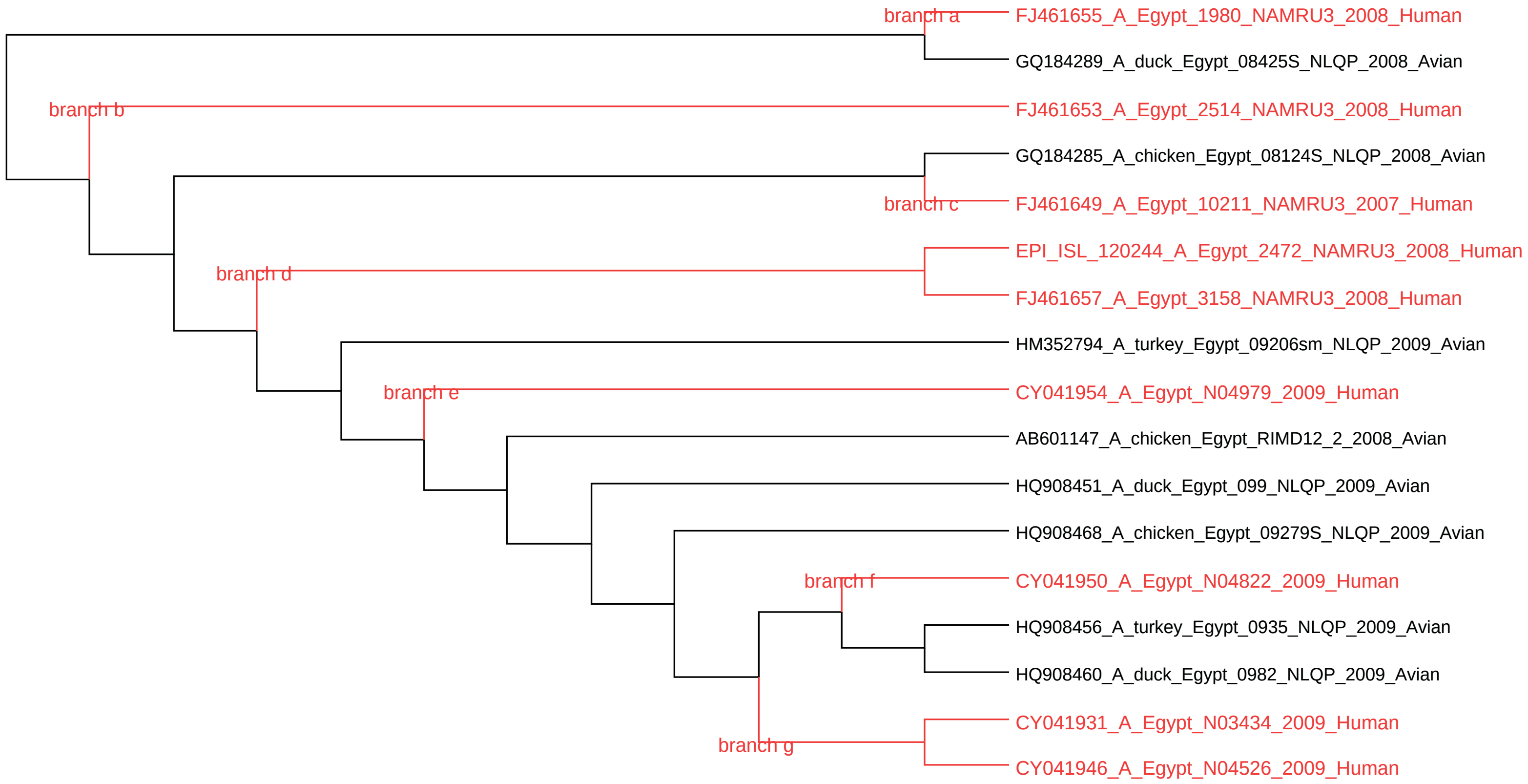
branch a
FJ461655_A_Egypt_1980_NAMRU3_2008_Human
GQ184289_A_duck_Egypt_08425S_NLQP_2008_Avian
branch b
FJ461653_A_Egypt_2514_NAMRU3_2008_Human
GQ184285_A_chicken_Egypt_08124S_NLQP_2008_Avian
branch c
FJ461649_A_Egypt_10211_NAMRU3_2007_Human
EPI_ISL_120244_A_Egypt_2472_NAMRU3_2008_Human
branch d
FJ461657_A_Egypt_3158_NAMRU3_2008_Human
HM352794_A_turkey_Egypt_09206sm_NLQP_2009_Avian
branch e
CY041954_A_Egypt_N04979_2009_Human
AB601147_A_chicken_Egypt_RIMD12_2_2008_Avian
HQ908451_A_duck_Egypt_099_NLQP_2009_Avian
HQ908468_A_chicken_Egypt_09279S_NLQP_2009_Avian
branch f
CY041950_A_Egypt_N04822_2009_Human
HQ908456_A_turkey_Egypt_0935_NLQP_2009_Avian
HQ908460_A_duck_Egypt_0982_NLQP_2009_Avian
CY041931_A_Egypt_N03434_2009_Human
branch g
CY041946_A_Egypt_N04526_2009_Human

NA1-Group19

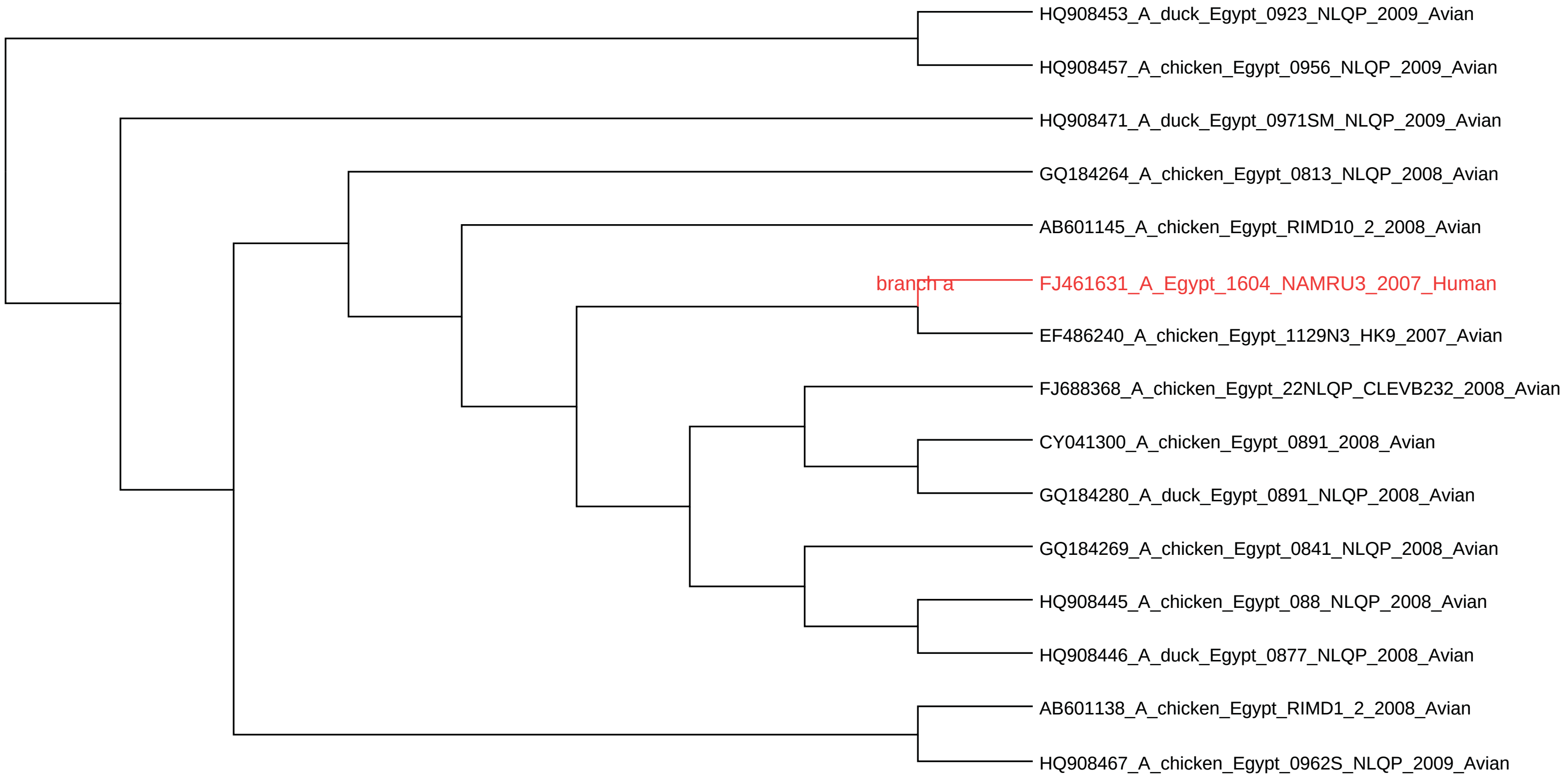

HQ908453_A_duck_Egypt_0923_NLQP_2009_Avian
HQ908457_A_chicken_Egypt_0956_NLQP_2009_Avian
HQ908471_A_duck_Egypt_0971SM_NLQP_2009_Avian
GQ184264_A_chicken_Egypt_0813_NLQP_2008_Avian
AB601145_A_chicken_Egypt_RIMD10_2_2008_Avian
branch a
FJ461631_A_Egypt_1604_NAMRU3_2007_Human
EF486240_A_chicken_Egypt_1129N3_HK9_2007_Avian
FJ688368_A_chicken_Egypt_22NLQP_CLEVB232_2008_Avian
CY041300_A_chicken_Egypt_0891_2008_Avian
GQ184280_A_duck_Egypt_0891_NLQP_2008_Avian
GQ184269_A_chicken_Egypt_0841_NLQP_2008_Avian
HQ908445_A_chicken_Egypt_088_NLQP_2008_Avian
HQ908446_A_duck_Egypt_0877_NLQP_2008_Avian
AB601138_A_chicken_Egypt_RIMD1_2_2008_Avian
HQ908467_A_chicken_Egypt_0962S_NLQP_2009_Avian

NA1-Group20

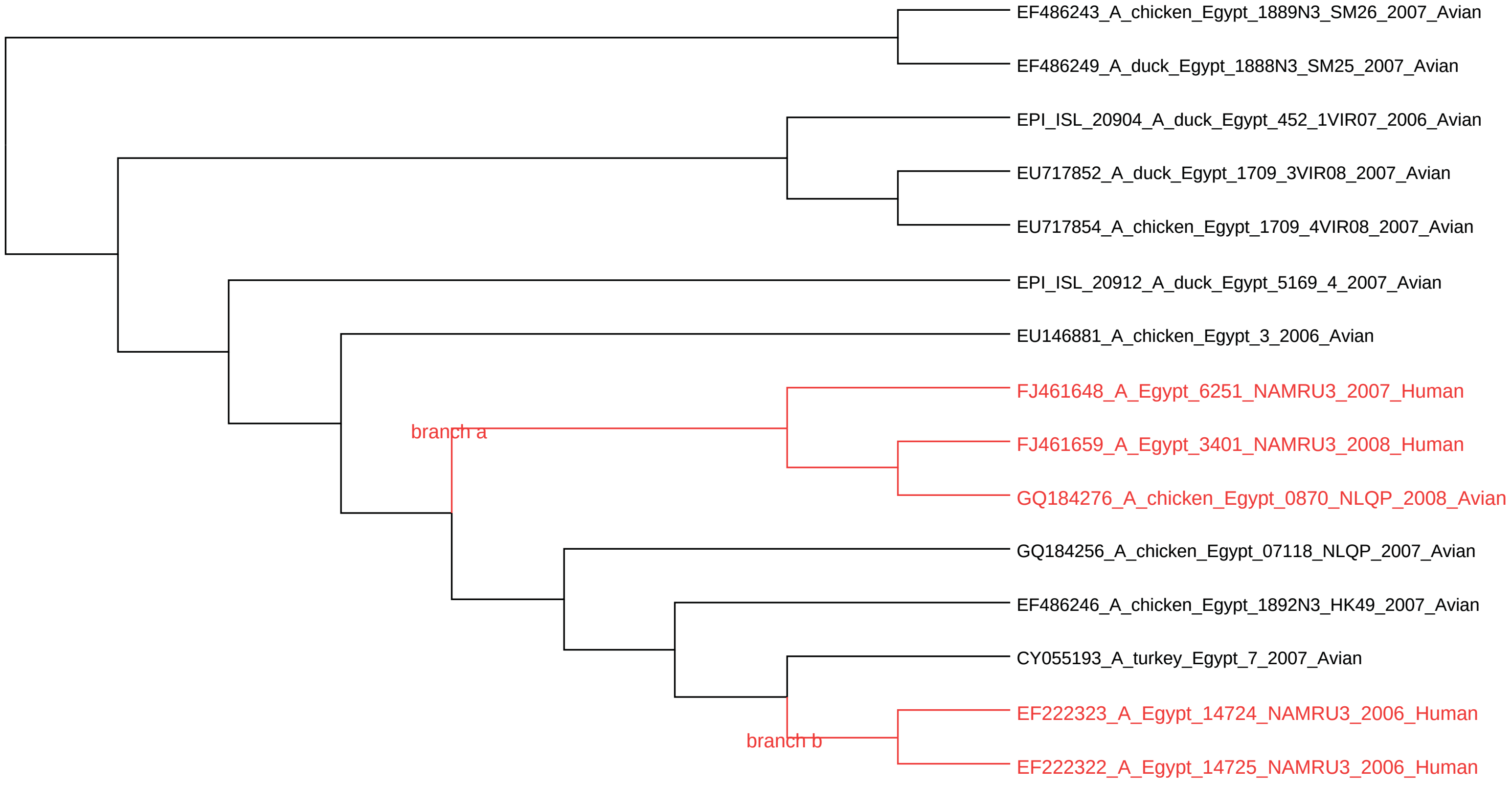

EF486243_A_chicken_Egypt_1889N3_SM26_2007_Avian
EF486249_A_duck_Egypt_1888N3_SM25_2007_Avian
EPI_ISL_20904_A_duck_Egypt_452_1VIR07_2006_Avian
EU717852_A_duck_Egypt_1709_3VIR08_2007_Avian
EU717854_A_chicken_Egypt_1709_4VIR08_2007_Avian
EPI_ISL_20912_A_duck_Egypt_5169_4_2007_Avian
EU146881_A_chicken_Egypt_3_2006_Avian
FJ461648_A_Egypt_6251_NAMRU3_2007_Human
branch a
FJ461659_A_Egypt_3401_NAMRU3_2008_Human
GQ184276_A_chicken_Egypt_0870_NLQP_2008_Avian
GQ184256_A_chicken_Egypt_07118_NLQP_2007_Avian
EF486246_A_chicken_Egypt_1892N3_HK49_2007_Avian
CY055193_A_turkey_Egypt_7_2007_Avian
EF222323_A_Egypt_14724_NAMRU3_2006_Human
branch b
EF222322_A_Egypt_14725_NAMRU3_2006_Human

NA1-Group21

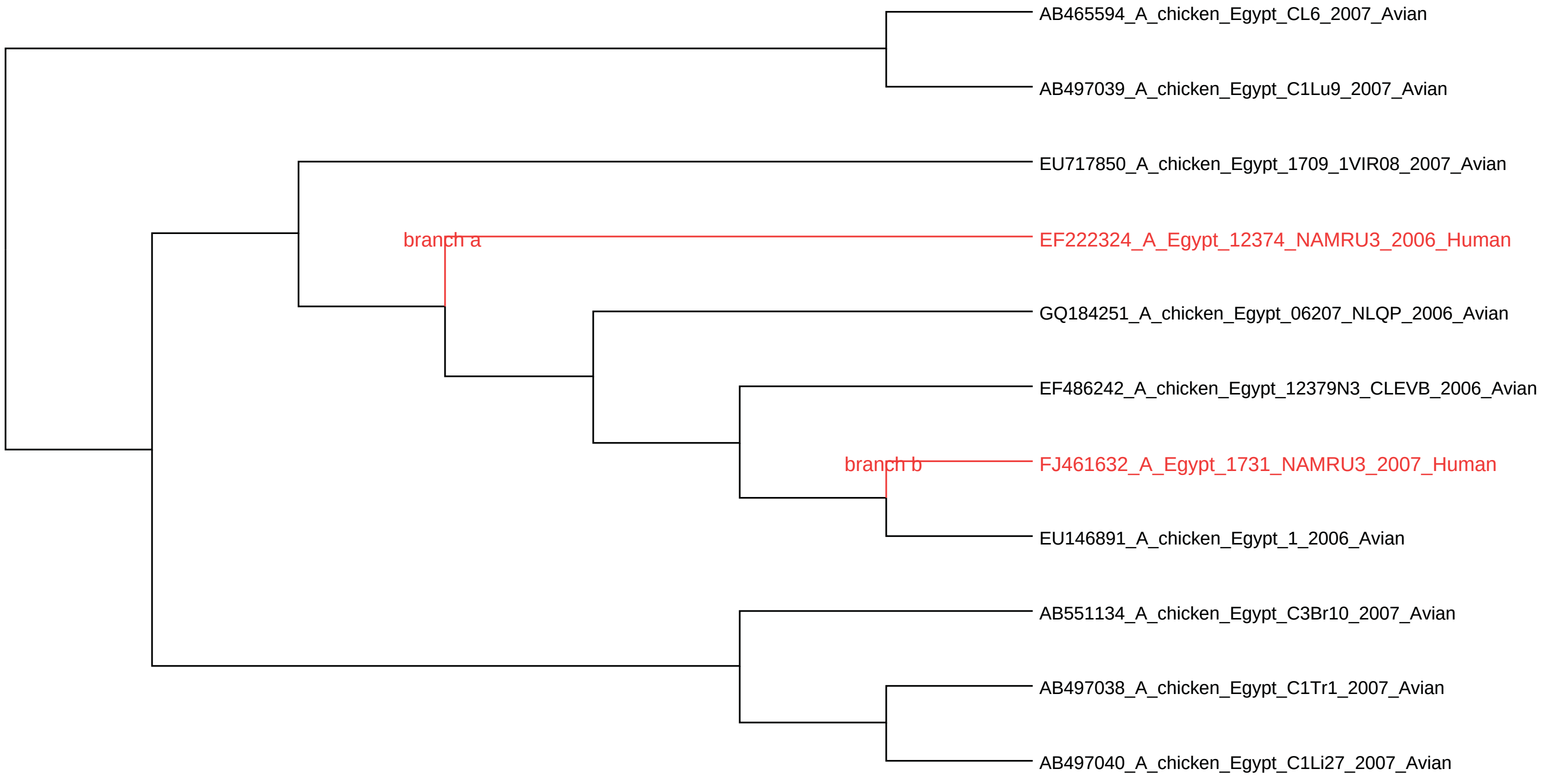

AB465594_A_chicken_Egypt_CL6_2007_Avian
AB497039_A_chicken_Egypt_C1Lu9_2007_Avian
EU717850_A_chicken_Egypt_1709_1VIR08_2007_Avian
branch a
EF222324_A_Egypt_12374_NAMRU3_2006_Human
GQ184251_A_chicken_Egypt_06207_NLQP_2006_Avian
EF486242_A_chicken_Egypt_12379N3_CLEVB_2006_Avian
branch b
FJ461632_A_Egypt_1731_NAMRU3_2007_Human
EU146891_A_chicken_Egypt_1_2006_Avian
AB551134_A_chicken_Egypt_C3Br10_2007_Avian
AB497038_A_chicken_Egypt_C1Tr1_2007_Avian
AB497040_A_chicken_Egypt_C1Li27_2007_Avian

NA1-Group22

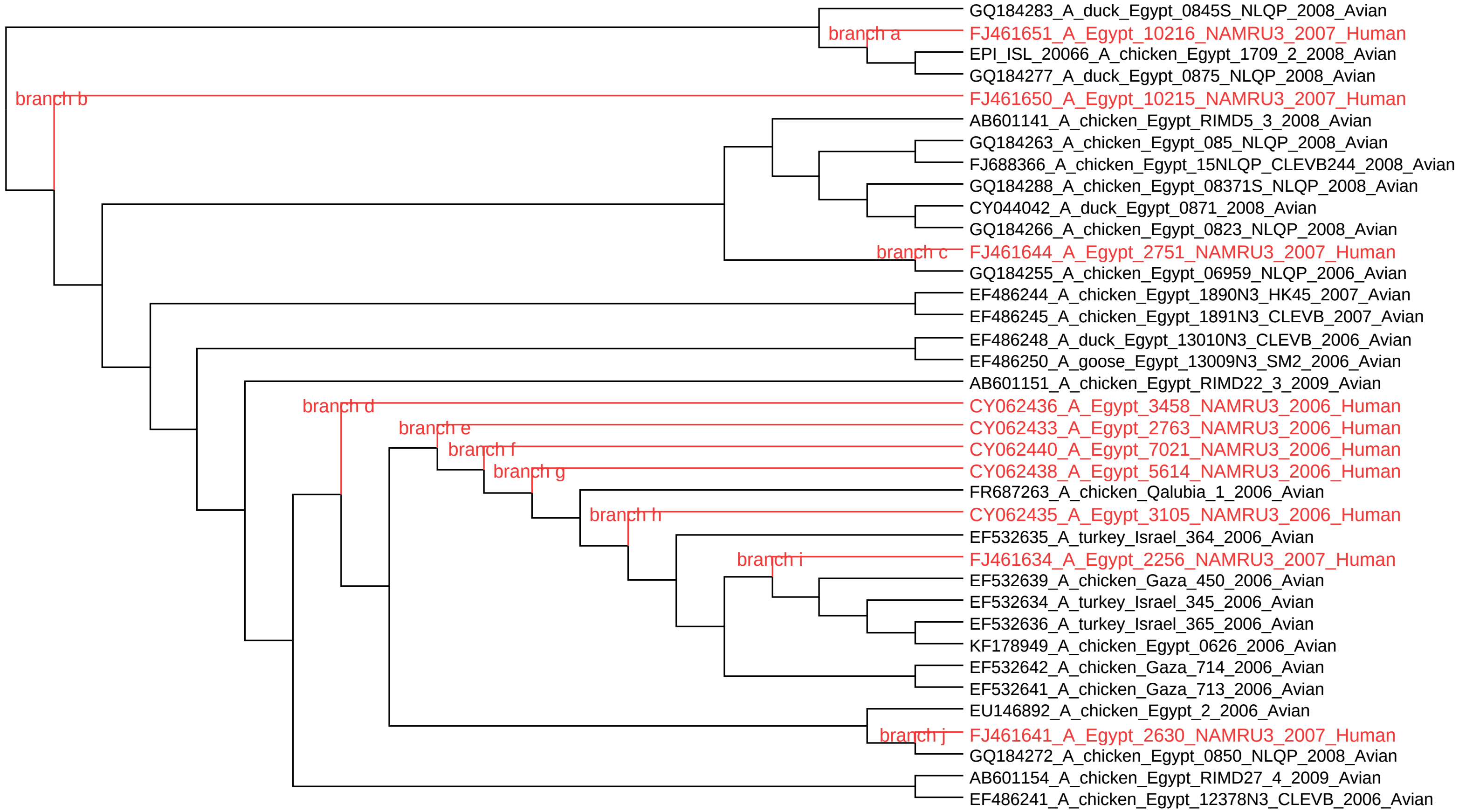

GQ184283_A_duck_Egypt_0845S_NLQP_2008_Avian
branch a
FJ461651_A_Egypt_10216_NAMRU3_2007_Human
EPI_ISL_20066_A_chicken_Egypt_1709_2_2008_Avian
GQ184277_A_duck_Egypt_0875_NLQP_2008_Avian
branch b
FJ461650_A_Egypt_10215_NAMRU3_2007_Human
AB601141_A_chicken_Egypt_RIMD5_3_2008_Avian
GQ184263_A_chicken_Egypt_085_NLQP_2008_Avian
FJ688366_A_chicken_Egypt_15NLQP_CLEVB244_2008_Avian
GQ184288_A_chicken_Egypt_08371S_NLQP_2008_Avian
CY044042_A_duck_Egypt_0871_2008_Avian
GQ184266_A_chicken_Egypt_0823_NLQP_2008_Avian
branch c
FJ461644_A_Egypt_2751_NAMRU3_2007_Human
GQ184255_A_chicken_Egypt_06959_NLQP_2006_Avian
EF486244_A_chicken_Egypt_1890N3_HK45_2007_Avian
EF486245_A_chicken_Egypt_1891N3_CLEVB_2007_Avian
EF486248_A_duck_Egypt_13010N3_CLEVB_2006_Avian
EF486250_A_goose_Egypt_13009N3_SM2_2006_Avian
AB601151_A_chicken_Egypt_RIMD22_3_2009_Avian
branch d
CY062436_A_Egypt_3458_NAMRU3_2006_Human
branch e
CY062433_A_Egypt_2763_NAMRU3_2006_Human
branch f
CY062440_A_Egypt_7021_NAMRU3_2006_Human
branch g
CY062438_A_Egypt_5614_NAMRU3_2006_Human
FR687263_A_chicken_Qalubia_1_2006_Avian
branch h
CY062435_A_Egypt_3105_NAMRU3_2006_Human
EF532635_A_turkey_Israel_364_2006_Avian
branch i
FJ461634_A_Egypt_2256_NAMRU3_2007_Human
EF532639_A_chicken_Gaza_450_2006_Avian
EF532634_A_turkey_Israel_345_2006_Avian
EF532636_A_turkey_Israel_365_2006_Avian
KF178949_A_chicken_Egypt_0626_2006_Avian
EF532642_A_chicken_Gaza_714_2006_Avian
EF532641_A_chicken_Gaza_713_2006_Avian
EU146892_A_chicken_Egypt_2_2006_Avian
branch j
FJ461641_A_Egypt_2630_NAMRU3_2007_Human
GQ184272_A_chicken_Egypt_0850_NLQP_2008_Avian
AB601154_A_chicken_Egypt_RIMD27_4_2009_Avian
EF486241_A_chicken_Egypt_12378N3_CLEVB_2006_Avian

NA1-Group23

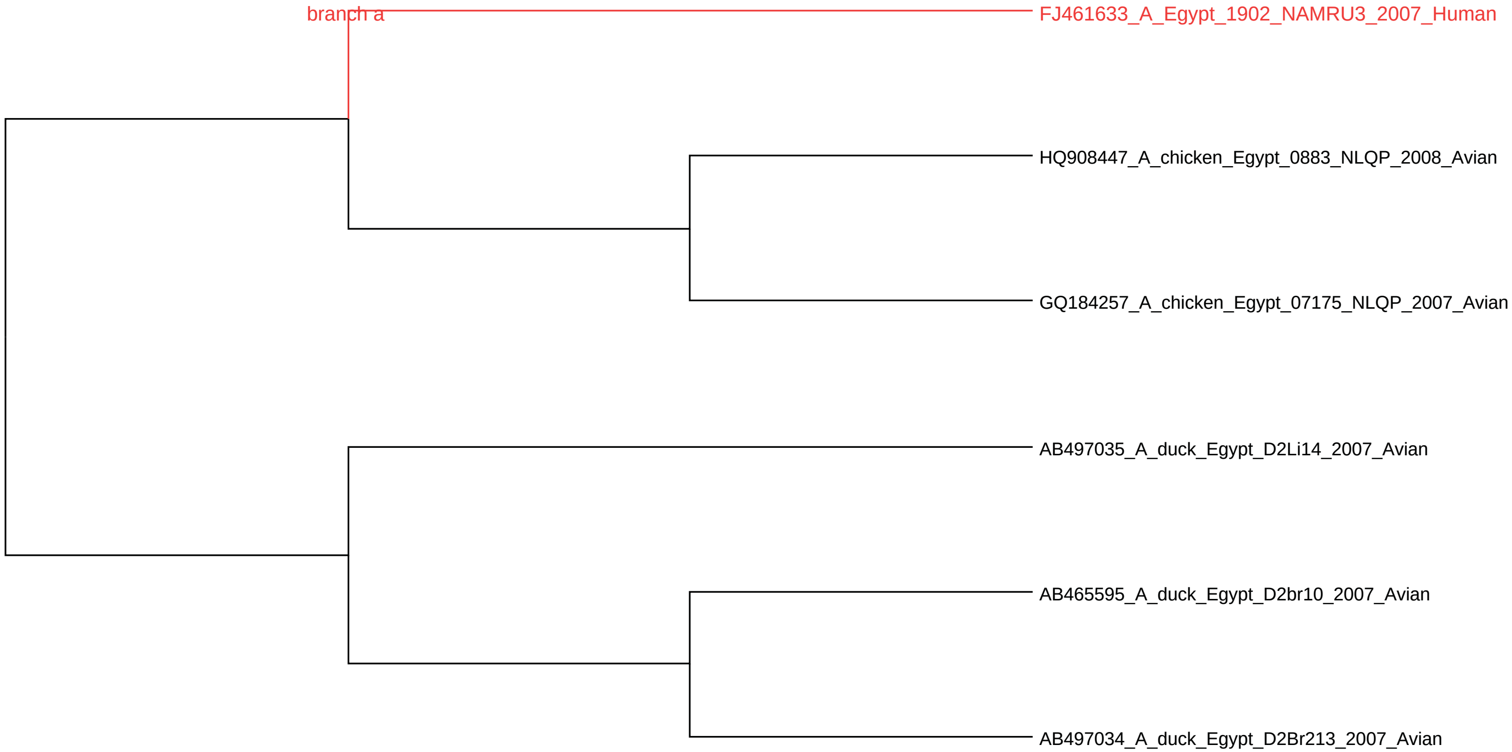
branch a
FJ461633_A_Egypt_1902_NAMRU3_2007_Human
HQ908447_A_chicken_Egypt_0883_NLQP_2008_Avian
GQ184257_A_chicken_Egypt_07175_NLQP_2007_Avian
AB497035_A_duck_Egypt_D2Li14_2007_Avian
AB465595_A_duck_Egypt_D2br10_2007_Avian
AB497034_A_duck_Egypt_D2Br213_2007_Avian

NA1-Group24

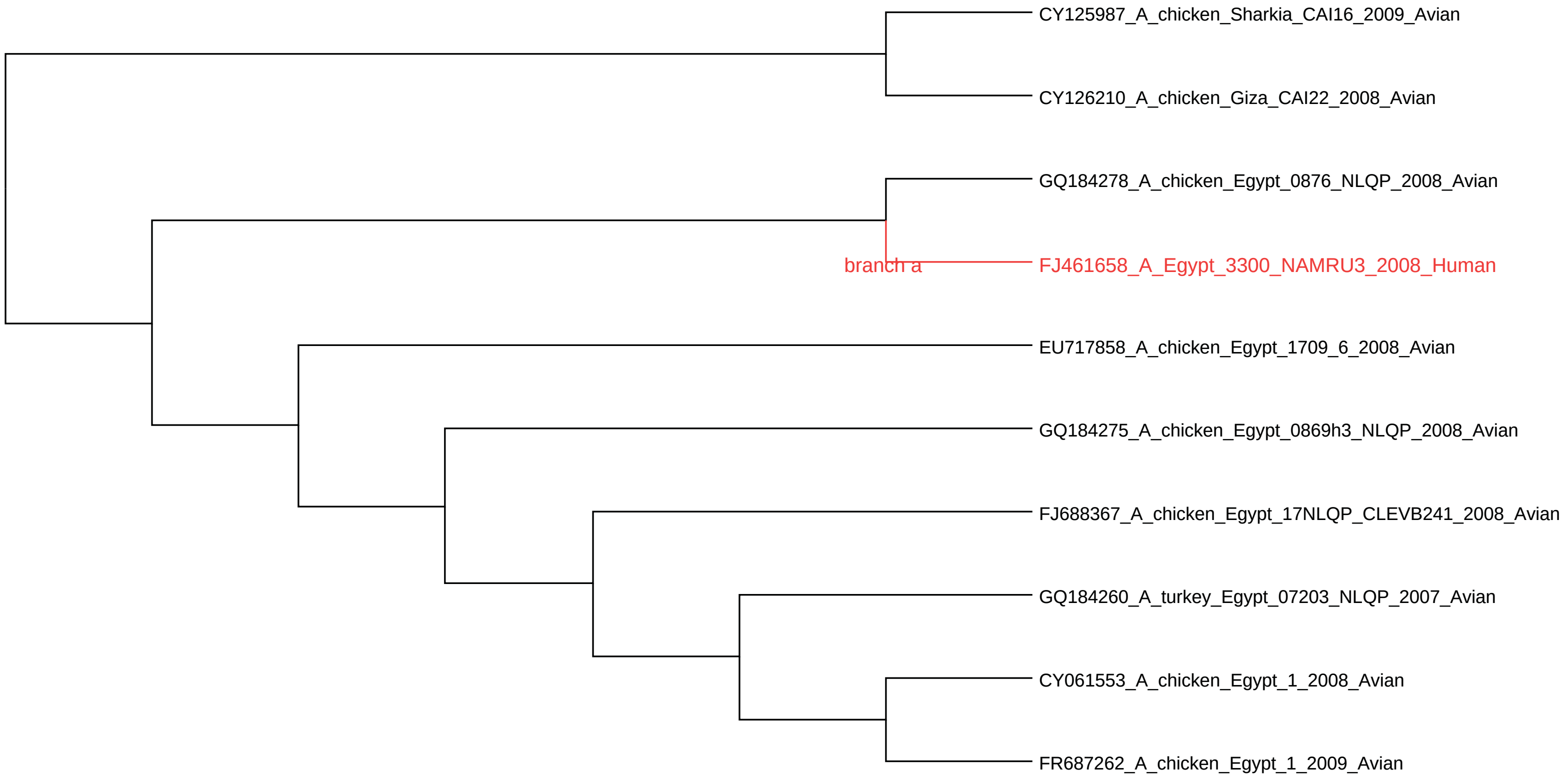
CY125987_A_chicken_Sharkia_CAI16_2009_Avian
CY126210_A_chicken_Giza_CAI22_2008_Avian
GQ184278_A_chicken_Egypt_0876_NLQP_2008_Avian
branch a
FJ461658_A_Egypt_3300_NAMRU3_2008_Human
EU717858_A_chicken_Egypt_1709_6_2008_Avian
GQ184275_A_chicken_Egypt_0869h3_NLQP_2008_Avian
FJ688367_A_chicken_Egypt_17NLQP_CLEVB241_2008_Avian
GQ184260_A_turkey_Egypt_07203_NLQP_2007_Avian
CY061553_A_chicken_Egypt_1_2008_Avian
FR687262_A_chicken_Egypt_1_2009_Avian

NA1-Group25

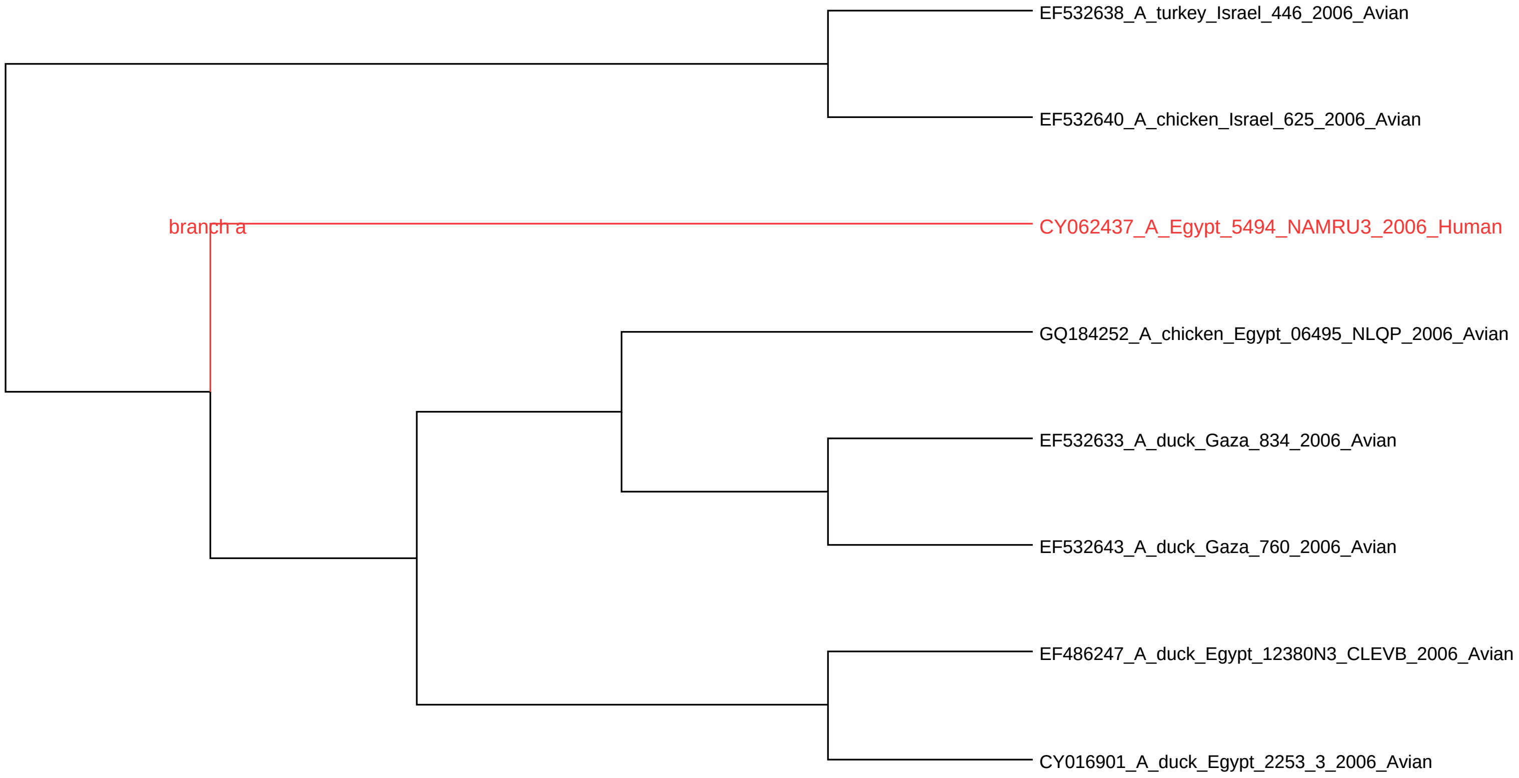

EF532638_A_turkey_Israel_446_2006_Avian
EF532640_A_chicken_Israel_625_2006_Avian
branch a
CY062437_A_Egypt_5494_NAMRU3_2006_Human
GQ184252_A_chicken_Egypt_06495_NLQP_2006_Avian
EF532633_A_duck_Gaza_834_2006_Avian
EF532643_A_duck_Gaza_760_2006_Avian
EF486247_A_duck_Egypt_12380N3_CLEVB_2006_Avian
CY016901_A_duck_Egypt_2253_3_2006_Avian

NA1-Group26

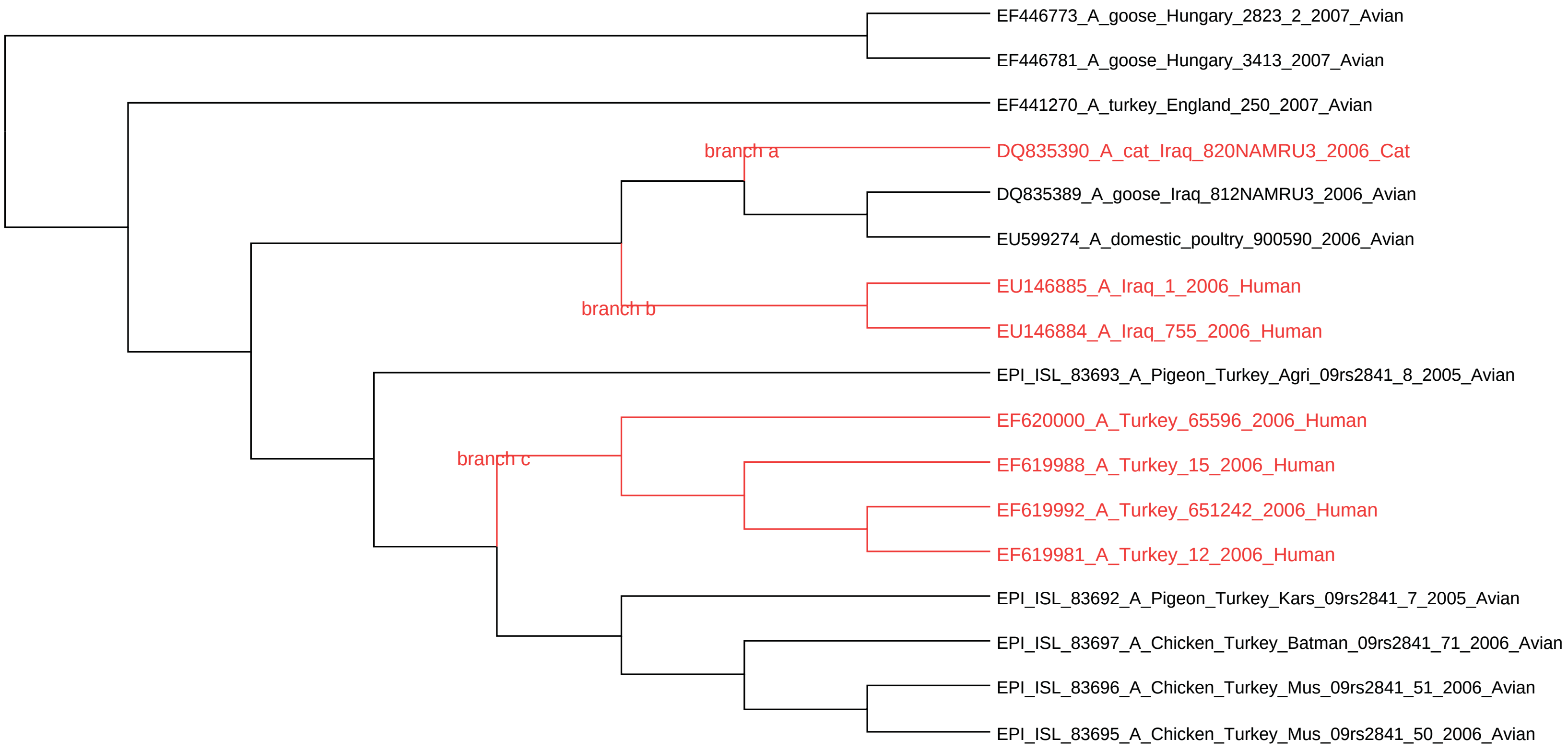
EF446773_A_goose_Hungary_2823_2_2007_Avian
EF446781_A_goose_Hungary_3413_2007_Avian
EF441270_A_turkey_England_250_2007_Avian
branch a
DQ835390_A_cat_Iraq_820NAMRU3_2006_Cat
DQ835389_A_goose_Iraq_812NAMRU3_2006_Avian
EU599274_A_domestic_poultry_900590_2006_Avian
EU146885_A_Iraq_1_2006_Human
branch b
EU146884_A_Iraq_755_2006_Human
EPI_ISL_83693_A_Pigeon_Turkey_Agri_09rs2841_8_2005_Avian
EF620000_A_Turkey_65596_2006_Human
branch c
EF619988_A_Turkey_15_2006_Human
EF619992_A_Turkey_651242_2006_Human
EF619981_A_Turkey_12_2006_Human
EPI_ISL_83692_A_Pigeon_Turkey_Kars_09rs2841_7_2005_Avian
EPI_ISL_83697_A_Chicken_Turkey_Batman_09rs2841_71_2006_Avian
EPI_ISL_83696_A_Chicken_Turkey_Mus_09rs2841_51_2006_Avian
EPI_ISL_83695_A_Chicken_Turkey_Mus_09rs2841_50_2006_Avian

NA1-Group27

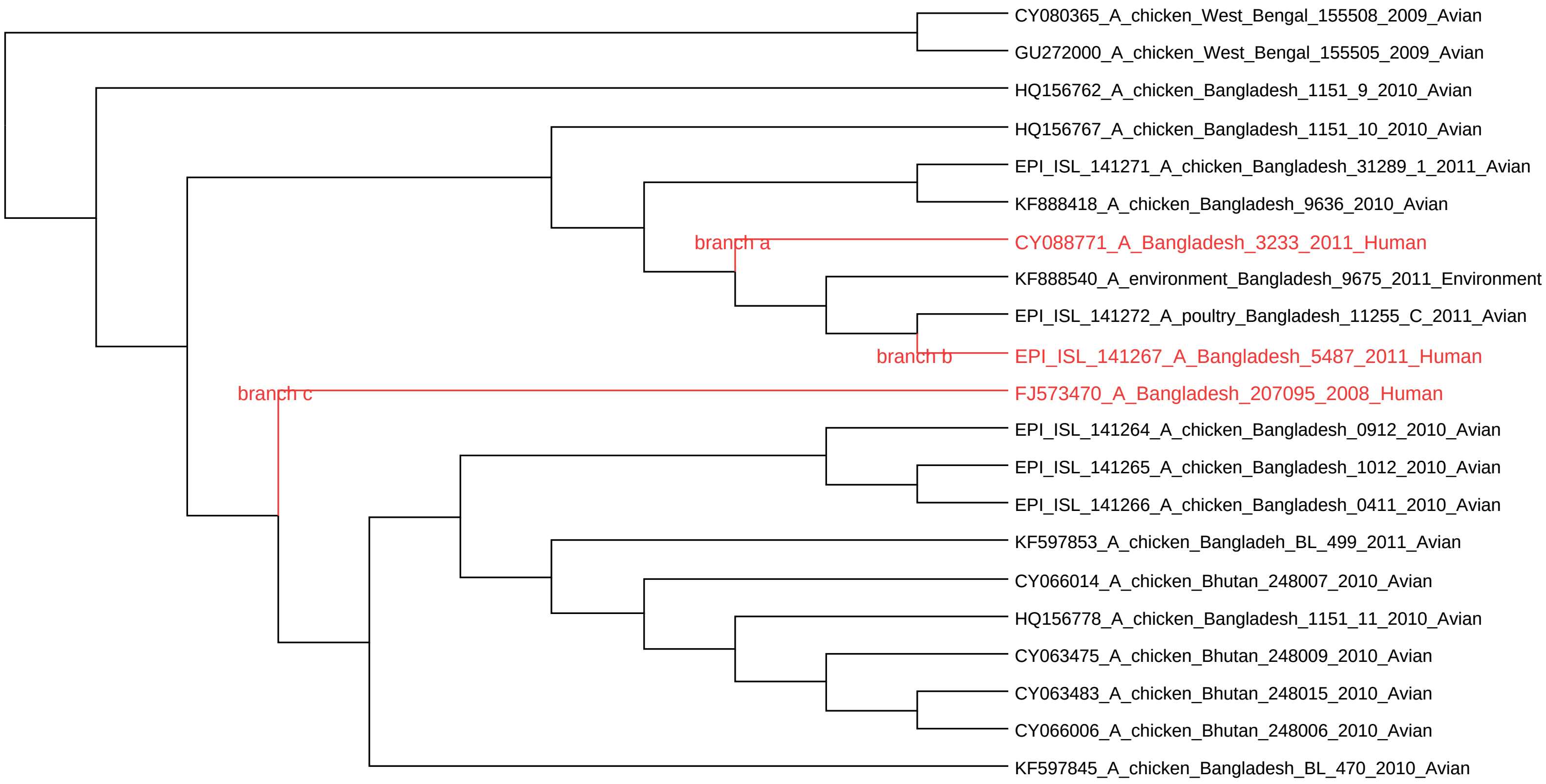

CY080365_A_chicken_West_Bengal_155508_2009_Avian
GU272000_A_chicken_West_Bengal_155505_2009_Avian
HQ156762_A_chicken_Bangladesh_1151_9_2010_Avian
HQ156767_A_chicken_Bangladesh_1151_10_2010_Avian
EPI_ISL_141271_A_chicken_Bangladesh_31289_1_2011_Avian
KF888418_A_chicken_Bangladesh_9636_2010_Avian
branch a
CY088771_A_Bangladesh_3233_2011_Human
KF888540_A_environment_Bangladesh_9675_2011_Environment
EPI_ISL_141272_A_poultry_Bangladesh_11255_C_2011_Avian
branch b
EPI_ISL_141267_A_Bangladesh_5487_2011_Human
branch c
FJ573470_A_Bangladesh_207095_2008_Human
EPI_ISL_141264_A_chicken_Bangladesh_0912_2010_Avian
EPI_ISL_141265_A_chicken_Bangladesh_1012_2010_Avian
EPI_ISL_141266_A_chicken_Bangladesh_0411_2010_Avian
KF597853_A_chicken_Bangladeh_BL_499_2011_Avian
CY066014_A_chicken_Bhutan_248007_2010_Avian
HQ156778_A_chicken_Bangladesh_1151_11_2010_Avian
CY063475_A_chicken_Bhutan_248009_2010_Avian
CY063483_A_chicken_Bhutan_248015_2010_Avian
CY066006_A_chicken_Bhutan_248006_2010_Avian
KF597845_A_chicken_Bangladesh_BL_470_2010_Avian

NA1-Group28

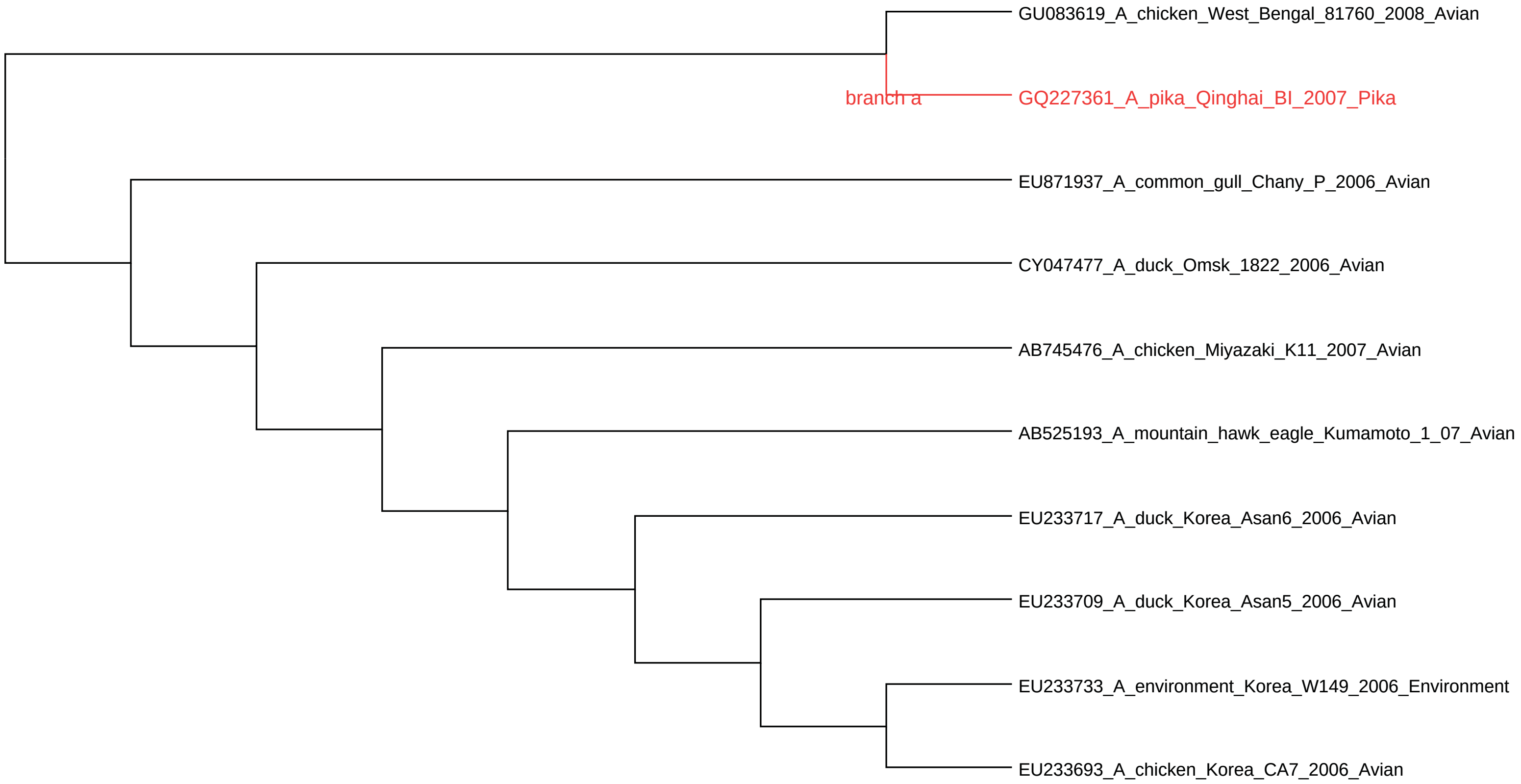

GU083619_A_chicken_West_Bengal_81760_2008_Avian
branch a
GQ227361_A_pika_Qinghai_BI_2007_Pika
EU871937_A_common_gull_Chany_P_2006_Avian
CY047477_A_duck_Omsk_1822_2006_Avian
AB745476_A_chicken_Miyazaki_K11_2007_Avian
AB525193_A_mountain_hawk_eagle_Kumamoto_1_07_Avian
EU233717_A_duck_Korea_Asan6_2006_Avian
EU233709_A_duck_Korea_Asan5_2006_Avian
EU233733_A_environment_Korea_W149_2006_Environment
EU233693_A_chicken_Korea_CA7_2006_Avian

NA1-Group29

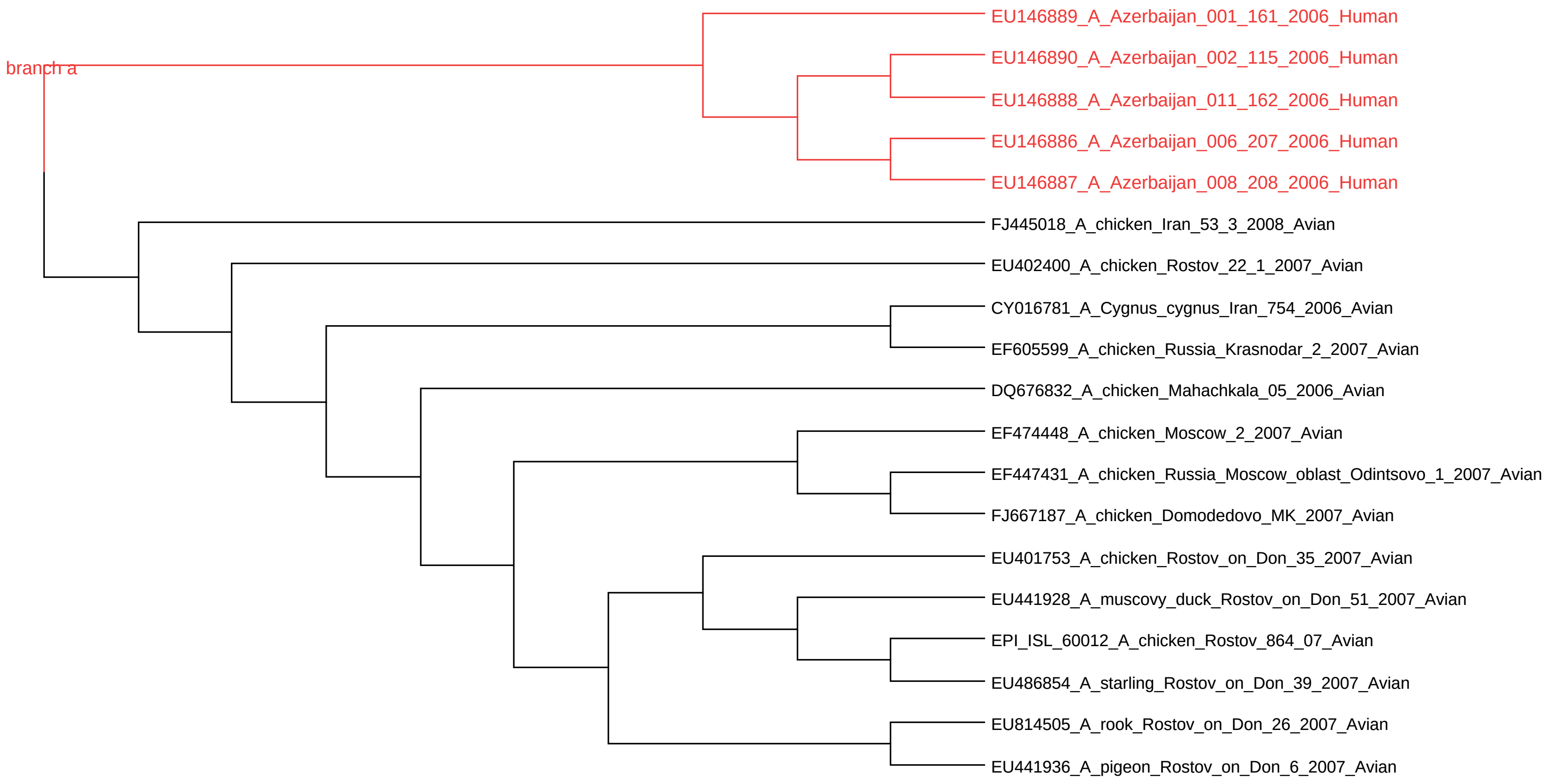
branch a
EU146889_A_Azerbaijan_001_161_2006_Human
EU146890_A_Azerbaijan_002_115_2006_Human
EU146888_A_Azerbaijan_011_162_2006_Human
EU146886_A_Azerbaijan_006_207_2006_Human
EU146887_A_Azerbaijan_008_208_2006_Human
FJ445018_A_chicken_Iran_53_3_2008_Avian
EU402400_A_chicken_Rostov_22_1_2007_Avian
CY016781_A_Cygnus_cygnus_Iran_754_2006_Avian
EF605599_A_chicken_Russia_Krasnodar_2_2007_Avian
DQ676832_A_chicken_Mahachkala_05_2006_Avian
EF474448_A_chicken_Moscow_2_2007_Avian
EF447431_A_chicken_Russia_Moscow_oblast_Odintsovo_1_2007_Avian
FJ667187_A_chicken_Domodedovo_MK_2007_Avian
EU401753_A_chicken_Rostov_on_Don_35_2007_Avian
EU441928_A_muscovy_duck_Rostov_on_Don_51_2007_Avian
EPI_ISL_60012_A_chicken_Rostov_864_07_Avian
EU486854_A_starling_Rostov_on_Don_39_2007_Avian
EU814505_A_rook_Rostov_on_Don_26_2007_Avian
EU441936_A_pigeon_Rostov_on_Don_6_2007_Avian

NA1-Group30

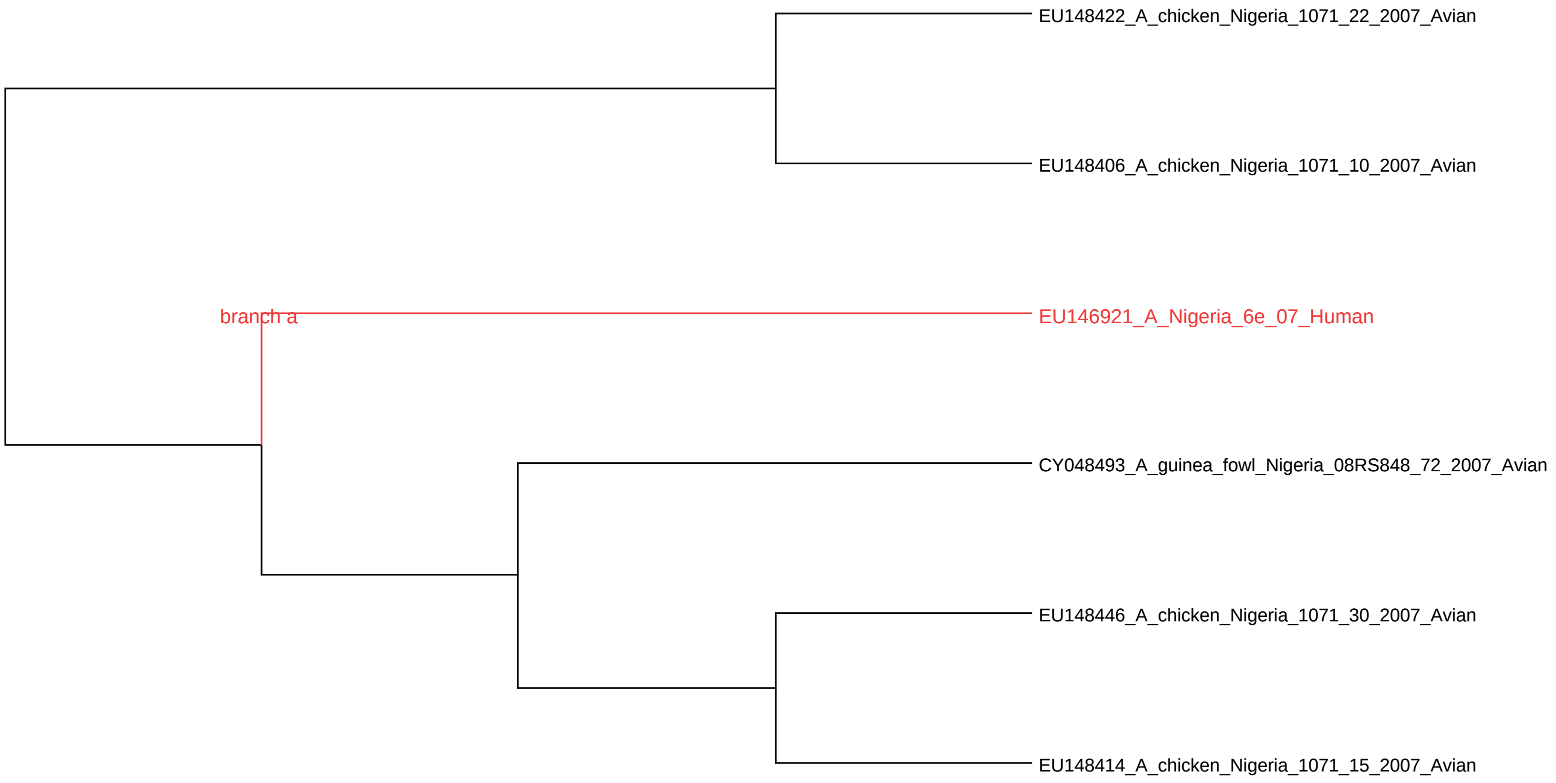
EU148422_A_chicken_Nigeria_1071_22_2007_Avian
EU148406_A_chicken_Nigeria_1071_10_2007_Avian
branch a
EU146921_A_Nigeria_6e_07_Human
CY048493_A_guinea_fowl_Nigeria_08RS848_72_2007_Avian
EU148446_A_chicken_Nigeria_1071_30_2007_Avian
EU148414_A_chicken_Nigeria_1071_15_2007_Avian

NA1-Group31

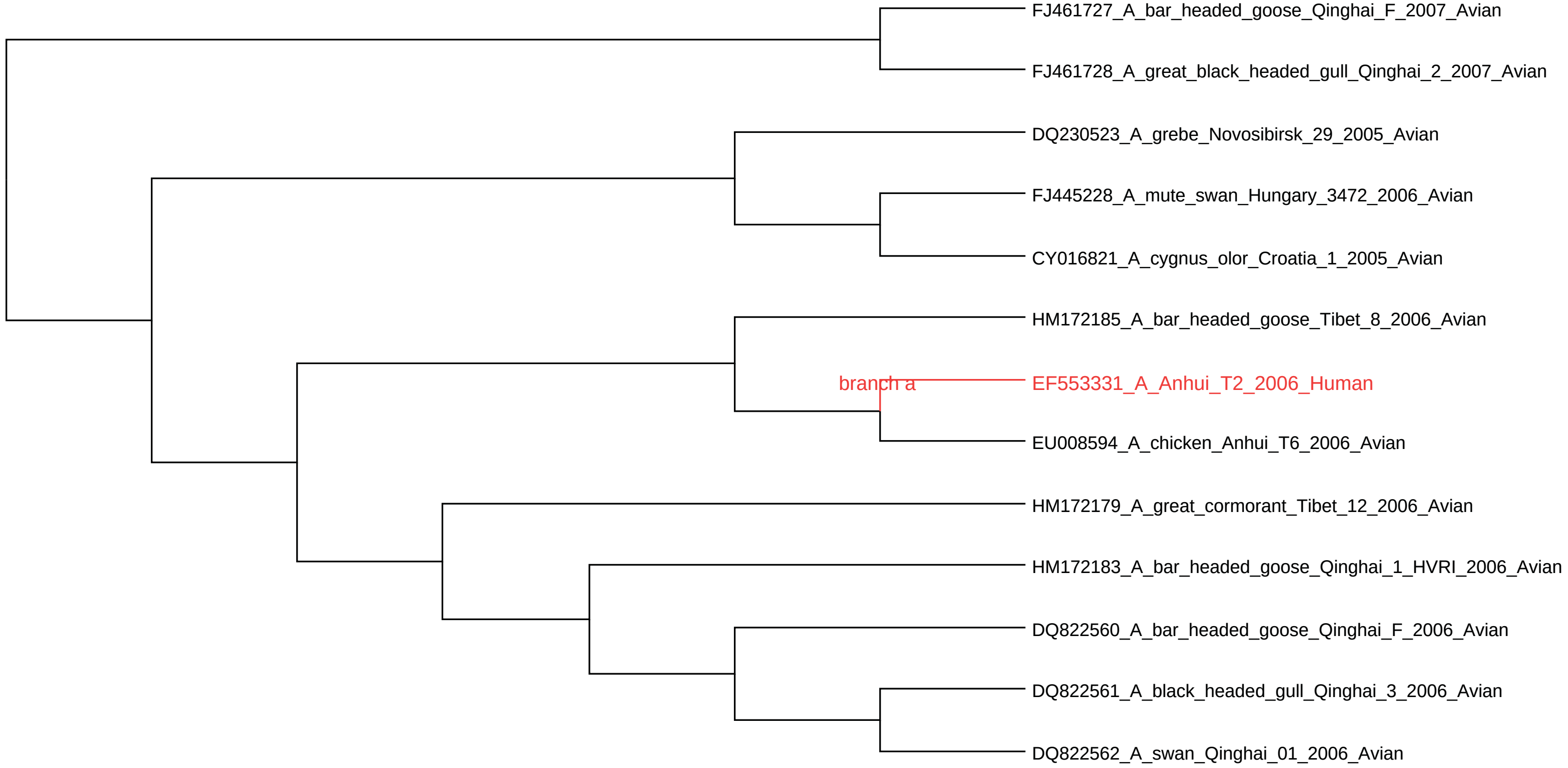
FJ461727_A_bar_headed_goose_Qinghai_F_2007_Avian
FJ461728_A_great_black_headed_gull_Qinghai_2_2007_Avian
DQ230523_A_grebe_Novosibirsk_29_2005_Avian
FJ445228_A_mute_swan_Hungary_3472_2006_Avian
CY016821_A_cygnus_olor_Croatia_1_2005_Avian
HM172185_A_bar_headed_goose_Tibet_8_2006_Avian
branch a
EF553331_A_Anhui_T2_2006_Human
EU008594_A_chicken_Anhui_T6_2006_Avian
HM172179_A_great_cormorant_Tibet_12_2006_Avian
HM172183_A_bar_headed_goose_Qinghai_1_HVRI_2006_Avian
DQ822560_A_bar_headed_goose_Qinghai_F_2006_Avian
DQ822561_A_black_headed_gull_Qinghai_3_2006_Avian
DQ822562_A_swan_Qinghai_01_2006_Avian

NA1-Group32

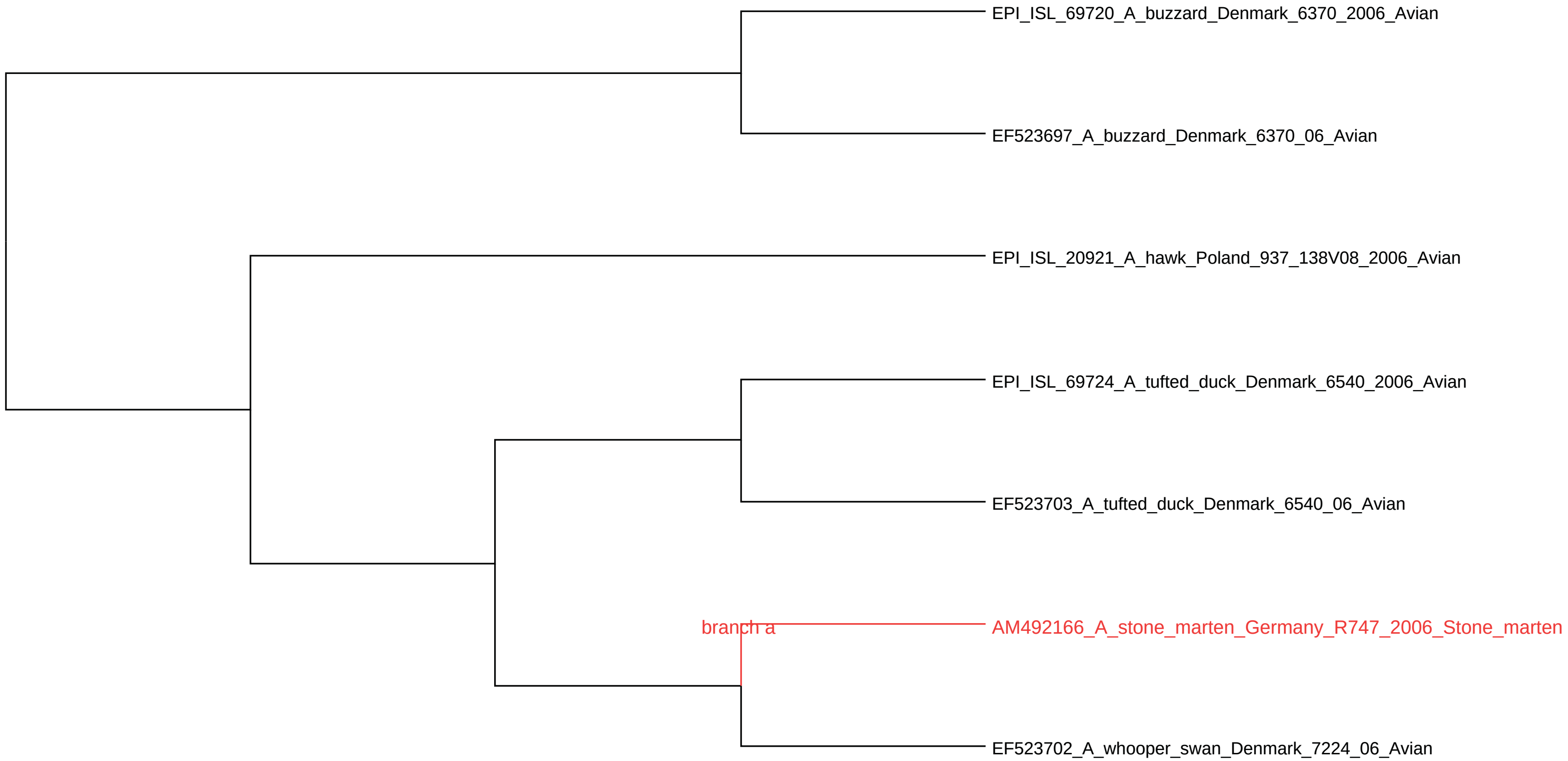
EPI_ISL_69720_A_buzzard_Denmark_6370_2006_Avian
EF523697_A_buzzard_Denmark_6370_06_Avian
EPI_ISL_20921_A_hawk_Poland_937_138V08_2006_Avian
EPI_ISL_69724_A_tufted_duck_Denmark_6540_2006_Avian
EF523703_A_tufted_duck_Denmark_6540_06_Avian
branch a
AM492166_A_stone_marten_Germany_R747_2006_Stone_marten
EF523702_A_whooper_swan_Denmark_7224_06_Avian

NA1-Group33

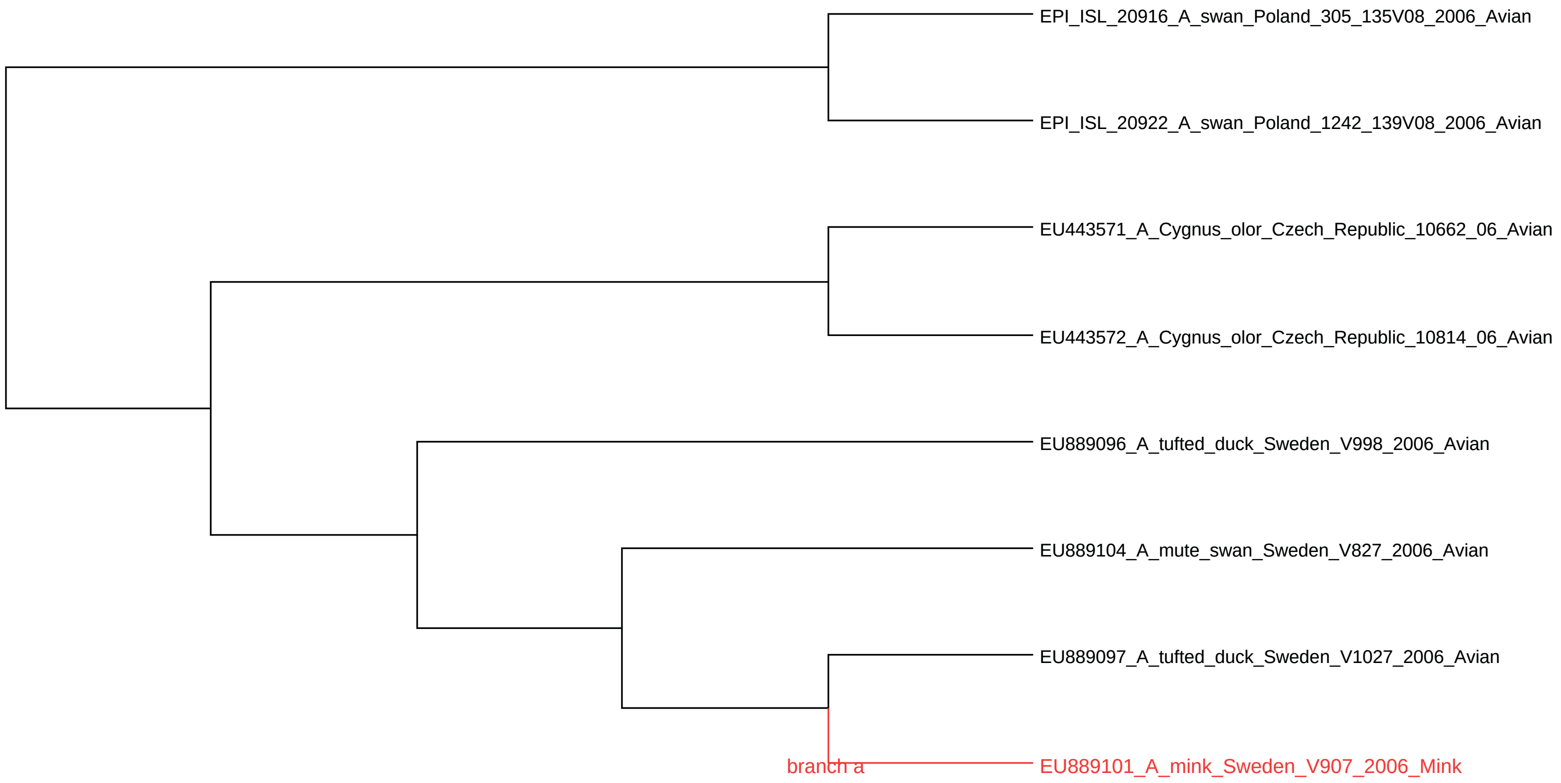

EPI_ISL_20916_A_swan_Poland_305_135V08_2006_Avian
EPI_ISL_20922_A_swan_Poland_1242_139V08_2006_Avian
EU443571_A_Cygnus_olor_Czech_Republic_10662_06_Avian
EU443572_A_Cygnus_olor_Czech_Republic_10814_06_Avian
EU889096_A_tufted_duck_Sweden_V998_2006_Avian
EU889104_A_mute_swan_Sweden_V827_2006_Avian
EU889097_A_tufted_duck_Sweden_V1027_2006_Avian
branch a
EU889101_A_mink_Sweden_V907_2006_Mink

NA1-Group34

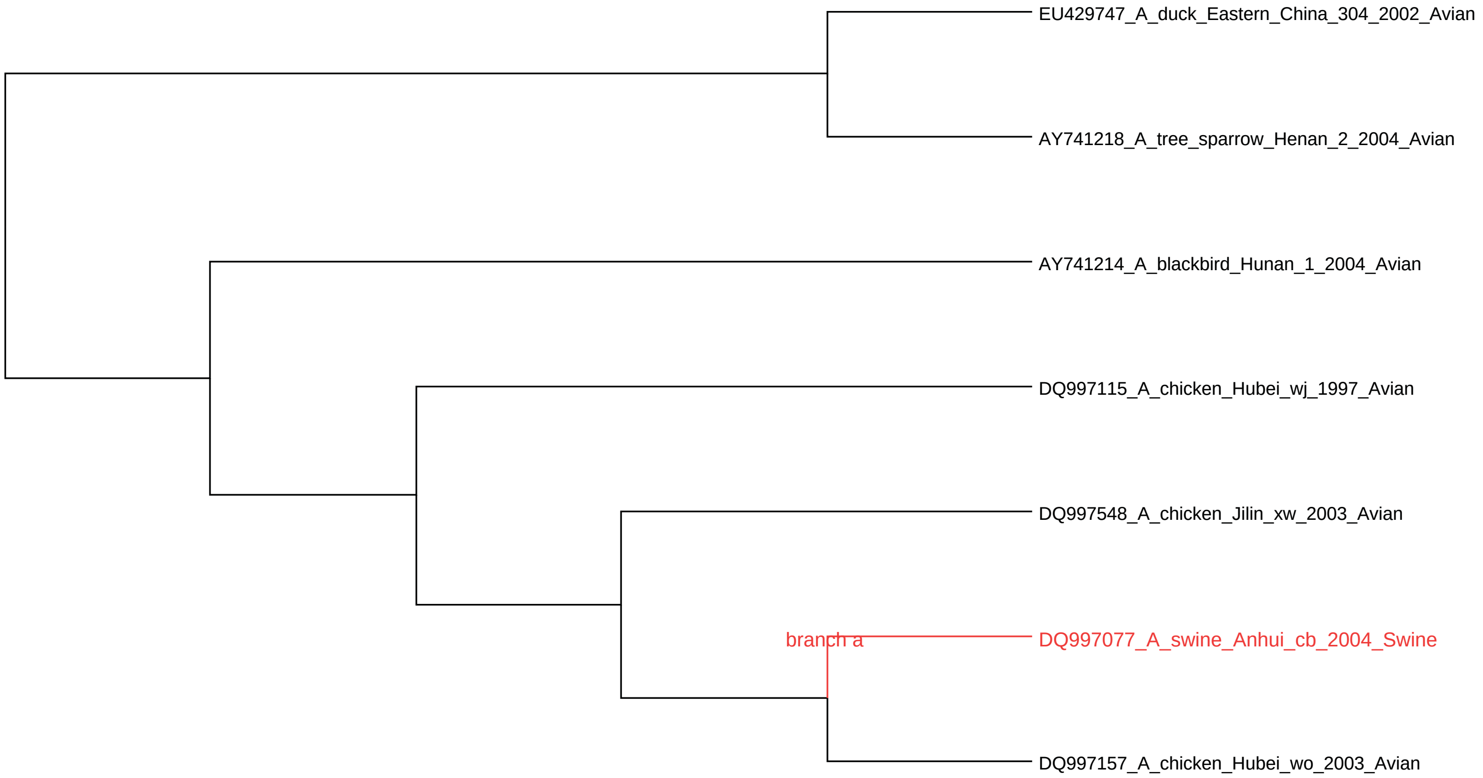
EU429747_A_duck_Eastern_China_304_2002_Avian
AY741218_A_tree_sparrow_Henan_2_2004_Avian
AY741214_A_blackbird_Hunan_1_2004_Avian
DQ997115_A_chicken_Hubei_wj_1997_Avian
DQ997548_A_chicken_Jilin_xw_2003_Avian
branch a
DQ997077_A_swine_Anhui_cb_2004_Swine
DQ997157_A_chicken_Hubei_wo_2003_Avian

NA1-Group35

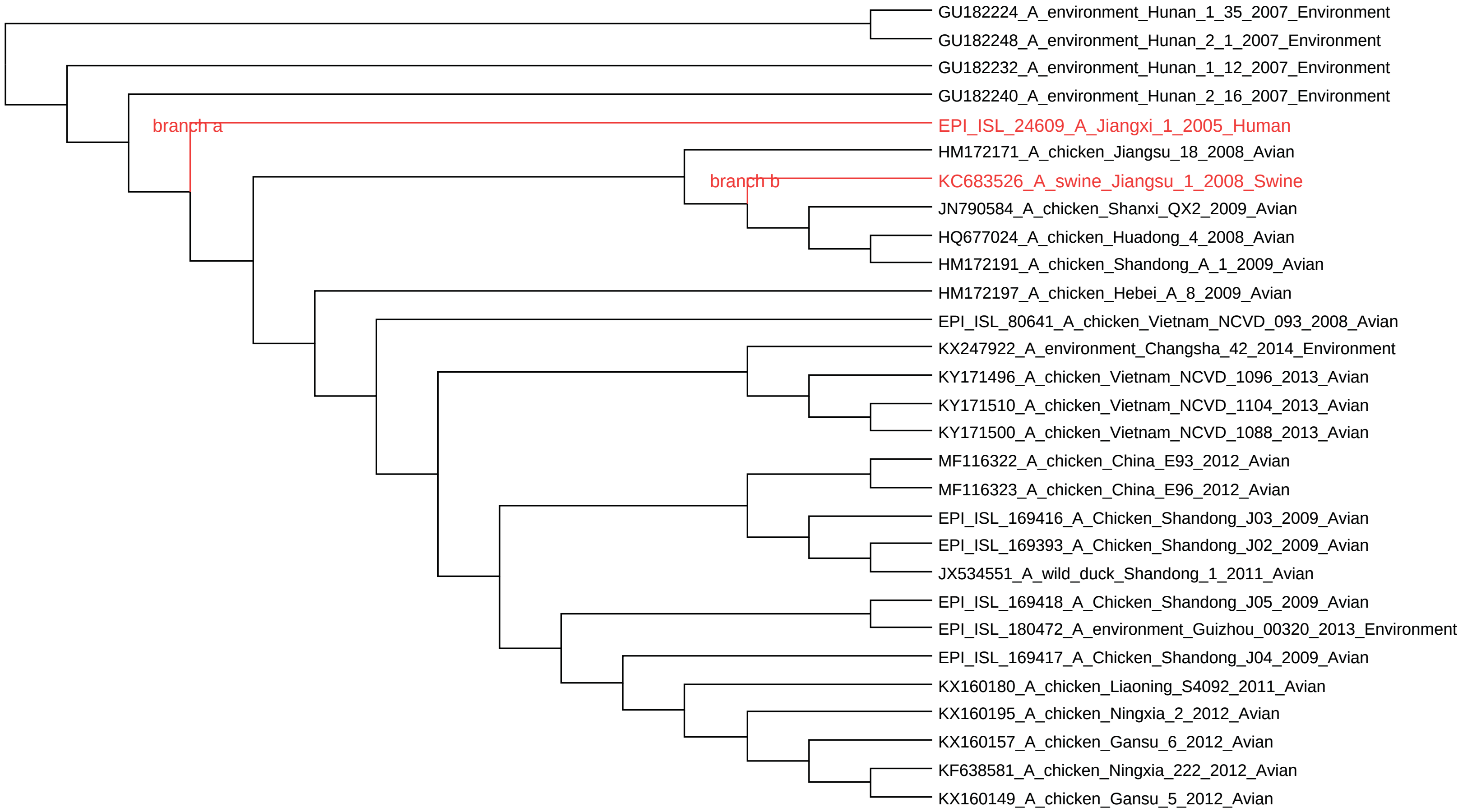

GU182224_A_environment_Hunan_1_35_2007_Environment
GU182248_A_environment_Hunan_2_1_2007_Environment
GU182232_A_environment_Hunan_1_12_2007_Environment
GU182240_A_environment_Hunan_2_16_2007_Environment
branch a
EPI_ISL_24609_A_Jiangxi_1_2005_Human
HM172171_A_chicken_Jiangsu_18_2008_Avian
branch b
KC683526_A_swine_Jiangsu_1_2008_Swine
JN790584_A_chicken_Shanxi_QX2_2009_Avian
HQ677024_A_chicken_Huadong_4_2008_Avian
HM172191_A_chicken_Shandong_A_1_2009_Avian
HM172197_A_chicken_Hebei_A_8_2009_Avian
EPI_ISL_80641_A_chicken_Vietnam_NCVD_093_2008_Avian
KX247922_A_environment_Changsha_42_2014_Environment
KY171496_A_chicken_Vietnam_NCVD_1096_2013_Avian
KY171510_A_chicken_Vietnam_NCVD_1104_2013_Avian
KY171500_A_chicken_Vietnam_NCVD_1088_2013_Avian
MF116322_A_chicken_China_E93_2012_Avian
MF116323_A_chicken_China_E96_2012_Avian
EPI_ISL_169416_A_Chicken_Shandong_J03_2009_Avian
EPI_ISL_169393_A_Chicken_Shandong_J02_2009_Avian
JX534551_A_wild_duck_Shandong_1_2011_Avian
EPI_ISL_169418_A_Chicken_Shandong_J05_2009_Avian
EPI_ISL_180472_A_environment_Guizhou_00320_2013_Environment
EPI_ISL_169417_A_Chicken_Shandong_J04_2009_Avian
KX160180_A_chicken_Liaoning_S4092_2011_Avian
KX160195_A_chicken_Ningxia_2_2012_Avian
KX160157_A_chicken_Gansu_6_2012_Avian
KF638581_A_chicken_Ningxia_222_2012_Avian
KX160149_A_chicken_Gansu_5_2012_Avian

NA1-Group36

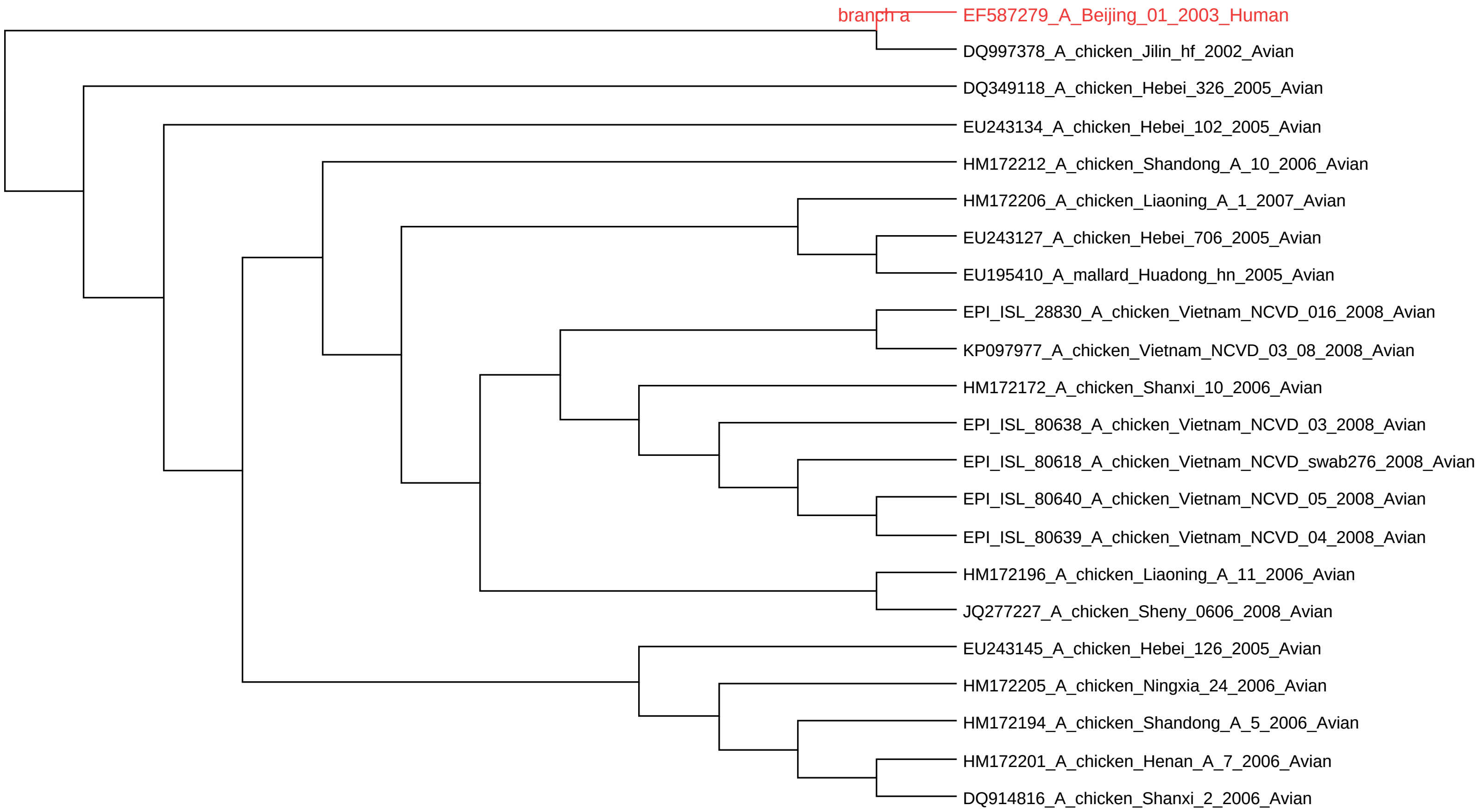

branch a
EF587279_A_Beijing_01_2003_Human
DQ997378_A_chicken_Jilin_hf_2002_Avian
DQ349118_A_chicken_Hebei_326_2005_Avian
EU243134_A_chicken_Hebei_102_2005_Avian
HM172212_A_chicken_Shandong_A_10_2006_Avian
HM172206_A_chicken_Liaoning_A_1_2007_Avian
EU243127_A_chicken_Hebei_706_2005_Avian
EU195410_A_mallard_Huadong_hn_2005_Avian
EPI_ISL_28830_A_chicken_Vietnam_NCVD_016_2008_Avian
KP097977_A_chicken_Vietnam_NCVD_03_08_2008_Avian
HM172172_A_chicken_Shanxi_10_2006_Avian
EPI_ISL_80638_A_chicken_Vietnam_NCVD_03_2008_Avian
EPI_ISL_80618_A_chicken_Vietnam_NCVD_swab276_2008_Avian
EPI_ISL_80640_A_chicken_Vietnam_NCVD_05_2008_Avian
EPI_ISL_80639_A_chicken_Vietnam_NCVD_04_2008_Avian
HM172196_A_chicken_Liaoning_A_11_2006_Avian
JQ277227_A_chicken_Sheny_0606_2008_Avian
EU243145_A_chicken_Hebei_126_2005_Avian
HM172205_A_chicken_Ningxia_24_2006_Avian
HM172194_A_chicken_Shandong_A_5_2006_Avian
HM172201_A_chicken_Henan_A_7_2006_Avian
DQ914816_A_chicken_Shanxi_2_2006_Avian

NA1-Group37

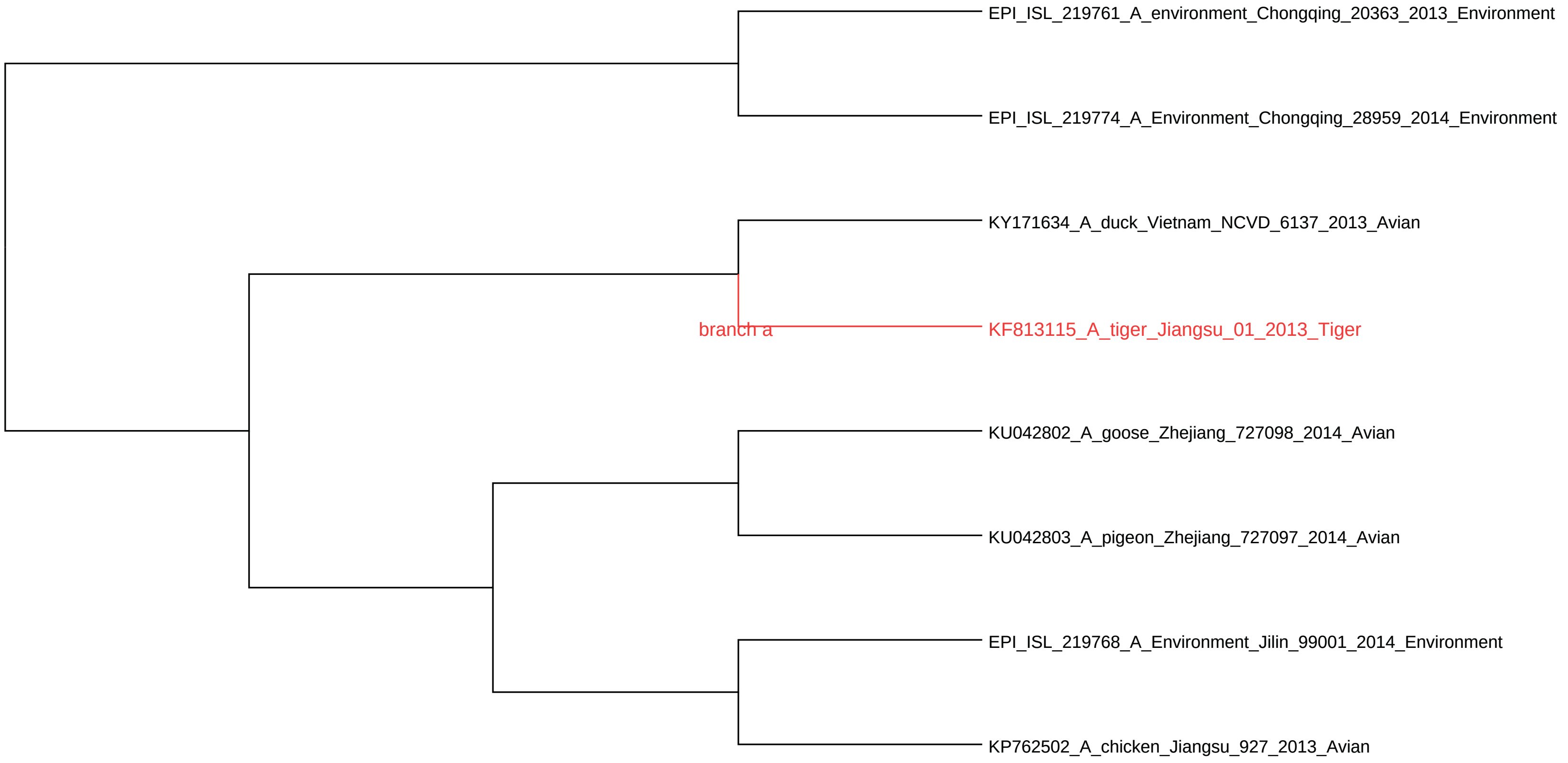

EPI_ISL_219761_A_environment_Chongqing_20363_2013_Environment
EPI_ISL_219774_A_Environment_Chongqing_28959_2014_Environment
KY171634_A_duck_Vietnam_NCVD_6137_2013_Avian
branch a
KF813115_A_tiger_Jiangsu_01_2013_Tiger
KU042802_A_goose_Zhejiang_727098_2014_Avian
KU042803_A_pigeon_Zhejiang_727097_2014_Avian
EPI_ISL_219768_A_Environment_Jilin_99001_2014_Environment
KP762502_A_chicken_Jiangsu_927_2013_Avian

NA1-Group38

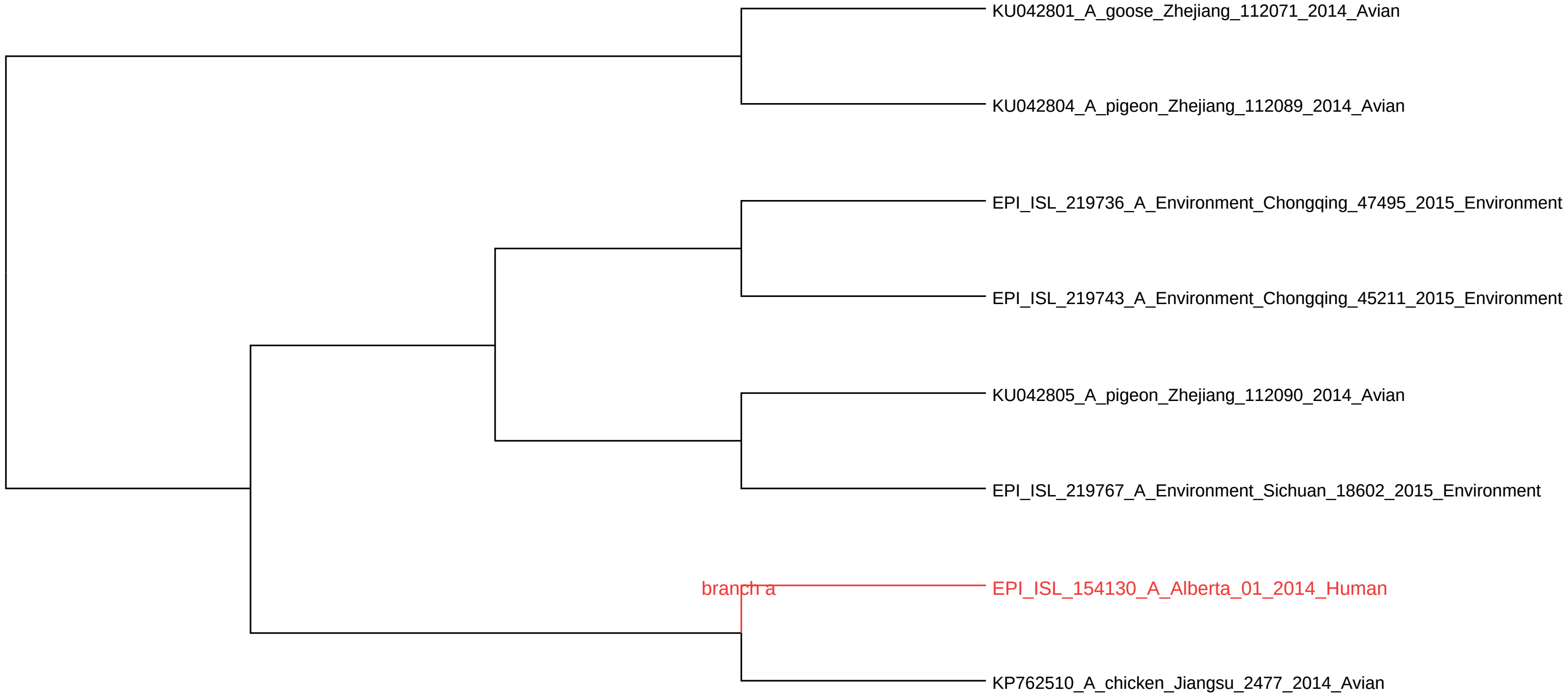

KU042801_A_goose_Zhejiang_112071_2014_Avian
KU042804_A_pigeon_Zhejiang_112089_2014_Avian
EPI_ISL_219736_A_Environment_Chongqing_47495_2015_Environment
EPI_ISL_219743_A_Environment_Chongqing_45211_2015_Environment
KU042805_A_pigeon_Zhejiang_112090_2014_Avian
EPI_ISL_219767_A_Environment_Sichuan_18602_2015_Environment
branch a
EPI_ISL_154130_A_Alberta_01_2014_Human
KP762510_A_chicken_Jiangsu_2477_2014_Avian

NA1-Group39

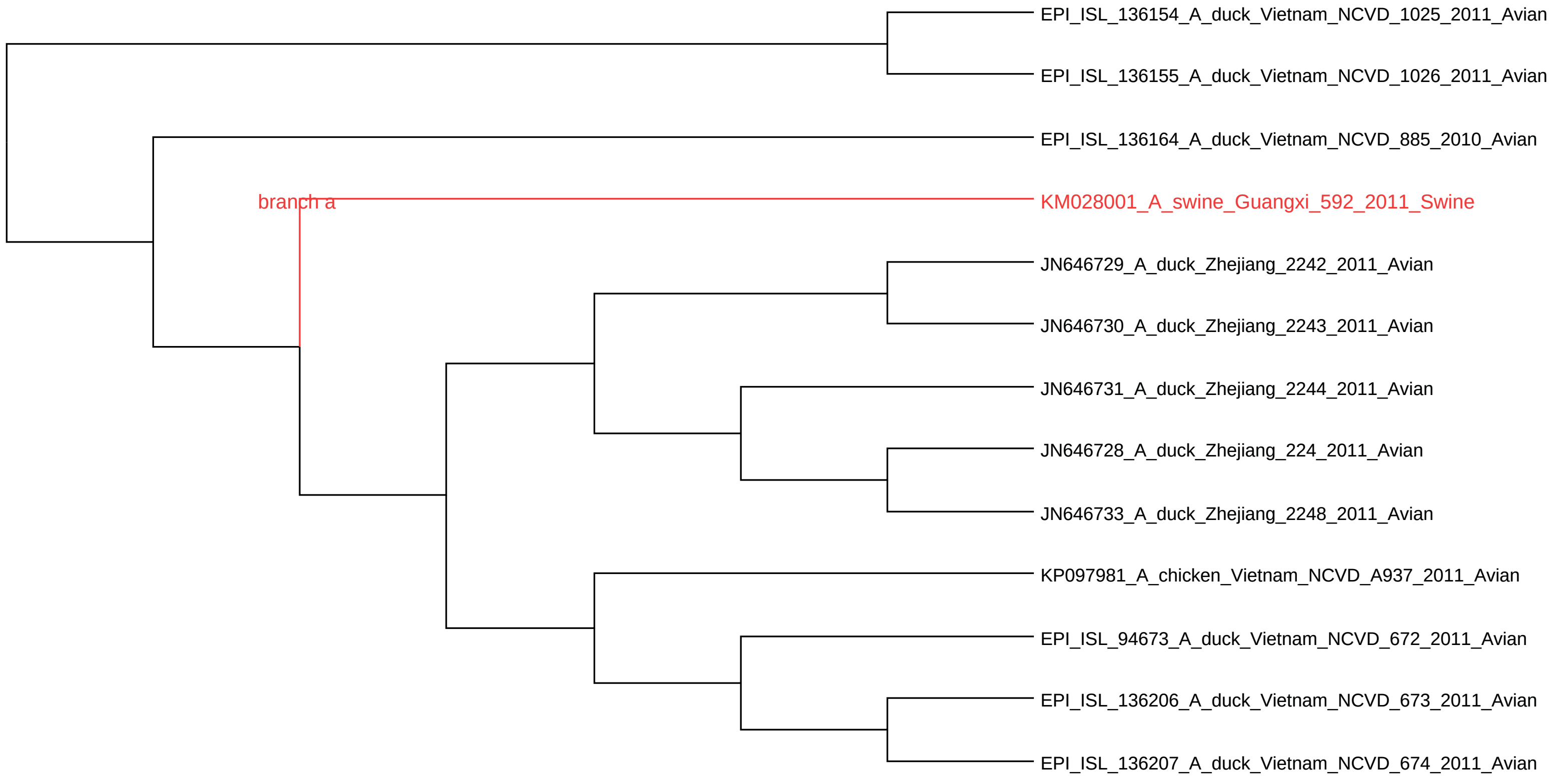
EPI_ISL_136154_A_duck_Vietnam_NCVD_1025_2011_Avian
EPI_ISL_136155_A_duck_Vietnam_NCVD_1026_2011_Avian
EPI_ISL_136164_A_duck_Vietnam_NCVD_885_2010_Avian
branch a
KM028001_A_swine_Guangxi_592_2011_Swine
JN646729_A_duck_Zhejiang_2242_2011_Avian
JN646730_A_duck_Zhejiang_2243_2011_Avian
JN646731_A_duck_Zhejiang_2244_2011_Avian
JN646728_A_duck_Zhejiang_224_2011_Avian
JN646733_A_duck_Zhejiang_2248_2011_Avian
KP097981_A_chicken_Vietnam_NCVD_A937_2011_Avian
EPI_ISL_94673_A_duck_Vietnam_NCVD_672_2011_Avian
EPI_ISL_136206_A_duck_Vietnam_NCVD_673_2011_Avian
EPI_ISL_136207_A_duck_Vietnam_NCVD_674_2011_Avian

NA1-Group40

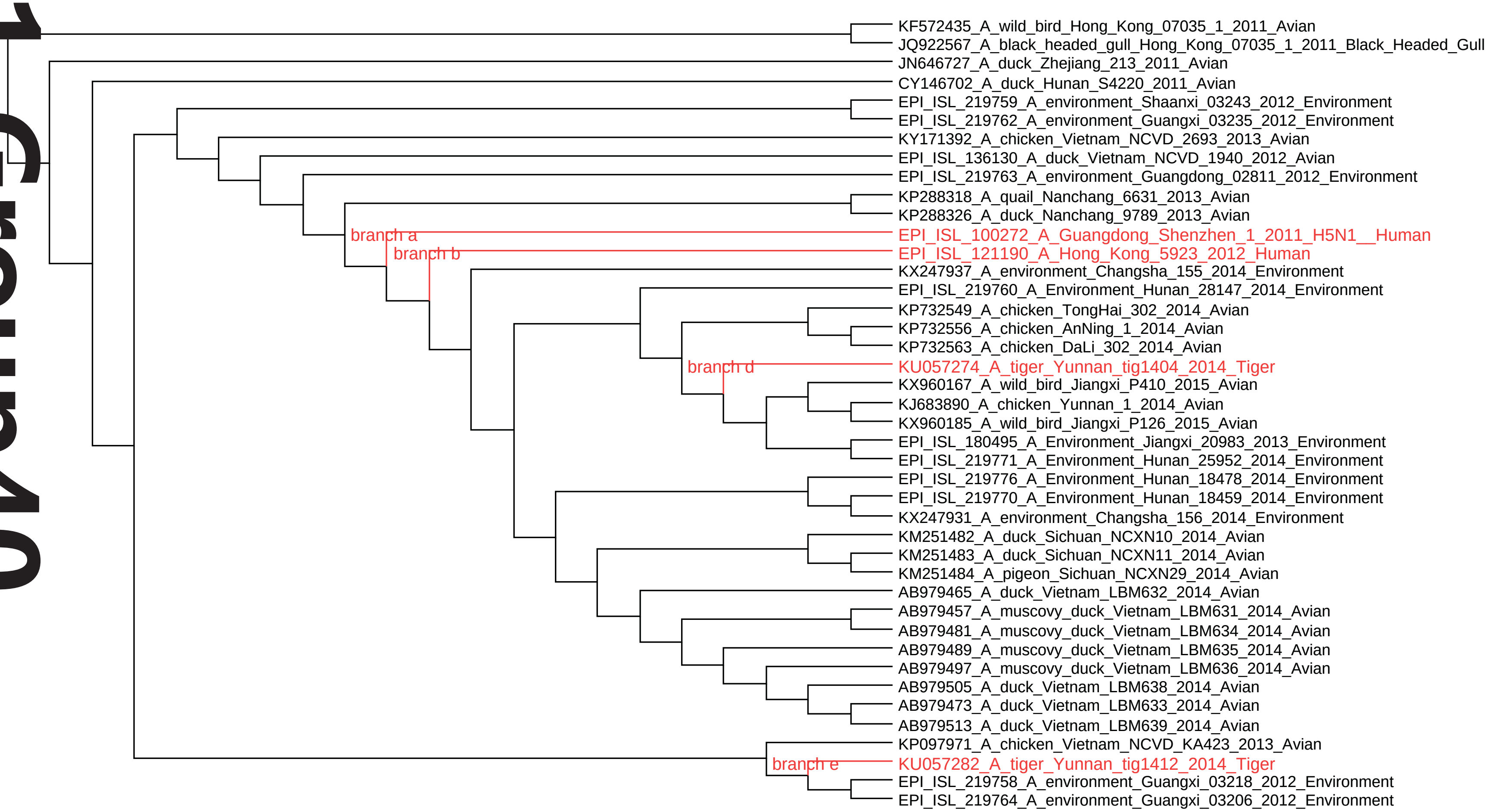
KF572435_A_wild_bird_Hong_Kong_07035_1_2011_Avian
JQ922567_A_black_headed_gull_Hong_Kong_07035_1_2011_Black_Headed_Gull
JN646727_A_duck_Zhejiang_213_2011_Avian
CY146702_A_duck_Hunan_S4220_2011_Avian
EPI_ISL_219759_A_environment_Shaanxi_03243_2012_Environment
EPI_ISL_219762_A_environment_Guangxi_03235_2012_Environment
KY171392_A_chicken_Vietnam_NCVD_2693_2013_Avian
EPI_ISL_136130_A_duck_Vietnam_NCVD_1940_2012_Avian
EPI_ISL_219763_A_environment_Guangdong_02811_2012_Environment
KP288318_A_quail_Nanchang_6631_2013_Avian
KP288326_A_duck_Nanchang_9789_2013_Avian
branch a
EPI_ISL_100272_A_Guangdong_Shenzhen_1_2011_H5N1__Human
branch b
EPI_ISL_121190_A_Hong_Kong_5923_2012_Human
KX247937_A_environment_Changsha_155_2014_Environment
EPI_ISL_219760_A_Environment_Hunan_28147_2014_Environment
KP732549_A_chicken_TongHai_302_2014_Avian
KP732556_A_chicken_AnNing_1_2014_Avian
KP732563_A_chicken_DaLi_302_2014_Avian
branch d
KU057274_A_tiger_Yunnan_tig1404_2014_Tiger
KX960167_A_wild_bird_Jiangxi_P410_2015_Avian
KJ683890_A_chicken_Yunnan_1_2014_Avian
KX960185_A_wild_bird_Jiangxi_P126_2015_Avian
EPI_ISL_180495_A_Environment_Jiangxi_20983_2013_Environment
EPI_ISL_219771_A_Environment_Hunan_25952_2014_Environment
EPI_ISL_219776_A_Environment_Hunan_18478_2014_Environment
EPI_ISL_219770_A_Environment_Hunan_18459_2014_Environment
KX247931_A_environment_Changsha_156_2014_Environment
KM251482_A_duck_Sichuan_NCXN10_2014_Avian
KM251483_A_duck_Sichuan_NCXN11_2014_Avian
KM251484_A_pigeon_Sichuan_NCXN29_2014_Avian
AB979465_A_duck_Vietnam_LBM632_2014_Avian
AB979457_A_muscovy_duck_Vietnam_LBM631_2014_Avian
AB979481_A_muscovy_duck_Vietnam_LBM634_2014_Avian
AB979489_A_muscovy_duck_Vietnam_LBM635_2014_Avian
AB979497_A_muscovy_duck_Vietnam_LBM636_2014_Avian
AB979505_A_duck_Vietnam_LBM638_2014_Avian
AB979473_A_duck_Vietnam_LBM633_2014_Avian
AB979513_A_duck_Vietnam_LBM639_2014_Avian
KP097971_A_chicken_Vietnam_NCVD_KA423_2013_Avian
branch e
KU057282_A_tiger_Yunnan_tig1412_2014_Tiger
EPI_ISL_219758_A_environment_Guangxi_03218_2012_Environment
EPI_ISL_219764_A_environment_Guangxi_03206_2012_Environment

NA1-Group41

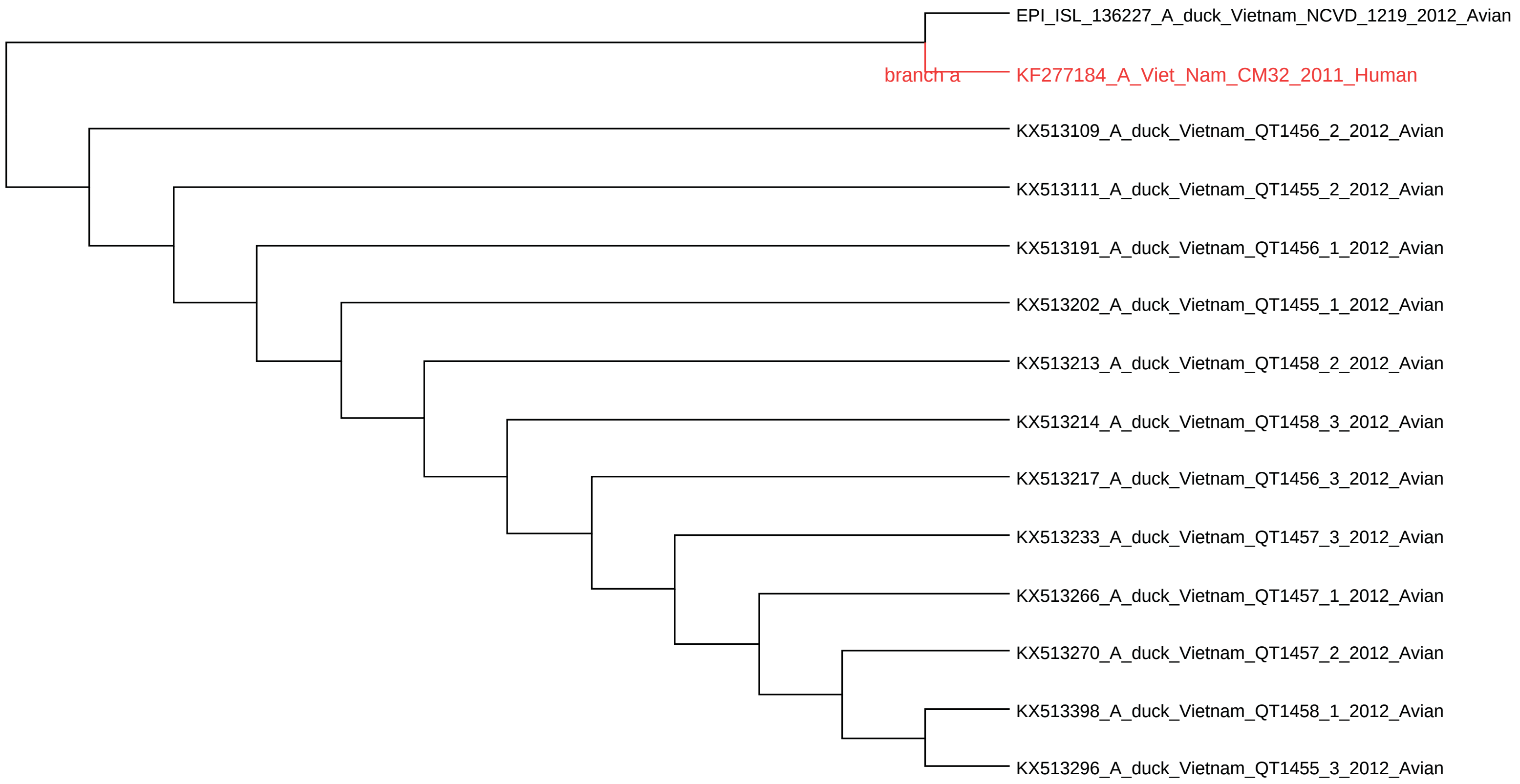
EPI_ISL_136227_A_duck_Vietnam_NCVD_1219_2012_Avian
branch a
KF277184_A_Viet_Nam_CM32_2011_Human
KX513109_A_duck_Vietnam_QT1456_2_2012_Avian
KX513111_A_duck_Vietnam_QT1455_2_2012_Avian
KX513191_A_duck_Vietnam_QT1456_1_2012_Avian
KX513202_A_duck_Vietnam_QT1455_1_2012_Avian
KX513213_A_duck_Vietnam_QT1458_2_2012_Avian
KX513214_A_duck_Vietnam_QT1458_3_2012_Avian
KX513217_A_duck_Vietnam_QT1456_3_2012_Avian
KX513233_A_duck_Vietnam_QT1457_3_2012_Avian
KX513266_A_duck_Vietnam_QT1457_1_2012_Avian
KX513270_A_duck_Vietnam_QT1457_2_2012_Avian
KX513398_A_duck_Vietnam_QT1458_1_2012_Avian
KX513296_A_duck_Vietnam_QT1455_3_2012_Avian

NA1-Group42

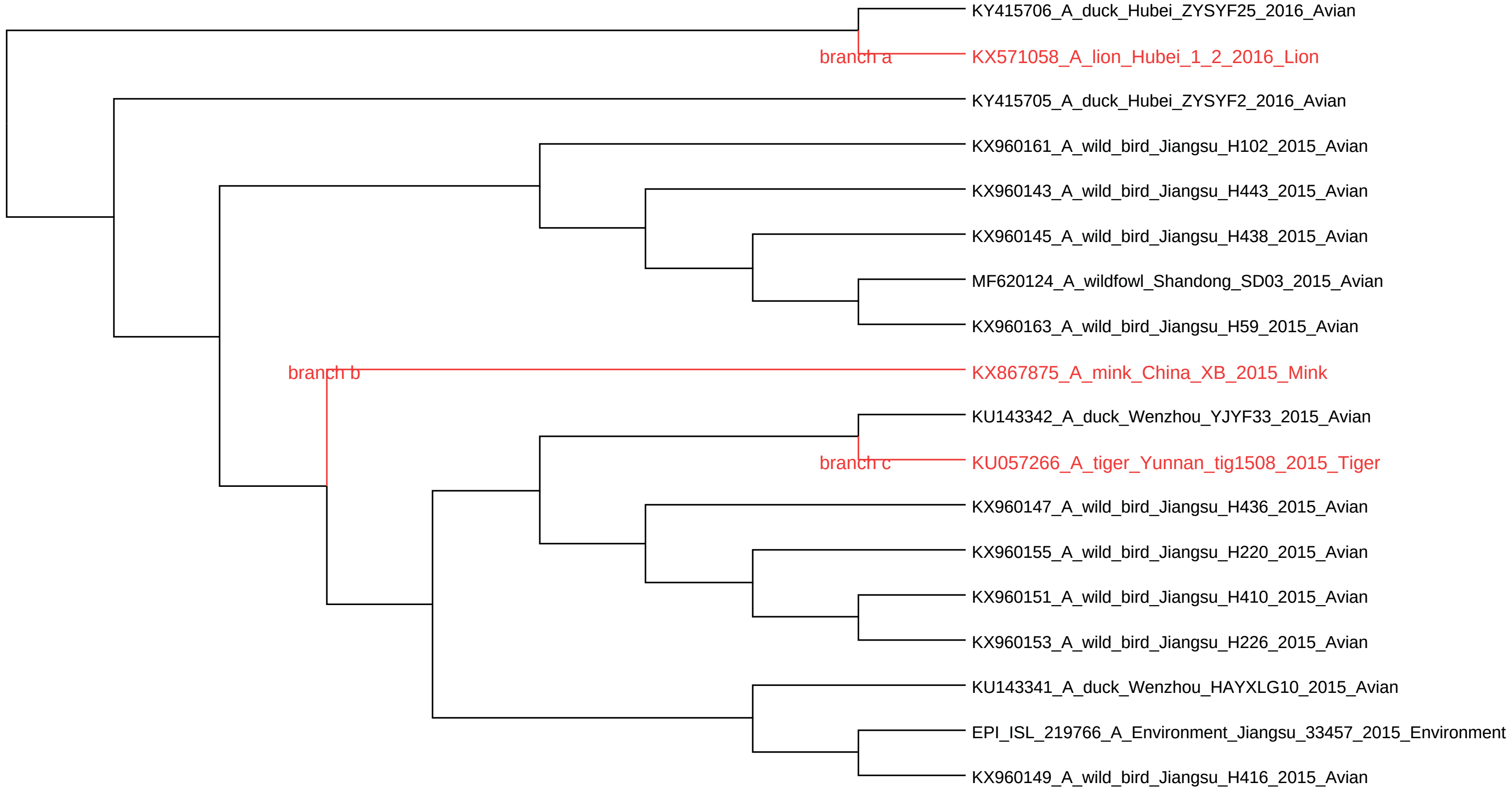
KY415706_A_duck_Hubei_ZYSYF25_2016_Avian
branch a
KX571058_A_lion_Hubei_1_2_2016_Lion
KY415705_A_duck_Hubei_ZYSYF2_2016_Avian
KX960161_A_wild_bird_Jiangsu_H102_2015_Avian
KX960143_A_wild_bird_Jiangsu_H443_2015_Avian
KX960145_A_wild_bird_Jiangsu_H438_2015_Avian
MF620124_A_wildfowl_Shandong_SD03_2015_Avian
KX960163_A_wild_bird_Jiangsu_H59_2015_Avian
branch b
KX867875_A_mink_China_XB_2015_Mink
KU143342_A_duck_Wenzhou_YJYF33_2015_Avian
branch c
KU057266_A_tiger_Yunnan_tig1508_2015_Tiger
KX960147_A_wild_bird_Jiangsu_H436_2015_Avian
KX960155_A_wild_bird_Jiangsu_H220_2015_Avian
KX960151_A_wild_bird_Jiangsu_H410_2015_Avian
KX960153_A_wild_bird_Jiangsu_H226_2015_Avian
KU143341_A_duck_Wenzhou_HAYXLG10_2015_Avian
EPI_ISL_219766_A_Environment_Jiangsu_33457_2015_Environment
KX960149_A_wild_bird_Jiangsu_H416_2015_Avian

NA1-Group43

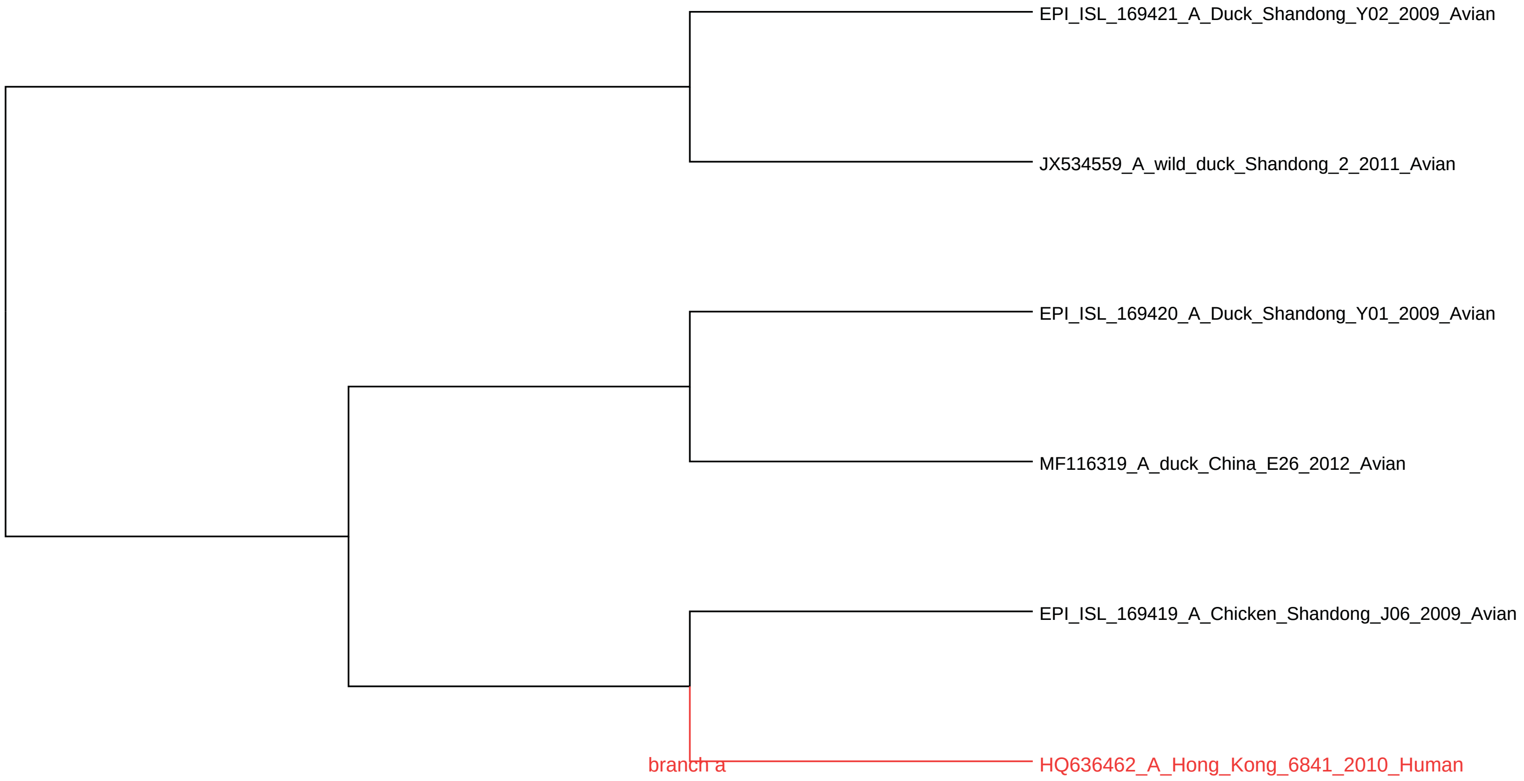

EPI_ISL_169421_A_Duck_Shandong_Y02_2009_Avian
JX534559_A_wild_duck_Shandong_2_2011_Avian
EPI_ISL_169420_A_Duck_Shandong_Y01_2009_Avian
MF116319_A_duck_China_E26_2012_Avian
EPI_ISL_169419_A_Chicken_Shandong_J06_2009_Avian
branch a
HQ636462_A_Hong_Kong_6841_2010_Human

NA1-Group44

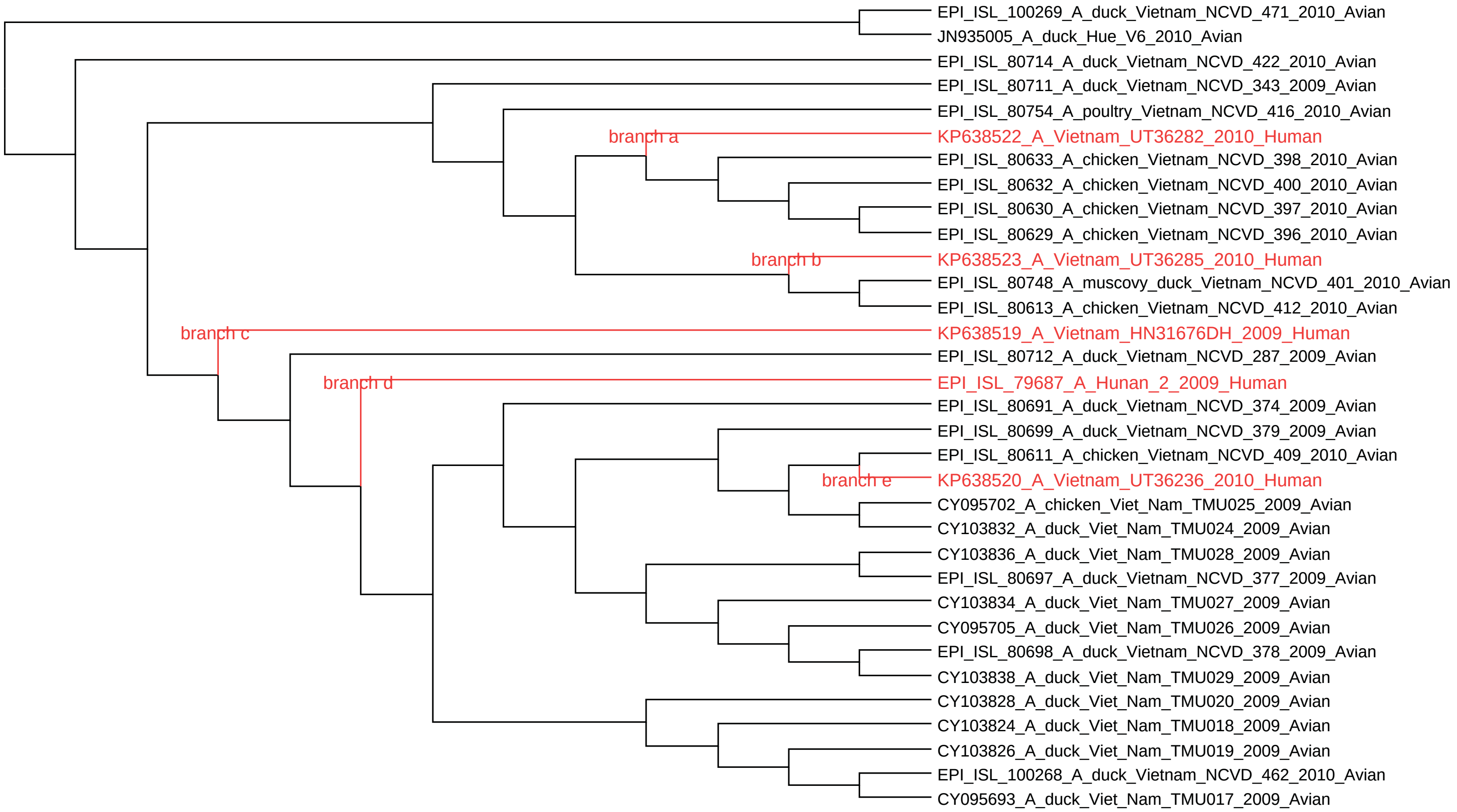
EPI_ISL_100269_A_duck_Vietnam_NCVD_471_2010_Avian
JN935005_A_duck_Hue_V6_2010_Avian
EPI_ISL_80714_A_duck_Vietnam_NCVD_422_2010_Avian
EPI_ISL_80711_A_duck_Vietnam_NCVD_343_2009_Avian
EPI_ISL_80754_A_poultry_Vietnam_NCVD_416_2010_Avian
branch a
KP638522_A_Vietnam_UT36282_2010_Human
EPI_ISL_80633_A_chicken_Vietnam_NCVD_398_2010_Avian
EPI_ISL_80632_A_chicken_Vietnam_NCVD_400_2010_Avian
EPI_ISL_80630_A_chicken_Vietnam_NCVD_397_2010_Avian
EPI_ISL_80629_A_chicken_Vietnam_NCVD_396_2010_Avian
branch b
KP638523_A_Vietnam_UT36285_2010_Human
EPI_ISL_80748_A_muscovy_duck_Vietnam_NCVD_401_2010_Avian
EPI_ISL_80613_A_chicken_Vietnam_NCVD_412_2010_Avian
branch c
KP638519_A_Vietnam_HN31676DH_2009_Human
EPI_ISL_80712_A_duck_Vietnam_NCVD_287_2009_Avian
branch d
EPI_ISL_79687_A_Hunan_2_2009_Human
EPI_ISL_80691_A_duck_Vietnam_NCVD_374_2009_Avian
EPI_ISL_80699_A_duck_Vietnam_NCVD_379_2009_Avian
EPI_ISL_80611_A_chicken_Vietnam_NCVD_409_2010_Avian
branch e
KP638520_A_Vietnam_UT36236_2010_Human
CY095702_A_chicken_Viet_Nam_TMU025_2009_Avian
CY103832_A_duck_Viet_Nam_TMU024_2009_Avian
CY103836_A_duck_Viet_Nam_TMU028_2009_Avian
EPI_ISL_80697_A_duck_Vietnam_NCVD_377_2009_Avian
CY103834_A_duck_Viet_Nam_TMU027_2009_Avian
CY095705_A_duck_Viet_Nam_TMU026_2009_Avian
EPI_ISL_80698_A_duck_Vietnam_NCVD_378_2009_Avian
CY103838_A_duck_Viet_Nam_TMU029_2009_Avian
CY103828_A_duck_Viet_Nam_TMU020_2009_Avian
CY103824_A_duck_Viet_Nam_TMU018_2009_Avian
CY103826_A_duck_Viet_Nam_TMU019_2009_Avian
EPI_ISL_100268_A_duck_Vietnam_NCVD_462_2010_Avian
CY095693_A_duck_Viet_Nam_TMU017_2009_Avian

NA1-Group45

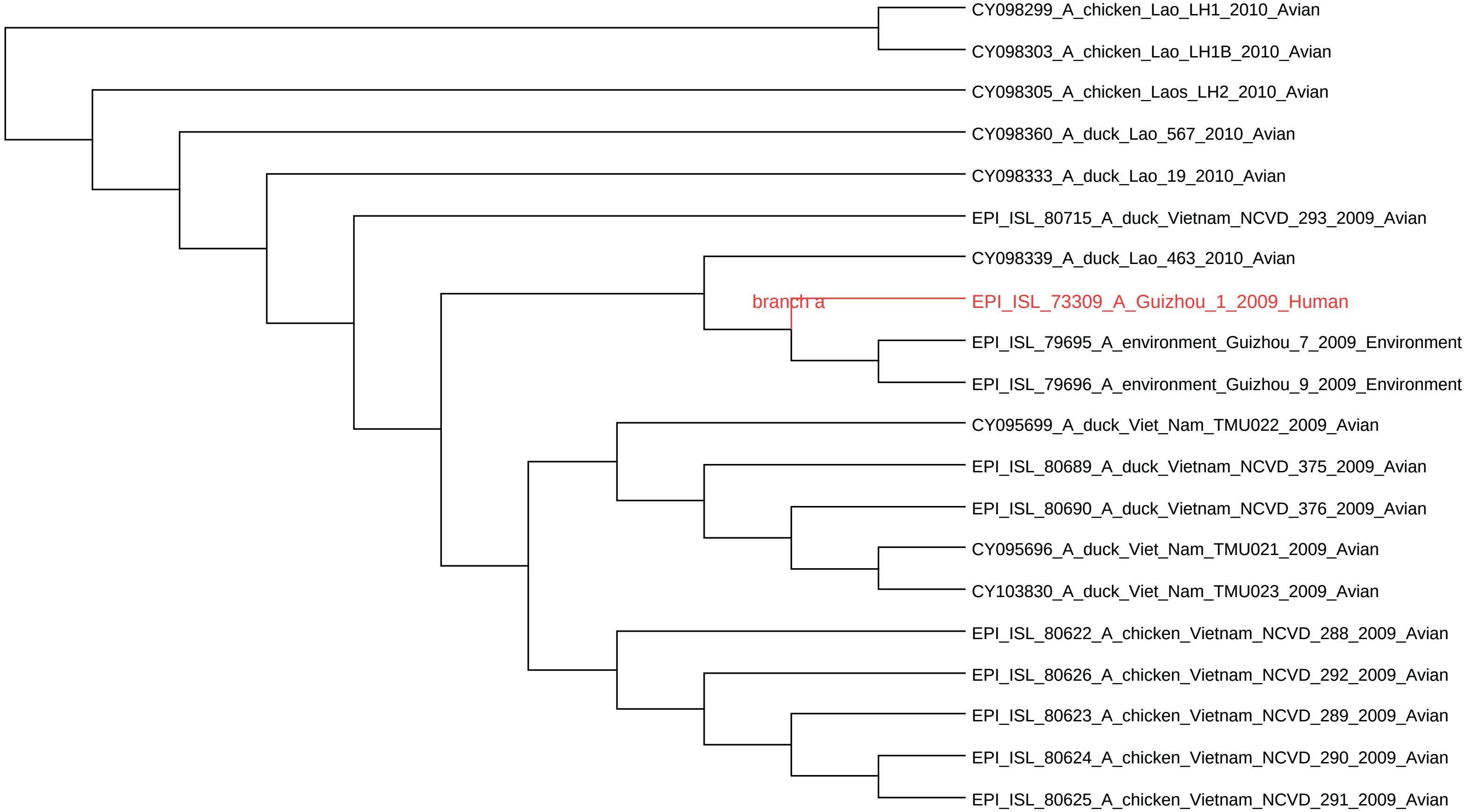
CY098299_A_chicken_Lao_LH1_2010_Avian
CY098303_A_chicken_Lao_LH1B_2010_Avian
CY098305_A_chicken_Laos_LH2_2010_Avian
CY098360_A_duck_Lao_567_2010_Avian
CY098333_A_duck_Lao_19_2010_Avian
EPI_ISL_80715_A_duck_Vietnam_NCVD_293_2009_Avian
CY098339_A_duck_Lao_463_2010_Avian
branch a
EPI_ISL_73309_A_Guizhou_1_2009_Human
EPI_ISL_79695_A_environment_Guizhou_7_2009_Environment
EPI_ISL_79696_A_environment_Guizhou_9_2009_Environment
CY095699_A_duck_Viet_Nam_TMU022_2009_Avian
EPI_ISL_80689_A_duck_Vietnam_NCVD_375_2009_Avian
EPI_ISL_80690_A_duck_Vietnam_NCVD_376_2009_Avian
CY095696_A_duck_Viet_Nam_TMU021_2009_Avian
CY103830_A_duck_Viet_Nam_TMU023_2009_Avian
EPI_ISL_80622_A_chicken_Vietnam_NCVD_288_2009_Avian
EPI_ISL_80626_A_chicken_Vietnam_NCVD_292_2009_Avian
EPI_ISL_80623_A_chicken_Vietnam_NCVD_289_2009_Avian
EPI_ISL_80624_A_chicken_Vietnam_NCVD_290_2009_Avian
EPI_ISL_80625_A_chicken_Vietnam_NCVD_291_2009_Avian

NA1-Group46

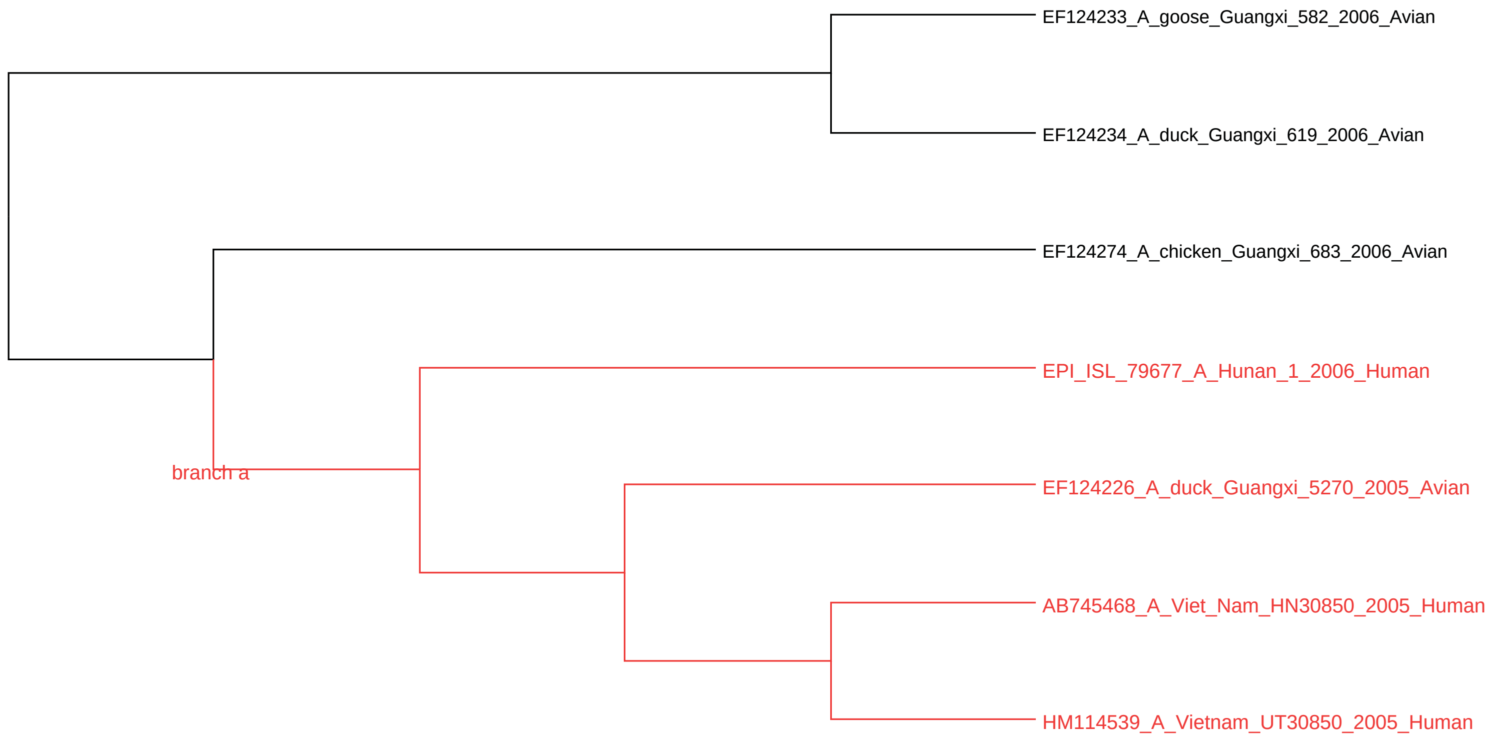
EF124233_A_goose_Guangxi_582_2006_Avian
EF124234_A_duck_Guangxi_619_2006_Avian
EF124274_A_chicken_Guangxi_683_2006_Avian
EPI_ISL_79677_A_Hunan_1_2006_Human
branch a
EF124226_A_duck_Guangxi_5270_2005_Avian
AB745468_A_Viet_Nam_HN30850_2005_Human
HM114539_A_Vietnam_UT30850_2005_Human

NA1-Group47

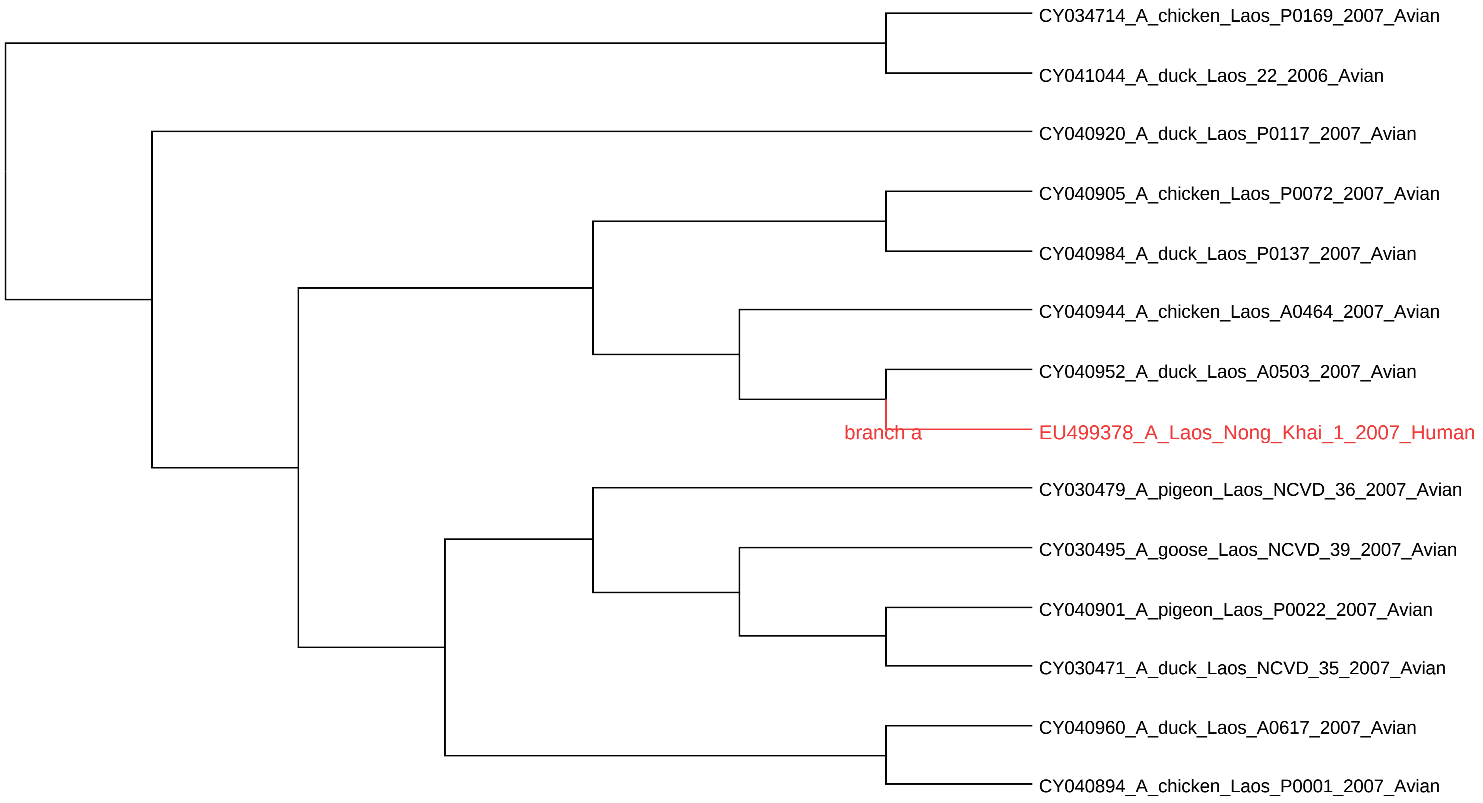

CY034714_A_chicken_Laos_P0169_2007_Avian
CY041044_A_duck_Laos_22_2006_Avian
CY040920_A_duck_Laos_P0117_2007_Avian
CY040905_A_chicken_Laos_P0072_2007_Avian
CY040984_A_duck_Laos_P0137_2007_Avian
CY040944_A_chicken_Laos_A0464_2007_Avian
CY040952_A_duck_Laos_A0503_2007_Avian
branch a
EU499378_A_Laos_Nong_Khai_1_2007_Human
CY030479_A_pigeon_Laos_NCVD_36_2007_Avian
CY030495_A_goose_Laos_NCVD_39_2007_Avian
CY040901_A_pigeon_Laos_P0022_2007_Avian
CY030471_A_duck_Laos_NCVD_35_2007_Avian
CY040960_A_duck_Laos_A0617_2007_Avian
CY040894_A_chicken_Laos_P0001_2007_Avian

NA1-Group48

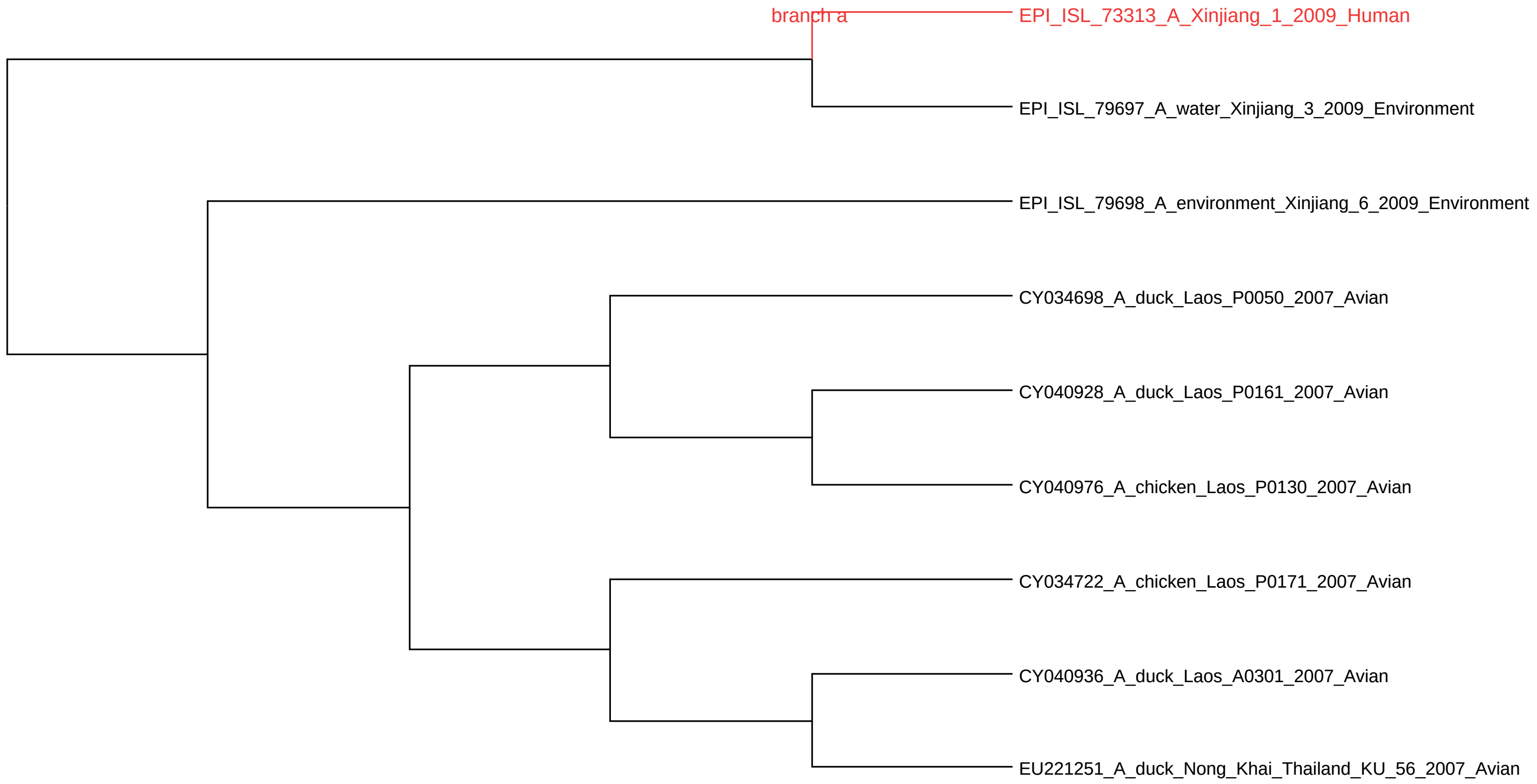
branch a
EPI_ISL_73313_A_Xinjiang_1_2009_Human
EPI_ISL_79697_A_water_Xinjiang_3_2009_Environment
EPI_ISL_79698_A_environment_Xinjiang_6_2009_Environment
CY034698_A_duck_Laos_P0050_2007_Avian
CY040928_A_duck_Laos_P0161_2007_Avian
CY040976_A_chicken_Laos_P0130_2007_Avian
CY034722_A_chicken_Laos_P0171_2007_Avian
CY040936_A_duck_Laos_A0301_2007_Avian
EU221251_A_duck_Nong_Khai_Thailand_KU_56_2007_Avian

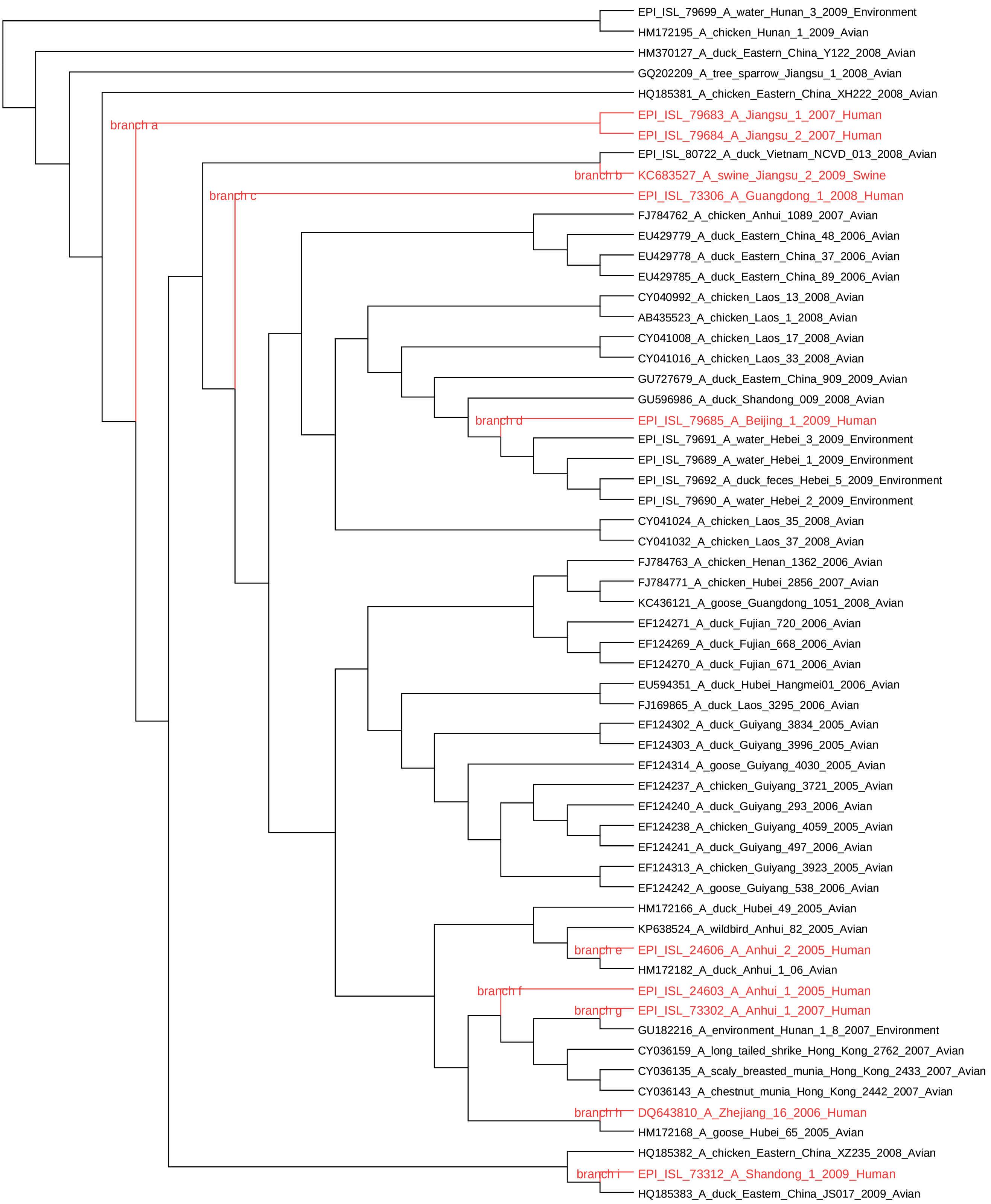

NA1-Group49

NA1-Group49

NA1-Group50

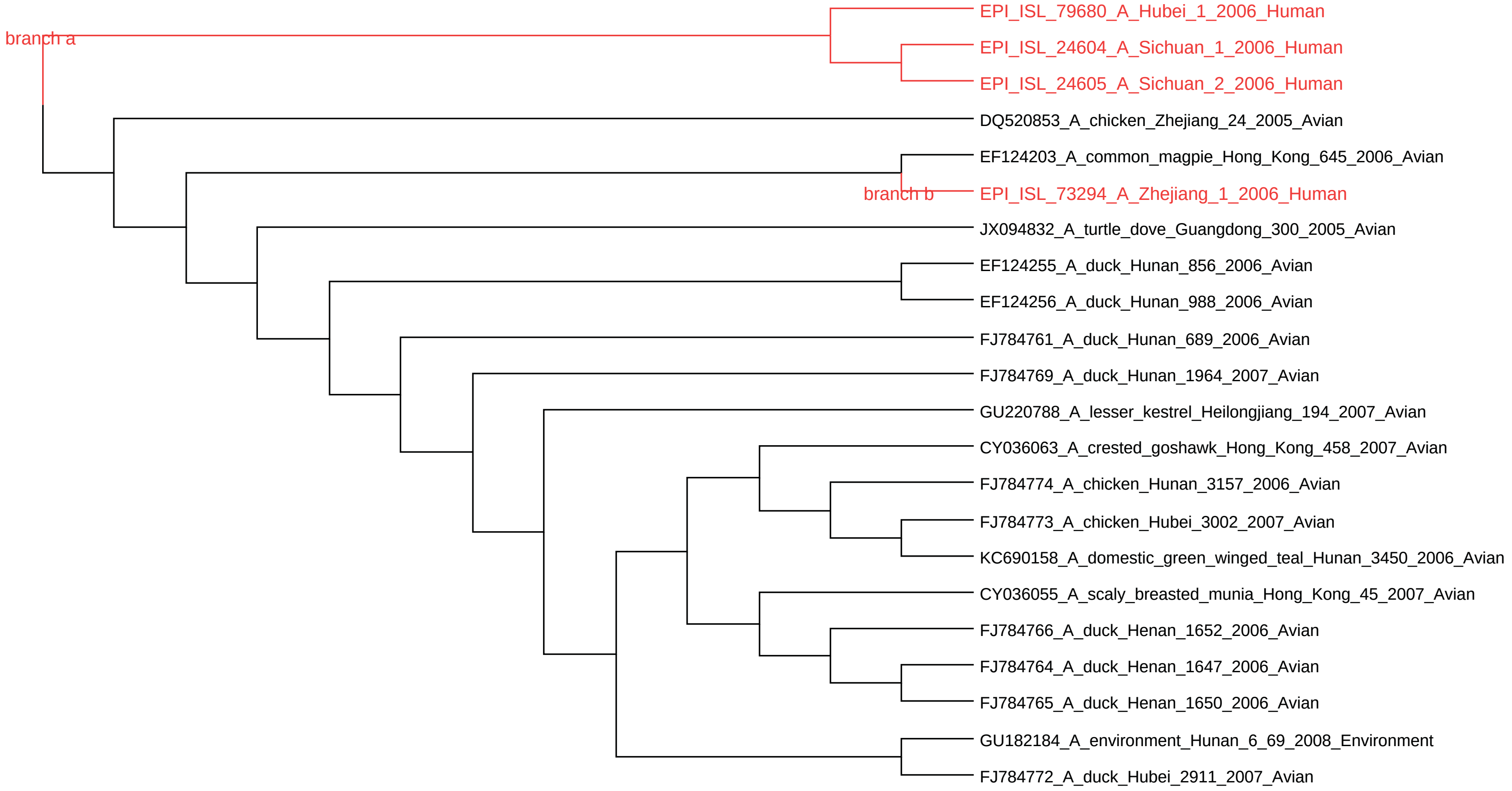
branch a
EPI_ISL_79680_A_Hubei_1_2006_Human
EPI_ISL_24604_A_Sichuan_1_2006_Human
EPI_ISL_24605_A_Sichuan_2_2006_Human
DQ520853_A_chicken_Zhejiang_24_2005_Avian
EF124203_A_common_magpie_Hong_Kong_645_2006_Avian
branch b
EPI_ISL_73294_A_Zhejiang_1_2006_Human
JX094832_A_turtle_dove_Guangdong_300_2005_Avian
EF124255_A_duck_Hunan_856_2006_Avian
EF124256_A_duck_Hunan_988_2006_Avian
FJ784761_A_duck_Hunan_689_2006_Avian
FJ784769_A_duck_Hunan_1964_2007_Avian
GU220788_A_lesser_kestrel_Heilongjiang_194_2007_Avian
CY036063_A_crested_goshawk_Hong_Kong_458_2007_Avian
FJ784774_A_chicken_Hunan_3157_2006_Avian
FJ784773_A_chicken_Hubei_3002_2007_Avian
KC690158_A_domestic_green_winged_teal_Hunan_3450_2006_Avian
CY036055_A_scaly_breasted_munia_Hong_Kong_45_2007_Avian
FJ784766_A_duck_Henan_1652_2006_Avian
FJ784764_A_duck_Henan_1647_2006_Avian
FJ784765_A_duck_Henan_1650_2006_Avian
GU182184_A_environment_Hunan_6_69_2008_Environment
FJ784772_A_duck_Hubei_2911_2007_Avian

NA1-Group51

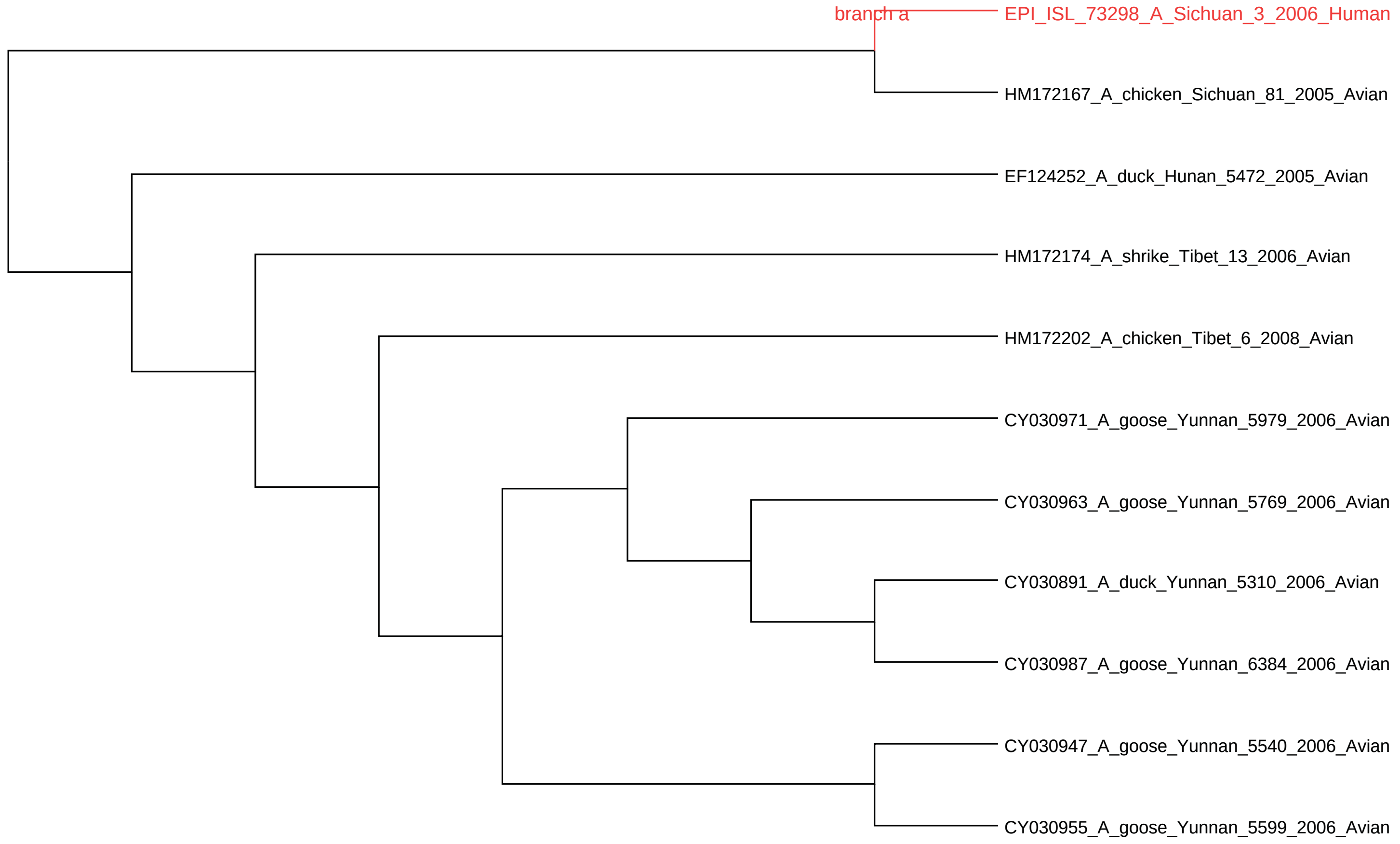

branch a
EPI_ISL_73298_A_Sichuan_3_2006_Human
HM172167_A_chicken_Sichuan_81_2005_Avian
EF124252_A_duck_Hunan_5472_2005_Avian
HM172174_A_shrike_Tibet_13_2006_Avian
HM172202_A_chicken_Tibet_6_2008_Avian
CY030971_A_goose_Yunnan_5979_2006_Avian
CY030963_A_goose_Yunnan_5769_2006_Avian
CY030891_A_duck_Yunnan_5310_2006_Avian
CY030987_A_goose_Yunnan_6384_2006_Avian
CY030947_A_goose_Yunnan_5540_2006_Avian
CY030955_A_goose_Yunnan_5599_2006_Avian

NA1-Group52

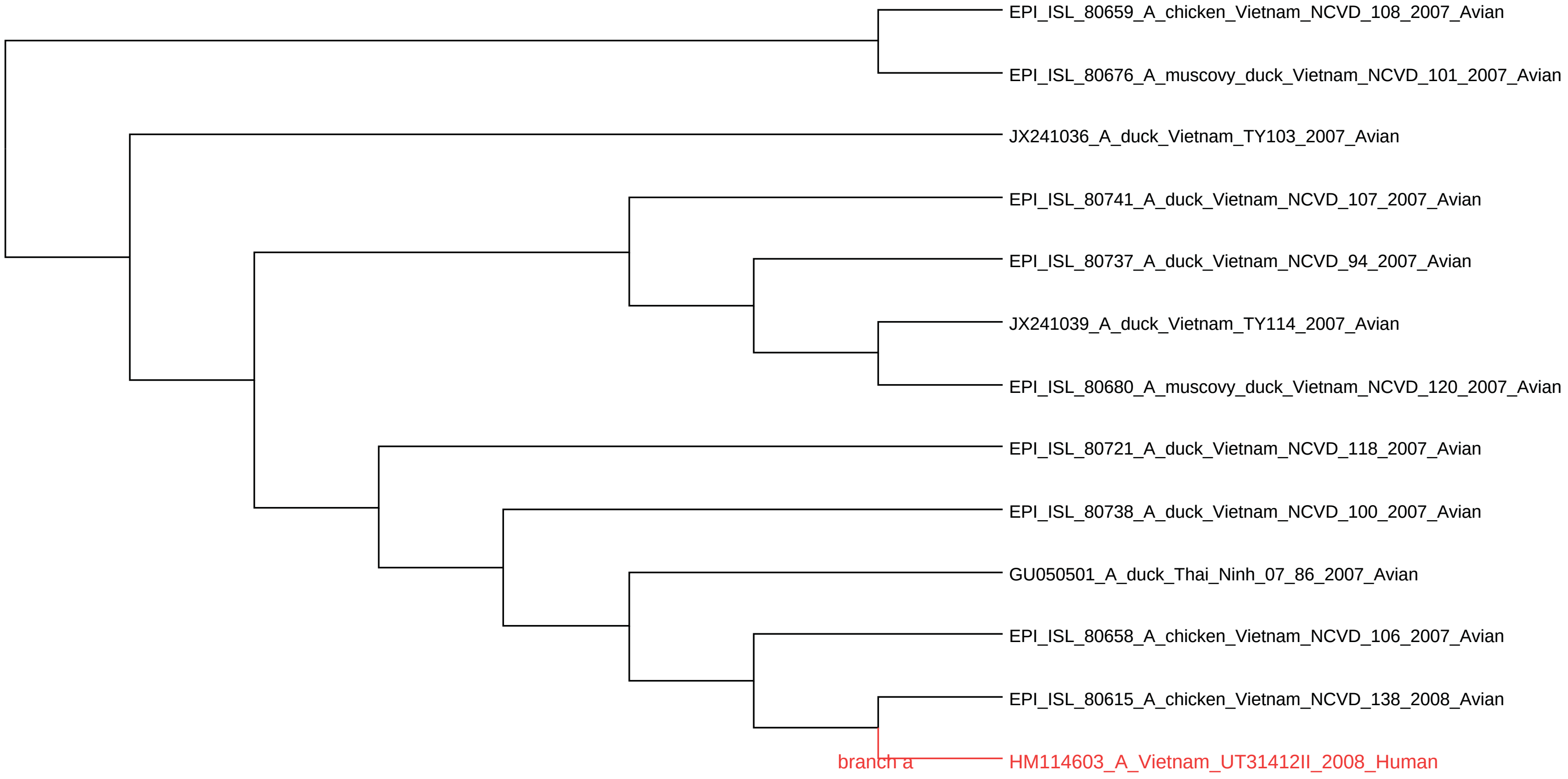
EPI_ISL_80659_A_chicken_Vietnam_NCVD_108_2007_Avian
EPI_ISL_80676_A_muscovy_duck_Vietnam_NCVD_101_2007_Avian
JX241036_A_duck_Vietnam_TY103_2007_Avian
EPI_ISL_80741_A_duck_Vietnam_NCVD_107_2007_Avian
EPI_ISL_80737_A_duck_Vietnam_NCVD_94_2007_Avian
JX241039_A_duck_Vietnam_TY114_2007_Avian
EPI_ISL_80680_A_muscovy_duck_Vietnam_NCVD_120_2007_Avian
EPI_ISL_80721_A_duck_Vietnam_NCVD_118_2007_Avian
EPI_ISL_80738_A_duck_Vietnam_NCVD_100_2007_Avian
GU050501_A_duck_Thai_Ninh_07_86_2007_Avian
EPI_ISL_80658_A_chicken_Vietnam_NCVD_106_2007_Avian
EPI_ISL_80615_A_chicken_Vietnam_NCVD_138_2008_Avian
branch a
HM114603_A_Vietnam_UT31412II_2008_Human

NA1-Group53

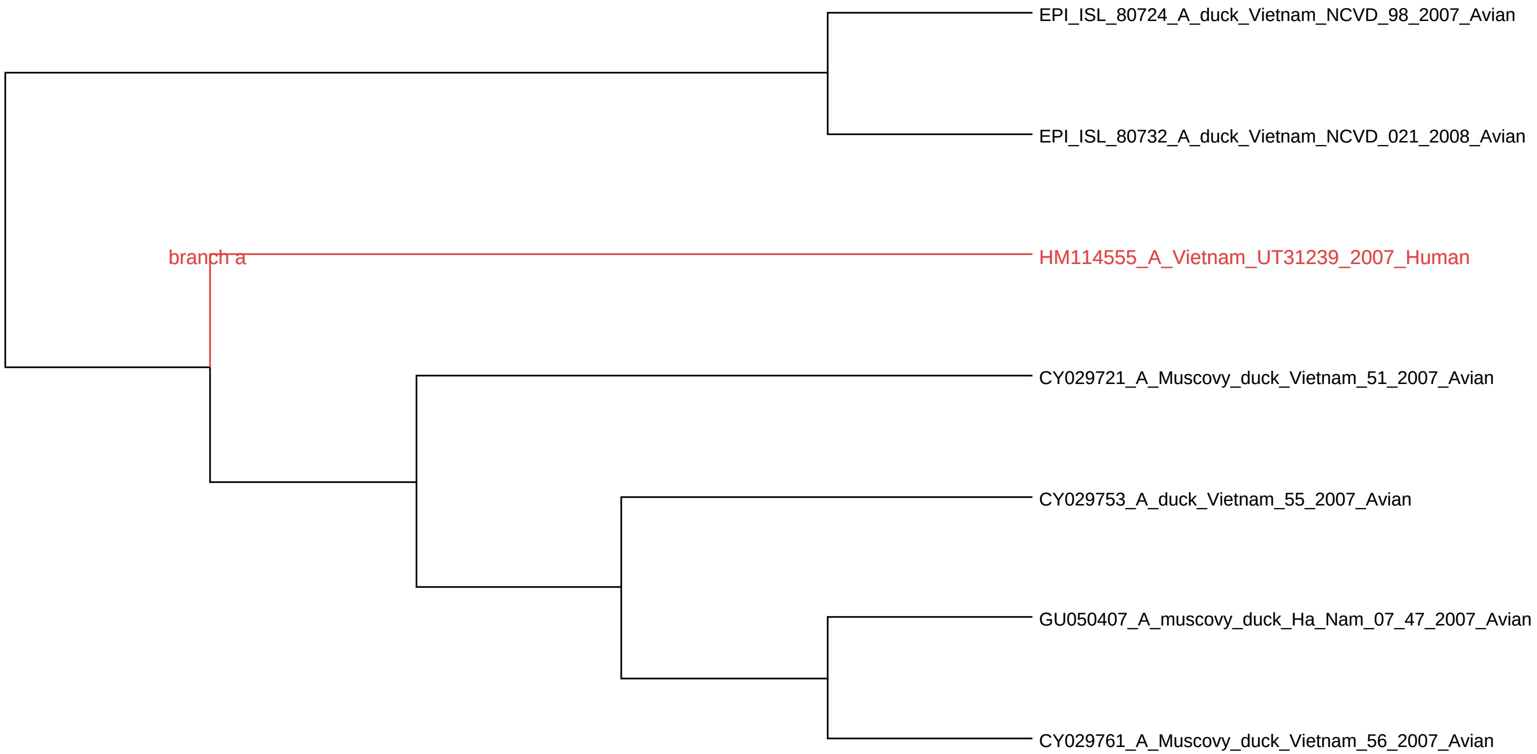

EPI_ISL_80724_A_duck_Vietnam_NCVD_98_2007_Avian
EPI_ISL_80732_A_duck_Vietnam_NCVD_021_2008_Avian
branch a
HM114555_A_Vietnam_UT31239_2007_Human
CY029721_A_Muscovy_duck_Vietnam_51_2007_Avian
CY029753_A_duck_Vietnam_55_2007_Avian
GU050407_A_muscovy_duck_Ha_Nam_07_47_2007_Avian
CY029761_A_Muscovy_duck_Vietnam_56_2007_Avian

NA1-Group54

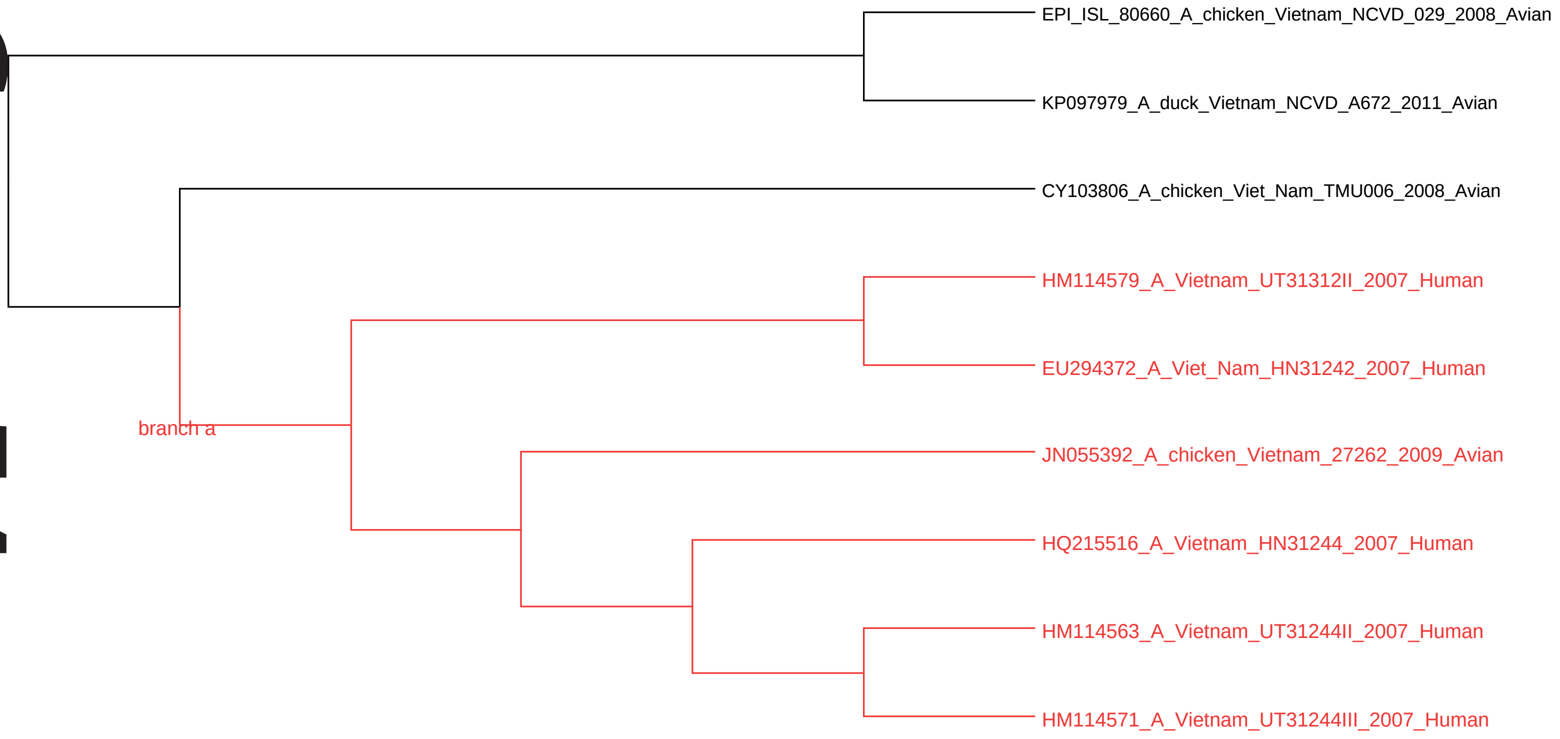

EPI_ISL_80660_A_chicken_Vietnam_NCVD_029_2008_Avian
KP097979_A_duck_Vietnam_NCVD_A672_2011_Avian
CY103806_A_chicken_Viet_Nam_TMU006_2008_Avian
HM114579_A_Vietnam_UT31312II_2007_Human
EU294372_A_Viet_Nam_HN31242_2007_Human
branch a
JN055392_A_chicken_Vietnam_27262_2009_Avian
HQ215516_A_Vietnam_HN31244_2007_Human
HM114563_A_Vietnam_UT31244II_2007_Human
HM114571_A_Vietnam_UT31244III_2007_Human

NA1-Group55

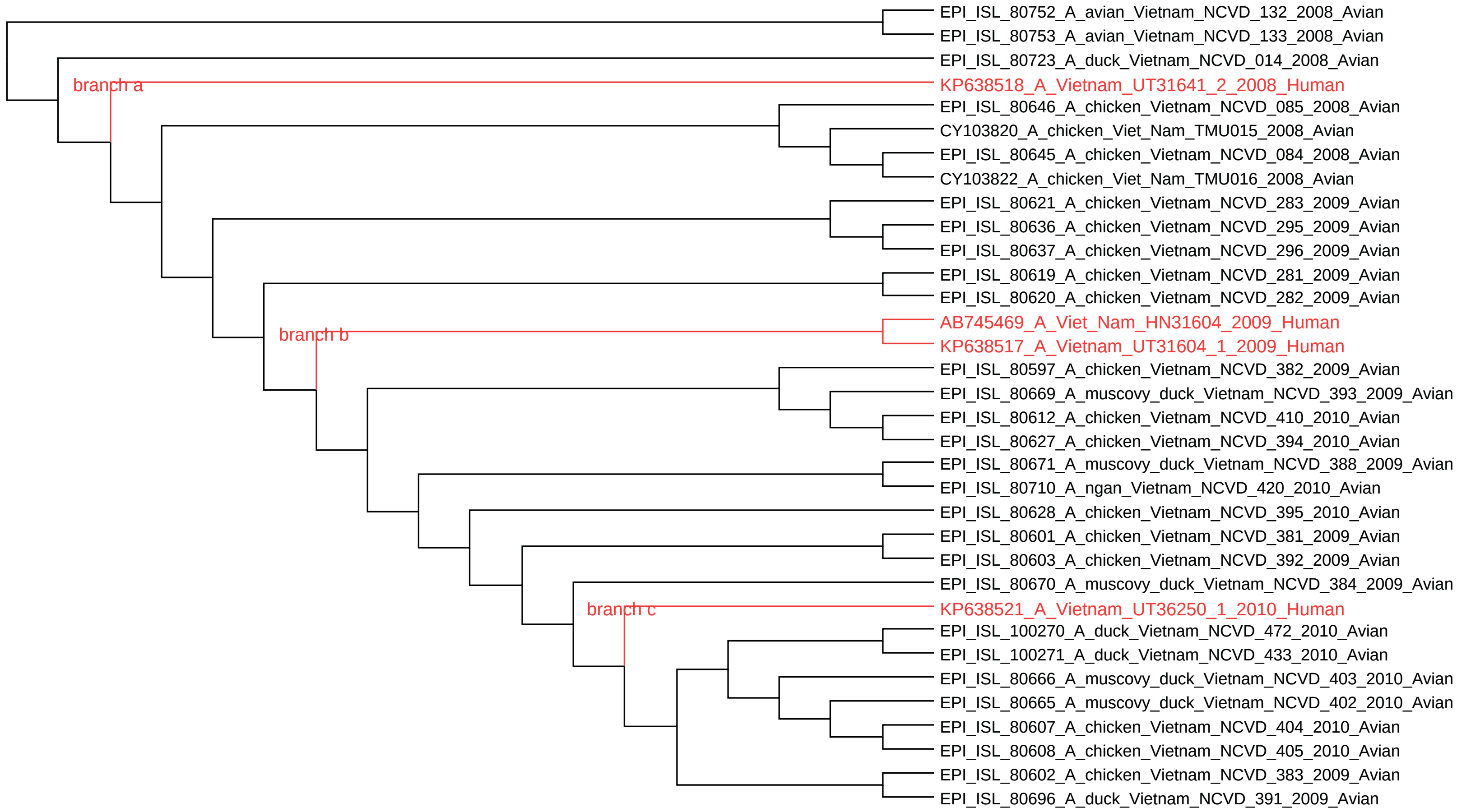

EPI_ISL_80752_A_avian_Vietnam_NCVD_132_2008_Avian
EPI_ISL_80753_A_avian_Vietnam_NCVD_133_2008_Avian
EPI_ISL_80723_A_duck_Vietnam_NCVD_014_2008_Avian
branch a
KP638518_A_Vietnam_UT31641_2_2008_Human
EPI_ISL_80646_A_chicken_Vietnam_NCVD_085_2008_Avian
CY103820_A_chicken_Viet_Nam_TMU015_2008_Avian
EPI_ISL_80645_A_chicken_Vietnam_NCVD_084_2008_Avian
CY103822_A_chicken_Viet_Nam_TMU016_2008_Avian
EPI_ISL_80621_A_chicken_Vietnam_NCVD_283_2009_Avian
EPI_ISL_80636_A_chicken_Vietnam_NCVD_295_2009_Avian
EPI_ISL_80637_A_chicken_Vietnam_NCVD_296_2009_Avian
EPI_ISL_80619_A_chicken_Vietnam_NCVD_281_2009_Avian
EPI_ISL_80620_A_chicken_Vietnam_NCVD_282_2009_Avian
branch b
AB745469_A_Viet_Nam_HN31604_2009_Human
KP638517_A_Vietnam_UT31604_1_2009_Human
EPI_ISL_80597_A_chicken_Vietnam_NCVD_382_2009_Avian
EPI_ISL_80669_A_muscovy_duck_Vietnam_NCVD_393_2009_Avian
EPI_ISL_80612_A_chicken_Vietnam_NCVD_410_2010_Avian
EPI_ISL_80627_A_chicken_Vietnam_NCVD_394_2010_Avian
EPI_ISL_80671_A_muscovy_duck_Vietnam_NCVD_388_2009_Avian
EPI_ISL_80710_A_ngan_Vietnam_NCVD_420_2010_Avian
EPI_ISL_80628_A_chicken_Vietnam_NCVD_395_2010_Avian
EPI_ISL_80601_A_chicken_Vietnam_NCVD_381_2009_Avian
EPI_ISL_80603_A_chicken_Vietnam_NCVD_392_2009_Avian
EPI_ISL_80670_A_muscovy_duck_Vietnam_NCVD_384_2009_Avian
branch c
KP638521_A_Vietnam_UT36250_1_2010_Human
EPI_ISL_100270_A_duck_Vietnam_NCVD_472_2010_Avian
EPI_ISL_100271_A_duck_Vietnam_NCVD_433_2010_Avian
EPI_ISL_80666_A_muscovy_duck_Vietnam_NCVD_403_2010_Avian
EPI_ISL_80665_A_muscovy_duck_Vietnam_NCVD_402_2010_Avian
EPI_ISL_80607_A_chicken_Vietnam_NCVD_404_2010_Avian
EPI_ISL_80608_A_chicken_Vietnam_NCVD_405_2010_Avian
EPI_ISL_80602_A_chicken_Vietnam_NCVD_383_2009_Avian
EPI_ISL_80696_A_duck_Vietnam_NCVD_391_2009_Avian

NA1-Group56

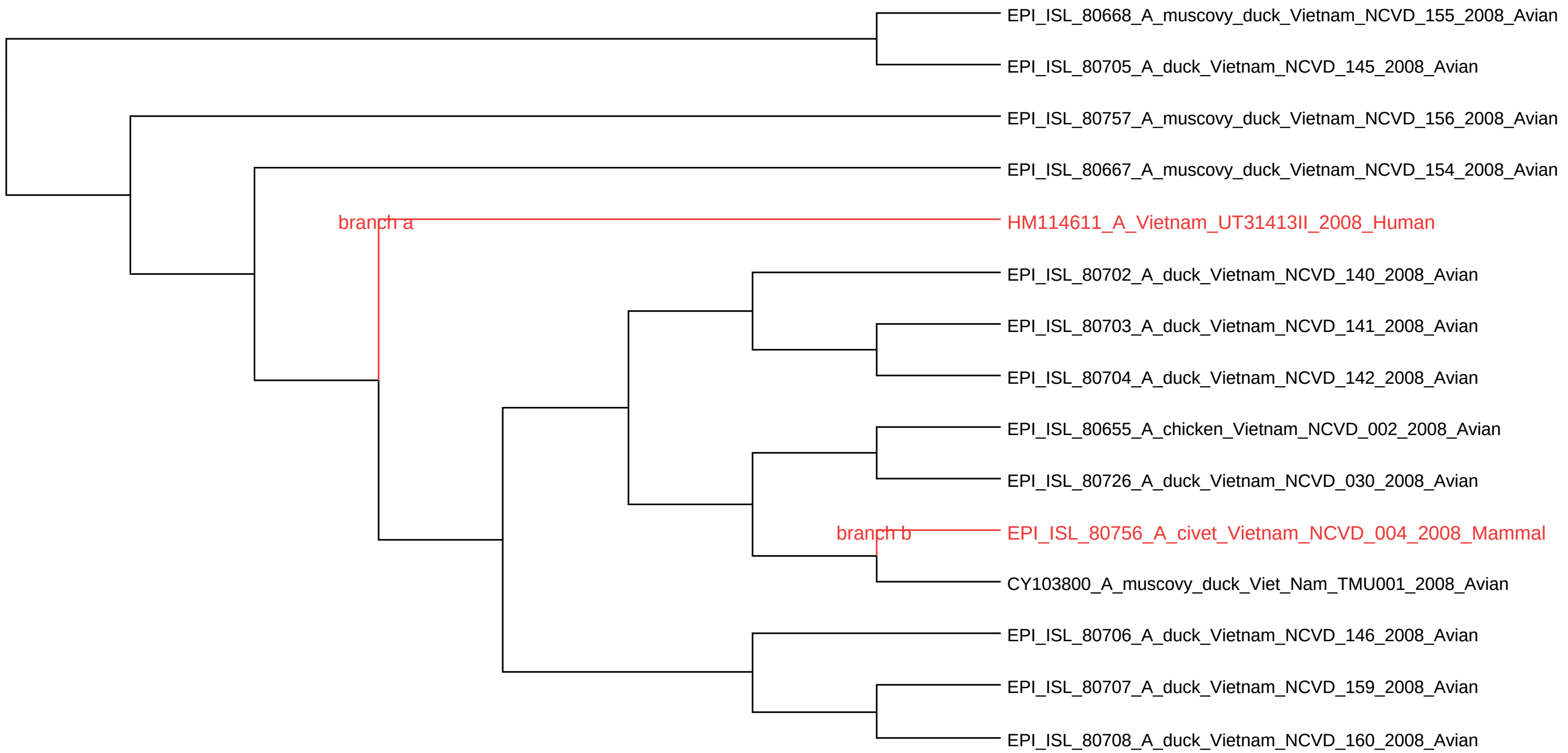
EPI_ISL_80668_A_muscovy_duck_Vietnam_NCVD_155_2008_Avian
EPI_ISL_80705_A_duck_Vietnam_NCVD_145_2008_Avian
EPI_ISL_80757_A_muscovy_duck_Vietnam_NCVD_156_2008_Avian
EPI_ISL_80667_A_muscovy_duck_Vietnam_NCVD_154_2008_Avian
branch a
HM114611_A_Vietnam_UT31413II_2008_Human
EPI_ISL_80702_A_duck_Vietnam_NCVD_140_2008_Avian
EPI_ISL_80703_A_duck_Vietnam_NCVD_141_2008_Avian
EPI_ISL_80704_A_duck_Vietnam_NCVD_142_2008_Avian
EPI_ISL_80655_A_chicken_Vietnam_NCVD_002_2008_Avian
EPI_ISL_80726_A_duck_Vietnam_NCVD_030_2008_Avian
branch b
EPI_ISL_80756_A_civet_Vietnam_NCVD_004_2008_Mammal
CY103800_A_muscovy_duck_Viet_Nam_TMU001_2008_Avian
EPI_ISL_80706_A_duck_Vietnam_NCVD_146_2008_Avian
EPI_ISL_80707_A_duck_Vietnam_NCVD_159_2008_Avian
EPI_ISL_80708_A_duck_Vietnam_NCVD_160_2008_Avian

NA1-Group57

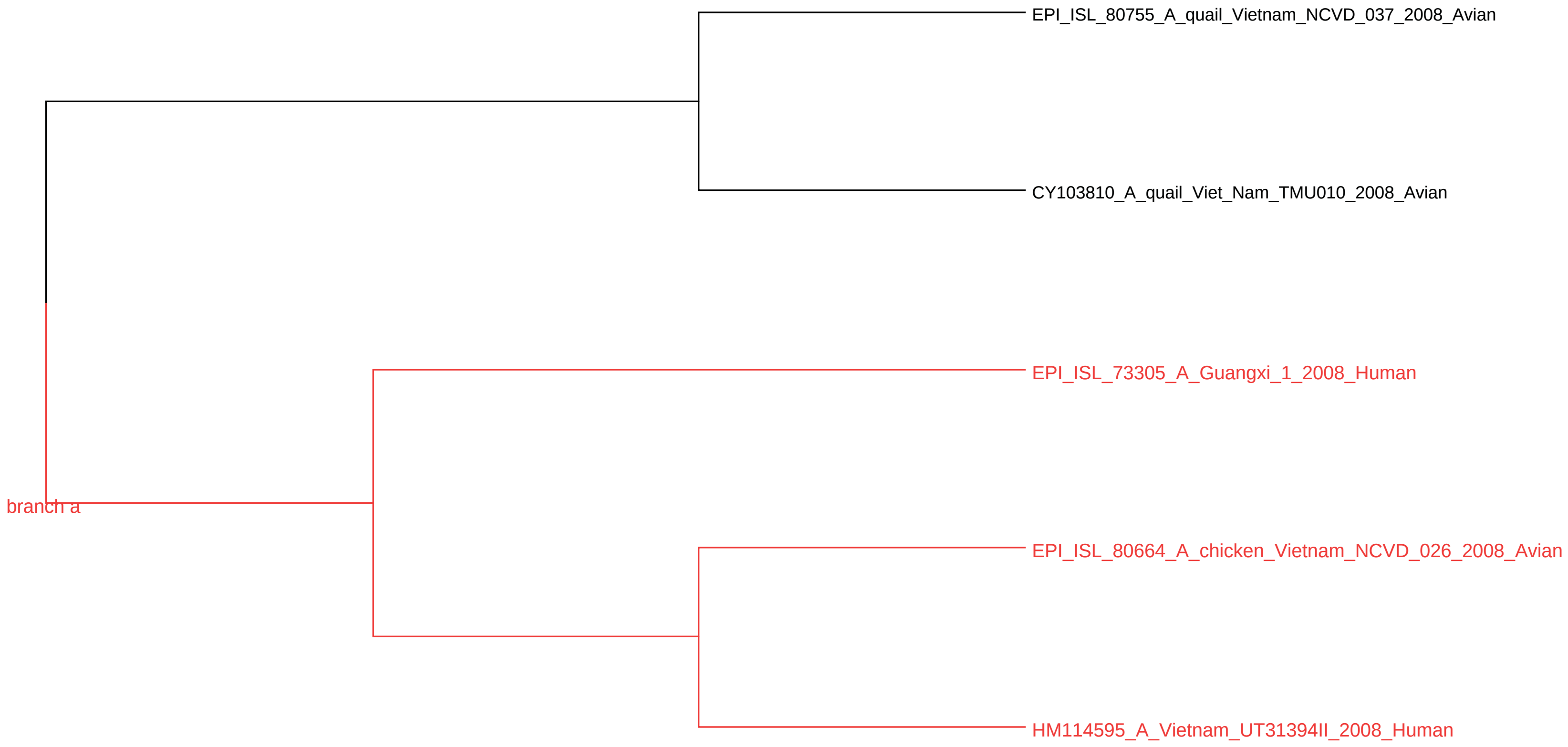
EPI_ISL_80755_A_quail_Vietnam_NCVD_037_2008_Avian
CY103810_A_quail_Viet_Nam_TMU010_2008_Avian
EPI_ISL_73305_A_Guangxi_1_2008_Human
branch a
EPI_ISL_80664_A_chicken_Vietnam_NCVD_026_2008_Avian
HM114595_A_Vietnam_UT31394II_2008_Human

NA1-Group58

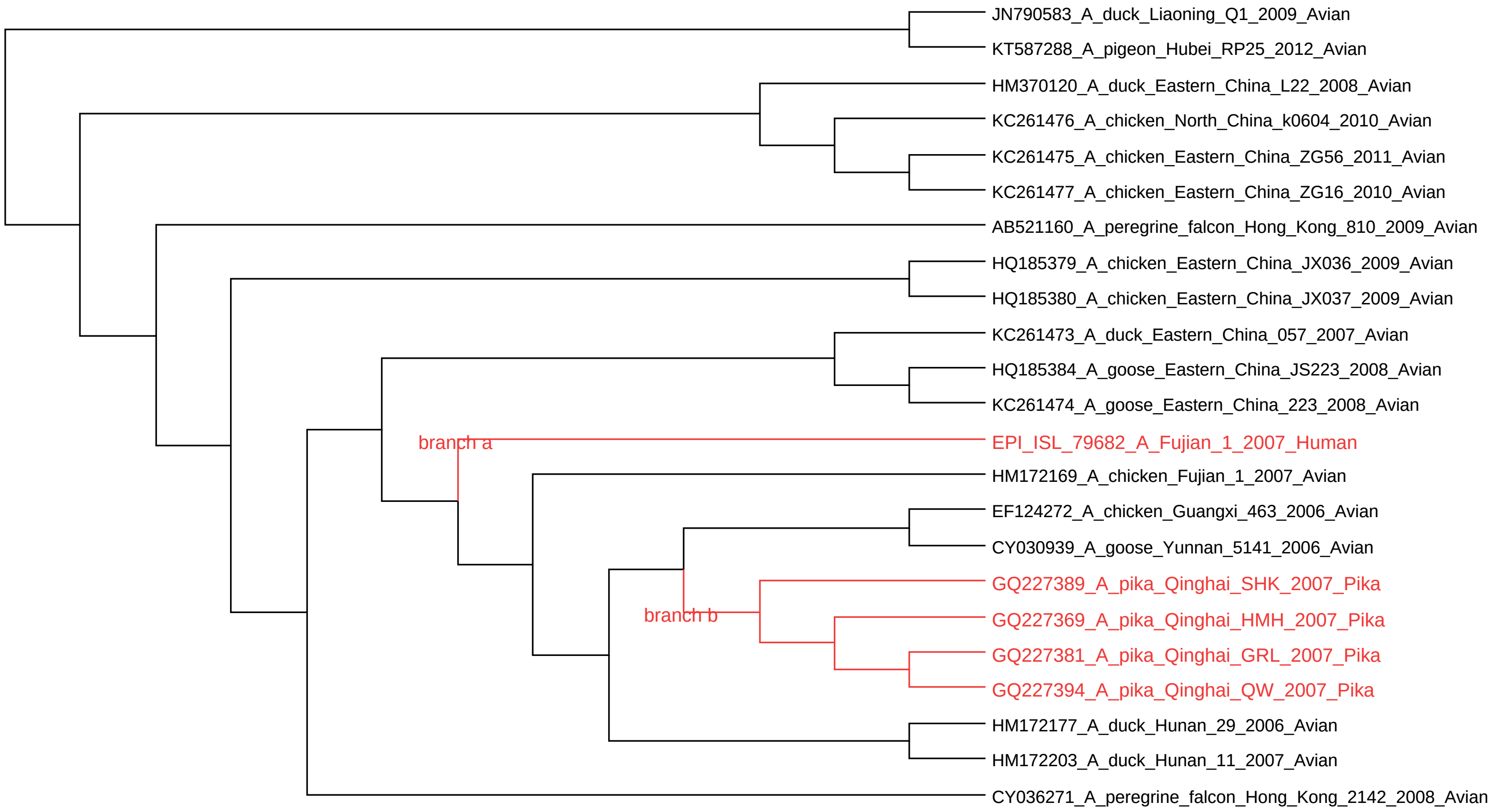

JN790583_A_duck_Liaoning_Q1_2009_Avian
KT587288_A_pigeon_Hubei_RP25_2012_Avian
HM370120_A_duck_Eastern_China_L22_2008_Avian
KC261476_A_chicken_North_China_k0604_2010_Avian
KC261475_A_chicken_Eastern_China_ZG56_2011_Avian
KC261477_A_chicken_Eastern_China_ZG16_2010_Avian
AB521160_A_peregrine_falcon_Hong_Kong_810_2009_Avian
HQ185379_A_chicken_Eastern_China_JX036_2009_Avian
HQ185380_A_chicken_Eastern_China_JX037_2009_Avian
KC261473_A_duck_Eastern_China_057_2007_Avian
HQ185384_A_goose_Eastern_China_JS223_2008_Avian
KC261474_A_goose_Eastern_China_223_2008_Avian
branch a
EPI_ISL_79682_A_Fujian_1_2007_Human
HM172169_A_chicken_Fujian_1_2007_Avian
EF124272_A_chicken_Guangxi_463_2006_Avian
CY030939_A_goose_Yunnan_5141_2006_Avian
GQ227389_A_pika_Qinghai_SHK_2007_Pika
branch b
GQ227369_A_pika_Qinghai_HMH_2007_Pika
GQ227381_A_pika_Qinghai_GRL_2007_Pika
GQ227394_A_pika_Qinghai_QW_2007_Pika
HM172177_A_duck_Hunan_29_2006_Avian
HM172203_A_duck_Hunan_11_2007_Avian
CY036271_A_peregrine_falcon_Hong_Kong_2142_2008_Avian

NA1-Group59

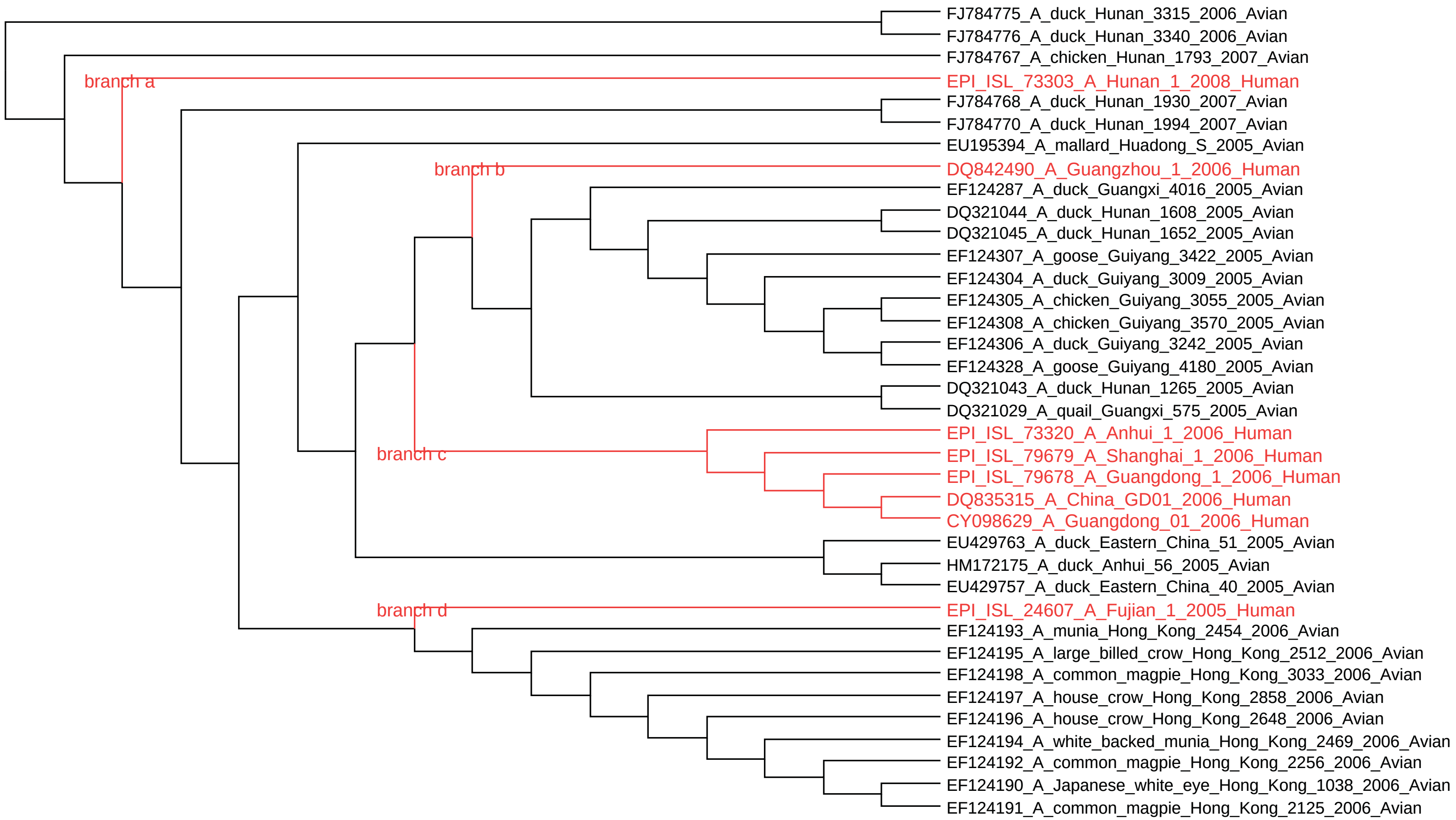
FJ784775_A_duck_Hunan_3315_2006_Avian
FJ784776_A_duck_Hunan_3340_2006_Avian
FJ784767_A_chicken_Hunan_1793_2007_Avian
branch a
EPI_ISL_73303_A_Hunan_1_2008_Human
FJ784768_A_duck_Hunan_1930_2007_Avian
FJ784770_A_duck_Hunan_1994_2007_Avian
EU195394_A_mallard_Huadong_S_2005_Avian
branch b
DQ842490_A_Guangzhou_1_2006_Human
EF124287_A_duck_Guangxi_4016_2005_Avian
DQ321044_A_duck_Hunan_1608_2005_Avian
DQ321045_A_duck_Hunan_1652_2005_Avian
EF124307_A_goose_Guiyang_3422_2005_Avian
EF124304_A_duck_Guiyang_3009_2005_Avian
EF124305_A_chicken_Guiyang_3055_2005_Avian
EF124308_A_chicken_Guiyang_3570_2005_Avian
EF124306_A_duck_Guiyang_3242_2005_Avian
EF124328_A_goose_Guiyang_4180_2005_Avian
DQ321043_A_duck_Hunan_1265_2005_Avian
DQ321029_A_quail_Guangxi_575_2005_Avian
EPI_ISL_73320_A_Anhui_1_2006_Human
branch c
EPI_ISL_79679_A_Shanghai_1_2006_Human
EPI_ISL_79678_A_Guangdong_1_2006_Human
DQ835315_A_China_GD01_2006_Human
CY098629_A_Guangdong_01_2006_Human
EU429763_A_duck_Eastern_China_51_2005_Avian
HM172175_A_duck_Anhui_56_2005_Avian
EU429757_A_duck_Eastern_China_40_2005_Avian
branch d
EPI_ISL_24607_A_Fujian_1_2005_Human
EF124193_A_munia_Hong_Kong_2454_2006_Avian
EF124195_A_large_billed_crow_Hong_Kong_2512_2006_Avian
EF124198_A_common_magpie_Hong_Kong_3033_2006_Avian
EF124197_A_house_crow_Hong_Kong_2858_2006_Avian
EF124196_A_house_crow_Hong_Kong_2648_2006_Avian
EF124194_A_white_backed_munia_Hong_Kong_2469_2006_Avian
EF124192_A_common_magpie_Hong_Kong_2256_2006_Avian
EF124190_A_Japanese_white_eye_Hong_Kong_1038_2006_Avian
EF124191_A_common_magpie_Hong_Kong_2125_2006_Avian

NA1-Group60

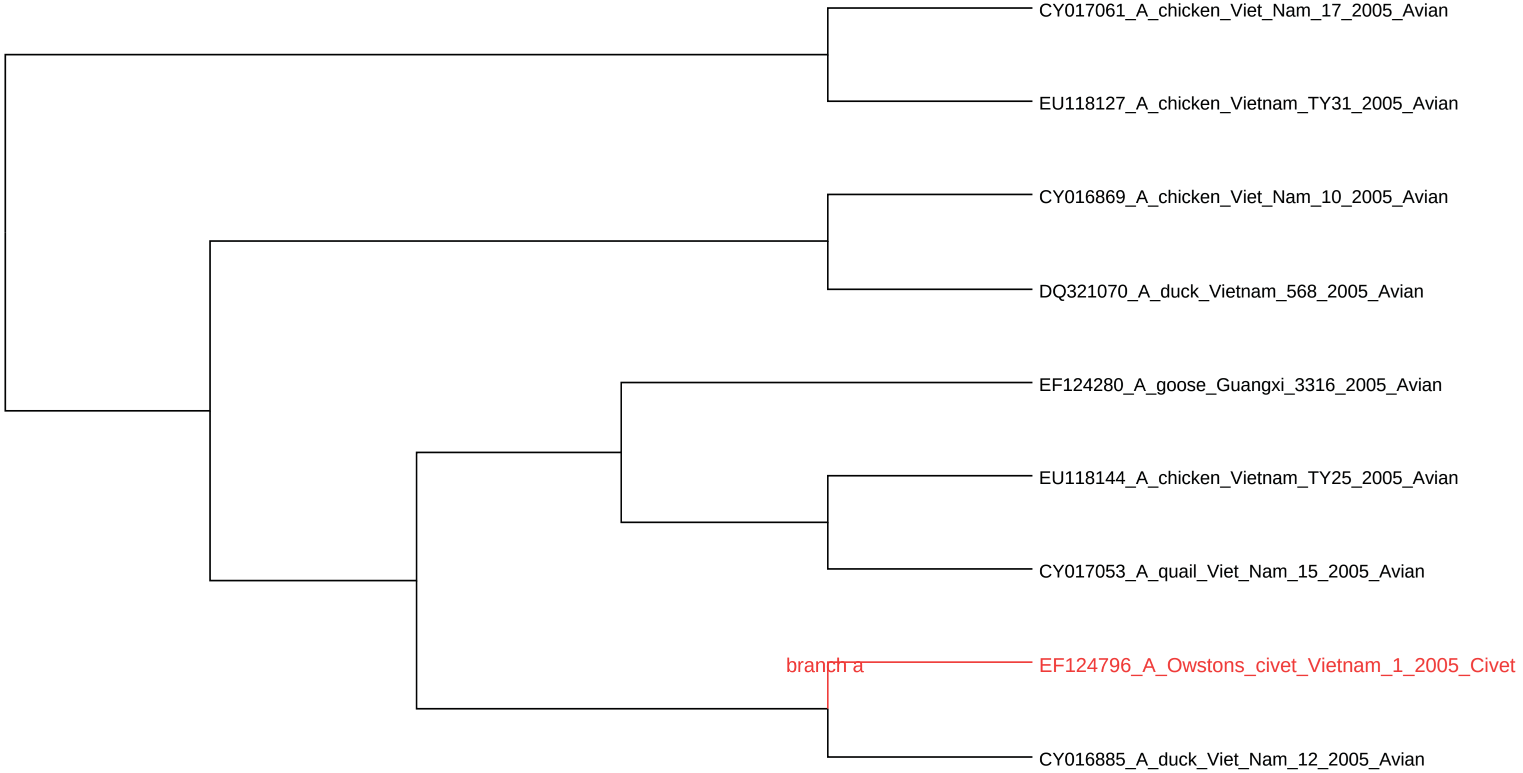
CY017061_A_chicken_Viet_Nam_17_2005_Avian
EU118127_A_chicken_Vietnam_TY31_2005_Avian
CY016869_A_chicken_Viet_Nam_10_2005_Avian
DQ321070_A_duck_Vietnam_568_2005_Avian
EF124280_A_goose_Guangxi_3316_2005_Avian
EU118144_A_chicken_Vietnam_TY25_2005_Avian
CY017053_A_quail_Viet_Nam_15_2005_Avian
branch a
EF124796_A_Owstons_civet_Vietnam_1_2005_Civet
CY016885_A_duck_Viet_Nam_12_2005_Avian

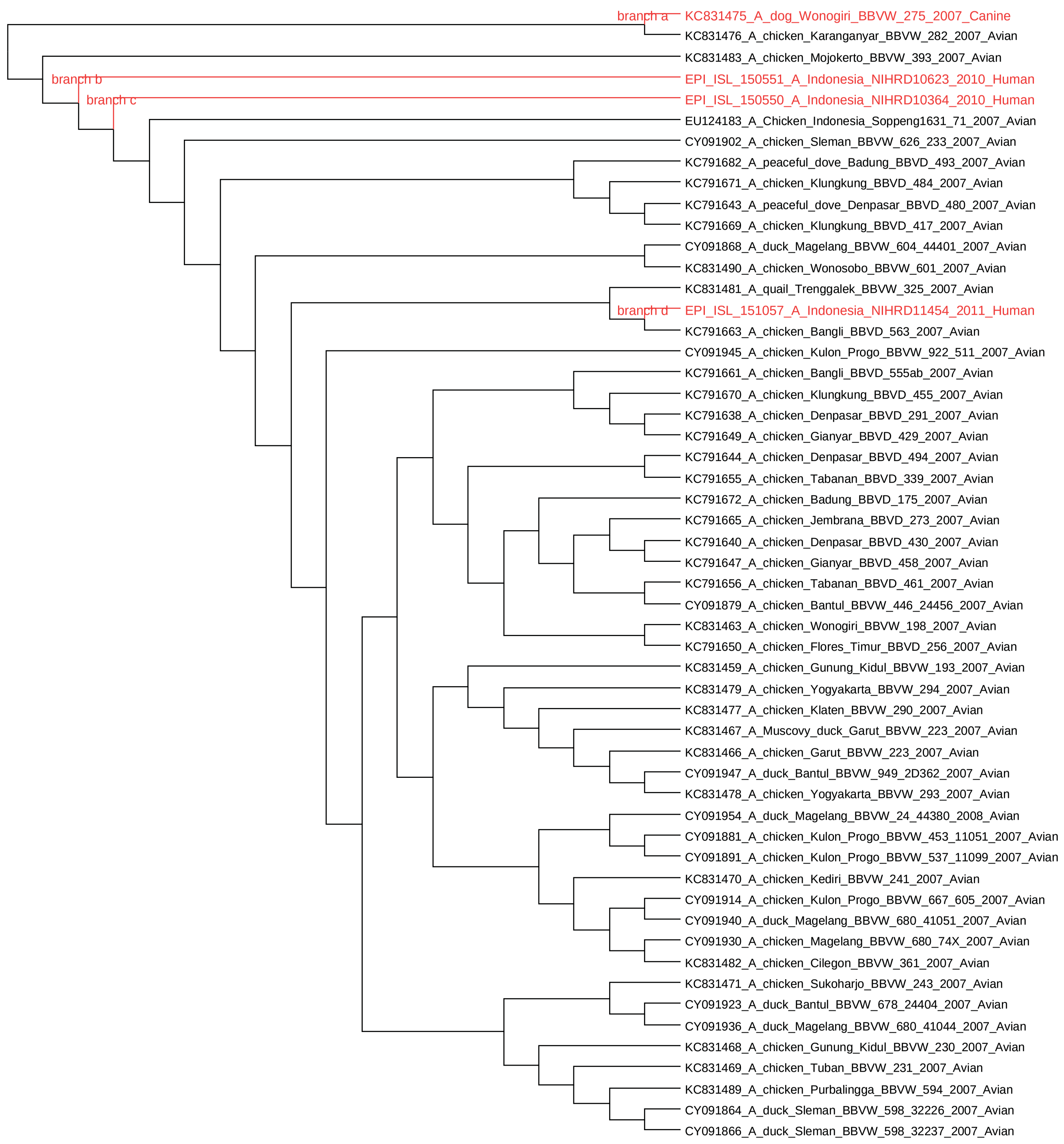

NA1-Group61

NA1-Group61

NA1-Group62

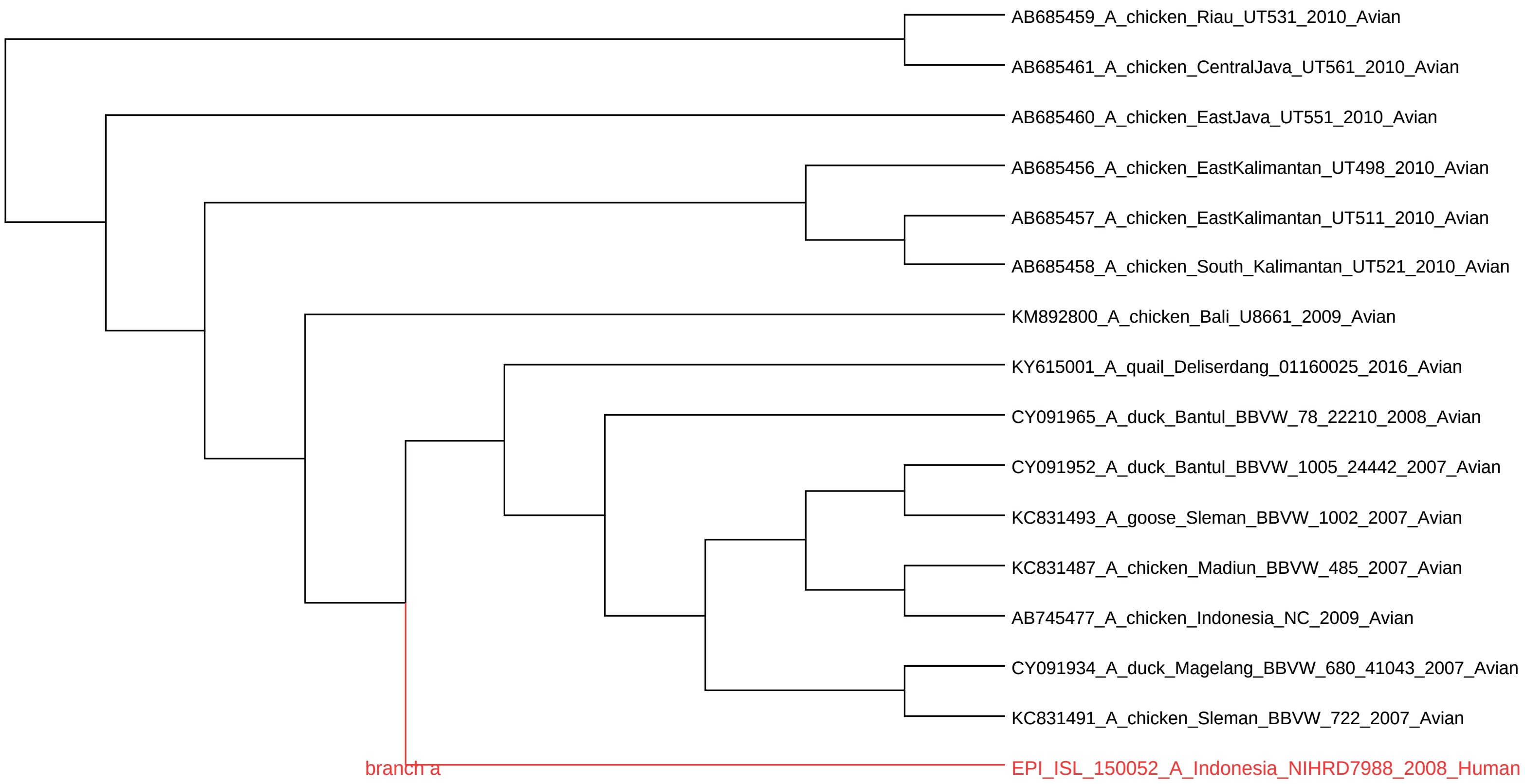

AB685459_A_chicken_Riau_UT531_2010_Avian
AB685461_A_chicken_CentralJava_UT561_2010_Avian
AB685460_A_chicken_EastJava_UT551_2010_Avian
AB685456_A_chicken_EastKalimantan_UT498_2010_Avian
AB685457_A_chicken_EastKalimantan_UT511_2010_Avian
AB685458_A_chicken_South_Kalimantan_UT521_2010_Avian
KM892800_A_chicken_Bali_U8661_2009_Avian
KY615001_A_quail_Deliserdang_01160025_2016_Avian
CY091965_A_duck_Bantul_BBVW_78_22210_2008_Avian
CY091952_A_duck_Bantul_BBVW_1005_24442_2007_Avian
KC831493_A_goose_Sleman_BBVW_1002_2007_Avian
KC831487_A_chicken_Madiun_BBVW_485_2007_Avian
AB745477_A_chicken_Indonesia_NC_2009_Avian
CY091934_A_duck_Magelang_BBVW_680_41043_2007_Avian
KC831491_A_chicken_Sleman_BBVW_722_2007_Avian
branch a
EPI_ISL_150052_A_Indonesia_NIHRD7988_2008_Human

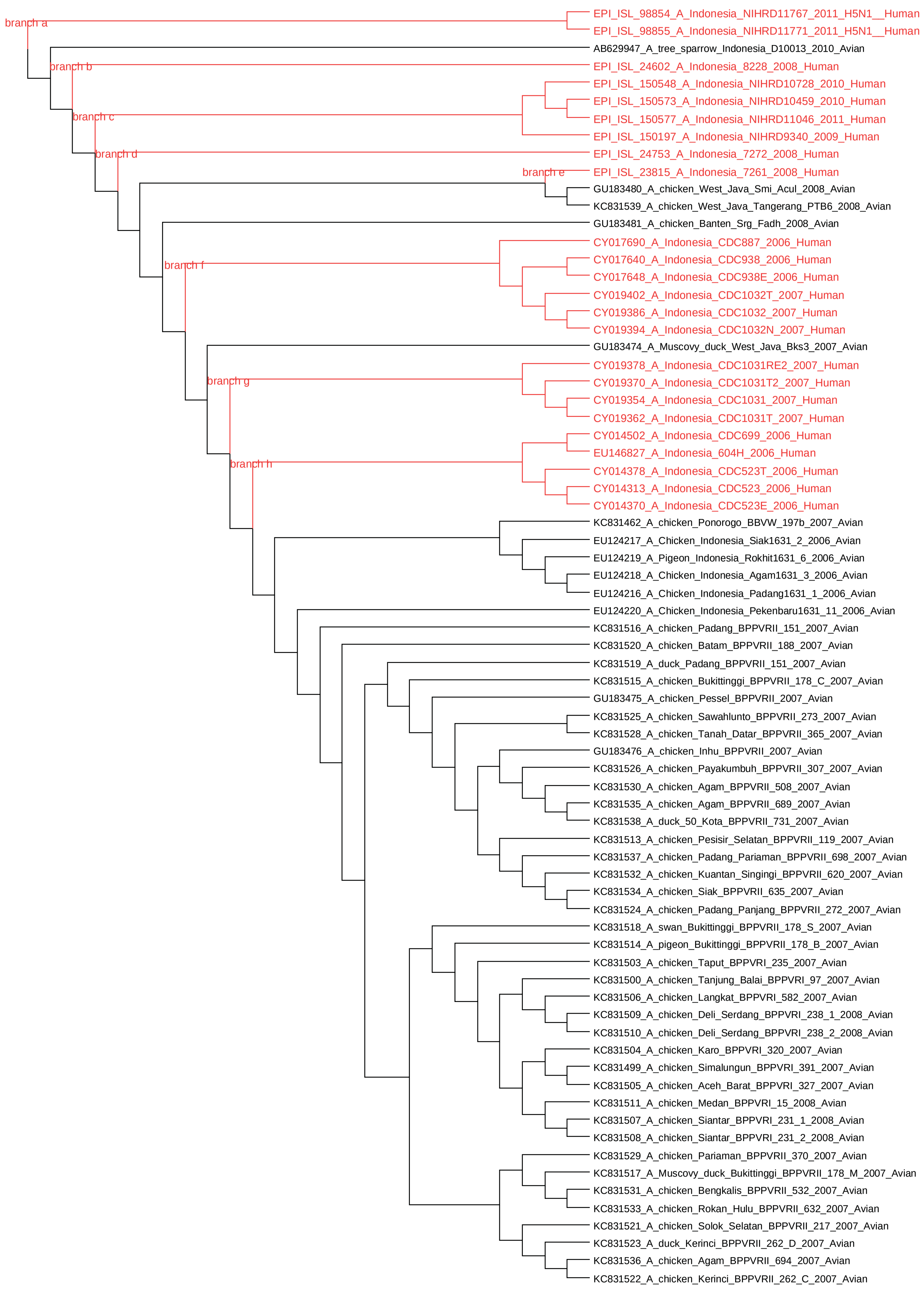

NA1-Group63

NA1-Group63

NA1-Group64

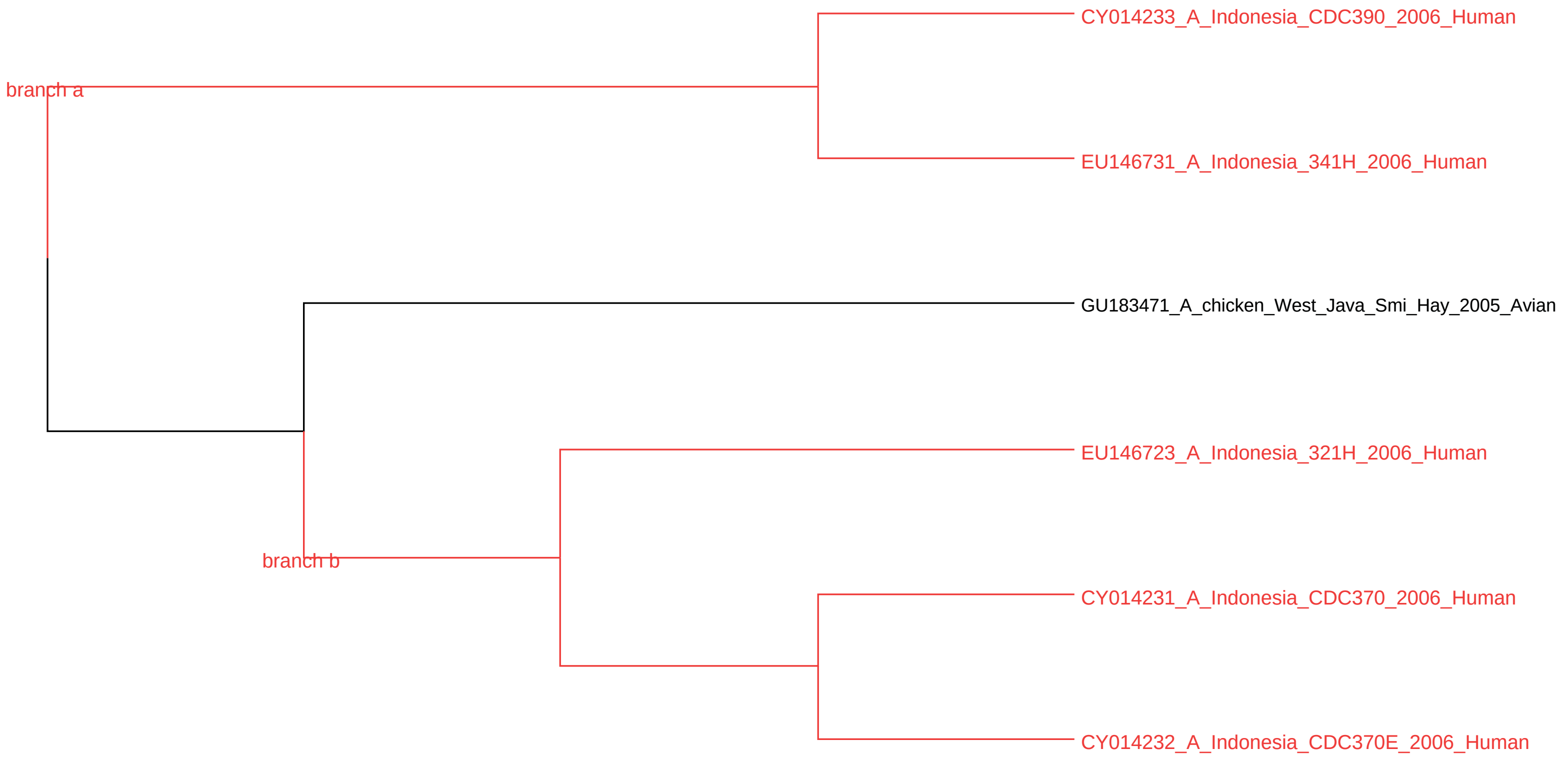

CY014233_A_Indonesia_CDC390_2006_Human
branch a
EU146731_A_Indonesia_341H_2006_Human
GU183471_A_chicken_West_Java_Smi_Hay_2005_Avian
EU146723_A_Indonesia_321H_2006_Human
branch b
CY014231_A_Indonesia_CDC370_2006_Human
CY014232_A_Indonesia_CDC370E_2006_Human

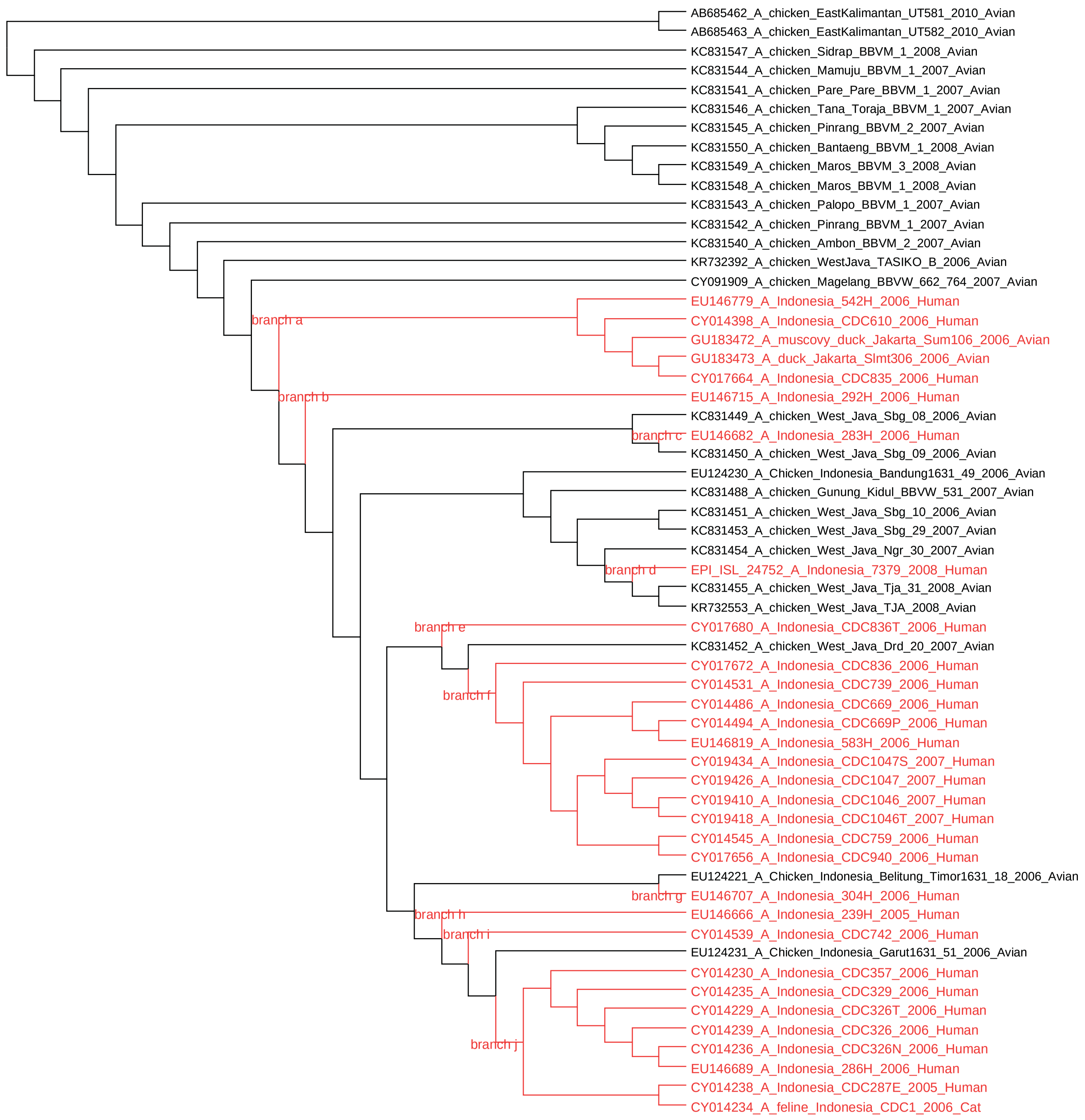

NA1-Group65

NA1-Group65

NA1-Group66

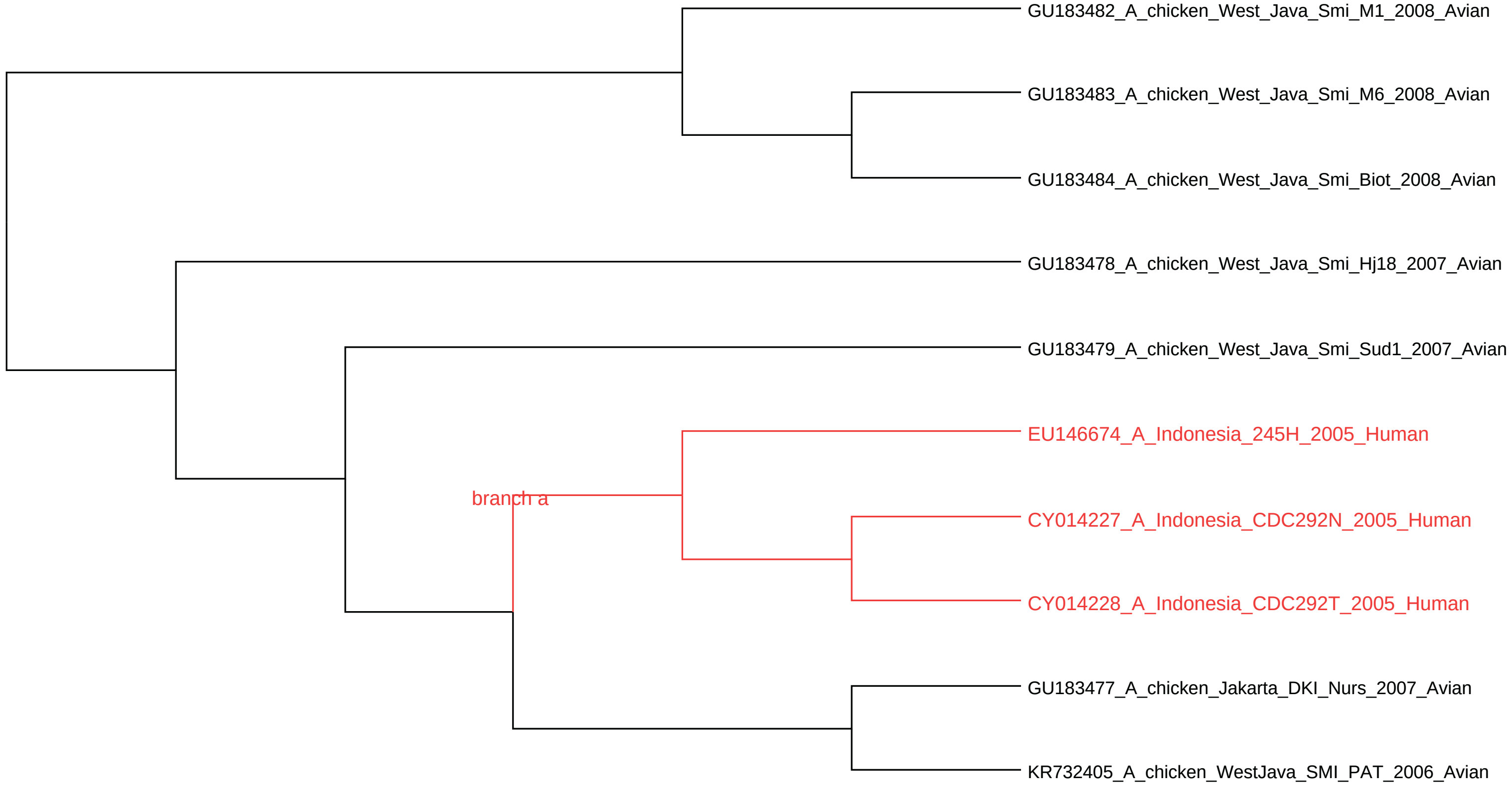
GU183482_A_chicken_West_Java_Smi_M1_2008_Avian
GU183483_A_chicken_West_Java_Smi_M6_2008_Avian
GU183484_A_chicken_West_Java_Smi_Biot_2008_Avian
GU183478_A_chicken_West_Java_Smi_Hj18_2007_Avian
GU183479_A_chicken_West_Java_Smi_Sud1_2007_Avian
EU146674_A_Indonesia_245H_2005_Human
branch a
CY014227_A_Indonesia_CDC292N_2005_Human
CY014228_A_Indonesia_CDC292T_2005_Human
GU183477_A_chicken_Jakarta_DKI_Nurs_2007_Avian
KR732405_A_chicken_WestJava_SMI_PAT_2006_Avian

NA1-Group67

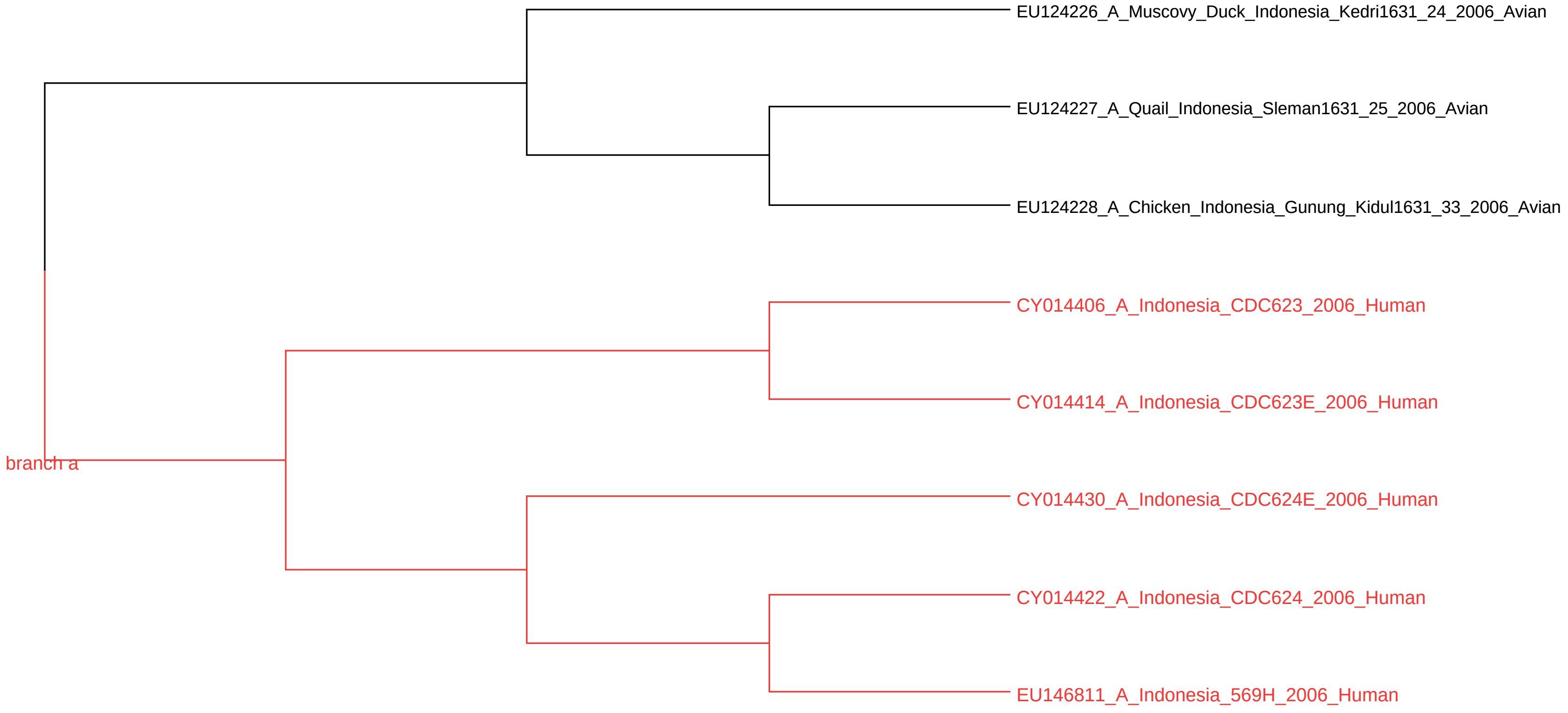

EU124226_A_Muscovy_Duck_Indonesia_Kedri1631_24_2006_Avian
EU124227_A_Quail_Indonesia_Sleman1631_25_2006_Avian
EU124228_A_Chicken_Indonesia_Gunung_Kidul1631_33_2006_Avian
CY014406_A_Indonesia_CDC623_2006_Human
CY014414_A_Indonesia_CDC623E_2006_Human
branch a
CY014430_A_Indonesia_CDC624E_2006_Human
CY014422_A_Indonesia_CDC624_2006_Human
EU146811_A_Indonesia_569H_2006_Human

NA1-Group68

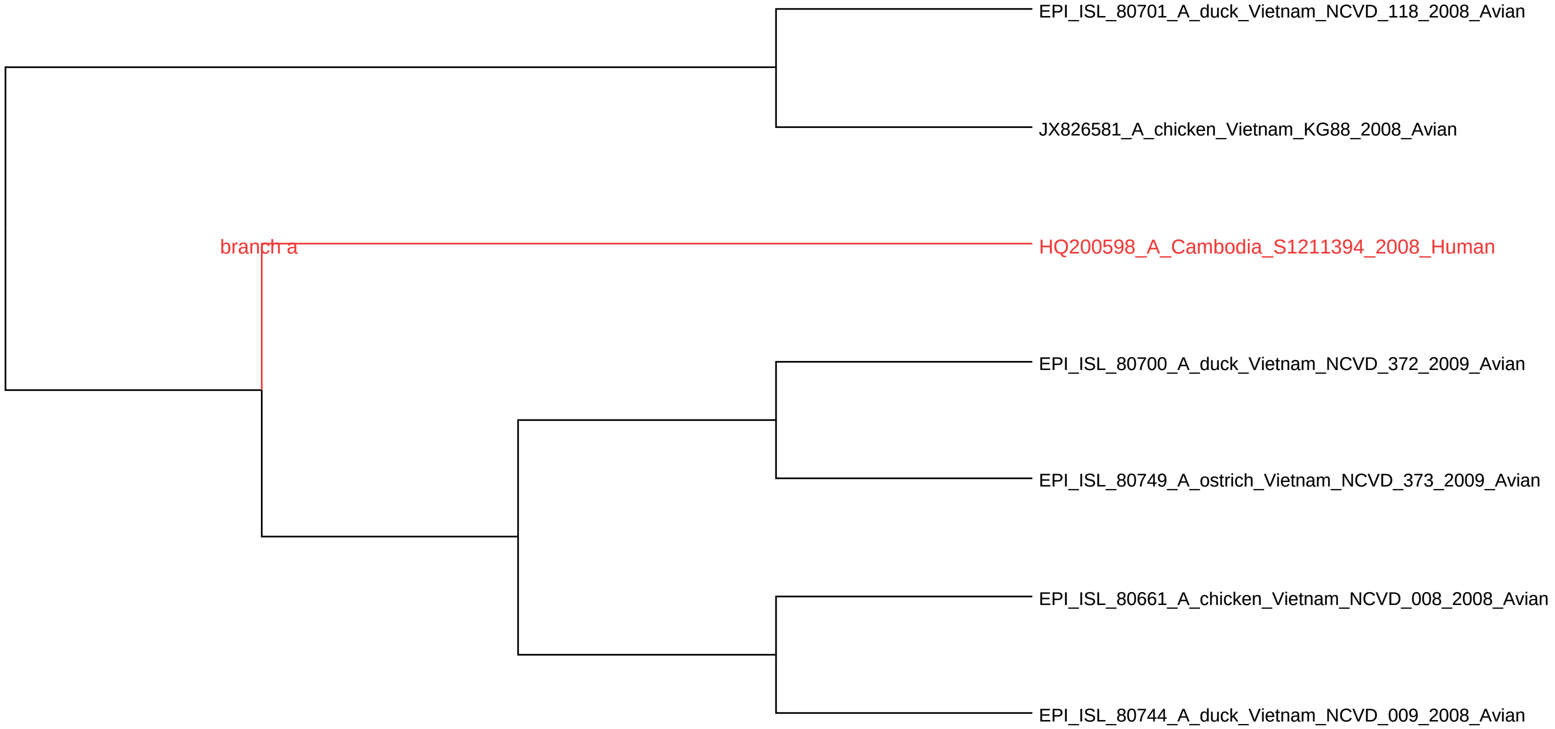

EPI_ISL_80701_A_duck_Vietnam_NCVD_118_2008_Avian
JX826581_A_chicken_Vietnam_KG88_2008_Avian
branch a
HQ200598_A_Cambodia_S1211394_2008_Human
EPI_ISL_80700_A_duck_Vietnam_NCVD_372_2009_Avian
EPI_ISL_80749_A_ostrich_Vietnam_NCVD_373_2009_Avian
EPI_ISL_80661_A_chicken_Vietnam_NCVD_008_2008_Avian
EPI_ISL_80744_A_duck_Vietnam_NCVD_009_2008_Avian

NA1-Group69

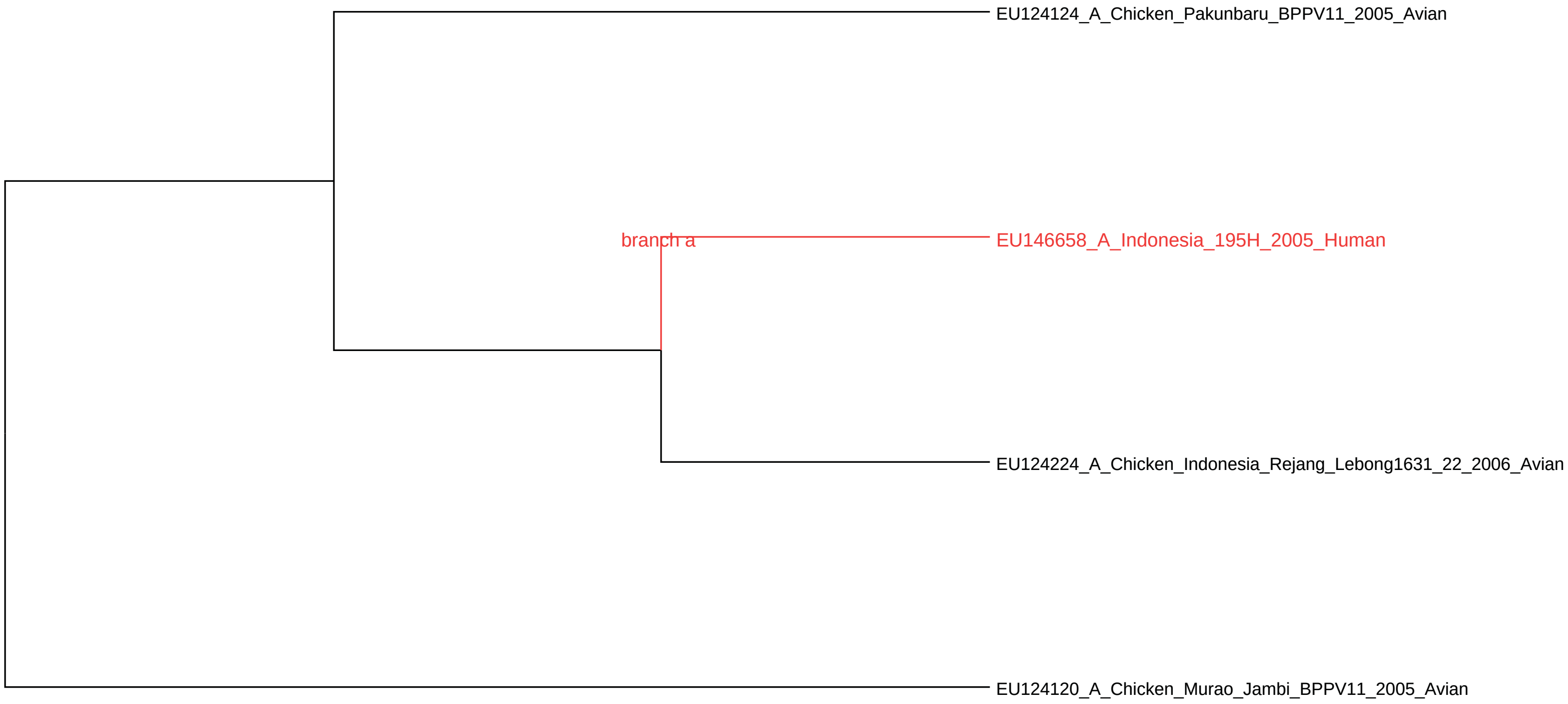
EU124124_A_Chicken_Pakunbaru_BPPV11_2005_Avian
branch a
EU146658_A_Indonesia_195H_2005_Human
EU124224_A_Chicken_Indonesia_Rejang_Lebong1631_22_2006_Avian
EU124120_A_Chicken_Murao_Jambi_BPPV11_2005_Avian

NA1-Group70

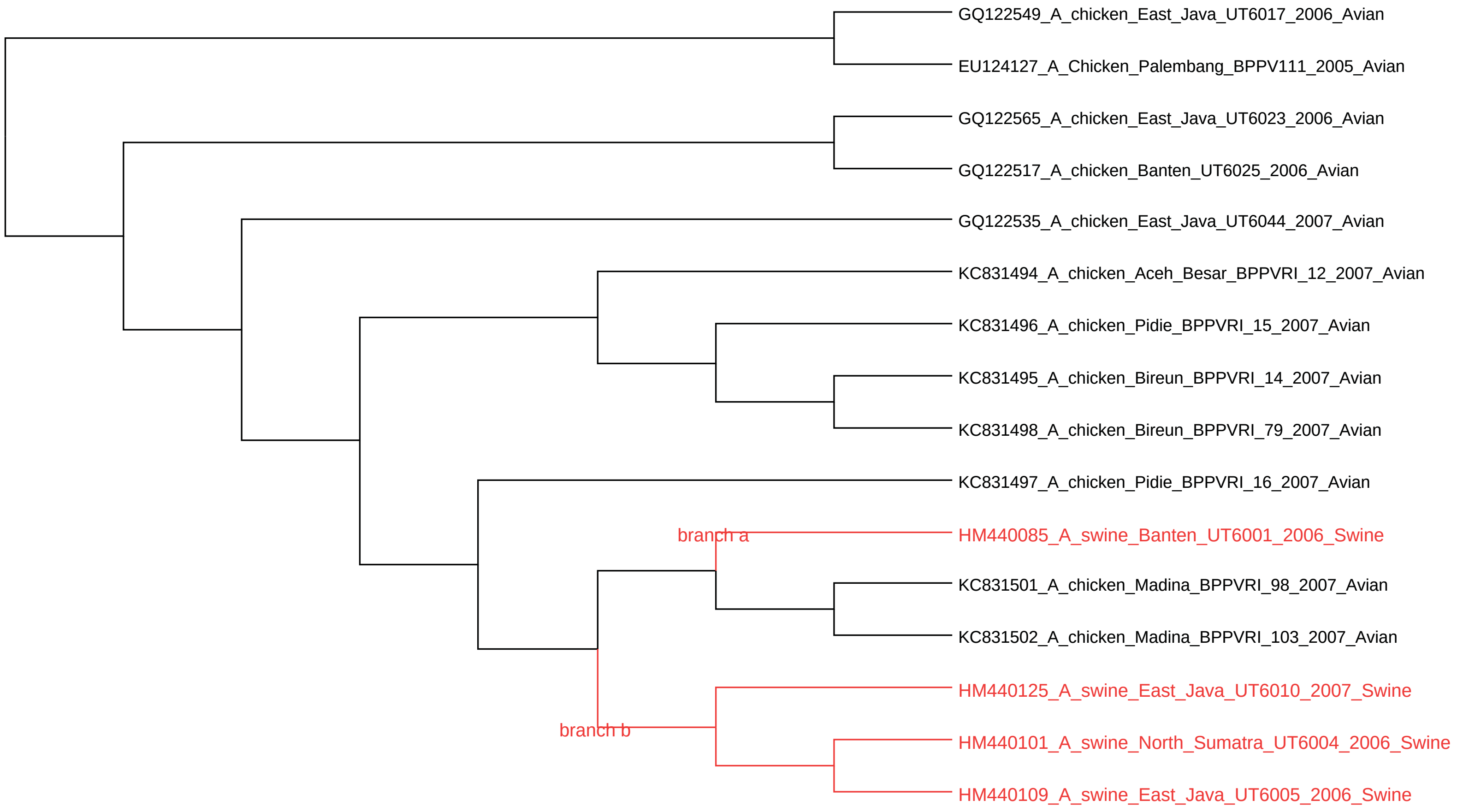
GQ122549_A_chicken_East_Java_UT6017_2006_Avian
EU124127_A_Chicken_Palembang_BPPV111_2005_Avian
GQ122565_A_chicken_East_Java_UT6023_2006_Avian
GQ122517_A_chicken_Banten_UT6025_2006_Avian
GQ122535_A_chicken_East_Java_UT6044_2007_Avian
KC831494_A_chicken_Aceh_Besar_BPPVRI_12_2007_Avian
KC831496_A_chicken_Pidie_BPPVRI_15_2007_Avian
KC831495_A_chicken_Bireun_BPPVRI_14_2007_Avian
KC831498_A_chicken_Bireun_BPPVRI_79_2007_Avian
KC831497_A_chicken_Pidie_BPPVRI_16_2007_Avian
branch a
HM440085_A_swine_Banten_UT6001_2006_Swine
KC831501_A_chicken_Madina_BPPVRI_98_2007_Avian
KC831502_A_chicken_Madina_BPPVRI_103_2007_Avian
HM440125_A_swine_East_Java_UT6010_2007_Swine
branch b
HM440101_A_swine_North_Sumatra_UT6004_2006_Swine
HM440109_A_swine_East_Java_UT6005_2006_Swine

NA1-Group71

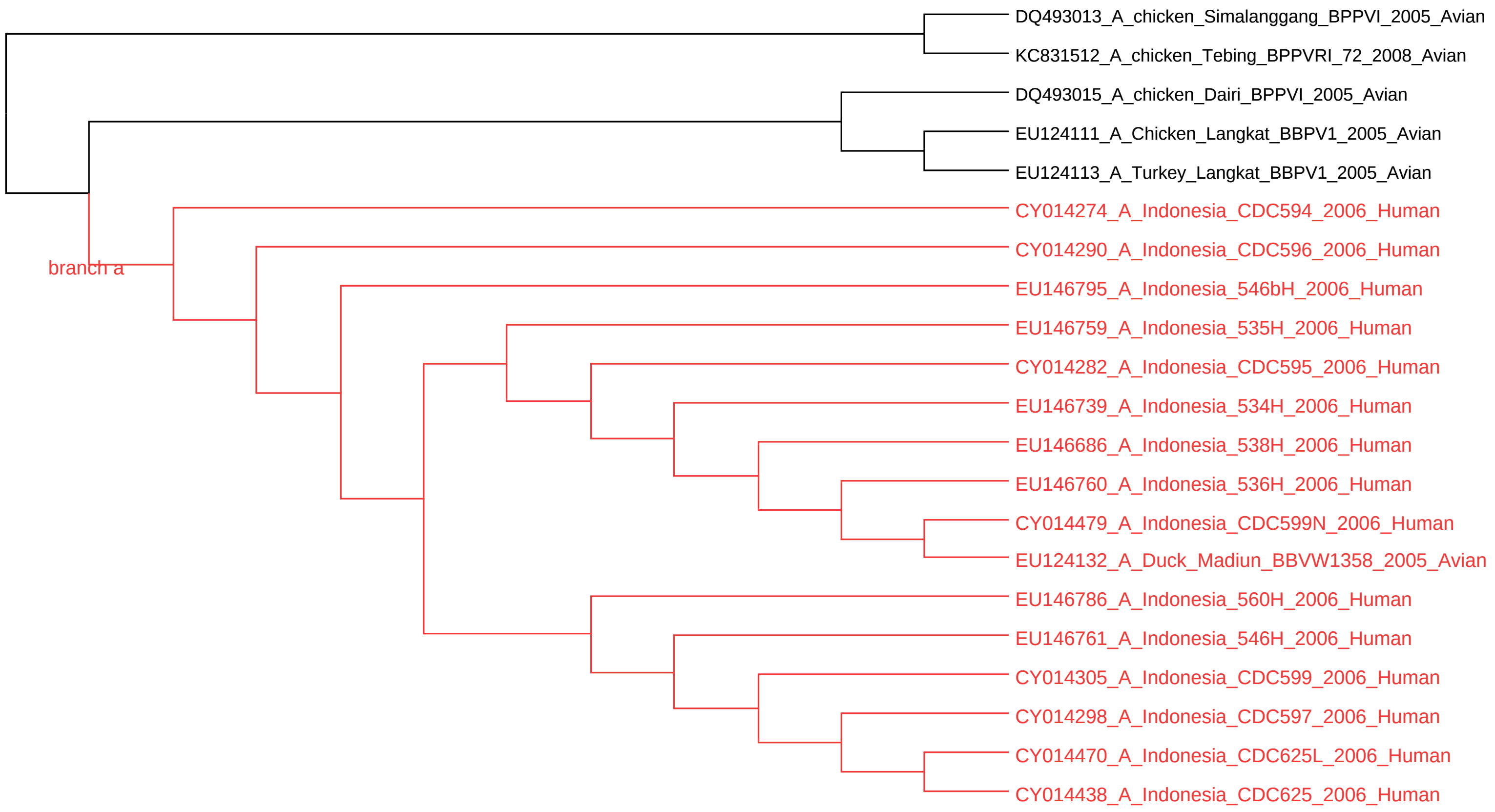
DQ493013_A_chicken_Simalanggang_BPPVI_2005_Avian
KC831512_A_chicken_Tebing_BPPVRI_72_2008_Avian
DQ493015_A_chicken_Dairi_BPPVI_2005_Avian
EU124111_A_Chicken_Langkat_BBPV1_2005_Avian
EU124113_A_Turkey_Langkat_BBPV1_2005_Avian
CY014274_A_Indonesia_CDC594_2006_Human
CY014290_A_Indonesia_CDC596_2006_Human
EU146795_A_Indonesia_546bH_2006_Human
EU146759_A_Indonesia_535H_2006_Human
CY014282_A_Indonesia_CDC595_2006_Human
EU146739_A_Indonesia_534H_2006_Human
EU146686_A_Indonesia_538H_2006_Human
EU146760_A_Indonesia_536H_2006_Human
CY014479_A_Indonesia_CDC599N_2006_Human
EU124132_A_Duck_Madiun_BBVW1358_2005_Avian
EU146786_A_Indonesia_560H_2006_Human
EU146761_A_Indonesia_546H_2006_Human
CY014305_A_Indonesia_CDC599_2006_Human
CY014298_A_Indonesia_CDC597_2006_Human
CY014470_A_Indonesia_CDC625L_2006_Human
CY014438_A_Indonesia_CDC625_2006_Human
branch a

NA1-Group72

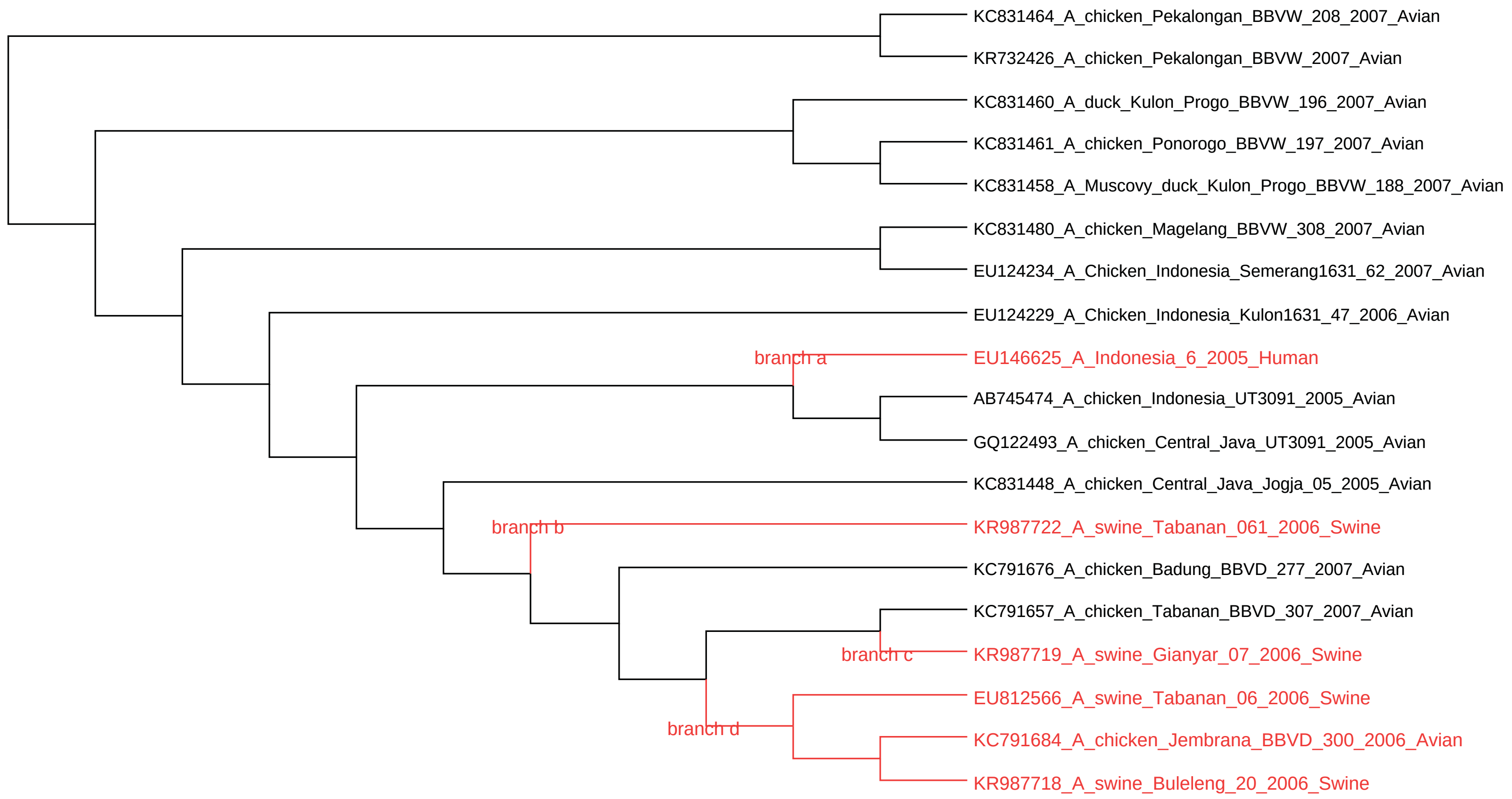

KC831464_A_chicken_Pekalongan_BBVW_208_2007_Avian
KR732426_A_chicken_Pekalongan_BBVW_2007_Avian
KC831460_A_duck_Kulon_Progo_BBVW_196_2007_Avian
KC831461_A_chicken_Ponorogo_BBVW_197_2007_Avian
KC831458_A_Muscovy_duck_Kulon_Progo_BBVW_188_2007_Avian
KC831480_A_chicken_Magelang_BBVW_308_2007_Avian
EU124234_A_Chicken_Indonesia_Semerang1631_62_2007_Avian
EU124229_A_Chicken_Indonesia_Kulon1631_47_2006_Avian
branch a
EU146625_A_Indonesia_6_2005_Human
AB745474_A_chicken_Indonesia_UT3091_2005_Avian
GQ122493_A_chicken_Central_Java_UT3091_2005_Avian
KC831448_A_chicken_Central_Java_Jogja_05_2005_Avian
branch b
KR987722_A_swine_Tabanan_061_2006_Swine
KC791676_A_chicken_Badung_BBVD_277_2007_Avian
KC791657_A_chicken_Tabanan_BBVD_307_2007_Avian
branch c
KR987719_A_swine_Gianyar_07_2006_Swine
EU812566_A_swine_Tabanan_06_2006_Swine
branch d
KC791684_A_chicken_Jembrana_BBVD_300_2006_Avian
KR987718_A_swine_Buleleng_20_2006_Swine

NA1-Group73

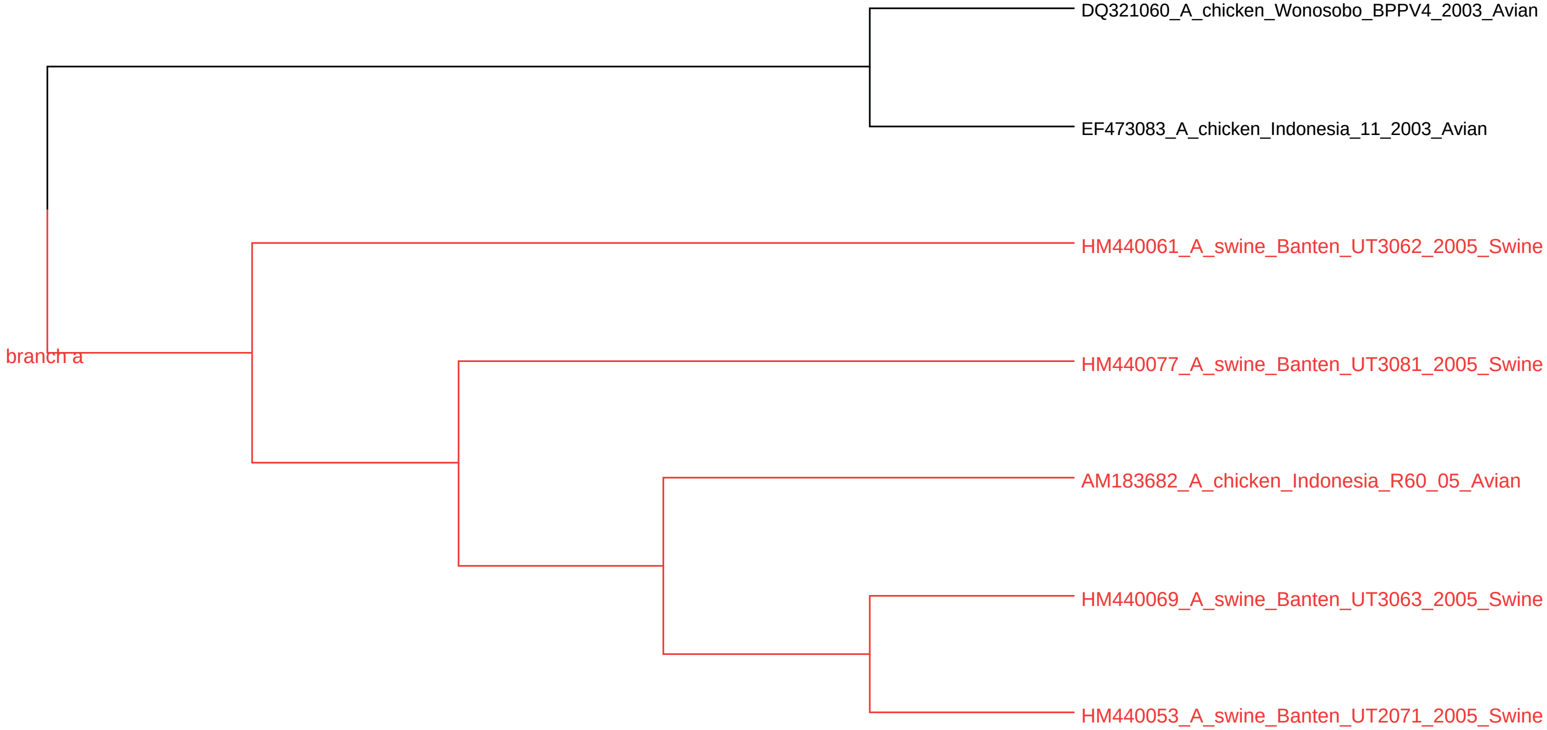
DQ321060_A_chicken_Wonosobo_BPPV4_2003_Avian
EF473083_A_chicken_Indonesia_11_2003_Avian
HM440061_A_swine_Banten_UT3062_2005_Swine
branch a
HM440077_A_swine_Banten_UT3081_2005_Swine
AM183682_A_chicken_Indonesia_R60_05_Avian
HM440069_A_swine_Banten_UT3063_2005_Swine
HM440053_A_swine_Banten_UT2071_2005_Swine

NA1-Group74

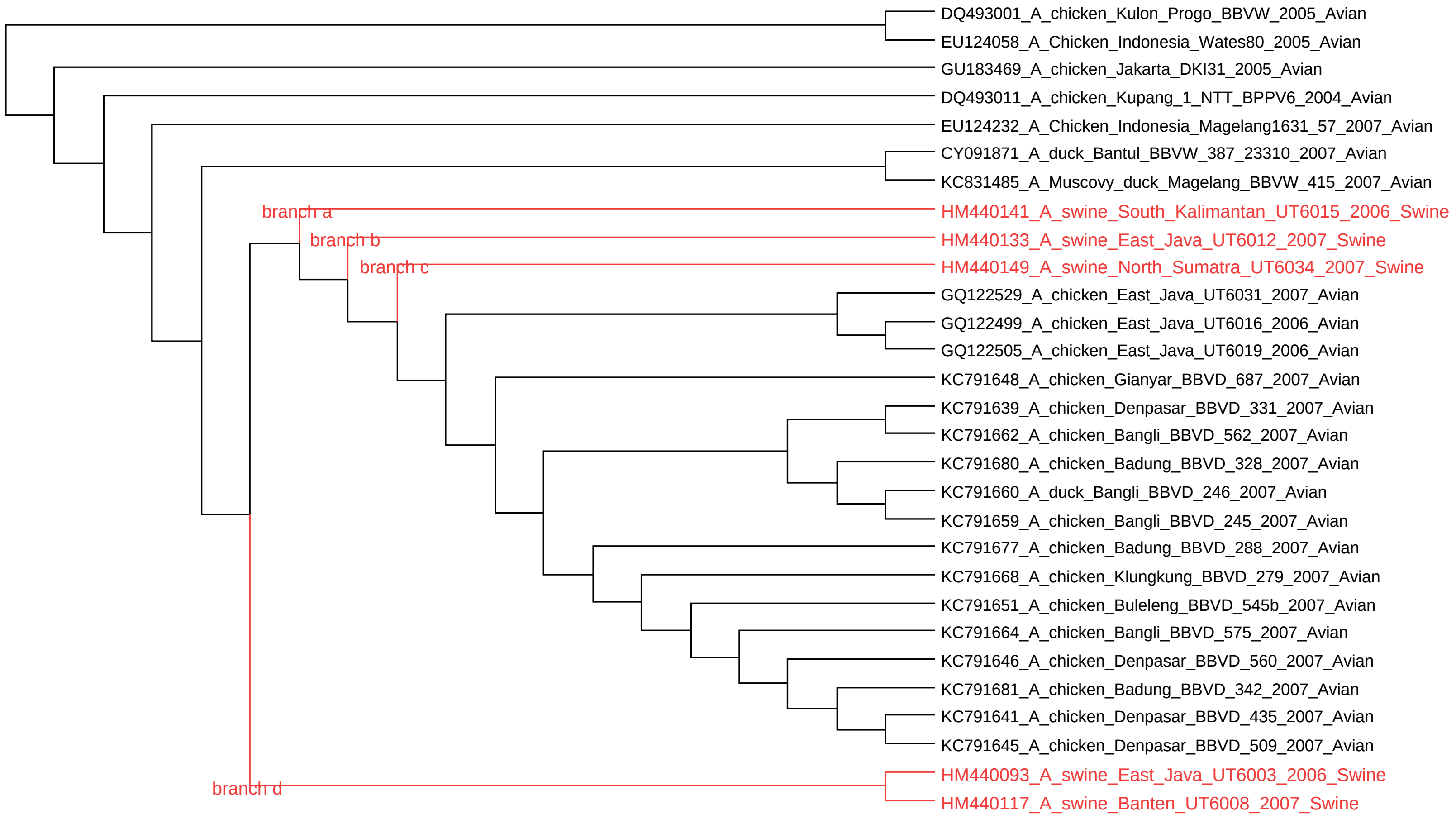
DQ493001_A_chicken_Kulon_Progo_BBVW_2005_Avian
EU124058_A_Chicken_Indonesia_Wates80_2005_Avian
GU183469_A_chicken_Jakarta_DKI31_2005_Avian
DQ493011_A_chicken_Kupang_1_NTT_BPPV6_2004_Avian
EU124232_A_Chicken_Indonesia_Magelang1631_57_2007_Avian
CY091871_A_duck_Bantul_BBVW_387_23310_2007_Avian
KC831485_A_Muscovy_duck_Magelang_BBVW_415_2007_Avian
branch a
HM440141_A_swine_South_Kalimantan_UT6015_2006_Swine
branch b
HM440133_A_swine_East_Java_UT6012_2007_Swine
branch c
HM440149_A_swine_North_Sumatra_UT6034_2007_Swine
GQ122529_A_chicken_East_Java_UT6031_2007_Avian
GQ122499_A_chicken_East_Java_UT6016_2006_Avian
GQ122505_A_chicken_East_Java_UT6019_2006_Avian
KC791648_A_chicken_Gianyar_BBVD_687_2007_Avian
KC791639_A_chicken_Denpasar_BBVD_331_2007_Avian
KC791662_A_chicken_Bangli_BBVD_562_2007_Avian
KC791680_A_chicken_Badung_BBVD_328_2007_Avian
KC791660_A_duck_Bangli_BBVD_246_2007_Avian
KC791659_A_chicken_Bangli_BBVD_245_2007_Avian
KC791677_A_chicken_Badung_BBVD_288_2007_Avian
KC791668_A_chicken_Klungkung_BBVD_279_2007_Avian
KC791651_A_chicken_Buleleng_BBVD_545b_2007_Avian
KC791664_A_chicken_Bangli_BBVD_575_2007_Avian
KC791646_A_chicken_Denpasar_BBVD_560_2007_Avian
KC791681_A_chicken_Badung_BBVD_342_2007_Avian
KC791641_A_chicken_Denpasar_BBVD_435_2007_Avian
KC791645_A_chicken_Denpasar_BBVD_509_2007_Avian
HM440093_A_swine_East_Java_UT6003_2006_Swine
branch d
HM440117_A_swine_Banten_UT6008_2007_Swine

NA1-Group75

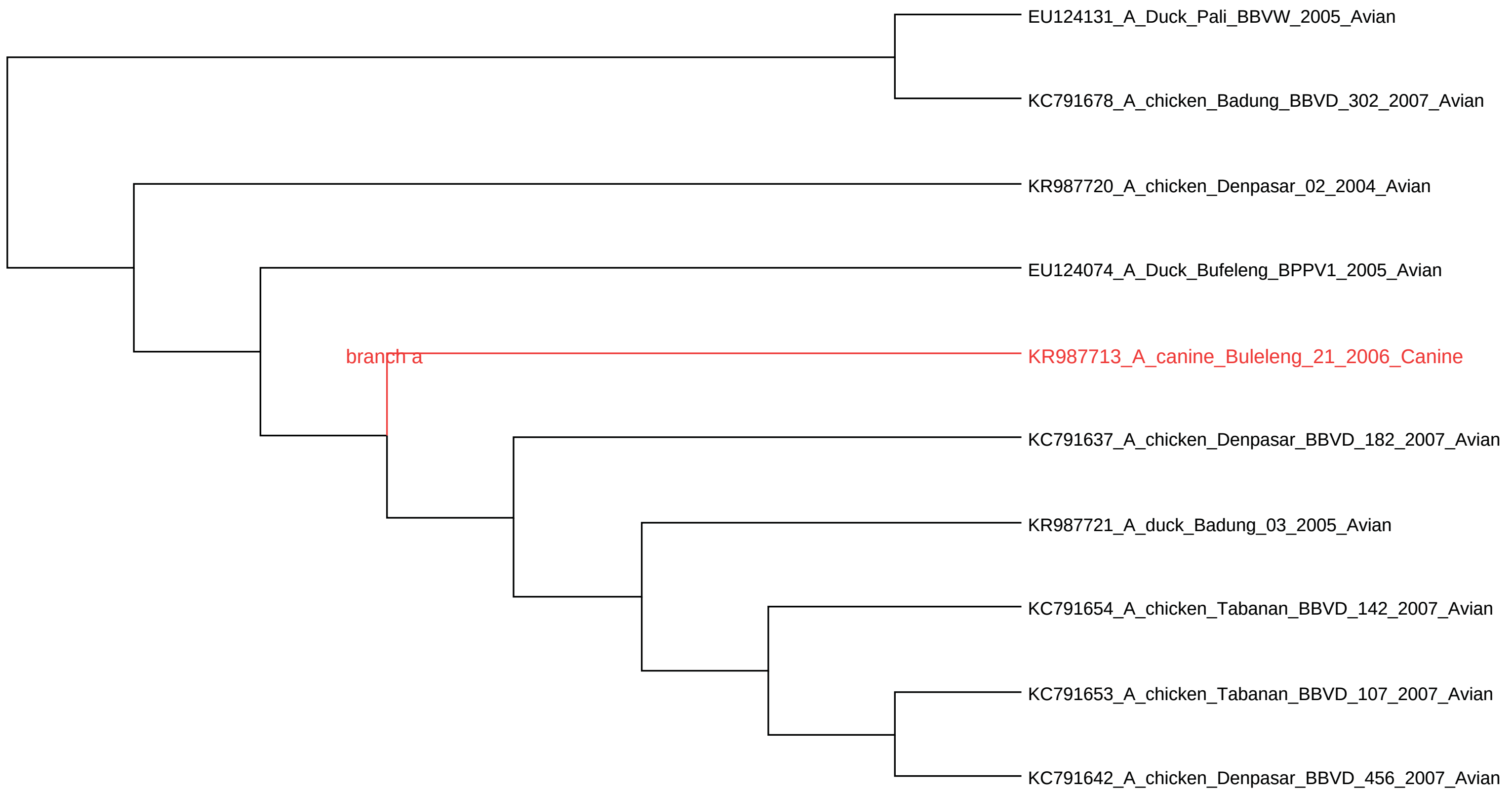

EU124131_A_Duck_Pali_BBVW_2005_Avian
KC791678_A_chicken_Badung_BBVD_302_2007_Avian
KR987720_A_chicken_Denpasar_02_2004_Avian
EU124074_A_Duck_Bufeleng_BPPV1_2005_Avian
branch a
KR987713_A_canine_Buleleng_21_2006_Canine
KC791637_A_chicken_Denpasar_BBVD_182_2007_Avian
KR987721_A_duck_Badung_03_2005_Avian
KC791654_A_chicken_Tabanan_BBVD_142_2007_Avian
KC791653_A_chicken_Tabanan_BBVD_107_2007_Avian
KC791642_A_chicken_Denpasar_BBVD_456_2007_Avian

NA1-Group76

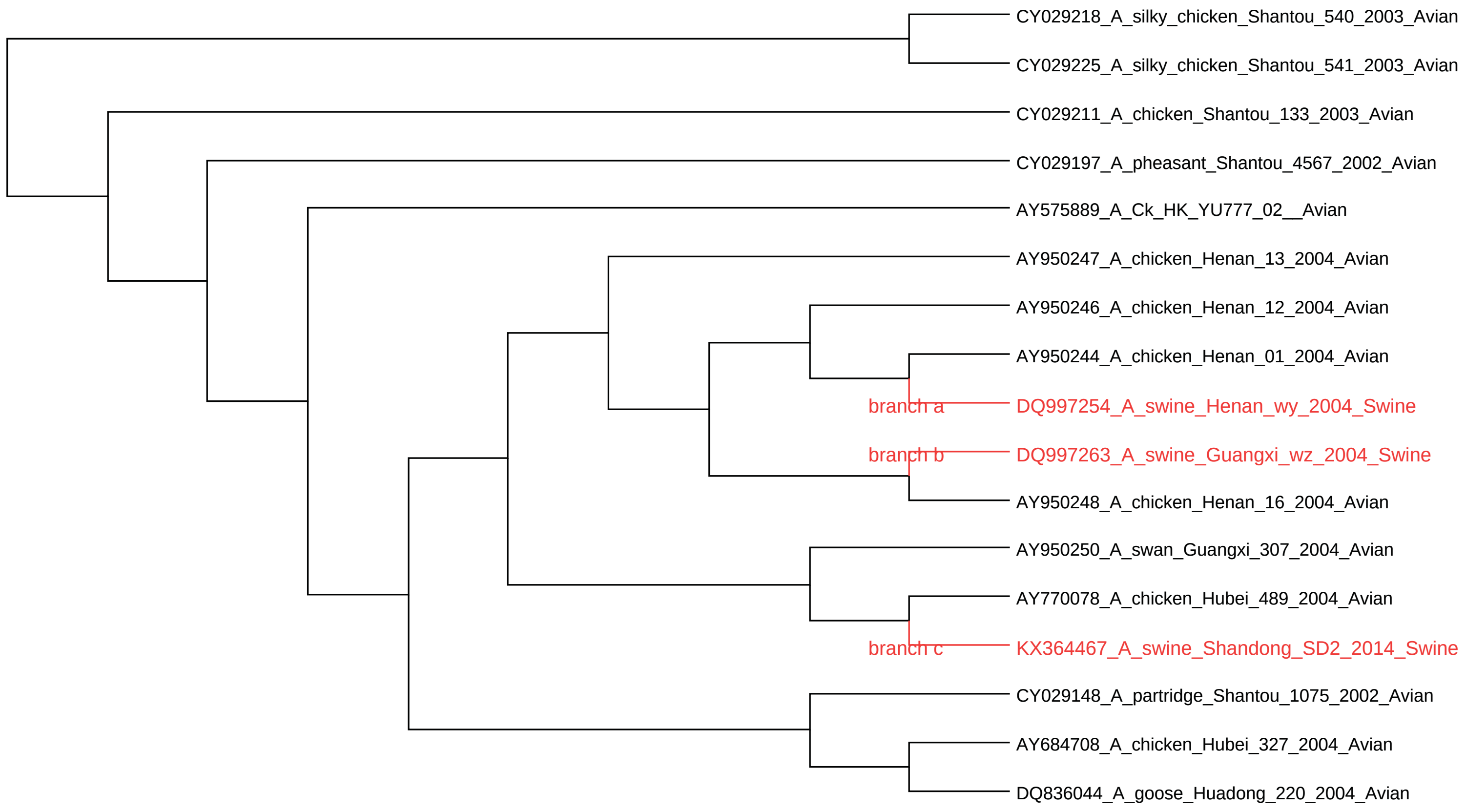
CY029218_A_silky_chicken_Shantou_540_2003_Avian
CY029225_A_silky_chicken_Shantou_541_2003_Avian
CY029211_A_chicken_Shantou_133_2003_Avian
CY029197_A_pheasant_Shantou_4567_2002_Avian
AY575889_A_Ck_HK_YU777_02__Avian
AY950247_A_chicken_Henan_13_2004_Avian
AY950246_A_chicken_Henan_12_2004_Avian
AY950244_A_chicken_Henan_01_2004_Avian
branch a
DQ997254_A_swine_Henan_wy_2004_Swine
branch b
DQ997263_A_swine_Guangxi_wz_2004_Swine
AY950248_A_chicken_Henan_16_2004_Avian
AY950250_A_swan_Guangxi_307_2004_Avian
AY770078_A_chicken_Hubei_489_2004_Avian
branch c
KX364467_A_swine_Shandong_SD2_2014_Swine
CY029148_A_partridge_Shantou_1075_2002_Avian
AY684708_A_chicken_Hubei_327_2004_Avian
DQ836044_A_goose_Huadong_220_2004_Avian

NA1-Group77

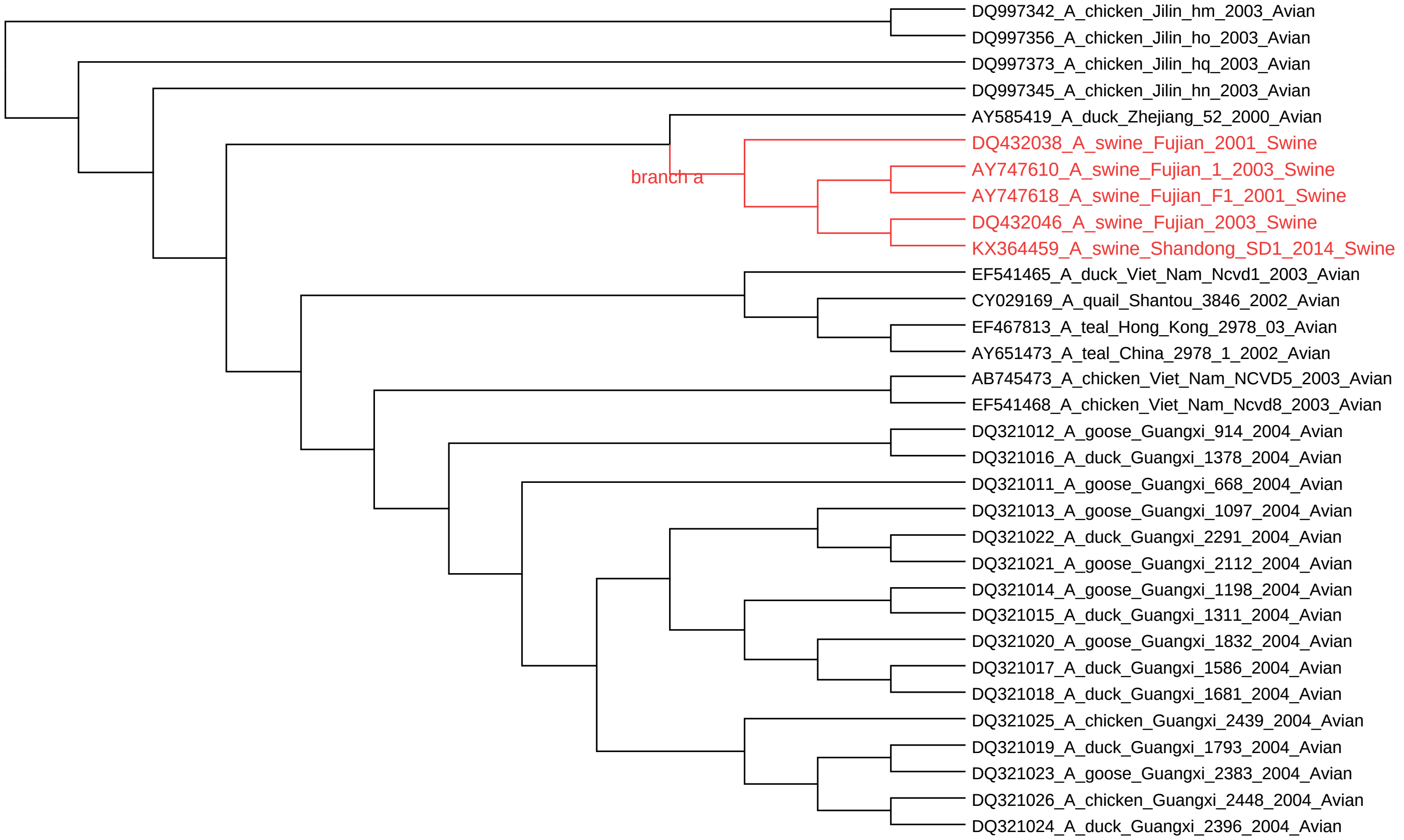

DQ997342_A_chicken_Jilin_hm_2003_Avian
DQ997356_A_chicken_Jilin_ho_2003_Avian
DQ997373_A_chicken_Jilin_hq_2003_Avian
DQ997345_A_chicken_Jilin_hn_2003_Avian
AY585419_A_duck_Zhejiang_52_2000_Avian
DQ432038_A_swine_Fujian_2001_Swine
branch a
AY747610_A_swine_Fujian_1_2003_Swine
AY747618_A_swine_Fujian_F1_2001_Swine
DQ432046_A_swine_Fujian_2003_Swine
KX364459_A_swine_Shandong_SD1_2014_Swine
EF541465_A_duck_Viet_Nam_Ncvd1_2003_Avian
CY029169_A_quail_Shantou_3846_2002_Avian
EF467813_A_teal_Hong_Kong_2978_03_Avian
AY651473_A_teal_China_2978_1_2002_Avian
AB745473_A_chicken_Viet_Nam_NCVD5_2003_Avian
EF541468_A_chicken_Viet_Nam_Ncvd8_2003_Avian
DQ321012_A_goose_Guangxi_914_2004_Avian
DQ321016_A_duck_Guangxi_1378_2004_Avian
DQ321011_A_goose_Guangxi_668_2004_Avian
DQ321013_A_goose_Guangxi_1097_2004_Avian
DQ321022_A_duck_Guangxi_2291_2004_Avian
DQ321021_A_goose_Guangxi_2112_2004_Avian
DQ321014_A_goose_Guangxi_1198_2004_Avian
DQ321015_A_duck_Guangxi_1311_2004_Avian
DQ321020_A_goose_Guangxi_1832_2004_Avian
DQ321017_A_duck_Guangxi_1586_2004_Avian
DQ321018_A_duck_Guangxi_1681_2004_Avian
DQ321025_A_chicken_Guangxi_2439_2004_Avian
DQ321019_A_duck_Guangxi_1793_2004_Avian
DQ321023_A_goose_Guangxi_2383_2004_Avian
DQ321026_A_chicken_Guangxi_2448_2004_Avian
DQ321024_A_duck_Guangxi_2396_2004_Avian

NA1-Group78

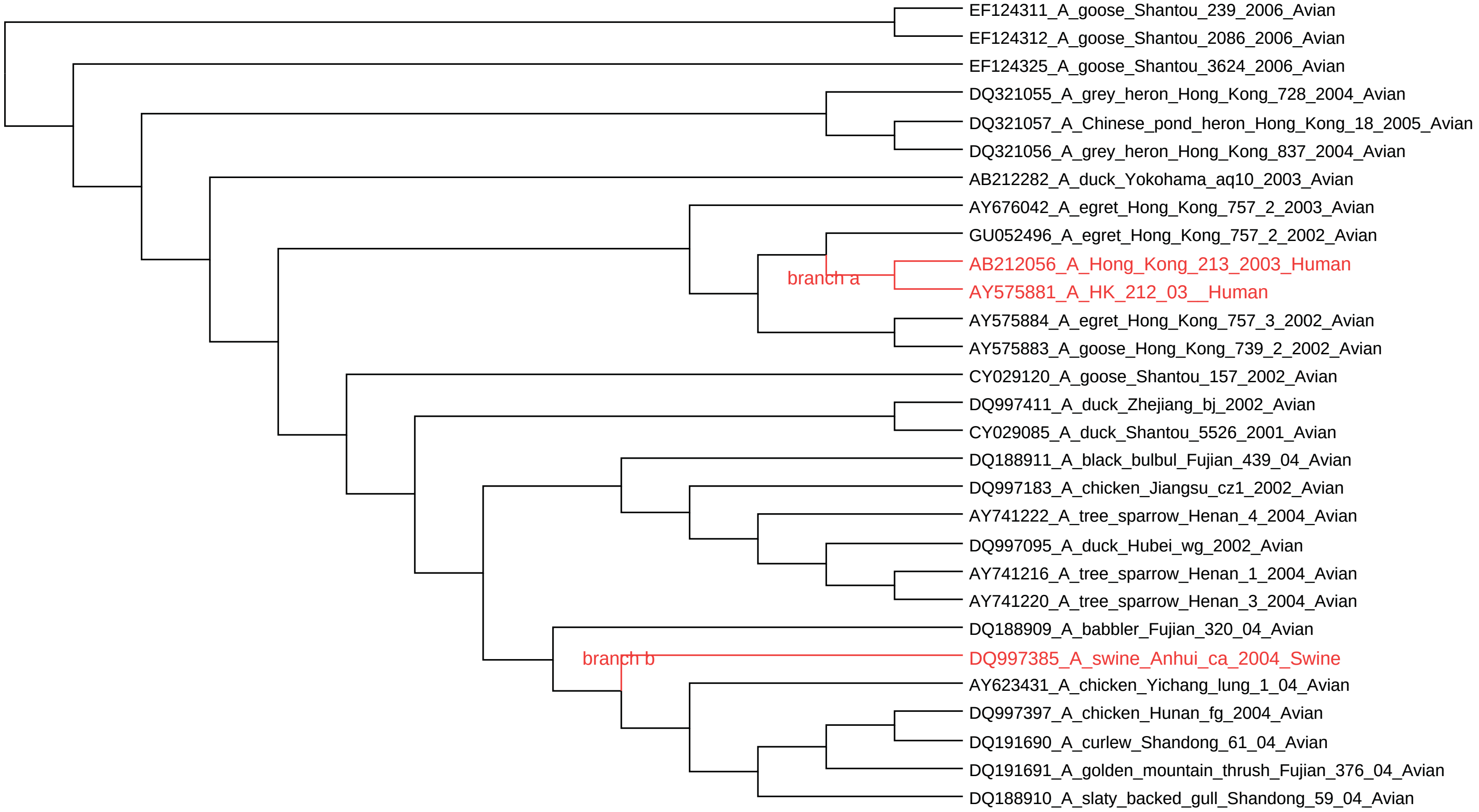
EF124311_A_goose_Shantou_239_2006_Avian
EF124312_A_goose_Shantou_2086_2006_Avian
EF124325_A_goose_Shantou_3624_2006_Avian
DQ321055_A_grey_heron_Hong_Kong_728_2004_Avian
DQ321057_A_Chinese_pond_heron_Hong_Kong_18_2005_Avian
DQ321056_A_grey_heron_Hong_Kong_837_2004_Avian
AB212282_A_duck_Yokohama_aq10_2003_Avian
AY676042_A_egret_Hong_Kong_757_2_2003_Avian
GU052496_A_egret_Hong_Kong_757_2_2002_Avian
AB212056_A_Hong_Kong_213_2003_Human
branch a
AY575881_A_HK_212_03__Human
AY575884_A_egret_Hong_Kong_757_3_2002_Avian
AY575883_A_goose_Hong_Kong_739_2_2002_Avian
CY029120_A_goose_Shantou_157_2002_Avian
DQ997411_A_duck_Zhejiang_bj_2002_Avian
CY029085_A_duck_Shantou_5526_2001_Avian
DQ188911_A_black_bulbul_Fujian_439_04_Avian
DQ997183_A_chicken_Jiangsu_cz1_2002_Avian
AY741222_A_tree_sparrow_Henan_4_2004_Avian
DQ997095_A_duck_Hubei_wg_2002_Avian
AY741216_A_tree_sparrow_Henan_1_2004_Avian
AY741220_A_tree_sparrow_Henan_3_2004_Avian
DQ188909_A_babbler_Fujian_320_04_Avian
branch b
DQ997385_A_swine_Anhui_ca_2004_Swine
AY623431_A_chicken_Yichang_lung_1_04_Avian
DQ997397_A_chicken_Hunan_fg_2004_Avian
DQ191690_A_curlew_Shandong_61_04_Avian
DQ191691_A_golden_mountain_thrush_Fujian_376_04_Avian
DQ188910_A_slaty_backed_gull_Shandong_59_04_Avian

NA1-Group79

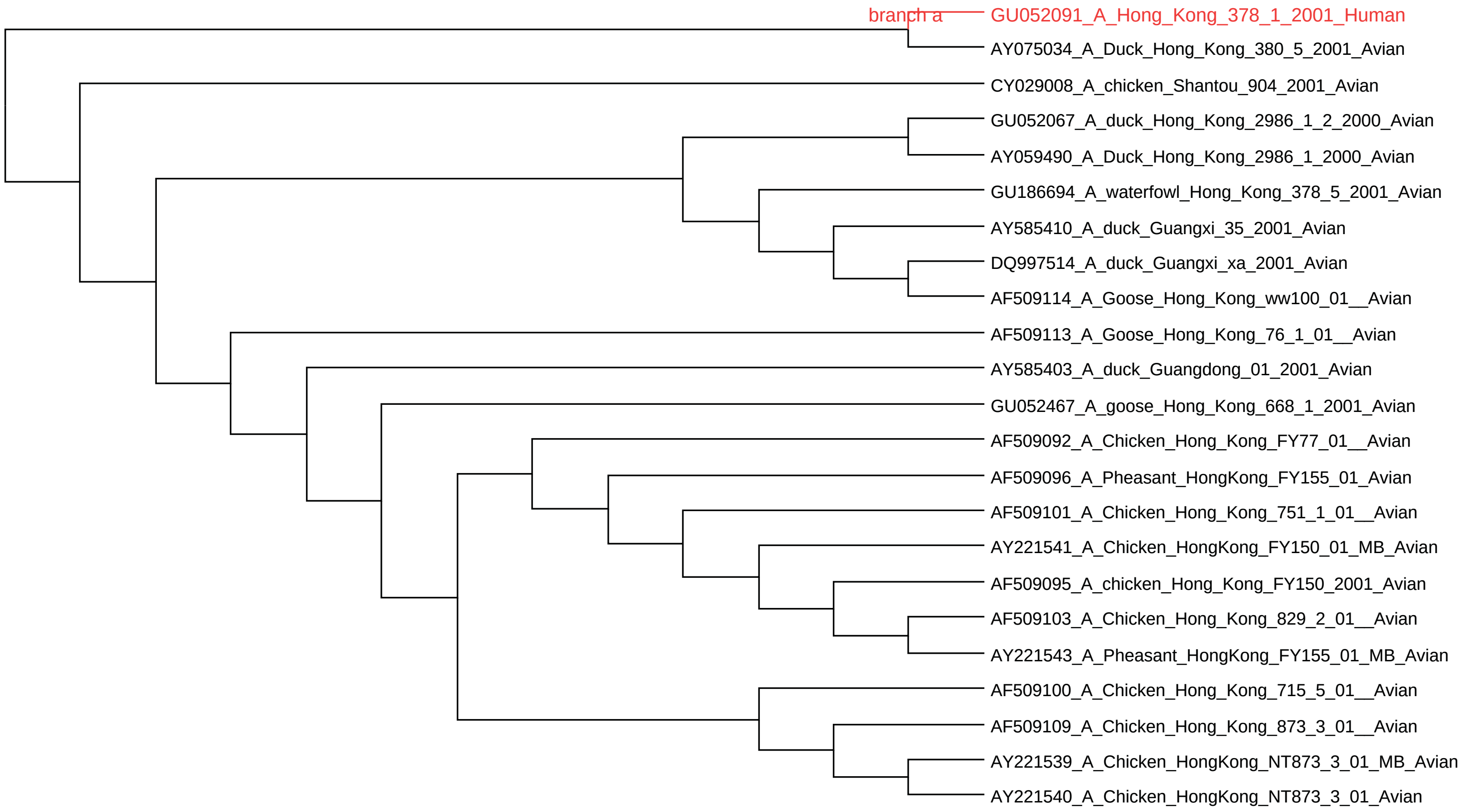

branch a
GU052091_A_Hong_Kong_378_1_2001_Human
AY075034_A_Duck_Hong_Kong_380_5_2001_Avian
CY029008_A_chicken_Shantou_904_2001_Avian
GU052067_A_duck_Hong_Kong_2986_1_2_2000_Avian
AY059490_A_Duck_Hong_Kong_2986_1_2000_Avian
GU186694_A_waterfowl_Hong_Kong_378_5_2001_Avian
AY585410_A_duck_Guangxi_35_2001_Avian
DQ997514_A_duck_Guangxi_xa_2001_Avian
AF509114_A_Goose_Hong_Kong_ww100_01__Avian
AF509113_A_Goose_Hong_Kong_76_1_01__Avian
AY585403_A_duck_Guangdong_01_2001_Avian
GU052467_A_goose_Hong_Kong_668_1_2001_Avian
AF509092_A_Chicken_Hong_Kong_FY77_01__Avian
AF509096_A_Pheasant_HongKong_FY155_01_Avian
AF509101_A_Chicken_Hong_Kong_751_1_01__Avian
AY221541_A_Chicken_HongKong_FY150_01_MB_Avian
AF509095_A_chicken_Hong_Kong_FY150_2001_Avian
AF509103_A_Chicken_Hong_Kong_829_2_01__Avian
AY221543_A_Pheasant_HongKong_FY155_01_MB_Avian
AF509100_A_Chicken_Hong_Kong_715_5_01__Avian
AF509109_A_Chicken_Hong_Kong_873_3_01__Avian
AY221539_A_Chicken_HongKong_NT873_3_01_MB_Avian
AY221540_A_Chicken_HongKong_NT873_3_01_Avian

NA1-Group80

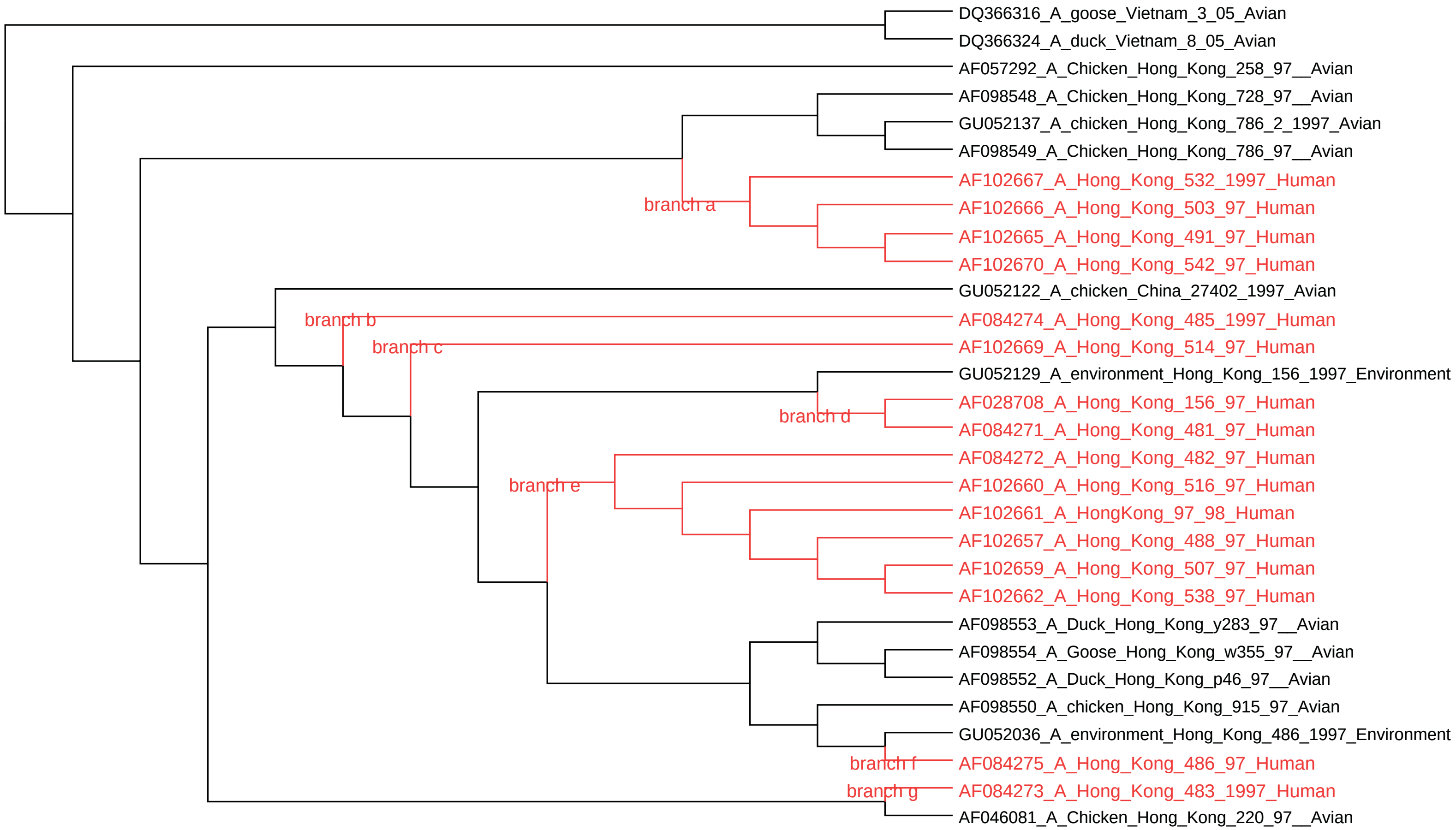
DQ366316_A_goose_Vietnam_3_05_Avian
DQ366324_A_duck_Vietnam_8_05_Avian
AF057292_A_Chicken_Hong_Kong_258_97__Avian
AF098548_A_Chicken_Hong_Kong_728_97__Avian
GU052137_A_chicken_Hong_Kong_786_2_1997_Avian
AF098549_A_Chicken_Hong_Kong_786_97__Avian
AF102667_A_Hong_Kong_532_1997_Human
branch a
AF102666_A_Hong_Kong_503_97_Human
AF102665_A_Hong_Kong_491_97_Human
AF102670_A_Hong_Kong_542_97_Human
GU052122_A_chicken_China_27402_1997_Avian
branch b
AF084274_A_Hong_Kong_485_1997_Human
branch c
AF102669_A_Hong_Kong_514_97_Human
GU052129_A_environment_Hong_Kong_156_1997_Environment
AF028708_A_Hong_Kong_156_97_Human
branch d
AF084271_A_Hong_Kong_481_97_Human
AF084272_A_Hong_Kong_482_97_Human
branch e
AF102660_A_Hong_Kong_516_97_Human
AF102661_A_HongKong_97_98_Human
AF102657_A_Hong_Kong_488_97_Human
AF102659_A_Hong_Kong_507_97_Human
AF102662_A_Hong_Kong_538_97_Human
AF098553_A_Duck_Hong_Kong_y283_97__Avian
AF098554_A_Goose_Hong_Kong_w355_97__Avian
AF098552_A_Duck_Hong_Kong_p46_97__Avian
AF098550_A_chicken_Hong_Kong_915_97_Avian
GU052036_A_environment_Hong_Kong_486_1997_Environment
branch f
AF084275_A_Hong_Kong_486_97_Human
branch g
AF084273_A_Hong_Kong_483_1997_Human
AF046081_A_Chicken_Hong_Kong_220_97__Avian

NA1-Group81

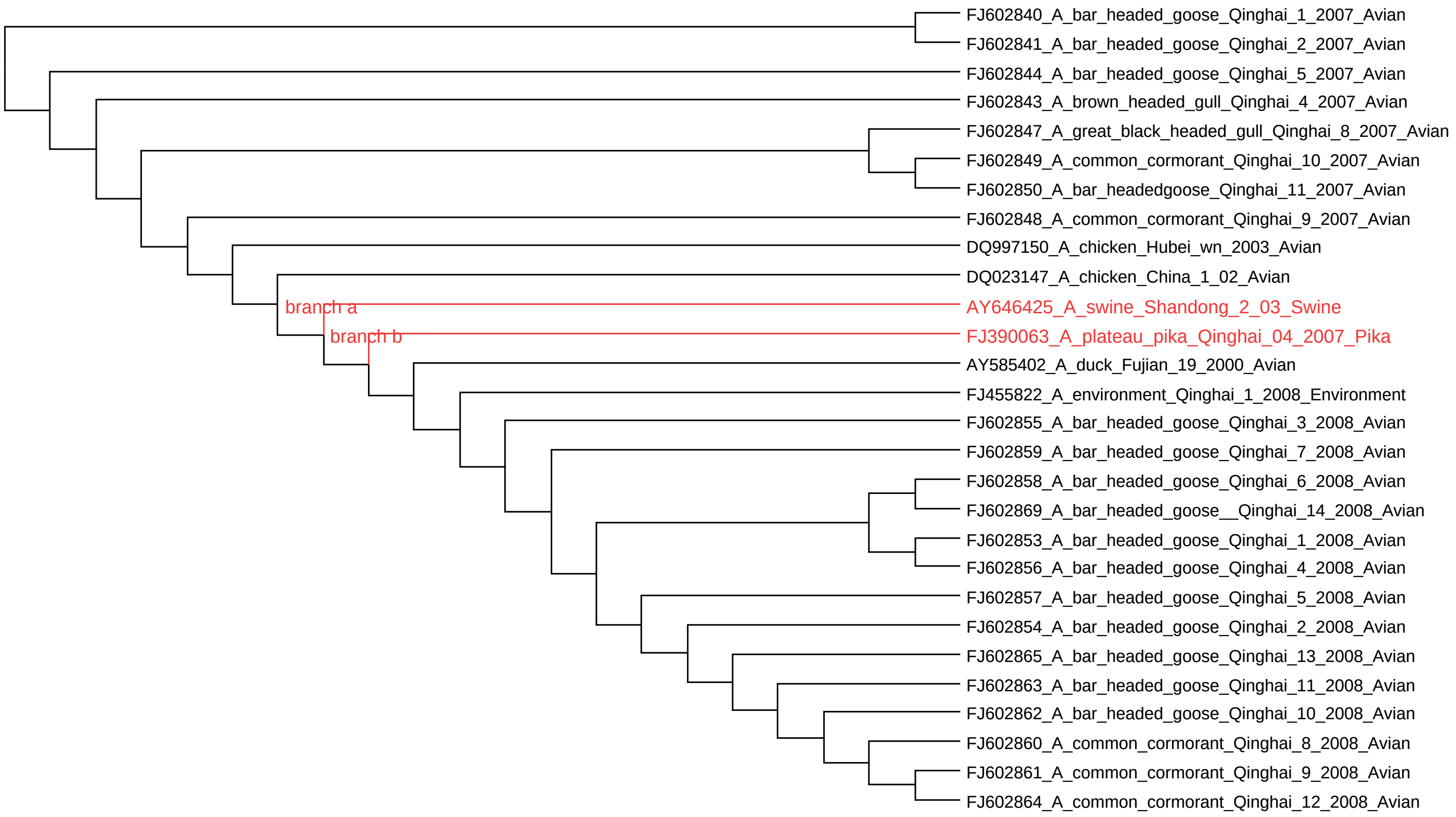

FJ602840_A_bar_headed_goose_Qinghai_1_2007_Avian
FJ602841_A_bar_headed_goose_Qinghai_2_2007_Avian
FJ602844_A_bar_headed_goose_Qinghai_5_2007_Avian
FJ602843_A_brown_headed_gull_Qinghai_4_2007_Avian
FJ602847_A_great_black_headed_gull_Qinghai_8_2007_Avian
FJ602849_A_common_cormorant_Qinghai_10_2007_Avian
FJ602850_A_bar_headedgoose_Qinghai_11_2007_Avian
FJ602848_A_common_cormorant_Qinghai_9_2007_Avian
DQ997150_A_chicken_Hubei_wn_2003_Avian
DQ023147_A_chicken_China_1_02_Avian
branch a
AY646425_A_swine_Shandong_2_03_Swine
branch b
FJ390063_A_plateau_pika_Qinghai_04_2007_Pika
AY585402_A_duck_Fujian_19_2000_Avian
FJ455822_A_environment_Qinghai_1_2008_Environment
FJ602855_A_bar_headed_goose_Qinghai_3_2008_Avian
FJ602859_A_bar_headed_goose_Qinghai_7_2008_Avian
FJ602858_A_bar_headed_goose_Qinghai_6_2008_Avian
FJ602869_A_bar_headed_goose__Qinghai_14_2008_Avian
FJ602853_A_bar_headed_goose_Qinghai_1_2008_Avian
FJ602856_A_bar_headed_goose_Qinghai_4_2008_Avian
FJ602857_A_bar_headed_goose_Qinghai_5_2008_Avian
FJ602854_A_bar_headed_goose_Qinghai_2_2008_Avian
FJ602865_A_bar_headed_goose_Qinghai_13_2008_Avian
FJ602863_A_bar_headed_goose_Qinghai_11_2008_Avian
FJ602862_A_bar_headed_goose_Qinghai_10_2008_Avian
FJ602860_A_common_cormorant_Qinghai_8_2008_Avian
FJ602861_A_common_cormorant_Qinghai_9_2008_Avian
FJ602864_A_common_cormorant_Qinghai_12_2008_Avian

NA1-Group82

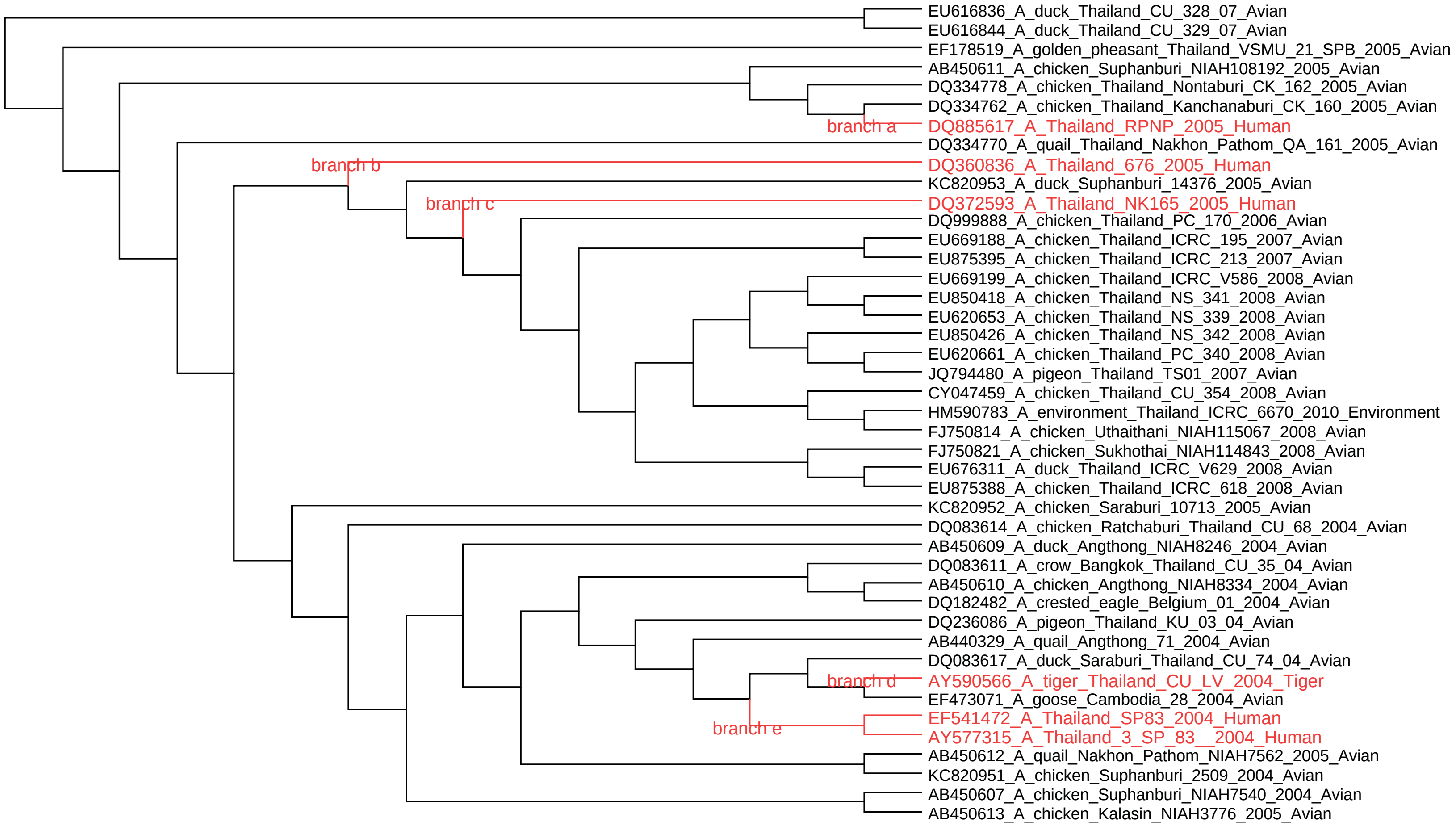
EU616836_A_duck_Thailand_CU_328_07_Avian
EU616844_A_duck_Thailand_CU_329_07_Avian
EF178519_A_golden_pheasant_Thailand_VSMU_21_SPB_2005_Avian
AB450611_A_chicken_Suphanburi_NIAH108192_2005_Avian
DQ334778_A_chicken_Thailand_Nontaburi_CK_162_2005_Avian
DQ334762_A_chicken_Thailand_Kanchanaburi_CK_160_2005_Avian
branch a
DQ885617_A_Thailand_RPNP_2005_Human
DQ334770_A_quail_Thailand_Nakhon_Pathom_QA_161_2005_Avian
branch b
DQ360836_A_Thailand_676_2005_Human
KC820953_A_duck_Suphanburi_14376_2005_Avian
branch c
DQ372593_A_Thailand_NK165_2005_Human
DQ999888_A_chicken_Thailand_PC_170_2006_Avian
EU669188_A_chicken_Thailand_ICRC_195_2007_Avian
EU875395_A_chicken_Thailand_ICRC_213_2007_Avian
EU669199_A_chicken_Thailand_ICRC_V586_2008_Avian
EU850418_A_chicken_Thailand_NS_341_2008_Avian
EU620653_A_chicken_Thailand_NS_339_2008_Avian
EU850426_A_chicken_Thailand_NS_342_2008_Avian
EU620661_A_chicken_Thailand_PC_340_2008_Avian
JQ794480_A_pigeon_Thailand_TS01_2007_Avian
CY047459_A_chicken_Thailand_CU_354_2008_Avian
HM590783_A_environment_Thailand_ICRC_6670_2010_Environment
FJ750814_A_chicken_Uthaithani_NIAH115067_2008_Avian
FJ750821_A_chicken_Sukhothai_NIAH114843_2008_Avian
EU676311_A_duck_Thailand_ICRC_V629_2008_Avian
EU875388_A_chicken_Thailand_ICRC_618_2008_Avian
KC820952_A_chicken_Saraburi_10713_2005_Avian
DQ083614_A_chicken_Ratchaburi_Thailand_CU_68_2004_Avian
AB450609_A_duck_Angthong_NIAH8246_2004_Avian
DQ083611_A_crow_Bangkok_Thailand_CU_35_04_Avian
AB450610_A_chicken_Angthong_NIAH8334_2004_Avian
DQ182482_A_crested_eagle_Belgium_01_2004_Avian
DQ236086_A_pigeon_Thailand_KU_03_04_Avian
AB440329_A_quail_Angthong_71_2004_Avian
DQ083617_A_duck_Saraburi_Thailand_CU_74_04_Avian
branch d
AY590566_A_tiger_Thailand_CU_LV_2004_Tiger
EF473071_A_goose_Cambodia_28_2004_Avian
branch e
EF541472_A_Thailand_SP83_2004_Human
AY577315_A_Thailand_3_SP_83__2004_Human
AB450612_A_quail_Nakhon_Pathom_NIAH7562_2005_Avian
KC820951_A_chicken_Suphanburi_2509_2004_Avian
AB450607_A_chicken_Suphanburi_NIAH7540_2004_Avian
AB450613_A_chicken_Kalasin_NIAH3776_2005_Avian

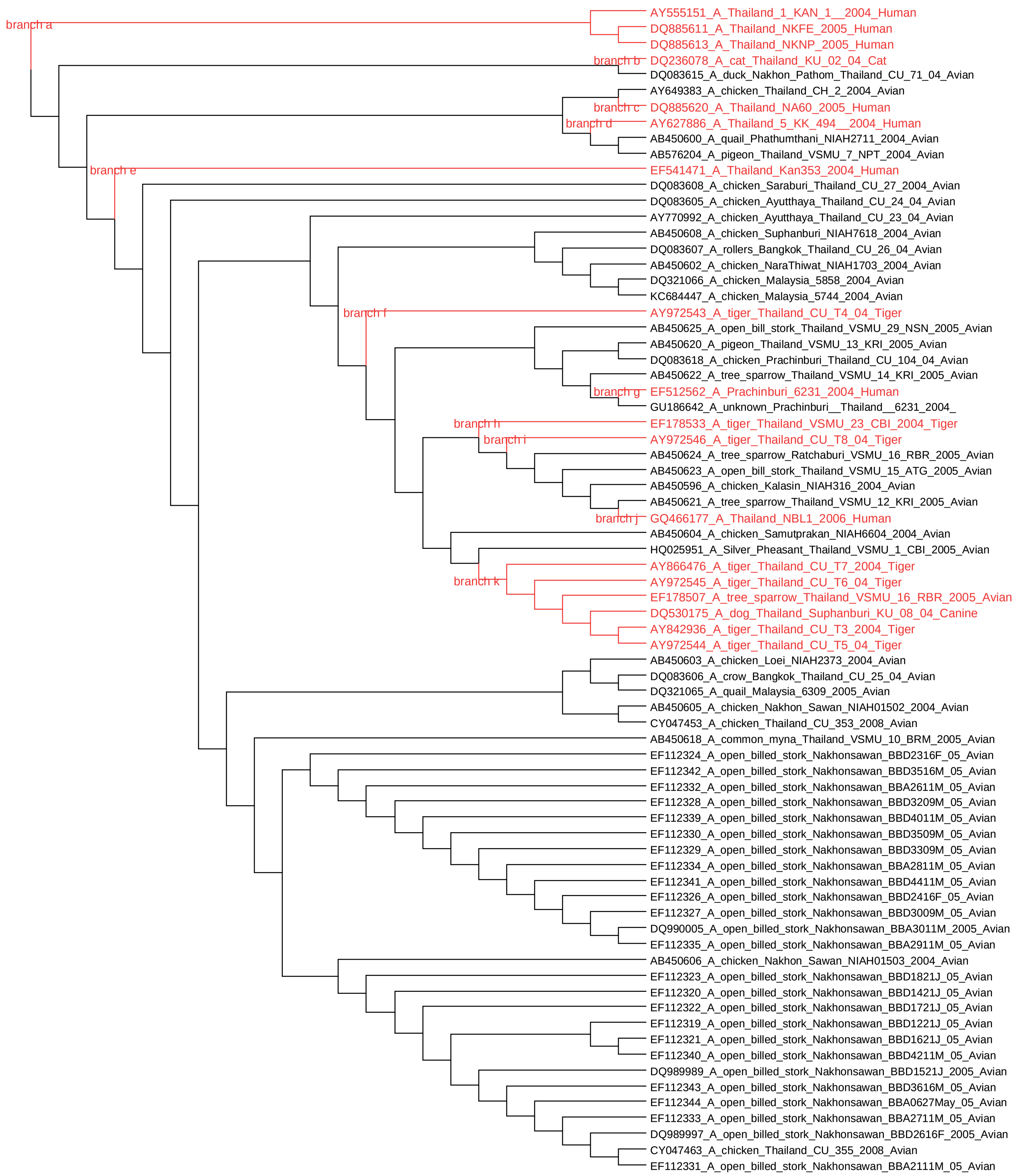

NA1-Group83

NA1-Group83

NA1-Group84

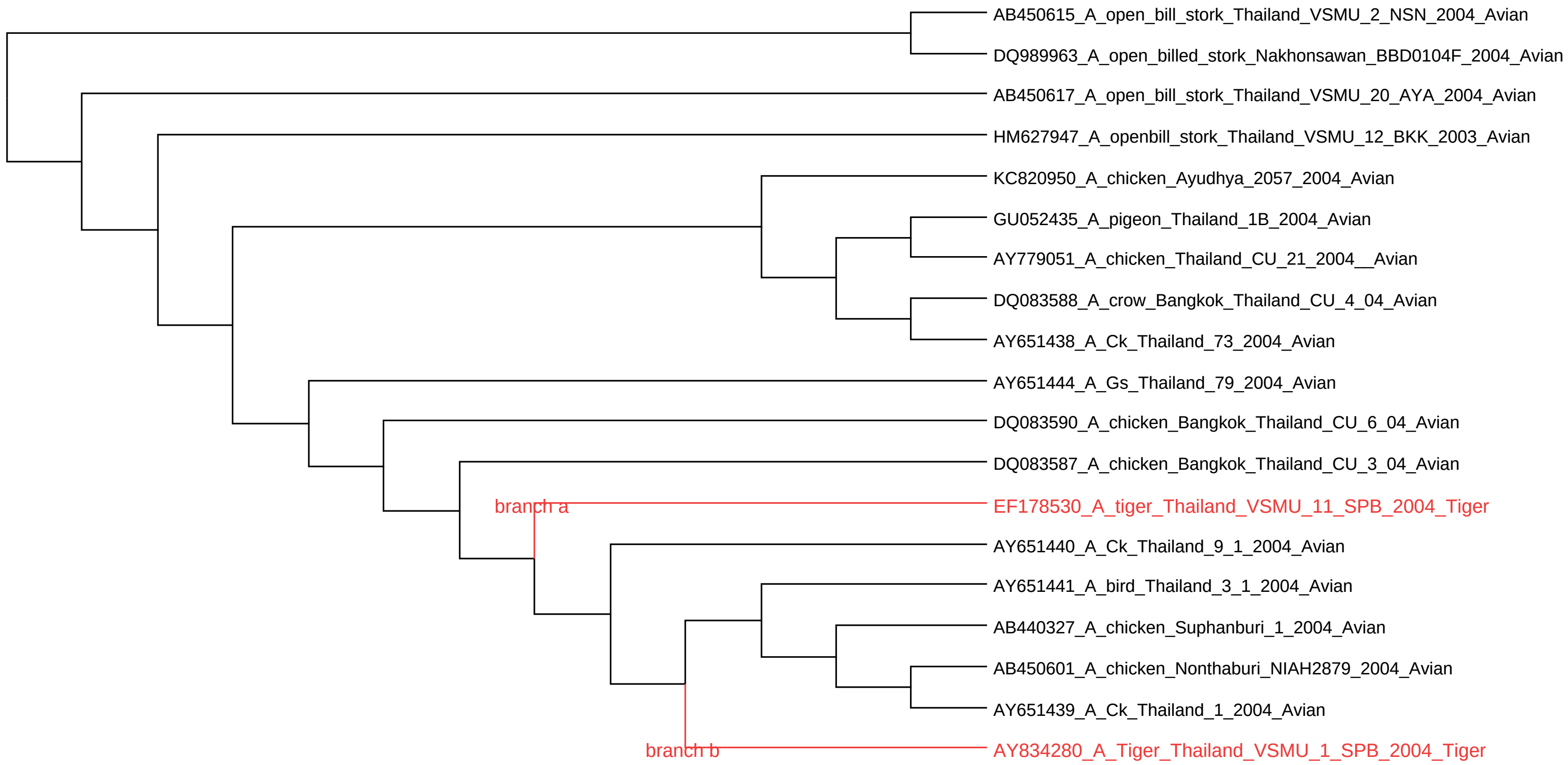

AB450615_A_open_bill_stork_Thailand_VSMU_2_NSN_2004_Avian
DQ989963_A_open_billed_stork_Nakhonsawan_BBD0104F_2004_Avian
AB450617_A_open_bill_stork_Thailand_VSMU_20_AYA_2004_Avian
HM627947_A_openbill_stork_Thailand_VSMU_12_BKK_2003_Avian
KC820950_A_chicken_Ayudhya_2057_2004_Avian
GU052435_A_pigeon_Thailand_1B_2004_Avian
AY779051_A_chicken_Thailand_CU_21_2004__Avian
DQ083588_A_crow_Bangkok_Thailand_CU_4_04_Avian
AY651438_A_Ck_Thailand_73_2004_Avian
AY651444_A_Gs_Thailand_79_2004_Avian
DQ083590_A_chicken_Bangkok_Thailand_CU_6_04_Avian
DQ083587_A_chicken_Bangkok_Thailand_CU_3_04_Avian
branch a
EF178530_A_tiger_Thailand_VSMU_11_SPB_2004_Tiger
AY651440_A_Ck_Thailand_9_1_2004_Avian
AY651441_A_bird_Thailand_3_1_2004_Avian
AB440327_A_chicken_Suphanburi_1_2004_Avian
AB450601_A_chicken_Nonthaburi_NIAH2879_2004_Avian
AY651439_A_Ck_Thailand_1_2004_Avian
branch b
AY834280_A_Tiger_Thailand_VSMU_1_SPB_2004_Tiger

NA1-Group85

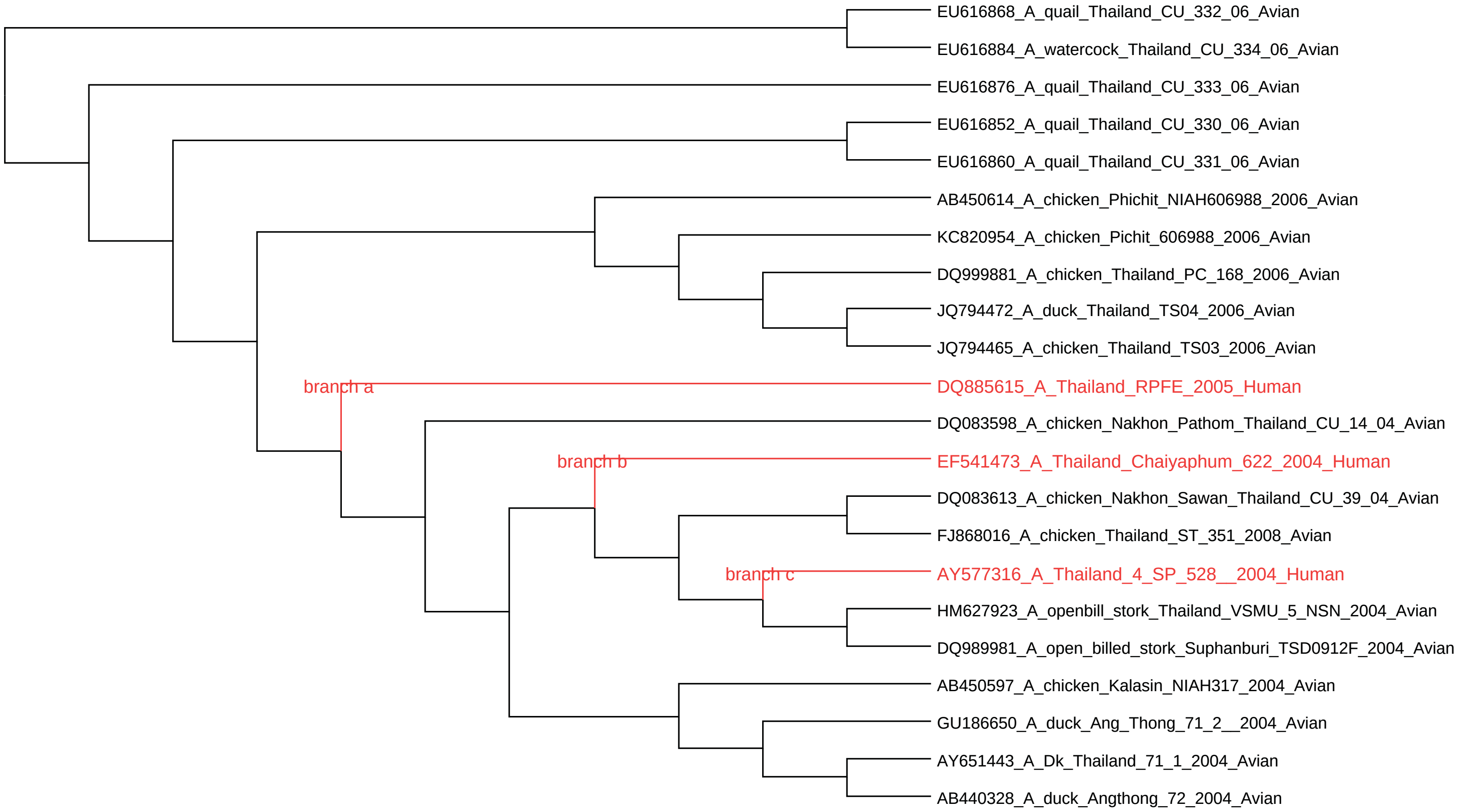
EU616868_A_quail_Thailand_CU_332_06_Avian
EU616884_A_watercock_Thailand_CU_334_06_Avian
EU616876_A_quail_Thailand_CU_333_06_Avian
EU616852_A_quail_Thailand_CU_330_06_Avian
EU616860_A_quail_Thailand_CU_331_06_Avian
AB450614_A_chicken_Phichit_NIAH606988_2006_Avian
KC820954_A_chicken_Pichit_606988_2006_Avian
DQ999881_A_chicken_Thailand_PC_168_2006_Avian
JQ794472_A_duck_Thailand_TS04_2006_Avian
JQ794465_A_chicken_Thailand_TS03_2006_Avian
branch a
DQ885615_A_Thailand_RPFE_2005_Human
DQ083598_A_chicken_Nakhon_Pathom_Thailand_CU_14_04_Avian
branch b
EF541473_A_Thailand_Chaiyaphum_622_2004_Human
DQ083613_A_chicken_Nakhon_Sawan_Thailand_CU_39_04_Avian
FJ868016_A_chicken_Thailand_ST_351_2008_Avian
branch c
AY577316_A_Thailand_4_SP_528__2004_Human
HM627923_A_openbill_stork_Thailand_VSMU_5_NSN_2004_Avian
DQ989981_A_open_billed_stork_Suphanburi_TSD0912F_2004_Avian
AB450597_A_chicken_Kalasin_NIAH317_2004_Avian
GU186650_A_duck_Ang_Thong_71_2__2004_Avian
AY651443_A_Dk_Thailand_71_1_2004_Avian
AB440328_A_duck_Angthong_72_2004_Avian

NA1-Group86

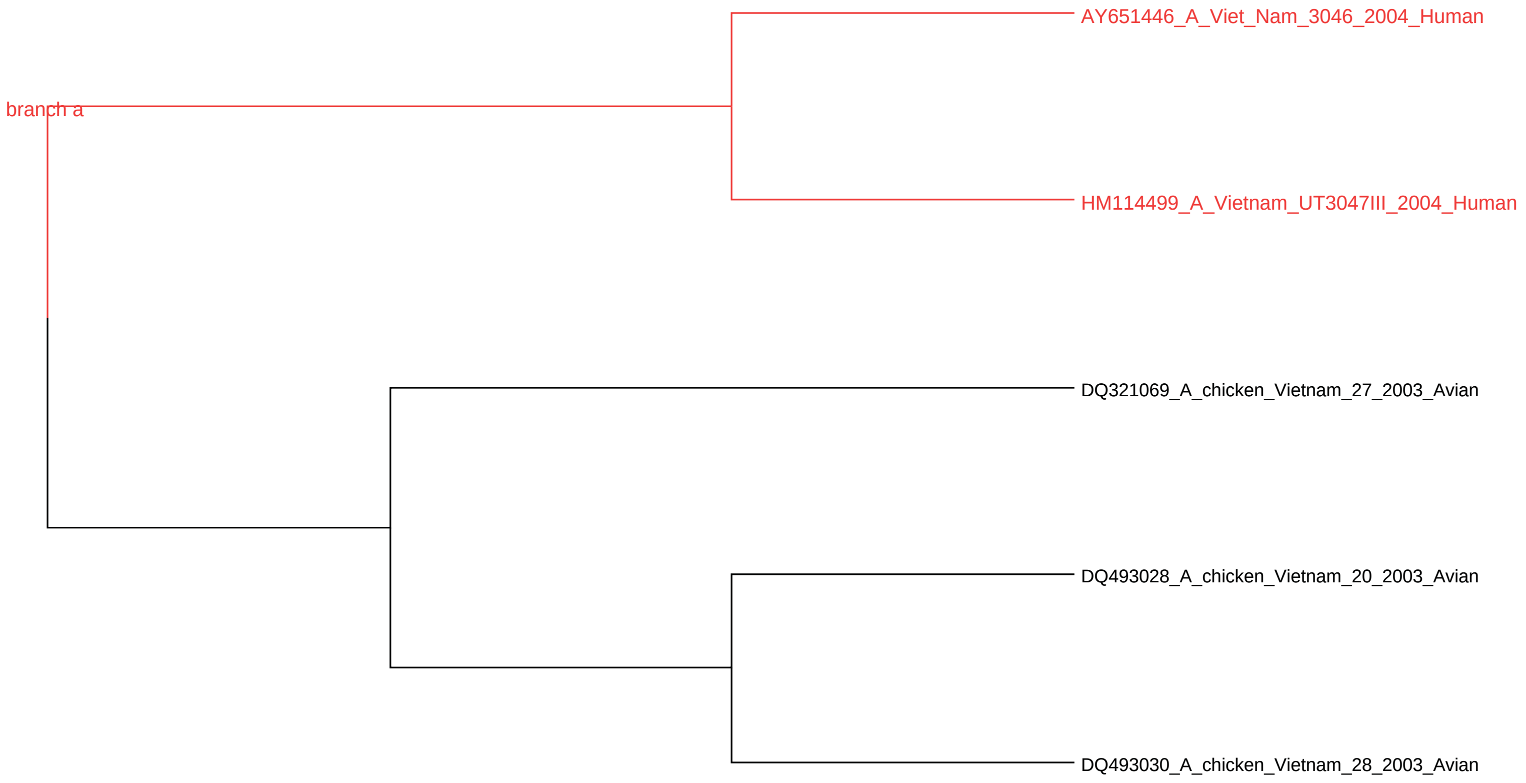
branch a
AY651446_A_Viet_Nam_3046_2004_Human
HM114499_A_Vietnam_UT3047III_2004_Human
DQ321069_A_chicken_Vietnam_27_2003_Avian
DQ493028_A_chicken_Vietnam_20_2003_Avian
DQ493030_A_chicken_Vietnam_28_2003_Avian

NA1-Group87

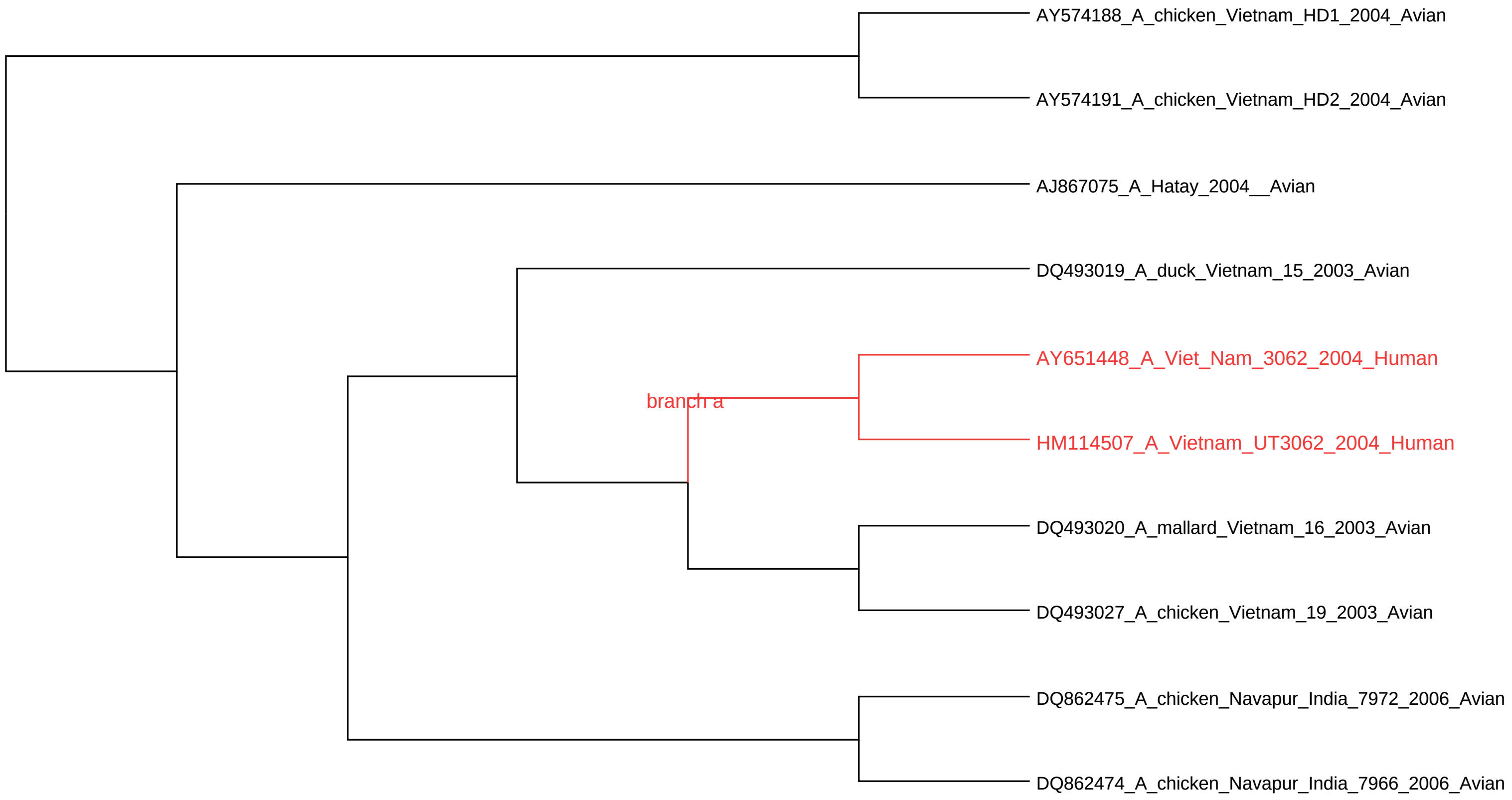

AY574188_A_chicken_Vietnam_HD1_2004_Avian
AY574191_A_chicken_Vietnam_HD2_2004_Avian
AJ867075_A_Hatay_2004__Avian
DQ493019_A_duck_Vietnam_15_2003_Avian
AY651448_A_Viet_Nam_3062_2004_Human
branch a
HM114507_A_Vietnam_UT3062_2004_Human
DQ493020_A_mallard_Vietnam_16_2003_Avian
DQ493027_A_chicken_Vietnam_19_2003_Avian
DQ862475_A_chicken_Navapur_India_7972_2006_Avian
DQ862474_A_chicken_Navapur_India_7966_2006_Avian

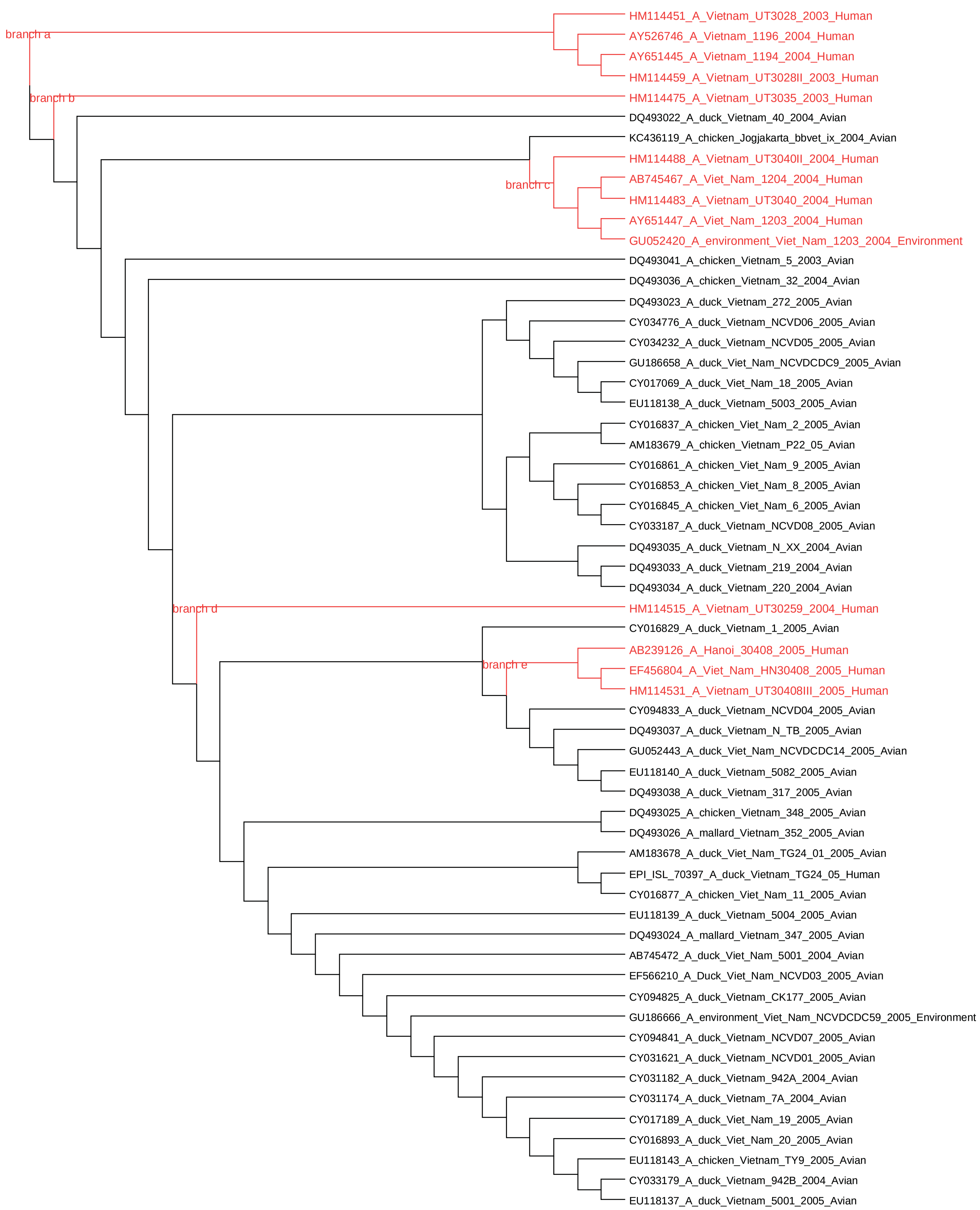

NA1-Group88

NA1-Group88

NA1-Group89

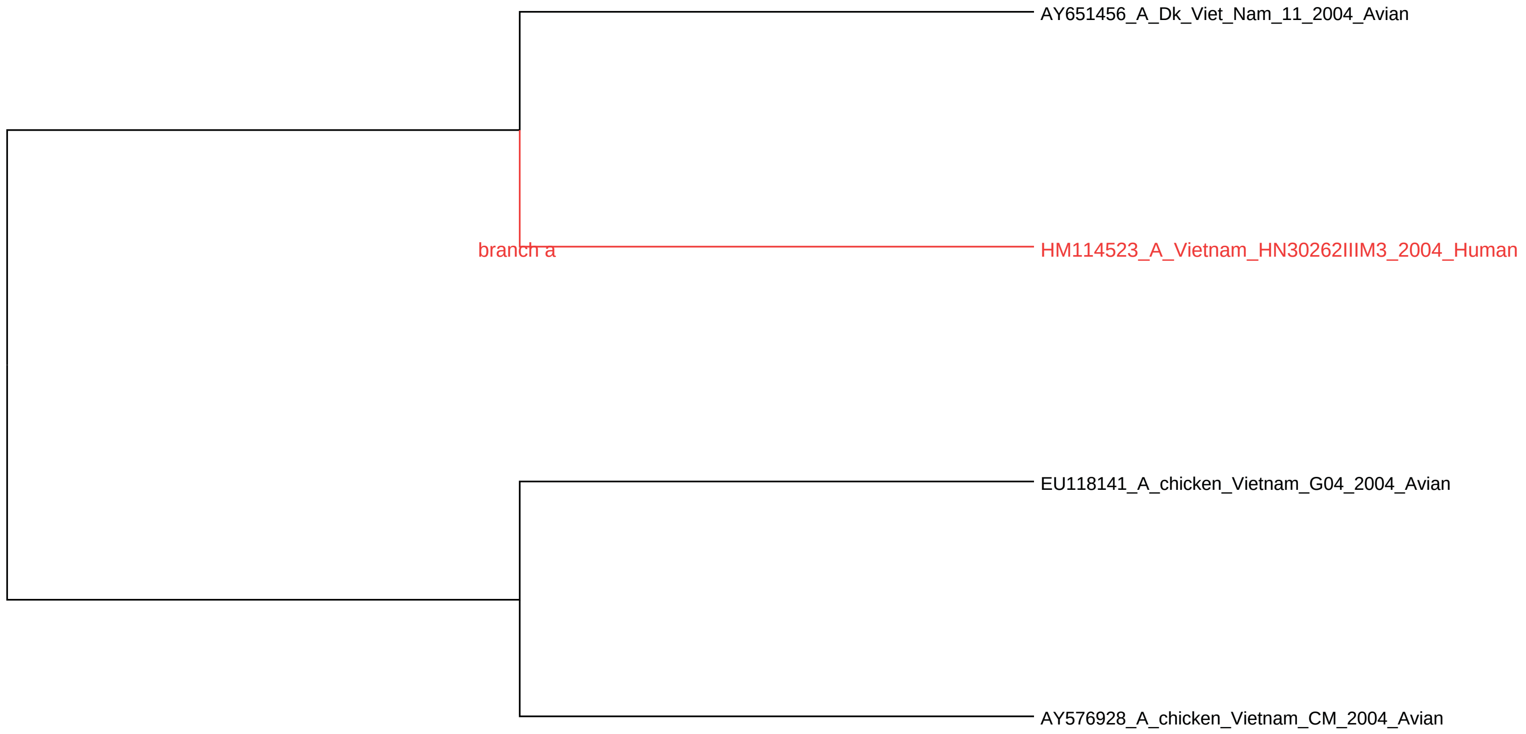

AY651456_A_Dk_Viet_Nam_11_2004_Avian
branch a
HM114523_A_Vietnam_HN30262IIIM3_2004_Human
EU118141_A_chicken_Vietnam_G04_2004_Avian
AY576928_A_chicken_Vietnam_CM_2004_Avian

NA1-Group90

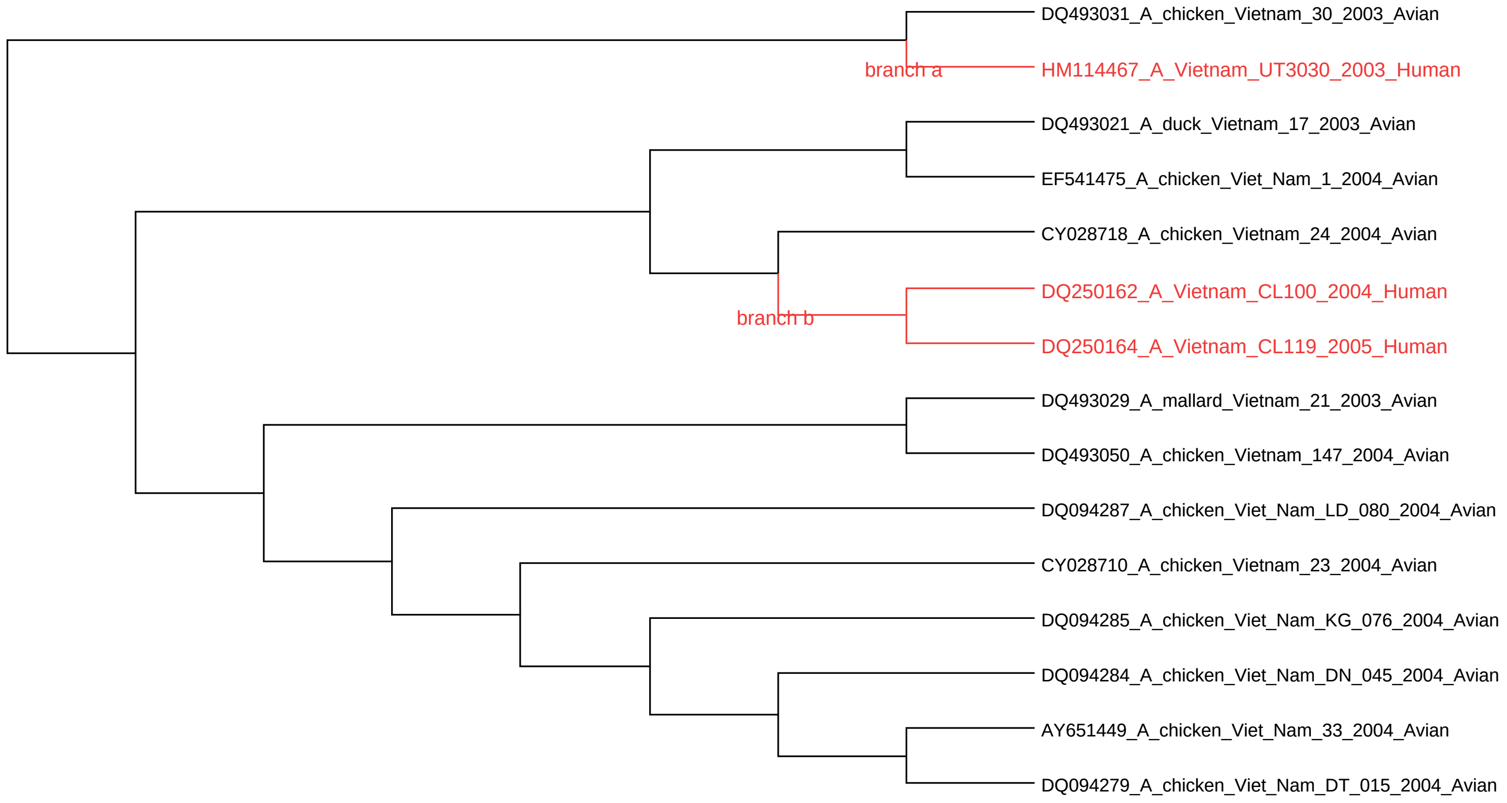
DQ493031_A_chicken_Vietnam_30_2003_Avian
branch a
HM114467_A_Vietnam_UT3030_2003_Human
DQ493021_A_duck_Vietnam_17_2003_Avian
EF541475_A_chicken_Viet_Nam_1_2004_Avian
CY028718_A_chicken_Vietnam_24_2004_Avian
DQ250162_A_Vietnam_CL100_2004_Human
branch b
DQ250164_A_Vietnam_CL119_2005_Human
DQ493029_A_mallard_Vietnam_21_2003_Avian
DQ493050_A_chicken_Vietnam_147_2004_Avian
DQ094287_A_chicken_Viet_Nam_LD_080_2004_Avian
CY028710_A_chicken_Vietnam_23_2004_Avian
DQ094285_A_chicken_Viet_Nam_KG_076_2004_Avian
DQ094284_A_chicken_Viet_Nam_DN_045_2004_Avian
AY651449_A_chicken_Viet_Nam_33_2004_Avian
DQ094279_A_chicken_Viet_Nam_DT_015_2004_Avian

NA1-Group91

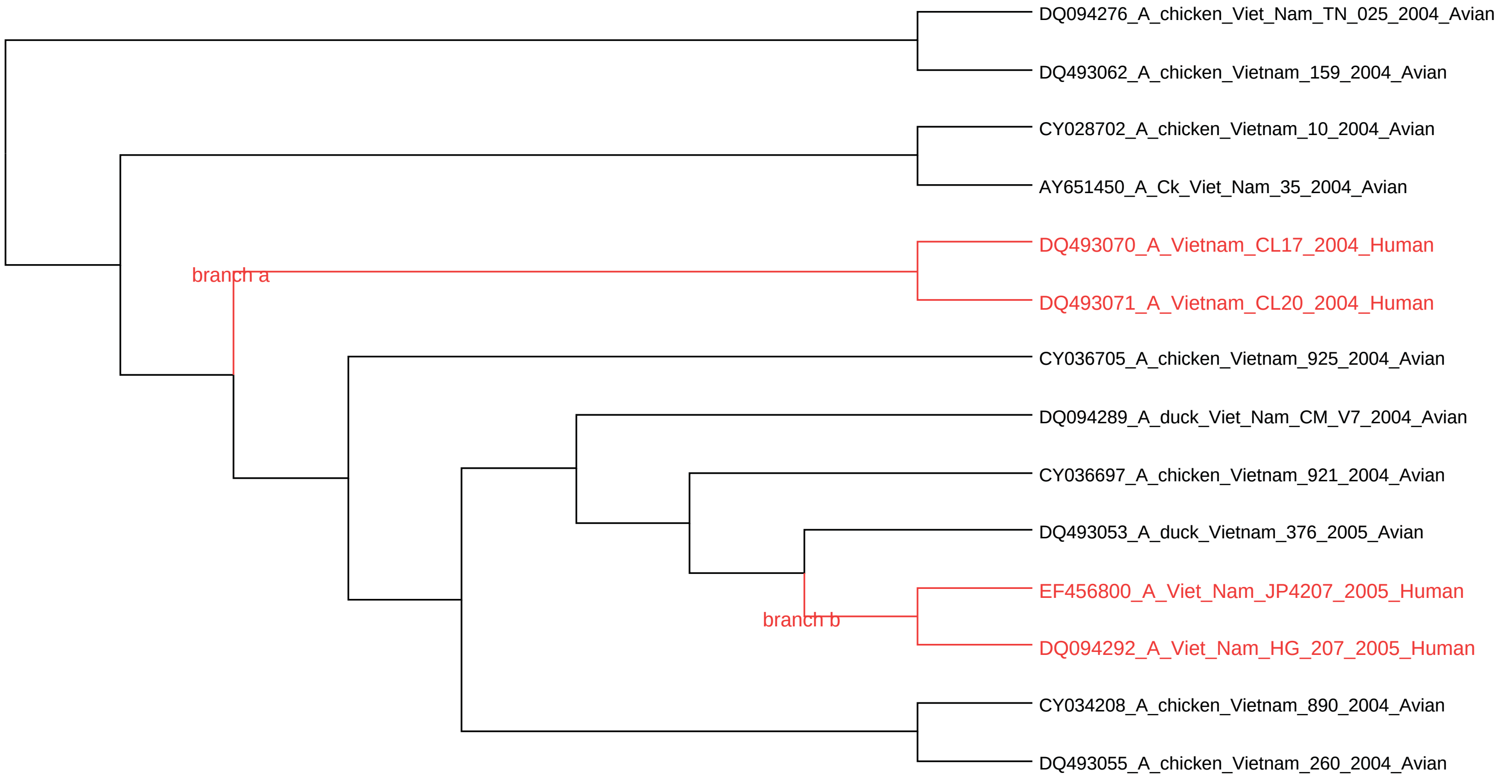

DQ094276_A_chicken_Viet_Nam_TN_025_2004_Avian
DQ493062_A_chicken_Vietnam_159_2004_Avian
CY028702_A_chicken_Vietnam_10_2004_Avian
AY651450_A_Ck_Viet_Nam_35_2004_Avian
branch a
DQ493070_A_Vietnam_CL17_2004_Human
DQ493071_A_Vietnam_CL20_2004_Human
CY036705_A_chicken_Vietnam_925_2004_Avian
DQ094289_A_duck_Viet_Nam_CM_V7_2004_Avian
CY036697_A_chicken_Vietnam_921_2004_Avian
DQ493053_A_duck_Vietnam_376_2005_Avian
branch b
EF456800_A_Viet_Nam_JP4207_2005_Human
DQ094292_A_Viet_Nam_HG_207_2005_Human
CY034208_A_chicken_Vietnam_890_2004_Avian
DQ493055_A_chicken_Vietnam_260_2004_Avian

NA1-Group92

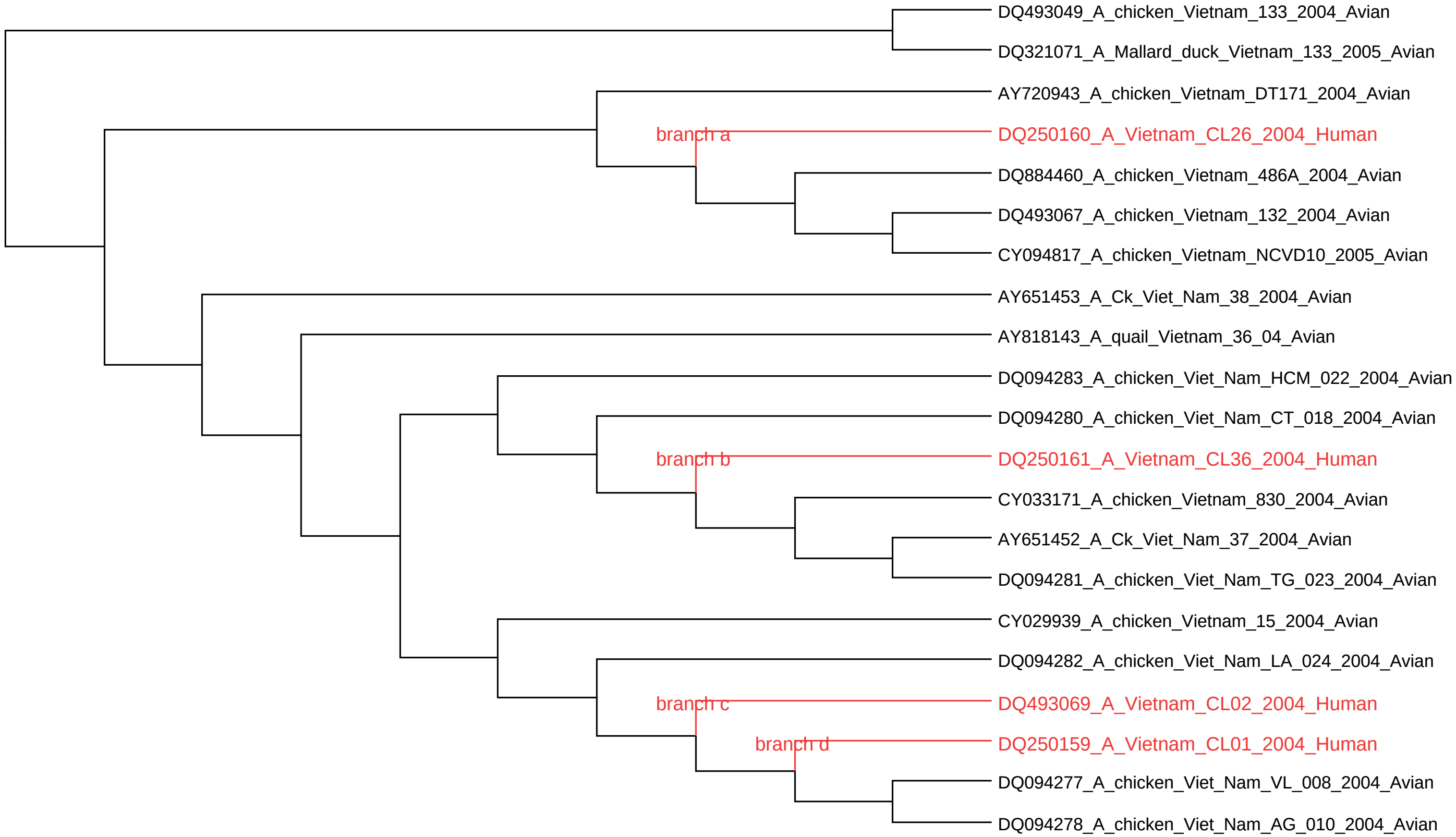
DQ493049_A_chicken_Vietnam_133_2004_Avian
DQ321071_A_Mallard_duck_Vietnam_133_2005_Avian
AY720943_A_chicken_Vietnam_DT171_2004_Avian
branch a
DQ250160_A_Vietnam_CL26_2004_Human
DQ884460_A_chicken_Vietnam_486A_2004_Avian
DQ493067_A_chicken_Vietnam_132_2004_Avian
CY094817_A_chicken_Vietnam_NCVD10_2005_Avian
AY651453_A_Ck_Viet_Nam_38_2004_Avian
AY818143_A_quail_Vietnam_36_04_Avian
DQ094283_A_chicken_Viet_Nam_HCM_022_2004_Avian
DQ094280_A_chicken_Viet_Nam_CT_018_2004_Avian
branch b
DQ250161_A_Vietnam_CL36_2004_Human
CY033171_A_chicken_Vietnam_830_2004_Avian
AY651452_A_Ck_Viet_Nam_37_2004_Avian
DQ094281_A_chicken_Viet_Nam_TG_023_2004_Avian
CY029939_A_chicken_Vietnam_15_2004_Avian
DQ094282_A_chicken_Viet_Nam_LA_024_2004_Avian
branch c
DQ493069_A_Vietnam_CL02_2004_Human
branch d
DQ250159_A_Vietnam_CL01_2004_Human
DQ094277_A_chicken_Viet_Nam_VL_008_2004_Avian
DQ094278_A_chicken_Viet_Nam_AG_010_2004_Avian

NA1-Group93

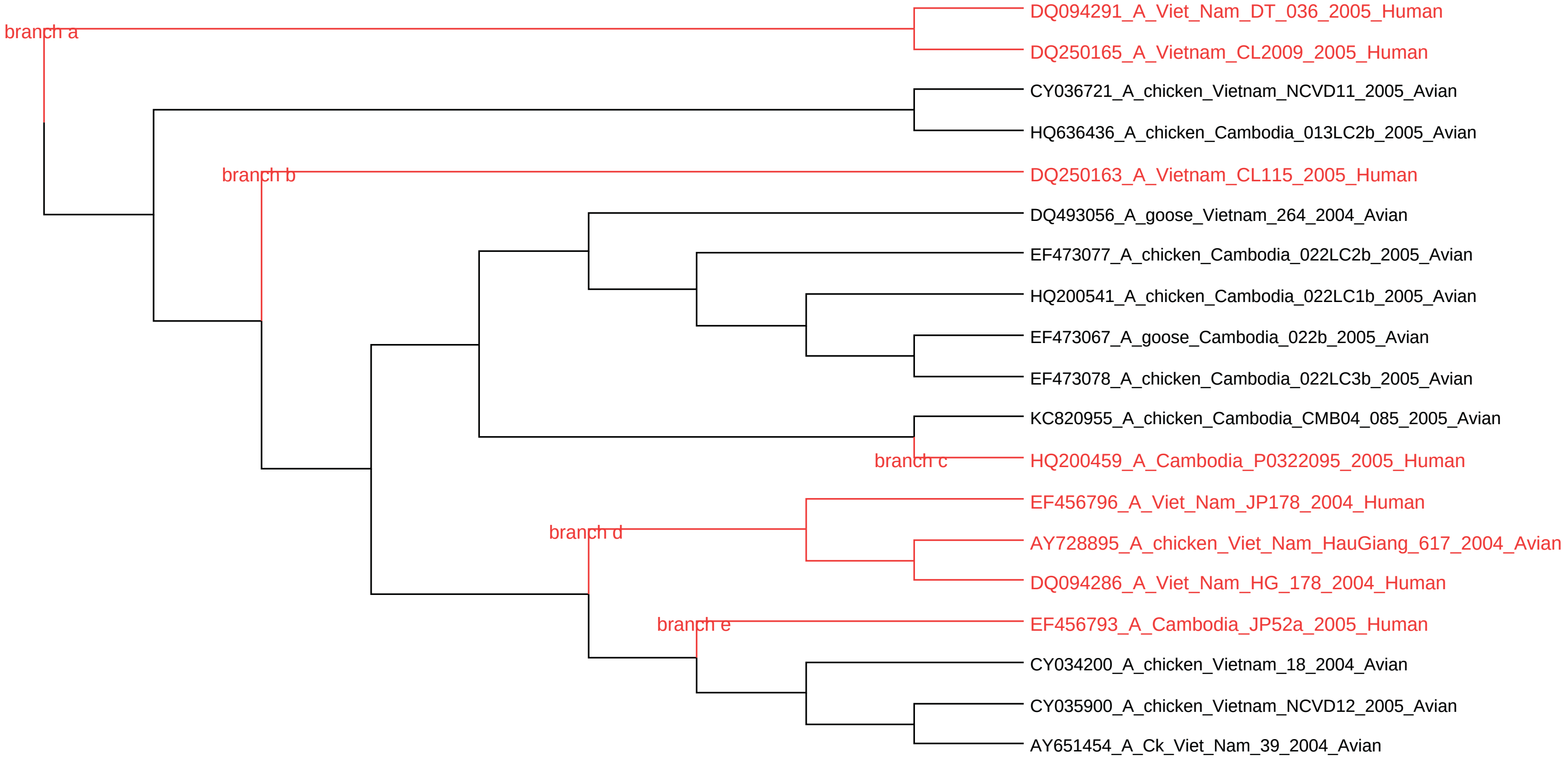
branch a
branch b
branch c
branch d
branch e
DQ094291_A_Viet_Nam_DT_036_2005_Human
DQ250165_A_Vietnam_CL2009_2005_Human
CY036721_A_chicken_Vietnam_NCVD11_2005_Avian
HQ636436_A_chicken_Cambodia_013LC2b_2005_Avian
DQ250163_A_Vietnam_CL115_2005_Human
DQ493056_A_goose_Vietnam_264_2004_Avian
EF473077_A_chicken_Cambodia_022LC2b_2005_Avian
HQ200541_A_chicken_Cambodia_022LC1b_2005_Avian
EF473067_A_goose_Cambodia_022b_2005_Avian
EF473078_A_chicken_Cambodia_022LC3b_2005_Avian
KC820955_A_chicken_Cambodia_CMB04_085_2005_Avian
HQ200459_A_Cambodia_P0322095_2005_Human
EF456796_A_Viet_Nam_JP178_2004_Human
AY728895_A_chicken_Viet_Nam_HauGiang_617_2004_Avian
DQ094286_A_Viet_Nam_HG_178_2004_Human
EF456793_A_Cambodia_JP52a_2005_Human
CY034200_A_chicken_Vietnam_18_2004_Avian
CY035900_A_chicken_Vietnam_NCVD12_2005_Avian
AY651454_A_Ck_Viet_Nam_39_2004_Avian

NA1-Group94

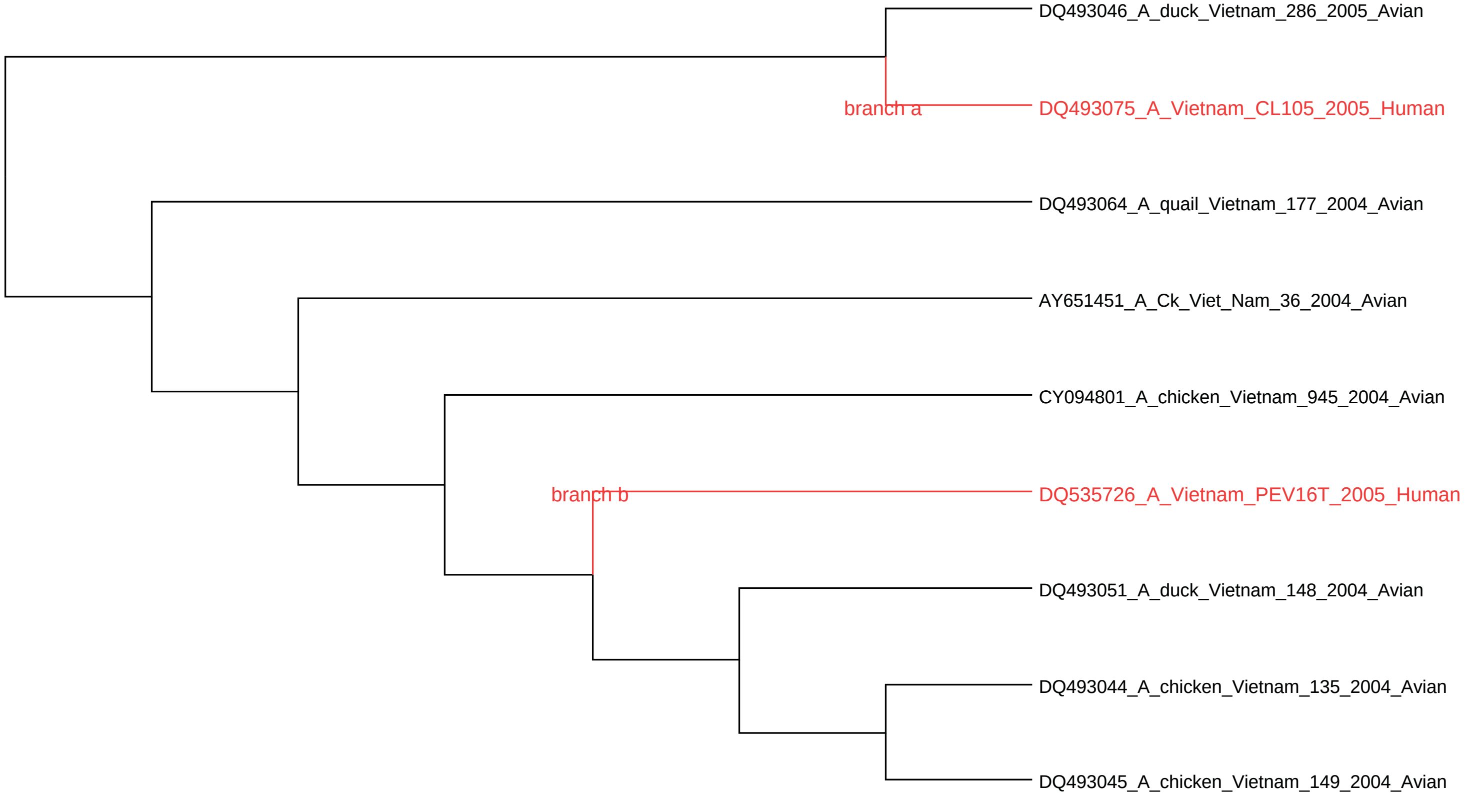
DQ493046_A_duck_Vietnam_286_2005_Avian
branch a
DQ493075_A_Vietnam_CL105_2005_Human
DQ493064_A_quail_Vietnam_177_2004_Avian
AY651451_A_Ck_Viet_Nam_36_2004_Avian
CY094801_A_chicken_Vietnam_945_2004_Avian
branch b
DQ535726_A_Vietnam_PEV16T_2005_Human
DQ493051_A_duck_Vietnam_148_2004_Avian
DQ493044_A_chicken_Vietnam_135_2004_Avian
DQ493045_A_chicken_Vietnam_149_2004_Avian

NA1-Group95

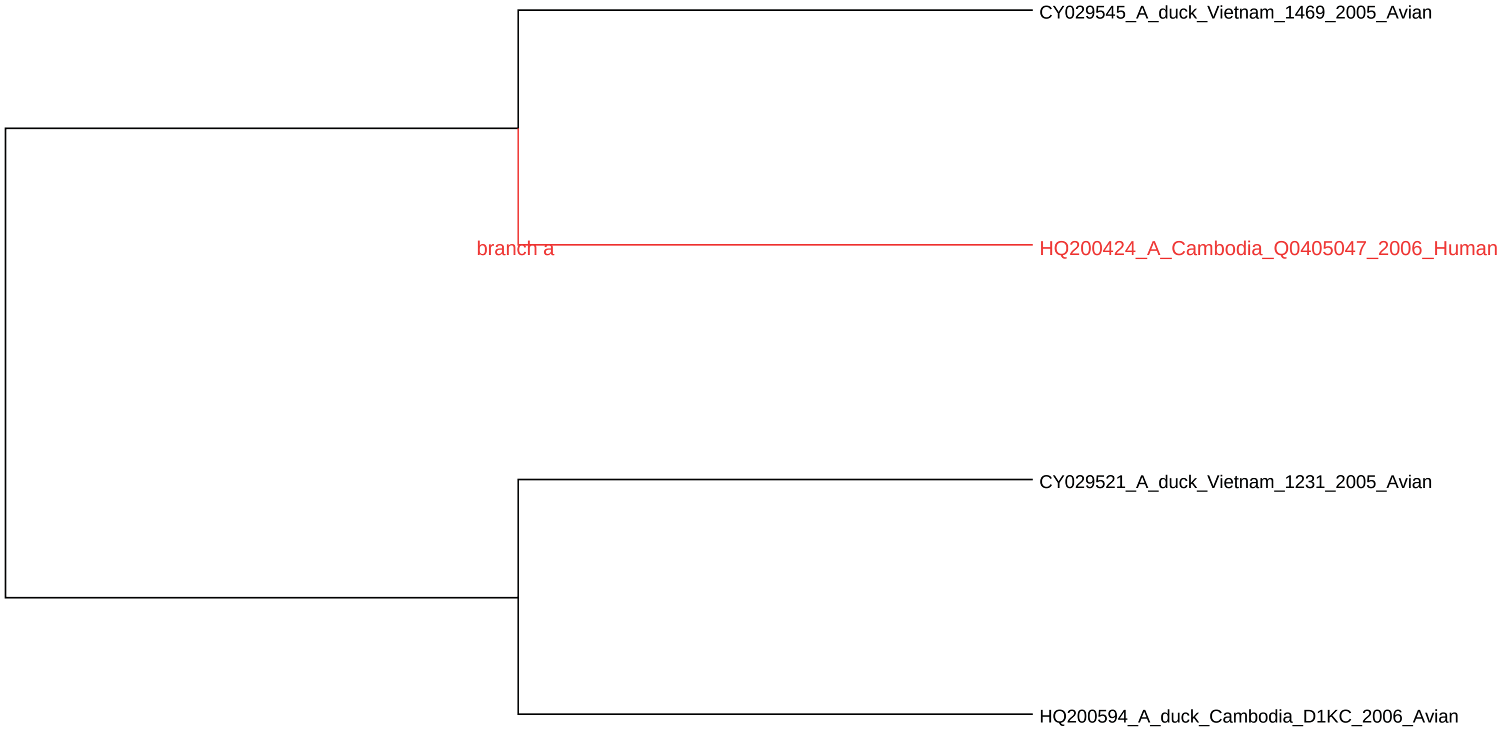

CY029545_A_duck_Vietnam_1469_2005_Avian
branch a
HQ200424_A_Cambodia_Q0405047_2006_Human
CY029521_A_duck_Vietnam_1231_2005_Avian
HQ200594_A_duck_Cambodia_D1KC_2006_Avian

NA1-Group96

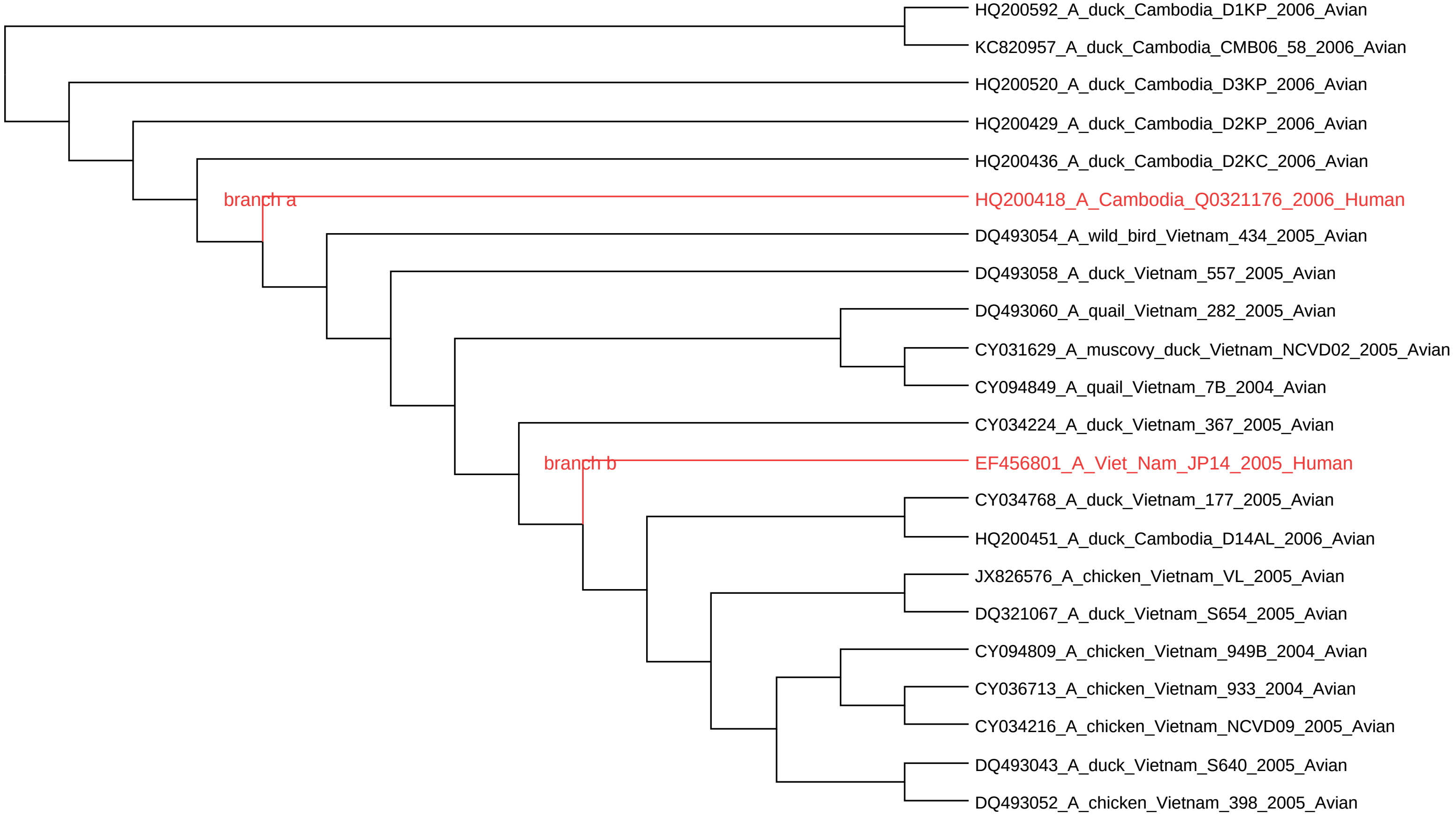
HQ200592_A_duck_Cambodia_D1KP_2006_Avian
KC820957_A_duck_Cambodia_CMB06_58_2006_Avian
HQ200520_A_duck_Cambodia_D3KP_2006_Avian
HQ200429_A_duck_Cambodia_D2KP_2006_Avian
HQ200436_A_duck_Cambodia_D2KC_2006_Avian
branch a
HQ200418_A_Cambodia_Q0321176_2006_Human
DQ493054_A_wild_bird_Vietnam_434_2005_Avian
DQ493058_A_duck_Vietnam_557_2005_Avian
DQ493060_A_quail_Vietnam_282_2005_Avian
CY031629_A_muscovy_duck_Vietnam_NCVD02_2005_Avian
CY094849_A_quail_Vietnam_7B_2004_Avian
CY034224_A_duck_Vietnam_367_2005_Avian
branch b
EF456801_A_Viet_Nam_JP14_2005_Human
CY034768_A_duck_Vietnam_177_2005_Avian
HQ200451_A_duck_Cambodia_D14AL_2006_Avian
JX826576_A_chicken_Vietnam_VL_2005_Avian
DQ321067_A_duck_Vietnam_S654_2005_Avian
CY094809_A_chicken_Vietnam_949B_2004_Avian
CY036713_A_chicken_Vietnam_933_2004_Avian
CY034216_A_chicken_Vietnam_NCVD09_2005_Avian
DQ493043_A_duck_Vietnam_S640_2005_Avian
DQ493052_A_chicken_Vietnam_398_2005_Avian

NA1-Group97

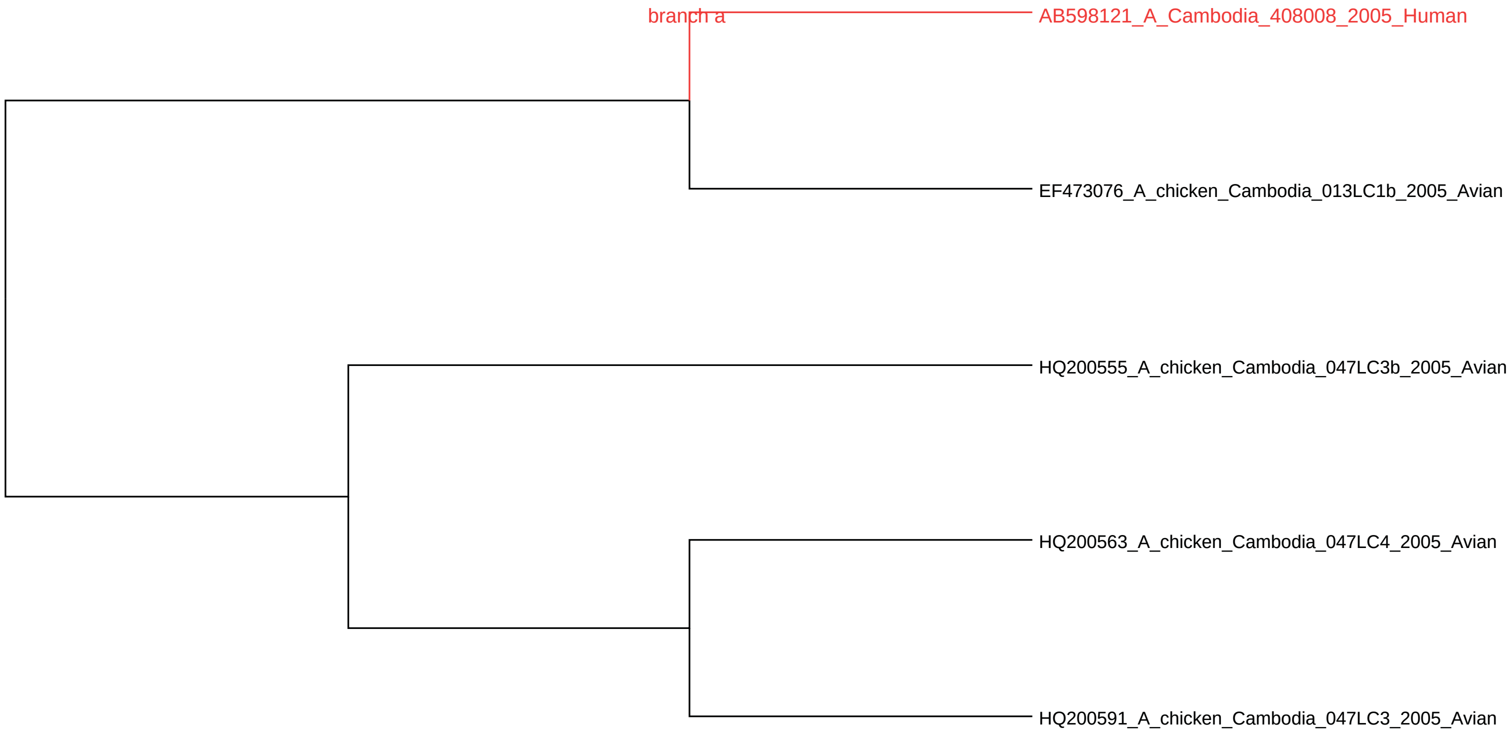

branch a
AB598121_A_Cambodia_408008_2005_Human
EF473076_A_chicken_Cambodia_013LC1b_2005_Avian
HQ200555_A_chicken_Cambodia_047LC3b_2005_Avian
HQ200563_A_chicken_Cambodia_047LC4_2005_Avian
HQ200591_A_chicken_Cambodia_047LC3_2005_Avian

NA1-Group98

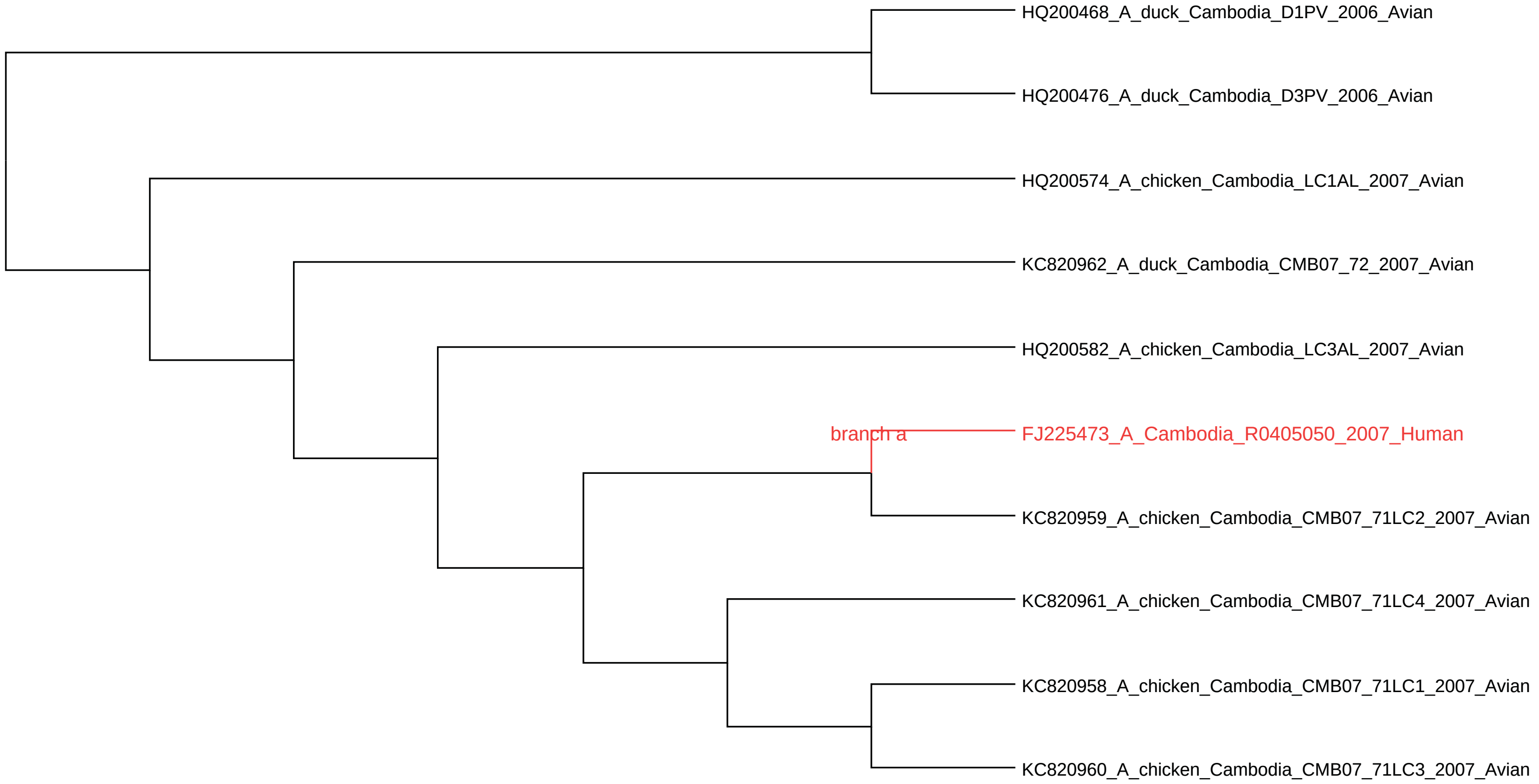

HQ200468_A_duck_Cambodia_D1PV_2006_Avian
HQ200476_A_duck_Cambodia_D3PV_2006_Avian
HQ200574_A_chicken_Cambodia_LC1AL_2007_Avian
KC820962_A_duck_Cambodia_CMB07_72_2007_Avian
HQ200582_A_chicken_Cambodia_LC3AL_2007_Avian
branch a
FJ225473_A_Cambodia_R0405050_2007_Human
KC820959_A_chicken_Cambodia_CMB07_71LC2_2007_Avian
KC820961_A_chicken_Cambodia_CMB07_71LC4_2007_Avian
KC820958_A_chicken_Cambodia_CMB07_71LC1_2007_Avian
KC820960_A_chicken_Cambodia_CMB07_71LC3_2007_Avian

NA1-Group99

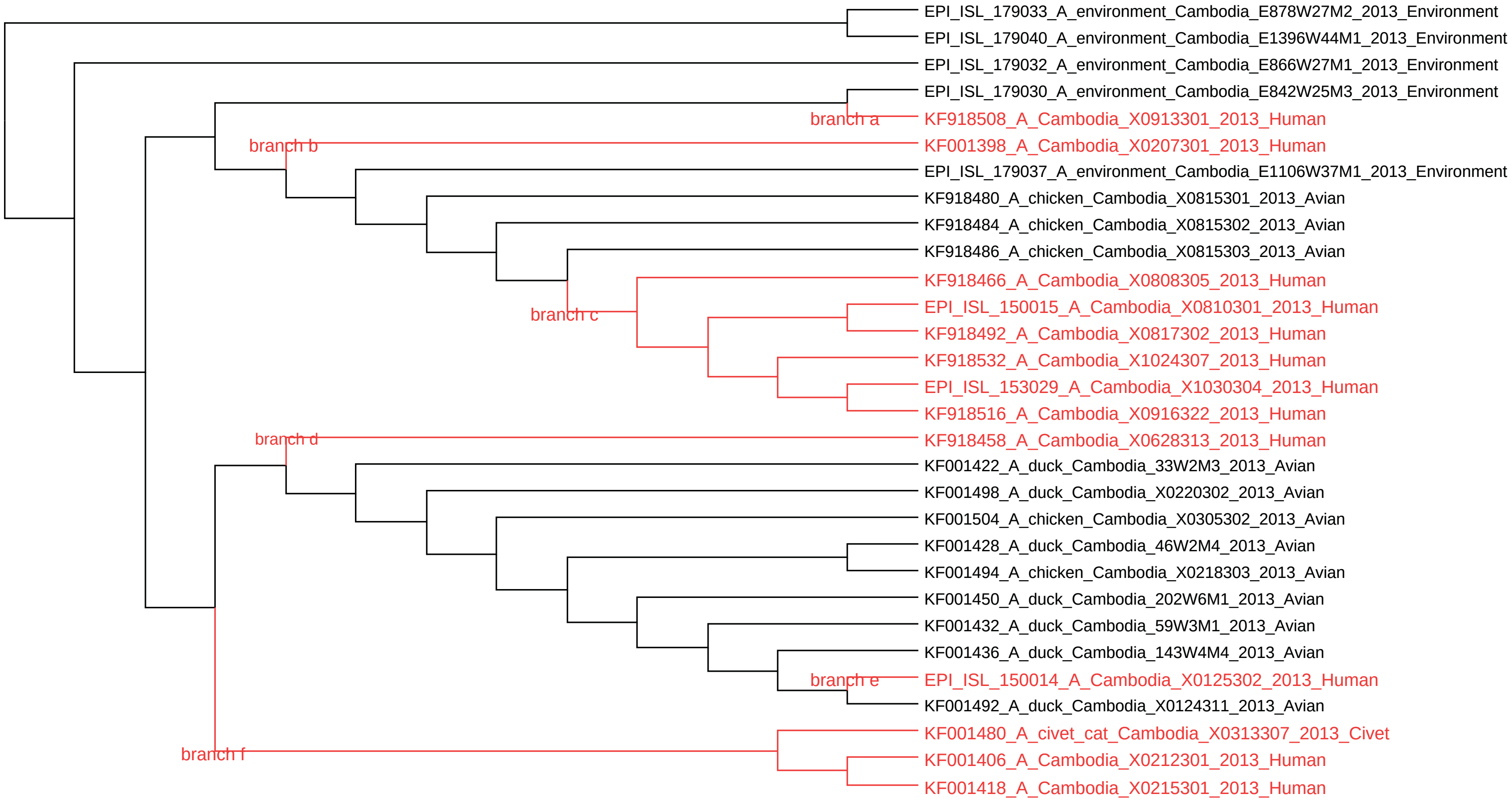

EPI_ISL_179033_A_environment_Cambodia_E878W27M2_2013_Environment
EPI_ISL_179040_A_environment_Cambodia_E1396W44M1_2013_Environment
EPI_ISL_179032_A_environment_Cambodia_E866W27M1_2013_Environment
EPI_ISL_179030_A_environment_Cambodia_E842W25M3_2013_Environment
branch a
KF918508_A_Cambodia_X0913301_2013_Human
branch b
KF001398_A_Cambodia_X0207301_2013_Human
EPI_ISL_179037_A_environment_Cambodia_E1106W37M1_2013_Environment
KF918480_A_chicken_Cambodia_X0815301_2013_Avian
KF918484_A_chicken_Cambodia_X0815302_2013_Avian
KF918486_A_chicken_Cambodia_X0815303_2013_Avian
KF918466_A_Cambodia_X0808305_2013_Human
branch c
EPI_ISL_150015_A_Cambodia_X0810301_2013_Human
KF918492_A_Cambodia_X0817302_2013_Human
KF918532_A_Cambodia_X1024307_2013_Human
EPI_ISL_153029_A_Cambodia_X1030304_2013_Human
KF918516_A_Cambodia_X0916322_2013_Human
branch d
KF918458_A_Cambodia_X0628313_2013_Human
KF001422_A_duck_Cambodia_33W2M3_2013_Avian
KF001498_A_duck_Cambodia_X0220302_2013_Avian
KF001504_A_chicken_Cambodia_X0305302_2013_Avian
KF001428_A_duck_Cambodia_46W2M4_2013_Avian
KF001494_A_chicken_Cambodia_X0218303_2013_Avian
KF001450_A_duck_Cambodia_202W6M1_2013_Avian
KF001432_A_duck_Cambodia_59W3M1_2013_Avian
KF001436_A_duck_Cambodia_143W4M4_2013_Avian
branch e
EPI_ISL_150014_A_Cambodia_X0125302_2013_Human
KF001492_A_duck_Cambodia_X0124311_2013_Avian
KF001480_A_civet_cat_Cambodia_X0313307_2013_Civet
branch f
KF001406_A_Cambodia_X0212301_2013_Human
KF001418_A_Cambodia_X0215301_2013_Human

NA1-Group100

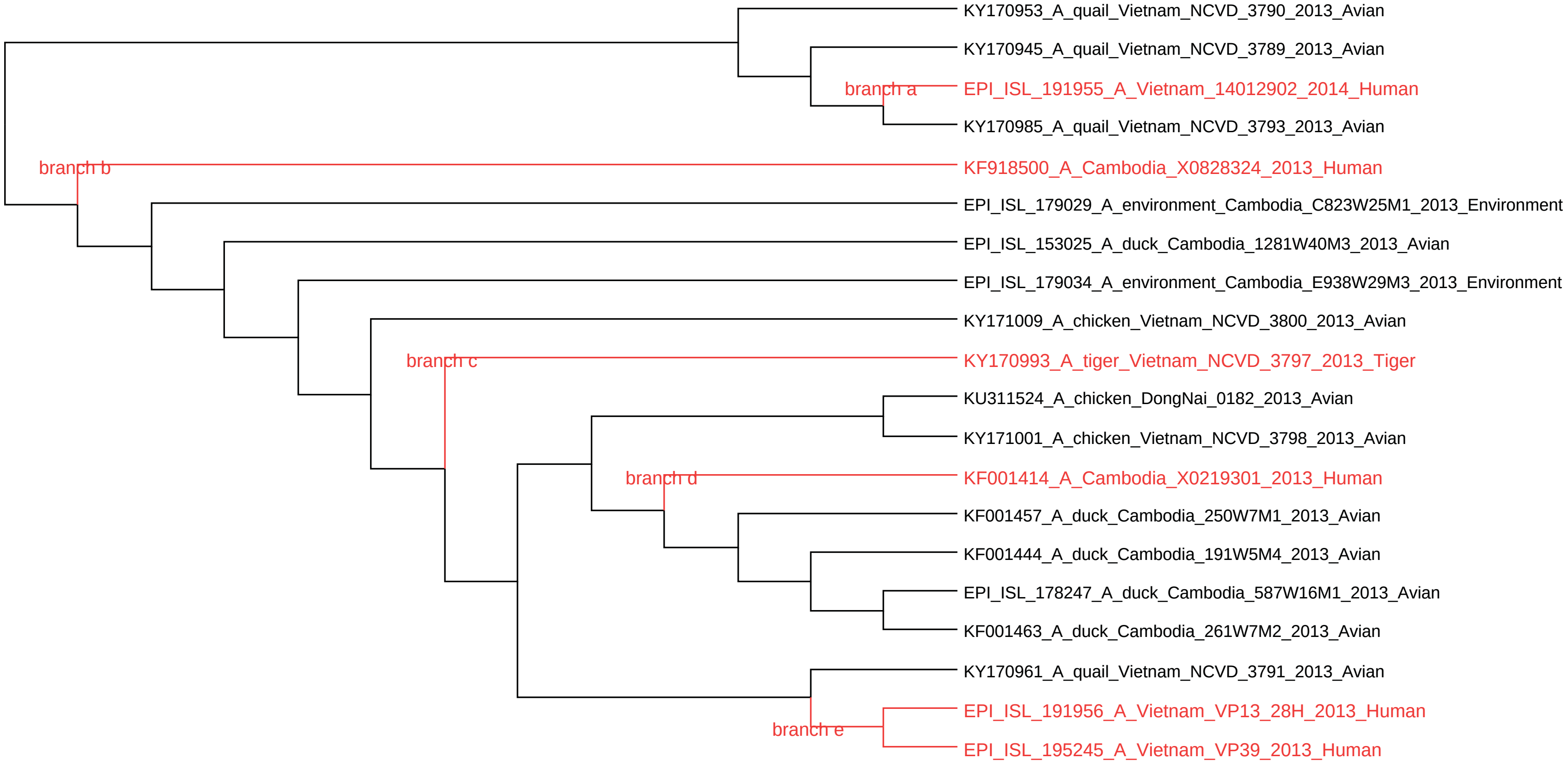

NA1-Group101

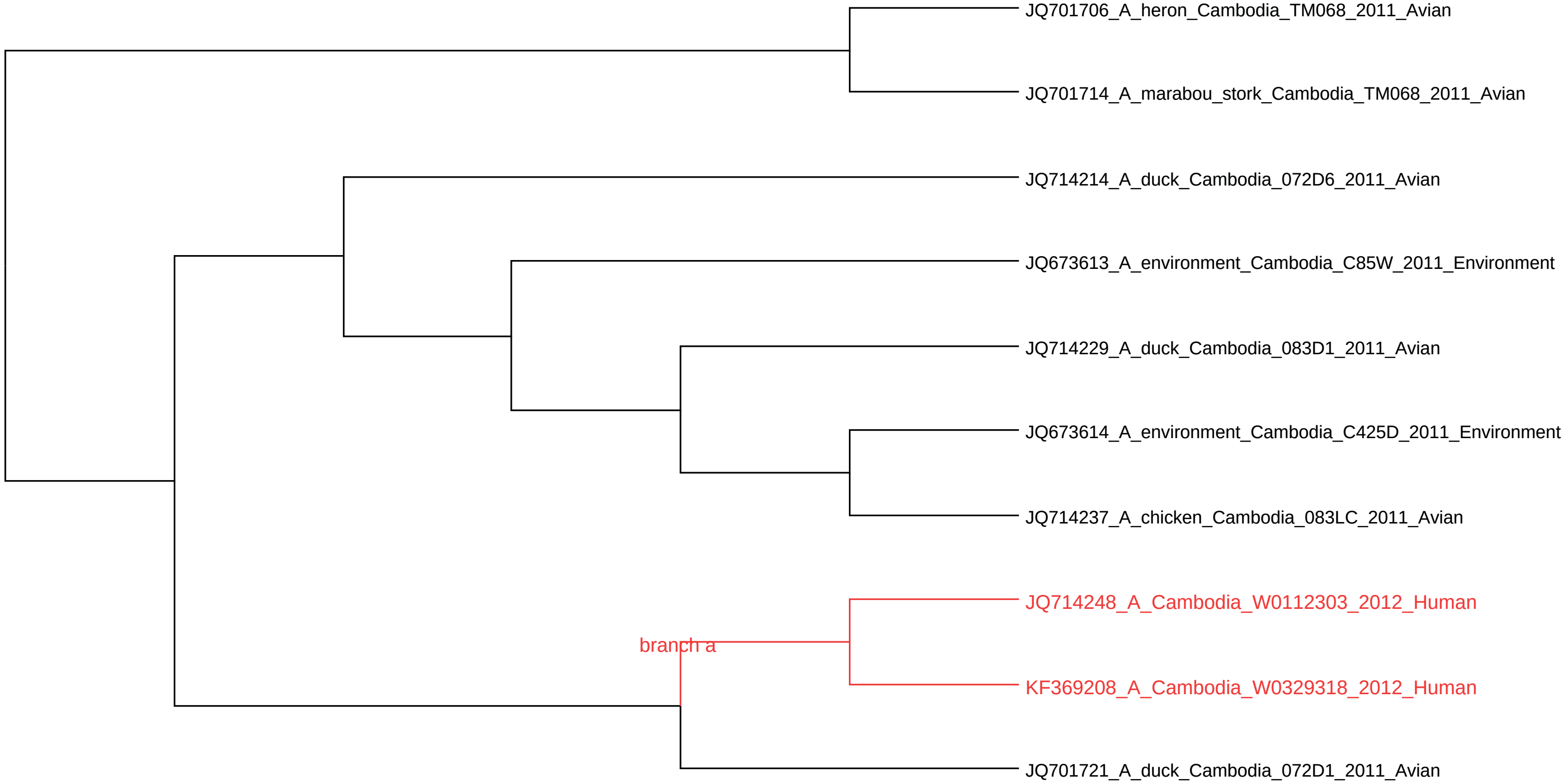

NA1-Group102

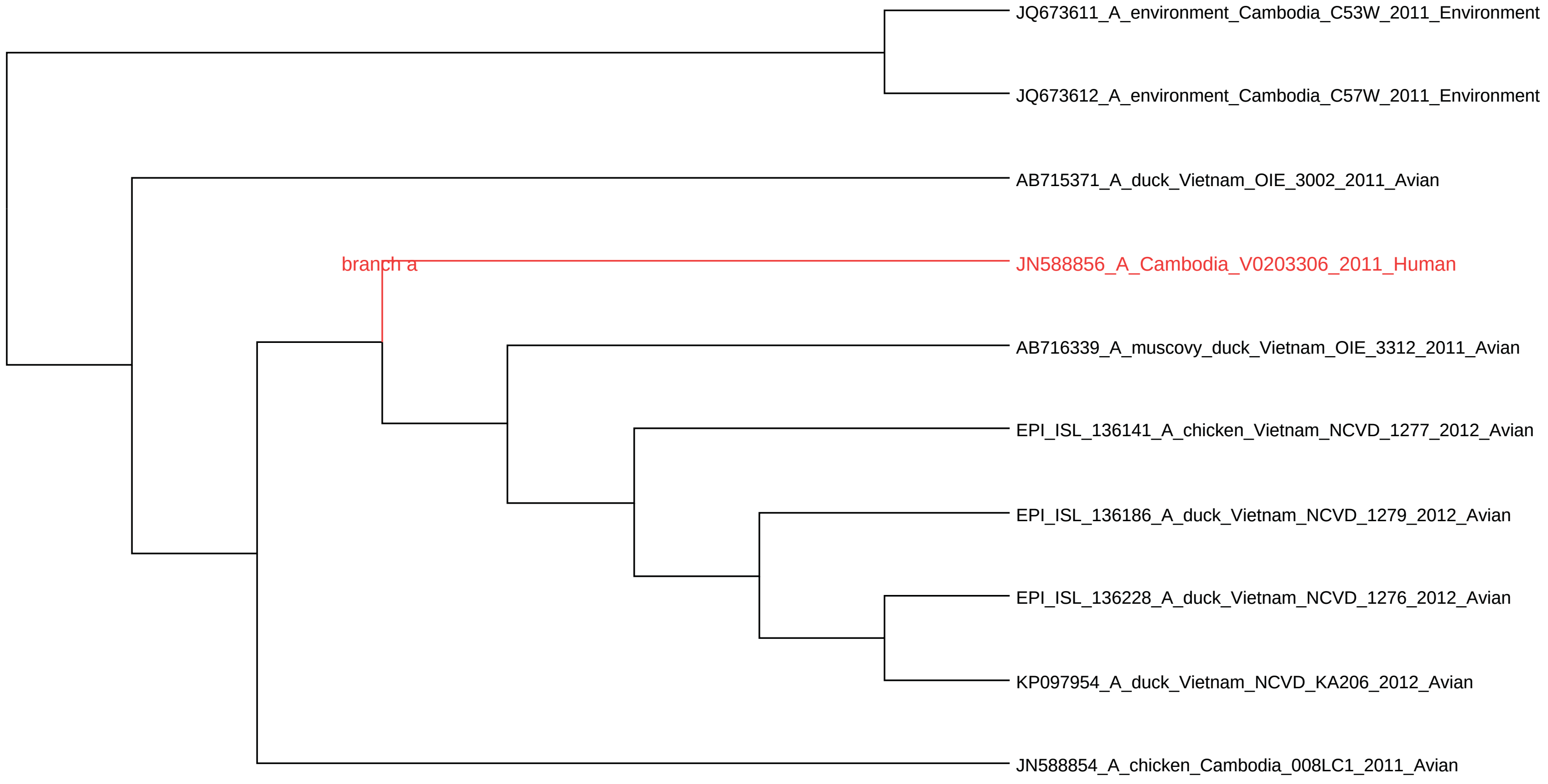

NA1-Group103

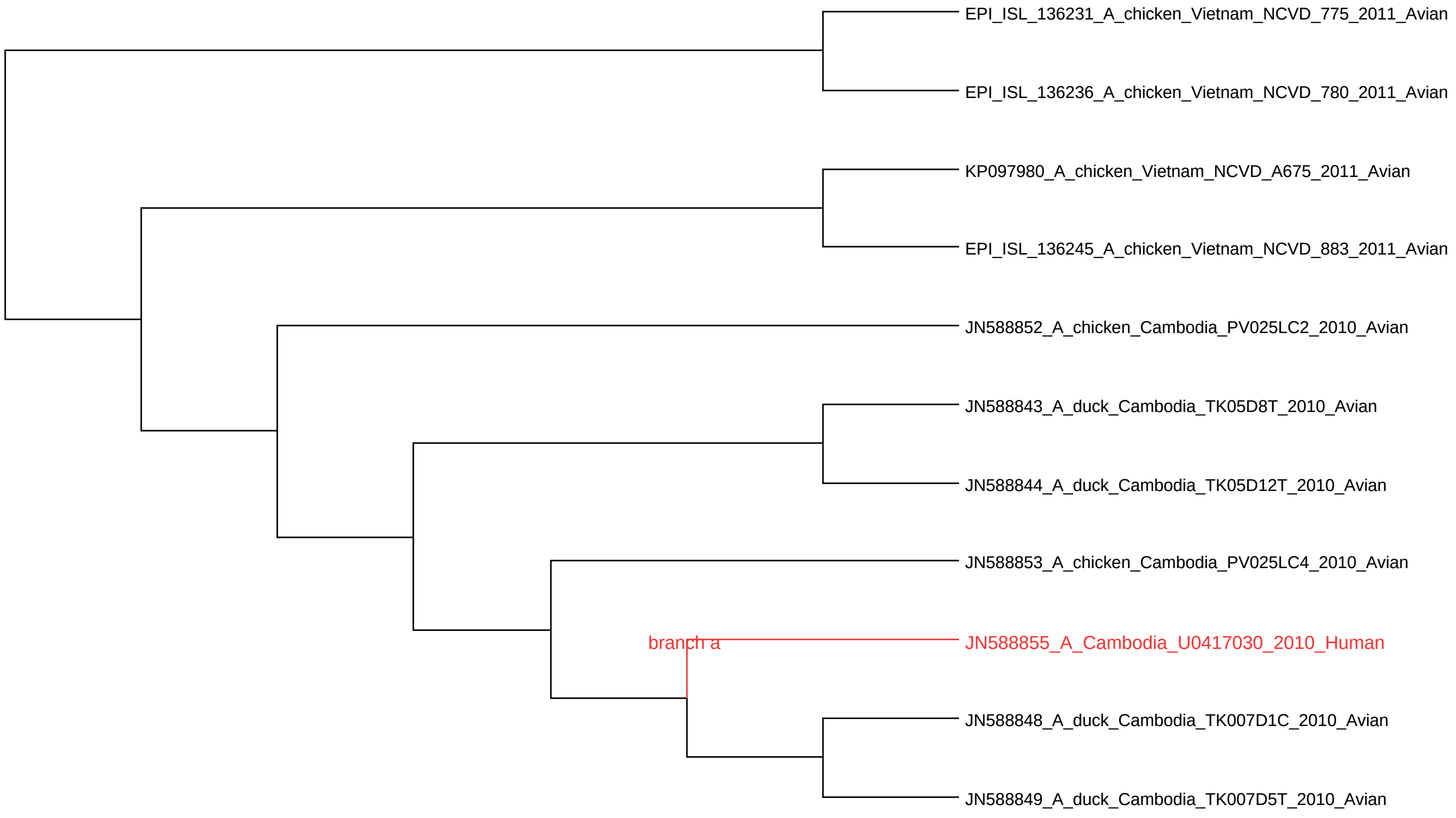

NA1-Group104

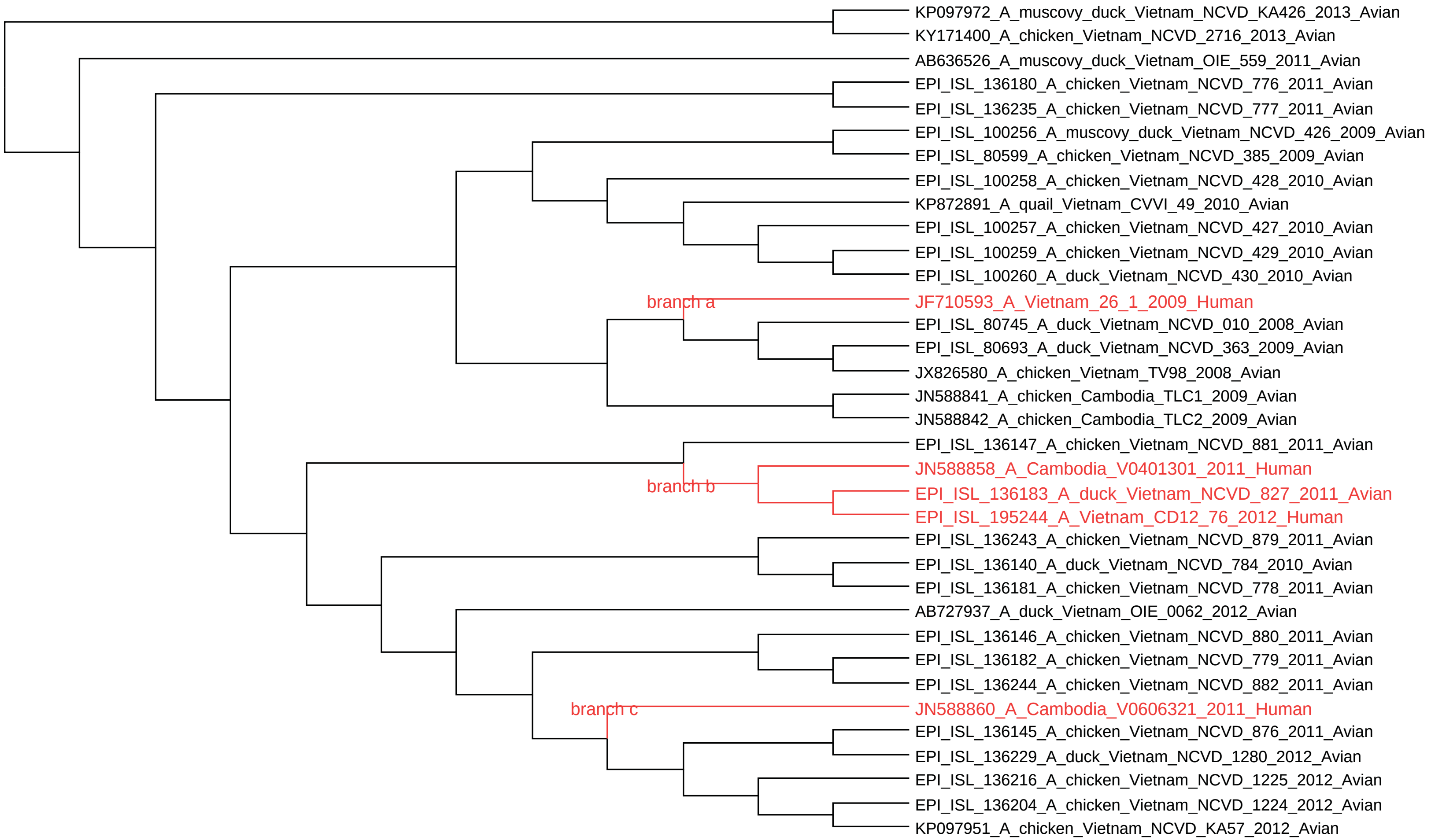

NA6_Group1

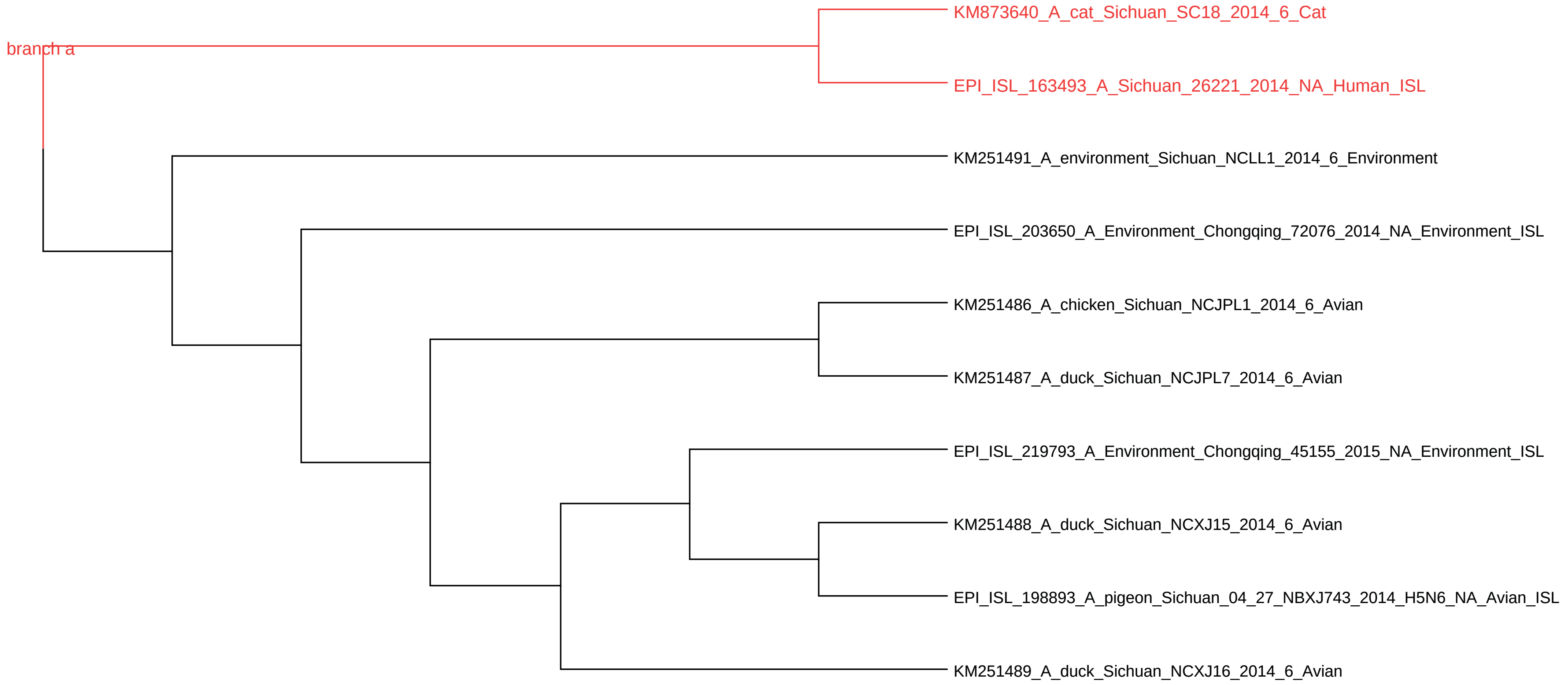
branch a
KM873640_A_cat_Sichuan_SC18_2014_6_Cat
EPI_ISL_163493_A_Sichuan_26221_2014_NA_Human_ISL
KM251491_A_environment_Sichuan_NCLL1_2014_6_Environment
EPI_ISL_203650_A_Environment_Chongqing_72076_2014_NA_Environment_ISL
KM251486_A_chicken_Sichuan_NCJPL1_2014_6_Avian
KM251487_A_duck_Sichuan_NCJPL7_2014_6_Avian
EPI_ISL_219793_A_Environment_Chongqing_45155_2015_NA_Environment_ISL
KM251488_A_duck_Sichuan_NCXJ15_2014_6_Avian
EPI_ISL_198893_A_pigeon_Sichuan_04_27_NBXJ743_2014_H5N6_NA_Avian_ISL
KM251489_A_duck_Sichuan_NCXJ16_2014_6_Avian

NA6_Group2

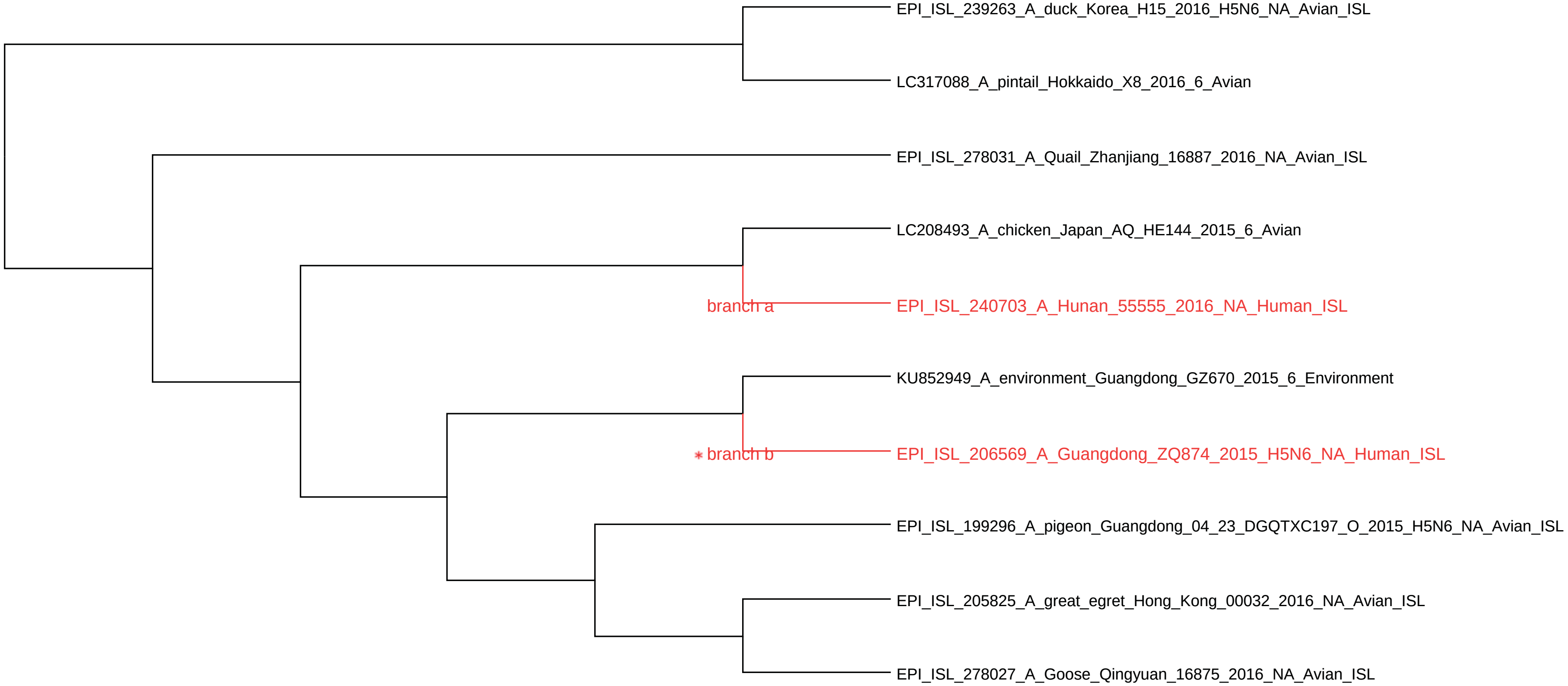

EPI_ISL_239263_A_duck_Korea_H15_2016_H5N6_NA_Avian_ISL
LC317088_A_pintail_Hokkaido_X8_2016_6_Avian
EPI_ISL_278031_A_Quail_Zhanjiang_16887_2016_NA_Avian_ISL
LC208493_A_chicken_Japan_AQ_HE144_2015_6_Avian
branch a
EPI_ISL_240703_A_Hunan_55555_2016_NA_Human_ISL
KU852949_A_environment_Guangdong_GZ670_2015_6_Environment
* branch b
EPI_ISL_206569_A_Guangdong_ZQ874_2015_H5N6_NA_Human_ISL
EPI_ISL_199296_A_pigeon_Guangdong_04_23_DGQTXC197_O_2015_H5N6_NA_Avian_ISL
EPI_ISL_205825_A_great_egret_Hong_Kong_00032_2016_NA_Avian_ISL
EPI_ISL_278027_A_Goose_Qingyuan_16875_2016_NA_Avian_ISL

NA6_Group3

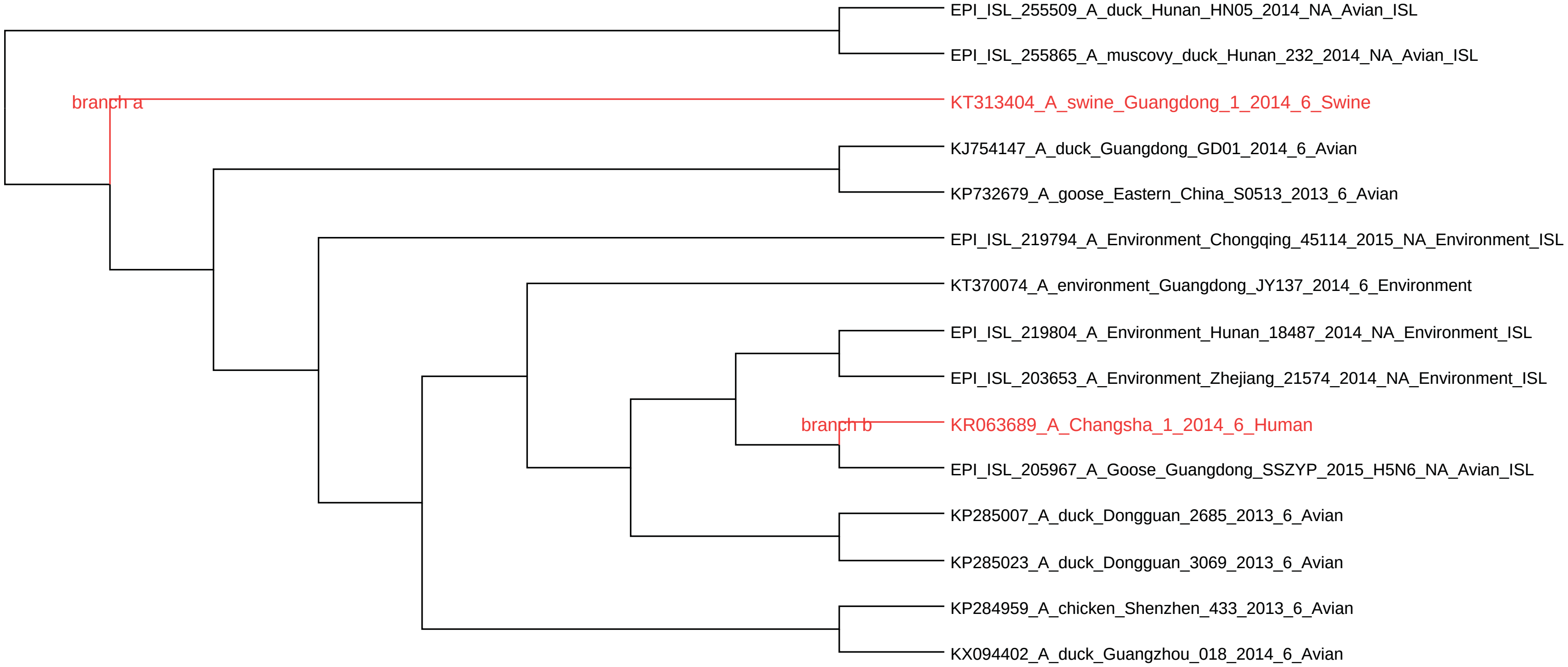
EPI_ISL_255509_A_duck_Hunan_HN05_2014_NA_Avian_ISL
EPI_ISL_255865_A_muscovy_duck_Hunan_232_2014_NA_Avian_ISL
branch a
KT313404_A_swine_Guangdong_1_2014_6_Swine
KJ754147_A_duck_Guangdong_GD01_2014_6_Avian
KP732679_A_goose_Eastern_China_S0513_2013_6_Avian
EPI_ISL_219794_A_Environment_Chongqing_45114_2015_NA_Environment_ISL
KT370074_A_environment_Guangdong_JY137_2014_6_Environment
EPI_ISL_219804_A_Environment_Hunan_18487_2014_NA_Environment_ISL
EPI_ISL_203653_A_Environment_Zhejiang_21574_2014_NA_Environment_ISL
branch b
KR063689_A_Changsha_1_2014_6_Human
EPI_ISL_205967_A_Goose_Guangdong_SSZYP_2015_H5N6_NA_Avian_ISL
KP285007_A_duck_Dongguan_2685_2013_6_Avian
KP285023_A_duck_Dongguan_3069_2013_6_Avian
KP284959_A_chicken_Shenzhen_433_2013_6_Avian
KX094402_A_duck_Guangzhou_018_2014_6_Avian

NA6_Group4

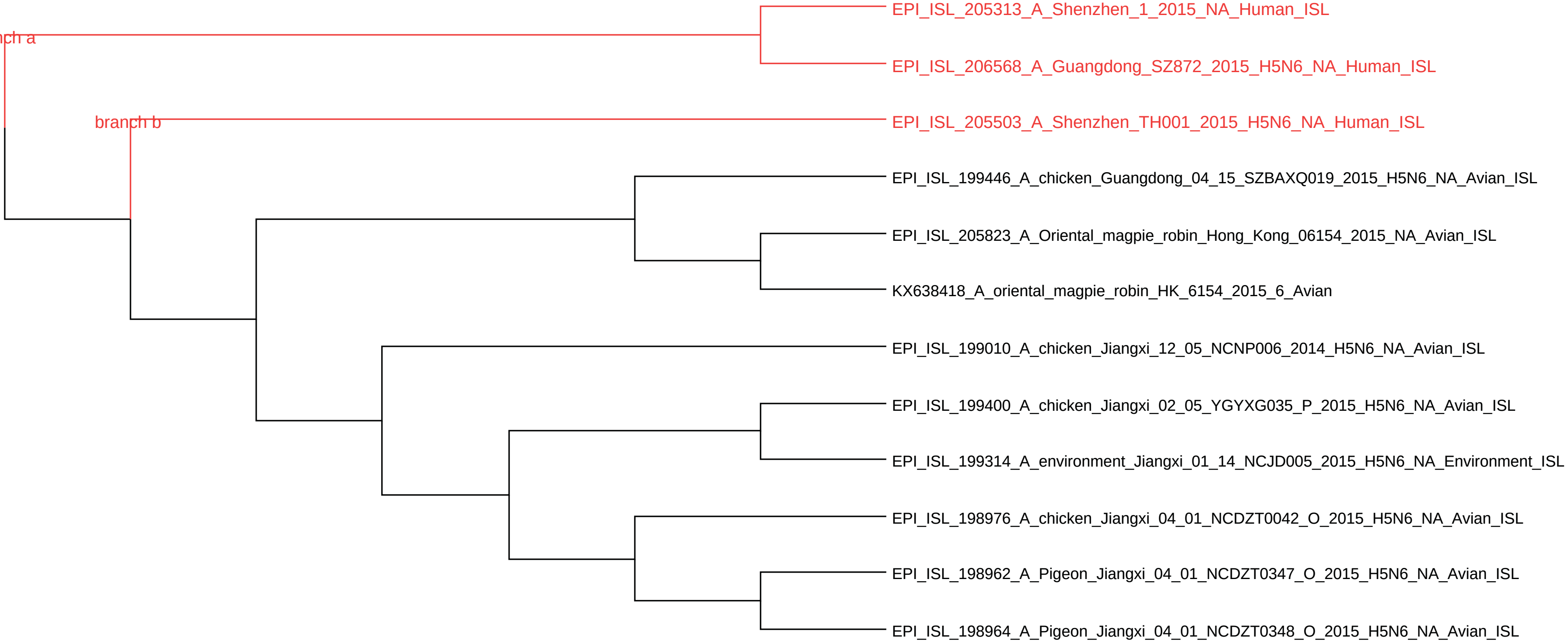
branch b
EPI_ISL_205313_A_Shenzhen_1_2015_NA_Human_ISL
EPI_ISL_206568_A_Guangdong_SZ872_2015_H5N6_NA_Human_ISL
EPI_ISL_205503_A_Shenzhen_TH001_2015_H5N6_NA_Human_ISL
EPI_ISL_199446_A_chicken_Guangdong_04_15_SZBAXQ019_2015_H5N6_NA_Avian_ISL
EPI_ISL_205823_A_Oriental_magpie_robin_Hong_Kong_06154_2015_NA_Avian_ISL
KX638418_A_oriental_magpie_robin_HK_6154_2015_6_Avian
EPI_ISL_199010_A_chicken_Jiangxi_12_05_NCNP006_2014_H5N6_NA_Avian_ISL
EPI_ISL_199400_A_chicken_Jiangxi_02_05_YGYXG035_P_2015_H5N6_NA_Avian_ISL
EPI_ISL_199314_A_environment_Jiangxi_01_14_NCJD005_2015_H5N6_NA_Environment_ISL
EPI_ISL_198976_A_chicken_Jiangxi_04_01_NCDZT0042_O_2015_H5N6_NA_Avian_ISL
EPI_ISL_198962_A_Pigeon_Jiangxi_04_01_NCDZT0347_O_2015_H5N6_NA_Avian_ISL
EPI_ISL_198964_A_Pigeon_Jiangxi_04_01_NCDZT0348_O_2015_H5N6_NA_Avian_ISL

NA6_Group5

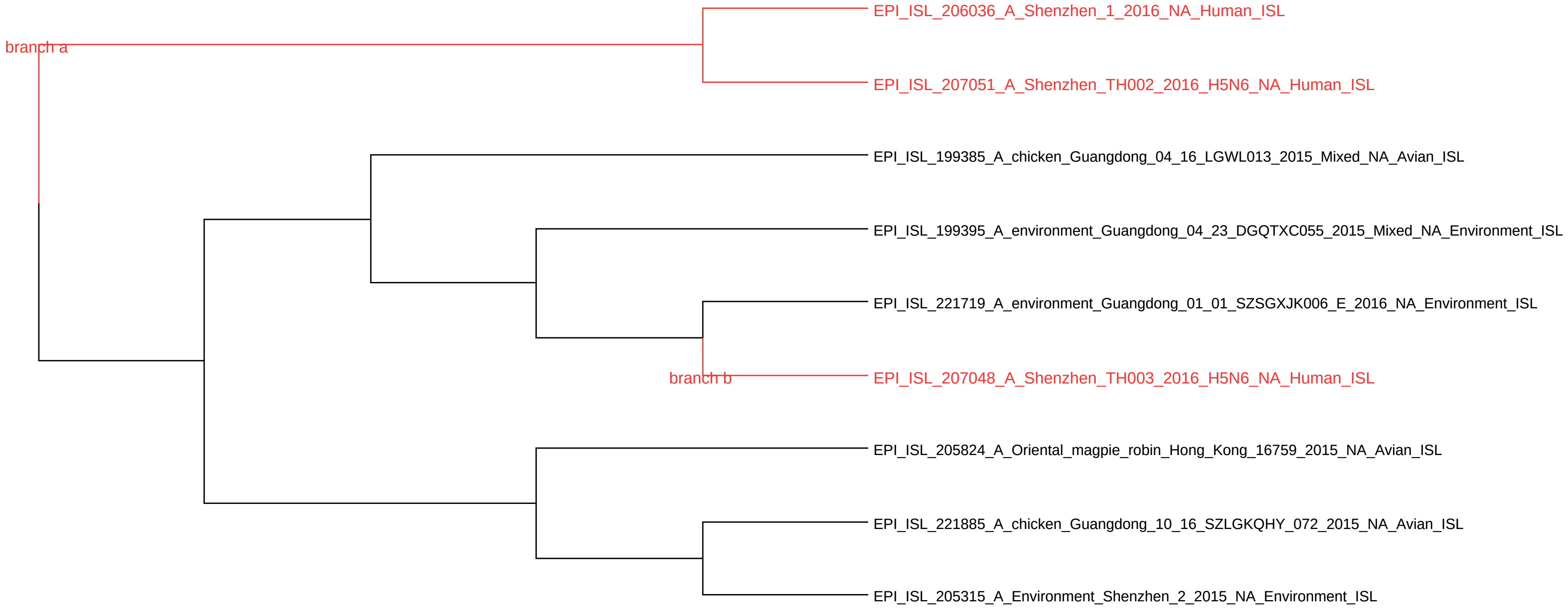

branch a
EPI_ISL_206036_A_Shenzhen_1_2016_NA_Human_ISL
EPI_ISL_207051_A_Shenzhen_TH002_2016_H5N6_NA_Human_ISL
EPI_ISL_199385_A_chicken_Guangdong_04_16_LGWL013_2015_Mixed_NA_Avian_ISL
EPI_ISL_199395_A_environment_Guangdong_04_23_DGQTXC055_2015_Mixed_NA_Environment_ISL
EPI_ISL_221719_A_environment_Guangdong_01_01_SZSGXJK006_E_2016_NA_Environment_ISL
branch b
EPI_ISL_207048_A_Shenzhen_TH003_2016_H5N6_NA_Human_ISL
EPI_ISL_205824_A_Oriental_magpie_robin_Hong_Kong_16759_2015_NA_Avian_ISL
EPI_ISL_221885_A_chicken_Guangdong_10_16_SZLGKQHY_072_2015_NA_Avian_ISL
EPI_ISL_205315_A_Environment_Shenzhen_2_2015_NA_Environment_ISL

NA6_Group6

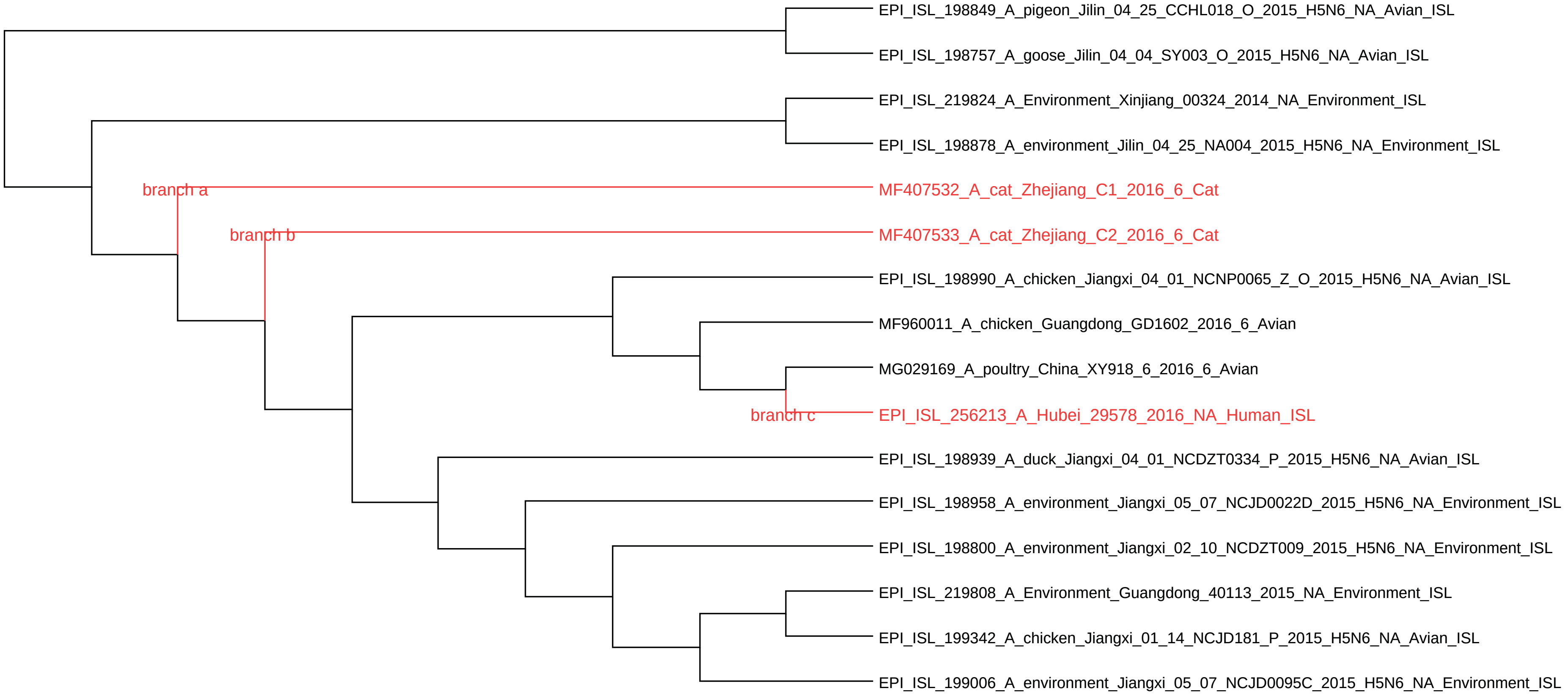
EPI_ISL_198849_A_pigeon_Jilin_04_25_CCHL018_O_2015_H5N6_NA_Avian_ISL
EPI_ISL_198757_A_goose_Jilin_04_04_SY003_O_2015_H5N6_NA_Avian_ISL
EPI_ISL_219824_A_Environment_Xinjiang_00324_2014_NA_Environment_ISL
EPI_ISL_198878_A_environment_Jilin_04_25_NA004_2015_H5N6_NA_Environment_ISL
branch a
MF407532_A_cat_Zhejiang_C1_2016_6_Cat
branch b
MF407533_A_cat_Zhejiang_C2_2016_6_Cat
EPI_ISL_198990_A_chicken_Jiangxi_04_01_NCNP0065_Z_O_2015_H5N6_NA_Avian_ISL
MF960011_A_chicken_Guangdong_GD1602_2016_6_Avian
MG029169_A_poultry_China_XY918_6_2016_6_Avian
branch c
EPI_ISL_256213_A_Hubei_29578_2016_NA_Human_ISL
EPI_ISL_198939_A_duck_Jiangxi_04_01_NCDZT0334_P_2015_H5N6_NA_Avian_ISL
EPI_ISL_198958_A_environment_Jiangxi_05_07_NCJD0022D_2015_H5N6_NA_Environment_ISL
EPI_ISL_198800_A_environment_Jiangxi_02_10_NCDZT009_2015_H5N6_NA_Environment_ISL
EPI_ISL_219808_A_Environment_Guangdong_40113_2015_NA_Environment_ISL
EPI_ISL_199342_A_chicken_Jiangxi_01_14_NCJD181_P_2015_H5N6_NA_Avian_ISL
EPI_ISL_199006_A_environment_Jiangxi_05_07_NCJD0095C_2015_H5N6_NA_Environment_ISL

NA6_Group7

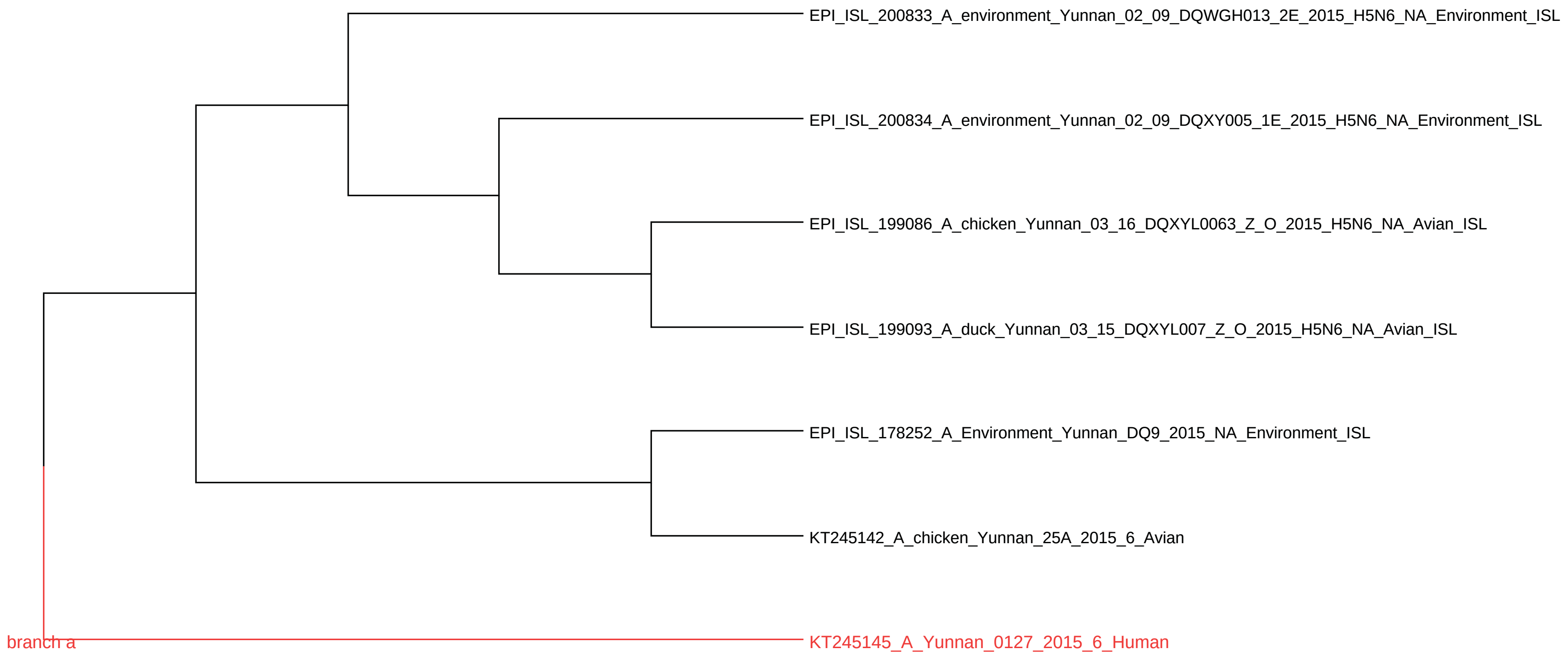
EPI_ISL_200833_A_environment_Yunnan_02_09_DQWGH013_2E_2015_H5N6_NA_Environment_ISL
EPI_ISL_200834_A_environment_Yunnan_02_09_DQXY005_1E_2015_H5N6_NA_Environment_ISL
EPI_ISL_199086_A_chicken_Yunnan_03_16_DQXYL0063_Z_O_2015_H5N6_NA_Avian_ISL
EPI_ISL_199093_A_duck_Yunnan_03_15_DQXYL007_Z_O_2015_H5N6_NA_Avian_ISL
EPI_ISL_178252_A_Environment_Yunnan_DQ9_2015_NA_Environment_ISL
KT245142_A_chicken_Yunnan_25A_2015_6_Avian
branch a
KT245145_A_Yunnan_0127_2015_6_Human

NA6_Group8

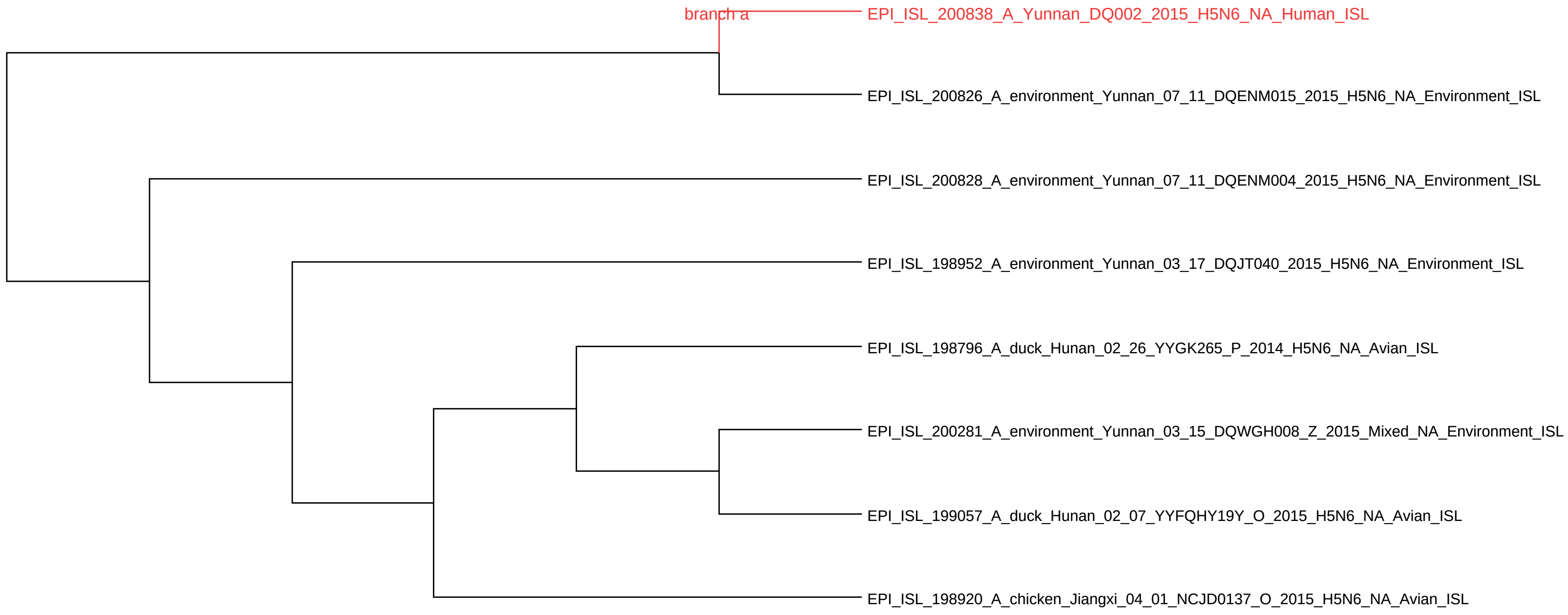

branch a
EPI_ISL_200838_A_Yunnan_DQ002_2015_H5N6_NA_Human_ISL
EPI_ISL_200826_A_environment_Yunnan_07_11_DQENM015_2015_H5N6_NA_Environment_ISL
EPI_ISL_200828_A_environment_Yunnan_07_11_DQENM004_2015_H5N6_NA_Environment_ISL
EPI_ISL_198952_A_environment_Yunnan_03_17_DQJT040_2015_H5N6_NA_Environment_ISL
EPI_ISL_198796_A_duck_Hunan_02_26_YYGK265_P_2014_H5N6_NA_Avian_ISL
EPI_ISL_200281_A_environment_Yunnan_03_15_DQWGH008_Z_2015_Mixed_NA_Environment_ISL
EPI_ISL_199057_A_duck_Hunan_02_07_YYFQHY19Y_O_2015_H5N6_NA_Avian_ISL
EPI_ISL_198920_A_chicken_Jiangxi_04_01_NCJD0137_O_2015_H5N6_NA_Avian_ISL

NA6_Group9

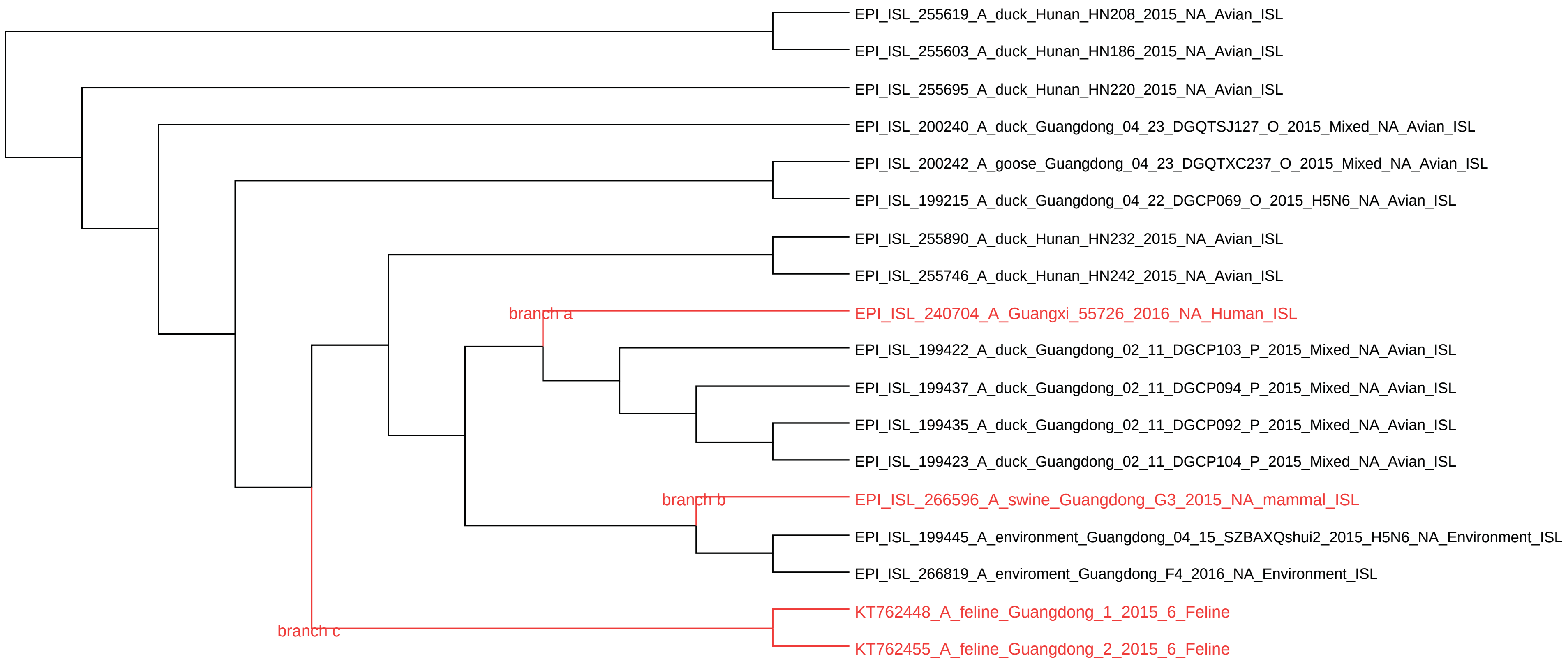

EPI_ISL_255619_A_duck_Hunan_HN208_2015_NA_Avian_ISL
EPI_ISL_255603_A_duck_Hunan_HN186_2015_NA_Avian_ISL
EPI_ISL_255695_A_duck_Hunan_HN220_2015_NA_Avian_ISL
EPI_ISL_200240_A_duck_Guangdong_04_23_DGQTSJ127_O_2015_Mixed_NA_Avian_ISL
EPI_ISL_200242_A_goose_Guangdong_04_23_DGQTXC237_O_2015_Mixed_NA_Avian_ISL
EPI_ISL_199215_A_duck_Guangdong_04_22_DGCP069_O_2015_H5N6_NA_Avian_ISL
EPI_ISL_255890_A_duck_Hunan_HN232_2015_NA_Avian_ISL
EPI_ISL_255746_A_duck_Hunan_HN242_2015_NA_Avian_ISL
branch a
EPI_ISL_240704_A_Guangxi_55726_2016_NA_Human_ISL
EPI_ISL_199422_A_duck_Guangdong_02_11_DGCP103_P_2015_Mixed_NA_Avian_ISL
EPI_ISL_199437_A_duck_Guangdong_02_11_DGCP094_P_2015_Mixed_NA_Avian_ISL
EPI_ISL_199435_A_duck_Guangdong_02_11_DGCP092_P_2015_Mixed_NA_Avian_ISL
EPI_ISL_199423_A_duck_Guangdong_02_11_DGCP104_P_2015_Mixed_NA_Avian_ISL
branch b
EPI_ISL_266596_A_swine_Guangdong_G3_2015_NA_mammal_ISL
EPI_ISL_199445_A_environment_Guangdong_04_15_SZBAXQshui2_2015_H5N6_NA_Environment_ISL
EPI_ISL_266819_A_enviroment_Guangdong_F4_2016_NA_Environment_ISL
KT762448_A_feline_Guangdong_1_2015_6_Feline
branch c
KT762455_A_feline_Guangdong_2_2015_6_Feline

NA6_Group10

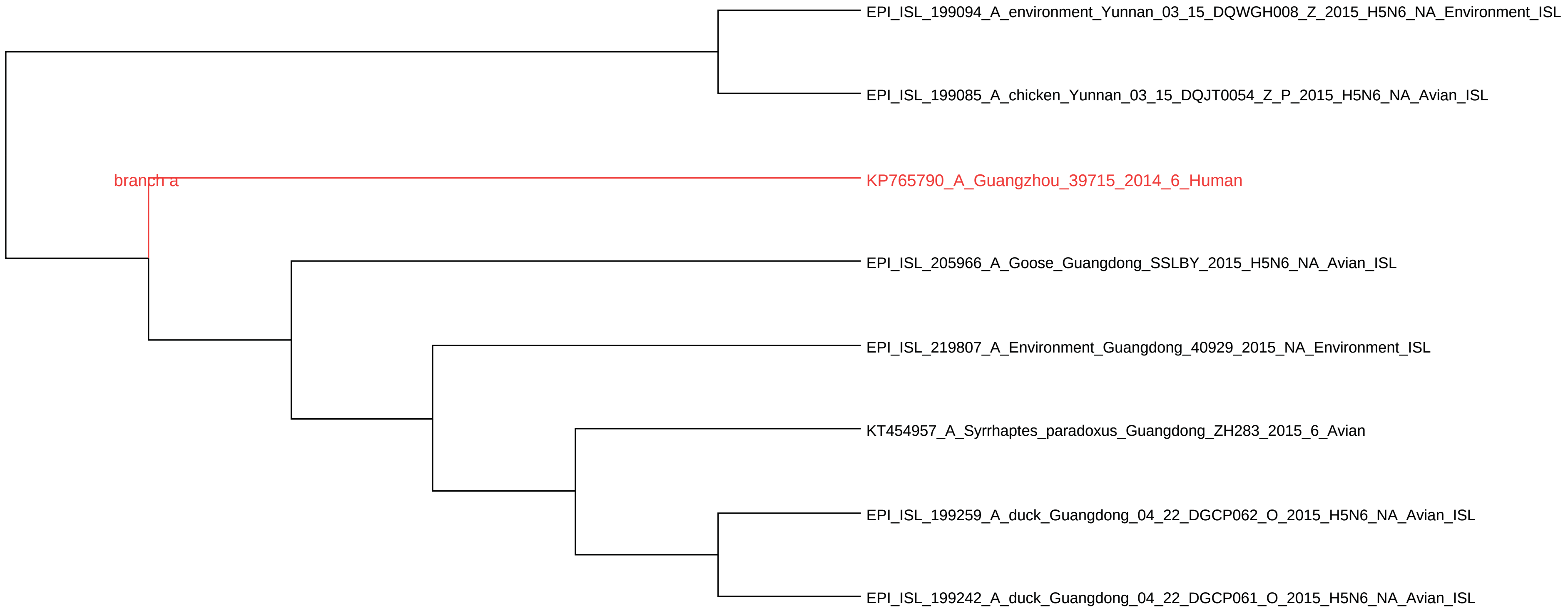
EPI_ISL_199094_A_environment_Yunnan_03_15_DQWGH008_Z_2015_H5N6_NA_Environment_ISL
EPI_ISL_199085_A_chicken_Yunnan_03_15_DQJT0054_Z_P_2015_H5N6_NA_Avian_ISL
branch a
KP765790_A_Guangzhou_39715_2014_6_Human
EPI_ISL_205966_A_Goose_Guangdong_SSLBY_2015_H5N6_NA_Avian_ISL
EPI_ISL_219807_A_Environment_Guangdong_40929_2015_NA_Environment_ISL
KT454957_A_Syrrhaptes_paradoxus_Guangdong_ZH283_2015_6_Avian
EPI_ISL_199259_A_duck_Guangdong_04_22_DGCP062_O_2015_H5N6_NA_Avian_ISL
EPI_ISL_199242_A_duck_Guangdong_04_22_DGCP061_O_2015_H5N6_NA_Avian_ISL
