# Supplementary material for: Adaptive Evolution of Human-Isolated H5Nx Avian Influenza A Viruses
Source: Front Microbiol. 2019 Jun 12;10:1328. doi: 10.3389/fmicb.2019.01328 (PMC6582624; doi:10.3389/fmicb.2019.01328)
Supplement: Supplementary file 5 [file Data_Sheet_5.PDF]

Supplementary Figure 5. 98 phylogenetic trees of MP used for the adaptive evolution analyses. Human strains are marked in red. Branches which have significant signals of positive selection are marked with *.

MP-Group1

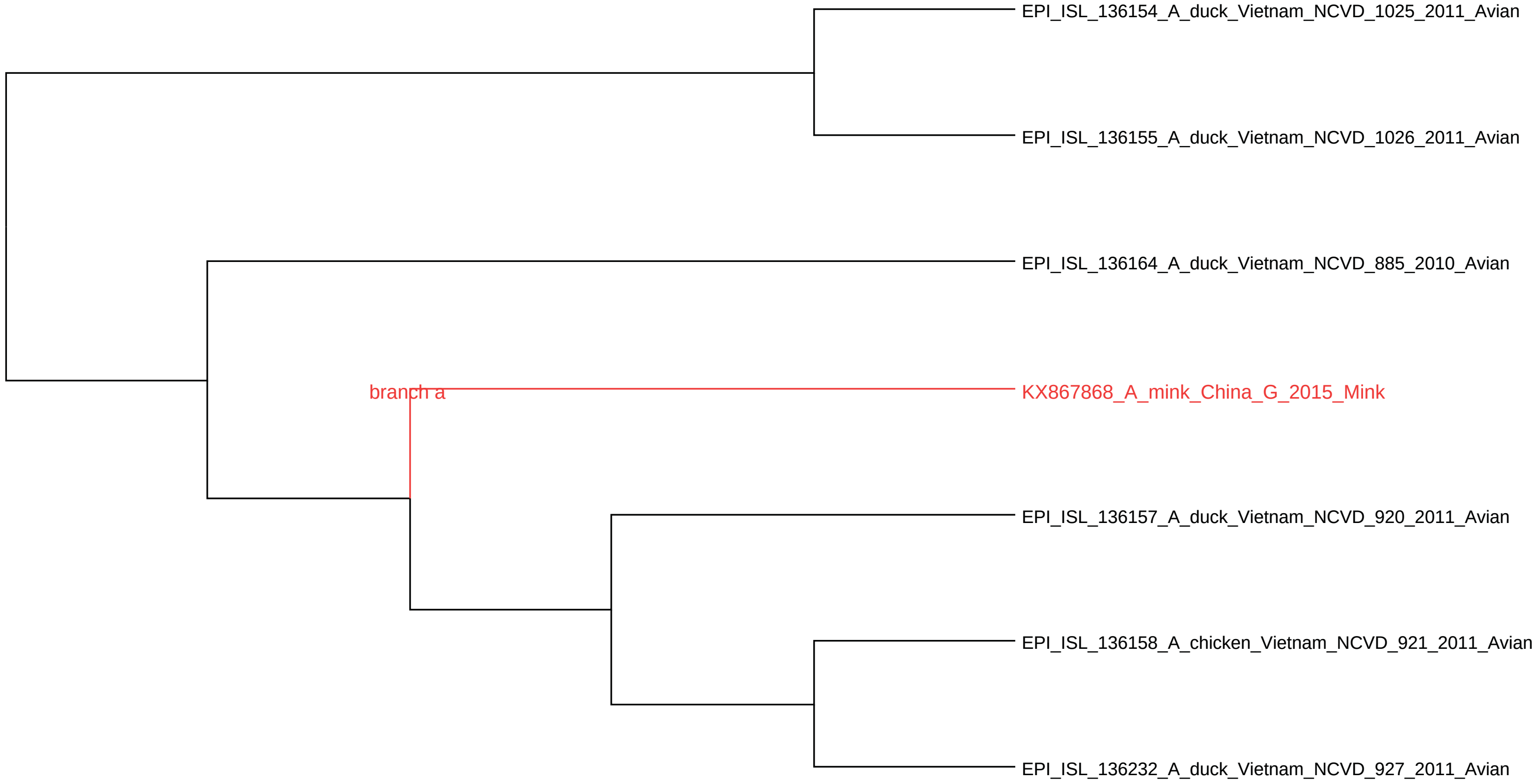

MP-Group2

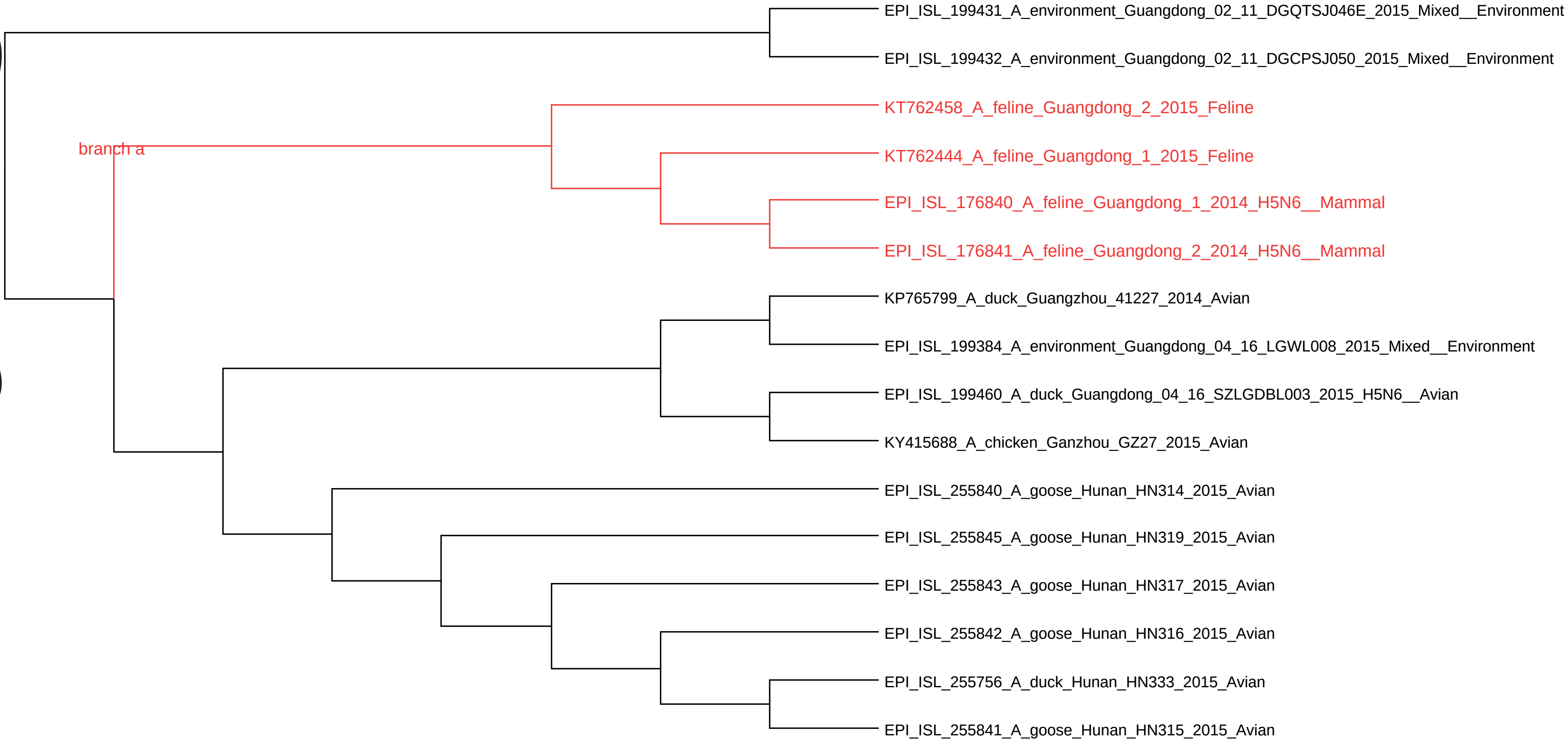
EPI_ISL_199431_A_environment_Guangdong_02_11_DGQTSJ046E_2015_Mixed__Environment
EPI_ISL_199432_A_environment_Guangdong_02_11_DGCPSJ050_2015_Mixed__Environment
KT762458_A_feline_Guangdong_2_2015_Feline
branch a
KT762444_A_feline_Guangdong_1_2015_Feline
EPI_ISL_176840_A_feline_Guangdong_1_2014_H5N6__Mammal
EPI_ISL_176841_A_feline_Guangdong_2_2014_H5N6__Mammal
KP765799_A_duck_Guangzhou_41227_2014_Avian
EPI_ISL_199384_A_environment_Guangdong_04_16_LGWL008_2015_Mixed__Environment
EPI_ISL_199460_A_duck_Guangdong_04_16_SZLGDBL003_2015_H5N6__Avian
KY415688_A_chicken_Ganzhou_GZ27_2015_Avian
EPI_ISL_255840_A_goose_Hunan_HN314_2015_Avian
EPI_ISL_255845_A_goose_Hunan_HN319_2015_Avian
EPI_ISL_255843_A_goose_Hunan_HN317_2015_Avian
EPI_ISL_255842_A_goose_Hunan_HN316_2015_Avian
EPI_ISL_255756_A_duck_Hunan_HN333_2015_Avian
EPI_ISL_255841_A_goose_Hunan_HN315_2015_Avian

MP-Group3

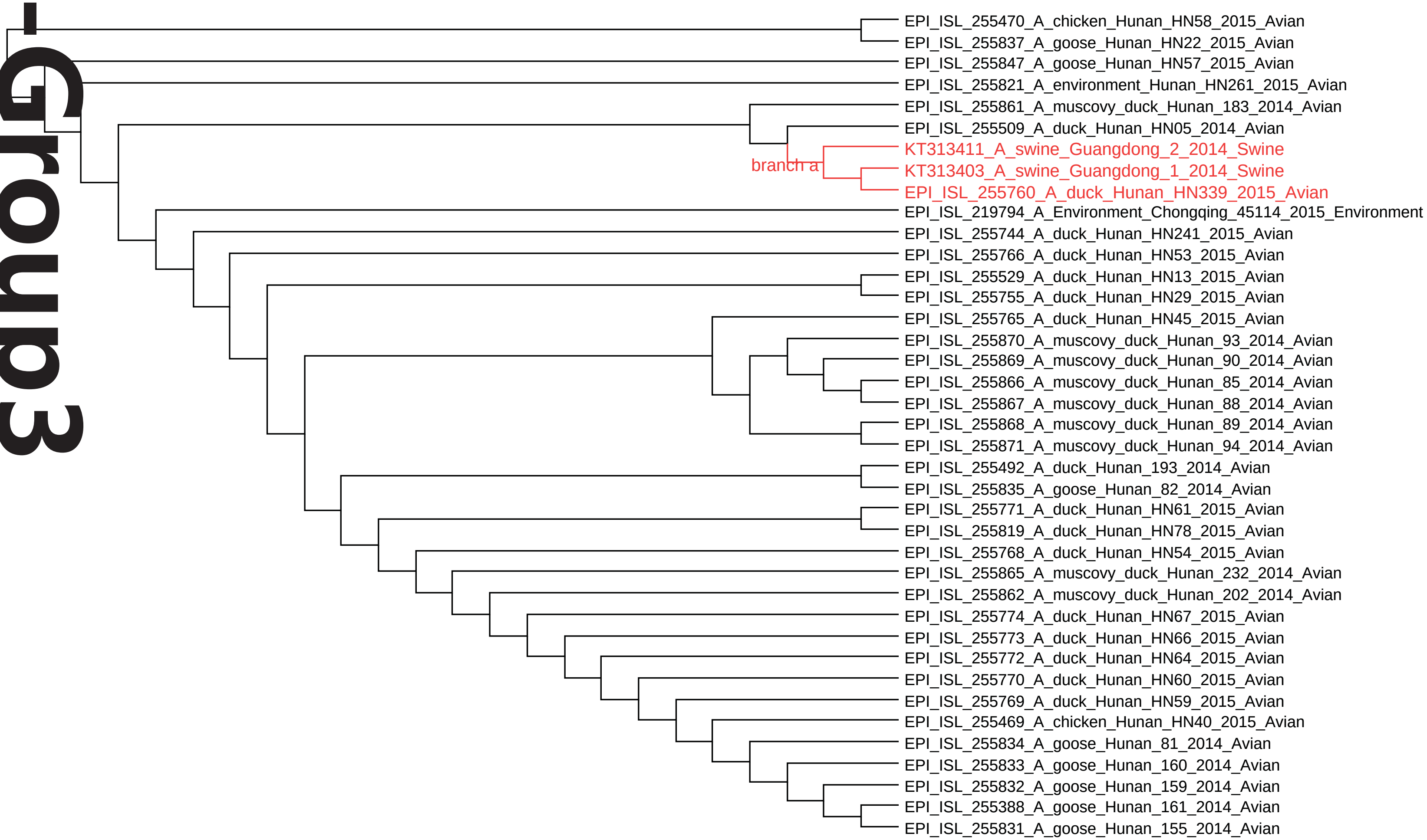

EPI_ISL_255470_A_chicken_Hunan_HN58_2015_Avian
EPI_ISL_255837_A_goose_Hunan_HN22_2015_Avian
EPI_ISL_255847_A_goose_Hunan_HN57_2015_Avian
EPI_ISL_255821_A_environment_Hunan_HN261_2015_Avian
EPI_ISL_255861_A_muscovy_duck_Hunan_183_2014_Avian
EPI_ISL_255509_A_duck_Hunan_HN05_2014_Avian
KT313411_A_swine_Guangdong_2_2014_Swine
branch a
KT313403_A_swine_Guangdong_1_2014_Swine
EPI_ISL_255760_A_duck_Hunan_HN339_2015_Avian
EPI_ISL_219794_A_Environment_Chongqing_45114_2015_Environment
EPI_ISL_255744_A_duck_Hunan_HN241_2015_Avian
EPI_ISL_255766_A_duck_Hunan_HN53_2015_Avian
EPI_ISL_255529_A_duck_Hunan_HN13_2015_Avian
EPI_ISL_255755_A_duck_Hunan_HN29_2015_Avian
EPI_ISL_255765_A_duck_Hunan_HN45_2015_Avian
EPI_ISL_255870_A_muscovy_duck_Hunan_93_2014_Avian
EPI_ISL_255869_A_muscovy_duck_Hunan_90_2014_Avian
EPI_ISL_255866_A_muscovy_duck_Hunan_85_2014_Avian
EPI_ISL_255867_A_muscovy_duck_Hunan_88_2014_Avian
EPI_ISL_255868_A_muscovy_duck_Hunan_89_2014_Avian
EPI_ISL_255871_A_muscovy_duck_Hunan_94_2014_Avian
EPI_ISL_255492_A_duck_Hunan_193_2014_Avian
EPI_ISL_255835_A_goose_Hunan_82_2014_Avian
EPI_ISL_255771_A_duck_Hunan_HN61_2015_Avian
EPI_ISL_255819_A_duck_Hunan_HN78_2015_Avian
EPI_ISL_255768_A_duck_Hunan_HN54_2015_Avian
EPI_ISL_255865_A_muscovy_duck_Hunan_232_2014_Avian
EPI_ISL_255862_A_muscovy_duck_Hunan_202_2014_Avian
EPI_ISL_255774_A_duck_Hunan_HN67_2015_Avian
EPI_ISL_255773_A_duck_Hunan_HN66_2015_Avian
EPI_ISL_255772_A_duck_Hunan_HN64_2015_Avian
EPI_ISL_255770_A_duck_Hunan_HN60_2015_Avian
EPI_ISL_255769_A_duck_Hunan_HN59_2015_Avian
EPI_ISL_255469_A_chicken_Hunan_HN40_2015_Avian
EPI_ISL_255834_A_goose_Hunan_81_2014_Avian
EPI_ISL_255833_A_goose_Hunan_160_2014_Avian
EPI_ISL_255832_A_goose_Hunan_159_2014_Avian
EPI_ISL_255388_A_goose_Hunan_161_2014_Avian
EPI_ISL_255831_A_goose_Hunan_155_2014_Avian

MP-Group4

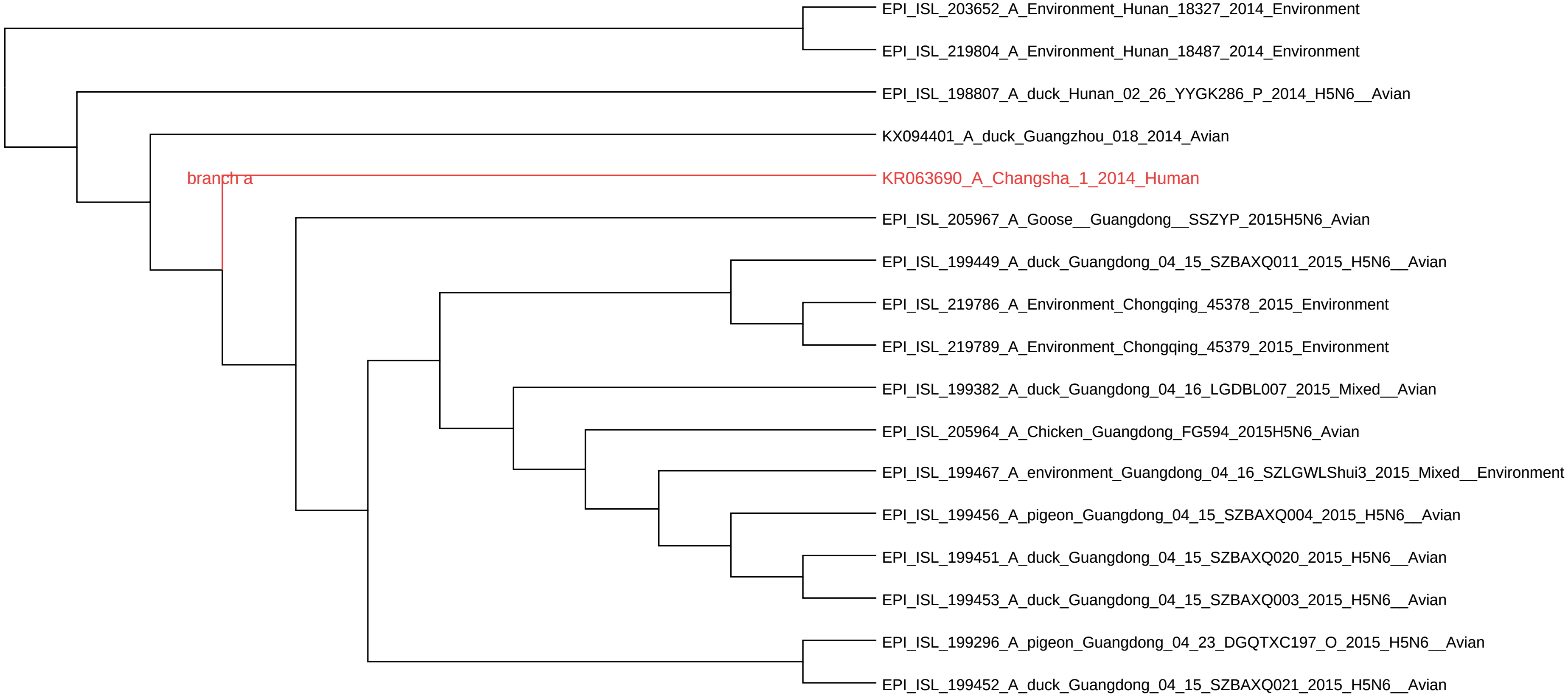
EPI_ISL_203652_A_Environment_Hunan_18327_2014_Environment
EPI_ISL_219804_A_Environment_Hunan_18487_2014_Environment
EPI_ISL_198807_A_duck_Hunan_02_26_YYGK286_P_2014_H5N6__Avian
KX094401_A_duck_Guangzhou_018_2014_Avian
branch a
KR063690_A_Changsha_1_2014_Human
EPI_ISL_205967_A_Goose__Guangdong__SSZYP_2015H5N6_Avian
EPI_ISL_199449_A_duck_Guangdong_04_15_SZBAXQ011_2015_H5N6__Avian
EPI_ISL_219786_A_Environment_Chongqing_45378_2015_Environment
EPI_ISL_219789_A_Environment_Chongqing_45379_2015_Environment
EPI_ISL_199382_A_duck_Guangdong_04_16_LGDBL007_2015_Mixed__Avian
EPI_ISL_205964_A_Chicken_Guangdong_FG594_2015H5N6_Avian
EPI_ISL_199467_A_environment_Guangdong_04_16_SZLGWLShui3_2015_Mixed__Environment
EPI_ISL_199456_A_pigeon_Guangdong_04_15_SZBAXQ004_2015_H5N6__Avian
EPI_ISL_199451_A_duck_Guangdong_04_15_SZBAXQ020_2015_H5N6__Avian
EPI_ISL_199453_A_duck_Guangdong_04_15_SZBAXQ003_2015_H5N6__Avian
EPI_ISL_199296_A_pigeon_Guangdong_04_23_DGQTXC197_O_2015_H5N6__Avian
EPI_ISL_199452_A_duck_Guangdong_04_15_SZBAXQ021_2015_H5N6__Avian

MP-Group5

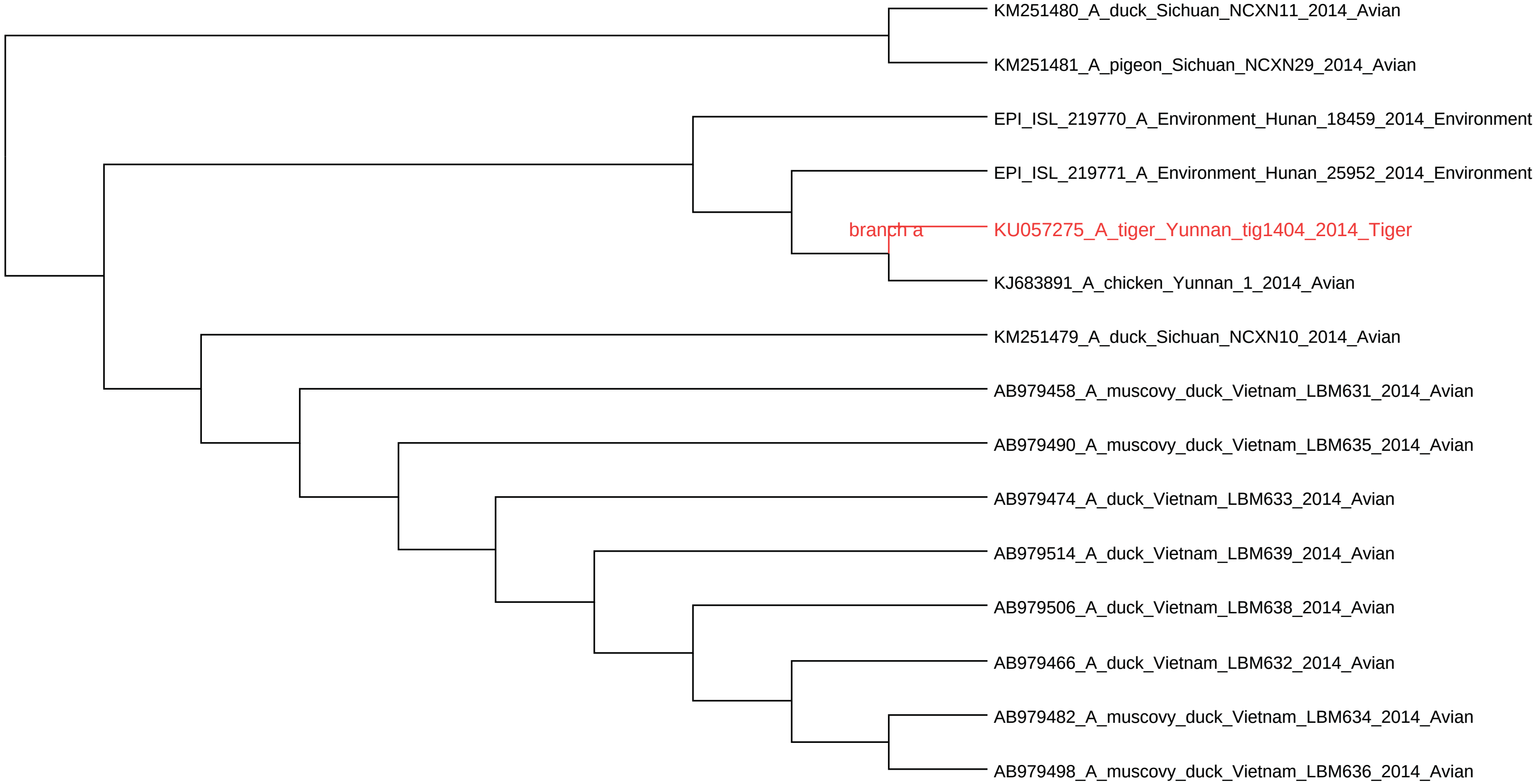
KM251480_A_duck_Sichuan_NCXN11_2014_Avian
KM251481_A_pigeon_Sichuan_NCXN29_2014_Avian
EPI_ISL_219770_A_Environment_Hunan_18459_2014_Environment
EPI_ISL_219771_A_Environment_Hunan_25952_2014_Environment
branch a
KU057275_A_tiger_Yunnan_tig1404_2014_Tiger
KJ683891_A_chicken_Yunnan_1_2014_Avian
KM251479_A_duck_Sichuan_NCXN10_2014_Avian
AB979458_A_muscovy_duck_Vietnam_LBM631_2014_Avian
AB979490_A_muscovy_duck_Vietnam_LBM635_2014_Avian
AB979474_A_duck_Vietnam_LBM633_2014_Avian
AB979514_A_duck_Vietnam_LBM639_2014_Avian
AB979506_A_duck_Vietnam_LBM638_2014_Avian
AB979466_A_duck_Vietnam_LBM632_2014_Avian
AB979482_A_muscovy_duck_Vietnam_LBM634_2014_Avian
AB979498_A_muscovy_duck_Vietnam_LBM636_2014_Avian

MP-Group6

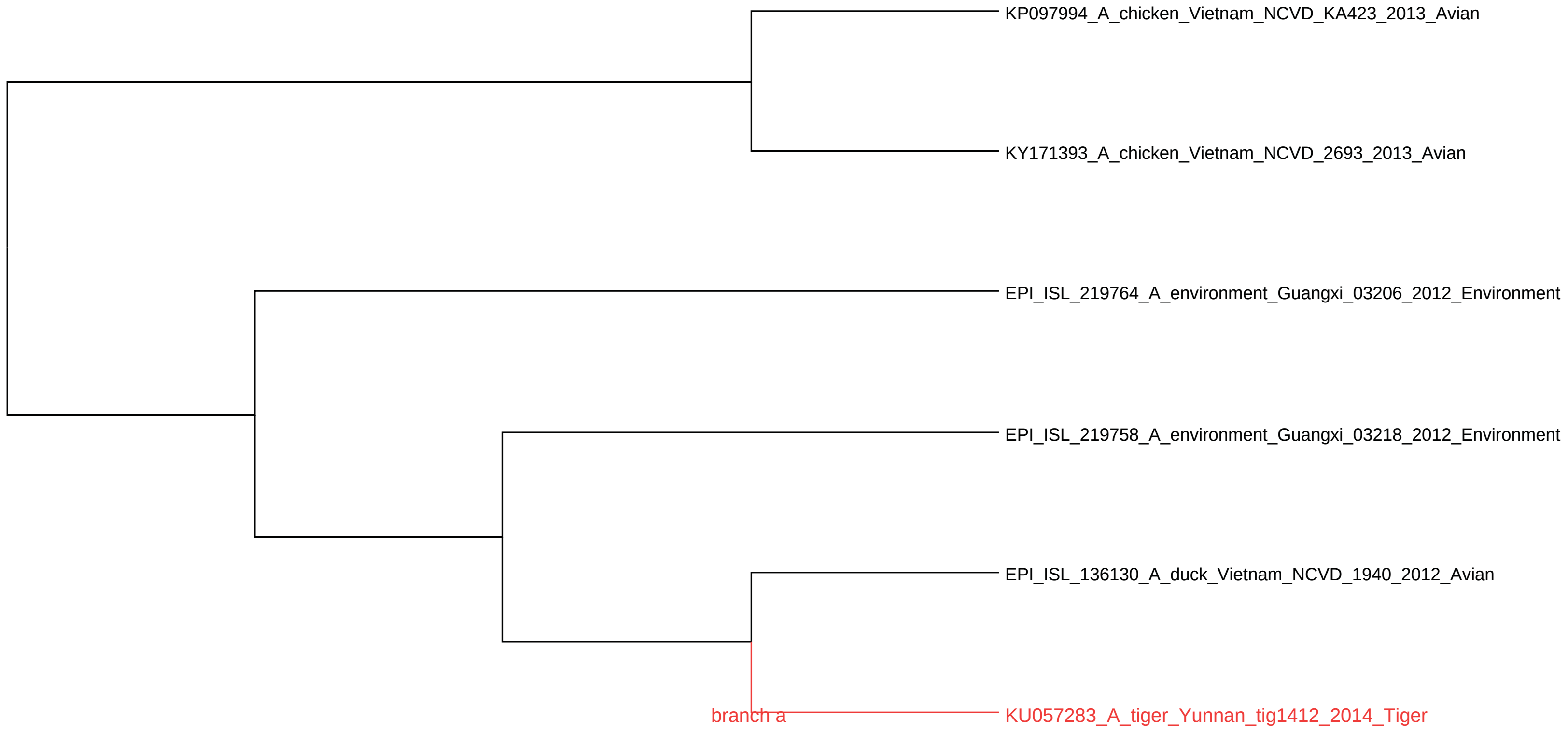
KP097994_A_chicken_Vietnam_NCVD_KA423_2013_Avian
KY171393_A_chicken_Vietnam_NCVD_2693_2013_Avian
EPI_ISL_219764_A_environment_Guangxi_03206_2012_Environment
EPI_ISL_219758_A_environment_Guangxi_03218_2012_Environment
EPI_ISL_136130_A_duck_Vietnam_NCVD_1940_2012_Avian
branch a
KU057283_A_tiger_Yunnan_tig1412_2014_Tiger

MP-Group7

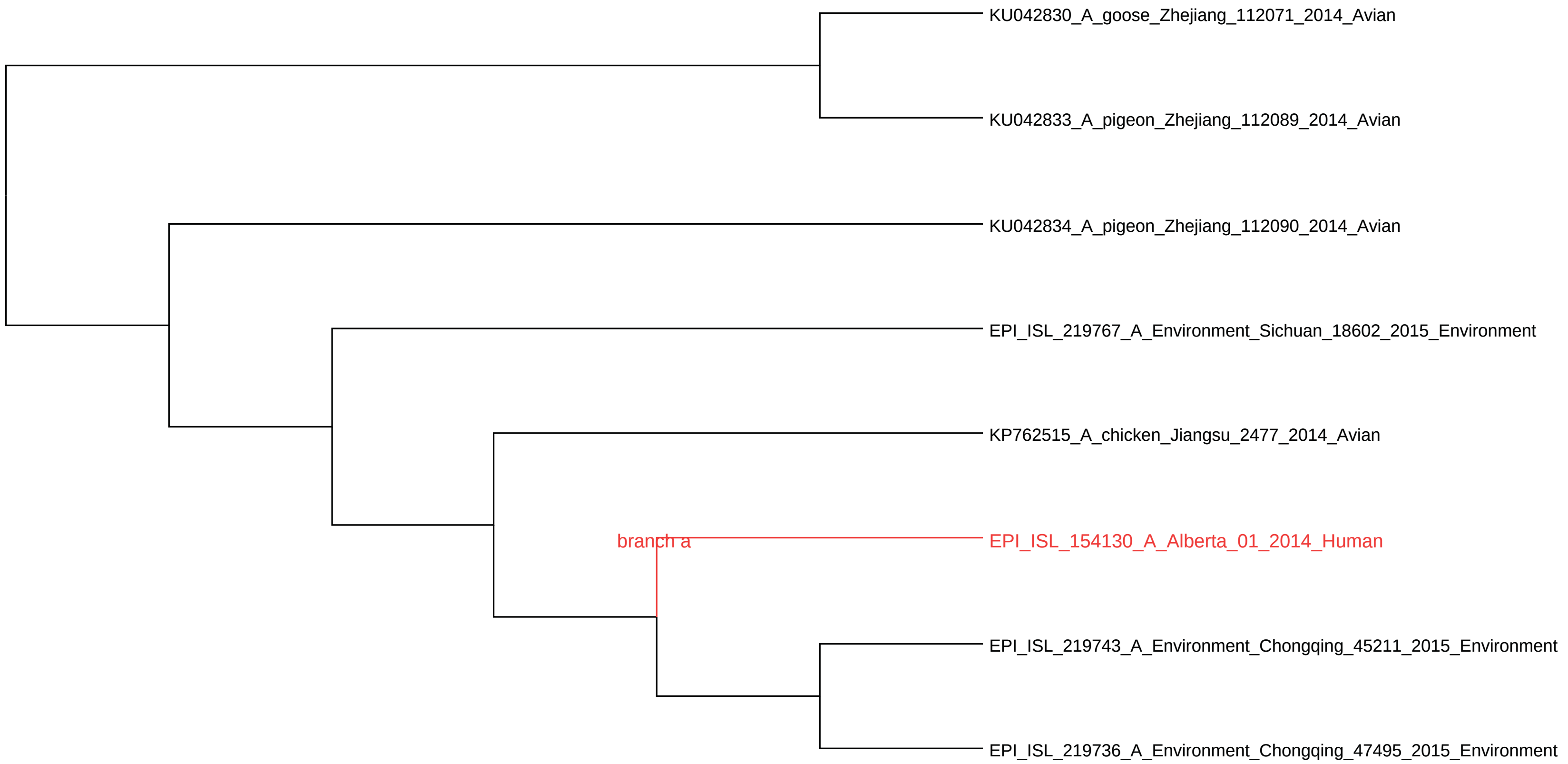
KU042830_A_goose_Zhejiang_112071_2014_Avian
KU042833_A_pigeon_Zhejiang_112089_2014_Avian
KU042834_A_pigeon_Zhejiang_112090_2014_Avian
EPI_ISL_219767_A_Environment_Sichuan_18602_2015_Environment
KP762515_A_chicken_Jiangsu_2477_2014_Avian
branch a
EPI_ISL_154130_A_Alberta_01_2014_Human
EPI_ISL_219743_A_Environment_Chongqing_45211_2015_Environment
EPI_ISL_219736_A_Environment_Chongqing_47495_2015_Environment

MP-Group8

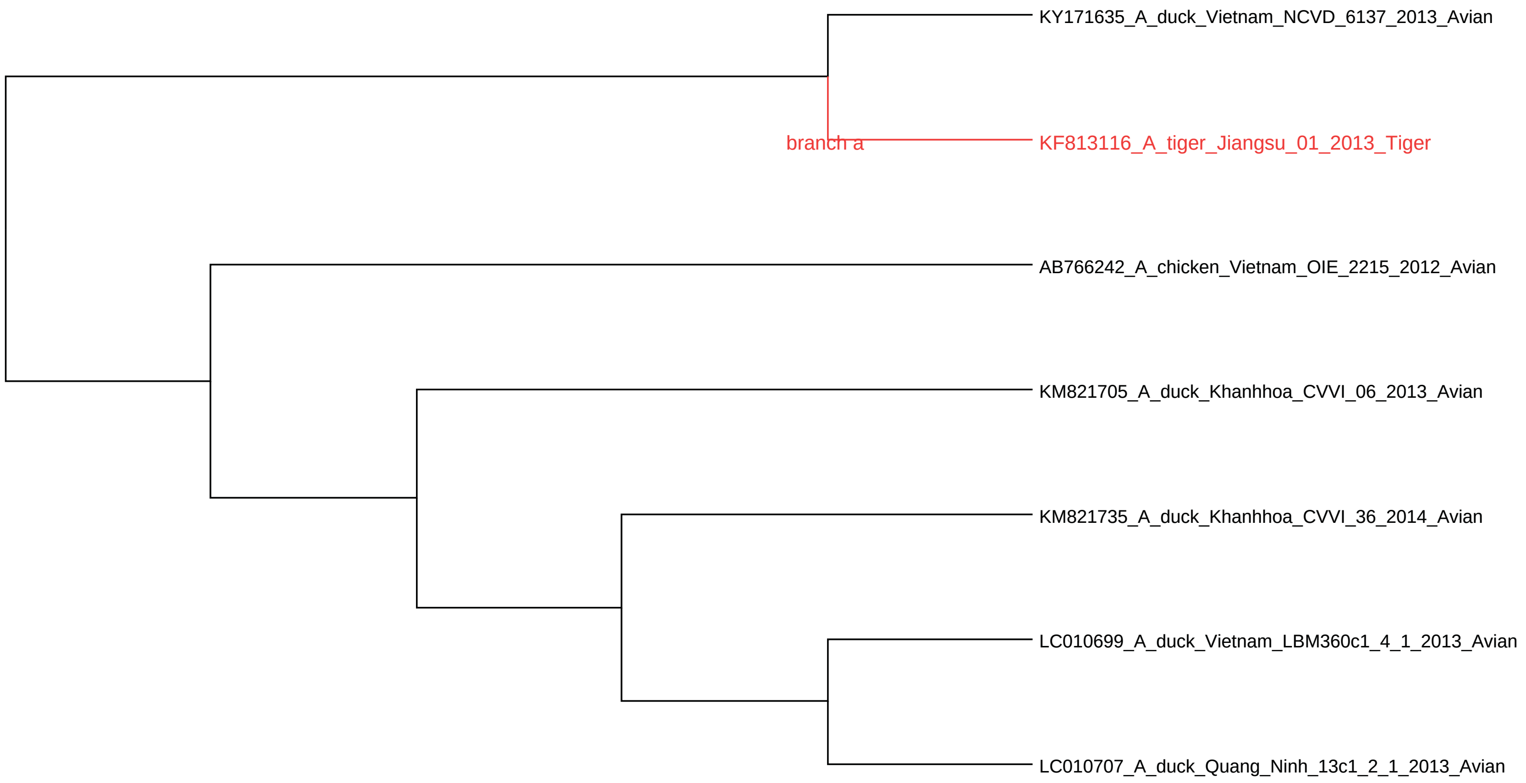

KY171635_A_duck_Vietnam_NCVD_6137_2013_Avian
branch a
KF813116_A_tiger_Jiangsu_01_2013_Tiger
AB766242_A_chicken_Vietnam_OIE_2215_2012_Avian
KM821705_A_duck_Khanhhoa_CVVI_06_2013_Avian
KM821735_A_duck_Khanhhoa_CVVI_36_2014_Avian
LC010699_A_duck_Vietnam_LBM360c1_4_1_2013_Avian
LC010707_A_duck_Quang_Ninh_13c1_2_1_2013_Avian

MP-Group9

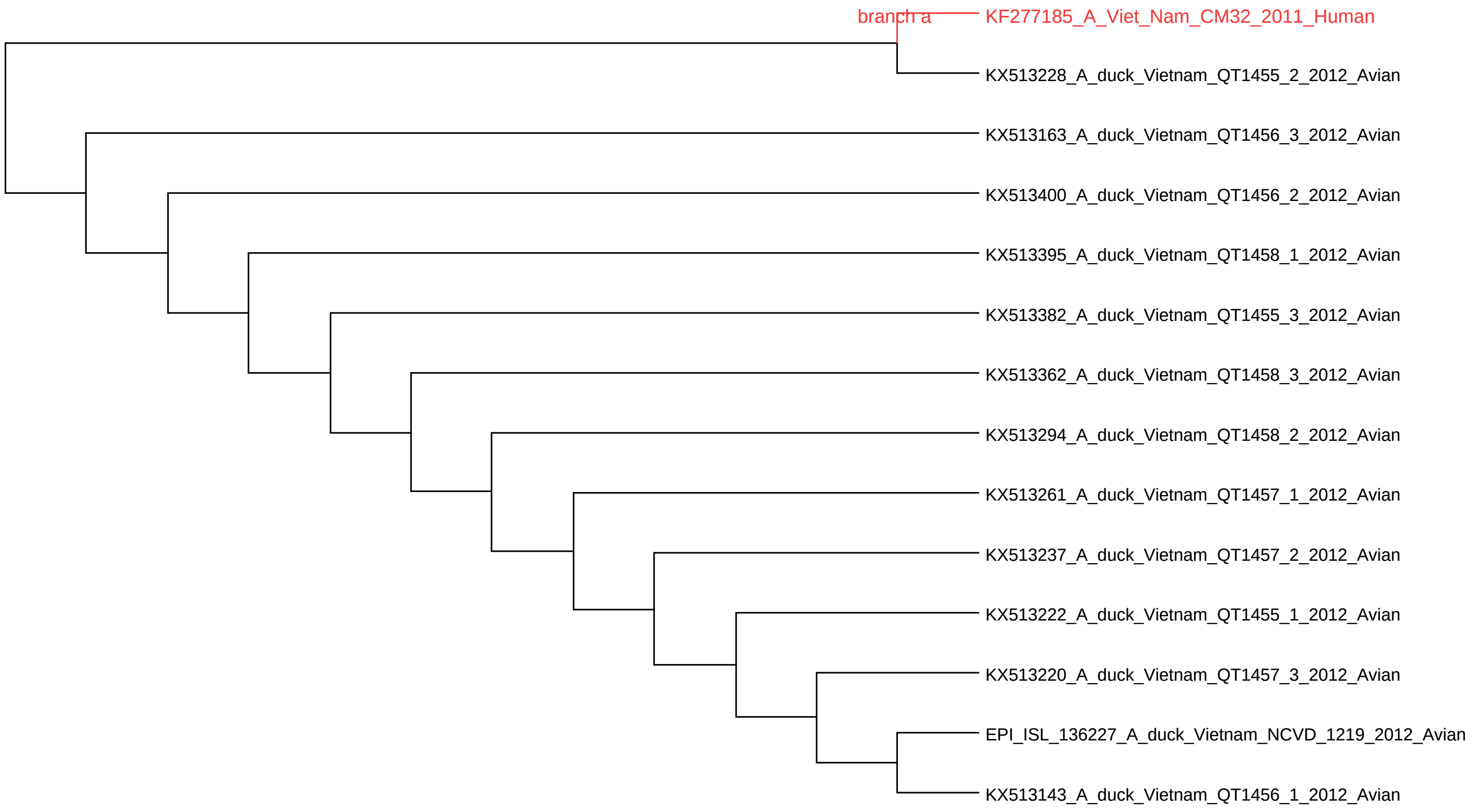
branch a
KF277185_A_Viet_Nam_CM32_2011_Human
KX513228_A_duck_Vietnam_QT1455_2_2012_Avian
KX513163_A_duck_Vietnam_QT1456_3_2012_Avian
KX513400_A_duck_Vietnam_QT1456_2_2012_Avian
KX513395_A_duck_Vietnam_QT1458_1_2012_Avian
KX513382_A_duck_Vietnam_QT1455_3_2012_Avian
KX513362_A_duck_Vietnam_QT1458_3_2012_Avian
KX513294_A_duck_Vietnam_QT1458_2_2012_Avian
KX513261_A_duck_Vietnam_QT1457_1_2012_Avian
KX513237_A_duck_Vietnam_QT1457_2_2012_Avian
KX513222_A_duck_Vietnam_QT1455_1_2012_Avian
KX513220_A_duck_Vietnam_QT1457_3_2012_Avian
EPI_ISL_136227_A_duck_Vietnam_NCVD_1219_2012_Avian
KX513143_A_duck_Vietnam_QT1456_1_2012_Avian

MP-Group10

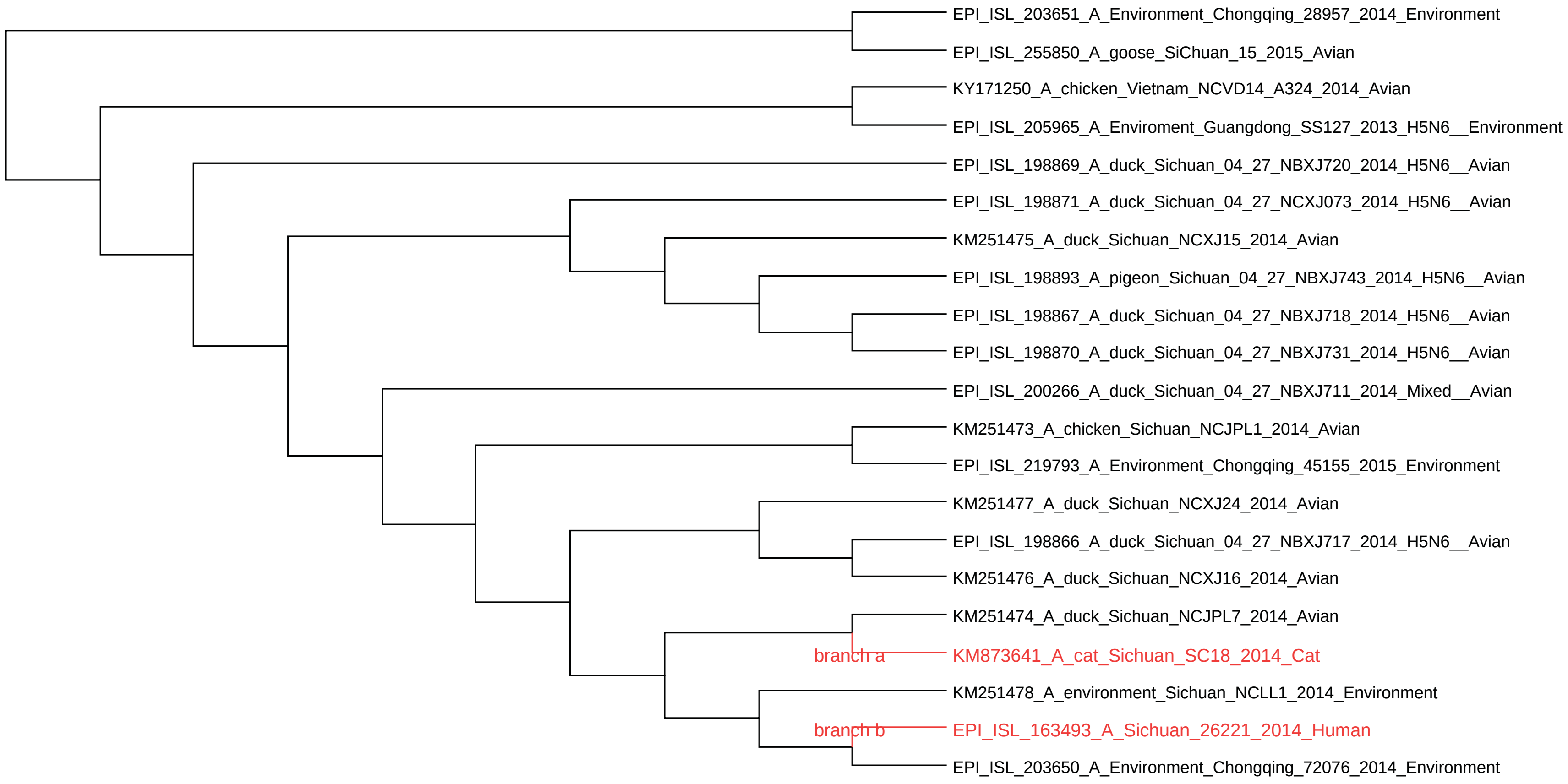
EPI_ISL_203651_A_Environment_Chongqing_28957_2014_Environment
EPI_ISL_255850_A_goose_SiChuan_15_2015_Avian
KY171250_A_chicken_Vietnam_NCVD14_A324_2014_Avian
EPI_ISL_205965_A_Enviroment_Guangdong_SS127_2013_H5N6__Environment
EPI_ISL_198869_A_duck_Sichuan_04_27_NBXJ720_2014_H5N6__Avian
EPI_ISL_198871_A_duck_Sichuan_04_27_NCXJ073_2014_H5N6__Avian
KM251475_A_duck_Sichuan_NCXJ15_2014_Avian
EPI_ISL_198893_A_pigeon_Sichuan_04_27_NBXJ743_2014_H5N6__Avian
EPI_ISL_198867_A_duck_Sichuan_04_27_NBXJ718_2014_H5N6__Avian
EPI_ISL_198870_A_duck_Sichuan_04_27_NBXJ731_2014_H5N6__Avian
EPI_ISL_200266_A_duck_Sichuan_04_27_NBXJ711_2014_Mixed__Avian
KM251473_A_chicken_Sichuan_NCJPL1_2014_Avian
EPI_ISL_219793_A_Environment_Chongqing_45155_2015_Environment
KM251477_A_duck_Sichuan_NCXJ24_2014_Avian
EPI_ISL_198866_A_duck_Sichuan_04_27_NBXJ717_2014_H5N6__Avian
KM251476_A_duck_Sichuan_NCXJ16_2014_Avian
KM251474_A_duck_Sichuan_NCJPL7_2014_Avian
branch a
KM873641_A_cat_Sichuan_SC18_2014_Cat
KM251478_A_environment_Sichuan_NCLL1_2014_Environment
branch b
EPI_ISL_163493_A_Sichuan_26221_2014_Human
EPI_ISL_203650_A_Environment_Chongqing_72076_2014_Environment

MP-Group11

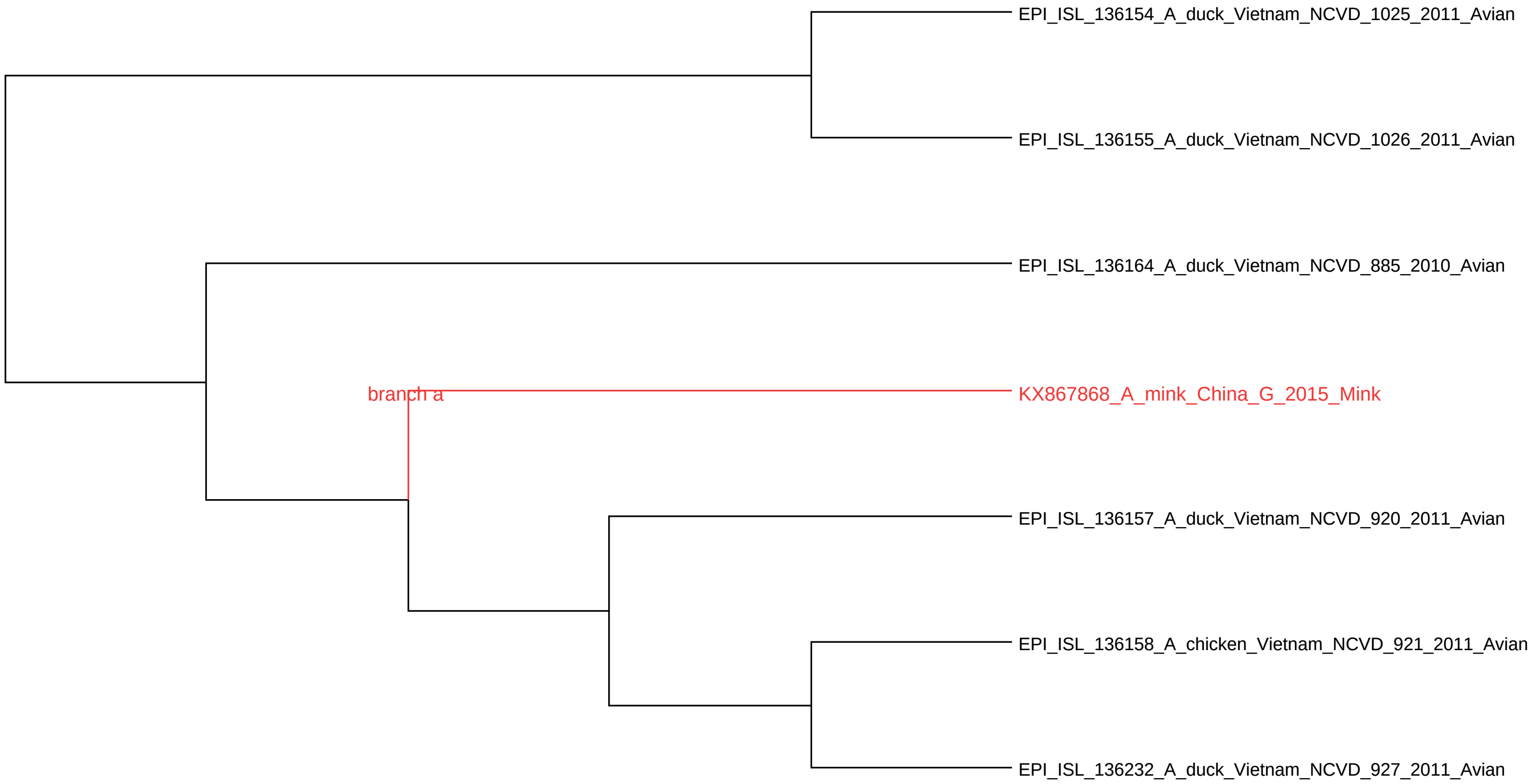

EPI_ISL_136154_A_duck_Vietnam_NCVD_1025_2011_Avian
EPI_ISL_136155_A_duck_Vietnam_NCVD_1026_2011_Avian
EPI_ISL_136164_A_duck_Vietnam_NCVD_885_2010_Avian
branch a
KX867868_A_mink_China_G_2015_Mink
EPI_ISL_136157_A_duck_Vietnam_NCVD_920_2011_Avian
EPI_ISL_136158_A_chicken_Vietnam_NCVD_921_2011_Avian
EPI_ISL_136232_A_duck_Vietnam_NCVD_927_2011_Avian

MP-Group12

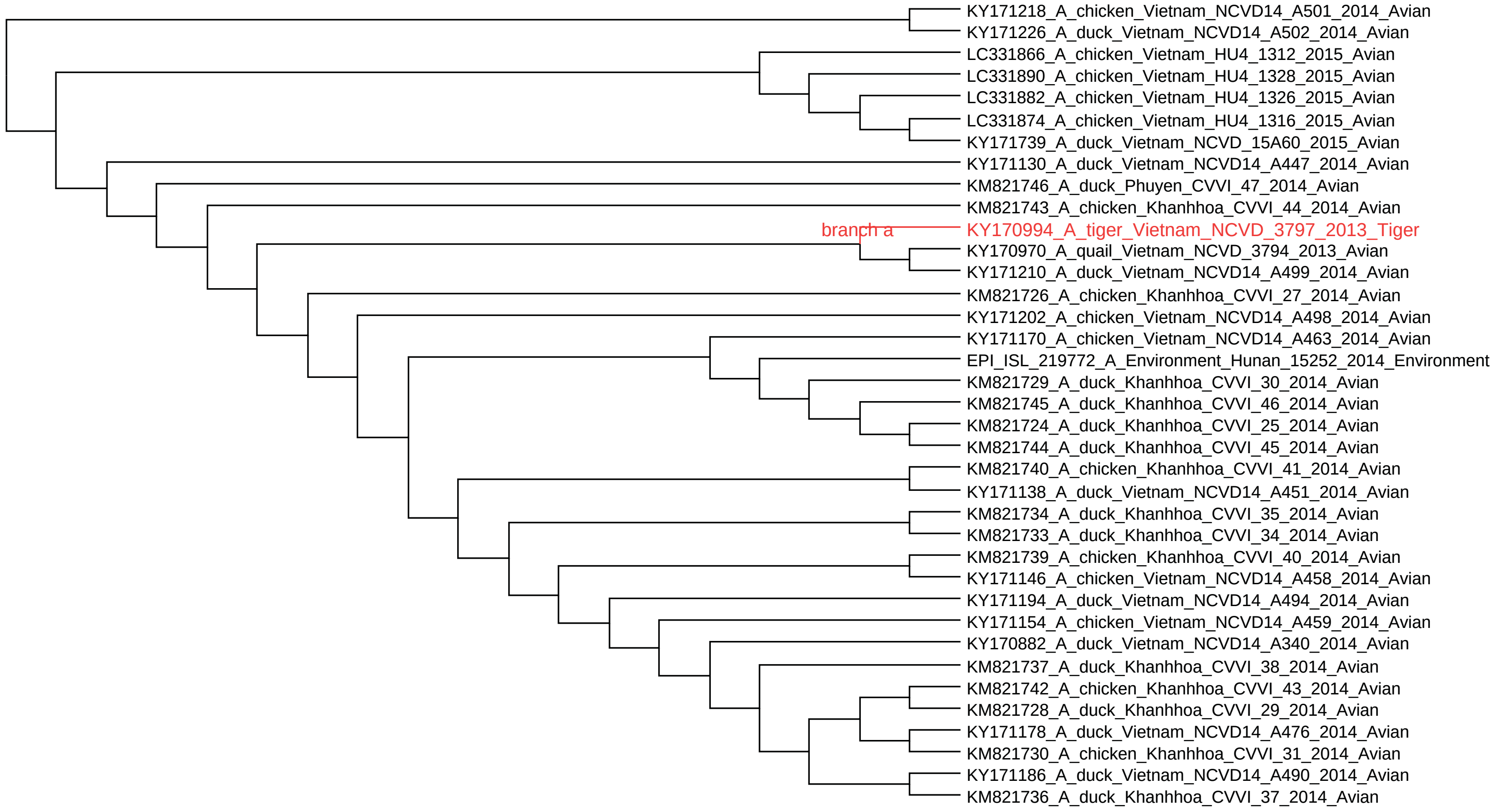
KY171218_A_chicken_Vietnam_NCVD14_A501_2014_Avian
KY171226_A_duck_Vietnam_NCVD14_A502_2014_Avian
LC331866_A_chicken_Vietnam_HU4_1312_2015_Avian
LC331890_A_chicken_Vietnam_HU4_1328_2015_Avian
LC331882_A_chicken_Vietnam_HU4_1326_2015_Avian
LC331874_A_chicken_Vietnam_HU4_1316_2015_Avian
KY171739_A_duck_Vietnam_NCVD_15A60_2015_Avian
KY171130_A_duck_Vietnam_NCVD14_A447_2014_Avian
KM821746_A_duck_Phuyen_CVVI_47_2014_Avian
KM821743_A_chicken_Khanhhoa_CVVI_44_2014_Avian
branch a
KY170994_A_tiger_Vietnam_NCVD_3797_2013_Tiger
KY170970_A_quail_Vietnam_NCVD_3794_2013_Avian
KY171210_A_duck_Vietnam_NCVD14_A499_2014_Avian
KM821726_A_chicken_Khanhhoa_CVVI_27_2014_Avian
KY171202_A_chicken_Vietnam_NCVD14_A498_2014_Avian
KY171170_A_chicken_Vietnam_NCVD14_A463_2014_Avian
EPI_ISL_219772_A_Environment_Hunan_15252_2014_Environment
KM821729_A_duck_Khanhhoa_CVVI_30_2014_Avian
KM821745_A_duck_Khanhhoa_CVVI_46_2014_Avian
KM821724_A_duck_Khanhhoa_CVVI_25_2014_Avian
KM821744_A_duck_Khanhhoa_CVVI_45_2014_Avian
KM821740_A_chicken_Khanhhoa_CVVI_41_2014_Avian
KY171138_A_duck_Vietnam_NCVD14_A451_2014_Avian
KM821734_A_duck_Khanhhoa_CVVI_35_2014_Avian
KM821733_A_duck_Khanhhoa_CVVI_34_2014_Avian
KM821739_A_chicken_Khanhhoa_CVVI_40_2014_Avian
KY171146_A_chicken_Vietnam_NCVD14_A458_2014_Avian
KY171194_A_duck_Vietnam_NCVD14_A494_2014_Avian
KY171154_A_chicken_Vietnam_NCVD14_A459_2014_Avian
KY170882_A_duck_Vietnam_NCVD14_A340_2014_Avian
KM821737_A_duck_Khanhhoa_CVVI_38_2014_Avian
KM821742_A_chicken_Khanhhoa_CVVI_43_2014_Avian
KM821728_A_duck_Khanhhoa_CVVI_29_2014_Avian
KY171178_A_duck_Vietnam_NCVD14_A476_2014_Avian
KM821730_A_chicken_Khanhhoa_CVVI_31_2014_Avian
KY171186_A_duck_Vietnam_NCVD14_A490_2014_Avian
KM821736_A_duck_Khanhhoa_CVVI_37_2014_Avian

MP-Group13

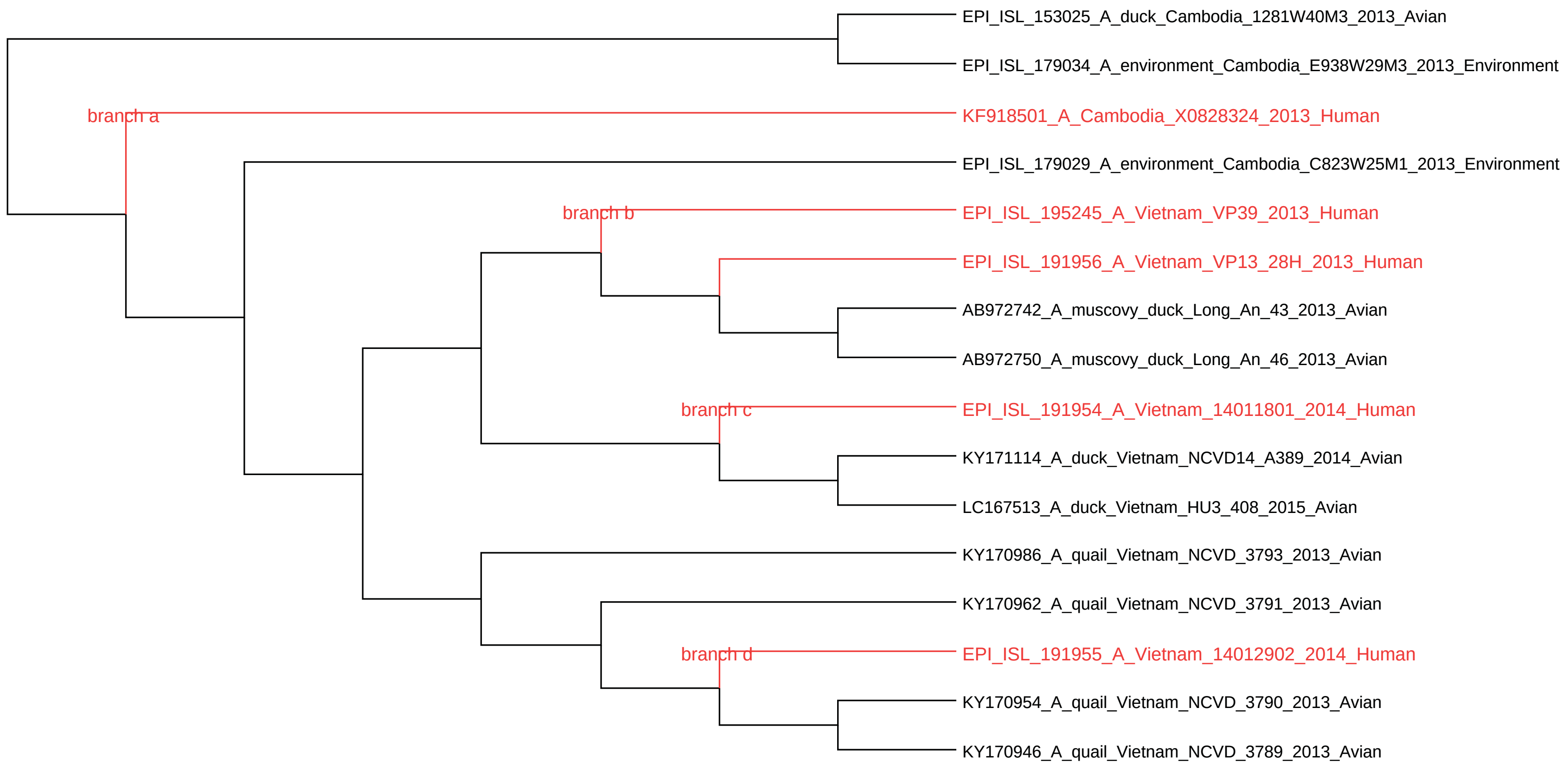

EPI_ISL_153025_A_duck_Cambodia_1281W40M3_2013_Avian
EPI_ISL_179034_A_environment_Cambodia_E938W29M3_2013_Environment
branch a
KF918501_A_Cambodia_X0828324_2013_Human
EPI_ISL_179029_A_environment_Cambodia_C823W25M1_2013_Environment
branch b
EPI_ISL_195245_A_Vietnam_VP39_2013_Human
EPI_ISL_191956_A_Vietnam_VP13_28H_2013_Human
AB972742_A_muscovy_duck_Long_An_43_2013_Avian
AB972750_A_muscovy_duck_Long_An_46_2013_Avian
branch c
EPI_ISL_191954_A_Vietnam_14011801_2014_Human
KY171114_A_duck_Vietnam_NCVD14_A389_2014_Avian
LC167513_A_duck_Vietnam_HU3_408_2015_Avian
KY170986_A_quail_Vietnam_NCVD_3793_2013_Avian
KY170962_A_quail_Vietnam_NCVD_3791_2013_Avian
branch d
EPI_ISL_191955_A_Vietnam_14012902_2014_Human
KY170954_A_quail_Vietnam_NCVD_3790_2013_Avian
KY170946_A_quail_Vietnam_NCVD_3789_2013_Avian

MP-Group14

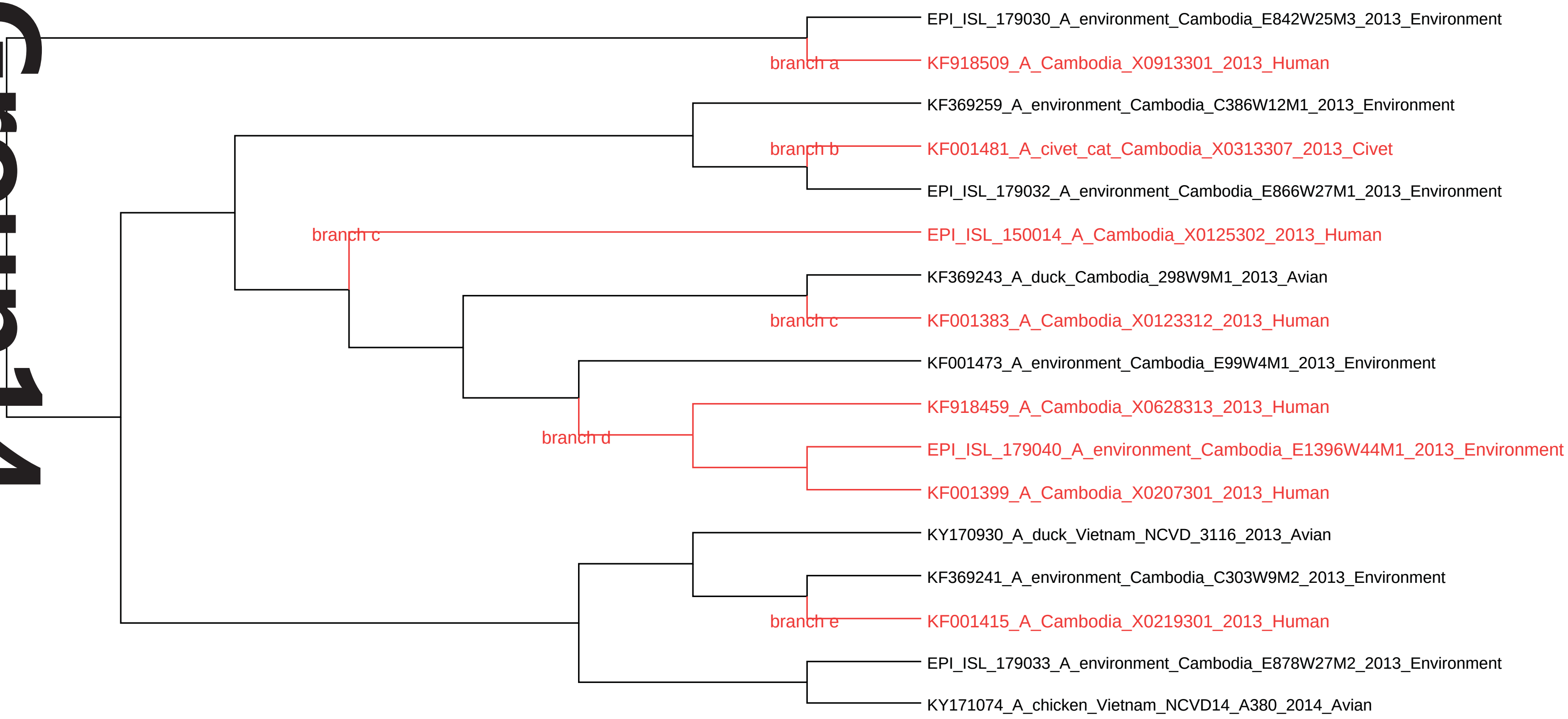
EPI_ISL_179030_A_environment_Cambodia_E842W25M3_2013_Environment
branch a
KF918509_A_Cambodia_X0913301_2013_Human
KF369259_A_environment_Cambodia_C386W12M1_2013_Environment
branch b
KF001481_A_civet_cat_Cambodia_X0313307_2013_Civet
EPI_ISL_179032_A_environment_Cambodia_E866W27M1_2013_Environment
branch c
EPI_ISL_150014_A_Cambodia_X0125302_2013_Human
KF369243_A_duck_Cambodia_298W9M1_2013_Avian
branch c
KF001383_A_Cambodia_X0123312_2013_Human
KF001473_A_environment_Cambodia_E99W4M1_2013_Environment
KF918459_A_Cambodia_X0628313_2013_Human
branch d
EPI_ISL_179040_A_environment_Cambodia_E1396W44M1_2013_Environment
KF001399_A_Cambodia_X0207301_2013_Human
KY170930_A_duck_Vietnam_NCVD_3116_2013_Avian
KF369241_A_environment_Cambodia_C303W9M2_2013_Environment
branch e
KF001415_A_Cambodia_X0219301_2013_Human
EPI_ISL_179033_A_environment_Cambodia_E878W27M2_2013_Environment
KY171074_A_chicken_Vietnam_NCVD14_A380_2014_Avian

MP-Group15

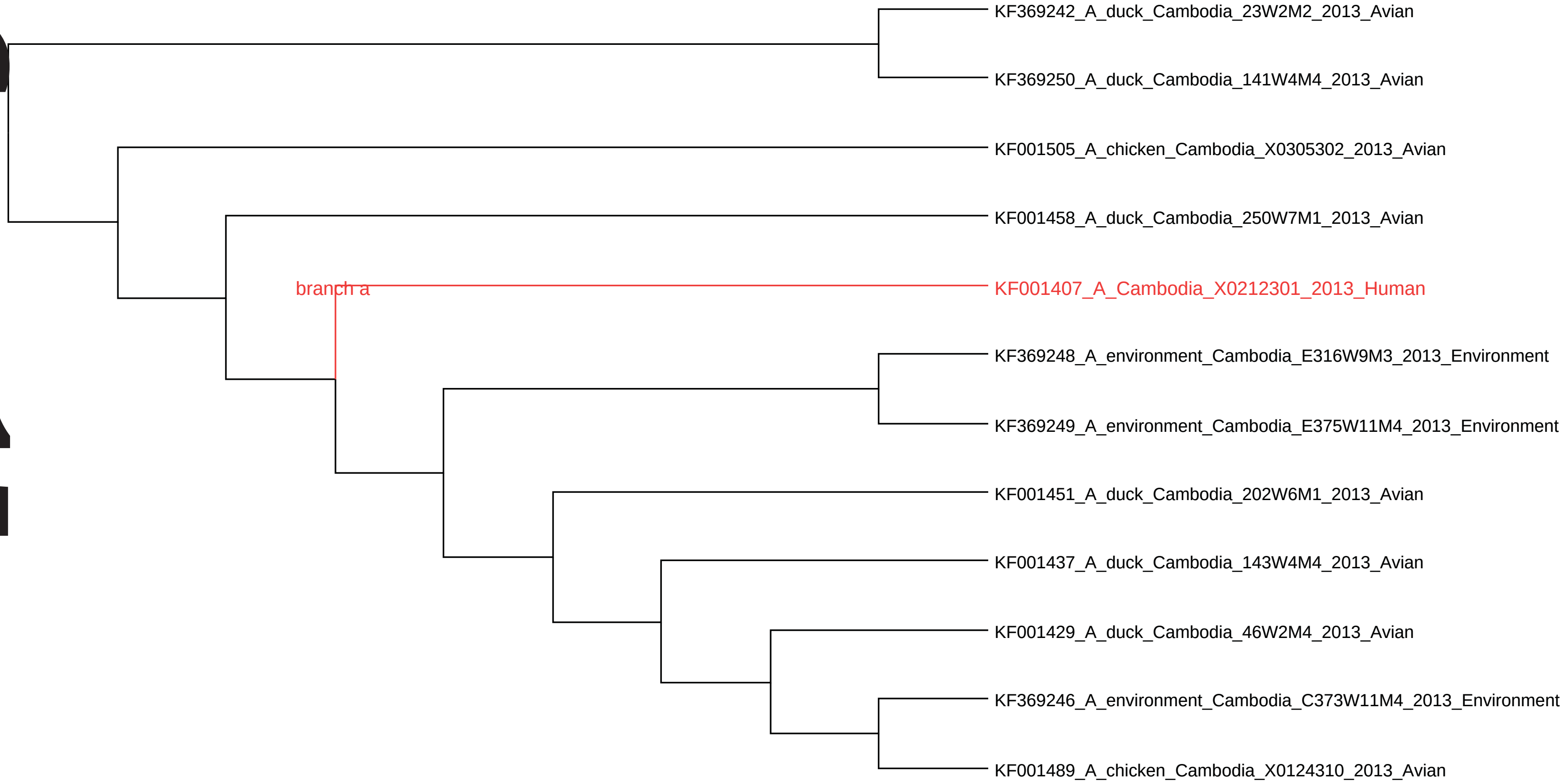
KF369242_A_duck_Cambodia_23W2M2_2013_Avian
KF369250_A_duck_Cambodia_141W4M4_2013_Avian
KF001505_A_chicken_Cambodia_X0305302_2013_Avian
KF001458_A_duck_Cambodia_250W7M1_2013_Avian
branch a
KF001407_A_Cambodia_X0212301_2013_Human
KF369248_A_environment_Cambodia_E316W9M3_2013_Environment
KF369249_A_environment_Cambodia_E375W11M4_2013_Environment
KF001451_A_duck_Cambodia_202W6M1_2013_Avian
KF001437_A_duck_Cambodia_143W4M4_2013_Avian
KF001429_A_duck_Cambodia_46W2M4_2013_Avian
KF369246_A_environment_Cambodia_C373W11M4_2013_Environment
KF001489_A_chicken_Cambodia_X0124310_2013_Avian

MP-Group16

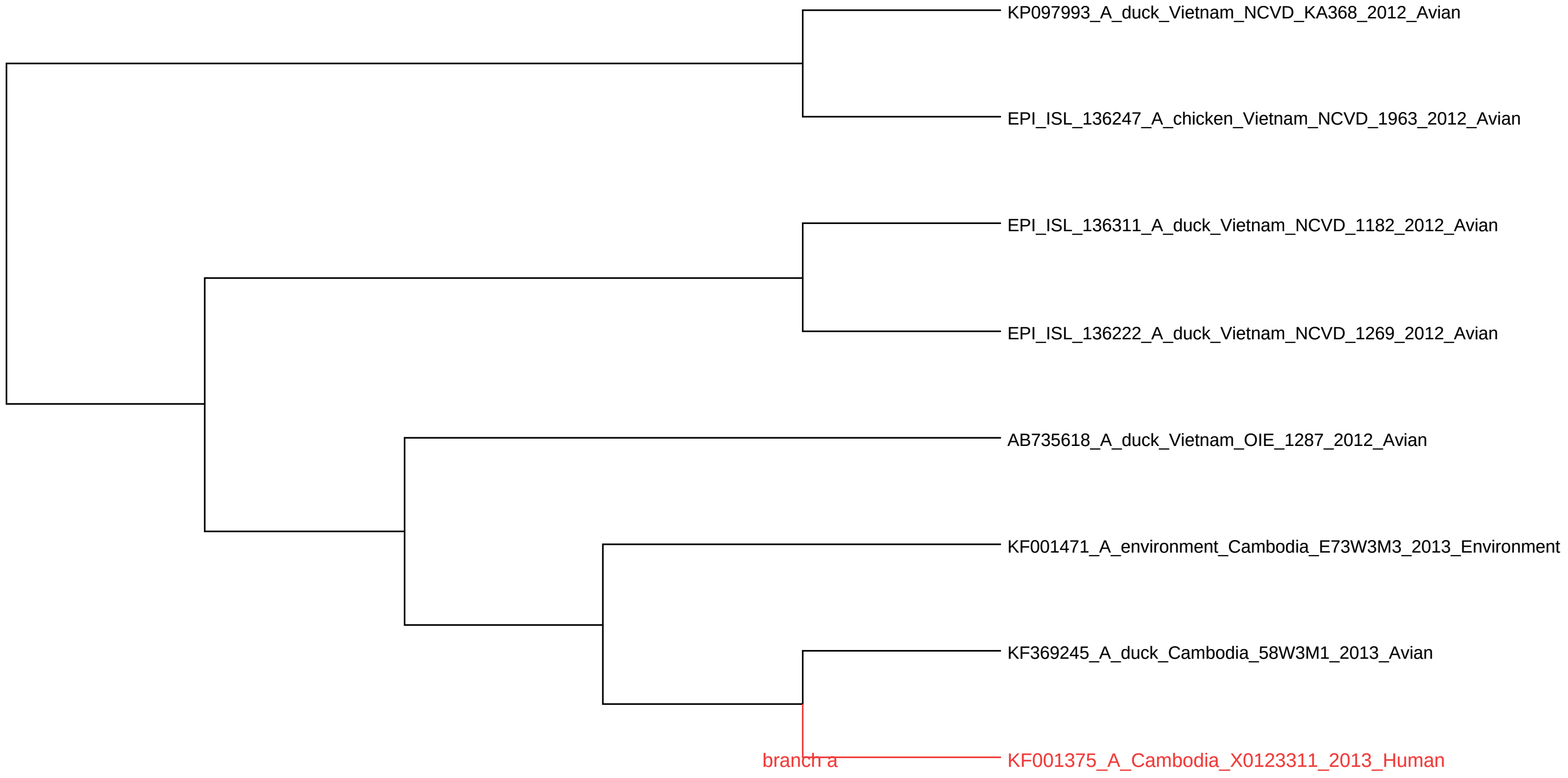

KP097993_A_duck_Vietnam_NCVD_KA368_2012_Avian
EPI_ISL_136247_A_chicken_Vietnam_NCVD_1963_2012_Avian
EPI_ISL_136311_A_duck_Vietnam_NCVD_1182_2012_Avian
EPI_ISL_136222_A_duck_Vietnam_NCVD_1269_2012_Avian
AB735618_A_duck_Vietnam_OIE_1287_2012_Avian
KF001471_A_environment_Cambodia_E73W3M3_2013_Environment
KF369245_A_duck_Cambodia_58W3M1_2013_Avian
branch a
KF001375_A_Cambodia_X0123311_2013_Human

MP-Group17

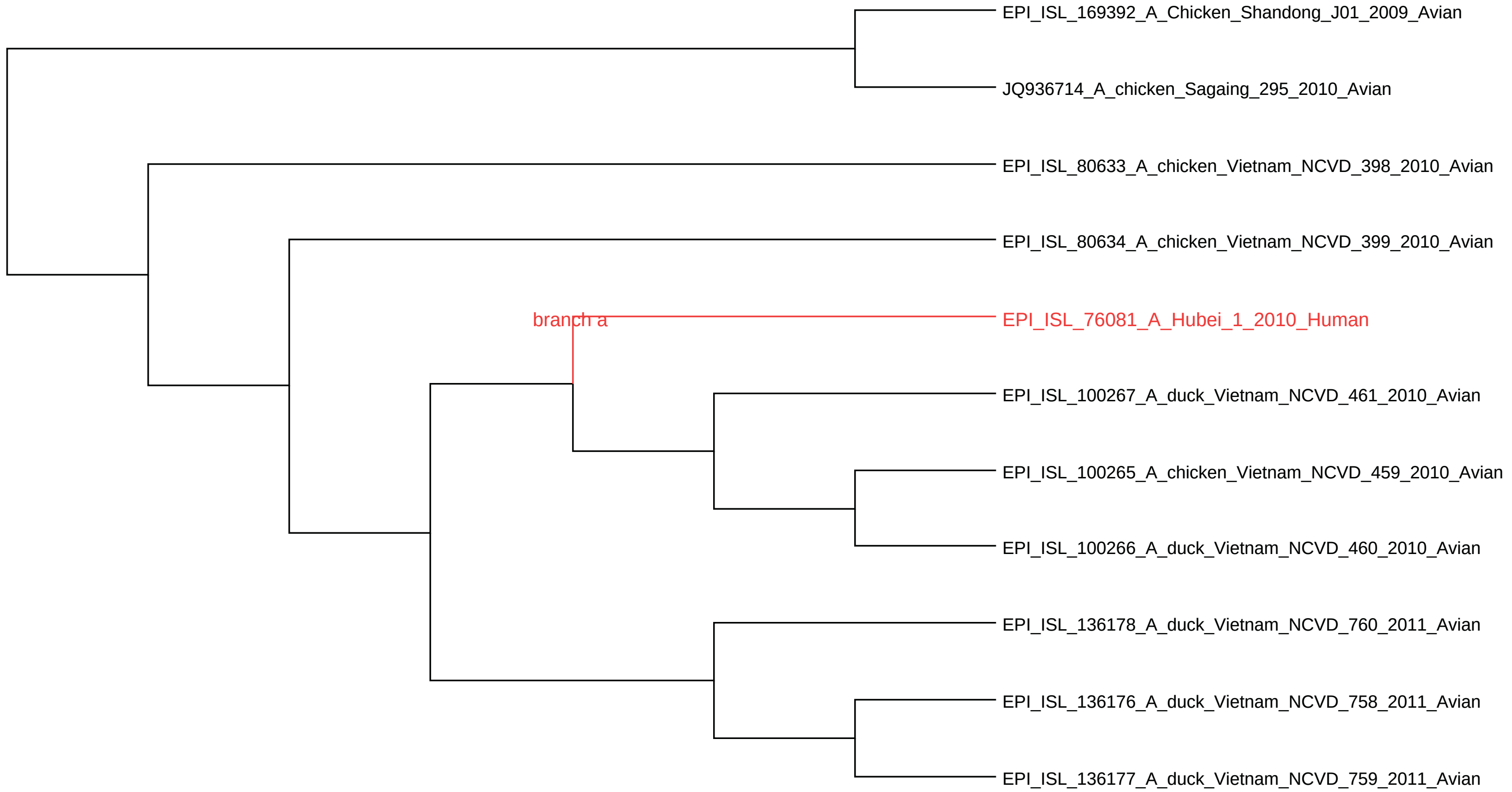
EPI_ISL_169392_A_Chicken_Shandong_J01_2009_Avian
JQ936714_A_chicken_Sagaing_295_2010_Avian
EPI_ISL_80633_A_chicken_Vietnam_NCVD_398_2010_Avian
EPI_ISL_80634_A_chicken_Vietnam_NCVD_399_2010_Avian
branch a
EPI_ISL_76081_A_Hubei_1_2010_Human
EPI_ISL_100267_A_duck_Vietnam_NCVD_461_2010_Avian
EPI_ISL_100265_A_chicken_Vietnam_NCVD_459_2010_Avian
EPI_ISL_100266_A_duck_Vietnam_NCVD_460_2010_Avian
EPI_ISL_136178_A_duck_Vietnam_NCVD_760_2011_Avian
EPI_ISL_136176_A_duck_Vietnam_NCVD_758_2011_Avian
EPI_ISL_136177_A_duck_Vietnam_NCVD_759_2011_Avian

MP-Group18

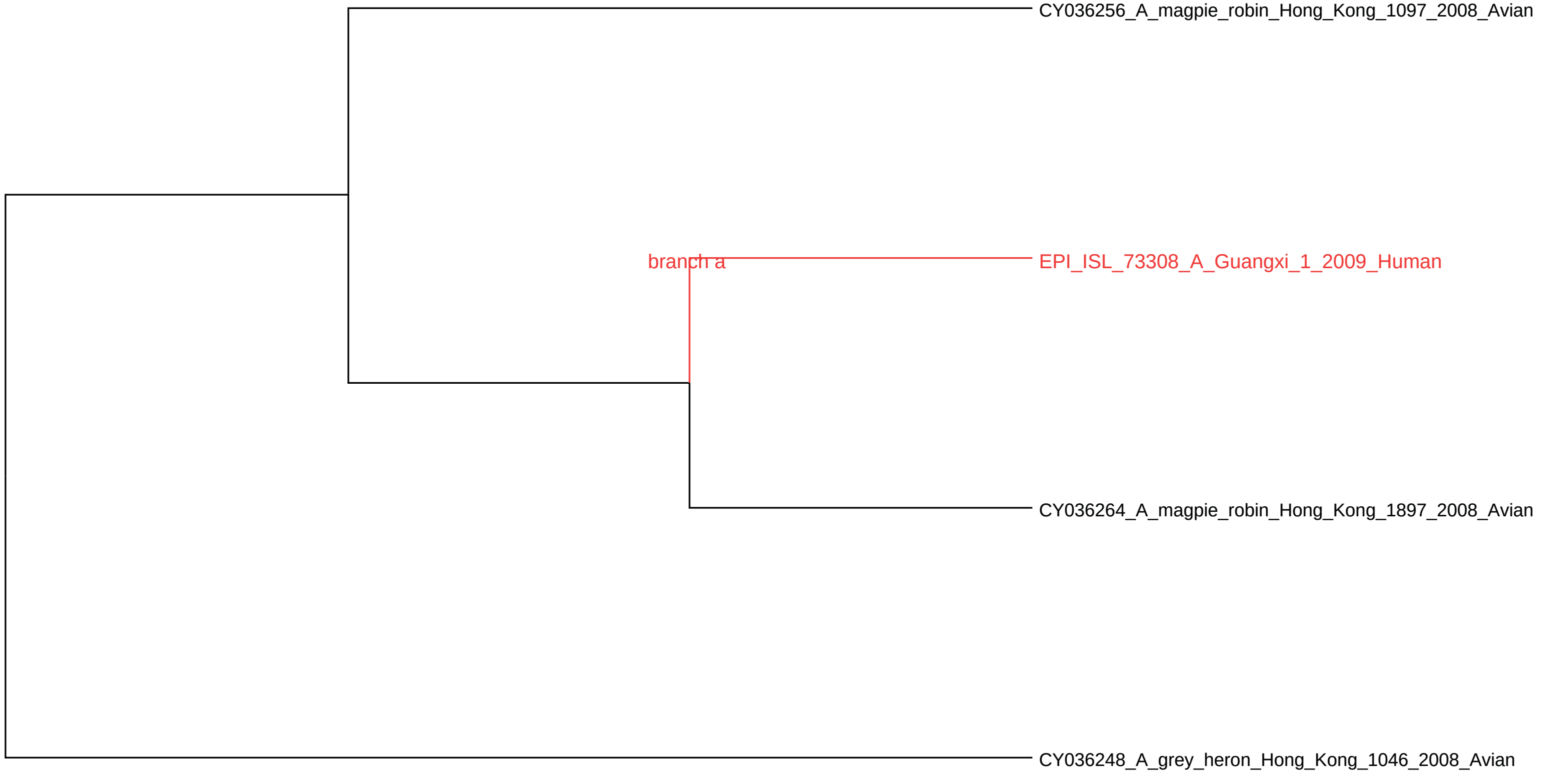
CY036256_A_magpie_robin_Hong_Kong_1097_2008_Avian
branch a
EPI_ISL_73308_A_Guangxi_1_2009_Human
CY036264_A_magpie_robin_Hong_Kong_1897_2008_Avian
CY036248_A_grey_heron_Hong_Kong_1046_2008_Avian

MP-Group19

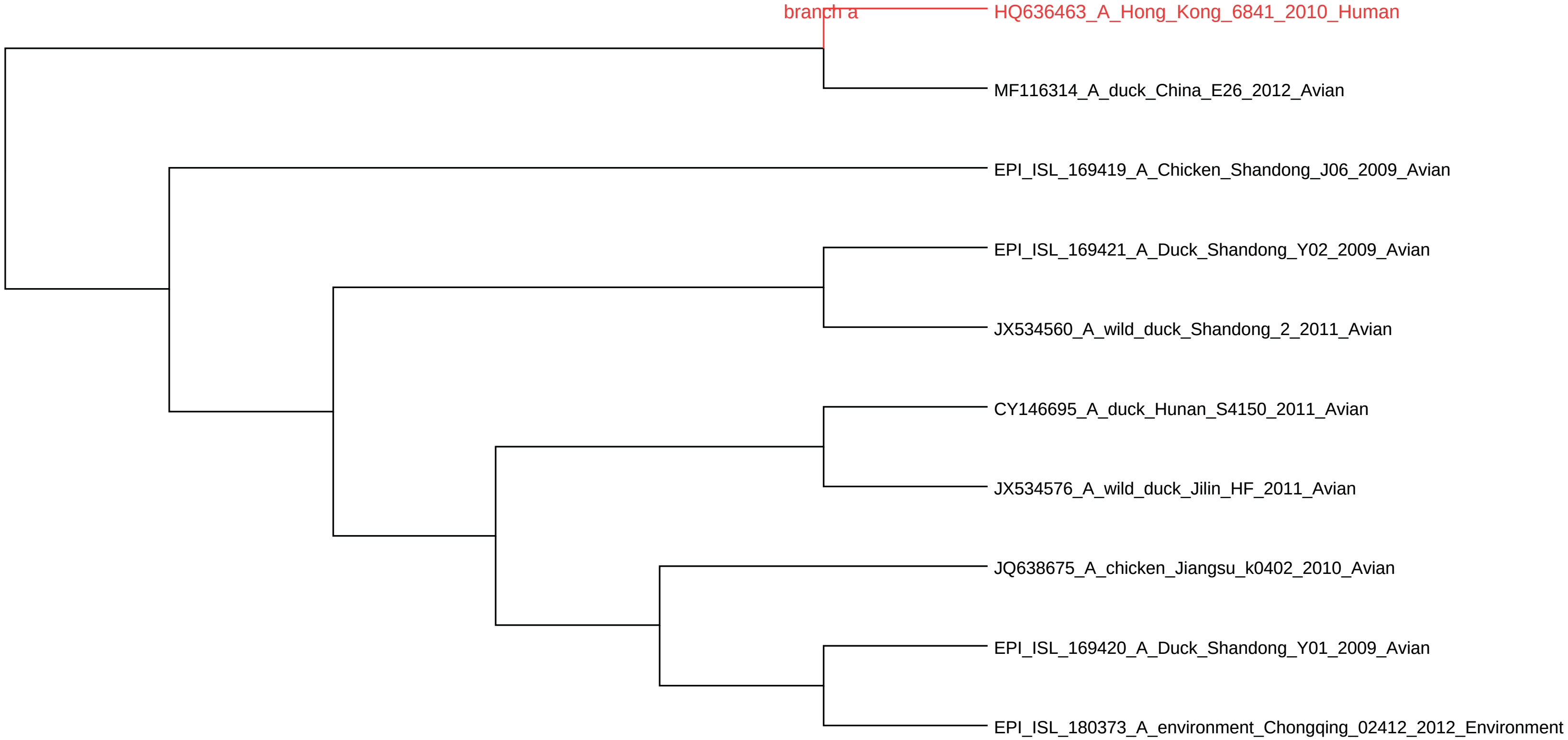
branch a
HQ636463_A_Hong_Kong_6841_2010_Human
MF116314_A_duck_China_E26_2012_Avian
EPI_ISL_169419_A_Chicken_Shandong_J06_2009_Avian
EPI_ISL_169421_A_Duck_Shandong_Y02_2009_Avian
JX534560_A_wild_duck_Shandong_2_2011_Avian
CY146695_A_duck_Hunan_S4150_2011_Avian
JX534576_A_wild_duck_Jilin_HF_2011_Avian
JQ638675_A_chicken_Jiangsu_k0402_2010_Avian
EPI_ISL_169420_A_Duck_Shandong_Y01_2009_Avian
EPI_ISL_180373_A_environment_Chongqing_02412_2012_Environment

MP-Group20

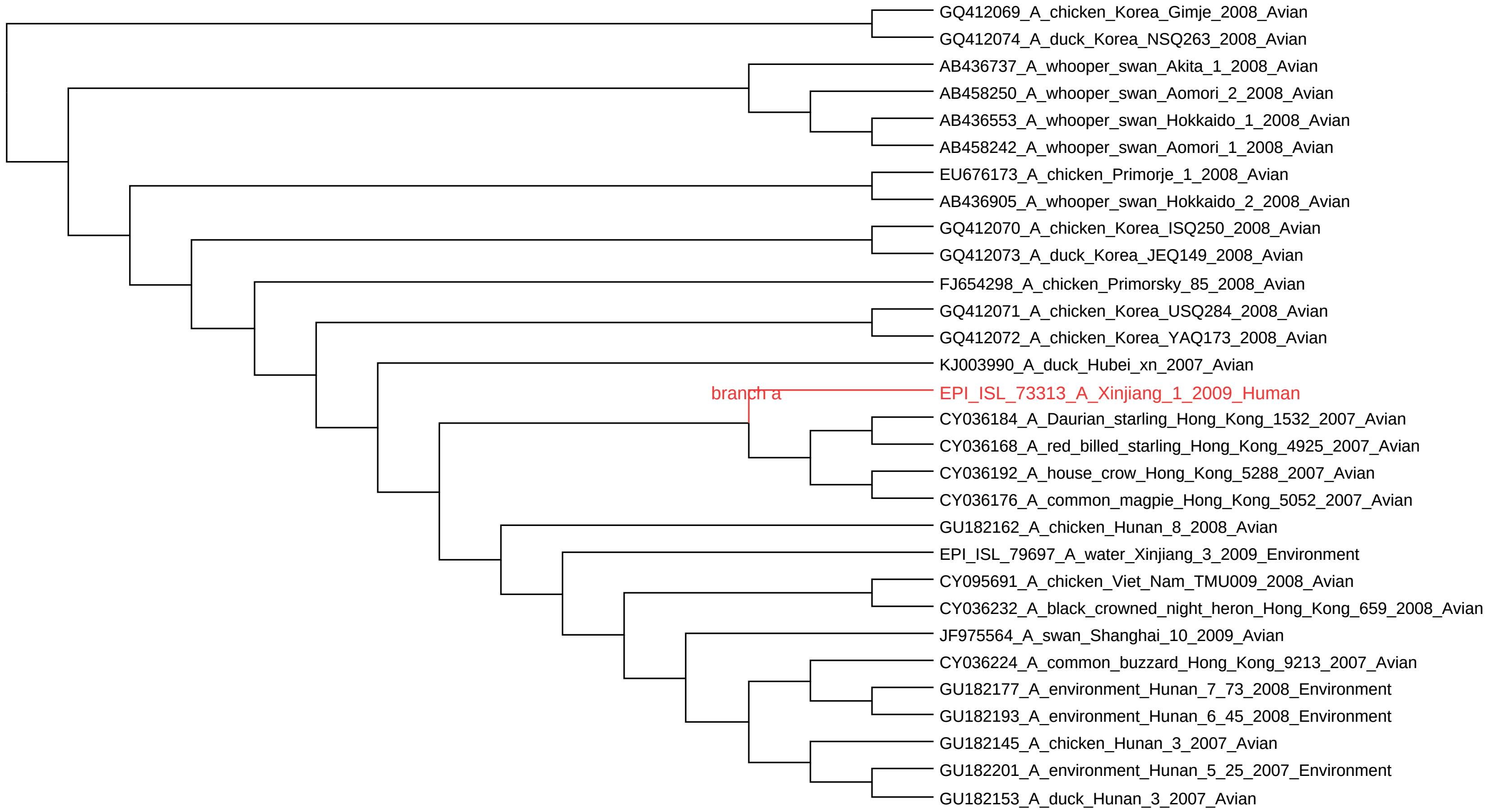
GQ412069_A_chicken_Korea_Gimje_2008_Avian
GQ412074_A_duck_Korea_NSQ263_2008_Avian
AB436737_A_whooper_swan_Akita_1_2008_Avian
AB458250_A_whooper_swan_Aomori_2_2008_Avian
AB436553_A_whooper_swan_Hokkaido_1_2008_Avian
AB458242_A_whooper_swan_Aomori_1_2008_Avian
EU676173_A_chicken_Primorje_1_2008_Avian
AB436905_A_whooper_swan_Hokkaido_2_2008_Avian
GQ412070_A_chicken_Korea_ISQ250_2008_Avian
GQ412073_A_duck_Korea_JEQ149_2008_Avian
FJ654298_A_chicken_Primorsky_85_2008_Avian
GQ412071_A_chicken_Korea_USQ284_2008_Avian
GQ412072_A_chicken_Korea_YAQ173_2008_Avian
KJ003990_A_duck_Hubei_xn_2007_Avian
branch a
EPI_ISL_73313_A_Xinjiang_1_2009_Human
CY036184_A_Daurian_starling_Hong_Kong_1532_2007_Avian
CY036168_A_red_billed_starling_Hong_Kong_4925_2007_Avian
CY036192_A_house_crow_Hong_Kong_5288_2007_Avian
CY036176_A_common_magpie_Hong_Kong_5052_2007_Avian
GU182162_A_chicken_Hunan_8_2008_Avian
EPI_ISL_79697_A_water_Xinjiang_3_2009_Environment
CY095691_A_chicken_Viet_Nam_TMU009_2008_Avian
CY036232_A_black_crowned_night_heron_Hong_Kong_659_2008_Avian
JF975564_A_swan_Shanghai_10_2009_Avian
CY036224_A_common_buzzard_Hong_Kong_9213_2007_Avian
GU182177_A_environment_Hunan_7_73_2008_Environment
GU182193_A_environment_Hunan_6_45_2008_Environment
GU182145_A_chicken_Hunan_3_2007_Avian
GU182201_A_environment_Hunan_5_25_2007_Environment
GU182153_A_duck_Hunan_3_2007_Avian

MP-Group21

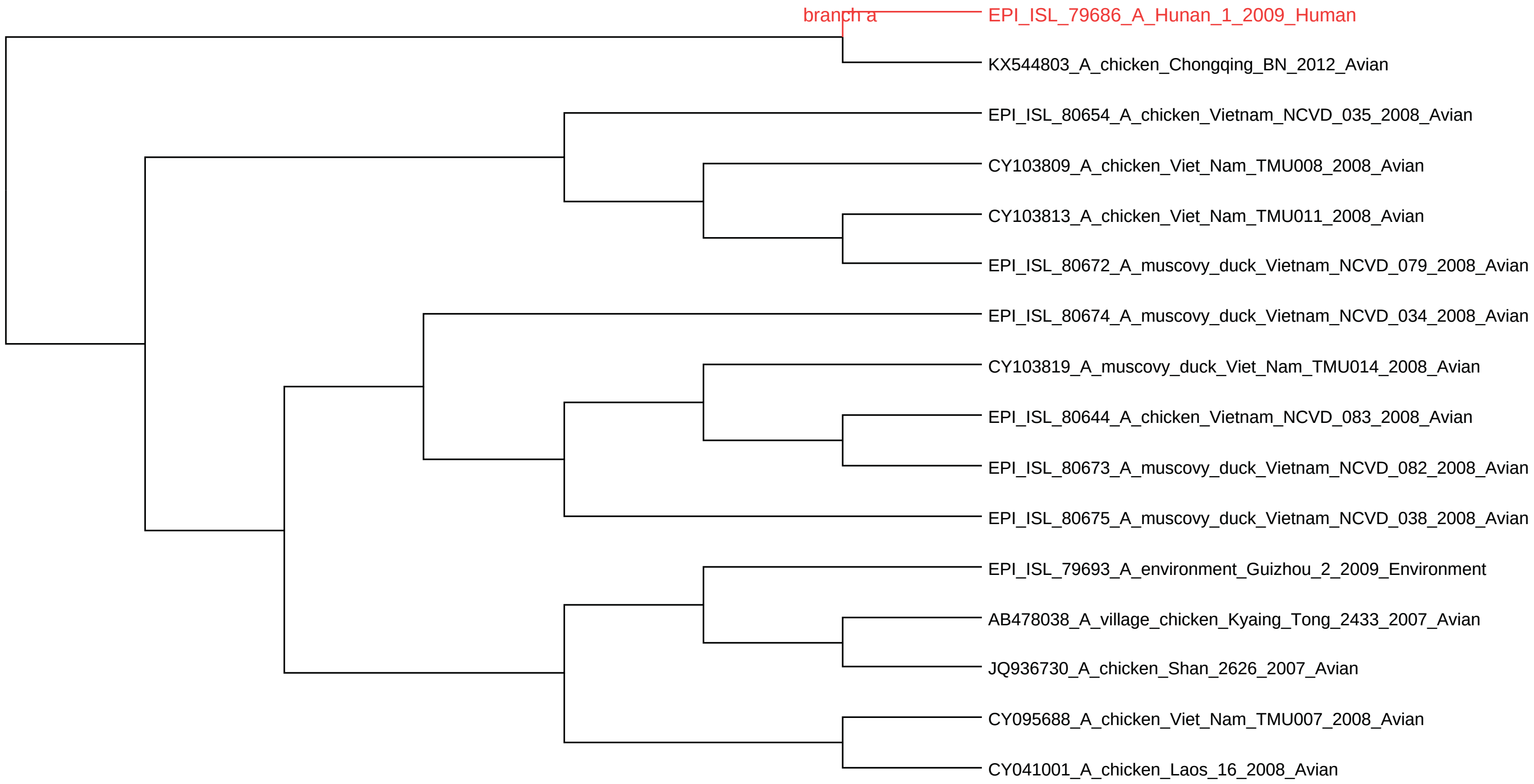
branch a
EPI_ISL_79686_A_Hunan_1_2009_Human
KX544803_A_chicken_Chongqing_BN_2012_Avian
EPI_ISL_80654_A_chicken_Vietnam_NCVD_035_2008_Avian
CY103809_A_chicken_Viet_Nam_TMU008_2008_Avian
CY103813_A_chicken_Viet_Nam_TMU011_2008_Avian
EPI_ISL_80672_A_muscovy_duck_Vietnam_NCVD_079_2008_Avian
EPI_ISL_80674_A_muscovy_duck_Vietnam_NCVD_034_2008_Avian
CY103819_A_muscovy_duck_Viet_Nam_TMU014_2008_Avian
EPI_ISL_80644_A_chicken_Vietnam_NCVD_083_2008_Avian
EPI_ISL_80673_A_muscovy_duck_Vietnam_NCVD_082_2008_Avian
EPI_ISL_80675_A_muscovy_duck_Vietnam_NCVD_038_2008_Avian
EPI_ISL_79693_A_environment_Guizhou_2_2009_Environment
AB478038_A_village_chicken_Kyaing_Tong_2433_2007_Avian
JQ936730_A_chicken_Shan_2626_2007_Avian
CY095688_A_chicken_Viet_Nam_TMU007_2008_Avian
CY041001_A_chicken_Laos_16_2008_Avian

MP-Group22

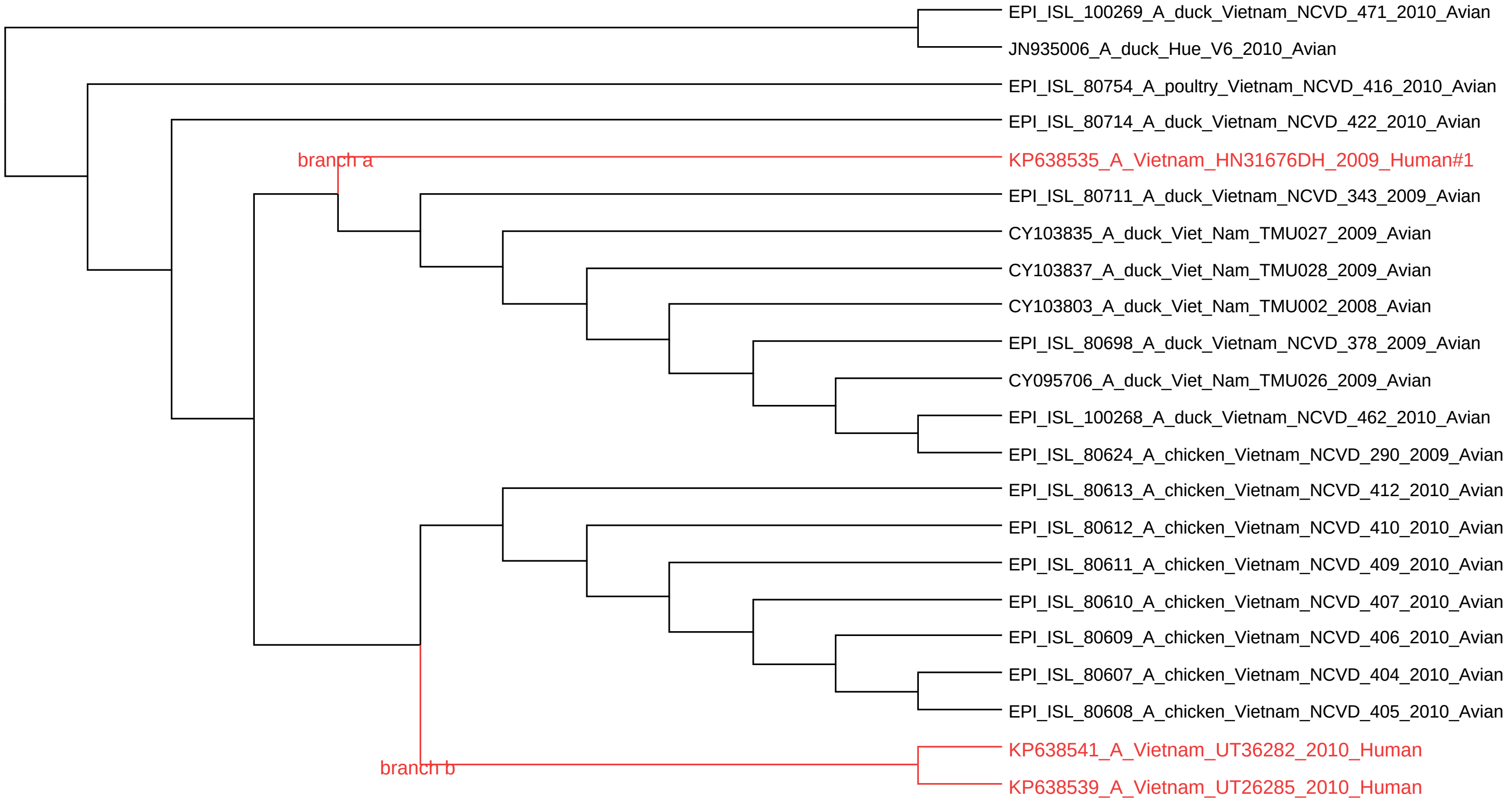
EPI_ISL_100269_A_duck_Vietnam_NCVD_471_2010_Avian
JN935006_A_duck_Hue_V6_2010_Avian
EPI_ISL_80754_A_poultry_Vietnam_NCVD_416_2010_Avian
EPI_ISL_80714_A_duck_Vietnam_NCVD_422_2010_Avian
branch a
KP638535_A_Vietnam_HN31676DH_2009_Human#1
EPI_ISL_80711_A_duck_Vietnam_NCVD_343_2009_Avian
CY103835_A_duck_Viet_Nam_TMU027_2009_Avian
CY103837_A_duck_Viet_Nam_TMU028_2009_Avian
CY103803_A_duck_Viet_Nam_TMU002_2008_Avian
EPI_ISL_80698_A_duck_Vietnam_NCVD_378_2009_Avian
CY095706_A_duck_Viet_Nam_TMU026_2009_Avian
EPI_ISL_100268_A_duck_Vietnam_NCVD_462_2010_Avian
EPI_ISL_80624_A_chicken_Vietnam_NCVD_290_2009_Avian
EPI_ISL_80613_A_chicken_Vietnam_NCVD_412_2010_Avian
EPI_ISL_80612_A_chicken_Vietnam_NCVD_410_2010_Avian
EPI_ISL_80611_A_chicken_Vietnam_NCVD_409_2010_Avian
EPI_ISL_80610_A_chicken_Vietnam_NCVD_407_2010_Avian
EPI_ISL_80609_A_chicken_Vietnam_NCVD_406_2010_Avian
EPI_ISL_80607_A_chicken_Vietnam_NCVD_404_2010_Avian
EPI_ISL_80608_A_chicken_Vietnam_NCVD_405_2010_Avian
branch b
KP638541_A_Vietnam_UT36282_2010_Human
KP638539_A_Vietnam_UT26285_2010_Human

MP-Group23

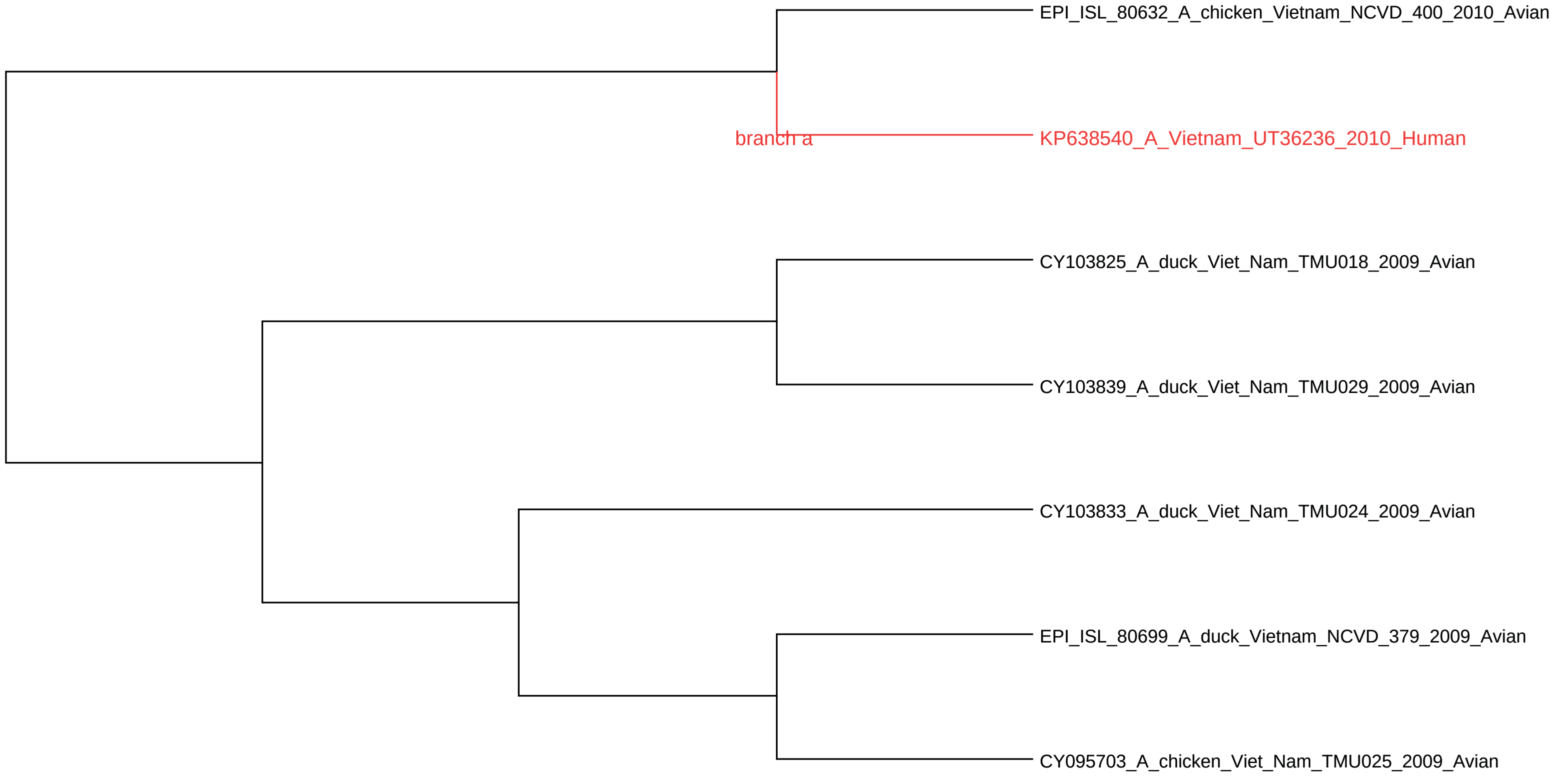
EPI_ISL_80632_A_chicken_Vietnam_NCVD_400_2010_Avian
branch a
KP638540_A_Vietnam_UT36236_2010_Human
CY103825_A_duck_Viet_Nam_TMU018_2009_Avian
CY103839_A_duck_Viet_Nam_TMU029_2009_Avian
CY103833_A_duck_Viet_Nam_TMU024_2009_Avian
EPI_ISL_80699_A_duck_Vietnam_NCVD_379_2009_Avian
CY095703_A_chicken_Viet_Nam_TMU025_2009_Avian

MP-Group24

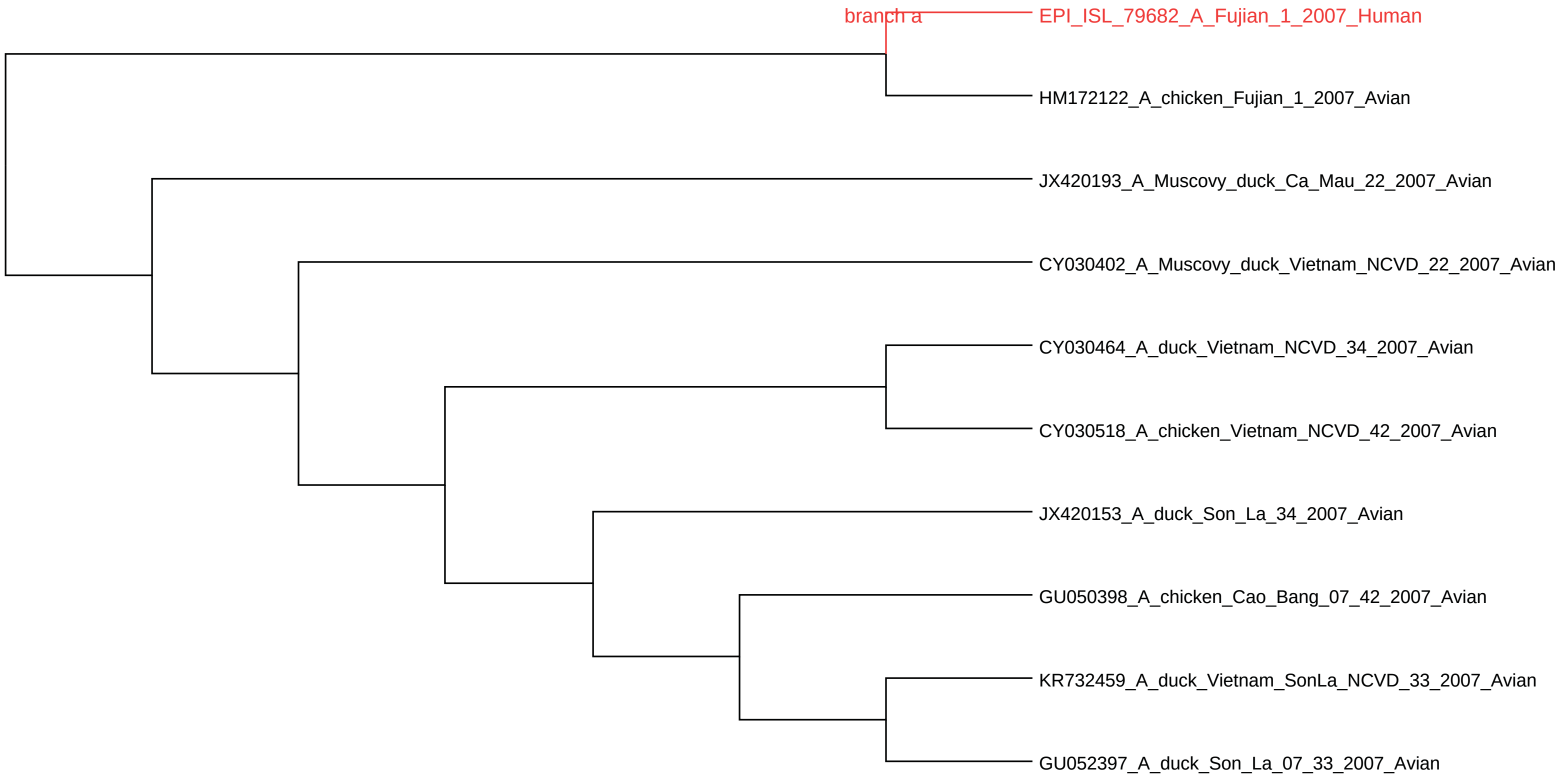
branch a
EPI_ISL_79682_A_Fujian_1_2007_Human
HM172122_A_chicken_Fujian_1_2007_Avian
JX420193_A_Muscovy_duck_Ca_Mau_22_2007_Avian
CY030402_A_Muscovy_duck_Vietnam_NCVD_22_2007_Avian
CY030464_A_duck_Vietnam_NCVD_34_2007_Avian
CY030518_A_chicken_Vietnam_NCVD_42_2007_Avian
JX420153_A_duck_Son_La_34_2007_Avian
GU050398_A_chicken_Cao_Bang_07_42_2007_Avian
KR732459_A_duck_Vietnam_SonLa_NCVD_33_2007_Avian
GU052397_A_duck_Son_La_07_33_2007_Avian

MP-Group25

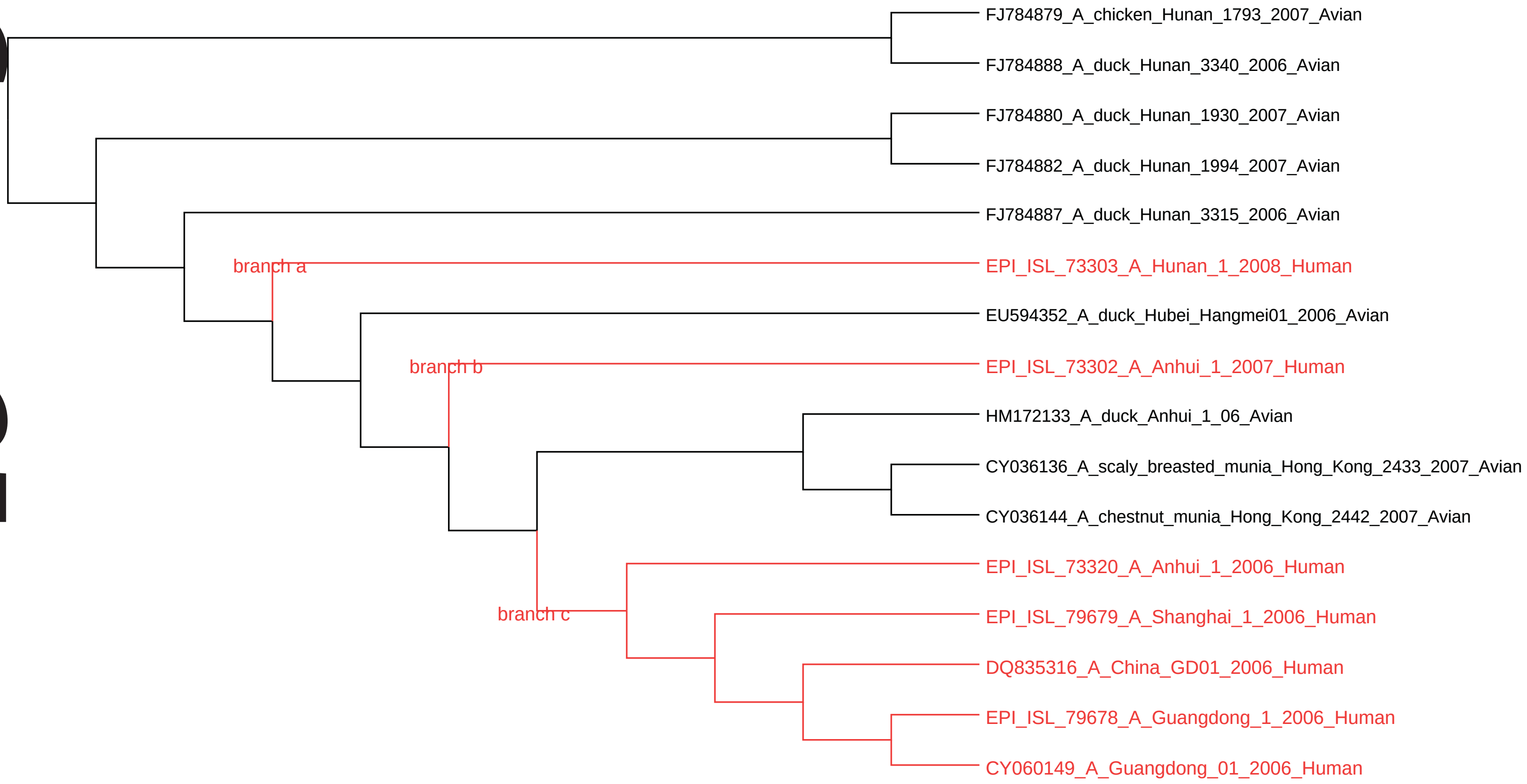

FJ784879_A_chicken_Hunan_1793_2007_Avian
FJ784888_A_duck_Hunan_3340_2006_Avian
FJ784880_A_duck_Hunan_1930_2007_Avian
FJ784882_A_duck_Hunan_1994_2007_Avian
FJ784887_A_duck_Hunan_3315_2006_Avian
branch a
EPI_ISL_73303_A_Hunan_1_2008_Human
EU594352_A_duck_Hubei_Hangmei01_2006_Avian
branch b
EPI_ISL_73302_A_Anhui_1_2007_Human
HM172133_A_duck_Anhui_1_06_Avian
CY036136_A_scaly_breasted_munia_Hong_Kong_2433_2007_Avian
CY036144_A_chestnut_munia_Hong_Kong_2442_2007_Avian
EPI_ISL_73320_A_Anhui_1_2006_Human
branch c
EPI_ISL_79679_A_Shanghai_1_2006_Human
DQ835316_A_China_GD01_2006_Human
EPI_ISL_79678_A_Guangdong_1_2006_Human
CY060149_A_Guangdong_01_2006_Human

MP-Group26

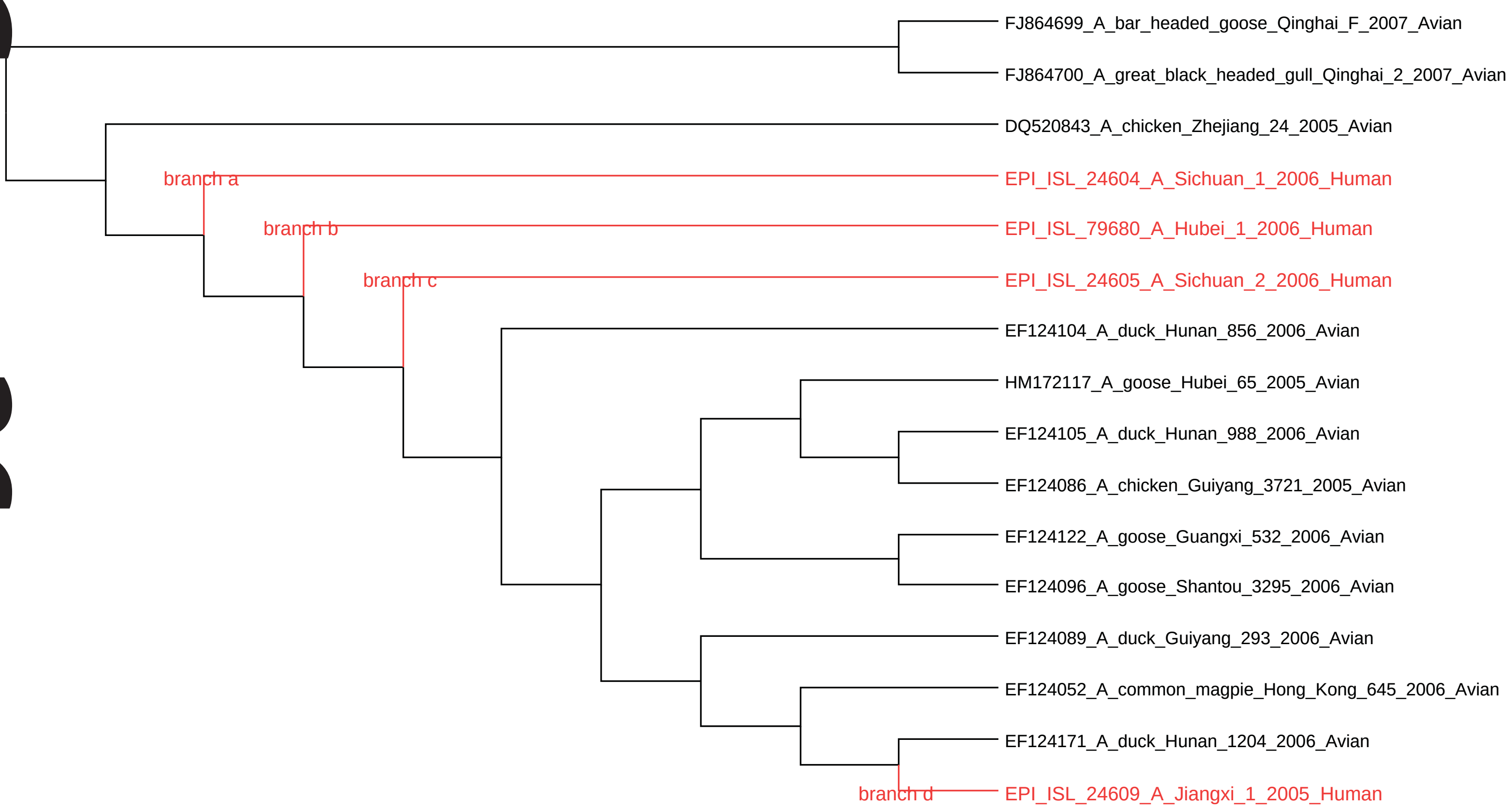
FJ864699_A_bar_headed_goose_Qinghai_F_2007_Avian
FJ864700_A_great_black_headed_gull_Qinghai_2_2007_Avian
DQ520843_A_chicken_Zhejiang_24_2005_Avian
branch a
EPI_ISL_24604_A_Sichuan_1_2006_Human
branch b
EPI_ISL_79680_A_Hubei_1_2006_Human
branch c
EPI_ISL_24605_A_Sichuan_2_2006_Human
EF124104_A_duck_Hunan_856_2006_Avian
HM172117_A_goose_Hubei_65_2005_Avian
EF124105_A_duck_Hunan_988_2006_Avian
EF124086_A_chicken_Guiyang_3721_2005_Avian
EF124122_A_goose_Guangxi_532_2006_Avian
EF124096_A_goose_Shantou_3295_2006_Avian
EF124089_A_duck_Guiyang_293_2006_Avian
EF124052_A_common_magpie_Hong_Kong_645_2006_Avian
EF124171_A_duck_Hunan_1204_2006_Avian
branch d
EPI_ISL_24609_A_Jiangxi_1_2005_Human

MP-Group27

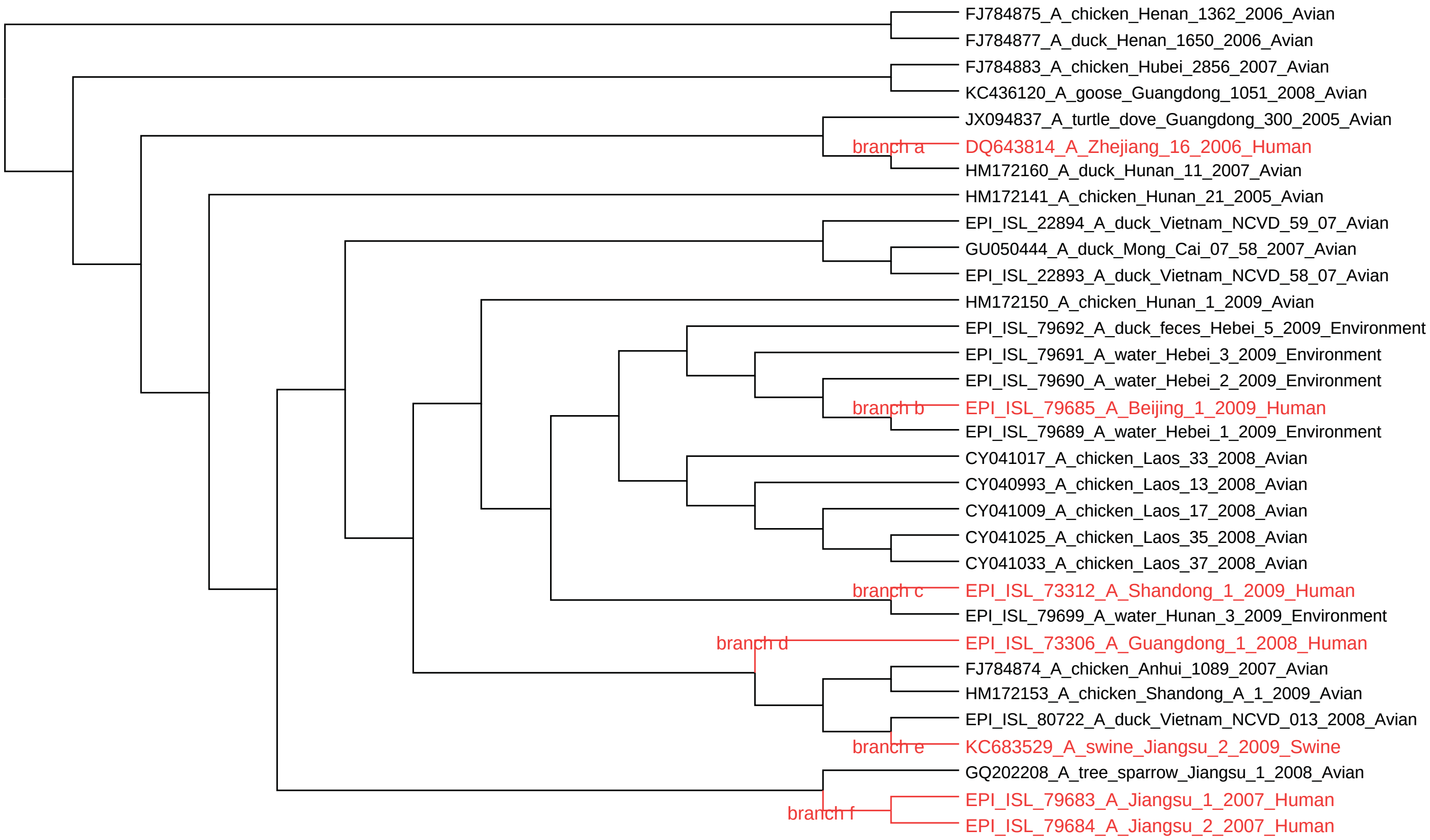
FJ784875_A_chicken_Henan_1362_2006_Avian
FJ784877_A_duck_Henan_1650_2006_Avian
FJ784883_A_chicken_Hubei_2856_2007_Avian
KC436120_A_goose_Guangdong_1051_2008_Avian
JX094837_A_turtle_dove_Guangdong_300_2005_Avian
branch a
DQ643814_A_Zhejiang_16_2006_Human
HM172160_A_duck_Hunan_11_2007_Avian
HM172141_A_chicken_Hunan_21_2005_Avian
EPI_ISL_22894_A_duck_Vietnam_NCVD_59_07_Avian
GU050444_A_duck_Mong_Cai_07_58_2007_Avian
EPI_ISL_22893_A_duck_Vietnam_NCVD_58_07_Avian
HM172150_A_chicken_Hunan_1_2009_Avian
EPI_ISL_79692_A_duck_feces_Hebei_5_2009_Environment
EPI_ISL_79691_A_water_Hebei_3_2009_Environment
EPI_ISL_79690_A_water_Hebei_2_2009_Environment
branch b
EPI_ISL_79685_A_Beijing_1_2009_Human
EPI_ISL_79689_A_water_Hebei_1_2009_Environment
CY041017_A_chicken_Laos_33_2008_Avian
CY040993_A_chicken_Laos_13_2008_Avian
CY041009_A_chicken_Laos_17_2008_Avian
CY041025_A_chicken_Laos_35_2008_Avian
CY041033_A_chicken_Laos_37_2008_Avian
branch c
EPI_ISL_73312_A_Shandong_1_2009_Human
EPI_ISL_79699_A_water_Hunan_3_2009_Environment
branch d
EPI_ISL_73306_A_Guangdong_1_2008_Human
FJ784874_A_chicken_Anhui_1089_2007_Avian
HM172153_A_chicken_Shandong_A_1_2009_Avian
EPI_ISL_80722_A_duck_Vietnam_NCVD_013_2008_Avian
branch e
KC683529_A_swine_Jiangsu_2_2009_Swine
GQ202208_A_tree_sparrow_Jiangsu_1_2008_Avian
branch f
EPI_ISL_79683_A_Jiangsu_1_2007_Human
EPI_ISL_79684_A_Jiangsu_2_2007_Human

MP-Group28

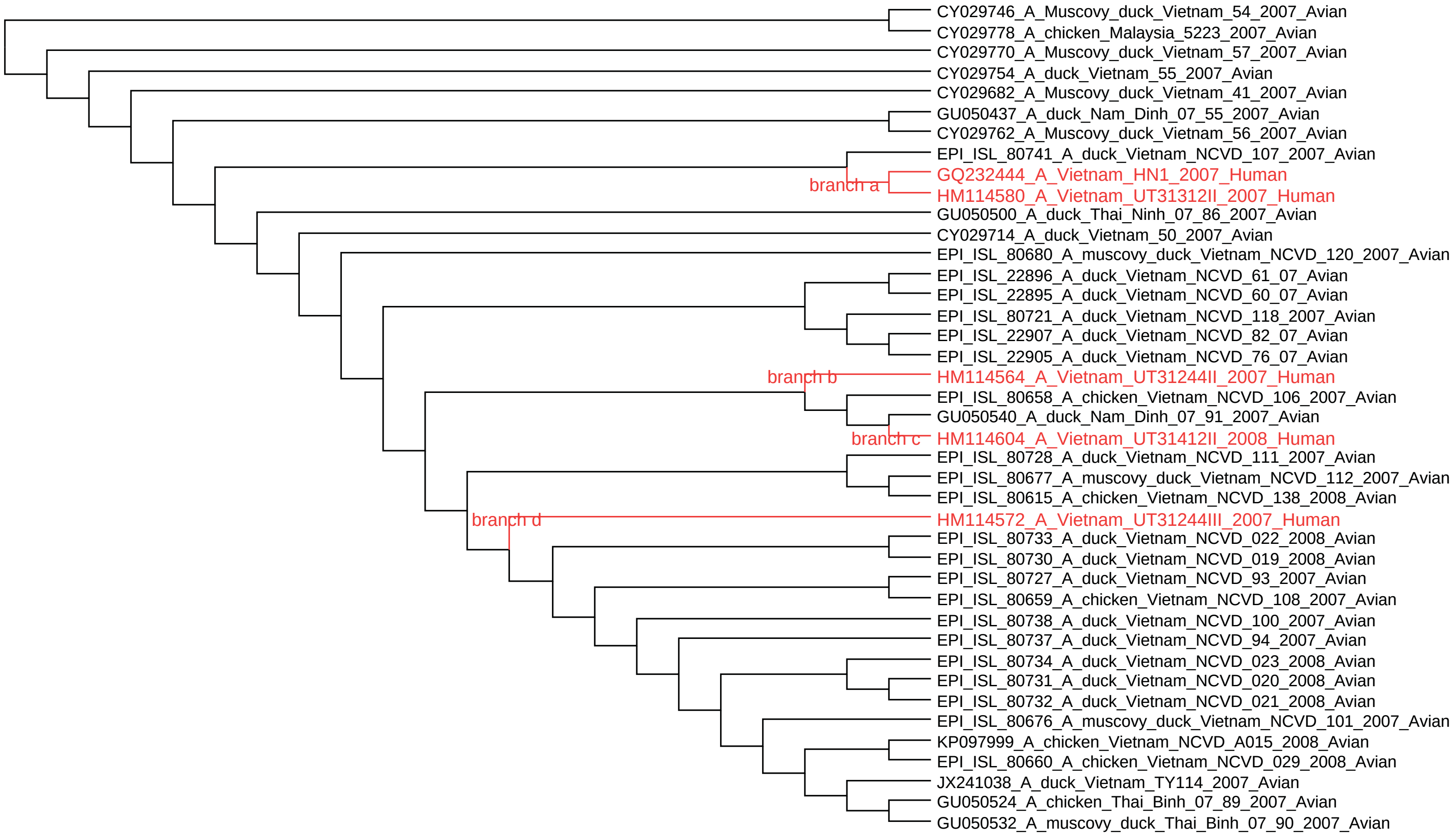
CY029746_A_Muscovy_duck_Vietnam_54_2007_Avian
CY029778_A_chicken_Malaysia_5223_2007_Avian
CY029770_A_Muscovy_duck_Vietnam_57_2007_Avian
CY029754_A_duck_Vietnam_55_2007_Avian
CY029682_A_Muscovy_duck_Vietnam_41_2007_Avian
GU050437_A_duck_Nam_Dinh_07_55_2007_Avian
CY029762_A_Muscovy_duck_Vietnam_56_2007_Avian
EPI_ISL_80741_A_duck_Vietnam_NCVD_107_2007_Avian
branch a
GQ232444_A_Vietnam_HN1_2007_Human
HM114580_A_Vietnam_UT31312II_2007_Human
GU050500_A_duck_Thai_Ninh_07_86_2007_Avian
CY029714_A_duck_Vietnam_50_2007_Avian
EPI_ISL_80680_A_muscovy_duck_Vietnam_NCVD_120_2007_Avian
EPI_ISL_22896_A_duck_Vietnam_NCVD_61_07_Avian
EPI_ISL_22895_A_duck_Vietnam_NCVD_60_07_Avian
EPI_ISL_80721_A_duck_Vietnam_NCVD_118_2007_Avian
EPI_ISL_22907_A_duck_Vietnam_NCVD_82_07_Avian
EPI_ISL_22905_A_duck_Vietnam_NCVD_76_07_Avian
branch b
HM114564_A_Vietnam_UT31244II_2007_Human
EPI_ISL_80658_A_chicken_Vietnam_NCVD_106_2007_Avian
GU050540_A_duck_Nam_Dinh_07_91_2007_Avian
branch c
HM114604_A_Vietnam_UT31412II_2008_Human
EPI_ISL_80728_A_duck_Vietnam_NCVD_111_2007_Avian
EPI_ISL_80677_A_muscovy_duck_Vietnam_NCVD_112_2007_Avian
EPI_ISL_80615_A_chicken_Vietnam_NCVD_138_2008_Avian
branch d
HM114572_A_Vietnam_UT31244III_2007_Human
EPI_ISL_80733_A_duck_Vietnam_NCVD_022_2008_Avian
EPI_ISL_80730_A_duck_Vietnam_NCVD_019_2008_Avian
EPI_ISL_80727_A_duck_Vietnam_NCVD_93_2007_Avian
EPI_ISL_80659_A_chicken_Vietnam_NCVD_108_2007_Avian
EPI_ISL_80738_A_duck_Vietnam_NCVD_100_2007_Avian
EPI_ISL_80737_A_duck_Vietnam_NCVD_94_2007_Avian
EPI_ISL_80734_A_duck_Vietnam_NCVD_023_2008_Avian
EPI_ISL_80731_A_duck_Vietnam_NCVD_020_2008_Avian
EPI_ISL_80732_A_duck_Vietnam_NCVD_021_2008_Avian
EPI_ISL_80676_A_muscovy_duck_Vietnam_NCVD_101_2007_Avian
KP097999_A_chicken_Vietnam_NCVD_A015_2008_Avian
EPI_ISL_80660_A_chicken_Vietnam_NCVD_029_2008_Avian
JX241038_A_duck_Vietnam_TY114_2007_Avian
GU050524_A_chicken_Thai_Binh_07_89_2007_Avian
GU050532_A_muscovy_duck_Thai_Binh_07_90_2007_Avian

MP-Group29

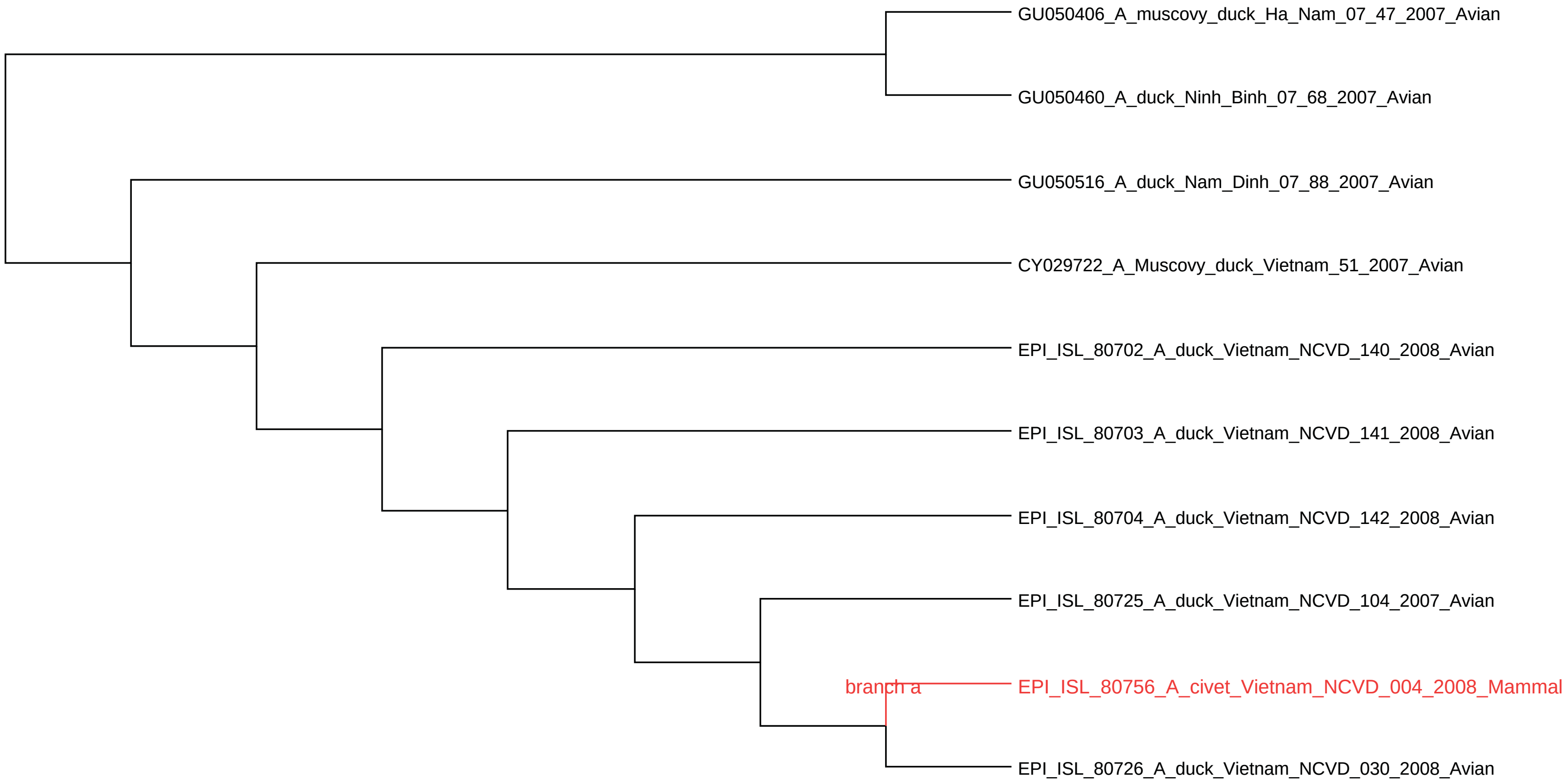
GU050406_A_muscovy_duck_Ha_Nam_07_47_2007_Avian
GU050460_A_duck_Ninh_Binh_07_68_2007_Avian
GU050516_A_duck_Nam_Dinh_07_88_2007_Avian
CY029722_A_Muscovy_duck_Vietnam_51_2007_Avian
EPI_ISL_80702_A_duck_Vietnam_NCVD_140_2008_Avian
EPI_ISL_80703_A_duck_Vietnam_NCVD_141_2008_Avian
EPI_ISL_80704_A_duck_Vietnam_NCVD_142_2008_Avian
EPI_ISL_80725_A_duck_Vietnam_NCVD_104_2007_Avian
branch a
EPI_ISL_80756_A_civet_Vietnam_NCVD_004_2008_Mammal
EPI_ISL_80726_A_duck_Vietnam_NCVD_030_2008_Avian

MP-Group30

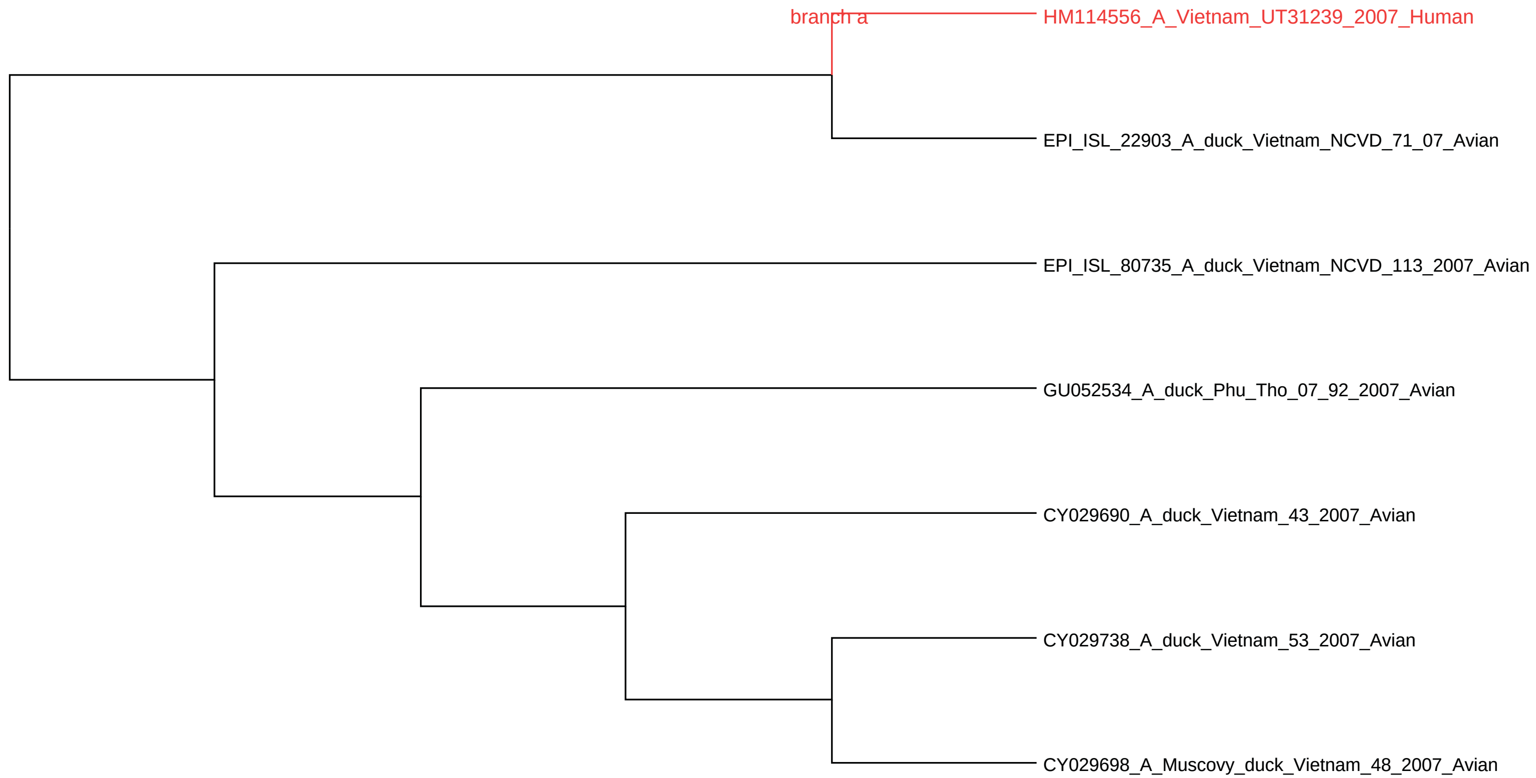
branch a
HM114556_A_Vietnam_UT31239_2007_Human
EPI_ISL_22903_A_duck_Vietnam_NCVD_71_07_Avian
EPI_ISL_80735_A_duck_Vietnam_NCVD_113_2007_Avian
GU052534_A_duck_Phu_Tho_07_92_2007_Avian
CY029690_A_duck_Vietnam_43_2007_Avian
CY029738_A_duck_Vietnam_53_2007_Avian
CY029698_A_Muscovy_duck_Vietnam_48_2007_Avian

MP-Group31

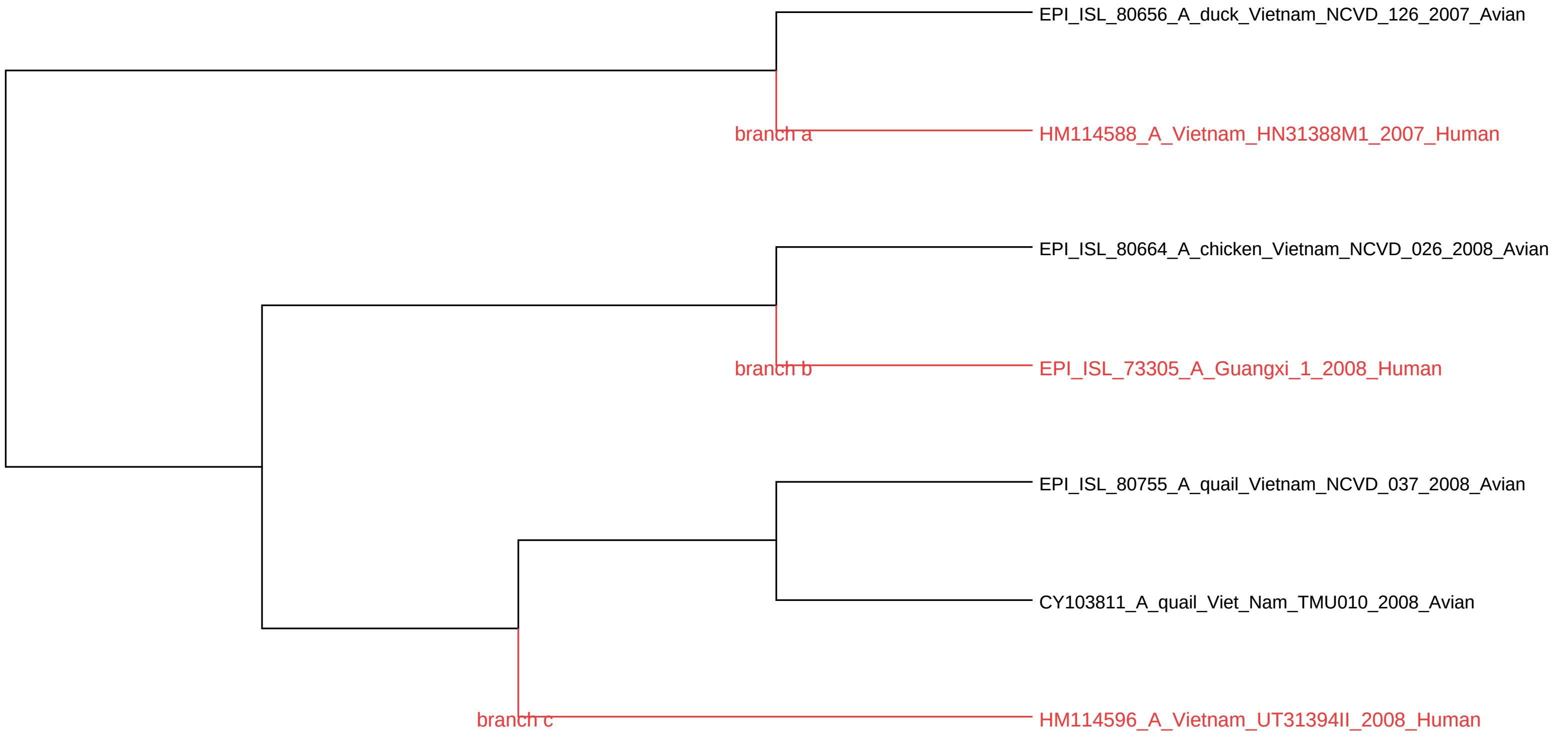
EPI_ISL_80656_A_duck_Vietnam_NCVD_126_2007_Avian
branch a
HM114588_A_Vietnam_HN31388M1_2007_Human
EPI_ISL_80664_A_chicken_Vietnam_NCVD_026_2008_Avian
branch b
EPI_ISL_73305_A_Guangxi_1_2008_Human
EPI_ISL_80755_A_quail_Vietnam_NCVD_037_2008_Avian
CY103811_A_quail_Viet_Nam_TMU010_2008_Avian
branch c
HM114596_A_Vietnam_UT31394II_2008_Human

MP-Group32

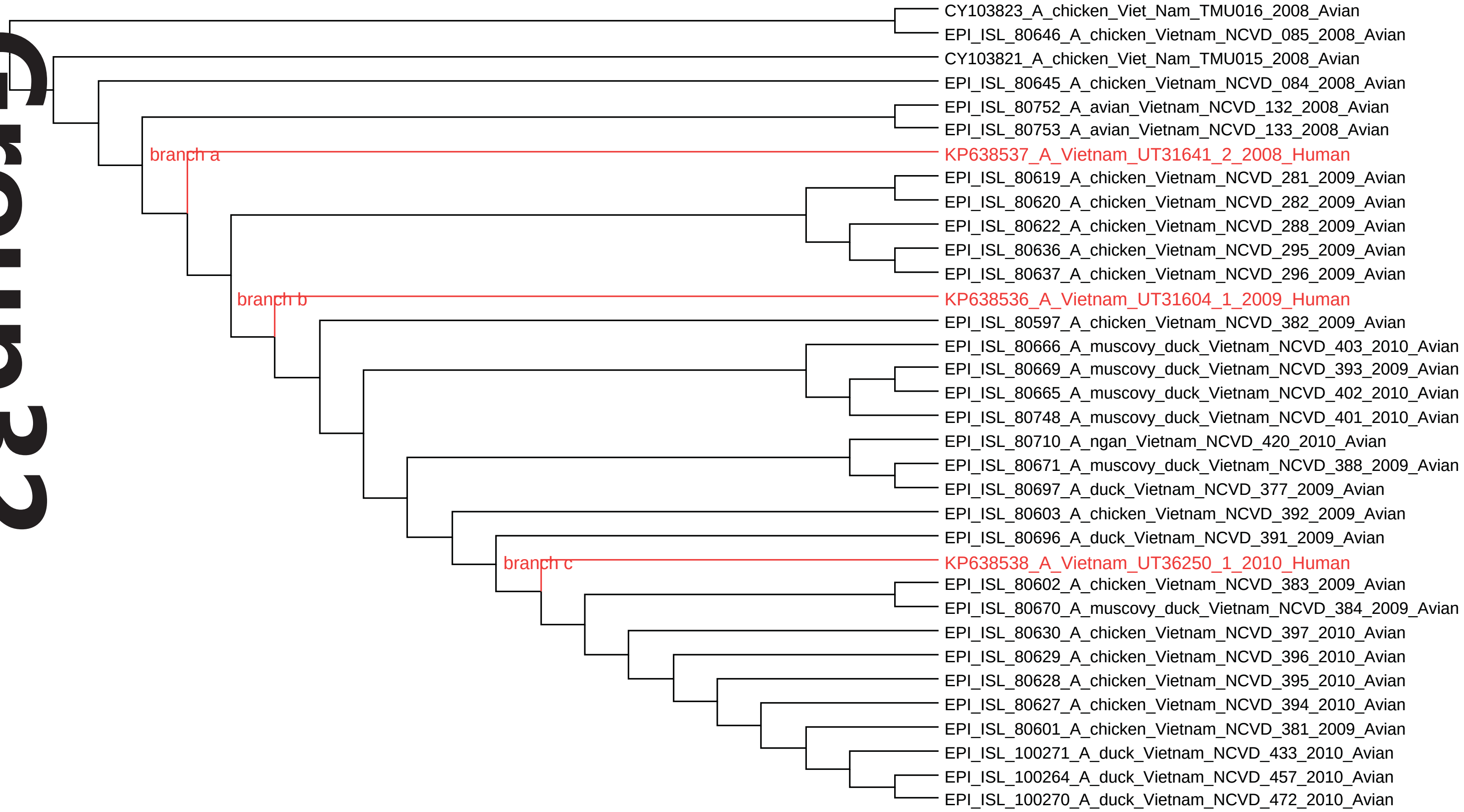
CY103823_A_chicken_Viet_Nam_TMU016_2008_Avian
EPI_ISL_80646_A_chicken_Vietnam_NCVD_085_2008_Avian
CY103821_A_chicken_Viet_Nam_TMU015_2008_Avian
EPI_ISL_80645_A_chicken_Vietnam_NCVD_084_2008_Avian
EPI_ISL_80752_A_avian_Vietnam_NCVD_132_2008_Avian
EPI_ISL_80753_A_avian_Vietnam_NCVD_133_2008_Avian
branch a
KP638537_A_Vietnam_UT31641_2_2008_Human
EPI_ISL_80619_A_chicken_Vietnam_NCVD_281_2009_Avian
EPI_ISL_80620_A_chicken_Vietnam_NCVD_282_2009_Avian
EPI_ISL_80622_A_chicken_Vietnam_NCVD_288_2009_Avian
EPI_ISL_80636_A_chicken_Vietnam_NCVD_295_2009_Avian
EPI_ISL_80637_A_chicken_Vietnam_NCVD_296_2009_Avian
branch b
KP638536_A_Vietnam_UT31604_1_2009_Human
EPI_ISL_80597_A_chicken_Vietnam_NCVD_382_2009_Avian
EPI_ISL_80666_A_muscovy_duck_Vietnam_NCVD_403_2010_Avian
EPI_ISL_80669_A_muscovy_duck_Vietnam_NCVD_393_2009_Avian
EPI_ISL_80665_A_muscovy_duck_Vietnam_NCVD_402_2010_Avian
EPI_ISL_80748_A_muscovy_duck_Vietnam_NCVD_401_2010_Avian
EPI_ISL_80710_A_ngan_Vietnam_NCVD_420_2010_Avian
EPI_ISL_80671_A_muscovy_duck_Vietnam_NCVD_388_2009_Avian
EPI_ISL_80697_A_duck_Vietnam_NCVD_377_2009_Avian
EPI_ISL_80603_A_chicken_Vietnam_NCVD_392_2009_Avian
EPI_ISL_80696_A_duck_Vietnam_NCVD_391_2009_Avian
branch c
KP638538_A_Vietnam_UT36250_1_2010_Human
EPI_ISL_80602_A_chicken_Vietnam_NCVD_383_2009_Avian
EPI_ISL_80670_A_muscovy_duck_Vietnam_NCVD_384_2009_Avian
EPI_ISL_80630_A_chicken_Vietnam_NCVD_397_2010_Avian
EPI_ISL_80629_A_chicken_Vietnam_NCVD_396_2010_Avian
EPI_ISL_80628_A_chicken_Vietnam_NCVD_395_2010_Avian
EPI_ISL_80627_A_chicken_Vietnam_NCVD_394_2010_Avian
EPI_ISL_80601_A_chicken_Vietnam_NCVD_381_2009_Avian
EPI_ISL_100271_A_duck_Vietnam_NCVD_433_2010_Avian
EPI_ISL_100264_A_duck_Vietnam_NCVD_457_2010_Avian
EPI_ISL_100270_A_duck_Vietnam_NCVD_472_2010_Avian

MP-Group33

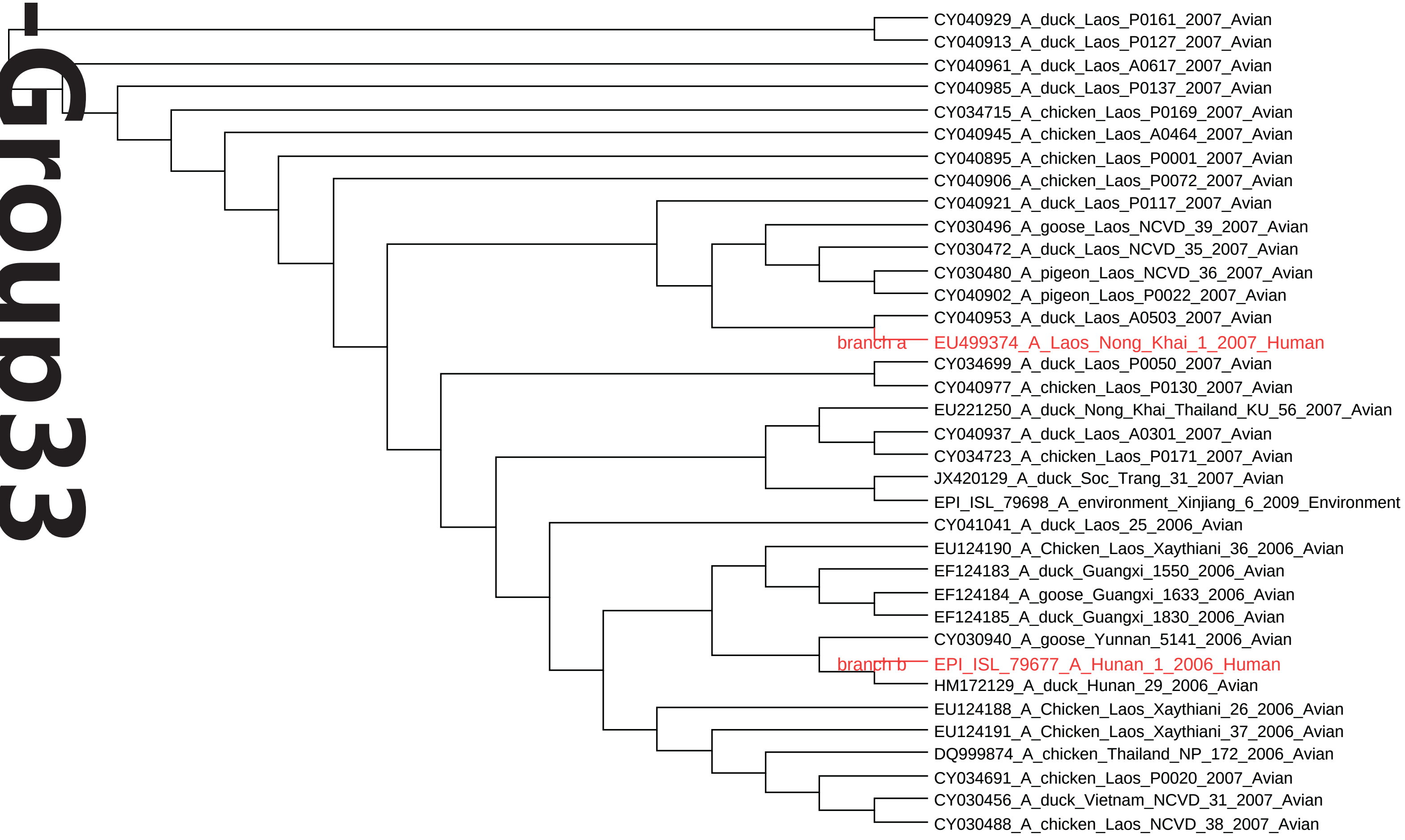
CY040929_A_duck_Laos_P0161_2007_Avian
CY040913_A_duck_Laos_P0127_2007_Avian
CY040961_A_duck_Laos_A0617_2007_Avian
CY040985_A_duck_Laos_P0137_2007_Avian
CY034715_A_chicken_Laos_P0169_2007_Avian
CY040945_A_chicken_Laos_A0464_2007_Avian
CY040895_A_chicken_Laos_P0001_2007_Avian
CY040906_A_chicken_Laos_P0072_2007_Avian
CY040921_A_duck_Laos_P0117_2007_Avian
CY030496_A_goose_Laos_NCVD_39_2007_Avian
CY030472_A_duck_Laos_NCVD_35_2007_Avian
CY030480_A_pigeon_Laos_NCVD_36_2007_Avian
CY040902_A_pigeon_Laos_P0022_2007_Avian
CY040953_A_duck_Laos_A0503_2007_Avian
branch a
EU499374_A_Laos_Nong_Khai_1_2007_Human
CY034699_A_duck_Laos_P0050_2007_Avian
CY040977_A_chicken_Laos_P0130_2007_Avian
EU221250_A_duck_Nong_Khai_Thailand_KU_56_2007_Avian
CY040937_A_duck_Laos_A0301_2007_Avian
CY034723_A_chicken_Laos_P0171_2007_Avian
JX420129_A_duck_Soc_Trang_31_2007_Avian
EPI_ISL_79698_A_environment_Xinjiang_6_2009_Environment
CY041041_A_duck_Laos_25_2006_Avian
EU124190_A_Chicken_Laos_Xaythiani_36_2006_Avian
EF124183_A_duck_Guangxi_1550_2006_Avian
EF124184_A_goose_Guangxi_1633_2006_Avian
EF124185_A_duck_Guangxi_1830_2006_Avian
CY030940_A_goose_Yunnan_5141_2006_Avian
branch b
EPI_ISL_79677_A_Hunan_1_2006_Human
HM172129_A_duck_Hunan_29_2006_Avian
EU124188_A_Chicken_Laos_Xaythiani_26_2006_Avian
EU124191_A_Chicken_Laos_Xaythiani_37_2006_Avian
DQ999874_A_chicken_Thailand_NP_172_2006_Avian
CY034691_A_chicken_Laos_P0020_2007_Avian
CY030456_A_duck_Vietnam_NCVD_31_2007_Avian
CY030488_A_chicken_Laos_NCVD_38_2007_Avian

MP-Group34

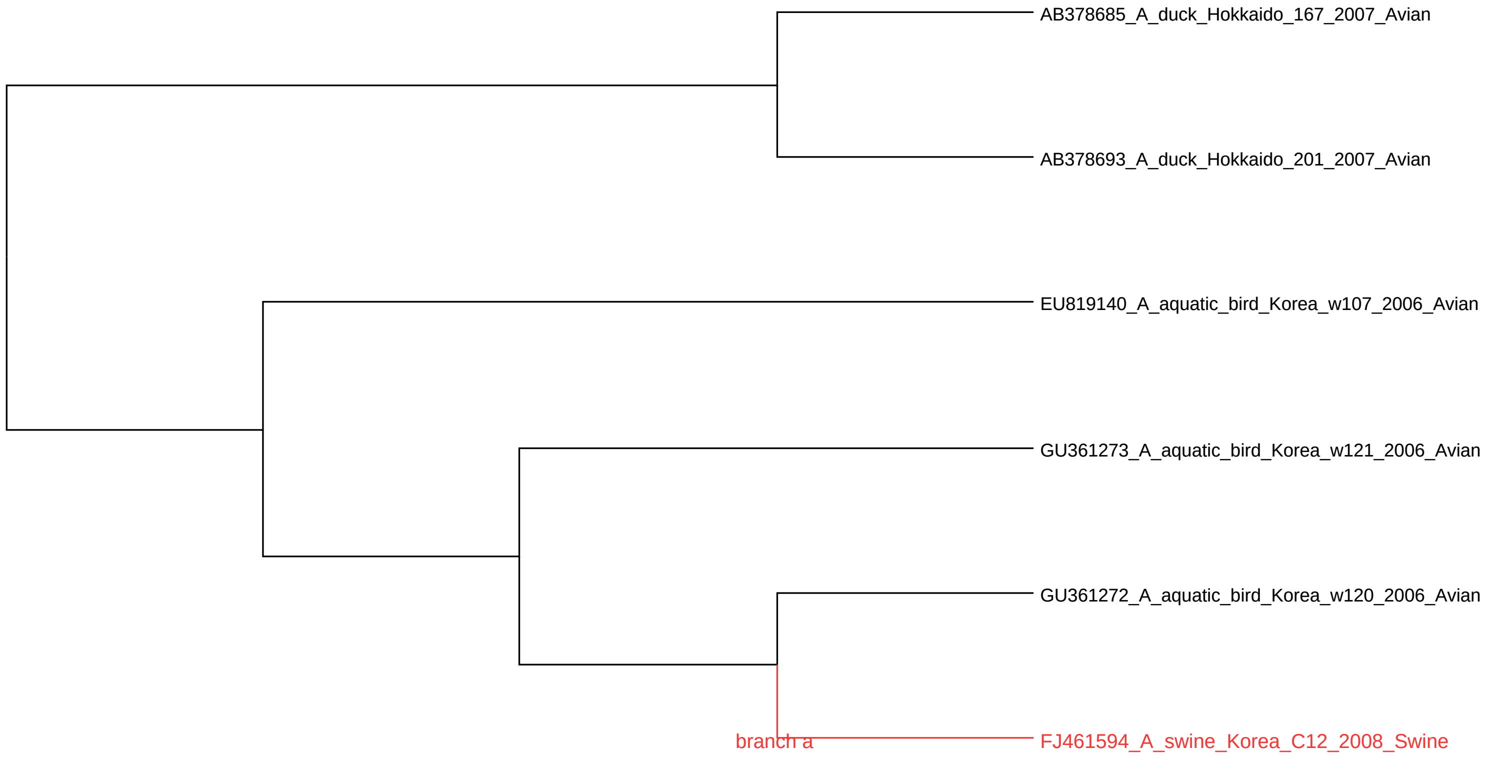
AB378685_A_duck_Hokkaido_167_2007_Avian
AB378693_A_duck_Hokkaido_201_2007_Avian
EU819140_A_aquatic_bird_Korea_w107_2006_Avian
GU361273_A_aquatic_bird_Korea_w121_2006_Avian
GU361272_A_aquatic_bird_Korea_w120_2006_Avian
branch a
FJ461594_A_swine_Korea_C12_2008_Swine

MP-Group35

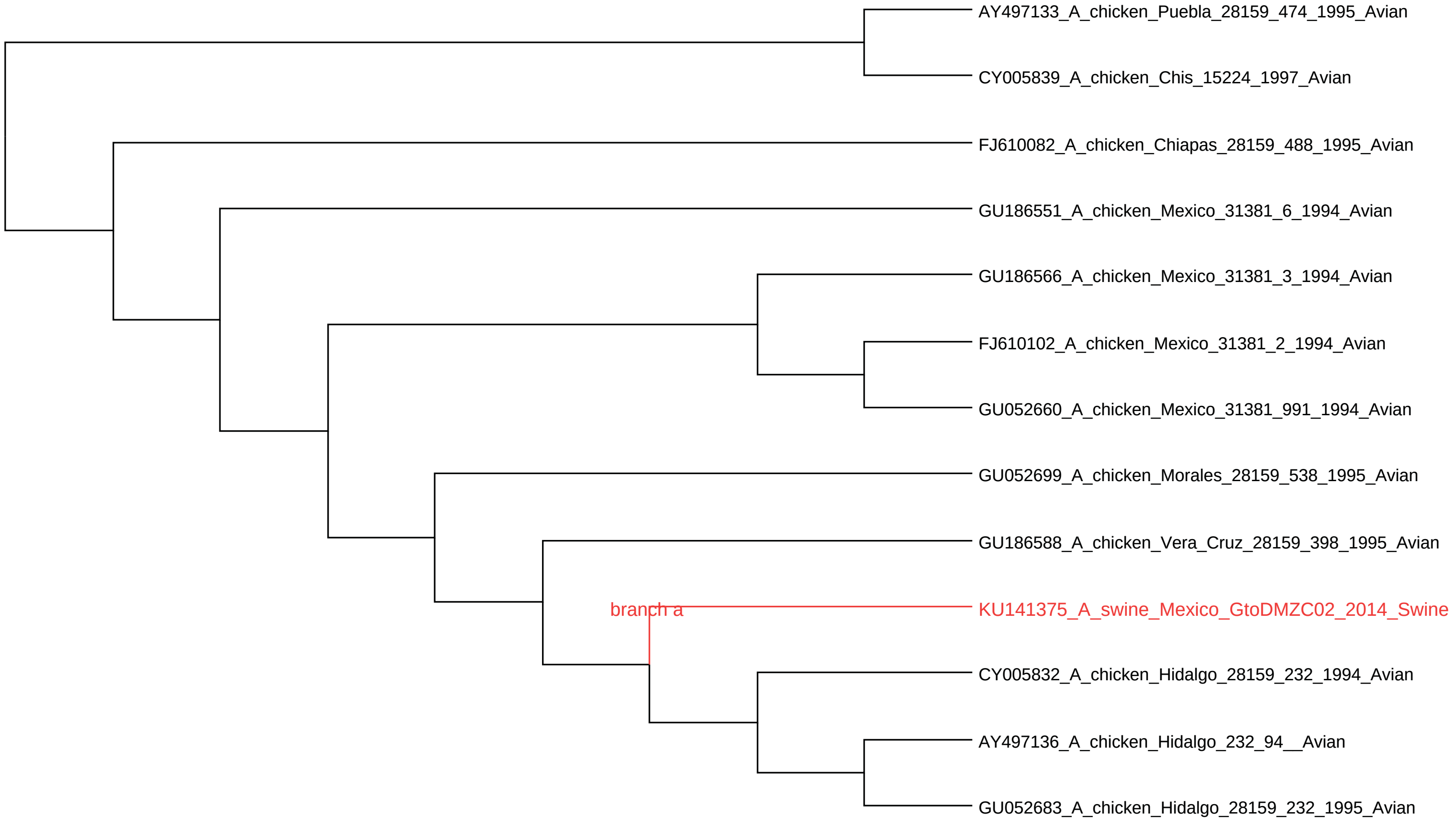
AY497133_A_chicken_Puebla_28159_474_1995_Avian
CY005839_A_chicken_Chis_15224_1997_Avian
FJ610082_A_chicken_Chiapas_28159_488_1995_Avian
GU186551_A_chicken_Mexico_31381_6_1994_Avian
GU186566_A_chicken_Mexico_31381_3_1994_Avian
FJ610102_A_chicken_Mexico_31381_2_1994_Avian
GU052660_A_chicken_Mexico_31381_991_1994_Avian
GU052699_A_chicken_Morales_28159_538_1995_Avian
GU186588_A_chicken_Vera_Cruz_28159_398_1995_Avian
branch a
KU141375_A_swine_Mexico_GtoDMZC02_2014_Swine
CY005832_A_chicken_Hidalgo_28159_232_1994_Avian
AY497136_A_chicken_Hidalgo_232_94__Avian
GU052683_A_chicken_Hidalgo_28159_232_1995_Avian

MP-Group36

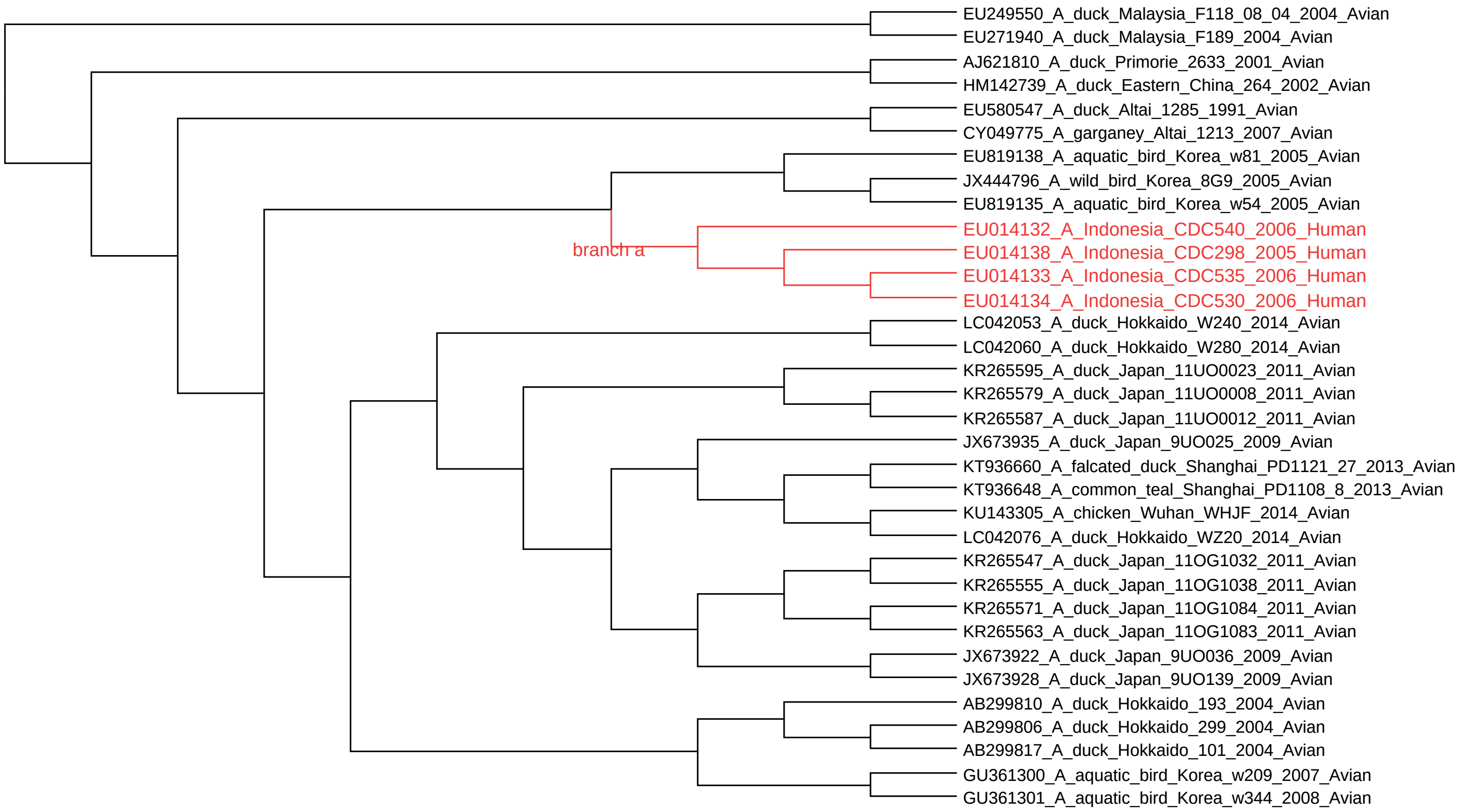
EU249550_A_duck_Malaysia_F118_08_04_2004_Avian
EU271940_A_duck_Malaysia_F189_2004_Avian
AJ621810_A_duck_Primorie_2633_2001_Avian
HM142739_A_duck_Eastern_China_264_2002_Avian
EU580547_A_duck_Altai_1285_1991_Avian
CY049775_A_garganey_Altai_1213_2007_Avian
EU819138_A_aquatic_bird_Korea_w81_2005_Avian
JX444796_A_wild_bird_Korea_8G9_2005_Avian
EU819135_A_aquatic_bird_Korea_w54_2005_Avian
branch a
EU014132_A_Indonesia_CDC540_2006_Human
EU014138_A_Indonesia_CDC298_2005_Human
EU014133_A_Indonesia_CDC535_2006_Human
EU014134_A_Indonesia_CDC530_2006_Human
LC042053_A_duck_Hokkaido_W240_2014_Avian
LC042060_A_duck_Hokkaido_W280_2014_Avian
KR265595_A_duck_Japan_11UO0023_2011_Avian
KR265579_A_duck_Japan_11UO0008_2011_Avian
KR265587_A_duck_Japan_11UO0012_2011_Avian
JX673935_A_duck_Japan_9UO025_2009_Avian
KT936660_A_falcated_duck_Shanghai_PD1121_27_2013_Avian
KT936648_A_common_teal_Shanghai_PD1108_8_2013_Avian
KU143305_A_chicken_Wuhan_WHJF_2014_Avian
LC042076_A_duck_Hokkaido_WZ20_2014_Avian
KR265547_A_duck_Japan_11OG1032_2011_Avian
KR265555_A_duck_Japan_11OG1038_2011_Avian
KR265571_A_duck_Japan_11OG1084_2011_Avian
KR265563_A_duck_Japan_11OG1083_2011_Avian
JX673922_A_duck_Japan_9UO036_2009_Avian
JX673928_A_duck_Japan_9UO139_2009_Avian
AB299810_A_duck_Hokkaido_193_2004_Avian
AB299806_A_duck_Hokkaido_299_2004_Avian
AB299817_A_duck_Hokkaido_101_2004_Avian
GU361300_A_aquatic_bird_Korea_w209_2007_Avian
GU361301_A_aquatic_bird_Korea_w344_2008_Avian

MP-Group37

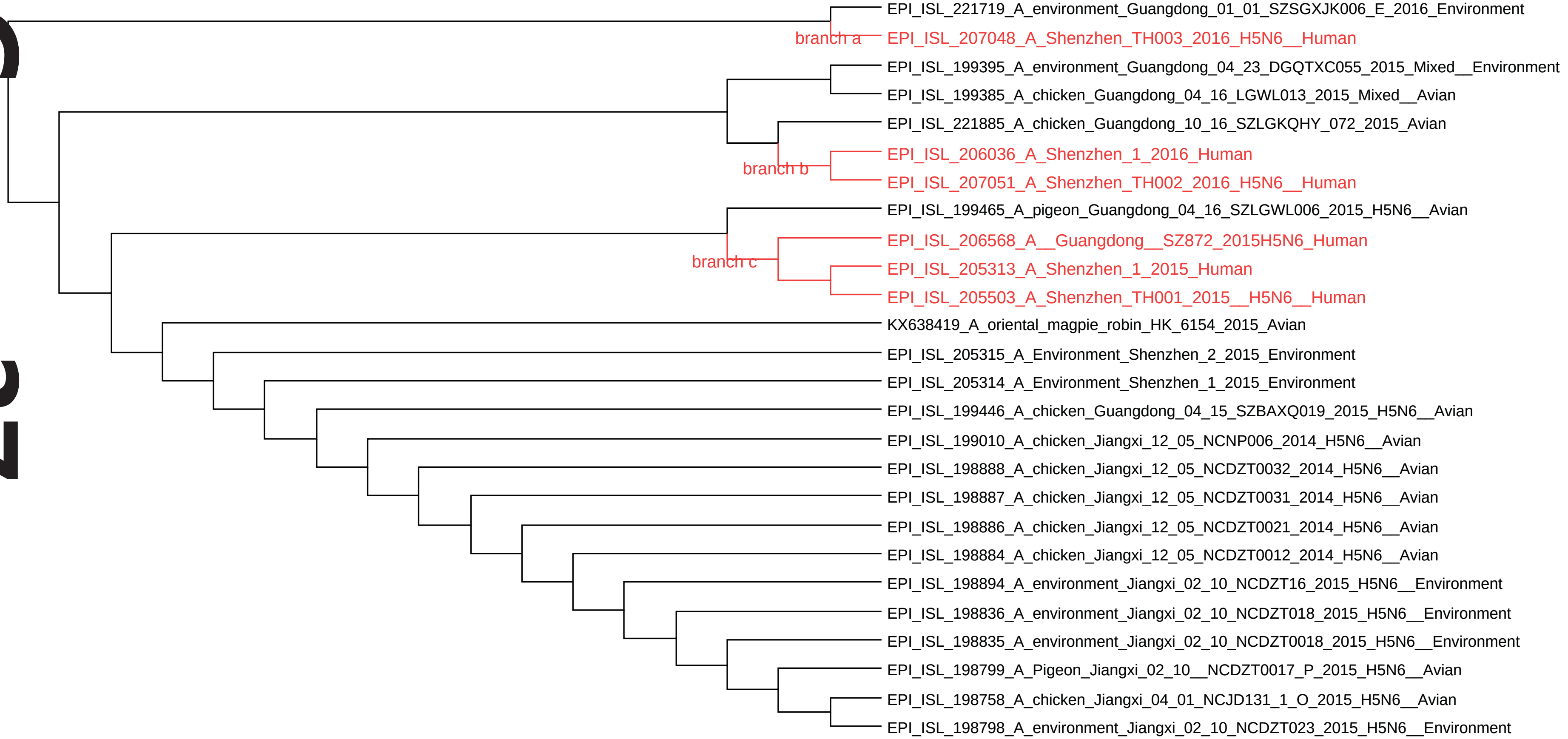
EPI_ISL_221719_A_environment_Guangdong_01_01_SZSGXJK006_E_2016_Environment
branch a
EPI_ISL_207048_A_Shenzhen_TH003_2016_H5N6__Human
EPI_ISL_199395_A_environment_Guangdong_04_23_DGQTXC055_2015_Mixed__Environment
EPI_ISL_199385_A_chicken_Guangdong_04_16_LGWL013_2015_Mixed__Avian
EPI_ISL_221885_A_chicken_Guangdong_10_16_SZLGKQHY_072_2015_Avian
EPI_ISL_206036_A_Shenzhen_1_2016_Human
branch b
EPI_ISL_207051_A_Shenzhen_TH002_2016_H5N6__Human
EPI_ISL_199465_A_pigeon_Guangdong_04_16_SZLGWL006_2015_H5N6__Avian
EPI_ISL_206568_A__Guangdong__SZ872_2015H5N6_Human
branch c
EPI_ISL_205313_A_Shenzhen_1_2015_Human
EPI_ISL_205503_A_Shenzhen_TH001_2015__H5N6__Human
KX638419_A_oriental_magpie_robin_HK_6154_2015_Avian
EPI_ISL_205315_A_Environment_Shenzhen_2_2015_Environment
EPI_ISL_205314_A_Environment_Shenzhen_1_2015_Environment
EPI_ISL_199446_A_chicken_Guangdong_04_15_SZBAXQ019_2015_H5N6__Avian
EPI_ISL_199010_A_chicken_Jiangxi_12_05_NCNP006_2014_H5N6__Avian
EPI_ISL_198888_A_chicken_Jiangxi_12_05_NCDZT0032_2014_H5N6__Avian
EPI_ISL_198887_A_chicken_Jiangxi_12_05_NCDZT0031_2014_H5N6__Avian
EPI_ISL_198886_A_chicken_Jiangxi_12_05_NCDZT0021_2014_H5N6__Avian
EPI_ISL_198884_A_chicken_Jiangxi_12_05_NCDZT0012_2014_H5N6__Avian
EPI_ISL_198894_A_environment_Jiangxi_02_10_NCDZT16_2015_H5N6__Environment
EPI_ISL_198836_A_environment_Jiangxi_02_10_NCDZT018_2015_H5N6__Environment
EPI_ISL_198835_A_environment_Jiangxi_02_10_NCDZT0018_2015_H5N6__Environment
EPI_ISL_198799_A_Pigeon_Jiangxi_02_10__NCDZT0017_P_2015_H5N6__Avian
EPI_ISL_198758_A_chicken_Jiangxi_04_01_NCJD131_1_O_2015_H5N6__Avian
EPI_ISL_198798_A_environment_Jiangxi_02_10_NCDZT023_2015_H5N6__Environment

MP-Group38

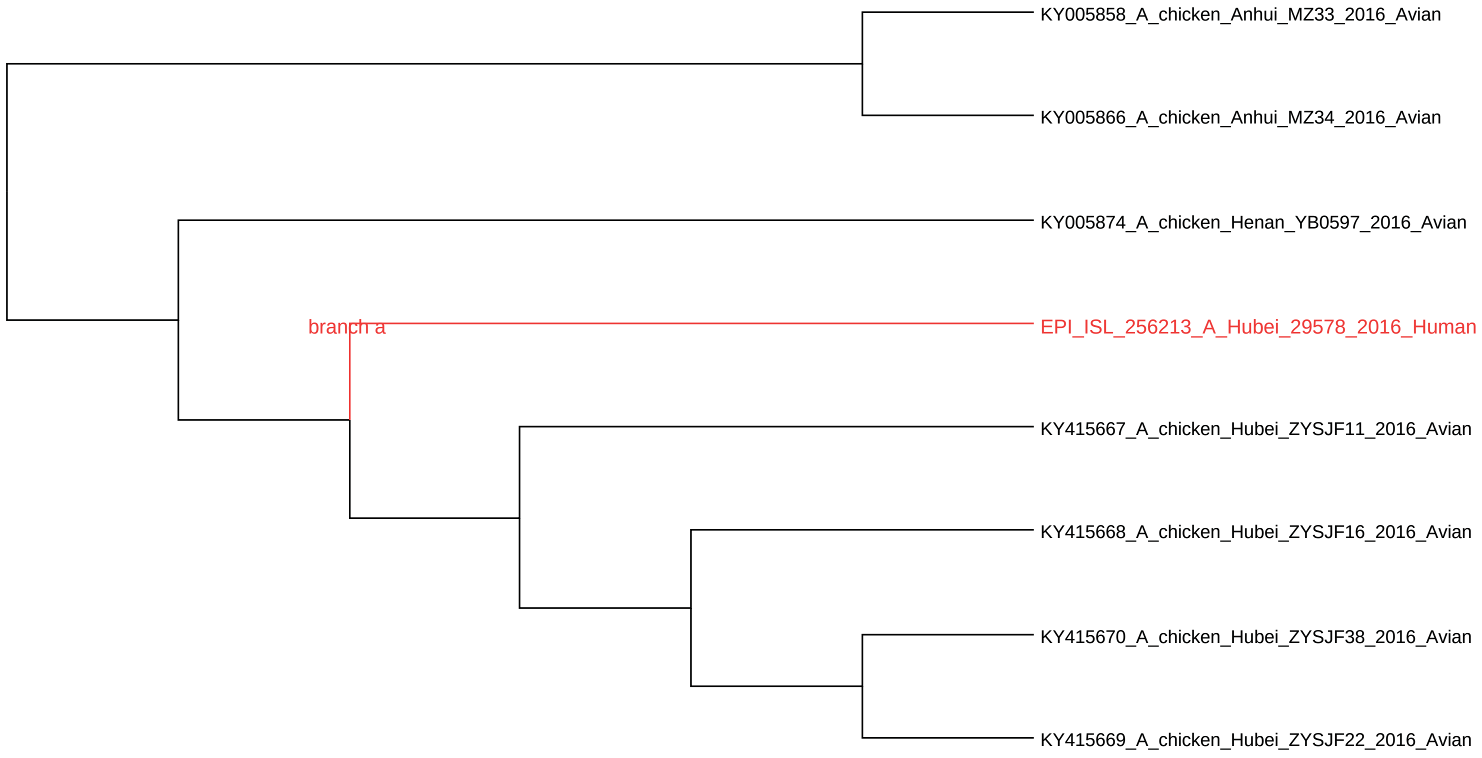
KY005858_A_chicken_Anhui_MZ33_2016_Avian
KY005866_A_chicken_Anhui_MZ34_2016_Avian
KY005874_A_chicken_Henan_YB0597_2016_Avian
branch a
EPI_ISL_256213_A_Hubei_29578_2016_Human
KY415667_A_chicken_Hubei_ZYSJF11_2016_Avian
KY415668_A_chicken_Hubei_ZYSJF16_2016_Avian
KY415670_A_chicken_Hubei_ZYSJF38_2016_Avian
KY415669_A_chicken_Hubei_ZYSJF22_2016_Avian

MP-Group39

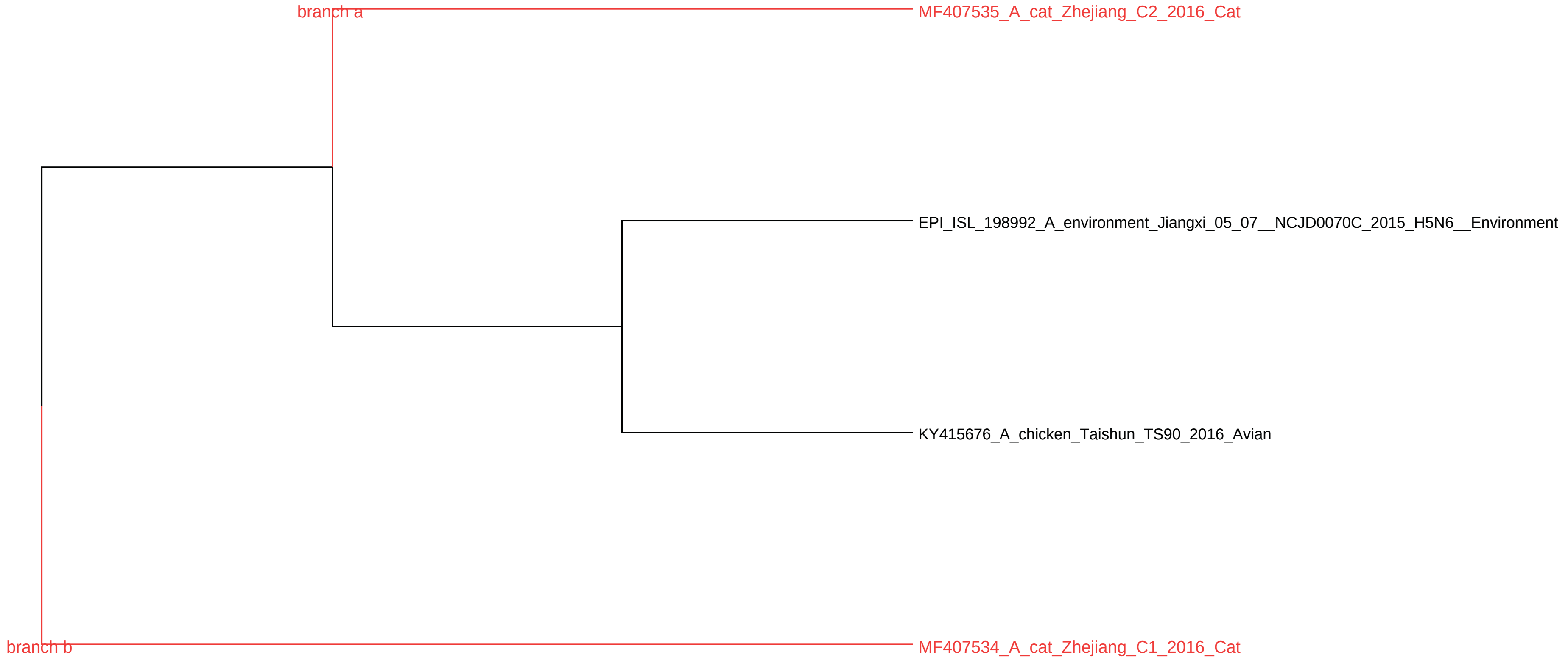
branch a
MF407535_A_cat_Zhejiang_C2_2016_Cat
EPI_ISL_198992_A_environment_Jiangxi_05_07__NCJD0070C_2015_H5N6__Environment
KY415676_A_chicken_Taishun_TS90_2016_Avian
branch b
MF407534_A_cat_Zhejiang_C1_2016_Cat

MP-Group40

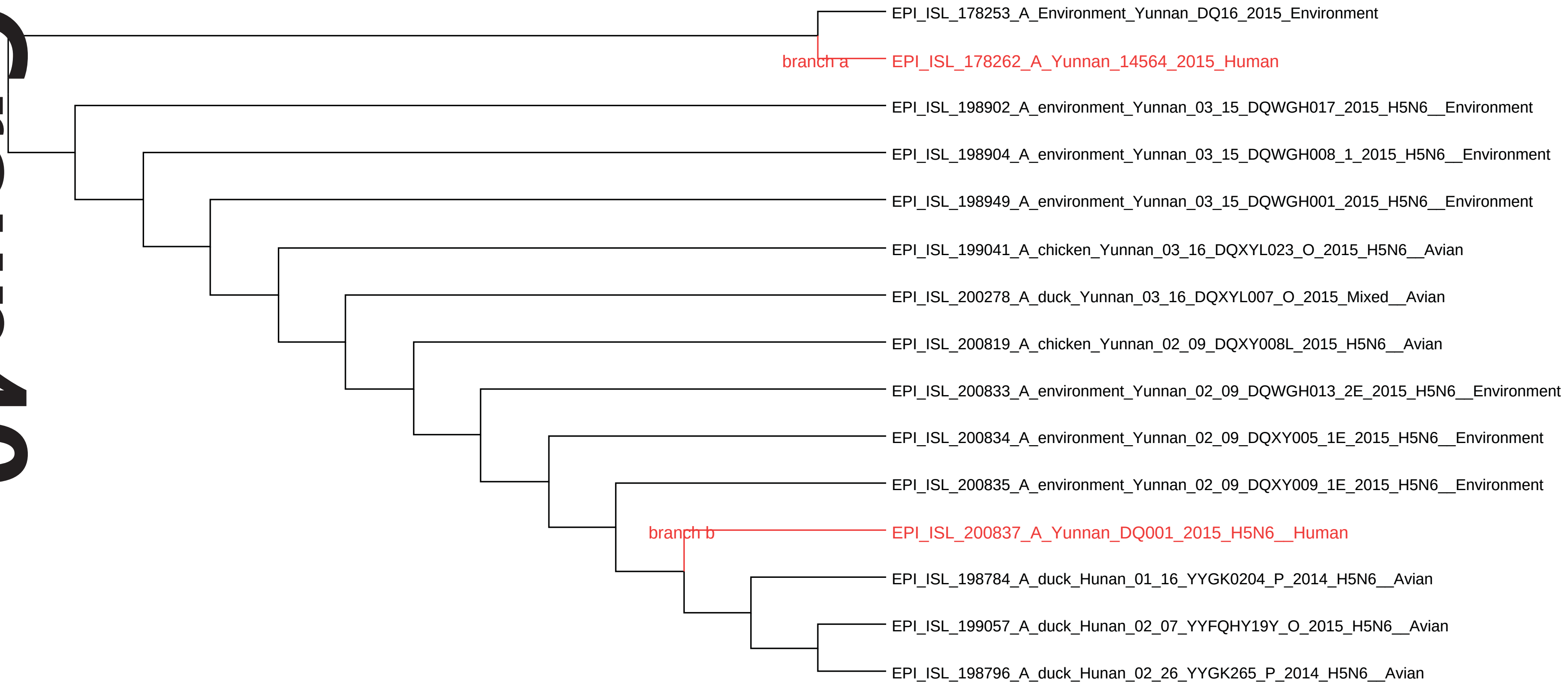
EPI_ISL_178253_A_Environment_Yunnan_DQ16_2015_Environment
branch a
EPI_ISL_178262_A_Yunnan_14564_2015_Human
EPI_ISL_198902_A_environment_Yunnan_03_15_DQWGH017_2015_H5N6__Environment
EPI_ISL_198904_A_environment_Yunnan_03_15_DQWGH008_1_2015_H5N6__Environment
EPI_ISL_198949_A_environment_Yunnan_03_15_DQWGH001_2015_H5N6__Environment
EPI_ISL_199041_A_chicken_Yunnan_03_16_DQXYL023_O_2015_H5N6__Avian
EPI_ISL_200278_A_duck_Yunnan_03_16_DQXYL007_O_2015_Mixed__Avian
EPI_ISL_200819_A_chicken_Yunnan_02_09_DQXY008L_2015_H5N6__Avian
EPI_ISL_200833_A_environment_Yunnan_02_09_DQWGH013_2E_2015_H5N6__Environment
EPI_ISL_200834_A_environment_Yunnan_02_09_DQXY005_1E_2015_H5N6__Environment
EPI_ISL_200835_A_environment_Yunnan_02_09_DQXY009_1E_2015_H5N6__Environment
branch b
EPI_ISL_200837_A_Yunnan_DQ001_2015_H5N6__Human
EPI_ISL_198784_A_duck_Hunan_01_16_YYGK0204_P_2014_H5N6__Avian
EPI_ISL_199057_A_duck_Hunan_02_07_YYFQHY19Y_O_2015_H5N6__Avian
EPI_ISL_198796_A_duck_Hunan_02_26_YYGK265_P_2014_H5N6__Avian

MP-Group41

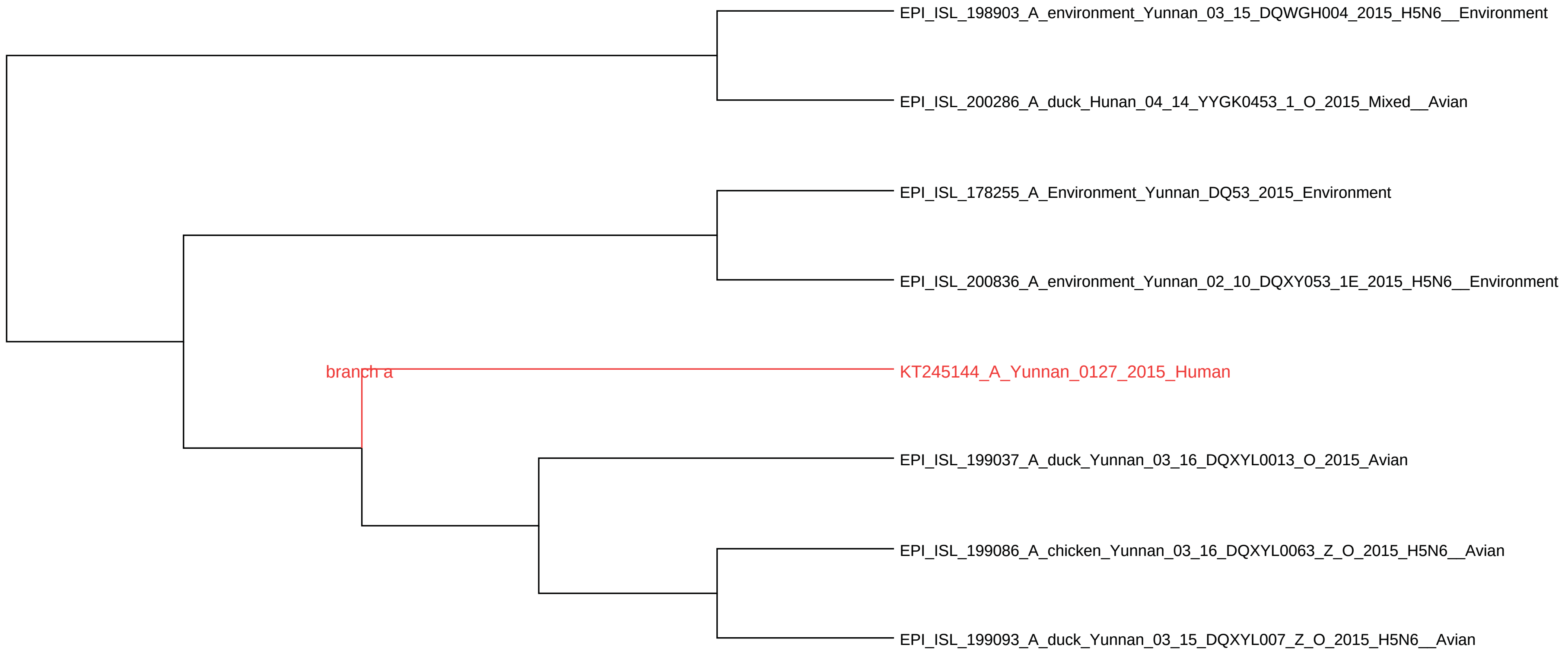
EPI_ISL_198903_A_environment_Yunnan_03_15_DQWGH004_2015_H5N6__Environment
EPI_ISL_200286_A_duck_Hunan_04_14_YYGK0453_1_O_2015_Mixed__Avian
EPI_ISL_178255_A_Environment_Yunnan_DQ53_2015_Environment
EPI_ISL_200836_A_environment_Yunnan_02_10_DQXY053_1E_2015_H5N6__Environment
branch a
KT245144_A_Yunnan_0127_2015_Human
EPI_ISL_199037_A_duck_Yunnan_03_16_DQXYL0013_O_2015_Avian
EPI_ISL_199086_A_chicken_Yunnan_03_16_DQXYL0063_Z_O_2015_H5N6__Avian
EPI_ISL_199093_A_duck_Yunnan_03_15_DQXYL007_Z_O_2015_H5N6__Avian

MP-Group42

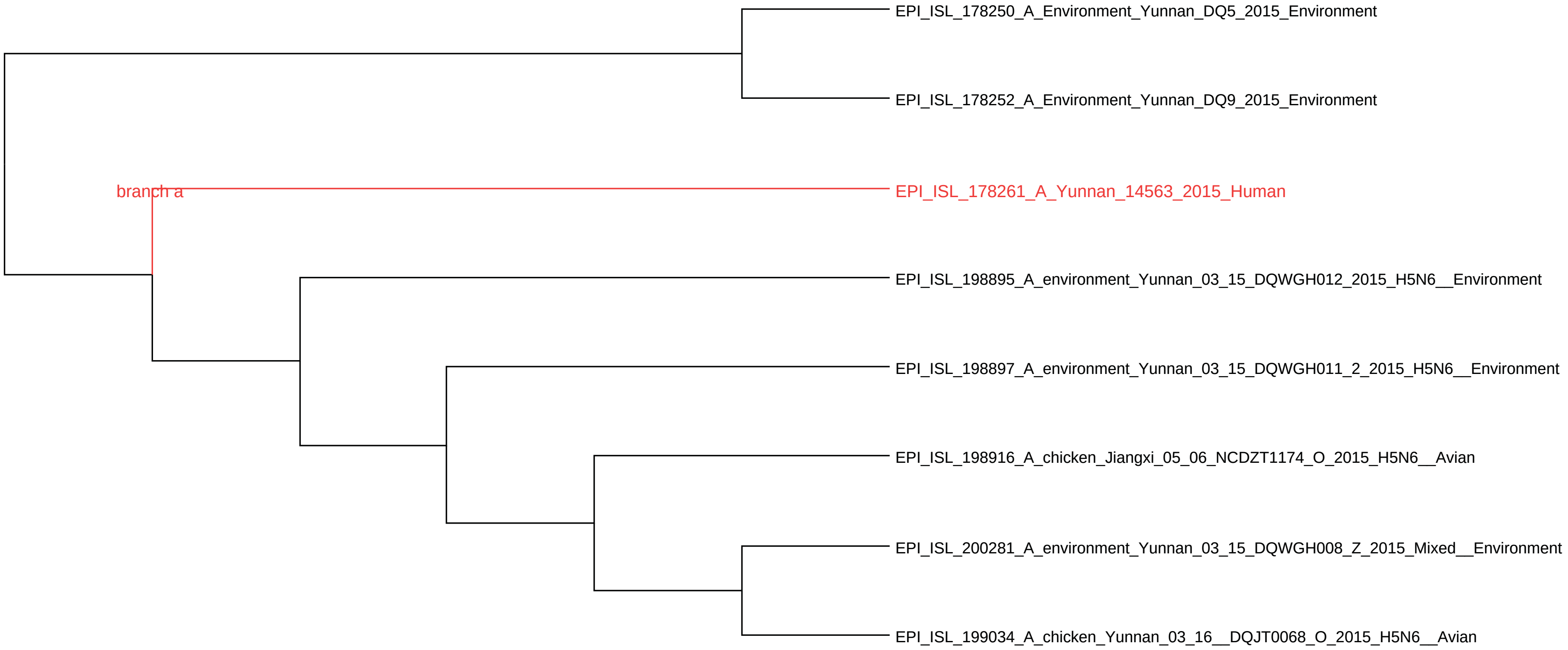
EPI_ISL_178250_A_Environment_Yunnan_DQ5_2015_Environment
EPI_ISL_178252_A_Environment_Yunnan_DQ9_2015_Environment
branch a
EPI_ISL_178261_A_Yunnan_14563_2015_Human
EPI_ISL_198895_A_environment_Yunnan_03_15_DQWGH012_2015_H5N6__Environment
EPI_ISL_198897_A_environment_Yunnan_03_15_DQWGH011_2_2015_H5N6__Environment
EPI_ISL_198916_A_chicken_Jiangxi_05_06_NCDZT1174_O_2015_H5N6__Avian
EPI_ISL_200281_A_environment_Yunnan_03_15_DQWGH008_Z_2015_Mixed__Environment
EPI_ISL_199034_A_chicken_Yunnan_03_16__DQJT0068_O_2015_H5N6__Avian

MP-Group43

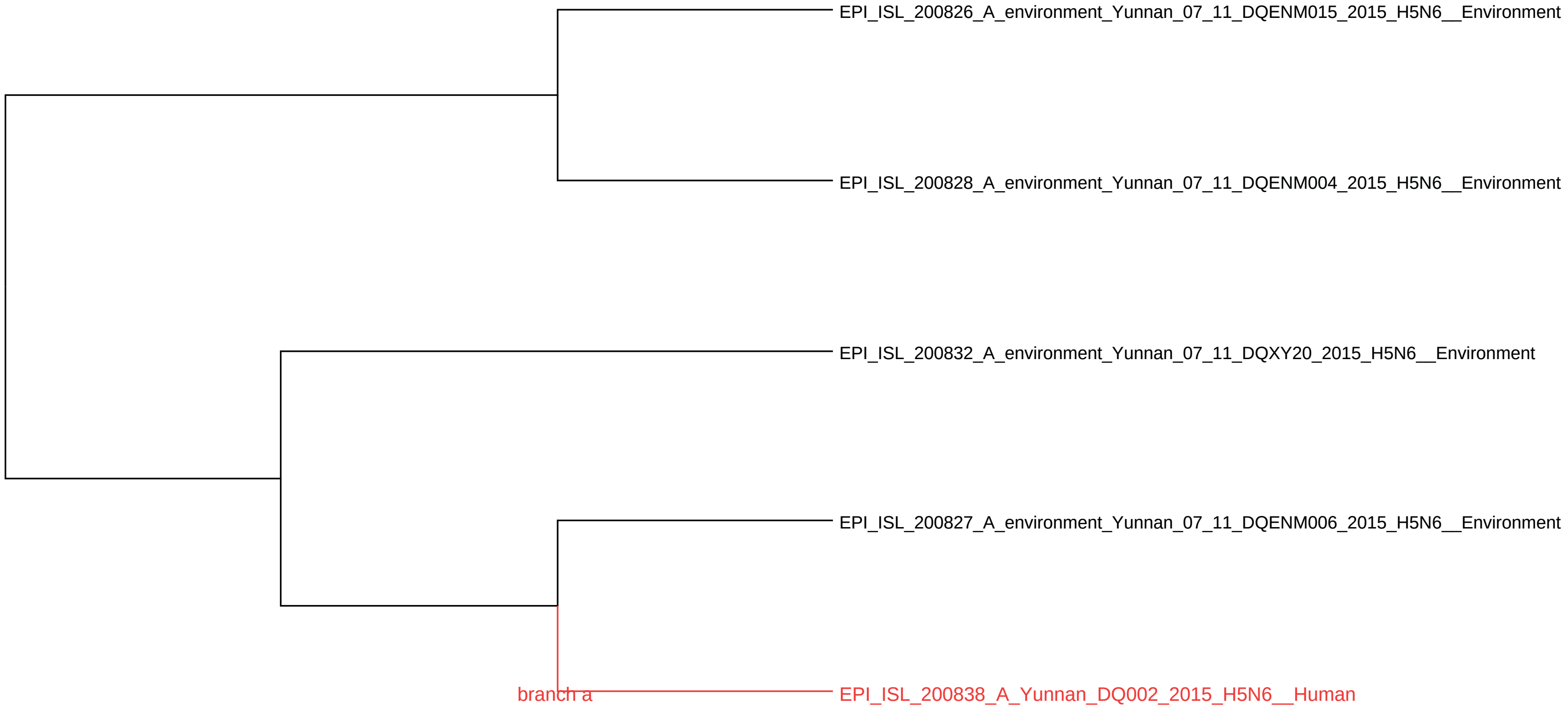
EPI_ISL_200826_A_environment_Yunnan_07_11_DQENM015_2015_H5N6__Environment
EPI_ISL_200828_A_environment_Yunnan_07_11_DQENM004_2015_H5N6__Environment
EPI_ISL_200832_A_environment_Yunnan_07_11_DQXY20_2015_H5N6__Environment
EPI_ISL_200827_A_environment_Yunnan_07_11_DQENM006_2015_H5N6__Environment
branch a
EPI_ISL_200838_A_Yunnan_DQ002_2015_H5N6__Human

MP-Group44

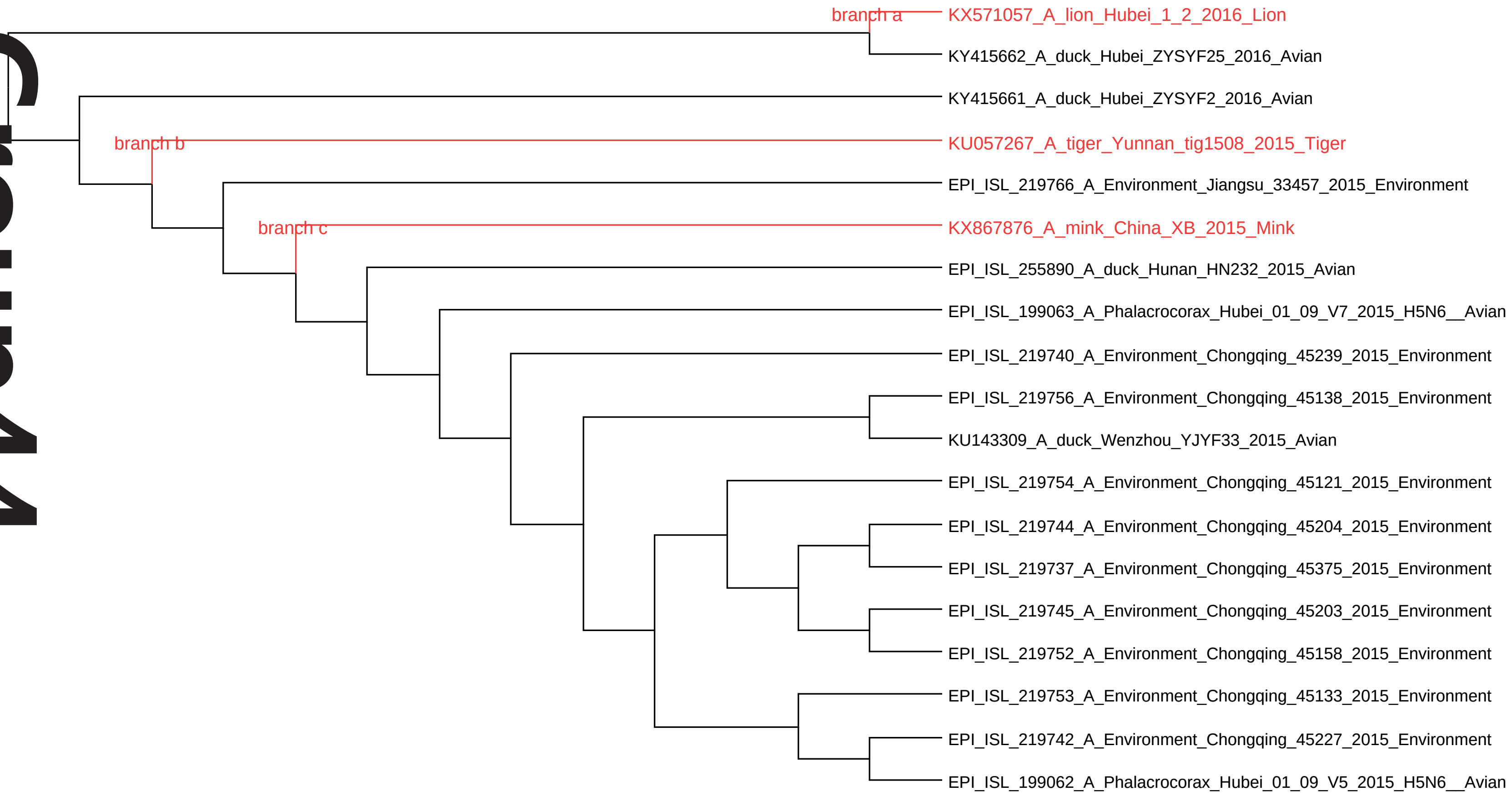

branch a
KX571057_A_lion_Hubei_1_2_2016_Lion
KY415662_A_duck_Hubei_ZYSYF25_2016_Avian
KY415661_A_duck_Hubei_ZYSYF2_2016_Avian
branch b
KU057267_A_tiger_Yunnan_tig1508_2015_Tiger
EPI_ISL_219766_A_Environment_Jiangsu_33457_2015_Environment
branch c
KX867876_A_mink_China_XB_2015_Mink
EPI_ISL_255890_A_duck_Hunan_HN232_2015_Avian
EPI_ISL_199063_A_Phalacrocorax_Hubei_01_09_V7_2015_H5N6__Avian
EPI_ISL_219740_A_Environment_Chongqing_45239_2015_Environment
EPI_ISL_219756_A_Environment_Chongqing_45138_2015_Environment
KU143309_A_duck_Wenzhou_YJYF33_2015_Avian
EPI_ISL_219754_A_Environment_Chongqing_45121_2015_Environment
EPI_ISL_219744_A_Environment_Chongqing_45204_2015_Environment
EPI_ISL_219737_A_Environment_Chongqing_45375_2015_Environment
EPI_ISL_219745_A_Environment_Chongqing_45203_2015_Environment
EPI_ISL_219752_A_Environment_Chongqing_45158_2015_Environment
EPI_ISL_219753_A_Environment_Chongqing_45133_2015_Environment
EPI_ISL_219742_A_Environment_Chongqing_45227_2015_Environment
EPI_ISL_199062_A_Phalacrocorax_Hubei_01_09_V5_2015_H5N6__Avian

MP-Group45

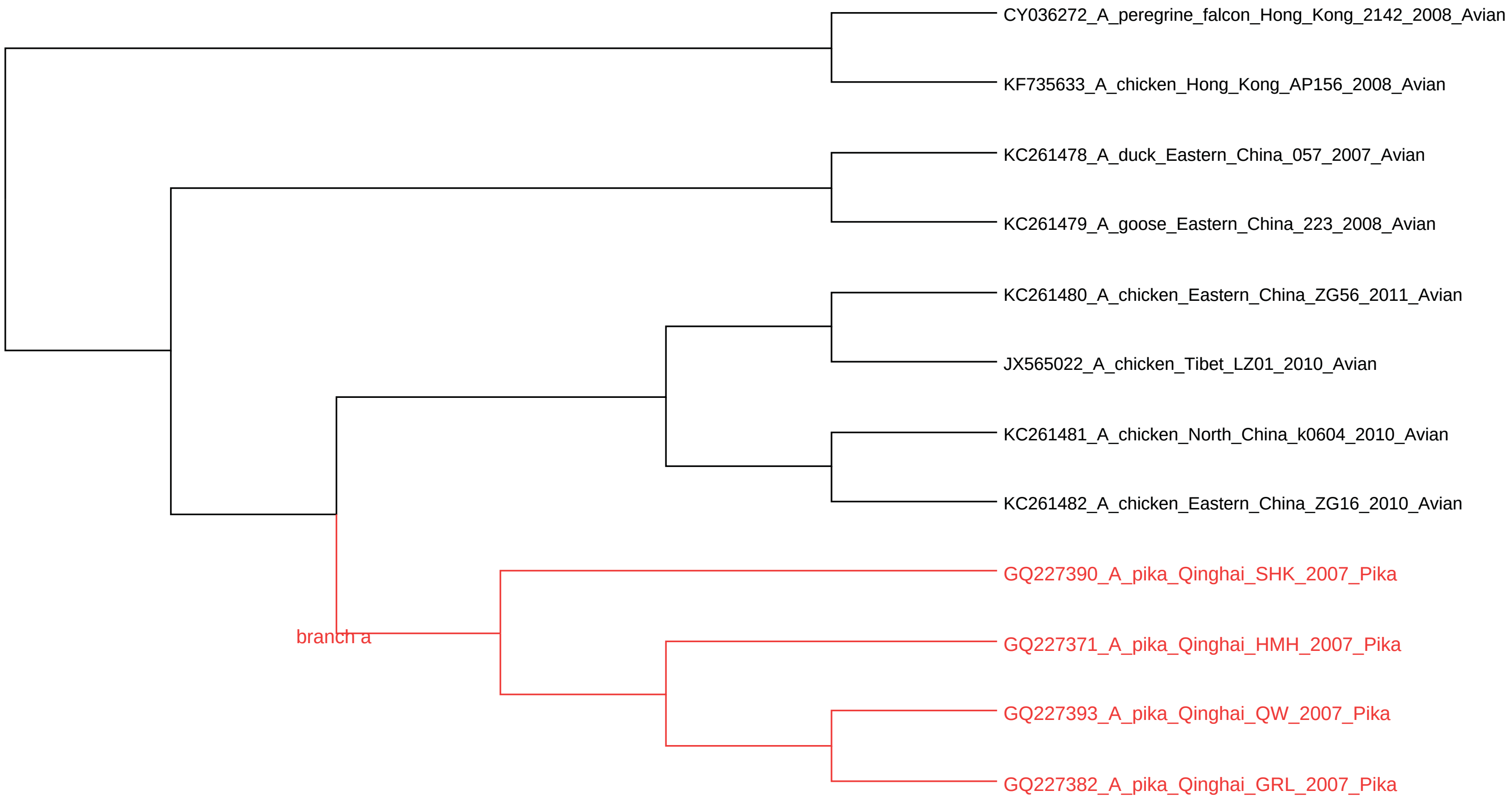
CY036272_A_peregrine_falcon_Hong_Kong_2142_2008_Avian
KF735633_A_chicken_Hong_Kong_AP156_2008_Avian
KC261478_A_duck_Eastern_China_057_2007_Avian
KC261479_A_goose_Eastern_China_223_2008_Avian
KC261480_A_chicken_Eastern_China_ZG56_2011_Avian
JX565022_A_chicken_Tibet_LZ01_2010_Avian
KC261481_A_chicken_North_China_k0604_2010_Avian
KC261482_A_chicken_Eastern_China_ZG16_2010_Avian
GQ227390_A_pika_Qinghai_SHK_2007_Pika
branch a
GQ227371_A_pika_Qinghai_HMH_2007_Pika
GQ227393_A_pika_Qinghai_QW_2007_Pika
GQ227382_A_pika_Qinghai_GRL_2007_Pika

MP-Group46

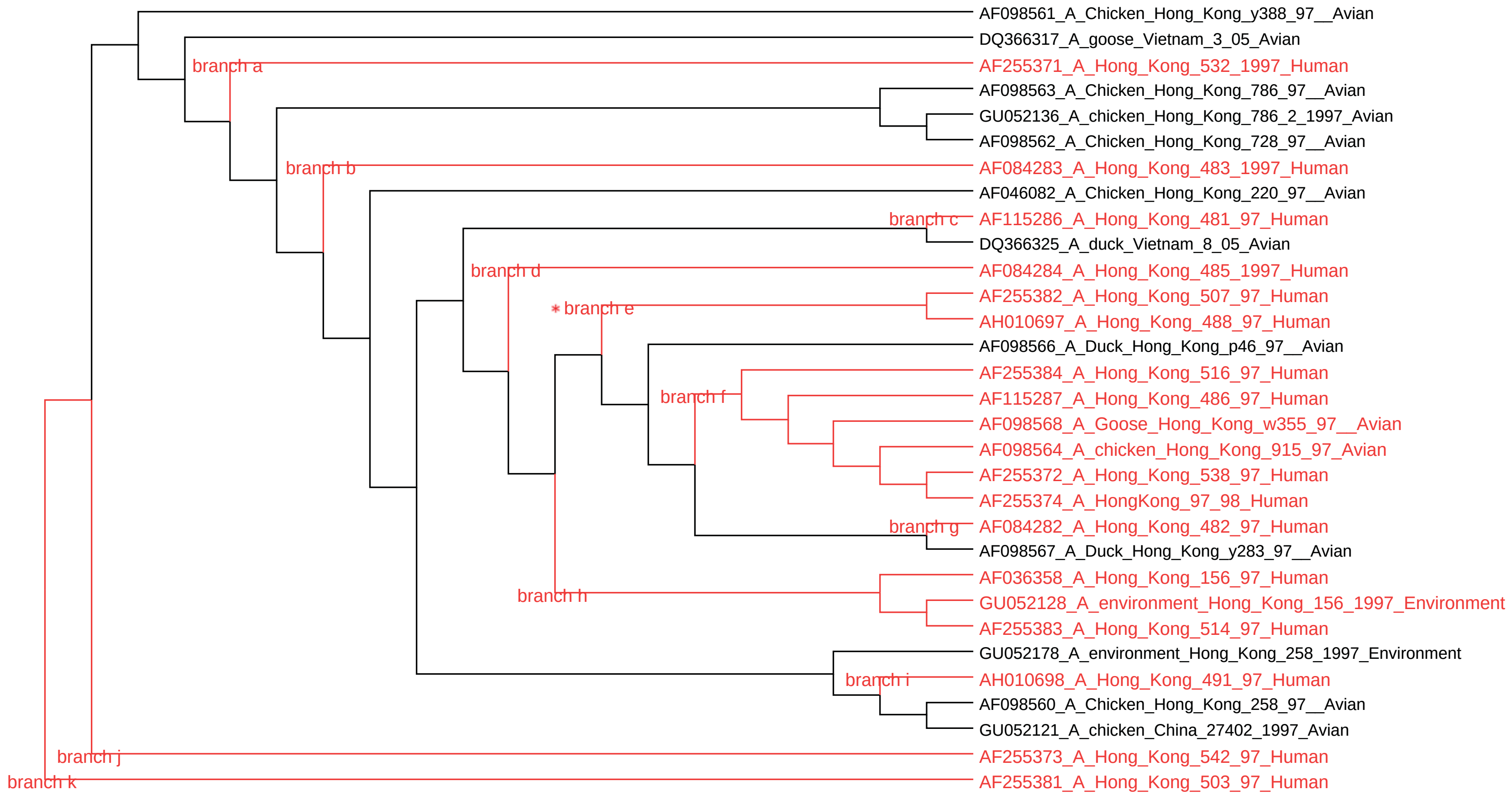
AF098561_A_Chicken_Hong_Kong_y388_97__Avian
DQ366317_A_goose_Vietnam_3_05_Avian
branch a
AF255371_A_Hong_Kong_532_1997_Human
AF098563_A_Chicken_Hong_Kong_786_97__Avian
GU052136_A_chicken_Hong_Kong_786_2_1997_Avian
AF098562_A_Chicken_Hong_Kong_728_97__Avian
branch b
AF084283_A_Hong_Kong_483_1997_Human
AF046082_A_Chicken_Hong_Kong_220_97__Avian
branch c
AF115286_A_Hong_Kong_481_97_Human
DQ366325_A_duck_Vietnam_8_05_Avian
branch d
AF084284_A_Hong_Kong_485_1997_Human
AF255382_A_Hong_Kong_507_97_Human
* branch e
AH010697_A_Hong_Kong_488_97_Human
AF098566_A_Duck_Hong_Kong_p46_97__Avian
AF255384_A_Hong_Kong_516_97_Human
branch f
AF115287_A_Hong_Kong_486_97_Human
AF098568_A_Goose_Hong_Kong_w355_97__Avian
AF098564_A_chicken_Hong_Kong_915_97_Avian
AF255372_A_Hong_Kong_538_97_Human
AF255374_A_HongKong_97_98_Human
branch g
AF084282_A_Hong_Kong_482_97_Human
AF098567_A_Duck_Hong_Kong_y283_97__Avian
AF036358_A_Hong_Kong_156_97_Human
branch h
GU052128_A_environment_Hong_Kong_156_1997_Environment
AF255383_A_Hong_Kong_514_97_Human
GU052178_A_environment_Hong_Kong_258_1997_Environment
branch i
AH010698_A_Hong_Kong_491_97_Human
AF098560_A_Chicken_Hong_Kong_258_97__Avian
GU052121_A_chicken_China_27402_1997_Avian
branch j
AF255373_A_Hong_Kong_542_97_Human
branch k
AF255381_A_Hong_Kong_503_97_Human

MP-Group47

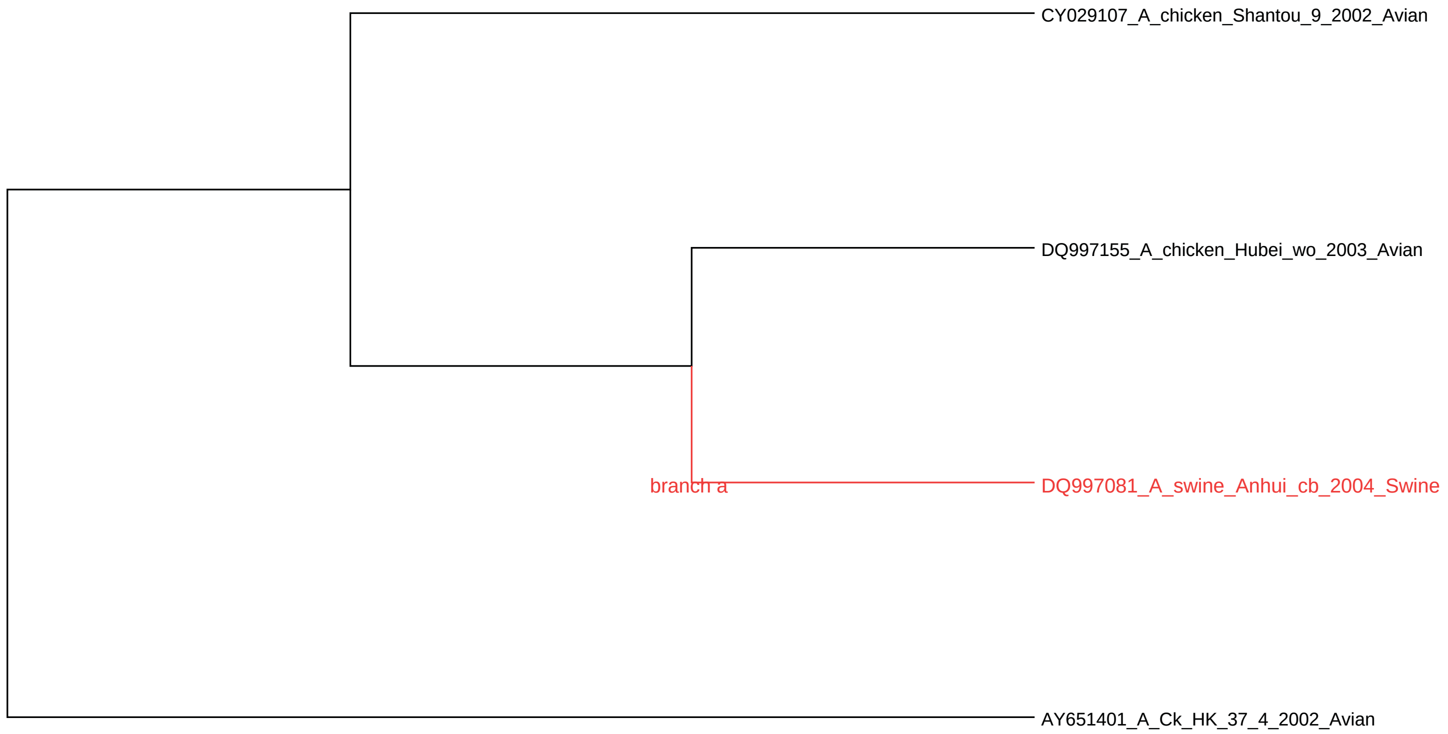

CY029107_A_chicken_Shantou_9_2002_Avian
DQ997155_A_chicken_Hubei_wo_2003_Avian
branch a
DQ997081_A_swine_Anhui_cb_2004_Swine
AY651401_A_Ck_HK_37_4_2002_Avian

MP-Group48

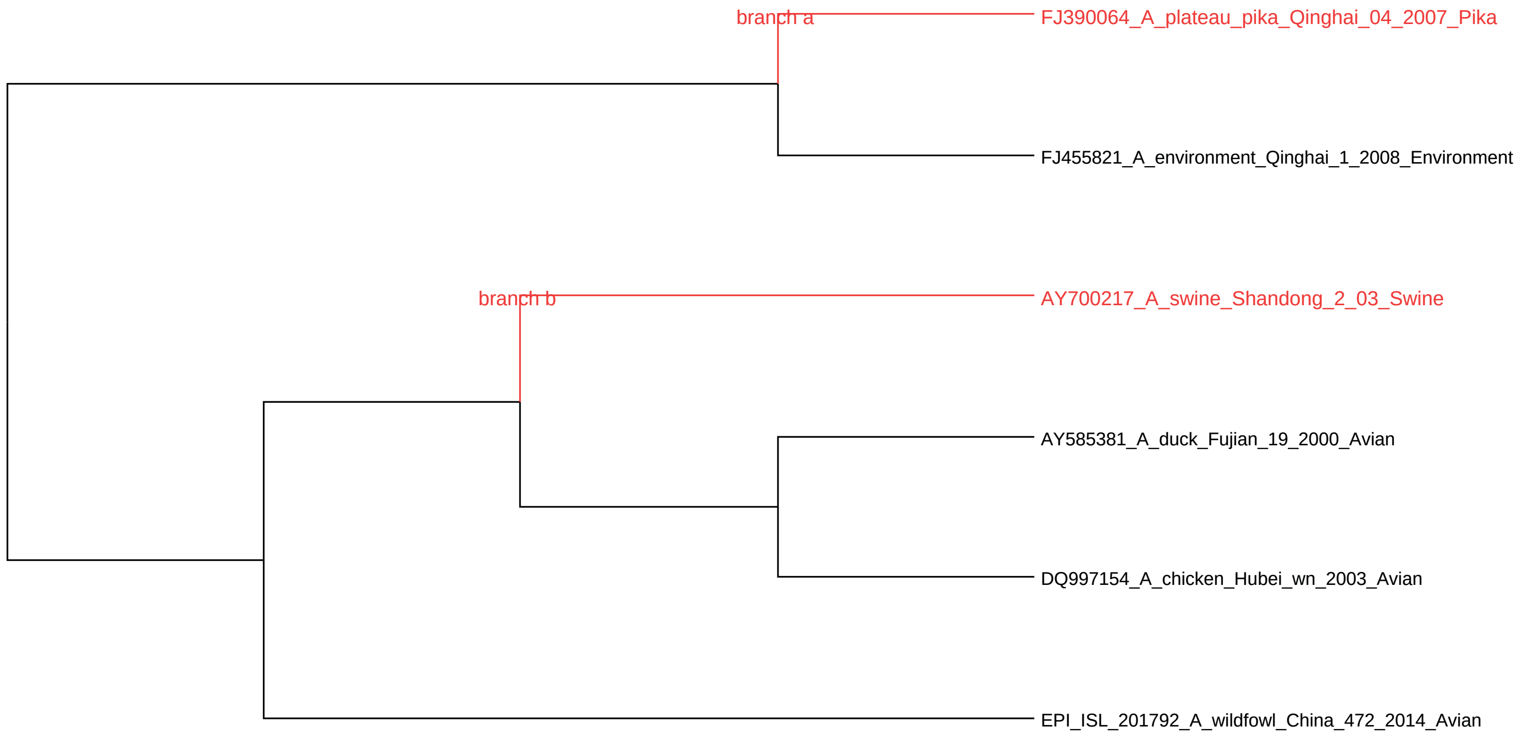

branch a
FJ390064_A_plateau_pika_Qinghai_04_2007_Pika
FJ455821_A_environment_Qinghai_1_2008_Environment
branch b
AY700217_A_swine_Shandong_2_03_Swine
AY585381_A_duck_Fujian_19_2000_Avian
DQ997154_A_chicken_Hubei_wn_2003_Avian
EPI_ISL_201792_A_wildfowl_China_472_2014_Avian

MP-Group49

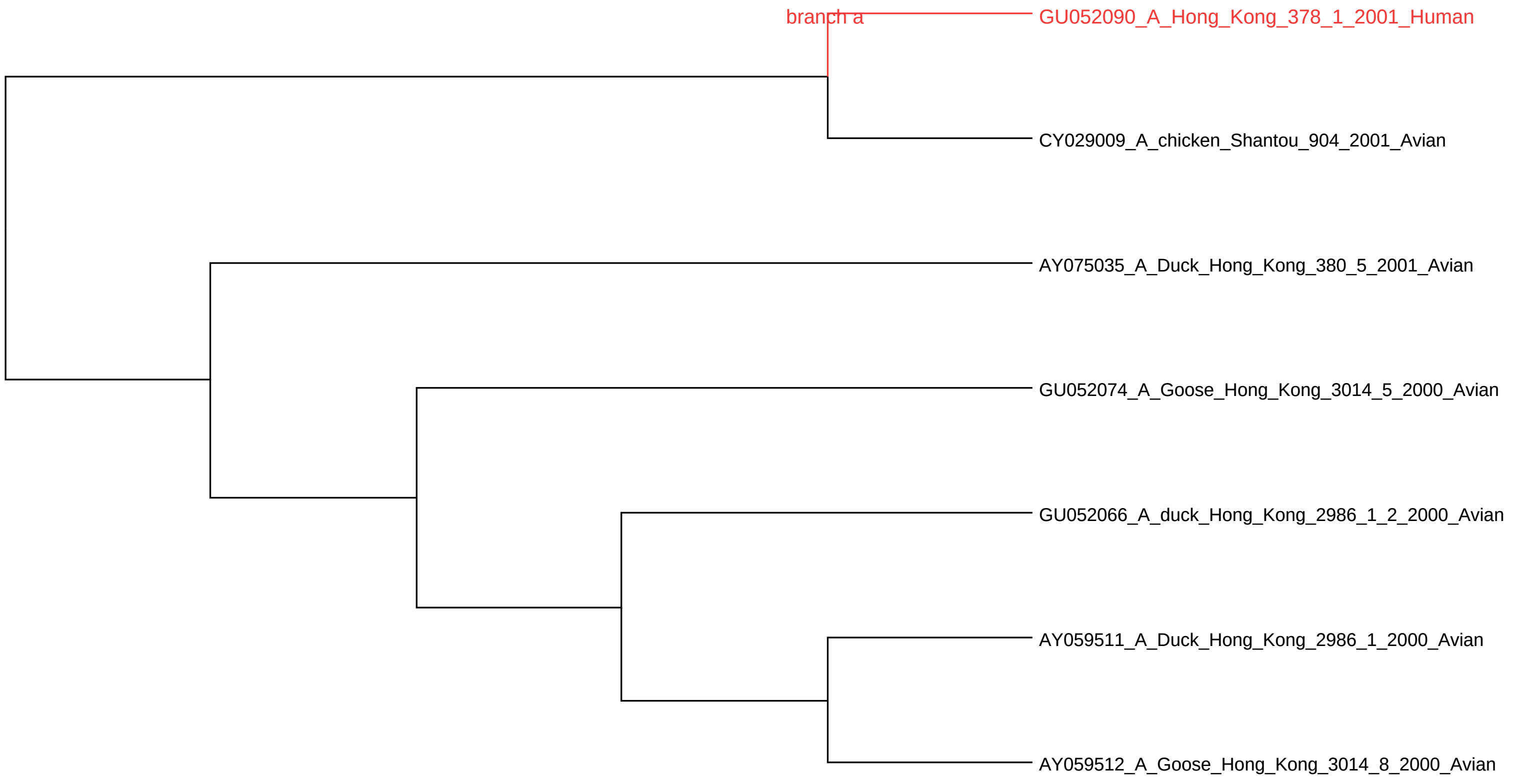

branch a
GU052090_A_Hong_Kong_378_1_2001_Human
CY029009_A_chicken_Shantou_904_2001_Avian
AY075035_A_Duck_Hong_Kong_380_5_2001_Avian
GU052074_A_Goose_Hong_Kong_3014_5_2000_Avian
GU052066_A_duck_Hong_Kong_2986_1_2_2000_Avian
AY059511_A_Duck_Hong_Kong_2986_1_2000_Avian
AY059512_A_Goose_Hong_Kong_3014_8_2000_Avian

MP-Group50

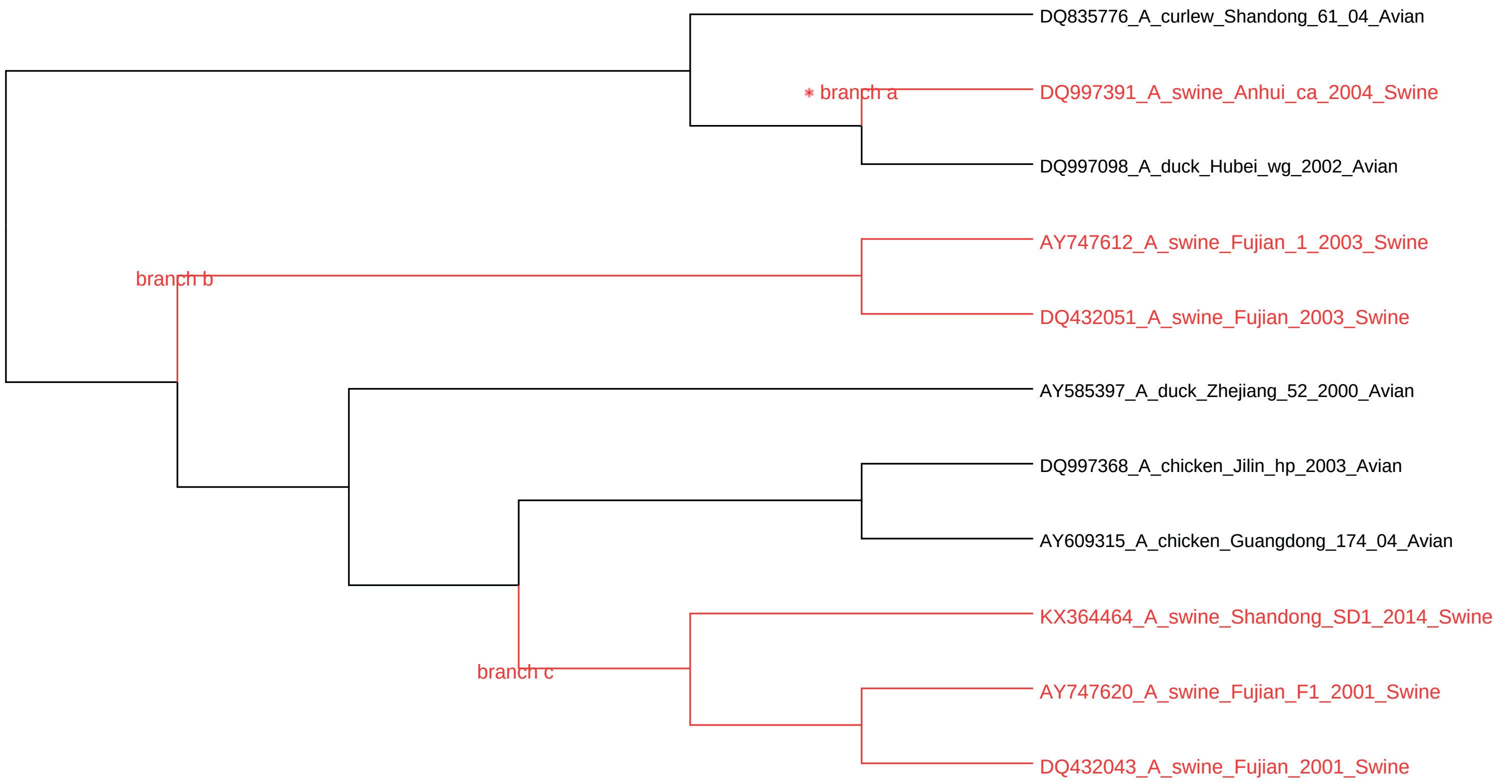
DQ835776_A_curlew_Shandong_61_04_Avian
* branch a
DQ997391_A_swine_Anhui_ca_2004_Swine
DQ997098_A_duck_Hubei_wg_2002_Avian
AY747612_A_swine_Fujian_1_2003_Swine
branch b
DQ432051_A_swine_Fujian_2003_Swine
AY585397_A_duck_Zhejiang_52_2000_Avian
DQ997368_A_chicken_Jilin_hp_2003_Avian
AY609315_A_chicken_Guangdong_174_04_Avian
KX364464_A_swine_Shandong_SD1_2014_Swine
branch c
AY747620_A_swine_Fujian_F1_2001_Swine
DQ432043_A_swine_Fujian_2001_Swine

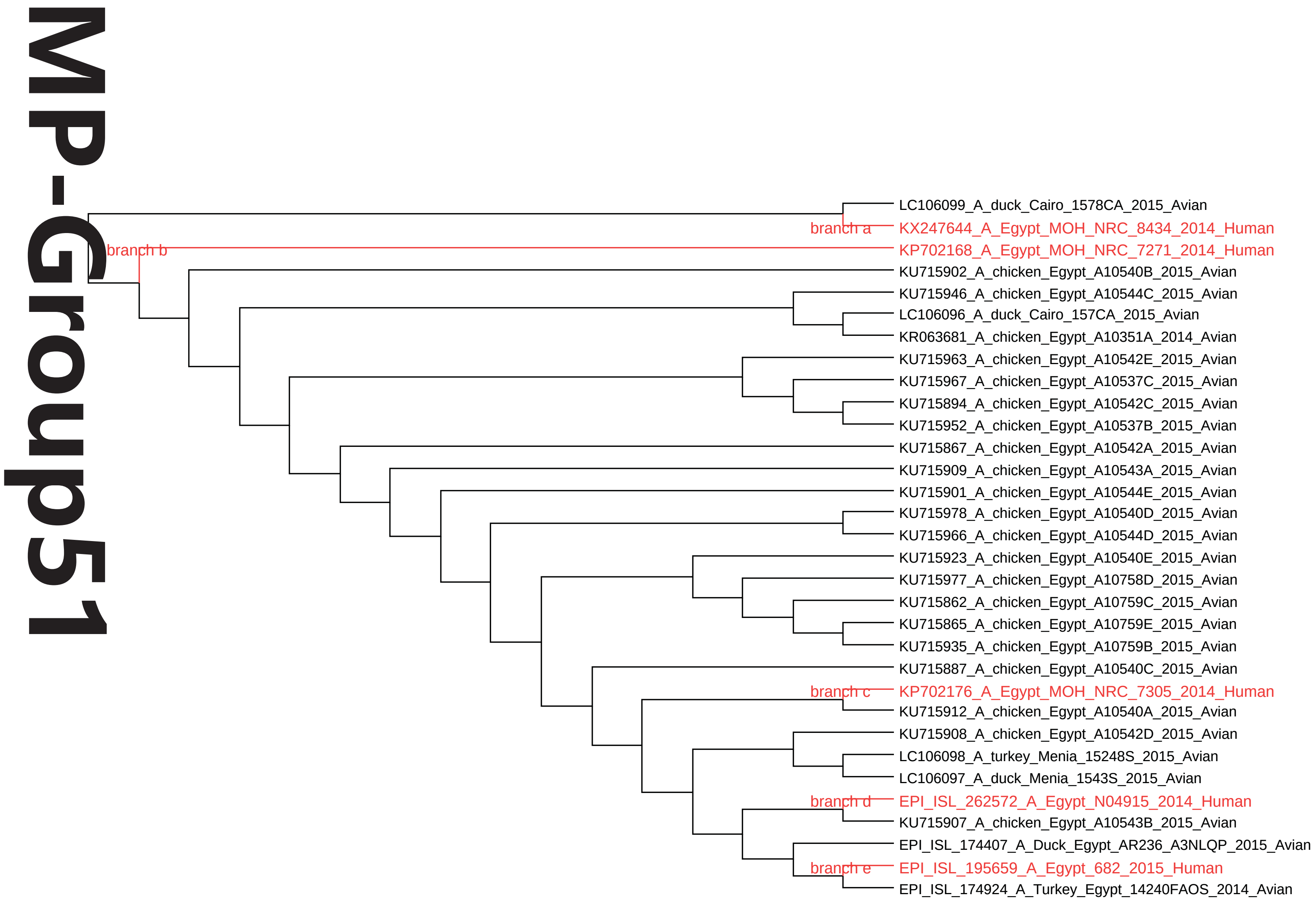
MP-Group51
LC106099_A_duck_Cairo_1578CA_2015_Avian
branch a
KX247644_A_Egypt_MOH_NRC_8434_2014_Human
branch b
KP702168_A_Egypt_MOH_NRC_7271_2014_Human
KU715902_A_chicken_Egypt_A10540B_2015_Avian
KU715946_A_chicken_Egypt_A10544C_2015_Avian
LC106096_A_duck_Cairo_157CA_2015_Avian
KR063681_A_chicken_Egypt_A10351A_2014_Avian
KU715963_A_chicken_Egypt_A10542E_2015_Avian
KU715967_A_chicken_Egypt_A10537C_2015_Avian
KU715894_A_chicken_Egypt_A10542C_2015_Avian
KU715952_A_chicken_Egypt_A10537B_2015_Avian
KU715867_A_chicken_Egypt_A10542A_2015_Avian
KU715909_A_chicken_Egypt_A10543A_2015_Avian
KU715901_A_chicken_Egypt_A10544E_2015_Avian
KU715978_A_chicken_Egypt_A10540D_2015_Avian
KU715966_A_chicken_Egypt_A10544D_2015_Avian
KU715923_A_chicken_Egypt_A10540E_2015_Avian
KU715977_A_chicken_Egypt_A10758D_2015_Avian
KU715862_A_chicken_Egypt_A10759C_2015_Avian
KU715865_A_chicken_Egypt_A10759E_2015_Avian
KU715935_A_chicken_Egypt_A10759B_2015_Avian
KU715887_A_chicken_Egypt_A10540C_2015_Avian
branch c
KP702176_A_Egypt_MOH_NRC_7305_2014_Human
KU715912_A_chicken_Egypt_A10540A_2015_Avian
KU715908_A_chicken_Egypt_A10542D_2015_Avian
LC106098_A_turkey_Menia_15248S_2015_Avian
LC106097_A_duck_Menia_1543S_2015_Avian
branch d
EPI_ISL_262572_A_Egypt_N04915_2014_Human
KU715907_A_chicken_Egypt_A10543B_2015_Avian
EPI_ISL_174407_A_Duck_Egypt_AR236_A3NLQP_2015_Avian
branch e
EPI_ISL_195659_A_Egypt_682_2015_Human
EPI_ISL_174924_A_Turkey_Egypt_14240FAOS_2014_Avian

MP-Group52

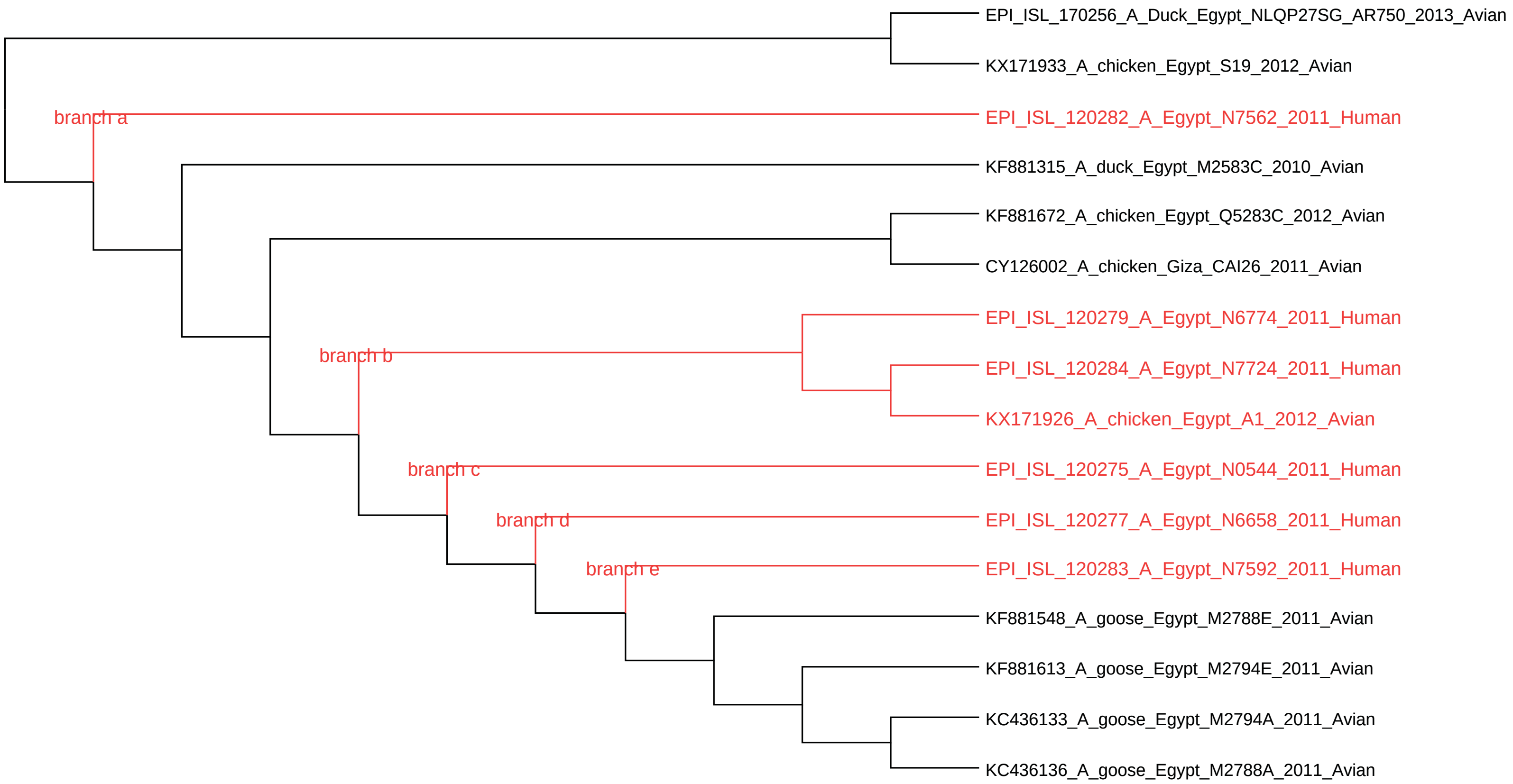

EPI_ISL_170256_A_Duck_Egypt_NLQP27SG_AR750_2013_Avian
KX171933_A_chicken_Egypt_S19_2012_Avian
branch a
EPI_ISL_120282_A_Egypt_N7562_2011_Human
KF881315_A_duck_Egypt_M2583C_2010_Avian
KF881672_A_chicken_Egypt_Q5283C_2012_Avian
CY126002_A_chicken_Giza_CAI26_2011_Avian
EPI_ISL_120279_A_Egypt_N6774_2011_Human
branch b
EPI_ISL_120284_A_Egypt_N7724_2011_Human
KX171926_A_chicken_Egypt_A1_2012_Avian
branch c
EPI_ISL_120275_A_Egypt_N0544_2011_Human
branch d
EPI_ISL_120277_A_Egypt_N6658_2011_Human
branch e
EPI_ISL_120283_A_Egypt_N7592_2011_Human
KF881548_A_goose_Egypt_M2788E_2011_Avian
KF881613_A_goose_Egypt_M2794E_2011_Avian
KC436133_A_goose_Egypt_M2794A_2011_Avian
KC436136_A_goose_Egypt_M2788A_2011_Avian

MP-Group53

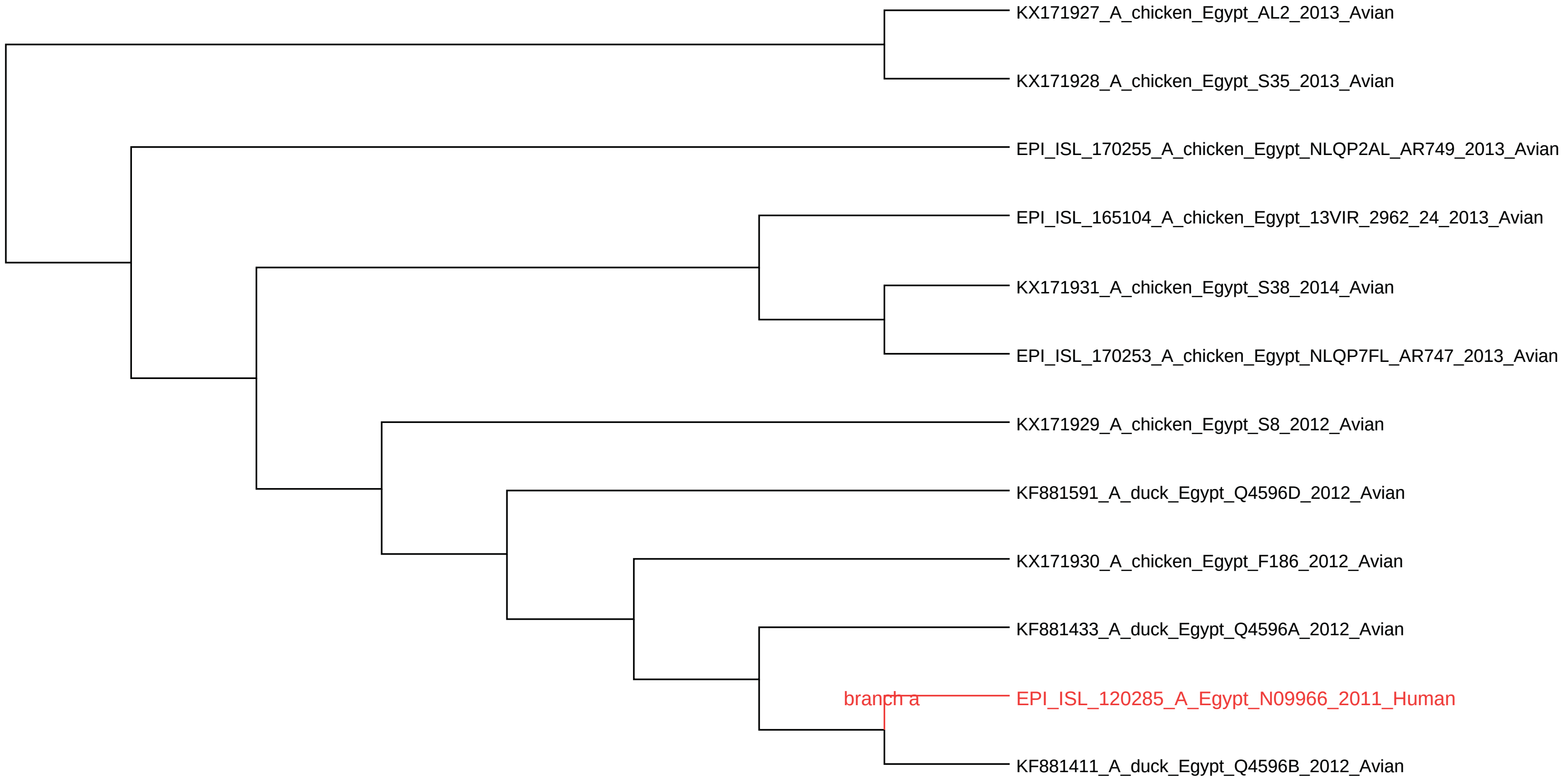
KX171927_A_chicken_Egypt_AL2_2013_Avian
KX171928_A_chicken_Egypt_S35_2013_Avian
EPI_ISL_170255_A_chicken_Egypt_NLQP2AL_AR749_2013_Avian
EPI_ISL_165104_A_chicken_Egypt_13VIR_2962_24_2013_Avian
KX171931_A_chicken_Egypt_S38_2014_Avian
EPI_ISL_170253_A_chicken_Egypt_NLQP7FL_AR747_2013_Avian
KX171929_A_chicken_Egypt_S8_2012_Avian
KF881591_A_duck_Egypt_Q4596D_2012_Avian
KX171930_A_chicken_Egypt_F186_2012_Avian
KF881433_A_duck_Egypt_Q4596A_2012_Avian
branch a
EPI_ISL_120285_A_Egypt_N09966_2011_Human
KF881411_A_duck_Egypt_Q4596B_2012_Avian

MP-Group54

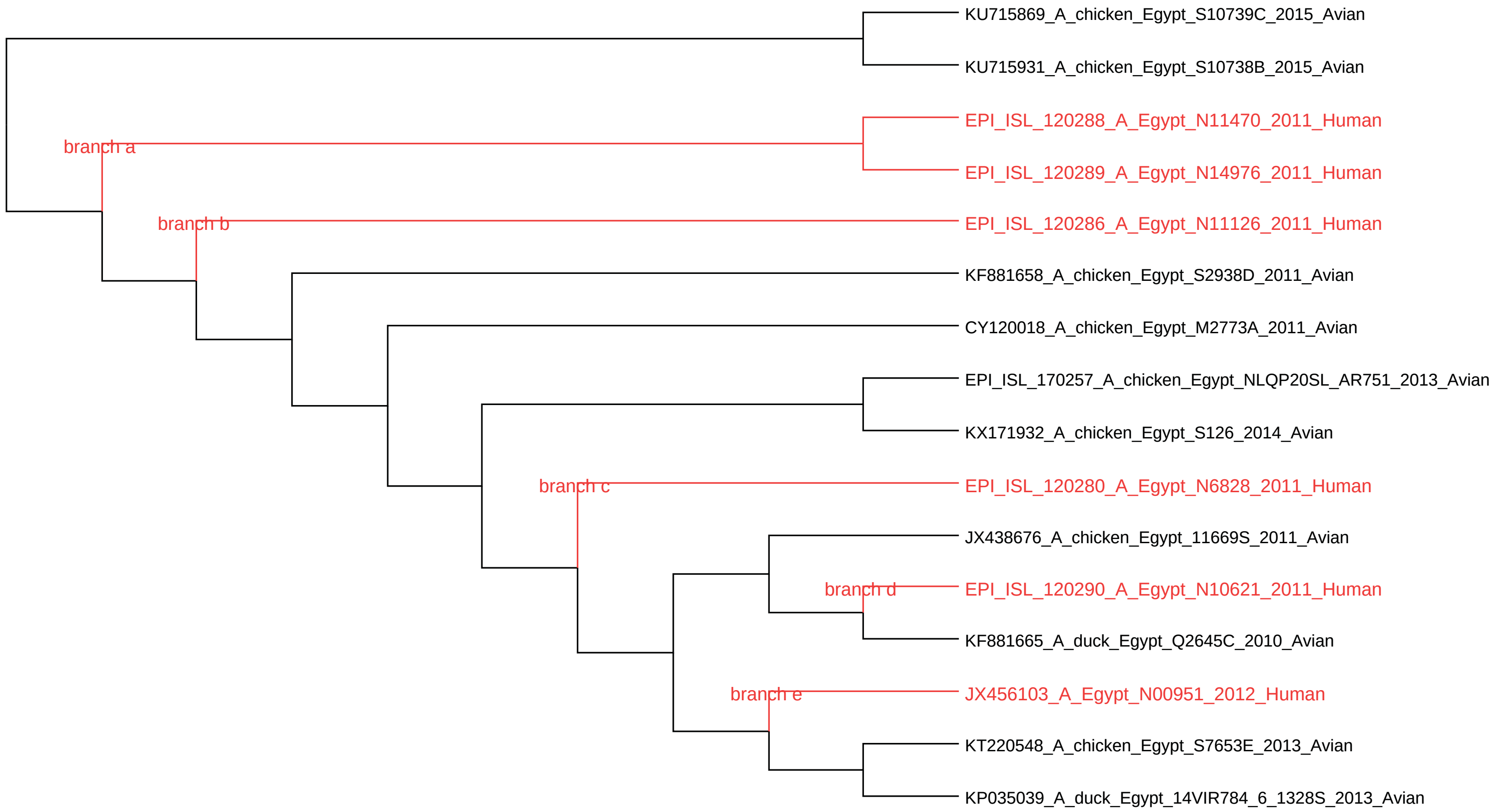

KU715869_A_chicken_Egypt_S10739C_2015_Avian
KU715931_A_chicken_Egypt_S10738B_2015_Avian
branch a
EPI_ISL_120288_A_Egypt_N11470_2011_Human
EPI_ISL_120289_A_Egypt_N14976_2011_Human
branch b
EPI_ISL_120286_A_Egypt_N11126_2011_Human
KF881658_A_chicken_Egypt_S2938D_2011_Avian
CY120018_A_chicken_Egypt_M2773A_2011_Avian
EPI_ISL_170257_A_chicken_Egypt_NLQP20SL_AR751_2013_Avian
KX171932_A_chicken_Egypt_S126_2014_Avian
branch c
EPI_ISL_120280_A_Egypt_N6828_2011_Human
JX438676_A_chicken_Egypt_11669S_2011_Avian
branch d
EPI_ISL_120290_A_Egypt_N10621_2011_Human
KF881665_A_duck_Egypt_Q2645C_2010_Avian
branch e
JX456103_A_Egypt_N00951_2012_Human
KT220548_A_chicken_Egypt_S7653E_2013_Avian
KP035039_A_duck_Egypt_14VIR784_6_1328S_2013_Avian

MP-Group55

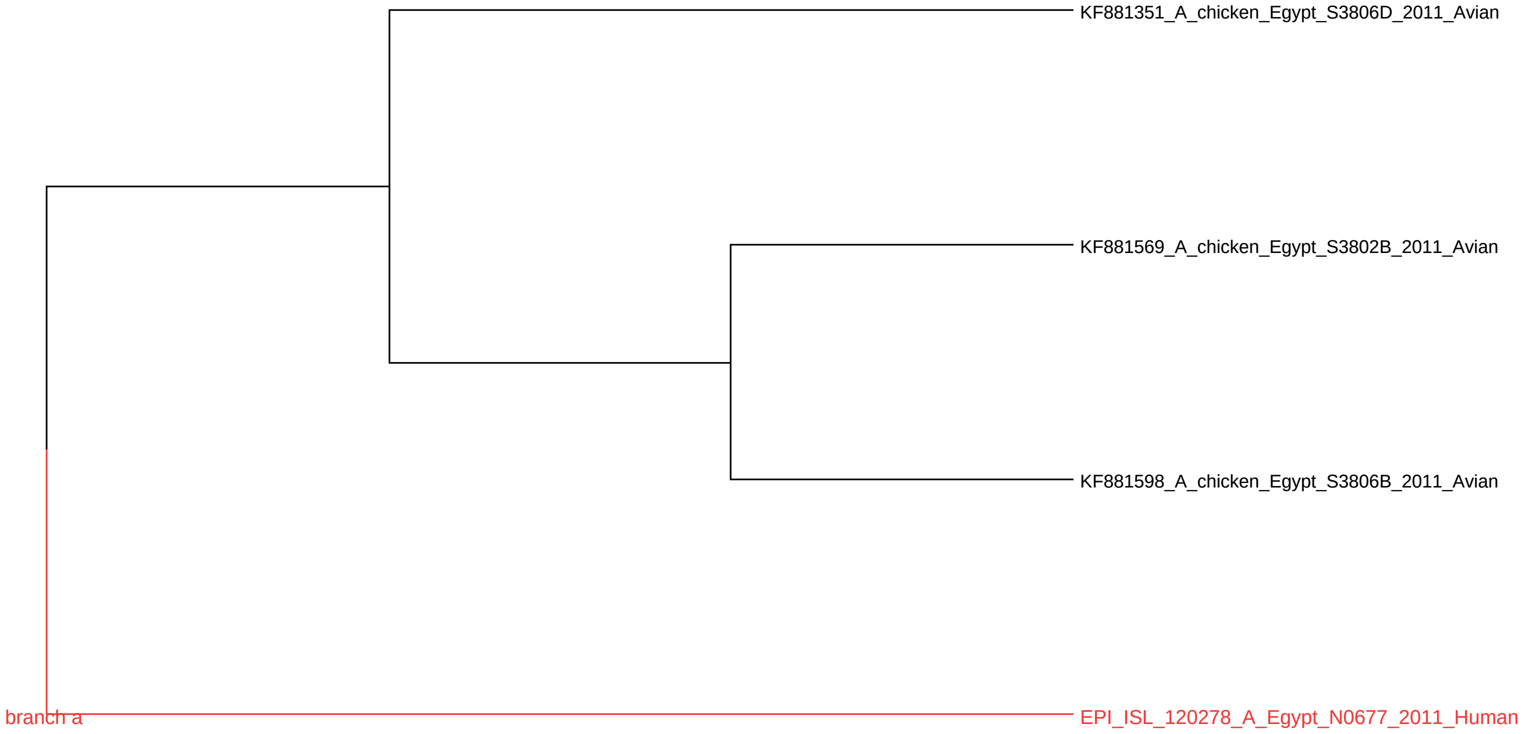
KF881351_A_chicken_Egypt_S3806D_2011_Avian
KF881569_A_chicken_Egypt_S3802B_2011_Avian
KF881598_A_chicken_Egypt_S3806B_2011_Avian
branch a
EPI_ISL_120278_A_Egypt_N0677_2011_Human

MP-Group56

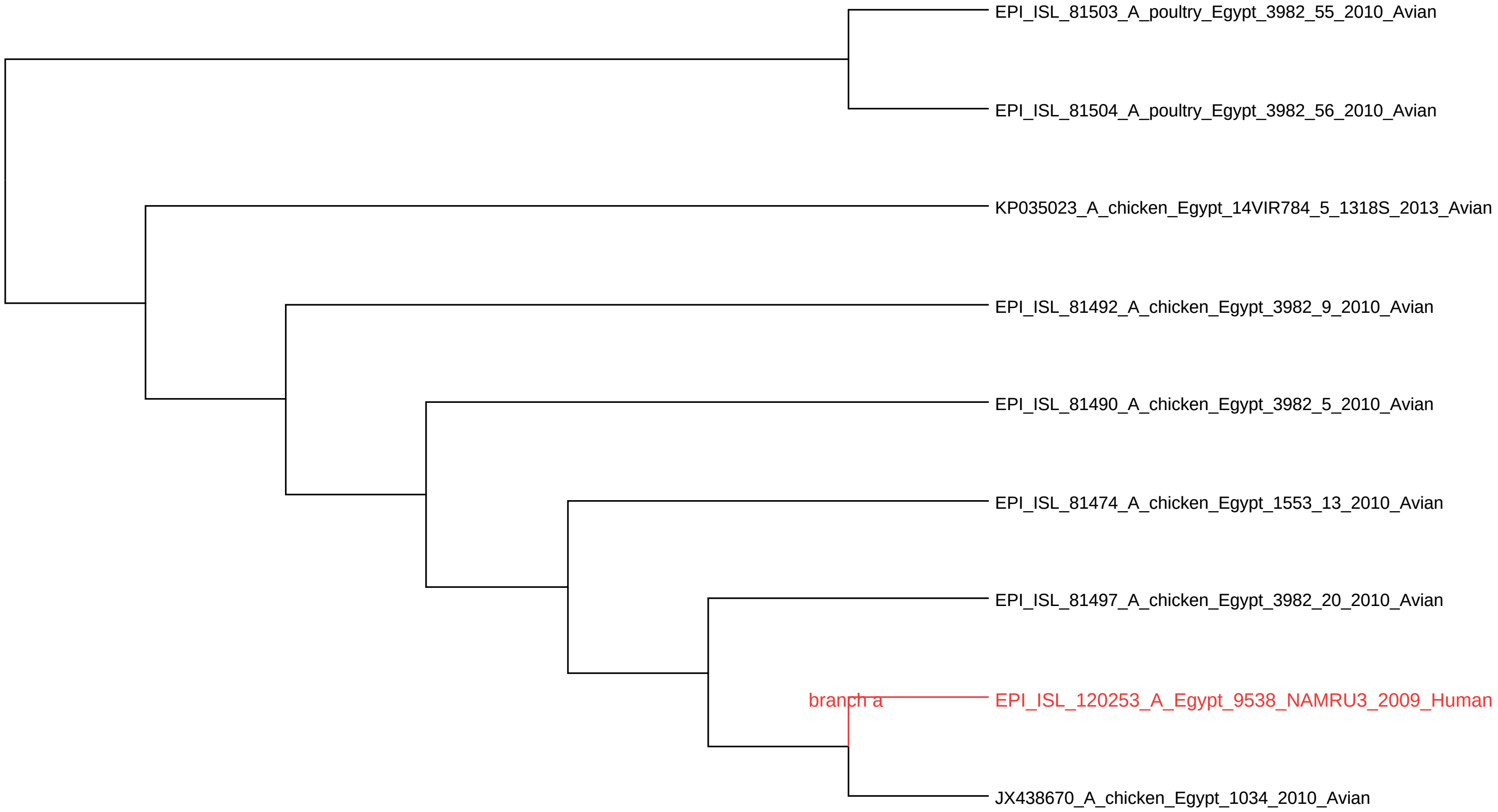
EPI_ISL_81503_A_poultry_Egypt_3982_55_2010_Avian
EPI_ISL_81504_A_poultry_Egypt_3982_56_2010_Avian
KP035023_A_chicken_Egypt_14VIR784_5_1318S_2013_Avian
EPI_ISL_81492_A_chicken_Egypt_3982_9_2010_Avian
EPI_ISL_81490_A_chicken_Egypt_3982_5_2010_Avian
EPI_ISL_81474_A_chicken_Egypt_1553_13_2010_Avian
EPI_ISL_81497_A_chicken_Egypt_3982_20_2010_Avian
branch a
EPI_ISL_120253_A_Egypt_9538_NAMRU3_2009_Human
JX438670_A_chicken_Egypt_1034_2010_Avian

MP-Group57

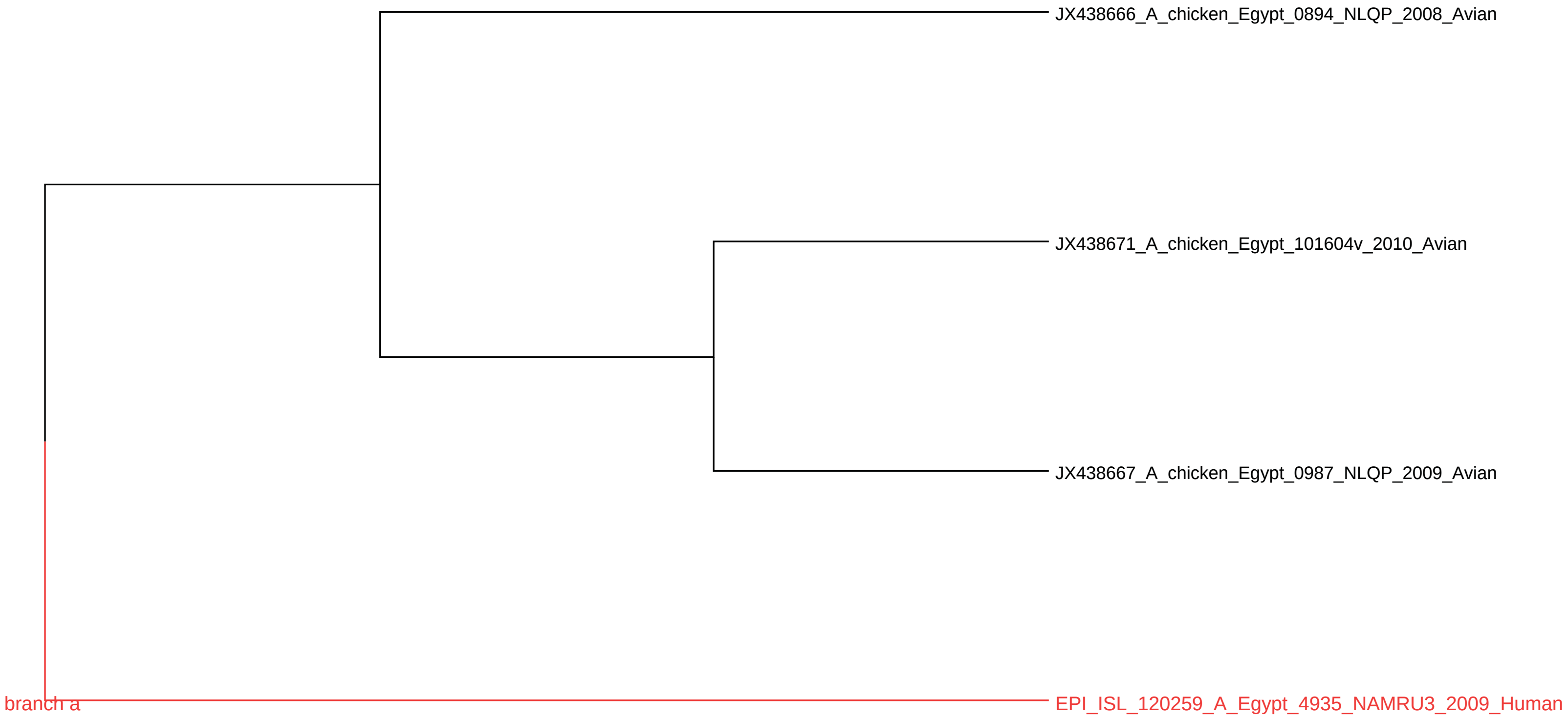
JX438666_A_chicken_Egypt_0894_NLQP_2008_Avian
JX438671_A_chicken_Egypt_101604v_2010_Avian
JX438667_A_chicken_Egypt_0987_NLQP_2009_Avian
branch a
EPI_ISL_120259_A_Egypt_4935_NAMRU3_2009_Human

MP-Group58

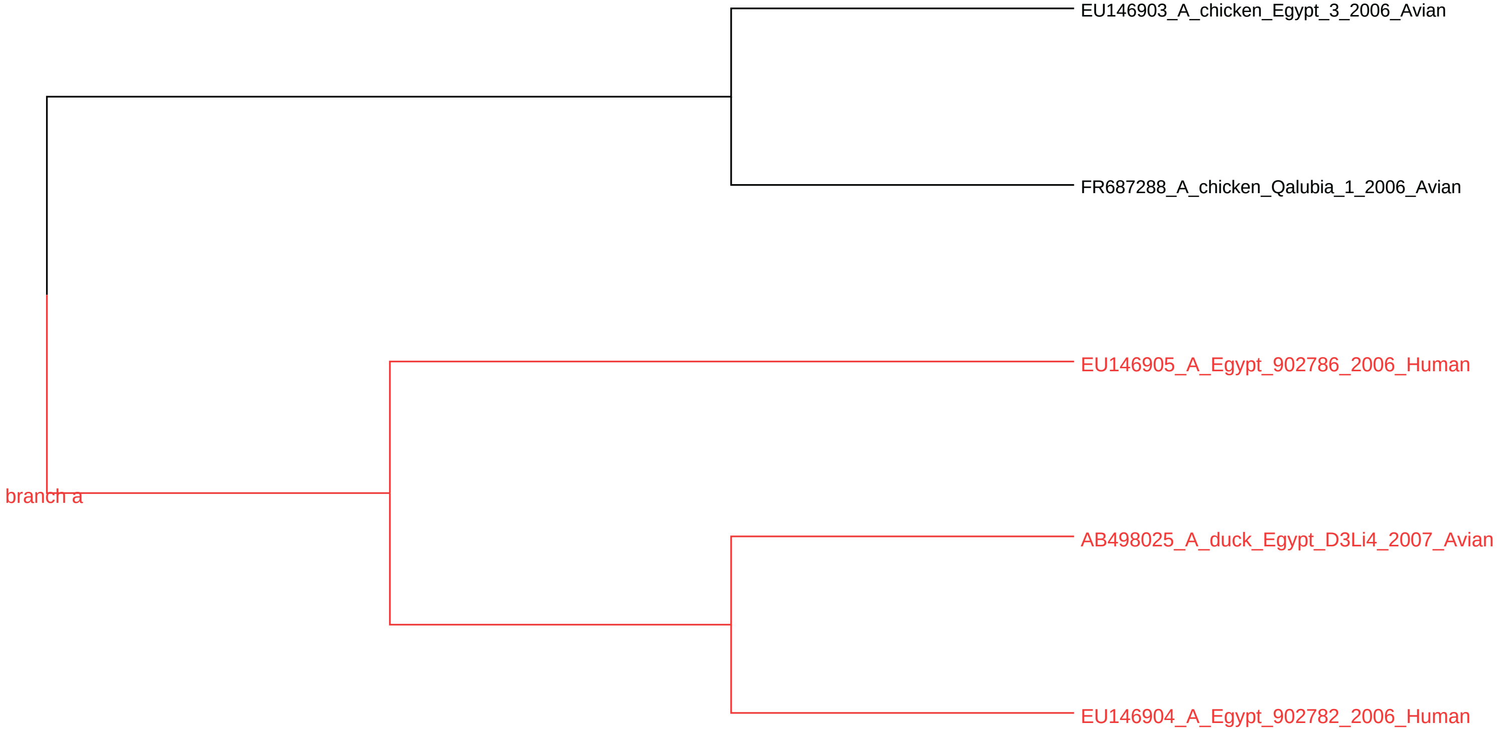

EU146903_A_chicken_Egypt_3_2006_Avian
FR687288_A_chicken_Qalubia_1_2006_Avian
EU146905_A_Egypt_902786_2006_Human
branch a
AB498025_A_duck_Egypt_D3Li4_2007_Avian
EU146904_A_Egypt_902782_2006_Human

MP-Group59

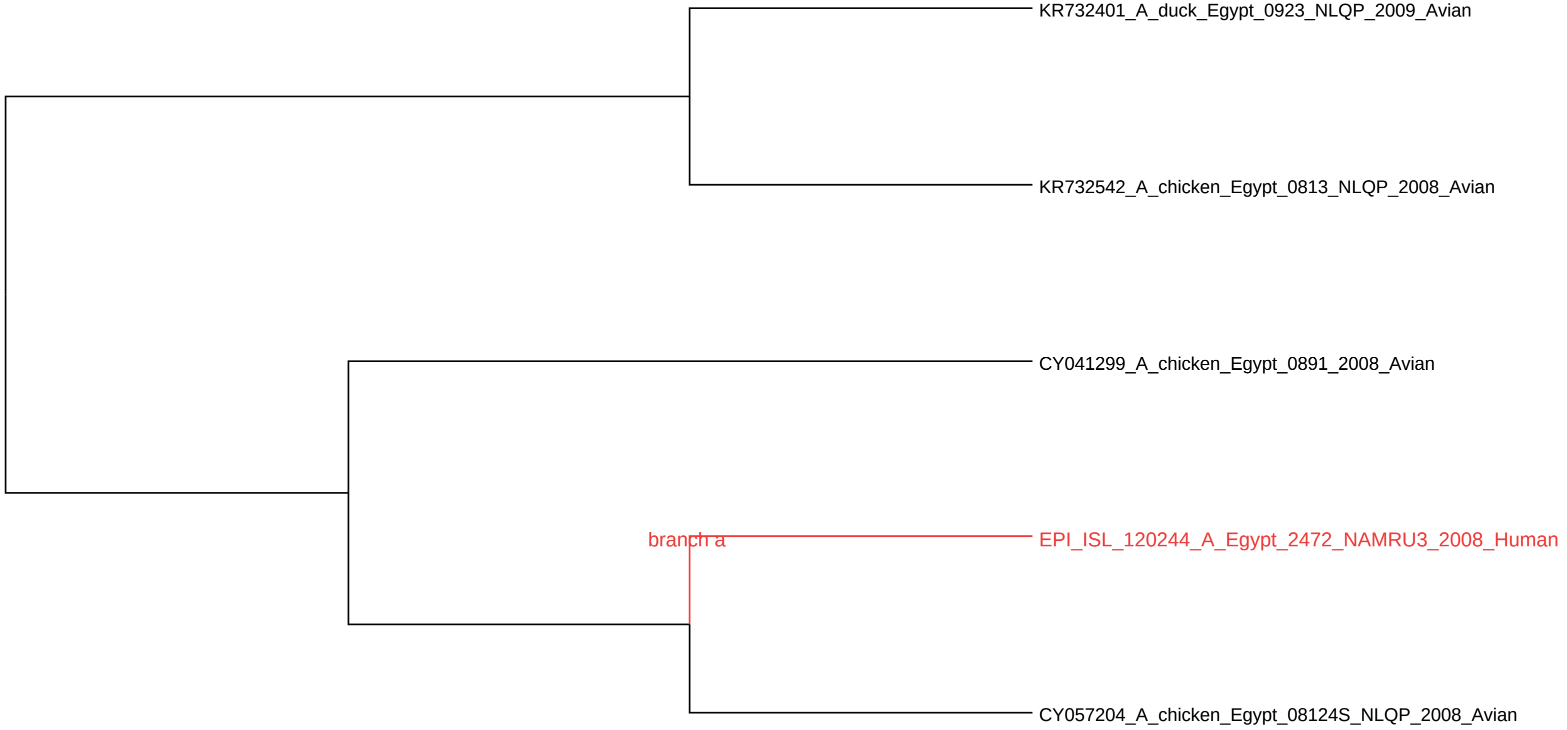
KR732401_A_duck_Egypt_0923_NLQP_2009_Avian
KR732542_A_chicken_Egypt_0813_NLQP_2008_Avian
CY041299_A_chicken_Egypt_0891_2008_Avian
branch a
EPI_ISL_120244_A_Egypt_2472_NAMRU3_2008_Human
CY057204_A_chicken_Egypt_08124S_NLQP_2008_Avian

MP-Group60

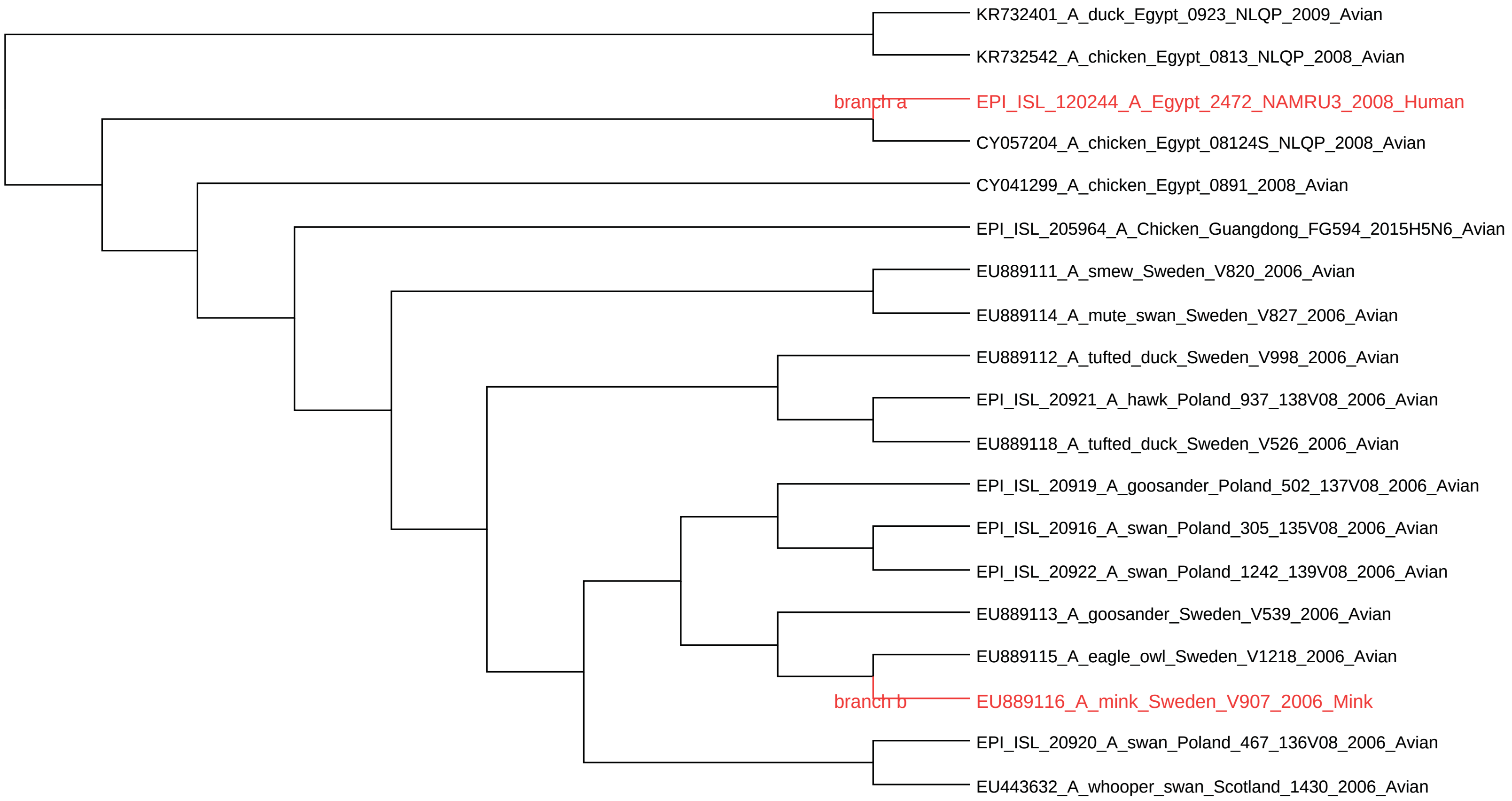
KR732401_A_duck_Egypt_0923_NLQP_2009_Avian
KR732542_A_chicken_Egypt_0813_NLQP_2008_Avian
branch a
EPI_ISL_120244_A_Egypt_2472_NAMRU3_2008_Human
CY057204_A_chicken_Egypt_08124S_NLQP_2008_Avian
CY041299_A_chicken_Egypt_0891_2008_Avian
EPI_ISL_205964_A_Chicken_Guangdong_FG594_2015H5N6_Avian
EU889111_A_smew_Sweden_V820_2006_Avian
EU889114_A_mute_swan_Sweden_V827_2006_Avian
EU889112_A_tufted_duck_Sweden_V998_2006_Avian
EPI_ISL_20921_A_hawk_Poland_937_138V08_2006_Avian
EU889118_A_tufted_duck_Sweden_V526_2006_Avian
EPI_ISL_20919_A_goosander_Poland_502_137V08_2006_Avian
EPI_ISL_20916_A_swan_Poland_305_135V08_2006_Avian
EPI_ISL_20922_A_swan_Poland_1242_139V08_2006_Avian
EU889113_A_goosander_Sweden_V539_2006_Avian
EU889115_A_eagle_owl_Sweden_V1218_2006_Avian
branch b
EU889116_A_mink_Sweden_V907_2006_Mink
EPI_ISL_20920_A_swan_Poland_467_136V08_2006_Avian
EU443632_A_whooper_swan_Scotland_1430_2006_Avian

MP-Group61

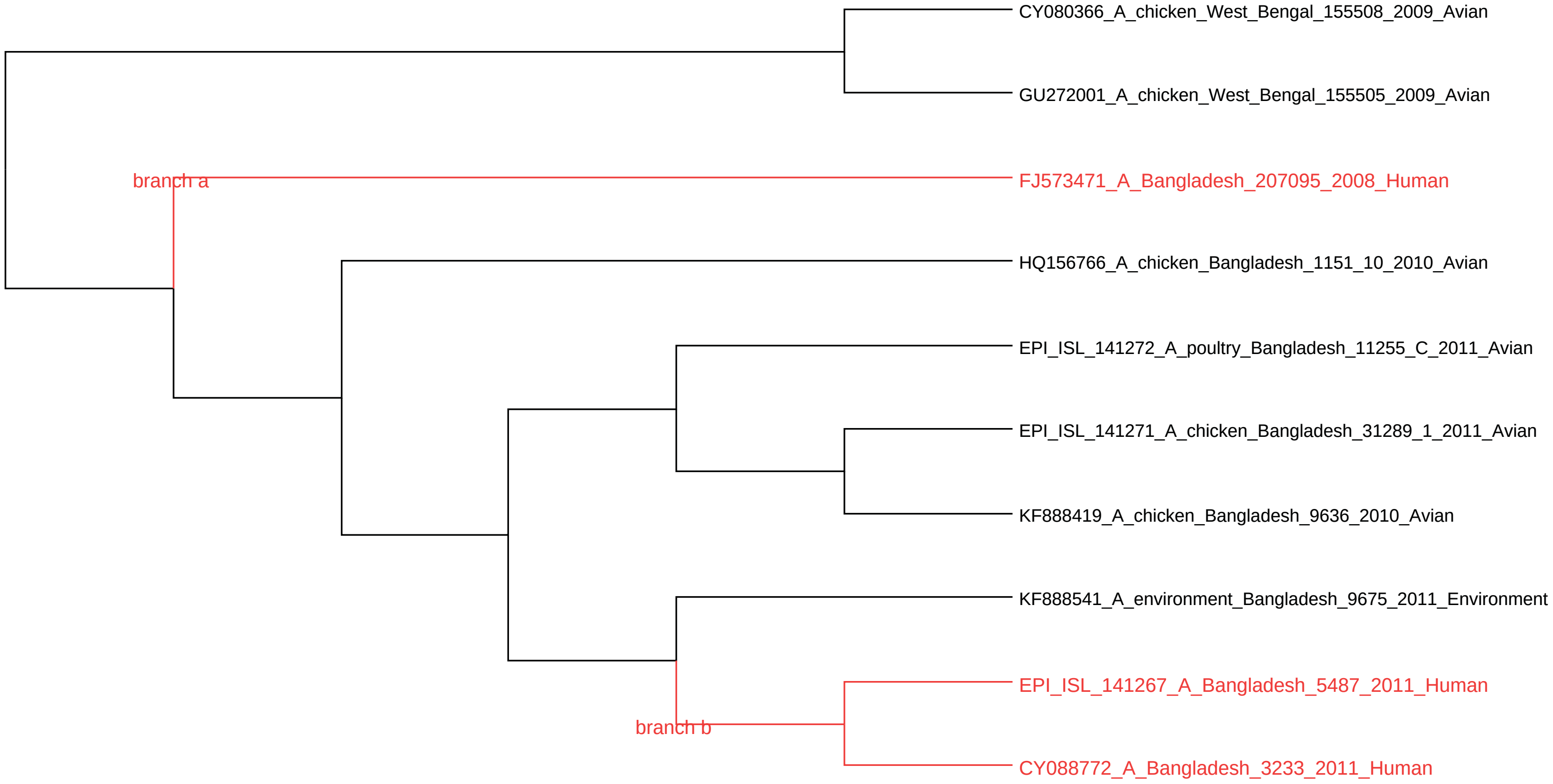
CY080366_A_chicken_West_Bengal_155508_2009_Avian
GU272001_A_chicken_West_Bengal_155505_2009_Avian
branch a
FJ573471_A_Bangladesh_207095_2008_Human
HQ156766_A_chicken_Bangladesh_1151_10_2010_Avian
EPI_ISL_141272_A_poultry_Bangladesh_11255_C_2011_Avian
EPI_ISL_141271_A_chicken_Bangladesh_31289_1_2011_Avian
KF888419_A_chicken_Bangladesh_9636_2010_Avian
KF888541_A_environment_Bangladesh_9675_2011_Environment
EPI_ISL_141267_A_Bangladesh_5487_2011_Human
branch b
CY088772_A_Bangladesh_3233_2011_Human

MP-Group62

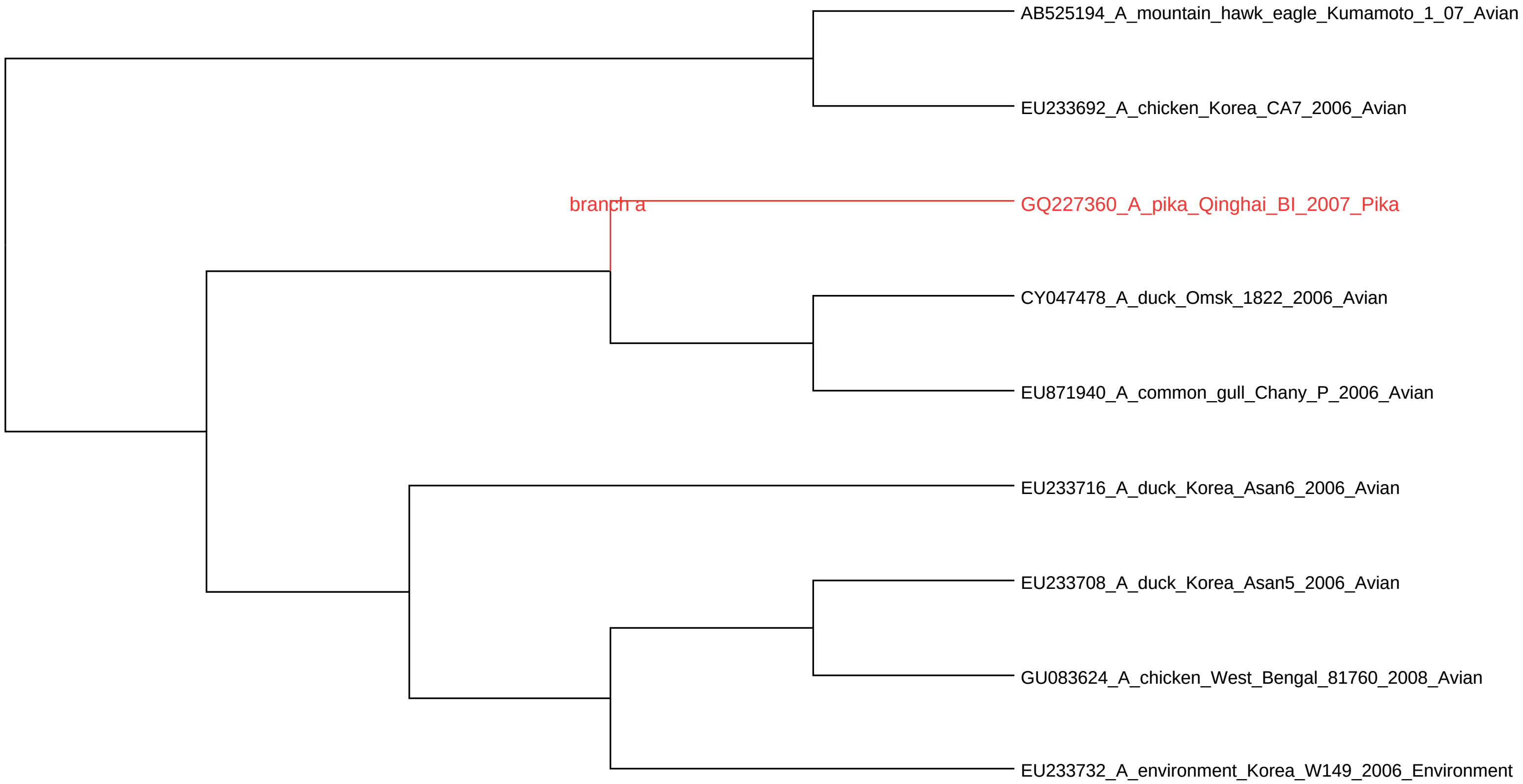

AB525194_A_mountain_hawk_eagle_Kumamoto_1_07_Avian
EU233692_A_chicken_Korea_CA7_2006_Avian
branch a
GQ227360_A_pika_Qinghai_BI_2007_Pika
CY047478_A_duck_Omsk_1822_2006_Avian
EU871940_A_common_gull_Chany_P_2006_Avian
EU233716_A_duck_Korea_Asan6_2006_Avian
EU233708_A_duck_Korea_Asan5_2006_Avian
GU083624_A_chicken_West_Bengal_81760_2008_Avian
EU233732_A_environment_Korea_W149_2006_Environment

MP-Group63

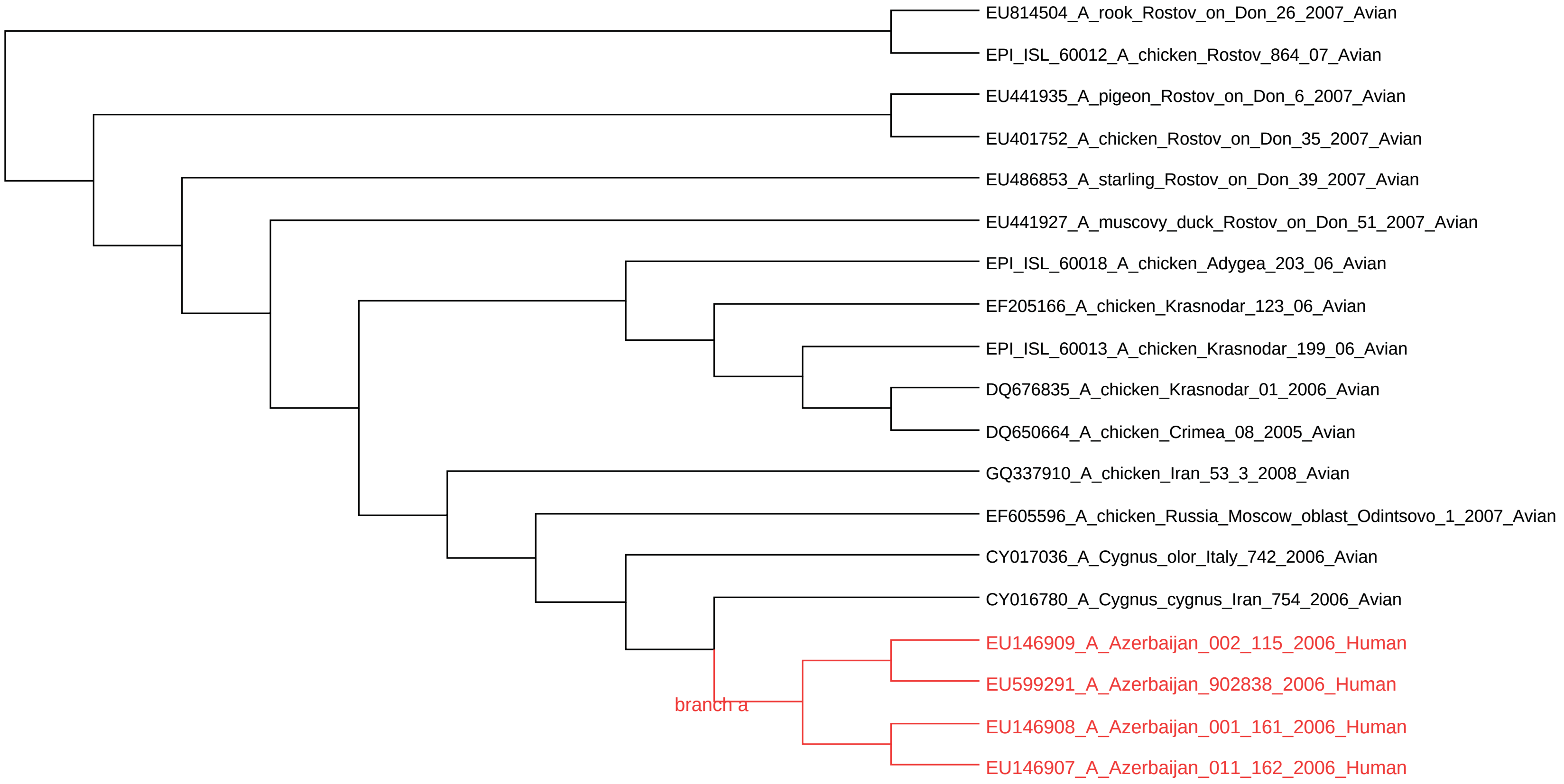

EU814504_A_rook_Rostov_on_Don_26_2007_Avian
EPI_ISL_60012_A_chicken_Rostov_864_07_Avian
EU441935_A_pigeon_Rostov_on_Don_6_2007_Avian
EU401752_A_chicken_Rostov_on_Don_35_2007_Avian
EU486853_A_starling_Rostov_on_Don_39_2007_Avian
EU441927_A_muscovy_duck_Rostov_on_Don_51_2007_Avian
EPI_ISL_60018_A_chicken_Adygea_203_06_Avian
EF205166_A_chicken_Krasnodar_123_06_Avian
EPI_ISL_60013_A_chicken_Krasnodar_199_06_Avian
DQ676835_A_chicken_Krasnodar_01_2006_Avian
DQ650664_A_chicken_Crimea_08_2005_Avian
GQ337910_A_chicken_Iran_53_3_2008_Avian
EF605596_A_chicken_Russia_Moscow_oblast_Odintsovo_1_2007_Avian
CY017036_A_Cygnus_olor_Italy_742_2006_Avian
CY016780_A_Cygnus_cygnus_Iran_754_2006_Avian
EU146909_A_Azerbaijan_002_115_2006_Human
EU599291_A_Azerbaijan_902838_2006_Human
branch a
EU146908_A_Azerbaijan_001_161_2006_Human
EU146907_A_Azerbaijan_011_162_2006_Human

MP-Group64

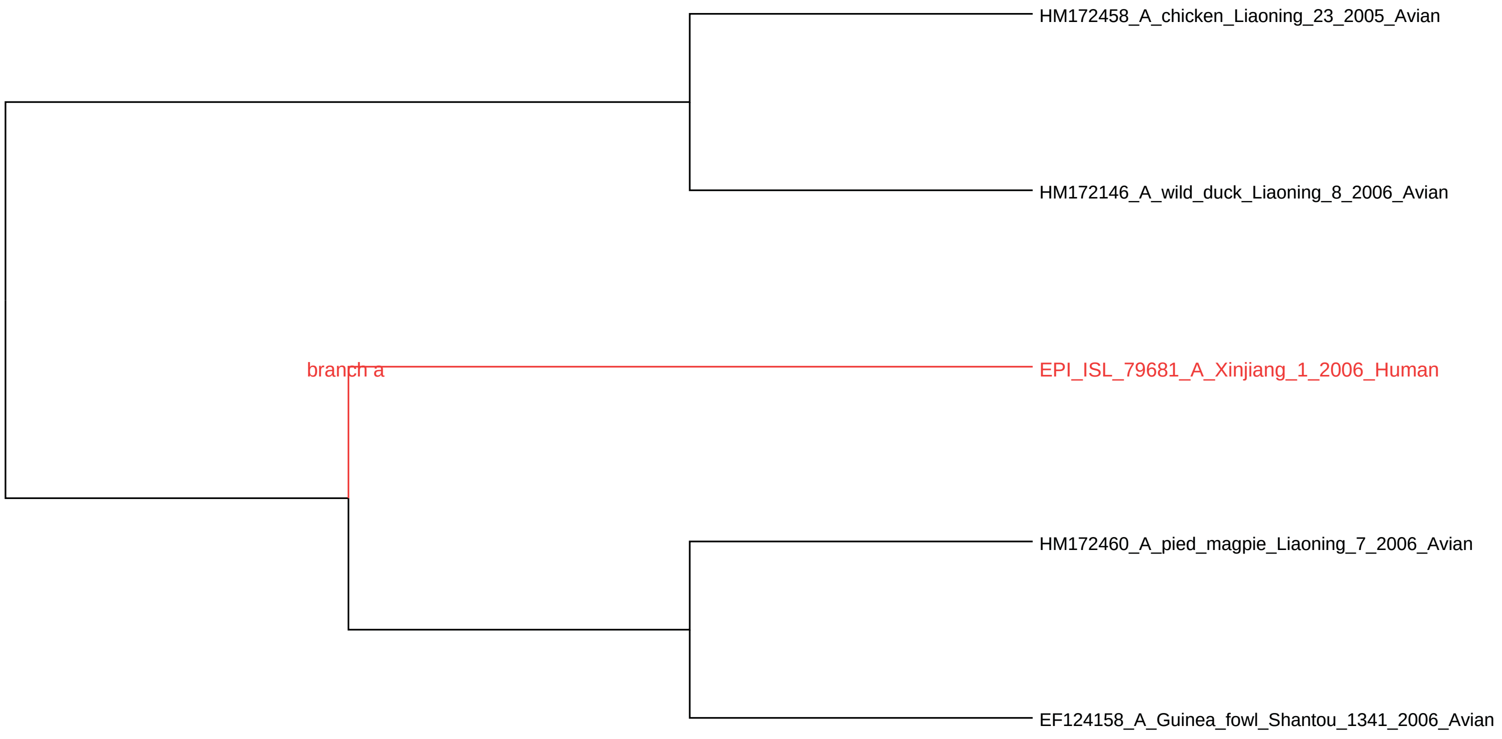
HM172458_A_chicken_Liaoning_23_2005_Avian
HM172146_A_wild_duck_Liaoning_8_2006_Avian
branch a
EPI_ISL_79681_A_Xinjiang_1_2006_Human
HM172460_A_pied_magpie_Liaoning_7_2006_Avian
EF124158_A_Guinea_fowl_Shantou_1341_2006_Avian

MP-Group65

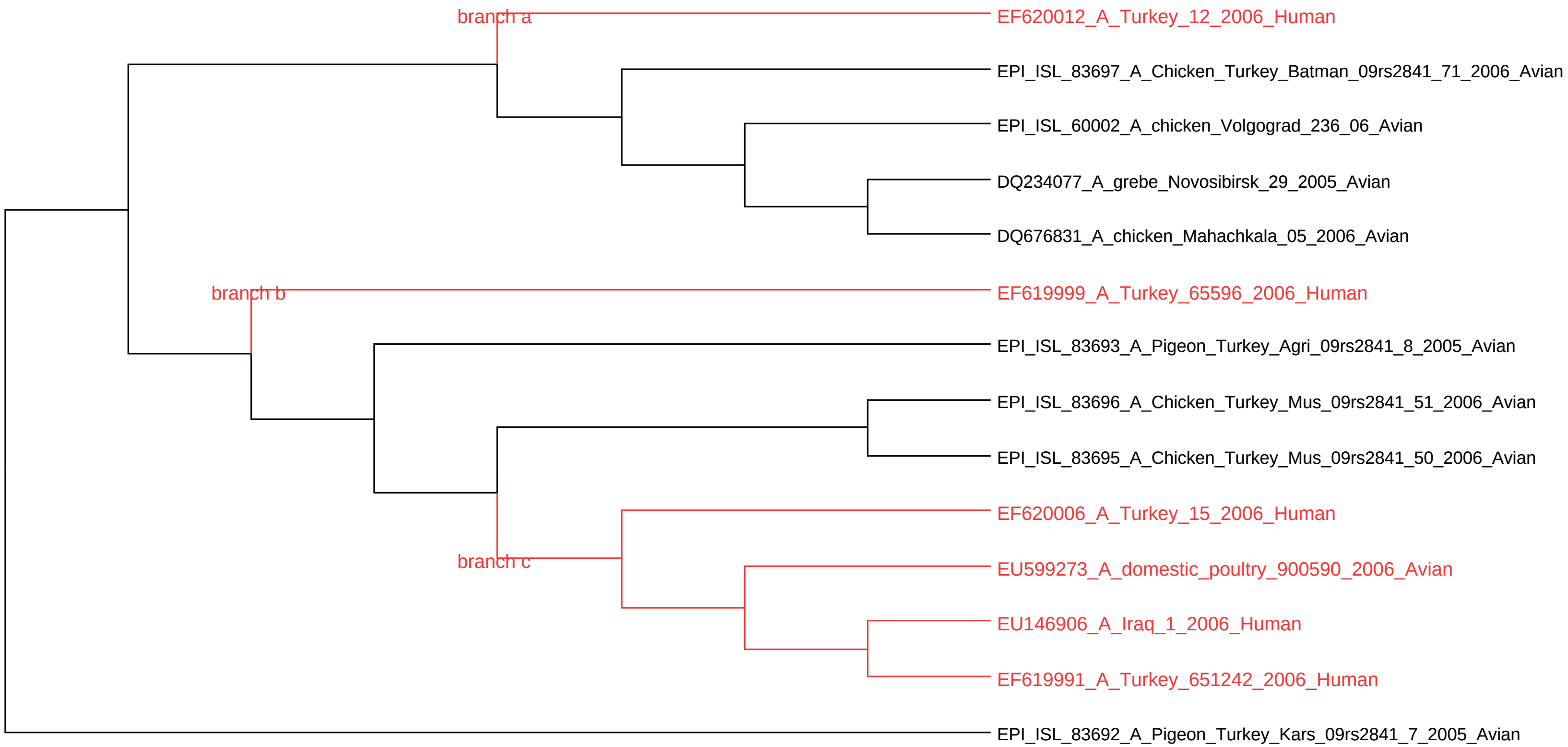

branch a
EF620012_A_Turkey_12_2006_Human
EPI_ISL_83697_A_Chicken_Turkey_Batman_09rs2841_71_2006_Avian
EPI_ISL_60002_A_chicken_Volgograd_236_06_Avian
DQ234077_A_grebe_Novosibirsk_29_2005_Avian
DQ676831_A_chicken_Mahachkala_05_2006_Avian
branch b
EF619999_A_Turkey_65596_2006_Human
EPI_ISL_83693_A_Pigeon_Turkey_Agri_09rs2841_8_2005_Avian
EPI_ISL_83696_A_Chicken_Turkey_Mus_09rs2841_51_2006_Avian
EPI_ISL_83695_A_Chicken_Turkey_Mus_09rs2841_50_2006_Avian
EF620006_A_Turkey_15_2006_Human
branch c
EU599273_A_domestic_poultry_900590_2006_Avian
EU146906_A_Iraq_1_2006_Human
EF619991_A_Turkey_651242_2006_Human
EPI_ISL_83692_A_Pigeon_Turkey_Kars_09rs2841_7_2005_Avian

MP-Group66

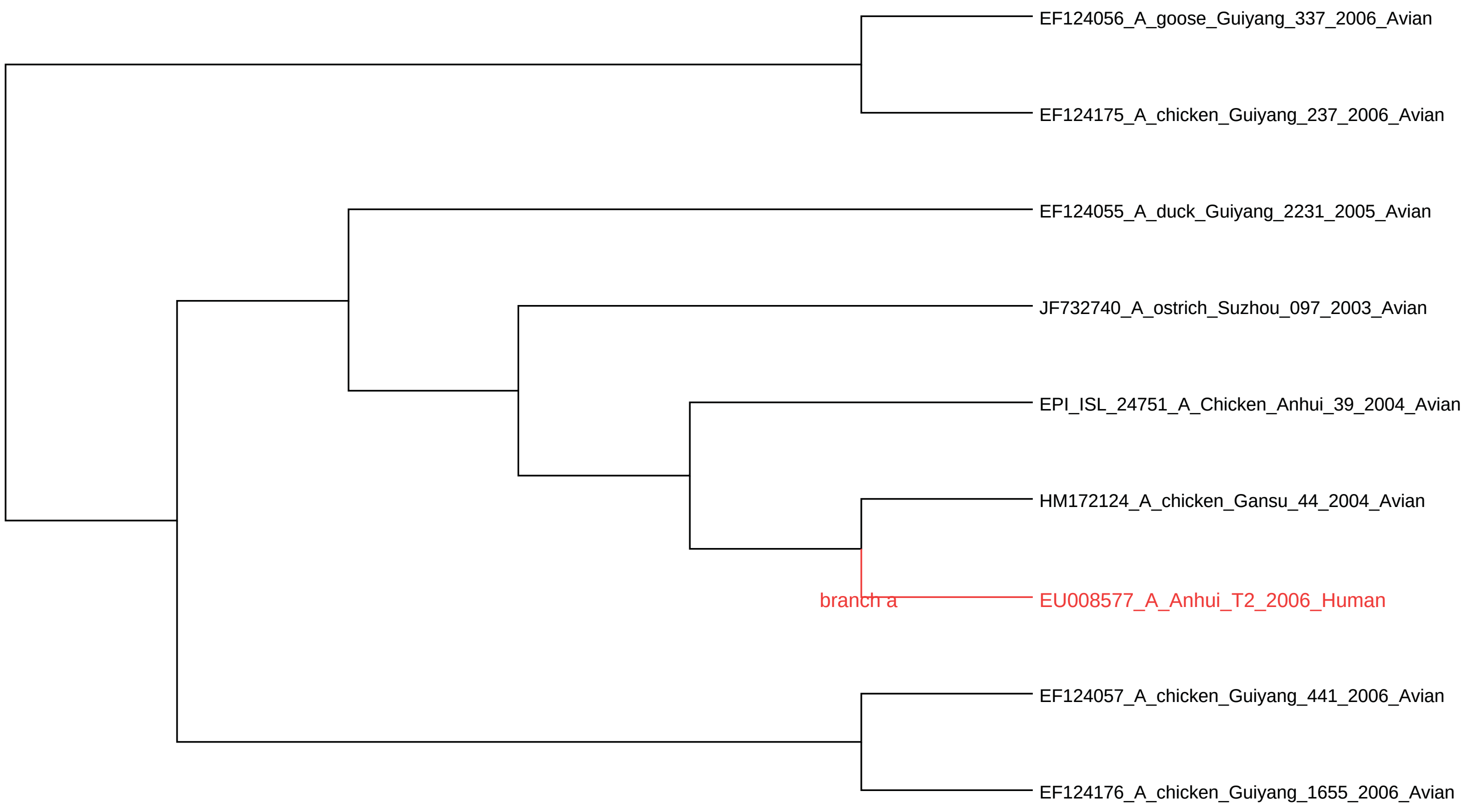
EF124056_A_goose_Guiyang_337_2006_Avian
EF124175_A_chicken_Guiyang_237_2006_Avian
EF124055_A_duck_Guiyang_2231_2005_Avian
JF732740_A_ostrich_Suzhou_097_2003_Avian
EPI_ISL_24751_A_Chicken_Anhui_39_2004_Avian
HM172124_A_chicken_Gansu_44_2004_Avian
branch a
EU008577_A_Anhui_T2_2006_Human
EF124057_A_chicken_Guiyang_441_2006_Avian
EF124176_A_chicken_Guiyang_1655_2006_Avian

MP-Group67

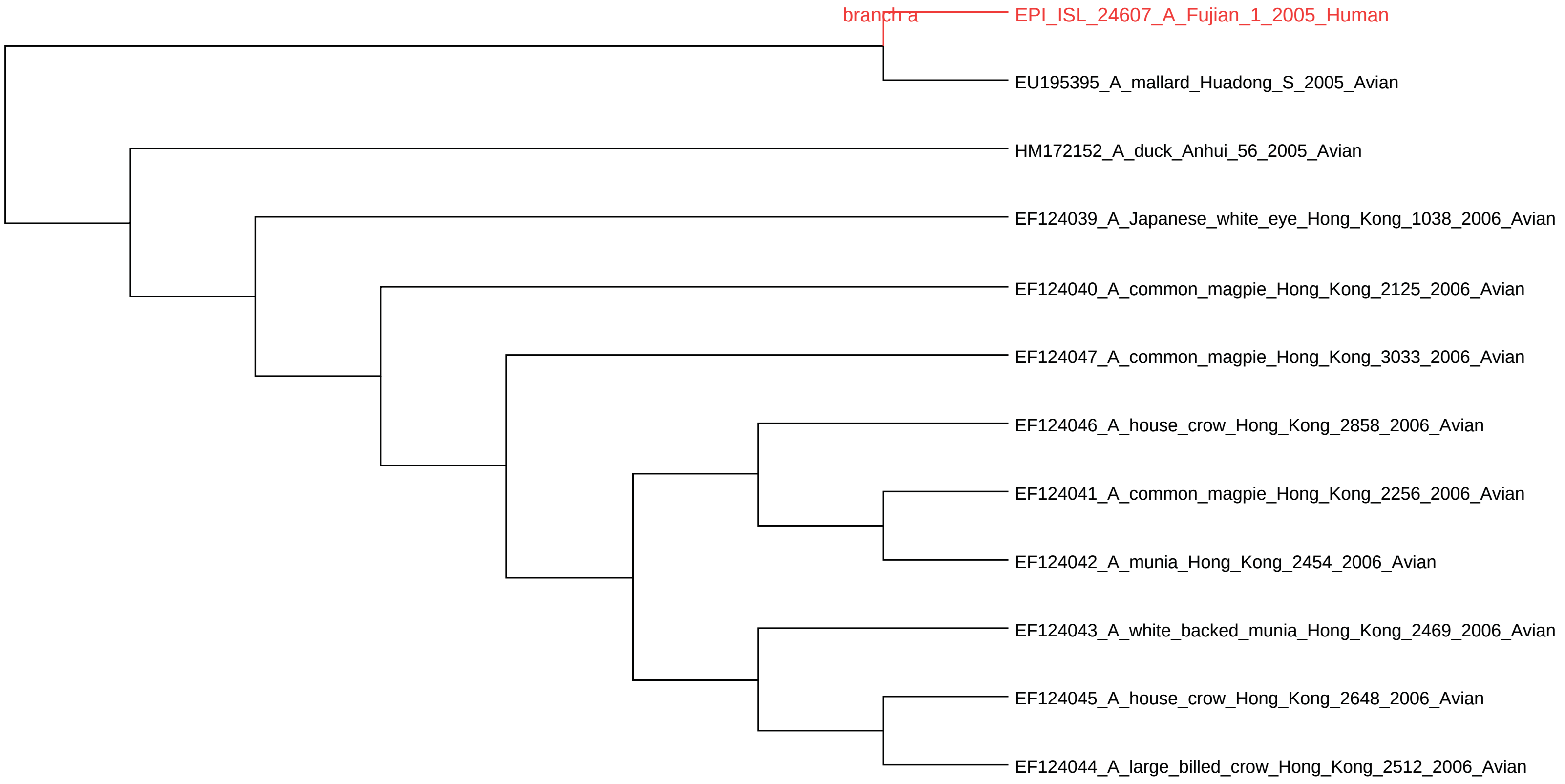
branch a
EPI_ISL_24607_A_Fujian_1_2005_Human
EU195395_A_mallard_Huadong_S_2005_Avian
HM172152_A_duck_Anhui_56_2005_Avian
EF124039_A_Japanese_white_eye_Hong_Kong_1038_2006_Avian
EF124040_A_common_magpie_Hong_Kong_2125_2006_Avian
EF124047_A_common_magpie_Hong_Kong_3033_2006_Avian
EF124046_A_house_crow_Hong_Kong_2858_2006_Avian
EF124041_A_common_magpie_Hong_Kong_2256_2006_Avian
EF124042_A_munia_Hong_Kong_2454_2006_Avian
EF124043_A_white_backed_munia_Hong_Kong_2469_2006_Avian
EF124045_A_house_crow_Hong_Kong_2648_2006_Avian
EF124044_A_large_billed_crow_Hong_Kong_2512_2006_Avian

MP-Group68

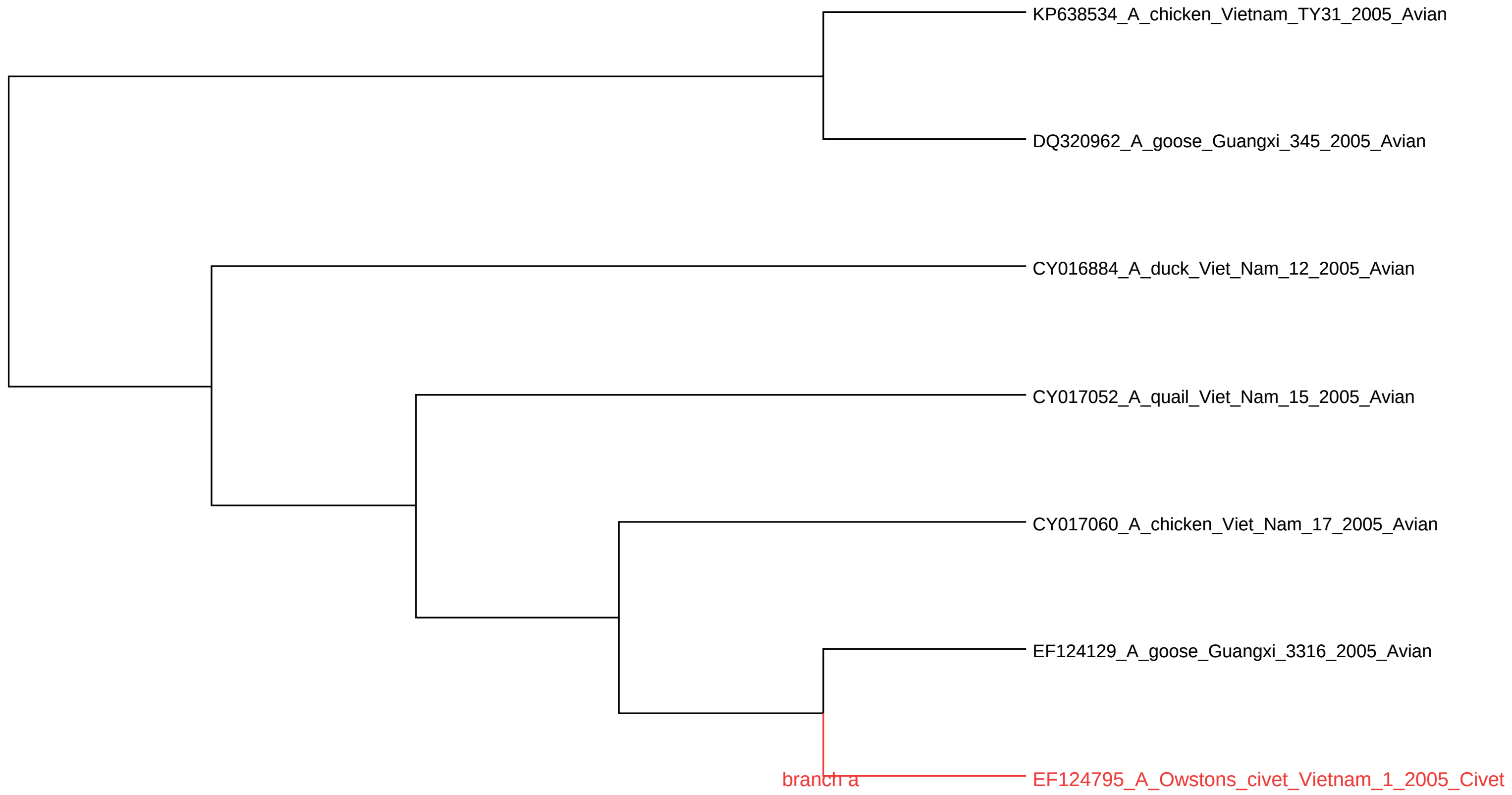
KP638534_A_chicken_Vietnam_TY31_2005_Avian
DQ320962_A_goose_Guangxi_345_2005_Avian
CY016884_A_duck_Viet_Nam_12_2005_Avian
CY017052_A_quail_Viet_Nam_15_2005_Avian
CY017060_A_chicken_Viet_Nam_17_2005_Avian
EF124129_A_goose_Guangxi_3316_2005_Avian
branch a
EF124795_A_Owstons_civet_Vietnam_1_2005_Civet

MP-Group69

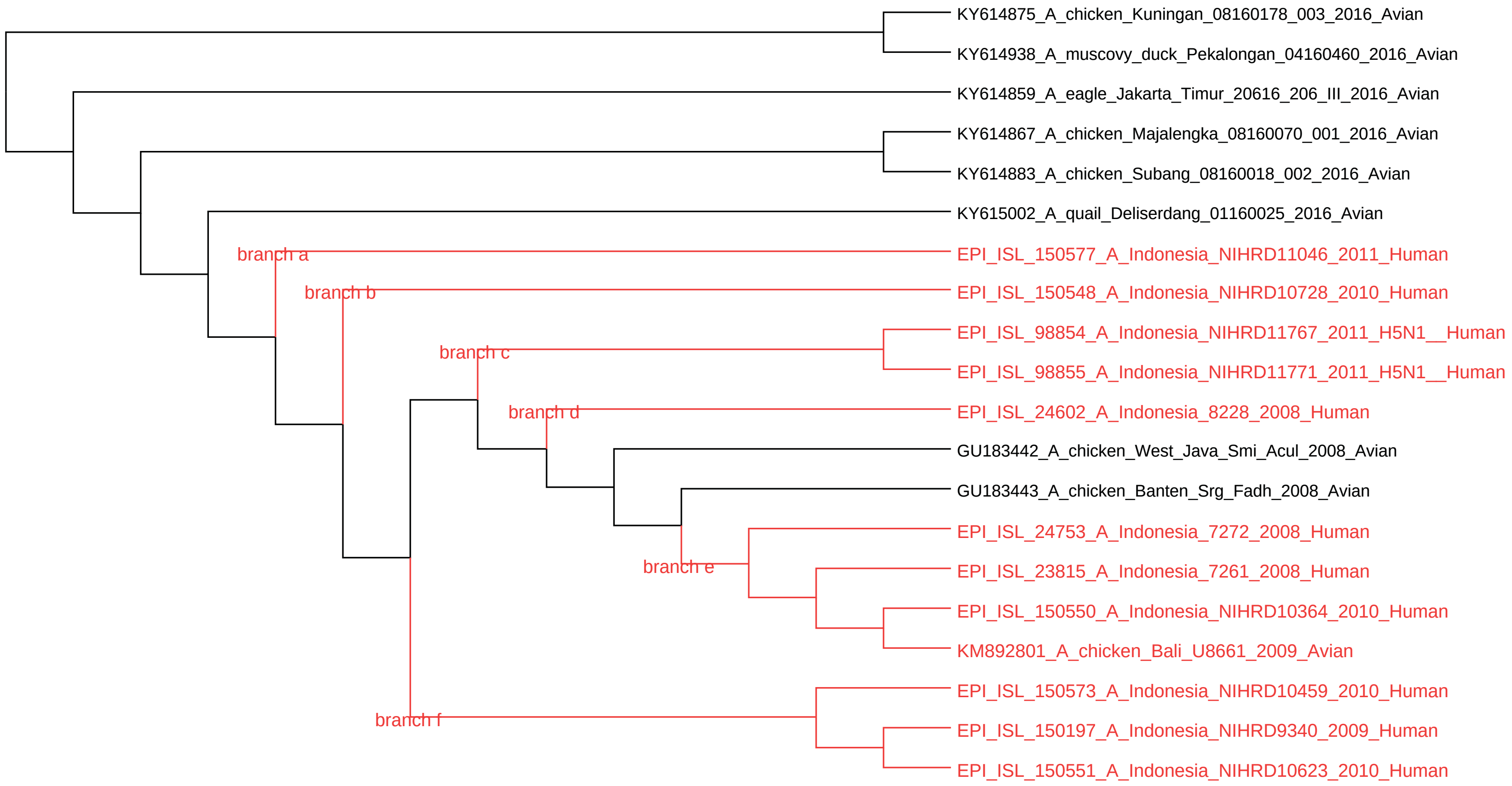
KY614875_A_chicken_Kuningan_08160178_003_2016_Avian
KY614938_A_muscovy_duck_Pekalongan_04160460_2016_Avian
KY614859_A_eagle_Jakarta_Timur_20616_206_III_2016_Avian
KY614867_A_chicken_Majalengka_08160070_001_2016_Avian
KY614883_A_chicken_Subang_08160018_002_2016_Avian
KY615002_A_quail_Deliserdang_01160025_2016_Avian
branch a
EPI_ISL_150577_A_Indonesia_NIHRD11046_2011_Human
branch b
EPI_ISL_150548_A_Indonesia_NIHRD10728_2010_Human
EPI_ISL_98854_A_Indonesia_NIHRD11767_2011_H5N1__Human
branch c
EPI_ISL_98855_A_Indonesia_NIHRD11771_2011_H5N1__Human
branch d
EPI_ISL_24602_A_Indonesia_8228_2008_Human
GU183442_A_chicken_West_Java_Smi_Acul_2008_Avian
GU183443_A_chicken_Banten_Srg_Fadh_2008_Avian
EPI_ISL_24753_A_Indonesia_7272_2008_Human
branch e
EPI_ISL_23815_A_Indonesia_7261_2008_Human
EPI_ISL_150550_A_Indonesia_NIHRD10364_2010_Human
KM892801_A_chicken_Bali_U8661_2009_Avian
EPI_ISL_150573_A_Indonesia_NIHRD10459_2010_Human
branch f
EPI_ISL_150197_A_Indonesia_NIHRD9340_2009_Human
EPI_ISL_150551_A_Indonesia_NIHRD10623_2010_Human

MP-Group70

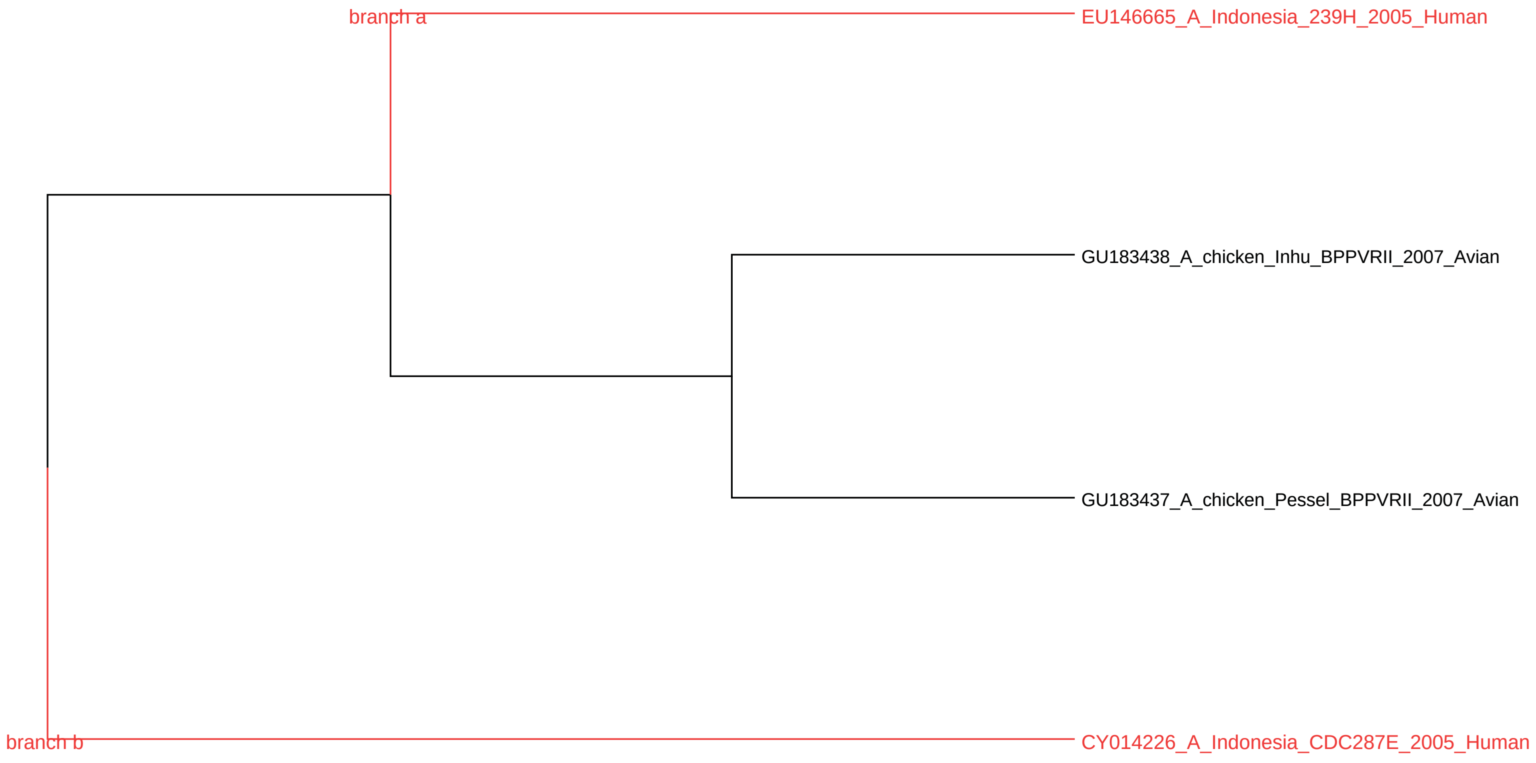
branch a
EU146665_A_Indonesia_239H_2005_Human
GU183438_A_chicken_Inhu_BPPVRII_2007_Avian
GU183437_A_chicken_Pessel_BPPVRII_2007_Avian
branch b
CY014226_A_Indonesia_CDC287E_2005_Human

MP-Group71

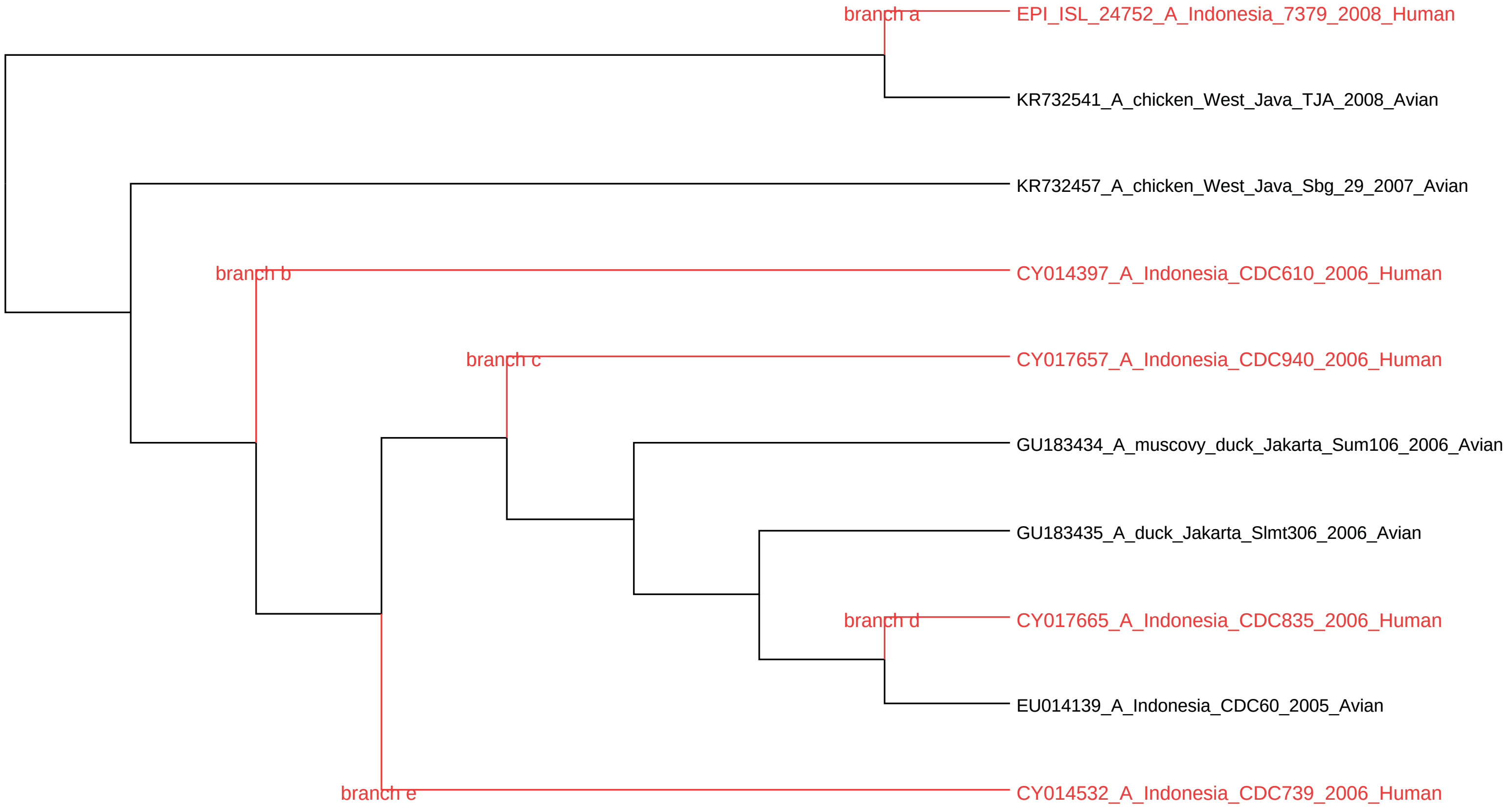
branch a
EPI_ISL_24752_A_Indonesia_7379_2008_Human
KR732541_A_chicken_West_Java_TJA_2008_Avian
KR732457_A_chicken_West_Java_Sbg_29_2007_Avian
branch b
CY014397_A_Indonesia_CDC610_2006_Human
branch c
CY017657_A_Indonesia_CDC940_2006_Human
GU183434_A_muscovy_duck_Jakarta_Sum106_2006_Avian
GU183435_A_duck_Jakarta_Slmt306_2006_Avian
branch d
CY017665_A_Indonesia_CDC835_2006_Human
EU014139_A_Indonesia_CDC60_2005_Avian
branch e
CY014532_A_Indonesia_CDC739_2006_Human

MP-Group72

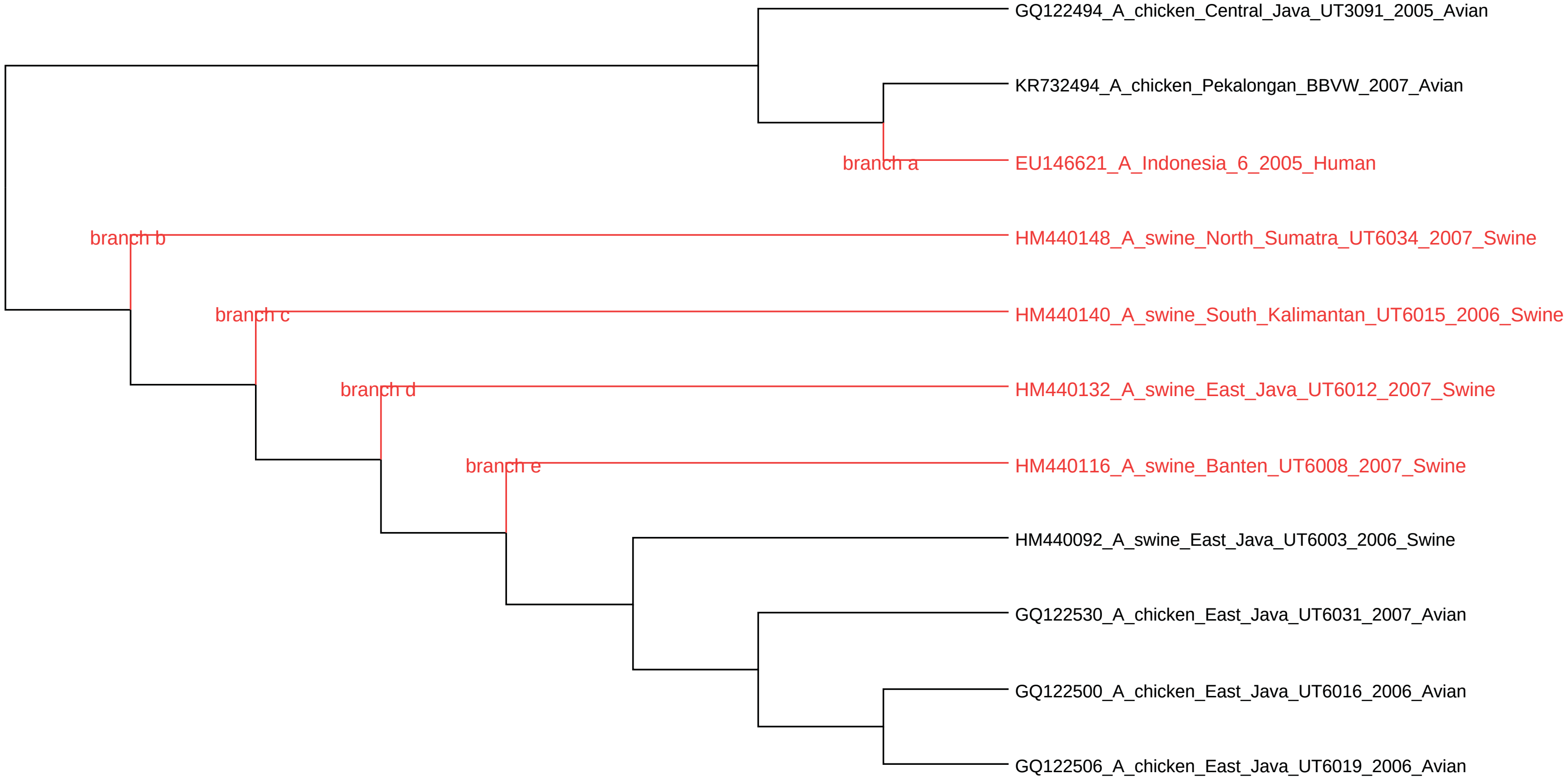
GQ122494_A_chicken_Central_Java_UT3091_2005_Avian
KR732494_A_chicken_Pekalongan_BBVW_2007_Avian
branch a
EU146621_A_Indonesia_6_2005_Human
branch b
HM440148_A_swine_North_Sumatra_UT6034_2007_Swine
branch c
HM440140_A_swine_South_Kalimantan_UT6015_2006_Swine
branch d
HM440132_A_swine_East_Java_UT6012_2007_Swine
branch e
HM440116_A_swine_Banten_UT6008_2007_Swine
HM440092_A_swine_East_Java_UT6003_2006_Swine
GQ122530_A_chicken_East_Java_UT6031_2007_Avian
GQ122500_A_chicken_East_Java_UT6016_2006_Avian
GQ122506_A_chicken_East_Java_UT6019_2006_Avian

MP-Group73

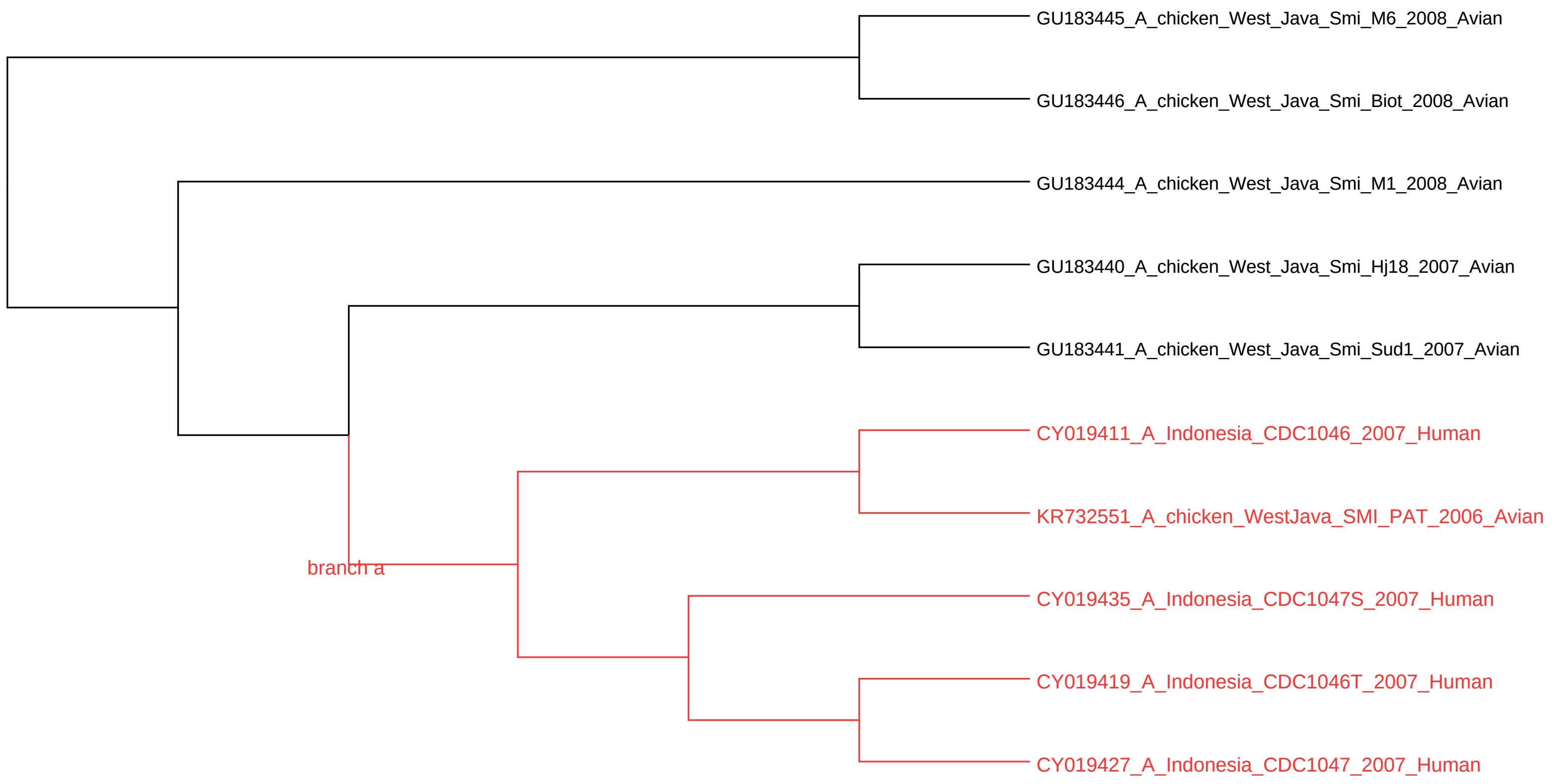
GU183445_A_chicken_West_Java_Smi_M6_2008_Avian
GU183446_A_chicken_West_Java_Smi_Biot_2008_Avian
GU183444_A_chicken_West_Java_Smi_M1_2008_Avian
GU183440_A_chicken_West_Java_Smi_Hj18_2007_Avian
GU183441_A_chicken_West_Java_Smi_Sud1_2007_Avian
CY019411_A_Indonesia_CDC1046_2007_Human
KR732551_A_chicken_WestJava_SMI_PAT_2006_Avian
branch a
CY019435_A_Indonesia_CDC1047S_2007_Human
CY019419_A_Indonesia_CDC1046T_2007_Human
CY019427_A_Indonesia_CDC1047_2007_Human

MP-Group74

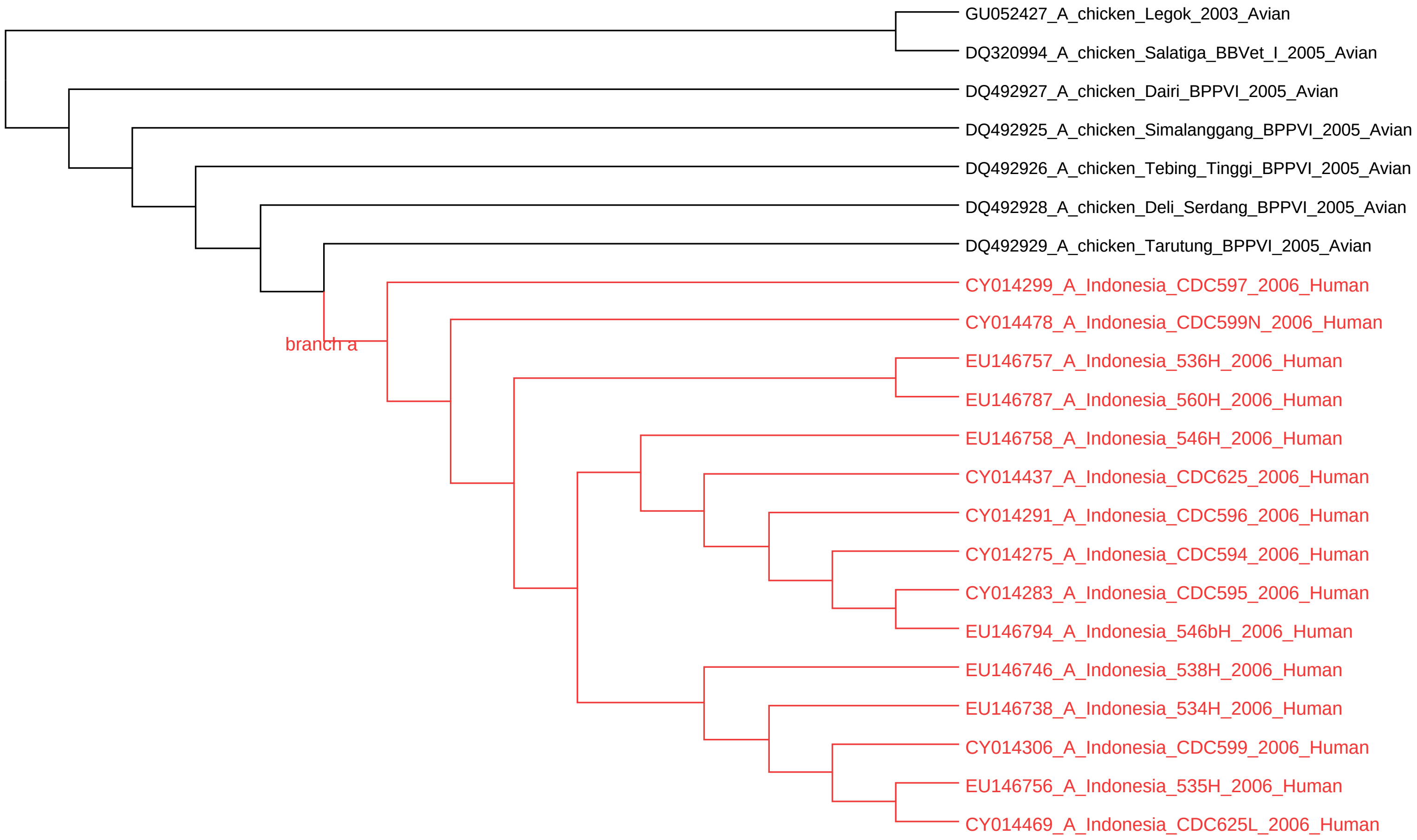

GU052427_A_chicken_Legok_2003_Avian
DQ320994_A_chicken_Salatiga_BBVet_I_2005_Avian
DQ492927_A_chicken_Dairi_BPPVI_2005_Avian
DQ492925_A_chicken_Simalanggang_BPPVI_2005_Avian
DQ492926_A_chicken_Tebing_Tinggi_BPPVI_2005_Avian
DQ492928_A_chicken_Deli_Serdang_BPPVI_2005_Avian
DQ492929_A_chicken_Tarutung_BPPVI_2005_Avian
CY014299_A_Indonesia_CDC597_2006_Human
CY014478_A_Indonesia_CDC599N_2006_Human
branch a
EU146757_A_Indonesia_536H_2006_Human
EU146787_A_Indonesia_560H_2006_Human
EU146758_A_Indonesia_546H_2006_Human
CY014437_A_Indonesia_CDC625_2006_Human
CY014291_A_Indonesia_CDC596_2006_Human
CY014275_A_Indonesia_CDC594_2006_Human
CY014283_A_Indonesia_CDC595_2006_Human
EU146794_A_Indonesia_546bH_2006_Human
EU146746_A_Indonesia_538H_2006_Human
EU146738_A_Indonesia_534H_2006_Human
CY014306_A_Indonesia_CDC599_2006_Human
EU146756_A_Indonesia_535H_2006_Human
CY014469_A_Indonesia_CDC625L_2006_Human

MP-Group75

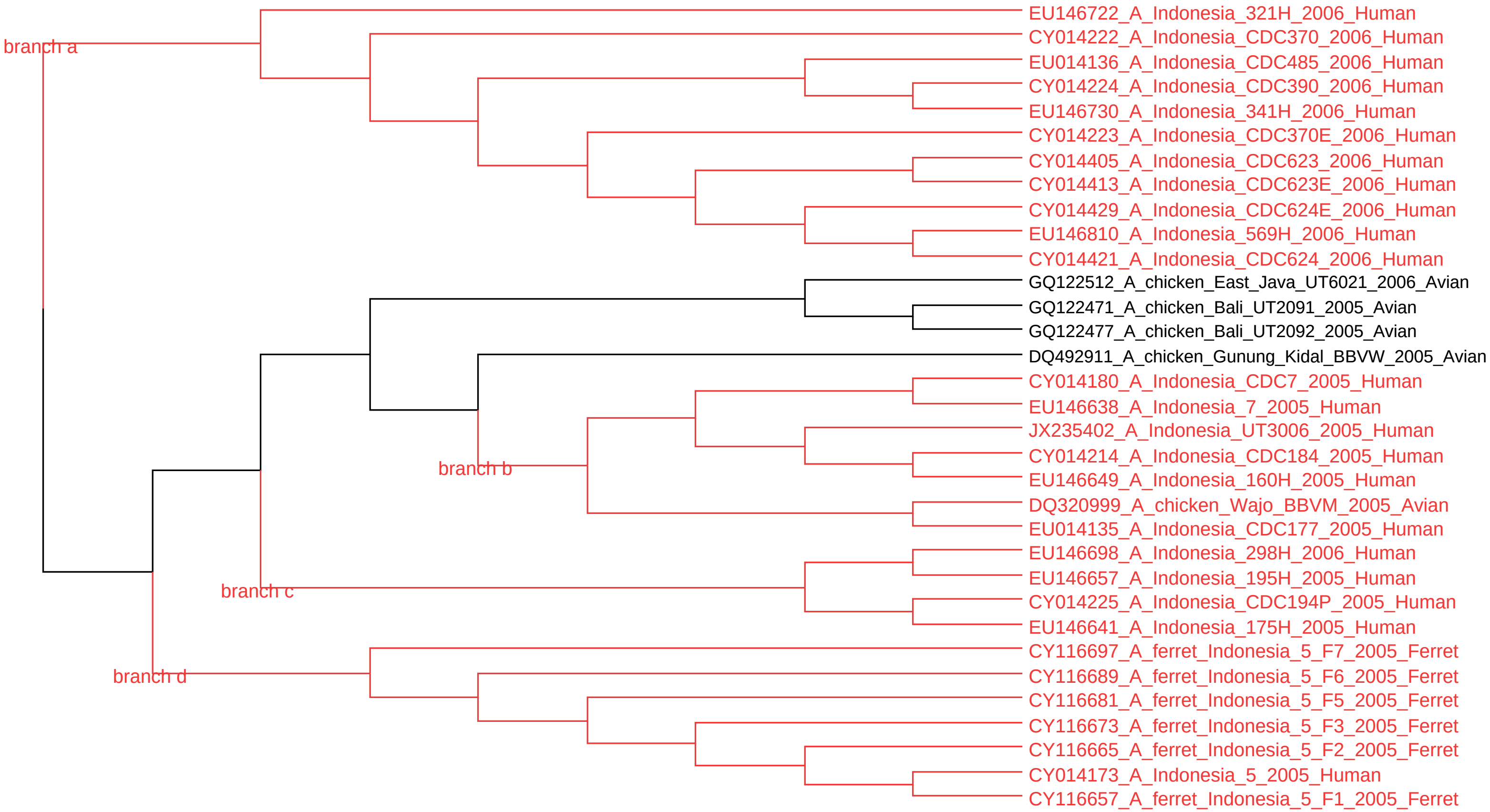
branch a
branch b
branch c
branch d
EU146722_A_Indonesia_321H_2006_Human
CY014222_A_Indonesia_CDC370_2006_Human
EU014136_A_Indonesia_CDC485_2006_Human
CY014224_A_Indonesia_CDC390_2006_Human
EU146730_A_Indonesia_341H_2006_Human
CY014223_A_Indonesia_CDC370E_2006_Human
CY014405_A_Indonesia_CDC623_2006_Human
CY014413_A_Indonesia_CDC623E_2006_Human
CY014429_A_Indonesia_CDC624E_2006_Human
EU146810_A_Indonesia_569H_2006_Human
CY014421_A_Indonesia_CDC624_2006_Human
GQ122512_A_chicken_East_Java_UT6021_2006_Avian
GQ122471_A_chicken_Bali_UT2091_2005_Avian
GQ122477_A_chicken_Bali_UT2092_2005_Avian
DQ492911_A_chicken_Gunung_Kidal_BBVW_2005_Avian
CY014180_A_Indonesia_CDC7_2005_Human
EU146638_A_Indonesia_7_2005_Human
JX235402_A_Indonesia_UT3006_2005_Human
CY014214_A_Indonesia_CDC184_2005_Human
EU146649_A_Indonesia_160H_2005_Human
DQ320999_A_chicken_Wajo_BBVM_2005_Avian
EU014135_A_Indonesia_CDC177_2005_Human
EU146698_A_Indonesia_298H_2006_Human
EU146657_A_Indonesia_195H_2005_Human
CY014225_A_Indonesia_CDC194P_2005_Human
EU146641_A_Indonesia_175H_2005_Human
CY116697_A_ferret_Indonesia_5_F7_2005_Ferret
CY116689_A_ferret_Indonesia_5_F6_2005_Ferret
CY116681_A_ferret_Indonesia_5_F5_2005_Ferret
CY116673_A_ferret_Indonesia_5_F3_2005_Ferret
CY116665_A_ferret_Indonesia_5_F2_2005_Ferret
CY014173_A_Indonesia_5_2005_Human
CY116657_A_ferret_Indonesia_5_F1_2005_Ferret

MP-Group76

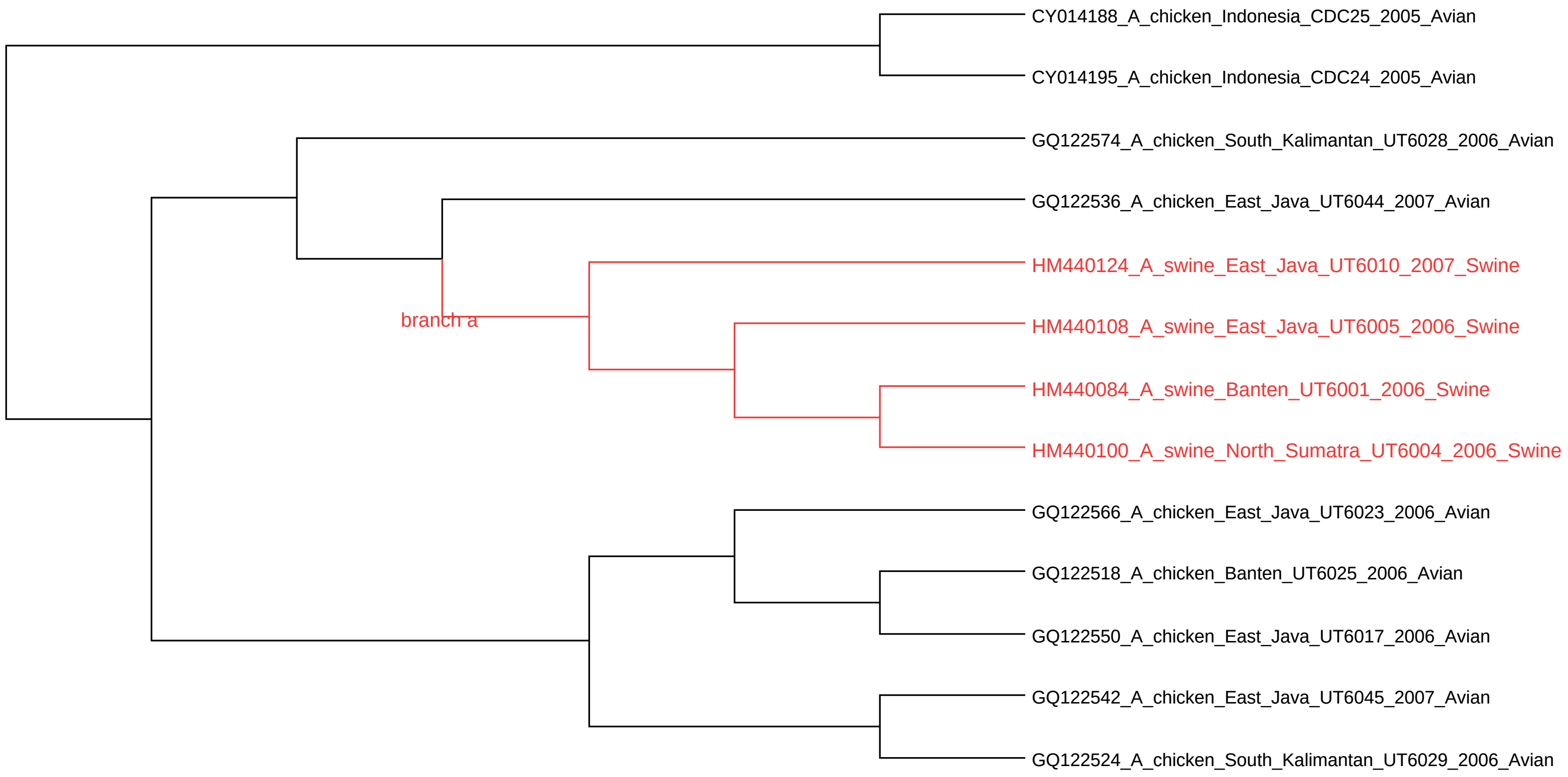
CY014188_A_chicken_Indonesia_CDC25_2005_Avian
CY014195_A_chicken_Indonesia_CDC24_2005_Avian
GQ122574_A_chicken_South_Kalimantan_UT6028_2006_Avian
GQ122536_A_chicken_East_Java_UT6044_2007_Avian
HM440124_A_swine_East_Java_UT6010_2007_Swine
branch a
HM440108_A_swine_East_Java_UT6005_2006_Swine
HM440084_A_swine_Banten_UT6001_2006_Swine
HM440100_A_swine_North_Sumatra_UT6004_2006_Swine
GQ122566_A_chicken_East_Java_UT6023_2006_Avian
GQ122518_A_chicken_Banten_UT6025_2006_Avian
GQ122550_A_chicken_East_Java_UT6017_2006_Avian
GQ122542_A_chicken_East_Java_UT6045_2007_Avian
GQ122524_A_chicken_South_Kalimantan_UT6029_2006_Avian

MP-Group77

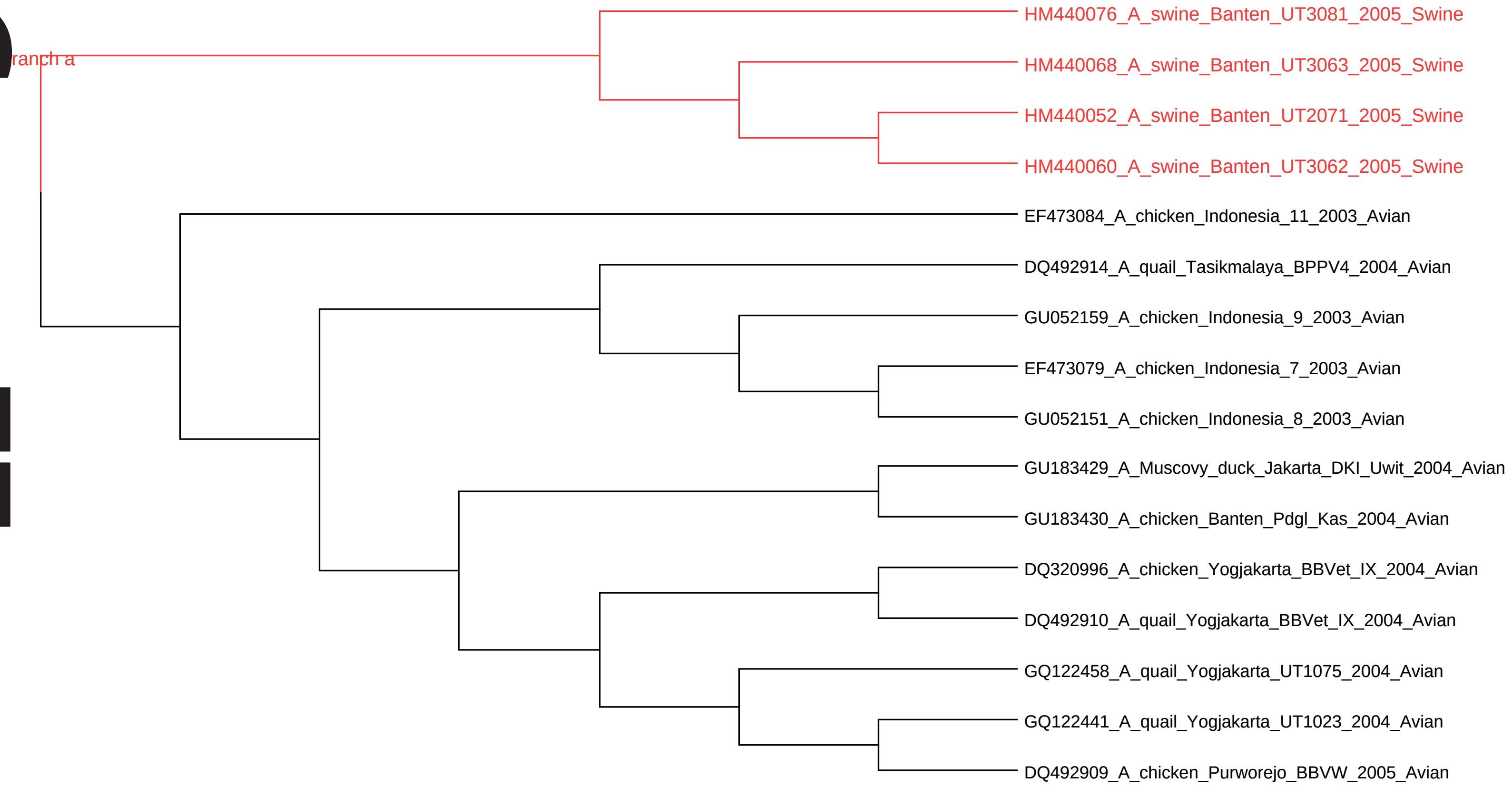
ranch a
HM440076_A_swine_Banten_UT3081_2005_Swine
HM440068_A_swine_Banten_UT3063_2005_Swine
HM440052_A_swine_Banten_UT2071_2005_Swine
HM440060_A_swine_Banten_UT3062_2005_Swine
EF473084_A_chicken_Indonesia_11_2003_Avian
DQ492914_A_quail_Tasikmalaya_BPPV4_2004_Avian
GU052159_A_chicken_Indonesia_9_2003_Avian
EF473079_A_chicken_Indonesia_7_2003_Avian
GU052151_A_chicken_Indonesia_8_2003_Avian
GU183429_A_Muscovy_duck_Jakarta_DKI_Uwit_2004_Avian
GU183430_A_chicken_Banten_Pdgl_Kas_2004_Avian
DQ320996_A_chicken_Yogjakarta_BBVet_IX_2004_Avian
DQ492910_A_quail_Yogjakarta_BBVet_IX_2004_Avian
GQ122458_A_quail_Yogjakarta_UT1075_2004_Avian
GQ122441_A_quail_Yogjakarta_UT1023_2004_Avian
DQ492909_A_chicken_Purworejo_BBVW_2005_Avian

MP-Group78

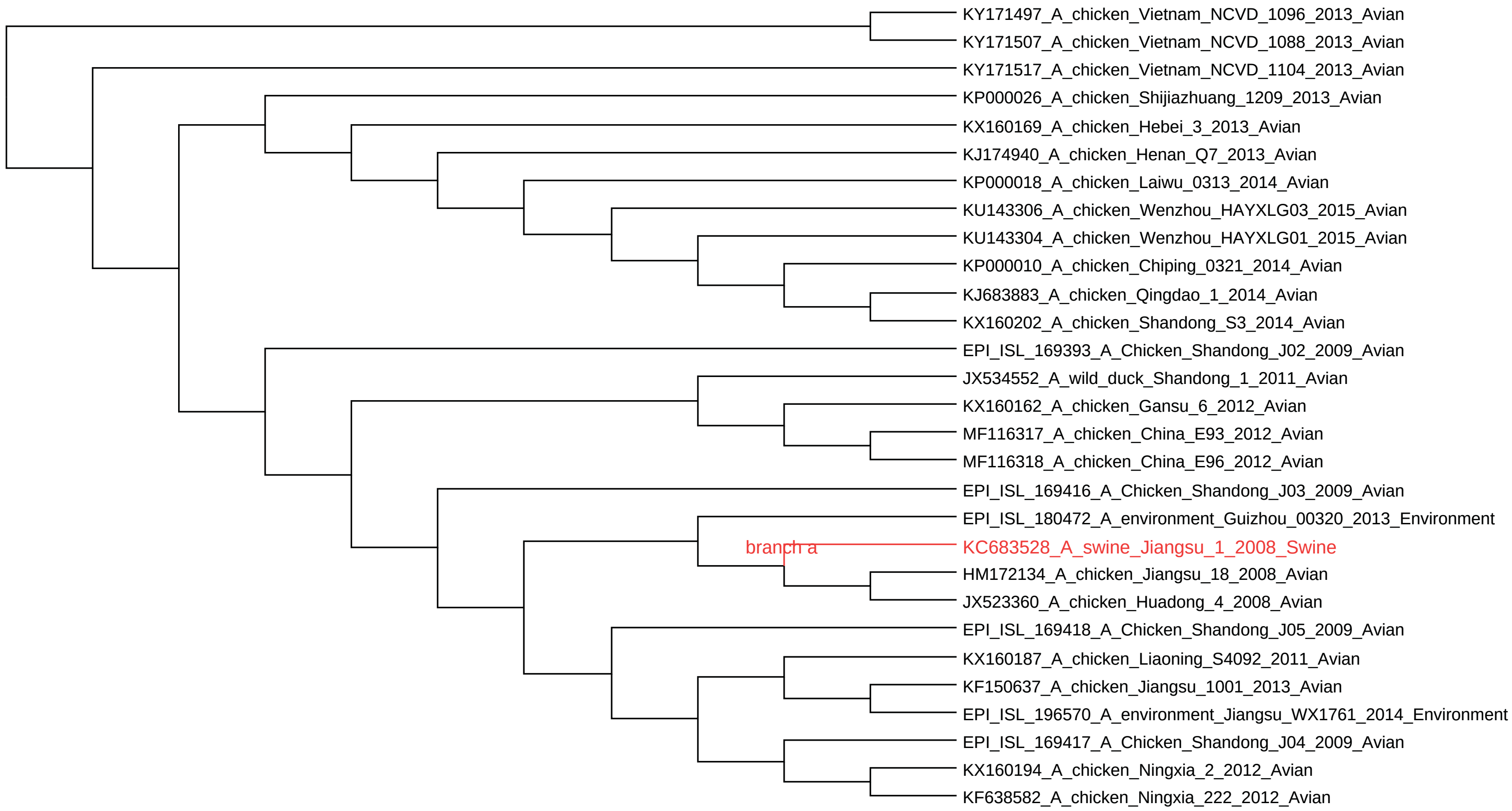
KY171497_A_chicken_Vietnam_NCVD_1096_2013_Avian
KY171507_A_chicken_Vietnam_NCVD_1088_2013_Avian
KY171517_A_chicken_Vietnam_NCVD_1104_2013_Avian
KP000026_A_chicken_Shijiazhuang_1209_2013_Avian
KX160169_A_chicken_Hebei_3_2013_Avian
KJ174940_A_chicken_Henan_Q7_2013_Avian
KP000018_A_chicken_Laiwu_0313_2014_Avian
KU143306_A_chicken_Wenzhou_HAYXLG03_2015_Avian
KU143304_A_chicken_Wenzhou_HAYXLG01_2015_Avian
KP000010_A_chicken_Chiping_0321_2014_Avian
KJ683883_A_chicken_Qingdao_1_2014_Avian
KX160202_A_chicken_Shandong_S3_2014_Avian
EPI_ISL_169393_A_Chicken_Shandong_J02_2009_Avian
JX534552_A_wild_duck_Shandong_1_2011_Avian
KX160162_A_chicken_Gansu_6_2012_Avian
MF116317_A_chicken_China_E93_2012_Avian
MF116318_A_chicken_China_E96_2012_Avian
EPI_ISL_169416_A_Chicken_Shandong_J03_2009_Avian
EPI_ISL_180472_A_environment_Guizhou_00320_2013_Environment
branch a
KC683528_A_swine_Jiangsu_1_2008_Swine
HM172134_A_chicken_Jiangsu_18_2008_Avian
JX523360_A_chicken_Huadong_4_2008_Avian
EPI_ISL_169418_A_Chicken_Shandong_J05_2009_Avian
KX160187_A_chicken_Liaoning_S4092_2011_Avian
KF150637_A_chicken_Jiangsu_1001_2013_Avian
EPI_ISL_196570_A_environment_Jiangsu_WX1761_2014_Environment
EPI_ISL_169417_A_Chicken_Shandong_J04_2009_Avian
KX160194_A_chicken_Ningxia_2_2012_Avian
KF638582_A_chicken_Ningxia_222_2012_Avian

MP-Group79

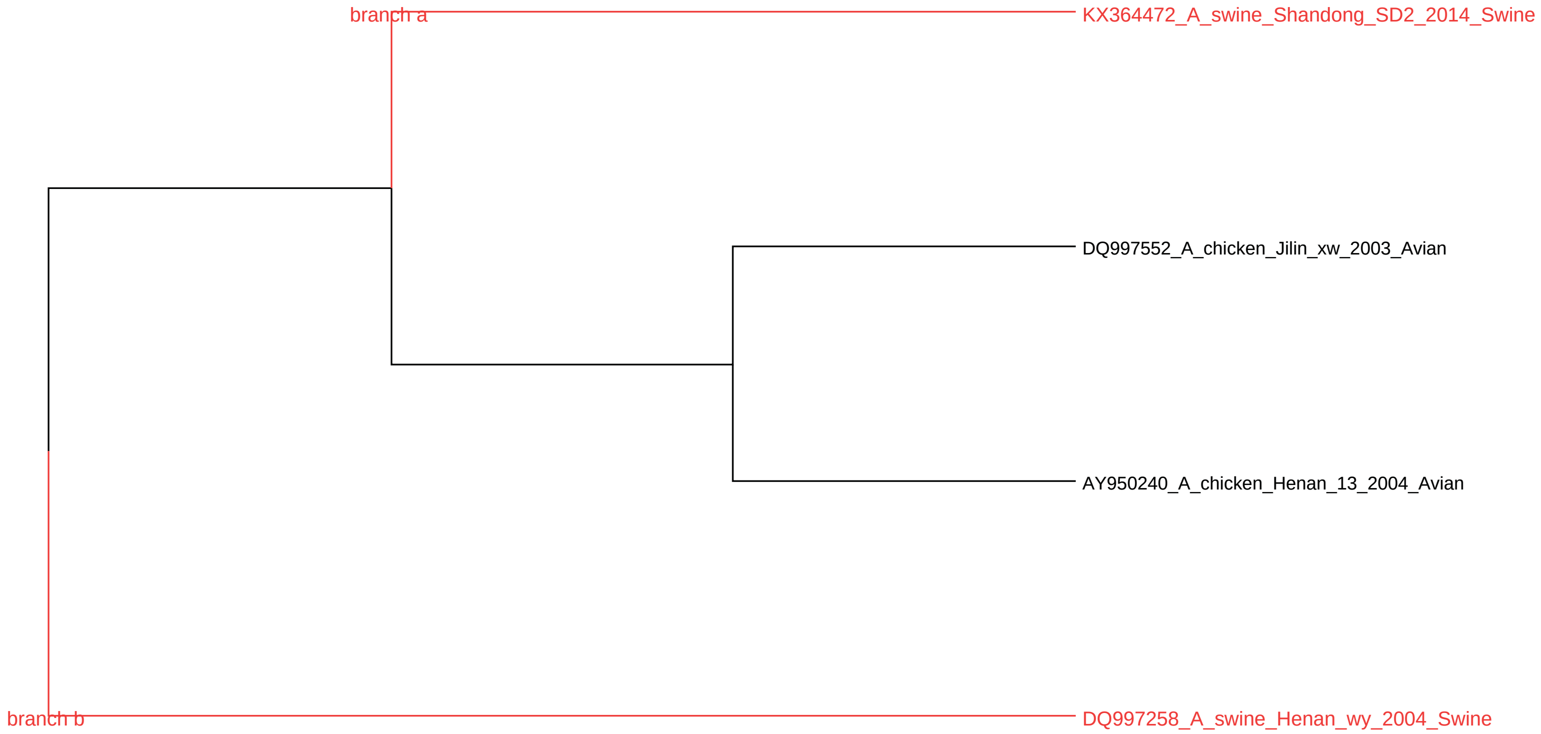

branch a
KX364472_A_swine_Shandong_SD2_2014_Swine
DQ997552_A_chicken_Jilin_xw_2003_Avian
AY950240_A_chicken_Henan_13_2004_Avian
branch b
DQ997258_A_swine_Henan_wy_2004_Swine

MP-Group80

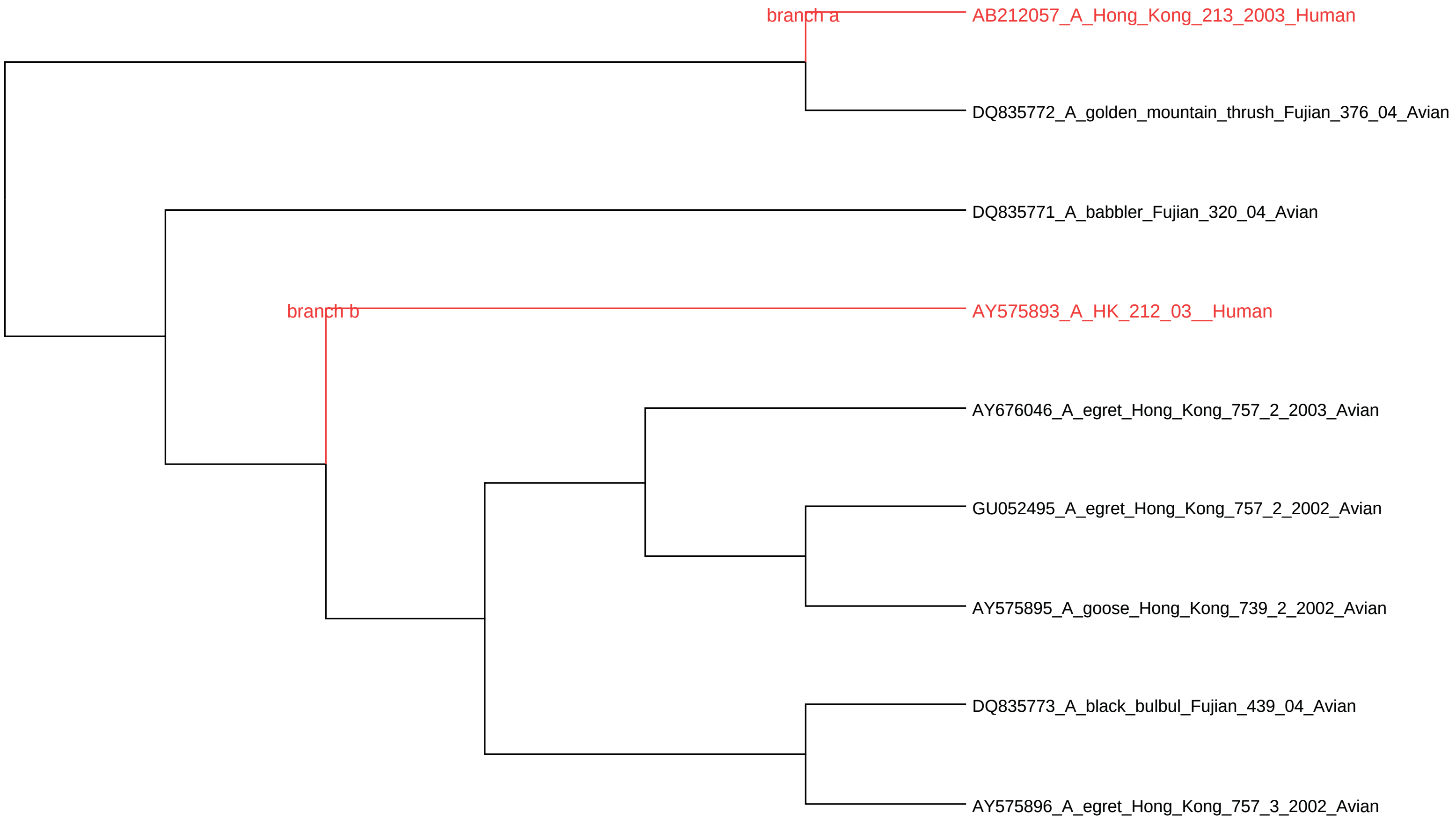
branch a
AB212057_A_Hong_Kong_213_2003_Human
DQ835772_A_golden_mountain_thrush_Fujian_376_04_Avian
DQ835771_A_babbler_Fujian_320_04_Avian
branch b
AY575893_A_HK_212_03__Human
AY676046_A_egret_Hong_Kong_757_2_2003_Avian
GU052495_A_egret_Hong_Kong_757_2_2002_Avian
AY575895_A_goose_Hong_Kong_739_2_2002_Avian
DQ835773_A_black_bulbul_Fujian_439_04_Avian
AY575896_A_egret_Hong_Kong_757_3_2002_Avian

MP-Group81

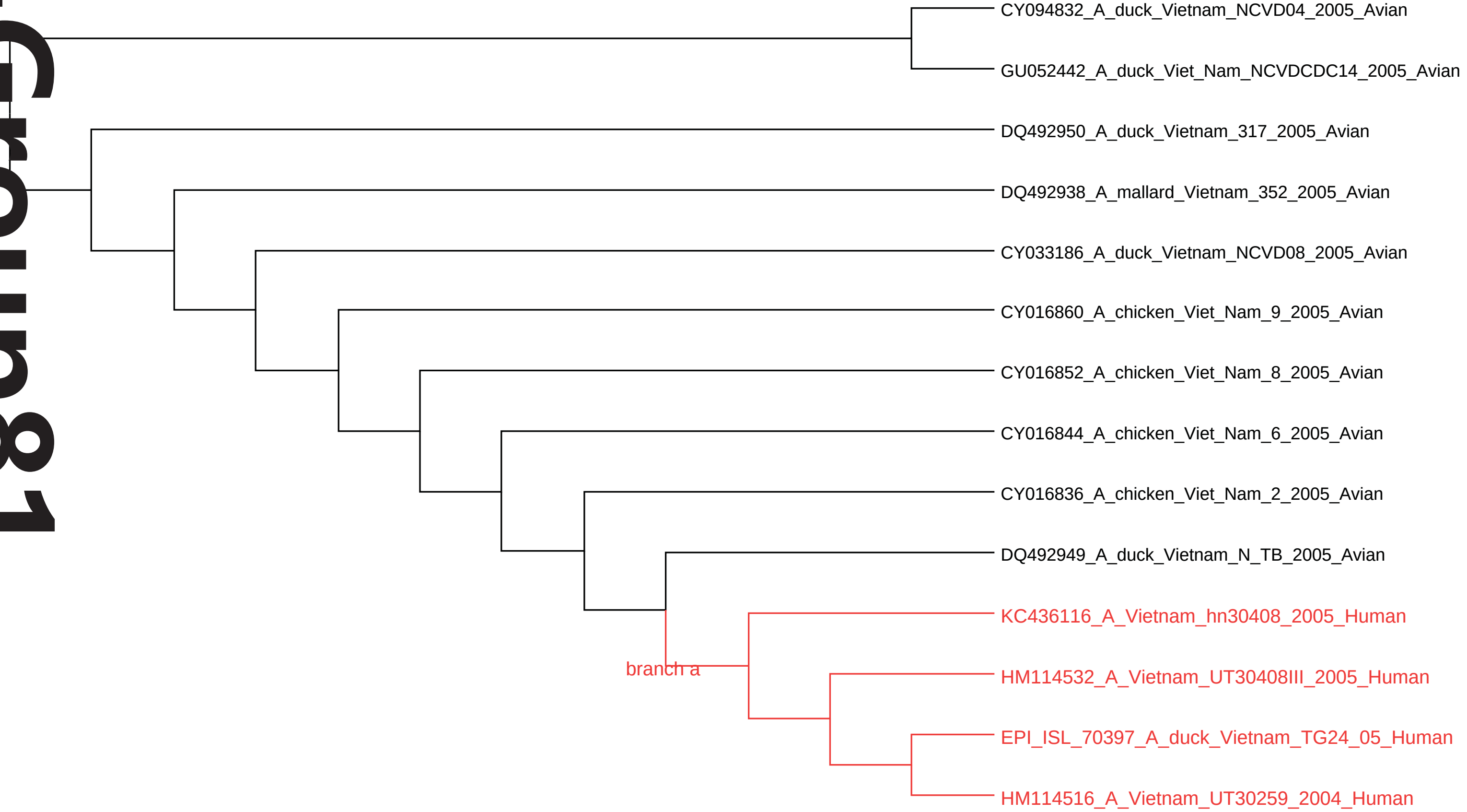

CY094832_A_duck_Vietnam_NCVD04_2005_Avian
GU052442_A_duck_Viet_Nam_NCVDCDC14_2005_Avian
DQ492950_A_duck_Vietnam_317_2005_Avian
DQ492938_A_mallard_Vietnam_352_2005_Avian
CY033186_A_duck_Vietnam_NCVD08_2005_Avian
CY016860_A_chicken_Viet_Nam_9_2005_Avian
CY016852_A_chicken_Viet_Nam_8_2005_Avian
CY016844_A_chicken_Viet_Nam_6_2005_Avian
CY016836_A_chicken_Viet_Nam_2_2005_Avian
DQ492949_A_duck_Vietnam_N_TB_2005_Avian
KC436116_A_Vietnam_hn30408_2005_Human
branch a
HM114532_A_Vietnam_UT30408III_2005_Human
EPI_ISL_70397_A_duck_Vietnam_TG24_05_Human
HM114516_A_Vietnam_UT30259_2004_Human

MP-Group82

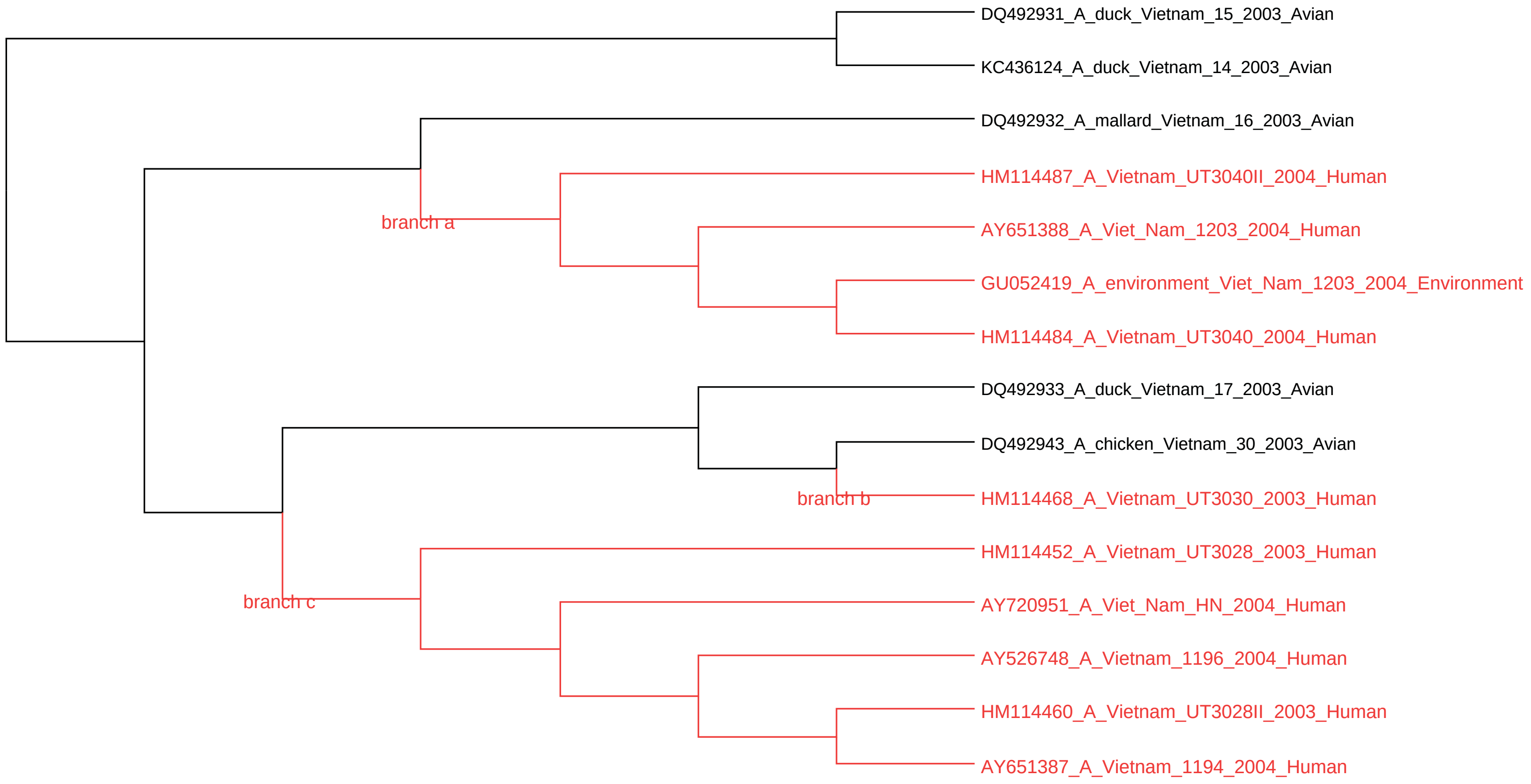
DQ492931_A_duck_Vietnam_15_2003_Avian
KC436124_A_duck_Vietnam_14_2003_Avian
DQ492932_A_mallard_Vietnam_16_2003_Avian
HM114487_A_Vietnam_UT3040II_2004_Human
branch a
AY651388_A_Viet_Nam_1203_2004_Human
GU052419_A_environment_Viet_Nam_1203_2004_Environment
HM114484_A_Vietnam_UT3040_2004_Human
DQ492933_A_duck_Vietnam_17_2003_Avian
DQ492943_A_chicken_Vietnam_30_2003_Avian
branch b
HM114468_A_Vietnam_UT3030_2003_Human
HM114452_A_Vietnam_UT3028_2003_Human
branch c
AY720951_A_Viet_Nam_HN_2004_Human
AY526748_A_Vietnam_1196_2004_Human
HM114460_A_Vietnam_UT3028II_2003_Human
AY651387_A_Vietnam_1194_2004_Human

MP-Group83

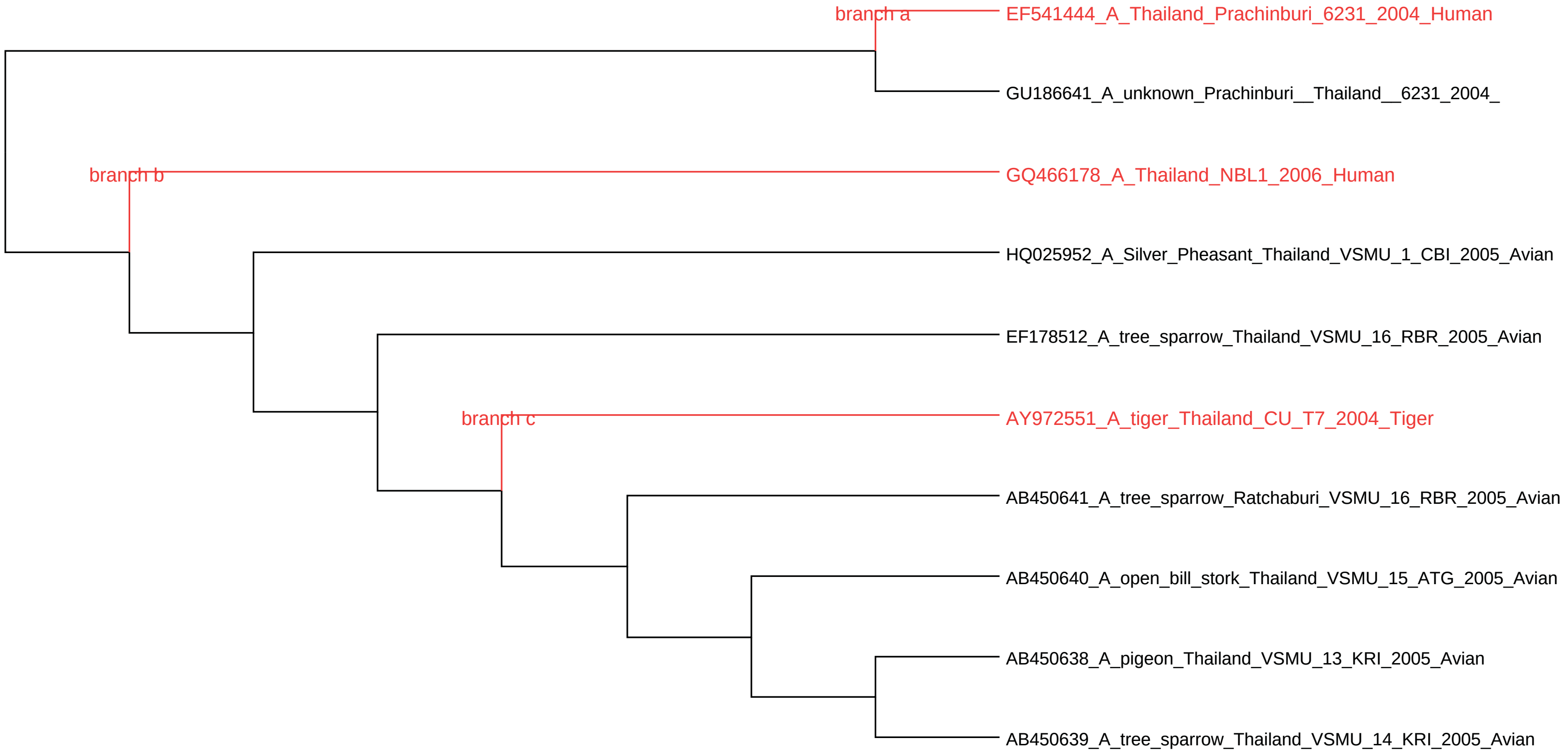
branch a
EF541444_A_Thailand_Prachinburi_6231_2004_Human
GU186641_A_unknown_Prachinburi__Thailand__6231_2004_
branch b
GQ466178_A_Thailand_NBL1_2006_Human
HQ025952_A_Silver_Pheasant_Thailand_VSMU_1_CBI_2005_Avian
EF178512_A_tree_sparrow_Thailand_VSMU_16_RBR_2005_Avian
branch c
AY972551_A_tiger_Thailand_CU_T7_2004_Tiger
AB450641_A_tree_sparrow_Ratchaburi_VSMU_16_RBR_2005_Avian
AB450640_A_open_bill_stork_Thailand_VSMU_15_ATG_2005_Avian
AB450638_A_pigeon_Thailand_VSMU_13_KRI_2005_Avian
AB450639_A_tree_sparrow_Thailand_VSMU_14_KRI_2005_Avian

MP-Group84

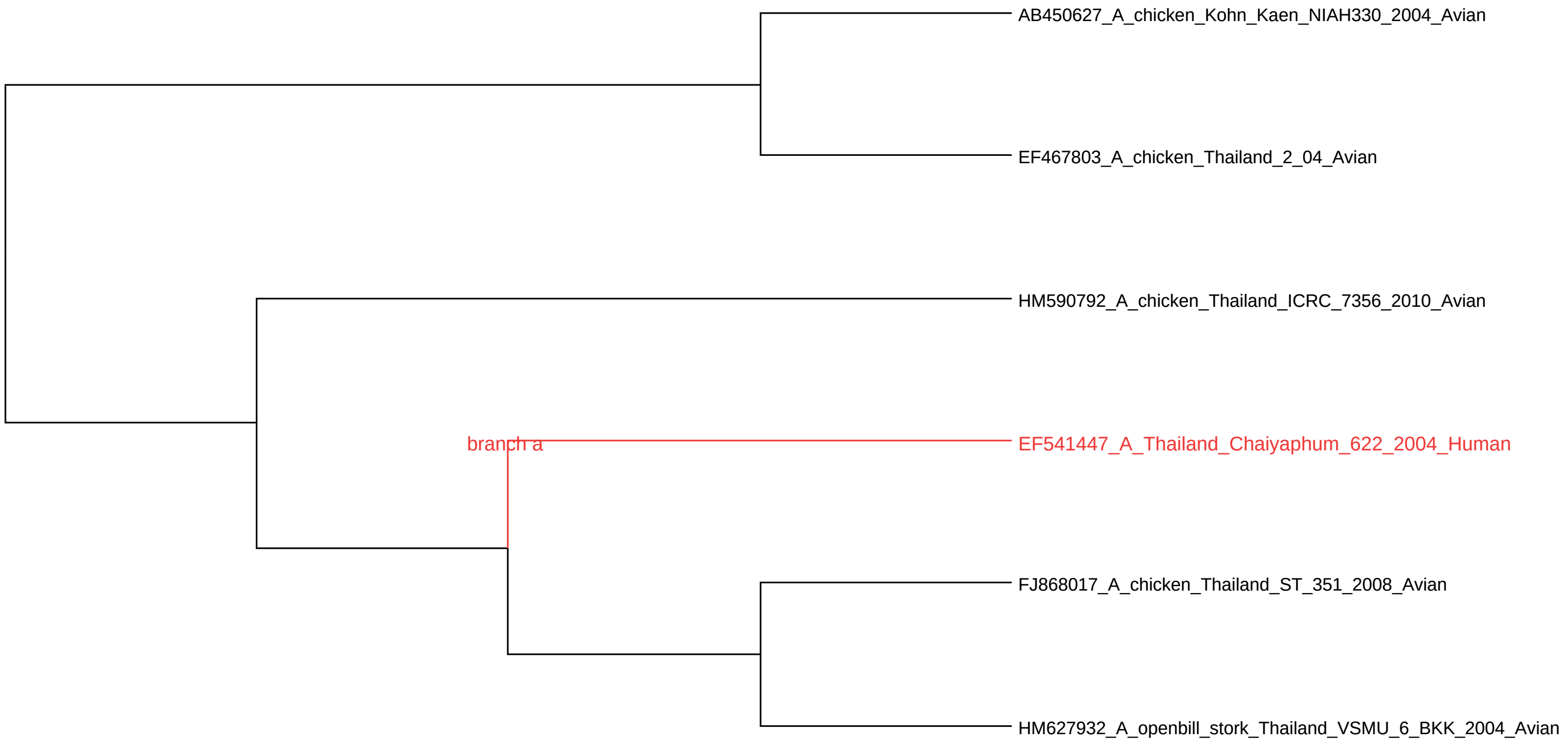
AB450627_A_chicken_Kohn_Kaen_NIAH330_2004_Avian
EF467803_A_chicken_Thailand_2_04_Avian
HM590792_A_chicken_Thailand_ICRC_7356_2010_Avian
branch a
EF541447_A_Thailand_Chaiyaphum_622_2004_Human
FJ868017_A_chicken_Thailand_ST_351_2008_Avian
HM627932_A_openbill_stork_Thailand_VSMU_6_BKK_2004_Avian

MP-Group85

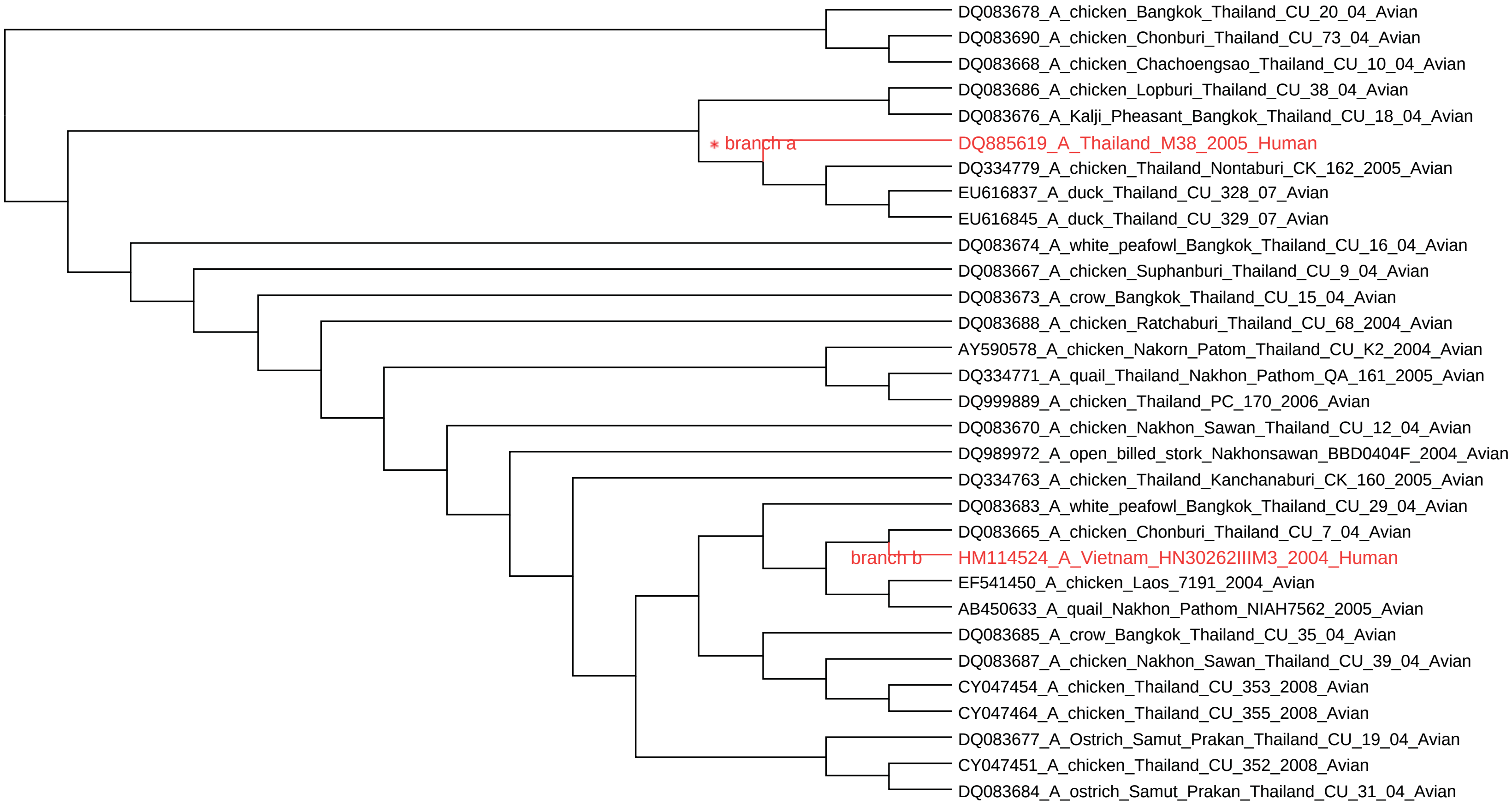
DQ083678_A_chicken_Bangkok_Thailand_CU_20_04_Avian
DQ083690_A_chicken_Chonburi_Thailand_CU_73_04_Avian
DQ083668_A_chicken_Chachoengsao_Thailand_CU_10_04_Avian
DQ083686_A_chicken_Lopburi_Thailand_CU_38_04_Avian
DQ083676_A_Kalji_Pheasant_Bangkok_Thailand_CU_18_04_Avian
* branch a
DQ885619_A_Thailand_M38_2005_Human
DQ334779_A_chicken_Thailand_Nontaburi_CK_162_2005_Avian
EU616837_A_duck_Thailand_CU_328_07_Avian
EU616845_A_duck_Thailand_CU_329_07_Avian
DQ083674_A_white_peafowl_Bangkok_Thailand_CU_16_04_Avian
DQ083667_A_chicken_Suphanburi_Thailand_CU_9_04_Avian
DQ083673_A_crow_Bangkok_Thailand_CU_15_04_Avian
DQ083688_A_chicken_Ratchaburi_Thailand_CU_68_2004_Avian
AY590578_A_chicken_Nakorn_Patom_Thailand_CU_K2_2004_Avian
DQ334771_A_quail_Thailand_Nakhon_Pathom_QA_161_2005_Avian
DQ999889_A_chicken_Thailand_PC_170_2006_Avian
DQ083670_A_chicken_Nakhon_Sawan_Thailand_CU_12_04_Avian
DQ989972_A_open_billed_stork_Nakhonsawan_BBD0404F_2004_Avian
DQ334763_A_chicken_Thailand_Kanchanaburi_CK_160_2005_Avian
DQ083683_A_white_peafowl_Bangkok_Thailand_CU_29_04_Avian
DQ083665_A_chicken_Chonburi_Thailand_CU_7_04_Avian
branch b
HM114524_A_Vietnam_HN30262IIIM3_2004_Human
EF541450_A_chicken_Laos_7191_2004_Avian
AB450633_A_quail_Nakhon_Pathom_NIAH7562_2005_Avian
DQ083685_A_crow_Bangkok_Thailand_CU_35_04_Avian
DQ083687_A_chicken_Nakhon_Sawan_Thailand_CU_39_04_Avian
CY047454_A_chicken_Thailand_CU_353_2008_Avian
CY047464_A_chicken_Thailand_CU_355_2008_Avian
DQ083677_A_Ostrich_Samut_Prakan_Thailand_CU_19_04_Avian
CY047451_A_chicken_Thailand_CU_352_2008_Avian
DQ083684_A_ostrich_Samut_Prakan_Thailand_CU_31_04_Avian

MP-Group86

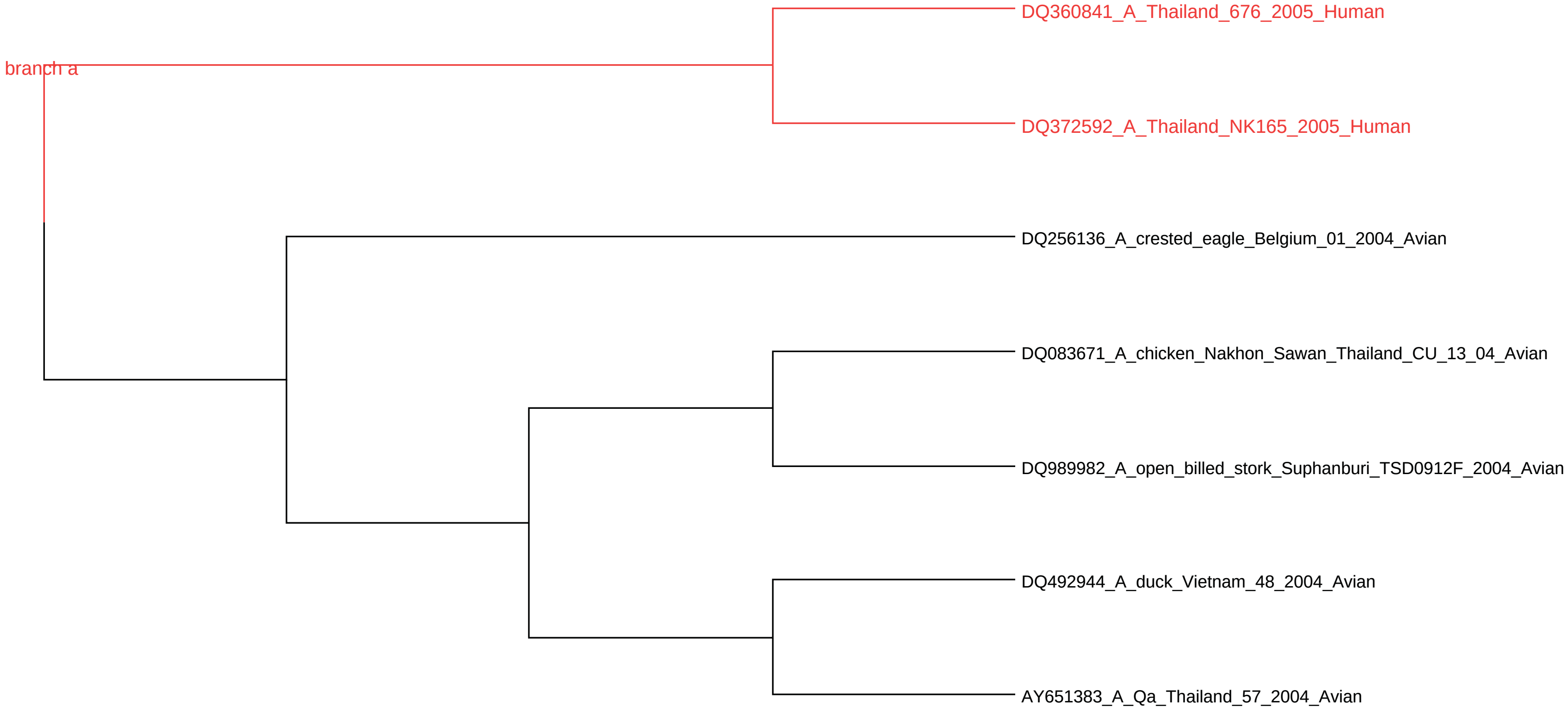
branch a
DQ360841_A_Thailand_676_2005_Human
DQ372592_A_Thailand_NK165_2005_Human
DQ256136_A_crested_eagle_Belgium_01_2004_Avian
DQ083671_A_chicken_Nakhon_Sawan_Thailand_CU_13_04_Avian
DQ989982_A_open_billed_stork_Suphanburi_TSD0912F_2004_Avian
DQ492944_A_duck_Vietnam_48_2004_Avian
AY651383_A_Qa_Thailand_57_2004_Avian

MP-Group87

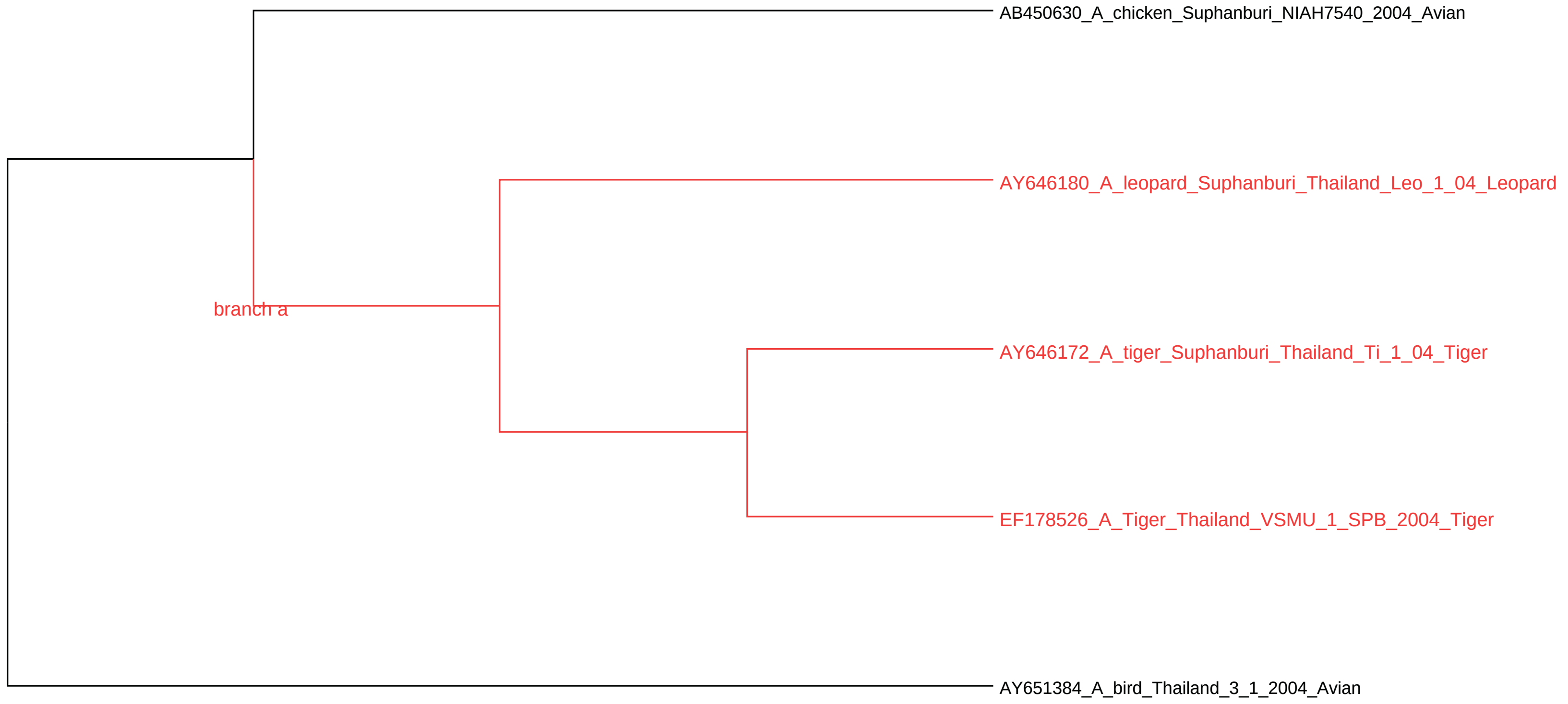

AB450630_A_chicken_Suphanburi_NIAH7540_2004_Avian
AY646180_A_leopard_Suphanburi_Thailand_Leo_1_04_Leopard
branch a
AY646172_A_tiger_Suphanburi_Thailand_Ti_1_04_Tiger
EF178526_A_Tiger_Thailand_VSMU_1_SPB_2004_Tiger
AY651384_A_bird_Thailand_3_1_2004_Avian

MP-Group88

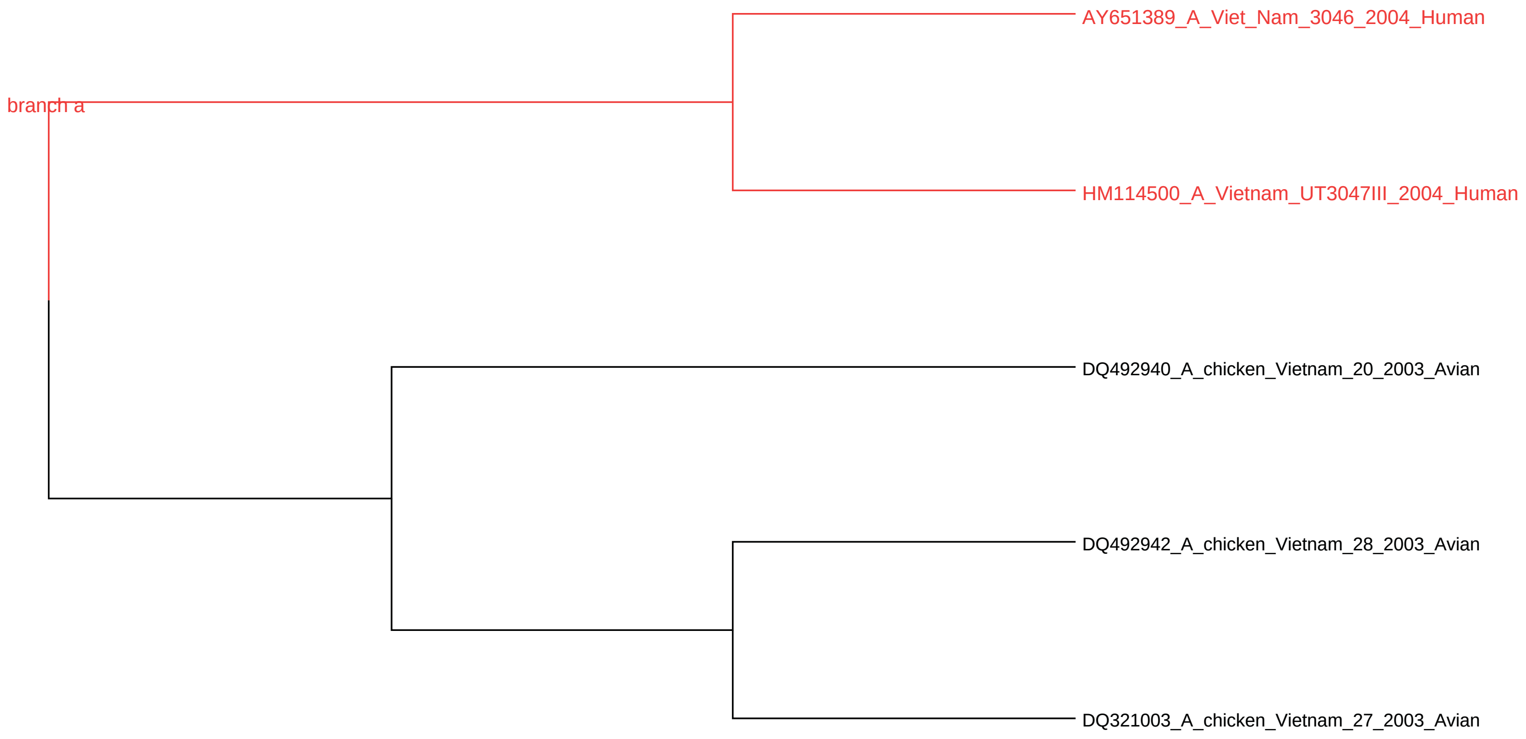
AY651389_A_Viet_Nam_3046_2004_Human
branch a
HM114500_A_Vietnam_UT3047III_2004_Human
DQ492940_A_chicken_Vietnam_20_2003_Avian
DQ492942_A_chicken_Vietnam_28_2003_Avian
DQ321003_A_chicken_Vietnam_27_2003_Avian

MP-Group89

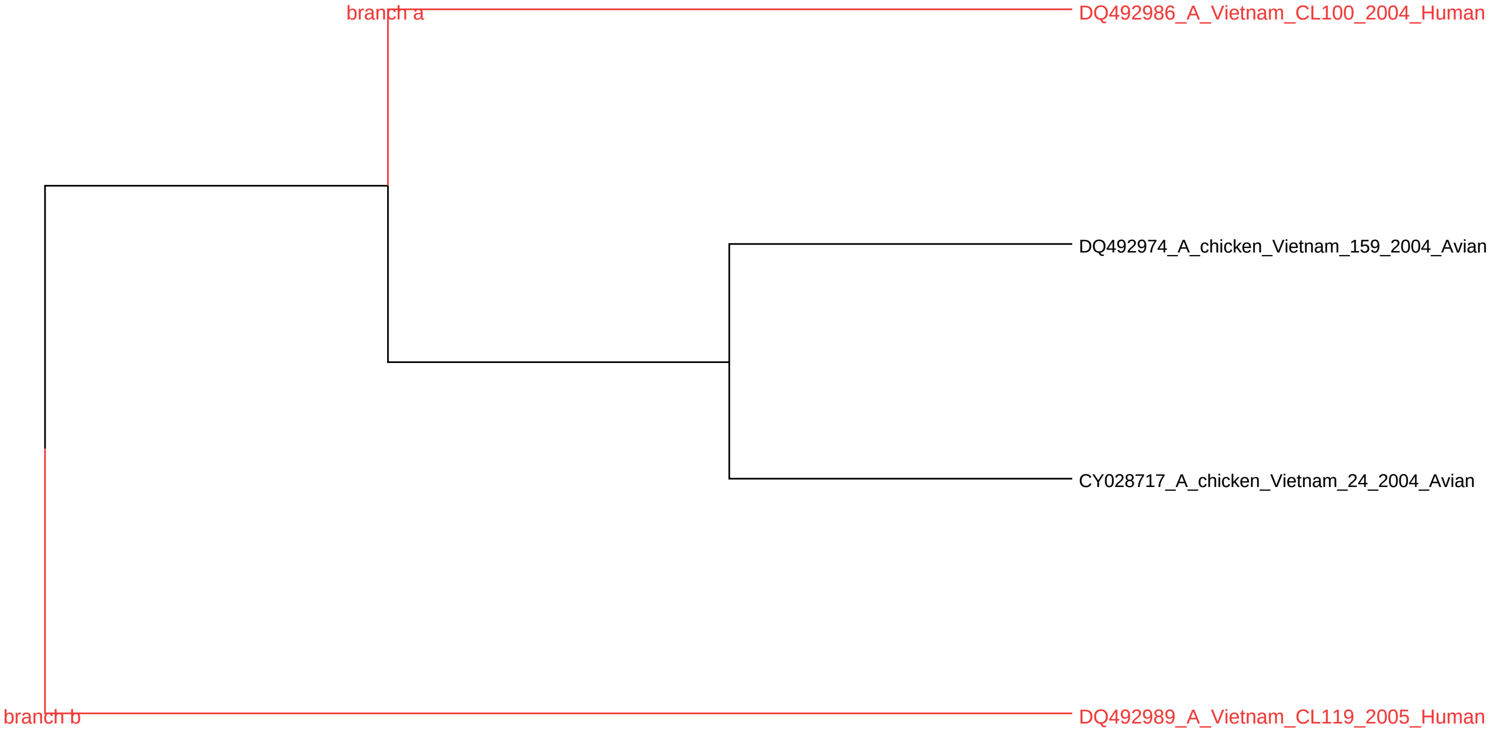
branch a
DQ492986_A_Vietnam_CL100_2004_Human
DQ492974_A_chicken_Vietnam_159_2004_Avian
CY028717_A_chicken_Vietnam_24_2004_Avian
branch b
DQ492989_A_Vietnam_CL119_2005_Human

MP-Group90

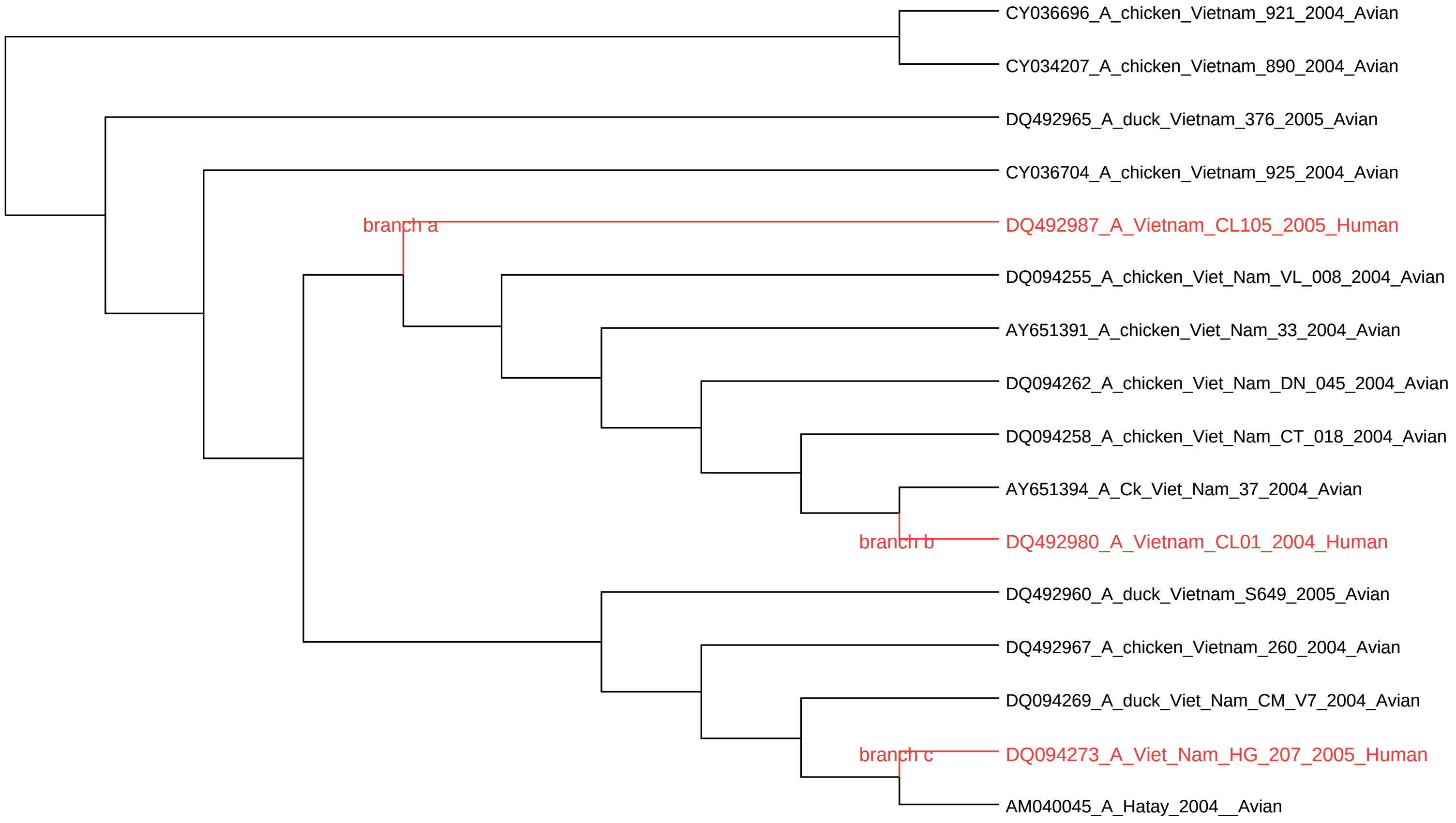

CY036696_A_chicken_Vietnam_921_2004_Avian
CY034207_A_chicken_Vietnam_890_2004_Avian
DQ492965_A_duck_Vietnam_376_2005_Avian
CY036704_A_chicken_Vietnam_925_2004_Avian
branch a
DQ492987_A_Vietnam_CL105_2005_Human
DQ094255_A_chicken_Viet_Nam_VL_008_2004_Avian
AY651391_A_chicken_Viet_Nam_33_2004_Avian
DQ094262_A_chicken_Viet_Nam_DN_045_2004_Avian
DQ094258_A_chicken_Viet_Nam_CT_018_2004_Avian
AY651394_A_Ck_Viet_Nam_37_2004_Avian
branch b
DQ492980_A_Vietnam_CL01_2004_Human
DQ492960_A_duck_Vietnam_S649_2005_Avian
DQ492967_A_chicken_Vietnam_260_2004_Avian
DQ094269_A_duck_Viet_Nam_CM_V7_2004_Avian
branch c
DQ094273_A_Viet_Nam_HG_207_2005_Human
AM040045_A_Hatay_2004__Avian

MP-Group91

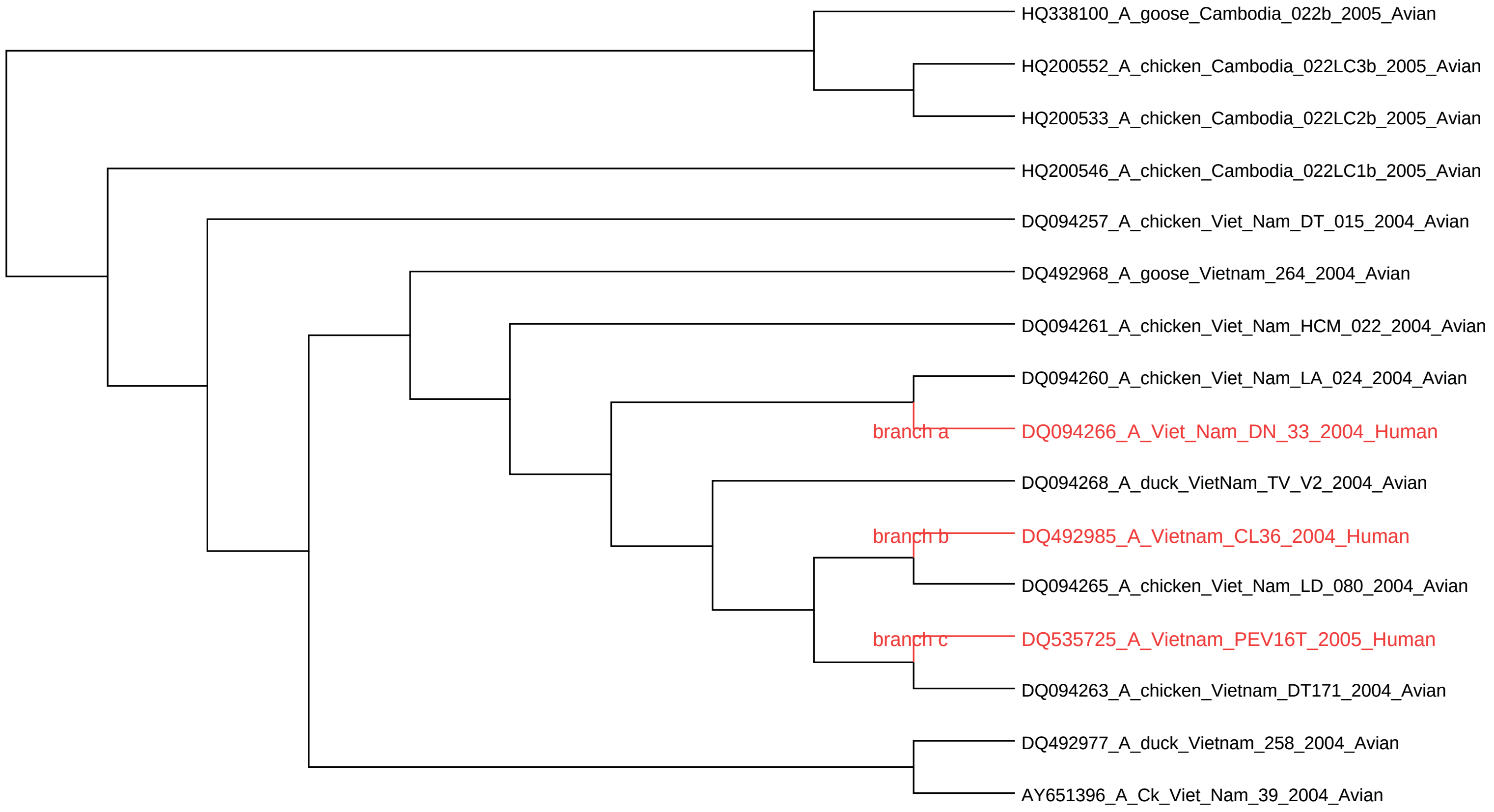
HQ338100_A_goose_Cambodia_022b_2005_Avian
HQ200552_A_chicken_Cambodia_022LC3b_2005_Avian
HQ200533_A_chicken_Cambodia_022LC2b_2005_Avian
HQ200546_A_chicken_Cambodia_022LC1b_2005_Avian
DQ094257_A_chicken_Viet_Nam_DT_015_2004_Avian
DQ492968_A_goose_Vietnam_264_2004_Avian
DQ094261_A_chicken_Viet_Nam_HCM_022_2004_Avian
DQ094260_A_chicken_Viet_Nam_LA_024_2004_Avian
branch a
DQ094266_A_Viet_Nam_DN_33_2004_Human
DQ094268_A_duck_VietNam_TV_V2_2004_Avian
branch b
DQ492985_A_Vietnam_CL36_2004_Human
DQ094265_A_chicken_Viet_Nam_LD_080_2004_Avian
branch c
DQ535725_A_Vietnam_PEV16T_2005_Human
DQ094263_A_chicken_Vietnam_DT171_2004_Avian
DQ492977_A_duck_Vietnam_258_2004_Avian
AY651396_A_Ck_Viet_Nam_39_2004_Avian

MP-Group92

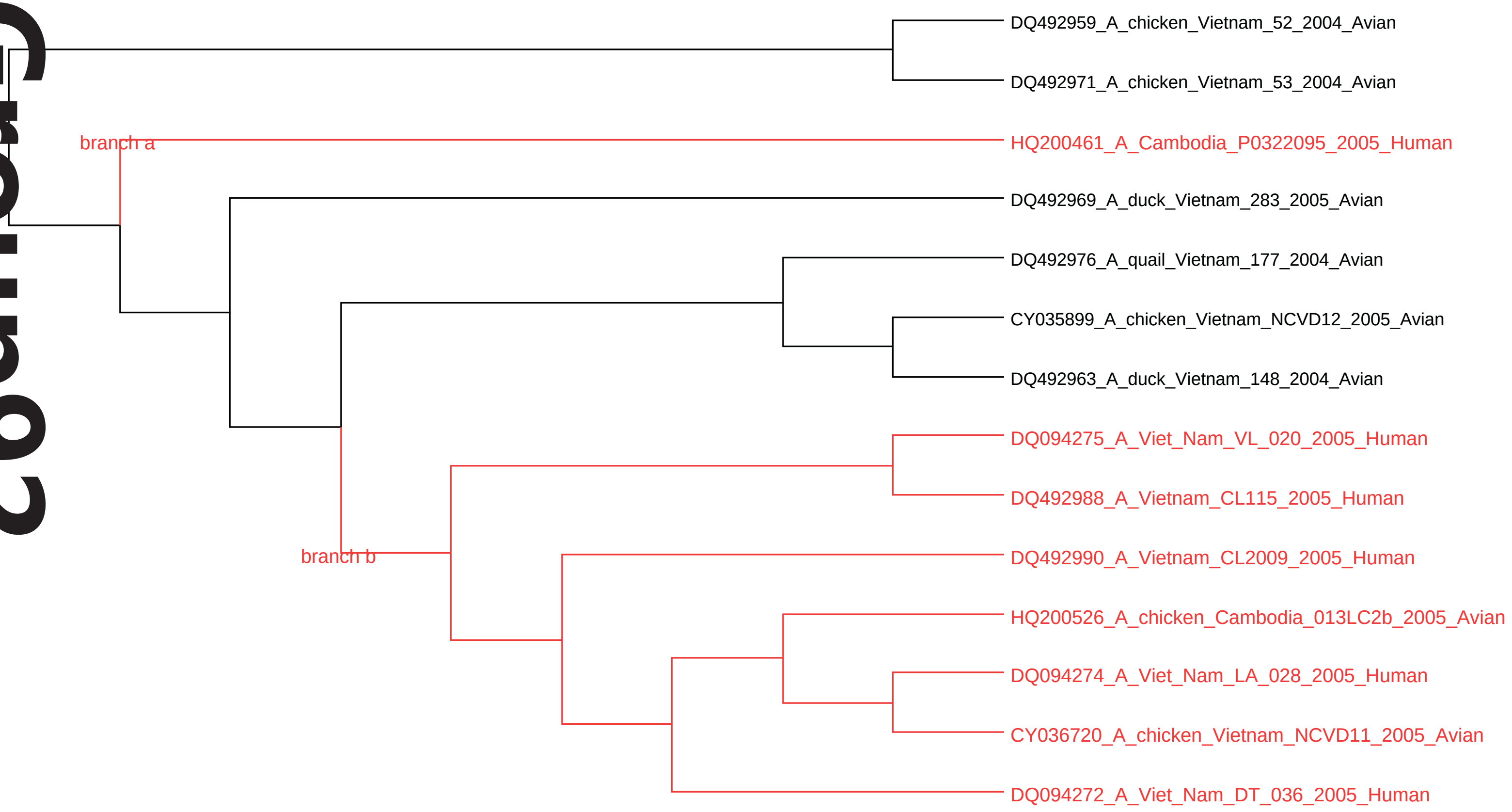

DQ492959_A_chicken_Vietnam_52_2004_Avian
DQ492971_A_chicken_Vietnam_53_2004_Avian
branch a
HQ200461_A_Cambodia_P0322095_2005_Human
DQ492969_A_duck_Vietnam_283_2005_Avian
DQ492976_A_quail_Vietnam_177_2004_Avian
CY035899_A_chicken_Vietnam_NCVD12_2005_Avian
DQ492963_A_duck_Vietnam_148_2004_Avian
DQ094275_A_Viet_Nam_VL_020_2005_Human
DQ492988_A_Vietnam_CL115_2005_Human
branch b
DQ492990_A_Vietnam_CL2009_2005_Human
HQ200526_A_chicken_Cambodia_013LC2b_2005_Avian
DQ094274_A_Viet_Nam_LA_028_2005_Human
CY036720_A_chicken_Vietnam_NCVD11_2005_Avian
DQ094272_A_Viet_Nam_DT_036_2005_Human

MP-Group93

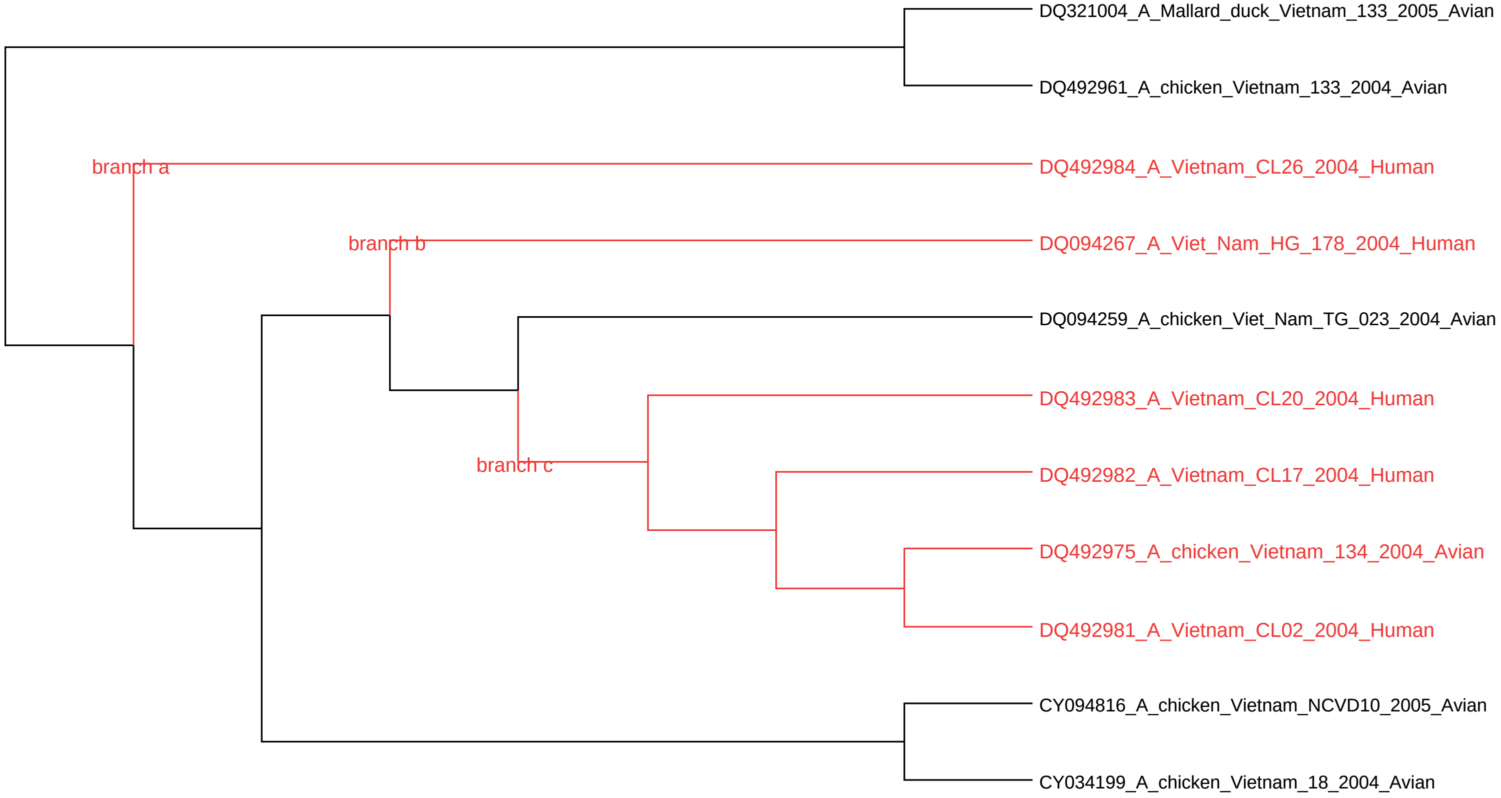
DQ321004_A_Mallard_duck_Vietnam_133_2005_Avian
DQ492961_A_chicken_Vietnam_133_2004_Avian
branch a
DQ492984_A_Vietnam_CL26_2004_Human
branch b
DQ094267_A_Viet_Nam_HG_178_2004_Human
DQ094259_A_chicken_Viet_Nam_TG_023_2004_Avian
DQ492983_A_Vietnam_CL20_2004_Human
branch c
DQ492982_A_Vietnam_CL17_2004_Human
DQ492975_A_chicken_Vietnam_134_2004_Avian
DQ492981_A_Vietnam_CL02_2004_Human
CY094816_A_chicken_Vietnam_NCVD10_2005_Avian
CY034199_A_chicken_Vietnam_18_2004_Avian

MP-Group94

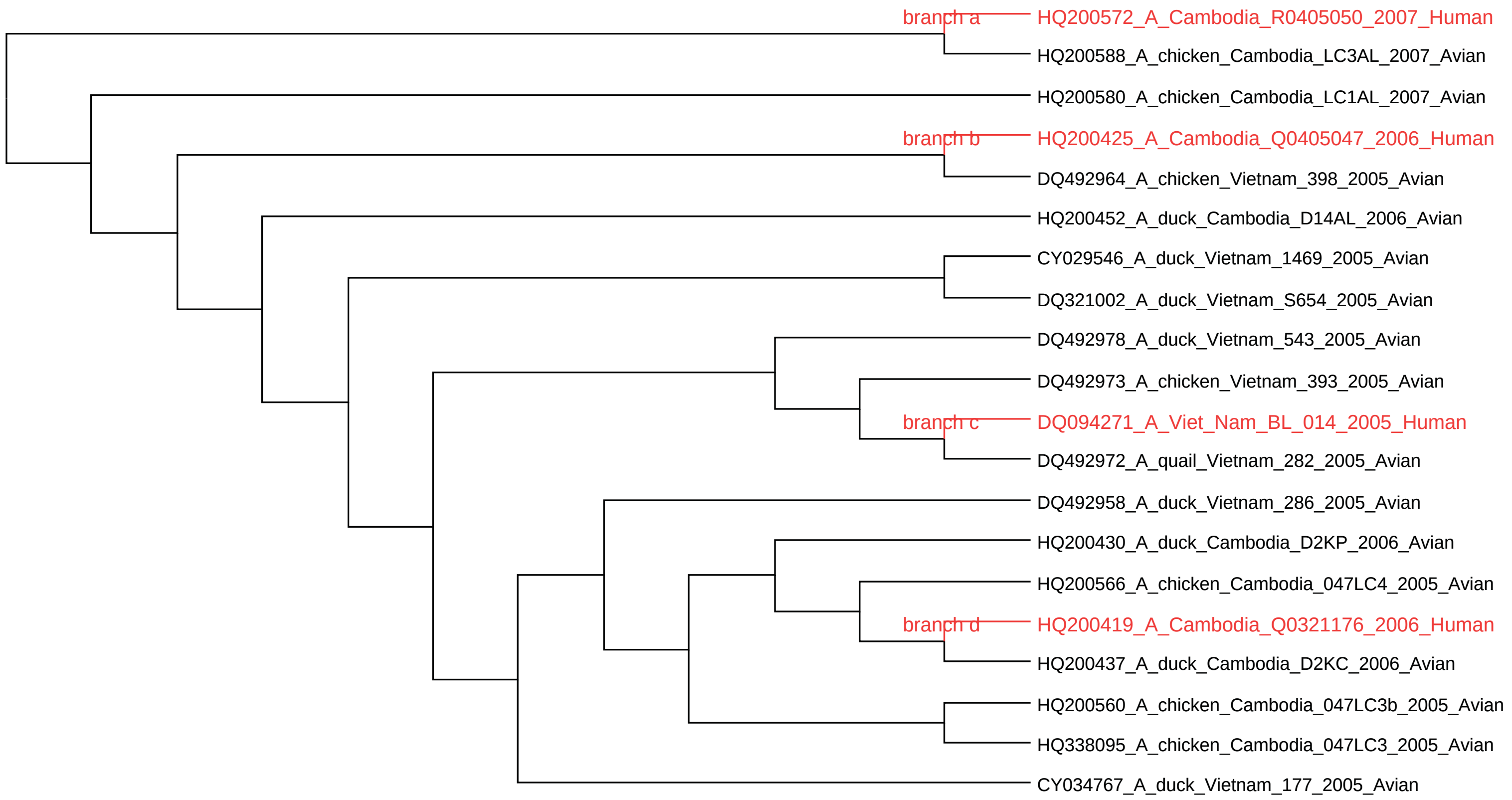
branch a
HQ200572_A_Cambodia_R0405050_2007_Human
HQ200588_A_chicken_Cambodia_LC3AL_2007_Avian
HQ200580_A_chicken_Cambodia_LC1AL_2007_Avian
branch b
HQ200425_A_Cambodia_Q0405047_2006_Human
DQ492964_A_chicken_Vietnam_398_2005_Avian
HQ200452_A_duck_Cambodia_D14AL_2006_Avian
CY029546_A_duck_Vietnam_1469_2005_Avian
DQ321002_A_duck_Vietnam_S654_2005_Avian
DQ492978_A_duck_Vietnam_543_2005_Avian
DQ492973_A_chicken_Vietnam_393_2005_Avian
branch c
DQ094271_A_Viet_Nam_BL_014_2005_Human
DQ492972_A_quail_Vietnam_282_2005_Avian
DQ492958_A_duck_Vietnam_286_2005_Avian
HQ200430_A_duck_Cambodia_D2KP_2006_Avian
HQ200566_A_chicken_Cambodia_047LC4_2005_Avian
branch d
HQ200419_A_Cambodia_Q0321176_2006_Human
HQ200437_A_duck_Cambodia_D2KC_2006_Avian
HQ200560_A_chicken_Cambodia_047LC3b_2005_Avian
HQ338095_A_chicken_Cambodia_047LC3_2005_Avian
CY034767_A_duck_Vietnam_177_2005_Avian

MP-Group95

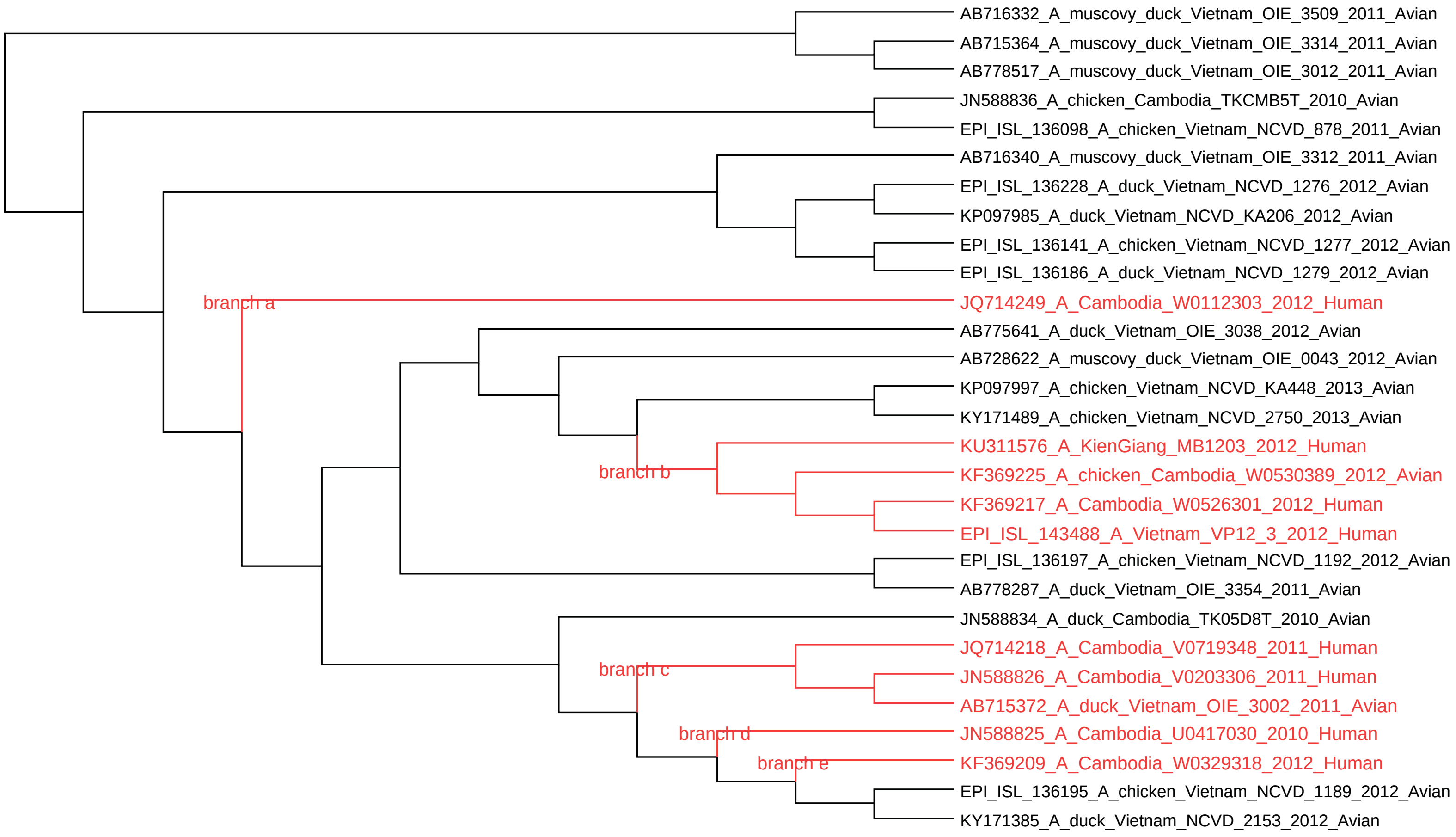
AB716332_A_muscovy_duck_Vietnam_OIE_3509_2011_Avian
AB715364_A_muscovy_duck_Vietnam_OIE_3314_2011_Avian
AB778517_A_muscovy_duck_Vietnam_OIE_3012_2011_Avian
JN588836_A_chicken_Cambodia_TKCMB5T_2010_Avian
EPI_ISL_136098_A_chicken_Vietnam_NCVD_878_2011_Avian
AB716340_A_muscovy_duck_Vietnam_OIE_3312_2011_Avian
EPI_ISL_136228_A_duck_Vietnam_NCVD_1276_2012_Avian
KP097985_A_duck_Vietnam_NCVD_KA206_2012_Avian
EPI_ISL_136141_A_chicken_Vietnam_NCVD_1277_2012_Avian
EPI_ISL_136186_A_duck_Vietnam_NCVD_1279_2012_Avian
branch a
JQ714249_A_Cambodia_W0112303_2012_Human
AB775641_A_duck_Vietnam_OIE_3038_2012_Avian
AB728622_A_muscovy_duck_Vietnam_OIE_0043_2012_Avian
KP097997_A_chicken_Vietnam_NCVD_KA448_2013_Avian
KY171489_A_chicken_Vietnam_NCVD_2750_2013_Avian
KU311576_A_KienGiang_MB1203_2012_Human
branch b
KF369225_A_chicken_Cambodia_W0530389_2012_Avian
KF369217_A_Cambodia_W0526301_2012_Human
EPI_ISL_143488_A_Vietnam_VP12_3_2012_Human
EPI_ISL_136197_A_chicken_Vietnam_NCVD_1192_2012_Avian
AB778287_A_duck_Vietnam_OIE_3354_2011_Avian
JN588834_A_duck_Cambodia_TK05D8T_2010_Avian
JQ714218_A_Cambodia_V0719348_2011_Human
branch c
JN588826_A_Cambodia_V0203306_2011_Human
AB715372_A_duck_Vietnam_OIE_3002_2011_Avian
branch d
JN588825_A_Cambodia_U0417030_2010_Human
branch e
KF369209_A_Cambodia_W0329318_2012_Human
EPI_ISL_136195_A_chicken_Vietnam_NCVD_1189_2012_Avian
KY171385_A_duck_Vietnam_NCVD_2153_2012_Avian

MP-Group96

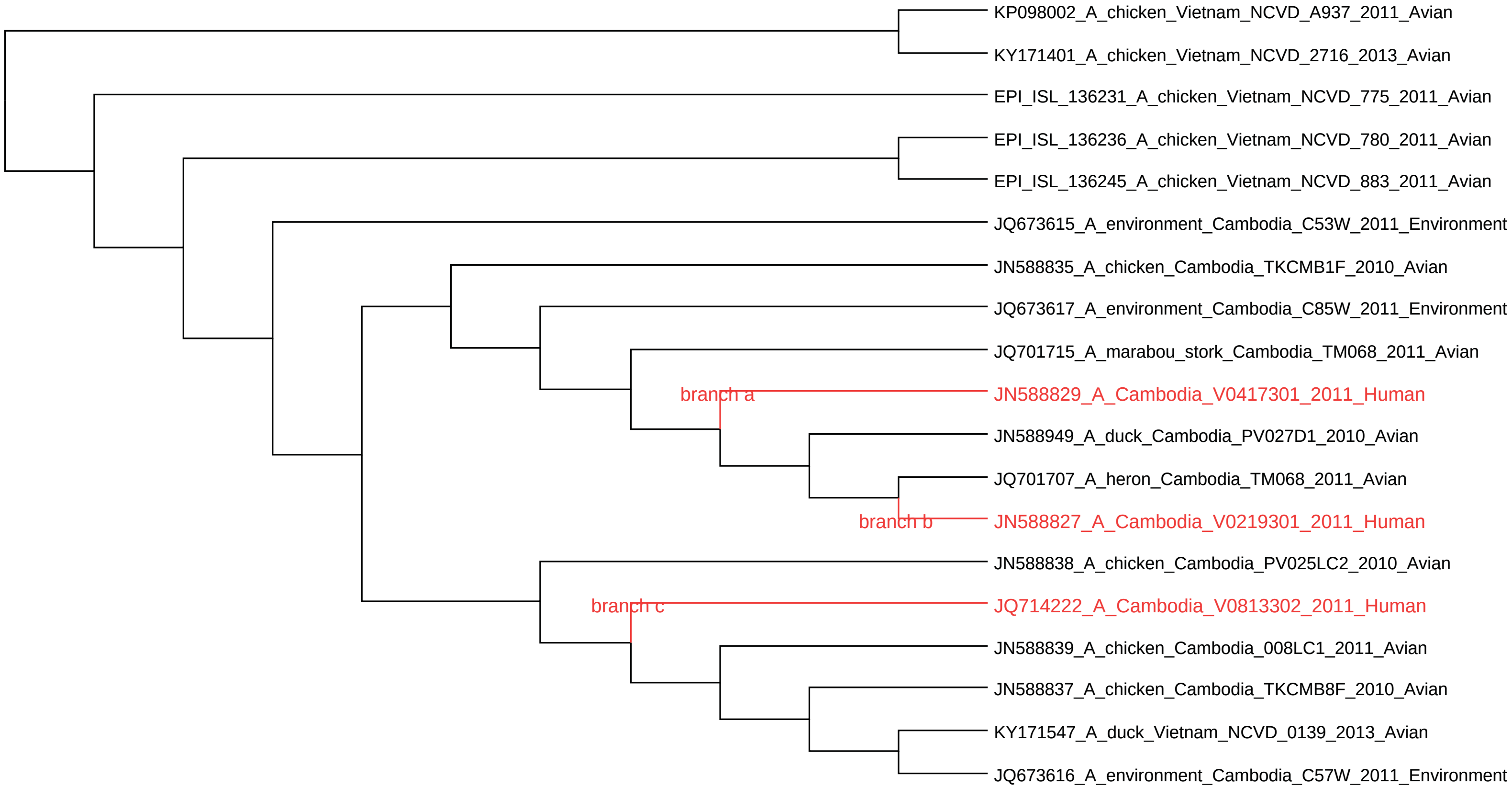
KP098002_A_chicken_Vietnam_NCVD_A937_2011_Avian
KY171401_A_chicken_Vietnam_NCVD_2716_2013_Avian
EPI_ISL_136231_A_chicken_Vietnam_NCVD_775_2011_Avian
EPI_ISL_136236_A_chicken_Vietnam_NCVD_780_2011_Avian
EPI_ISL_136245_A_chicken_Vietnam_NCVD_883_2011_Avian
JQ673615_A_environment_Cambodia_C53W_2011_Environment
JN588835_A_chicken_Cambodia_TKCMB1F_2010_Avian
JQ673617_A_environment_Cambodia_C85W_2011_Environment
JQ701715_A_marabou_stork_Cambodia_TM068_2011_Avian
branch a
JN588829_A_Cambodia_V0417301_2011_Human
JN588949_A_duck_Cambodia_PV027D1_2010_Avian
JQ701707_A_heron_Cambodia_TM068_2011_Avian
branch b
JN588827_A_Cambodia_V0219301_2011_Human
JN588838_A_chicken_Cambodia_PV025LC2_2010_Avian
branch c
JQ714222_A_Cambodia_V0813302_2011_Human
JN588839_A_chicken_Cambodia_008LC1_2011_Avian
JN588837_A_chicken_Cambodia_TKCMB8F_2010_Avian
KY171547_A_duck_Vietnam_NCVD_0139_2013_Avian
JQ673616_A_environment_Cambodia_C57W_2011_Environment

MP-Group97

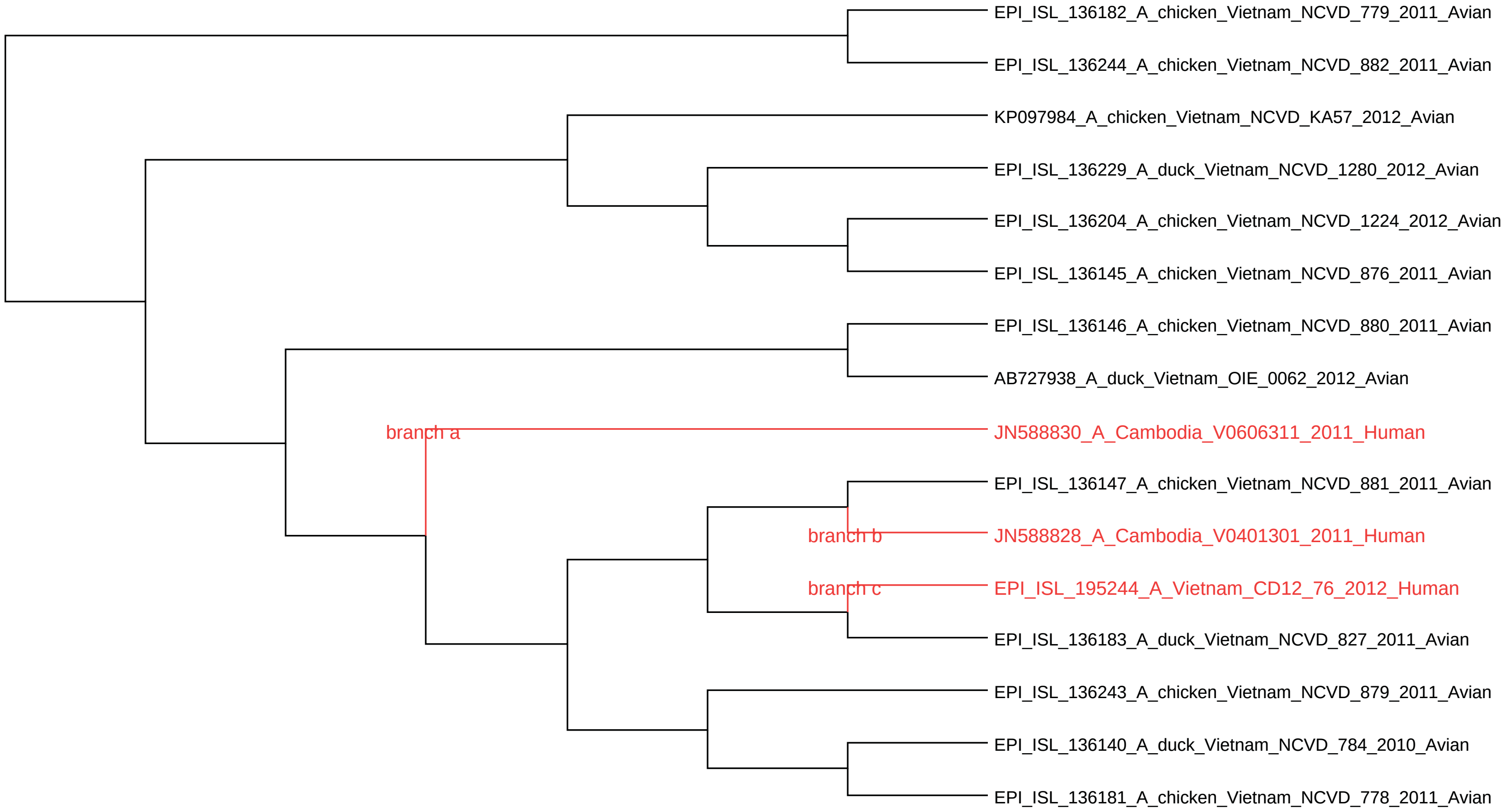
EPI_ISL_136182_A_chicken_Vietnam_NCVD_779_2011_Avian
EPI_ISL_136244_A_chicken_Vietnam_NCVD_882_2011_Avian
KP097984_A_chicken_Vietnam_NCVD_KA57_2012_Avian
EPI_ISL_136229_A_duck_Vietnam_NCVD_1280_2012_Avian
EPI_ISL_136204_A_chicken_Vietnam_NCVD_1224_2012_Avian
EPI_ISL_136145_A_chicken_Vietnam_NCVD_876_2011_Avian
EPI_ISL_136146_A_chicken_Vietnam_NCVD_880_2011_Avian
AB727938_A_duck_Vietnam_OIE_0062_2012_Avian
branch a
JN588830_A_Cambodia_V0606311_2011_Human
EPI_ISL_136147_A_chicken_Vietnam_NCVD_881_2011_Avian
branch b
JN588828_A_Cambodia_V0401301_2011_Human
branch c
EPI_ISL_195244_A_Vietnam_CD12_76_2012_Human
EPI_ISL_136183_A_duck_Vietnam_NCVD_827_2011_Avian
EPI_ISL_136243_A_chicken_Vietnam_NCVD_879_2011_Avian
EPI_ISL_136140_A_duck_Vietnam_NCVD_784_2010_Avian
EPI_ISL_136181_A_chicken_Vietnam_NCVD_778_2011_Avian

MP-Group98

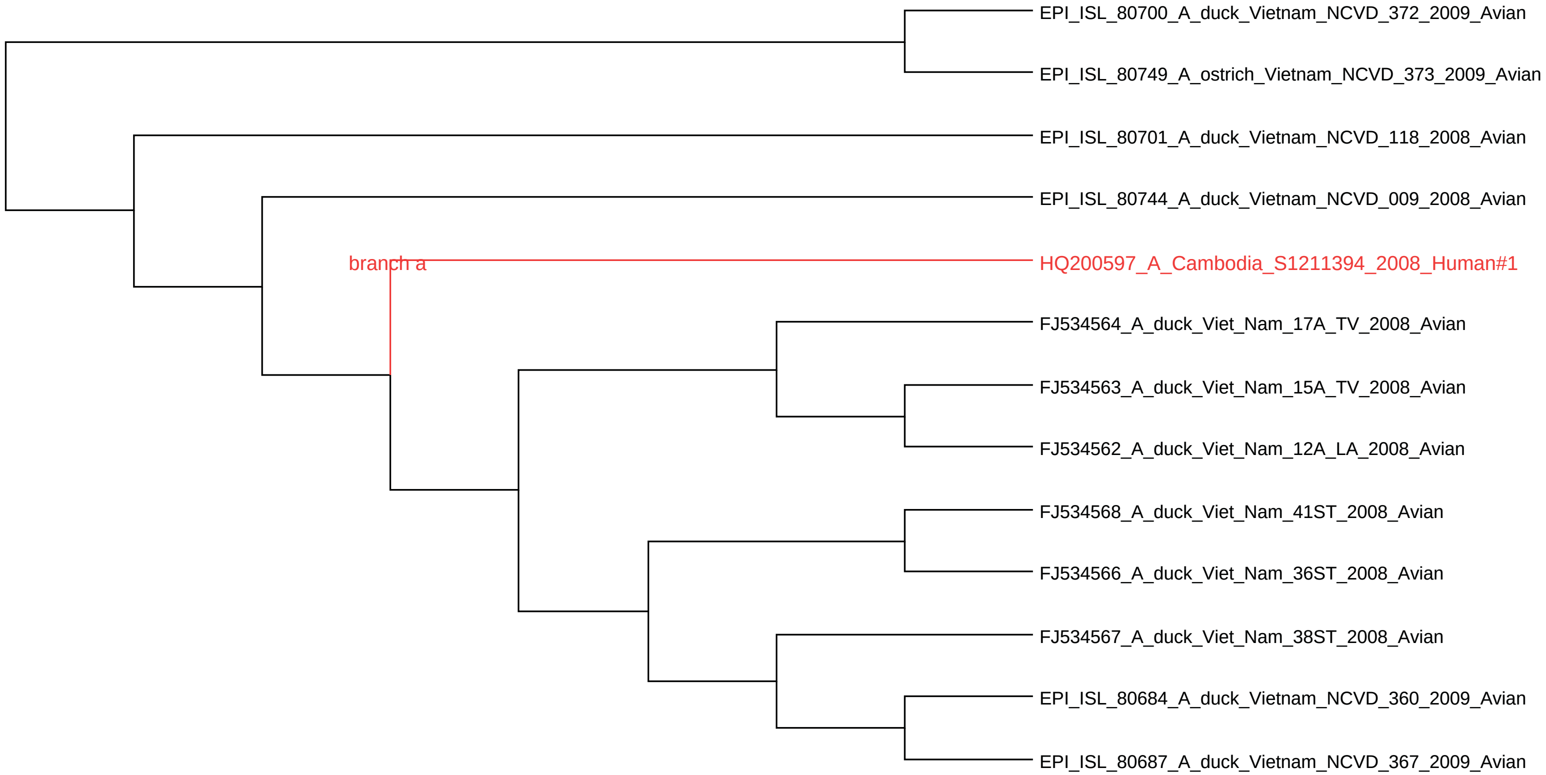
EPI_ISL_80700_A_duck_Vietnam_NCVD_372_2009_Avian
EPI_ISL_80749_A_ostrich_Vietnam_NCVD_373_2009_Avian
EPI_ISL_80701_A_duck_Vietnam_NCVD_118_2008_Avian
EPI_ISL_80744_A_duck_Vietnam_NCVD_009_2008_Avian
branch a
HQ200597_A_Cambodia_S1211394_2008_Human#1
FJ534564_A_duck_Viet_Nam_17A_TV_2008_Avian
FJ534563_A_duck_Viet_Nam_15A_TV_2008_Avian
FJ534562_A_duck_Viet_Nam_12A_LA_2008_Avian
FJ534568_A_duck_Viet_Nam_41ST_2008_Avian
FJ534566_A_duck_Viet_Nam_36ST_2008_Avian
FJ534567_A_duck_Viet_Nam_38ST_2008_Avian
EPI_ISL_80684_A_duck_Vietnam_NCVD_360_2009_Avian
EPI_ISL_80687_A_duck_Vietnam_NCVD_367_2009_Avian
