# Supplementary material for: Adaptive Evolution of Human-Isolated H5Nx Avian Influenza A Viruses
Source: Front Microbiol. 2019 Jun 12;10:1328. doi: 10.3389/fmicb.2019.01328 (PMC6582624; doi:10.3389/fmicb.2019.01328)
Supplement: Supplementary file 6 [file Data_Sheet_6.PDF]

Supplementary Figure 6. 87 phylogenetic trees of NP used for the adaptive evolution analyses. Human strains are marked in red. Branches which have significant signals of positive selection are marked with *.

NP-Group1

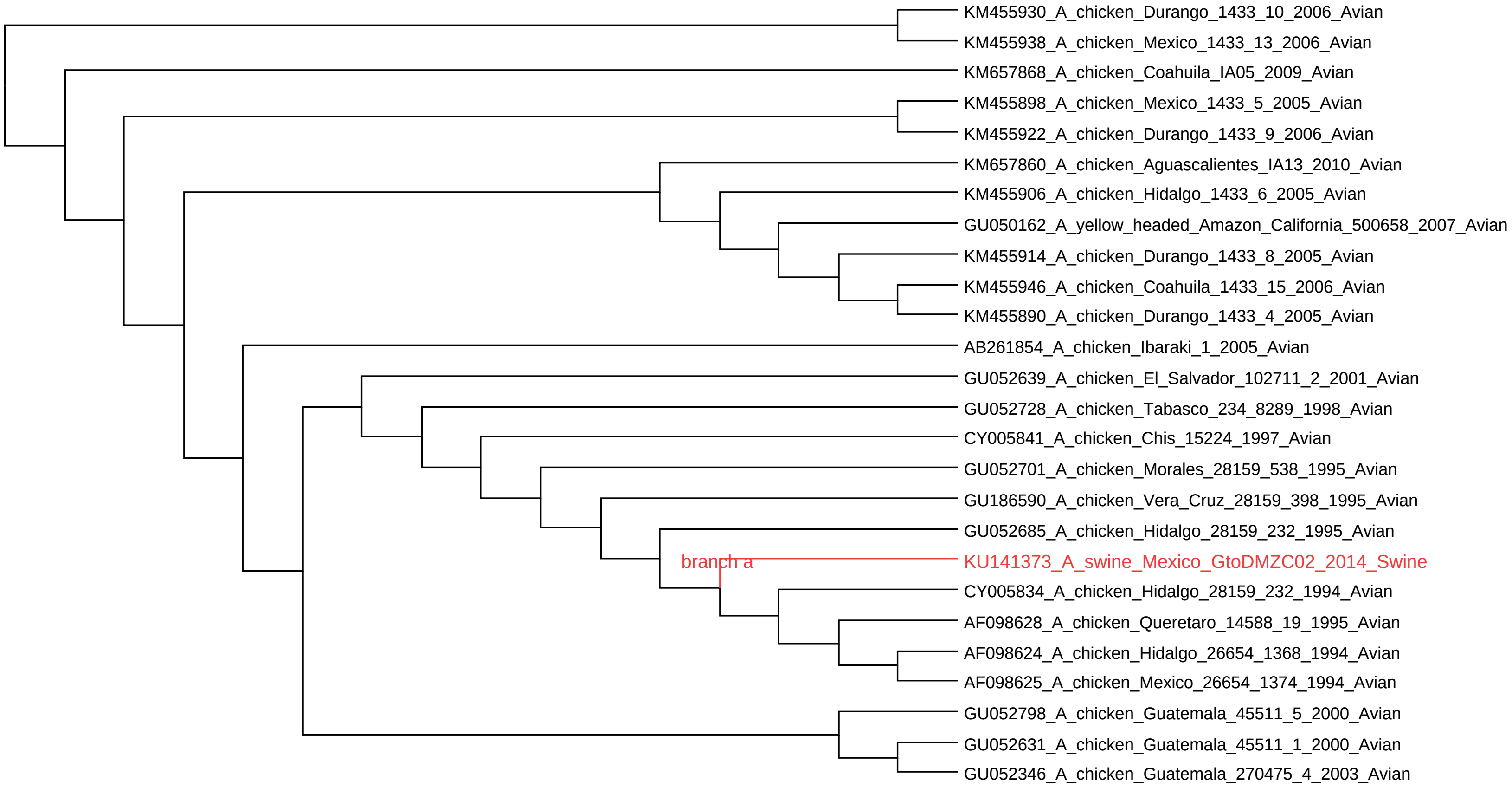

NP-Group2

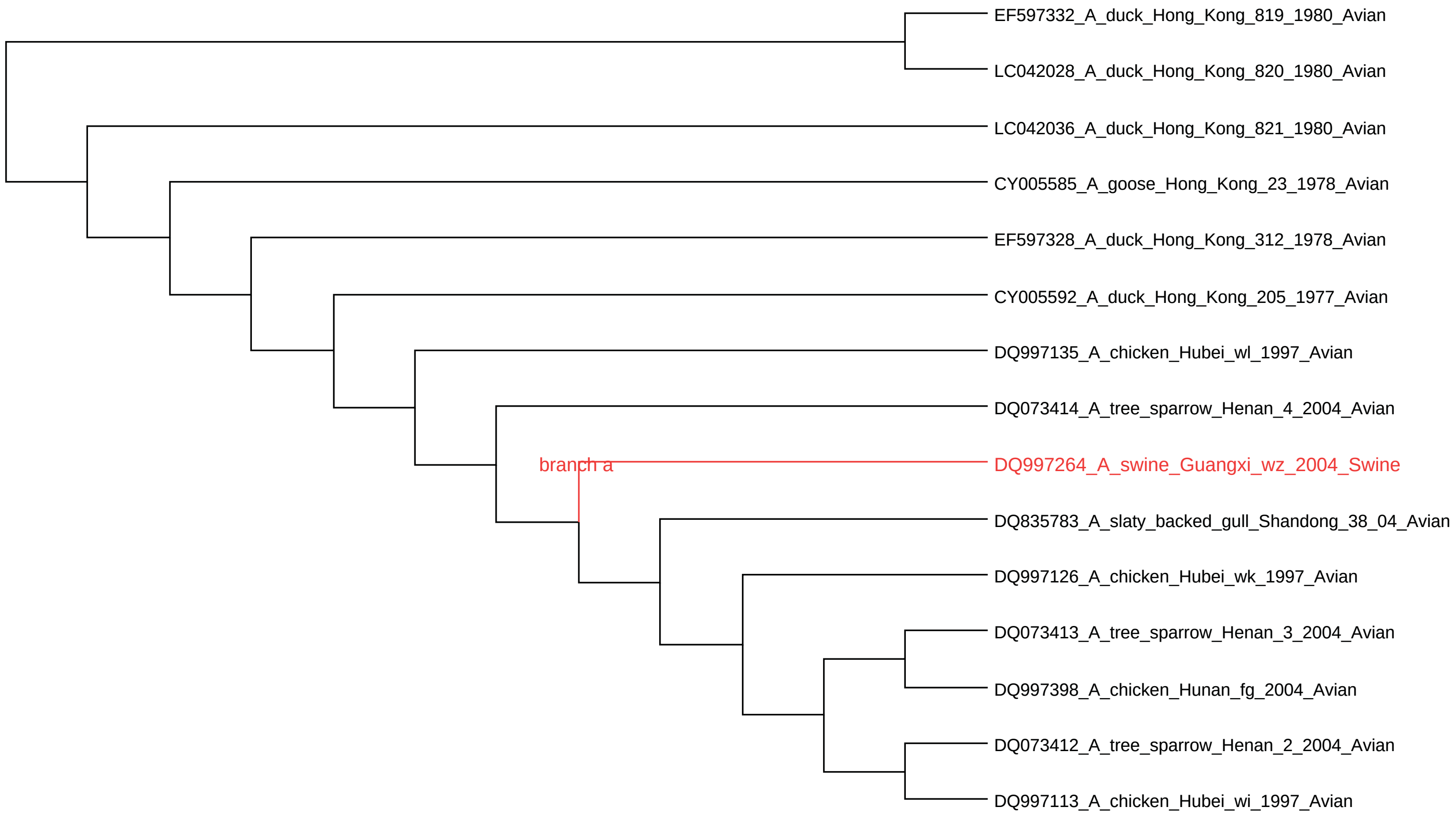
EF597332_A_duck_Hong_Kong_819_1980_Avian
LC042028_A_duck_Hong_Kong_820_1980_Avian
LC042036_A_duck_Hong_Kong_821_1980_Avian
CY005585_A_goose_Hong_Kong_23_1978_Avian
EF597328_A_duck_Hong_Kong_312_1978_Avian
CY005592_A_duck_Hong_Kong_205_1977_Avian
DQ997135_A_chicken_Hubei_wl_1997_Avian
DQ073414_A_tree_sparrow_Henan_4_2004_Avian
branch a
DQ997264_A_swine_Guangxi_wz_2004_Swine
DQ835783_A_slaty_backed_gull_Shandong_38_04_Avian
DQ997126_A_chicken_Hubei_wk_1997_Avian
DQ073413_A_tree_sparrow_Henan_3_2004_Avian
DQ997398_A_chicken_Hunan_fg_2004_Avian
DQ073412_A_tree_sparrow_Henan_2_2004_Avian
DQ997113_A_chicken_Hubei_wi_1997_Avian

NP-Group3

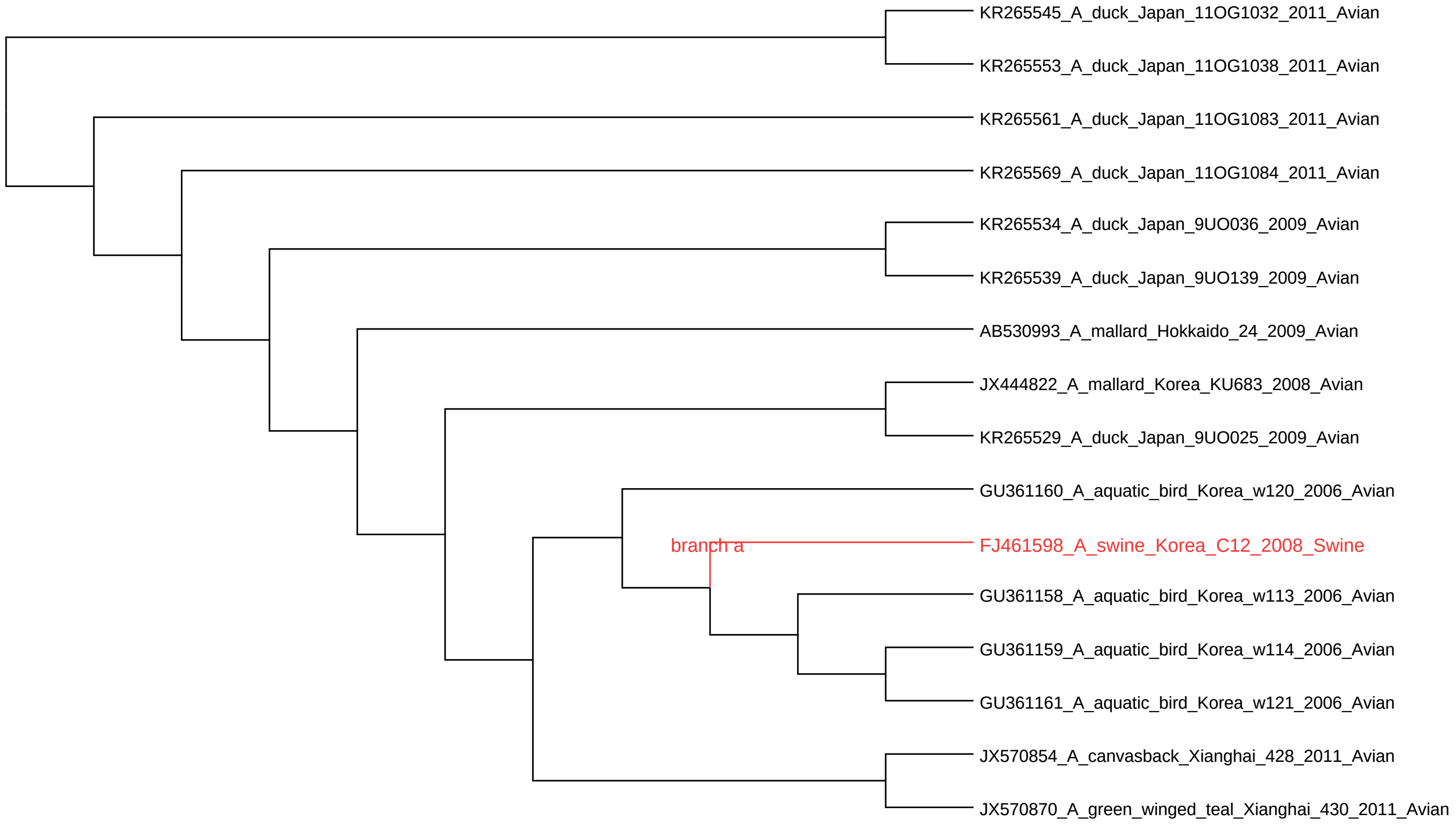
KR265545_A_duck_Japan_11OG1032_2011_Avian
KR265553_A_duck_Japan_11OG1038_2011_Avian
KR265561_A_duck_Japan_11OG1083_2011_Avian
KR265569_A_duck_Japan_11OG1084_2011_Avian
KR265534_A_duck_Japan_9UO036_2009_Avian
KR265539_A_duck_Japan_9UO139_2009_Avian
AB530993_A_mallard_Hokkaido_24_2009_Avian
JX444822_A_mallard_Korea_KU683_2008_Avian
KR265529_A_duck_Japan_9UO025_2009_Avian
GU361160_A_aquatic_bird_Korea_w120_2006_Avian
branch a
FJ461598_A_swine_Korea_C12_2008_Swine
GU361158_A_aquatic_bird_Korea_w113_2006_Avian
GU361159_A_aquatic_bird_Korea_w114_2006_Avian
GU361161_A_aquatic_bird_Korea_w121_2006_Avian
JX570854_A_canvasback_Xianghai_428_2011_Avian
JX570870_A_green_winged_teal_Xianghai_430_2011_Avian

NP-Group4

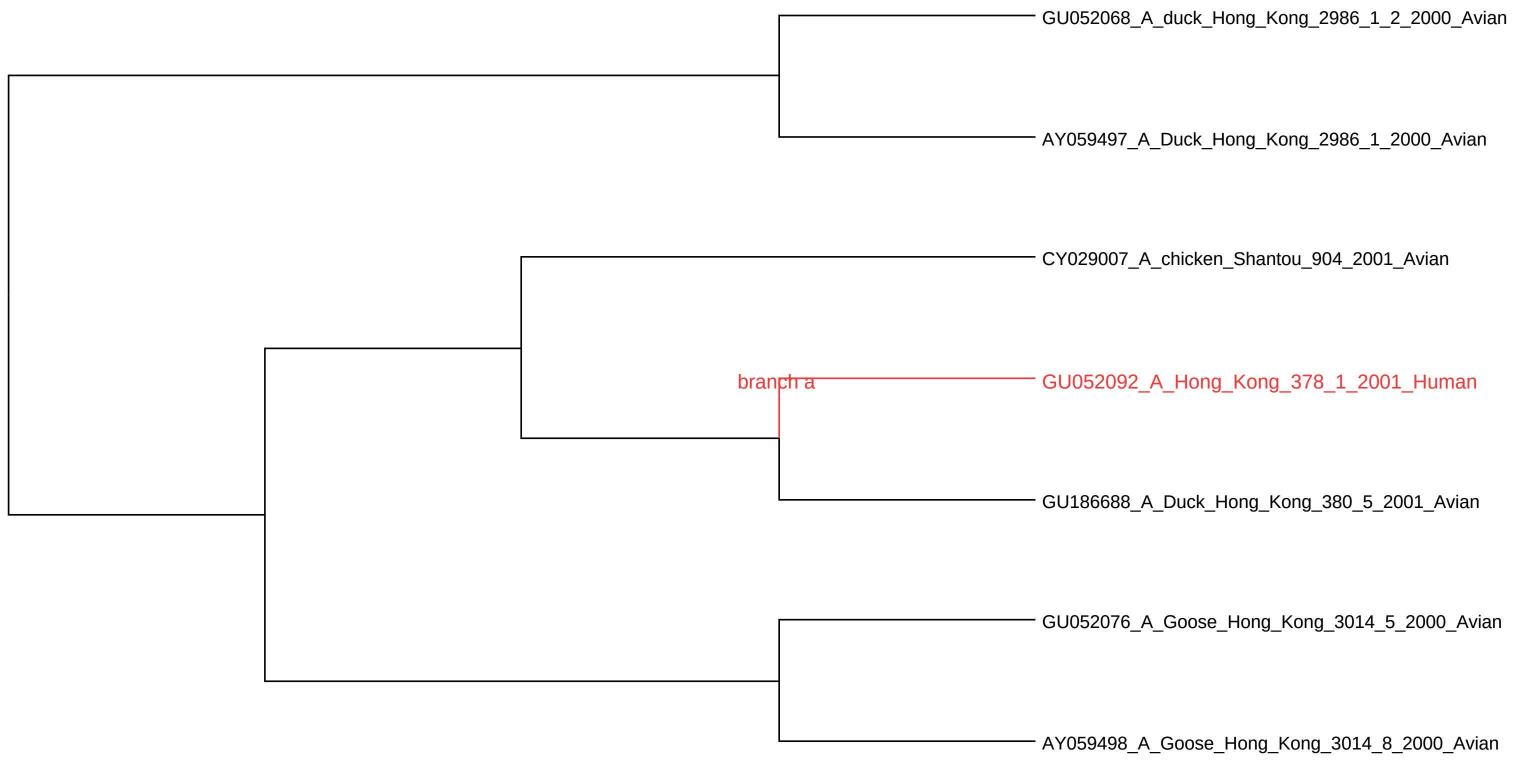
GU052068_A_duck_Hong_Kong_2986_1_2_2000_Avian
AY059497_A_Duck_Hong_Kong_2986_1_2000_Avian
CY029007_A_chicken_Shantou_904_2001_Avian
branch a
GU052092_A_Hong_Kong_378_1_2001_Human
GU186688_A_Duck_Hong_Kong_380_5_2001_Avian
GU052076_A_Goose_Hong_Kong_3014_5_2000_Avian
AY059498_A_Goose_Hong_Kong_3014_8_2000_Avian

NP-Group5

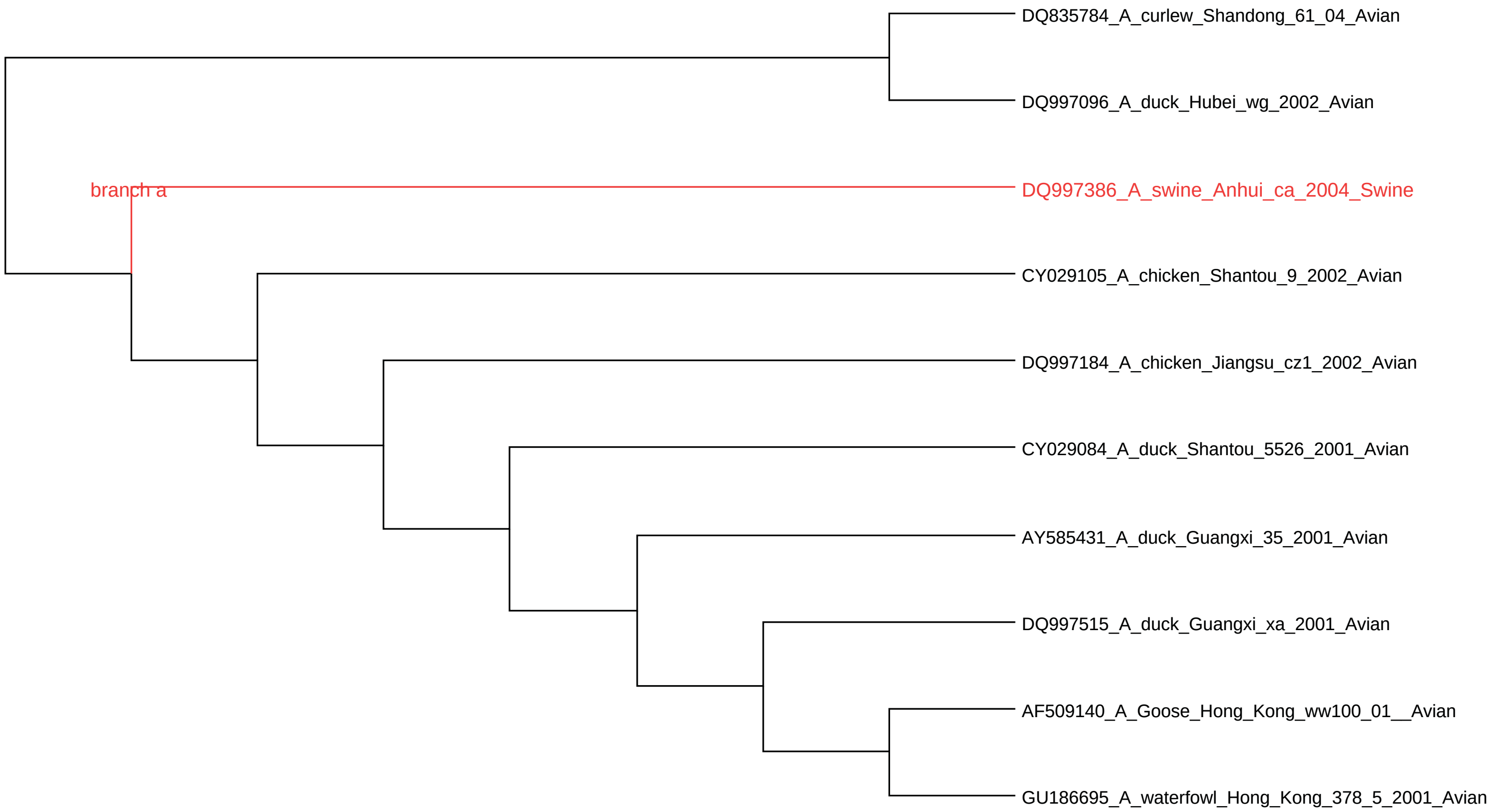
DQ835784_A_curlew_Shandong_61_04_Avian
DQ997096_A_duck_Hubei_wg_2002_Avian
branch a
DQ997386_A_swine_Anhui_ca_2004_Swine
CY029105_A_chicken_Shantou_9_2002_Avian
DQ997184_A_chicken_Jiangsu_cz1_2002_Avian
CY029084_A_duck_Shantou_5526_2001_Avian
AY585431_A_duck_Guangxi_35_2001_Avian
DQ997515_A_duck_Guangxi_xa_2001_Avian
AF509140_A_Goose_Hong_Kong_ww100_01__Avian
GU186695_A_waterfowl_Hong_Kong_378_5_2001_Avian

NP-Group6

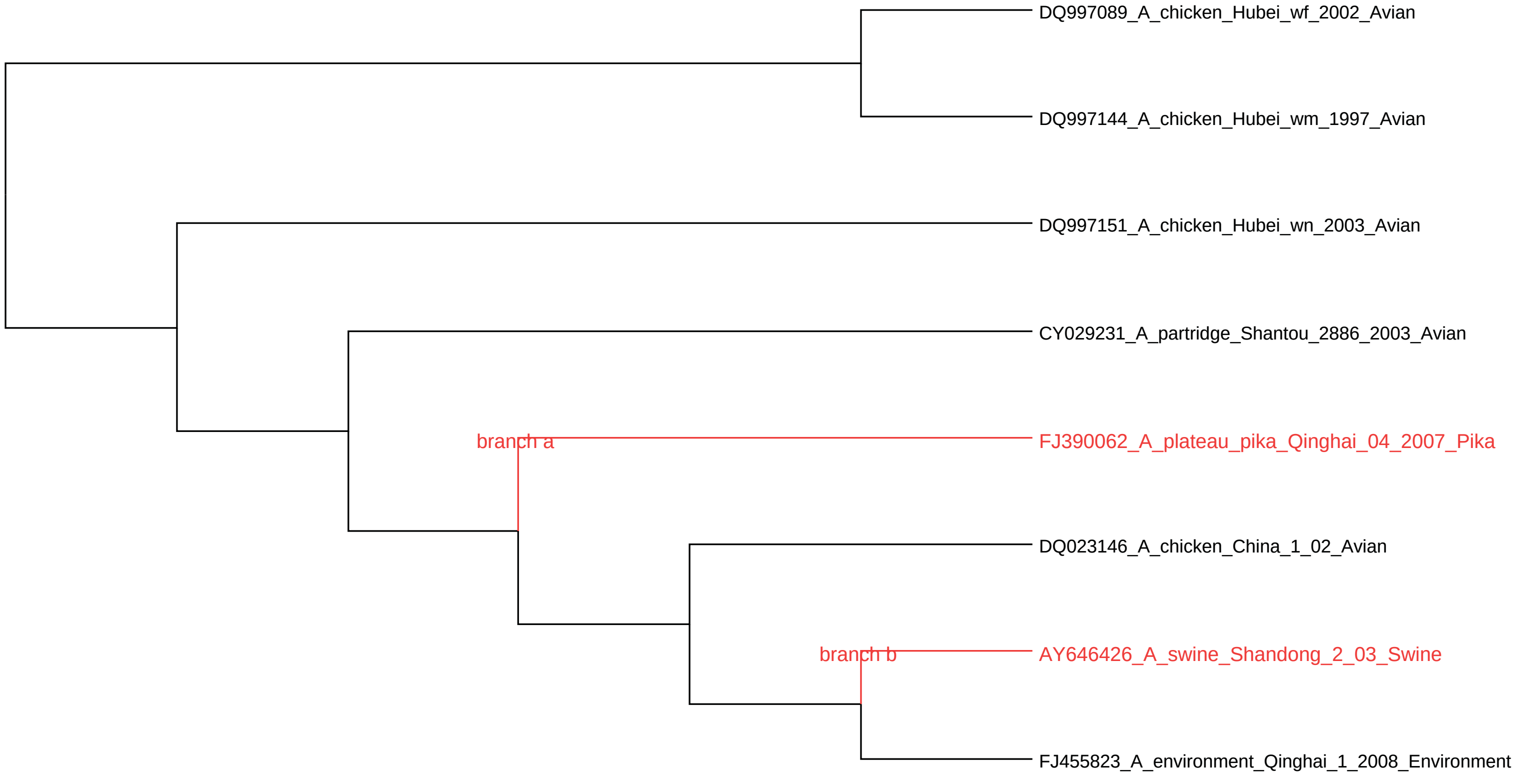
DQ997089_A_chicken_Hubei_wf_2002_Avian
DQ997144_A_chicken_Hubei_wm_1997_Avian
DQ997151_A_chicken_Hubei_wn_2003_Avian
CY029231_A_partridge_Shantou_2886_2003_Avian
branch a
FJ390062_A_plateau_pika_Qinghai_04_2007_Pika
DQ023146_A_chicken_China_1_02_Avian
branch b
AY646426_A_swine_Shandong_2_03_Swine
FJ455823_A_environment_Qinghai_1_2008_Environment

NP-Group7

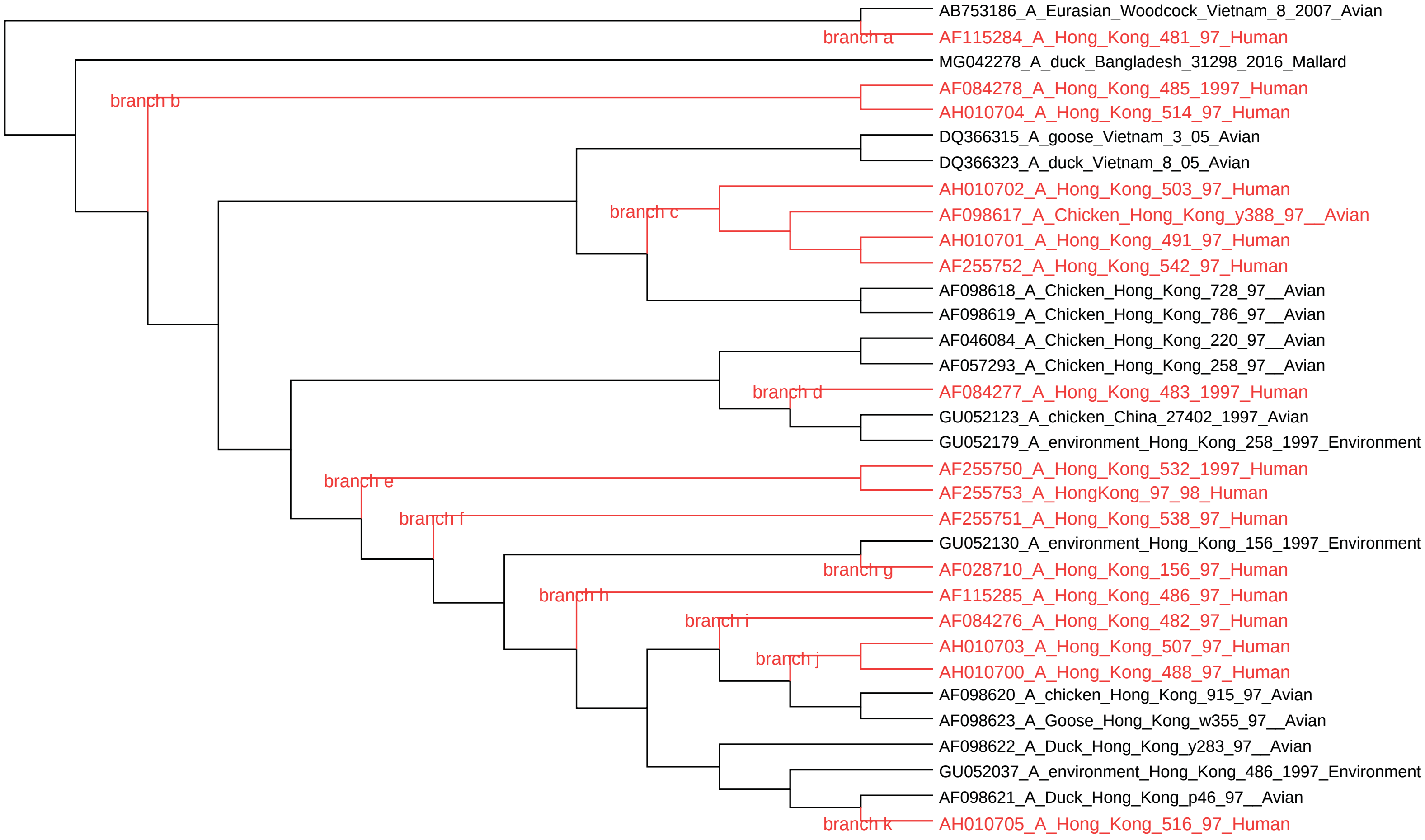

AB753186_A_Eurasian_Woodcock_Vietnam_8_2007_Avian
branch a
AF115284_A_Hong_Kong_481_97_Human
MG042278_A_duck_Bangladesh_31298_2016_Mallard
branch b
AF084278_A_Hong_Kong_485_1997_Human
AH010704_A_Hong_Kong_514_97_Human
DQ366315_A_goose_Vietnam_3_05_Avian
DQ366323_A_duck_Vietnam_8_05_Avian
AH010702_A_Hong_Kong_503_97_Human
branch c
AF098617_A_Chicken_Hong_Kong_y388_97__Avian
AH010701_A_Hong_Kong_491_97_Human
AF255752_A_Hong_Kong_542_97_Human
AF098618_A_Chicken_Hong_Kong_728_97__Avian
AF098619_A_Chicken_Hong_Kong_786_97__Avian
AF046084_A_Chicken_Hong_Kong_220_97__Avian
AF057293_A_Chicken_Hong_Kong_258_97__Avian
branch d
AF084277_A_Hong_Kong_483_1997_Human
GU052123_A_chicken_China_27402_1997_Avian
GU052179_A_environment_Hong_Kong_258_1997_Environment
AF255750_A_Hong_Kong_532_1997_Human
branch e
AF255753_A_HongKong_97_98_Human
branch f
AF255751_A_Hong_Kong_538_97_Human
GU052130_A_environment_Hong_Kong_156_1997_Environment
branch g
AF028710_A_Hong_Kong_156_97_Human
branch h
AF115285_A_Hong_Kong_486_97_Human
branch i
AF084276_A_Hong_Kong_482_97_Human
AH010703_A_Hong_Kong_507_97_Human
branch j
AH010700_A_Hong_Kong_488_97_Human
AF098620_A_chicken_Hong_Kong_915_97_Avian
AF098623_A_Goose_Hong_Kong_w355_97__Avian
AF098622_A_Duck_Hong_Kong_y283_97__Avian
GU052037_A_environment_Hong_Kong_486_1997_Environment
AF098621_A_Duck_Hong_Kong_p46_97__Avian
branch k
AH010705_A_Hong_Kong_516_97_Human

NP-Group8

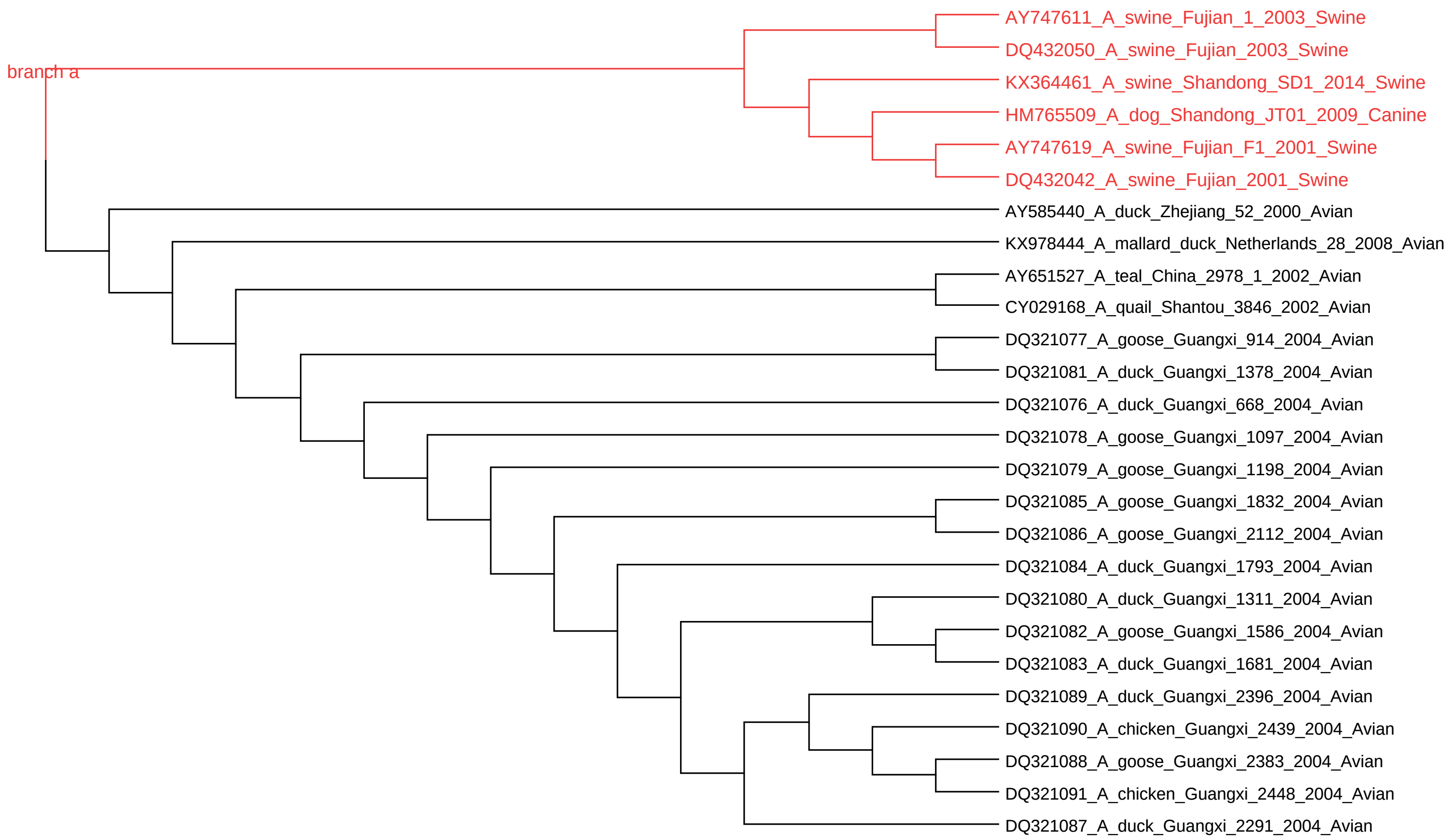
branch a
AY747611_A_swine_Fujian_1_2003_Swine
DQ432050_A_swine_Fujian_2003_Swine
KX364461_A_swine_Shandong_SD1_2014_Swine
HM765509_A_dog_Shandong_JT01_2009_Canine
AY747619_A_swine_Fujian_F1_2001_Swine
DQ432042_A_swine_Fujian_2001_Swine
AY585440_A_duck_Zhejiang_52_2000_Avian
KX978444_A_mallard_duck_Netherlands_28_2008_Avian
AY651527_A_teal_China_2978_1_2002_Avian
CY029168_A_quail_Shantou_3846_2002_Avian
DQ321077_A_goose_Guangxi_914_2004_Avian
DQ321081_A_duck_Guangxi_1378_2004_Avian
DQ321076_A_duck_Guangxi_668_2004_Avian
DQ321078_A_goose_Guangxi_1097_2004_Avian
DQ321079_A_goose_Guangxi_1198_2004_Avian
DQ321085_A_goose_Guangxi_1832_2004_Avian
DQ321086_A_goose_Guangxi_2112_2004_Avian
DQ321084_A_duck_Guangxi_1793_2004_Avian
DQ321080_A_duck_Guangxi_1311_2004_Avian
DQ321082_A_goose_Guangxi_1586_2004_Avian
DQ321083_A_duck_Guangxi_1681_2004_Avian
DQ321089_A_duck_Guangxi_2396_2004_Avian
DQ321090_A_chicken_Guangxi_2439_2004_Avian
DQ321088_A_goose_Guangxi_2383_2004_Avian
DQ321091_A_chicken_Guangxi_2448_2004_Avian
DQ321087_A_duck_Guangxi_2291_2004_Avian

NP-Group9

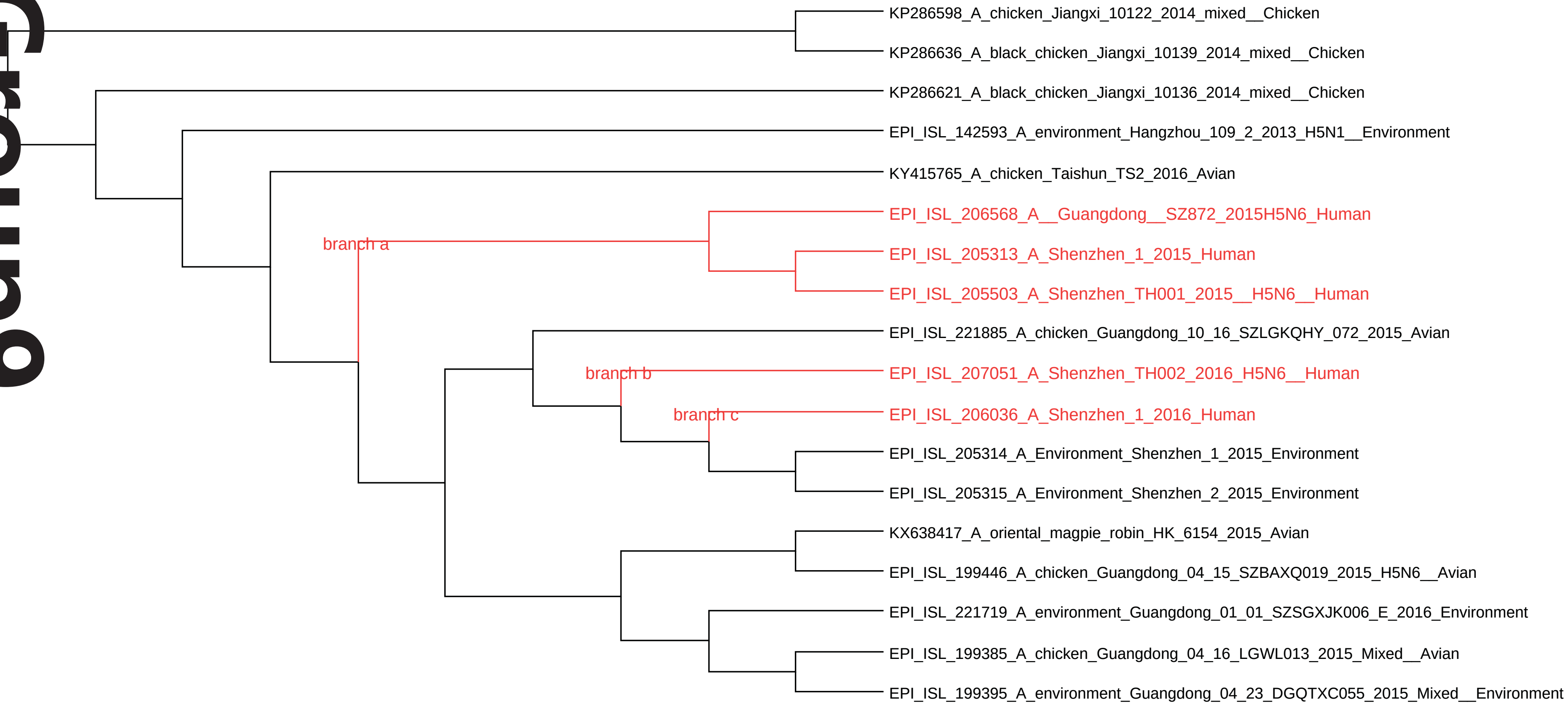
KP286598_A_chicken_Jiangxi_10122_2014_mixed__Chicken
KP286636_A_black_chicken_Jiangxi_10139_2014_mixed__Chicken
KP286621_A_black_chicken_Jiangxi_10136_2014_mixed__Chicken
EPI_ISL_142593_A_environment_Hangzhou_109_2_2013_H5N1__Environment
KY415765_A_chicken_Taishun_TS2_2016_Avian
branch a
EPI_ISL_206568_A__Guangdong__SZ872_2015H5N6_Human
EPI_ISL_205313_A_Shenzhen_1_2015_Human
EPI_ISL_205503_A_Shenzhen_TH001_2015__H5N6__Human
EPI_ISL_221885_A_chicken_Guangdong_10_16_SZLGKQHY_072_2015_Avian
branch b
EPI_ISL_207051_A_Shenzhen_TH002_2016_H5N6__Human
branch c
EPI_ISL_206036_A_Shenzhen_1_2016_Human
EPI_ISL_205314_A_Environment_Shenzhen_1_2015_Environment
EPI_ISL_205315_A_Environment_Shenzhen_2_2015_Environment
KX638417_A_oriental_magpie_robin_HK_6154_2015_Avian
EPI_ISL_199446_A_chicken_Guangdong_04_15_SZBAXQ019_2015_H5N6__Avian
EPI_ISL_221719_A_environment_Guangdong_01_01_SZSGXJK006_E_2016_Environment
EPI_ISL_199385_A_chicken_Guangdong_04_16_LGWL013_2015_Mixed__Avian
EPI_ISL_199395_A_environment_Guangdong_04_23_DGQTXC055_2015_Mixed__Environment

NP-Group10

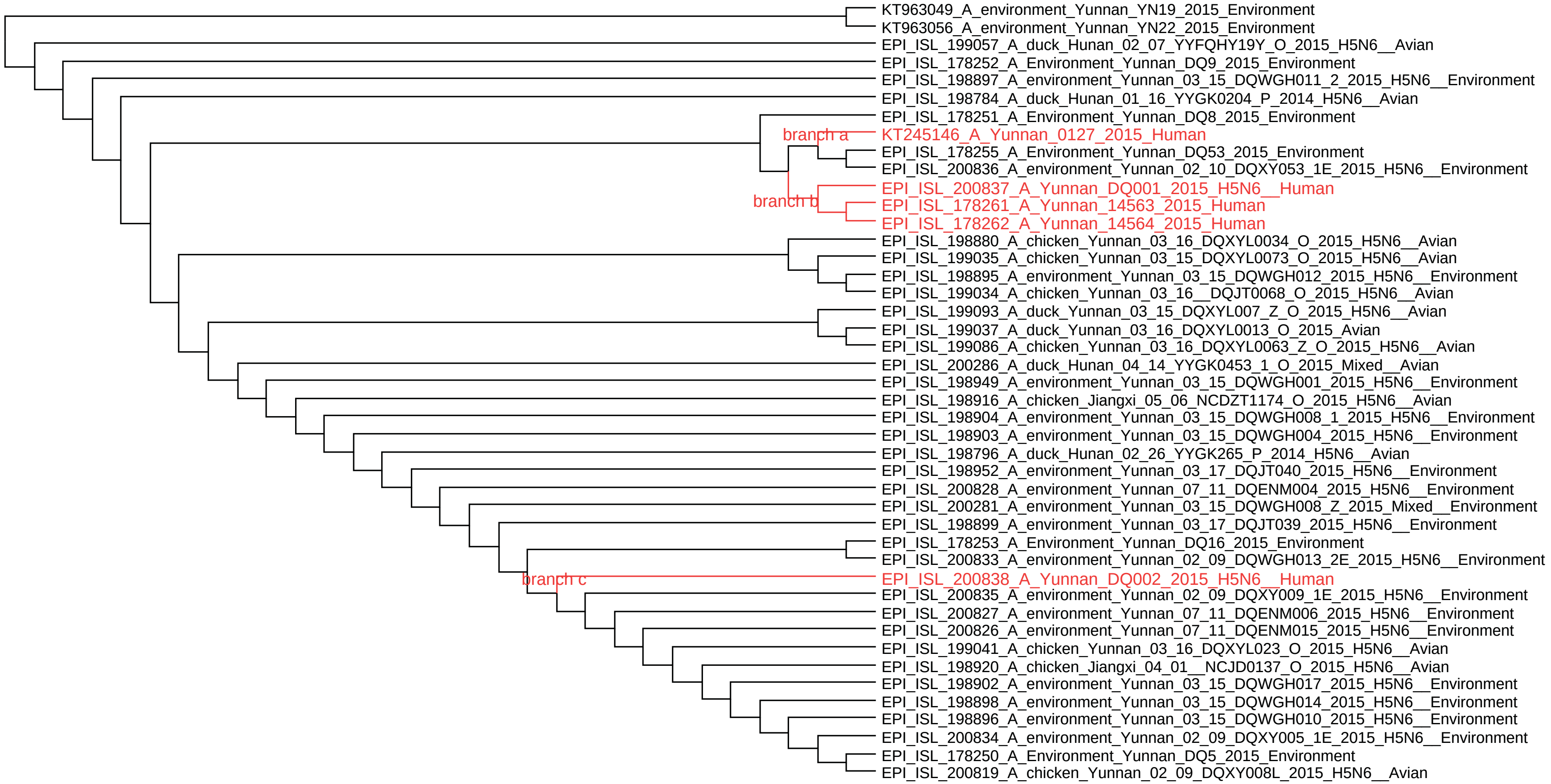
KT963049_A_environment_Yunnan_YN19_2015_Environment
KT963056_A_environment_Yunnan_YN22_2015_Environment
EPI_ISL_199057_A_duck_Hunan_02_07_YYFQHY19Y_O_2015_H5N6__Avian
EPI_ISL_178252_A_Environment_Yunnan_DQ9_2015_Environment
EPI_ISL_198897_A_environment_Yunnan_03_15_DQWGH011_2_2015_H5N6__Environment
EPI_ISL_198784_A_duck_Hunan_01_16_YYGK0204_P_2014_H5N6__Avian
EPI_ISL_178251_A_Environment_Yunnan_DQ8_2015_Environment
branch a
KT245146_A_Yunnan_0127_2015_Human
EPI_ISL_178255_A_Environment_Yunnan_DQ53_2015_Environment
EPI_ISL_200836_A_environment_Yunnan_02_10_DQXY053_1E_2015_H5N6__Environment
EPI_ISL_200837_A_Yunnan_DQ001_2015_H5N6__Human
branch b
EPI_ISL_178261_A_Yunnan_14563_2015_Human
EPI_ISL_178262_A_Yunnan_14564_2015_Human
EPI_ISL_198880_A_chicken_Yunnan_03_16_DQXYL0034_O_2015_H5N6__Avian
EPI_ISL_199035_A_chicken_Yunnan_03_15_DQXYL0073_O_2015_H5N6__Avian
EPI_ISL_198895_A_environment_Yunnan_03_15_DQWGH012_2015_H5N6__Environment
EPI_ISL_199034_A_chicken_Yunnan_03_16__DQJT0068_O_2015_H5N6__Avian
EPI_ISL_199093_A_duck_Yunnan_03_15_DQXYL007_Z_O_2015_H5N6__Avian
EPI_ISL_199037_A_duck_Yunnan_03_16_DQXYL0013_O_2015_Avian
EPI_ISL_199086_A_chicken_Yunnan_03_16_DQXYL0063_Z_O_2015_H5N6__Avian
EPI_ISL_200286_A_duck_Hunan_04_14_YYGK0453_1_O_2015_Mixed__Avian
EPI_ISL_198949_A_environment_Yunnan_03_15_DQWGH001_2015_H5N6__Environment
EPI_ISL_198916_A_chicken_Jiangxi_05_06_NCDZT1174_O_2015_H5N6__Avian
EPI_ISL_198904_A_environment_Yunnan_03_15_DQWGH008_1_2015_H5N6__Environment
EPI_ISL_198903_A_environment_Yunnan_03_15_DQWGH004_2015_H5N6__Environment
EPI_ISL_198796_A_duck_Hunan_02_26_YYGK265_P_2014_H5N6__Avian
EPI_ISL_198952_A_environment_Yunnan_03_17_DQJT040_2015_H5N6__Environment
EPI_ISL_200828_A_environment_Yunnan_07_11_DQENM004_2015_H5N6__Environment
EPI_ISL_200281_A_environment_Yunnan_03_15_DQWGH008_Z_2015_Mixed__Environment
EPI_ISL_198899_A_environment_Yunnan_03_17_DQJT039_2015_H5N6__Environment
EPI_ISL_178253_A_Environment_Yunnan_DQ16_2015_Environment
EPI_ISL_200833_A_environment_Yunnan_02_09_DQWGH013_2E_2015_H5N6__Environment
branch c
EPI_ISL_200838_A_Yunnan_DQ002_2015_H5N6__Human
EPI_ISL_200835_A_environment_Yunnan_02_09_DQXY009_1E_2015_H5N6__Environment
EPI_ISL_200827_A_environment_Yunnan_07_11_DQENM006_2015_H5N6__Environment
EPI_ISL_200826_A_environment_Yunnan_07_11_DQENM015_2015_H5N6__Environment
EPI_ISL_199041_A_chicken_Yunnan_03_16_DQXYL023_O_2015_H5N6__Avian
EPI_ISL_198920_A_chicken_Jiangxi_04_01__NCJD0137_O_2015_H5N6__Avian
EPI_ISL_198902_A_environment_Yunnan_03_15_DQWGH017_2015_H5N6__Environment
EPI_ISL_198898_A_environment_Yunnan_03_15_DQWGH014_2015_H5N6__Environment
EPI_ISL_198896_A_environment_Yunnan_03_15_DQWGH010_2015_H5N6__Environment
EPI_ISL_200834_A_environment_Yunnan_02_09_DQXY005_1E_2015_H5N6__Environment
EPI_ISL_178250_A_Environment_Yunnan_DQ5_2015_Environment
EPI_ISL_200819_A_chicken_Yunnan_02_09_DQXY008L_2015_H5N6__Avian

NP-Group11

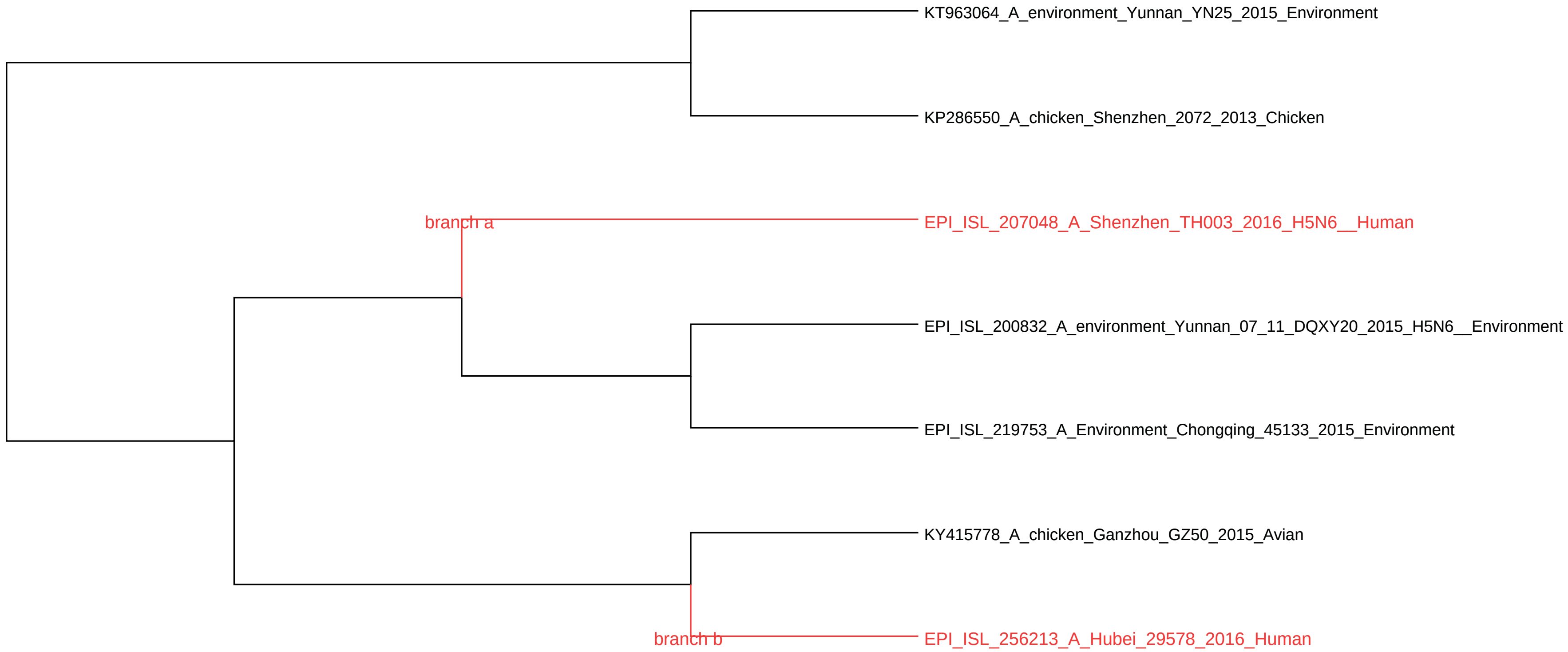

KT963064_A_environment_Yunnan_YN25_2015_Environment
KP286550_A_chicken_Shenzhen_2072_2013_Chicken
branch a
EPI_ISL_207048_A_Shenzhen_TH003_2016_H5N6__Human
EPI_ISL_200832_A_environment_Yunnan_07_11_DQXY20_2015_H5N6__Environment
EPI_ISL_219753_A_Environment_Chongqing_45133_2015_Environment
KY415778_A_chicken_Ganzhou_GZ50_2015_Avian
branch b
EPI_ISL_256213_A_Hubei_29578_2016_Human

NP-Group12

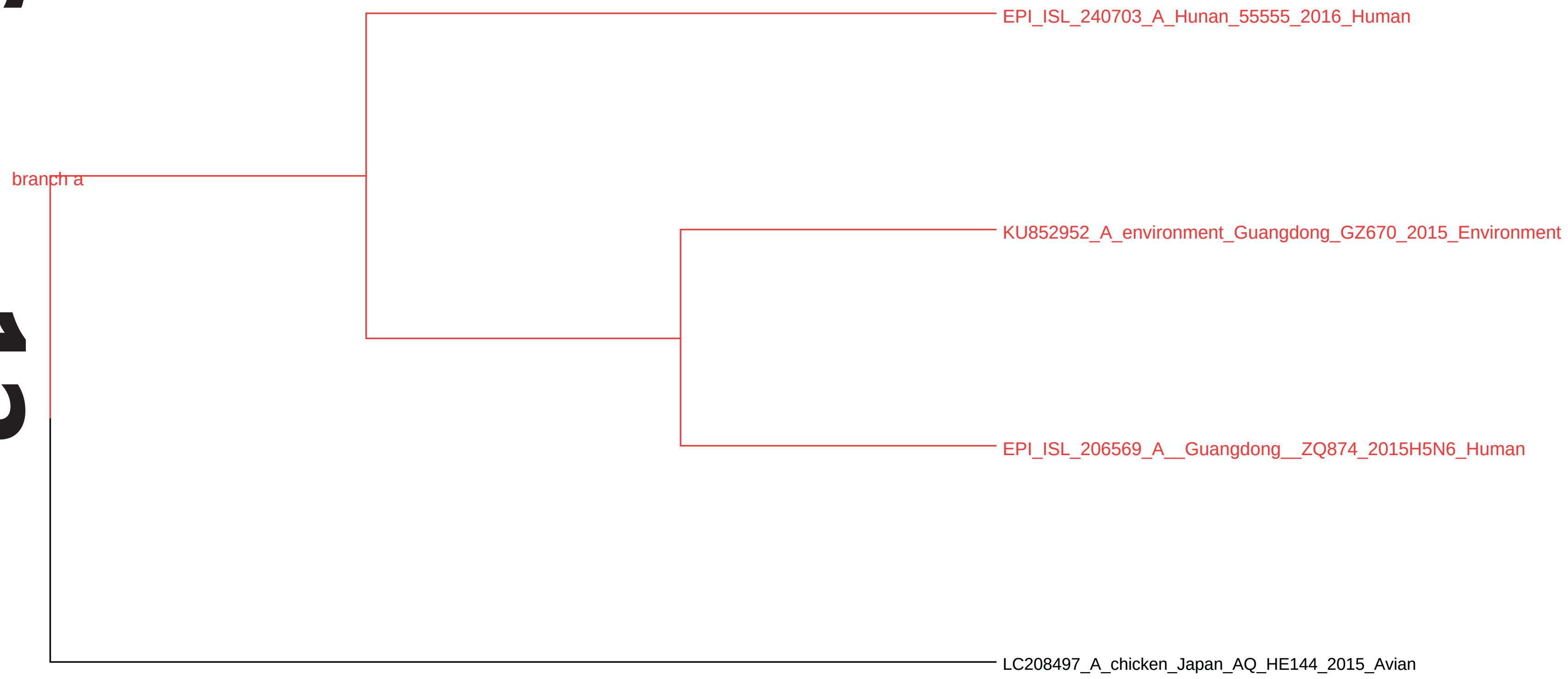

branch a
EPI_ISL_240703_A_Hunan_55555_2016_Human
KU852952_A_environment_Guangdong_GZ670_2015_Environment
EPI_ISL_206569_A__Guangdong__ZQ874_2015H5N6_Human
LC208497_A_chicken_Japan_AQ_HE144_2015_Avian

NP-Group13

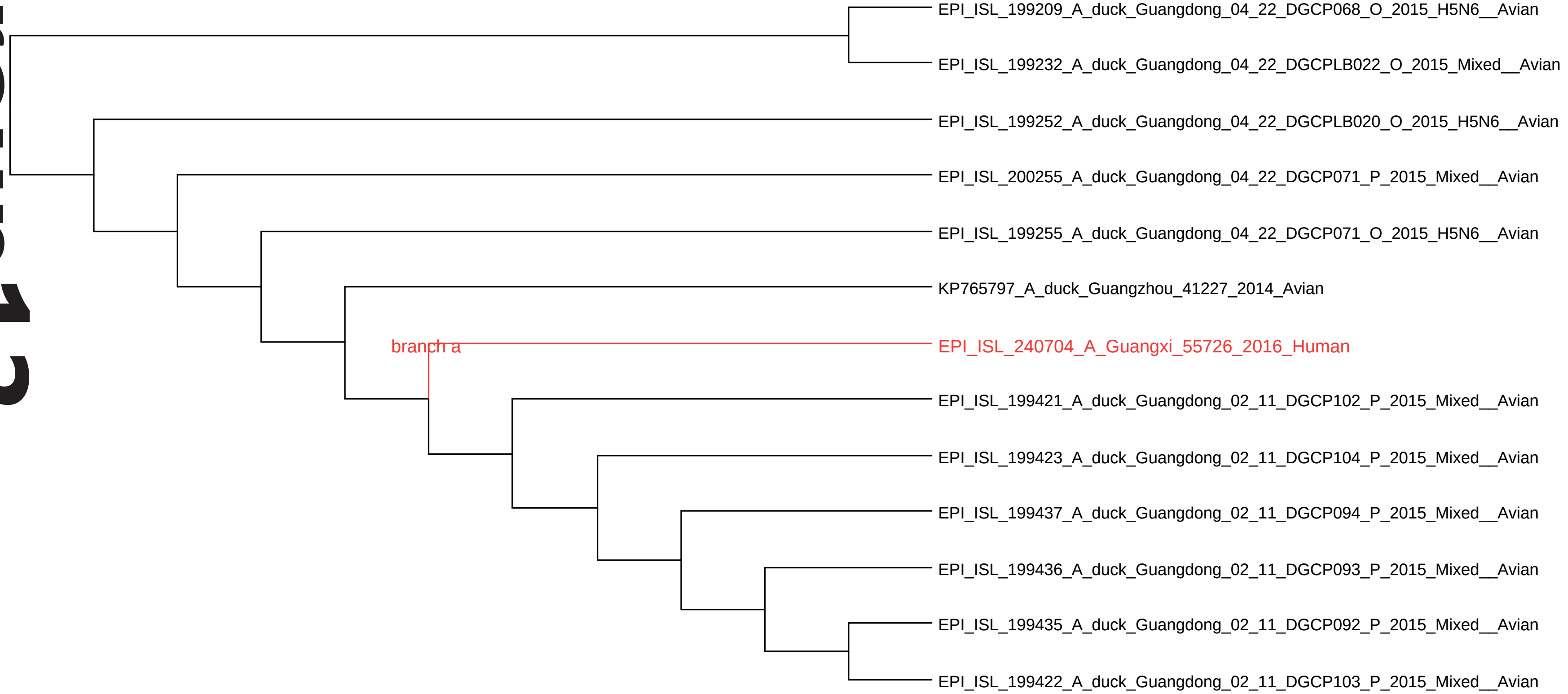
EPI_ISL_199209_A_duck_Guangdong_04_22_DGCP068_O_2015_H5N6__Avian
EPI_ISL_199232_A_duck_Guangdong_04_22_DGCPLB022_O_2015_Mixed__Avian
EPI_ISL_199252_A_duck_Guangdong_04_22_DGCPLB020_O_2015_H5N6__Avian
EPI_ISL_200255_A_duck_Guangdong_04_22_DGCP071_P_2015_Mixed__Avian
EPI_ISL_199255_A_duck_Guangdong_04_22_DGCP071_O_2015_H5N6__Avian
KP765797_A_duck_Guangzhou_41227_2014_Avian
branch a
EPI_ISL_240704_A_Guangxi_55726_2016_Human
EPI_ISL_199421_A_duck_Guangdong_02_11_DGCP102_P_2015_Mixed__Avian
EPI_ISL_199423_A_duck_Guangdong_02_11_DGCP104_P_2015_Mixed__Avian
EPI_ISL_199437_A_duck_Guangdong_02_11_DGCP094_P_2015_Mixed__Avian
EPI_ISL_199436_A_duck_Guangdong_02_11_DGCP093_P_2015_Mixed__Avian
EPI_ISL_199435_A_duck_Guangdong_02_11_DGCP092_P_2015_Mixed__Avian
EPI_ISL_199422_A_duck_Guangdong_02_11_DGCP103_P_2015_Mixed__Avian

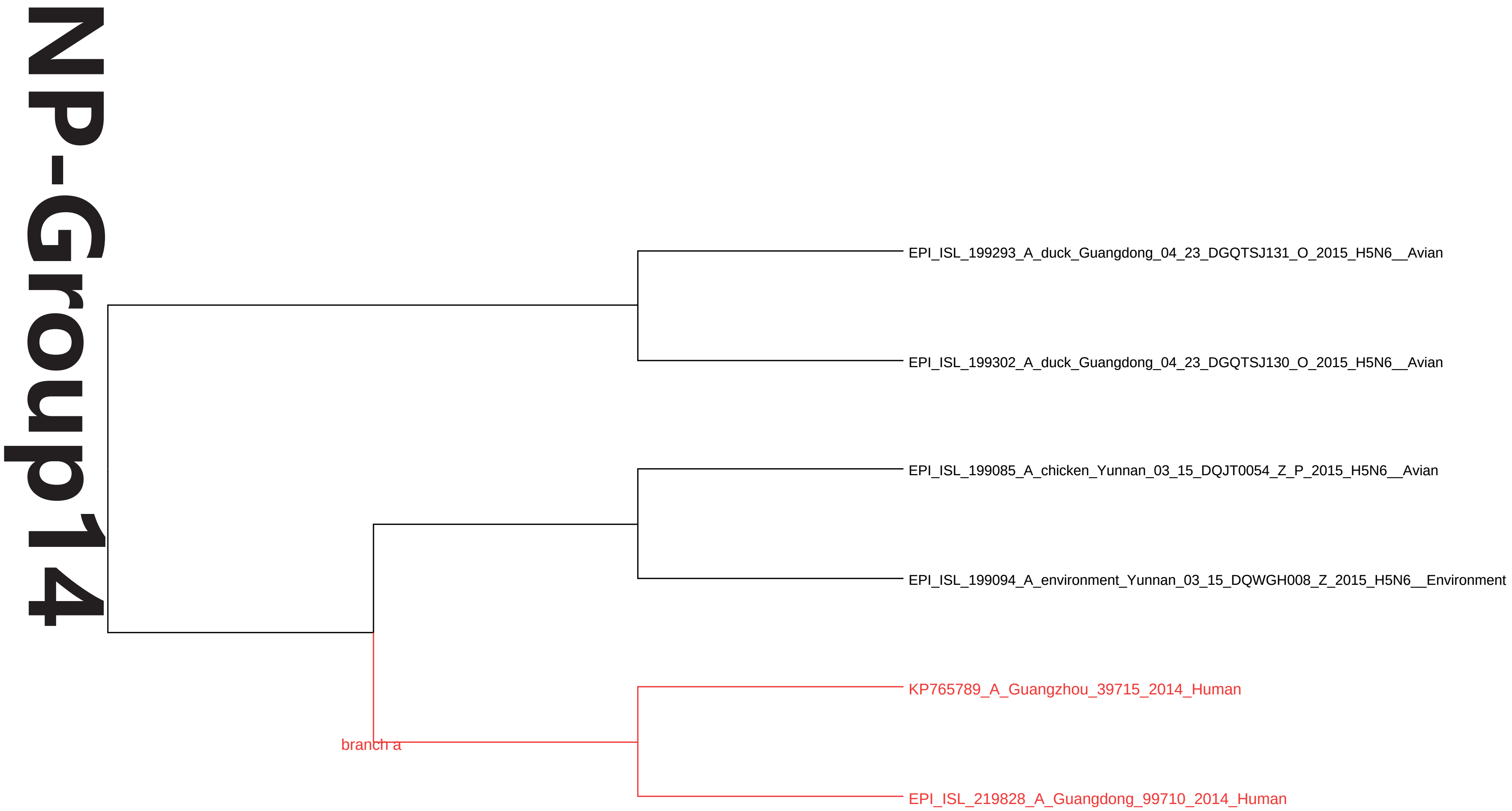
NP-Group14
EPI_ISL_199293_A_duck_Guangdong_04_23_DGQTSJ131_O_2015_H5N6__Avian
EPI_ISL_199302_A_duck_Guangdong_04_23_DGQTSJ130_O_2015_H5N6__Avian
EPI_ISL_199085_A_chicken_Yunnan_03_15_DQJT0054_Z_P_2015_H5N6__Avian
EPI_ISL_199094_A_environment_Yunnan_03_15_DQWGH008_Z_2015_H5N6__Environment
KP765789_A_Guangzhou_39715_2014_Human
branch a
EPI_ISL_219828_A_Guangdong_99710_2014_Human

NP-Group15

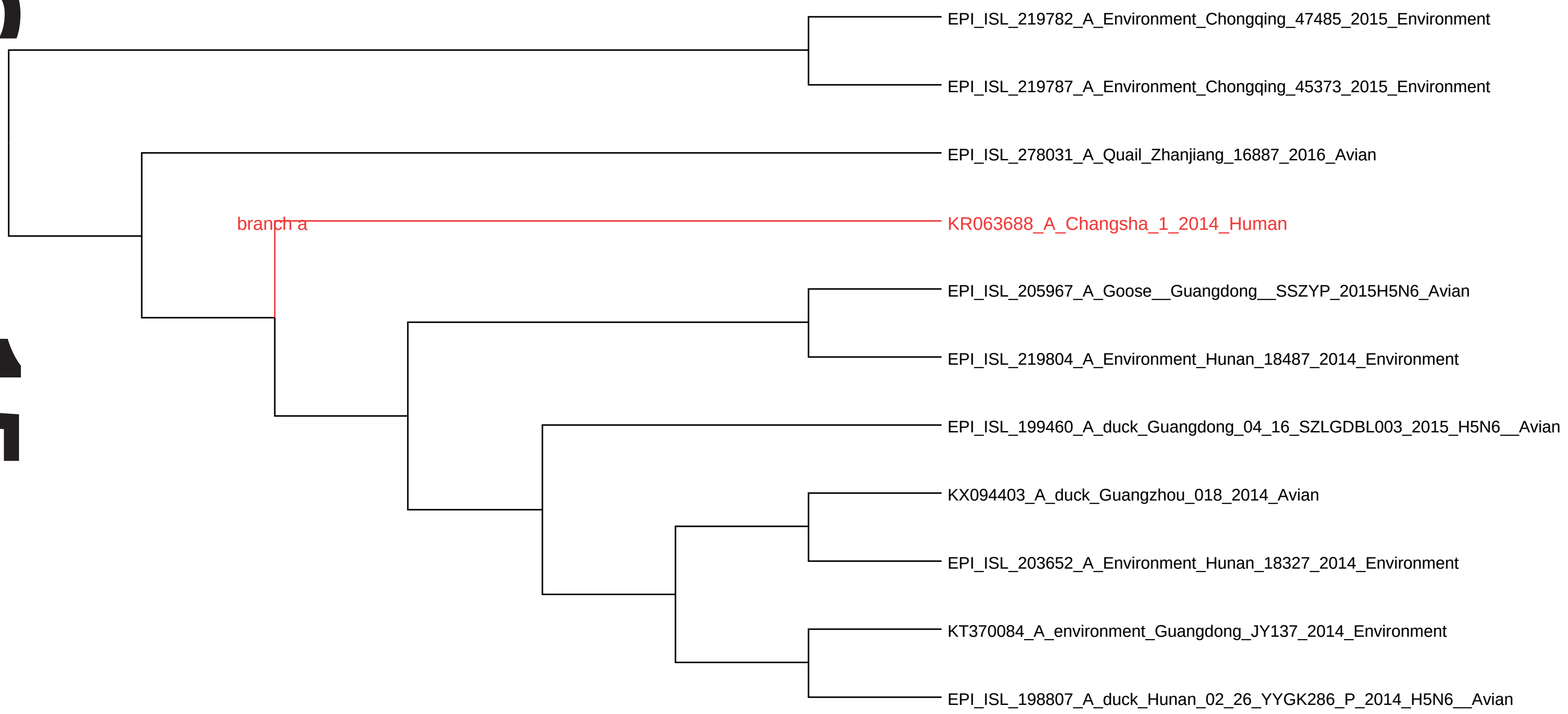
EPI_ISL_219782_A_Environment_Chongqing_47485_2015_Environment
EPI_ISL_219787_A_Environment_Chongqing_45373_2015_Environment
EPI_ISL_278031_A_Quail_Zhanjiang_16887_2016_Avian
branch a
KR063688_A_Changsha_1_2014_Human
EPI_ISL_205967_A_Goose__Guangdong__SSZYP_2015H5N6_Avian
EPI_ISL_219804_A_Environment_Hunan_18487_2014_Environment
EPI_ISL_199460_A_duck_Guangdong_04_16_SZLGDBL003_2015_H5N6__Avian
KX094403_A_duck_Guangzhou_018_2014_Avian
EPI_ISL_203652_A_Environment_Hunan_18327_2014_Environment
KT370084_A_environment_Guangdong_JY137_2014_Environment
EPI_ISL_198807_A_duck_Hunan_02_26_YYGK286_P_2014_H5N6__Avian

NP-Group16

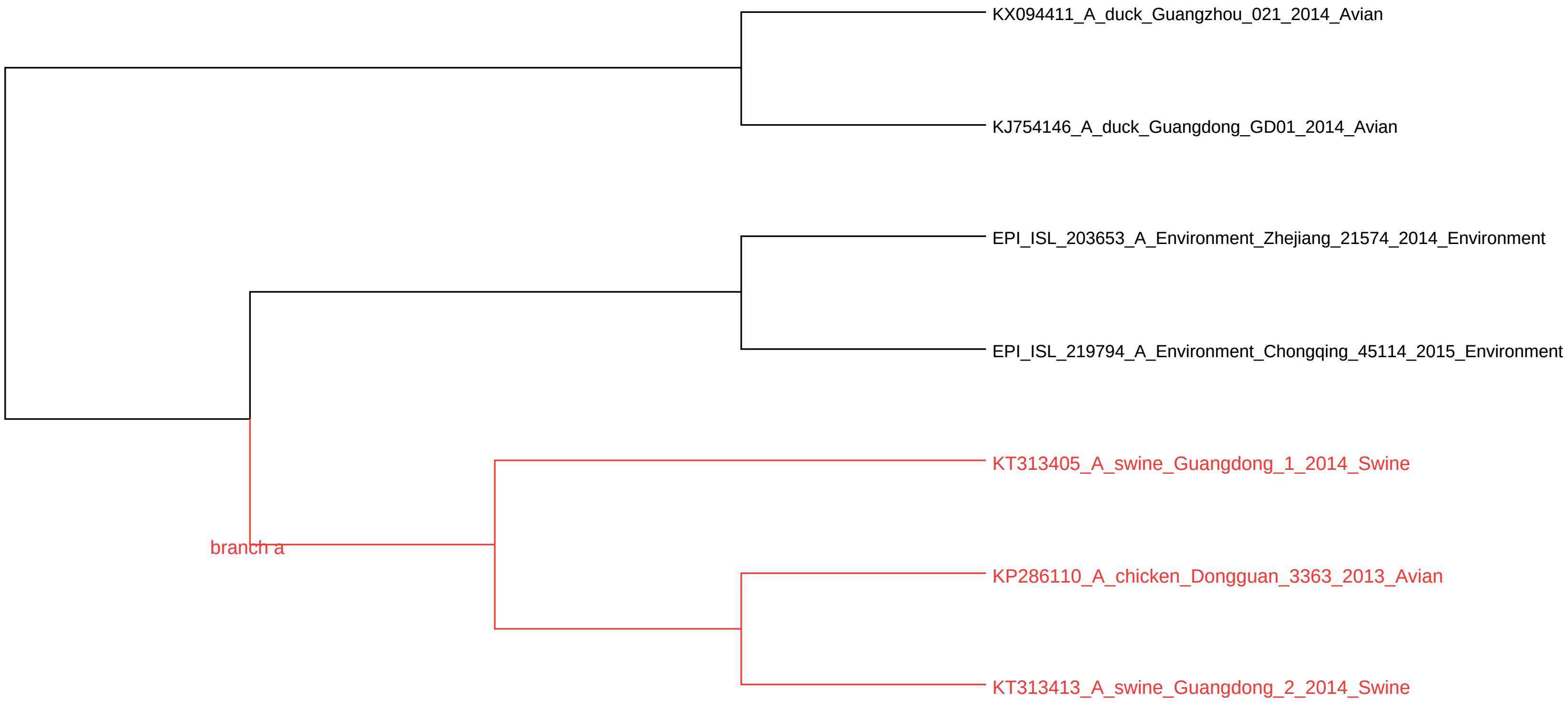
KX094411_A_duck_Guangzhou_021_2014_Avian
KJ754146_A_duck_Guangdong_GD01_2014_Avian
EPI_ISL_203653_A_Environment_Zhejiang_21574_2014_Environment
EPI_ISL_219794_A_Environment_Chongqing_45114_2015_Environment
KT313405_A_swine_Guangdong_1_2014_Swine
branch a
KP286110_A_chicken_Dongguan_3363_2013_Avian
KT313413_A_swine_Guangdong_2_2014_Swine

NP-Group17

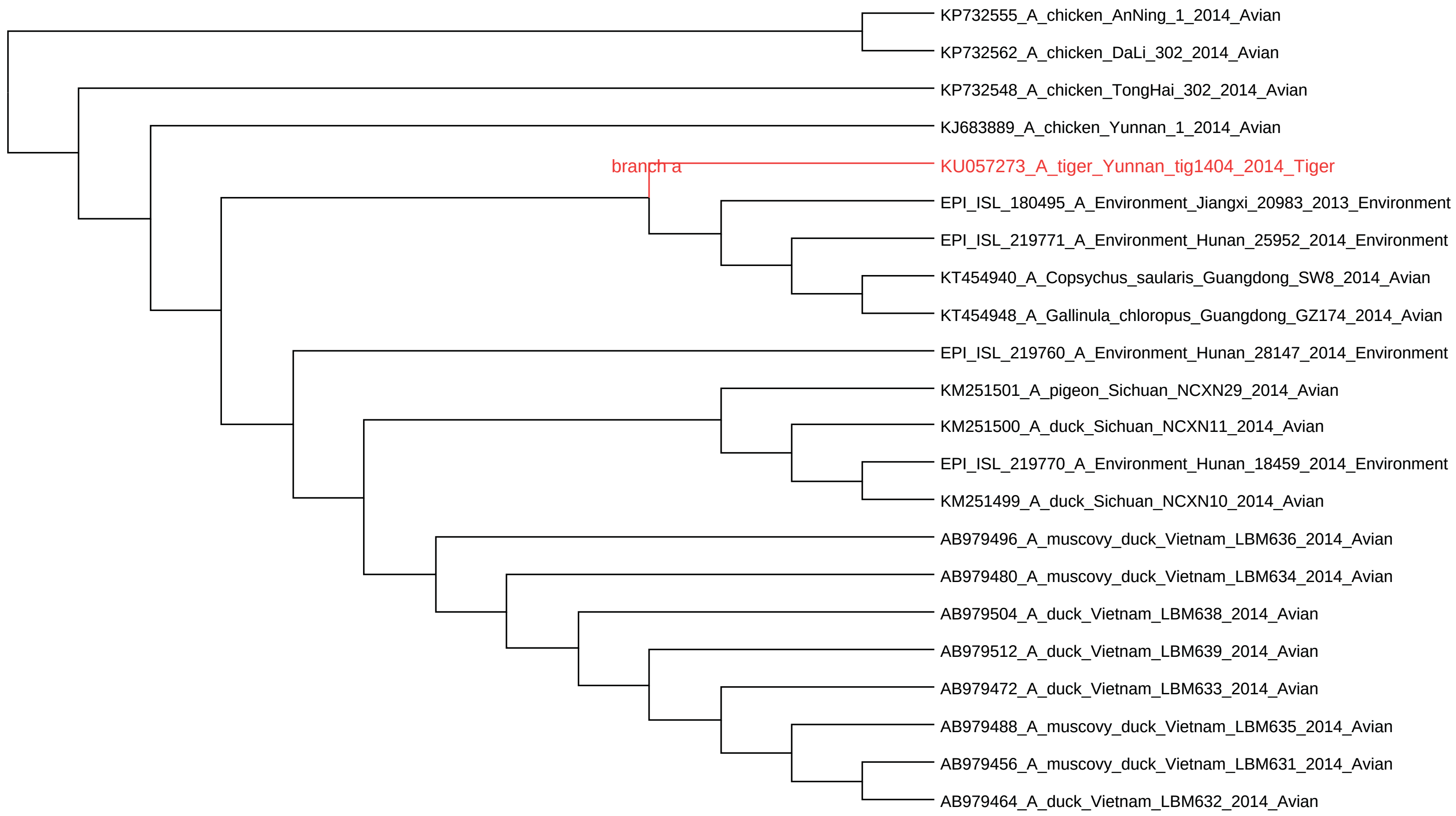
KP732555_A_chicken_AnNing_1_2014_Avian
KP732562_A_chicken_DaLi_302_2014_Avian
KP732548_A_chicken_TongHai_302_2014_Avian
KJ683889_A_chicken_Yunnan_1_2014_Avian
branch a
KU057273_A_tiger_Yunnan_tig1404_2014_Tiger
EPI_ISL_180495_A_Environment_Jiangxi_20983_2013_Environment
EPI_ISL_219771_A_Environment_Hunan_25952_2014_Environment
KT454940_A_Copsychus_saularis_Guangdong_SW8_2014_Avian
KT454948_A_Gallinula_chloropus_Guangdong_GZ174_2014_Avian
EPI_ISL_219760_A_Environment_Hunan_28147_2014_Environment
KM251501_A_pigeon_Sichuan_NCXN29_2014_Avian
KM251500_A_duck_Sichuan_NCXN11_2014_Avian
EPI_ISL_219770_A_Environment_Hunan_18459_2014_Environment
KM251499_A_duck_Sichuan_NCXN10_2014_Avian
AB979496_A_muscovy_duck_Vietnam_LBM636_2014_Avian
AB979480_A_muscovy_duck_Vietnam_LBM634_2014_Avian
AB979504_A_duck_Vietnam_LBM638_2014_Avian
AB979512_A_duck_Vietnam_LBM639_2014_Avian
AB979472_A_duck_Vietnam_LBM633_2014_Avian
AB979488_A_muscovy_duck_Vietnam_LBM635_2014_Avian
AB979456_A_muscovy_duck_Vietnam_LBM631_2014_Avian
AB979464_A_duck_Vietnam_LBM632_2014_Avian

NP-Group18

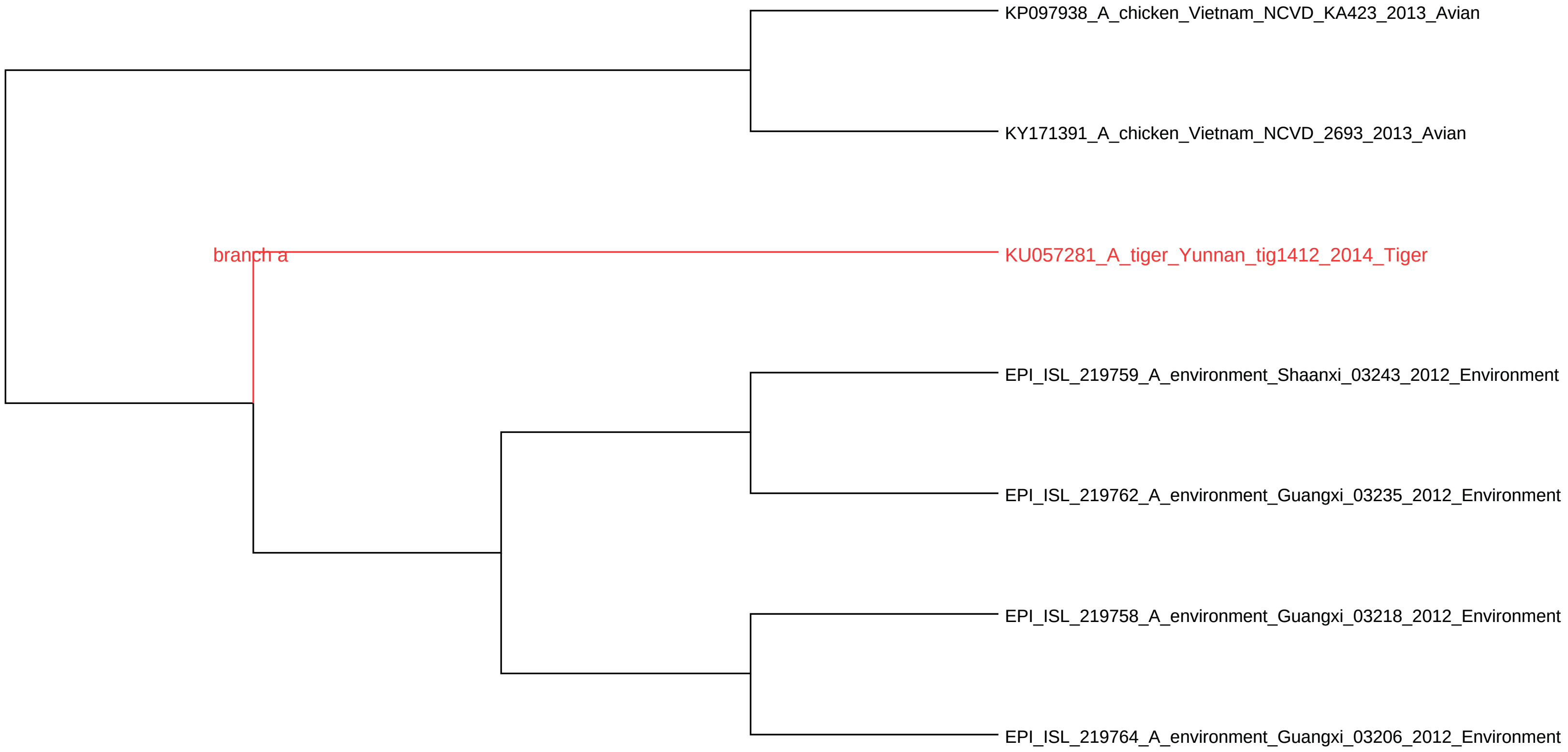
KP097938_A_chicken_Vietnam_NCVD_KA423_2013_Avian
KY171391_A_chicken_Vietnam_NCVD_2693_2013_Avian
branch a
KU057281_A_tiger_Yunnan_tig1412_2014_Tiger
EPI_ISL_219759_A_environment_Shaanxi_03243_2012_Environment
EPI_ISL_219762_A_environment_Guangxi_03235_2012_Environment
EPI_ISL_219758_A_environment_Guangxi_03218_2012_Environment
EPI_ISL_219764_A_environment_Guangxi_03206_2012_Environment

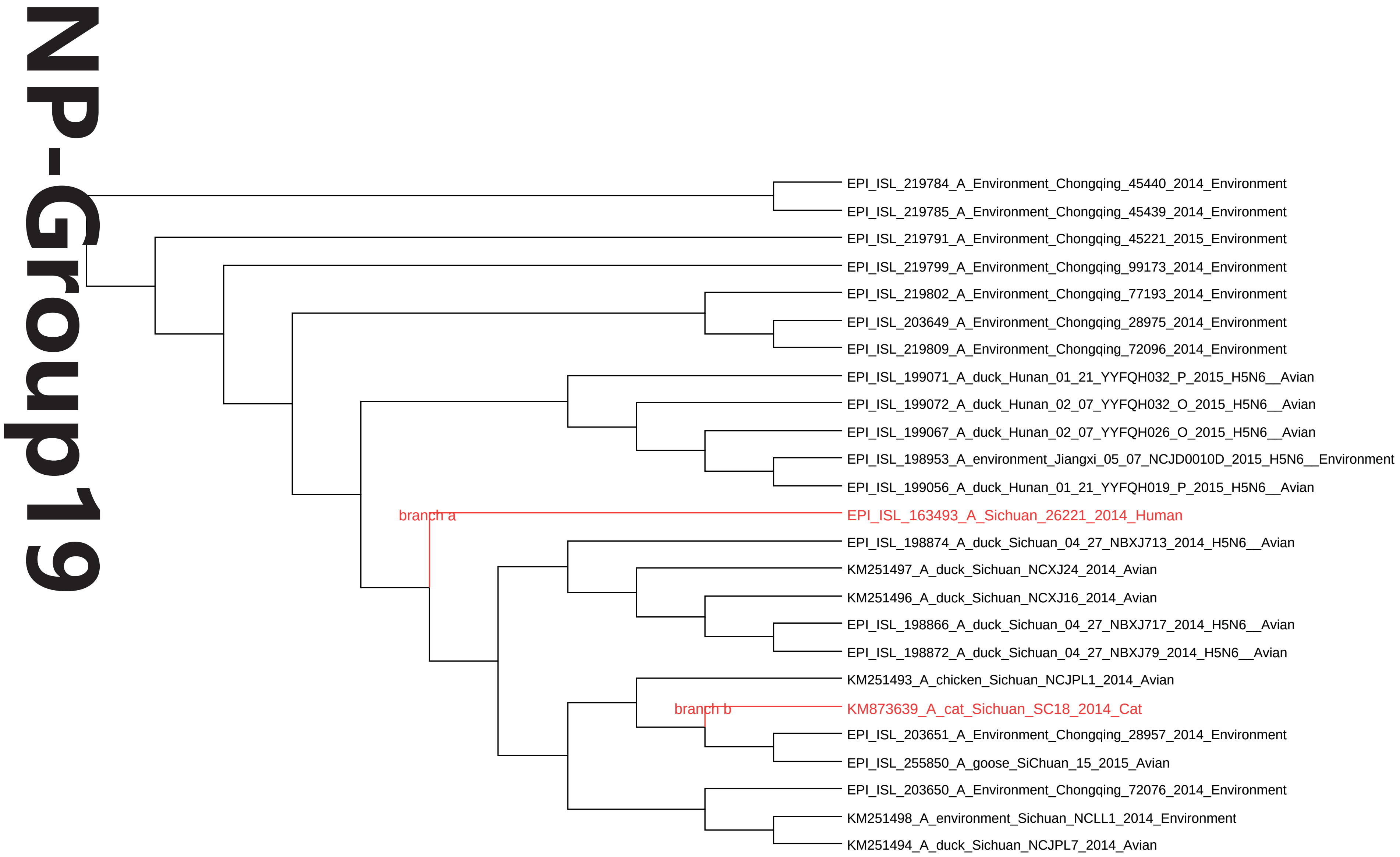
NP-Group19
EPI_ISL_219784_A_Environment_Chongqing_45440_2014_Environment
EPI_ISL_219785_A_Environment_Chongqing_45439_2014_Environment
EPI_ISL_219791_A_Environment_Chongqing_45221_2015_Environment
EPI_ISL_219799_A_Environment_Chongqing_99173_2014_Environment
EPI_ISL_219802_A_Environment_Chongqing_77193_2014_Environment
EPI_ISL_203649_A_Environment_Chongqing_28975_2014_Environment
EPI_ISL_219809_A_Environment_Chongqing_72096_2014_Environment
EPI_ISL_199071_A_duck_Hunan_01_21_YYFQH032_P_2015_H5N6__Avian
EPI_ISL_199072_A_duck_Hunan_02_07_YYFQH032_O_2015_H5N6__Avian
EPI_ISL_199067_A_duck_Hunan_02_07_YYFQH026_O_2015_H5N6__Avian
EPI_ISL_198953_A_environment_Jiangxi_05_07_NCJD0010D_2015_H5N6__Environment
EPI_ISL_199056_A_duck_Hunan_01_21_YYFQH019_P_2015_H5N6__Avian
branch a
EPI_ISL_163493_A_Sichuan_26221_2014_Human
EPI_ISL_198874_A_duck_Sichuan_04_27_NBXJ713_2014_H5N6__Avian
KM251497_A_duck_Sichuan_NCXJ24_2014_Avian
KM251496_A_duck_Sichuan_NCXJ16_2014_Avian
EPI_ISL_198866_A_duck_Sichuan_04_27_NBXJ717_2014_H5N6__Avian
EPI_ISL_198872_A_duck_Sichuan_04_27_NBXJ79_2014_H5N6__Avian
KM251493_A_chicken_Sichuan_NCJPL1_2014_Avian
branch b
KM873639_A_cat_Sichuan_SC18_2014_Cat
EPI_ISL_203651_A_Environment_Chongqing_28957_2014_Environment
EPI_ISL_255850_A_goose_SiChuan_15_2015_Avian
EPI_ISL_203650_A_Environment_Chongqing_72076_2014_Environment
KM251498_A_environment_Sichuan_NCLL1_2014_Environment
KM251494_A_duck_Sichuan_NCJPL7_2014_Avian

NP-Group20

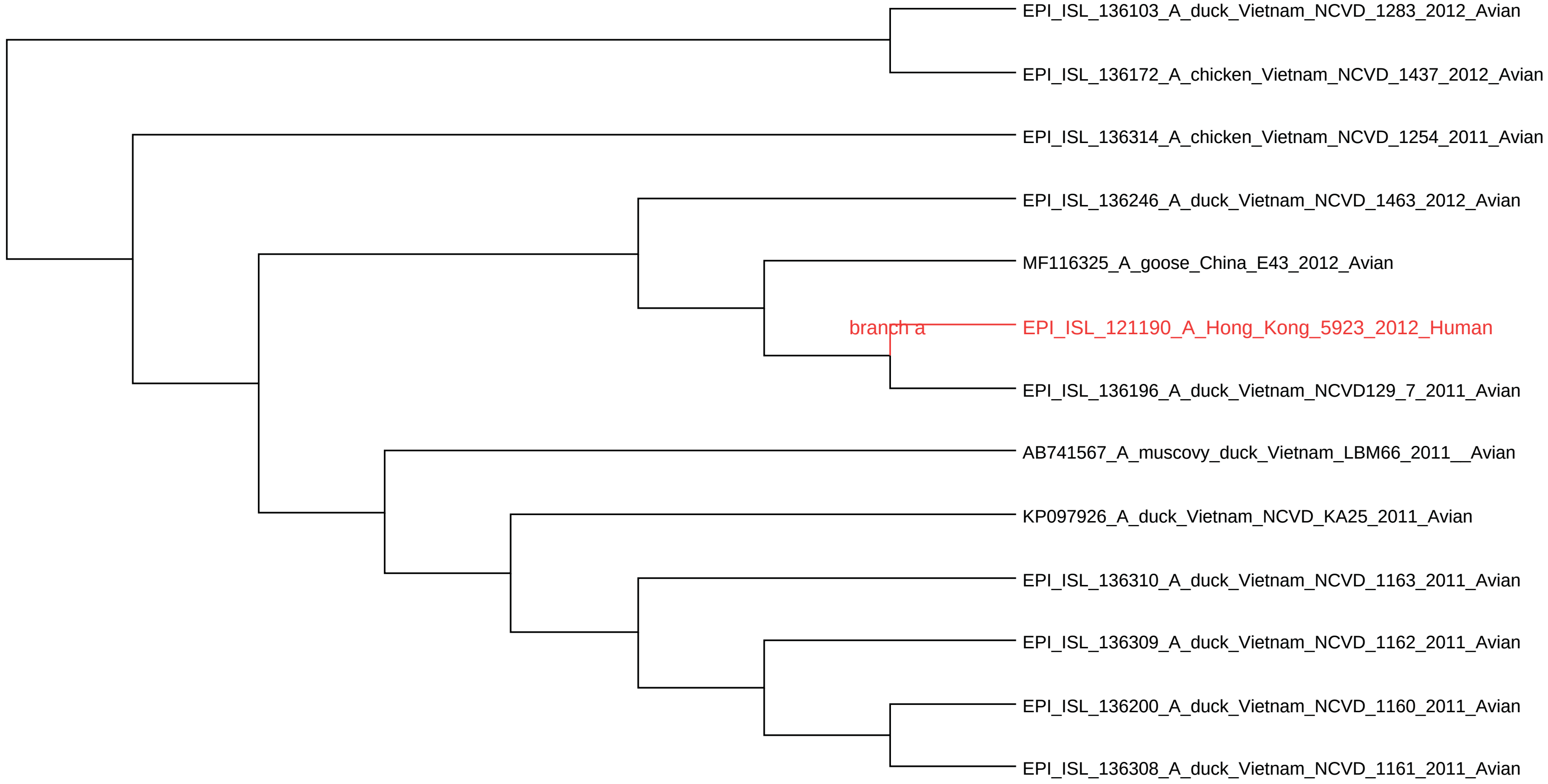
EPI_ISL_136103_A_duck_Vietnam_NCVD_1283_2012_Avian
EPI_ISL_136172_A_chicken_Vietnam_NCVD_1437_2012_Avian
EPI_ISL_136314_A_chicken_Vietnam_NCVD_1254_2011_Avian
EPI_ISL_136246_A_duck_Vietnam_NCVD_1463_2012_Avian
MF116325_A_goose_China_E43_2012_Avian
branch a
EPI_ISL_121190_A_Hong_Kong_5923_2012_Human
EPI_ISL_136196_A_duck_Vietnam_NCVD129_7_2011_Avian
AB741567_A_muscovy_duck_Vietnam_LBM66_2011__Avian
KP097926_A_duck_Vietnam_NCVD_KA25_2011_Avian
EPI_ISL_136310_A_duck_Vietnam_NCVD_1163_2011_Avian
EPI_ISL_136309_A_duck_Vietnam_NCVD_1162_2011_Avian
EPI_ISL_136200_A_duck_Vietnam_NCVD_1160_2011_Avian
EPI_ISL_136308_A_duck_Vietnam_NCVD_1161_2011_Avian

NP-Group21

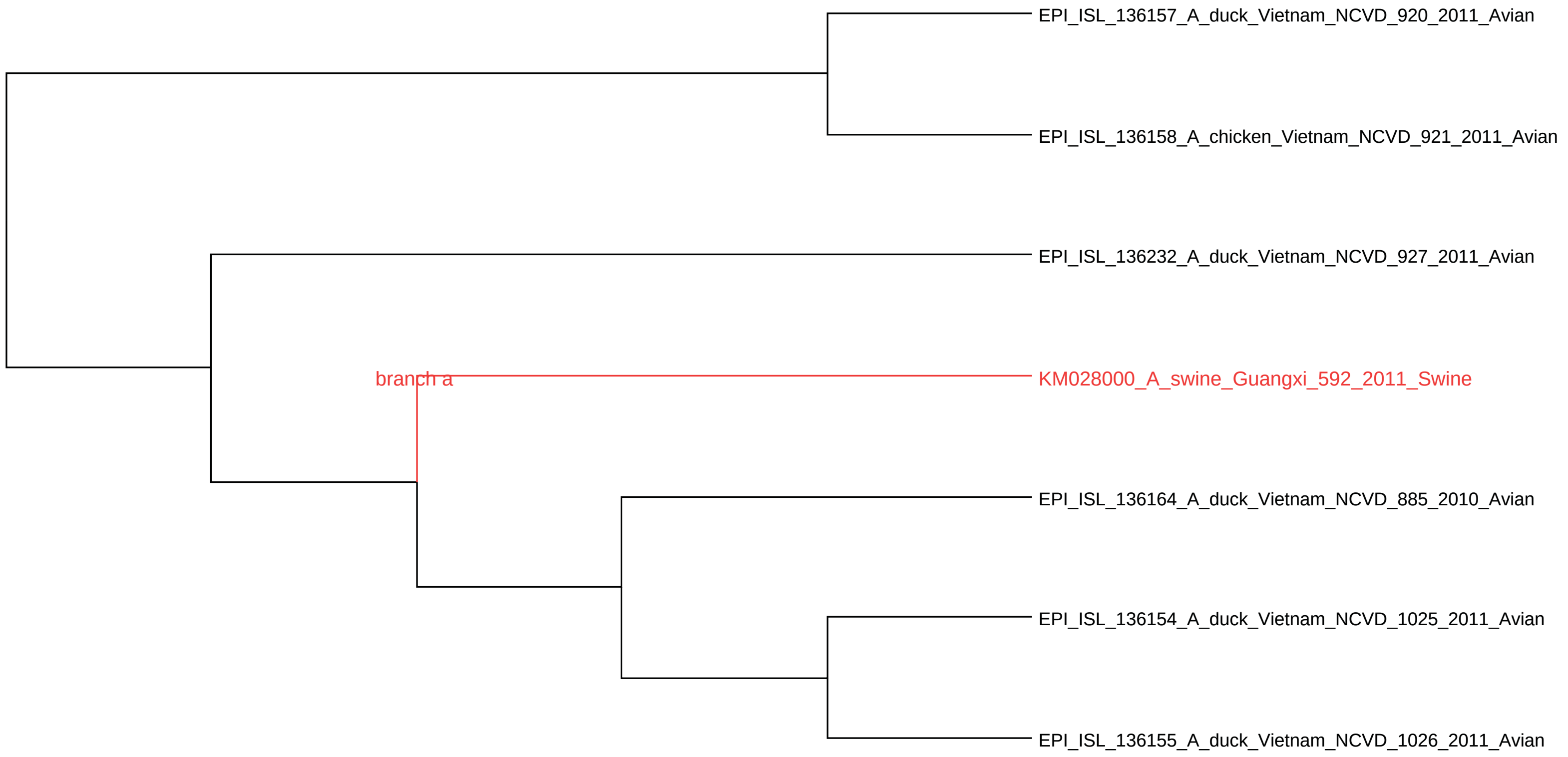
EPI_ISL_136157_A_duck_Vietnam_NCVD_920_2011_Avian
EPI_ISL_136158_A_chicken_Vietnam_NCVD_921_2011_Avian
EPI_ISL_136232_A_duck_Vietnam_NCVD_927_2011_Avian
branch a
KM028000_A_swine_Guangxi_592_2011_Swine
EPI_ISL_136164_A_duck_Vietnam_NCVD_885_2010_Avian
EPI_ISL_136154_A_duck_Vietnam_NCVD_1025_2011_Avian
EPI_ISL_136155_A_duck_Vietnam_NCVD_1026_2011_Avian

NP-Group22

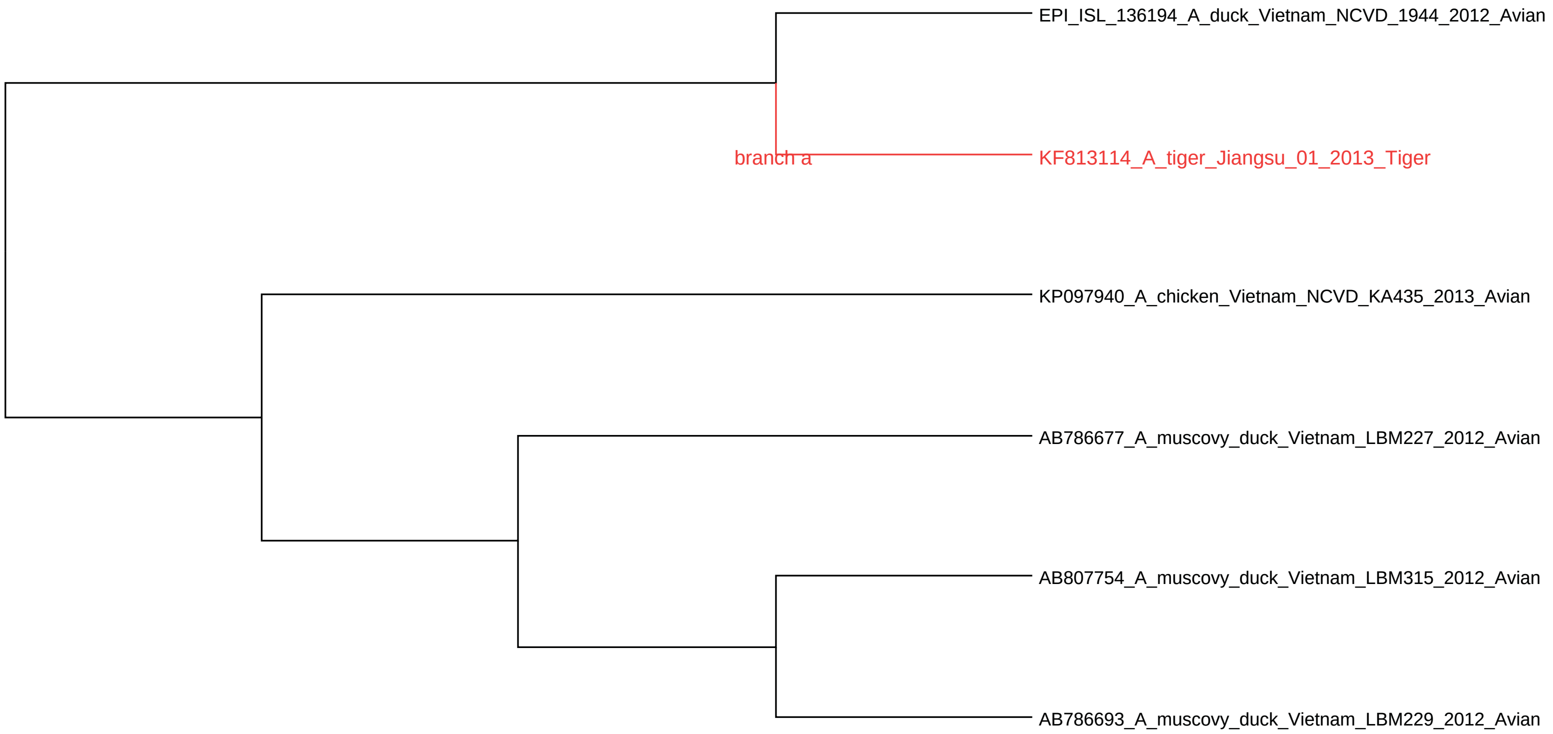
EPI_ISL_136194_A_duck_Vietnam_NCVD_1944_2012_Avian
branch a
KF813114_A_tiger_Jiangsu_01_2013_Tiger
KP097940_A_chicken_Vietnam_NCVD_KA435_2013_Avian
AB786677_A_muscovy_duck_Vietnam_LBM227_2012_Avian
AB807754_A_muscovy_duck_Vietnam_LBM315_2012_Avian
AB786693_A_muscovy_duck_Vietnam_LBM229_2012_Avian

NP-Group23

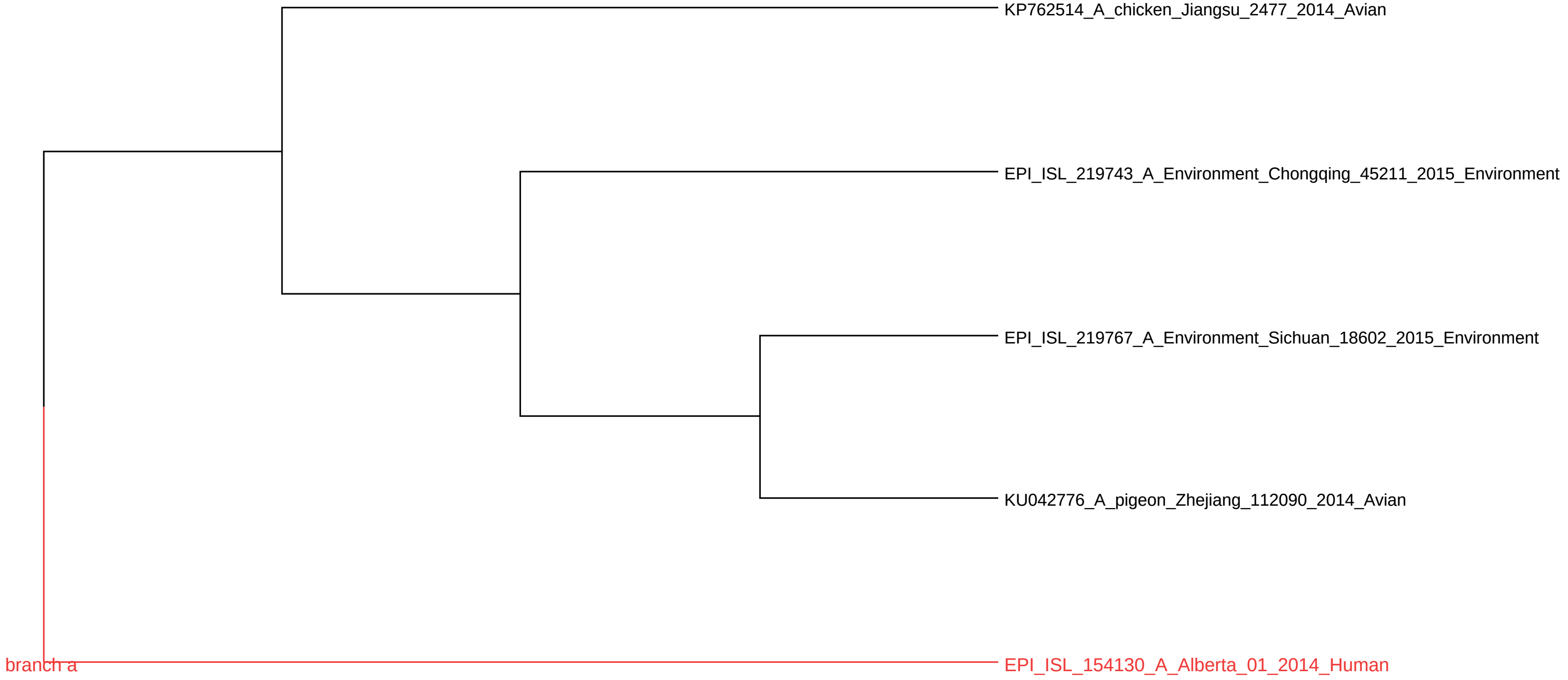
KP762514_A_chicken_Jiangsu_2477_2014_Avian
EPI_ISL_219743_A_Environment_Chongqing_45211_2015_Environment
EPI_ISL_219767_A_Environment_Sichuan_18602_2015_Environment
KU042776_A_pigeon_Zhejiang_112090_2014_Avian
branch a
EPI_ISL_154130_A_Alberta_01_2014_Human

NP-Group24

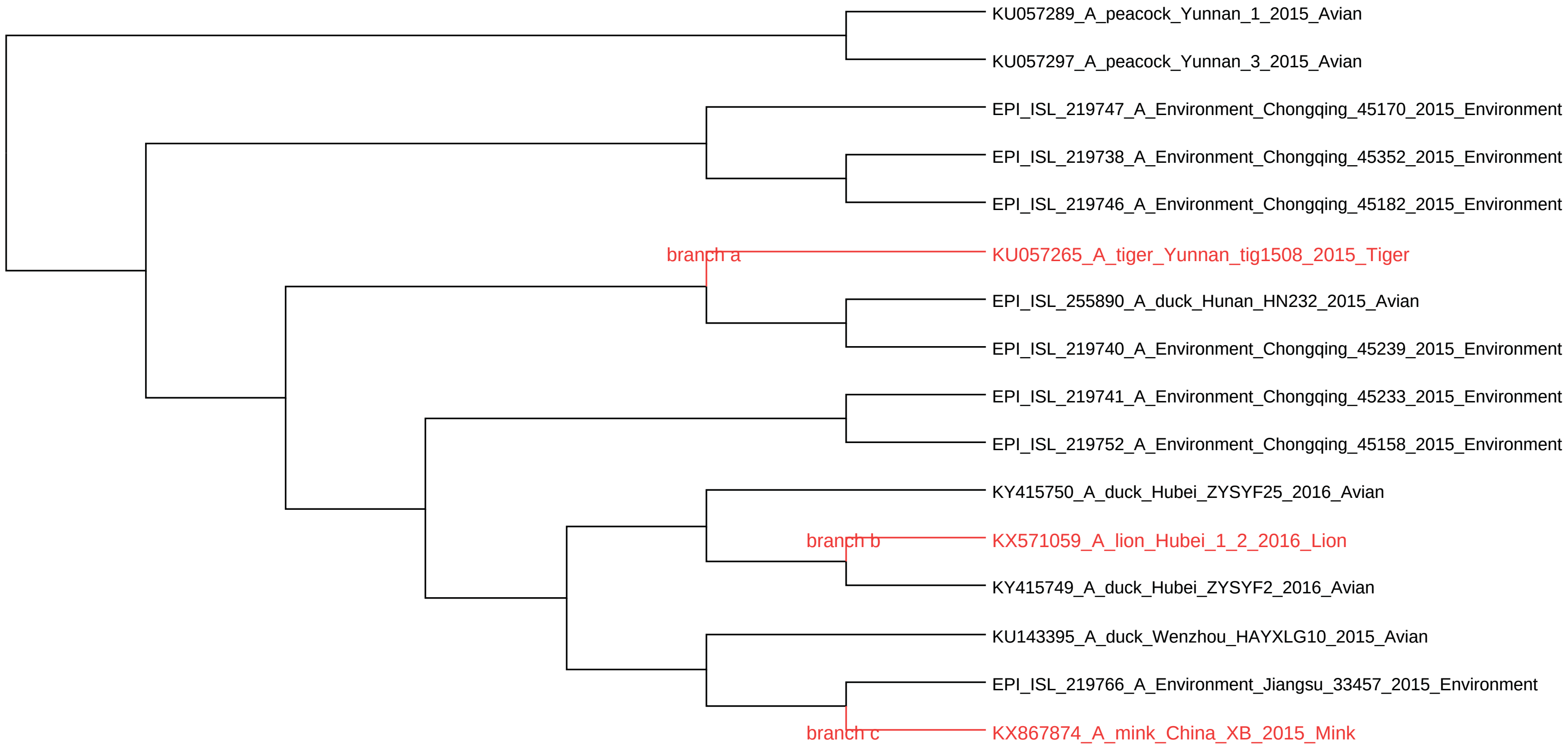
KU057289_A_peacock_Yunnan_1_2015_Avian
KU057297_A_peacock_Yunnan_3_2015_Avian
EPI_ISL_219747_A_Environment_Chongqing_45170_2015_Environment
EPI_ISL_219738_A_Environment_Chongqing_45352_2015_Environment
EPI_ISL_219746_A_Environment_Chongqing_45182_2015_Environment
branch a
KU057265_A_tiger_Yunnan_tig1508_2015_Tiger
EPI_ISL_255890_A_duck_Hunan_HN232_2015_Avian
EPI_ISL_219740_A_Environment_Chongqing_45239_2015_Environment
EPI_ISL_219741_A_Environment_Chongqing_45233_2015_Environment
EPI_ISL_219752_A_Environment_Chongqing_45158_2015_Environment
KY415750_A_duck_Hubei_ZYSYF25_2016_Avian
branch b
KX571059_A_lion_Hubei_1_2_2016_Lion
KY415749_A_duck_Hubei_ZYSYF2_2016_Avian
KU143395_A_duck_Wenzhou_HAYXLG10_2015_Avian
EPI_ISL_219766_A_Environment_Jiangsu_33457_2015_Environment
branch c
KX867874_A_mink_China_XB_2015_Mink

NP-Group25

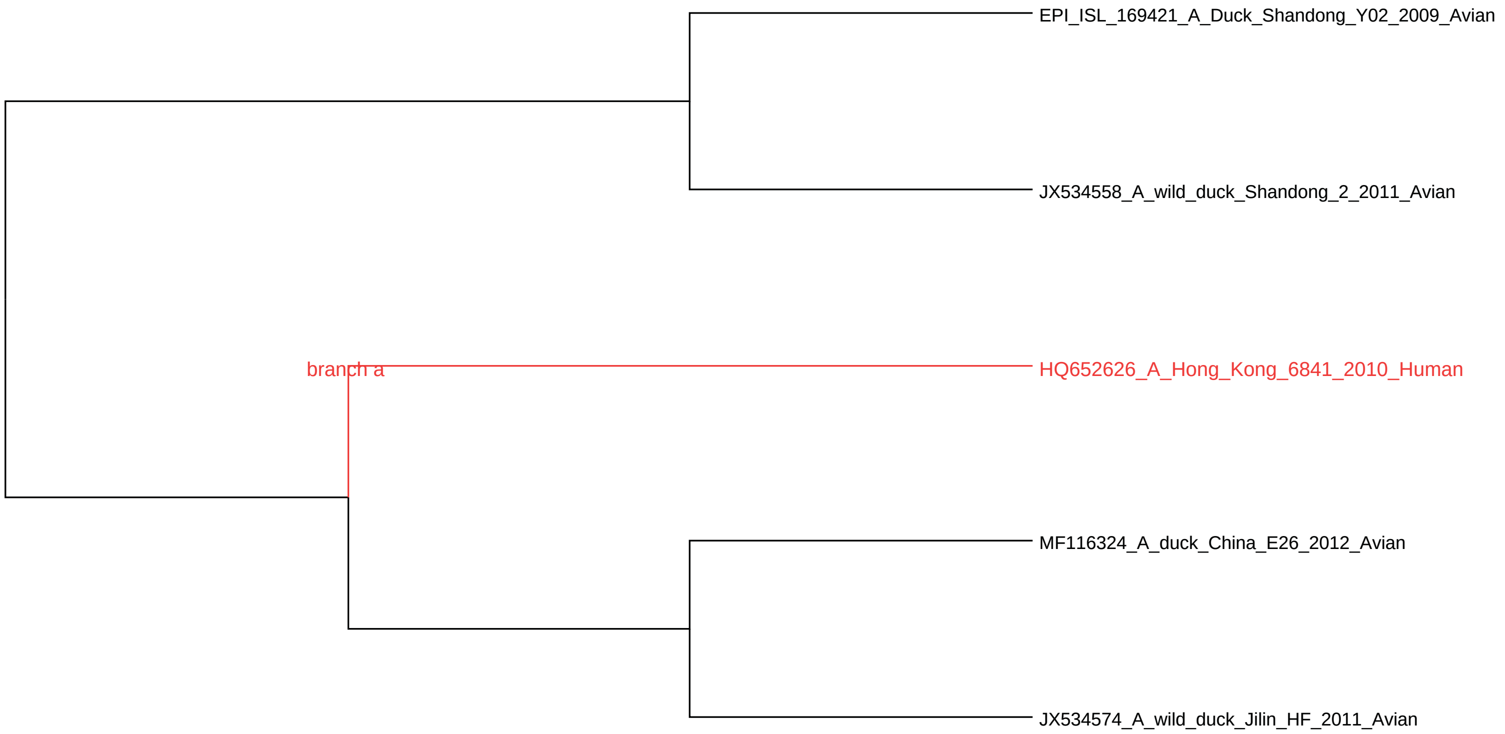
EPI_ISL_169421_A_Duck_Shandong_Y02_2009_Avian
JX534558_A_wild_duck_Shandong_2_2011_Avian
branch a
HQ652626_A_Hong_Kong_6841_2010_Human
MF116324_A_duck_China_E26_2012_Avian
JX534574_A_wild_duck_Jilin_HF_2011_Avian

NP-Group26

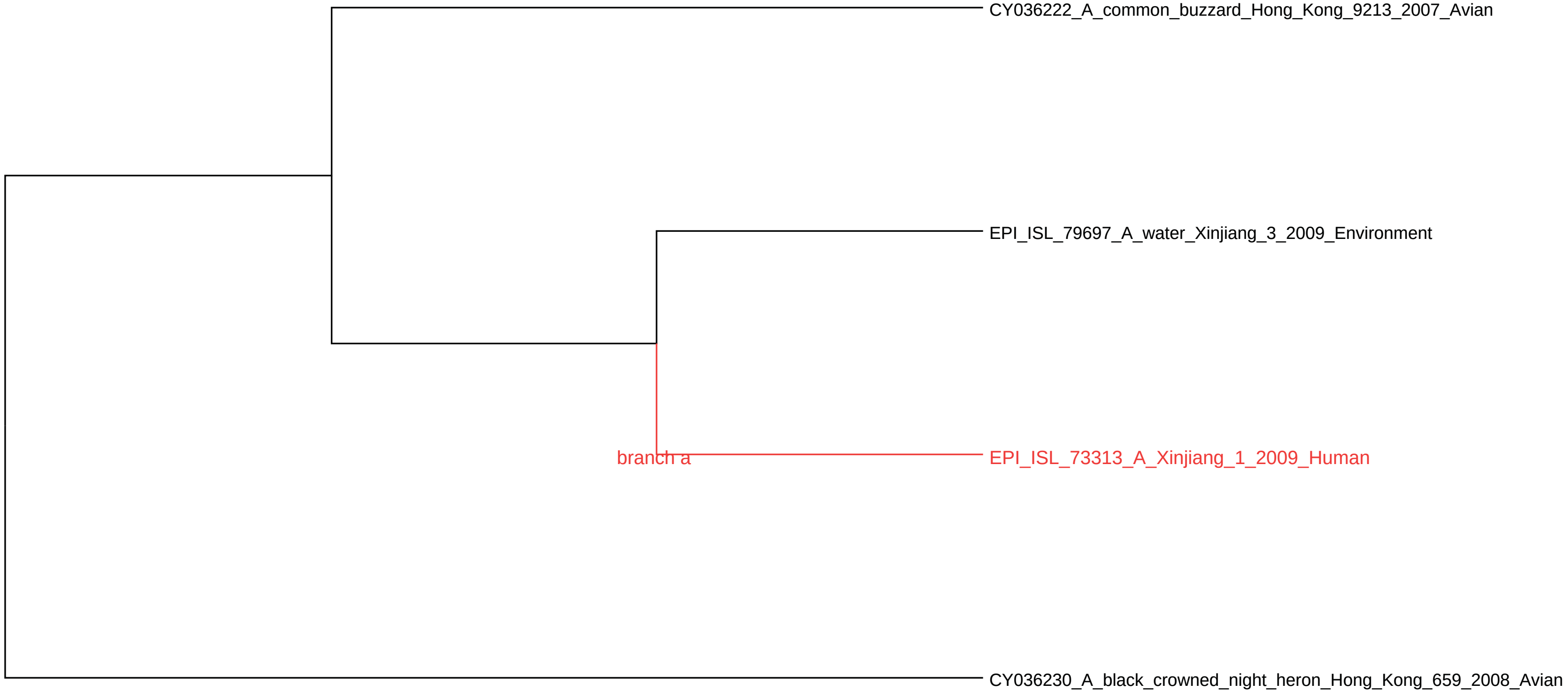
CY036222_A_common_buzzard_Hong_Kong_9213_2007_Avian
EPI_ISL_79697_A_water_Xinjiang_3_2009_Environment
branch a
EPI_ISL_73313_A_Xinjiang_1_2009_Human
CY036230_A_black_crowned_night_heron_Hong_Kong_659_2008_Avian

NP-Group27

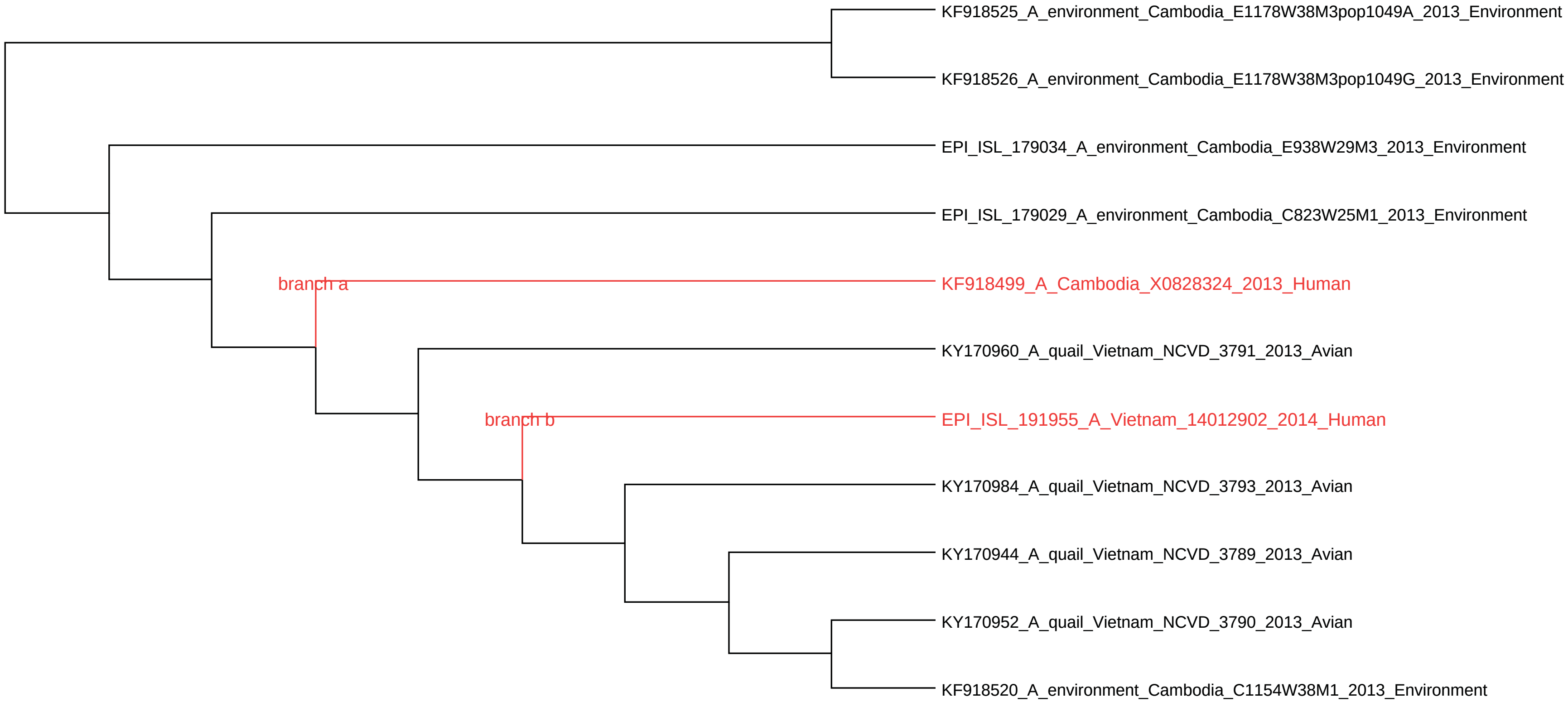
KF918525_A_environment_Cambodia_E1178W38M3pop1049A_2013_Environment
KF918526_A_environment_Cambodia_E1178W38M3pop1049G_2013_Environment
EPI_ISL_179034_A_environment_Cambodia_E938W29M3_2013_Environment
EPI_ISL_179029_A_environment_Cambodia_C823W25M1_2013_Environment
branch a
KF918499_A_Cambodia_X0828324_2013_Human
KY170960_A_quail_Vietnam_NCVD_3791_2013_Avian
branch b
EPI_ISL_191955_A_Vietnam_14012902_2014_Human
KY170984_A_quail_Vietnam_NCVD_3793_2013_Avian
KY170944_A_quail_Vietnam_NCVD_3789_2013_Avian
KY170952_A_quail_Vietnam_NCVD_3790_2013_Avian
KF918520_A_environment_Cambodia_C1154W38M1_2013_Environment

NP-Group28

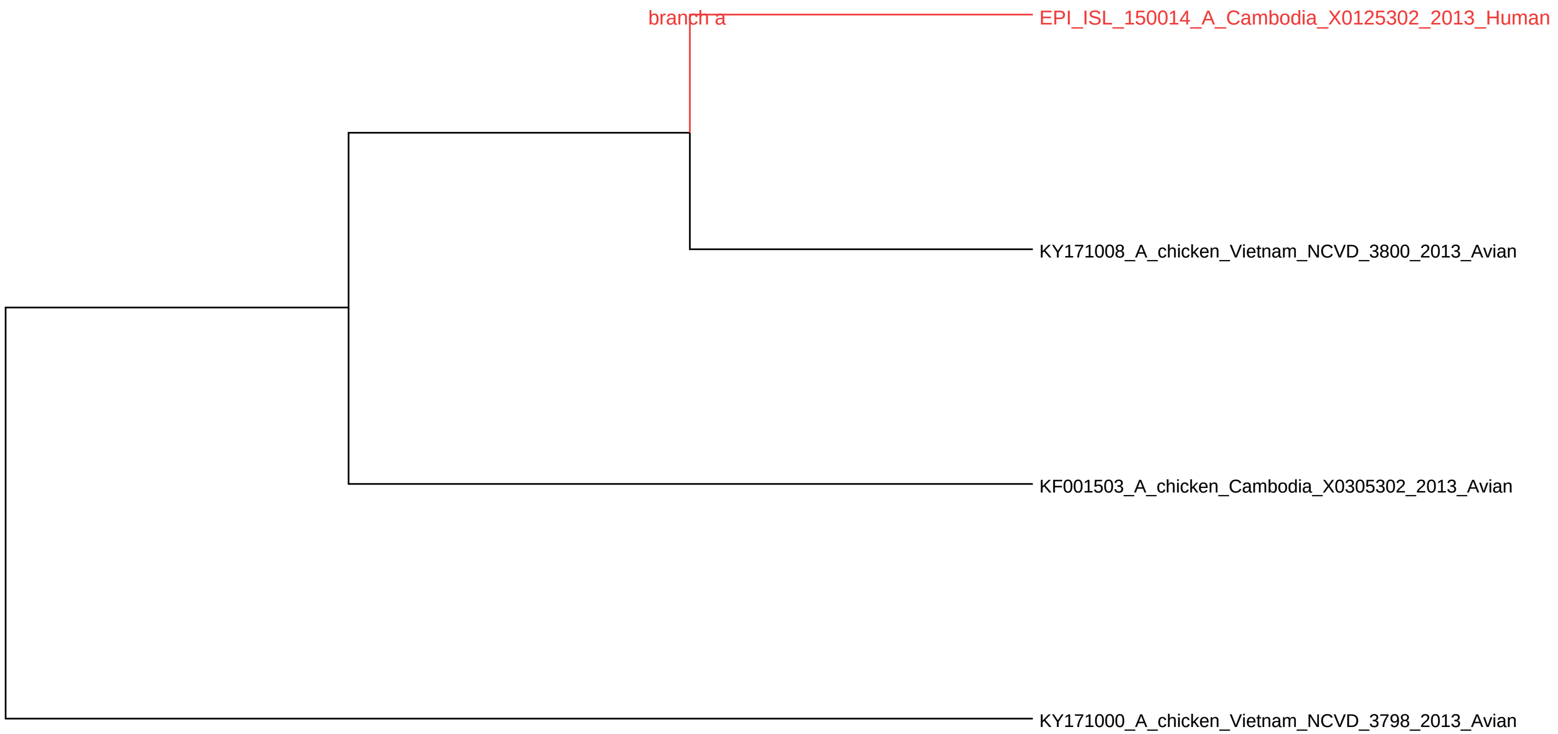
branch a
EPI_ISL_150014_A_Cambodia_X0125302_2013_Human
KY171008_A_chicken_Vietnam_NCVD_3800_2013_Avian
KF001503_A_chicken_Cambodia_X0305302_2013_Avian
KY171000_A_chicken_Vietnam_NCVD_3798_2013_Avian

NP-Group29

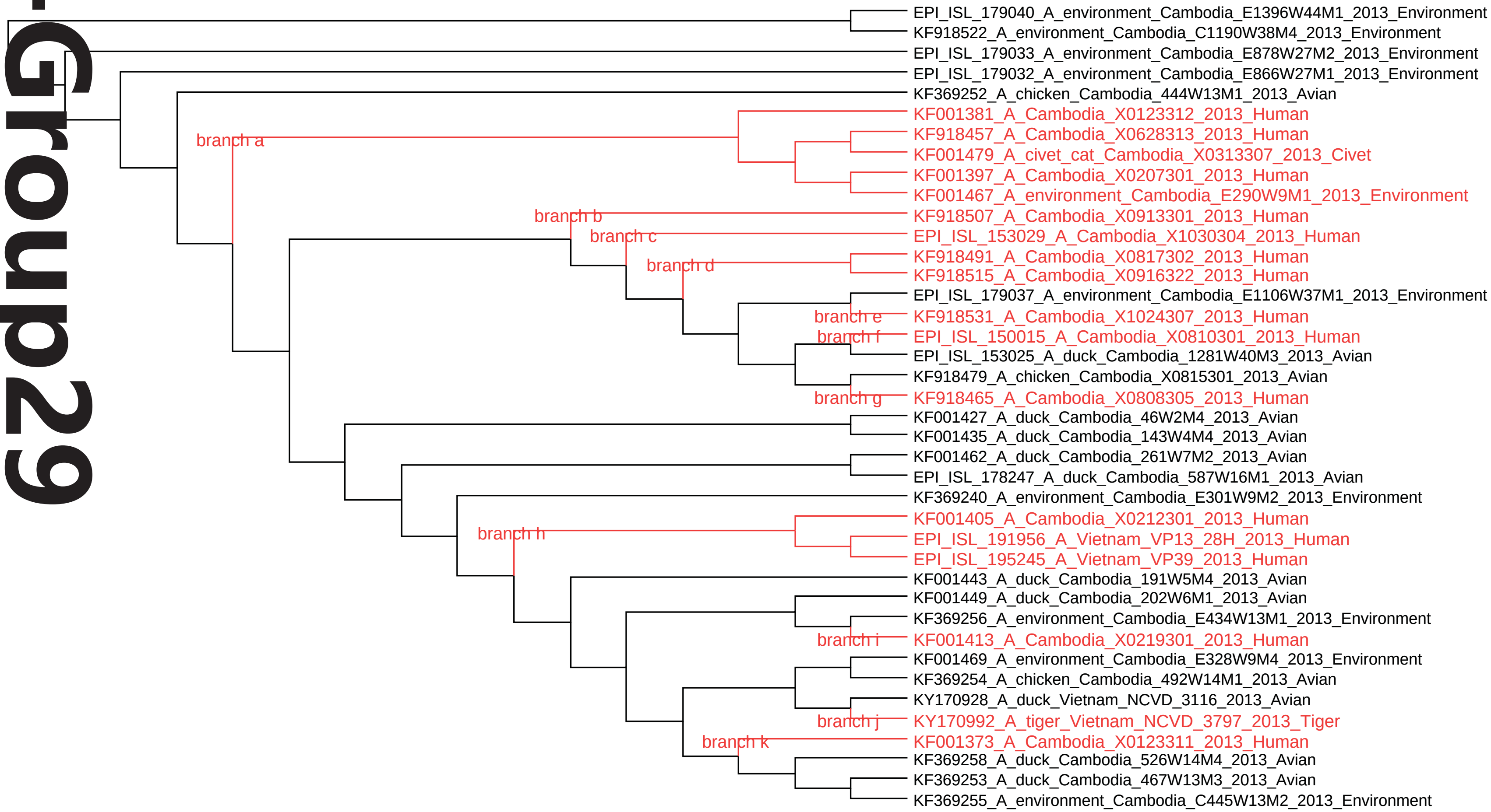

EPI_ISL_179040_A_environment_Cambodia_E1396W44M1_2013_Environment
KF918522_A_environment_Cambodia_C1190W38M4_2013_Environment
EPI_ISL_179033_A_environment_Cambodia_E878W27M2_2013_Environment
EPI_ISL_179032_A_environment_Cambodia_E866W27M1_2013_Environment
KF369252_A_chicken_Cambodia_444W13M1_2013_Avian
KF001381_A_Cambodia_X0123312_2013_Human
KF918457_A_Cambodia_X0628313_2013_Human
KF001479_A_civet_cat_Cambodia_X0313307_2013_Civet
KF001397_A_Cambodia_X0207301_2013_Human
KF001467_A_environment_Cambodia_E290W9M1_2013_Environment
KF918507_A_Cambodia_X0913301_2013_Human
EPI_ISL_153029_A_Cambodia_X1030304_2013_Human
KF918491_A_Cambodia_X0817302_2013_Human
KF918515_A_Cambodia_X0916322_2013_Human
EPI_ISL_179037_A_environment_Cambodia_E1106W37M1_2013_Environment
KF918531_A_Cambodia_X1024307_2013_Human
EPI_ISL_150015_A_Cambodia_X0810301_2013_Human
EPI_ISL_153025_A_duck_Cambodia_1281W40M3_2013_Avian
KF918479_A_chicken_Cambodia_X0815301_2013_Avian
KF918465_A_Cambodia_X0808305_2013_Human
KF001427_A_duck_Cambodia_46W2M4_2013_Avian
KF001435_A_duck_Cambodia_143W4M4_2013_Avian
KF001462_A_duck_Cambodia_261W7M2_2013_Avian
EPI_ISL_178247_A_duck_Cambodia_587W16M1_2013_Avian
KF369240_A_environment_Cambodia_E301W9M2_2013_Environment
KF001405_A_Cambodia_X0212301_2013_Human
EPI_ISL_191956_A_Vietnam_VP13_28H_2013_Human
EPI_ISL_195245_A_Vietnam_VP39_2013_Human
KF001443_A_duck_Cambodia_191W5M4_2013_Avian
KF001449_A_duck_Cambodia_202W6M1_2013_Avian
KF369256_A_environment_Cambodia_E434W13M1_2013_Environment
KF001413_A_Cambodia_X0219301_2013_Human
KF001469_A_environment_Cambodia_E328W9M4_2013_Environment
KF369254_A_chicken_Cambodia_492W14M1_2013_Avian
KY170928_A_duck_Vietnam_NCVD_3116_2013_Avian
KY170992_A_tiger_Vietnam_NCVD_3797_2013_Tiger
KF001373_A_Cambodia_X0123311_2013_Human
KF369258_A_duck_Cambodia_526W14M4_2013_Avian
KF369253_A_duck_Cambodia_467W13M3_2013_Avian
KF369255_A_environment_Cambodia_C445W13M2_2013_Environment
branch a
branch b
branch c
branch d
branch e
branch f
branch g
branch h
branch i
branch j
branch k

NP-Group30

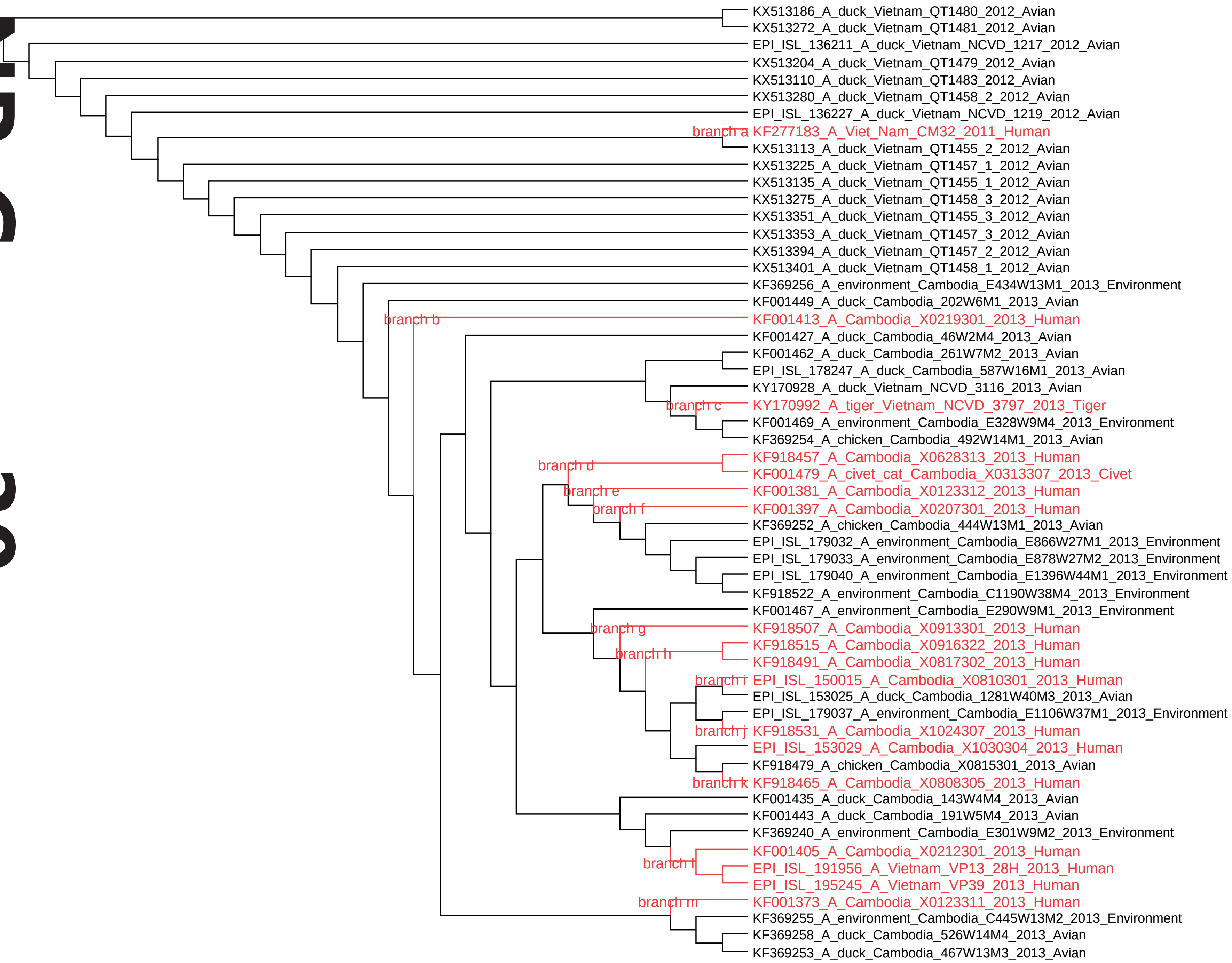

N -Group3

NP-Group31

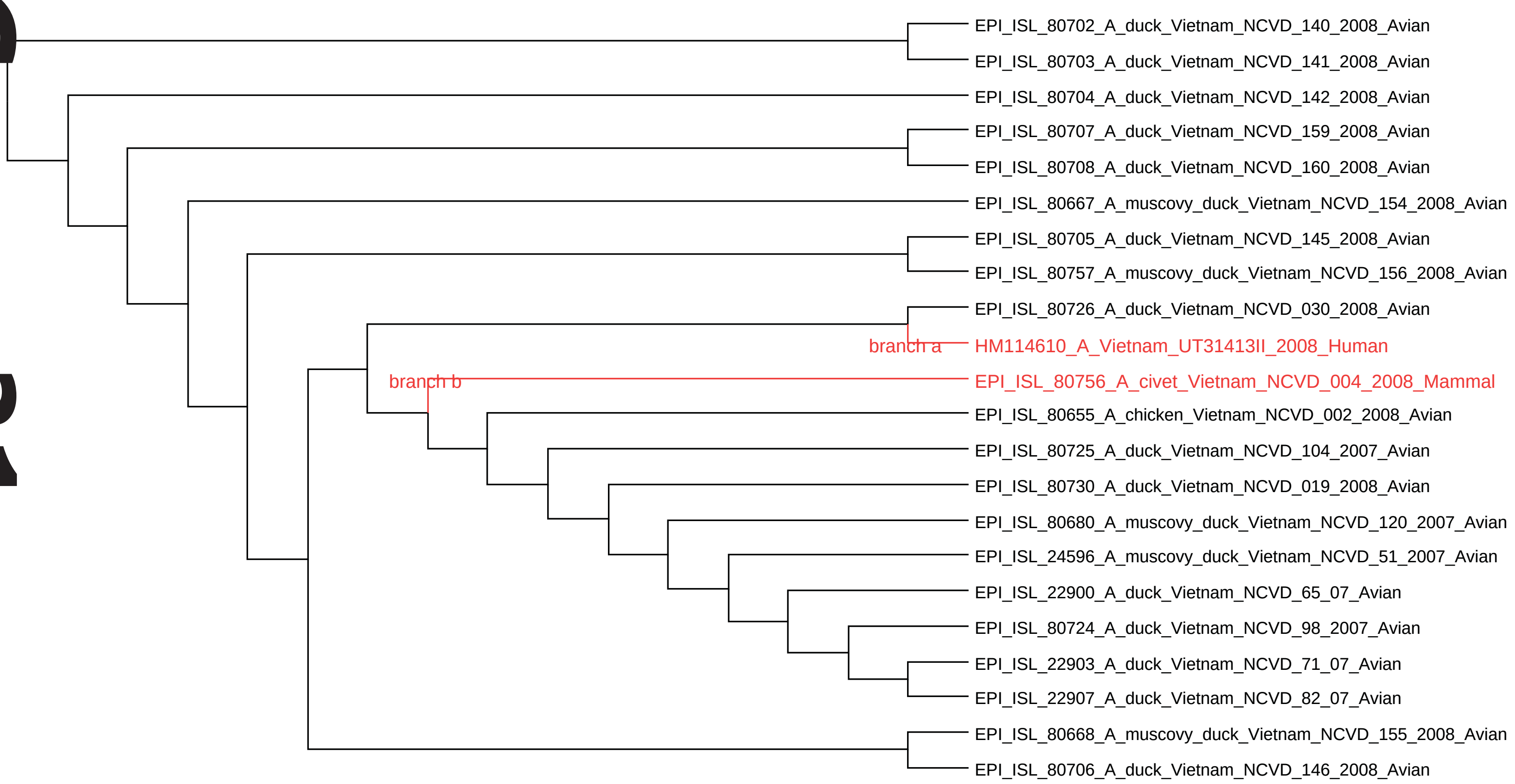
EPI_ISL_80702_A_duck_Vietnam_NCVD_140_2008_Avian
EPI_ISL_80703_A_duck_Vietnam_NCVD_141_2008_Avian
EPI_ISL_80704_A_duck_Vietnam_NCVD_142_2008_Avian
EPI_ISL_80707_A_duck_Vietnam_NCVD_159_2008_Avian
EPI_ISL_80708_A_duck_Vietnam_NCVD_160_2008_Avian
EPI_ISL_80667_A_muscovy_duck_Vietnam_NCVD_154_2008_Avian
EPI_ISL_80705_A_duck_Vietnam_NCVD_145_2008_Avian
EPI_ISL_80757_A_muscovy_duck_Vietnam_NCVD_156_2008_Avian
EPI_ISL_80726_A_duck_Vietnam_NCVD_030_2008_Avian
branch a
HM114610_A_Vietnam_UT31413II_2008_Human
branch b
EPI_ISL_80756_A_civet_Vietnam_NCVD_004_2008_Mammal
EPI_ISL_80655_A_chicken_Vietnam_NCVD_002_2008_Avian
EPI_ISL_80725_A_duck_Vietnam_NCVD_104_2007_Avian
EPI_ISL_80730_A_duck_Vietnam_NCVD_019_2008_Avian
EPI_ISL_80680_A_muscovy_duck_Vietnam_NCVD_120_2007_Avian
EPI_ISL_24596_A_muscovy_duck_Vietnam_NCVD_51_2007_Avian
EPI_ISL_22900_A_duck_Vietnam_NCVD_65_07_Avian
EPI_ISL_80724_A_duck_Vietnam_NCVD_98_2007_Avian
EPI_ISL_22903_A_duck_Vietnam_NCVD_71_07_Avian
EPI_ISL_22907_A_duck_Vietnam_NCVD_82_07_Avian
EPI_ISL_80668_A_muscovy_duck_Vietnam_NCVD_155_2008_Avian
EPI_ISL_80706_A_duck_Vietnam_NCVD_146_2008_Avian

NP-Group32

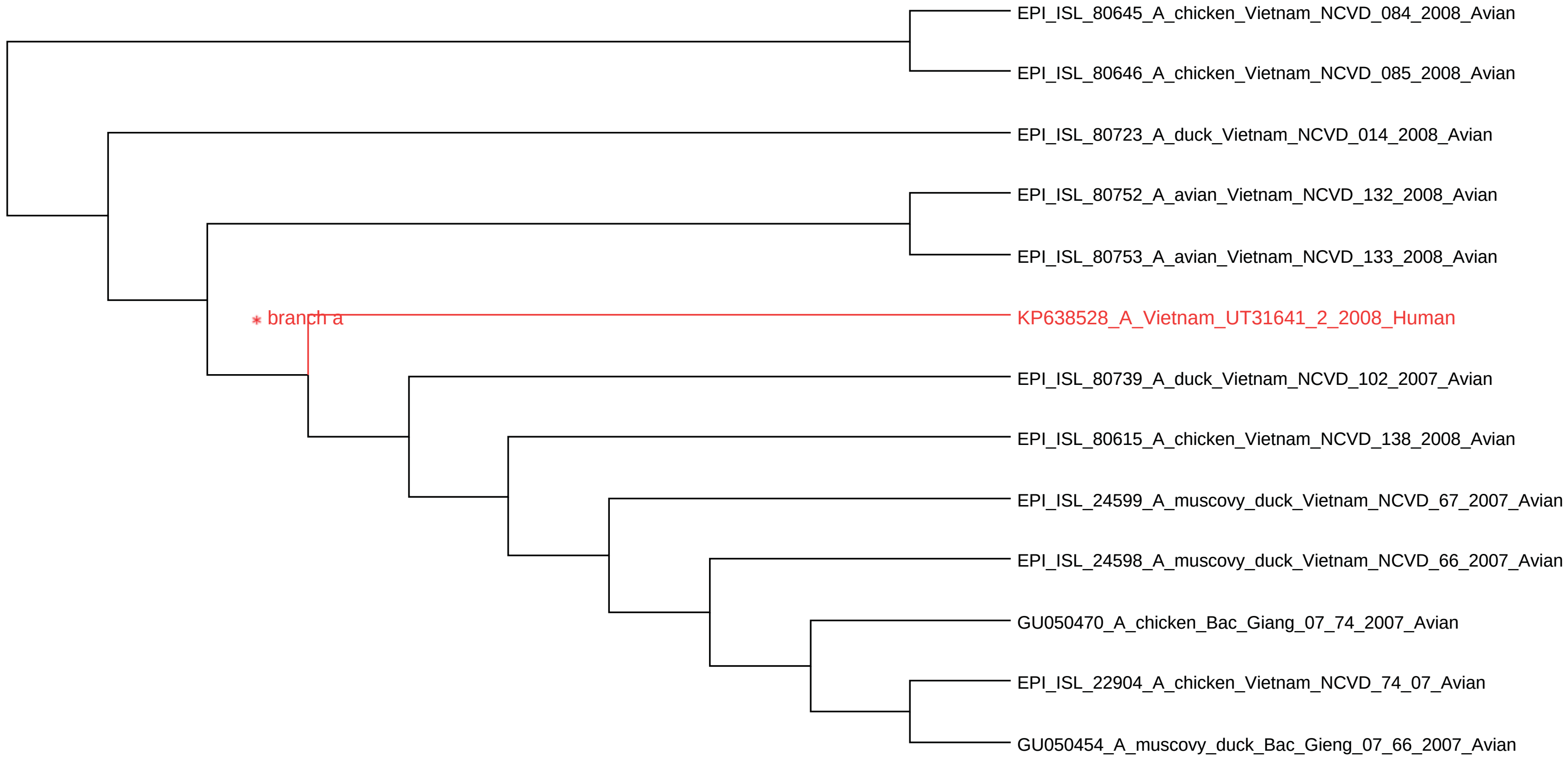
EPI_ISL_80645_A_chicken_Vietnam_NCVD_084_2008_Avian
EPI_ISL_80646_A_chicken_Vietnam_NCVD_085_2008_Avian
EPI_ISL_80723_A_duck_Vietnam_NCVD_014_2008_Avian
EPI_ISL_80752_A_avian_Vietnam_NCVD_132_2008_Avian
EPI_ISL_80753_A_avian_Vietnam_NCVD_133_2008_Avian
* branch a
KP638528_A_Vietnam_UT31641_2_2008_Human
EPI_ISL_80739_A_duck_Vietnam_NCVD_102_2007_Avian
EPI_ISL_80615_A_chicken_Vietnam_NCVD_138_2008_Avian
EPI_ISL_24599_A_muscovy_duck_Vietnam_NCVD_67_2007_Avian
EPI_ISL_24598_A_muscovy_duck_Vietnam_NCVD_66_2007_Avian
GU050470_A_chicken_Bac_Giang_07_74_2007_Avian
EPI_ISL_22904_A_chicken_Vietnam_NCVD_74_07_Avian
GU050454_A_muscovy_duck_Bac_Gieng_07_66_2007_Avian

NP-Group33

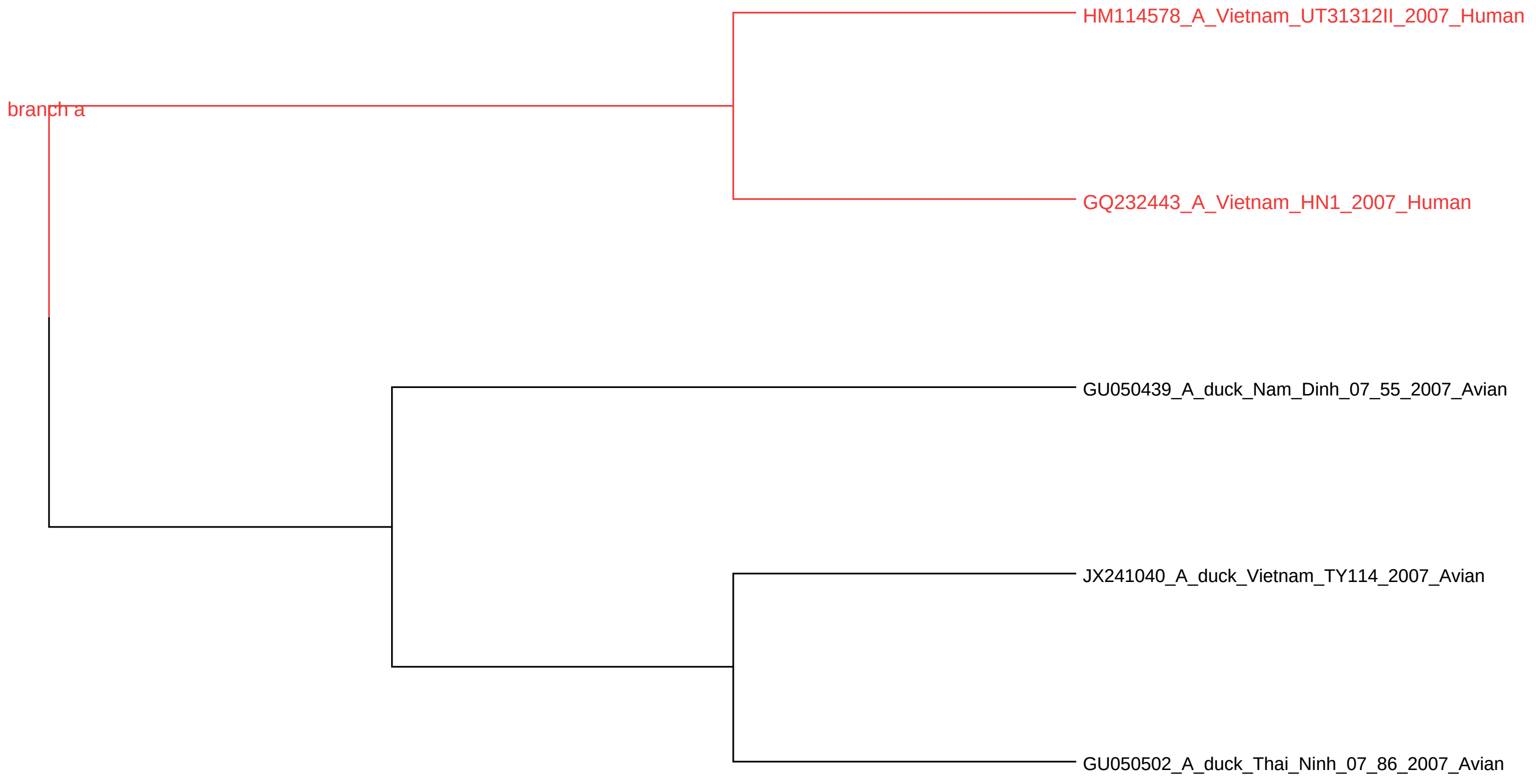
branch a
HM114578_A_Vietnam_UT31312II_2007_Human
GQ232443_A_Vietnam_HN1_2007_Human
GU050439_A_duck_Nam_Dinh_07_55_2007_Avian
JX241040_A_duck_Vietnam_TY114_2007_Avian
GU050502_A_duck_Thai_Ninh_07_86_2007_Avian

NP-Group34

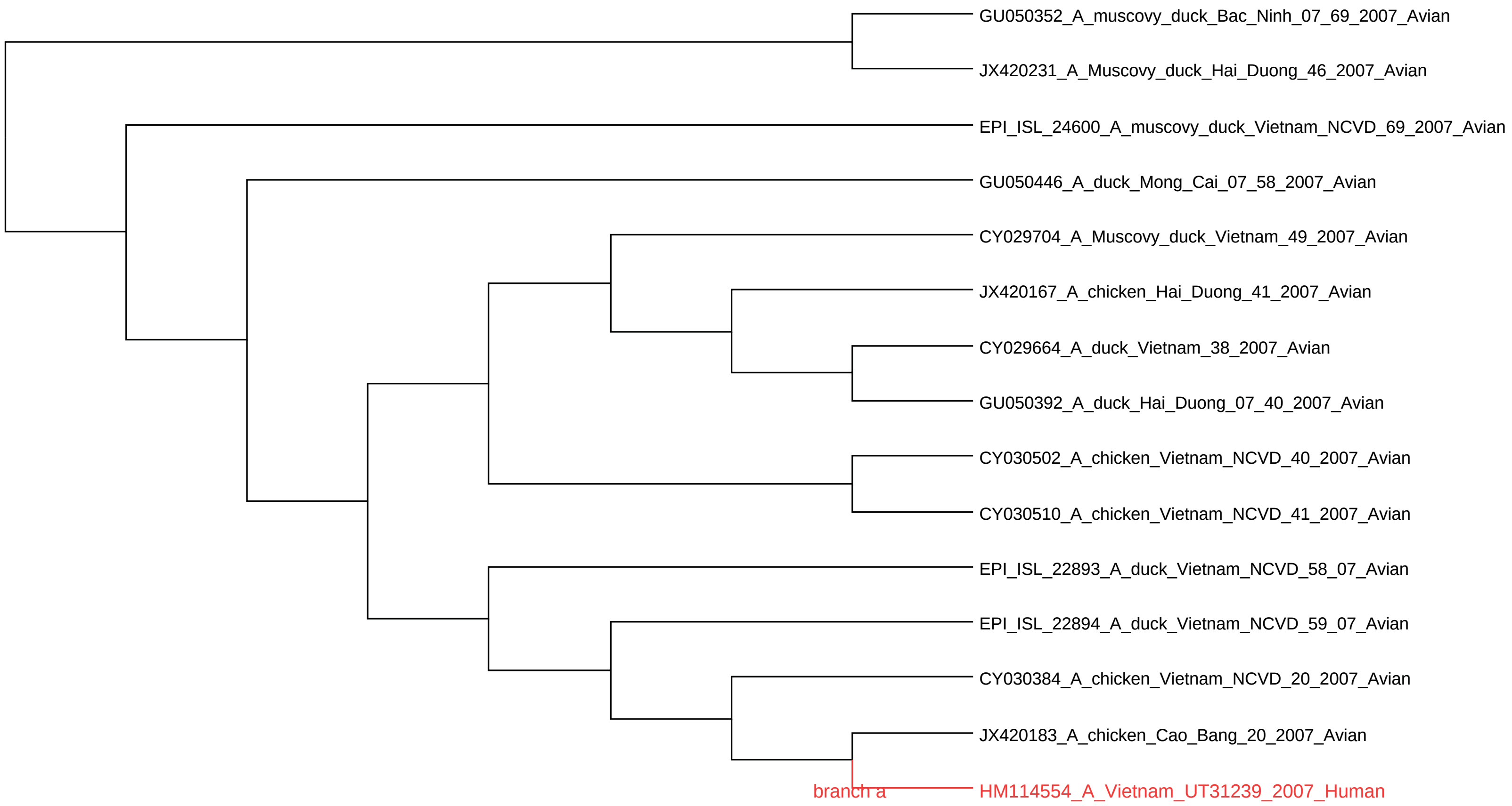
GU050352_A_muscovy_duck_Bac_Ninh_07_69_2007_Avian
JX420231_A_Muscovy_duck_Hai_Duong_46_2007_Avian
EPI_ISL_24600_A_muscovy_duck_Vietnam_NCVD_69_2007_Avian
GU050446_A_duck_Mong_Cai_07_58_2007_Avian
CY029704_A_Muscovy_duck_Vietnam_49_2007_Avian
JX420167_A_chicken_Hai_Duong_41_2007_Avian
CY029664_A_duck_Vietnam_38_2007_Avian
GU050392_A_duck_Hai_Duong_07_40_2007_Avian
CY030502_A_chicken_Vietnam_NCVD_40_2007_Avian
CY030510_A_chicken_Vietnam_NCVD_41_2007_Avian
EPI_ISL_22893_A_duck_Vietnam_NCVD_58_07_Avian
EPI_ISL_22894_A_duck_Vietnam_NCVD_59_07_Avian
CY030384_A_chicken_Vietnam_NCVD_20_2007_Avian
JX420183_A_chicken_Cao_Bang_20_2007_Avian
branch a
HM114554_A_Vietnam_UT31239_2007_Human

NP-Group35

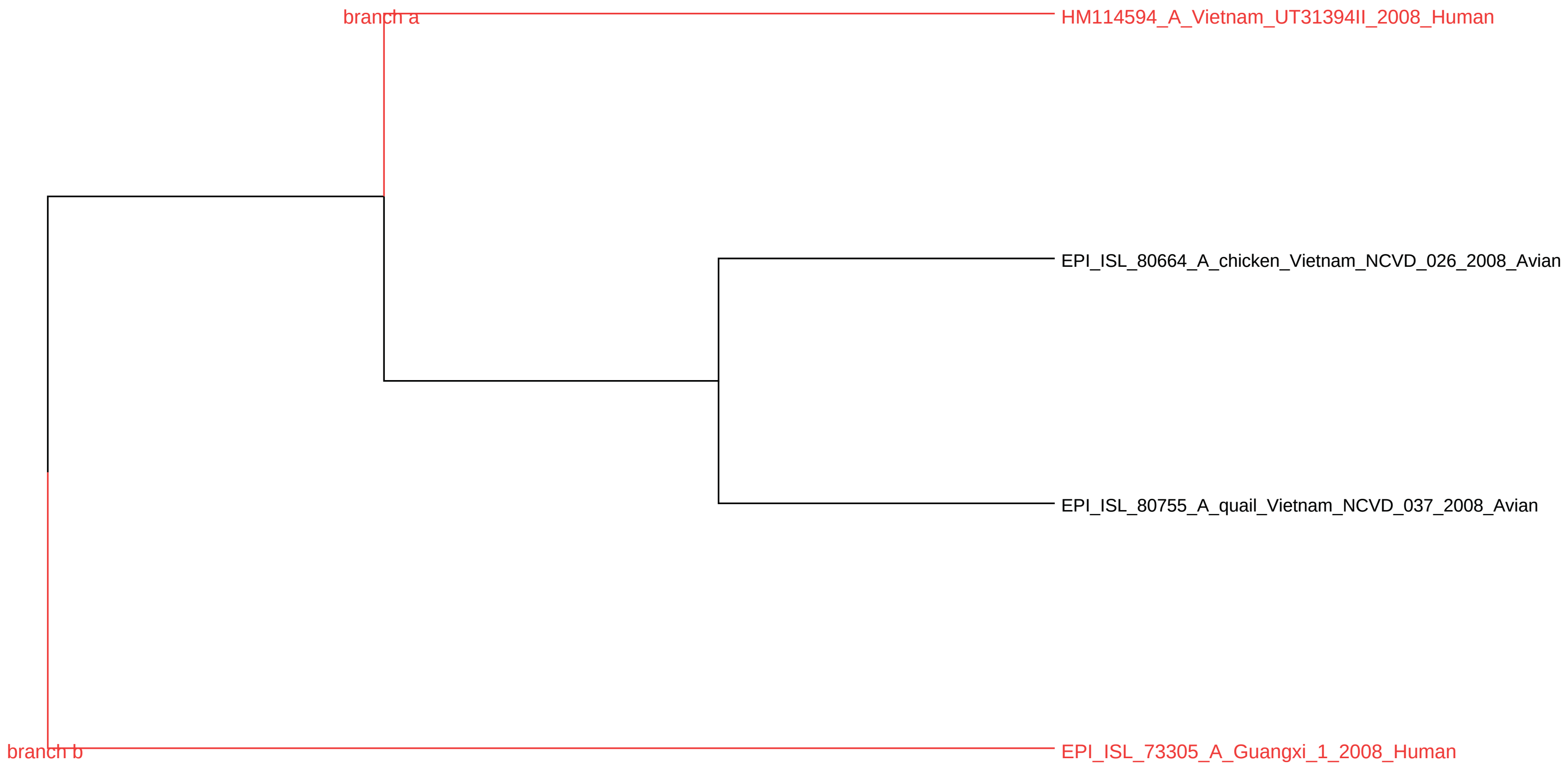
branch a
HM114594_A_Vietnam_UT31394II_2008_Human
EPI_ISL_80664_A_chicken_Vietnam_NCVD_026_2008_Avian
EPI_ISL_80755_A_quail_Vietnam_NCVD_037_2008_Avian
branch b
EPI_ISL_73305_A_Guangxi_1_2008_Human

NP-Group36

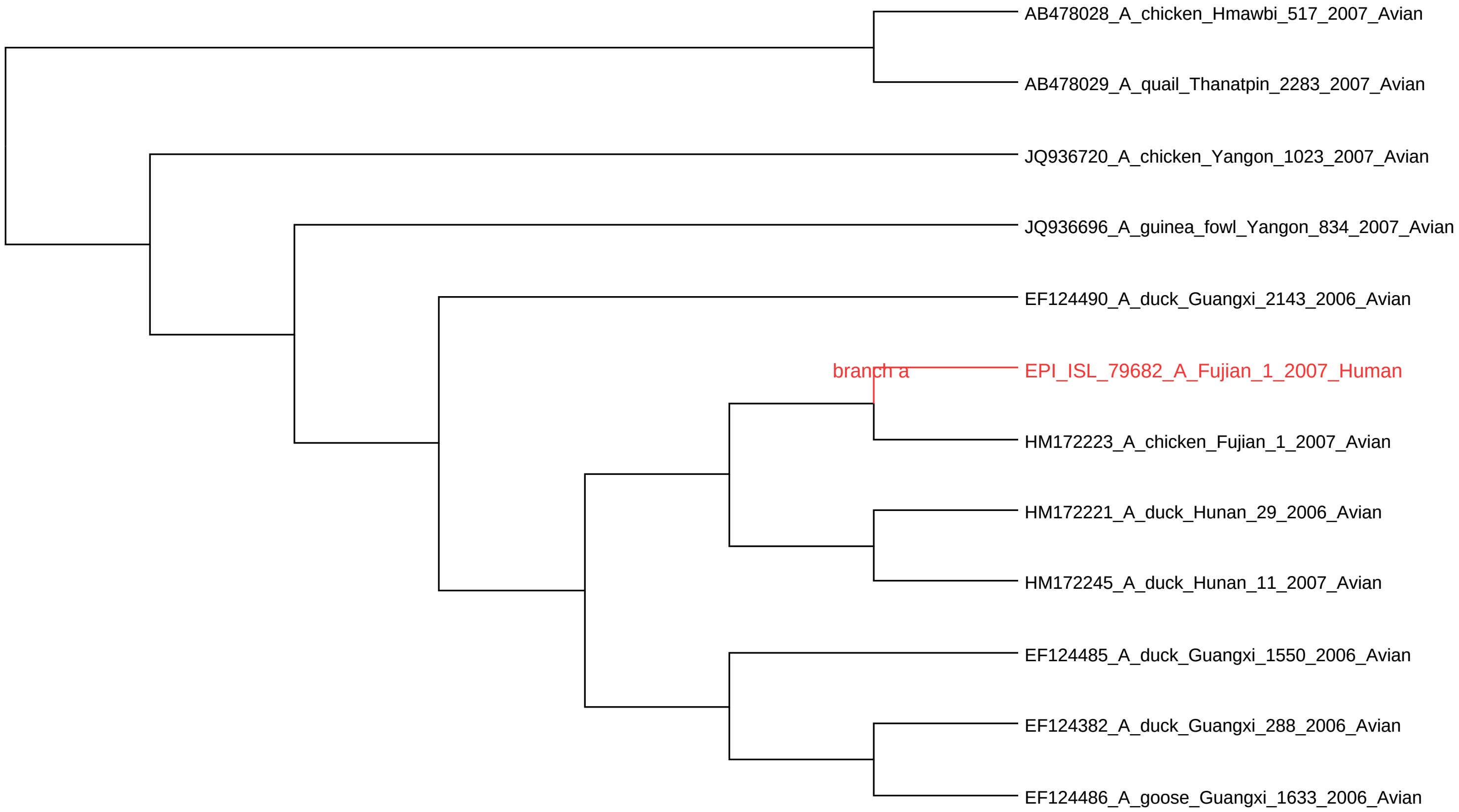
AB478028_A_chicken_Hmawbi_517_2007_Avian
AB478029_A_quail_Thanatpin_2283_2007_Avian
JQ936720_A_chicken_Yangon_1023_2007_Avian
JQ936696_A_guinea_fowl_Yangon_834_2007_Avian
EF124490_A_duck_Guangxi_2143_2006_Avian
branch a
EPI_ISL_79682_A_Fujian_1_2007_Human
HM172223_A_chicken_Fujian_1_2007_Avian
HM172221_A_duck_Hunan_29_2006_Avian
HM172245_A_duck_Hunan_11_2007_Avian
EF124485_A_duck_Guangxi_1550_2006_Avian
EF124382_A_duck_Guangxi_288_2006_Avian
EF124486_A_goose_Guangxi_1633_2006_Avian

NP-Group37

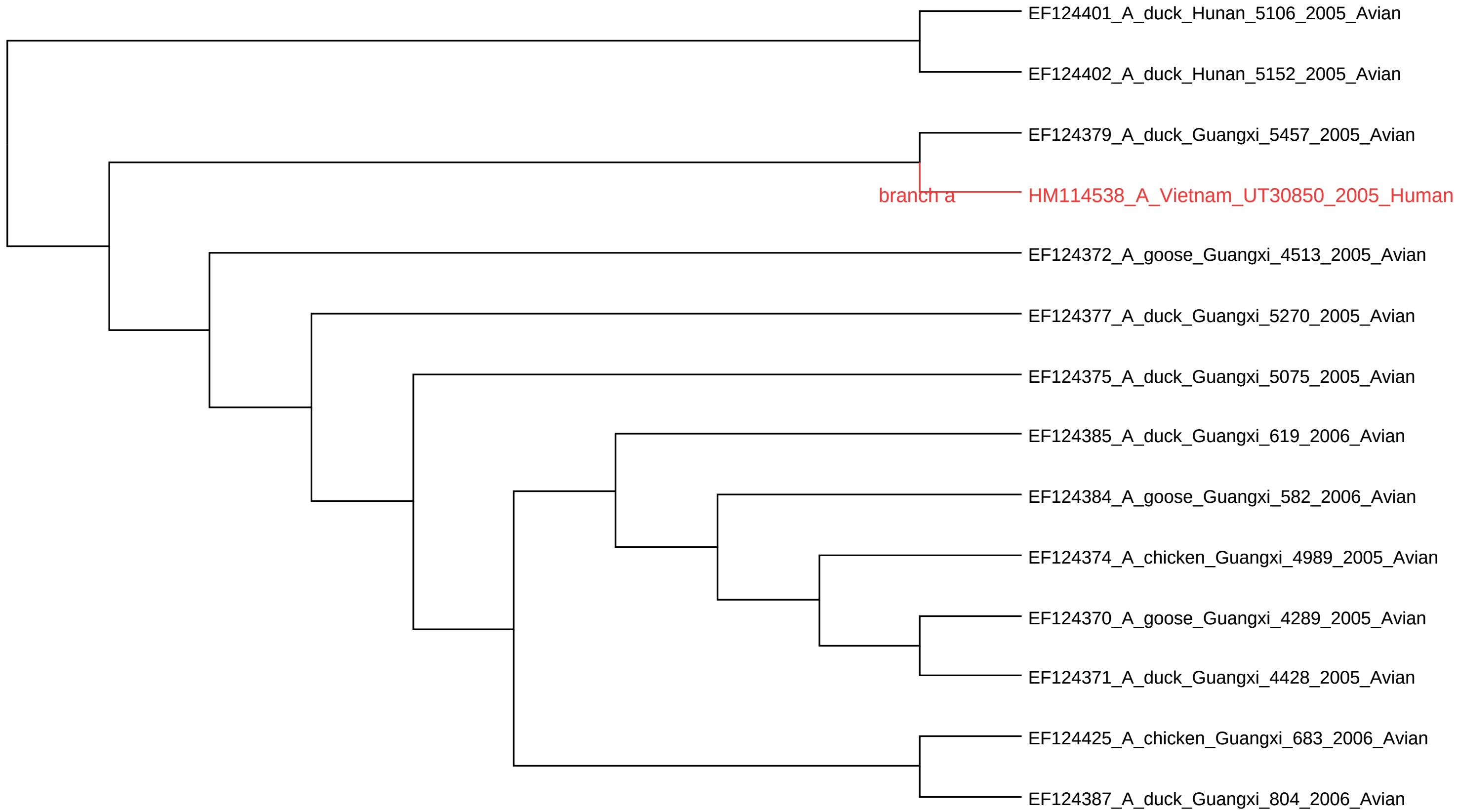
EF124401_A_duck_Hunan_5106_2005_Avian
EF124402_A_duck_Hunan_5152_2005_Avian
EF124379_A_duck_Guangxi_5457_2005_Avian
branch a
HM114538_A_Vietnam_UT30850_2005_Human
EF124372_A_goose_Guangxi_4513_2005_Avian
EF124377_A_duck_Guangxi_5270_2005_Avian
EF124375_A_duck_Guangxi_5075_2005_Avian
EF124385_A_duck_Guangxi_619_2006_Avian
EF124384_A_goose_Guangxi_582_2006_Avian
EF124374_A_chicken_Guangxi_4989_2005_Avian
EF124370_A_goose_Guangxi_4289_2005_Avian
EF124371_A_duck_Guangxi_4428_2005_Avian
EF124425_A_chicken_Guangxi_683_2006_Avian
EF124387_A_duck_Guangxi_804_2006_Avian

NP-Group38

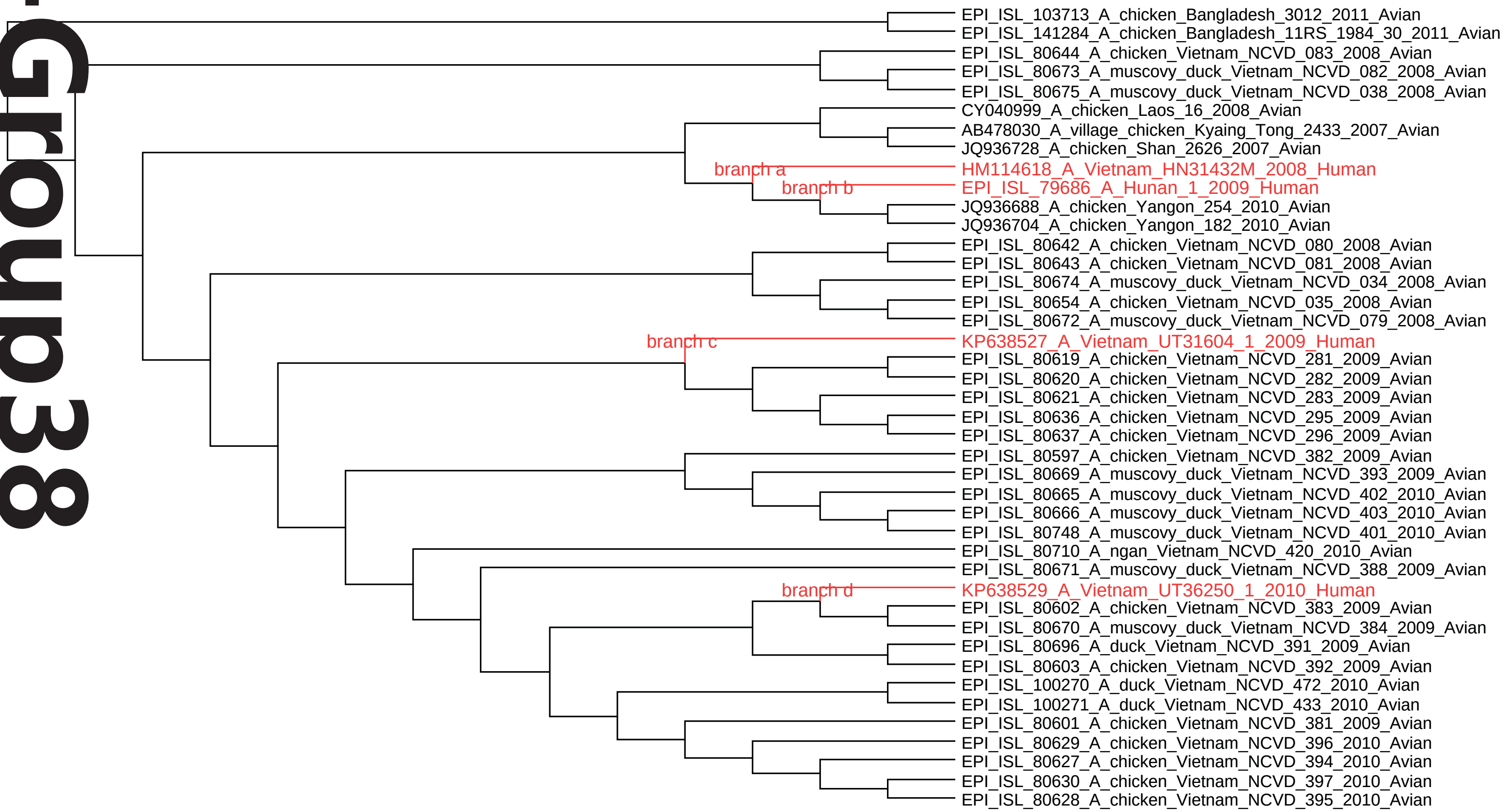
EPI_ISL_103713_A_chicken_Bangladesh_3012_2011_Avian
EPI_ISL_141284_A_chicken_Bangladesh_11RS_1984_30_2011_Avian
EPI_ISL_80644_A_chicken_Vietnam_NCVD_083_2008_Avian
EPI_ISL_80673_A_muscovy_duck_Vietnam_NCVD_082_2008_Avian
EPI_ISL_80675_A_muscovy_duck_Vietnam_NCVD_038_2008_Avian
CY040999_A_chicken_Laos_16_2008_Avian
AB478030_A_village_chicken_Kyaing_Tong_2433_2007_Avian
JQ936728_A_chicken_Shan_2626_2007_Avian
branch a
HM114618_A_Vietnam_HN31432M_2008_Human
branch b
EPI_ISL_79686_A_Hunan_1_2009_Human
JQ936688_A_chicken_Yangon_254_2010_Avian
JQ936704_A_chicken_Yangon_182_2010_Avian
EPI_ISL_80642_A_chicken_Vietnam_NCVD_080_2008_Avian
EPI_ISL_80643_A_chicken_Vietnam_NCVD_081_2008_Avian
EPI_ISL_80674_A_muscovy_duck_Vietnam_NCVD_034_2008_Avian
EPI_ISL_80654_A_chicken_Vietnam_NCVD_035_2008_Avian
EPI_ISL_80672_A_muscovy_duck_Vietnam_NCVD_079_2008_Avian
branch c
KP638527_A_Vietnam_UT31604_1_2009_Human
EPI_ISL_80619_A_chicken_Vietnam_NCVD_281_2009_Avian
EPI_ISL_80620_A_chicken_Vietnam_NCVD_282_2009_Avian
EPI_ISL_80621_A_chicken_Vietnam_NCVD_283_2009_Avian
EPI_ISL_80636_A_chicken_Vietnam_NCVD_295_2009_Avian
EPI_ISL_80637_A_chicken_Vietnam_NCVD_296_2009_Avian
EPI_ISL_80597_A_chicken_Vietnam_NCVD_382_2009_Avian
EPI_ISL_80669_A_muscovy_duck_Vietnam_NCVD_393_2009_Avian
EPI_ISL_80665_A_muscovy_duck_Vietnam_NCVD_402_2010_Avian
EPI_ISL_80666_A_muscovy_duck_Vietnam_NCVD_403_2010_Avian
EPI_ISL_80748_A_muscovy_duck_Vietnam_NCVD_401_2010_Avian
EPI_ISL_80710_A_ngan_Vietnam_NCVD_420_2010_Avian
EPI_ISL_80671_A_muscovy_duck_Vietnam_NCVD_388_2009_Avian
branch d
KP638529_A_Vietnam_UT36250_1_2010_Human
EPI_ISL_80602_A_chicken_Vietnam_NCVD_383_2009_Avian
EPI_ISL_80670_A_muscovy_duck_Vietnam_NCVD_384_2009_Avian
EPI_ISL_80696_A_duck_Vietnam_NCVD_391_2009_Avian
EPI_ISL_80603_A_chicken_Vietnam_NCVD_392_2009_Avian
EPI_ISL_100270_A_duck_Vietnam_NCVD_472_2010_Avian
EPI_ISL_100271_A_duck_Vietnam_NCVD_433_2010_Avian
EPI_ISL_80601_A_chicken_Vietnam_NCVD_381_2009_Avian
EPI_ISL_80629_A_chicken_Vietnam_NCVD_396_2010_Avian
EPI_ISL_80627_A_chicken_Vietnam_NCVD_394_2010_Avian
EPI_ISL_80630_A_chicken_Vietnam_NCVD_397_2010_Avian
EPI_ISL_80628_A_chicken_Vietnam_NCVD_395_2010_Avian

NP-Group39

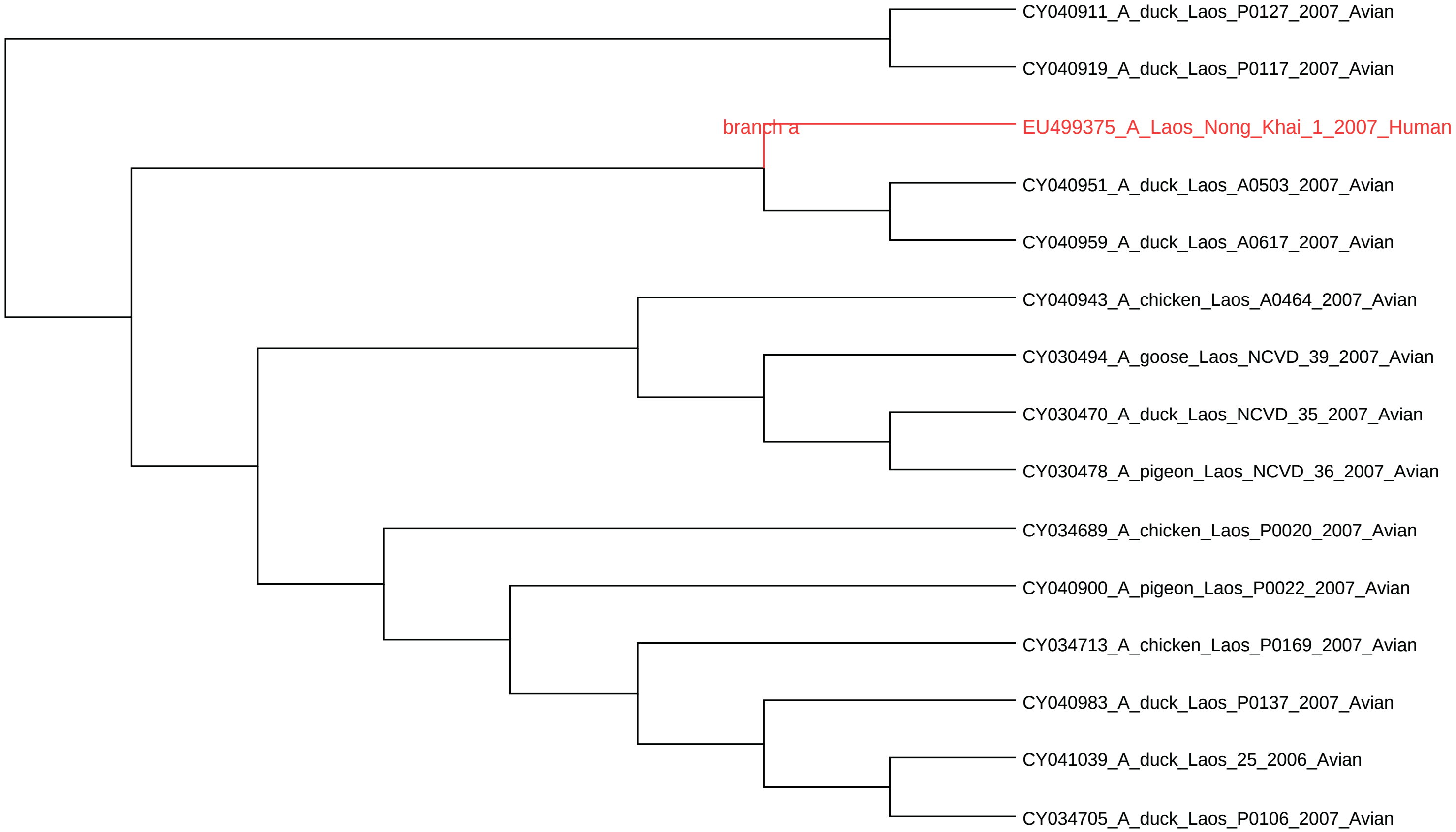
CY040911_A_duck_Laos_P0127_2007_Avian
CY040919_A_duck_Laos_P0117_2007_Avian
branch a
EU499375_A_Laos_Nong_Khai_1_2007_Human
CY040951_A_duck_Laos_A0503_2007_Avian
CY040959_A_duck_Laos_A0617_2007_Avian
CY040943_A_chicken_Laos_A0464_2007_Avian
CY030494_A_goose_Laos_NCVD_39_2007_Avian
CY030470_A_duck_Laos_NCVD_35_2007_Avian
CY030478_A_pigeon_Laos_NCVD_36_2007_Avian
CY034689_A_chicken_Laos_P0020_2007_Avian
CY040900_A_pigeon_Laos_P0022_2007_Avian
CY034713_A_chicken_Laos_P0169_2007_Avian
CY040983_A_duck_Laos_P0137_2007_Avian
CY041039_A_duck_Laos_25_2006_Avian
CY034705_A_duck_Laos_P0106_2007_Avian

NP-Group40

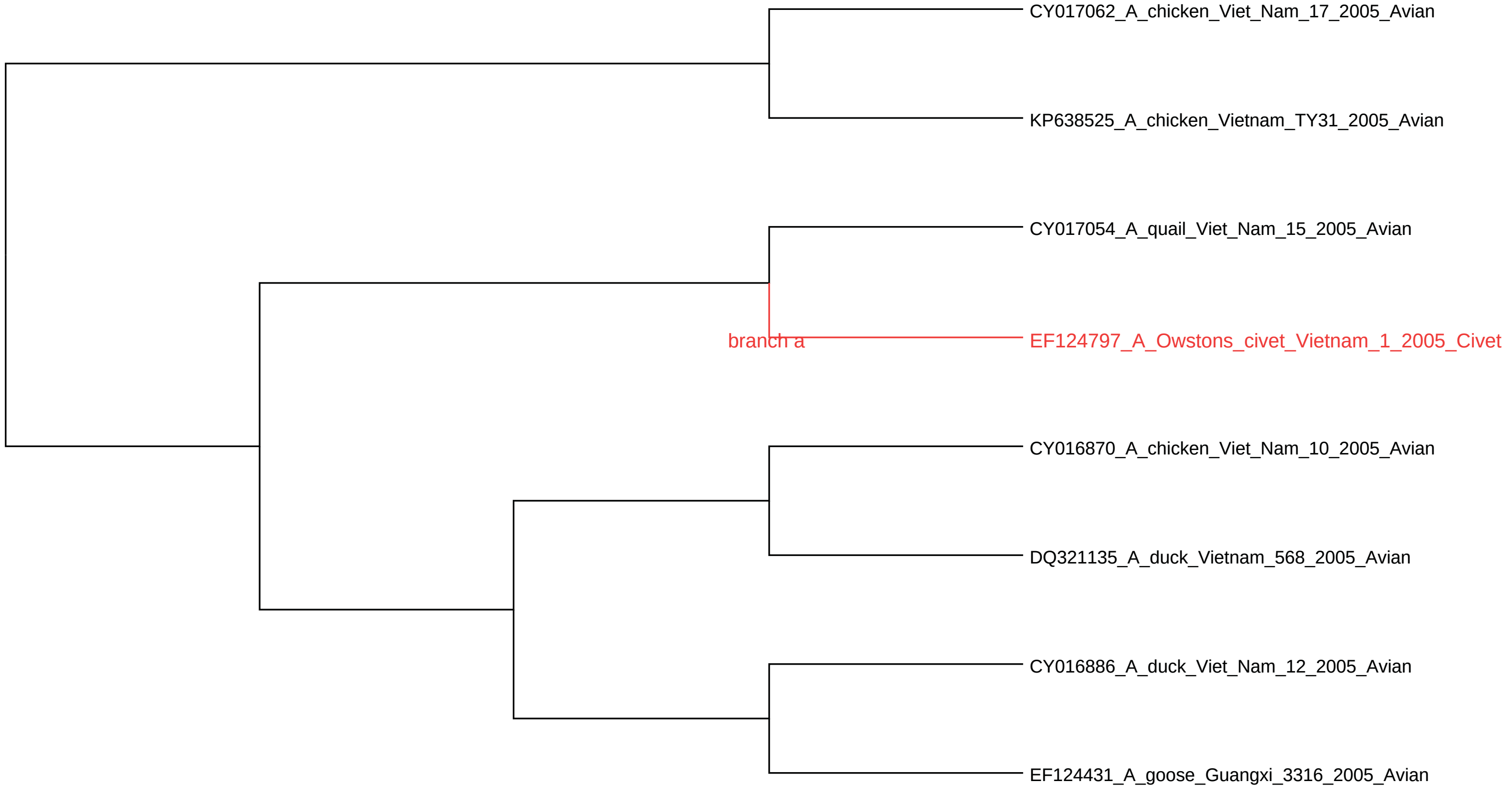
CY017062_A_chicken_Viet_Nam_17_2005_Avian
KP638525_A_chicken_Vietnam_TY31_2005_Avian
CY017054_A_quail_Viet_Nam_15_2005_Avian
branch a
EF124797_A_Owstons_civet_Vietnam_1_2005_Civet
CY016870_A_chicken_Viet_Nam_10_2005_Avian
DQ321135_A_duck_Vietnam_568_2005_Avian
CY016886_A_duck_Viet_Nam_12_2005_Avian
EF124431_A_goose_Guangxi_3316_2005_Avian

NP-Group41

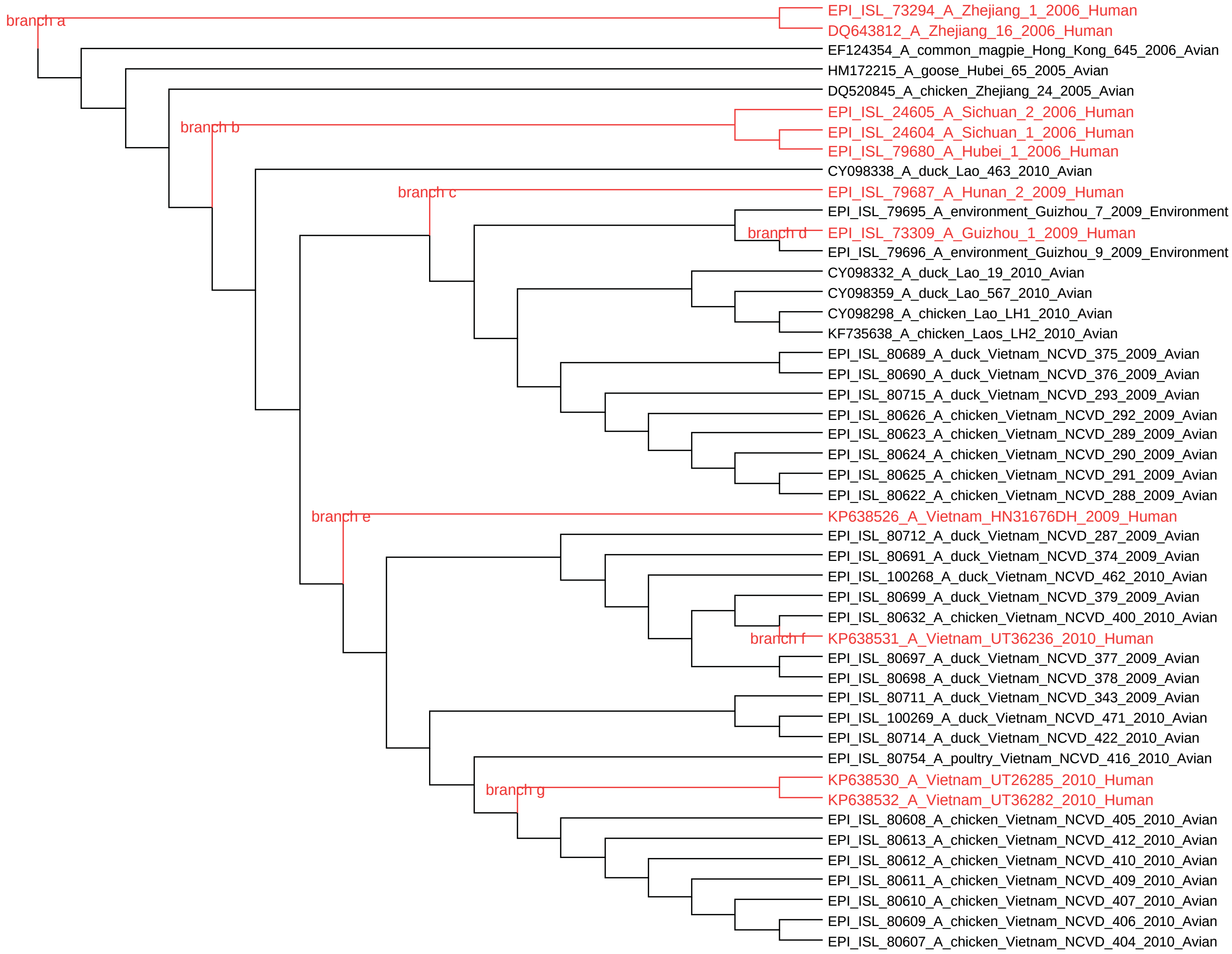

branch a
EPI_ISL_73294_A_Zhejiang_1_2006_Human
DQ643812_A_Zhejiang_16_2006_Human
EF124354_A_common_magpie_Hong_Kong_645_2006_Avian
HM172215_A_goose_Hubei_65_2005_Avian
DQ520845_A_chicken_Zhejiang_24_2005_Avian
branch b
EPI_ISL_24605_A_Sichuan_2_2006_Human
EPI_ISL_24604_A_Sichuan_1_2006_Human
EPI_ISL_79680_A_Hubei_1_2006_Human
CY098338_A_duck_Lao_463_2010_Avian
branch c
EPI_ISL_79687_A_Hunan_2_2009_Human
EPI_ISL_79695_A_environment_Guizhou_7_2009_Environment
branch d
EPI_ISL_73309_A_Guizhou_1_2009_Human
EPI_ISL_79696_A_environment_Guizhou_9_2009_Environment
CY098332_A_duck_Lao_19_2010_Avian
CY098359_A_duck_Lao_567_2010_Avian
CY098298_A_chicken_Lao_LH1_2010_Avian
KF735638_A_chicken_Laos_LH2_2010_Avian
EPI_ISL_80689_A_duck_Vietnam_NCVD_375_2009_Avian
EPI_ISL_80690_A_duck_Vietnam_NCVD_376_2009_Avian
EPI_ISL_80715_A_duck_Vietnam_NCVD_293_2009_Avian
EPI_ISL_80626_A_chicken_Vietnam_NCVD_292_2009_Avian
EPI_ISL_80623_A_chicken_Vietnam_NCVD_289_2009_Avian
EPI_ISL_80624_A_chicken_Vietnam_NCVD_290_2009_Avian
EPI_ISL_80625_A_chicken_Vietnam_NCVD_291_2009_Avian
EPI_ISL_80622_A_chicken_Vietnam_NCVD_288_2009_Avian
branch e
KP638526_A_Vietnam_HN31676DH_2009_Human
EPI_ISL_80712_A_duck_Vietnam_NCVD_287_2009_Avian
EPI_ISL_80691_A_duck_Vietnam_NCVD_374_2009_Avian
EPI_ISL_100268_A_duck_Vietnam_NCVD_462_2010_Avian
EPI_ISL_80699_A_duck_Vietnam_NCVD_379_2009_Avian
EPI_ISL_80632_A_chicken_Vietnam_NCVD_400_2010_Avian
branch f
KP638531_A_Vietnam_UT36236_2010_Human
EPI_ISL_80697_A_duck_Vietnam_NCVD_377_2009_Avian
EPI_ISL_80698_A_duck_Vietnam_NCVD_378_2009_Avian
EPI_ISL_80711_A_duck_Vietnam_NCVD_343_2009_Avian
EPI_ISL_100269_A_duck_Vietnam_NCVD_471_2010_Avian
EPI_ISL_80714_A_duck_Vietnam_NCVD_422_2010_Avian
EPI_ISL_80754_A_poultry_Vietnam_NCVD_416_2010_Avian
branch g
KP638530_A_Vietnam_UT26285_2010_Human
KP638532_A_Vietnam_UT36282_2010_Human
EPI_ISL_80608_A_chicken_Vietnam_NCVD_405_2010_Avian
EPI_ISL_80613_A_chicken_Vietnam_NCVD_412_2010_Avian
EPI_ISL_80612_A_chicken_Vietnam_NCVD_410_2010_Avian
EPI_ISL_80611_A_chicken_Vietnam_NCVD_409_2010_Avian
EPI_ISL_80610_A_chicken_Vietnam_NCVD_407_2010_Avian
EPI_ISL_80609_A_chicken_Vietnam_NCVD_406_2010_Avian
EPI_ISL_80607_A_chicken_Vietnam_NCVD_404_2010_Avian

NP-Group42

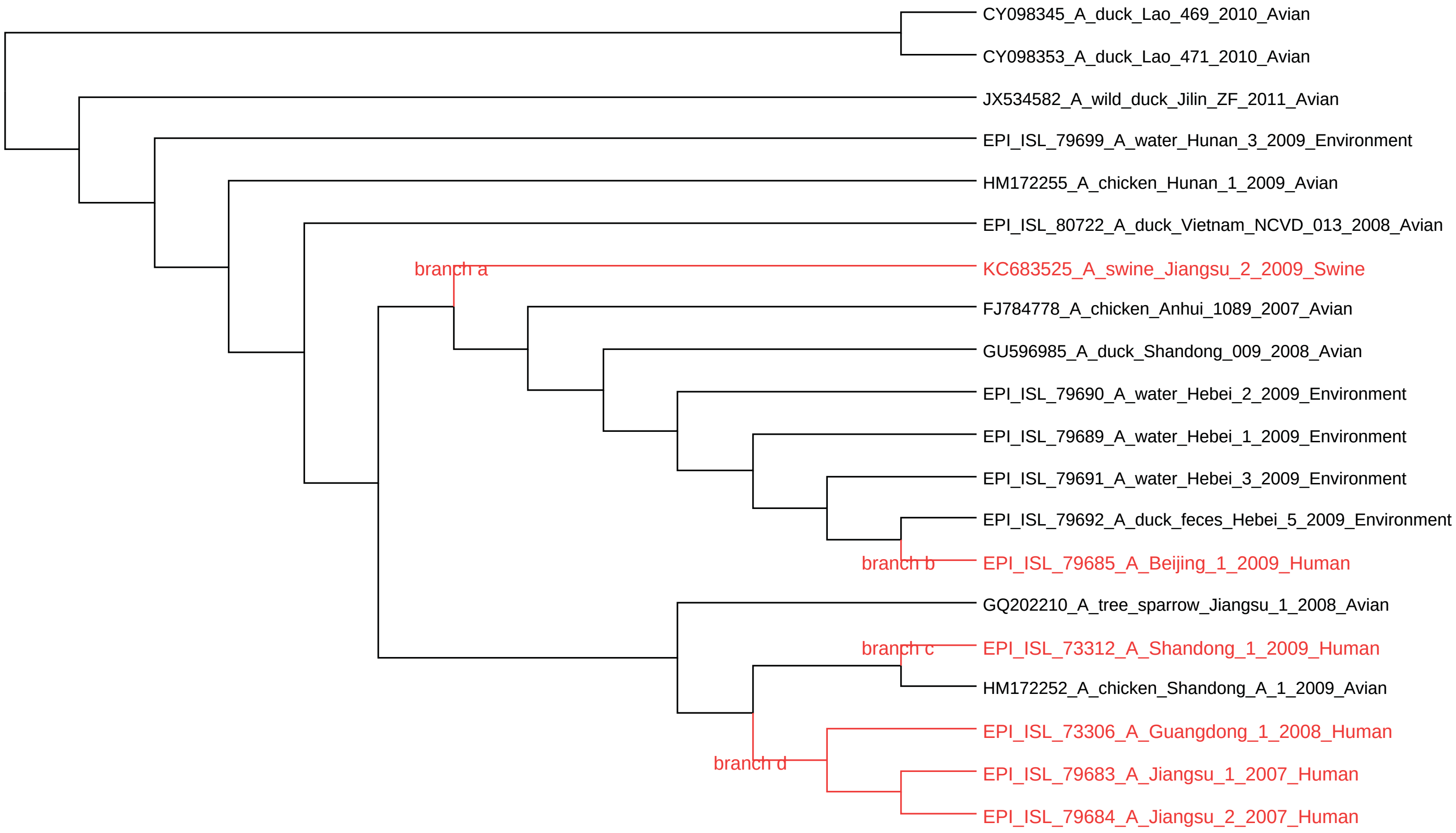
CY098345_A_duck_Lao_469_2010_Avian
CY098353_A_duck_Lao_471_2010_Avian
JX534582_A_wild_duck_Jilin_ZF_2011_Avian
EPI_ISL_79699_A_water_Hunan_3_2009_Environment
HM172255_A_chicken_Hunan_1_2009_Avian
EPI_ISL_80722_A_duck_Vietnam_NCVD_013_2008_Avian
branch a
KC683525_A_swine_Jiangsu_2_2009_Swine
FJ784778_A_chicken_Anhui_1089_2007_Avian
GU596985_A_duck_Shandong_009_2008_Avian
EPI_ISL_79690_A_water_Hebei_2_2009_Environment
EPI_ISL_79689_A_water_Hebei_1_2009_Environment
EPI_ISL_79691_A_water_Hebei_3_2009_Environment
EPI_ISL_79692_A_duck_feces_Hebei_5_2009_Environment
branch b
EPI_ISL_79685_A_Beijing_1_2009_Human
GQ202210_A_tree_sparrow_Jiangsu_1_2008_Avian
branch c
EPI_ISL_73312_A_Shandong_1_2009_Human
HM172252_A_chicken_Shandong_A_1_2009_Avian
EPI_ISL_73306_A_Guangdong_1_2008_Human
branch d
EPI_ISL_79683_A_Jiangsu_1_2007_Human
EPI_ISL_79684_A_Jiangsu_2_2007_Human

NP-Group43

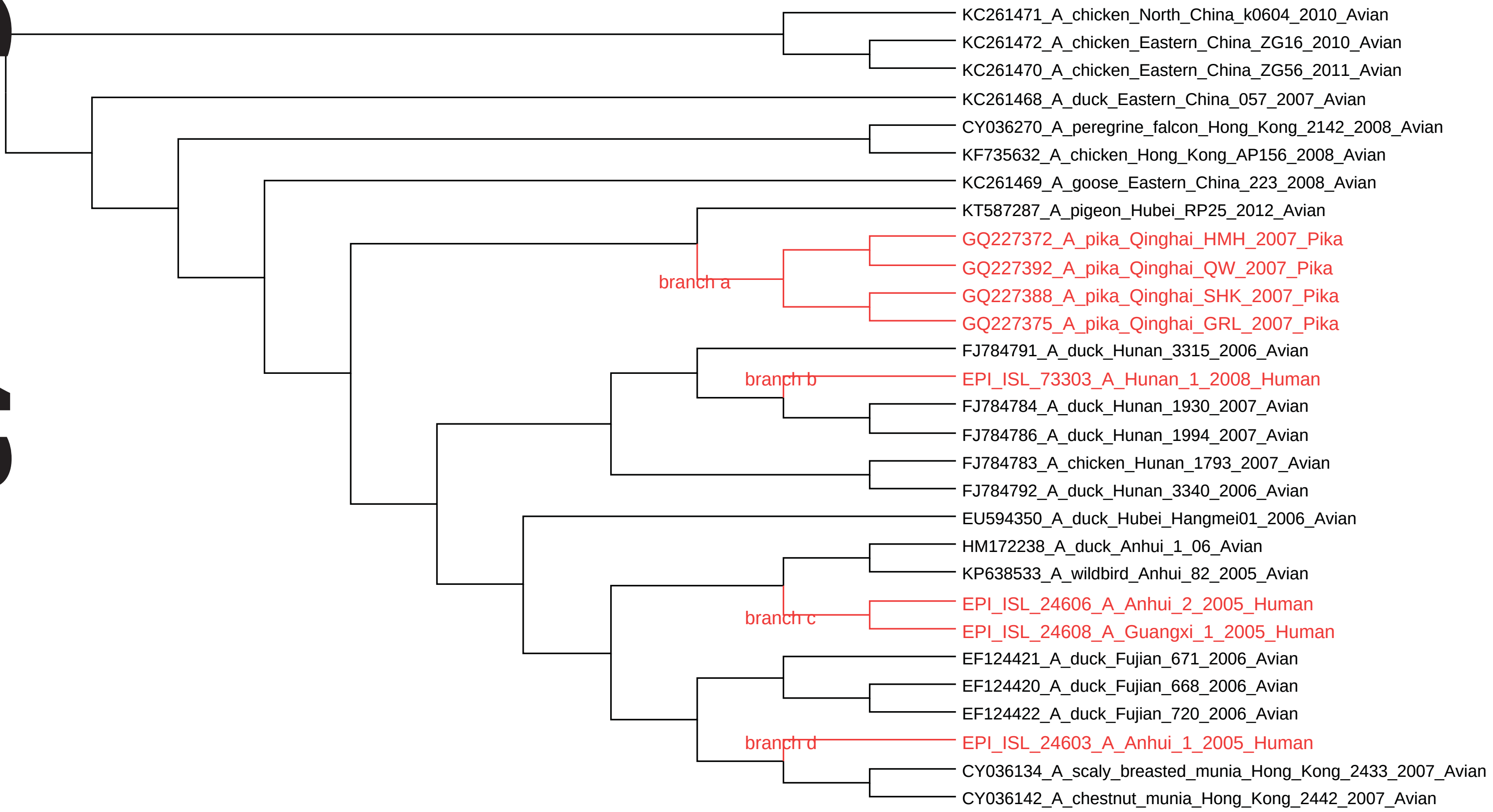
KC261471_A_chicken_North_China_k0604_2010_Avian
KC261472_A_chicken_Eastern_China_ZG16_2010_Avian
KC261470_A_chicken_Eastern_China_ZG56_2011_Avian
KC261468_A_duck_Eastern_China_057_2007_Avian
CY036270_A_peregrine_falcon_Hong_Kong_2142_2008_Avian
KF735632_A_chicken_Hong_Kong_AP156_2008_Avian
KC261469_A_goose_Eastern_China_223_2008_Avian
KT587287_A_pigeon_Hubei_RP25_2012_Avian
GQ227372_A_pika_Qinghai_HMH_2007_Pika
GQ227392_A_pika_Qinghai_QW_2007_Pika
branch a
GQ227388_A_pika_Qinghai_SHK_2007_Pika
GQ227375_A_pika_Qinghai_GRL_2007_Pika
FJ784791_A_duck_Hunan_3315_2006_Avian
branch b
EPI_ISL_73303_A_Hunan_1_2008_Human
FJ784784_A_duck_Hunan_1930_2007_Avian
FJ784786_A_duck_Hunan_1994_2007_Avian
FJ784783_A_chicken_Hunan_1793_2007_Avian
FJ784792_A_duck_Hunan_3340_2006_Avian
EU594350_A_duck_Hubei_Hangmei01_2006_Avian
HM172238_A_duck_Anhui_1_06_Avian
KP638533_A_wildbird_Anhui_82_2005_Avian
EPI_ISL_24606_A_Anhui_2_2005_Human
branch c
EPI_ISL_24608_A_Guangxi_1_2005_Human
EF124421_A_duck_Fujian_671_2006_Avian
EF124420_A_duck_Fujian_668_2006_Avian
EF124422_A_duck_Fujian_720_2006_Avian
branch d
EPI_ISL_24603_A_Anhui_1_2005_Human
CY036134_A_scaly_breasted_munia_Hong_Kong_2433_2007_Avian
CY036142_A_chestnut_munia_Hong_Kong_2442_2007_Avian

NP-Group44

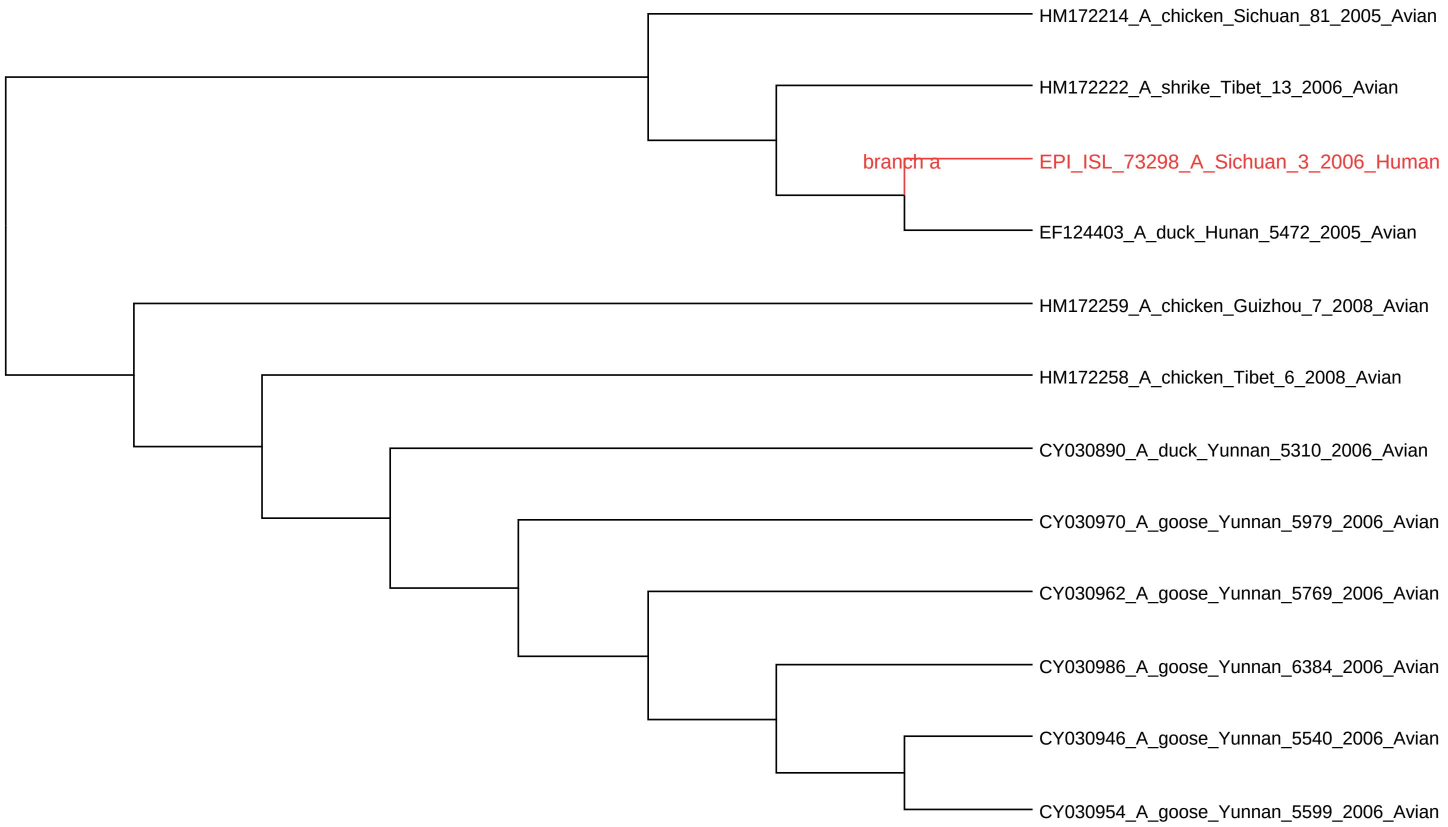
HM172214_A_chicken_Sichuan_81_2005_Avian
HM172222_A_shrike_Tibet_13_2006_Avian
branch a
EPI_ISL_73298_A_Sichuan_3_2006_Human
EF124403_A_duck_Hunan_5472_2005_Avian
HM172259_A_chicken_Guizhou_7_2008_Avian
HM172258_A_chicken_Tibet_6_2008_Avian
CY030890_A_duck_Yunnan_5310_2006_Avian
CY030970_A_goose_Yunnan_5979_2006_Avian
CY030962_A_goose_Yunnan_5769_2006_Avian
CY030986_A_goose_Yunnan_6384_2006_Avian
CY030946_A_goose_Yunnan_5540_2006_Avian
CY030954_A_goose_Yunnan_5599_2006_Avian

NP-Group45

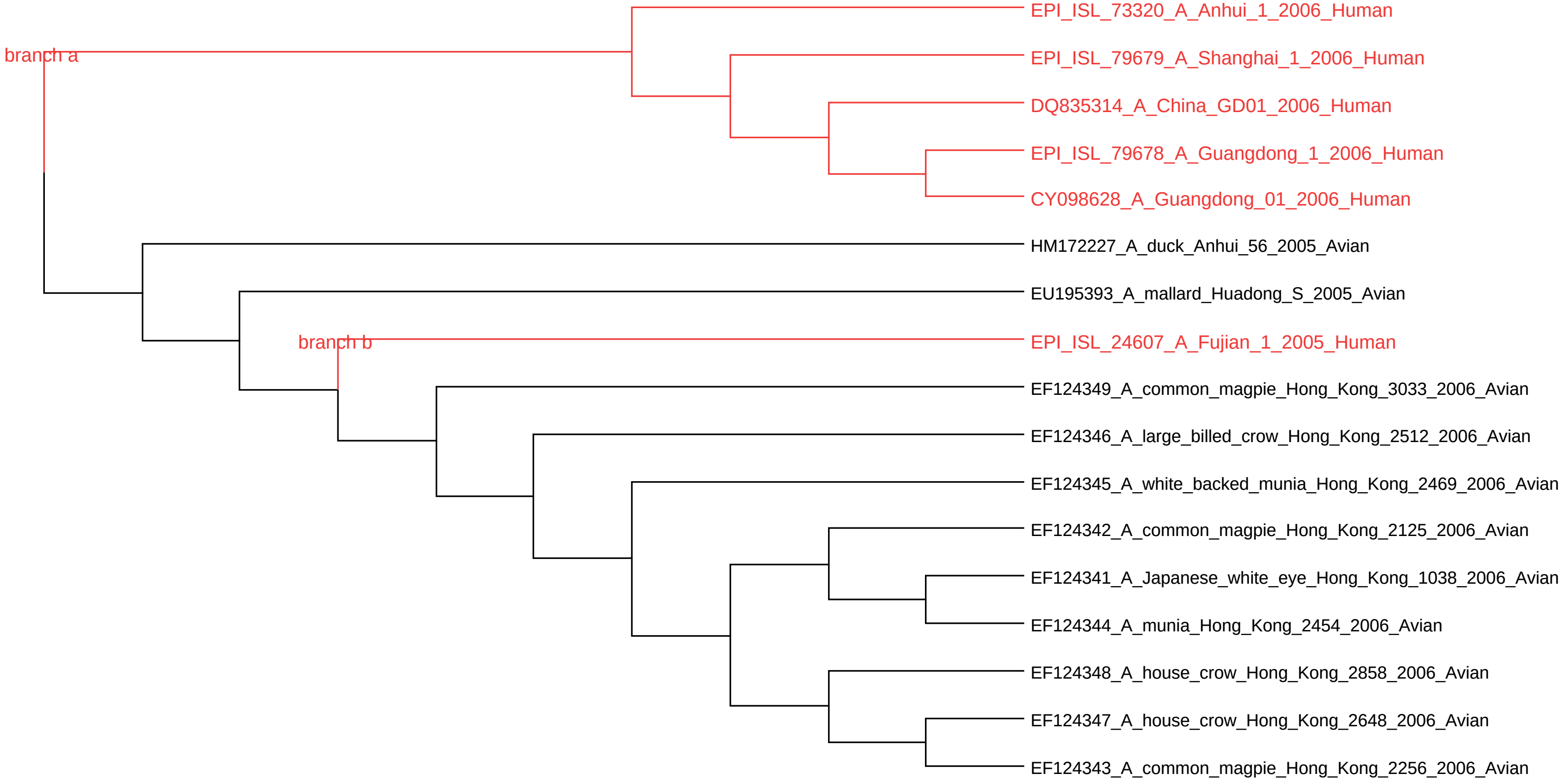

branch a
EPI_ISL_73320_A_Anhui_1_2006_Human
EPI_ISL_79679_A_Shanghai_1_2006_Human
DQ835314_A_China_GD01_2006_Human
EPI_ISL_79678_A_Guangdong_1_2006_Human
CY098628_A_Guangdong_01_2006_Human
HM172227_A_duck_Anhui_56_2005_Avian
EU195393_A_mallard_Huadong_S_2005_Avian
branch b
EPI_ISL_24607_A_Fujian_1_2005_Human
EF124349_A_common_magpie_Hong_Kong_3033_2006_Avian
EF124346_A_large_billed_crow_Hong_Kong_2512_2006_Avian
EF124345_A_white_backed_munia_Hong_Kong_2469_2006_Avian
EF124342_A_common_magpie_Hong_Kong_2125_2006_Avian
EF124341_A_Japanese_white_eye_Hong_Kong_1038_2006_Avian
EF124344_A_munia_Hong_Kong_2454_2006_Avian
EF124348_A_house_crow_Hong_Kong_2858_2006_Avian
EF124347_A_house_crow_Hong_Kong_2648_2006_Avian
EF124343_A_common_magpie_Hong_Kong_2256_2006_Avian

NP-Group46

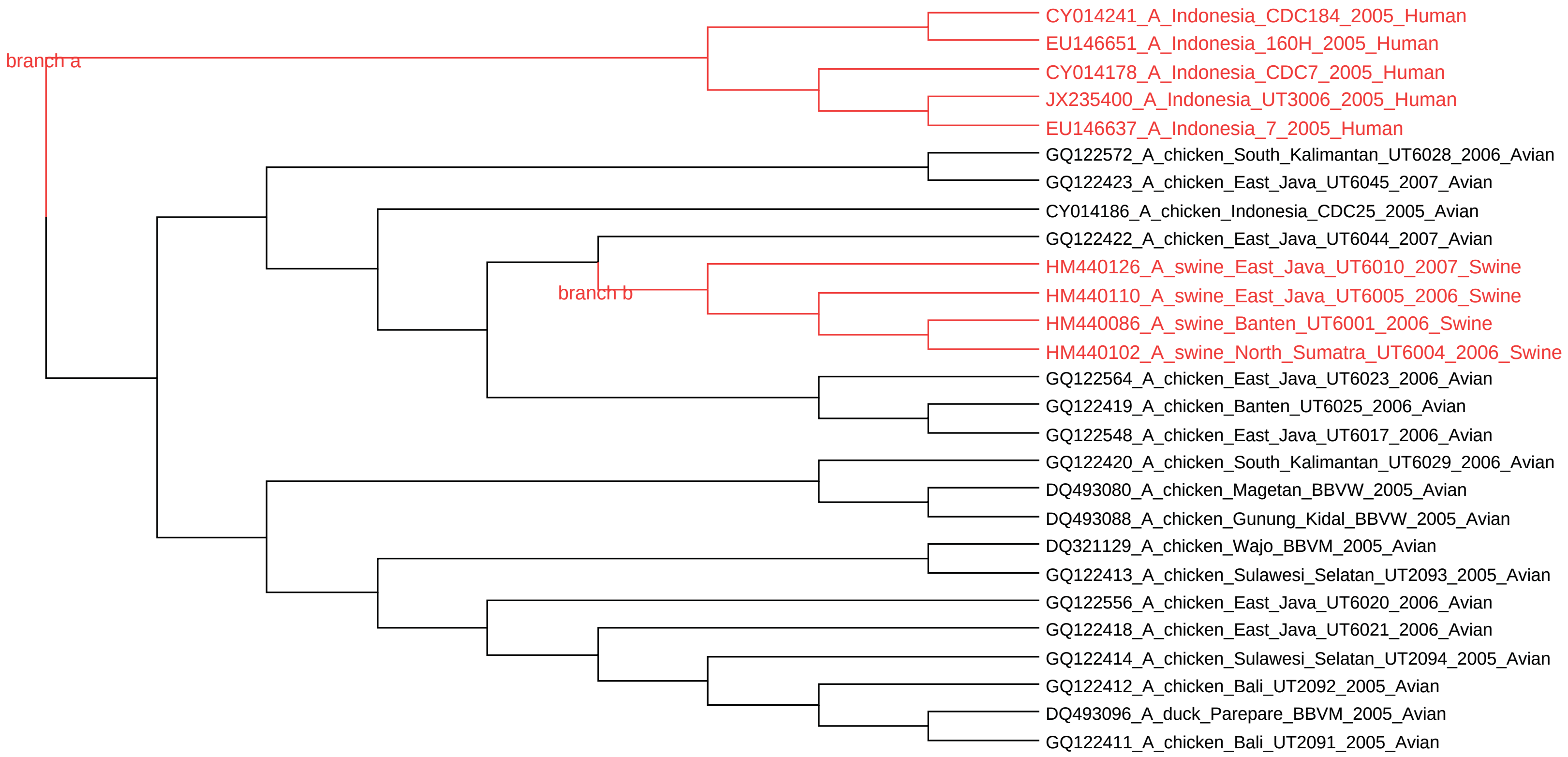
branch a
branch b
CY014241_A_Indonesia_CDC184_2005_Human
EU146651_A_Indonesia_160H_2005_Human
CY014178_A_Indonesia_CDC7_2005_Human
JX235400_A_Indonesia_UT3006_2005_Human
EU146637_A_Indonesia_7_2005_Human
GQ122572_A_chicken_South_Kalimantan_UT6028_2006_Avian
GQ122423_A_chicken_East_Java_UT6045_2007_Avian
CY014186_A_chicken_Indonesia_CDC25_2005_Avian
GQ122422_A_chicken_East_Java_UT6044_2007_Avian
HM440126_A_swine_East_Java_UT6010_2007_Swine
HM440110_A_swine_East_Java_UT6005_2006_Swine
HM440086_A_swine_Banten_UT6001_2006_Swine
HM440102_A_swine_North_Sumatra_UT6004_2006_Swine
GQ122564_A_chicken_East_Java_UT6023_2006_Avian
GQ122419_A_chicken_Banten_UT6025_2006_Avian
GQ122548_A_chicken_East_Java_UT6017_2006_Avian
GQ122420_A_chicken_South_Kalimantan_UT6029_2006_Avian
DQ493080_A_chicken_Magetan_BBVW_2005_Avian
DQ493088_A_chicken_Gunung_Kidal_BBVW_2005_Avian
DQ321129_A_chicken_Wajo_BBVM_2005_Avian
GQ122413_A_chicken_Sulawesi_Selatan_UT2093_2005_Avian
GQ122556_A_chicken_East_Java_UT6020_2006_Avian
GQ122418_A_chicken_East_Java_UT6021_2006_Avian
GQ122414_A_chicken_Sulawesi_Selatan_UT2094_2005_Avian
GQ122412_A_chicken_Bali_UT2092_2005_Avian
DQ493096_A_duck_Parepare_BBVM_2005_Avian
GQ122411_A_chicken_Bali_UT2091_2005_Avian

NP-Group47

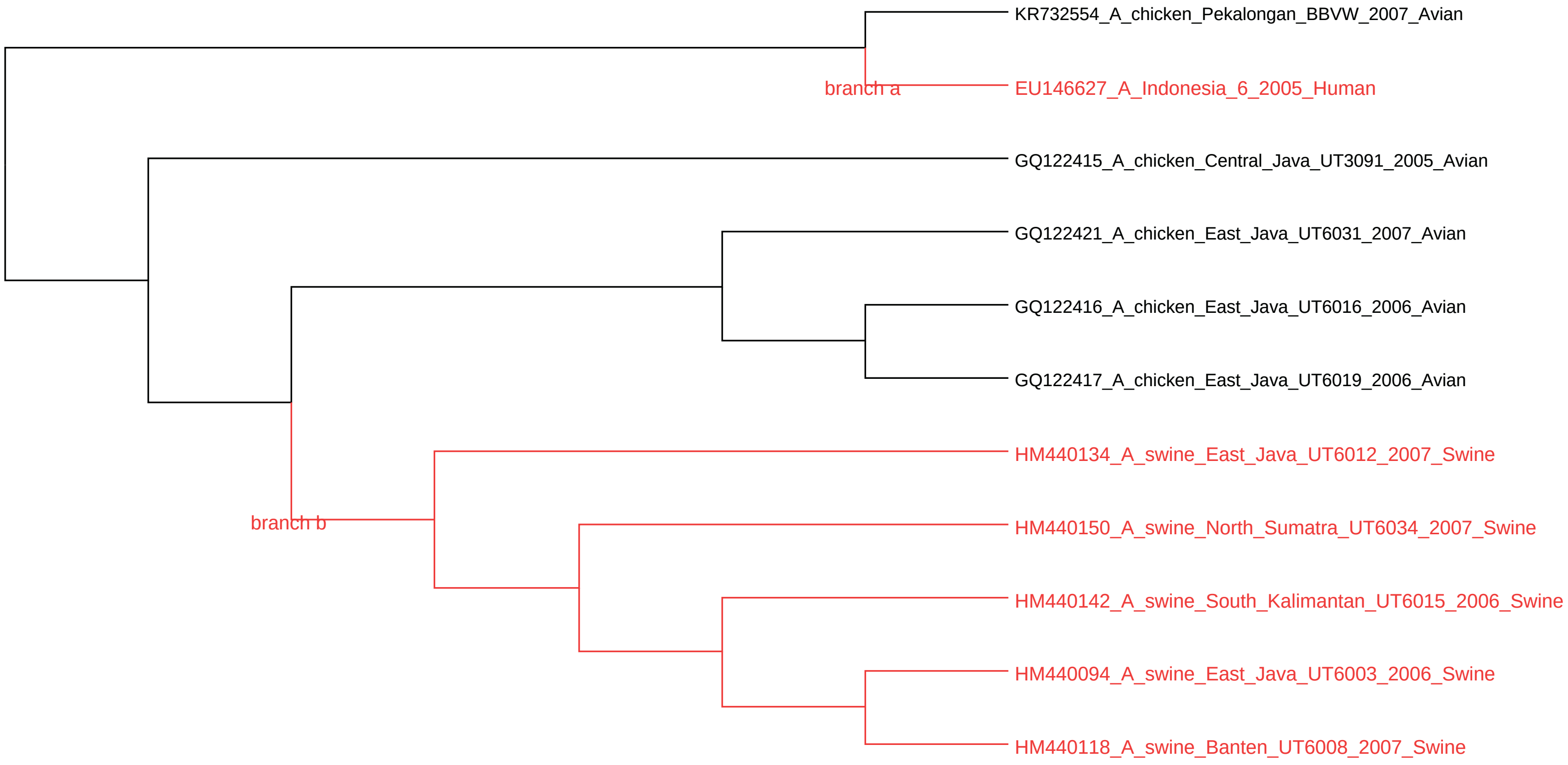
KR732554_A_chicken_Pekalongan_BBVW_2007_Avian
branch a
EU146627_A_Indonesia_6_2005_Human
GQ122415_A_chicken_Central_Java_UT3091_2005_Avian
GQ122421_A_chicken_East_Java_UT6031_2007_Avian
GQ122416_A_chicken_East_Java_UT6016_2006_Avian
GQ122417_A_chicken_East_Java_UT6019_2006_Avian
HM440134_A_swine_East_Java_UT6012_2007_Swine
branch b
HM440150_A_swine_North_Sumatra_UT6034_2007_Swine
HM440142_A_swine_South_Kalimantan_UT6015_2006_Swine
HM440094_A_swine_East_Java_UT6003_2006_Swine
HM440118_A_swine_Banten_UT6008_2007_Swine

NP-Group48

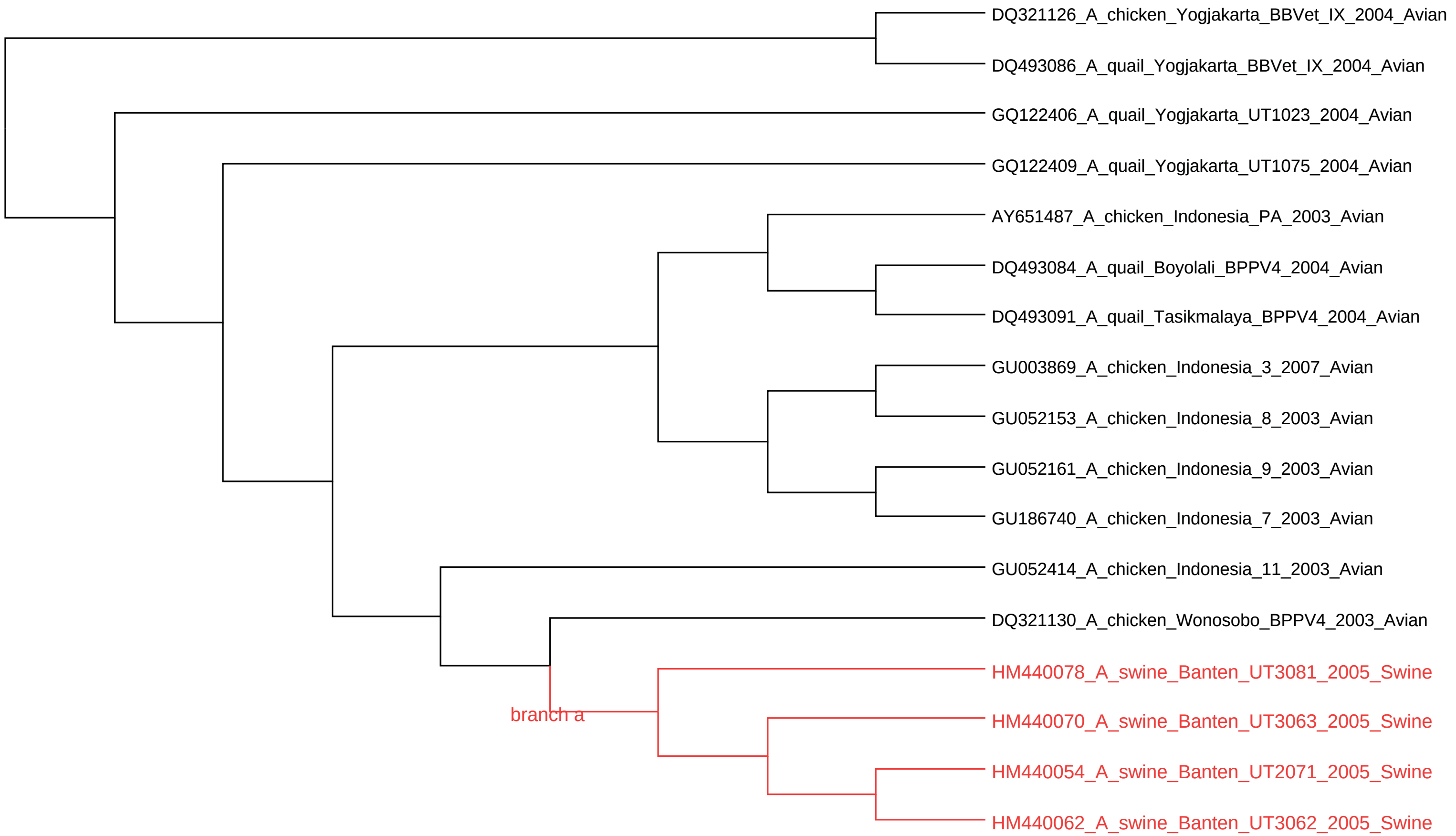
DQ321126_A_chicken_Yogjakarta_BBVet_IX_2004_Avian
DQ493086_A_quail_Yogjakarta_BBVet_IX_2004_Avian
GQ122406_A_quail_Yogjakarta_UT1023_2004_Avian
GQ122409_A_quail_Yogjakarta_UT1075_2004_Avian
AY651487_A_chicken_Indonesia_PA_2003_Avian
DQ493084_A_quail_Boyolali_BPPV4_2004_Avian
DQ493091_A_quail_Tasikmalaya_BPPV4_2004_Avian
GU003869_A_chicken_Indonesia_3_2007_Avian
GU052153_A_chicken_Indonesia_8_2003_Avian
GU052161_A_chicken_Indonesia_9_2003_Avian
GU186740_A_chicken_Indonesia_7_2003_Avian
GU052414_A_chicken_Indonesia_11_2003_Avian
DQ321130_A_chicken_Wonosobo_BPPV4_2003_Avian
branch a
HM440078_A_swine_Banten_UT3081_2005_Swine
HM440070_A_swine_Banten_UT3063_2005_Swine
HM440054_A_swine_Banten_UT2071_2005_Swine
HM440062_A_swine_Banten_UT3062_2005_Swine

NP-Group49

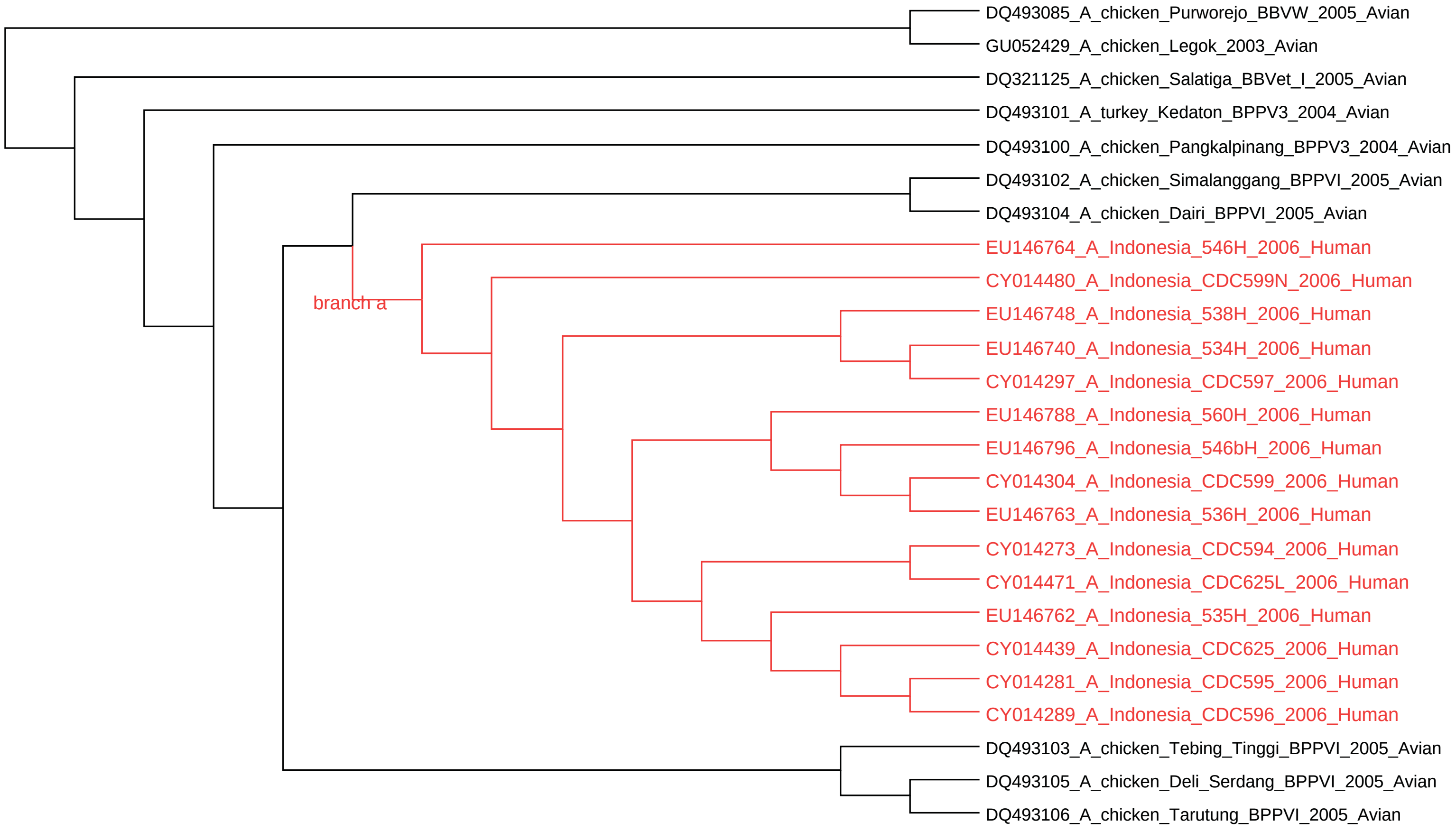
DQ493085_A_chicken_Purworejo_BBVW_2005_Avian
GU052429_A_chicken_Legok_2003_Avian
DQ321125_A_chicken_Salatiga_BBVet_I_2005_Avian
DQ493101_A_turkey_Kedaton_BPPV3_2004_Avian
DQ493100_A_chicken_Pangkalpinang_BPPV3_2004_Avian
DQ493102_A_chicken_Simalanggang_BPPVI_2005_Avian
DQ493104_A_chicken_Dairi_BPPVI_2005_Avian
branch a
EU146764_A_Indonesia_546H_2006_Human
CY014480_A_Indonesia_CDC599N_2006_Human
EU146748_A_Indonesia_538H_2006_Human
EU146740_A_Indonesia_534H_2006_Human
CY014297_A_Indonesia_CDC597_2006_Human
EU146788_A_Indonesia_560H_2006_Human
EU146796_A_Indonesia_546bH_2006_Human
CY014304_A_Indonesia_CDC599_2006_Human
EU146763_A_Indonesia_536H_2006_Human
CY014273_A_Indonesia_CDC594_2006_Human
CY014471_A_Indonesia_CDC625L_2006_Human
EU146762_A_Indonesia_535H_2006_Human
CY014439_A_Indonesia_CDC625_2006_Human
CY014281_A_Indonesia_CDC595_2006_Human
CY014289_A_Indonesia_CDC596_2006_Human
DQ493103_A_chicken_Tebing_Tinggi_BPPVI_2005_Avian
DQ493105_A_chicken_Deli_Serdang_BPPVI_2005_Avian
DQ493106_A_chicken_Tarutung_BPPVI_2005_Avian

NP-Group50

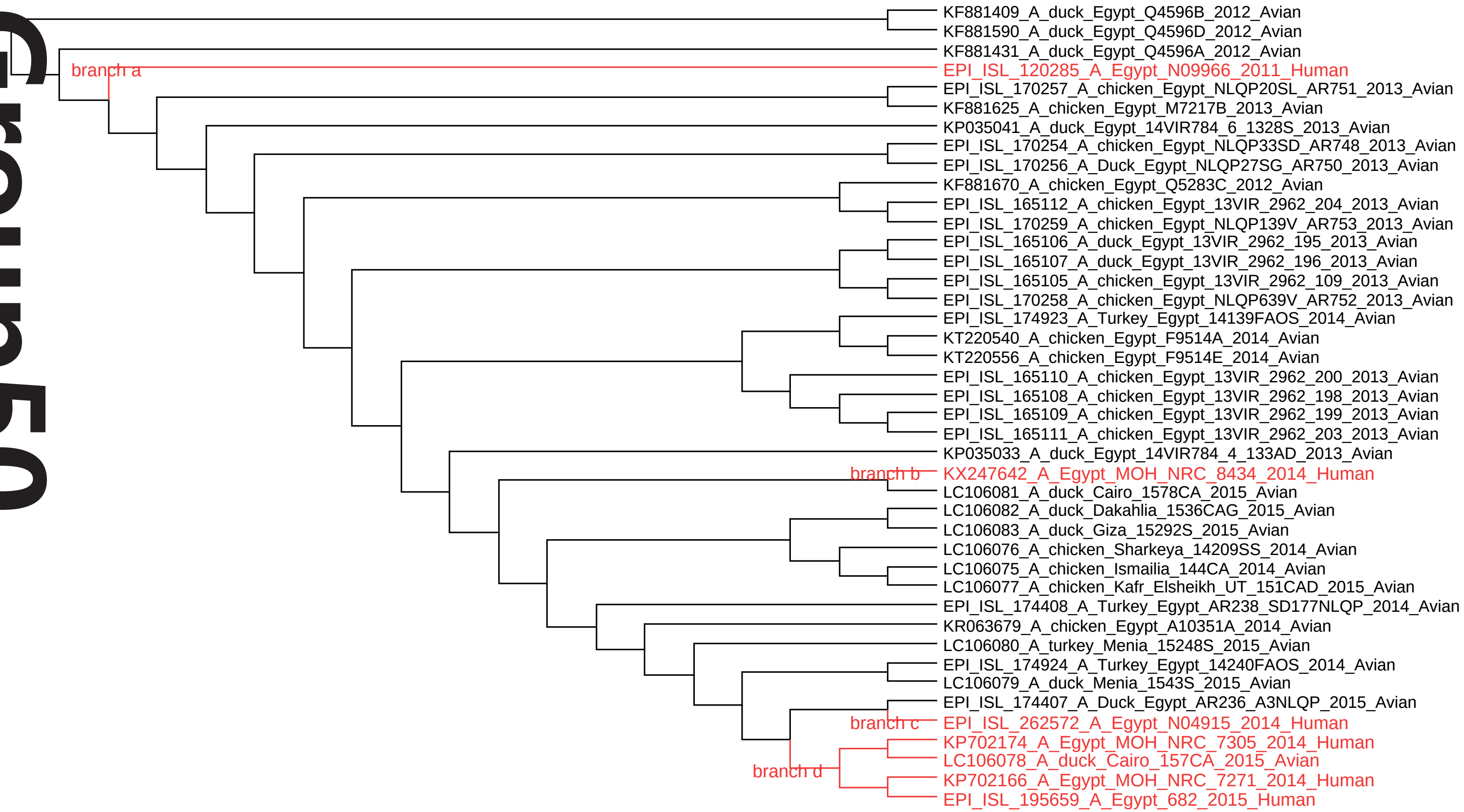

KF881409_A_duck_Egypt_Q4596B_2012_Avian
KF881590_A_duck_Egypt_Q4596D_2012_Avian
KF881431_A_duck_Egypt_Q4596A_2012_Avian
branch a
EPI_ISL_120285_A_Egypt_N09966_2011_Human
EPI_ISL_170257_A_chicken_Egypt_NLQP20SL_AR751_2013_Avian
KF881625_A_chicken_Egypt_M7217B_2013_Avian
KP035041_A_duck_Egypt_14VIR784_6_1328S_2013_Avian
EPI_ISL_170254_A_chicken_Egypt_NLQP33SD_AR748_2013_Avian
EPI_ISL_170256_A_Duck_Egypt_NLQP27SG_AR750_2013_Avian
KF881670_A_chicken_Egypt_Q5283C_2012_Avian
EPI_ISL_165112_A_chicken_Egypt_13VIR_2962_204_2013_Avian
EPI_ISL_170259_A_chicken_Egypt_NLQP139V_AR753_2013_Avian
EPI_ISL_165106_A_duck_Egypt_13VIR_2962_195_2013_Avian
EPI_ISL_165107_A_duck_Egypt_13VIR_2962_196_2013_Avian
EPI_ISL_165105_A_chicken_Egypt_13VIR_2962_109_2013_Avian
EPI_ISL_170258_A_chicken_Egypt_NLQP639V_AR752_2013_Avian
EPI_ISL_174923_A_Turkey_Egypt_14139FAOS_2014_Avian
KT220540_A_chicken_Egypt_F9514A_2014_Avian
KT220556_A_chicken_Egypt_F9514E_2014_Avian
EPI_ISL_165110_A_chicken_Egypt_13VIR_2962_200_2013_Avian
EPI_ISL_165108_A_chicken_Egypt_13VIR_2962_198_2013_Avian
EPI_ISL_165109_A_chicken_Egypt_13VIR_2962_199_2013_Avian
EPI_ISL_165111_A_chicken_Egypt_13VIR_2962_203_2013_Avian
KP035033_A_duck_Egypt_14VIR784_4_133AD_2013_Avian
branch b
KX247642_A_Egypt_MOH_NRC_8434_2014_Human
LC106081_A_duck_Cairo_1578CA_2015_Avian
LC106082_A_duck_Dakahlia_1536CAG_2015_Avian
LC106083_A_duck_Giza_15292S_2015_Avian
LC106076_A_chicken_Sharkeya_14209SS_2014_Avian
LC106075_A_chicken_Ismailia_144CA_2014_Avian
LC106077_A_chicken_Kafr_Elsheikh_UT_151CAD_2015_Avian
EPI_ISL_174408_A_Turkey_Egypt_AR238_SD177NLQP_2014_Avian
KR063679_A_chicken_Egypt_A10351A_2014_Avian
LC106080_A_turkey_Menia_15248S_2015_Avian
EPI_ISL_174924_A_Turkey_Egypt_14240FAOS_2014_Avian
LC106079_A_duck_Menia_1543S_2015_Avian
EPI_ISL_174407_A_Duck_Egypt_AR236_A3NLQP_2015_Avian
branch c
EPI_ISL_262572_A_Egypt_N04915_2014_Human
KP702174_A_Egypt_MOH_NRC_7305_2014_Human
branch d
LC106078_A_duck_Cairo_157CA_2015_Avian
KP702166_A_Egypt_MOH_NRC_7271_2014_Human
EPI_ISL_195659_A_Egypt_682_2015_Human

NP-Group51

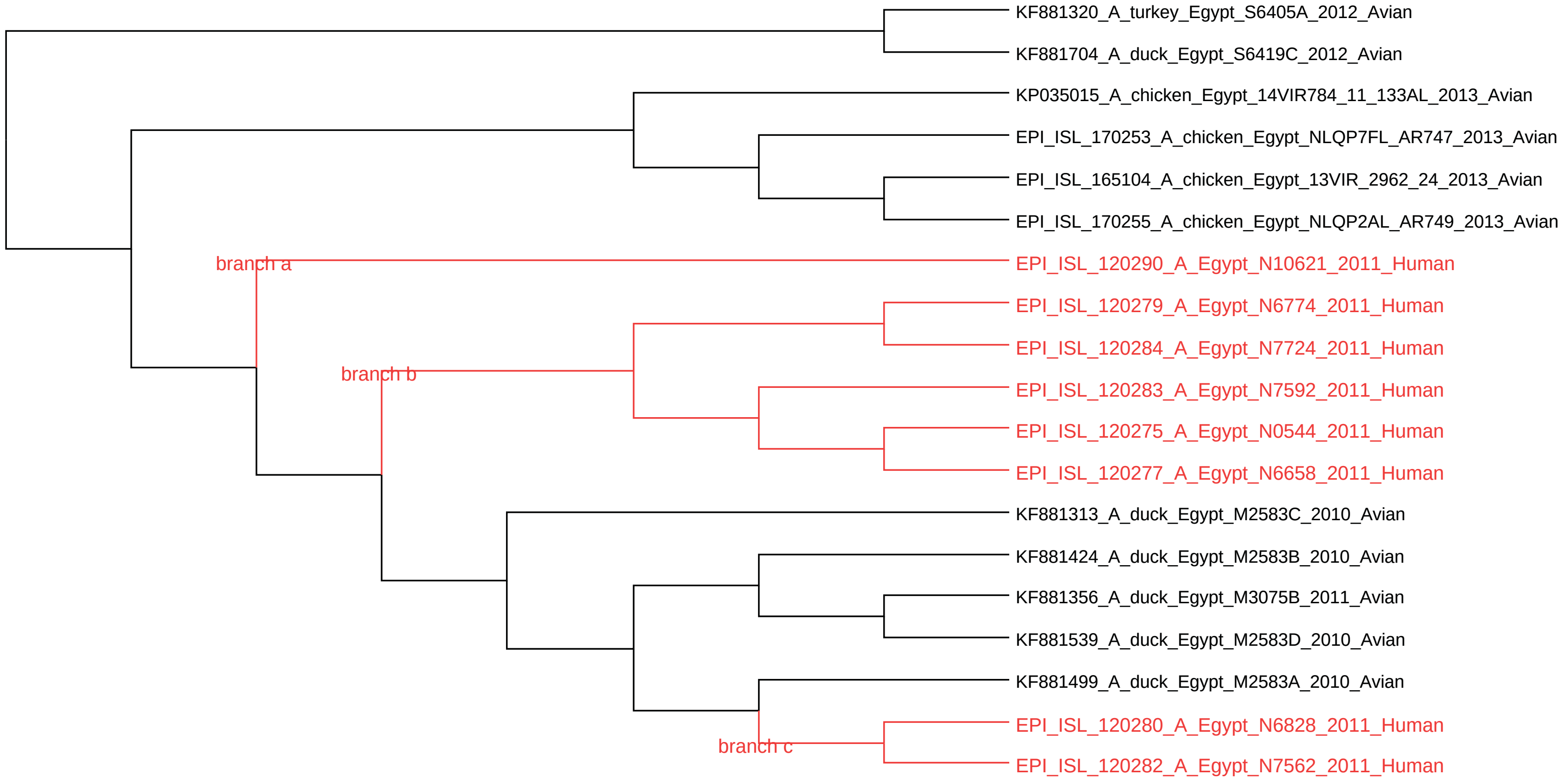
KF881320_A_turkey_Egypt_S6405A_2012_Avian
KF881704_A_duck_Egypt_S6419C_2012_Avian
KP035015_A_chicken_Egypt_14VIR784_11_133AL_2013_Avian
EPI_ISL_170253_A_chicken_Egypt_NLQP7FL_AR747_2013_Avian
EPI_ISL_165104_A_chicken_Egypt_13VIR_2962_24_2013_Avian
EPI_ISL_170255_A_chicken_Egypt_NLQP2AL_AR749_2013_Avian
branch a
EPI_ISL_120290_A_Egypt_N10621_2011_Human
EPI_ISL_120279_A_Egypt_N6774_2011_Human
EPI_ISL_120284_A_Egypt_N7724_2011_Human
branch b
EPI_ISL_120283_A_Egypt_N7592_2011_Human
EPI_ISL_120275_A_Egypt_N0544_2011_Human
EPI_ISL_120277_A_Egypt_N6658_2011_Human
KF881313_A_duck_Egypt_M2583C_2010_Avian
KF881424_A_duck_Egypt_M2583B_2010_Avian
KF881356_A_duck_Egypt_M3075B_2011_Avian
KF881539_A_duck_Egypt_M2583D_2010_Avian
KF881499_A_duck_Egypt_M2583A_2010_Avian
EPI_ISL_120280_A_Egypt_N6828_2011_Human
branch c
EPI_ISL_120282_A_Egypt_N7562_2011_Human

NP-Group52

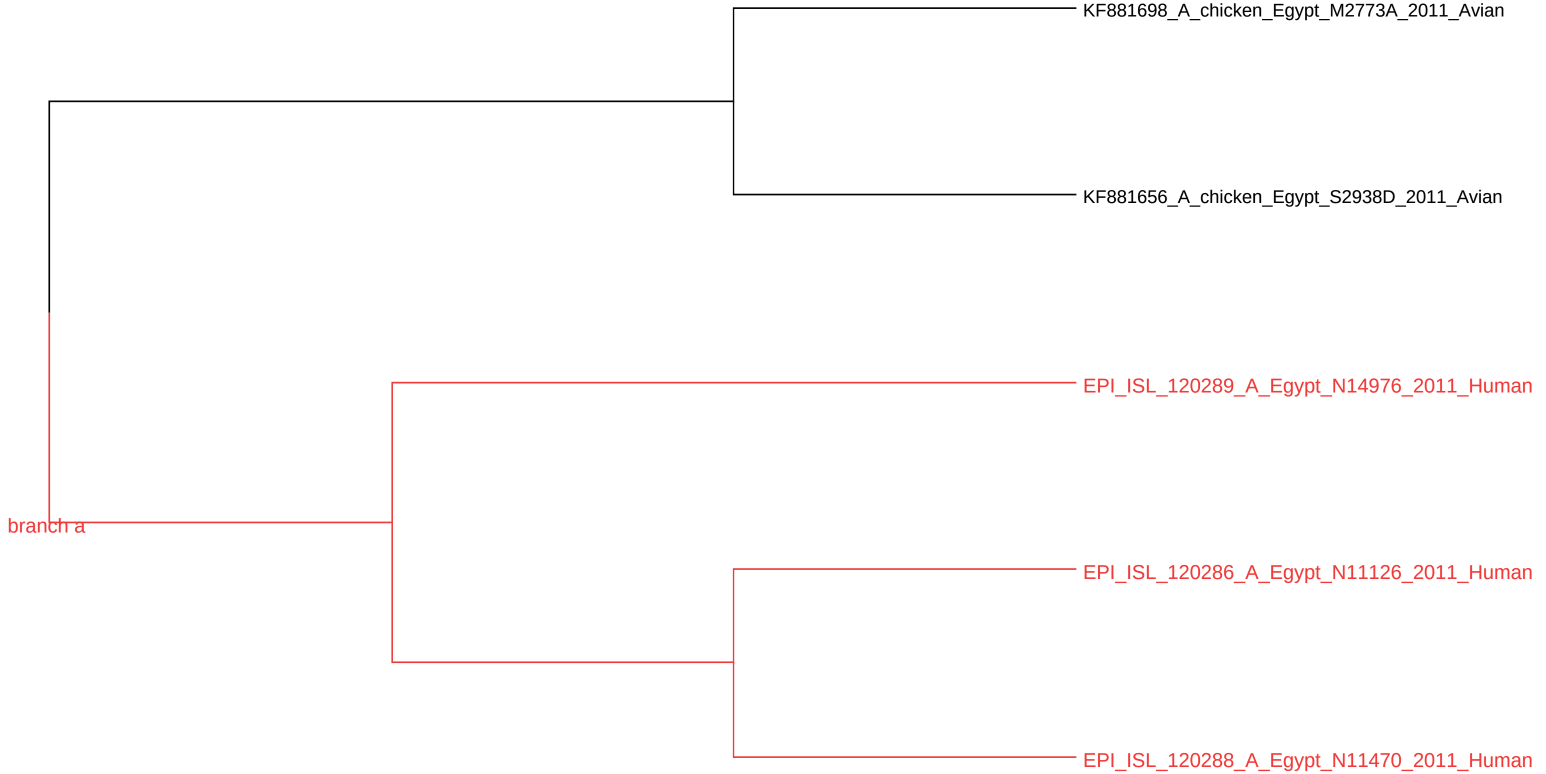
KF881698_A_chicken_Egypt_M2773A_2011_Avian
KF881656_A_chicken_Egypt_S2938D_2011_Avian
EPI_ISL_120289_A_Egypt_N14976_2011_Human
branch a
EPI_ISL_120286_A_Egypt_N11126_2011_Human
EPI_ISL_120288_A_Egypt_N11470_2011_Human

NP-Group53

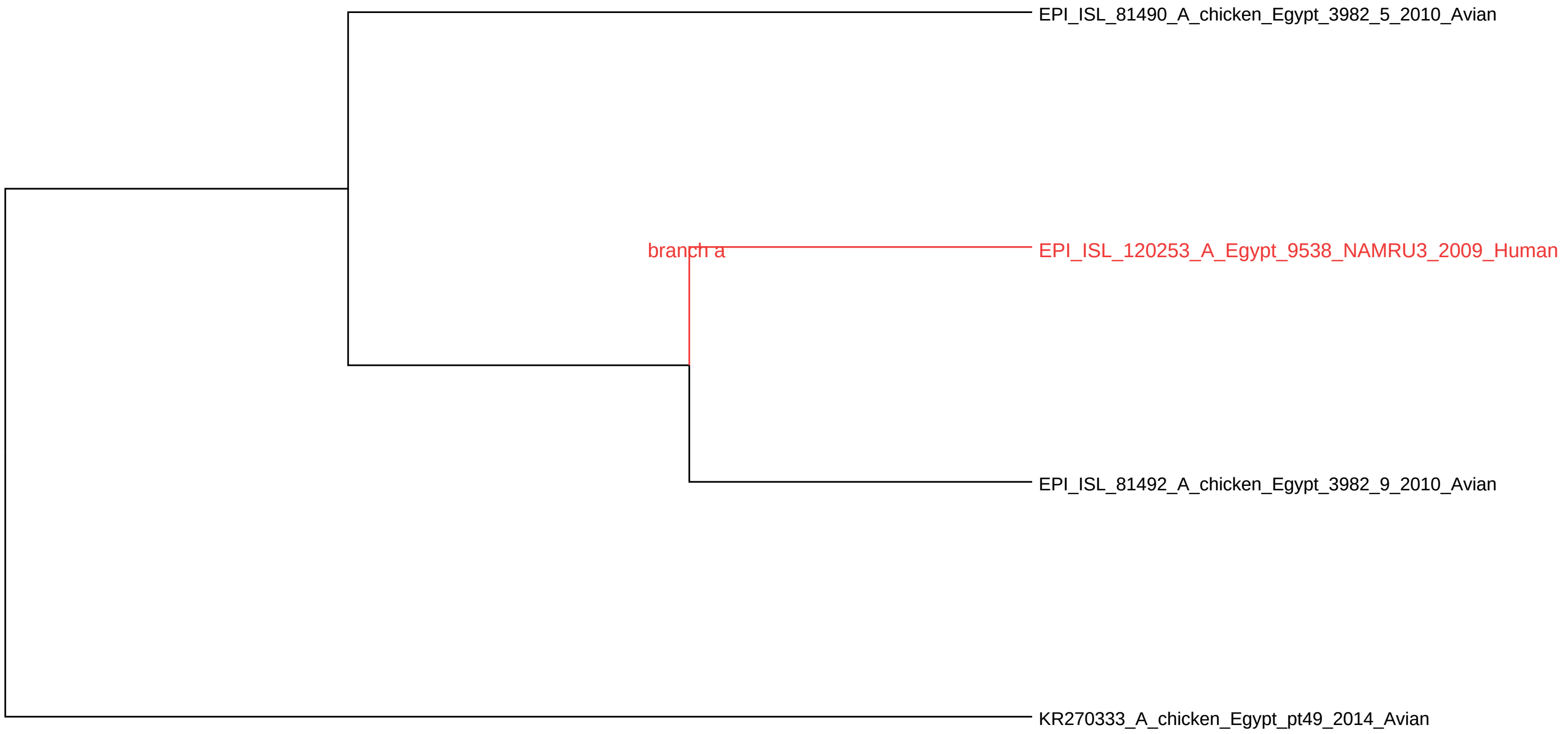
EPI_ISL_81490_A_chicken_Egypt_3982_5_2010_Avian
branch a
EPI_ISL_120253_A_Egypt_9538_NAMRU3_2009_Human
EPI_ISL_81492_A_chicken_Egypt_3982_9_2010_Avian
KR270333_A_chicken_Egypt_pt49_2014_Avian

NP-Group54

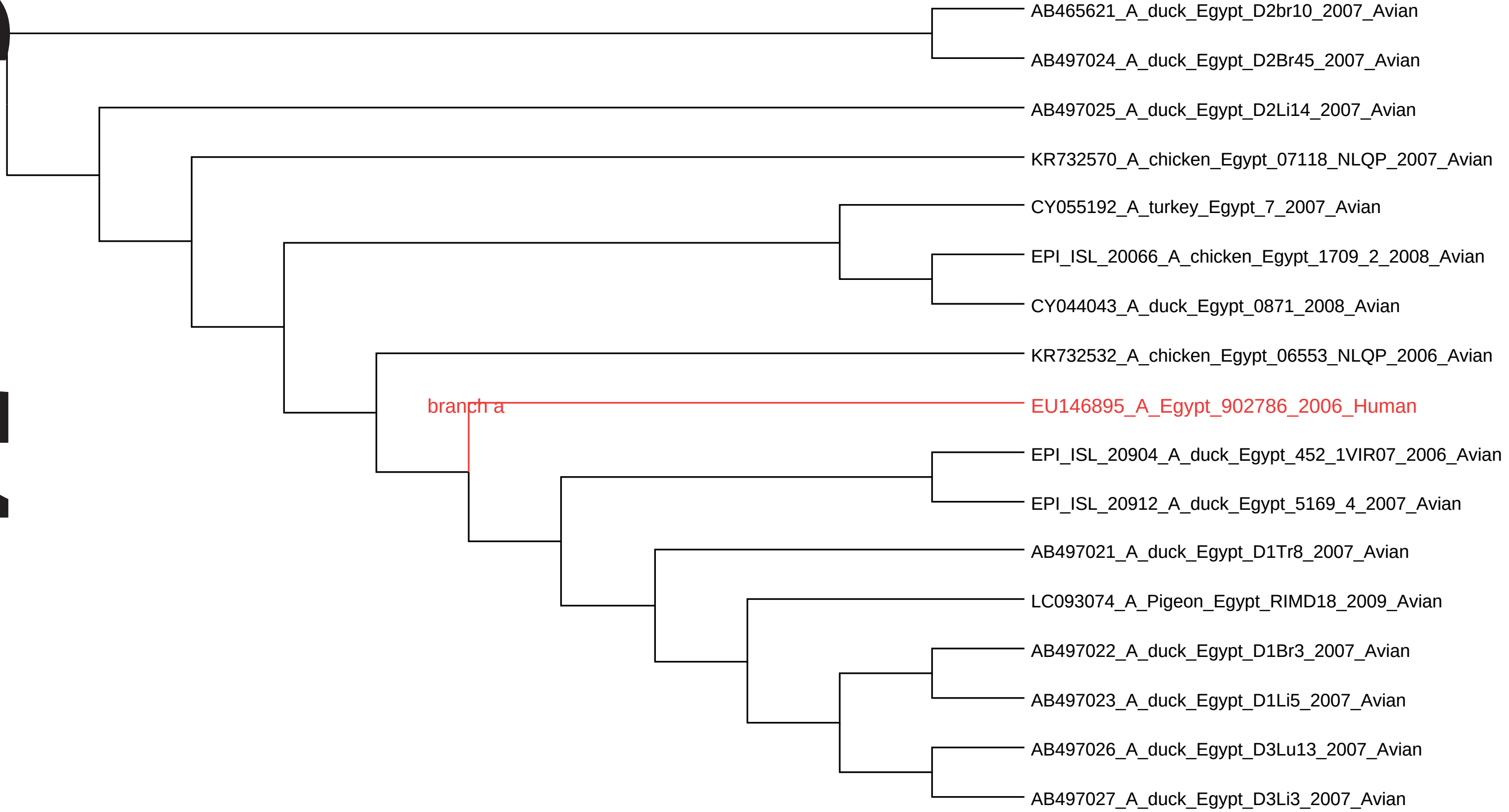

AB465621_A_duck_Egypt_D2br10_2007_Avian
AB497024_A_duck_Egypt_D2Br45_2007_Avian
AB497025_A_duck_Egypt_D2Li14_2007_Avian
KR732570_A_chicken_Egypt_07118_NLQP_2007_Avian
CY055192_A_turkey_Egypt_7_2007_Avian
EPI_ISL_20066_A_chicken_Egypt_1709_2_2008_Avian
CY044043_A_duck_Egypt_0871_2008_Avian
KR732532_A_chicken_Egypt_06553_NLQP_2006_Avian
branch a
EU146895_A_Egypt_902786_2006_Human
EPI_ISL_20904_A_duck_Egypt_452_1VIR07_2006_Avian
EPI_ISL_20912_A_duck_Egypt_5169_4_2007_Avian
AB497021_A_duck_Egypt_D1Tr8_2007_Avian
LC093074_A_Pigeon_Egypt_RIMD18_2009_Avian
AB497022_A_duck_Egypt_D1Br3_2007_Avian
AB497023_A_duck_Egypt_D1Li5_2007_Avian
AB497026_A_duck_Egypt_D3Lu13_2007_Avian
AB497027_A_duck_Egypt_D3Li3_2007_Avian

NP-Group55

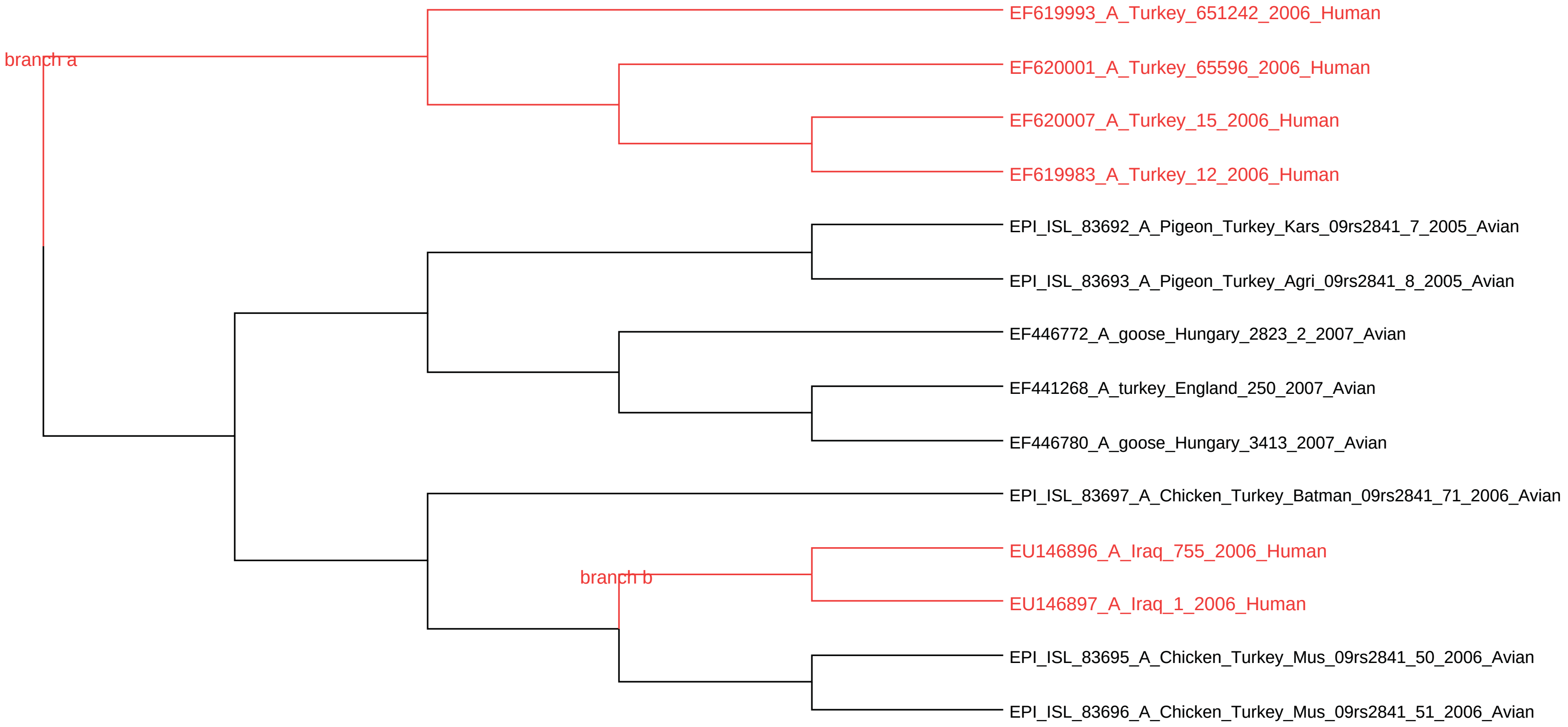
branch a
EF619993_A_Turkey_651242_2006_Human
EF620001_A_Turkey_65596_2006_Human
EF620007_A_Turkey_15_2006_Human
EF619983_A_Turkey_12_2006_Human
EPI_ISL_83692_A_Pigeon_Turkey_Kars_09rs2841_7_2005_Avian
EPI_ISL_83693_A_Pigeon_Turkey_Agri_09rs2841_8_2005_Avian
EF446772_A_goose_Hungary_2823_2_2007_Avian
EF441268_A_turkey_England_250_2007_Avian
EF446780_A_goose_Hungary_3413_2007_Avian
EPI_ISL_83697_A_Chicken_Turkey_Batman_09rs2841_71_2006_Avian
branch b
EU146896_A_Iraq_755_2006_Human
EU146897_A_Iraq_1_2006_Human
EPI_ISL_83695_A_Chicken_Turkey_Mus_09rs2841_50_2006_Avian
EPI_ISL_83696_A_Chicken_Turkey_Mus_09rs2841_51_2006_Avian

NP-Group56

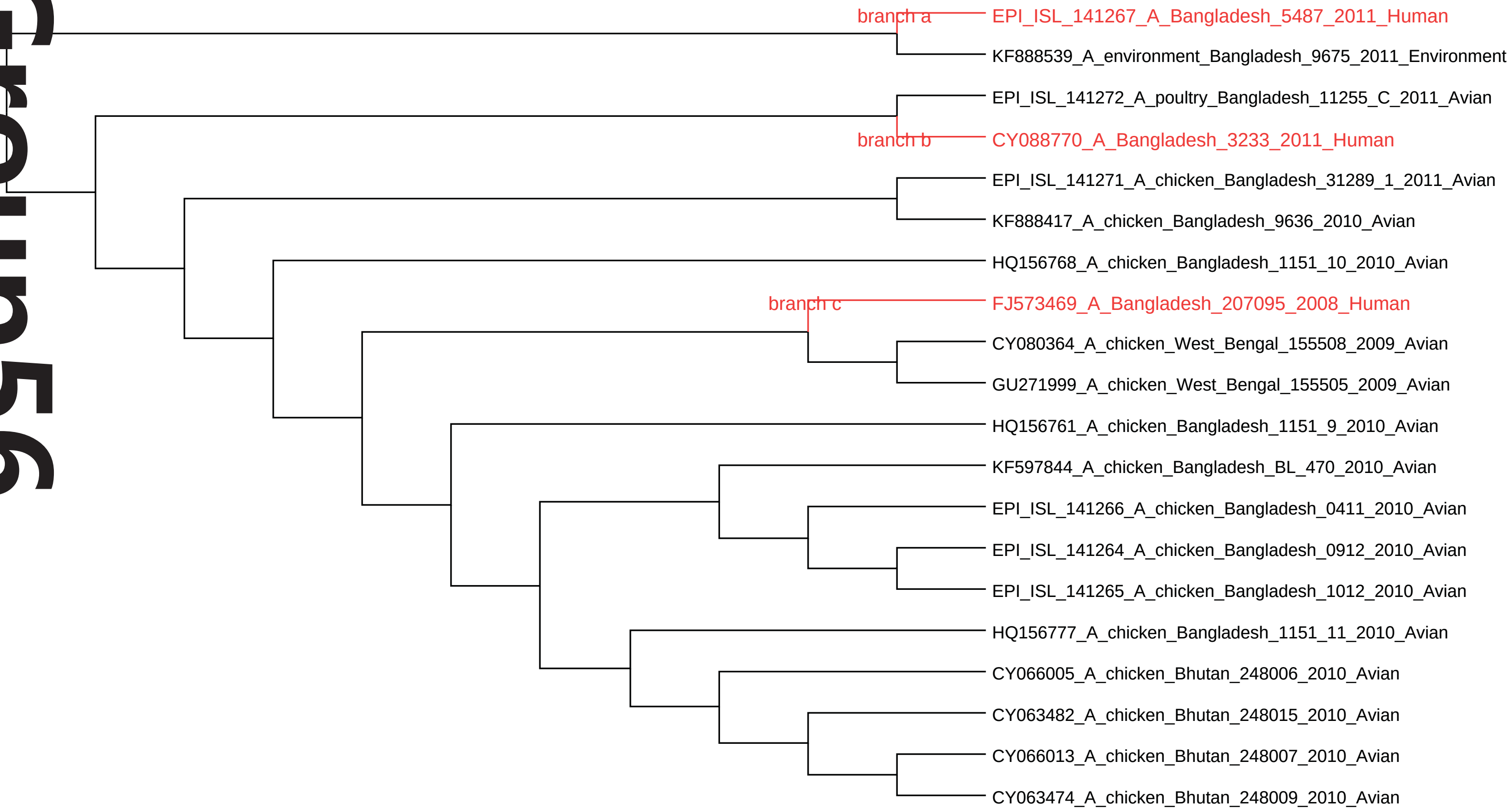
branch a
EPI_ISL_141267_A_Bangladesh_5487_2011_Human
KF888539_A_environment_Bangladesh_9675_2011_Environment
EPI_ISL_141272_A_poultry_Bangladesh_11255_C_2011_Avian
branch b
CY088770_A_Bangladesh_3233_2011_Human
EPI_ISL_141271_A_chicken_Bangladesh_31289_1_2011_Avian
KF888417_A_chicken_Bangladesh_9636_2010_Avian
HQ156768_A_chicken_Bangladesh_1151_10_2010_Avian
branch c
FJ573469_A_Bangladesh_207095_2008_Human
CY080364_A_chicken_West_Bengal_155508_2009_Avian
GU271999_A_chicken_West_Bengal_155505_2009_Avian
HQ156761_A_chicken_Bangladesh_1151_9_2010_Avian
KF597844_A_chicken_Bangladesh_BL_470_2010_Avian
EPI_ISL_141266_A_chicken_Bangladesh_0411_2010_Avian
EPI_ISL_141264_A_chicken_Bangladesh_0912_2010_Avian
EPI_ISL_141265_A_chicken_Bangladesh_1012_2010_Avian
HQ156777_A_chicken_Bangladesh_1151_11_2010_Avian
CY066005_A_chicken_Bhutan_248006_2010_Avian
CY063482_A_chicken_Bhutan_248015_2010_Avian
CY066013_A_chicken_Bhutan_248007_2010_Avian
CY063474_A_chicken_Bhutan_248009_2010_Avian

NP-Group57

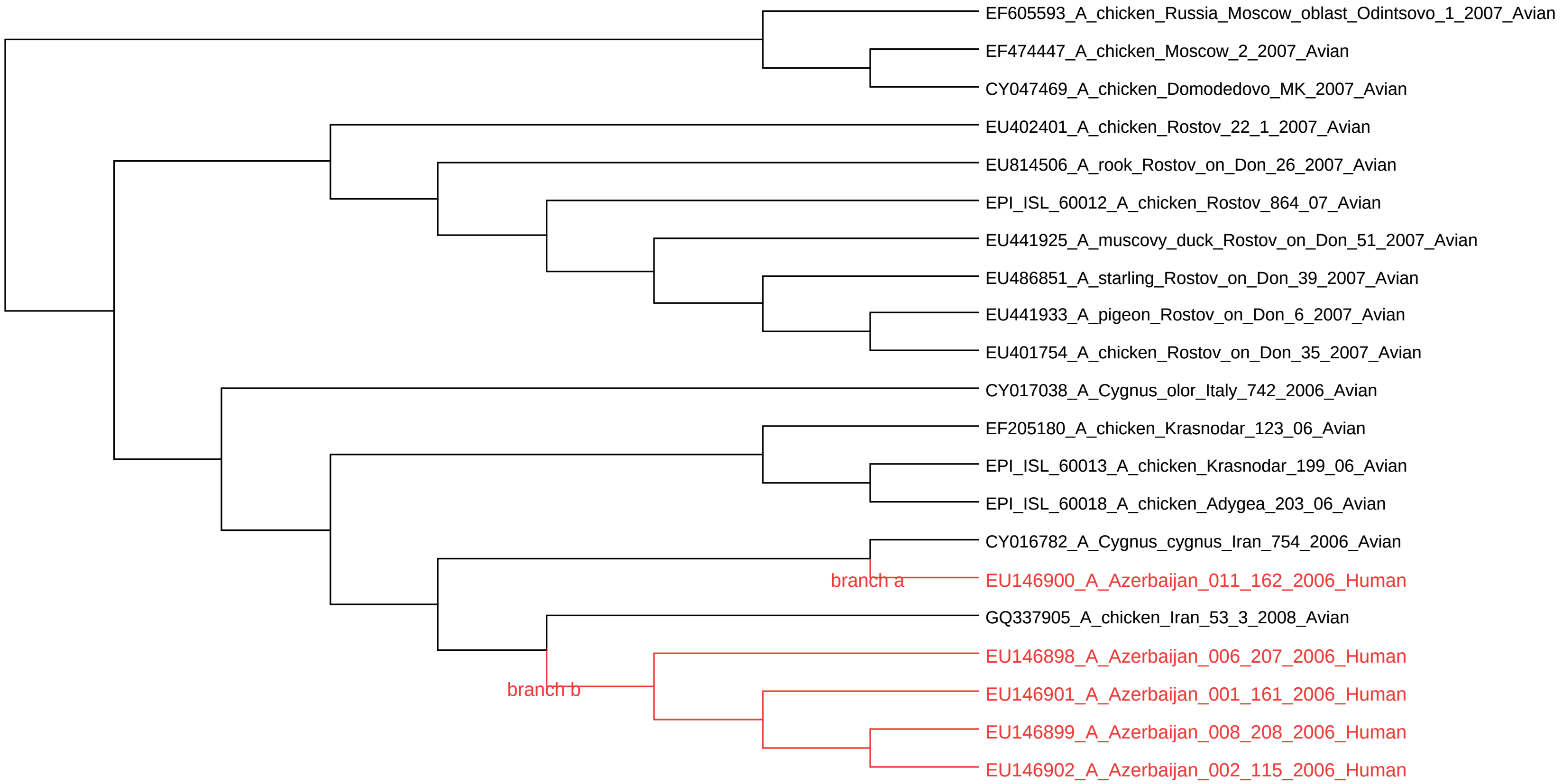
EF605593_A_chicken_Russia_Moscow_oblast_Odintsovo_1_2007_Avian
EF474447_A_chicken_Moscow_2_2007_Avian
CY047469_A_chicken_Domodedovo_MK_2007_Avian
EU402401_A_chicken_Rostov_22_1_2007_Avian
EU814506_A_rook_Rostov_on_Don_26_2007_Avian
EPI_ISL_60012_A_chicken_Rostov_864_07_Avian
EU441925_A_muscovy_duck_Rostov_on_Don_51_2007_Avian
EU486851_A_starling_Rostov_on_Don_39_2007_Avian
EU441933_A_pigeon_Rostov_on_Don_6_2007_Avian
EU401754_A_chicken_Rostov_on_Don_35_2007_Avian
CY017038_A_Cygnus_olor_Italy_742_2006_Avian
EF205180_A_chicken_Krasnodar_123_06_Avian
EPI_ISL_60013_A_chicken_Krasnodar_199_06_Avian
EPI_ISL_60018_A_chicken_Adygea_203_06_Avian
CY016782_A_Cygnus_cygnus_Iran_754_2006_Avian
branch a
EU146900_A_Azerbaijan_011_162_2006_Human
GQ337905_A_chicken_Iran_53_3_2008_Avian
EU146898_A_Azerbaijan_006_207_2006_Human
branch b
EU146901_A_Azerbaijan_001_161_2006_Human
EU146899_A_Azerbaijan_008_208_2006_Human
EU146902_A_Azerbaijan_002_115_2006_Human

NP-Group58

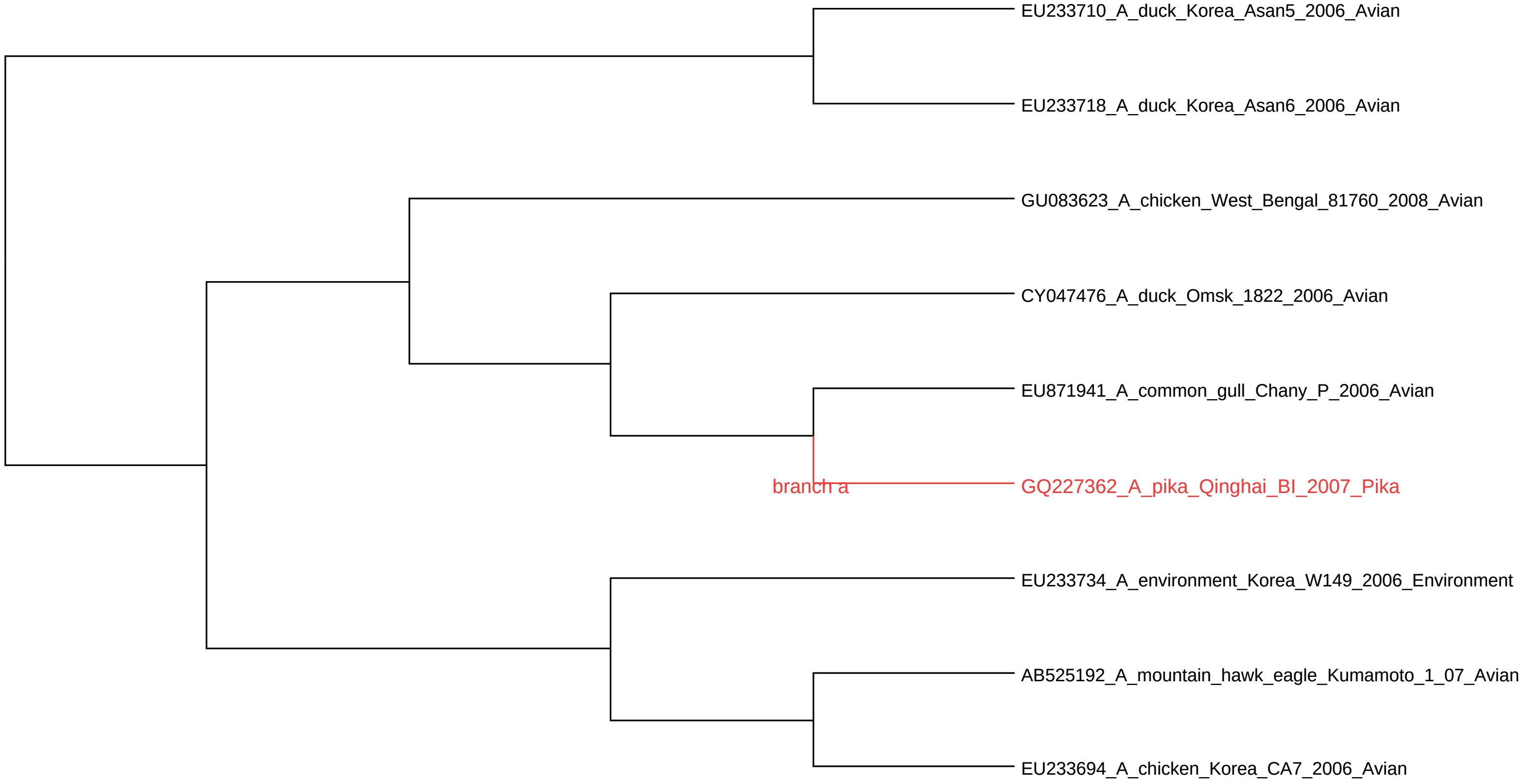
EU233710_A_duck_Korea_Asan5_2006_Avian
EU233718_A_duck_Korea_Asan6_2006_Avian
GU083623_A_chicken_West_Bengal_81760_2008_Avian
CY047476_A_duck_Omsk_1822_2006_Avian
EU871941_A_common_gull_Chany_P_2006_Avian
branch a
GQ227362_A_pika_Qinghai_BI_2007_Pika
EU233734_A_environment_Korea_W149_2006_Environment
AB525192_A_mountain_hawk_eagle_Kumamoto_1_07_Avian
EU233694_A_chicken_Korea_CA7_2006_Avian

NP-Group59

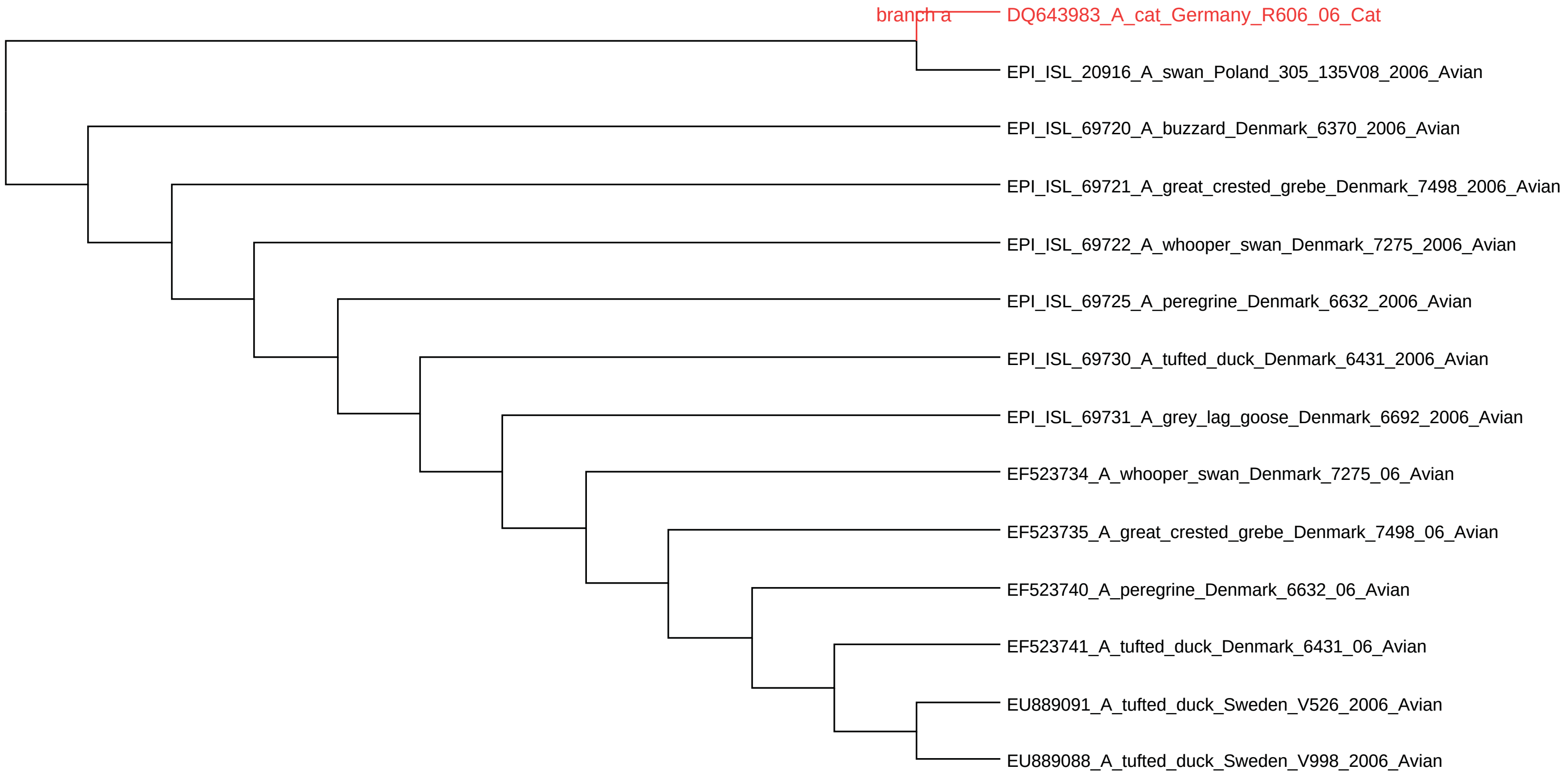
branch a
DQ643983_A_cat_Germany_R606_06_Cat
EPI_ISL_20916_A_swan_Poland_305_135V08_2006_Avian
EPI_ISL_69720_A_buzzard_Denmark_6370_2006_Avian
EPI_ISL_69721_A_great_crested_grebe_Denmark_7498_2006_Avian
EPI_ISL_69722_A_whooper_swan_Denmark_7275_2006_Avian
EPI_ISL_69725_A_peregrine_Denmark_6632_2006_Avian
EPI_ISL_69730_A_tufted_duck_Denmark_6431_2006_Avian
EPI_ISL_69731_A_grey_lag_goose_Denmark_6692_2006_Avian
EF523734_A_whooper_swan_Denmark_7275_06_Avian
EF523735_A_great_crested_grebe_Denmark_7498_06_Avian
EF523740_A_peregrine_Denmark_6632_06_Avian
EF523741_A_tufted_duck_Denmark_6431_06_Avian
EU889091_A_tufted_duck_Sweden_V526_2006_Avian
EU889088_A_tufted_duck_Sweden_V998_2006_Avian

NP-Group60

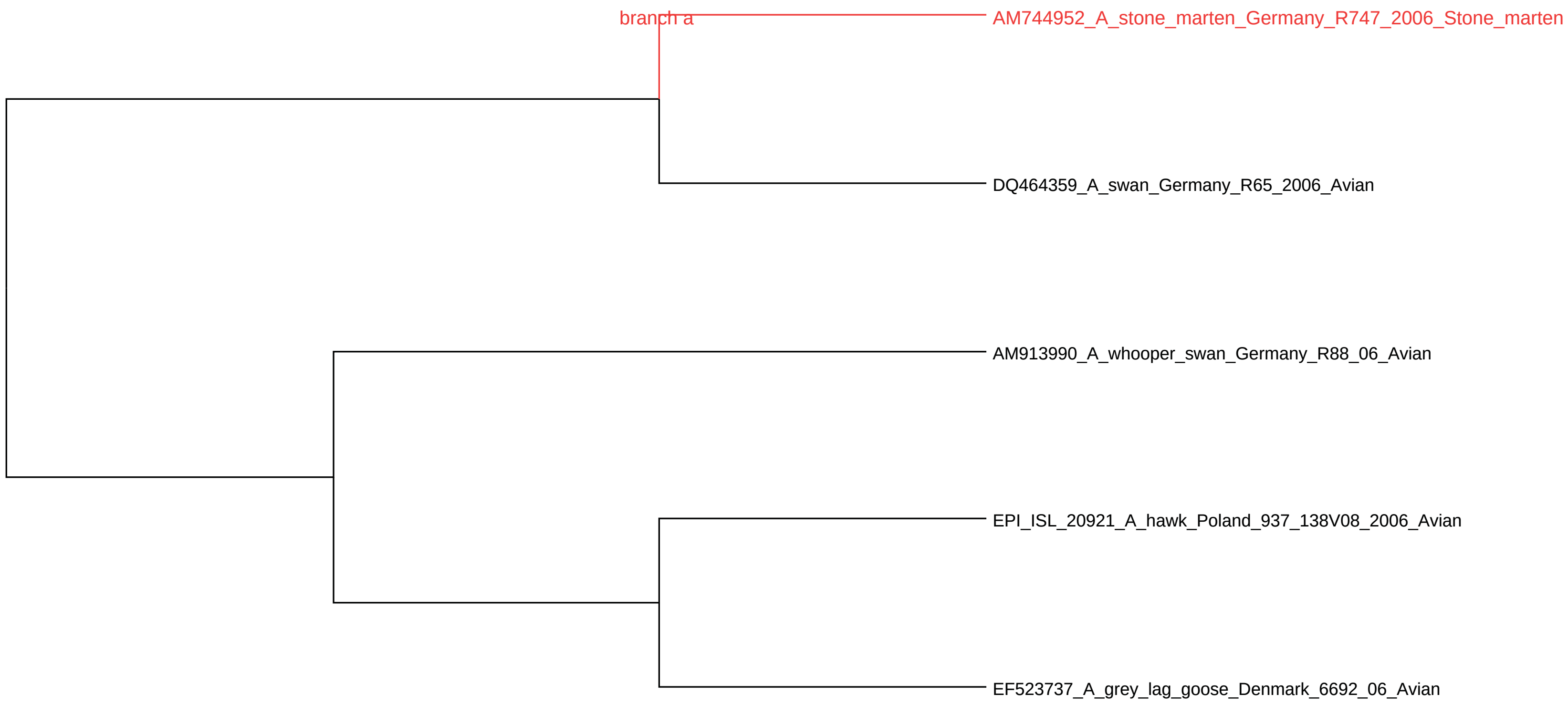
branch a
AM744952_A_stone_marten_Germany_R747_2006_Stone_marten
DQ464359_A_swan_Germany_R65_2006_Avian
AM913990_A_whooper_swan_Germany_R88_06_Avian
EPI_ISL_20921_A_hawk_Poland_937_138V08_2006_Avian
EF523737_A_grey_lag_goose_Denmark_6692_06_Avian

NP-Group61

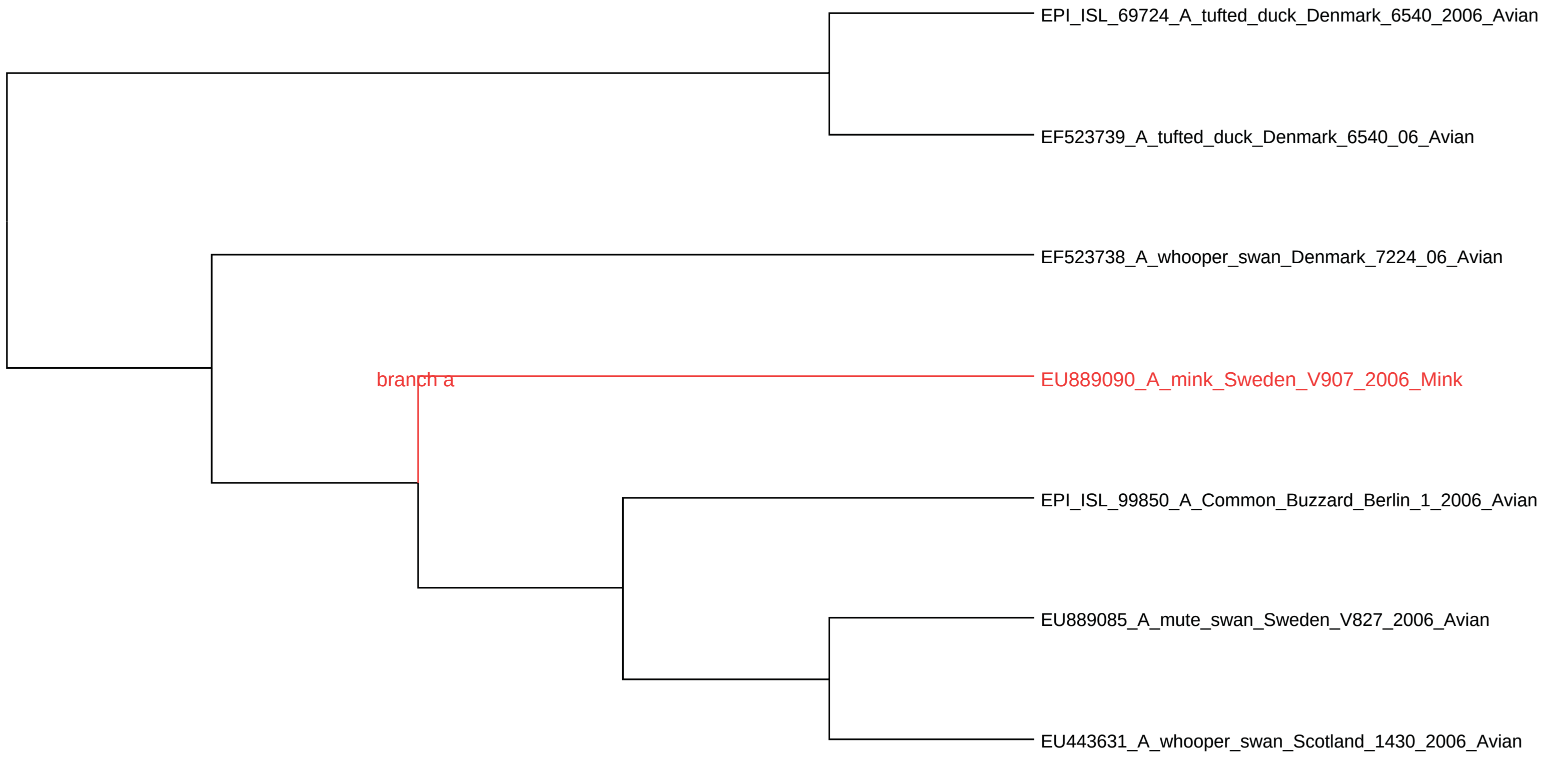
EPI_ISL_69724_A_tufted_duck_Denmark_6540_2006_Avian
EF523739_A_tufted_duck_Denmark_6540_06_Avian
EF523738_A_whooper_swan_Denmark_7224_06_Avian
branch a
EU889090_A_mink_Sweden_V907_2006_Mink
EPI_ISL_99850_A_Common_Buzzard_Berlin_1_2006_Avian
EU889085_A_mute_swan_Sweden_V827_2006_Avian
EU443631_A_whooper_swan_Scotland_1430_2006_Avian

NP-Group62

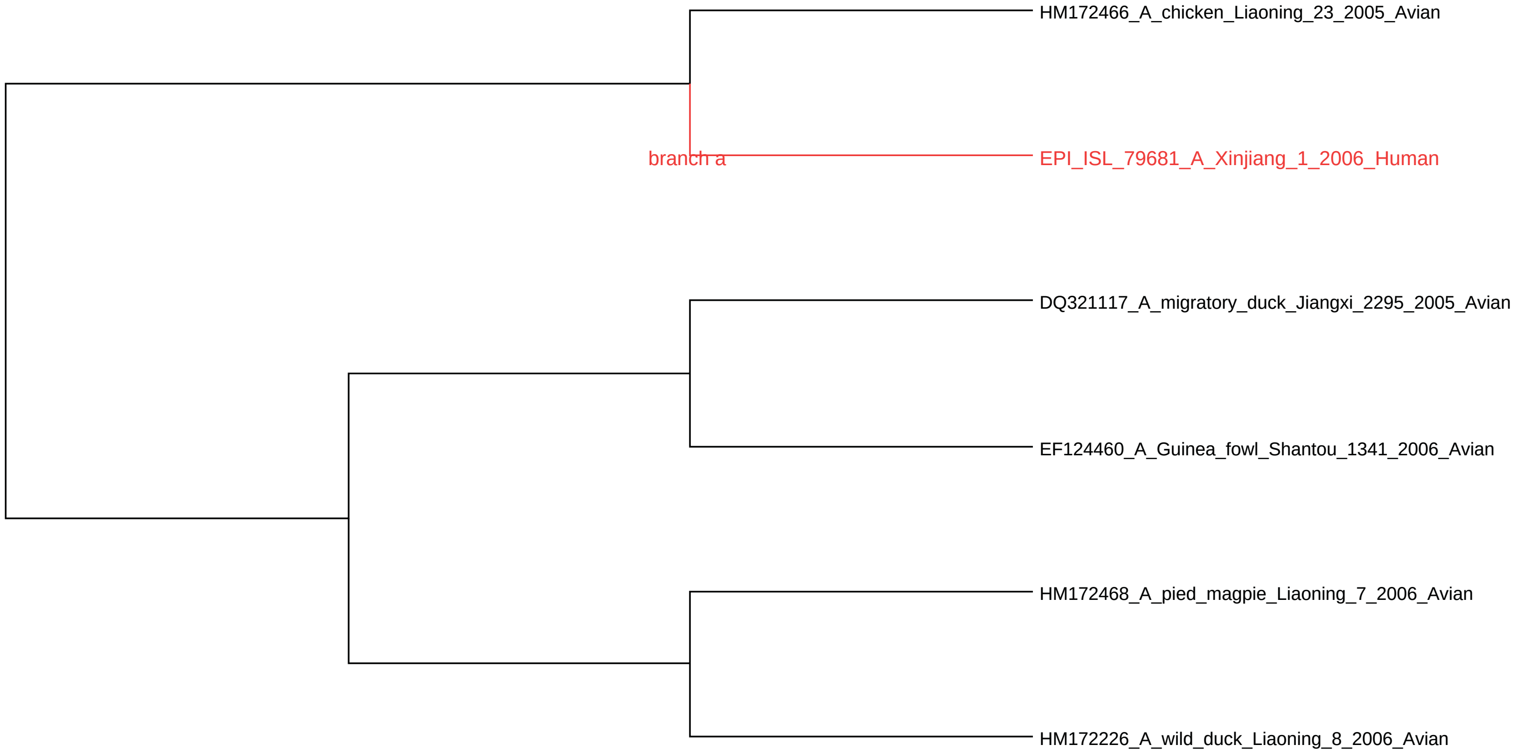

HM172466_A_chicken_Liaoning_23_2005_Avian
branch a
EPI_ISL_79681_A_Xinjiang_1_2006_Human
DQ321117_A_migratory_duck_Jiangxi_2295_2005_Avian
EF124460_A_Guinea_fowl_Shantou_1341_2006_Avian
HM172468_A_pied_magpie_Liaoning_7_2006_Avian
HM172226_A_wild_duck_Liaoning_8_2006_Avian

NP-Group63

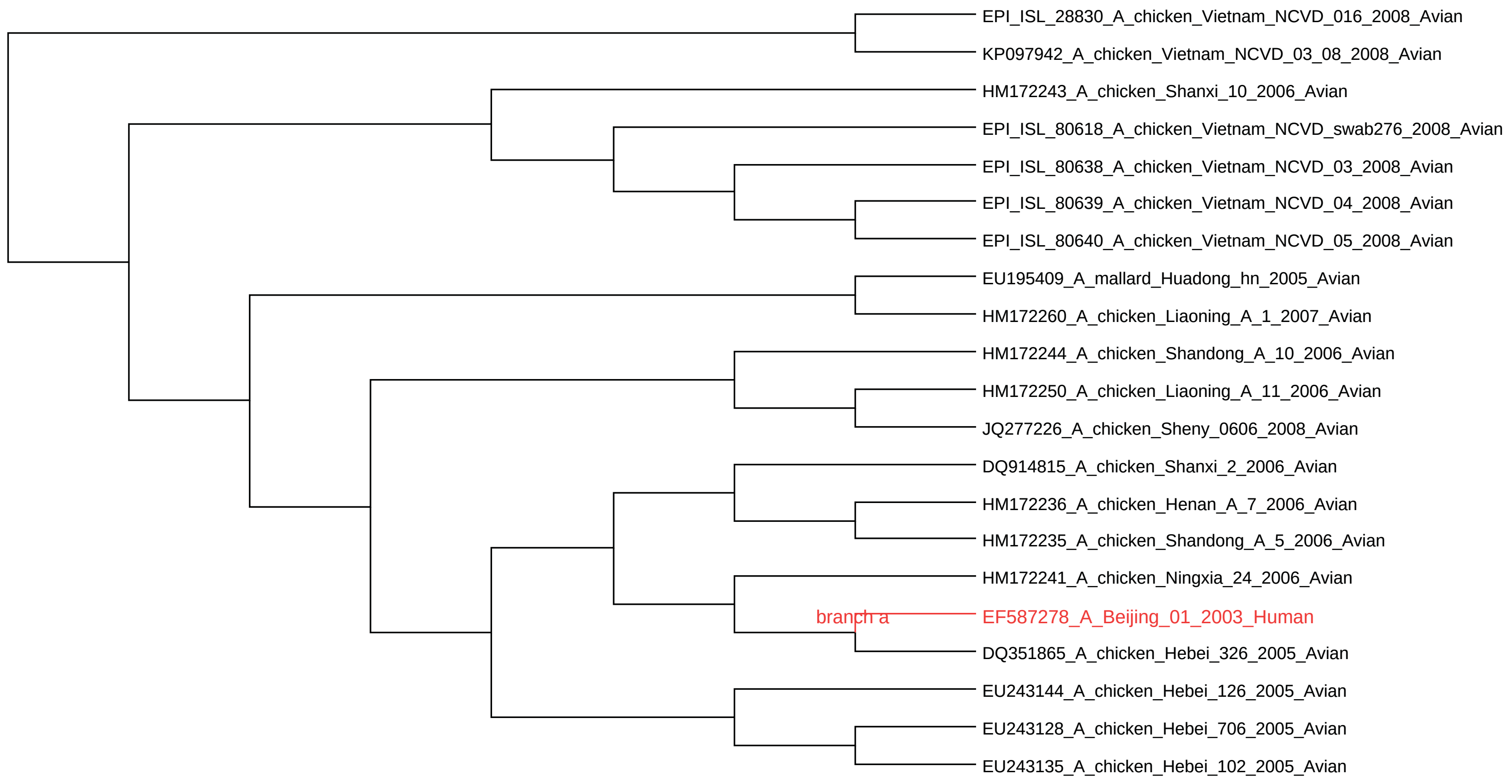
EPI_ISL_28830_A_chicken_Vietnam_NCVD_016_2008_Avian
KP097942_A_chicken_Vietnam_NCVD_03_08_2008_Avian
HM172243_A_chicken_Shanxi_10_2006_Avian
EPI_ISL_80618_A_chicken_Vietnam_NCVD_swab276_2008_Avian
EPI_ISL_80638_A_chicken_Vietnam_NCVD_03_2008_Avian
EPI_ISL_80639_A_chicken_Vietnam_NCVD_04_2008_Avian
EPI_ISL_80640_A_chicken_Vietnam_NCVD_05_2008_Avian
EU195409_A_mallard_Huadong_hn_2005_Avian
HM172260_A_chicken_Liaoning_A_1_2007_Avian
HM172244_A_chicken_Shandong_A_10_2006_Avian
HM172250_A_chicken_Liaoning_A_11_2006_Avian
JQ277226_A_chicken_Sheny_0606_2008_Avian
DQ914815_A_chicken_Shanxi_2_2006_Avian
HM172236_A_chicken_Henan_A_7_2006_Avian
HM172235_A_chicken_Shandong_A_5_2006_Avian
HM172241_A_chicken_Ningxia_24_2006_Avian
branch a
EF587278_A_Beijing_01_2003_Human
DQ351865_A_chicken_Hebei_326_2005_Avian
EU243144_A_chicken_Hebei_126_2005_Avian
EU243128_A_chicken_Hebei_706_2005_Avian
EU243135_A_chicken_Hebei_102_2005_Avian

NP-Group64

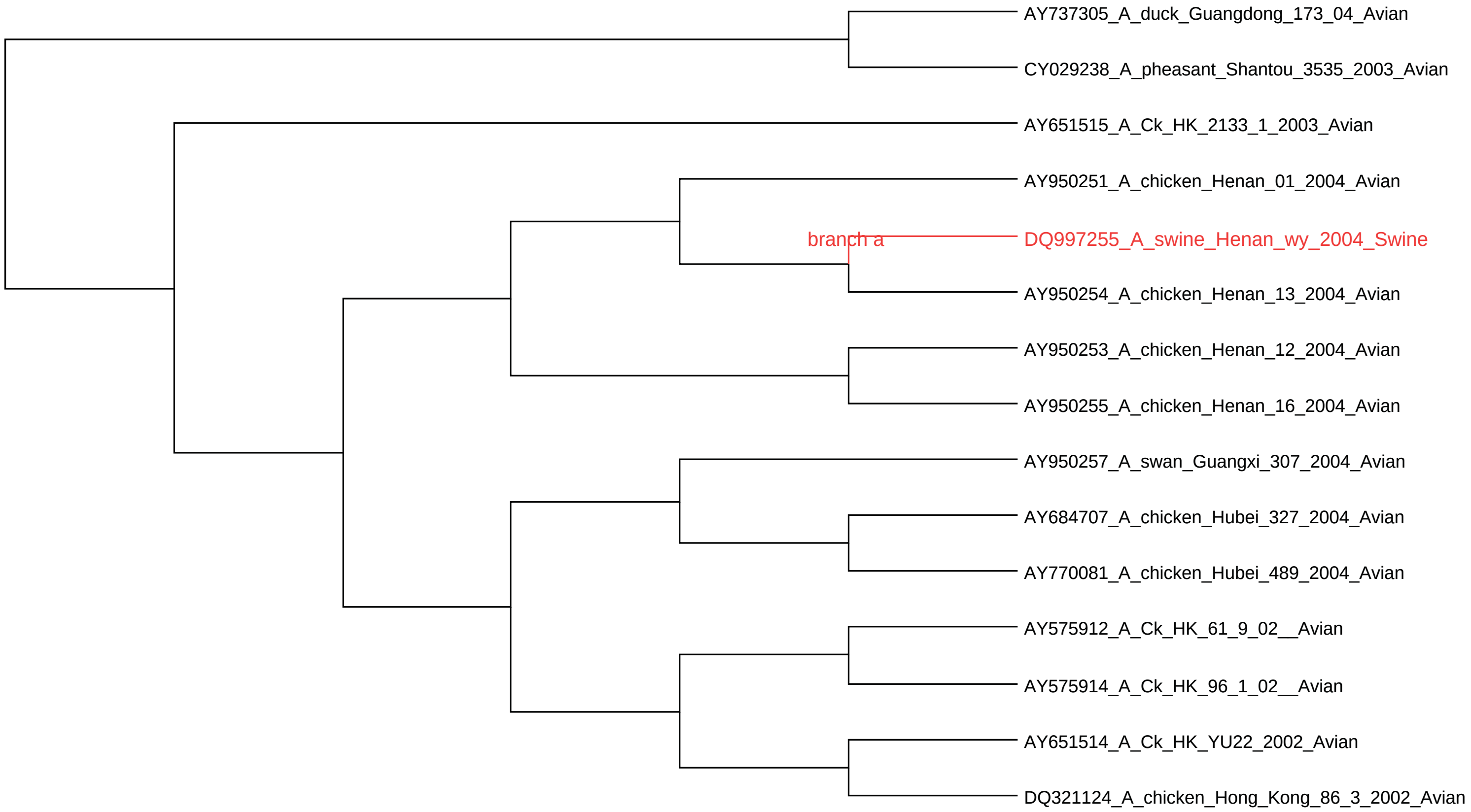
AY737305_A_duck_Guangdong_173_04_Avian
CY029238_A_pheasant_Shantou_3535_2003_Avian
AY651515_A_Ck_HK_2133_1_2003_Avian
AY950251_A_chicken_Henan_01_2004_Avian
branch a
DQ997255_A_swine_Henan_wy_2004_Swine
AY950254_A_chicken_Henan_13_2004_Avian
AY950253_A_chicken_Henan_12_2004_Avian
AY950255_A_chicken_Henan_16_2004_Avian
AY950257_A_swan_Guangxi_307_2004_Avian
AY684707_A_chicken_Hubei_327_2004_Avian
AY770081_A_chicken_Hubei_489_2004_Avian
AY575912_A_Ck_HK_61_9_02__Avian
AY575914_A_Ck_HK_96_1_02__Avian
AY651514_A_Ck_HK_YU22_2002_Avian
DQ321124_A_chicken_Hong_Kong_86_3_2002_Avian

NP-Group65

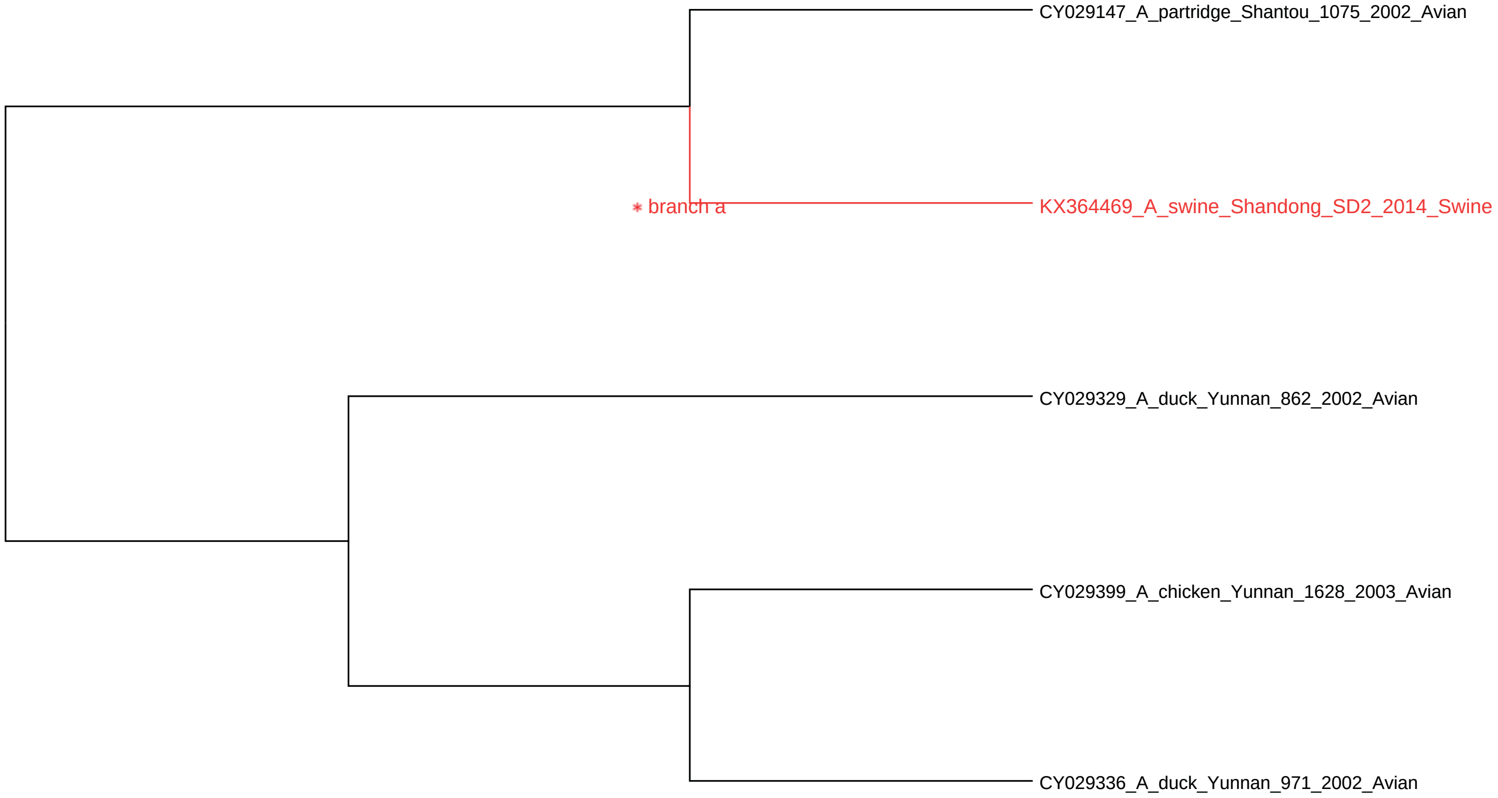
CY029147_A_partridge_Shantou_1075_2002_Avian
* branch a
KX364469_A_swine_Shandong_SD2_2014_Swine
CY029329_A_duck_Yunnan_862_2002_Avian
CY029399_A_chicken_Yunnan_1628_2003_Avian
CY029336_A_duck_Yunnan_971_2002_Avian

NP-Group66

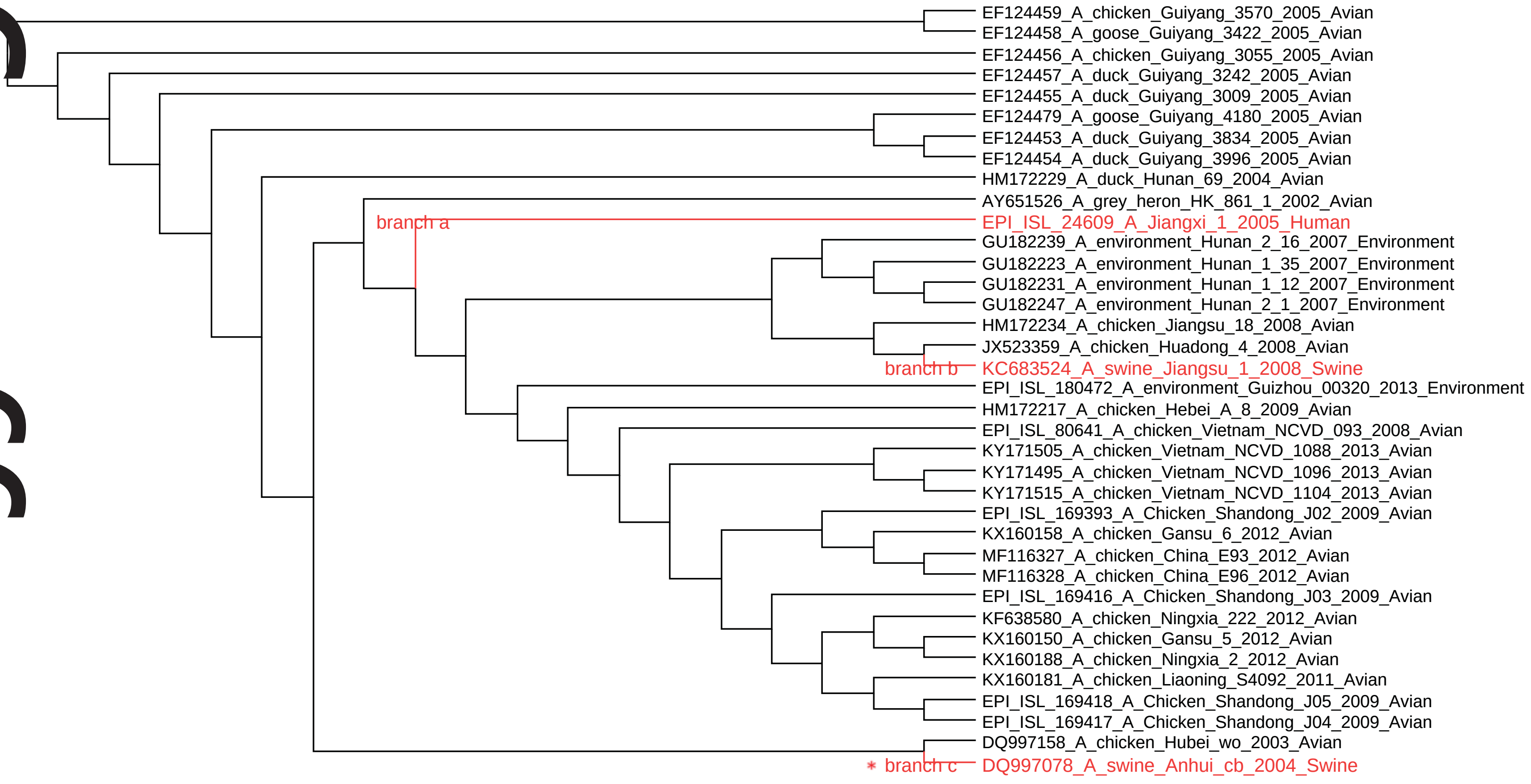
EF124459_A_chicken_Guiyang_3570_2005_Avian
EF124458_A_goose_Guiyang_3422_2005_Avian
EF124456_A_chicken_Guiyang_3055_2005_Avian
EF124457_A_duck_Guiyang_3242_2005_Avian
EF124455_A_duck_Guiyang_3009_2005_Avian
EF124479_A_goose_Guiyang_4180_2005_Avian
EF124453_A_duck_Guiyang_3834_2005_Avian
EF124454_A_duck_Guiyang_3996_2005_Avian
HM172229_A_duck_Hunan_69_2004_Avian
AY651526_A_grey_heron_HK_861_1_2002_Avian
branch a
EPI_ISL_24609_A_Jiangxi_1_2005_Human
GU182239_A_environment_Hunan_2_16_2007_Environment
GU182223_A_environment_Hunan_1_35_2007_Environment
GU182231_A_environment_Hunan_1_12_2007_Environment
GU182247_A_environment_Hunan_2_1_2007_Environment
HM172234_A_chicken_Jiangsu_18_2008_Avian
JX523359_A_chicken_Huadong_4_2008_Avian
branch b
KC683524_A_swine_Jiangsu_1_2008_Swine
EPI_ISL_180472_A_environment_Guizhou_00320_2013_Environment
HM172217_A_chicken_Hebei_A_8_2009_Avian
EPI_ISL_80641_A_chicken_Vietnam_NCVD_093_2008_Avian
KY171505_A_chicken_Vietnam_NCVD_1088_2013_Avian
KY171495_A_chicken_Vietnam_NCVD_1096_2013_Avian
KY171515_A_chicken_Vietnam_NCVD_1104_2013_Avian
EPI_ISL_169393_A_Chicken_Shandong_J02_2009_Avian
KX160158_A_chicken_Gansu_6_2012_Avian
MF116327_A_chicken_China_E93_2012_Avian
MF116328_A_chicken_China_E96_2012_Avian
EPI_ISL_169416_A_Chicken_Shandong_J03_2009_Avian
KF638580_A_chicken_Ningxia_222_2012_Avian
KX160150_A_chicken_Gansu_5_2012_Avian
KX160188_A_chicken_Ningxia_2_2012_Avian
KX160181_A_chicken_Liaoning_S4092_2011_Avian
EPI_ISL_169418_A_Chicken_Shandong_J05_2009_Avian
EPI_ISL_169417_A_Chicken_Shandong_J04_2009_Avian
DQ997158_A_chicken_Hubei_wo_2003_Avian
* branch c
DQ997078_A_swine_Anhui_cb_2004_Swine

NP-Group67

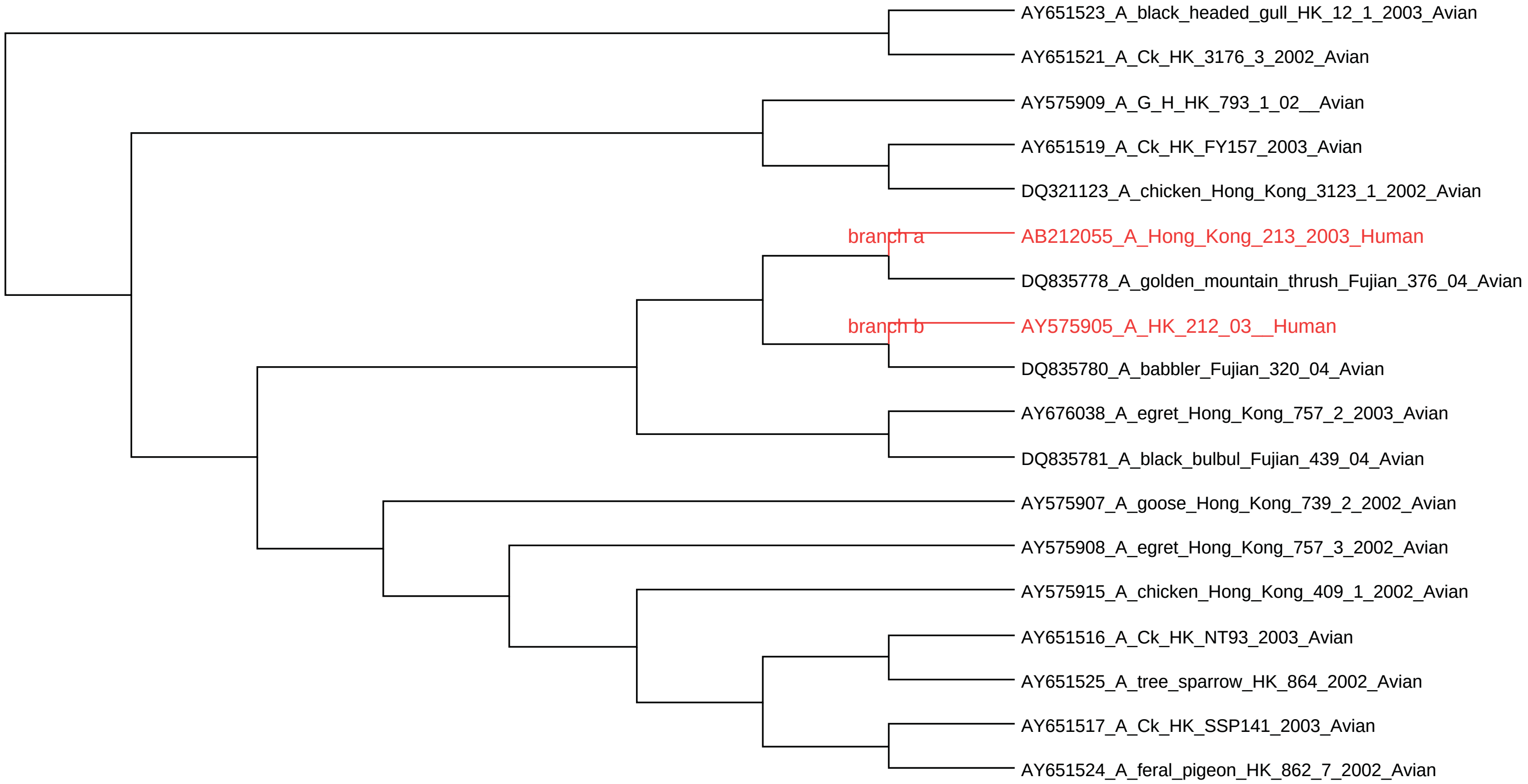
AY651523_A_black_headed_gull_HK_12_1_2003_Avian
AY651521_A_Ck_HK_3176_3_2002_Avian
AY575909_A_G_H_HK_793_1_02__Avian
AY651519_A_Ck_HK_FY157_2003_Avian
DQ321123_A_chicken_Hong_Kong_3123_1_2002_Avian
branch a
AB212055_A_Hong_Kong_213_2003_Human
DQ835778_A_golden_mountain_thrush_Fujian_376_04_Avian
branch b
AY575905_A_HK_212_03__Human
DQ835780_A_babbler_Fujian_320_04_Avian
AY676038_A_egret_Hong_Kong_757_2_2003_Avian
DQ835781_A_black_bulbul_Fujian_439_04_Avian
AY575907_A_goose_Hong_Kong_739_2_2002_Avian
AY575908_A_egret_Hong_Kong_757_3_2002_Avian
AY575915_A_chicken_Hong_Kong_409_1_2002_Avian
AY651516_A_Ck_HK_NT93_2003_Avian
AY651525_A_tree_sparrow_HK_864_2002_Avian
AY651517_A_Ck_HK_SSP141_2003_Avian
AY651524_A_feral_pigeon_HK_862_7_2002_Avian

NP-Group68

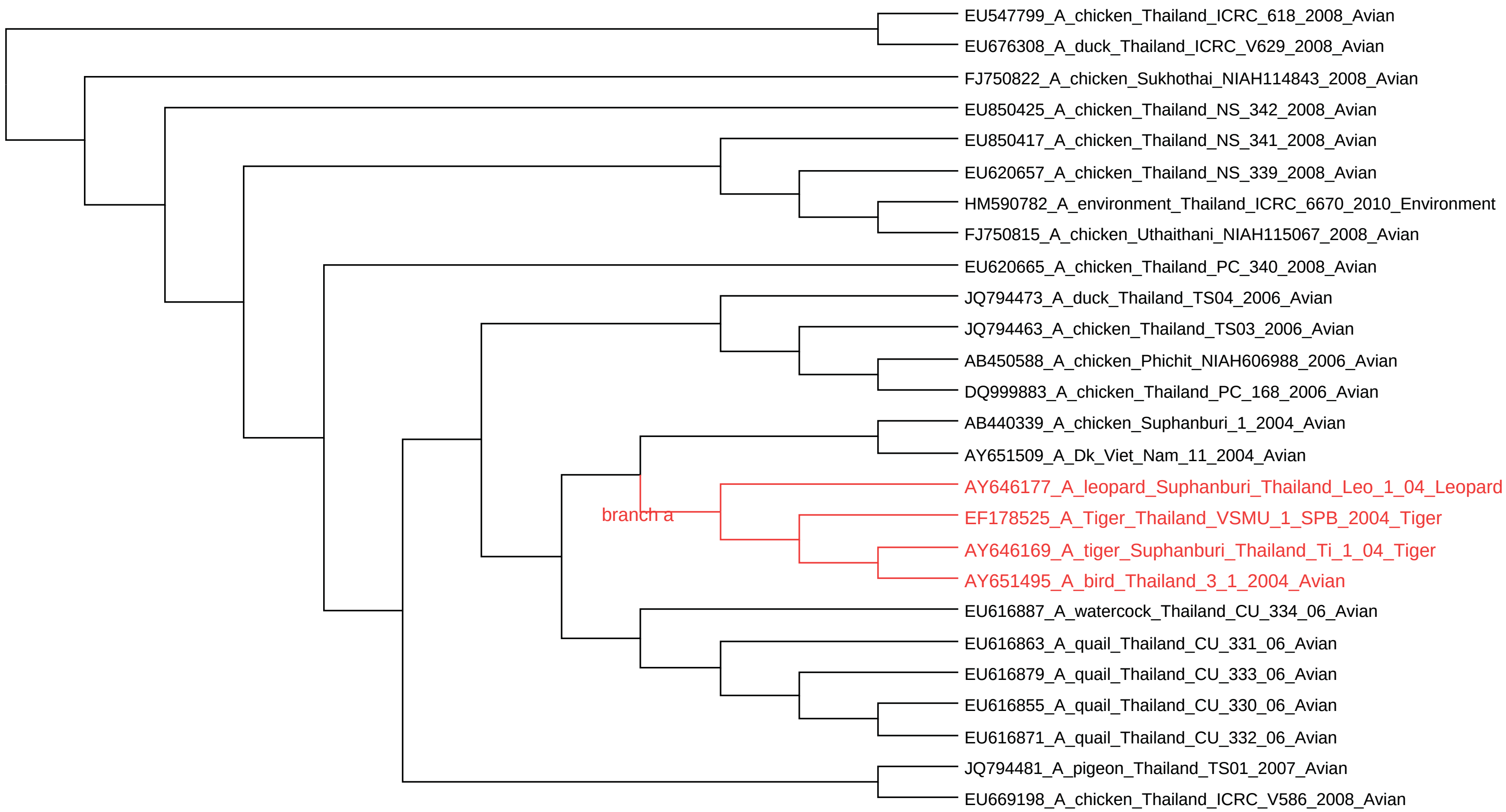

EU547799_A_chicken_Thailand_ICRC_618_2008_Avian
EU676308_A_duck_Thailand_ICRC_V629_2008_Avian
FJ750822_A_chicken_Sukhothai_NIAH114843_2008_Avian
EU850425_A_chicken_Thailand_NS_342_2008_Avian
EU850417_A_chicken_Thailand_NS_341_2008_Avian
EU620657_A_chicken_Thailand_NS_339_2008_Avian
HM590782_A_environment_Thailand_ICRC_6670_2010_Environment
FJ750815_A_chicken_Uthaithani_NIAH115067_2008_Avian
EU620665_A_chicken_Thailand_PC_340_2008_Avian
JQ794473_A_duck_Thailand_TS04_2006_Avian
JQ794463_A_chicken_Thailand_TS03_2006_Avian
AB450588_A_chicken_Phichit_NIAH606988_2006_Avian
DQ999883_A_chicken_Thailand_PC_168_2006_Avian
AB440339_A_chicken_Suphanburi_1_2004_Avian
AY651509_A_Dk_Viet_Nam_11_2004_Avian
AY646177_A_leopard_Suphanburi_Thailand_Leo_1_04_Leopard
branch a
EF178525_A_Tiger_Thailand_VSMU_1_SPB_2004_Tiger
AY646169_A_tiger_Suphanburi_Thailand_Ti_1_04_Tiger
AY651495_A_bird_Thailand_3_1_2004_Avian
EU616887_A_watercock_Thailand_CU_334_06_Avian
EU616863_A_quail_Thailand_CU_331_06_Avian
EU616879_A_quail_Thailand_CU_333_06_Avian
EU616855_A_quail_Thailand_CU_330_06_Avian
EU616871_A_quail_Thailand_CU_332_06_Avian
JQ794481_A_pigeon_Thailand_TS01_2007_Avian
EU669198_A_chicken_Thailand_ICRC_V586_2008_Avian

NP-Group69

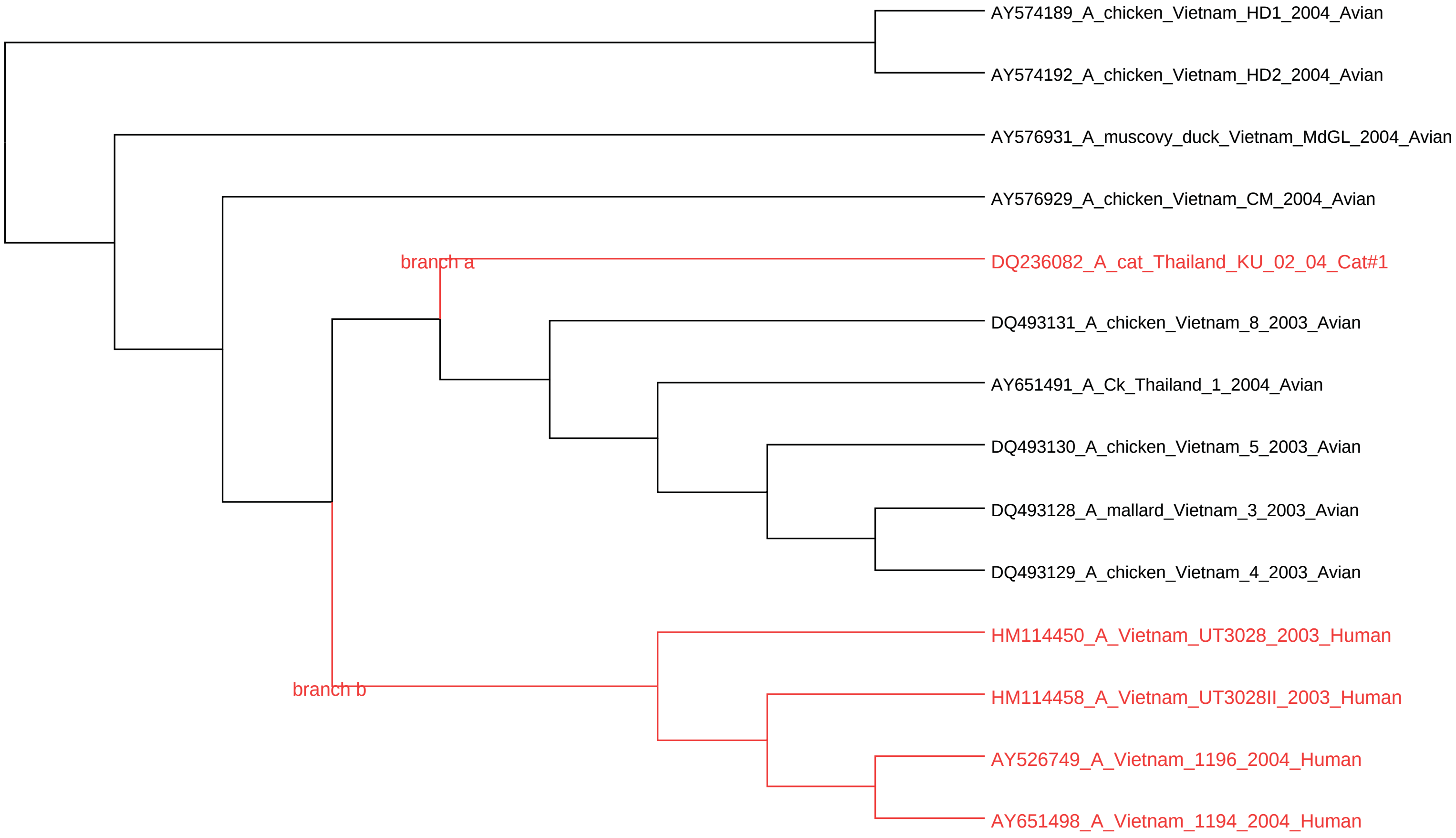

AY574189_A_chicken_Vietnam_HD1_2004_Avian
AY574192_A_chicken_Vietnam_HD2_2004_Avian
AY576931_A_muscovy_duck_Vietnam_MdGL_2004_Avian
AY576929_A_chicken_Vietnam_CM_2004_Avian
branch a
DQ236082_A_cat_Thailand_KU_02_04_Cat#1
DQ493131_A_chicken_Vietnam_8_2003_Avian
AY651491_A_Ck_Thailand_1_2004_Avian
DQ493130_A_chicken_Vietnam_5_2003_Avian
DQ493128_A_mallard_Vietnam_3_2003_Avian
DQ493129_A_chicken_Vietnam_4_2003_Avian
HM114450_A_Vietnam_UT3028_2003_Human
branch b
HM114458_A_Vietnam_UT3028II_2003_Human
AY526749_A_Vietnam_1196_2004_Human
AY651498_A_Vietnam_1194_2004_Human

NP-Group70

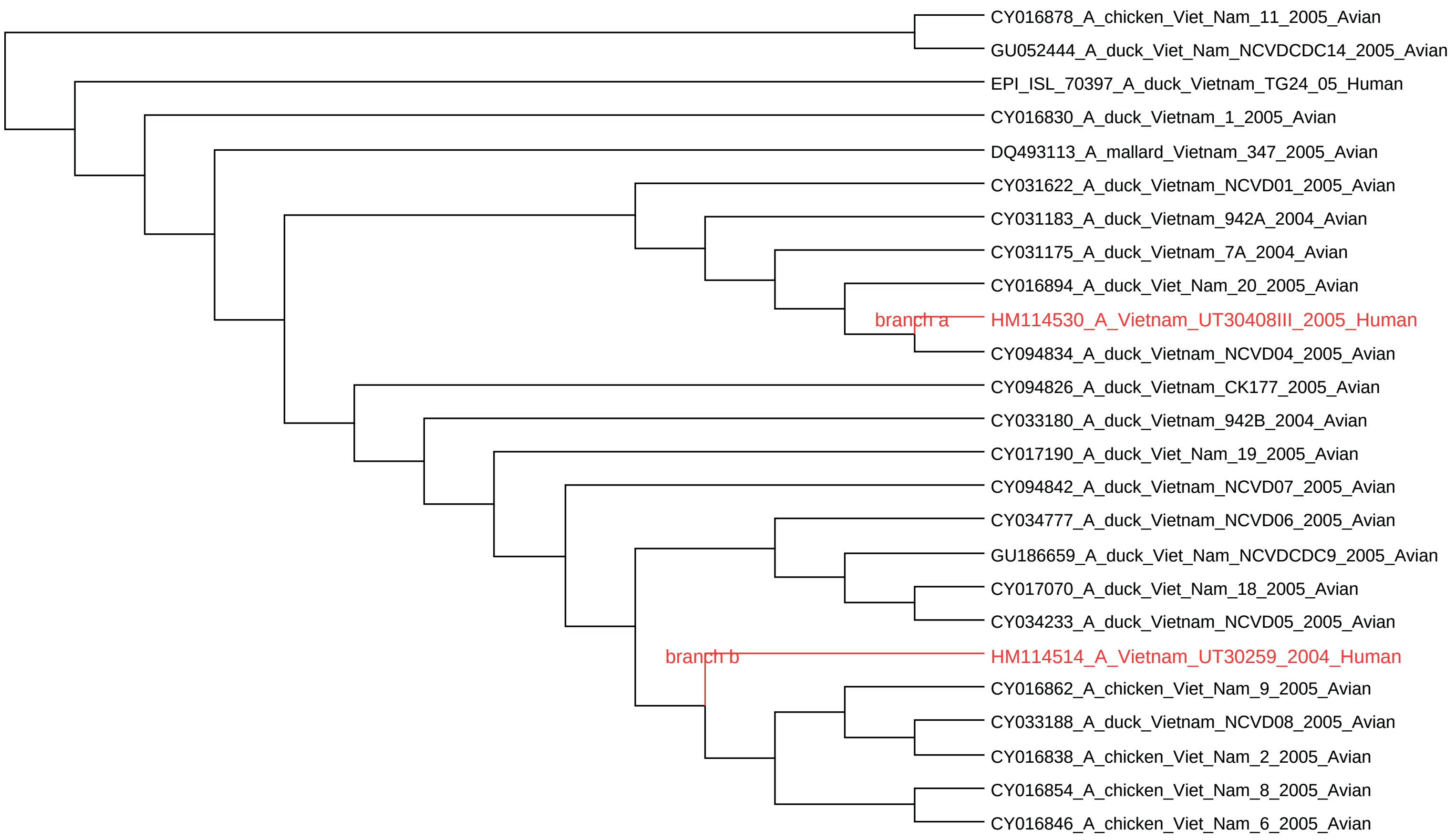
CY016878_A_chicken_Viet_Nam_11_2005_Avian
GU052444_A_duck_Viet_Nam_NCVDCDC14_2005_Avian
EPI_ISL_70397_A_duck_Vietnam_TG24_05_Human
CY016830_A_duck_Vietnam_1_2005_Avian
DQ493113_A_mallard_Vietnam_347_2005_Avian
CY031622_A_duck_Vietnam_NCVD01_2005_Avian
CY031183_A_duck_Vietnam_942A_2004_Avian
CY031175_A_duck_Vietnam_7A_2004_Avian
CY016894_A_duck_Viet_Nam_20_2005_Avian
branch a
HM114530_A_Vietnam_UT30408III_2005_Human
CY094834_A_duck_Vietnam_NCVD04_2005_Avian
CY094826_A_duck_Vietnam_CK177_2005_Avian
CY033180_A_duck_Vietnam_942B_2004_Avian
CY017190_A_duck_Viet_Nam_19_2005_Avian
CY094842_A_duck_Vietnam_NCVD07_2005_Avian
CY034777_A_duck_Vietnam_NCVD06_2005_Avian
GU186659_A_duck_Viet_Nam_NCVDCDC9_2005_Avian
CY017070_A_duck_Viet_Nam_18_2005_Avian
CY034233_A_duck_Vietnam_NCVD05_2005_Avian
branch b
HM114514_A_Vietnam_UT30259_2004_Human
CY016862_A_chicken_Viet_Nam_9_2005_Avian
CY033188_A_duck_Vietnam_NCVD08_2005_Avian
CY016838_A_chicken_Viet_Nam_2_2005_Avian
CY016854_A_chicken_Viet_Nam_8_2005_Avian
CY016846_A_chicken_Viet_Nam_6_2005_Avian

NP-Group71

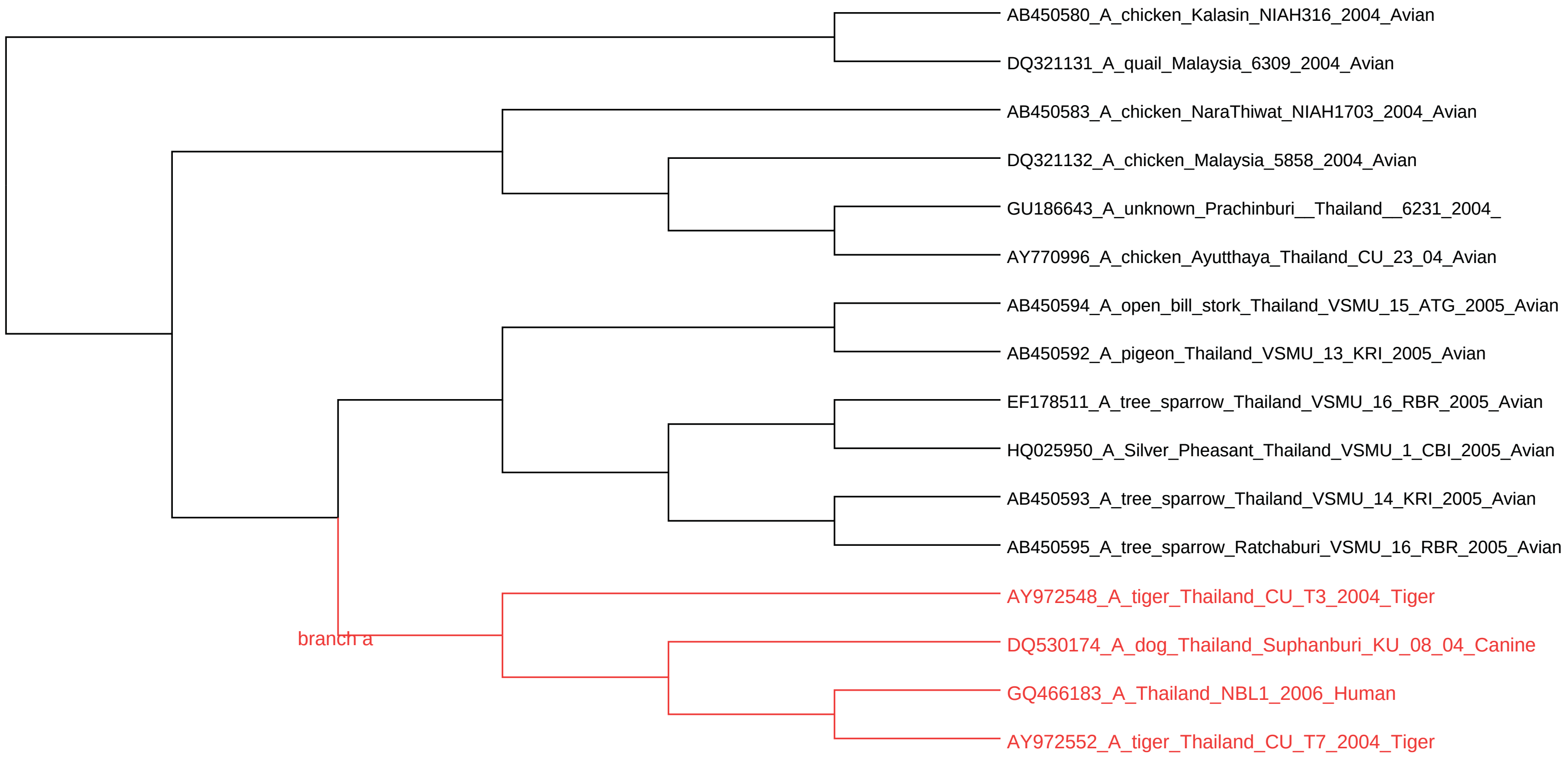
AB450580_A_chicken_Kalasin_NIAH316_2004_Avian
DQ321131_A_quail_Malaysia_6309_2004_Avian
AB450583_A_chicken_NaraThiwat_NIAH1703_2004_Avian
DQ321132_A_chicken_Malaysia_5858_2004_Avian
GU186643_A_unknown_Prachinburi__Thailand__6231_2004_
AY770996_A_chicken_Ayutthaya_Thailand_CU_23_04_Avian
AB450594_A_open_bill_stork_Thailand_VSMU_15_ATG_2005_Avian
AB450592_A_pigeon_Thailand_VSMU_13_KRI_2005_Avian
EF178511_A_tree_sparrow_Thailand_VSMU_16_RBR_2005_Avian
HQ025950_A_Silver_Pheasant_Thailand_VSMU_1_CBI_2005_Avian
AB450593_A_tree_sparrow_Thailand_VSMU_14_KRI_2005_Avian
AB450595_A_tree_sparrow_Ratchaburi_VSMU_16_RBR_2005_Avian
AY972548_A_tiger_Thailand_CU_T3_2004_Tiger
branch a
DQ530174_A_dog_Thailand_Suphanburi_KU_08_04_Canine
GQ466183_A_Thailand_NBL1_2006_Human
AY972552_A_tiger_Thailand_CU_T7_2004_Tiger

NP-Group72

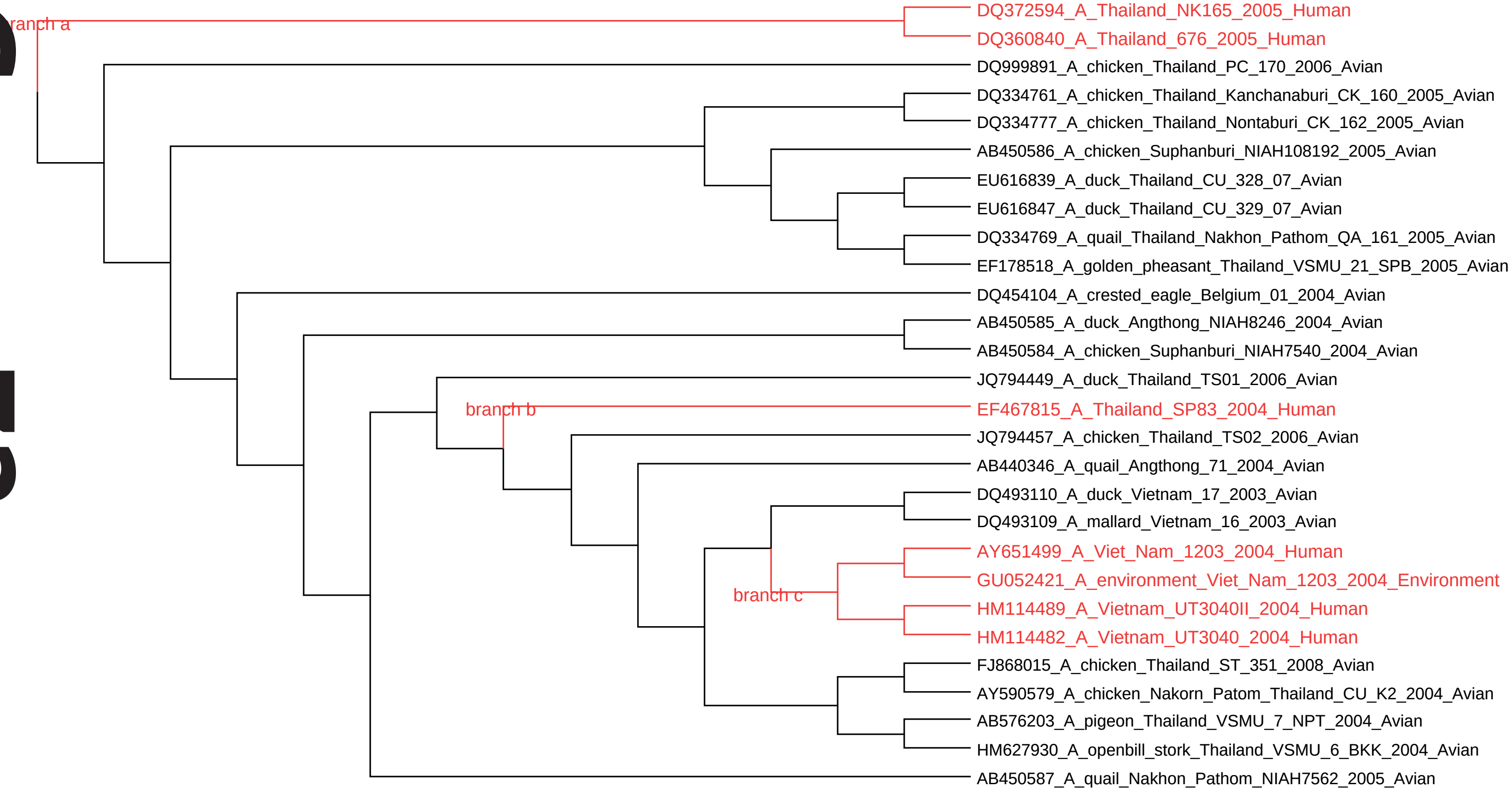
branch a
DQ372594_A_Thailand_NK165_2005_Human
DQ360840_A_Thailand_676_2005_Human
DQ999891_A_chicken_Thailand_PC_170_2006_Avian
DQ334761_A_chicken_Thailand_Kanchanaburi_CK_160_2005_Avian
DQ334777_A_chicken_Thailand_Nontaburi_CK_162_2005_Avian
AB450586_A_chicken_Suphanburi_NIAH108192_2005_Avian
EU616839_A_duck_Thailand_CU_328_07_Avian
EU616847_A_duck_Thailand_CU_329_07_Avian
DQ334769_A_quail_Thailand_Nakhon_Pathom_QA_161_2005_Avian
EF178518_A_golden_pheasant_Thailand_VSMU_21_SPB_2005_Avian
DQ454104_A_crested_eagle_Belgium_01_2004_Avian
AB450585_A_duck_Angthong_NIAH8246_2004_Avian
AB450584_A_chicken_Suphanburi_NIAH7540_2004_Avian
JQ794449_A_duck_Thailand_TS01_2006_Avian
branch b
EF467815_A_Thailand_SP83_2004_Human
JQ794457_A_chicken_Thailand_TS02_2006_Avian
AB440346_A_quail_Angthong_71_2004_Avian
DQ493110_A_duck_Vietnam_17_2003_Avian
DQ493109_A_mallard_Vietnam_16_2003_Avian
AY651499_A_Viet_Nam_1203_2004_Human
GU052421_A_environment_Viet_Nam_1203_2004_Environment
branch c
HM114489_A_Vietnam_UT3040II_2004_Human
HM114482_A_Vietnam_UT3040_2004_Human
FJ868015_A_chicken_Thailand_ST_351_2008_Avian
AY590579_A_chicken_Nakorn_Patom_Thailand_CU_K2_2004_Avian
AB576203_A_pigeon_Thailand_VSMU_7_NPT_2004_Avian
HM627930_A_openbill_stork_Thailand_VSMU_6_BKK_2004_Avian
AB450587_A_quail_Nakhon_Pathom_NIAH7562_2005_Avian

NP-Group73

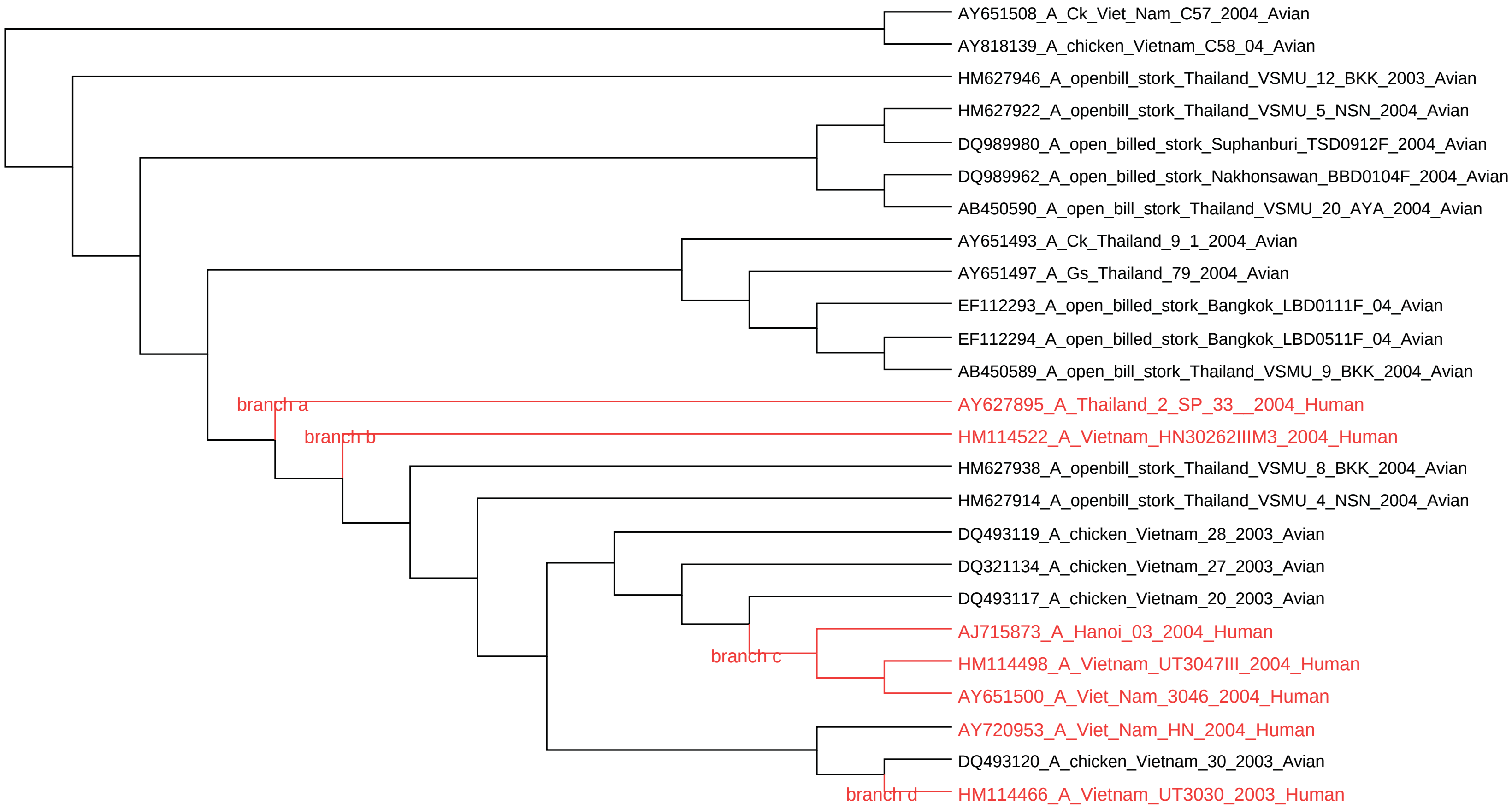
AY651508_A_Ck_Viet_Nam_C57_2004_Avian
AY818139_A_chicken_Vietnam_C58_04_Avian
HM627946_A_openbill_stork_Thailand_VSMU_12_BKK_2003_Avian
HM627922_A_openbill_stork_Thailand_VSMU_5_NSN_2004_Avian
DQ989980_A_open_billed_stork_Suphanburi_TSD0912F_2004_Avian
DQ989962_A_open_billed_stork_Nakhonsawan_BBD0104F_2004_Avian
AB450590_A_open_bill_stork_Thailand_VSMU_20_AYA_2004_Avian
AY651493_A_Ck_Thailand_9_1_2004_Avian
AY651497_A_Gs_Thailand_79_2004_Avian
EF112293_A_open_billed_stork_Bangkok_LBD0111F_04_Avian
EF112294_A_open_billed_stork_Bangkok_LBD0511F_04_Avian
AB450589_A_open_bill_stork_Thailand_VSMU_9_BKK_2004_Avian
branch a
AY627895_A_Thailand_2_SP_33__2004_Human
branch b
HM114522_A_Vietnam_HN30262IIIM3_2004_Human
HM627938_A_openbill_stork_Thailand_VSMU_8_BKK_2004_Avian
HM627914_A_openbill_stork_Thailand_VSMU_4_NSN_2004_Avian
DQ493119_A_chicken_Vietnam_28_2003_Avian
DQ321134_A_chicken_Vietnam_27_2003_Avian
DQ493117_A_chicken_Vietnam_20_2003_Avian
AJ715873_A_Hanoi_03_2004_Human
branch c
HM114498_A_Vietnam_UT3047III_2004_Human
AY651500_A_Viet_Nam_3046_2004_Human
AY720953_A_Viet_Nam_HN_2004_Human
DQ493120_A_chicken_Vietnam_30_2003_Avian
branch d
HM114466_A_Vietnam_UT3030_2003_Human

NP-Group74

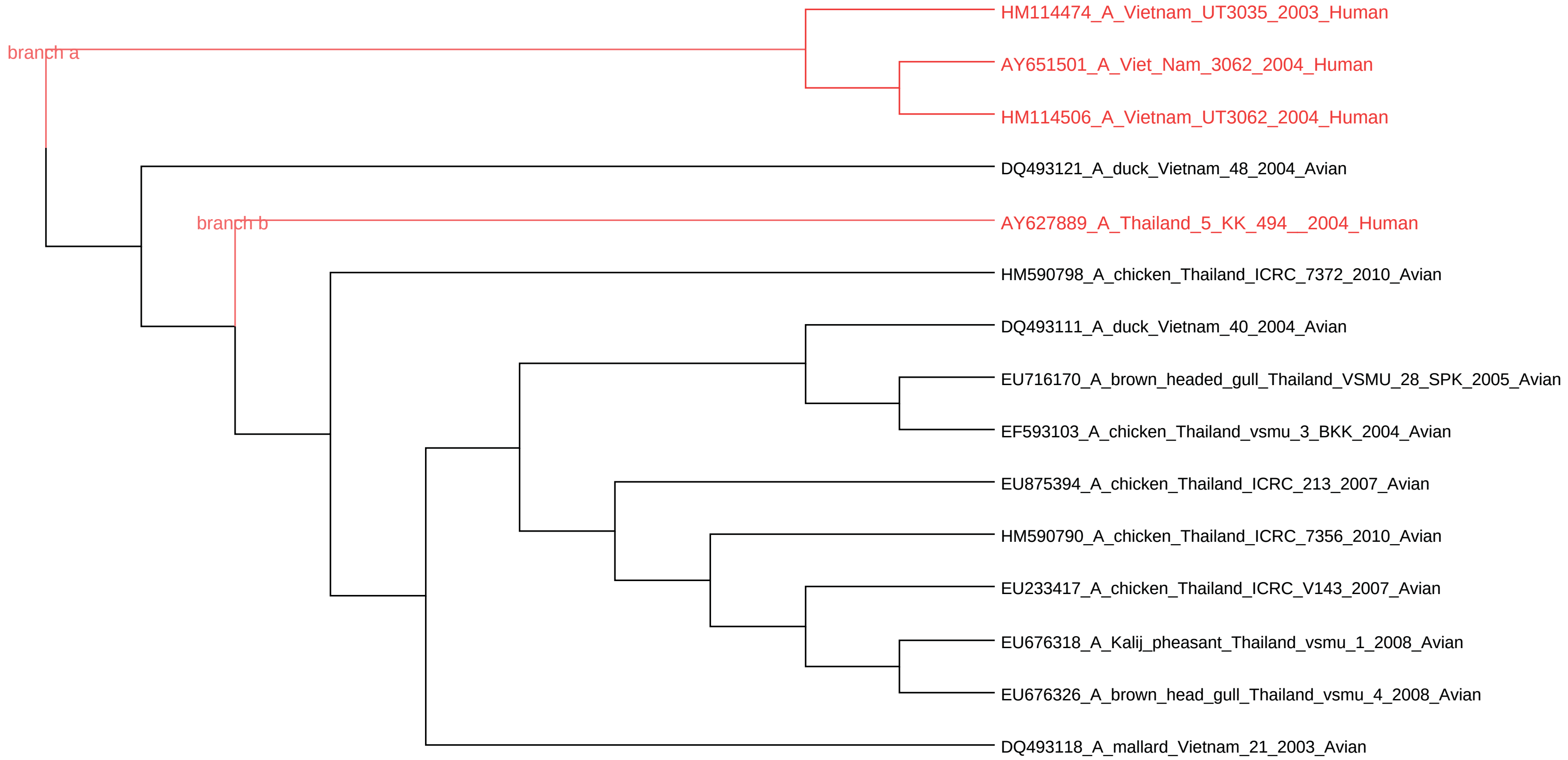

branch a
HM114474_A_Vietnam_UT3035_2003_Human
AY651501_A_Viet_Nam_3062_2004_Human
HM114506_A_Vietnam_UT3062_2004_Human
DQ493121_A_duck_Vietnam_48_2004_Avian
branch b
AY627889_A_Thailand_5_KK_494__2004_Human
HM590798_A_chicken_Thailand_ICRC_7372_2010_Avian
DQ493111_A_duck_Vietnam_40_2004_Avian
EU716170_A_brown_headed_gull_Thailand_VSMU_28_SPK_2005_Avian
EF593103_A_chicken_Thailand_vsmu_3_BKK_2004_Avian
EU875394_A_chicken_Thailand_ICRC_213_2007_Avian
HM590790_A_chicken_Thailand_ICRC_7356_2010_Avian
EU233417_A_chicken_Thailand_ICRC_V143_2007_Avian
EU676318_A_Kalij_pheasant_Thailand_vsmu_1_2008_Avian
EU676326_A_brown_head_gull_Thailand_vsmu_4_2008_Avian
DQ493118_A_mallard_Vietnam_21_2003_Avian

NP-Group75

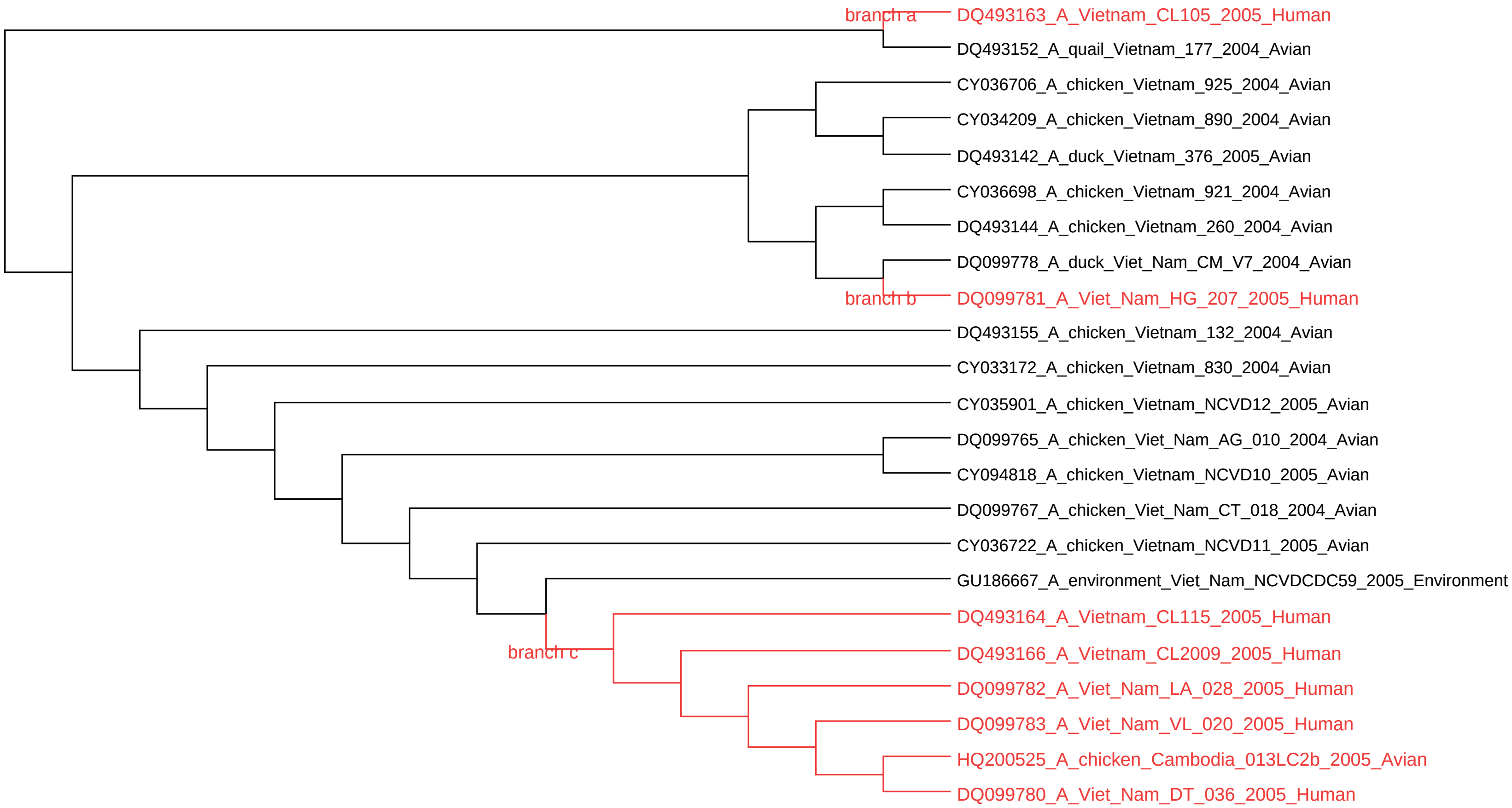

branch a
DQ493163_A_Vietnam_CL105_2005_Human
DQ493152_A_quail_Vietnam_177_2004_Avian
CY036706_A_chicken_Vietnam_925_2004_Avian
CY034209_A_chicken_Vietnam_890_2004_Avian
DQ493142_A_duck_Vietnam_376_2005_Avian
CY036698_A_chicken_Vietnam_921_2004_Avian
DQ493144_A_chicken_Vietnam_260_2004_Avian
DQ099778_A_duck_Viet_Nam_CM_V7_2004_Avian
branch b
DQ099781_A_Viet_Nam_HG_207_2005_Human
DQ493155_A_chicken_Vietnam_132_2004_Avian
CY033172_A_chicken_Vietnam_830_2004_Avian
CY035901_A_chicken_Vietnam_NCVD12_2005_Avian
DQ099765_A_chicken_Viet_Nam_AG_010_2004_Avian
CY094818_A_chicken_Vietnam_NCVD10_2005_Avian
DQ099767_A_chicken_Viet_Nam_CT_018_2004_Avian
CY036722_A_chicken_Vietnam_NCVD11_2005_Avian
GU186667_A_environment_Viet_Nam_NCVDCDC59_2005_Environment
DQ493164_A_Vietnam_CL115_2005_Human
branch c
DQ493166_A_Vietnam_CL2009_2005_Human
DQ099782_A_Viet_Nam_LA_028_2005_Human
DQ099783_A_Viet_Nam_VL_020_2005_Human
HQ200525_A_chicken_Cambodia_013LC2b_2005_Avian
DQ099780_A_Viet_Nam_DT_036_2005_Human

NP-Group76

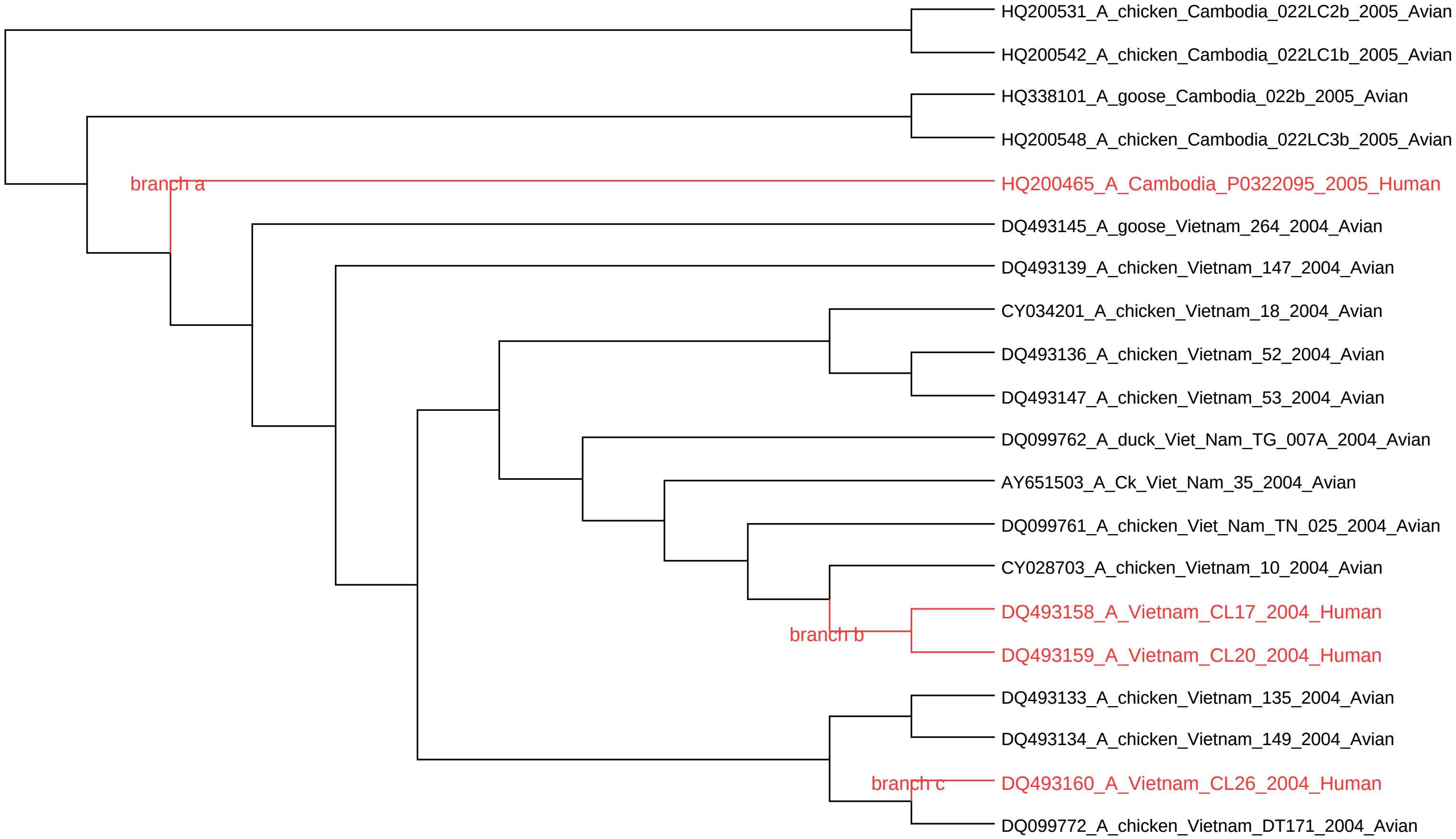
HQ200531_A_chicken_Cambodia_022LC2b_2005_Avian
HQ200542_A_chicken_Cambodia_022LC1b_2005_Avian
HQ338101_A_goose_Cambodia_022b_2005_Avian
HQ200548_A_chicken_Cambodia_022LC3b_2005_Avian
branch a
HQ200465_A_Cambodia_P0322095_2005_Human
DQ493145_A_goose_Vietnam_264_2004_Avian
DQ493139_A_chicken_Vietnam_147_2004_Avian
CY034201_A_chicken_Vietnam_18_2004_Avian
DQ493136_A_chicken_Vietnam_52_2004_Avian
DQ493147_A_chicken_Vietnam_53_2004_Avian
DQ099762_A_duck_Viet_Nam_TG_007A_2004_Avian
AY651503_A_Ck_Viet_Nam_35_2004_Avian
DQ099761_A_chicken_Viet_Nam_TN_025_2004_Avian
CY028703_A_chicken_Vietnam_10_2004_Avian
DQ493158_A_Vietnam_CL17_2004_Human
branch b
DQ493159_A_Vietnam_CL20_2004_Human
DQ493133_A_chicken_Vietnam_135_2004_Avian
DQ493134_A_chicken_Vietnam_149_2004_Avian
branch c
DQ493160_A_Vietnam_CL26_2004_Human
DQ099772_A_chicken_Vietnam_DT171_2004_Avian

NP-Group77

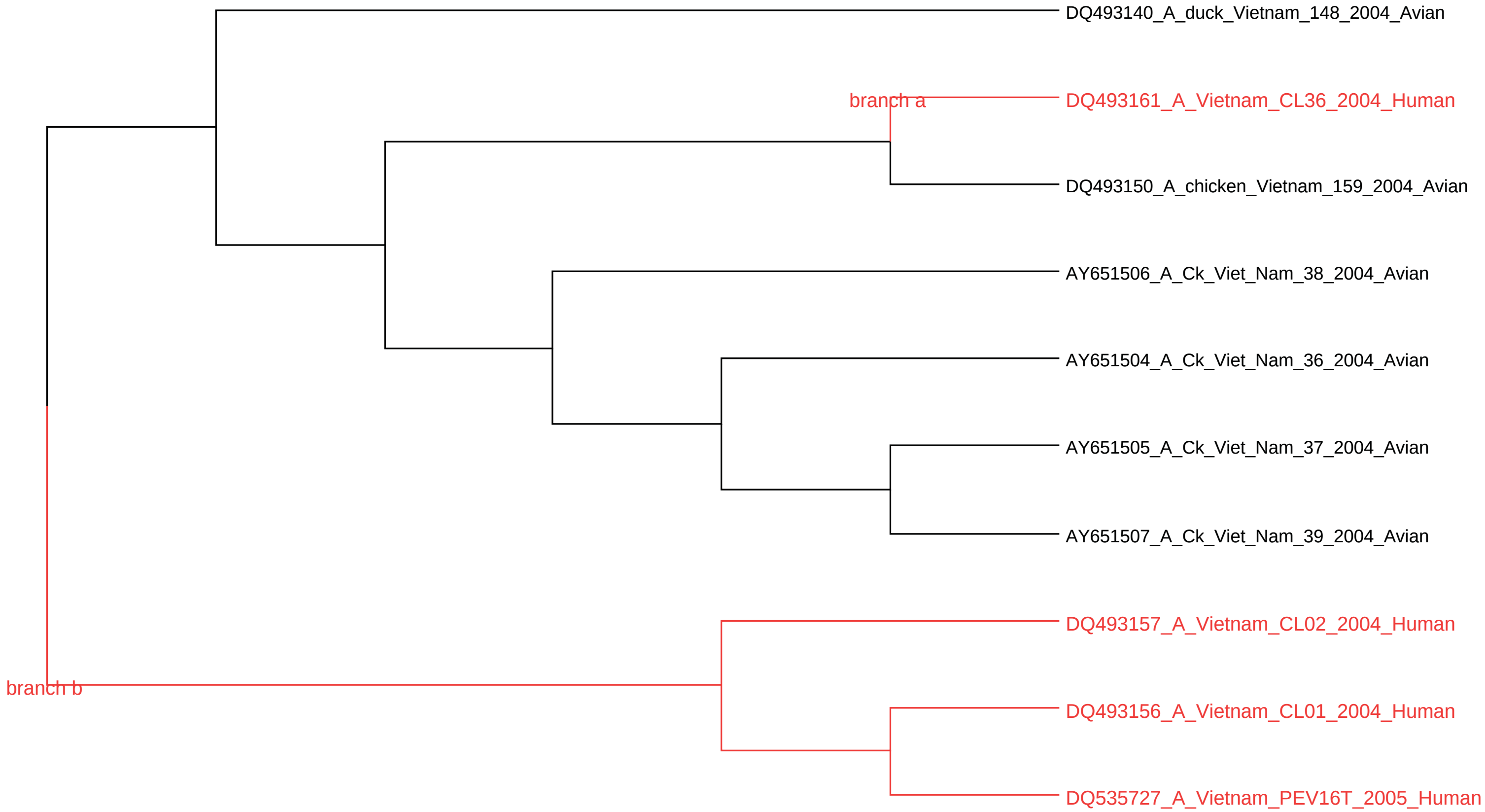
DQ493140_A_duck_Vietnam_148_2004_Avian
branch a
DQ493161_A_Vietnam_CL36_2004_Human
DQ493150_A_chicken_Vietnam_159_2004_Avian
AY651506_A_Ck_Viet_Nam_38_2004_Avian
AY651504_A_Ck_Viet_Nam_36_2004_Avian
AY651505_A_Ck_Viet_Nam_37_2004_Avian
AY651507_A_Ck_Viet_Nam_39_2004_Avian
DQ493157_A_Vietnam_CL02_2004_Human
branch b
DQ493156_A_Vietnam_CL01_2004_Human
DQ535727_A_Vietnam_PEV16T_2005_Human

NP-Group78

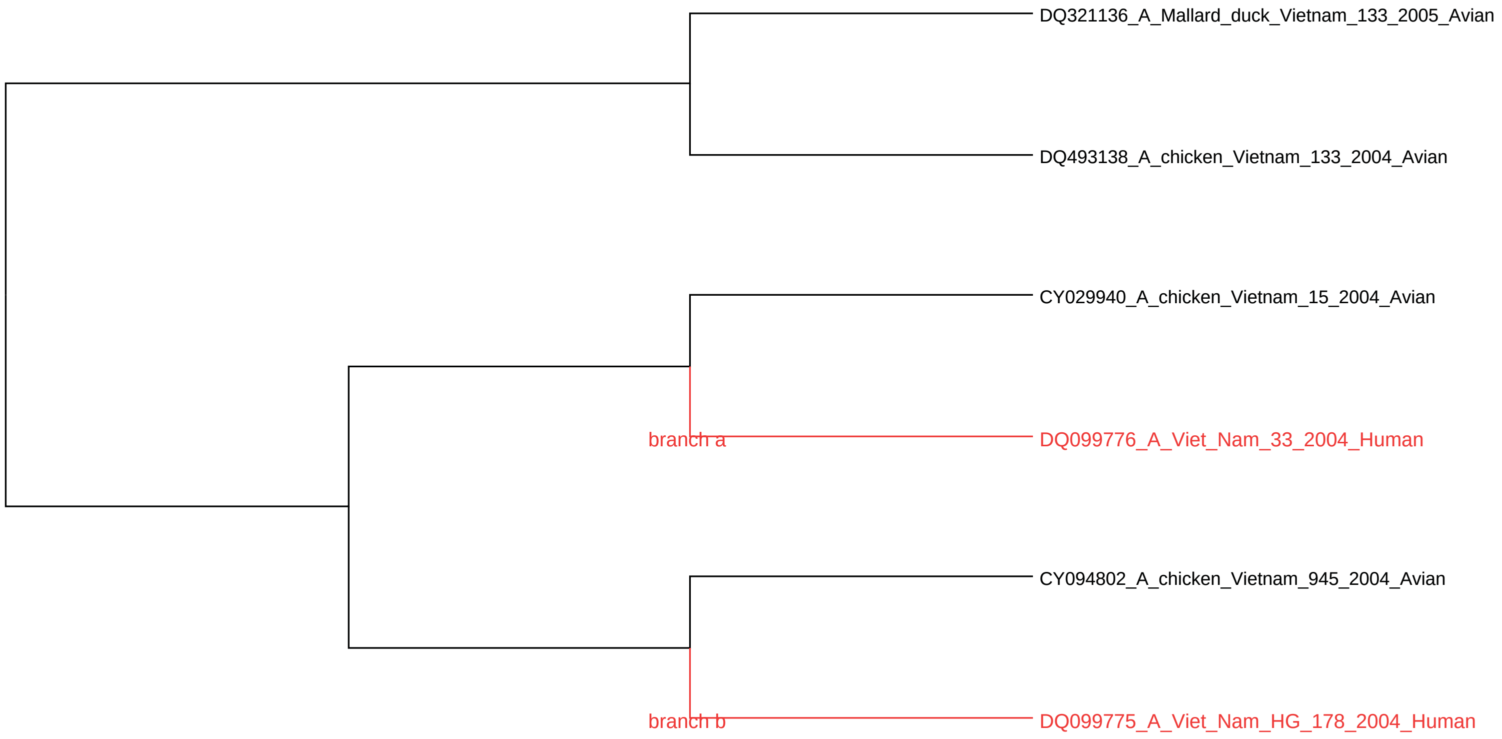
DQ321136_A_Mallard_duck_Vietnam_133_2005_Avian
DQ493138_A_chicken_Vietnam_133_2004_Avian
CY029940_A_chicken_Vietnam_15_2004_Avian
branch a
DQ099776_A_Viet_Nam_33_2004_Human
CY094802_A_chicken_Vietnam_945_2004_Avian
branch b
DQ099775_A_Viet_Nam_HG_178_2004_Human

NP-Group79

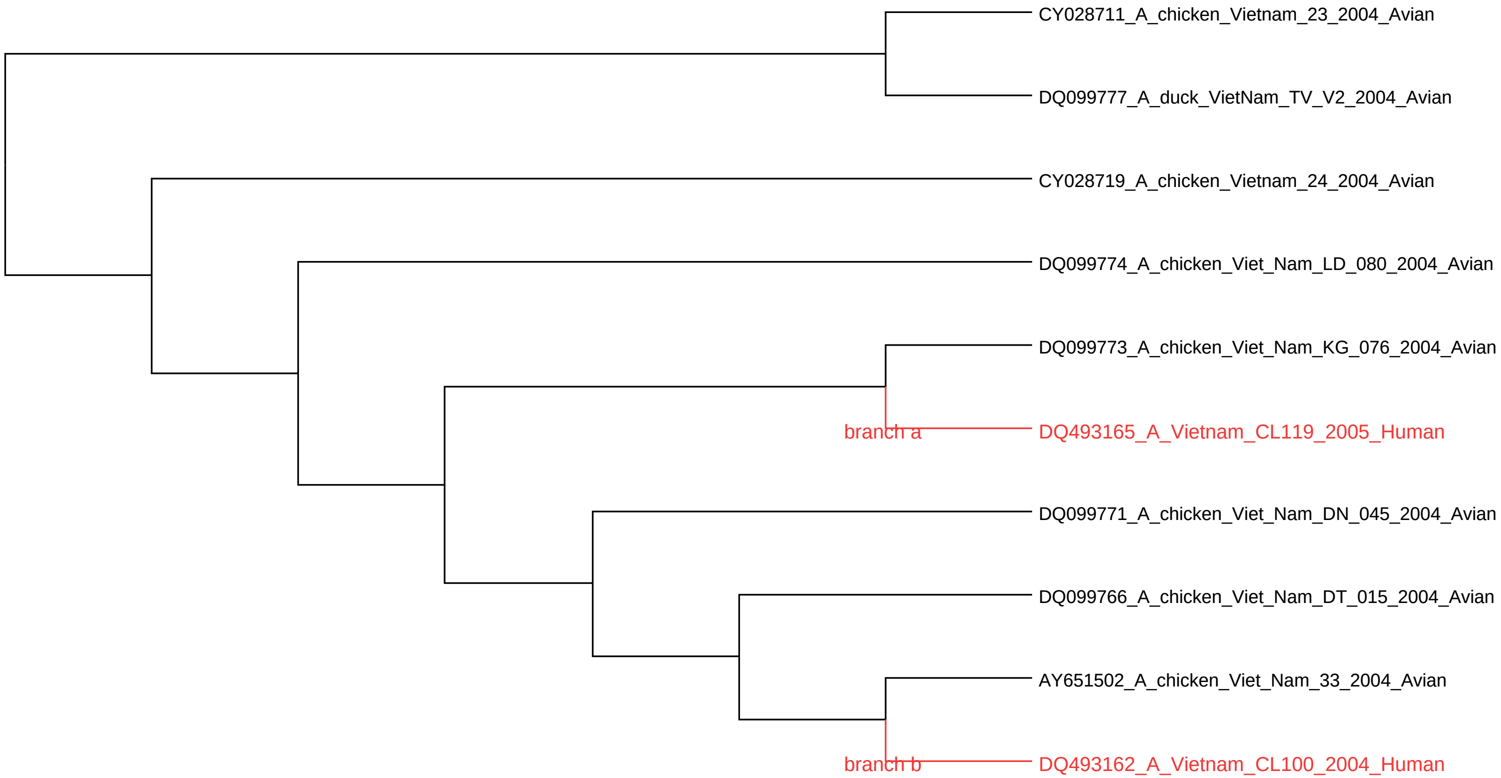

CY028711_A_chicken_Vietnam_23_2004_Avian
DQ099777_A_duck_VietNam_TV_V2_2004_Avian
CY028719_A_chicken_Vietnam_24_2004_Avian
DQ099774_A_chicken_Viet_Nam_LD_080_2004_Avian
DQ099773_A_chicken_Viet_Nam_KG_076_2004_Avian
branch a
DQ493165_A_Vietnam_CL119_2005_Human
DQ099771_A_chicken_Viet_Nam_DN_045_2004_Avian
DQ099766_A_chicken_Viet_Nam_DT_015_2004_Avian
AY651502_A_chicken_Viet_Nam_33_2004_Avian
branch b
DQ493162_A_Vietnam_CL100_2004_Human

NP-Group80

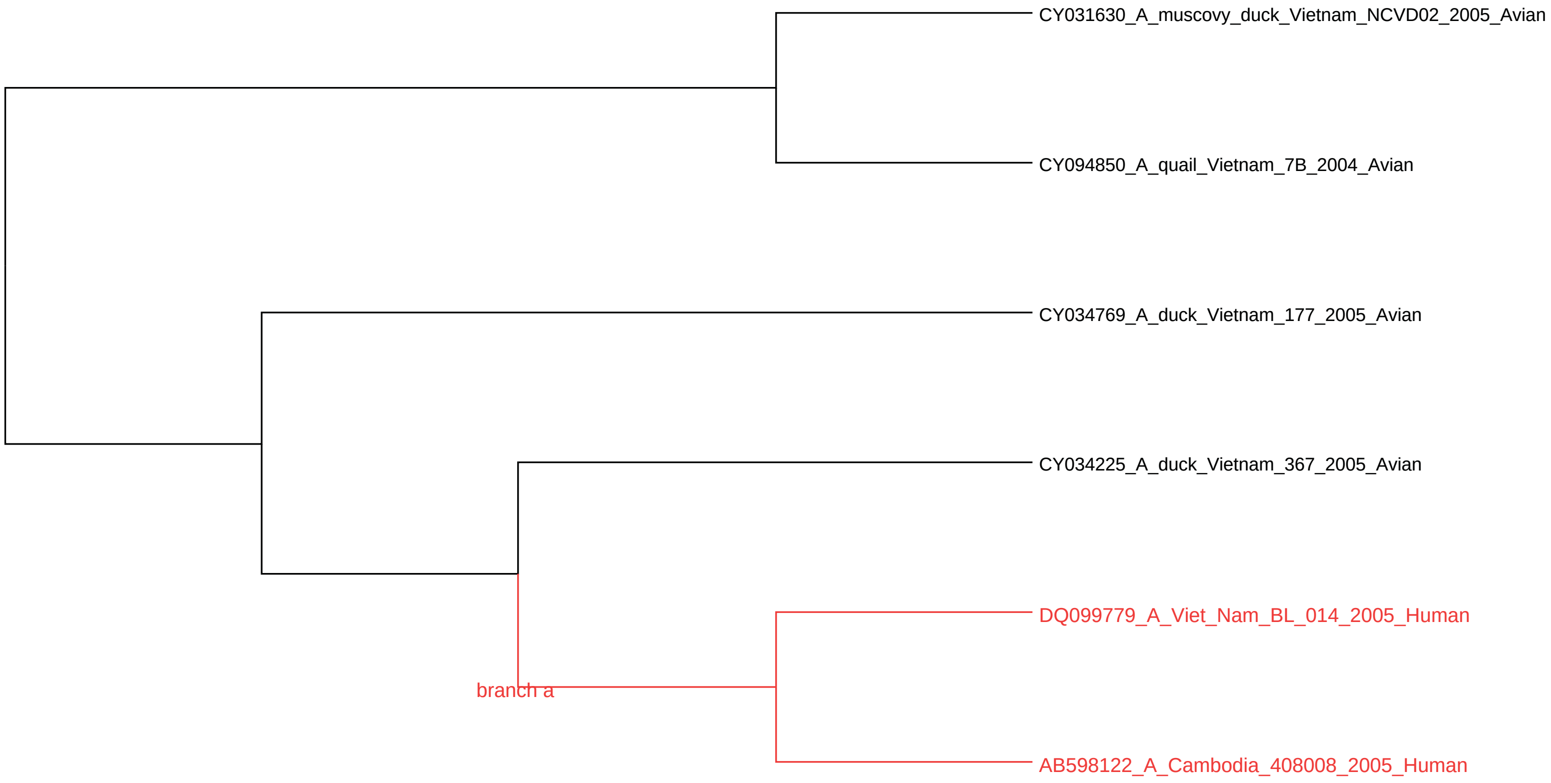
CY031630_A_muscovy_duck_Vietnam_NCVD02_2005_Avian
CY094850_A_quail_Vietnam_7B_2004_Avian
CY034769_A_duck_Vietnam_177_2005_Avian
CY034225_A_duck_Vietnam_367_2005_Avian
DQ099779_A_Viet_Nam_BL_014_2005_Human
branch a
AB598122_A_Cambodia_408008_2005_Human

NP-Group81

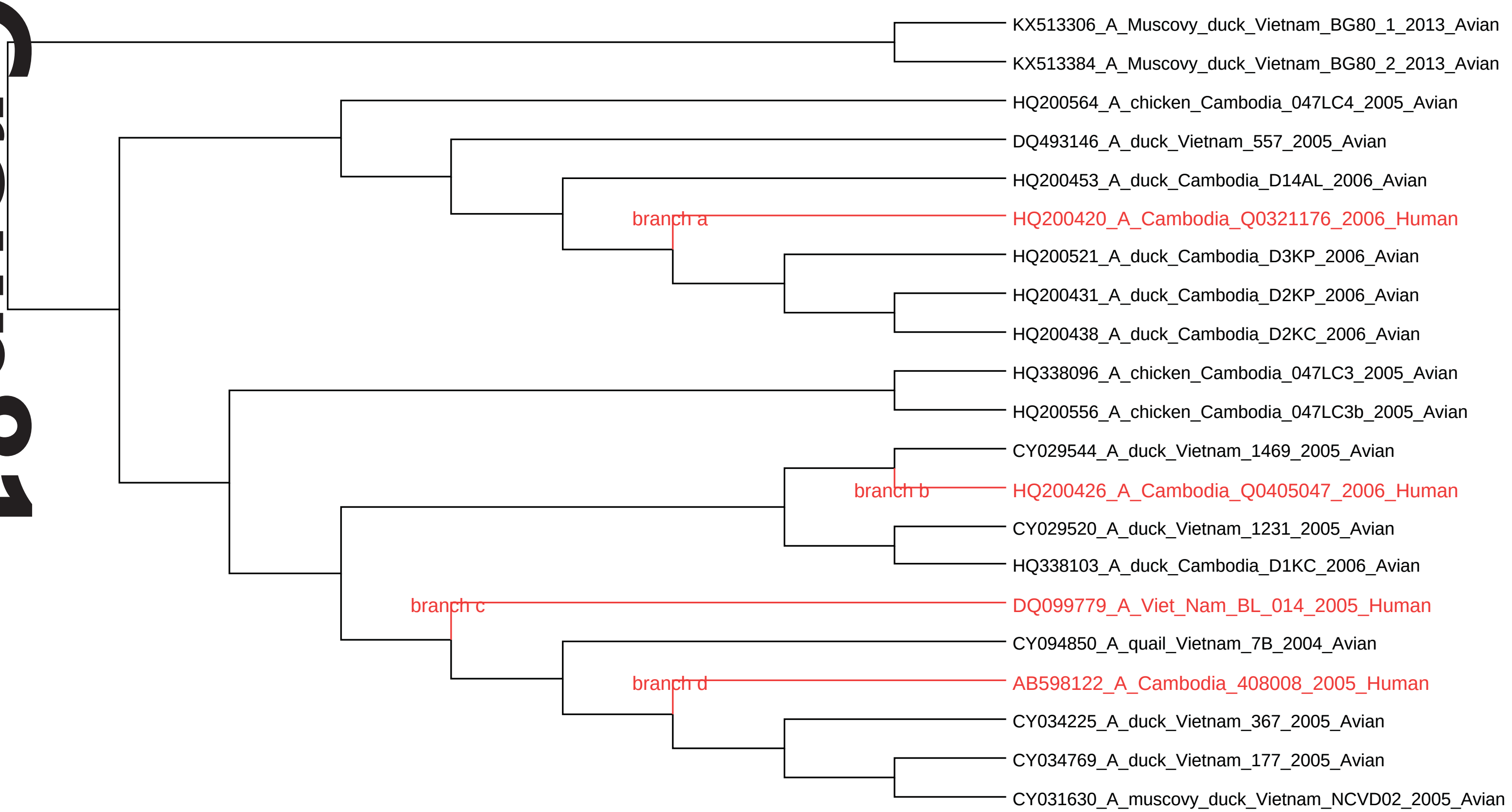

KX513306_A_Muscovy_duck_Vietnam_BG80_1_2013_Avian
KX513384_A_Muscovy_duck_Vietnam_BG80_2_2013_Avian
HQ200564_A_chicken_Cambodia_047LC4_2005_Avian
DQ493146_A_duck_Vietnam_557_2005_Avian
HQ200453_A_duck_Cambodia_D14AL_2006_Avian
branch a
HQ200420_A_Cambodia_Q0321176_2006_Human
HQ200521_A_duck_Cambodia_D3KP_2006_Avian
HQ200431_A_duck_Cambodia_D2KP_2006_Avian
HQ200438_A_duck_Cambodia_D2KC_2006_Avian
HQ338096_A_chicken_Cambodia_047LC3_2005_Avian
HQ200556_A_chicken_Cambodia_047LC3b_2005_Avian
CY029544_A_duck_Vietnam_1469_2005_Avian
branch b
HQ200426_A_Cambodia_Q0405047_2006_Human
CY029520_A_duck_Vietnam_1231_2005_Avian
HQ338103_A_duck_Cambodia_D1KC_2006_Avian
branch c
DQ099779_A_Viet_Nam_BL_014_2005_Human
CY094850_A_quail_Vietnam_7B_2004_Avian
branch d
AB598122_A_Cambodia_408008_2005_Human
CY034225_A_duck_Vietnam_367_2005_Avian
CY034769_A_duck_Vietnam_177_2005_Avian
CY031630_A_muscovy_duck_Vietnam_NCVD02_2005_Avian

NP-Group82

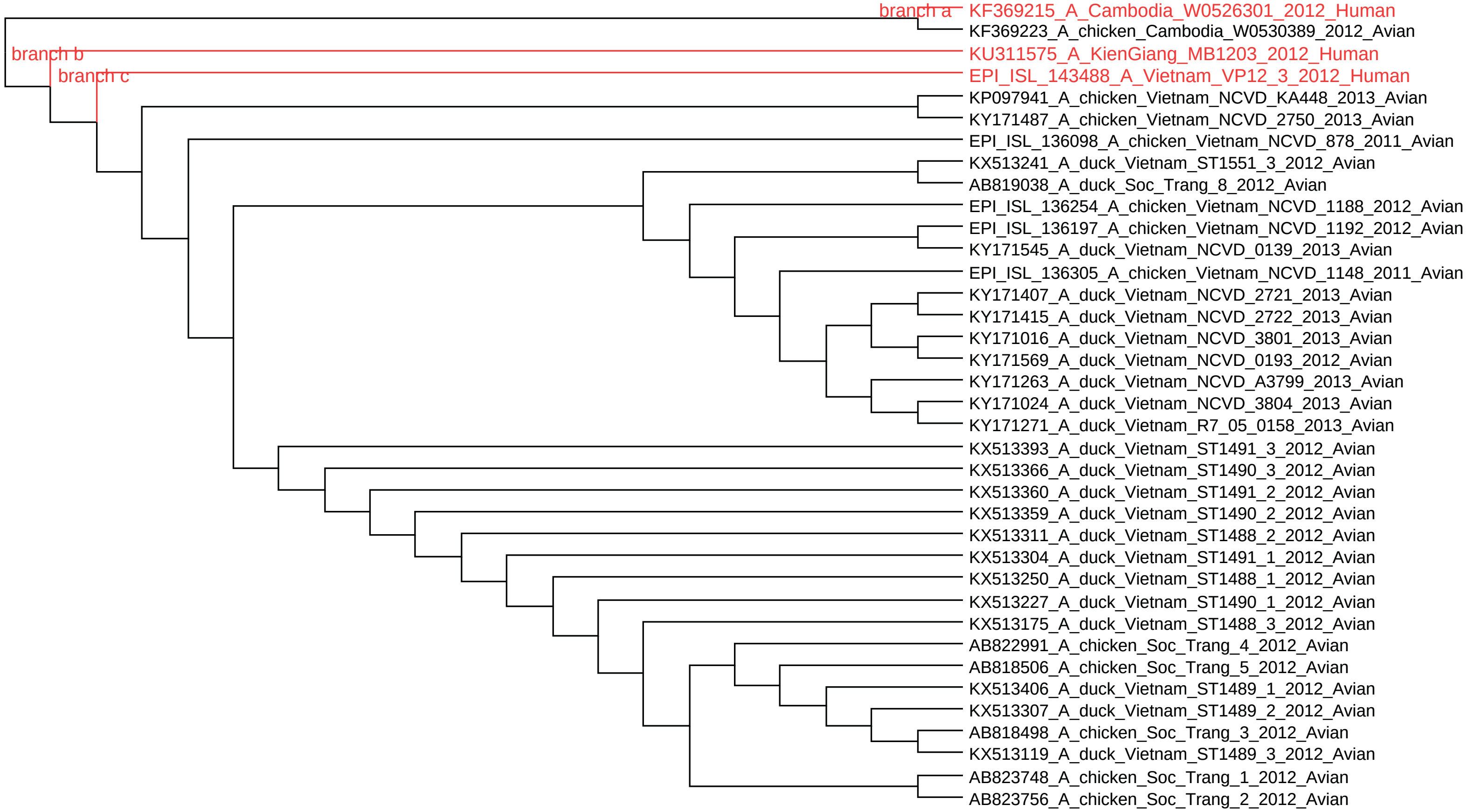
branch a
KF369215_A_Cambodia_W0526301_2012_Human
KF369223_A_chicken_Cambodia_W0530389_2012_Avian
branch b
KU311575_A_KienGiang_MB1203_2012_Human
branch c
EPI_ISL_143488_A_Vietnam_VP12_3_2012_Human
KP097941_A_chicken_Vietnam_NCVD_KA448_2013_Avian
KY171487_A_chicken_Vietnam_NCVD_2750_2013_Avian
EPI_ISL_136098_A_chicken_Vietnam_NCVD_878_2011_Avian
KX513241_A_duck_Vietnam_ST1551_3_2012_Avian
AB819038_A_duck_Soc_Trang_8_2012_Avian
EPI_ISL_136254_A_chicken_Vietnam_NCVD_1188_2012_Avian
EPI_ISL_136197_A_chicken_Vietnam_NCVD_1192_2012_Avian
KY171545_A_duck_Vietnam_NCVD_0139_2013_Avian
EPI_ISL_136305_A_chicken_Vietnam_NCVD_1148_2011_Avian
KY171407_A_duck_Vietnam_NCVD_2721_2013_Avian
KY171415_A_duck_Vietnam_NCVD_2722_2013_Avian
KY171016_A_duck_Vietnam_NCVD_3801_2013_Avian
KY171569_A_duck_Vietnam_NCVD_0193_2012_Avian
KY171263_A_duck_Vietnam_NCVD_A3799_2013_Avian
KY171024_A_duck_Vietnam_NCVD_3804_2013_Avian
KY171271_A_duck_Vietnam_R7_05_0158_2013_Avian
KX513393_A_duck_Vietnam_ST1491_3_2012_Avian
KX513366_A_duck_Vietnam_ST1490_3_2012_Avian
KX513360_A_duck_Vietnam_ST1491_2_2012_Avian
KX513359_A_duck_Vietnam_ST1490_2_2012_Avian
KX513311_A_duck_Vietnam_ST1488_2_2012_Avian
KX513304_A_duck_Vietnam_ST1491_1_2012_Avian
KX513250_A_duck_Vietnam_ST1488_1_2012_Avian
KX513227_A_duck_Vietnam_ST1490_1_2012_Avian
KX513175_A_duck_Vietnam_ST1488_3_2012_Avian
AB822991_A_chicken_Soc_Trang_4_2012_Avian
AB818506_A_chicken_Soc_Trang_5_2012_Avian
KX513406_A_duck_Vietnam_ST1489_1_2012_Avian
KX513307_A_duck_Vietnam_ST1489_2_2012_Avian
AB818498_A_chicken_Soc_Trang_3_2012_Avian
KX513119_A_duck_Vietnam_ST1489_3_2012_Avian
AB823748_A_chicken_Soc_Trang_1_2012_Avian
AB823756_A_chicken_Soc_Trang_2_2012_Avian

NP-Group83

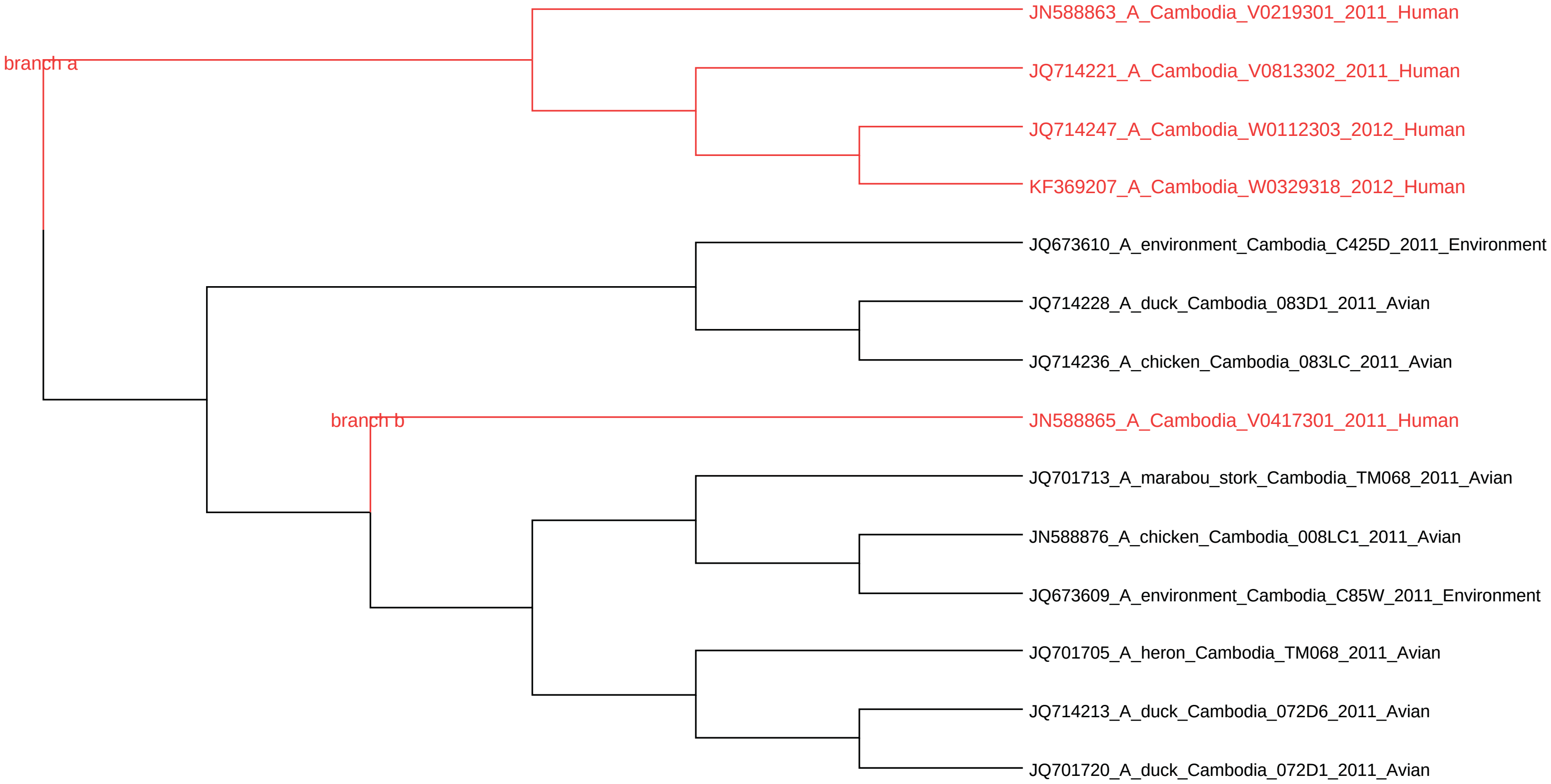
branch a
branch b
JN588863_A_Cambodia_V0219301_2011_Human
JQ714221_A_Cambodia_V0813302_2011_Human
JQ714247_A_Cambodia_W0112303_2012_Human
KF369207_A_Cambodia_W0329318_2012_Human
JQ673610_A_environment_Cambodia_C425D_2011_Environment
JQ714228_A_duck_Cambodia_083D1_2011_Avian
JQ714236_A_chicken_Cambodia_083LC_2011_Avian
JN588865_A_Cambodia_V0417301_2011_Human
JQ701713_A_marabou_stork_Cambodia_TM068_2011_Avian
JN588876_A_chicken_Cambodia_008LC1_2011_Avian
JQ673609_A_environment_Cambodia_C85W_2011_Environment
JQ701705_A_heron_Cambodia_TM068_2011_Avian
JQ714213_A_duck_Cambodia_072D6_2011_Avian
JQ701720_A_duck_Cambodia_072D1_2011_Avian

NP-Group84

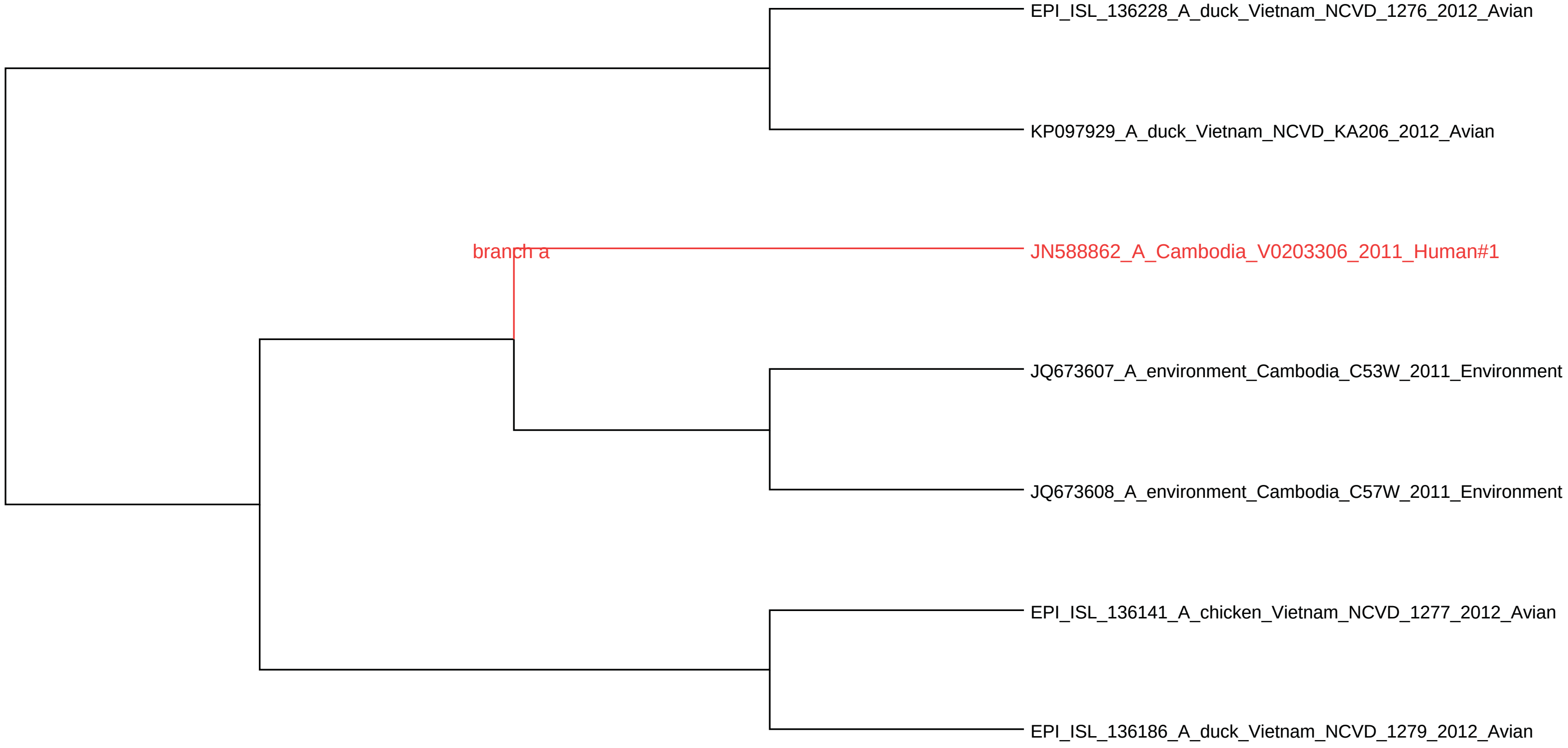
EPI_ISL_136228_A_duck_Vietnam_NCVD_1276_2012_Avian
KP097929_A_duck_Vietnam_NCVD_KA206_2012_Avian
branch a
JN588862_A_Cambodia_V0203306_2011_Human#1
JQ673607_A_environment_Cambodia_C53W_2011_Environment
JQ673608_A_environment_Cambodia_C57W_2011_Environment
EPI_ISL_136141_A_chicken_Vietnam_NCVD_1277_2012_Avian
EPI_ISL_136186_A_duck_Vietnam_NCVD_1279_2012_Avian

NP-Group85

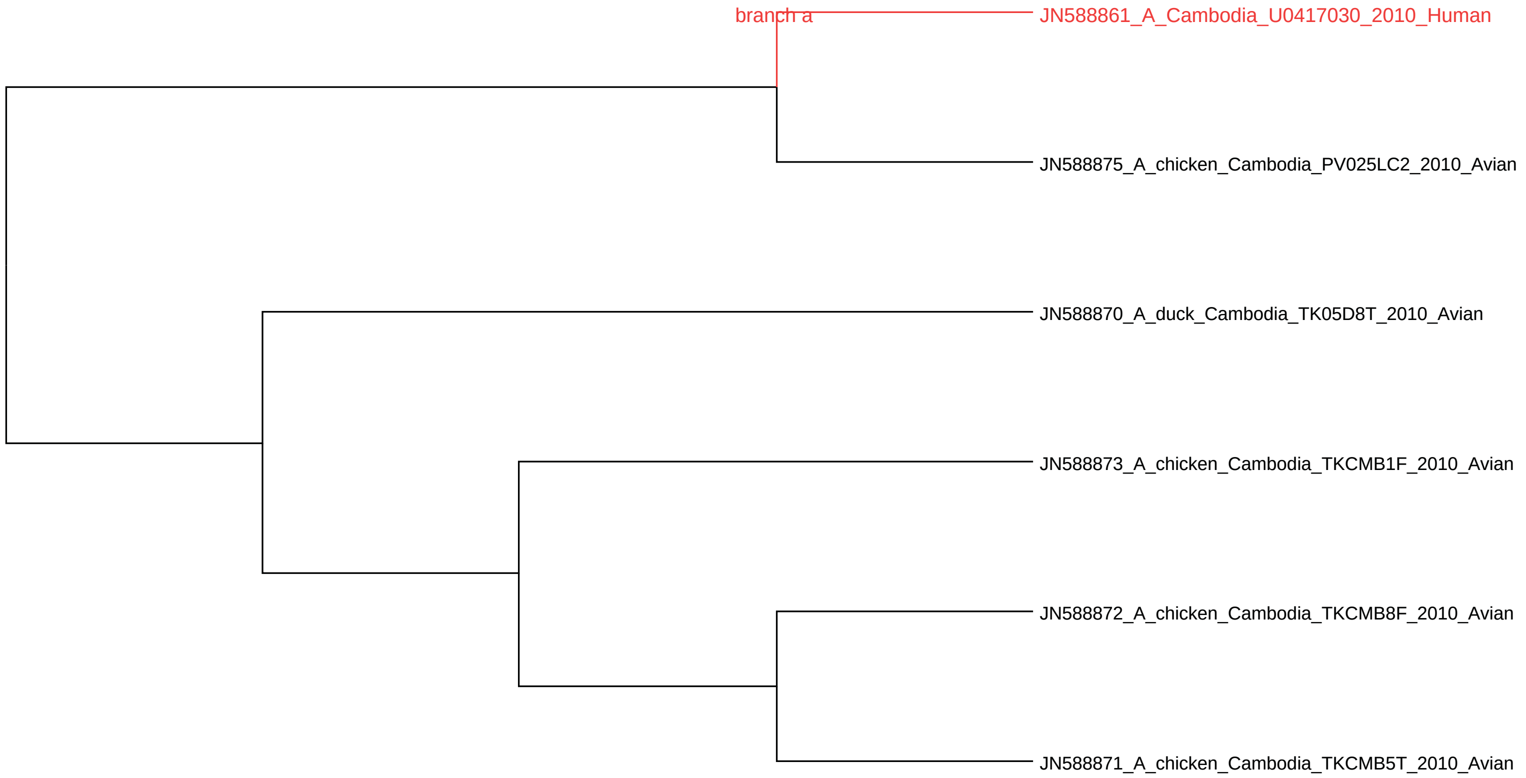
branch a
JN588861_A_Cambodia_U0417030_2010_Human
JN588875_A_chicken_Cambodia_PV025LC2_2010_Avian
JN588870_A_duck_Cambodia_TK05D8T_2010_Avian
JN588873_A_chicken_Cambodia_TKCMB1F_2010_Avian
JN588872_A_chicken_Cambodia_TKCMB8F_2010_Avian
JN588871_A_chicken_Cambodia_TKCMB5T_2010_Avian

NP-Group86

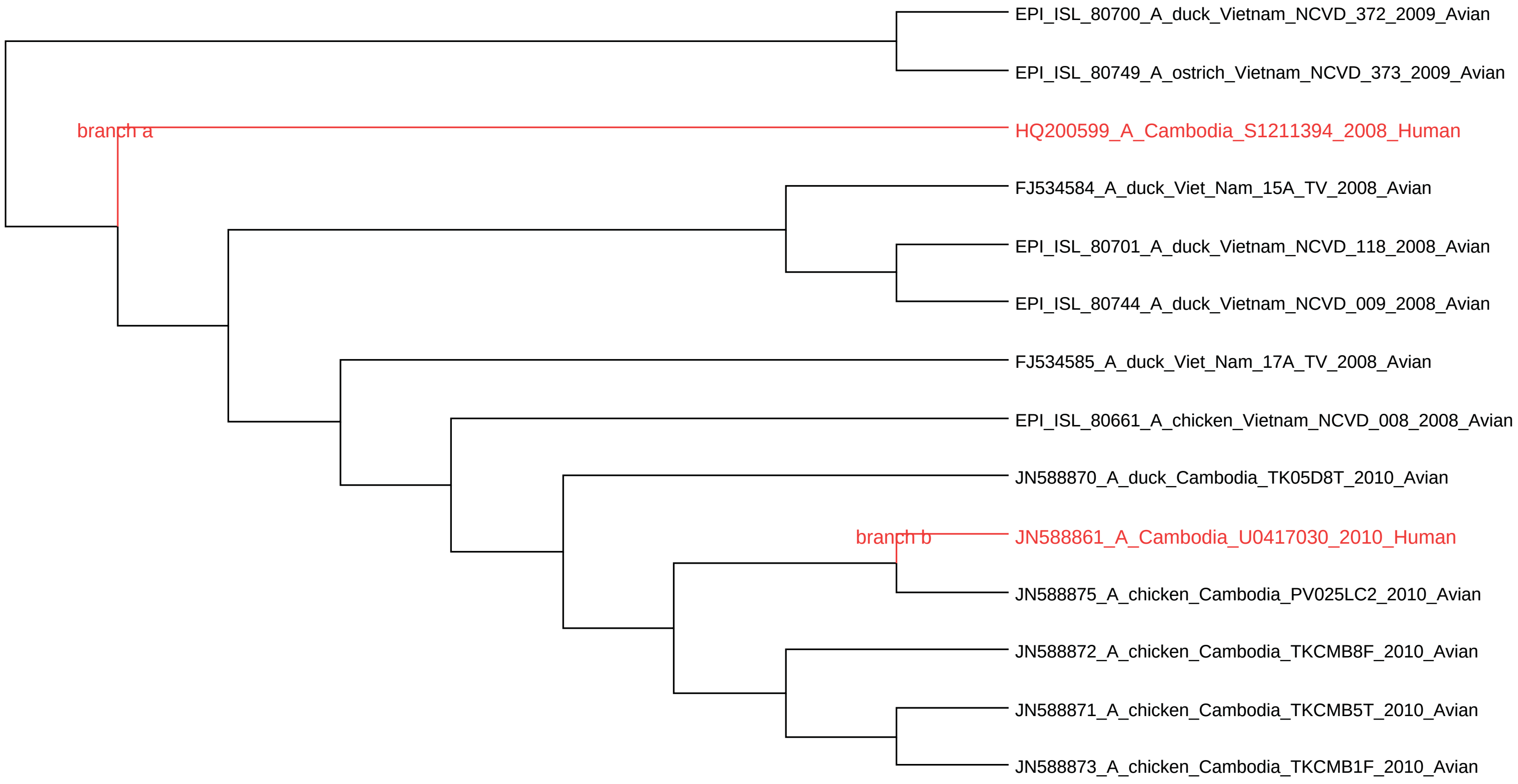
EPI_ISL_80700_A_duck_Vietnam_NCVD_372_2009_Avian
EPI_ISL_80749_A_ostrich_Vietnam_NCVD_373_2009_Avian
branch a
HQ200599_A_Cambodia_S1211394_2008_Human
FJ534584_A_duck_Viet_Nam_15A_TV_2008_Avian
EPI_ISL_80701_A_duck_Vietnam_NCVD_118_2008_Avian
EPI_ISL_80744_A_duck_Vietnam_NCVD_009_2008_Avian
FJ534585_A_duck_Viet_Nam_17A_TV_2008_Avian
EPI_ISL_80661_A_chicken_Vietnam_NCVD_008_2008_Avian
JN588870_A_duck_Cambodia_TK05D8T_2010_Avian
branch b
JN588861_A_Cambodia_U0417030_2010_Human
JN588875_A_chicken_Cambodia_PV025LC2_2010_Avian
JN588872_A_chicken_Cambodia_TKCMB8F_2010_Avian
JN588871_A_chicken_Cambodia_TKCMB5T_2010_Avian
JN588873_A_chicken_Cambodia_TKCMB1F_2010_Avian

NP-Group87

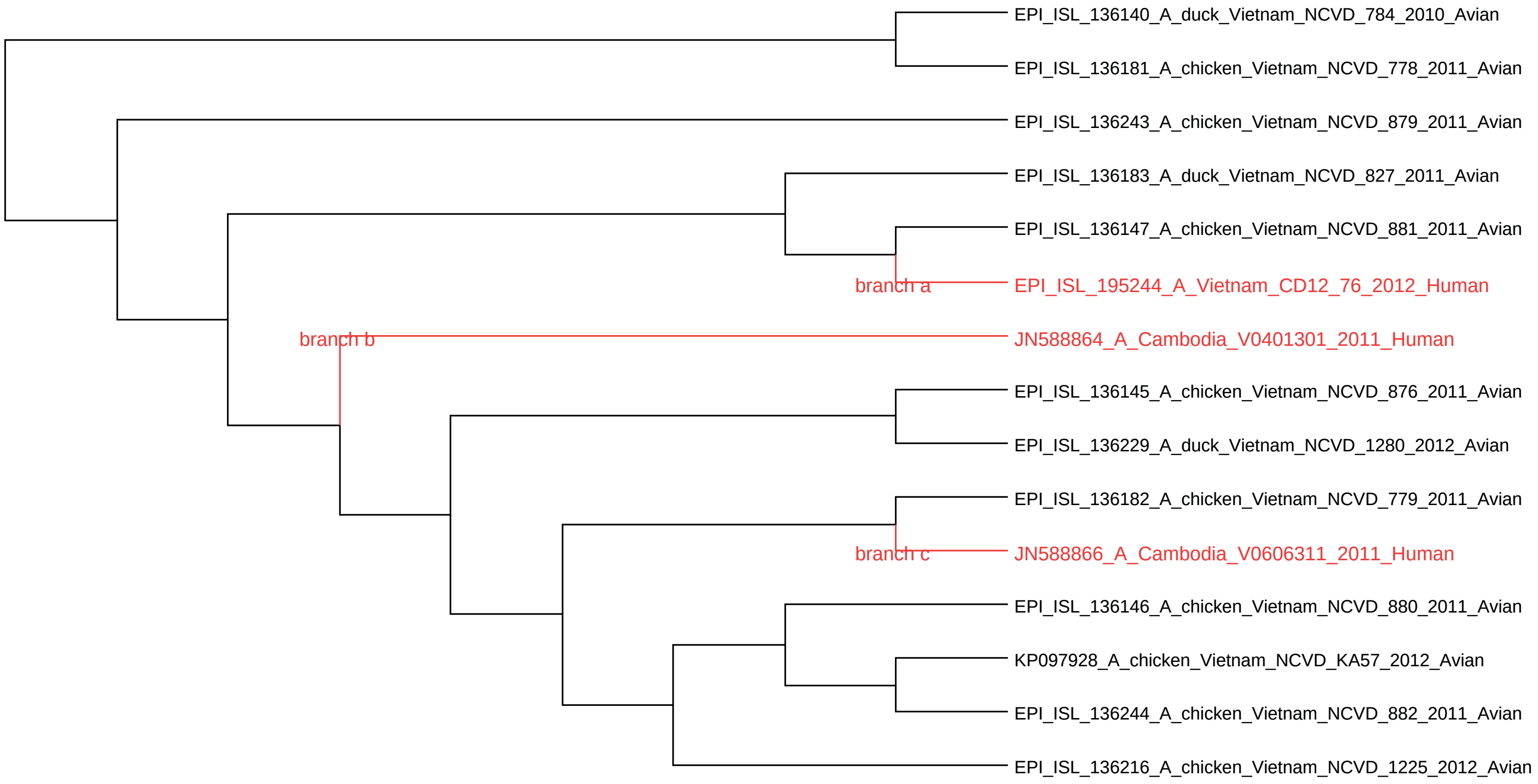

EPI_ISL_136140_A_duck_Vietnam_NCVD_784_2010_Avian
EPI_ISL_136181_A_chicken_Vietnam_NCVD_778_2011_Avian
EPI_ISL_136243_A_chicken_Vietnam_NCVD_879_2011_Avian
EPI_ISL_136183_A_duck_Vietnam_NCVD_827_2011_Avian
EPI_ISL_136147_A_chicken_Vietnam_NCVD_881_2011_Avian
branch a
EPI_ISL_195244_A_Vietnam_CD12_76_2012_Human
branch b
JN588864_A_Cambodia_V0401301_2011_Human
EPI_ISL_136145_A_chicken_Vietnam_NCVD_876_2011_Avian
EPI_ISL_136229_A_duck_Vietnam_NCVD_1280_2012_Avian
EPI_ISL_136182_A_chicken_Vietnam_NCVD_779_2011_Avian
branch c
JN588866_A_Cambodia_V0606311_2011_Human
EPI_ISL_136146_A_chicken_Vietnam_NCVD_880_2011_Avian
KP097928_A_chicken_Vietnam_NCVD_KA57_2012_Avian
EPI_ISL_136244_A_chicken_Vietnam_NCVD_882_2011_Avian
EPI_ISL_136216_A_chicken_Vietnam_NCVD_1225_2012_Avian
