# Supplementary material for: Adaptive Evolution of Human-Isolated H5Nx Avian Influenza A Viruses
Source: Front Microbiol. 2019 Jun 12;10:1328. doi: 10.3389/fmicb.2019.01328 (PMC6582624; doi:10.3389/fmicb.2019.01328)
Supplement: Supplementary file 7 [file Data_Sheet_7.PDF]

Supplementary Figure 7. 101 phylogenetic trees of NS used for the adaptive evolution analyses. Human strains are marked in red. Branches which have significant signals of positive selection are marked with *.

NS-Group1

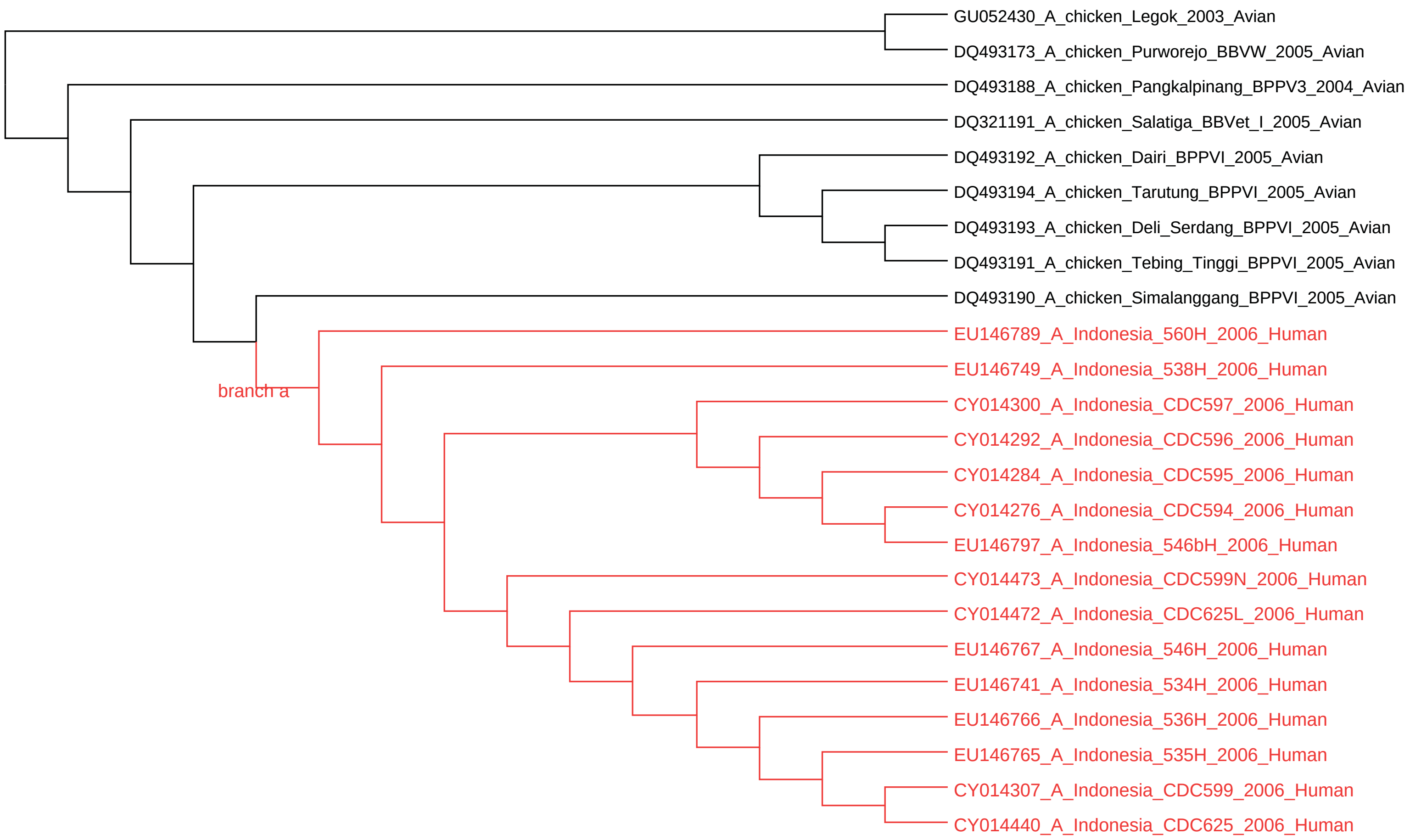

NS-Group2

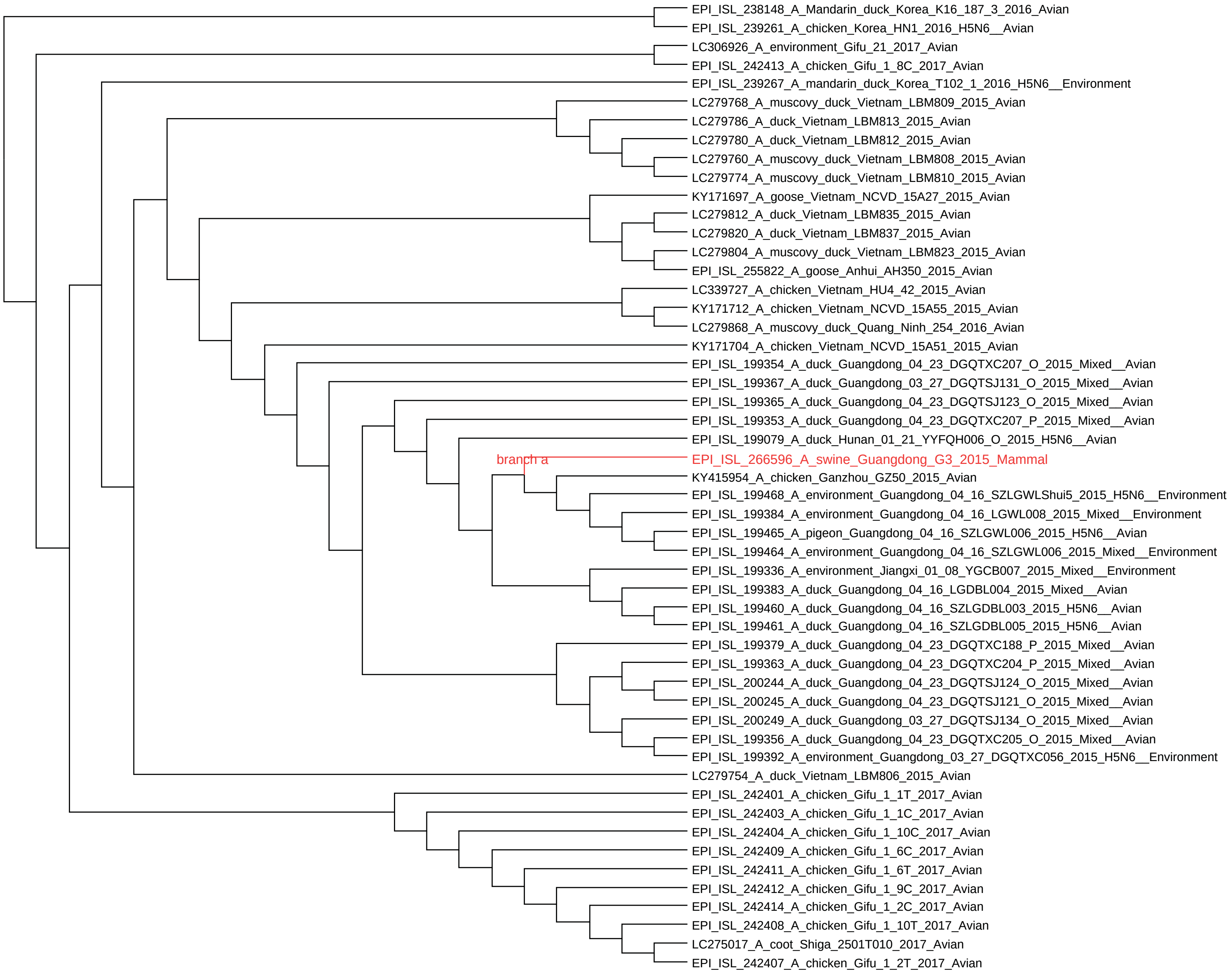

N -Group

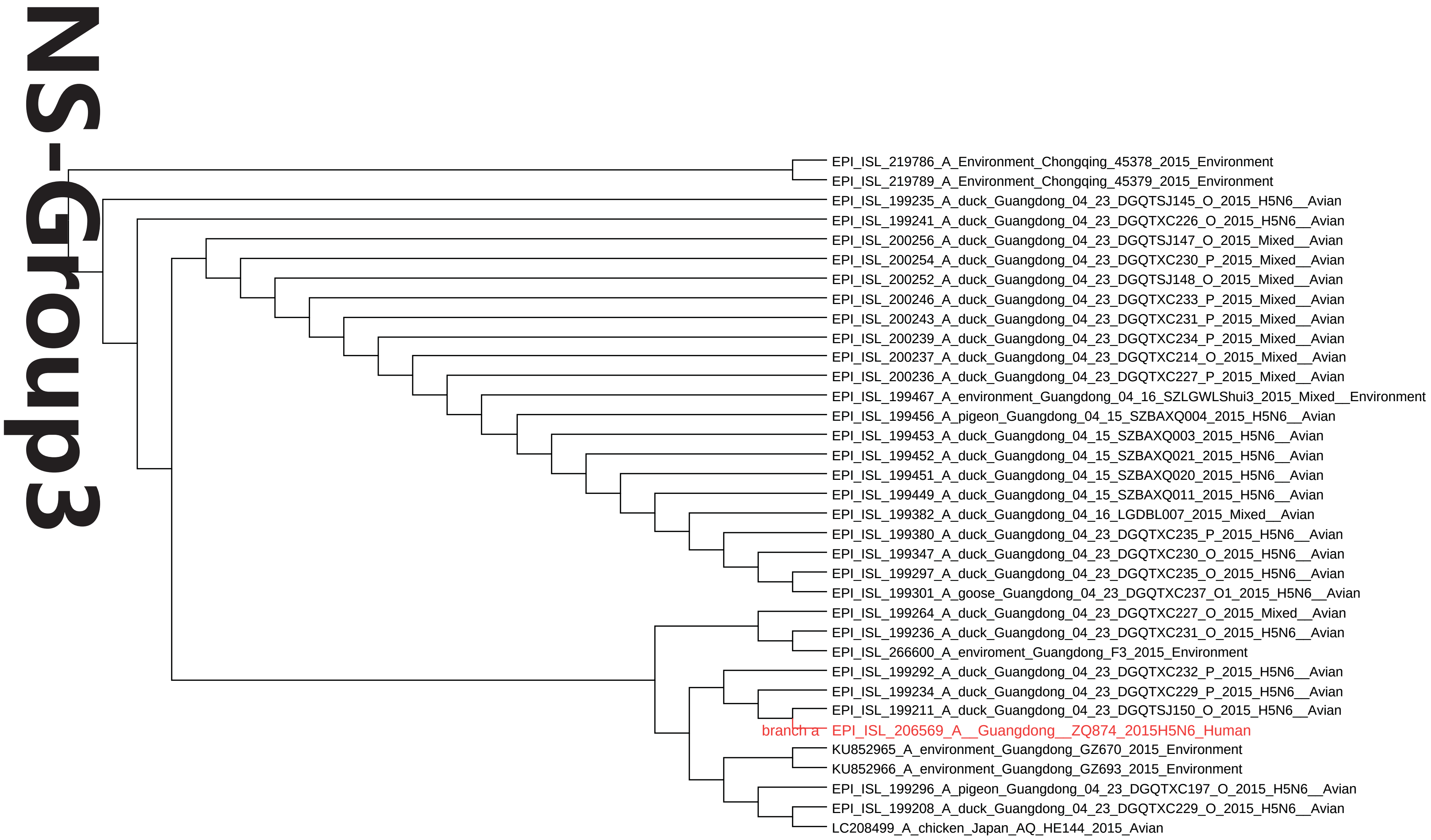
NS-Group3
EPI_ISL_219786_A_Environment_Chongqing_45378_2015_Environment
EPI_ISL_219789_A_Environment_Chongqing_45379_2015_Environment
EPI_ISL_199235_A_duck_Guangdong_04_23_DGQTSJ145_O_2015_H5N6__Avian
EPI_ISL_199241_A_duck_Guangdong_04_23_DGQTXC226_O_2015_H5N6__Avian
EPI_ISL_200256_A_duck_Guangdong_04_23_DGQTSJ147_O_2015_Mixed__Avian
EPI_ISL_200254_A_duck_Guangdong_04_23_DGQTXC230_P_2015_Mixed__Avian
EPI_ISL_200252_A_duck_Guangdong_04_23_DGQTSJ148_O_2015_Mixed__Avian
EPI_ISL_200246_A_duck_Guangdong_04_23_DGQTXC233_P_2015_Mixed__Avian
EPI_ISL_200243_A_duck_Guangdong_04_23_DGQTXC231_P_2015_Mixed__Avian
EPI_ISL_200239_A_duck_Guangdong_04_23_DGQTXC234_P_2015_Mixed__Avian
EPI_ISL_200237_A_duck_Guangdong_04_23_DGQTXC214_O_2015_Mixed__Avian
EPI_ISL_200236_A_duck_Guangdong_04_23_DGQTXC227_P_2015_Mixed__Avian
EPI_ISL_199467_A_environment_Guangdong_04_16_SZLGWLShui3_2015_Mixed__Environment
EPI_ISL_199456_A_pigeon_Guangdong_04_15_SZBAXQ004_2015_H5N6__Avian
EPI_ISL_199453_A_duck_Guangdong_04_15_SZBAXQ003_2015_H5N6__Avian
EPI_ISL_199452_A_duck_Guangdong_04_15_SZBAXQ021_2015_H5N6__Avian
EPI_ISL_199451_A_duck_Guangdong_04_15_SZBAXQ020_2015_H5N6__Avian
EPI_ISL_199449_A_duck_Guangdong_04_15_SZBAXQ011_2015_H5N6__Avian
EPI_ISL_199382_A_duck_Guangdong_04_16_LGDBL007_2015_Mixed__Avian
EPI_ISL_199380_A_duck_Guangdong_04_23_DGQTXC235_P_2015_H5N6__Avian
EPI_ISL_199347_A_duck_Guangdong_04_23_DGQTXC230_O_2015_H5N6__Avian
EPI_ISL_199297_A_duck_Guangdong_04_23_DGQTXC235_O_2015_H5N6__Avian
EPI_ISL_199301_A_goose_Guangdong_04_23_DGQTXC237_O1_2015_H5N6__Avian
EPI_ISL_199264_A_duck_Guangdong_04_23_DGQTXC227_O_2015_Mixed__Avian
EPI_ISL_199236_A_duck_Guangdong_04_23_DGQTXC231_O_2015_H5N6__Avian
EPI_ISL_266600_A_enviroment_Guangdong_F3_2015_Environment
EPI_ISL_199292_A_duck_Guangdong_04_23_DGQTXC232_P_2015_H5N6__Avian
EPI_ISL_199234_A_duck_Guangdong_04_23_DGQTXC229_P_2015_H5N6__Avian
EPI_ISL_199211_A_duck_Guangdong_04_23_DGQTSJ150_O_2015_H5N6__Avian
branch a
EPI_ISL_206569_A__Guangdong__ZQ874_2015H5N6_Human
KU852965_A_environment_Guangdong_GZ670_2015_Environment
KU852966_A_environment_Guangdong_GZ693_2015_Environment
EPI_ISL_199296_A_pigeon_Guangdong_04_23_DGQTXC197_O_2015_H5N6__Avian
EPI_ISL_199208_A_duck_Guangdong_04_23_DGQTXC229_O_2015_H5N6__Avian
LC208499_A_chicken_Japan_AQ_HE144_2015_Avian

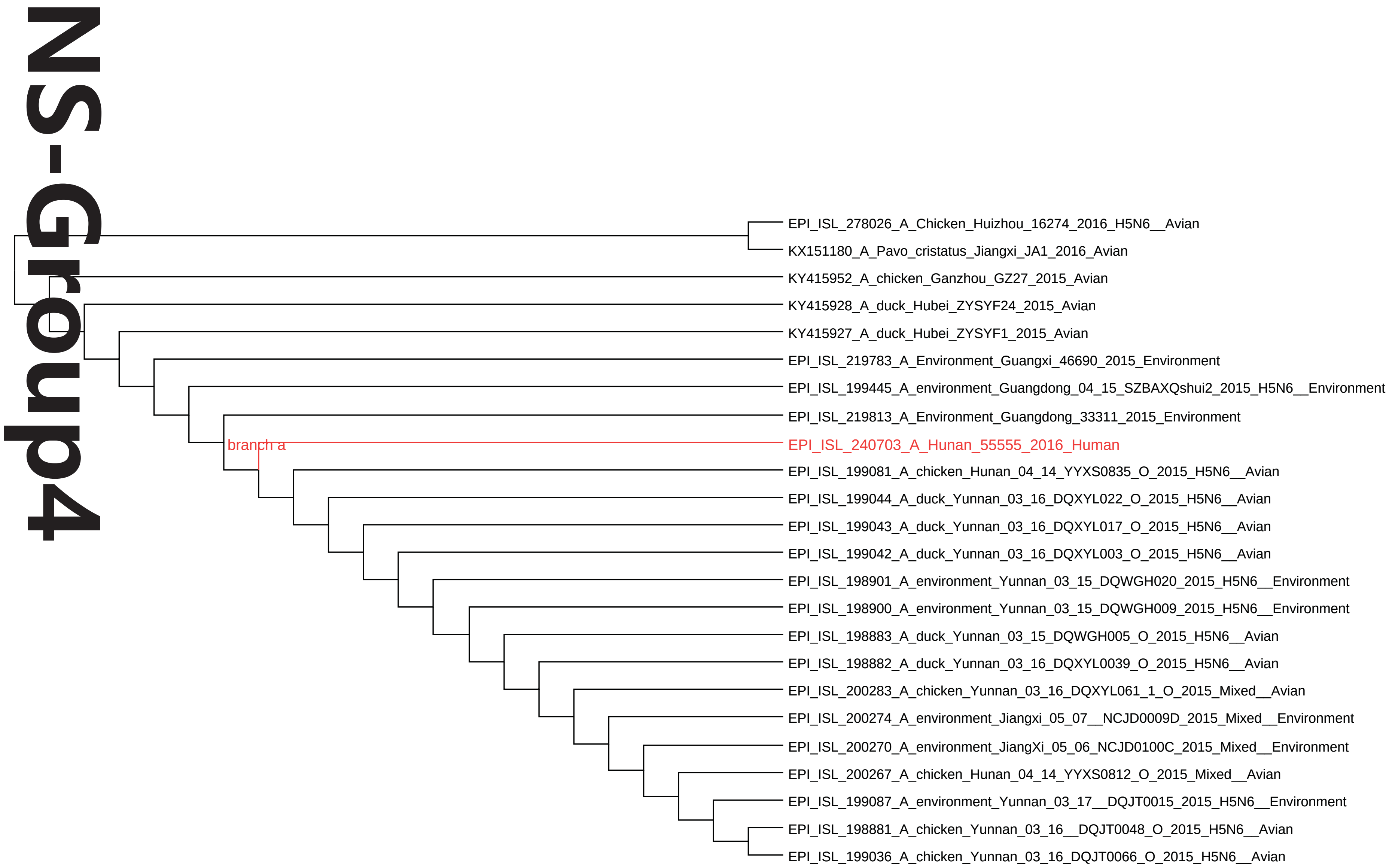
NS-Group4
EPI_ISL_278026_A_Chicken_Huizhou_16274_2016_H5N6__Avian
KX151180_A_Pavo_cristatus_Jiangxi_JA1_2016_Avian
KY415952_A_chicken_Ganzhou_GZ27_2015_Avian
KY415928_A_duck_Hubei_ZYSYF24_2015_Avian
KY415927_A_duck_Hubei_ZYSYF1_2015_Avian
EPI_ISL_219783_A_Environment_Guangxi_46690_2015_Environment
EPI_ISL_199445_A_environment_Guangdong_04_15_SZBAXQshui2_2015_H5N6__Environment
EPI_ISL_219813_A_Environment_Guangdong_33311_2015_Environment
branch a
EPI_ISL_240703_A_Hunan_55555_2016_Human
EPI_ISL_199081_A_chicken_Hunan_04_14_YYXS0835_O_2015_H5N6__Avian
EPI_ISL_199044_A_duck_Yunnan_03_16_DQXYL022_O_2015_H5N6__Avian
EPI_ISL_199043_A_duck_Yunnan_03_16_DQXYL017_O_2015_H5N6__Avian
EPI_ISL_199042_A_duck_Yunnan_03_16_DQXYL003_O_2015_H5N6__Avian
EPI_ISL_198901_A_environment_Yunnan_03_15_DQWGH020_2015_H5N6__Environment
EPI_ISL_198900_A_environment_Yunnan_03_15_DQWGH009_2015_H5N6__Environment
EPI_ISL_198883_A_duck_Yunnan_03_15_DQWGH005_O_2015_H5N6__Avian
EPI_ISL_198882_A_duck_Yunnan_03_16_DQXYL0039_O_2015_H5N6__Avian
EPI_ISL_200283_A_chicken_Yunnan_03_16_DQXYL061_1_O_2015_Mixed__Avian
EPI_ISL_200274_A_environment_Jiangxi_05_07__NCJD0009D_2015_Mixed__Environment
EPI_ISL_200270_A_environment_JiangXi_05_06_NCJD0100C_2015_Mixed__Environment
EPI_ISL_200267_A_chicken_Hunan_04_14_YYXS0812_O_2015_Mixed__Avian
EPI_ISL_199087_A_environment_Yunnan_03_17__DQJT0015_2015_H5N6__Environment
EPI_ISL_198881_A_chicken_Yunnan_03_16__DQJT0048_O_2015_H5N6__Avian
EPI_ISL_199036_A_chicken_Yunnan_03_16_DQJT0066_O_2015_H5N6__Avian

NS-Group5

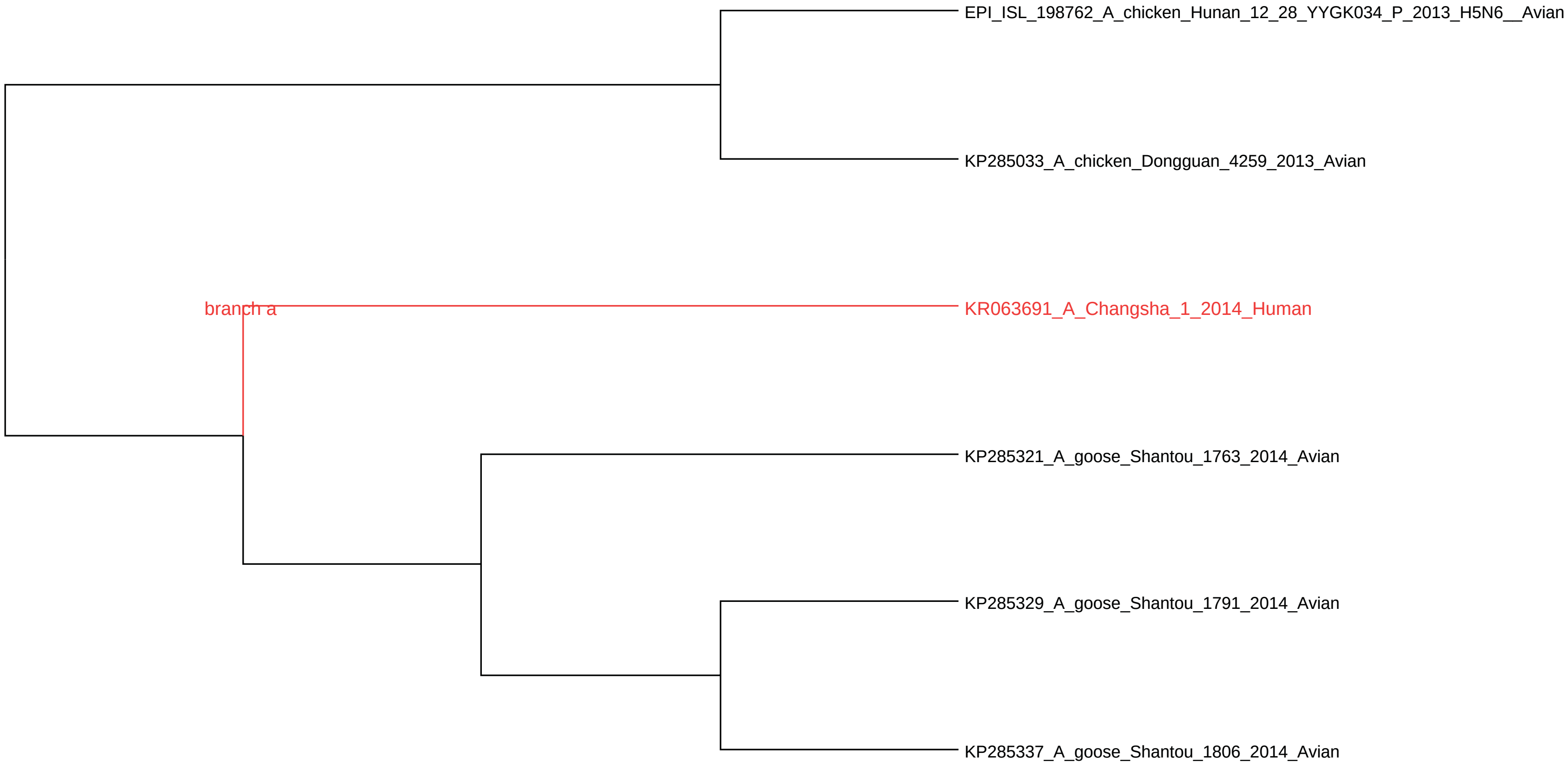
EPI_ISL_198762_A_chicken_Hunan_12_28_YYGK034_P_2013_H5N6__Avian
KP285033_A_chicken_Dongguan_4259_2013_Avian
branch a
KR063691_A_Changsha_1_2014_Human
KP285321_A_goose_Shantou_1763_2014_Avian
KP285329_A_goose_Shantou_1791_2014_Avian
KP285337_A_goose_Shantou_1806_2014_Avian

NS-Group6

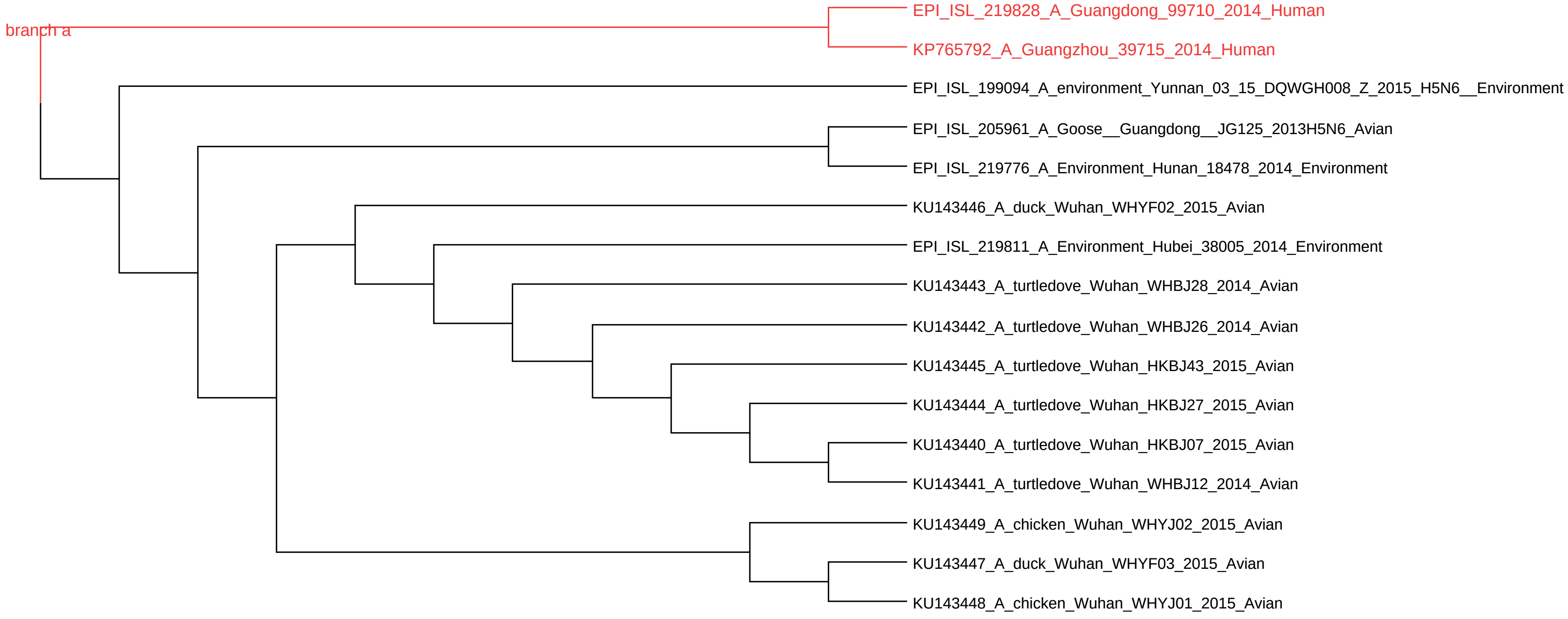
branch a
EPI_ISL_219828_A_Guangdong_99710_2014_Human
KP765792_A_Guangzhou_39715_2014_Human
EPI_ISL_199094_A_environment_Yunnan_03_15_DQWGH008_Z_2015_H5N6__Environment
EPI_ISL_205961_A_Goose__Guangdong__JG125_2013H5N6_Avian
EPI_ISL_219776_A_Environment_Hunan_18478_2014_Environment
KU143446_A_duck_Wuhan_WHYF02_2015_Avian
EPI_ISL_219811_A_Environment_Hubei_38005_2014_Environment
KU143443_A_turtledove_Wuhan_WHBJ28_2014_Avian
KU143442_A_turtledove_Wuhan_WHBJ26_2014_Avian
KU143445_A_turtledove_Wuhan_HKBJ43_2015_Avian
KU143444_A_turtledove_Wuhan_HKBJ27_2015_Avian
KU143440_A_turtledove_Wuhan_HKBJ07_2015_Avian
KU143441_A_turtledove_Wuhan_WHBJ12_2014_Avian
KU143449_A_chicken_Wuhan_WHYJ02_2015_Avian
KU143447_A_duck_Wuhan_WHYF03_2015_Avian
KU143448_A_chicken_Wuhan_WHYJ01_2015_Avian

NS-Group7

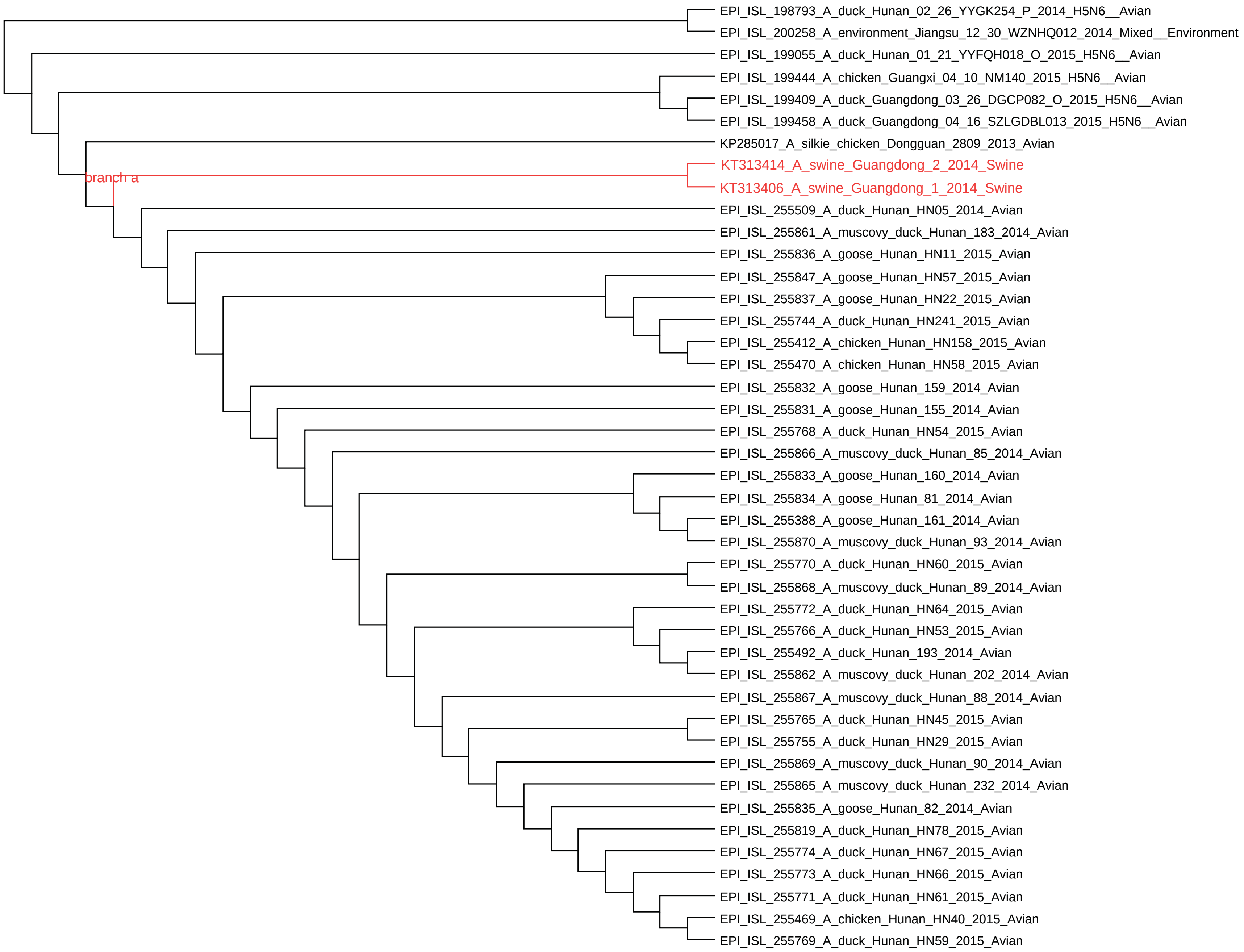

N -Group

NS-Group8

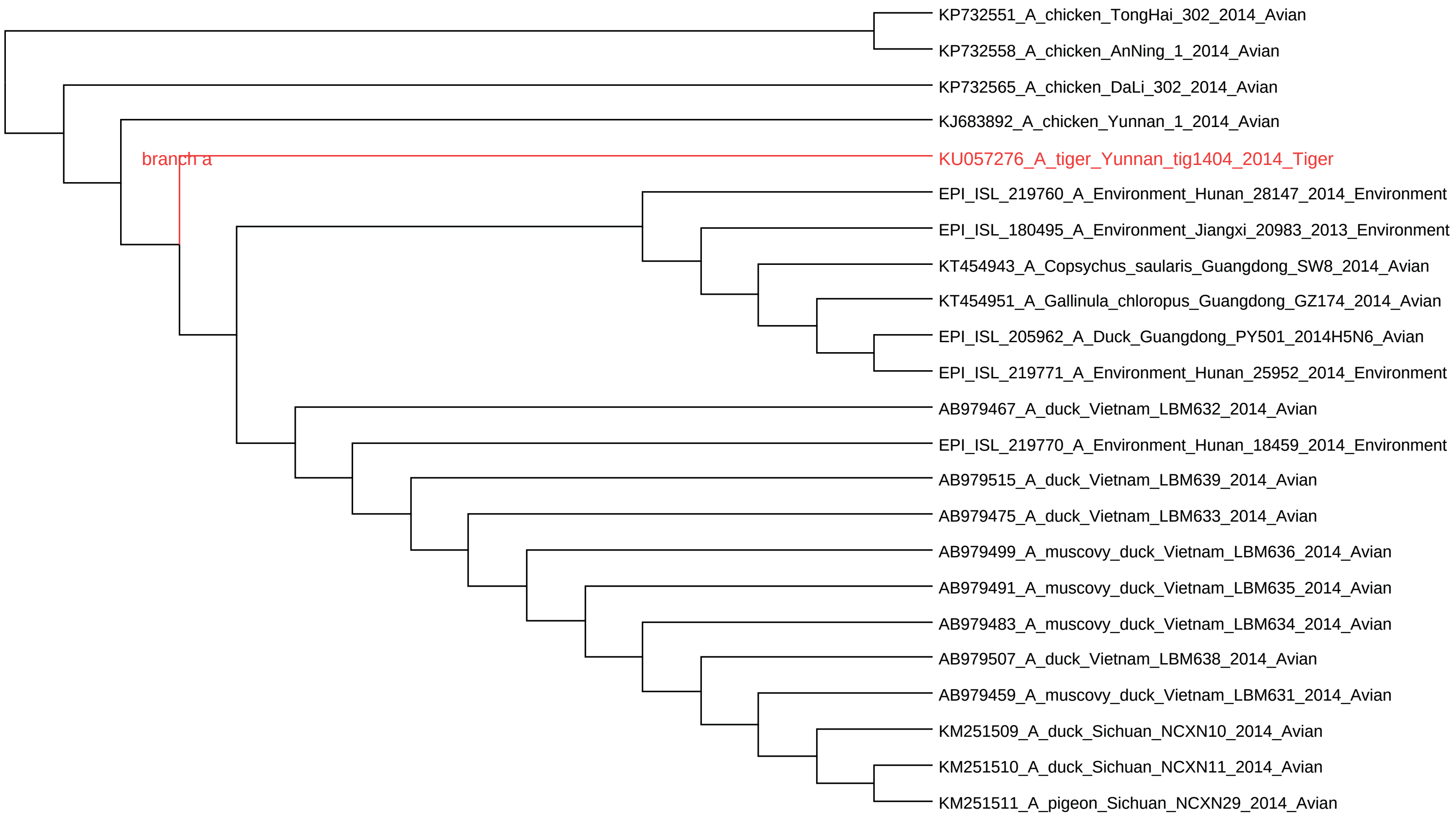

KP732551_A_chicken_TongHai_302_2014_Avian
KP732558_A_chicken_AnNing_1_2014_Avian
KP732565_A_chicken_DaLi_302_2014_Avian
KJ683892_A_chicken_Yunnan_1_2014_Avian
branch a
KU057276_A_tiger_Yunnan_tig1404_2014_Tiger
EPI_ISL_219760_A_Environment_Hunan_28147_2014_Environment
EPI_ISL_180495_A_Environment_Jiangxi_20983_2013_Environment
KT454943_A_Copsychus_saularis_Guangdong_SW8_2014_Avian
KT454951_A_Gallinula_chloropus_Guangdong_GZ174_2014_Avian
EPI_ISL_205962_A_Duck_Guangdong_PY501_2014H5N6_Avian
EPI_ISL_219771_A_Environment_Hunan_25952_2014_Environment
AB979467_A_duck_Vietnam_LBM632_2014_Avian
EPI_ISL_219770_A_Environment_Hunan_18459_2014_Environment
AB979515_A_duck_Vietnam_LBM639_2014_Avian
AB979475_A_duck_Vietnam_LBM633_2014_Avian
AB979499_A_muscovy_duck_Vietnam_LBM636_2014_Avian
AB979491_A_muscovy_duck_Vietnam_LBM635_2014_Avian
AB979483_A_muscovy_duck_Vietnam_LBM634_2014_Avian
AB979507_A_duck_Vietnam_LBM638_2014_Avian
AB979459_A_muscovy_duck_Vietnam_LBM631_2014_Avian
KM251509_A_duck_Sichuan_NCXN10_2014_Avian
KM251510_A_duck_Sichuan_NCXN11_2014_Avian
KM251511_A_pigeon_Sichuan_NCXN29_2014_Avian

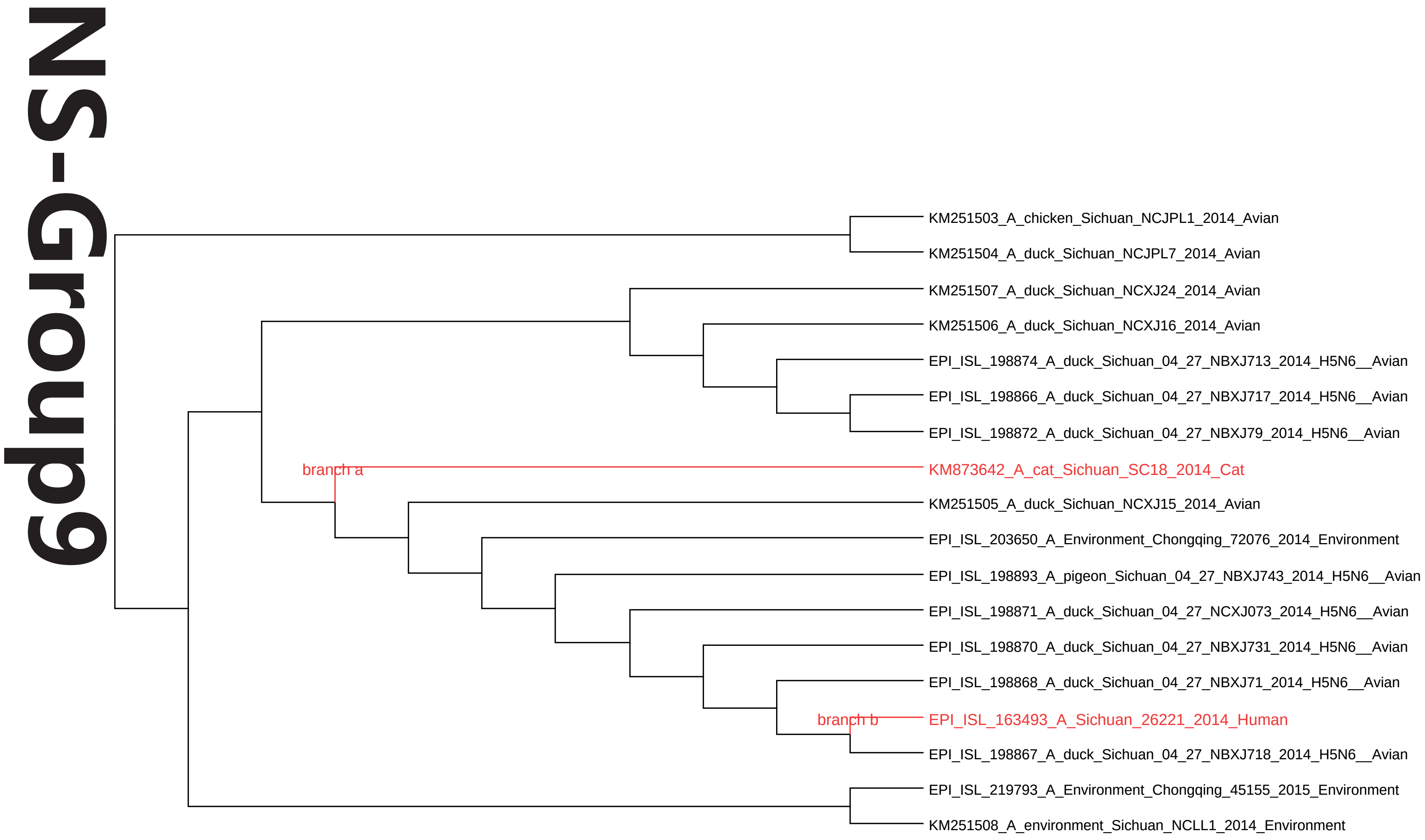
NS-Group9
KM251503_A_chicken_Sichuan_NCJPL1_2014_Avian
KM251504_A_duck_Sichuan_NCJPL7_2014_Avian
KM251507_A_duck_Sichuan_NCXJ24_2014_Avian
KM251506_A_duck_Sichuan_NCXJ16_2014_Avian
EPI_ISL_198874_A_duck_Sichuan_04_27_NBXJ713_2014_H5N6__Avian
EPI_ISL_198866_A_duck_Sichuan_04_27_NBXJ717_2014_H5N6__Avian
EPI_ISL_198872_A_duck_Sichuan_04_27_NBXJ79_2014_H5N6__Avian
branch a
KM873642_A_cat_Sichuan_SC18_2014_Cat
KM251505_A_duck_Sichuan_NCXJ15_2014_Avian
EPI_ISL_203650_A_Environment_Chongqing_72076_2014_Environment
EPI_ISL_198893_A_pigeon_Sichuan_04_27_NBXJ743_2014_H5N6__Avian
EPI_ISL_198871_A_duck_Sichuan_04_27_NCXJ073_2014_H5N6__Avian
EPI_ISL_198870_A_duck_Sichuan_04_27_NBXJ731_2014_H5N6__Avian
EPI_ISL_198868_A_duck_Sichuan_04_27_NBXJ71_2014_H5N6__Avian
branch b
EPI_ISL_163493_A_Sichuan_26221_2014_Human
EPI_ISL_198867_A_duck_Sichuan_04_27_NBXJ718_2014_H5N6__Avian
EPI_ISL_219793_A_Environment_Chongqing_45155_2015_Environment
KM251508_A_environment_Sichuan_NCLL1_2014_Environment

NS-Group10

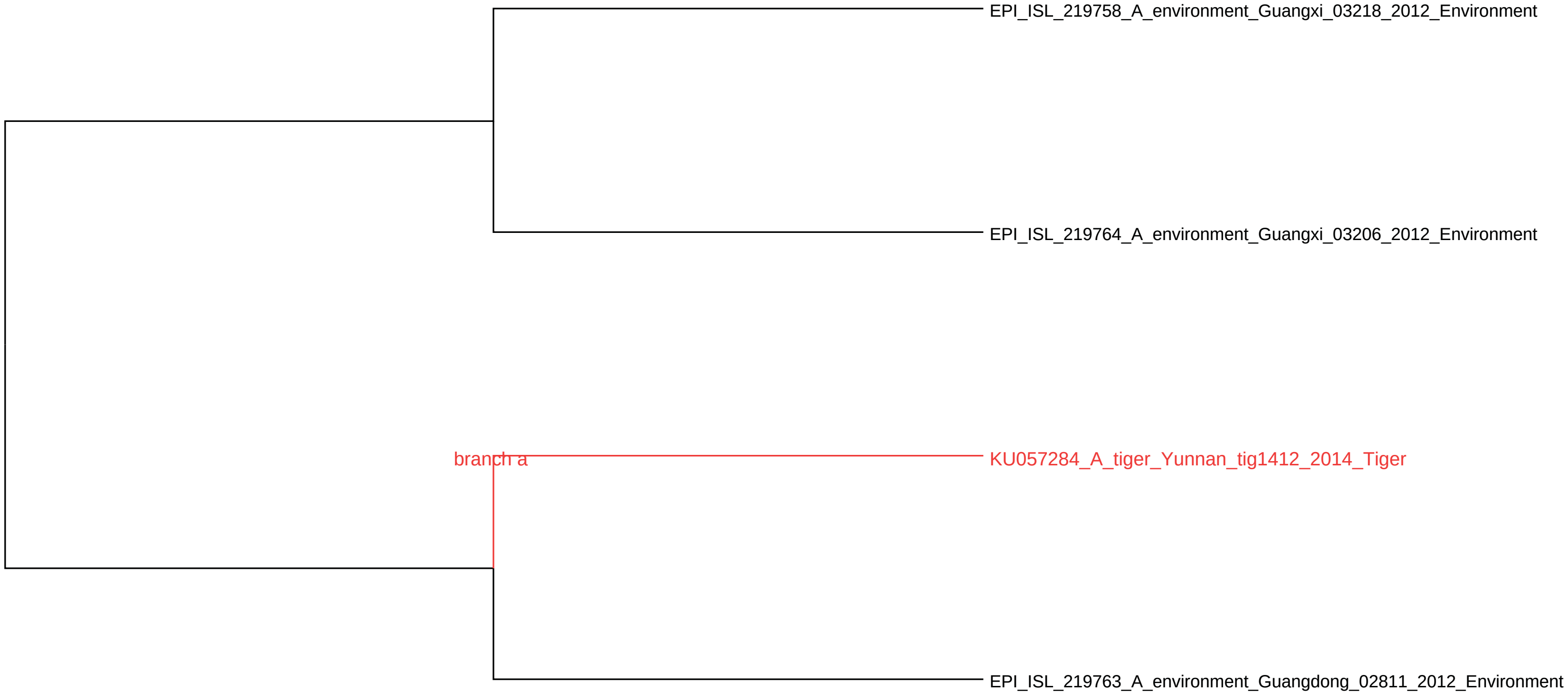
EPI_ISL_219758_A_environment_Guangxi_03218_2012_Environment
EPI_ISL_219764_A_environment_Guangxi_03206_2012_Environment
branch a
KU057284_A_tiger_Yunnan_tig1412_2014_Tiger
EPI_ISL_219763_A_environment_Guangdong_02811_2012_Environment

NS-Group11

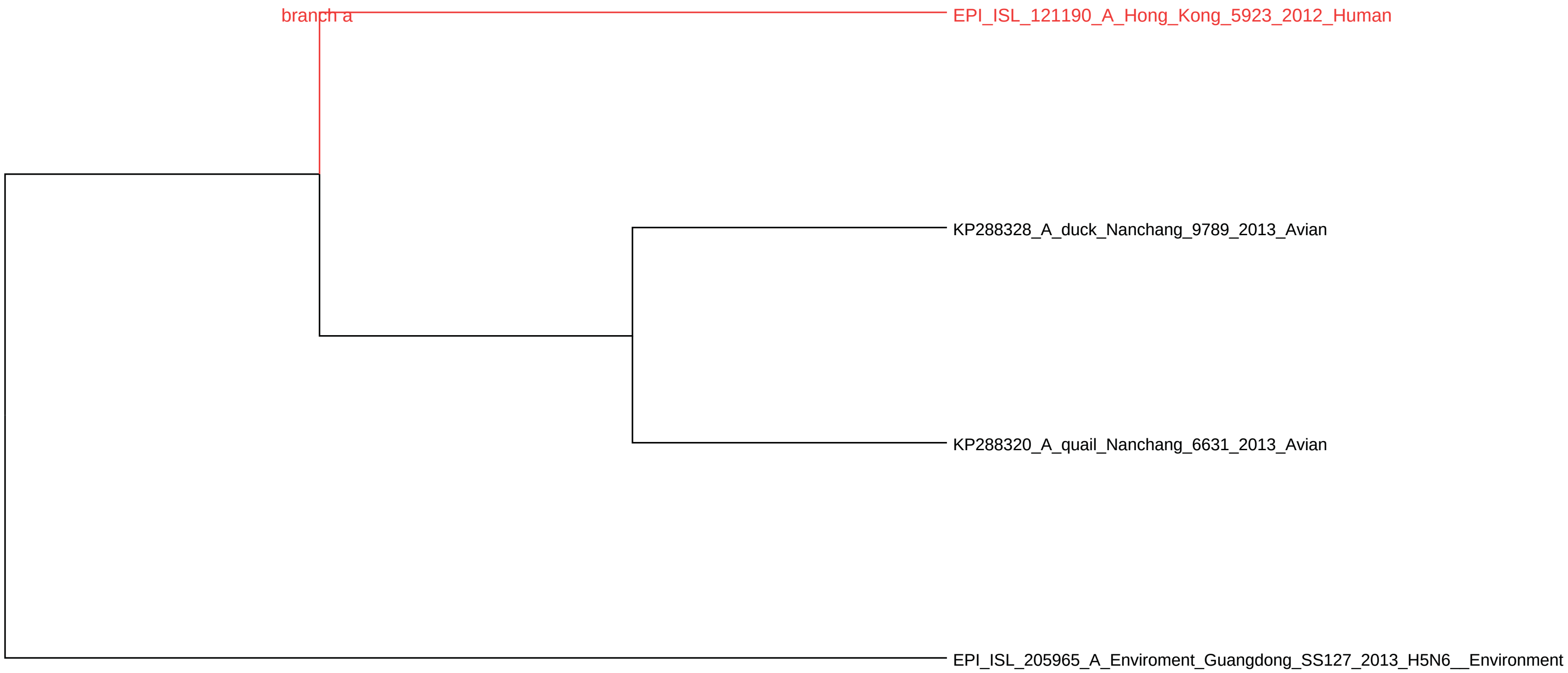
branch a
EPI_ISL_121190_A_Hong_Kong_5923_2012_Human
KP288328_A_duck_Nanchang_9789_2013_Avian
KP288320_A_quail_Nanchang_6631_2013_Avian
EPI_ISL_205965_A_Enviroment_Guangdong_SS127_2013_H5N6__Environment

NS-Group12

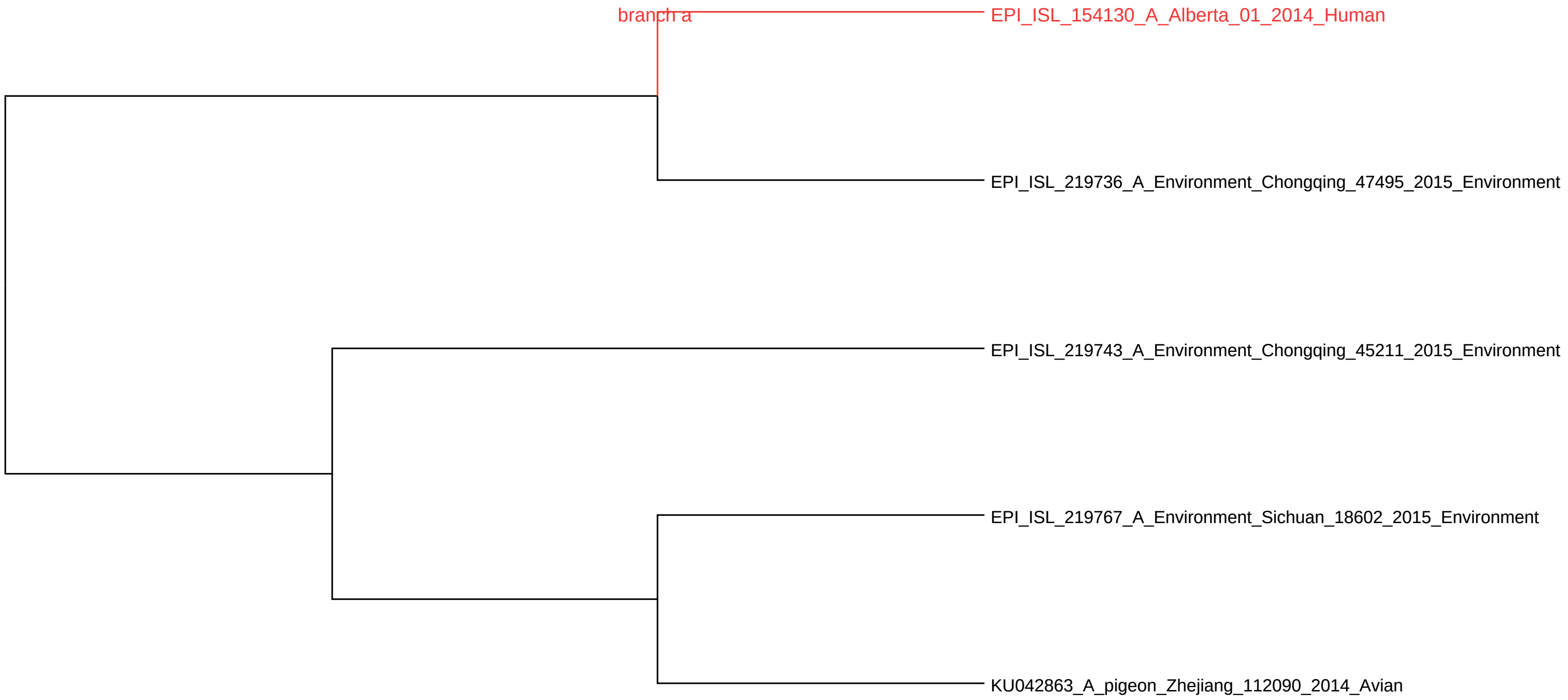
branch a
EPI_ISL_154130_A_Alberta_01_2014_Human
EPI_ISL_219736_A_Environment_Chongqing_47495_2015_Environment
EPI_ISL_219743_A_Environment_Chongqing_45211_2015_Environment
EPI_ISL_219767_A_Environment_Sichuan_18602_2015_Environment
KU042863_A_pigeon_Zhejiang_112090_2014_Avian

NS-Group13

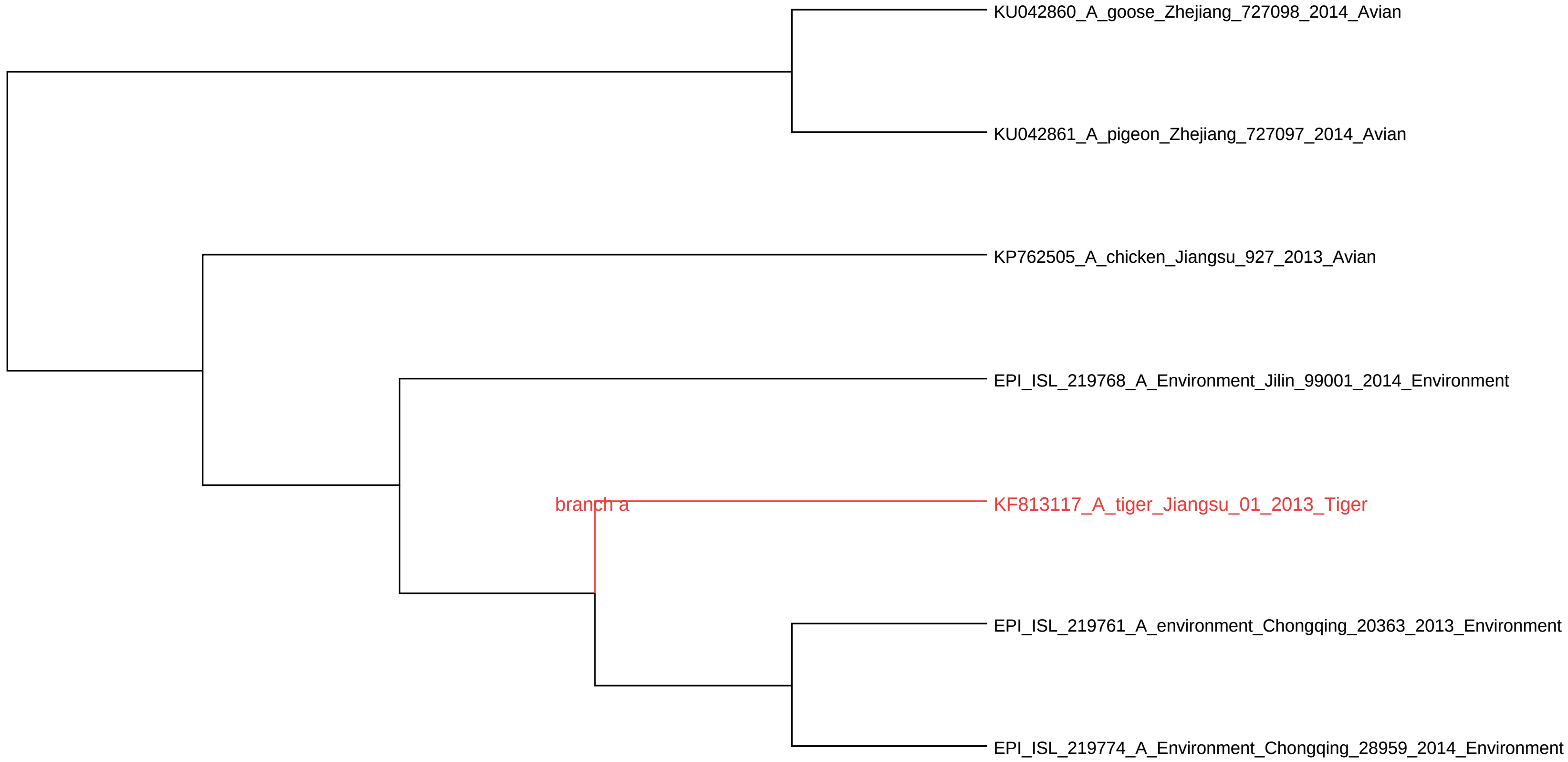
KU042860_A_goose_Zhejiang_727098_2014_Avian
KU042861_A_pigeon_Zhejiang_727097_2014_Avian
KP762505_A_chicken_Jiangsu_927_2013_Avian
EPI_ISL_219768_A_Environment_Jilin_99001_2014_Environment
branch a
KF813117_A_tiger_Jiangsu_01_2013_Tiger
EPI_ISL_219761_A_environment_Chongqing_20363_2013_Environment
EPI_ISL_219774_A_Environment_Chongqing_28959_2014_Environment

NS-Group14

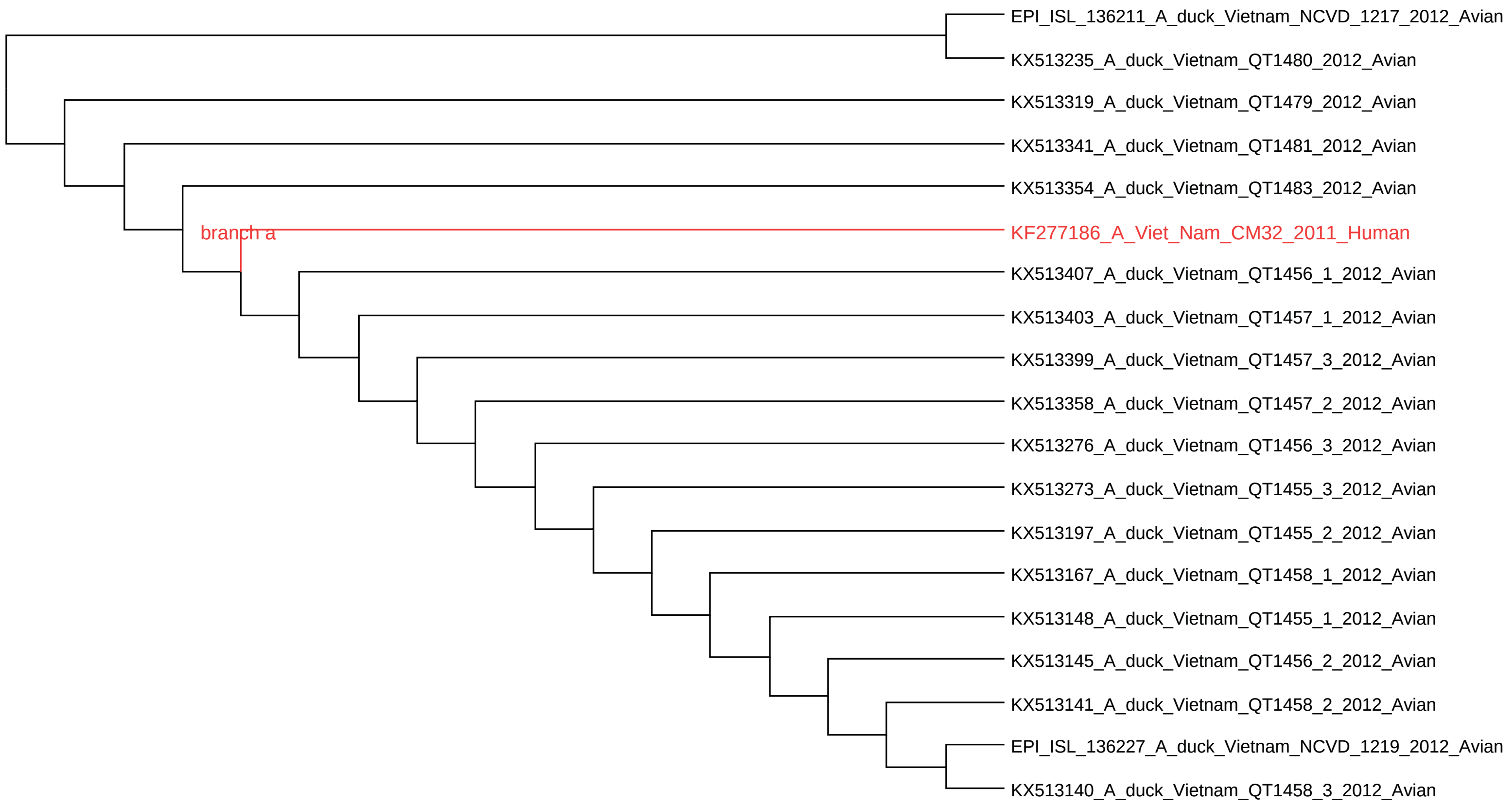

EPI_ISL_136211_A_duck_Vietnam_NCVD_1217_2012_Avian
KX513235_A_duck_Vietnam_QT1480_2012_Avian
KX513319_A_duck_Vietnam_QT1479_2012_Avian
KX513341_A_duck_Vietnam_QT1481_2012_Avian
KX513354_A_duck_Vietnam_QT1483_2012_Avian
branch a
KF277186_A_Viet_Nam_CM32_2011_Human
KX513407_A_duck_Vietnam_QT1456_1_2012_Avian
KX513403_A_duck_Vietnam_QT1457_1_2012_Avian
KX513399_A_duck_Vietnam_QT1457_3_2012_Avian
KX513358_A_duck_Vietnam_QT1457_2_2012_Avian
KX513276_A_duck_Vietnam_QT1456_3_2012_Avian
KX513273_A_duck_Vietnam_QT1455_3_2012_Avian
KX513197_A_duck_Vietnam_QT1455_2_2012_Avian
KX513167_A_duck_Vietnam_QT1458_1_2012_Avian
KX513148_A_duck_Vietnam_QT1455_1_2012_Avian
KX513145_A_duck_Vietnam_QT1456_2_2012_Avian
KX513141_A_duck_Vietnam_QT1458_2_2012_Avian
EPI_ISL_136227_A_duck_Vietnam_NCVD_1219_2012_Avian
KX513140_A_duck_Vietnam_QT1458_3_2012_Avian

NS-Group15

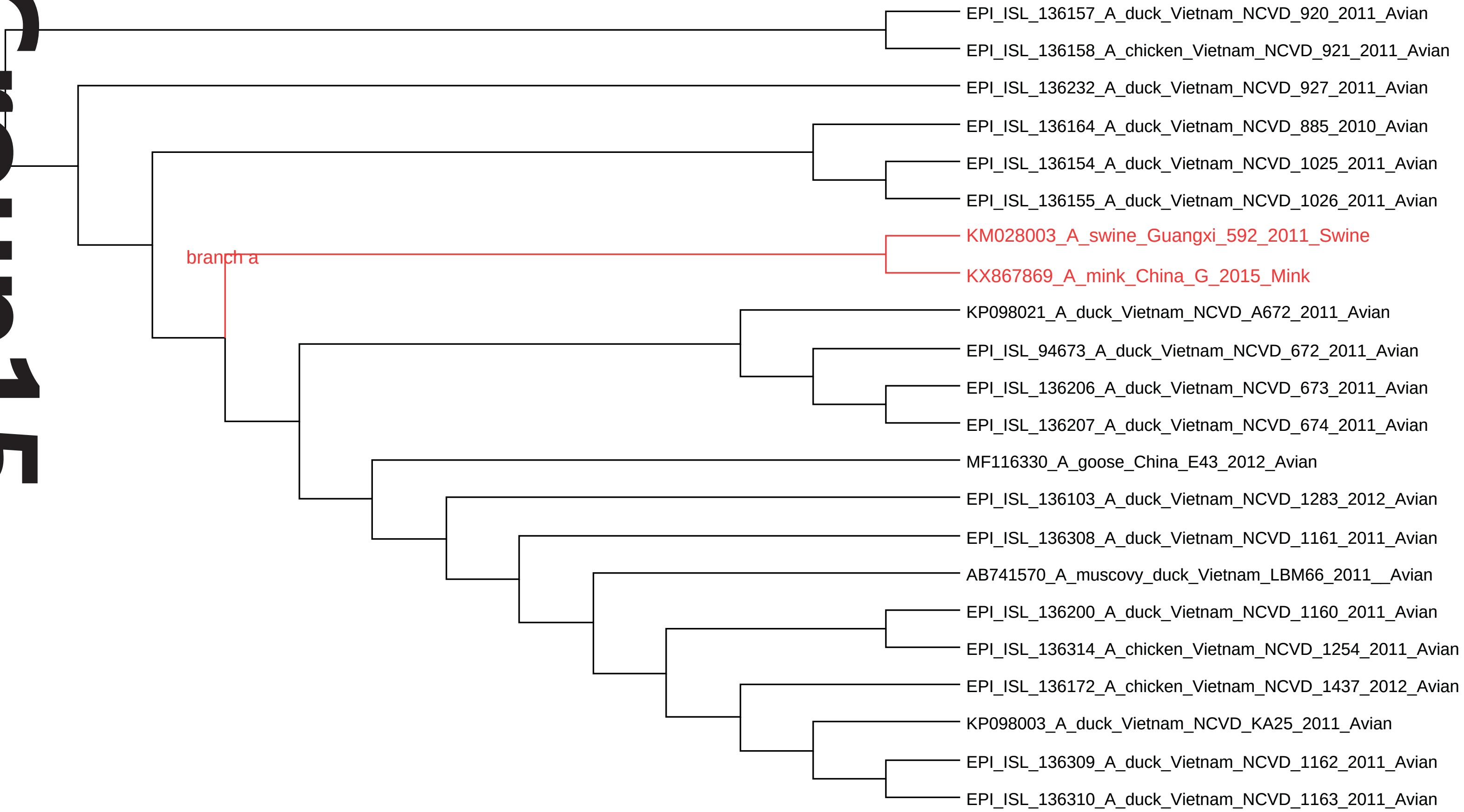

EPI_ISL_136157_A_duck_Vietnam_NCVD_920_2011_Avian
EPI_ISL_136158_A_chicken_Vietnam_NCVD_921_2011_Avian
EPI_ISL_136232_A_duck_Vietnam_NCVD_927_2011_Avian
EPI_ISL_136164_A_duck_Vietnam_NCVD_885_2010_Avian
EPI_ISL_136154_A_duck_Vietnam_NCVD_1025_2011_Avian
EPI_ISL_136155_A_duck_Vietnam_NCVD_1026_2011_Avian
KM028003_A_swine_Guangxi_592_2011_Swine
branch a
KX867869_A_mink_China_G_2015_Mink
KP098021_A_duck_Vietnam_NCVD_A672_2011_Avian
EPI_ISL_94673_A_duck_Vietnam_NCVD_672_2011_Avian
EPI_ISL_136206_A_duck_Vietnam_NCVD_673_2011_Avian
EPI_ISL_136207_A_duck_Vietnam_NCVD_674_2011_Avian
MF116330_A_goose_China_E43_2012_Avian
EPI_ISL_136103_A_duck_Vietnam_NCVD_1283_2012_Avian
EPI_ISL_136308_A_duck_Vietnam_NCVD_1161_2011_Avian
AB741570_A_muscovy_duck_Vietnam_LBM66_2011__Avian
EPI_ISL_136200_A_duck_Vietnam_NCVD_1160_2011_Avian
EPI_ISL_136314_A_chicken_Vietnam_NCVD_1254_2011_Avian
EPI_ISL_136172_A_chicken_Vietnam_NCVD_1437_2012_Avian
KP098003_A_duck_Vietnam_NCVD_KA25_2011_Avian
EPI_ISL_136309_A_duck_Vietnam_NCVD_1162_2011_Avian
EPI_ISL_136310_A_duck_Vietnam_NCVD_1163_2011_Avian

NS-Group16

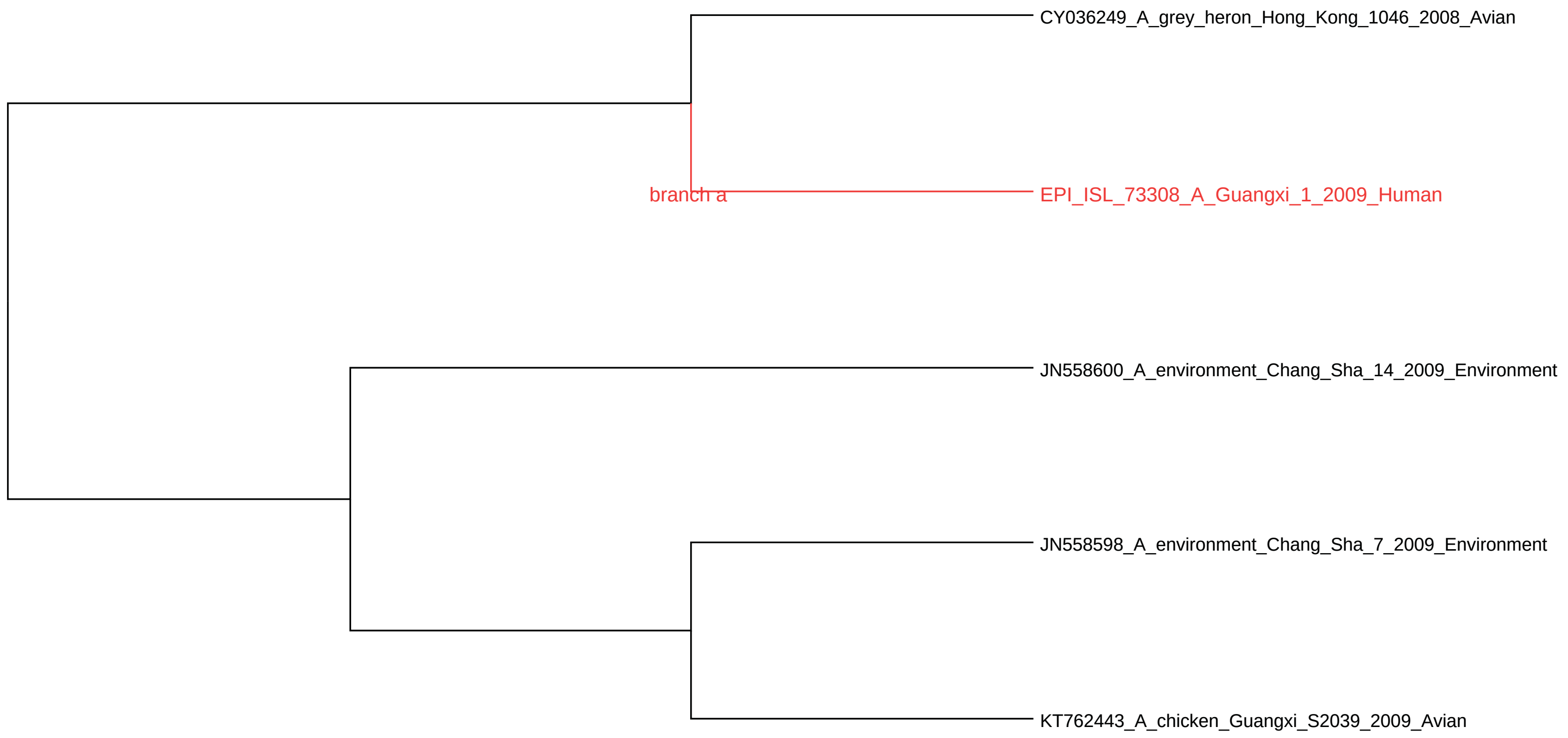
CY036249_A_grey_heron_Hong_Kong_1046_2008_Avian
branch a
EPI_ISL_73308_A_Guangxi_1_2009_Human
JN558600_A_environment_Chang_Sha_14_2009_Environment
JN558598_A_environment_Chang_Sha_7_2009_Environment
KT762443_A_chicken_Guangxi_S2039_2009_Avian

NS-Group17

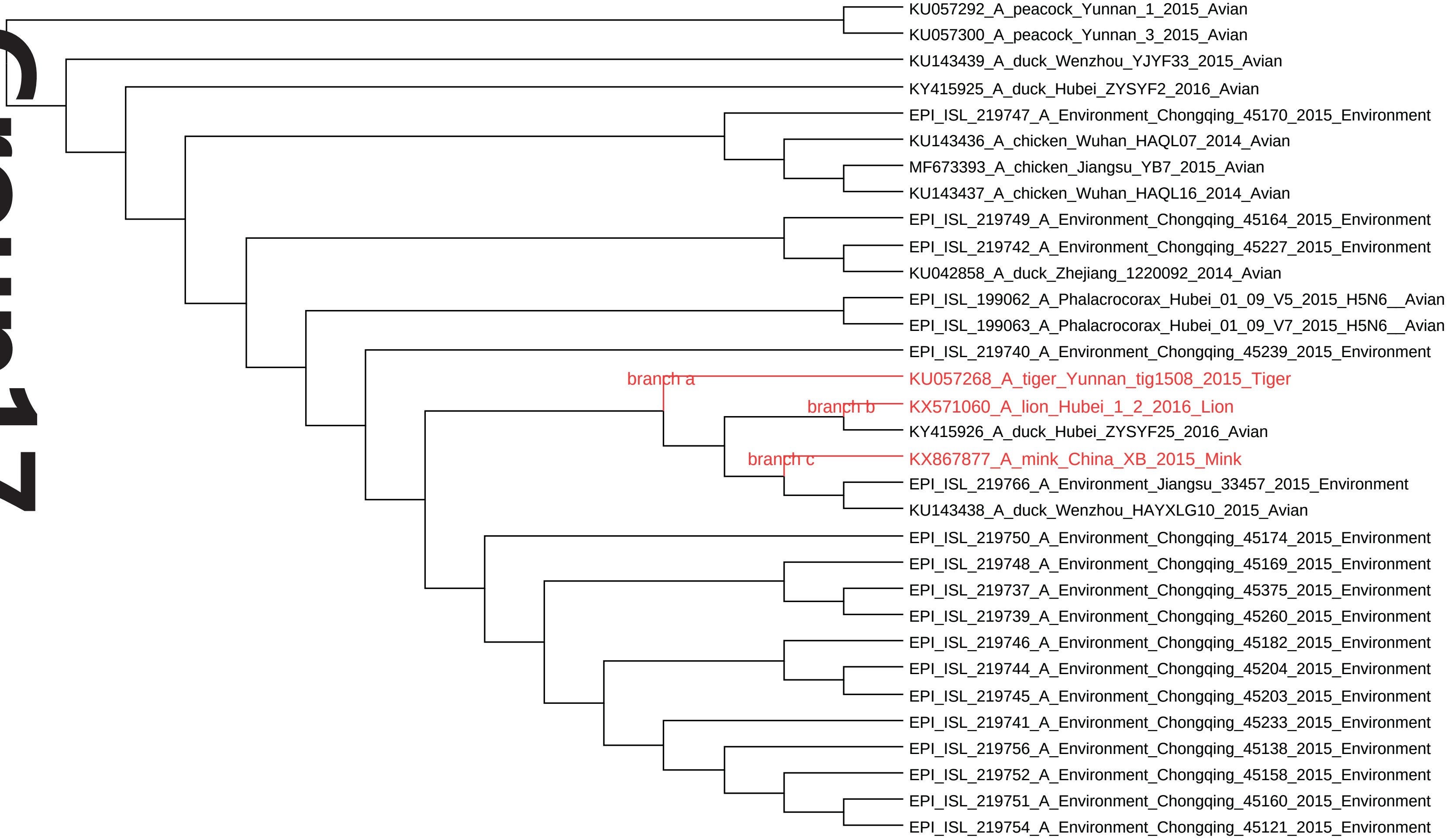
KU057292_A_peacock_Yunnan_1_2015_Avian
KU057300_A_peacock_Yunnan_3_2015_Avian
KU143439_A_duck_Wenzhou_YJYF33_2015_Avian
KY415925_A_duck_Hubei_ZYSYF2_2016_Avian
EPI_ISL_219747_A_Environment_Chongqing_45170_2015_Environment
KU143436_A_chicken_Wuhan_HAQL07_2014_Avian
MF673393_A_chicken_Jiangsu_YB7_2015_Avian
KU143437_A_chicken_Wuhan_HAQL16_2014_Avian
EPI_ISL_219749_A_Environment_Chongqing_45164_2015_Environment
EPI_ISL_219742_A_Environment_Chongqing_45227_2015_Environment
KU042858_A_duck_Zhejiang_1220092_2014_Avian
EPI_ISL_199062_A_Phalacrocorax_Hubei_01_09_V5_2015_H5N6__Avian
EPI_ISL_199063_A_Phalacrocorax_Hubei_01_09_V7_2015_H5N6__Avian
EPI_ISL_219740_A_Environment_Chongqing_45239_2015_Environment
branch a
KU057268_A_tiger_Yunnan_tig1508_2015_Tiger
branch b
KX571060_A_lion_Hubei_1_2_2016_Lion
KY415926_A_duck_Hubei_ZYSYF25_2016_Avian
branch c
KX867877_A_mink_China_XB_2015_Mink
EPI_ISL_219766_A_Environment_Jiangsu_33457_2015_Environment
KU143438_A_duck_Wenzhou_HAYXLG10_2015_Avian
EPI_ISL_219750_A_Environment_Chongqing_45174_2015_Environment
EPI_ISL_219748_A_Environment_Chongqing_45169_2015_Environment
EPI_ISL_219737_A_Environment_Chongqing_45375_2015_Environment
EPI_ISL_219739_A_Environment_Chongqing_45260_2015_Environment
EPI_ISL_219746_A_Environment_Chongqing_45182_2015_Environment
EPI_ISL_219744_A_Environment_Chongqing_45204_2015_Environment
EPI_ISL_219745_A_Environment_Chongqing_45203_2015_Environment
EPI_ISL_219741_A_Environment_Chongqing_45233_2015_Environment
EPI_ISL_219756_A_Environment_Chongqing_45138_2015_Environment
EPI_ISL_219752_A_Environment_Chongqing_45158_2015_Environment
EPI_ISL_219751_A_Environment_Chongqing_45160_2015_Environment
EPI_ISL_219754_A_Environment_Chongqing_45121_2015_Environment

NS-Group18

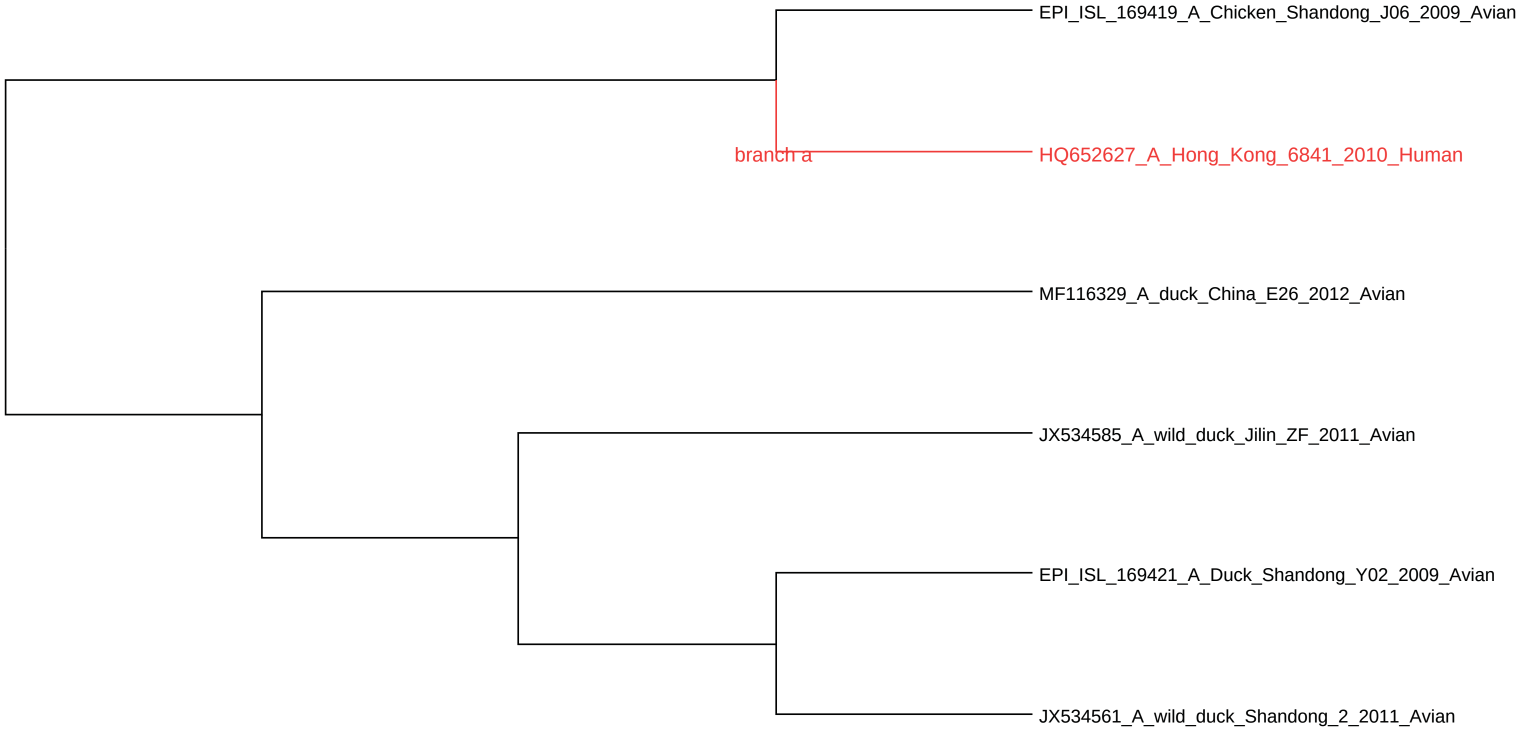
EPI_ISL_169419_A_Chicken_Shandong_J06_2009_Avian
branch a
HQ652627_A_Hong_Kong_6841_2010_Human
MF116329_A_duck_China_E26_2012_Avian
JX534585_A_wild_duck_Jilin_ZF_2011_Avian
EPI_ISL_169421_A_Duck_Shandong_Y02_2009_Avian
JX534561_A_wild_duck_Shandong_2_2011_Avian

NS-Group19

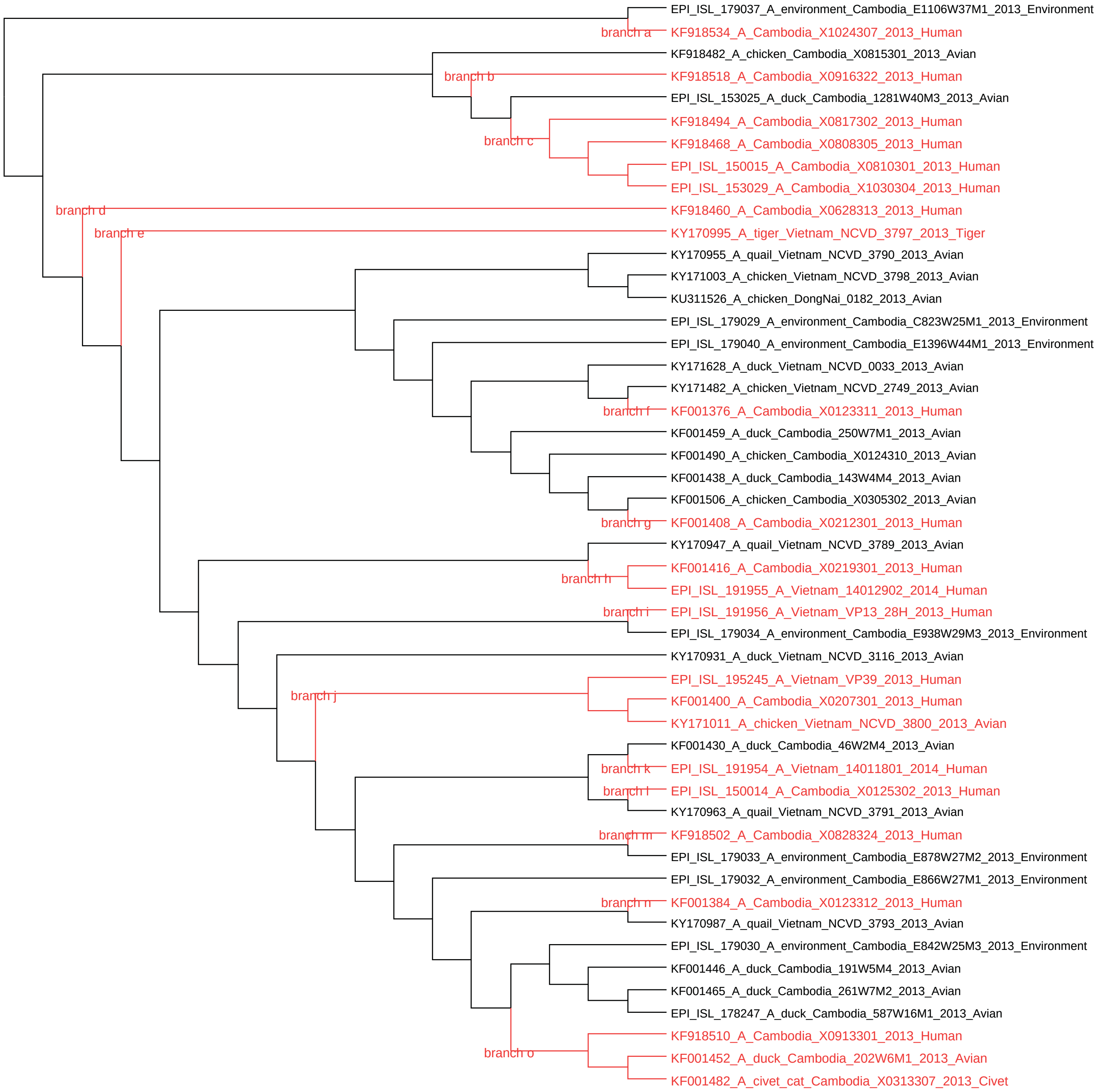

N -Group19

NS-Group20

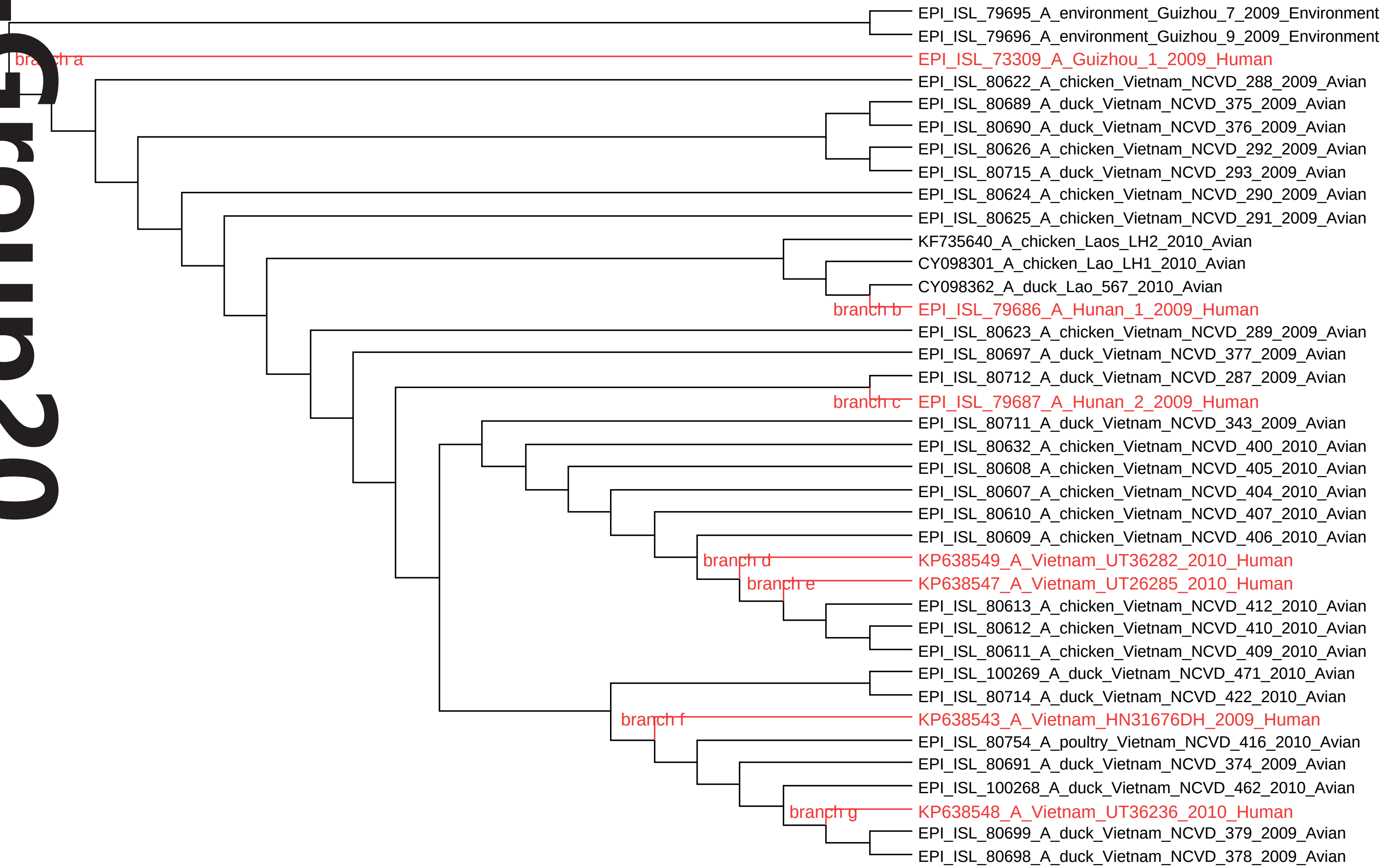

EPI_ISL_79695_A_environment_Guizhou_7_2009_Environment
EPI_ISL_79696_A_environment_Guizhou_9_2009_Environment
branch a
EPI_ISL_73309_A_Guizhou_1_2009_Human
EPI_ISL_80622_A_chicken_Vietnam_NCVD_288_2009_Avian
EPI_ISL_80689_A_duck_Vietnam_NCVD_375_2009_Avian
EPI_ISL_80690_A_duck_Vietnam_NCVD_376_2009_Avian
EPI_ISL_80626_A_chicken_Vietnam_NCVD_292_2009_Avian
EPI_ISL_80715_A_duck_Vietnam_NCVD_293_2009_Avian
EPI_ISL_80624_A_chicken_Vietnam_NCVD_290_2009_Avian
EPI_ISL_80625_A_chicken_Vietnam_NCVD_291_2009_Avian
KF735640_A_chicken_Laos_LH2_2010_Avian
CY098301_A_chicken_Lao_LH1_2010_Avian
CY098362_A_duck_Lao_567_2010_Avian
branch b
EPI_ISL_79686_A_Hunan_1_2009_Human
EPI_ISL_80623_A_chicken_Vietnam_NCVD_289_2009_Avian
EPI_ISL_80697_A_duck_Vietnam_NCVD_377_2009_Avian
EPI_ISL_80712_A_duck_Vietnam_NCVD_287_2009_Avian
branch c
EPI_ISL_79687_A_Hunan_2_2009_Human
EPI_ISL_80711_A_duck_Vietnam_NCVD_343_2009_Avian
EPI_ISL_80632_A_chicken_Vietnam_NCVD_400_2010_Avian
EPI_ISL_80608_A_chicken_Vietnam_NCVD_405_2010_Avian
EPI_ISL_80607_A_chicken_Vietnam_NCVD_404_2010_Avian
EPI_ISL_80610_A_chicken_Vietnam_NCVD_407_2010_Avian
EPI_ISL_80609_A_chicken_Vietnam_NCVD_406_2010_Avian
branch d
KP638549_A_Vietnam_UT36282_2010_Human
branch e
KP638547_A_Vietnam_UT26285_2010_Human
EPI_ISL_80613_A_chicken_Vietnam_NCVD_412_2010_Avian
EPI_ISL_80612_A_chicken_Vietnam_NCVD_410_2010_Avian
EPI_ISL_80611_A_chicken_Vietnam_NCVD_409_2010_Avian
EPI_ISL_100269_A_duck_Vietnam_NCVD_471_2010_Avian
EPI_ISL_80714_A_duck_Vietnam_NCVD_422_2010_Avian
branch f
KP638543_A_Vietnam_HN31676DH_2009_Human
EPI_ISL_80754_A_poultry_Vietnam_NCVD_416_2010_Avian
EPI_ISL_80691_A_duck_Vietnam_NCVD_374_2009_Avian
EPI_ISL_100268_A_duck_Vietnam_NCVD_462_2010_Avian
branch g
KP638548_A_Vietnam_UT36236_2010_Human
EPI_ISL_80699_A_duck_Vietnam_NCVD_379_2009_Avian
EPI_ISL_80698_A_duck_Vietnam_NCVD_378_2009_Avian

NS-Group21

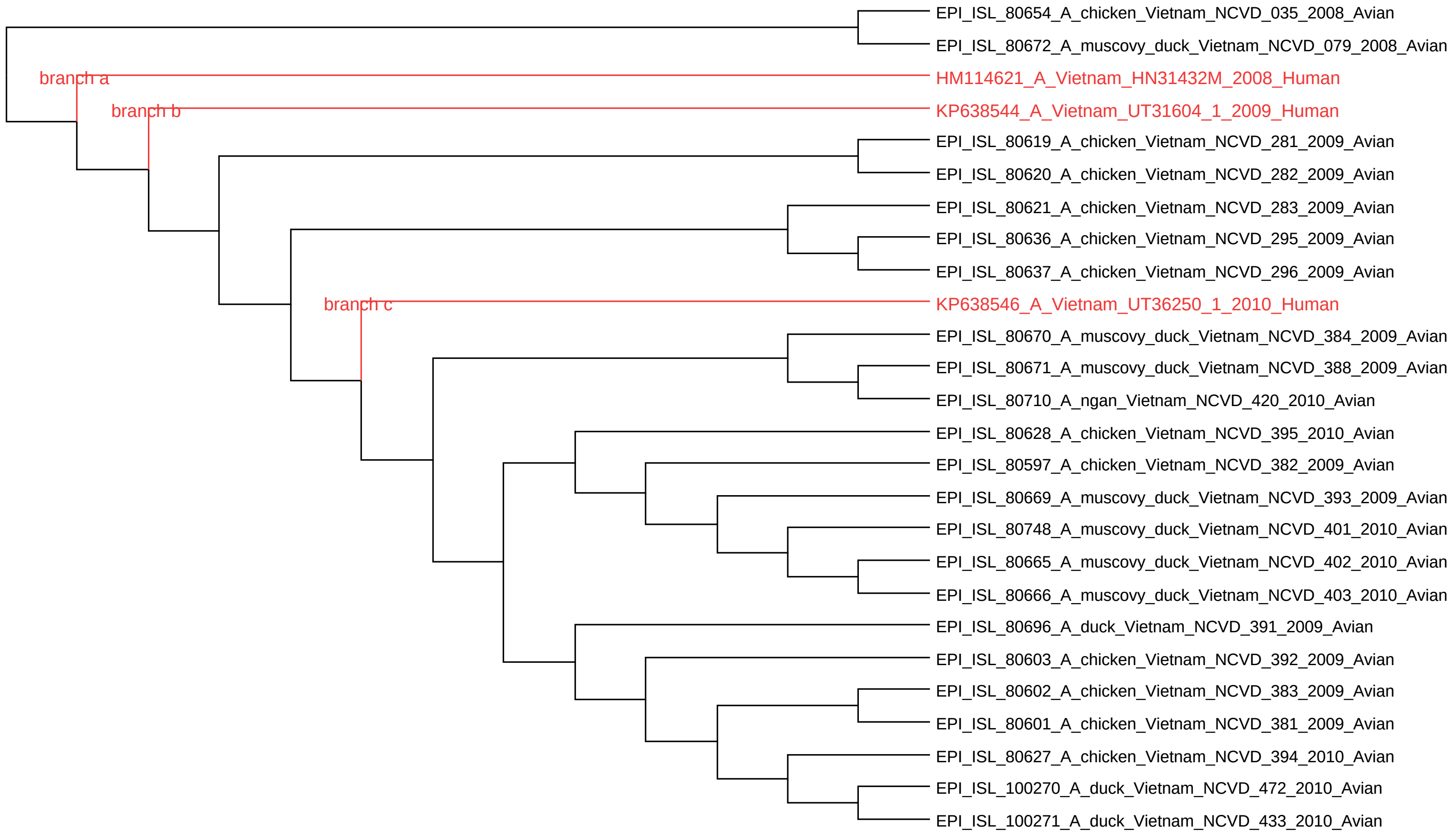

EPI_ISL_80654_A_chicken_Vietnam_NCVD_035_2008_Avian
EPI_ISL_80672_A_muscovy_duck_Vietnam_NCVD_079_2008_Avian
branch a
HM114621_A_Vietnam_HN31432M_2008_Human
branch b
KP638544_A_Vietnam_UT31604_1_2009_Human
EPI_ISL_80619_A_chicken_Vietnam_NCVD_281_2009_Avian
EPI_ISL_80620_A_chicken_Vietnam_NCVD_282_2009_Avian
EPI_ISL_80621_A_chicken_Vietnam_NCVD_283_2009_Avian
EPI_ISL_80636_A_chicken_Vietnam_NCVD_295_2009_Avian
EPI_ISL_80637_A_chicken_Vietnam_NCVD_296_2009_Avian
branch c
KP638546_A_Vietnam_UT36250_1_2010_Human
EPI_ISL_80670_A_muscovy_duck_Vietnam_NCVD_384_2009_Avian
EPI_ISL_80671_A_muscovy_duck_Vietnam_NCVD_388_2009_Avian
EPI_ISL_80710_A_ngan_Vietnam_NCVD_420_2010_Avian
EPI_ISL_80628_A_chicken_Vietnam_NCVD_395_2010_Avian
EPI_ISL_80597_A_chicken_Vietnam_NCVD_382_2009_Avian
EPI_ISL_80669_A_muscovy_duck_Vietnam_NCVD_393_2009_Avian
EPI_ISL_80748_A_muscovy_duck_Vietnam_NCVD_401_2010_Avian
EPI_ISL_80665_A_muscovy_duck_Vietnam_NCVD_402_2010_Avian
EPI_ISL_80666_A_muscovy_duck_Vietnam_NCVD_403_2010_Avian
EPI_ISL_80696_A_duck_Vietnam_NCVD_391_2009_Avian
EPI_ISL_80603_A_chicken_Vietnam_NCVD_392_2009_Avian
EPI_ISL_80602_A_chicken_Vietnam_NCVD_383_2009_Avian
EPI_ISL_80601_A_chicken_Vietnam_NCVD_381_2009_Avian
EPI_ISL_80627_A_chicken_Vietnam_NCVD_394_2010_Avian
EPI_ISL_100270_A_duck_Vietnam_NCVD_472_2010_Avian
EPI_ISL_100271_A_duck_Vietnam_NCVD_433_2010_Avian

NS-Group22

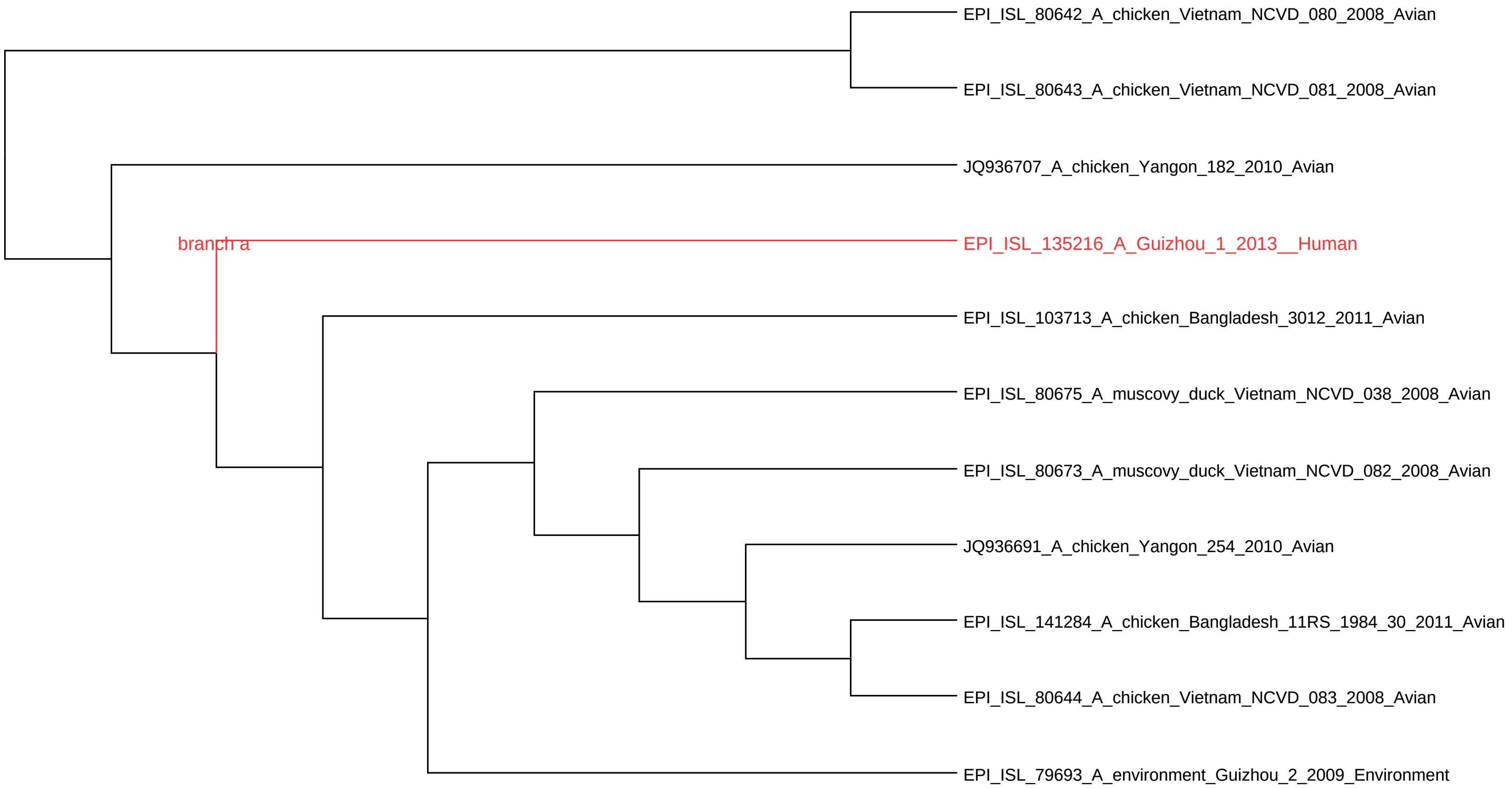

EPI_ISL_80642_A_chicken_Vietnam_NCVD_080_2008_Avian
EPI_ISL_80643_A_chicken_Vietnam_NCVD_081_2008_Avian
JQ936707_A_chicken_Yangon_182_2010_Avian
branch a
EPI_ISL_135216_A_Guizhou_1_2013__Human
EPI_ISL_103713_A_chicken_Bangladesh_3012_2011_Avian
EPI_ISL_80675_A_muscovy_duck_Vietnam_NCVD_038_2008_Avian
EPI_ISL_80673_A_muscovy_duck_Vietnam_NCVD_082_2008_Avian
JQ936691_A_chicken_Yangon_254_2010_Avian
EPI_ISL_141284_A_chicken_Bangladesh_11RS_1984_30_2011_Avian
EPI_ISL_80644_A_chicken_Vietnam_NCVD_083_2008_Avian
EPI_ISL_79693_A_environment_Guizhou_2_2009_Environment

NS-Group23

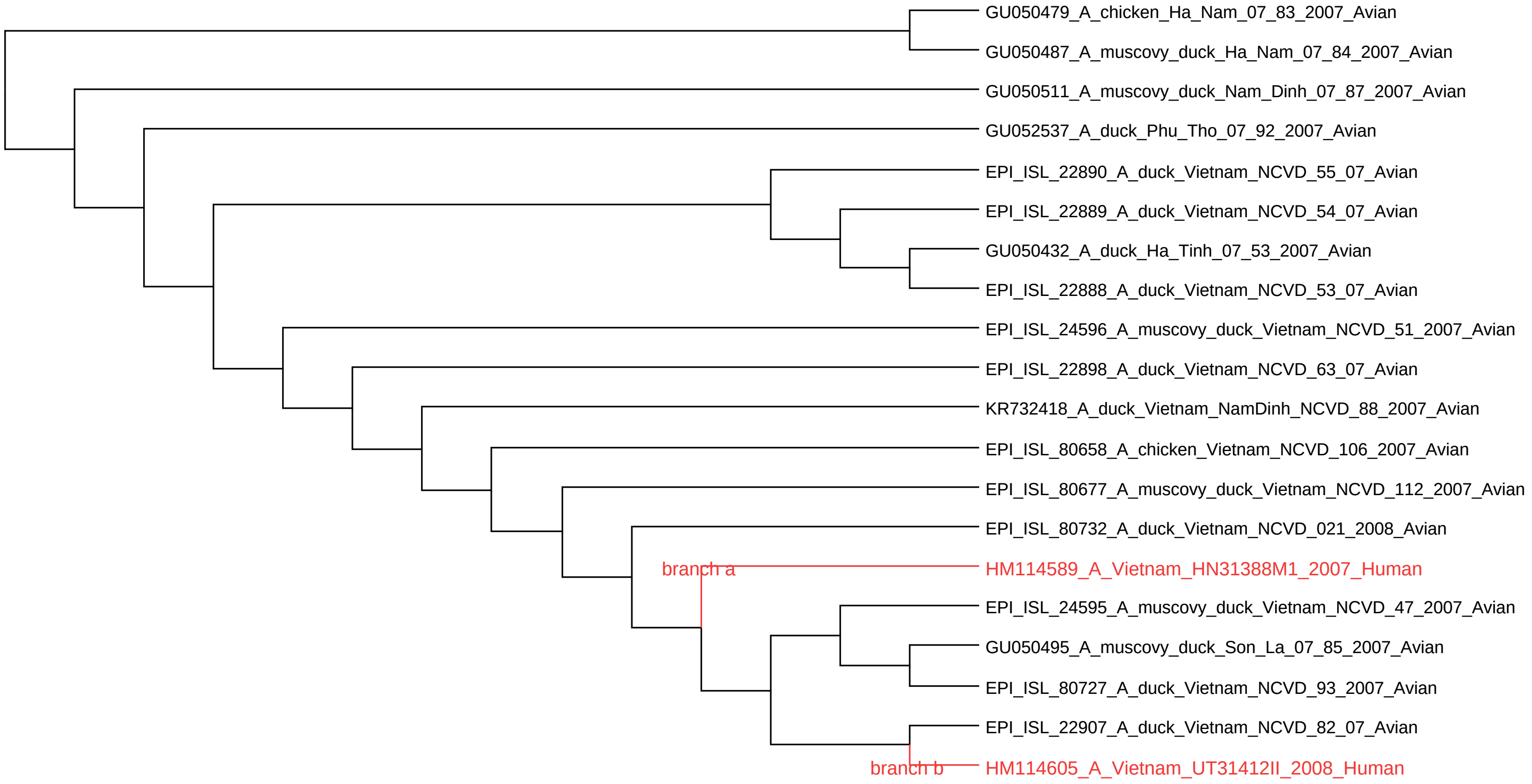

GU050479_A_chicken_Ha_Nam_07_83_2007_Avian
GU050487_A_muscovy_duck_Ha_Nam_07_84_2007_Avian
GU050511_A_muscovy_duck_Nam_Dinh_07_87_2007_Avian
GU052537_A_duck_Phu_Tho_07_92_2007_Avian
EPI_ISL_22890_A_duck_Vietnam_NCVD_55_07_Avian
EPI_ISL_22889_A_duck_Vietnam_NCVD_54_07_Avian
GU050432_A_duck_Ha_Tinh_07_53_2007_Avian
EPI_ISL_22888_A_duck_Vietnam_NCVD_53_07_Avian
EPI_ISL_24596_A_muscovy_duck_Vietnam_NCVD_51_2007_Avian
EPI_ISL_22898_A_duck_Vietnam_NCVD_63_07_Avian
KR732418_A_duck_Vietnam_NamDinh_NCVD_88_2007_Avian
EPI_ISL_80658_A_chicken_Vietnam_NCVD_106_2007_Avian
EPI_ISL_80677_A_muscovy_duck_Vietnam_NCVD_112_2007_Avian
EPI_ISL_80732_A_duck_Vietnam_NCVD_021_2008_Avian
branch a
HM114589_A_Vietnam_HN31388M1_2007_Human
EPI_ISL_24595_A_muscovy_duck_Vietnam_NCVD_47_2007_Avian
GU050495_A_muscovy_duck_Son_La_07_85_2007_Avian
EPI_ISL_80727_A_duck_Vietnam_NCVD_93_2007_Avian
EPI_ISL_22907_A_duck_Vietnam_NCVD_82_07_Avian
branch b
HM114605_A_Vietnam_UT31412II_2008_Human

NS-Group24

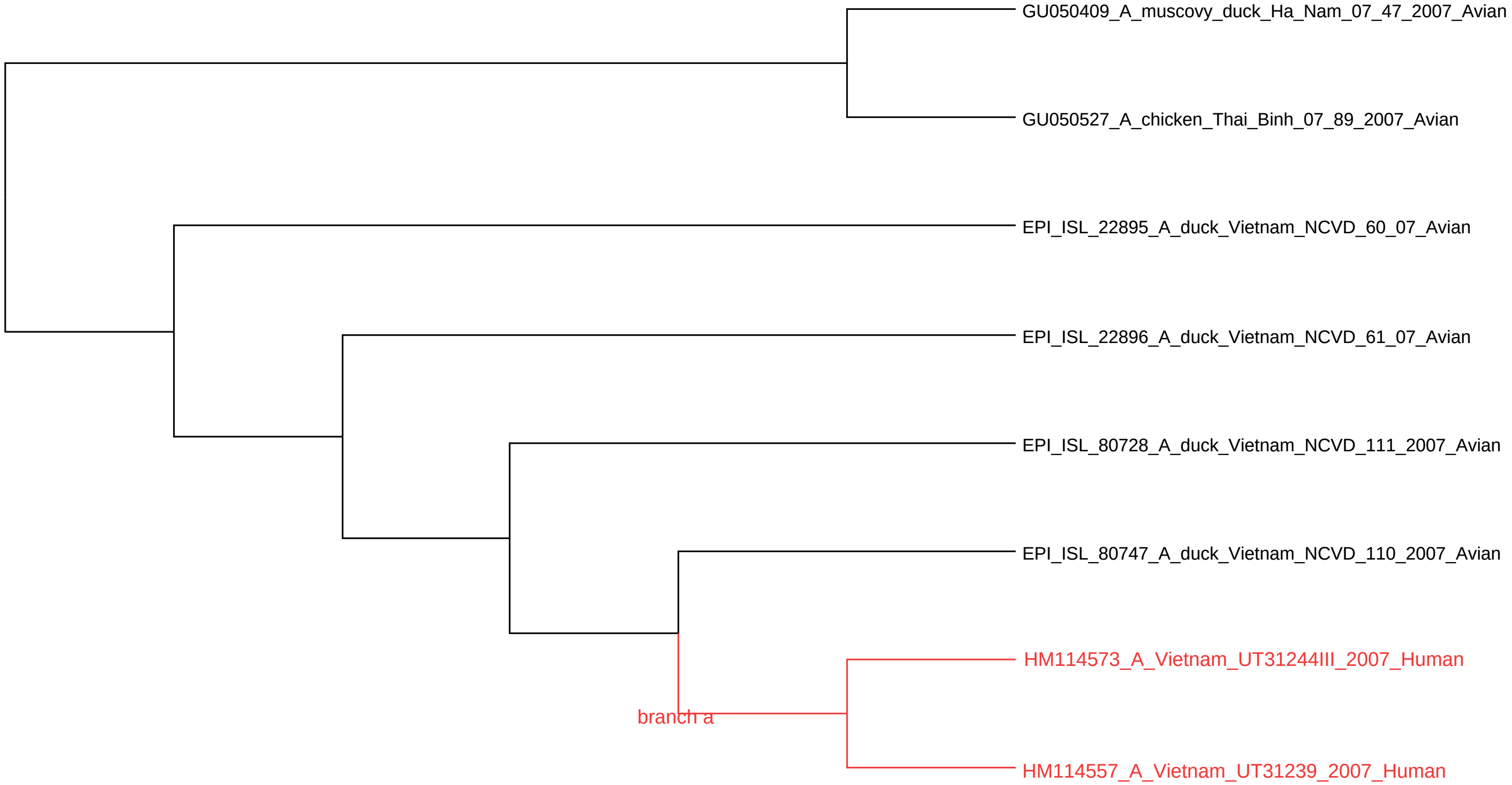
GU050409_A_muscovy_duck_Ha_Nam_07_47_2007_Avian
GU050527_A_chicken_Thai_Binh_07_89_2007_Avian
EPI_ISL_22895_A_duck_Vietnam_NCVD_60_07_Avian
EPI_ISL_22896_A_duck_Vietnam_NCVD_61_07_Avian
EPI_ISL_80728_A_duck_Vietnam_NCVD_111_2007_Avian
EPI_ISL_80747_A_duck_Vietnam_NCVD_110_2007_Avian
HM114573_A_Vietnam_UT31244III_2007_Human
branch a
HM114557_A_Vietnam_UT31239_2007_Human

NS-Group25

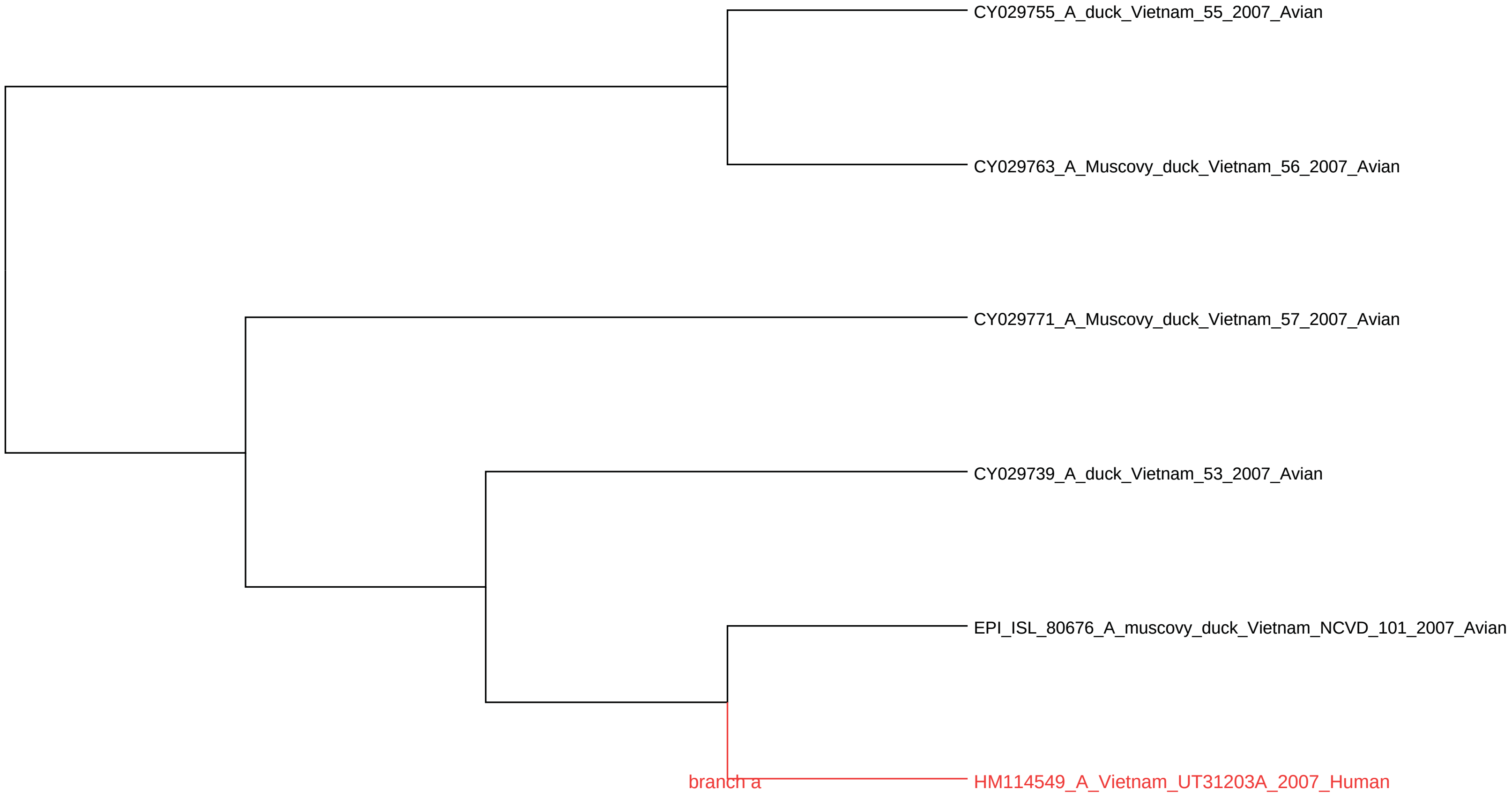
CY029755_A_duck_Vietnam_55_2007_Avian
CY029763_A_Muscovy_duck_Vietnam_56_2007_Avian
CY029771_A_Muscovy_duck_Vietnam_57_2007_Avian
CY029739_A_duck_Vietnam_53_2007_Avian
EPI_ISL_80676_A_muscovy_duck_Vietnam_NCVD_101_2007_Avian
branch a
HM114549_A_Vietnam_UT31203A_2007_Human

NS-Group26

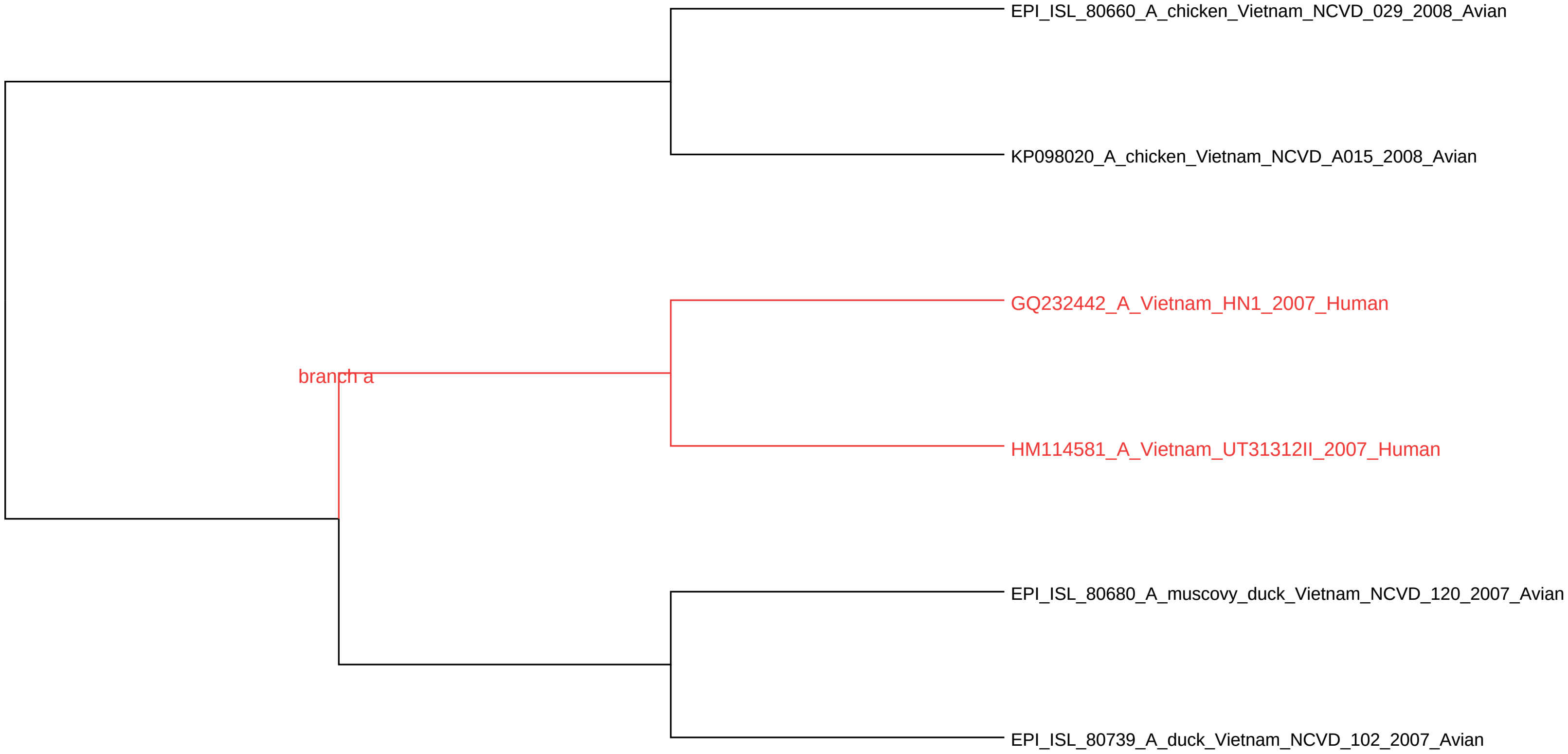
EPI_ISL_80660_A_chicken_Vietnam_NCVD_029_2008_Avian
KP098020_A_chicken_Vietnam_NCVD_A015_2008_Avian
branch a
GQ232442_A_Vietnam_HN1_2007_Human
HM114581_A_Vietnam_UT31312II_2007_Human
EPI_ISL_80680_A_muscovy_duck_Vietnam_NCVD_120_2007_Avian
EPI_ISL_80739_A_duck_Vietnam_NCVD_102_2007_Avian

NS-Group27

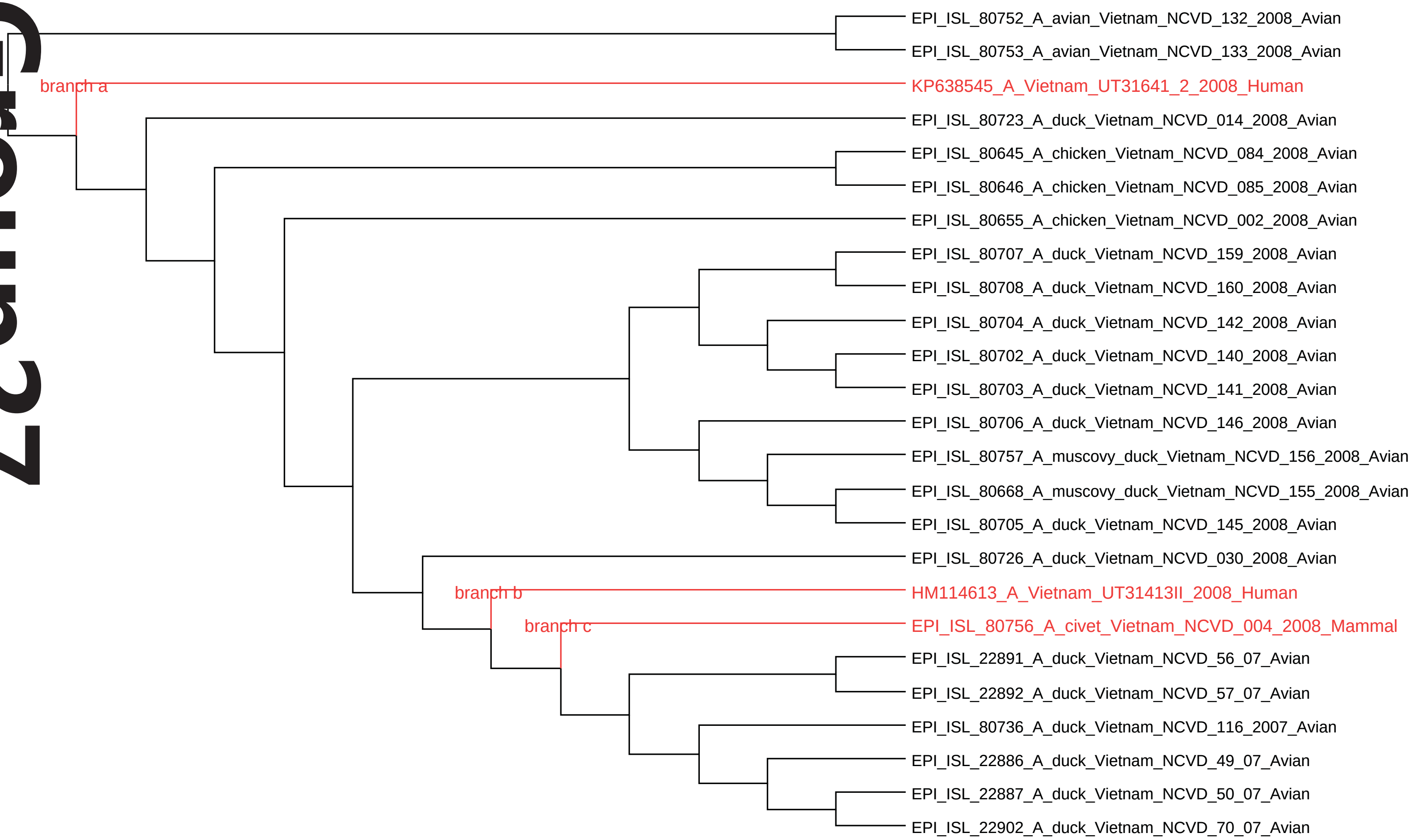

EPI_ISL_80752_A_avian_Vietnam_NCVD_132_2008_Avian
EPI_ISL_80753_A_avian_Vietnam_NCVD_133_2008_Avian
branch a
KP638545_A_Vietnam_UT31641_2_2008_Human
EPI_ISL_80723_A_duck_Vietnam_NCVD_014_2008_Avian
EPI_ISL_80645_A_chicken_Vietnam_NCVD_084_2008_Avian
EPI_ISL_80646_A_chicken_Vietnam_NCVD_085_2008_Avian
EPI_ISL_80655_A_chicken_Vietnam_NCVD_002_2008_Avian
EPI_ISL_80707_A_duck_Vietnam_NCVD_159_2008_Avian
EPI_ISL_80708_A_duck_Vietnam_NCVD_160_2008_Avian
EPI_ISL_80704_A_duck_Vietnam_NCVD_142_2008_Avian
EPI_ISL_80702_A_duck_Vietnam_NCVD_140_2008_Avian
EPI_ISL_80703_A_duck_Vietnam_NCVD_141_2008_Avian
EPI_ISL_80706_A_duck_Vietnam_NCVD_146_2008_Avian
EPI_ISL_80757_A_muscovy_duck_Vietnam_NCVD_156_2008_Avian
EPI_ISL_80668_A_muscovy_duck_Vietnam_NCVD_155_2008_Avian
EPI_ISL_80705_A_duck_Vietnam_NCVD_145_2008_Avian
EPI_ISL_80726_A_duck_Vietnam_NCVD_030_2008_Avian
branch b
HM114613_A_Vietnam_UT31413II_2008_Human
branch c
EPI_ISL_80756_A_civet_Vietnam_NCVD_004_2008_Mammal
EPI_ISL_22891_A_duck_Vietnam_NCVD_56_07_Avian
EPI_ISL_22892_A_duck_Vietnam_NCVD_57_07_Avian
EPI_ISL_80736_A_duck_Vietnam_NCVD_116_2007_Avian
EPI_ISL_22886_A_duck_Vietnam_NCVD_49_07_Avian
EPI_ISL_22887_A_duck_Vietnam_NCVD_50_07_Avian
EPI_ISL_22902_A_duck_Vietnam_NCVD_70_07_Avian

NS-Group28

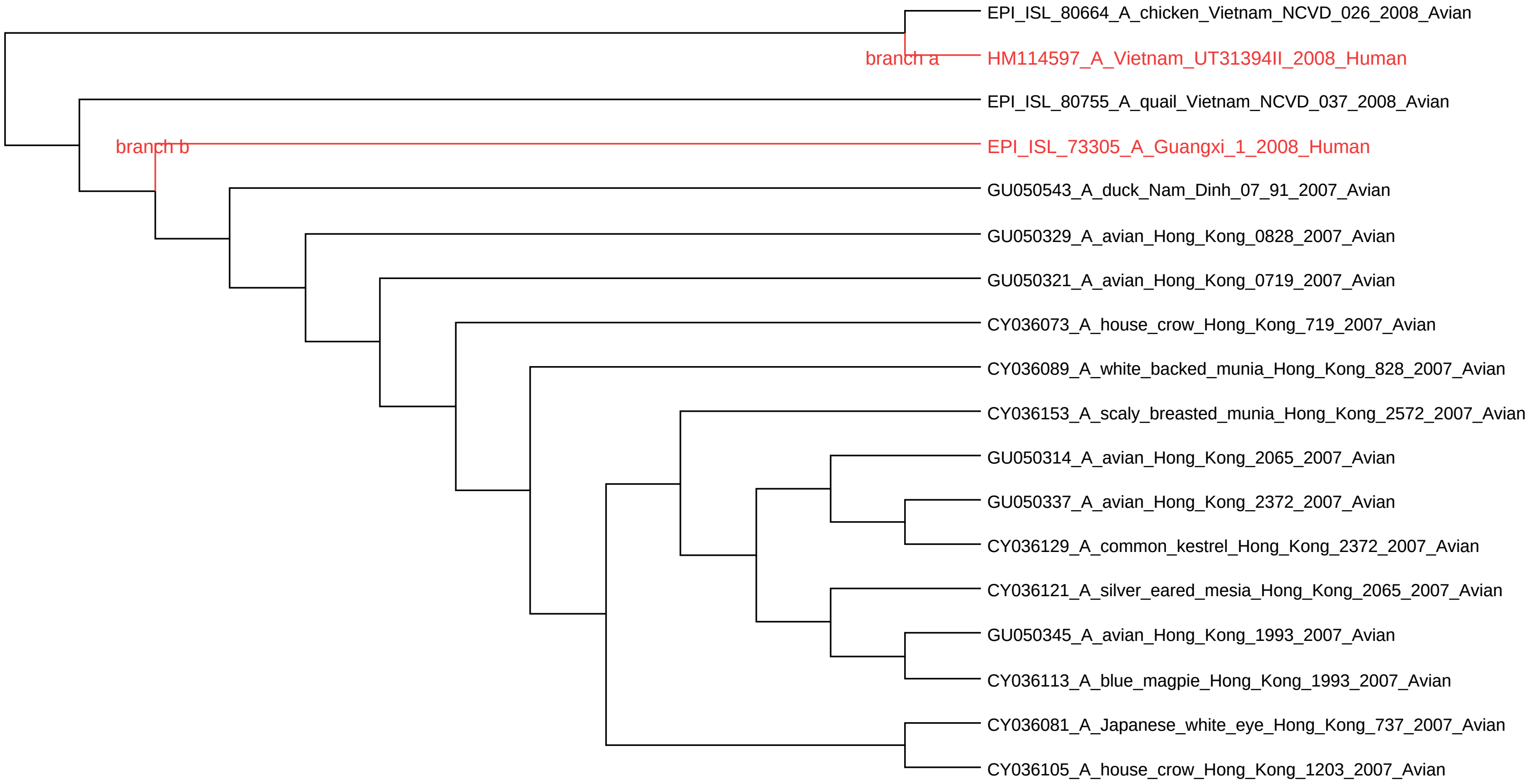

EPI_ISL_80664_A_chicken_Vietnam_NCVD_026_2008_Avian
branch a
HM114597_A_Vietnam_UT31394II_2008_Human
EPI_ISL_80755_A_quail_Vietnam_NCVD_037_2008_Avian
branch b
EPI_ISL_73305_A_Guangxi_1_2008_Human
GU050543_A_duck_Nam_Dinh_07_91_2007_Avian
GU050329_A_avian_Hong_Kong_0828_2007_Avian
GU050321_A_avian_Hong_Kong_0719_2007_Avian
CY036073_A_house_crow_Hong_Kong_719_2007_Avian
CY036089_A_white_backed_munia_Hong_Kong_828_2007_Avian
CY036153_A_scaly_breasted_munia_Hong_Kong_2572_2007_Avian
GU050314_A_avian_Hong_Kong_2065_2007_Avian
GU050337_A_avian_Hong_Kong_2372_2007_Avian
CY036129_A_common_kestrel_Hong_Kong_2372_2007_Avian
CY036121_A_silver_eared_mesia_Hong_Kong_2065_2007_Avian
GU050345_A_avian_Hong_Kong_1993_2007_Avian
CY036113_A_blue_magpie_Hong_Kong_1993_2007_Avian
CY036081_A_Japanese_white_eye_Hong_Kong_737_2007_Avian
CY036105_A_house_crow_Hong_Kong_1203_2007_Avian

NS-Group29

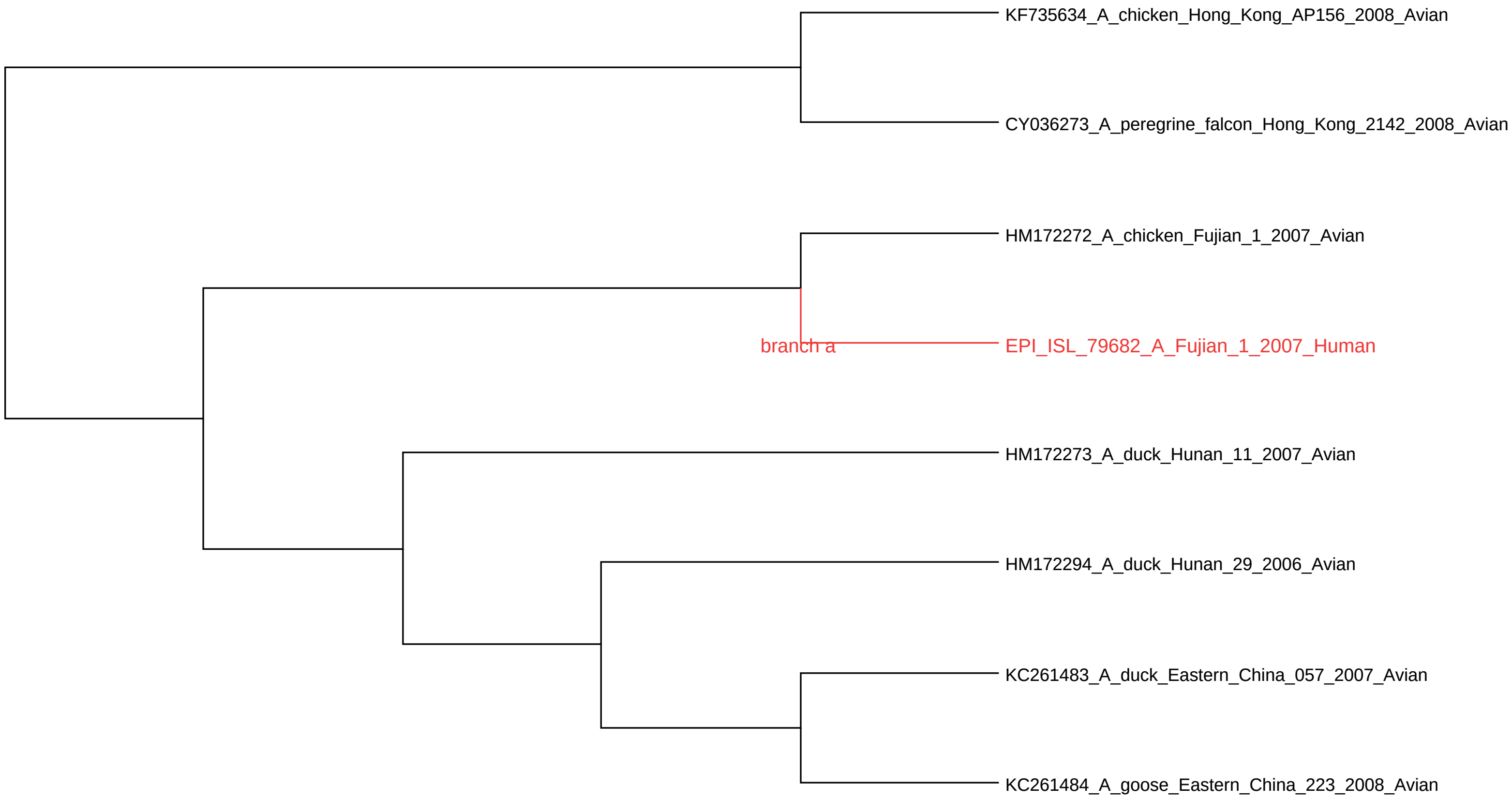
KF735634_A_chicken_Hong_Kong_AP156_2008_Avian
CY036273_A_peregrine_falcon_Hong_Kong_2142_2008_Avian
HM172272_A_chicken_Fujian_1_2007_Avian
branch a
EPI_ISL_79682_A_Fujian_1_2007_Human
HM172273_A_duck_Hunan_11_2007_Avian
HM172294_A_duck_Hunan_29_2006_Avian
KC261483_A_duck_Eastern_China_057_2007_Avian
KC261484_A_goose_Eastern_China_223_2008_Avian

NS-Group30

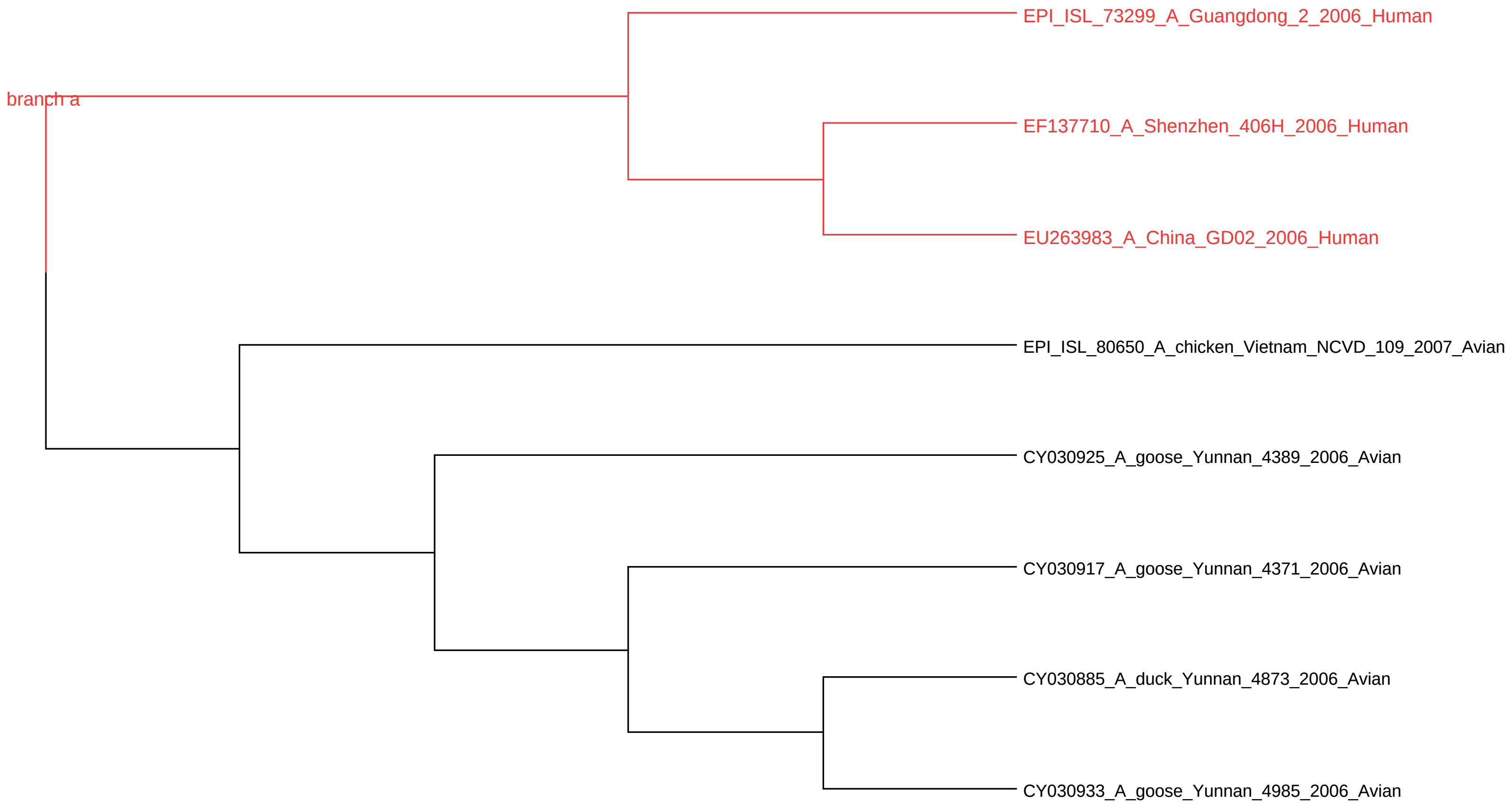

branch a
EPI_ISL_73299_A_Guangdong_2_2006_Human
EF137710_A_Shenzhen_406H_2006_Human
EU263983_A_China_GD02_2006_Human
EPI_ISL_80650_A_chicken_Vietnam_NCVD_109_2007_Avian
CY030925_A_goose_Yunnan_4389_2006_Avian
CY030917_A_goose_Yunnan_4371_2006_Avian
CY030885_A_duck_Yunnan_4873_2006_Avian
CY030933_A_goose_Yunnan_4985_2006_Avian

NS-Group31

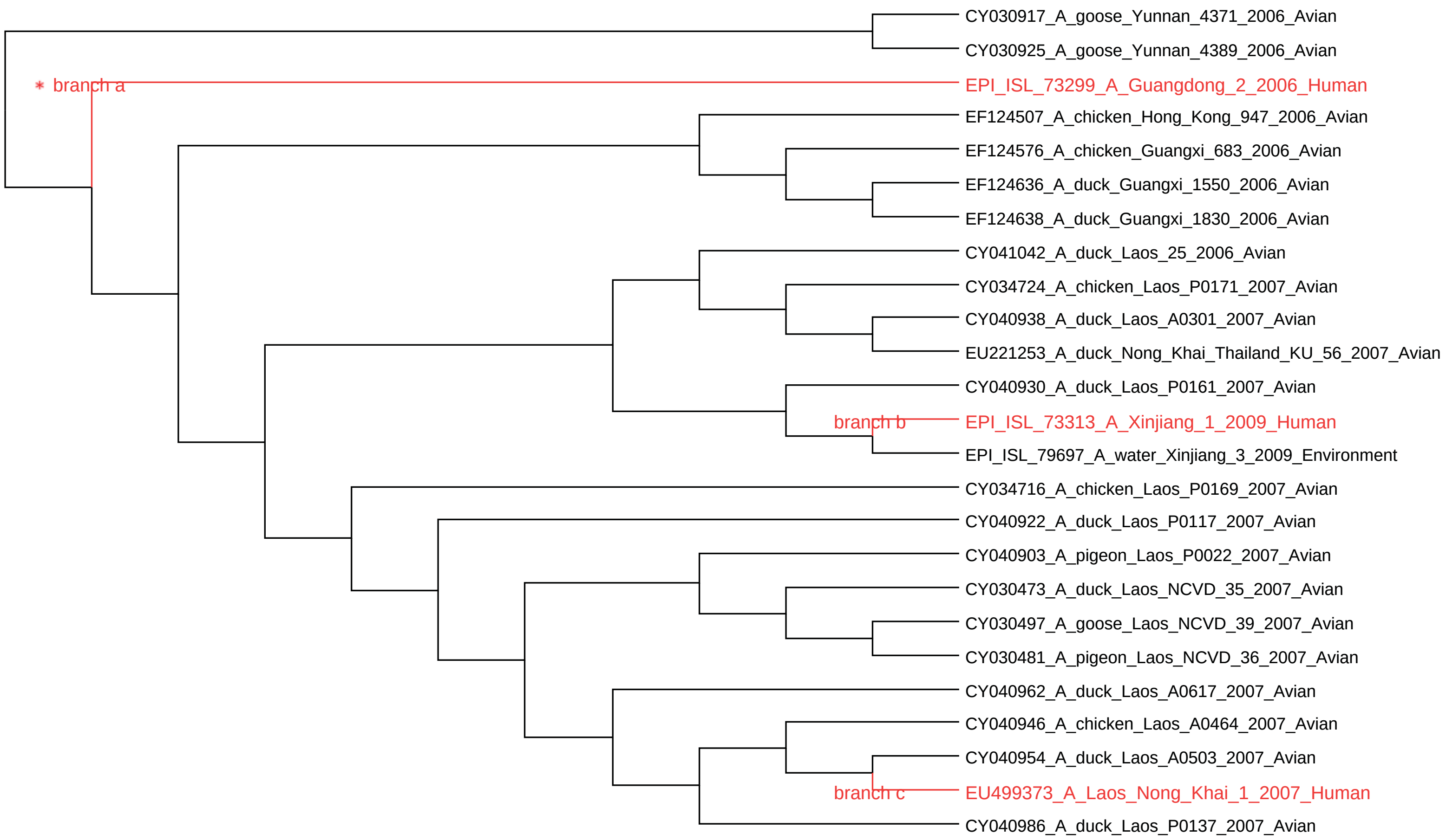
CY030917_A_goose_Yunnan_4371_2006_Avian
CY030925_A_goose_Yunnan_4389_2006_Avian
* branch a
EPI_ISL_73299_A_Guangdong_2_2006_Human
EF124507_A_chicken_Hong_Kong_947_2006_Avian
EF124576_A_chicken_Guangxi_683_2006_Avian
EF124636_A_duck_Guangxi_1550_2006_Avian
EF124638_A_duck_Guangxi_1830_2006_Avian
CY041042_A_duck_Laos_25_2006_Avian
CY034724_A_chicken_Laos_P0171_2007_Avian
CY040938_A_duck_Laos_A0301_2007_Avian
EU221253_A_duck_Nong_Khai_Thailand_KU_56_2007_Avian
CY040930_A_duck_Laos_P0161_2007_Avian
branch b
EPI_ISL_73313_A_Xinjiang_1_2009_Human
EPI_ISL_79697_A_water_Xinjiang_3_2009_Environment
CY034716_A_chicken_Laos_P0169_2007_Avian
CY040922_A_duck_Laos_P0117_2007_Avian
CY040903_A_pigeon_Laos_P0022_2007_Avian
CY030473_A_duck_Laos_NCVD_35_2007_Avian
CY030497_A_goose_Laos_NCVD_39_2007_Avian
CY030481_A_pigeon_Laos_NCVD_36_2007_Avian
CY040962_A_duck_Laos_A0617_2007_Avian
CY040946_A_chicken_Laos_A0464_2007_Avian
CY040954_A_duck_Laos_A0503_2007_Avian
branch c
EU499373_A_Laos_Nong_Khai_1_2007_Human
CY040986_A_duck_Laos_P0137_2007_Avian

NS-Group32

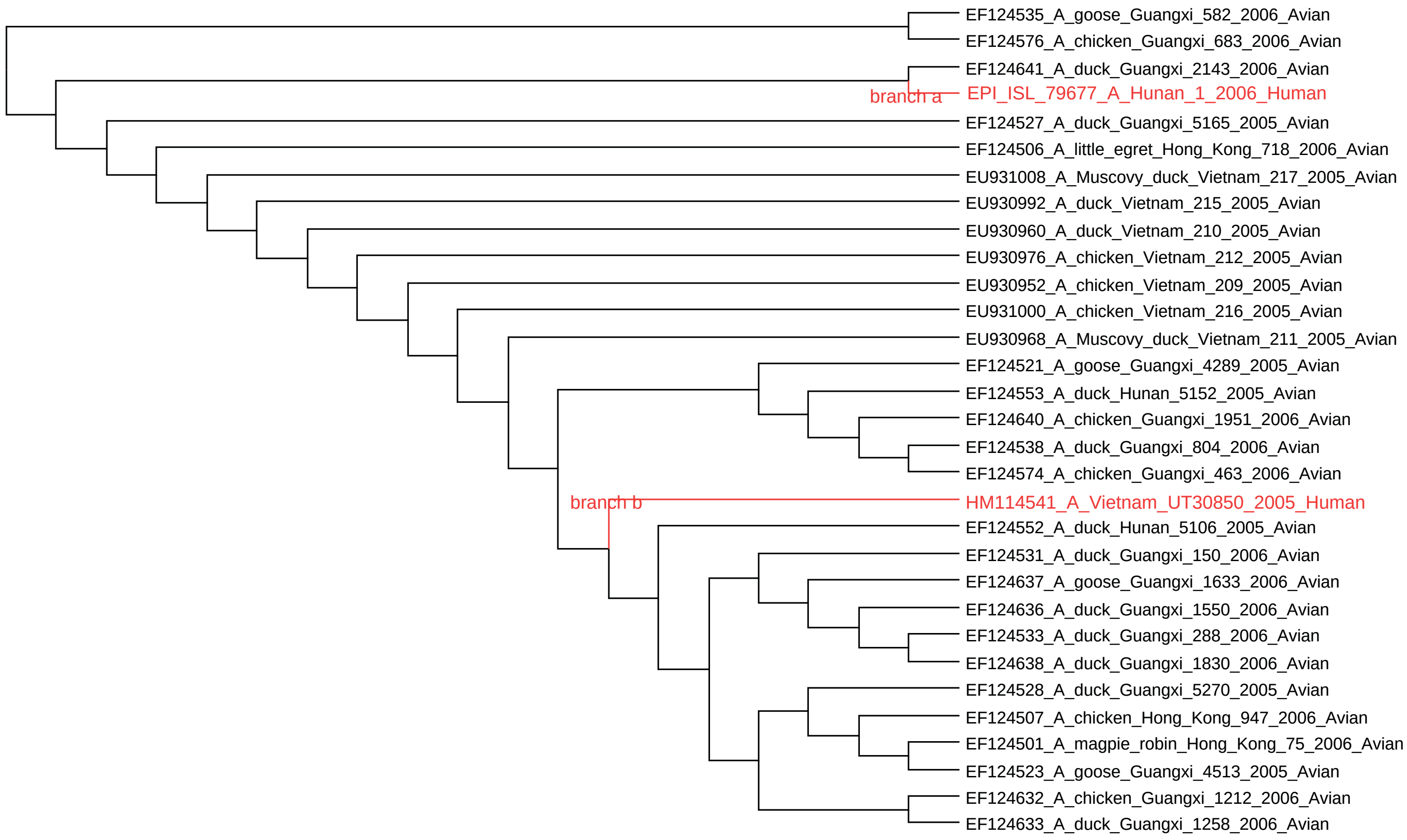
EF124535_A_goose_Guangxi_582_2006_Avian
EF124576_A_chicken_Guangxi_683_2006_Avian
EF124641_A_duck_Guangxi_2143_2006_Avian
branch a
EPI_ISL_79677_A_Hunan_1_2006_Human
EF124527_A_duck_Guangxi_5165_2005_Avian
EF124506_A_little_egret_Hong_Kong_718_2006_Avian
EU931008_A_Muscovy_duck_Vietnam_217_2005_Avian
EU930992_A_duck_Vietnam_215_2005_Avian
EU930960_A_duck_Vietnam_210_2005_Avian
EU930976_A_chicken_Vietnam_212_2005_Avian
EU930952_A_chicken_Vietnam_209_2005_Avian
EU931000_A_chicken_Vietnam_216_2005_Avian
EU930968_A_Muscovy_duck_Vietnam_211_2005_Avian
EF124521_A_goose_Guangxi_4289_2005_Avian
EF124553_A_duck_Hunan_5152_2005_Avian
EF124640_A_chicken_Guangxi_1951_2006_Avian
EF124538_A_duck_Guangxi_804_2006_Avian
EF124574_A_chicken_Guangxi_463_2006_Avian
branch b
HM114541_A_Vietnam_UT30850_2005_Human
EF124552_A_duck_Hunan_5106_2005_Avian
EF124531_A_duck_Guangxi_150_2006_Avian
EF124637_A_goose_Guangxi_1633_2006_Avian
EF124636_A_duck_Guangxi_1550_2006_Avian
EF124533_A_duck_Guangxi_288_2006_Avian
EF124638_A_duck_Guangxi_1830_2006_Avian
EF124528_A_duck_Guangxi_5270_2005_Avian
EF124507_A_chicken_Hong_Kong_947_2006_Avian
EF124501_A_magpie_robin_Hong_Kong_75_2006_Avian
EF124523_A_goose_Guangxi_4513_2005_Avian
EF124632_A_chicken_Guangxi_1212_2006_Avian
EF124633_A_duck_Guangxi_1258_2006_Avian

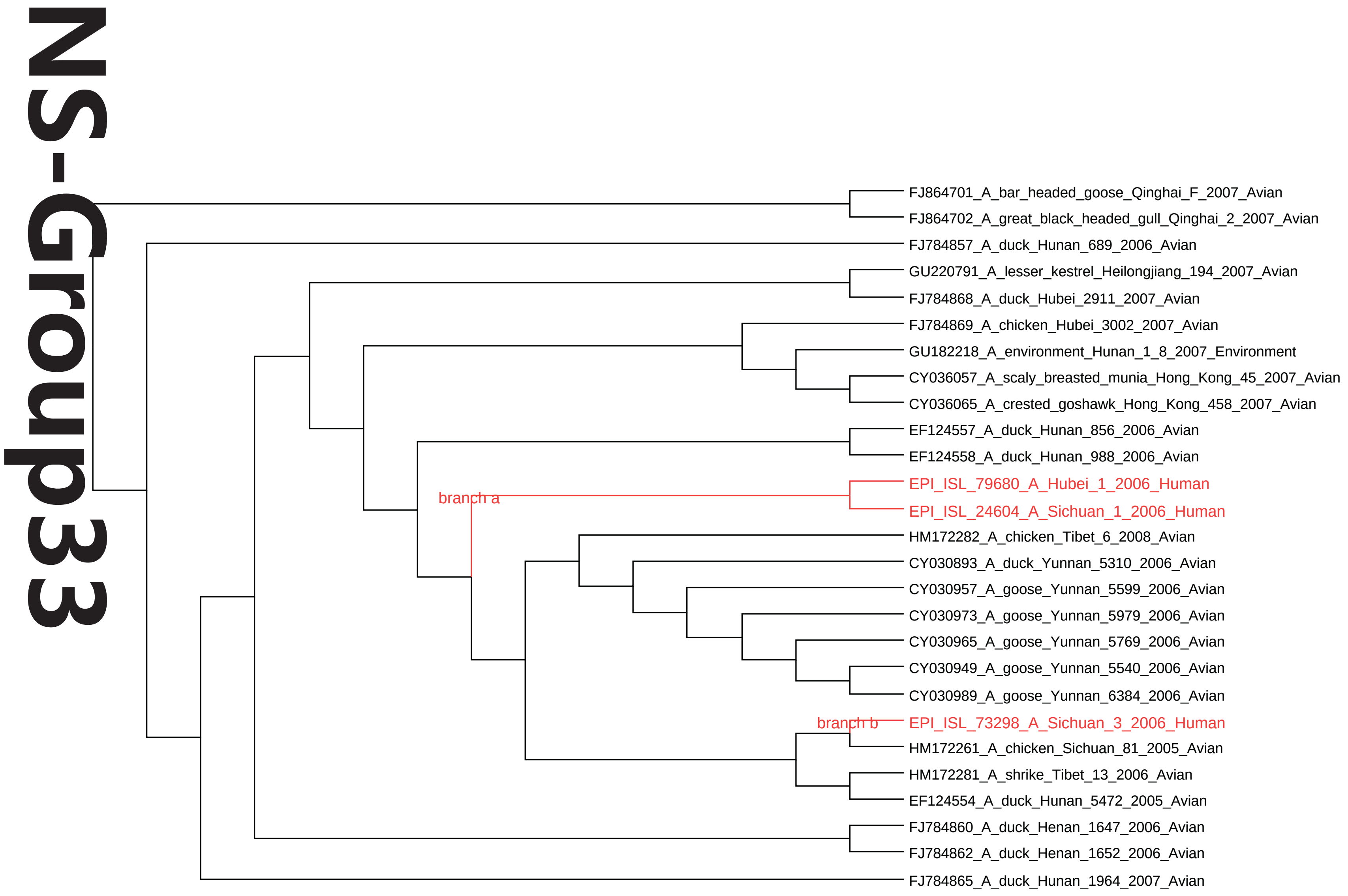
NS-Group33
FJ864701_A_bar_headed_goose_Qinghai_F_2007_Avian
FJ864702_A_great_black_headed_gull_Qinghai_2_2007_Avian
FJ784857_A_duck_Hunan_689_2006_Avian
GU220791_A_lesser_kestrel_Heilongjiang_194_2007_Avian
FJ784868_A_duck_Hubei_2911_2007_Avian
FJ784869_A_chicken_Hubei_3002_2007_Avian
GU182218_A_environment_Hunan_1_8_2007_Environment
CY036057_A_scaly_breasted_munia_Hong_Kong_45_2007_Avian
CY036065_A_crested_goshawk_Hong_Kong_458_2007_Avian
EF124557_A_duck_Hunan_856_2006_Avian
EF124558_A_duck_Hunan_988_2006_Avian
branch a
EPI_ISL_79680_A_Hubei_1_2006_Human
EPI_ISL_24604_A_Sichuan_1_2006_Human
HM172282_A_chicken_Tibet_6_2008_Avian
CY030893_A_duck_Yunnan_5310_2006_Avian
CY030957_A_goose_Yunnan_5599_2006_Avian
CY030973_A_goose_Yunnan_5979_2006_Avian
CY030965_A_goose_Yunnan_5769_2006_Avian
CY030949_A_goose_Yunnan_5540_2006_Avian
CY030989_A_goose_Yunnan_6384_2006_Avian
branch b
EPI_ISL_73298_A_Sichuan_3_2006_Human
HM172261_A_chicken_Sichuan_81_2005_Avian
HM172281_A_shrike_Tibet_13_2006_Avian
EF124554_A_duck_Hunan_5472_2005_Avian
FJ784860_A_duck_Henan_1647_2006_Avian
FJ784862_A_duck_Henan_1652_2006_Avian
FJ784865_A_duck_Hunan_1964_2007_Avian

NS-Group34

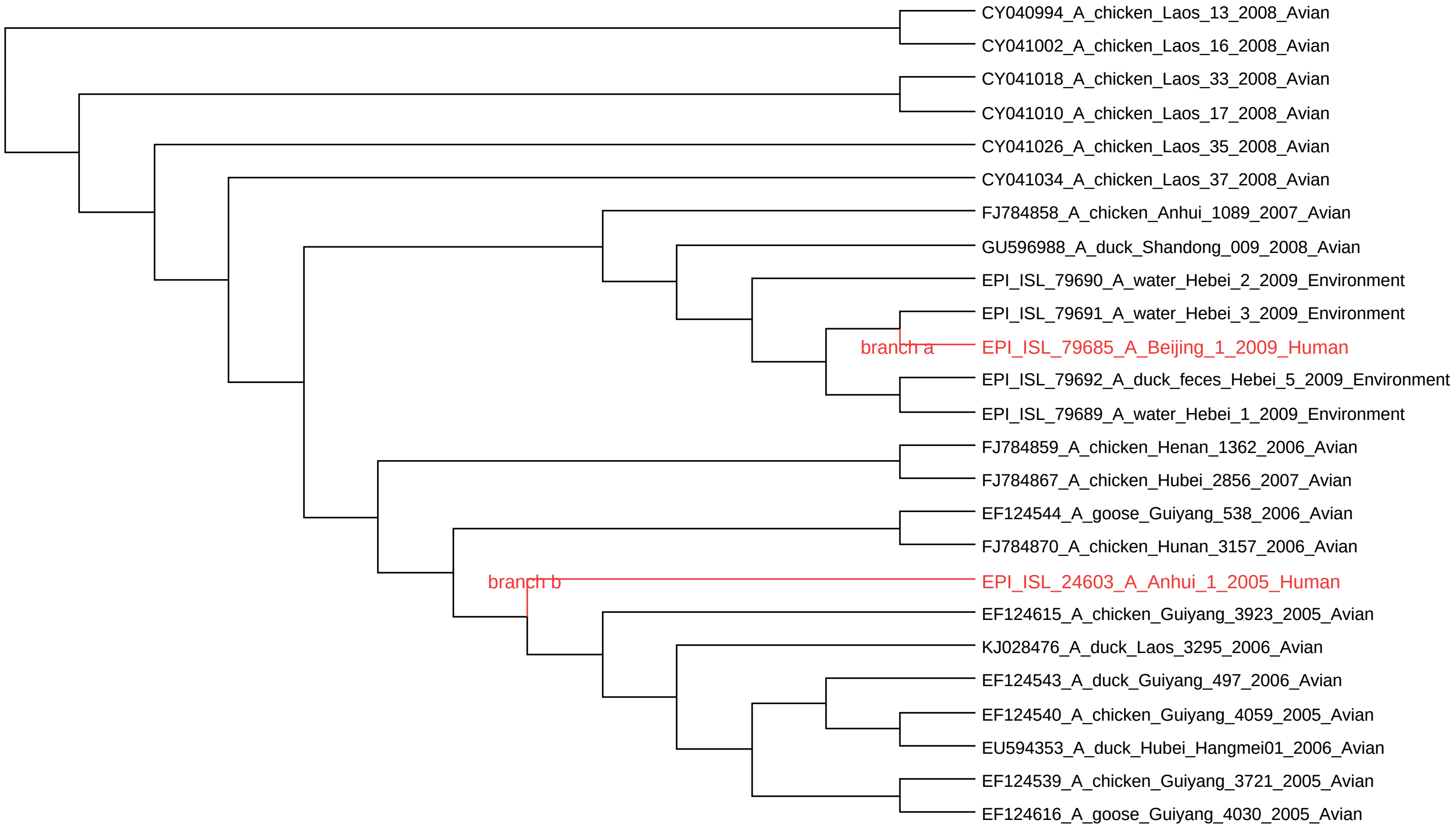
CY040994_A_chicken_Laos_13_2008_Avian
CY041002_A_chicken_Laos_16_2008_Avian
CY041018_A_chicken_Laos_33_2008_Avian
CY041010_A_chicken_Laos_17_2008_Avian
CY041026_A_chicken_Laos_35_2008_Avian
CY041034_A_chicken_Laos_37_2008_Avian
FJ784858_A_chicken_Anhui_1089_2007_Avian
GU596988_A_duck_Shandong_009_2008_Avian
EPI_ISL_79690_A_water_Hebei_2_2009_Environment
EPI_ISL_79691_A_water_Hebei_3_2009_Environment
branch a
EPI_ISL_79685_A_Beijing_1_2009_Human
EPI_ISL_79692_A_duck_feces_Hebei_5_2009_Environment
EPI_ISL_79689_A_water_Hebei_1_2009_Environment
FJ784859_A_chicken_Henan_1362_2006_Avian
FJ784867_A_chicken_Hubei_2856_2007_Avian
EF124544_A_goose_Guiyang_538_2006_Avian
FJ784870_A_chicken_Hunan_3157_2006_Avian
branch b
EPI_ISL_24603_A_Anhui_1_2005_Human
EF124615_A_chicken_Guiyang_3923_2005_Avian
KJ028476_A_duck_Laos_3295_2006_Avian
EF124543_A_duck_Guiyang_497_2006_Avian
EF124540_A_chicken_Guiyang_4059_2005_Avian
EU594353_A_duck_Hubei_Hangmei01_2006_Avian
EF124539_A_chicken_Guiyang_3721_2005_Avian
EF124616_A_goose_Guiyang_4030_2005_Avian

NS-Group35

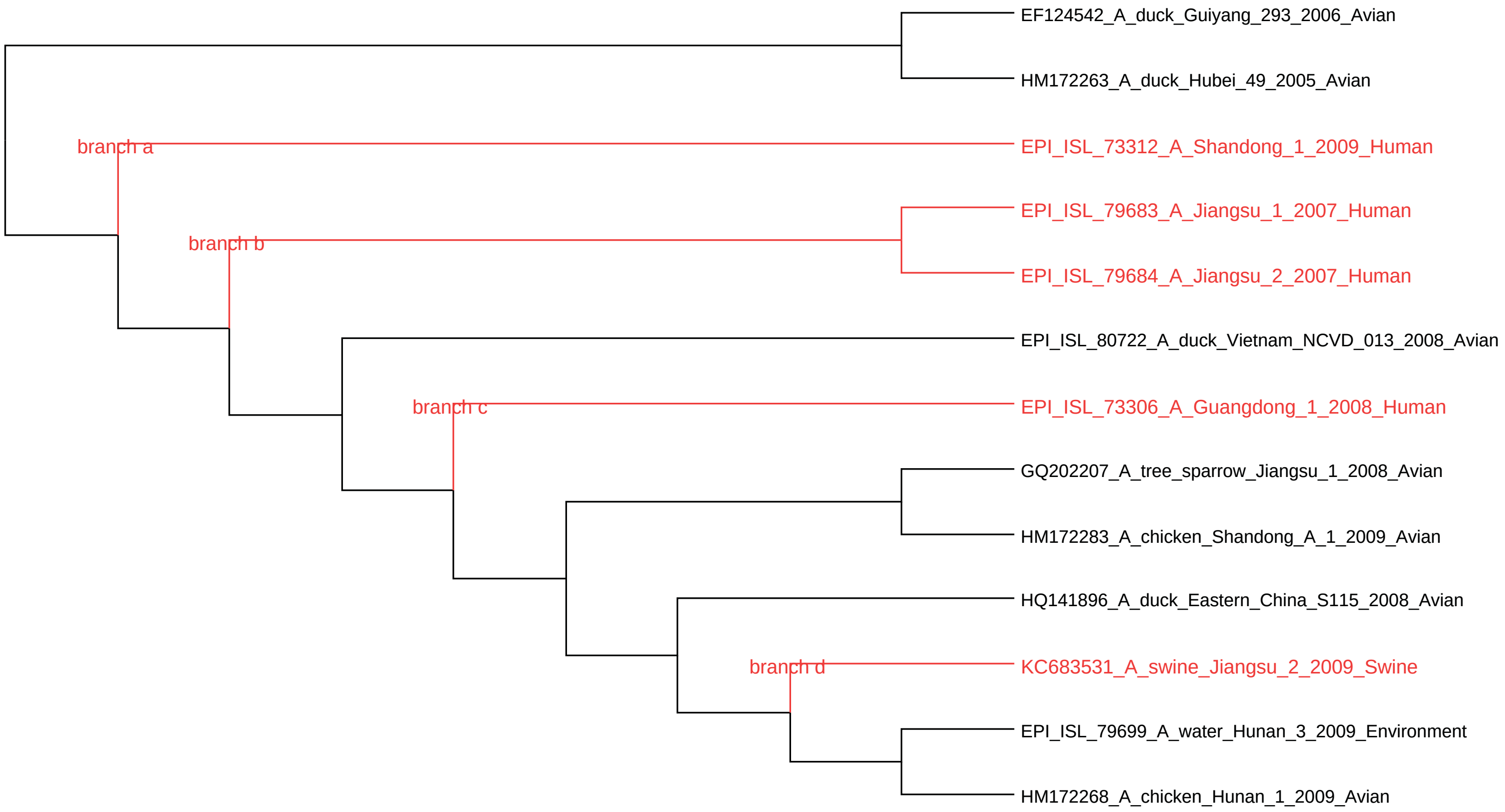
EF124542_A_duck_Guiyang_293_2006_Avian
HM172263_A_duck_Hubei_49_2005_Avian
branch a
EPI_ISL_73312_A_Shandong_1_2009_Human
branch b
EPI_ISL_79683_A_Jiangsu_1_2007_Human
EPI_ISL_79684_A_Jiangsu_2_2007_Human
EPI_ISL_80722_A_duck_Vietnam_NCVD_013_2008_Avian
branch c
EPI_ISL_73306_A_Guangdong_1_2008_Human
GQ202207_A_tree_sparrow_Jiangsu_1_2008_Avian
HM172283_A_chicken_Shandong_A_1_2009_Avian
HQ141896_A_duck_Eastern_China_S115_2008_Avian
branch d
KC683531_A_swine_Jiangsu_2_2009_Swine
EPI_ISL_79699_A_water_Hunan_3_2009_Environment
HM172268_A_chicken_Hunan_1_2009_Avian

NS-Group36

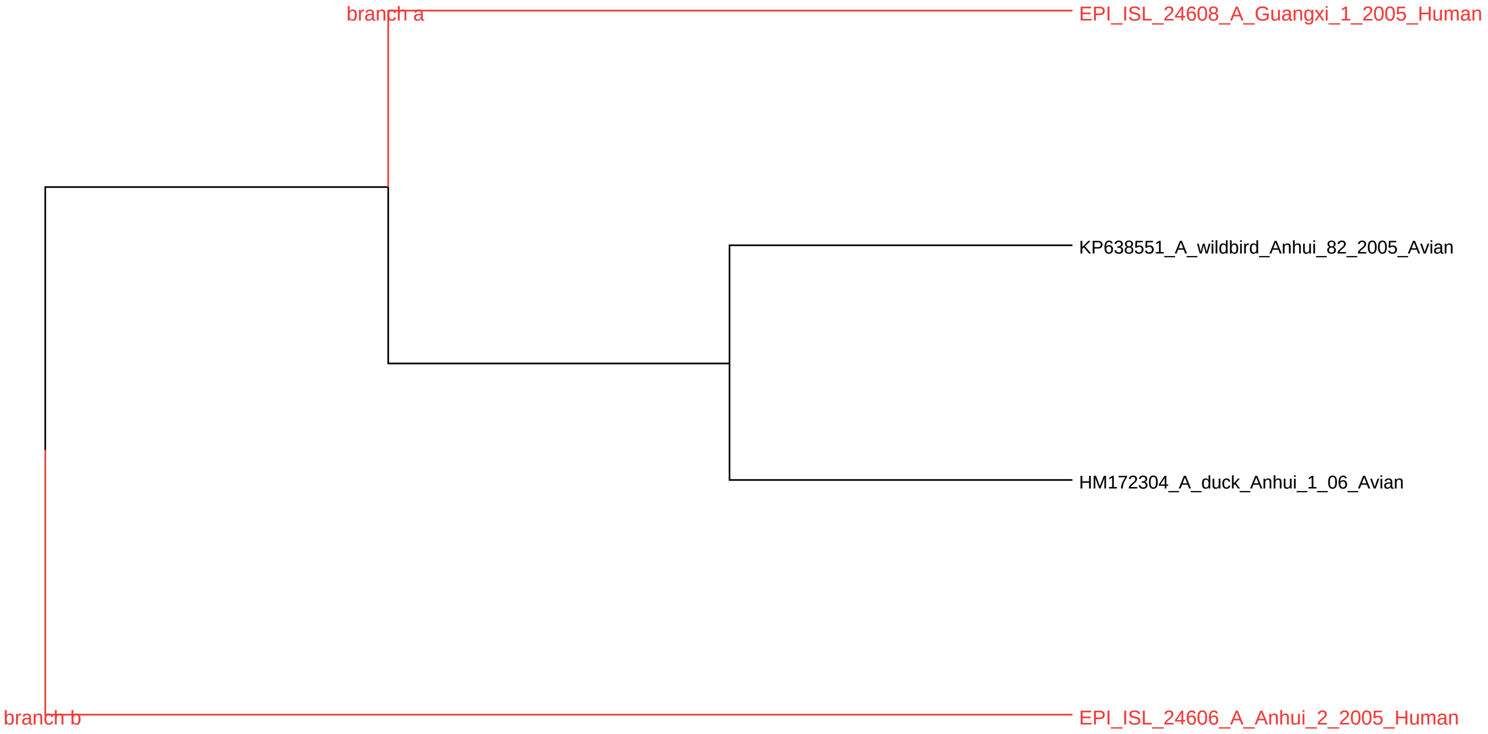
branch a
EPI_ISL_24608_A_Guangxi_1_2005_Human
KP638551_A_wildbird_Anhui_82_2005_Avian
HM172304_A_duck_Anhui_1_06_Avian
branch b
EPI_ISL_24606_A_Anhui_2_2005_Human

NS-Group37

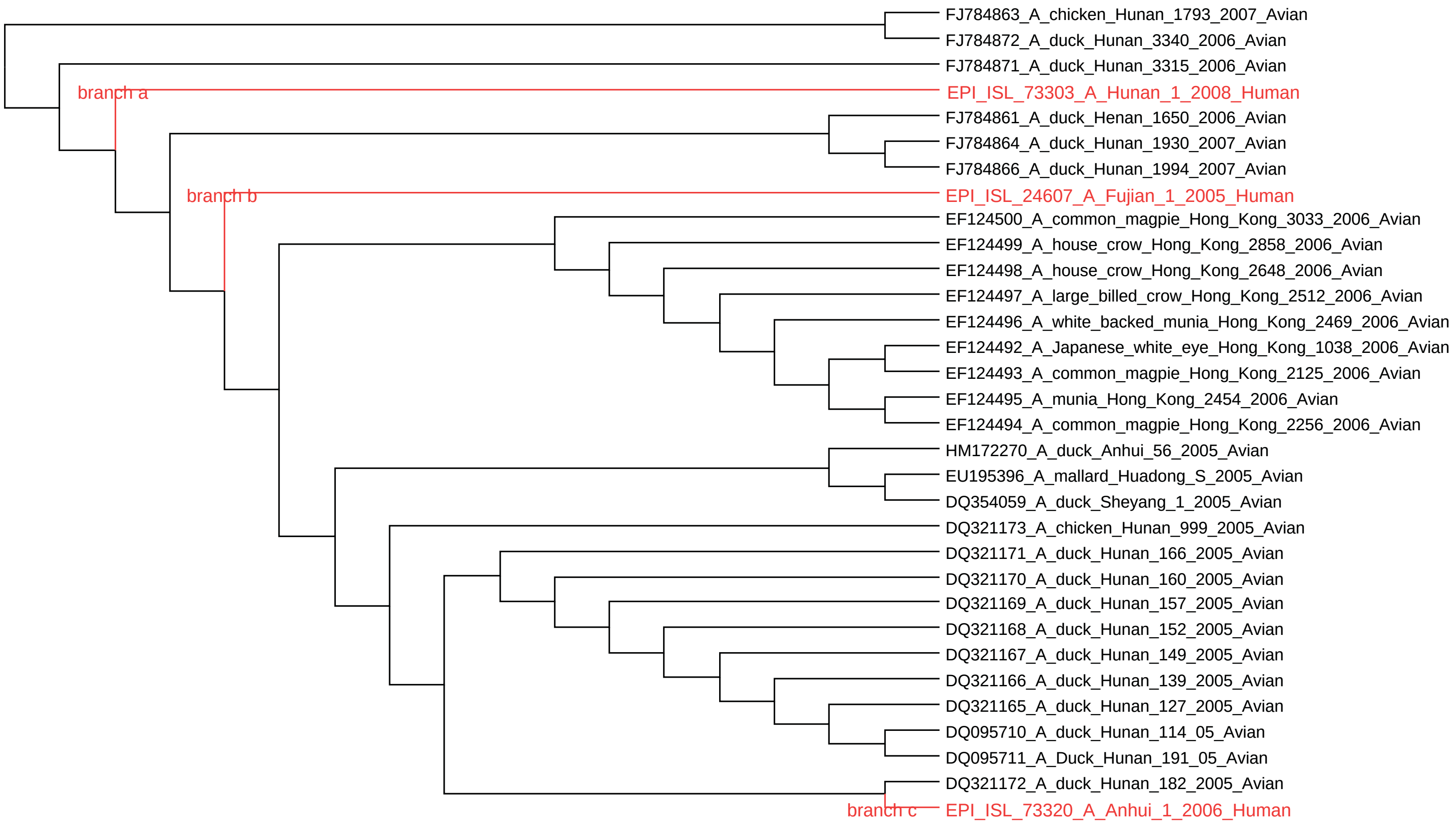

FJ784863_A_chicken_Hunan_1793_2007_Avian
FJ784872_A_duck_Hunan_3340_2006_Avian
FJ784871_A_duck_Hunan_3315_2006_Avian
branch a
EPI_ISL_73303_A_Hunan_1_2008_Human
FJ784861_A_duck_Henan_1650_2006_Avian
FJ784864_A_duck_Hunan_1930_2007_Avian
FJ784866_A_duck_Hunan_1994_2007_Avian
branch b
EPI_ISL_24607_A_Fujian_1_2005_Human
EF124500_A_common_magpie_Hong_Kong_3033_2006_Avian
EF124499_A_house_crow_Hong_Kong_2858_2006_Avian
EF124498_A_house_crow_Hong_Kong_2648_2006_Avian
EF124497_A_large_billed_crow_Hong_Kong_2512_2006_Avian
EF124496_A_white_backed_munia_Hong_Kong_2469_2006_Avian
EF124492_A_Japanese_white_eye_Hong_Kong_1038_2006_Avian
EF124493_A_common_magpie_Hong_Kong_2125_2006_Avian
EF124495_A_munia_Hong_Kong_2454_2006_Avian
EF124494_A_common_magpie_Hong_Kong_2256_2006_Avian
HM172270_A_duck_Anhui_56_2005_Avian
EU195396_A_mallard_Huadong_S_2005_Avian
DQ354059_A_duck_Sheyang_1_2005_Avian
DQ321173_A_chicken_Hunan_999_2005_Avian
DQ321171_A_duck_Hunan_166_2005_Avian
DQ321170_A_duck_Hunan_160_2005_Avian
DQ321169_A_duck_Hunan_157_2005_Avian
DQ321168_A_duck_Hunan_152_2005_Avian
DQ321167_A_duck_Hunan_149_2005_Avian
DQ321166_A_duck_Hunan_139_2005_Avian
DQ321165_A_duck_Hunan_127_2005_Avian
DQ095710_A_duck_Hunan_114_05_Avian
DQ095711_A_Duck_Hunan_191_05_Avian
DQ321172_A_duck_Hunan_182_2005_Avian
branch c
EPI_ISL_73320_A_Anhui_1_2006_Human

NS-Group38

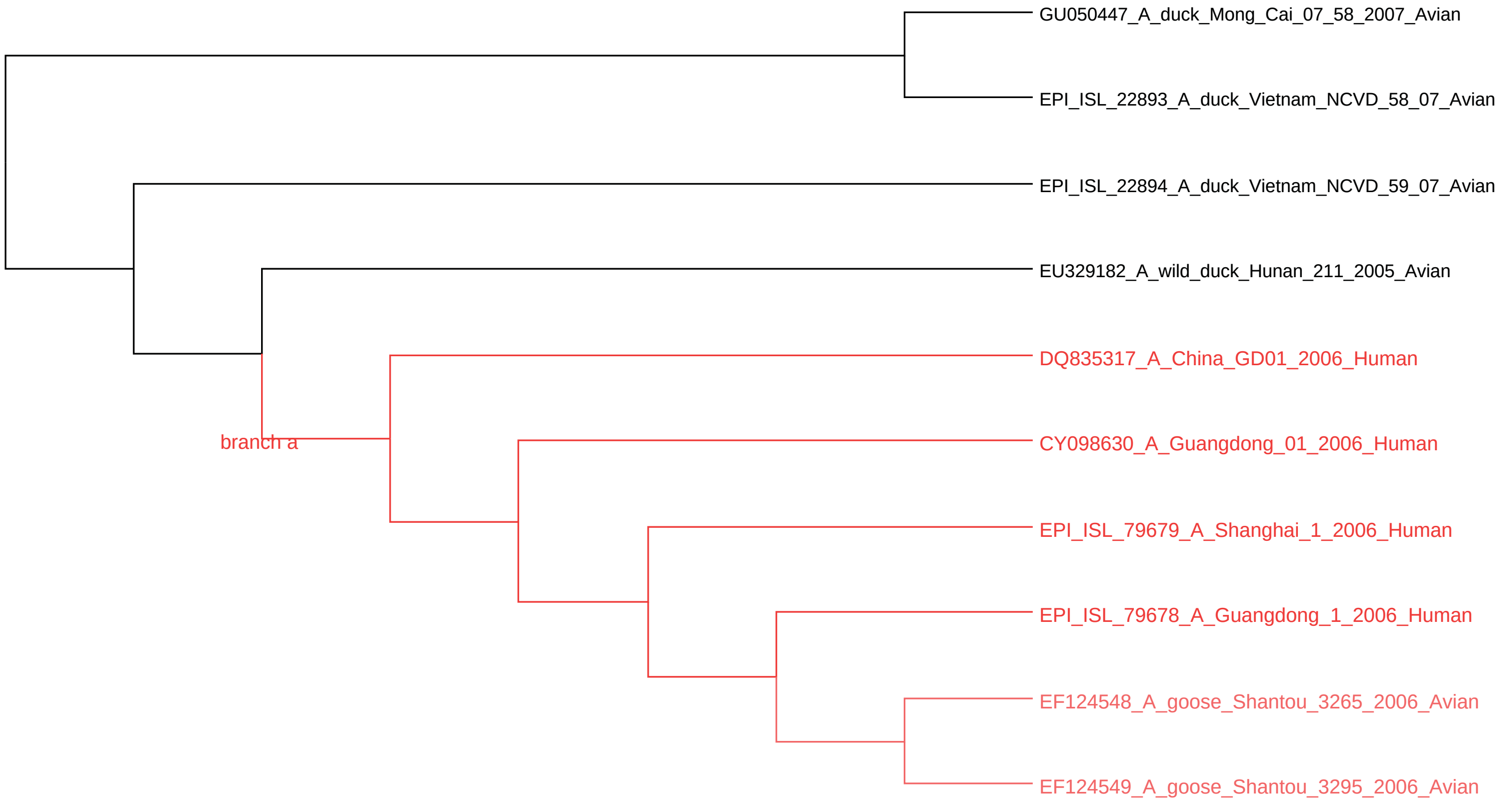
GU050447_A_duck_Mong_Cai_07_58_2007_Avian
EPI_ISL_22893_A_duck_Vietnam_NCVD_58_07_Avian
EPI_ISL_22894_A_duck_Vietnam_NCVD_59_07_Avian
EU329182_A_wild_duck_Hunan_211_2005_Avian
DQ835317_A_China_GD01_2006_Human
branch a
CY098630_A_Guangdong_01_2006_Human
EPI_ISL_79679_A_Shanghai_1_2006_Human
EPI_ISL_79678_A_Guangdong_1_2006_Human
EF124548_A_goose_Shantou_3265_2006_Avian
EF124549_A_goose_Shantou_3295_2006_Avian

NS-Group39

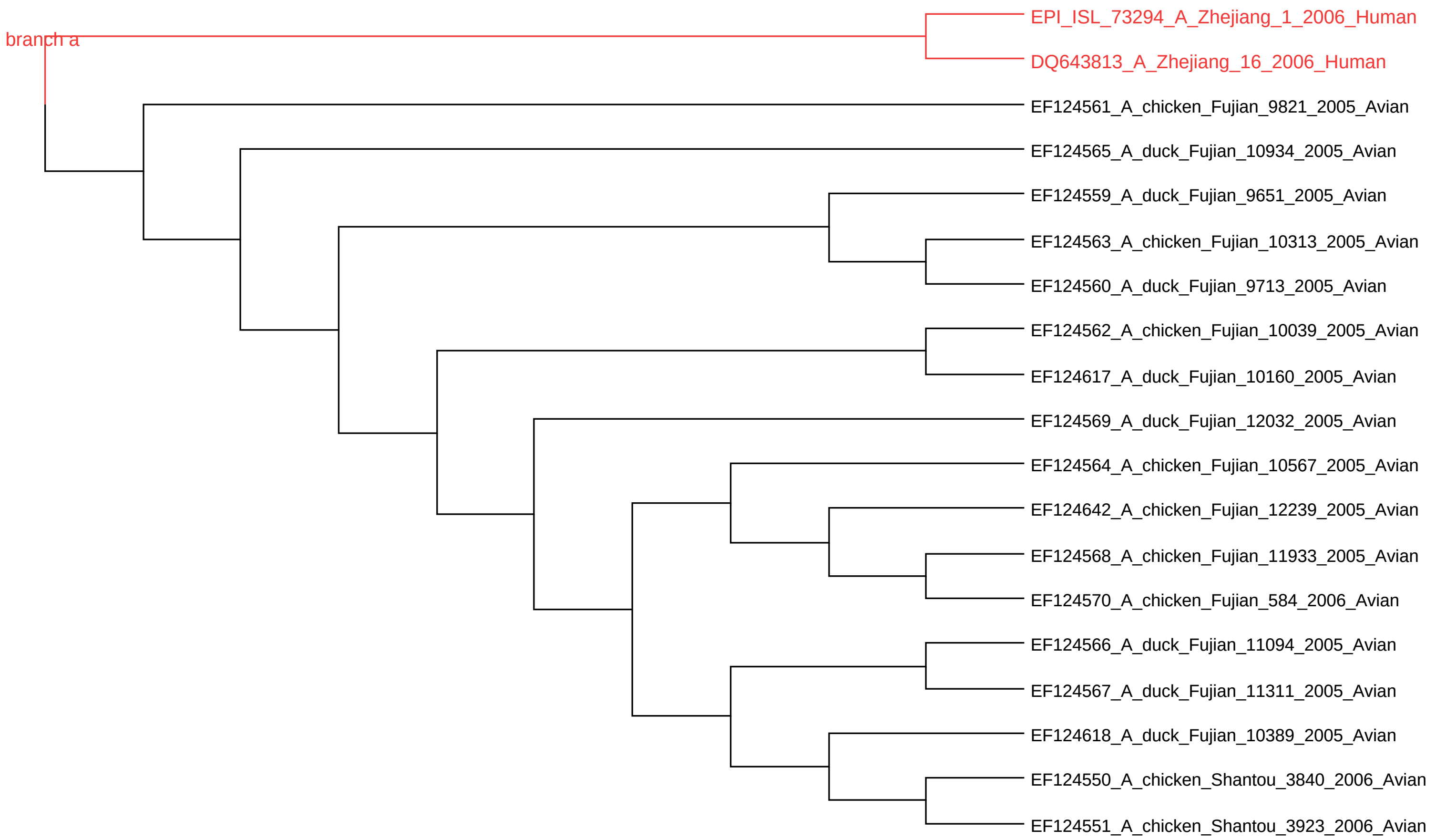

branch a
EPI_ISL_73294_A_Zhejiang_1_2006_Human
DQ643813_A_Zhejiang_16_2006_Human
EF124561_A_chicken_Fujian_9821_2005_Avian
EF124565_A_duck_Fujian_10934_2005_Avian
EF124559_A_duck_Fujian_9651_2005_Avian
EF124563_A_chicken_Fujian_10313_2005_Avian
EF124560_A_duck_Fujian_9713_2005_Avian
EF124562_A_chicken_Fujian_10039_2005_Avian
EF124617_A_duck_Fujian_10160_2005_Avian
EF124569_A_duck_Fujian_12032_2005_Avian
EF124564_A_chicken_Fujian_10567_2005_Avian
EF124642_A_chicken_Fujian_12239_2005_Avian
EF124568_A_chicken_Fujian_11933_2005_Avian
EF124570_A_chicken_Fujian_584_2006_Avian
EF124566_A_duck_Fujian_11094_2005_Avian
EF124567_A_duck_Fujian_11311_2005_Avian
EF124618_A_duck_Fujian_10389_2005_Avian
EF124550_A_chicken_Shantou_3840_2006_Avian
EF124551_A_chicken_Shantou_3923_2006_Avian

NS-Group40

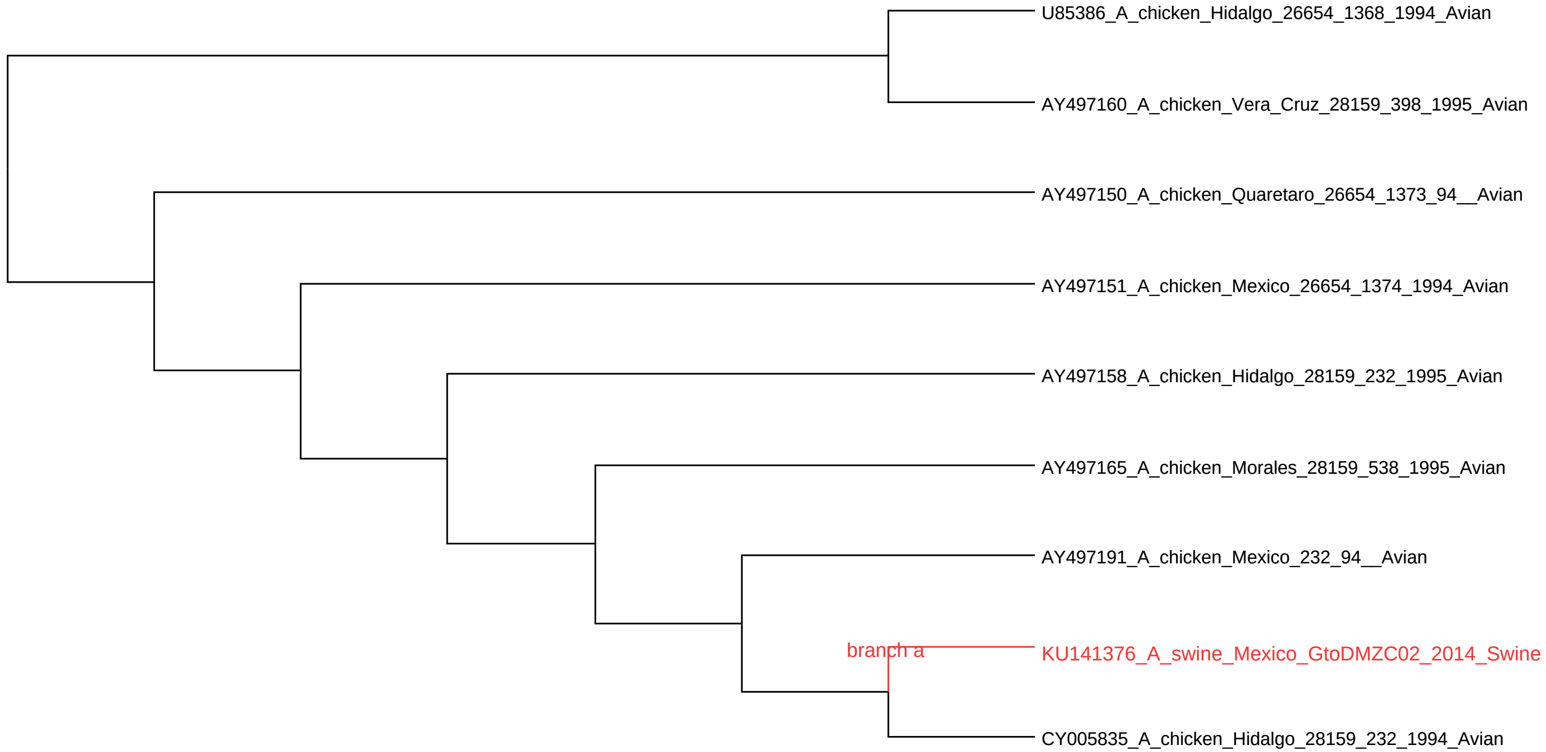
U85386_A_chicken_Hidalgo_26654_1368_1994_Avian
AY497160_A_chicken_Vera_Cruz_28159_398_1995_Avian
AY497150_A_chicken_Quaretaro_26654_1373_94__Avian
AY497151_A_chicken_Mexico_26654_1374_1994_Avian
AY497158_A_chicken_Hidalgo_28159_232_1995_Avian
AY497165_A_chicken_Morales_28159_538_1995_Avian
AY497191_A_chicken_Mexico_232_94__Avian
branch a
KU141376_A_swine_Mexico_GtoDMZC02_2014_Swine
CY005835_A_chicken_Hidalgo_28159_232_1994_Avian

NS-Group41

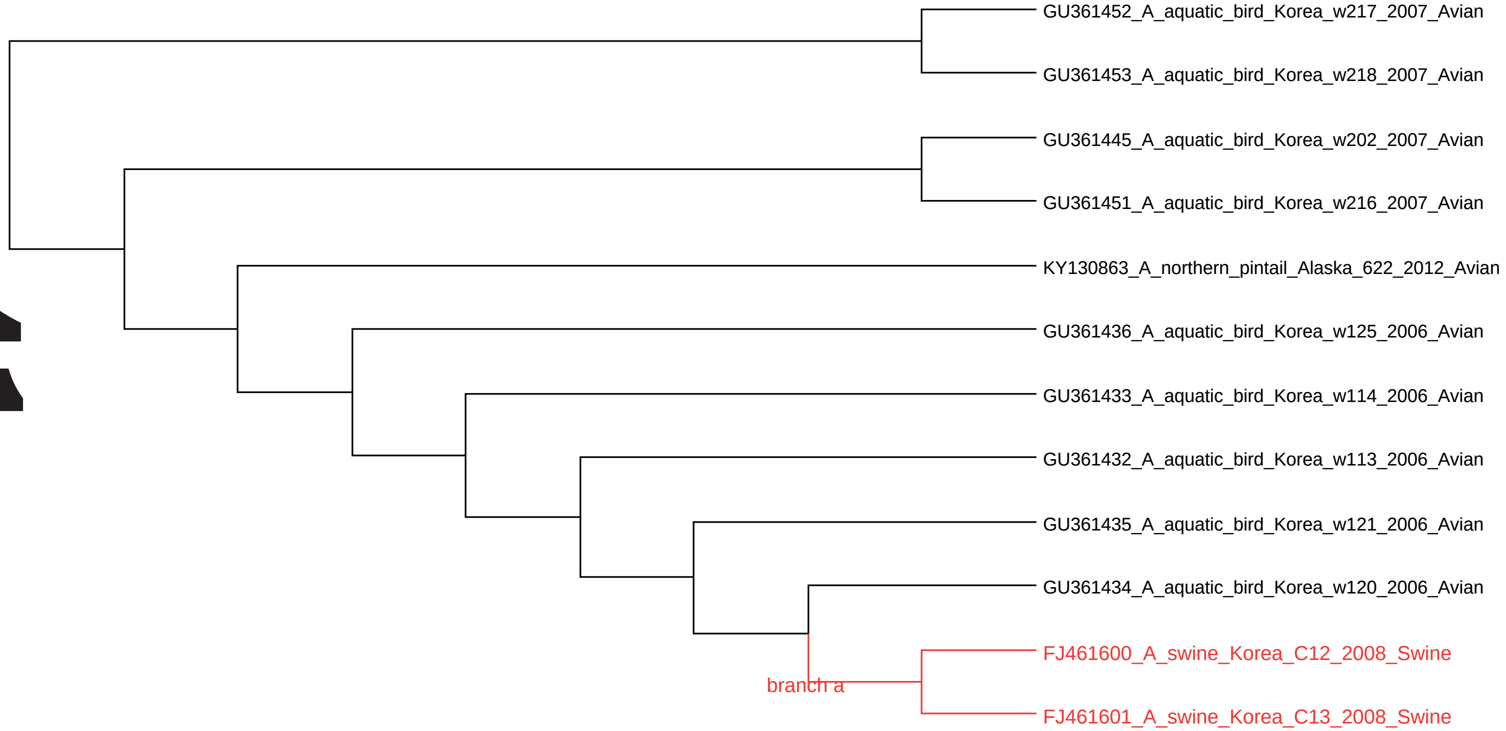

GU361452_A_aquatic_bird_Korea_w217_2007_Avian
GU361453_A_aquatic_bird_Korea_w218_2007_Avian
GU361445_A_aquatic_bird_Korea_w202_2007_Avian
GU361451_A_aquatic_bird_Korea_w216_2007_Avian
KY130863_A_northern_pintail_Alaska_622_2012_Avian
GU361436_A_aquatic_bird_Korea_w125_2006_Avian
GU361433_A_aquatic_bird_Korea_w114_2006_Avian
GU361432_A_aquatic_bird_Korea_w113_2006_Avian
GU361435_A_aquatic_bird_Korea_w121_2006_Avian
GU361434_A_aquatic_bird_Korea_w120_2006_Avian
FJ461600_A_swine_Korea_C12_2008_Swine
branch a
FJ461601_A_swine_Korea_C13_2008_Swine

NS-Group42

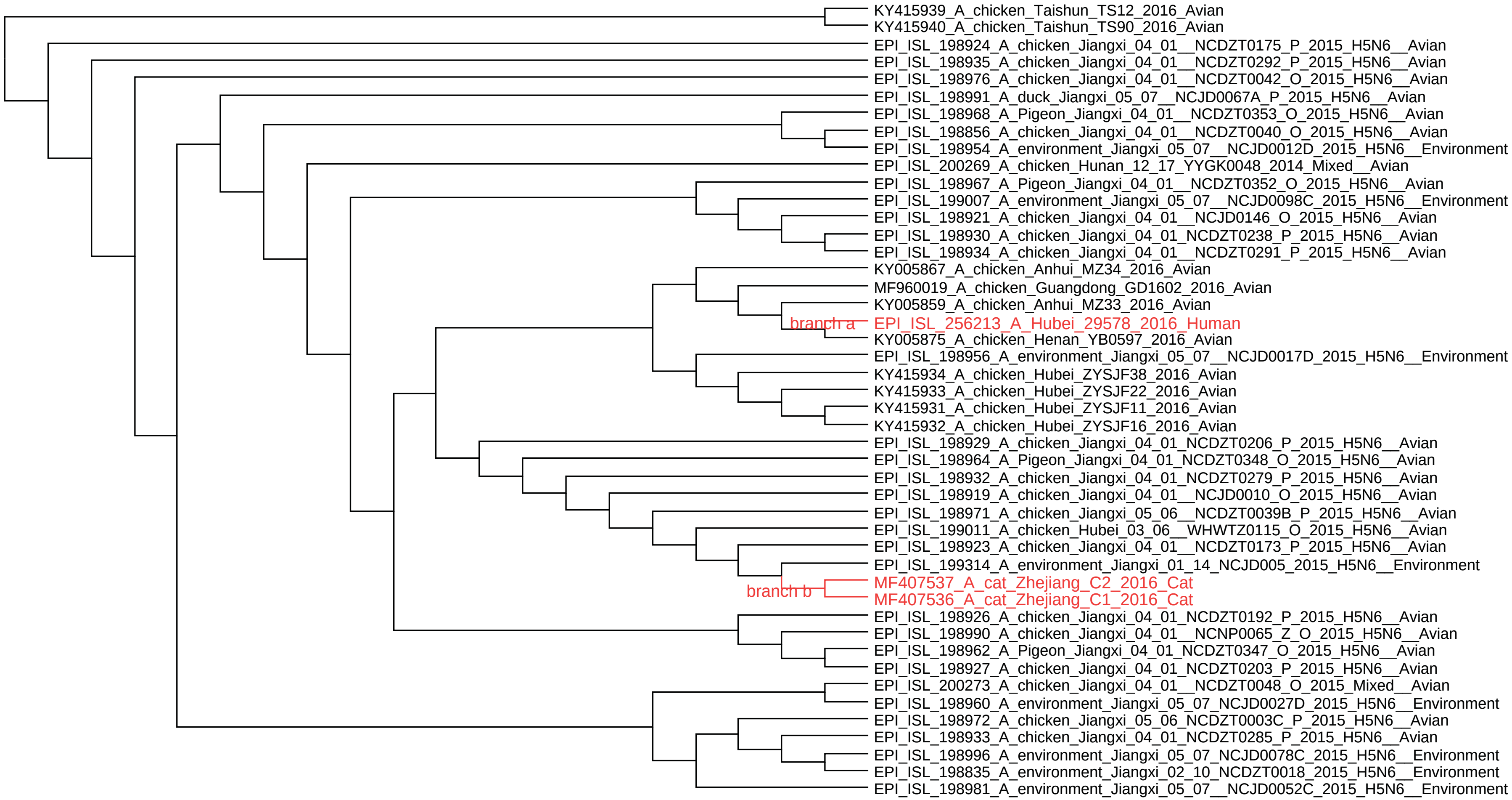

KY415939_A_chicken_Taishun_TS12_2016_Avian
KY415940_A_chicken_Taishun_TS90_2016_Avian
EPI_ISL_198924_A_chicken_Jiangxi_04_01__NCDZT0175_P_2015_H5N6__Avian
EPI_ISL_198935_A_chicken_Jiangxi_04_01__NCDZT0292_P_2015_H5N6__Avian
EPI_ISL_198976_A_chicken_Jiangxi_04_01__NCDZT0042_O_2015_H5N6__Avian
EPI_ISL_198991_A_duck_Jiangxi_05_07__NCJD0067A_P_2015_H5N6__Avian
EPI_ISL_198968_A_Pigeon_Jiangxi_04_01__NCDZT0353_O_2015_H5N6__Avian
EPI_ISL_198856_A_chicken_Jiangxi_04_01__NCDZT0040_O_2015_H5N6__Avian
EPI_ISL_198954_A_environment_Jiangxi_05_07__NCJD0012D_2015_H5N6__Environment
EPI_ISL_200269_A_chicken_Hunan_12_17_YYGK0048_2014_Mixed__Avian
EPI_ISL_198967_A_Pigeon_Jiangxi_04_01__NCDZT0352_O_2015_H5N6__Avian
EPI_ISL_199007_A_environment_Jiangxi_05_07__NCJD0098C_2015_H5N6__Environment
EPI_ISL_198921_A_chicken_Jiangxi_04_01__NCJD0146_O_2015_H5N6__Avian
EPI_ISL_198930_A_chicken_Jiangxi_04_01_NCDZT0238_P_2015_H5N6__Avian
EPI_ISL_198934_A_chicken_Jiangxi_04_01__NCDZT0291_P_2015_H5N6__Avian
KY005867_A_chicken_Anhui_MZ34_2016_Avian
MF960019_A_chicken_Guangdong_GD1602_2016_Avian
KY005859_A_chicken_Anhui_MZ33_2016_Avian
branch a
EPI_ISL_256213_A_Hubei_29578_2016_Human
KY005875_A_chicken_Henan_YB0597_2016_Avian
EPI_ISL_198956_A_environment_Jiangxi_05_07__NCJD0017D_2015_H5N6__Environment
KY415934_A_chicken_Hubei_ZYSJF38_2016_Avian
KY415933_A_chicken_Hubei_ZYSJF22_2016_Avian
KY415931_A_chicken_Hubei_ZYSJF11_2016_Avian
KY415932_A_chicken_Hubei_ZYSJF16_2016_Avian
EPI_ISL_198929_A_chicken_Jiangxi_04_01_NCDZT0206_P_2015_H5N6__Avian
EPI_ISL_198964_A_Pigeon_Jiangxi_04_01_NCDZT0348_O_2015_H5N6__Avian
EPI_ISL_198932_A_chicken_Jiangxi_04_01_NCDZT0279_P_2015_H5N6__Avian
EPI_ISL_198919_A_chicken_Jiangxi_04_01__NCJD0010_O_2015_H5N6__Avian
EPI_ISL_198971_A_chicken_Jiangxi_05_06__NCDZT0039B_P_2015_H5N6__Avian
EPI_ISL_199011_A_chicken_Hubei_03_06__WHWTZ0115_O_2015_H5N6__Avian
EPI_ISL_198923_A_chicken_Jiangxi_04_01__NCDZT0173_P_2015_H5N6__Avian
EPI_ISL_199314_A_environment_Jiangxi_01_14_NCJD005_2015_H5N6__Environment
MF407537_A_cat_Zhejiang_C2_2016_Cat
branch b
MF407536_A_cat_Zhejiang_C1_2016_Cat
EPI_ISL_198926_A_chicken_Jiangxi_04_01_NCDZT0192_P_2015_H5N6__Avian
EPI_ISL_198990_A_chicken_Jiangxi_04_01__NCNP0065_Z_O_2015_H5N6__Avian
EPI_ISL_198962_A_Pigeon_Jiangxi_04_01_NCDZT0347_O_2015_H5N6__Avian
EPI_ISL_198927_A_chicken_Jiangxi_04_01_NCDZT0203_P_2015_H5N6__Avian
EPI_ISL_200273_A_chicken_Jiangxi_04_01__NCDZT0048_O_2015_Mixed__Avian
EPI_ISL_198960_A_environment_Jiangxi_05_07_NCJD0027D_2015_H5N6__Environment
EPI_ISL_198972_A_chicken_Jiangxi_05_06_NCDZT0003C_P_2015_H5N6__Avian
EPI_ISL_198933_A_chicken_Jiangxi_04_01_NCDZT0285_P_2015_H5N6__Avian
EPI_ISL_198996_A_environment_Jiangxi_05_07_NCJD0078C_2015_H5N6__Environment
EPI_ISL_198835_A_environment_Jiangxi_02_10_NCDZT0018_2015_H5N6__Environment
EPI_ISL_198981_A_environment_Jiangxi_05_07__NCJD0052C_2015_H5N6__Environment

NS-Group43

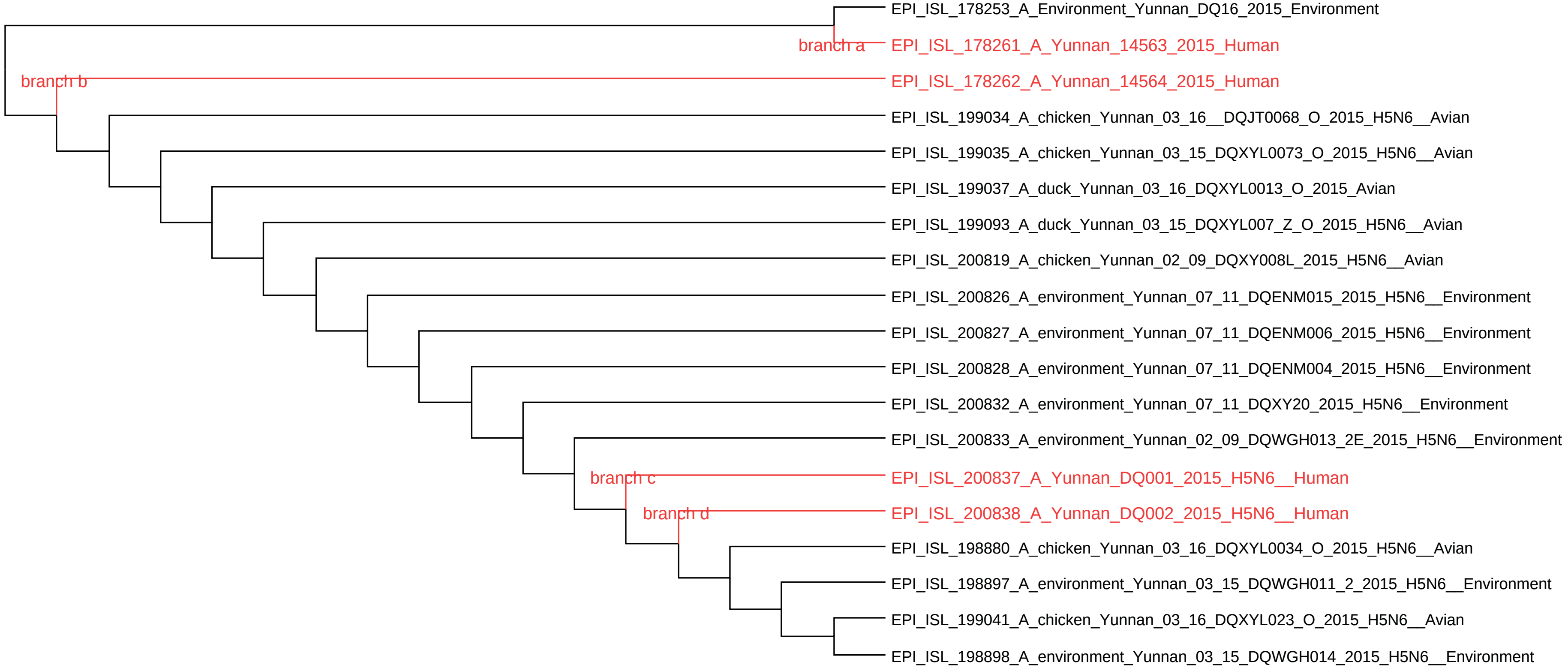

EPI_ISL_178253_A_Environment_Yunnan_DQ16_2015_Environment
branch a
EPI_ISL_178261_A_Yunnan_14563_2015_Human
branch b
EPI_ISL_178262_A_Yunnan_14564_2015_Human
EPI_ISL_199034_A_chicken_Yunnan_03_16__DQJT0068_O_2015_H5N6__Avian
EPI_ISL_199035_A_chicken_Yunnan_03_15_DQXYL0073_O_2015_H5N6__Avian
EPI_ISL_199037_A_duck_Yunnan_03_16_DQXYL0013_O_2015_Avian
EPI_ISL_199093_A_duck_Yunnan_03_15_DQXYL007_Z_O_2015_H5N6__Avian
EPI_ISL_200819_A_chicken_Yunnan_02_09_DQXY008L_2015_H5N6__Avian
EPI_ISL_200826_A_environment_Yunnan_07_11_DQENM015_2015_H5N6__Environment
EPI_ISL_200827_A_environment_Yunnan_07_11_DQENM006_2015_H5N6__Environment
EPI_ISL_200828_A_environment_Yunnan_07_11_DQENM004_2015_H5N6__Environment
EPI_ISL_200832_A_environment_Yunnan_07_11_DQXY20_2015_H5N6__Environment
EPI_ISL_200833_A_environment_Yunnan_02_09_DQWGH013_2E_2015_H5N6__Environment
branch c
EPI_ISL_200837_A_Yunnan_DQ001_2015_H5N6__Human
branch d
EPI_ISL_200838_A_Yunnan_DQ002_2015_H5N6__Human
EPI_ISL_198880_A_chicken_Yunnan_03_16_DQXYL0034_O_2015_H5N6__Avian
EPI_ISL_198897_A_environment_Yunnan_03_15_DQWGH011_2_2015_H5N6__Environment
EPI_ISL_199041_A_chicken_Yunnan_03_16_DQXYL023_O_2015_H5N6__Avian
EPI_ISL_198898_A_environment_Yunnan_03_15_DQWGH014_2015_H5N6__Environment

NS-Group44

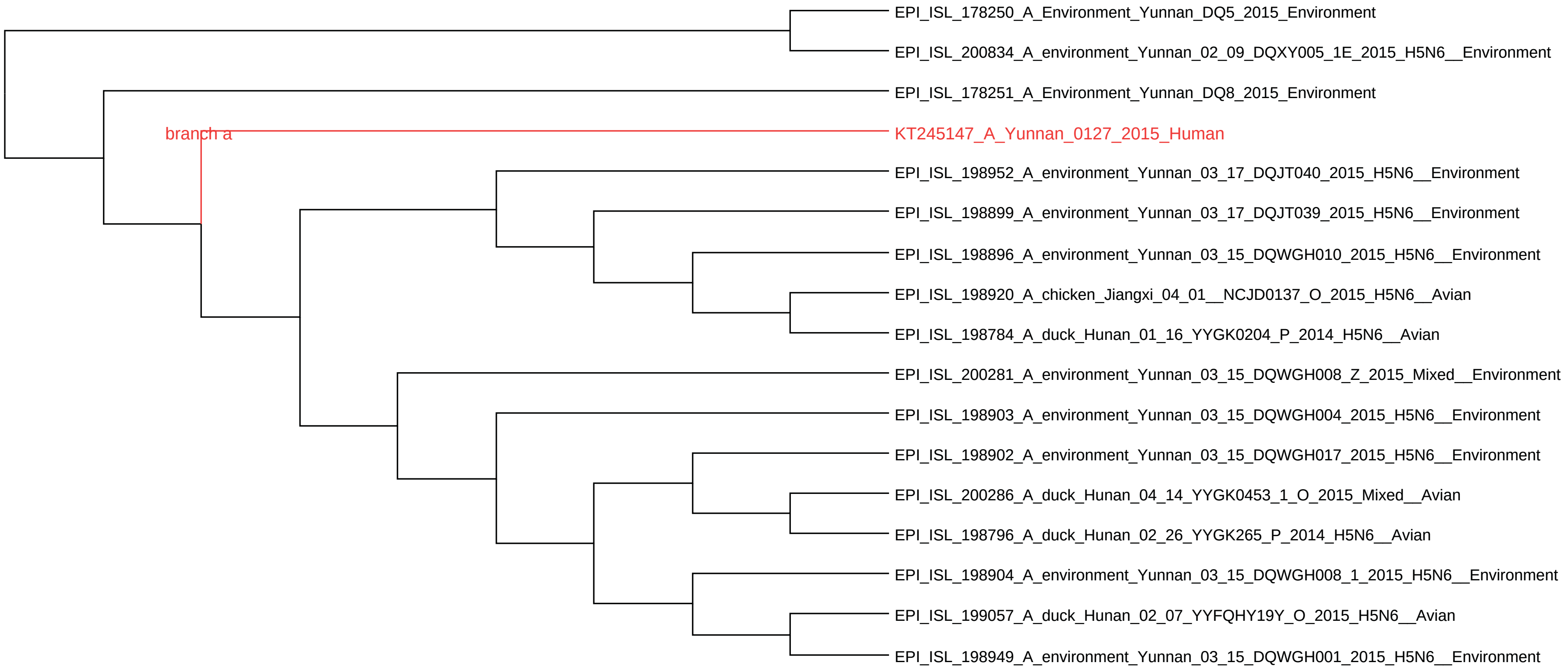

branch a
EPI_ISL_178250_A_Environment_Yunnan_DQ5_2015_Environment
EPI_ISL_200834_A_environment_Yunnan_02_09_DQXY005_1E_2015_H5N6__Environment
EPI_ISL_178251_A_Environment_Yunnan_DQ8_2015_Environment
KT245147_A_Yunnan_0127_2015_Human
EPI_ISL_198952_A_environment_Yunnan_03_17_DQJT040_2015_H5N6__Environment
EPI_ISL_198899_A_environment_Yunnan_03_17_DQJT039_2015_H5N6__Environment
EPI_ISL_198896_A_environment_Yunnan_03_15_DQWGH010_2015_H5N6__Environment
EPI_ISL_198920_A_chicken_Jiangxi_04_01__NCJD0137_O_2015_H5N6__Avian
EPI_ISL_198784_A_duck_Hunan_01_16_YYGK0204_P_2014_H5N6__Avian
EPI_ISL_200281_A_environment_Yunnan_03_15_DQWGH008_Z_2015_Mixed__Environment
EPI_ISL_198903_A_environment_Yunnan_03_15_DQWGH004_2015_H5N6__Environment
EPI_ISL_198902_A_environment_Yunnan_03_15_DQWGH017_2015_H5N6__Environment
EPI_ISL_200286_A_duck_Hunan_04_14_YYGK0453_1_O_2015_Mixed__Avian
EPI_ISL_198796_A_duck_Hunan_02_26_YYGK265_P_2014_H5N6__Avian
EPI_ISL_198904_A_environment_Yunnan_03_15_DQWGH008_1_2015_H5N6__Environment
EPI_ISL_199057_A_duck_Hunan_02_07_YYFQHY19Y_O_2015_H5N6__Avian
EPI_ISL_198949_A_environment_Yunnan_03_15_DQWGH001_2015_H5N6__Environment

NS-Group45

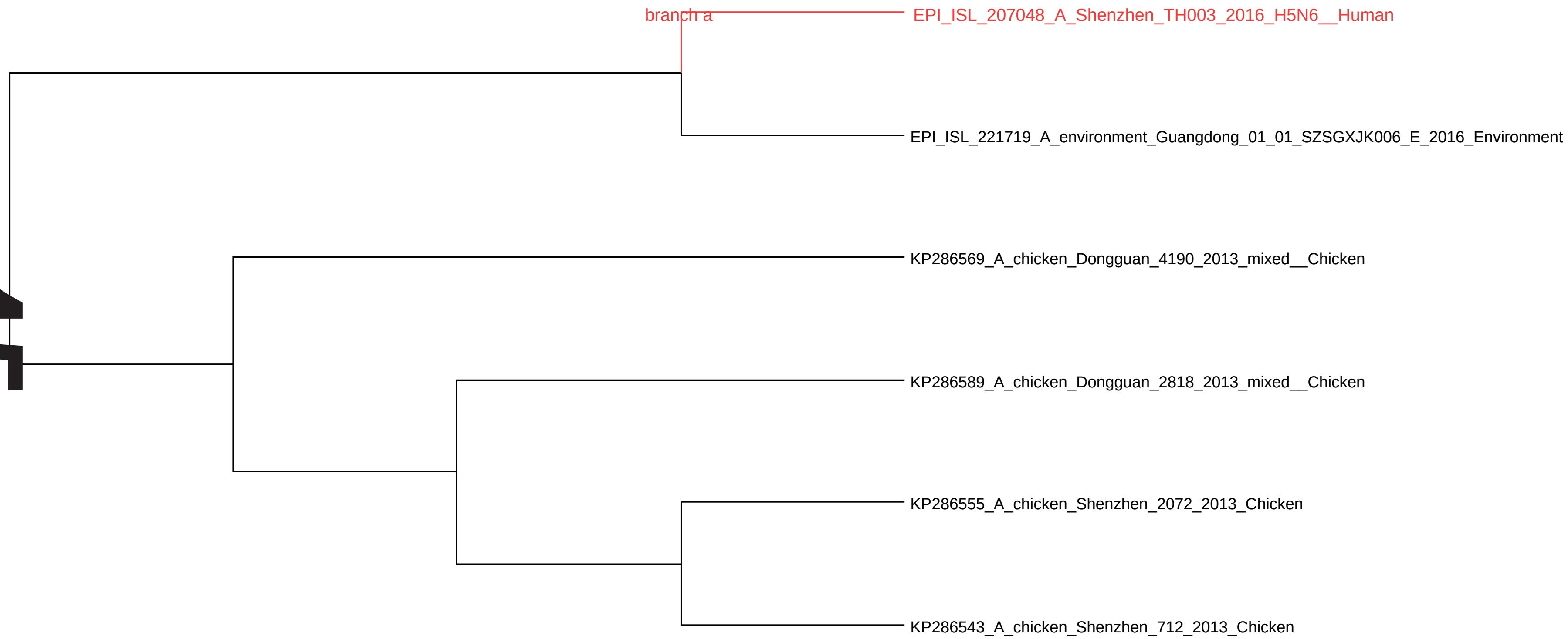

branch a
EPI_ISL_207048_A_Shenzhen_TH003_2016_H5N6__Human
EPI_ISL_221719_A_environment_Guangdong_01_01_SZSGXJK006_E_2016_Environment
KP286569_A_chicken_Dongguan_4190_2013_mixed__Chicken
KP286589_A_chicken_Dongguan_2818_2013_mixed__Chicken
KP286555_A_chicken_Shenzhen_2072_2013_Chicken
KP286543_A_chicken_Shenzhen_712_2013_Chicken

NS-Group46

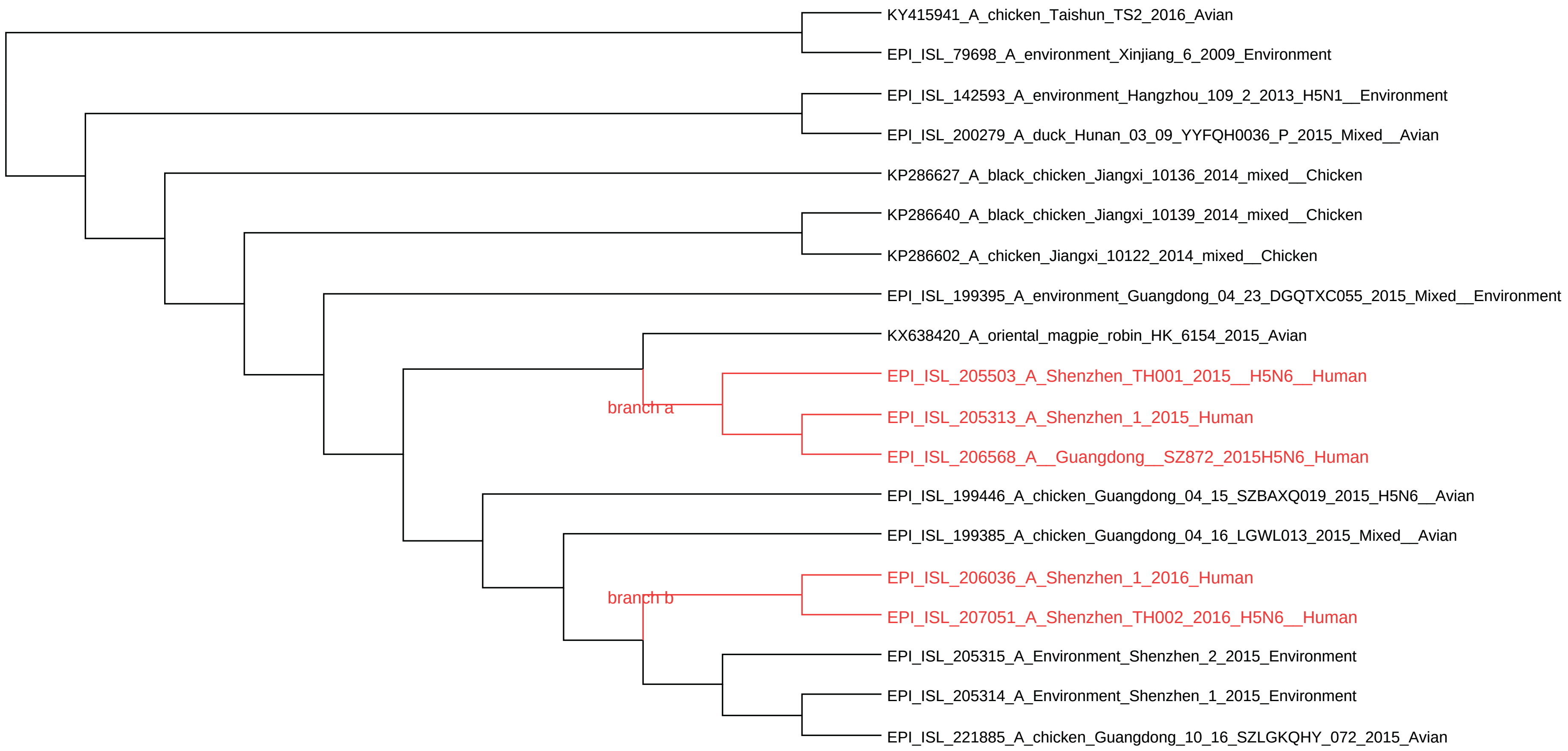

KY415941_A_chicken_Taishun_TS2_2016_Avian
EPI_ISL_79698_A_environment_Xinjiang_6_2009_Environment
EPI_ISL_142593_A_environment_Hangzhou_109_2_2013_H5N1__Environment
EPI_ISL_200279_A_duck_Hunan_03_09_YYFQH0036_P_2015_Mixed__Avian
KP286627_A_black_chicken_Jiangxi_10136_2014_mixed__Chicken
KP286640_A_black_chicken_Jiangxi_10139_2014_mixed__Chicken
KP286602_A_chicken_Jiangxi_10122_2014_mixed__Chicken
EPI_ISL_199395_A_environment_Guangdong_04_23_DGQTXC055_2015_Mixed__Environment
KX638420_A_oriental_magpie_robin_HK_6154_2015_Avian
EPI_ISL_205503_A_Shenzhen_TH001_2015__H5N6__Human
branch a
EPI_ISL_205313_A_Shenzhen_1_2015_Human
EPI_ISL_206568_A__Guangdong__SZ872_2015H5N6_Human
EPI_ISL_199446_A_chicken_Guangdong_04_15_SZBAXQ019_2015_H5N6__Avian
EPI_ISL_199385_A_chicken_Guangdong_04_16_LGWL013_2015_Mixed__Avian
EPI_ISL_206036_A_Shenzhen_1_2016_Human
branch b
EPI_ISL_207051_A_Shenzhen_TH002_2016_H5N6__Human
EPI_ISL_205315_A_Environment_Shenzhen_2_2015_Environment
EPI_ISL_205314_A_Environment_Shenzhen_1_2015_Environment
EPI_ISL_221885_A_chicken_Guangdong_10_16_SZLGKQHY_072_2015_Avian

NS-Group47

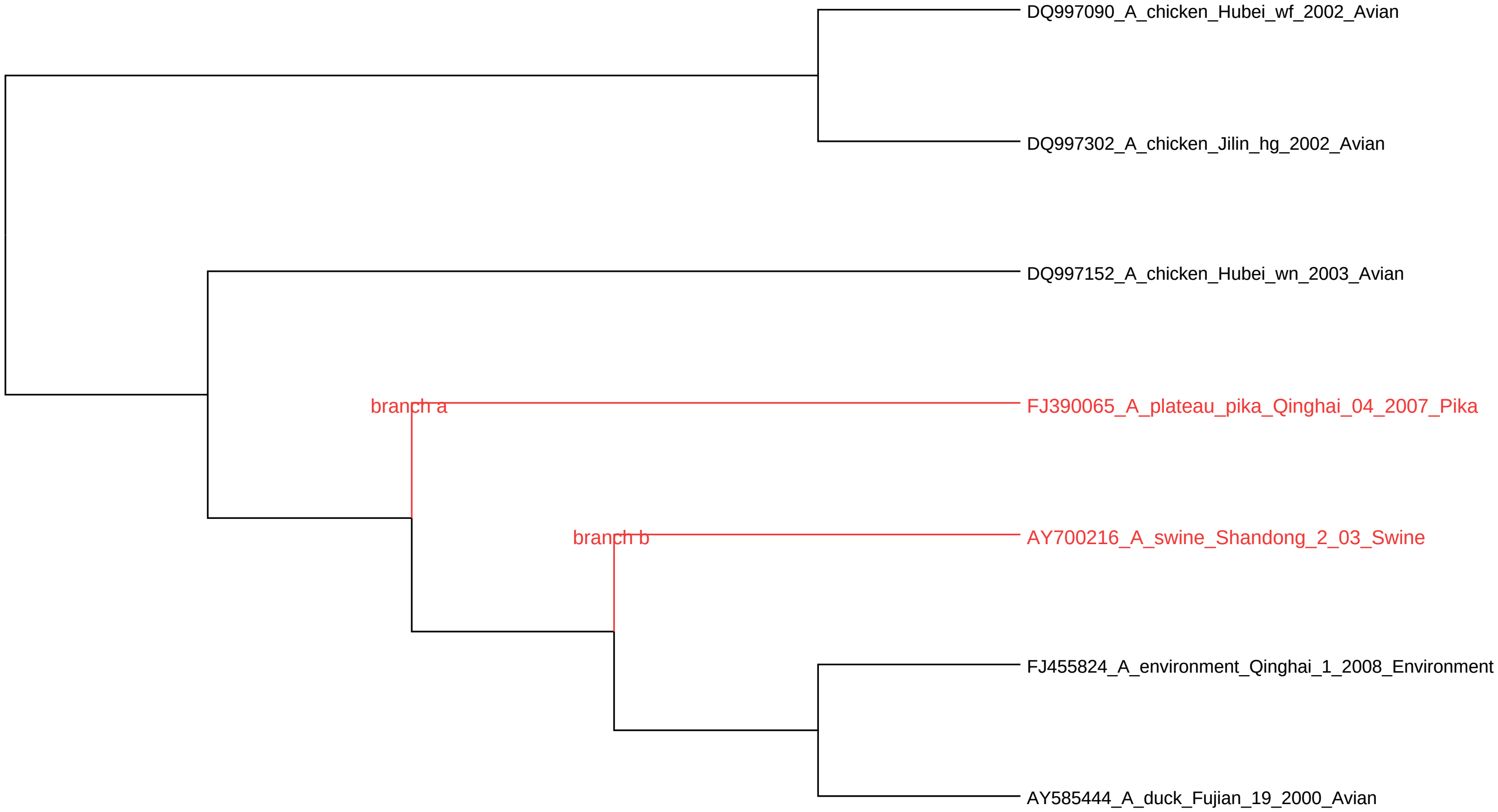

DQ997090_A_chicken_Hubei_wf_2002_Avian
DQ997302_A_chicken_Jilin_hg_2002_Avian
DQ997152_A_chicken_Hubei_wn_2003_Avian
branch a
FJ390065_A_plateau_pika_Qinghai_04_2007_Pika
branch b
AY700216_A_swine_Shandong_2_03_Swine
FJ455824_A_environment_Qinghai_1_2008_Environment
AY585444_A_duck_Fujian_19_2000_Avian

NS-Group48

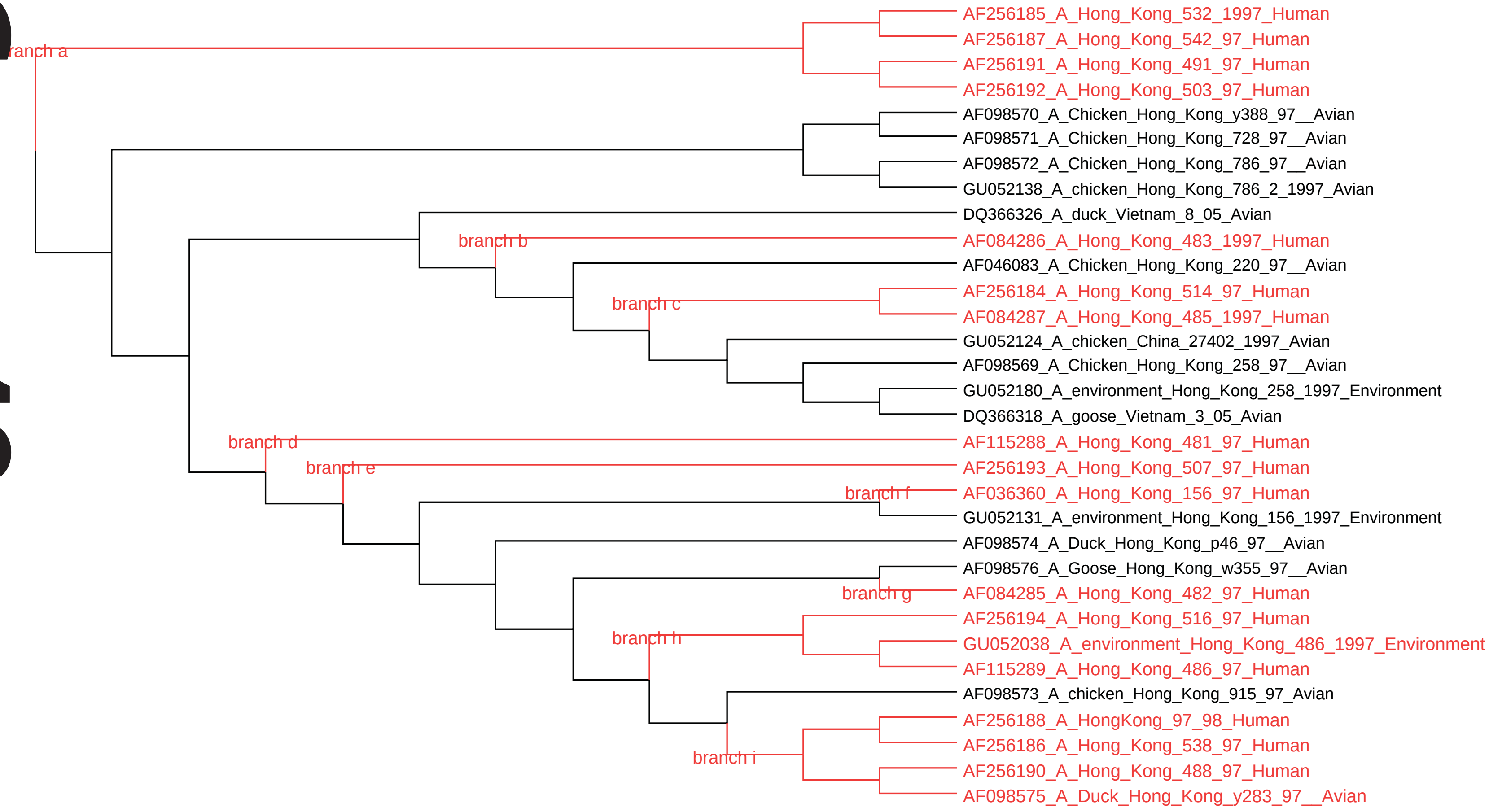

branch a
AF256185_A_Hong_Kong_532_1997_Human
AF256187_A_Hong_Kong_542_97_Human
AF256191_A_Hong_Kong_491_97_Human
AF256192_A_Hong_Kong_503_97_Human
AF098570_A_Chicken_Hong_Kong_y388_97__Avian
AF098571_A_Chicken_Hong_Kong_728_97__Avian
AF098572_A_Chicken_Hong_Kong_786_97__Avian
GU052138_A_chicken_Hong_Kong_786_2_1997_Avian
DQ366326_A_duck_Vietnam_8_05_Avian
branch b
AF084286_A_Hong_Kong_483_1997_Human
AF046083_A_Chicken_Hong_Kong_220_97__Avian
branch c
AF256184_A_Hong_Kong_514_97_Human
AF084287_A_Hong_Kong_485_1997_Human
GU052124_A_chicken_China_27402_1997_Avian
AF098569_A_Chicken_Hong_Kong_258_97__Avian
GU052180_A_environment_Hong_Kong_258_1997_Environment
DQ366318_A_goose_Vietnam_3_05_Avian
branch d
AF115288_A_Hong_Kong_481_97_Human
branch e
AF256193_A_Hong_Kong_507_97_Human
branch f
AF036360_A_Hong_Kong_156_97_Human
GU052131_A_environment_Hong_Kong_156_1997_Environment
AF098574_A_Duck_Hong_Kong_p46_97__Avian
AF098576_A_Goose_Hong_Kong_w355_97__Avian
branch g
AF084285_A_Hong_Kong_482_97_Human
branch h
AF256194_A_Hong_Kong_516_97_Human
GU052038_A_environment_Hong_Kong_486_1997_Environment
AF115289_A_Hong_Kong_486_97_Human
AF098573_A_chicken_Hong_Kong_915_97_Avian
AF256188_A_HongKong_97_98_Human
branch i
AF256186_A_Hong_Kong_538_97_Human
AF256190_A_Hong_Kong_488_97_Human
AF098575_A_Duck_Hong_Kong_y283_97__Avian

NS-Group49

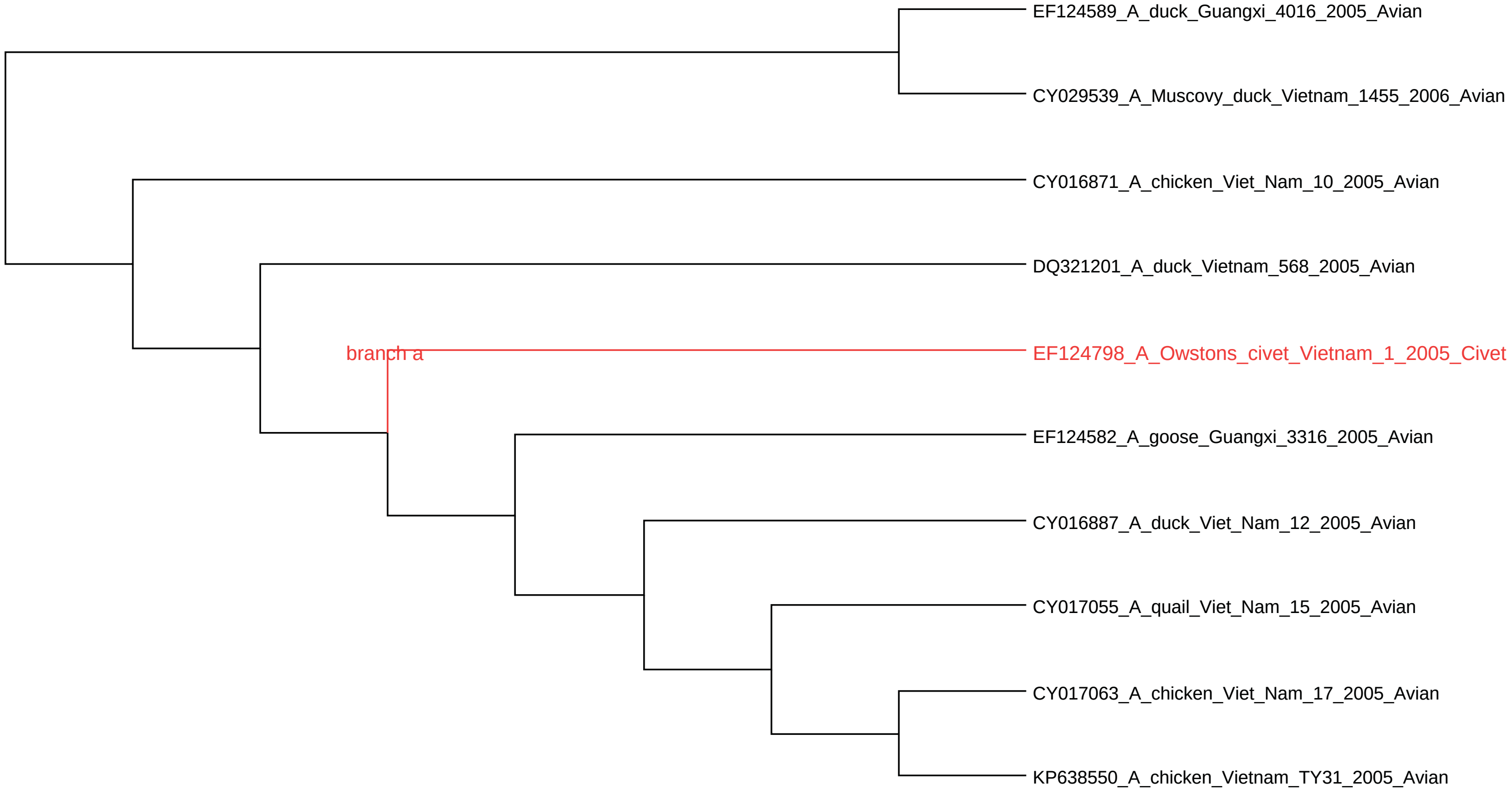

EF124589_A_duck_Guangxi_4016_2005_Avian
CY029539_A_Muscovy_duck_Vietnam_1455_2006_Avian
CY016871_A_chicken_Viet_Nam_10_2005_Avian
DQ321201_A_duck_Vietnam_568_2005_Avian
branch a
EF124798_A_Owstons_civet_Vietnam_1_2005_Civet
EF124582_A_goose_Guangxi_3316_2005_Avian
CY016887_A_duck_Viet_Nam_12_2005_Avian
CY017055_A_quail_Viet_Nam_15_2005_Avian
CY017063_A_chicken_Viet_Nam_17_2005_Avian
KP638550_A_chicken_Vietnam_TY31_2005_Avian

NS-Group50

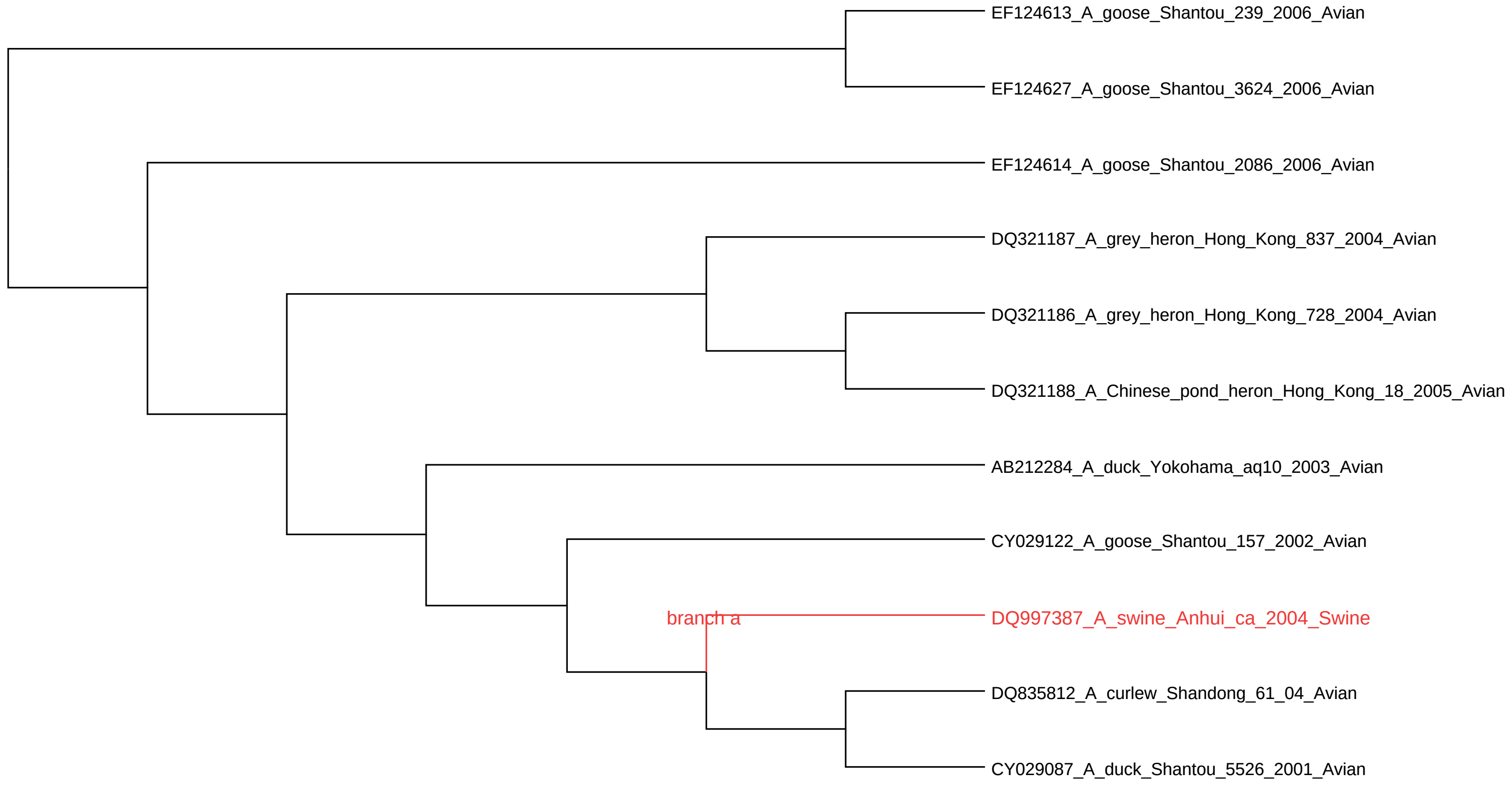

EF124613_A_goose_Shantou_239_2006_Avian
EF124627_A_goose_Shantou_3624_2006_Avian
EF124614_A_goose_Shantou_2086_2006_Avian
DQ321187_A_grey_heron_Hong_Kong_837_2004_Avian
DQ321186_A_grey_heron_Hong_Kong_728_2004_Avian
DQ321188_A_Chinese_pond_heron_Hong_Kong_18_2005_Avian
AB212284_A_duck_Yokohama_aq10_2003_Avian
CY029122_A_goose_Shantou_157_2002_Avian
branch a
DQ997387_A_swine_Anhui_ca_2004_Swine
DQ835812_A_curlew_Shandong_61_04_Avian
CY029087_A_duck_Shantou_5526_2001_Avian

NS-Group51

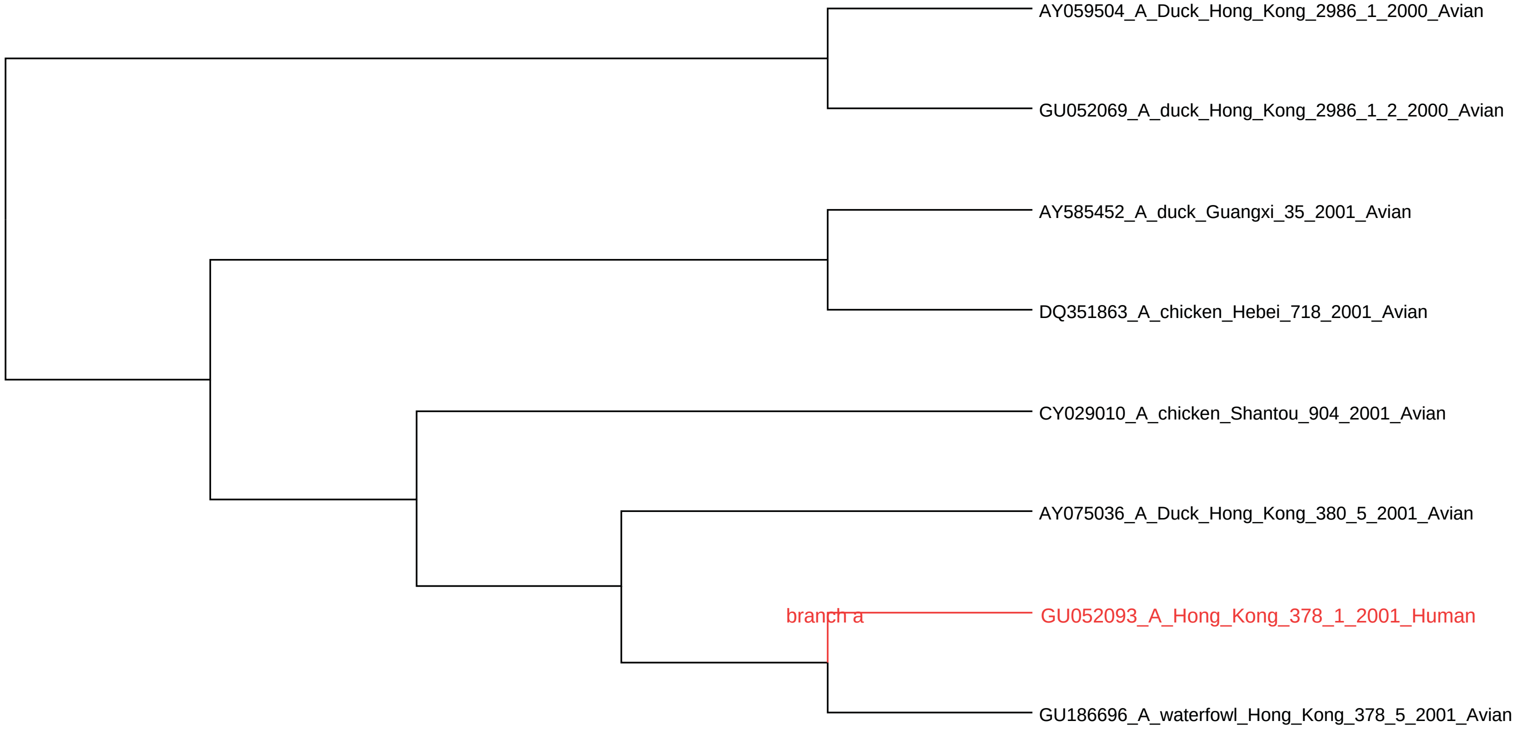
AY059504_A_Duck_Hong_Kong_2986_1_2000_Avian
GU052069_A_duck_Hong_Kong_2986_1_2_2000_Avian
AY585452_A_duck_Guangxi_35_2001_Avian
DQ351863_A_chicken_Hebei_718_2001_Avian
CY029010_A_chicken_Shantou_904_2001_Avian
AY075036_A_Duck_Hong_Kong_380_5_2001_Avian
branch a
GU052093_A_Hong_Kong_378_1_2001_Human
GU186696_A_waterfowl_Hong_Kong_378_5_2001_Avian

NS-Group52

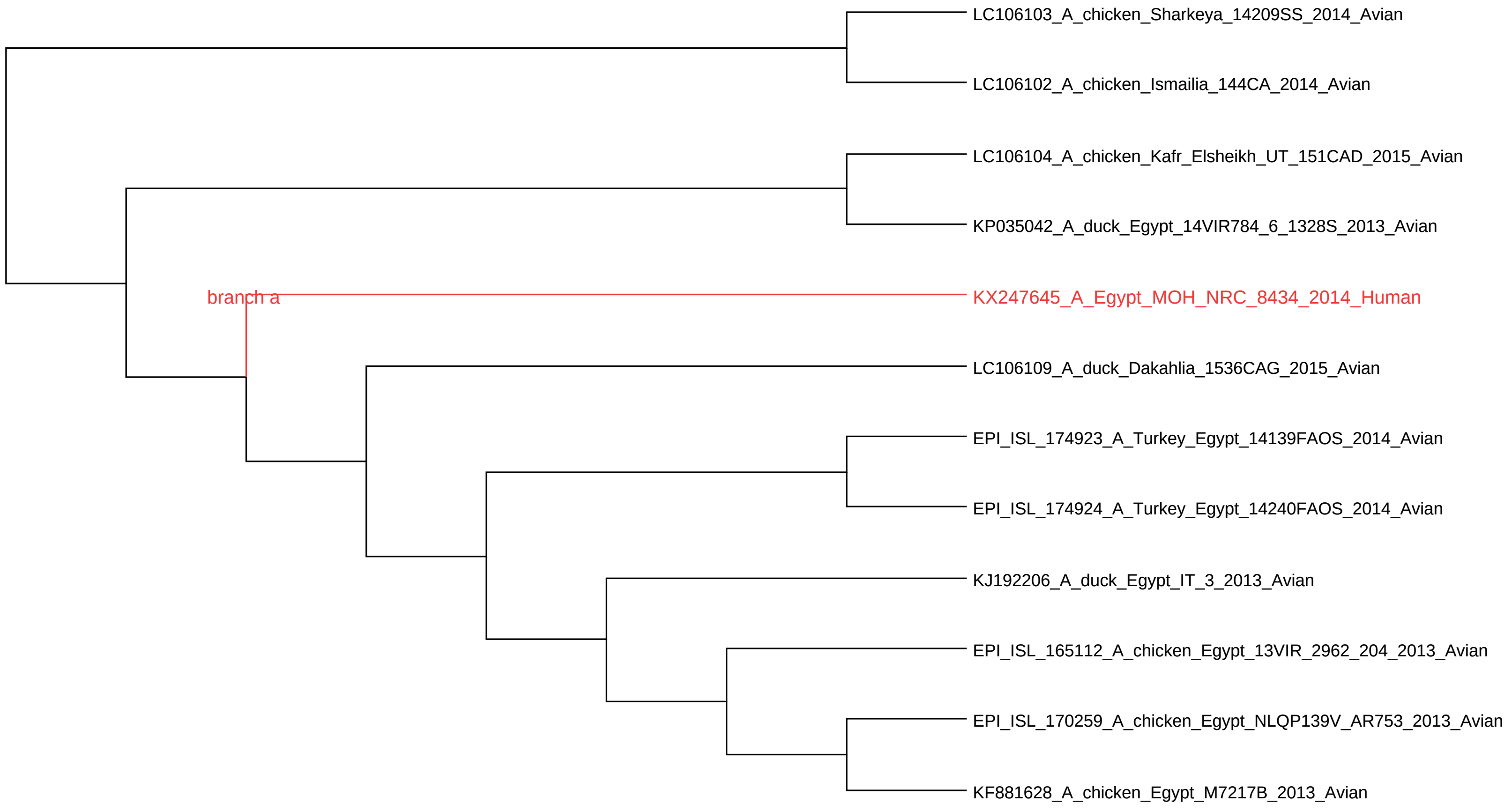

LC106103_A_chicken_Sharkeya_14209SS_2014_Avian
LC106102_A_chicken_Ismailia_144CA_2014_Avian
LC106104_A_chicken_Kafr_Elsheikh_UT_151CAD_2015_Avian
KP035042_A_duck_Egypt_14VIR784_6_1328S_2013_Avian
branch a
KX247645_A_Egypt_MOH_NRC_8434_2014_Human
LC106109_A_duck_Dakahlia_1536CAG_2015_Avian
EPI_ISL_174923_A_Turkey_Egypt_14139FAOS_2014_Avian
EPI_ISL_174924_A_Turkey_Egypt_14240FAOS_2014_Avian
KJ192206_A_duck_Egypt_IT_3_2013_Avian
EPI_ISL_165112_A_chicken_Egypt_13VIR_2962_204_2013_Avian
EPI_ISL_170259_A_chicken_Egypt_NLQP139V_AR753_2013_Avian
KF881628_A_chicken_Egypt_M7217B_2013_Avian

NS-Group53

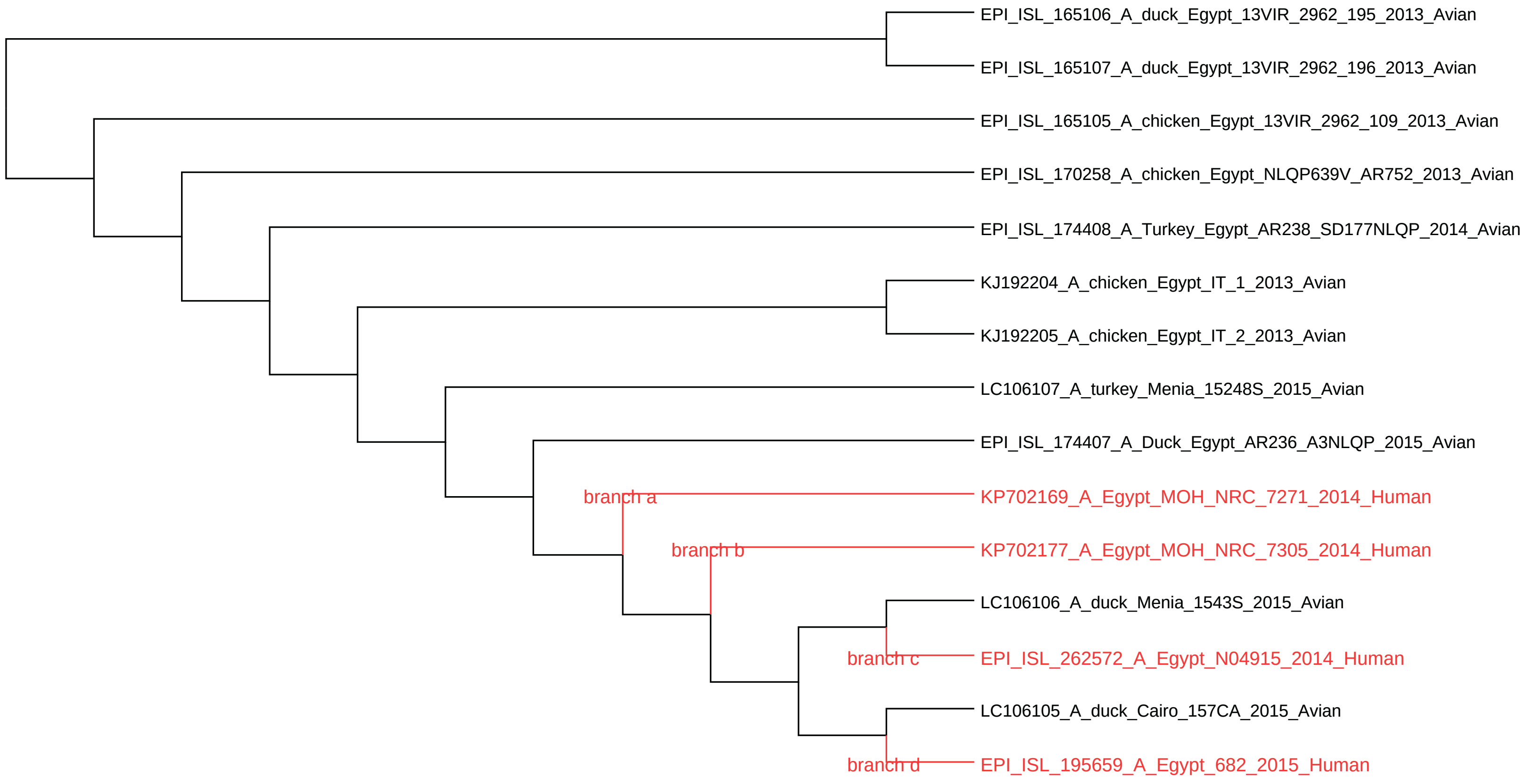
EPI_ISL_165106_A_duck_Egypt_13VIR_2962_195_2013_Avian
EPI_ISL_165107_A_duck_Egypt_13VIR_2962_196_2013_Avian
EPI_ISL_165105_A_chicken_Egypt_13VIR_2962_109_2013_Avian
EPI_ISL_170258_A_chicken_Egypt_NLQP639V_AR752_2013_Avian
EPI_ISL_174408_A_Turkey_Egypt_AR238_SD177NLQP_2014_Avian
KJ192204_A_chicken_Egypt_IT_1_2013_Avian
KJ192205_A_chicken_Egypt_IT_2_2013_Avian
LC106107_A_turkey_Menia_15248S_2015_Avian
EPI_ISL_174407_A_Duck_Egypt_AR236_A3NLQP_2015_Avian
branch a
KP702169_A_Egypt_MOH_NRC_7271_2014_Human
branch b
KP702177_A_Egypt_MOH_NRC_7305_2014_Human
LC106106_A_duck_Menia_1543S_2015_Avian
branch c
EPI_ISL_262572_A_Egypt_N04915_2014_Human
LC106105_A_duck_Cairo_157CA_2015_Avian
branch d
EPI_ISL_195659_A_Egypt_682_2015_Human

NS-Group54

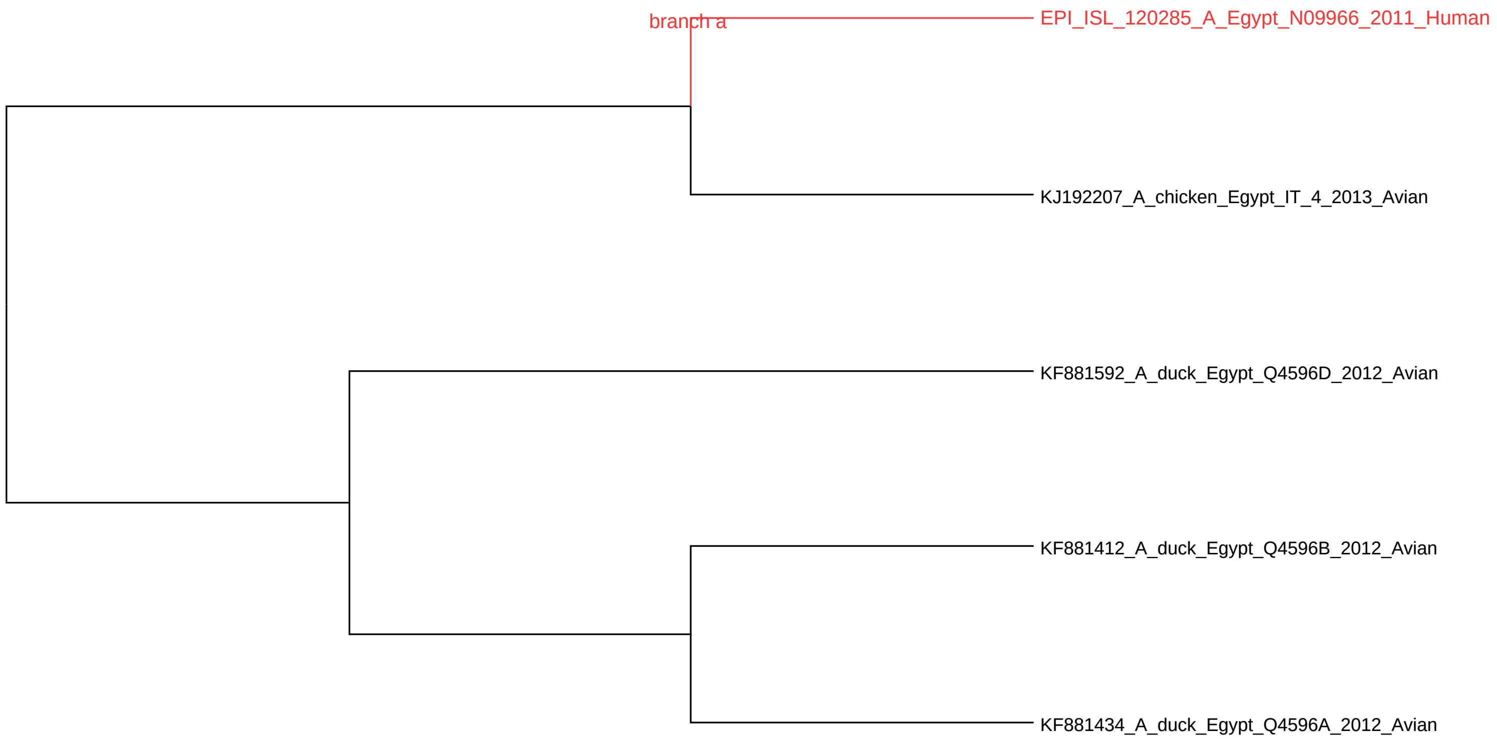
branch a
EPI_ISL_120285_A_Egypt_N09966_2011_Human
KJ192207_A_chicken_Egypt_IT_4_2013_Avian
KF881592_A_duck_Egypt_Q4596D_2012_Avian
KF881412_A_duck_Egypt_Q4596B_2012_Avian
KF881434_A_duck_Egypt_Q4596A_2012_Avian

NS-Group55

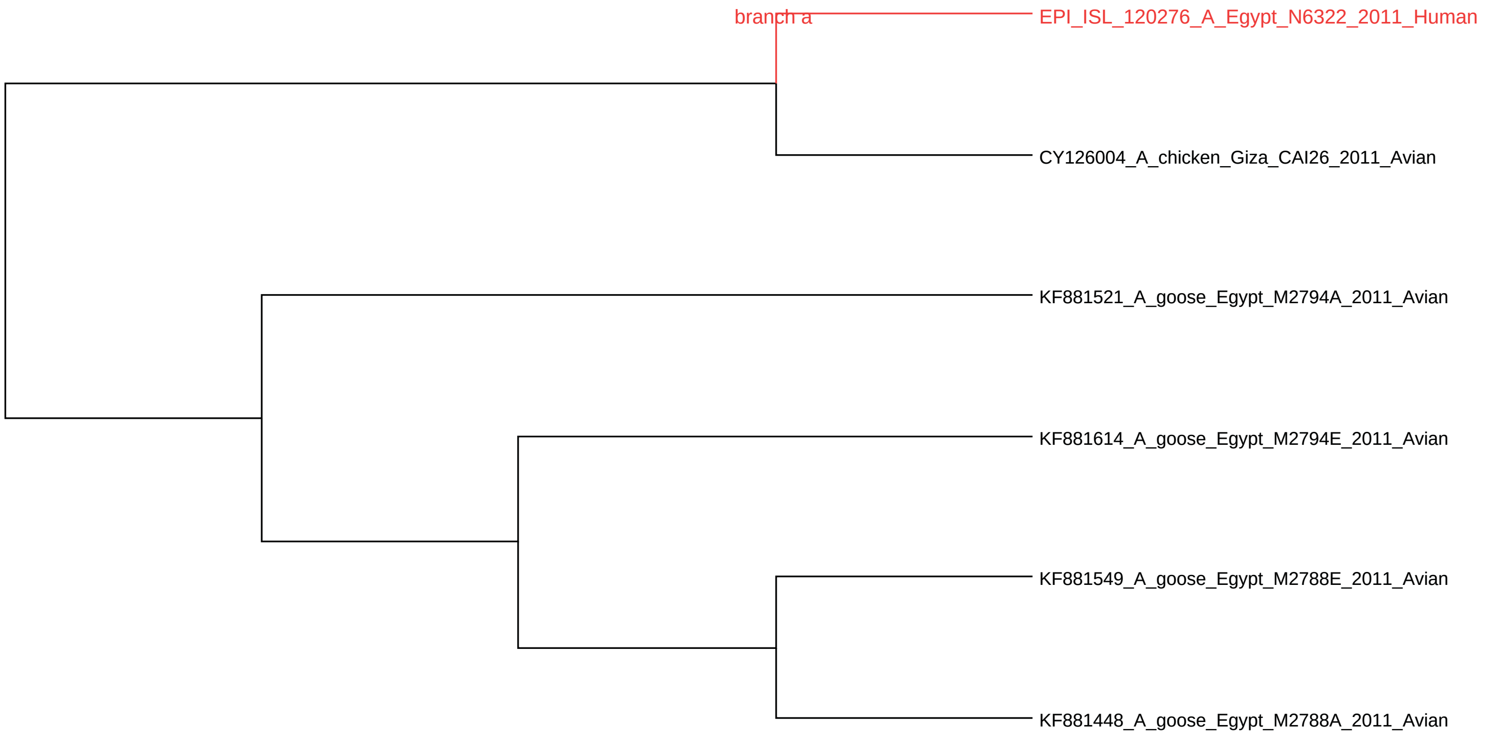

branch a
EPI_ISL_120276_A_Egypt_N6322_2011_Human
CY126004_A_chicken_Giza_CAI26_2011_Avian
KF881521_A_goose_Egypt_M2794A_2011_Avian
KF881614_A_goose_Egypt_M2794E_2011_Avian
KF881549_A_goose_Egypt_M2788E_2011_Avian
KF881448_A_goose_Egypt_M2788A_2011_Avian

NS-Group56

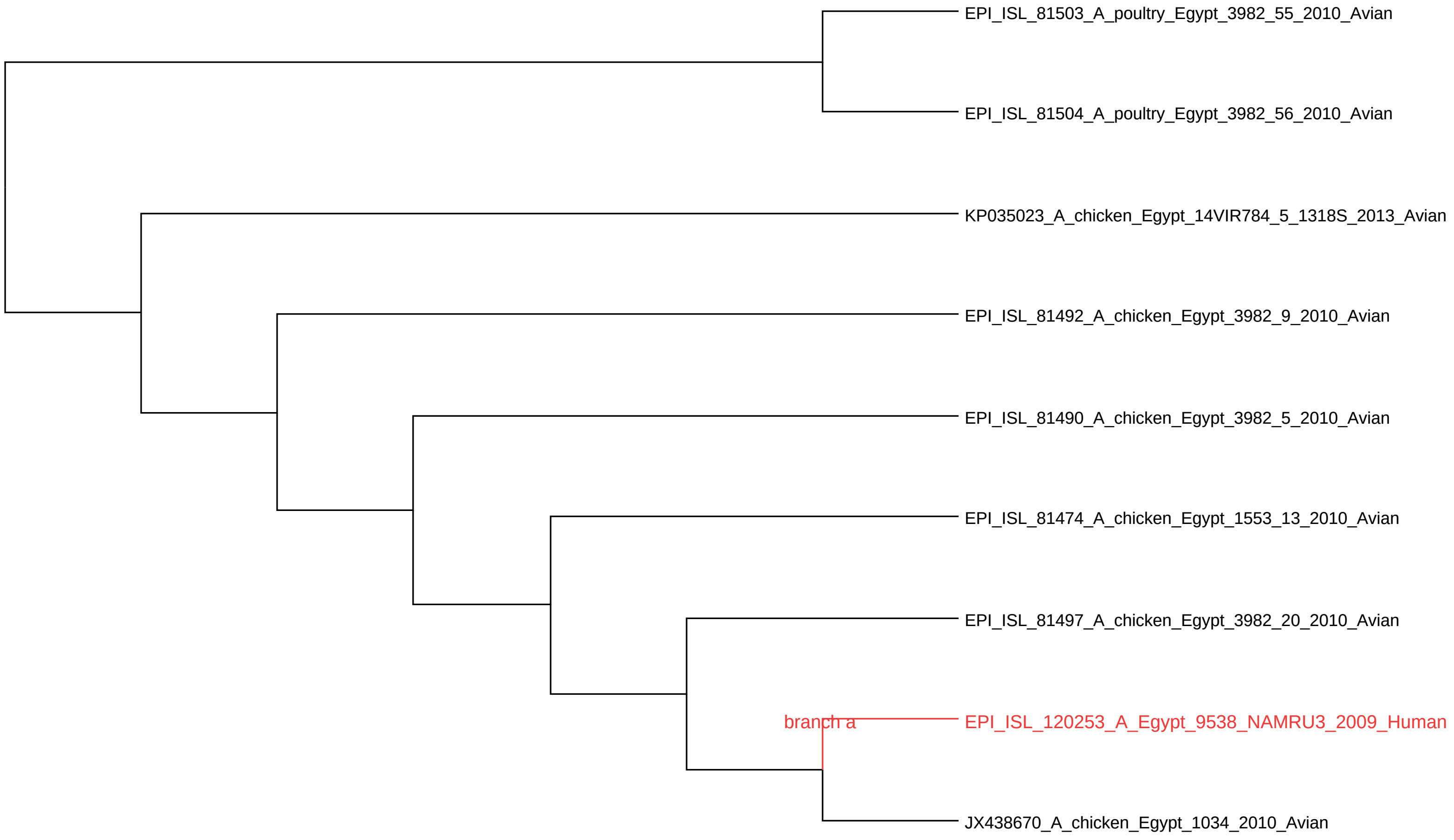

EPI_ISL_81503_A_poultry_Egypt_3982_55_2010_Avian
EPI_ISL_81504_A_poultry_Egypt_3982_56_2010_Avian
KP035023_A_chicken_Egypt_14VIR784_5_1318S_2013_Avian
EPI_ISL_81492_A_chicken_Egypt_3982_9_2010_Avian
EPI_ISL_81490_A_chicken_Egypt_3982_5_2010_Avian
EPI_ISL_81474_A_chicken_Egypt_1553_13_2010_Avian
EPI_ISL_81497_A_chicken_Egypt_3982_20_2010_Avian
branch a
EPI_ISL_120253_A_Egypt_9538_NAMRU3_2009_Human
JX438670_A_chicken_Egypt_1034_2010_Avian

NS-Group57

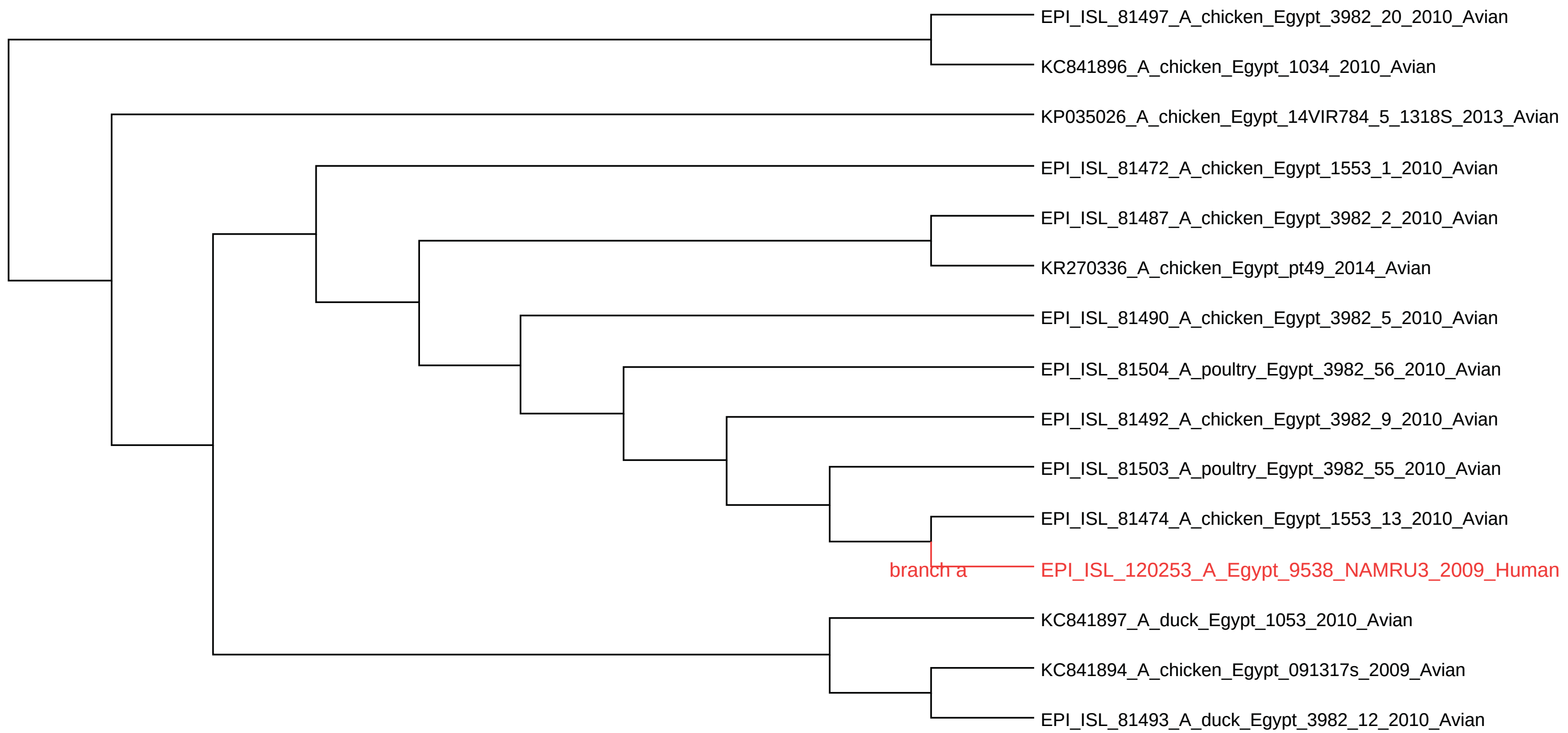

EPI_ISL_81497_A_chicken_Egypt_3982_20_2010_Avian
KC841896_A_chicken_Egypt_1034_2010_Avian
KP035026_A_chicken_Egypt_14VIR784_5_1318S_2013_Avian
EPI_ISL_81472_A_chicken_Egypt_1553_1_2010_Avian
EPI_ISL_81487_A_chicken_Egypt_3982_2_2010_Avian
KR270336_A_chicken_Egypt_pt49_2014_Avian
EPI_ISL_81490_A_chicken_Egypt_3982_5_2010_Avian
EPI_ISL_81504_A_poultry_Egypt_3982_56_2010_Avian
EPI_ISL_81492_A_chicken_Egypt_3982_9_2010_Avian
EPI_ISL_81503_A_poultry_Egypt_3982_55_2010_Avian
EPI_ISL_81474_A_chicken_Egypt_1553_13_2010_Avian
branch a
EPI_ISL_120253_A_Egypt_9538_NAMRU3_2009_Human
KC841897_A_duck_Egypt_1053_2010_Avian
KC841894_A_chicken_Egypt_091317s_2009_Avian
EPI_ISL_81493_A_duck_Egypt_3982_12_2010_Avian

NS-Group58

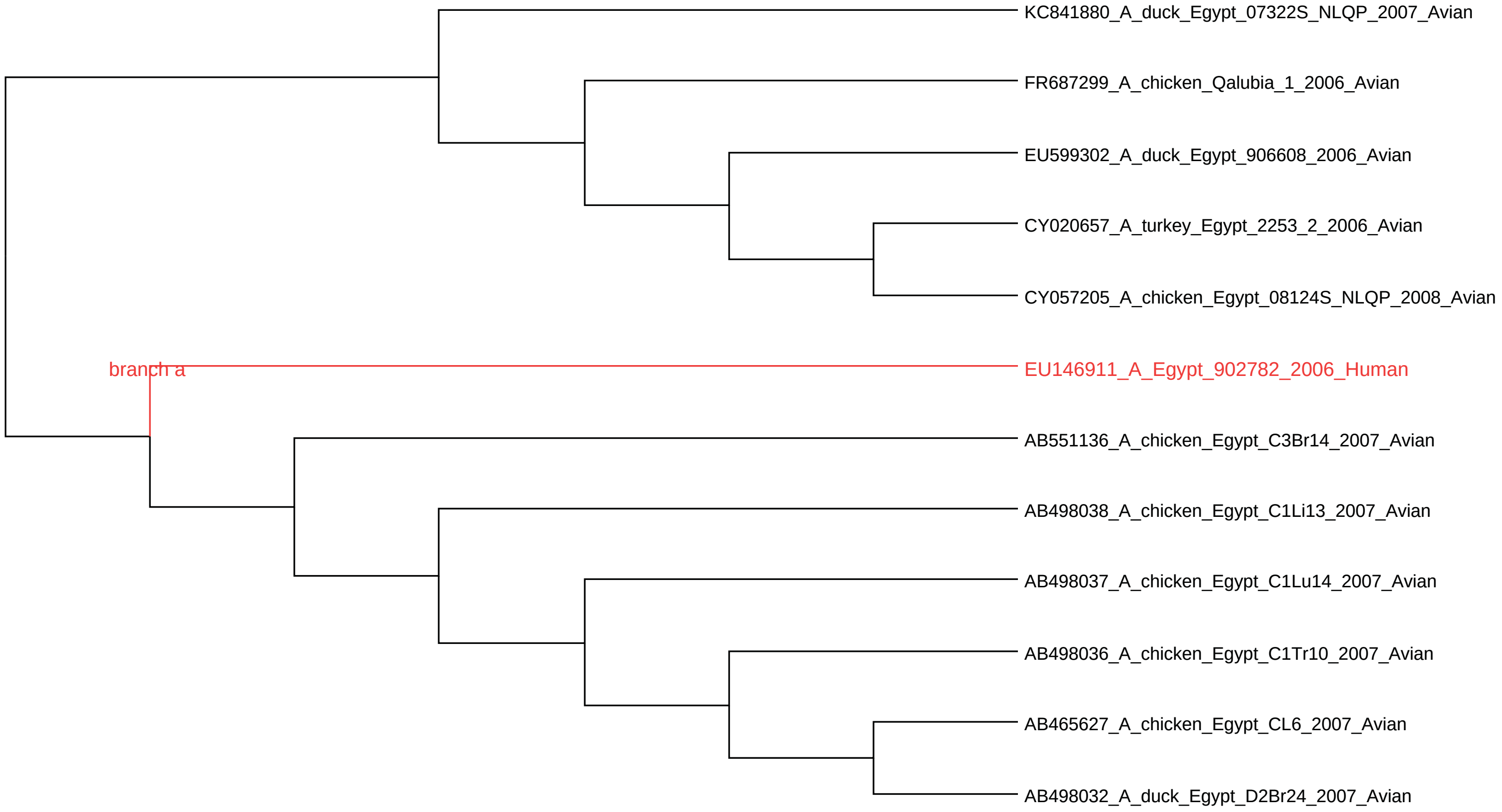
KC841880_A_duck_Egypt_07322S_NLQP_2007_Avian
FR687299_A_chicken_Qalubia_1_2006_Avian
EU599302_A_duck_Egypt_906608_2006_Avian
CY020657_A_turkey_Egypt_2253_2_2006_Avian
CY057205_A_chicken_Egypt_08124S_NLQP_2008_Avian
branch a
EU146911_A_Egypt_902782_2006_Human
AB551136_A_chicken_Egypt_C3Br14_2007_Avian
AB498038_A_chicken_Egypt_C1Li13_2007_Avian
AB498037_A_chicken_Egypt_C1Lu14_2007_Avian
AB498036_A_chicken_Egypt_C1Tr10_2007_Avian
AB465627_A_chicken_Egypt_CL6_2007_Avian
AB498032_A_duck_Egypt_D2Br24_2007_Avian

NS-Group59

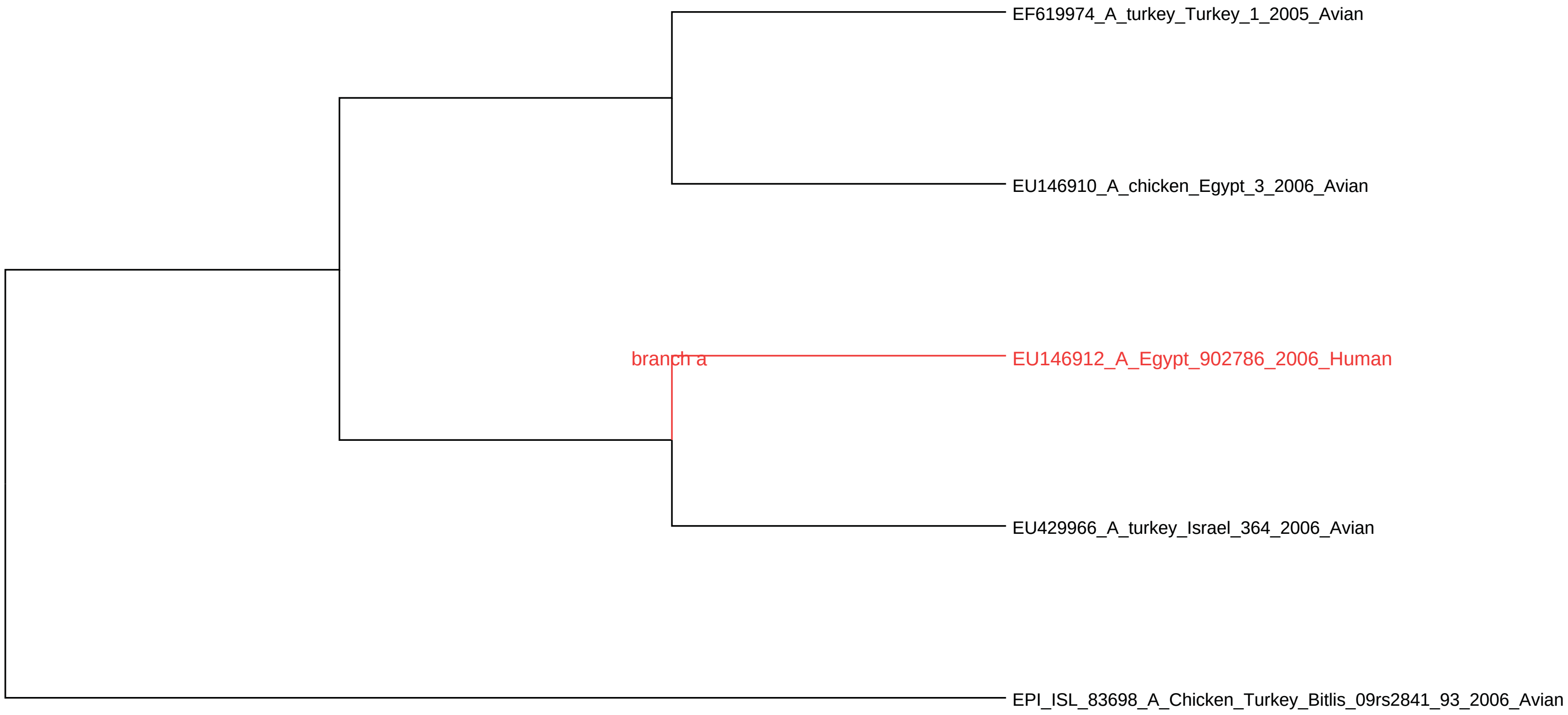
EF619974_A_turkey_Turkey_1_2005_Avian
EU146910_A_chicken_Egypt_3_2006_Avian
branch a
EU146912_A_Egypt_902786_2006_Human
EU429966_A_turkey_Israel_364_2006_Avian
EPI_ISL_83698_A_Chicken_Turkey_Bitlis_09rs2841_93_2006_Avian

NS-Group60

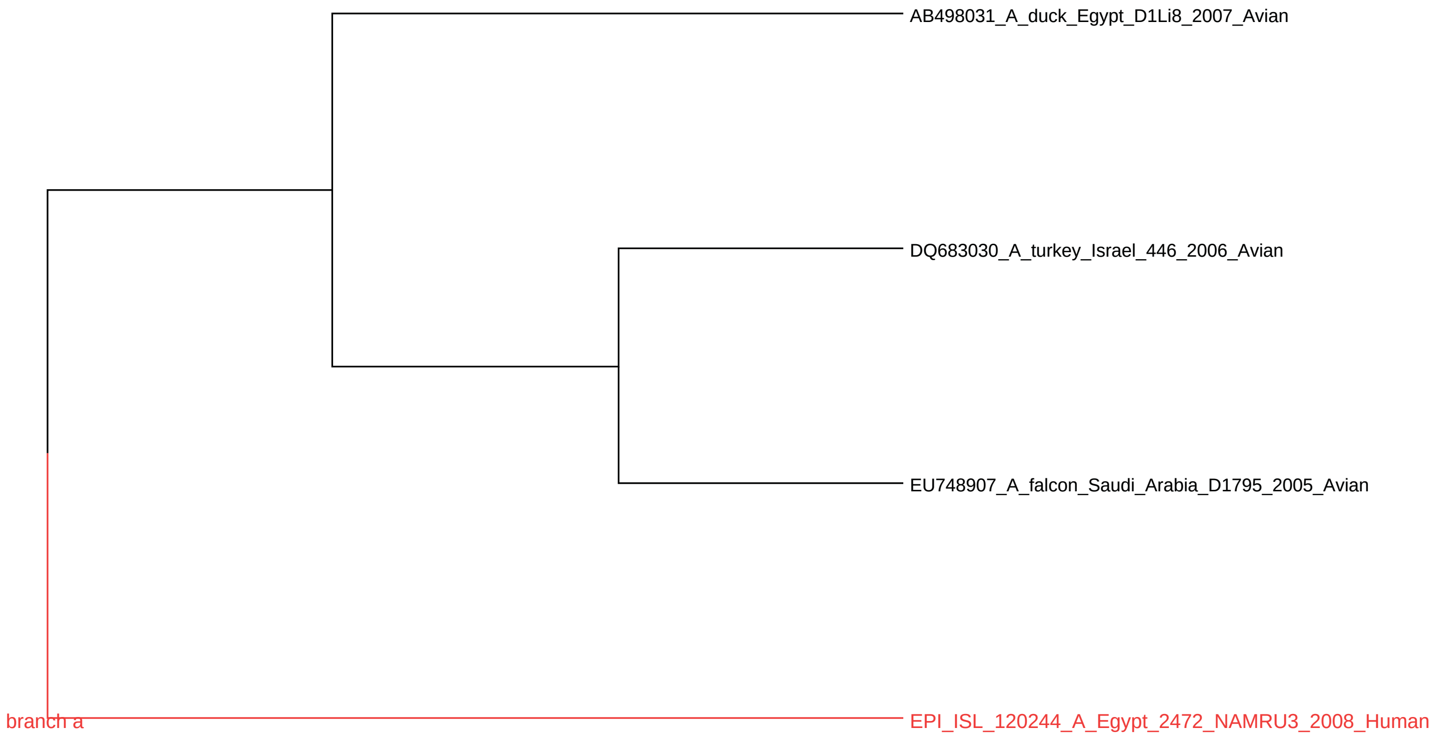
AB498031_A_duck_Egypt_D1Li8_2007_Avian
DQ683030_A_turkey_Israel_446_2006_Avian
EU748907_A_falcon_Saudi_Arabia_D1795_2005_Avian
branch a
EPI_ISL_120244_A_Egypt_2472_NAMRU3_2008_Human

NS-Group61

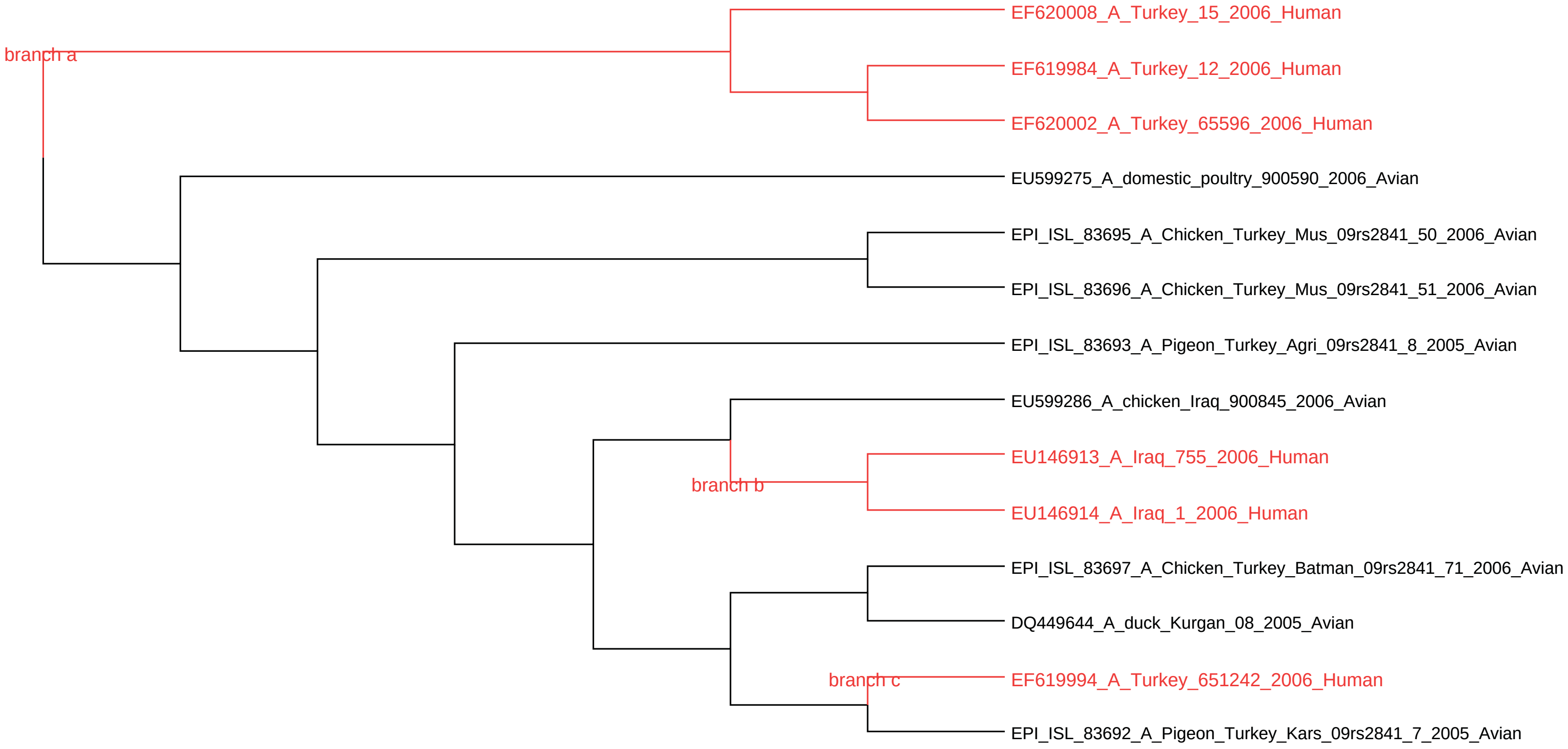
branch a
EF620008_A_Turkey_15_2006_Human
EF619984_A_Turkey_12_2006_Human
EF620002_A_Turkey_65596_2006_Human
EU599275_A_domestic_poultry_900590_2006_Avian
EPI_ISL_83695_A_Chicken_Turkey_Mus_09rs2841_50_2006_Avian
EPI_ISL_83696_A_Chicken_Turkey_Mus_09rs2841_51_2006_Avian
EPI_ISL_83693_A_Pigeon_Turkey_Agri_09rs2841_8_2005_Avian
EU599286_A_chicken_Iraq_900845_2006_Avian
branch b
EU146913_A_Iraq_755_2006_Human
EU146914_A_Iraq_1_2006_Human
EPI_ISL_83697_A_Chicken_Turkey_Batman_09rs2841_71_2006_Avian
DQ449644_A_duck_Kurgan_08_2005_Avian
branch c
EF619994_A_Turkey_651242_2006_Human
EPI_ISL_83692_A_Pigeon_Turkey_Kars_09rs2841_7_2005_Avian

NS-Group62

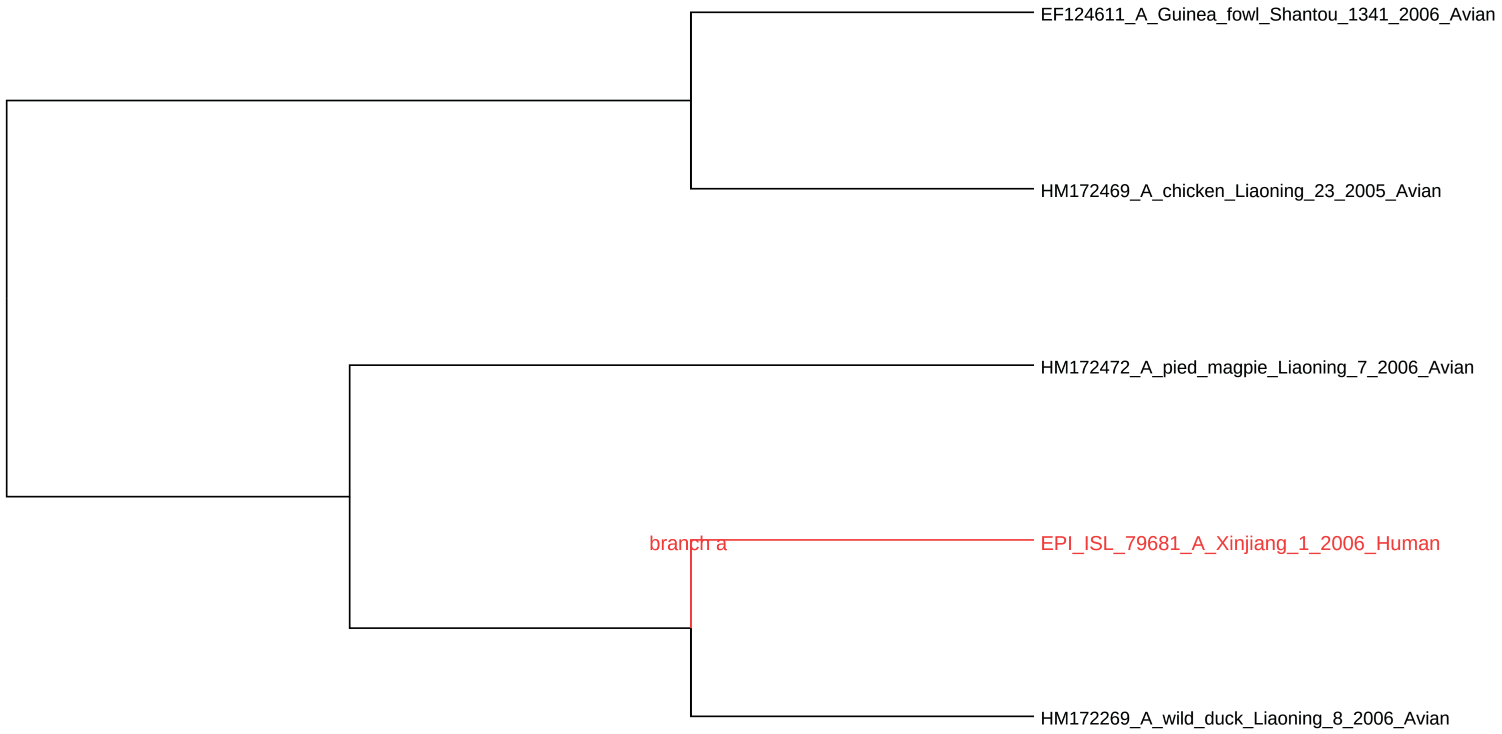
EF124611_A_Guinea_fowl_Shantou_1341_2006_Avian
HM172469_A_chicken_Liaoning_23_2005_Avian
HM172472_A_pied_magpie_Liaoning_7_2006_Avian
branch a
EPI_ISL_79681_A_Xinjiang_1_2006_Human
HM172269_A_wild_duck_Liaoning_8_2006_Avian

NS-Group63

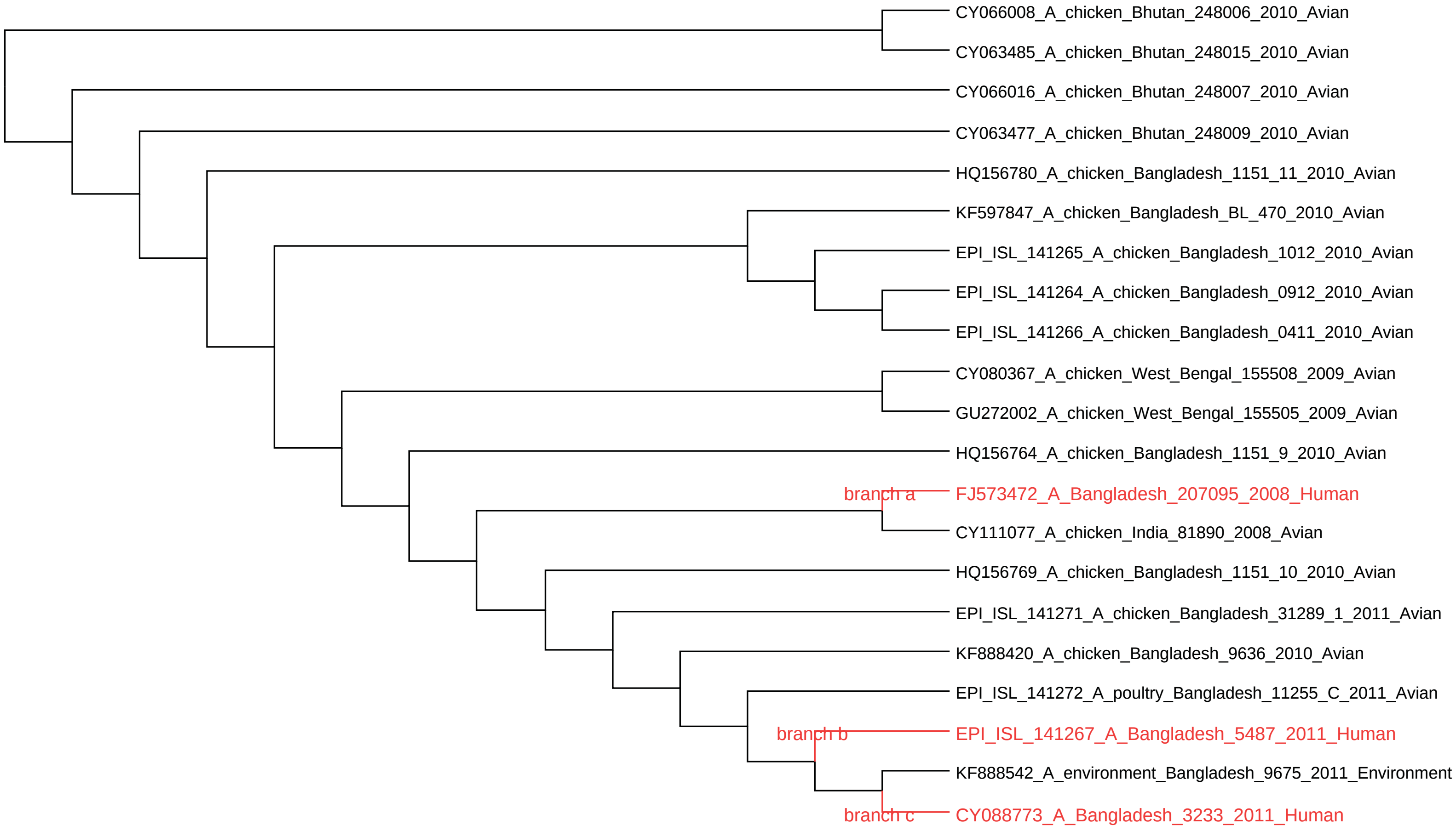

CY066008_A_chicken_Bhutan_248006_2010_Avian
CY063485_A_chicken_Bhutan_248015_2010_Avian
CY066016_A_chicken_Bhutan_248007_2010_Avian
CY063477_A_chicken_Bhutan_248009_2010_Avian
HQ156780_A_chicken_Bangladesh_1151_11_2010_Avian
KF597847_A_chicken_Bangladesh_BL_470_2010_Avian
EPI_ISL_141265_A_chicken_Bangladesh_1012_2010_Avian
EPI_ISL_141264_A_chicken_Bangladesh_0912_2010_Avian
EPI_ISL_141266_A_chicken_Bangladesh_0411_2010_Avian
CY080367_A_chicken_West_Bengal_155508_2009_Avian
GU272002_A_chicken_West_Bengal_155505_2009_Avian
HQ156764_A_chicken_Bangladesh_1151_9_2010_Avian
branch a
FJ573472_A_Bangladesh_207095_2008_Human
CY111077_A_chicken_India_81890_2008_Avian
HQ156769_A_chicken_Bangladesh_1151_10_2010_Avian
EPI_ISL_141271_A_chicken_Bangladesh_31289_1_2011_Avian
KF888420_A_chicken_Bangladesh_9636_2010_Avian
EPI_ISL_141272_A_poultry_Bangladesh_11255_C_2011_Avian
branch b
EPI_ISL_141267_A_Bangladesh_5487_2011_Human
KF888542_A_environment_Bangladesh_9675_2011_Environment
branch c
CY088773_A_Bangladesh_3233_2011_Human

NS-Group64

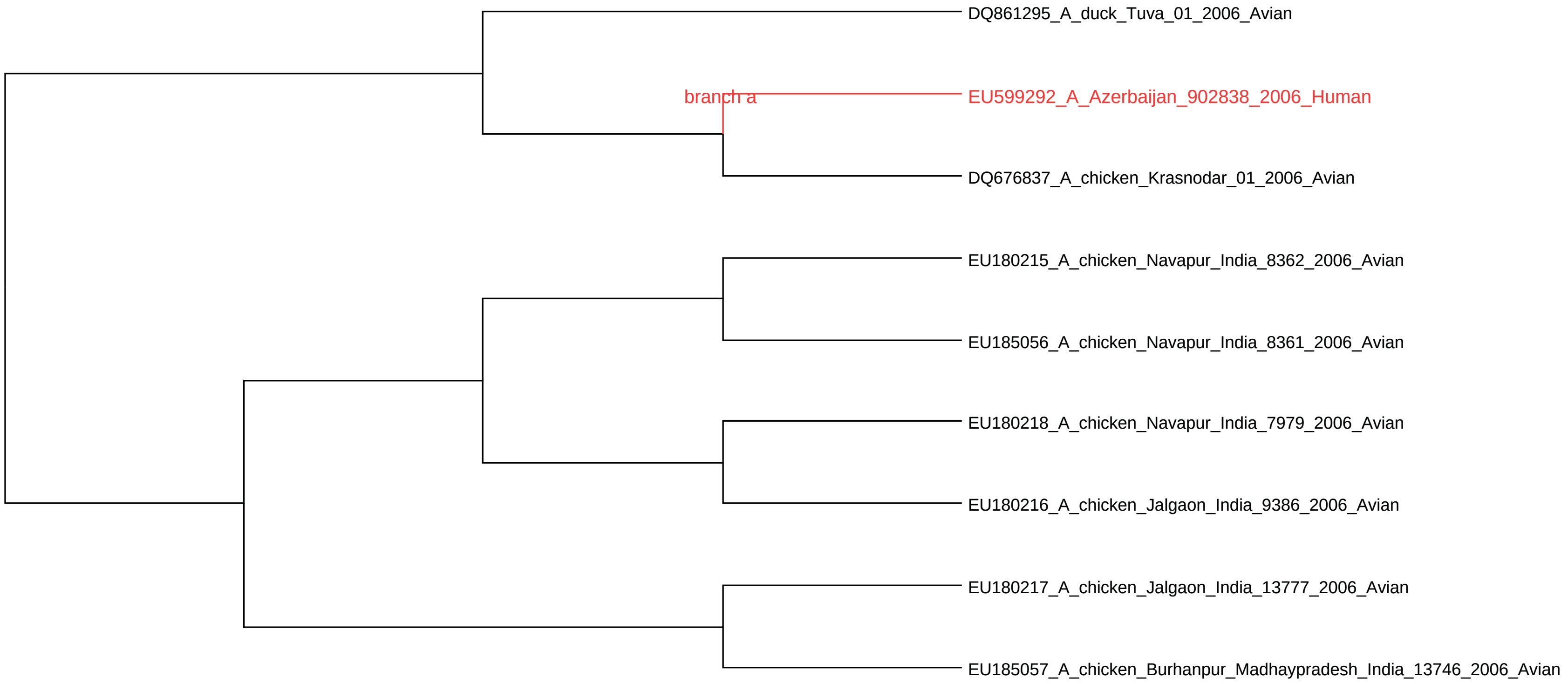

DQ861295_A_duck_Tuva_01_2006_Avian
branch a
EU599292_A_Azerbaijan_902838_2006_Human
DQ676837_A_chicken_Krasnodar_01_2006_Avian
EU180215_A_chicken_Navapur_India_8362_2006_Avian
EU185056_A_chicken_Navapur_India_8361_2006_Avian
EU180218_A_chicken_Navapur_India_7979_2006_Avian
EU180216_A_chicken_Jalgaon_India_9386_2006_Avian
EU180217_A_chicken_Jalgaon_India_13777_2006_Avian
EU185057_A_chicken_Burhanpur_Madhaypradesh_India_13746_2006_Avian

NS-Group65

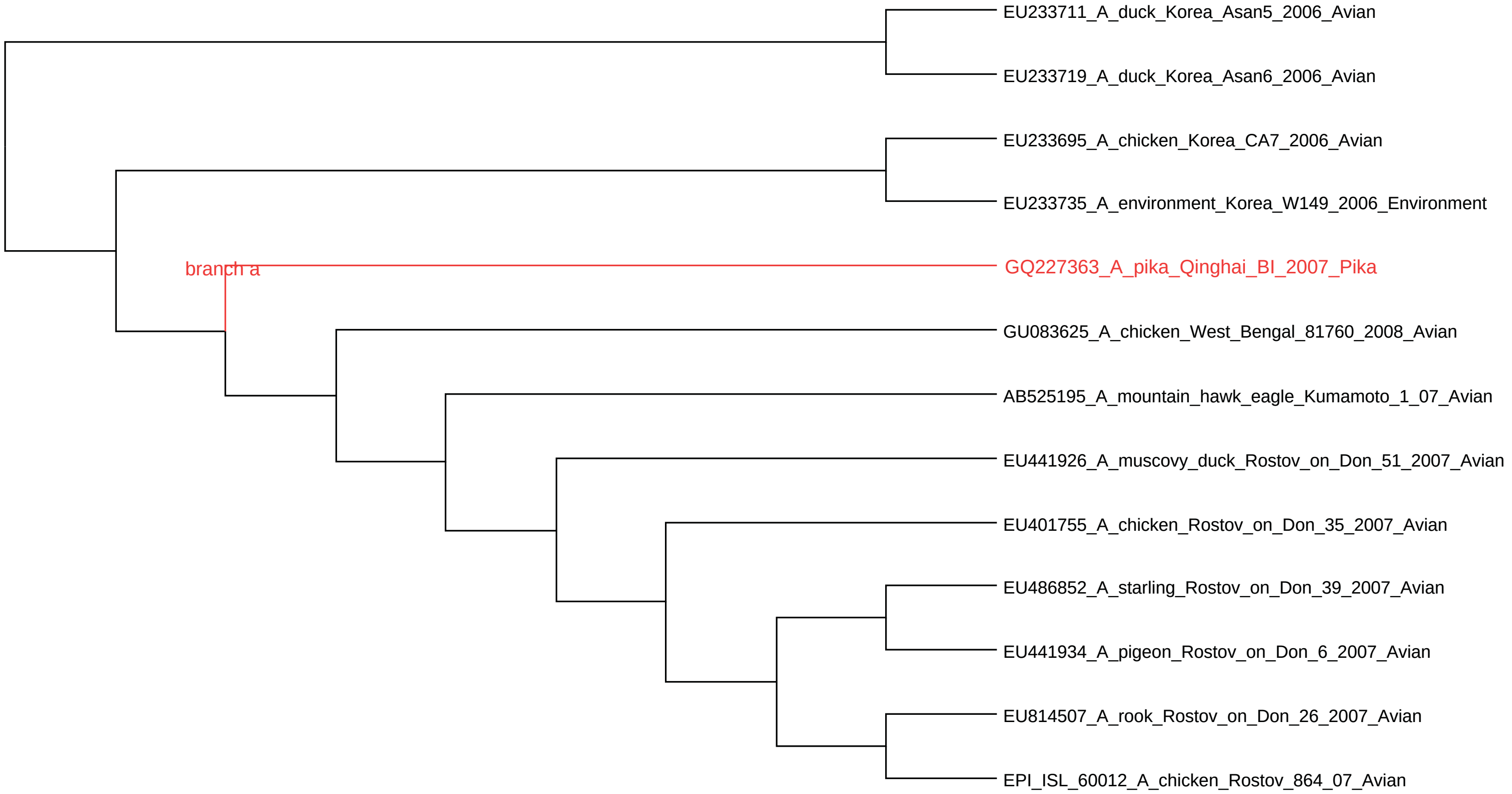
EU233711_A_duck_Korea_Asan5_2006_Avian
EU233719_A_duck_Korea_Asan6_2006_Avian
EU233695_A_chicken_Korea_CA7_2006_Avian
EU233735_A_environment_Korea_W149_2006_Environment
branch a
GQ227363_A_pika_Qinghai_BI_2007_Pika
GU083625_A_chicken_West_Bengal_81760_2008_Avian
AB525195_A_mountain_hawk_eagle_Kumamoto_1_07_Avian
EU441926_A_muscovy_duck_Rostov_on_Don_51_2007_Avian
EU401755_A_chicken_Rostov_on_Don_35_2007_Avian
EU486852_A_starling_Rostov_on_Don_39_2007_Avian
EU441934_A_pigeon_Rostov_on_Don_6_2007_Avian
EU814507_A_rook_Rostov_on_Don_26_2007_Avian
EPI_ISL_60012_A_chicken_Rostov_864_07_Avian

NS-Group66

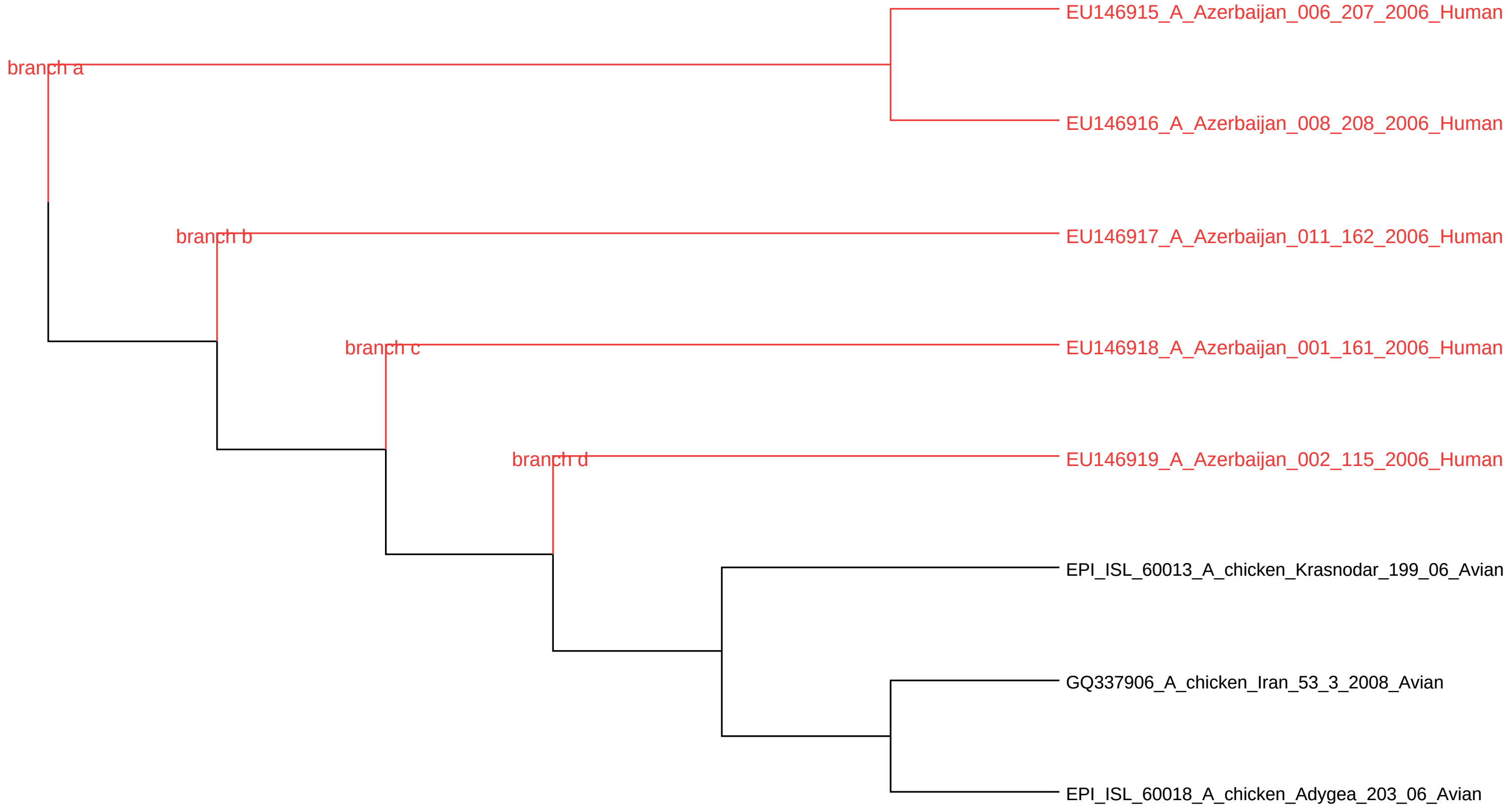
branch a
branch b
branch c
branch d
EU146915_A_Azerbaijan_006_207_2006_Human
EU146916_A_Azerbaijan_008_208_2006_Human
EU146917_A_Azerbaijan_011_162_2006_Human
EU146918_A_Azerbaijan_001_161_2006_Human
EU146919_A_Azerbaijan_002_115_2006_Human
EPI_ISL_60013_A_chicken_Krasnodar_199_06_Avian
GQ337906_A_chicken_Iran_53_3_2008_Avian
EPI_ISL_60018_A_chicken_Adygea_203_06_Avian

NS-Group67

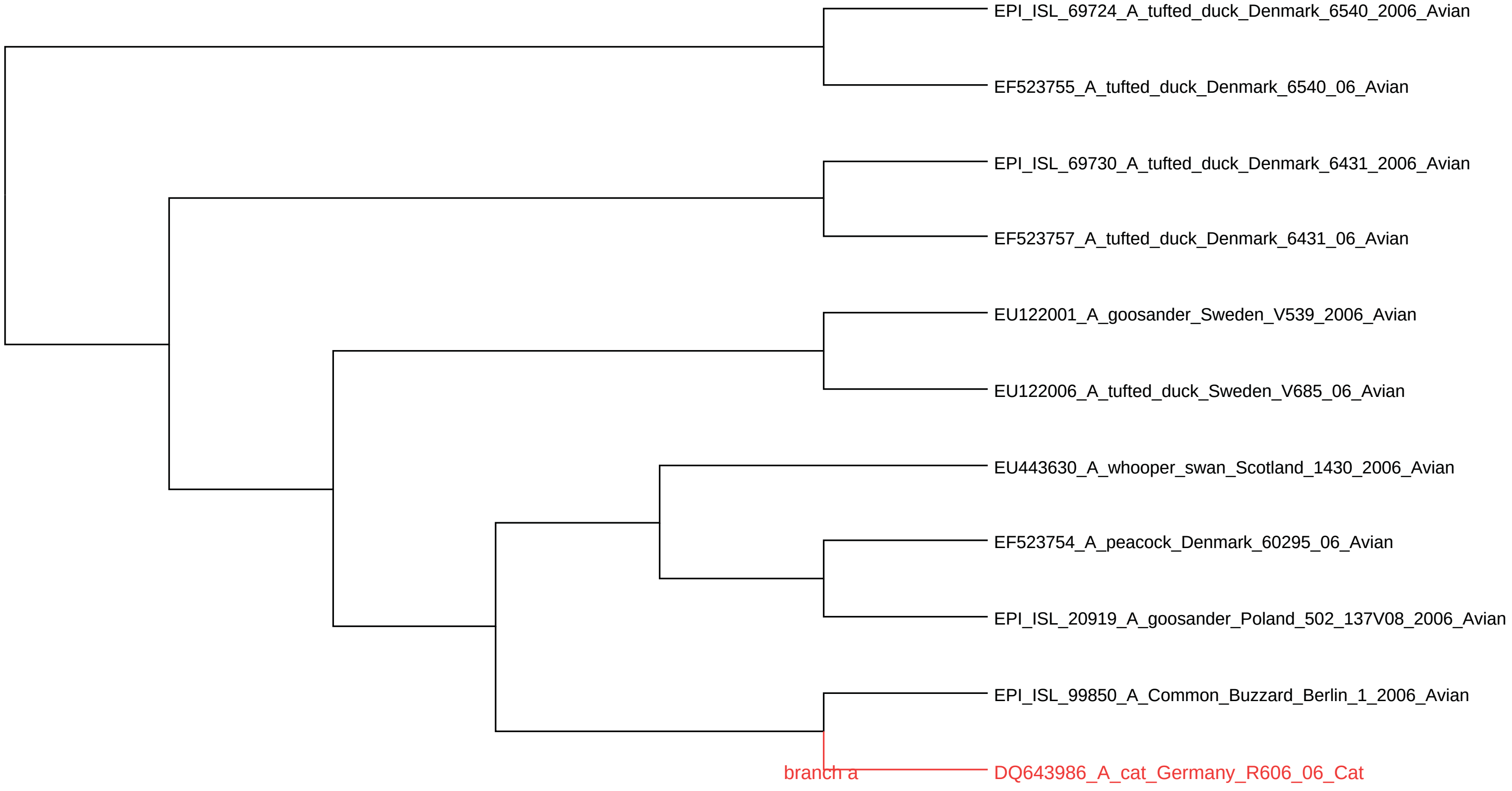

EPI_ISL_69724_A_tufted_duck_Denmark_6540_2006_Avian
EF523755_A_tufted_duck_Denmark_6540_06_Avian
EPI_ISL_69730_A_tufted_duck_Denmark_6431_2006_Avian
EF523757_A_tufted_duck_Denmark_6431_06_Avian
EU122001_A_goosander_Sweden_V539_2006_Avian
EU122006_A_tufted_duck_Sweden_V685_06_Avian
EU443630_A_whooper_swan_Scotland_1430_2006_Avian
EF523754_A_peacock_Denmark_60295_06_Avian
EPI_ISL_20919_A_goosander_Poland_502_137V08_2006_Avian
EPI_ISL_99850_A_Common_Buzzard_Berlin_1_2006_Avian
branch a
DQ643986_A_cat_Germany_R606_06_Cat

NS-Group68

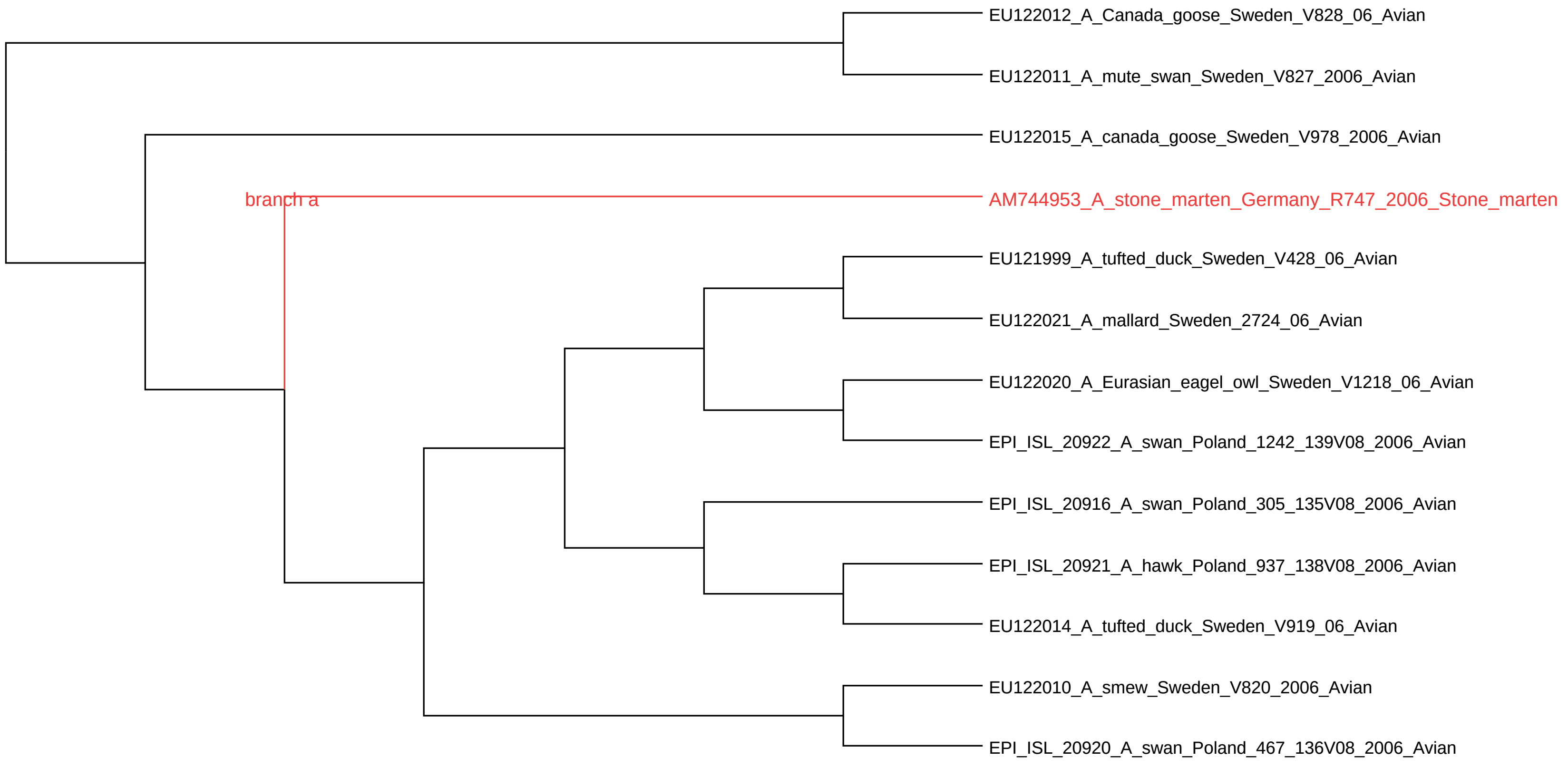
EU122012_A_Canada_goose_Sweden_V828_06_Avian
EU122011_A_mute_swan_Sweden_V827_2006_Avian
EU122015_A_canada_goose_Sweden_V978_2006_Avian
branch a
AM744953_A_stone_marten_Germany_R747_2006_Stone_marten
EU121999_A_tufted_duck_Sweden_V428_06_Avian
EU122021_A_mallard_Sweden_2724_06_Avian
EU122020_A_Eurasian_eagel_owl_Sweden_V1218_06_Avian
EPI_ISL_20922_A_swan_Poland_1242_139V08_2006_Avian
EPI_ISL_20916_A_swan_Poland_305_135V08_2006_Avian
EPI_ISL_20921_A_hawk_Poland_937_138V08_2006_Avian
EU122014_A_tufted_duck_Sweden_V919_06_Avian
EU122010_A_smew_Sweden_V820_2006_Avian
EPI_ISL_20920_A_swan_Poland_467_136V08_2006_Avian

NS-Group69

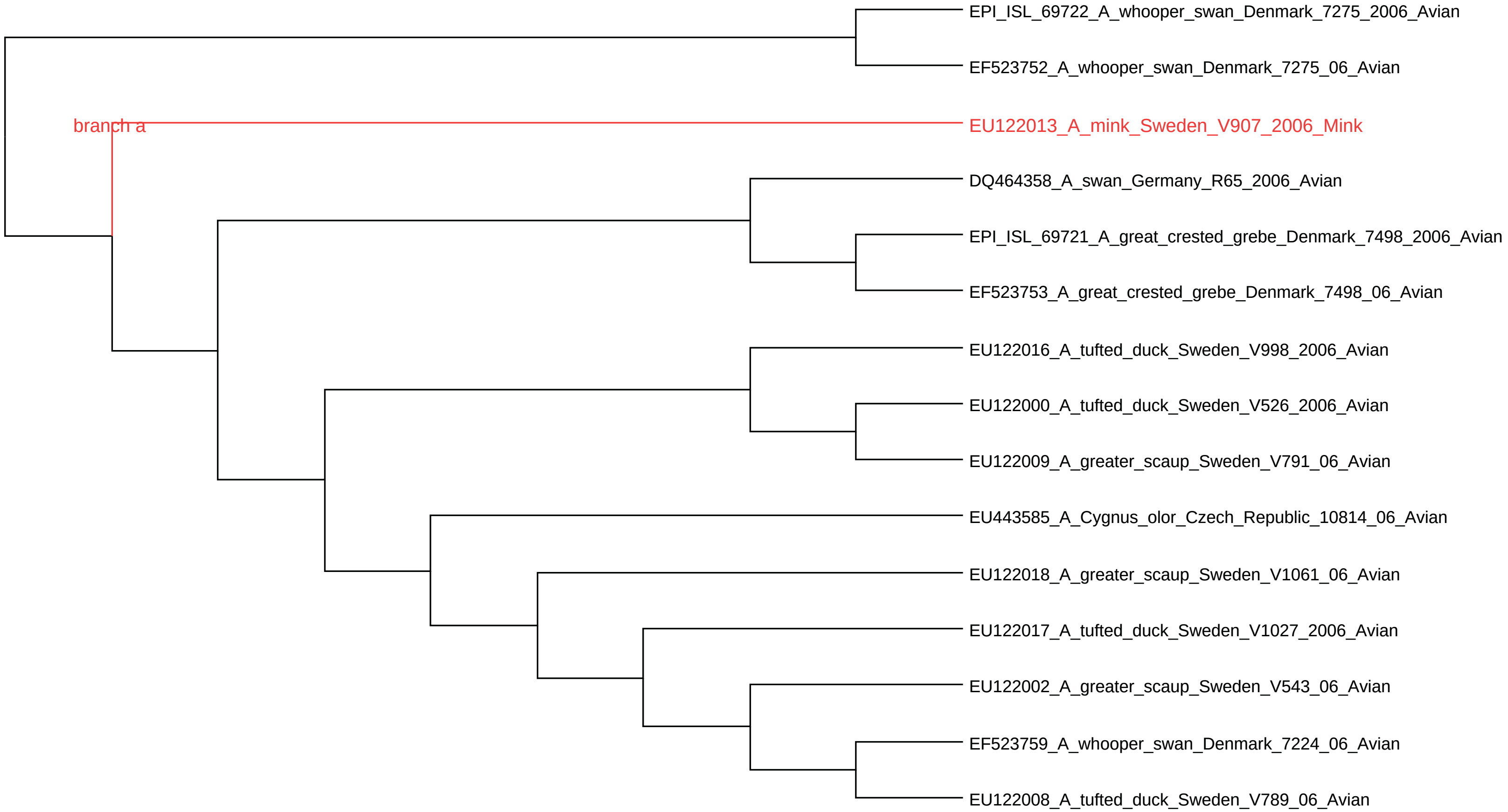
EPI_ISL_69722_A_whooper_swan_Denmark_7275_2006_Avian
EF523752_A_whooper_swan_Denmark_7275_06_Avian
branch a
EU122013_A_mink_Sweden_V907_2006_Mink
DQ464358_A_swan_Germany_R65_2006_Avian
EPI_ISL_69721_A_great_crested_grebe_Denmark_7498_2006_Avian
EF523753_A_great_crested_grebe_Denmark_7498_06_Avian
EU122016_A_tufted_duck_Sweden_V998_2006_Avian
EU122000_A_tufted_duck_Sweden_V526_2006_Avian
EU122009_A_greater_scaup_Sweden_V791_06_Avian
EU443585_A_Cygnus_olor_Czech_Republic_10814_06_Avian
EU122018_A_greater_scaup_Sweden_V1061_06_Avian
EU122017_A_tufted_duck_Sweden_V1027_2006_Avian
EU122002_A_greater_scaup_Sweden_V543_06_Avian
EF523759_A_whooper_swan_Denmark_7224_06_Avian
EU122008_A_tufted_duck_Sweden_V789_06_Avian

NS-Group70

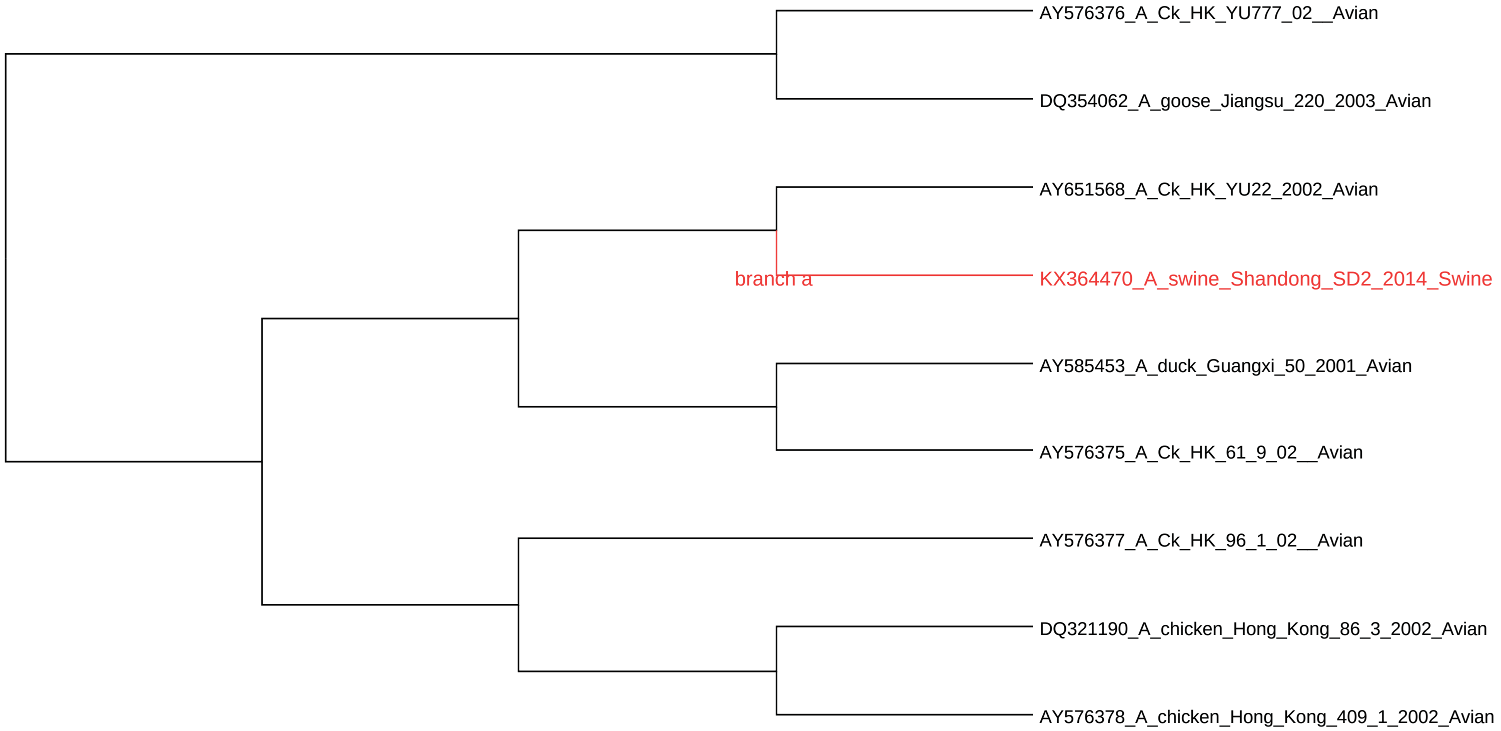

AY576376_A_Ck_HK_YU777_02__Avian
DQ354062_A_goose_Jiangsu_220_2003_Avian
AY651568_A_Ck_HK_YU22_2002_Avian
branch a
KX364470_A_swine_Shandong_SD2_2014_Swine
AY585453_A_duck_Guangxi_50_2001_Avian
AY576375_A_Ck_HK_61_9_02__Avian
AY576377_A_Ck_HK_96_1_02__Avian
DQ321190_A_chicken_Hong_Kong_86_3_2002_Avian
AY576378_A_chicken_Hong_Kong_409_1_2002_Avian

NS-Group71

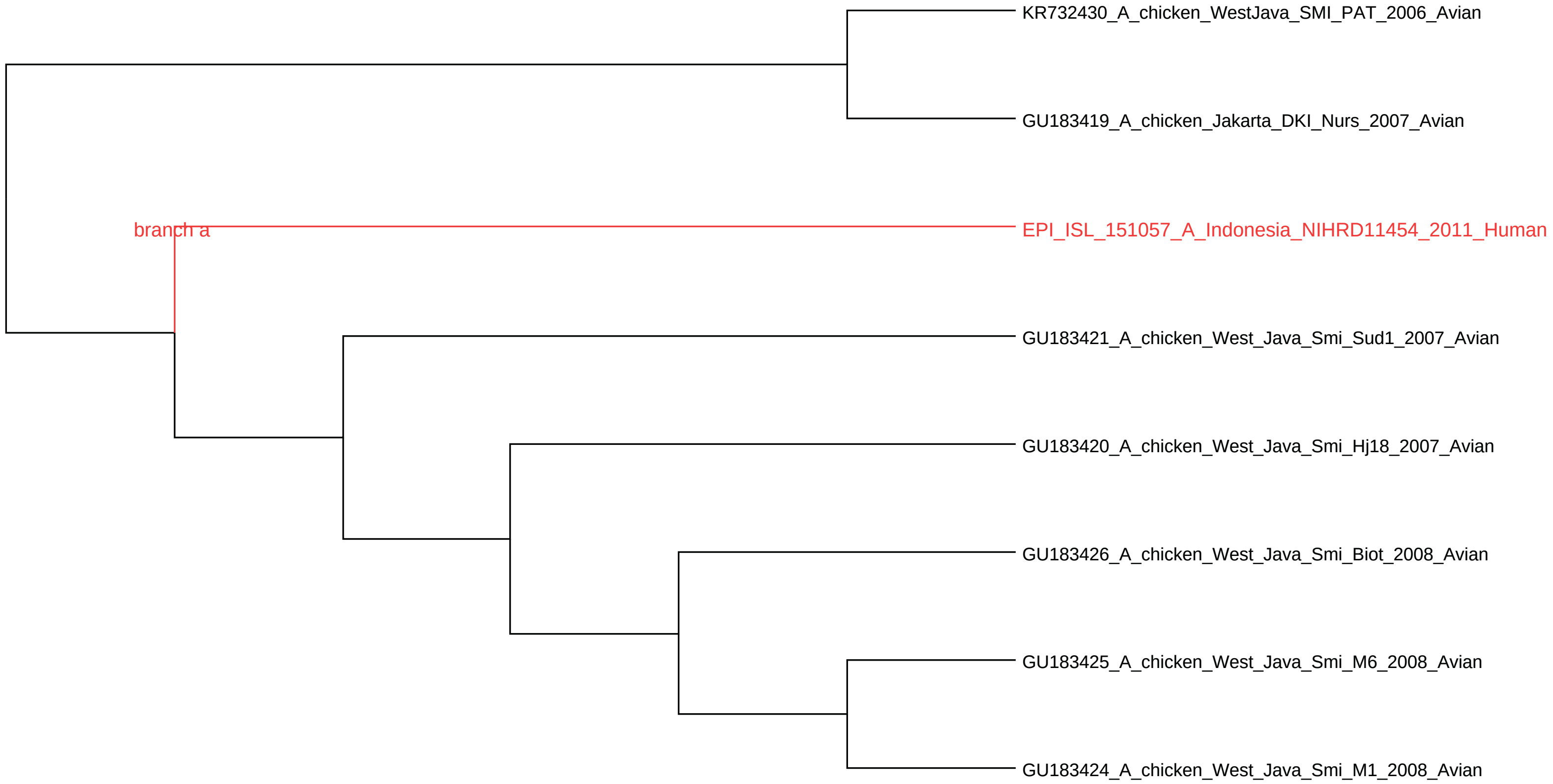
KR732430_A_chicken_WestJava_SMI_PAT_2006_Avian
GU183419_A_chicken_Jakarta_DKI_Nurs_2007_Avian
branch a
EPI_ISL_151057_A_Indonesia_NIHRD11454_2011_Human
GU183421_A_chicken_West_Java_Smi_Sud1_2007_Avian
GU183420_A_chicken_West_Java_Smi_Hj18_2007_Avian
GU183426_A_chicken_West_Java_Smi_Biot_2008_Avian
GU183425_A_chicken_West_Java_Smi_M6_2008_Avian
GU183424_A_chicken_West_Java_Smi_M1_2008_Avian

NS-Group72

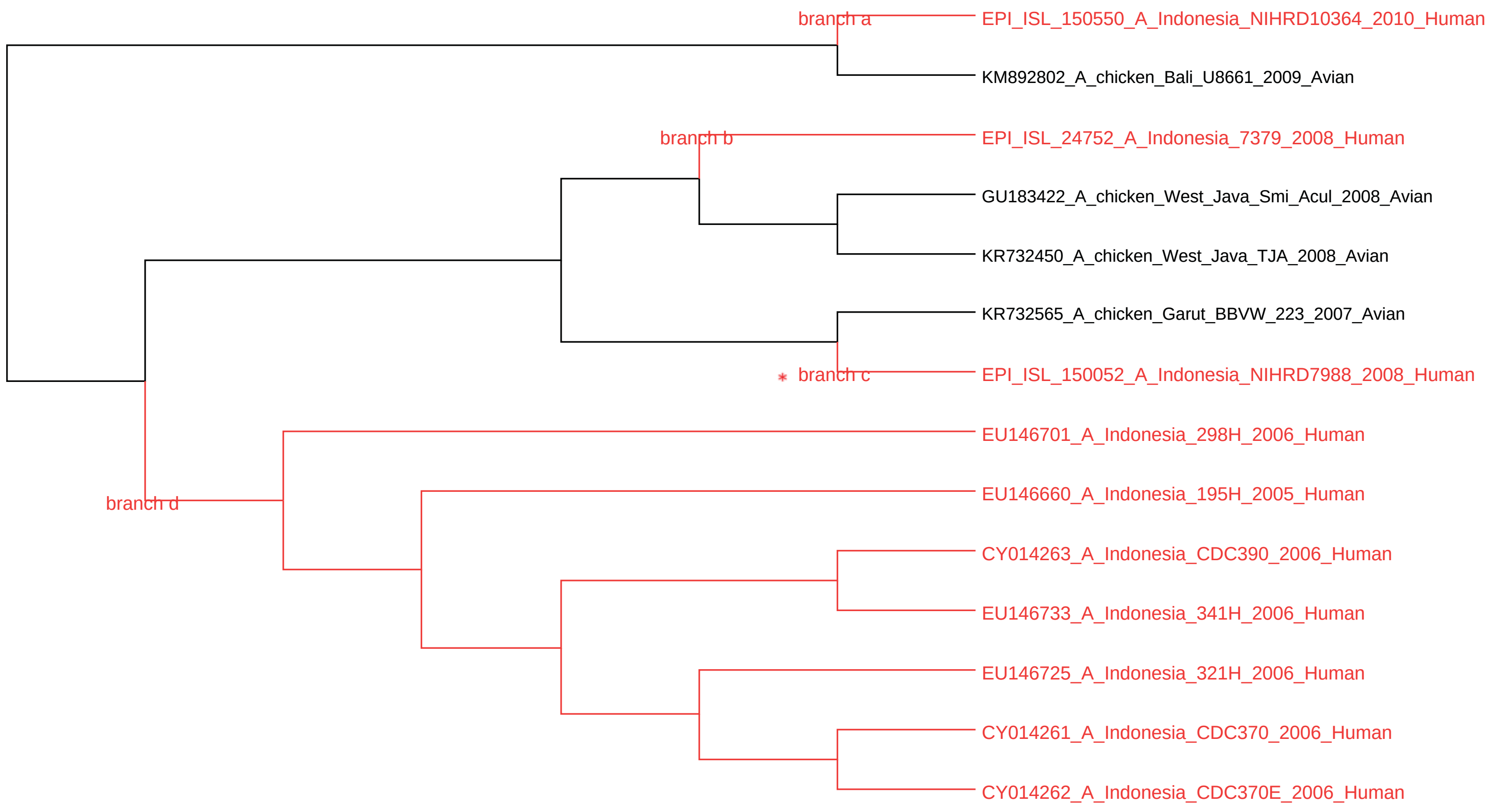
branch a
EPI_ISL_150550_A_Indonesia_NIHRD10364_2010_Human
KM892802_A_chicken_Bali_U8661_2009_Avian
branch b
EPI_ISL_24752_A_Indonesia_7379_2008_Human
GU183422_A_chicken_West_Java_Smi_Acul_2008_Avian
KR732450_A_chicken_West_Java_TJA_2008_Avian
KR732565_A_chicken_Garut_BBVW_223_2007_Avian
* branch c
EPI_ISL_150052_A_Indonesia_NIHRD7988_2008_Human
EU146701_A_Indonesia_298H_2006_Human
branch d
EU146660_A_Indonesia_195H_2005_Human
CY014263_A_Indonesia_CDC390_2006_Human
EU146733_A_Indonesia_341H_2006_Human
EU146725_A_Indonesia_321H_2006_Human
CY014261_A_Indonesia_CDC370_2006_Human
CY014262_A_Indonesia_CDC370E_2006_Human

NS-Group73

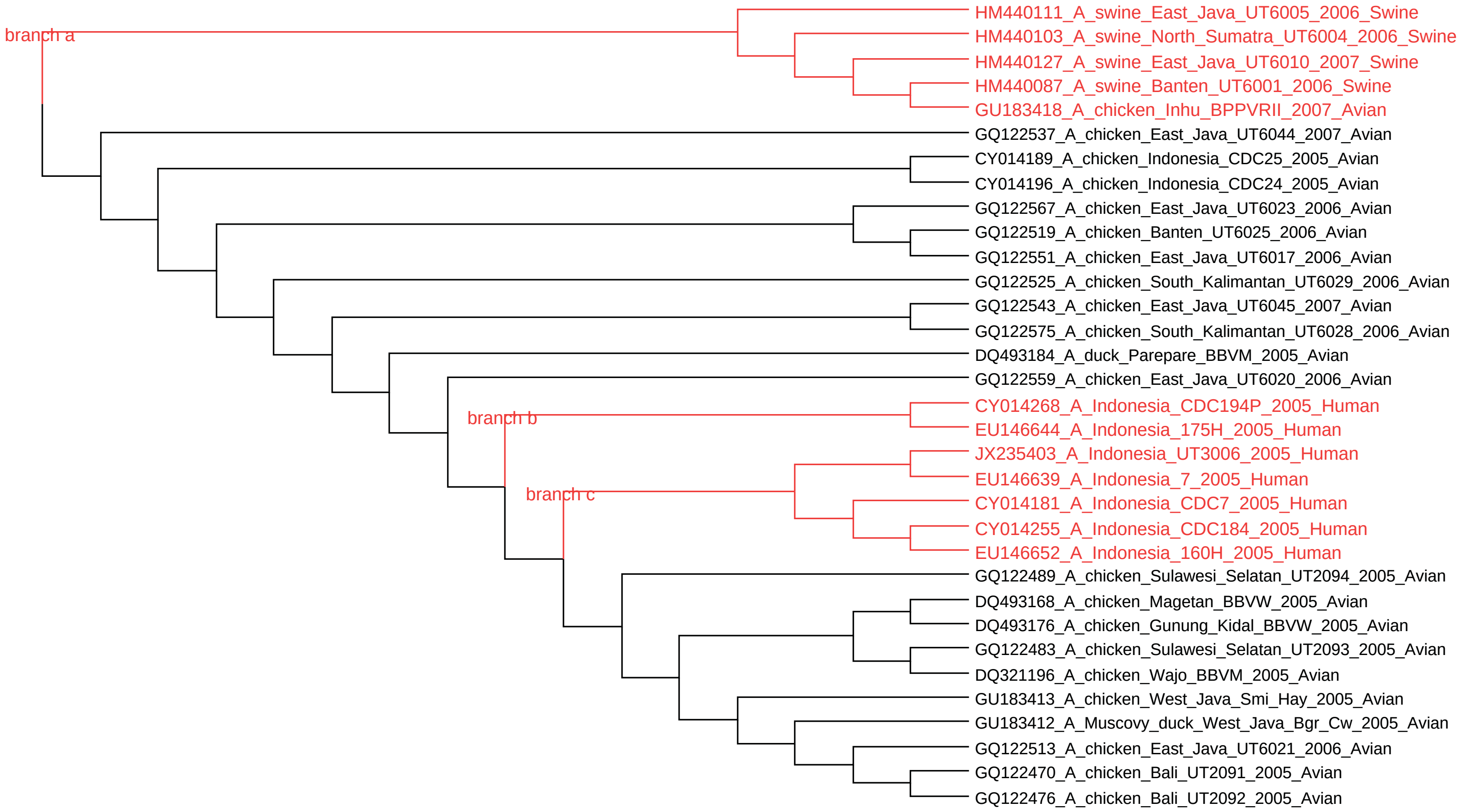
branch a
branch b
branch c
HM440111_A_swine_East_Java_UT6005_2006_Swine
HM440103_A_swine_North_Sumatra_UT6004_2006_Swine
HM440127_A_swine_East_Java_UT6010_2007_Swine
HM440087_A_swine_Banten_UT6001_2006_Swine
GU183418_A_chicken_Inhu_BPPVRII_2007_Avian
GQ122537_A_chicken_East_Java_UT6044_2007_Avian
CY014189_A_chicken_Indonesia_CDC25_2005_Avian
CY014196_A_chicken_Indonesia_CDC24_2005_Avian
GQ122567_A_chicken_East_Java_UT6023_2006_Avian
GQ122519_A_chicken_Banten_UT6025_2006_Avian
GQ122551_A_chicken_East_Java_UT6017_2006_Avian
GQ122525_A_chicken_South_Kalimantan_UT6029_2006_Avian
GQ122543_A_chicken_East_Java_UT6045_2007_Avian
GQ122575_A_chicken_South_Kalimantan_UT6028_2006_Avian
DQ493184_A_duck_Parepare_BBVM_2005_Avian
GQ122559_A_chicken_East_Java_UT6020_2006_Avian
CY014268_A_Indonesia_CDC194P_2005_Human
EU146644_A_Indonesia_175H_2005_Human
JX235403_A_Indonesia_UT3006_2005_Human
EU146639_A_Indonesia_7_2005_Human
CY014181_A_Indonesia_CDC7_2005_Human
CY014255_A_Indonesia_CDC184_2005_Human
EU146652_A_Indonesia_160H_2005_Human
GQ122489_A_chicken_Sulawesi_Selatan_UT2094_2005_Avian
DQ493168_A_chicken_Magetan_BBVW_2005_Avian
DQ493176_A_chicken_Gunung_Kidal_BBVW_2005_Avian
GQ122483_A_chicken_Sulawesi_Selatan_UT2093_2005_Avian
DQ321196_A_chicken_Wajo_BBVM_2005_Avian
GU183413_A_chicken_West_Java_Smi_Hay_2005_Avian
GU183412_A_Muscovy_duck_West_Java_Bgr_Cw_2005_Avian
GQ122513_A_chicken_East_Java_UT6021_2006_Avian
GQ122470_A_chicken_Bali_UT2091_2005_Avian
GQ122476_A_chicken_Bali_UT2092_2005_Avian

NS-Group74

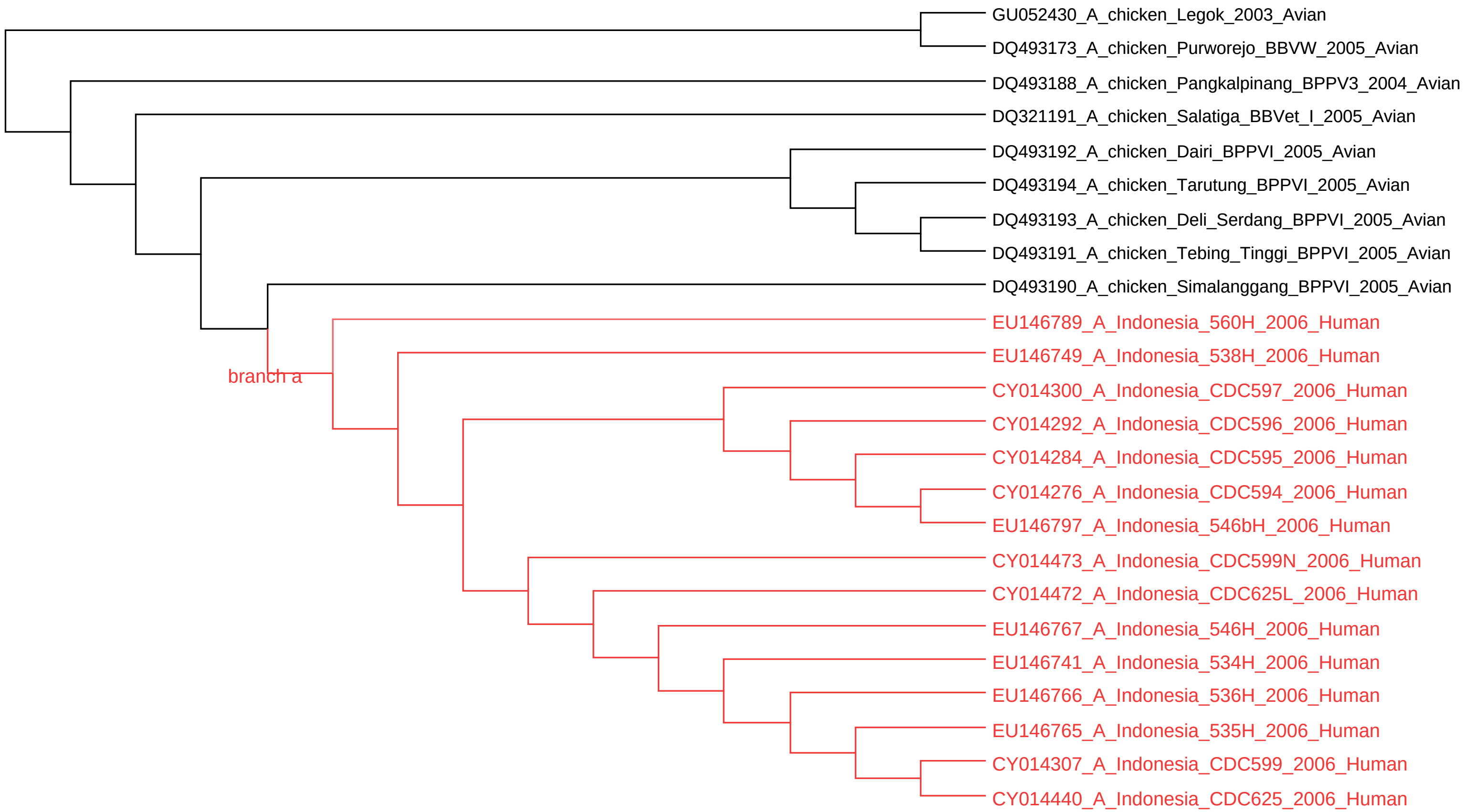
GU052430_A_chicken_Legok_2003_Avian
DQ493173_A_chicken_Purworejo_BBVW_2005_Avian
DQ493188_A_chicken_Pangkalpinang_BPPV3_2004_Avian
DQ321191_A_chicken_Salatiga_BBVet_I_2005_Avian
DQ493192_A_chicken_Dairi_BPPVI_2005_Avian
DQ493194_A_chicken_Tarutung_BPPVI_2005_Avian
DQ493193_A_chicken_Deli_Serdang_BPPVI_2005_Avian
DQ493191_A_chicken_Tebing_Tinggi_BPPVI_2005_Avian
DQ493190_A_chicken_Simalanggang_BPPVI_2005_Avian
EU146789_A_Indonesia_560H_2006_Human
branch a
EU146749_A_Indonesia_538H_2006_Human
CY014300_A_Indonesia_CDC597_2006_Human
CY014292_A_Indonesia_CDC596_2006_Human
CY014284_A_Indonesia_CDC595_2006_Human
CY014276_A_Indonesia_CDC594_2006_Human
EU146797_A_Indonesia_546bH_2006_Human
CY014473_A_Indonesia_CDC599N_2006_Human
CY014472_A_Indonesia_CDC625L_2006_Human
EU146767_A_Indonesia_546H_2006_Human
EU146741_A_Indonesia_534H_2006_Human
EU146766_A_Indonesia_536H_2006_Human
EU146765_A_Indonesia_535H_2006_Human
CY014307_A_Indonesia_CDC599_2006_Human
CY014440_A_Indonesia_CDC625_2006_Human

NS-Group75

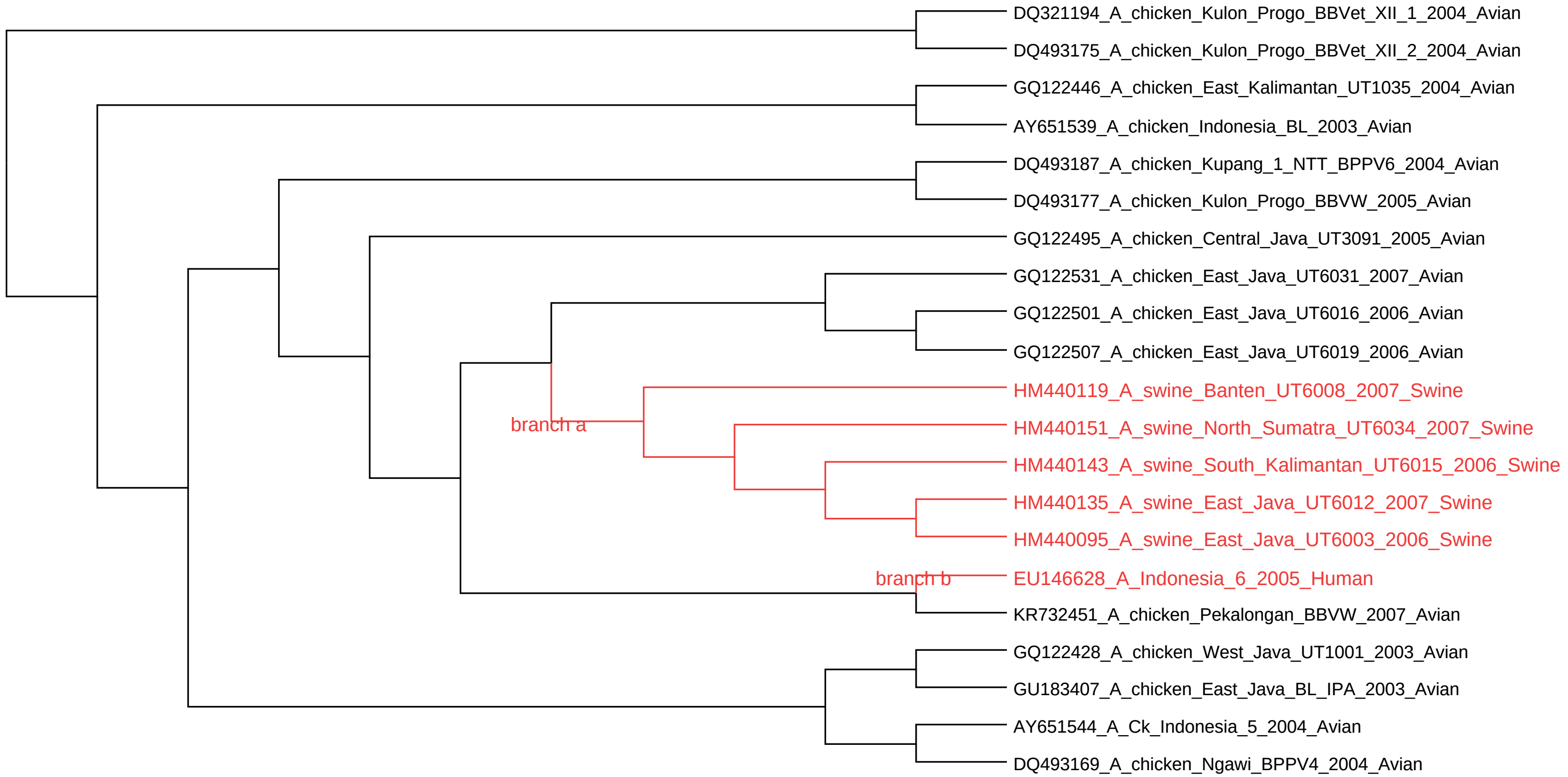

DQ321194_A_chicken_Kulon_Progo_BBVet_XII_1_2004_Avian
DQ493175_A_chicken_Kulon_Progo_BBVet_XII_2_2004_Avian
GQ122446_A_chicken_East_Kalimantan_UT1035_2004_Avian
AY651539_A_chicken_Indonesia_BL_2003_Avian
DQ493187_A_chicken_Kupang_1_NTT_BPPV6_2004_Avian
DQ493177_A_chicken_Kulon_Progo_BBVW_2005_Avian
GQ122495_A_chicken_Central_Java_UT3091_2005_Avian
GQ122531_A_chicken_East_Java_UT6031_2007_Avian
GQ122501_A_chicken_East_Java_UT6016_2006_Avian
GQ122507_A_chicken_East_Java_UT6019_2006_Avian
HM440119_A_swine_Banten_UT6008_2007_Swine
branch a
HM440151_A_swine_North_Sumatra_UT6034_2007_Swine
HM440143_A_swine_South_Kalimantan_UT6015_2006_Swine
HM440135_A_swine_East_Java_UT6012_2007_Swine
HM440095_A_swine_East_Java_UT6003_2006_Swine
branch b
EU146628_A_Indonesia_6_2005_Human
KR732451_A_chicken_Pekalongan_BBVW_2007_Avian
GQ122428_A_chicken_West_Java_UT1001_2003_Avian
GU183407_A_chicken_East_Java_BL_IPA_2003_Avian
AY651544_A_Ck_Indonesia_5_2004_Avian
DQ493169_A_chicken_Ngawi_BPPV4_2004_Avian

NS-Group76

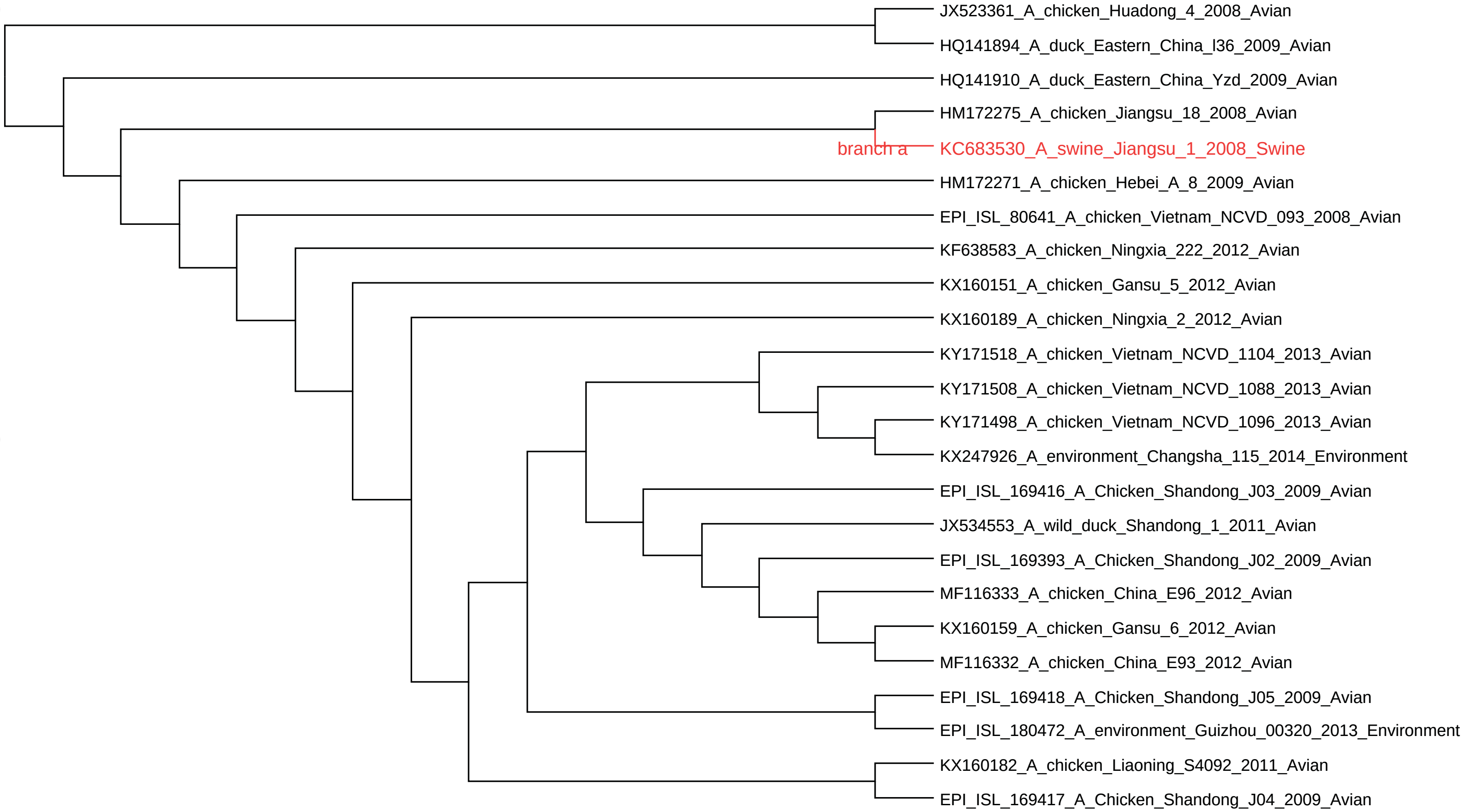
JX523361_A_chicken_Huadong_4_2008_Avian
HQ141894_A_duck_Eastern_China_l36_2009_Avian
HQ141910_A_duck_Eastern_China_Yzd_2009_Avian
HM172275_A_chicken_Jiangsu_18_2008_Avian
branch a
KC683530_A_swine_Jiangsu_1_2008_Swine
HM172271_A_chicken_Hebei_A_8_2009_Avian
EPI_ISL_80641_A_chicken_Vietnam_NCVD_093_2008_Avian
KF638583_A_chicken_Ningxia_222_2012_Avian
KX160151_A_chicken_Gansu_5_2012_Avian
KX160189_A_chicken_Ningxia_2_2012_Avian
KY171518_A_chicken_Vietnam_NCVD_1104_2013_Avian
KY171508_A_chicken_Vietnam_NCVD_1088_2013_Avian
KY171498_A_chicken_Vietnam_NCVD_1096_2013_Avian
KX247926_A_environment_Changsha_115_2014_Environment
EPI_ISL_169416_A_Chicken_Shandong_J03_2009_Avian
JX534553_A_wild_duck_Shandong_1_2011_Avian
EPI_ISL_169393_A_Chicken_Shandong_J02_2009_Avian
MF116333_A_chicken_China_E96_2012_Avian
KX160159_A_chicken_Gansu_6_2012_Avian
MF116332_A_chicken_China_E93_2012_Avian
EPI_ISL_169418_A_Chicken_Shandong_J05_2009_Avian
EPI_ISL_180472_A_environment_Guizhou_00320_2013_Environment
KX160182_A_chicken_Liaoning_S4092_2011_Avian
EPI_ISL_169417_A_Chicken_Shandong_J04_2009_Avian

NS-Group77

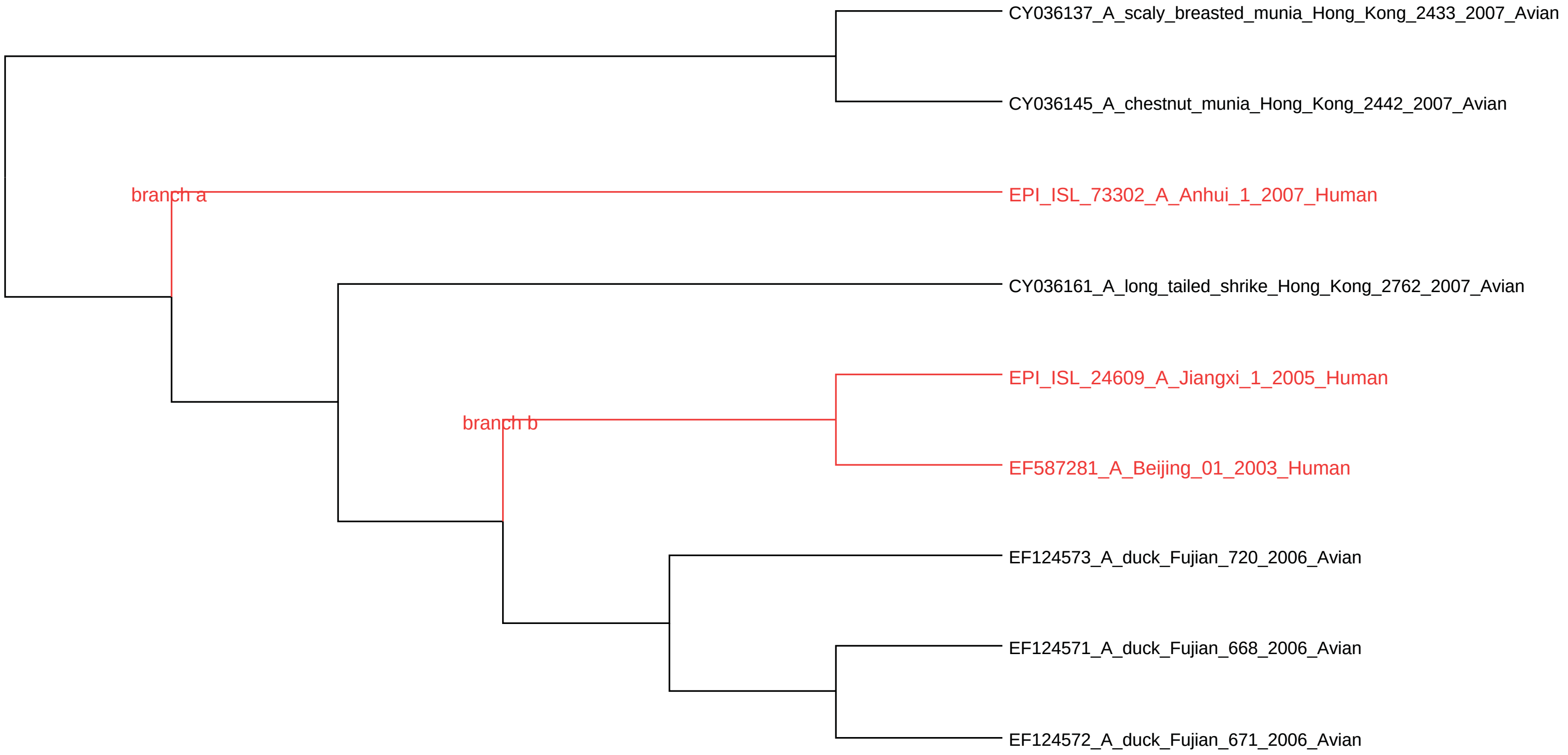

CY036137_A_scaly_breasted_munia_Hong_Kong_2433_2007_Avian
CY036145_A_chestnut_munia_Hong_Kong_2442_2007_Avian
branch a
EPI_ISL_73302_A_Anhui_1_2007_Human
CY036161_A_long_tailed_shrike_Hong_Kong_2762_2007_Avian
EPI_ISL_24609_A_Jiangxi_1_2005_Human
branch b
EF587281_A_Beijing_01_2003_Human
EF124573_A_duck_Fujian_720_2006_Avian
EF124571_A_duck_Fujian_668_2006_Avian
EF124572_A_duck_Fujian_671_2006_Avian

NS-Group78

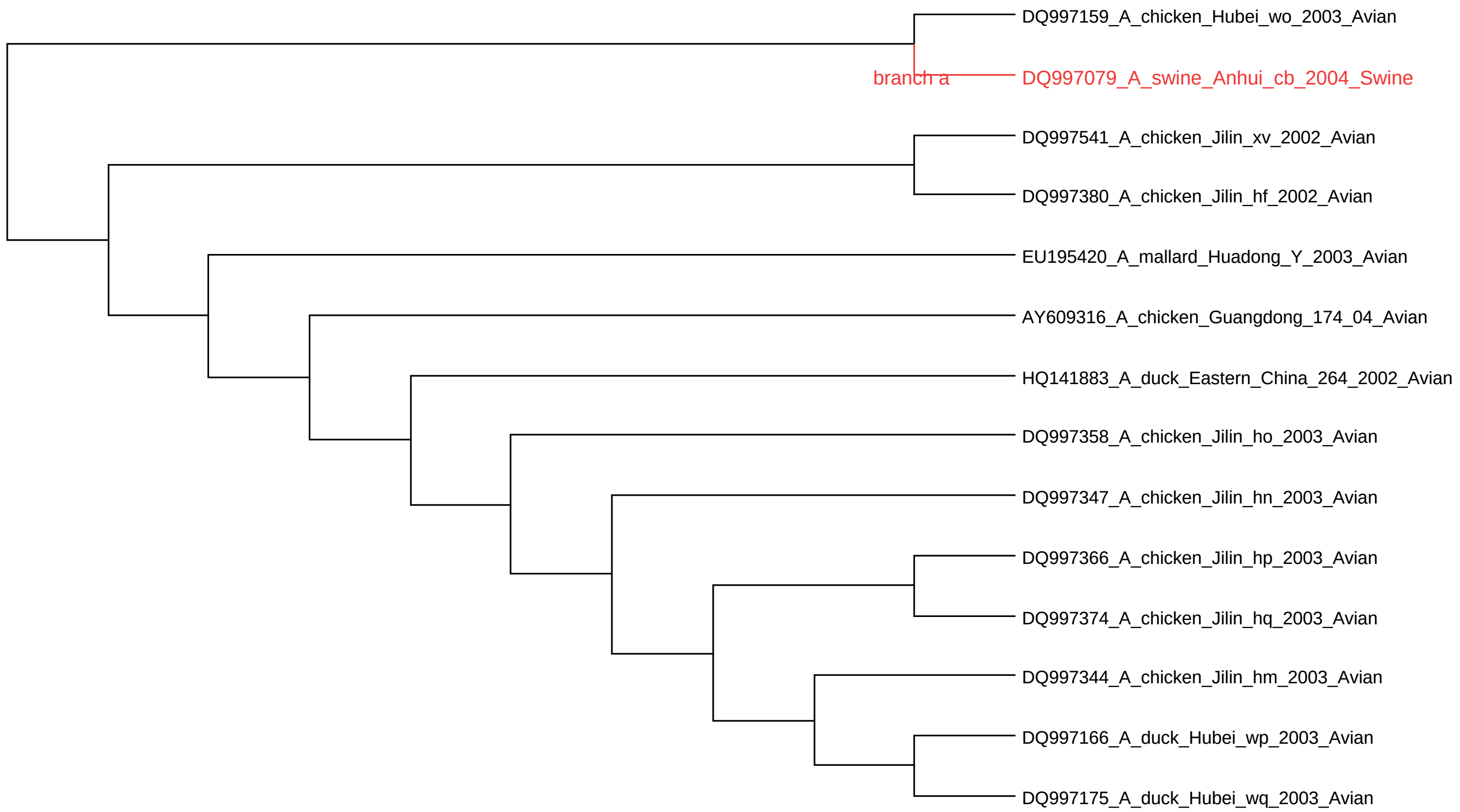
DQ997159_A_chicken_Hubei_wo_2003_Avian
branch a
DQ997079_A_swine_Anhui_cb_2004_Swine
DQ997541_A_chicken_Jilin_xv_2002_Avian
DQ997380_A_chicken_Jilin_hf_2002_Avian
EU195420_A_mallard_Huadong_Y_2003_Avian
AY609316_A_chicken_Guangdong_174_04_Avian
HQ141883_A_duck_Eastern_China_264_2002_Avian
DQ997358_A_chicken_Jilin_ho_2003_Avian
DQ997347_A_chicken_Jilin_hn_2003_Avian
DQ997366_A_chicken_Jilin_hp_2003_Avian
DQ997374_A_chicken_Jilin_hq_2003_Avian
DQ997344_A_chicken_Jilin_hm_2003_Avian
DQ997166_A_duck_Hubei_wp_2003_Avian
DQ997175_A_duck_Hubei_wq_2003_Avian

NS-Group79

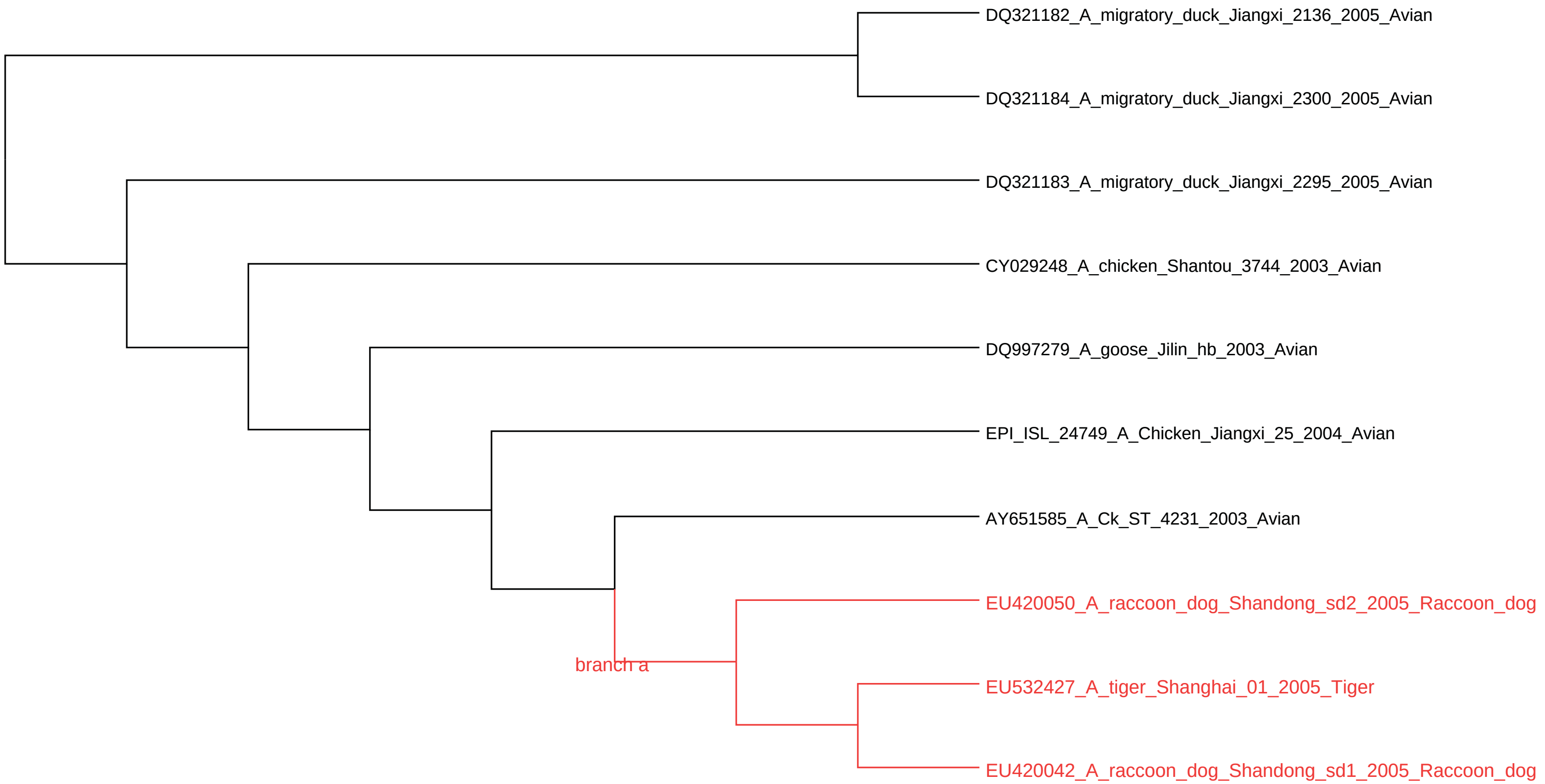
DQ321182_A_migratory_duck_Jiangxi_2136_2005_Avian
DQ321184_A_migratory_duck_Jiangxi_2300_2005_Avian
DQ321183_A_migratory_duck_Jiangxi_2295_2005_Avian
CY029248_A_chicken_Shantou_3744_2003_Avian
DQ997279_A_goose_Jilin_hb_2003_Avian
EPI_ISL_24749_A_Chicken_Jiangxi_25_2004_Avian
AY651585_A_Ck_ST_4231_2003_Avian
EU420050_A_raccoon_dog_Shandong_sd2_2005_Raccoon_dog
branch a
EU532427_A_tiger_Shanghai_01_2005_Tiger
EU420042_A_raccoon_dog_Shandong_sd1_2005_Raccoon_dog

NS-Group80

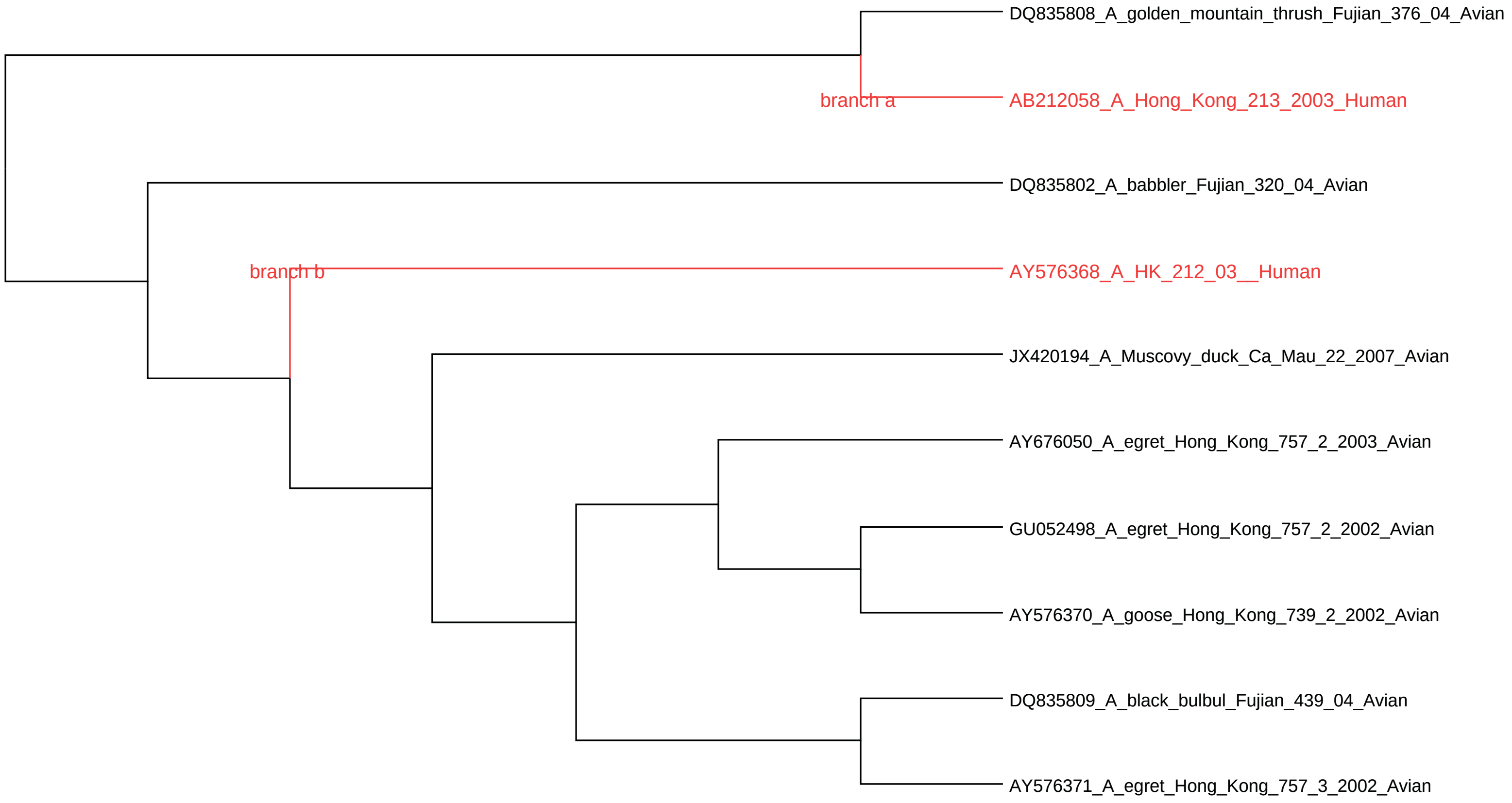
DQ835808_A_golden_mountain_thrush_Fujian_376_04_Avian
branch a
AB212058_A_Hong_Kong_213_2003_Human
DQ835802_A_babbler_Fujian_320_04_Avian
branch b
AY576368_A_HK_212_03__Human
JX420194_A_Muscovy_duck_Ca_Mau_22_2007_Avian
AY676050_A_egret_Hong_Kong_757_2_2003_Avian
GU052498_A_egret_Hong_Kong_757_2_2002_Avian
AY576370_A_goose_Hong_Kong_739_2_2002_Avian
DQ835809_A_black_bulbul_Fujian_439_04_Avian
AY576371_A_egret_Hong_Kong_757_3_2002_Avian

NS-Group81

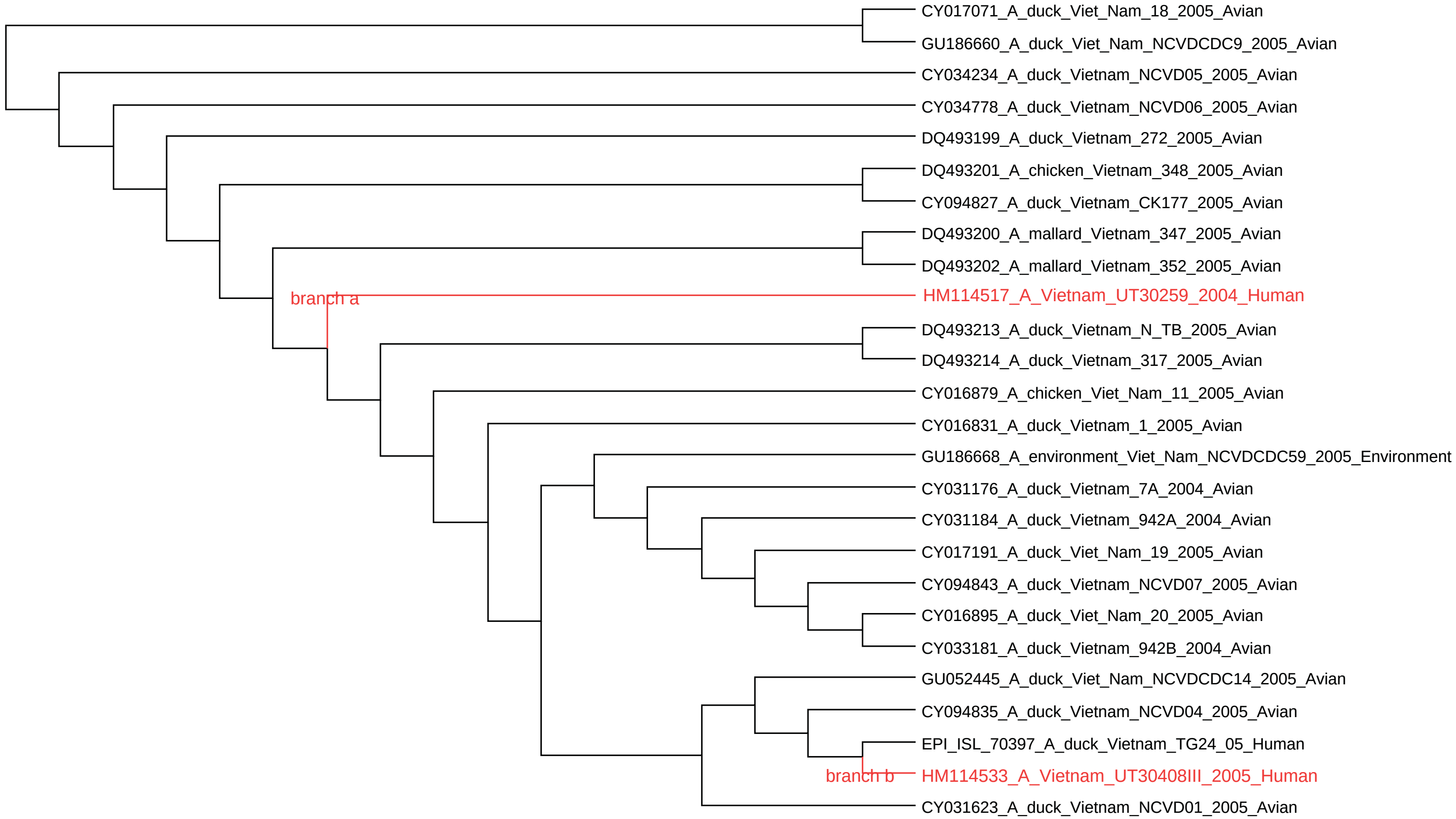

CY017071_A_duck_Viet_Nam_18_2005_Avian
GU186660_A_duck_Viet_Nam_NCVDCDC9_2005_Avian
CY034234_A_duck_Vietnam_NCVD05_2005_Avian
CY034778_A_duck_Vietnam_NCVD06_2005_Avian
DQ493199_A_duck_Vietnam_272_2005_Avian
DQ493201_A_chicken_Vietnam_348_2005_Avian
CY094827_A_duck_Vietnam_CK177_2005_Avian
DQ493200_A_mallard_Vietnam_347_2005_Avian
DQ493202_A_mallard_Vietnam_352_2005_Avian
branch a
HM114517_A_Vietnam_UT30259_2004_Human
DQ493213_A_duck_Vietnam_N_TB_2005_Avian
DQ493214_A_duck_Vietnam_317_2005_Avian
CY016879_A_chicken_Viet_Nam_11_2005_Avian
CY016831_A_duck_Vietnam_1_2005_Avian
GU186668_A_environment_Viet_Nam_NCVDCDC59_2005_Environment
CY031176_A_duck_Vietnam_7A_2004_Avian
CY031184_A_duck_Vietnam_942A_2004_Avian
CY017191_A_duck_Viet_Nam_19_2005_Avian
CY094843_A_duck_Vietnam_NCVD07_2005_Avian
CY016895_A_duck_Viet_Nam_20_2005_Avian
CY033181_A_duck_Vietnam_942B_2004_Avian
GU052445_A_duck_Viet_Nam_NCVDCDC14_2005_Avian
CY094835_A_duck_Vietnam_NCVD04_2005_Avian
EPI_ISL_70397_A_duck_Vietnam_TG24_05_Human
branch b
HM114533_A_Vietnam_UT30408III_2005_Human
CY031623_A_duck_Vietnam_NCVD01_2005_Avian

NS-Group82

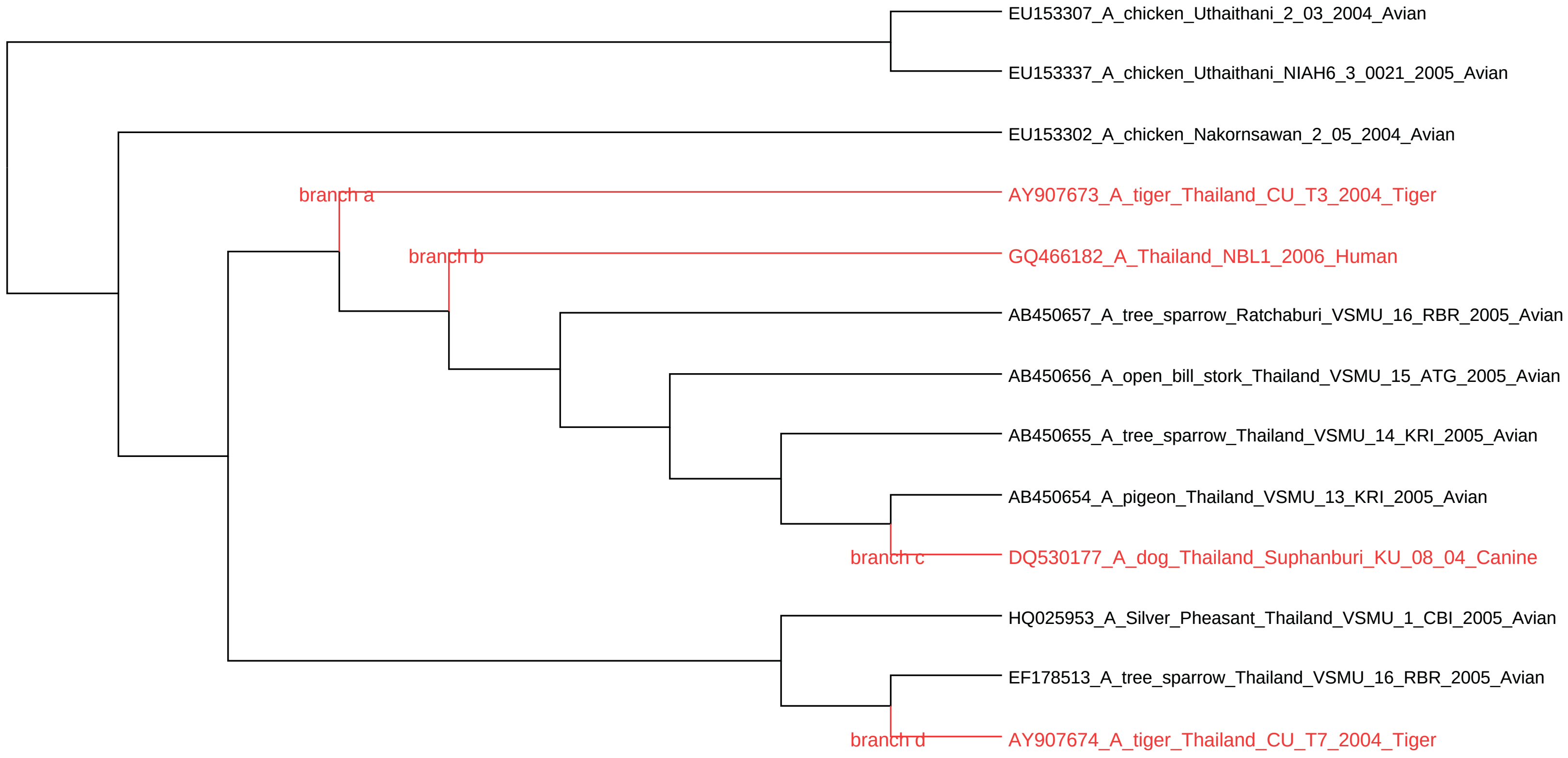

EU153307_A_chicken_Uthaithani_2_03_2004_Avian
EU153337_A_chicken_Uthaithani_NIAH6_3_0021_2005_Avian
EU153302_A_chicken_Nakornsawan_2_05_2004_Avian
branch a
AY907673_A_tiger_Thailand_CU_T3_2004_Tiger
branch b
GQ466182_A_Thailand_NBL1_2006_Human
AB450657_A_tree_sparrow_Ratchaburi_VSMU_16_RBR_2005_Avian
AB450656_A_open_bill_stork_Thailand_VSMU_15_ATG_2005_Avian
AB450655_A_tree_sparrow_Thailand_VSMU_14_KRI_2005_Avian
AB450654_A_pigeon_Thailand_VSMU_13_KRI_2005_Avian
branch c
DQ530177_A_dog_Thailand_Suphanburi_KU_08_04_Canine
HQ025953_A_Silver_Pheasant_Thailand_VSMU_1_CBI_2005_Avian
EF178513_A_tree_sparrow_Thailand_VSMU_16_RBR_2005_Avian
branch d
AY907674_A_tiger_Thailand_CU_T7_2004_Tiger

NS-Group83

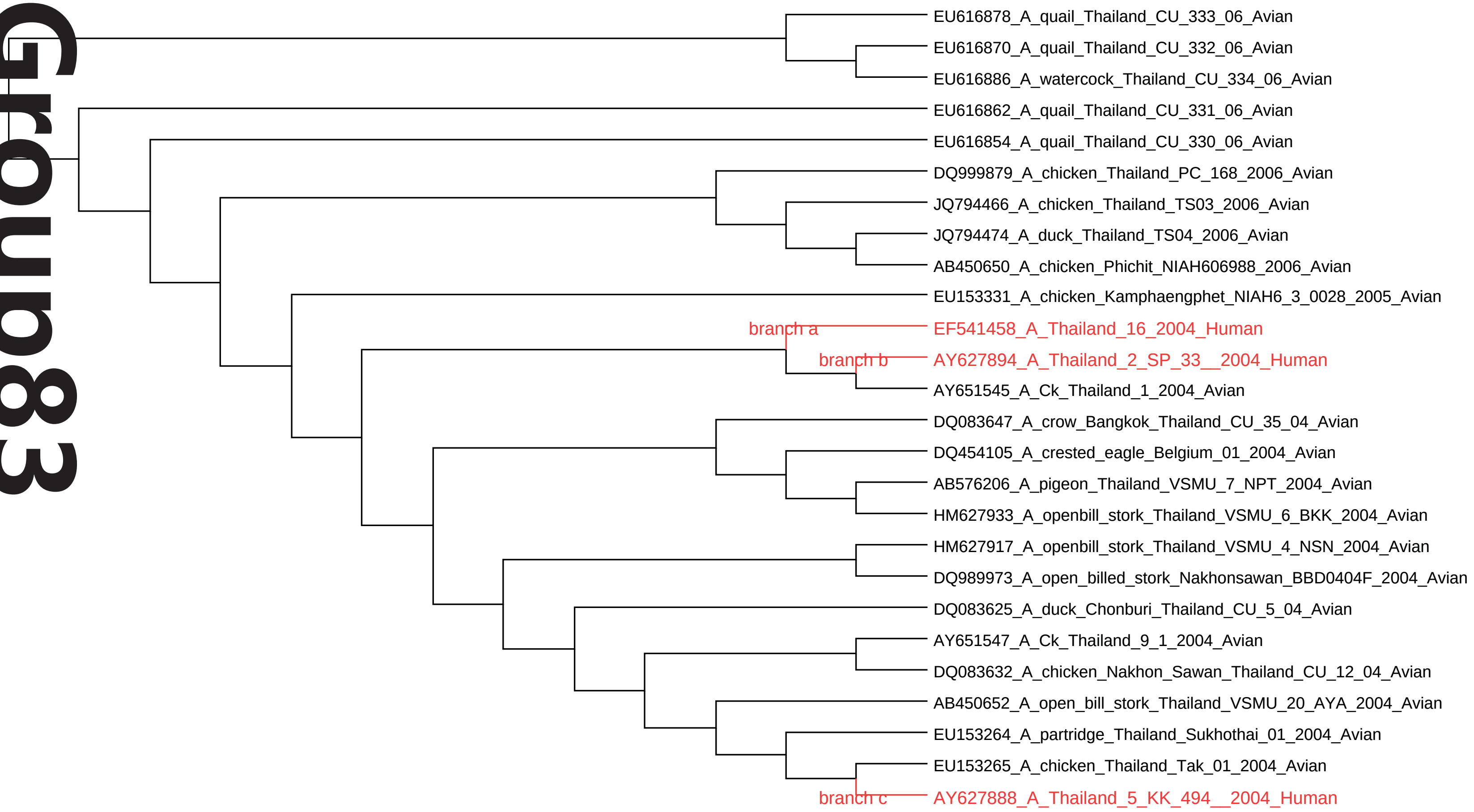

EU616878_A_quail_Thailand_CU_333_06_Avian
EU616870_A_quail_Thailand_CU_332_06_Avian
EU616886_A_watercock_Thailand_CU_334_06_Avian
EU616862_A_quail_Thailand_CU_331_06_Avian
EU616854_A_quail_Thailand_CU_330_06_Avian
DQ999879_A_chicken_Thailand_PC_168_2006_Avian
JQ794466_A_chicken_Thailand_TS03_2006_Avian
JQ794474_A_duck_Thailand_TS04_2006_Avian
AB450650_A_chicken_Phichit_NIAH606988_2006_Avian
EU153331_A_chicken_Kamphaengphet_NIAH6_3_0028_2005_Avian
branch a
EF541458_A_Thailand_16_2004_Human
branch b
AY627894_A_Thailand_2_SP_33__2004_Human
AY651545_A_Ck_Thailand_1_2004_Avian
DQ083647_A_crow_Bangkok_Thailand_CU_35_04_Avian
DQ454105_A_crested_eagle_Belgium_01_2004_Avian
AB576206_A_pigeon_Thailand_VSMU_7_NPT_2004_Avian
HM627933_A_openbill_stork_Thailand_VSMU_6_BKK_2004_Avian
HM627917_A_openbill_stork_Thailand_VSMU_4_NSN_2004_Avian
DQ989973_A_open_billed_stork_Nakhonsawan_BBD0404F_2004_Avian
DQ083625_A_duck_Chonburi_Thailand_CU_5_04_Avian
AY651547_A_Ck_Thailand_9_1_2004_Avian
DQ083632_A_chicken_Nakhon_Sawan_Thailand_CU_12_04_Avian
AB450652_A_open_bill_stork_Thailand_VSMU_20_AYA_2004_Avian
EU153264_A_partridge_Thailand_Sukhothai_01_2004_Avian
EU153265_A_chicken_Thailand_Tak_01_2004_Avian
branch c
AY627888_A_Thailand_5_KK_494__2004_Human

NS-Group84

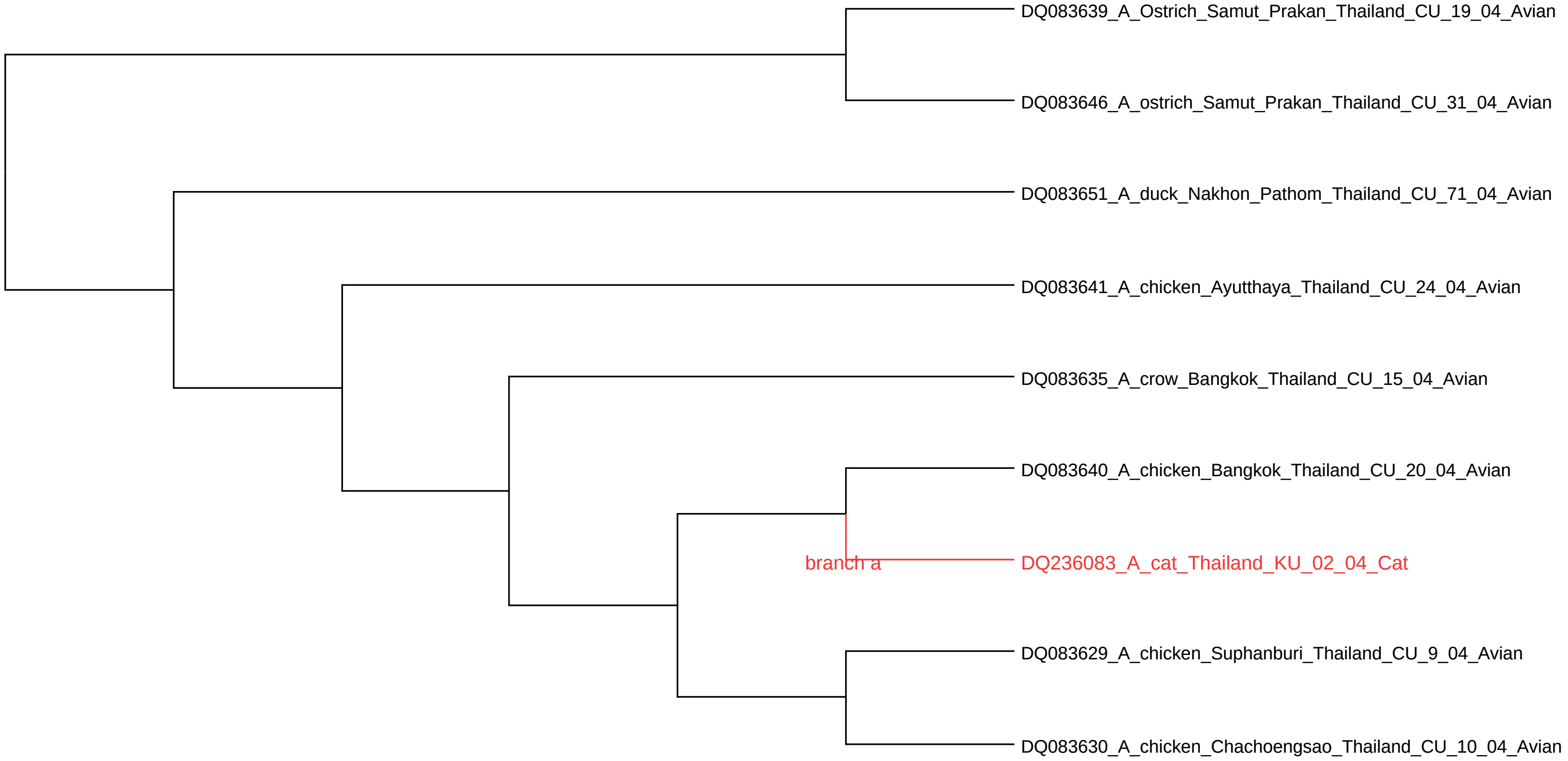
DQ083639_A_Ostrich_Samut_Prakan_Thailand_CU_19_04_Avian
DQ083646_A_ostrich_Samut_Prakan_Thailand_CU_31_04_Avian
DQ083651_A_duck_Nakhon_Pathom_Thailand_CU_71_04_Avian
DQ083641_A_chicken_Ayutthaya_Thailand_CU_24_04_Avian
DQ083635_A_crow_Bangkok_Thailand_CU_15_04_Avian
DQ083640_A_chicken_Bangkok_Thailand_CU_20_04_Avian
branch a
DQ236083_A_cat_Thailand_KU_02_04_Cat
DQ083629_A_chicken_Suphanburi_Thailand_CU_9_04_Avian
DQ083630_A_chicken_Chachoengsao_Thailand_CU_10_04_Avian

NS-Group85

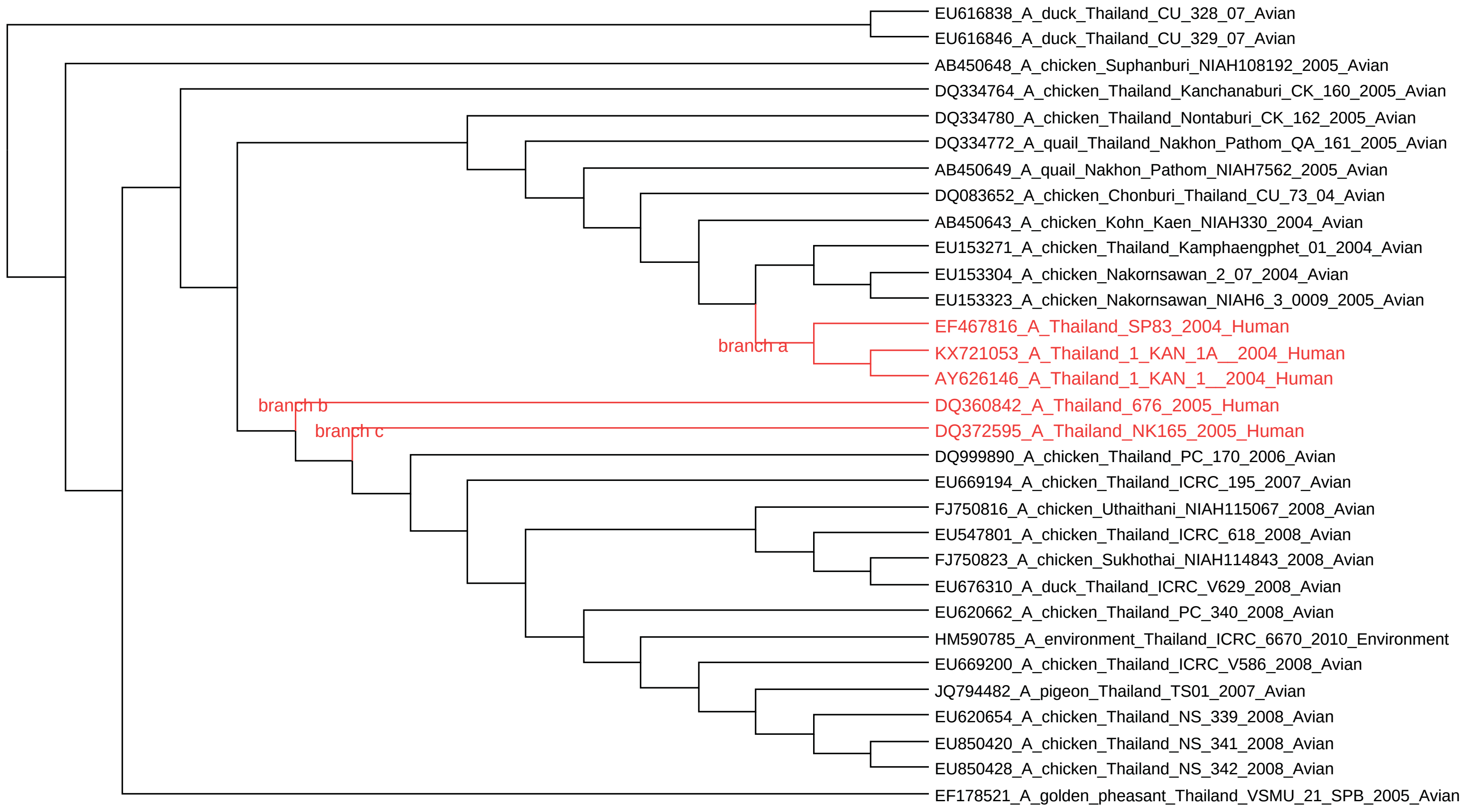
EU616838_A_duck_Thailand_CU_328_07_Avian
EU616846_A_duck_Thailand_CU_329_07_Avian
AB450648_A_chicken_Suphanburi_NIAH108192_2005_Avian
DQ334764_A_chicken_Thailand_Kanchanaburi_CK_160_2005_Avian
DQ334780_A_chicken_Thailand_Nontaburi_CK_162_2005_Avian
DQ334772_A_quail_Thailand_Nakhon_Pathom_QA_161_2005_Avian
AB450649_A_quail_Nakhon_Pathom_NIAH7562_2005_Avian
DQ083652_A_chicken_Chonburi_Thailand_CU_73_04_Avian
AB450643_A_chicken_Kohn_Kaen_NIAH330_2004_Avian
EU153271_A_chicken_Thailand_Kamphaengphet_01_2004_Avian
EU153304_A_chicken_Nakornsawan_2_07_2004_Avian
EU153323_A_chicken_Nakornsawan_NIAH6_3_0009_2005_Avian
EF467816_A_Thailand_SP83_2004_Human
branch a
KX721053_A_Thailand_1_KAN_1A__2004_Human
AY626146_A_Thailand_1_KAN_1__2004_Human
branch b
DQ360842_A_Thailand_676_2005_Human
branch c
DQ372595_A_Thailand_NK165_2005_Human
DQ999890_A_chicken_Thailand_PC_170_2006_Avian
EU669194_A_chicken_Thailand_ICRC_195_2007_Avian
FJ750816_A_chicken_Uthaithani_NIAH115067_2008_Avian
EU547801_A_chicken_Thailand_ICRC_618_2008_Avian
FJ750823_A_chicken_Sukhothai_NIAH114843_2008_Avian
EU676310_A_duck_Thailand_ICRC_V629_2008_Avian
EU620662_A_chicken_Thailand_PC_340_2008_Avian
HM590785_A_environment_Thailand_ICRC_6670_2010_Environment
EU669200_A_chicken_Thailand_ICRC_V586_2008_Avian
JQ794482_A_pigeon_Thailand_TS01_2007_Avian
EU620654_A_chicken_Thailand_NS_339_2008_Avian
EU850420_A_chicken_Thailand_NS_341_2008_Avian
EU850428_A_chicken_Thailand_NS_342_2008_Avian
EF178521_A_golden_pheasant_Thailand_VSMU_21_SPB_2005_Avian

NS-Group86

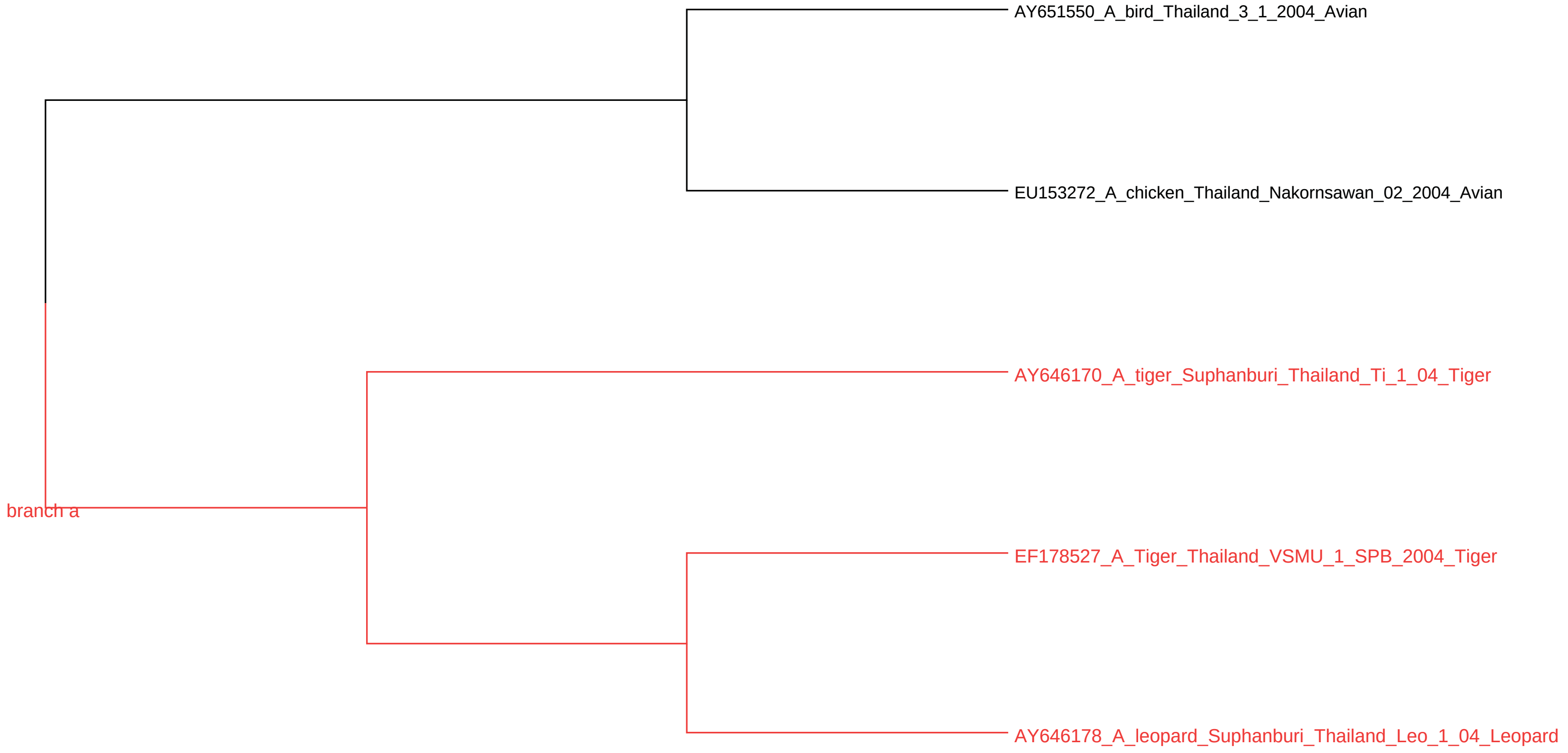

AY651550_A_bird_Thailand_3_1_2004_Avian
EU153272_A_chicken_Thailand_Nakornsawan_02_2004_Avian
AY646170_A_tiger_Suphanburi_Thailand_Ti_1_04_Tiger
branch a
EF178527_A_Tiger_Thailand_VSMU_1_SPB_2004_Tiger
AY646178_A_leopard_Suphanburi_Thailand_Leo_1_04_Leopard

NS-Group87

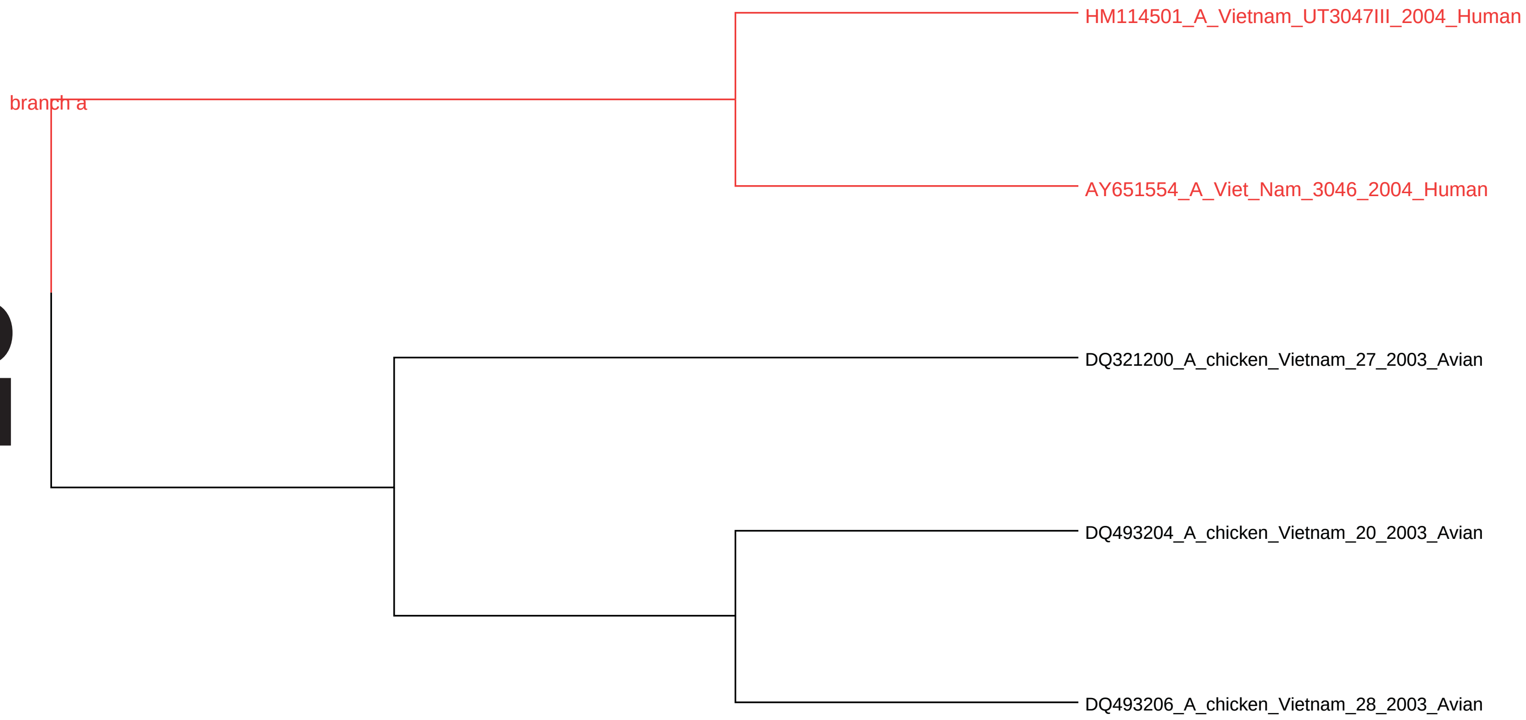

branch a
HM114501_A_Vietnam_UT3047III_2004_Human
AY651554_A_Viet_Nam_3046_2004_Human
DQ321200_A_chicken_Vietnam_27_2003_Avian
DQ493204_A_chicken_Vietnam_20_2003_Avian
DQ493206_A_chicken_Vietnam_28_2003_Avian

NS-Group88

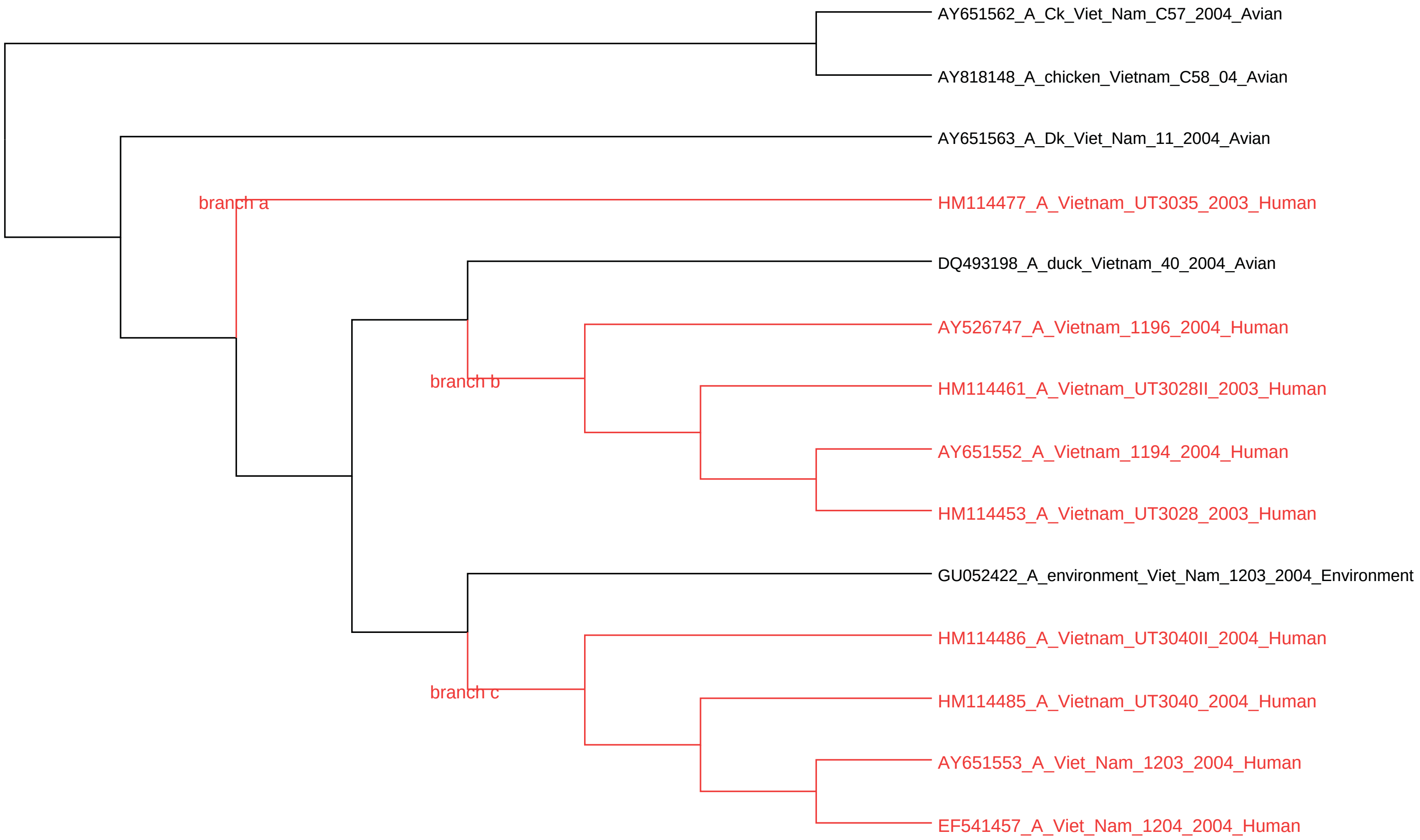
AY651562_A_Ck_Viet_Nam_C57_2004_Avian
AY818148_A_chicken_Vietnam_C58_04_Avian
AY651563_A_Dk_Viet_Nam_11_2004_Avian
branch a
HM114477_A_Vietnam_UT3035_2003_Human
DQ493198_A_duck_Vietnam_40_2004_Avian
AY526747_A_Vietnam_1196_2004_Human
branch b
HM114461_A_Vietnam_UT3028II_2003_Human
AY651552_A_Vietnam_1194_2004_Human
HM114453_A_Vietnam_UT3028_2003_Human
GU052422_A_environment_Viet_Nam_1203_2004_Environment
HM114486_A_Vietnam_UT3040II_2004_Human
branch c
HM114485_A_Vietnam_UT3040_2004_Human
AY651553_A_Viet_Nam_1203_2004_Human
EF541457_A_Viet_Nam_1204_2004_Human

NS-Group89

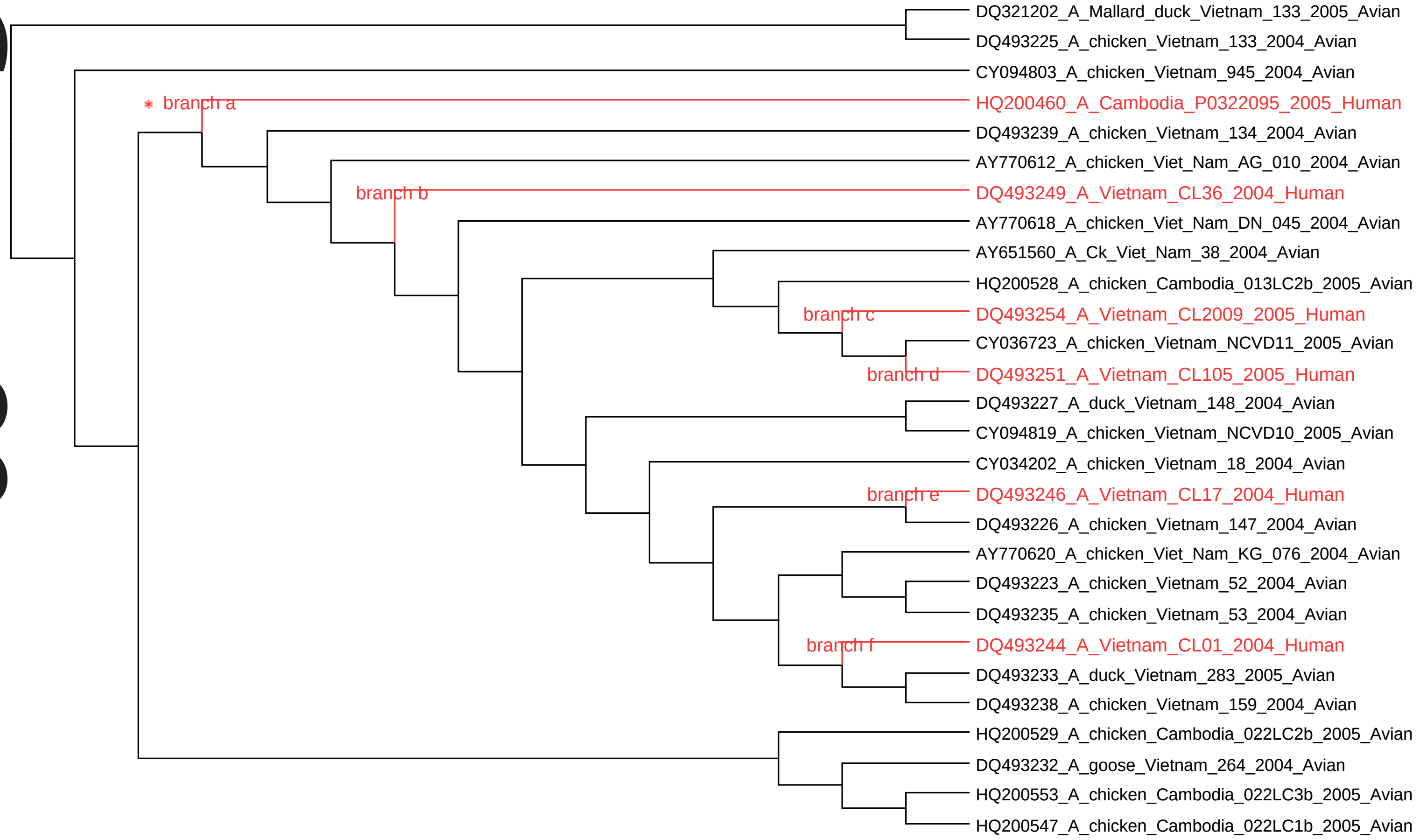
DQ321202_A_Mallard_duck_Vietnam_133_2005_Avian
DQ493225_A_chicken_Vietnam_133_2004_Avian
CY094803_A_chicken_Vietnam_945_2004_Avian
* branch a
HQ200460_A_Cambodia_P0322095_2005_Human
DQ493239_A_chicken_Vietnam_134_2004_Avian
AY770612_A_chicken_Viet_Nam_AG_010_2004_Avian
branch b
DQ493249_A_Vietnam_CL36_2004_Human
AY770618_A_chicken_Viet_Nam_DN_045_2004_Avian
AY651560_A_Ck_Viet_Nam_38_2004_Avian
HQ200528_A_chicken_Cambodia_013LC2b_2005_Avian
branch c
DQ493254_A_Vietnam_CL2009_2005_Human
CY036723_A_chicken_Vietnam_NCVD11_2005_Avian
branch d
DQ493251_A_Vietnam_CL105_2005_Human
DQ493227_A_duck_Vietnam_148_2004_Avian
CY094819_A_chicken_Vietnam_NCVD10_2005_Avian
CY034202_A_chicken_Vietnam_18_2004_Avian
branch e
DQ493246_A_Vietnam_CL17_2004_Human
DQ493226_A_chicken_Vietnam_147_2004_Avian
AY770620_A_chicken_Viet_Nam_KG_076_2004_Avian
DQ493223_A_chicken_Vietnam_52_2004_Avian
DQ493235_A_chicken_Vietnam_53_2004_Avian
branch f
DQ493244_A_Vietnam_CL01_2004_Human
DQ493233_A_duck_Vietnam_283_2005_Avian
DQ493238_A_chicken_Vietnam_159_2004_Avian
HQ200529_A_chicken_Cambodia_022LC2b_2005_Avian
DQ493232_A_goose_Vietnam_264_2004_Avian
HQ200553_A_chicken_Cambodia_022LC3b_2005_Avian
HQ200547_A_chicken_Cambodia_022LC1b_2005_Avian

NS-Group90

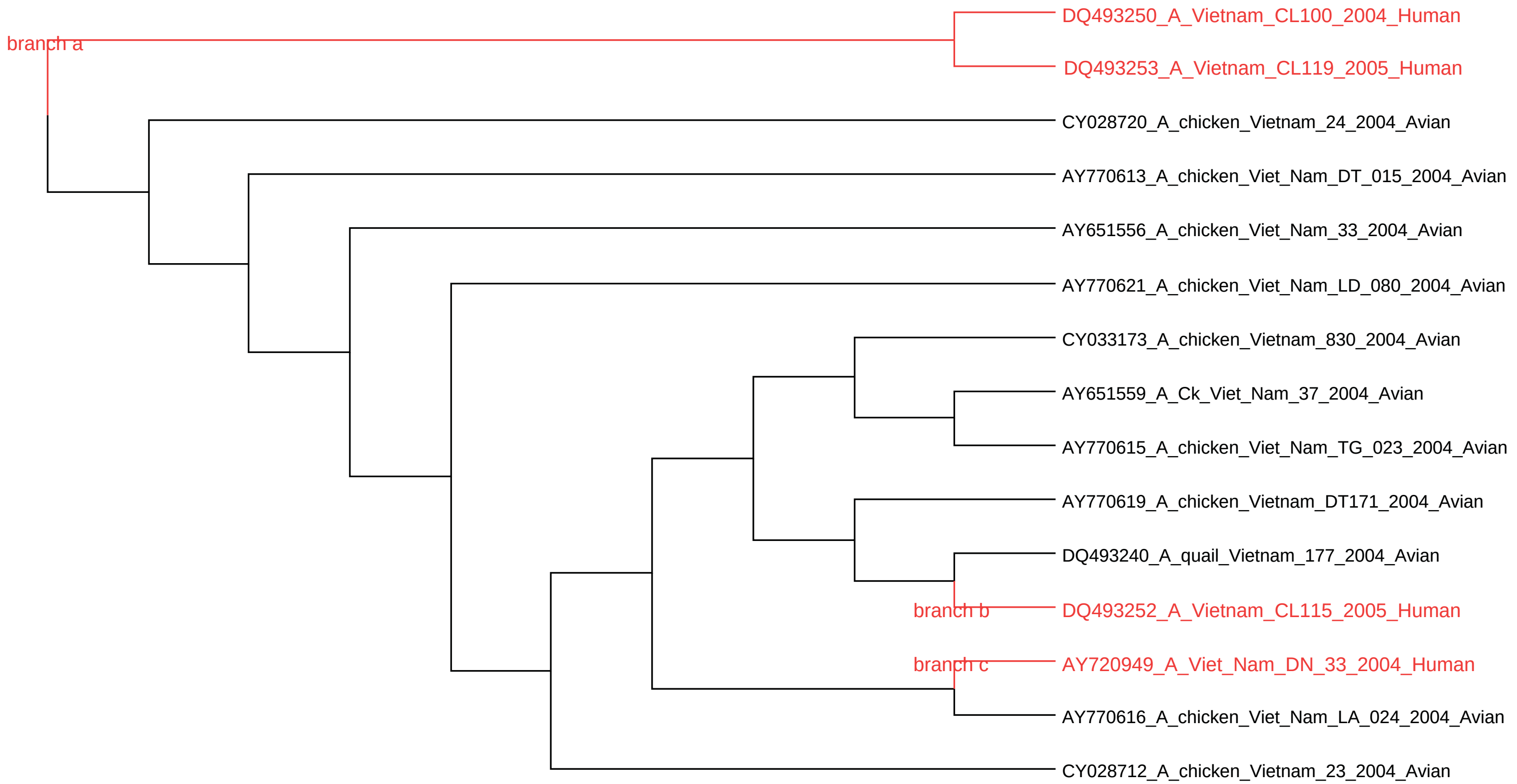

branch a
DQ493250_A_Vietnam_CL100_2004_Human
DQ493253_A_Vietnam_CL119_2005_Human
CY028720_A_chicken_Vietnam_24_2004_Avian
AY770613_A_chicken_Viet_Nam_DT_015_2004_Avian
AY651556_A_chicken_Viet_Nam_33_2004_Avian
AY770621_A_chicken_Viet_Nam_LD_080_2004_Avian
CY033173_A_chicken_Vietnam_830_2004_Avian
AY651559_A_Ck_Viet_Nam_37_2004_Avian
AY770615_A_chicken_Viet_Nam_TG_023_2004_Avian
AY770619_A_chicken_Vietnam_DT171_2004_Avian
DQ493240_A_quail_Vietnam_177_2004_Avian
branch b
DQ493252_A_Vietnam_CL115_2005_Human
branch c
AY720949_A_Viet_Nam_DN_33_2004_Human
AY770616_A_chicken_Viet_Nam_LA_024_2004_Avian
CY028712_A_chicken_Vietnam_23_2004_Avian

NS-Group91

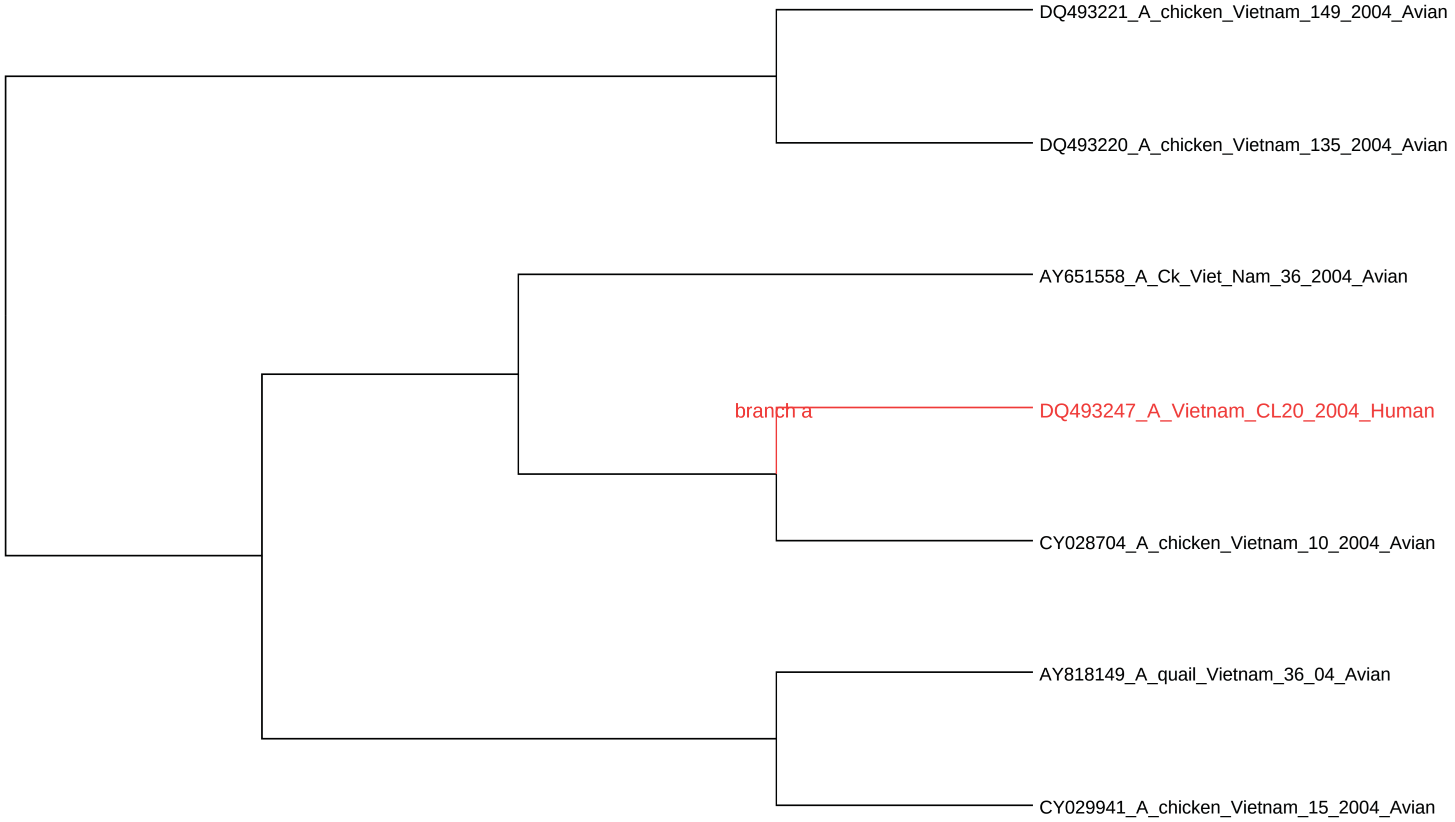

DQ493221_A_chicken_Vietnam_149_2004_Avian
DQ493220_A_chicken_Vietnam_135_2004_Avian
AY651558_A_Ck_Viet_Nam_36_2004_Avian
branch a
DQ493247_A_Vietnam_CL20_2004_Human
CY028704_A_chicken_Vietnam_10_2004_Avian
AY818149_A_quail_Vietnam_36_04_Avian
CY029941_A_chicken_Vietnam_15_2004_Avian

NS-Group92

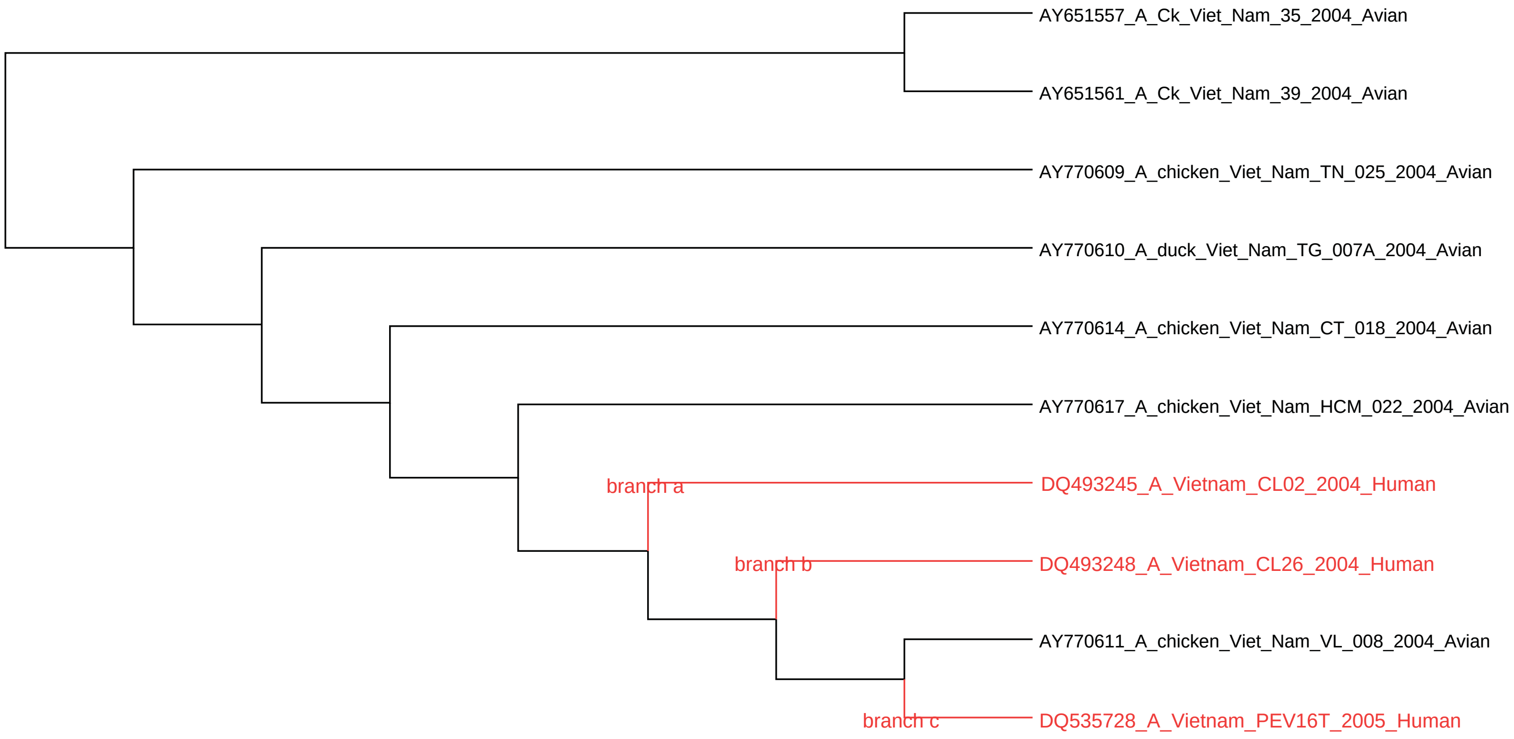

AY651557_A_Ck_Viet_Nam_35_2004_Avian
AY651561_A_Ck_Viet_Nam_39_2004_Avian
AY770609_A_chicken_Viet_Nam_TN_025_2004_Avian
AY770610_A_duck_Viet_Nam_TG_007A_2004_Avian
AY770614_A_chicken_Viet_Nam_CT_018_2004_Avian
AY770617_A_chicken_Viet_Nam_HCM_022_2004_Avian
branch a
DQ493245_A_Vietnam_CL02_2004_Human
branch b
DQ493248_A_Vietnam_CL26_2004_Human
AY770611_A_chicken_Viet_Nam_VL_008_2004_Avian
branch c
DQ535728_A_Vietnam_PEV16T_2005_Human

NS-Group93

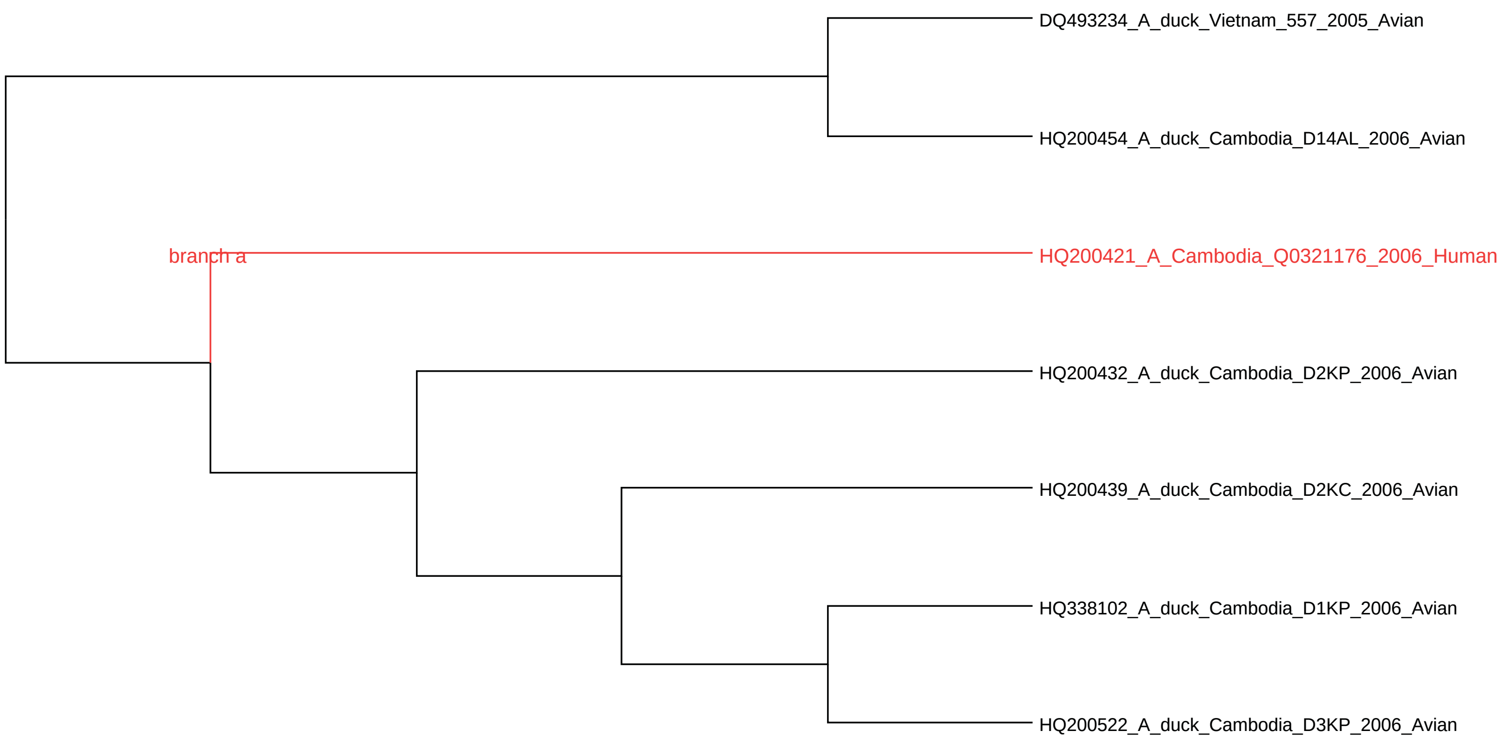
DQ493234_A_duck_Vietnam_557_2005_Avian
HQ200454_A_duck_Cambodia_D14AL_2006_Avian
branch a
HQ200421_A_Cambodia_Q0321176_2006_Human
HQ200432_A_duck_Cambodia_D2KP_2006_Avian
HQ200439_A_duck_Cambodia_D2KC_2006_Avian
HQ338102_A_duck_Cambodia_D1KP_2006_Avian
HQ200522_A_duck_Cambodia_D3KP_2006_Avian

NS-Group94

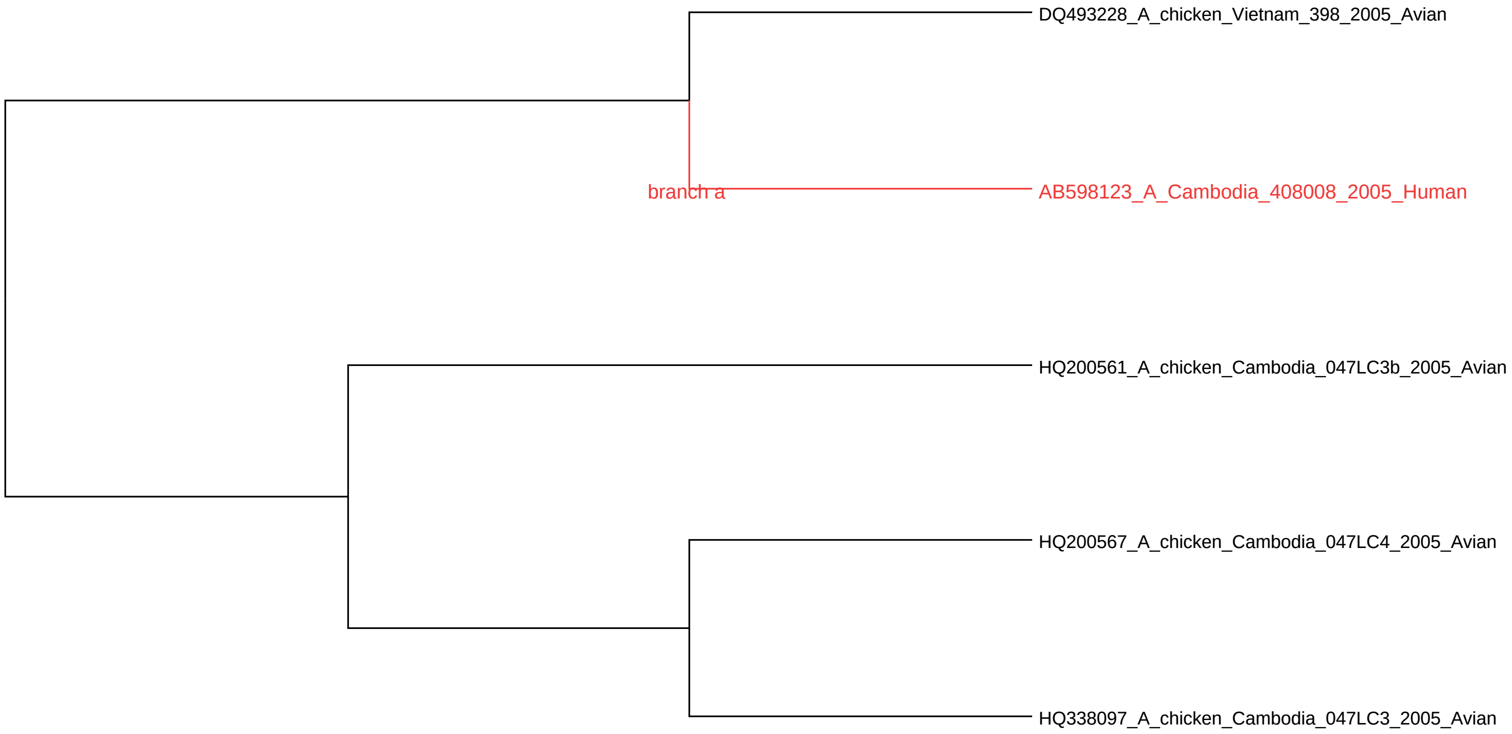

DQ493228_A_chicken_Vietnam_398_2005_Avian
branch a
AB598123_A_Cambodia_408008_2005_Human
HQ200561_A_chicken_Cambodia_047LC3b_2005_Avian
HQ200567_A_chicken_Cambodia_047LC4_2005_Avian
HQ338097_A_chicken_Cambodia_047LC3_2005_Avian

NS-Group95

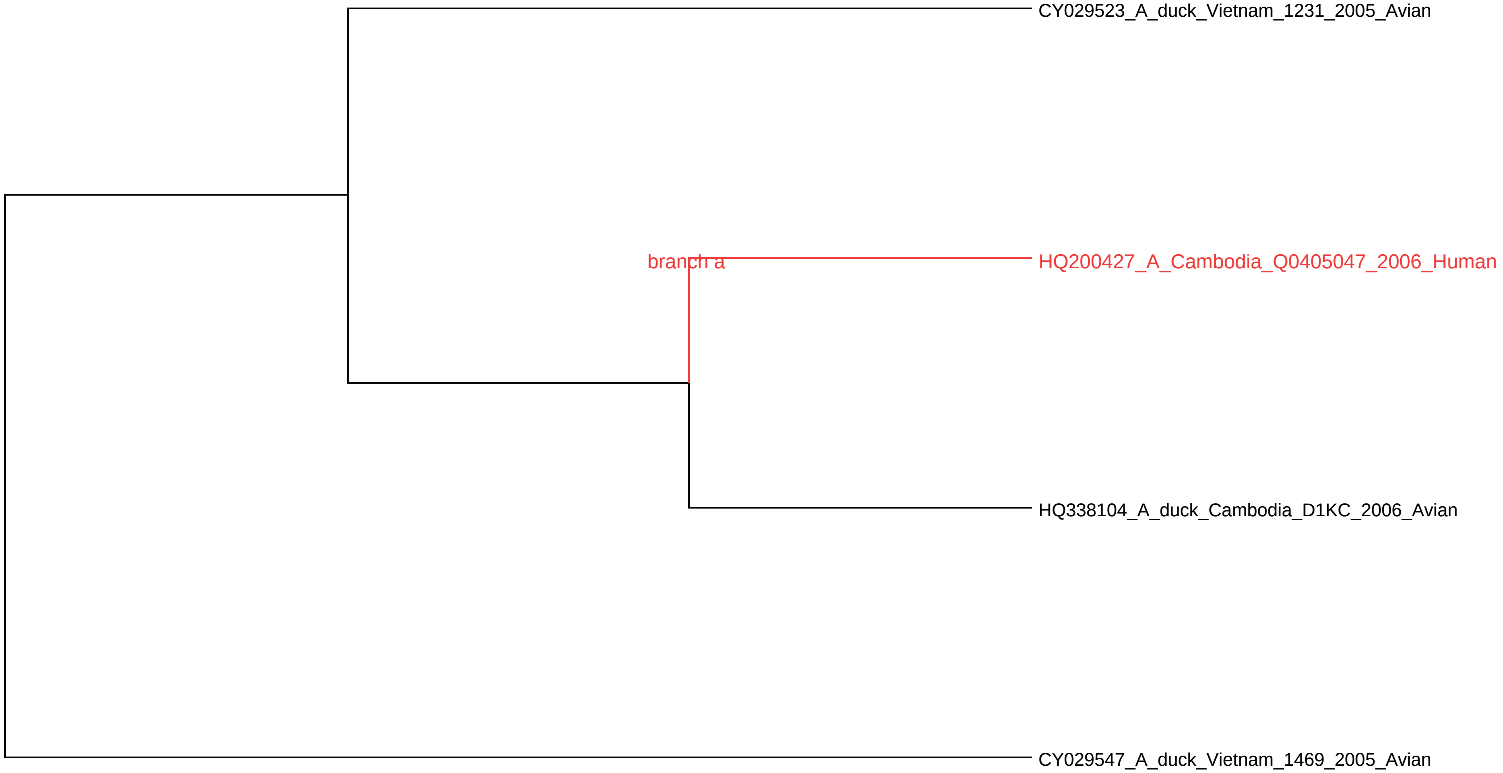

CY029523_A_duck_Vietnam_1231_2005_Avian
branch a
HQ200427_A_Cambodia_Q0405047_2006_Human
HQ338104_A_duck_Cambodia_D1KC_2006_Avian
CY029547_A_duck_Vietnam_1469_2005_Avian

NS-Group6

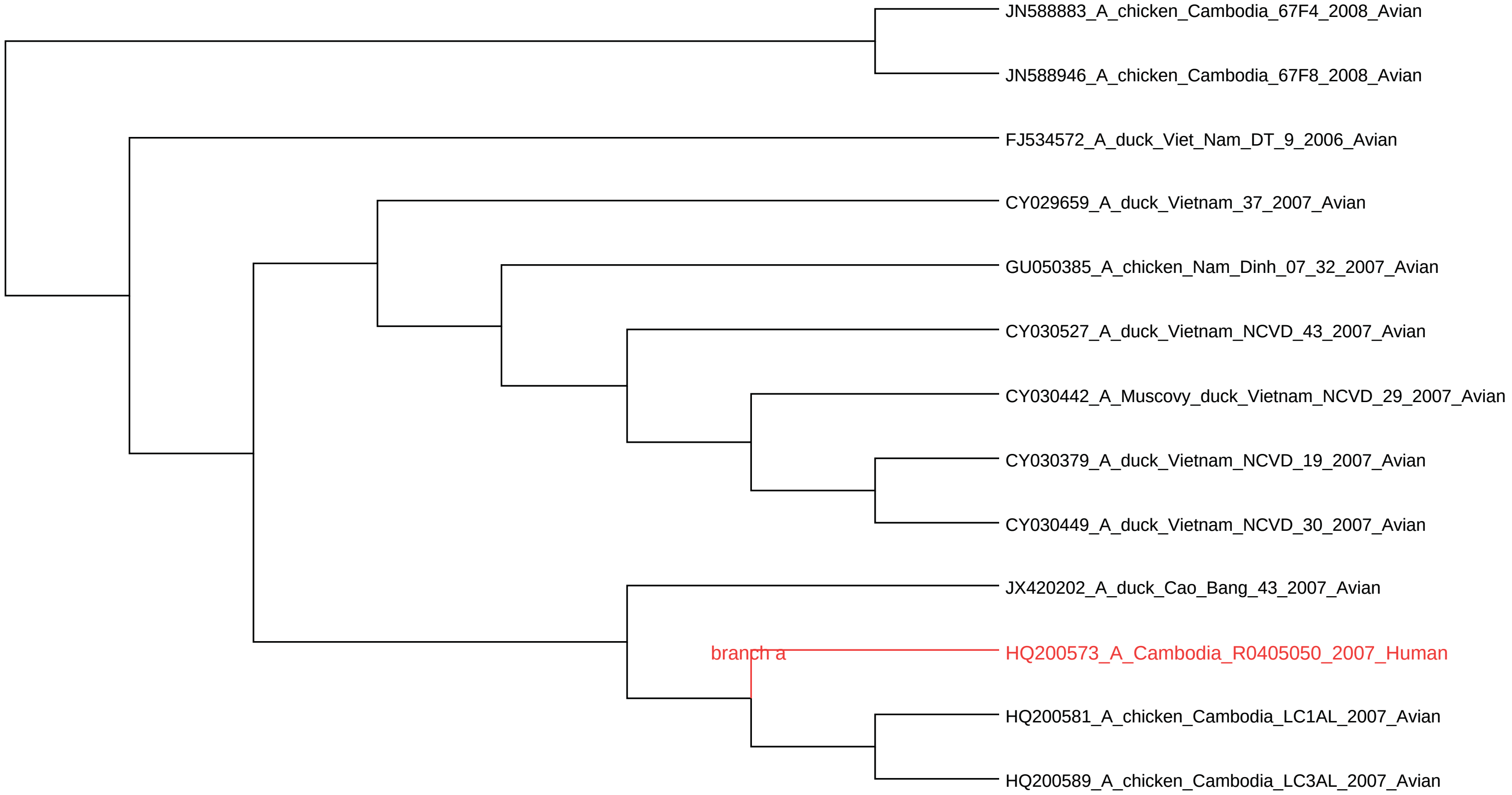

JN588883_A_chicken_Cambodia_67F4_2008_Avian
JN588946_A_chicken_Cambodia_67F8_2008_Avian
FJ534572_A_duck_Viet_Nam_DT_9_2006_Avian
CY029659_A_duck_Vietnam_37_2007_Avian
GU050385_A_chicken_Nam_Dinh_07_32_2007_Avian
CY030527_A_duck_Vietnam_NCVD_43_2007_Avian
CY030442_A_Muscovy_duck_Vietnam_NCVD_29_2007_Avian
CY030379_A_duck_Vietnam_NCVD_19_2007_Avian
CY030449_A_duck_Vietnam_NCVD_30_2007_Avian
JX420202_A_duck_Cao_Bang_43_2007_Avian
branch a
HQ200573_A_Cambodia_R0405050_2007_Human
HQ200581_A_chicken_Cambodia_LC1AL_2007_Avian
HQ200589_A_chicken_Cambodia_LC3AL_2007_Avian

NS-Group7

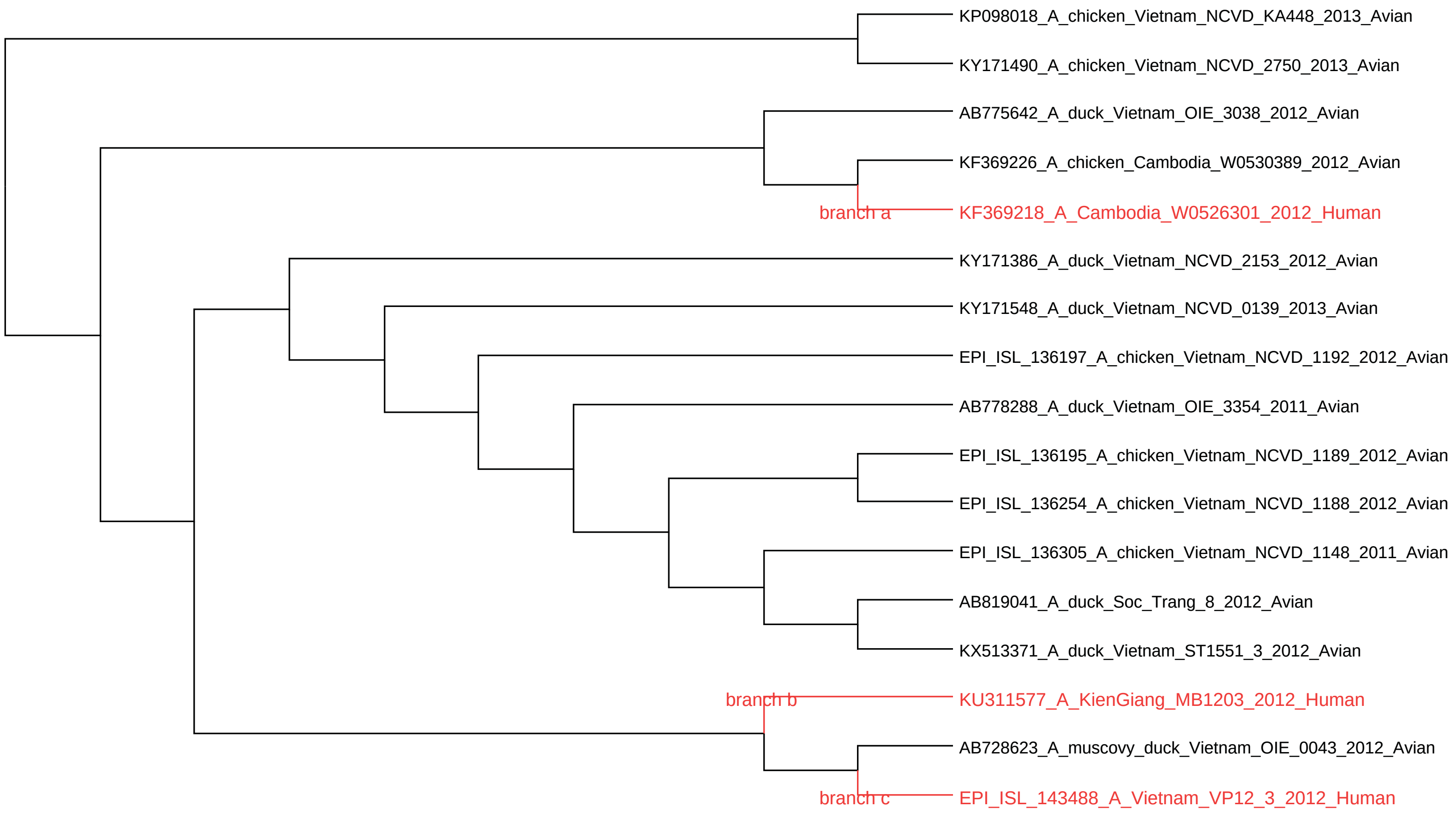

KP098018_A_chicken_Vietnam_NCVD_KA448_2013_Avian
KY171490_A_chicken_Vietnam_NCVD_2750_2013_Avian
AB775642_A_duck_Vietnam_OIE_3038_2012_Avian
KF369226_A_chicken_Cambodia_W0530389_2012_Avian
branch a
KF369218_A_Cambodia_W0526301_2012_Human
KY171386_A_duck_Vietnam_NCVD_2153_2012_Avian
KY171548_A_duck_Vietnam_NCVD_0139_2013_Avian
EPI_ISL_136197_A_chicken_Vietnam_NCVD_1192_2012_Avian
AB778288_A_duck_Vietnam_OIE_3354_2011_Avian
EPI_ISL_136195_A_chicken_Vietnam_NCVD_1189_2012_Avian
EPI_ISL_136254_A_chicken_Vietnam_NCVD_1188_2012_Avian
EPI_ISL_136305_A_chicken_Vietnam_NCVD_1148_2011_Avian
AB819041_A_duck_Soc_Trang_8_2012_Avian
KX513371_A_duck_Vietnam_ST1551_3_2012_Avian
branch b
KU311577_A_KienGiang_MB1203_2012_Human
AB728623_A_muscovy_duck_Vietnam_OIE_0043_2012_Avian
branch c
EPI_ISL_143488_A_Vietnam_VP12_3_2012_Human

NS-Group98

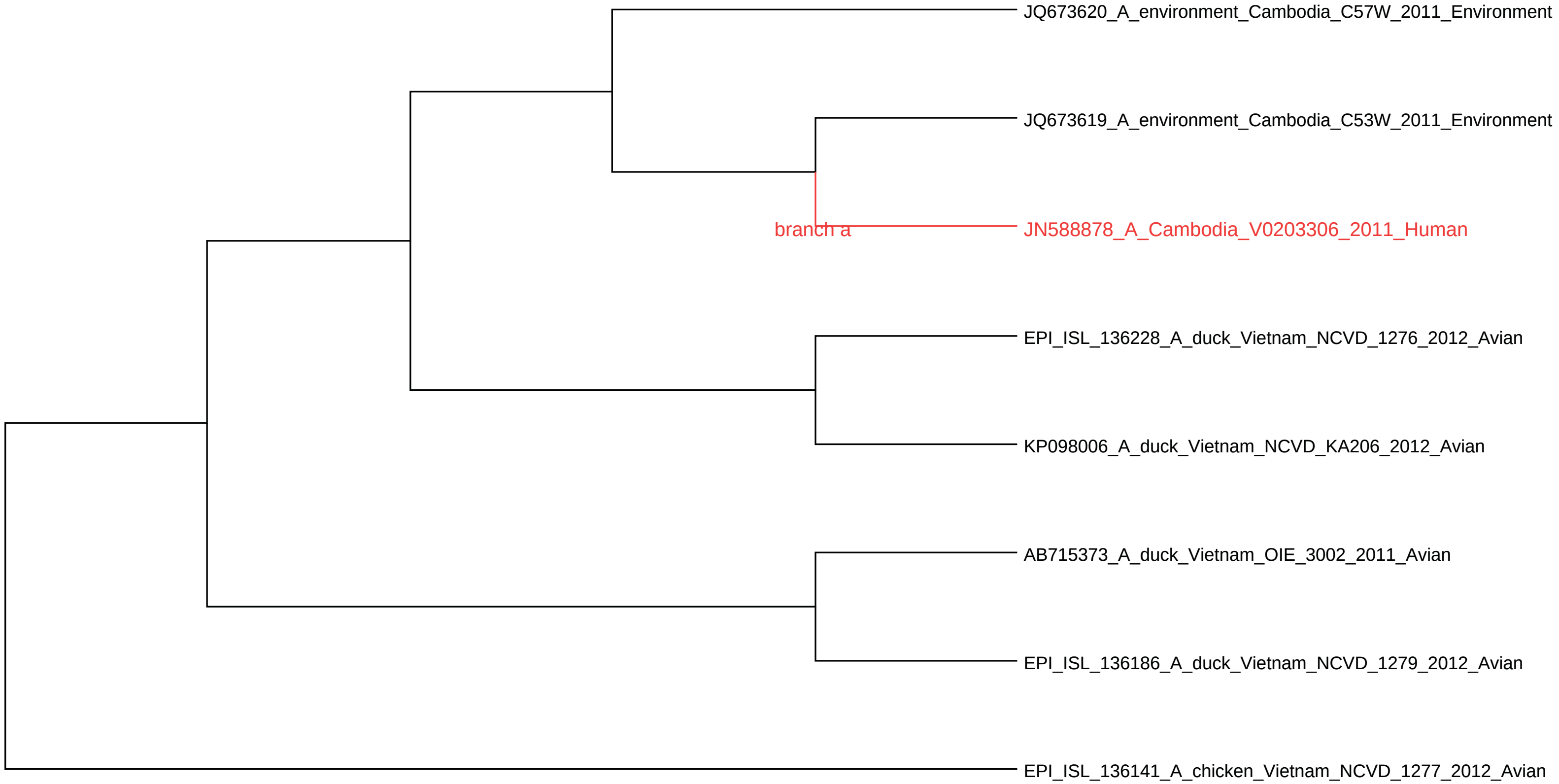
JQ673620_A_environment_Cambodia_C57W_2011_Environment
JQ673619_A_environment_Cambodia_C53W_2011_Environment
branch a
JN588878_A_Cambodia_V0203306_2011_Human
EPI_ISL_136228_A_duck_Vietnam_NCVD_1276_2012_Avian
KP098006_A_duck_Vietnam_NCVD_KA206_2012_Avian
AB715373_A_duck_Vietnam_OIE_3002_2011_Avian
EPI_ISL_136186_A_duck_Vietnam_NCVD_1279_2012_Avian
EPI_ISL_136141_A_chicken_Vietnam_NCVD_1277_2012_Avian

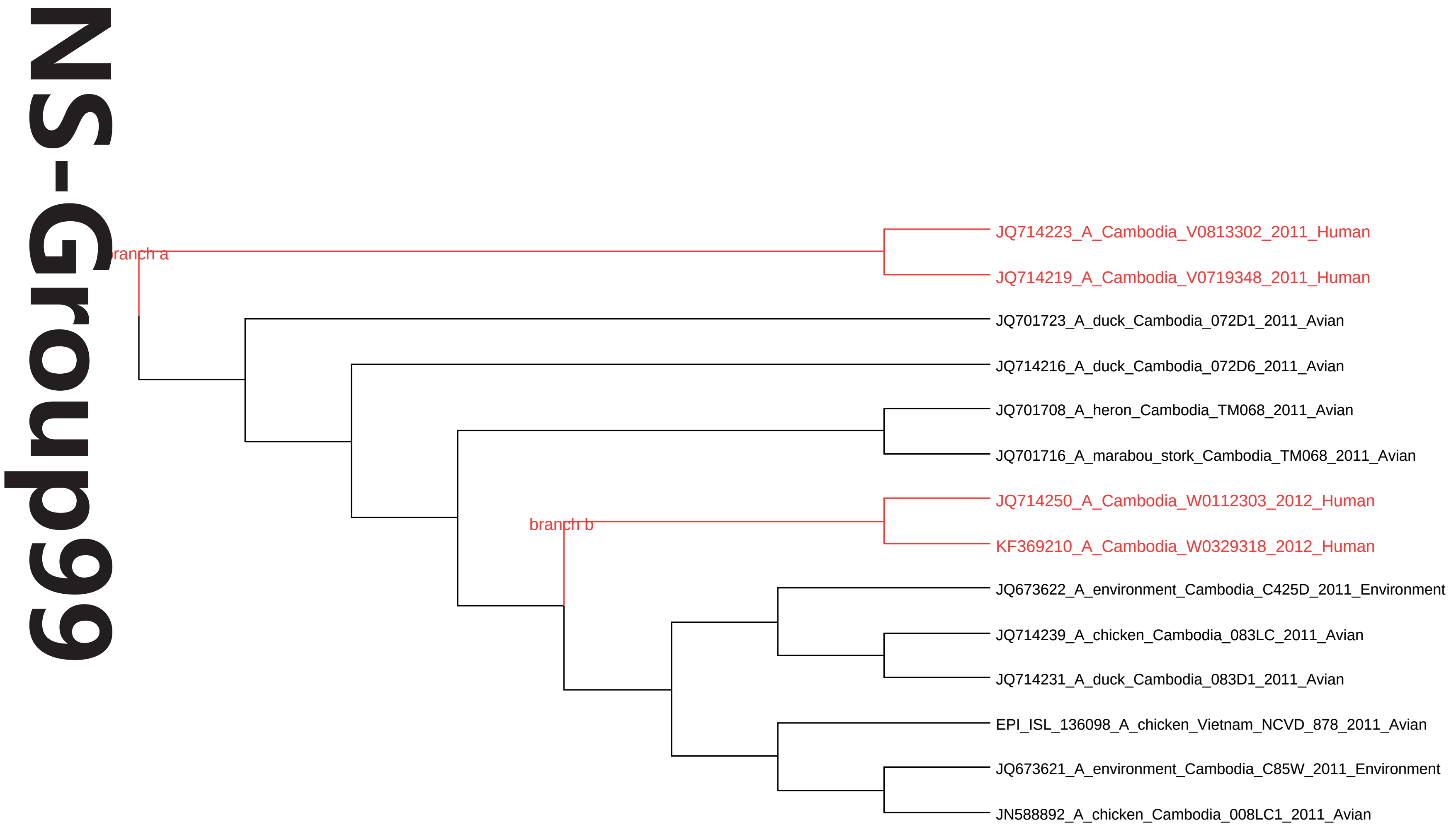

NS-Group9
branch a
branch b
JQ714223_A_Cambodia_V0813302_2011_Human
JQ714219_A_Cambodia_V0719348_2011_Human
JQ701723_A_duck_Cambodia_072D1_2011_Avian
JQ714216_A_duck_Cambodia_072D6_2011_Avian
JQ701708_A_heron_Cambodia_TM068_2011_Avian
JQ701716_A_marabou_stork_Cambodia_TM068_2011_Avian
JQ714250_A_Cambodia_W0112303_2012_Human
KF369210_A_Cambodia_W0329318_2012_Human
JQ673622_A_environment_Cambodia_C425D_2011_Environment
JQ714239_A_chicken_Cambodia_083LC_2011_Avian
JQ714231_A_duck_Cambodia_083D1_2011_Avian
EPI_ISL_136098_A_chicken_Vietnam_NCVD_878_2011_Avian
JQ673621_A_environment_Cambodia_C85W_2011_Environment
JN588892_A_chicken_Cambodia_008LC1_2011_Avian

NS-Group100

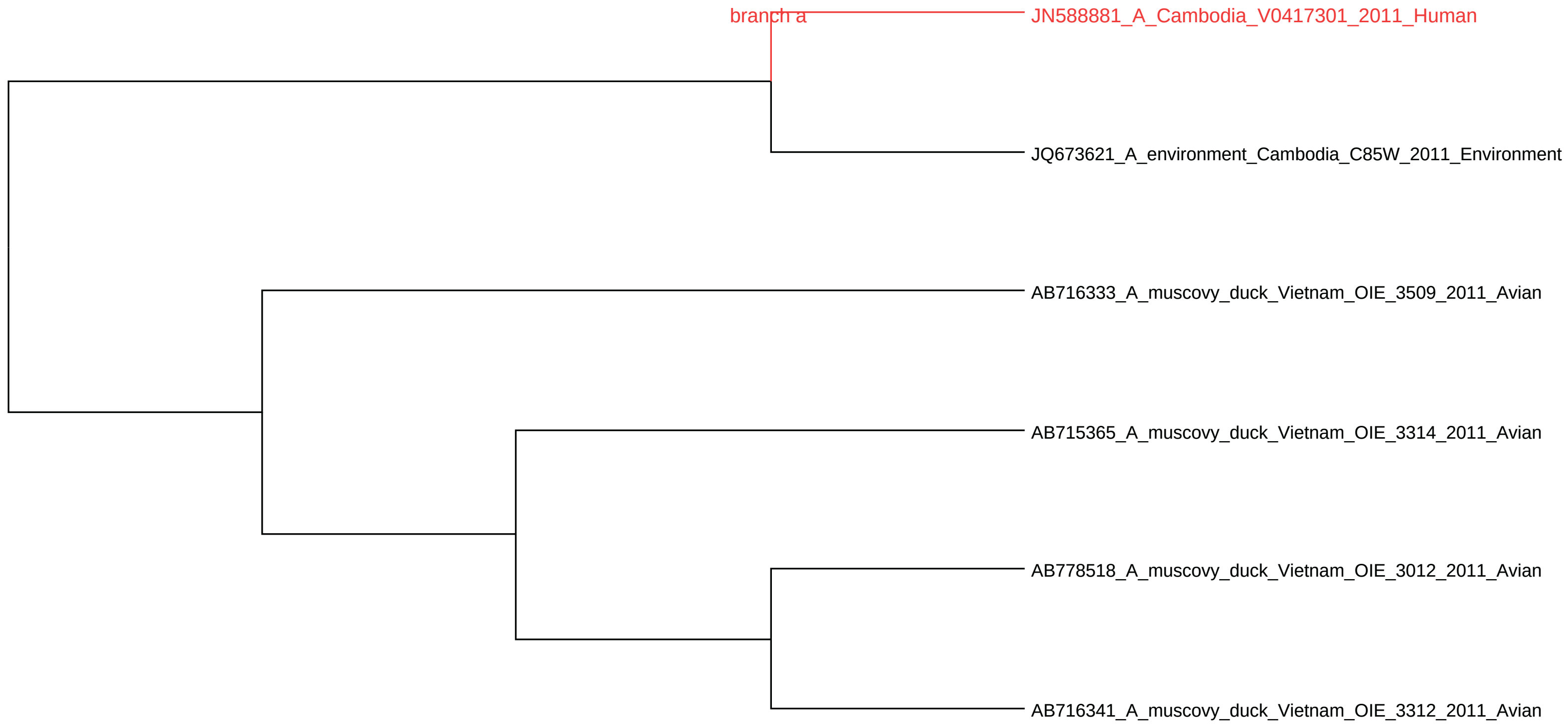

branch a
JN588881_A_Cambodia_V0417301_2011_Human
JQ673621_A_environment_Cambodia_C85W_2011_Environment
AB716333_A_muscovy_duck_Vietnam_OIE_3509_2011_Avian
AB715365_A_muscovy_duck_Vietnam_OIE_3314_2011_Avian
AB778518_A_muscovy_duck_Vietnam_OIE_3012_2011_Avian
AB716341_A_muscovy_duck_Vietnam_OIE_3312_2011_Avian

NS-Group101

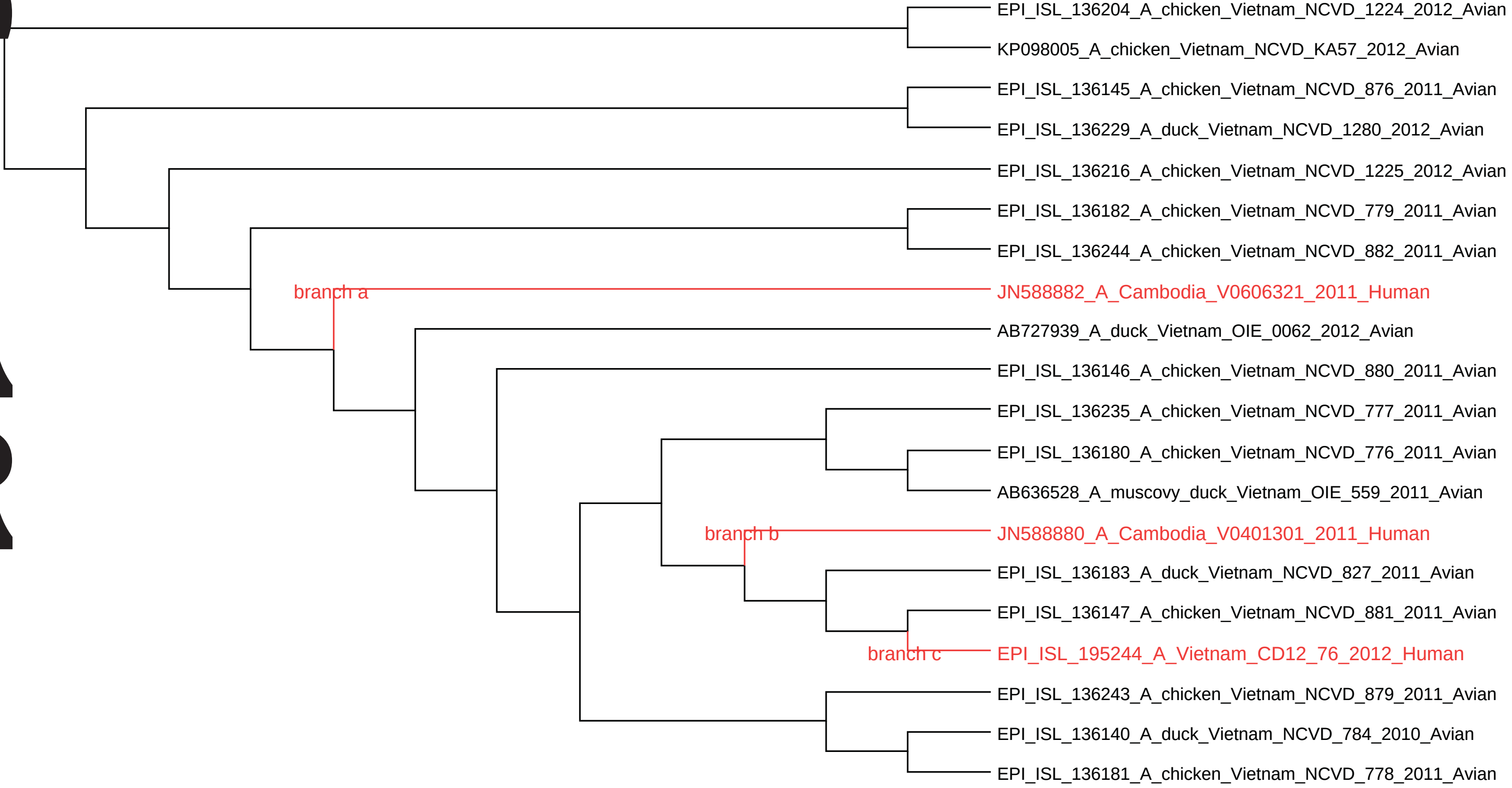

EPI_ISL_136204_A_chicken_Vietnam_NCVD_1224_2012_Avian
KP098005_A_chicken_Vietnam_NCVD_KA57_2012_Avian
EPI_ISL_136145_A_chicken_Vietnam_NCVD_876_2011_Avian
EPI_ISL_136229_A_duck_Vietnam_NCVD_1280_2012_Avian
EPI_ISL_136216_A_chicken_Vietnam_NCVD_1225_2012_Avian
EPI_ISL_136182_A_chicken_Vietnam_NCVD_779_2011_Avian
EPI_ISL_136244_A_chicken_Vietnam_NCVD_882_2011_Avian
branch a
JN588882_A_Cambodia_V0606321_2011_Human
AB727939_A_duck_Vietnam_OIE_0062_2012_Avian
EPI_ISL_136146_A_chicken_Vietnam_NCVD_880_2011_Avian
EPI_ISL_136235_A_chicken_Vietnam_NCVD_777_2011_Avian
EPI_ISL_136180_A_chicken_Vietnam_NCVD_776_2011_Avian
AB636528_A_muscovy_duck_Vietnam_OIE_559_2011_Avian
branch b
JN588880_A_Cambodia_V0401301_2011_Human
EPI_ISL_136183_A_duck_Vietnam_NCVD_827_2011_Avian
EPI_ISL_136147_A_chicken_Vietnam_NCVD_881_2011_Avian
branch c
EPI_ISL_195244_A_Vietnam_CD12_76_2012_Human
EPI_ISL_136243_A_chicken_Vietnam_NCVD_879_2011_Avian
EPI_ISL_136140_A_duck_Vietnam_NCVD_784_2010_Avian
EPI_ISL_136181_A_chicken_Vietnam_NCVD_778_2011_Avian
