# Supplementary material for: Adaptive Evolution of Human-Isolated H5Nx Avian Influenza A Viruses
Source: Front Microbiol. 2019 Jun 12;10:1328. doi: 10.3389/fmicb.2019.01328 (PMC6582624; doi:10.3389/fmicb.2019.01328)
Supplement: Supplementary file 8 [file Data_Sheet_8.PDF]

Supplementary Figure 8. 80 phylogenetic trees of PA used for the adaptive evolution analyses. Human strains are marked in red. Branches which have significant signals of positive selection are marked with *.

PA-Group1

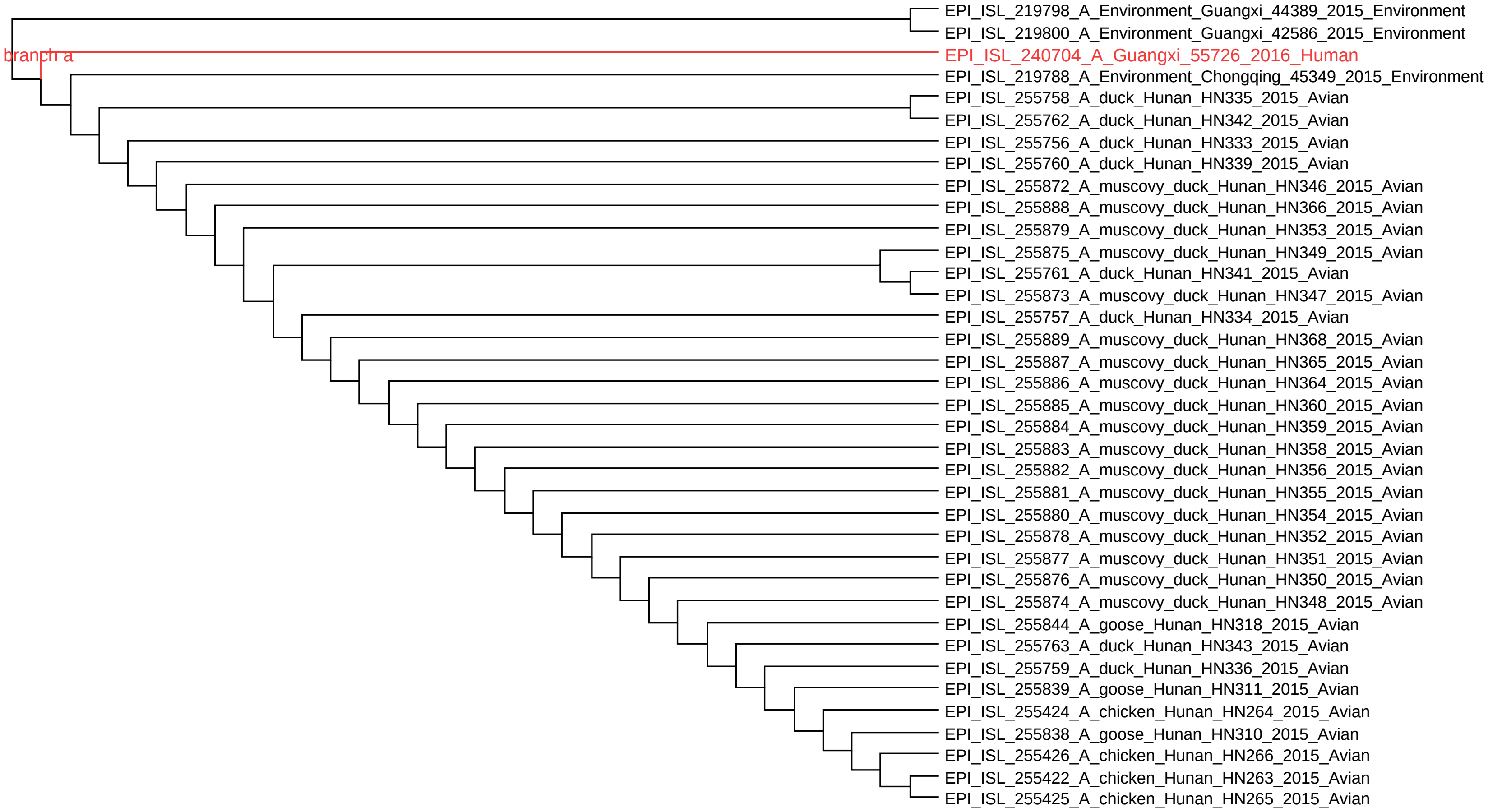

PA-Group2

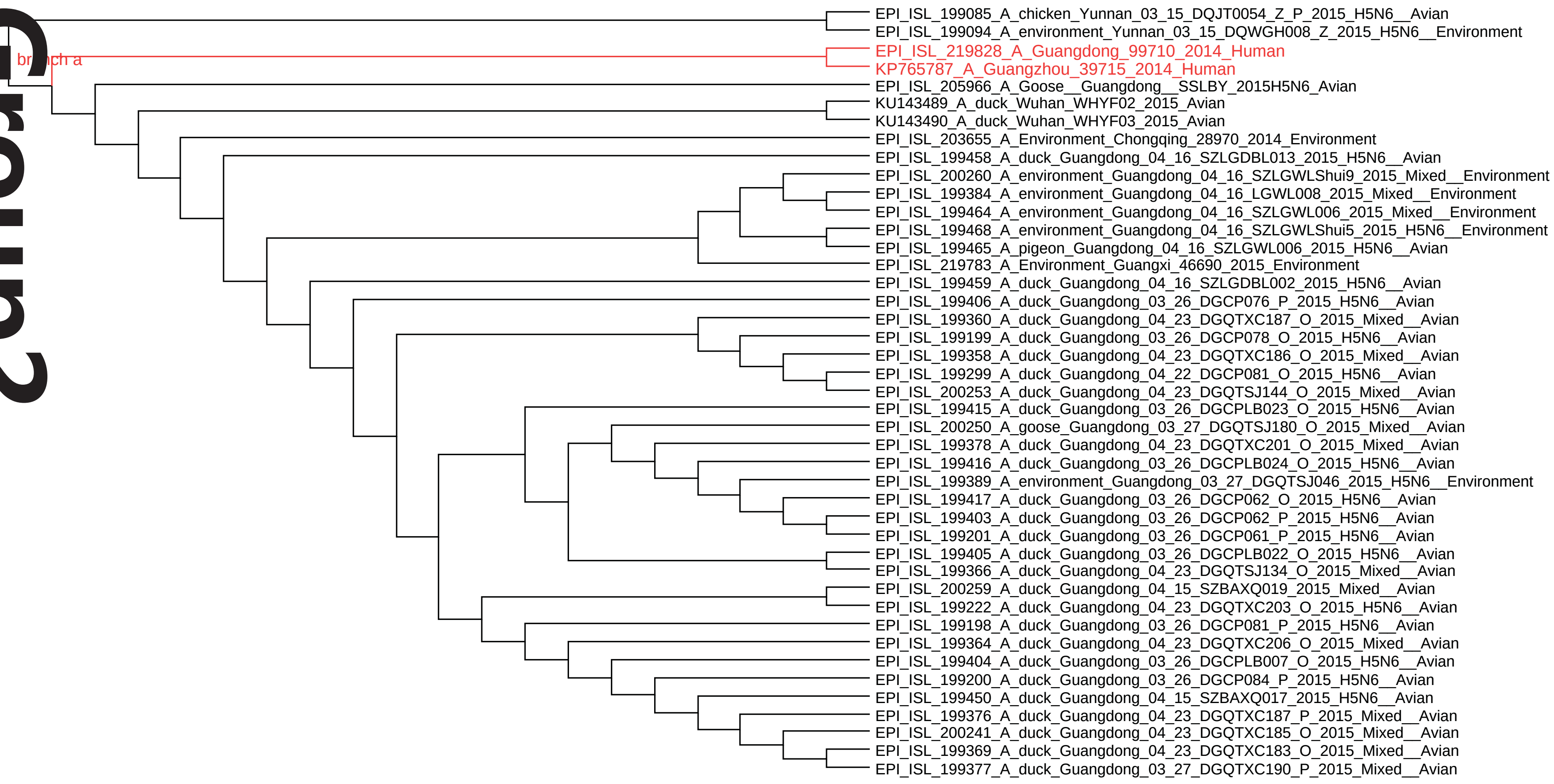
branch a
EPI_ISL_199085_A_chicken_Yunnan_03_15_DQJT0054_Z_P_2015_H5N6__Avian
EPI_ISL_199094_A_environment_Yunnan_03_15_DQWGH008_Z_2015_H5N6__Environment
EPI_ISL_219828_A_Guangdong_99710_2014_Human
KP765787_A_Guangzhou_39715_2014_Human
EPI_ISL_205966_A_Goose__Guangdong__SSLBY_2015H5N6_Avian
KU143489_A_duck_Wuhan_WHYF02_2015_Avian
KU143490_A_duck_Wuhan_WHYF03_2015_Avian
EPI_ISL_203655_A_Environment_Chongqing_28970_2014_Environment
EPI_ISL_199458_A_duck_Guangdong_04_16_SZLGDBL013_2015_H5N6__Avian
EPI_ISL_200260_A_environment_Guangdong_04_16_SZLGWLShui9_2015_Mixed__Environment
EPI_ISL_199384_A_environment_Guangdong_04_16_LGWL008_2015_Mixed__Environment
EPI_ISL_199464_A_environment_Guangdong_04_16_SZLGWL006_2015_Mixed__Environment
EPI_ISL_199468_A_environment_Guangdong_04_16_SZLGWLShui5_2015_H5N6__Environment
EPI_ISL_199465_A_pigeon_Guangdong_04_16_SZLGWL006_2015_H5N6__Avian
EPI_ISL_219783_A_Environment_Guangxi_46690_2015_Environment
EPI_ISL_199459_A_duck_Guangdong_04_16_SZLGDBL002_2015_H5N6__Avian
EPI_ISL_199406_A_duck_Guangdong_03_26_DGCP076_P_2015_H5N6__Avian
EPI_ISL_199360_A_duck_Guangdong_04_23_DGQTXC187_O_2015_Mixed__Avian
EPI_ISL_199199_A_duck_Guangdong_03_26_DGCP078_O_2015_H5N6__Avian
EPI_ISL_199358_A_duck_Guangdong_04_23_DGQTXC186_O_2015_Mixed__Avian
EPI_ISL_199299_A_duck_Guangdong_04_22_DGCP081_O_2015_H5N6__Avian
EPI_ISL_200253_A_duck_Guangdong_04_23_DGQTSJ144_O_2015_Mixed__Avian
EPI_ISL_199415_A_duck_Guangdong_03_26_DGCPLB023_O_2015_H5N6__Avian
EPI_ISL_200250_A_goose_Guangdong_03_27_DGQTSJ180_O_2015_Mixed__Avian
EPI_ISL_199378_A_duck_Guangdong_04_23_DGQTXC201_O_2015_Mixed__Avian
EPI_ISL_199416_A_duck_Guangdong_03_26_DGCPLB024_O_2015_H5N6__Avian
EPI_ISL_199389_A_environment_Guangdong_03_27_DGQTSJ046_2015_H5N6__Environment
EPI_ISL_199417_A_duck_Guangdong_03_26_DGCP062_O_2015_H5N6__Avian
EPI_ISL_199403_A_duck_Guangdong_03_26_DGCP062_P_2015_H5N6__Avian
EPI_ISL_199201_A_duck_Guangdong_03_26_DGCP061_P_2015_H5N6__Avian
EPI_ISL_199405_A_duck_Guangdong_03_26_DGCPLB022_O_2015_H5N6__Avian
EPI_ISL_199366_A_duck_Guangdong_04_23_DGQTSJ134_O_2015_Mixed__Avian
EPI_ISL_200259_A_duck_Guangdong_04_15_SZBAXQ019_2015_Mixed__Avian
EPI_ISL_199222_A_duck_Guangdong_04_23_DGQTXC203_O_2015_H5N6__Avian
EPI_ISL_199198_A_duck_Guangdong_03_26_DGCP081_P_2015_H5N6__Avian
EPI_ISL_199364_A_duck_Guangdong_04_23_DGQTXC206_O_2015_Mixed__Avian
EPI_ISL_199404_A_duck_Guangdong_03_26_DGCPLB007_O_2015_H5N6__Avian
EPI_ISL_199200_A_duck_Guangdong_03_26_DGCP084_P_2015_H5N6__Avian
EPI_ISL_199450_A_duck_Guangdong_04_15_SZBAXQ017_2015_H5N6__Avian
EPI_ISL_199376_A_duck_Guangdong_04_23_DGQTXC187_P_2015_Mixed__Avian
EPI_ISL_200241_A_duck_Guangdong_04_23_DGQTXC185_O_2015_Mixed__Avian
EPI_ISL_199369_A_duck_Guangdong_04_23_DGQTXC183_O_2015_Mixed__Avian
EPI_ISL_199377_A_duck_Guangdong_03_27_DGQTXC190_P_2015_Mixed__Avian

PA-Group3

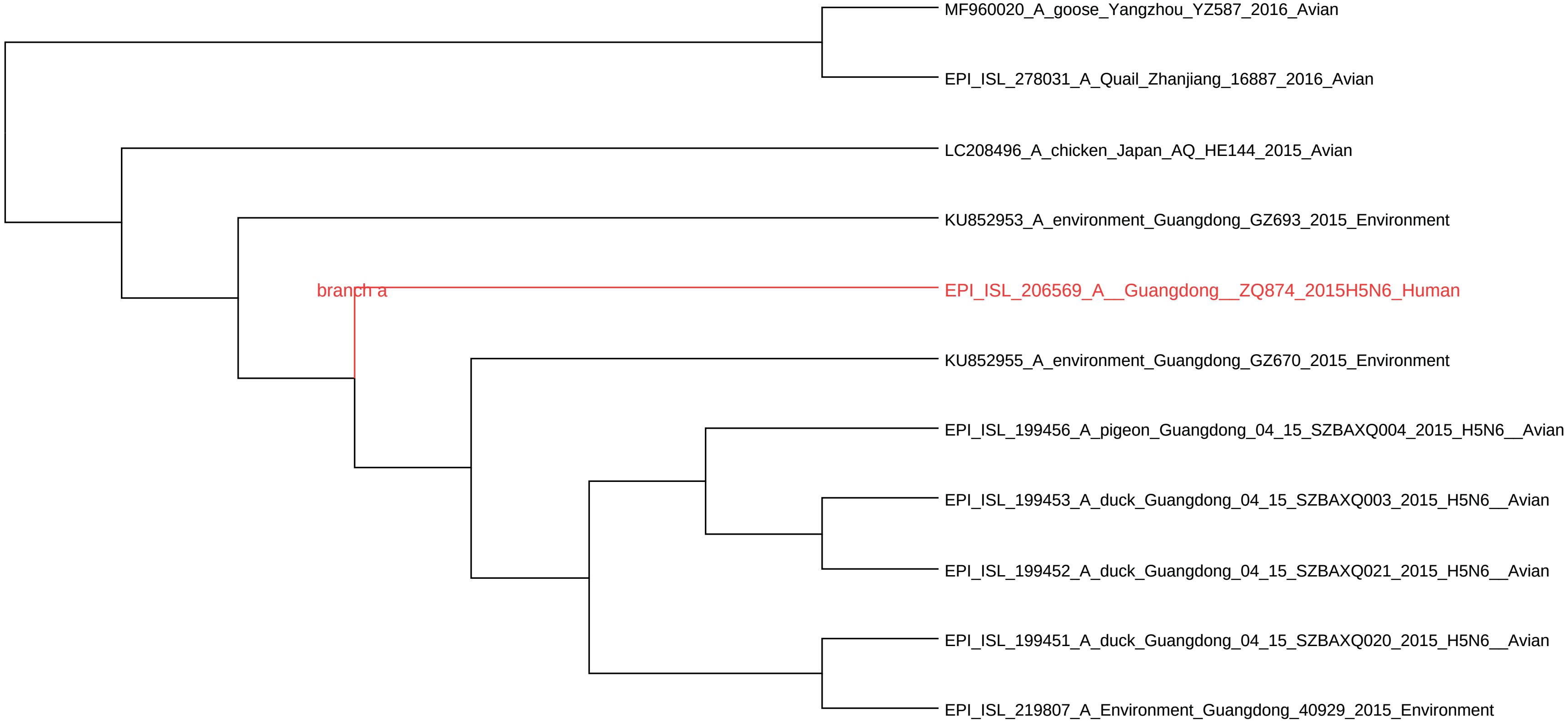
MF960020_A_goose_Yangzhou_YZ587_2016_Avian
EPI_ISL_278031_A_Quail_Zhanjiang_16887_2016_Avian
LC208496_A_chicken_Japan_AQ_HE144_2015_Avian
KU852953_A_environment_Guangdong_GZ693_2015_Environment
branch a
EPI_ISL_206569_A__Guangdong__ZQ874_2015H5N6_Human
KU852955_A_environment_Guangdong_GZ670_2015_Environment
EPI_ISL_199456_A_pigeon_Guangdong_04_15_SZBAXQ004_2015_H5N6__Avian
EPI_ISL_199453_A_duck_Guangdong_04_15_SZBAXQ003_2015_H5N6__Avian
EPI_ISL_199452_A_duck_Guangdong_04_15_SZBAXQ021_2015_H5N6__Avian
EPI_ISL_199451_A_duck_Guangdong_04_15_SZBAXQ020_2015_H5N6__Avian
EPI_ISL_219807_A_Environment_Guangdong_40929_2015_Environment

PA-Group4

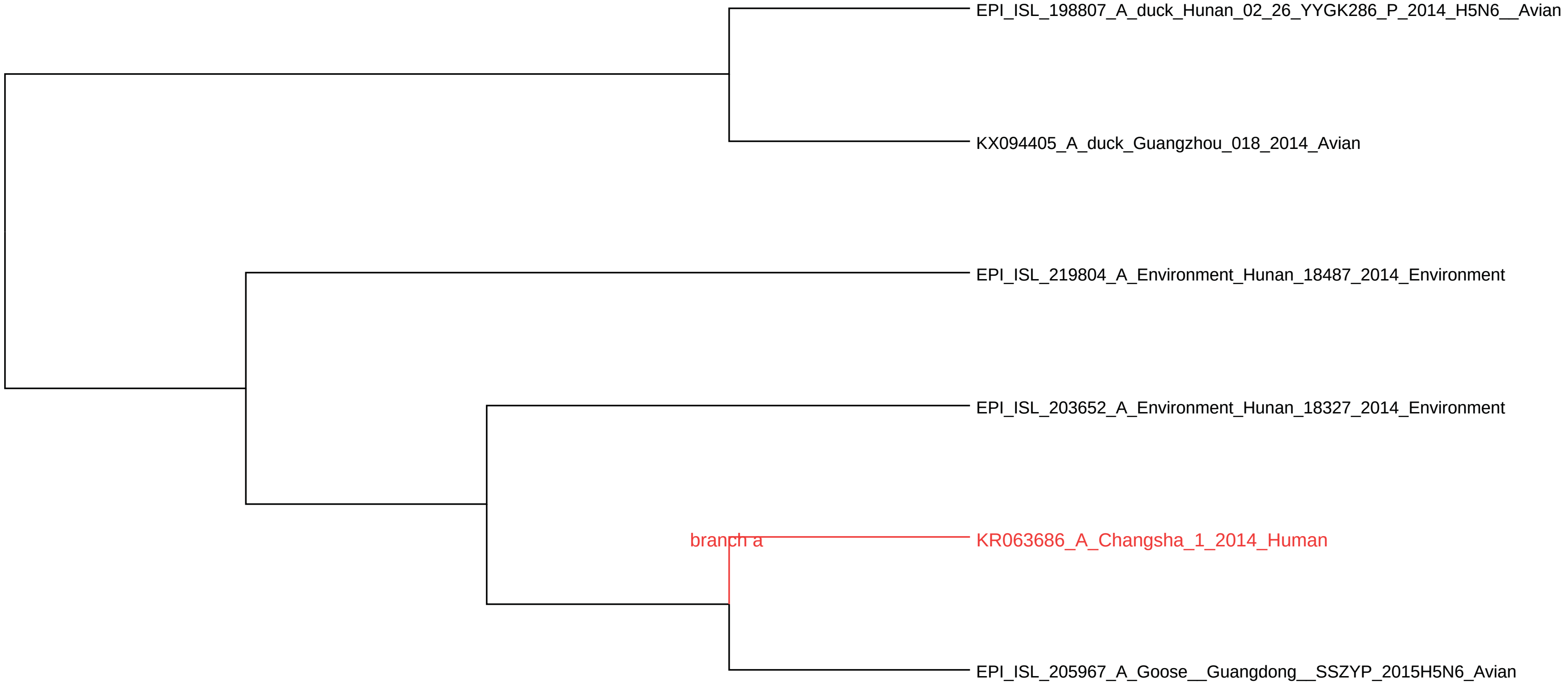
EPI_ISL_198807_A_duck_Hunan_02_26_YYGK286_P_2014_H5N6__Avian
KX094405_A_duck_Guangzhou_018_2014_Avian
EPI_ISL_219804_A_Environment_Hunan_18487_2014_Environment
EPI_ISL_203652_A_Environment_Hunan_18327_2014_Environment
branch a
KR063686_A_Changsha_1_2014_Human
EPI_ISL_205967_A_Goose__Guangdong__SSZYP_2015H5N6_Avian

PA-Group5

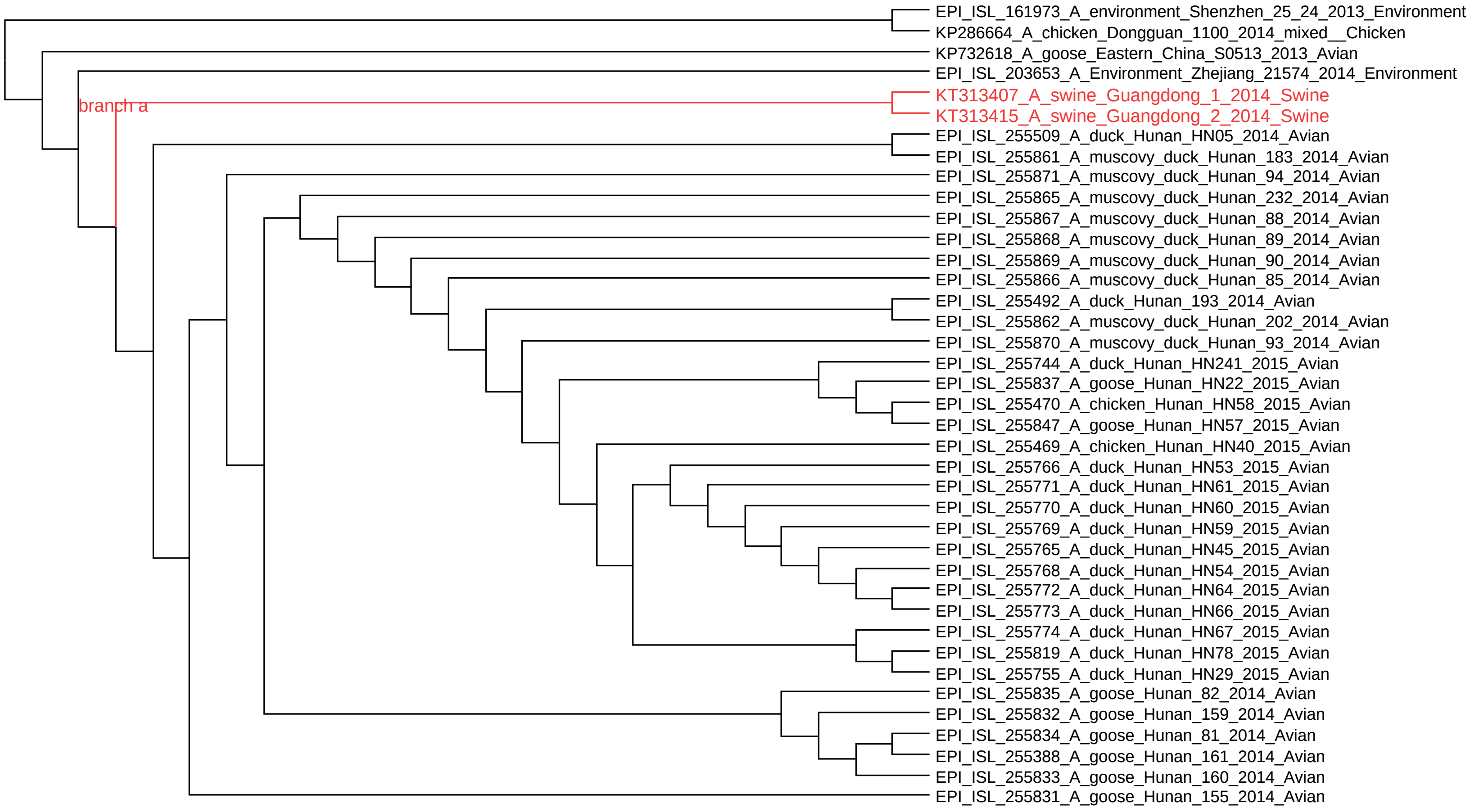

EPI_ISL_161973_A_environment_Shenzhen_25_24_2013_Environment
KP286664_A_chicken_Dongguan_1100_2014_mixed__Chicken
KP732618_A_goose_Eastern_China_S0513_2013_Avian
EPI_ISL_203653_A_Environment_Zhejiang_21574_2014_Environment
branch a
KT313407_A_swine_Guangdong_1_2014_Swine
KT313415_A_swine_Guangdong_2_2014_Swine
EPI_ISL_255509_A_duck_Hunan_HN05_2014_Avian
EPI_ISL_255861_A_muscovy_duck_Hunan_183_2014_Avian
EPI_ISL_255871_A_muscovy_duck_Hunan_94_2014_Avian
EPI_ISL_255865_A_muscovy_duck_Hunan_232_2014_Avian
EPI_ISL_255867_A_muscovy_duck_Hunan_88_2014_Avian
EPI_ISL_255868_A_muscovy_duck_Hunan_89_2014_Avian
EPI_ISL_255869_A_muscovy_duck_Hunan_90_2014_Avian
EPI_ISL_255866_A_muscovy_duck_Hunan_85_2014_Avian
EPI_ISL_255492_A_duck_Hunan_193_2014_Avian
EPI_ISL_255862_A_muscovy_duck_Hunan_202_2014_Avian
EPI_ISL_255870_A_muscovy_duck_Hunan_93_2014_Avian
EPI_ISL_255744_A_duck_Hunan_HN241_2015_Avian
EPI_ISL_255837_A_goose_Hunan_HN22_2015_Avian
EPI_ISL_255470_A_chicken_Hunan_HN58_2015_Avian
EPI_ISL_255847_A_goose_Hunan_HN57_2015_Avian
EPI_ISL_255469_A_chicken_Hunan_HN40_2015_Avian
EPI_ISL_255766_A_duck_Hunan_HN53_2015_Avian
EPI_ISL_255771_A_duck_Hunan_HN61_2015_Avian
EPI_ISL_255770_A_duck_Hunan_HN60_2015_Avian
EPI_ISL_255769_A_duck_Hunan_HN59_2015_Avian
EPI_ISL_255765_A_duck_Hunan_HN45_2015_Avian
EPI_ISL_255768_A_duck_Hunan_HN54_2015_Avian
EPI_ISL_255772_A_duck_Hunan_HN64_2015_Avian
EPI_ISL_255773_A_duck_Hunan_HN66_2015_Avian
EPI_ISL_255774_A_duck_Hunan_HN67_2015_Avian
EPI_ISL_255819_A_duck_Hunan_HN78_2015_Avian
EPI_ISL_255755_A_duck_Hunan_HN29_2015_Avian
EPI_ISL_255835_A_goose_Hunan_82_2014_Avian
EPI_ISL_255832_A_goose_Hunan_159_2014_Avian
EPI_ISL_255834_A_goose_Hunan_81_2014_Avian
EPI_ISL_255388_A_goose_Hunan_161_2014_Avian
EPI_ISL_255833_A_goose_Hunan_160_2014_Avian
EPI_ISL_255831_A_goose_Hunan_155_2014_Avian

PA-Group6

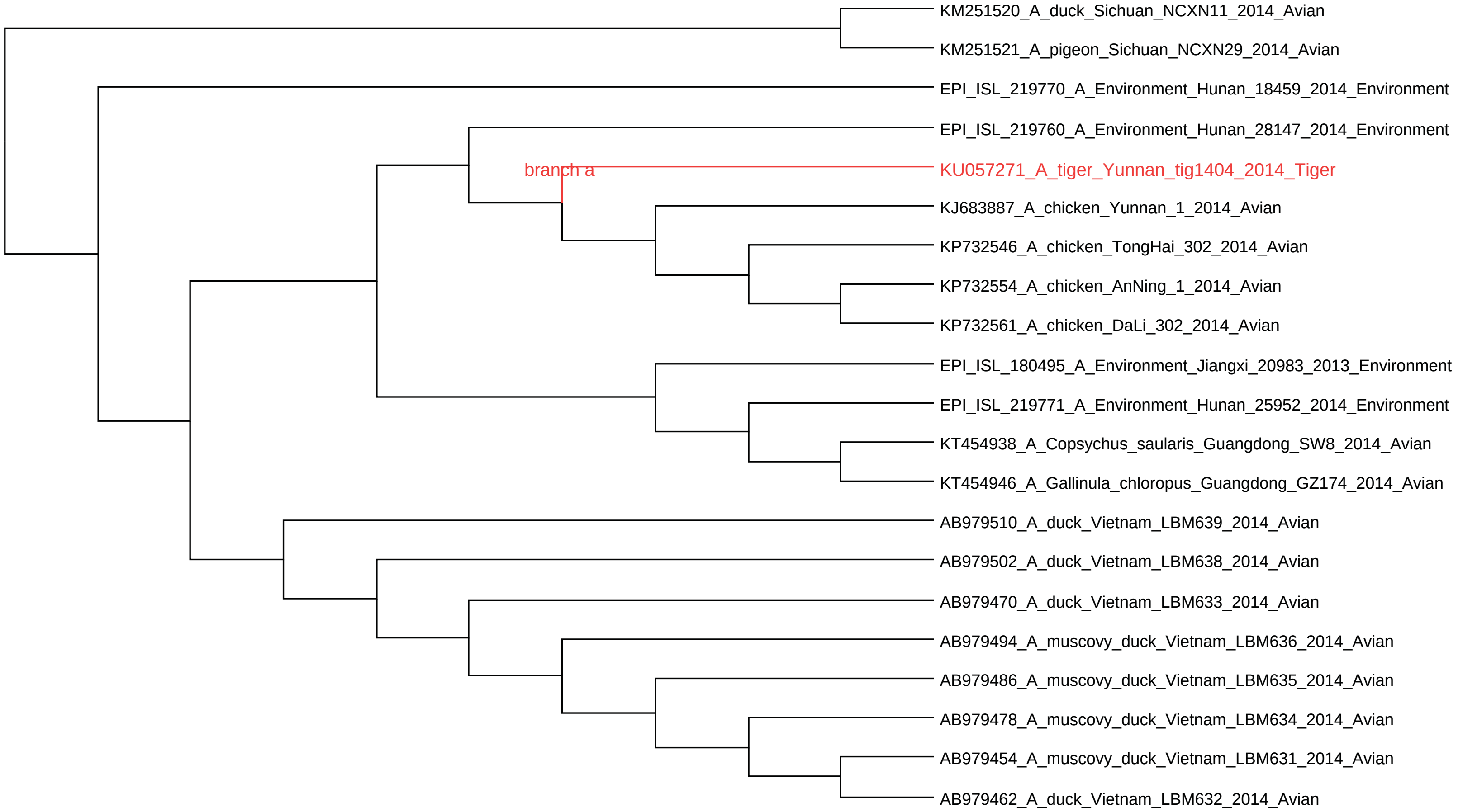
KM251520_A_duck_Sichuan_NCXN11_2014_Avian
KM251521_A_pigeon_Sichuan_NCXN29_2014_Avian
EPI_ISL_219770_A_Environment_Hunan_18459_2014_Environment
EPI_ISL_219760_A_Environment_Hunan_28147_2014_Environment
branch a
KU057271_A_tiger_Yunnan_tig1404_2014_Tiger
KJ683887_A_chicken_Yunnan_1_2014_Avian
KP732546_A_chicken_TongHai_302_2014_Avian
KP732554_A_chicken_AnNing_1_2014_Avian
KP732561_A_chicken_DaLi_302_2014_Avian
EPI_ISL_180495_A_Environment_Jiangxi_20983_2013_Environment
EPI_ISL_219771_A_Environment_Hunan_25952_2014_Environment
KT454938_A_Copsychus_saularis_Guangdong_SW8_2014_Avian
KT454946_A_Gallinula_chloropus_Guangdong_GZ174_2014_Avian
AB979510_A_duck_Vietnam_LBM639_2014_Avian
AB979502_A_duck_Vietnam_LBM638_2014_Avian
AB979470_A_duck_Vietnam_LBM633_2014_Avian
AB979494_A_muscovy_duck_Vietnam_LBM636_2014_Avian
AB979486_A_muscovy_duck_Vietnam_LBM635_2014_Avian
AB979478_A_muscovy_duck_Vietnam_LBM634_2014_Avian
AB979454_A_muscovy_duck_Vietnam_LBM631_2014_Avian
AB979462_A_duck_Vietnam_LBM632_2014_Avian

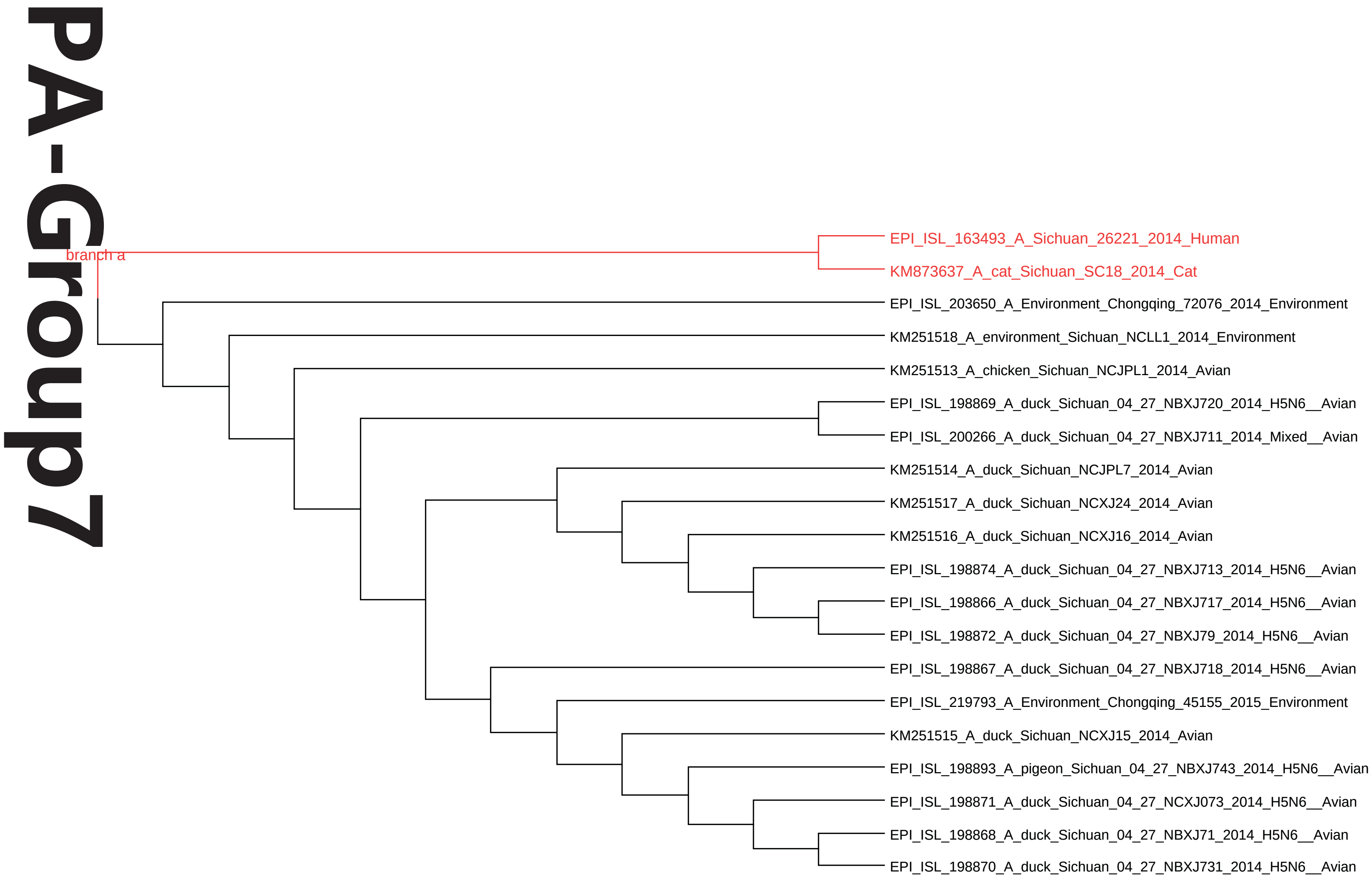
PA-Group7
branch a
EPI_ISL_163493_A_Sichuan_26221_2014_Human
KM873637_A_cat_Sichuan_SC18_2014_Cat
EPI_ISL_203650_A_Environment_Chongqing_72076_2014_Environment
KM251518_A_environment_Sichuan_NCLL1_2014_Environment
KM251513_A_chicken_Sichuan_NCJPL1_2014_Avian
EPI_ISL_198869_A_duck_Sichuan_04_27_NBXJ720_2014_H5N6__Avian
EPI_ISL_200266_A_duck_Sichuan_04_27_NBXJ711_2014_Mixed__Avian
KM251514_A_duck_Sichuan_NCJPL7_2014_Avian
KM251517_A_duck_Sichuan_NCXJ24_2014_Avian
KM251516_A_duck_Sichuan_NCXJ16_2014_Avian
EPI_ISL_198874_A_duck_Sichuan_04_27_NBXJ713_2014_H5N6__Avian
EPI_ISL_198866_A_duck_Sichuan_04_27_NBXJ717_2014_H5N6__Avian
EPI_ISL_198872_A_duck_Sichuan_04_27_NBXJ79_2014_H5N6__Avian
EPI_ISL_198867_A_duck_Sichuan_04_27_NBXJ718_2014_H5N6__Avian
EPI_ISL_219793_A_Environment_Chongqing_45155_2015_Environment
KM251515_A_duck_Sichuan_NCXJ15_2014_Avian
EPI_ISL_198893_A_pigeon_Sichuan_04_27_NBXJ743_2014_H5N6__Avian
EPI_ISL_198871_A_duck_Sichuan_04_27_NCXJ073_2014_H5N6__Avian
EPI_ISL_198868_A_duck_Sichuan_04_27_NBXJ71_2014_H5N6__Avian
EPI_ISL_198870_A_duck_Sichuan_04_27_NBXJ731_2014_H5N6__Avian

PA-Group8

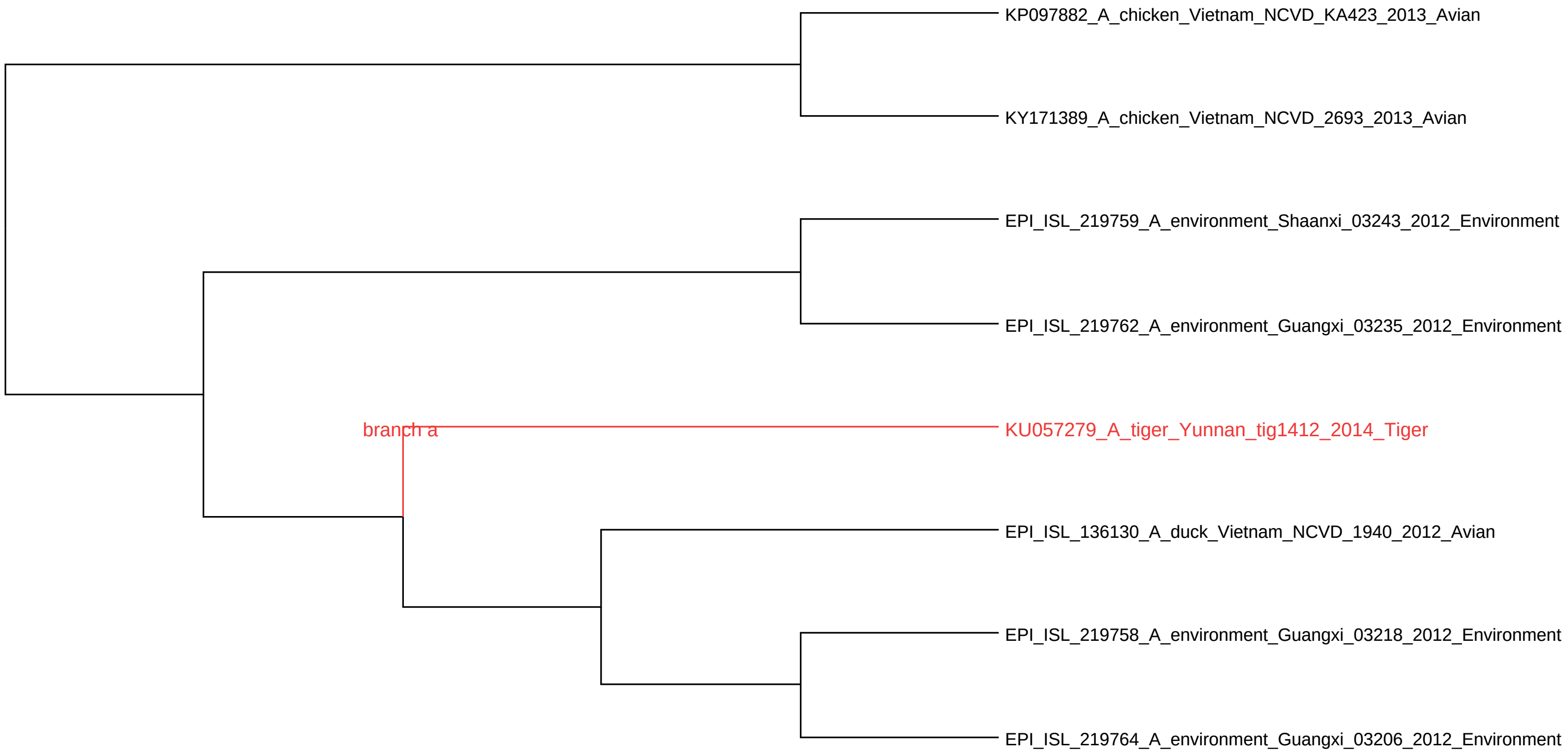
KP097882_A_chicken_Vietnam_NCVD_KA423_2013_Avian
KY171389_A_chicken_Vietnam_NCVD_2693_2013_Avian
EPI_ISL_219759_A_environment_Shaanxi_03243_2012_Environment
EPI_ISL_219762_A_environment_Guangxi_03235_2012_Environment
branch a
KU057279_A_tiger_Yunnan_tig1412_2014_Tiger
EPI_ISL_136130_A_duck_Vietnam_NCVD_1940_2012_Avian
EPI_ISL_219758_A_environment_Guangxi_03218_2012_Environment
EPI_ISL_219764_A_environment_Guangxi_03206_2012_Environment

PA-Group9

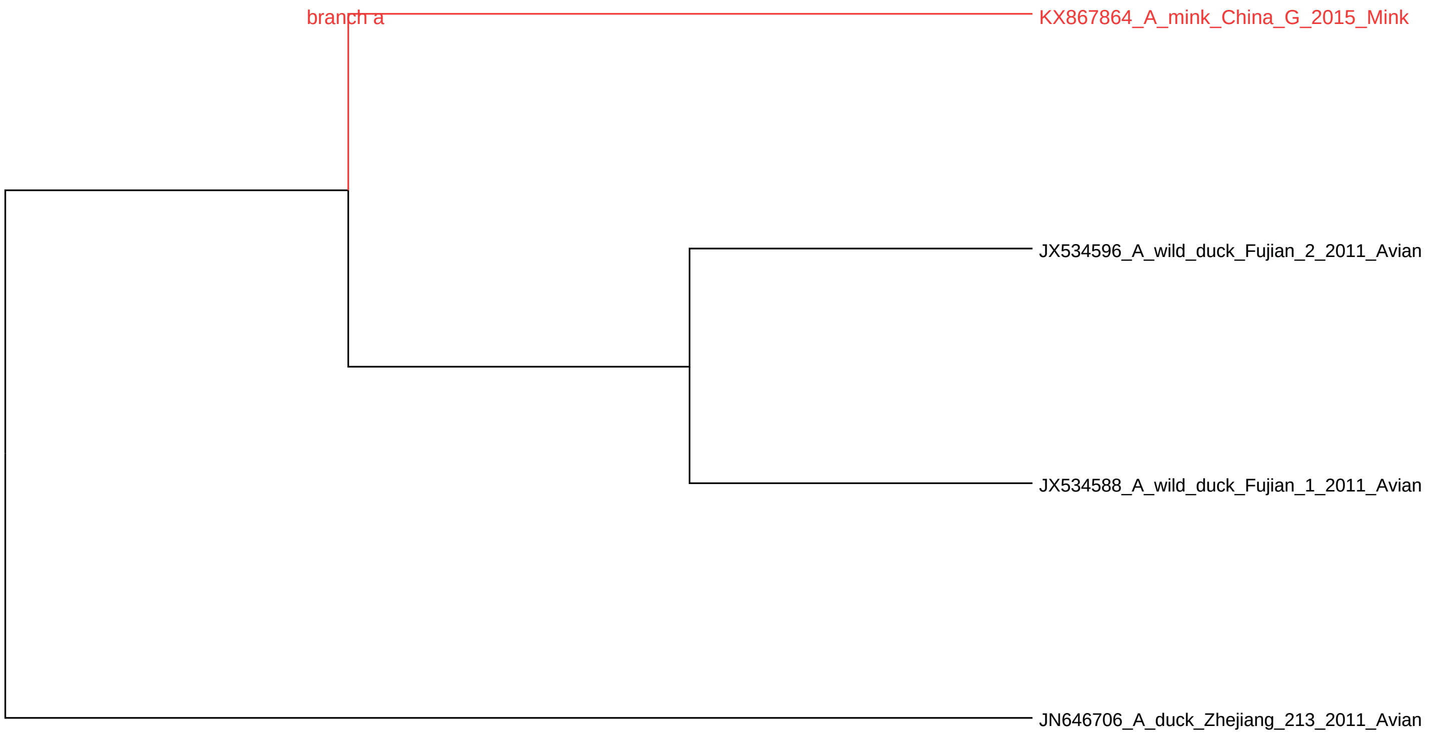
branch a
KX867864_A_mink_China_G_2015_Mink
JX534596_A_wild_duck_Fujian_2_2011_Avian
JX534588_A_wild_duck_Fujian_1_2011_Avian
JN646706_A_duck_Zhejiang_213_2011_Avian

PA-Group10

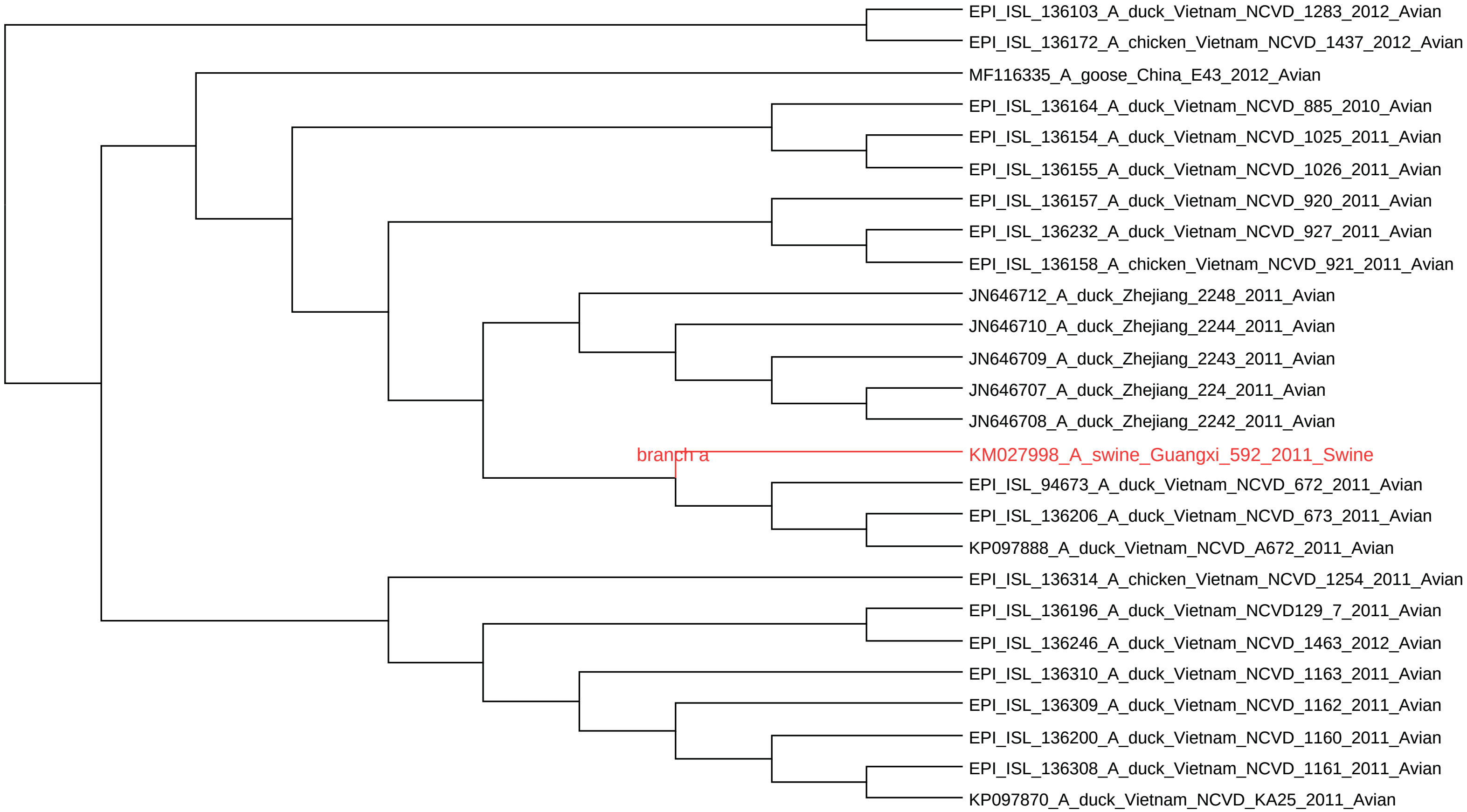
EPI_ISL_136103_A_duck_Vietnam_NCVD_1283_2012_Avian
EPI_ISL_136172_A_chicken_Vietnam_NCVD_1437_2012_Avian
MF116335_A_goose_China_E43_2012_Avian
EPI_ISL_136164_A_duck_Vietnam_NCVD_885_2010_Avian
EPI_ISL_136154_A_duck_Vietnam_NCVD_1025_2011_Avian
EPI_ISL_136155_A_duck_Vietnam_NCVD_1026_2011_Avian
EPI_ISL_136157_A_duck_Vietnam_NCVD_920_2011_Avian
EPI_ISL_136232_A_duck_Vietnam_NCVD_927_2011_Avian
EPI_ISL_136158_A_chicken_Vietnam_NCVD_921_2011_Avian
JN646712_A_duck_Zhejiang_2248_2011_Avian
JN646710_A_duck_Zhejiang_2244_2011_Avian
JN646709_A_duck_Zhejiang_2243_2011_Avian
JN646707_A_duck_Zhejiang_224_2011_Avian
JN646708_A_duck_Zhejiang_2242_2011_Avian
branch a
KM027998_A_swine_Guangxi_592_2011_Swine
EPI_ISL_94673_A_duck_Vietnam_NCVD_672_2011_Avian
EPI_ISL_136206_A_duck_Vietnam_NCVD_673_2011_Avian
KP097888_A_duck_Vietnam_NCVD_A672_2011_Avian
EPI_ISL_136314_A_chicken_Vietnam_NCVD_1254_2011_Avian
EPI_ISL_136196_A_duck_Vietnam_NCVD129_7_2011_Avian
EPI_ISL_136246_A_duck_Vietnam_NCVD_1463_2012_Avian
EPI_ISL_136310_A_duck_Vietnam_NCVD_1163_2011_Avian
EPI_ISL_136309_A_duck_Vietnam_NCVD_1162_2011_Avian
EPI_ISL_136200_A_duck_Vietnam_NCVD_1160_2011_Avian
EPI_ISL_136308_A_duck_Vietnam_NCVD_1161_2011_Avian
KP097870_A_duck_Vietnam_NCVD_KA25_2011_Avian

PA-Group11

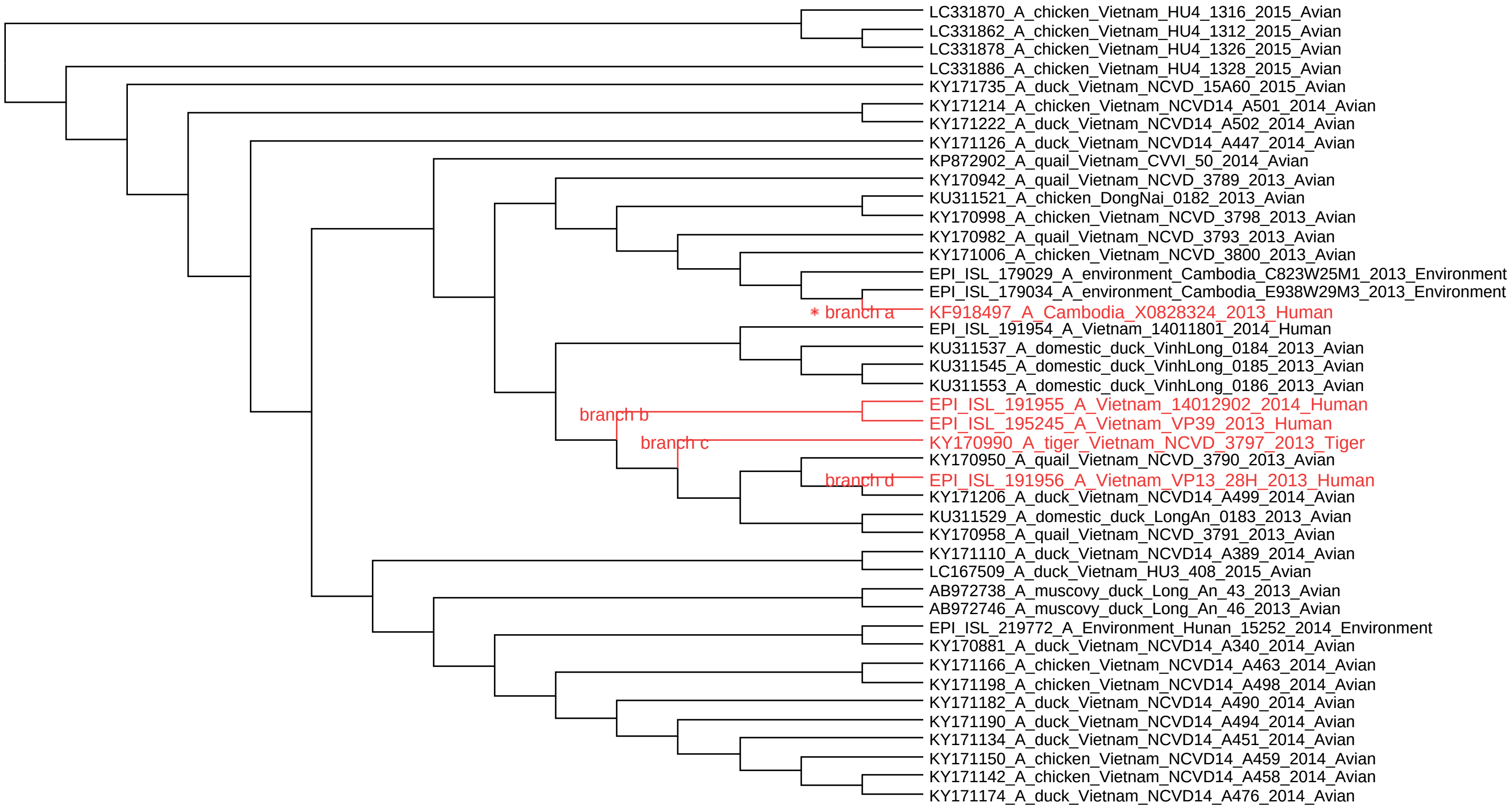
LC331870_A_chicken_Vietnam_HU4_1316_2015_Avian
LC331862_A_chicken_Vietnam_HU4_1312_2015_Avian
LC331878_A_chicken_Vietnam_HU4_1326_2015_Avian
LC331886_A_chicken_Vietnam_HU4_1328_2015_Avian
KY171735_A_duck_Vietnam_NCVD_15A60_2015_Avian
KY171214_A_chicken_Vietnam_NCVD14_A501_2014_Avian
KY171222_A_duck_Vietnam_NCVD14_A502_2014_Avian
KY171126_A_duck_Vietnam_NCVD14_A447_2014_Avian
KP872902_A_quail_Vietnam_CVVI_50_2014_Avian
KY170942_A_quail_Vietnam_NCVD_3789_2013_Avian
KU311521_A_chicken_DongNai_0182_2013_Avian
KY170998_A_chicken_Vietnam_NCVD_3798_2013_Avian
KY170982_A_quail_Vietnam_NCVD_3793_2013_Avian
KY171006_A_chicken_Vietnam_NCVD_3800_2013_Avian
EPI_ISL_179029_A_environment_Cambodia_C823W25M1_2013_Environment
EPI_ISL_179034_A_environment_Cambodia_E938W29M3_2013_Environment
* branch a
KF918497_A_Cambodia_X0828324_2013_Human
EPI_ISL_191954_A_Vietnam_14011801_2014_Human
KU311537_A_domestic_duck_VinhLong_0184_2013_Avian
KU311545_A_domestic_duck_VinhLong_0185_2013_Avian
KU311553_A_domestic_duck_VinhLong_0186_2013_Avian
branch b
EPI_ISL_191955_A_Vietnam_14012902_2014_Human
EPI_ISL_195245_A_Vietnam_VP39_2013_Human
branch c
KY170990_A_tiger_Vietnam_NCVD_3797_2013_Tiger
KY170950_A_quail_Vietnam_NCVD_3790_2013_Avian
branch d
EPI_ISL_191956_A_Vietnam_VP13_28H_2013_Human
KY171206_A_duck_Vietnam_NCVD14_A499_2014_Avian
KU311529_A_domestic_duck_LongAn_0183_2013_Avian
KY170958_A_quail_Vietnam_NCVD_3791_2013_Avian
KY171110_A_duck_Vietnam_NCVD14_A389_2014_Avian
LC167509_A_duck_Vietnam_HU3_408_2015_Avian
AB972738_A_muscovy_duck_Long_An_43_2013_Avian
AB972746_A_muscovy_duck_Long_An_46_2013_Avian
EPI_ISL_219772_A_Environment_Hunan_15252_2014_Environment
KY170881_A_duck_Vietnam_NCVD14_A340_2014_Avian
KY171166_A_chicken_Vietnam_NCVD14_A463_2014_Avian
KY171198_A_chicken_Vietnam_NCVD14_A498_2014_Avian
KY171182_A_duck_Vietnam_NCVD14_A490_2014_Avian
KY171190_A_duck_Vietnam_NCVD14_A494_2014_Avian
KY171134_A_duck_Vietnam_NCVD14_A451_2014_Avian
KY171150_A_chicken_Vietnam_NCVD14_A459_2014_Avian
KY171142_A_chicken_Vietnam_NCVD14_A458_2014_Avian
KY171174_A_duck_Vietnam_NCVD14_A476_2014_Avian

PA-Group12

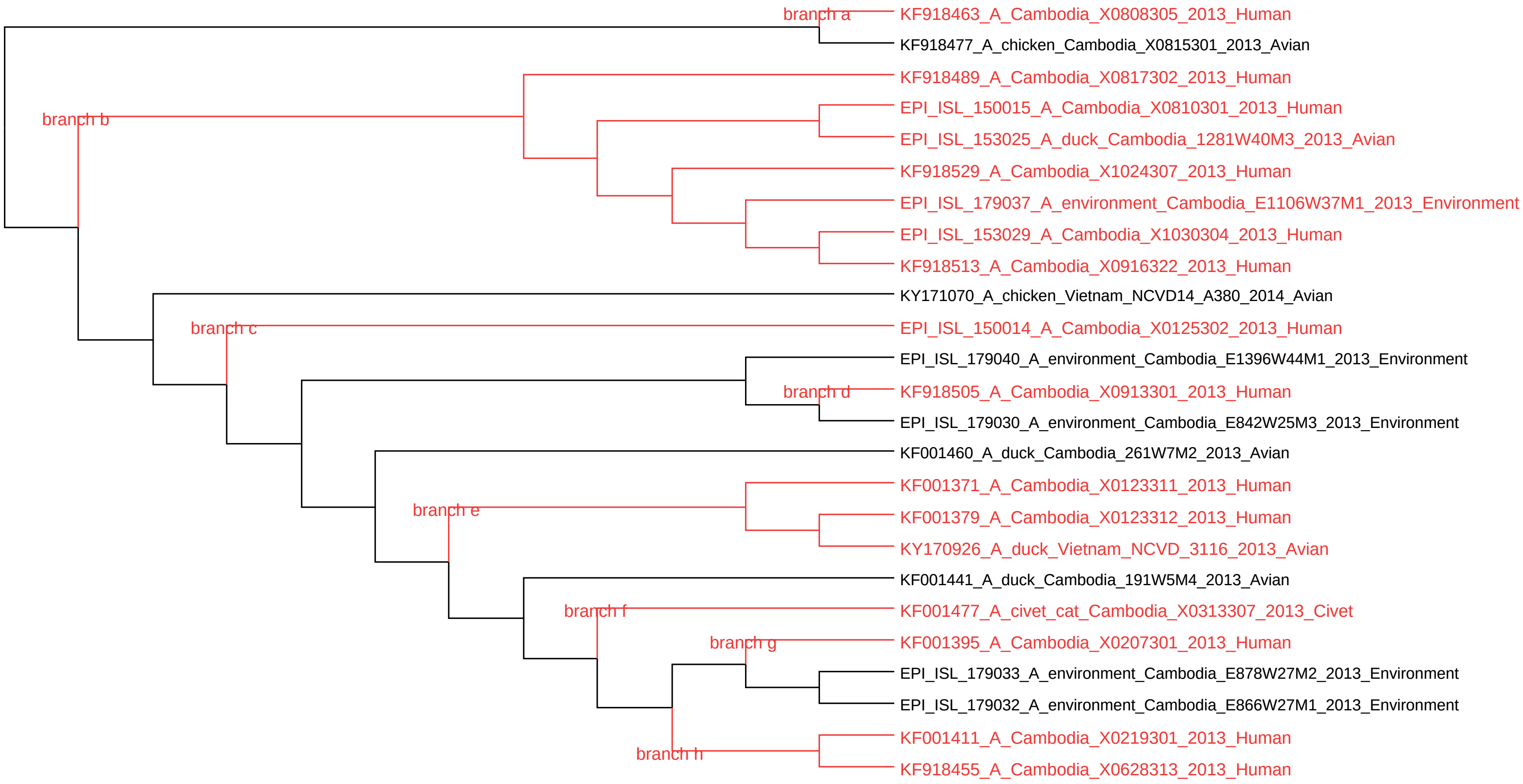

branch a
KF918463_A_Cambodia_X0808305_2013_Human
KF918477_A_chicken_Cambodia_X0815301_2013_Avian
KF918489_A_Cambodia_X0817302_2013_Human
branch b
EPI_ISL_150015_A_Cambodia_X0810301_2013_Human
EPI_ISL_153025_A_duck_Cambodia_1281W40M3_2013_Avian
KF918529_A_Cambodia_X1024307_2013_Human
EPI_ISL_179037_A_environment_Cambodia_E1106W37M1_2013_Environment
EPI_ISL_153029_A_Cambodia_X1030304_2013_Human
KF918513_A_Cambodia_X0916322_2013_Human
KY171070_A_chicken_Vietnam_NCVD14_A380_2014_Avian
branch c
EPI_ISL_150014_A_Cambodia_X0125302_2013_Human
EPI_ISL_179040_A_environment_Cambodia_E1396W44M1_2013_Environment
branch d
KF918505_A_Cambodia_X0913301_2013_Human
EPI_ISL_179030_A_environment_Cambodia_E842W25M3_2013_Environment
KF001460_A_duck_Cambodia_261W7M2_2013_Avian
KF001371_A_Cambodia_X0123311_2013_Human
branch e
KF001379_A_Cambodia_X0123312_2013_Human
KY170926_A_duck_Vietnam_NCVD_3116_2013_Avian
KF001441_A_duck_Cambodia_191W5M4_2013_Avian
branch f
KF001477_A_civet_cat_Cambodia_X0313307_2013_Civet
branch g
KF001395_A_Cambodia_X0207301_2013_Human
EPI_ISL_179033_A_environment_Cambodia_E878W27M2_2013_Environment
EPI_ISL_179032_A_environment_Cambodia_E866W27M1_2013_Environment
KF001411_A_Cambodia_X0219301_2013_Human
branch h
KF918455_A_Cambodia_X0628313_2013_Human

PA-Group13

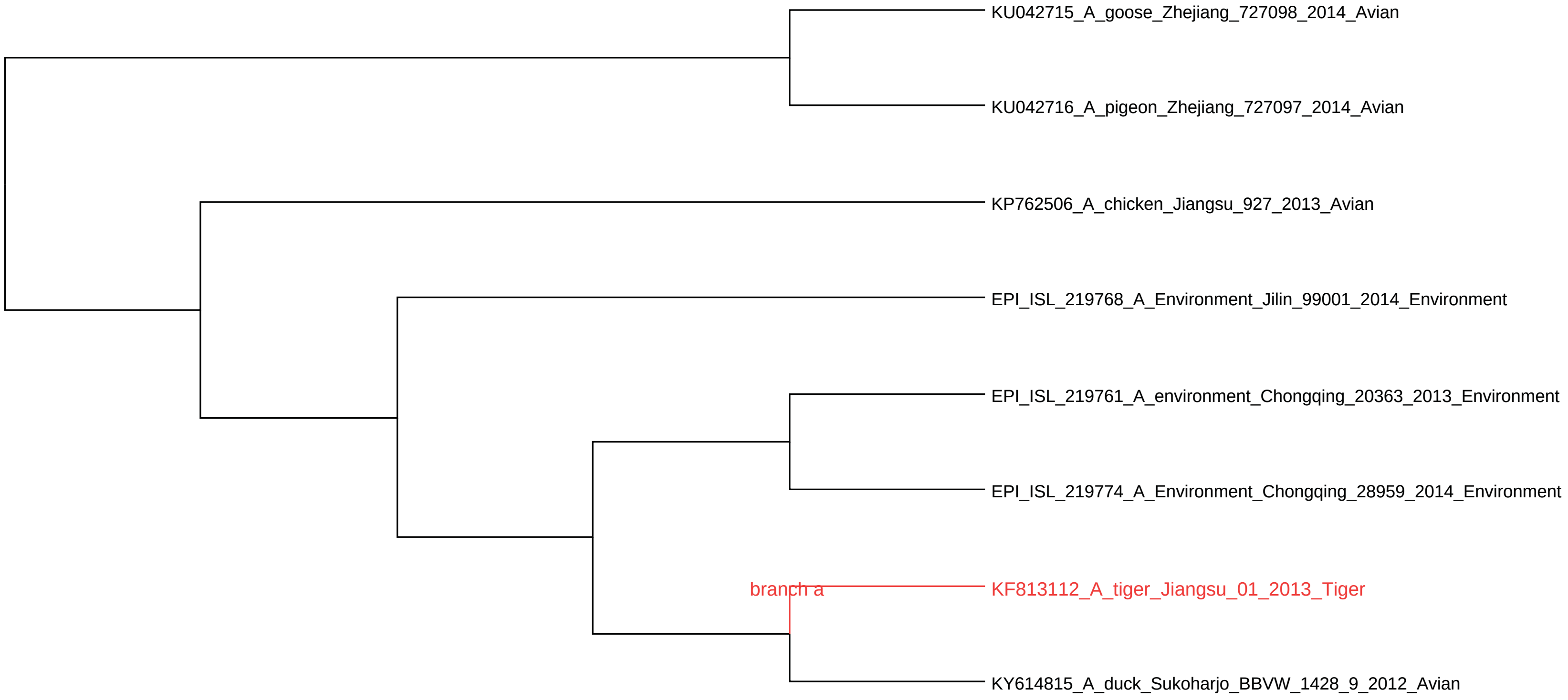
KU042715_A_goose_Zhejiang_727098_2014_Avian
KU042716_A_pigeon_Zhejiang_727097_2014_Avian
KP762506_A_chicken_Jiangsu_927_2013_Avian
EPI_ISL_219768_A_Environment_Jilin_99001_2014_Environment
EPI_ISL_219761_A_environment_Chongqing_20363_2013_Environment
EPI_ISL_219774_A_Environment_Chongqing_28959_2014_Environment
branch a
KF813112_A_tiger_Jiangsu_01_2013_Tiger
KY614815_A_duck_Sukoharjo_BBVW_1428_9_2012_Avian

PA-Group14

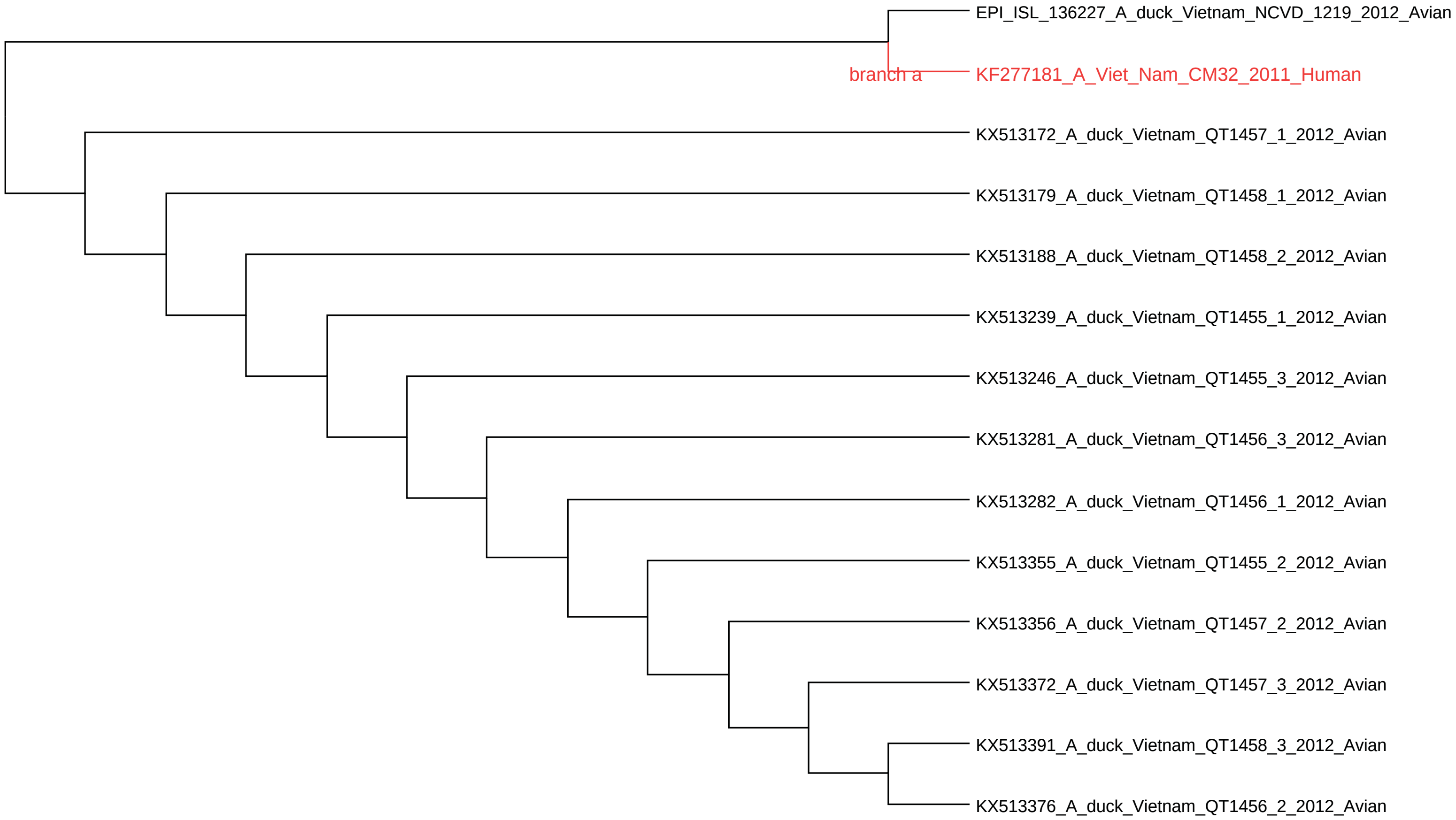
EPI_ISL_136227_A_duck_Vietnam_NCVD_1219_2012_Avian
branch a
KF277181_A_Viet_Nam_CM32_2011_Human
KX513172_A_duck_Vietnam_QT1457_1_2012_Avian
KX513179_A_duck_Vietnam_QT1458_1_2012_Avian
KX513188_A_duck_Vietnam_QT1458_2_2012_Avian
KX513239_A_duck_Vietnam_QT1455_1_2012_Avian
KX513246_A_duck_Vietnam_QT1455_3_2012_Avian
KX513281_A_duck_Vietnam_QT1456_3_2012_Avian
KX513282_A_duck_Vietnam_QT1456_1_2012_Avian
KX513355_A_duck_Vietnam_QT1455_2_2012_Avian
KX513356_A_duck_Vietnam_QT1457_2_2012_Avian
KX513372_A_duck_Vietnam_QT1457_3_2012_Avian
KX513391_A_duck_Vietnam_QT1458_3_2012_Avian
KX513376_A_duck_Vietnam_QT1456_2_2012_Avian

PA-Group15

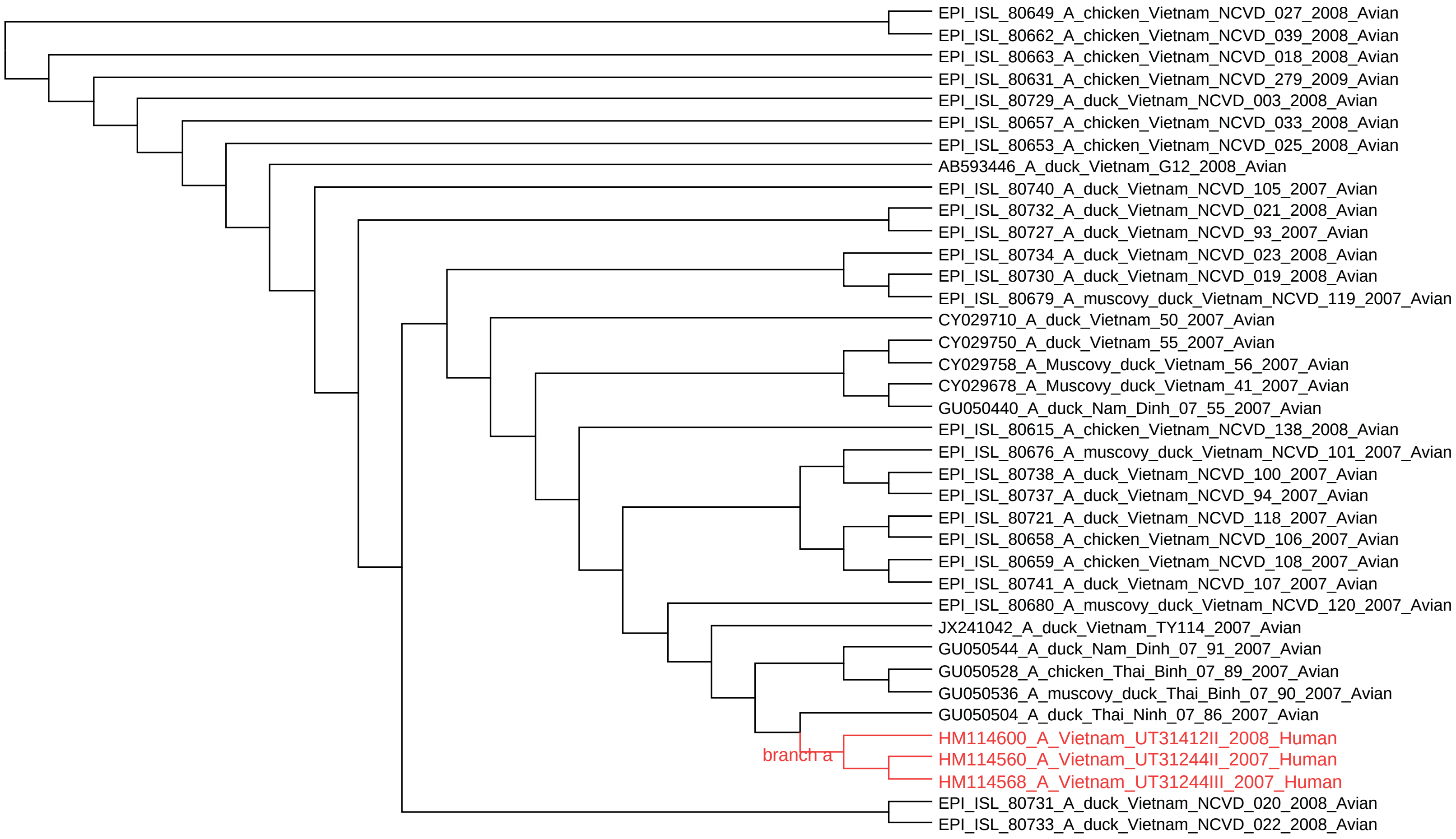
EPI_ISL_80649_A_chicken_Vietnam_NCVD_027_2008_Avian
EPI_ISL_80662_A_chicken_Vietnam_NCVD_039_2008_Avian
EPI_ISL_80663_A_chicken_Vietnam_NCVD_018_2008_Avian
EPI_ISL_80631_A_chicken_Vietnam_NCVD_279_2009_Avian
EPI_ISL_80729_A_duck_Vietnam_NCVD_003_2008_Avian
EPI_ISL_80657_A_chicken_Vietnam_NCVD_033_2008_Avian
EPI_ISL_80653_A_chicken_Vietnam_NCVD_025_2008_Avian
AB593446_A_duck_Vietnam_G12_2008_Avian
EPI_ISL_80740_A_duck_Vietnam_NCVD_105_2007_Avian
EPI_ISL_80732_A_duck_Vietnam_NCVD_021_2008_Avian
EPI_ISL_80727_A_duck_Vietnam_NCVD_93_2007_Avian
EPI_ISL_80734_A_duck_Vietnam_NCVD_023_2008_Avian
EPI_ISL_80730_A_duck_Vietnam_NCVD_019_2008_Avian
EPI_ISL_80679_A_muscovy_duck_Vietnam_NCVD_119_2007_Avian
CY029710_A_duck_Vietnam_50_2007_Avian
CY029750_A_duck_Vietnam_55_2007_Avian
CY029758_A_Muscovy_duck_Vietnam_56_2007_Avian
CY029678_A_Muscovy_duck_Vietnam_41_2007_Avian
GU050440_A_duck_Nam_Dinh_07_55_2007_Avian
EPI_ISL_80615_A_chicken_Vietnam_NCVD_138_2008_Avian
EPI_ISL_80676_A_muscovy_duck_Vietnam_NCVD_101_2007_Avian
EPI_ISL_80738_A_duck_Vietnam_NCVD_100_2007_Avian
EPI_ISL_80737_A_duck_Vietnam_NCVD_94_2007_Avian
EPI_ISL_80721_A_duck_Vietnam_NCVD_118_2007_Avian
EPI_ISL_80658_A_chicken_Vietnam_NCVD_106_2007_Avian
EPI_ISL_80659_A_chicken_Vietnam_NCVD_108_2007_Avian
EPI_ISL_80741_A_duck_Vietnam_NCVD_107_2007_Avian
EPI_ISL_80680_A_muscovy_duck_Vietnam_NCVD_120_2007_Avian
JX241042_A_duck_Vietnam_TY114_2007_Avian
GU050544_A_duck_Nam_Dinh_07_91_2007_Avian
GU050528_A_chicken_Thai_Binh_07_89_2007_Avian
GU050536_A_muscovy_duck_Thai_Binh_07_90_2007_Avian
GU050504_A_duck_Thai_Ninh_07_86_2007_Avian
branch a
HM114600_A_Vietnam_UT31412II_2008_Human
HM114560_A_Vietnam_UT31244II_2007_Human
HM114568_A_Vietnam_UT31244III_2007_Human
EPI_ISL_80731_A_duck_Vietnam_NCVD_020_2008_Avian
EPI_ISL_80733_A_duck_Vietnam_NCVD_022_2008_Avian

PA-Group16

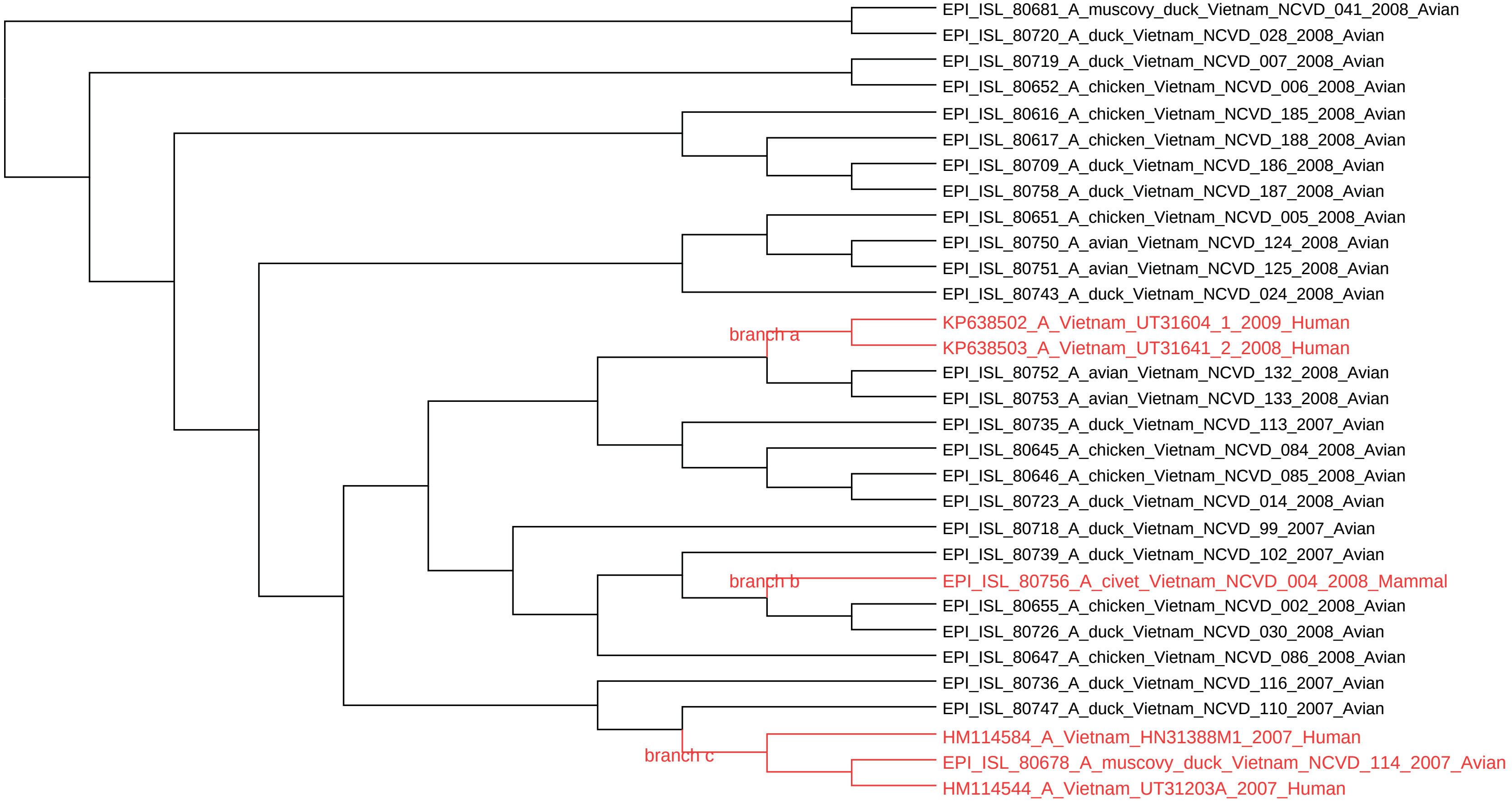
EPI_ISL_80681_A_muscovy_duck_Vietnam_NCVD_041_2008_Avian
EPI_ISL_80720_A_duck_Vietnam_NCVD_028_2008_Avian
EPI_ISL_80719_A_duck_Vietnam_NCVD_007_2008_Avian
EPI_ISL_80652_A_chicken_Vietnam_NCVD_006_2008_Avian
EPI_ISL_80616_A_chicken_Vietnam_NCVD_185_2008_Avian
EPI_ISL_80617_A_chicken_Vietnam_NCVD_188_2008_Avian
EPI_ISL_80709_A_duck_Vietnam_NCVD_186_2008_Avian
EPI_ISL_80758_A_duck_Vietnam_NCVD_187_2008_Avian
EPI_ISL_80651_A_chicken_Vietnam_NCVD_005_2008_Avian
EPI_ISL_80750_A_avian_Vietnam_NCVD_124_2008_Avian
EPI_ISL_80751_A_avian_Vietnam_NCVD_125_2008_Avian
EPI_ISL_80743_A_duck_Vietnam_NCVD_024_2008_Avian
branch a
KP638502_A_Vietnam_UT31604_1_2009_Human
KP638503_A_Vietnam_UT31641_2_2008_Human
EPI_ISL_80752_A_avian_Vietnam_NCVD_132_2008_Avian
EPI_ISL_80753_A_avian_Vietnam_NCVD_133_2008_Avian
EPI_ISL_80735_A_duck_Vietnam_NCVD_113_2007_Avian
EPI_ISL_80645_A_chicken_Vietnam_NCVD_084_2008_Avian
EPI_ISL_80646_A_chicken_Vietnam_NCVD_085_2008_Avian
EPI_ISL_80723_A_duck_Vietnam_NCVD_014_2008_Avian
EPI_ISL_80718_A_duck_Vietnam_NCVD_99_2007_Avian
EPI_ISL_80739_A_duck_Vietnam_NCVD_102_2007_Avian
branch b
EPI_ISL_80756_A_civet_Vietnam_NCVD_004_2008_Mammal
EPI_ISL_80655_A_chicken_Vietnam_NCVD_002_2008_Avian
EPI_ISL_80726_A_duck_Vietnam_NCVD_030_2008_Avian
EPI_ISL_80647_A_chicken_Vietnam_NCVD_086_2008_Avian
EPI_ISL_80736_A_duck_Vietnam_NCVD_116_2007_Avian
EPI_ISL_80747_A_duck_Vietnam_NCVD_110_2007_Avian
HM114584_A_Vietnam_HN31388M1_2007_Human
branch c
EPI_ISL_80678_A_muscovy_duck_Vietnam_NCVD_114_2007_Avian
HM114544_A_Vietnam_UT31203A_2007_Human

PA-Group17

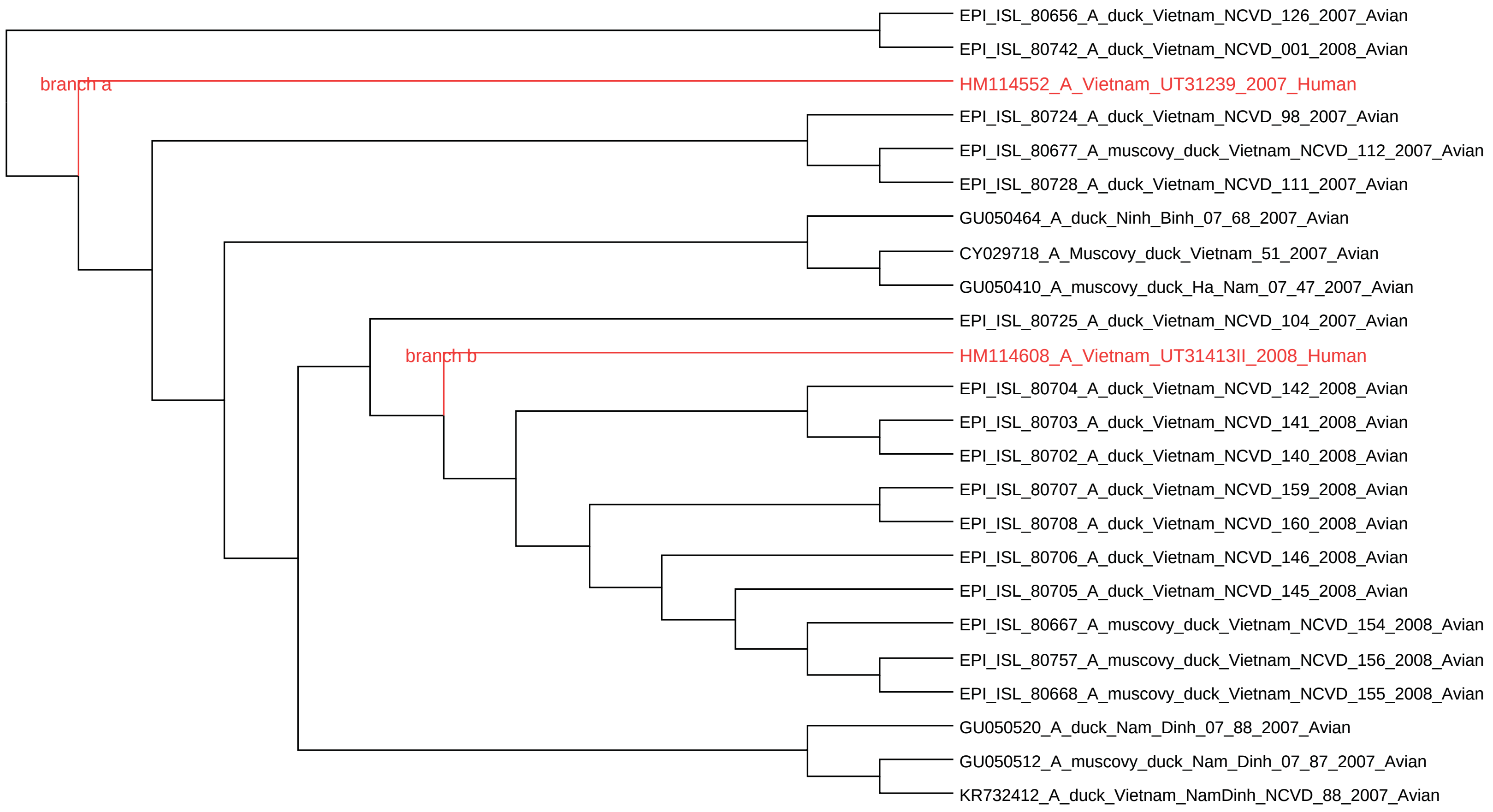
EPI_ISL_80656_A_duck_Vietnam_NCVD_126_2007_Avian
EPI_ISL_80742_A_duck_Vietnam_NCVD_001_2008_Avian
branch a
HM114552_A_Vietnam_UT31239_2007_Human
EPI_ISL_80724_A_duck_Vietnam_NCVD_98_2007_Avian
EPI_ISL_80677_A_muscovy_duck_Vietnam_NCVD_112_2007_Avian
EPI_ISL_80728_A_duck_Vietnam_NCVD_111_2007_Avian
GU050464_A_duck_Ninh_Binh_07_68_2007_Avian
CY029718_A_Muscovy_duck_Vietnam_51_2007_Avian
GU050410_A_muscovy_duck_Ha_Nam_07_47_2007_Avian
EPI_ISL_80725_A_duck_Vietnam_NCVD_104_2007_Avian
branch b
HM114608_A_Vietnam_UT31413II_2008_Human
EPI_ISL_80704_A_duck_Vietnam_NCVD_142_2008_Avian
EPI_ISL_80703_A_duck_Vietnam_NCVD_141_2008_Avian
EPI_ISL_80702_A_duck_Vietnam_NCVD_140_2008_Avian
EPI_ISL_80707_A_duck_Vietnam_NCVD_159_2008_Avian
EPI_ISL_80708_A_duck_Vietnam_NCVD_160_2008_Avian
EPI_ISL_80706_A_duck_Vietnam_NCVD_146_2008_Avian
EPI_ISL_80705_A_duck_Vietnam_NCVD_145_2008_Avian
EPI_ISL_80667_A_muscovy_duck_Vietnam_NCVD_154_2008_Avian
EPI_ISL_80757_A_muscovy_duck_Vietnam_NCVD_156_2008_Avian
EPI_ISL_80668_A_muscovy_duck_Vietnam_NCVD_155_2008_Avian
GU050520_A_duck_Nam_Dinh_07_88_2007_Avian
GU050512_A_muscovy_duck_Nam_Dinh_07_87_2007_Avian
KR732412_A_duck_Vietnam_NamDinh_NCVD_88_2007_Avian

PA-Group18

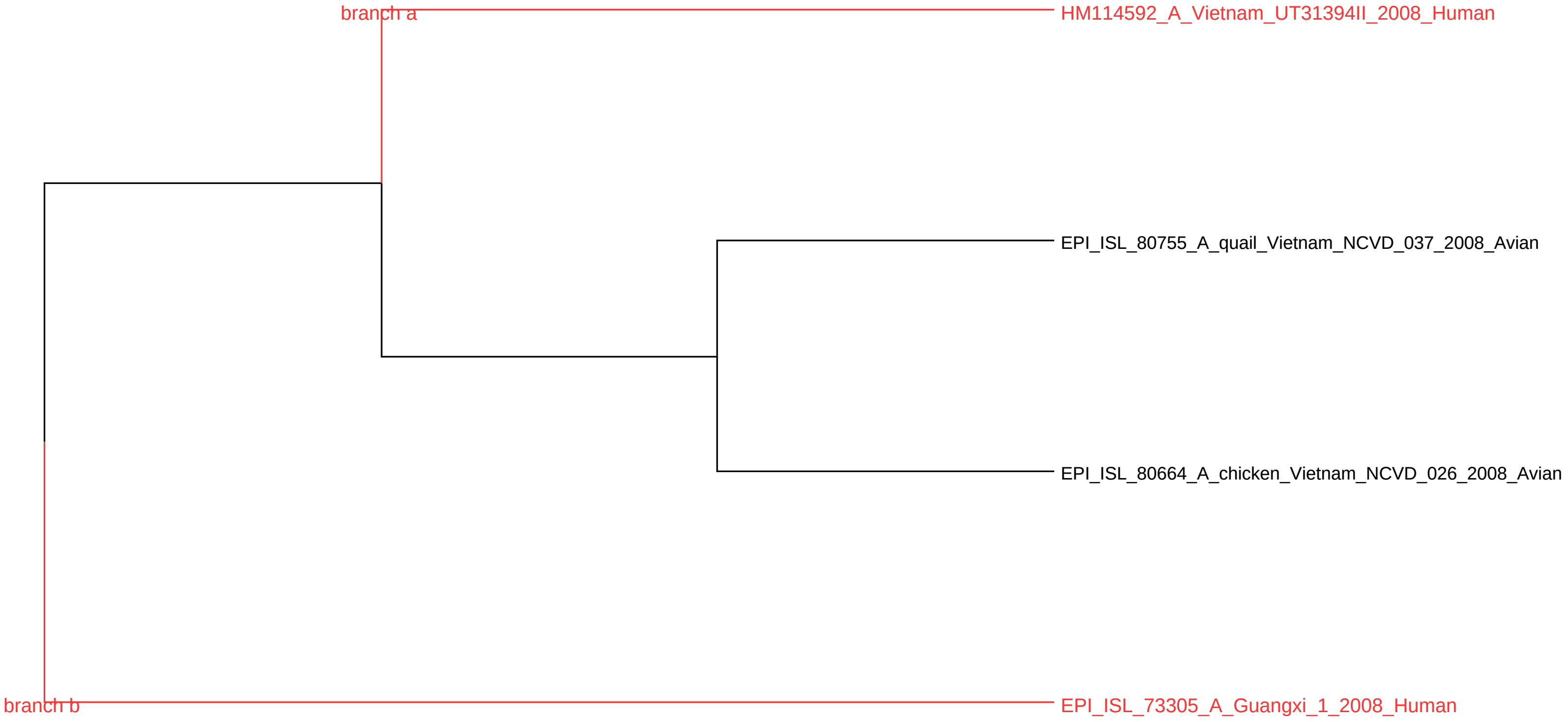

branch a
HM114592_A_Vietnam_UT31394II_2008_Human
EPI_ISL_80755_A_quail_Vietnam_NCVD_037_2008_Avian
EPI_ISL_80664_A_chicken_Vietnam_NCVD_026_2008_Avian
branch b
EPI_ISL_73305_A_Guangxi_1_2008_Human

PA-Group19

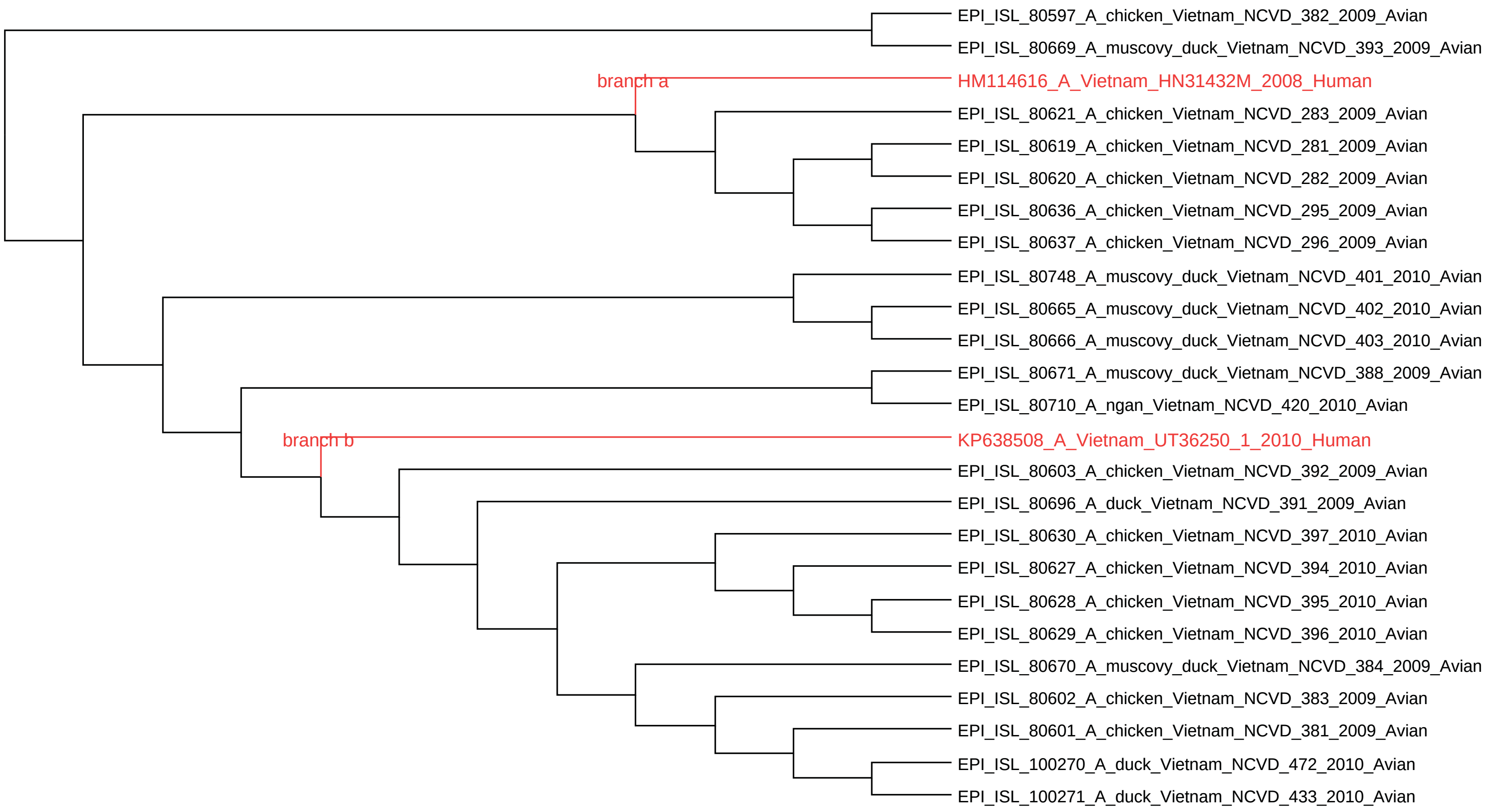
EPI_ISL_80597_A_chicken_Vietnam_NCVD_382_2009_Avian
EPI_ISL_80669_A_muscovy_duck_Vietnam_NCVD_393_2009_Avian
branch a
HM114616_A_Vietnam_HN31432M_2008_Human
EPI_ISL_80621_A_chicken_Vietnam_NCVD_283_2009_Avian
EPI_ISL_80619_A_chicken_Vietnam_NCVD_281_2009_Avian
EPI_ISL_80620_A_chicken_Vietnam_NCVD_282_2009_Avian
EPI_ISL_80636_A_chicken_Vietnam_NCVD_295_2009_Avian
EPI_ISL_80637_A_chicken_Vietnam_NCVD_296_2009_Avian
EPI_ISL_80748_A_muscovy_duck_Vietnam_NCVD_401_2010_Avian
EPI_ISL_80665_A_muscovy_duck_Vietnam_NCVD_402_2010_Avian
EPI_ISL_80666_A_muscovy_duck_Vietnam_NCVD_403_2010_Avian
EPI_ISL_80671_A_muscovy_duck_Vietnam_NCVD_388_2009_Avian
EPI_ISL_80710_A_ngan_Vietnam_NCVD_420_2010_Avian
branch b
KP638508_A_Vietnam_UT36250_1_2010_Human
EPI_ISL_80603_A_chicken_Vietnam_NCVD_392_2009_Avian
EPI_ISL_80696_A_duck_Vietnam_NCVD_391_2009_Avian
EPI_ISL_80630_A_chicken_Vietnam_NCVD_397_2010_Avian
EPI_ISL_80627_A_chicken_Vietnam_NCVD_394_2010_Avian
EPI_ISL_80628_A_chicken_Vietnam_NCVD_395_2010_Avian
EPI_ISL_80629_A_chicken_Vietnam_NCVD_396_2010_Avian
EPI_ISL_80670_A_muscovy_duck_Vietnam_NCVD_384_2009_Avian
EPI_ISL_80602_A_chicken_Vietnam_NCVD_383_2009_Avian
EPI_ISL_80601_A_chicken_Vietnam_NCVD_381_2009_Avian
EPI_ISL_100270_A_duck_Vietnam_NCVD_472_2010_Avian
EPI_ISL_100271_A_duck_Vietnam_NCVD_433_2010_Avian

PA-Group20

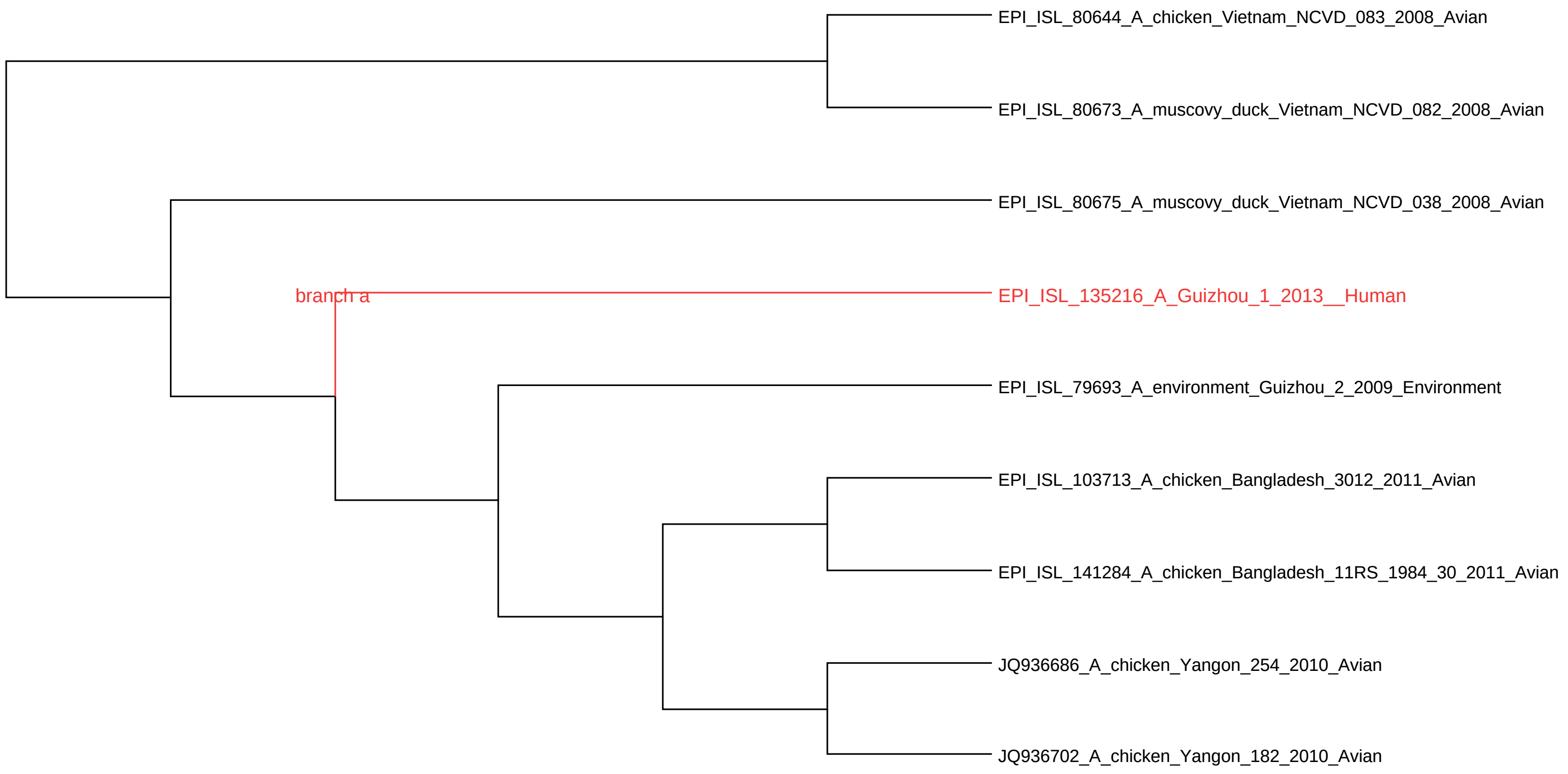

EPI_ISL_80644_A_chicken_Vietnam_NCVD_083_2008_Avian
EPI_ISL_80673_A_muscovy_duck_Vietnam_NCVD_082_2008_Avian
EPI_ISL_80675_A_muscovy_duck_Vietnam_NCVD_038_2008_Avian
branch a
EPI_ISL_135216_A_Guizhou_1_2013__Human
EPI_ISL_79693_A_environment_Guizhou_2_2009_Environment
EPI_ISL_103713_A_chicken_Bangladesh_3012_2011_Avian
EPI_ISL_141284_A_chicken_Bangladesh_11RS_1984_30_2011_Avian
JQ936686_A_chicken_Yangon_254_2010_Avian
JQ936702_A_chicken_Yangon_182_2010_Avian

PA-Group21

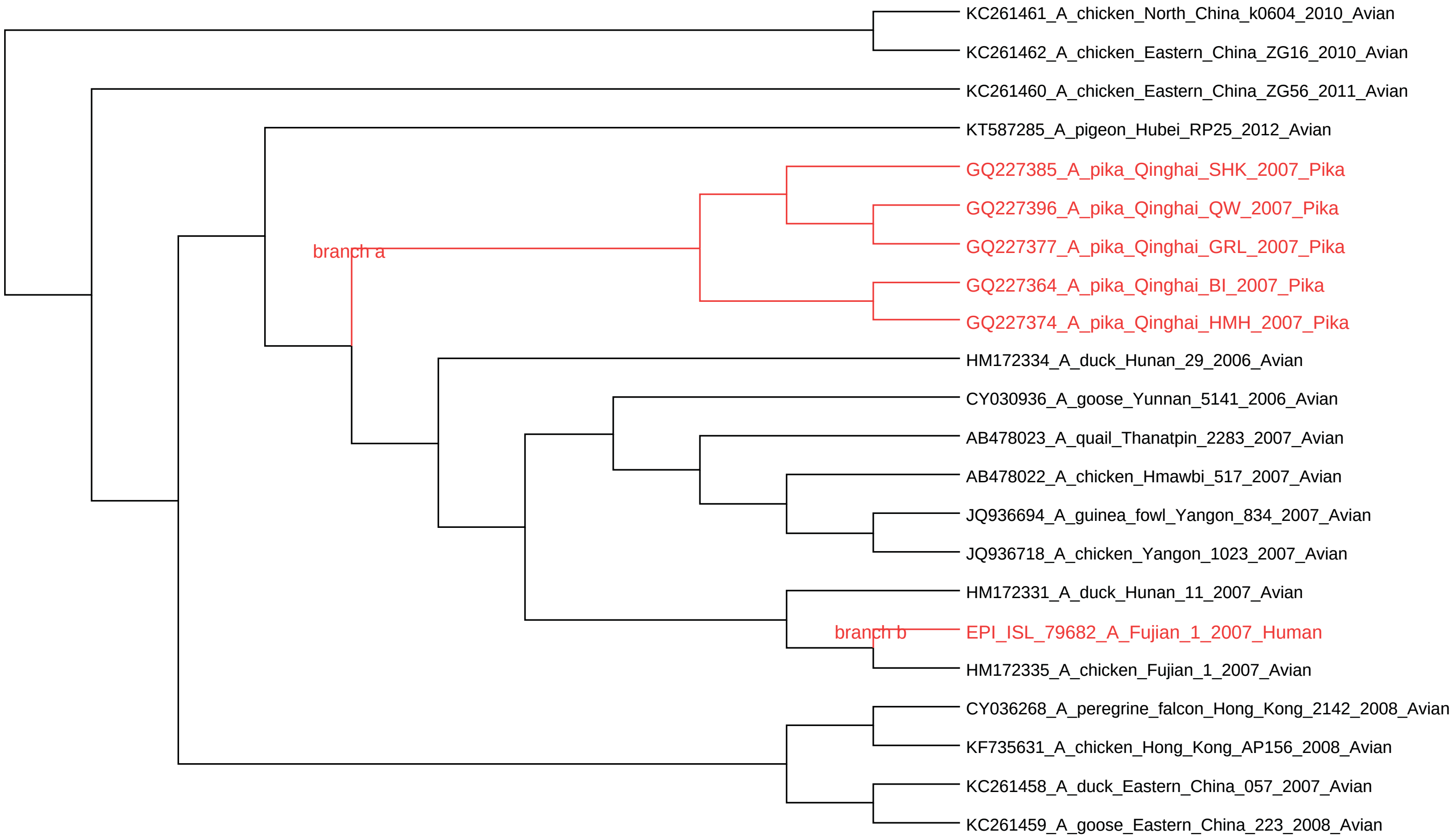

KC261461_A_chicken_North_China_k0604_2010_Avian
KC261462_A_chicken_Eastern_China_ZG16_2010_Avian
KC261460_A_chicken_Eastern_China_ZG56_2011_Avian
KT587285_A_pigeon_Hubei_RP25_2012_Avian
GQ227385_A_pika_Qinghai_SHK_2007_Pika
GQ227396_A_pika_Qinghai_QW_2007_Pika
GQ227377_A_pika_Qinghai_GRL_2007_Pika
GQ227364_A_pika_Qinghai_BI_2007_Pika
GQ227374_A_pika_Qinghai_HMH_2007_Pika
branch a
HM172334_A_duck_Hunan_29_2006_Avian
CY030936_A_goose_Yunnan_5141_2006_Avian
AB478023_A_quail_Thanatpin_2283_2007_Avian
AB478022_A_chicken_Hmawbi_517_2007_Avian
JQ936694_A_guinea_fowl_Yangon_834_2007_Avian
JQ936718_A_chicken_Yangon_1023_2007_Avian
HM172331_A_duck_Hunan_11_2007_Avian
branch b
EPI_ISL_79682_A_Fujian_1_2007_Human
HM172335_A_chicken_Fujian_1_2007_Avian
CY036268_A_peregrine_falcon_Hong_Kong_2142_2008_Avian
KF735631_A_chicken_Hong_Kong_AP156_2008_Avian
KC261458_A_duck_Eastern_China_057_2007_Avian
KC261459_A_goose_Eastern_China_223_2008_Avian

PA-Group22

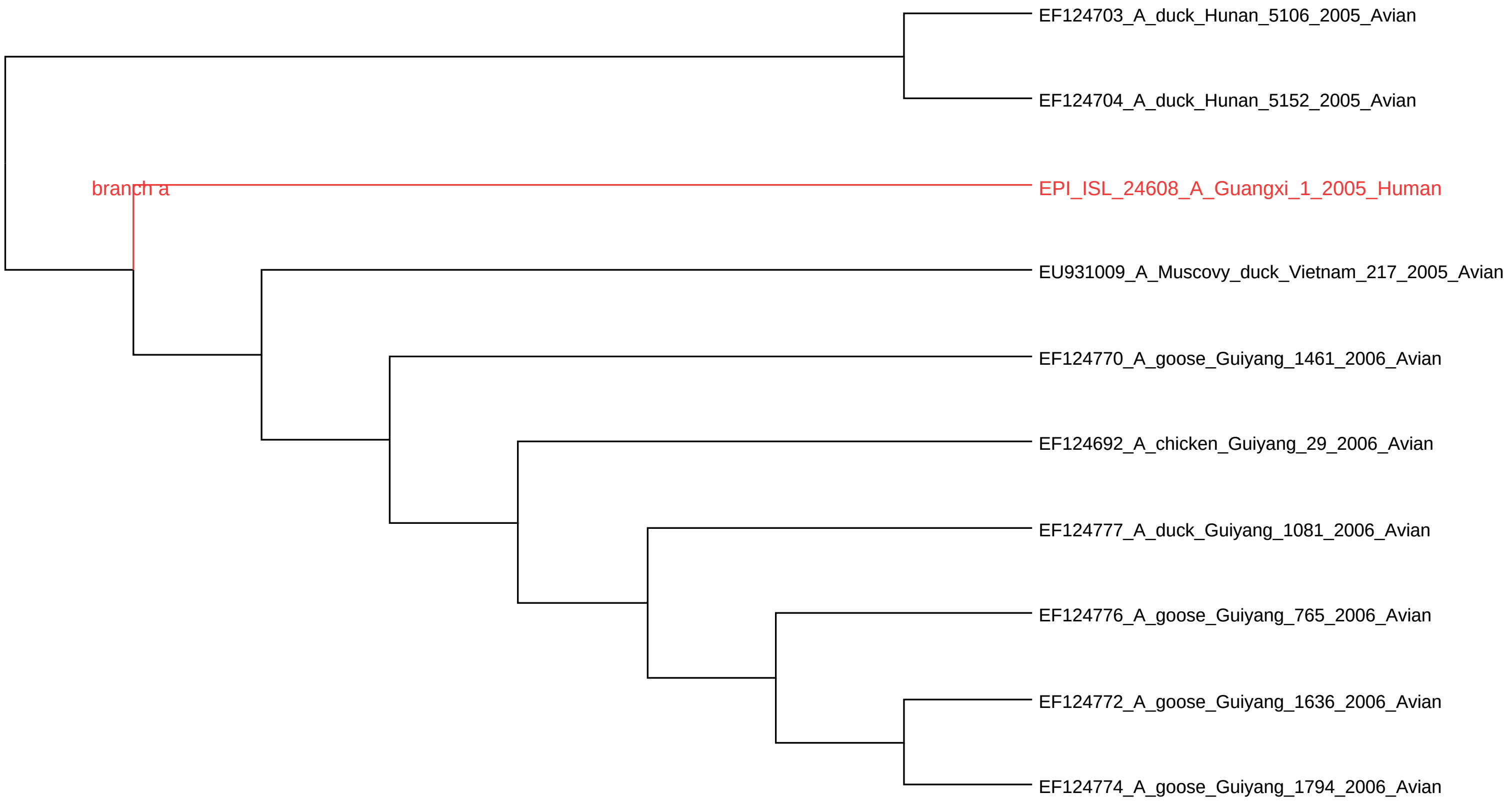

EF124703_A_duck_Hunan_5106_2005_Avian
EF124704_A_duck_Hunan_5152_2005_Avian
branch a
EPI_ISL_24608_A_Guangxi_1_2005_Human
EU931009_A_Muscovy_duck_Vietnam_217_2005_Avian
EF124770_A_goose_Guiyang_1461_2006_Avian
EF124692_A_chicken_Guiyang_29_2006_Avian
EF124777_A_duck_Guiyang_1081_2006_Avian
EF124776_A_goose_Guiyang_765_2006_Avian
EF124772_A_goose_Guiyang_1636_2006_Avian
EF124774_A_goose_Guiyang_1794_2006_Avian

PA-Group23

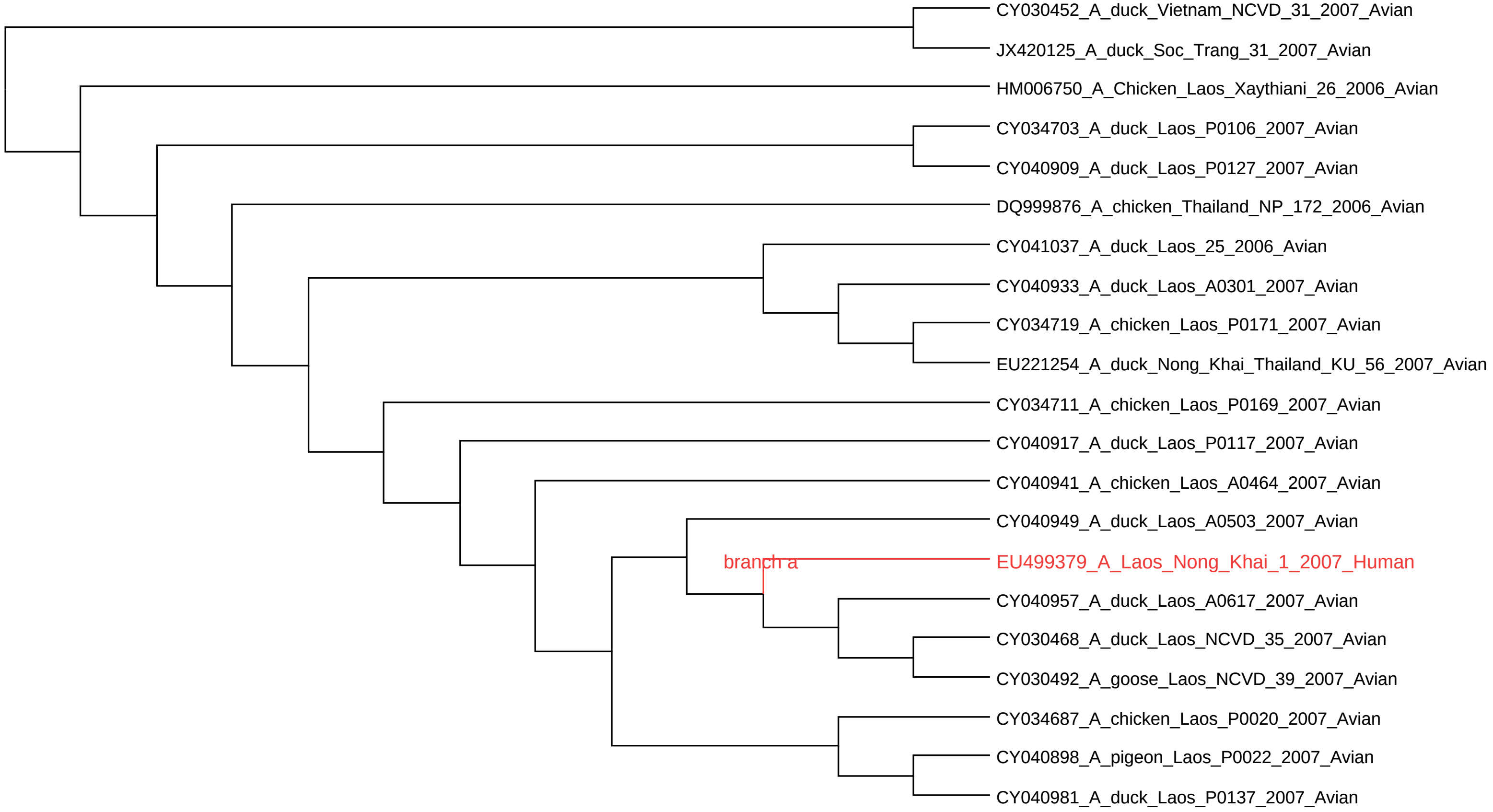
CY030452_A_duck_Vietnam_NCVD_31_2007_Avian
JX420125_A_duck_Soc_Trang_31_2007_Avian
HM006750_A_Chicken_Laos_Xaythiani_26_2006_Avian
CY034703_A_duck_Laos_P0106_2007_Avian
CY040909_A_duck_Laos_P0127_2007_Avian
DQ999876_A_chicken_Thailand_NP_172_2006_Avian
CY041037_A_duck_Laos_25_2006_Avian
CY040933_A_duck_Laos_A0301_2007_Avian
CY034719_A_chicken_Laos_P0171_2007_Avian
EU221254_A_duck_Nong_Khai_Thailand_KU_56_2007_Avian
CY034711_A_chicken_Laos_P0169_2007_Avian
CY040917_A_duck_Laos_P0117_2007_Avian
CY040941_A_chicken_Laos_A0464_2007_Avian
CY040949_A_duck_Laos_A0503_2007_Avian
branch a
EU499379_A_Laos_Nong_Khai_1_2007_Human
CY040957_A_duck_Laos_A0617_2007_Avian
CY030468_A_duck_Laos_NCVD_35_2007_Avian
CY030492_A_goose_Laos_NCVD_39_2007_Avian
CY034687_A_chicken_Laos_P0020_2007_Avian
CY040898_A_pigeon_Laos_P0022_2007_Avian
CY040981_A_duck_Laos_P0137_2007_Avian

PA-Group24

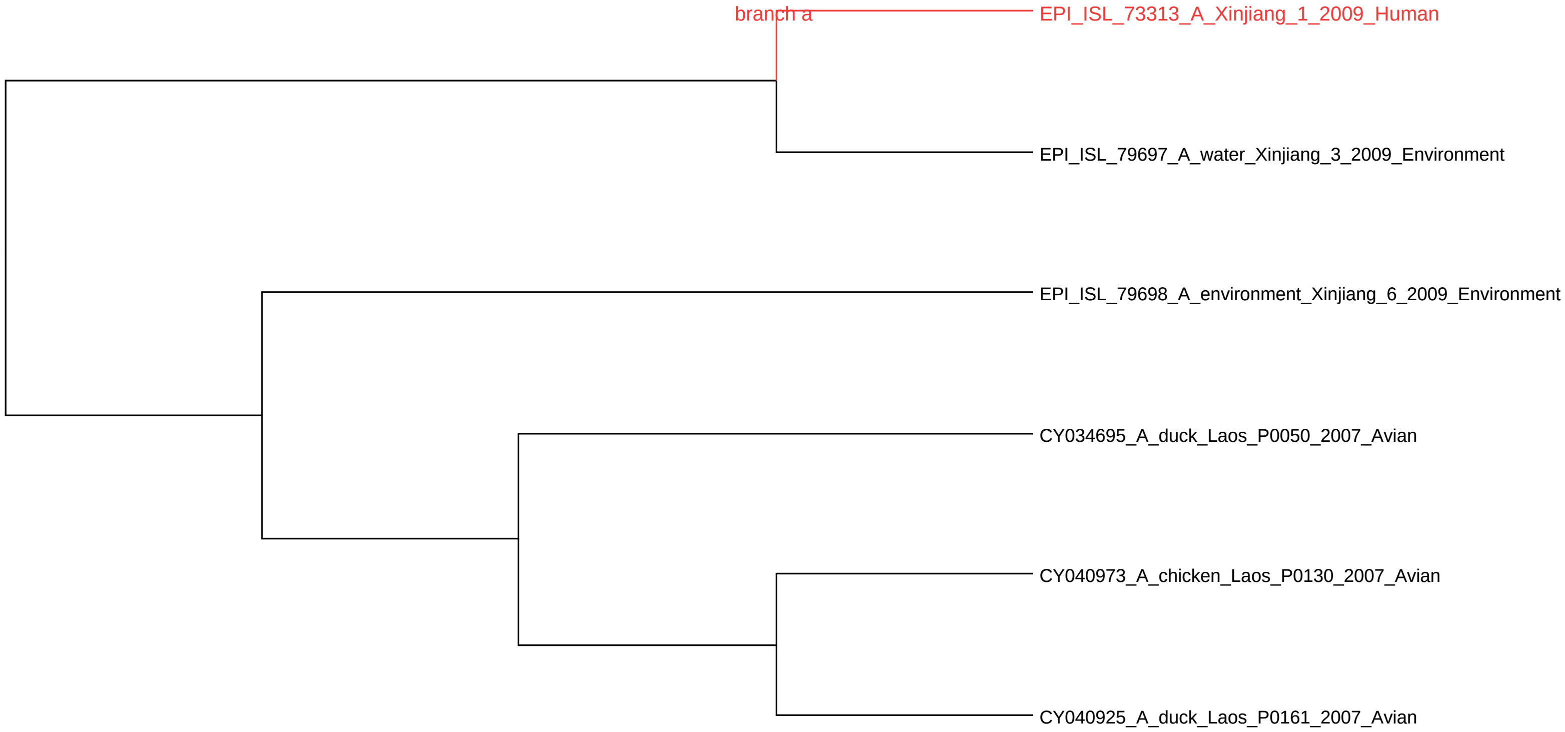
branch a
EPI_ISL_73313_A_Xinjiang_1_2009_Human
EPI_ISL_79697_A_water_Xinjiang_3_2009_Environment
EPI_ISL_79698_A_environment_Xinjiang_6_2009_Environment
CY034695_A_duck_Laos_P0050_2007_Avian
CY040973_A_chicken_Laos_P0130_2007_Avian
CY040925_A_duck_Laos_P0161_2007_Avian

PA-Group25

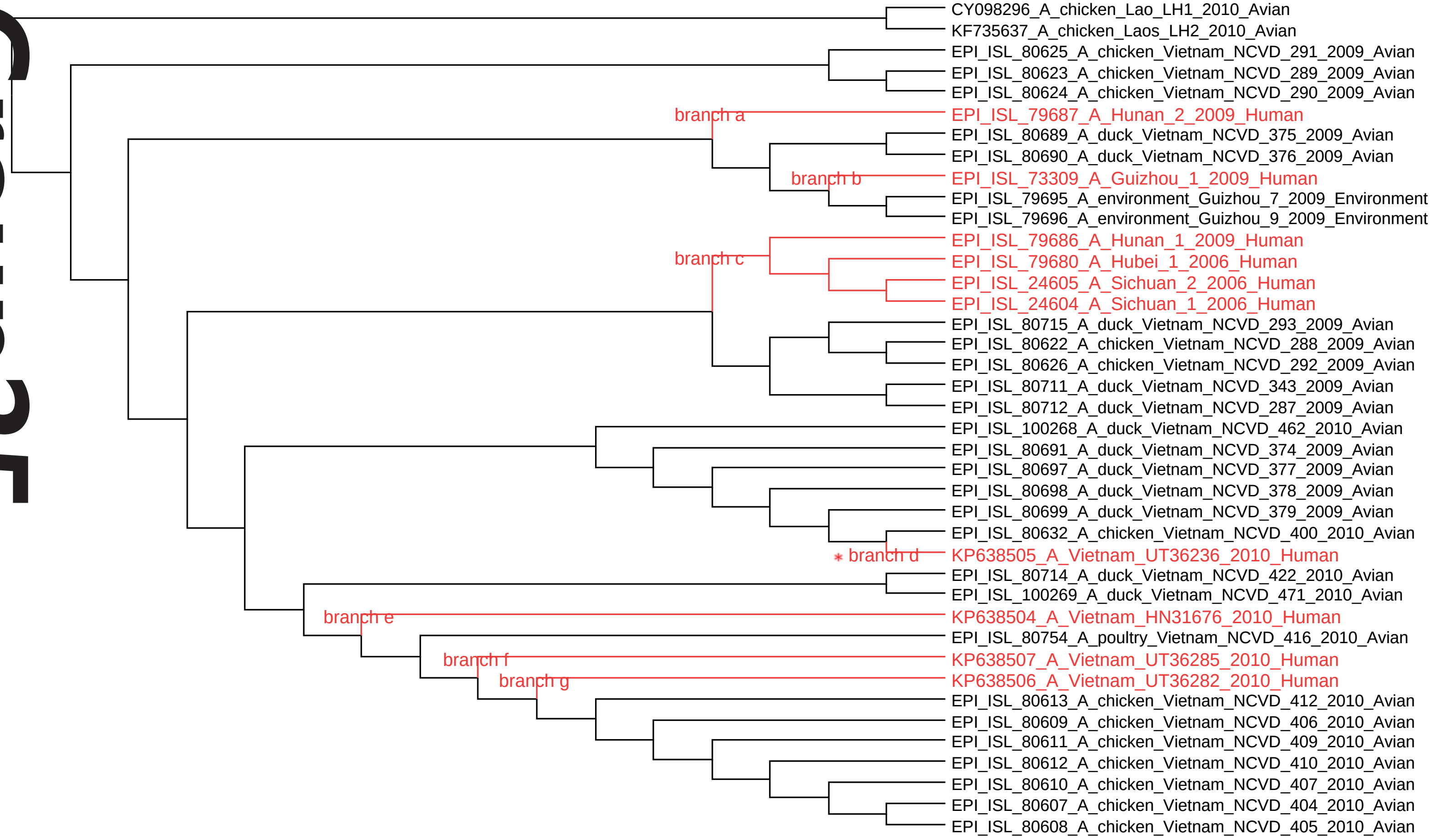

CY098296_A_chicken_Lao_LH1_2010_Avian
KF735637_A_chicken_Laos_LH2_2010_Avian
EPI_ISL_80625_A_chicken_Vietnam_NCVD_291_2009_Avian
EPI_ISL_80623_A_chicken_Vietnam_NCVD_289_2009_Avian
EPI_ISL_80624_A_chicken_Vietnam_NCVD_290_2009_Avian
branch a
EPI_ISL_79687_A_Hunan_2_2009_Human
EPI_ISL_80689_A_duck_Vietnam_NCVD_375_2009_Avian
EPI_ISL_80690_A_duck_Vietnam_NCVD_376_2009_Avian
branch b
EPI_ISL_73309_A_Guizhou_1_2009_Human
EPI_ISL_79695_A_environment_Guizhou_7_2009_Environment
EPI_ISL_79696_A_environment_Guizhou_9_2009_Environment
branch c
EPI_ISL_79686_A_Hunan_1_2009_Human
EPI_ISL_79680_A_Hubei_1_2006_Human
EPI_ISL_24605_A_Sichuan_2_2006_Human
EPI_ISL_24604_A_Sichuan_1_2006_Human
EPI_ISL_80715_A_duck_Vietnam_NCVD_293_2009_Avian
EPI_ISL_80622_A_chicken_Vietnam_NCVD_288_2009_Avian
EPI_ISL_80626_A_chicken_Vietnam_NCVD_292_2009_Avian
EPI_ISL_80711_A_duck_Vietnam_NCVD_343_2009_Avian
EPI_ISL_80712_A_duck_Vietnam_NCVD_287_2009_Avian
EPI_ISL_100268_A_duck_Vietnam_NCVD_462_2010_Avian
EPI_ISL_80691_A_duck_Vietnam_NCVD_374_2009_Avian
EPI_ISL_80697_A_duck_Vietnam_NCVD_377_2009_Avian
EPI_ISL_80698_A_duck_Vietnam_NCVD_378_2009_Avian
EPI_ISL_80699_A_duck_Vietnam_NCVD_379_2009_Avian
EPI_ISL_80632_A_chicken_Vietnam_NCVD_400_2010_Avian
* branch d
KP638505_A_Vietnam_UT36236_2010_Human
EPI_ISL_80714_A_duck_Vietnam_NCVD_422_2010_Avian
EPI_ISL_100269_A_duck_Vietnam_NCVD_471_2010_Avian
branch e
KP638504_A_Vietnam_HN31676_2010_Human
EPI_ISL_80754_A_poultry_Vietnam_NCVD_416_2010_Avian
branch f
KP638507_A_Vietnam_UT36285_2010_Human
branch g
KP638506_A_Vietnam_UT36282_2010_Human
EPI_ISL_80613_A_chicken_Vietnam_NCVD_412_2010_Avian
EPI_ISL_80609_A_chicken_Vietnam_NCVD_406_2010_Avian
EPI_ISL_80611_A_chicken_Vietnam_NCVD_409_2010_Avian
EPI_ISL_80612_A_chicken_Vietnam_NCVD_410_2010_Avian
EPI_ISL_80610_A_chicken_Vietnam_NCVD_407_2010_Avian
EPI_ISL_80607_A_chicken_Vietnam_NCVD_404_2010_Avian
EPI_ISL_80608_A_chicken_Vietnam_NCVD_405_2010_Avian

PA-Group26

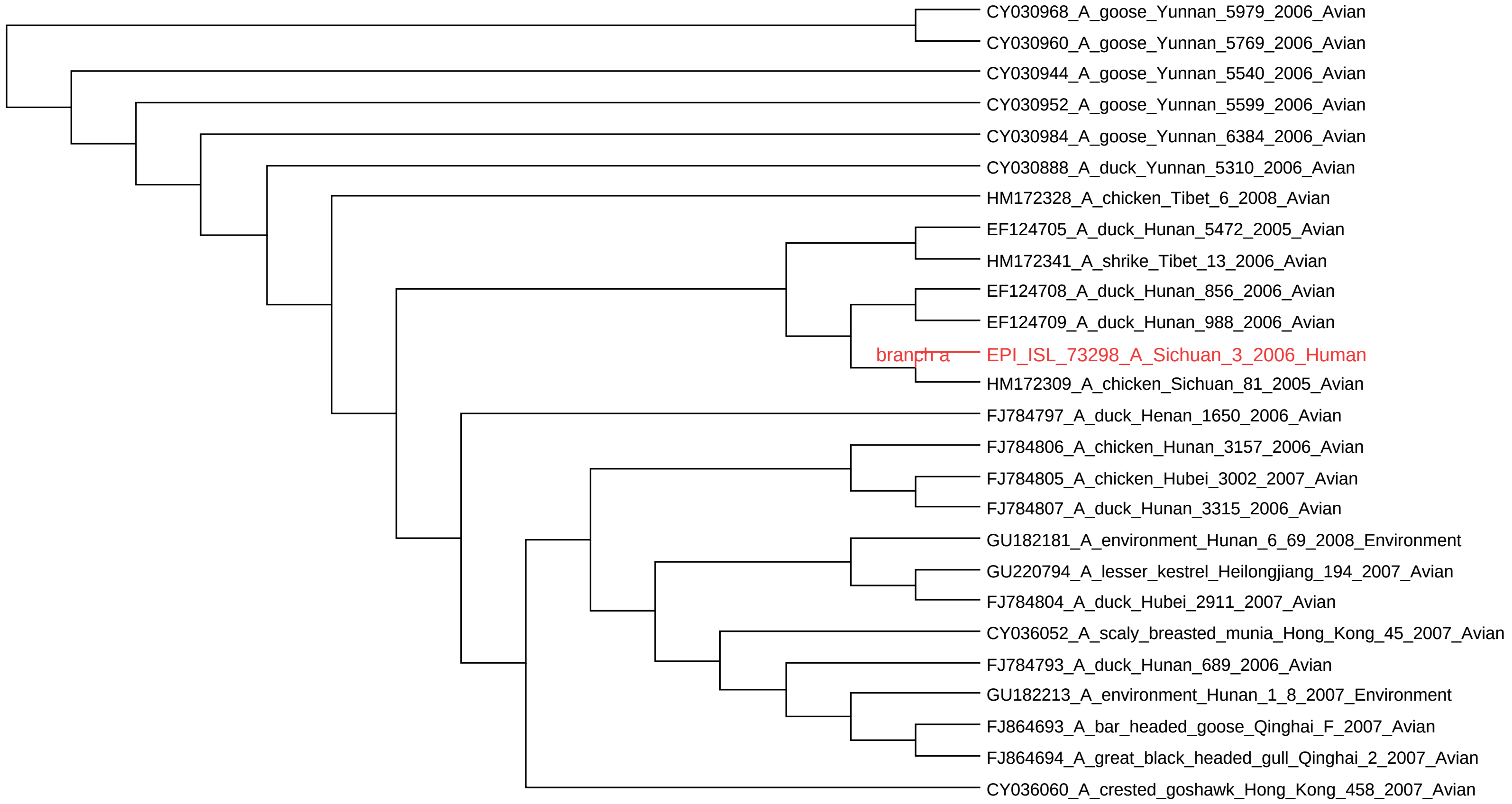

CY030968_A_goose_Yunnan_5979_2006_Avian
CY030960_A_goose_Yunnan_5769_2006_Avian
CY030944_A_goose_Yunnan_5540_2006_Avian
CY030952_A_goose_Yunnan_5599_2006_Avian
CY030984_A_goose_Yunnan_6384_2006_Avian
CY030888_A_duck_Yunnan_5310_2006_Avian
HM172328_A_chicken_Tibet_6_2008_Avian
EF124705_A_duck_Hunan_5472_2005_Avian
HM172341_A_shrike_Tibet_13_2006_Avian
EF124708_A_duck_Hunan_856_2006_Avian
EF124709_A_duck_Hunan_988_2006_Avian
branch a
EPI_ISL_73298_A_Sichuan_3_2006_Human
HM172309_A_chicken_Sichuan_81_2005_Avian
FJ784797_A_duck_Henan_1650_2006_Avian
FJ784806_A_chicken_Hunan_3157_2006_Avian
FJ784805_A_chicken_Hubei_3002_2007_Avian
FJ784807_A_duck_Hunan_3315_2006_Avian
GU182181_A_environment_Hunan_6_69_2008_Environment
GU220794_A_lesser_kestrel_Heilongjiang_194_2007_Avian
FJ784804_A_duck_Hubei_2911_2007_Avian
CY036052_A_scaly_breasted_munia_Hong_Kong_45_2007_Avian
FJ784793_A_duck_Hunan_689_2006_Avian
GU182213_A_environment_Hunan_1_8_2007_Environment
FJ864693_A_bar_headed_goose_Qinghai_F_2007_Avian
FJ864694_A_great_black_headed_gull_Qinghai_2_2007_Avian
CY036060_A_crested_goshawk_Hong_Kong_458_2007_Avian

PA-Group27

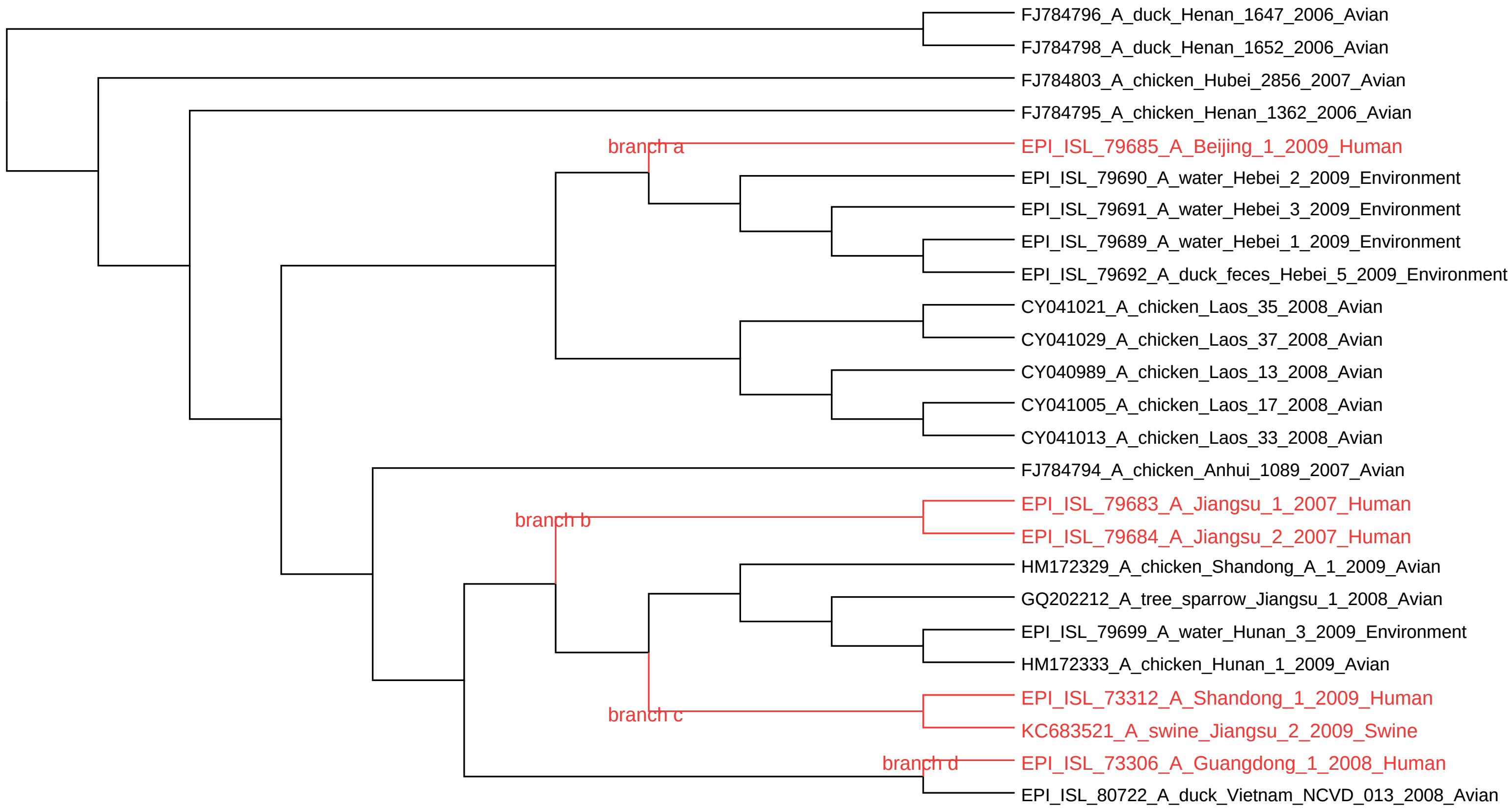
FJ784796_A_duck_Henan_1647_2006_Avian
FJ784798_A_duck_Henan_1652_2006_Avian
FJ784803_A_chicken_Hubei_2856_2007_Avian
FJ784795_A_chicken_Henan_1362_2006_Avian
branch a
EPI_ISL_79685_A_Beijing_1_2009_Human
EPI_ISL_79690_A_water_Hebei_2_2009_Environment
EPI_ISL_79691_A_water_Hebei_3_2009_Environment
EPI_ISL_79689_A_water_Hebei_1_2009_Environment
EPI_ISL_79692_A_duck_feces_Hebei_5_2009_Environment
CY041021_A_chicken_Laos_35_2008_Avian
CY041029_A_chicken_Laos_37_2008_Avian
CY040989_A_chicken_Laos_13_2008_Avian
CY041005_A_chicken_Laos_17_2008_Avian
CY041013_A_chicken_Laos_33_2008_Avian
FJ784794_A_chicken_Anhui_1089_2007_Avian
branch b
EPI_ISL_79683_A_Jiangsu_1_2007_Human
EPI_ISL_79684_A_Jiangsu_2_2007_Human
HM172329_A_chicken_Shandong_A_1_2009_Avian
GQ202212_A_tree_sparrow_Jiangsu_1_2008_Avian
EPI_ISL_79699_A_water_Hunan_3_2009_Environment
HM172333_A_chicken_Hunan_1_2009_Avian
branch c
EPI_ISL_73312_A_Shandong_1_2009_Human
KC683521_A_swine_Jiangsu_2_2009_Swine
branch d
EPI_ISL_73306_A_Guangdong_1_2008_Human
EPI_ISL_80722_A_duck_Vietnam_NCVD_013_2008_Avian

PA-Group28

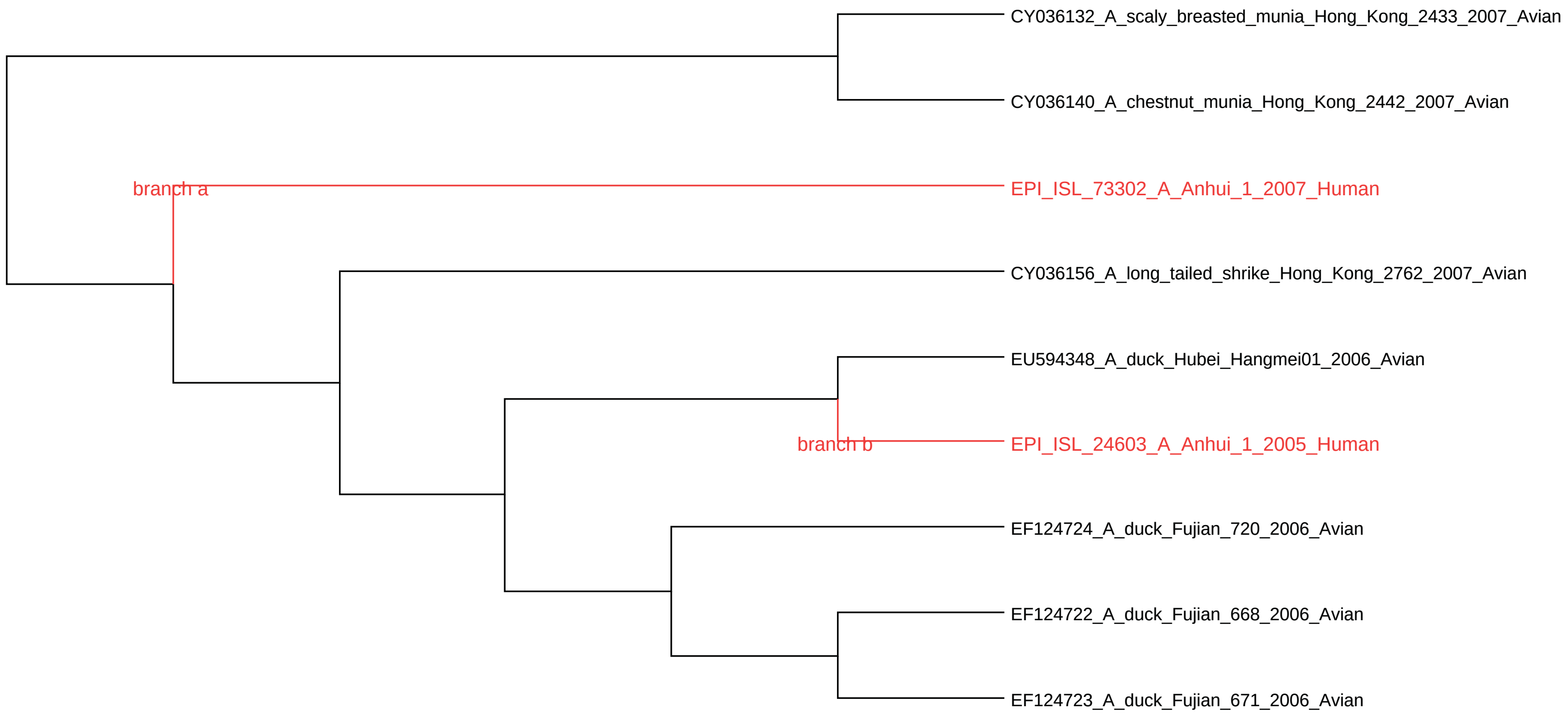
CY036132_A_scaly_breasted_munia_Hong_Kong_2433_2007_Avian
CY036140_A_chestnut_munia_Hong_Kong_2442_2007_Avian
branch a
EPI_ISL_73302_A_Anhui_1_2007_Human
CY036156_A_long_tailed_shrike_Hong_Kong_2762_2007_Avian
EU594348_A_duck_Hubei_Hangmei01_2006_Avian
branch b
EPI_ISL_24603_A_Anhui_1_2005_Human
EF124724_A_duck_Fujian_720_2006_Avian
EF124722_A_duck_Fujian_668_2006_Avian
EF124723_A_duck_Fujian_671_2006_Avian

PA-Group29

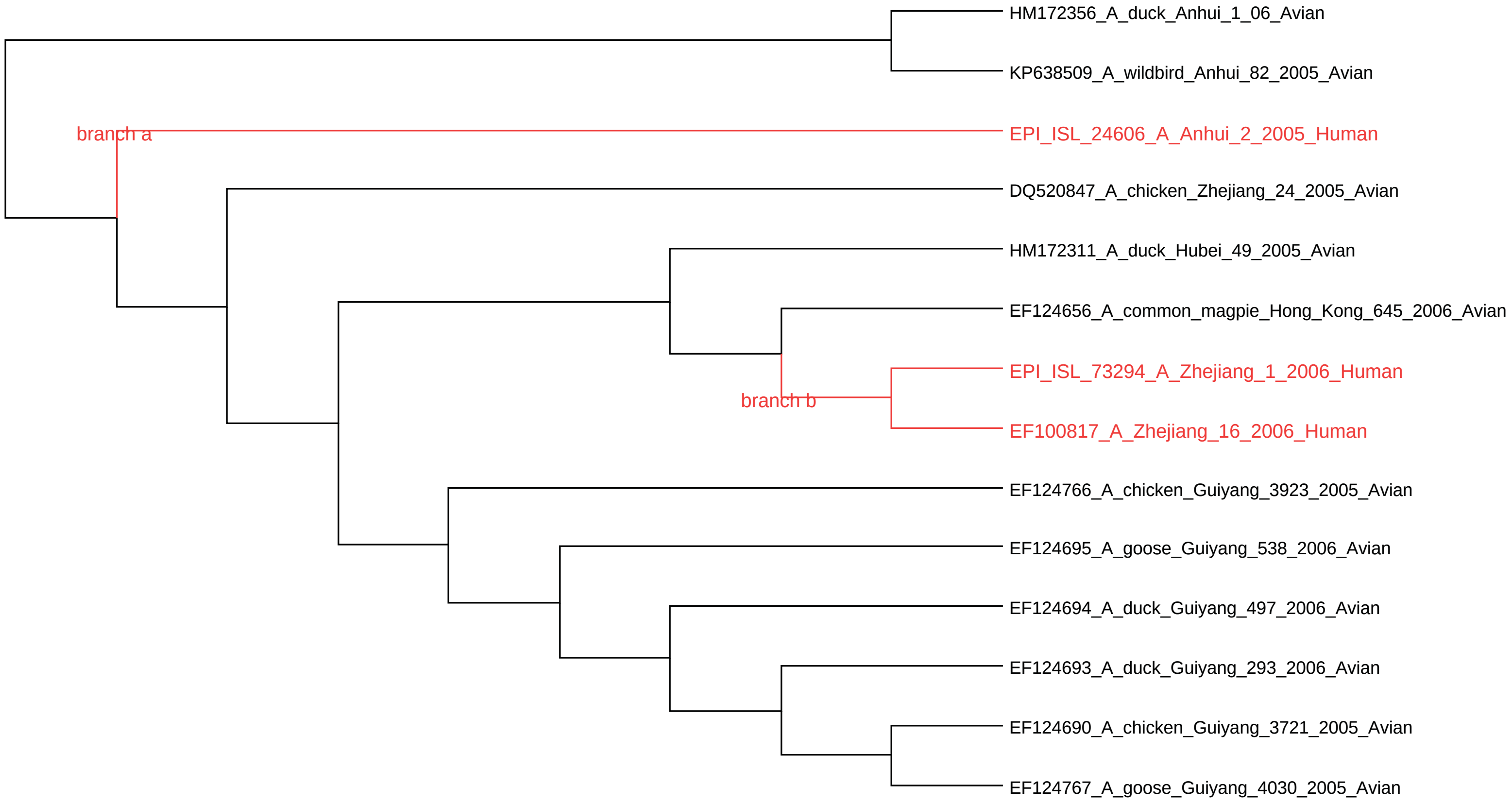
HM172356_A_duck_Anhui_1_06_Avian
KP638509_A_wildbird_Anhui_82_2005_Avian
branch a
EPI_ISL_24606_A_Anhui_2_2005_Human
DQ520847_A_chicken_Zhejiang_24_2005_Avian
HM172311_A_duck_Hubei_49_2005_Avian
EF124656_A_common_magpie_Hong_Kong_645_2006_Avian
EPI_ISL_73294_A_Zhejiang_1_2006_Human
branch b
EF100817_A_Zhejiang_16_2006_Human
EF124766_A_chicken_Guiyang_3923_2005_Avian
EF124695_A_goose_Guiyang_538_2006_Avian
EF124694_A_duck_Guiyang_497_2006_Avian
EF124693_A_duck_Guiyang_293_2006_Avian
EF124690_A_chicken_Guiyang_3721_2005_Avian
EF124767_A_goose_Guiyang_4030_2005_Avian

PA-Group30

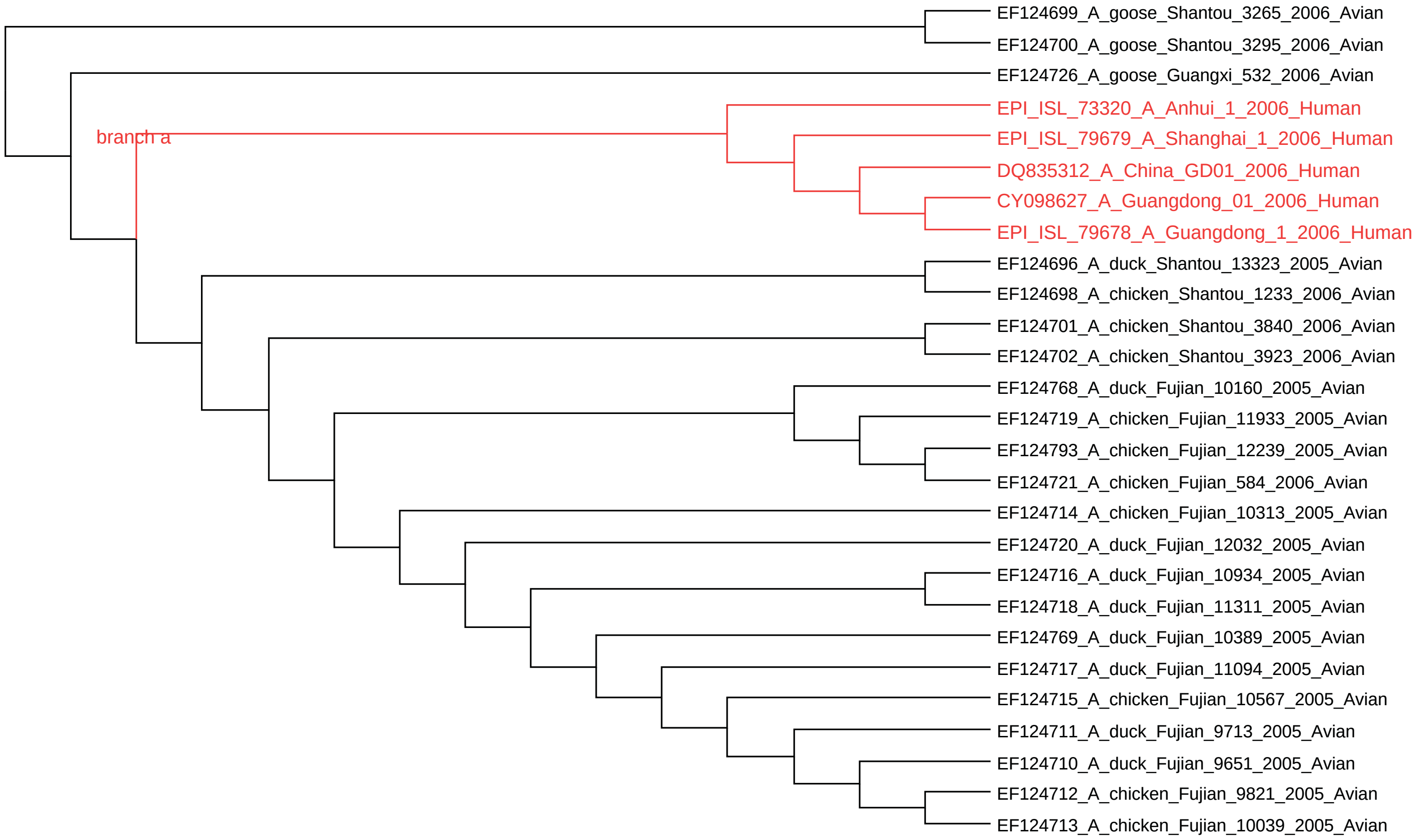
EF124699_A_goose_Shantou_3265_2006_Avian
EF124700_A_goose_Shantou_3295_2006_Avian
EF124726_A_goose_Guangxi_532_2006_Avian
branch a
EPI_ISL_73320_A_Anhui_1_2006_Human
EPI_ISL_79679_A_Shanghai_1_2006_Human
DQ835312_A_China_GD01_2006_Human
CY098627_A_Guangdong_01_2006_Human
EPI_ISL_79678_A_Guangdong_1_2006_Human
EF124696_A_duck_Shantou_13323_2005_Avian
EF124698_A_chicken_Shantou_1233_2006_Avian
EF124701_A_chicken_Shantou_3840_2006_Avian
EF124702_A_chicken_Shantou_3923_2006_Avian
EF124768_A_duck_Fujian_10160_2005_Avian
EF124719_A_chicken_Fujian_11933_2005_Avian
EF124793_A_chicken_Fujian_12239_2005_Avian
EF124721_A_chicken_Fujian_584_2006_Avian
EF124714_A_chicken_Fujian_10313_2005_Avian
EF124720_A_duck_Fujian_12032_2005_Avian
EF124716_A_duck_Fujian_10934_2005_Avian
EF124718_A_duck_Fujian_11311_2005_Avian
EF124769_A_duck_Fujian_10389_2005_Avian
EF124717_A_duck_Fujian_11094_2005_Avian
EF124715_A_chicken_Fujian_10567_2005_Avian
EF124711_A_duck_Fujian_9713_2005_Avian
EF124710_A_duck_Fujian_9651_2005_Avian
EF124712_A_chicken_Fujian_9821_2005_Avian
EF124713_A_chicken_Fujian_10039_2005_Avian

PA-Group31

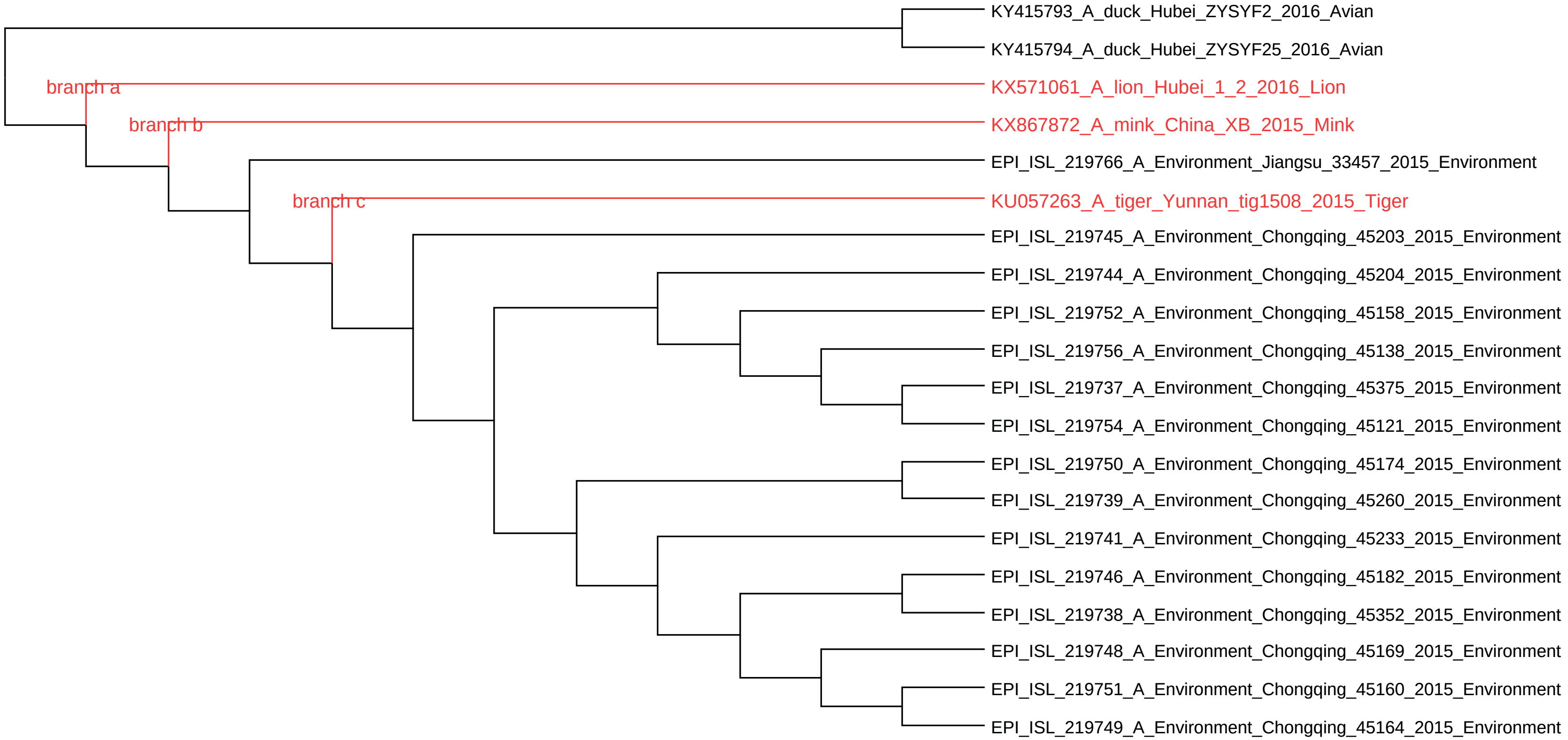

KY415793_A_duck_Hubei_ZYSYF2_2016_Avian
KY415794_A_duck_Hubei_ZYSYF25_2016_Avian
branch a
KX571061_A_lion_Hubei_1_2_2016_Lion
branch b
KX867872_A_mink_China_XB_2015_Mink
EPI_ISL_219766_A_Environment_Jiangsu_33457_2015_Environment
branch c
KU057263_A_tiger_Yunnan_tig1508_2015_Tiger
EPI_ISL_219745_A_Environment_Chongqing_45203_2015_Environment
EPI_ISL_219744_A_Environment_Chongqing_45204_2015_Environment
EPI_ISL_219752_A_Environment_Chongqing_45158_2015_Environment
EPI_ISL_219756_A_Environment_Chongqing_45138_2015_Environment
EPI_ISL_219737_A_Environment_Chongqing_45375_2015_Environment
EPI_ISL_219754_A_Environment_Chongqing_45121_2015_Environment
EPI_ISL_219750_A_Environment_Chongqing_45174_2015_Environment
EPI_ISL_219739_A_Environment_Chongqing_45260_2015_Environment
EPI_ISL_219741_A_Environment_Chongqing_45233_2015_Environment
EPI_ISL_219746_A_Environment_Chongqing_45182_2015_Environment
EPI_ISL_219738_A_Environment_Chongqing_45352_2015_Environment
EPI_ISL_219748_A_Environment_Chongqing_45169_2015_Environment
EPI_ISL_219751_A_Environment_Chongqing_45160_2015_Environment
EPI_ISL_219749_A_Environment_Chongqing_45164_2015_Environment

PA-Group32

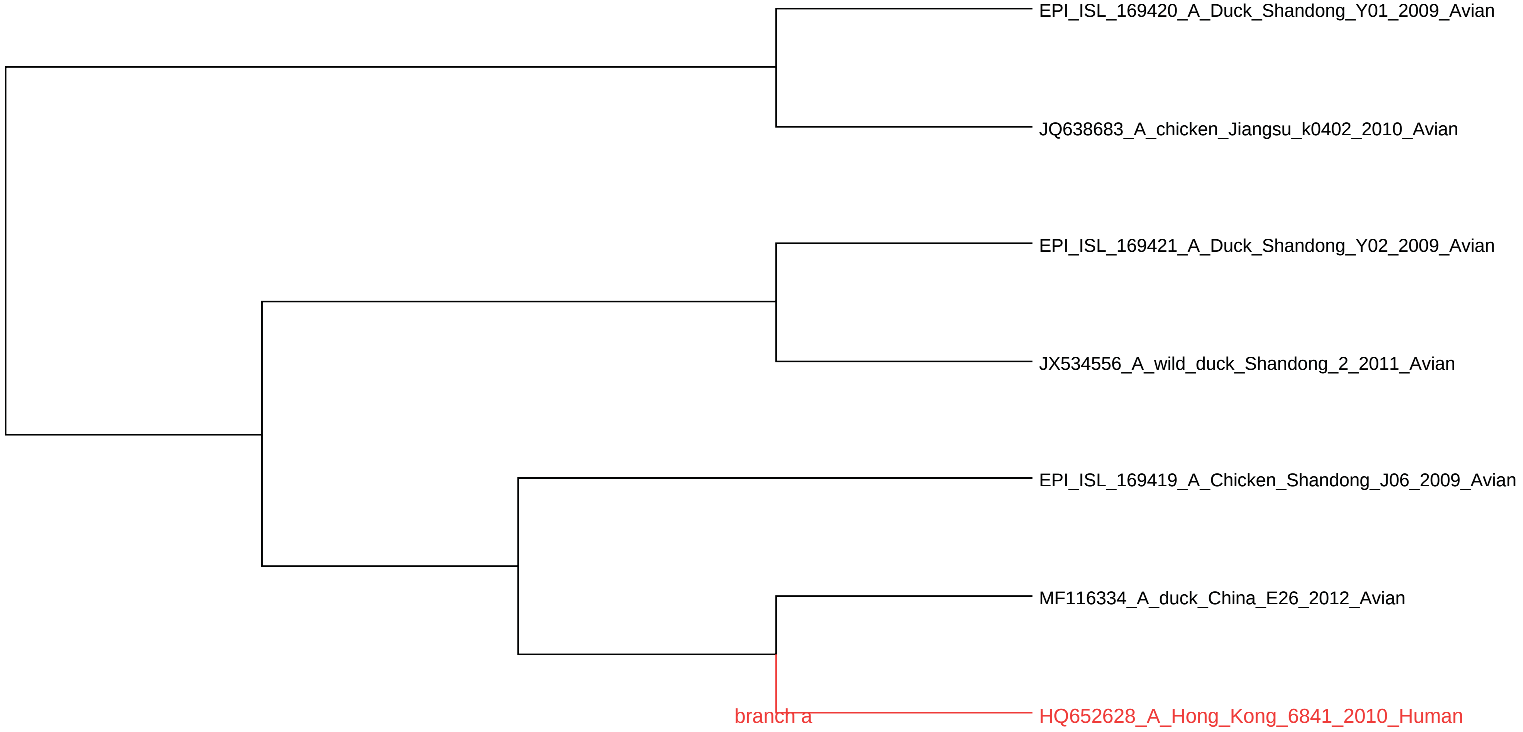
EPI_ISL_169420_A_Duck_Shandong_Y01_2009_Avian
JQ638683_A_chicken_Jiangsu_k0402_2010_Avian
EPI_ISL_169421_A_Duck_Shandong_Y02_2009_Avian
JX534556_A_wild_duck_Shandong_2_2011_Avian
EPI_ISL_169419_A_Chicken_Shandong_J06_2009_Avian
MF116334_A_duck_China_E26_2012_Avian
branch a
HQ652628_A_Hong_Kong_6841_2010_Human

PA-Group33

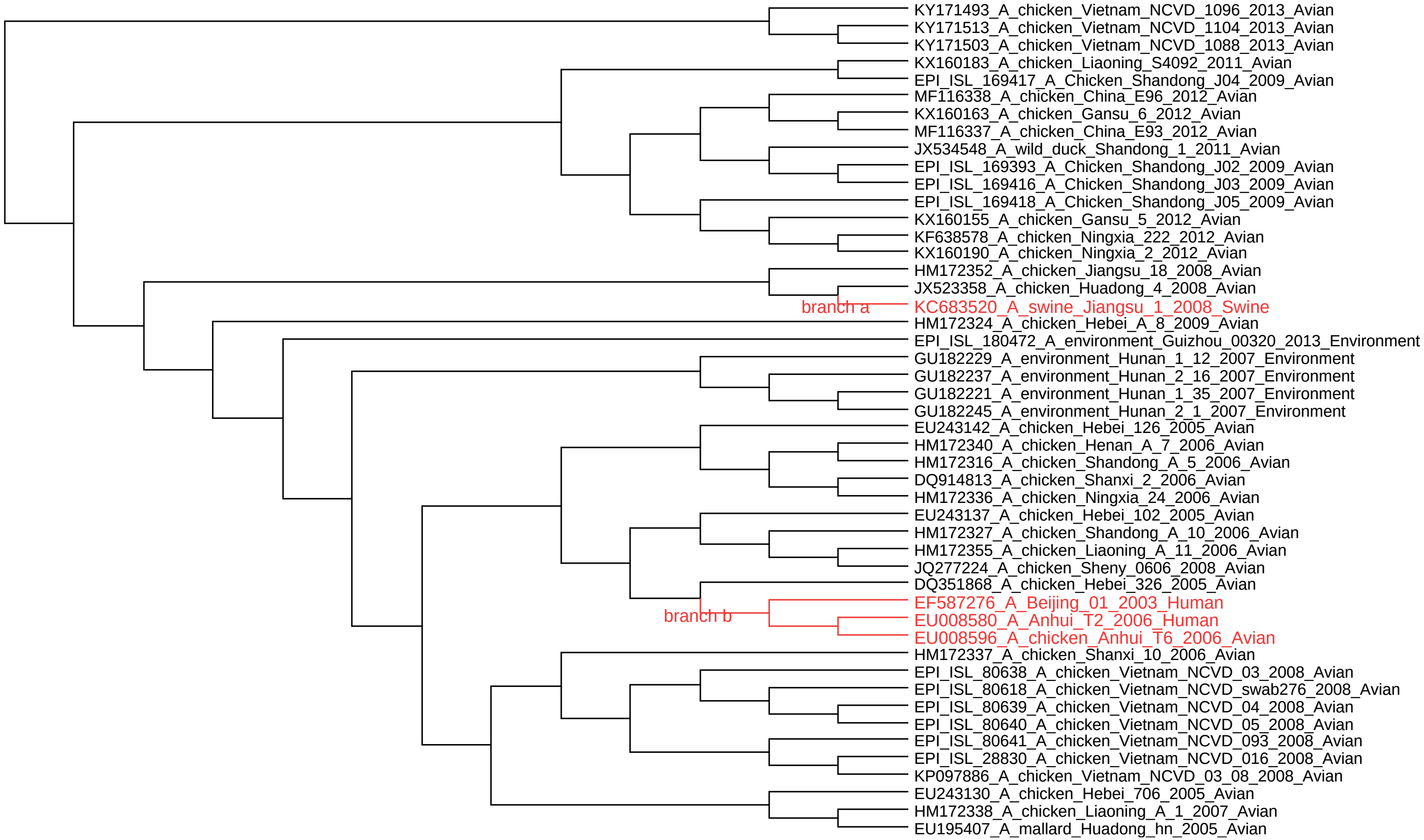
KY171493_A_chicken_Vietnam_NCVD_1096_2013_Avian
KY171513_A_chicken_Vietnam_NCVD_1104_2013_Avian
KY171503_A_chicken_Vietnam_NCVD_1088_2013_Avian
KX160183_A_chicken_Liaoning_S4092_2011_Avian
EPI_ISL_169417_A_Chicken_Shandong_J04_2009_Avian
MF116338_A_chicken_China_E96_2012_Avian
KX160163_A_chicken_Gansu_6_2012_Avian
MF116337_A_chicken_China_E93_2012_Avian
JX534548_A_wild_duck_Shandong_1_2011_Avian
EPI_ISL_169393_A_Chicken_Shandong_J02_2009_Avian
EPI_ISL_169416_A_Chicken_Shandong_J03_2009_Avian
EPI_ISL_169418_A_Chicken_Shandong_J05_2009_Avian
KX160155_A_chicken_Gansu_5_2012_Avian
KF638578_A_chicken_Ningxia_222_2012_Avian
KX160190_A_chicken_Ningxia_2_2012_Avian
HM172352_A_chicken_Jiangsu_18_2008_Avian
JX523358_A_chicken_Huadong_4_2008_Avian
branch a
KC683520_A_swine_Jiangsu_1_2008_Swine
HM172324_A_chicken_Hebei_A_8_2009_Avian
EPI_ISL_180472_A_environment_Guizhou_00320_2013_Environment
GU182229_A_environment_Hunan_1_12_2007_Environment
GU182237_A_environment_Hunan_2_16_2007_Environment
GU182221_A_environment_Hunan_1_35_2007_Environment
GU182245_A_environment_Hunan_2_1_2007_Environment
EU243142_A_chicken_Hebei_126_2005_Avian
HM172340_A_chicken_Henan_A_7_2006_Avian
HM172316_A_chicken_Shandong_A_5_2006_Avian
DQ914813_A_chicken_Shanxi_2_2006_Avian
HM172336_A_chicken_Ningxia_24_2006_Avian
EU243137_A_chicken_Hebei_102_2005_Avian
HM172327_A_chicken_Shandong_A_10_2006_Avian
HM172355_A_chicken_Liaoning_A_11_2006_Avian
JQ277224_A_chicken_Sheny_0606_2008_Avian
DQ351868_A_chicken_Hebei_326_2005_Avian
EF587276_A_Beijing_01_2003_Human
branch b
EU008580_A_Anhui_T2_2006_Human
EU008596_A_chicken_Anhui_T6_2006_Avian
HM172337_A_chicken_Shanxi_10_2006_Avian
EPI_ISL_80638_A_chicken_Vietnam_NCVD_03_2008_Avian
EPI_ISL_80618_A_chicken_Vietnam_NCVD_swab276_2008_Avian
EPI_ISL_80639_A_chicken_Vietnam_NCVD_04_2008_Avian
EPI_ISL_80640_A_chicken_Vietnam_NCVD_05_2008_Avian
EPI_ISL_80641_A_chicken_Vietnam_NCVD_093_2008_Avian
EPI_ISL_28830_A_chicken_Vietnam_NCVD_016_2008_Avian
KP097886_A_chicken_Vietnam_NCVD_03_08_2008_Avian
EU243130_A_chicken_Hebei_706_2005_Avian
HM172338_A_chicken_Liaoning_A_1_2007_Avian
EU195407_A_mallard_Huadong_hn_2005_Avian

PA-Group34

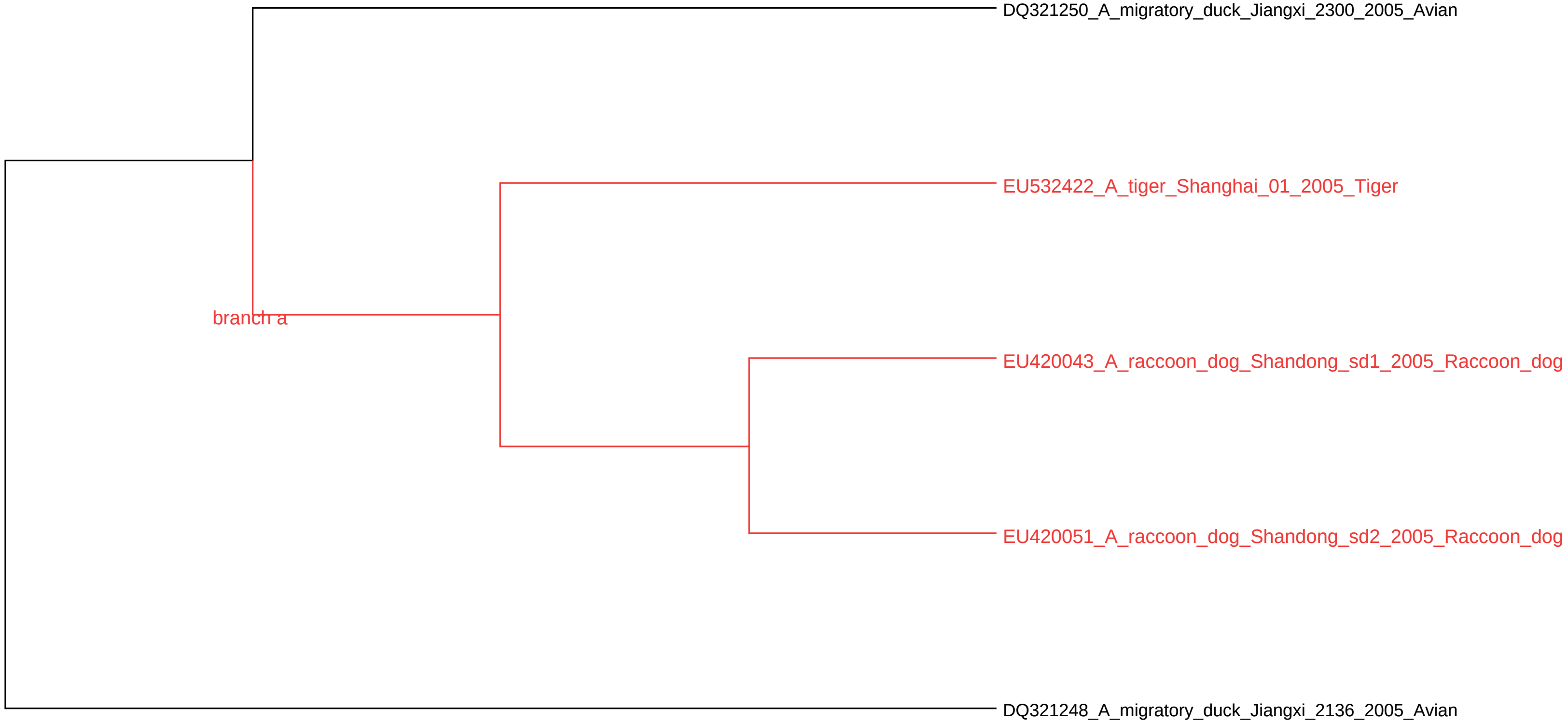
DQ321250_A_migratory_duck_Jiangxi_2300_2005_Avian
EU532422_A_tiger_Shanghai_01_2005_Tiger
branch a
EU420043_A_raccoon_dog_Shandong_sd1_2005_Raccoon_dog
EU420051_A_raccoon_dog_Shandong_sd2_2005_Raccoon_dog
DQ321248_A_migratory_duck_Jiangxi_2136_2005_Avian

PA-Group35

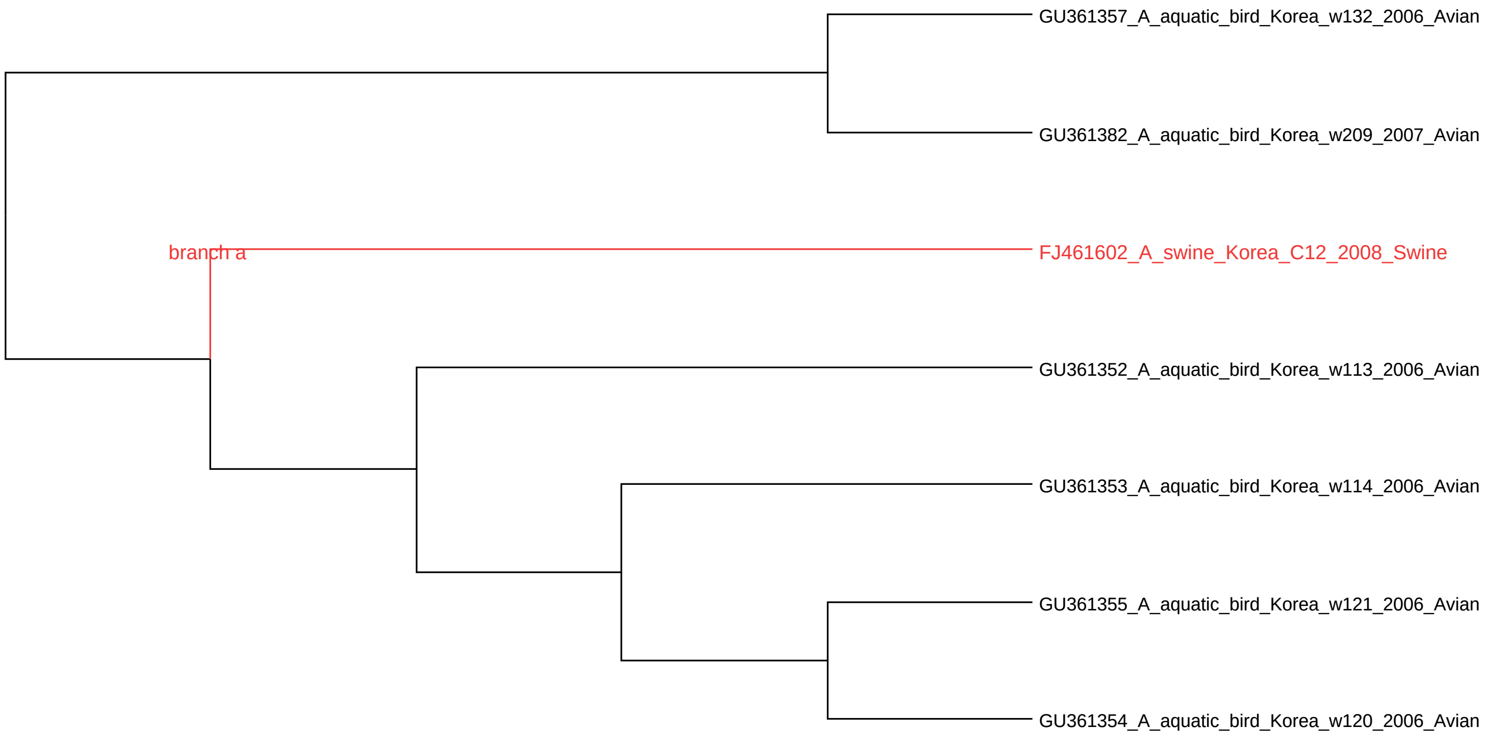
GU361357_A_aquatic_bird_Korea_w132_2006_Avian
GU361382_A_aquatic_bird_Korea_w209_2007_Avian
branch a
FJ461602_A_swine_Korea_C12_2008_Swine
GU361352_A_aquatic_bird_Korea_w113_2006_Avian
GU361353_A_aquatic_bird_Korea_w114_2006_Avian
GU361355_A_aquatic_bird_Korea_w121_2006_Avian
GU361354_A_aquatic_bird_Korea_w120_2006_Avian

PA-Group36

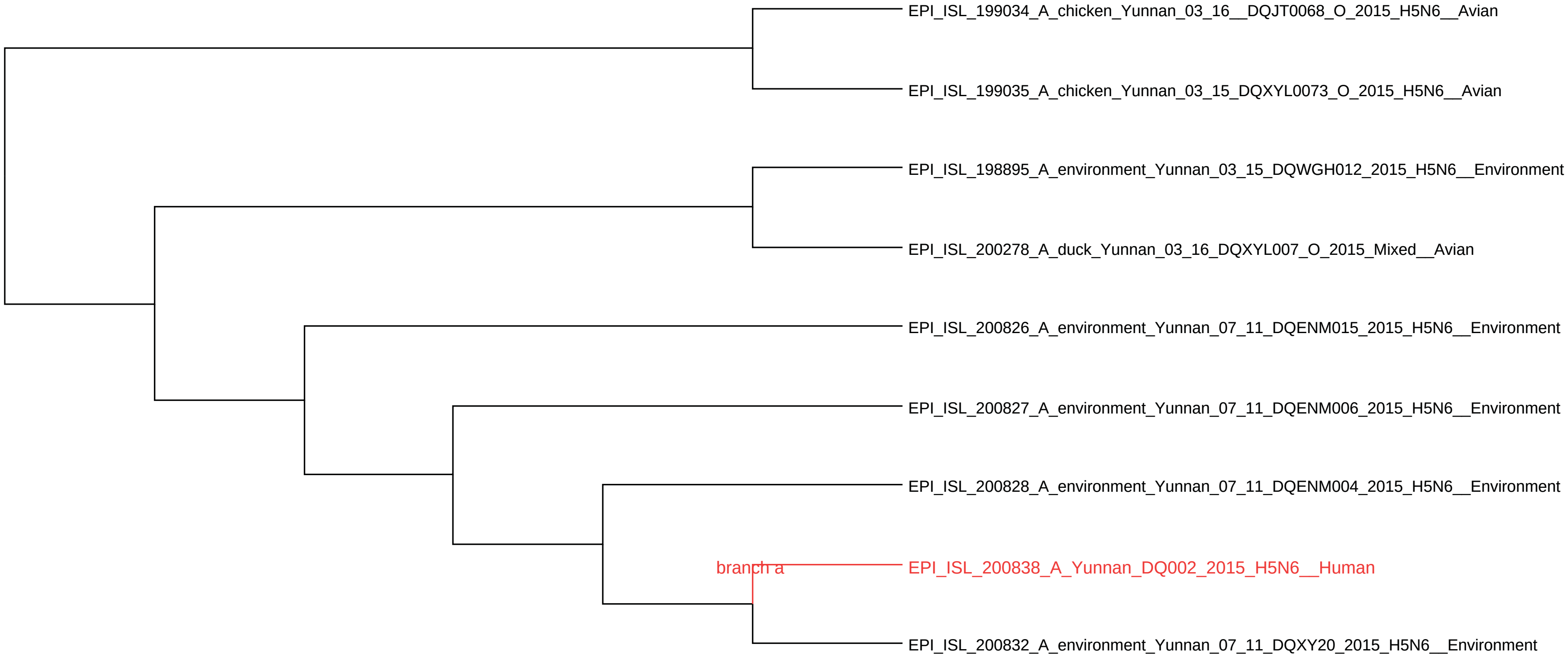
EPI_ISL_199034_A_chicken_Yunnan_03_16__DQJT0068_O_2015_H5N6__Avian
EPI_ISL_199035_A_chicken_Yunnan_03_15_DQXYL0073_O_2015_H5N6__Avian
EPI_ISL_198895_A_environment_Yunnan_03_15_DQWGH012_2015_H5N6__Environment
EPI_ISL_200278_A_duck_Yunnan_03_16_DQXYL007_O_2015_Mixed__Avian
EPI_ISL_200826_A_environment_Yunnan_07_11_DQENM015_2015_H5N6__Environment
EPI_ISL_200827_A_environment_Yunnan_07_11_DQENM006_2015_H5N6__Environment
EPI_ISL_200828_A_environment_Yunnan_07_11_DQENM004_2015_H5N6__Environment
branch a
EPI_ISL_200838_A_Yunnan_DQ002_2015_H5N6__Human
EPI_ISL_200832_A_environment_Yunnan_07_11_DQXY20_2015_H5N6__Environment

PA-Group37

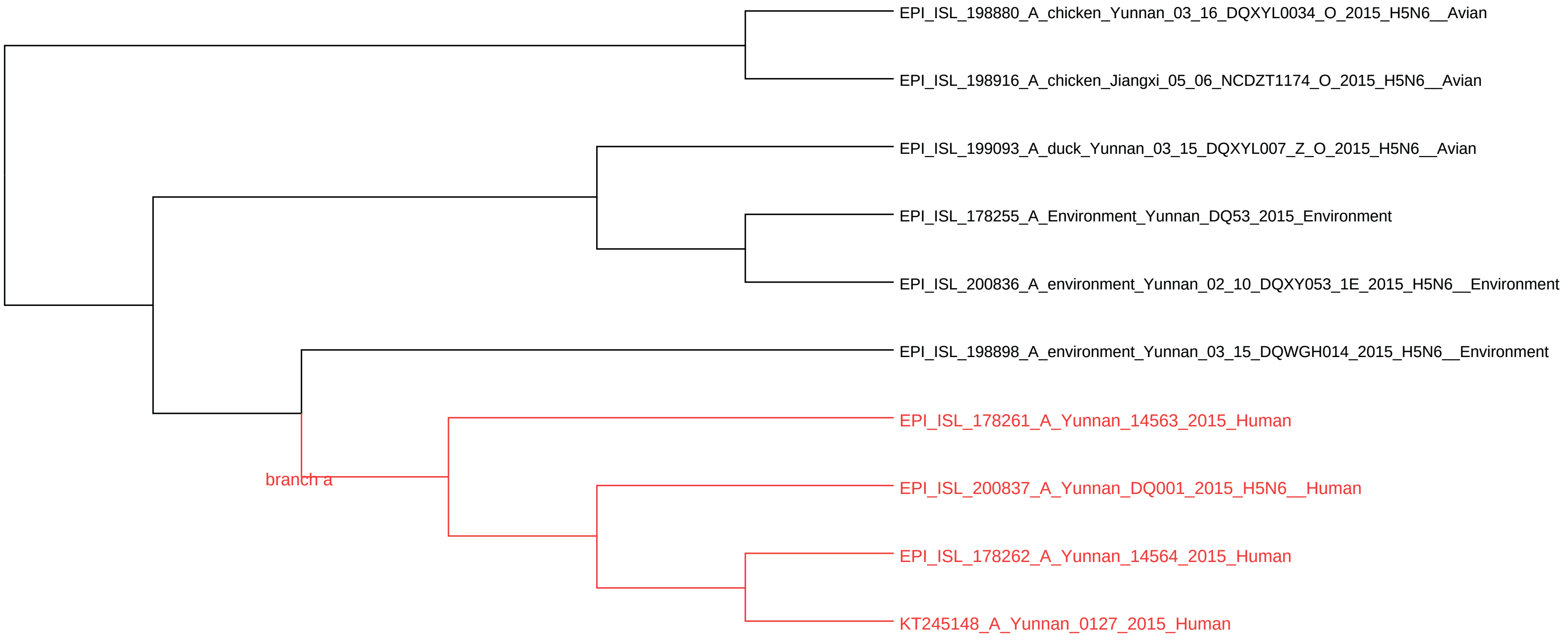
EPI_ISL_198880_A_chicken_Yunnan_03_16_DQXYL0034_O_2015_H5N6__Avian
EPI_ISL_198916_A_chicken_Jiangxi_05_06_NCDZT1174_O_2015_H5N6__Avian
EPI_ISL_199093_A_duck_Yunnan_03_15_DQXYL007_Z_O_2015_H5N6__Avian
EPI_ISL_178255_A_Environment_Yunnan_DQ53_2015_Environment
EPI_ISL_200836_A_environment_Yunnan_02_10_DQXY053_1E_2015_H5N6__Environment
EPI_ISL_198898_A_environment_Yunnan_03_15_DQWGH014_2015_H5N6__Environment
EPI_ISL_178261_A_Yunnan_14563_2015_Human
branch a
EPI_ISL_200837_A_Yunnan_DQ001_2015_H5N6__Human
EPI_ISL_178262_A_Yunnan_14564_2015_Human
KT245148_A_Yunnan_0127_2015_Human

PA-Group38

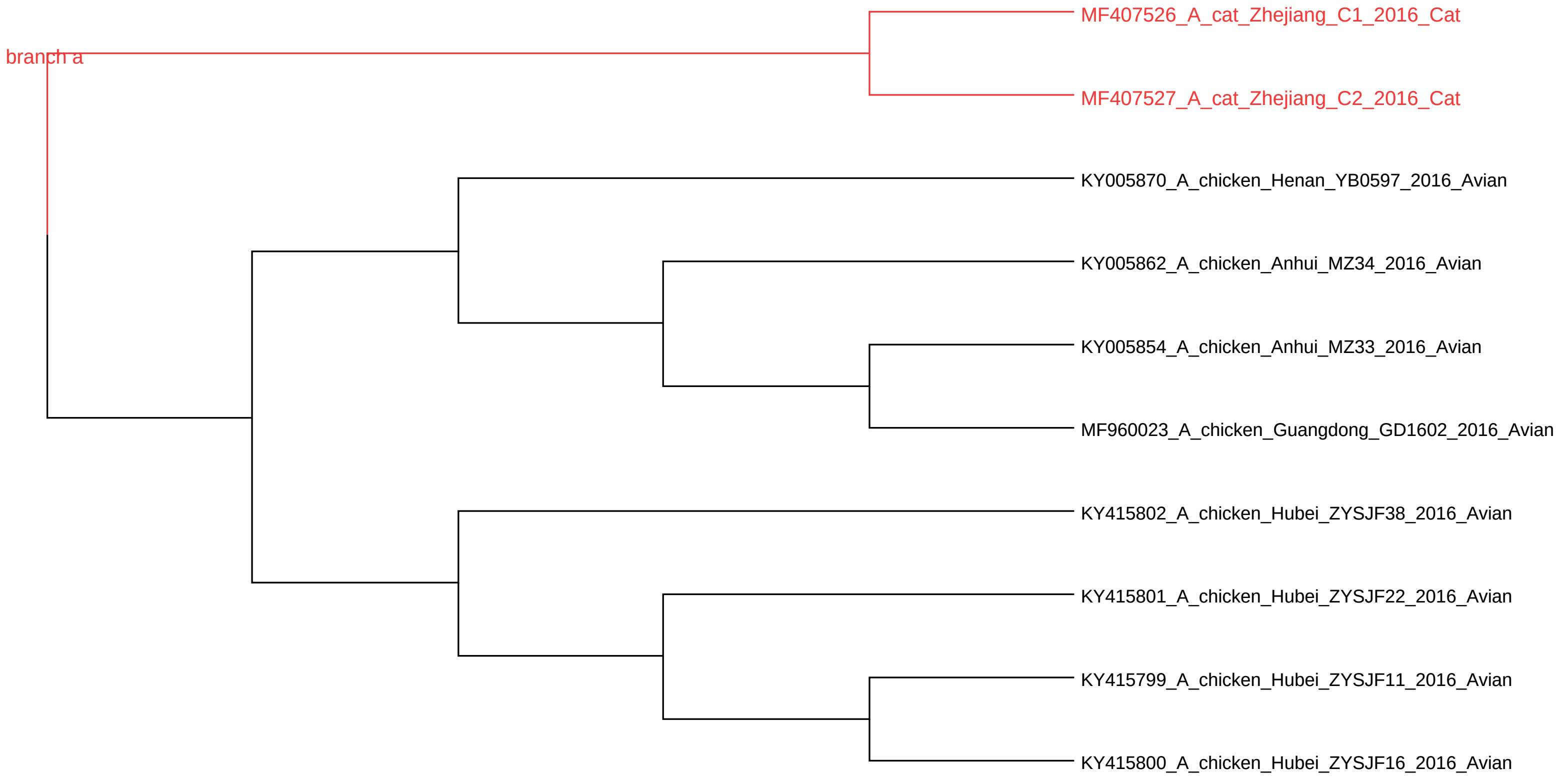
branch a
MF407526_A_cat_Zhejiang_C1_2016_Cat
MF407527_A_cat_Zhejiang_C2_2016_Cat
KY005870_A_chicken_Henan_YB0597_2016_Avian
KY005862_A_chicken_Anhui_MZ34_2016_Avian
KY005854_A_chicken_Anhui_MZ33_2016_Avian
MF960023_A_chicken_Guangdong_GD1602_2016_Avian
KY415802_A_chicken_Hubei_ZYSJF38_2016_Avian
KY415801_A_chicken_Hubei_ZYSJF22_2016_Avian
KY415799_A_chicken_Hubei_ZYSJF11_2016_Avian
KY415800_A_chicken_Hubei_ZYSJF16_2016_Avian

PA-Group39

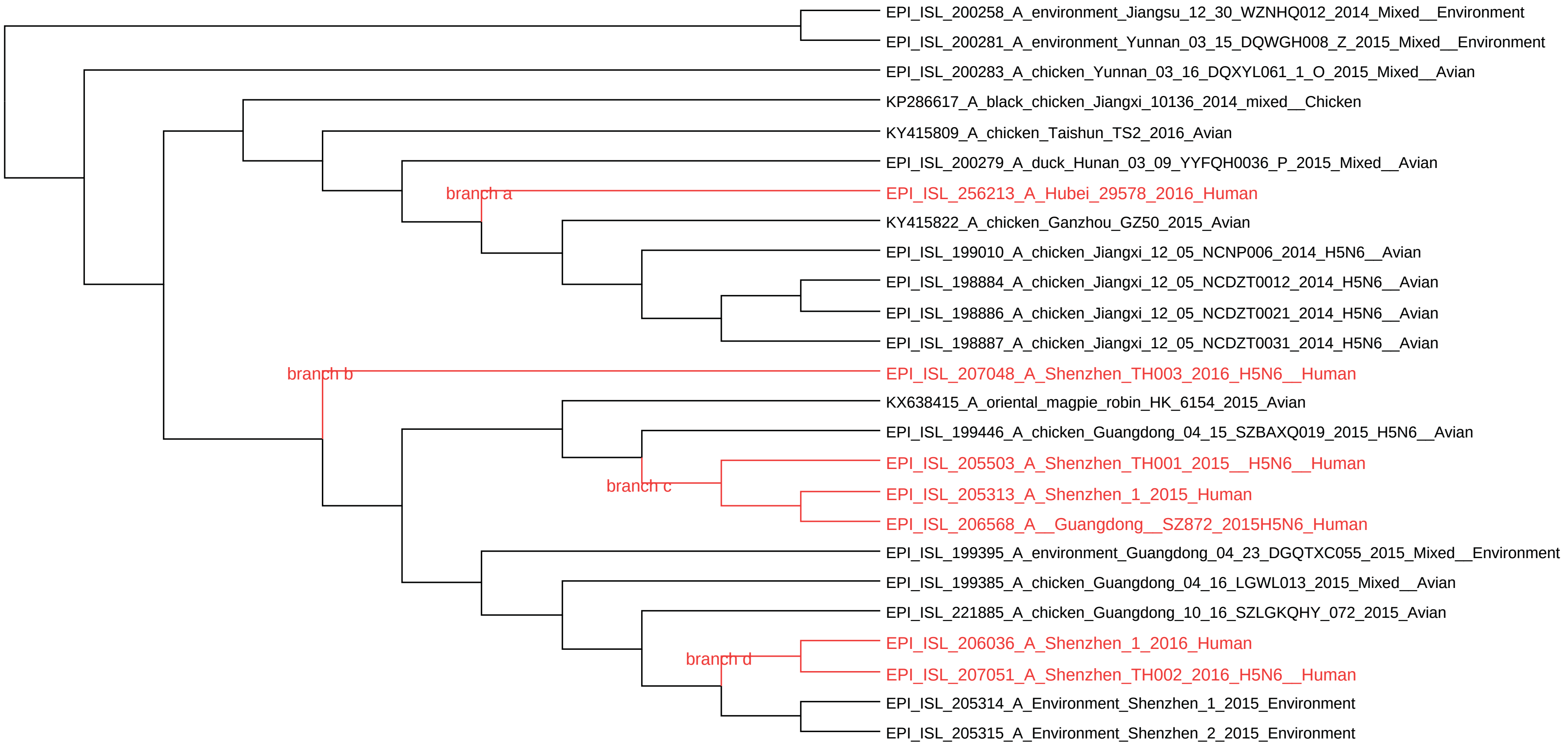
EPI_ISL_200258_A_environment_Jiangsu_12_30_WZNHQ012_2014_Mixed__Environment
EPI_ISL_200281_A_environment_Yunnan_03_15_DQWGH008_Z_2015_Mixed__Environment
EPI_ISL_200283_A_chicken_Yunnan_03_16_DQXYL061_1_O_2015_Mixed__Avian
KP286617_A_black_chicken_Jiangxi_10136_2014_mixed__Chicken
KY415809_A_chicken_Taishun_TS2_2016_Avian
EPI_ISL_200279_A_duck_Hunan_03_09_YYFQH0036_P_2015_Mixed__Avian
branch a
EPI_ISL_256213_A_Hubei_29578_2016_Human
KY415822_A_chicken_Ganzhou_GZ50_2015_Avian
EPI_ISL_199010_A_chicken_Jiangxi_12_05_NCNP006_2014_H5N6__Avian
EPI_ISL_198884_A_chicken_Jiangxi_12_05_NCDZT0012_2014_H5N6__Avian
EPI_ISL_198886_A_chicken_Jiangxi_12_05_NCDZT0021_2014_H5N6__Avian
EPI_ISL_198887_A_chicken_Jiangxi_12_05_NCDZT0031_2014_H5N6__Avian
branch b
EPI_ISL_207048_A_Shenzhen_TH003_2016_H5N6__Human
KX638415_A_oriental_magpie_robin_HK_6154_2015_Avian
EPI_ISL_199446_A_chicken_Guangdong_04_15_SZBAXQ019_2015_H5N6__Avian
EPI_ISL_205503_A_Shenzhen_TH001_2015__H5N6__Human
branch c
EPI_ISL_205313_A_Shenzhen_1_2015_Human
EPI_ISL_206568_A__Guangdong__SZ872_2015H5N6_Human
EPI_ISL_199395_A_environment_Guangdong_04_23_DGQTXC055_2015_Mixed__Environment
EPI_ISL_199385_A_chicken_Guangdong_04_16_LGWL013_2015_Mixed__Avian
EPI_ISL_221885_A_chicken_Guangdong_10_16_SZLGKQHY_072_2015_Avian
EPI_ISL_206036_A_Shenzhen_1_2016_Human
branch d
EPI_ISL_207051_A_Shenzhen_TH002_2016_H5N6__Human
EPI_ISL_205314_A_Environment_Shenzhen_1_2015_Environment
EPI_ISL_205315_A_Environment_Shenzhen_2_2015_Environment

PA-Group40

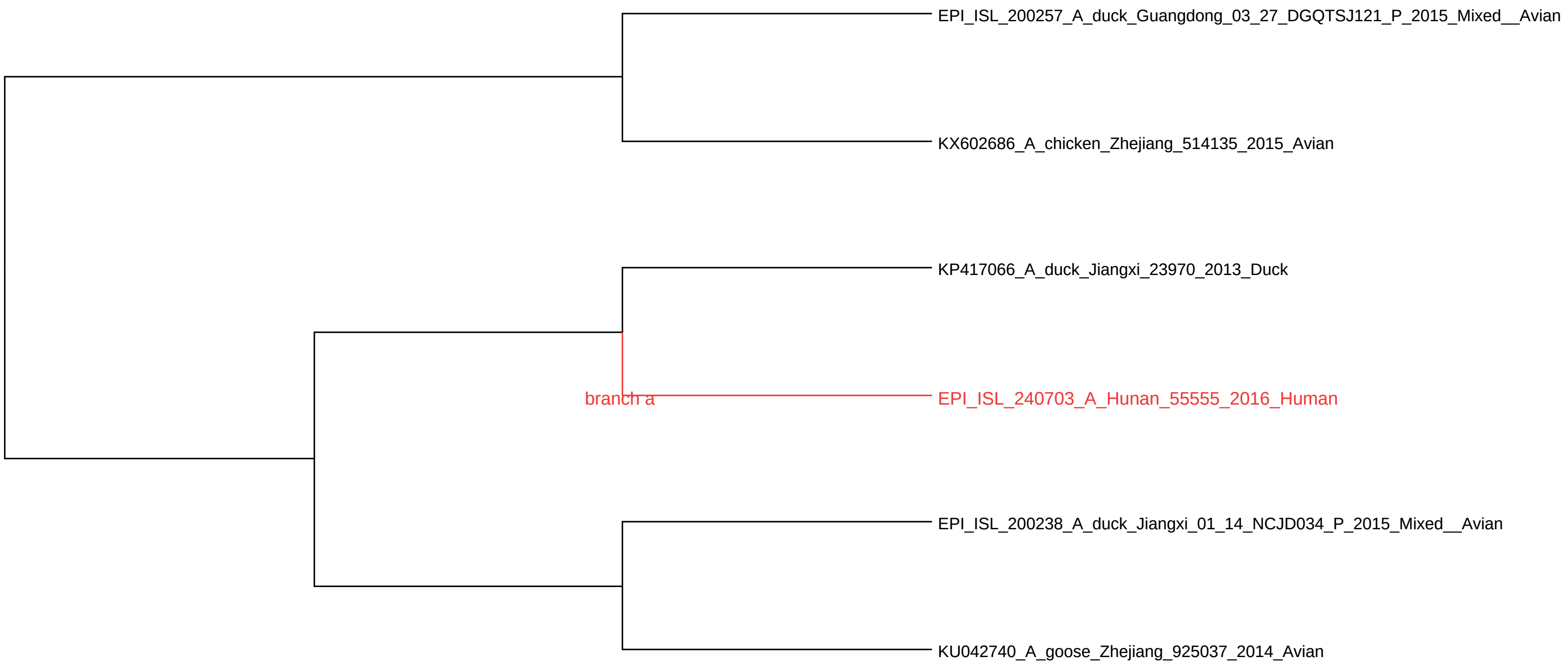
EPI_ISL_200257_A_duck_Guangdong_03_27_DGQTSJ121_P_2015_Mixed__Avian
KX602686_A_chicken_Zhejiang_514135_2015_Avian
KP417066_A_duck_Jiangxi_23970_2013_Duck
branch a
EPI_ISL_240703_A_Hunan_55555_2016_Human
EPI_ISL_200238_A_duck_Jiangxi_01_14_NCJD034_P_2015_Mixed__Avian
KU042740_A_goose_Zhejiang_925037_2014_Avian

PA-Group41

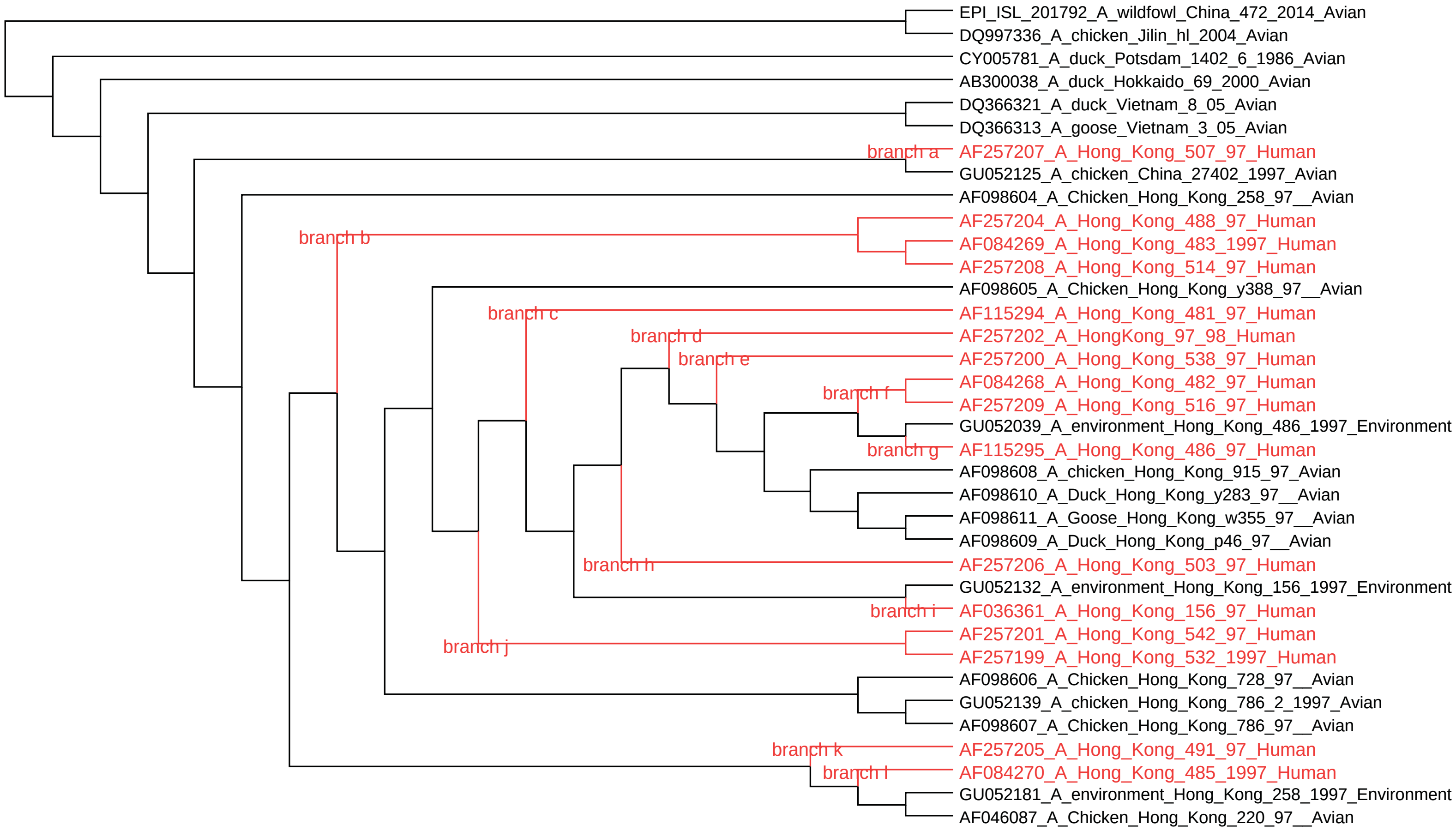
EPI_ISL_201792_A_wildfowl_China_472_2014_Avian
DQ997336_A_chicken_Jilin_hl_2004_Avian
CY005781_A_duck_Potsdam_1402_6_1986_Avian
AB300038_A_duck_Hokkaido_69_2000_Avian
DQ366321_A_duck_Vietnam_8_05_Avian
DQ366313_A_goose_Vietnam_3_05_Avian
branch a
AF257207_A_Hong_Kong_507_97_Human
GU052125_A_chicken_China_27402_1997_Avian
AF098604_A_Chicken_Hong_Kong_258_97__Avian
branch b
AF257204_A_Hong_Kong_488_97_Human
AF084269_A_Hong_Kong_483_1997_Human
AF257208_A_Hong_Kong_514_97_Human
AF098605_A_Chicken_Hong_Kong_y388_97__Avian
branch c
AF115294_A_Hong_Kong_481_97_Human
branch d
AF257202_A_HongKong_97_98_Human
branch e
AF257200_A_Hong_Kong_538_97_Human
branch f
AF084268_A_Hong_Kong_482_97_Human
AF257209_A_Hong_Kong_516_97_Human
GU052039_A_environment_Hong_Kong_486_1997_Environment
branch g
AF115295_A_Hong_Kong_486_97_Human
AF098608_A_chicken_Hong_Kong_915_97_Avian
AF098610_A_Duck_Hong_Kong_y283_97__Avian
AF098611_A_Goose_Hong_Kong_w355_97__Avian
AF098609_A_Duck_Hong_Kong_p46_97__Avian
branch h
AF257206_A_Hong_Kong_503_97_Human
GU052132_A_environment_Hong_Kong_156_1997_Environment
branch i
AF036361_A_Hong_Kong_156_97_Human
AF257201_A_Hong_Kong_542_97_Human
branch j
AF257199_A_Hong_Kong_532_1997_Human
AF098606_A_Chicken_Hong_Kong_728_97__Avian
GU052139_A_chicken_Hong_Kong_786_2_1997_Avian
AF098607_A_Chicken_Hong_Kong_786_97__Avian
branch k
AF257205_A_Hong_Kong_491_97_Human
branch l
AF084270_A_Hong_Kong_485_1997_Human
GU052181_A_environment_Hong_Kong_258_1997_Environment
AF046087_A_Chicken_Hong_Kong_220_97__Avian

PA-Group42

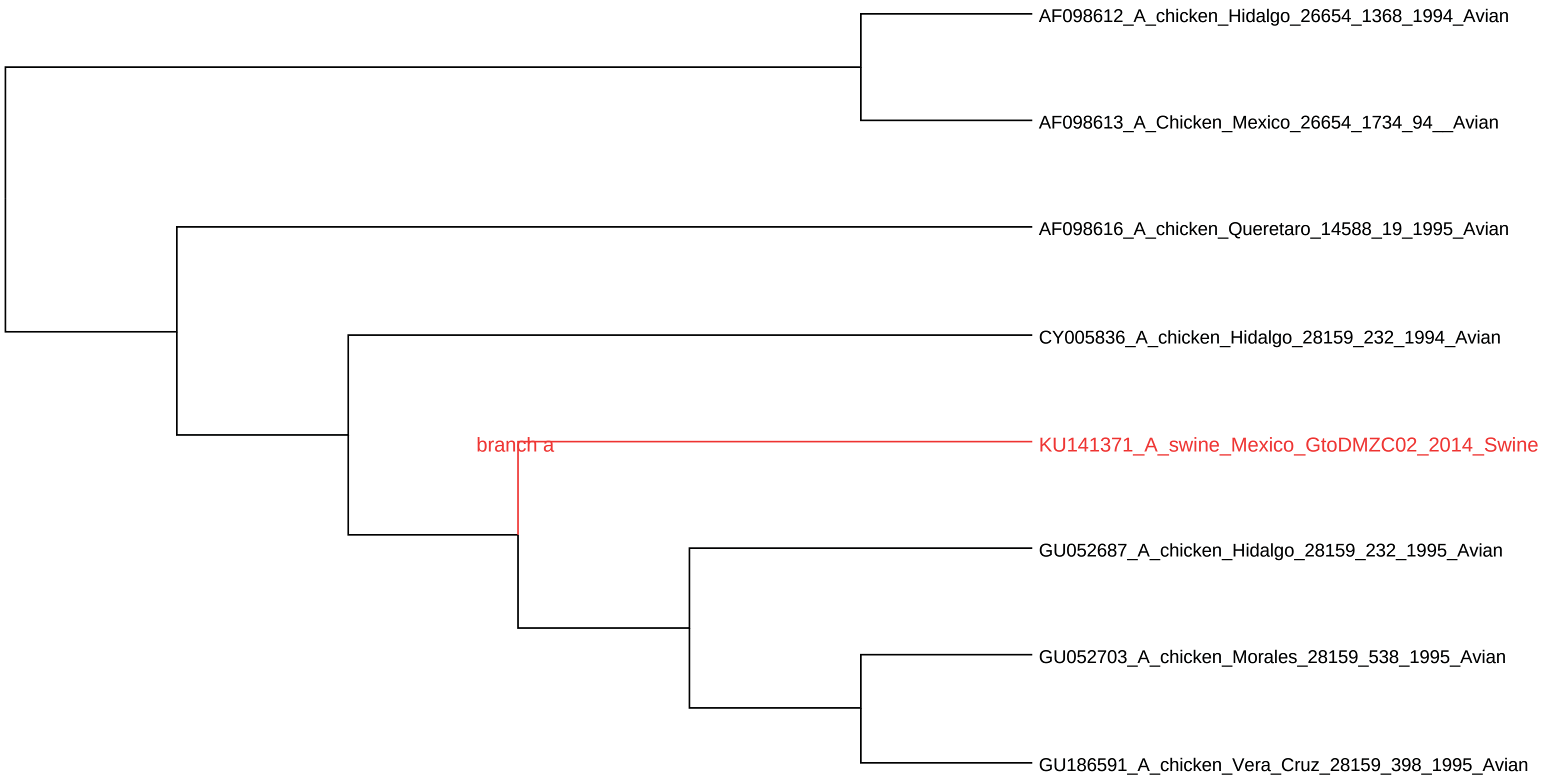
AF098612_A_chicken_Hidalgo_26654_1368_1994_Avian
AF098613_A_Chicken_Mexico_26654_1734_94__Avian
AF098616_A_chicken_Queretaro_14588_19_1995_Avian
CY005836_A_chicken_Hidalgo_28159_232_1994_Avian
branch a
KU141371_A_swine_Mexico_GtoDMZC02_2014_Swine
GU052687_A_chicken_Hidalgo_28159_232_1995_Avian
GU052703_A_chicken_Morales_28159_538_1995_Avian
GU186591_A_chicken_Vera_Cruz_28159_398_1995_Avian

PA-Group43

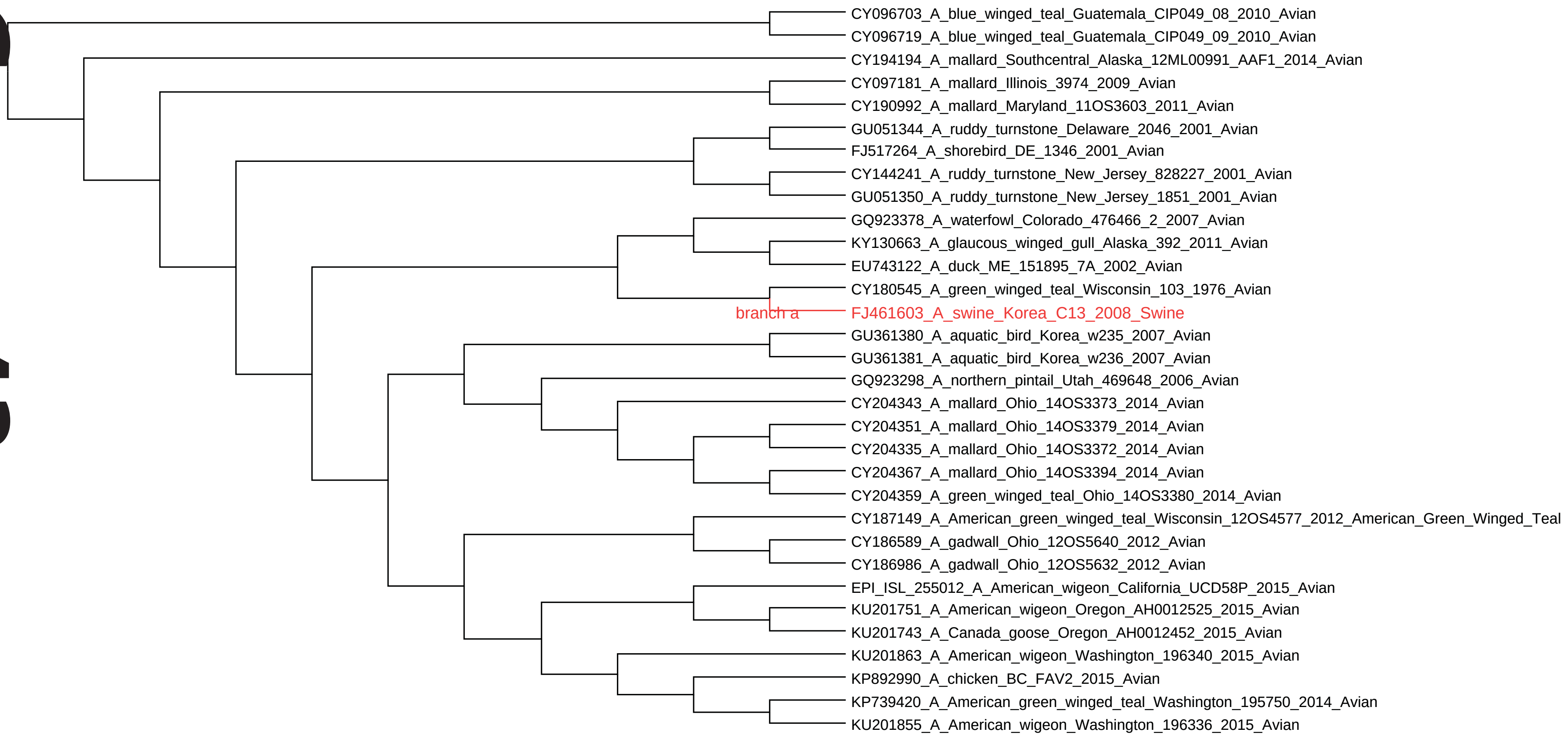

CY096703_A_blue_winged_teal_Guatemala_CIP049_08_2010_Avian
CY096719_A_blue_winged_teal_Guatemala_CIP049_09_2010_Avian
CY194194_A_mallard_Southcentral_Alaska_12ML00991_AAF1_2014_Avian
CY097181_A_mallard_Illinois_3974_2009_Avian
CY190992_A_mallard_Maryland_11OS3603_2011_Avian
GU051344_A_ruddy_turnstone_Delaware_2046_2001_Avian
FJ517264_A_shorebird_DE_1346_2001_Avian
CY144241_A_ruddy_turnstone_New_Jersey_828227_2001_Avian
GU051350_A_ruddy_turnstone_New_Jersey_1851_2001_Avian
GQ923378_A_waterfowl_Colorado_476466_2_2007_Avian
KY130663_A_glaucous_winged_gull_Alaska_392_2011_Avian
EU743122_A_duck_ME_151895_7A_2002_Avian
CY180545_A_green_winged_teal_Wisconsin_103_1976_Avian
branch a
FJ461603_A_swine_Korea_C13_2008_Swine
GU361380_A_aquatic_bird_Korea_w235_2007_Avian
GU361381_A_aquatic_bird_Korea_w236_2007_Avian
GQ923298_A_northern_pintail_Utah_469648_2006_Avian
CY204343_A_mallard_Ohio_14OS3373_2014_Avian
CY204351_A_mallard_Ohio_14OS3379_2014_Avian
CY204335_A_mallard_Ohio_14OS3372_2014_Avian
CY204367_A_mallard_Ohio_14OS3394_2014_Avian
CY204359_A_green_winged_teal_Ohio_14OS3380_2014_Avian
CY187149_A_American_green_winged_teal_Wisconsin_12OS4577_2012_American_Green_Winged_Teal
CY186589_A_gadwall_Ohio_12OS5640_2012_Avian
CY186986_A_gadwall_Ohio_12OS5632_2012_Avian
EPI_ISL_255012_A_American_wigeon_California_UCD58P_2015_Avian
KU201751_A_American_wigeon_Oregon_AH0012525_2015_Avian
KU201743_A_Canada_goose_Oregon_AH0012452_2015_Avian
KU201863_A_American_wigeon_Washington_196340_2015_Avian
KP892990_A_chicken_BC_FAV2_2015_Avian
KP739420_A_American_green_winged_teal_Washington_195750_2014_Avian
KU201855_A_American_wigeon_Washington_196336_2015_Avian

PA-Group44

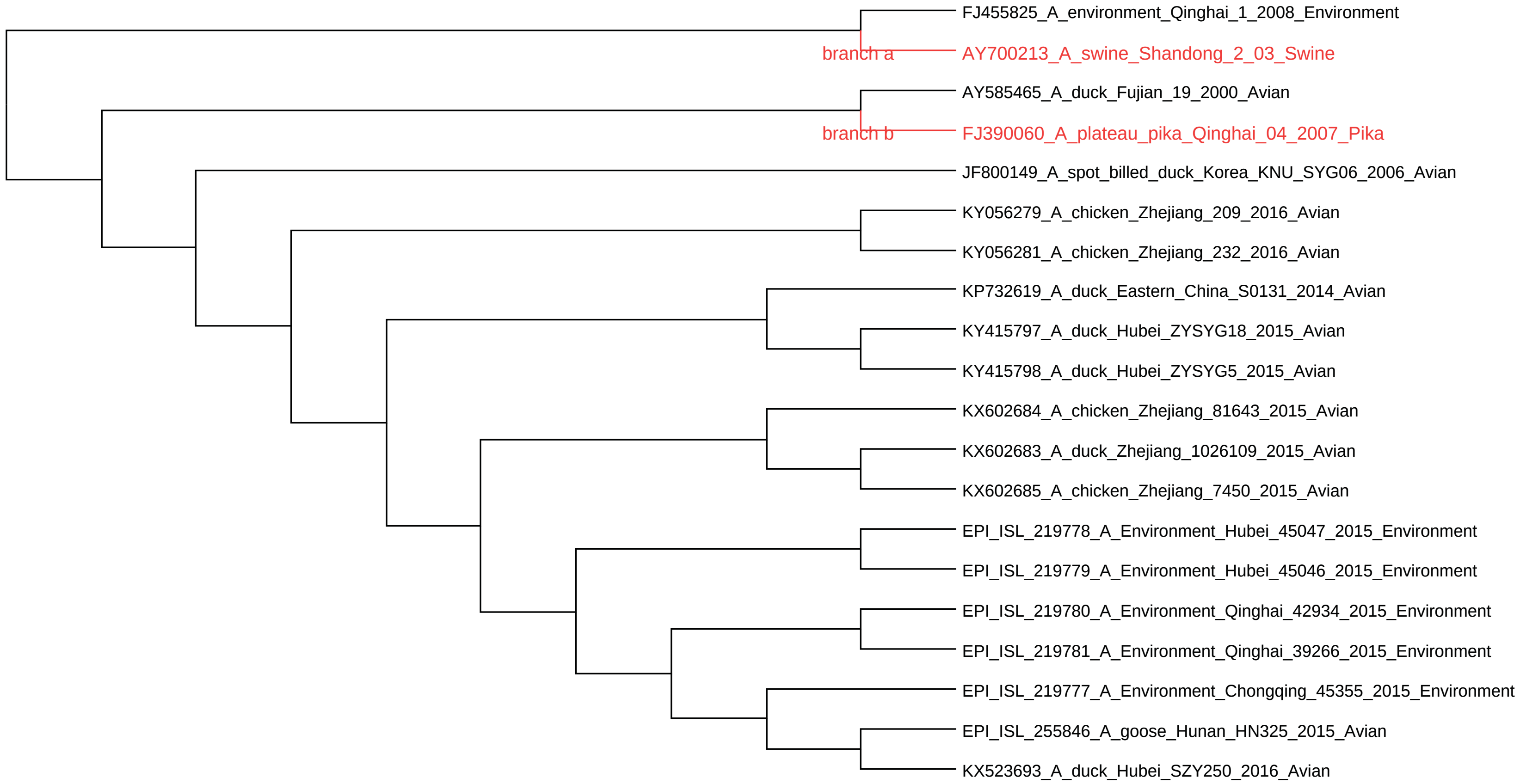
FJ455825_A_environment_Qinghai_1_2008_Environment
branch a
AY700213_A_swine_Shandong_2_03_Swine
AY585465_A_duck_Fujian_19_2000_Avian
branch b
FJ390060_A_plateau_pika_Qinghai_04_2007_Pika
JF800149_A_spot_billed_duck_Korea_KNU_SYG06_2006_Avian
KY056279_A_chicken_Zhejiang_209_2016_Avian
KY056281_A_chicken_Zhejiang_232_2016_Avian
KP732619_A_duck_Eastern_China_S0131_2014_Avian
KY415797_A_duck_Hubei_ZYSYG18_2015_Avian
KY415798_A_duck_Hubei_ZYSYG5_2015_Avian
KX602684_A_chicken_Zhejiang_81643_2015_Avian
KX602683_A_duck_Zhejiang_1026109_2015_Avian
KX602685_A_chicken_Zhejiang_7450_2015_Avian
EPI_ISL_219778_A_Environment_Hubei_45047_2015_Environment
EPI_ISL_219779_A_Environment_Hubei_45046_2015_Environment
EPI_ISL_219780_A_Environment_Qinghai_42934_2015_Environment
EPI_ISL_219781_A_Environment_Qinghai_39266_2015_Environment
EPI_ISL_219777_A_Environment_Chongqing_45355_2015_Environment
EPI_ISL_255846_A_goose_Hunan_HN325_2015_Avian
KX523693_A_duck_Hubei_SZY250_2016_Avian

PA-Group45

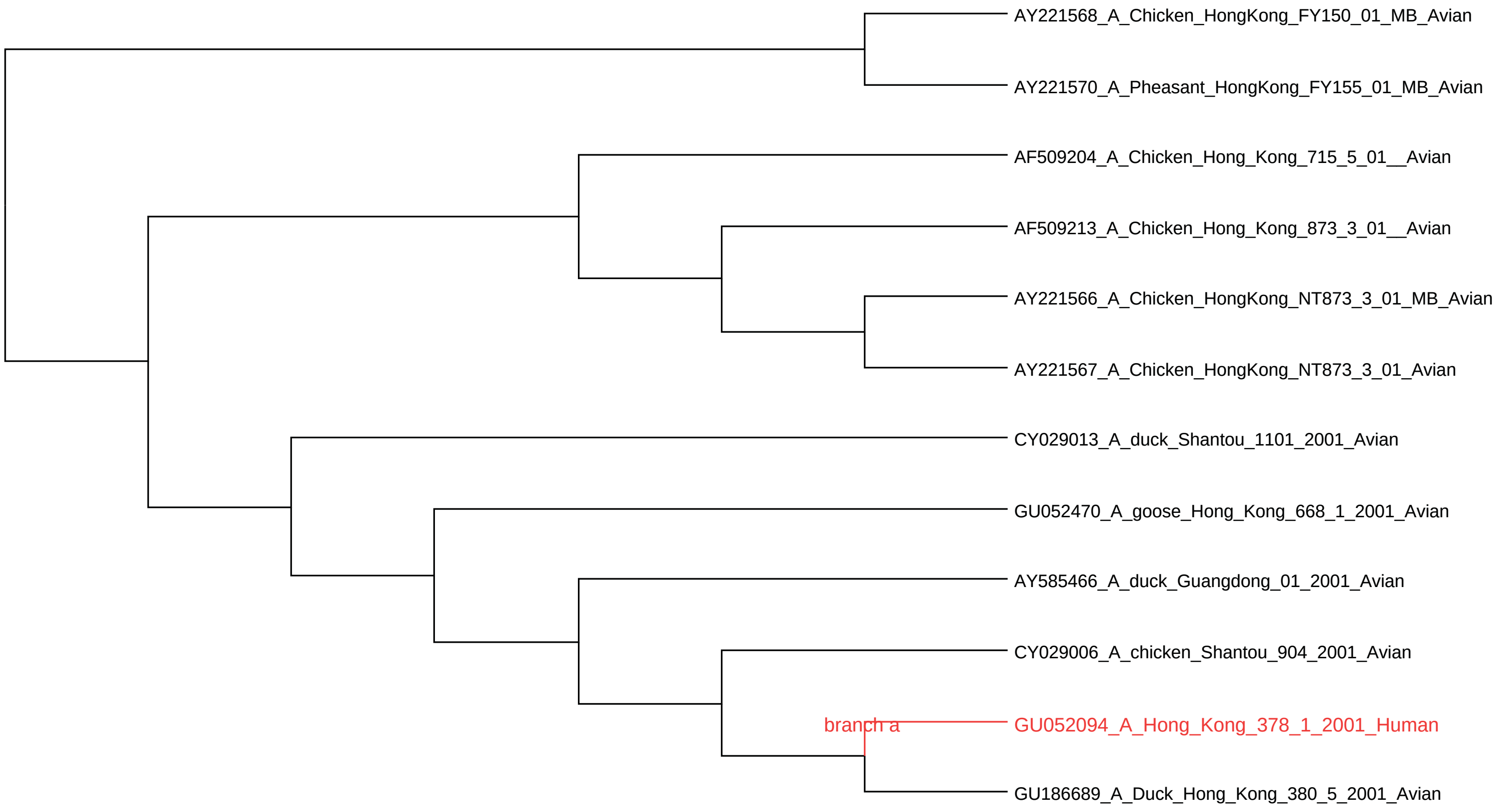
AY221568_A_Chicken_HongKong_FY150_01_MB_Avian
AY221570_A_Pheasant_HongKong_FY155_01_MB_Avian
AF509204_A_Chicken_Hong_Kong_715_5_01__Avian
AF509213_A_Chicken_Hong_Kong_873_3_01__Avian
AY221566_A_Chicken_HongKong_NT873_3_01_MB_Avian
AY221567_A_Chicken_HongKong_NT873_3_01_Avian
CY029013_A_duck_Shantou_1101_2001_Avian
GU052470_A_goose_Hong_Kong_668_1_2001_Avian
AY585466_A_duck_Guangdong_01_2001_Avian
CY029006_A_chicken_Shantou_904_2001_Avian
branch a
GU052094_A_Hong_Kong_378_1_2001_Human
GU186689_A_Duck_Hong_Kong_380_5_2001_Avian

PA-Group46

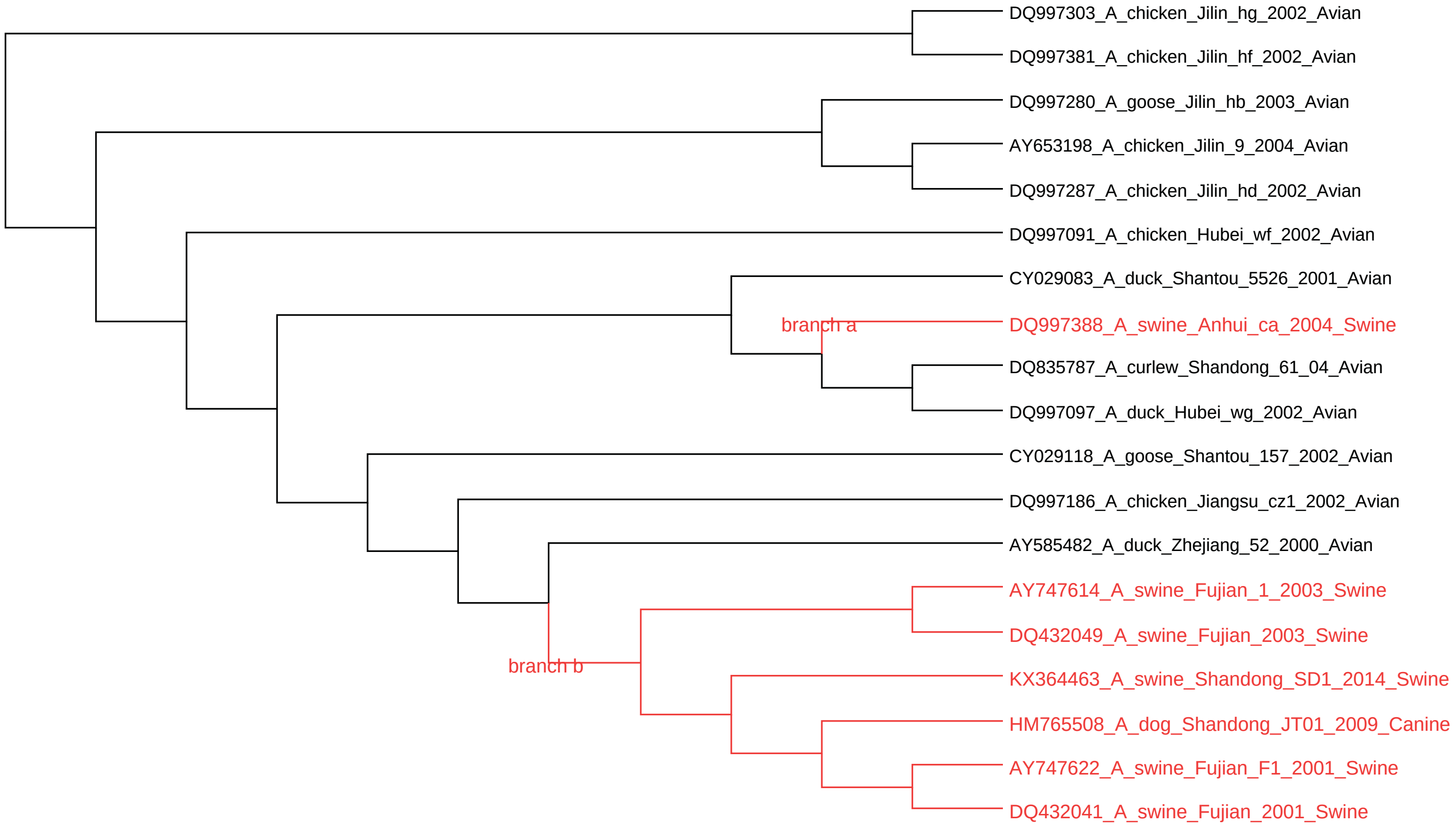

DQ997303_A_chicken_Jilin_hg_2002_Avian
DQ997381_A_chicken_Jilin_hf_2002_Avian
DQ997280_A_goose_Jilin_hb_2003_Avian
AY653198_A_chicken_Jilin_9_2004_Avian
DQ997287_A_chicken_Jilin_hd_2002_Avian
DQ997091_A_chicken_Hubei_wf_2002_Avian
CY029083_A_duck_Shantou_5526_2001_Avian
branch a
DQ997388_A_swine_Anhui_ca_2004_Swine
DQ835787_A_curlew_Shandong_61_04_Avian
DQ997097_A_duck_Hubei_wg_2002_Avian
CY029118_A_goose_Shantou_157_2002_Avian
DQ997186_A_chicken_Jiangsu_cz1_2002_Avian
AY585482_A_duck_Zhejiang_52_2000_Avian
AY747614_A_swine_Fujian_1_2003_Swine
DQ432049_A_swine_Fujian_2003_Swine
branch b
KX364463_A_swine_Shandong_SD1_2014_Swine
HM765508_A_dog_Shandong_JT01_2009_Canine
AY747622_A_swine_Fujian_F1_2001_Swine
DQ432041_A_swine_Fujian_2001_Swine

PA-Group47

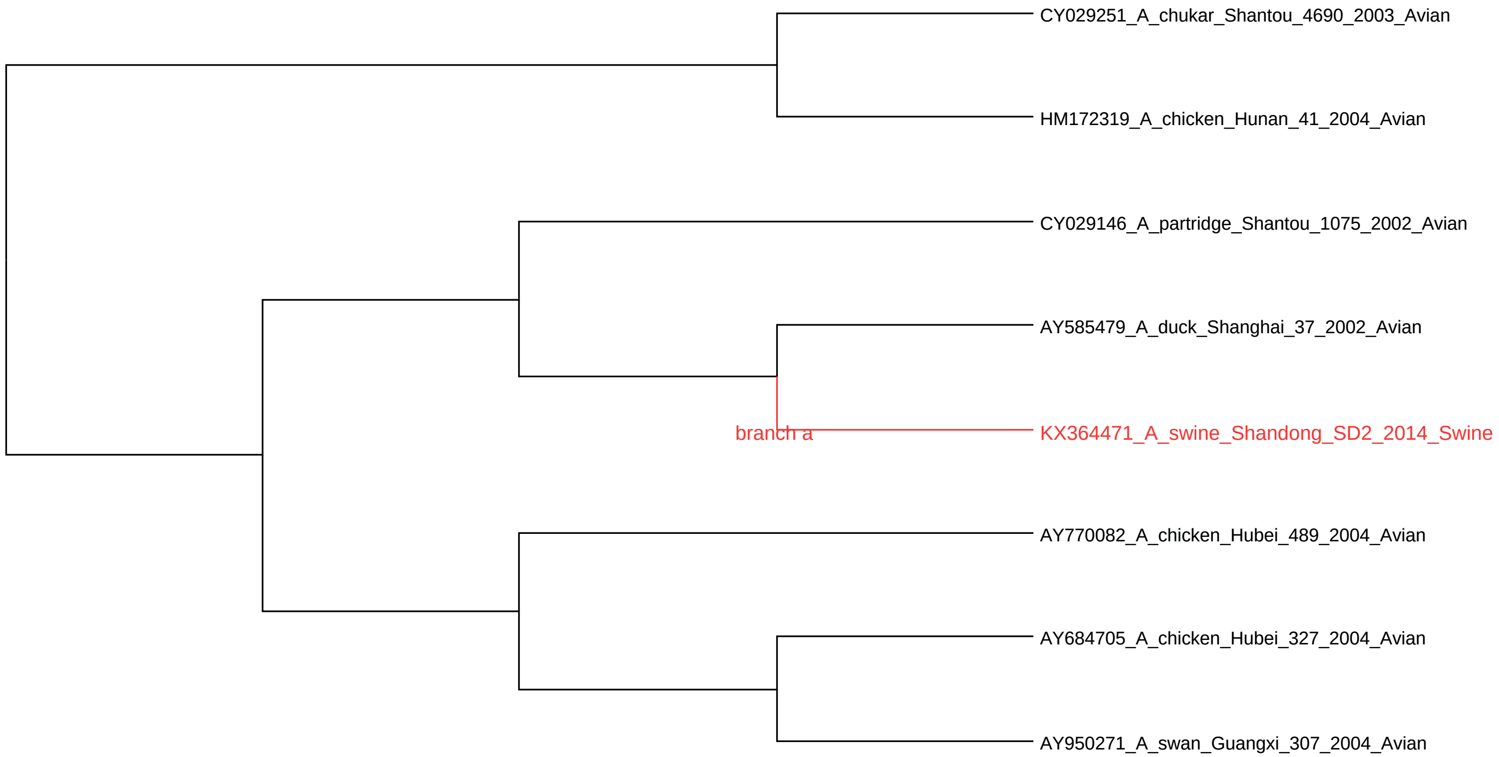

CY029251_A_chukar_Shantou_4690_2003_Avian
HM172319_A_chicken_Hunan_41_2004_Avian
CY029146_A_partridge_Shantou_1075_2002_Avian
AY585479_A_duck_Shanghai_37_2002_Avian
branch a
KX364471_A_swine_Shandong_SD2_2014_Swine
AY770082_A_chicken_Hubei_489_2004_Avian
AY684705_A_chicken_Hubei_327_2004_Avian
AY950271_A_swan_Guangxi_307_2004_Avian

PA-Group48

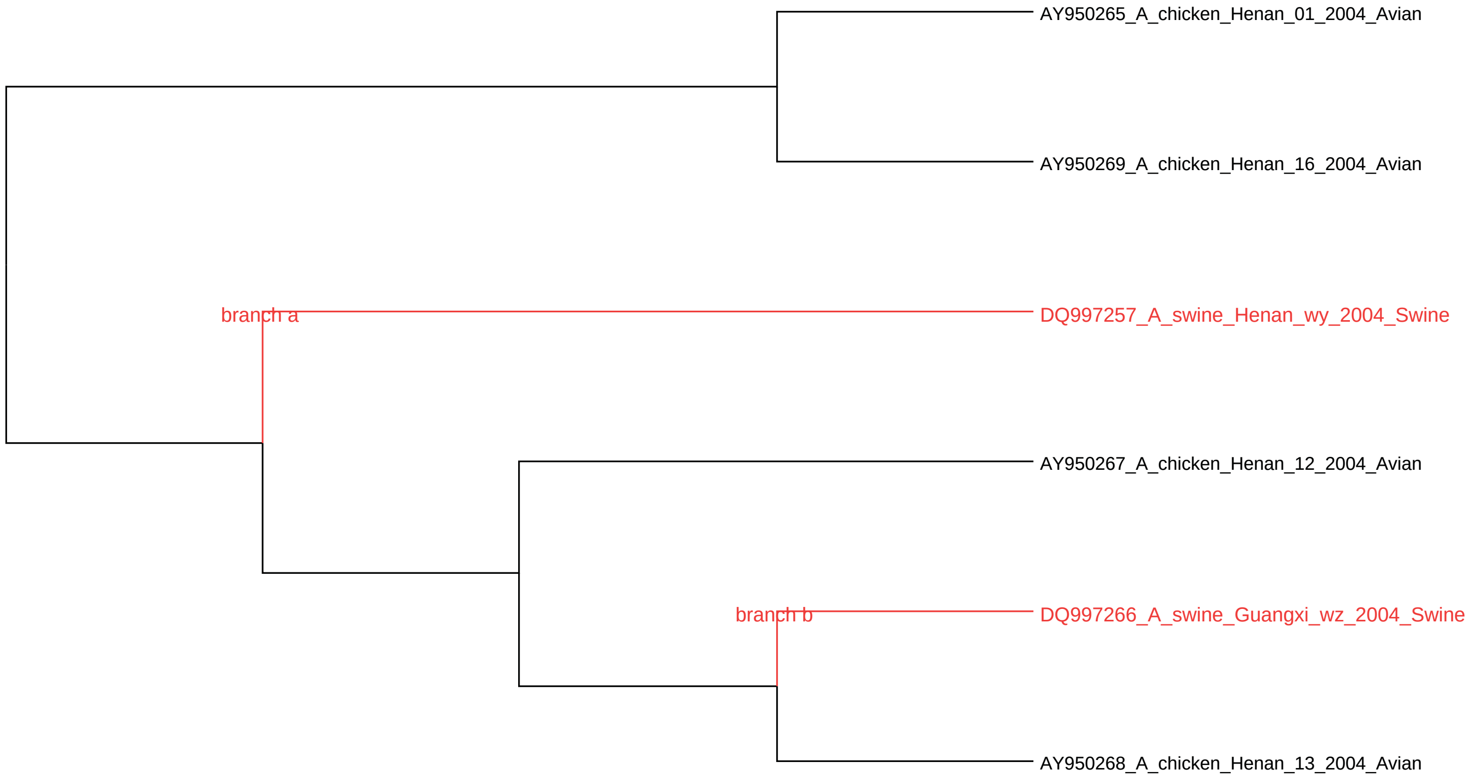
AY950265_A_chicken_Henan_01_2004_Avian
AY950269_A_chicken_Henan_16_2004_Avian
branch a
DQ997257_A_swine_Henan_wy_2004_Swine
AY950267_A_chicken_Henan_12_2004_Avian
branch b
DQ997266_A_swine_Guangxi_wz_2004_Swine
AY950268_A_chicken_Henan_13_2004_Avian

PA-Group49

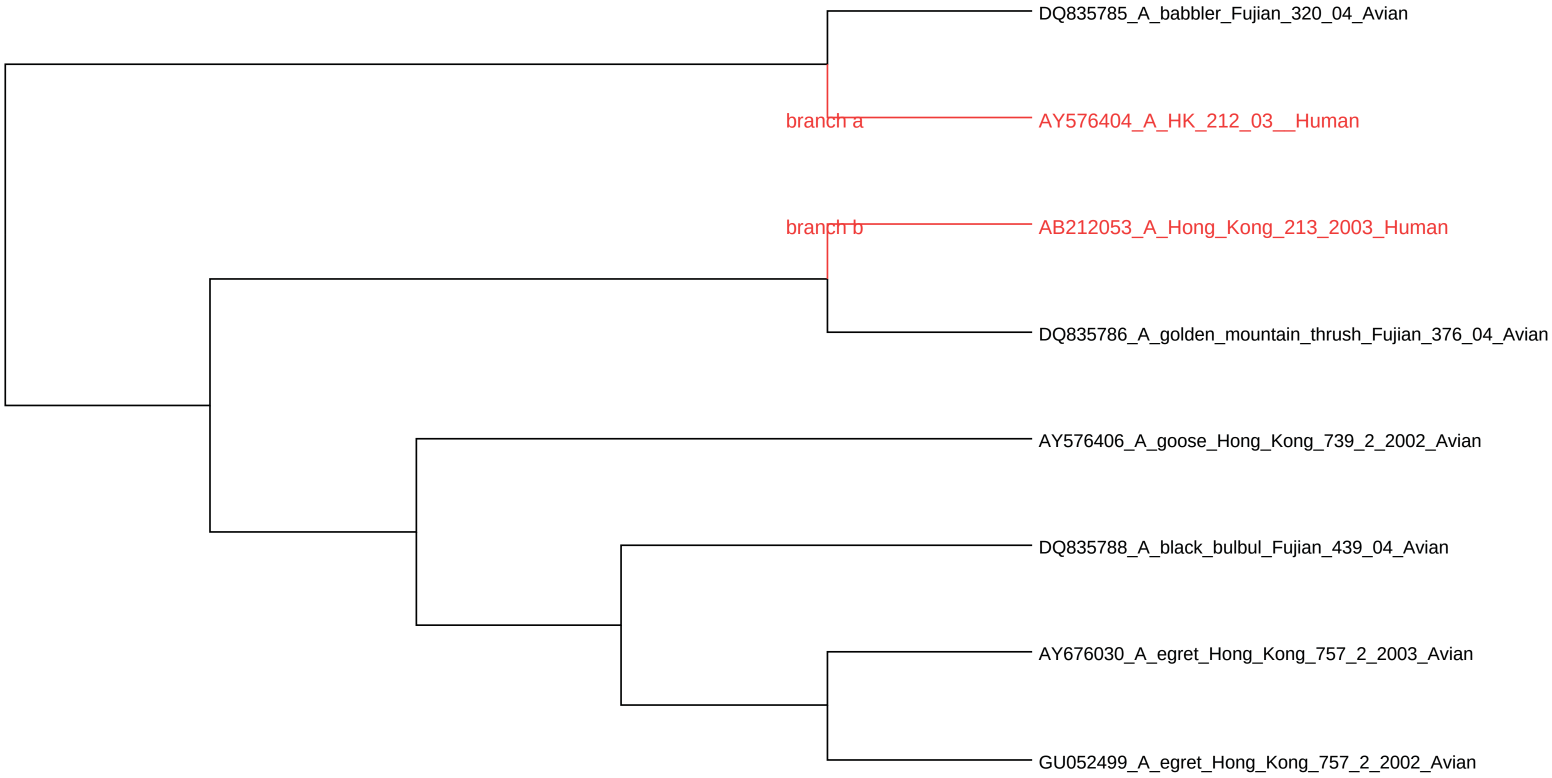

DQ835785_A_babbler_Fujian_320_04_Avian
branch a
AY576404_A_HK_212_03__Human
branch b
AB212053_A_Hong_Kong_213_2003_Human
DQ835786_A_golden_mountain_thrush_Fujian_376_04_Avian
AY576406_A_goose_Hong_Kong_739_2_2002_Avian
DQ835788_A_black_bulbul_Fujian_439_04_Avian
AY676030_A_egret_Hong_Kong_757_2_2003_Avian
GU052499_A_egret_Hong_Kong_757_2_2002_Avian

PA-Group50

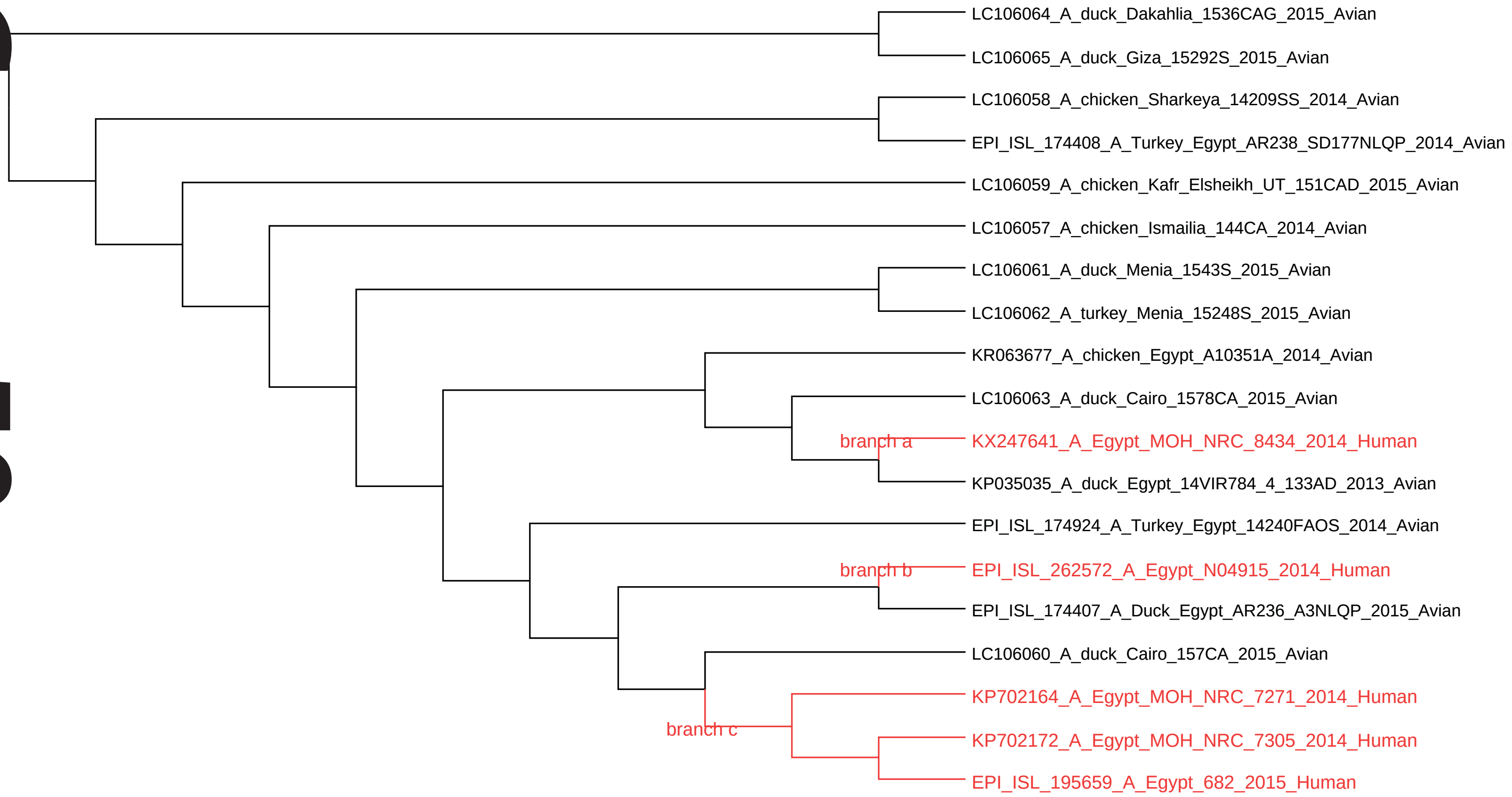

LC106064_A_duck_Dakahlia_1536CAG_2015_Avian
LC106065_A_duck_Giza_15292S_2015_Avian
LC106058_A_chicken_Sharkeya_14209SS_2014_Avian
EPI_ISL_174408_A_Turkey_Egypt_AR238_SD177NLQP_2014_Avian
LC106059_A_chicken_Kafr_Elsheikh_UT_151CAD_2015_Avian
LC106057_A_chicken_Ismailia_144CA_2014_Avian
LC106061_A_duck_Menia_1543S_2015_Avian
LC106062_A_turkey_Menia_15248S_2015_Avian
KR063677_A_chicken_Egypt_A10351A_2014_Avian
LC106063_A_duck_Cairo_1578CA_2015_Avian
branch a
KX247641_A_Egypt_MOH_NRC_8434_2014_Human
KP035035_A_duck_Egypt_14VIR784_4_133AD_2013_Avian
EPI_ISL_174924_A_Turkey_Egypt_14240FAOS_2014_Avian
branch b
EPI_ISL_262572_A_Egypt_N04915_2014_Human
EPI_ISL_174407_A_Duck_Egypt_AR236_A3NLQP_2015_Avian
LC106060_A_duck_Cairo_157CA_2015_Avian
KP702164_A_Egypt_MOH_NRC_7271_2014_Human
branch c
KP702172_A_Egypt_MOH_NRC_7305_2014_Human
EPI_ISL_195659_A_Egypt_682_2015_Human

PA-Group51

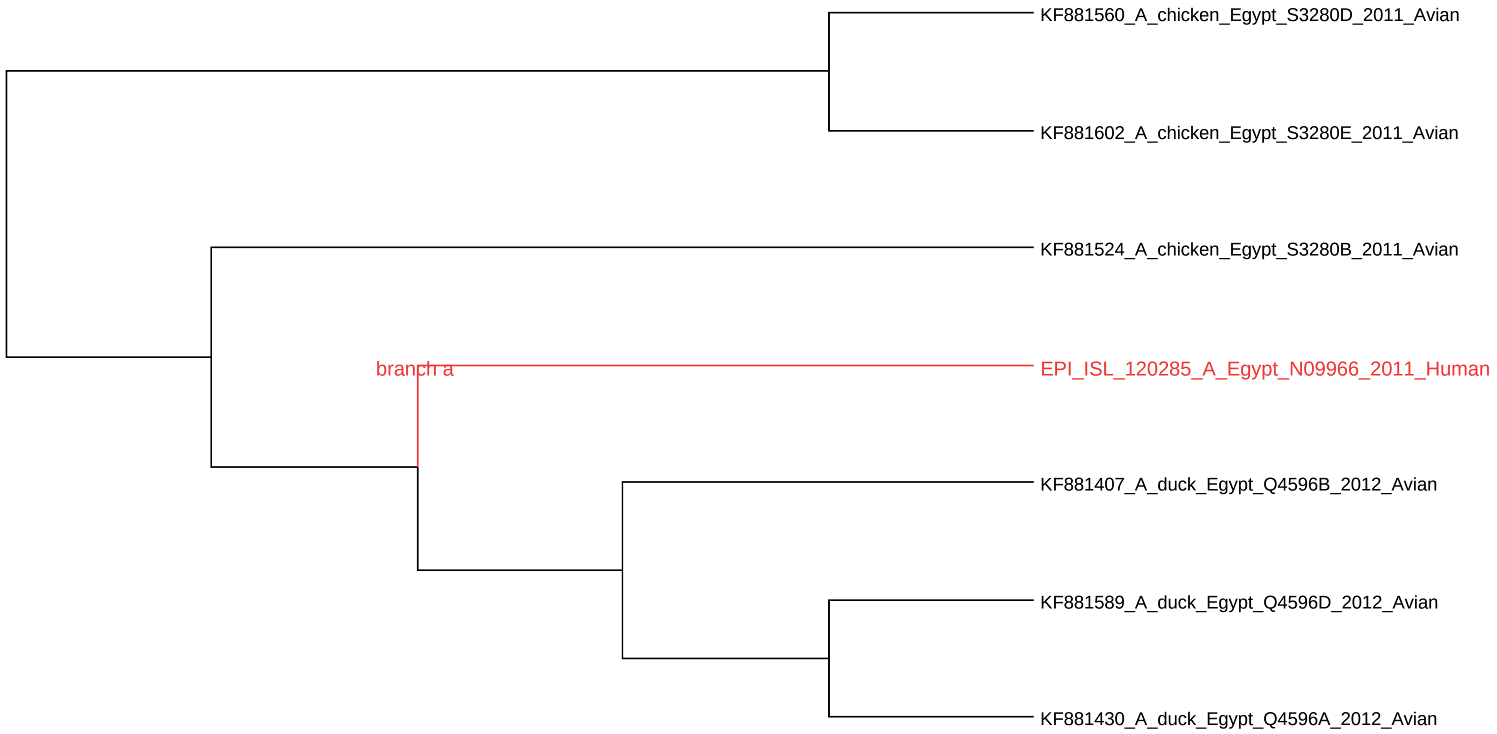
KF881560_A_chicken_Egypt_S3280D_2011_Avian
KF881602_A_chicken_Egypt_S3280E_2011_Avian
KF881524_A_chicken_Egypt_S3280B_2011_Avian
branch a
EPI_ISL_120285_A_Egypt_N09966_2011_Human
KF881407_A_duck_Egypt_Q4596B_2012_Avian
KF881589_A_duck_Egypt_Q4596D_2012_Avian
KF881430_A_duck_Egypt_Q4596A_2012_Avian

PA-Group52

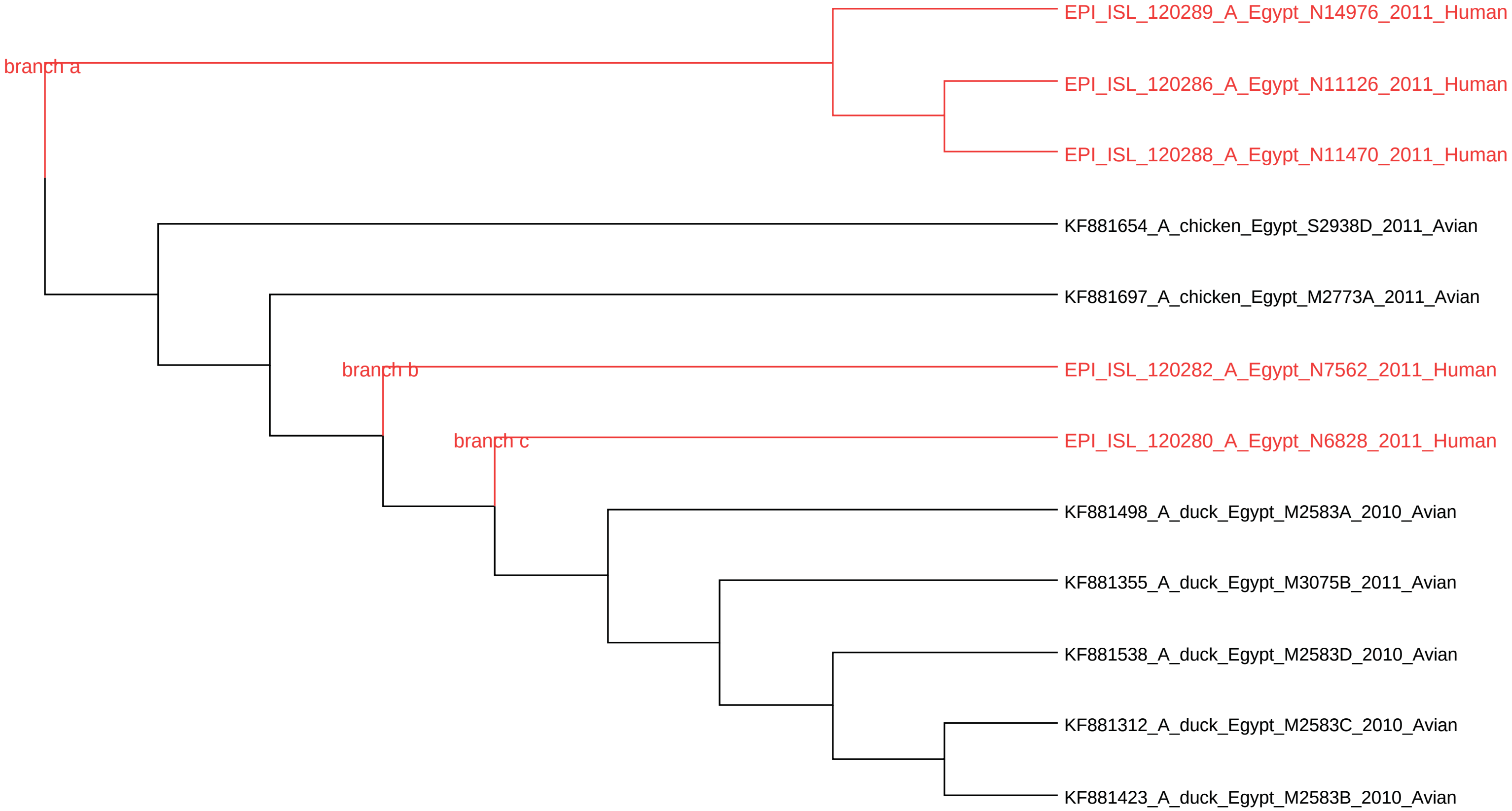
branch a
branch b
branch c
EPI_ISL_120289_A_Egypt_N14976_2011_Human
EPI_ISL_120286_A_Egypt_N11126_2011_Human
EPI_ISL_120288_A_Egypt_N11470_2011_Human
KF881654_A_chicken_Egypt_S2938D_2011_Avian
KF881697_A_chicken_Egypt_M2773A_2011_Avian
EPI_ISL_120282_A_Egypt_N7562_2011_Human
EPI_ISL_120280_A_Egypt_N6828_2011_Human
KF881498_A_duck_Egypt_M2583A_2010_Avian
KF881355_A_duck_Egypt_M3075B_2011_Avian
KF881538_A_duck_Egypt_M2583D_2010_Avian
KF881312_A_duck_Egypt_M2583C_2010_Avian
KF881423_A_duck_Egypt_M2583B_2010_Avian

PA-Group53

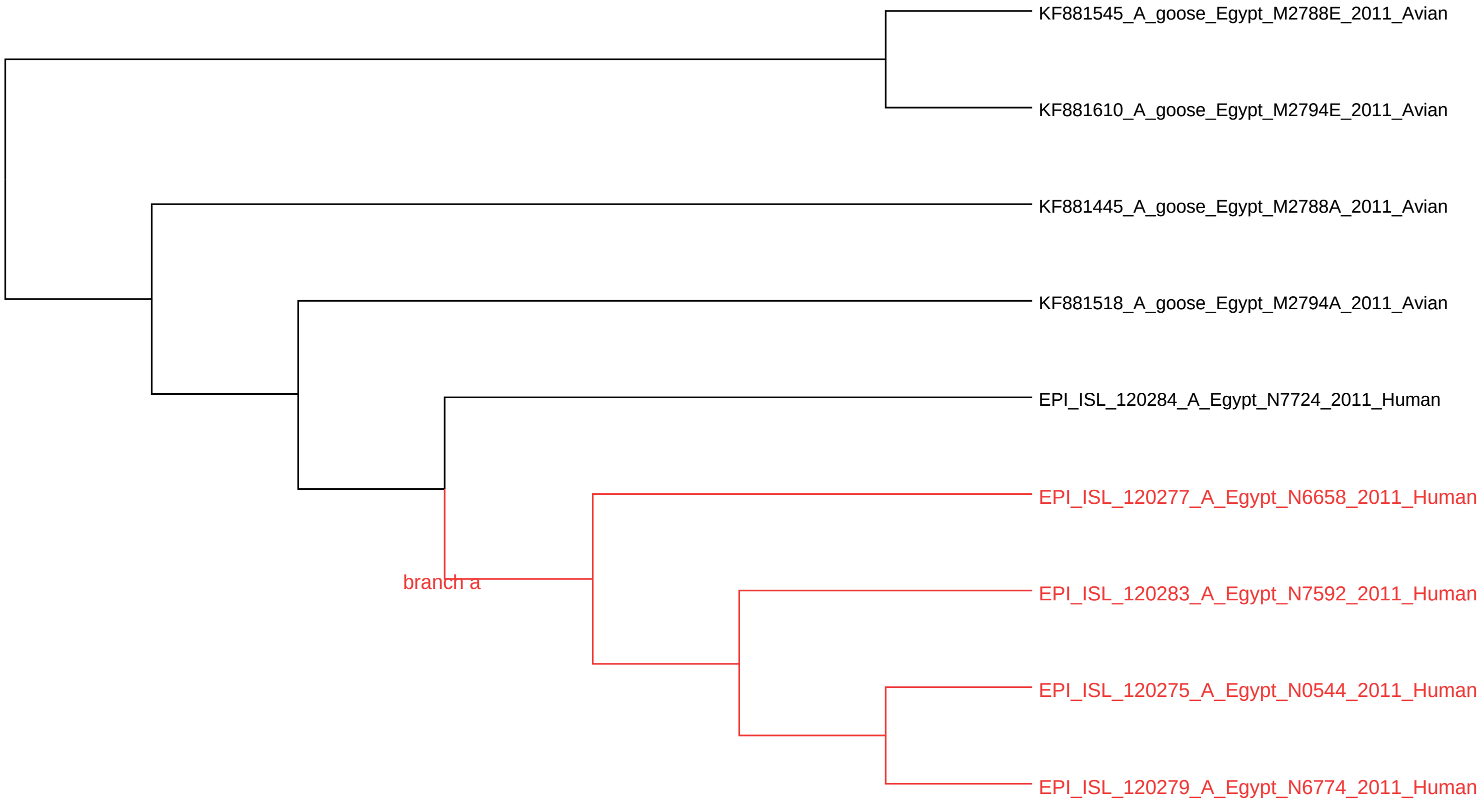

KF881545_A_goose_Egypt_M2788E_2011_Avian
KF881610_A_goose_Egypt_M2794E_2011_Avian
KF881445_A_goose_Egypt_M2788A_2011_Avian
KF881518_A_goose_Egypt_M2794A_2011_Avian
EPI_ISL_120284_A_Egypt_N7724_2011_Human
EPI_ISL_120277_A_Egypt_N6658_2011_Human
branch a
EPI_ISL_120283_A_Egypt_N7592_2011_Human
EPI_ISL_120275_A_Egypt_N0544_2011_Human
EPI_ISL_120279_A_Egypt_N6774_2011_Human

PA-Group54

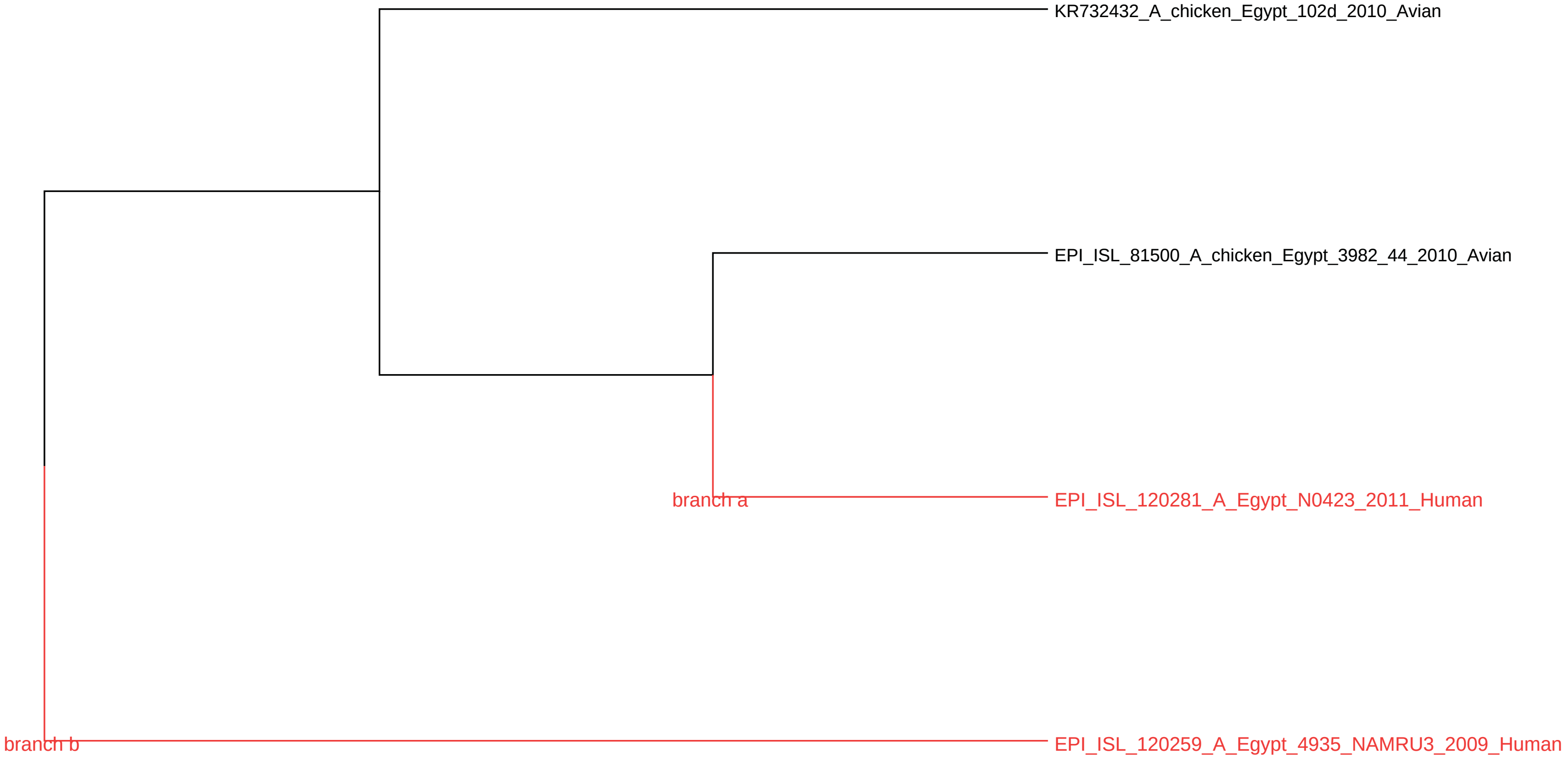
KR732432_A_chicken_Egypt_102d_2010_Avian
EPI_ISL_81500_A_chicken_Egypt_3982_44_2010_Avian
branch a
EPI_ISL_120281_A_Egypt_N0423_2011_Human
branch b
EPI_ISL_120259_A_Egypt_4935_NAMRU3_2009_Human

PA-Group55

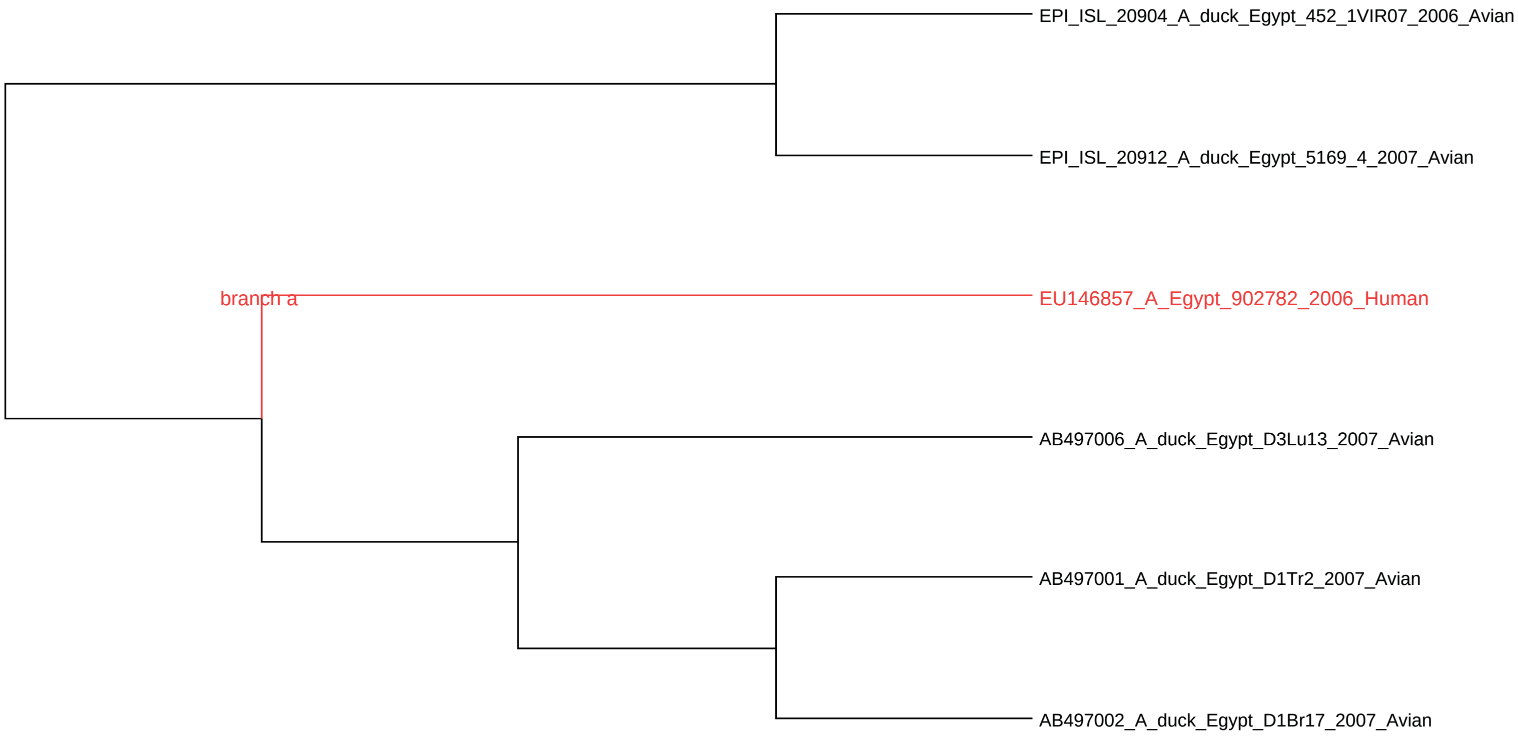
EPI_ISL_20904_A_duck_Egypt_452_1VIR07_2006_Avian
EPI_ISL_20912_A_duck_Egypt_5169_4_2007_Avian
branch a
EU146857_A_Egypt_902782_2006_Human
AB497006_A_duck_Egypt_D3Lu13_2007_Avian
AB497001_A_duck_Egypt_D1Tr2_2007_Avian
AB497002_A_duck_Egypt_D1Br17_2007_Avian

PA-Group56

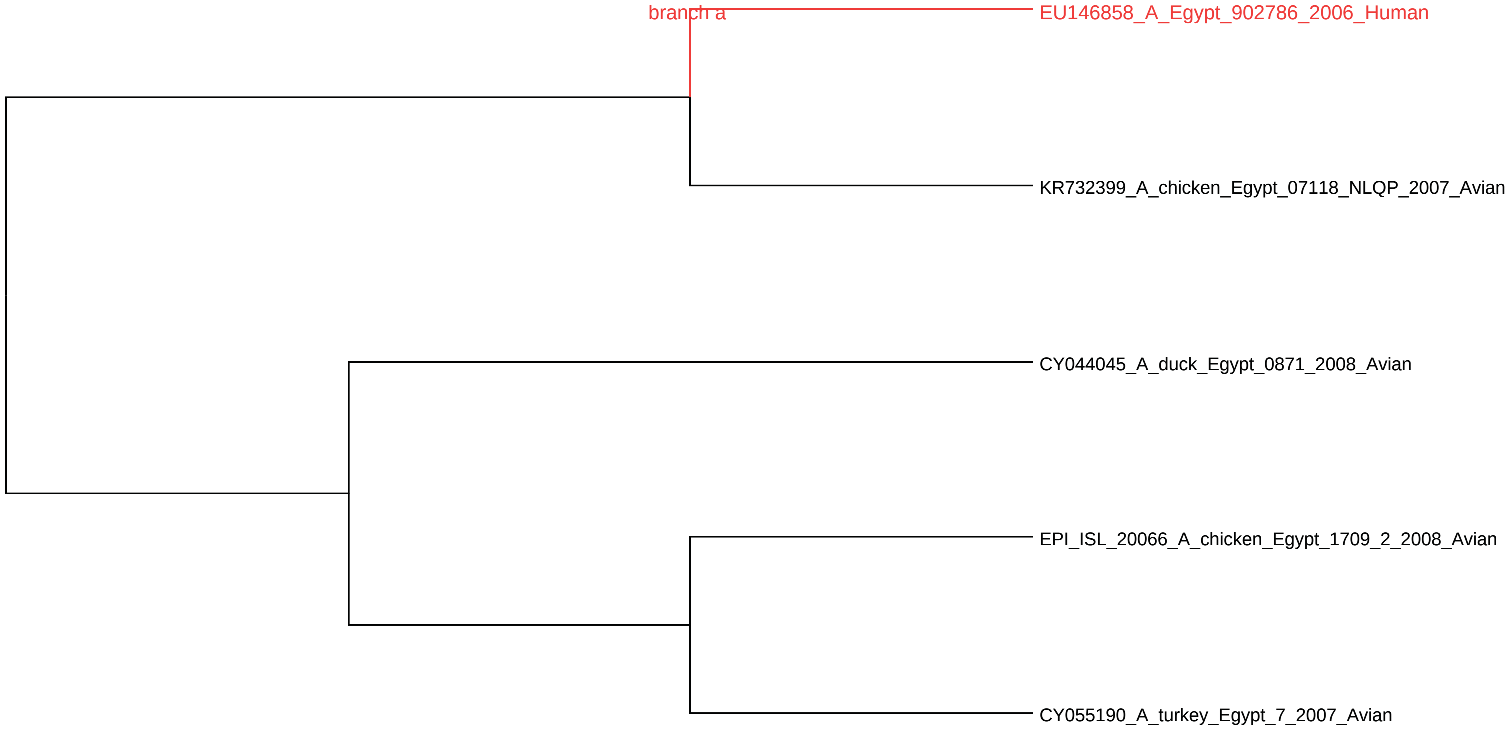

branch a
EU146858_A_Egypt_902786_2006_Human
KR732399_A_chicken_Egypt_07118_NLQP_2007_Avian
CY044045_A_duck_Egypt_0871_2008_Avian
EPI_ISL_20066_A_chicken_Egypt_1709_2_2008_Avian
CY055190_A_turkey_Egypt_7_2007_Avian

PA-Group57

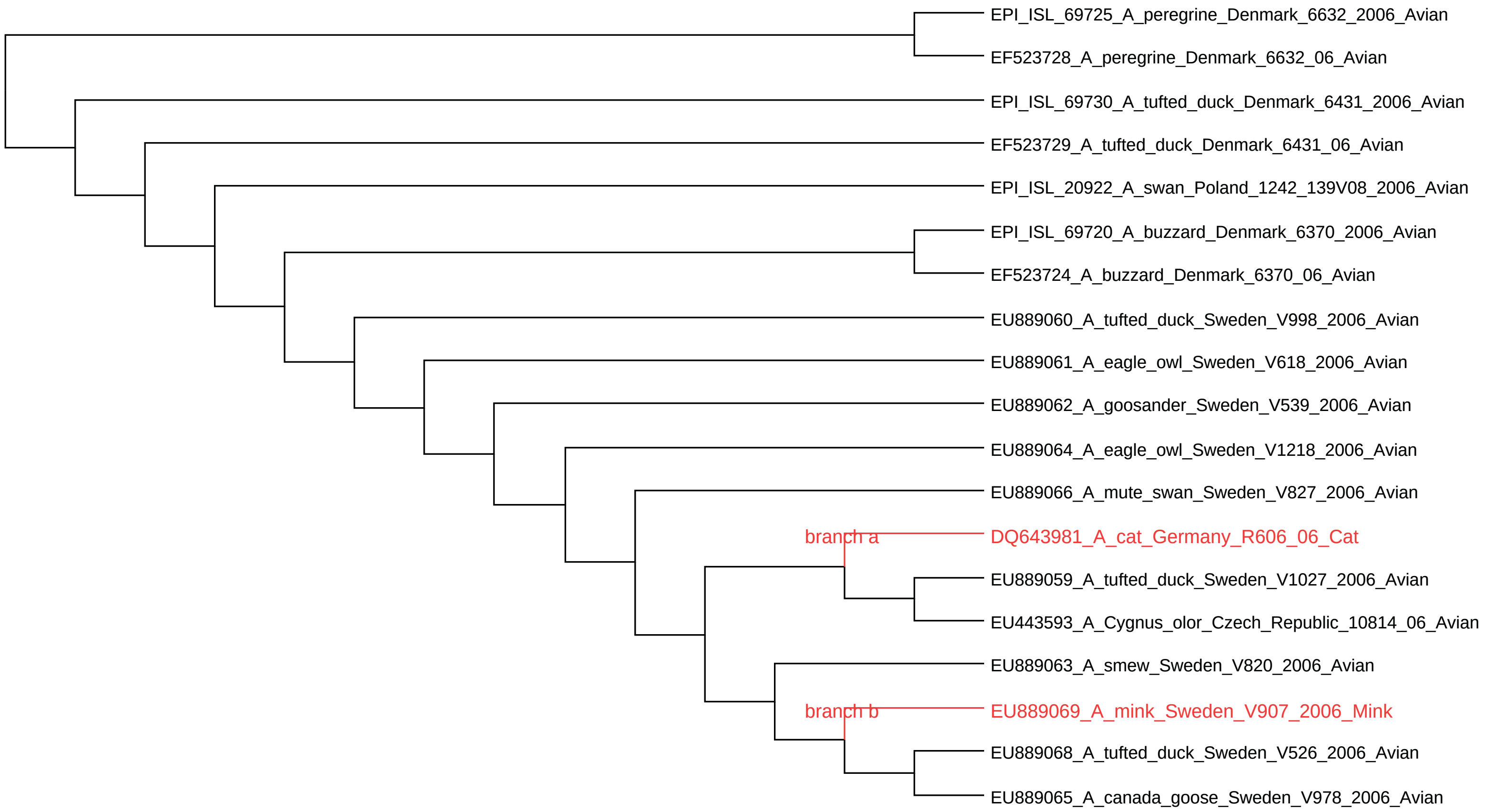
EPI_ISL_69725_A_peregrine_Denmark_6632_2006_Avian
EF523728_A_peregrine_Denmark_6632_06_Avian
EPI_ISL_69730_A_tufted_duck_Denmark_6431_2006_Avian
EF523729_A_tufted_duck_Denmark_6431_06_Avian
EPI_ISL_20922_A_swan_Poland_1242_139V08_2006_Avian
EPI_ISL_69720_A_buzzard_Denmark_6370_2006_Avian
EF523724_A_buzzard_Denmark_6370_06_Avian
EU889060_A_tufted_duck_Sweden_V998_2006_Avian
EU889061_A_eagle_owl_Sweden_V618_2006_Avian
EU889062_A_goosander_Sweden_V539_2006_Avian
EU889064_A_eagle_owl_Sweden_V1218_2006_Avian
EU889066_A_mute_swan_Sweden_V827_2006_Avian
branch a
DQ643981_A_cat_Germany_R606_06_Cat
EU889059_A_tufted_duck_Sweden_V1027_2006_Avian
EU443593_A_Cygnus_olor_Czech_Republic_10814_06_Avian
EU889063_A_smew_Sweden_V820_2006_Avian
branch b
EU889069_A_mink_Sweden_V907_2006_Mink
EU889068_A_tufted_duck_Sweden_V526_2006_Avian
EU889065_A_canada_goose_Sweden_V978_2006_Avian

PA-Group58

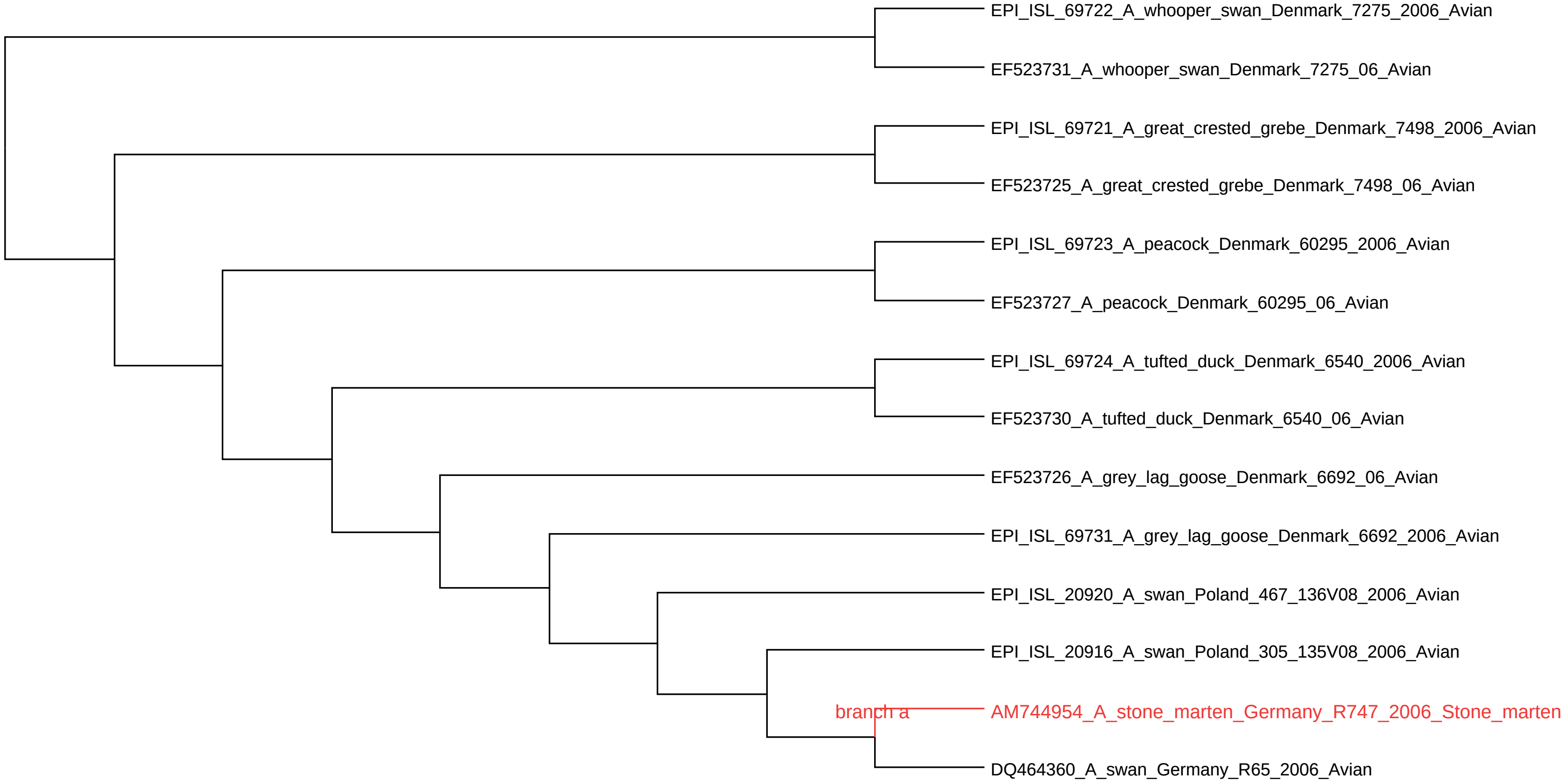
EPI_ISL_69722_A_whooper_swan_Denmark_7275_2006_Avian
EF523731_A_whooper_swan_Denmark_7275_06_Avian
EPI_ISL_69721_A_great_crested_grebe_Denmark_7498_2006_Avian
EF523725_A_great_crested_grebe_Denmark_7498_06_Avian
EPI_ISL_69723_A_peacock_Denmark_60295_2006_Avian
EF523727_A_peacock_Denmark_60295_06_Avian
EPI_ISL_69724_A_tufted_duck_Denmark_6540_2006_Avian
EF523730_A_tufted_duck_Denmark_6540_06_Avian
EF523726_A_grey_lag_goose_Denmark_6692_06_Avian
EPI_ISL_69731_A_grey_lag_goose_Denmark_6692_2006_Avian
EPI_ISL_20920_A_swan_Poland_467_136V08_2006_Avian
EPI_ISL_20916_A_swan_Poland_305_135V08_2006_Avian
branch a
AM744954_A_stone_marten_Germany_R747_2006_Stone_marten
DQ464360_A_swan_Germany_R65_2006_Avian

PA-Group59

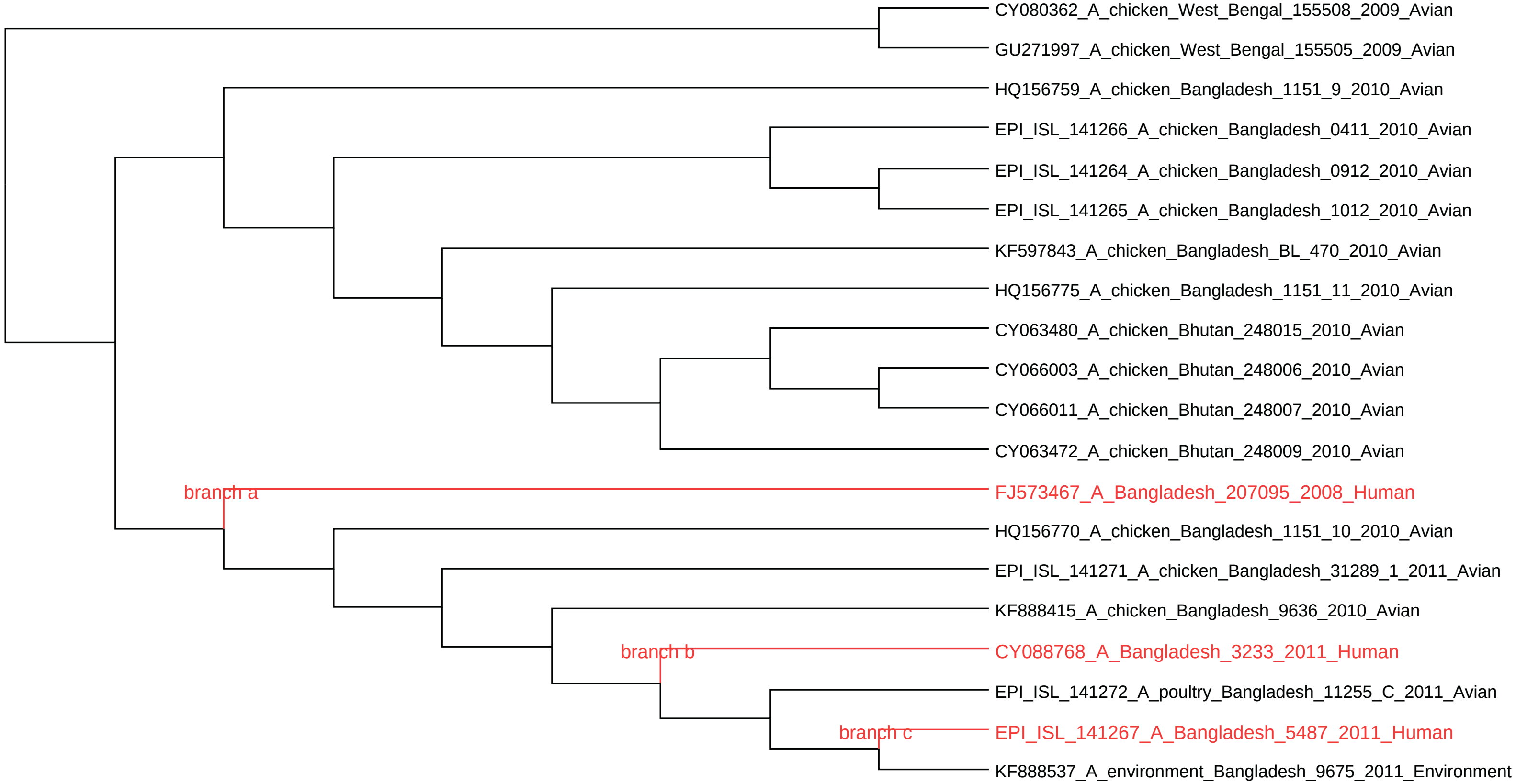

CY080362_A_chicken_West_Bengal_155508_2009_Avian
GU271997_A_chicken_West_Bengal_155505_2009_Avian
HQ156759_A_chicken_Bangladesh_1151_9_2010_Avian
EPI_ISL_141266_A_chicken_Bangladesh_0411_2010_Avian
EPI_ISL_141264_A_chicken_Bangladesh_0912_2010_Avian
EPI_ISL_141265_A_chicken_Bangladesh_1012_2010_Avian
KF597843_A_chicken_Bangladesh_BL_470_2010_Avian
HQ156775_A_chicken_Bangladesh_1151_11_2010_Avian
CY063480_A_chicken_Bhutan_248015_2010_Avian
CY066003_A_chicken_Bhutan_248006_2010_Avian
CY066011_A_chicken_Bhutan_248007_2010_Avian
CY063472_A_chicken_Bhutan_248009_2010_Avian
branch a
FJ573467_A_Bangladesh_207095_2008_Human
HQ156770_A_chicken_Bangladesh_1151_10_2010_Avian
EPI_ISL_141271_A_chicken_Bangladesh_31289_1_2011_Avian
KF888415_A_chicken_Bangladesh_9636_2010_Avian
branch b
CY088768_A_Bangladesh_3233_2011_Human
EPI_ISL_141272_A_poultry_Bangladesh_11255_C_2011_Avian
branch c
EPI_ISL_141267_A_Bangladesh_5487_2011_Human
KF888537_A_environment_Bangladesh_9675_2011_Environment

PA-Group60

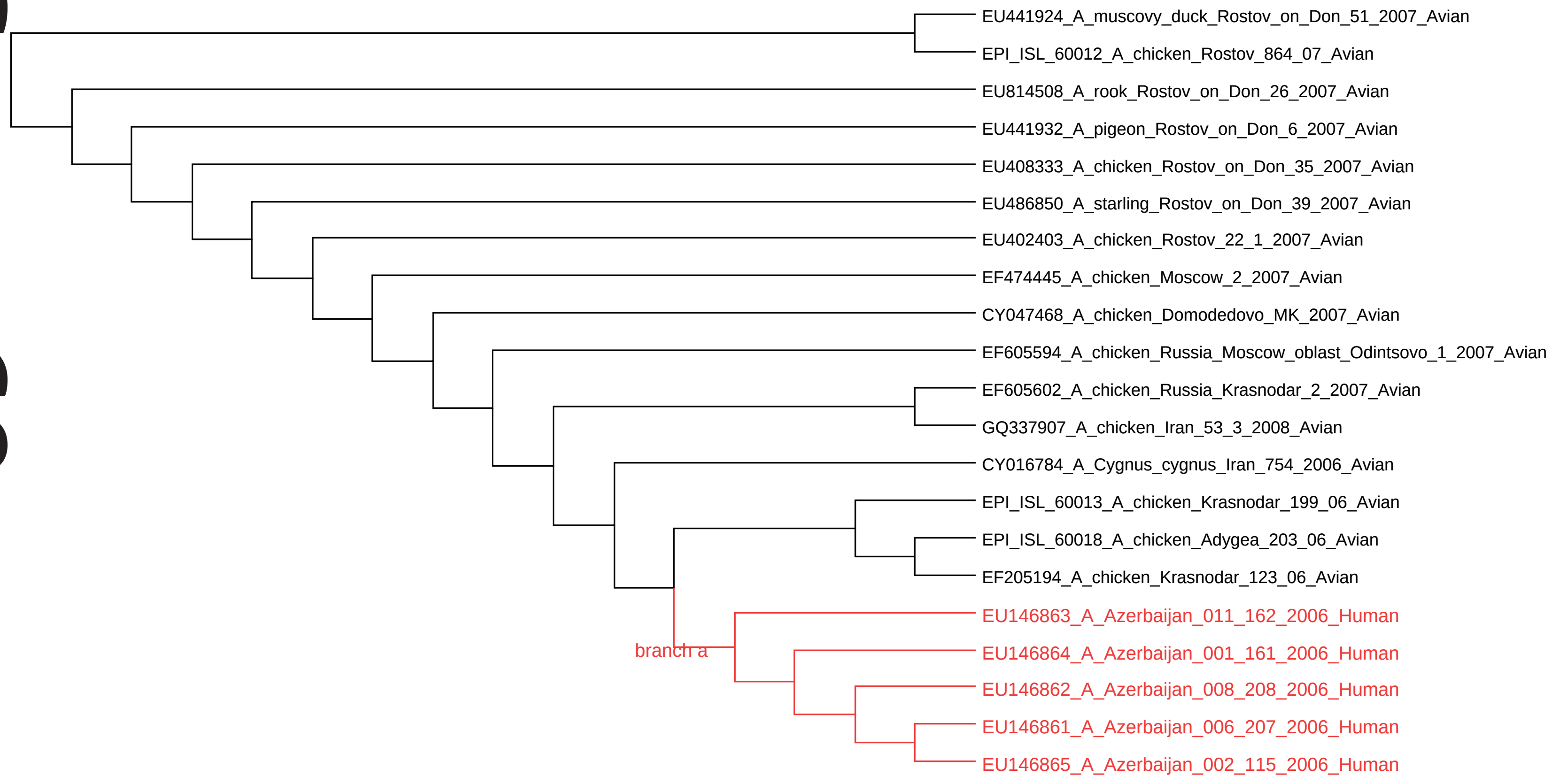

EU441924_A_muscovy_duck_Rostov_on_Don_51_2007_Avian
EPI_ISL_60012_A_chicken_Rostov_864_07_Avian
EU814508_A_rook_Rostov_on_Don_26_2007_Avian
EU441932_A_pigeon_Rostov_on_Don_6_2007_Avian
EU408333_A_chicken_Rostov_on_Don_35_2007_Avian
EU486850_A_starling_Rostov_on_Don_39_2007_Avian
EU402403_A_chicken_Rostov_22_1_2007_Avian
EF474445_A_chicken_Moscow_2_2007_Avian
CY047468_A_chicken_Domodedovo_MK_2007_Avian
EF605594_A_chicken_Russia_Moscow_oblast_Odintsovo_1_2007_Avian
EF605602_A_chicken_Russia_Krasnodar_2_2007_Avian
GQ337907_A_chicken_Iran_53_3_2008_Avian
CY016784_A_Cygnus_cygnus_Iran_754_2006_Avian
EPI_ISL_60013_A_chicken_Krasnodar_199_06_Avian
EPI_ISL_60018_A_chicken_Adygea_203_06_Avian
EF205194_A_chicken_Krasnodar_123_06_Avian
branch a
EU146863_A_Azerbaijan_011_162_2006_Human
EU146864_A_Azerbaijan_001_161_2006_Human
EU146862_A_Azerbaijan_008_208_2006_Human
EU146861_A_Azerbaijan_006_207_2006_Human
EU146865_A_Azerbaijan_002_115_2006_Human

PA-Group61

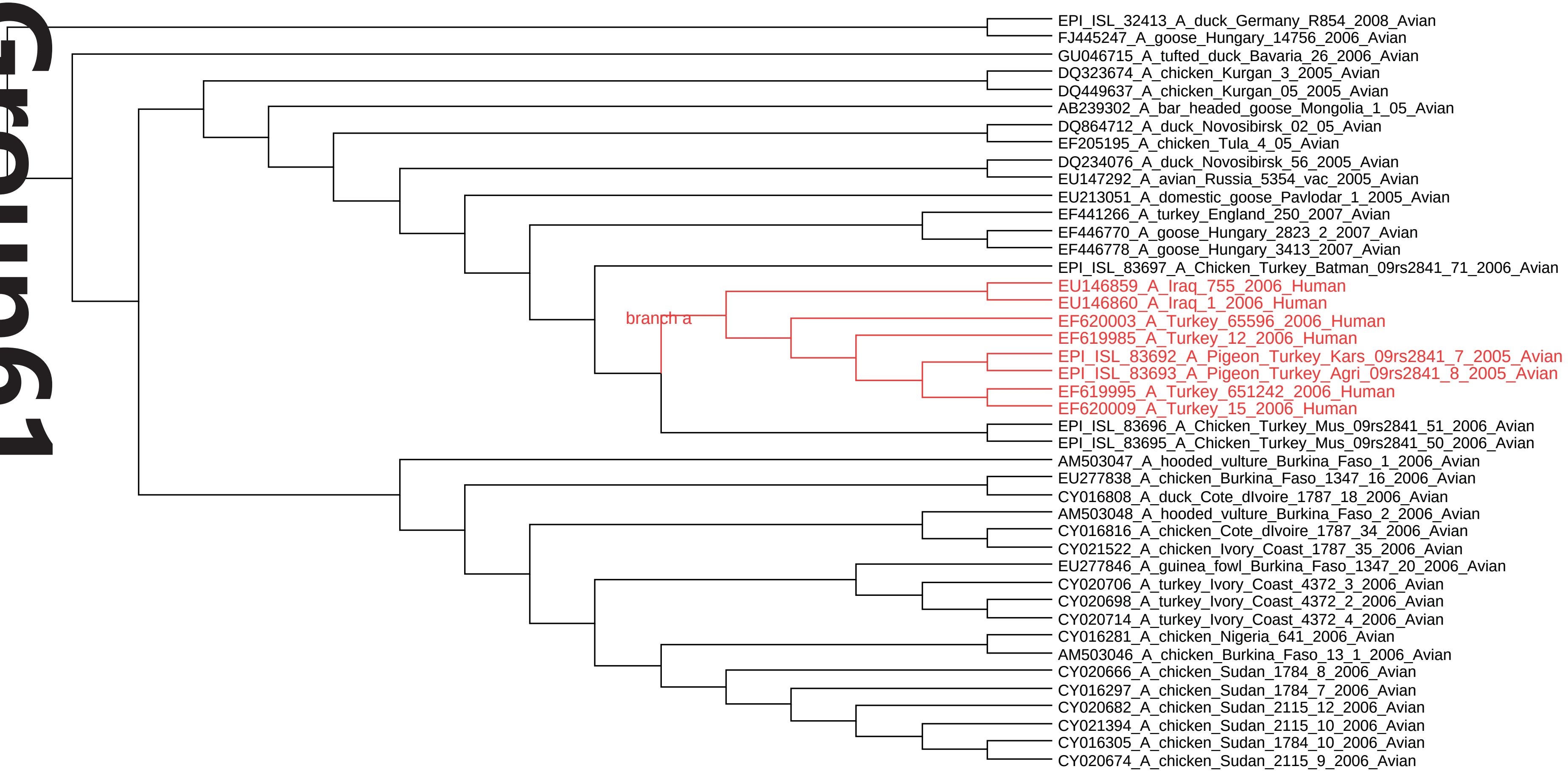

EPI_ISL_32413_A_duck_Germany_R854_2008_Avian
FJ445247_A_goose_Hungary_14756_2006_Avian
GU046715_A_tufted_duck_Bavaria_26_2006_Avian
DQ323674_A_chicken_Kurgan_3_2005_Avian
DQ449637_A_chicken_Kurgan_05_2005_Avian
AB239302_A_bar_headed_goose_Mongolia_1_05_Avian
DQ864712_A_duck_Novosibirsk_02_05_Avian
EF205195_A_chicken_Tula_4_05_Avian
DQ234076_A_duck_Novosibirsk_56_2005_Avian
EU147292_A_avian_Russia_5354_vac_2005_Avian
EU213051_A_domestic_goose_Pavlodar_1_2005_Avian
EF441266_A_turkey_England_250_2007_Avian
EF446770_A_goose_Hungary_2823_2_2007_Avian
EF446778_A_goose_Hungary_3413_2007_Avian
EPI_ISL_83697_A_Chicken_Turkey_Batman_09rs2841_71_2006_Avian
branch a
EU146859_A_Iraq_755_2006_Human
EU146860_A_Iraq_1_2006_Human
EF620003_A_Turkey_65596_2006_Human
EF619985_A_Turkey_12_2006_Human
EPI_ISL_83692_A_Pigeon_Turkey_Kars_09rs2841_7_2005_Avian
EPI_ISL_83693_A_Pigeon_Turkey_Agri_09rs2841_8_2005_Avian
EF619995_A_Turkey_651242_2006_Human
EF620009_A_Turkey_15_2006_Human
EPI_ISL_83696_A_Chicken_Turkey_Mus_09rs2841_51_2006_Avian
EPI_ISL_83695_A_Chicken_Turkey_Mus_09rs2841_50_2006_Avian
AM503047_A_hooded_vulture_Burkina_Faso_1_2006_Avian
EU277838_A_chicken_Burkina_Faso_1347_16_2006_Avian
CY016808_A_duck_Cote_dIvoire_1787_18_2006_Avian
AM503048_A_hooded_vulture_Burkina_Faso_2_2006_Avian
CY016816_A_chicken_Cote_dIvoire_1787_34_2006_Avian
CY021522_A_chicken_Ivory_Coast_1787_35_2006_Avian
EU277846_A_guinea_fowl_Burkina_Faso_1347_20_2006_Avian
CY020706_A_turkey_Ivory_Coast_4372_3_2006_Avian
CY020698_A_turkey_Ivory_Coast_4372_2_2006_Avian
CY020714_A_turkey_Ivory_Coast_4372_4_2006_Avian
CY016281_A_chicken_Nigeria_641_2006_Avian
AM503046_A_chicken_Burkina_Faso_13_1_2006_Avian
CY020666_A_chicken_Sudan_1784_8_2006_Avian
CY016297_A_chicken_Sudan_1784_7_2006_Avian
CY020682_A_chicken_Sudan_2115_12_2006_Avian
CY021394_A_chicken_Sudan_2115_10_2006_Avian
CY016305_A_chicken_Sudan_1784_10_2006_Avian
CY020674_A_chicken_Sudan_2115_9_2006_Avian

PA-Group62

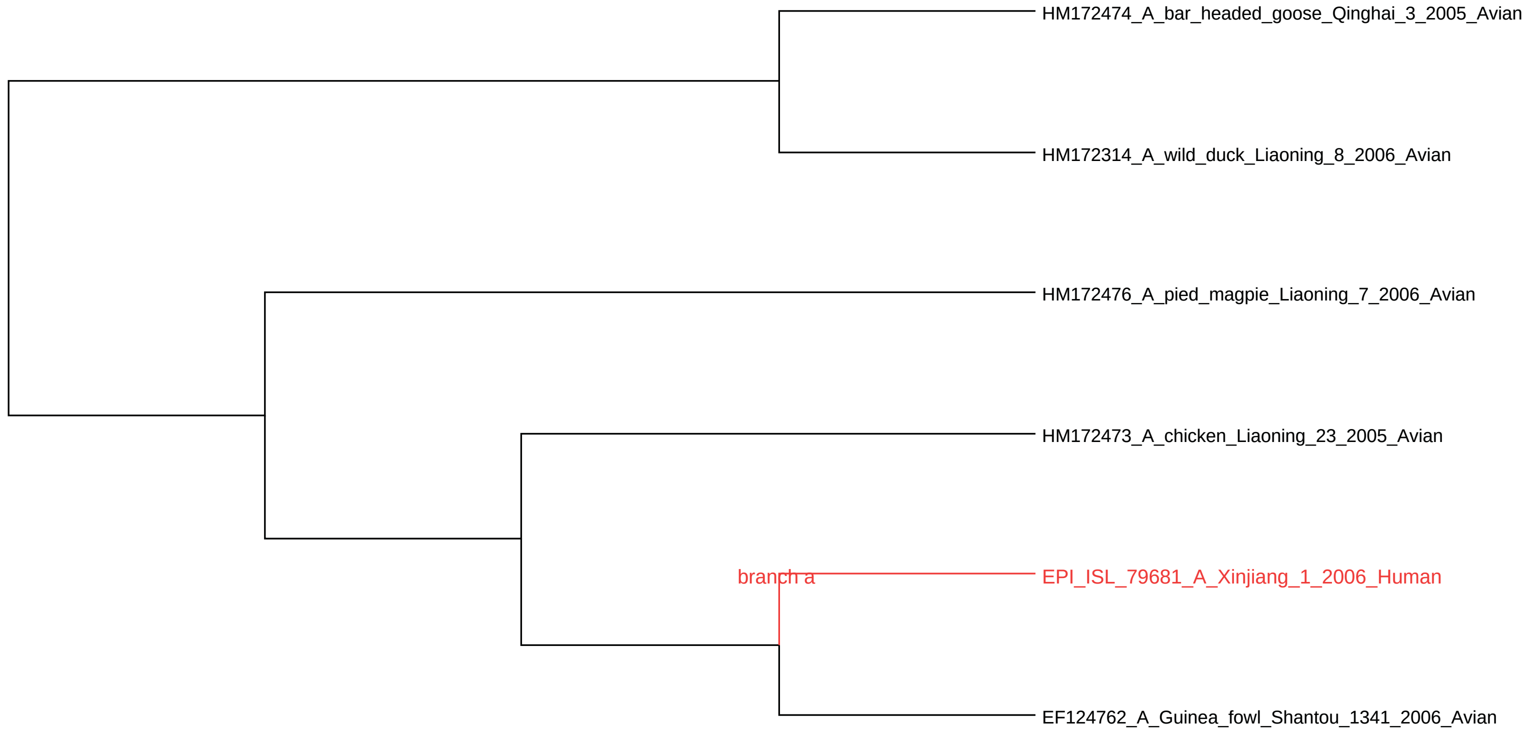
HM172474_A_bar_headed_goose_Qinghai_3_2005_Avian
HM172314_A_wild_duck_Liaoning_8_2006_Avian
HM172476_A_pied_magpie_Liaoning_7_2006_Avian
HM172473_A_chicken_Liaoning_23_2005_Avian
branch a
EPI_ISL_79681_A_Xinjiang_1_2006_Human
EF124762_A_Guinea_fowl_Shantou_1341_2006_Avian

PA-Group63

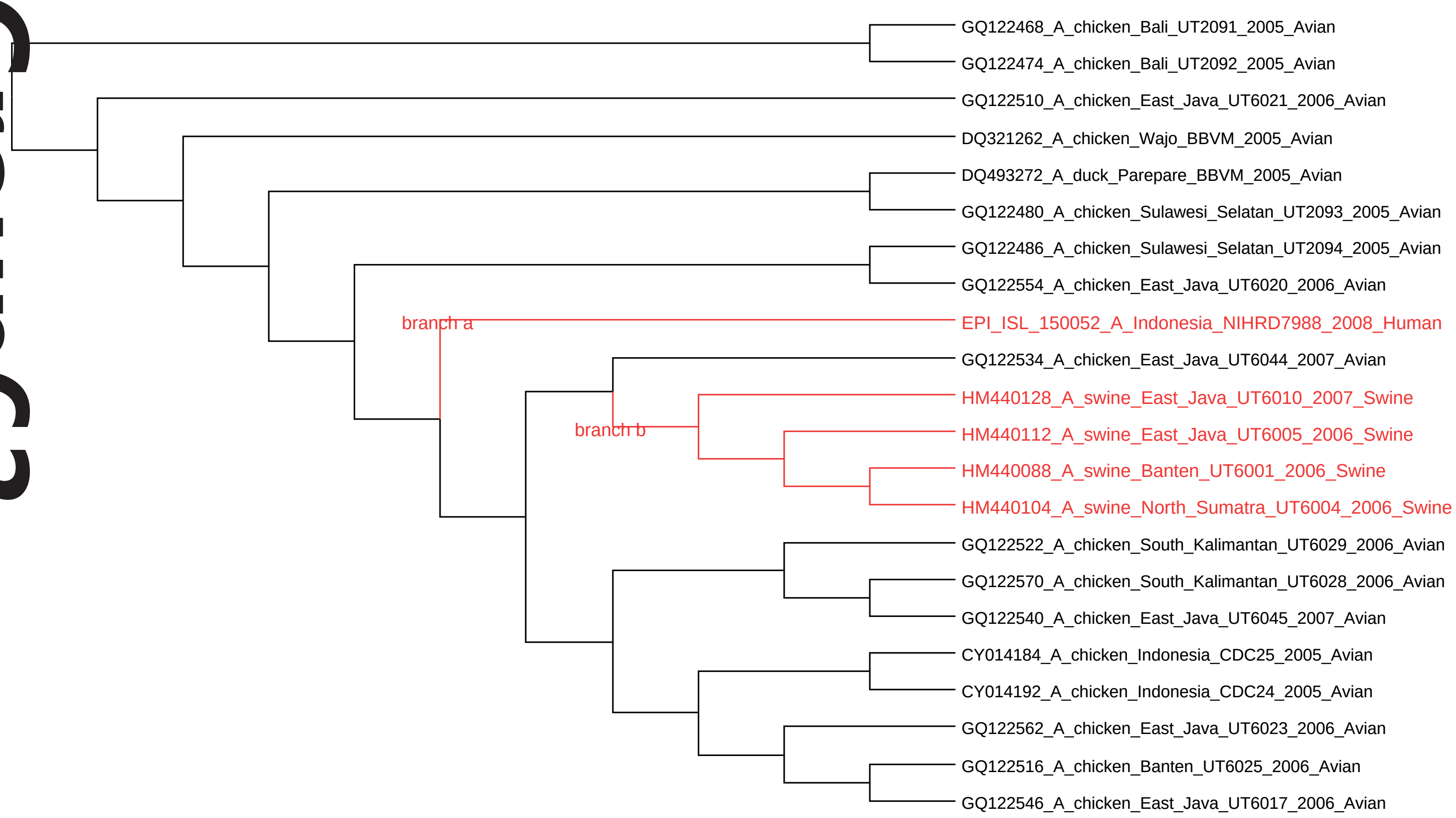
GQ122468_A_chicken_Bali_UT2091_2005_Avian
GQ122474_A_chicken_Bali_UT2092_2005_Avian
GQ122510_A_chicken_East_Java_UT6021_2006_Avian
DQ321262_A_chicken_Wajo_BBVM_2005_Avian
DQ493272_A_duck_Parepare_BBVM_2005_Avian
GQ122480_A_chicken_Sulawesi_Selatan_UT2093_2005_Avian
GQ122486_A_chicken_Sulawesi_Selatan_UT2094_2005_Avian
GQ122554_A_chicken_East_Java_UT6020_2006_Avian
branch a
EPI_ISL_150052_A_Indonesia_NIHRD7988_2008_Human
GQ122534_A_chicken_East_Java_UT6044_2007_Avian
branch b
HM440128_A_swine_East_Java_UT6010_2007_Swine
HM440112_A_swine_East_Java_UT6005_2006_Swine
HM440088_A_swine_Banten_UT6001_2006_Swine
HM440104_A_swine_North_Sumatra_UT6004_2006_Swine
GQ122522_A_chicken_South_Kalimantan_UT6029_2006_Avian
GQ122570_A_chicken_South_Kalimantan_UT6028_2006_Avian
GQ122540_A_chicken_East_Java_UT6045_2007_Avian
CY014184_A_chicken_Indonesia_CDC25_2005_Avian
CY014192_A_chicken_Indonesia_CDC24_2005_Avian
GQ122562_A_chicken_East_Java_UT6023_2006_Avian
GQ122516_A_chicken_Banten_UT6025_2006_Avian
GQ122546_A_chicken_East_Java_UT6017_2006_Avian

PA-Group64

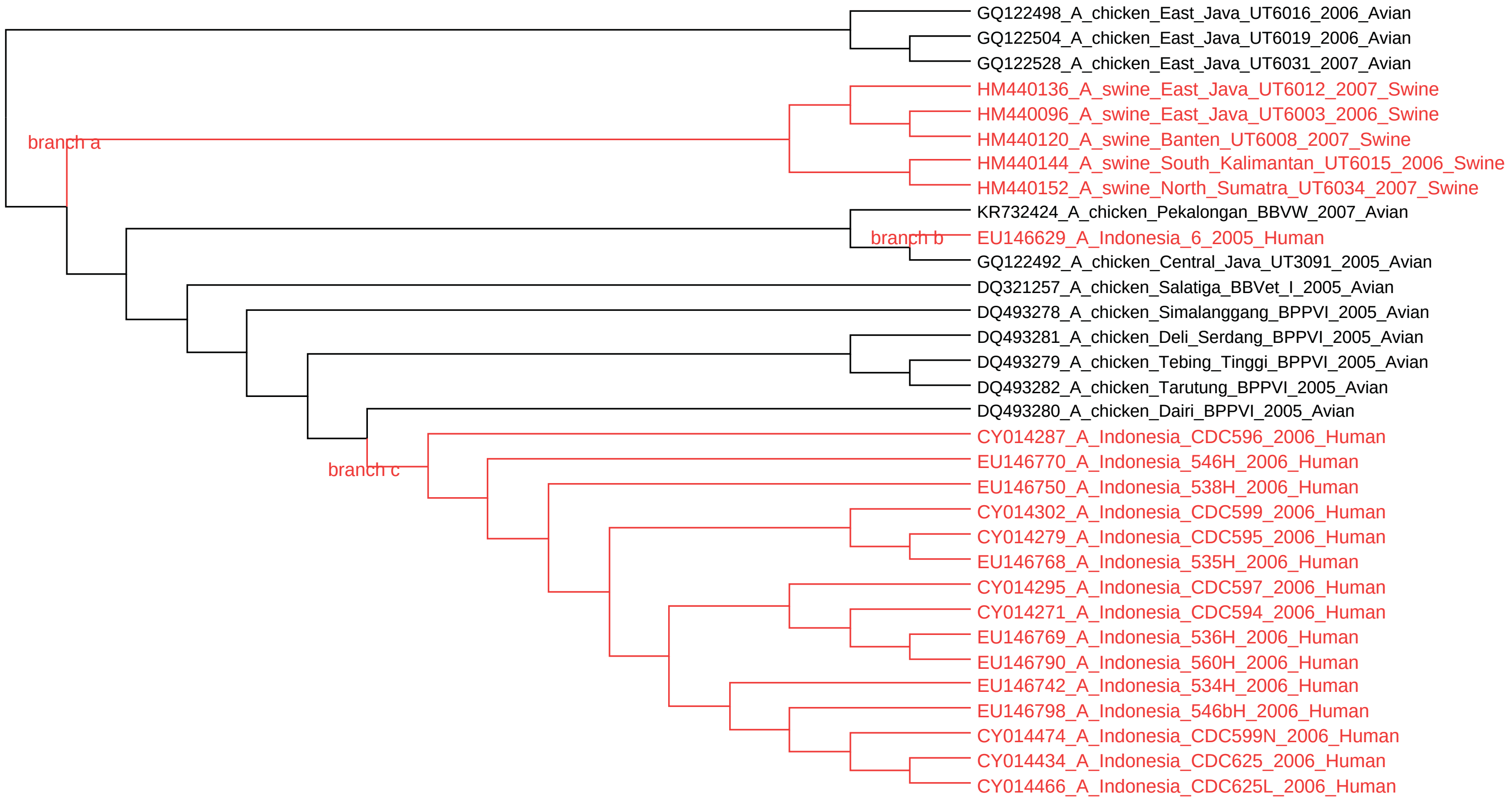

GQ122498_A_chicken_East_Java_UT6016_2006_Avian
GQ122504_A_chicken_East_Java_UT6019_2006_Avian
GQ122528_A_chicken_East_Java_UT6031_2007_Avian
HM440136_A_swine_East_Java_UT6012_2007_Swine
HM440096_A_swine_East_Java_UT6003_2006_Swine
HM440120_A_swine_Banten_UT6008_2007_Swine
HM440144_A_swine_South_Kalimantan_UT6015_2006_Swine
HM440152_A_swine_North_Sumatra_UT6034_2007_Swine
branch a
KR732424_A_chicken_Pekalongan_BBVW_2007_Avian
branch b
EU146629_A_Indonesia_6_2005_Human
GQ122492_A_chicken_Central_Java_UT3091_2005_Avian
DQ321257_A_chicken_Salatiga_BBVet_I_2005_Avian
DQ493278_A_chicken_Simalanggang_BPPVI_2005_Avian
DQ493281_A_chicken_Deli_Serdang_BPPVI_2005_Avian
DQ493279_A_chicken_Tebing_Tinggi_BPPVI_2005_Avian
DQ493282_A_chicken_Tarutung_BPPVI_2005_Avian
DQ493280_A_chicken_Dairi_BPPVI_2005_Avian
branch c
CY014287_A_Indonesia_CDC596_2006_Human
EU146770_A_Indonesia_546H_2006_Human
EU146750_A_Indonesia_538H_2006_Human
CY014302_A_Indonesia_CDC599_2006_Human
CY014279_A_Indonesia_CDC595_2006_Human
EU146768_A_Indonesia_535H_2006_Human
CY014295_A_Indonesia_CDC597_2006_Human
CY014271_A_Indonesia_CDC594_2006_Human
EU146769_A_Indonesia_536H_2006_Human
EU146790_A_Indonesia_560H_2006_Human
EU146742_A_Indonesia_534H_2006_Human
EU146798_A_Indonesia_546bH_2006_Human
CY014474_A_Indonesia_CDC599N_2006_Human
CY014434_A_Indonesia_CDC625_2006_Human
CY014466_A_Indonesia_CDC625L_2006_Human

PA-Group65

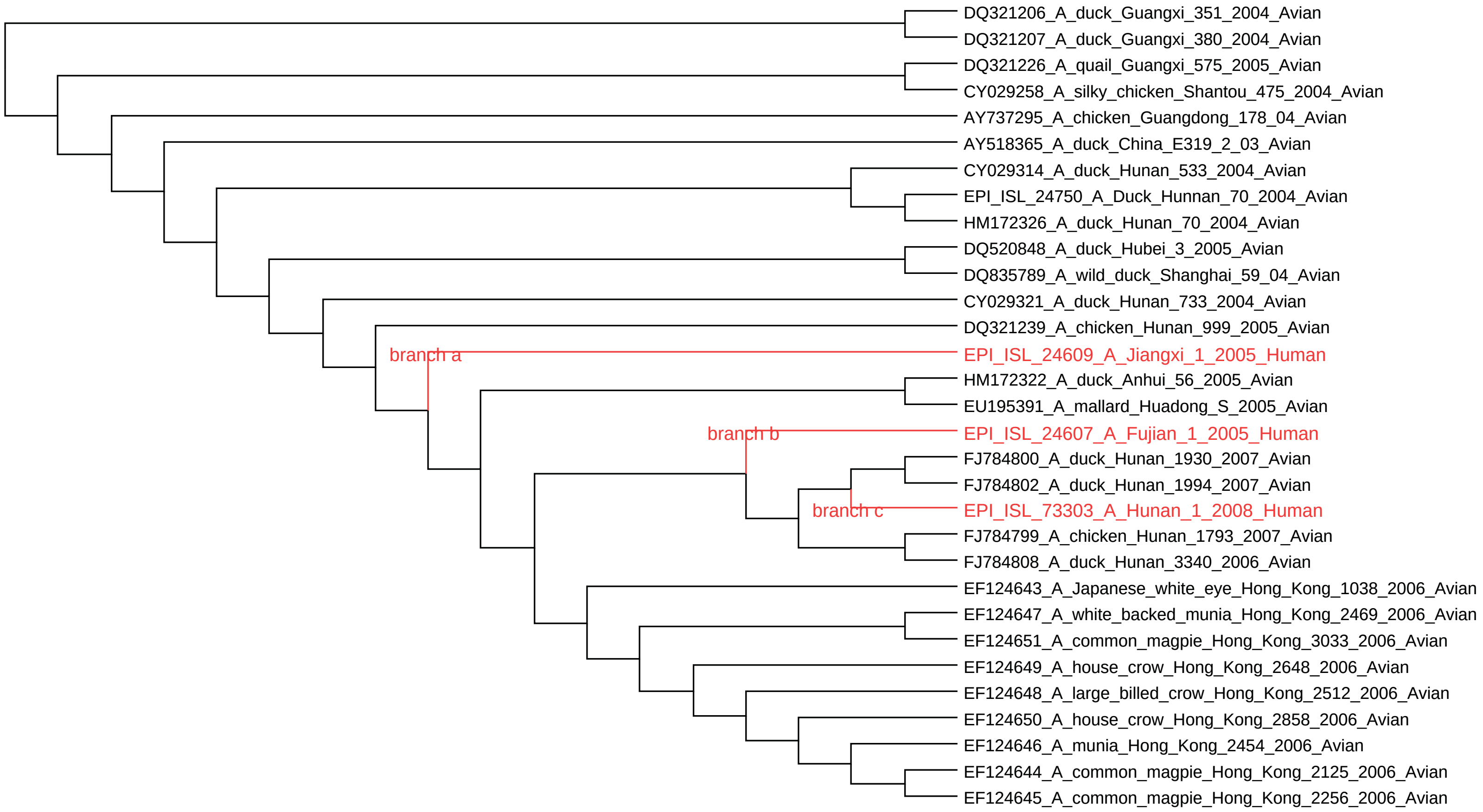
DQ321206_A_duck_Guangxi_351_2004_Avian
DQ321207_A_duck_Guangxi_380_2004_Avian
DQ321226_A_quail_Guangxi_575_2005_Avian
CY029258_A_silky_chicken_Shantou_475_2004_Avian
AY737295_A_chicken_Guangdong_178_04_Avian
AY518365_A_duck_China_E319_2_03_Avian
CY029314_A_duck_Hunan_533_2004_Avian
EPI_ISL_24750_A_Duck_Hunnan_70_2004_Avian
HM172326_A_duck_Hunan_70_2004_Avian
DQ520848_A_duck_Hubei_3_2005_Avian
DQ835789_A_wild_duck_Shanghai_59_04_Avian
CY029321_A_duck_Hunan_733_2004_Avian
DQ321239_A_chicken_Hunan_999_2005_Avian
branch a
EPI_ISL_24609_A_Jiangxi_1_2005_Human
HM172322_A_duck_Anhui_56_2005_Avian
EU195391_A_mallard_Huadong_S_2005_Avian
branch b
EPI_ISL_24607_A_Fujian_1_2005_Human
FJ784800_A_duck_Hunan_1930_2007_Avian
FJ784802_A_duck_Hunan_1994_2007_Avian
branch c
EPI_ISL_73303_A_Hunan_1_2008_Human
FJ784799_A_chicken_Hunan_1793_2007_Avian
FJ784808_A_duck_Hunan_3340_2006_Avian
EF124643_A_Japanese_white_eye_Hong_Kong_1038_2006_Avian
EF124647_A_white_backed_munia_Hong_Kong_2469_2006_Avian
EF124651_A_common_magpie_Hong_Kong_3033_2006_Avian
EF124649_A_house_crow_Hong_Kong_2648_2006_Avian
EF124648_A_large_billed_crow_Hong_Kong_2512_2006_Avian
EF124650_A_house_crow_Hong_Kong_2858_2006_Avian
EF124646_A_munia_Hong_Kong_2454_2006_Avian
EF124644_A_common_magpie_Hong_Kong_2125_2006_Avian
EF124645_A_common_magpie_Hong_Kong_2256_2006_Avian

PA-Group66

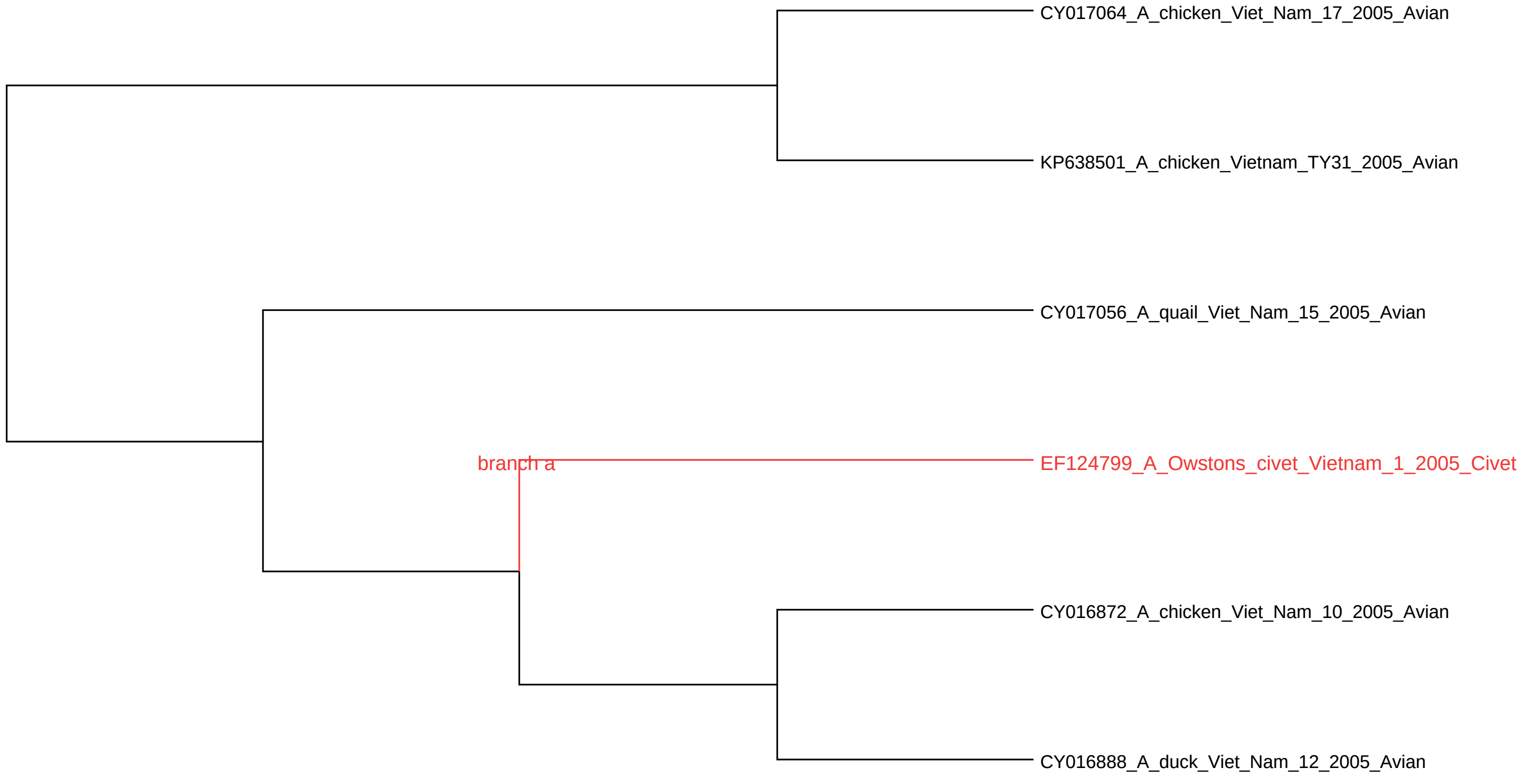

CY017064_A_chicken_Viet_Nam_17_2005_Avian
KP638501_A_chicken_Vietnam_TY31_2005_Avian
CY017056_A_quail_Viet_Nam_15_2005_Avian
branch a
EF124799_A_Owstons_civet_Vietnam_1_2005_Civet
CY016872_A_chicken_Viet_Nam_10_2005_Avian
CY016888_A_duck_Viet_Nam_12_2005_Avian

PA-Group67

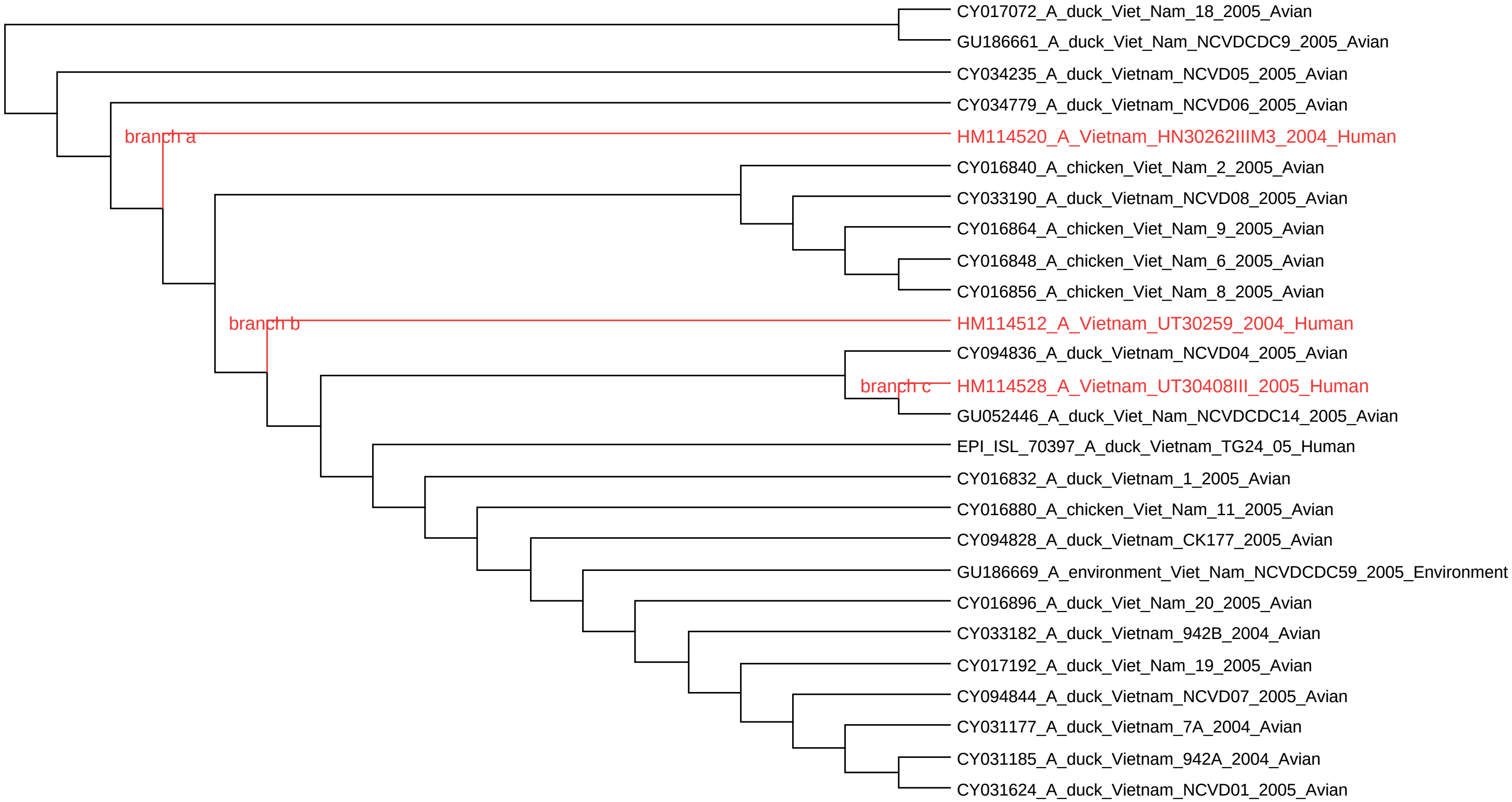

CY017072_A_duck_Viet_Nam_18_2005_Avian
GU186661_A_duck_Viet_Nam_NCVDCDC9_2005_Avian
CY034235_A_duck_Vietnam_NCVD05_2005_Avian
CY034779_A_duck_Vietnam_NCVD06_2005_Avian
branch a
HM114520_A_Vietnam_HN30262IIIM3_2004_Human
CY016840_A_chicken_Viet_Nam_2_2005_Avian
CY033190_A_duck_Vietnam_NCVD08_2005_Avian
CY016864_A_chicken_Viet_Nam_9_2005_Avian
CY016848_A_chicken_Viet_Nam_6_2005_Avian
CY016856_A_chicken_Viet_Nam_8_2005_Avian
branch b
HM114512_A_Vietnam_UT30259_2004_Human
CY094836_A_duck_Vietnam_NCVD04_2005_Avian
branch c
HM114528_A_Vietnam_UT30408III_2005_Human
GU052446_A_duck_Viet_Nam_NCVDCDC14_2005_Avian
EPI_ISL_70397_A_duck_Vietnam_TG24_05_Human
CY016832_A_duck_Vietnam_1_2005_Avian
CY016880_A_chicken_Viet_Nam_11_2005_Avian
CY094828_A_duck_Vietnam_CK177_2005_Avian
GU186669_A_environment_Viet_Nam_NCVDCDC59_2005_Environment
CY016896_A_duck_Viet_Nam_20_2005_Avian
CY033182_A_duck_Vietnam_942B_2004_Avian
CY017192_A_duck_Viet_Nam_19_2005_Avian
CY094844_A_duck_Vietnam_NCVD07_2005_Avian
CY031177_A_duck_Vietnam_7A_2004_Avian
CY031185_A_duck_Vietnam_942A_2004_Avian
CY031624_A_duck_Vietnam_NCVD01_2005_Avian

PA-Group68

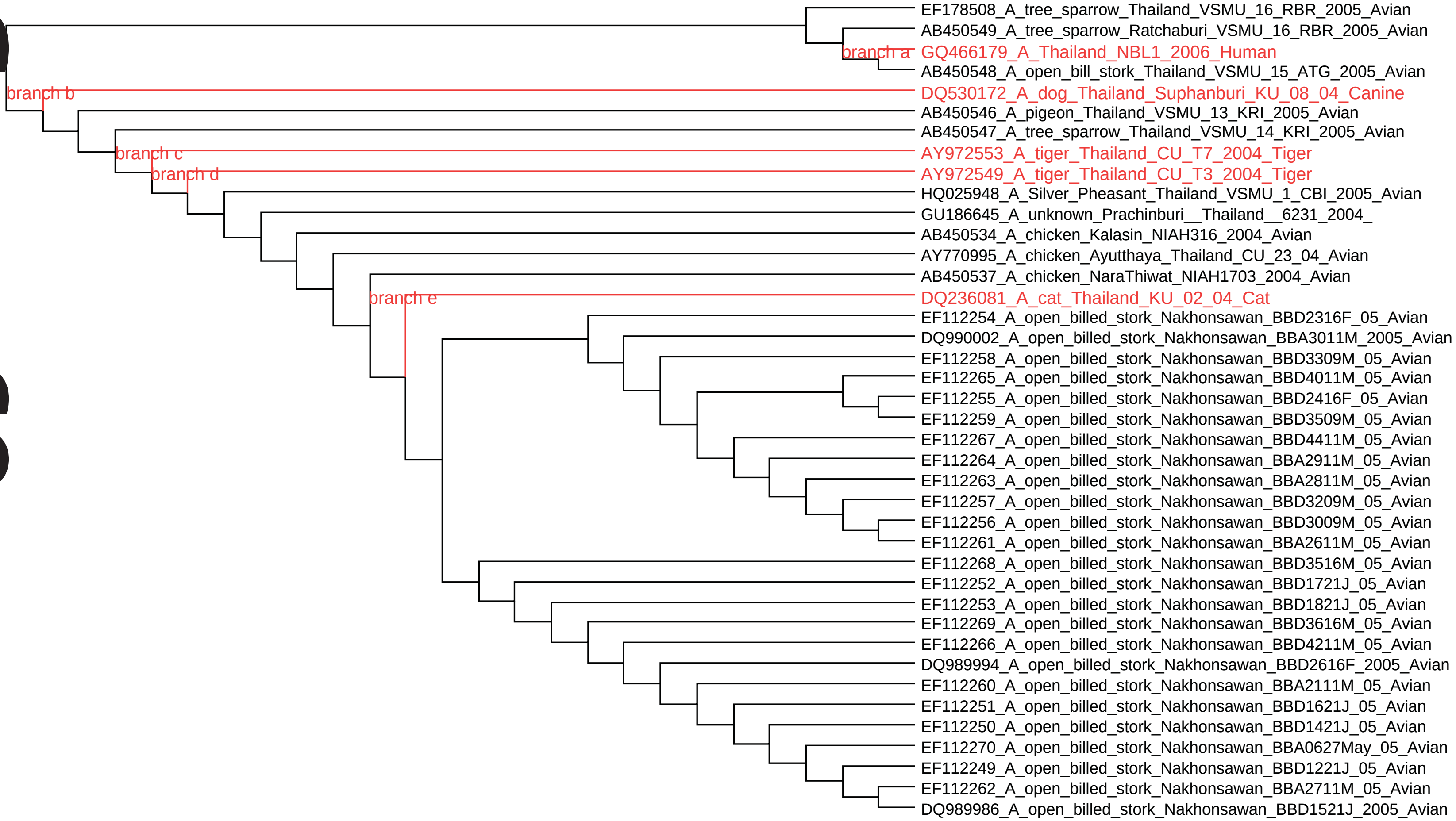
EF178508_A_tree_sparrow_Thailand_VSMU_16_RBR_2005_Avian
AB450549_A_tree_sparrow_Ratchaburi_VSMU_16_RBR_2005_Avian
branch a
GQ466179_A_Thailand_NBL1_2006_Human
AB450548_A_open_bill_stork_Thailand_VSMU_15_ATG_2005_Avian
branch b
DQ530172_A_dog_Thailand_Suphanburi_KU_08_04_Canine
AB450546_A_pigeon_Thailand_VSMU_13_KRI_2005_Avian
AB450547_A_tree_sparrow_Thailand_VSMU_14_KRI_2005_Avian
branch c
AY972553_A_tiger_Thailand_CU_T7_2004_Tiger
branch d
AY972549_A_tiger_Thailand_CU_T3_2004_Tiger
HQ025948_A_Silver_Pheasant_Thailand_VSMU_1_CBI_2005_Avian
GU186645_A_unknown_Prachinburi__Thailand__6231_2004_
AB450534_A_chicken_Kalasin_NIAH316_2004_Avian
AY770995_A_chicken_Ayutthaya_Thailand_CU_23_04_Avian
AB450537_A_chicken_NaraThiwat_NIAH1703_2004_Avian
branch e
DQ236081_A_cat_Thailand_KU_02_04_Cat
EF112254_A_open_billed_stork_Nakhonsawan_BBD2316F_05_Avian
DQ990002_A_open_billed_stork_Nakhonsawan_BBA3011M_2005_Avian
EF112258_A_open_billed_stork_Nakhonsawan_BBD3309M_05_Avian
EF112265_A_open_billed_stork_Nakhonsawan_BBD4011M_05_Avian
EF112255_A_open_billed_stork_Nakhonsawan_BBD2416F_05_Avian
EF112259_A_open_billed_stork_Nakhonsawan_BBD3509M_05_Avian
EF112267_A_open_billed_stork_Nakhonsawan_BBD4411M_05_Avian
EF112264_A_open_billed_stork_Nakhonsawan_BBA2911M_05_Avian
EF112263_A_open_billed_stork_Nakhonsawan_BBA2811M_05_Avian
EF112257_A_open_billed_stork_Nakhonsawan_BBD3209M_05_Avian
EF112256_A_open_billed_stork_Nakhonsawan_BBD3009M_05_Avian
EF112261_A_open_billed_stork_Nakhonsawan_BBA2611M_05_Avian
EF112268_A_open_billed_stork_Nakhonsawan_BBD3516M_05_Avian
EF112252_A_open_billed_stork_Nakhonsawan_BBD1721J_05_Avian
EF112253_A_open_billed_stork_Nakhonsawan_BBD1821J_05_Avian
EF112269_A_open_billed_stork_Nakhonsawan_BBD3616M_05_Avian
EF112266_A_open_billed_stork_Nakhonsawan_BBD4211M_05_Avian
DQ989994_A_open_billed_stork_Nakhonsawan_BBD2616F_2005_Avian
EF112260_A_open_billed_stork_Nakhonsawan_BBA2111M_05_Avian
EF112251_A_open_billed_stork_Nakhonsawan_BBD1621J_05_Avian
EF112250_A_open_billed_stork_Nakhonsawan_BBD1421J_05_Avian
EF112270_A_open_billed_stork_Nakhonsawan_BBA0627May_05_Avian
EF112249_A_open_billed_stork_Nakhonsawan_BBD1221J_05_Avian
EF112262_A_open_billed_stork_Nakhonsawan_BBA2711M_05_Avian
DQ989986_A_open_billed_stork_Nakhonsawan_BBD1521J_2005_Avian

PA-Group69

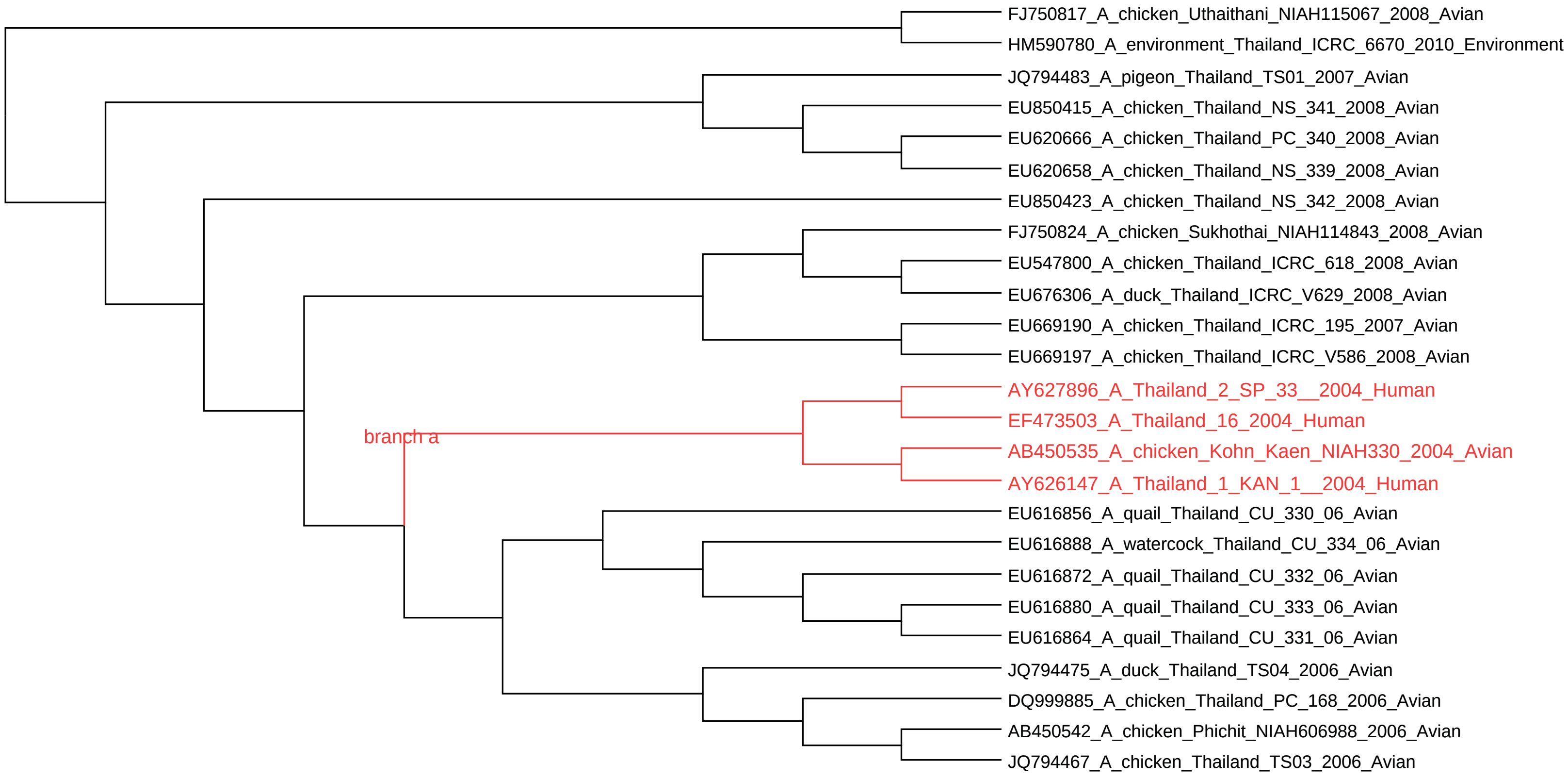
FJ750817_A_chicken_Uthaithani_NIAH115067_2008_Avian
HM590780_A_environment_Thailand_ICRC_6670_2010_Environment
JQ794483_A_pigeon_Thailand_TS01_2007_Avian
EU850415_A_chicken_Thailand_NS_341_2008_Avian
EU620666_A_chicken_Thailand_PC_340_2008_Avian
EU620658_A_chicken_Thailand_NS_339_2008_Avian
EU850423_A_chicken_Thailand_NS_342_2008_Avian
FJ750824_A_chicken_Sukhothai_NIAH114843_2008_Avian
EU547800_A_chicken_Thailand_ICRC_618_2008_Avian
EU676306_A_duck_Thailand_ICRC_V629_2008_Avian
EU669190_A_chicken_Thailand_ICRC_195_2007_Avian
EU669197_A_chicken_Thailand_ICRC_V586_2008_Avian
branch a
AY627896_A_Thailand_2_SP_33__2004_Human
EF473503_A_Thailand_16_2004_Human
AB450535_A_chicken_Kohn_Kaen_NIAH330_2004_Avian
AY626147_A_Thailand_1_KAN_1__2004_Human
EU616856_A_quail_Thailand_CU_330_06_Avian
EU616888_A_watercock_Thailand_CU_334_06_Avian
EU616872_A_quail_Thailand_CU_332_06_Avian
EU616880_A_quail_Thailand_CU_333_06_Avian
EU616864_A_quail_Thailand_CU_331_06_Avian
JQ794475_A_duck_Thailand_TS04_2006_Avian
DQ999885_A_chicken_Thailand_PC_168_2006_Avian
AB450542_A_chicken_Phichit_NIAH606988_2006_Avian
JQ794467_A_chicken_Thailand_TS03_2006_Avian

PA-Grup70

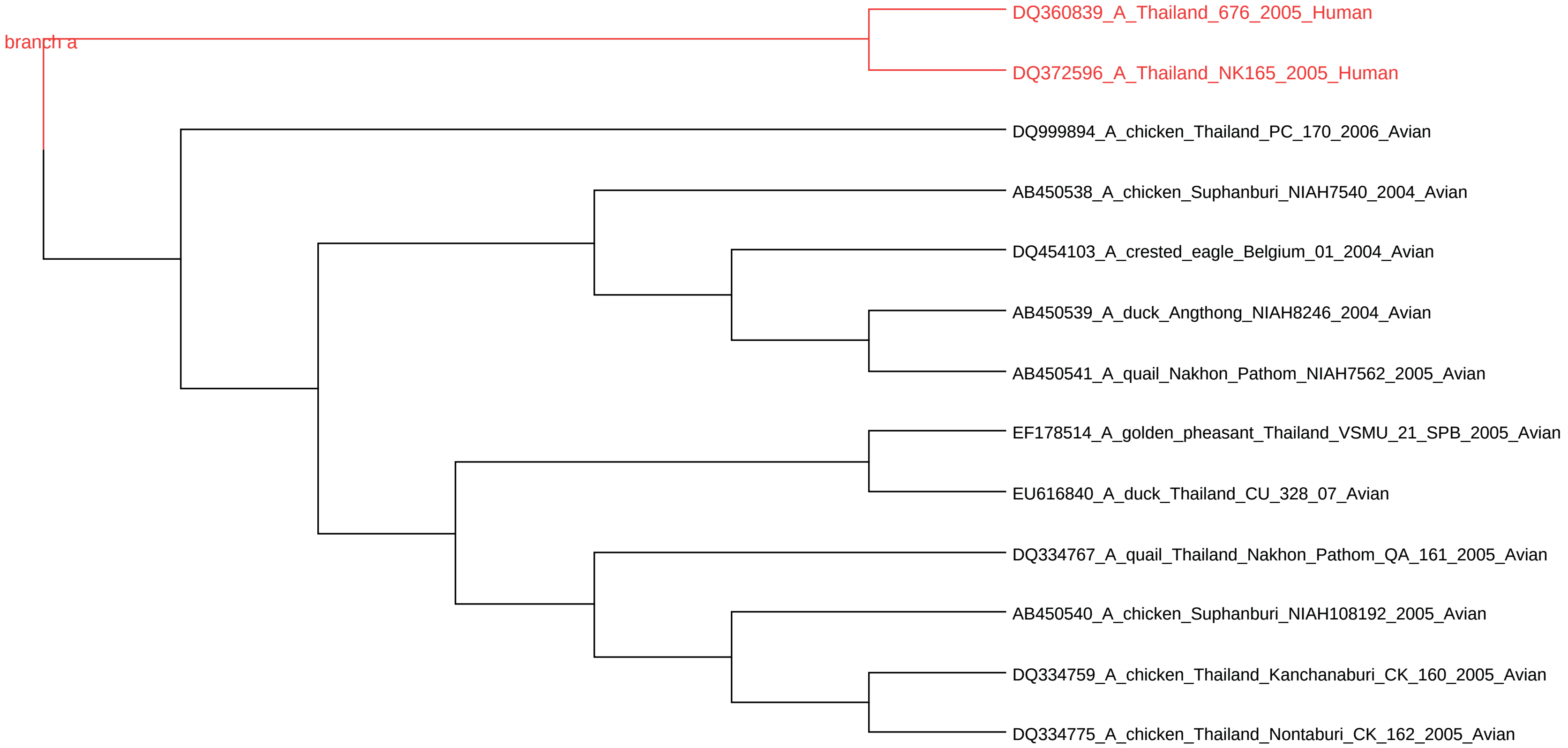

branch a
DQ360839_A_Thailand_676_2005_Human
DQ372596_A_Thailand_NK165_2005_Human
DQ999894_A_chicken_Thailand_PC_170_2006_Avian
AB450538_A_chicken_Suphanburi_NIAH7540_2004_Avian
DQ454103_A_crested_eagle_Belgium_01_2004_Avian
AB450539_A_duck_Angthong_NIAH8246_2004_Avian
AB450541_A_quail_Nakhon_Pathom_NIAH7562_2005_Avian
EF178514_A_golden_pheasant_Thailand_VSMU_21_SPB_2005_Avian
EU616840_A_duck_Thailand_CU_328_07_Avian
DQ334767_A_quail_Thailand_Nakhon_Pathom_QA_161_2005_Avian
AB450540_A_chicken_Suphanburi_NIAH108192_2005_Avian
DQ334759_A_chicken_Thailand_Kanchanaburi_CK_160_2005_Avian
DQ334775_A_chicken_Thailand_Nontaburi_CK_162_2005_Avian

PA-Group71

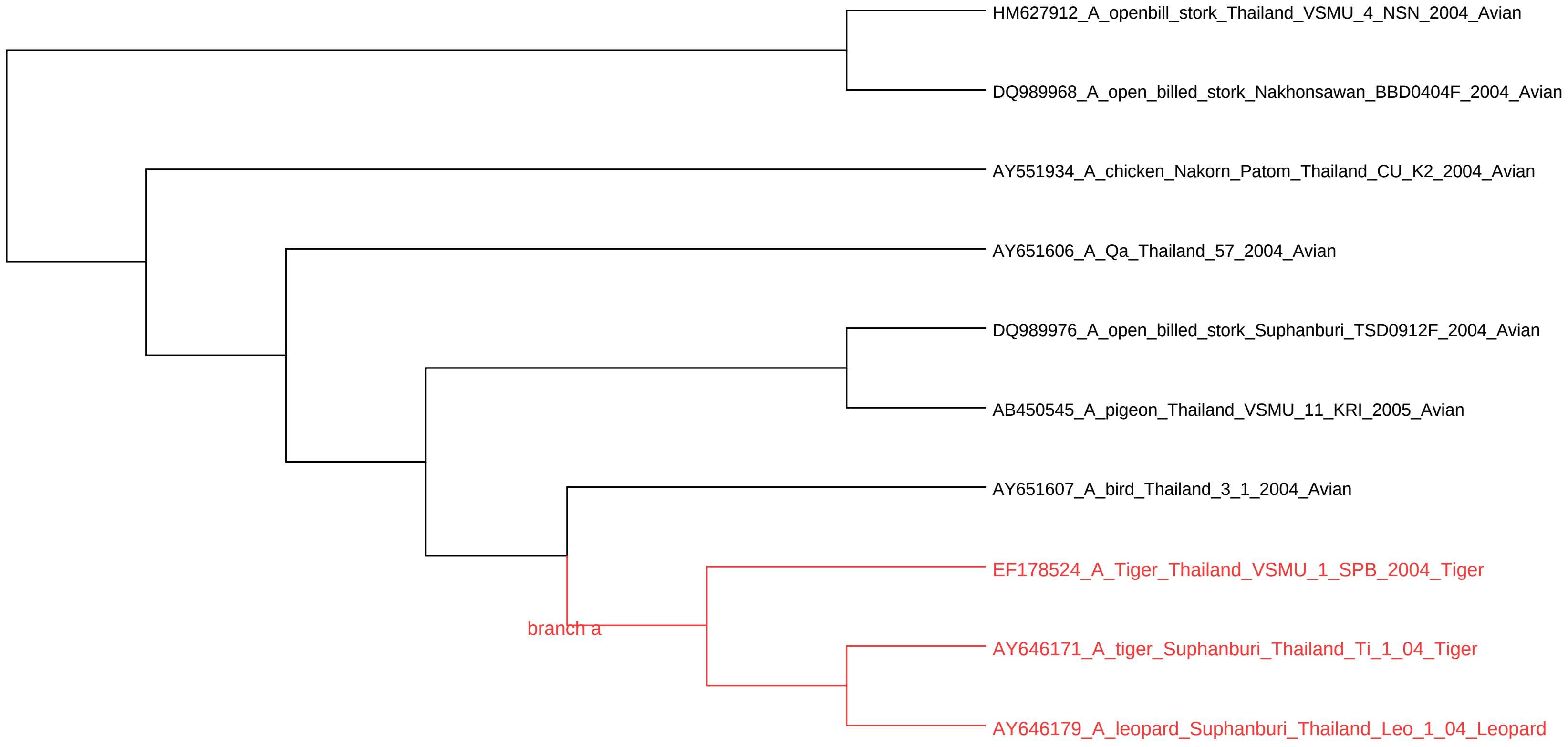
HM627912_A_openbill_stork_Thailand_VSMU_4_NSN_2004_Avian
DQ989968_A_open_billed_stork_Nakhonsawan_BBD0404F_2004_Avian
AY551934_A_chicken_Nakorn_Patom_Thailand_CU_K2_2004_Avian
AY651606_A_Qa_Thailand_57_2004_Avian
DQ989976_A_open_billed_stork_Suphanburi_TSD0912F_2004_Avian
AB450545_A_pigeon_Thailand_VSMU_11_KRI_2005_Avian
AY651607_A_bird_Thailand_3_1_2004_Avian
EF178524_A_Tiger_Thailand_VSMU_1_SPB_2004_Tiger
branch a
AY646171_A_tiger_Suphanburi_Thailand_Ti_1_04_Tiger
AY646179_A_leopard_Suphanburi_Thailand_Leo_1_04_Leopard

PA-Group72

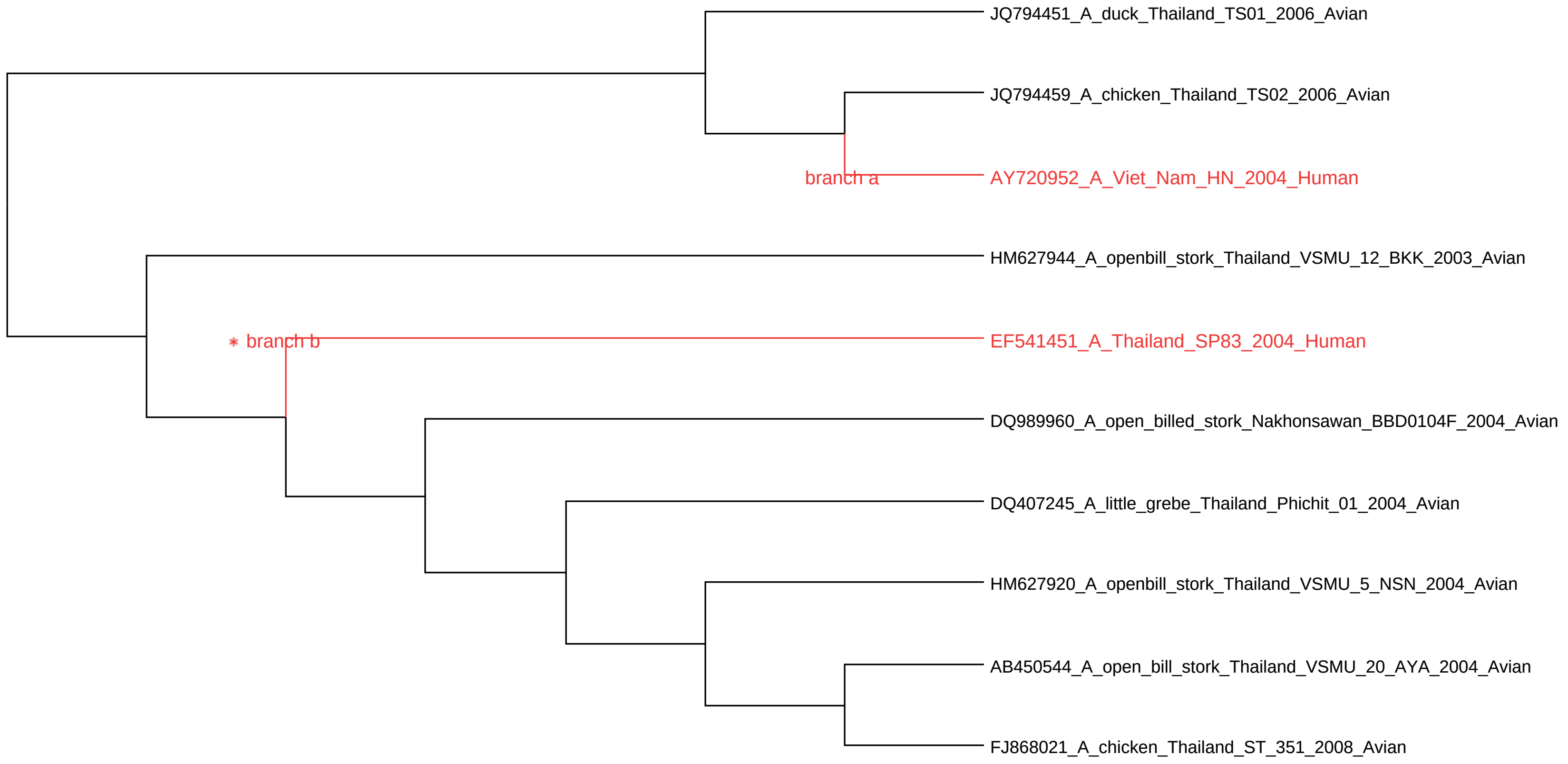
JQ794451_A_duck_Thailand_TS01_2006_Avian
JQ794459_A_chicken_Thailand_TS02_2006_Avian
branch a
AY720952_A_Viet_Nam_HN_2004_Human
HM627944_A_openbill_stork_Thailand_VSMU_12_BKK_2003_Avian
* branch b
EF541451_A_Thailand_SP83_2004_Human
DQ989960_A_open_billed_stork_Nakhonsawan_BBD0104F_2004_Avian
DQ407245_A_little_grebe_Thailand_Phichit_01_2004_Avian
HM627920_A_openbill_stork_Thailand_VSMU_5_NSN_2004_Avian
AB450544_A_open_bill_stork_Thailand_VSMU_20_AYA_2004_Avian
FJ868021_A_chicken_Thailand_ST_351_2008_Avian

PA-Group73

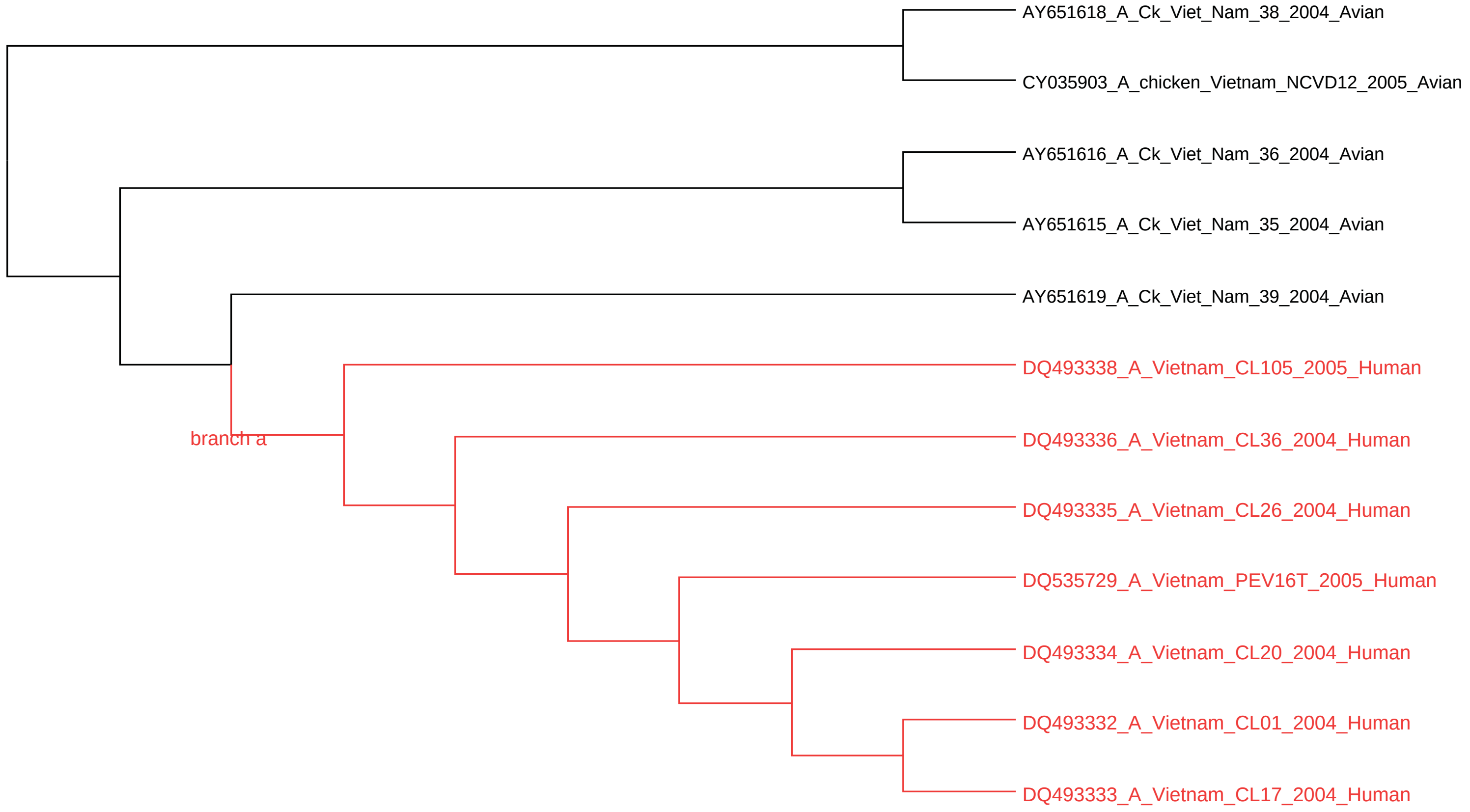
AY651618_A_Ck_Viet_Nam_38_2004_Avian
CY035903_A_chicken_Vietnam_NCVD12_2005_Avian
AY651616_A_Ck_Viet_Nam_36_2004_Avian
AY651615_A_Ck_Viet_Nam_35_2004_Avian
AY651619_A_Ck_Viet_Nam_39_2004_Avian
DQ493338_A_Vietnam_CL105_2005_Human
branch a
DQ493336_A_Vietnam_CL36_2004_Human
DQ493335_A_Vietnam_CL26_2004_Human
DQ535729_A_Vietnam_PEV16T_2005_Human
DQ493334_A_Vietnam_CL20_2004_Human
DQ493332_A_Vietnam_CL01_2004_Human
DQ493333_A_Vietnam_CL17_2004_Human

PA-Group74

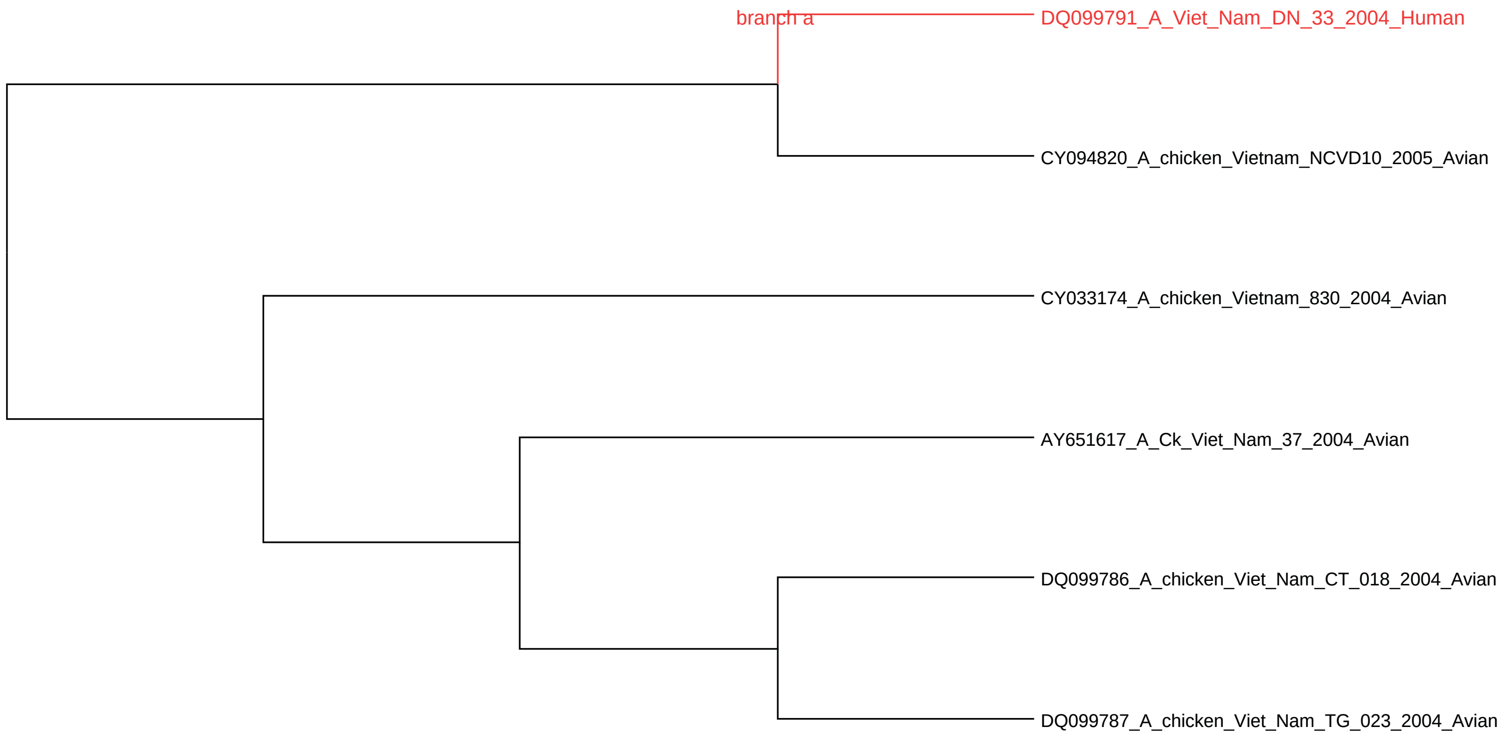
branch a
DQ099791_A_Viet_Nam_DN_33_2004_Human
CY094820_A_chicken_Vietnam_NCVD10_2005_Avian
CY033174_A_chicken_Vietnam_830_2004_Avian
AY651617_A_Ck_Viet_Nam_37_2004_Avian
DQ099786_A_chicken_Viet_Nam_CT_018_2004_Avian
DQ099787_A_chicken_Viet_Nam_TG_023_2004_Avian

PA-Group75

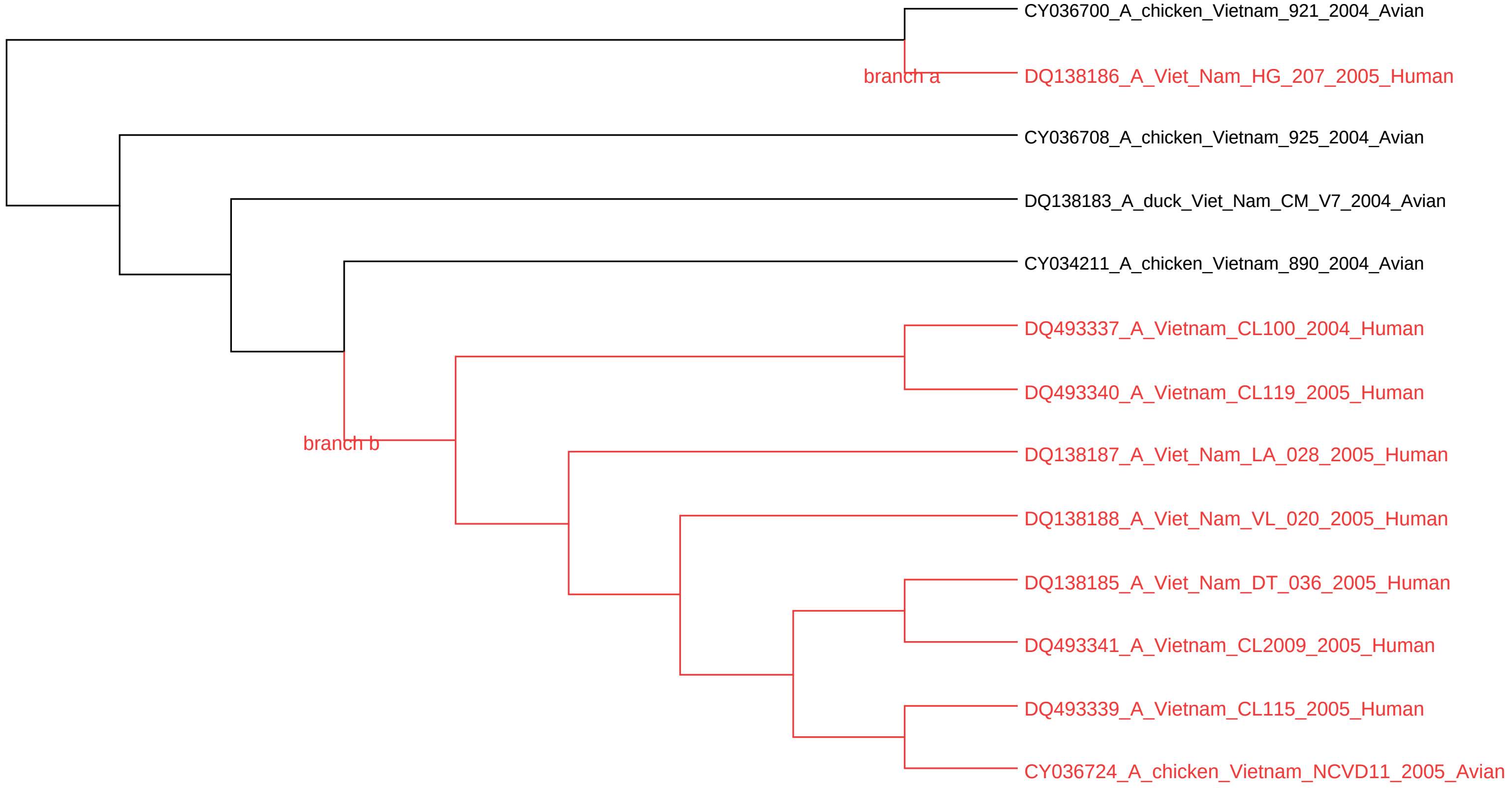
CY036700_A_chicken_Vietnam_921_2004_Avian
branch a
DQ138186_A_Viet_Nam_HG_207_2005_Human
CY036708_A_chicken_Vietnam_925_2004_Avian
DQ138183_A_duck_Viet_Nam_CM_V7_2004_Avian
CY034211_A_chicken_Vietnam_890_2004_Avian
DQ493337_A_Vietnam_CL100_2004_Human
DQ493340_A_Vietnam_CL119_2005_Human
branch b
DQ138187_A_Viet_Nam_LA_028_2005_Human
DQ138188_A_Viet_Nam_VL_020_2005_Human
DQ138185_A_Viet_Nam_DT_036_2005_Human
DQ493341_A_Vietnam_CL2009_2005_Human
DQ493339_A_Vietnam_CL115_2005_Human
CY036724_A_chicken_Vietnam_NCVD11_2005_Avian

PA-Group76

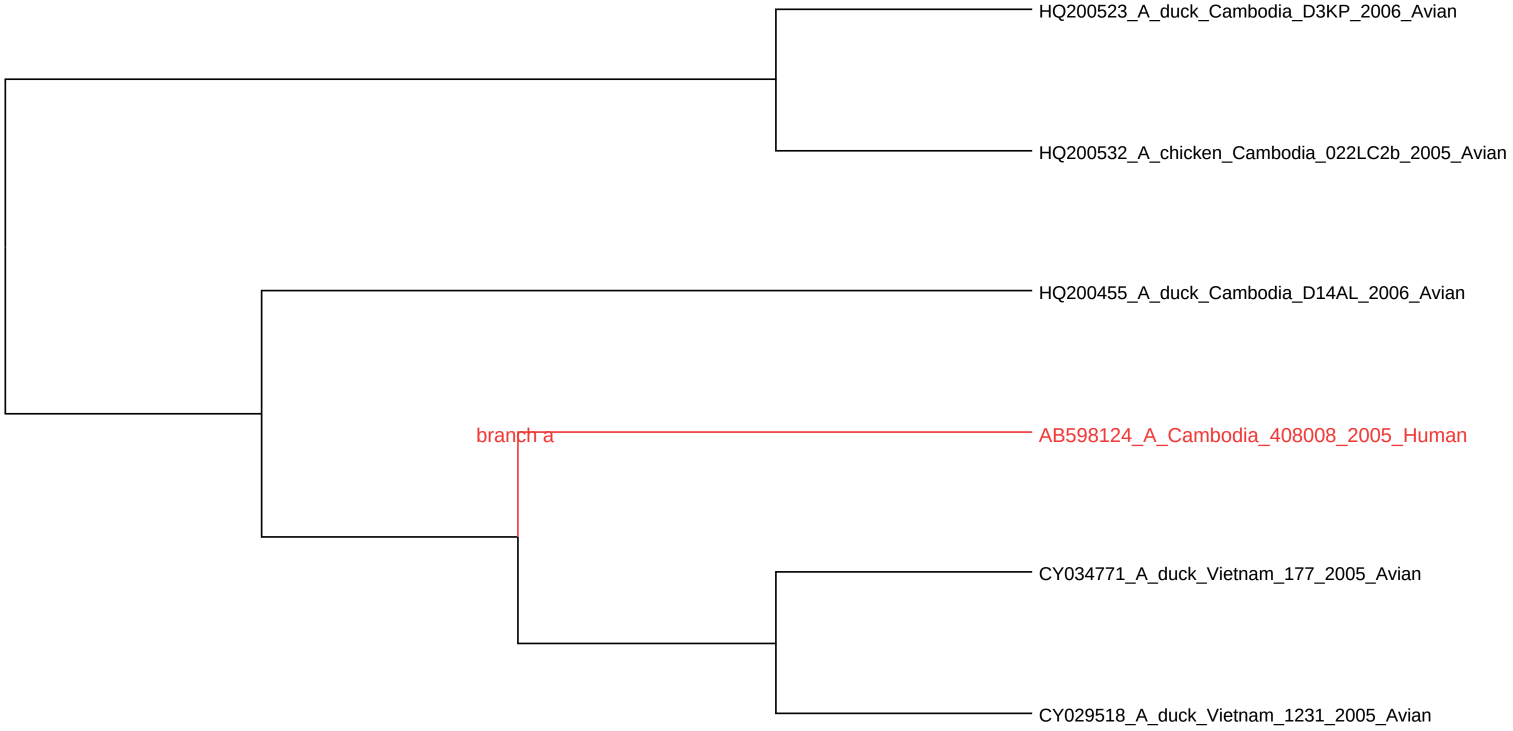

HQ200523_A_duck_Cambodia_D3KP_2006_Avian
HQ200532_A_chicken_Cambodia_022LC2b_2005_Avian
HQ200455_A_duck_Cambodia_D14AL_2006_Avian
branch a
AB598124_A_Cambodia_408008_2005_Human
CY034771_A_duck_Vietnam_177_2005_Avian
CY029518_A_duck_Vietnam_1231_2005_Avian

PA-Group77

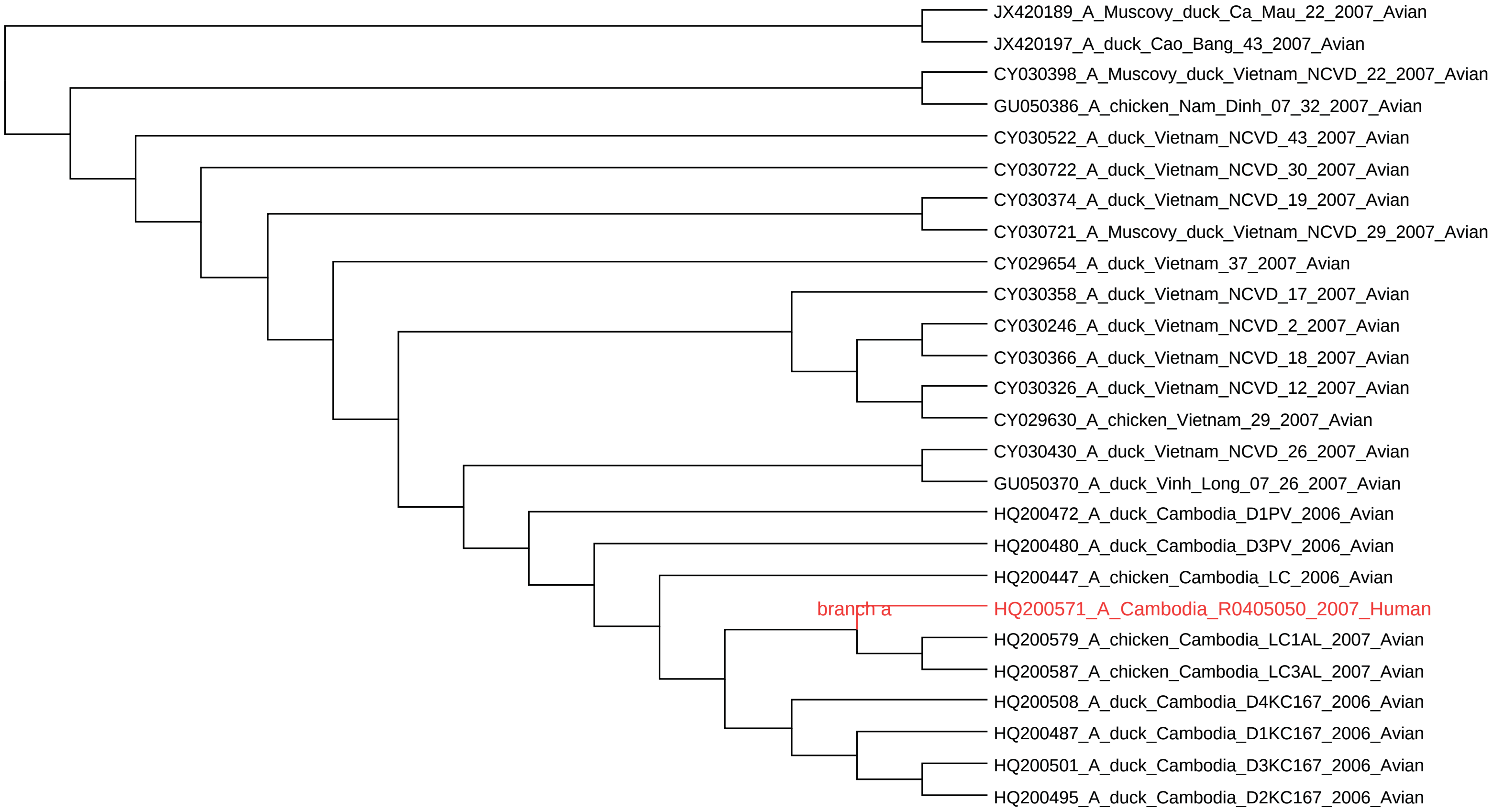
JX420189_A_Muscovy_duck_Ca_Mau_22_2007_Avian
JX420197_A_duck_Cao_Bang_43_2007_Avian
CY030398_A_Muscovy_duck_Vietnam_NCVD_22_2007_Avian
GU050386_A_chicken_Nam_Dinh_07_32_2007_Avian
CY030522_A_duck_Vietnam_NCVD_43_2007_Avian
CY030722_A_duck_Vietnam_NCVD_30_2007_Avian
CY030374_A_duck_Vietnam_NCVD_19_2007_Avian
CY030721_A_Muscovy_duck_Vietnam_NCVD_29_2007_Avian
CY029654_A_duck_Vietnam_37_2007_Avian
CY030358_A_duck_Vietnam_NCVD_17_2007_Avian
CY030246_A_duck_Vietnam_NCVD_2_2007_Avian
CY030366_A_duck_Vietnam_NCVD_18_2007_Avian
CY030326_A_duck_Vietnam_NCVD_12_2007_Avian
CY029630_A_chicken_Vietnam_29_2007_Avian
CY030430_A_duck_Vietnam_NCVD_26_2007_Avian
GU050370_A_duck_Vinh_Long_07_26_2007_Avian
HQ200472_A_duck_Cambodia_D1PV_2006_Avian
HQ200480_A_duck_Cambodia_D3PV_2006_Avian
HQ200447_A_chicken_Cambodia_LC_2006_Avian
branch a
HQ200571_A_Cambodia_R0405050_2007_Human
HQ200579_A_chicken_Cambodia_LC1AL_2007_Avian
HQ200587_A_chicken_Cambodia_LC3AL_2007_Avian
HQ200508_A_duck_Cambodia_D4KC167_2006_Avian
HQ200487_A_duck_Cambodia_D1KC167_2006_Avian
HQ200501_A_duck_Cambodia_D3KC167_2006_Avian
HQ200495_A_duck_Cambodia_D2KC167_2006_Avian

PA-Group78

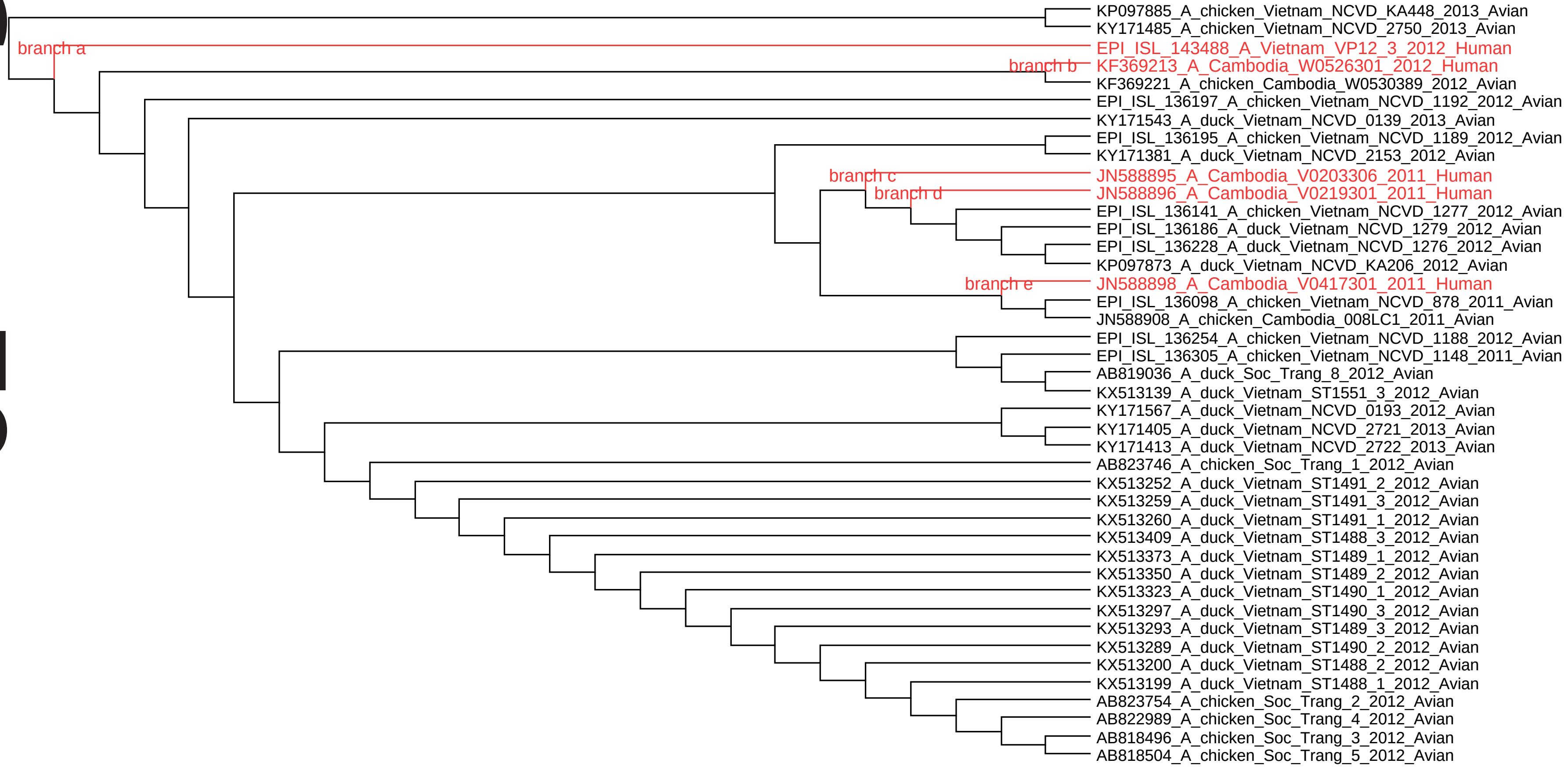

branch a
branch b
branch c
branch d
branch e
KP097885_A_chicken_Vietnam_NCVD_KA448_2013_Avian
KY171485_A_chicken_Vietnam_NCVD_2750_2013_Avian
EPI_ISL_143488_A_Vietnam_VP12_3_2012_Human
KF369213_A_Cambodia_W0526301_2012_Human
KF369221_A_chicken_Cambodia_W0530389_2012_Avian
EPI_ISL_136197_A_chicken_Vietnam_NCVD_1192_2012_Avian
KY171543_A_duck_Vietnam_NCVD_0139_2013_Avian
EPI_ISL_136195_A_chicken_Vietnam_NCVD_1189_2012_Avian
KY171381_A_duck_Vietnam_NCVD_2153_2012_Avian
JN588895_A_Cambodia_V0203306_2011_Human
JN588896_A_Cambodia_V0219301_2011_Human
EPI_ISL_136141_A_chicken_Vietnam_NCVD_1277_2012_Avian
EPI_ISL_136186_A_duck_Vietnam_NCVD_1279_2012_Avian
EPI_ISL_136228_A_duck_Vietnam_NCVD_1276_2012_Avian
KP097873_A_duck_Vietnam_NCVD_KA206_2012_Avian
JN588898_A_Cambodia_V0417301_2011_Human
EPI_ISL_136098_A_chicken_Vietnam_NCVD_878_2011_Avian
JN588908_A_chicken_Cambodia_008LC1_2011_Avian
EPI_ISL_136254_A_chicken_Vietnam_NCVD_1188_2012_Avian
EPI_ISL_136305_A_chicken_Vietnam_NCVD_1148_2011_Avian
AB819036_A_duck_Soc_Trang_8_2012_Avian
KX513139_A_duck_Vietnam_ST1551_3_2012_Avian
KY171567_A_duck_Vietnam_NCVD_0193_2012_Avian
KY171405_A_duck_Vietnam_NCVD_2721_2013_Avian
KY171413_A_duck_Vietnam_NCVD_2722_2013_Avian
AB823746_A_chicken_Soc_Trang_1_2012_Avian
KX513252_A_duck_Vietnam_ST1491_2_2012_Avian
KX513259_A_duck_Vietnam_ST1491_3_2012_Avian
KX513260_A_duck_Vietnam_ST1491_1_2012_Avian
KX513409_A_duck_Vietnam_ST1488_3_2012_Avian
KX513373_A_duck_Vietnam_ST1489_1_2012_Avian
KX513350_A_duck_Vietnam_ST1489_2_2012_Avian
KX513323_A_duck_Vietnam_ST1490_1_2012_Avian
KX513297_A_duck_Vietnam_ST1490_3_2012_Avian
KX513293_A_duck_Vietnam_ST1489_3_2012_Avian
KX513289_A_duck_Vietnam_ST1490_2_2012_Avian
KX513200_A_duck_Vietnam_ST1488_2_2012_Avian
KX513199_A_duck_Vietnam_ST1488_1_2012_Avian
AB823754_A_chicken_Soc_Trang_2_2012_Avian
AB822989_A_chicken_Soc_Trang_4_2012_Avian
AB818496_A_chicken_Soc_Trang_3_2012_Avian
AB818504_A_chicken_Soc_Trang_5_2012_Avian

PA-Group79

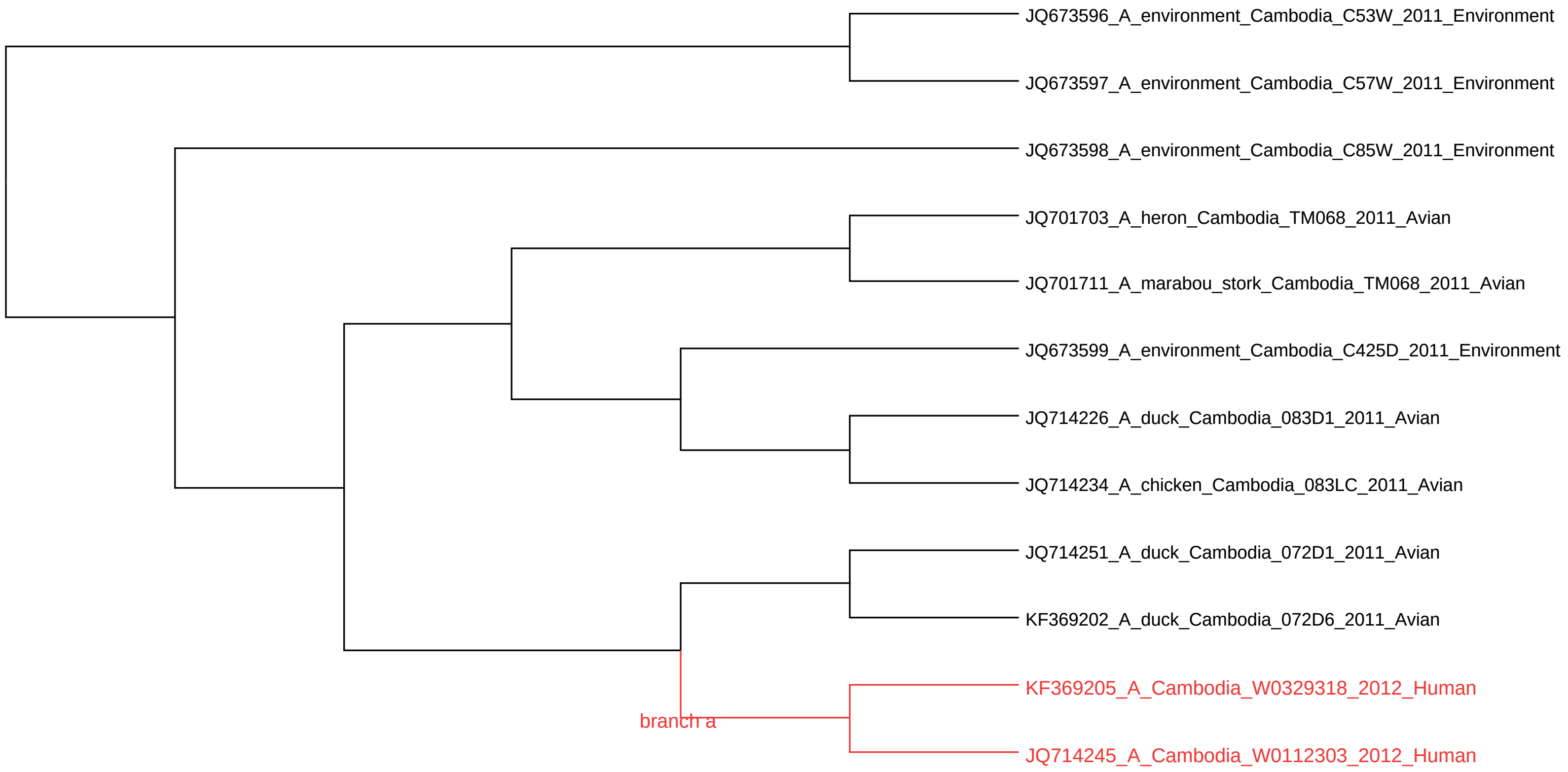

JQ673596_A_environment_Cambodia_C53W_2011_Environment
JQ673597_A_environment_Cambodia_C57W_2011_Environment
JQ673598_A_environment_Cambodia_C85W_2011_Environment
JQ701703_A_heron_Cambodia_TM068_2011_Avian
JQ701711_A_marabou_stork_Cambodia_TM068_2011_Avian
JQ673599_A_environment_Cambodia_C425D_2011_Environment
JQ714226_A_duck_Cambodia_083D1_2011_Avian
JQ714234_A_chicken_Cambodia_083LC_2011_Avian
JQ714251_A_duck_Cambodia_072D1_2011_Avian
KF369202_A_duck_Cambodia_072D6_2011_Avian
KF369205_A_Cambodia_W0329318_2012_Human
branch a
JQ714245_A_Cambodia_W0112303_2012_Human

PA-Group80

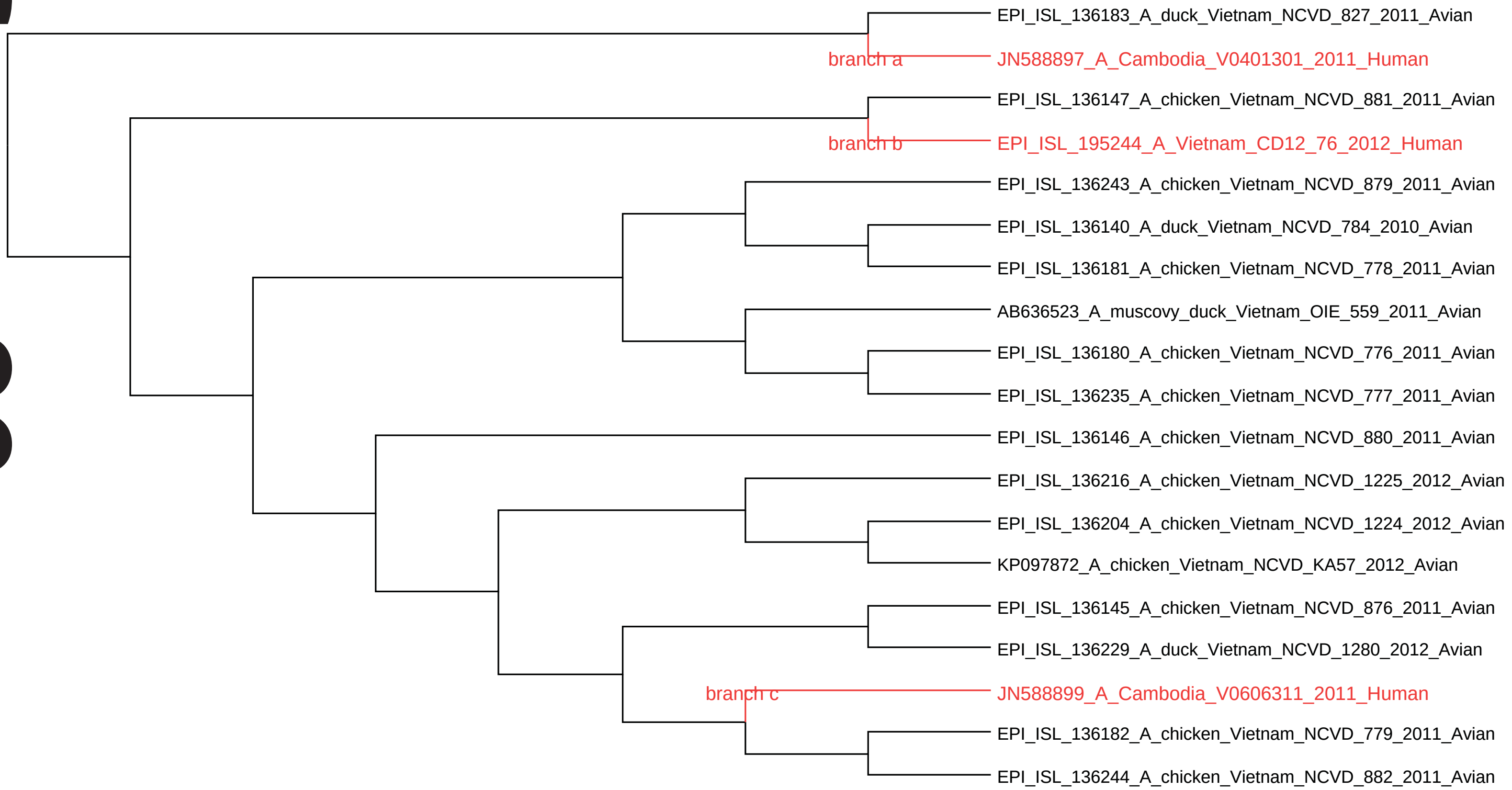
EPI_ISL_136183_A_duck_Vietnam_NCVD_827_2011_Avian
branch a
JN588897_A_Cambodia_V0401301_2011_Human
EPI_ISL_136147_A_chicken_Vietnam_NCVD_881_2011_Avian
branch b
EPI_ISL_195244_A_Vietnam_CD12_76_2012_Human
EPI_ISL_136243_A_chicken_Vietnam_NCVD_879_2011_Avian
EPI_ISL_136140_A_duck_Vietnam_NCVD_784_2010_Avian
EPI_ISL_136181_A_chicken_Vietnam_NCVD_778_2011_Avian
AB636523_A_muscovy_duck_Vietnam_OIE_559_2011_Avian
EPI_ISL_136180_A_chicken_Vietnam_NCVD_776_2011_Avian
EPI_ISL_136235_A_chicken_Vietnam_NCVD_777_2011_Avian
EPI_ISL_136146_A_chicken_Vietnam_NCVD_880_2011_Avian
EPI_ISL_136216_A_chicken_Vietnam_NCVD_1225_2012_Avian
EPI_ISL_136204_A_chicken_Vietnam_NCVD_1224_2012_Avian
KP097872_A_chicken_Vietnam_NCVD_KA57_2012_Avian
EPI_ISL_136145_A_chicken_Vietnam_NCVD_876_2011_Avian
EPI_ISL_136229_A_duck_Vietnam_NCVD_1280_2012_Avian
branch c
JN588899_A_Cambodia_V0606311_2011_Human
EPI_ISL_136182_A_chicken_Vietnam_NCVD_779_2011_Avian
EPI_ISL_136244_A_chicken_Vietnam_NCVD_882_2011_Avian
