# Supplementary material for: Adaptive Evolution of Human-Isolated H5Nx Avian Influenza A Viruses
Source: Front Microbiol. 2019 Jun 12;10:1328. doi: 10.3389/fmicb.2019.01328 (PMC6582624; doi:10.3389/fmicb.2019.01328)
Supplement: Supplementary file 9 [file Data_Sheet_9.PDF]

PB1-Group1

Supplementary Figure 9. 90 phylogenetic trees of PB1 used for the adaptive evolution analyses. Human strains are marked in red. Branches which have significant signals of positive selection are marked with *.

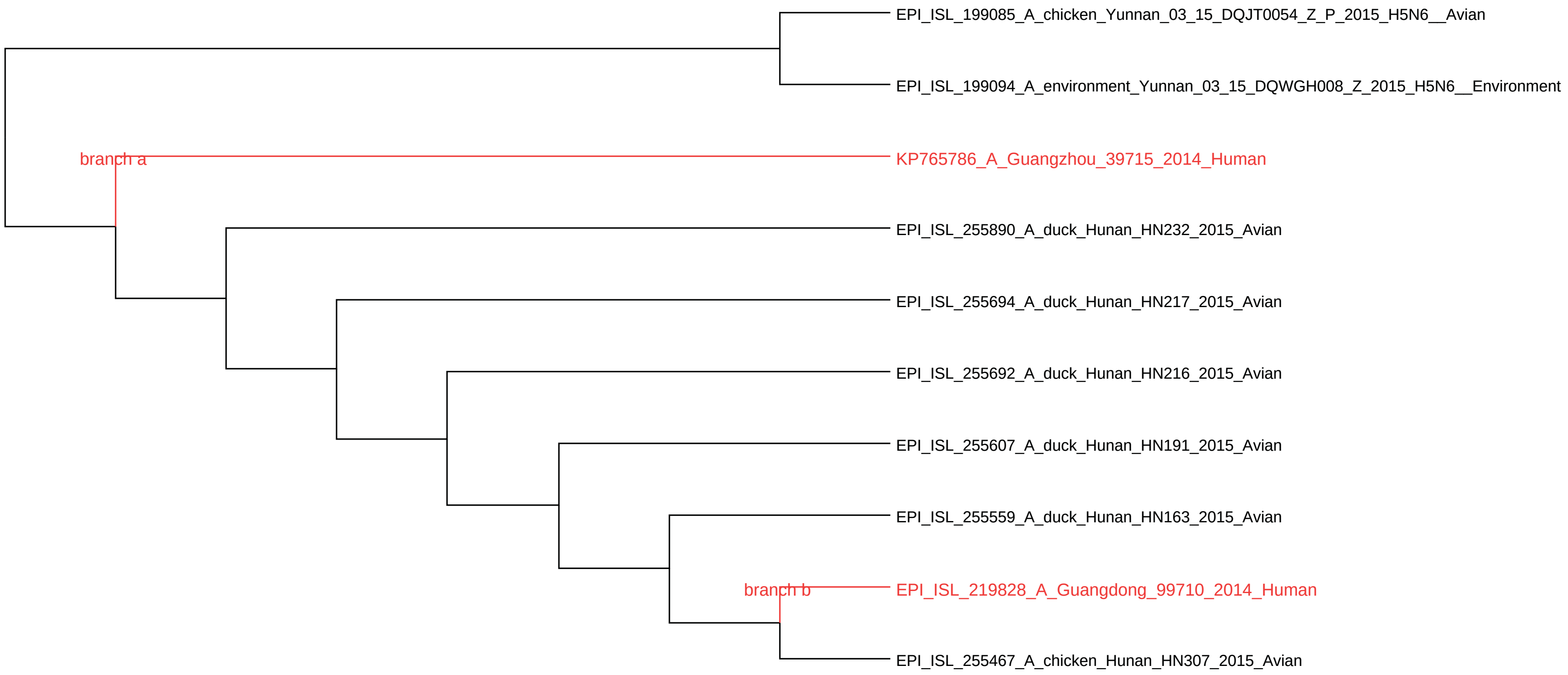

PB1-Group2

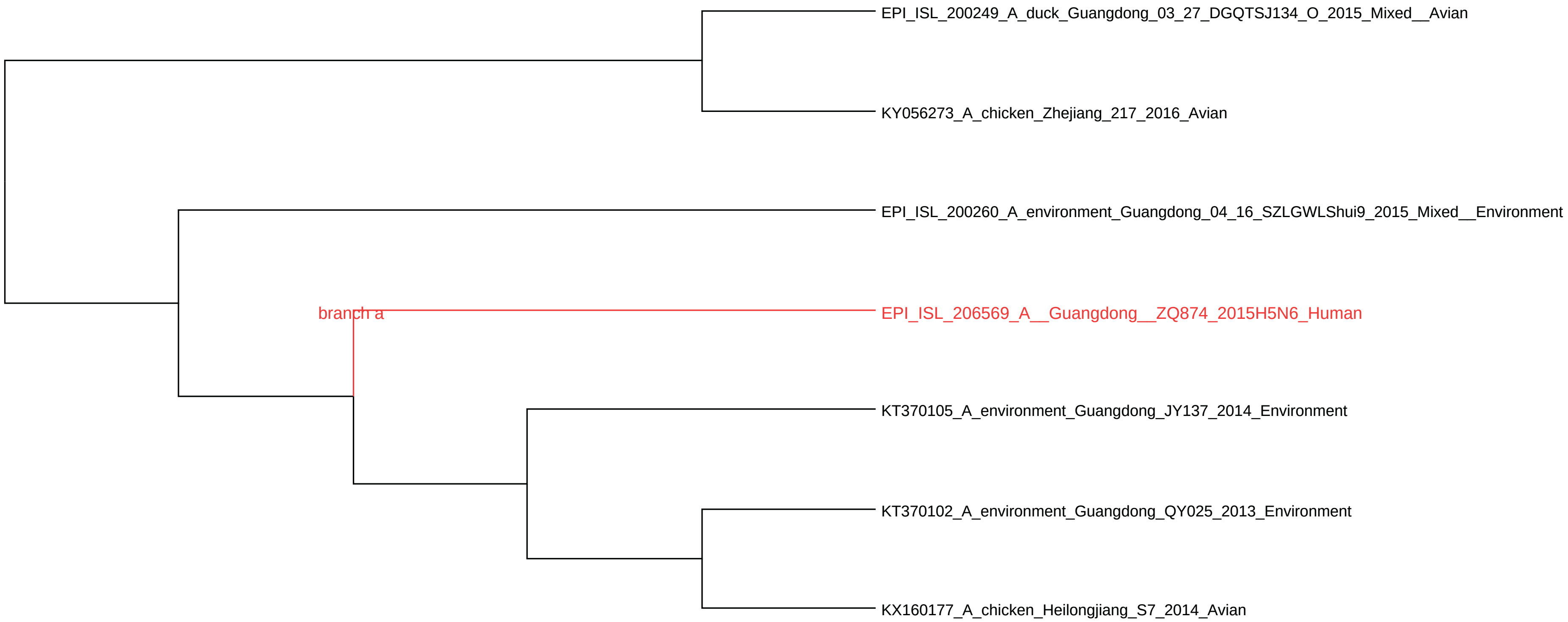
EPI_ISL_200249_A_duck_Guangdong_03_27_DGQTSJ134_O_2015_Mixed__Avian
KY056273_A_chicken_Zhejiang_217_2016_Avian
EPI_ISL_200260_A_environment_Guangdong_04_16_SZLGWLShui9_2015_Mixed__Environment
branch a
EPI_ISL_206569_A__Guangdong__ZQ874_2015H5N6_Human
KT370105_A_environment_Guangdong_JY137_2014_Environment
KT370102_A_environment_Guangdong_QY025_2013_Environment
KX160177_A_chicken_Heilongjiang_S7_2014_Avian

PB1-Group3

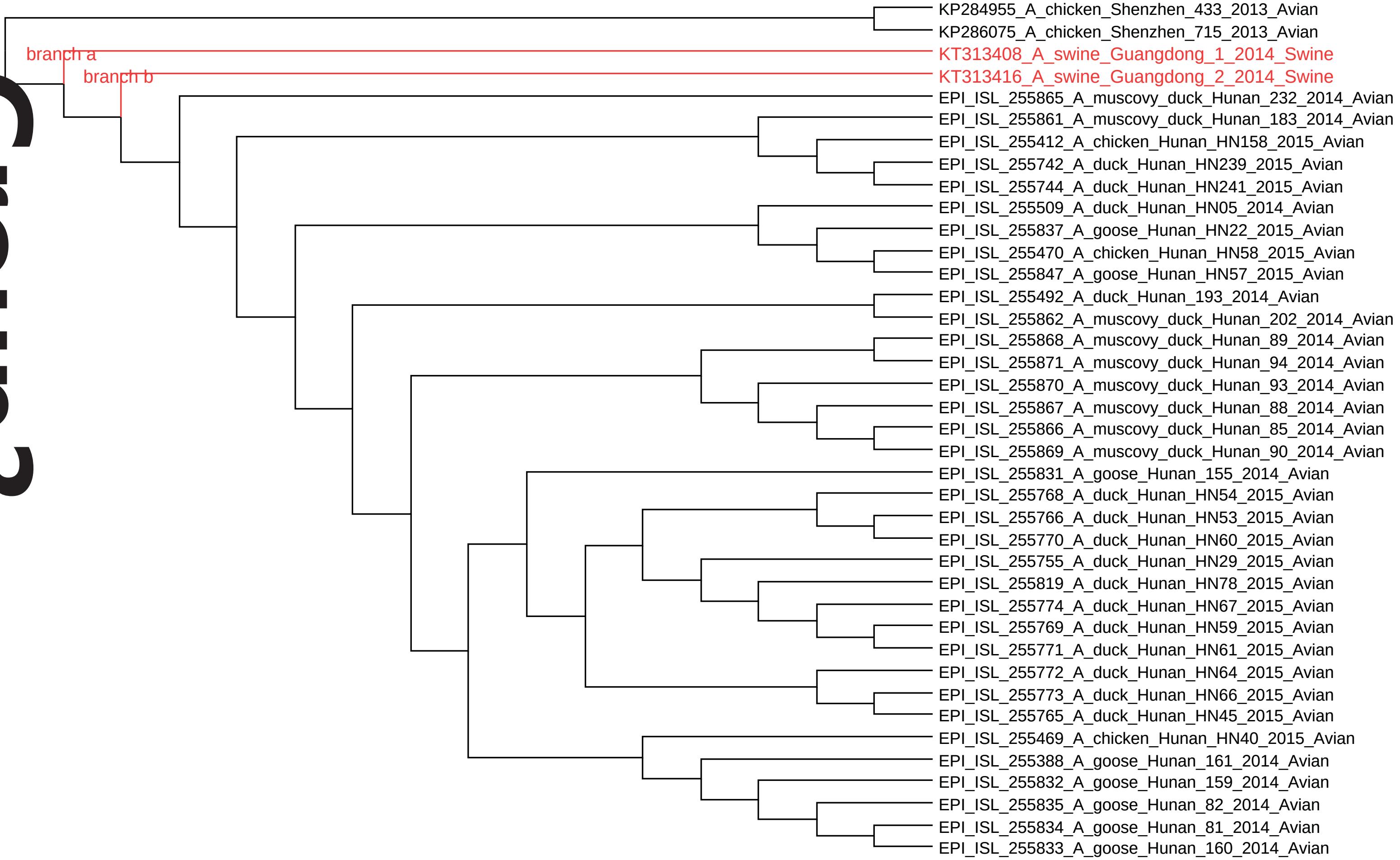
KP284955_A_chicken_Shenzhen_433_2013_Avian
KP286075_A_chicken_Shenzhen_715_2013_Avian
branch a
KT313408_A_swine_Guangdong_1_2014_Swine
branch b
KT313416_A_swine_Guangdong_2_2014_Swine
EPI_ISL_255865_A_muscovy_duck_Hunan_232_2014_Avian
EPI_ISL_255861_A_muscovy_duck_Hunan_183_2014_Avian
EPI_ISL_255412_A_chicken_Hunan_HN158_2015_Avian
EPI_ISL_255742_A_duck_Hunan_HN239_2015_Avian
EPI_ISL_255744_A_duck_Hunan_HN241_2015_Avian
EPI_ISL_255509_A_duck_Hunan_HN05_2014_Avian
EPI_ISL_255837_A_goose_Hunan_HN22_2015_Avian
EPI_ISL_255470_A_chicken_Hunan_HN58_2015_Avian
EPI_ISL_255847_A_goose_Hunan_HN57_2015_Avian
EPI_ISL_255492_A_duck_Hunan_193_2014_Avian
EPI_ISL_255862_A_muscovy_duck_Hunan_202_2014_Avian
EPI_ISL_255868_A_muscovy_duck_Hunan_89_2014_Avian
EPI_ISL_255871_A_muscovy_duck_Hunan_94_2014_Avian
EPI_ISL_255870_A_muscovy_duck_Hunan_93_2014_Avian
EPI_ISL_255867_A_muscovy_duck_Hunan_88_2014_Avian
EPI_ISL_255866_A_muscovy_duck_Hunan_85_2014_Avian
EPI_ISL_255869_A_muscovy_duck_Hunan_90_2014_Avian
EPI_ISL_255831_A_goose_Hunan_155_2014_Avian
EPI_ISL_255768_A_duck_Hunan_HN54_2015_Avian
EPI_ISL_255766_A_duck_Hunan_HN53_2015_Avian
EPI_ISL_255770_A_duck_Hunan_HN60_2015_Avian
EPI_ISL_255755_A_duck_Hunan_HN29_2015_Avian
EPI_ISL_255819_A_duck_Hunan_HN78_2015_Avian
EPI_ISL_255774_A_duck_Hunan_HN67_2015_Avian
EPI_ISL_255769_A_duck_Hunan_HN59_2015_Avian
EPI_ISL_255771_A_duck_Hunan_HN61_2015_Avian
EPI_ISL_255772_A_duck_Hunan_HN64_2015_Avian
EPI_ISL_255773_A_duck_Hunan_HN66_2015_Avian
EPI_ISL_255765_A_duck_Hunan_HN45_2015_Avian
EPI_ISL_255469_A_chicken_Hunan_HN40_2015_Avian
EPI_ISL_255388_A_goose_Hunan_161_2014_Avian
EPI_ISL_255832_A_goose_Hunan_159_2014_Avian
EPI_ISL_255835_A_goose_Hunan_82_2014_Avian
EPI_ISL_255834_A_goose_Hunan_81_2014_Avian
EPI_ISL_255833_A_goose_Hunan_160_2014_Avian

PB1-Group4

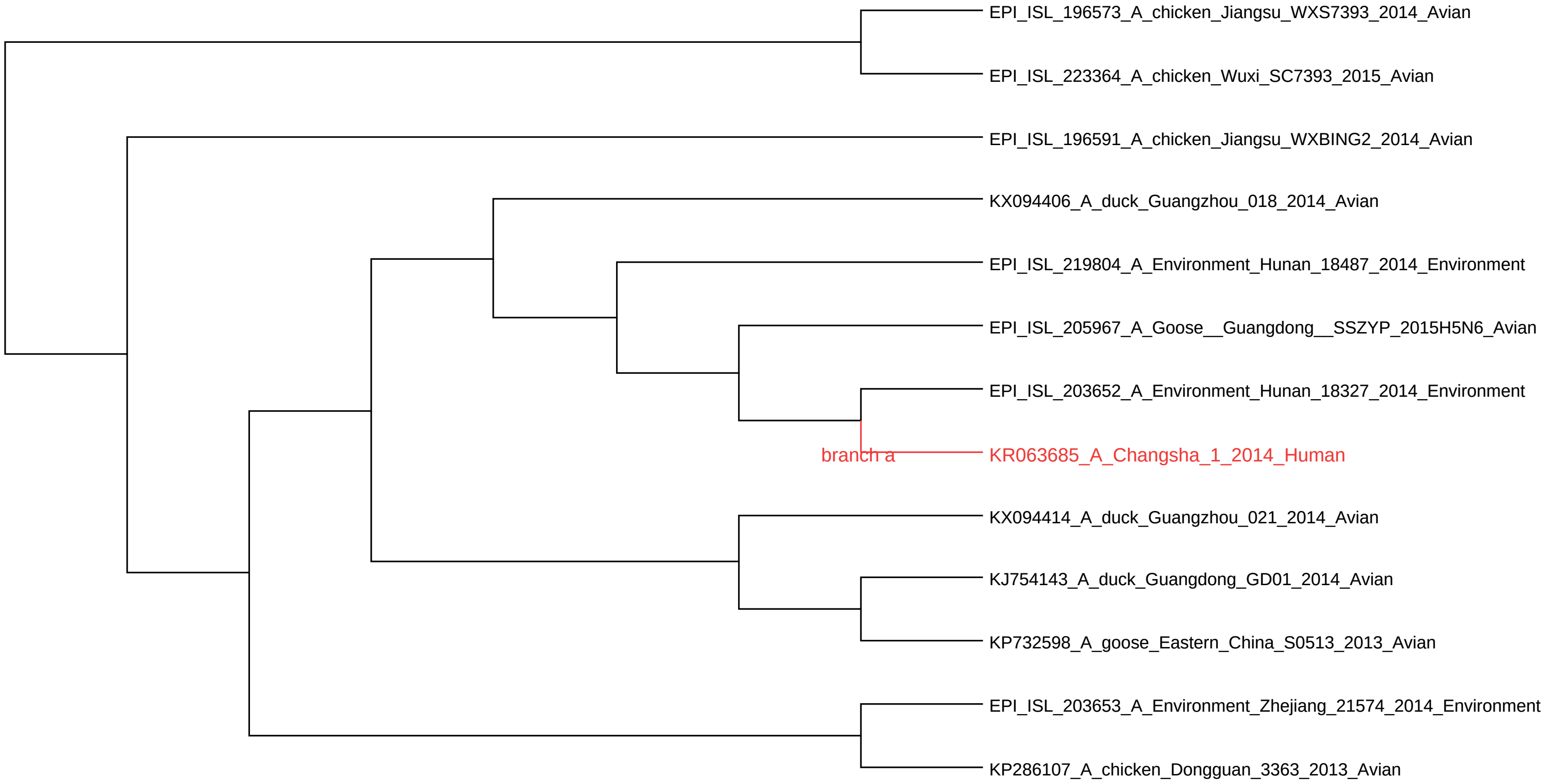
EPI_ISL_196573_A_chicken_Jiangsu_WXS7393_2014_Avian
EPI_ISL_223364_A_chicken_Wuxi_SC7393_2015_Avian
EPI_ISL_196591_A_chicken_Jiangsu_WXBING2_2014_Avian
KX094406_A_duck_Guangzhou_018_2014_Avian
EPI_ISL_219804_A_Environment_Hunan_18487_2014_Environment
EPI_ISL_205967_A_Goose__Guangdong__SSZYP_2015H5N6_Avian
EPI_ISL_203652_A_Environment_Hunan_18327_2014_Environment
branch a
KR063685_A_Changsha_1_2014_Human
KX094414_A_duck_Guangzhou_021_2014_Avian
KJ754143_A_duck_Guangdong_GD01_2014_Avian
KP732598_A_goose_Eastern_China_S0513_2013_Avian
EPI_ISL_203653_A_Environment_Zhejiang_21574_2014_Environment
KP286107_A_chicken_Dongguan_3363_2013_Avian

PB1-Group5

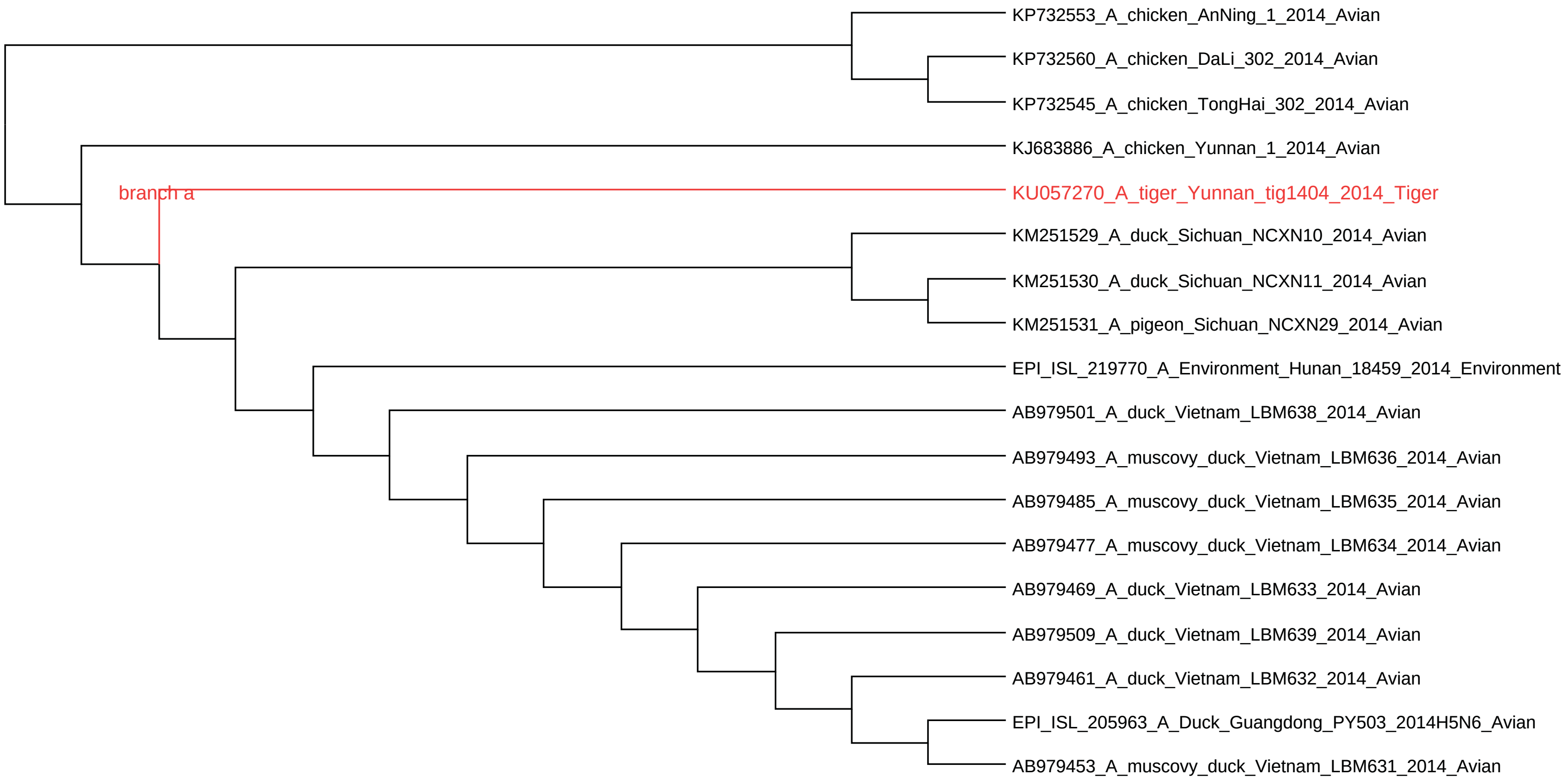
KP732553_A_chicken_AnNing_1_2014_Avian
KP732560_A_chicken_DaLi_302_2014_Avian
KP732545_A_chicken_TongHai_302_2014_Avian
KJ683886_A_chicken_Yunnan_1_2014_Avian
branch a
KU057270_A_tiger_Yunnan_tig1404_2014_Tiger
KM251529_A_duck_Sichuan_NCXN10_2014_Avian
KM251530_A_duck_Sichuan_NCXN11_2014_Avian
KM251531_A_pigeon_Sichuan_NCXN29_2014_Avian
EPI_ISL_219770_A_Environment_Hunan_18459_2014_Environment
AB979501_A_duck_Vietnam_LBM638_2014_Avian
AB979493_A_muscovy_duck_Vietnam_LBM636_2014_Avian
AB979485_A_muscovy_duck_Vietnam_LBM635_2014_Avian
AB979477_A_muscovy_duck_Vietnam_LBM634_2014_Avian
AB979469_A_duck_Vietnam_LBM633_2014_Avian
AB979509_A_duck_Vietnam_LBM639_2014_Avian
AB979461_A_duck_Vietnam_LBM632_2014_Avian
EPI_ISL_205963_A_Duck_Guangdong_PY503_2014H5N6_Avian
AB979453_A_muscovy_duck_Vietnam_LBM631_2014_Avian

PB1-Group6

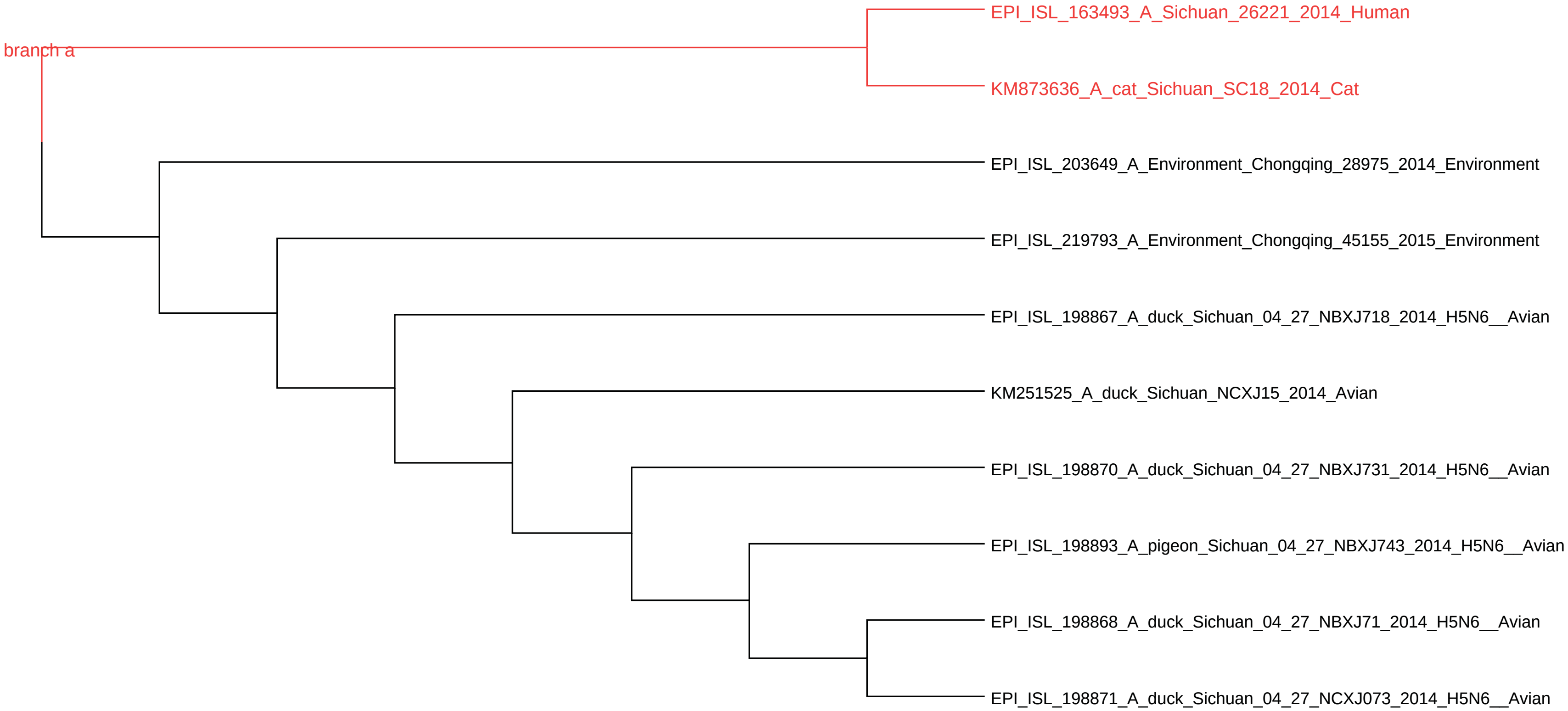
branch a
EPI_ISL_163493_A_Sichuan_26221_2014_Human
KM873636_A_cat_Sichuan_SC18_2014_Cat
EPI_ISL_203649_A_Environment_Chongqing_28975_2014_Environment
EPI_ISL_219793_A_Environment_Chongqing_45155_2015_Environment
EPI_ISL_198867_A_duck_Sichuan_04_27_NBXJ718_2014_H5N6__Avian
KM251525_A_duck_Sichuan_NCXJ15_2014_Avian
EPI_ISL_198870_A_duck_Sichuan_04_27_NBXJ731_2014_H5N6__Avian
EPI_ISL_198893_A_pigeon_Sichuan_04_27_NBXJ743_2014_H5N6__Avian
EPI_ISL_198868_A_duck_Sichuan_04_27_NBXJ71_2014_H5N6__Avian
EPI_ISL_198871_A_duck_Sichuan_04_27_NCXJ073_2014_H5N6__Avian

PB1-Group7

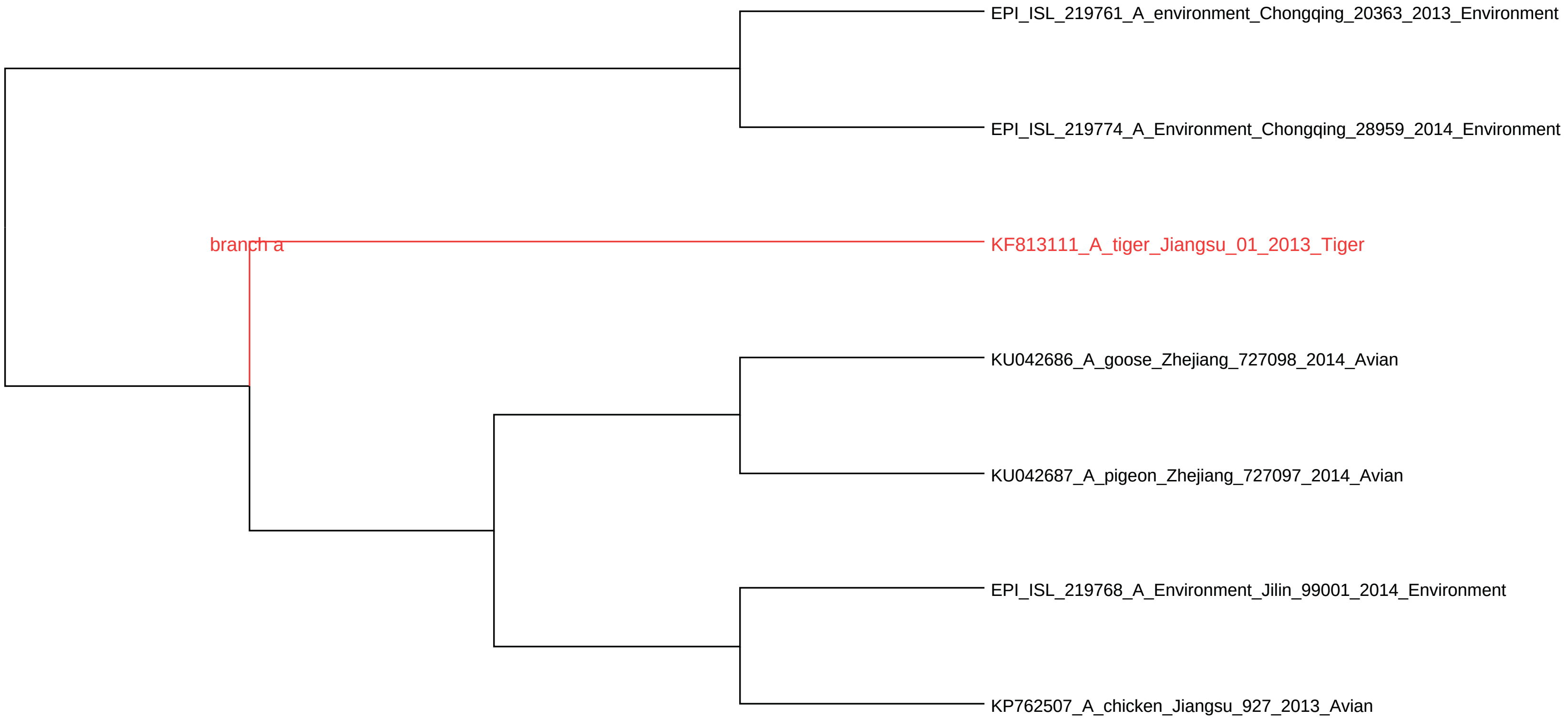
EPI_ISL_219761_A_environment_Chongqing_20363_2013_Environment
EPI_ISL_219774_A_Environment_Chongqing_28959_2014_Environment
branch a
KF813111_A_tiger_Jiangsu_01_2013_Tiger
KU042686_A_goose_Zhejiang_727098_2014_Avian
KU042687_A_pigeon_Zhejiang_727097_2014_Avian
EPI_ISL_219768_A_Environment_Jilin_99001_2014_Environment
KP762507_A_chicken_Jiangsu_927_2013_Avian

PB1-Group8

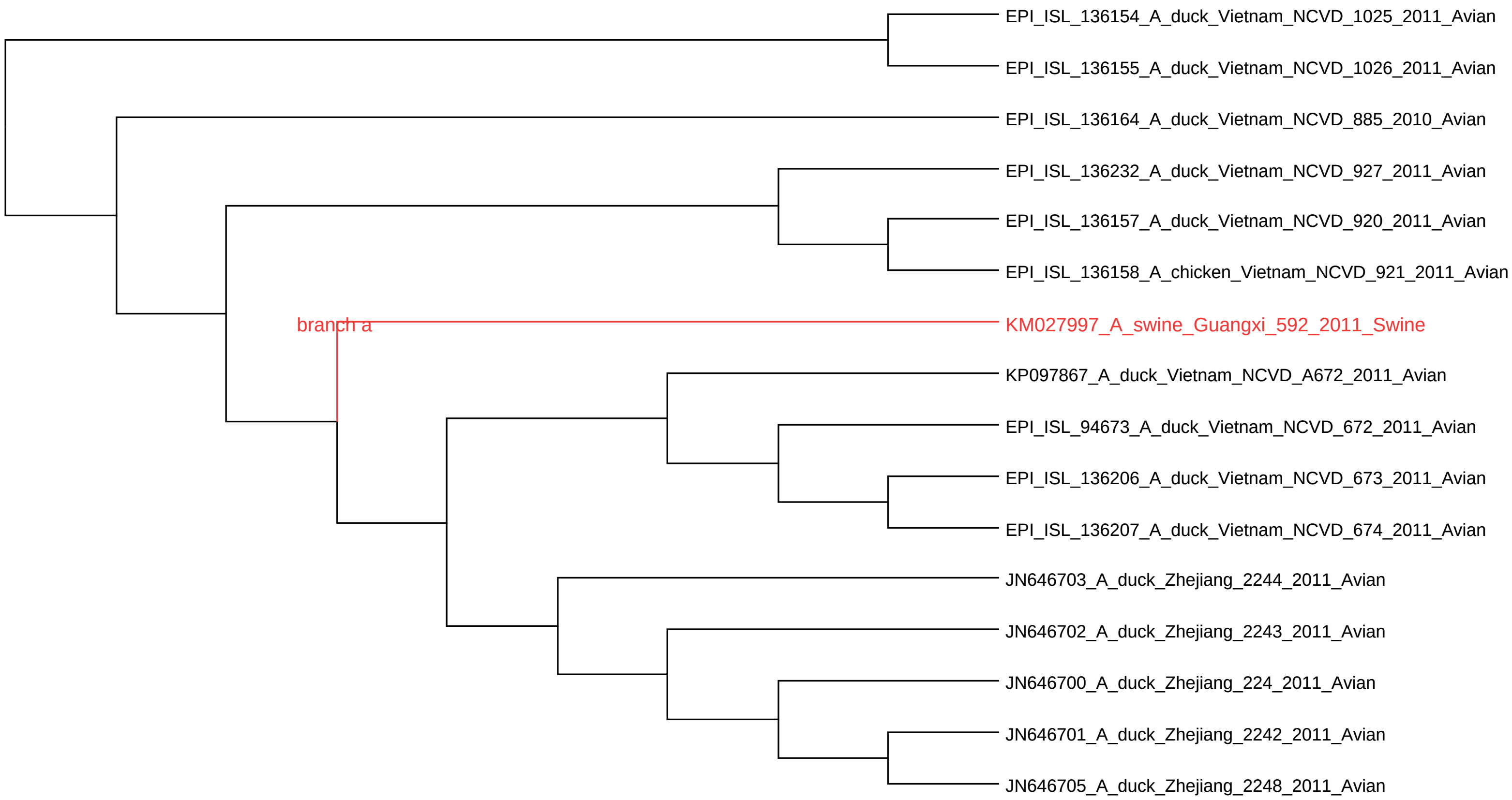
EPI_ISL_136154_A_duck_Vietnam_NCVD_1025_2011_Avian
EPI_ISL_136155_A_duck_Vietnam_NCVD_1026_2011_Avian
EPI_ISL_136164_A_duck_Vietnam_NCVD_885_2010_Avian
EPI_ISL_136232_A_duck_Vietnam_NCVD_927_2011_Avian
EPI_ISL_136157_A_duck_Vietnam_NCVD_920_2011_Avian
EPI_ISL_136158_A_chicken_Vietnam_NCVD_921_2011_Avian
branch a
KM027997_A_swine_Guangxi_592_2011_Swine
KP097867_A_duck_Vietnam_NCVD_A672_2011_Avian
EPI_ISL_94673_A_duck_Vietnam_NCVD_672_2011_Avian
EPI_ISL_136206_A_duck_Vietnam_NCVD_673_2011_Avian
EPI_ISL_136207_A_duck_Vietnam_NCVD_674_2011_Avian
JN646703_A_duck_Zhejiang_2244_2011_Avian
JN646702_A_duck_Zhejiang_2243_2011_Avian
JN646700_A_duck_Zhejiang_224_2011_Avian
JN646701_A_duck_Zhejiang_2242_2011_Avian
JN646705_A_duck_Zhejiang_2248_2011_Avian

PB1-Group9

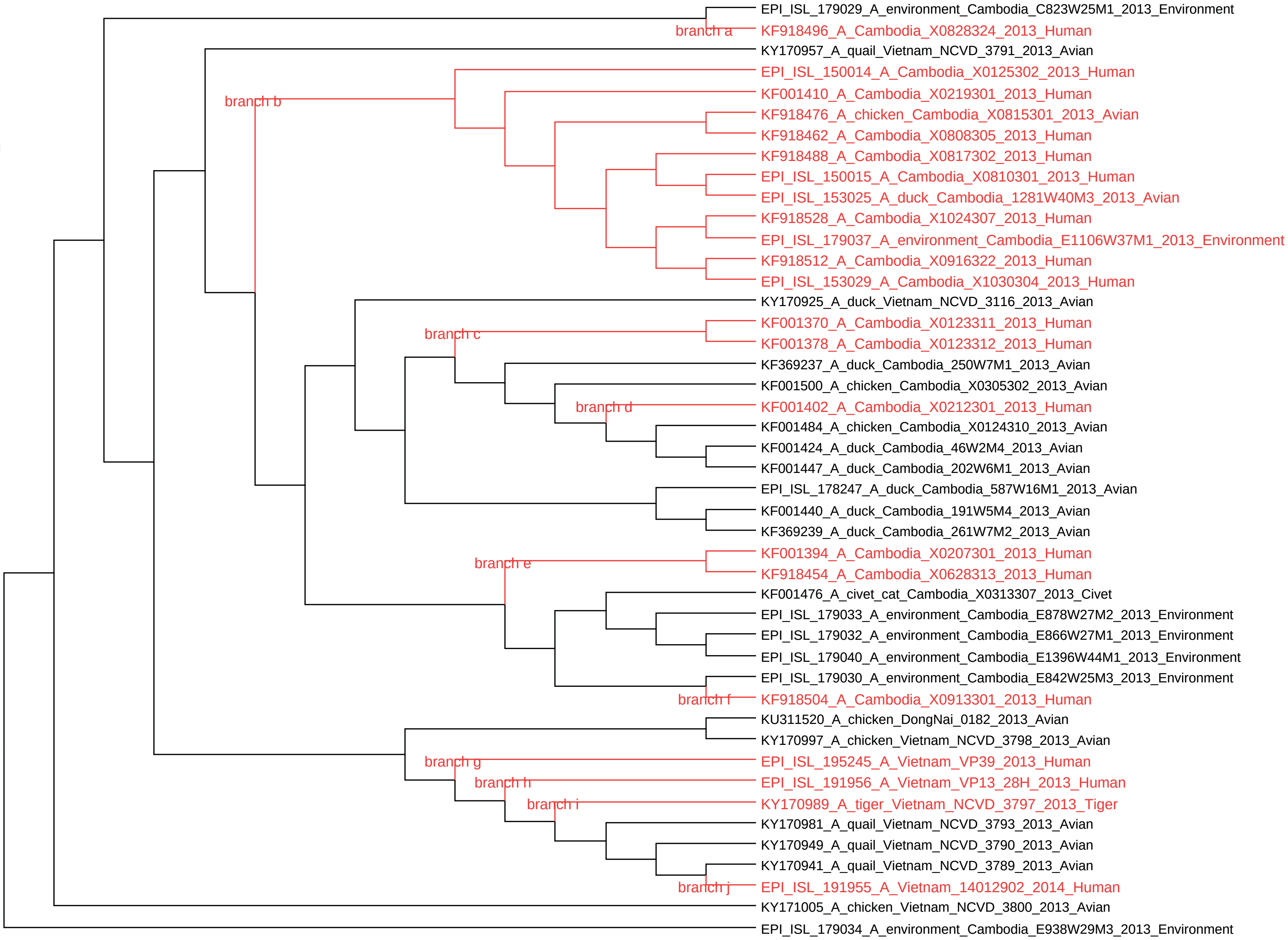

1-Group9

PB1-Group10

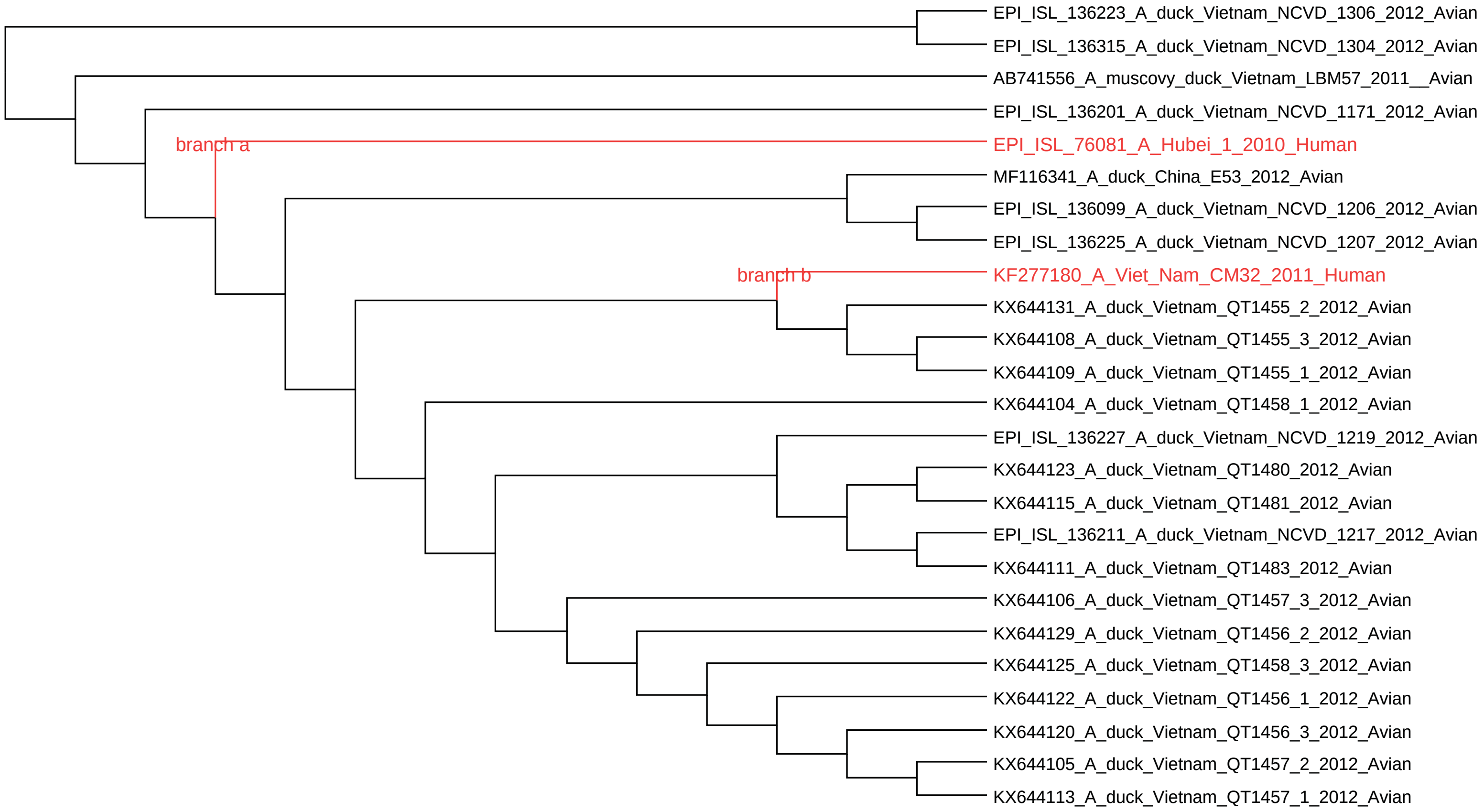
EPI_ISL_136223_A_duck_Vietnam_NCVD_1306_2012_Avian
EPI_ISL_136315_A_duck_Vietnam_NCVD_1304_2012_Avian
AB741556_A_muscovy_duck_Vietnam_LBM57_2011__Avian
EPI_ISL_136201_A_duck_Vietnam_NCVD_1171_2012_Avian
branch a
EPI_ISL_76081_A_Hubei_1_2010_Human
MF116341_A_duck_China_E53_2012_Avian
EPI_ISL_136099_A_duck_Vietnam_NCVD_1206_2012_Avian
EPI_ISL_136225_A_duck_Vietnam_NCVD_1207_2012_Avian
branch b
KF277180_A_Viet_Nam_CM32_2011_Human
KX644131_A_duck_Vietnam_QT1455_2_2012_Avian
KX644108_A_duck_Vietnam_QT1455_3_2012_Avian
KX644109_A_duck_Vietnam_QT1455_1_2012_Avian
KX644104_A_duck_Vietnam_QT1458_1_2012_Avian
EPI_ISL_136227_A_duck_Vietnam_NCVD_1219_2012_Avian
KX644123_A_duck_Vietnam_QT1480_2012_Avian
KX644115_A_duck_Vietnam_QT1481_2012_Avian
EPI_ISL_136211_A_duck_Vietnam_NCVD_1217_2012_Avian
KX644111_A_duck_Vietnam_QT1483_2012_Avian
KX644106_A_duck_Vietnam_QT1457_3_2012_Avian
KX644129_A_duck_Vietnam_QT1456_2_2012_Avian
KX644125_A_duck_Vietnam_QT1458_3_2012_Avian
KX644122_A_duck_Vietnam_QT1456_1_2012_Avian
KX644120_A_duck_Vietnam_QT1456_3_2012_Avian
KX644105_A_duck_Vietnam_QT1457_2_2012_Avian
KX644113_A_duck_Vietnam_QT1457_1_2012_Avian

PB1-Group11

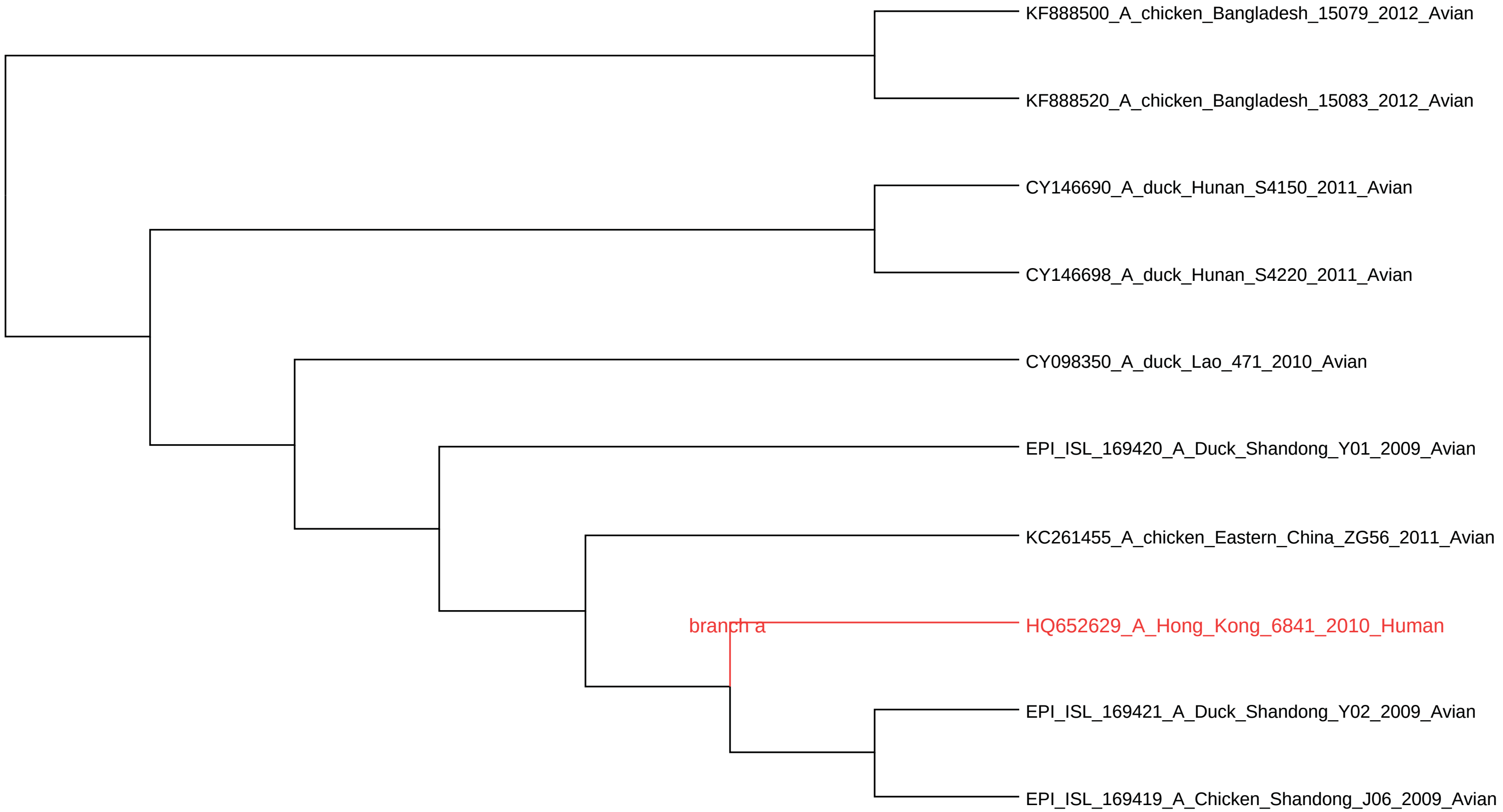

KF888500_A_chicken_Bangladesh_15079_2012_Avian
KF888520_A_chicken_Bangladesh_15083_2012_Avian
CY146690_A_duck_Hunan_S4150_2011_Avian
CY146698_A_duck_Hunan_S4220_2011_Avian
CY098350_A_duck_Lao_471_2010_Avian
EPI_ISL_169420_A_Duck_Shandong_Y01_2009_Avian
KC261455_A_chicken_Eastern_China_ZG56_2011_Avian
branch a
HQ652629_A_Hong_Kong_6841_2010_Human
EPI_ISL_169421_A_Duck_Shandong_Y02_2009_Avian
EPI_ISL_169419_A_Chicken_Shandong_J06_2009_Avian

PB1-Group12

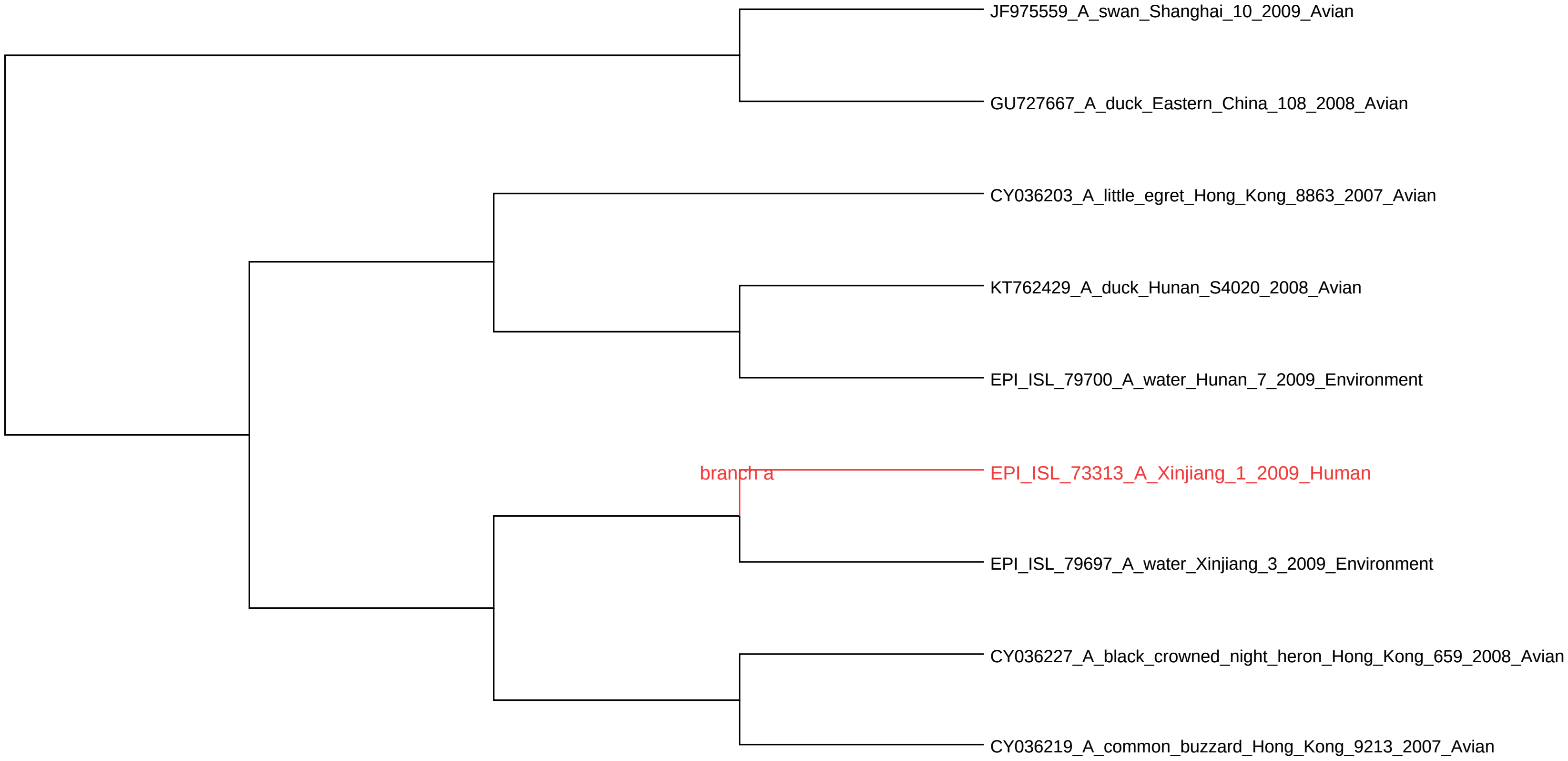
JF975559_A_swan_Shanghai_10_2009_Avian
GU727667_A_duck_Eastern_China_108_2008_Avian
CY036203_A_little_egret_Hong_Kong_8863_2007_Avian
KT762429_A_duck_Hunan_S4020_2008_Avian
EPI_ISL_79700_A_water_Hunan_7_2009_Environment
branch a
EPI_ISL_73313_A_Xinjiang_1_2009_Human
EPI_ISL_79697_A_water_Xinjiang_3_2009_Environment
CY036227_A_black_crowned_night_heron_Hong_Kong_659_2008_Avian
CY036219_A_common_buzzard_Hong_Kong_9213_2007_Avian

PB1-Group13

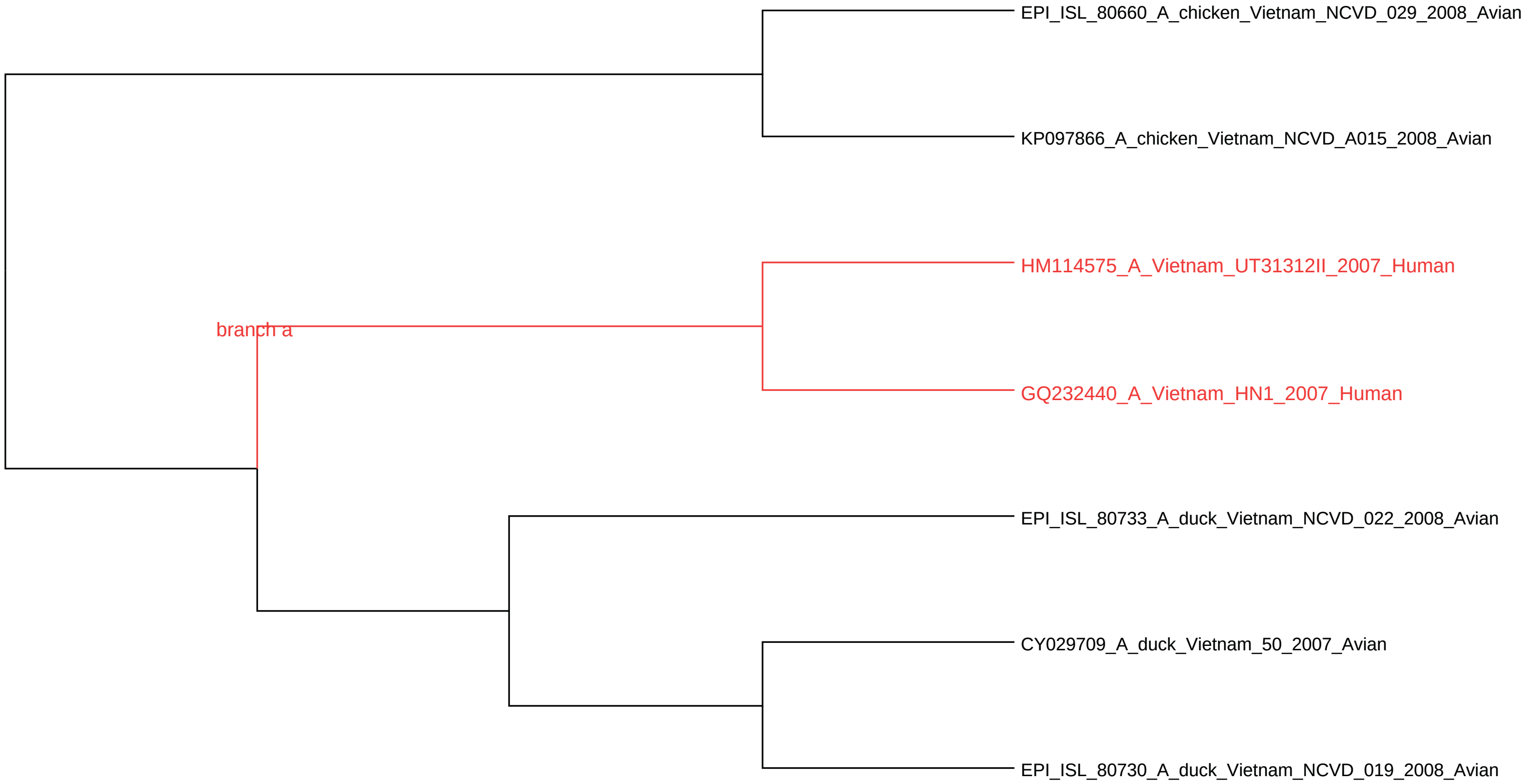
EPI_ISL_80660_A_chicken_Vietnam_NCVD_029_2008_Avian
KP097866_A_chicken_Vietnam_NCVD_A015_2008_Avian
HM114575_A_Vietnam_UT31312II_2007_Human
branch a
GQ232440_A_Vietnam_HN1_2007_Human
EPI_ISL_80733_A_duck_Vietnam_NCVD_022_2008_Avian
CY029709_A_duck_Vietnam_50_2007_Avian
EPI_ISL_80730_A_duck_Vietnam_NCVD_019_2008_Avian

PB1-Group14

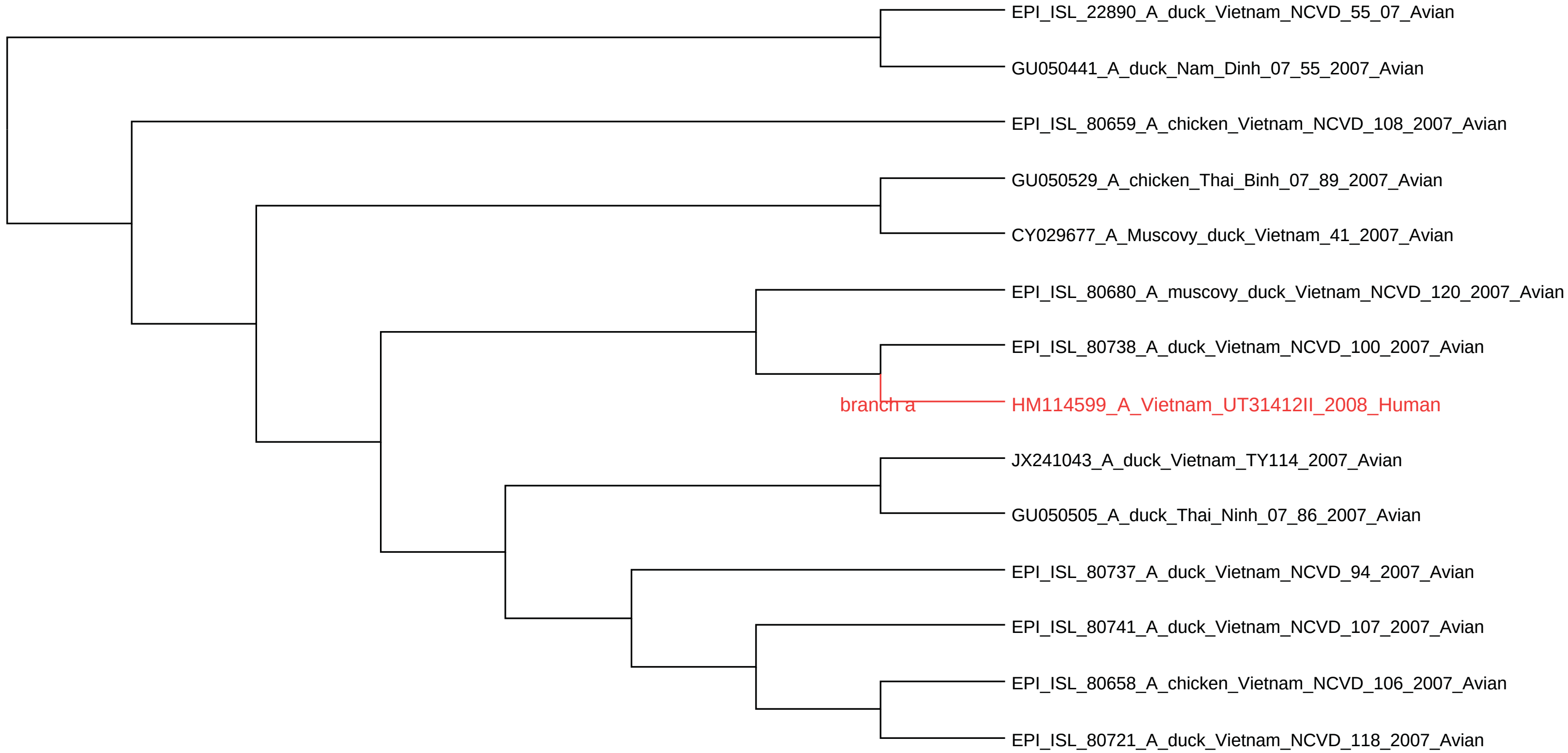
EPI_ISL_22890_A_duck_Vietnam_NCVD_55_07_Avian
GU050441_A_duck_Nam_Dinh_07_55_2007_Avian
EPI_ISL_80659_A_chicken_Vietnam_NCVD_108_2007_Avian
GU050529_A_chicken_Thai_Binh_07_89_2007_Avian
CY029677_A_Muscovy_duck_Vietnam_41_2007_Avian
EPI_ISL_80680_A_muscovy_duck_Vietnam_NCVD_120_2007_Avian
EPI_ISL_80738_A_duck_Vietnam_NCVD_100_2007_Avian
branch a
HM114599_A_Vietnam_UT31412II_2008_Human
JX241043_A_duck_Vietnam_TY114_2007_Avian
GU050505_A_duck_Thai_Ninh_07_86_2007_Avian
EPI_ISL_80737_A_duck_Vietnam_NCVD_94_2007_Avian
EPI_ISL_80741_A_duck_Vietnam_NCVD_107_2007_Avian
EPI_ISL_80658_A_chicken_Vietnam_NCVD_106_2007_Avian
EPI_ISL_80721_A_duck_Vietnam_NCVD_118_2007_Avian

PB1-Group15

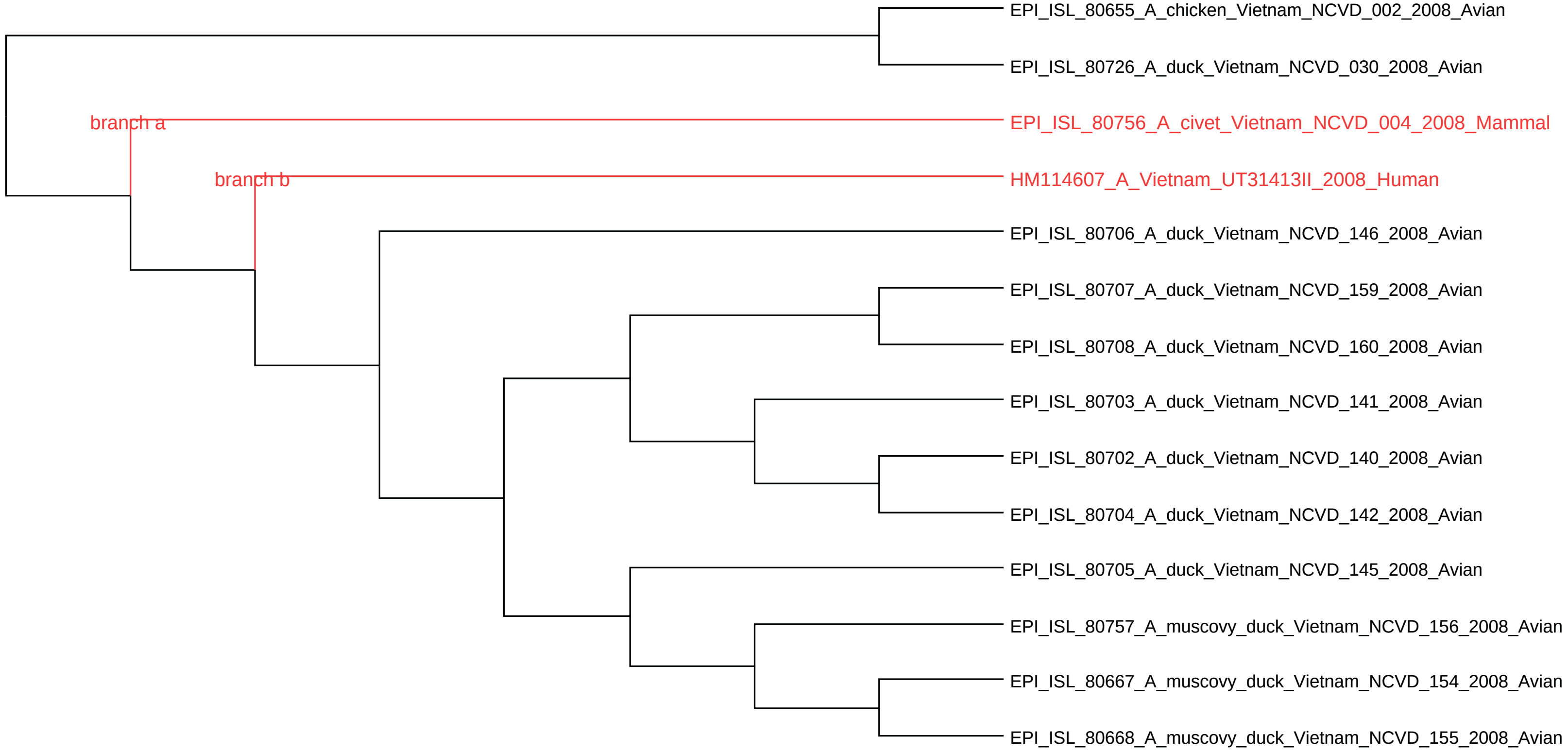
EPI_ISL_80655_A_chicken_Vietnam_NCVD_002_2008_Avian
EPI_ISL_80726_A_duck_Vietnam_NCVD_030_2008_Avian
branch a
EPI_ISL_80756_A_civet_Vietnam_NCVD_004_2008_Mammal
branch b
HM114607_A_Vietnam_UT31413II_2008_Human
EPI_ISL_80706_A_duck_Vietnam_NCVD_146_2008_Avian
EPI_ISL_80707_A_duck_Vietnam_NCVD_159_2008_Avian
EPI_ISL_80708_A_duck_Vietnam_NCVD_160_2008_Avian
EPI_ISL_80703_A_duck_Vietnam_NCVD_141_2008_Avian
EPI_ISL_80702_A_duck_Vietnam_NCVD_140_2008_Avian
EPI_ISL_80704_A_duck_Vietnam_NCVD_142_2008_Avian
EPI_ISL_80705_A_duck_Vietnam_NCVD_145_2008_Avian
EPI_ISL_80757_A_muscovy_duck_Vietnam_NCVD_156_2008_Avian
EPI_ISL_80667_A_muscovy_duck_Vietnam_NCVD_154_2008_Avian
EPI_ISL_80668_A_muscovy_duck_Vietnam_NCVD_155_2008_Avian

PB1-Group16

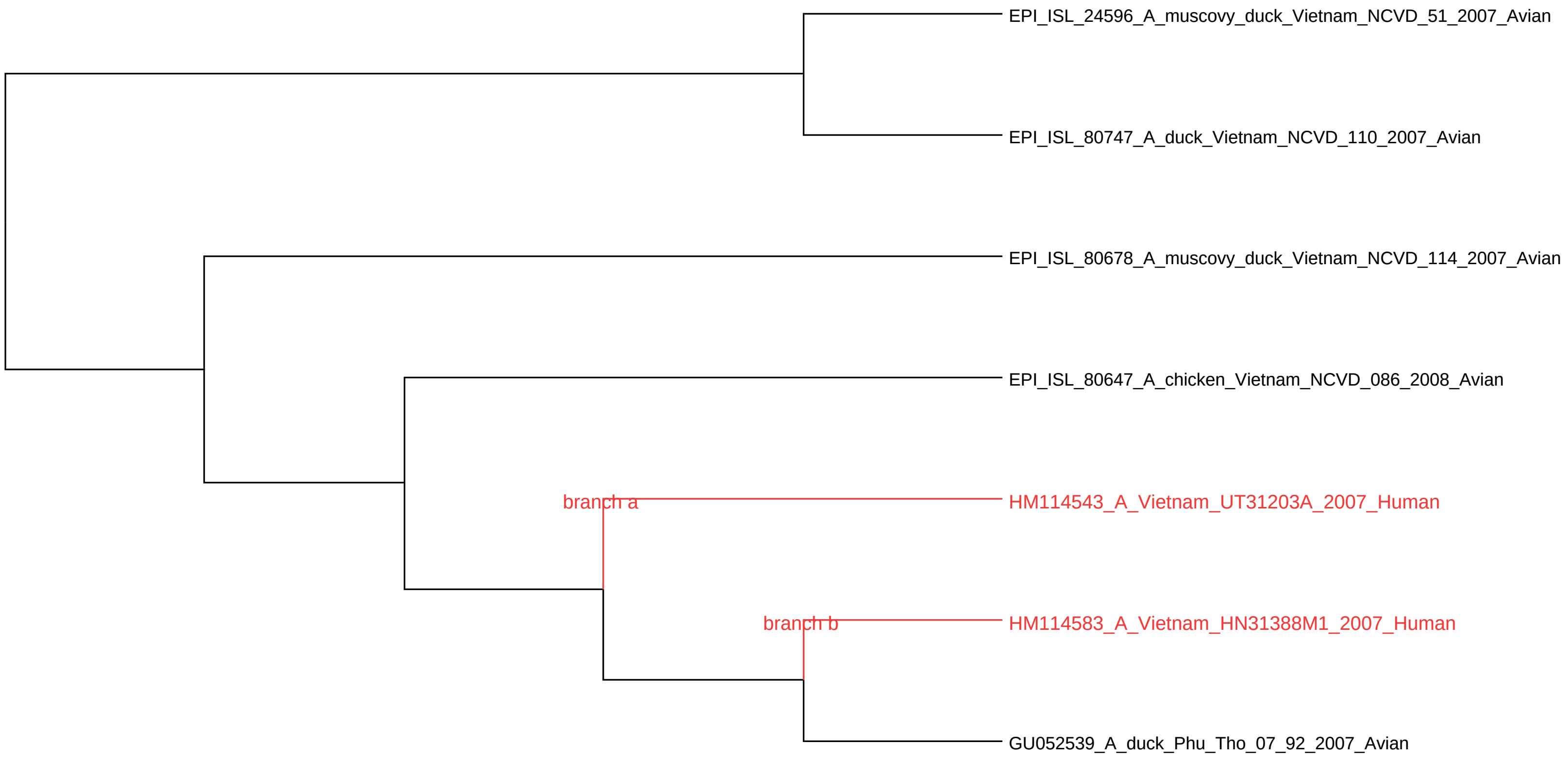
EPI_ISL_24596_A_muscovy_duck_Vietnam_NCVD_51_2007_Avian
EPI_ISL_80747_A_duck_Vietnam_NCVD_110_2007_Avian
EPI_ISL_80678_A_muscovy_duck_Vietnam_NCVD_114_2007_Avian
EPI_ISL_80647_A_chicken_Vietnam_NCVD_086_2008_Avian
branch a
HM114543_A_Vietnam_UT31203A_2007_Human
branch b
HM114583_A_Vietnam_HN31388M1_2007_Human
GU052539_A_duck_Phu_Tho_07_92_2007_Avian

PB1-Group17

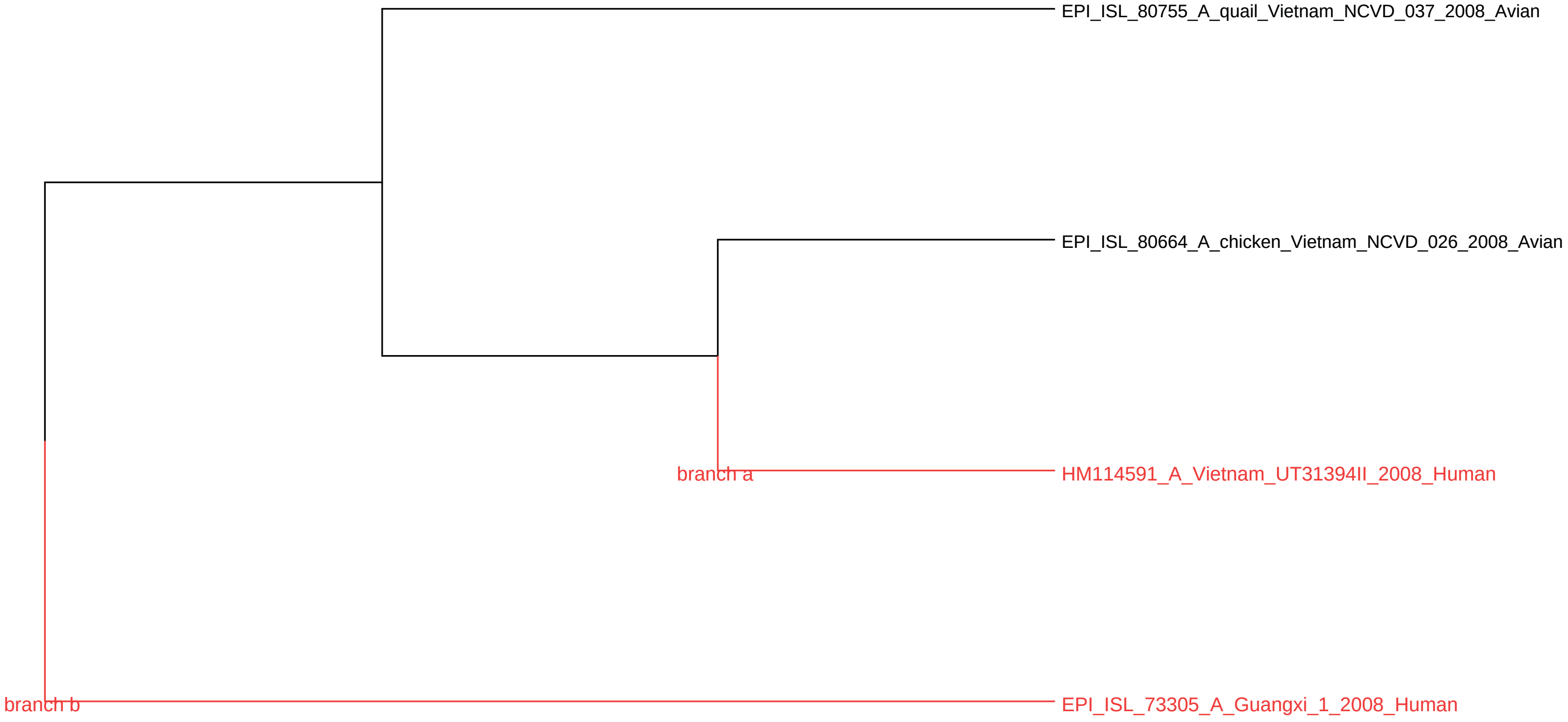
EPI_ISL_80755_A_quail_Vietnam_NCVD_037_2008_Avian
EPI_ISL_80664_A_chicken_Vietnam_NCVD_026_2008_Avian
branch a
HM114591_A_Vietnam_UT31394II_2008_Human
branch b
EPI_ISL_73305_A_Guangxi_1_2008_Human

PB1-Group18

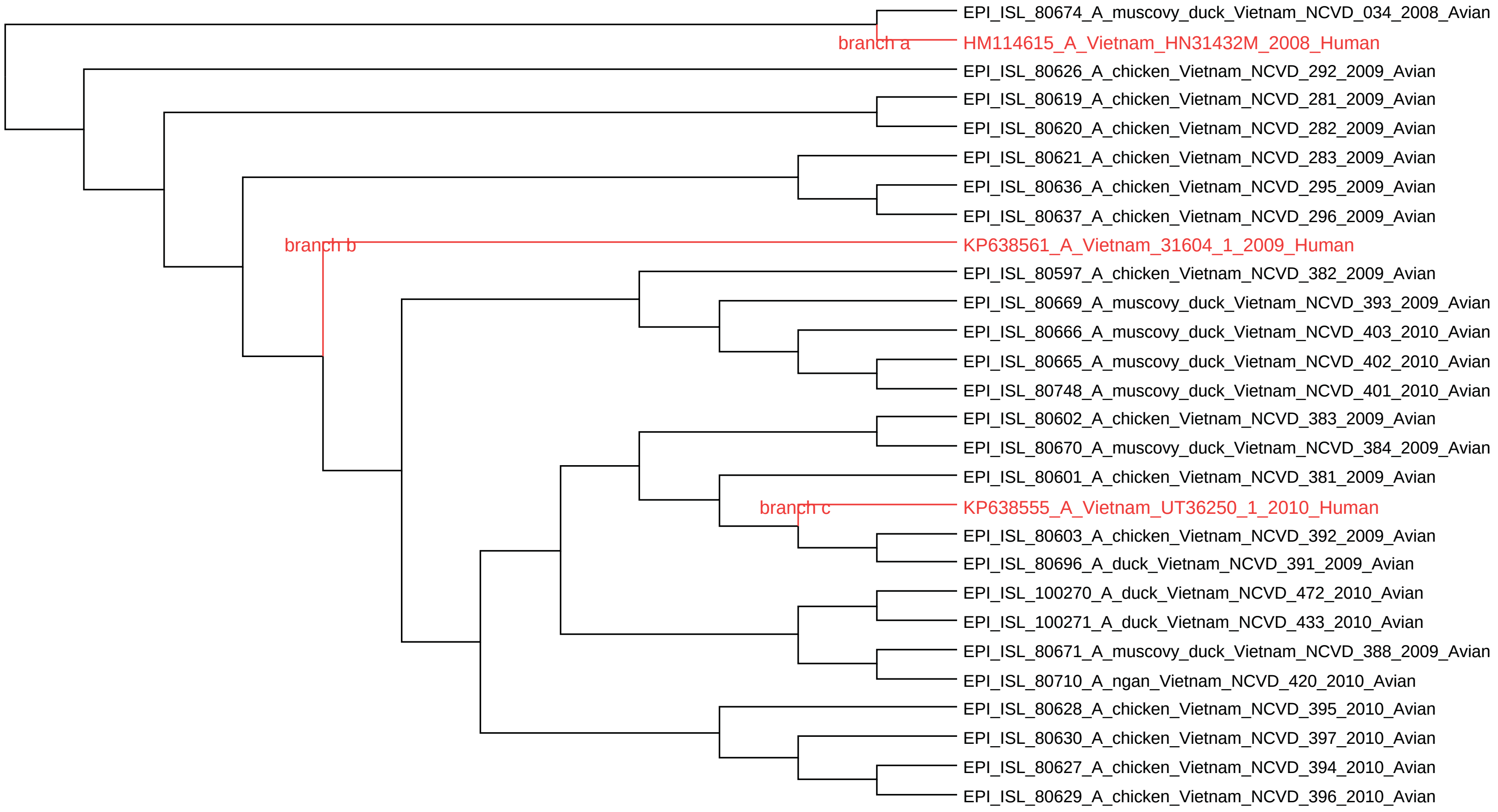
EPI_ISL_80674_A_muscovy_duck_Vietnam_NCVD_034_2008_Avian
branch a
HM114615_A_Vietnam_HN31432M_2008_Human
EPI_ISL_80626_A_chicken_Vietnam_NCVD_292_2009_Avian
EPI_ISL_80619_A_chicken_Vietnam_NCVD_281_2009_Avian
EPI_ISL_80620_A_chicken_Vietnam_NCVD_282_2009_Avian
EPI_ISL_80621_A_chicken_Vietnam_NCVD_283_2009_Avian
EPI_ISL_80636_A_chicken_Vietnam_NCVD_295_2009_Avian
EPI_ISL_80637_A_chicken_Vietnam_NCVD_296_2009_Avian
branch b
KP638561_A_Vietnam_31604_1_2009_Human
EPI_ISL_80597_A_chicken_Vietnam_NCVD_382_2009_Avian
EPI_ISL_80669_A_muscovy_duck_Vietnam_NCVD_393_2009_Avian
EPI_ISL_80666_A_muscovy_duck_Vietnam_NCVD_403_2010_Avian
EPI_ISL_80665_A_muscovy_duck_Vietnam_NCVD_402_2010_Avian
EPI_ISL_80748_A_muscovy_duck_Vietnam_NCVD_401_2010_Avian
EPI_ISL_80602_A_chicken_Vietnam_NCVD_383_2009_Avian
EPI_ISL_80670_A_muscovy_duck_Vietnam_NCVD_384_2009_Avian
EPI_ISL_80601_A_chicken_Vietnam_NCVD_381_2009_Avian
branch c
KP638555_A_Vietnam_UT36250_1_2010_Human
EPI_ISL_80603_A_chicken_Vietnam_NCVD_392_2009_Avian
EPI_ISL_80696_A_duck_Vietnam_NCVD_391_2009_Avian
EPI_ISL_100270_A_duck_Vietnam_NCVD_472_2010_Avian
EPI_ISL_100271_A_duck_Vietnam_NCVD_433_2010_Avian
EPI_ISL_80671_A_muscovy_duck_Vietnam_NCVD_388_2009_Avian
EPI_ISL_80710_A_ngan_Vietnam_NCVD_420_2010_Avian
EPI_ISL_80628_A_chicken_Vietnam_NCVD_395_2010_Avian
EPI_ISL_80630_A_chicken_Vietnam_NCVD_397_2010_Avian
EPI_ISL_80627_A_chicken_Vietnam_NCVD_394_2010_Avian
EPI_ISL_80629_A_chicken_Vietnam_NCVD_396_2010_Avian

PB1-Group19

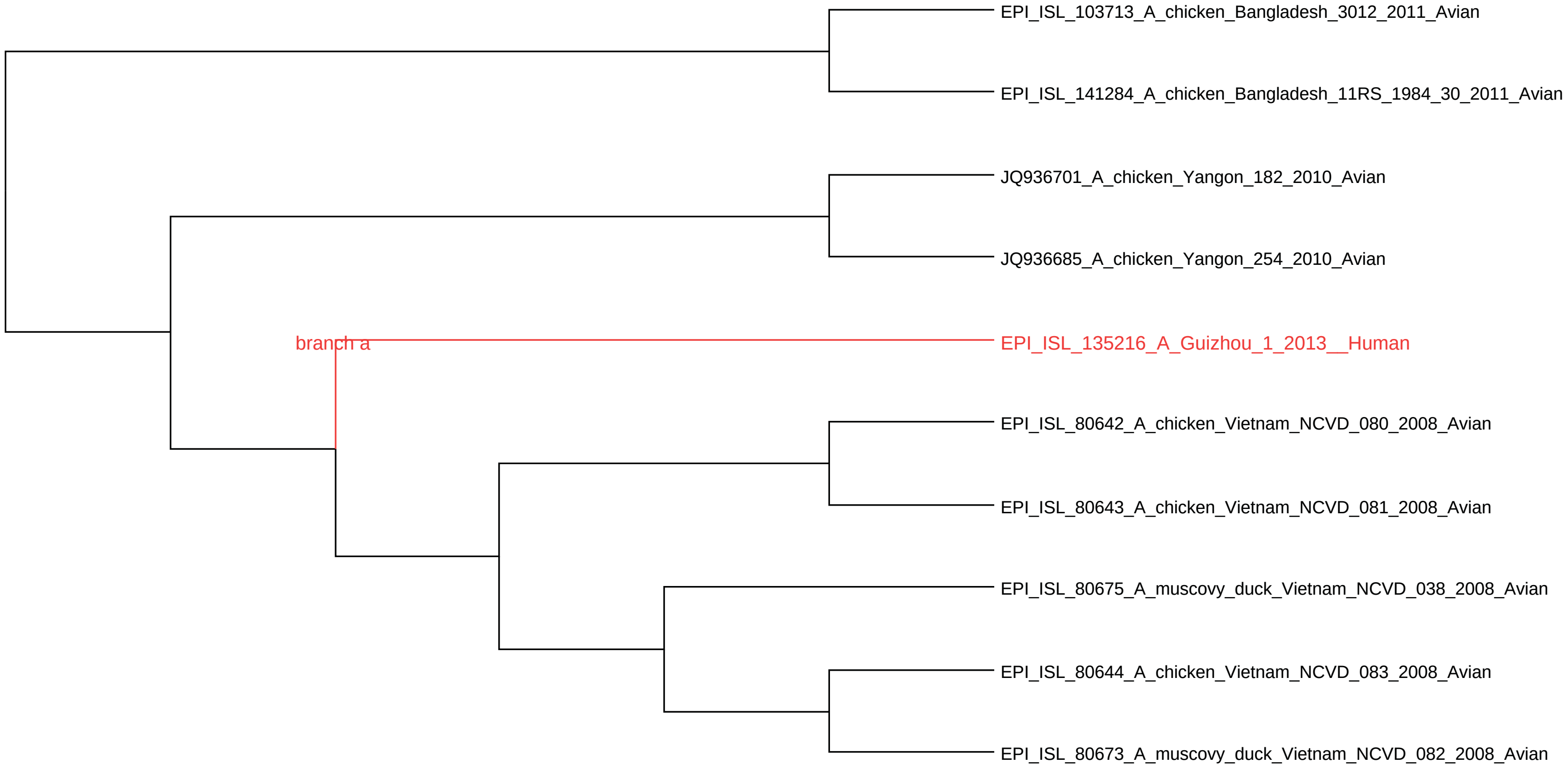
EPI_ISL_103713_A_chicken_Bangladesh_3012_2011_Avian
EPI_ISL_141284_A_chicken_Bangladesh_11RS_1984_30_2011_Avian
JQ936701_A_chicken_Yangon_182_2010_Avian
JQ936685_A_chicken_Yangon_254_2010_Avian
branch a
EPI_ISL_135216_A_Guizhou_1_2013__Human
EPI_ISL_80642_A_chicken_Vietnam_NCVD_080_2008_Avian
EPI_ISL_80643_A_chicken_Vietnam_NCVD_081_2008_Avian
EPI_ISL_80675_A_muscovy_duck_Vietnam_NCVD_038_2008_Avian
EPI_ISL_80644_A_chicken_Vietnam_NCVD_083_2008_Avian
EPI_ISL_80673_A_muscovy_duck_Vietnam_NCVD_082_2008_Avian

PB1-Group20

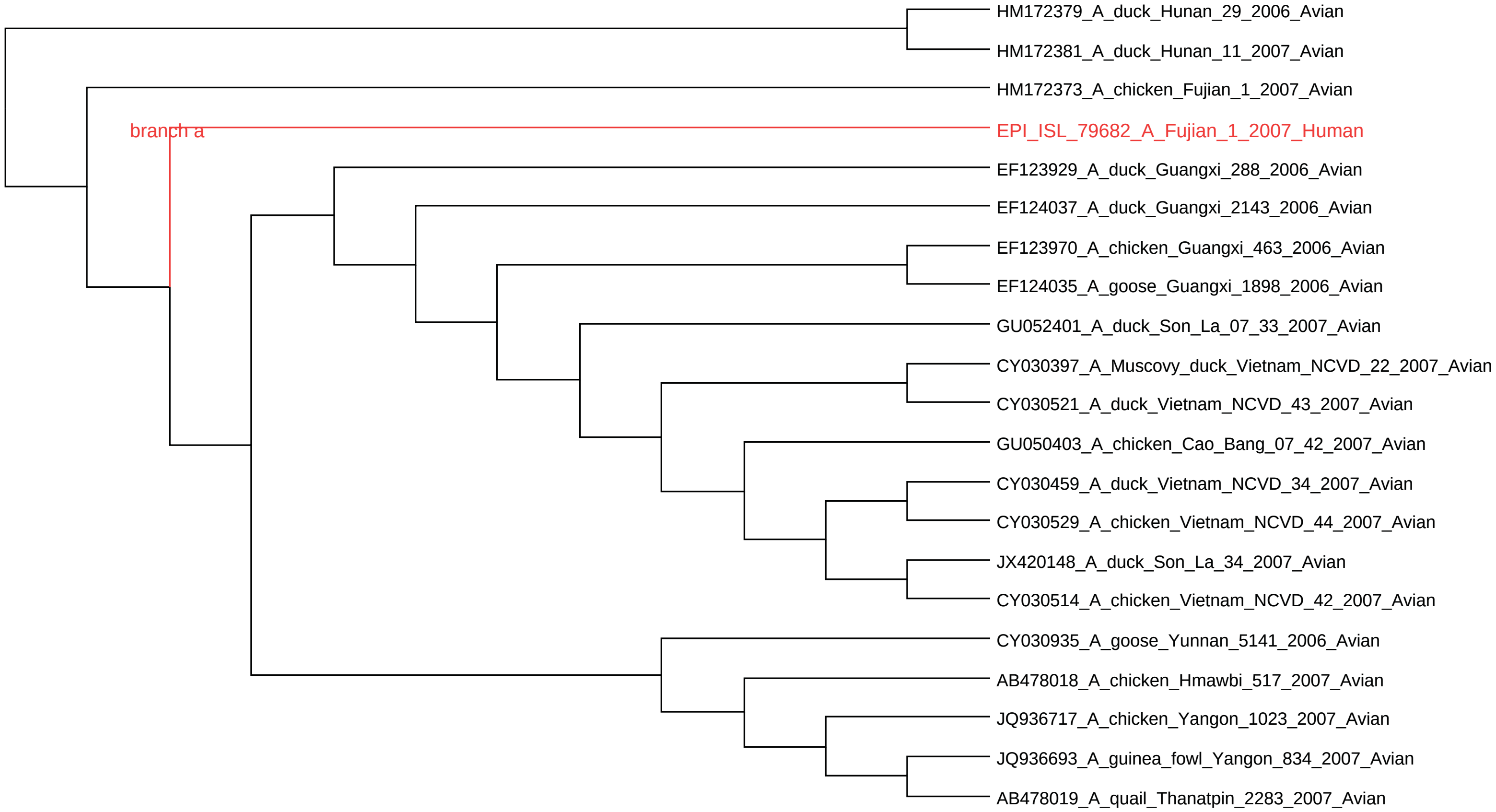
HM172379_A_duck_Hunan_29_2006_Avian
HM172381_A_duck_Hunan_11_2007_Avian
HM172373_A_chicken_Fujian_1_2007_Avian
branch a
EPI_ISL_79682_A_Fujian_1_2007_Human
EF123929_A_duck_Guangxi_288_2006_Avian
EF124037_A_duck_Guangxi_2143_2006_Avian
EF123970_A_chicken_Guangxi_463_2006_Avian
EF124035_A_goose_Guangxi_1898_2006_Avian
GU052401_A_duck_Son_La_07_33_2007_Avian
CY030397_A_Muscovy_duck_Vietnam_NCVD_22_2007_Avian
CY030521_A_duck_Vietnam_NCVD_43_2007_Avian
GU050403_A_chicken_Cao_Bang_07_42_2007_Avian
CY030459_A_duck_Vietnam_NCVD_34_2007_Avian
CY030529_A_chicken_Vietnam_NCVD_44_2007_Avian
JX420148_A_duck_Son_La_34_2007_Avian
CY030514_A_chicken_Vietnam_NCVD_42_2007_Avian
CY030935_A_goose_Yunnan_5141_2006_Avian
AB478018_A_chicken_Hmawbi_517_2007_Avian
JQ936717_A_chicken_Yangon_1023_2007_Avian
JQ936693_A_guinea_fowl_Yangon_834_2007_Avian
AB478019_A_quail_Thanatpin_2283_2007_Avian

PB1-Group21

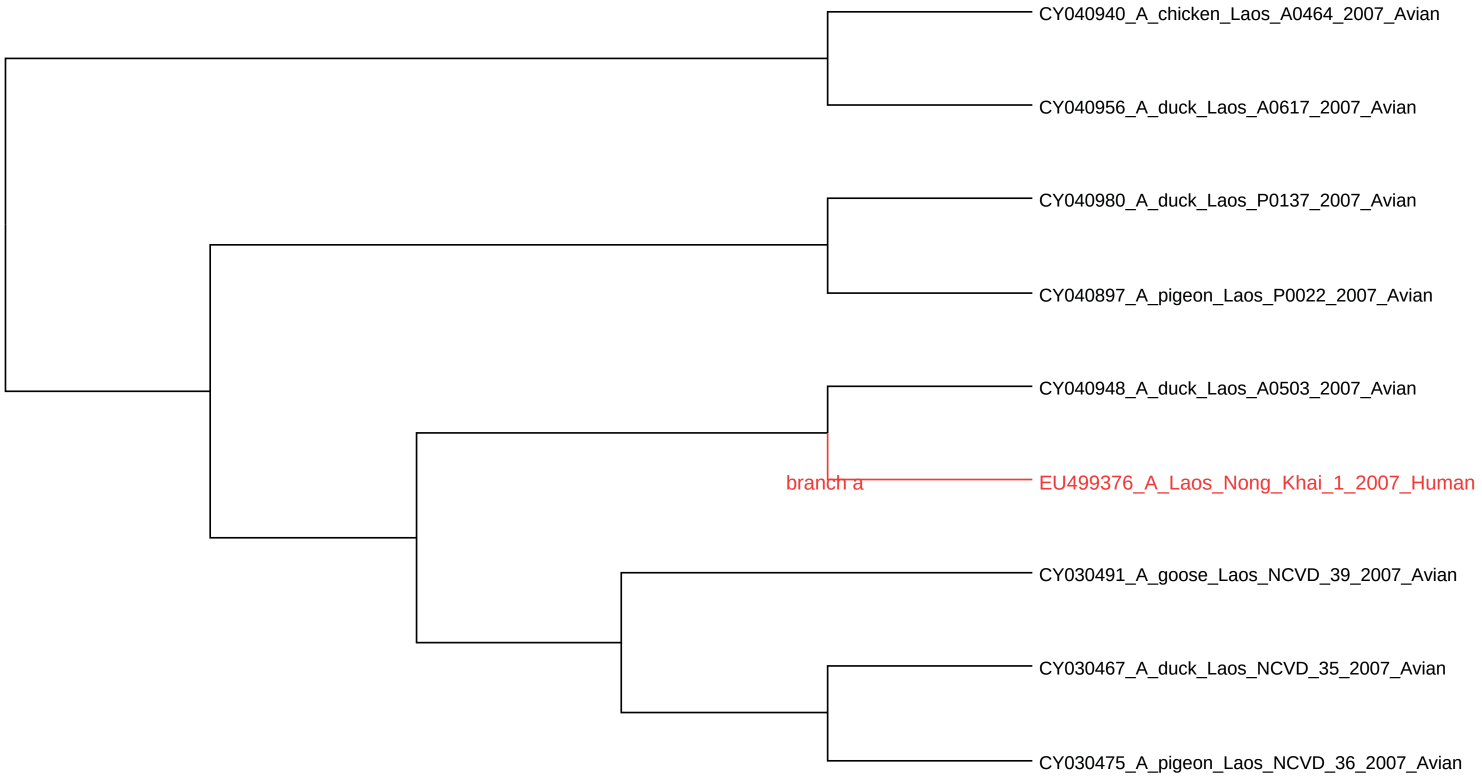
CY040940_A_chicken_Laos_A0464_2007_Avian
CY040956_A_duck_Laos_A0617_2007_Avian
CY040980_A_duck_Laos_P0137_2007_Avian
CY040897_A_pigeon_Laos_P0022_2007_Avian
CY040948_A_duck_Laos_A0503_2007_Avian
branch a
EU499376_A_Laos_Nong_Khai_1_2007_Human
CY030491_A_goose_Laos_NCVD_39_2007_Avian
CY030467_A_duck_Laos_NCVD_35_2007_Avian
CY030475_A_pigeon_Laos_NCVD_36_2007_Avian

PB1-Group22

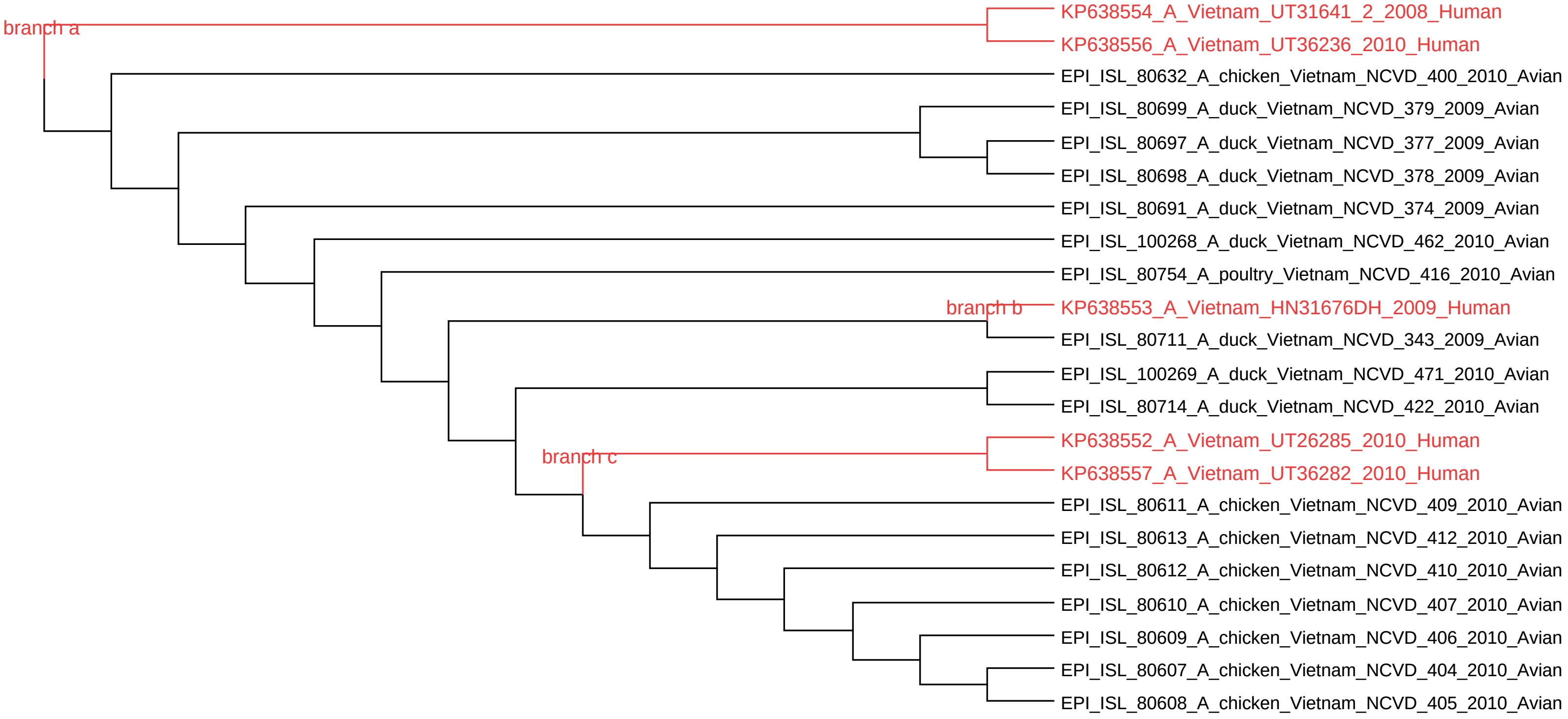
branch a
KP638554_A_Vietnam_UT31641_2_2008_Human
KP638556_A_Vietnam_UT36236_2010_Human
EPI_ISL_80632_A_chicken_Vietnam_NCVD_400_2010_Avian
EPI_ISL_80699_A_duck_Vietnam_NCVD_379_2009_Avian
EPI_ISL_80697_A_duck_Vietnam_NCVD_377_2009_Avian
EPI_ISL_80698_A_duck_Vietnam_NCVD_378_2009_Avian
EPI_ISL_80691_A_duck_Vietnam_NCVD_374_2009_Avian
EPI_ISL_100268_A_duck_Vietnam_NCVD_462_2010_Avian
EPI_ISL_80754_A_poultry_Vietnam_NCVD_416_2010_Avian
branch b
KP638553_A_Vietnam_HN31676DH_2009_Human
EPI_ISL_80711_A_duck_Vietnam_NCVD_343_2009_Avian
EPI_ISL_100269_A_duck_Vietnam_NCVD_471_2010_Avian
EPI_ISL_80714_A_duck_Vietnam_NCVD_422_2010_Avian
branch c
KP638552_A_Vietnam_UT26285_2010_Human
KP638557_A_Vietnam_UT36282_2010_Human
EPI_ISL_80611_A_chicken_Vietnam_NCVD_409_2010_Avian
EPI_ISL_80613_A_chicken_Vietnam_NCVD_412_2010_Avian
EPI_ISL_80612_A_chicken_Vietnam_NCVD_410_2010_Avian
EPI_ISL_80610_A_chicken_Vietnam_NCVD_407_2010_Avian
EPI_ISL_80609_A_chicken_Vietnam_NCVD_406_2010_Avian
EPI_ISL_80607_A_chicken_Vietnam_NCVD_404_2010_Avian
EPI_ISL_80608_A_chicken_Vietnam_NCVD_405_2010_Avian

PB1-Group23

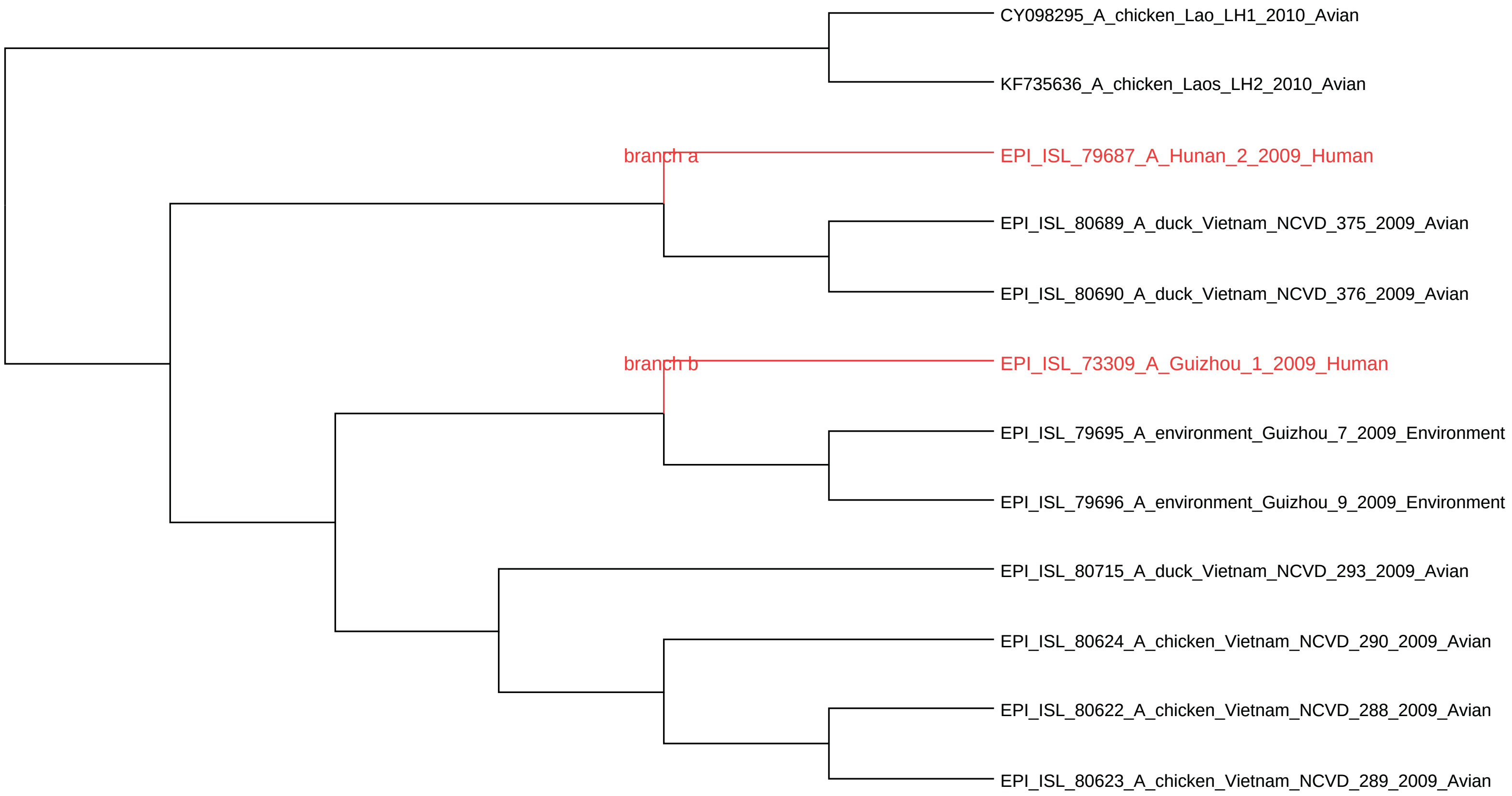
CY098295_A_chicken_Lao_LH1_2010_Avian
KF735636_A_chicken_Laos_LH2_2010_Avian
branch a
EPI_ISL_79687_A_Hunan_2_2009_Human
EPI_ISL_80689_A_duck_Vietnam_NCVD_375_2009_Avian
EPI_ISL_80690_A_duck_Vietnam_NCVD_376_2009_Avian
branch b
EPI_ISL_73309_A_Guizhou_1_2009_Human
EPI_ISL_79695_A_environment_Guizhou_7_2009_Environment
EPI_ISL_79696_A_environment_Guizhou_9_2009_Environment
EPI_ISL_80715_A_duck_Vietnam_NCVD_293_2009_Avian
EPI_ISL_80624_A_chicken_Vietnam_NCVD_290_2009_Avian
EPI_ISL_80622_A_chicken_Vietnam_NCVD_288_2009_Avian
EPI_ISL_80623_A_chicken_Vietnam_NCVD_289_2009_Avian

PB1-Group24

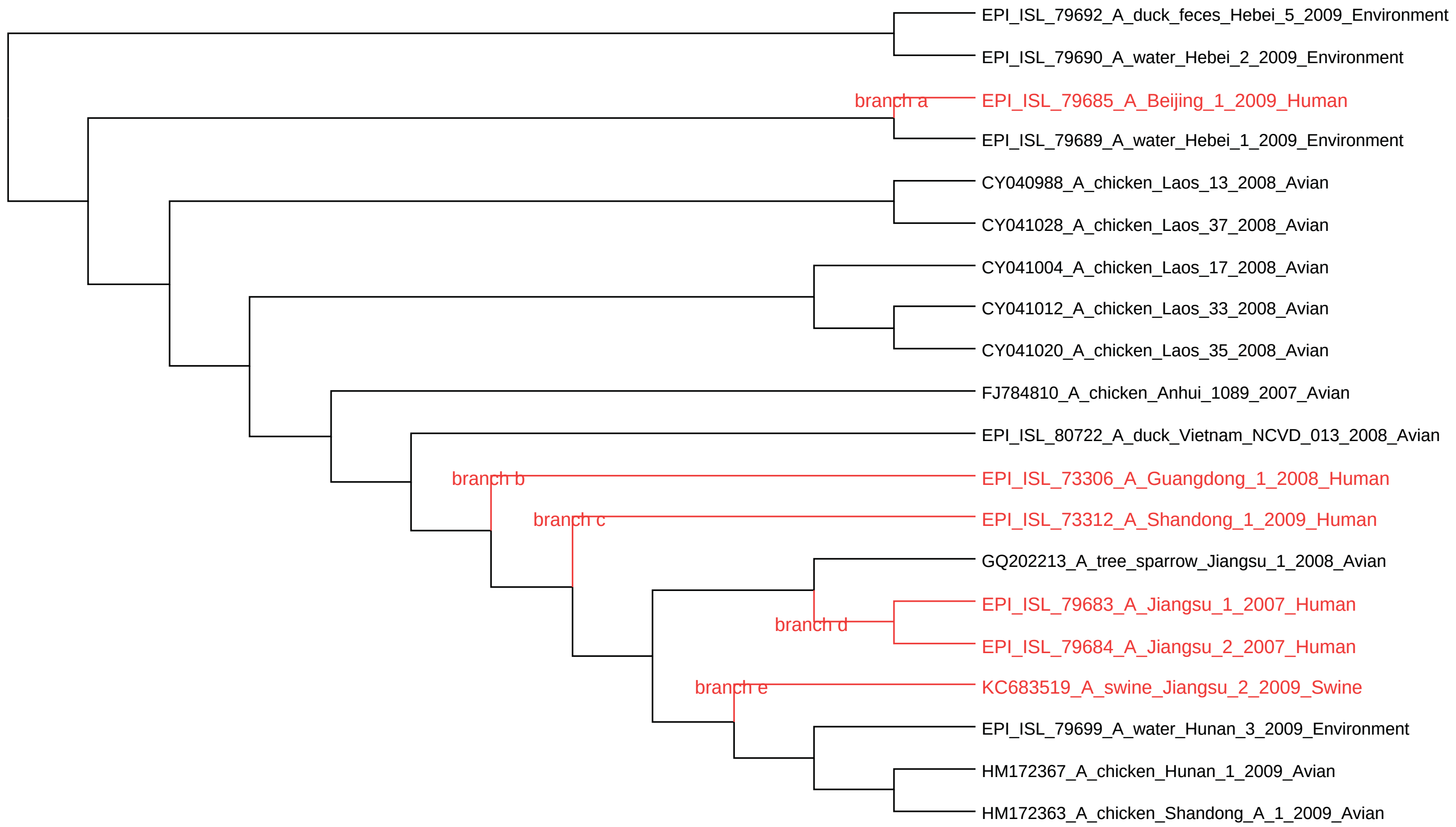
EPI_ISL_79692_A_duck_feces_Hebei_5_2009_Environment
EPI_ISL_79690_A_water_Hebei_2_2009_Environment
branch a
EPI_ISL_79685_A_Beijing_1_2009_Human
EPI_ISL_79689_A_water_Hebei_1_2009_Environment
CY040988_A_chicken_Laos_13_2008_Avian
CY041028_A_chicken_Laos_37_2008_Avian
CY041004_A_chicken_Laos_17_2008_Avian
CY041012_A_chicken_Laos_33_2008_Avian
CY041020_A_chicken_Laos_35_2008_Avian
FJ784810_A_chicken_Anhui_1089_2007_Avian
EPI_ISL_80722_A_duck_Vietnam_NCVD_013_2008_Avian
branch b
EPI_ISL_73306_A_Guangdong_1_2008_Human
branch c
EPI_ISL_73312_A_Shandong_1_2009_Human
GQ202213_A_tree_sparrow_Jiangsu_1_2008_Avian
EPI_ISL_79683_A_Jiangsu_1_2007_Human
branch d
EPI_ISL_79684_A_Jiangsu_2_2007_Human
branch e
KC683519_A_swine_Jiangsu_2_2009_Swine
EPI_ISL_79699_A_water_Hunan_3_2009_Environment
HM172367_A_chicken_Hunan_1_2009_Avian
HM172363_A_chicken_Shandong_A_1_2009_Avian

PB1-Group25

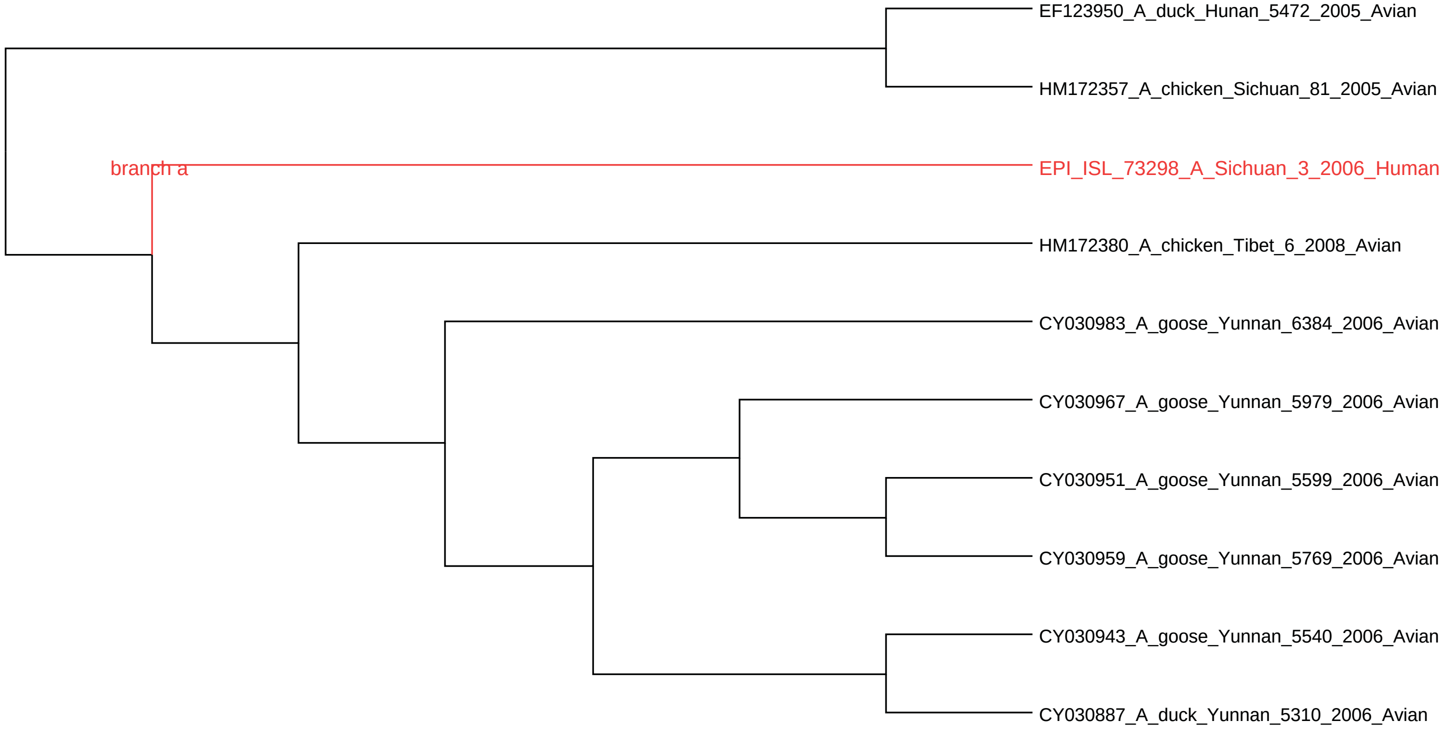
EF123950_A_duck_Hunan_5472_2005_Avian
HM172357_A_chicken_Sichuan_81_2005_Avian
branch a
EPI_ISL_73298_A_Sichuan_3_2006_Human
HM172380_A_chicken_Tibet_6_2008_Avian
CY030983_A_goose_Yunnan_6384_2006_Avian
CY030967_A_goose_Yunnan_5979_2006_Avian
CY030951_A_goose_Yunnan_5599_2006_Avian
CY030959_A_goose_Yunnan_5769_2006_Avian
CY030943_A_goose_Yunnan_5540_2006_Avian
CY030887_A_duck_Yunnan_5310_2006_Avian

PB1-Group26

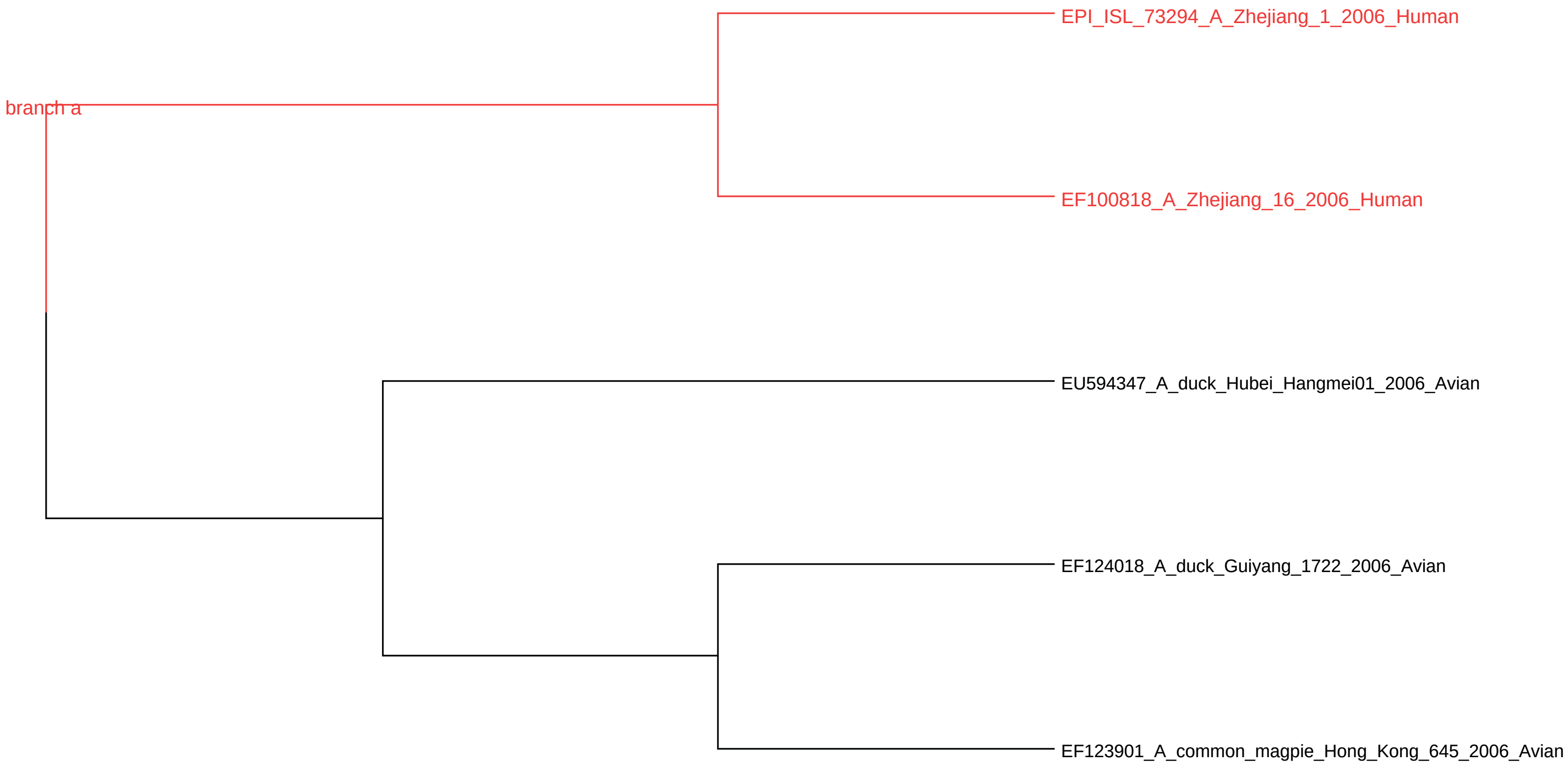
branch a
EPI_ISL_73294_A_Zhejiang_1_2006_Human
EF100818_A_Zhejiang_16_2006_Human
EU594347_A_duck_Hubei_Hangmei01_2006_Avian
EF124018_A_duck_Guiyang_1722_2006_Avian
EF123901_A_common_magpie_Hong_Kong_645_2006_Avian

PB1-Group27

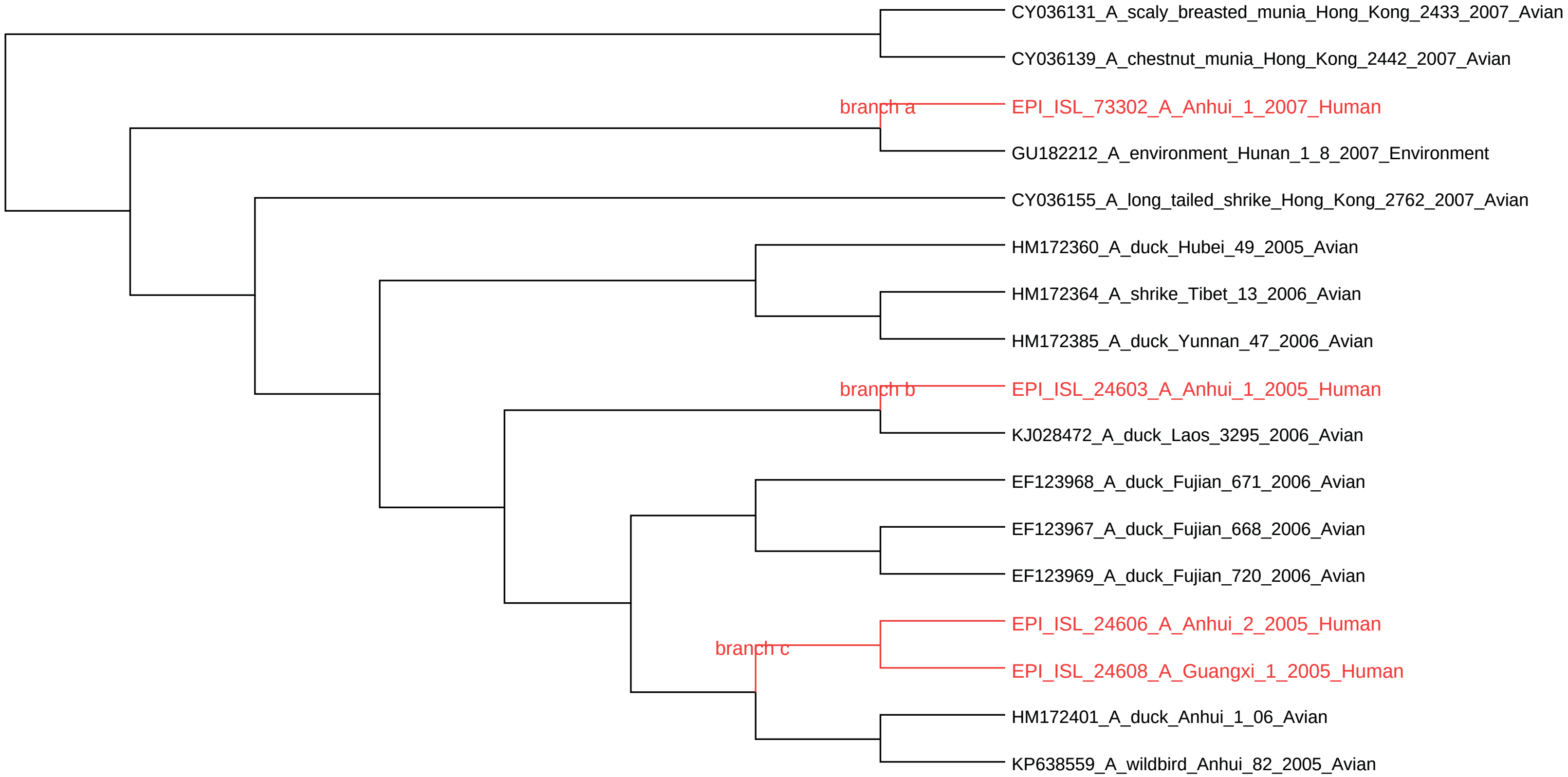

CY036131_A_scaly_breasted_munia_Hong_Kong_2433_2007_Avian
CY036139_A_chestnut_munia_Hong_Kong_2442_2007_Avian
branch a
EPI_ISL_73302_A_Anhui_1_2007_Human
GU182212_A_environment_Hunan_1_8_2007_Environment
CY036155_A_long_tailed_shrike_Hong_Kong_2762_2007_Avian
HM172360_A_duck_Hubei_49_2005_Avian
HM172364_A_shrike_Tibet_13_2006_Avian
HM172385_A_duck_Yunnan_47_2006_Avian
branch b
EPI_ISL_24603_A_Anhui_1_2005_Human
KJ028472_A_duck_Laos_3295_2006_Avian
EF123968_A_duck_Fujian_671_2006_Avian
EF123967_A_duck_Fujian_668_2006_Avian
EF123969_A_duck_Fujian_720_2006_Avian
EPI_ISL_24606_A_Anhui_2_2005_Human
branch c
EPI_ISL_24608_A_Guangxi_1_2005_Human
HM172401_A_duck_Anhui_1_06_Avian
KP638559_A_wildbird_Anhui_82_2005_Avian

PB1-Group28

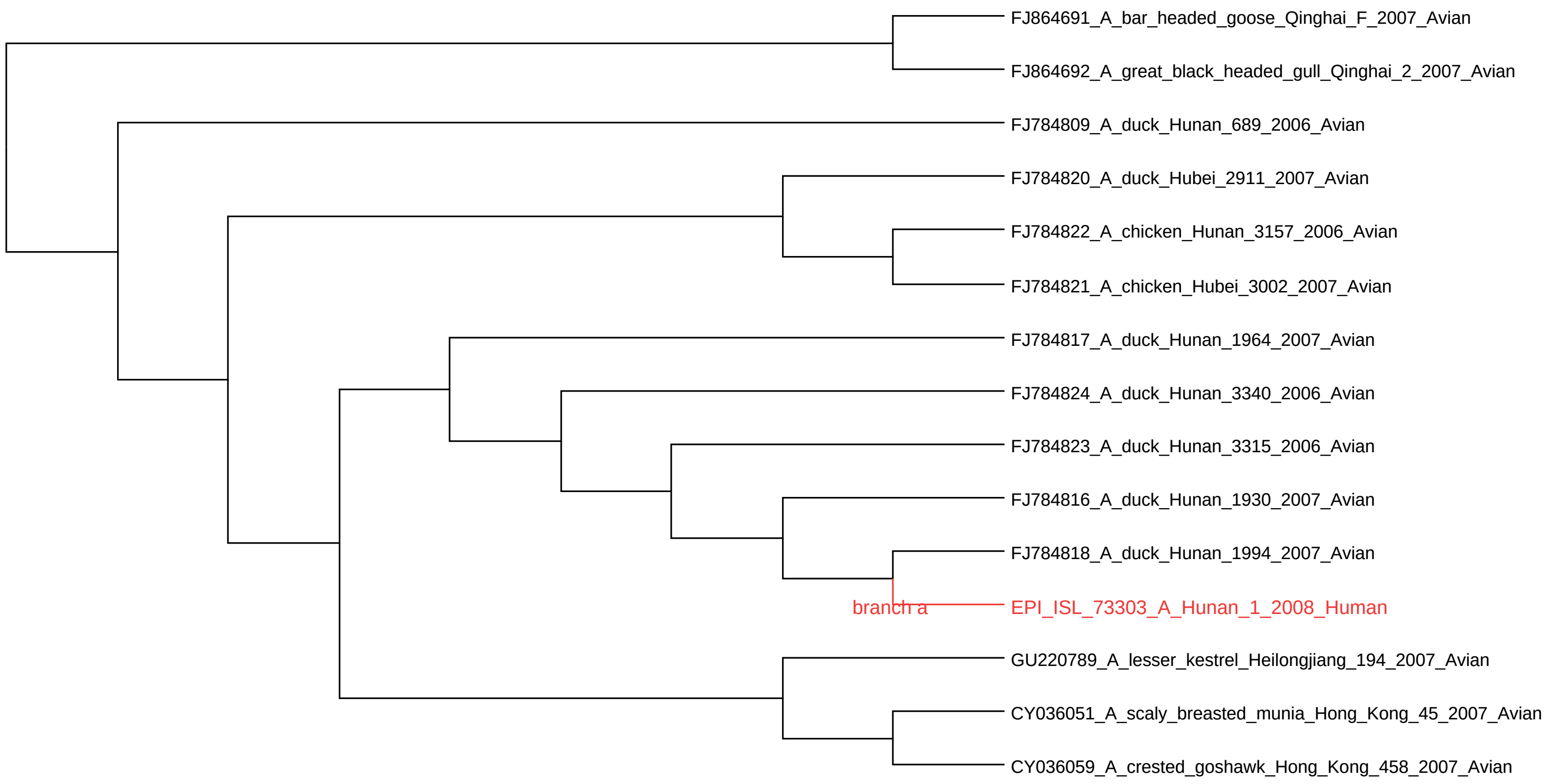
FJ864691_A_bar_headed_goose_Qinghai_F_2007_Avian
FJ864692_A_great_black_headed_gull_Qinghai_2_2007_Avian
FJ784809_A_duck_Hunan_689_2006_Avian
FJ784820_A_duck_Hubei_2911_2007_Avian
FJ784822_A_chicken_Hunan_3157_2006_Avian
FJ784821_A_chicken_Hubei_3002_2007_Avian
FJ784817_A_duck_Hunan_1964_2007_Avian
FJ784824_A_duck_Hunan_3340_2006_Avian
FJ784823_A_duck_Hunan_3315_2006_Avian
FJ784816_A_duck_Hunan_1930_2007_Avian
FJ784818_A_duck_Hunan_1994_2007_Avian
branch a
EPI_ISL_73303_A_Hunan_1_2008_Human
GU220789_A_lesser_kestrel_Heilongjiang_194_2007_Avian
CY036051_A_scaly_breasted_munia_Hong_Kong_45_2007_Avian
CY036059_A_crested_goshawk_Hong_Kong_458_2007_Avian

PB1-Group29

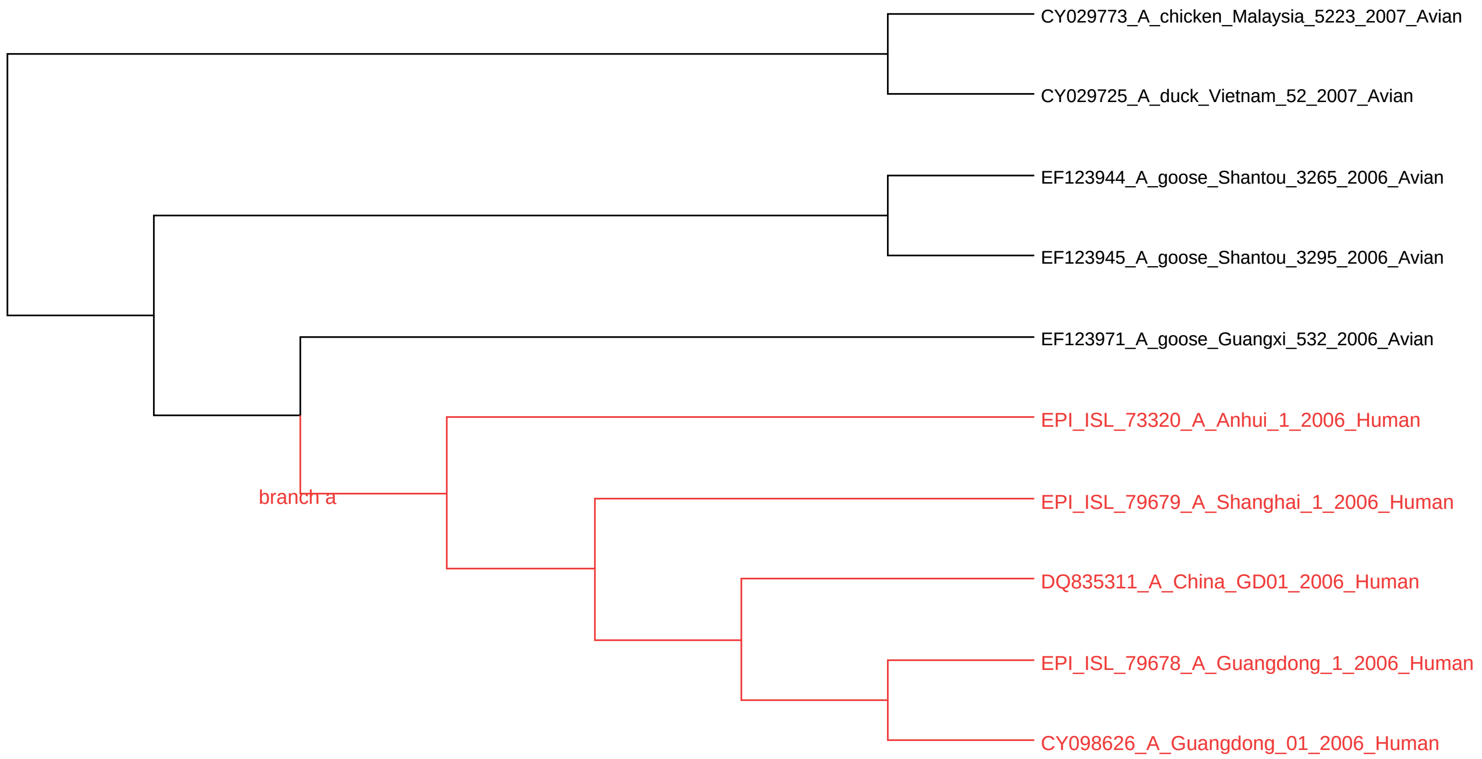

CY029773_A_chicken_Malaysia_5223_2007_Avian
CY029725_A_duck_Vietnam_52_2007_Avian
EF123944_A_goose_Shantou_3265_2006_Avian
EF123945_A_goose_Shantou_3295_2006_Avian
EF123971_A_goose_Guangxi_532_2006_Avian
EPI_ISL_73320_A_Anhui_1_2006_Human
branch a
EPI_ISL_79679_A_Shanghai_1_2006_Human
DQ835311_A_China_GD01_2006_Human
EPI_ISL_79678_A_Guangdong_1_2006_Human
CY098626_A_Guangdong_01_2006_Human

PB1-Group30

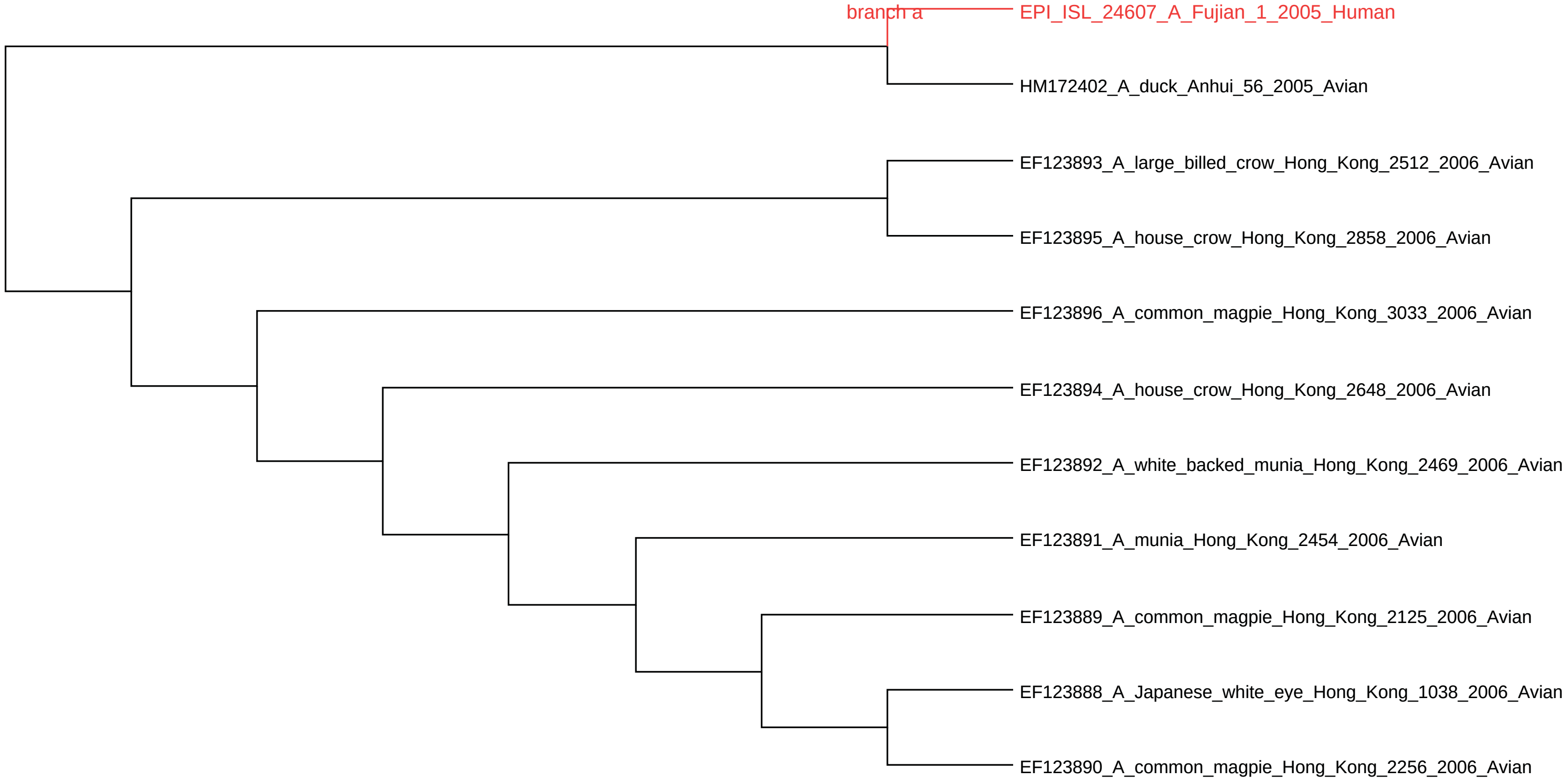

branch a
EPI_ISL_24607_A_Fujian_1_2005_Human
HM172402_A_duck_Anhui_56_2005_Avian
EF123893_A_large_billed_crow_Hong_Kong_2512_2006_Avian
EF123895_A_house_crow_Hong_Kong_2858_2006_Avian
EF123896_A_common_magpie_Hong_Kong_3033_2006_Avian
EF123894_A_house_crow_Hong_Kong_2648_2006_Avian
EF123892_A_white_backed_munia_Hong_Kong_2469_2006_Avian
EF123891_A_munia_Hong_Kong_2454_2006_Avian
EF123889_A_common_magpie_Hong_Kong_2125_2006_Avian
EF123888_A_Japanese_white_eye_Hong_Kong_1038_2006_Avian
EF123890_A_common_magpie_Hong_Kong_2256_2006_Avian

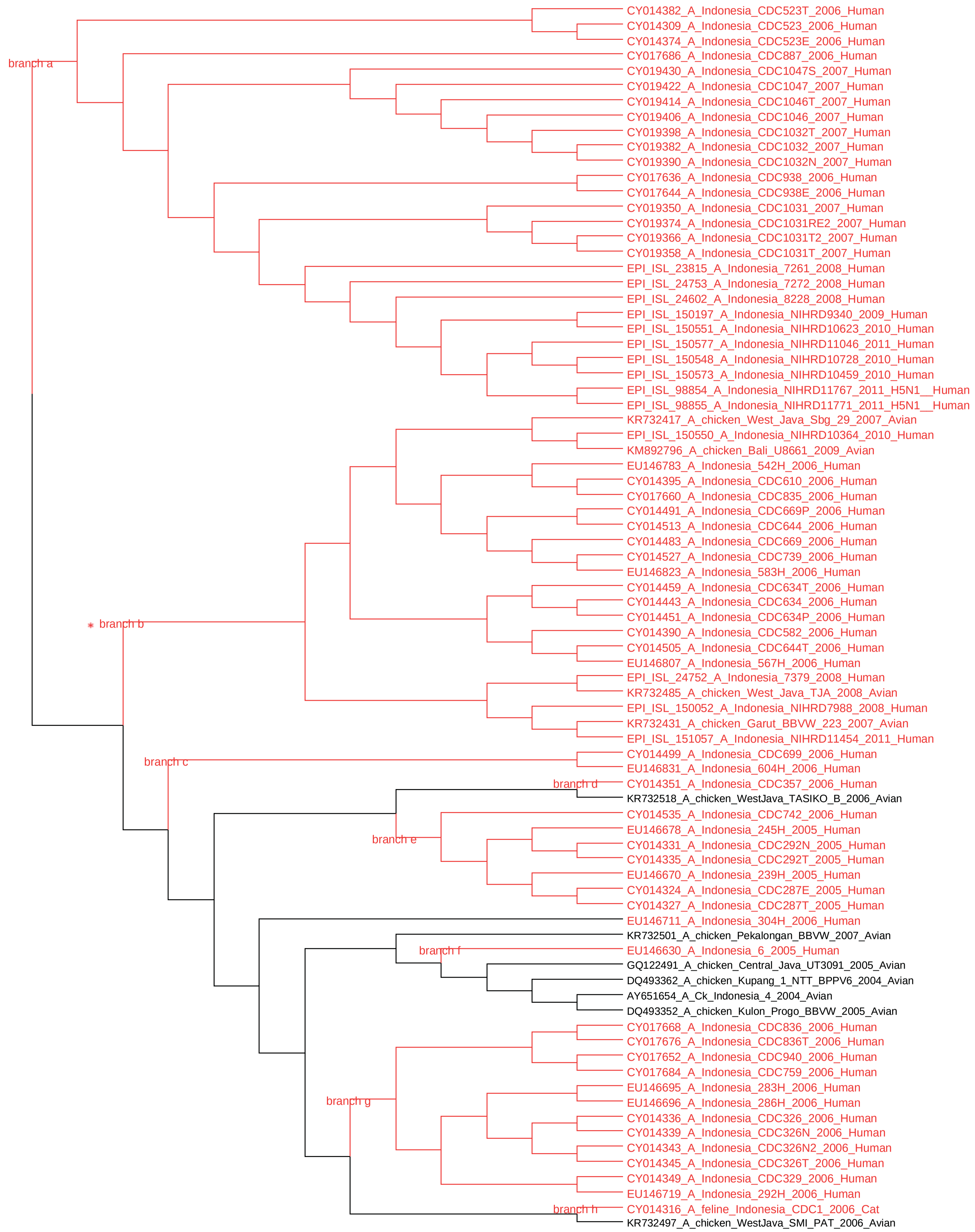

branch a
CY014382_A_Indonesia_CDC523T_2006_Human
CY014309_A_Indonesia_CDC523_2006_Human
CY014374_A_Indonesia_CDC523E_2006_Human
CY017686_A_Indonesia_CDC887_2006_Human
CY019430_A_Indonesia_CDC1047S_2007_Human
CY019422_A_Indonesia_CDC1047_2007_Human
CY019414_A_Indonesia_CDC1046T_2007_Human
CY019406_A_Indonesia_CDC1046_2007_Human
CY019398_A_Indonesia_CDC1032T_2007_Human
CY019382_A_Indonesia_CDC1032_2007_Human
CY019390_A_Indonesia_CDC1032N_2007_Human
CY017636_A_Indonesia_CDC938_2006_Human
CY017644_A_Indonesia_CDC938E_2006_Human
CY019350_A_Indonesia_CDC1031_2007_Human
CY019374_A_Indonesia_CDC1031RE2_2007_Human
CY019366_A_Indonesia_CDC1031T2_2007_Human
CY019358_A_Indonesia_CDC1031T_2007_Human
EPI_ISL_23815_A_Indonesia_7261_2008_Human
EPI_ISL_24753_A_Indonesia_7272_2008_Human
EPI_ISL_24602_A_Indonesia_8228_2008_Human
EPI_ISL_150197_A_Indonesia_NIHRD9340_2009_Human
EPI_ISL_150551_A_Indonesia_NIHRD10623_2010_Human
EPI_ISL_150577_A_Indonesia_NIHRD11046_2011_Human
EPI_ISL_150548_A_Indonesia_NIHRD10728_2010_Human
EPI_ISL_150573_A_Indonesia_NIHRD10459_2010_Human
EPI_ISL_98854_A_Indonesia_NIHRD11767_2011_H5N1__Human
EPI_ISL_98855_A_Indonesia_NIHRD11771_2011_H5N1__Human
KR732417_A_chicken_West_Java_Sbg_29_2007_Avian
EPI_ISL_150550_A_Indonesia_NIHRD10364_2010_Human
KM892796_A_chicken_Bali_U8661_2009_Avian
EU146783_A_Indonesia_542H_2006_Human
CY014395_A_Indonesia_CDC610_2006_Human
CY017660_A_Indonesia_CDC835_2006_Human
CY014491_A_Indonesia_CDC669P_2006_Human
CY014513_A_Indonesia_CDC644_2006_Human
CY014483_A_Indonesia_CDC669_2006_Human
CY014527_A_Indonesia_CDC739_2006_Human
EU146823_A_Indonesia_583H_2006_Human
CY014459_A_Indonesia_CDC634T_2006_Human
CY014443_A_Indonesia_CDC634_2006_Human
CY014451_A_Indonesia_CDC634P_2006_Human
CY014390_A_Indonesia_CDC582_2006_Human
CY014505_A_Indonesia_CDC644T_2006_Human
EU146807_A_Indonesia_567H_2006_Human
* branch b
EPI_ISL_24752_A_Indonesia_7379_2008_Human
KR732485_A_chicken_West_Java_TJA_2008_Avian
EPI_ISL_150052_A_Indonesia_NIHRD7988_2008_Human
KR732431_A_chicken_Garut_BBVW_223_2007_Avian
EPI_ISL_151057_A_Indonesia_NIHRD11454_2011_Human
branch c
CY014499_A_Indonesia_CDC699_2006_Human
EU146831_A_Indonesia_604H_2006_Human
branch d
CY014351_A_Indonesia_CDC357_2006_Human
KR732518_A_chicken_WestJava_TASIKO_B_2006_Avian
CY014535_A_Indonesia_CDC742_2006_Human
branch e
EU146678_A_Indonesia_245H_2005_Human
CY014331_A_Indonesia_CDC292N_2005_Human
CY014335_A_Indonesia_CDC292T_2005_Human
EU146670_A_Indonesia_239H_2005_Human
CY014324_A_Indonesia_CDC287E_2005_Human
CY014327_A_Indonesia_CDC287T_2005_Human
EU146711_A_Indonesia_304H_2006_Human
KR732501_A_chicken_Pekalongan_BBVW_2007_Avian
branch f
EU146630_A_Indonesia_6_2005_Human
GQ122491_A_chicken_Central_Java_UT3091_2005_Avian
DQ493362_A_chicken_Kupang_1_NTT_BPPV6_2004_Avian
AY651654_A_Ck_Indonesia_4_2004_Avian
DQ493352_A_chicken_Kulon_Progo_BBVW_2005_Avian
CY017668_A_Indonesia_CDC836_2006_Human
CY017676_A_Indonesia_CDC836T_2006_Human
CY017652_A_Indonesia_CDC940_2006_Human
CY017684_A_Indonesia_CDC759_2006_Human
EU146695_A_Indonesia_283H_2006_Human
branch g
EU146696_A_Indonesia_286H_2006_Human
CY014336_A_Indonesia_CDC326_2006_Human
CY014339_A_Indonesia_CDC326N_2006_Human
CY014343_A_Indonesia_CDC326N2_2006_Human
CY014345_A_Indonesia_CDC326T_2006_Human
CY014349_A_Indonesia_CDC329_2006_Human
EU146719_A_Indonesia_292H_2006_Human
branch h
CY014316_A_feline_Indonesia_CDC1_2006_Cat
KR732497_A_chicken_WestJava_SMI_PAT_2006_Avian

PB1-Group31

PB1-Group32

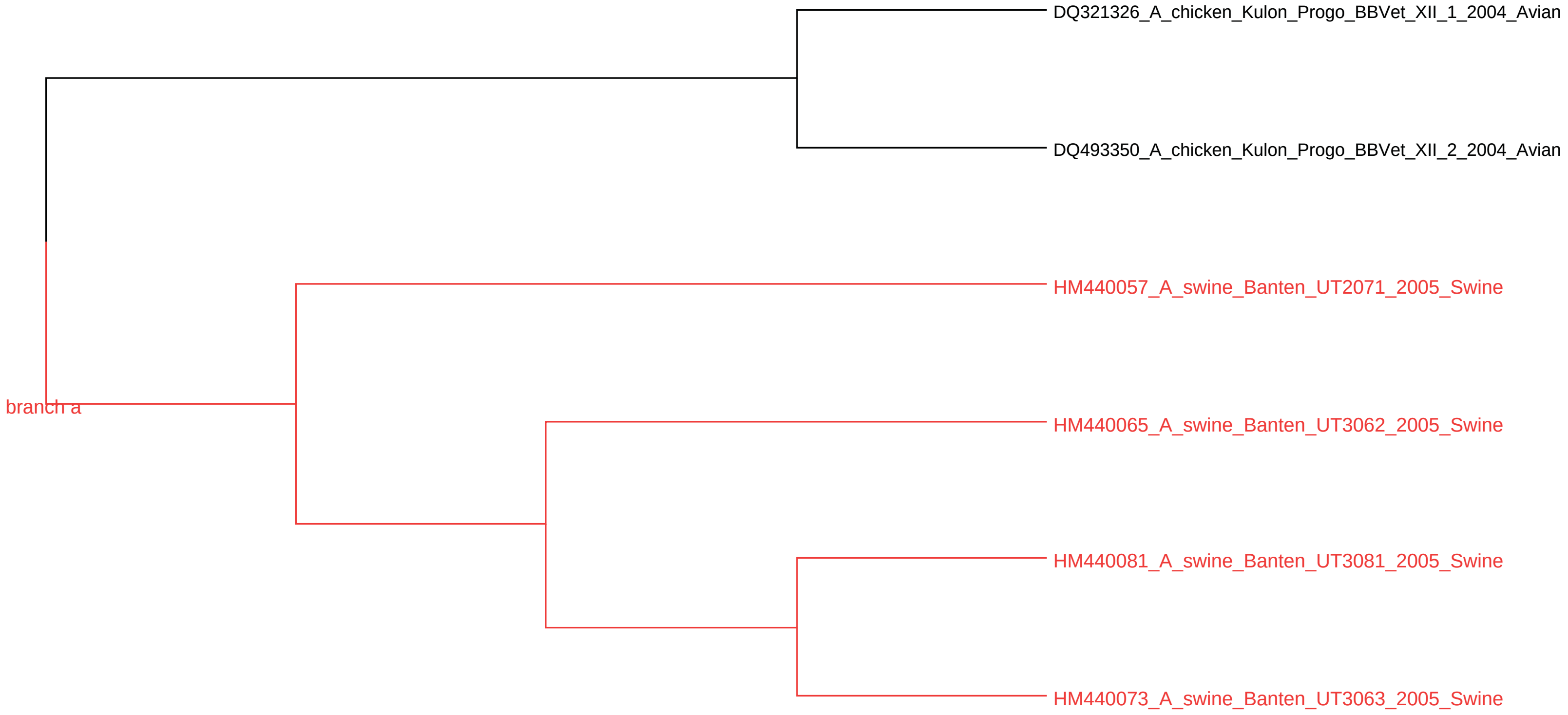
DQ321326_A_chicken_Kulon_Progo_BBVet_XII_1_2004_Avian
DQ493350_A_chicken_Kulon_Progo_BBVet_XII_2_2004_Avian
HM440057_A_swine_Banten_UT2071_2005_Swine
branch a
HM440065_A_swine_Banten_UT3062_2005_Swine
HM440081_A_swine_Banten_UT3081_2005_Swine
HM440073_A_swine_Banten_UT3063_2005_Swine

PB1-Group33

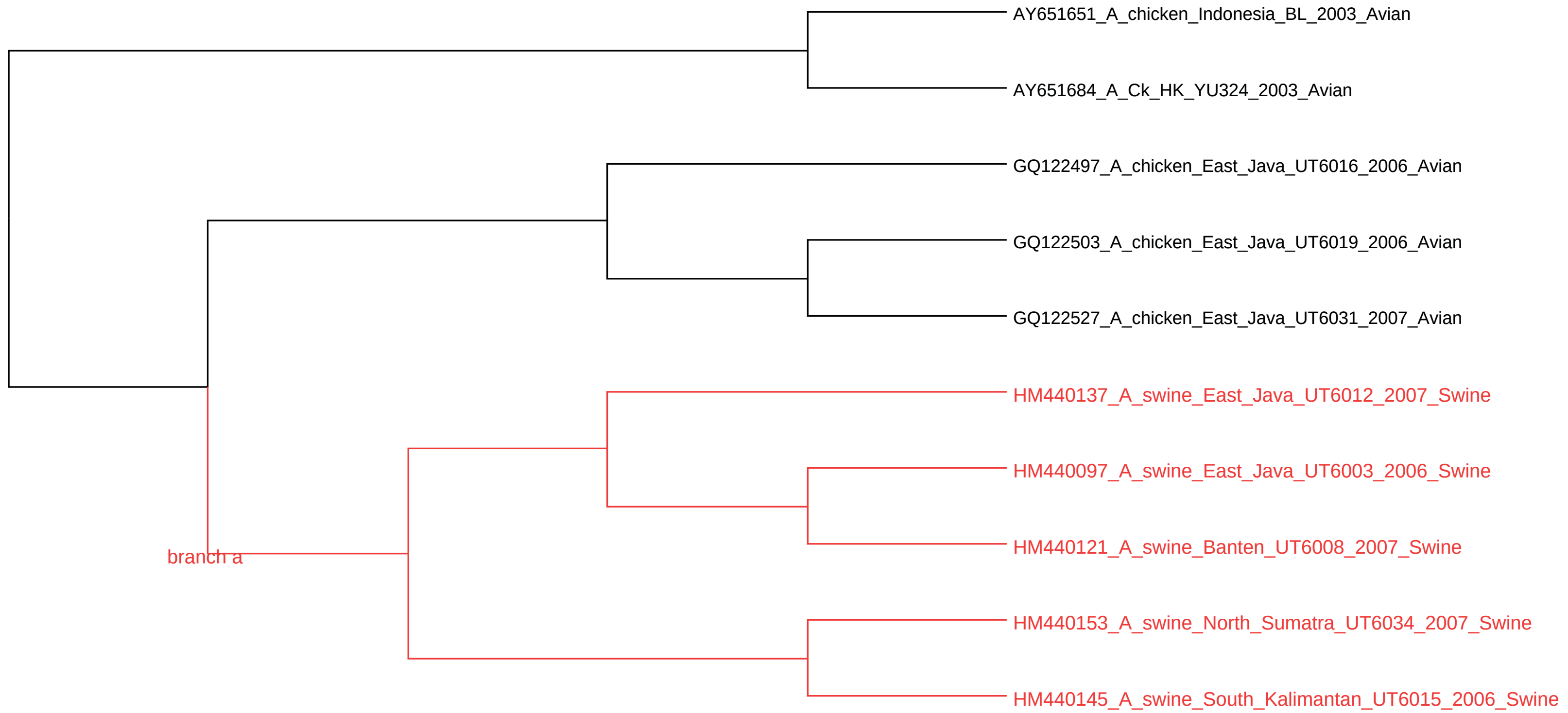
AY651651_A_chicken_Indonesia_BL_2003_Avian
AY651684_A_Ck_HK_YU324_2003_Avian
GQ122497_A_chicken_East_Java_UT6016_2006_Avian
GQ122503_A_chicken_East_Java_UT6019_2006_Avian
GQ122527_A_chicken_East_Java_UT6031_2007_Avian
HM440137_A_swine_East_Java_UT6012_2007_Swine
HM440097_A_swine_East_Java_UT6003_2006_Swine
HM440121_A_swine_Banten_UT6008_2007_Swine
HM440153_A_swine_North_Sumatra_UT6034_2007_Swine
HM440145_A_swine_South_Kalimantan_UT6015_2006_Swine
branch a

PB1-Group34

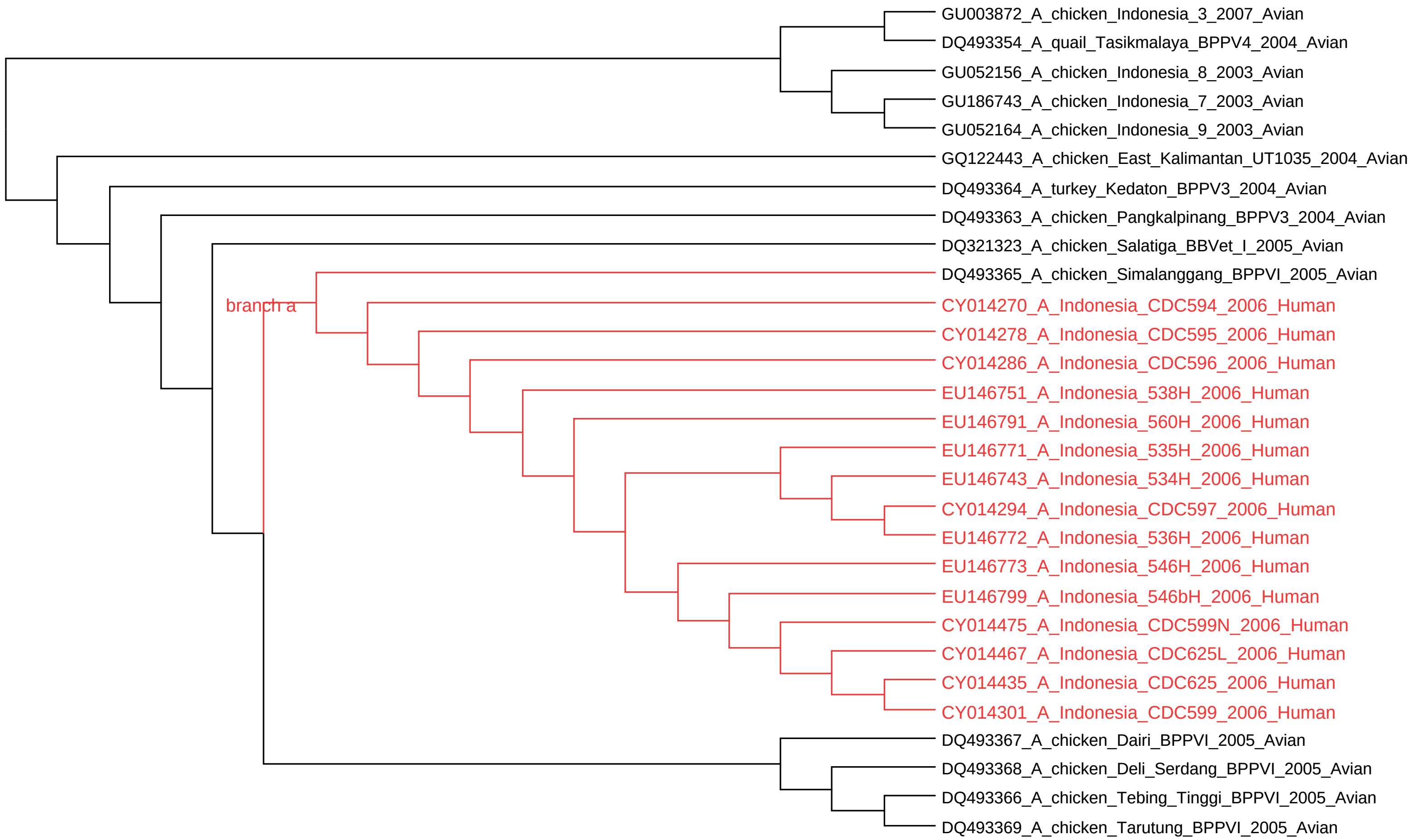
GU003872_A_chicken_Indonesia_3_2007_Avian
DQ493354_A_quail_Tasikmalaya_BPPV4_2004_Avian
GU052156_A_chicken_Indonesia_8_2003_Avian
GU186743_A_chicken_Indonesia_7_2003_Avian
GU052164_A_chicken_Indonesia_9_2003_Avian
GQ122443_A_chicken_East_Kalimantan_UT1035_2004_Avian
DQ493364_A_turkey_Kedaton_BPPV3_2004_Avian
DQ493363_A_chicken_Pangkalpinang_BPPV3_2004_Avian
DQ321323_A_chicken_Salatiga_BBVet_I_2005_Avian
DQ493365_A_chicken_Simalanggang_BPPVI_2005_Avian
branch a
CY014270_A_Indonesia_CDC594_2006_Human
CY014278_A_Indonesia_CDC595_2006_Human
CY014286_A_Indonesia_CDC596_2006_Human
EU146751_A_Indonesia_538H_2006_Human
EU146791_A_Indonesia_560H_2006_Human
EU146771_A_Indonesia_535H_2006_Human
EU146743_A_Indonesia_534H_2006_Human
CY014294_A_Indonesia_CDC597_2006_Human
EU146772_A_Indonesia_536H_2006_Human
EU146773_A_Indonesia_546H_2006_Human
EU146799_A_Indonesia_546bH_2006_Human
CY014475_A_Indonesia_CDC599N_2006_Human
CY014467_A_Indonesia_CDC625L_2006_Human
CY014435_A_Indonesia_CDC625_2006_Human
CY014301_A_Indonesia_CDC599_2006_Human
DQ493367_A_chicken_Dairi_BPPVI_2005_Avian
DQ493368_A_chicken_Deli_Serdang_BPPVI_2005_Avian
DQ493366_A_chicken_Tebing_Tinggi_BPPVI_2005_Avian
DQ493369_A_chicken_Tarutung_BPPVI_2005_Avian

PB1-Group35

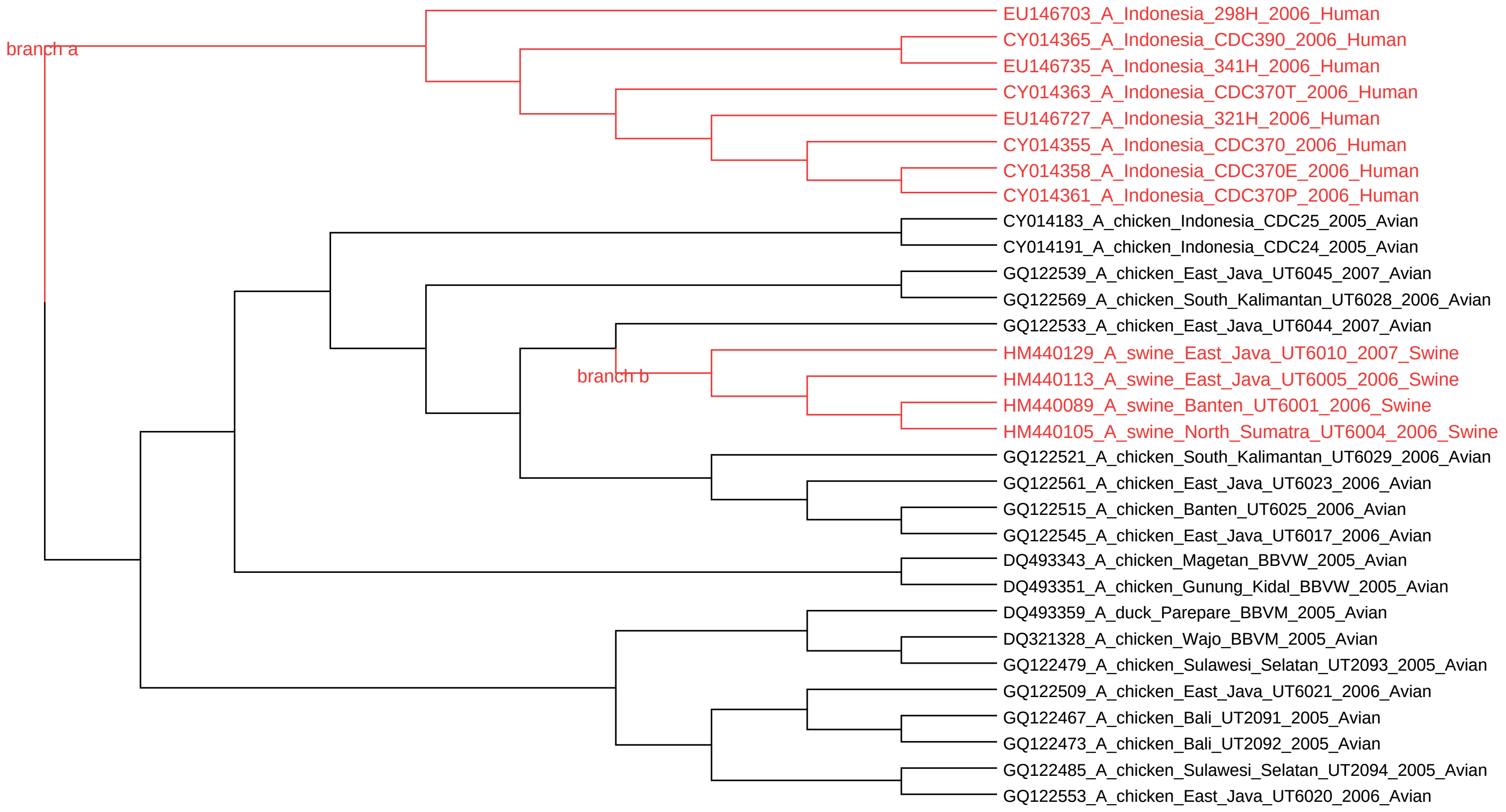
branch a
branch b
EU146703_A_Indonesia_298H_2006_Human
CY014365_A_Indonesia_CDC390_2006_Human
EU146735_A_Indonesia_341H_2006_Human
CY014363_A_Indonesia_CDC370T_2006_Human
EU146727_A_Indonesia_321H_2006_Human
CY014355_A_Indonesia_CDC370_2006_Human
CY014358_A_Indonesia_CDC370E_2006_Human
CY014361_A_Indonesia_CDC370P_2006_Human
CY014183_A_chicken_Indonesia_CDC25_2005_Avian
CY014191_A_chicken_Indonesia_CDC24_2005_Avian
GQ122539_A_chicken_East_Java_UT6045_2007_Avian
GQ122569_A_chicken_South_Kalimantan_UT6028_2006_Avian
GQ122533_A_chicken_East_Java_UT6044_2007_Avian
HM440129_A_swine_East_Java_UT6010_2007_Swine
HM440113_A_swine_East_Java_UT6005_2006_Swine
HM440089_A_swine_Banten_UT6001_2006_Swine
HM440105_A_swine_North_Sumatra_UT6004_2006_Swine
GQ122521_A_chicken_South_Kalimantan_UT6029_2006_Avian
GQ122561_A_chicken_East_Java_UT6023_2006_Avian
GQ122515_A_chicken_Banten_UT6025_2006_Avian
GQ122545_A_chicken_East_Java_UT6017_2006_Avian
DQ493343_A_chicken_Magetan_BBVW_2005_Avian
DQ493351_A_chicken_Gunung_Kidal_BBVW_2005_Avian
DQ493359_A_duck_Parepare_BBVM_2005_Avian
DQ321328_A_chicken_Wajo_BBVM_2005_Avian
GQ122479_A_chicken_Sulawesi_Selatan_UT2093_2005_Avian
GQ122509_A_chicken_East_Java_UT6021_2006_Avian
GQ122467_A_chicken_Bali_UT2091_2005_Avian
GQ122473_A_chicken_Bali_UT2092_2005_Avian
GQ122485_A_chicken_Sulawesi_Selatan_UT2094_2005_Avian
GQ122553_A_chicken_East_Java_UT6020_2006_Avian

PB1-Group36

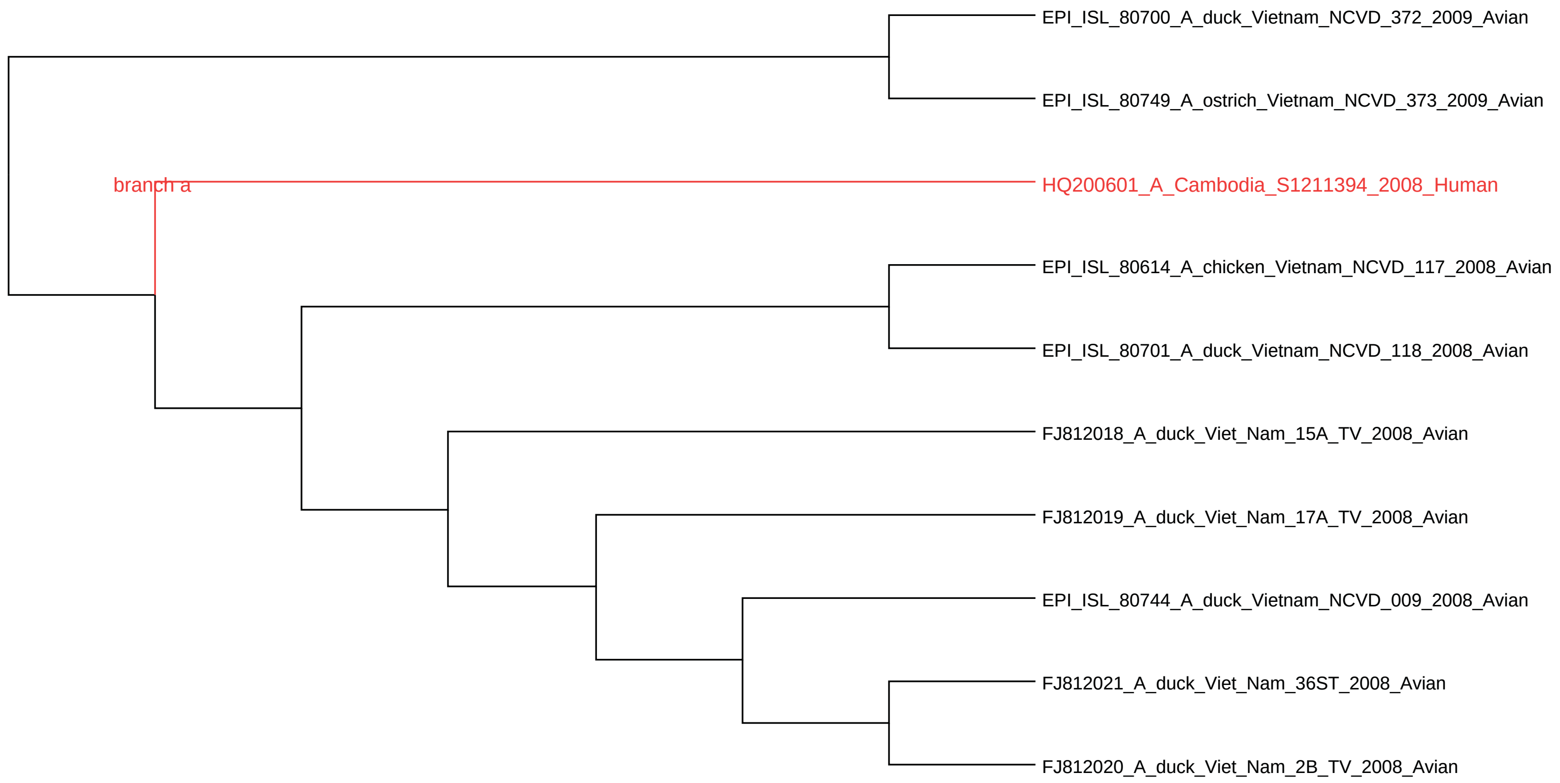

branch a
EPI_ISL_80700_A_duck_Vietnam_NCVD_372_2009_Avian
EPI_ISL_80749_A_ostrich_Vietnam_NCVD_373_2009_Avian
HQ200601_A_Cambodia_S1211394_2008_Human
EPI_ISL_80614_A_chicken_Vietnam_NCVD_117_2008_Avian
EPI_ISL_80701_A_duck_Vietnam_NCVD_118_2008_Avian
FJ812018_A_duck_Viet_Nam_15A_TV_2008_Avian
FJ812019_A_duck_Viet_Nam_17A_TV_2008_Avian
EPI_ISL_80744_A_duck_Vietnam_NCVD_009_2008_Avian
FJ812021_A_duck_Viet_Nam_36ST_2008_Avian
FJ812020_A_duck_Viet_Nam_2B_TV_2008_Avian

PB1-Group37

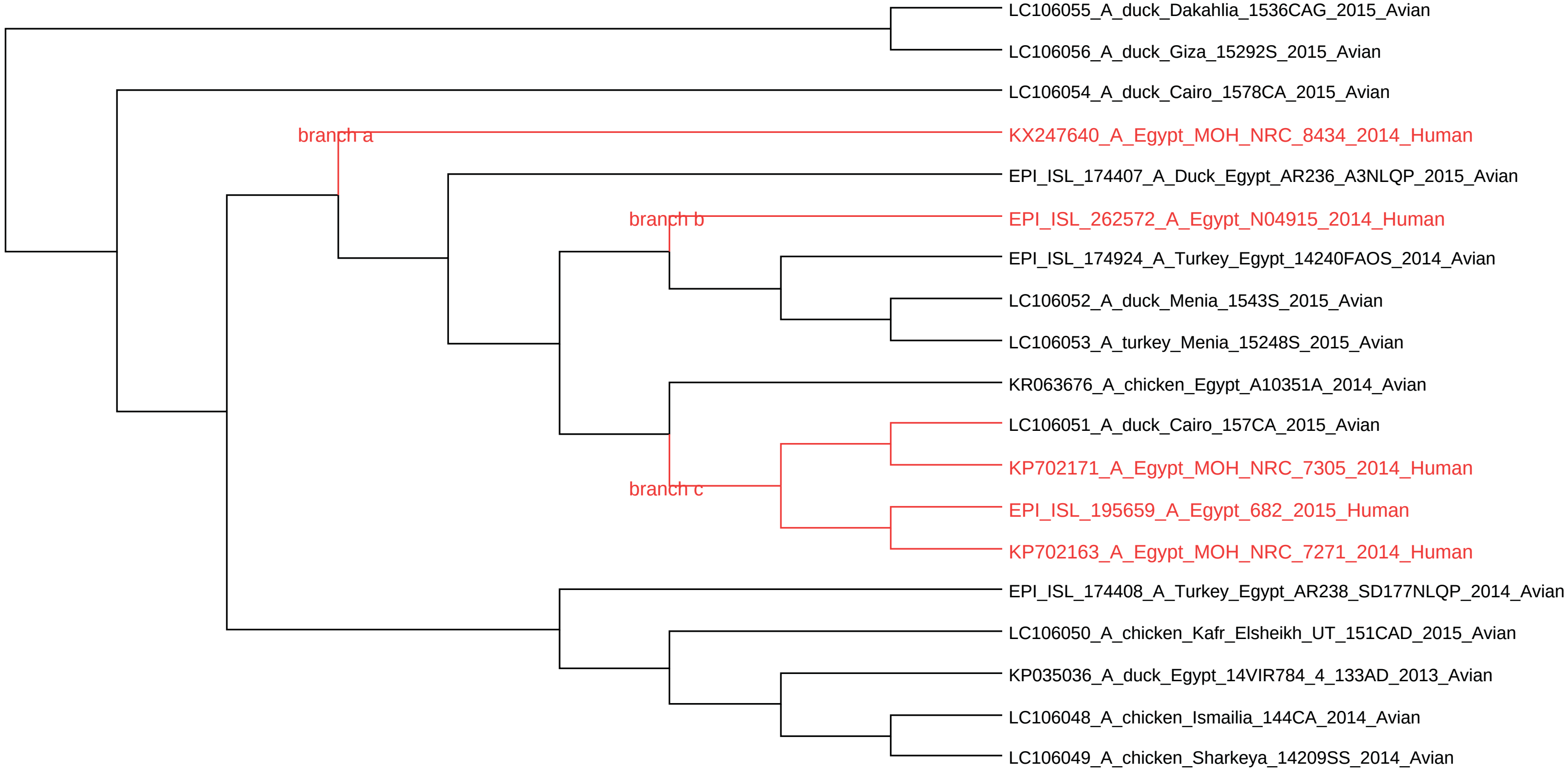
LC106055_A_duck_Dakahlia_1536CAG_2015_Avian
LC106056_A_duck_Giza_15292S_2015_Avian
LC106054_A_duck_Cairo_1578CA_2015_Avian
branch a
KX247640_A_Egypt_MOH_NRC_8434_2014_Human
EPI_ISL_174407_A_Duck_Egypt_AR236_A3NLQP_2015_Avian
branch b
EPI_ISL_262572_A_Egypt_N04915_2014_Human
EPI_ISL_174924_A_Turkey_Egypt_14240FAOS_2014_Avian
LC106052_A_duck_Menia_1543S_2015_Avian
LC106053_A_turkey_Menia_15248S_2015_Avian
KR063676_A_chicken_Egypt_A10351A_2014_Avian
LC106051_A_duck_Cairo_157CA_2015_Avian
KP702171_A_Egypt_MOH_NRC_7305_2014_Human
branch c
EPI_ISL_195659_A_Egypt_682_2015_Human
KP702163_A_Egypt_MOH_NRC_7271_2014_Human
EPI_ISL_174408_A_Turkey_Egypt_AR238_SD177NLQP_2014_Avian
LC106050_A_chicken_Kafr_Elsheikh_UT_151CAD_2015_Avian
KP035036_A_duck_Egypt_14VIR784_4_133AD_2013_Avian
LC106048_A_chicken_Ismailia_144CA_2014_Avian
LC106049_A_chicken_Sharkeya_14209SS_2014_Avian

PB1-Group38

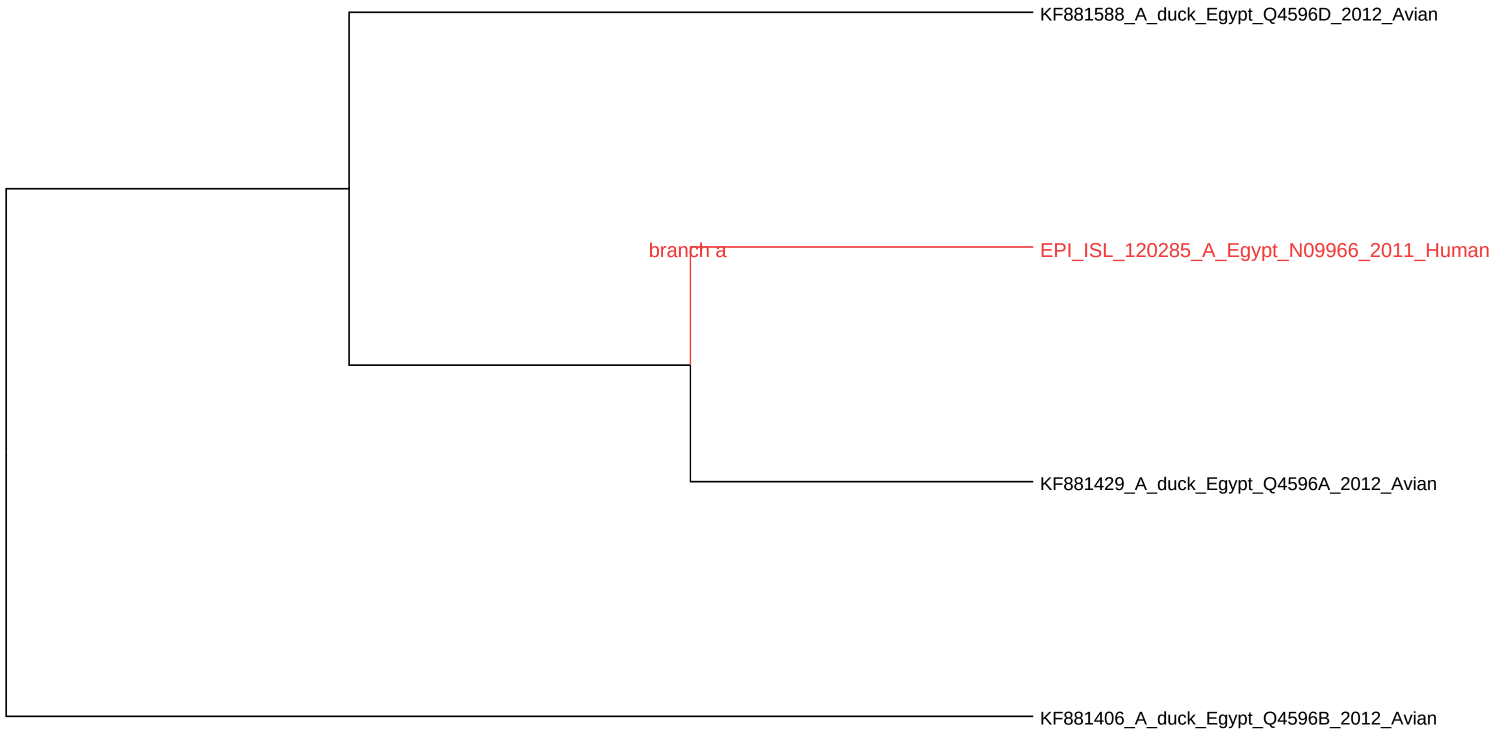
KF881588_A_duck_Egypt_Q4596D_2012_Avian
branch a
EPI_ISL_120285_A_Egypt_N09966_2011_Human
KF881429_A_duck_Egypt_Q4596A_2012_Avian
KF881406_A_duck_Egypt_Q4596B_2012_Avian

PB1-Group39

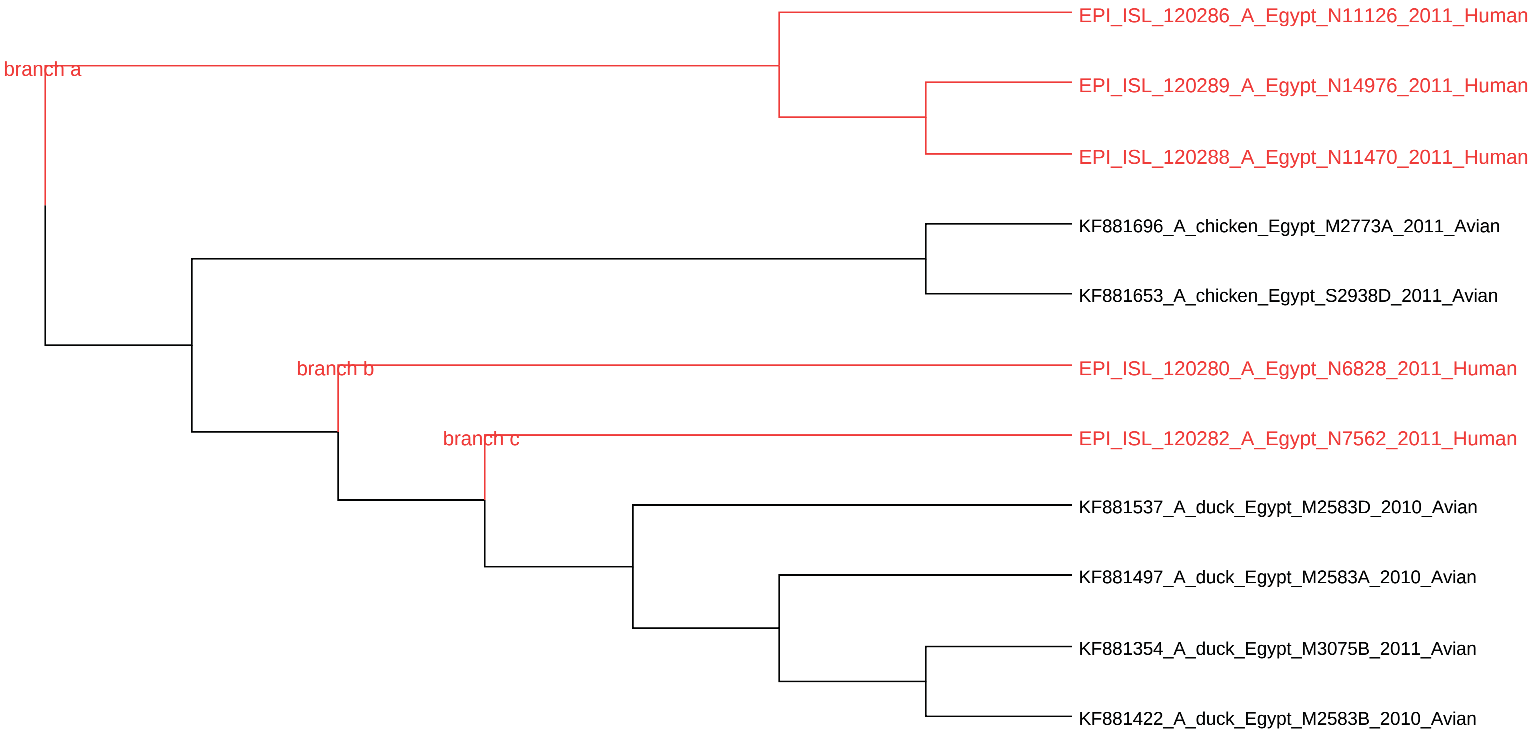
branch a
EPI_ISL_120286_A_Egypt_N11126_2011_Human
EPI_ISL_120289_A_Egypt_N14976_2011_Human
EPI_ISL_120288_A_Egypt_N11470_2011_Human
KF881696_A_chicken_Egypt_M2773A_2011_Avian
KF881653_A_chicken_Egypt_S2938D_2011_Avian
branch b
EPI_ISL_120280_A_Egypt_N6828_2011_Human
branch c
EPI_ISL_120282_A_Egypt_N7562_2011_Human
KF881537_A_duck_Egypt_M2583D_2010_Avian
KF881497_A_duck_Egypt_M2583A_2010_Avian
KF881354_A_duck_Egypt_M3075B_2011_Avian
KF881422_A_duck_Egypt_M2583B_2010_Avian

PB1-Group40

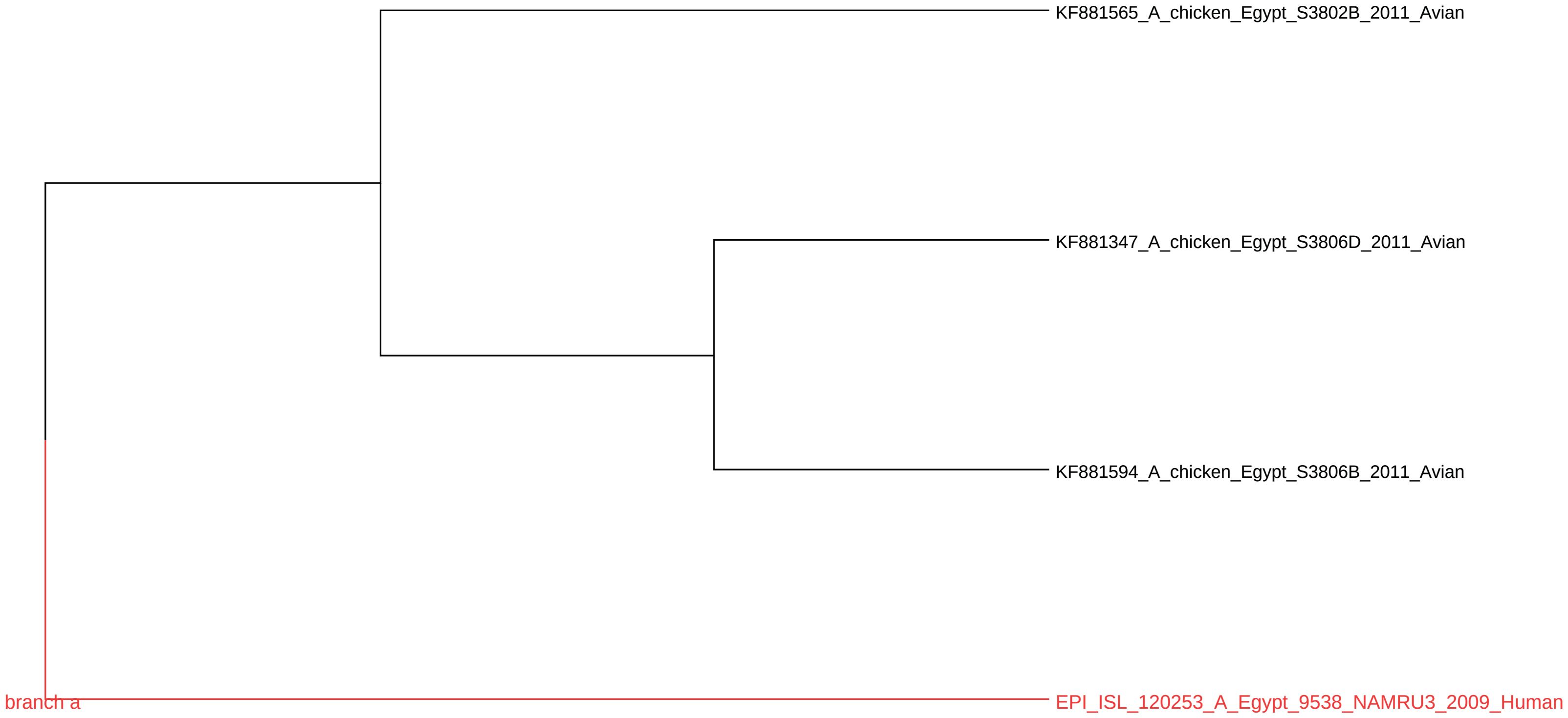
KF881565_A_chicken_Egypt_S3802B_2011_Avian
KF881347_A_chicken_Egypt_S3806D_2011_Avian
KF881594_A_chicken_Egypt_S3806B_2011_Avian
branch a
EPI_ISL_120253_A_Egypt_9538_NAMRU3_2009_Human

PB1-Group41

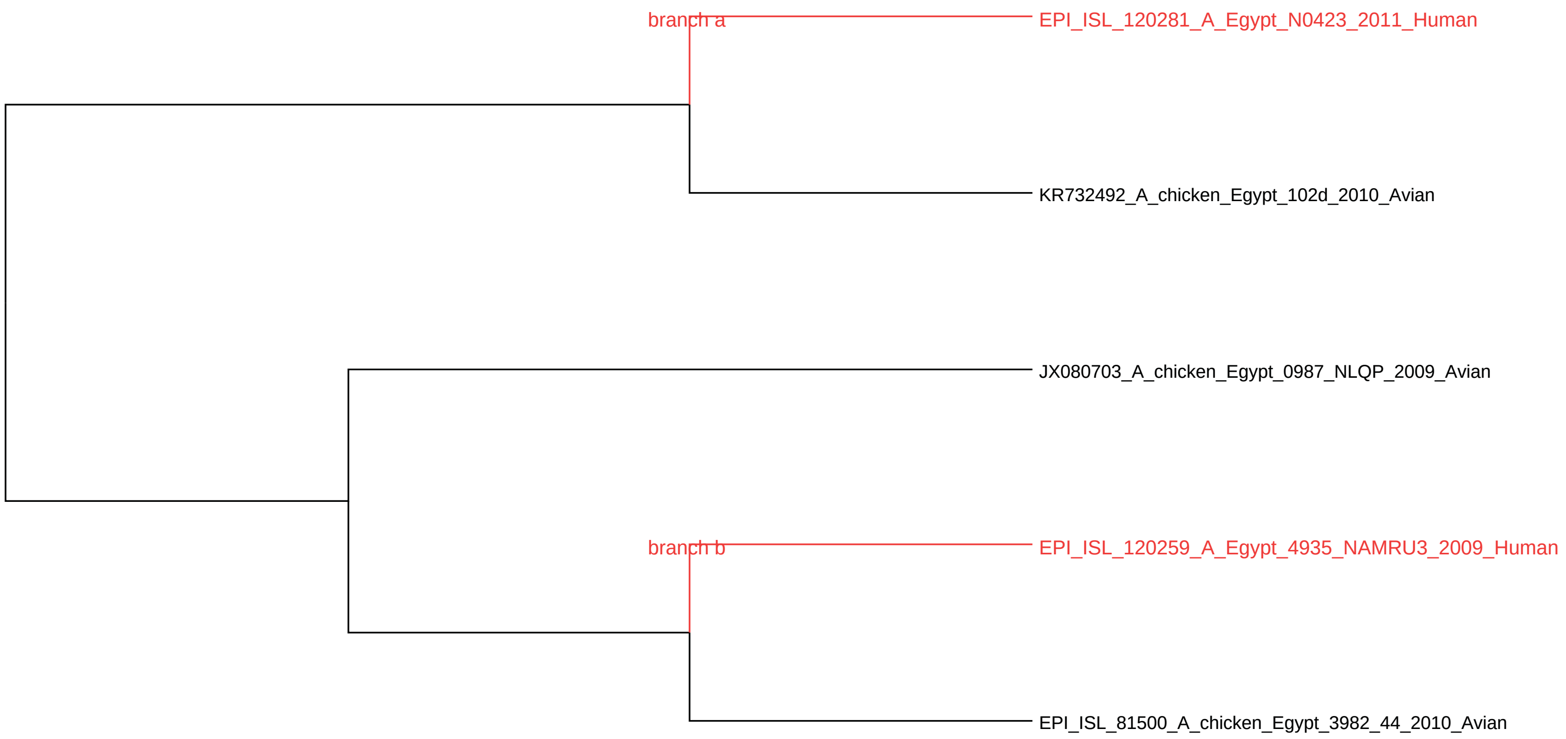
branch a
EPI_ISL_120281_A_Egypt_N0423_2011_Human
KR732492_A_chicken_Egypt_102d_2010_Avian
JX080703_A_chicken_Egypt_0987_NLQP_2009_Avian
branch b
EPI_ISL_120259_A_Egypt_4935_NAMRU3_2009_Human
EPI_ISL_81500_A_chicken_Egypt_3982_44_2010_Avian

PB1-Group42

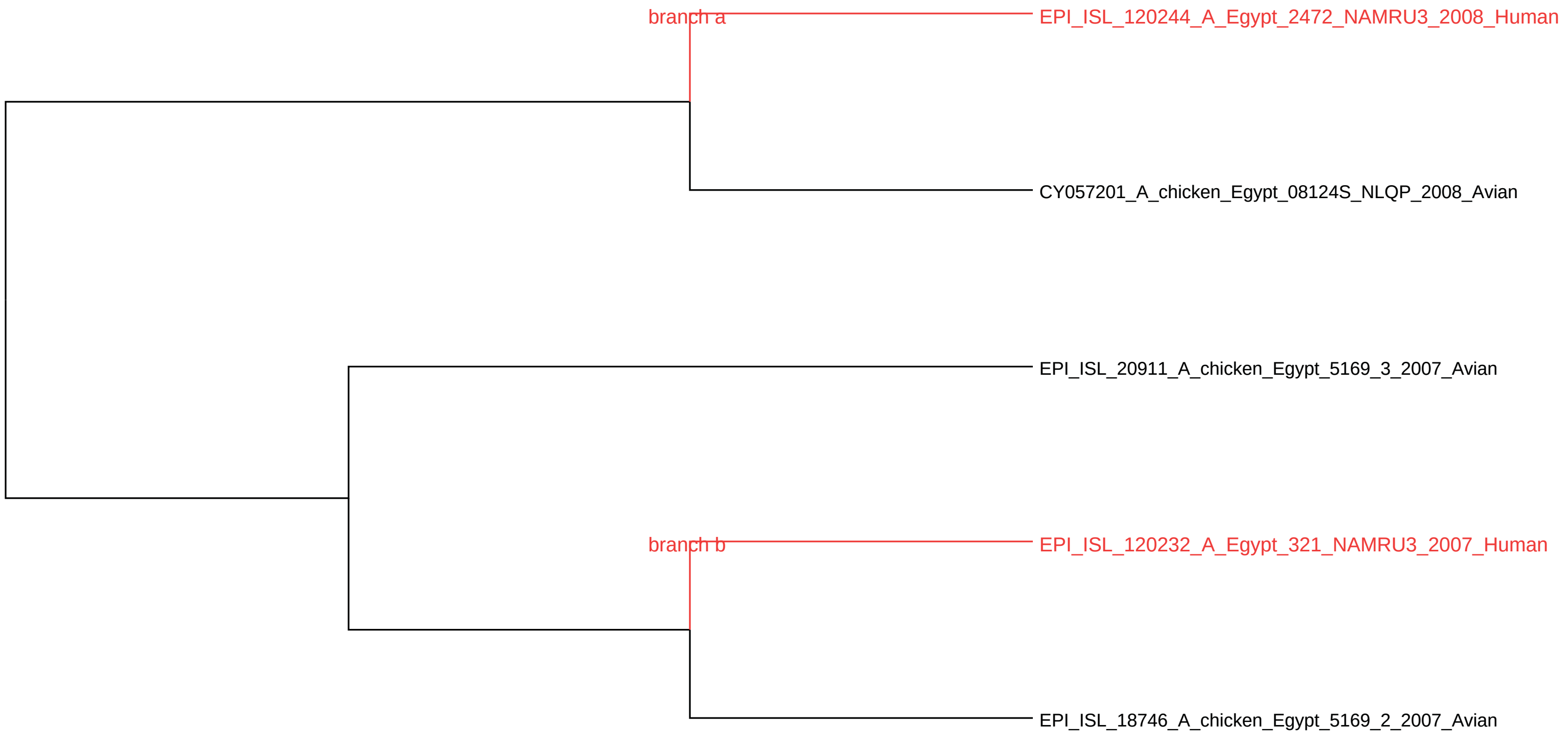
branch a
EPI_ISL_120244_A_Egypt_2472_NAMRU3_2008_Human
CY057201_A_chicken_Egypt_08124S_NLQP_2008_Avian
EPI_ISL_20911_A_chicken_Egypt_5169_3_2007_Avian
branch b
EPI_ISL_120232_A_Egypt_321_NAMRU3_2007_Human
EPI_ISL_18746_A_chicken_Egypt_5169_2_2007_Avian

PB1-Group43

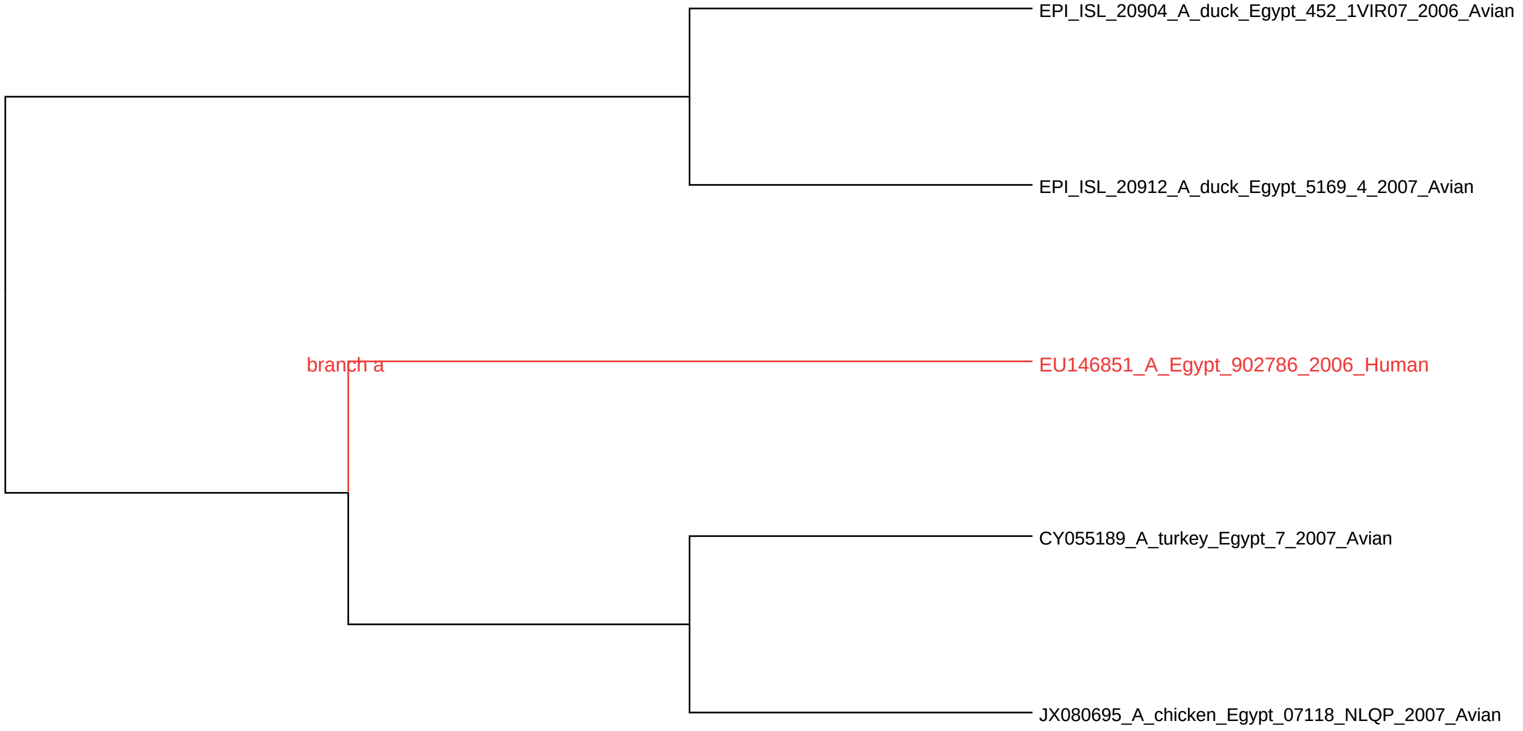

EPI_ISL_20904_A_duck_Egypt_452_1VIR07_2006_Avian
EPI_ISL_20912_A_duck_Egypt_5169_4_2007_Avian
branch a
EU146851_A_Egypt_902786_2006_Human
CY055189_A_turkey_Egypt_7_2007_Avian
JX080695_A_chicken_Egypt_07118_NLQP_2007_Avian

PB1-Group44

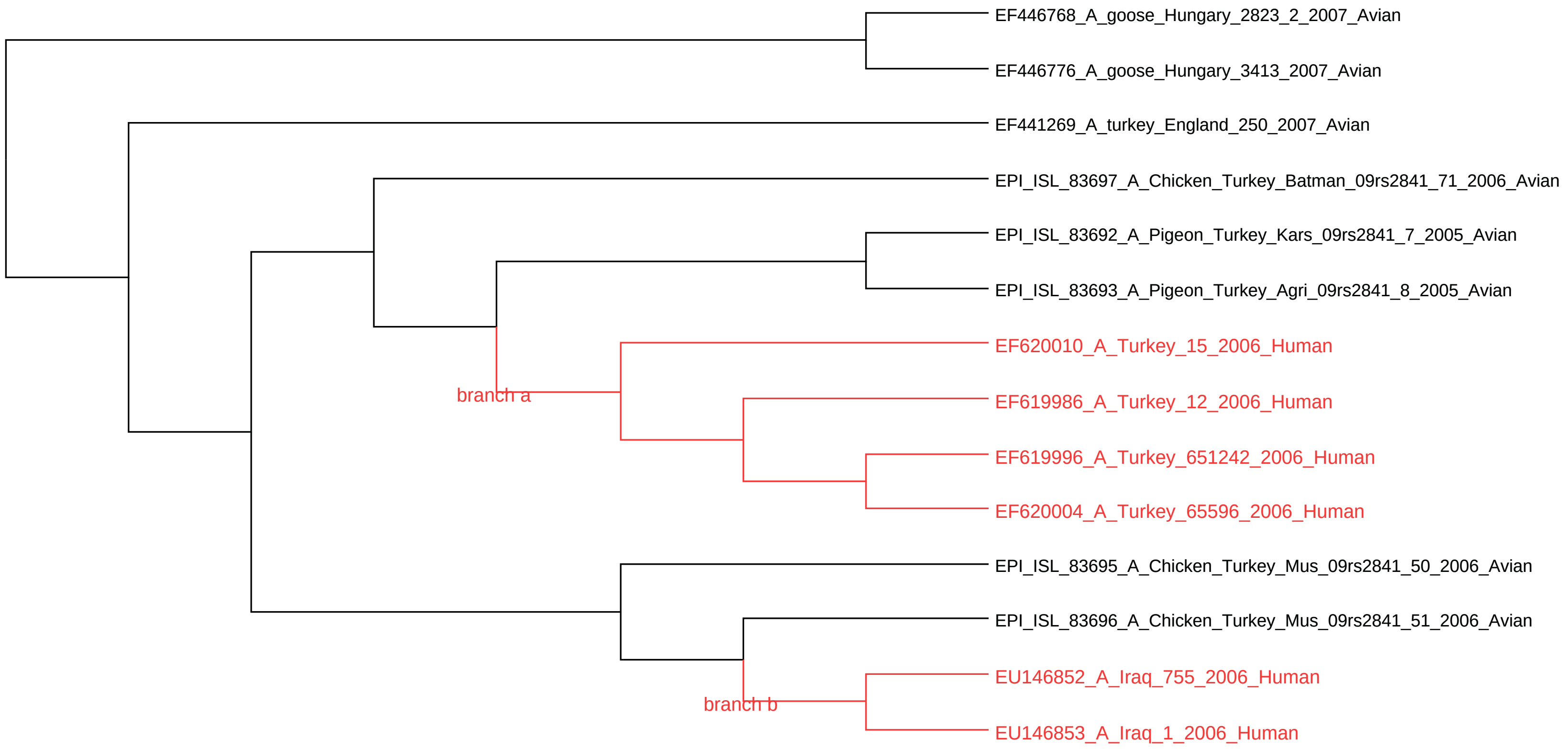
EF446768_A_goose_Hungary_2823_2_2007_Avian
EF446776_A_goose_Hungary_3413_2007_Avian
EF441269_A_turkey_England_250_2007_Avian
EPI_ISL_83697_A_Chicken_Turkey_Batman_09rs2841_71_2006_Avian
EPI_ISL_83692_A_Pigeon_Turkey_Kars_09rs2841_7_2005_Avian
EPI_ISL_83693_A_Pigeon_Turkey_Agri_09rs2841_8_2005_Avian
EF620010_A_Turkey_15_2006_Human
branch a
EF619986_A_Turkey_12_2006_Human
EF619996_A_Turkey_651242_2006_Human
EF620004_A_Turkey_65596_2006_Human
EPI_ISL_83695_A_Chicken_Turkey_Mus_09rs2841_50_2006_Avian
EPI_ISL_83696_A_Chicken_Turkey_Mus_09rs2841_51_2006_Avian
EU146852_A_Iraq_755_2006_Human
branch b
EU146853_A_Iraq_1_2006_Human

PB1-Group45

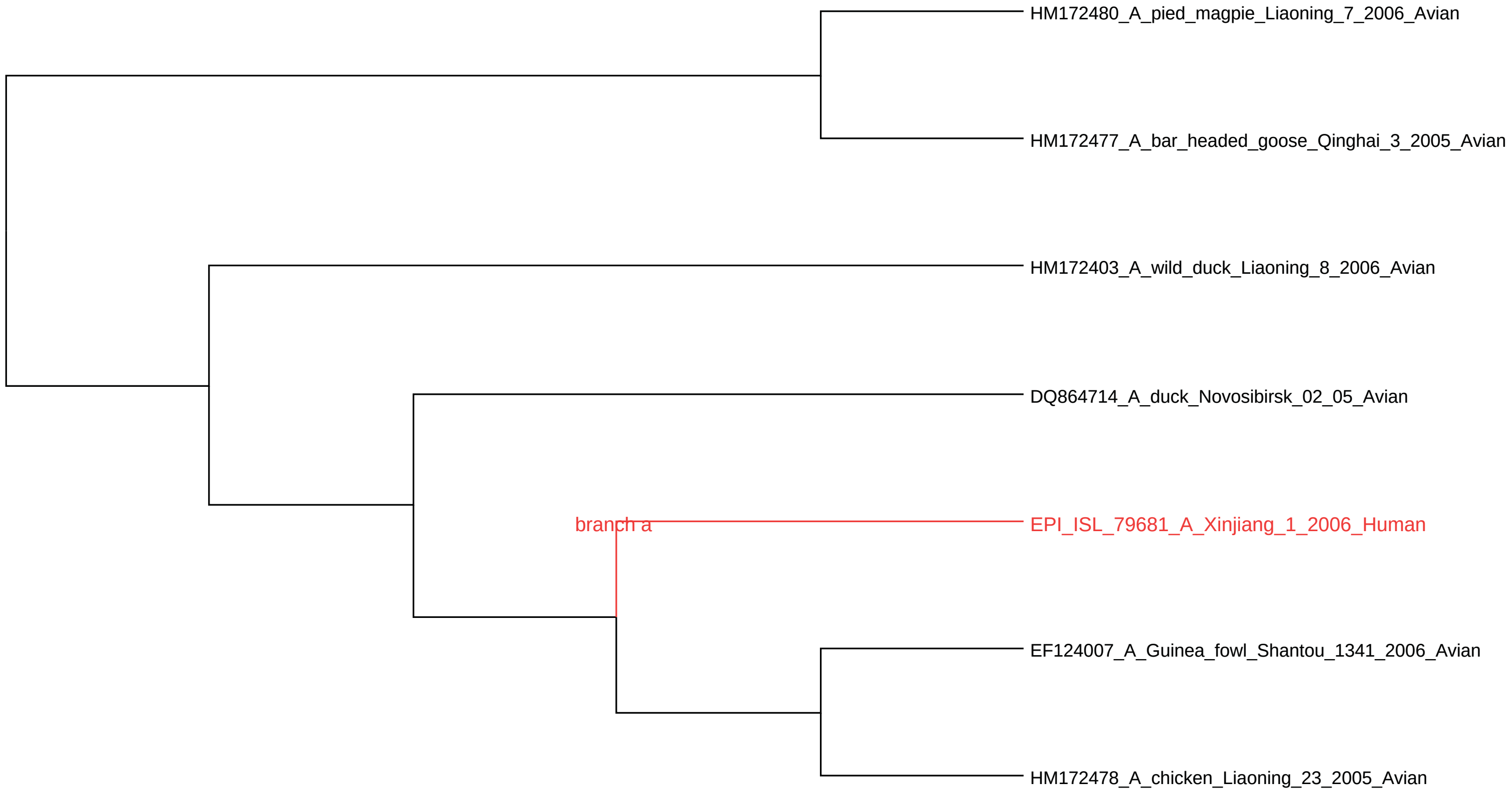
HM172480_A_pied_magpie_Liaoning_7_2006_Avian
HM172477_A_bar_headed_goose_Qinghai_3_2005_Avian
HM172403_A_wild_duck_Liaoning_8_2006_Avian
DQ864714_A_duck_Novosibirsk_02_05_Avian
branch a
EPI_ISL_79681_A_Xinjiang_1_2006_Human
EF124007_A_Guinea_fowl_Shantou_1341_2006_Avian
HM172478_A_chicken_Liaoning_23_2005_Avian

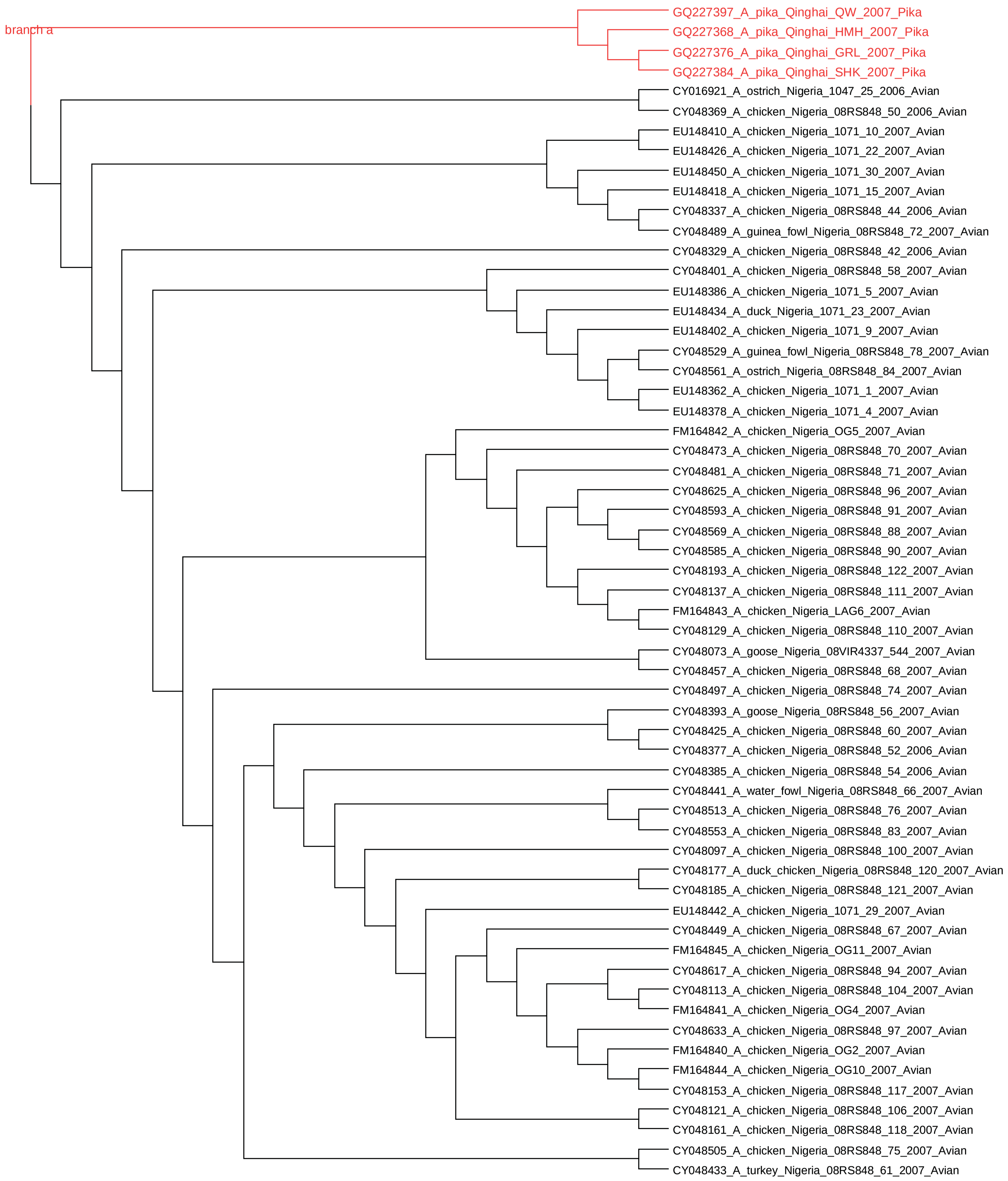

1-Group46

PB1-Group46

PB1-Group47

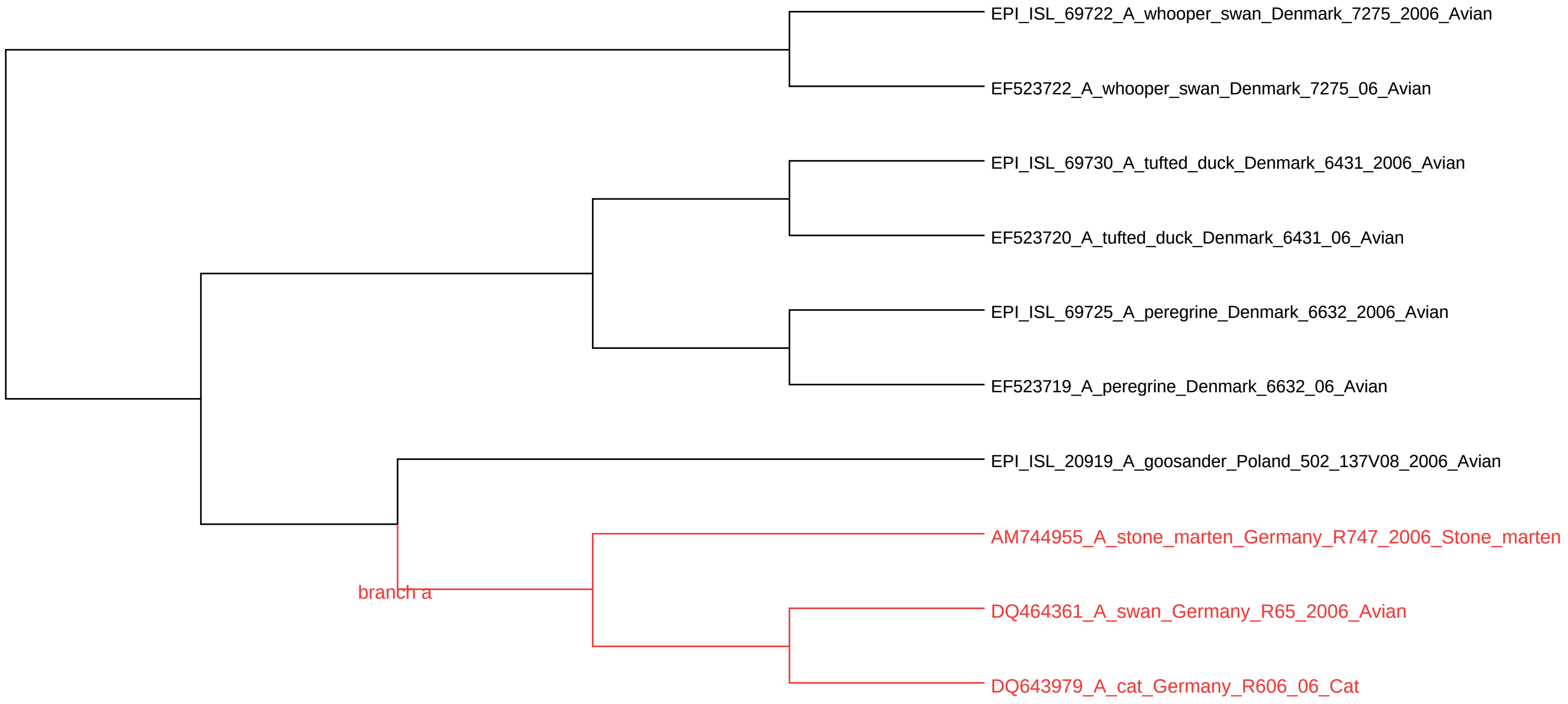
EPI_ISL_69722_A_whooper_swan_Denmark_7275_2006_Avian
EF523722_A_whooper_swan_Denmark_7275_06_Avian
EPI_ISL_69730_A_tufted_duck_Denmark_6431_2006_Avian
EF523720_A_tufted_duck_Denmark_6431_06_Avian
EPI_ISL_69725_A_peregrine_Denmark_6632_2006_Avian
EF523719_A_peregrine_Denmark_6632_06_Avian
EPI_ISL_20919_A_goosander_Poland_502_137V08_2006_Avian
AM744955_A_stone_marten_Germany_R747_2006_Stone_marten
branch a
DQ464361_A_swan_Germany_R65_2006_Avian
DQ643979_A_cat_Germany_R606_06_Cat

PB1-Group48

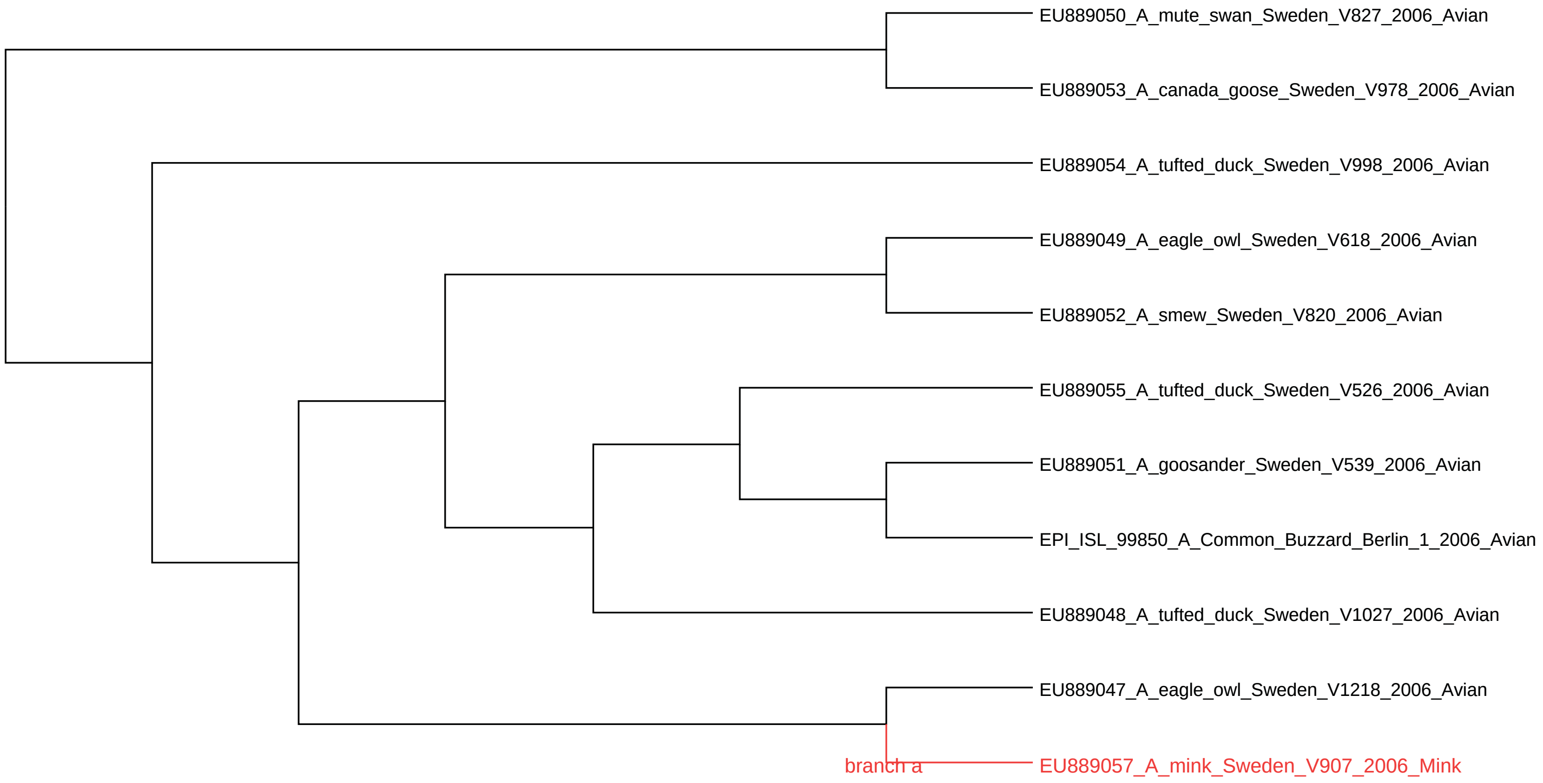
EU889050_A_mute_swan_Sweden_V827_2006_Avian
EU889053_A_canada_goose_Sweden_V978_2006_Avian
EU889054_A_tufted_duck_Sweden_V998_2006_Avian
EU889049_A_eagle_owl_Sweden_V618_2006_Avian
EU889052_A_smew_Sweden_V820_2006_Avian
EU889055_A_tufted_duck_Sweden_V526_2006_Avian
EU889051_A_goosander_Sweden_V539_2006_Avian
EPI_ISL_99850_A_Common_Buzzard_Berlin_1_2006_Avian
EU889048_A_tufted_duck_Sweden_V1027_2006_Avian
EU889047_A_eagle_owl_Sweden_V1218_2006_Avian
branch a
EU889057_A_mink_Sweden_V907_2006_Mink

PB1-Group49

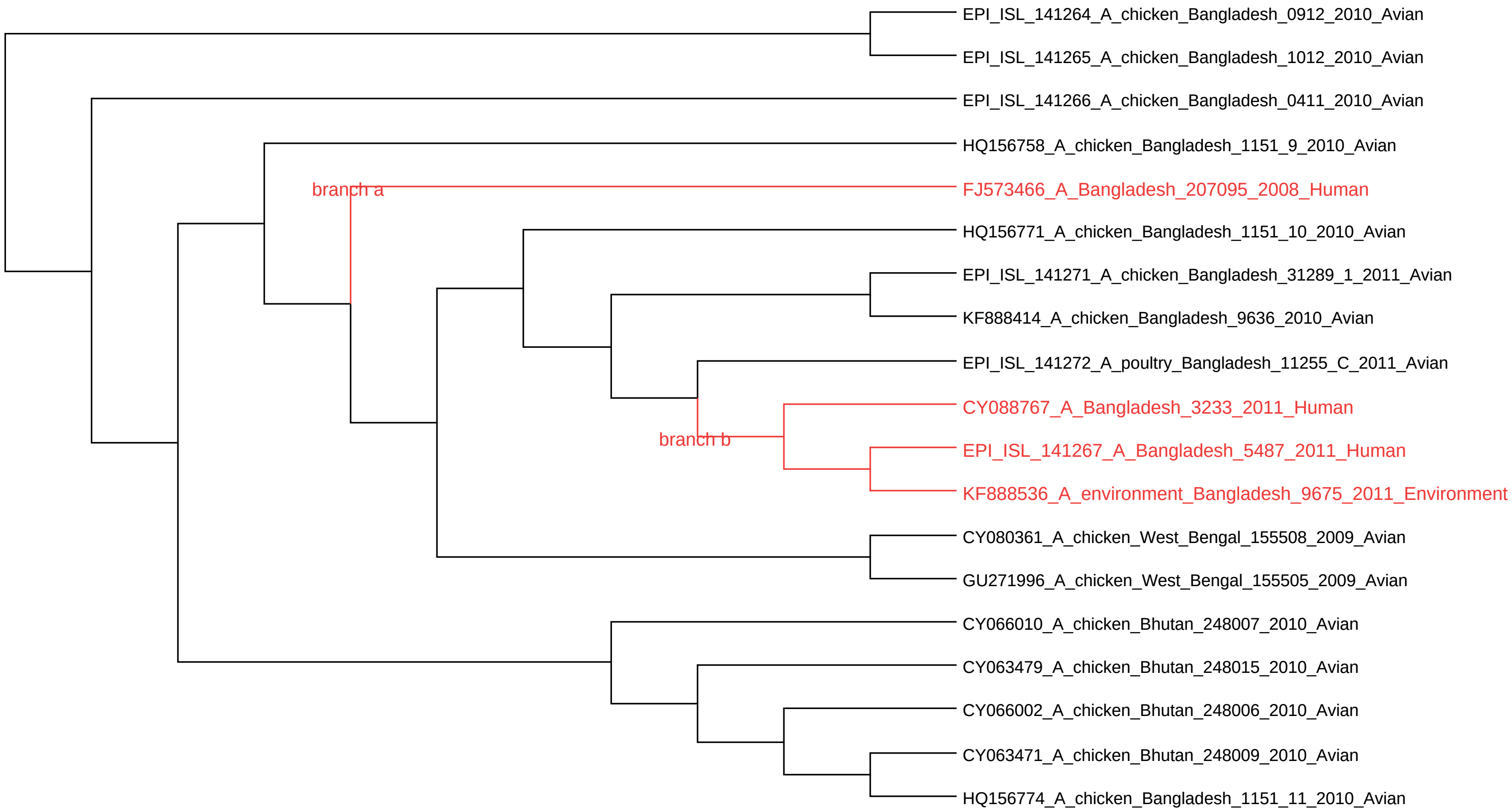
EPI_ISL_141264_A_chicken_Bangladesh_0912_2010_Avian
EPI_ISL_141265_A_chicken_Bangladesh_1012_2010_Avian
EPI_ISL_141266_A_chicken_Bangladesh_0411_2010_Avian
HQ156758_A_chicken_Bangladesh_1151_9_2010_Avian
branch a
FJ573466_A_Bangladesh_207095_2008_Human
HQ156771_A_chicken_Bangladesh_1151_10_2010_Avian
EPI_ISL_141271_A_chicken_Bangladesh_31289_1_2011_Avian
KF888414_A_chicken_Bangladesh_9636_2010_Avian
EPI_ISL_141272_A_poultry_Bangladesh_11255_C_2011_Avian
CY088767_A_Bangladesh_3233_2011_Human
branch b
EPI_ISL_141267_A_Bangladesh_5487_2011_Human
KF888536_A_environment_Bangladesh_9675_2011_Environment
CY080361_A_chicken_West_Bengal_155508_2009_Avian
GU271996_A_chicken_West_Bengal_155505_2009_Avian
CY066010_A_chicken_Bhutan_248007_2010_Avian
CY063479_A_chicken_Bhutan_248015_2010_Avian
CY066002_A_chicken_Bhutan_248006_2010_Avian
CY063471_A_chicken_Bhutan_248009_2010_Avian
HQ156774_A_chicken_Bangladesh_1151_11_2010_Avian

PB1-Group50

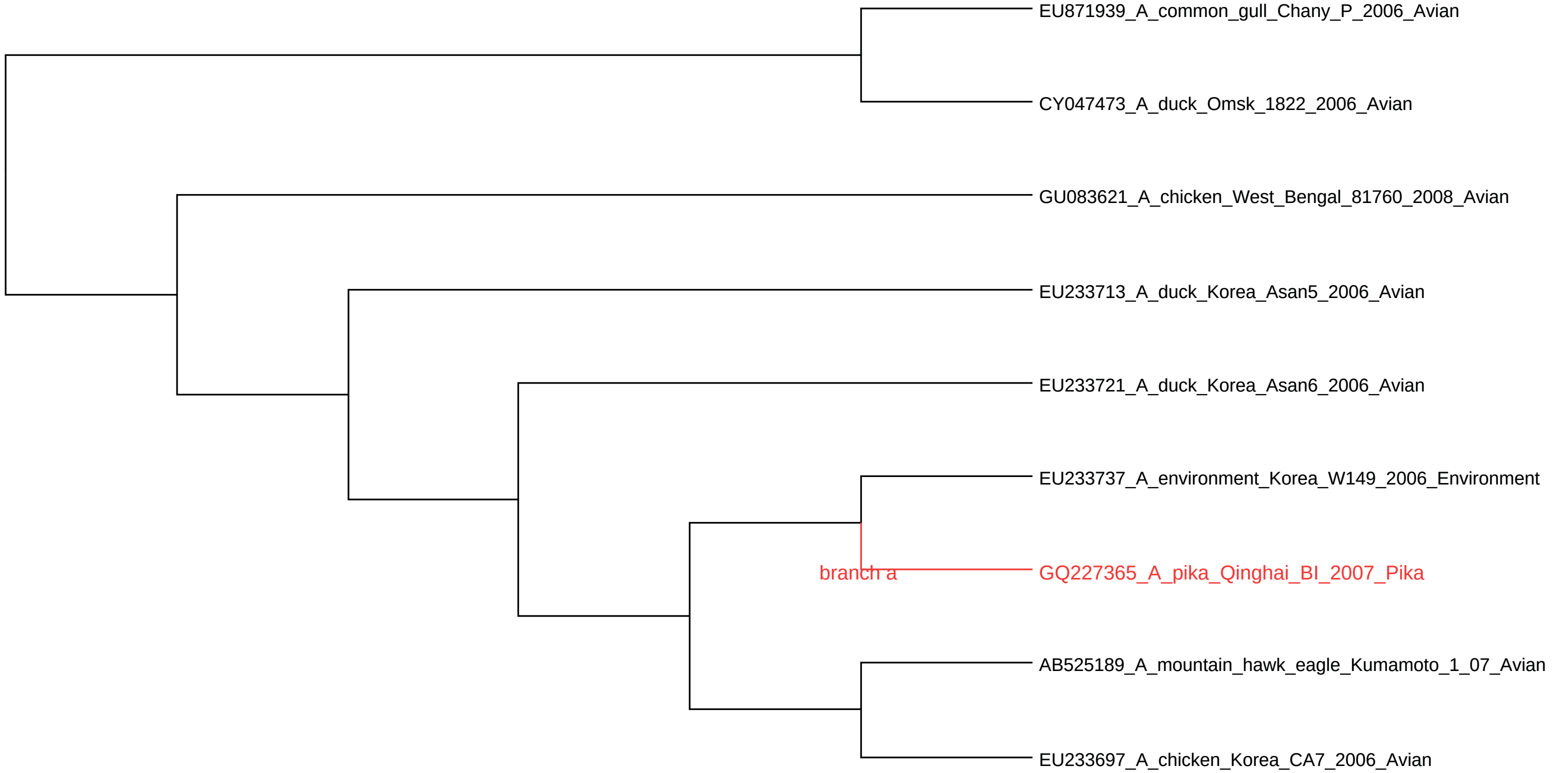
EU871939_A_common_gull_Chany_P_2006_Avian
CY047473_A_duck_Omsk_1822_2006_Avian
GU083621_A_chicken_West_Bengal_81760_2008_Avian
EU233713_A_duck_Korea_Asan5_2006_Avian
EU233721_A_duck_Korea_Asan6_2006_Avian
EU233737_A_environment_Korea_W149_2006_Environment
branch a
GQ227365_A_pika_Qinghai_BI_2007_Pika
AB525189_A_mountain_hawk_eagle_Kumamoto_1_07_Avian
EU233697_A_chicken_Korea_CA7_2006_Avian

PB1-Group51

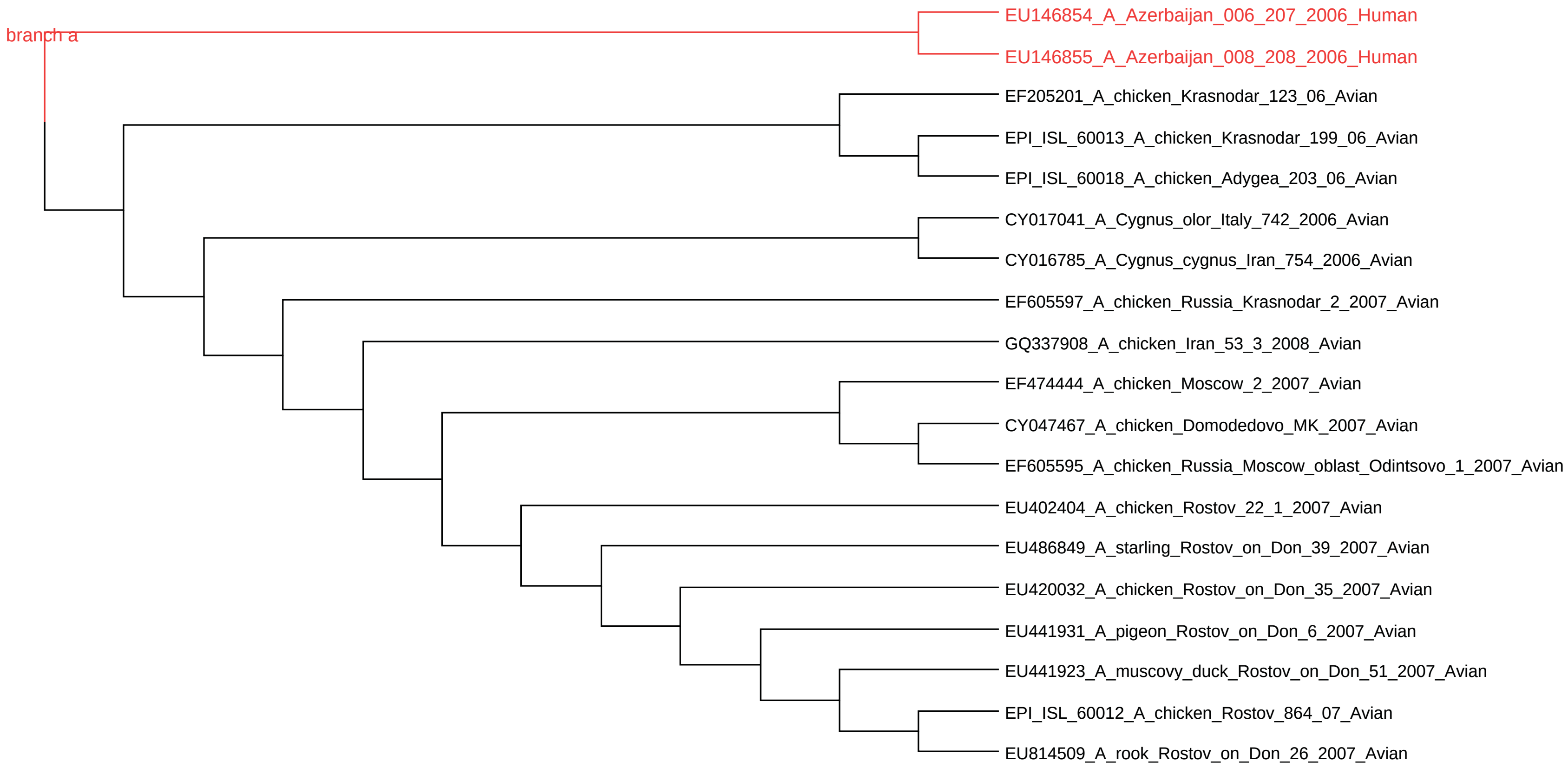
branch a
EU146854_A_Azerbaijan_006_207_2006_Human
EU146855_A_Azerbaijan_008_208_2006_Human
EF205201_A_chicken_Krasnodar_123_06_Avian
EPI_ISL_60013_A_chicken_Krasnodar_199_06_Avian
EPI_ISL_60018_A_chicken_Adygea_203_06_Avian
CY017041_A_Cygnus_olor_Italy_742_2006_Avian
CY016785_A_Cygnus_cygnus_Iran_754_2006_Avian
EF605597_A_chicken_Russia_Krasnodar_2_2007_Avian
GQ337908_A_chicken_Iran_53_3_2008_Avian
EF474444_A_chicken_Moscow_2_2007_Avian
CY047467_A_chicken_Domodedovo_MK_2007_Avian
EF605595_A_chicken_Russia_Moscow_oblast_Odintsovo_1_2007_Avian
EU402404_A_chicken_Rostov_22_1_2007_Avian
EU486849_A_starling_Rostov_on_Don_39_2007_Avian
EU420032_A_chicken_Rostov_on_Don_35_2007_Avian
EU441931_A_pigeon_Rostov_on_Don_6_2007_Avian
EU441923_A_muscovy_duck_Rostov_on_Don_51_2007_Avian
EPI_ISL_60012_A_chicken_Rostov_864_07_Avian
EU814509_A_rook_Rostov_on_Don_26_2007_Avian

PB1-Group52

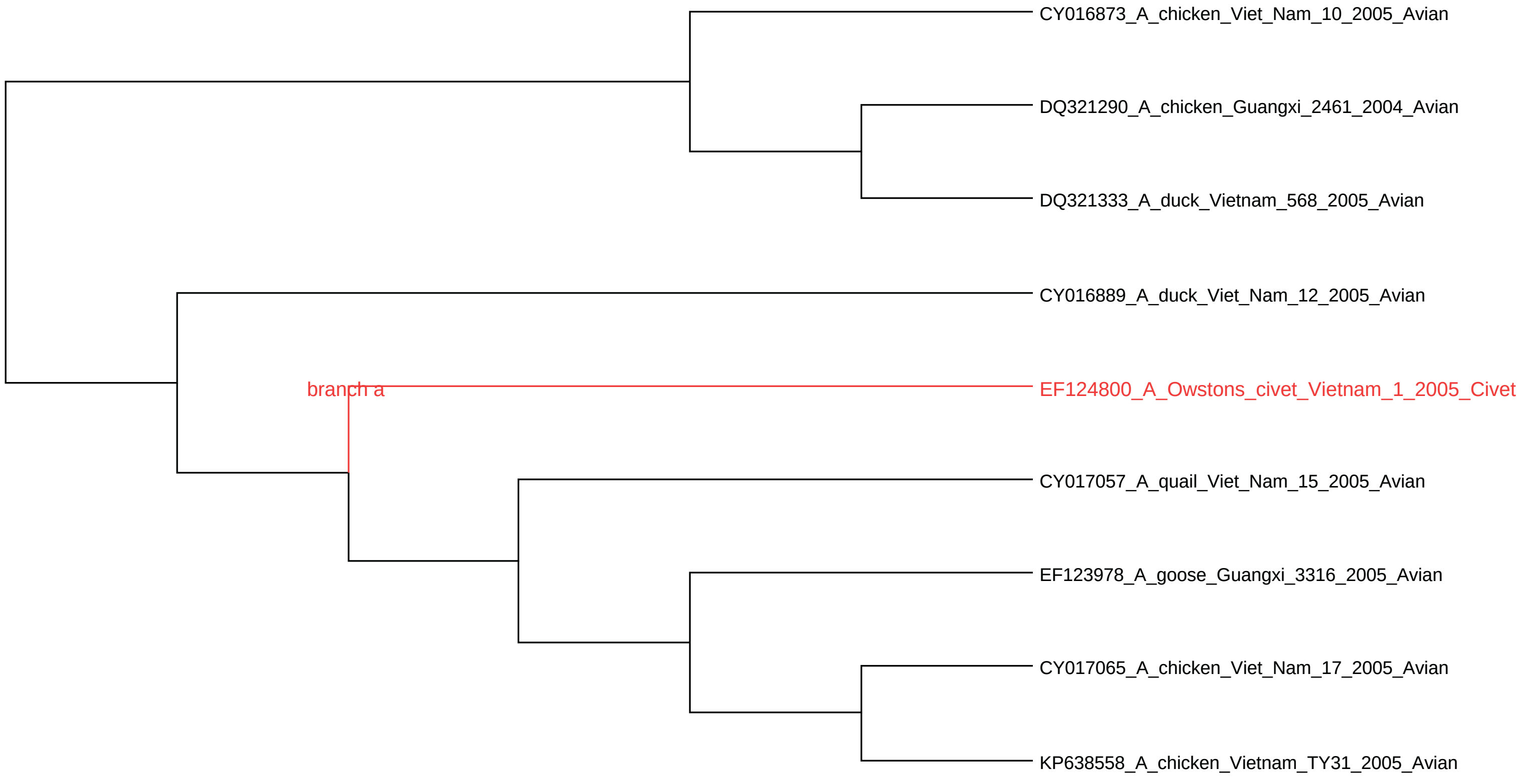
CY016873_A_chicken_Viet_Nam_10_2005_Avian
DQ321290_A_chicken_Guangxi_2461_2004_Avian
DQ321333_A_duck_Vietnam_568_2005_Avian
CY016889_A_duck_Viet_Nam_12_2005_Avian
branch a
EF124800_A_Owstons_civet_Vietnam_1_2005_Civet
CY017057_A_quail_Viet_Nam_15_2005_Avian
EF123978_A_goose_Guangxi_3316_2005_Avian
CY017065_A_chicken_Viet_Nam_17_2005_Avian
KP638558_A_chicken_Vietnam_TY31_2005_Avian

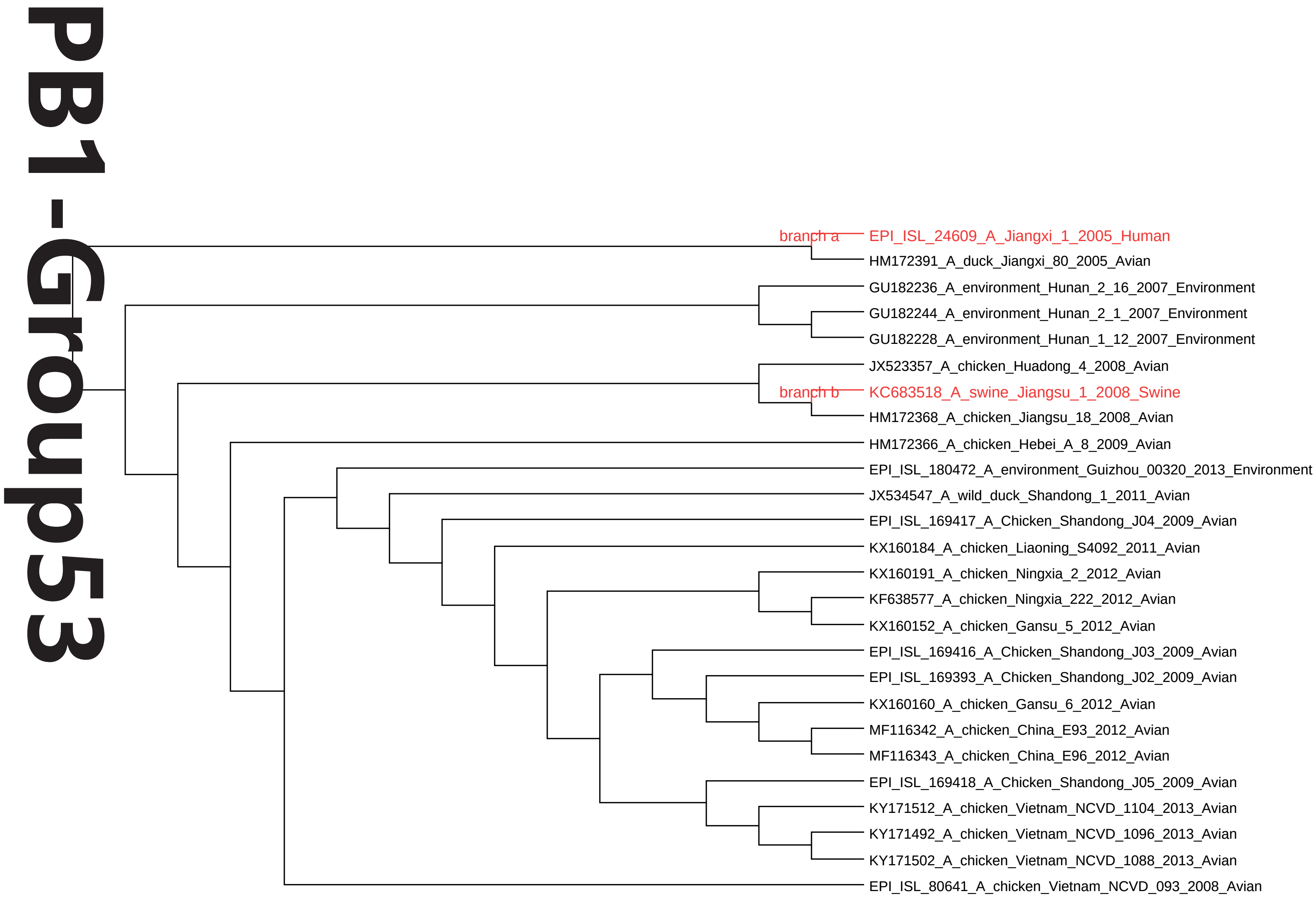
PB1-Group53
branch a
EPI_ISL_24609_A_Jiangxi_1_2005_Human
HM172391_A_duck_Jiangxi_80_2005_Avian
GU182236_A_environment_Hunan_2_16_2007_Environment
GU182244_A_environment_Hunan_2_1_2007_Environment
GU182228_A_environment_Hunan_1_12_2007_Environment
JX523357_A_chicken_Huadong_4_2008_Avian
branch b
KC683518_A_swine_Jiangsu_1_2008_Swine
HM172368_A_chicken_Jiangsu_18_2008_Avian
HM172366_A_chicken_Hebei_A_8_2009_Avian
EPI_ISL_180472_A_environment_Guizhou_00320_2013_Environment
JX534547_A_wild_duck_Shandong_1_2011_Avian
EPI_ISL_169417_A_Chicken_Shandong_J04_2009_Avian
KX160184_A_chicken_Liaoning_S4092_2011_Avian
KX160191_A_chicken_Ningxia_2_2012_Avian
KF638577_A_chicken_Ningxia_222_2012_Avian
KX160152_A_chicken_Gansu_5_2012_Avian
EPI_ISL_169416_A_Chicken_Shandong_J03_2009_Avian
EPI_ISL_169393_A_Chicken_Shandong_J02_2009_Avian
KX160160_A_chicken_Gansu_6_2012_Avian
MF116342_A_chicken_China_E93_2012_Avian
MF116343_A_chicken_China_E96_2012_Avian
EPI_ISL_169418_A_Chicken_Shandong_J05_2009_Avian
KY171512_A_chicken_Vietnam_NCVD_1104_2013_Avian
KY171492_A_chicken_Vietnam_NCVD_1096_2013_Avian
KY171502_A_chicken_Vietnam_NCVD_1088_2013_Avian
EPI_ISL_80641_A_chicken_Vietnam_NCVD_093_2008_Avian

PB1-Group54

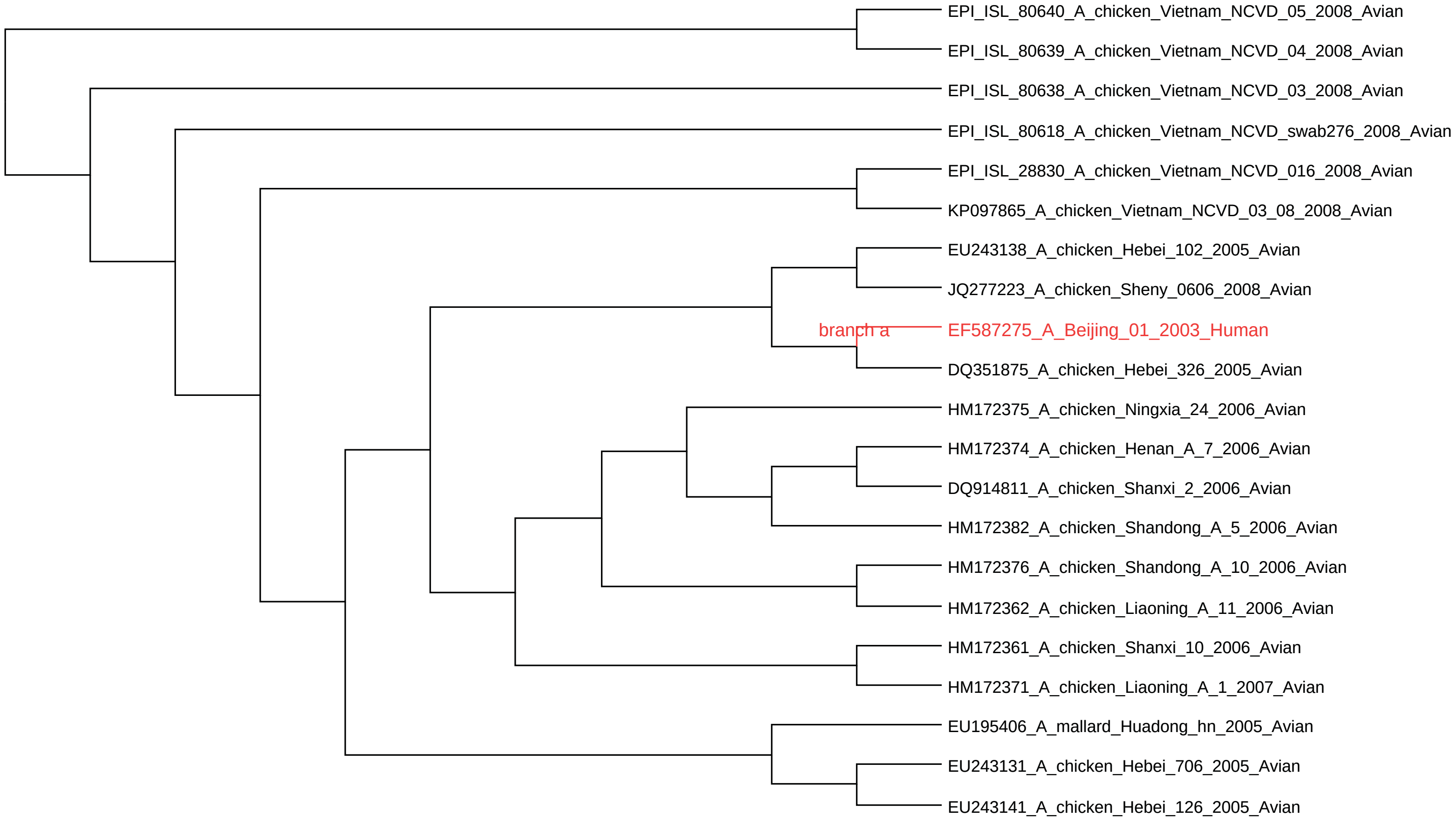
EPI_ISL_80640_A_chicken_Vietnam_NCVD_05_2008_Avian
EPI_ISL_80639_A_chicken_Vietnam_NCVD_04_2008_Avian
EPI_ISL_80638_A_chicken_Vietnam_NCVD_03_2008_Avian
EPI_ISL_80618_A_chicken_Vietnam_NCVD_swab276_2008_Avian
EPI_ISL_28830_A_chicken_Vietnam_NCVD_016_2008_Avian
KP097865_A_chicken_Vietnam_NCVD_03_08_2008_Avian
EU243138_A_chicken_Hebei_102_2005_Avian
JQ277223_A_chicken_Sheny_0606_2008_Avian
branch a
EF587275_A_Beijing_01_2003_Human
DQ351875_A_chicken_Hebei_326_2005_Avian
HM172375_A_chicken_Ningxia_24_2006_Avian
HM172374_A_chicken_Henan_A_7_2006_Avian
DQ914811_A_chicken_Shanxi_2_2006_Avian
HM172382_A_chicken_Shandong_A_5_2006_Avian
HM172376_A_chicken_Shandong_A_10_2006_Avian
HM172362_A_chicken_Liaoning_A_11_2006_Avian
HM172361_A_chicken_Shanxi_10_2006_Avian
HM172371_A_chicken_Liaoning_A_1_2007_Avian
EU195406_A_mallard_Huadong_hn_2005_Avian
EU243131_A_chicken_Hebei_706_2005_Avian
EU243141_A_chicken_Hebei_126_2005_Avian

PB1-Group55

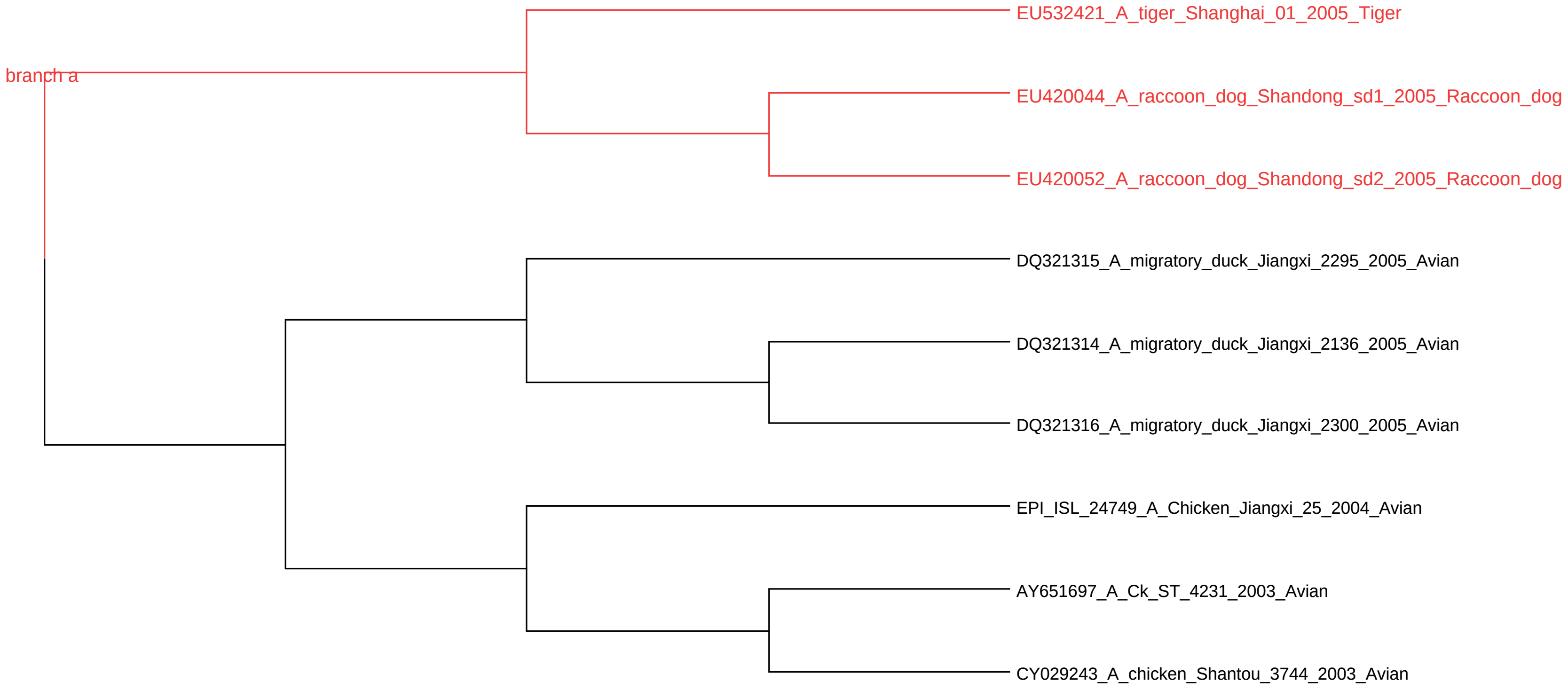
branch a
EU532421_A_tiger_Shanghai_01_2005_Tiger
EU420044_A_raccoon_dog_Shandong_sd1_2005_Raccoon_dog
EU420052_A_raccoon_dog_Shandong_sd2_2005_Raccoon_dog
DQ321315_A_migratory_duck_Jiangxi_2295_2005_Avian
DQ321314_A_migratory_duck_Jiangxi_2136_2005_Avian
DQ321316_A_migratory_duck_Jiangxi_2300_2005_Avian
EPI_ISL_24749_A_Chicken_Jiangxi_25_2004_Avian
AY651697_A_Ck_ST_4231_2003_Avian
CY029243_A_chicken_Shantou_3744_2003_Avian

PB1-Group56

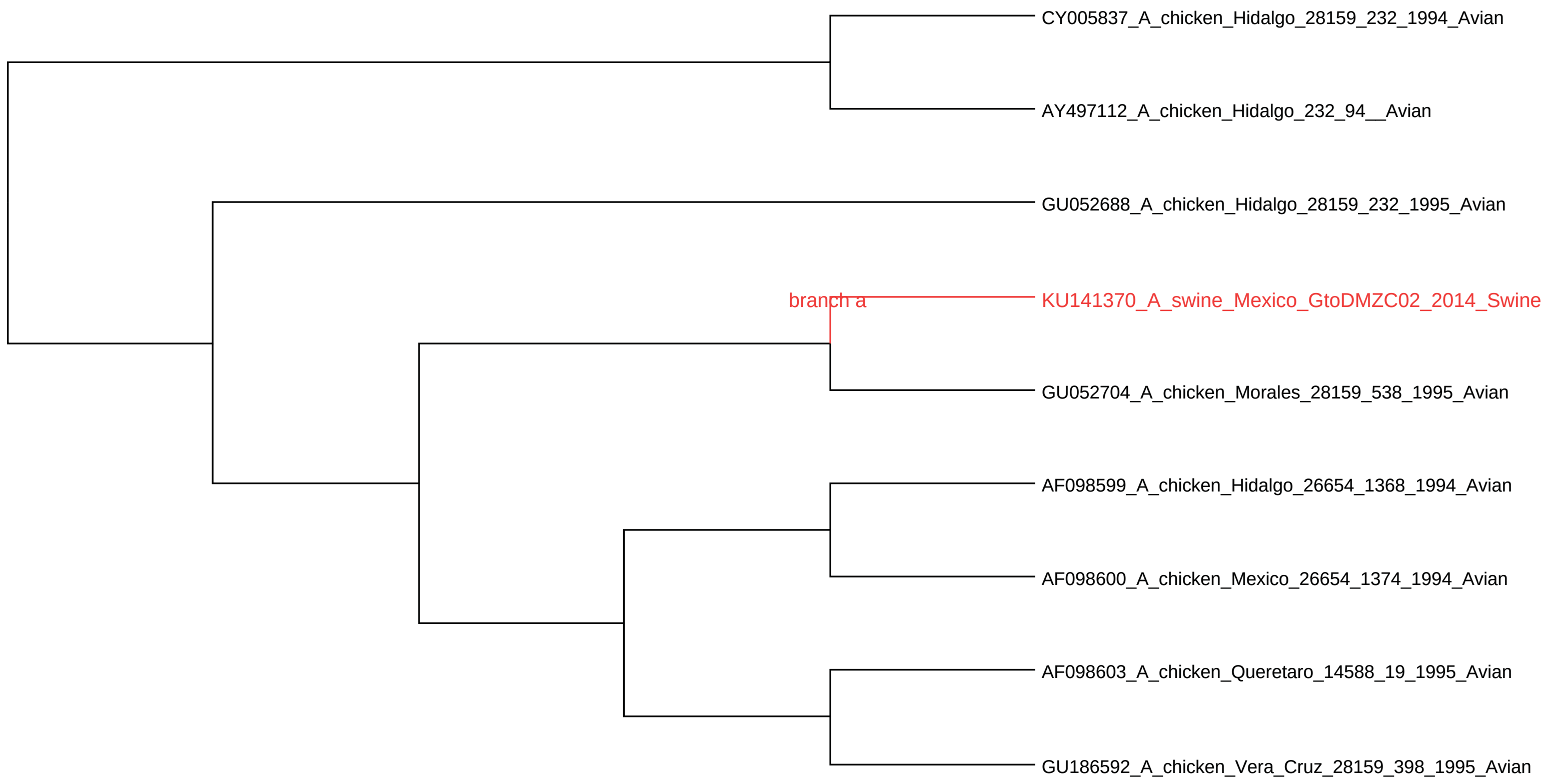
CY005837_A_chicken_Hidalgo_28159_232_1994_Avian
AY497112_A_chicken_Hidalgo_232_94__Avian
GU052688_A_chicken_Hidalgo_28159_232_1995_Avian
branch a
KU141370_A_swine_Mexico_GtoDMZC02_2014_Swine
GU052704_A_chicken_Morales_28159_538_1995_Avian
AF098599_A_chicken_Hidalgo_26654_1368_1994_Avian
AF098600_A_chicken_Mexico_26654_1374_1994_Avian
AF098603_A_chicken_Queretaro_14588_19_1995_Avian
GU186592_A_chicken_Vera_Cruz_28159_398_1995_Avian

PB1-Group57

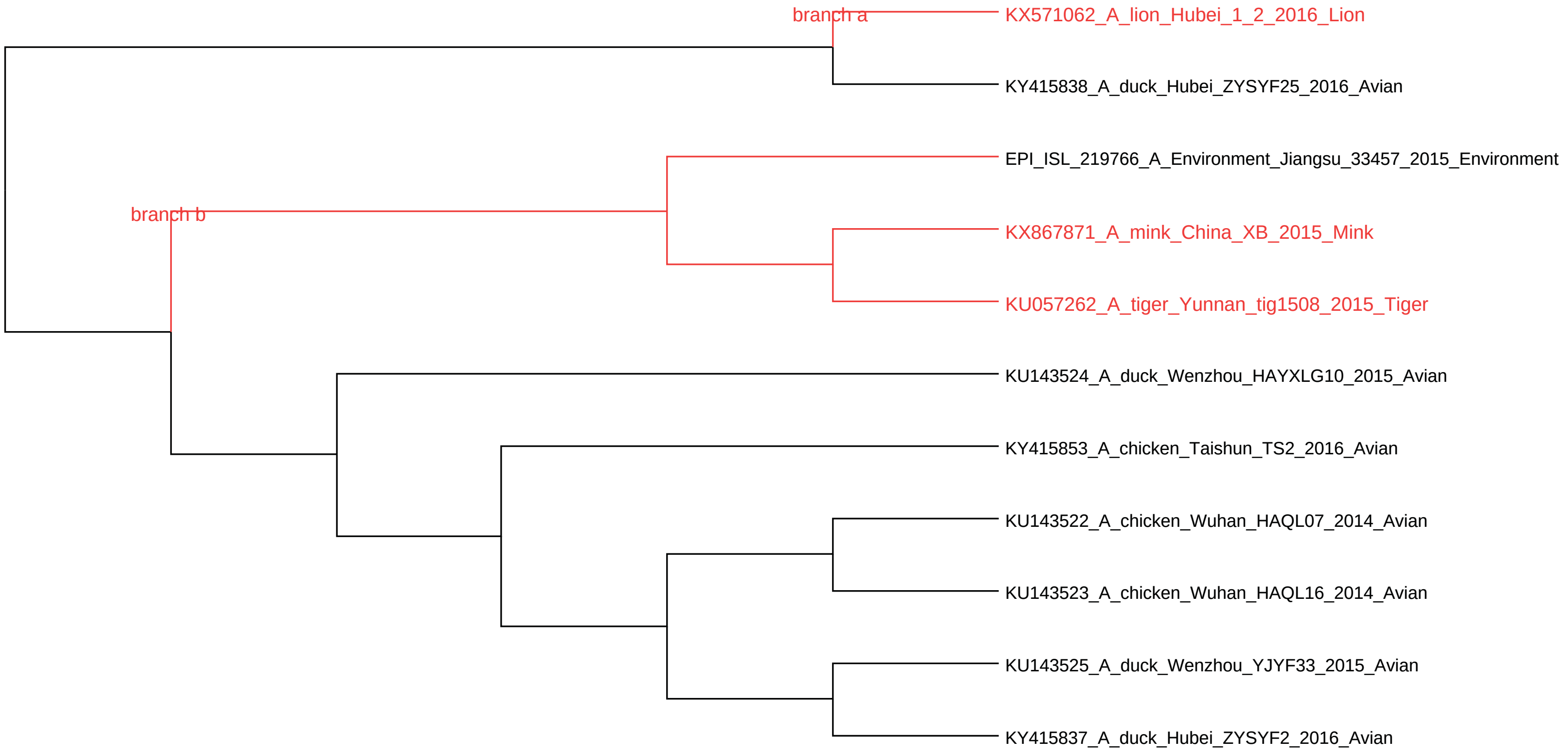

branch a
KX571062_A_lion_Hubei_1_2_2016_Lion
KY415838_A_duck_Hubei_ZYSYF25_2016_Avian
EPI_ISL_219766_A_Environment_Jiangsu_33457_2015_Environment
branch b
KX867871_A_mink_China_XB_2015_Mink
KU057262_A_tiger_Yunnan_tig1508_2015_Tiger
KU143524_A_duck_Wenzhou_HAYXLG10_2015_Avian
KY415853_A_chicken_Taishun_TS2_2016_Avian
KU143522_A_chicken_Wuhan_HAQL07_2014_Avian
KU143523_A_chicken_Wuhan_HAQL16_2014_Avian
KU143525_A_duck_Wenzhou_YJYF33_2015_Avian
KY415837_A_duck_Hubei_ZYSYF2_2016_Avian

PB1-Group58

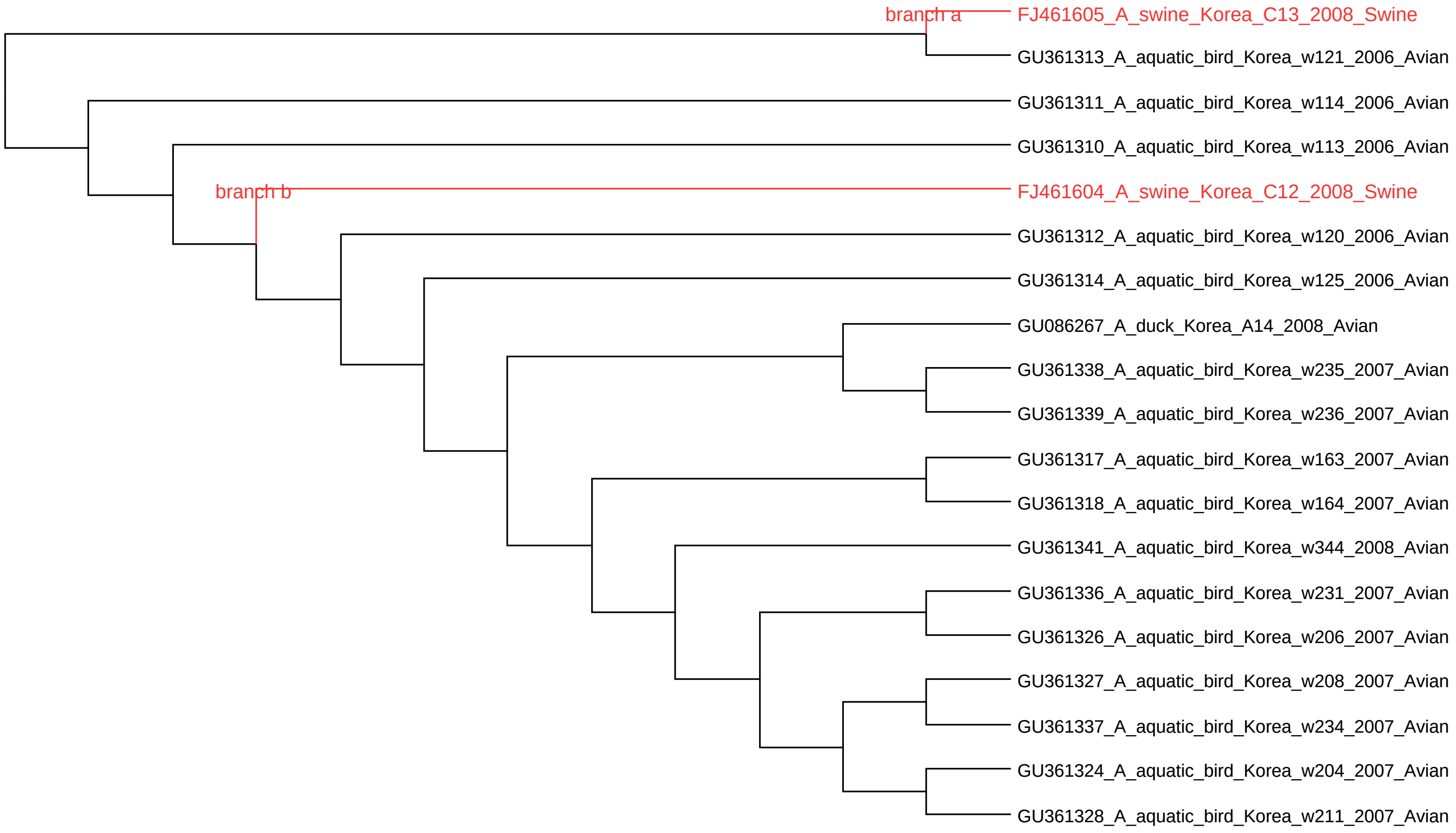
branch a
FJ461605_A_swine_Korea_C13_2008_Swine
GU361313_A_aquatic_bird_Korea_w121_2006_Avian
GU361311_A_aquatic_bird_Korea_w114_2006_Avian
GU361310_A_aquatic_bird_Korea_w113_2006_Avian
branch b
FJ461604_A_swine_Korea_C12_2008_Swine
GU361312_A_aquatic_bird_Korea_w120_2006_Avian
GU361314_A_aquatic_bird_Korea_w125_2006_Avian
GU086267_A_duck_Korea_A14_2008_Avian
GU361338_A_aquatic_bird_Korea_w235_2007_Avian
GU361339_A_aquatic_bird_Korea_w236_2007_Avian
GU361317_A_aquatic_bird_Korea_w163_2007_Avian
GU361318_A_aquatic_bird_Korea_w164_2007_Avian
GU361341_A_aquatic_bird_Korea_w344_2008_Avian
GU361336_A_aquatic_bird_Korea_w231_2007_Avian
GU361326_A_aquatic_bird_Korea_w206_2007_Avian
GU361327_A_aquatic_bird_Korea_w208_2007_Avian
GU361337_A_aquatic_bird_Korea_w234_2007_Avian
GU361324_A_aquatic_bird_Korea_w204_2007_Avian
GU361328_A_aquatic_bird_Korea_w211_2007_Avian

PB1-Group59

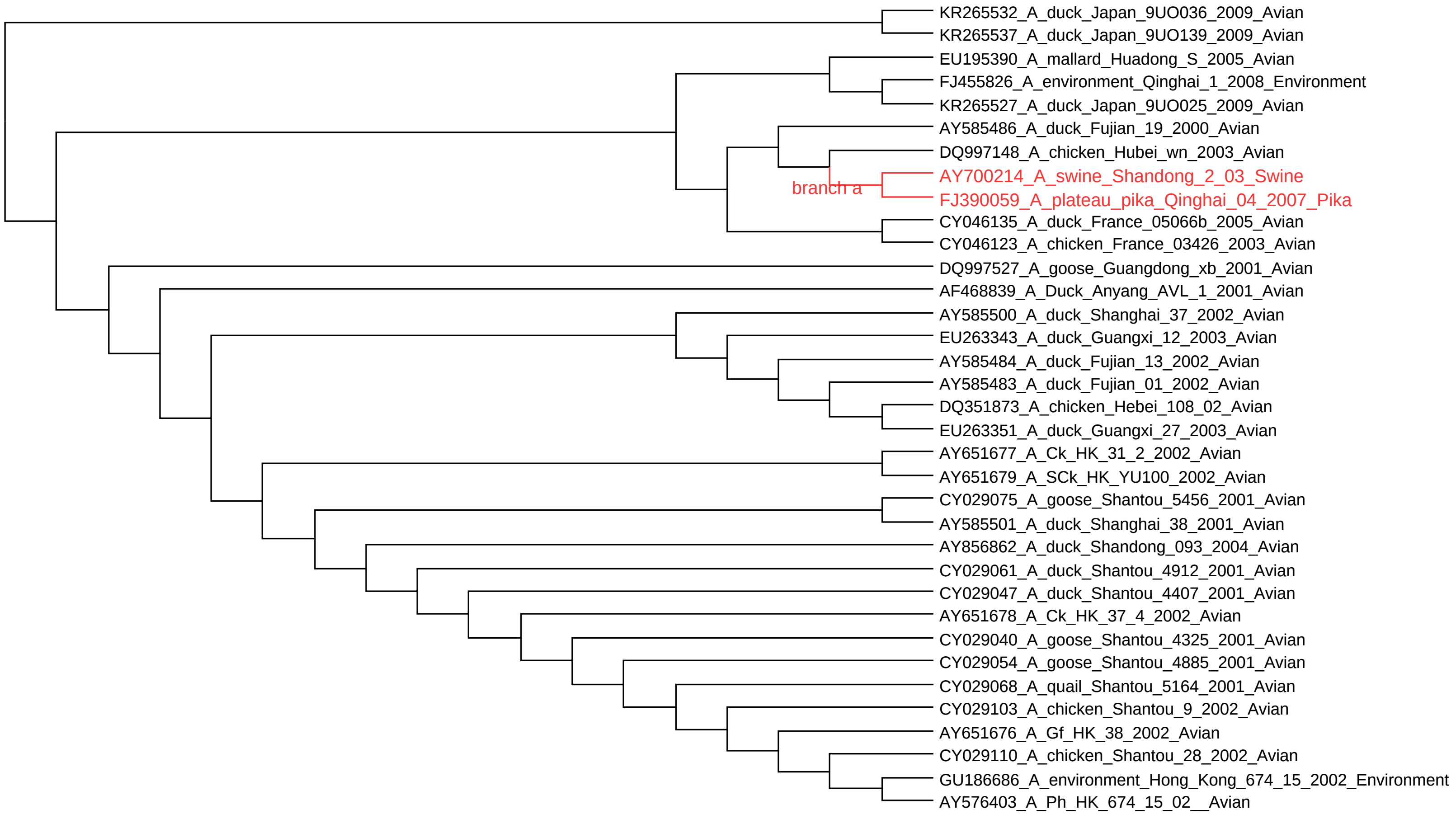
KR265532_A_duck_Japan_9UO036_2009_Avian
KR265537_A_duck_Japan_9UO139_2009_Avian
EU195390_A_mallard_Huadong_S_2005_Avian
FJ455826_A_environment_Qinghai_1_2008_Environment
KR265527_A_duck_Japan_9UO025_2009_Avian
AY585486_A_duck_Fujian_19_2000_Avian
DQ997148_A_chicken_Hubei_wn_2003_Avian
AY700214_A_swine_Shandong_2_03_Swine
branch a
FJ390059_A_plateau_pika_Qinghai_04_2007_Pika
CY046135_A_duck_France_05066b_2005_Avian
CY046123_A_chicken_France_03426_2003_Avian
DQ997527_A_goose_Guangdong_xb_2001_Avian
AF468839_A_Duck_Anyang_AVL_1_2001_Avian
AY585500_A_duck_Shanghai_37_2002_Avian
EU263343_A_duck_Guangxi_12_2003_Avian
AY585484_A_duck_Fujian_13_2002_Avian
AY585483_A_duck_Fujian_01_2002_Avian
DQ351873_A_chicken_Hebei_108_02_Avian
EU263351_A_duck_Guangxi_27_2003_Avian
AY651677_A_Ck_HK_31_2_2002_Avian
AY651679_A_SCk_HK_YU100_2002_Avian
CY029075_A_goose_Shantou_5456_2001_Avian
AY585501_A_duck_Shanghai_38_2001_Avian
AY856862_A_duck_Shandong_093_2004_Avian
CY029061_A_duck_Shantou_4912_2001_Avian
CY029047_A_duck_Shantou_4407_2001_Avian
AY651678_A_Ck_HK_37_4_2002_Avian
CY029040_A_goose_Shantou_4325_2001_Avian
CY029054_A_goose_Shantou_4885_2001_Avian
CY029068_A_quail_Shantou_5164_2001_Avian
CY029103_A_chicken_Shantou_9_2002_Avian
AY651676_A_Gf_HK_38_2002_Avian
CY029110_A_chicken_Shantou_28_2002_Avian
GU186686_A_environment_Hong_Kong_674_15_2002_Environment
AY576403_A_Ph_HK_674_15_02__Avian

PB1-Group60

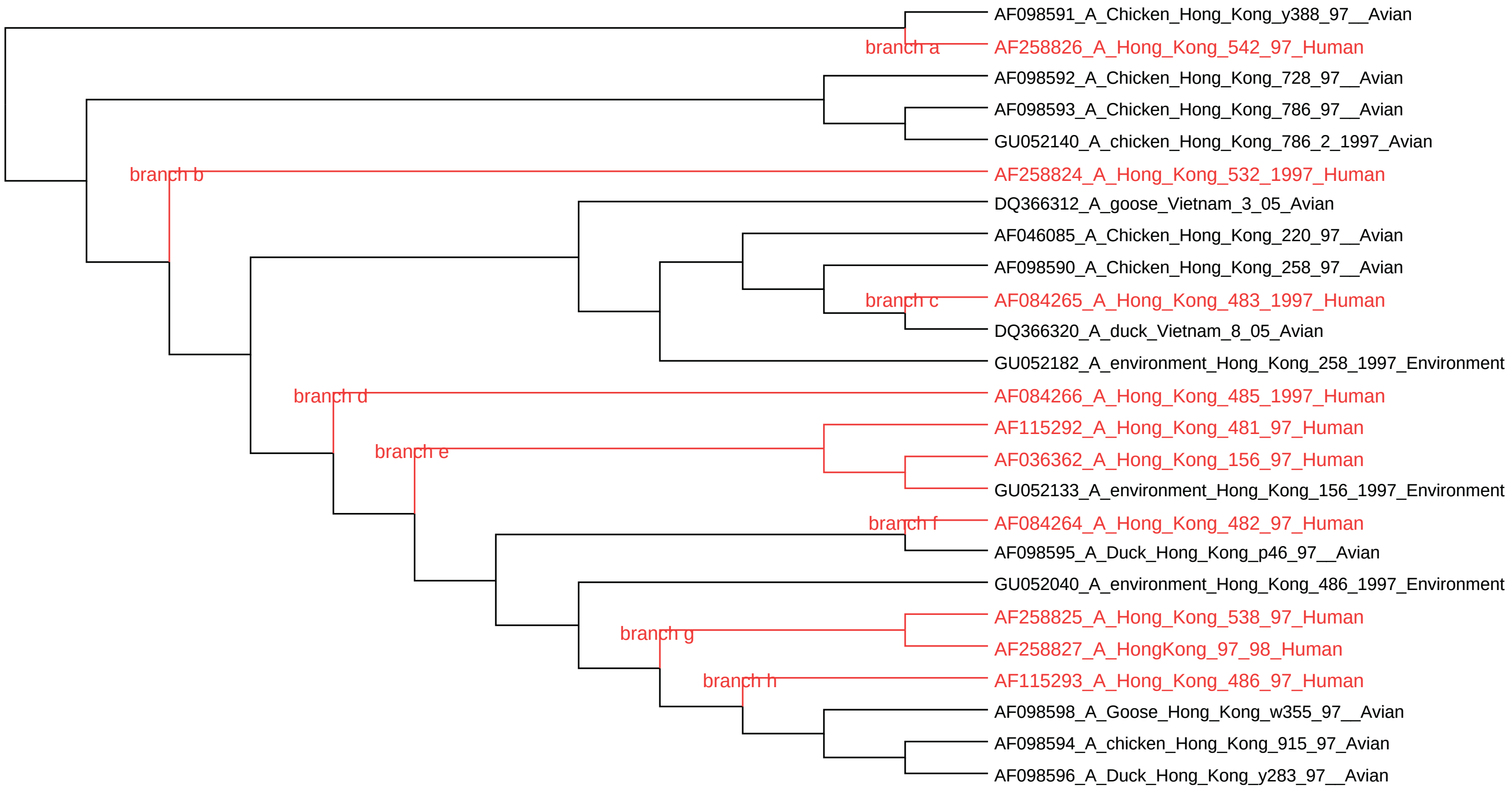

AF098591_A_Chicken_Hong_Kong_y388_97__Avian
branch a
AF258826_A_Hong_Kong_542_97_Human
AF098592_A_Chicken_Hong_Kong_728_97__Avian
AF098593_A_Chicken_Hong_Kong_786_97__Avian
GU052140_A_chicken_Hong_Kong_786_2_1997_Avian
branch b
AF258824_A_Hong_Kong_532_1997_Human
DQ366312_A_goose_Vietnam_3_05_Avian
AF046085_A_Chicken_Hong_Kong_220_97__Avian
AF098590_A_Chicken_Hong_Kong_258_97__Avian
branch c
AF084265_A_Hong_Kong_483_1997_Human
DQ366320_A_duck_Vietnam_8_05_Avian
GU052182_A_environment_Hong_Kong_258_1997_Environment
branch d
AF084266_A_Hong_Kong_485_1997_Human
AF115292_A_Hong_Kong_481_97_Human
branch e
AF036362_A_Hong_Kong_156_97_Human
GU052133_A_environment_Hong_Kong_156_1997_Environment
branch f
AF084264_A_Hong_Kong_482_97_Human
AF098595_A_Duck_Hong_Kong_p46_97__Avian
GU052040_A_environment_Hong_Kong_486_1997_Environment
AF258825_A_Hong_Kong_538_97_Human
branch g
AF258827_A_HongKong_97_98_Human
branch h
AF115293_A_Hong_Kong_486_97_Human
AF098598_A_Goose_Hong_Kong_w355_97__Avian
AF098594_A_chicken_Hong_Kong_915_97_Avian
AF098596_A_Duck_Hong_Kong_y283_97__Avian

PB1-Group61

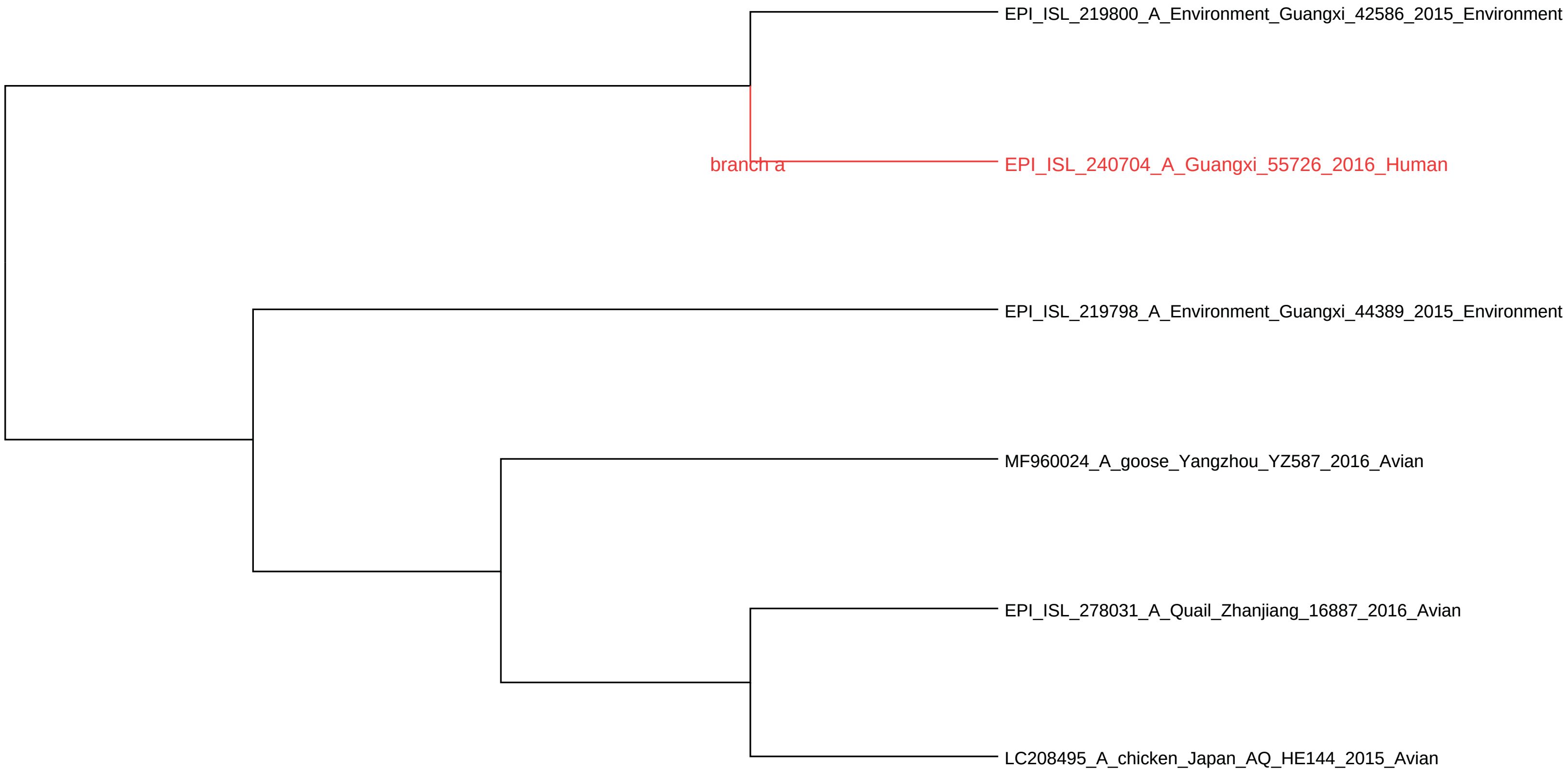
EPI_ISL_219800_A_Environment_Guangxi_42586_2015_Environment
branch a
EPI_ISL_240704_A_Guangxi_55726_2016_Human
EPI_ISL_219798_A_Environment_Guangxi_44389_2015_Environment
MF960024_A_goose_Yangzhou_YZ587_2016_Avian
EPI_ISL_278031_A_Quail_Zhanjiang_16887_2016_Avian
LC208495_A_chicken_Japan_AQ_HE144_2015_Avian

PB1-Group62

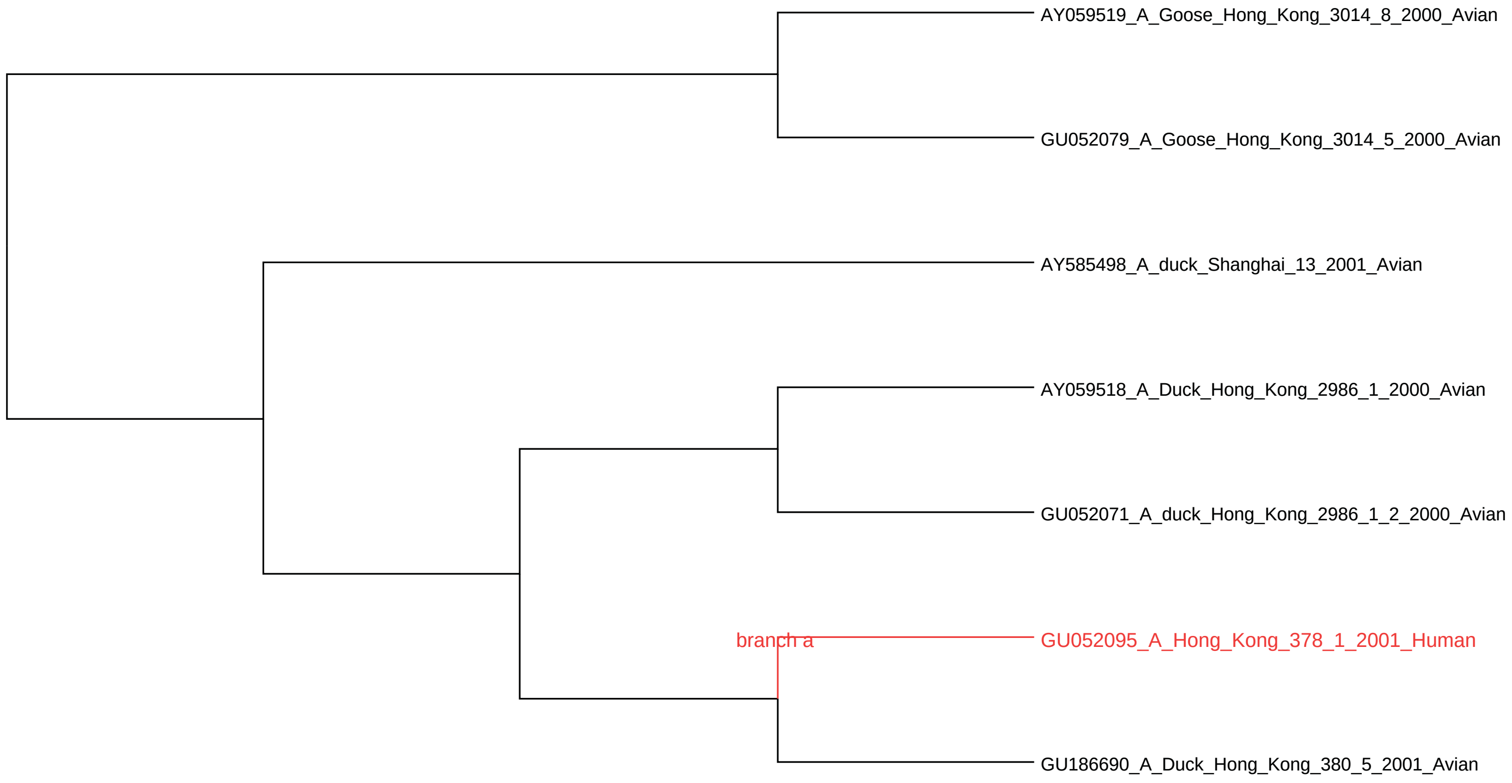
AY059519_A_Goose_Hong_Kong_3014_8_2000_Avian
GU052079_A_Goose_Hong_Kong_3014_5_2000_Avian
AY585498_A_duck_Shanghai_13_2001_Avian
AY059518_A_Duck_Hong_Kong_2986_1_2000_Avian
GU052071_A_duck_Hong_Kong_2986_1_2_2000_Avian
branch a
GU052095_A_Hong_Kong_378_1_2001_Human
GU186690_A_Duck_Hong_Kong_380_5_2001_Avian

PB1-Group63

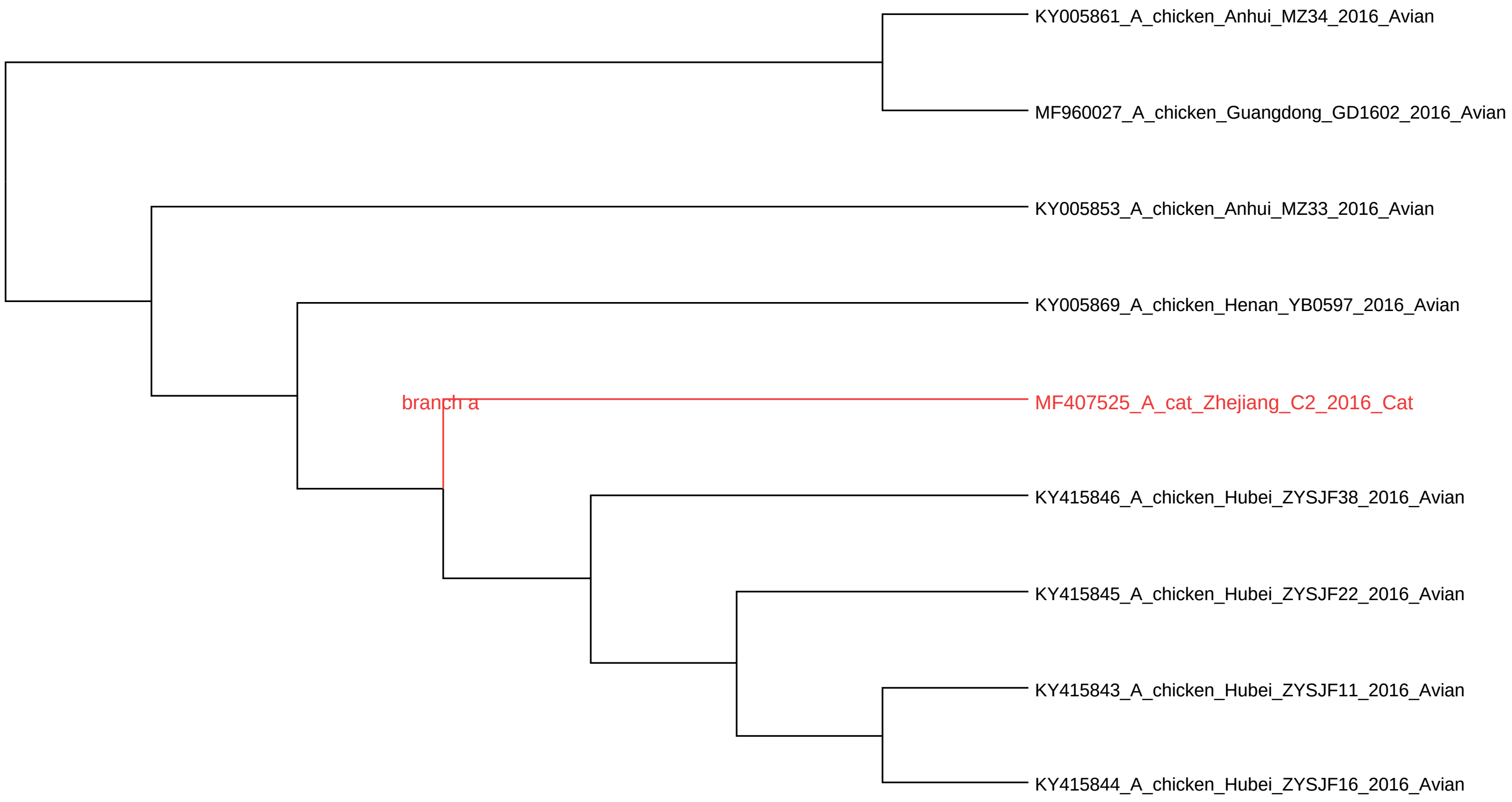
KY005861_A_chicken_Anhui_MZ34_2016_Avian
MF960027_A_chicken_Guangdong_GD1602_2016_Avian
KY005853_A_chicken_Anhui_MZ33_2016_Avian
KY005869_A_chicken_Henan_YB0597_2016_Avian
branch a
MF407525_A_cat_Zhejiang_C2_2016_Cat
KY415846_A_chicken_Hubei_ZYSJF38_2016_Avian
KY415845_A_chicken_Hubei_ZYSJF22_2016_Avian
KY415843_A_chicken_Hubei_ZYSJF11_2016_Avian
KY415844_A_chicken_Hubei_ZYSJF16_2016_Avian

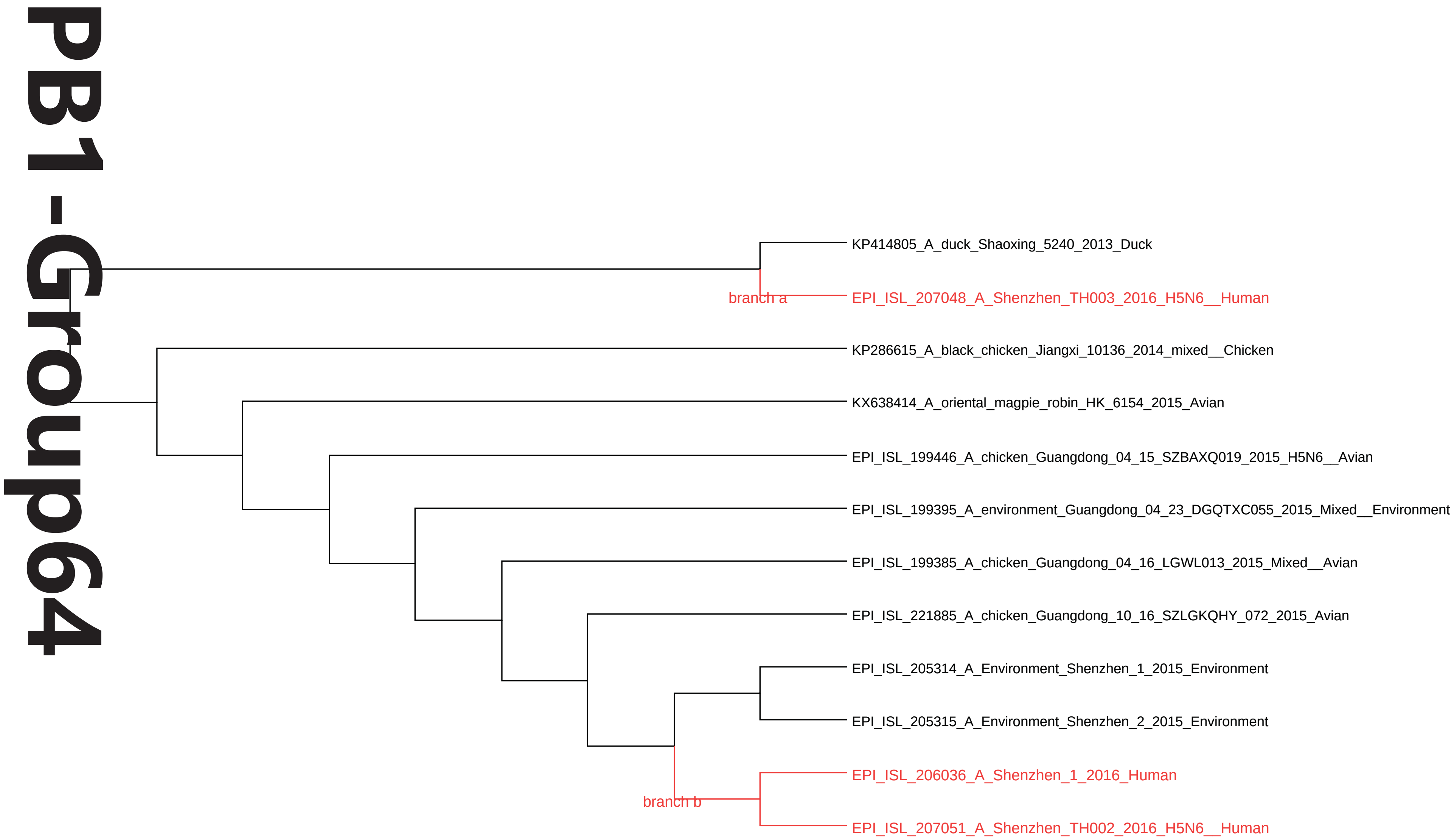
PB1-Group64
KP414805_A_duck_Shaoxing_5240_2013_Duck
branch a
EPI_ISL_207048_A_Shenzhen_TH003_2016_H5N6__Human
KP286615_A_black_chicken_Jiangxi_10136_2014_mixed__Chicken
KX638414_A_oriental_magpie_robin_HK_6154_2015_Avian
EPI_ISL_199446_A_chicken_Guangdong_04_15_SZBAXQ019_2015_H5N6__Avian
EPI_ISL_199395_A_environment_Guangdong_04_23_DGQTXC055_2015_Mixed__Environment
EPI_ISL_199385_A_chicken_Guangdong_04_16_LGWL013_2015_Mixed__Avian
EPI_ISL_221885_A_chicken_Guangdong_10_16_SZLGKQHY_072_2015_Avian
EPI_ISL_205314_A_Environment_Shenzhen_1_2015_Environment
EPI_ISL_205315_A_Environment_Shenzhen_2_2015_Environment
EPI_ISL_206036_A_Shenzhen_1_2016_Human
branch b
EPI_ISL_207051_A_Shenzhen_TH002_2016_H5N6__Human

PB1-Group65

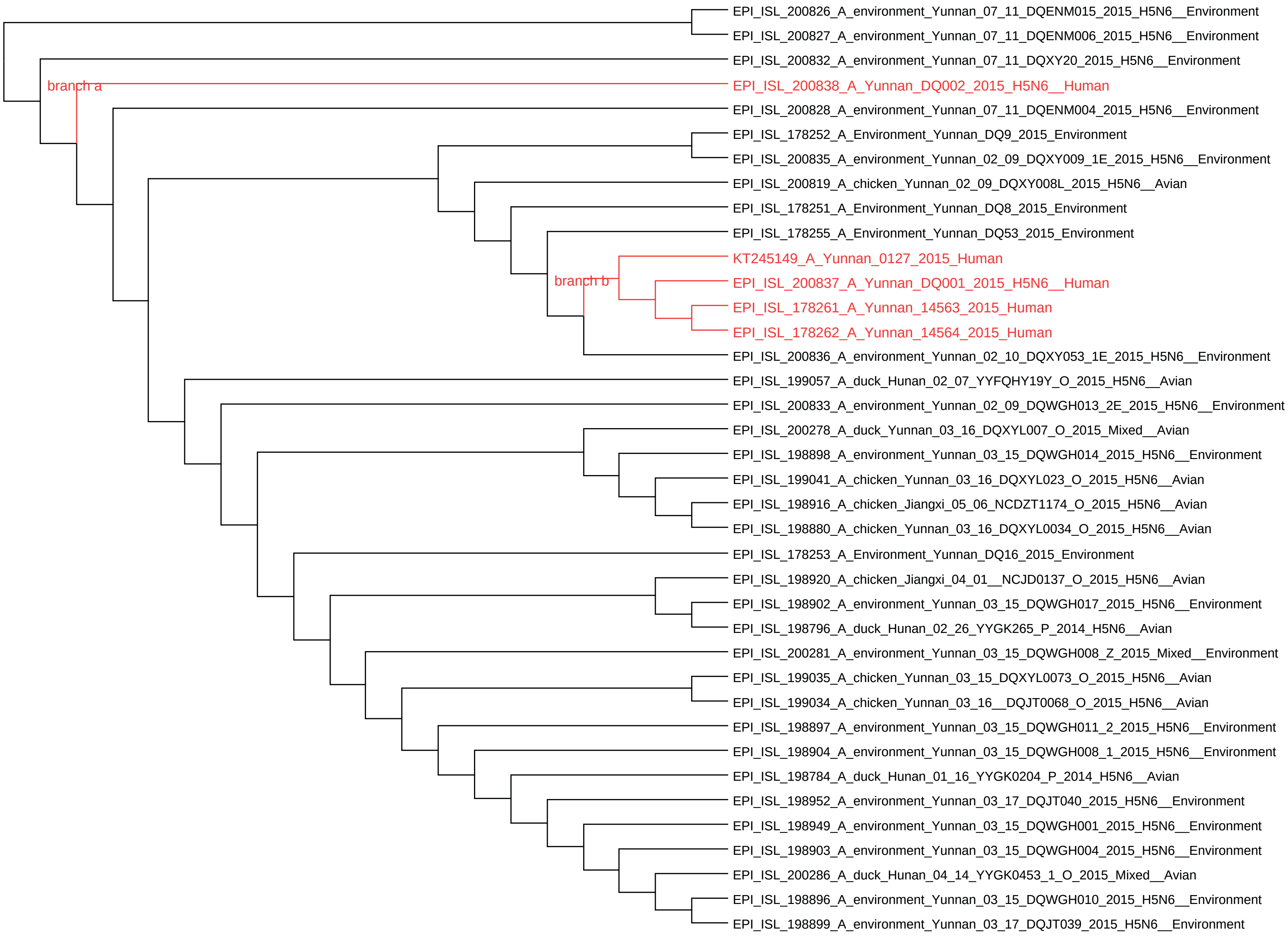

EPI_ISL_200826_A_environment_Yunnan_07_11_DQENM015_2015_H5N6__Environment
EPI_ISL_200827_A_environment_Yunnan_07_11_DQENM006_2015_H5N6__Environment
EPI_ISL_200832_A_environment_Yunnan_07_11_DQXY20_2015_H5N6__Environment
branch a
EPI_ISL_200838_A_Yunnan_DQ002_2015_H5N6__Human
EPI_ISL_200828_A_environment_Yunnan_07_11_DQENM004_2015_H5N6__Environment
EPI_ISL_178252_A_Environment_Yunnan_DQ9_2015_Environment
EPI_ISL_200835_A_environment_Yunnan_02_09_DQXY009_1E_2015_H5N6__Environment
EPI_ISL_200819_A_chicken_Yunnan_02_09_DQXY008L_2015_H5N6__Avian
EPI_ISL_178251_A_Environment_Yunnan_DQ8_2015_Environment
EPI_ISL_178255_A_Environment_Yunnan_DQ53_2015_Environment
KT245149_A_Yunnan_0127_2015_Human
branch b
EPI_ISL_200837_A_Yunnan_DQ001_2015_H5N6__Human
EPI_ISL_178261_A_Yunnan_14563_2015_Human
EPI_ISL_178262_A_Yunnan_14564_2015_Human
EPI_ISL_200836_A_environment_Yunnan_02_10_DQXY053_1E_2015_H5N6__Environment
EPI_ISL_199057_A_duck_Hunan_02_07_YYFQHY19Y_O_2015_H5N6__Avian
EPI_ISL_200833_A_environment_Yunnan_02_09_DQWGH013_2E_2015_H5N6__Environment
EPI_ISL_200278_A_duck_Yunnan_03_16_DQXYL007_O_2015_Mixed__Avian
EPI_ISL_198898_A_environment_Yunnan_03_15_DQWGH014_2015_H5N6__Environment
EPI_ISL_199041_A_chicken_Yunnan_03_16_DQXYL023_O_2015_H5N6__Avian
EPI_ISL_198916_A_chicken_Jiangxi_05_06_NCDZT1174_O_2015_H5N6__Avian
EPI_ISL_198880_A_chicken_Yunnan_03_16_DQXYL0034_O_2015_H5N6__Avian
EPI_ISL_178253_A_Environment_Yunnan_DQ16_2015_Environment
EPI_ISL_198920_A_chicken_Jiangxi_04_01__NCJD0137_O_2015_H5N6__Avian
EPI_ISL_198902_A_environment_Yunnan_03_15_DQWGH017_2015_H5N6__Environment
EPI_ISL_198796_A_duck_Hunan_02_26_YYGK265_P_2014_H5N6__Avian
EPI_ISL_200281_A_environment_Yunnan_03_15_DQWGH008_Z_2015_Mixed__Environment
EPI_ISL_199035_A_chicken_Yunnan_03_15_DQXYL0073_O_2015_H5N6__Avian
EPI_ISL_199034_A_chicken_Yunnan_03_16__DQJT0068_O_2015_H5N6__Avian
EPI_ISL_198897_A_environment_Yunnan_03_15_DQWGH011_2_2015_H5N6__Environment
EPI_ISL_198904_A_environment_Yunnan_03_15_DQWGH008_1_2015_H5N6__Environment
EPI_ISL_198784_A_duck_Hunan_01_16_YYGK0204_P_2014_H5N6__Avian
EPI_ISL_198952_A_environment_Yunnan_03_17_DQJT040_2015_H5N6__Environment
EPI_ISL_198949_A_environment_Yunnan_03_15_DQWGH001_2015_H5N6__Environment
EPI_ISL_198903_A_environment_Yunnan_03_15_DQWGH004_2015_H5N6__Environment
EPI_ISL_200286_A_duck_Hunan_04_14_YYGK0453_1_O_2015_Mixed__Avian
EPI_ISL_198896_A_environment_Yunnan_03_15_DQWGH010_2015_H5N6__Environment
EPI_ISL_198899_A_environment_Yunnan_03_17_DQJT039_2015_H5N6__Environment

PB1-Group66

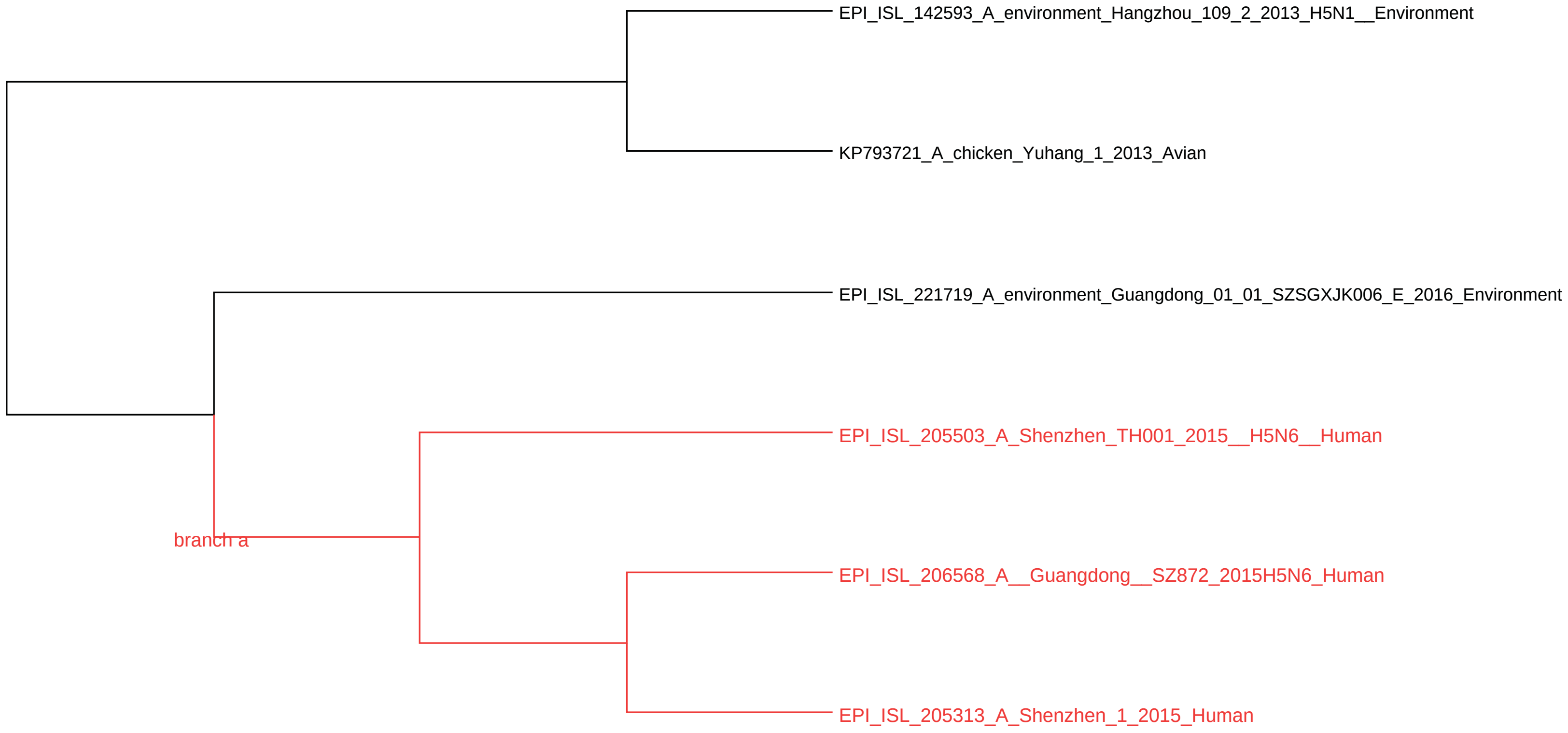

EPI_ISL_142593_A_environment_Hangzhou_109_2_2013_H5N1__Environment
KP793721_A_chicken_Yuhang_1_2013_Avian
EPI_ISL_221719_A_environment_Guangdong_01_01_SZSGXJK006_E_2016_Environment
EPI_ISL_205503_A_Shenzhen_TH001_2015__H5N6__Human
branch a
EPI_ISL_206568_A__Guangdong__SZ872_2015H5N6_Human
EPI_ISL_205313_A_Shenzhen_1_2015_Human

PB1-Group67

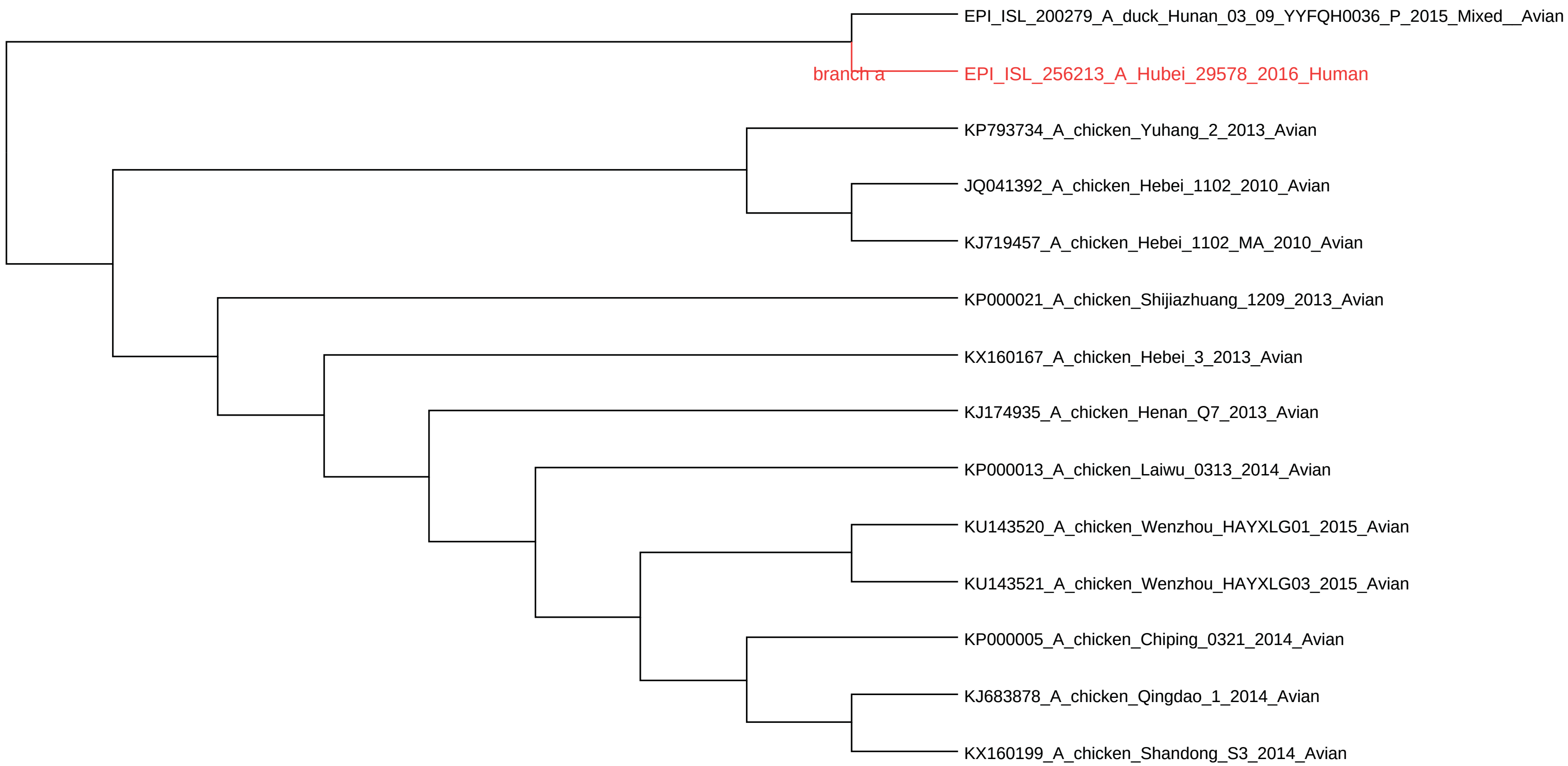
EPI_ISL_200279_A_duck_Hunan_03_09_YYFQH0036_P_2015_Mixed__Avian
branch a
EPI_ISL_256213_A_Hubei_29578_2016_Human
KP793734_A_chicken_Yuhang_2_2013_Avian
JQ041392_A_chicken_Hebei_1102_2010_Avian
KJ719457_A_chicken_Hebei_1102_MA_2010_Avian
KP000021_A_chicken_Shijiazhuang_1209_2013_Avian
KX160167_A_chicken_Hebei_3_2013_Avian
KJ174935_A_chicken_Henan_Q7_2013_Avian
KP000013_A_chicken_Laiwu_0313_2014_Avian
KU143520_A_chicken_Wenzhou_HAYXLG01_2015_Avian
KU143521_A_chicken_Wenzhou_HAYXLG03_2015_Avian
KP000005_A_chicken_Chiping_0321_2014_Avian
KJ683878_A_chicken_Qingdao_1_2014_Avian
KX160199_A_chicken_Shandong_S3_2014_Avian

PB1-Group68

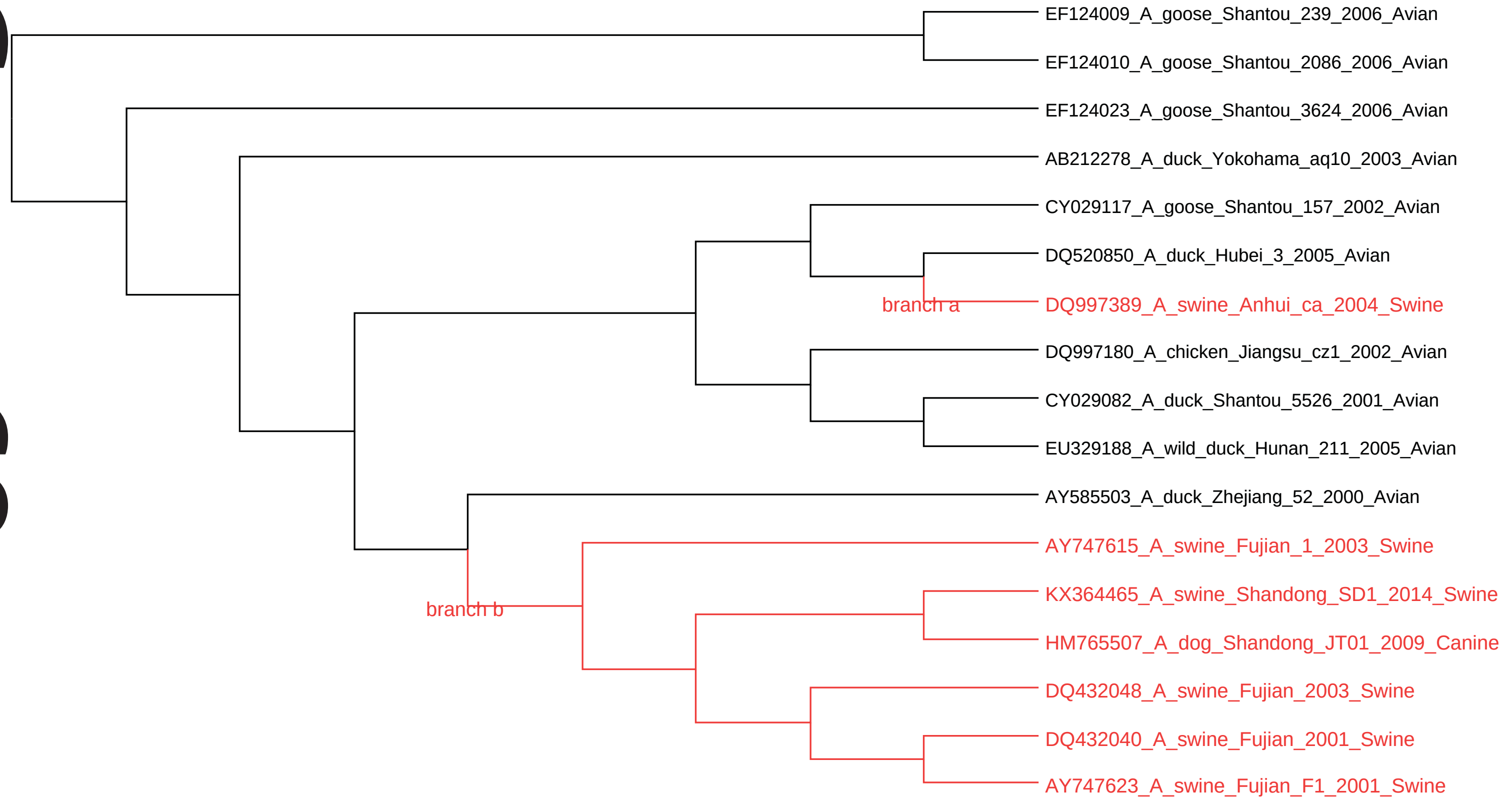
EF124009_A_goose_Shantou_239_2006_Avian
EF124010_A_goose_Shantou_2086_2006_Avian
EF124023_A_goose_Shantou_3624_2006_Avian
AB212278_A_duck_Yokohama_aq10_2003_Avian
CY029117_A_goose_Shantou_157_2002_Avian
DQ520850_A_duck_Hubei_3_2005_Avian
branch a
DQ997389_A_swine_Anhui_ca_2004_Swine
DQ997180_A_chicken_Jiangsu_cz1_2002_Avian
CY029082_A_duck_Shantou_5526_2001_Avian
EU329188_A_wild_duck_Hunan_211_2005_Avian
AY585503_A_duck_Zhejiang_52_2000_Avian
AY747615_A_swine_Fujian_1_2003_Swine
branch b
KX364465_A_swine_Shandong_SD1_2014_Swine
HM765507_A_dog_Shandong_JT01_2009_Canine
DQ432048_A_swine_Fujian_2003_Swine
DQ432040_A_swine_Fujian_2001_Swine
AY747623_A_swine_Fujian_F1_2001_Swine

PB1-Group69

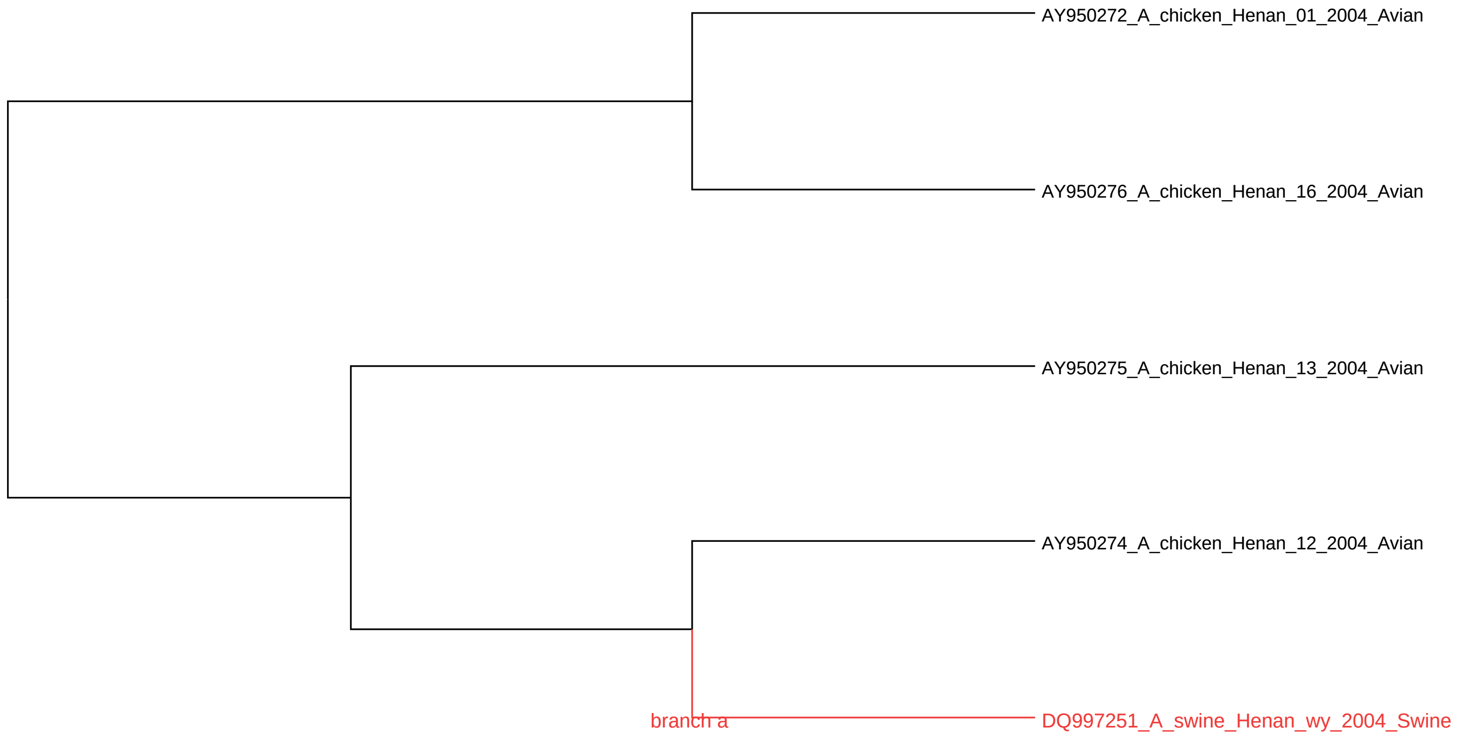
AY950272_A_chicken_Henan_01_2004_Avian
AY950276_A_chicken_Henan_16_2004_Avian
AY950275_A_chicken_Henan_13_2004_Avian
AY950274_A_chicken_Henan_12_2004_Avian
branch a
DQ997251_A_swine_Henan_wy_2004_Swine

PB1-Group70

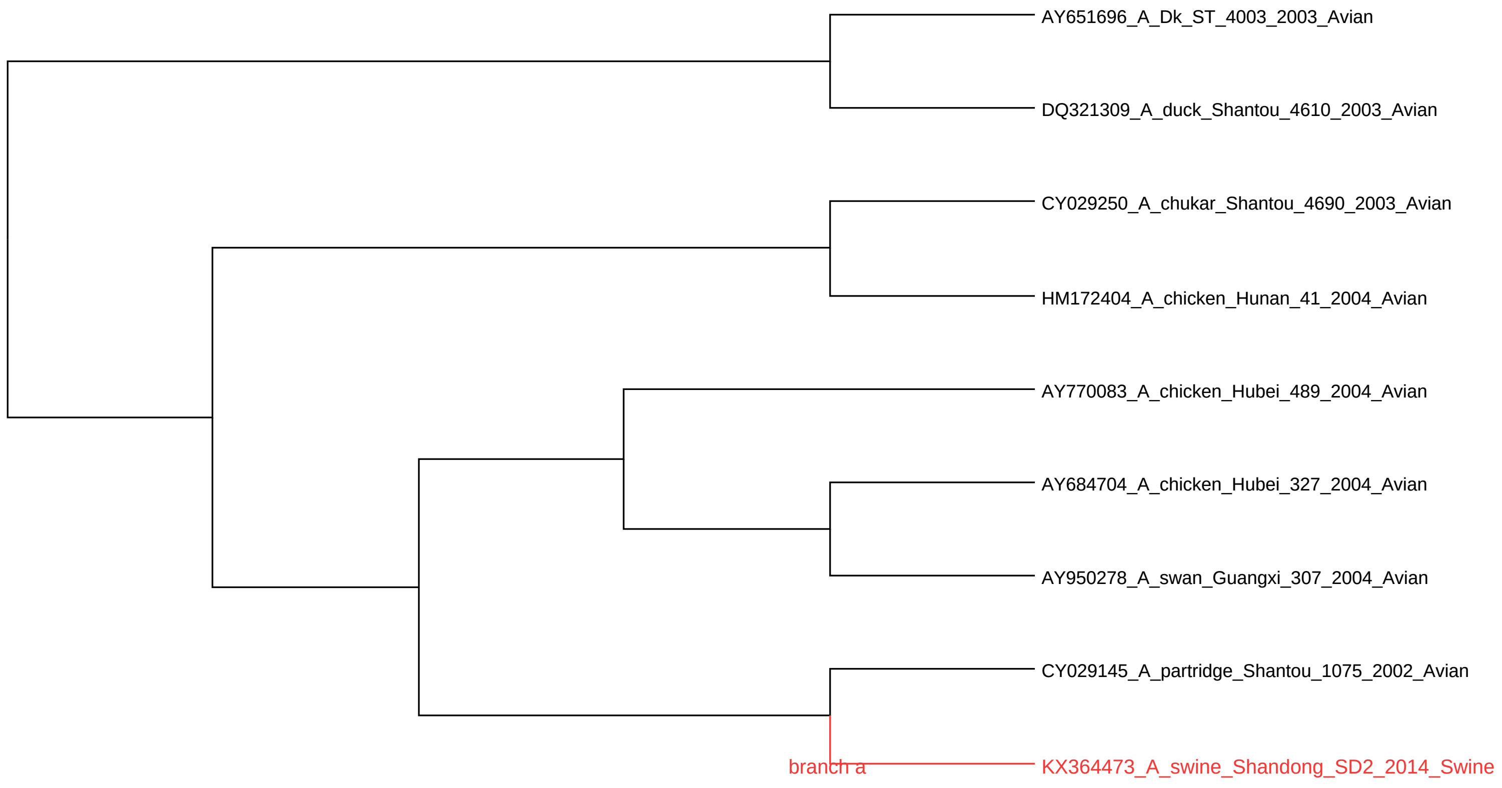
AY651696_A_Dk_ST_4003_2003_Avian
DQ321309_A_duck_Shantou_4610_2003_Avian
CY029250_A_chukar_Shantou_4690_2003_Avian
HM172404_A_chicken_Hunan_41_2004_Avian
AY770083_A_chicken_Hubei_489_2004_Avian
AY684704_A_chicken_Hubei_327_2004_Avian
AY950278_A_swan_Guangxi_307_2004_Avian
CY029145_A_partridge_Shantou_1075_2002_Avian
branch a
KX364473_A_swine_Shandong_SD2_2014_Swine

PB1-Group71

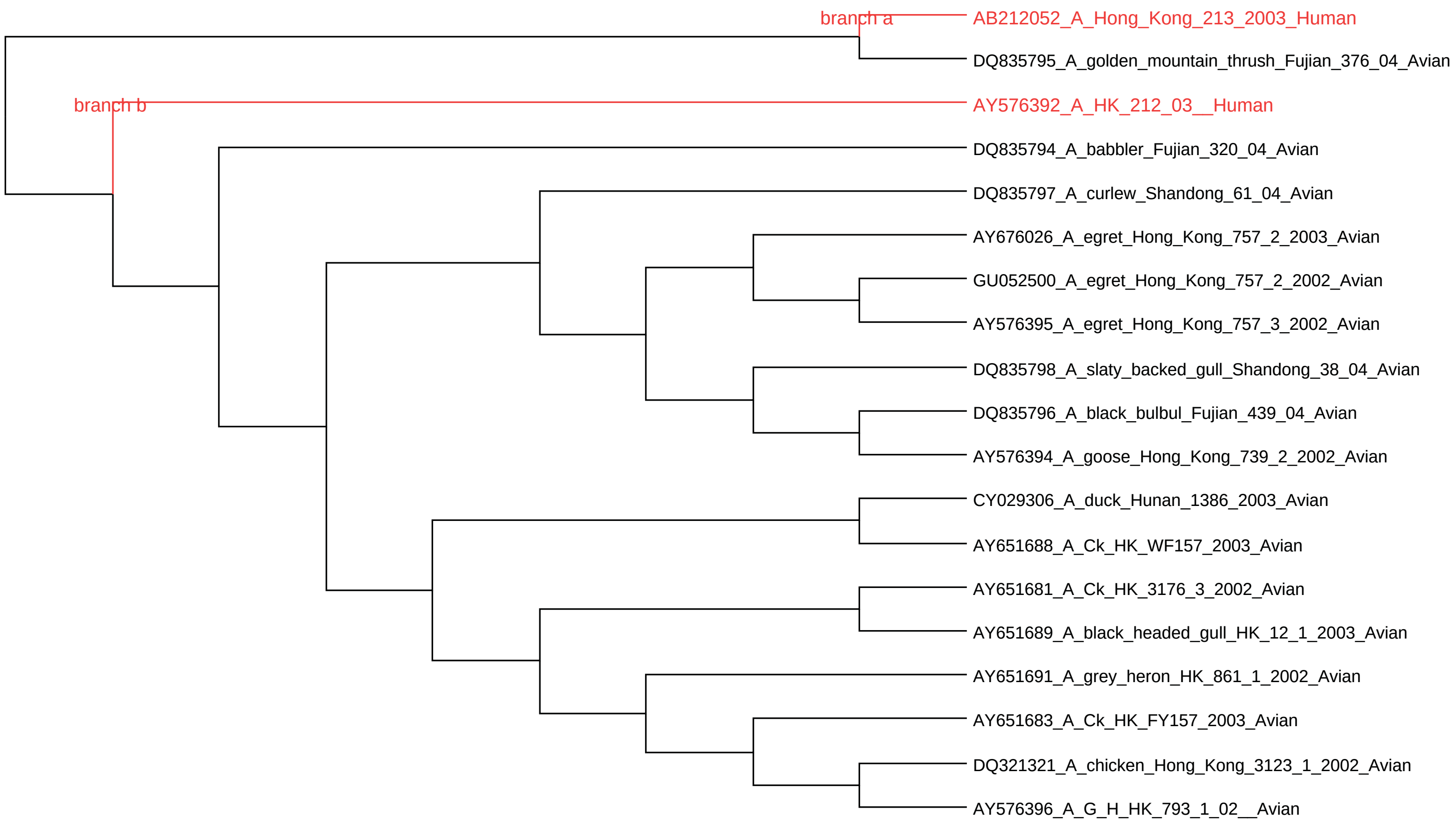
branch a
AB212052_A_Hong_Kong_213_2003_Human
DQ835795_A_golden_mountain_thrush_Fujian_376_04_Avian
branch b
AY576392_A_HK_212_03__Human
DQ835794_A_babbler_Fujian_320_04_Avian
DQ835797_A_curlew_Shandong_61_04_Avian
AY676026_A_egret_Hong_Kong_757_2_2003_Avian
GU052500_A_egret_Hong_Kong_757_2_2002_Avian
AY576395_A_egret_Hong_Kong_757_3_2002_Avian
DQ835798_A_slaty_backed_gull_Shandong_38_04_Avian
DQ835796_A_black_bulbul_Fujian_439_04_Avian
AY576394_A_goose_Hong_Kong_739_2_2002_Avian
CY029306_A_duck_Hunan_1386_2003_Avian
AY651688_A_Ck_HK_WF157_2003_Avian
AY651681_A_Ck_HK_3176_3_2002_Avian
AY651689_A_black_headed_gull_HK_12_1_2003_Avian
AY651691_A_grey_heron_HK_861_1_2002_Avian
AY651683_A_Ck_HK_FY157_2003_Avian
DQ321321_A_chicken_Hong_Kong_3123_1_2002_Avian
AY576396_A_G_H_HK_793_1_02__Avian

PB1-Group72

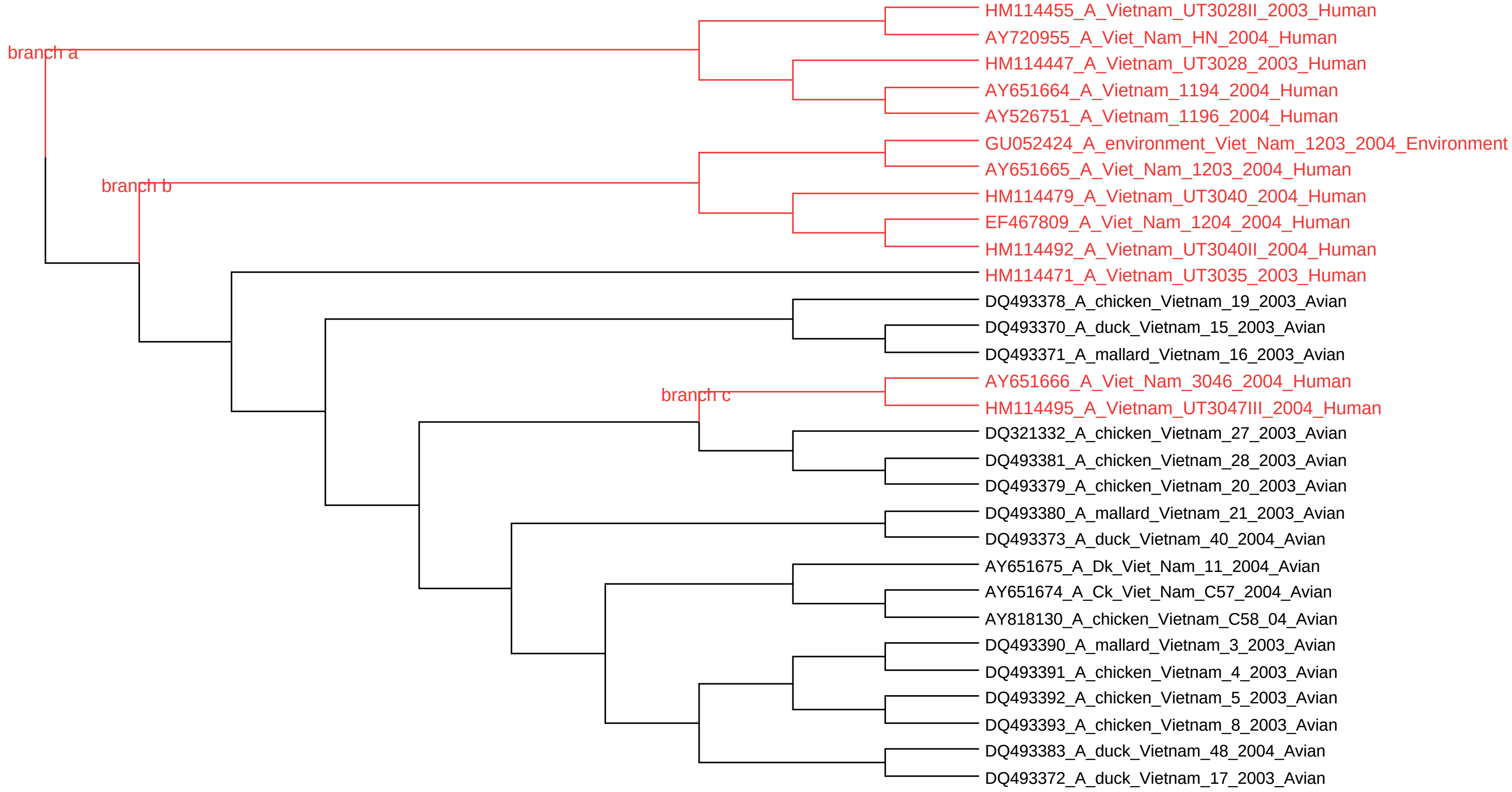

branch a
branch b
branch c
HM114455_A_Vietnam_UT3028II_2003_Human
AY720955_A_Viet_Nam_HN_2004_Human
HM114447_A_Vietnam_UT3028_2003_Human
AY651664_A_Vietnam_1194_2004_Human
AY526751_A_Vietnam_1196_2004_Human
GU052424_A_environment_Viet_Nam_1203_2004_Environment
AY651665_A_Viet_Nam_1203_2004_Human
HM114479_A_Vietnam_UT3040_2004_Human
EF467809_A_Viet_Nam_1204_2004_Human
HM114492_A_Vietnam_UT3040II_2004_Human
HM114471_A_Vietnam_UT3035_2003_Human
DQ493378_A_chicken_Vietnam_19_2003_Avian
DQ493370_A_duck_Vietnam_15_2003_Avian
DQ493371_A_mallard_Vietnam_16_2003_Avian
AY651666_A_Viet_Nam_3046_2004_Human
HM114495_A_Vietnam_UT3047III_2004_Human
DQ321332_A_chicken_Vietnam_27_2003_Avian
DQ493381_A_chicken_Vietnam_28_2003_Avian
DQ493379_A_chicken_Vietnam_20_2003_Avian
DQ493380_A_mallard_Vietnam_21_2003_Avian
DQ493373_A_duck_Vietnam_40_2004_Avian
AY651675_A_Dk_Viet_Nam_11_2004_Avian
AY651674_A_Ck_Viet_Nam_C57_2004_Avian
AY818130_A_chicken_Vietnam_C58_04_Avian
DQ493390_A_mallard_Vietnam_3_2003_Avian
DQ493391_A_chicken_Vietnam_4_2003_Avian
DQ493392_A_chicken_Vietnam_5_2003_Avian
DQ493393_A_chicken_Vietnam_8_2003_Avian
DQ493383_A_duck_Vietnam_48_2004_Avian
DQ493372_A_duck_Vietnam_17_2003_Avian

PB1-Group73

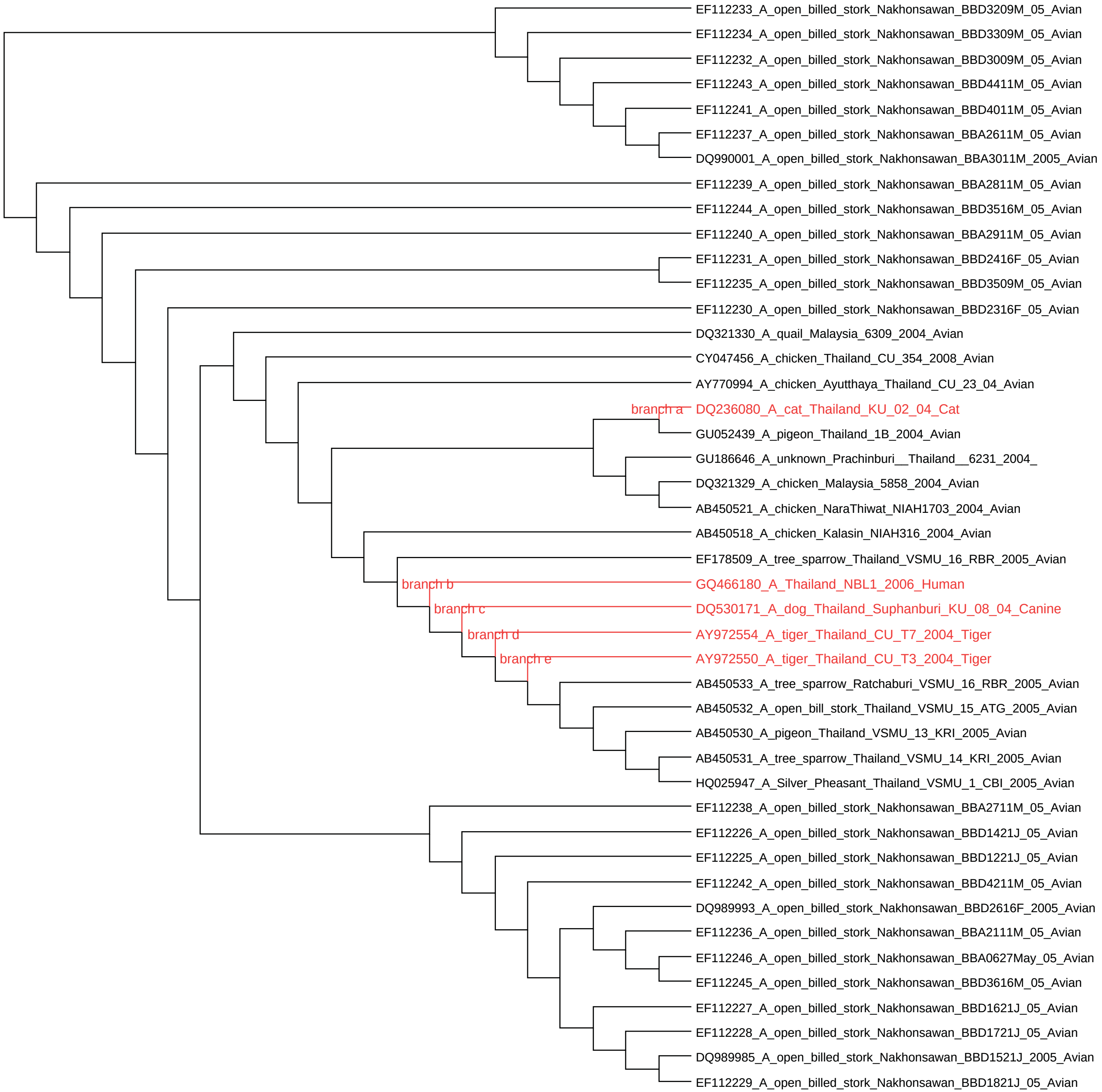

1-Group 3

PB1-Group74

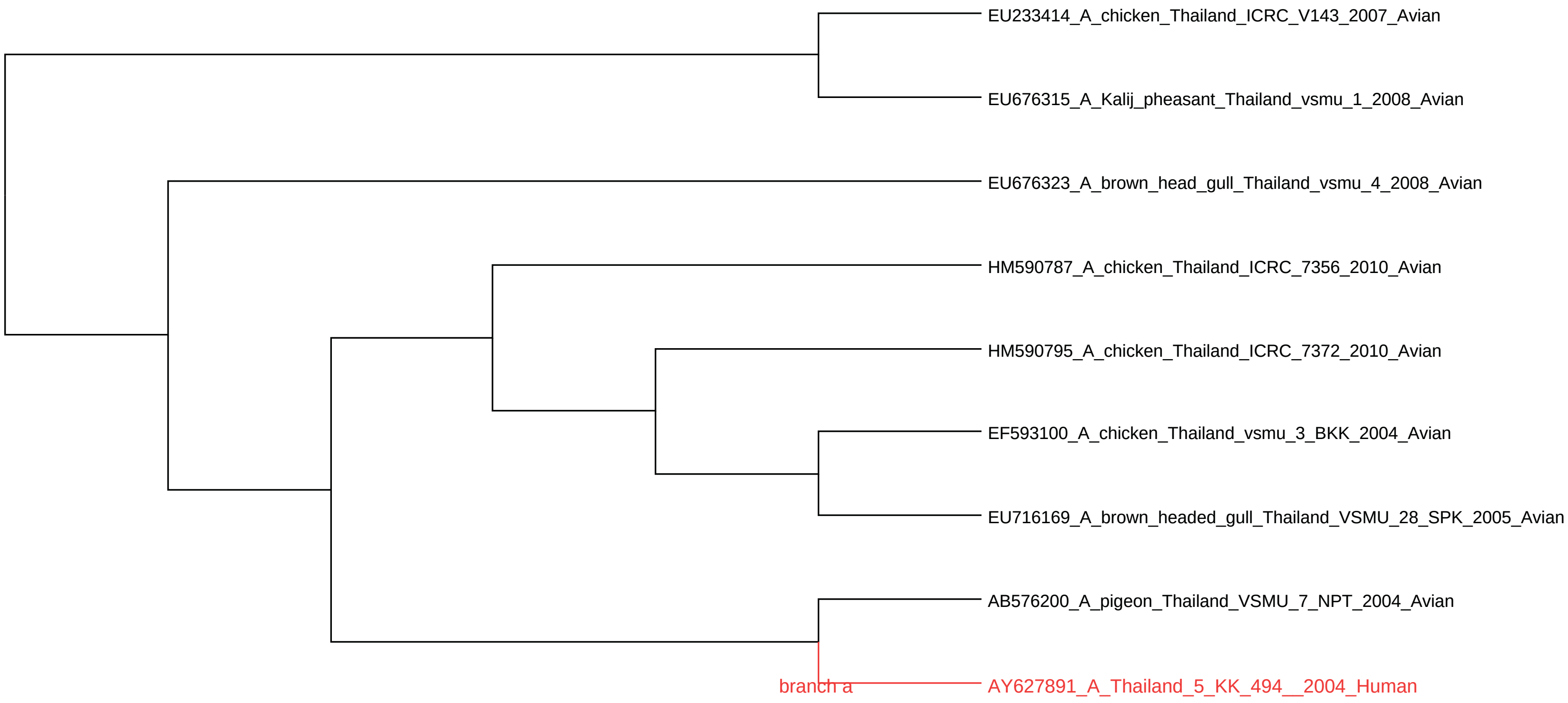
EU233414_A_chicken_Thailand_ICRC_V143_2007_Avian
EU676315_A_Kalij_pheasant_Thailand_vsmu_1_2008_Avian
EU676323_A_brown_head_gull_Thailand_vsmu_4_2008_Avian
HM590787_A_chicken_Thailand_ICRC_7356_2010_Avian
HM590795_A_chicken_Thailand_ICRC_7372_2010_Avian
EF593100_A_chicken_Thailand_vsmu_3_BKK_2004_Avian
EU716169_A_brown_headed_gull_Thailand_VSMU_28_SPK_2005_Avian
AB576200_A_pigeon_Thailand_VSMU_7_NPT_2004_Avian
branch a
AY627891_A_Thailand_5_KK_494__2004_Human

PB1-Group75

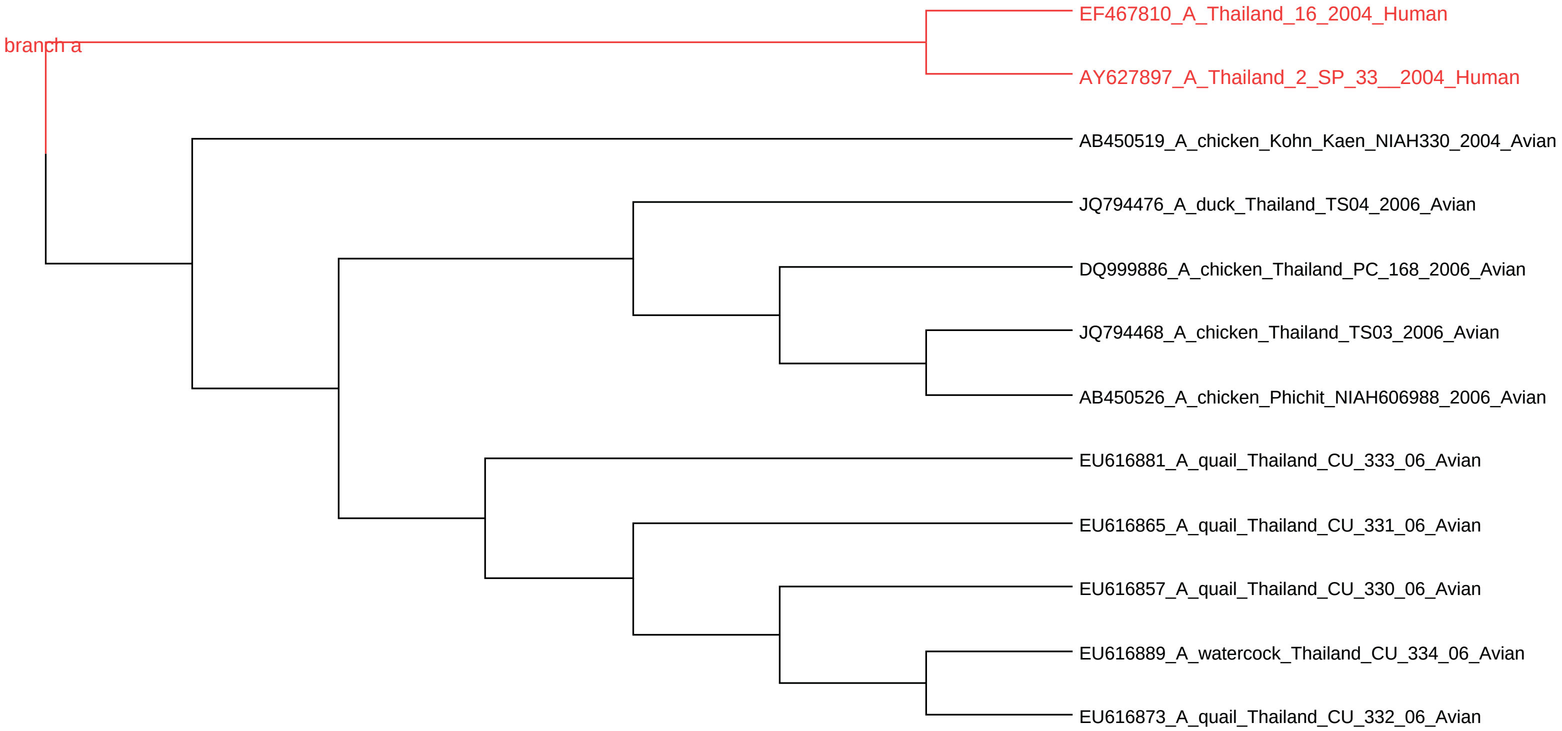
branch a
EF467810_A_Thailand_16_2004_Human
AY627897_A_Thailand_2_SP_33__2004_Human
AB450519_A_chicken_Kohn_Kaen_NIAH330_2004_Avian
JQ794476_A_duck_Thailand_TS04_2006_Avian
DQ999886_A_chicken_Thailand_PC_168_2006_Avian
JQ794468_A_chicken_Thailand_TS03_2006_Avian
AB450526_A_chicken_Phichit_NIAH606988_2006_Avian
EU616881_A_quail_Thailand_CU_333_06_Avian
EU616865_A_quail_Thailand_CU_331_06_Avian
EU616857_A_quail_Thailand_CU_330_06_Avian
EU616889_A_watercock_Thailand_CU_334_06_Avian
EU616873_A_quail_Thailand_CU_332_06_Avian

PB1-Group76

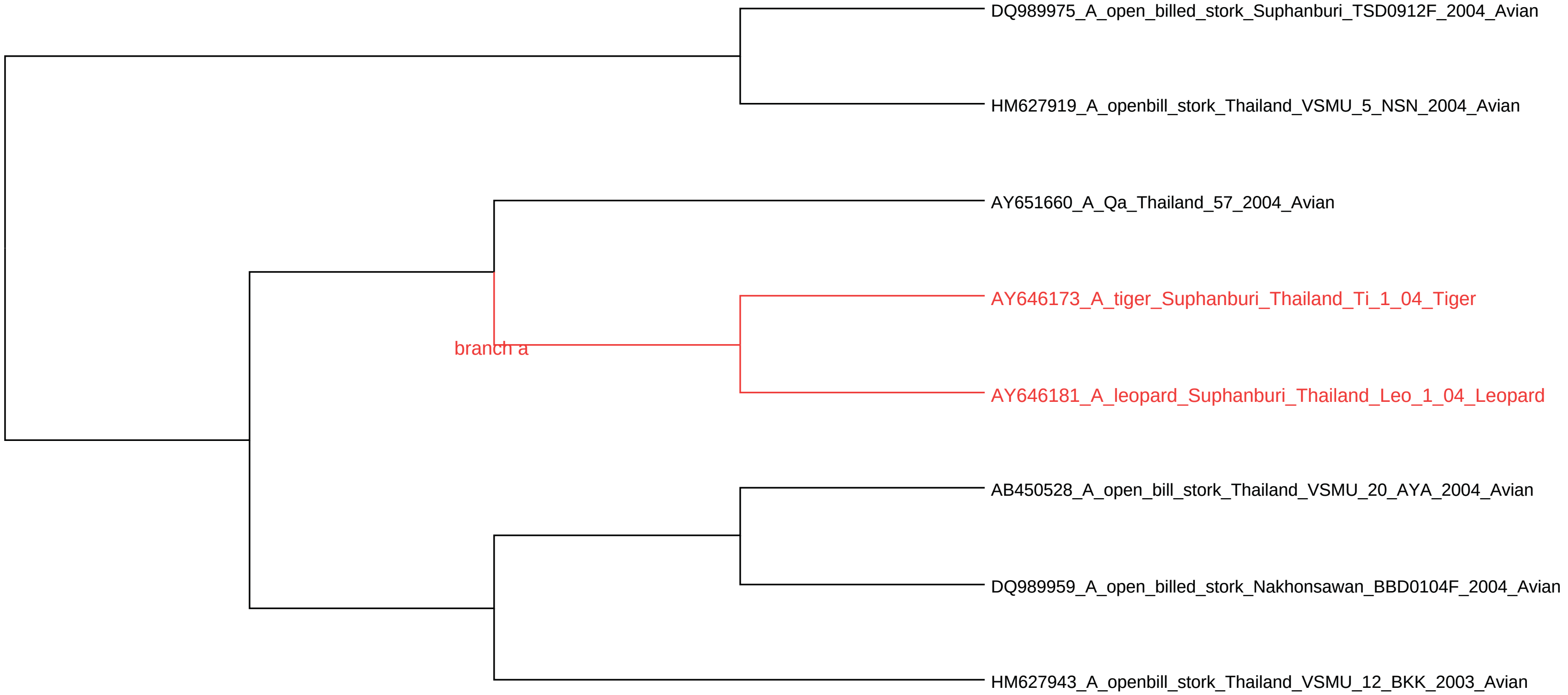

DQ989975_A_open_billed_stork_Suphanburi_TSD0912F_2004_Avian
HM627919_A_openbill_stork_Thailand_VSMU_5_NSN_2004_Avian
AY651660_A_Qa_Thailand_57_2004_Avian
AY646173_A_tiger_Suphanburi_Thailand_Ti_1_04_Tiger
branch a
AY646181_A_leopard_Suphanburi_Thailand_Leo_1_04_Leopard
AB450528_A_open_bill_stork_Thailand_VSMU_20_AYA_2004_Avian
DQ989959_A_open_billed_stork_Nakhonsawan_BBD0104F_2004_Avian
HM627943_A_openbill_stork_Thailand_VSMU_12_BKK_2003_Avian

PB1-Group77

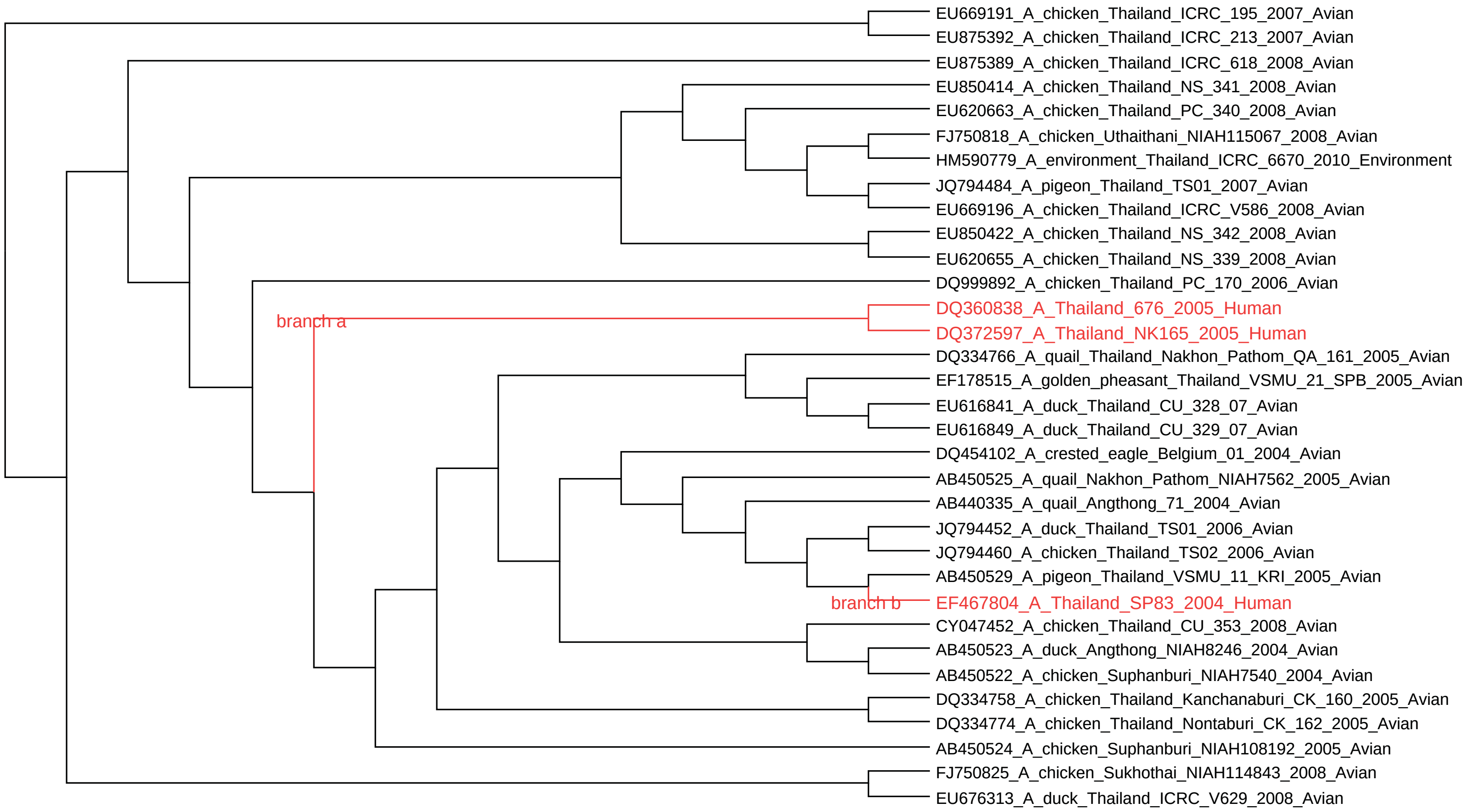
EU669191_A_chicken_Thailand_ICRC_195_2007_Avian
EU875392_A_chicken_Thailand_ICRC_213_2007_Avian
EU875389_A_chicken_Thailand_ICRC_618_2008_Avian
EU850414_A_chicken_Thailand_NS_341_2008_Avian
EU620663_A_chicken_Thailand_PC_340_2008_Avian
FJ750818_A_chicken_Uthaithani_NIAH115067_2008_Avian
HM590779_A_environment_Thailand_ICRC_6670_2010_Environment
JQ794484_A_pigeon_Thailand_TS01_2007_Avian
EU669196_A_chicken_Thailand_ICRC_V586_2008_Avian
EU850422_A_chicken_Thailand_NS_342_2008_Avian
EU620655_A_chicken_Thailand_NS_339_2008_Avian
DQ999892_A_chicken_Thailand_PC_170_2006_Avian
branch a
DQ360838_A_Thailand_676_2005_Human
DQ372597_A_Thailand_NK165_2005_Human
DQ334766_A_quail_Thailand_Nakhon_Pathom_QA_161_2005_Avian
EF178515_A_golden_pheasant_Thailand_VSMU_21_SPB_2005_Avian
EU616841_A_duck_Thailand_CU_328_07_Avian
EU616849_A_duck_Thailand_CU_329_07_Avian
DQ454102_A_crested_eagle_Belgium_01_2004_Avian
AB450525_A_quail_Nakhon_Pathom_NIAH7562_2005_Avian
AB440335_A_quail_Angthong_71_2004_Avian
JQ794452_A_duck_Thailand_TS01_2006_Avian
JQ794460_A_chicken_Thailand_TS02_2006_Avian
AB450529_A_pigeon_Thailand_VSMU_11_KRI_2005_Avian
branch b
EF467804_A_Thailand_SP83_2004_Human
CY047452_A_chicken_Thailand_CU_353_2008_Avian
AB450523_A_duck_Angthong_NIAH8246_2004_Avian
AB450522_A_chicken_Suphanburi_NIAH7540_2004_Avian
DQ334758_A_chicken_Thailand_Kanchanaburi_CK_160_2005_Avian
DQ334774_A_chicken_Thailand_Nontaburi_CK_162_2005_Avian
AB450524_A_chicken_Suphanburi_NIAH108192_2005_Avian
FJ750825_A_chicken_Sukhothai_NIAH114843_2008_Avian
EU676313_A_duck_Thailand_ICRC_V629_2008_Avian

PB1-Group78

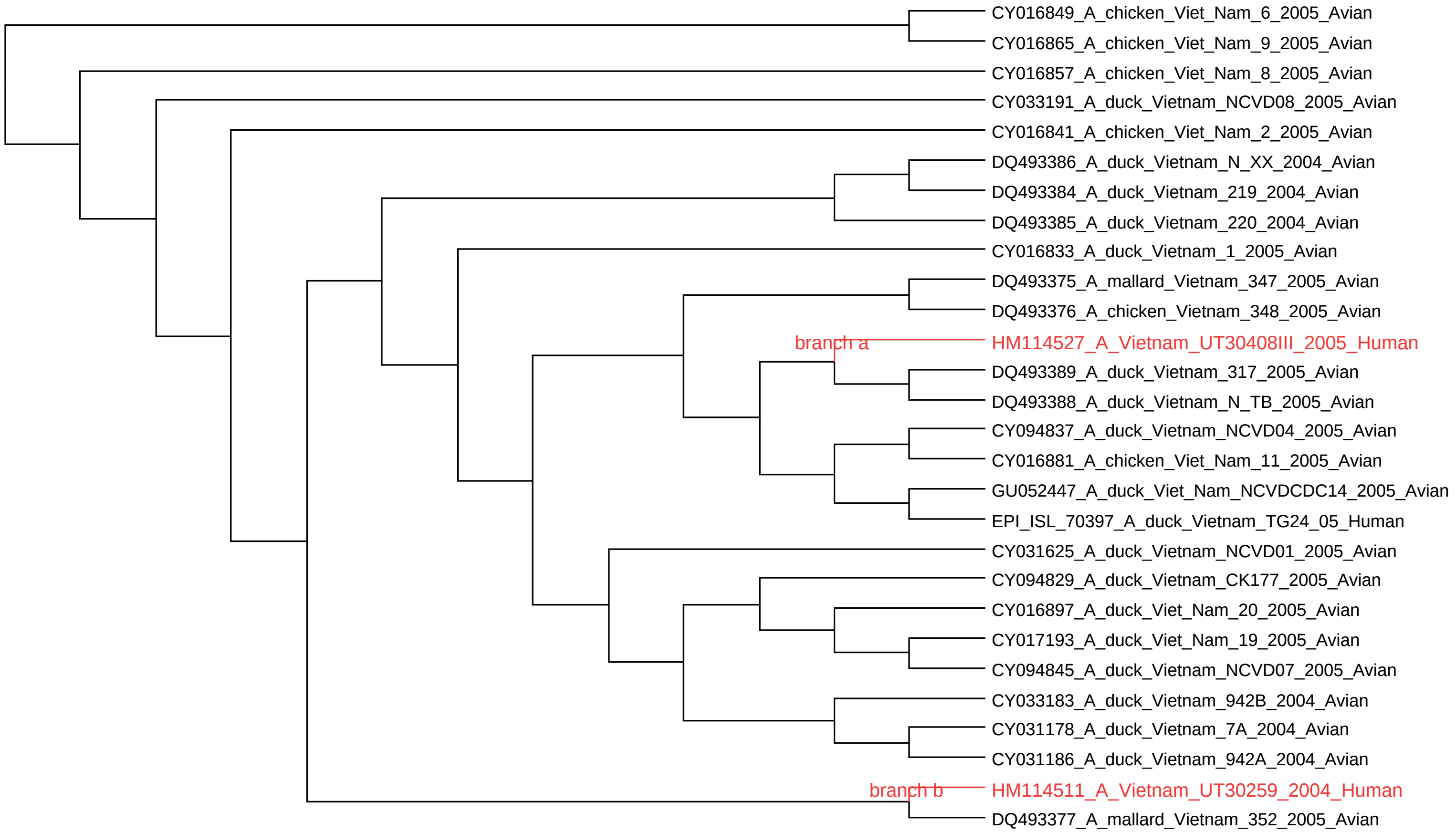

CY016849_A_chicken_Viet_Nam_6_2005_Avian
CY016865_A_chicken_Viet_Nam_9_2005_Avian
CY016857_A_chicken_Viet_Nam_8_2005_Avian
CY033191_A_duck_Vietnam_NCVD08_2005_Avian
CY016841_A_chicken_Viet_Nam_2_2005_Avian
DQ493386_A_duck_Vietnam_N_XX_2004_Avian
DQ493384_A_duck_Vietnam_219_2004_Avian
DQ493385_A_duck_Vietnam_220_2004_Avian
CY016833_A_duck_Vietnam_1_2005_Avian
DQ493375_A_mallard_Vietnam_347_2005_Avian
DQ493376_A_chicken_Vietnam_348_2005_Avian
branch a
HM114527_A_Vietnam_UT30408III_2005_Human
DQ493389_A_duck_Vietnam_317_2005_Avian
DQ493388_A_duck_Vietnam_N_TB_2005_Avian
CY094837_A_duck_Vietnam_NCVD04_2005_Avian
CY016881_A_chicken_Viet_Nam_11_2005_Avian
GU052447_A_duck_Viet_Nam_NCVDCDC14_2005_Avian
EPI_ISL_70397_A_duck_Vietnam_TG24_05_Human
CY031625_A_duck_Vietnam_NCVD01_2005_Avian
CY094829_A_duck_Vietnam_CK177_2005_Avian
CY016897_A_duck_Viet_Nam_20_2005_Avian
CY017193_A_duck_Viet_Nam_19_2005_Avian
CY094845_A_duck_Vietnam_NCVD07_2005_Avian
CY033183_A_duck_Vietnam_942B_2004_Avian
CY031178_A_duck_Vietnam_7A_2004_Avian
CY031186_A_duck_Vietnam_942A_2004_Avian
branch b
HM114511_A_Vietnam_UT30259_2004_Human
DQ493377_A_mallard_Vietnam_352_2005_Avian

PB1-Group79

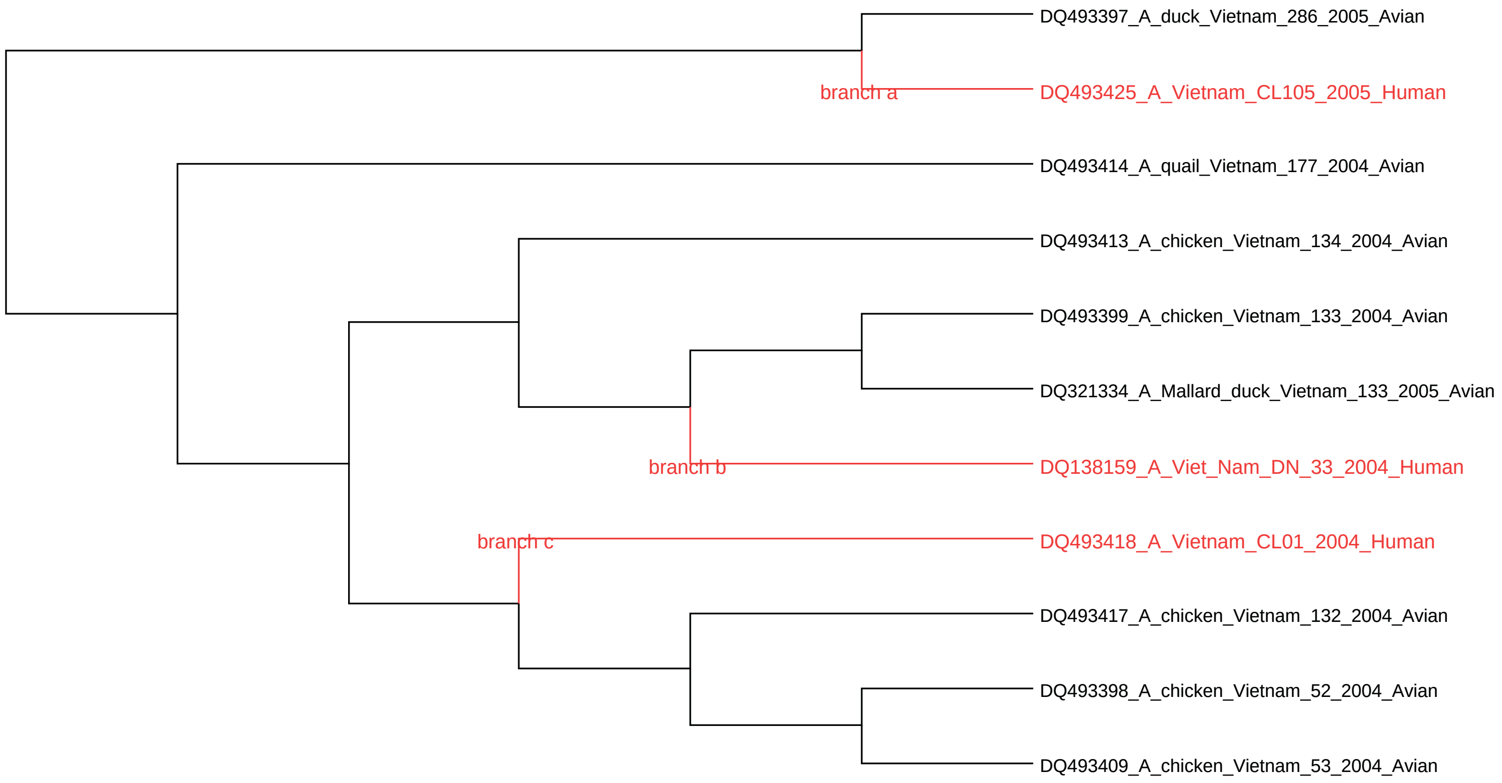
DQ493397_A_duck_Vietnam_286_2005_Avian
branch a
DQ493425_A_Vietnam_CL105_2005_Human
DQ493414_A_quail_Vietnam_177_2004_Avian
DQ493413_A_chicken_Vietnam_134_2004_Avian
DQ493399_A_chicken_Vietnam_133_2004_Avian
DQ321334_A_Mallard_duck_Vietnam_133_2005_Avian
branch b
DQ138159_A_Viet_Nam_DN_33_2004_Human
branch c
DQ493418_A_Vietnam_CL01_2004_Human
DQ493417_A_chicken_Vietnam_132_2004_Avian
DQ493398_A_chicken_Vietnam_52_2004_Avian
DQ493409_A_chicken_Vietnam_53_2004_Avian

PB1-Group80

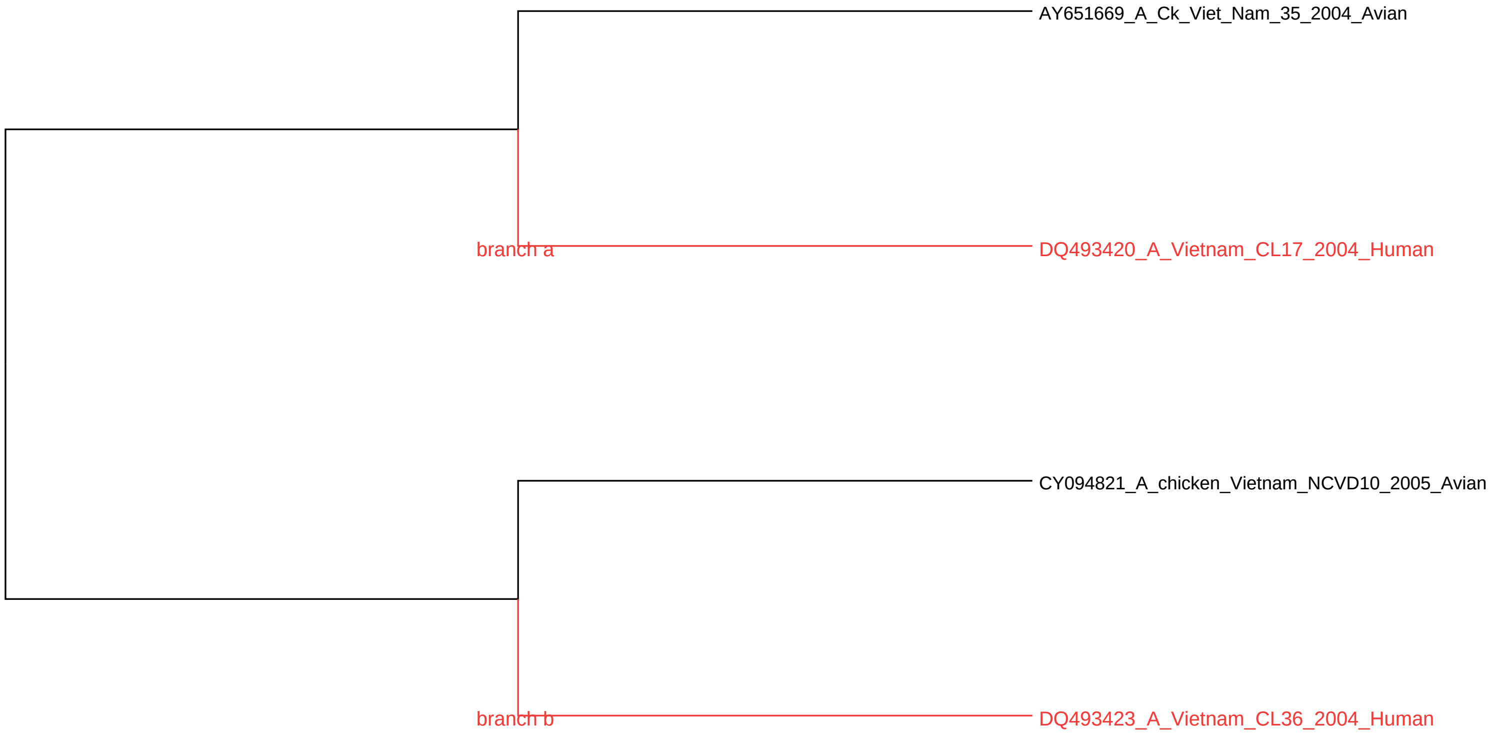
AY651669_A_Ck_Viet_Nam_35_2004_Avian
branch a
DQ493420_A_Vietnam_CL17_2004_Human
CY094821_A_chicken_Vietnam_NCVD10_2005_Avian
branch b
DQ493423_A_Vietnam_CL36_2004_Human

PB1-Group81

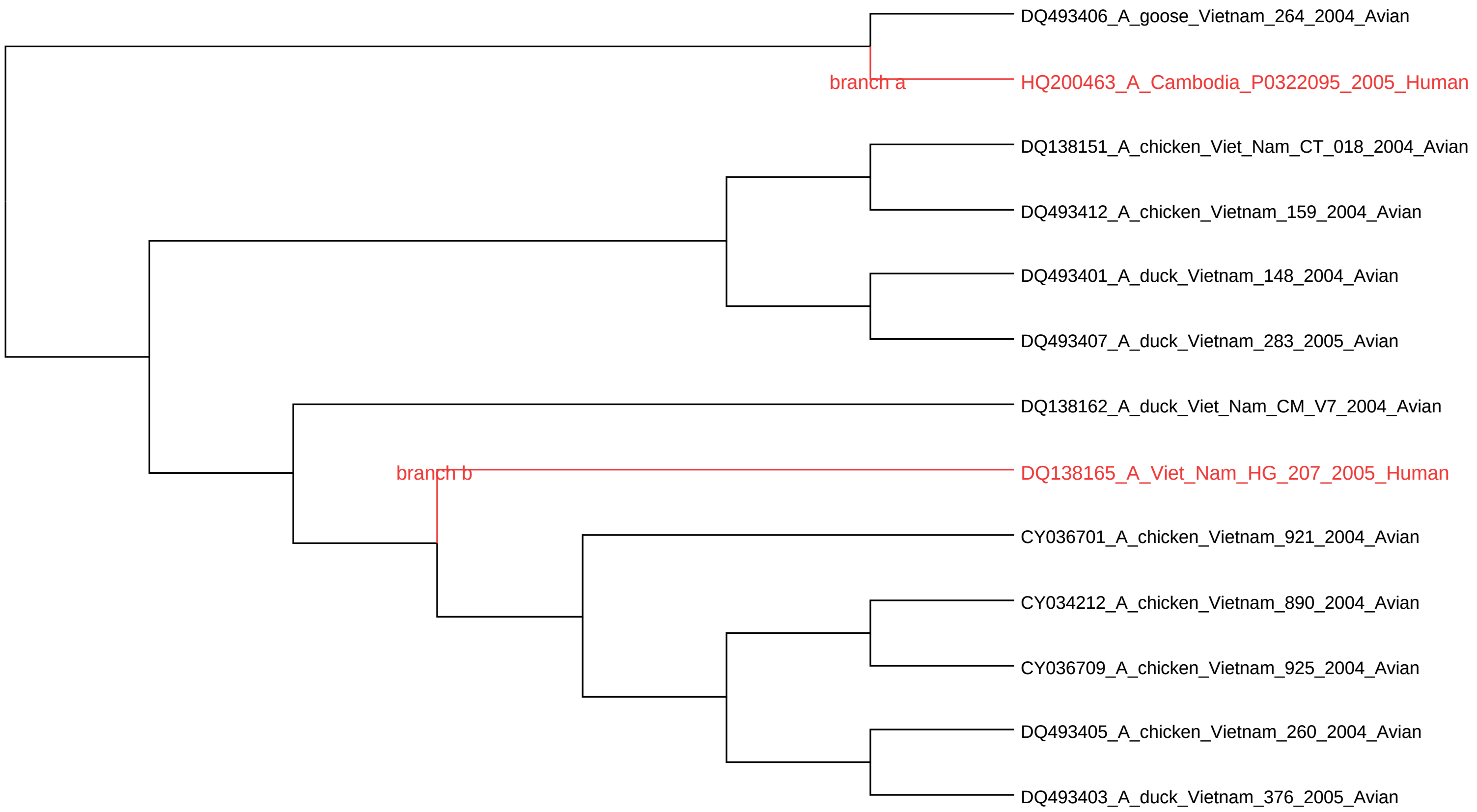
DQ493406_A_goose_Vietnam_264_2004_Avian
branch a
HQ200463_A_Cambodia_P0322095_2005_Human
DQ138151_A_chicken_Viet_Nam_CT_018_2004_Avian
DQ493412_A_chicken_Vietnam_159_2004_Avian
DQ493401_A_duck_Vietnam_148_2004_Avian
DQ493407_A_duck_Vietnam_283_2005_Avian
DQ138162_A_duck_Viet_Nam_CM_V7_2004_Avian
branch b
DQ138165_A_Viet_Nam_HG_207_2005_Human
CY036701_A_chicken_Vietnam_921_2004_Avian
CY034212_A_chicken_Vietnam_890_2004_Avian
CY036709_A_chicken_Vietnam_925_2004_Avian
DQ493405_A_chicken_Vietnam_260_2004_Avian
DQ493403_A_duck_Vietnam_376_2005_Avian

PB1-Group82

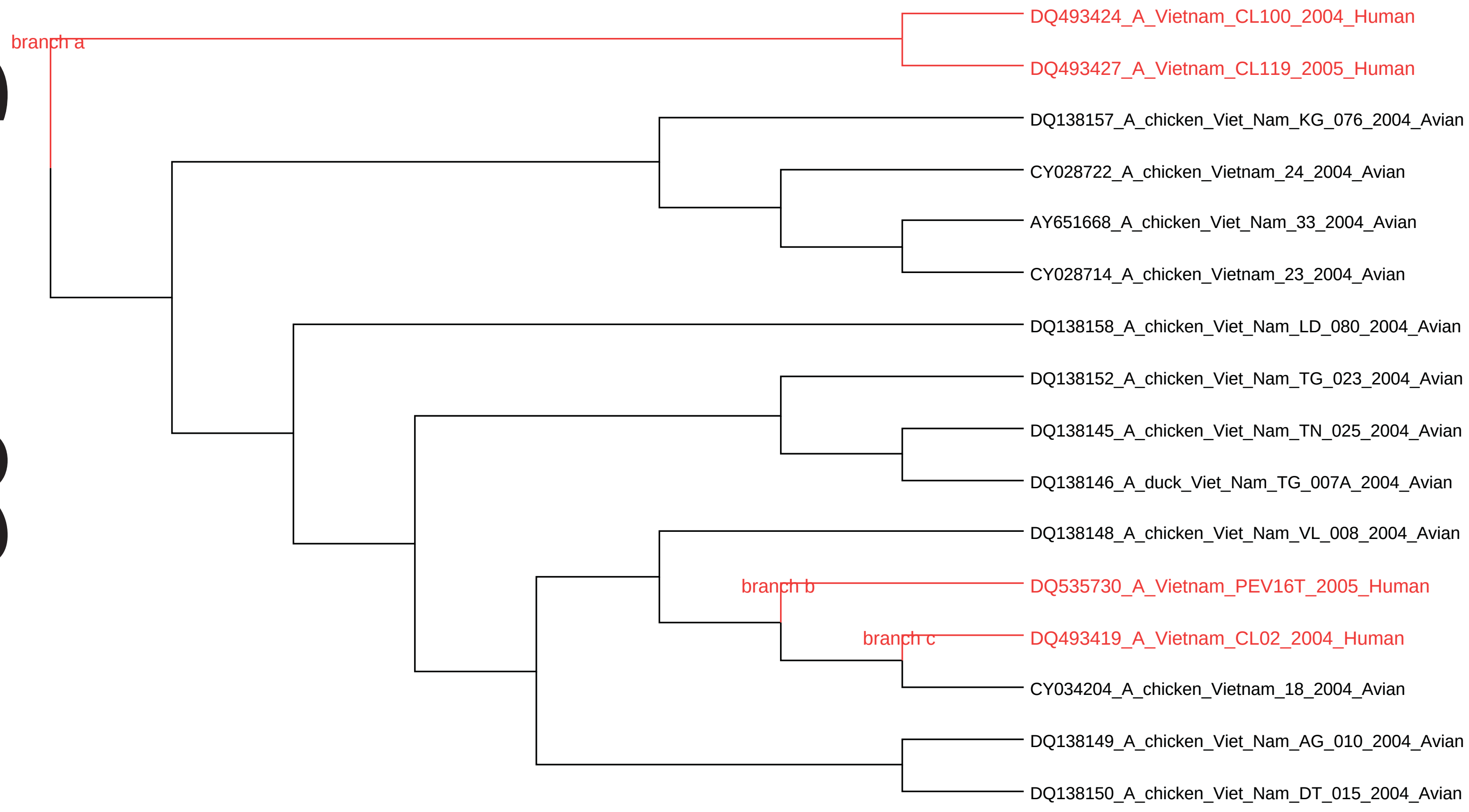

branch a
DQ493424_A_Vietnam_CL100_2004_Human
DQ493427_A_Vietnam_CL119_2005_Human
DQ138157_A_chicken_Viet_Nam_KG_076_2004_Avian
CY028722_A_chicken_Vietnam_24_2004_Avian
AY651668_A_chicken_Viet_Nam_33_2004_Avian
CY028714_A_chicken_Vietnam_23_2004_Avian
DQ138158_A_chicken_Viet_Nam_LD_080_2004_Avian
DQ138152_A_chicken_Viet_Nam_TG_023_2004_Avian
DQ138145_A_chicken_Viet_Nam_TN_025_2004_Avian
DQ138146_A_duck_Viet_Nam_TG_007A_2004_Avian
DQ138148_A_chicken_Viet_Nam_VL_008_2004_Avian
branch b
DQ535730_A_Vietnam_PEV16T_2005_Human
branch c
DQ493419_A_Vietnam_CL02_2004_Human
CY034204_A_chicken_Vietnam_18_2004_Avian
DQ138149_A_chicken_Viet_Nam_AG_010_2004_Avian
DQ138150_A_chicken_Viet_Nam_DT_015_2004_Avian

PB1-Group83

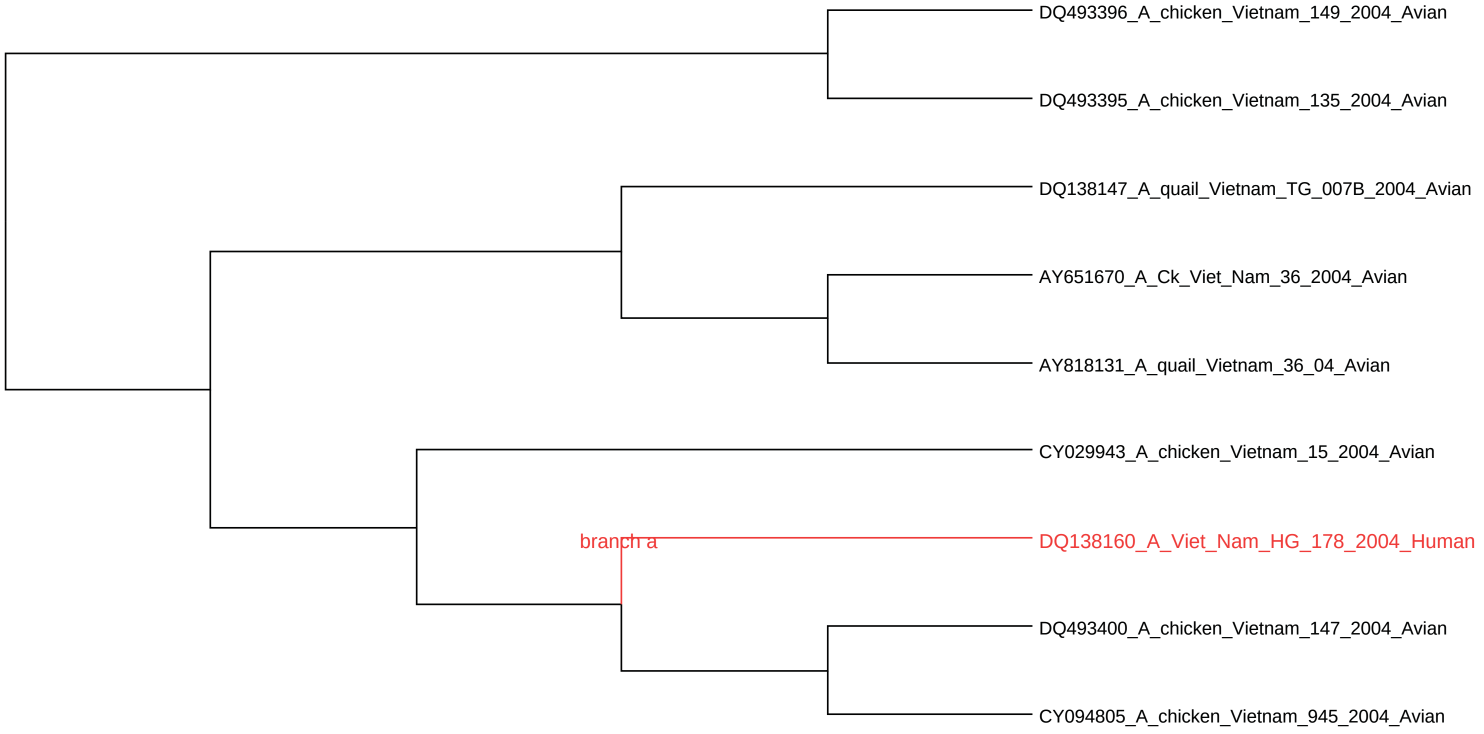

DQ493396_A_chicken_Vietnam_149_2004_Avian
DQ493395_A_chicken_Vietnam_135_2004_Avian
DQ138147_A_quail_Vietnam_TG_007B_2004_Avian
AY651670_A_Ck_Viet_Nam_36_2004_Avian
AY818131_A_quail_Vietnam_36_04_Avian
CY029943_A_chicken_Vietnam_15_2004_Avian
branch a
DQ138160_A_Viet_Nam_HG_178_2004_Human
DQ493400_A_chicken_Vietnam_147_2004_Avian
CY094805_A_chicken_Vietnam_945_2004_Avian

PB1-Group84

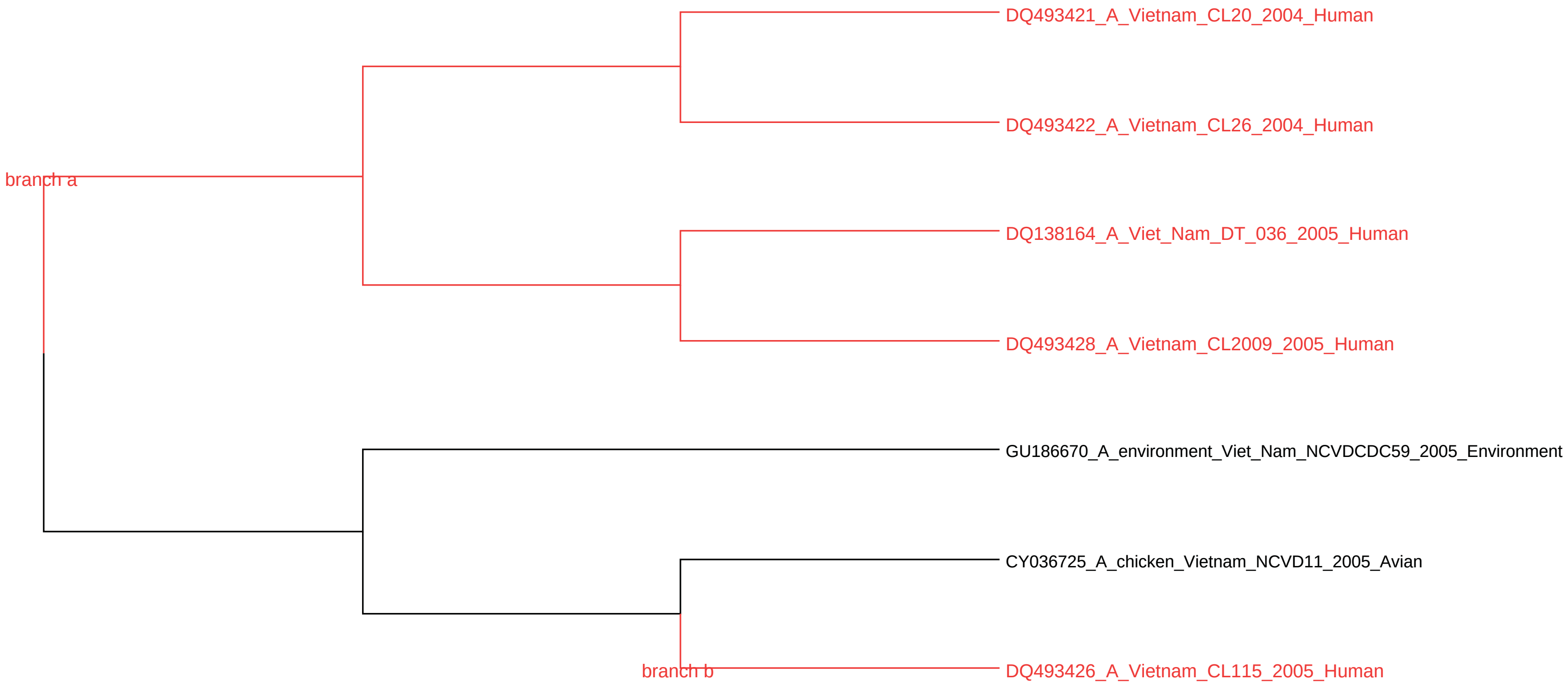

DQ493421_A_Vietnam_CL20_2004_Human
DQ493422_A_Vietnam_CL26_2004_Human
branch a
DQ138164_A_Viet_Nam_DT_036_2005_Human
DQ493428_A_Vietnam_CL2009_2005_Human
GU186670_A_environment_Viet_Nam_NCVDCDC59_2005_Environment
CY036725_A_chicken_Vietnam_NCVD11_2005_Avian
branch b
DQ493426_A_Vietnam_CL115_2005_Human

PB1-Group85

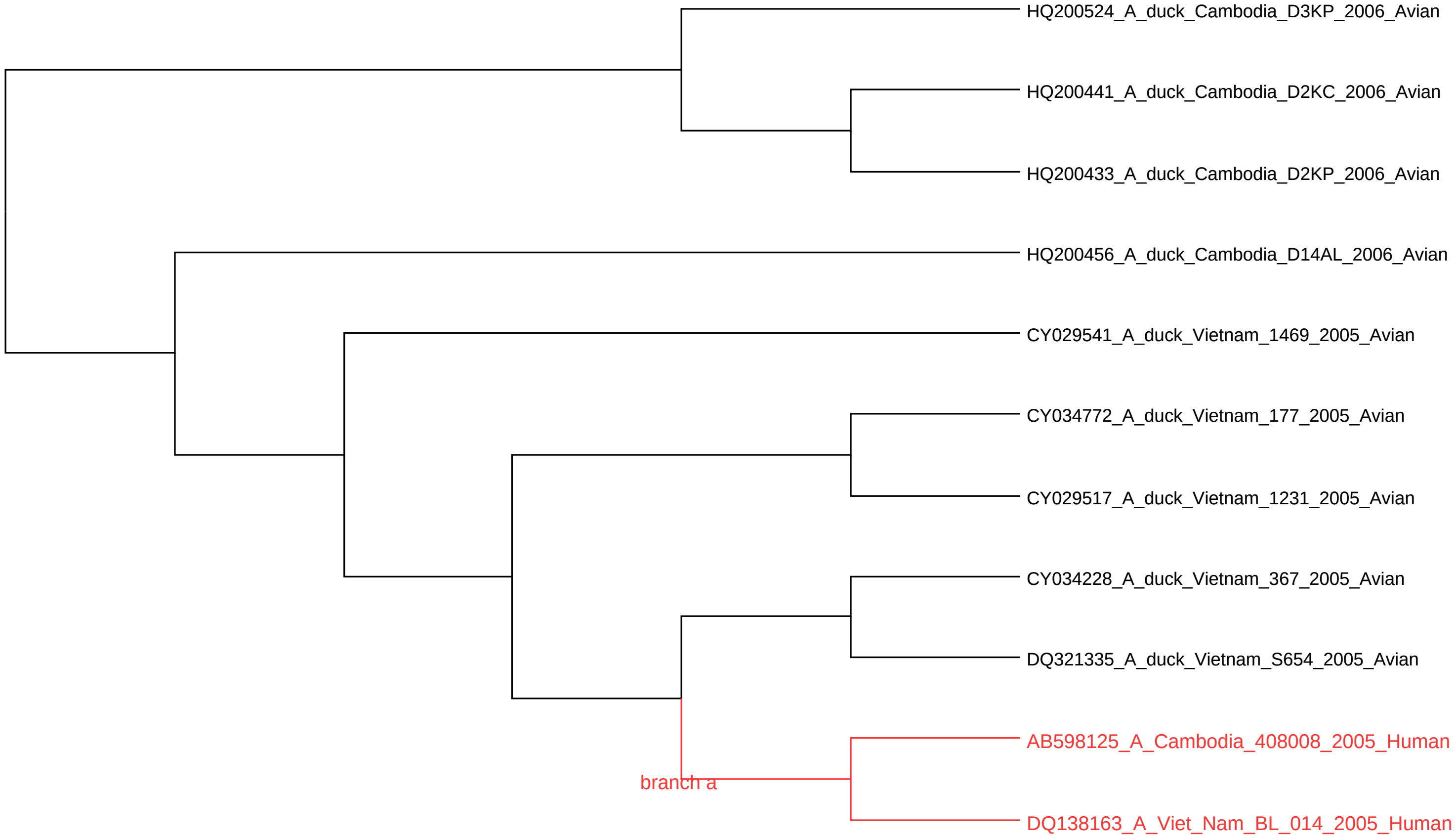
HQ200524_A_duck_Cambodia_D3KP_2006_Avian
HQ200441_A_duck_Cambodia_D2KC_2006_Avian
HQ200433_A_duck_Cambodia_D2KP_2006_Avian
HQ200456_A_duck_Cambodia_D14AL_2006_Avian
CY029541_A_duck_Vietnam_1469_2005_Avian
CY034772_A_duck_Vietnam_177_2005_Avian
CY029517_A_duck_Vietnam_1231_2005_Avian
CY034228_A_duck_Vietnam_367_2005_Avian
DQ321335_A_duck_Vietnam_S654_2005_Avian
AB598125_A_Cambodia_408008_2005_Human
branch a
DQ138163_A_Viet_Nam_BL_014_2005_Human

PB1-Group86

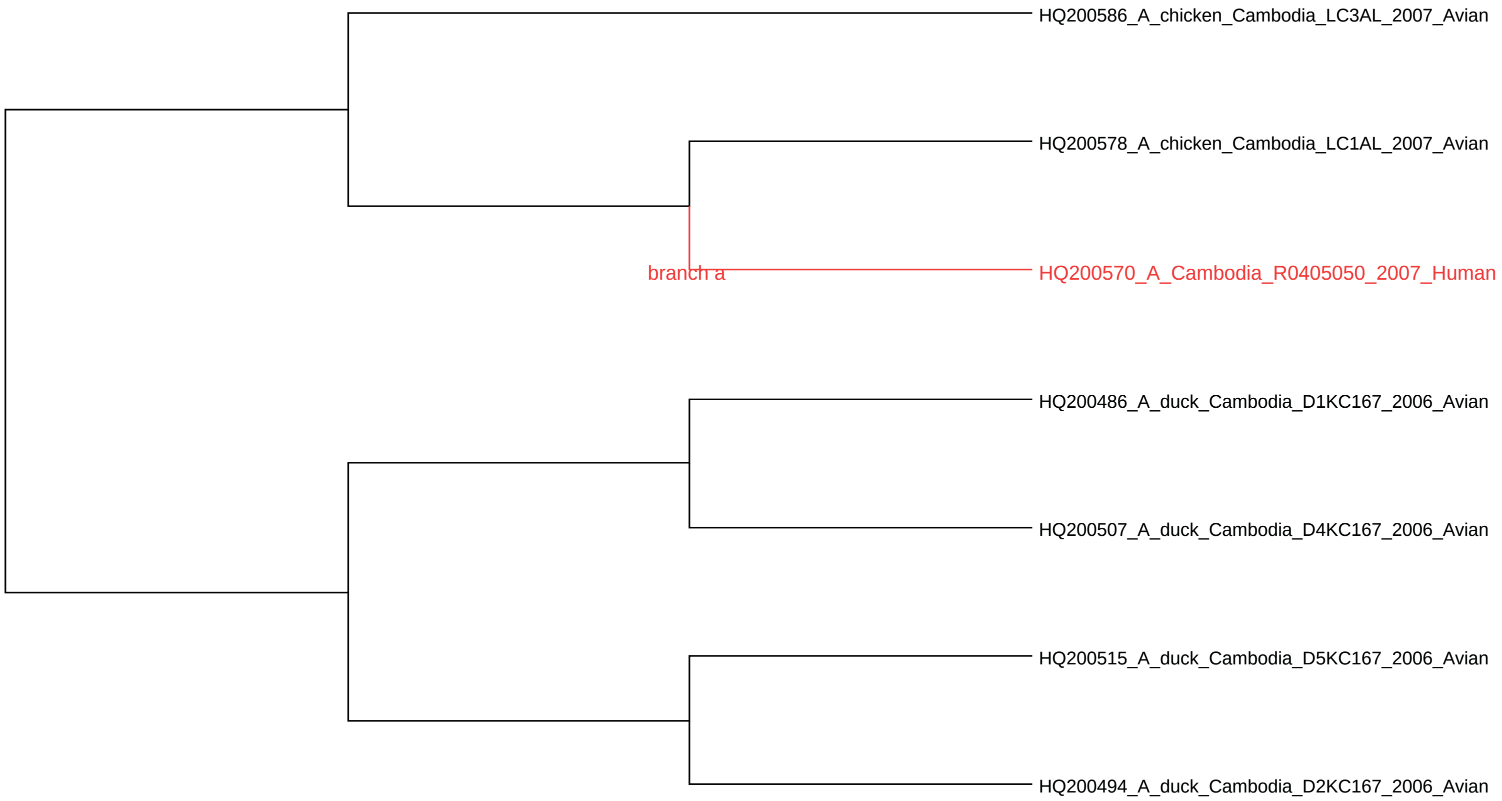
HQ200586_A_chicken_Cambodia_LC3AL_2007_Avian
HQ200578_A_chicken_Cambodia_LC1AL_2007_Avian
branch a
HQ200570_A_Cambodia_R0405050_2007_Human
HQ200486_A_duck_Cambodia_D1KC167_2006_Avian
HQ200507_A_duck_Cambodia_D4KC167_2006_Avian
HQ200515_A_duck_Cambodia_D5KC167_2006_Avian
HQ200494_A_duck_Cambodia_D2KC167_2006_Avian

PB1-Group87

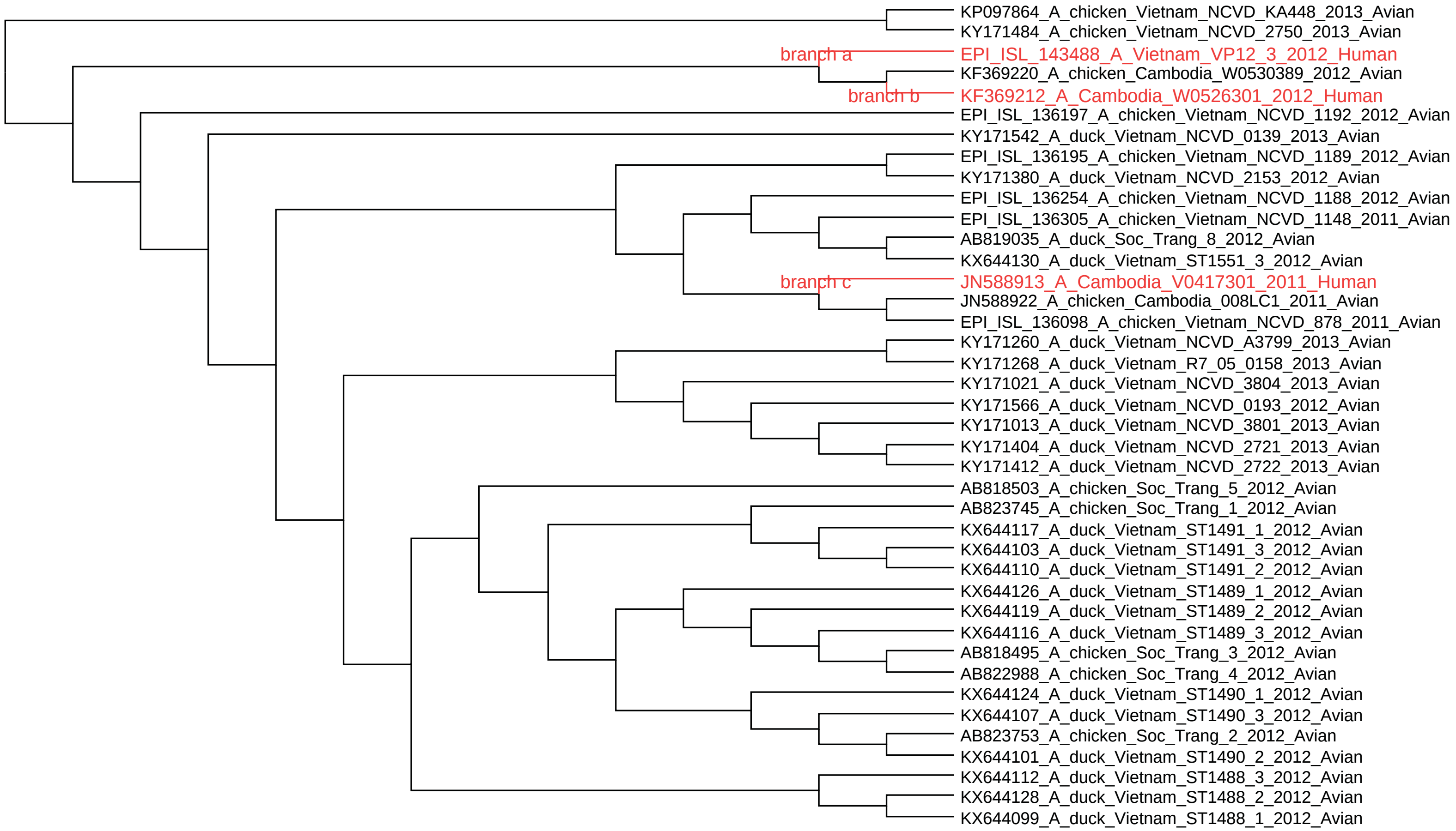
KP097864_A_chicken_Vietnam_NCVD_KA448_2013_Avian
KY171484_A_chicken_Vietnam_NCVD_2750_2013_Avian
branch a
EPI_ISL_143488_A_Vietnam_VP12_3_2012_Human
KF369220_A_chicken_Cambodia_W0530389_2012_Avian
branch b
KF369212_A_Cambodia_W0526301_2012_Human
EPI_ISL_136197_A_chicken_Vietnam_NCVD_1192_2012_Avian
KY171542_A_duck_Vietnam_NCVD_0139_2013_Avian
EPI_ISL_136195_A_chicken_Vietnam_NCVD_1189_2012_Avian
KY171380_A_duck_Vietnam_NCVD_2153_2012_Avian
EPI_ISL_136254_A_chicken_Vietnam_NCVD_1188_2012_Avian
EPI_ISL_136305_A_chicken_Vietnam_NCVD_1148_2011_Avian
AB819035_A_duck_Soc_Trang_8_2012_Avian
KX644130_A_duck_Vietnam_ST1551_3_2012_Avian
branch c
JN588913_A_Cambodia_V0417301_2011_Human
JN588922_A_chicken_Cambodia_008LC1_2011_Avian
EPI_ISL_136098_A_chicken_Vietnam_NCVD_878_2011_Avian
KY171260_A_duck_Vietnam_NCVD_A3799_2013_Avian
KY171268_A_duck_Vietnam_R7_05_0158_2013_Avian
KY171021_A_duck_Vietnam_NCVD_3804_2013_Avian
KY171566_A_duck_Vietnam_NCVD_0193_2012_Avian
KY171013_A_duck_Vietnam_NCVD_3801_2013_Avian
KY171404_A_duck_Vietnam_NCVD_2721_2013_Avian
KY171412_A_duck_Vietnam_NCVD_2722_2013_Avian
AB818503_A_chicken_Soc_Trang_5_2012_Avian
AB823745_A_chicken_Soc_Trang_1_2012_Avian
KX644117_A_duck_Vietnam_ST1491_1_2012_Avian
KX644103_A_duck_Vietnam_ST1491_3_2012_Avian
KX644110_A_duck_Vietnam_ST1491_2_2012_Avian
KX644126_A_duck_Vietnam_ST1489_1_2012_Avian
KX644119_A_duck_Vietnam_ST1489_2_2012_Avian
KX644116_A_duck_Vietnam_ST1489_3_2012_Avian
AB818495_A_chicken_Soc_Trang_3_2012_Avian
AB822988_A_chicken_Soc_Trang_4_2012_Avian
KX644124_A_duck_Vietnam_ST1490_1_2012_Avian
KX644107_A_duck_Vietnam_ST1490_3_2012_Avian
AB823753_A_chicken_Soc_Trang_2_2012_Avian
KX644101_A_duck_Vietnam_ST1490_2_2012_Avian
KX644112_A_duck_Vietnam_ST1488_3_2012_Avian
KX644128_A_duck_Vietnam_ST1488_2_2012_Avian
KX644099_A_duck_Vietnam_ST1488_1_2012_Avian

PB1-Group88

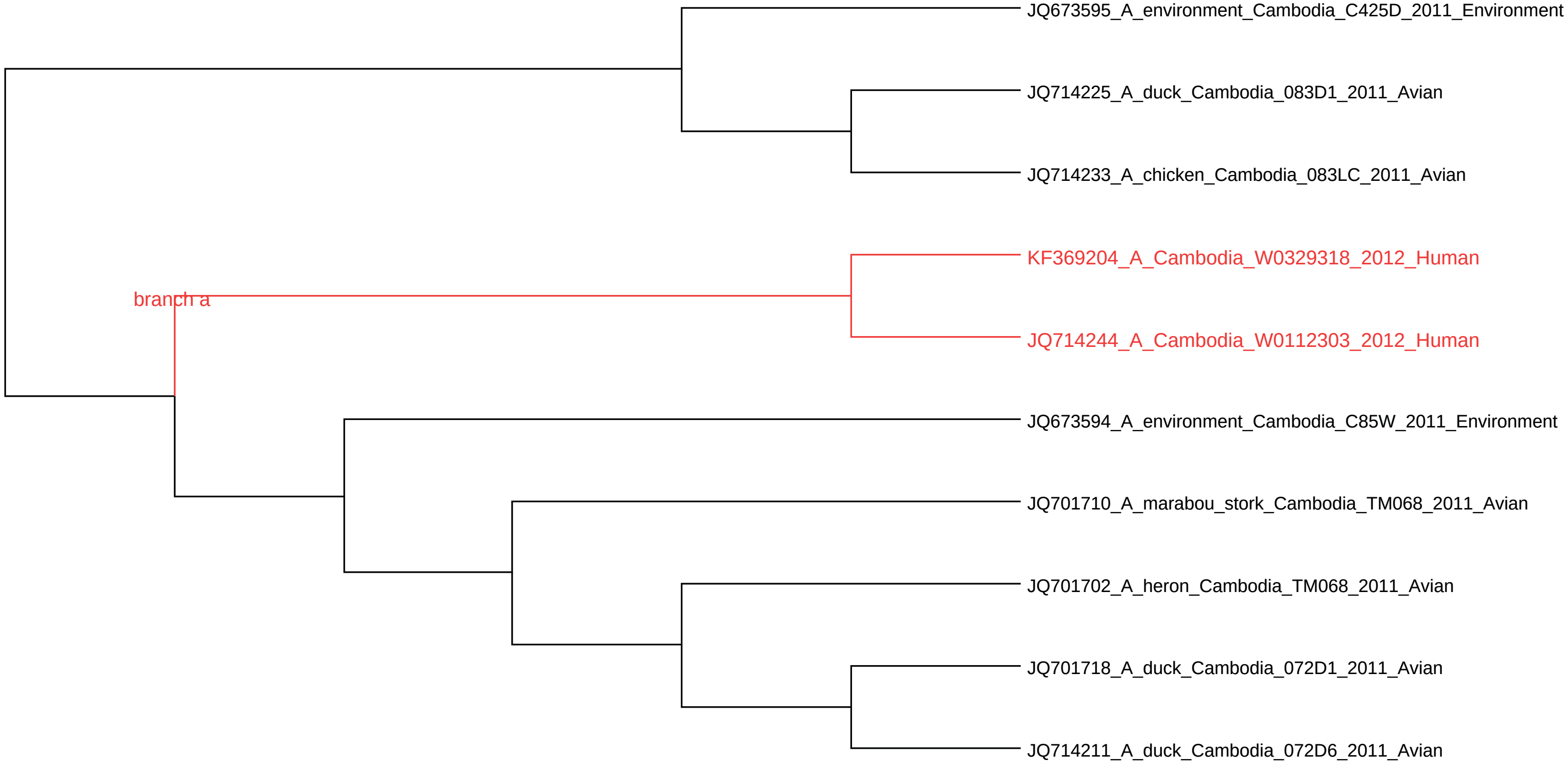
JQ673595_A_environment_Cambodia_C425D_2011_Environment
JQ714225_A_duck_Cambodia_083D1_2011_Avian
JQ714233_A_chicken_Cambodia_083LC_2011_Avian
KF369204_A_Cambodia_W0329318_2012_Human
branch a
JQ714244_A_Cambodia_W0112303_2012_Human
JQ673594_A_environment_Cambodia_C85W_2011_Environment
JQ701710_A_marabou_stork_Cambodia_TM068_2011_Avian
JQ701702_A_heron_Cambodia_TM068_2011_Avian
JQ701718_A_duck_Cambodia_072D1_2011_Avian
JQ714211_A_duck_Cambodia_072D6_2011_Avian

PB1-Group89

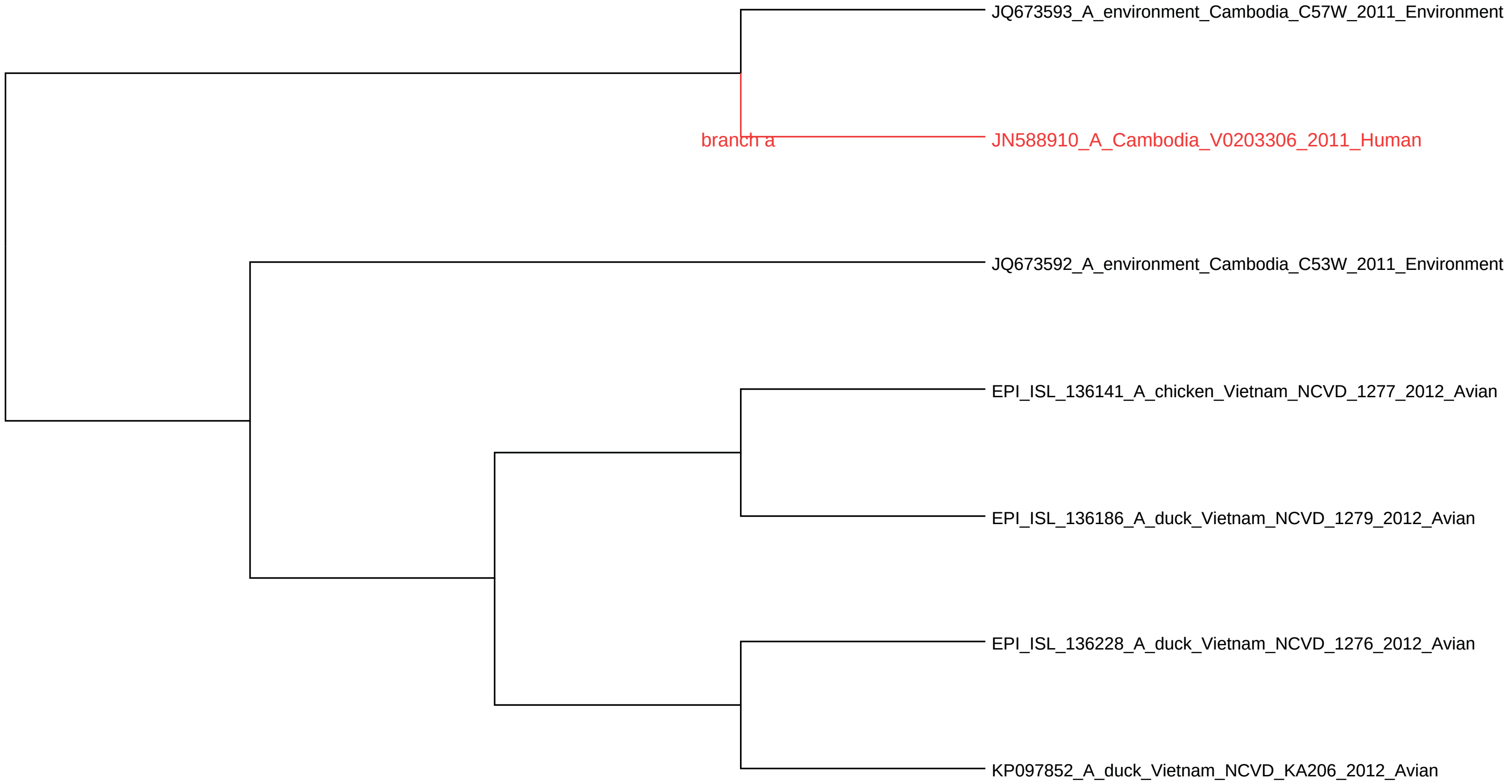
JQ673593_A_environment_Cambodia_C57W_2011_Environment
branch a
JN588910_A_Cambodia_V0203306_2011_Human
JQ673592_A_environment_Cambodia_C53W_2011_Environment
EPI_ISL_136141_A_chicken_Vietnam_NCVD_1277_2012_Avian
EPI_ISL_136186_A_duck_Vietnam_NCVD_1279_2012_Avian
EPI_ISL_136228_A_duck_Vietnam_NCVD_1276_2012_Avian
KP097852_A_duck_Vietnam_NCVD_KA206_2012_Avian

PB1-Group90

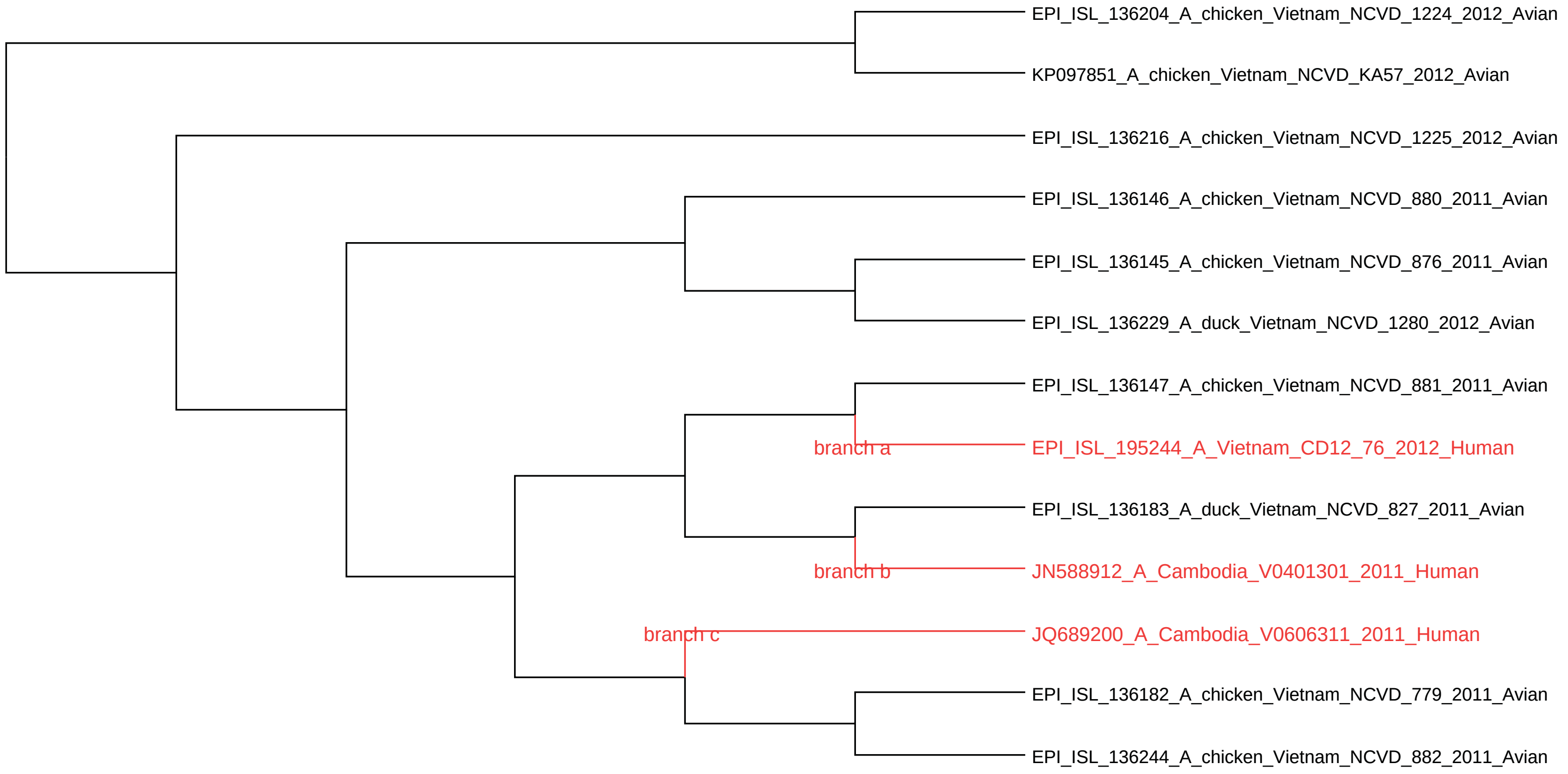
EPI_ISL_136204_A_chicken_Vietnam_NCVD_1224_2012_Avian
KP097851_A_chicken_Vietnam_NCVD_KA57_2012_Avian
EPI_ISL_136216_A_chicken_Vietnam_NCVD_1225_2012_Avian
EPI_ISL_136146_A_chicken_Vietnam_NCVD_880_2011_Avian
EPI_ISL_136145_A_chicken_Vietnam_NCVD_876_2011_Avian
EPI_ISL_136229_A_duck_Vietnam_NCVD_1280_2012_Avian
EPI_ISL_136147_A_chicken_Vietnam_NCVD_881_2011_Avian
branch a
EPI_ISL_195244_A_Vietnam_CD12_76_2012_Human
EPI_ISL_136183_A_duck_Vietnam_NCVD_827_2011_Avian
branch b
JN588912_A_Cambodia_V0401301_2011_Human
branch c
JQ689200_A_Cambodia_V0606311_2011_Human
EPI_ISL_136182_A_chicken_Vietnam_NCVD_779_2011_Avian
EPI_ISL_136244_A_chicken_Vietnam_NCVD_882_2011_Avian
