# Supplementary material for: Adaptive Evolution of Human-Isolated H5Nx Avian Influenza A Viruses
Source: Front Microbiol. 2019 Jun 12;10:1328. doi: 10.3389/fmicb.2019.01328 (PMC6582624; doi:10.3389/fmicb.2019.01328)
Supplement: Supplementary file 10 [file Data_Sheet_10.PDF]

Supplementary Figure 10. 92 phylogenetic trees of PB2 used for the adaptive evolution analyses. Human strains are marked in red. Branches which have significant signals of positive selection are marked with *.

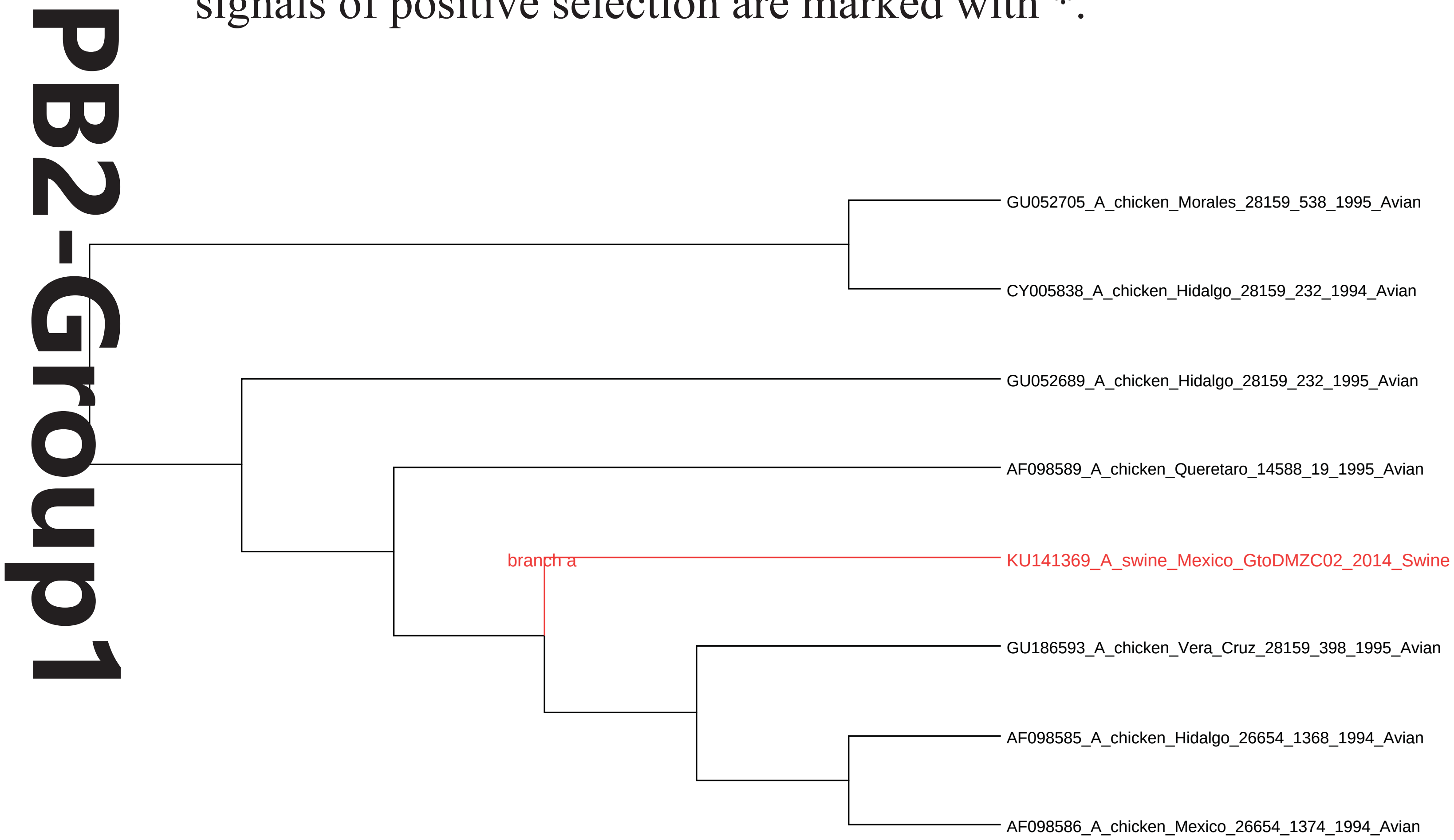

PB2-Group2

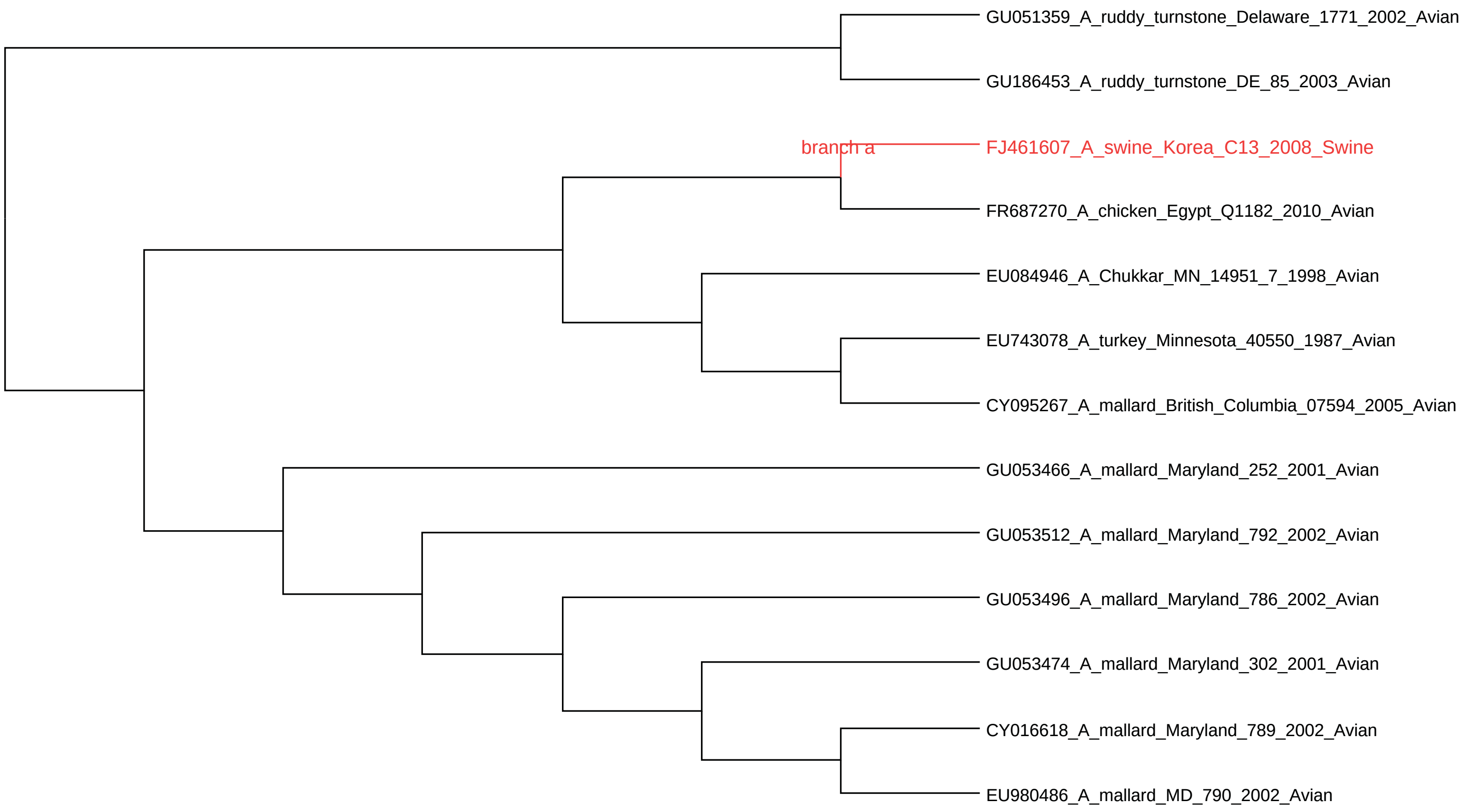

GU051359_A_ruddy_turnstone_Delaware_1771_2002_Avian
GU186453_A_ruddy_turnstone_DE_85_2003_Avian
branch a
FJ461607_A_swine_Korea_C13_2008_Swine
FR687270_A_chicken_Egypt_Q1182_2010_Avian
EU084946_A_Chukkar_MN_14951_7_1998_Avian
EU743078_A_turkey_Minnesota_40550_1987_Avian
CY095267_A_mallard_British_Columbia_07594_2005_Avian
GU053466_A_mallard_Maryland_252_2001_Avian
GU053512_A_mallard_Maryland_792_2002_Avian
GU053496_A_mallard_Maryland_786_2002_Avian
GU053474_A_mallard_Maryland_302_2001_Avian
CY016618_A_mallard_Maryland_789_2002_Avian
EU980486_A_mallard_MD_790_2002_Avian

PB2-Group3

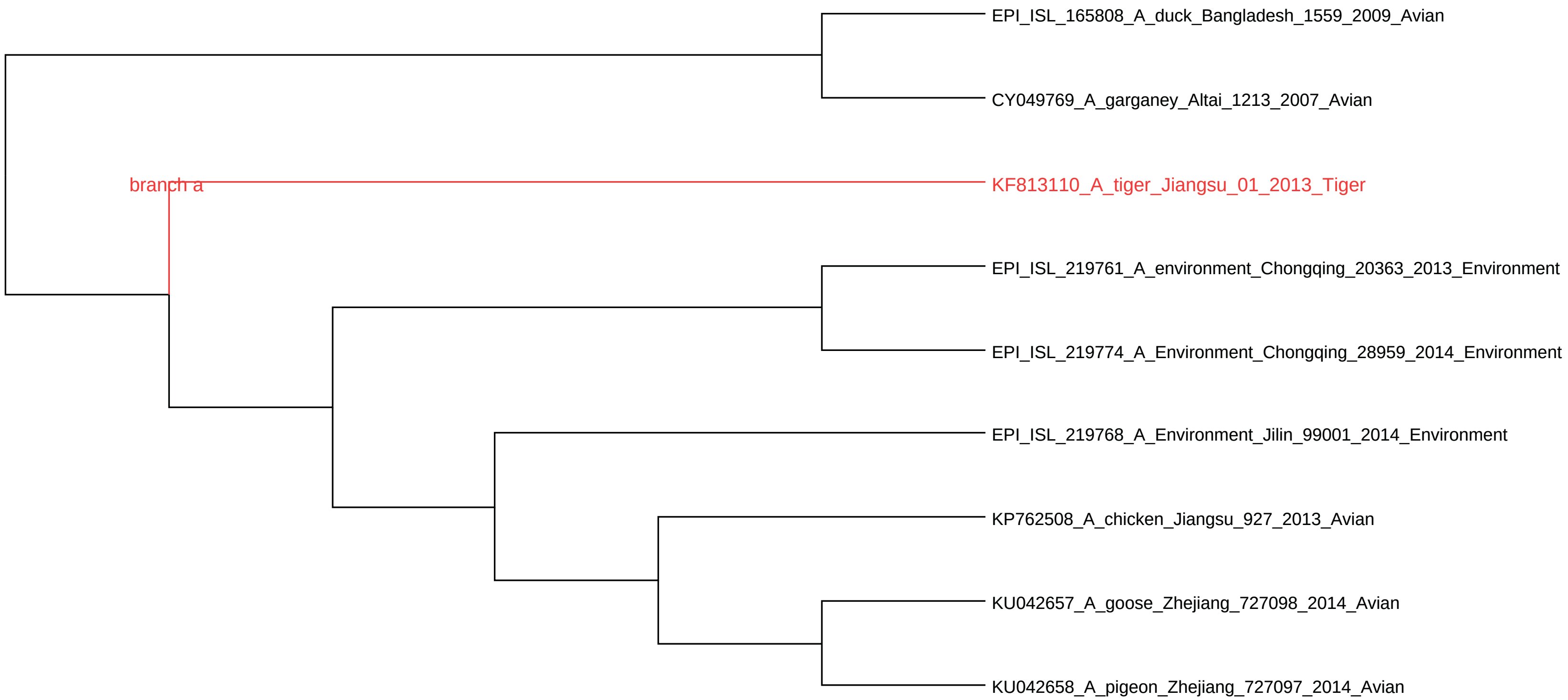
EPI_ISL_165808_A_duck_Bangladesh_1559_2009_Avian
CY049769_A_garganey_Altai_1213_2007_Avian
branch a
KF813110_A_tiger_Jiangsu_01_2013_Tiger
EPI_ISL_219761_A_environment_Chongqing_20363_2013_Environment
EPI_ISL_219774_A_Environment_Chongqing_28959_2014_Environment
EPI_ISL_219768_A_Environment_Jilin_99001_2014_Environment
KP762508_A_chicken_Jiangsu_927_2013_Avian
KU042657_A_goose_Zhejiang_727098_2014_Avian
KU042658_A_pigeon_Zhejiang_727097_2014_Avian

PB2-Group4

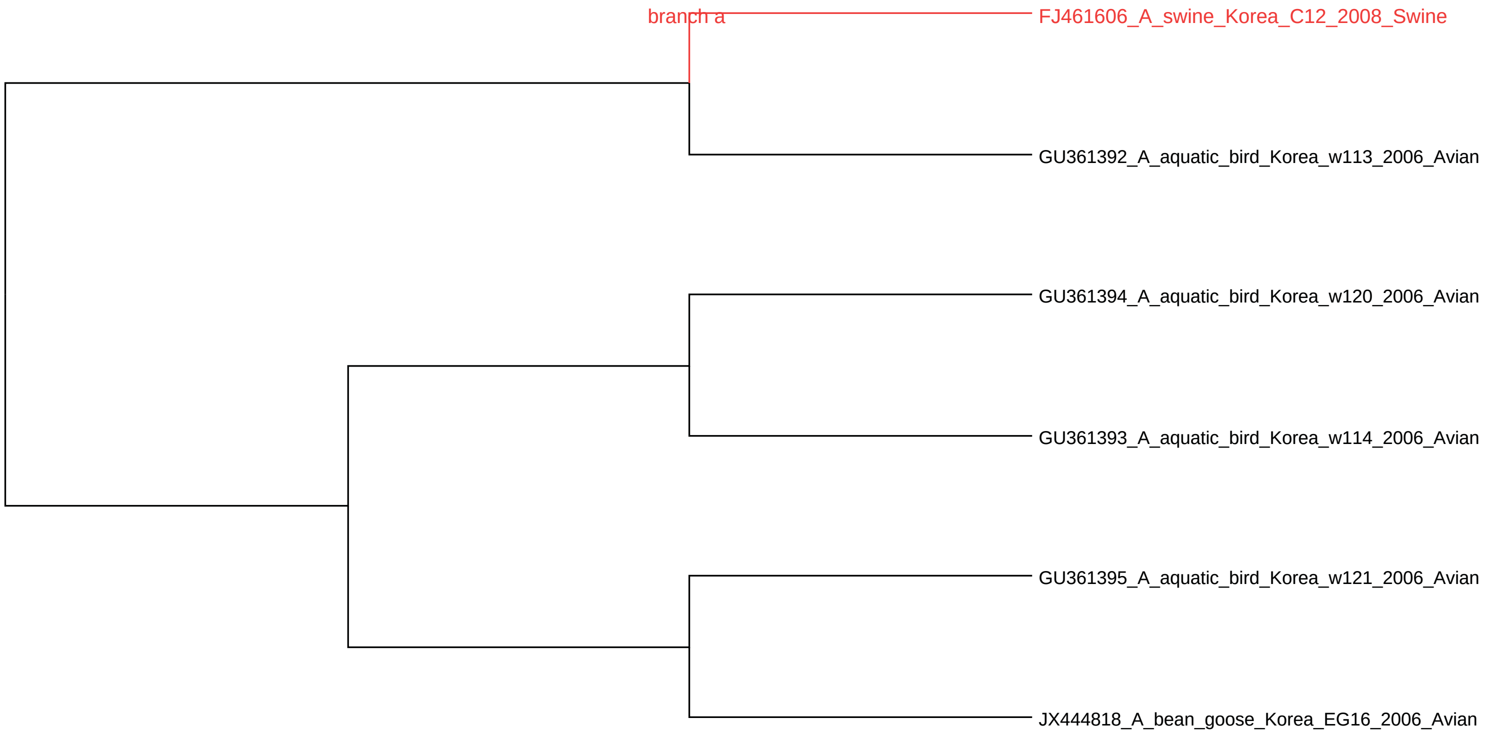
branch a
FJ461606_A_swine_Korea_C12_2008_Swine
GU361392_A_aquatic_bird_Korea_w113_2006_Avian
GU361394_A_aquatic_bird_Korea_w120_2006_Avian
GU361393_A_aquatic_bird_Korea_w114_2006_Avian
GU361395_A_aquatic_bird_Korea_w121_2006_Avian
JX444818_A_bean_goose_Korea_EG16_2006_Avian

PB2-Group5

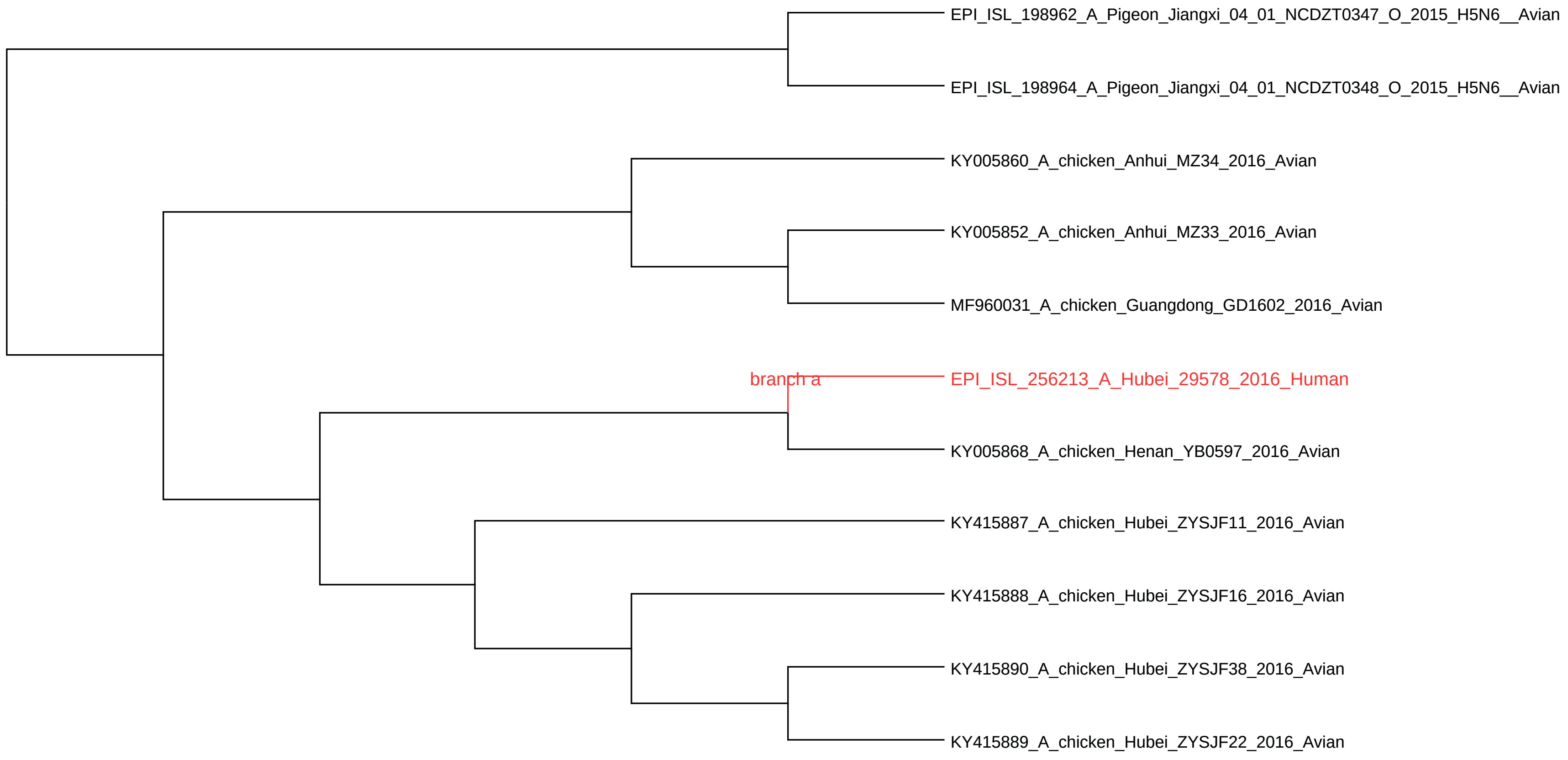

EPI_ISL_198962_A_Pigeon_Jiangxi_04_01_NCDZT0347_O_2015_H5N6__Avian
EPI_ISL_198964_A_Pigeon_Jiangxi_04_01_NCDZT0348_O_2015_H5N6__Avian
KY005860_A_chicken_Anhui_MZ34_2016_Avian
KY005852_A_chicken_Anhui_MZ33_2016_Avian
MF960031_A_chicken_Guangdong_GD1602_2016_Avian
branch a
EPI_ISL_256213_A_Hubei_29578_2016_Human
KY005868_A_chicken_Henan_YB0597_2016_Avian
KY415887_A_chicken_Hubei_ZYSJF11_2016_Avian
KY415888_A_chicken_Hubei_ZYSJF16_2016_Avian
KY415890_A_chicken_Hubei_ZYSJF38_2016_Avian
KY415889_A_chicken_Hubei_ZYSJF22_2016_Avian

PB2-Group6

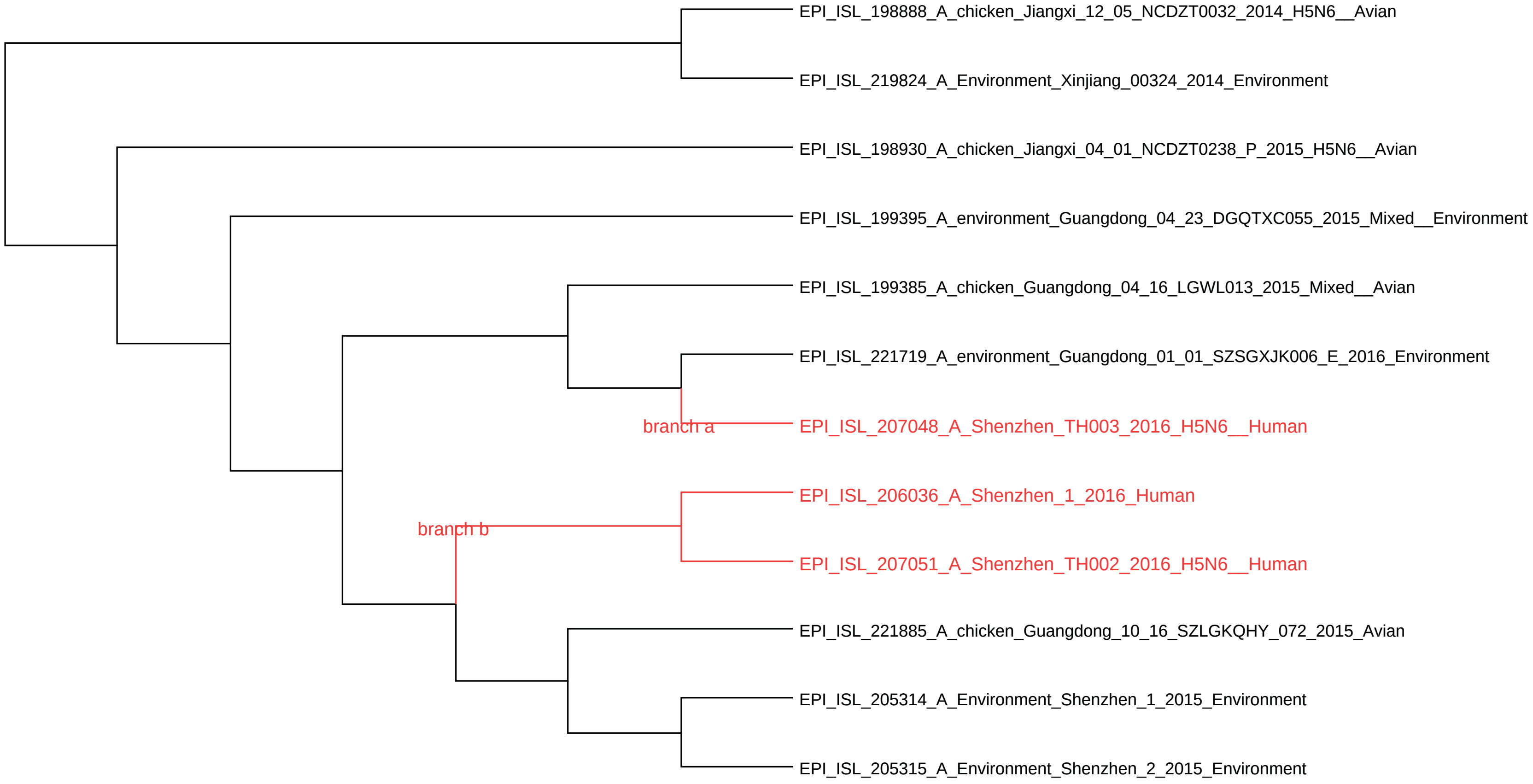

EPI_ISL_198888_A_chicken_Jiangxi_12_05_NCDZT0032_2014_H5N6__Avian
EPI_ISL_219824_A_Environment_Xinjiang_00324_2014_Environment
EPI_ISL_198930_A_chicken_Jiangxi_04_01_NCDZT0238_P_2015_H5N6__Avian
EPI_ISL_199395_A_environment_Guangdong_04_23_DGQTXC055_2015_Mixed__Environment
EPI_ISL_199385_A_chicken_Guangdong_04_16_LGWL013_2015_Mixed__Avian
EPI_ISL_221719_A_environment_Guangdong_01_01_SZSGXJK006_E_2016_Environment
branch a
EPI_ISL_207048_A_Shenzhen_TH003_2016_H5N6__Human
EPI_ISL_206036_A_Shenzhen_1_2016_Human
branch b
EPI_ISL_207051_A_Shenzhen_TH002_2016_H5N6__Human
EPI_ISL_221885_A_chicken_Guangdong_10_16_SZLGKQHY_072_2015_Avian
EPI_ISL_205314_A_Environment_Shenzhen_1_2015_Environment
EPI_ISL_205315_A_Environment_Shenzhen_2_2015_Environment

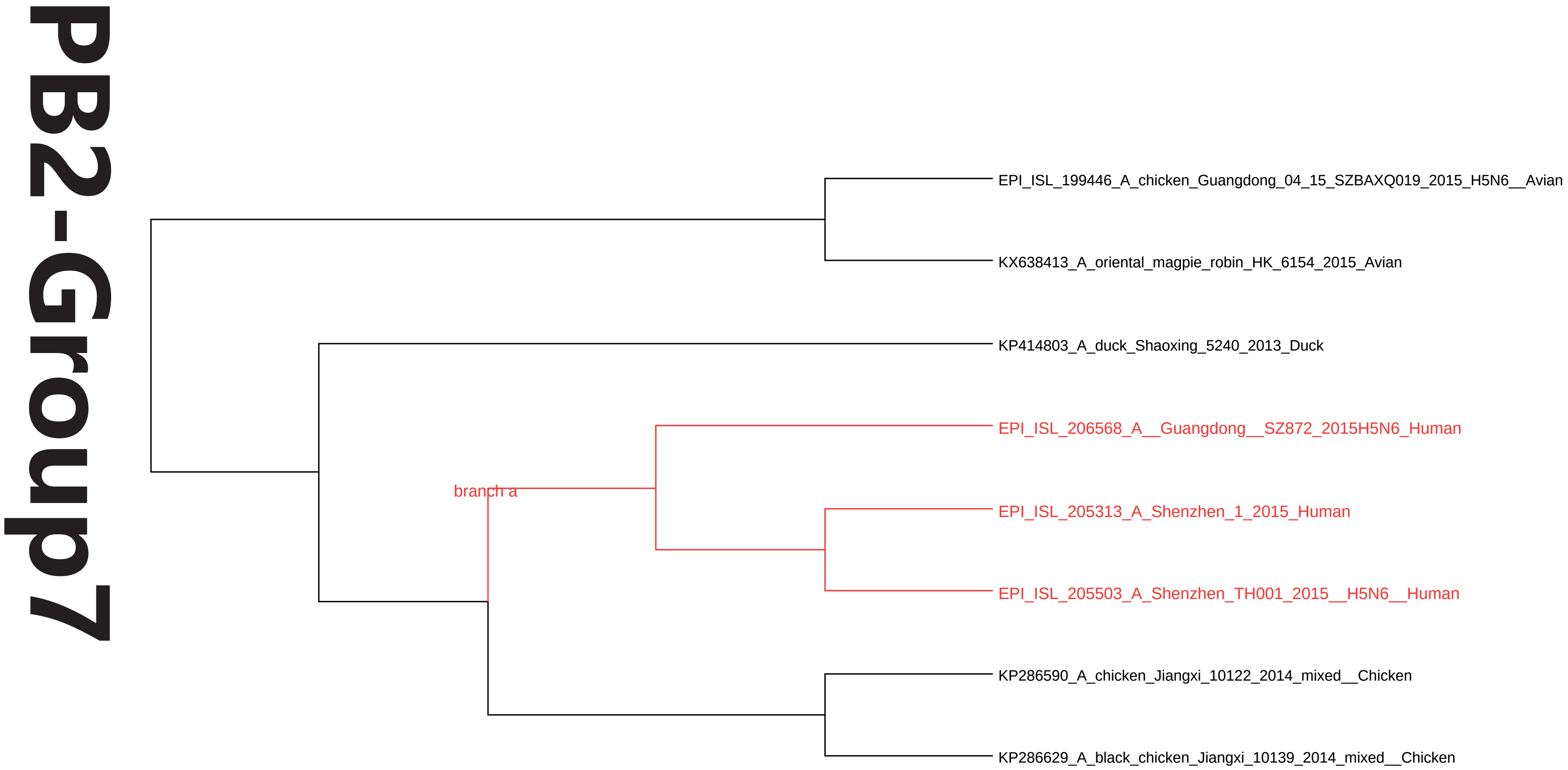
PB2-Group7
EPI_ISL_199446_A_chicken_Guangdong_04_15_SZBAXQ019_2015_H5N6__Avian
KX638413_A_oriental_magpie_robin_HK_6154_2015_Avian
KP414803_A_duck_Shaoxing_5240_2013_Duck
EPI_ISL_206568_A__Guangdong__SZ872_2015H5N6_Human
branch a
EPI_ISL_205313_A_Shenzhen_1_2015_Human
EPI_ISL_205503_A_Shenzhen_TH001_2015__H5N6__Human
KP286590_A_chicken_Jiangxi_10122_2014_mixed__Chicken
KP286629_A_black_chicken_Jiangxi_10139_2014_mixed__Chicken

PB2-Group8

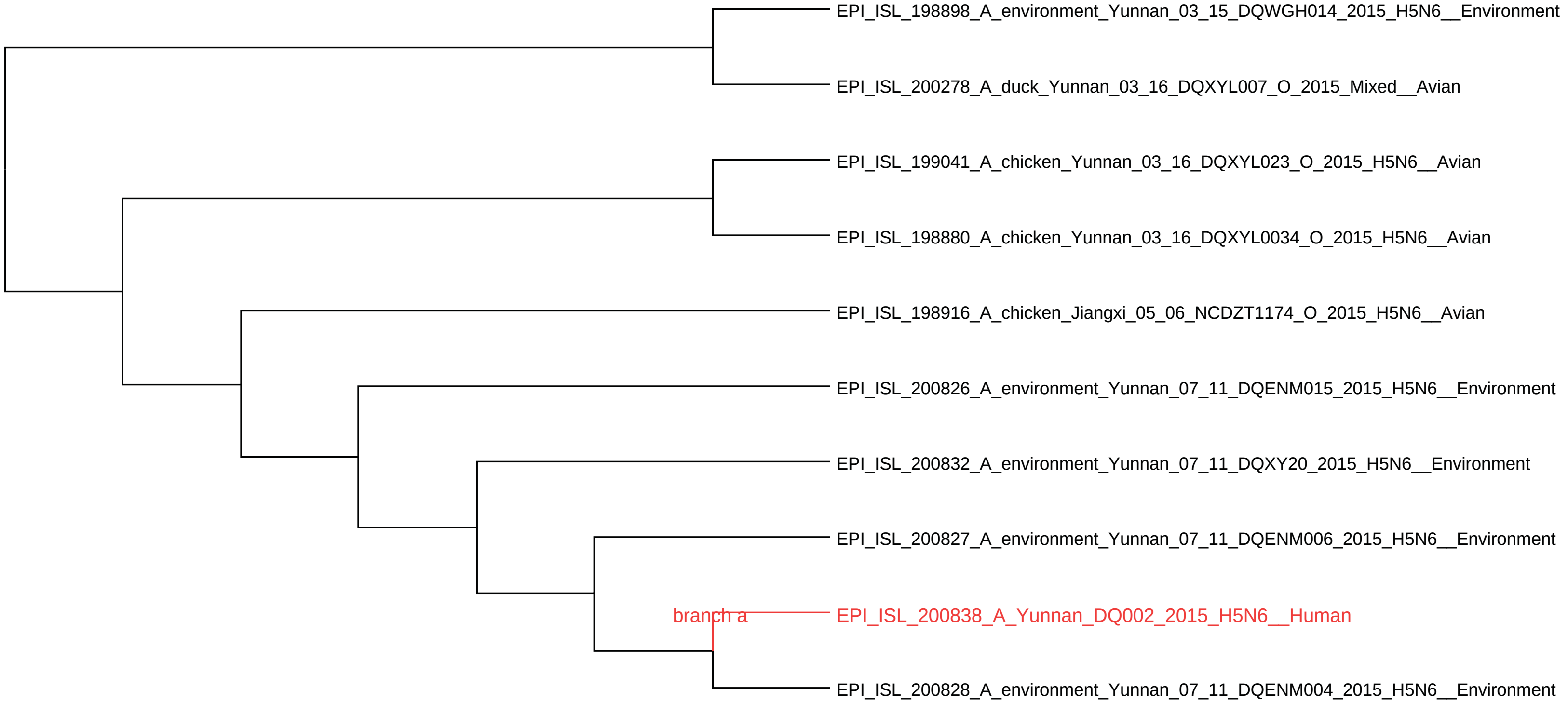

EPI_ISL_198898_A_environment_Yunnan_03_15_DQWGH014_2015_H5N6__Environment
EPI_ISL_200278_A_duck_Yunnan_03_16_DQXYL007_O_2015_Mixed__Avian
EPI_ISL_199041_A_chicken_Yunnan_03_16_DQXYL023_O_2015_H5N6__Avian
EPI_ISL_198880_A_chicken_Yunnan_03_16_DQXYL0034_O_2015_H5N6__Avian
EPI_ISL_198916_A_chicken_Jiangxi_05_06_NCDZT1174_O_2015_H5N6__Avian
EPI_ISL_200826_A_environment_Yunnan_07_11_DQENM015_2015_H5N6__Environment
EPI_ISL_200832_A_environment_Yunnan_07_11_DQXY20_2015_H5N6__Environment
EPI_ISL_200827_A_environment_Yunnan_07_11_DQENM006_2015_H5N6__Environment
branch a
EPI_ISL_200838_A_Yunnan_DQ002_2015_H5N6__Human
EPI_ISL_200828_A_environment_Yunnan_07_11_DQENM004_2015_H5N6__Environment

PB2-Group9

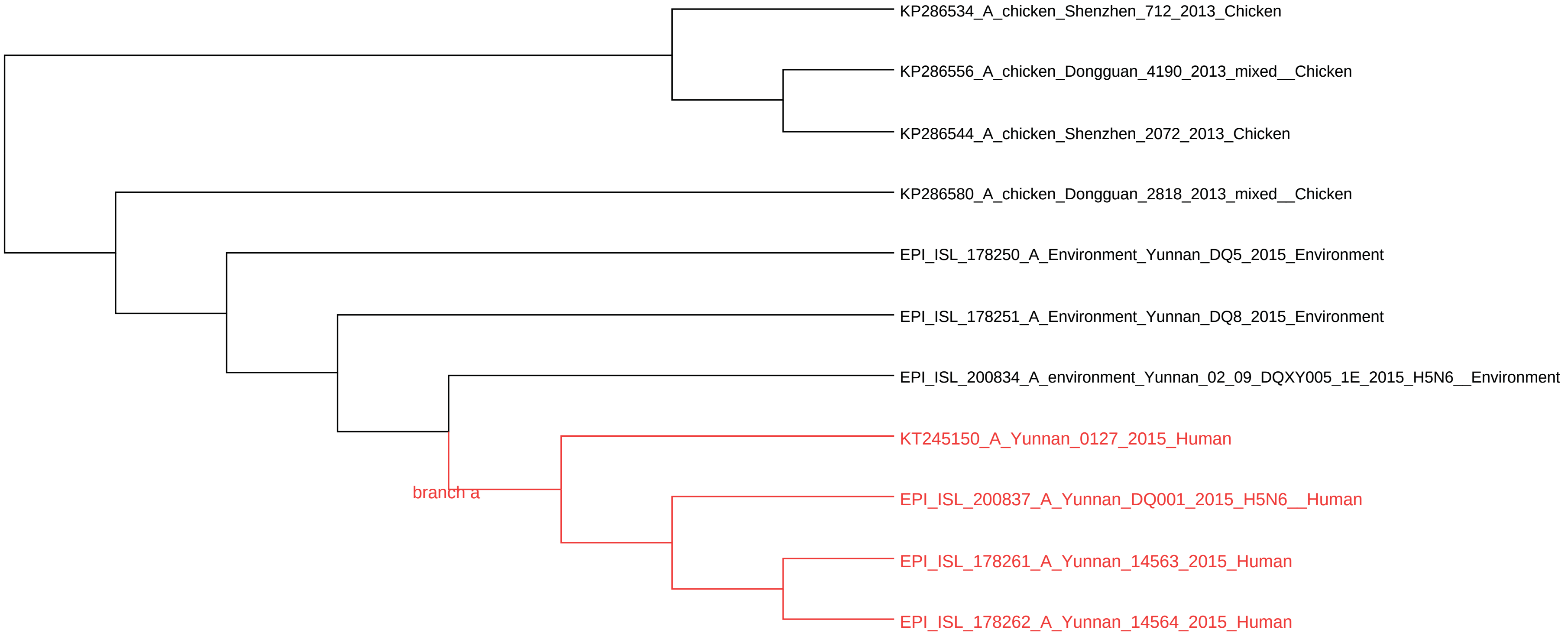

KP286534_A_chicken_Shenzhen_712_2013_Chicken
KP286556_A_chicken_Dongguan_4190_2013_mixed__Chicken
KP286544_A_chicken_Shenzhen_2072_2013_Chicken
KP286580_A_chicken_Dongguan_2818_2013_mixed__Chicken
EPI_ISL_178250_A_Environment_Yunnan_DQ5_2015_Environment
EPI_ISL_178251_A_Environment_Yunnan_DQ8_2015_Environment
EPI_ISL_200834_A_environment_Yunnan_02_09_DQXY005_1E_2015_H5N6__Environment
KT245150_A_Yunnan_0127_2015_Human
branch a
EPI_ISL_200837_A_Yunnan_DQ001_2015_H5N6__Human
EPI_ISL_178261_A_Yunnan_14563_2015_Human
EPI_ISL_178262_A_Yunnan_14564_2015_Human

PB2-Group10

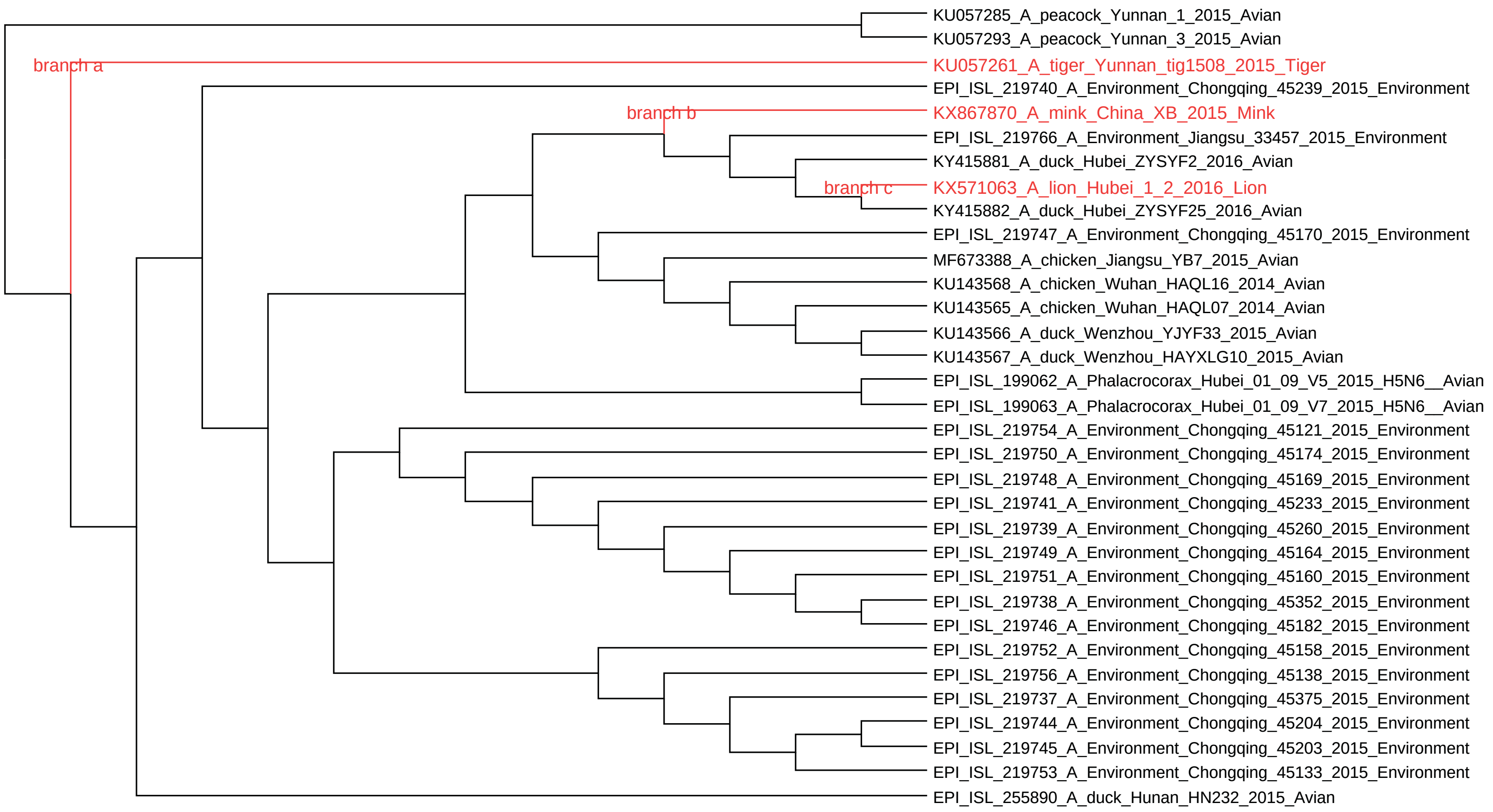
KU057285_A_peacock_Yunnan_1_2015_Avian
KU057293_A_peacock_Yunnan_3_2015_Avian
branch a
KU057261_A_tiger_Yunnan_tig1508_2015_Tiger
EPI_ISL_219740_A_Environment_Chongqing_45239_2015_Environment
branch b
KX867870_A_mink_China_XB_2015_Mink
EPI_ISL_219766_A_Environment_Jiangsu_33457_2015_Environment
KY415881_A_duck_Hubei_ZYSYF2_2016_Avian
branch c
KX571063_A_lion_Hubei_1_2_2016_Lion
KY415882_A_duck_Hubei_ZYSYF25_2016_Avian
EPI_ISL_219747_A_Environment_Chongqing_45170_2015_Environment
MF673388_A_chicken_Jiangsu_YB7_2015_Avian
KU143568_A_chicken_Wuhan_HAQL16_2014_Avian
KU143565_A_chicken_Wuhan_HAQL07_2014_Avian
KU143566_A_duck_Wenzhou_YJYF33_2015_Avian
KU143567_A_duck_Wenzhou_HAYXLG10_2015_Avian
EPI_ISL_199062_A_Phalacrocorax_Hubei_01_09_V5_2015_H5N6__Avian
EPI_ISL_199063_A_Phalacrocorax_Hubei_01_09_V7_2015_H5N6__Avian
EPI_ISL_219754_A_Environment_Chongqing_45121_2015_Environment
EPI_ISL_219750_A_Environment_Chongqing_45174_2015_Environment
EPI_ISL_219748_A_Environment_Chongqing_45169_2015_Environment
EPI_ISL_219741_A_Environment_Chongqing_45233_2015_Environment
EPI_ISL_219739_A_Environment_Chongqing_45260_2015_Environment
EPI_ISL_219749_A_Environment_Chongqing_45164_2015_Environment
EPI_ISL_219751_A_Environment_Chongqing_45160_2015_Environment
EPI_ISL_219738_A_Environment_Chongqing_45352_2015_Environment
EPI_ISL_219746_A_Environment_Chongqing_45182_2015_Environment
EPI_ISL_219752_A_Environment_Chongqing_45158_2015_Environment
EPI_ISL_219756_A_Environment_Chongqing_45138_2015_Environment
EPI_ISL_219737_A_Environment_Chongqing_45375_2015_Environment
EPI_ISL_219744_A_Environment_Chongqing_45204_2015_Environment
EPI_ISL_219745_A_Environment_Chongqing_45203_2015_Environment
EPI_ISL_219753_A_Environment_Chongqing_45133_2015_Environment
EPI_ISL_255890_A_duck_Hunan_HN232_2015_Avian

PB2-Group11

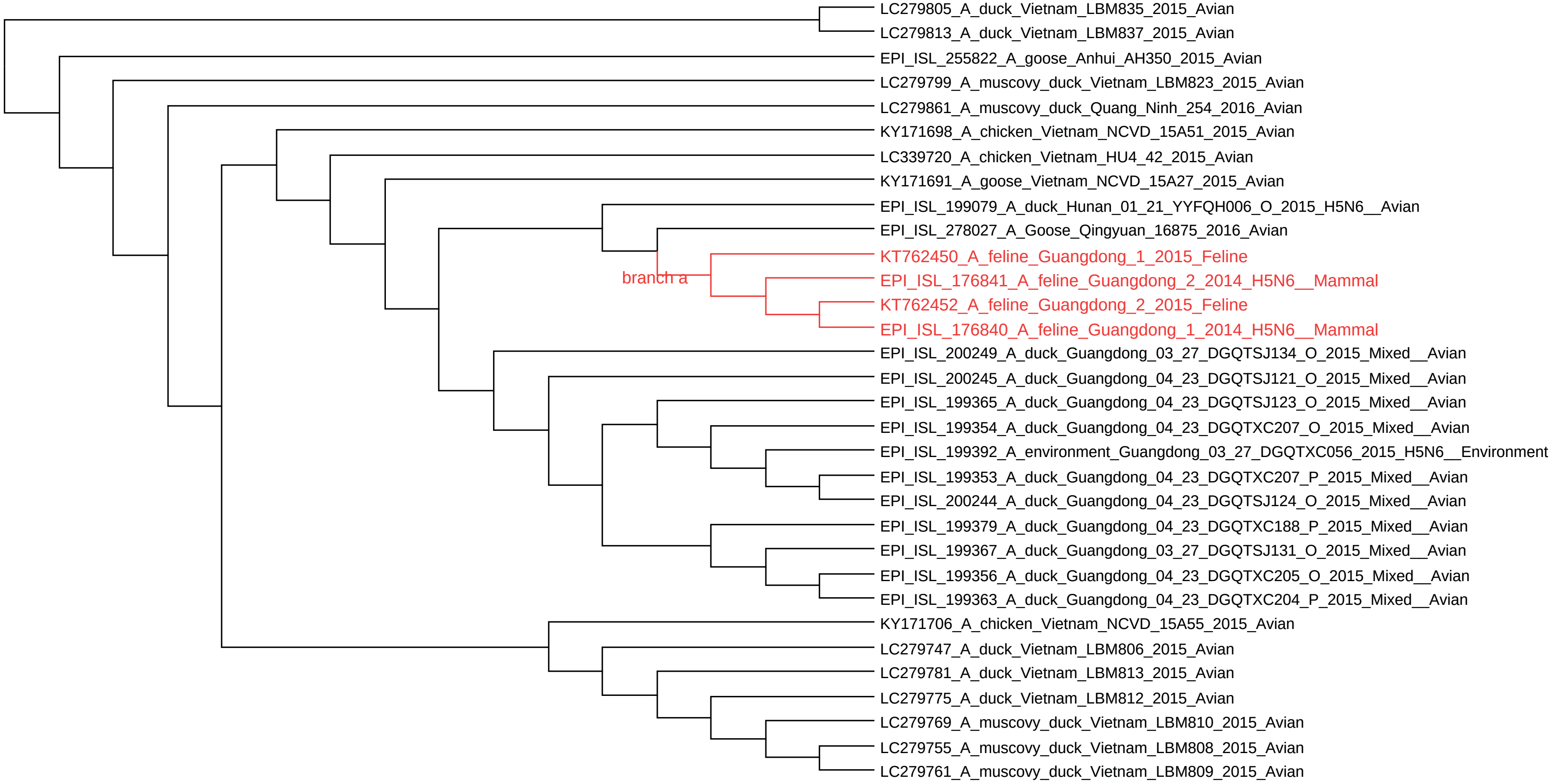
LC279805_A_duck_Vietnam_LBM835_2015_Avian
LC279813_A_duck_Vietnam_LBM837_2015_Avian
EPI_ISL_255822_A_goose_Anhui_AH350_2015_Avian
LC279799_A_muscovy_duck_Vietnam_LBM823_2015_Avian
LC279861_A_muscovy_duck_Quang_Ninh_254_2016_Avian
KY171698_A_chicken_Vietnam_NCVD_15A51_2015_Avian
LC339720_A_chicken_Vietnam_HU4_42_2015_Avian
KY171691_A_goose_Vietnam_NCVD_15A27_2015_Avian
EPI_ISL_199079_A_duck_Hunan_01_21_YYFQH006_O_2015_H5N6__Avian
EPI_ISL_278027_A_Goose_Qingyuan_16875_2016_Avian
branch a
KT762450_A_feline_Guangdong_1_2015_Feline
EPI_ISL_176841_A_feline_Guangdong_2_2014_H5N6__Mammal
KT762452_A_feline_Guangdong_2_2015_Feline
EPI_ISL_176840_A_feline_Guangdong_1_2014_H5N6__Mammal
EPI_ISL_200249_A_duck_Guangdong_03_27_DGQTSJ134_O_2015_Mixed__Avian
EPI_ISL_200245_A_duck_Guangdong_04_23_DGQTSJ121_O_2015_Mixed__Avian
EPI_ISL_199365_A_duck_Guangdong_04_23_DGQTSJ123_O_2015_Mixed__Avian
EPI_ISL_199354_A_duck_Guangdong_04_23_DGQTXC207_O_2015_Mixed__Avian
EPI_ISL_199392_A_environment_Guangdong_03_27_DGQTXC056_2015_H5N6__Environment
EPI_ISL_199353_A_duck_Guangdong_04_23_DGQTXC207_P_2015_Mixed__Avian
EPI_ISL_200244_A_duck_Guangdong_04_23_DGQTSJ124_O_2015_Mixed__Avian
EPI_ISL_199379_A_duck_Guangdong_04_23_DGQTXC188_P_2015_Mixed__Avian
EPI_ISL_199367_A_duck_Guangdong_03_27_DGQTSJ131_O_2015_Mixed__Avian
EPI_ISL_199356_A_duck_Guangdong_04_23_DGQTXC205_O_2015_Mixed__Avian
EPI_ISL_199363_A_duck_Guangdong_04_23_DGQTXC204_P_2015_Mixed__Avian
KY171706_A_chicken_Vietnam_NCVD_15A55_2015_Avian
LC279747_A_duck_Vietnam_LBM806_2015_Avian
LC279781_A_duck_Vietnam_LBM813_2015_Avian
LC279775_A_duck_Vietnam_LBM812_2015_Avian
LC279769_A_muscovy_duck_Vietnam_LBM810_2015_Avian
LC279755_A_muscovy_duck_Vietnam_LBM808_2015_Avian
LC279761_A_muscovy_duck_Vietnam_LBM809_2015_Avian

PB2-Group12

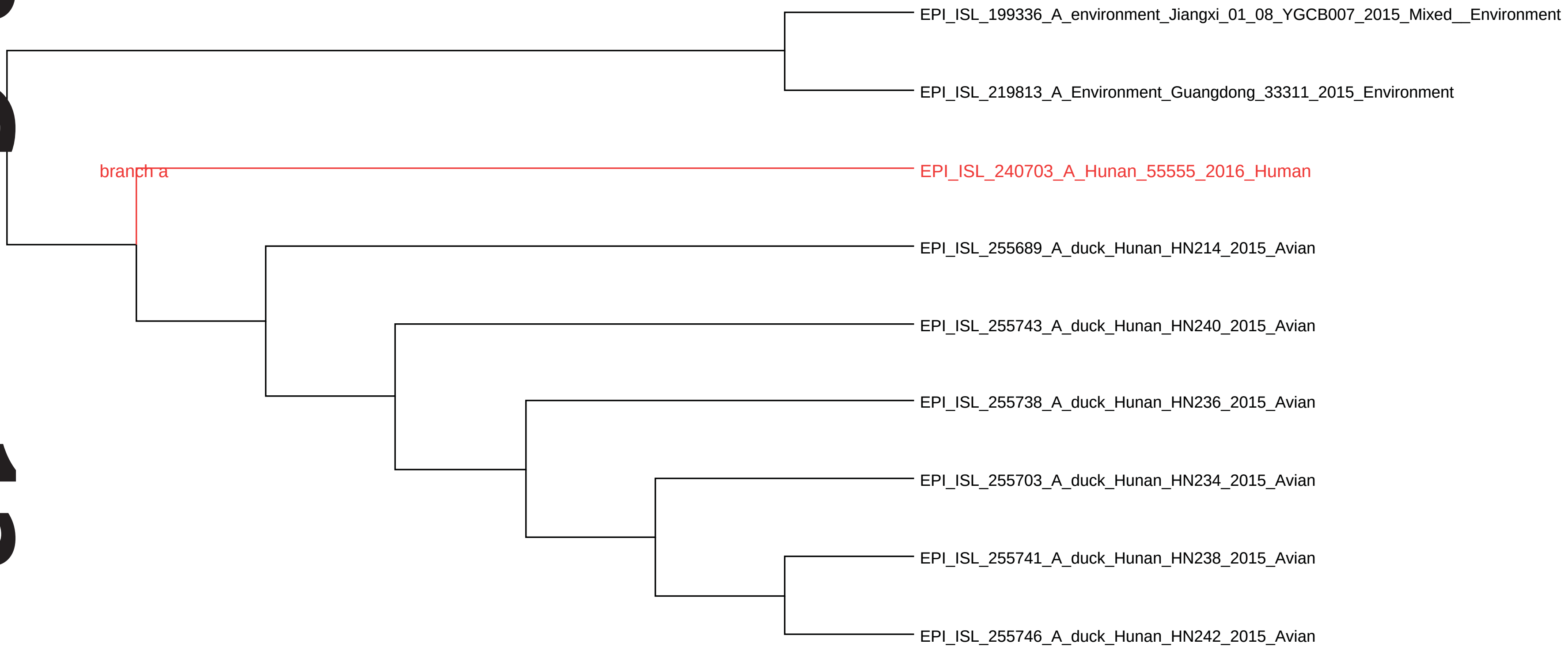
EPI_ISL_199336_A_environment_Jiangxi_01_08_YGCB007_2015_Mixed__Environment
EPI_ISL_219813_A_Environment_Guangdong_33311_2015_Environment
branch a
EPI_ISL_240703_A_Hunan_55555_2016_Human
EPI_ISL_255689_A_duck_Hunan_HN214_2015_Avian
EPI_ISL_255743_A_duck_Hunan_HN240_2015_Avian
EPI_ISL_255738_A_duck_Hunan_HN236_2015_Avian
EPI_ISL_255703_A_duck_Hunan_HN234_2015_Avian
EPI_ISL_255741_A_duck_Hunan_HN238_2015_Avian
EPI_ISL_255746_A_duck_Hunan_HN242_2015_Avian

PB2-Group13

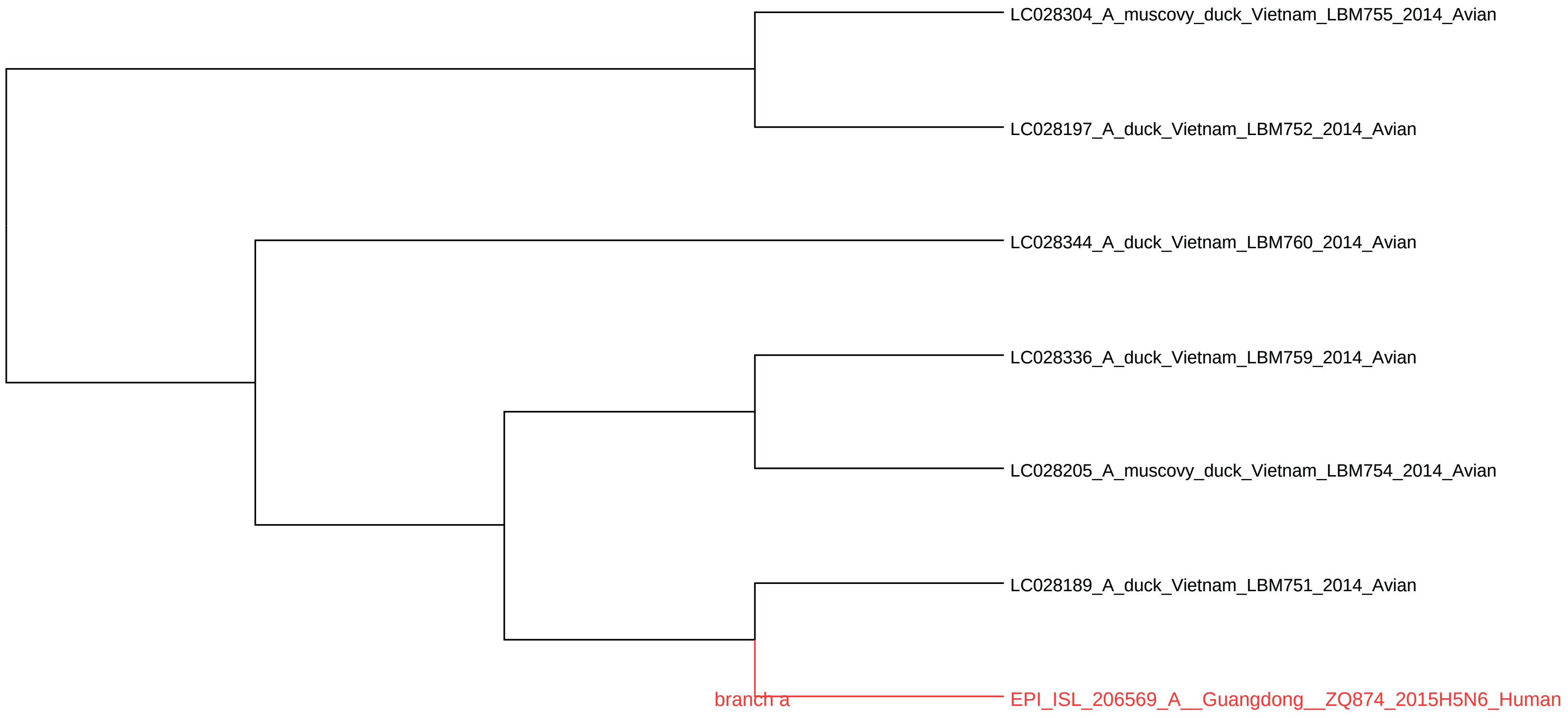
LC028304_A_muscovy_duck_Vietnam_LBM755_2014_Avian
LC028197_A_duck_Vietnam_LBM752_2014_Avian
LC028344_A_duck_Vietnam_LBM760_2014_Avian
LC028336_A_duck_Vietnam_LBM759_2014_Avian
LC028205_A_muscovy_duck_Vietnam_LBM754_2014_Avian
LC028189_A_duck_Vietnam_LBM751_2014_Avian
branch a
EPI_ISL_206569_A__Guangdong__ZQ874_2015H5N6_Human

PB2-Group14

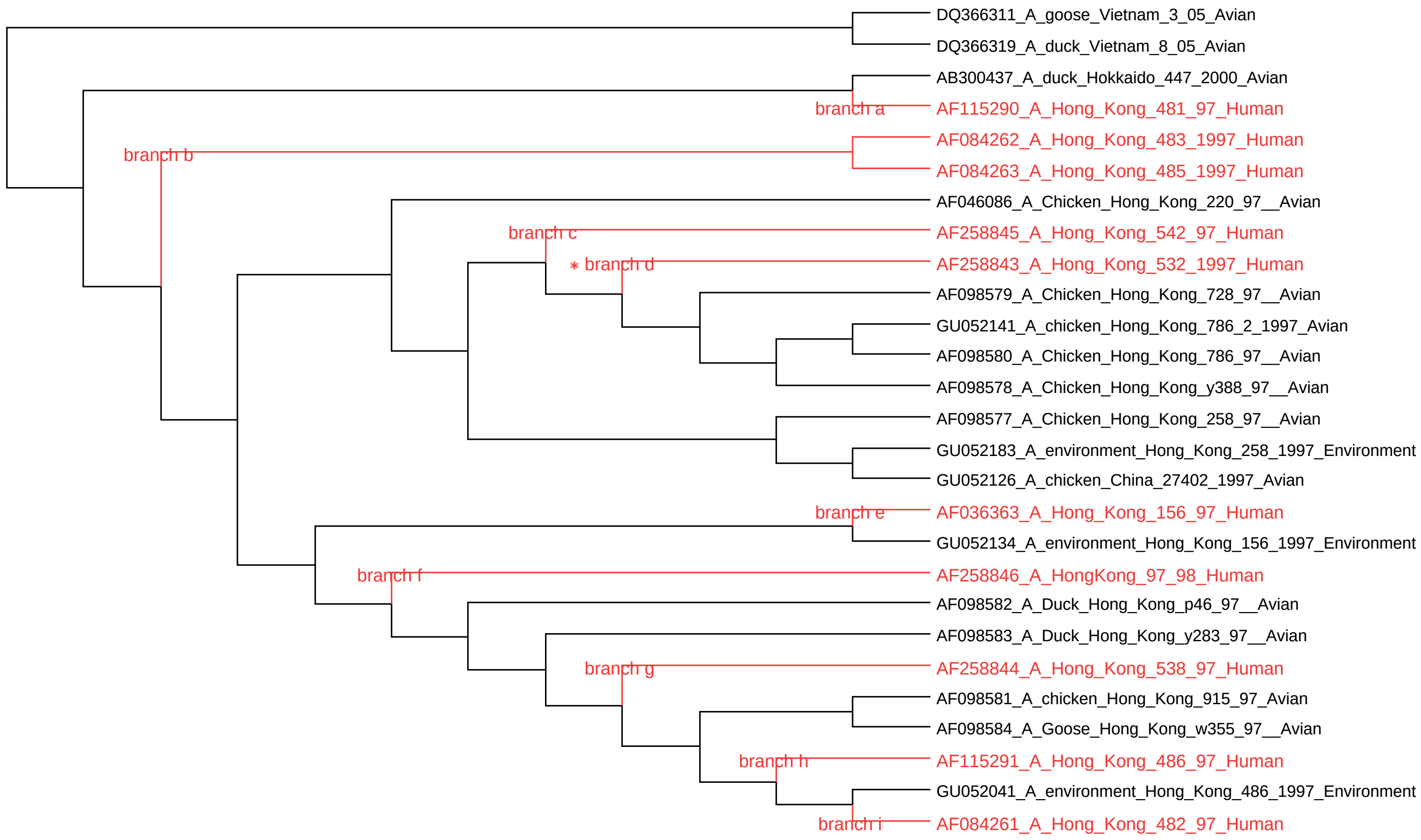
DQ366311_A_goose_Vietnam_3_05_Avian
DQ366319_A_duck_Vietnam_8_05_Avian
AB300437_A_duck_Hokkaido_447_2000_Avian
branch a
AF115290_A_Hong_Kong_481_97_Human
branch b
AF084262_A_Hong_Kong_483_1997_Human
AF084263_A_Hong_Kong_485_1997_Human
AF046086_A_Chicken_Hong_Kong_220_97__Avian
branch c
AF258845_A_Hong_Kong_542_97_Human
* branch d
AF258843_A_Hong_Kong_532_1997_Human
AF098579_A_Chicken_Hong_Kong_728_97__Avian
GU052141_A_chicken_Hong_Kong_786_2_1997_Avian
AF098580_A_Chicken_Hong_Kong_786_97__Avian
AF098578_A_Chicken_Hong_Kong_y388_97__Avian
AF098577_A_Chicken_Hong_Kong_258_97__Avian
GU052183_A_environment_Hong_Kong_258_1997_Environment
GU052126_A_chicken_China_27402_1997_Avian
branch e
AF036363_A_Hong_Kong_156_97_Human
GU052134_A_environment_Hong_Kong_156_1997_Environment
branch f
AF258846_A_HongKong_97_98_Human
AF098582_A_Duck_Hong_Kong_p46_97__Avian
AF098583_A_Duck_Hong_Kong_y283_97__Avian
branch g
AF258844_A_Hong_Kong_538_97_Human
AF098581_A_chicken_Hong_Kong_915_97_Avian
AF098584_A_Goose_Hong_Kong_w355_97__Avian
branch h
AF115291_A_Hong_Kong_486_97_Human
GU052041_A_environment_Hong_Kong_486_1997_Environment
branch i
AF084261_A_Hong_Kong_482_97_Human

PB2-Group15

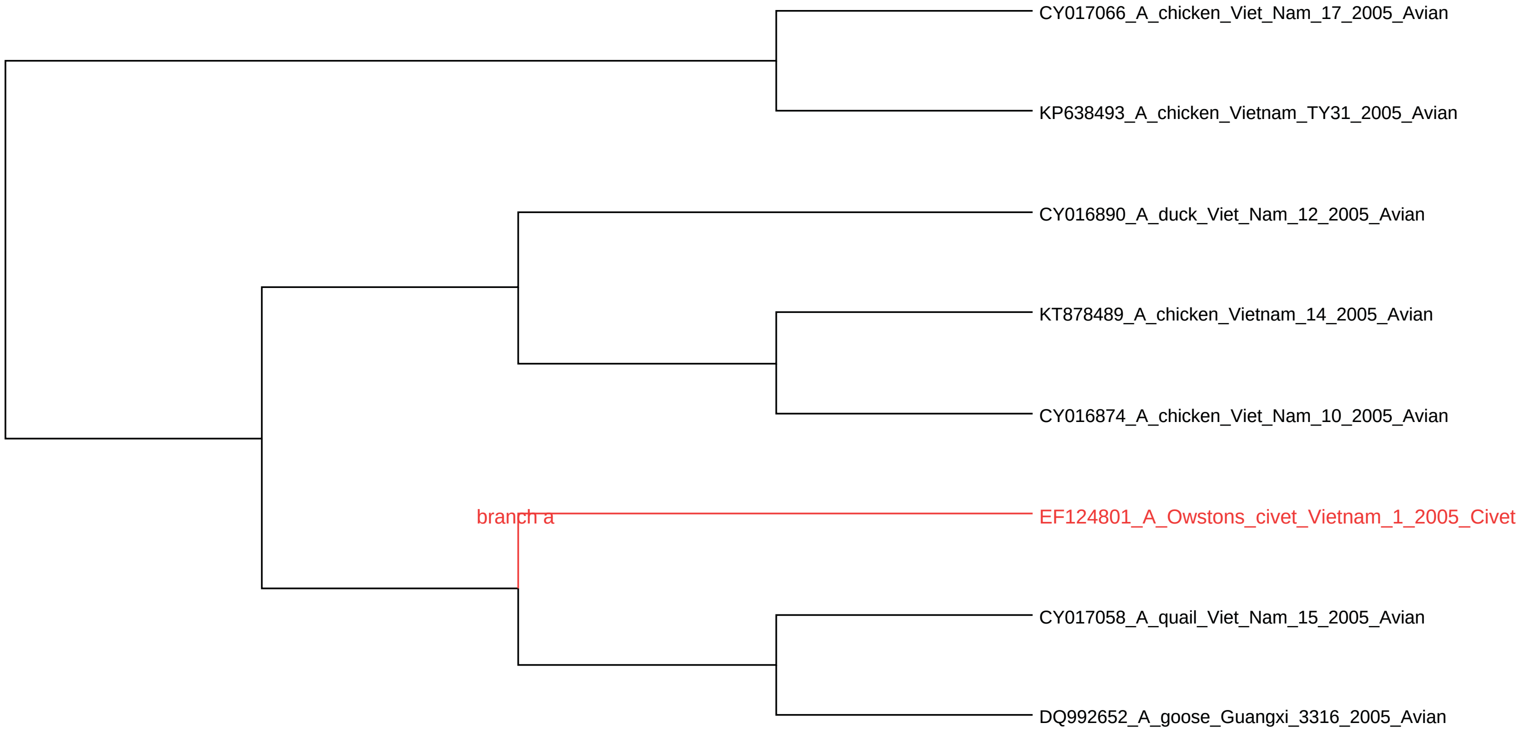
CY017066_A_chicken_Viet_Nam_17_2005_Avian
KP638493_A_chicken_Vietnam_TY31_2005_Avian
CY016890_A_duck_Viet_Nam_12_2005_Avian
KT878489_A_chicken_Vietnam_14_2005_Avian
CY016874_A_chicken_Viet_Nam_10_2005_Avian
branch a
EF124801_A_Owstons_civet_Vietnam_1_2005_Civet
CY017058_A_quail_Viet_Nam_15_2005_Avian
DQ992652_A_goose_Guangxi_3316_2005_Avian

PB2-Group16

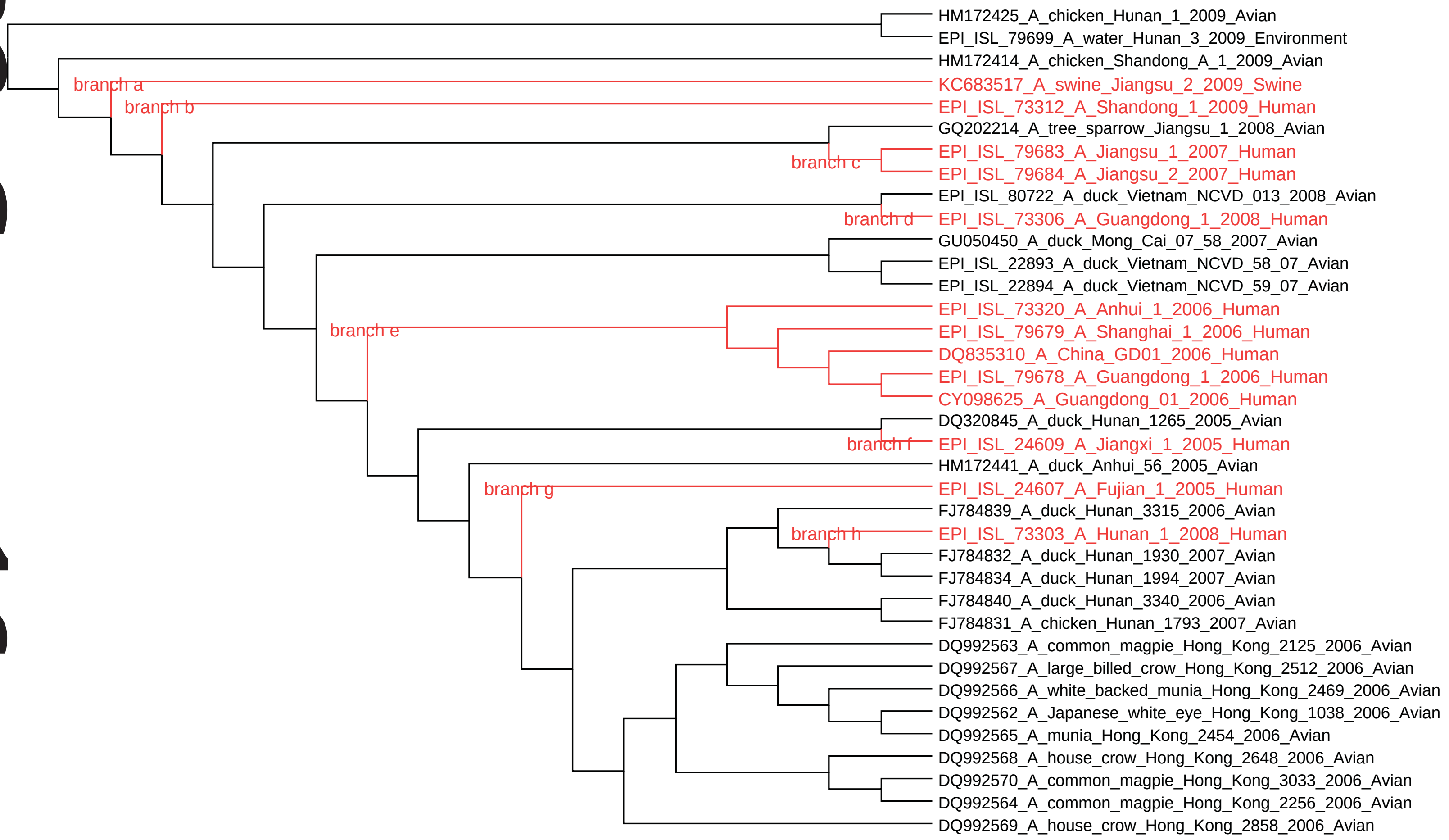

HM172425_A_chicken_Hunan_1_2009_Avian
EPI_ISL_79699_A_water_Hunan_3_2009_Environment
HM172414_A_chicken_Shandong_A_1_2009_Avian
branch a
KC683517_A_swine_Jiangsu_2_2009_Swine
branch b
EPI_ISL_73312_A_Shandong_1_2009_Human
GQ202214_A_tree_sparrow_Jiangsu_1_2008_Avian
EPI_ISL_79683_A_Jiangsu_1_2007_Human
branch c
EPI_ISL_79684_A_Jiangsu_2_2007_Human
EPI_ISL_80722_A_duck_Vietnam_NCVD_013_2008_Avian
branch d
EPI_ISL_73306_A_Guangdong_1_2008_Human
GU050450_A_duck_Mong_Cai_07_58_2007_Avian
EPI_ISL_22893_A_duck_Vietnam_NCVD_58_07_Avian
EPI_ISL_22894_A_duck_Vietnam_NCVD_59_07_Avian
EPI_ISL_73320_A_Anhui_1_2006_Human
branch e
EPI_ISL_79679_A_Shanghai_1_2006_Human
DQ835310_A_China_GD01_2006_Human
EPI_ISL_79678_A_Guangdong_1_2006_Human
CY098625_A_Guangdong_01_2006_Human
DQ320845_A_duck_Hunan_1265_2005_Avian
branch f
EPI_ISL_24609_A_Jiangxi_1_2005_Human
HM172441_A_duck_Anhui_56_2005_Avian
branch g
EPI_ISL_24607_A_Fujian_1_2005_Human
FJ784839_A_duck_Hunan_3315_2006_Avian
branch h
EPI_ISL_73303_A_Hunan_1_2008_Human
FJ784832_A_duck_Hunan_1930_2007_Avian
FJ784834_A_duck_Hunan_1994_2007_Avian
FJ784840_A_duck_Hunan_3340_2006_Avian
FJ784831_A_chicken_Hunan_1793_2007_Avian
DQ992563_A_common_magpie_Hong_Kong_2125_2006_Avian
DQ992567_A_large_billed_crow_Hong_Kong_2512_2006_Avian
DQ992566_A_white_backed_munia_Hong_Kong_2469_2006_Avian
DQ992562_A_Japanese_white_eye_Hong_Kong_1038_2006_Avian
DQ992565_A_munia_Hong_Kong_2454_2006_Avian
DQ992568_A_house_crow_Hong_Kong_2648_2006_Avian
DQ992570_A_common_magpie_Hong_Kong_3033_2006_Avian
DQ992564_A_common_magpie_Hong_Kong_2256_2006_Avian
DQ992569_A_house_crow_Hong_Kong_2858_2006_Avian

PB2-Group17

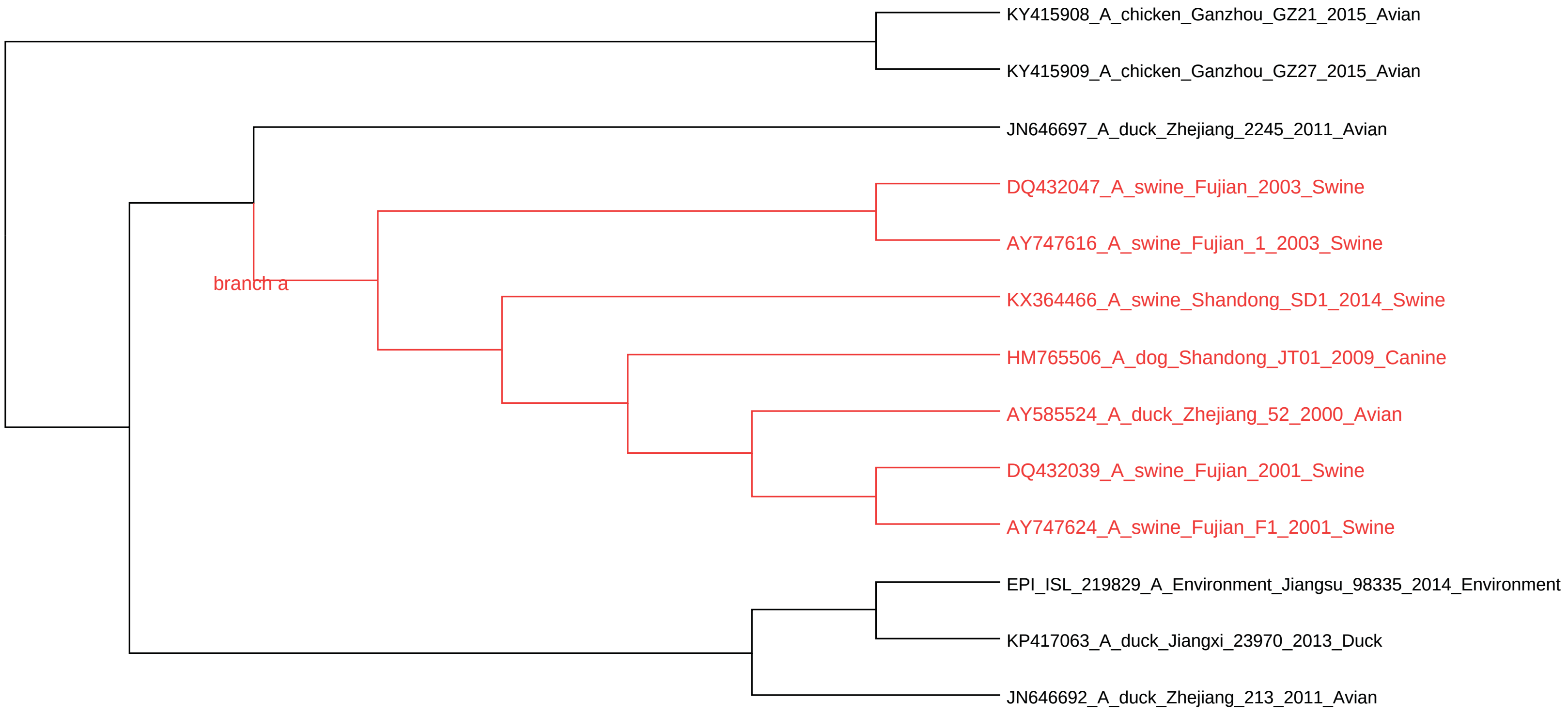
KY415908_A_chicken_Ganzhou_GZ21_2015_Avian
KY415909_A_chicken_Ganzhou_GZ27_2015_Avian
JN646697_A_duck_Zhejiang_2245_2011_Avian
DQ432047_A_swine_Fujian_2003_Swine
AY747616_A_swine_Fujian_1_2003_Swine
branch a
KX364466_A_swine_Shandong_SD1_2014_Swine
HM765506_A_dog_Shandong_JT01_2009_Canine
AY585524_A_duck_Zhejiang_52_2000_Avian
DQ432039_A_swine_Fujian_2001_Swine
AY747624_A_swine_Fujian_F1_2001_Swine
EPI_ISL_219829_A_Environment_Jiangsu_98335_2014_Environment
KP417063_A_duck_Jiangxi_23970_2013_Duck
JN646692_A_duck_Zhejiang_213_2011_Avian

PB2-Group18

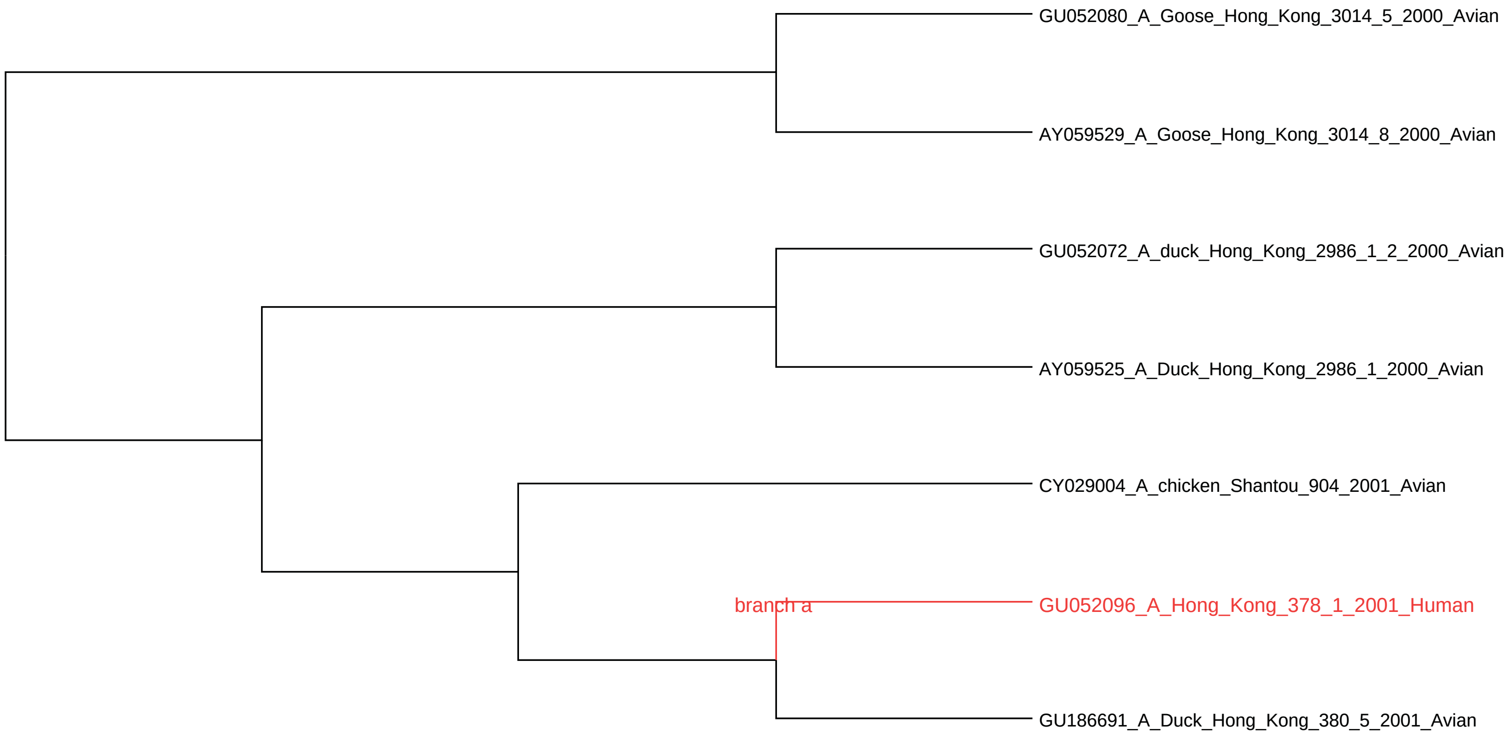

GU052080_A_Goose_Hong_Kong_3014_5_2000_Avian
AY059529_A_Goose_Hong_Kong_3014_8_2000_Avian
GU052072_A_duck_Hong_Kong_2986_1_2_2000_Avian
AY059525_A_Duck_Hong_Kong_2986_1_2000_Avian
CY029004_A_chicken_Shantou_904_2001_Avian
branch a
GU052096_A_Hong_Kong_378_1_2001_Human
GU186691_A_Duck_Hong_Kong_380_5_2001_Avian

PB2-Group19

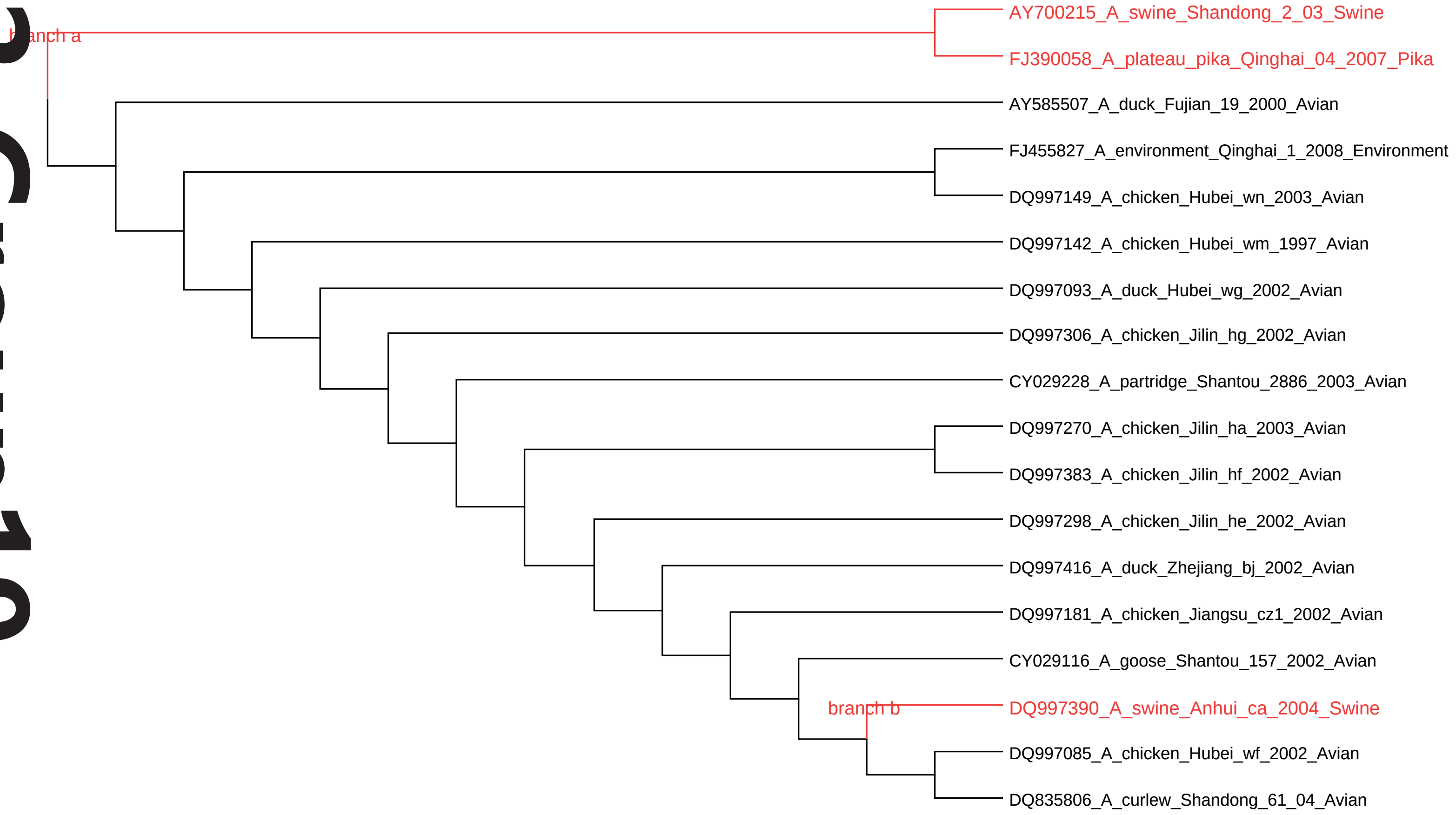

branch a
AY700215_A_swine_Shandong_2_03_Swine
FJ390058_A_plateau_pika_Qinghai_04_2007_Pika
AY585507_A_duck_Fujian_19_2000_Avian
FJ455827_A_environment_Qinghai_1_2008_Environment
DQ997149_A_chicken_Hubei_wn_2003_Avian
DQ997142_A_chicken_Hubei_wm_1997_Avian
DQ997093_A_duck_Hubei_wg_2002_Avian
DQ997306_A_chicken_Jilin_hg_2002_Avian
CY029228_A_partridge_Shantou_2886_2003_Avian
DQ997270_A_chicken_Jilin_ha_2003_Avian
DQ997383_A_chicken_Jilin_hf_2002_Avian
DQ997298_A_chicken_Jilin_he_2002_Avian
DQ997416_A_duck_Zhejiang_bj_2002_Avian
DQ997181_A_chicken_Jiangsu_cz1_2002_Avian
CY029116_A_goose_Shantou_157_2002_Avian
branch b
DQ997390_A_swine_Anhui_ca_2004_Swine
DQ997085_A_chicken_Hubei_wf_2002_Avian
DQ835806_A_curlew_Shandong_61_04_Avian

PB2-Group20

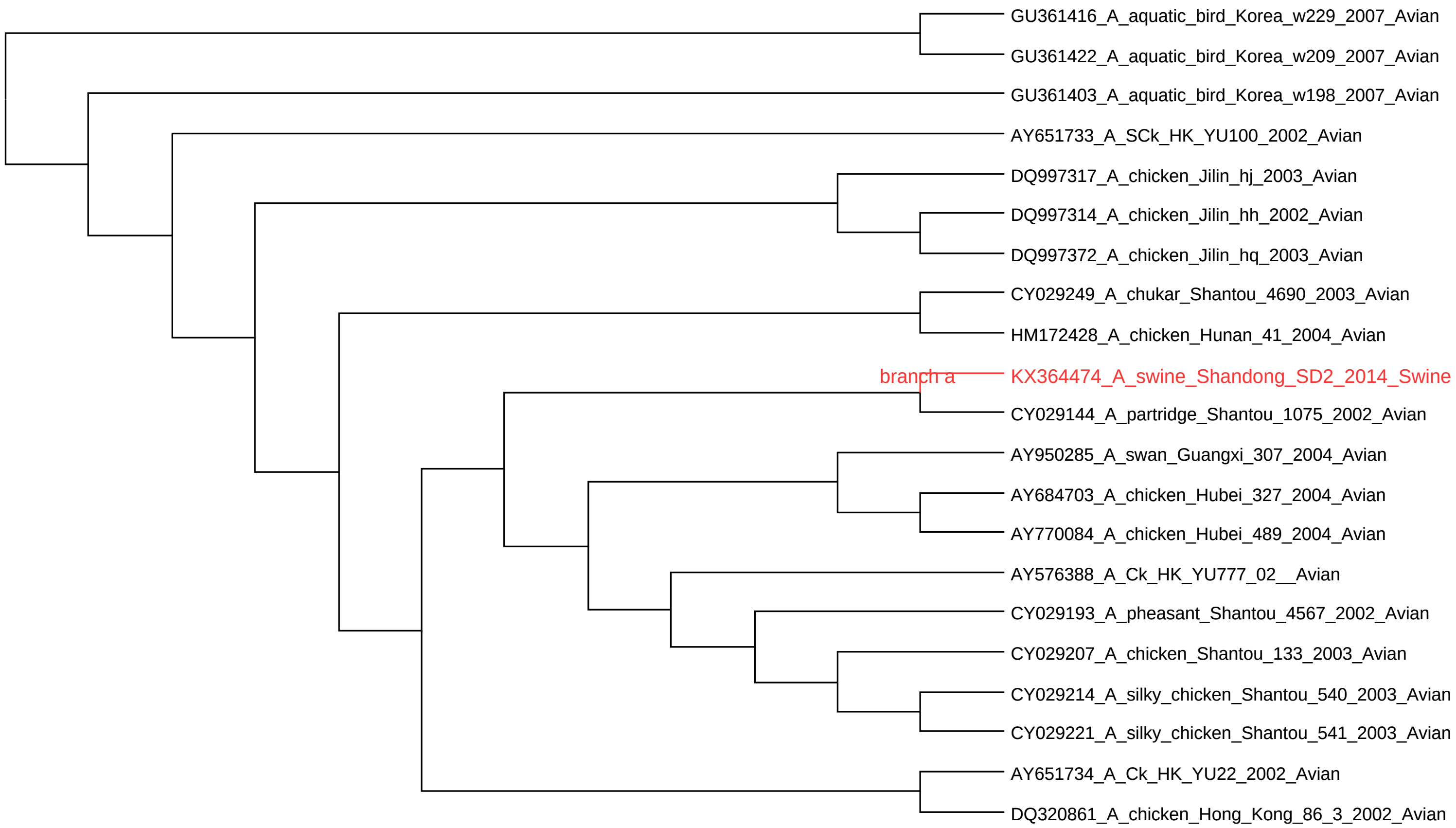

GU361416_A_aquatic_bird_Korea_w229_2007_Avian
GU361422_A_aquatic_bird_Korea_w209_2007_Avian
GU361403_A_aquatic_bird_Korea_w198_2007_Avian
AY651733_A_SCk_HK_YU100_2002_Avian
DQ997317_A_chicken_Jilin_hj_2003_Avian
DQ997314_A_chicken_Jilin_hh_2002_Avian
DQ997372_A_chicken_Jilin_hq_2003_Avian
CY029249_A_chukar_Shantou_4690_2003_Avian
HM172428_A_chicken_Hunan_41_2004_Avian
branch a
KX364474_A_swine_Shandong_SD2_2014_Swine
CY029144_A_partridge_Shantou_1075_2002_Avian
AY950285_A_swan_Guangxi_307_2004_Avian
AY684703_A_chicken_Hubei_327_2004_Avian
AY770084_A_chicken_Hubei_489_2004_Avian
AY576388_A_Ck_HK_YU777_02__Avian
CY029193_A_pheasant_Shantou_4567_2002_Avian
CY029207_A_chicken_Shantou_133_2003_Avian
CY029214_A_silky_chicken_Shantou_540_2003_Avian
CY029221_A_silky_chicken_Shantou_541_2003_Avian
AY651734_A_Ck_HK_YU22_2002_Avian
DQ320861_A_chicken_Hong_Kong_86_3_2002_Avian

PB2-Group21

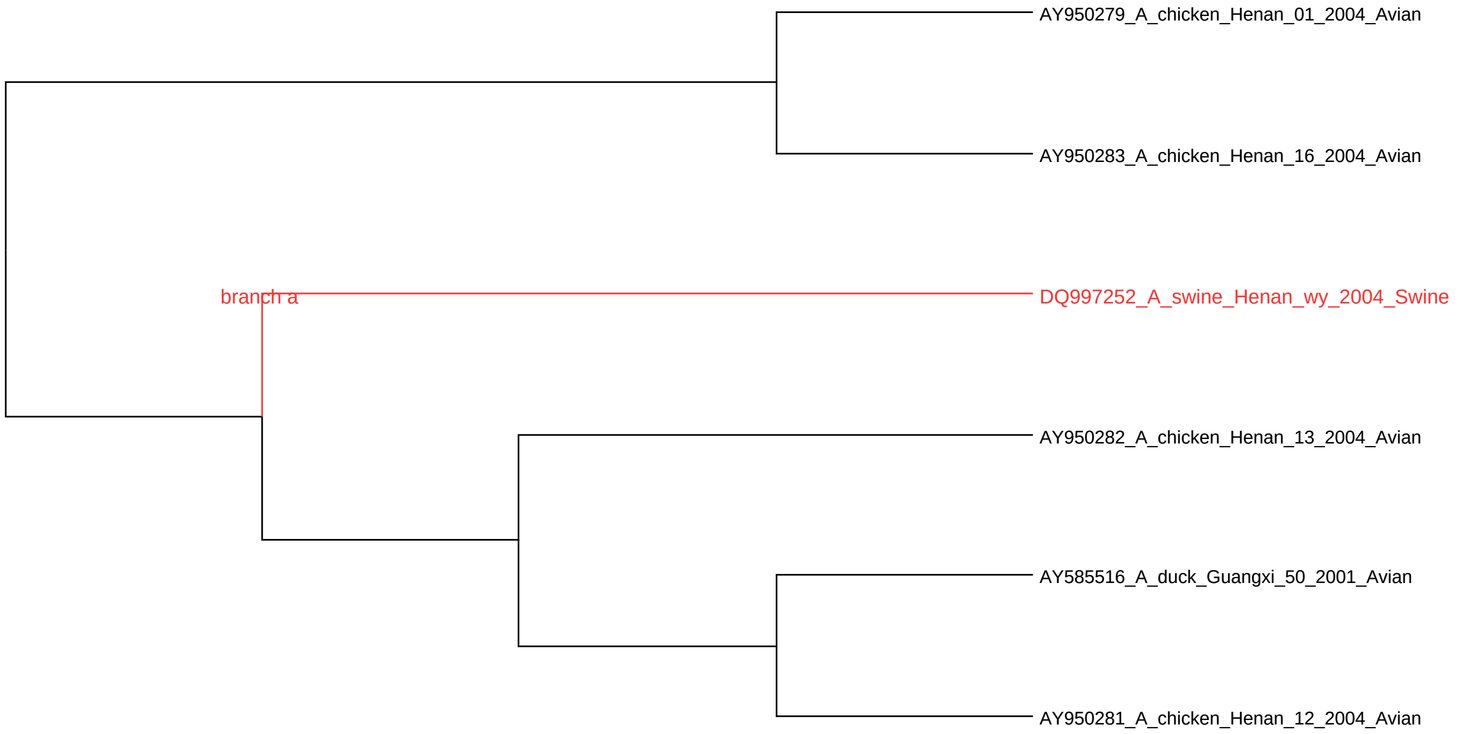
AY950279_A_chicken_Henan_01_2004_Avian
AY950283_A_chicken_Henan_16_2004_Avian
branch a
DQ997252_A_swine_Henan_wy_2004_Swine
AY950282_A_chicken_Henan_13_2004_Avian
AY585516_A_duck_Guangxi_50_2001_Avian
AY950281_A_chicken_Henan_12_2004_Avian

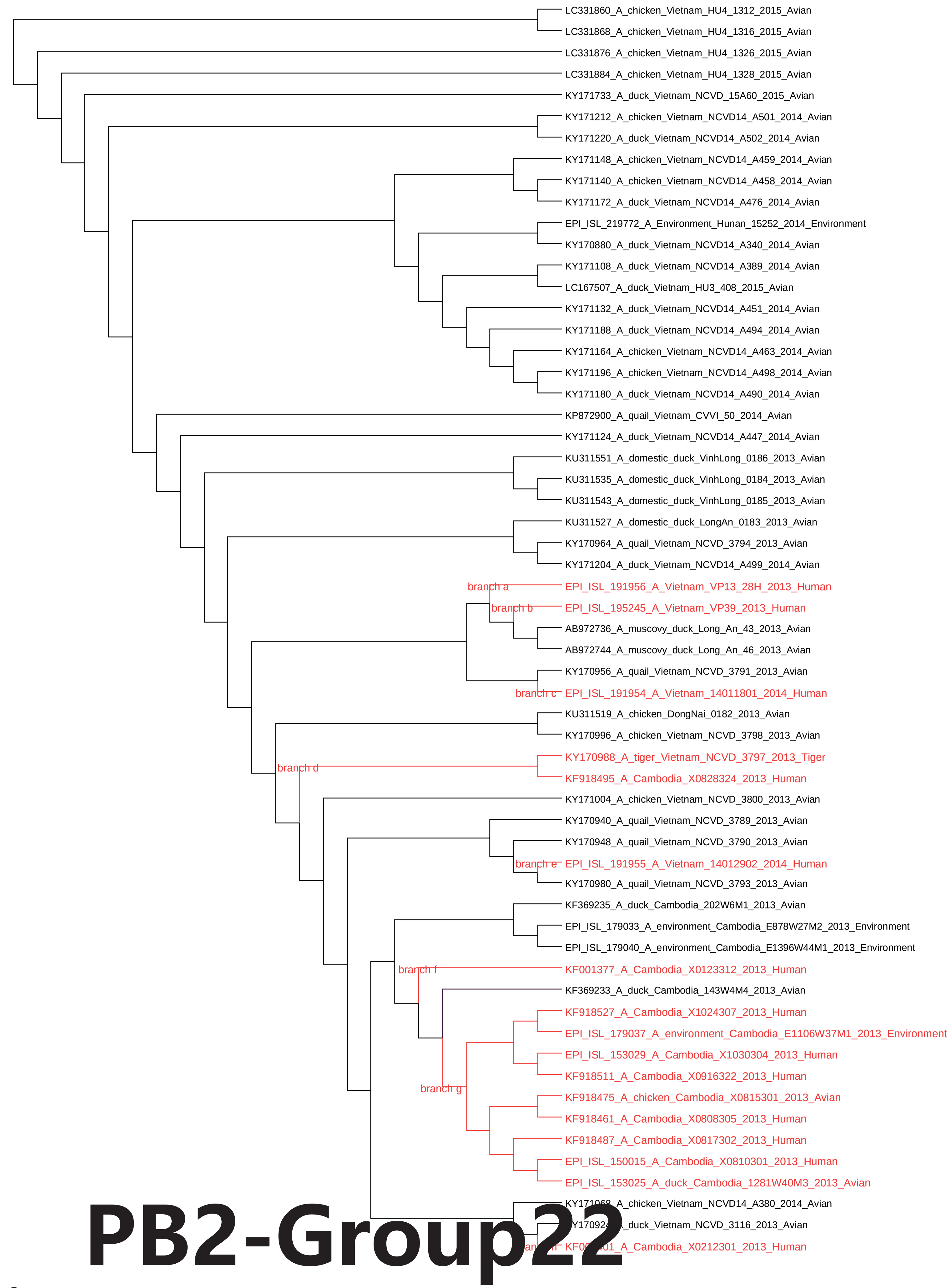

PB2-Group22

-Group

PB2-Group23

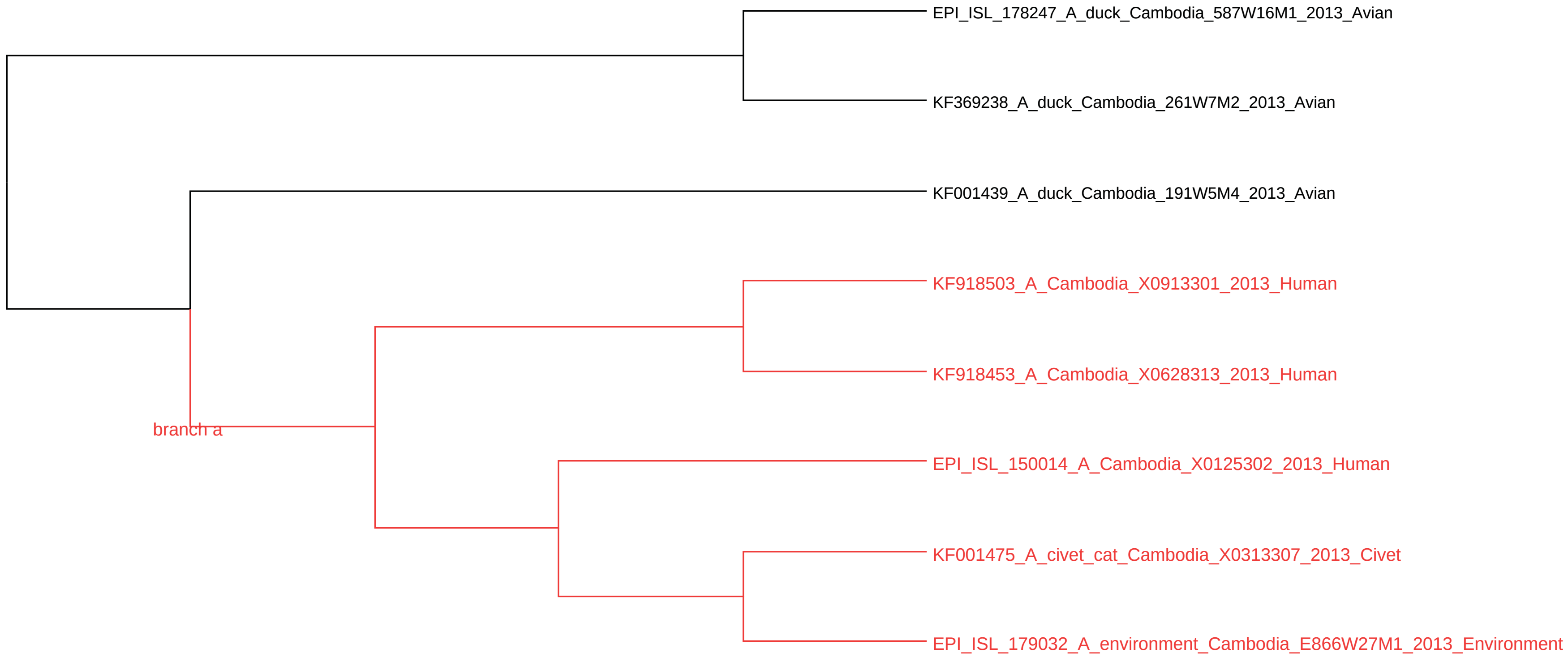

EPI_ISL_178247_A_duck_Cambodia_587W16M1_2013_Avian
KF369238_A_duck_Cambodia_261W7M2_2013_Avian
KF001439_A_duck_Cambodia_191W5M4_2013_Avian
KF918503_A_Cambodia_X0913301_2013_Human
KF918453_A_Cambodia_X0628313_2013_Human
branch a
EPI_ISL_150014_A_Cambodia_X0125302_2013_Human
KF001475_A_civet_cat_Cambodia_X0313307_2013_Civet
EPI_ISL_179032_A_environment_Cambodia_E866W27M1_2013_Environment

PB2-Group24

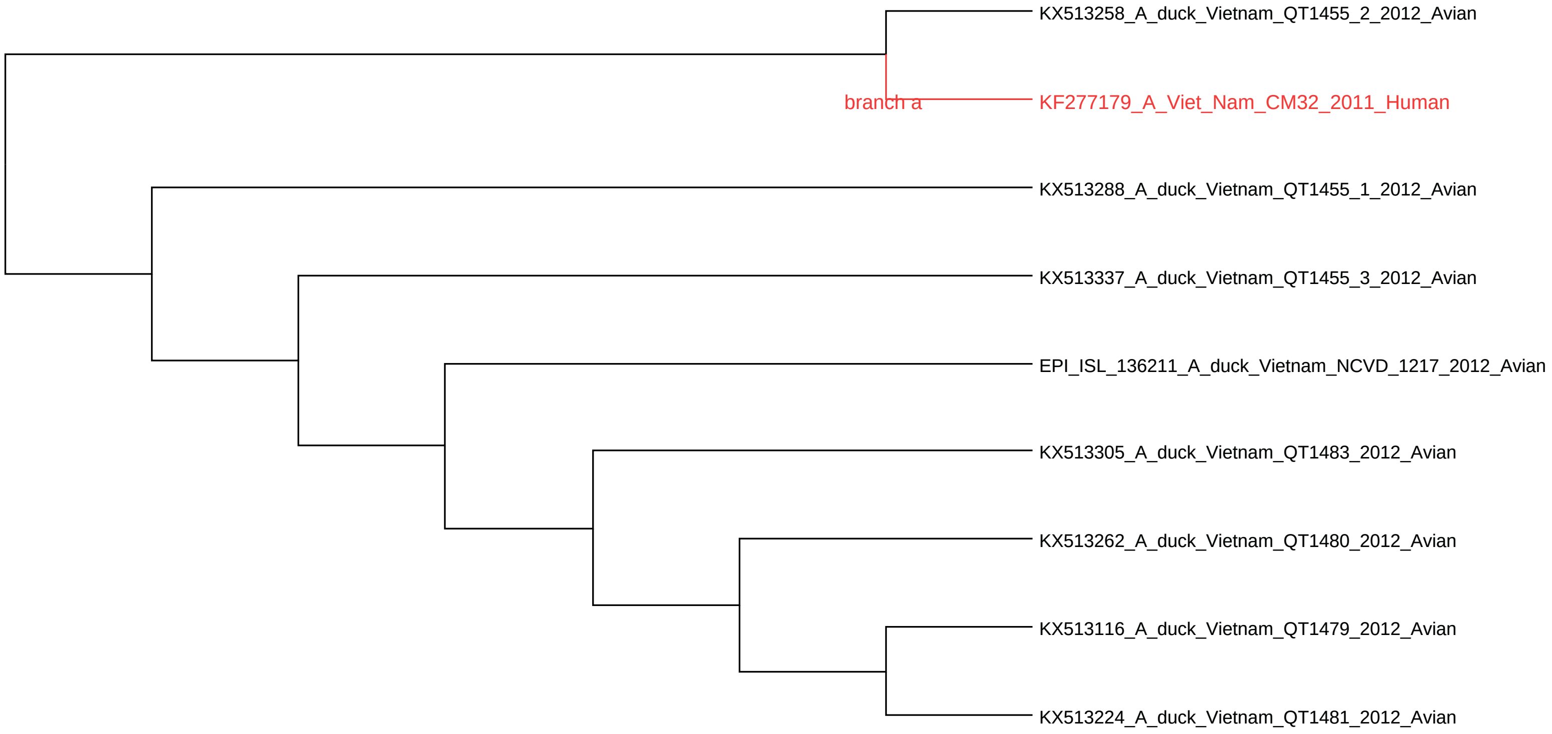
KX513258_A_duck_Vietnam_QT1455_2_2012_Avian
branch a
KF277179_A_Viet_Nam_CM32_2011_Human
KX513288_A_duck_Vietnam_QT1455_1_2012_Avian
KX513337_A_duck_Vietnam_QT1455_3_2012_Avian
EPI_ISL_136211_A_duck_Vietnam_NCVD_1217_2012_Avian
KX513305_A_duck_Vietnam_QT1483_2012_Avian
KX513262_A_duck_Vietnam_QT1480_2012_Avian
KX513116_A_duck_Vietnam_QT1479_2012_Avian
KX513224_A_duck_Vietnam_QT1481_2012_Avian

PB2-Group25

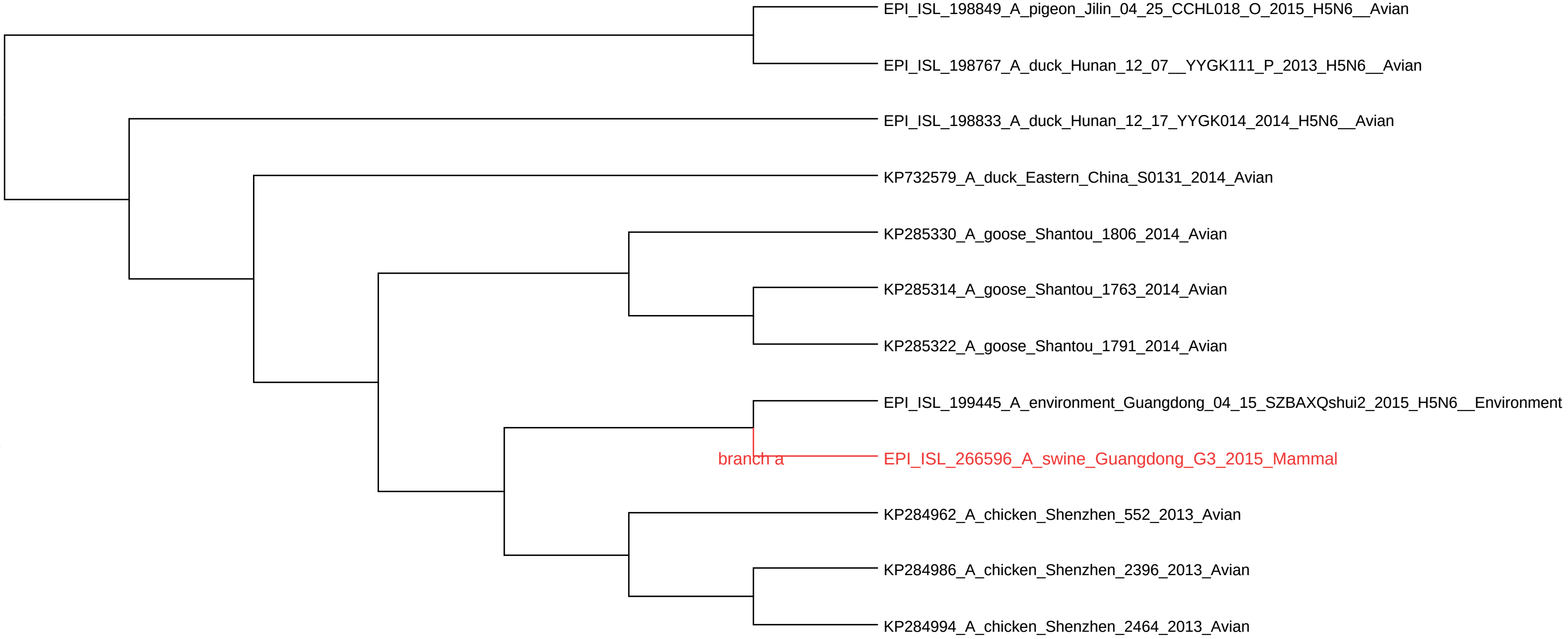
EPI_ISL_198849_A_pigeon_Jilin_04_25_CCHL018_O_2015_H5N6__Avian
EPI_ISL_198767_A_duck_Hunan_12_07__YYGK111_P_2013_H5N6__Avian
EPI_ISL_198833_A_duck_Hunan_12_17_YYGK014_2014_H5N6__Avian
KP732579_A_duck_Eastern_China_S0131_2014_Avian
KP285330_A_goose_Shantou_1806_2014_Avian
KP285314_A_goose_Shantou_1763_2014_Avian
KP285322_A_goose_Shantou_1791_2014_Avian
EPI_ISL_199445_A_environment_Guangdong_04_15_SZBAXQshui2_2015_H5N6__Environment
branch a
EPI_ISL_266596_A_swine_Guangdong_G3_2015_Mammal
KP284962_A_chicken_Shenzhen_552_2013_Avian
KP284986_A_chicken_Shenzhen_2396_2013_Avian
KP284994_A_chicken_Shenzhen_2464_2013_Avian

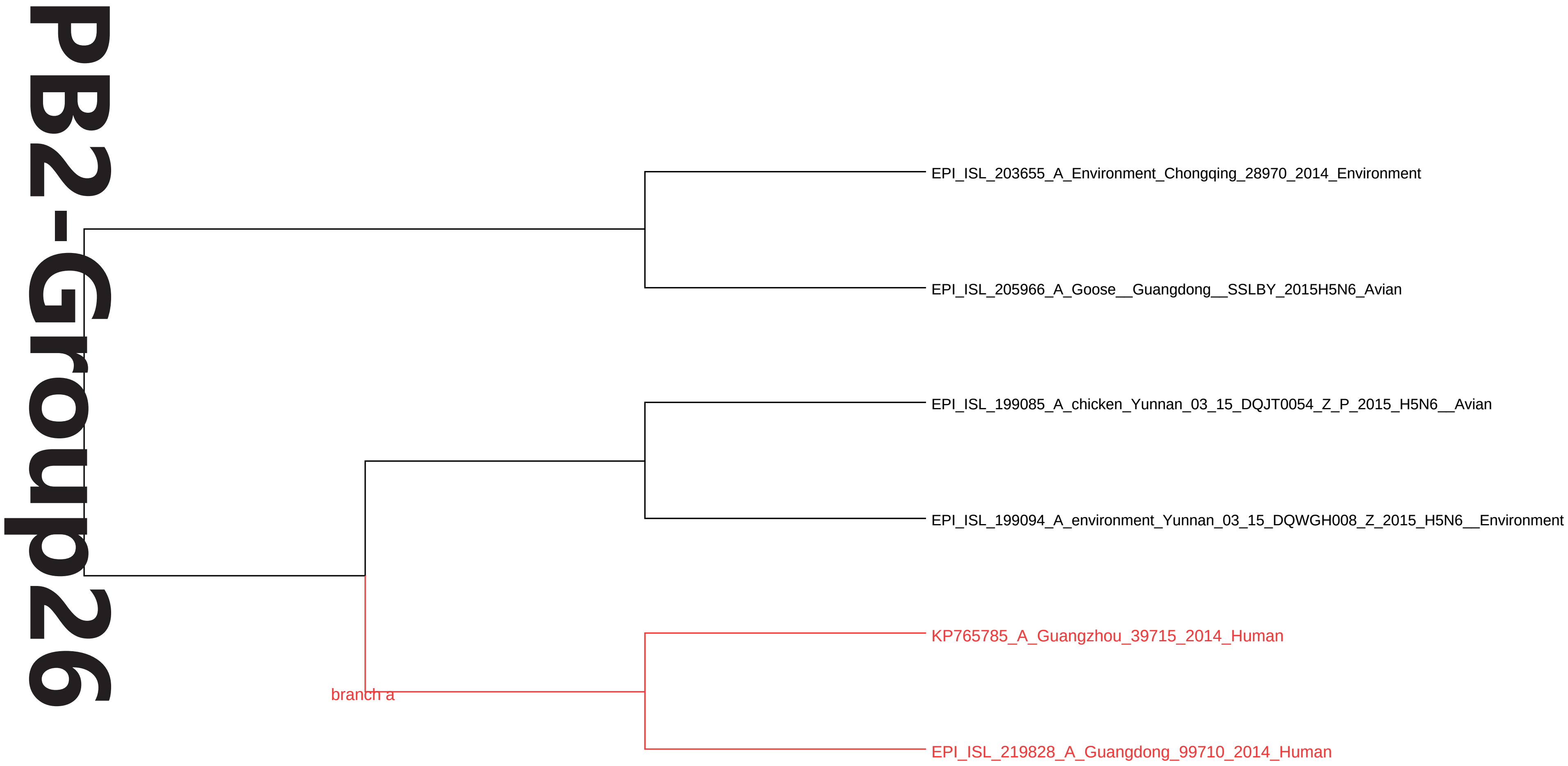
PB2-Group26
EPI_ISL_203655_A_Environment_Chongqing_28970_2014_Environment
EPI_ISL_205966_A_Goose__Guangdong__SSLBY_2015H5N6_Avian
EPI_ISL_199085_A_chicken_Yunnan_03_15_DQJT0054_Z_P_2015_H5N6__Avian
EPI_ISL_199094_A_environment_Yunnan_03_15_DQWGH008_Z_2015_H5N6__Environment
KP765785_A_Guangzhou_39715_2014_Human
branch a
EPI_ISL_219828_A_Guangdong_99710_2014_Human

PB2-Group27

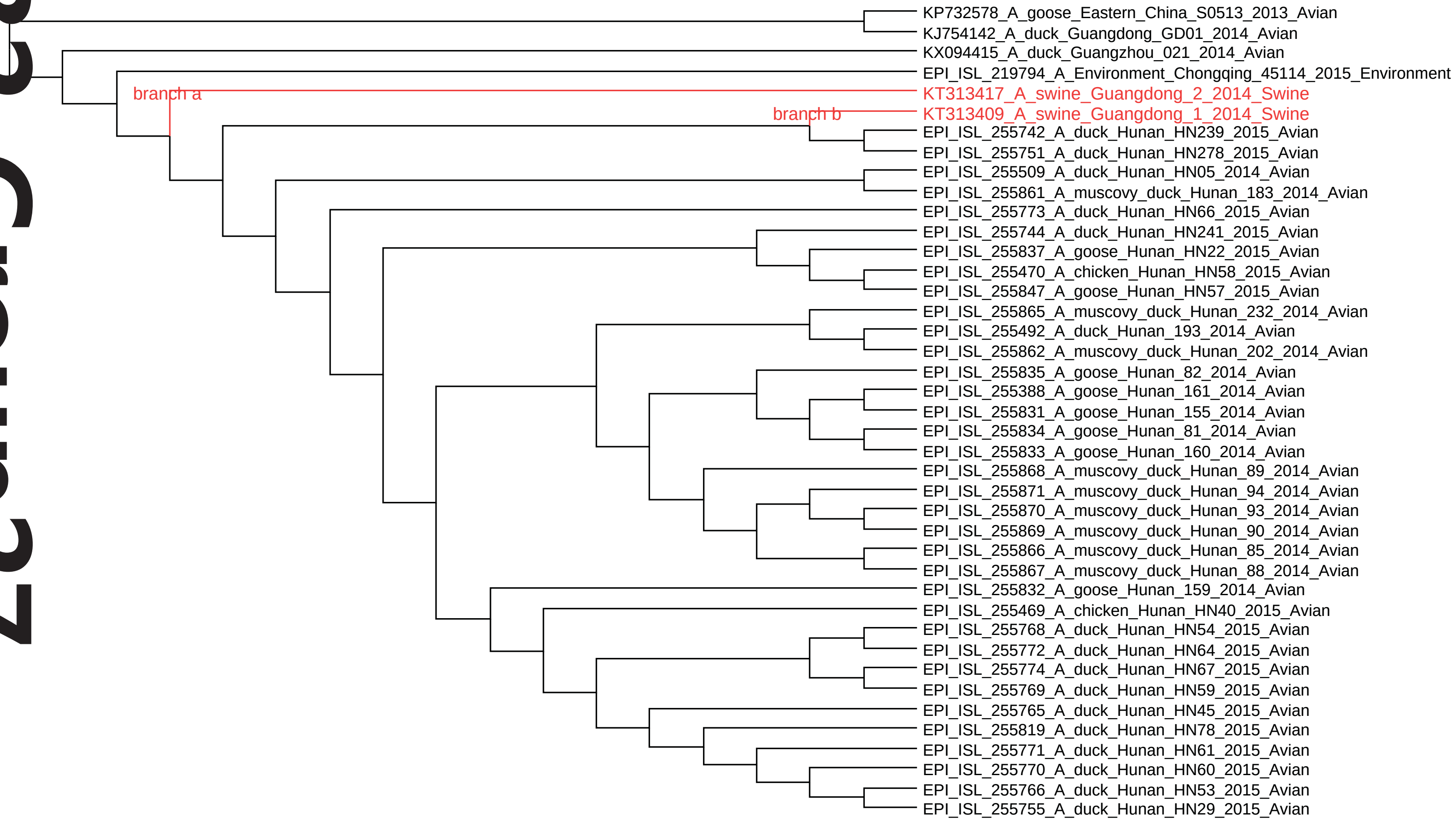
KP732578_A_goose_Eastern_China_S0513_2013_Avian
KJ754142_A_duck_Guangdong_GD01_2014_Avian
KX094415_A_duck_Guangzhou_021_2014_Avian
EPI_ISL_219794_A_Environment_Chongqing_45114_2015_Environment
branch a
KT313417_A_swine_Guangdong_2_2014_Swine
branch b
KT313409_A_swine_Guangdong_1_2014_Swine
EPI_ISL_255742_A_duck_Hunan_HN239_2015_Avian
EPI_ISL_255751_A_duck_Hunan_HN278_2015_Avian
EPI_ISL_255509_A_duck_Hunan_HN05_2014_Avian
EPI_ISL_255861_A_muscovy_duck_Hunan_183_2014_Avian
EPI_ISL_255773_A_duck_Hunan_HN66_2015_Avian
EPI_ISL_255744_A_duck_Hunan_HN241_2015_Avian
EPI_ISL_255837_A_goose_Hunan_HN22_2015_Avian
EPI_ISL_255470_A_chicken_Hunan_HN58_2015_Avian
EPI_ISL_255847_A_goose_Hunan_HN57_2015_Avian
EPI_ISL_255865_A_muscovy_duck_Hunan_232_2014_Avian
EPI_ISL_255492_A_duck_Hunan_193_2014_Avian
EPI_ISL_255862_A_muscovy_duck_Hunan_202_2014_Avian
EPI_ISL_255835_A_goose_Hunan_82_2014_Avian
EPI_ISL_255388_A_goose_Hunan_161_2014_Avian
EPI_ISL_255831_A_goose_Hunan_155_2014_Avian
EPI_ISL_255834_A_goose_Hunan_81_2014_Avian
EPI_ISL_255833_A_goose_Hunan_160_2014_Avian
EPI_ISL_255868_A_muscovy_duck_Hunan_89_2014_Avian
EPI_ISL_255871_A_muscovy_duck_Hunan_94_2014_Avian
EPI_ISL_255870_A_muscovy_duck_Hunan_93_2014_Avian
EPI_ISL_255869_A_muscovy_duck_Hunan_90_2014_Avian
EPI_ISL_255866_A_muscovy_duck_Hunan_85_2014_Avian
EPI_ISL_255867_A_muscovy_duck_Hunan_88_2014_Avian
EPI_ISL_255832_A_goose_Hunan_159_2014_Avian
EPI_ISL_255469_A_chicken_Hunan_HN40_2015_Avian
EPI_ISL_255768_A_duck_Hunan_HN54_2015_Avian
EPI_ISL_255772_A_duck_Hunan_HN64_2015_Avian
EPI_ISL_255774_A_duck_Hunan_HN67_2015_Avian
EPI_ISL_255769_A_duck_Hunan_HN59_2015_Avian
EPI_ISL_255765_A_duck_Hunan_HN45_2015_Avian
EPI_ISL_255819_A_duck_Hunan_HN78_2015_Avian
EPI_ISL_255771_A_duck_Hunan_HN61_2015_Avian
EPI_ISL_255770_A_duck_Hunan_HN60_2015_Avian
EPI_ISL_255766_A_duck_Hunan_HN53_2015_Avian
EPI_ISL_255755_A_duck_Hunan_HN29_2015_Avian

PB2-Group28

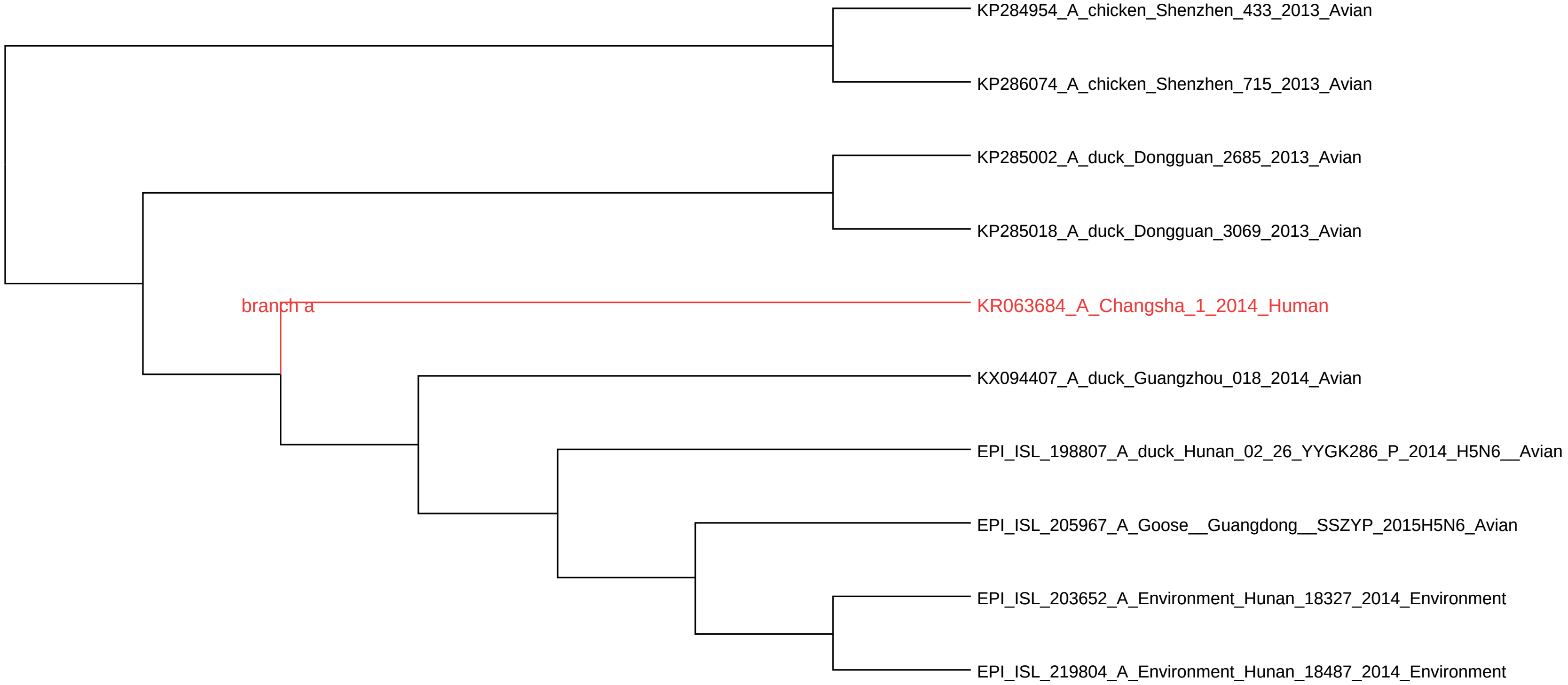

KP284954_A_chicken_Shenzhen_433_2013_Avian
KP286074_A_chicken_Shenzhen_715_2013_Avian
KP285002_A_duck_Dongguan_2685_2013_Avian
KP285018_A_duck_Dongguan_3069_2013_Avian
branch a
KR063684_A_Changsha_1_2014_Human
KX094407_A_duck_Guangzhou_018_2014_Avian
EPI_ISL_198807_A_duck_Hunan_02_26_YYGK286_P_2014_H5N6__Avian
EPI_ISL_205967_A_Goose__Guangdong__SSZYP_2015H5N6_Avian
EPI_ISL_203652_A_Environment_Hunan_18327_2014_Environment
EPI_ISL_219804_A_Environment_Hunan_18487_2014_Environment

PB2-Group29

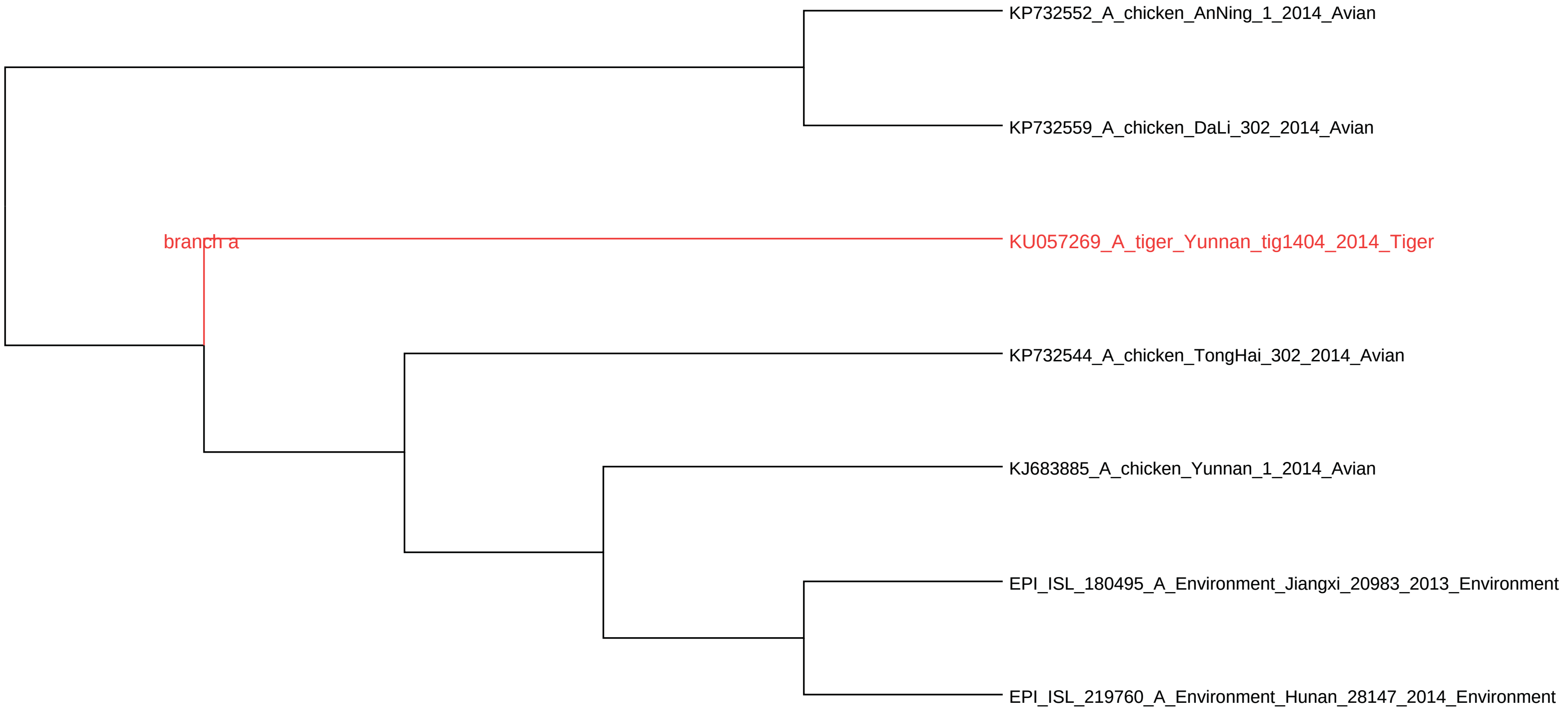
KP732552_A_chicken_AnNing_1_2014_Avian
KP732559_A_chicken_DaLi_302_2014_Avian
branch a
KU057269_A_tiger_Yunnan_tig1404_2014_Tiger
KP732544_A_chicken_TongHai_302_2014_Avian
KJ683885_A_chicken_Yunnan_1_2014_Avian
EPI_ISL_180495_A_Environment_Jiangxi_20983_2013_Environment
EPI_ISL_219760_A_Environment_Hunan_28147_2014_Environment

PB2-Group30

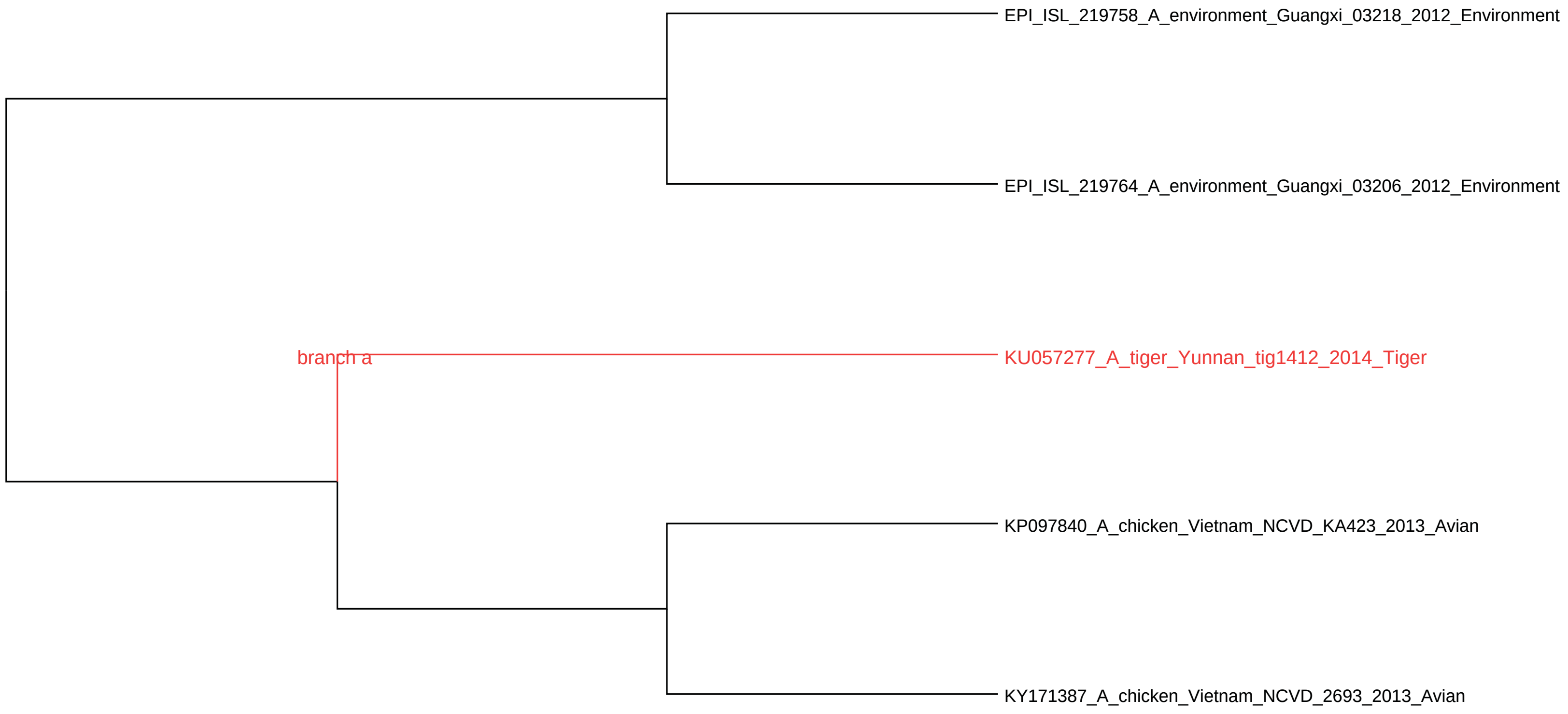
EPI_ISL_219758_A_environment_Guangxi_03218_2012_Environment
EPI_ISL_219764_A_environment_Guangxi_03206_2012_Environment
branch a
KU057277_A_tiger_Yunnan_tig1412_2014_Tiger
KP097840_A_chicken_Vietnam_NCVD_KA423_2013_Avian
KY171387_A_chicken_Vietnam_NCVD_2693_2013_Avian

PB2-Group31

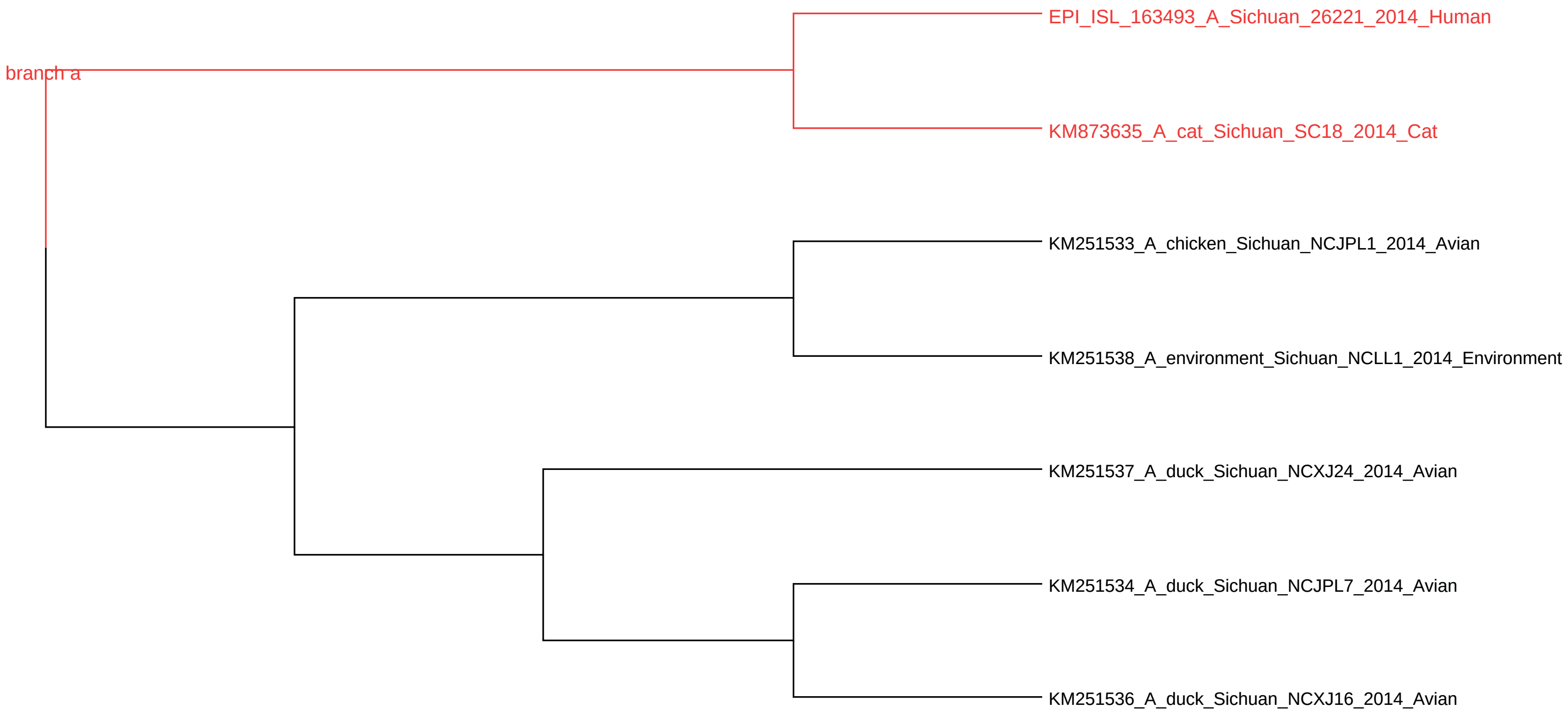

branch a
EPI_ISL_163493_A_Sichuan_26221_2014_Human
KM873635_A_cat_Sichuan_SC18_2014_Cat
KM251533_A_chicken_Sichuan_NCJPL1_2014_Avian
KM251538_A_environment_Sichuan_NCLL1_2014_Environment
KM251537_A_duck_Sichuan_NCXJ24_2014_Avian
KM251534_A_duck_Sichuan_NCJPL7_2014_Avian
KM251536_A_duck_Sichuan_NCXJ16_2014_Avian

PB2-Group32

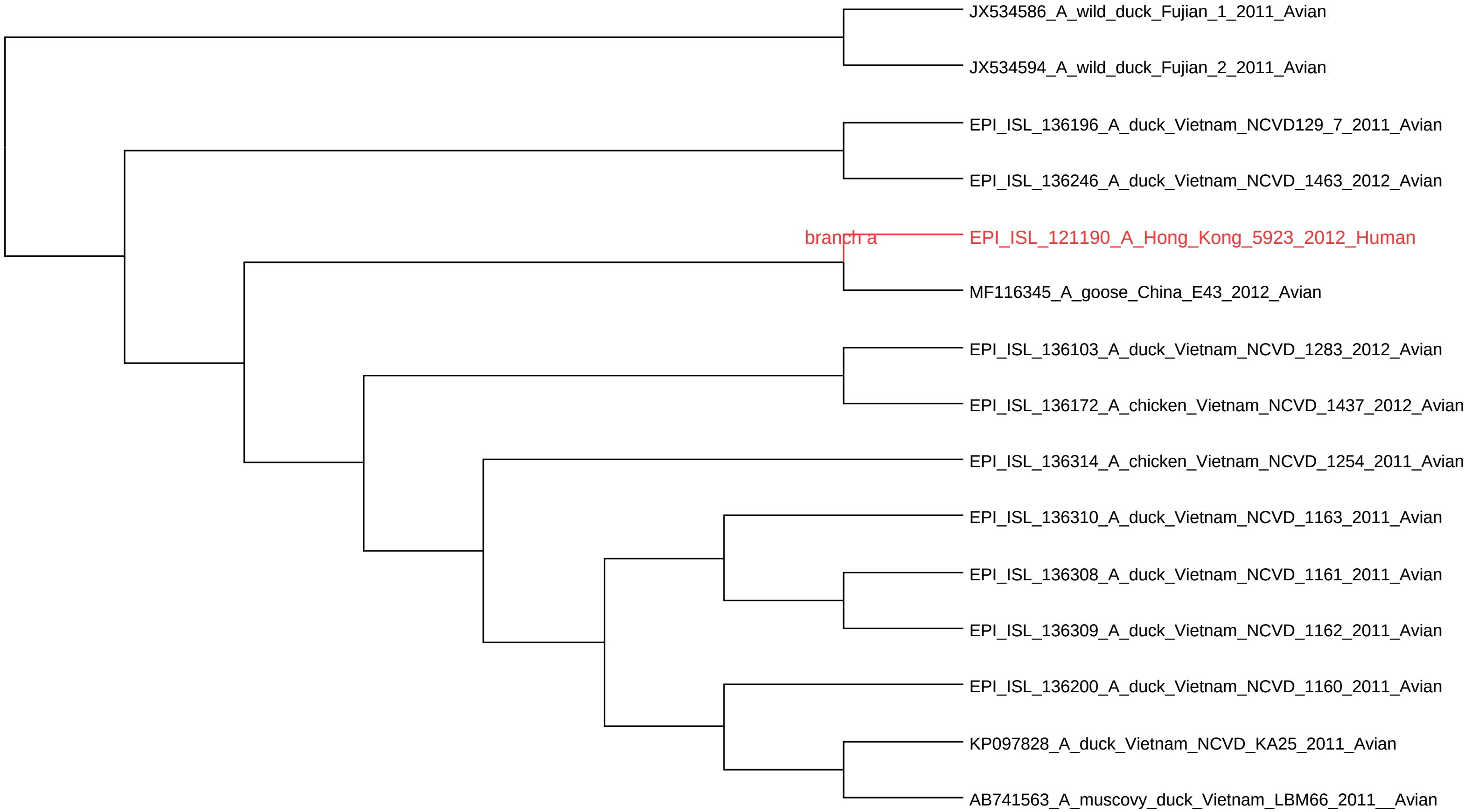
JX534586_A_wild_duck_Fujian_1_2011_Avian
JX534594_A_wild_duck_Fujian_2_2011_Avian
EPI_ISL_136196_A_duck_Vietnam_NCVD129_7_2011_Avian
EPI_ISL_136246_A_duck_Vietnam_NCVD_1463_2012_Avian
branch a
EPI_ISL_121190_A_Hong_Kong_5923_2012_Human
MF116345_A_goose_China_E43_2012_Avian
EPI_ISL_136103_A_duck_Vietnam_NCVD_1283_2012_Avian
EPI_ISL_136172_A_chicken_Vietnam_NCVD_1437_2012_Avian
EPI_ISL_136314_A_chicken_Vietnam_NCVD_1254_2011_Avian
EPI_ISL_136310_A_duck_Vietnam_NCVD_1163_2011_Avian
EPI_ISL_136308_A_duck_Vietnam_NCVD_1161_2011_Avian
EPI_ISL_136309_A_duck_Vietnam_NCVD_1162_2011_Avian
EPI_ISL_136200_A_duck_Vietnam_NCVD_1160_2011_Avian
KP097828_A_duck_Vietnam_NCVD_KA25_2011_Avian
AB741563_A_muscovy_duck_Vietnam_LBM66_2011__Avian

PB2-Group33

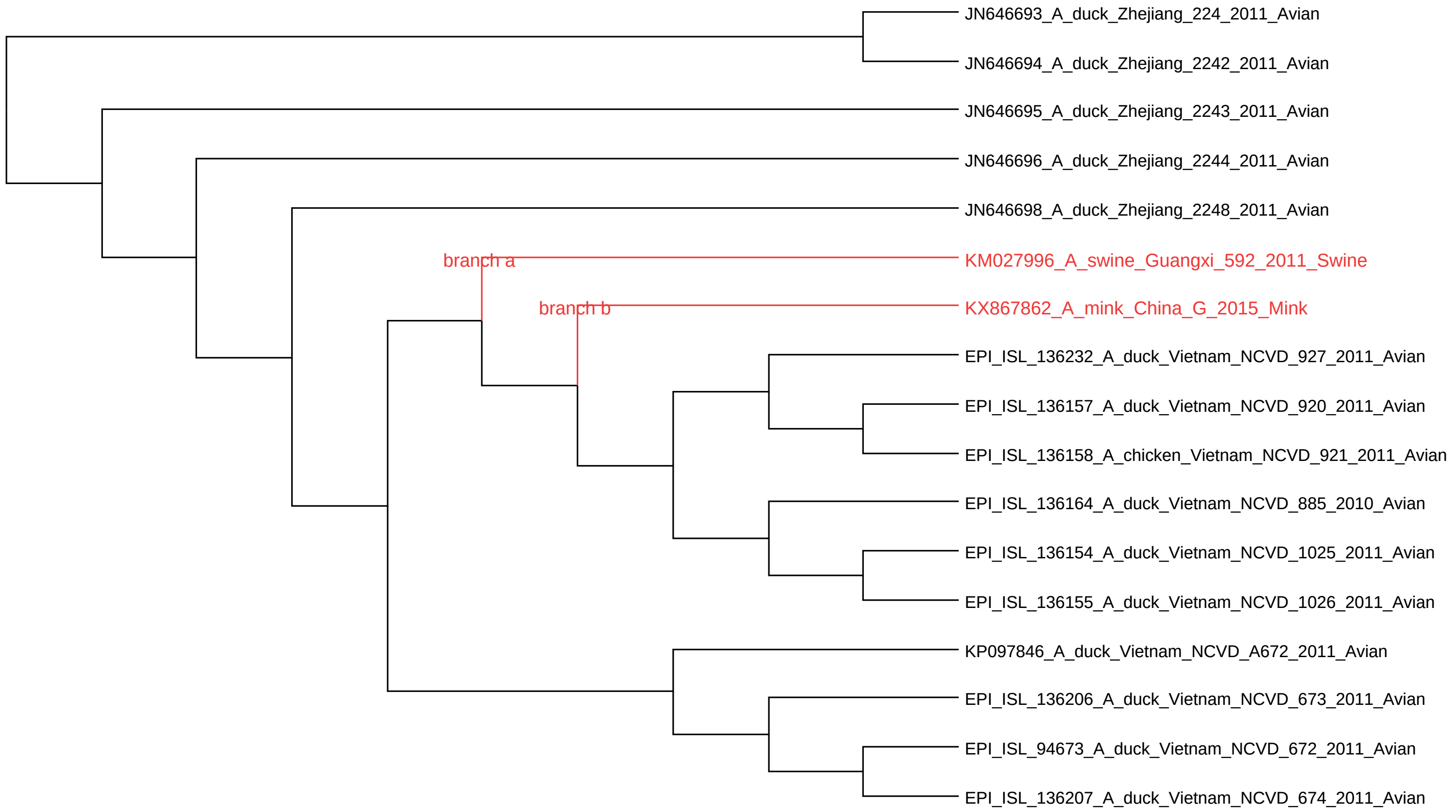

JN646693_A_duck_Zhejiang_224_2011_Avian
JN646694_A_duck_Zhejiang_2242_2011_Avian
JN646695_A_duck_Zhejiang_2243_2011_Avian
JN646696_A_duck_Zhejiang_2244_2011_Avian
JN646698_A_duck_Zhejiang_2248_2011_Avian
branch a
KM027996_A_swine_Guangxi_592_2011_Swine
branch b
KX867862_A_mink_China_G_2015_Mink
EPI_ISL_136232_A_duck_Vietnam_NCVD_927_2011_Avian
EPI_ISL_136157_A_duck_Vietnam_NCVD_920_2011_Avian
EPI_ISL_136158_A_chicken_Vietnam_NCVD_921_2011_Avian
EPI_ISL_136164_A_duck_Vietnam_NCVD_885_2010_Avian
EPI_ISL_136154_A_duck_Vietnam_NCVD_1025_2011_Avian
EPI_ISL_136155_A_duck_Vietnam_NCVD_1026_2011_Avian
KP097846_A_duck_Vietnam_NCVD_A672_2011_Avian
EPI_ISL_136206_A_duck_Vietnam_NCVD_673_2011_Avian
EPI_ISL_94673_A_duck_Vietnam_NCVD_672_2011_Avian
EPI_ISL_136207_A_duck_Vietnam_NCVD_674_2011_Avian

PB2-Group34

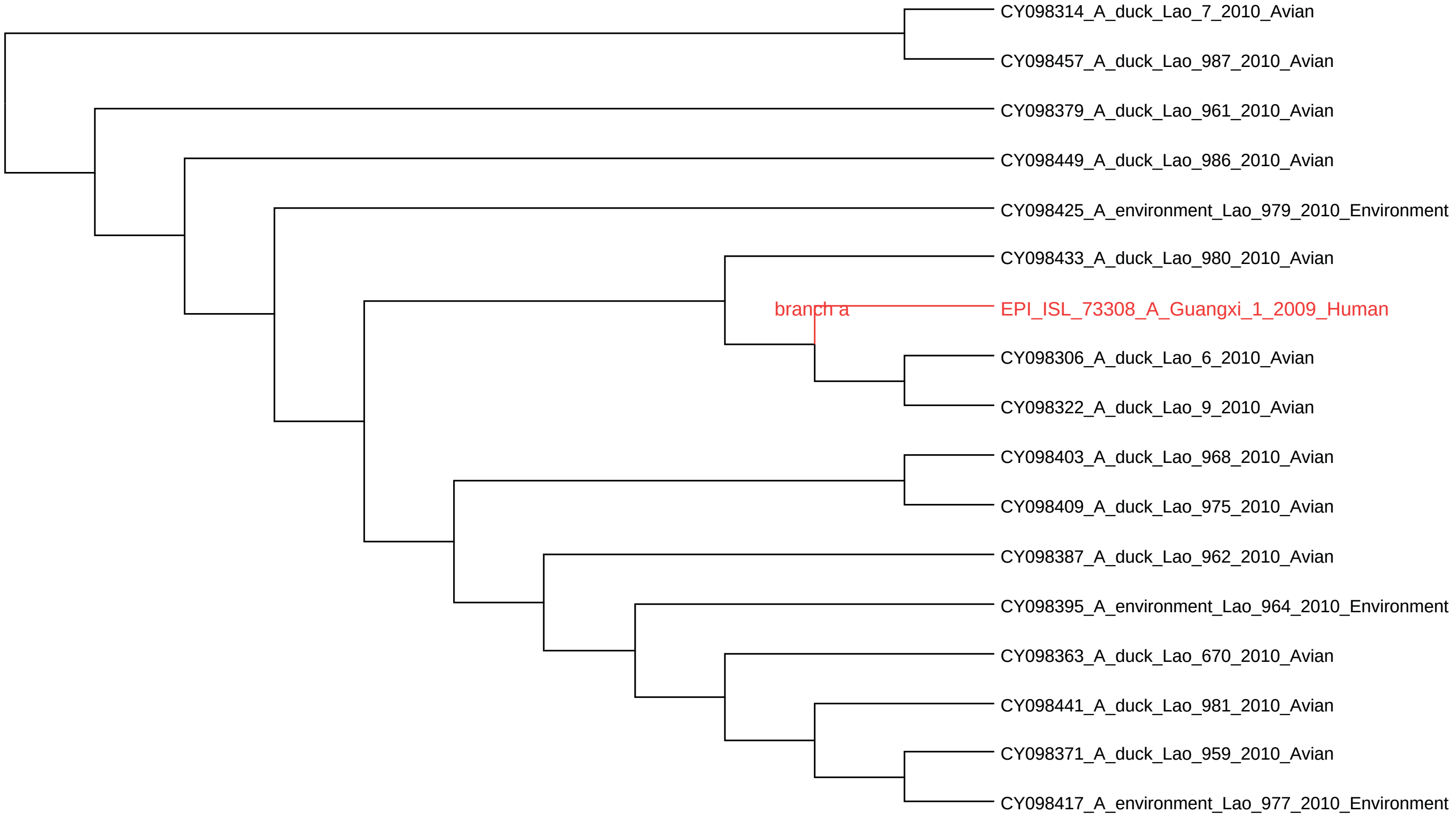
CY098314_A_duck_Lao_7_2010_Avian
CY098457_A_duck_Lao_987_2010_Avian
CY098379_A_duck_Lao_961_2010_Avian
CY098449_A_duck_Lao_986_2010_Avian
CY098425_A_environment_Lao_979_2010_Environment
CY098433_A_duck_Lao_980_2010_Avian
branch a
EPI_ISL_73308_A_Guangxi_1_2009_Human
CY098306_A_duck_Lao_6_2010_Avian
CY098322_A_duck_Lao_9_2010_Avian
CY098403_A_duck_Lao_968_2010_Avian
CY098409_A_duck_Lao_975_2010_Avian
CY098387_A_duck_Lao_962_2010_Avian
CY098395_A_environment_Lao_964_2010_Environment
CY098363_A_duck_Lao_670_2010_Avian
CY098441_A_duck_Lao_981_2010_Avian
CY098371_A_duck_Lao_959_2010_Avian
CY098417_A_environment_Lao_977_2010_Environment

PB2-Group35

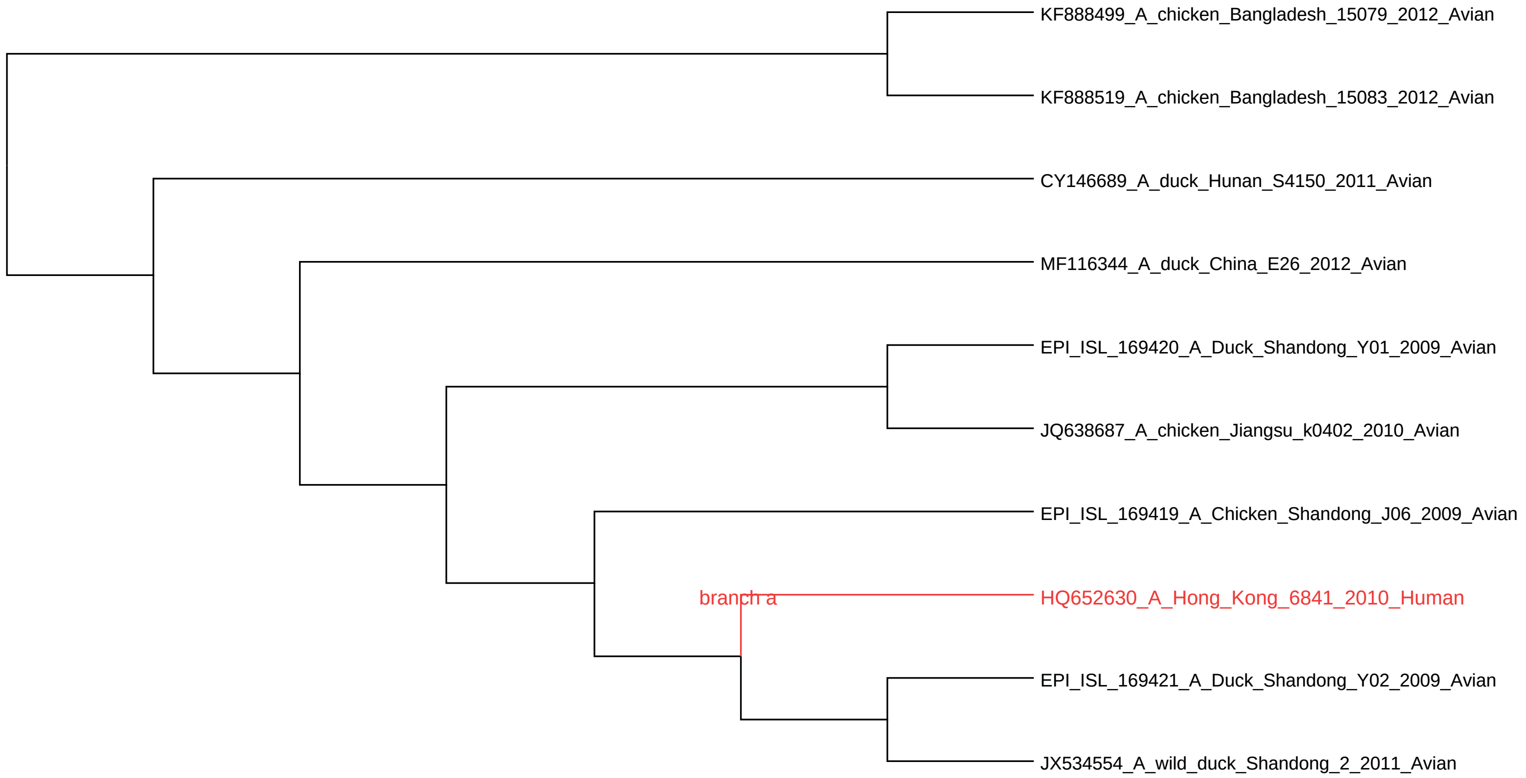
KF888499_A_chicken_Bangladesh_15079_2012_Avian
KF888519_A_chicken_Bangladesh_15083_2012_Avian
CY146689_A_duck_Hunan_S4150_2011_Avian
MF116344_A_duck_China_E26_2012_Avian
EPI_ISL_169420_A_Duck_Shandong_Y01_2009_Avian
JQ638687_A_chicken_Jiangsu_k0402_2010_Avian
EPI_ISL_169419_A_Chicken_Shandong_J06_2009_Avian
branch a
HQ652630_A_Hong_Kong_6841_2010_Human
EPI_ISL_169421_A_Duck_Shandong_Y02_2009_Avian
JX534554_A_wild_duck_Shandong_2_2011_Avian

PB2-Group36

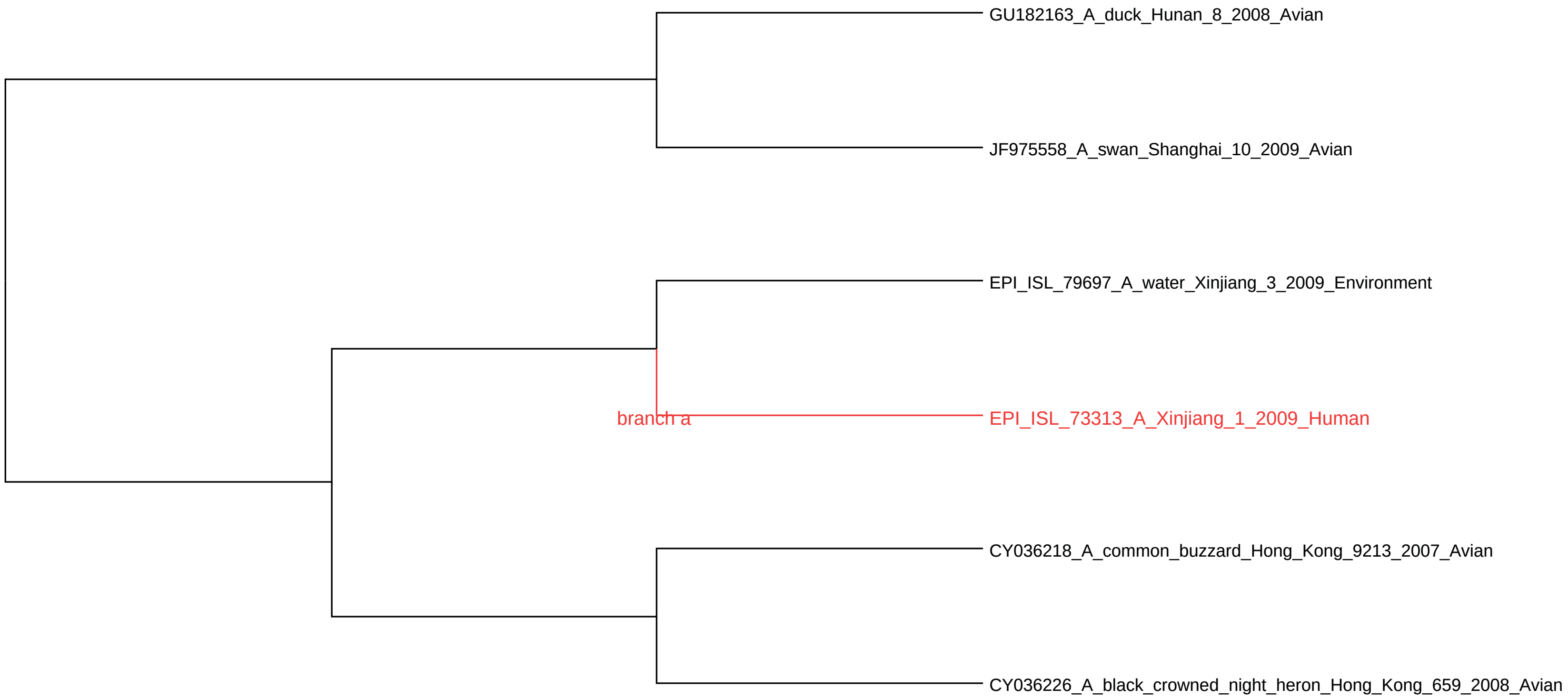
GU182163_A_duck_Hunan_8_2008_Avian
JF975558_A_swan_Shanghai_10_2009_Avian
EPI_ISL_79697_A_water_Xinjiang_3_2009_Environment
branch a
EPI_ISL_73313_A_Xinjiang_1_2009_Human
CY036218_A_common_buzzard_Hong_Kong_9213_2007_Avian
CY036226_A_black_crowned_night_heron_Hong_Kong_659_2008_Avian

PB2-Group37

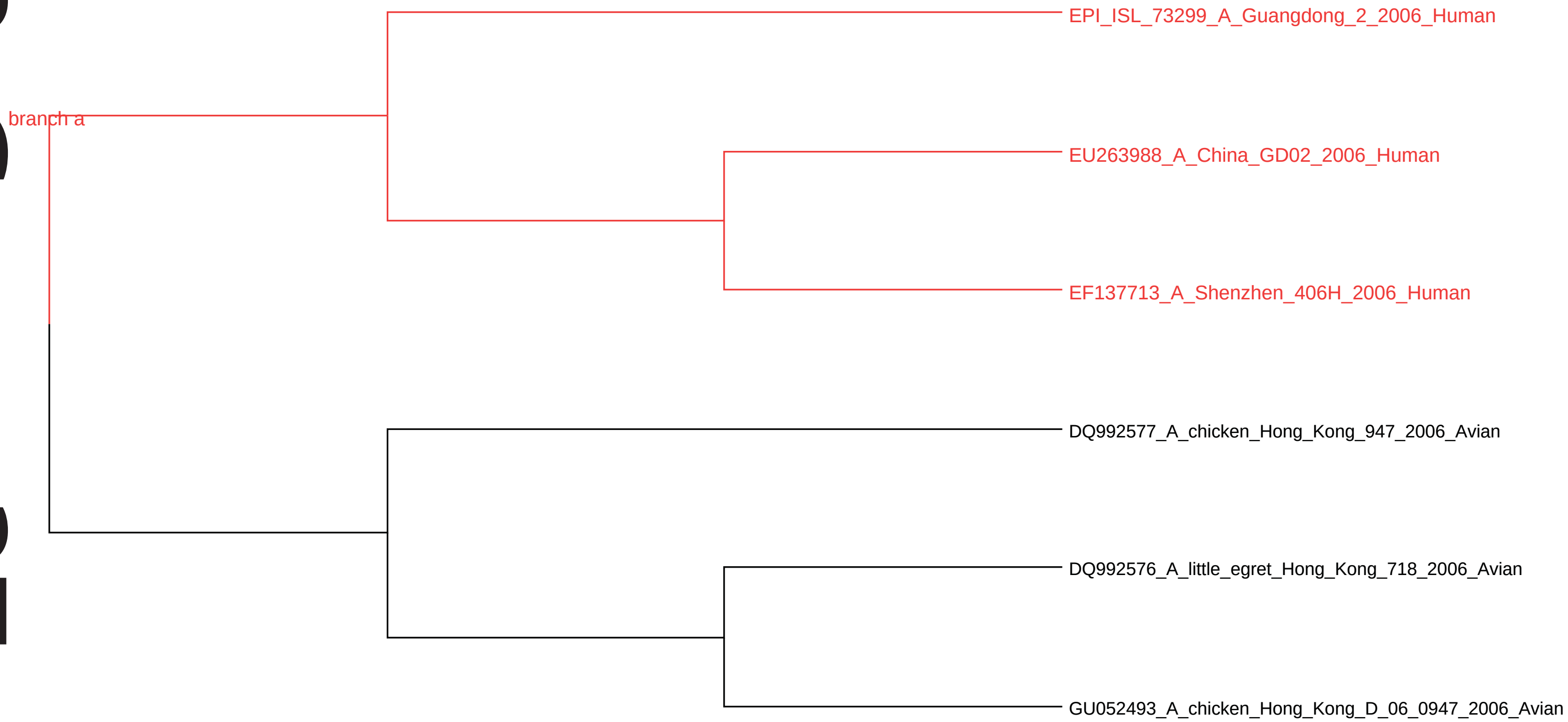
EPI_ISL_73299_A_Guangdong_2_2006_Human
branch a
EU263988_A_China_GD02_2006_Human
EF137713_A_Shenzhen_406H_2006_Human
DQ992577_A_chicken_Hong_Kong_947_2006_Avian
DQ992576_A_little_egret_Hong_Kong_718_2006_Avian
GU052493_A_chicken_Hong_Kong_D_06_0947_2006_Avian

PB2-Group38

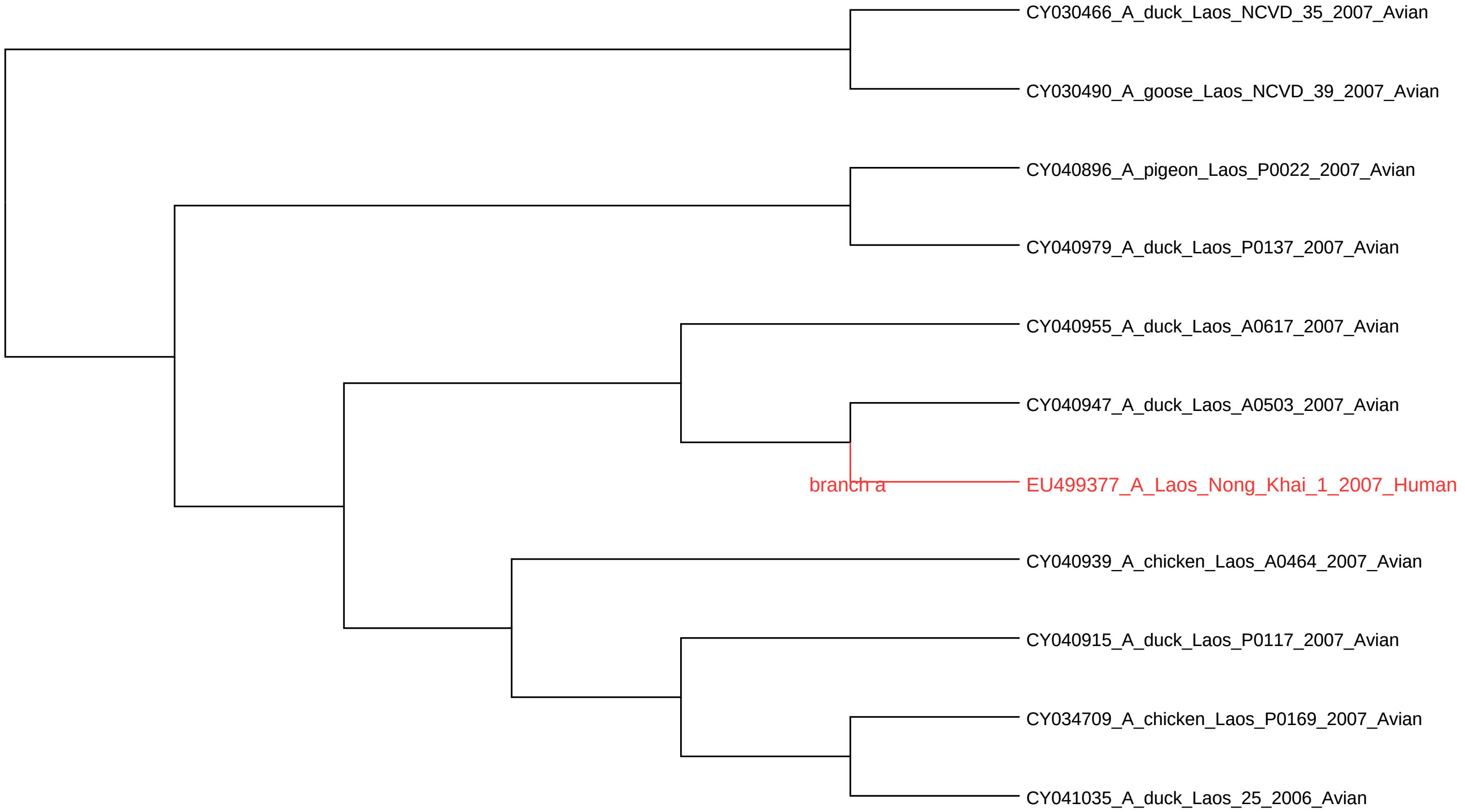

CY030466_A_duck_Laos_NCVD_35_2007_Avian
CY030490_A_goose_Laos_NCVD_39_2007_Avian
CY040896_A_pigeon_Laos_P0022_2007_Avian
CY040979_A_duck_Laos_P0137_2007_Avian
CY040955_A_duck_Laos_A0617_2007_Avian
CY040947_A_duck_Laos_A0503_2007_Avian
branch a
EU499377_A_Laos_Nong_Khai_1_2007_Human
CY040939_A_chicken_Laos_A0464_2007_Avian
CY040915_A_duck_Laos_P0117_2007_Avian
CY034709_A_chicken_Laos_P0169_2007_Avian
CY041035_A_duck_Laos_25_2006_Avian

PB2-Group39

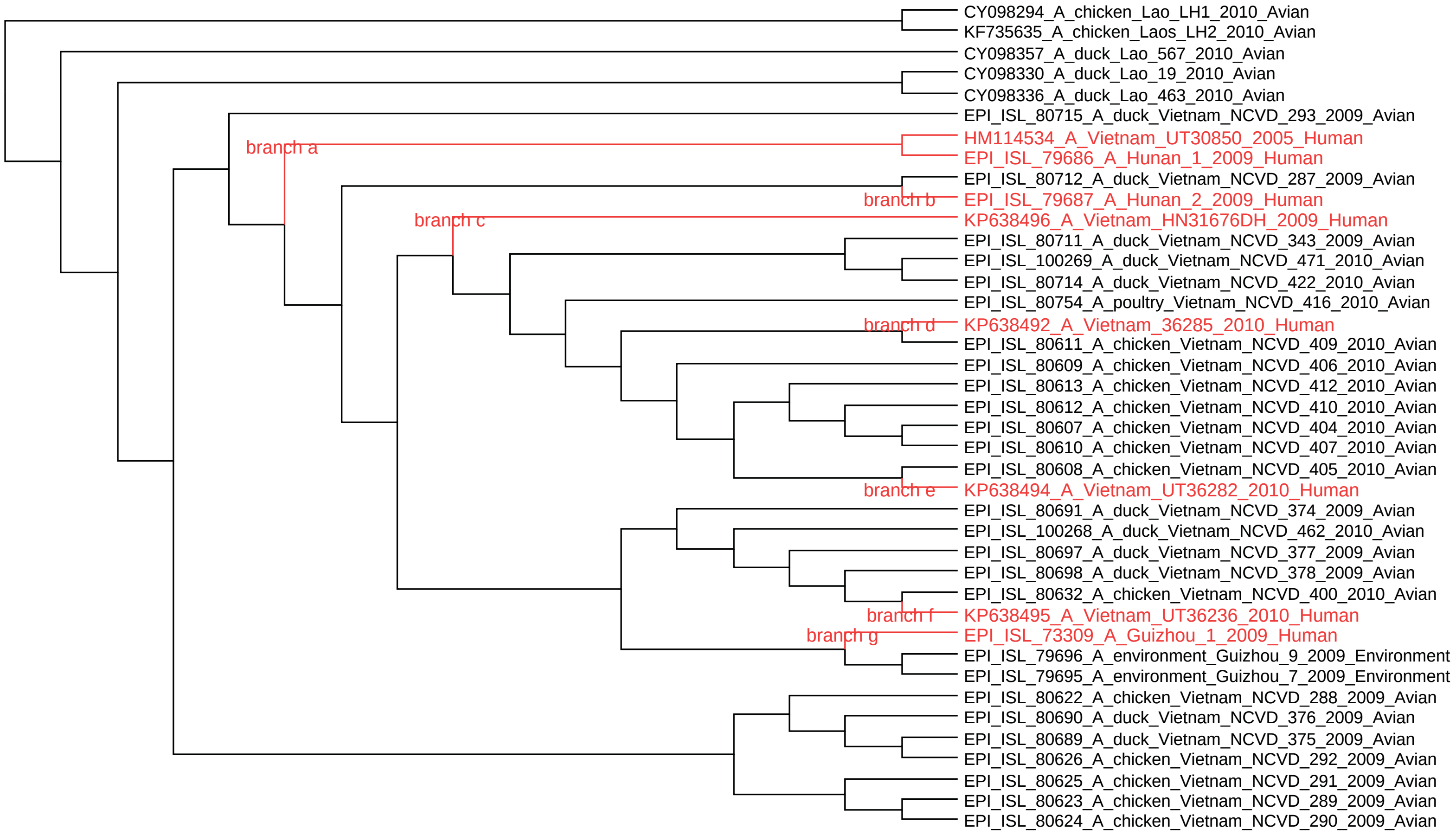

CY098294_A_chicken_Lao_LH1_2010_Avian
KF735635_A_chicken_Laos_LH2_2010_Avian
CY098357_A_duck_Lao_567_2010_Avian
CY098330_A_duck_Lao_19_2010_Avian
CY098336_A_duck_Lao_463_2010_Avian
EPI_ISL_80715_A_duck_Vietnam_NCVD_293_2009_Avian
branch a
HM114534_A_Vietnam_UT30850_2005_Human
EPI_ISL_79686_A_Hunan_1_2009_Human
EPI_ISL_80712_A_duck_Vietnam_NCVD_287_2009_Avian
branch b
EPI_ISL_79687_A_Hunan_2_2009_Human
branch c
KP638496_A_Vietnam_HN31676DH_2009_Human
EPI_ISL_80711_A_duck_Vietnam_NCVD_343_2009_Avian
EPI_ISL_100269_A_duck_Vietnam_NCVD_471_2010_Avian
EPI_ISL_80714_A_duck_Vietnam_NCVD_422_2010_Avian
EPI_ISL_80754_A_poultry_Vietnam_NCVD_416_2010_Avian
branch d
KP638492_A_Vietnam_36285_2010_Human
EPI_ISL_80611_A_chicken_Vietnam_NCVD_409_2010_Avian
EPI_ISL_80609_A_chicken_Vietnam_NCVD_406_2010_Avian
EPI_ISL_80613_A_chicken_Vietnam_NCVD_412_2010_Avian
EPI_ISL_80612_A_chicken_Vietnam_NCVD_410_2010_Avian
EPI_ISL_80607_A_chicken_Vietnam_NCVD_404_2010_Avian
EPI_ISL_80610_A_chicken_Vietnam_NCVD_407_2010_Avian
EPI_ISL_80608_A_chicken_Vietnam_NCVD_405_2010_Avian
branch e
KP638494_A_Vietnam_UT36282_2010_Human
EPI_ISL_80691_A_duck_Vietnam_NCVD_374_2009_Avian
EPI_ISL_100268_A_duck_Vietnam_NCVD_462_2010_Avian
EPI_ISL_80697_A_duck_Vietnam_NCVD_377_2009_Avian
EPI_ISL_80698_A_duck_Vietnam_NCVD_378_2009_Avian
EPI_ISL_80632_A_chicken_Vietnam_NCVD_400_2010_Avian
branch f
KP638495_A_Vietnam_UT36236_2010_Human
branch g
EPI_ISL_73309_A_Guizhou_1_2009_Human
EPI_ISL_79696_A_environment_Guizhou_9_2009_Environment
EPI_ISL_79695_A_environment_Guizhou_7_2009_Environment
EPI_ISL_80622_A_chicken_Vietnam_NCVD_288_2009_Avian
EPI_ISL_80690_A_duck_Vietnam_NCVD_376_2009_Avian
EPI_ISL_80689_A_duck_Vietnam_NCVD_375_2009_Avian
EPI_ISL_80626_A_chicken_Vietnam_NCVD_292_2009_Avian
EPI_ISL_80625_A_chicken_Vietnam_NCVD_291_2009_Avian
EPI_ISL_80623_A_chicken_Vietnam_NCVD_289_2009_Avian
EPI_ISL_80624_A_chicken_Vietnam_NCVD_290_2009_Avian

PB2-Group40

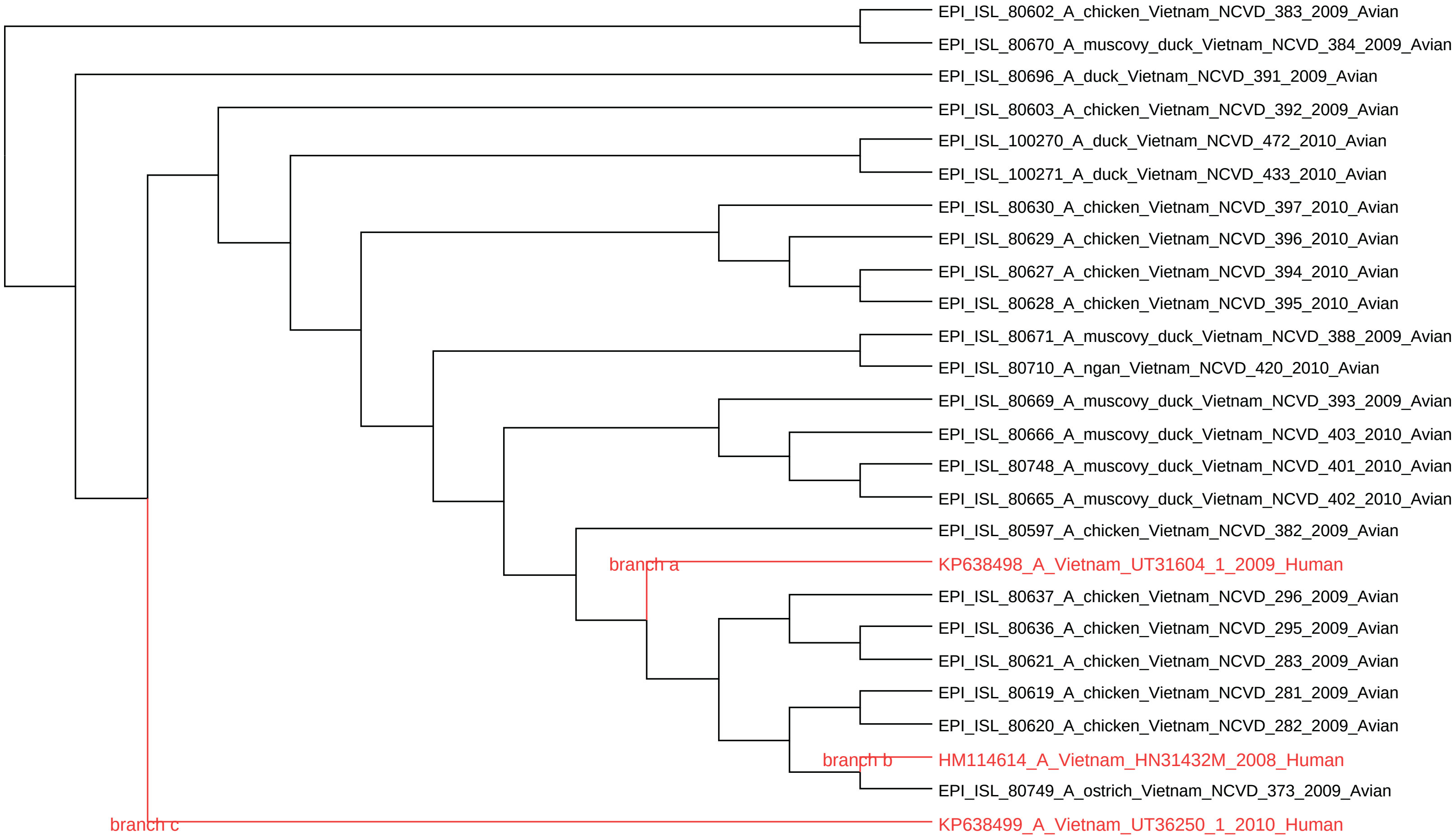
EPI_ISL_80602_A_chicken_Vietnam_NCVD_383_2009_Avian
EPI_ISL_80670_A_muscovy_duck_Vietnam_NCVD_384_2009_Avian
EPI_ISL_80696_A_duck_Vietnam_NCVD_391_2009_Avian
EPI_ISL_80603_A_chicken_Vietnam_NCVD_392_2009_Avian
EPI_ISL_100270_A_duck_Vietnam_NCVD_472_2010_Avian
EPI_ISL_100271_A_duck_Vietnam_NCVD_433_2010_Avian
EPI_ISL_80630_A_chicken_Vietnam_NCVD_397_2010_Avian
EPI_ISL_80629_A_chicken_Vietnam_NCVD_396_2010_Avian
EPI_ISL_80627_A_chicken_Vietnam_NCVD_394_2010_Avian
EPI_ISL_80628_A_chicken_Vietnam_NCVD_395_2010_Avian
EPI_ISL_80671_A_muscovy_duck_Vietnam_NCVD_388_2009_Avian
EPI_ISL_80710_A_ngan_Vietnam_NCVD_420_2010_Avian
EPI_ISL_80669_A_muscovy_duck_Vietnam_NCVD_393_2009_Avian
EPI_ISL_80666_A_muscovy_duck_Vietnam_NCVD_403_2010_Avian
EPI_ISL_80748_A_muscovy_duck_Vietnam_NCVD_401_2010_Avian
EPI_ISL_80665_A_muscovy_duck_Vietnam_NCVD_402_2010_Avian
EPI_ISL_80597_A_chicken_Vietnam_NCVD_382_2009_Avian
branch a
KP638498_A_Vietnam_UT31604_1_2009_Human
EPI_ISL_80637_A_chicken_Vietnam_NCVD_296_2009_Avian
EPI_ISL_80636_A_chicken_Vietnam_NCVD_295_2009_Avian
EPI_ISL_80621_A_chicken_Vietnam_NCVD_283_2009_Avian
EPI_ISL_80619_A_chicken_Vietnam_NCVD_281_2009_Avian
EPI_ISL_80620_A_chicken_Vietnam_NCVD_282_2009_Avian
branch b
HM114614_A_Vietnam_HN31432M_2008_Human
EPI_ISL_80749_A_ostrich_Vietnam_NCVD_373_2009_Avian
branch c
KP638499_A_Vietnam_UT36250_1_2010_Human

PB2-Group41

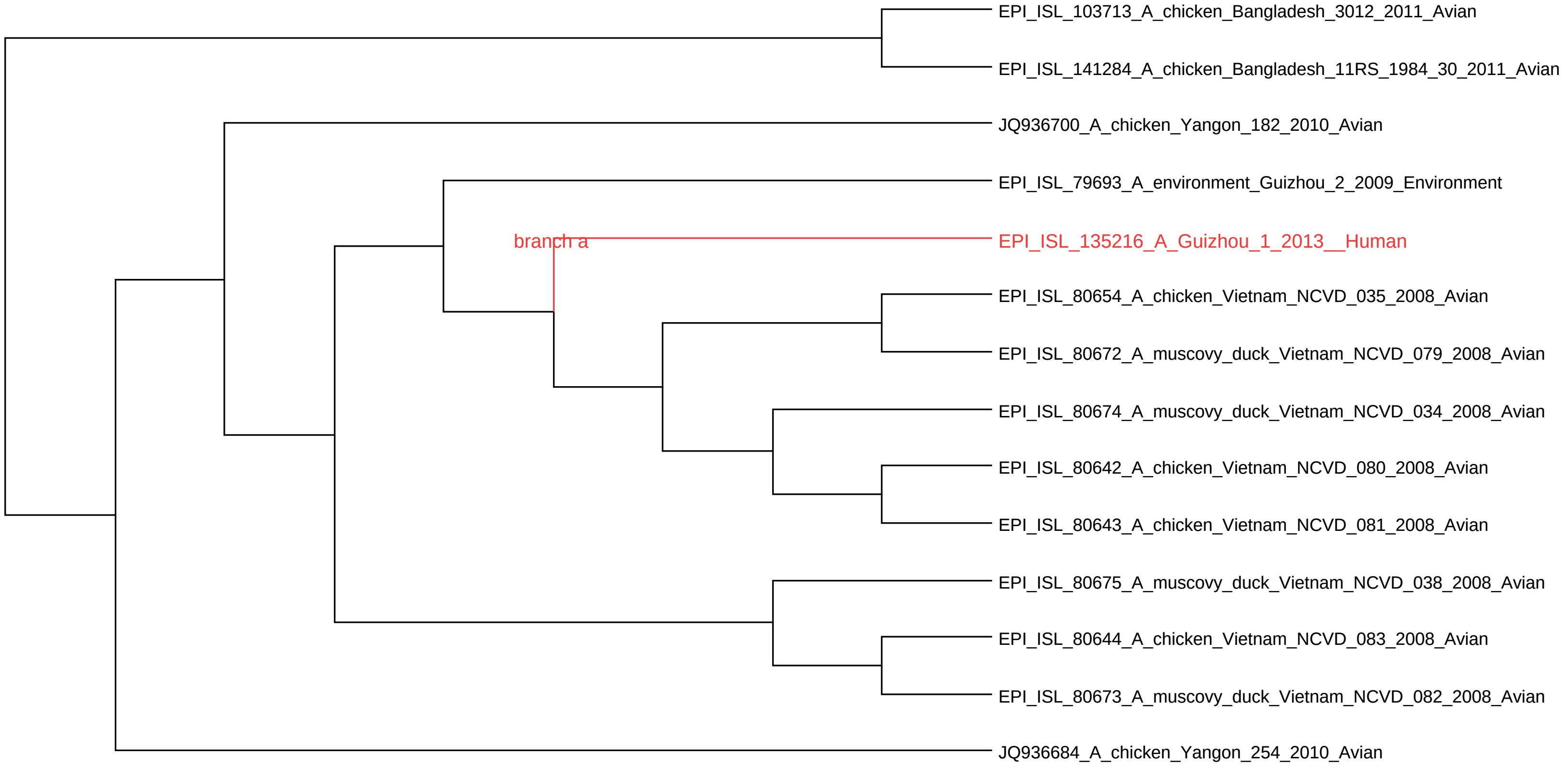
EPI_ISL_103713_A_chicken_Bangladesh_3012_2011_Avian
EPI_ISL_141284_A_chicken_Bangladesh_11RS_1984_30_2011_Avian
JQ936700_A_chicken_Yangon_182_2010_Avian
EPI_ISL_79693_A_environment_Guizhou_2_2009_Environment
branch a
EPI_ISL_135216_A_Guizhou_1_2013__Human
EPI_ISL_80654_A_chicken_Vietnam_NCVD_035_2008_Avian
EPI_ISL_80672_A_muscovy_duck_Vietnam_NCVD_079_2008_Avian
EPI_ISL_80674_A_muscovy_duck_Vietnam_NCVD_034_2008_Avian
EPI_ISL_80642_A_chicken_Vietnam_NCVD_080_2008_Avian
EPI_ISL_80643_A_chicken_Vietnam_NCVD_081_2008_Avian
EPI_ISL_80675_A_muscovy_duck_Vietnam_NCVD_038_2008_Avian
EPI_ISL_80644_A_chicken_Vietnam_NCVD_083_2008_Avian
EPI_ISL_80673_A_muscovy_duck_Vietnam_NCVD_082_2008_Avian
JQ936684_A_chicken_Yangon_254_2010_Avian

PB2-Group42

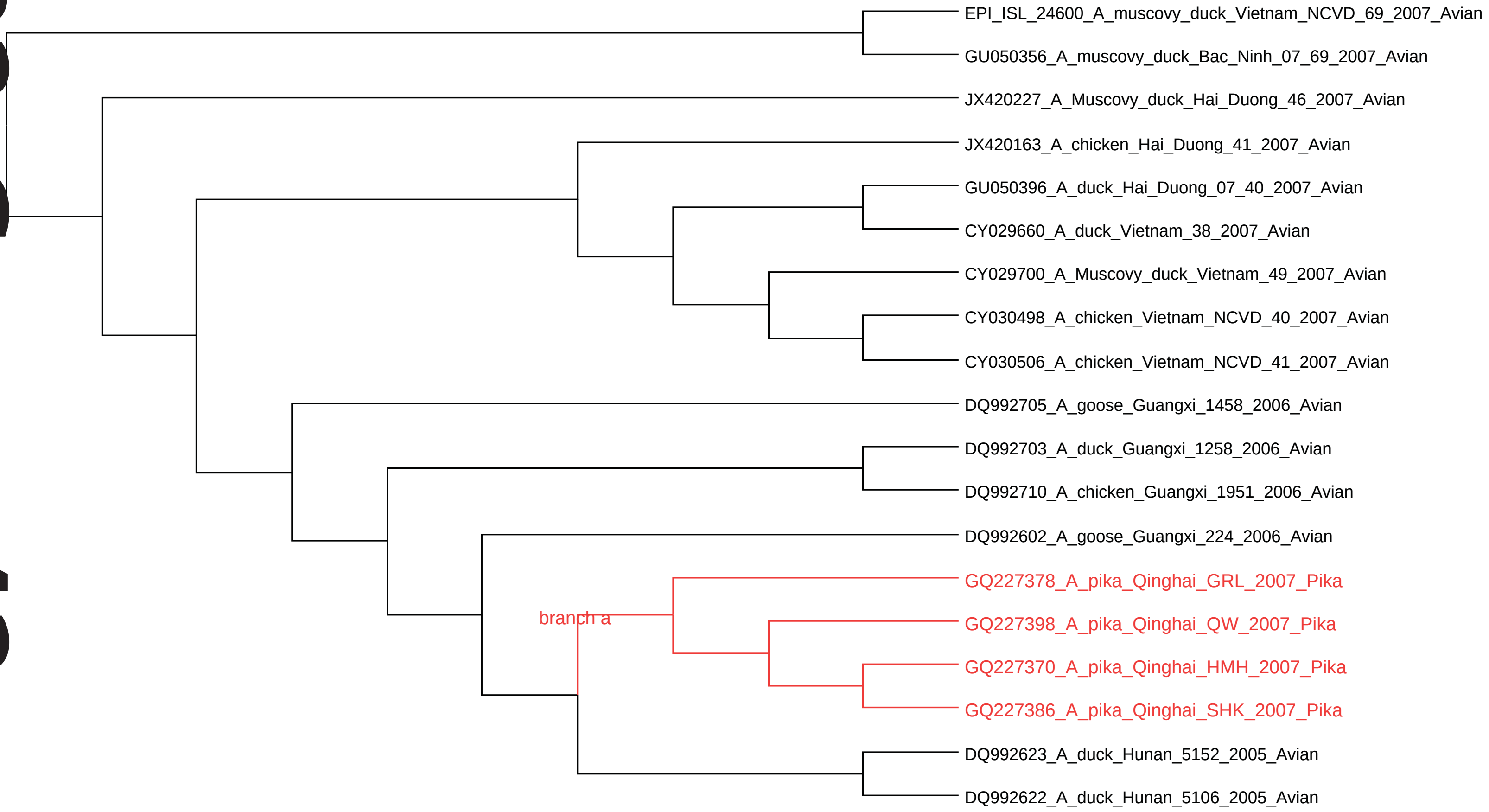

EPI_ISL_24600_A_muscovy_duck_Vietnam_NCVD_69_2007_Avian
GU050356_A_muscovy_duck_Bac_Ninh_07_69_2007_Avian
JX420227_A_Muscovy_duck_Hai_Duong_46_2007_Avian
JX420163_A_chicken_Hai_Duong_41_2007_Avian
GU050396_A_duck_Hai_Duong_07_40_2007_Avian
CY029660_A_duck_Vietnam_38_2007_Avian
CY029700_A_Muscovy_duck_Vietnam_49_2007_Avian
CY030498_A_chicken_Vietnam_NCVD_40_2007_Avian
CY030506_A_chicken_Vietnam_NCVD_41_2007_Avian
DQ992705_A_goose_Guangxi_1458_2006_Avian
DQ992703_A_duck_Guangxi_1258_2006_Avian
DQ992710_A_chicken_Guangxi_1951_2006_Avian
DQ992602_A_goose_Guangxi_224_2006_Avian
branch a
GQ227378_A_pika_Qinghai_GRL_2007_Pika
GQ227398_A_pika_Qinghai_QW_2007_Pika
GQ227370_A_pika_Qinghai_HMH_2007_Pika
GQ227386_A_pika_Qinghai_SHK_2007_Pika
DQ992623_A_duck_Hunan_5152_2005_Avian
DQ992622_A_duck_Hunan_5106_2005_Avian

PB2-Group43

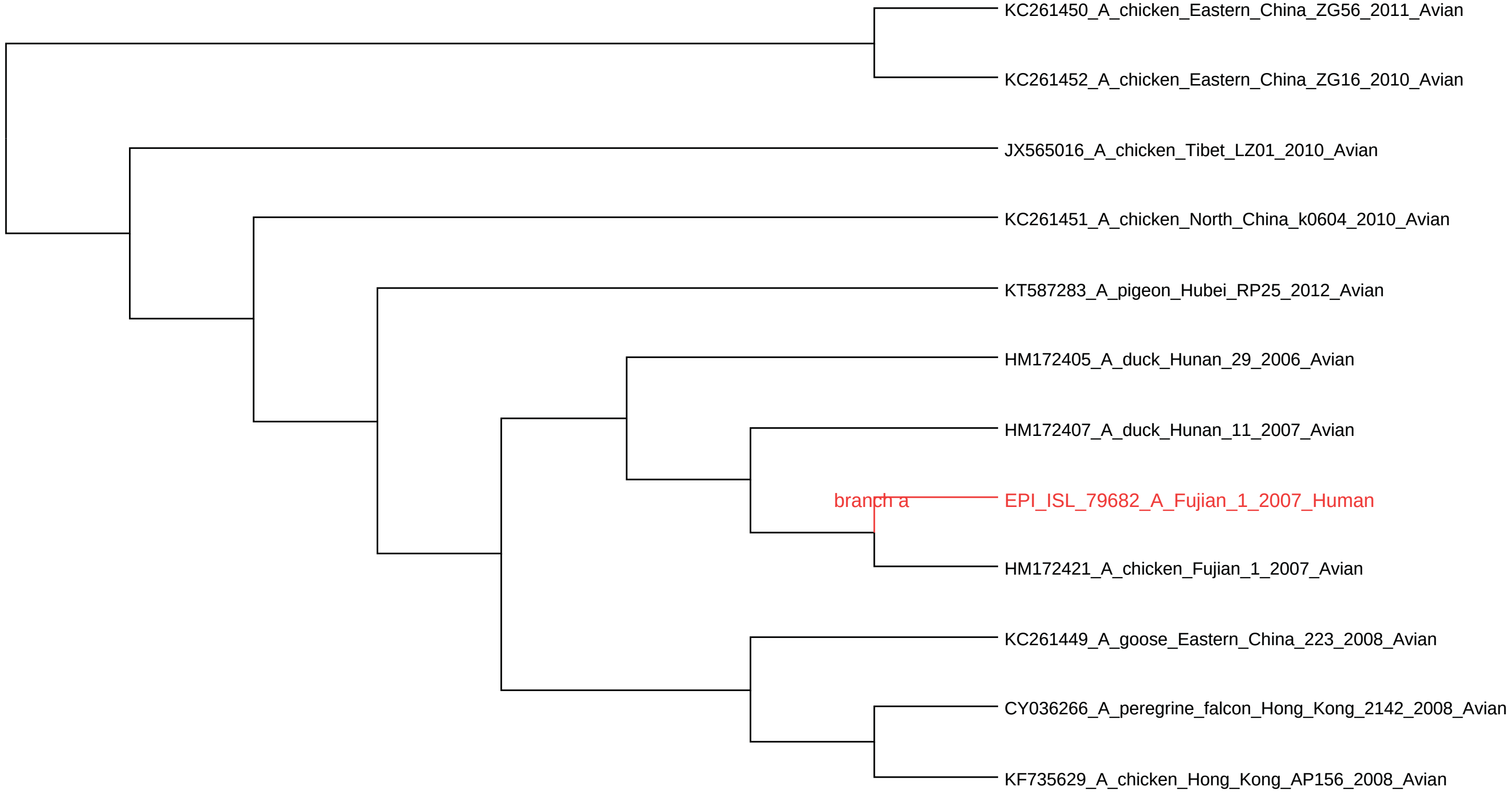

KC261450_A_chicken_Eastern_China_ZG56_2011_Avian
KC261452_A_chicken_Eastern_China_ZG16_2010_Avian
JX565016_A_chicken_Tibet_LZ01_2010_Avian
KC261451_A_chicken_North_China_k0604_2010_Avian
KT587283_A_pigeon_Hubei_RP25_2012_Avian
HM172405_A_duck_Hunan_29_2006_Avian
HM172407_A_duck_Hunan_11_2007_Avian
branch a
EPI_ISL_79682_A_Fujian_1_2007_Human
HM172421_A_chicken_Fujian_1_2007_Avian
KC261449_A_goose_Eastern_China_223_2008_Avian
CY036266_A_peregrine_falcon_Hong_Kong_2142_2008_Avian
KF735629_A_chicken_Hong_Kong_AP156_2008_Avian

PB2-Group44

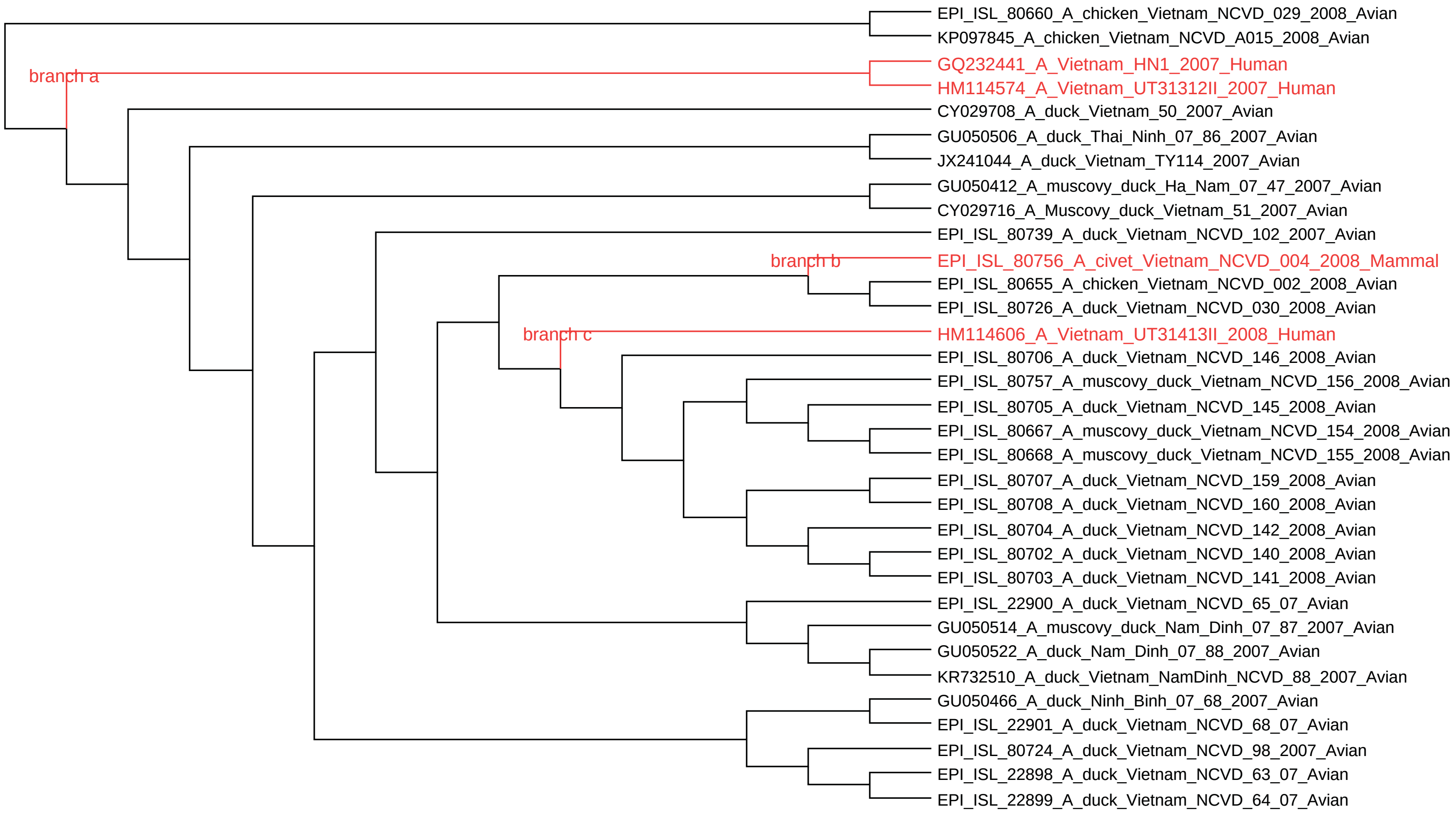

branch a
branch b
branch c
EPI_ISL_80660_A_chicken_Vietnam_NCVD_029_2008_Avian
KP097845_A_chicken_Vietnam_NCVD_A015_2008_Avian
GQ232441_A_Vietnam_HN1_2007_Human
HM114574_A_Vietnam_UT31312II_2007_Human
CY029708_A_duck_Vietnam_50_2007_Avian
GU050506_A_duck_Thai_Ninh_07_86_2007_Avian
JX241044_A_duck_Vietnam_TY114_2007_Avian
GU050412_A_muscovy_duck_Ha_Nam_07_47_2007_Avian
CY029716_A_Muscovy_duck_Vietnam_51_2007_Avian
EPI_ISL_80739_A_duck_Vietnam_NCVD_102_2007_Avian
EPI_ISL_80756_A_civet_Vietnam_NCVD_004_2008_Mammal
EPI_ISL_80655_A_chicken_Vietnam_NCVD_002_2008_Avian
EPI_ISL_80726_A_duck_Vietnam_NCVD_030_2008_Avian
HM114606_A_Vietnam_UT31413II_2008_Human
EPI_ISL_80706_A_duck_Vietnam_NCVD_146_2008_Avian
EPI_ISL_80757_A_muscovy_duck_Vietnam_NCVD_156_2008_Avian
EPI_ISL_80705_A_duck_Vietnam_NCVD_145_2008_Avian
EPI_ISL_80667_A_muscovy_duck_Vietnam_NCVD_154_2008_Avian
EPI_ISL_80668_A_muscovy_duck_Vietnam_NCVD_155_2008_Avian
EPI_ISL_80707_A_duck_Vietnam_NCVD_159_2008_Avian
EPI_ISL_80708_A_duck_Vietnam_NCVD_160_2008_Avian
EPI_ISL_80704_A_duck_Vietnam_NCVD_142_2008_Avian
EPI_ISL_80702_A_duck_Vietnam_NCVD_140_2008_Avian
EPI_ISL_80703_A_duck_Vietnam_NCVD_141_2008_Avian
EPI_ISL_22900_A_duck_Vietnam_NCVD_65_07_Avian
GU050514_A_muscovy_duck_Nam_Dinh_07_87_2007_Avian
GU050522_A_duck_Nam_Dinh_07_88_2007_Avian
KR732510_A_duck_Vietnam_NamDinh_NCVD_88_2007_Avian
GU050466_A_duck_Ninh_Binh_07_68_2007_Avian
EPI_ISL_22901_A_duck_Vietnam_NCVD_68_07_Avian
EPI_ISL_80724_A_duck_Vietnam_NCVD_98_2007_Avian
EPI_ISL_22898_A_duck_Vietnam_NCVD_63_07_Avian
EPI_ISL_22899_A_duck_Vietnam_NCVD_64_07_Avian

PB2-Group45

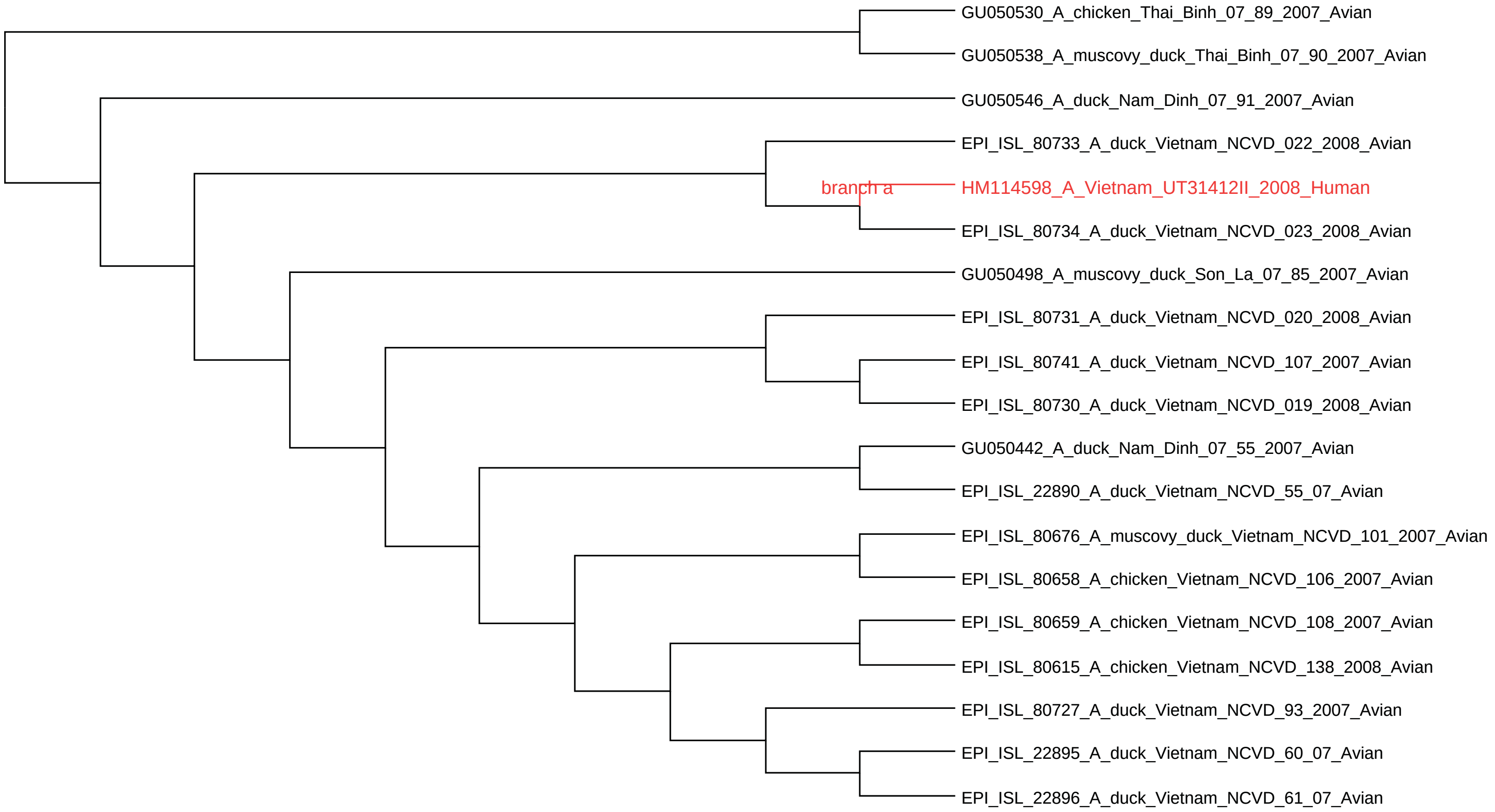

GU050530_A_chicken_Thai_Binh_07_89_2007_Avian
GU050538_A_muscovy_duck_Thai_Binh_07_90_2007_Avian
GU050546_A_duck_Nam_Dinh_07_91_2007_Avian
EPI_ISL_80733_A_duck_Vietnam_NCVD_022_2008_Avian
branch a
HM114598_A_Vietnam_UT31412II_2008_Human
EPI_ISL_80734_A_duck_Vietnam_NCVD_023_2008_Avian
GU050498_A_muscovy_duck_Son_La_07_85_2007_Avian
EPI_ISL_80731_A_duck_Vietnam_NCVD_020_2008_Avian
EPI_ISL_80741_A_duck_Vietnam_NCVD_107_2007_Avian
EPI_ISL_80730_A_duck_Vietnam_NCVD_019_2008_Avian
GU050442_A_duck_Nam_Dinh_07_55_2007_Avian
EPI_ISL_22890_A_duck_Vietnam_NCVD_55_07_Avian
EPI_ISL_80676_A_muscovy_duck_Vietnam_NCVD_101_2007_Avian
EPI_ISL_80658_A_chicken_Vietnam_NCVD_106_2007_Avian
EPI_ISL_80659_A_chicken_Vietnam_NCVD_108_2007_Avian
EPI_ISL_80615_A_chicken_Vietnam_NCVD_138_2008_Avian
EPI_ISL_80727_A_duck_Vietnam_NCVD_93_2007_Avian
EPI_ISL_22895_A_duck_Vietnam_NCVD_60_07_Avian
EPI_ISL_22896_A_duck_Vietnam_NCVD_61_07_Avian

PB2-Group46

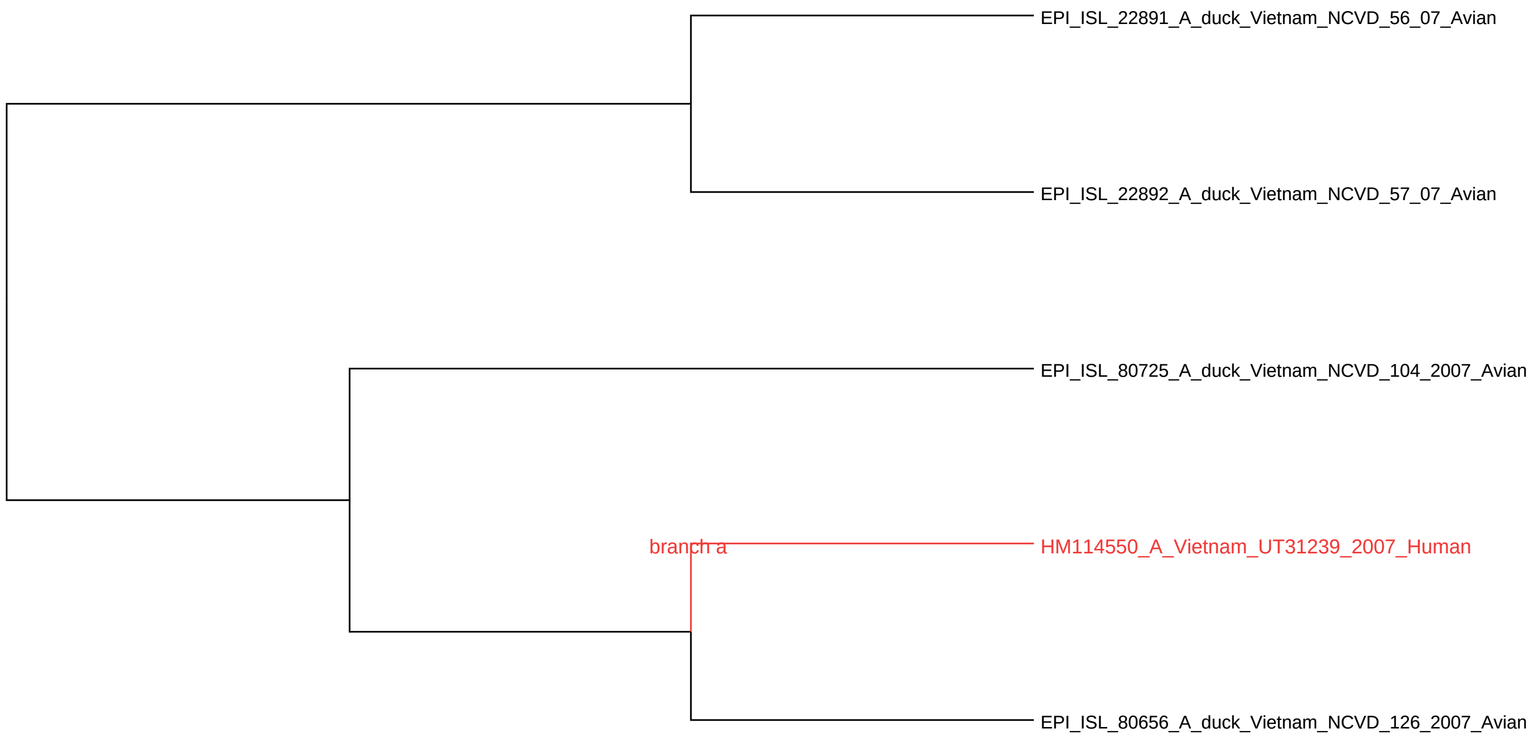
EPI_ISL_22891_A_duck_Vietnam_NCVD_56_07_Avian
EPI_ISL_22892_A_duck_Vietnam_NCVD_57_07_Avian
EPI_ISL_80725_A_duck_Vietnam_NCVD_104_2007_Avian
branch a
HM114550_A_Vietnam_UT31239_2007_Human
EPI_ISL_80656_A_duck_Vietnam_NCVD_126_2007_Avian

PB2-Group47

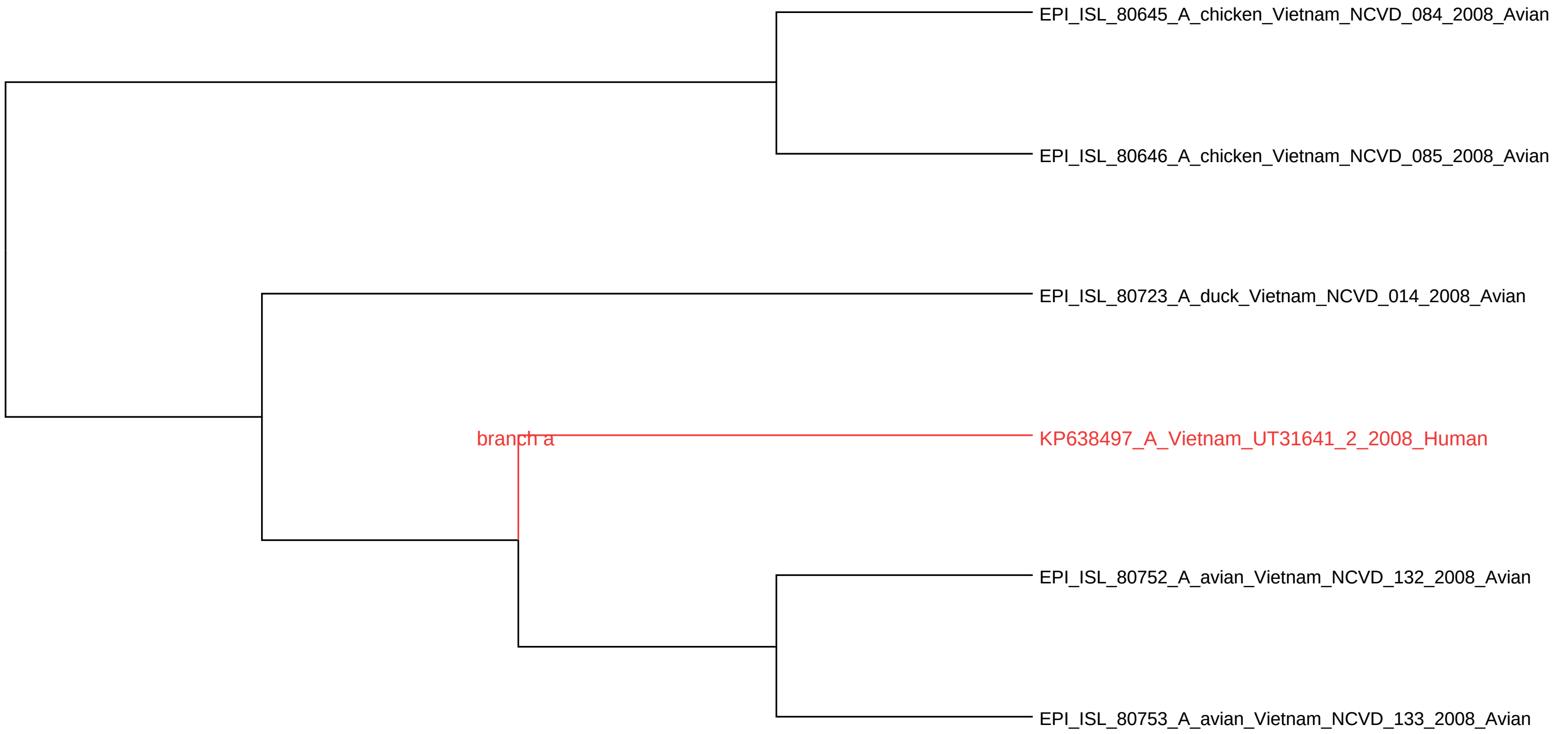
EPI_ISL_80645_A_chicken_Vietnam_NCVD_084_2008_Avian
EPI_ISL_80646_A_chicken_Vietnam_NCVD_085_2008_Avian
EPI_ISL_80723_A_duck_Vietnam_NCVD_014_2008_Avian
branch a
KP638497_A_Vietnam_UT31641_2_2008_Human
EPI_ISL_80752_A_avian_Vietnam_NCVD_132_2008_Avian
EPI_ISL_80753_A_avian_Vietnam_NCVD_133_2008_Avian

PB2-Group48

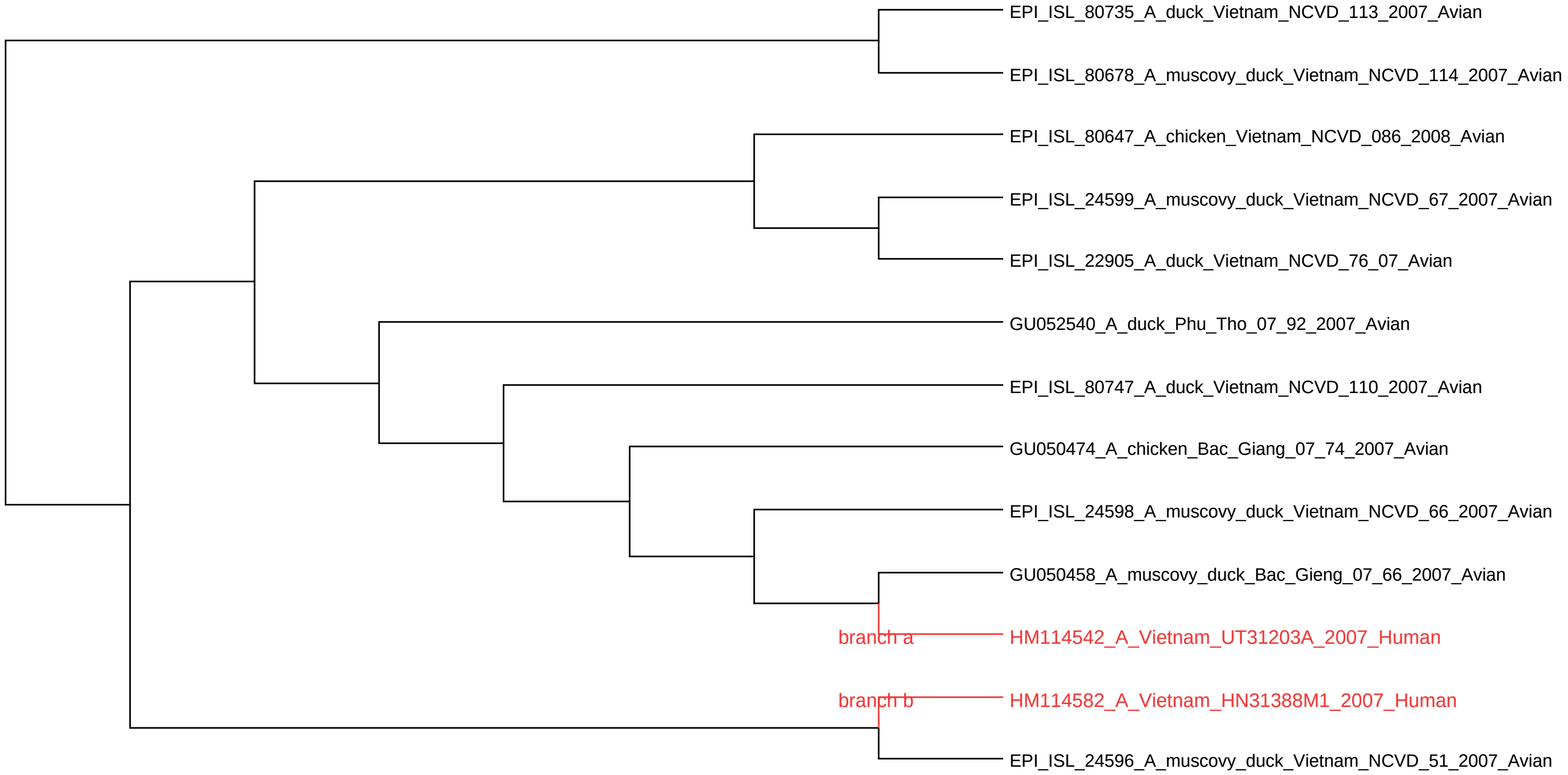

EPI_ISL_80735_A_duck_Vietnam_NCVD_113_2007_Avian
EPI_ISL_80678_A_muscovy_duck_Vietnam_NCVD_114_2007_Avian
EPI_ISL_80647_A_chicken_Vietnam_NCVD_086_2008_Avian
EPI_ISL_24599_A_muscovy_duck_Vietnam_NCVD_67_2007_Avian
EPI_ISL_22905_A_duck_Vietnam_NCVD_76_07_Avian
GU052540_A_duck_Phu_Tho_07_92_2007_Avian
EPI_ISL_80747_A_duck_Vietnam_NCVD_110_2007_Avian
GU050474_A_chicken_Bac_Giang_07_74_2007_Avian
EPI_ISL_24598_A_muscovy_duck_Vietnam_NCVD_66_2007_Avian
GU050458_A_muscovy_duck_Bac_Gieng_07_66_2007_Avian
branch a
HM114542_A_Vietnam_UT31203A_2007_Human
branch b
HM114582_A_Vietnam_HN31388M1_2007_Human
EPI_ISL_24596_A_muscovy_duck_Vietnam_NCVD_51_2007_Avian

PB2-Group49

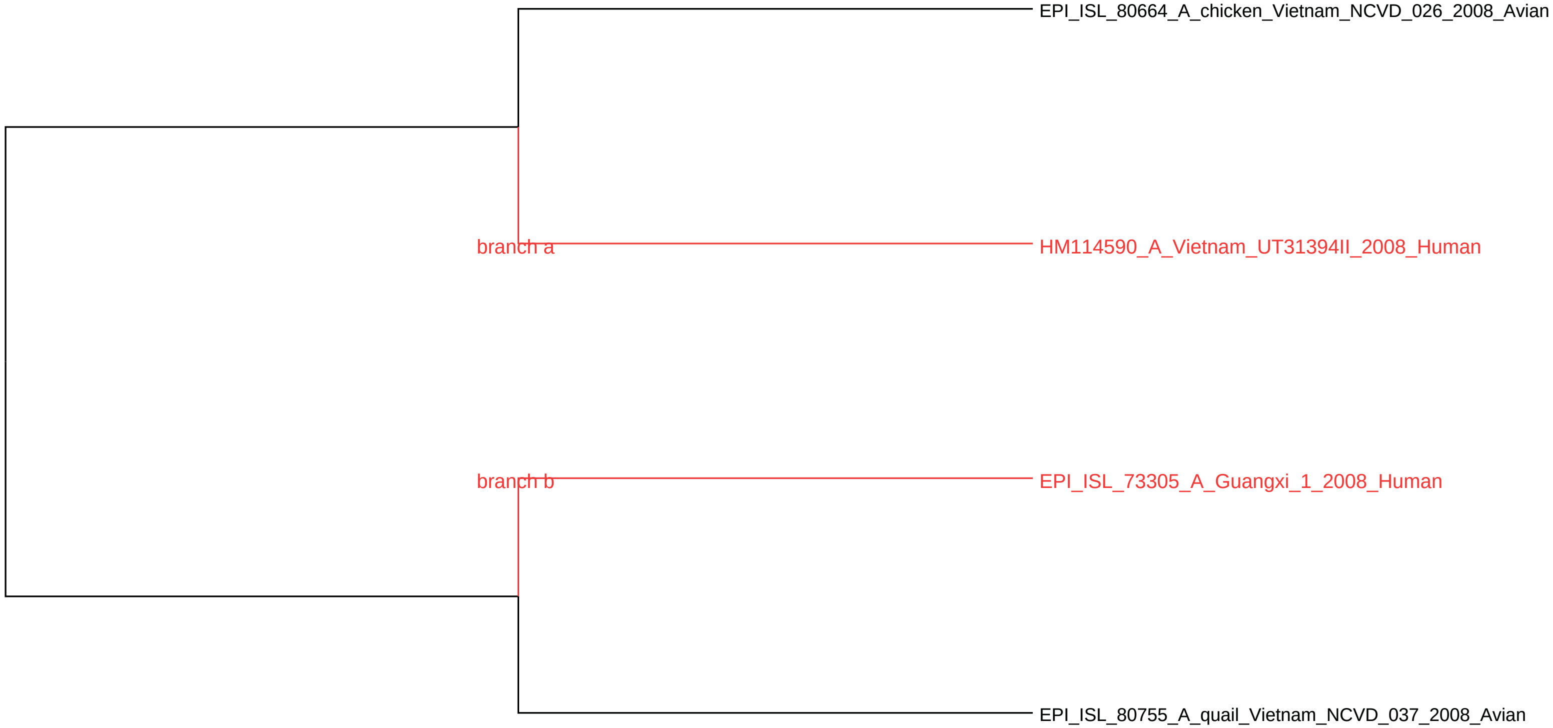

EPI_ISL_80664_A_chicken_Vietnam_NCVD_026_2008_Avian
branch a
HM114590_A_Vietnam_UT31394II_2008_Human
branch b
EPI_ISL_73305_A_Guangxi_1_2008_Human
EPI_ISL_80755_A_quail_Vietnam_NCVD_037_2008_Avian

PB2-Group50

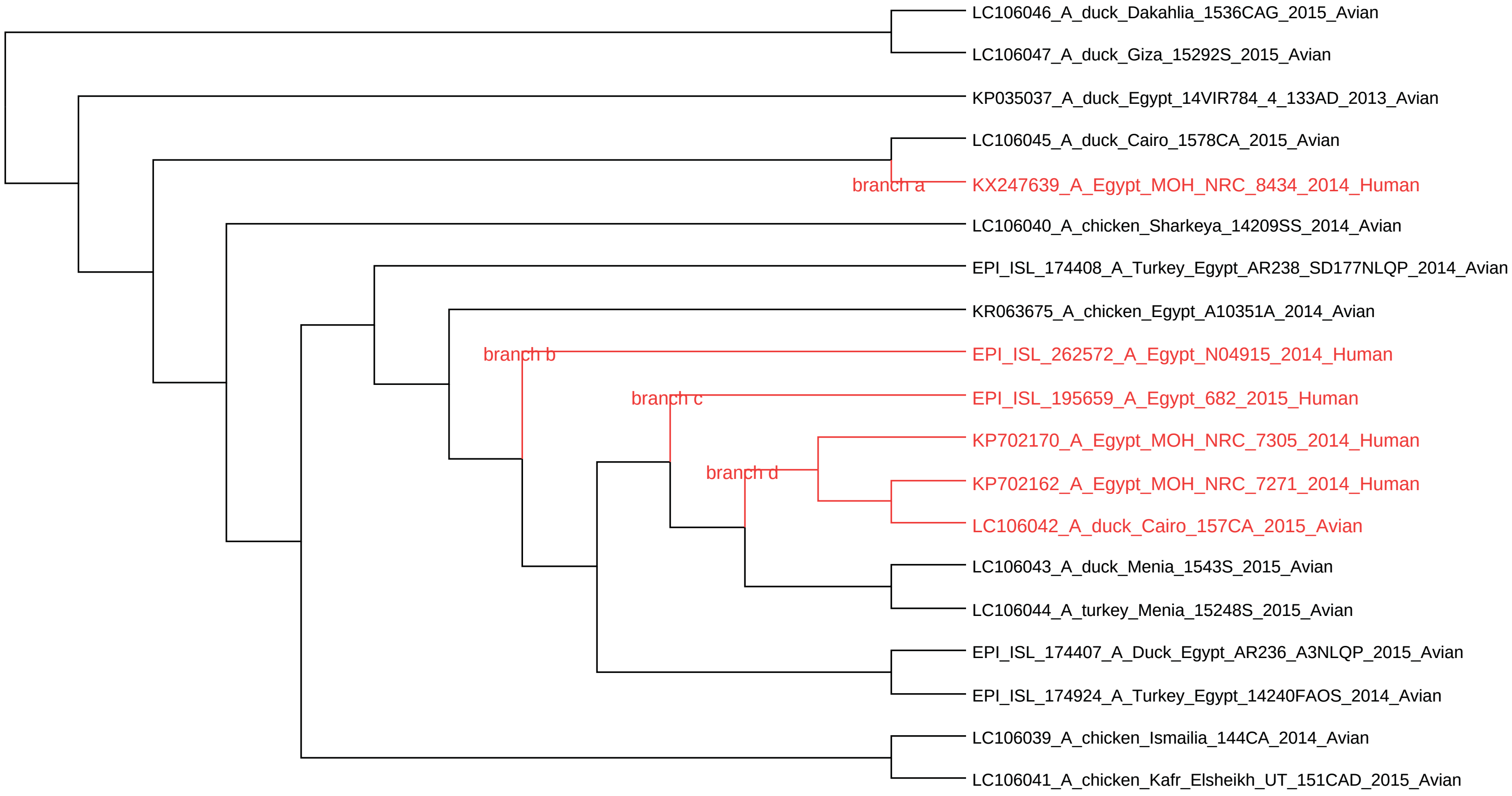
LC106046_A_duck_Dakahlia_1536CAG_2015_Avian
LC106047_A_duck_Giza_15292S_2015_Avian
KP035037_A_duck_Egypt_14VIR784_4_133AD_2013_Avian
LC106045_A_duck_Cairo_1578CA_2015_Avian
branch a
KX247639_A_Egypt_MOH_NRC_8434_2014_Human
LC106040_A_chicken_Sharkeya_14209SS_2014_Avian
EPI_ISL_174408_A_Turkey_Egypt_AR238_SD177NLQP_2014_Avian
KR063675_A_chicken_Egypt_A10351A_2014_Avian
branch b
EPI_ISL_262572_A_Egypt_N04915_2014_Human
branch c
EPI_ISL_195659_A_Egypt_682_2015_Human
KP702170_A_Egypt_MOH_NRC_7305_2014_Human
branch d
KP702162_A_Egypt_MOH_NRC_7271_2014_Human
LC106042_A_duck_Cairo_157CA_2015_Avian
LC106043_A_duck_Menia_1543S_2015_Avian
LC106044_A_turkey_Menia_15248S_2015_Avian
EPI_ISL_174407_A_Duck_Egypt_AR236_A3NLQP_2015_Avian
EPI_ISL_174924_A_Turkey_Egypt_14240FAOS_2014_Avian
LC106039_A_chicken_Ismailia_144CA_2014_Avian
LC106041_A_chicken_Kafr_Elsheikh_UT_151CAD_2015_Avian

PB2-Group51

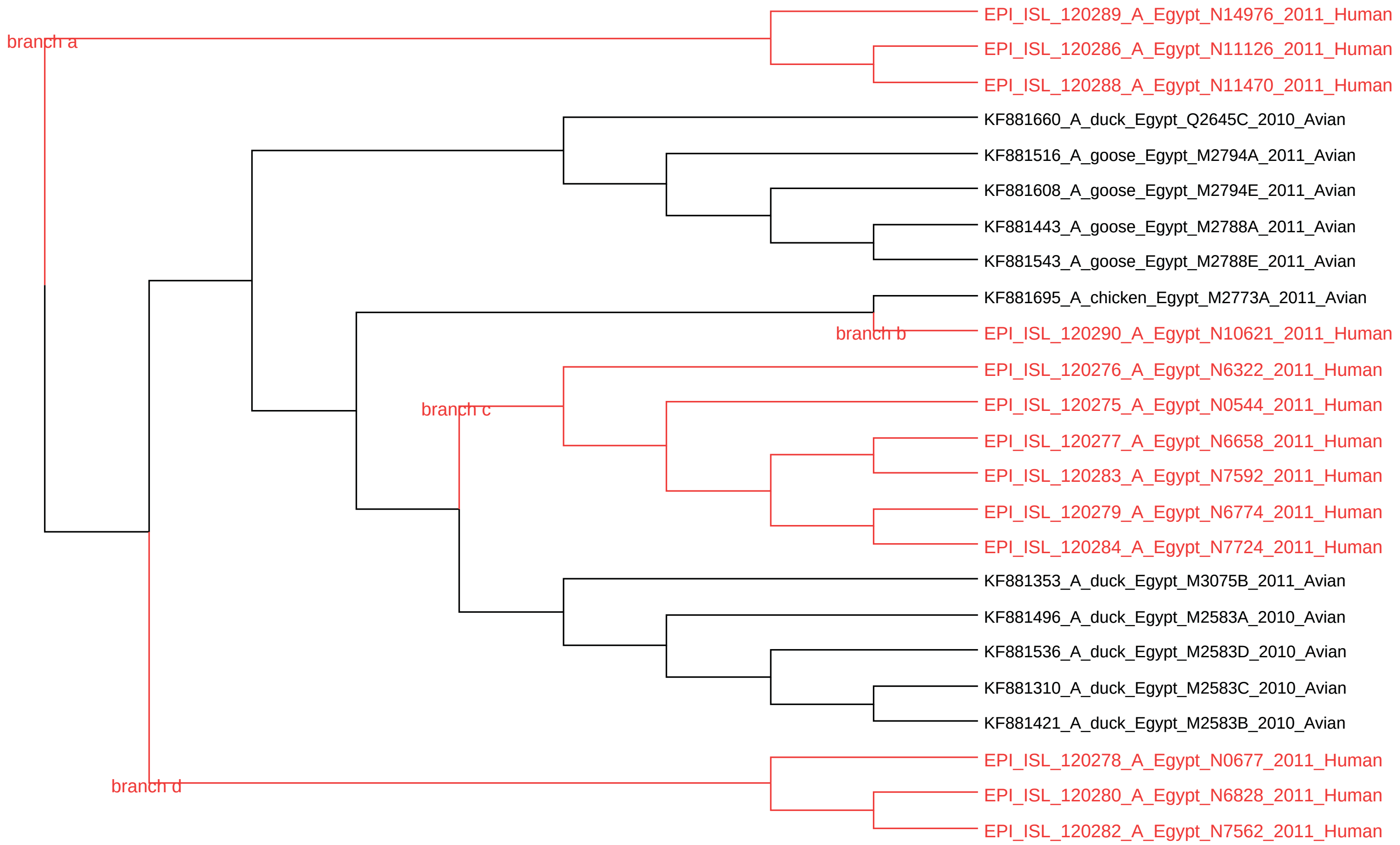

EPI_ISL_120289_A_Egypt_N14976_2011_Human
branch a
EPI_ISL_120286_A_Egypt_N11126_2011_Human
EPI_ISL_120288_A_Egypt_N11470_2011_Human
KF881660_A_duck_Egypt_Q2645C_2010_Avian
KF881516_A_goose_Egypt_M2794A_2011_Avian
KF881608_A_goose_Egypt_M2794E_2011_Avian
KF881443_A_goose_Egypt_M2788A_2011_Avian
KF881543_A_goose_Egypt_M2788E_2011_Avian
KF881695_A_chicken_Egypt_M2773A_2011_Avian
branch b
EPI_ISL_120290_A_Egypt_N10621_2011_Human
EPI_ISL_120276_A_Egypt_N6322_2011_Human
branch c
EPI_ISL_120275_A_Egypt_N0544_2011_Human
EPI_ISL_120277_A_Egypt_N6658_2011_Human
EPI_ISL_120283_A_Egypt_N7592_2011_Human
EPI_ISL_120279_A_Egypt_N6774_2011_Human
EPI_ISL_120284_A_Egypt_N7724_2011_Human
KF881353_A_duck_Egypt_M3075B_2011_Avian
KF881496_A_duck_Egypt_M2583A_2010_Avian
KF881536_A_duck_Egypt_M2583D_2010_Avian
KF881310_A_duck_Egypt_M2583C_2010_Avian
KF881421_A_duck_Egypt_M2583B_2010_Avian
EPI_ISL_120278_A_Egypt_N0677_2011_Human
branch d
EPI_ISL_120280_A_Egypt_N6828_2011_Human
EPI_ISL_120282_A_Egypt_N7562_2011_Human

PB2-Group52

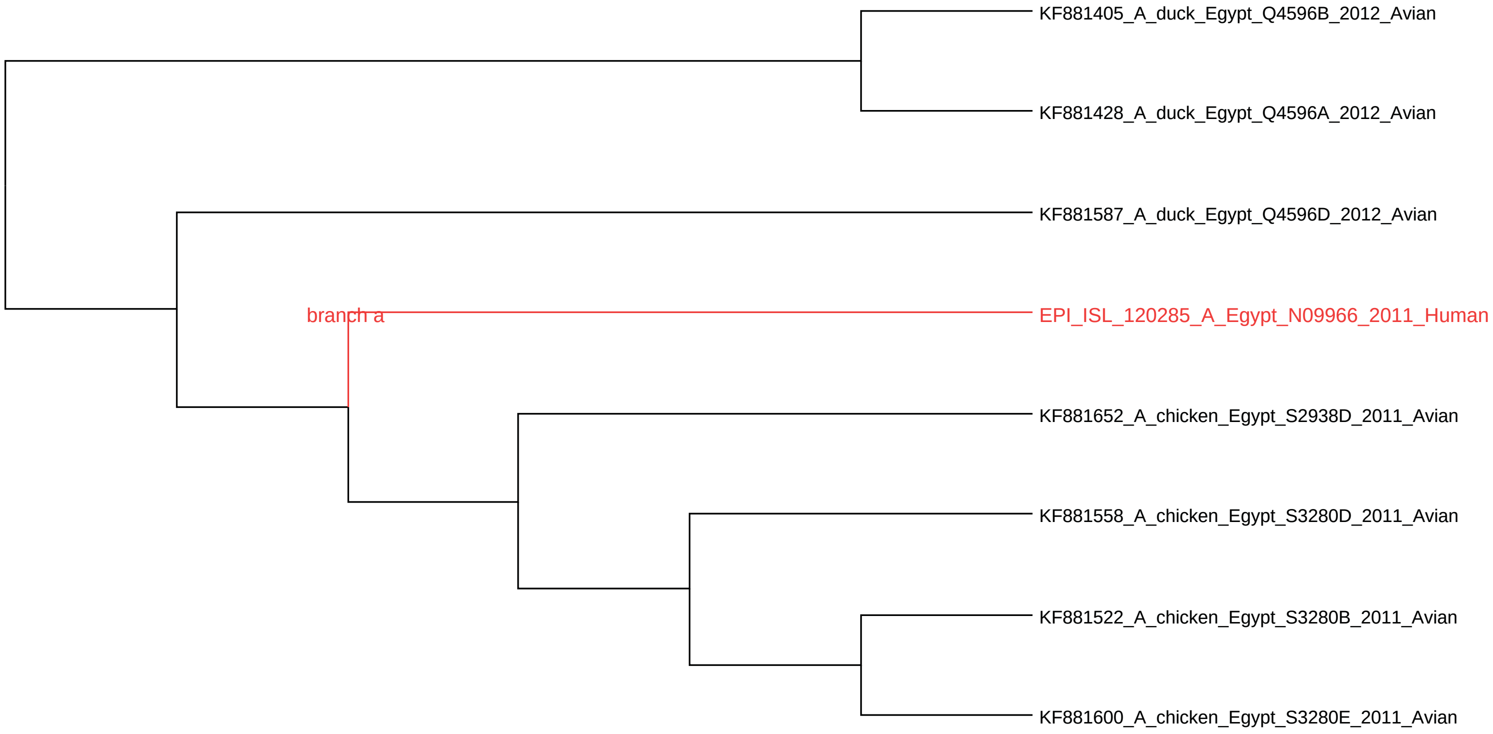
KF881405_A_duck_Egypt_Q4596B_2012_Avian
KF881428_A_duck_Egypt_Q4596A_2012_Avian
KF881587_A_duck_Egypt_Q4596D_2012_Avian
branch a
EPI_ISL_120285_A_Egypt_N09966_2011_Human
KF881652_A_chicken_Egypt_S2938D_2011_Avian
KF881558_A_chicken_Egypt_S3280D_2011_Avian
KF881522_A_chicken_Egypt_S3280B_2011_Avian
KF881600_A_chicken_Egypt_S3280E_2011_Avian

PB2-Group53

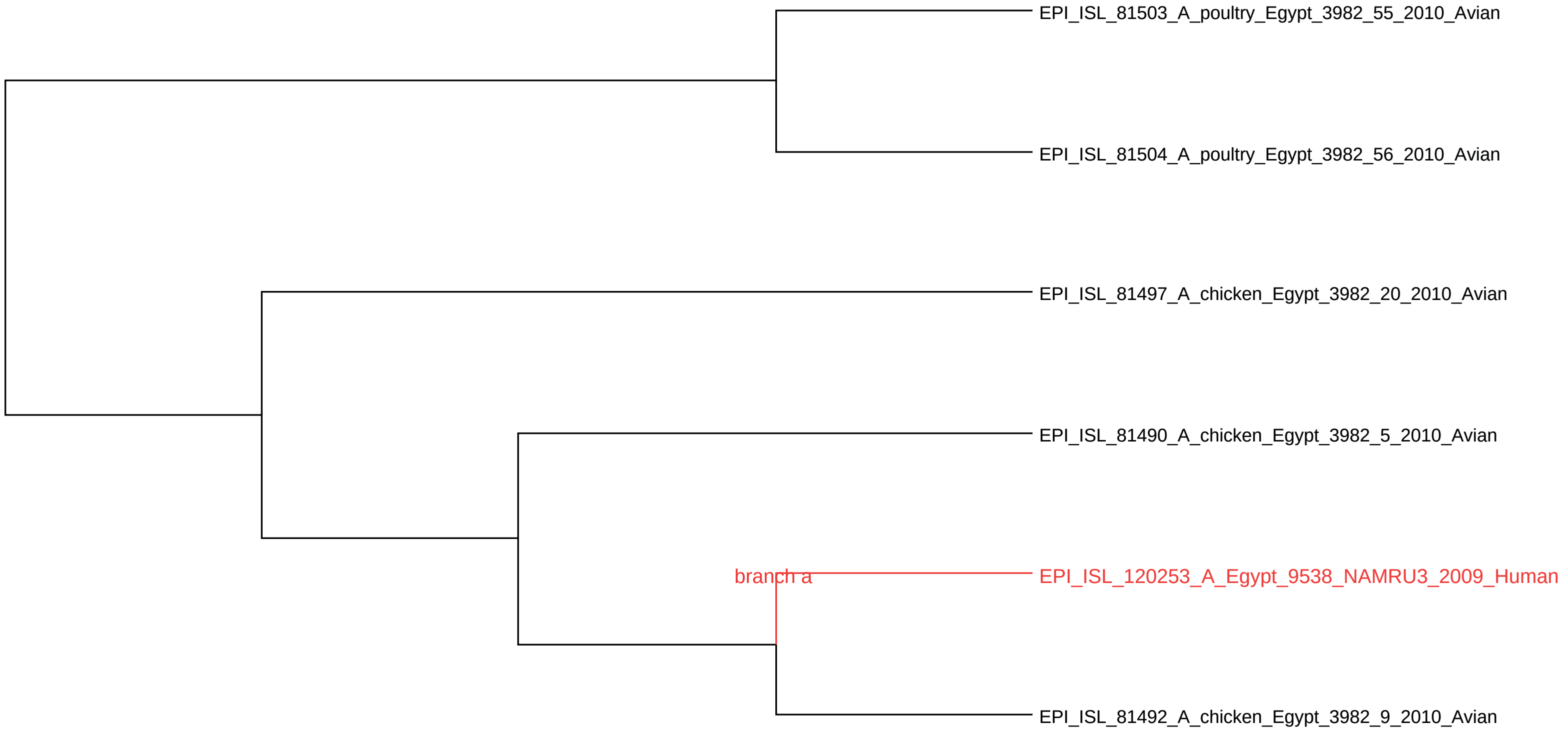

EPI_ISL_81503_A_poultry_Egypt_3982_55_2010_Avian
EPI_ISL_81504_A_poultry_Egypt_3982_56_2010_Avian
EPI_ISL_81497_A_chicken_Egypt_3982_20_2010_Avian
EPI_ISL_81490_A_chicken_Egypt_3982_5_2010_Avian
branch a
EPI_ISL_120253_A_Egypt_9538_NAMRU3_2009_Human
EPI_ISL_81492_A_chicken_Egypt_3982_9_2010_Avian

PB2-Group54

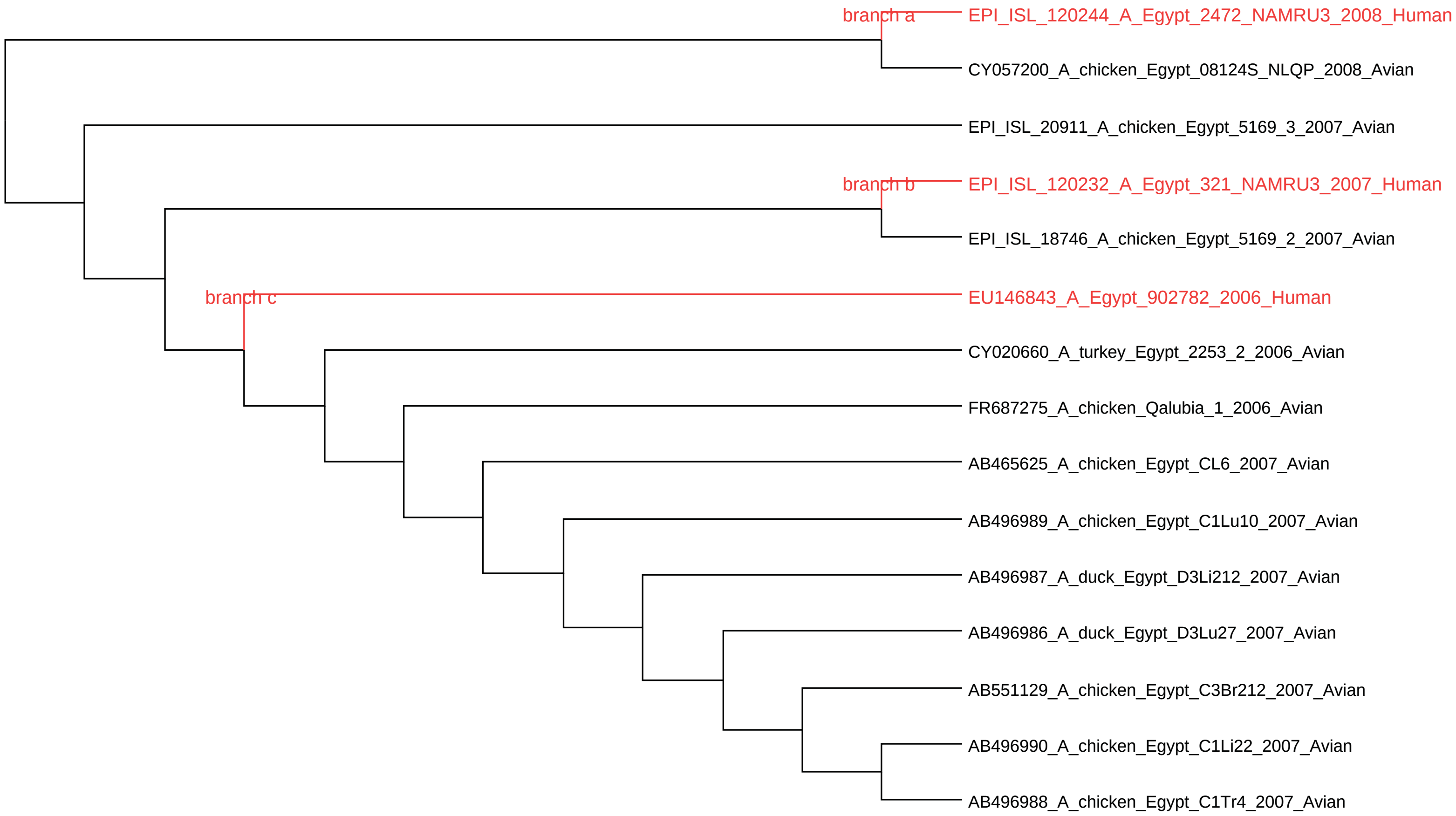
branch a
EPI_ISL_120244_A_Egypt_2472_NAMRU3_2008_Human
CY057200_A_chicken_Egypt_08124S_NLQP_2008_Avian
EPI_ISL_20911_A_chicken_Egypt_5169_3_2007_Avian
branch b
EPI_ISL_120232_A_Egypt_321_NAMRU3_2007_Human
EPI_ISL_18746_A_chicken_Egypt_5169_2_2007_Avian
branch c
EU146843_A_Egypt_902782_2006_Human
CY020660_A_turkey_Egypt_2253_2_2006_Avian
FR687275_A_chicken_Qalubia_1_2006_Avian
AB465625_A_chicken_Egypt_CL6_2007_Avian
AB496989_A_chicken_Egypt_C1Lu10_2007_Avian
AB496987_A_duck_Egypt_D3Li212_2007_Avian
AB496986_A_duck_Egypt_D3Lu27_2007_Avian
AB551129_A_chicken_Egypt_C3Br212_2007_Avian
AB496990_A_chicken_Egypt_C1Li22_2007_Avian
AB496988_A_chicken_Egypt_C1Tr4_2007_Avian

PB2-Group55

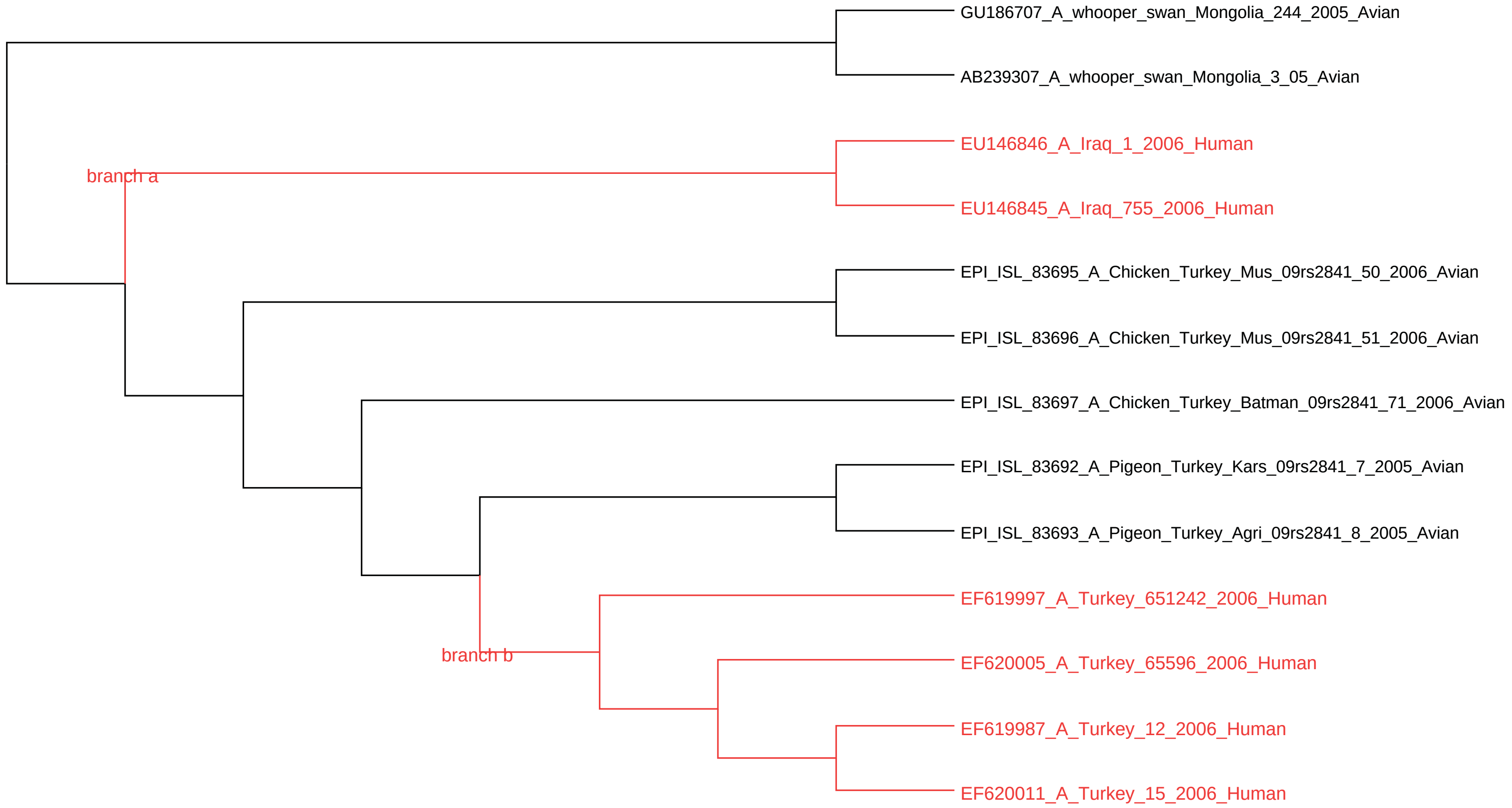

GU186707_A_whooper_swan_Mongolia_244_2005_Avian
AB239307_A_whooper_swan_Mongolia_3_05_Avian
branch a
EU146846_A_Iraq_1_2006_Human
EU146845_A_Iraq_755_2006_Human
EPI_ISL_83695_A_Chicken_Turkey_Mus_09rs2841_50_2006_Avian
EPI_ISL_83696_A_Chicken_Turkey_Mus_09rs2841_51_2006_Avian
EPI_ISL_83697_A_Chicken_Turkey_Batman_09rs2841_71_2006_Avian
EPI_ISL_83692_A_Pigeon_Turkey_Kars_09rs2841_7_2005_Avian
EPI_ISL_83693_A_Pigeon_Turkey_Agri_09rs2841_8_2005_Avian
EF619997_A_Turkey_651242_2006_Human
branch b
EF620005_A_Turkey_65596_2006_Human
EF619987_A_Turkey_12_2006_Human
EF620011_A_Turkey_15_2006_Human

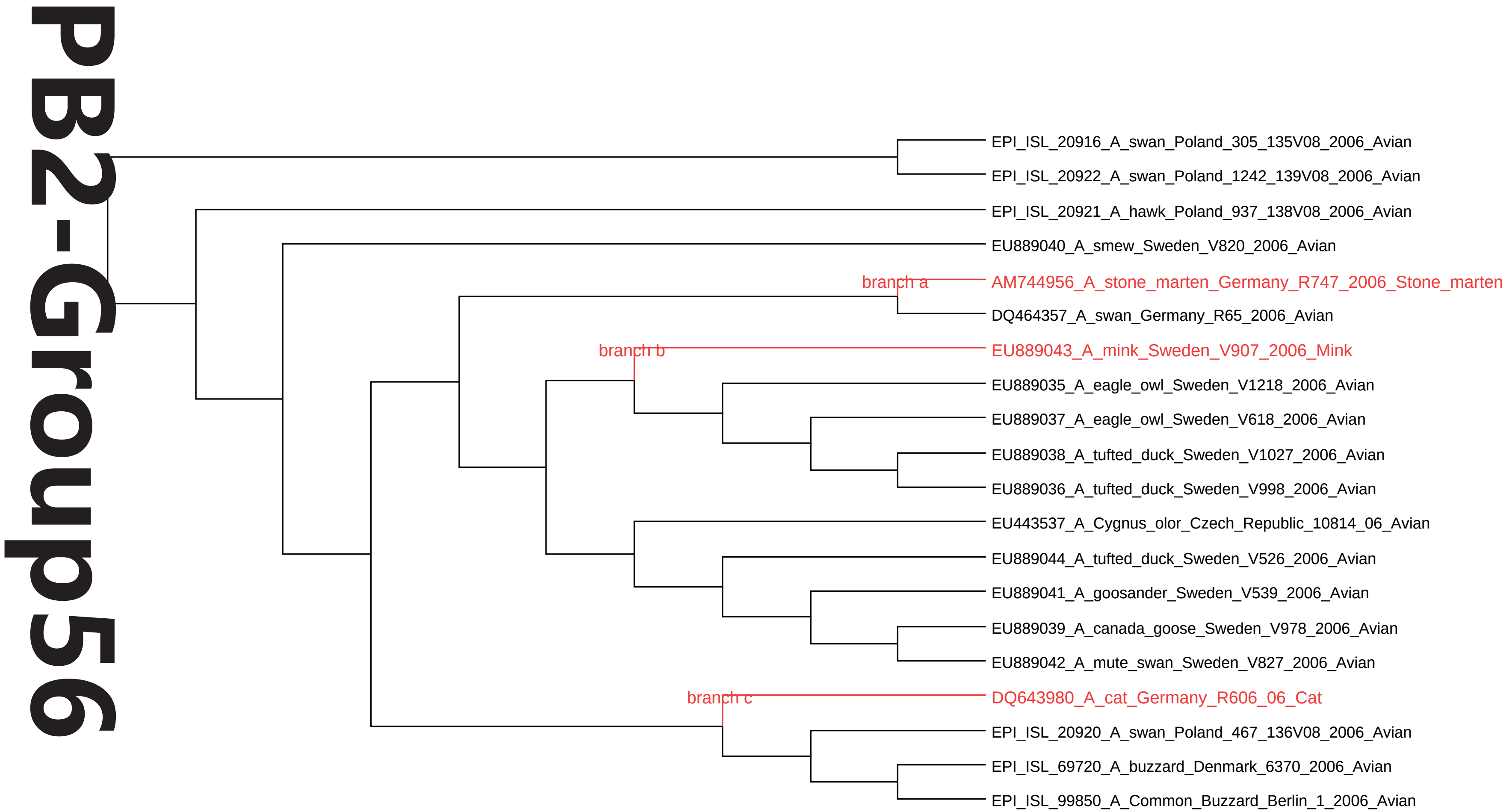
PB2-Group56
EPI_ISL_20916_A_swan_Poland_305_135V08_2006_Avian
EPI_ISL_20922_A_swan_Poland_1242_139V08_2006_Avian
EPI_ISL_20921_A_hawk_Poland_937_138V08_2006_Avian
EU889040_A_smew_Sweden_V820_2006_Avian
branch a
AM744956_A_stone_marten_Germany_R747_2006_Stone_marten
DQ464357_A_swan_Germany_R65_2006_Avian
branch b
EU889043_A_mink_Sweden_V907_2006_Mink
EU889035_A_eagle_owl_Sweden_V1218_2006_Avian
EU889037_A_eagle_owl_Sweden_V618_2006_Avian
EU889038_A_tufted_duck_Sweden_V1027_2006_Avian
EU889036_A_tufted_duck_Sweden_V998_2006_Avian
EU443537_A_Cygnus_olor_Czech_Republic_10814_06_Avian
EU889044_A_tufted_duck_Sweden_V526_2006_Avian
EU889041_A_goosander_Sweden_V539_2006_Avian
EU889039_A_canada_goose_Sweden_V978_2006_Avian
EU889042_A_mute_swan_Sweden_V827_2006_Avian
branch c
DQ643980_A_cat_Germany_R606_06_Cat
EPI_ISL_20920_A_swan_Poland_467_136V08_2006_Avian
EPI_ISL_69720_A_buzzard_Denmark_6370_2006_Avian
EPI_ISL_99850_A_Common_Buzzard_Berlin_1_2006_Avian

PB2-Group57

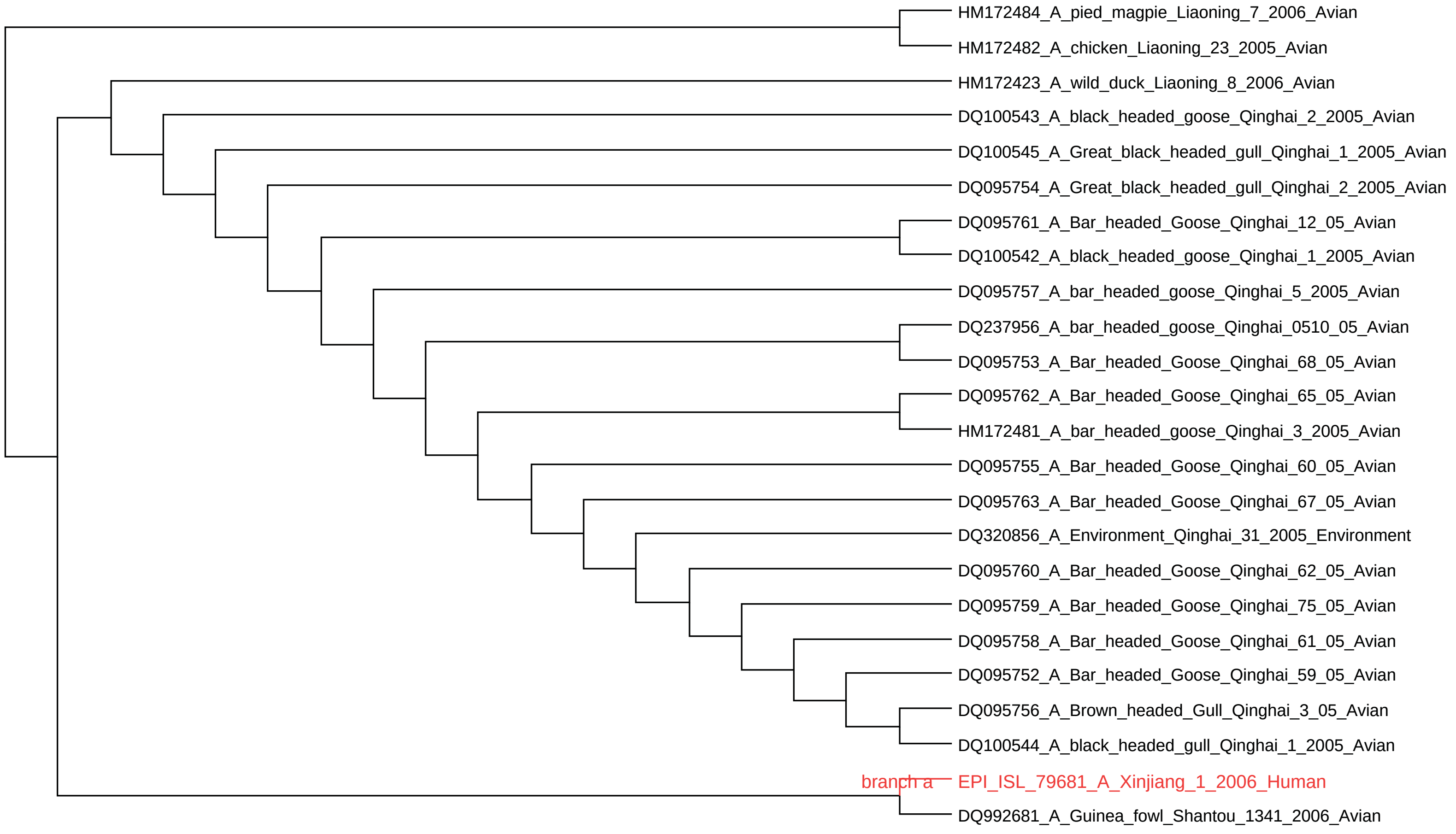

HM172484_A_pied_magpie_Liaoning_7_2006_Avian
HM172482_A_chicken_Liaoning_23_2005_Avian
HM172423_A_wild_duck_Liaoning_8_2006_Avian
DQ100543_A_black_headed_goose_Qinghai_2_2005_Avian
DQ100545_A_Great_black_headed_gull_Qinghai_1_2005_Avian
DQ095754_A_Great_black_headed_gull_Qinghai_2_2005_Avian
DQ095761_A_Bar_headed_Goose_Qinghai_12_05_Avian
DQ100542_A_black_headed_goose_Qinghai_1_2005_Avian
DQ095757_A_bar_headed_goose_Qinghai_5_2005_Avian
DQ237956_A_bar_headed_goose_Qinghai_0510_05_Avian
DQ095753_A_Bar_headed_Goose_Qinghai_68_05_Avian
DQ095762_A_Bar_headed_Goose_Qinghai_65_05_Avian
HM172481_A_bar_headed_goose_Qinghai_3_2005_Avian
DQ095755_A_Bar_headed_Goose_Qinghai_60_05_Avian
DQ095763_A_Bar_headed_Goose_Qinghai_67_05_Avian
DQ320856_A_Environment_Qinghai_31_2005_Environment
DQ095760_A_Bar_headed_Goose_Qinghai_62_05_Avian
DQ095759_A_Bar_headed_Goose_Qinghai_75_05_Avian
DQ095758_A_Bar_headed_Goose_Qinghai_61_05_Avian
DQ095752_A_Bar_headed_Goose_Qinghai_59_05_Avian
DQ095756_A_Brown_headed_Gull_Qinghai_3_05_Avian
DQ100544_A_black_headed_gull_Qinghai_1_2005_Avian
branch a
EPI_ISL_79681_A_Xinjiang_1_2006_Human
DQ992681_A_Guinea_fowl_Shantou_1341_2006_Avian

PB2-Group58

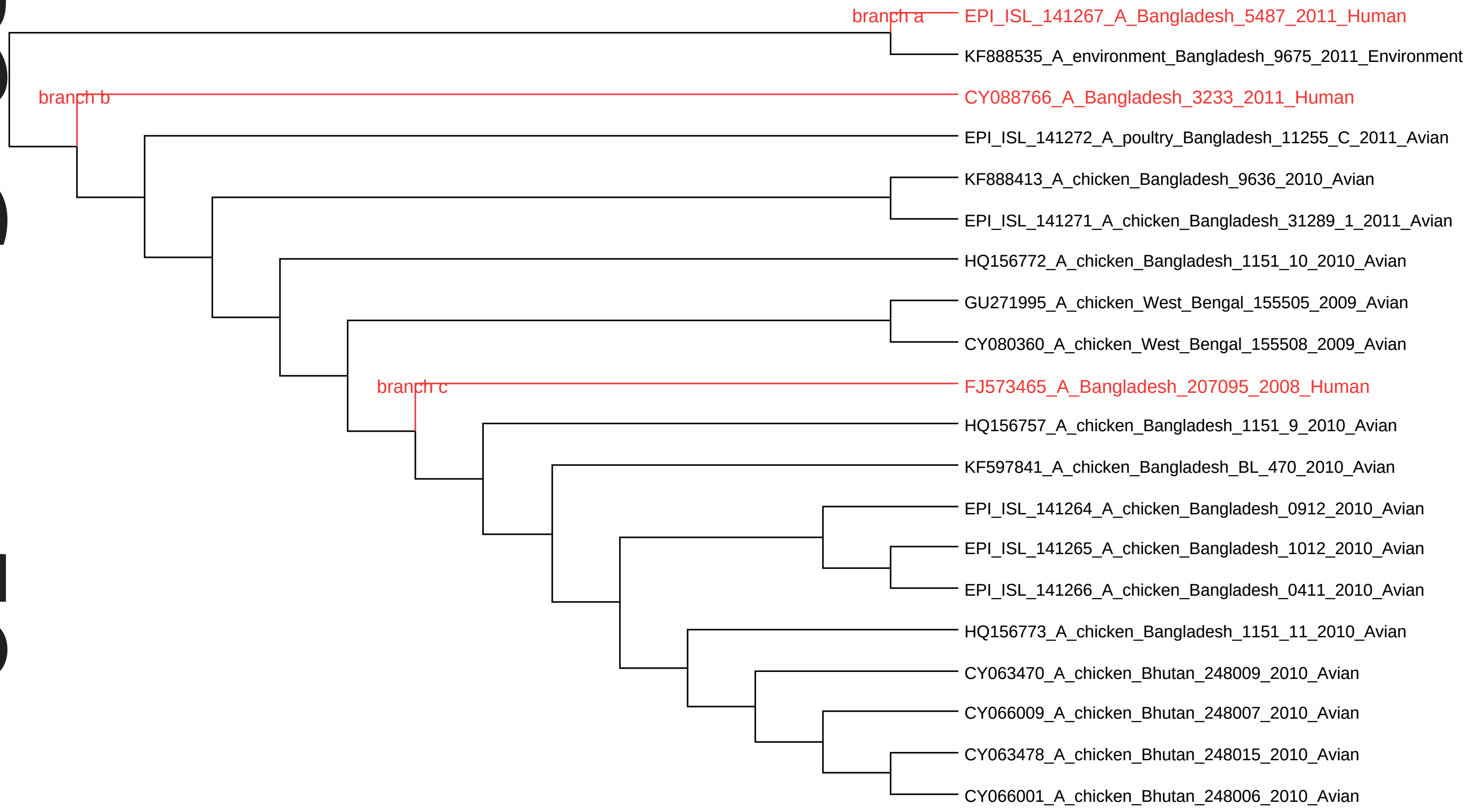
branch a
EPI_ISL_141267_A_Bangladesh_5487_2011_Human
KF888535_A_environment_Bangladesh_9675_2011_Environment
branch b
CY088766_A_Bangladesh_3233_2011_Human
EPI_ISL_141272_A_poultry_Bangladesh_11255_C_2011_Avian
KF888413_A_chicken_Bangladesh_9636_2010_Avian
EPI_ISL_141271_A_chicken_Bangladesh_31289_1_2011_Avian
HQ156772_A_chicken_Bangladesh_1151_10_2010_Avian
GU271995_A_chicken_West_Bengal_155505_2009_Avian
CY080360_A_chicken_West_Bengal_155508_2009_Avian
branch c
FJ573465_A_Bangladesh_207095_2008_Human
HQ156757_A_chicken_Bangladesh_1151_9_2010_Avian
KF597841_A_chicken_Bangladesh_BL_470_2010_Avian
EPI_ISL_141264_A_chicken_Bangladesh_0912_2010_Avian
EPI_ISL_141265_A_chicken_Bangladesh_1012_2010_Avian
EPI_ISL_141266_A_chicken_Bangladesh_0411_2010_Avian
HQ156773_A_chicken_Bangladesh_1151_11_2010_Avian
CY063470_A_chicken_Bhutan_248009_2010_Avian
CY066009_A_chicken_Bhutan_248007_2010_Avian
CY063478_A_chicken_Bhutan_248015_2010_Avian
CY066001_A_chicken_Bhutan_248006_2010_Avian

PB2-Group59

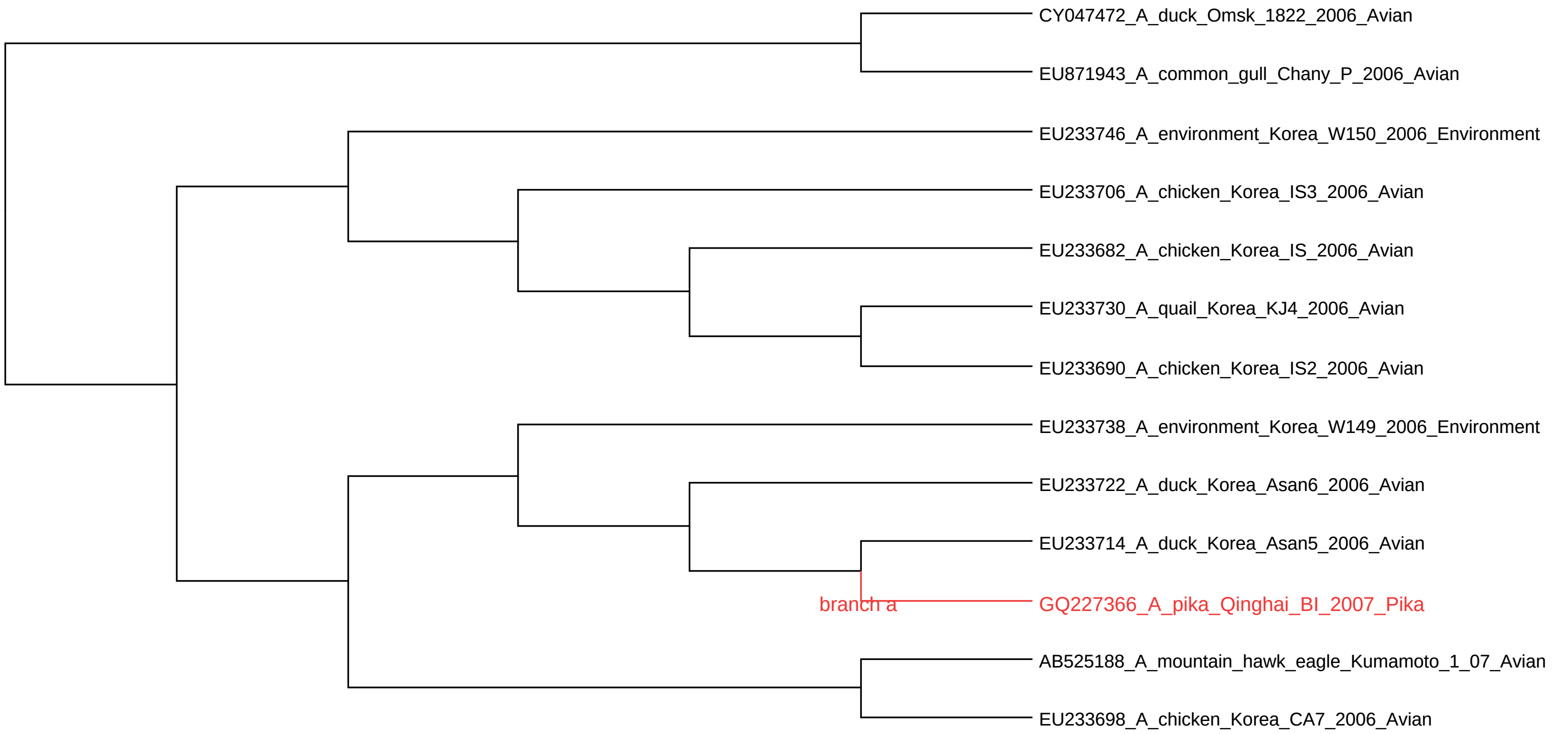
CY047472_A_duck_Omsk_1822_2006_Avian
EU871943_A_common_gull_Chany_P_2006_Avian
EU233746_A_environment_Korea_W150_2006_Environment
EU233706_A_chicken_Korea_IS3_2006_Avian
EU233682_A_chicken_Korea_IS_2006_Avian
EU233730_A_quail_Korea_KJ4_2006_Avian
EU233690_A_chicken_Korea_IS2_2006_Avian
EU233738_A_environment_Korea_W149_2006_Environment
EU233722_A_duck_Korea_Asan6_2006_Avian
EU233714_A_duck_Korea_Asan5_2006_Avian
branch a
GQ227366_A_pika_Qinghai_BI_2007_Pika
AB525188_A_mountain_hawk_eagle_Kumamoto_1_07_Avian
EU233698_A_chicken_Korea_CA7_2006_Avian

PB2-Group60

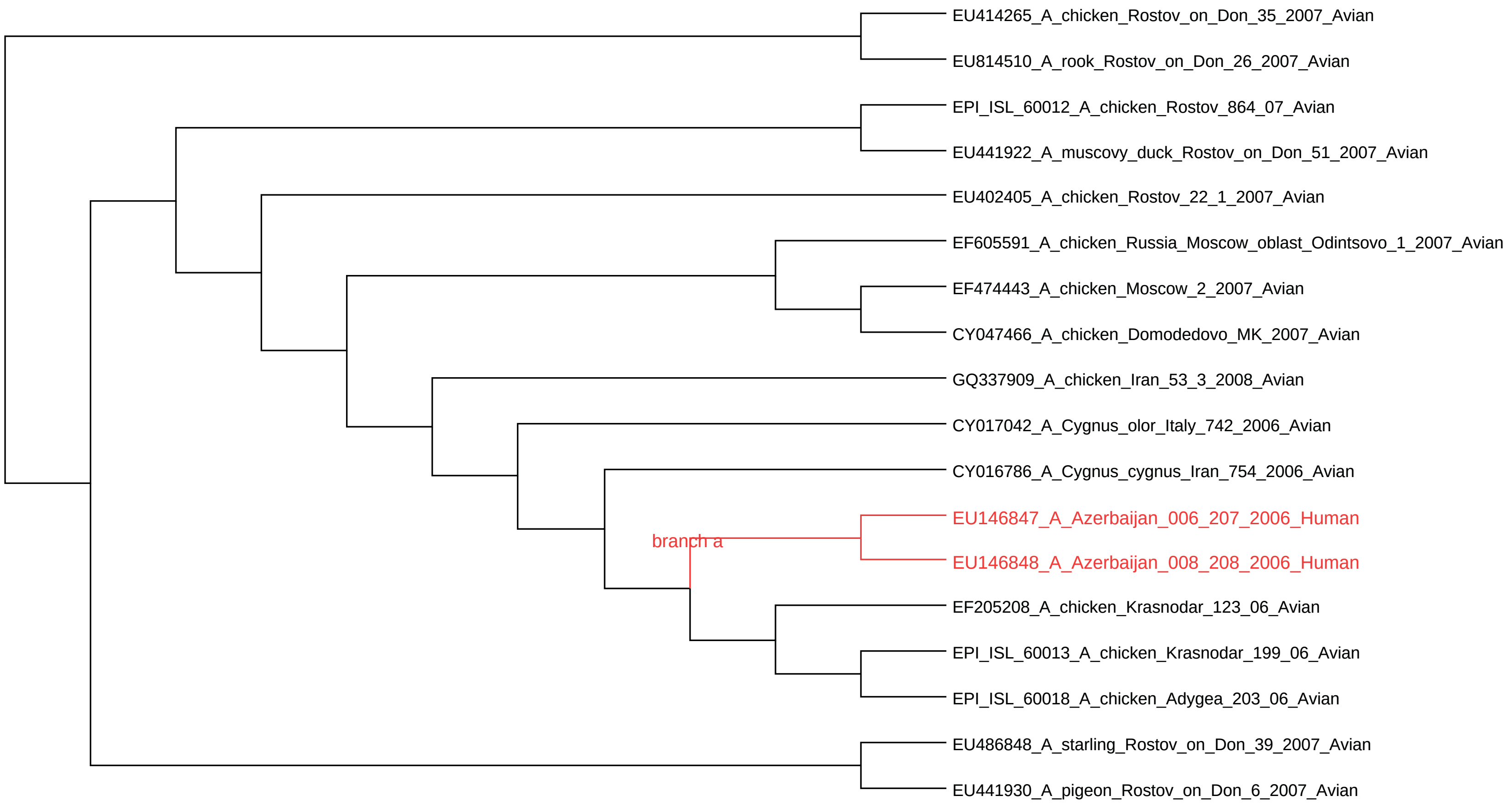
EU414265_A_chicken_Rostov_on_Don_35_2007_Avian
EU814510_A_rook_Rostov_on_Don_26_2007_Avian
EPI_ISL_60012_A_chicken_Rostov_864_07_Avian
EU441922_A_muscovy_duck_Rostov_on_Don_51_2007_Avian
EU402405_A_chicken_Rostov_22_1_2007_Avian
EF605591_A_chicken_Russia_Moscow_oblast_Odintsovo_1_2007_Avian
EF474443_A_chicken_Moscow_2_2007_Avian
CY047466_A_chicken_Domodedovo_MK_2007_Avian
GQ337909_A_chicken_Iran_53_3_2008_Avian
CY017042_A_Cygnus_olor_Italy_742_2006_Avian
CY016786_A_Cygnus_cygnus_Iran_754_2006_Avian
EU146847_A_Azerbaijan_006_207_2006_Human
branch a
EU146848_A_Azerbaijan_008_208_2006_Human
EF205208_A_chicken_Krasnodar_123_06_Avian
EPI_ISL_60013_A_chicken_Krasnodar_199_06_Avian
EPI_ISL_60018_A_chicken_Adygea_203_06_Avian
EU486848_A_starling_Rostov_on_Don_39_2007_Avian
EU441930_A_pigeon_Rostov_on_Don_6_2007_Avian

PB2-Group61

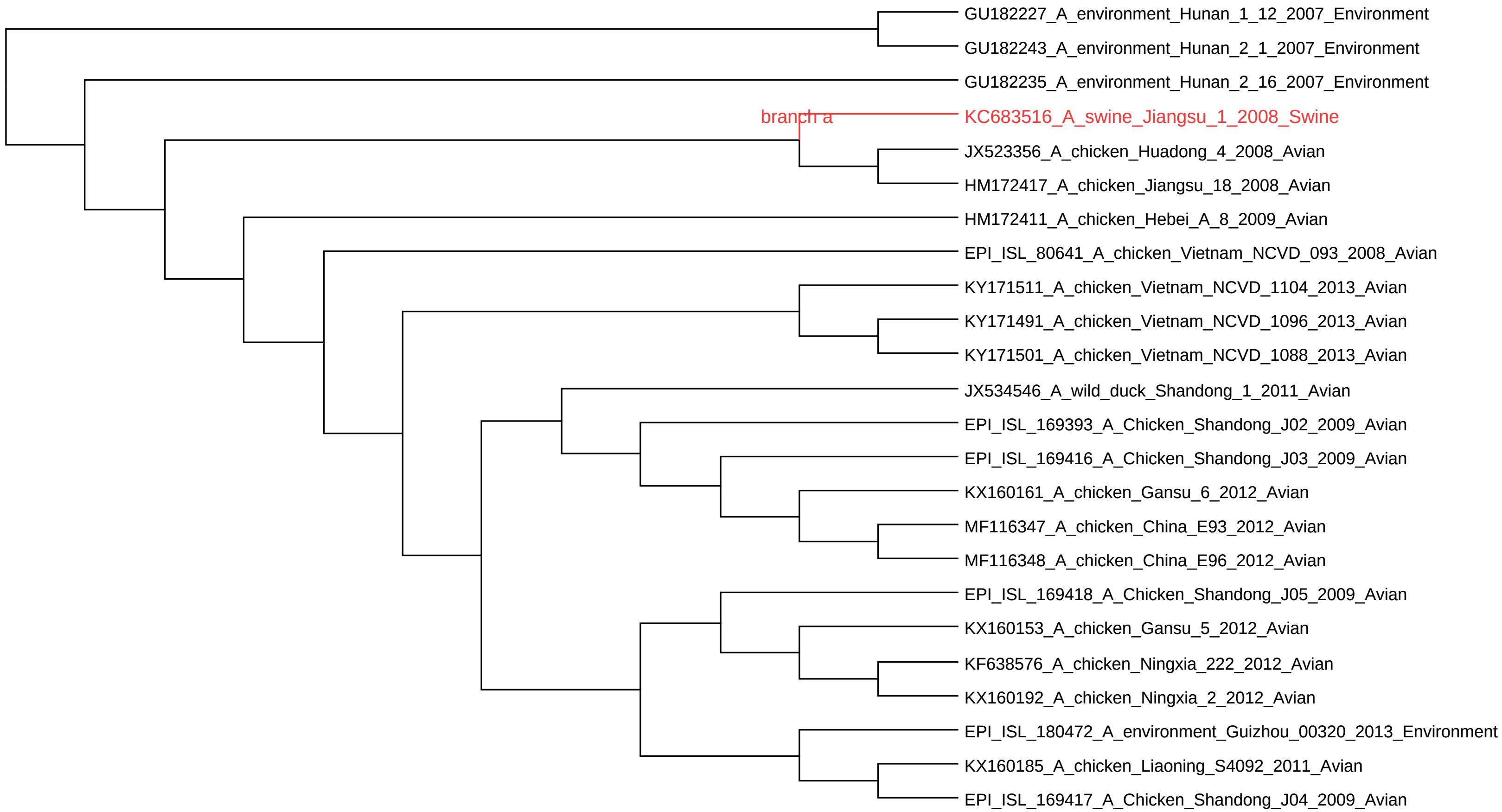
GU182227_A_environment_Hunan_1_12_2007_Environment
GU182243_A_environment_Hunan_2_1_2007_Environment
GU182235_A_environment_Hunan_2_16_2007_Environment
branch a
KC683516_A_swine_Jiangsu_1_2008_Swine
JX523356_A_chicken_Huadong_4_2008_Avian
HM172417_A_chicken_Jiangsu_18_2008_Avian
HM172411_A_chicken_Hebei_A_8_2009_Avian
EPI_ISL_80641_A_chicken_Vietnam_NCVD_093_2008_Avian
KY171511_A_chicken_Vietnam_NCVD_1104_2013_Avian
KY171491_A_chicken_Vietnam_NCVD_1096_2013_Avian
KY171501_A_chicken_Vietnam_NCVD_1088_2013_Avian
JX534546_A_wild_duck_Shandong_1_2011_Avian
EPI_ISL_169393_A_Chicken_Shandong_J02_2009_Avian
EPI_ISL_169416_A_Chicken_Shandong_J03_2009_Avian
KX160161_A_chicken_Gansu_6_2012_Avian
MF116347_A_chicken_China_E93_2012_Avian
MF116348_A_chicken_China_E96_2012_Avian
EPI_ISL_169418_A_Chicken_Shandong_J05_2009_Avian
KX160153_A_chicken_Gansu_5_2012_Avian
KF638576_A_chicken_Ningxia_222_2012_Avian
KX160192_A_chicken_Ningxia_2_2012_Avian
EPI_ISL_180472_A_environment_Guizhou_00320_2013_Environment
KX160185_A_chicken_Liaoning_S4092_2011_Avian
EPI_ISL_169417_A_Chicken_Shandong_J04_2009_Avian

PB2-Group62

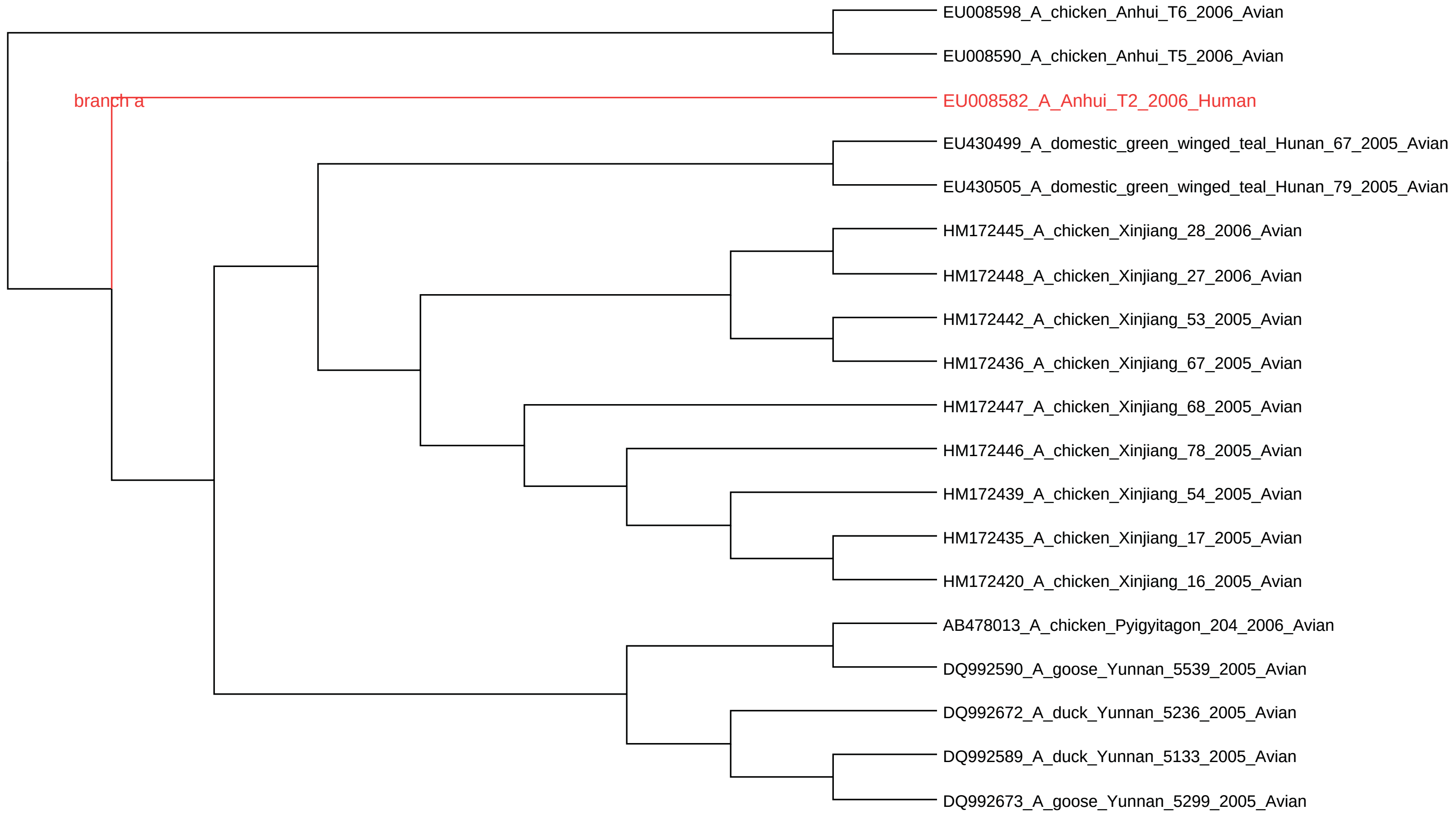

EU008598_A_chicken_Anhui_T6_2006_Avian
EU008590_A_chicken_Anhui_T5_2006_Avian
branch a
EU008582_A_Anhui_T2_2006_Human
EU430499_A_domestic_green_winged_teal_Hunan_67_2005_Avian
EU430505_A_domestic_green_winged_teal_Hunan_79_2005_Avian
HM172445_A_chicken_Xinjiang_28_2006_Avian
HM172448_A_chicken_Xinjiang_27_2006_Avian
HM172442_A_chicken_Xinjiang_53_2005_Avian
HM172436_A_chicken_Xinjiang_67_2005_Avian
HM172447_A_chicken_Xinjiang_68_2005_Avian
HM172446_A_chicken_Xinjiang_78_2005_Avian
HM172439_A_chicken_Xinjiang_54_2005_Avian
HM172435_A_chicken_Xinjiang_17_2005_Avian
HM172420_A_chicken_Xinjiang_16_2005_Avian
AB478013_A_chicken_Pyigyitagon_204_2006_Avian
DQ992590_A_goose_Yunnan_5539_2005_Avian
DQ992672_A_duck_Yunnan_5236_2005_Avian
DQ992589_A_duck_Yunnan_5133_2005_Avian
DQ992673_A_goose_Yunnan_5299_2005_Avian

PB2-Group63

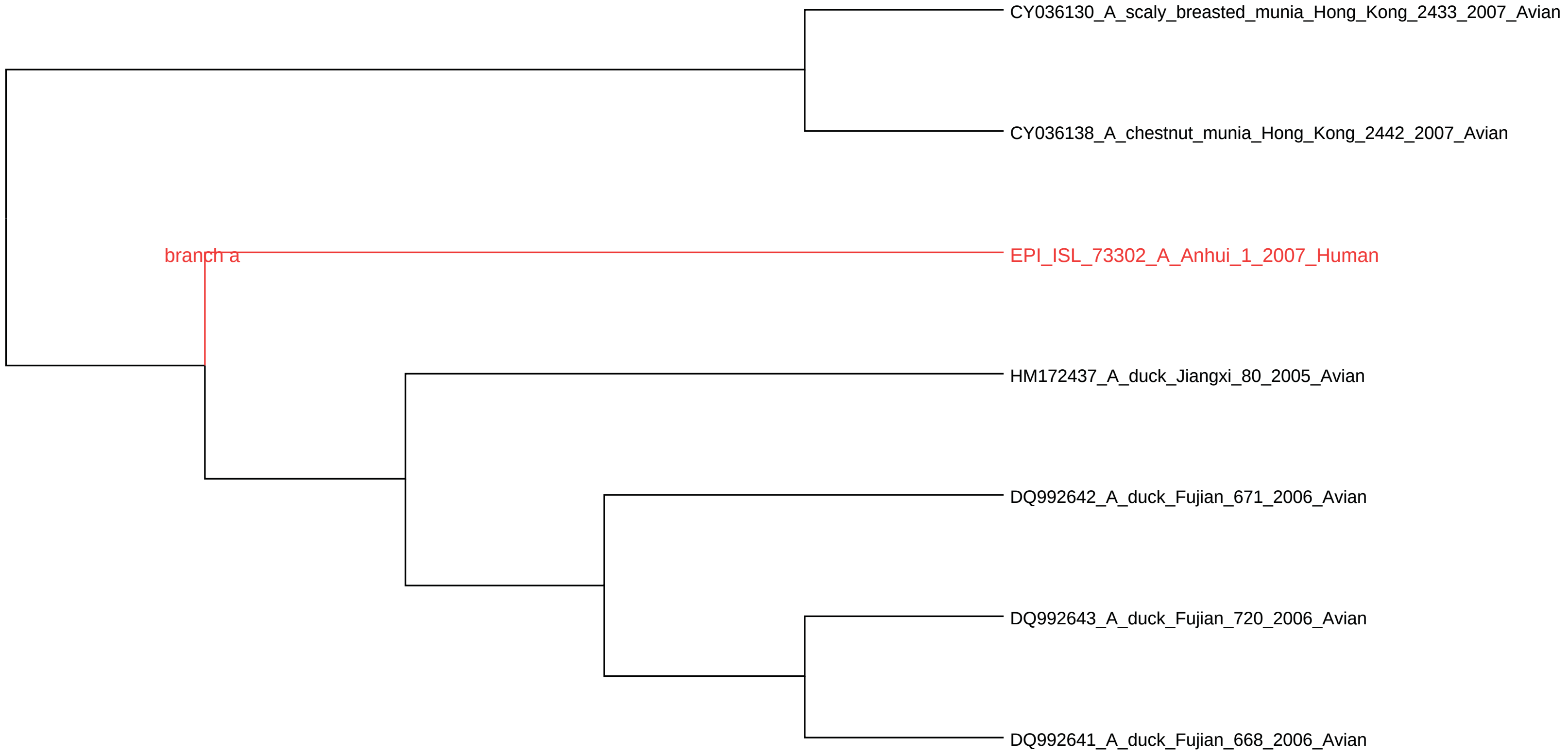

CY036130_A_scaly_breasted_munia_Hong_Kong_2433_2007_Avian
CY036138_A_chestnut_munia_Hong_Kong_2442_2007_Avian
branch a
EPI_ISL_73302_A_Anhui_1_2007_Human
HM172437_A_duck_Jiangxi_80_2005_Avian
DQ992642_A_duck_Fujian_671_2006_Avian
DQ992643_A_duck_Fujian_720_2006_Avian
DQ992641_A_duck_Fujian_668_2006_Avian

PB2-Group64

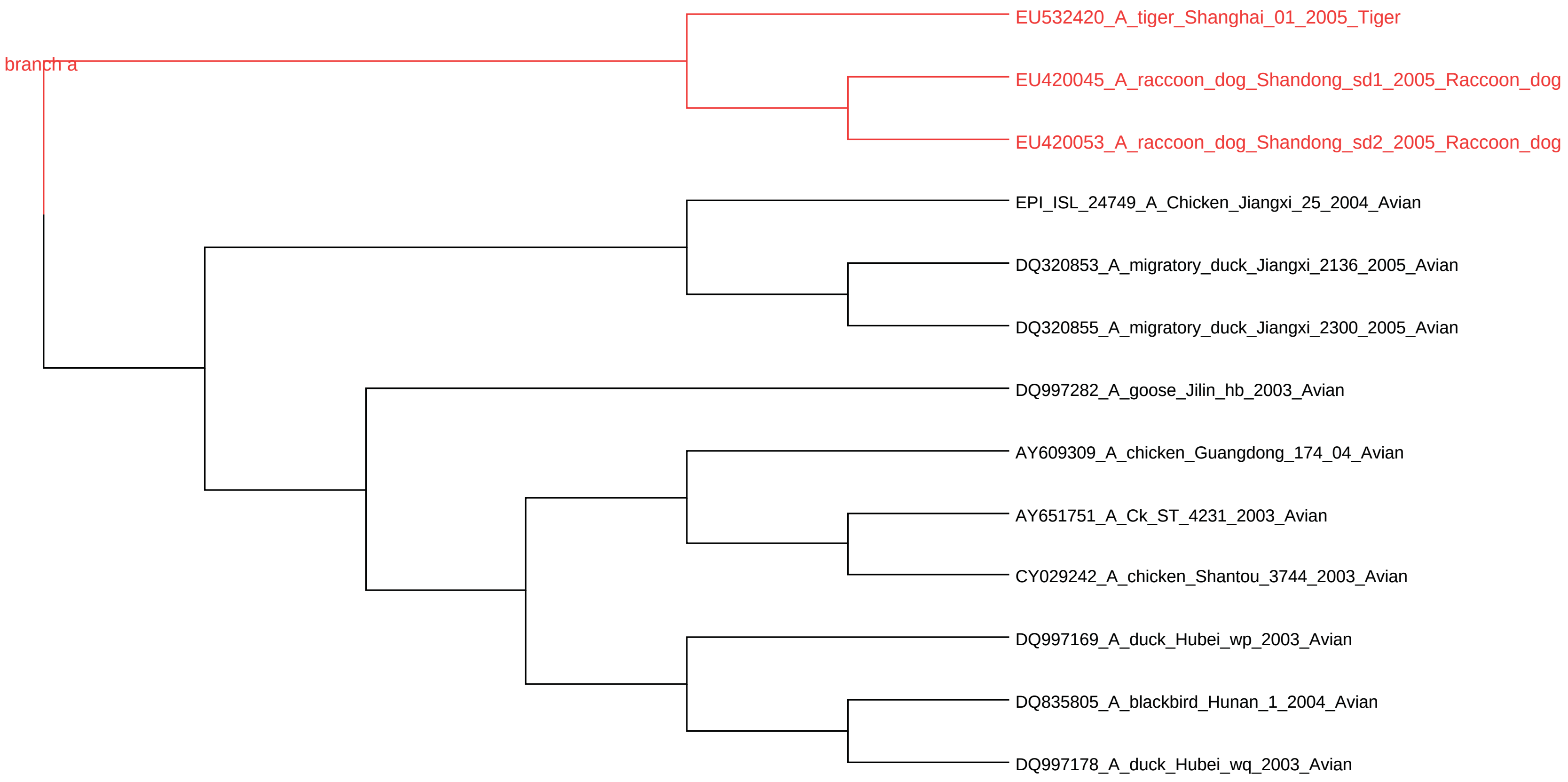

branch a
EU532420_A_tiger_Shanghai_01_2005_Tiger
EU420045_A_raccoon_dog_Shandong_sd1_2005_Raccoon_dog
EU420053_A_raccoon_dog_Shandong_sd2_2005_Raccoon_dog
EPI_ISL_24749_A_Chicken_Jiangxi_25_2004_Avian
DQ320853_A_migratory_duck_Jiangxi_2136_2005_Avian
DQ320855_A_migratory_duck_Jiangxi_2300_2005_Avian
DQ997282_A_goose_Jilin_hb_2003_Avian
AY609309_A_chicken_Guangdong_174_04_Avian
AY651751_A_Ck_ST_4231_2003_Avian
CY029242_A_chicken_Shantou_3744_2003_Avian
DQ997169_A_duck_Hubei_wp_2003_Avian
DQ835805_A_blackbird_Hunan_1_2004_Avian
DQ997178_A_duck_Hubei_wq_2003_Avian

PB2-Group65

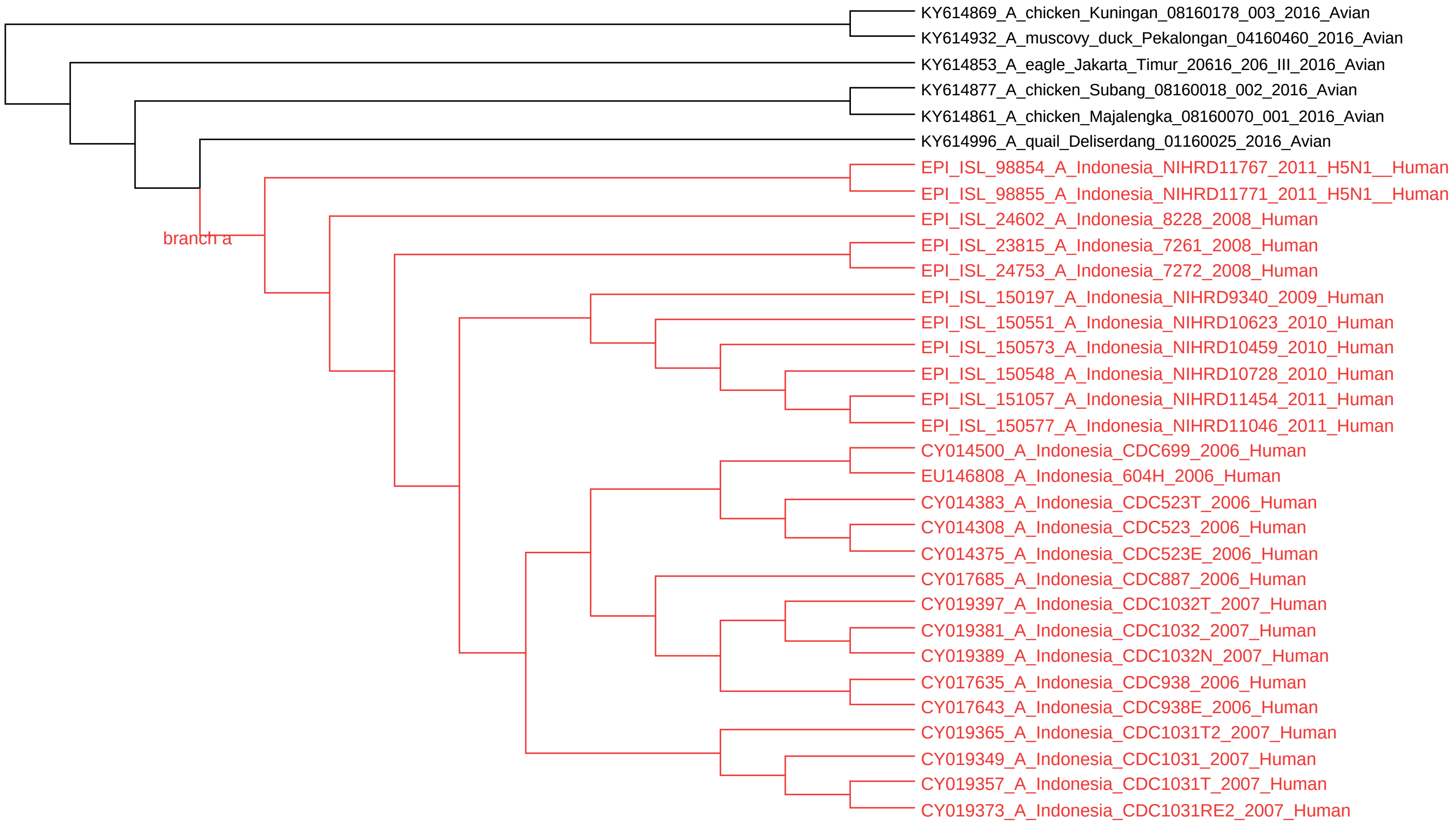

KY614869_A_chicken_Kuningan_08160178_003_2016_Avian
KY614932_A_muscovy_duck_Pekalongan_04160460_2016_Avian
KY614853_A_eagle_Jakarta_Timur_20616_206_III_2016_Avian
KY614877_A_chicken_Subang_08160018_002_2016_Avian
KY614861_A_chicken_Majalengka_08160070_001_2016_Avian
KY614996_A_quail_Deliserdang_01160025_2016_Avian
branch a
EPI_ISL_98854_A_Indonesia_NIHRD11767_2011_H5N1__Human
EPI_ISL_98855_A_Indonesia_NIHRD11771_2011_H5N1__Human
EPI_ISL_24602_A_Indonesia_8228_2008_Human
EPI_ISL_23815_A_Indonesia_7261_2008_Human
EPI_ISL_24753_A_Indonesia_7272_2008_Human
EPI_ISL_150197_A_Indonesia_NIHRD9340_2009_Human
EPI_ISL_150551_A_Indonesia_NIHRD10623_2010_Human
EPI_ISL_150573_A_Indonesia_NIHRD10459_2010_Human
EPI_ISL_150548_A_Indonesia_NIHRD10728_2010_Human
EPI_ISL_151057_A_Indonesia_NIHRD11454_2011_Human
EPI_ISL_150577_A_Indonesia_NIHRD11046_2011_Human
CY014500_A_Indonesia_CDC699_2006_Human
EU146808_A_Indonesia_604H_2006_Human
CY014383_A_Indonesia_CDC523T_2006_Human
CY014308_A_Indonesia_CDC523_2006_Human
CY014375_A_Indonesia_CDC523E_2006_Human
CY017685_A_Indonesia_CDC887_2006_Human
CY019397_A_Indonesia_CDC1032T_2007_Human
CY019381_A_Indonesia_CDC1032_2007_Human
CY019389_A_Indonesia_CDC1032N_2007_Human
CY017635_A_Indonesia_CDC938_2006_Human
CY017643_A_Indonesia_CDC938E_2006_Human
CY019365_A_Indonesia_CDC1031T2_2007_Human
CY019349_A_Indonesia_CDC1031_2007_Human
CY019357_A_Indonesia_CDC1031T_2007_Human
CY019373_A_Indonesia_CDC1031RE2_2007_Human

PB2-Group66

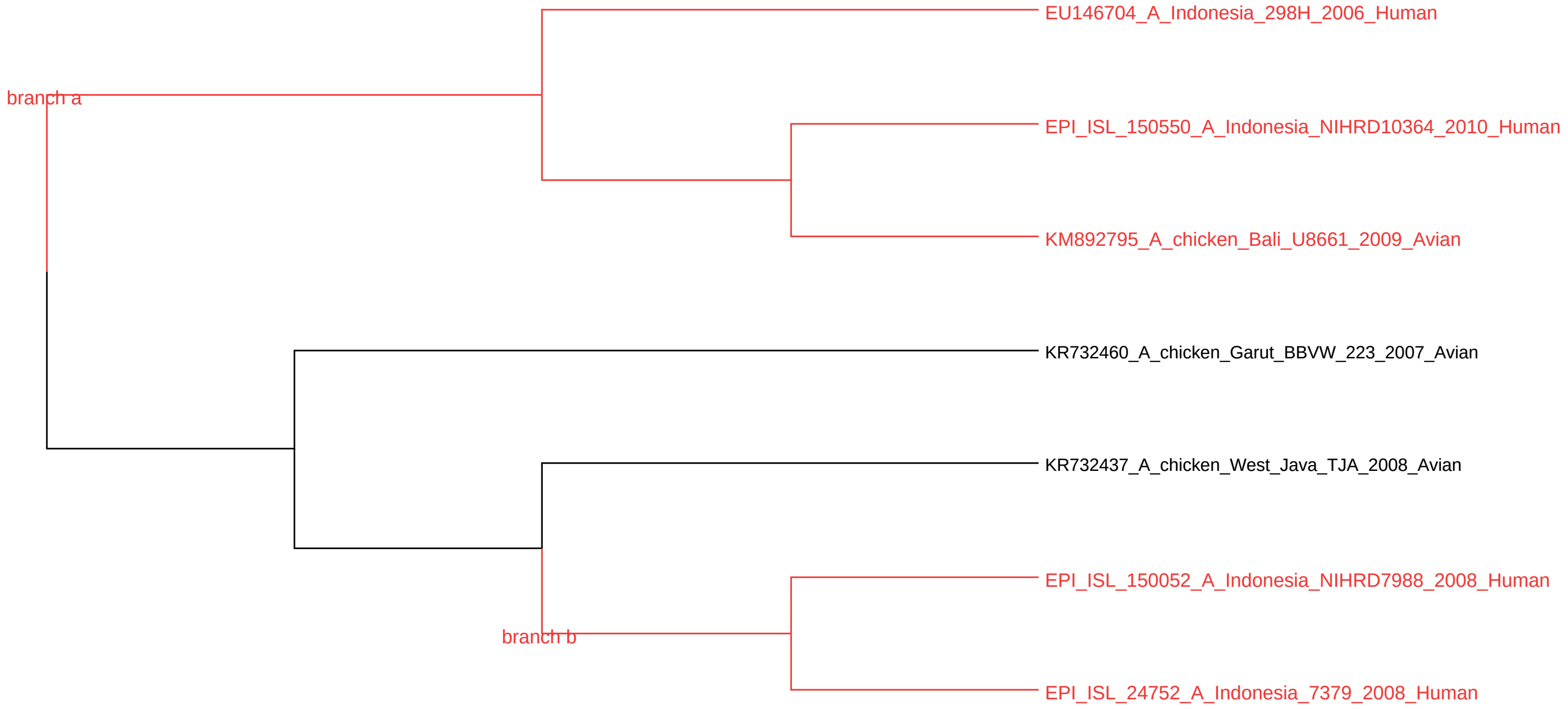

branch a
EU146704_A_Indonesia_298H_2006_Human
EPI_ISL_150550_A_Indonesia_NIHRD10364_2010_Human
KM892795_A_chicken_Bali_U8661_2009_Avian
KR732460_A_chicken_Garut_BBVW_223_2007_Avian
KR732437_A_chicken_West_Java_TJA_2008_Avian
EPI_ISL_150052_A_Indonesia_NIHRD7988_2008_Human
branch b
EPI_ISL_24752_A_Indonesia_7379_2008_Human

PB2-Group67

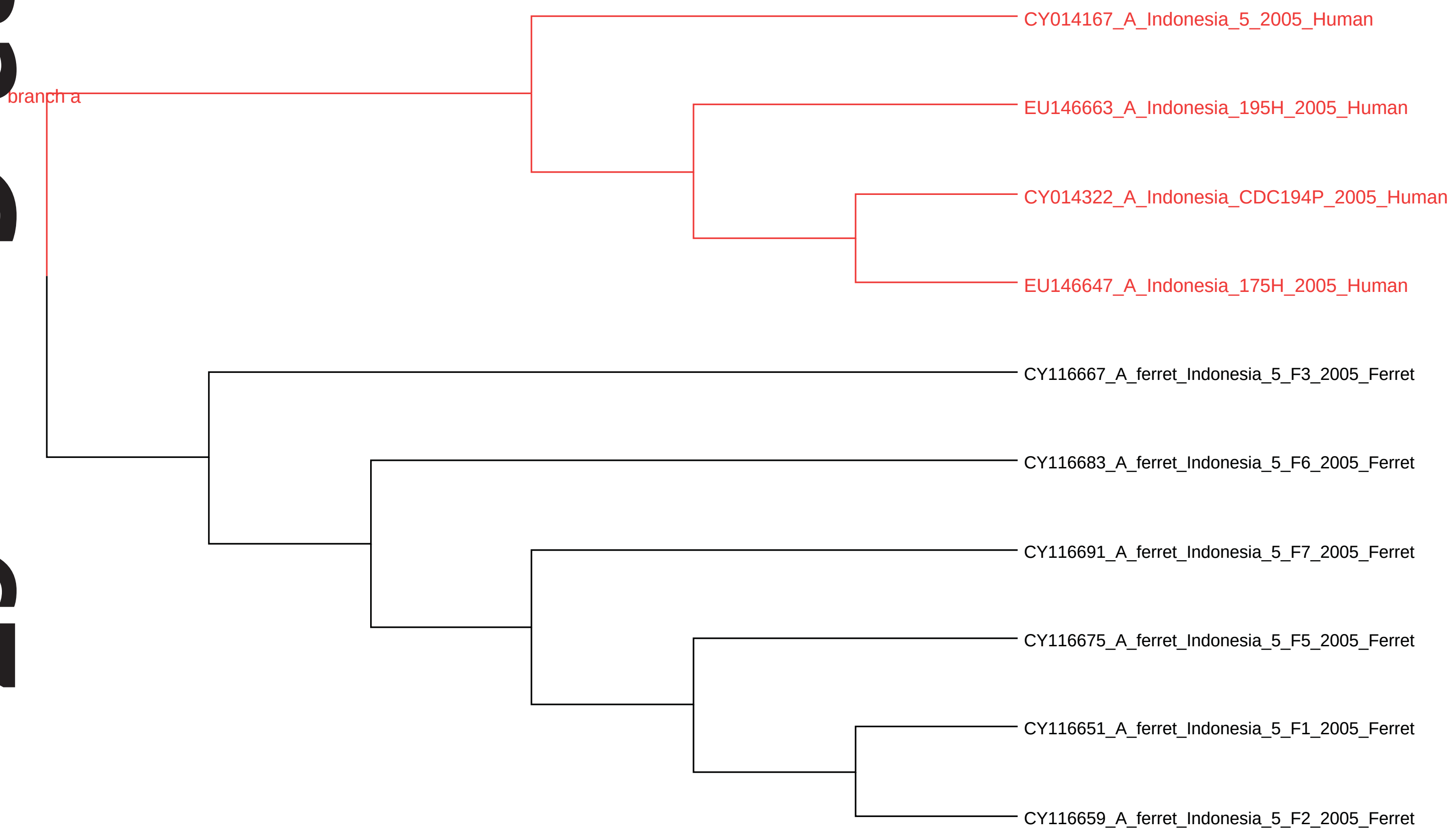

branch a
CY014167_A_Indonesia_5_2005_Human
EU146663_A_Indonesia_195H_2005_Human
CY014322_A_Indonesia_CDC194P_2005_Human
EU146647_A_Indonesia_175H_2005_Human
CY116667_A_ferret_Indonesia_5_F3_2005_Ferret
CY116683_A_ferret_Indonesia_5_F6_2005_Ferret
CY116691_A_ferret_Indonesia_5_F7_2005_Ferret
CY116675_A_ferret_Indonesia_5_F5_2005_Ferret
CY116651_A_ferret_Indonesia_5_F1_2005_Ferret
CY116659_A_ferret_Indonesia_5_F2_2005_Ferret

PB2-Group68

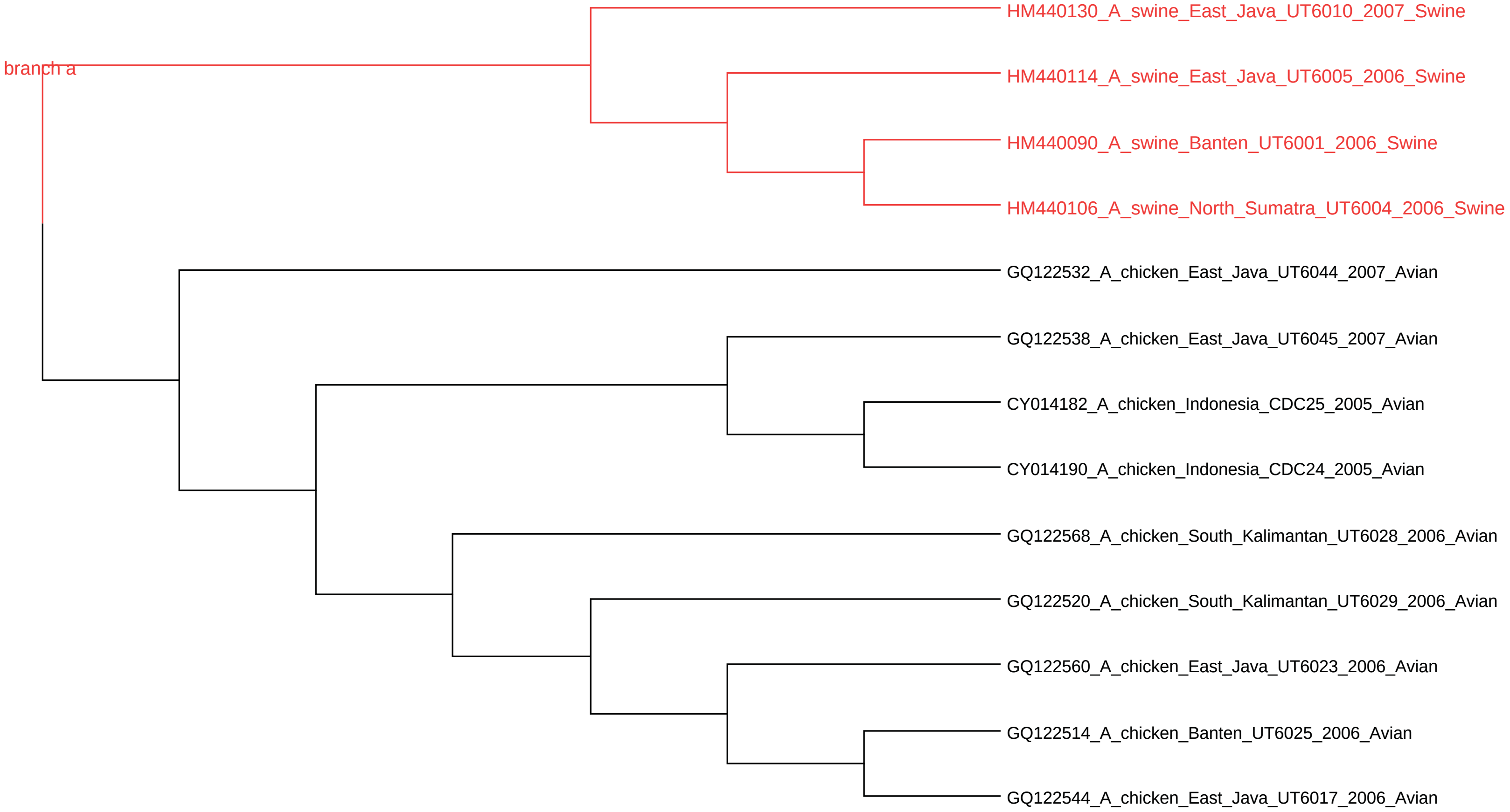

branch a
HM440130_A_swine_East_Java_UT6010_2007_Swine
HM440114_A_swine_East_Java_UT6005_2006_Swine
HM440090_A_swine_Banten_UT6001_2006_Swine
HM440106_A_swine_North_Sumatra_UT6004_2006_Swine
GQ122532_A_chicken_East_Java_UT6044_2007_Avian
GQ122538_A_chicken_East_Java_UT6045_2007_Avian
CY014182_A_chicken_Indonesia_CDC25_2005_Avian
CY014190_A_chicken_Indonesia_CDC24_2005_Avian
GQ122568_A_chicken_South_Kalimantan_UT6028_2006_Avian
GQ122520_A_chicken_South_Kalimantan_UT6029_2006_Avian
GQ122560_A_chicken_East_Java_UT6023_2006_Avian
GQ122514_A_chicken_Banten_UT6025_2006_Avian
GQ122544_A_chicken_East_Java_UT6017_2006_Avian

PB2-Group69

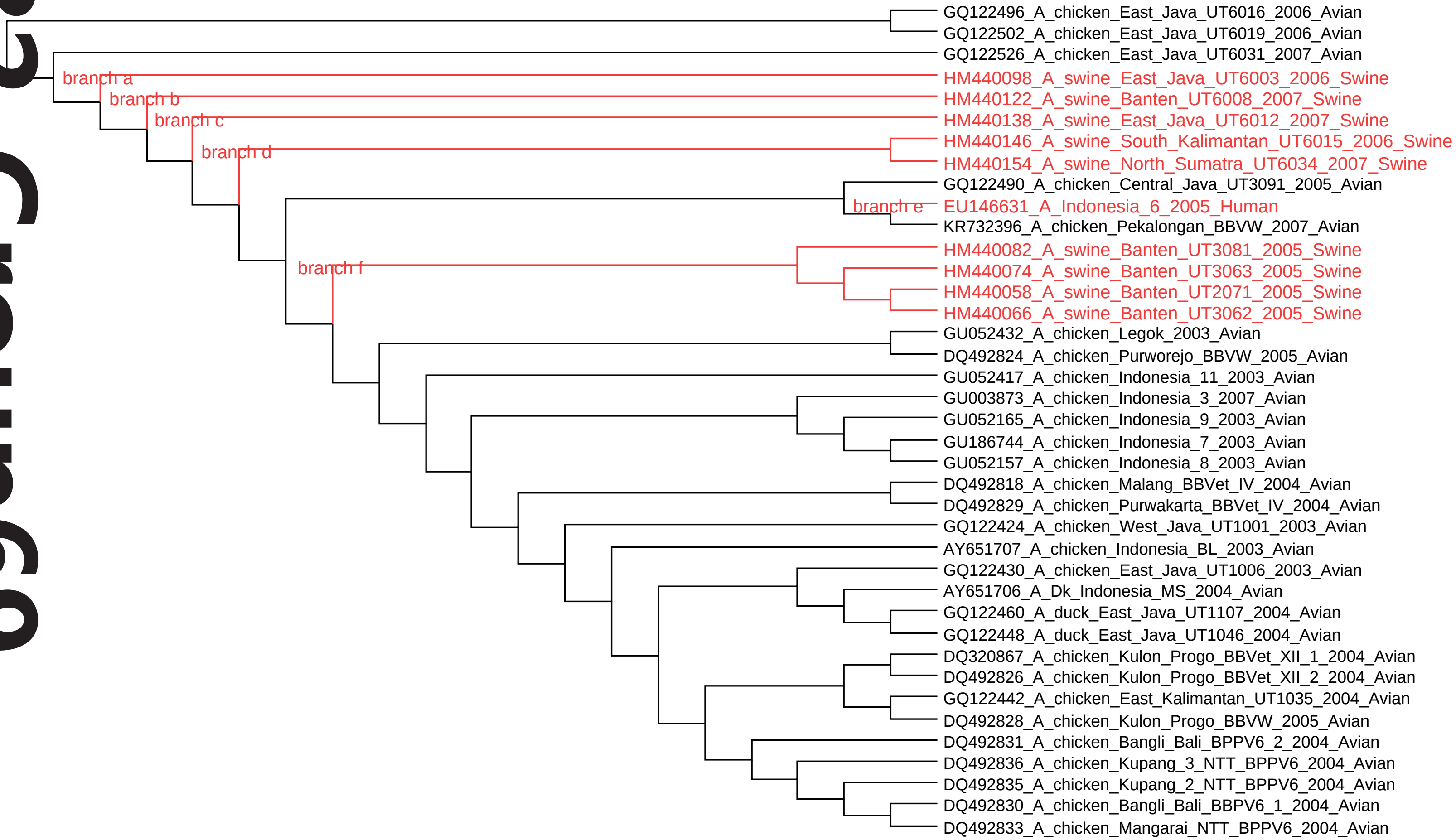
GQ122496_A_chicken_East_Java_UT6016_2006_Avian
GQ122502_A_chicken_East_Java_UT6019_2006_Avian
GQ122526_A_chicken_East_Java_UT6031_2007_Avian
branch a
HM440098_A_swine_East_Java_UT6003_2006_Swine
branch b
HM440122_A_swine_Banten_UT6008_2007_Swine
branch c
HM440138_A_swine_East_Java_UT6012_2007_Swine
HM440146_A_swine_South_Kalimantan_UT6015_2006_Swine
branch d
HM440154_A_swine_North_Sumatra_UT6034_2007_Swine
GQ122490_A_chicken_Central_Java_UT3091_2005_Avian
branch e
EU146631_A_Indonesia_6_2005_Human
KR732396_A_chicken_Pekalongan_BBVW_2007_Avian
HM440082_A_swine_Banten_UT3081_2005_Swine
branch f
HM440074_A_swine_Banten_UT3063_2005_Swine
HM440058_A_swine_Banten_UT2071_2005_Swine
HM440066_A_swine_Banten_UT3062_2005_Swine
GU052432_A_chicken_Legok_2003_Avian
DQ492824_A_chicken_Purworejo_BBVW_2005_Avian
GU052417_A_chicken_Indonesia_11_2003_Avian
GU003873_A_chicken_Indonesia_3_2007_Avian
GU052165_A_chicken_Indonesia_9_2003_Avian
GU186744_A_chicken_Indonesia_7_2003_Avian
GU052157_A_chicken_Indonesia_8_2003_Avian
DQ492818_A_chicken_Malang_BBVet_IV_2004_Avian
DQ492829_A_chicken_Purwakarta_BBVet_IV_2004_Avian
GQ122424_A_chicken_West_Java_UT1001_2003_Avian
AY651707_A_chicken_Indonesia_BL_2003_Avian
GQ122430_A_chicken_East_Java_UT1006_2003_Avian
AY651706_A_Dk_Indonesia_MS_2004_Avian
GQ122460_A_duck_East_Java_UT1107_2004_Avian
GQ122448_A_duck_East_Java_UT1046_2004_Avian
DQ320867_A_chicken_Kulon_Progo_BBVet_XII_1_2004_Avian
DQ492826_A_chicken_Kulon_Progo_BBVet_XII_2_2004_Avian
GQ122442_A_chicken_East_Kalimantan_UT1035_2004_Avian
DQ492828_A_chicken_Kulon_Progo_BBVW_2005_Avian
DQ492831_A_chicken_Bangli_Bali_BPPV6_2_2004_Avian
DQ492836_A_chicken_Kupang_3_NTT_BPPV6_2004_Avian
DQ492835_A_chicken_Kupang_2_NTT_BPPV6_2004_Avian
DQ492830_A_chicken_Bangli_Bali_BBPV6_1_2004_Avian
DQ492833_A_chicken_Mangarai_NTT_BPPV6_2004_Avian

PB2-Group70

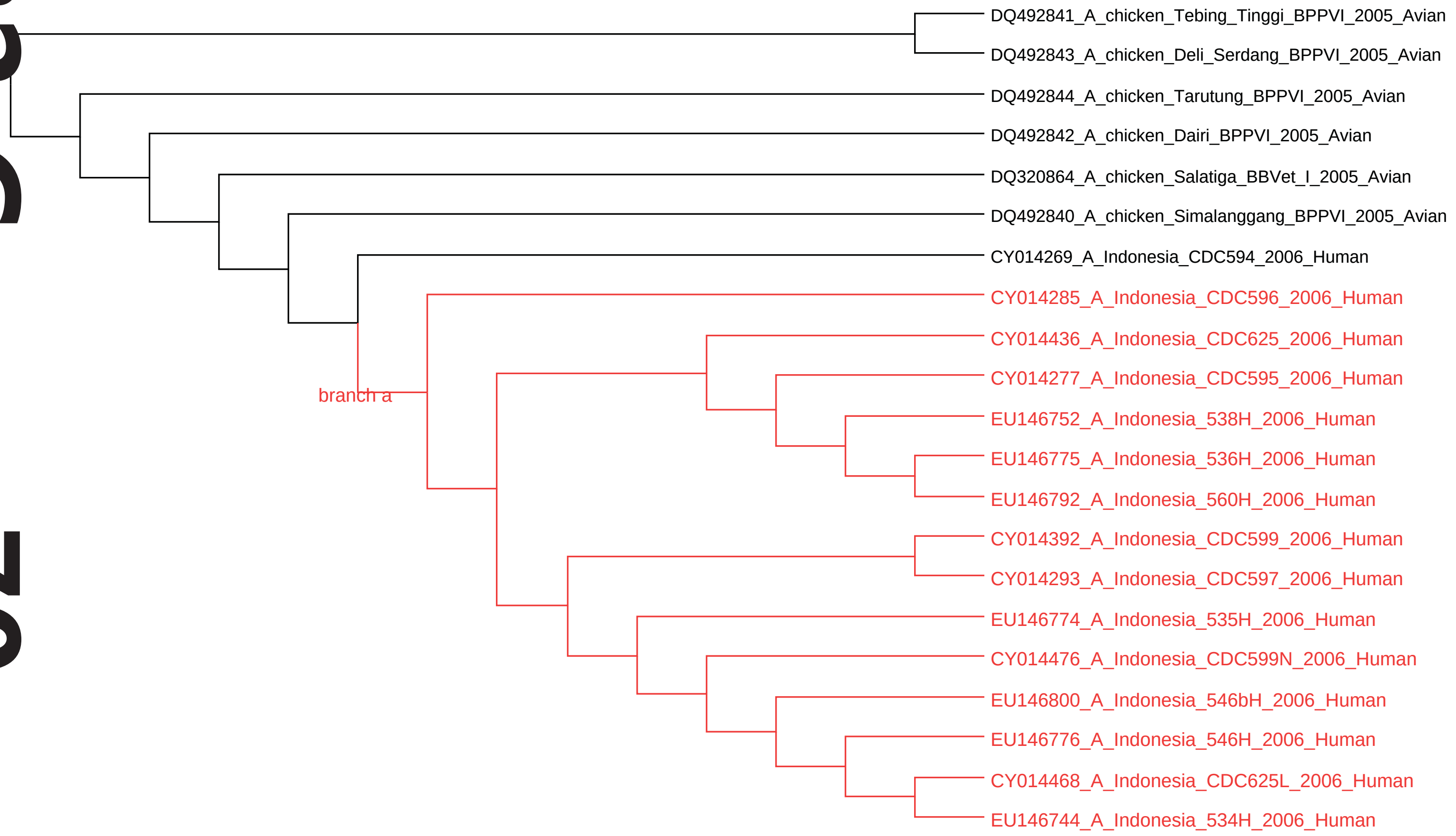

DQ492841_A_chicken_Tebing_Tinggi_BPPVI_2005_Avian
DQ492843_A_chicken_Deli_Serdang_BPPVI_2005_Avian
DQ492844_A_chicken_Tarutung_BPPVI_2005_Avian
DQ492842_A_chicken_Dairi_BPPVI_2005_Avian
DQ320864_A_chicken_Salatiga_BBVet_I_2005_Avian
DQ492840_A_chicken_Simalanggang_BPPVI_2005_Avian
CY014269_A_Indonesia_CDC594_2006_Human
CY014285_A_Indonesia_CDC596_2006_Human
CY014436_A_Indonesia_CDC625_2006_Human
CY014277_A_Indonesia_CDC595_2006_Human
EU146752_A_Indonesia_538H_2006_Human
EU146775_A_Indonesia_536H_2006_Human
EU146792_A_Indonesia_560H_2006_Human
CY014392_A_Indonesia_CDC599_2006_Human
CY014293_A_Indonesia_CDC597_2006_Human
EU146774_A_Indonesia_535H_2006_Human
CY014476_A_Indonesia_CDC599N_2006_Human
EU146800_A_Indonesia_546bH_2006_Human
EU146776_A_Indonesia_546H_2006_Human
CY014468_A_Indonesia_CDC625L_2006_Human
EU146744_A_Indonesia_534H_2006_Human
branch a

PB2-Group71

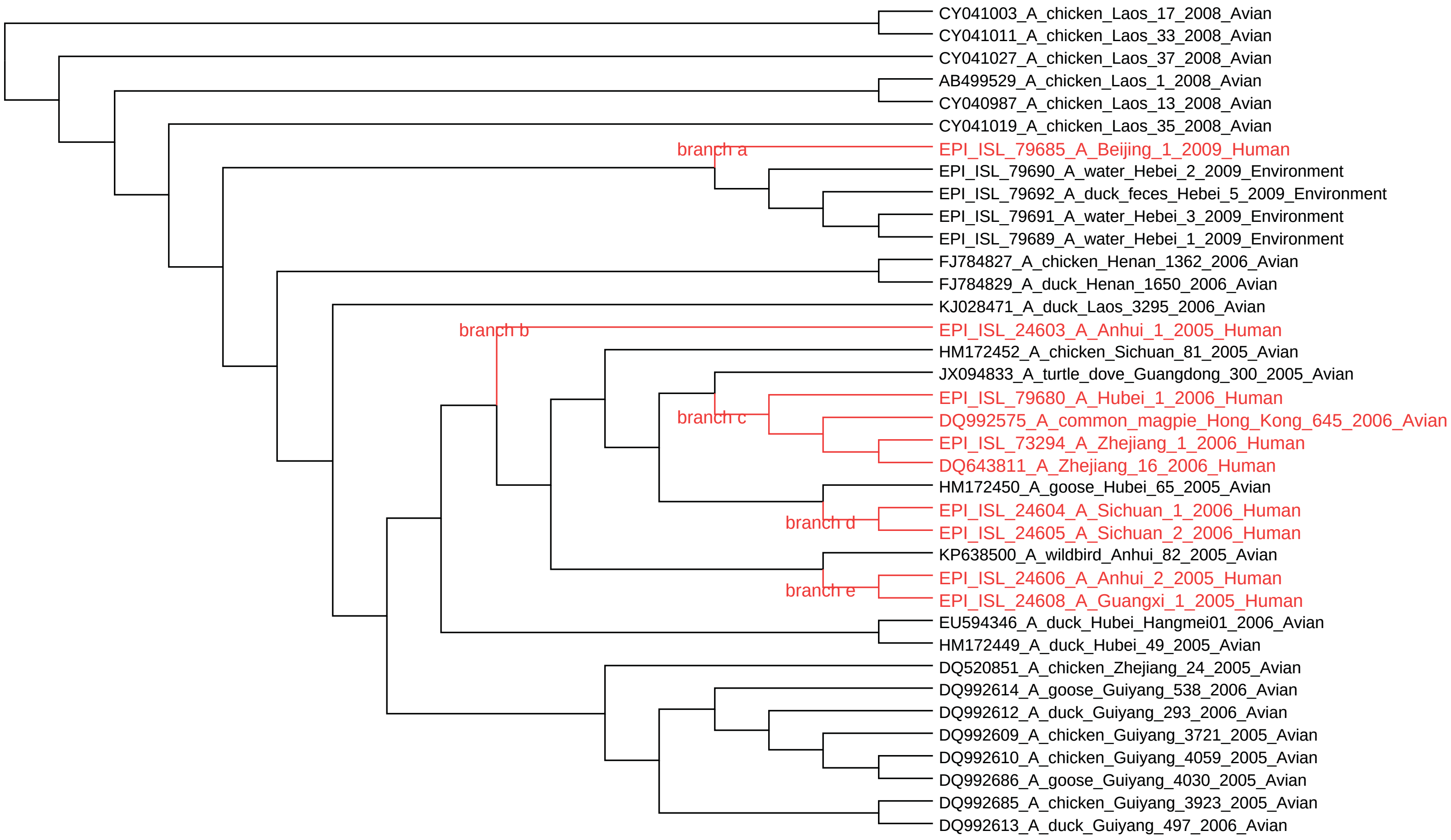
CY041003_A_chicken_Laos_17_2008_Avian
CY041011_A_chicken_Laos_33_2008_Avian
CY041027_A_chicken_Laos_37_2008_Avian
AB499529_A_chicken_Laos_1_2008_Avian
CY040987_A_chicken_Laos_13_2008_Avian
CY041019_A_chicken_Laos_35_2008_Avian
branch a
EPI_ISL_79685_A_Beijing_1_2009_Human
EPI_ISL_79690_A_water_Hebei_2_2009_Environment
EPI_ISL_79692_A_duck_feces_Hebei_5_2009_Environment
EPI_ISL_79691_A_water_Hebei_3_2009_Environment
EPI_ISL_79689_A_water_Hebei_1_2009_Environment
FJ784827_A_chicken_Henan_1362_2006_Avian
FJ784829_A_duck_Henan_1650_2006_Avian
KJ028471_A_duck_Laos_3295_2006_Avian
branch b
EPI_ISL_24603_A_Anhui_1_2005_Human
HM172452_A_chicken_Sichuan_81_2005_Avian
JX094833_A_turtle_dove_Guangdong_300_2005_Avian
EPI_ISL_79680_A_Hubei_1_2006_Human
branch c
DQ992575_A_common_magpie_Hong_Kong_645_2006_Avian
EPI_ISL_73294_A_Zhejiang_1_2006_Human
DQ643811_A_Zhejiang_16_2006_Human
HM172450_A_goose_Hubei_65_2005_Avian
EPI_ISL_24604_A_Sichuan_1_2006_Human
branch d
EPI_ISL_24605_A_Sichuan_2_2006_Human
KP638500_A_wildbird_Anhui_82_2005_Avian
EPI_ISL_24606_A_Anhui_2_2005_Human
branch e
EPI_ISL_24608_A_Guangxi_1_2005_Human
EU594346_A_duck_Hubei_Hangmei01_2006_Avian
HM172449_A_duck_Hubei_49_2005_Avian
DQ520851_A_chicken_Zhejiang_24_2005_Avian
DQ992614_A_goose_Guiyang_538_2006_Avian
DQ992612_A_duck_Guiyang_293_2006_Avian
DQ992609_A_chicken_Guiyang_3721_2005_Avian
DQ992610_A_chicken_Guiyang_4059_2005_Avian
DQ992686_A_goose_Guiyang_4030_2005_Avian
DQ992685_A_chicken_Guiyang_3923_2005_Avian
DQ992613_A_duck_Guiyang_497_2006_Avian

PB2-Group72

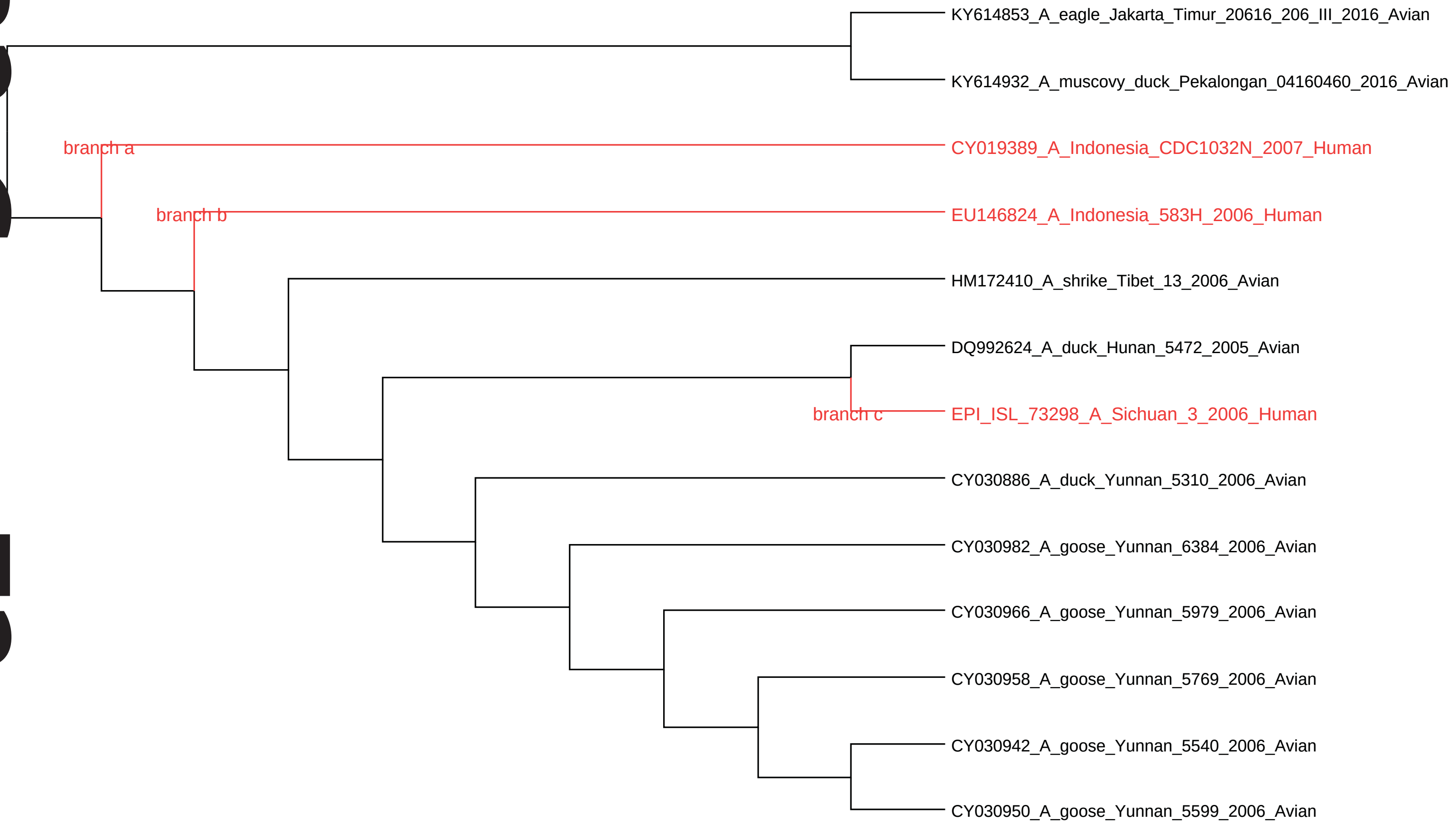
KY614853_A_eagle_Jakarta_Timur_20616_206_III_2016_Avian
KY614932_A_muscovy_duck_Pekalongan_04160460_2016_Avian
branch a
CY019389_A_Indonesia_CDC1032N_2007_Human
branch b
EU146824_A_Indonesia_583H_2006_Human
HM172410_A_shrike_Tibet_13_2006_Avian
DQ992624_A_duck_Hunan_5472_2005_Avian
branch c
EPI_ISL_73298_A_Sichuan_3_2006_Human
CY030886_A_duck_Yunnan_5310_2006_Avian
CY030982_A_goose_Yunnan_6384_2006_Avian
CY030966_A_goose_Yunnan_5979_2006_Avian
CY030958_A_goose_Yunnan_5769_2006_Avian
CY030942_A_goose_Yunnan_5540_2006_Avian
CY030950_A_goose_Yunnan_5599_2006_Avian

PB2-Group73

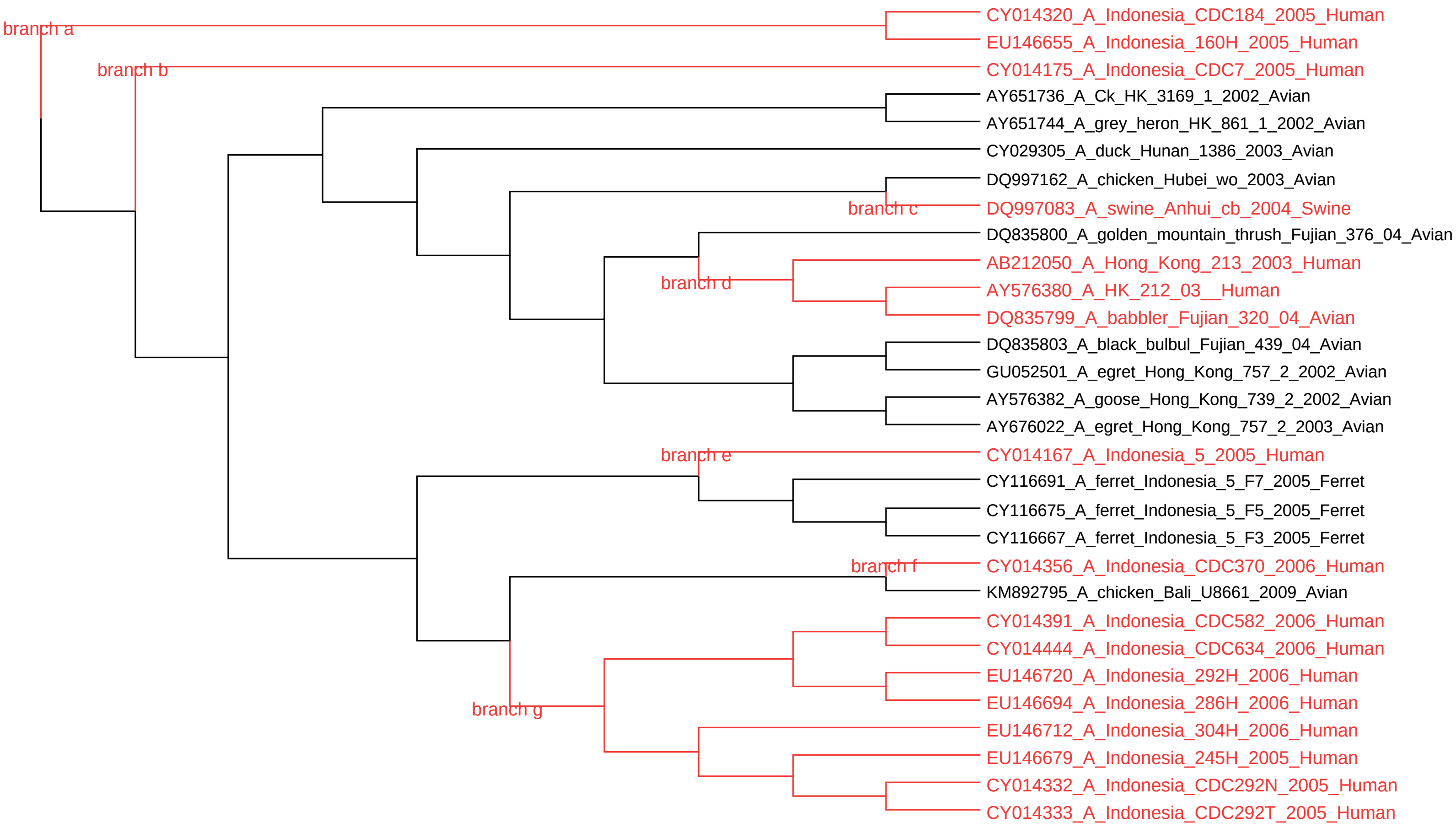
branch a
branch b
branch c
branch d
branch e
branch f
branch g
CY014320_A_Indonesia_CDC184_2005_Human
EU146655_A_Indonesia_160H_2005_Human
CY014175_A_Indonesia_CDC7_2005_Human
AY651736_A_Ck_HK_3169_1_2002_Avian
AY651744_A_grey_heron_HK_861_1_2002_Avian
CY029305_A_duck_Hunan_1386_2003_Avian
DQ997162_A_chicken_Hubei_wo_2003_Avian
DQ997083_A_swine_Anhui_cb_2004_Swine
DQ835800_A_golden_mountain_thrush_Fujian_376_04_Avian
AB212050_A_Hong_Kong_213_2003_Human
AY576380_A_HK_212_03__Human
DQ835799_A_babbler_Fujian_320_04_Avian
DQ835803_A_black_bulbul_Fujian_439_04_Avian
GU052501_A_egret_Hong_Kong_757_2_2002_Avian
AY576382_A_goose_Hong_Kong_739_2_2002_Avian
AY676022_A_egret_Hong_Kong_757_2_2003_Avian
CY014167_A_Indonesia_5_2005_Human
CY116691_A_ferret_Indonesia_5_F7_2005_Ferret
CY116675_A_ferret_Indonesia_5_F5_2005_Ferret
CY116667_A_ferret_Indonesia_5_F3_2005_Ferret
CY014356_A_Indonesia_CDC370_2006_Human
KM892795_A_chicken_Bali_U8661_2009_Avian
CY014391_A_Indonesia_CDC582_2006_Human
CY014444_A_Indonesia_CDC634_2006_Human
EU146720_A_Indonesia_292H_2006_Human
EU146694_A_Indonesia_286H_2006_Human
EU146712_A_Indonesia_304H_2006_Human
EU146679_A_Indonesia_245H_2005_Human
CY014332_A_Indonesia_CDC292N_2005_Human
CY014333_A_Indonesia_CDC292T_2005_Human

PB2-Group74

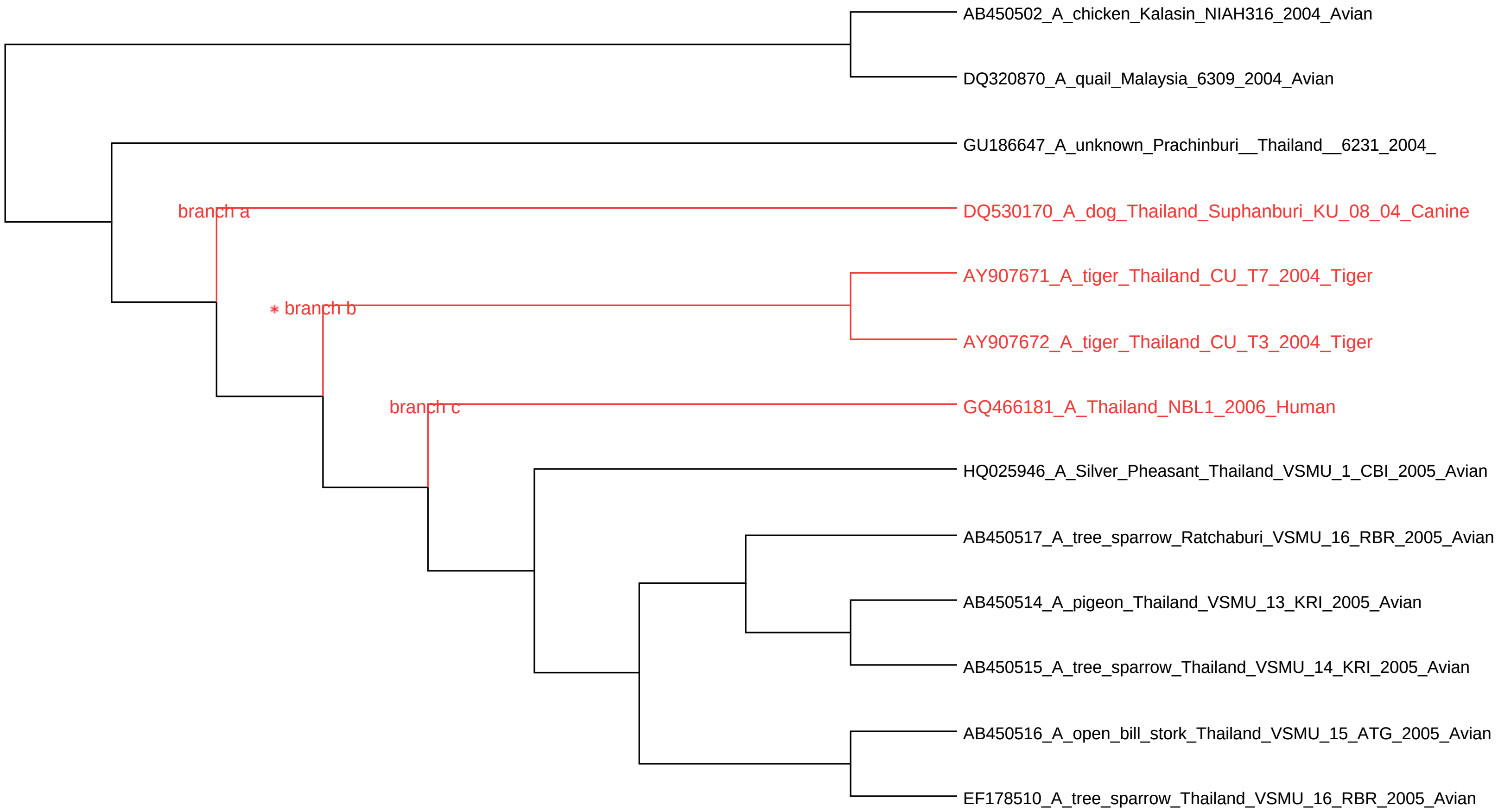

AB450502_A_chicken_Kalasin_NIAH316_2004_Avian
DQ320870_A_quail_Malaysia_6309_2004_Avian
GU186647_A_unknown_Prachinburi__Thailand__6231_2004_
branch a
DQ530170_A_dog_Thailand_Suphanburi_KU_08_04_Canine
* branch b
AY907671_A_tiger_Thailand_CU_T7_2004_Tiger
AY907672_A_tiger_Thailand_CU_T3_2004_Tiger
branch c
GQ466181_A_Thailand_NBL1_2006_Human
HQ025946_A_Silver_Pheasant_Thailand_VSMU_1_CBI_2005_Avian
AB450517_A_tree_sparrow_Ratchaburi_VSMU_16_RBR_2005_Avian
AB450514_A_pigeon_Thailand_VSMU_13_KRI_2005_Avian
AB450515_A_tree_sparrow_Thailand_VSMU_14_KRI_2005_Avian
AB450516_A_open_bill_stork_Thailand_VSMU_15_ATG_2005_Avian
EF178510_A_tree_sparrow_Thailand_VSMU_16_RBR_2005_Avian

PB2-Group75

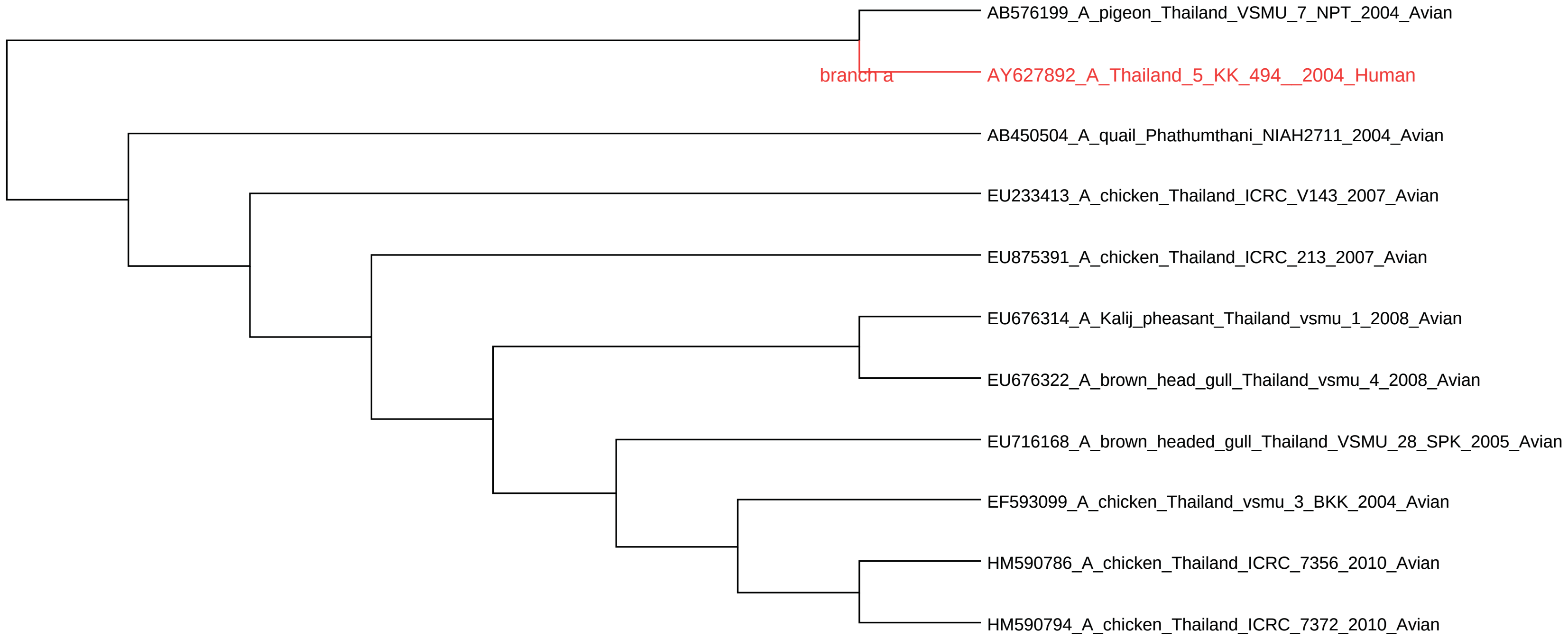

AB576199_A_pigeon_Thailand_VSMU_7_NPT_2004_Avian
branch a
AY627892_A_Thailand_5_KK_494__2004_Human
AB450504_A_quail_Phathumthani_NIAH2711_2004_Avian
EU233413_A_chicken_Thailand_ICRC_V143_2007_Avian
EU875391_A_chicken_Thailand_ICRC_213_2007_Avian
EU676314_A_Kalij_pheasant_Thailand_vsmu_1_2008_Avian
EU676322_A_brown_head_gull_Thailand_vsmu_4_2008_Avian
EU716168_A_brown_headed_gull_Thailand_VSMU_28_SPK_2005_Avian
EF593099_A_chicken_Thailand_vsmu_3_BKK_2004_Avian
HM590786_A_chicken_Thailand_ICRC_7356_2010_Avian
HM590794_A_chicken_Thailand_ICRC_7372_2010_Avian

PB2-Group76

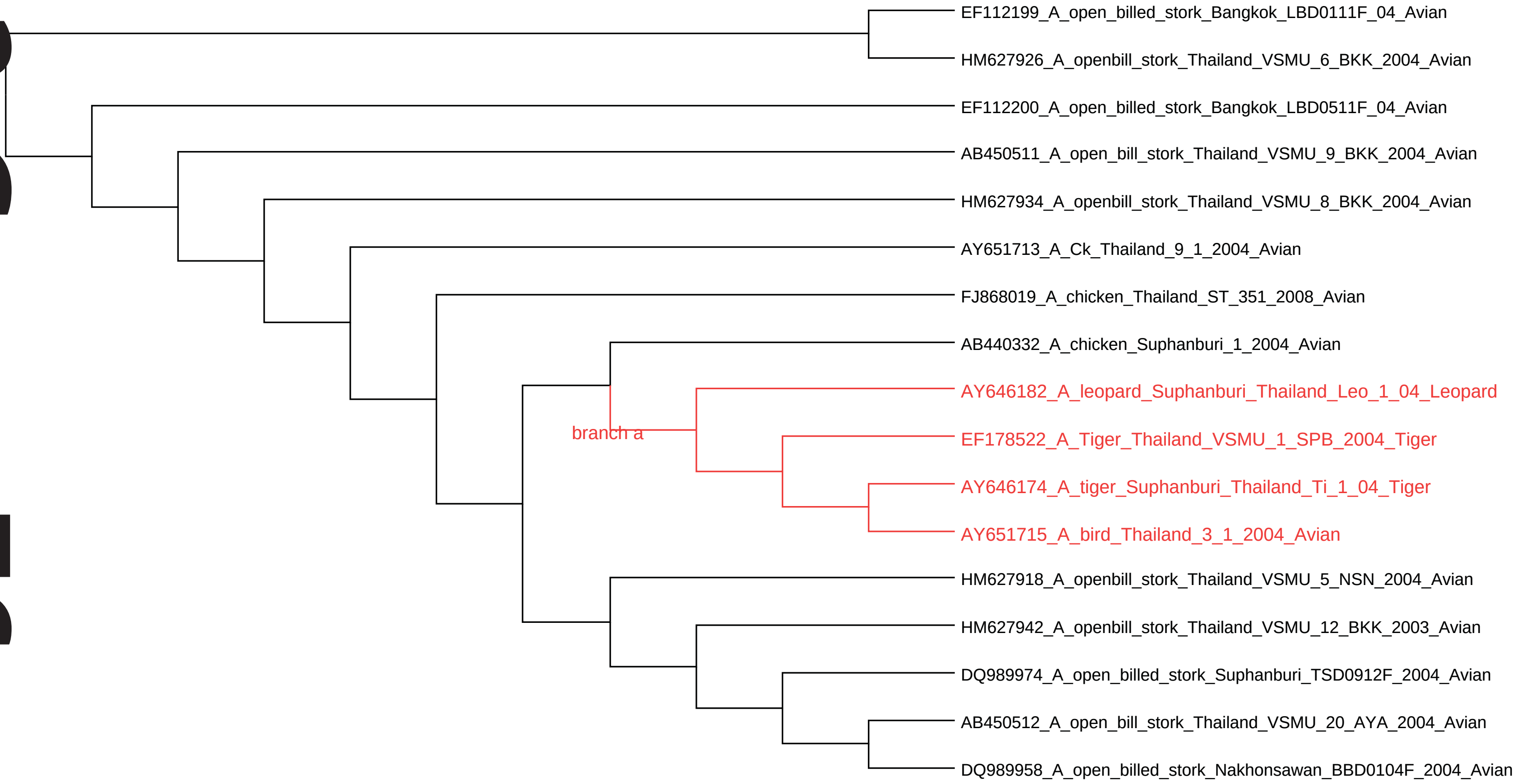

EF112199_A_open_billed_stork_Bangkok_LBD0111F_04_Avian
HM627926_A_openbill_stork_Thailand_VSMU_6_BKK_2004_Avian
EF112200_A_open_billed_stork_Bangkok_LBD0511F_04_Avian
AB450511_A_open_bill_stork_Thailand_VSMU_9_BKK_2004_Avian
HM627934_A_openbill_stork_Thailand_VSMU_8_BKK_2004_Avian
AY651713_A_Ck_Thailand_9_1_2004_Avian
FJ868019_A_chicken_Thailand_ST_351_2008_Avian
AB440332_A_chicken_Suphanburi_1_2004_Avian
AY646182_A_leopard_Suphanburi_Thailand_Leo_1_04_Leopard
branch a
EF178522_A_Tiger_Thailand_VSMU_1_SPB_2004_Tiger
AY646174_A_tiger_Suphanburi_Thailand_Ti_1_04_Tiger
AY651715_A_bird_Thailand_3_1_2004_Avian
HM627918_A_openbill_stork_Thailand_VSMU_5_NSN_2004_Avian
HM627942_A_openbill_stork_Thailand_VSMU_12_BKK_2003_Avian
DQ989974_A_open_billed_stork_Suphanburi_TSD0912F_2004_Avian
AB450512_A_open_bill_stork_Thailand_VSMU_20_AYA_2004_Avian
DQ989958_A_open_billed_stork_Nakhonsawan_BBD0104F_2004_Avian

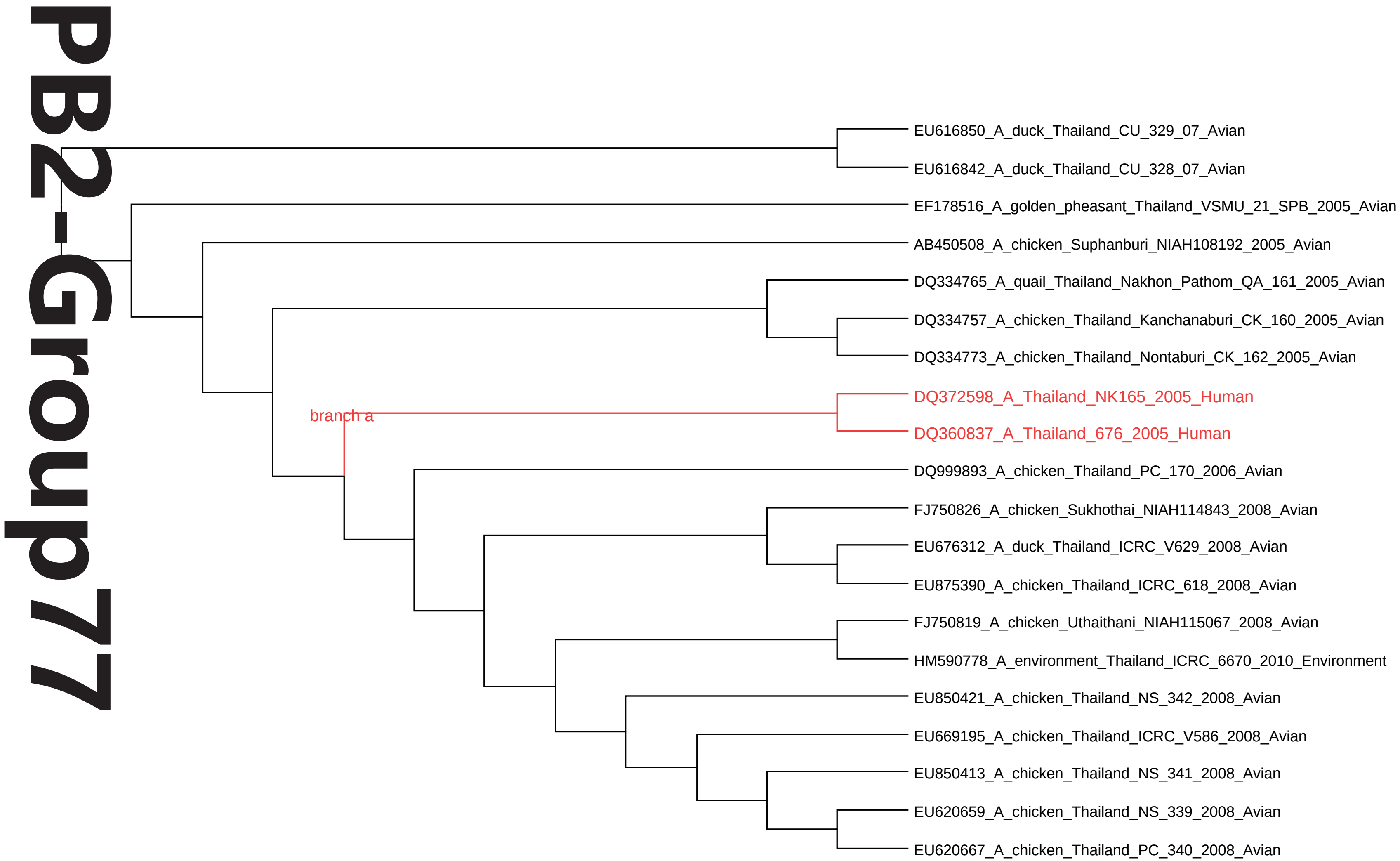
PB2-Group77
EU616850_A_duck_Thailand_CU_329_07_Avian
EU616842_A_duck_Thailand_CU_328_07_Avian
EF178516_A_golden_pheasant_Thailand_VSMU_21_SPB_2005_Avian
AB450508_A_chicken_Suphanburi_NIAH108192_2005_Avian
DQ334765_A_quail_Thailand_Nakhon_Pathom_QA_161_2005_Avian
DQ334757_A_chicken_Thailand_Kanchanaburi_CK_160_2005_Avian
DQ334773_A_chicken_Thailand_Nontaburi_CK_162_2005_Avian
branch a
DQ372598_A_Thailand_NK165_2005_Human
DQ360837_A_Thailand_676_2005_Human
DQ999893_A_chicken_Thailand_PC_170_2006_Avian
FJ750826_A_chicken_Sukhothai_NIAH114843_2008_Avian
EU676312_A_duck_Thailand_ICRC_V629_2008_Avian
EU875390_A_chicken_Thailand_ICRC_618_2008_Avian
FJ750819_A_chicken_Uthaithani_NIAH115067_2008_Avian
HM590778_A_environment_Thailand_ICRC_6670_2010_Environment
EU850421_A_chicken_Thailand_NS_342_2008_Avian
EU669195_A_chicken_Thailand_ICRC_V586_2008_Avian
EU850413_A_chicken_Thailand_NS_341_2008_Avian
EU620659_A_chicken_Thailand_NS_339_2008_Avian
EU620667_A_chicken_Thailand_PC_340_2008_Avian

PB2-Group78

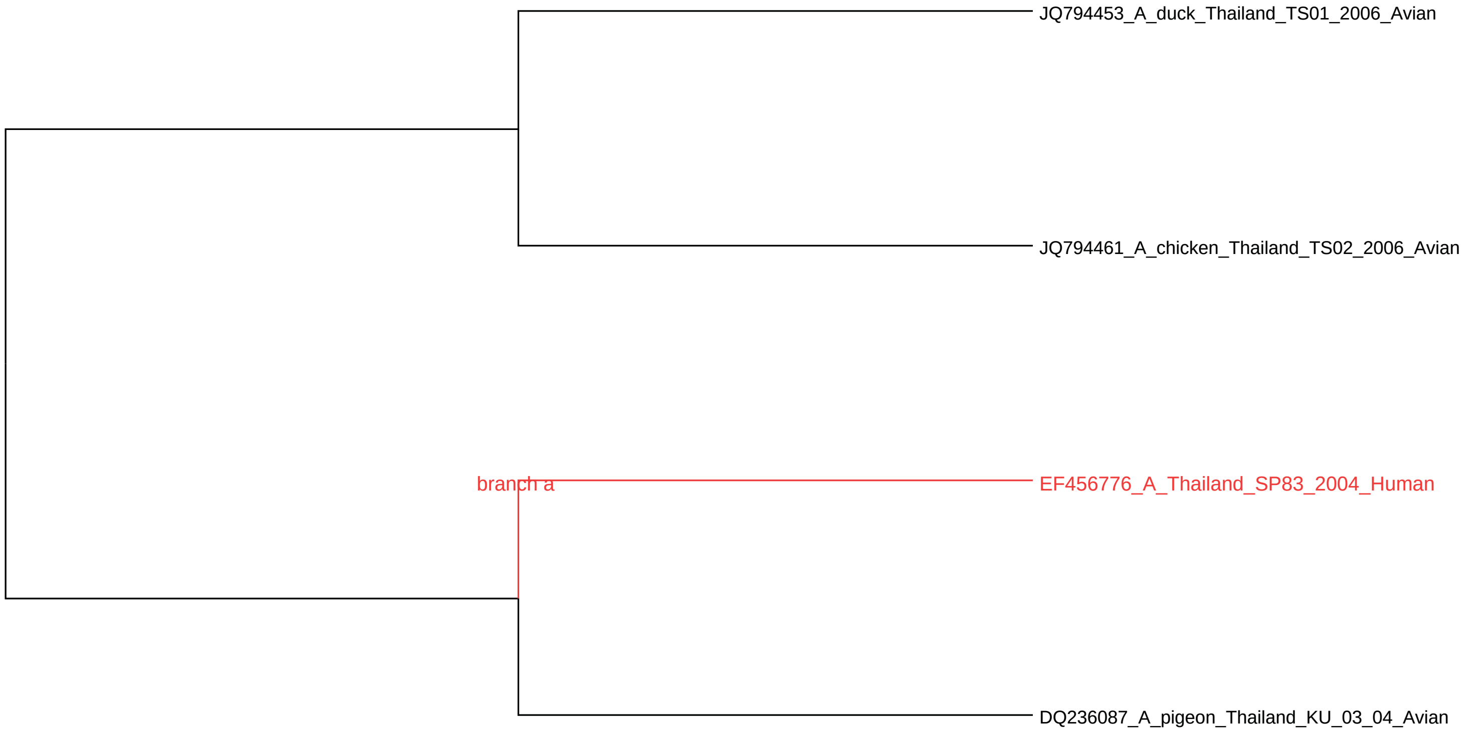

JQ794453_A_duck_Thailand_TS01_2006_Avian
JQ794461_A_chicken_Thailand_TS02_2006_Avian
branch a
EF456776_A_Thailand_SP83_2004_Human
DQ236087_A_pigeon_Thailand_KU_03_04_Avian

PB2-Group79

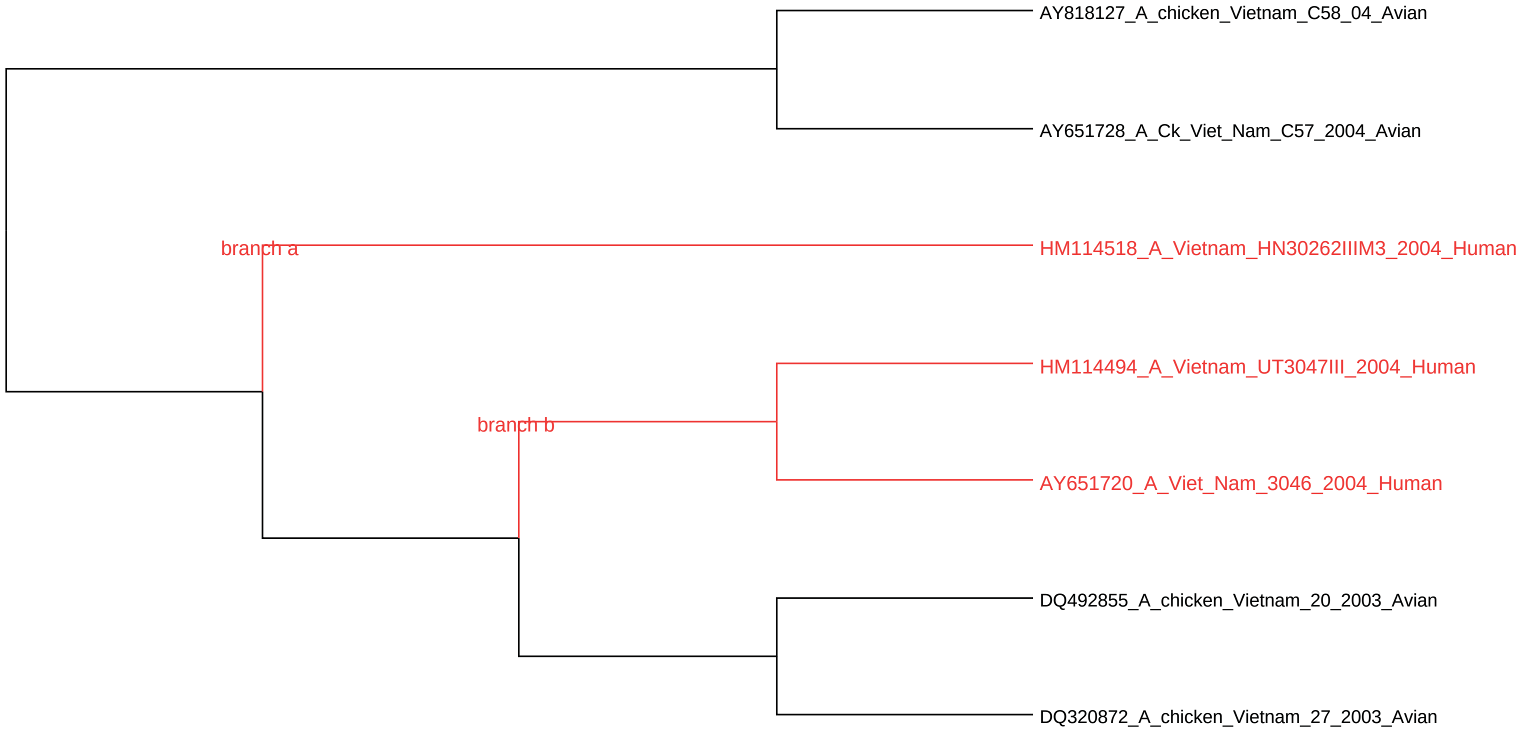

AY818127_A_chicken_Vietnam_C58_04_Avian
AY651728_A_Ck_Viet_Nam_C57_2004_Avian
branch a
HM114518_A_Vietnam_HN30262IIIM3_2004_Human
HM114494_A_Vietnam_UT3047III_2004_Human
branch b
AY651720_A_Viet_Nam_3046_2004_Human
DQ492855_A_chicken_Vietnam_20_2003_Avian
DQ320872_A_chicken_Vietnam_27_2003_Avian

PB2-Group80

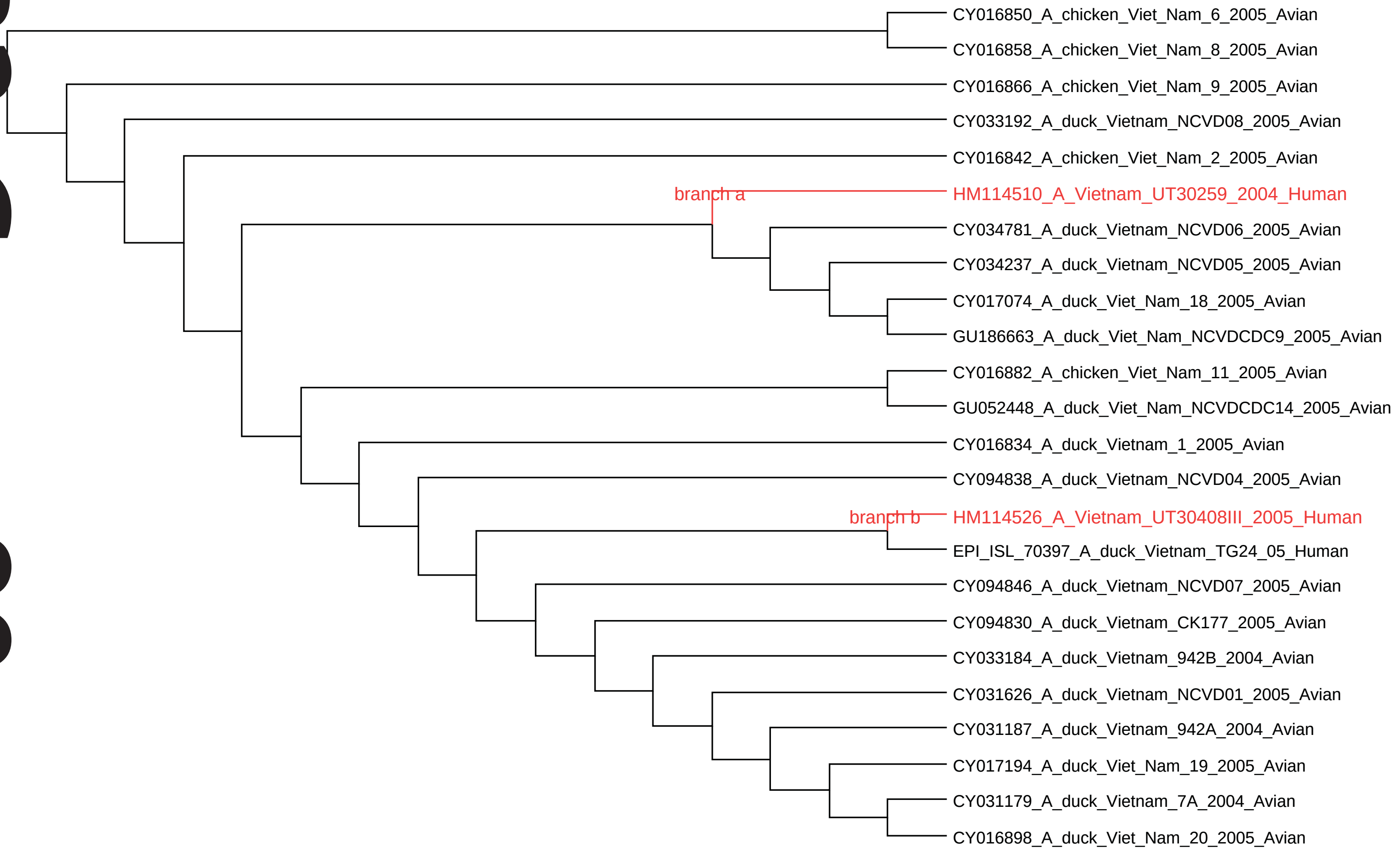

CY016850_A_chicken_Viet_Nam_6_2005_Avian
CY016858_A_chicken_Viet_Nam_8_2005_Avian
CY016866_A_chicken_Viet_Nam_9_2005_Avian
CY033192_A_duck_Vietnam_NCVD08_2005_Avian
CY016842_A_chicken_Viet_Nam_2_2005_Avian
branch a
HM114510_A_Vietnam_UT30259_2004_Human
CY034781_A_duck_Vietnam_NCVD06_2005_Avian
CY034237_A_duck_Vietnam_NCVD05_2005_Avian
CY017074_A_duck_Viet_Nam_18_2005_Avian
GU186663_A_duck_Viet_Nam_NCVDCDC9_2005_Avian
CY016882_A_chicken_Viet_Nam_11_2005_Avian
GU052448_A_duck_Viet_Nam_NCVDCDC14_2005_Avian
CY016834_A_duck_Vietnam_1_2005_Avian
CY094838_A_duck_Vietnam_NCVD04_2005_Avian
branch b
HM114526_A_Vietnam_UT30408III_2005_Human
EPI_ISL_70397_A_duck_Vietnam_TG24_05_Human
CY094846_A_duck_Vietnam_NCVD07_2005_Avian
CY094830_A_duck_Vietnam_CK177_2005_Avian
CY033184_A_duck_Vietnam_942B_2004_Avian
CY031626_A_duck_Vietnam_NCVD01_2005_Avian
CY031187_A_duck_Vietnam_942A_2004_Avian
CY017194_A_duck_Viet_Nam_19_2005_Avian
CY031179_A_duck_Vietnam_7A_2004_Avian
CY016898_A_duck_Viet_Nam_20_2005_Avian

PB2-Group81

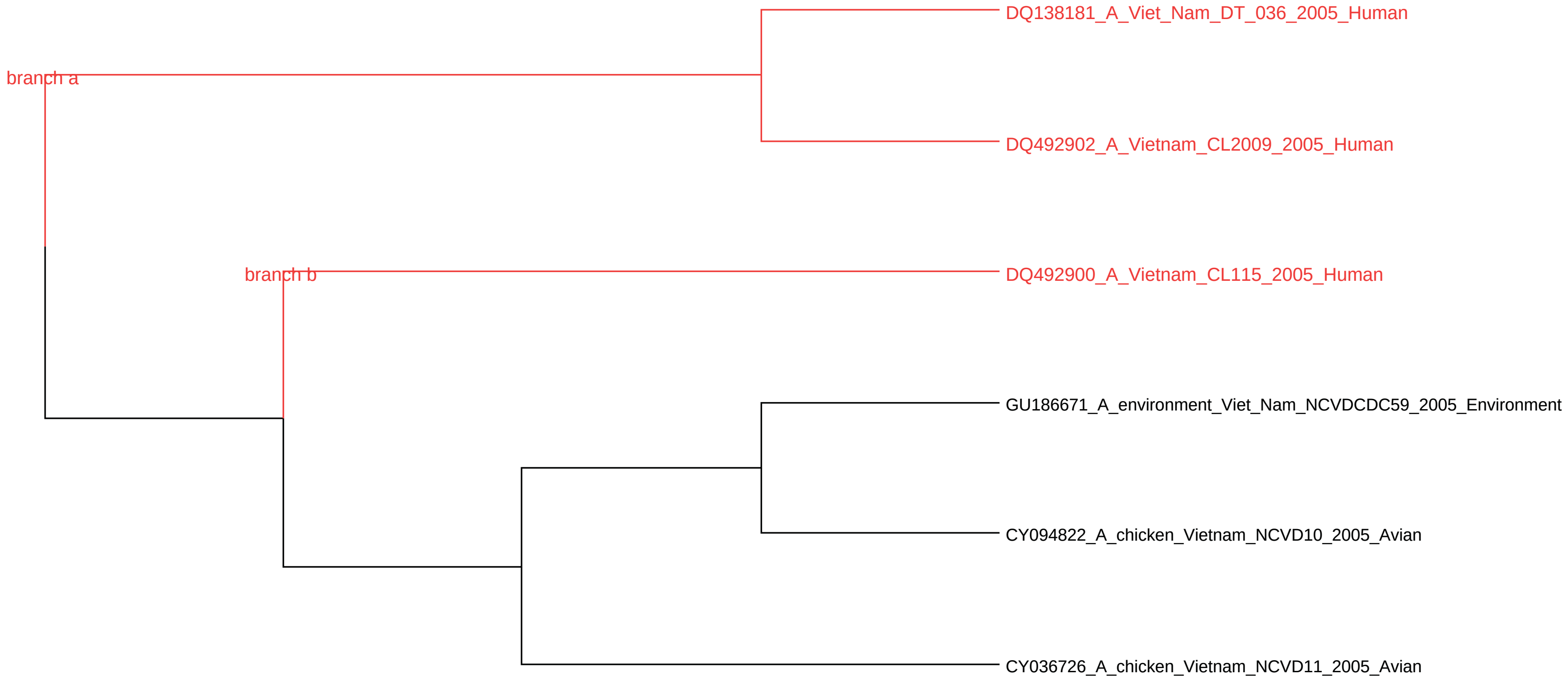

branch a
branch b
DQ138181_A_Viet_Nam_DT_036_2005_Human
DQ492902_A_Vietnam_CL2009_2005_Human
DQ492900_A_Vietnam_CL115_2005_Human
GU186671_A_environment_Viet_Nam_NCVDCDC59_2005_Environment
CY094822_A_chicken_Vietnam_NCVD10_2005_Avian
CY036726_A_chicken_Vietnam_NCVD11_2005_Avian

PB2-Group82

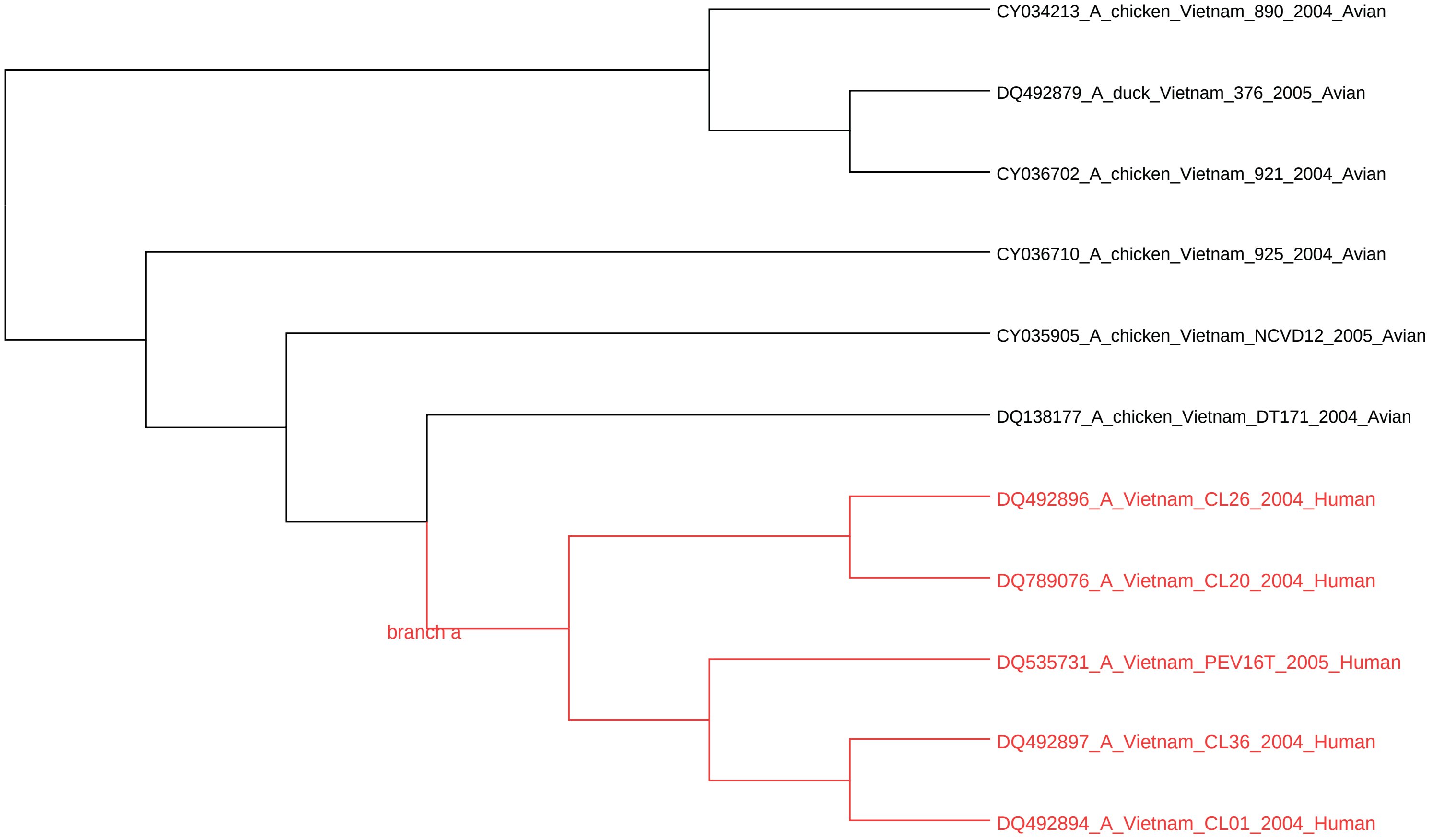

CY034213_A_chicken_Vietnam_890_2004_Avian
DQ492879_A_duck_Vietnam_376_2005_Avian
CY036702_A_chicken_Vietnam_921_2004_Avian
CY036710_A_chicken_Vietnam_925_2004_Avian
CY035905_A_chicken_Vietnam_NCVD12_2005_Avian
DQ138177_A_chicken_Vietnam_DT171_2004_Avian
DQ492896_A_Vietnam_CL26_2004_Human
DQ789076_A_Vietnam_CL20_2004_Human
branch a
DQ535731_A_Vietnam_PEV16T_2005_Human
DQ492897_A_Vietnam_CL36_2004_Human
DQ492894_A_Vietnam_CL01_2004_Human

PB2-Group83

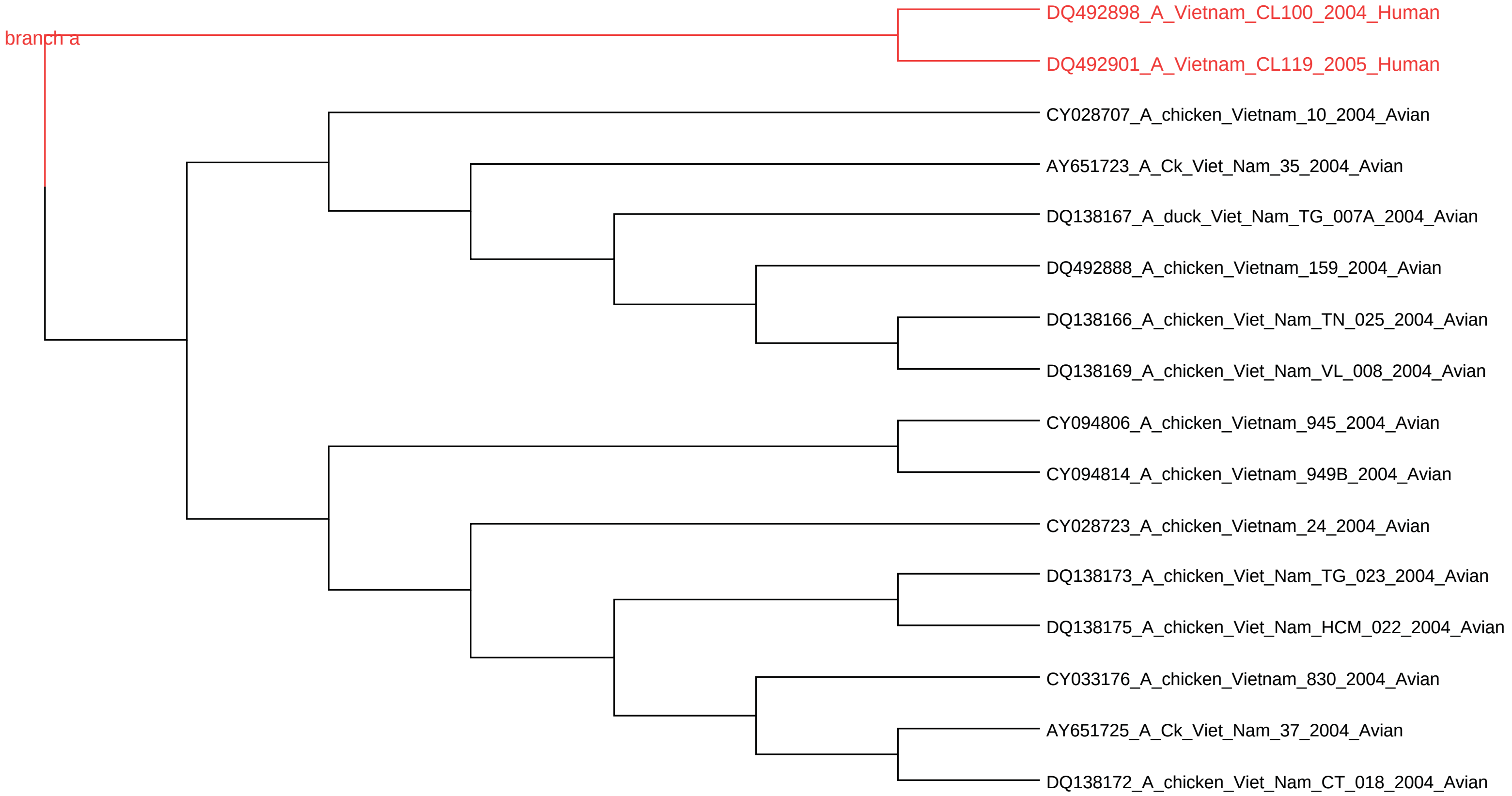
branch a
DQ492898_A_Vietnam_CL100_2004_Human
DQ492901_A_Vietnam_CL119_2005_Human
CY028707_A_chicken_Vietnam_10_2004_Avian
AY651723_A_Ck_Viet_Nam_35_2004_Avian
DQ138167_A_duck_Viet_Nam_TG_007A_2004_Avian
DQ492888_A_chicken_Vietnam_159_2004_Avian
DQ138166_A_chicken_Viet_Nam_TN_025_2004_Avian
DQ138169_A_chicken_Viet_Nam_VL_008_2004_Avian
CY094806_A_chicken_Vietnam_945_2004_Avian
CY094814_A_chicken_Vietnam_949B_2004_Avian
CY028723_A_chicken_Vietnam_24_2004_Avian
DQ138173_A_chicken_Viet_Nam_TG_023_2004_Avian
DQ138175_A_chicken_Viet_Nam_HCM_022_2004_Avian
CY033176_A_chicken_Vietnam_830_2004_Avian
AY651725_A_Ck_Viet_Nam_37_2004_Avian
DQ138172_A_chicken_Viet_Nam_CT_018_2004_Avian

PB2-Group84

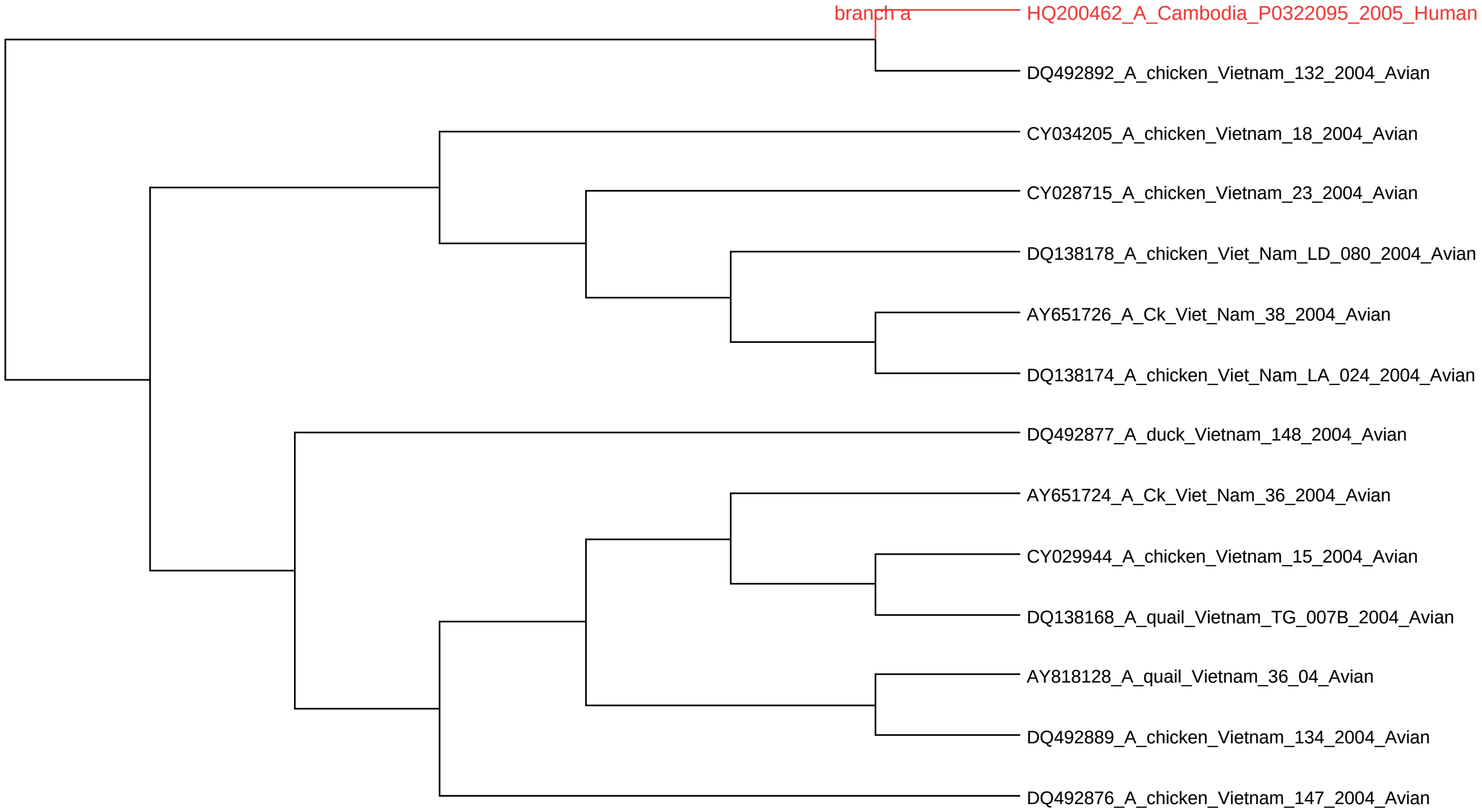

branch a
HQ200462_A_Cambodia_P0322095_2005_Human
DQ492892_A_chicken_Vietnam_132_2004_Avian
CY034205_A_chicken_Vietnam_18_2004_Avian
CY028715_A_chicken_Vietnam_23_2004_Avian
DQ138178_A_chicken_Viet_Nam_LD_080_2004_Avian
AY651726_A_Ck_Viet_Nam_38_2004_Avian
DQ138174_A_chicken_Viet_Nam_LA_024_2004_Avian
DQ492877_A_duck_Vietnam_148_2004_Avian
AY651724_A_Ck_Viet_Nam_36_2004_Avian
CY029944_A_chicken_Vietnam_15_2004_Avian
DQ138168_A_quail_Vietnam_TG_007B_2004_Avian
AY818128_A_quail_Vietnam_36_04_Avian
DQ492889_A_chicken_Vietnam_134_2004_Avian
DQ492876_A_chicken_Vietnam_147_2004_Avian

PB2-Group85

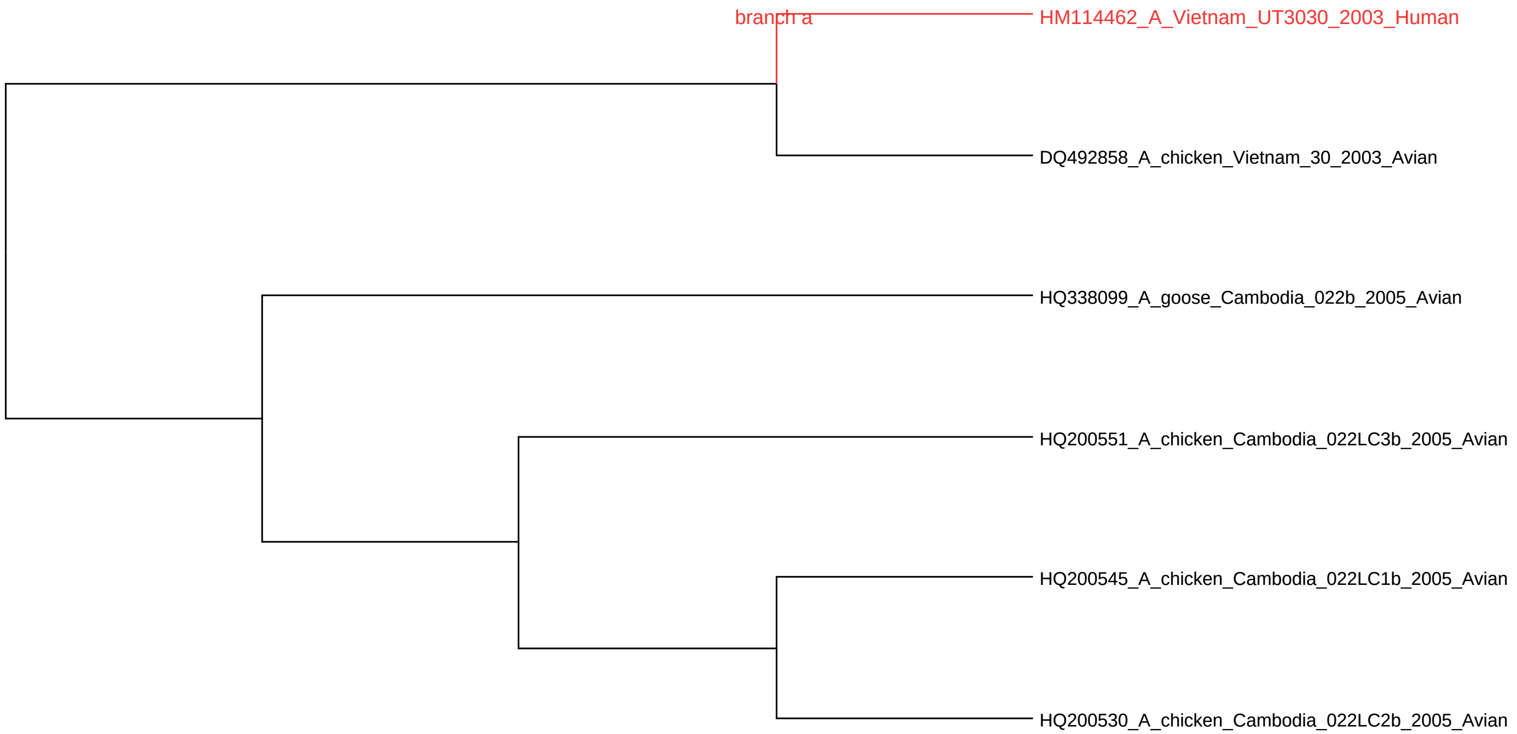

branch a
HM114462_A_Vietnam_UT3030_2003_Human
DQ492858_A_chicken_Vietnam_30_2003_Avian
HQ338099_A_goose_Cambodia_022b_2005_Avian
HQ200551_A_chicken_Cambodia_022LC3b_2005_Avian
HQ200545_A_chicken_Cambodia_022LC1b_2005_Avian
HQ200530_A_chicken_Cambodia_022LC2b_2005_Avian

PB2-Group86

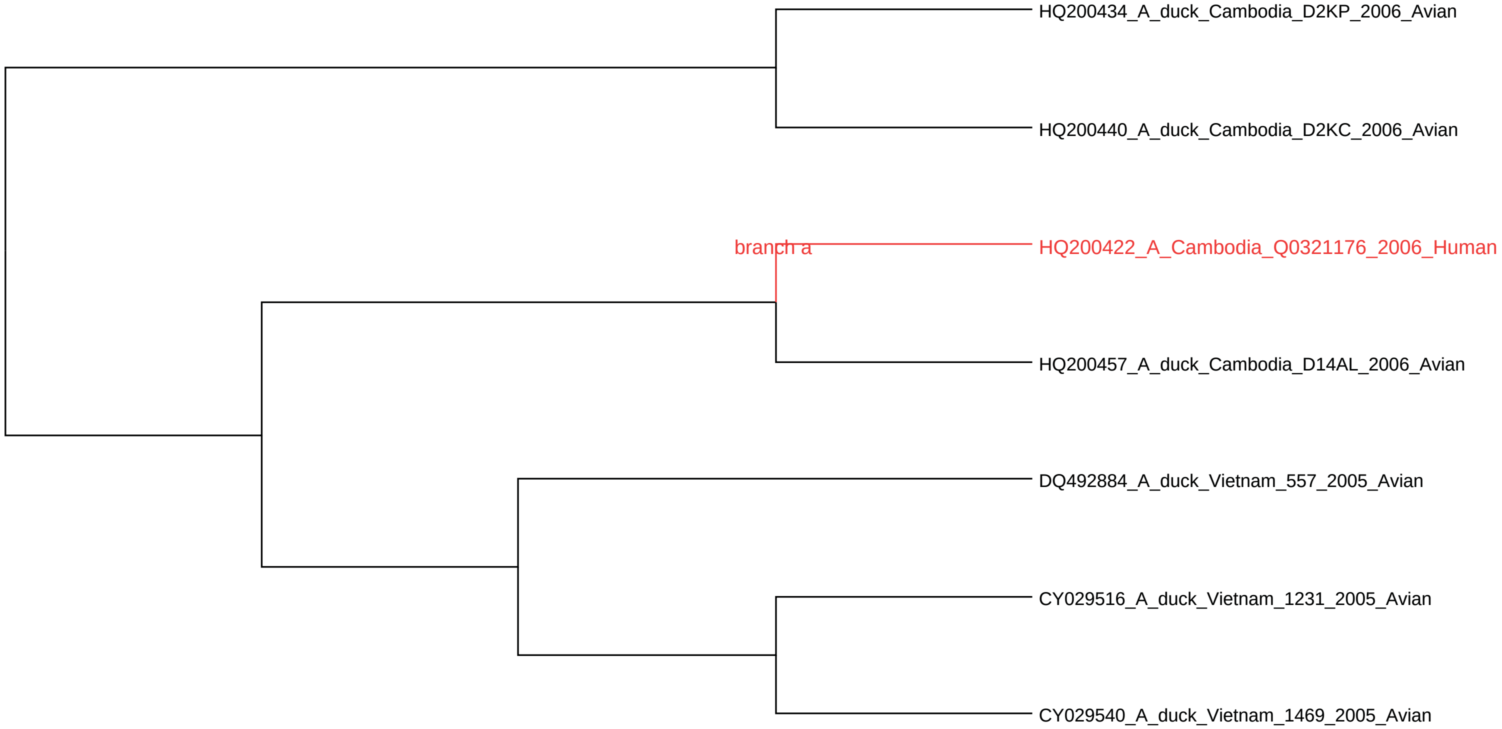

HQ200434_A_duck_Cambodia_D2KP_2006_Avian
HQ200440_A_duck_Cambodia_D2KC_2006_Avian
branch a
HQ200422_A_Cambodia_Q0321176_2006_Human
HQ200457_A_duck_Cambodia_D14AL_2006_Avian
DQ492884_A_duck_Vietnam_557_2005_Avian
CY029516_A_duck_Vietnam_1231_2005_Avian
CY029540_A_duck_Vietnam_1469_2005_Avian

PB2-Group87

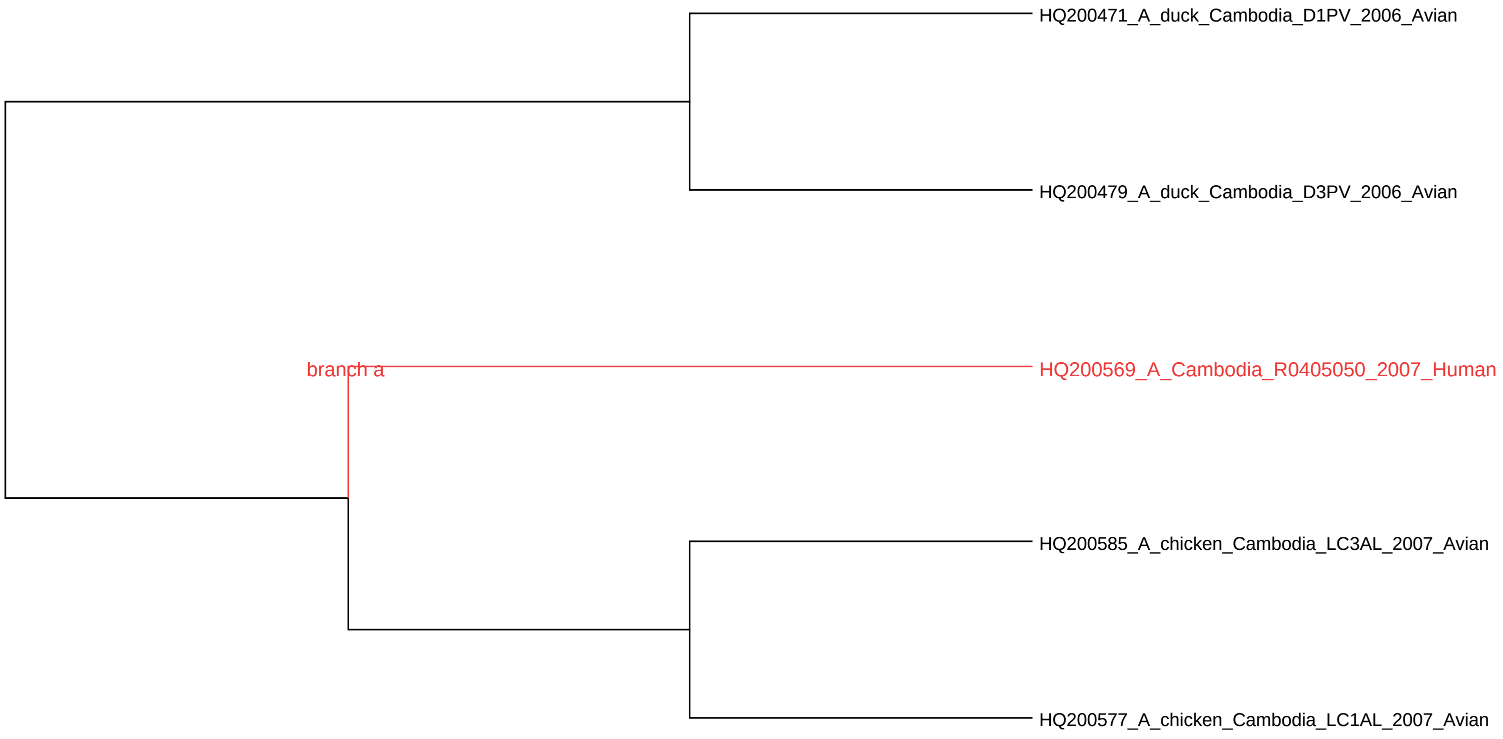
HQ200471_A_duck_Cambodia_D1PV_2006_Avian
HQ200479_A_duck_Cambodia_D3PV_2006_Avian
branch a
HQ200569_A_Cambodia_R0405050_2007_Human
HQ200585_A_chicken_Cambodia_LC3AL_2007_Avian
HQ200577_A_chicken_Cambodia_LC1AL_2007_Avian

PB2-Group88

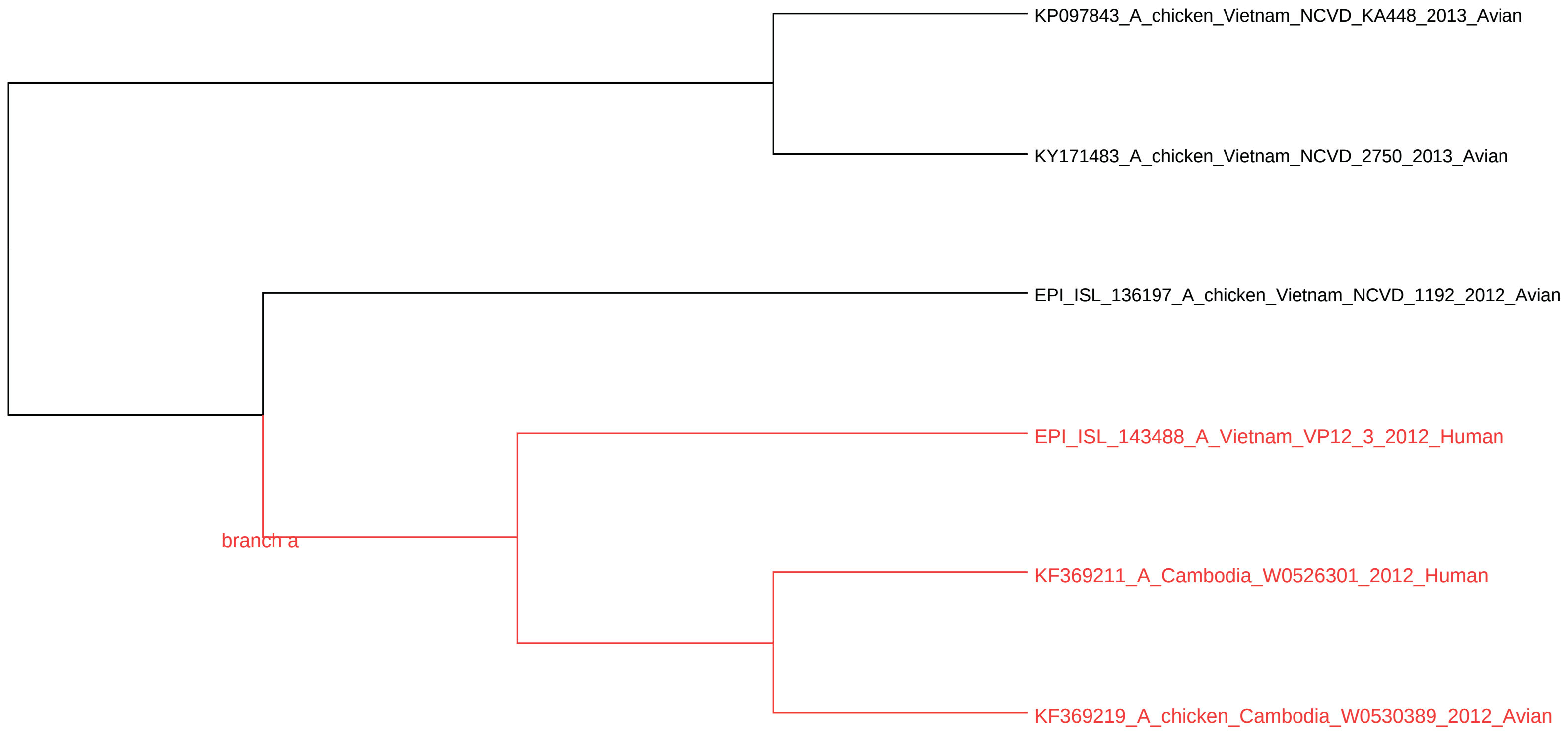
KP097843_A_chicken_Vietnam_NCVD_KA448_2013_Avian
KY171483_A_chicken_Vietnam_NCVD_2750_2013_Avian
EPI_ISL_136197_A_chicken_Vietnam_NCVD_1192_2012_Avian
EPI_ISL_143488_A_Vietnam_VP12_3_2012_Human
branch a
KF369211_A_Cambodia_W0526301_2012_Human
KF369219_A_chicken_Cambodia_W0530389_2012_Avian

PB2-Group89

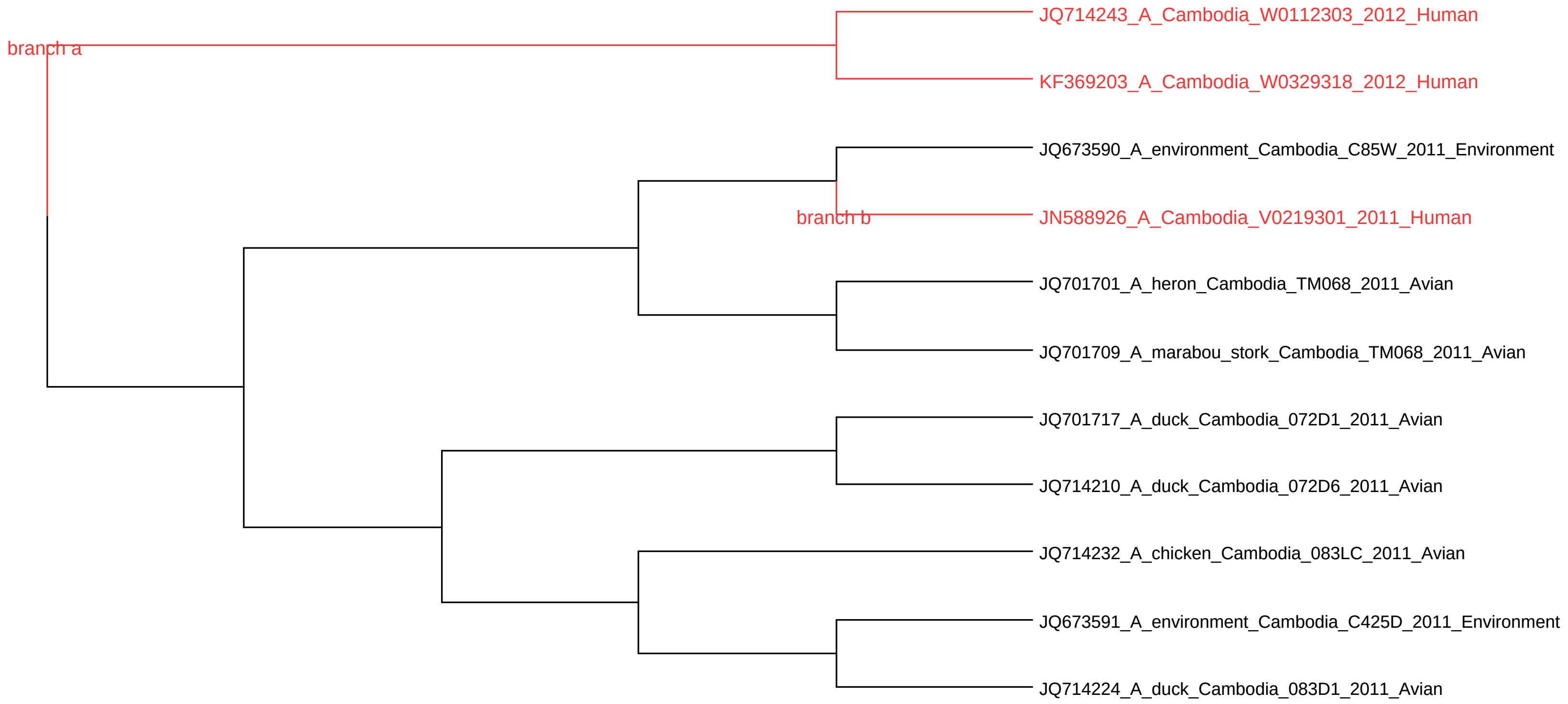
branch a
branch b
JQ714243_A_Cambodia_W0112303_2012_Human
KF369203_A_Cambodia_W0329318_2012_Human
JQ673590_A_environment_Cambodia_C85W_2011_Environment
JN588926_A_Cambodia_V0219301_2011_Human
JQ701701_A_heron_Cambodia_TM068_2011_Avian
JQ701709_A_marabou_stork_Cambodia_TM068_2011_Avian
JQ701717_A_duck_Cambodia_072D1_2011_Avian
JQ714210_A_duck_Cambodia_072D6_2011_Avian
JQ714232_A_chicken_Cambodia_083LC_2011_Avian
JQ673591_A_environment_Cambodia_C425D_2011_Environment
JQ714224_A_duck_Cambodia_083D1_2011_Avian

PB2-Group90

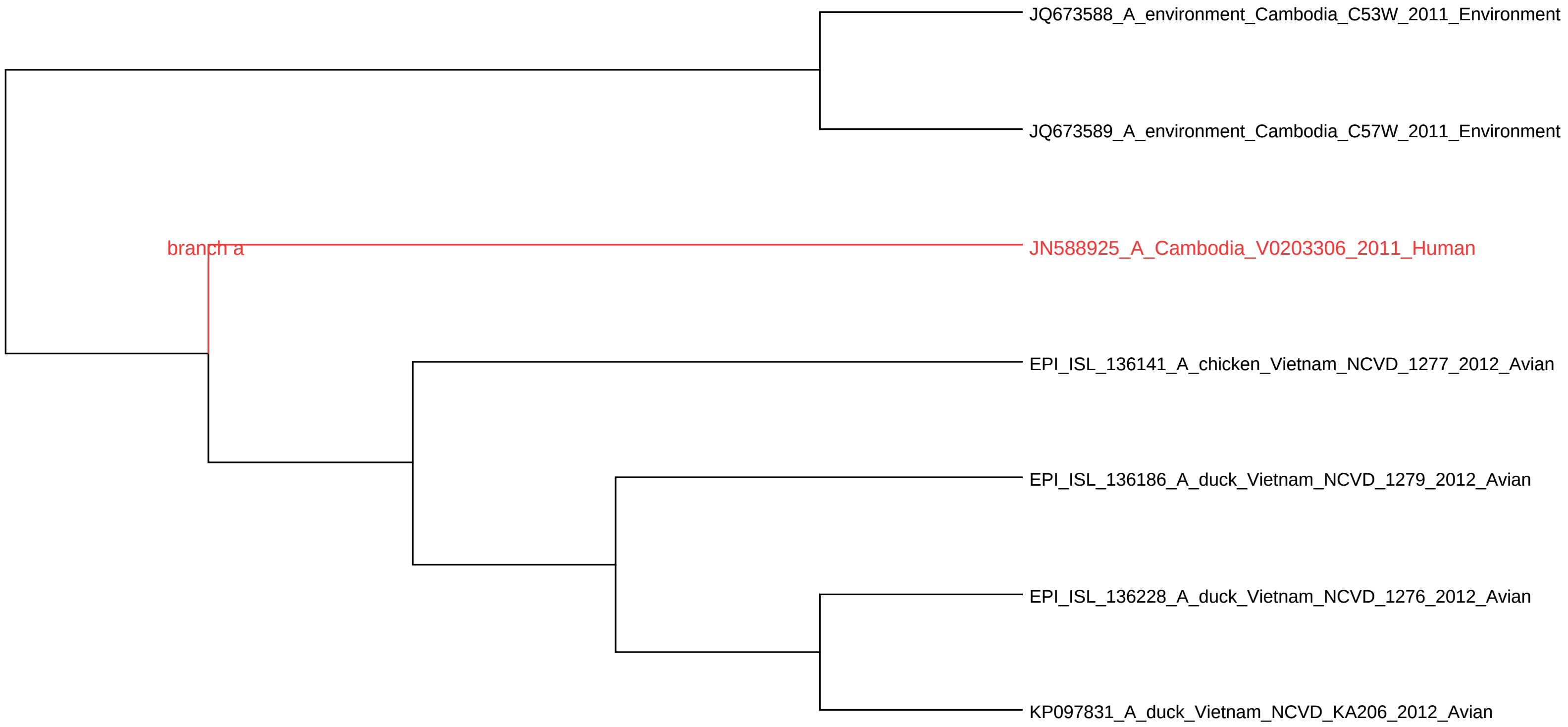
JQ673588_A_environment_Cambodia_C53W_2011_Environment
JQ673589_A_environment_Cambodia_C57W_2011_Environment
branch a
JN588925_A_Cambodia_V0203306_2011_Human
EPI_ISL_136141_A_chicken_Vietnam_NCVD_1277_2012_Avian
EPI_ISL_136186_A_duck_Vietnam_NCVD_1279_2012_Avian
EPI_ISL_136228_A_duck_Vietnam_NCVD_1276_2012_Avian
KP097831_A_duck_Vietnam_NCVD_KA206_2012_Avian

PB2-Group91

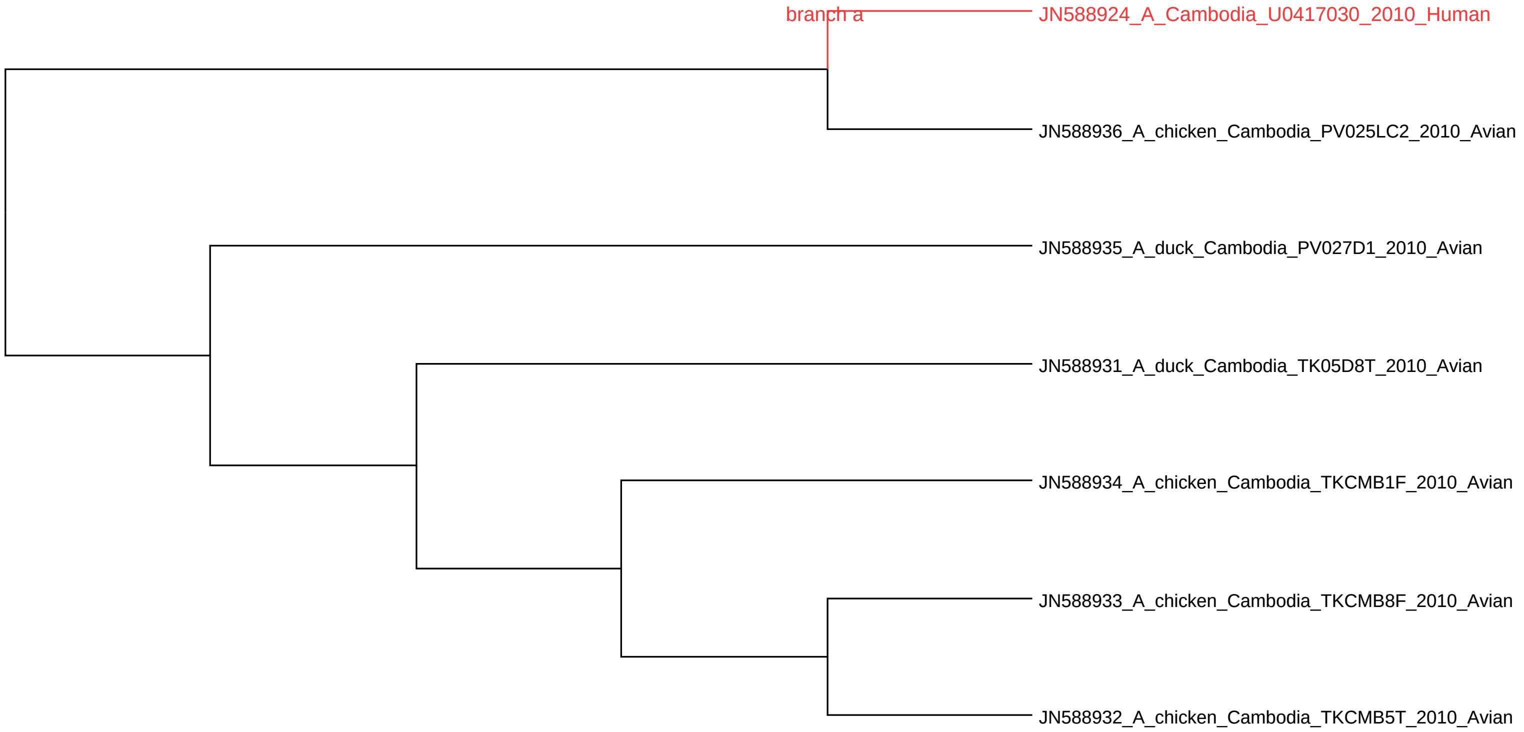
branch a
JN588924_A_Cambodia_U0417030_2010_Human
JN588936_A_chicken_Cambodia_PV025LC2_2010_Avian
JN588935_A_duck_Cambodia_PV027D1_2010_Avian
JN588931_A_duck_Cambodia_TK05D8T_2010_Avian
JN588934_A_chicken_Cambodia_TKCMB1F_2010_Avian
JN588933_A_chicken_Cambodia_TKCMB8F_2010_Avian
JN588932_A_chicken_Cambodia_TKCMB5T_2010_Avian

PB2-Group92

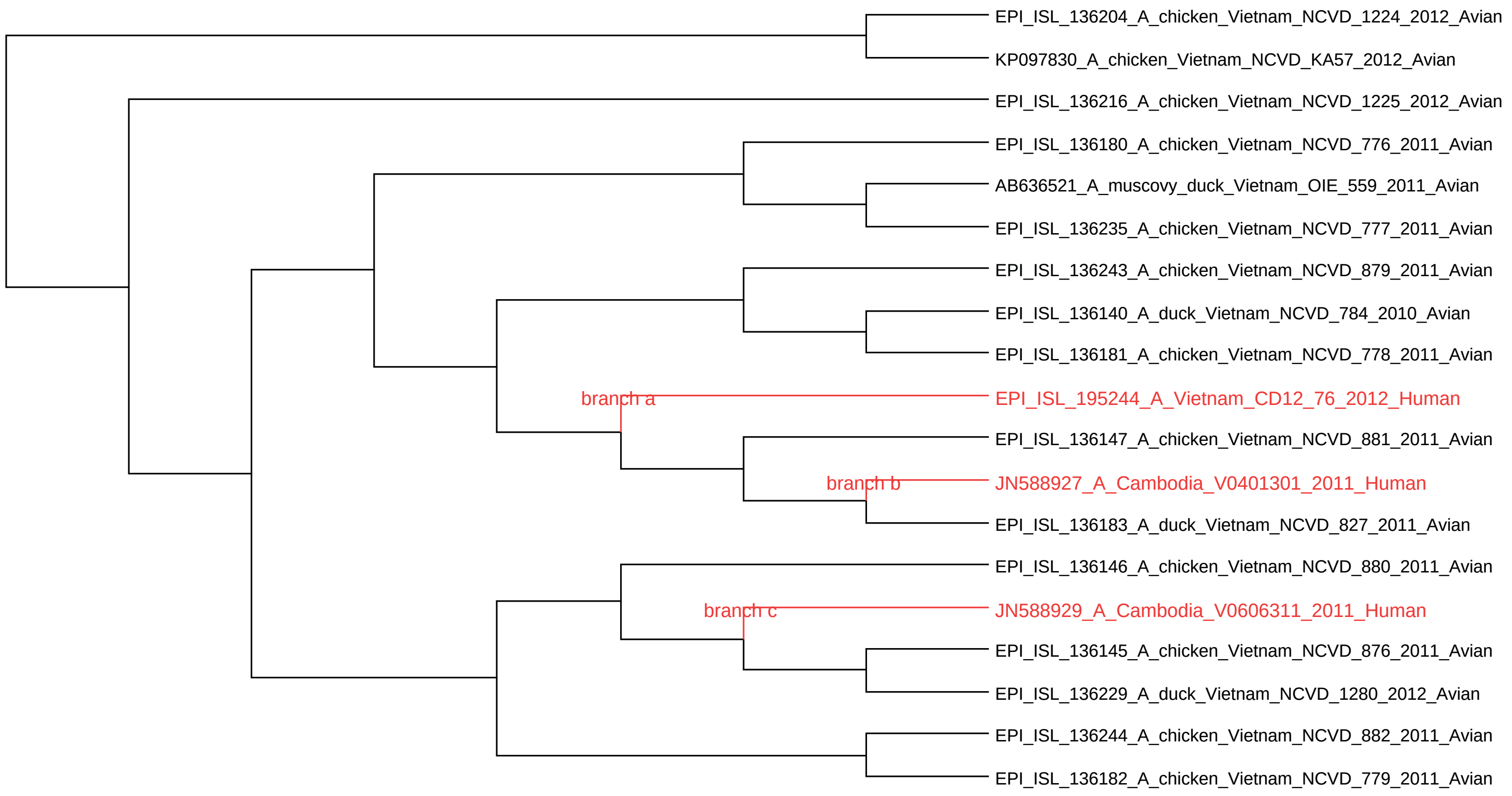

EPI_ISL_136204_A_chicken_Vietnam_NCVD_1224_2012_Avian
KP097830_A_chicken_Vietnam_NCVD_KA57_2012_Avian
EPI_ISL_136216_A_chicken_Vietnam_NCVD_1225_2012_Avian
EPI_ISL_136180_A_chicken_Vietnam_NCVD_776_2011_Avian
AB636521_A_muscovy_duck_Vietnam_OIE_559_2011_Avian
EPI_ISL_136235_A_chicken_Vietnam_NCVD_777_2011_Avian
EPI_ISL_136243_A_chicken_Vietnam_NCVD_879_2011_Avian
EPI_ISL_136140_A_duck_Vietnam_NCVD_784_2010_Avian
EPI_ISL_136181_A_chicken_Vietnam_NCVD_778_2011_Avian
branch a
EPI_ISL_195244_A_Vietnam_CD12_76_2012_Human
EPI_ISL_136147_A_chicken_Vietnam_NCVD_881_2011_Avian
branch b
JN588927_A_Cambodia_V0401301_2011_Human
EPI_ISL_136183_A_duck_Vietnam_NCVD_827_2011_Avian
EPI_ISL_136146_A_chicken_Vietnam_NCVD_880_2011_Avian
branch c
JN588929_A_Cambodia_V0606311_2011_Human
EPI_ISL_136145_A_chicken_Vietnam_NCVD_876_2011_Avian
EPI_ISL_136229_A_duck_Vietnam_NCVD_1280_2012_Avian
EPI_ISL_136244_A_chicken_Vietnam_NCVD_882_2011_Avian
EPI_ISL_136182_A_chicken_Vietnam_NCVD_779_2011_Avian
