# Supplementary material for: Adaptive Evolution of Human-Isolated H5Nx Avian Influenza A Viruses
Source: Front Microbiol. 2019 Jun 12;10:1328. doi: 10.3389/fmicb.2019.01328 (PMC6582624; doi:10.3389/fmicb.2019.01328)
Supplement: Supplementary file 12 [file Table_2.DOCX]

**Supplementary table 2:** Clade distribution of human-isolated H5Nx sequences.

| **Human-isolated Strains of H5Nx** | **Clade** |
| --- | --- |
| AF028709_A_Hong_Kong_156_97 | 0 |
| AF046096_A_Hong_Kong_481_97 | 0 |
| AF046097_A_Hong_Kong_483_1997 | 0 |
| AF046098_A_Hong_Kong_482_97 | 0 |
| AF084281_A_Hong_Kong_486_97 | 0 |
| AF084532_A_Hong_Kong_485_1997 | 0 |
| AF102672_A_Hong_Kong_488_97 | 0 |
| AF102673_A_Hong_Kong_516_97 | 0 |
| AF102674_A_Hong_Kong_538_97 | 0 |
| AF102675_A_Hong_Kong_507_97 | 0 |
| AF102676_A_HongKong_97_98 | 0 |
| AF102677_A_Hong_Kong_491_97 | 0 |
| AF102678_A_Hong_Kong_542_97 | 0 |
| AF102679_A_Hong_Kong_503_97 | 0 |
| AF102680_A_Hong_Kong_532_1997 | 0 |
| AF102682_A_Hong_Kong_514_97 | 0 |
| AB212054_A_Hong_Kong_213_2003 | 1 |
| AB239125_A_Hanoi_30408_2005 | 1 |
| AB598119_A_Cambodia_408008_2005 | 1 |
| AJ715872_A_Hanoi_03_2004 | 1 |
| AY555150_A_Thailand_1_KAN_1_2004 | 1 |
| AY555153_A_Thailand_2_SP_33_2004 | 1 |
| AY575869_A_HK_212_03_ | 1 |
| AY577314_A_Thailand_3_SP_83_2004 | 1 |
| AY626143_A_Thailand_4_SP_528_2004 | 1 |
| AY627885_A_Thailand_5_KK_494_2004 | 1 |
| AY651333_A_Vietnam_1194_2004 | 1 |
| AY651334_A_Viet_Nam_1203_2004 | 1 |
| AY651335_A_Viet_Nam_3046_2004 | 1 |
| AY651336_A_Viet_Nam_3062_2004 | 1 |
| AY679514_A_Thailand_LFPN_2004_2004 | 1 |
| AY720950_A_Viet_Nam_DN_33_2004 | 1 |
| DQ360835_A_Thailand_676_2005 | 1 |
| DQ372591_A_Thailand_NK165_2005 | 1 |
| DQ497719_A_Vietnam_CL01_2004 | 1 |
| DQ497720_A_Vietnam_CL02_2004 | 1 |
| DQ497721_A_Vietnam_CL17_2004 | 1 |
| DQ497722_A_Vietnam_CL20_2004 | 1 |
| DQ497723_A_Vietnam_CL26_2004 | 1 |
| DQ497724_A_Vietnam_CL36_2004 | 1 |
| DQ497725_A_Vietnam_CL100_2004 | 1 |
| DQ497726_A_Vietnam_CL105_2005 | 1 |
| DQ497727_A_Vietnam_CL115_2005 | 1 |
| DQ497728_A_Vietnam_CL119_2005 | 1 |
| DQ497729_A_Vietnam_CL2009_2005 | 1 |
| DQ535724_A_Vietnam_PEV16T_2005 | 1 |
| DQ885610_A_Thailand_NKFE_2005 | 1 |
| DQ885612_A_Thailand_NKNP_2005 | 1 |
| DQ885614_A_Thailand_RPFE_2005 | 1 |
| DQ885616_A_Thailand_RPNP_2005 | 1 |
| DQ885618_A_Thailand_HA20_2005 | 1 |
| EF107522_A_Thailand_1_KAN_1A_2004 | 1 |
| EF451059_A_Viet_Nam_3212_2004 | 1 |
| EF456795_A_Viet_Nam_JP178_2004 | 1 |
| EF456798_A_Viet_Nam_JP4207_2005 | 1 |
| EF456799_A_Viet_Nam_JP14_2005 | 1 |
| EF456802_A_Viet_Nam_JPHN30321_2005 | 1 |
| EF456803_A_Viet_Nam_HN30408_2005 | 1 |
| EF456805_A_Cambodia_JP52a_2005 | 1 |
| EF541392_A_Prachinburi_6231_2004 | 1 |
| EF541404_A_Viet_Nam_1204_2004 | 1 |
| EF541408_A_Thailand_16_2004 | 1 |
| EF541410_A_Thailand_SP83_2004 | 1 |
| EF541411_A_Thailand_Kan353_2004 | 1 |
| EF541417_A_Thailand_Chaiyaphum_622_2004 | 1 |
| EU311220_A_India_m777_2007 | 1 |
| GQ466176_A_Thailand_NBL1_2006 | 1 |
| HM114449_A_Vietnam_UT3028_2003 | 1 |
| HM114457_A_Vietnam_UT3028II_2003 | 1 |
| HM114465_A_Vietnam_UT3030_2003 | 1 |
| HM114473_A_Vietnam_UT3035_2003 | 1 |
| HM114481_A_Vietnam_UT3040_2004 | 1 |
| HM114490_A_Vietnam_UT3040II_2004 | 1 |
| HM114497_A_Vietnam_UT3047III_2004 | 1 |
| HM114505_A_Vietnam_UT3062_2004 | 1 |
| HM114513_A_Vietnam_UT30259_2004 | 1 |
| HM114521_A_Vietnam_HN30262IIIM3_2004 | 1 |
| HM114529_A_Vietnam_UT30408III_2005 | 1 |
| HQ200417_A_Cambodia_Q0321176_2006 | 1 |
| HQ200423_A_Cambodia_Q0405047_2006 | 1 |
| HQ200458_A_Cambodia_P0322095_2005 | 1 |
| HQ200466_A_Cambodia_VN05_103_2005 | 1 |
| FJ225472_A_Cambodia_R0405050_2007 | 1.1 |
| DQ435202_A_Iraq_207_NAMRU3_2006 | 2.2 |
| EF619982_A_Turkey_12_2006 | 2.2 |
| EF619989_A_Turkey_15_2006 | 2.2 |
| EF619990_A_Turkey_651242_2006 | 2.2 |
| EF619998_A_Turkey_65596_2006 | 2.2 |
| EPI_ISL_79681_A_Xinjiang_1_2006 | 2.2 |
| EU146869_A_Iraq_755_2006 | 2.2 |
| EU146870_A_Iraq_1_2006 | 2.2 |
| EU146871_A_Azerbaijan_006_207_2006 | 2.2 |
| EU146872_A_Azerbaijan_008_208_2006 | 2.2 |
| EU146873_A_Azerbaijan_011_162_2006 | 2.2 |
| EU146874_A_Azerbaijan_001_161_2006 | 2.2 |
| EU146875_A_Azerbaijan_002_115_2006 | 2.2 |
| EU146876_A_Iraq_659_2006 | 2.2 |
| EU146877_A_Iraq_754_2006 | 2.2 |
| EU146878_A_Iraq_756_2006 | 2.2 |
| EU146920_A_Nigeria_6e_07 | 2.2 |
| GU052089_A_Hong_Kong_378_1_2001 | 3 |
| EF553329_A_Anhui_T2_2006 | 7 |
| EF587277_A_Beijing_01_2003 | 7 |
| EPI_ISL_195244_A_Vietnam_CD12_76_2012 | 1.1.1 |
| HQ200596_A_Cambodia_S1211394_2008 | 1.1.1 |
| JN588807_A_Cambodia_V0401301_2011 | 1.1.1 |
| JN588809_A_Cambodia_V0606311_2011 | 1.1.1 |
| EPI_ISL_143488_A_Vietnam_VP12_3_2012 | 1.1.2 |
| EPI_ISL_150014_A_Cambodia_X0125302_2013 | 1.1.2 |
| EPI_ISL_150015_A_Cambodia_X0810301_2013 | 1.1.2 |
| EPI_ISL_153029_A_Cambodia_X1030304_2013 | 1.1.2 |
| EPI_ISL_191955_A_Vietnam_14012902_2014 | 1.1.2 |
| EPI_ISL_191956_A_Vietnam_VP13_28H_2013 | 1.1.2 |
| EPI_ISL_195245_A_Vietnam_VP39_2013 | 1.1.2 |
| JN588804_A_Cambodia_U0417030_2010 | 1.1.2 |
| JN588805_A_Cambodia_V0203306_2011 | 1.1.2 |
| JN588806_A_Cambodia_V0219301_2011 | 1.1.2 |
| JN588808_A_Cambodia_V0417301_2011 | 1.1.2 |
| JQ714217_A_Cambodia_V0719348_2011 | 1.1.2 |
| JQ714220_A_Cambodia_V0813302_2011 | 1.1.2 |
| JQ714246_A_Cambodia_W0112303_2012 | 1.1.2 |
| KF001372_A_Cambodia_X0123311_2013 | 1.1.2 |
| KF001380_A_Cambodia_X0123312_2013 | 1.1.2 |
| KF001396_A_Cambodia_X0207301_2013 | 1.1.2 |
| KF001404_A_Cambodia_X0212301_2013 | 1.1.2 |
| KF001412_A_Cambodia_X0219301_2013 | 1.1.2 |
| KF001417_A_Cambodia_X0215301_2013 | 1.1.2 |
| KF369206_A_Cambodia_W0329318_2012 | 1.1.2 |
| KF369214_A_Cambodia_W0526301_2012 | 1.1.2 |
| KF369257_A_Cambodia_X0502302_2013 | 1.1.2 |
| KF918456_A_Cambodia_X0628313_2013 | 1.1.2 |
| KF918464_A_Cambodia_X0808305_2013 | 1.1.2 |
| KF918490_A_Cambodia_X0817302_2013 | 1.1.2 |
| KF918498_A_Cambodia_X0828324_2013 | 1.1.2 |
| KF918506_A_Cambodia_X0913301_2013 | 1.1.2 |
| KF918514_A_Cambodia_X0916322_2013 | 1.1.2 |
| KF918530_A_Cambodia_X1024307_2013 | 1.1.2 |
| CY014272_A_Indonesia_CDC594_2006 | 2.1.2 |
| CY014280_A_Indonesia_CDC595_2006 | 2.1.2 |
| CY014288_A_Indonesia_CDC596_2006 | 2.1.2 |
| CY014296_A_Indonesia_CDC597_2006 | 2.1.2 |
| CY014303_A_Indonesia_CDC599_2006 | 2.1.2 |
| CY014433_A_Indonesia_CDC625_2006 | 2.1.2 |
| CY014465_A_Indonesia_CDC625L_2006 | 2.1.2 |
| CY014477_A_Indonesia_CDC599N_2006 | 2.1.2 |
| EU146737_A_Indonesia_534H_2006 | 2.1.2 |
| EU146745_A_Indonesia_538H_2006 | 2.1.2 |
| EU146753_A_Indonesia_535H_2006 | 2.1.2 |
| EU146754_A_Indonesia_536H_2006 | 2.1.2 |
| EU146755_A_Indonesia_546H_2006 | 2.1.2 |
| EU146785_A_Indonesia_560H_2006 | 2.1.2 |
| EU146793_A_Indonesia_546bH_2006 | 2.1.2 |
| EU146624_A_Indonesia_6_2005 | 2.1.3 |
| CY014168_A_Indonesia_CDC194P_2005 | 2.1.3.2 |
| CY014177_A_Indonesia_CDC7_2005 | 2.1.3.2 |
| CY014197_A_Indonesia_CDC184_2005 | 2.1.3.2 |
| CY014198_A_Indonesia_CDC287E_2005 | 2.1.3.2 |
| CY014199_A_Indonesia_CDC287T_2005 | 2.1.3.2 |
| CY014200_A_Indonesia_CDC292N_2005 | 2.1.3.2 |
| CY014201_A_Indonesia_CDC292T_2005 | 2.1.3.2 |
| CY014202_A_Indonesia_CDC326N_2006 | 2.1.3.2 |
| CY014203_A_Indonesia_CDC326N2_2006 | 2.1.3.2 |
| CY014204_A_Indonesia_CDC326_2006 | 2.1.3.2 |
| CY014205_A_Indonesia_CDC326T_2006 | 2.1.3.2 |
| CY014206_A_Indonesia_CDC329_2006 | 2.1.3.2 |
| CY014207_A_Indonesia_CDC357_2006 | 2.1.3.2 |
| CY014209_A_Indonesia_CDC370_2006 | 2.1.3.2 |
| CY014210_A_Indonesia_CDC370E_2006 | 2.1.3.2 |
| CY014211_A_Indonesia_CDC370P_2006 | 2.1.3.2 |
| CY014212_A_Indonesia_CDC370T_2006 | 2.1.3.2 |
| CY014213_A_Indonesia_CDC390_2006 | 2.1.3.2 |
| CY014311_A_Indonesia_CDC523_2006 | 2.1.3.2 |
| CY014368_A_Indonesia_CDC523E_2006 | 2.1.3.2 |
| CY014376_A_Indonesia_CDC523T_2006 | 2.1.3.2 |
| CY014384_A_Indonesia_CDC582_2006 | 2.1.3.2 |
| CY014393_A_Indonesia_CDC610_2006 | 2.1.3.2 |
| CY014401_A_Indonesia_CDC623_2006 | 2.1.3.2 |
| CY014409_A_Indonesia_CDC623E_2006 | 2.1.3.2 |
| CY014417_A_Indonesia_CDC624_2006 | 2.1.3.2 |
| CY014425_A_Indonesia_CDC624E_2006 | 2.1.3.2 |
| CY014441_A_Indonesia_CDC634_2006 | 2.1.3.2 |
| CY014449_A_Indonesia_CDC634P_2006 | 2.1.3.2 |
| CY014457_A_Indonesia_CDC634T_2006 | 2.1.3.2 |
| CY014481_A_Indonesia_CDC669_2006 | 2.1.3.2 |
| CY014489_A_Indonesia_CDC669P_2006 | 2.1.3.2 |
| CY014497_A_Indonesia_CDC699_2006 | 2.1.3.2 |
| CY014510_A_Indonesia_CDC644T_2006 | 2.1.3.2 |
| CY014518_A_Indonesia_CDC644_2006 | 2.1.3.2 |
| CY014529_A_Indonesia_CDC739_2006 | 2.1.3.2 |
| CY014537_A_Indonesia_CDC742_2006 | 2.1.3.2 |
| CY014543_A_Indonesia_CDC759_2006 | 2.1.3.2 |
| CY017638_A_Indonesia_CDC938_2006 | 2.1.3.2 |
| CY017646_A_Indonesia_CDC938E_2006 | 2.1.3.2 |
| CY017654_A_Indonesia_CDC940_2006 | 2.1.3.2 |
| CY017662_A_Indonesia_CDC835_2006 | 2.1.3.2 |
| CY017670_A_Indonesia_CDC836_2006 | 2.1.3.2 |
| CY017678_A_Indonesia_CDC836T_2006 | 2.1.3.2 |
| CY017688_A_Indonesia_CDC887_2006 | 2.1.3.2 |
| CY019352_A_Indonesia_CDC1031_2007 | 2.1.3.2 |
| CY019360_A_Indonesia_CDC1031T_2007 | 2.1.3.2 |
| CY019368_A_Indonesia_CDC1031T2_2007 | 2.1.3.2 |
| CY019376_A_Indonesia_CDC1031RE2_2007 | 2.1.3.2 |
| CY019384_A_Indonesia_CDC1032_2007 | 2.1.3.2 |
| CY019392_A_Indonesia_CDC1032N_2007 | 2.1.3.2 |
| CY019400_A_Indonesia_CDC1032T_2007 | 2.1.3.2 |
| CY019408_A_Indonesia_CDC1046_2007 | 2.1.3.2 |
| CY019416_A_Indonesia_CDC1046T_2007 | 2.1.3.2 |
| CY019424_A_Indonesia_CDC1047_2007 | 2.1.3.2 |
| CY019432_A_Indonesia_CDC1047S_2007 | 2.1.3.2 |
| CY116646_A_Indonesia_5_2005 | 2.1.3.2 |
| EPI_ISL_150052_A_Indonesia_NIHRD7988_2008 | 2.1.3.2 |
| EPI_ISL_151057_A_Indonesia_NIHRD11454_2011 | 2.1.3.2 |
| EPI_ISL_24752_A_Indonesia_7379_2008 | 2.1.3.2 |
| EU015403_A_Indonesia_TLL001_2006 | 2.1.3.2 |
| EU015404_A_Indonesia_TLL002_2006 | 2.1.3.2 |
| EU015405_A_Indonesia_TLL003_2006 | 2.1.3.2 |
| EU015406_A_Indonesia_TLL004_2006 | 2.1.3.2 |
| EU015407_A_Indonesia_TLL005_2006 | 2.1.3.2 |
| EU015408_A_Indonesia_TLL006_2006 | 2.1.3.2 |
| EU015409_A_Indonesia_TLL007_2006 | 2.1.3.2 |
| EU015410_A_Indonesia_TLL008_2006 | 2.1.3.2 |
| EU015411_A_Indonesia_TLL009_2006 | 2.1.3.2 |
| EU015412_A_Indonesia_TLL010_2006 | 2.1.3.2 |
| EU015413_A_Indonesia_TLL011_2006 | 2.1.3.2 |
| EU015414_A_Indonesia_TLL012_2006 | 2.1.3.2 |
| EU015415_A_Indonesia_TLL013_2006 | 2.1.3.2 |
| EU015416_A_Indonesia_TLL014_2006 | 2.1.3.2 |
| EU146632_A_Indonesia_7_2005 | 2.1.3.2 |
| EU146640_A_Indonesia_175H_2005 | 2.1.3.2 |
| EU146648_A_Indonesia_160H_2005 | 2.1.3.2 |
| EU146656_A_Indonesia_195H_2005 | 2.1.3.2 |
| EU146664_A_Indonesia_239H_2005 | 2.1.3.2 |
| EU146672_A_Indonesia_245H_2005 | 2.1.3.2 |
| EU146680_A_Indonesia_177H_2005 | 2.1.3.2 |
| EU146681_A_Indonesia_283H_2006 | 2.1.3.2 |
| EU146688_A_Indonesia_286H_2006 | 2.1.3.2 |
| EU146697_A_Indonesia_298H_2006 | 2.1.3.2 |
| EU146705_A_Indonesia_304H_2006 | 2.1.3.2 |
| EU146713_A_Indonesia_292H_2006 | 2.1.3.2 |
| EU146721_A_Indonesia_321H_2006 | 2.1.3.2 |
| EU146729_A_Indonesia_341H_2006 | 2.1.3.2 |
| EU146777_A_Indonesia_542H_2006 | 2.1.3.2 |
| EU146801_A_Indonesia_567H_2006 | 2.1.3.2 |
| EU146809_A_Indonesia_569H_2006 | 2.1.3.2 |
| EU146817_A_Indonesia_583H_2006 | 2.1.3.2 |
| EU146825_A_Indonesia_604H_2006 | 2.1.3.2 |
| JX235399_A_Indonesia_UT3006_2005 | 2.1.3.2 |
| EPI_ISL_150197_A_Indonesia_NIHRD9340_2009 | 2.1.3.2a |
| EPI_ISL_150548_A_Indonesia_NIHRD10728_2010 | 2.1.3.2a |
| EPI_ISL_150550_A_Indonesia_NIHRD10364_2010 | 2.1.3.2a |
| EPI_ISL_150551_A_Indonesia_NIHRD10623_2010 | 2.1.3.2a |
| EPI_ISL_150573_A_Indonesia_NIHRD10459_2010 | 2.1.3.2a |
| EPI_ISL_150577_A_Indonesia_NIHRD11046_2011 | 2.1.3.2a |
| EPI_ISL_23815_A_Indonesia_7261_2008 | 2.1.3.2a |
| EPI_ISL_24602_A_Indonesia_8228_2008 | 2.1.3.2a |
| EPI_ISL_24753_A_Indonesia_7272_2008 | 2.1.3.2a |
| EPI_ISL_98854_A_Indonesia_NIHRD11767_2011 | 2.1.3.2a |
| EPI_ISL_98855_A_Indonesia_NIHRD11771_2011 | 2.1.3.2a |
| CY041908_A_Egypt_N00001_2009 | 2.2.1 |
| CY041910_A_Egypt_N00585_2009 | 2.2.1 |
| CY041912_A_Egypt_N00605_2009 | 2.2.1 |
| CY041914_A_Egypt_N00606_2009 | 2.2.1 |
| CY041916_A_Egypt_N01310_2009 | 2.2.1 |
| CY041918_A_Egypt_N02039_2009 | 2.2.1 |
| CY041920_A_Egypt_N02407_2009 | 2.2.1 |
| CY041922_A_Egypt_N02563_2009 | 2.2.1 |
| CY041924_A_Egypt_N02752_2009 | 2.2.1 |
| CY041926_A_Egypt_N03228_2009 | 2.2.1 |
| CY041928_A_Egypt_N03272_2009 | 2.2.1 |
| CY041930_A_Egypt_N03434_2009 | 2.2.1 |
| CY041932_A_Egypt_N03438_2009 | 2.2.1 |
| CY041934_A_Egypt_N03439_2009 | 2.2.1 |
| CY041936_A_Egypt_N03450_2009 | 2.2.1 |
| CY041938_A_Egypt_N04316_2009 | 2.2.1 |
| CY041940_A_Egypt_N04394_2009 | 2.2.1 |
| CY041941_A_Egypt_N04395_2009 | 2.2.1 |
| CY041943_A_Egypt_N04396_2009 | 2.2.1 |
| CY041945_A_Egypt_N04526_2009 | 2.2.1 |
| CY041947_A_Egypt_N04527_2009 | 2.2.1 |
| CY041949_A_Egypt_N04822_2009 | 2.2.1 |
| CY041951_A_Egypt_N04823_2009 | 2.2.1 |
| CY041953_A_Egypt_N04979_2009 | 2.2.1 |
| CY041955_A_Egypt_N05056_2009 | 2.2.1 |
| CY062439_A_Egypt_7021_NAMRU3_2006 | 2.2.1 |
| CY062442_A_Egypt_10217_NAMRU3_2007 | 2.2.1 |
| CY062444_A_Egypt_N04830_2009 | 2.2.1 |
| CY062447_A_Egypt_N05912_2009 | 2.2.1 |
| CY062449_A_Egypt_N07392_2009 | 2.2.1 |
| CY062451_A_Egypt_N07908_2009 | 2.2.1 |
| CY062453_A_Egypt_N08835_2009 | 2.2.1 |
| CY062455_A_Egypt_N09174_2009 | 2.2.1 |
| CY062457_A_Egypt_N09539_2009 | 2.2.1 |
| CY062459_A_Egypt_N11981_2009 | 2.2.1 |
| CY062462_A_Egypt_N15262_2009 | 2.2.1 |
| CY062464_A_Egypt_N00269_2010 | 2.2.1 |
| CY062466_A_Egypt_N00270_2010 | 2.2.1 |
| CY062468_A_Egypt_N01360_2010 | 2.2.1 |
| CY062470_A_Egypt_N01644_2010 | 2.2.1 |
| CY062472_A_Egypt_N01982_2010 | 2.2.1 |
| CY062474_A_Egypt_N02038_2010 | 2.2.1 |
| CY062476_A_Egypt_N02127_2010 | 2.2.1 |
| CY062478_A_Egypt_N02554_2010 | 2.2.1 |
| CY062480_A_Egypt_N02770_2010 | 2.2.1 |
| CY062482_A_Egypt_N03071_2010 | 2.2.1 |
| CY062484_A_Egypt_N03072_2010 | 2.2.1 |
| CY062486_A_Egypt_N04434_2010 | 2.2.1 |
| DQ464377_A_Egypt_2782_NAMRU3_2006 | 2.2.1 |
| DQ666146_A_Djibouti_5691NAMRU3_2006 | 2.2.1 |
| EF042614_A_Egypt_2763_NAMRU3_2006 | 2.2.1 |
| EF042615_A_Egypt_2783_NAMRU3_2006 | 2.2.1 |
| EF042616_A_Egypt_2786_NAMRU3_2006 | 2.2.1 |
| EF042617_A_Egypt_2947_NAMRU3_2006 | 2.2.1 |
| EF042618_A_Egypt_3105_NAMRU3_2006 | 2.2.1 |
| EF042619_A_Egypt_3458_NAMRU3_2006 | 2.2.1 |
| EF042620_A_Egypt_5494_NAMRU3_2006 | 2.2.1 |
| EF042621_A_Egypt_5614_NAMRU3_2006 | 2.2.1 |
| EF061116_A_Egypt_12374_NAMRU3_2006 | 2.2.1 |
| EF200512_A_Egypt_14724_NAMRU3_2006 | 2.2.1 |
| EF200513_A_Egypt_14725_NAMRU3_2006 | 2.2.1 |
| EF382359_A_Egypt_0636_NAMRU3_2007 | 2.2.1 |
| EF535817_A_Egypt_1394_NAMRU3_2007 | 2.2.1 |
| EF535818_A_Egypt_1604_NAMRU3_2007 | 2.2.1 |
| EF535819_A_Egypt_1731_NAMRU3_2007 | 2.2.1 |
| EF535820_A_Egypt_1902_NAMRU3_2007 | 2.2.1 |
| EF535821_A_Egypt_2256_NAMRU3_2007 | 2.2.1 |
| EF535822_A_Egypt_2321_NAMRU3_2007 | 2.2.1 |
| EF535823_A_Egypt_2331_NAMRU3_2007 | 2.2.1 |
| EF535824_A_Egypt_2616_NAMRU3_2007 | 2.2.1 |
| EF535825_A_Egypt_2620_NAMRU3_2007 | 2.2.1 |
| EF535826_A_Egypt_2621_NAMRU3_2007 | 2.2.1 |
| EPI_ISL_120232_A_Egypt_321_NAMRU3_2007 | 2.2.1 |
| EPI_ISL_120244_A_Egypt_2472_NAMRU3_2008 | 2.2.1 |
| EPI_ISL_120253_A_Egypt_9538_NAMRU3_2009 | 2.2.1 |
| EPI_ISL_120259_A_Egypt_4935_NAMRU3_2009 | 2.2.1 |
| EPI_ISL_120275_A_Egypt_N0544_2011 | 2.2.1 |
| EPI_ISL_120276_A_Egypt_N6322_2011 | 2.2.1 |
| EPI_ISL_120277_A_Egypt_N6658_2011 | 2.2.1 |
| EPI_ISL_120278_A_Egypt_N0677_2011 | 2.2.1 |
| EPI_ISL_120279_A_Egypt_N6774_2011 | 2.2.1 |
| EPI_ISL_120280_A_Egypt_N6828_2011 | 2.2.1 |
| EPI_ISL_120281_A_Egypt_N0423_2011 | 2.2.1 |
| EPI_ISL_120282_A_Egypt_N7562_2011 | 2.2.1 |
| EPI_ISL_120283_A_Egypt_N7592_2011 | 2.2.1 |
| EPI_ISL_120284_A_Egypt_N7724_2011 | 2.2.1 |
| EPI_ISL_120285_A_Egypt_N09966_2011 | 2.2.1 |
| EPI_ISL_120286_A_Egypt_N11126_2011 | 2.2.1 |
| EPI_ISL_120288_A_Egypt_N11470_2011 | 2.2.1 |
| EPI_ISL_120289_A_Egypt_N14976_2011 | 2.2.1 |
| EPI_ISL_120290_A_Egypt_N10621_2011 | 2.2.1 |
| EPI_ISL_195659_A_Egypt_682_2015 | 2.2.1 |
| EPI_ISL_262572_A_Egypt_N04915_2014 | 2.2.1 |
| EU095023_A_Egypt_2991_NAMRU3_2006 | 2.2.1 |
| EU095024_A_Egypt_2992_NAMRU3_2006 | 2.2.1 |
| EU095025_A_Egypt_2629_NAMRU3_2007 | 2.2.1 |
| EU095026_A_Egypt_2630_NAMRU3_2007 | 2.2.1 |
| EU095027_A_Egypt_2631_NAMRU3_2007 | 2.2.1 |
| EU095028_A_Egypt_2750_NAMRU3_2007 | 2.2.1 |
| EU095029_A_Egypt_2751_NAMRU3_2007 | 2.2.1 |
| EU095030_A_Egypt_4081_NAMRU3_2007 | 2.2.1 |
| EU095031_A_Egypt_4082_NAMRU3_2007 | 2.2.1 |
| EU095032_A_Egypt_4226_NAMRU3_2007 | 2.2.1 |
| EU095033_A_Egypt_6251_NAMRU3_2007 | 2.2.1 |
| EU146867_A_Egypt_902782_2006 | 2.2.1 |
| EU146868_A_Egypt_902786_2006 | 2.2.1 |
| FJ226056_A_Egypt_1980_NAMRU3_2008 | 2.2.1 |
| FJ226057_A_Egypt_2289_NAMRU3_2008 | 2.2.1 |
| FJ226058_A_Egypt_2514_NAMRU3_2008 | 2.2.1 |
| FJ226059_A_Egypt_2546_NAMRU3_2008 | 2.2.1 |
| FJ226060_A_Egypt_3158_NAMRU3_2008 | 2.2.1 |
| FJ226062_A_Egypt_3401_NAMRU3_2008 | 2.2.1 |
| FJ226063_A_Egypt_10211_NAMRU3_2007 | 2.2.1 |
| FJ226064_A_Egypt_10215_NAMRU3_2007 | 2.2.1 |
| FJ226065_A_Egypt_10216_NAMRU3_2007 | 2.2.1 |
| JX456101_A_Egypt_N00951_2012 | 2.2.1 |
| JX456104_A_Egypt_N02137_2012 | 2.2.1 |
| KM392416_A_Egypt_N01753_2014 | 2.2.1 |
| KM392417_A_Egypt_N01754_2014 | 2.2.1 |
| KP702165_A_Egypt_MOH_NRC_7271_2014 | 2.2.1 |
| KP702173_A_Egypt_MOH_NRC_7305_2014 | 2.2.1 |
| KP864426_A_Egypt_N5560_2014 | 2.2.1 |
| KP864427_A_Egypt_N5561_2014 | 2.2.1 |
| KP864428_A_Egypt_N5563_2014 | 2.2.1 |
| KP864429_A_Egypt_N5564_2014 | 2.2.1 |
| KP864430_A_Egypt_N5565_2014 | 2.2.1 |
| KP864431_A_Egypt_N5566_2014 | 2.2.1 |
| KP864432_A_Egypt_N0001_2015 | 2.2.1 |
| KP864433_A_Egypt_N0002_2015 | 2.2.1 |
| KP864434_A_Egypt_N0004_2015 | 2.2.1 |
| KP864435_A_Egypt_N0005_2015 | 2.2.1 |
| KR063683_A_Egypt_MOH_NRC_8434_2014 | 2.2.1 |
| FJ226061_A_Egypt_3300_NAMRU3_2008 | 2.2.1.1 |
| CY088769_A_Bangladesh_3233_2011 | 2.2.2.1 |
| EPI_ISL_141267_A_Bangladesh_5487_2011 | 2.2.2.1 |
| FJ573468_A_Bangladesh_207095_2008 | 2.2.2.1 |
| EPI_ISL_73308_A_Guangxi_1_2009 | 2.3.2.1 |
| EPI_ISL_76081_A_Hubei_1_2010 | 2.3.2.1a |
| KF277182_A_Viet_Nam_CM32_2011 | 2.3.2.1a |
| EPI_ISL_100272_A_Guangdong_Shenzhen_1_2011 | 2.3.2.1b |
| EPI_ISL_121190_A_Hong_Kong_5923_2012 | 2.3.2.1b |
| EPI_ISL_154130_A_Alberta_01_2014 | 2.3.2.1c |
| EPI_ISL_191954_A_Vietnam_14011801_2014 | 2.3.2.1c |
| HQ636461_A_Hong_Kong_6841_2010 | 2.3.2.1c |
| DQ643809_A_Zhejiang_16_2006 | 2.3.4 |
| DQ835313_A_China_GD01_2006 | 2.3.4 |
| DQ842489_A_Guangzhou_1_2006 | 2.3.4 |
| EF137706_A_Shenzhen_406H_2006 | 2.3.4 |
| EF624256_A_China_2006 | 2.3.4 |
| EPI_ISL_24603_A_Anhui_1_2005 | 2.3.4 |
| EPI_ISL_24604_A_Sichuan_1_2006 | 2.3.4 |
| EPI_ISL_24605_A_Sichuan_2_2006 | 2.3.4 |
| EPI_ISL_24606_A_Anhui_2_2005 | 2.3.4 |
| EPI_ISL_24607_A_Fujian_1_2005 | 2.3.4 |
| EPI_ISL_24608_A_Guangxi_1_2005 | 2.3.4 |
| EPI_ISL_24609_A_Jiangxi_1_2005 | 2.3.4 |
| EPI_ISL_73294_A_Zhejiang_1_2006 | 2.3.4 |
| EPI_ISL_73298_A_Sichuan_3_2006 | 2.3.4 |
| EPI_ISL_73299_A_Guangdong_2_2006 | 2.3.4 |
| EPI_ISL_73302_A_Anhui_1_2007 | 2.3.4 |
| EPI_ISL_73303_A_Hunan_1_2008 | 2.3.4 |
| EPI_ISL_73306_A_Guangdong_1_2008 | 2.3.4 |
| EPI_ISL_73312_A_Shandong_1_2009 | 2.3.4 |
| EPI_ISL_73313_A_Xinjiang_1_2009 | 2.3.4 |
| EPI_ISL_73320_A_Anhui_1_2006 | 2.3.4 |
| EPI_ISL_79677_A_Hunan_1_2006 | 2.3.4 |
| EPI_ISL_79678_A_Guangdong_1_2006 | 2.3.4 |
| EPI_ISL_79679_A_Shanghai_1_2006 | 2.3.4 |
| EPI_ISL_79680_A_Hubei_1_2006 | 2.3.4 |
| EPI_ISL_79682_A_Fujian_1_2007 | 2.3.4 |
| EPI_ISL_79683_A_Jiangsu_1_2007 | 2.3.4 |
| EPI_ISL_79684_A_Jiangsu_2_2007 | 2.3.4 |
| EPI_ISL_79685_A_Beijing_1_2009 | 2.3.4 |
| EU263981_A_China_GD02_2006 | 2.3.4 |
| EU499372_A_Laos_Nong_Khai_1_2007 | 2.3.4 |
| FJ492884_A_Guangdong_01_2006 | 2.3.4 |
| HM114537_A_Vietnam_UT30850_2005 | 2.3.4 |
| KC784946_A_Jiangsu_6_2008 | 2.3.4 |
| KC784948_A_Jiangsu_4_2007 | 2.3.4 |
| EPI_ISL_73309_A_Guizhou_1_2009 | 2.3.4.1 |
| EPI_ISL_79686_A_Hunan_1_2009 | 2.3.4.1 |
| EPI_ISL_79687_A_Hunan_2_2009 | 2.3.4.1 |
| KP638513_A_Vietnam_HN31676DH_2009 | 2.3.4.1 |
| KP638514_A_Vietnam_UT36236_2010 | 2.3.4.1 |
| KP638515_A_Vietnam_UT36282_2010 | 2.3.4.1 |
| KP638560_A_Vietnam_36285_2010 | 2.3.4.1 |
| EPI_ISL_135216_A_Guizhou_1_2013_ | 2.3.4.2 |
| HM114617_A_Vietnam_HN31432M_2008 | 2.3.4.2 |
| KP638510_A_Vietnam_UT36250_1_2010 | 2.3.4.2 |
| KP638511_A_Vietnam_UT31604_1_2009 | 2.3.4.2 |
| EPI_ISL_73305_A_Guangxi_1_2008 | 2.3.4.3 |
| EU294369_A_Viet_Nam_HN31242_2007 | 2.3.4.3 |
| HM114545_A_Vietnam_UT31203A_2007 | 2.3.4.3 |
| HM114553_A_Vietnam_UT31239_2007 | 2.3.4.3 |
| HM114561_A_Vietnam_UT31244II_2007 | 2.3.4.3 |
| HM114569_A_Vietnam_UT31244III_2007 | 2.3.4.3 |
| HM114577_A_Vietnam_UT31312II_2007 | 2.3.4.3 |
| HM114585_A_Vietnam_HN31388M1_2007 | 2.3.4.3 |
| HM114593_A_Vietnam_UT31394II_2008 | 2.3.4.3 |
| HM114601_A_Vietnam_UT31412II_2008 | 2.3.4.3 |
| HM114609_A_Vietnam_UT31413II_2008 | 2.3.4.3 |
| HQ215515_A_Vietnam_HN31244_2007 | 2.3.4.3 |
| KP638512_A_Vietnam_UT31641_2_2008 | 2.3.4.3 |
| EPI_ISL_163493_A_Sichuan_26221_2014 | 2.3.4.4 |
| EPI_ISL_178261_A_Yunnan_14563_2015 | 2.3.4.4 |
| EPI_ISL_178262_A_Yunnan_14564_2015 | 2.3.4.4 |
| EPI_ISL_200837_A_Yunnan_DQ001_2015_H5N6 | 2.3.4.4 |
| EPI_ISL_200838_A_Yunnan_DQ002_2015_H5N6 | 2.3.4.4 |
| EPI_ISL_205313_A_Shenzhen_1_2015 | 2.3.4.4 |
| EPI_ISL_205503_A_Shenzhen_TH001_2015_H5N6 | 2.3.4.4 |
| EPI_ISL_206036_A_Shenzhen_1_2016 | 2.3.4.4 |
| EPI_ISL_206568_A_Guangdong_SZ872_2015H5N6 | 2.3.4.4 |
| EPI_ISL_206569_A_Guangdong_ZQ874_2015H5N6 | 2.3.4.4 |
| EPI_ISL_207048_A_Shenzhen_TH003_2016_H5N6 | 2.3.4.4 |
| EPI_ISL_207051_A_Shenzhen_TH002_2016_H5N6 | 2.3.4.4 |
| EPI_ISL_219828_A_Guangdong_99710_2014 | 2.3.4.4 |
| EPI_ISL_240703_A_Hunan_55555_2016 | 2.3.4.4 |
| EPI_ISL_240704_A_Guangxi_55726_2016 | 2.3.4.4 |
| EPI_ISL_256213_A_Hubei_29578_2016 | 2.3.4.4 |
| KP765788_A_Guangzhou_39715_2014 | 2.3.4.4 |
| KR063687_A_Changsha_1_2014 | 2.3.4.4 |
| KT245143_A_Yunnan_0127_2015 | 2.3.4.4 |
