# Supplementary material for: Adaptive Evolution of Human-Isolated H5Nx Avian Influenza A Viruses
Source: Front Microbiol. 2019 Jun 12;10:1328. doi: 10.3389/fmicb.2019.01328 (PMC6582624; doi:10.3389/fmicb.2019.01328)
Supplement: Supplementary file 13 [file Table_3.DOCX]

**Supplementary table 3:** Branches with signals of positive selection in eight gene sequences.

| **Model** | **Estimated parameters^a^** | **2⊿lnL^b^**  ***P* value^c^** | **Positively Selected Sites (BEB Analysis)^d^** |
| --- | --- | --- | --- |
| **Branch-site Model** | |  |  |
| Branch HA-107b | site class 0 1 2a 2b  proportion 0.99415 0.00000 0.00585 0.00000  background w 0.12406 1.00000 0.12406 1.00000  foreground w 0.12406 1.00000 999.00000 999.00000 | Test:  13.560  *P*=0 |  |
| Branch HA-107b  ω_a_ = 1 | site class 0 1 2a 2b  proportion 1.00000 0.00000 0.00000 0.00000  background w 0.16520 1.00000 0.16520 1.00000  foreground w 0.16520 1.00000 1.00000 1.00000 |  |  |
| Branch HA-18c | site class 0 1 2a 2b  proportion 0.84510 0.15088 0.00341 0.00061  background w 0.09295 1.00000 0.09295 1.00000  foreground w 0.09295 1.00000 647.45276 647.45276 | Test:  9.956  *P*=0 | 473 R 0.972* |
| Branch HA-18c  ω_a_ = 1 | site class 0 1 2a 2b  proportion 0.49899 0.09852 0.33613 0.06636  background w 0.07982 1.00000 0.07982 1.00000  foreground w 0.07982 1.00000 1.00000 1.00000 |  |  |
| Branch HA-6a | site class 0 1 2a 2b  proportion 0.82431 0.15986 0.01326 0.00257  background w 0.13025 1.00000 0.13025 1.00000  foreground w 0.13025 1.00000 80.55150 80.55150 | Test:  5.594  *P*=0.02 |  |
| Branch HA-6a  ω_a_ = 1 | site class 0 1 2a 2b  proportion 0.59876 0.22660 0.12669 0.04795  background w 0.00787 1.00000 0.00787 1.00000  foreground w 0.00787 1.00000 1.00000 1.00000 |  |  |
| Branch HA-64b | site class 0 1 2a 2b  proportion 0.77287 0.20678 0.01605 0.00429  background w 0.10471 1.00000 0.10471 1.00000  foreground w 0.10471 1.00000 859.72983 859.72983 | Test:  28.630  *P*=0 | 11 N 0.997**  15 Q 0.961*  20 M 0.963*  314 K 0.963*  315 T 0.962*  522 T 0.963*  529 L 0.961*  546 L 0.961*  547 Q 0.999**  548 C 0.961* |
| Branch HA-64b  ω_a_ = 1 | site class 0 1 2a 2b  proportion 0.00013 0.00003 0.81620 0.18365  background w 0.10947 1.00000 0.10947 1.00000  foreground w 0.10947 1.00000 1.00000 1.00000 |  |  |
| Branch HA-68a | site class 0 1 2a 2b  proportion 0.59479 0.37393 0.01921 0.01207  background w 0.00000 1.00000 0.00000 1.00000  foreground w 0.00000 1.00000 107.43740 107.43740 | Test:  12.692  *P*=0 | 212 R 0.986*  500 R 0.985* |
| Branch HA-68a  ω_a_ = 1 | site class 0 1 2a 2b  proportion 0.00002 0.00001 0.60933 0.39064  background w 0.00000 1.00000 0.00000 1.00000  foreground w 0.00000 1.00000 1.00000 1.00000 |  |  |
| Branch HA-72b | site class 0 1 2a 2b  proportion 0.00008 0.00006 0.56134 0.43853  background w 0.01096 1.00000 0.01096 1.00000  foreground w 0.01096 1.00000 999.00000 999.00000 | Test:  4359.326  *P*=0 |  |
| Branch HA-72b  ω_a_ = 1 | site class 0 1 2a 2b  proportion 0.00000 0.00000 0.56226 0.43774  background w 0.01153 1.00000 0.01153 1.00000  foreground w 0.01153 1.00000 1.00000 1.00000 |  |  |
| Branch HA-74a | site class 0 1 2a 2b  proportion 0.71585 0.28415 0.00000 0.00000  background w 0.00000 1.00000 0.00000 1.00000  foreground w 0.00000 1.00000 1.00000 1.00000 | Test:  1628.128  *P*=0 |  |
| Branch HA-74a  ω_a_ = 1 | site class 0 1 2a 2b  proportion 0.71585 0.28415 0.00000 0.00000  background w 0.00000 1.00000 0.00000 1.00000  foreground w 0.00000 1.00000 1.00000 1.00000 |  |  |
| Branch HA-75a | site class 0 1 2a 2b  proportion 0.00000 0.00000 1.00000 0.00000  background w 0.42303 1.00000 0.42303 1.00000  foreground w 0.42303 1.00000 999.00000 999.00000 | Test:  452.474  *P*=0 |  |
| Branch HA-75a  ω_a_ = 1 | site class 0 1 2a 2b  proportion 0.00000 0.00000 0.57329 0.42671  background w 0.00000 1.00000 0.00000 1.00000  foreground w 0.00000 1.00000 1.00000 1.00000 |  |  |
| Branch HA-76a | site class 0 1 2a 2b  proportion 0.00000 0.00000 1.00000 0.00000  background w 0.19922 1.00000 0.19922 1.00000  foreground w 0.19922 1.00000 999.00000 999.000000 | Test:  33.736  *P*=0.002 |  |
| Branch HA-76a  ω_a_ = 1 | site class 0 1 2a 2b  proportion 0.00000 0.00000 1.00000 0.00000  background w 0.19875 1.00000 0.19875 1.00000  foreground w 0.19875 1.00000 1.00000 1.00000 |  |  |
| Branch HA-77a | site class 0 1 2a 2b  proportion 0.76019 0.22786 0.00919 0.00275  background w 0.02593 1.00000 0.02593 1.00000  foreground w 0.02593 1.00000 1.00000 1.00000 | Test:  12899.222  *P*=0 |  |
| Branch HA-77a  ω_a_ = 1 | site class 0 1 2a 2b  proportion 0.29890 0.12375 0.40831 0.16904  background w 0.07218 1.00000 0.07218 1.00000  foreground w 0.07218 1.00000 1.00000 1.00000 |  |  |
| Branch HA-83a | site class 0 1 2a 2b  proportion 0.69778 0.29863 0.00252 0.00108  background w 0.00000 1.00000 0.00000 1.00000  foreground w 0.00000 1.00000 247.37715 247.37715 | Test:  6.486  *P*=0.01 |  |
| Branch HA-83a  ω_a_ = 1 | site class 0 1 2a 2b  proportion 0.69157 0.30843 0.00000 0.00000  background w 0.00000 1.00000 0.00000 1.00000  foreground w 0.00000 1.00000 1.00000 1.00000 |  |  |
| Branch HA-107b | ite class 0 1 2a 2b  proportion 0.99415 0.00000 0.00585 0.00000  background w 0.12406 1.00000 0.12406 1.00000  foreground w 0.12406 1.00000 999.00000 999.00000 | Test:  13.560  *P*=0 |  |
| Branch HA-107b  ω_a_ = 1 | site class 0 1 2a 2b  proportion 1.00000 0.00000 0.00000 0.00000  background w 0.16520 1.00000 0.16520 1.00000  foreground w 0.16520 1.00000 1.00000 1.000000 |  |  |
|  |  |  |  |
| Branch PB2-14d | site class 0 1 2a 2b  proportion 0.93312 0.06519 0.00158 0.00011  background w 0.05796 1.00000 0.05796 1.00000  foreground w 0.05796 1.00000 529.13909 529.13909 | Test:  9.229  *P*=0.002 |  |
| Branch PB2-14d  ω_a_ = 1 | site class 0 1 2a 2b  proportion 0.80684 0.05782 0.12629 0.00905  background w 0.05400 1.00000 0.05400 1.00000  foreground w 0.05400 1.00000 1.00000 1.00000 |  |  |
| Branch PB2-74b | site class 0 1 2a 2b  proportion 0.99579 0.00000 0.00421 0.00000  background w 0.22337 1.00000 0.22337 1.00000  foreground w 0.22337 1.00000 999.00000 999.00000 | Test:  16.618  *P*=0 |  |
| Branch PB2-74b  ω_a_ = 1 | site class 0 1 2a 2b  proportion 0.27051 0.00013 0.72900 0.00035  background w 0.23704 1.00000 0.23704 1.00000  foreground w 0.23704 1.00000 1.00000 1.00000 |  |  |
|  | | | |
| Branch MP-46e | site class 0 1 2a 2b  proportion 0.67481 0.14328 0.15006 0.03186  background w 0.00000 1.00000 0.00000 1.00000  foreground w 0.00000 1.00000 1.05007 1.05007 | Test:  5.576  *P*=0.02 |  |
| Branch MP-46e  ω_a_ = 1 | site class 0 1 2a 2b  proportion 0.29890 0.12375 0.40831 0.16904  background w 0.07218 1.00000 0.07218 1.00000  foreground w 0.07218 1.00000 1.00000 1.00000 |  |  |
| Branch MP-50a | site class 0 1 2a 2b  proportion 0.70511 0.26339 0.02293 0.00857  background w 0.00000 1.00000 0.00000 1.00000  foreground w 0.00000 1.00000 924.75072 924.75072 | Test:  57.268  *P*=0 | 5 T 0.990**  6 E 0.990**  7 V 0.990**  8 E 0.990**  257 T 0.990**  258 E 0.990**  259 V 0.990**  260 E 0.990** |
| Branch MP-50a  ω_a_ = 1 | site class 0 1 2a 2b  proportion 0.71765 0.27994 0.00173 0.00067  background w 0.00000 1.00000 0.00000 1.00000  foreground w 0.00000 1.00000 1.00000 1.000000 |  |  |
| Branch MP-85a | site class 0 1 2a 2b  proportion 0.73642 0.16764 0.07815 0.01779  background w 0.00000 1.00000 0.00000 1.00000  foreground w 0.00000 1.00000 655.43062 655.43062 | Test:  91.804  *P*=0 | 277 P 0.955*  279 V 0.997**  282 A 0.998**  283 N 1.000**  284 I 0.999**  285 I 0.995**  287 I 0.989*  292 L 0.999**  328 Y 0.995**  330 Q 0.999**  336 V 0.954*  339 D 0.991**  340 D 0.995**  344 V 0.995** |
| Branch MP-85a  ω_a_ = 1 | site class 0 1 2a 2b  proportion 0.65421 0.10306 0.20969 0.03304  background w 0.00000 1.00000 0.00000 1.00000  foreground w 0.00000 1.00000 1.00000 1.00000 |  |  |
|  | | | |
| Branch NA1-15b | site class 0 1 2a 2b  proportion 0.98862 0.00000 0.01138 0.00000  background w 0.11415 1.00000 0.11415 1.00000  foreground w 0.11415 1.00000 999.00000 999.00000 | Test:  17.266  *P*=0 | 188 N 0.984* |
| Branch NA1-15b  ω_a_ = 1 | site class 0 1 2a 2b  proportion 0.55775 0.00000 0.44225 0.00000  background w 0.10519 1.00000 0.10519 1.00000  foreground w 0.10519 1.00000 1.00000 1.00000 |  |  |
| Branch NA6-2b | site class 0 1 2a 2b  proportion 0.93057 0.06337 0.00567 0.00039  background w 0.23891 1.00000 0.23891 1.00000  foreground w 0.23891 1.00000 999.00000 999.00000 | Test:  18.223  *P*=0 |  |
| Branch NA6-2b  ω_a_ = 1 | site class 0 1 2a 2b  proportion 0.73859 0.15348 0.08936 0.01857  background w 0.15262 1.00000 0.15262 1.00000  foreground w 0.15262 1.00000 1.00000 1.00000 |  |  |
|  |  |  |  |
| Branch NP-32a | site class 0 1 2a 2b  proportion 0.94536 0.04697 0.00731 0.00036  background w 0.00000 1.00000 0.00000 1.00000  foreground w 0.00000 1.00000 62.57317 62.57317 | Test:  6.85  *P*=0.01 |  |
| Branch NP-32a  ω_a_ = 1 | site class 0 1 2a 2b  proportion 0.86463 0.04985 0.08086 0.00466  background w 0.00000 1.00000 0.00000 1.00000  foreground w 0.00000 1.00000 1.00000 1.00000 |  |  |
| Branch NP-65a | site class 0 1 2a 2b  proportion 0.99534 0.00000 0.00466 0.00000  background w 0.07274 1.00000 0.07274 1.00000  foreground w 0.07274 1.00000 999.00000 999.00000 | Test:  25.346  *P*=0 | 486 S 0.967*  487 Y 0.967* |
| Branch NP-65a  ω_a_ = 1 | site class 0 1 2a 2b  proportion 0.86641 0.08485 0.04439 0.00435  background w 0.00000 1.00000 0.00000 1.00000  foreground w 0.00000 1.00000 1.00000 1.00000 |  |  |
| Branch NP-66c | site class 0 1 2a 2b  proportion 0.91272 0.08433 0.00270 0.00025  background w 0.04090 1.00000 0.04090 1.00000  foreground w 0.04090 1.00000 511.35947 511.35947 | Test:  6.914  *P*=0.009 |  |
| Branch NP-66c  ω_a_ = 1 | site class 0 1 2a 2b  proportion 0.85846 0.08016 0.05613 0.00524  background w 0.04093 1.00000 0.04093 1.00000  foreground w 0.04093 1.00000 1.00000 1.00000 |  |  |
|  | | |  |
| Branch PA-11a | ite class 0 1 2a 2b  proportion 0.99688 0.00004 0.00308 0.00000  background w 0.14532 1.00000 0.14532 1.00000  foreground w 0.14532 1.00000 214.24716 214.24716 | Test:  5.975  *P*=0.014 |  |
| Branch PA-11a  ω_a_ = 1 | site class 0 1 2a 2b  proportion 0.72341 0.02341 0.24524 0.00794  background w 0.11664 1.00000 0.11664 1.00000  foreground w 0.11664 1.00000 1.00000 1.00000 |  |  |
| Branch PA-25d | site class 0 1 2a 2b  proportion 0.62240 0.15474 0.17848 0.04437  background w 0.00000 1.00000 0.00000 1.00000  foreground w 0.00000 1.00000 117.01600 117.01600 | Test:  4.774  *P*=0.029 |  |
| Branch PA-25d  ω_a_ = 1 | site class 0 1 2a 2b  proportion 0.60512 0.14684 0.19961 0.04844  background w 0.00000 1.00000 0.00000 1.00000  foreground w 0.00000 1.00000 1.00000 1.00000 |  |  |
| Branch PA-72b | site class 0 1 2a 2b  proportion 0.99814 0.00000 0.00186 0.00000  background w 0.34035 1.00000 0.34035 1.00000  foreground w 0.34035 1.00000 999.00000 999.00000 | Test:  4.772  *P*=0.029 |  |
| Branch PA-72b  ω_a_ = 1 | site class 0 1 2a 2b  proportion 0.67857 0.32142 0.00000 0.00000  background w 0.00000 1.00000 0.00000 1.00000  foreground w 0.00000 1.00000 1.00000 1.00000 |  |  |
|  | | | |
| Branch NS-31a | site class 0 1 2a 2b  proportion 0.99534 0.00000 0.00466 0.00000  background w 0.07274 1.00000 0.07274 1.00000  foreground w 0.07274 1.00000 999.00000 999.00000 | Test:  4.828  *P*=0.03 |  |
| Branch NS-31a  ω_a_ = 1 | site class 0 1 2a 2b  proportion 0.86641 0.08485 0.04439 0.00435  background w 0.00000 1.00000 0.00000 1.00000  foreground w 0.00000 1.00000 1.00000 1.00000 |  |  |
| Branch NS-72c | site class 0 1 2a 2b  proportion 0.98288 0.00005 0.01707 0.00000  background w 0.35600 1.00000 0.35600 1.00000  foreground w 0.35600 1.00000 139.80480 139.80480 | Test:  4.59  *P*=0.032 |  |
| Branch NS-72c  ω_a_ = 1 | site class 0 1 2a 2b  proportion 0.59809 0.40158 0.00020 0.00013  background w 0.00000 1.00000 0.00000 1.00000  foreground w 0.00000 1.00000 1.00000 1.00000 |  |  |
| Branch NS-89a | site class 0 1 2a 2b  proportion 0.47549 0.52451 0.00000 0.00000  background w 0.00000 1.00000 0.00000 1.00000  foreground w 0.00000 1.00000 1.00000 1.00000 | Test:  4.69  *P*=0.030 |  |
| Branch NS-89a  ω_a_ = 1 | site class 0 1 2a 2b  proportion 0.47480 0.52035 0.00232 0.00254  background w 0.00000 1.00000 0.00000 1.00000  foreground w 0.00000 1.00000 1.00000 1.00000 |  |  |
|  |  |  |  |
| Branch PB1-31b | site class 0 1 2a 2b  proportion 0.96440 0.03560 0.00000 0.00000  background w 0.05679 1.00000 0.05679 1.00000  foreground w 0.05679 1.00000 1.00000 1.00000 | Test:  19.716  *P*=0 |  |
| Branch PB1-31b  ω_a_ = 1 | site class 0 1 2a 2b  proportion 0.88210 0.02877 0.08632 0.00282  background w 0.05236 1.00000 0.05236 1.00000  foreground w 0.05236 1.00000 1.00000 1.00000 |  |  |
|  |  |  |  |
