# Supplementary material for: Adaptive Evolution of Human-Isolated H5Nx Avian Influenza A Viruses
Source: Front Microbiol. 2019 Jun 12;10:1328. doi: 10.3389/fmicb.2019.01328 (PMC6582624; doi:10.3389/fmicb.2019.01328)
Supplement: Supplementary file 14 [file Table_4.DOCX]

**Supplementary table 4:** Branches without signals of positive selection in eight gene sequences.

| Branch | lnL(Branch-site(w=0)) | lnL(Branch-site) | 2⊿lnL | *P* value |
| --- | --- | --- | --- | --- |
| HA-1a | -2873.423 | -2873.423 | 0 | 1.000 |
| HA-1b | -2873.514 | -2873.514 | 0 | 1.000 |
| HA-1c | -2873.514 | -2873.514 | 0 | 1.000 |
| HA-1d | -2873.514 | -2873.514 | 0 | 1.000 |
| HA-10a | -2483.761 | -2483.761 | 0 | 1.000 |
| HA-10b | -2483.234 | -2483.234 | 0 | 1.000 |
| HA-100a | -2703.493 | -2703.155 | 0.675 | 0.41 |
| HA-101a | -2376.026 | -2376.026 | 0 | 1.000 |
| HA-102a | -2402.082 | -2402.082 | 0 | 1.000 |
| HA-103a | -2982.880 | -2982.880 | 0 | 1.000 |
| HA-103b | -2983.813 | -2983.813 | 0 | 1.000 |
| HA-103c | -2983.813 | -2983.813 | 0 | 1.000 |
| HA-104a | -3029.408 | -3029.408 | 0 | 1.000 |
| HA-105a | -2942.588 | -2942.588 | 0 | 1.000 |
| HA-105b | -2942.776 | -2942.776 | 0 | 1.000 |
| HA-105c | -2942.093 | -2941.718 | 0.75 | 0.386 |
| HA-105d | -2942.776 | -2942.776 | 0 | 1.000 |
| HA-106a | -2618.873 | -2618.858 | 0.029 | 0.862 |
| HA-107a | -2467.139 | -2467.139 | 0 | 1.000 |
| HA-108a | -2477.044 | -2477.044 | 0 | 1.000 |
| HA-109a | -2737.840 | -2737.667 | 0.346 | 0.556 |
| HA-109b | -2737.682 | -2737.854 | 0 | 1.000 |
| HA-11a | -2694.485 | -2694.485 | 0 | 1.000 |
| HA-11b | -2694.486 | -2694.486 | 0 | 1.000 |
| HA-11c | -2694.485 | -2694.486 | 0 | 1.000 |
| HA-110a | -2658.171 | -2658.171 | 0 | 1.000 |
| HA-110b | -2658.168 | -2658.168 | 0 | 1.000 |
| HA-111a | -2512.740 | -2512.740 | 0 | 1.000 |
| HA-112a | -2548.177 | -2548.177 | 0 | 1.000 |
| HA-113a | -2548.177 | -2548.177 | 0 | 1.000 |
| HA-114a | -2594.276 | -2594.276 | 0 | 1.000 |
| HA-115a | -2738.256 | -2738.256 | 0 | 1.000 |
| HA-116a | -2258.507 | -2258.510 | 0.006 | 0.938 |
| HA-117a | -3066.769 | -3066.768 | 0.001 | 0.964 |
| HA-117b | -3066.778 | -3066.728 | 0.099 | 0.751 |
| HA-117c | -3066.801 | -3066.801 | 0 | 1.000 |
| HA-117d | -3066.825 | -3066.825 | 0 | 1.000 |
| HA-118a | -2481.556 | -2481.556 | 0 | 1.000 |
| HA-119a | -2362.605 | -2362.530 | 0.149 | 0.698 |
| HA-12a | -2767.347 | -2767.347 | 0 | 1.000 |
| HA-12b | -2766.399 | -2766.124 | 0.55 | 0.458 |
| HA-120a | -2507.240 | -2507.240 | 0 | 1.000 |
| HA-121a | -2439.295 | -2439.295 | 0 | 1.000 |
| HA-121b | -2438.484 | -2438.484 | 0 | 1.000 |
| HA-122a | -3746.625 | -3746.625 | 0 | 1.000 |
| HA-123a | -2378.846 | -2378.846 | 0 | 1.000 |
| HA-124a | -3149.656 | -3149.656 | 0 | 1.000 |
| HA-125a | -3544.311 | -3544.311 | 0 | 1.000 |
| HA-125b | -3544.311 | -3544.311 | 0 | 1.000 |
| HA-125c | -3544.311 | -3544.311 | 0 | 1.000 |
| HA-126a | -2377.680 | -2377.680 | 0 | 1.000 |
| HA-127a | -2515.611 | -2515.615 | 0.007 | 0.928 |
| HA-127b | -2514.957 | -2514.680 | 0 | 1.000 |
| HA-127c | -2515.840 | -2515.840 | 0 | 1.000 |
| HA-127d | -2515.840 | -2515.840 | 0 | 1.000 |
| HA-127e | -2515.840 | -2515.840 | 0 | 1.000 |
| HA-128a | -3332.552 | -3332.552 | 0 | 1.000 |
| HA-128b | -3332.552 | -3332.552 | 0 | 1.000 |
| HA-128c | -3332.552 | -3332.552 | 0 | 1.000 |
| HA-128d | -3332.552 | -3332.552 | 0 | 1.000 |
| HA-128e | -3332.430 | -3332.430 | 0 | 1.000 |
| HA-129a | -2436.348 | -2436.348 | 0 | 1.000 |
| HA-129b | -2436.261 | -2436.261 | 0 | 1.000 |
| HA-129c | -2436.348 | -2436.348 | 0 | 1.000 |
| HA-13a | -2542.018 | -2542.018 | 0 | 1.000 |
| HA-13b | -2542.018 | -2542.018 | 0 | 1.000 |
| HA-13c | -2541.385 | -2541.019 | 0.732 | 0.392 |
| HA-130a | -2925.293 | -2925.280 | 0.025 | 0.871 |
| HA-131a | -2494.705 | -2494.678 | 0.054 | 0.816 |
| HA-132a | -2844.248 | -2844.248 | 0 | 1.000 |
| HA-133a | -2700.351 | -2700.351 | 0 | 1.000 |
| HA-14a | -2319.605 | -2319.605 | 0 | 1.000 |
| HA-15a | -2775.718 | -2775.718 | 0 | 1.000 |
| HA-15b | -2775.718 | -2775.718 | 0 | 1.000 |
| HA-16a | -2876.765 | -2876.765 | 0 | 1.000 |
| HA-16b | -2877.084 | -2877.084 | 0 | 1.000 |
| HA-16c | -2876.765 | -2877.084 | 0 | 1.000 |
| HA-16d | -2876.765 | -2877.084 | 0 | 1.000 |
| HA-16e | -2876.765 | -2876.765 | 0 | 1.000 |
| HA-17a | -3068.367 | -3068.357 | 0 | 1.000 |
| HA-17b | -3068.373 | -3068.373 | 0 | 1.000 |
| HA-17c | -3068.373 | -3068.373 | 0 | 1.000 |
| HA-17d | -3067.349 | -3066.994 | 0.71 | 0.399 |
| HA-17e | -3068.373 | -3068.373 | 0 | 1.000 |
| HA-17f | -3068.086 | -3068.086 | 0 | 1.000 |
| HA-17g | -3068.265 | -3068.265 | 0 | 1.000 |
| HA-18a | -2843.732 | -2843.732 | 0 | 1.000 |
| HA-18b | -2844.189 | -2844.189 | 0 | 1.000 |
| HA-18d | -2844.666 | -2844.666 | 0 | 1.000 |
| HA-19a | -2420.946 | -2420.946 | 0 | 1.000 |
| HA-2a | -2473.862 | -2473.862 | 0 | 1.000 |
| HA-20a | -2576.984 | -2576.984 | 0 | 1.000 |
| HA-20b | -2576.712 | -2576.712 | 0 | 1.000 |
| HA-20c | -2576.984 | -2576.984 | 0 | 1.000 |
| HA-20d | -2576.984 | -2576.984 | 0 | 1.000 |
| HA-21a | -2555.876 | -2555.876 | 0 | 1.000 |
| HA-22a | -2495.632 | -2495.632 | 0 | 1.000 |
| HA-23a | -3497.214 | -3497.214 | 0 | 1.000 |
| HA-23j | -3497.088 | -3497.043 | 0.09 | 0.764 |
| HA-23b | -3496.567 | -3496.249 | 0.636 | 0.425 |
| HA-23c | -3497.214 | -3497.214 | 0 | 1.000 |
| HA-23d | -3495.563 | -3494.920 | 1.286 | 0.256 |
| HA-23e | -3497.214 | -3497.214 | 0 | 1.000 |
| HA-23f | -3497.023 | -3497.023 | 0 | 1.000 |
| HA-23g | -3497.214 | -3497.214 | 0 | 1.000 |
| HA-23h | -3497.214 | -3497.214 | 0 | 1.000 |
| HA-23i | -3497.214 | -3497.214 | 0 | 1.000 |
| HA-24a | -2466.526 | -2465.811 | 1.429 | 0.231 |
| HA-24b | -2467.655 | -2467.655 | 0 | 1.000 |
| HA-25a | -2458.946 | -2458.946 | 0 | 1.000 |
| HA-25b | -2458.946 | -2458.946 | 0 | 1.000 |
| HA-25c | -2458.946 | -2458.946 | 0 | 1.000 |
| HA-25d | -2458.946 | -2458.946 | 0 | 1.000 |
| HA-25e | -2458.730 | -2458.730 | 0 | 1.000 |
| HA-25f | -2458.946 | -2458.946 | 0 | 1.000 |
| HA-25g | -2458.161 | -2457.871 | 0.579 | 0.446 |
| HA-25h | -2458.063 | -2457.704 | 0.717 | 0.396 |
| HA-25i | -2458.946 | -2458.946 | 0 | 1.000 |
| HA-26a | -2888.970 | -2888.970 | 0 | 1.000 |
| HA-27a | -2825.085 | -2825.085 | 0 | 1.000 |
| HA-27b | -2825.085 | -2825.085 | 0 | 1.000 |
| HA-28a | -3136.522 | -3136.522 | 0 | 1.000 |
| HA-28b | -3136.297 | -3136.297 | 0 | 1.000 |
| HA-28c | -3135.829 | -3135.829 | 0 | 1.000 |
| HA-28d | -3136.522 | -3136.522 | 0 | 1.000 |
| HA-28e | -3136.522 | -3136.522 | 0 | 1.000 |
| HA-29a | -2457.605 | -2457.605 | 0 | 1.000 |
| HA-3a | -2552.149 | -2552.149 | 0 | 1.000 |
| HA-30a | -2597.894 | -2597.894 | 0 | 1.000 |
| HA-31a | -2444.489 | -2444.489 | 0 | 1.000 |
| HA-32a | -2453.672 | -2453.672 | 0 | 1.000 |
| HA-33a | -2497.967 | -2497.307 | 0 | 1.000 |
| HA-33b | -2499.480 | -2499.480 | 0 | 1.000 |
| HA-33c | -2498.843 | -2498.854 | 0.021 | 0.882 |
| HA-33d | -2499.480 | -2499.480 | 0 | 1.000 |
| HA-34a | -2665.178 | -2664.244 | 1.867 | 0.171 |
| HA-35a | -2764.193 | -2764.193 | 0 | 1.000 |
| HA-35b | -2764.266 | -2764.266 | 0 | 1.000 |
| HA-36a | -2704.557 | -2704.557 | 0 | 1.000 |
| HA-37a | -2527.844 | -2527.844 | 0 | 1.000 |
| HA-38a | -2622.969 | -2622.969 | 0 | 1.000 |
| HA-38b | -2622.797 | -2622.797 | 0 | 1.000 |
| HA-39a | -2362.025 | -2362.025 | 0 | 1.000 |
| HA-4a | -2366.642 | -2366.642 | 0 | 1.000 |
| HA-40a | -2383.595 | -2383.595 | 0 | 1.000 |
| HA-41a | -2447.687 | -2447.687 | 0 | 1.000 |
| HA-42a | -2888.424 | -2887.810 | 1.228 | 0.267 |
| HA-42j | -2889.669 | -2889.669 | 0 | 1.000 |
| HA-42b | -2889.152 | -2888.845 | 0.614 | 0.433 |
| HA-42c | -2889.669 | -2889.669 | 0 | 1.000 |
| HA-42d | -2889.669 | -2889.669 | 0 | 1.000 |
| HA-42e | -2889.584 | -2889.584 | 0 | 1.000 |
| HA-42f | -2889.136 | -2888.831 | 0.609 | 0.434 |
| HA-42g | -2889.669 | -2889.525 | 0 | 1.000 |
| HA-42h | -2889.669 | -2889.669 | 0 | 1.000 |
| HA-42i | -2889.669 | -2889.669 | 0 | 1.000 |
| HA-43a | -2763.246 | -2763.246 | 0 | 1.000 |
| HA-43b | -2762.456 | -2762.105 | 0.702 | 0.402 |
| HA-43c | -2763.246 | -2763.246 | 0 | 1.000 |
| HA-43d | -2762.470 | -2762.464 | 0.011 | 0.912 |
| HA-43e | -2763.246 | -2763.246 | 0 | 1.000 |
| HA-44a | -2335.109 | -2335.109 | 0 | 1.000 |
| HA-45a | -2523.913 | -2523.913 | 0 | 1.000 |
| HA-46a | -2630.452 | -2630.452 | 0 | 1.000 |
| HA-47a | -2335.956 | -2335.956 | 0 | 1.000 |
| HA-48a | -3141.176 | -3141.176 | 0 | 1.000 |
| HA-48b | -3141.171 | -3141.171 | 0 | 1.000 |
| HA-48c | -3139.357 | -3139.176 | 0.362 | 0.547 |
| HA-49a | -2554.023 | -2554.023 | 0 | 1.000 |
| HA-5a | -2457.588 | -2457.525 | 0.126 | 0.722 |
| HA-50a | -2666.693 | -2666.693 | 0 | 1.000 |
| HA-51a | -2387.708 | -2387.708 | 0 | 1.000 |
| HA-52a | -2552.224 | -2552.224 | 0 | 1.000 |
| HA-53a | -2476.804 | -2476.804 | 0 | 1.000 |
| HA-54a | -2367.544 | -2367.544 | 0 | 1.000 |
| HA-55a | -2921.005 | -2920.901 | 0.208 | 0.648 |
| HA-55b | -2921.810 | -2921.810 | 0 | 1.000 |
| HA-55c | -2920.317 | -2918.628 | 3.377 | 0.066 |
| HA-55d | -2921.810 | -2921.810 | 0 | 1.000 |
| HA-56a | -2369.025 | -2369.025 | 0 | 1.000 |
| HA-57a | -2678.207 | -2678.207 | 0 | 1.000 |
| HA-57b | -2678.599 | -2678.599 | 0 | 1.000 |
| HA-57c | -2678.570 | -2678.599 | 0 | 1.000 |
| HA-58a | -2512.806 | -2512.806 | 0 | 1.000 |
| HA-59a | -2433.781 | -2433.719 | 0.123 | 0.724 |
| HA-6a | -2640.442 | -2637.645 | 5.594 | 0.018 |
| HA-60a | -2362.475 | -2362.475 | 0 | 1.000 |
| HA-60b | -2362.475 | -2362.475 | 0 | 1.000 |
| HA-61a | -2443.539 | -2442.958 | 1.162 | 0.281 |
| HA-61b | -2443.535 | -2442.955 | 1.159 | 0.281 |
| HA-62a | -2515.196 | -2515.196 | 0 | 1.000 |
| HA-62b | -2515.196 | -2515.196 | 0 | 1.000 |
| HA-62c | -2514.676 | -2514.676 | 0 | 1.000 |
| HA-62d | -2515.196 | -2515.196 | 0 | 1.000 |
| HA-63a | -2666.257 | -2666.257 | 0 | 1.000 |
| HA-64a | -2923.658 | -2923.658 | 0 | 1.000 |
| HA-65a | -2621.513 | -2621.513 | 0 | 1.000 |
| HA-65b | -2622.401 | -2622.401 | 0 | 1.000 |
| HA-65c | -2622.401 | -2622.401 | 0 | 1.000 |
| HA-66a | -2497.249 | -2497.249 | 0 | 1.000 |
| HA-67a | -2688.211 | -2688.211 | 0 | 1.000 |
| HA-67b | -2688.211 | -2688.211 | 0 | 1.000 |
| HA-67c | -2688.211 | -2688.211 | 0 | 1.000 |
| HA-67d | -2688.211 | -2688.211 | 0 | 1.000 |
| HA-67e | -2688.211 | -2688.211 | 0 | 1.000 |
| HA-68b | -2775.123 | -2775.123 | 0 | 1.000 |
| HA-69a | -2333.627 | -2333.627 | 0 | 1.000 |
| HA-7a | -2315.202 | -2315.202 | 0 | 1.000 |
| HA-70a | -2659.545 | -2659.545 | 0 | 1.000 |
| HA-71a | -2651.979 | -2651.979 | 0 | 1.000 |
| HA-71b | -2651.159 | -2649.808 | 2.702 | 0.100 |
| HA-72a | -5647.030 | -5647.324 | 0.587 | 0.443 |
| HA-73a | -3468.215 | -3468.215 | 0 | 1.000 |
| HA-73b | -3468.214 | -3468.215 | 0.002 | 0.964 |
| HA-73c | -3468.214 | -3468.214 | 0 | 1.000 |
| HA-73d | -3465.944 | -3465.094 | 1.699 | 0.192 |
| HA-73e | -3468.476 | -3468.214 | 0.524 | 0.469 |
| HA-73f | -3468.214 | -3468.214 | 0 | 1.000 |
| HA-73g | -3468.214 | -3468.214 | 0 | 1.000 |
| HA-73h | -3468.246 | -3468.509 | 0.525 | 0.468 |
| HA-78a | -3180.932 | -3180.932 | 0 | 1.000 |
| HA-79a | -3820.237 | -3820.222 | 0.029 | 0.862 |
| HA-8a | -2795.193 | -2795.193 | 0 | 1 |
| HA-80a | -2640.357 | -2640.593 | 0.471 | 0.492 |
| HA-80b | -2640.593 | -2640.357 | 0.471 | 0.492 |
| HA-81a | -2615.585 | -2615.585 | 0 | 1.000 |
| HA-81b | -2615.585 | -2615.585 | 0 | 1.000 |
| HA-82a | -2692.531 | -2692.531 | 0 | 1.000 |
| HA-82b | -2692.478 | -2692.451 | 0.054 | 0.816 |
| HA-82c | -2692.442 | -2692.435 | 0.014 | 0.905 |
| HA-84a | -2796.601 | -2796.601 | 0 | 1.000 |
| HA-84b | -2796.601 | -2796.601 | 0 | 1.000 |
| HA-85a | -2673.293 | -2673.293 | 0 | 1.000 |
| HA-86a | -2934.498 | -2934.498 | 0 | 1.000 |
| HA-87a | -2496.468 | -2495.811 | 1.313 | 0.251 |
| HA-88a | -2466.187 | -2466.187 | 0 | 1.000 |
| HA-89a | -2521.723 | -2521.723 | 0 | 1.000 |
| HA-89b | -2521.538 | -2521.538 | 0 | 1.000 |
| HA-9a | -2769.489 | -2769.489 | 0 | 1.000 |
| HA-90a | -4661.262 | -4661.262 | 0 | 1.000 |
| HA-91a | -2561.855 | -2561.854 | 0 | 1.000 |
| HA-91b | -2561.112 | -2560.751 | 0.721 | 0.395 |
| HA-92a | -2794.892 | -2794.892 | 0 | 1.000 |
| HA-93a | -2491.629 | -2491.629 | 0 | 1.000 |
| HA-94a | -2447.844 | -2446.609 | 2.47 | 0.116 |
| HA-94b | -2453.345 | -2453.345 | 0 | 1.000 |
| HA-95a | -2393.305 | -2393.305 | 0 | 1.000 |
| HA-96a | -2315.868 | -2315.868 | 0 | 1.000 |
| HA-97a | -2466.617 | -2466.298 | 0.638 | 0.424 |
| HA-98a | -2367.850 | -2367.850 | 0 | 1.000 |
| HA-99a | -2713.391 | -2713.394 | 0 | 1.000 |
|  |  |  |  |  |
| PB2-1a | -3076.248 | -3076.248 | 0 | 1.000 |
| PB2-10a | -4049.920 | -4049.920 | 0 | 1.000 |
| PB2-10b | -4050.002 | -4050.002 | 0 | 1.000 |
| PB2-10c | -4048.504 | -4048.183 | 0.641 | 0.641 |
| PB2-11a | -3632.753 | -3632.753 | 0 | 1.000 |
| PB2-12a | -3194.507 | -3194.507 | 0 | 1.000 |
| PB2-13a | -3002.276 | -3002.276 | 0 | 1.000 |
| PB2-14a | -4466.328 | -4466.328 | 0 | 1.000 |
| PB2-14b | -4464.830 | -4464.528 | 0.603 | 0.437 |
| PB2-14c | -4466.310 | -4466.296 | 0.028 | 0.867 |
| PB2-14e | -4466.328 | -4466.328 | 0 | 1.000 |
| PB2-14f | -4463.178 | -4463.172 | 0.012 | 0.912 |
| PB2-14g | -4464.962 | -4464.656 | 0 | 1.000 |
| PB2-14h | -4466.328 | -4466.328 | 0 | 1.000 |
| PB2-14i | -4466.328 | -4466.328 | 0 | 1.000 |
| PB2-15a | -3242.959 | -3242.305 | 1.307 | 0.252 |
| PB2-16a | -4634.431 | -4634.431 | 0 | 1.000 |
| PB2-16b | -4634.688 | -4634.688 | 0 | 1.000 |
| PB2-16c | -4634.980 | -4634.980 | 0 | 1.000 |
| PB2-16d | -4634.980 | -4634.980 | 0 | 1.000 |
| PB2-16e | -4634.980 | -4634.980 | 0 | 1.000 |
| PB2-16f | -4634.472 | -4634.413 | 0.118 | 0.731 |
| PB2-16g | -4634.199 | -4633.915 | 0 | 1.000 |
| PB2-16h | -4634.980 | -4634.980 | 0 | 1.000 |
| PB2-17a | -4306.058 | -4306.058 | 0 | 1.000 |
| PB2-18a | -3247.522 | -3247.521 | 0.001 | 0.964 |
| PB2-19a | -5449.450 | -5449.450 | 0 | 1.000 |
| PB2-19b | -5448.540 | -5448.540 | 0 | 1.000 |
| PB2-2a | -5161.593 | -5161.550 | 0.085 | 0.769 |
| PB2-20a | -5174.140 | -5174.140 | 0 | 1.000 |
| PB2-21a | -3097.920 | -3097.920 | 0 | 1.000 |
| PB2-22a | -5230.952 | -5230.952 | 0 | 1.000 |
| PB2-22b | -5230.952 | -5230.952 | 0 | 1.000 |
| PB2-22c | -5230.952 | -5230.952 | 0 | 1.000 |
| PB2-22d | -5230.952 | -5230.952 | 0 | 1.000 |
| PB2-22e | -5230.952 | -5230.952 | 0 | 1.000 |
| PB2-22f | -5229.262 | -5229.262 | 0 | 1.000 |
| PB2-22g | -5230.905 | -5230.905 | 0 | 1.000 |
| PB2-22h | -5230.690 | -5230.648 | 0.083 | 0.771 |
| PB2-23a | -3300.420 | -3300.420 | 0 | 1.000 |
| PB2-24a | -3073.620 | -3073.624 | 0.007 | 0.928 |
| PB2-25a | -4578.198 | -4578.198 | 0 | 1.000 |
| PB2-26a | -3183.248 | -3183.248 | 0 | 1.000 |
| PB2-27a | -3838.258 | -3837.655 | 1.205 | 0.272 |
| PB2-27b | -3839.210 | -3839.501 | 0.582 | 0.445 |
| PB2-28a | -3275.008 | -3274.911 | 0 | 1.000 |
| PB2-29a | -3549.554 | -3549.554 | 0 | 1.000 |
| PB2-3a | -3518.224 | -3518.202 | 0.043 | 0.833 |
| PB2-30a | -3069.056 | -3069.056 | 0 | 1.000 |
| PB2-31a | -3054.491 | -3054.491 | 0 | 1.000 |
| PB2-32a | -3416.481 | -3416.481 | 0 | 1.000 |
| PB2-33a | -3395.925 | -3395.925 | 0 | 1.000 |
| PB2-33b | -3395.561 | -3395.561 | 0 | 1.000 |
| PB2-34a | -3375.078 | -3375.078 | 0 | 1.000 |
| PB2-35a | -3961.680 | -3961.364 | 0.631 | 0.426 |
| PB2-36a | -3357.453 | -3357.453 | 0 | 1.000 |
| PB2-37a | -3083.998 | -3084.018 | 0.039 | 0.841 |
| PB2-38a | -3437.712 | -3437.712 | 0 | 1.000 |
| PB2-39a | -5045.903 | -5045.903 | 0 | 1.000 |
| PB2-39b | -5045.884 | -5045.883 | 0 | 1.000 |
| PB2-39c | -5045.903 | -5045.903 | 0 | 1.000 |
| PB2-39d | -5045.903 | -5045.903 | 0 | 1.000 |
| PB2-39e | -5044.411 | -5044.093 | 0.636 | 0.425 |
| PB2-39f | -5044.762 | -5044.575 | 0.373 | 0.540 |
| PB2-39g | -5045.903 | -5045.903 | 0 | 1.000 |
| PB2-4a | -3267.585 | -3267.585 | 0 | 1.000 |
| PB2-40a | -4103.048 | -4102.956 | 0.183 | 0.667 |
| PB2-40b | -4103.038 | -4103.038 | 0 | 1.000 |
| PB2-40c | -4103.127 | -4103.127 | 0 | 1.000 |
| PB2-41a | -3815.320 | -3815.320 | 0 | 1.000 |
| PB2-42a | -3475.877 | -3475.877 | 0 | 1.000 |
| PB2-43a | -4178.839 | -4178.839 | 0 | 1.000 |
| PB2-44a | -3783.631 | -3783.932 | 0.601 | 0.437 |
| PB2-44b | -3783.631 | -3783.631 | 0 | 1.000 |
| PB2-44c | -3782.912 | -3782.694 | 0.435 | 0.509 |
| PB2-45a | -3505.688 | -3505.688 | 0 | 1.000 |
| PB2-46a | -3103.293 | -3102.937 | 0.712 | 0.398 |
| PB2-47a | -3214.591 | -3214.591 | 0 | 1.000 |
| PB2-48a | -3252.346 | -3252.345 | 0.002 | 0.964 |
| PB2-48b | -3253.860 | -3253.860 | 0 | 1.000 |
| PB2-49a | -3086.787 | -3086.783 | 0.007 | 0.928 |
| PB2-49b | -3086.787 | -3086.787 | 0 | 1.000 |
| PB2-5a | -3165.430 | -3165.430 | 0 | 1.000 |
| PB2-50a | -3420.036 | -3420.036 | 0 | 1.000 |
| PB2-50b | -3420.145 | -3420.145 | 0 | 1.000 |
| PB2-50c | -3419.856 | -3419.856 | 0 | 1.000 |
| PB2-50d | -3420.145 | -3420.145 | 0 | 1.000 |
| PB2-51a | -3530.422 | -3530.422 | 0 | 1.000 |
| PB2-51b | -3530.422 | -3530.422 | 0 | 1.000 |
| PB2-51c | -3529.228 | -3528.896 | 0.663 | 0.415 |
| PB2-51d | -3530.435 | -3530.422 | 0.025 | 0.871 |
| PB2-52a | -3090.990 | -3090.669 | 0.641 | 0.422 |
| PB2-53a | -3159.424 | -3159.107 | 0.634 | 0.425 |
| PB2-54a | -3383.161 | -3383.161 | 0 | 1.000 |
| PB2-54b | -3384.768 | -3384.768 | 0 | 1.000 |
| PB2-54c | -3384.768 | -3384.768 | 0 | 1.000 |
| PB2-55a | -3320.027 | -3320.027 | 0 | 1.000 |
| PB2-55b | -3320.027 | -3320.027 | 0 | 1.000 |
| PB2-56a | -3225.660 | -3225.677 | 0.634 | 0.425 |
| PB2-56b | -3226.622 | -3226.622 | 0 | 1.000 |
| PB2-56c | -3226.302 | -3226.622 | 0.639 | 0.423 |
| PB2-57a | -3474.679 | -3474.679 | 0 | 1.000 |
| PB2-58a | -3882.209 | -3882.209 | 0 | 1.000 |
| PB2-58b | -3883.920 | -3883.920 | 0 | 1.000 |
| PB2-58c | -3883.920 | -3883.920 | 0 | 1.000 |
| PB2-59a | -3229.369 | -3229.368 | 0 | 1.000 |
| PB2-6a | -3314.789 | -3314.789 | 0 | 1.000 |
| PB2-6b | -3314.789 | -3314.789 | 0 | 1.000 |
| PB2-60a | -3436.214 | -3436.214 | 0 | 1.000 |
| PB2-61a | -4455.209 | -4455.209 | 0 | 1.000 |
| PB2-62a | -4092.158 | -4091.847 | 0.621 | 0.430 |
| PB2-63a | -3301.617 | -3301.617 | 0 | 1.000 |
| PB2-64a | -3639.955 | -3639.955 | 0 | 1.000 |
| PB2-65a | -4725.984 | -4725.984 | 0 | 1.000 |
| PB2-66a | -3471.089 | -3471.089 | 0 | 1.000 |
| PB2-66b | -3471.177 | -3471.177 | 0 | 1.000 |
| PB2-67a | -3151.189 | -3150.502 | 1.373 | 0.241 |
| PB2-68a | -3350.026 | -3350.026 | 0 | 1.000 |
| PB2-69a | -4195.683 | -4195.683 | 0 | 1.000 |
| PB2-69b | -4195.683 | -4195.683 | 0 | 1.000 |
| PB2-69c | -4195.683 | -4195.683 | 0 | 1.000 |
| PB2-69d | -4195.683 | -4195.683 | 0 | 1.000 |
| PB2-69e | -4193.328 | -4192.681 | 1.294 | 0.255 |
| PB2-69f | -4195.683 | -4195.683 | 0 | 1.000 |
| PB2-7a | -3315.223 | -3315.223 | 0 | 1.000 |
| PB2-70a | -3391.516 | -3391.516 | 0 | 1.000 |
| PB2-71a | -4154.063 | -4154.063 | 0 | 1.000 |
| PB2-71b | -4154.368 | -4154.369 | 0.001 | 0.964 |
| PB2-71c | -4154.640 | -4154.911 | 0.541 | 0.461 |
| PB2-71d | -4154.376 | -4154.376 | 0 | 1.000 |
| PB2-71e | -4154.640 | -4154.911 | 0 | 1.000 |
| PB2-72a | -4258.271 | -4258.271 | 0 | 1.000 |
| PB2-72b | -4258.988 | -4258.983 | 0.010 | 0.920 |
| PB2-72c | -4257.632 | -4257.632 | 0 | 1.000 |
| PB2-73a | -4129.412 | -4129.394 | 0.036 | 0.849 |
| PB2-73b | -4129.447 | -4129.447 | 0 | 1.000 |
| PB2-73c | -4128.887 | -4128.887 | 0 | 1.000 |
| PB2-73d | -4129.456 | -4129.456 | 0 | 1.000 |
| PB2-73e | -4129.456 | -4129.456 | 0 | 1.000 |
| PB2-73f | -4129.456 | -4129.456 | 0 | 1.000 |
| PB2-73g | -4129.454 | -4129.454 | 0 | 1.000 |
| PB2-74a | -3181.740 | -3181.740 | 0 | 1.000 |
| PB2-74c | -3181.778 | -3181.778 | 0 | 1.000 |
| PB2-75a | -3211.979 | -3211.979 | 0 | 1.000 |
| PB2-76a | -3330.219 | -3330.219 | 0 | 1.000 |
| PB2-77a | -4622.521 | -4622.521 | 0 | 1.000 |
| PB2-78a | -3061.154 | -3061.155 | 0.002 | 0.964 |
| PB2-79a | -3212.411 | -3212.372 | 0.078 | 0.780 |
| PB2-79b | -3212.426 | -3212.426 | 0 | 1.000 |
| PB2-8a | -3097.290 | -3096.959 | 0.662 | 0.415 |
| PB2-80a | -3349.537 | -3349.537 | 0 | 1.000 |
| PB2-80b | -3349.537 | -3349.537 | 0 | 1.000 |
| PB2-81a | -3176.464 | -3176.464 | 0 | 1.000 |
| PB2-81b | -3176.464 | -3176.463 | 0 | 1.000 |
| PB2-82a | -3202.247 | -3201.545 | 1.403 | 0.236 |
| PB2-83a | -3294.747 | -3294.747 | 0 | 1.000 |
| PB2-84a | -3191.209 | -3190.813 | 0.791 | 0.373 |
| PB2-85a | -3157.362 | -3158.368 | 2.011 | 0.156 |
| PB2-86a | -3248.023 | -3247.933 | 0.180 | 0.671 |
| PB2-87a | -3103.717 | -3103.357 | 0 | 1.000 |
| PB2-88a | -3186.322 | -3186.322 | 0 | 1.000 |
| PB2-89a | -3267.112 | -3267.112 | 0 | 1.000 |
| PB2-89b | -3263.639 | -3263.639 | 0 | 1.000 |
| PB2-9a | -3587.216 | -3587.216 | 0 | 1.000 |
| PB2-90a | -3242.598 | -3242.599 | 0.002 | 0.964 |
| PB2-91a | -3112.267 | -3111.639 | 0 | 1.000 |
| PB2-92a | -3799.094 | -3799.094 | 0 | 1.000 |
| PB2-92b | -3799.093 | -3799.094 | 0 | 1.000 |
| PB2-92c | -3797.773 | -3797.773 | 0 | 1.000 |
|  |  |  |  |  |
| MP-1a | -1597.756 | -1597.934 | 0.355 | 0.550 |
| MP-10a | -1564.212 | -1564.212 | 0 | 1.000 |
| MP-10b | -1564.212 | -1564.212 | 0 | 1.000 |
| MP-11a | -1504.282 | -1504.282 | 0 | 1.000 |
| MP-12a | -1667.435 | -1667.435 | 0 | 1.000 |
| MP-13a | -1621.457 | -1621.458 | 0.001 | 0.964 |
| MP-13b | -1621.457 | -1621.457 | 0 | 1.000 |
| MP-13c | -1621.457 | -1621.457 | 0 | 1.000 |
| MP-13d | -1621.457 | -1621.457 | 0 | 1.000 |
| MP-13e | -1621.457 | -1621.458 | 0.001 | 0.964 |
| MP-14a | -1572.414 | -1571.908 | 1.012 | 0.314 |
| MP-14b | -1570.823 | -1570.703 | 0.24 | 0.624 |
| MP-14c | -1572.414 | -1572.414 | 0 | 1.000 |
| MP-14d | -1572.414 | -1572.414 | 0 | 1.000 |
| MP-14e | -1572.414 | -1572.414 | 0 | 1.000 |
| MP-14f | -1572.414 | -1572.414 | 0 | 1.000 |
| MP-15a | -1452.599 | -1452.599 | 0 | 1.000 |
| MP-16a | -1468.602 | -1468.602 | 0 | 1.000 |
| MP-17a | -1625.652 | -1625.652 | 0 | 1.000 |
| MP-18a | -1426.206 | -1426.206 | 0 | 1.000 |
| MP-19a | -1582.623 | -1582.623 | 0 | 1.000 |
| MP-2a | -1510.710 | -1510.710 | 0 | 1.000 |
| MP-20a | -1681.961 | -1681.961 | 0 | 1.000 |
| MP-21a | -1591.263 | -1591.569 | 0.612 | 0.434 |
| MP-22a | -1560.417 | -1560.417 | 0 | 1.000 |
| MP-22b | -1560.417 | -1560.417 | 0 | 1.000 |
| MP-23a | -1496.222 | -1496.222 | 0 | 1.000 |
| MP-24a | -1551.141 | -1551.141 | 0 | 1.000 |
| MP-25a | -1557.413 | -1557.049 | 0.728 | 0.393 |
| MP-25b | -1557.656 | -1557.656 | 0 | 1.000 |
| MP-25c | -1557.830 | -1557.656 | 0.347 | 0.555 |
| MP-26a | -1630.910 | -1630.910 | 0 | 1.000 |
| MP-26b | -1630.910 | -1630.910 | 0 | 1.000 |
| MP-26c | -1630.910 | -1630.910 | 0 | 1.000 |
| MP-26d | -1630.303 | -1629.982 | -1629 | 0.422 |
| MP-27a | -1890.566 | -1890.566 | 0 | 1.000 |
| MP-27b | -1890.566 | -1890.566 | 0 | 1.000 |
| MP-27c | -1890.566 | -1890.566 | 0 | 1.000 |
| MP-27d | -1890.566 | -1890.566 | 0 | 1.000 |
| MP-27e | -1889.838 | -1889.838 | 0 | 1.000 |
| MP-27f | -1890.586 | -1890.586 | 0 | 1.000 |
| MP-28a | -1798.367 | -1798.367 | 0 | 1.000 |
| MP-28b | -1798.367 | -1798.367 | 0 | 1.000 |
| MP-28c | -1798.367 | -1798.367 | 0 | 1.000 |
| MP-28d | -1797.025 | -1796.673 | 0.704 | 0.401 |
| MP-29a | -1411.868 | -1411.867 | 0.001 | 0.964 |
| MP-3a | -1664.249 | -1664.249 | 0 | 1.000 |
| MP-30a | -1462.843 | -1462.843 | 0 | 1.000 |
| MP-31a | -1520.335 | -1520.335 | 0 | 1.000 |
| MP-31b | -1520.335 | -1520.335 | 0 | 1.000 |
| MP-31c | -1520.335 | -1520.335 | 0 | 1.000 |
| MP-32a | -1699.039 | -1699.039 | 0 | 1.000 |
| MP-32b | -1699.039 | -1699.036 | 0.005 | 0.938 |
| MP-32c | -1699.039 | -1699.036 | 0.005 | 0.938 |
| MP-33a | -1938.499 | -1938.695 | 0.391 | 0.531 |
| MP-33b | -1938.714 | -1938.714 | 0 | 1.000 |
| MP-34a | -1518.010 | -1517.054 | 1.911 | 0.166 |
| MP-35a | -1758.482 | -1758.482 | 0 | 1.000 |
| MP-36a | -2460.426 | -2460.405 | 0.041 | 0.837 |
| MP-37a | -1538.909 | -1538.909 | 0 | 1.000 |
| MP-37b | -1538.909 | -1538.909 | 0 | 1.000 |
| MP-37c | -1538.909 | -1538.909 | 0 | 1.000 |
| MP-38a | -1424.484 | -1424.481 | 0.005 | 0.938 |
| MP-39a | -1537.404 | -1537.404 | 0 | 1.000 |
| MP-39b | -1536.915 | -1536.915 | 0 | 1.000 |
| MP-4a | -1553.200 | -1552.970 | 0.460 | 0.497 |
| MP-40a | -1411.578 | -1411.578 | 0 | 1.000 |
| MP-40b | -1411.578 | -1411.578 | 0 | 1.000 |
| MP-41a | -1462.761 | -1462.761 | 0 | 1.000 |
| MP-42a | -1411.623 | -1411.623 | 0 | 1.000 |
| MP-43a | -1425.007 | -1425.002 | 0.010 | 0.920 |
| MP-44a | -1640.28 | -1640.280 | 0 | 1.000 |
| MP-44b | -1640.528 | -1640.528 | 0 | 1.000 |
| MP-44c | -1640.528 | -1640.528 | 0 | 1.000 |
| MP-45a | -1795.884 | -1795.555 | 0 | 1.000 |
| MP-46a | -1779.930 | -1779.93 | 0 | 1.000 |
| MP-46j | -1779.930 | -1779.93 | 0 | 1.000 |
| MP-46k | -1778.928 | -1778.599 | 0 | 1.000 |
| MP-46b | -1779.930 | -1779.930 | 0 | 1.000 |
| MP-46c | -1779.930 | -1779.930 | 0 | 1.000 |
| MP-46d | -1779.930 | -1779.930 | 0 | 1.000 |
| MP-46f | -1779.930 | -1779.93 | 0 | 1.000 |
| MP-46g | -1779.930 | -1779.930 | 0 | 1.000 |
| MP-46h | -1779.930 | -1779.930 | 0 | 1.000 |
| MP-46i | -1779.797 | -1779.695 | 0.204 | 0.651 |
| MP-48a | -2458.772 | -2458.772 |  | 1.000 |
| MP-48b | -2458.681 | -2458.579 | 0.203 | 0.651 |
| MP-49a | -1473.478 | -1473.478 | 0 | 1.000 |
| MP-5a | -1521.482 | -1521.482 | 0 | 1.000 |
| MP-50b | -1868.661 | -1868.642 | 0.038 | 0.845 |
| MP-50c | -1868.894 | -1868.894 | 0 | 1.000 |
| MP-51b | -1729.659 | -1729.659 | 0 | 1.000 |
| MP-51c | -1729.552 | -1729.552 | 0 | 1.000 |
| MP-51d | -1729.659 | -1729.659 | 0 | 1.000 |
| MP-51e | -1729.659 | -1729.659 | 0 | 1.000 |
| MP-52a | -1594.529 | -1594.529 | 0 | 1.000 |
| MP-52b | -1593.964 | -1593.625 | 0.677 | 0.410 |
| MP-52c | -1594.529 | -1594.529 | 0 | 1.000 |
| MP-52d | -1594.529 | -1594.529 | 0 | 1.000 |
| MP-52e | -1594.529 | -1594.529 | 0 | 1.000 |
| MP-53a | -1529.316 | -1529.316 | 0 | 1.000 |
| MP-54a | -1712.019 | -1711.346 | 1.346 | 0.245 |
| MP-54b | -1713.942 | -1713.942 | 0 | 1.000 |
| MP-54c | -1713.743 | -1713.743 | 0 | 1.000 |
| MP-54d | -1713.930 | -1713.942 | 0.023 | 0.876 |
| MP-54e | -1713.930 | -1713.930 | 0 | 1.000 |
| MP-55a | -1441.851 | -1442.007 | 0.311 | 0.576 |
| MP-56a | -1498.134 | -1498.134 | 0 | 1.000 |
| MP-57a | -1492.043 | -1492.043 | 0 | 1.000 |
| MP-58a | -1440.788 | -1440.791 | 0.005 | 0.938 |
| MP-59a | -1478.600 | -1478.600 | 0 | 1.000 |
| MP-6a | -1459.996 | -1459.996 | 0 | 1.000 |
| MP-60a | -1896.369 | -1896.369 | 0 | 1.000 |
| MP-60b | -1896.214 | -1896.202 | 0.023 | 0.876 |
| MP-61a | -1529.535 | -1529.535 | 0 | 1.000 |
| MP-61b | -1529.535 | -1529.535 | 0 | 1.000 |
| MP-62a | -1502.718 | -1502.718 | 0 | 1.000 |
| MP-63a | -1574.287 | -1574.287 | 0 | 1.000 |
| MP-64a | -1527.652 | -1527.652 | 0 | 1.000 |
| MP-65a | -1536.447 | -1536.447 | 0 | 1.000 |
| MP-65b | -1536.447 | -1536.447 | 0 | 1.000 |
| MP-65c | -1536.447 | -1536.447 | 0 | 1.000 |
| MP-66a | -1622.203 | -1622.203 | 0 | 1.000 |
| MP-67a | -1512.569 | -1512.569 | 0 | 1.000 |
| MP-68a | -1437.470 | -1437.470 | 0 | 1.000 |
| MP-69a | -2016.099 | -2016.099 | 0 | 1.000 |
| MP-69b | -2016.099 | -2016.100 | 0.001 | 0.964 |
| MP-69c | -2016.099 | -2016.099 | 0 | 1.000 |
| MP-69d | -2015.880 | -2015.764 | 0.232 | 0.630 |
| MP-69e | -2016.099 | -2016.099 | 0 | 1.000 |
| MP-69f | -2016.099 | -2016.099 | 0 | 1.000 |
| MP-7a | -1536.379 | -1536.379 | 0 | 1.000 |
| MP-70a | -1466.308 | -1466.308 | 0 | 1.000 |
| MP-70b | -1466.308 | -1466.308 | 0 | 1.000 |
| MP-71a | -1707.525 | -1707.525 | 0 | 1.000 |
| MP-71b | -1707.525 | -1707.525 | 0 | 1.000 |
| MP-71c | -1707.525 | -1707.525 | 0 | 1.000 |
| MP-71d | -1707.525 | -1707.525 | 0 | 1.000 |
| MP-71e | -1707.525 | -1707.525 | 0 | 1.000 |
| MP-72a | -1484.966 | -1484.966 | 0 | 1.000 |
| MP-72b | -1485.659 | -1485.659 | 0 | 1.000 |
| MP-72c | -1485.659 | -1485.659 | 0 | 1.000 |
| MP-72d | -1485.659 | -1485.659 | 0 | 1.000 |
| MP-72e | -1485.659 | -1485.659 | 0 | 1.000 |
| MP-72f | -1485.659 | -1485.659 | 0 | 1.000 |
| MP-73a | -1640.192 | -1640.192 | 0 | 1.000 |
| MP-74a | -1556.553 | -1556.553 | 0 | 1.000 |
| MP-75a | -1598.472 | -1598.472 | 0 | 1.000 |
| MP-75b | -1598.472 | -1598.472 | 0 | 1.000 |
| MP-75c | -1598.472 | -1598.472 | 0 | 1.000 |
| MP-75d | -1598.472 | -1598.472 | 0 | 1.000 |
| MP-76a | -1535.102 | -1535.103 | 0.001 | 0.964 |
| MP-77a | -1673.293 | -1673.293 | 0 | 1.000 |
| MP-78a | -2194.056 | -2194.056 | 0 | 1.000 |
| MP-79a | -1518.752 | -1518.752 | 0 | 1.000 |
| MP-79b | -1518.752 | -1518.752 | 0 | 1.000 |
| MP-8a | -1460.586 | -1460.586 | 0 | 1.000 |
| MP-80a | -1446.524 | -1446.524 | 0 | 1.000 |
| MP-80b | -1446.524 | -1446.527 | 0.006 | 0.938 |
| MP-81a | -1425.539 | -1425.533 | 0 | 1.000 |
| MP-82a | -1466.210 | -1466.210 | 0 | 1.000 |
| MP-82b | -1466.210 | -1466.210 | 0 | 1.000 |
| MP-82c | -1466.210 | -1466.210 | 0 | 1.000 |
| MP-83a | -1417.956 | -1417.977 | 0.042 | 0.837 |
| MP-83b | -1417.956 | -1417.956 | 0 | 1.000 |
| MP-83c | -1417.956 | -1417.956 | 0 | 1.000 |
| MP-84a | -1410.568 | -1410.568 | 0 | 1.000 |
| MP-85b | -1968.779 | -1968.779 | 0 | 1.000 |
| MP-86a | -1485.399 | -1485.399 | 0 | 1.000 |
| MP-87a | -1434.155 | -1434.155 | 0 | 1.000 |
| MP-88a | -1457.764 | -1457.764 | 0 | 1.000 |
| MP-89a | -1457.959 | -1457.170 | 1.577 | 0.209 |
| MP-89b | -1459.415 | -1459.415 | 0 | 1.000 |
| MP-9a | -1426.295 | -1426.295 | 0 | 1.000 |
| MP-90a | -1539.519 | -1539.519 | 0 | 1.000 |
| MP-90b | -1539.519 | -1539.519 | 0 | 1.000 |
| MP-90c | -1539.302 | -1539.302 | 0 | 1.000 |
| MP-91a | -1512.131 | -1511.779 | 0.704 | 0.401 |
| MP-91b | -1512.430 | -1512.430 | 0 | 1.000 |
| MP-91c | -1512.430 | -1512.430 | 0 | 1.000 |
| MP-92a | -1545.293 | -1545.293 | 0 | 1.000 |
| MP-92b | -1544.508 | -1544.181 | 0.653 | 0.418 |
| MP-93a | -1459.215 | -1459.215 | 0 | 1.000 |
| MP-93b | -1459.215 | -1459.215 | 0 | 1.000 |
| MP-93c | -1459.215 | -1459.215 | 0 | 1.000 |
| MP-94a | -1545.025 | -1545.025 | 0 | 1.000 |
| MP-94b | -1545.025 | -1545.025 | 0 | 1.000 |
| MP-94c | -1545.025 | -1545.025 | 0 | 1.000 |
| MP-94d | -1545.025 | -1545.025 | 0 | 1.000 |
| MP-95a | -1709.905 | -1709.905 | 0 | 1.000 |
| MP-95b | -1709.905 | -1709.905 | 0 | 1.000 |
| MP-95c | -1709.905 | -1709.905 | 0 | 1.000 |
| MP-95d | -1709.905 | -1709.905 | 0 | 1.000 |
| MP-95e | -1709.370 | -1709.374 | 0.008 | 0.928 |
| MP-96a | -1624.221 | -1624.221 | 0 | 1.000 |
| MP-96b | -1624.221 | -1624.221 | 0 | 1.000 |
| MP-96c | -1624.221 | -1624.221 | 0 | 1.000 |
| MP-97a | -1604.759 | -1604.759 | 0 | 1.000 |
| MP-97b | -1604.759 | -1604.759 | 0 | 1.000 |
| MP-97c | -1603.860 | -1603.845 | 0.029 | 0.862 |
| MP-98a | -1585.739 | -1585.739 | 0 | 1.000 |
|  |  |  |  |  |
| NA1-1a | -1900.270 | -1900.270 | 0 | 1.000 |
| NA1-1b | -1900.270 | -1900.270 | 0 | 1.000 |
| NA1-1c | -1900.270 | -1900.270 | 0 | 1.000 |
| NA1-10a | -1866.386 | -1866.385 | 0.001 | 0.964 |
| NA1-100a | -2135.583 | -2135.583 | 0 | 1.000 |
| NA1-100b | -2135.583 | -2135.583 | 0 | 1.000 |
| NA1-100c | -2135.583 | -2135.583 | 0 | 1.000 |
| NA1-100d | -2135.583 | -2135.583 | 0 | 1.000 |
| NA1-100e | -2135.583 | -2135.583 | 0 | 1.000 |
| NA1-101a | -1937.604 | -1937.604 | 0 | 1.000 |
| NA1-102a | -2024.142 | -2024.142 | 0 | 1.000 |
| NA1-103a | -1988.107 | -1988.107 | 0 | 1.000 |
| NA1-104a | -2886.020 | -2886.020 | 0 | 1.000 |
| NA1-104b | -2886.058 | -2886.058 | 0 | 1.000 |
| NA1-104c | -2885.482 | -2884.834 | 1.296 | 0.254 |
| NA1-11a | -1896.556 | -1896.203 | 0.706 | 0.400 |
| NA1-11b | -1896.856 | -1896.856 | 0 | 1.000 |
| NA1-12a | -1970.569 | -1970.569 | 0 | 1.000 |
| NA1-13a | -1839.195 | -1838.820 | 0.75 | 0.386 |
| NA1-14a | -2263.194 | -2263.194 | 0 | 1.000 |
| NA1-14b | -2263.166 | -2263.166 | 0 | 1.000 |
| NA1-14c | -2263.325 | -2263.325 | 0 | 1.000 |
| NA1-14d | -2262.592 | -2262.290 | 0.604 | 0.437 |
| NA1-14e | -2263.325 | -2263.325 | 0 | 1.000 |
| NA1-14f | -2262.523 | -2262.186 | 0.673 | 0.411 |
| NA1-14g | -2263.325 | -2263.325 | 0 | 1.000 |
| NA1-15a | -2080.715 | -2080.715 | 0 | 1.000 |
| NA1-15c | -2080.715 | -2080.715 | 0 | 1.000 |
| NA1-16a | -1842.805 | -1842.805 | 0 | 1.000 |
| NA1-17a | -1892.582 | -1892.582 | 0 | 1.000 |
| NA1-17b | -1892.582 | -1892.582 | 0 | 1.000 |
| NA1-18a | -2188.619 | -2188.619 | 0 | 1.000 |
| NA1-18b | -2188.619 | -2188.619 | 0 | 1.000 |
| NA1-18c | -2188.619 | -2188.619 | 0 | 1.000 |
| NA1-18d | -2187.241 | -2187.240 | 0.002 | 0.964 |
| NA1-18e | -2188.619 | -2188.619 | 0 | 1.000 |
| NA1-18f | -2188.619 | -2188.619 | 0 | 1.000 |
| NA1-18g | -2188.619 | -2188.619 | 0 | 1.000 |
| NA1-19a | -2300.217 | -2300.217 | 0 | 1.000 |
| NA1-2a | -1987.507 | -1987.507 | 0 | 1.000 |
| NA1-20a | -2052.610 | -2052.610 | 0 | 1.000 |
| NA1-20b | -2052.610 | -2052.610 | 0 | 1.000 |
| NA1-21a | -1936.180 | -1936.181 | 0.001 | 0.964 |
| NA1-21b | -1934.971 | -1934.971 | 0 | 1.000 |
| NA1-22a | -2562.543 | -2562.543 | 0 | 1.000 |
| NA1-22j | -2562.348 | -2562.267 | 0.162 | 0.687 |
| NA1-22b | -2562.543 | -2562.543 | 0 | 1.000 |
| NA1-22c | -2561.464 | -2560.491 | 1.945 | 0.163 |
| NA1-22d | -2562.317 | -2562.317 | 0 | 1.000 |
| NA1-22e | -2562.543 | -2562.543 | 0 | 1.000 |
| NA1-22f | -2562.543 | -2562.543 | 0 | 1.000 |
| NA1-22g | -2562.543 | -2562.543 | 0 | 1.000 |
| NA1-22h | -2562.543 | -2562.543 | 0 | 1.000 |
| NA1-22i | -2562.543 | -2562.543 | 0 | 1.000 |
| NA1-23a | -1944.645 | -1944.645 | 0 | 1.000 |
| NA1-24a | -1868.212 | -1868.212 | 0 | 1.000 |
| NA1-25a | -1868.503 | -1868.503 | 0 | 1.000 |
| NA1-26a | -1956.670 | -1956.670 | 0 | 1.000 |
| NA1-26b | -1956.670 | -1956.670 | 0 | 1.000 |
| NA1-26c | -1956.670 | -1956.670 | 0 | 1.000 |
| NA1-27a | -2453.319 | -2453.319 | 0 | 1.000 |
| NA1-27b | -2453.319 | -2453.319 | 0 | 1.000 |
| NA1-27c | -2453.278 | -2453.278 | 0 | 1.000 |
| NA1-28a | -1935.135 | -1935.135 | 0 | 1.000 |
| NA1-29a | -2131.563 | -2131.563 | 0 | 1.000 |
| NA1-3a | -1913.415 | -1913.398 | 0.034 | 0.853 |
| NA1-30a | -1920.614 | -1920.614 | 0 | 1.000 |
| NA1-31a | -2134.156 | -2134.156 | 0 | 1.000 |
| NA1-32a | -1858.949 | -1858.771 | 0.356 | 0.550 |
| NA1-33a | -1899.644 | -1899.644 | 0 | 1.000 |
| NA1-34a | -2265.068 | -2265.068 | 0 | 1.000 |
| NA1-35a | -3189.271 | -3189.271 | 0 | 1.000 |
| NA1-35b | -3189.271 | -3189.271 | 0 | 1.000 |
| NA1-36a | -3047.695 | -3047.402 | 0.586 | 0.443 |
| NA1-37a | -2026.088 | -2026.088 | 0 | 1.000 |
| NA1-38a | -1978.808 | -1978.808 | 0 | 1.000 |
| NA1-39a | -1940.742 | -1940.742 | 0 | 1.000 |
| NA1-4a | -2033.124 | -2033.124 | 0 | 1.000 |
| NA1-40a | -2723.281 | -2723.245 | 0.072 | 0.788 |
| NA1-40b | -2723.086 | -2723.086 | 0 | 1.000 |
| NA1-40c | -2723.285 | -2723.284 | 0 | 1.000 |
| NA1-41a | -1807.139 | -1807.139 | 0 | 1.000 |
| NA1-42a | -2152.550 | -2152.550 | 0 | 1.000 |
| NA1-42b | -2152.550 | -2152.550 | 0 | 1.000 |
| NA1-42c | -2152.550 | -2152.550 | 0 | 1.000 |
| NA1-43a | -2002.823 | -2002.823 | 0 | 1.000 |
| NA1-44a | -2578.511 | -2578.511 | 0 | 1.000 |
| NA1-44b | -2577.896 | -2577.552 | 0.688 | 0.406 |
| NA1-44c | -2578.511 | -2578.511 | 0 | 1.000 |
| NA1-44d | -2578.511 | -2578.510 | 0.001 | 0.964 |
| NA1-44e | -2578.510 | -2578.510 | 0 | 1.000 |
| NA1-45a | -2214.424 | -2214.396 | 0.055 | 0.812 |
| NA1-46a | -1904.468 | -1904.468 | 0 | 1.000 |
| NA1-47a | -2118.315 | -2118.315 | 0 | 1.000 |
| NA1-48a | -2128.927 | -2128.927 | 0 | 1.000 |
| NA1-49a | -3373.078 | -3373.078 | 0 | 1.000 |
| NA1-49b | -3373.078 | -3373.078 | 0 | 1.000 |
| NA1-49c | -3373.078 | -3373.078 | 0 | 1.000 |
| NA1-49d | -3373.078 | -3373.078 | 0 | 1.000 |
| NA1-49e | -3373.078 | -3373.078 | 0 | 1.000 |
| NA1-49f | -3373.078 | -3373.078 | 0 | 1.000 |
| NA1-49g | -3373.059 | -3373.059 | 0 | 1.000 |
| NA1-49h | -3372.887 | -3372.559 | 0.655 | 0.417 |
| NA1-49i | -3373.078 | -3373.078 | 0 | 1.000 |
| NA1-5a | -2326.068 | -2326.068 | 0 | 1.000 |
| NA1-5b | -2326.068 | -2326.068 | 0 | 1.000 |
| NA1-50a | -2377.449 | -2377.449 | 0 | 1.000 |
| NA1-50b | -2376.941 | -2376.941 | 0 | 1.000 |
| NA1-51a | -2040.581 | -2040.581 | 0 | 1.000 |
| NA1-52a | -1958.826 | -1958.826 | 0 | 1.000 |
| NA1-53a | -1808.641 | -1808.642 | 0.001 | 0.964 |
| NA1-54a | -2033.854 | -2033.854 | 0 | 1.000 |
| NA1-55a | -2592.922 | -2592.922 | 0 | 1.000 |
| NA1-55b | -2592.959 | -2592.959 | 0 | 1.000 |
| NA1-55c | -2592.961 | -2592.961 | 0 | 1.000 |
| NA1-56a | -1997.757 | -1997.757 | 0 | 1.000 |
| NA1-56b | -1998.787 | -1998.108 | 1.358 | 0.243 |
| NA1-57a | -1880.146 | -1880.146 | 0 | 1.000 |
| NA1-58a | -2859.440 | -2859.440 | 0 | 1.000 |
| NA1-58b | -2859.465 | -2859.465 | 0 | 1.000 |
| NA1-59a | -2501.184 | -2501.184 | 0 | 1.000 |
| NA1-59b | -2501.184 | -2501.185 | 0.001 | 0.964 |
| NA1-59c | -2500.886 | -2500.886 | 0 | 1.000 |
| NA1-59d | -2500.763 | -2500.763 | 0 | 1.000 |
| NA1-6a | -2142.969 | -2142.969 | 0 | 1.000 |
| NA1-60a | -1962.153 | -1962.154 | 0.001 | 0.964 |
| NA1-61a | -2416.718 | -2416.718 | 0 | 1.000 |
| NA1-61b | -2416.718 | -2416.718 | 0 | 1.000 |
| NA1-61c | -2416.718 | -2416.718 | 0 | 1.000 |
| NA1-61d | -2416.718 | -2416.718 | 0 | 1.000 |
| NA1-62a | -2439.147 | -2439.148 | 0.002 | 0.964 |
| NA1-63a | -3052.051 | -3052.051 | 0 | 1.000 |
| NA1-63b | -3052.051 | -3052.051 | 0 | 1.000 |
| NA1-63c | -3052.051 | -3052.051 | 0 | 1.000 |
| NA1-63d | -3051.908 | -3051.908 | 0 | 1.000 |
| NA1-63e | -3051.665 | -3051.665 | 0 | 1.000 |
| NA1-63f | -3052.051 | -3052.051 | 0 | 1.000 |
| NA1-63g | -3052.051 | -3052.051 | 0 | 1.000 |
| NA1-63h | -3052.051 | -3052.051 | 0 | 1.000 |
| NA1-64a | -1941.934 | -1941.934 | 0 | 1.000 |
| NA1-64b | -1941.557 | -1941.564 | 0.014 | 0.905 |
| NA1-65a | -2813.774 | -2813.774 | 0 | 1.000 |
| NA1-65j | -2813.774 | -2813.774 | 0 | 1.000 |
| NA1-65b | -2813.774 | -2813.774 | 0 | 1.000 |
| NA1-65c | -2813.774 | -2813.774 | 0 | 1.000 |
| NA1-65d | -2813.774 | -2813.774 | 0 | 1.000 |
| NA1-65e | -2813.774 | -2813.774 | 0 | 1.000 |
| NA1-65f | -2813.774 | -2813.774 | 0 | 1.000 |
| NA1-65g | -2813.774 | -2813.774 | 0 | 1.000 |
| NA1-65h | -2813.774 | -2813.774 | 0 | 1.000 |
| NA1-65i | -2813.774 | -2813.774 | 0 | 1.000 |
| NA1-66a | -2022.319 | -2022.319 | 0 | 1.000 |
| NA1-67a | -1915.466 | -1915.466 | 0 | 1.000 |
| NA1-68a | -2108.997 | -2108.997 | 0 | 1.000 |
| NA1-69a | -1825.129 | -1825.121 | 0.015 | 0.899 |
| NA1-7a | -1910.166 | -1910.166 | 0 | 1.000 |
| NA1-7b | -1910.166 | -1910.166 | 0 | 1.000 |
| NA1-70a | -2020.754 | -2020.754 | 0 | 1.000 |
| NA1-70b | -2020.754 | -2020.754 | 0 | 1.000 |
| NA1-71a | -1998.957 | -1998.941 | 0.032 | 0.858 |
| NA1-72a | -2188.093 | -2188.093 | 0 | 1.000 |
| NA1-72b | -2188.093 | -2188.093 | 0 | 1.000 |
| NA1-72c | -2188.093 | -2188.093 | 0 | 1.000 |
| NA1-72d | -2188.093 | -2188.093 | 0 | 1.000 |
| NA1-73a | -1961.118 | -1961.118 | 0 | 1.000 |
| NA1-74a | -2254.571 | -2254.571 | 0 | 1.000 |
| NA1-74b | -2254.571 | -2254.571 | 0 | 1.000 |
| NA1-74c | -2254.571 | -2254.571 | 0 | 1.000 |
| NA1-74d | -2254.571 | -2254.571 | 0 | 1.000 |
| NA1-75a | -2179.030 | -2179.030 | 0 | 1.000 |
| NA1-76a | -2079.150 | -2079.150 | 0 | 1.000 |
| NA1-76b | -2078.607 | -2078.285 | 0.644 | 0.422 |
| NA1-76c | -2079.150 | -2079.150 | 0 | 1.000 |
| NA1-77a | -4097.773 | -4097.773 | 0 | 1.000 |
| NA1-78a | -3118.326 | -3118.326 | 0 | 1.000 |
| NA1-78b | -3118.326 | -3118.326 | 0 | 1.000 |
| NA1-79a | -2275.009 | -2275.009 | 0 | 1.000 |
| NA1-8a | -1993.425 | -1993.425 | 0 | 1.000 |
| NA1-80a | -2814.622 | -2814.622 | 0 | 1.000 |
| NA1-80b | -2814.210 | -2814.210 | 0 | 1.000 |
| NA1-80c | -2813.520 | -2813.520 | 0 | 1.000 |
| NA1-80d | -2814.622 | -2815.219 | 1.194 | 0.274 |
| NA1-80e | -2814.622 | -2814.622 | 0 | 1.000 |
| NA1-80f | -2814.622 | -2815.219 | 1.194 | 0.274 |
| NA1-80g | -2815.219 | -2815.219 | 0 | 1.000 |
| NA1-81a | -2134.720 | -2134.719 | 0.001 | 0.964 |
| NA1-81b | -2134.175 | -2133.710 | 0.930 | 0.334 |
| NA1-82a | -2879.923 | -2879.898 | 0.930 | 0.334 |
| NA1-82b | -2881.568 | -2881.568 | 0 | 1.000 |
| NA1-82c | -2881.568 | -2881.568 | 0 | 1.000 |
| NA1-83a | -2611.839 | -2611.839 | 0 | 1.000 |
| NA1-83j | -2610.636 | -2610.601 | 0.069 | 0.791 |
| NA1-83k | -2611.839 | -2611.839 | 0 | 1.000 |
| NA1-83b | -2611.840 | -2611.839 | 0.002 | 0.964 |
| NA1-83c | -2611.839 | -2611.839 | 0 | 1.000 |
| NA1-83d | -2611.839 | -2611.839 | 0 | 1.000 |
| NA1-83e | -2611.839 | -2611.839 | 0 | 1.000 |
| NA1-83f | -2611.839 | -2611.839 | 0 | 1.000 |
| NA1-83g | -2611.839 | -2611.839 | 0 | 1.000 |
| NA1-83h | -2611.839 | -2611.839 | 0 | 1.000 |
| NA1-83i | -2611.839 | -2611.840 | 0.002 | 0.002 |
| NA1-84a | -1919.901 | -1919.901 | 0 | 1.000 |
| NA1-84b | -1919.901 | -1919.901 | 0 | 1.000 |
| NA1-85a | -2305.525 | -2304.520 | 2.010 | 0.156 |
| NA1-85b | -2309.703 | -2309.703 | 0 | 1.000 |
| NA1-85c | -2309.703 | -2309.703 | 0 | 1.000 |
| NA1-86a | -1855.166 | -1855.166 | 0 | 1.000 |
| NA1-87a | -1966.893 | -1966.893 | 0 | 1.000 |
| NA1-88a | -2433.319 | -2433.319 | 0 | 1.000 |
| NA1-88b | -2433.172 | -2433.172 | 0 | 1.000 |
| NA1-88c | -2433.319 | -2433.319 | 0 | 1.000 |
| NA1-88d | -2433.319 | -2433.319 | 0 | 1.000 |
| NA1-88e | -2432.618 | -2432.327 | 0.581 | 0.445 |
| NA1-89a | -1913.648 | -1913.648 | 0 | 1.000 |
| NA1-9a | -1920.364 | -1920.364 | 0 | 1.000 |
| NA1-90a | -2046.765 | -2046.765 | 0 | 1.000 |
| NA1-90b | -2046.765 | -2046.765 | 0 | 1.000 |
| NA1-91a | -2034.104 | -2034.104 | 0 | 1.000 |
| NA1-91b | -2034.104 | -2034.104 | 0 | 1.000 |
| NA1-92a | -2037.091 | -2037.091 | 0 | 1.000 |
| NA1-92b | -2036.974 | -2036.646 | 0.655 | 0.417 |
| NA1-92c | -2037.091 | -2037.091 | 0 | 1.000 |
| NA1-92d | -2037.091 | -2037.091 | 0 | 1.000 |
| NA1-93a | -2205.176 | -2205.176 | 0 | 1.000 |
| NA1-93b | -2205.176 | -2205.176 | 0 | 1.000 |
| NA1-93c | -2204.312 | -2204.312 | 0 | 1.000 |
| NA1-93d | -2204.653 | -2204.333 | 0.639 | 0.423 |
| NA1-93e | -2205.109 | -2205.109 | 0 | 1.000 |
| NA1-94a | -2022.765 | -2022.765 | 0 | 1.000 |
| NA1-94b | -2022.366 | -2022.058 | 0.615 | 0.432 |
| NA1-95a | -1939.178 | -1939.178 | 0 | 1.000 |
| NA1-96a | -2089.077 | -2089.077 | 0 | 1.000 |
| NA1-96b | -2089.077 | -2089.077 | 0 | 1.000 |
| NA1-97a | -1870.605 | -1870.605 | 0 | 1.000 |
| NA1-98a | -1877.012 | -1877.012 | 0 | 1.000 |
| NA1-99a | -2307.974 | -2307.974 | 0 | 1.000 |
| NA1-99b | -2306.103 | -2305.444 | 1.318 | 0.250 |
| NA1-99c | -2307.974 | -2307.974 | 0 | 1.000 |
| NA1-99d | -2307.974 | -2307.974 | 0 | 1.000 |
| NA1-99e | -2307.780 | -2307.470 | 0.620 | 0.431 |
| NA1-99f | -2307.974 | -2307.974 | 0 | 1.000 |
|  |  |  |  |  |
| NA6-1a | -2003.841 | -2003.841 | 0 | 1.000 |
| NA6-10a | -2096.276 | -2096.276 | 0 | 1.000 |
| NA6-2a | -2205.834 | -2205.833 | 0 | 1.000 |
| NA6-3a | -2244.012 | -2244.012 | 0 | 1.000 |
| NA6-3b | -2243.736 | -2243.437 | 0 | 1.000 |
| NA6-4a | -2086.415 | -2086.415 | 0 | 1.000 |
| NA6-4b | -2086.415 | -2086.415 | 0 | 1.000 |
| NA6-5a | -2010.395 | -2010.394 | 0 | 1.000 |
| NA6-5b | -2006.658 | -2005.453 | 2.409 | 0.120 |
| NA6-6a | -2301.503 | -2301.503 | 0 | 1.000 |
| NA6-6b | -2302.000 | -2302.000 | 0 | 1.000 |
| NA6-6c | -2301.788 | -2301.788 | 0 | 1.000 |
| NA6-7a | -1882.414 | -1882.152 | 0 | 1.000 |
| NA6-8a | -1928.032 | -1928.032 | 0 | 1.000 |
| NA6-9a | -2462.794 | -2462.794 | 0 | 1.000 |
| NA6-9b | -2462.794 | -2462.794 | 0 | 1.000 |
| NA6-9c | -2462.794 | -2462.794 | 0 | 1.000 |
|  |  |  |  |  |
| NP-1a | -3670.053 | -3669.750 | 0.605 | 0.436 |
| NP-10a | -2157.975 | -2157.816 | 0 | 1.000 |
| NP-10b | -2158.093 | -2158.093 | 0 | 1.000 |
| NP-10c | -2158.093 | -2158.093 | 0 | 1.000 |
| NP-11a | -2559.141 | -2559.141 | 0 | 1.000 |
| NP-11b | -2559.033 | -2559.033 | 0 | 1.000 |
| NP-12a | -2144.476 | -2144.476 | 0 | 1.000 |
| NP-12b | -2141.500 | -2141.500 | 0 | 1.000 |
| NP-13a | -2113.460 | -2113.432 | 0 | 1.000 |
| NP-14a | -2093.659 | -2093.659 | 0 | 1.000 |
| NP-15a | -2307.539 | -2307.539 | 0 | 1.000 |
| NP-16a | -2143.250 | -2143.250 | 0 | 1.000 |
| NP-17a | -2351.554 | -2351.554 | 0 | 1.000 |
| NP-18a | -2085.671 | -2085.671 | 0 | 1.000 |
| NP-19a | -2213.984 | -2213.984 | 0 | 1.000 |
| NP-19b | -2213.984 | -2213.984 | 0 | 1.000 |
| NP-2a | -2727.848 | -2727.848 | 0 | 1.000 |
| NP-20a | -2178.897 | -2178.897 | 0 | 1.000 |
| NP-21a | -2122.987 | -2122.941 | 0.092 | 0.761 |
| NP-22a | -2037.873 | -2037.895 | 0.043 | 0.833 |
| NP-23a | -2088.050 | -2088.050 | 0 | 1.000 |
| NP-24a | -2449.556 | -2449.556 | 0 | 1.000 |
| NP-24b | -2449.556 | -2449.556 | 0 | 1.000 |
| NP-24c | -2449.556 | -2449.556 | 0 | 1.000 |
| NP-25a | -2153.723 | -2153.723 | 0 | 1.000 |
| NP-26a | -2093.500 | -2093.500 | 0 | 1.000 |
| NP-27a | -2174.412 | -2174.412 | 0 | 1.000 |
| NP-27b | -2174.412 | -2174.412 | 0 | 1.000 |
| NP-28a | -2054.279 | -2054.279 | 0 | 1.000 |
| NP-29a | -2685.702 | -2685.702 | 0 | 1.000 |
| NP-29j | -2685.702 | -2685.702 | 0 | 1.000 |
| NP-29k | -2685.702 | -2685.702 | 0 | 1.000 |
| NP-29b | -2685.702 | -2685.702 | 0 | 1.000 |
| NP-29c | -2685.702 | -2685.702 | 0 | 1.000 |
| NP-29d | -2685.702 | -2685.702 | 0 | 1.000 |
| NP-29e | -2685.374 | -2685.374 | 0 | 1.000 |
| NP-29f | -2685.702 | -2685.702 | 0 | 1.000 |
| NP-29g | -2685.702 | -2685.702 | 0 | 1.000 |
| NP-29h | -2685.702 | -2685.702 | 0 | 1.000 |
| NP-29i | -2685.702 | -2685.702 | 0 | 1.000 |
| NP-3a | -2328.792 | -2328.792 | 0 | 1.000 |
| NP-30a | -2824.569 | -2824.569 | 0 | 1.000 |
| NP-30j | -2824.131 | -2824.131 | 0 | 1.000 |
| NP-30k | -2824.569 | -2824.569 | 0 | 1.000 |
| NP-30l | -2824.569 | -2824.569 | 0 | 1.000 |
| NP-30m | -2824.569 | -2824.569 | 0 | 1.000 |
| NP-30b | -2824.569 | -2824.569 | 0 | 1.000 |
| NP-30c | -2824.569 | -2824.569 | 0 | 1.000 |
| NP-30d | -2824.569 | -2824.569 | 0 | 1.000 |
| NP-30e | -2824.430 | -2824.430 | 0 | 1.000 |
| NP-30f | -2824.569 | -2824.569 | 0 | 1.000 |
| NP-30g | -2824.569 | -2824.569 | 0 | 1.000 |
| NP-30h | -2824.569 | -2824.569 | 0 | 1.000 |
| NP-30i | -2824.569 | -2824.569 | 0 | 1.000 |
| NP-31a | -2199.877 | -2199.921 | 0.087 | 0.766 |
| NP-31b | -2199.921 | -2199.921 | 0 | 1.000 |
| NP-33a | -2082.333 | -2082.333 | 0 | 1.000 |
| NP-34a | -2179.050 | -2178.054 | 1.992 | 0.158 |
| NP-35a | -2149.173 | -2148.877 | 0.591 | 0.441 |
| NP-35b | -2150.163 | -2150.163 | 0 | 1.000 |
| NP-36a | -2336.283 | -2336.283 | 0 | 1.000 |
| NP-37a | -2090.546 | -2090.546 | 0 | 1.000 |
| NP-38a | -2936.909 | -2936.829 | 0.159 | 0.689 |
| NP-38b | -2938.032 | -2938.032 | 0 | 1.000 |
| NP-38c | -2934.176 | -2934.176 | 0 | 1.000 |
| NP-38d | -2938.032 | -2938.032 | 0 | 1.000 |
| NP-39a | -2247.414 | -2246.808 | 0 | 1.000 |
| NP-4a | -2079.271 | -2079.271 | 0 | 1.000 |
| NP-40a | -2149.324 | -2149.324 | 0 | 1.000 |
| NP-41a | -2984.327 | -2984.327 | 0 | 1.000 |
| NP-41b | -2984.492 | -2984.492 | 0 | 1.000 |
| NP-41c | -2984.066 | -2984.066 | 0 | 1.000 |
| NP-41d | -2982.744 | -2982.575 | 0 | 1.000 |
| NP-41e | -2984.327 | -2984.492 | 0 | 1.000 |
| NP-41f | -2984.492 | -2984.327 | 0 | 1.000 |
| NP-41g | -2984.492 | -2984.327 | 0 | 1.000 |
| NP-42a | -2543.218 | -2543.218 | 0 | 1.000 |
| NP-42b | -2542.254 | -2541.949 | 0.609 | 0.434 |
| NP-42c | -2542.638 | -2542.638 | 0 | 1.000 |
| NP-42d | -2543.218 | -2543.218 | 0 | 1.000 |
| NP-43a | -2955.732 | -2955.732 | 0 | 1.000 |
| NP-43b | -2955.893 | -2955.893 | 0 | 1.000 |
| NP-43c | -2955.379 | -2955.379 | 0 | 1.000 |
| NP-43d | -2955.419 | -2955.419 | 0 | 1.000 |
| NP-44a | -2384.999 | -2384.999 | 0 | 1.000 |
| NP-45a | -2222.068 | -2222.068 | 0 | 1.000 |
| NP-45b | -2221.810 | -2221.810 | 0 | 1.000 |
| NP-46a | -2345.065 | -2345.065 | 0 | 1.000 |
| NP-46b | -2345.065 | -2345.065 | 0 | 1.000 |
| NP-47a | -2173.016 | -2173.016 | 0 | 1.000 |
| NP-47b | -2170.423 | -2170.064 | 0.717 | 0.396 |
| NP-48a | -2228.434 | -2228.434 | 0 | 1.000 |
| NP-49a | -2283.109 | -2282.960 | 0.297 | 0.585 |
| NP-5a | -2278.128 | -2278.128 | 0 | 1.000 |
| NP-50a | -2891.303 | -2891.303 | 0 | 1.000 |
| NP-50b | -2891.303 | -2891.303 | 0 | 1.000 |
| NP-50c | -2891.303 | -2891.303 | 0 | 1.000 |
| NP-50d | -2891.303 | -2891.320 | 0 | 1.000 |
| NP-51a | -2301.095 | -2301.095 | 0 | 1.000 |
| NP-51b | -2300.840 | -2300.840 | 0 | 1.000 |
| NP-51c | -2301.101 | -2301.101 | 0 | 1.000 |
| NP-52a | -2064.825 | -2064.825 | 0 | 1.000 |
| NP-53a | -2134.594 | -2134.594 | 0 | 1.000 |
| NP-54a | -2302.239 | -2302.239 | 0 | 1.000 |
| NP-55a | -2060.812 | -2060.812 | 0 | 1.000 |
| NP-55b | -2057.997 | -2057.621 | 0.751 | 0.385 |
| NP-56a | -2498.704 | -2498.704 | 0 | 1.000 |
| NP-56b | -2498.235 | -2498.235 | 0 | 1.000 |
| NP-56c | -2497.389 | -2497.058 | -2497 | 0.415 |
| NP-57a | -2249.909 | -2249.803 | 0 | 1.000 |
| NP-57b | -2249.993 | -2249.993 | 0 | 1.000 |
| NP-58a | -2130.696 | -2130.355 | 0.681 | 0.408 |
| NP-59a | -2010.792 | -2010.795 | 0 | 1.000 |
| NP-6a | -2367.951 | -2367.951 | 0 | 1.000 |
| NP-6b | -2367.951 | -2367.951 | 0 | 1.000 |
| NP-60a | -2010.777 | -2010.776 | 0 | 1.000 |
| NP-61a | -2110.320 | -2110.320 | 0 | 1.000 |
| NP-62a | -2222.814 | -2222.814 | 0 | 1.000 |
| NP-63a | -3057.577 | -3057.577 | 0 | 1.000 |
| NP-64a | -2220.614 | -2220.614 | 0 | 1.000 |
| NP-66a | -3414.206 | -3414.040 | 0 | 1.000 |
| NP-66b | -3413.601 | -3413.579 | 0.043 | 0.833 |
| NP-67a | -2141.075 | -2141.075 | 0 | 1.000 |
| NP-67b | -2141.075 | -2141.075 | 0 | 1.000 |
| NP-68a | -2442.416 | -2442.416 | 0 | 1.000 |
| NP-69a | -2171.580 | -2171.580 | 0 | 1.000 |
| NP-69b | -2171.580 | -2171.580 | 0 | 1.000 |
| NP-7a | -3259.270 | -3259.270 | 0 | 1.000 |
| NP-7j | -3259.942 | -3259.942 | 0 | 1.000 |
| NP-7k | -3258.484 | -3258.166 | 0.635 | 0.425 |
| NP-7b | -3259.462 | -3259.273 | 0.377 | 0.538 |
| NP-7c | -3259.942 | -3259.942 | 0 | 1.000 |
| NP-7d | -3259.942 | -3259.942 | 0 | 1.000 |
| NP-7e | -3259.949 | -3259.942 | 0 | 1.000 |
| NP-7f | -3259.942 | -3259.942 | 0 | 1.000 |
| NP-7g | -3250.092 | -3249.608 | 0.967 | 0.325 |
| NP-7h | -3259.942 | -3259.942 | 0 | 1.000 |
| NP-7i | -3259.942 | -3259.942 | 0 | 1.000 |
| NP-70a | -2140.969 | -2141.017 | -2141 | 0.756 |
| NP-70b | -2140.983 | -2140.975 | 0.016 | 0.899 |
| NP-71a | -2134.739 | -2134.739 | 0 | 1.000 |
| NP-72a | -2380.782 | -2380.782 | 0 | 1.000 |
| NP-72b | -2379.477 | -2379.153 | 0.648 | 0.420 |
| NP-72c | -2380.782 | -2380.782 | 0 | 1.000 |
| NP-73a | -2279.841 | -2279.841 | 0 | 1.000 |
| NP-73b | -2279.841 | -2279.733 | 0.215 | 0.642 |
| NP-73c | -2278.558 | -2278.332 | 0 | 1.000 |
| NP-73d | -2279.841 | -2279.841 | 0 | 1.000 |
| NP-74a | -2229.488 | -2229.489 | 0 | 1.000 |
| NP-74b | -2229.617 | -2229.617 | 0 | 1.000 |
| NP-75a | -2280.599 | -2280.599 | 0 | 1.000 |
| NP-75b | -2281.104 | -2281.104 | 0 | 1.000 |
| NP-75c | -2281.104 | -2281.104 | 0 | 1.000 |
| NP-76a | -2199.974 | -2199.974 | 0 | 1.000 |
| NP-76b | -2199.974 | -2199.974 | 0 | 1.000 |
| NP-76c | -2199.974 | -2199.974 | 0 | 1.000 |
| NP-77a | -2052.132 | -2052.132 | 0 | 1.000 |
| NP-77b | -2052.132 | -2052.132 | 0 | 1.000 |
| NP-78a | -2108.448 | -2108.448 | 0 | 1.000 |
| NP-78b | -2108.448 | -2108.448 | 0 | 1.000 |
| NP-79a | -2109.473 | -2109.473 | 0 | 1.000 |
| NP-79b | -2109.473 | -2109.473 | 0 | 1.000 |
| NP-8a | -2686.787 | -2686.787 | 0 | 1.000 |
| NP-80a | -2024.195 | -2024.195 | 0 | 1.000 |
| NP-81a | -2506.691 | -2506.691 | 0 | 1.000 |
| NP-81b | -2506.320 | -2506.320 | 0 | 1.000 |
| NP-81c | -2506.691 | -2506.691 | 0 | 1.000 |
| NP-81d | -2506.691 | -2506.691 | 0 | 1.000 |
| NP-82a | -2418.827 | -2418.827 | 0 | 1.000 |
| NP-82b | -2418.072 | -2418.072 | 0 | 1.000 |
| NP-82c | -2418.827 | -2418.827 | 0 | 1.000 |
| NP-83a | -2176.598 | -2176.598 | 0 | 1.000 |
| NP-83b | -2176.598 | -2176.598 | 0 | 1.000 |
| NP-84a | -2112.547 | -2112.547 | 0 | 1.000 |
| NP-85a | -2071.410 | -2071.384 | 0.051 | 0.819 |
| NP-86a | -2232.849 | -2232.849 | 0 | 1.000 |
| NP-86b | -2232.408 | -2232.408 | 0 | 1.000 |
| NP-87a | -2316.950 | -2316.950 | 0 | 1.000 |
| NP-87b | -2318.836 | -2318.836 | 0 | 1.000 |
| NP-87c | -2318.836 | -2318.836 | 0 | 1.000 |
| NP-9a | -2312.665 | -2312.665 | 0 | 1.000 |
| NP-9b | -2312.665 | -2312.665 | 0 | 1.000 |
| NP-9c | -2312.665 | -2312.665 | 0 | 1.000 |
|  |  |  |  |  |
| NS-1a | -1455.784 | -1455.422 | 0.724 | 0.394 |
| NS-10a | -1402.048 | -1402.048 | 0 | 1.000 |
| NS-100a | -1416.972 | -1416.972 | 0 | 1.000 |
| NS-101a | -1745.990 | -1745.990 | 0 | 1.000 |
| NS-101b | -1745.990 | -1745.990 | 0 | 1.000 |
| NS-101c | -1745.990 | -1745.990 | 0 | 1.000 |
| NS-11a | -1419.304 | -1419.309 | 0.009 | 0.920 |
| NS-12a | -1466.108 | -1466.108 | 0 | 1.000 |
| NS-13a | -1583.204 | -1582.871 | 0.665 | 0.414 |
| NS-14a | -1402.713 | -1402.713 | 0 | 1.000 |
| NS-15a | -1604.531 | -1604.531 | 0 | 1.000 |
| NS-16a | -1527.769 | -1527.411 | 0.715 | 0.397 |
| NS-17a | -1740.002 | -1740.002 | 0 | 1.000 |
| NS-17b | -1740.168 | -1740.168 | 0 | 1.000 |
| NS-17c | -1740.168 | -1740.168 | 0 | 1.000 |
| NS-18a | -1541.947 | -1541.947 | 0 | 1.000 |
| NS-19a | -1986.455 | -1986.404 | 0.101 | 0.749 |
| NS-19j | -1986.567 | -1986.567 | 0 | 1.000 |
| NS-19k | -1985.138 | -1985.131 | 0.013 | 0.905 |
| NS-19l | -1986.517 | -1986.401 | 0.231 | 0.630 |
| NS-19m | -1986.567 | -1986.567 | 0 | 1.000 |
| NS-19n | -1986.486 | -1986.486 | 0 | 1.000 |
| NS-19o | -1986.567 | -1986.567 | 0 | 1.000 |
| NS-19b | -1986.567 | -1986.567 | 0 | 1.000 |
| NS-19c | -1986.567 | -1986.567 | 0 | 1.000 |
| NS-19d | -1986.567 | -1986.567 | 0 | 1.000 |
| NS-19e | -1986.470 | -1986.470 | 0 | 1.000 |
| NS-19f | -1986.567 | -1986.567 | 0 | 1.000 |
| NS-19g | -1985.891 | -1985.569 | 0.644 | 0.422 |
| NS-19h | -1986.567 | -1986.567 | 0 | 1.000 |
| NS-19i | -1986.567 | -1986.567 | 0 | 1.000 |
| NS-2a | -1955.068 | -1955.067 | 0.001 | 0.964 |
| NS-20a | -1818.952 | -1818.952 | 0 | 1.000 |
| NS-20b | -1818.952 | -1818.952 | 0 | 1.000 |
| NS-20c | -1818.924 | -1818.924 | 0 | 1.000 |
| NS-20d | -1818.952 | -1818.952 | 0 | 1.000 |
| NS-20e | -1818.952 | -1818.952 | 0 | 1.000 |
| NS-20f | -1818.952 | -1818.952 | 0 | 1.000 |
| NS-20g | -1818.392 | -1818.061 | 0.662 | 0.415 |
| NS-21a | -1671.872 | -1671.957 | 0.170 | 0.680 |
| NS-21b | -1671.957 | -1671.957 | 0 | 1.000 |
| NS-21c | -1671.872 | -1671.872 | 0 | 1.000 |
| NS-22a | -1658.924 | -1658.924 | 0 | 1.000 |
| NS-23a | -1471.565 | -1471.564 | 0.001 | 0.964 |
| NS-23b | -1471.564 | -1471.564 | 0 | 1.000 |
| NS-24a | -1387.029 | -1387.028 | 0.001 | 0.964 |
| NS-25a | -1463.379 | -1462.687 | 1.384 | 0.239 |
| NS-26a | -1465.280 | -1465.280 | 0 | 1.000 |
| NS-27a | -1693.824 | -1693.824 | 0 | 1.000 |
| NS-27b | -1693.322 | -1692.982 | 0.679 | 0.409 |
| NS-27c | -1693.824 | -1693.824 | 0 | 1.000 |
| NS-28a | -1542.813 | -1542.432 | 0.762 | 0.382 |
| NS-28b | -1543.097 | -1543.097 | 0 | 1.000 |
| NS-29a | -1556.526 | -1556.526 | 0 | 1.000 |
| NS-3a | -1526.925 | -1526.925 | 0 | 1.000 |
| NS-30a | -1511.025 | -1511.025 | 0 | 1.000 |
| NS-31b | -1816.401 | -1816.401 | 0 | 1.000 |
| NS-31c | -1816.999 | -1816.999 | 0 | 1.000 |
| NS-32a | -1532.522 | -1532.366 | 0.311 | 0.576 |
| NS-32b | -1532.366 | -1532.366 | 0 | 1.000 |
| NS-33a | -2005.936 | -2005.936 | 0 | 1.000 |
| NS-33b | -2005.936 | -2005.936 | 0 | 1.000 |
| NS-34a | -1678.561 | -1678.561 | 0 | 1.000 |
| NS-34b | -1678.561 | -1678.561 | 0 | 1.000 |
| NS-35a | -1957.198 | -1957.198 | 0 | 1.000 |
| NS-35b | -1959.586 | -1959.576 | 0.019 | 0.887 |
| NS-35c | -1958.979 | -1958.669 | 0.619 | 0.431 |
| NS-35d | -1959.514 | -1959.514 | 0 | 1.000 |
| NS-36a | -1425.947 | -1425.947 | 0 | 1.000 |
| NS-36b | -1425.947 | -1425.947 | 0 | 1.000 |
| NS-37a | -1787.004 | -1787.004 | 0 | 1.000 |
| NS-37b | -1787.004 | -1787.004 | 0 | 1.000 |
| NS-37c | -1787.004 | -1787.004 | 0 | 1.000 |
| NS-38a | -1427.858 | -1427.858 | 0 | 1.000 |
| NS-39a | -1524.511 | -1524.511 | 0 | 1.000 |
| NS-4a | -1585.292 | -1584.945 | 0.693 | 0.404 |
| NS-4b | -1585.541 | -1585.541 | 0 | 1.000 |
| NS-40a | -1448.803 | -1448.803 | 0 | 1.000 |
| NS-41a | -1591.006 | -1591.006 | 0 | 1.000 |
| NS-42a | -1826.379 | -1826.379 | 0 | 1.000 |
| NS-42b | -1826.379 | -1826.379 | 0 | 1.000 |
| NS-43a | -1382.908 | -1382.908 | 0 | 1.000 |
| NS-43b | -1382.908 | -1382.908 | 0 | 1.000 |
| NS-43c | -1382.908 | -1382.908 | 0 | 1.000 |
| NS-43d | -1382.911 | -1382.908 | 0.006 | 0.938 |
| NS-44a | -1445.954 | -1445.954 | 0 | 1.000 |
| NS-45a | -1572.775 | -1572.775 | 0 | 1.000 |
| NS-46a | -1733.452 | -1733.452 | 0 | 1.000 |
| NS-46b | -1733.399 | -1733.399 | 0 | 1.000 |
| NS-47a | -1653.172 | -1653.172 | 0 | 1.000 |
| NS-47b | -1653.172 | -1653.172 | 0 | 1.000 |
| NS-48a | -1932.462 | -1932.462 | 0 | 1.000 |
| NS-48b | -1931.688 | -1931.021 | 1.334 | 0.248 |
| NS-48c | -1932.462 | -1932.462 | 0 | 1.000 |
| NS-48d | -1932.123 | -1932.123 | 0 | 1.000 |
| NS-48e | -1932.097 | -1932.097 | 0 | 1.000 |
| NS-48f | -1932.462 | -1932.462 | 0 | 1.000 |
| NS-48g | -1932.462 | -1932.462 | 0 | 1.000 |
| NS-48h | -1932.462 | -1932.462 | 0 | 1.000 |
| NS-48i | -1932.462 | -1932.462 | 0 | 1.000 |
| NS-49a | -1571.986 | -1571.986 | 0 | 1.000 |
| NS-5a | -1484.885 | -1484.619 | 0.532 | 0.465 |
| NS-50a | -1682.579 | -1682.536 | 0.085 | 0.769 |
| NS-51a | -1552.963 | -1552.963 | 0 | 1.000 |
| NS-52a | -1537.847 | -1537.847 | 0 | 1.000 |
| NS-53a | -1569.818 | -1569.818 | 0 | 1.000 |
| NS-53b | -1569.818 | -1569.818 | 0 | 1.000 |
| NS-54a | -1415.015 | -1415.015 | 0 | 1.000 |
| NS-55a | -1418.558 | -1418.496 | 0.123 | 0.724 |
| NS-57a | -1672.522 | -1672.522 | 0 | 1.000 |
| NS-58a | -1554.985 | -1554.636 | 0.697 | 0.403 |
| NS-59a | -1456.700 | -1456.700 | 0 | 1.000 |
| NS-6a | -1530.092 | -1530.092 | 0 | 1.000 |
| NS-60a | -1385.045 | -1385.044 | 0.001 | 0.964 |
| NS-61a | -1450.468 | -1450.468 | 0 | 1.000 |
| NS-61b | -1450.468 | -1450.468 | 0 | 1.000 |
| NS-61c | -1450.469 | -1450.469 | 0 | 1.000 |
| NS-62a | -1401.183 | -1401.184 | 0.001 | 0.964 |
| NS-63a | -1689.136 | -1689.136 | 0 | 1.000 |
| NS-63b | -1689.136 | -1689.136 | 0 | 1.000 |
| NS-64a | -1519.679 | -1519.679 | 0 | 1.000 |
| NS-65a | -1531.735 | -1531.735 | 0 | 1.000 |
| NS-66a | -1388.252 | -1388.252 | 0 | 1.000 |
| NS-67a | -1418.421 | -1418.421 | 0 | 1.000 |
| NS-68a | -1525.542 | -1525.542 | 0 | 1.000 |
| NS-69a | -1503.575 | -1503.575 | 0 | 1.000 |
| NS-7a | -2254.773 | -2254.773 | 0 | 1.000 |
| NS-70a | -1529.613 | -1529.613 | 0 | 1.000 |
| NS-71a | -1603.758 | -1602.767 | 1.981 | 0.159 |
| NS-72a | -1714.781 | -1714.781 | 0 | 1.000 |
| NS-72b | -1714.781 | -1714.781 | 0 | 1.000 |
| NS-72d | -1714.376 | -1714.376 | 0 | 1.000 |
| NS-73a | -1794.914 | -1794.914 | 0 | 1.000 |
| NS-73b | -1794.925 | -1794.925 | 0 | 1.000 |
| NS-73c | -1794.925 | -1794.925 | 0 | 1.000 |
| NS-74a | -1638.506 | -1638.506 | 0 | 1.000 |
| NS-75a | -1766.746 | -1766.746 | 0 | 1.000 |
| NS-75b | -1766.746 | -1766.746 | 0 | 1.000 |
| NS-76a | -1828.175 | -1827.543 | 1.264 | 0.260 |
| NS-77a | -1661.881 | -1661.881 | 0 | 1.000 |
| NS-77b | -1662.626 | -1662.626 | 0 | 1.000 |
| NS-78a | -1752.939 | -1752.939 | 0 | 1.000 |
| NS-79a | -1575.125 | -1575.125 | 0 | 1.000 |
| NS-8a | -1756.916 | -1756.278 | 1.275 | 0.258 |
| NS-80a | -1511.205 | -1511.205 | 0 | 1.000 |
| NS-80b | -1511.205 | -1511.205 | 0 | 1.000 |
| NS-81a | -1542.144 | -1542.144 | 0 | 1.000 |
| NS-81b | -1541.693 | -1541.376 | 0.634 | 0.425 |
| NS-82a | -1412.764 | -1412.764 | 0 | 1.000 |
| NS-82b | -1412.764 | -1412.764 | 0 | 1.000 |
| NS-82c | -1412.764 | -1412.764 | 0 | 1.000 |
| NS-82d | -1412.764 | -1412.764 | 0 | 1.000 |
| NS-83a | -1677.337 | -1677.337 | 0 | 1.000 |
| NS-83b | -1676.895 | -1676.895 | 0 | 1.000 |
| NS-84a | -1397.345 | -1397.345 | 0 | 1.000 |
| NS-85a | -1836.313 | -1836.313 | 0 | 1.000 |
| NS-85b | -1836.313 | -1836.313 | 0 | 1.000 |
| NS-85c | -1836.313 | -1836.313 | 0 | 1.000 |
| NS-86a | -1416.747 | -1416.747 | 0 | 1.000 |
| NS-87a | -1464.168 | -1464.168 | 0 | 1.000 |
| NS-88a | -1519.637 | -1519.293 | 0.688 | 0.406 |
| NS-88b | -1519.801 | -1519.801 | 0 | 1.000 |
| NS-88c | -1519.801 | -1519.801 | 0 | 1.000 |
| NS-89b | -1742.651 | -1742.651 | 0 | 1.000 |
| NS-89c | -1742.651 | -1742.651 | 0 | 1.000 |
| NS-89d | -1742.652 | -1742.652 | 0 | 1.000 |
| NS-89e | -1742.652 | -1742.652 | 0 | 1.000 |
| NS-9a | -1426.756 | -1426.756 | 0 | 1.000 |
| NS-9b | -1426.756 | -1426.756 | 0 | 1.000 |
| NS-90a | -1575.811 | -1575.811 | 0 | 1.000 |
| NS-90b | -1575.864 | -1575.864 | 0 | 1.000 |
| NS-90c | -1575.835 | -1575.807 | 0.056 | 0.812 |
| NS-91a | -1483.276 | -1483.276 | 0 | 1.000 |
| NS-92a | -1386.138 | -1386.138 | 0 | 1.000 |
| NS-93a | -1493.488 | -1493.130 | 0.715 | 0.397 |
| NS-94a | -1424.096 | -1424.096 | 0 | 1.000 |
| NS-95a | -1465.865 | -1465.865 | 0 | 1.000 |
| NS-96a | -1616.767 | -1616.767 | 0 | 1.000 |
| NS-97a | -1690.404 | -1690.404 | 0 | 1.000 |
| NS-97b | -1690.404 | -1690.404 | 0 | 1.000 |
| NS-98a | -1452.621 | -1452.621 | 0 | 1.000 |
| NS-99a | -1608.154 | -1607.947 | 0.414 | 0.519 |
| NS-99b | -1608.185 | -1608.185 | 0 | 1.000 |
|  |  |  |  |  |
| PA-1a | -3260.808 | -3260.808 | 0 | 1.000 |
| PA-10a | -3313.689 | -3313.689 | 0 | 1.000 |
| PA-11b | -4003.834 | -4003.834 | 0 | 1.000 |
| PA-11c | -4003.834 | -4003.834 | 0 | 1.000 |
| PA-11d | -4002.832 | -4002.502 | 0.659 | 0.416 |
| PA-12a | -3457.081 | -3457.081 | 0 | 1.000 |
| PA-12b | -3457.081 | -3457.081 | 0 | 1.000 |
| PA-12c | -3457.081 | -3457.081 | 0 | 1.000 |
| PA-12d | -3457.081 | -3457.081 | 0 | 1.000 |
| PA-12e | -3456.417 | -3456.109 | 0.615 | 0.432 |
| PA-12f | -3457.081 | -3457.081 | 0 | 1.000 |
| PA-12g | -3456.928 | -3456.928 | 0 | 1.000 |
| PA-12h | -3457.081 | -3457.081 | 0 | 1.000 |
| PA-13a | -3205.847 | -3205.847 | 0 | 1.000 |
| PA-14a | -2882.074 | -2882.072 | 0.003 | 0.949 |
| PA-15a | -4408.903 | -4408.903 | 0 | 1.000 |
| PA-16a | -4447.299 | -4447.299 | 0 | 1.000 |
| PA-16b | -4447.094 | -4447.094 | 0 | 1.000 |
| PA-16c | -4447.299 | -4447.299 | 0 | 1.000 |
| PA-17a | -3502.374 | -3502.374 | 0 | 1.000 |
| PA-17b | -3502.403 | -3502.403 | 0 | 1.000 |
| PA-18a | -3014.623 | -3014.623 | 0 | 1.000 |
| PA-18b | -3014.623 | -3014.623 | 0 | 1.000 |
| PA-19a | -3669.324 | -3669.324 | 0 | 1.000 |
| PA-19b | -3670.730 | -3670.730 | 0 | 1.000 |
| PA-2a | -3890.098 | -3890.098 | 0 | 1.000 |
| PA-20a | -3511.953 | -3511.953 | 0 | 1.000 |
| PA-21a | -4633.779 | -4633.779 | 0 | 1.000 |
| PA-21b | -4633.779 | -4633.779 | 0 | 1.000 |
| PA-22a | -3028.643 | -3028.643 | 0 | 1.000 |
| PA-23a | -3900.362 | -3900.362 | 0 | 1.000 |
| PA-24a | -3216.405 | -3216.088 | 0.634 | 0.425 |
| PA-25a | -4302.157 | -4302.915 | 1.515 | 0.218 |
| PA-25b | -4304.818 | -4302.969 | 3.698 | 0.054 |
| PA-25c | -4306.118 | -4302.961 | 6.314 | 0.011 |
| PA-25e | -4301.424 | -4301.723 | 0 | 1.000 |
| PA-25f | -4303.380 | -4302.960 | 0 | 1.000 |
| PA-26a | -3926.596 | -3926.596 | 0 | 1.000 |
| PA-27a | -4229.964 | -4229.964 | 0 | 1.000 |
| PA-27b | -4229.956 | -4229.654 | 0.603 | 0.437 |
| PA-27c | -4229.964 | -4229.964 | 0 | 1.000 |
| PA-27d | -4229.964 | -4229.964 | 0 | 1.000 |
| PA-28a | -3140.326 | -3140.326 | 0 | 1.000 |
| PA-28b | -3140.326 | -3140.326 | 0 | 1.000 |
| PA-29a | -3254.204 | -3254.204 | 0 | 1.000 |
| PA-29b | -3253.011 | -3252.381 | 1.260 | 0.261 |
| PA-3a | -3175.215 | -3175.215 | 0 | 1.000 |
| PA-30a | -3476.715 | -3476.715 | 0 | 1.000 |
| PA-31a | -3256.162 | -3256.162 | 0 | 1.000 |
| PA-31b | -3257.198 | -3257.133 | 0.130 | 0.718 |
| PA-31c | -3257.742 | -3257.742 | 0 | 1.000 |
| PA-32a | -3130.131 | -3130.131 | 0 | 1.000 |
| PA-33a | -6634.609 | -6634.609 | 0 | 1.000 |
| PA-33b | -6634.609 | -6634.609 | 0 | 1.000 |
| PA-34a | -3050.783 | -3050.783 | 0 | 1.000 |
| PA-35a | -3157.280 | -3157.280 | 0 | 1.000 |
| PA-36a | -2964.535 | -2964.534 | 0.001 | 0.964 |
| PA-37a | -2993.896 | -2993.644 | 0.504 | 0.477 |
| PA-38a | -3263.104 | -3263.104 | 0 | 1.000 |
| PA-39a | -4282.160 | -4282.160 | 0 | 1.000 |
| PA-39b | -4282.160 | -4282.160 | 0 | 1.000 |
| PA-39c | -4280.949 | -4280.949 | 0 | 1.000 |
| PA-39d | -4282.161 | -4282.160 | 0.002 | 0.964 |
| PA-4a | -3039.318 | -3039.318 | 0 | 1.000 |
| PA-40a | -4293.984 | -4293.984 | 0 | 1.000 |
| PA-41a | -5442.357 | -5442.359 | 0.004 | 0.949 |
| PA-41j | -5439.913 | -5439.333 | 1.159 | 0.281 |
| PA-41k | -5442.358 | -5442.357 | 0.002 | 0.964 |
| PA-41l | -5442.357 | -5442.359 | 0.004 | 0.949 |
| PA-41b | -5440.356 | -5440.217 | 0.278 | 0.598 |
| PA-41c | -5442.358 | -5442.357 | 0.002 | 0.964 |
| PA-41d | -5437.275 | -5437.276 | 0.002 | 0.964 |
| PA-41e | -5441.658 | -5441.657 | 0.002 | 0.964 |
| PA-41f | -5442.357 | -5442.357 | 0 | 1.000 |
| PA-41g | -5442.357 | -5442.359 | 0.004 | 0.949 |
| PA-41h | -5442.358 | -5442.358 | 0 | 1.000 |
| PA-41i | -5441.148 | -5441.133 | 0.030 | 0.862 |
| PA-42a | -2898.606 | -2898.606 | 0 | 1.000 |
| PA-43a | -5970.087 | -5970.087 | 0 | 1.000 |
| PA-44a | -4538.832 | -4538.832 | 0 | 1.000 |
| PA-44b | -4535.323 | -4534.646 | 1.354 | 0.244 |
| PA-45a | -3348.172 | -3348.172 | 0 | 1.000 |
| PA-46a | -4503.586 | -4503.586 | 0 | 1.000 |
| PA-46b | -4503.586 | -4503.586 | 0 | 1.000 |
| PA-47a | -3399.705 | -3399.705 | 0 | 1.000 |
| PA-48a | -2905.002 | -2905.002 | 0 | 1.000 |
| PA-48b | -2905.002 | -2905.002 | 0 | 1.000 |
| PA-49a | -2960.233 | -2958.611 | 3.244 | 0.071 |
| PA-49b | -2958.101 | -2958.101 | 0 | 1.000 |
| PA-5a | -3785.388 | -3785.388 | 0 | 1.000 |
| PA-50a | -3352.907 | -3352.907 | 0 | 1.000 |
| PA-50b | -3353.507 | -3353.507 | 0 | 1.000 |
| PA-50c | -3353.507 | -3353.507 | 0 | 1.000 |
| PA-51a | -2941.649 | -2941.649 | 0 | 1.000 |
| PA-52a | -3116.367 | -3116.367 | 0 | 1.000 |
| PA-52b | -3115.747 | -3115.747 | 0 | 1.000 |
| PA-52c | -3116.367 | -3116.367 | 0 | 1.000 |
| PA-53a | -2993.052 | -2993.053 | 0.001 | 0.964 |
| PA-54a | -3168.629 | -3168.629 | 0 | 1.000 |
| PA-54b | -3168.520 | -3168.520 | 0 | 1.000 |
| PA-55a | -3012.520 | -3012.525 | 0.010 | 0.920 |
| PA-56a | -3159.602 | -3159.602 | 0 | 1.000 |
| PA-57a | -2984.978 | -2984.654 | 0.648 | 0.420 |
| PA-57b | -2985.659 | -2985.659 | 0 | 1.000 |
| PA-58a | -2947.755 | -2947.480 | 0.550 | 0.458 |
| PA-59a | -3760.181 | -3760.106 | 0.149 | 0.698 |
| PA-59b | -3764.594 | -3764.594 | 0 | 1.000 |
| PA-59c | -3766.481 | -3766.481 | 0 | 1.000 |
| PA-6a | -3335.730 | -3335.730 | 0 | 1.000 |
| PA-60a | -3248.198 | -3248.198 | 0 | 1.000 |
| PA-61a | -3698.881 | -3698.881 | 0 | 1.000 |
| PA-62a | -3195.306 | -3195.306 | 0 | 1.000 |
| PA-63a | -3424.935 | -3424.935 | 0 | 1.000 |
| PA-63b | -3424.935 | -3424.935 | 0 | 1.000 |
| PA-64a | -3579.810 | -3579.448 | 0.724 | 0.394 |
| PA-64b | -3581.123 | -3581.123 | 0 | 1.000 |
| PA-64c | -3581.123 | -3581.123 | 0 | 1.000 |
| PA-65a | -4206.526 | -4206.526 | 0 | 1.000 |
| PA-65b | -4206.214 | -4206.214 | 0 | 1.000 |
| PA-65c | -4206.526 | -4206.526 | 0 | 1.000 |
| PA-66a | -2997.352 | -2997.352 | 0 | 1.000 |
| PA-67a | -3324.988 | -3324.988 | 0 | 1.000 |
| PA-67b | -3324.310 | -3323.984 | 0.652 | 0.419 |
| PA-67c | -3324.988 | -3324.988 | 0 | 1.000 |
| PA-68a | -3236.571 | -3236.290 | 0.561 | 0.453 |
| PA-68b | -3237.270 | -3237.270 | 0 | 1.000 |
| PA-68c | -3237.270 | -3237.270 | 0 | 1.000 |
| PA-68d | -3237.270 | -3237.270 | 0 | 1.000 |
| PA-68e | -3236.609 | -3236.609 | 0 | 1.000 |
| PA-69a | -3789.113 | -3789.103 | 0.019 | 0.887 |
| PA-7a | -3093.157 | -3092.814 | 0 | 1.000 |
| PA-70a | -3343.698 | -3343.698 | 0 | 1.000 |
| PA-71a | -3023.335 | -3023.335 | 0 | 1.000 |
| PA-72a | -2957.709 | -2957.709 | 0 | 1.000 |
| PA-73a | -3073.049 | -3073.049 | 0 | 1.000 |
| PA-74a | -2955.693 | -2955.693 | 0 | 1.000 |
| PA-75a | -3274.211 | -3274.213 | 0.004 | 0.949 |
| PA-75b | -3274.282 | -3274.282 | 0 | 1.000 |
| PA-76a | -3110.305 | -3110.241 | 0.127 | 0.720 |
| PA-77a | -4041.005 | -4041.006 | 0 | 1.000 |
| PA-78a | -3831.227 | -3831.227 | 0 | 1.000 |
| PA-78b | -3830.110 | -3829.780 | 0.659 | 0.416 |
| PA-78c | -3829.631 | -3829.654 | 0.046 | 0.830 |
| PA-78d | -3831.227 | -3831.227 | 0 | 1.000 |
| PA-78e | -3830.896 | -3830.896 | 0 | 1.000 |
| PA-79a | -3069.452 | -3069.452 | 0 | 1.000 |
| PA-8a | -3013.338 | -3013.338 | 0 | 1.000 |
| PA-80a | -3489.777 | -3489.777 | 0 | 1.000 |
| PA-80b | -3489.299 | -3489.299 | 0 | 1.000 |
| PA-80c | -3489.777 | -3489.777 | 0 | 1.000 |
| PA-9a | -3113.102 | -3113.102 | 0 | 1.000 |
|  |  |  |  |  |
| PB1-1a | -3018.605 | -3018.605 | 0 | 1.000 |
| PB1-1b | -3018.605 | -3018.605 | 0 | 1.000 |
| PB1-10a | -3408.297 | -3408.297 | 0 | 1.000 |
| PB1-10b | -3408.756 | -3408.756 | 0 | 1.000 |
| PB1-11a | -3857.772 | -3857.772 | 0 | 1.000 |
| PB1-12a | -3609.684 | -3609.027 | 1.314 | 0.251 |
| PB1-13a | -3109.425 | -3109.425 | 0 | 1.000 |
| PB1-14a | -3182.319 | -3182.319 | 0 | 1.000 |
| PB1-15a | -3308.021 | -3308.021 | 0 | 1.000 |
| PB1-15b | -3307.506 | -3307.461 | 0.090 | 0.764 |
| PB1-16a | -3081.308 | -3081.308 | 0 | 1.000 |
| PB1-17a | -3083.104 | -3083.104 | 0 | 1.000 |
| PB1-17b | -3083.701 | -3083.701 | 0 | 1.000 |
| PB1-18a | -4125.970 | -4125.970 | 0 | 1.000 |
| PB1-18b | -4116.440 | -4116.440 | 0 | 1.000 |
| PB1-18c | -4125.858 | -4125.858 | 0 | 1.000 |
| PB1-19a | -3545.462 | -3545.462 | 0 | 1.000 |
| PB1-2a | -4014.865 | -4014.865 | 0 | 1.000 |
| PB1-20a | -3794.427 | -3794.427 | 0 | 1.000 |
| PB1-21a | -3222.463 | -3222.463 | 0 | 1.000 |
| PB1-22a | -3405.610 | -3405.572 | 0.076 | 0.782 |
| PB1-22b | -3405.173 | -3404.665 | 1.015 | 0.313 |
| PB1-22c | -3405.633 | -3405.633 | 0 | 1.000 |
| PB1-23a | -3231.749 | -3231.749 | 0 | 1.000 |
| PB1-23b | -3229.778 | -3229.477 | 0.601 | 0.437 |
| PB1-24a | -3784.450 | -3784.450 | 0 | 1.000 |
| PB1-24b | -3784.450 | -3784.450 | 0 | 1.000 |
| PB1-24c | -3783.683 | -3783.683 | 0 | 1.000 |
| PB1-24d | -3784.302 | -3784.302 | 0 | 1.000 |
| PB1-24e | -3784.450 | -3784.450 | 0 | 1.000 |
| PB1-25a | -3221.525 | -3221.525 | 0 | 1.000 |
| PB1-26a | -3085.796 | -3085.796 | 0 | 1.000 |
| PB1-27a | -3642.209 | -3642.209 | 0 | 1.000 |
| PB1-27b | -3642.209 | -3642.209 | 0 | 1.000 |
| PB1-27c | -3642.209 | -3642.209 | 0 | 1.000 |
| PB1-28a | -3709.272 | -3709.276 | 0.007 | 0.928 |
| PB1-29a | -3314.898 | -3314.897 | 0.002 | 0.964 |
| PB1-3a | -3792.322 | -3792.128 | 0.387 | 0.533 |
| PB1-3b | -3797.572 | -3797.572 | 0 | 1.000 |
| PB1-30a | -3078.330 | -3078.330 | 0 | 1.000 |
| PB1-31a | -5658.098 | -5658.194 | 0.192 | 0.661 |
| PB1-31c | -5658.026 | -5658.026 | 0 | 1.000 |
| PB1-31d | -5658.352 | -5658.171 | 0.361 | 0.547 |
| PB1-31e | -5658.267 | -5658.267 | 0 | 1.000 |
| PB1-31f | -5658.267 | -5658.267 | 0 | 1.000 |
| PB1-31g | -5658.098 | -5658.098 | 0 | 1.000 |
| PB1-31h | -5658.196 | -5658.671 | 0.950 | 0.329 |
| PB1-32a | -3044.087 | -3044.087 | 0 | 1.000 |
| PB1-33a | -3157.542 | -3157.542 | 0 | 1.000 |
| PB1-34a | -3413.633 | -3413.633 | 0 | 1.000 |
| PB1-35a | -3622.316 | -3622.316 | 0 | 1.000 |
| PB1-35b | -3622.316 | -3622.316 | 0 | 1.000 |
| PB1-36a | -3185.122 | -3185.122 | 0 | 1.000 |
| PB1-37a | -3505.119 | -3505.119 | 0 | 1.000 |
| PB1-37b | -3505.119 | -3505.119 | 0 | 1.000 |
| PB1-37c | -3505.179 | -3505.179 | 0 | 1.000 |
| PB1-38a | -2962.710 | -2962.710 | 0 | 1.000 |
| PB1-39a | -3201.807 | -3201.807 | 0 | 1.000 |
| PB1-39b | -3201.173 | -3201.173 | 0 | 1.000 |
| PB1-39c | -3201.807 | -3201.807 | 0 | 1.000 |
| PB1-4a | -3345.893 | -3345.893 | 0 | 1.000 |
| PB1-40a | -3036.246 | -3036.246 | 0 | 1.000 |
| PB1-41a | -3171.410 | -3171.410 | 0 | 1.000 |
| PB1-41b | -3170.612 | -3170.612 | 0 | 1.000 |
| PB1-42a | -3167.404 | -3167.404 | 0 | 1.000 |
| PB1-42b | -3171.664 | -3171.664 | 0 | 1.000 |
| PB1-43a | -3067.028 | -3067.028 | 0 | 1.000 |
| PB1-44a | -3026.506 | -3026.568 | 0.124 | 0.724 |
| PB1-44b | -3026.316 | -3026.622 | 0.612 | 0.434 |
| PB1-45a | -3419.064 | -3419.064 | 0 | 1.000 |
| PB1-46a | -4224.379 | -4224.210 | 0.337 | 0.560 |
| PB1-47a | -3032.098 | -3032.098 | 0 | 1.000 |
| PB1-48a | -3080.797 | -3080.532 | 0.529 | 0.466 |
| PB1-49a | -3484.195 | -3484.195 | 0 | 1.000 |
| PB1-49b | -3484.195 | -3484.195 | 0 | 1.000 |
| PB1-5a | -3276.253 | -3276.253 | 0 | 1.000 |
| PB1-50a | -3058.657 | -3058.657 | 0 | 1.000 |
| PB1-51a | -3353.820 | -3353.820 | 0 | 1.000 |
| PB1-52a | -3156.251 | -3156.252 | 0.001 | 0.964 |
| PB1-53a | -4790.845 | -4790.716 | 0.257 | 0.611 |
| PB1-53b | -4792.216 | -4792.216 | 0 | 1.000 |
| PB1-54a | -4812.042 | -4812.042 | 0 | 1.000 |
| PB1-55a | -3316.058 | -3316.058 | 0 | 1.000 |
| PB1-56a | -3001.834 | -3001.834 | 0 | 1.000 |
| PB1-57a | -3357.545 | -3357.545 | 0 | 1.000 |
| PB1-57b | -3357.545 | -3357.545 | 0 | 1.000 |
| PB1-58a | -3594.304 | -3594.304 | 0 | 1.000 |
| PB1-58b | -3594.304 | -3594.304 | 0 | 1.000 |
| PB1-59a | -5556.745 | -5556.830 | 0.170 | 0.680 |
| PB1-6a | -3024.480 | -3024.530 | 0.100 | 0.751 |
| PB1-60a | -3864.582 | -3864.581 | 0.001 | 0.964 |
| PB1-60b | -3864.699 | -3864.699 | 0 | 1.000 |
| PB1-60c | -3864.706 | -3864.706 | 0 | 1.000 |
| PB1-60d | -3864.706 | -3864.706 | 0 | 1.000 |
| PB1-60e | -3864.706 | -3864.706 | 0 | 1.000 |
| PB1-60f | -3864.706 | -3864.706 | 0 | 1.000 |
| PB1-60g | -3864.706 | -3864.706 | 0 | 1.000 |
| PB1-60h | -3864.706 | -3864.706 | 0 | 1.000 |
| PB1-61a | -3172.920 | -3172.920 | 0 | 1.000 |
| PB1-62a | -3181.092 | -3181.092 | 0 | 1.000 |
| PB1-63a | -3062.169 | -3062.169 | 0 | 1.000 |
| PB1-64a | -3443.846 | -3443.846 | 0 | 1.000 |
| PB1-64b | -3443.849 | -3443.846 | 0.006 | 0.938 |
| PB1-65a | -3203.292 | -3203.292 | 0 | 1.000 |
| PB1-65b | -3203.292 | -3203.292 | 0 | 1.000 |
| PB1-66a | -3226.212 | -3226.212 | 0 | 1.000 |
| PB1-67a | -4175.738 | -4175.738 | 0 | 1.000 |
| PB1-68a | -3624.848 | -3624.714 | 0.268 | 0.604 |
| PB1-68b | -3625.127 | -3625.127 | 0 | 1.000 |
| PB1-69a | -3153.262 | -3153.262 | 0 | 1.000 |
| PB1-7a | -3235.407 | -3235.414 | 0.014 | 0.905 |
| PB1-70a | -3393.515 | -3393.515 | 0 | 1.000 |
| PB1-71a | -3189.239 | -3189.239 | 0 | 1.000 |
| PB1-71b | -3189.239 | -3189.239 | 0 | 1.000 |
| PB1-72a | -3391.472 | -3391.471 | 0.002 | 0.964 |
| PB1-72b | -3391.471 | -3391.471 | 0 | 1.000 |
| PB1-72c | -3391.471 | -3391.471 | 0 | 1.000 |
| PB1-72d | -3391.471 | -3391.471 | 0 | 1.000 |
| PB1-73a | -3294.798 | -3294.798 | 0 | 1.000 |
| PB1-73b | -3294.798 | -3294.798 | 0 | 1.000 |
| PB1-73c | -3294.798 | -3294.798 | 0 | 1.000 |
| PB1-73d | -3294.798 | -3294.798 | 0 | 1.000 |
| PB1-73e | -3294.798 | -3294.798 | 0 | 1.000 |
| PB1-74a | -3162.196 | -3162.196 | 0 | 1.000 |
| PB1-75a | -3130.126 | -3130.126 | 0 | 1.000 |
| PB1-76a | -3011.968 | -3011.968 | 0 | 1.000 |
| PB1-77a | -4222.649 | -4222.649 | 0 | 1.000 |
| PB1-77b | -4222.585 | -4222.585 | 0 | 1.000 |
| PB1-78a | -3199.209 | -3199.209 | 0 | 1.000 |
| PB1-79a | -3154.409 | -3154.409 | 0 | 1.000 |
| PB1-79b | -3154.409 | -3154.409 | 0 | 1.000 |
| PB1-79c | -3154.409 | -3154.410 | 0.001 | 0.964 |
| PB1-8a | -3191.515 | -3191.515 | 0 | 1.000 |
| PB1-80a | -2963.443 | -2963.440 | 0.006 | 0.938 |
| PB1-80b | -2963.440 | -2963.440 | 0 | 1.000 |
| PB1-81a | -3242.559 | -3242.559 | 0 | 1.000 |
| PB1-81b | -3242.825 | -3242.825 | 0 | 1.000 |
| PB1-82a | -3101.665 | -3101.655 | 0.019 | 0.887 |
| PB1-82b | -3101.692 | -3101.966 | 0.547 | 0.459 |
| PB1-82c | -3101.966 | -3101.692 | 0.547 | 0.459 |
| PB1-83a | -3101.227 | -3101.227 | 0 | 1.000 |
| PB1-84a | -3042.196 | -3042.196 | 0 | 1.000 |
| PB1-84b | -3042.196 | -3042.196 | 0 | 1.000 |
| PB1-85a | -3144.636 | -3144.636 | 0 | 1.000 |
| PB1-86a | -3025.558 | -3025.558 | 0 | 1.000 |
| PB1-87a | -3985.743 | -3985.743 | 0 | 1.000 |
| PB1-87b | -3985.743 | -3985.743 | 0 | 1.000 |
| PB1-88a | -3104.124 | -3103.547 | 1.153 | 0.282 |
| PB1-89a | -3050.400 | -3050.305 | 0 | 1.000 |
| PB1-9a | -4442.256 | -4442.256 | 0 | 1.000 |
| PB1-9j | -4442.256 | -4442.256 | 0 | 1.000 |
| PB1-9b | -4442.256 | -4442.256 | 0 | 1.000 |
| PB1-9c | -4441.137 | -4440.818 | 0.637 | 0.424 |
| PB1-9d | -4442.256 | -4442.256 | 0 | 1.000 |
| PB1-9e | -4442.256 | -4442.256 | 0 | 1.000 |
| PB1-9f | -4442.256 | -4442.256 | 0 | 1.000 |
| PB1-9g | -4442.256 | -4442.256 | 0 | 1.000 |
| PB1-9h | -4441.130 | -4440.811 | 0.638 | 0.424 |
| PB1-9i | -4442.256 | -4442.256 | 0 | 1.000 |
| PB1-90a | -3377.369 | -3377.369 | 0 | 1.000 |
| PB1-90b | -3377.369 | -3377.369 | 0 | 1.000 |
| PB1-90c | -3377.021 | -3377.021 | 0 | 1.000 |
