# Supplementary material for: Adaptive Evolution of Human-Isolated H5Nx Avian Influenza A Viruses
Source: Front Microbiol. 2019 Jun 12;10:1328. doi: 10.3389/fmicb.2019.01328 (PMC6582624; doi:10.3389/fmicb.2019.01328)
Supplement: Supplementary file 15 [file Table_5.DOCX]

**Supplementary table 5:** Proportions of adaptive mutations (positive selection and convergent/parallel evolution sites) in the human- and avian-isolated strains.

| Gene |  | Amino acid changes | Avian isolates | Human isolates |
| --- | --- | --- | --- | --- |
| HA | Convergent / Parallet sites | D31N | 0.57%(47/8287) | 0.63%(3/480) |
|  |  | K35R | 1.79%(148/8287) | 5.00%(24/480) |
|  |  | D45N | 42.22%(3499/8287) | 9.79%(47/480) |
|  |  | D88G | 17.82%(1477/8287) | 4.17%(20/480) |
|  |  | A127T | 20.41%(1691/8287) | 93.54%(449/480) |
|  |  | A/T127S | 1.40%(116/8287) | 2.71%(13/480) |
|  |  | A134V | 0.01%(1/8287) | 2.08%(10/480) |
|  |  | R140K | 12.67%(1050/8287) | 16.67%(80/480) |
|  |  | M140T | 13.36%(1107/8287) | 10.42%(50/480) |
|  |  | S141P | 35.61%(2951/8287) | 38.33%(184/480) |
|  |  | D154N | 74.66%(6187/8287) | 81.67%(392/480) |
|  |  | R162I | 12.90%(1069/8287) | 2.50%(12/480) |
|  |  | V174I | 45.99%(3811/8287) | 17.92%(86/480) |
|  |  | N182S | 0.34%(28/8287) | 1.67%(8/480) |
|  |  | E184G | 0.18%(15/8287) | 1.46%(7/480) |
|  |  | T195A | 0.47%(39/8287) | 0.42%(2/480) |
|  |  | T/N195I | 1.47%(122/8287) | 1.04%(5/480) |
|  |  | K189R | 36.96%(3063/8287) | 57.08%(274/480) |
|  |  | V219I | 14.43%(1196/8287) | 2.29%(11/480) |
|  |  | S223N | 0.06%(5/8287) | 3.13%(15/480) |
|  |  | R310K | 55.42%(4593/8287) | 18.96%(91/480) |
|  |  | R323K | 1.57%(130/8287) | 0.63%(3/480) |
|  |  | R326K | 0.21%(17/8287) | 0.63%(3/480) |
|  |  | I375M | 0.08%(7/8287) | 0.83%(4/480) |
|  |  | D376N | 1.42%(118/8287) | 0.21%(1/480) |
|  |  | D387N | 2.21%(183/8287) | 5.00%(24/480) |
|  |  | E433G | 0.02%(2/8287) | 0.42%(2/480) |
|  |  | N476D | 4.22%(350/8287) | 1.88%(9/480) |
|  |  | E477K | 1.24%(103/8287) | 0.63%(3/480) |
|  |  | M479I | 1.04%(86/8287) | 1.46%(7/480) |
|  |  | R497K | 1.62%(134/8287) | 1.25%(6/480) |
|  |  | E502G | 0.08%(7/8287) | 0.21%(1/480) |
|  |  | M532I | 16.30%(1351/8287) | 4.17%(20/480) |
|  |  | V533I | 9.92%(822/8287) | 13.96%(67/480) |
|  | Positive selectived sites | 473R | 19.97%(1655/8287) | 31.25%(150/480) |
|  |  | 11N | 96.39%(7988/8287) | 97.92%(470/480) |
|  |  | 15Q | 95.32%(7899/8287) | 97.71%(469/480) |
|  |  | 20M | 97.18%(8053/8287) | 98.54%(473/480) |
|  |  | 304K | 98.35%(8150/8287) | 99.79%(479/480) |
|  |  | 315T | 99.28%(8227/8287) | 98.96%(475/480) |
|  |  | 522T | 91.19%(7557/8287) | 97.08%(466/480) |
|  |  | 529L | 90.84%(7528/8287) | 96.25%(462/480) |
|  |  | 546L | 85.66%(7099/8287) | 91.67%(440/480) |
|  |  | 547Q | 85.35%(7073/8287) | 91.04%(437/480) |
|  |  | 548C | 85.05%(7048/8287) | 90.63%(435/480) |
|  |  | 212R | 9.81%(813/8287) | 21.04%(101/480) |
|  |  | 500R | 91.53%(7585/8287) | 97.08%(466/480) |
| MP | Convergent / Parallet sites | T137A | 0.62%(37/5981) | 17.70%(60/339) |
|  |  | A239T | 0.20%(12/5981) | 0.59%(2/339) |
|  |  | C269Y | 0.55%(33/5981) | 0.29%(1/339) |
|  |  | V280I | 25.56% (1529/5981) | 11.80%(40/339) |
|  |  | S283N | 21.40%(1280/5981) | 32.15%(109/339) |
|  |  | D340N | 0.92%(55/5981) | 0.59%(2/339) |
|  | Positive selectived sites | 5T | 94.98% (5681/5981) | 89.97%(305/339) |
|  |  | 6E | 96.27% (5758/5981) | 90.86%(308/339) |
|  |  | 7V | 96.56% (5775/5981) | 93.81%(318/339) |
|  |  | 8E | 96.59% (5777/5981) | 93.81%(318/339) |
|  |  | 257T | 94.98% (5681/5981) | 89.97%(305/339) |
|  |  | 258E | 96.27% (5758/5981) | 90.86%(308/339) |
|  |  | 259V | 96.56% (5775/5981) | 93.81%(318/339) |
|  |  | 260E | 96.59% (5777/5981) | 93.81%(318/339) |
|  |  | 277P | 97.84% (5852/5981) | 99.12%(336/339) |
|  |  | 279V | 90.34% (5403/5981) | 78.47%(266/339) |
|  |  | 282A | 97.71% (5844/5981) | 99.12%(336/339) |
|  |  | 283N | 21.40% (1280/5981) | 32.15%(109/339) |
|  |  | 284I | 97.54% (5834/5981) | 98.82%(335/339) |
|  |  | 285I | 98.11% (5868/5981) | 99.71%(338/339) |
|  |  | 287I | 97.17% (5812/5981) | 99.12%(336/339) |
|  |  | 292L | 97.86% (5853/5981) | 98.82%(335/339) |
|  |  | 328Y | 97.27% (5818/5981) | 97.35%(330/339) |
|  |  | 330Q | 96.04% (5744/5981) | 97.35%(330/339) |
|  |  | 336V | 96.02% (5743/5981) | 96.46%(327/339) |
|  |  | 339D | 95.74% (5726/5981) | 95.58%(324/339) |
|  |  | 340D | 93.75% (5607/5981) | 94.69%(321/339) |
|  |  | 344V | 92.59% (5538/5981) | 93.22%(316/339) |
|  | Convergent / Parallet sites | I8T | 1.74%(74/4262) | 0.71%(3/422) |
|  |  | I/V16A | 2.58%(110/4262) | 4.98%(21/422) |
|  |  | V16I | 6.92%(295/4262) | 0.47%(2/422) |
|  |  | V33I | 4.69%(200/4262) | 10.90%(46/422) |
|  |  | N39S | 1.78%(76/4262) | 0.47%(2/422) |
|  |  | P45T | 1.45%(62/4262) | 0.47%(2/422) |
|  |  | K55R | 0.16%(7/4262) | 0.71%(3/422) |
|  |  | A58T | 1.78%(76/4262) | 0.71%(3/422) |
|  |  | K241R | 1.24%(53/4262) | 4.50%(19/422) |
|  |  | I243V | 85.95%(3663/4262) | 86.49%(365/422) |
|  |  | N305T | 1.34%(57/4262) | 2.13%(9/422) |
|  |  | G318S | 1.01%(43/4262) | 0.71%(3/422) |
|  |  | P323S | 31.32%(1335/4262) | 28.44%(120/422) |
|  |  | G365E | 25.93%(1105/4262) | 36.49%(154/422) |
|  |  | I380V | 0.23%(10/4262) | 0.71%(3/422) |
|  |  | V404I | 0.42%(18/4262) | 0.95%(4/422) |
|  |  | N430S | 5.14%(219/4262) | 8.53%(36/422) |
|  |  | N430D | 1.76%(75/4262) | 1.18%(5/422) |
|  |  | G435S | 69.12%(2946/4262) | 70.38%(297/422) |
|  |  | T441D | 0.38%(16/4262) | 5.92%(25/422) |
|  | Positive selectived sites | 188N | 97.39%(4151/4262) | 99.76%(421/422) |
| NP | Convergent / Parallet sites | R100I | 0.02%(1/5538) | 0.61%(2/327) |
|  |  | A284T | 0.04%(2/5538) | 2.14%(7/327) |
|  |  | V343I | 0.83%(46/5538) | 1.53%(5/327) |
|  |  | R384K | 0.65%(36/5538) | 0.92%(3/327) |
|  |  | S413L | 0%(0/5538) | 0.61%(2/327) |
|  |  | P419S | 0%(0/5538) | 0.61%(2/327) |
|  |  | R452K | 29.31%(1623/5538) | 7.65%(25/327) |
|  |  | P453S | 1.46%(81/5538) | 2.14%(7/327) |
|  | Positive selectived sites | 486S | 93.23%(5163/5538) | 95.11%(311/327) |
|  |  | 487Y | 92.56%(5126/5538) | 94.50%(309/327) |
| NS1 | Convergent / Parallet sites | G47S | 2.45%(144/5870) | 4.63%(15/324) |
|  |  | N48S | 29.60%(1738/5870) | 16.05%(52/324) |
|  |  | R59H | 1.91%(112/5870) | 5.25%(17/324) |
|  |  | R67Q | 0.65%(38/5870) | 1.23%(4/324) |
|  |  | E70K | 7.32%(430/5870) | 1.85%(6/324) |
|  |  | T81I | 0.17%(10/5870) | 0.62%(2/324) |
|  |  | R88C | 0.99%(58/5870) | 1.85%(6/324) |
|  |  | V136A | 0.05%(3/5870) | 1.54%(5/324) |
|  |  | I137V | 1.28%(75/5870) | 0.93%(3/324) |
|  |  | D139N | 13.81%(811/5870) | 7.72%(25/324) |
|  |  | L185F | 5.16%(303/5870) | 11.42%(37/324) |
|  |  | S205N | 9.54%(560/5870) | 3.40%(11/324) |
|  |  | D209N | 10.02%(588/5870) | 6.17%(20/324) |
|  |  | V209I | 0.09%(5/5870) | 0.93%(3/324) |
|  |  | L212F | 0.56%(33/5870) | 1.23%(4/324) |
| NEP/NS2 | Convergent / Parallet sites | M/A14V | 31.24%(1834/5870) | 68.83%(223/324) |
|  |  | A48T | 9.64%(566/5870) | 3.40%(11/324) |
|  |  | T/V115A | 46.10%(2706/5870) | 62.96%(204/324) |
| PA | Convergent / Parallet sites | F4C | 0.00%(0/5323) | 0.31%(1/324) |
|  |  | M12I | 0.43%(23/5323) | 0.62%(2/324) |
|  |  | M86V | 1.33%(71/5323) | 2.47%(8/324) |
|  |  | T97I | 0.36%(19/5323) | 97.84%(317/324) |
|  |  | F105L | 0.53%(28/5323) | 1.23%(4/324) |
|  |  | K142E | 0.02%(1/5323) | 1.23%(4/324) |
|  |  | L226F | 0.75%(40/5323) | 0.93%(3/324) |
|  |  | E237K | 16.01%(852/5323) | 8.95%(29/324) |
|  |  | C241Y | 2.67%(142/5323) | 3.70%(12/324) |
|  |  | P275L | 1.16%(62/5323) | 0.31%(1/324) |
|  |  | N321K | 1.47%(78/5323) | 1.23%(4/324) |
|  |  | T369A | 97.48%(5189/5323) | 97.22%(315/324) |
|  |  | V387I | 6.78%(361/5323) | 15.12%(49/324) |
|  |  | K615R | 5.43%(289/5323) | 11.42%(37/324) |
| PB1 | Convergent / Parallet sites | I57T | 93.73%(5159/5504) | 88.25%(278/315) |
|  |  | E172D | 4.34%(239/5504) | 4.44%(14/315) |
|  |  | M179I | 2.63%(145/5504) | 0.32%(1/315) |
|  |  | S361G | 0.22%(12/5504) | 1.27%(4/315) |
|  |  | N375S | 10.79%(594/5504) | 5.40%(17/315) |
|  |  | K387R | 6.56%(361/5504) | 1.27%(4/315) |
|  |  | L598P | 0.09%(5/5504) | 1.59%(5/315) |
| PB2 | Convergent / Parallet sites | I64M | 49.04%(2636/5375) | 17.04%(53/311) |
|  |  | V108A | 13.36%(718/5375) | 47.59%(148/311) |
|  |  | I147M | 0.26%(14/5375) | 4.18%(13/311) |
|  |  | E192K | 0.04%(2/5375) | 0.96%(3/311) |
|  |  | T271A | 0.06%(3/5375) | 0.32%(1/311) |
|  |  | A274T | 0.35%(19/5375) | 0.32%(1/311) |
|  |  | T339M | 1.43%(77/5375) | 3.54%(11/311) |
|  |  | R369K | 2.92%(157/5375) | 9.97%(31/311) |
|  |  | V451I | 69.17%(3718/5375) | 83.28%(259/311) |
|  |  | N456D | 0.41%(22/5375) | 2.89%(9/311) |
|  |  | I461V | 11.07%(595/5375) | 1.93%(6/311) |
|  |  | M570V | 0.37%(20/5375) | 0.96%(3/311) |
|  |  | Q591K | 0.06%(3/5375) | 0.96%(3/311) |
|  |  | I615V | 0.43%(23/5375) | 2.25%(7/311) |
|  |  | E627K | 11.14%(599/5375) | 34.73%(108/311) |
|  |  | V649I | 22.33%(1200/5375) | 16.72%(52/311) |
|  |  | D701N | 0.07%(4/5375) | 4.82%(15/311) |
|  |  | G727R | 0.30%(16/5375) | 1.29%(4/311) |
|  |  | D740N | 0.19%(10/5375) | 2.89%(9/311) |
|  |  | S741F | 0.30%(16/5375) | 0.64%(2/311) |
|  |  |  |  |  |

Note: Sites marked in yellow mean the proportions of these mutations are higher in the human- than avian-isolated strains.
